# Genome Profiling of SARS-CoV-2 in Indonesia, ASEAN and the Neighbouring East Asian Countries: Features, Challenges and Achievements

**DOI:** 10.3390/v14040778

**Published:** 2022-04-08

**Authors:** Inswasti Cahyani, Eko W. Putro, Asep M. Ridwanuloh, Satrio Wibowo, Hariyatun Hariyatun, Gita Syahputra, Gilang Akbariani, Ahmad R. Utomo, Mohammad Ilyas, Matthew Loose, Wien Kusharyoto, Susanti Susanti

**Affiliations:** 1 COMGen Division, School of Life Sciences, University of Nottingham, Nottingham NG7 2UH, UK; inswasti.cahyani@nottingham.ac.uk (I.C.); matt.loose@nottingham.ac.uk (M.L.); 2 Research Center for Biotechnology, National Research and Innovation Agency (Badan Riset dan Inovasi Nasional/BRIN), Bogor 16911, Indonesia; eko.wahyu.putro@brin.go.id (E.W.P.); asep.muhamad.ridwanuloh@brin.go.id (A.M.R.); hariyatun@brin.go.id (H.H.); gita.syahputra@brin.go.id (G.S.); wien.kusharyoto@brin.go.id (W.K.); 3 PathGen Diagnostik Teknologi, Center for Innovation and Utilization of Science and Technology, National Research and Innovation Agency (Badan Riset dan Inovasi Nasional/BRIN), Bogor 16911, Indonesia; satrio@pathgen.co.id (S.W.); gilang@pathgen.co.id (G.A.); 4 Graduate School of Biomedical Science, Universitas YARSI, Jakarta 10510, Indonesia; ahmad.utomo@gmail.com; 5 Molecular Pathology Research Group, Academic Unit of Translational Medical Science, Biodiscovery Institute, School of Medicine, University of Nottingham, Nottingham NG7 2UH, UK; mohammad.ilyas@nottingham.ac.uk; 6 Department of Pharmacology and Clinical Pharmacy, Faculty of Pharmacy, Universitas Muhammadiyah, Purwokerto 53182, Indonesia

**Keywords:** ASEAN, COVID-19, genomic surveillance, GISAID, nanopore, NICCRAT, SARS-CoV-2, variants of concern, whole-genome sequencing

## Abstract

Whole-genome sequencing (WGS) has played a significant role in understanding the epidemiology and biology of SARS-CoV-2 virus. Here, we investigate the use of SARS-CoV-2 WGS in Southeast and East Asian countries as a genomic surveillance during the COVID-19 pandemic. Nottingham–Indonesia Collaboration for Clinical Research and Training (NICCRAT) initiative has facilitated collaboration between the University of Nottingham and a team in the Research Center for Biotechnology, National Research and Innovation Agency (BRIN), to carry out a small number of SARS-CoV-2 WGS in Indonesia using Oxford Nanopore Technology (ONT). Analyses of SARS- CoV-2 genomes deposited on GISAID reveal the importance of clinical and demographic metadata collection and the importance of open access and data sharing. Lineage and phylogenetic analyses of two periods defined by the Delta variant outbreak reveal that: (1) B.1.466.2 variants were the most predominant in Indonesia before the Delta variant outbreak, having a unique spike gene mutation N439K at more than 98% frequency, (2) Delta variants AY.23 sub-lineage took over after June 2021, and (3) the highest rate of virus transmissions between Indonesia and other countries was through interactions with Singapore and Japan, two neighbouring countries with a high degree of access and travels to and from Indonesia.

## 1. Introduction

Coronaviruses belong to a large family of RNA viruses that usually cause respiratory illnesses. Most of the time, diseases caused by coronaviruses, such as HCoV-229E, OC43, NL63, and HKU1, are mild [1]. Nonetheless, in the past two decades, more deadly forms of coronaviruses have emerged, including severe acute respiratory syndrome coronavirus (SARS-CoV) [2] and the Middle East respiratory syndrome coronavirus (MERS) [3]. Most recently, coronavirus disease 2019 (COVID-19) emerged and was declared a global pandemic by the World Health Organization (WHO) on 11 March 2020 [4]. This disease is caused by a novel, previously unreported coronavirus strain subsequently named severe acute respiratory syndrome coronavirus-2 (SARS-CoV-2) [5].

COVID-19 was first reported in Wuhan in early December 2019 [6]. In early January 2020, the Chinese government released the first genome sequence of SARS-CoV-2 (i.e., labelled as WH-Human_1) [7,8]. By the end of February 2020, the infection had been reported in 51 countries, with almost 84,000 cases [9]. Despite being one of the world’s most populated countries, with more than 260 million people, and no travel restrictions in place at the time, Indonesia only diagnosed and announced its first case of infection on 2 March 2020 [10]. However, neighbouring countries, such as Singapore and Malaysia, had reported their first cases of infection on 23 and 25 January 2020, respectively. Before March 2020, Indonesia only carried out early testing and screening of individuals with symptoms and travel links [11,12].

Besides the utility of screening and tracing for containing and reducing transmission, there is also a need to pinpoint and follow variants of SARS-CoV-2 that might worsen the pandemic. The World Health Organization defines a variant to be a variant of interest (VoI) if it has changed phenotypically from the reference genome (or shown phenotypic implications) and has been identified to cause community transmissions or detected in multiple countries [13]. A VoI can become a variant of concern (VoC) if it has been demonstrated to (1) increase transmissibility or detrimental changes in COVID-19 epidemiology; (2) exhibit an increase in virulence or change in clinical disease presentation; or (3) decrease the effectiveness of public health and social measures or available diagnostics, vaccines, and therapeutics [13].

In the next-generation sequencing (NGS) era, whole-genome sequencing (WGS) for epidemiological monitoring should be straightforward, in principle, with the availability of many tools and relatively low cost of sequencing compared to one or two decades ago. Unfortunately, WGS, which uses cutting-edge sequencing technology, typically means that only developed countries with access and resources to technology can readily use it in a pandemic. There is a need for reliable, easy to set-up, rapid, and cost-effective genome sequencing technology in resource-limited countries, including Indonesia. In the relatively early days of the pandemic, on a small number of samples (*n* = 12), we showed that Oxford Nanopore Technology^®^ (ONT), Oxford, United Kingdom, can be used to perform whole-genome sequencing that fits with the Indonesian context.

The Association of Southeast Asian Nations (ASEAN) accommodates free cross-border travels for its members, with Indonesia having the largest area and population size. Indonesia also has bilateral travel agreements that make travel more accessible to other East Asian countries, such as Japan and China. With global travel shown to be a significant contributing factor to the rapid spread of COVID-19 [14], we sought to compare the genomic epidemiology and metadata of SARS-CoV-2 between ASEAN member countries and others in the region deposited on GISAID [15]. This report covers two defining periods in Indonesia: (1) pre-Delta variant outbreak (1 March 2020–1 June 2021) and (2) Delta variant outbreak (2 June–1 October 2021). We highlighted how the knowledge obtained from the WGS of SARS-CoV-2 is pivotal in the understanding and tackling of COVID-19 spread in Indonesia and its surrounding regions.

## 2. Materials and Methods

### 2.1. NICCRAT-BRIN Team Sample Collection and WGS

Samples (*n* = 12) were collected from nasopharyngeal and oropharyngeal swabs of patients in viral transport medium [16,17,18,19] or DNA/RNA ShieldTM (Zymo Research, Irvine, CA, USA) abiding by the Center for Disease Control’s (CDC) guidelines for pathogen inactivation [20]. These samples were collected between 7 September 2020 and 13 January 2021 by the Indonesian counterpart of Nottingham–Indonesia Collaboration for Clinical Research and Training (NICCRAT) in the Research Center for Biotechnology, National Research and Innovation Agency (the NICCRAT-BRIN team). RNA extraction was carried out using Viral Nucleic Acid Extraction Kit II (Geneaid Biotech Ltd., New Taipei City, Taiwan) according to the manufacturer’s instructions. All samples were tested for the presence of SARS-CoV-2 with an RT–qPCR assay [16,17,18,19,21] using Real-Q 2019-nCoV Detection Kit (Rev.2 (2020.03.25); BioSewoom, Seoul, Korea) according to the manufacturer’s instructions in a CFX96 Touch Real-Time PCR Detection System (BioRad, Hercules, CA, USA) machine. Samples with a Ct value of 11–30 were chosen for sequencing [22,23].

Sequencing libraries were prepared based on the ARTIC nCoV-2019 sequencing protocol v2 (GunIt) V.2 [22,23], with minor modifications, using reverse-transcribed cDNA. Primers were obtained from IDT, Coralville, IA, USA (ARTIC nCoV-2019 Amplicon Panel Version 3) [22,23]. Libraries were loaded on a MinION device and flow cell. ONT’s MinKNOW software v.4.1.23, ONT, Oxford, United Kingdom was used to run sequencing. The Rampart software v.1.2.0, ARTIC network, United Kingdom was applied to monitor coverage of each barcoded sample in real time by running fast base-calling.

### 2.2. Ethical Statements on the Use of Samples from Human Participants

The study protocol was reviewed and approved by the Health Research Ethics Committee, University of Indonesia and Cipto Mangunkusumo Hospital (HREC-FMUI/CMH) (20-10-1321_EXP). We confirm that all research was performed in accordance with the relevant guidelines/regulations stipulated in the approval. All oropharyngeal and nasopharyngeal swab samples used in this study were those accompanied by written informed consents signed by the patients, agreeing to donate their samples for research purposes.

### 2.3. NICCRAT-BRIN Team Bioinformatics Processes

A validated pipeline was followed to conduct high-accuracy base-calling, demultiplexing, trimming, alignment, and consensus establishment [24]. Medaka workflow was used in generating consensus genome sequences due to the speed and GPU compatibility. A minimum of 20× coverage was set in the pipeline [25]. Concatenated consensus sequences of all barcoded samples which also contained the number of ambiguous nucleotides (Ns) were then further analysed. Assignment of clade membership was conducted by PANGOLIN (version 28 September 2021) [26]. FASTA files of assembled sequences along with metadata in comma-separated values (.csv) files were prepared for submission to GISAID. The metadata included collection date, location, the origin of samples, passage history, and sequencing technology. Further analysis for possible missing metadata, FASTA sequences, certain information, and frameshift mutations was undertaken following the result of GISAID quality control checking after submission [27]. In total, 12 genomes were submitted to GISAID from the NICCRAT-BRIN team.

### 2.4. Lineage and Phylogenetic Analyses

Full-genome SARS-CoV-2 sequences from ASEAN member countries, China, Hong Kong, Japan, South Korea, Taiwan, and the United Kingdom, along with their metadata, were downloaded from the GISAID database per 1 October 2021. The metadata already contains annotations of genomes based on PANGOLIN [28]. These lineages were used to sub-divide the genomes in further analyses. The list of mutations in each VoC and Indonesian-associated variant were analysed and Venn diagrams were created using a web tool [29].

All Indonesian sequences and those transmitted from or historically linked to Indonesia were selected for phylogenetic analysis; only complete genomes with high coverage were chosen for phylogenetic analysis. Up to 1 October 2021, there were 4650 sequences that were aligned and the 5′ and 3′ ends trimmed using MAFFT v.7.475, Katoh, RIMD, Osaka, Japan with automatic flavour selection [30]. Maximum likelihood phylogenetic trees were produced from the trimmed sequences using IQ-TREE2 v.1.6.12, Minh, Canberra, ACT, Australia [31], employing the GTR+F+R3 model of nucleotide substitution as suggested by the software’s model finder [32], with 1000 SH-like approximate likelihood ratio test (SH-aLRT) Ultra-Fast Bootstrap [33]. The tree was visualised and annotated using FigTree v.1.4.4, Rambaut, Edinburgh, United Kingdom [34].

## 3. Results

### 3.1. Analyses of the Genomes Deposited by the NICCRAT-BRIN Team

The NICCRAT-BRIN team sequenced 12 samples collected from a period of 7 September 2020 to 13 January 2021. Nine of these samples were of high coverage quality (Table S1). Lineage assignation by PANGOLIN confirmed three variants that were frequently detected in Indonesia during the early period of the pandemic: B.1.398, B.1.459, and B.1.470 (Table S1). These variants are among those we term the Indonesian-associated variants. As well as a B.1.1 variant, all 12 genomes share common mutations in the spike protein D614G and NSP12_P323L (Table S1). These mutations were newly acquired by the variants and were not present in the original SARS-CoV-2 reference genome from Wuhan [7].

There was no recorded travel history accompanying the metadata of our sequenced genomes. The metadata record showed that five of the patients were symptomatic and another one was hospitalised (Table S1). The sex proportion was an equal 50:50 of females to males, while the age of these patients ranged from 12 to 67 years (Table S1).

Considering the small number of samples, it was not feasible to conduct further phylogenetic, mutation, and/or transmission pattern analyses. Therefore, we expanded our analyses by including WGS data from other institutions in Indonesia (Table S2). These institutions, and more afterwards, were to be included in the nationwide network for SARS-CoV-2 WGS for pandemic surveillance. In addition, we also looked at the genome data of ASEAN member countries and some of our East Asian neighbours to obtain more thorough insights on the Indonesian-associated variants and transmission pattern within the region.

### 3.2. Profiles of Genomic Sequences Deposited by Indonesia, Neigbouring Countries, and the World

#### 3.2.1. Sequencing Rates

**Figure 1:**
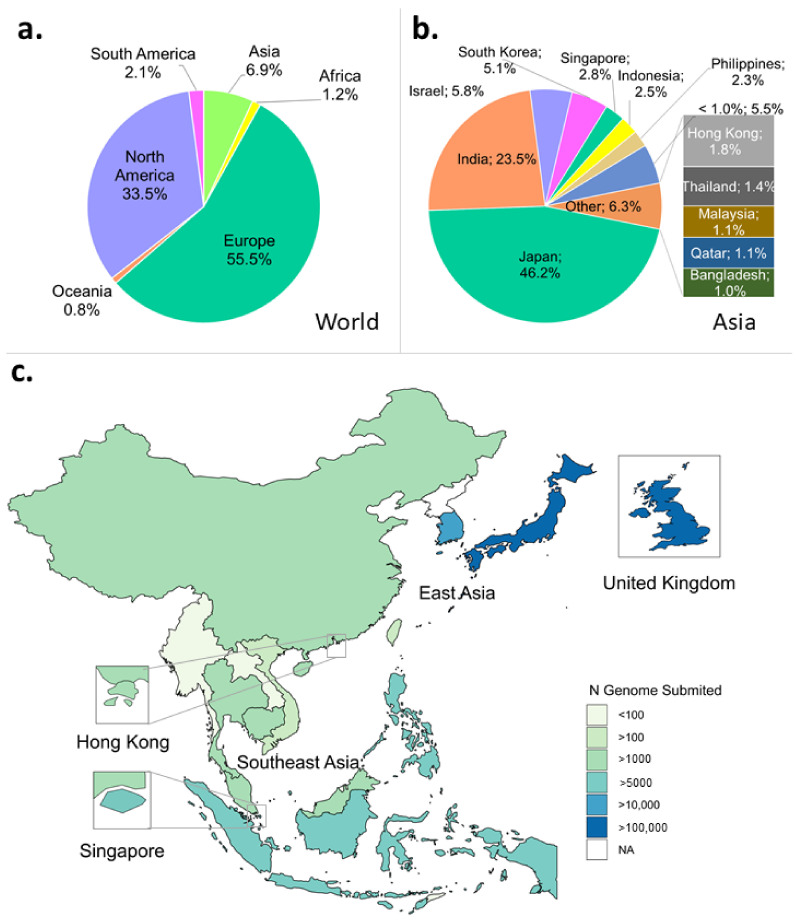
Profiles of SARS-CoV-2 WGS in the world, Asian continent, and East and Southeast Asian region submitted to GISAID. Metadata were downloaded per 1 October 2021. (**a**) Asia only accounts for almost 7% of the total number of submitted genomes compared to the rest of the world. (**b**) In Asia, Japan is the country with the highest number of submitted genomes. (**c**) Distribution map of the number of genomes submitted to GISAID in ASEAN, East Asia region, and the UK.

Despite being the largest continent in the world, Asia has deposited only about 7% of the total SARS-CoV-2 genomes on GISAID per 1 October 2021, with Japan submitting the most at just over 46% (Figure 1a,b). Indonesia contributed to 2.5% of genomes submitted from Asia, slightly behind its close neighbour, Singapore, at 2.8%, amounting to more than 5000 genomes per country (Figure 1b,c). Moreover, Indonesia experienced the highest number of positive cases in the Southeast and East Asian region, amounting to more than 4.2 million per 30 October 2021 (Figure S1A). This number was 2.5 times higher than the number of cases detected five months earlier (Figure S1B).

Sequencing rate, calculated as the ratio of genome number to positive cases, was still low in Indonesia per 1 October 2021 in contrast to some of ASEAN’s neighbours, such as Hong Kong, Japan, and South Korea (Figure S1C). These countries have a COVID-19 test rate proportional to their population size (Figure S2). However, despite Indonesia’s low sequencing rate, it has managed to increase sequencing from 0.10% pre-Delta to 0.17% during Delta outbreak within five months (compare Figure S1C,D).

#### 3.2.2. Metadata

In the pre-Delta outbreak, Indonesia performed well in terms of completeness of the clinical metadata recording compared to other ASEAN and neighbouring countries (Table S3). Over 70% of the Indonesian genomes have the corresponding clinical metadata. However, advancing to the Delta outbreak period, the overall proportion of recorded metadata has decreased to just over 37% (Table 1). Indonesia also recorded most sample sources used in WGS (i.e., nasal and oropharyngeal swab), which was lacking in many other large datasets, such as from COG-UK (Figure S3). Many countries, including Japan as the top contributor of SARS-CoV-2 genomes (Figure 1b), did not have accompanying clinical and/or demographic metadata submitted to GISAID (Table 1). Most likely, these metadata are stored in a local/national repository but not reported to GISAID to comply with their respective data-sharing regulations.

Recording metadata effectively proved to be challenging, as GISAID only provides one column for filling patient status. Furthermore, many of the categories in this column were overlapping and not mutually exclusive (e.g., live or dead vs. hospitalised patients) or non-standardised (e.g., live, recovered, or discharged could all be classed under the “live” category) (Table 2 and Table S3). Therefore, we may find the percentage of the recorded metadata in Table 1 to be over 100%, such as with Cambodia, as the identical genome IDs might have been categorised more than once. This resulted in a slight overestimation of recorded metadata and highlighted the need to create a better recording format.

**Figure 2:**
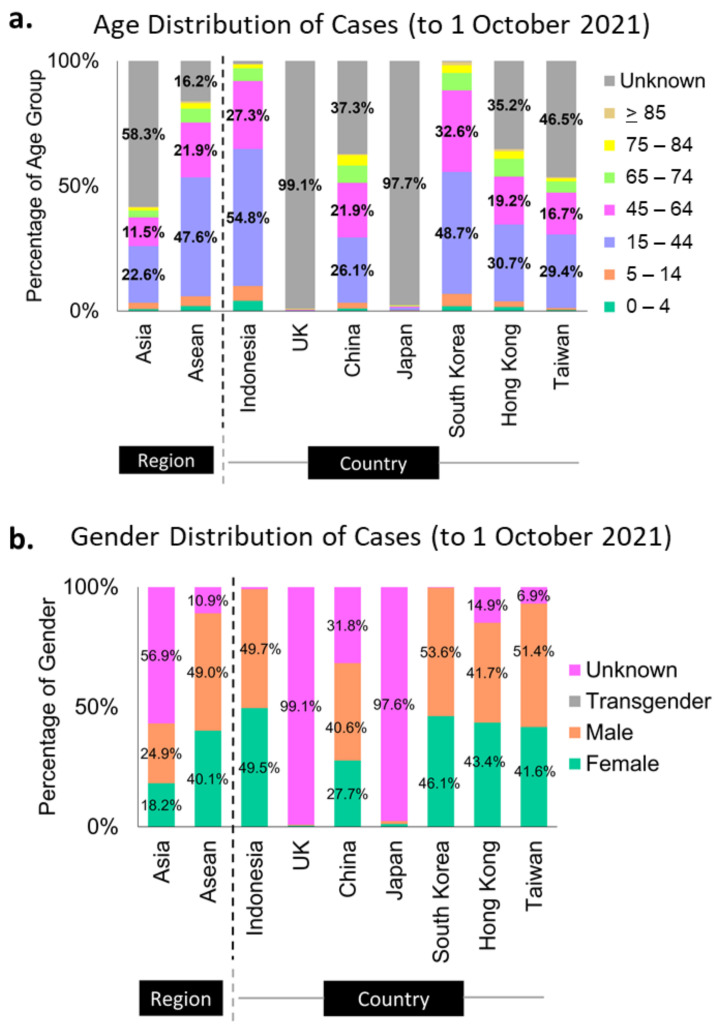
Metadata profiles of some Asian countries and the UK. Indonesia and South Korea submitted almost complete metadata of age (**a**) and sex (**b**) categories to GISAID.

More than half of the sequenced genomes in Indonesia (at almost 55%) belonged to the productive age group of 15- to 44-year-olds, while the second-largest group (29%) was the 45- to 64-year-olds (Figure 2a). These data can merely represent the age distribution of the Indonesian population (Table S4 [35,36,37]), as it is also reflected in the population age distribution of South Korea (Figure 2a, Table S4 [35,36,37]). However, it is hard to say for some countries, such as Hong Kong and China, as their unknown categories of these metadata are relatively large (Figure 2a). The same assumption could be applied to the metadata distribution of sex in Indonesia, where 49.5% of the infection came from female cases and 49.7% from males (Figure 2b), in correlation with the 50:50 sex distribution in the Indonesian population (Table S4 [38]).

#### 3.2.3. The Distribution of Variants by PANGO Lineages

**Figure 3:**
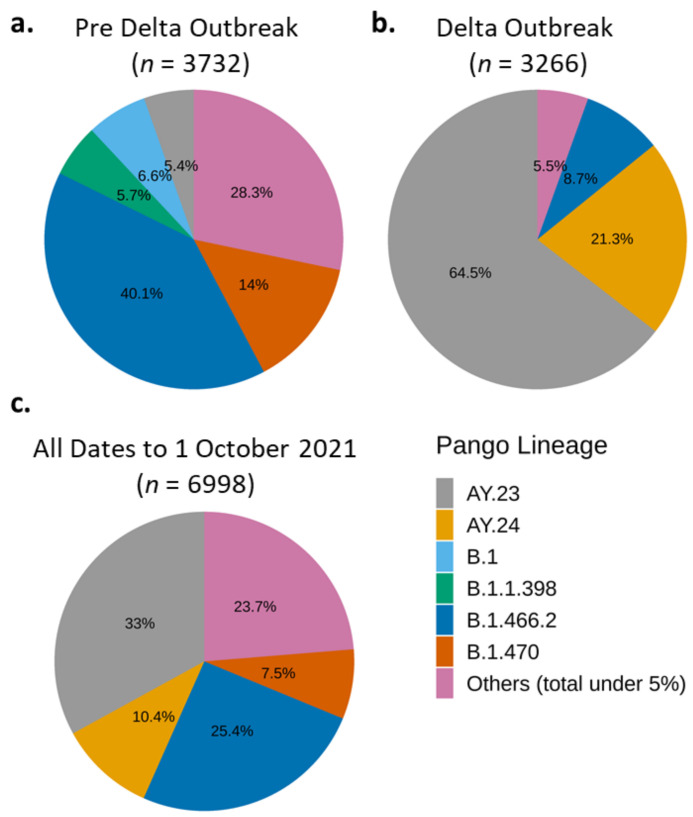
Predominant SARS-CoV-2 variants in Indonesia during pre-Delta (1 March–1 June 2020) and Delta (2 June–1 October 2022) periods. B.1.466.2 and AY.23 (Delta sub-lineage) were the dominant variant during pre-Delta (**a**) and Delta (**b**) period, respectively. Variant AY.23 followed by B.1.466.2 were the most dominant variant during all periods (**c**).

Lineage annotation by Phylogenetic Assignment of Named Global Outbreak Lineages (PANGO Lineages; PANGOLIN) presents a dynamic nomenclature of the SARS-CoV-2 [28]. Because it labels the circulating and active variants, it can track the transmission of SARS-CoV-2 based on sequence differences while also considering new virus diversity [28]. Therefore, we use the PANGOLIN annotations by GISAID included in the metadata of the respective genomes to analyse their distributions before and after the Delta outbreak (Figure 3 and Figure S4). In the following sections, we also use the WHO’s nomenclature of Greek alphabets [39] when generally discussing lineages that have become VoCs.

**Figure 4:**
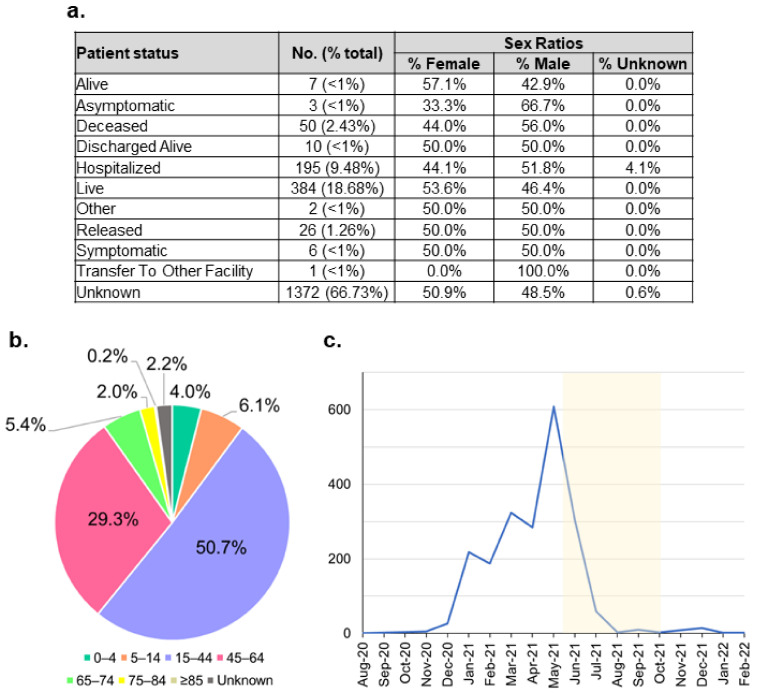
Indonesian B.1.466.2 variant metadata distribution. (**a**) Tabulation of patient status along with their sex ratios. (**b**) Age distribution shows that this variant mostly infected the productive age range of 15–64 years. (**c**) The variant case number peaked during April–May 2021, just before the outbreak of the Delta variant; highlighted in yellow is the Delta outbreak period.

Per 1 June 2021, the B.1.1.7 lineage (Alpha variant) was still dominating the world and Asia (Figure S4—left panels), although, interestingly, not in Indonesia (Figure 3a), despite its first detection in January 2021 (Table 2). At the pre-Delta outbreak period, the most dominant variant in Indonesia was B.1.466.2 [40], followed by B.1.470 [41] (Figure 3a). The B.1.470 variant was first detected in Indonesia on 9 April 2020, and then spread to the near- and far-neighbouring countries, such as Malaysia, South Korea, and Japan [41]. Other unique Indonesian-associated variants detected in the pre-Delta period were B.1.1.398 [42] at 5.7% (Figure 4a and Figure S5A) and B.1.468 [43] at less than 5% (Figure S5A). These unique Indonesian variants were no longer detected in significant amounts during the Delta outbreak, except for B.1.466.2 (Figure 3b and Figure S5B).

During the four months of the Delta outbreak, the Indonesian-associated variants were quickly outcompeted by the Delta sub-lineages, AY.23 [44] and AY.24 (Figure 3b). Like Alpha, the original Delta variant, B.1.617.2, was first detected in Indonesia in January 2021 (Table 2). Similar to the Indonesian-associated variants, this original Delta variant proportion was flatly below 5% of the total sequenced genomes during the Delta outbreak because it had been outcompeted by the Delta sub-lineages, AY.23 [44] and AY.24 [45] (Figure 3). Delta sub-lineages AY.23 and AY.24 overtook and radically changed the distribution of the Indonesian variants and defined the period of Delta outbreak (Figure 3b,c). Interestingly, AY.23 was already brewing at an observable frequency just before the overall Delta outbreak (Figure 3a and Figure S5A). There was an almost 100% increase in the number of WGS during the four months of the Delta outbreak compared to the 15-month preoutbreak period (compare Figure 3a to Figure 3b). This increase is a positive testimony to the working progress of the Indonesian SARS-CoV-2 Genomics Surveillance Network.

#### 3.2.4. Mutations and Metadata of the Indonesian Unique Variants

All B.1.466.2 and B.1.470 variants in Indonesia carried the D614G spike protein mutation, as do the other VoCs (Table 3). Another frequently shared mutation between the Indonesian variants and VoCs is the P323L mutation in the NSP12 gene locus in the interface of the viral RNA-dependent polymerase, RdRP [46] (Table 3). In Indonesia, this P323L mutation was found in all B.1.466.2, B.1.1.7, and B.1.617.2 variants (Table 3) and was co-present with the D614G mutation in many genomes in the world [46]. Several studies postulated that the two mutations play a role in higher infectivity [47] and severity of COVID-19 but not necessarily mortality [48] (Tables S5–S7).

The B.1.466.2 variant also shared the highest number of synonymous mutations (*n* = 334) with the collective genomes of the Delta AY.xx variants (Figure S6A). In Indonesia, both types of variants have accumulated a large number of distinct mutations, where the AY.xx variants had 1612 and B.1.466.2 had 1073 total mutations, respectively (Figure S6A). Specifically, over 98% of the B.1.466.2 genomes carried the spike protein mutation of N439K which was not found in other Indonesian variants (Table 4 and Table S5, Figure S6B). The uniqueness of the Indonesian B.1.466.2 variant suggests that it has accumulated these mutations during local transmissions from imported parental variants. Moreover, its similar mutations to the Delta sub-lineages might have conferred a more infectious and/or successful phenotype, and hence its prevalence in Indonesia prior to the takeover by the Delta variants (Figure 3). The Delta variants also significantly (*p*-value < 0.001) infected a younger demographic (i.e., median of 35-year-olds) compared to those infected with the local Indonesian variants (i.e., median of 38-year-olds) (Table 5).

Regarding the B.1.466.2 metadata, only less than 34% of the patients’ status was recorded (Figure 4a). Most of these patients recovered, where more than 53% were female patients. Interestingly, there were larger proportions of male patients infected with the B.1.466.2 variants who were hospitalised or ended up deceased, 51.8% and 56%, respectively. Indeed, a study shows that the male sex of higher age with comorbidity may have more prevalence in requiring intensive care treatment and mortality [49,50]. Patients infected with this variant mostly belonged to the productive age range of 15–64 years (Figure 4b). This mirrors the age distribution of all cases detected in Indonesia (Figure 2a). The number of the B.1.466.2 cases markedly increased from the end of 2020 and peaked during April–May 2021, before flattening significantly, coinciding with the Delta outbreak (Figure 4c).

#### 3.2.5. Phylogenetic Relationship Exists between Indonesian Variants and Those Exported to the Neighbouring Countries

**Figure 5:**
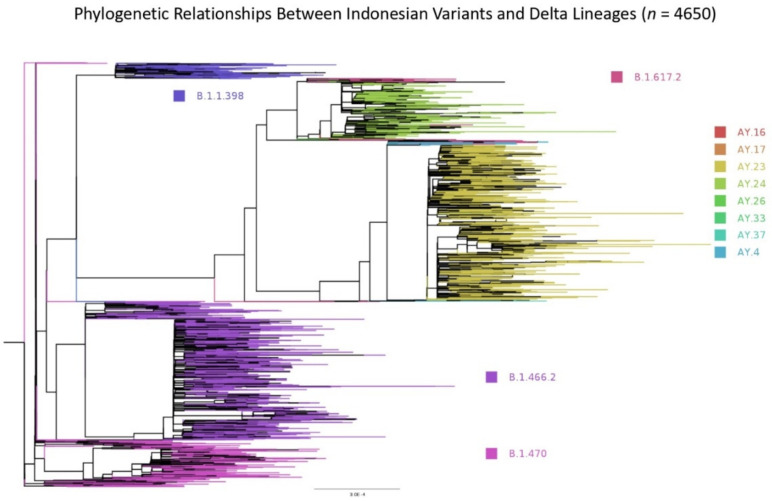
Phylogenetic relationships between the Indonesian variants (B.1.466.2, B.1.470, and B.1.398) and all Delta lineages per 1 October 2021 (*n* = 4650). The scale of branch length is given.

In Indonesia, the earliest dated sample for sequencing was collected by *Universitas Airlangga* (Airlangga University) in Surabaya on 12 March 2020. We used this sequence as the root in the phylogenetic tree analysis (Figure 5). Three Indonesian variants: B.1.466.2, B.1.470, and B.1.1.398 and all Delta lineages (including the exported variants) were analysed to measure the phylogenetic relationship between them (Figure 5; *n* = 4650). Most Indonesian variants formed distinct phylogenetic association groups and existed in the early onset of the pandemic, then disappeared with time, especially B.1.398 and B.1.470, when the Delta variants took over (Figure 5). The branches being further away from the root mainly consisted of the Delta variants and sub-lineages, confirming how dominant the Delta variants were in the spreading of COVID-19.

#### 3.2.6. Inter-Nation Transmissions

**Figure 6:**
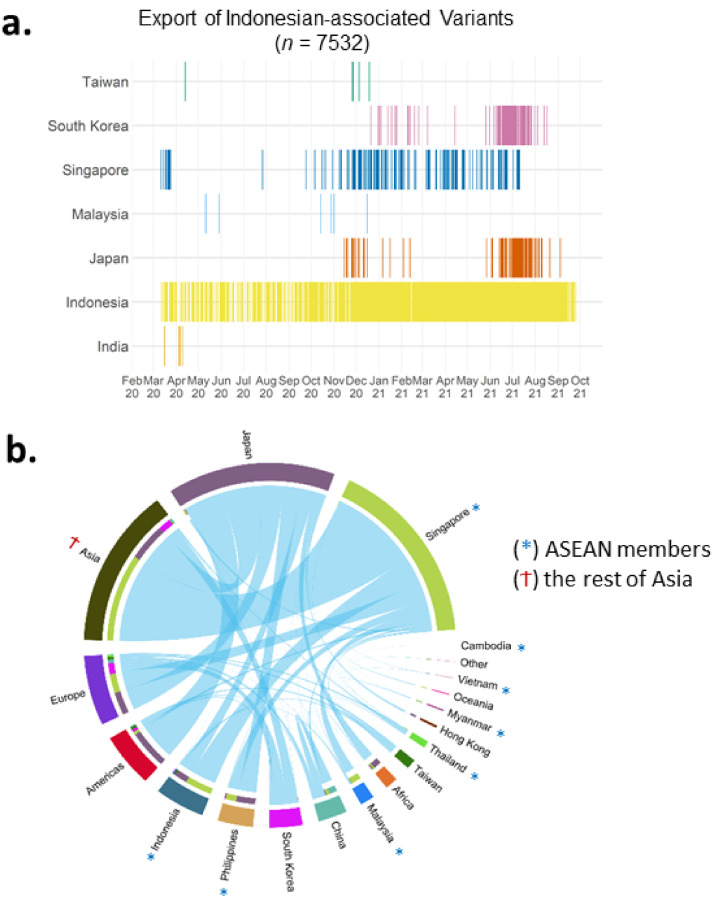
Inter-nation transmission of variants. (**a**) Timeline of the Indonesian-associated variants based on export to other countries, showing Singapore as the most frequent destination. (**b**) Worldwide transmission showing the highest events from/to Singapore and Japan.

The transmission data were extracted based on any existing travel history in the GISAID metadata. In the metadata up to 1 October 2021, a few near- and far-neighbouring countries mentioned Indonesia. In order of their frequencies, these were Singapore, Malaysia, South Korea, Taiwan, Japan, and, a bit further away in the region, India (Figure 6a). We term these Indonesian-associated variants as exported variants. These exported variants were detected in a relatively continuous manner because of travel and/or contact history with Indonesia (Figure 6a). Early during the pandemic, Indonesian-exported variants were found in Singapore in parallel to the local transmission of these variants within Indonesia (Figure 6a). Japan and South Korea received these Indonesian-exported variants too, albeit less frequently than Singapore, and were only detected late in 2020 and early 2021, respectively (Figure 6a).

Meanwhile, based on the downloaded data per 1 October 2021, there were 22 genomes imported into Indonesia (Figure S7A). Almost half of these were annotated as having travel history with Malaysia, a neighbouring country that shares part of its land borders with Indonesia, and around 27% had unknown countries of origin (Figure S7B). This small number of imported cases is likely due to a low rate of tracking and contact tracing within Indonesia instead of the actual lower rate of import versus export.

In a more general context, associations between inter-nation transmission of variants in different countries are visualised in Figure 6b (data per 1 June 2021). We filtered 3850 genomes from the metadata that had different country annotations compared to their countries of exposure, or other location information (Figure 6b). Worldwide associations show that Singapore had the most inter-nation transmission of variants, both in the total number of genomes and the total number of linked countries, followed by Japan (Figure 6b). Just four months earlier (end of February 2021), Japan was the country with the highest inter-nation transmission of variants, whilst Singapore data on this were minimum (Figure S8).

## 4. Discussion and Conclusions

The Nottingham–Indonesia Collaboration in Clinical Research and Training (NICCRAT) initiative, established in 2019, aims to foster partnership between the University of Nottingham and several Indonesian institutions in the field of health and clinical science [51,52]. As COVID-19 became a global pandemic in 2020, the initiative contributed some expertise in SARS-CoV-2 WGS in Indonesia. In collaboration with some researchers in the Indonesian Institute of Sciences (*Lembaga Ilmu Pengetahuan Indonesia*/LIPI) that had transformed into the National Research and Innovation Agency/BRIN, i.e., the NICCRAT-LIPI team, we utilised the MinION sequencing platform to sequence 12 SARS-CoV-2 genomes that have been published on GISAID in February and March 2021. The MinION sequencing platform offers speed, portability, relatively low cost, and established protocols. Our sequenced genomes were among the earliest few nanopore-sequenced genomes submitted from Indonesia. Meanwhile, as of 1 October 2021, a total of 389 whole genomes have been deposited by the LIPI-wide SARS-CoV-2 WGS team. The total number of SARS-CoV-2 genomes from Indonesia submitted to GISAID per 1 October 2021 was 6873.

In January 2021, the Indonesian Ministry of Health, working together with the Ministry of Research and Technology/National Agency for Research and Innovation, started a nationwide network for SARS-CoV-2 WGS for pandemic surveillance, bringing together laboratories and research and academic institutions [53] (Table S2). The network was expected to accelerate and scale-up SARS-CoV-2 WGS, informing the government on the genomic epidemiology of the pandemic in a timely manner [53]. The creation of nationwide consortia/networks for SARS-CoV-2 WGS has been shown to work in the United Kingdom with the COG-UK consortium [54]. At the time of writing, the UK was the second biggest contributor of SARS-CoV-2 genomes on GISAID after the United States of America [15], sequencing about 10% of its positive cases [54]. Therefore, emulating some aspects of this consortium in Indonesia is feasible while considering the local context, such as demography and logistics.

One of the difficulties in WGS within a pandemic context is sampling, i.e., obtaining an optimum number of samples representing cases in the population. It is clearly better if sequenced samples come from a wide range of subjects rather than only one group or a subset of individuals [55]. In Indonesia, this has been challenging, as samples were mostly nonrandomly taken from symptomatic and/or hospitalised patients (Table 1). Institutions were also more likely to sequence samples with low Ct values to ensure successful WGS. These biases will likely affect how the pandemic is observed and may not typify the events occurring in the broader community. It is also important to allocate resources for asymptomatic case research to study transmission patterns by asymptomatic individuals, which could influence case numbers [56,57,58].

Indonesia has recorded and shared its genomic metadata on GISAID on par with its effort to increase sequencing rate, despite the sporadic origins of the samples. However, during Delta outbreak, metadata recording was decreasing. We speculate that this may correlate to the large number of new cases that overwhelmed the Indonesian health system. Considering the different national and international rules governing personal information and data sharing, clinical metadata information may best be made available and accessible to selected relevant parties (e.g., health ministry, medical professionals, and epidemiological researchers). This way, the metadata will put the SARS-CoV-2 WGS output into context and draw as correct conclusions as possible to deal with the COVID-19 pandemic.

In the case of VoCs, only the Delta variants (B.1.617.2 and AY.xx sub-lineages) have been dominant in Indonesia (Figure 3). The frequencies of Alpha and Beta variant were low throughout the pandemic and were overridden by the Indonesian B.1.466.2 variant prior to the Delta outbreak (Figure 3). The Delta variants also tend to infect a younger demographic, as observed in Indonesia and in the UK [59]. The emergence of new variants that may become VoIs and VoCs highlights the importance of genomic surveillance of SARS-CoV-2 to control the COVID-19 pandemic.

Regarding Indonesian variants, functional studies of the highly prevalent variants, such as B.1.466.2 and B.1.470, can be pursued to understand their biological significance. Moreover, the unique mutation list of these variants can be utilised to develop genotyping diagnostic tests to quickly survey variant distribution in the population. Of note, the N439K mutation prevalent in the B.1.466.2 variant has been indicated to confer resistance to some monoclonal antibodies and evade some polyclonal ones [60]. Therefore, it may be necessary to track this variant and its response to vaccines used in Indonesia. However, such research efforts need to be approached with caution, as the prevalence of these variants will change with time as the pandemic progresses. This is shown already with the waning of B.1.466.2 occurrences when the Delta variant took over during the past few months (Figure 4c). Interestingly, this variant was detected again during the period of December 2021 to February 2022 (Figure 4c). We and a group supporting the PANGO designation of SARS-CoV-2 new (sub)lineages are currently monitoring the B.1.466.2 to be able to share any information should there be concern on its phenotype and circulation [61].

Lastly, based on our finding of the Indonesian exported cases to ASEAN countries and beyond, governments in the region could use the existing diplomatic relationships to set more synchronous efforts in border control, trace-and-track, and vaccination schemes, for example. In addition, reducing cross-border spread would conceivably reduce mutation opportunities and thus the emergence of new variants.

## Figures and Tables

**Table 1:** Clinical metadata of genomes in Indonesia, Southeast and East Asia (based on data downloaded from GISAID per 1 October 2021).

Country	Patient Status				No. Sequenced Genomes	Recorded Metadata (% Sequenced Genome)
	Alive	Asymptomatic	Deceased	Hospitalised		
Japan	114	62	16	9114	130,481	7.1%
South Korea	25	-	9	-	14,118	0.2%
Singapore	-	-	-	585	7979	7.3%
Philippines	5090	1	221	20	7099	75.1%
Indonesia	1689	24	159	764	7045	37.4%
Hong Kong	601	-	26	323	4997	19.0%
Thailand	32	-	42	10	3767	2.2%
Malaysia	1876	1	231	128	3207	69.7%
China	239	2	1	153	1347	29.3%
Cambodia	1325	-	-	-	1165	113.7%
Vietnam	312	-	6	68	569	67.8%
Timor-Leste	-	-	-	-	357	0.0%
Taiwan	25	-	-	74	245	40.4%
Myanmar	2	-	1	27	75	40.0%
Brunei	-	-	-	-	38	0.0%
Laos	-	-	-	-	23	0.0%
Total	11,330	90	712	11,266	182,512	12.8%

**Table 2:** VoCs before and during the Delta outbreak.

VoC	First Detected Case		Total Sequenced Genomes	
	Accession ID	Date	Pre-Outbreak (Until 1 June 2021)	Delta Outbreak (2 June–1 October 2021)
B.1.1.7 (Alpha)	hCoV-19/Indonesia/SS-NIHRD-WGS00427/2021	5 January 2021	23	44
B.1.351 (Beta)	hCoV-19/Indonesia/BA-NIHRD-WGS00725/2021	25 January 2021	4	18
B.1.617.2 (Delta) and AY.xx	hCoV-19/Indonesia/JK-NIHRD-WGS00007/2021	7 January 2021	29	3035

**Table 3:** Common mutations found in all listed variants (VoCs and Indonesian variants).

Pango Lineage	Other Name	Mutation Type (% Frequency)				Total Genomes
		NSP12_P323L	NSP4_K35R	NSP6_L37F	Spike_D614G	
AY.xx	Delta 2	99.9	1.8	2.7	100.0	2385
B.1.1.7	Alpha	100.0	1.7	1.7	100.0	59
B.1.351	Beta	80.0	10.0	10.0	100.0	10
B.1.466.2	-	100.0	2.8	5.2	100.0	1530
B.1.470	-	98.9	0.4	3.0	100.0	527
B.1.617.2	Delta (original)	100.0	6.8	4.1	100.0	74

**Table 4:** Unique mutations found in two of the Indonesian variants.

Pango Lineage	Top 10 Mutation Type (% Frequency)	
B.1.466.2	**Spike_N439K** (98.63%) NSP12_R889K (6.47%) NSP3_S692F (6.47%) NS3_T223I (4.58%) NSP13_I575V (3.92%)	6. NSP3_Q203H (3.66%) 7. NSP4_M324I (3.66%) 8. N_Q389H (3.4%) 9. Spike_A1078S (2.48%) 10. NSP14_L157F (1.9%)
B.1.470	1. **NS3_S220I** (70.59%) 2. NSP2_L217V (59.77%) 3. NSP1_E2G (3.61%) 4. E_D72G (2.66%) 5. Spike_M1237I (2.09%)	6. M_W75L (1.9%) 7. NSP3_E95D (1.9%) 8. NSP4_S137A (1.71%) 9. NS8_C102G (1.52%) 10. Spike_V1176F (1.52%)

**Table 5:** Age distribution between Indonesian variants and Delta (including sub-lineages) in Indonesia.

Variant Type (*n* = 4253)	Age				*t*-Test *p*-Value
	Minimum	Median	Mean	Maximum	
Indonesian	0.5	38	38.9	92	<0.001
Delta (and sub-lineages)	0.4	35	36.4	91	

## Data Availability

The datasets analysed during the current study are available for registered users in the GISAID repository, https://www.gisaid.org/ (accessed on 28 February 2022).

## Supplementary Materials

The following are available online at https://www.mdpi.com/article/10.3390/v14040778/s1, Figure S1: COVID-19 test rates of ASEAN, East Asian countries, and the United Kingdom per 1 October 2021, Figure S2: COVID-19 positive cases and SARS-CoV-2 sequencing rate, Figure S3: Proportion of sample sources used for sequencing, Figure S4: Proportion of PANGO lineages pre- and during Delta outbreak in Indonesia for the world and Asia, Figure S5: Proportions of Indonesian-associated variants in Indonesia, ASEAN, Asia and worldwide pre-Delta outbreak and during the outbreak, Figure S6: Venn diagrams showing the shared and unique total mutations of Indonesian variants versus Delta lineages [29], Figure S7: Data of genomes imported into Indonesia per 1 October 2021, Figure S8: Circular plot showing inter-nation transmissions of variants in the world per 1 February 2021, Table S1: Genomes submitted by NICCRAT-BRIN team, Table S2: List of Indonesian institutions that carried out SARS-CoV-2 WGS per 1 October 2021, Table S3: Clinical metadata of genomes in Indonesia, Southeast and East Asia Pre-Delta Outbreak (per 1 June 2021), Table S4: Age and Sex Distribution of Countries [35,36,37,38], Table S5: Functions of genes and their mutation effects [60,62,63,64,65,66,67], Table S6 Acknowledgement 1: Genome ID and submitting all institutions, excluding Japan, Table S7 Acknowledgement 2: Genome ID and submission date from Japan.

## Abbreviations

Indonesian SARS-CoV-2 Genomics Surveillance Network.

BRIN	National Research and Innovation Agency
EIJK	Eijkman Research Center for Molecular Biology
GSI Lab	Laboratory of Genomik Solidaritas Indonesia
IRCVS	Indonesian Research Center for Veterinary Science
ITB	Bandung Institute of technology
ITD-UNAIR	Research Center for Vaccine Technology and Development, Institute of Tropical Disease, Airlangga University
LIPI	Indonesian Institute of Sciences
MRINUPH	Mochtar Riady Institute for Nanotechnology-Universitas Pelita Harapan
NICCRAT	Nottingham Indonesia Collaboration for Clinical Research and Training
NIHRD	National Institute of Health Research and Development
RSRM	Raden Mattaher Regional General Hospital
SHSIU	Syarif Hidayatullah State Islamic University Jakarta
TFRIC	Padjadjaran University
TUH	Tanjungpura University Hospital
UA	Institute of Tropical Disease-Airlangga University
UGM	Gadjah Mada University
UHAS	Hasanuddin University
UI	University of Indonesia
UNS	Sebelas Maret University
UNSOED	Jenderal Soedirman University
UNSRI	Sriwijaya University
UNWAR	Warmadewa University
UPNVJ	University of Pembangunan Nasional Veteran Jakarta
USK	Syiah Kuala University
USU	Sumatera Utara University
WJHL	West Java Health Laboratory
