# Supplementary material for: Genome Profiling of SARS-CoV-2 in Indonesia, ASEAN and the Neighbouring East Asian Countries: Features, Challenges and Achievements
Source: Viruses. 2022 Apr 8;14(4):778. doi: 10.3390/v14040778 (PMC9027902; doi:10.3390/v14040778)

Supplementary Figure Legends

Figure S1. COVID-19 test rates of ASEAN, East Asian countries, and the United Kingdom per 1 October 2021

Figure S2. COVID-19 cases and SARS-CoV-2 sequencing rate in ASEAN and East Asia region. A-B) Indonesia currently holds the highest number of positive cases, followed by the Philippines. C-D) Hong Kong has the highest sequencing rate at over 40% of its positive cases.

Figure S3. Proportion of sample sources used for sequencing; Indonesia obtained most of its sequencing samples from swab while the UK did not provide this information to GISAID.

Figure S4. Proportion of PANGO lineages pre- and during Delta outbreak in Indonesia for the world (a) and Asia (b).

Figure S5. Proportions of Indonesian-associated variants in Indonesia, ASEAN, Asia and worldwide pre-Delta outbreak (a) and during the outbreak (b).

Figure S6. Venn diagrams showing the shared and unique total mutations of Indonesian variants versus Delta lineages (a) and compared to just the spike protein mutation (b).

Figure S7. Data of genomes imported into Indonesia per 1 October 2021; most cases came from Malaysia followed by non-specific category where only travel history was mentioned but no recorded location/country.

Figure S8 Circular plot showing inter-nation transmissions of variants in the world per 1 February 2021; transmission events were the highest in Japan.

Figure S1

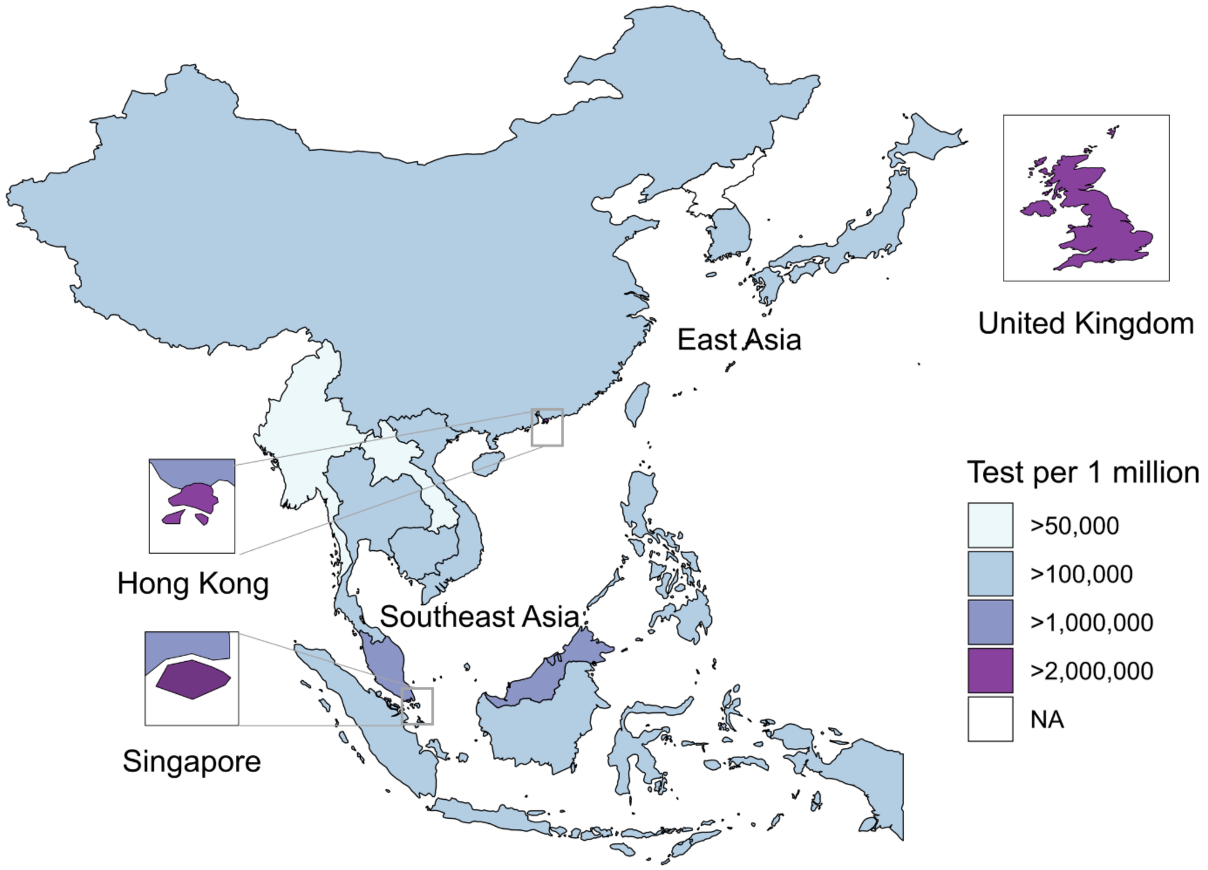
East Asia
United Kingdom
Hong Kong
Southeast Asia
Singapore
Test per 1 million
>50,000
>100,000
>1,000,000
>2,000,000
NA

Figure S2

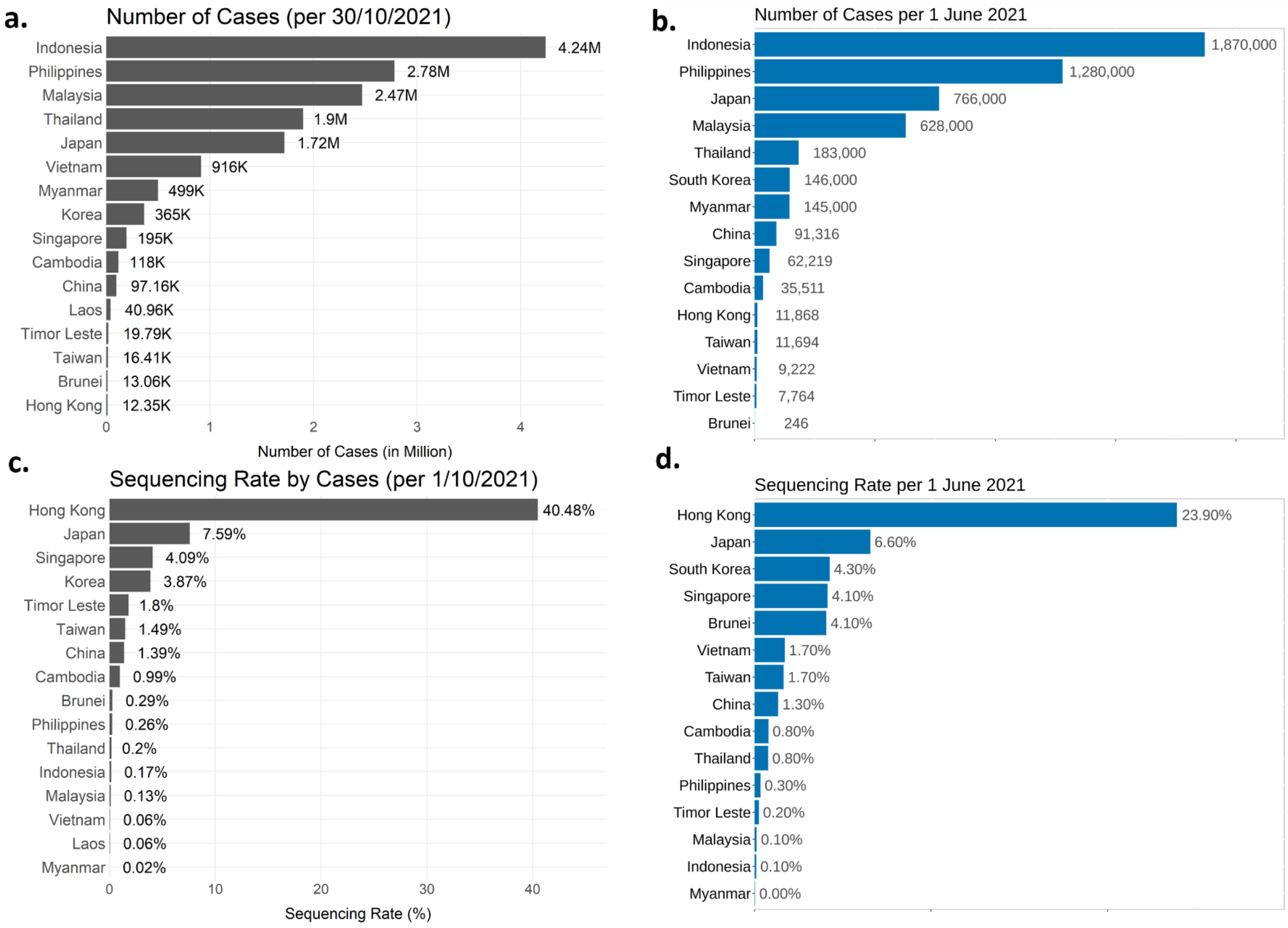

a.
Number of Cases (per 30/10/2021)
Indonesia 4.24M
Philippines 2.78M
Malaysia 2.47M
Thailand 1.9M
Japan 1.72M
Vietnam 916K
Myanmar 499K
Korea 365K
Singapore 195K
Cambodia 118K
China 97.16K
Laos 40.96K
Timor Leste 19.79K
Taiwan 16.41K
Brunei 13.06K
Hong Kong 12.35K
0 1 2 3 4
Number of Cases (in Million)
b.
Number of Cases per 1 June 2021
Indonesia 1,870,000
Philippines 1,280,000
Japan 766,000
Malaysia 628,000
Thailand 183,000
South Korea 146,000
Myanmar 145,000
China 91,316
Singapore 62,219
Cambodia 35,511
Hong Kong 11,868
Taiwan 11,694
Vietnam 9,222
Timor Leste 7,764
Brunei 246
c.
Sequencing Rate by Cases (per 1/10/2021)
Hong Kong 40.48%
Japan 7.59%
Singapore 4.09%
Korea 3.87%
Timor Leste 1.8%
Taiwan 1.49%
China 1.39%
Cambodia 0.99%
Brunei 0.29%
Philippines 0.26%
Thailand 0.2%
Indonesia 0.17%
Malaysia 0.13%
Vietnam 0.06%
Laos 0.06%
Myanmar 0.02%
0 10 20 30 40
Sequencing Rate (%)
d.
Sequencing Rate per 1 June 2021
Hong Kong 23.90%
Japan 6.60%
South Korea 4.30%
Singapore 4.10%
Brunei 4.10%
Vietnam 1.70%
Taiwan 1.70%
China 1.30%
Cambodia 0.80%
Thailand 0.80%
Philippines 0.30%
Timor Leste 0.20%
Malaysia 0.10%
Indonesia 0.10%
Myanmar 0.00%

Figure S3

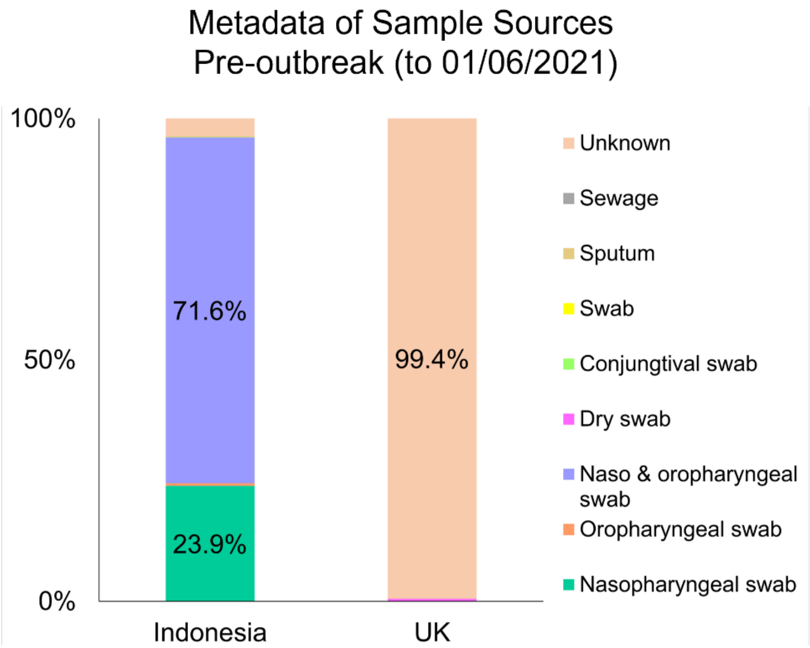

Figure S4

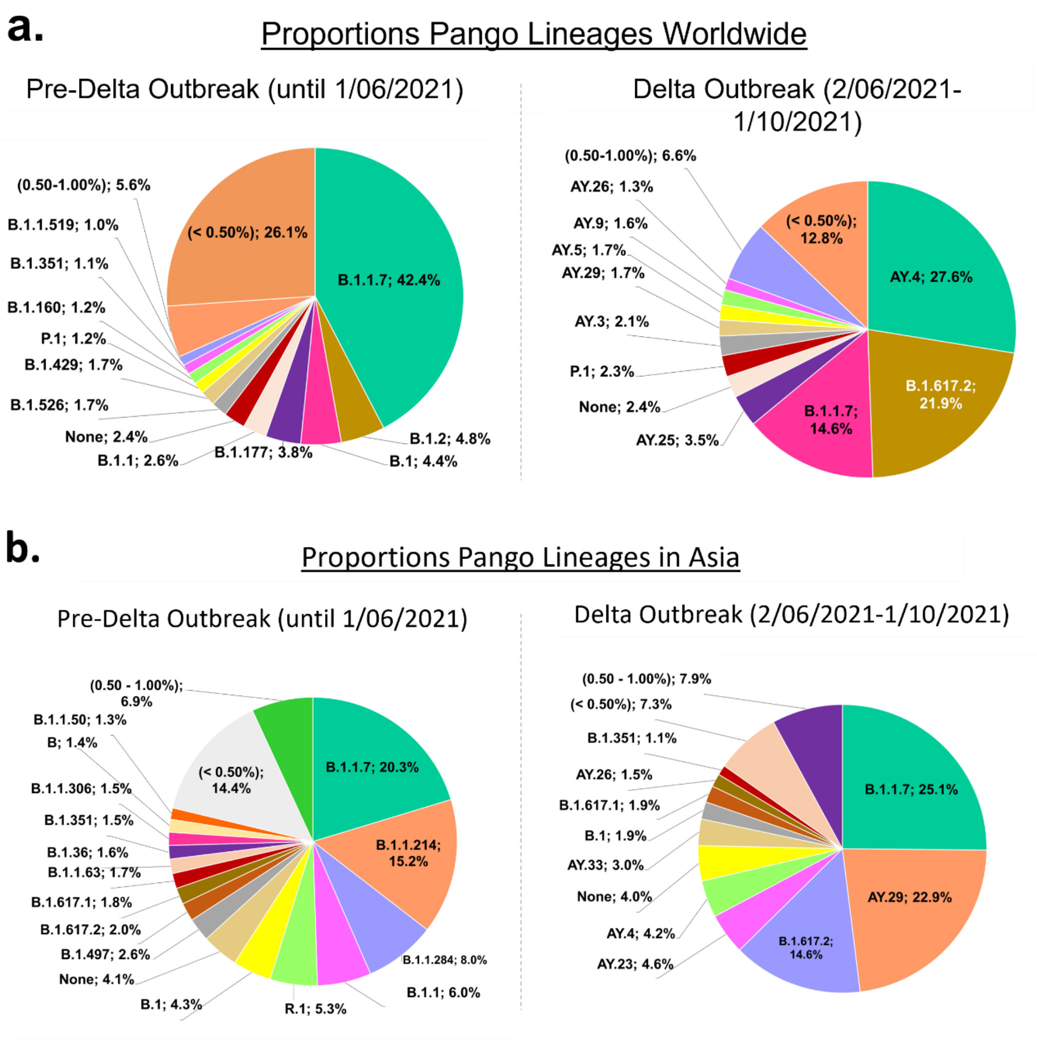

Figure S5

a. Pre Delta Outbreak

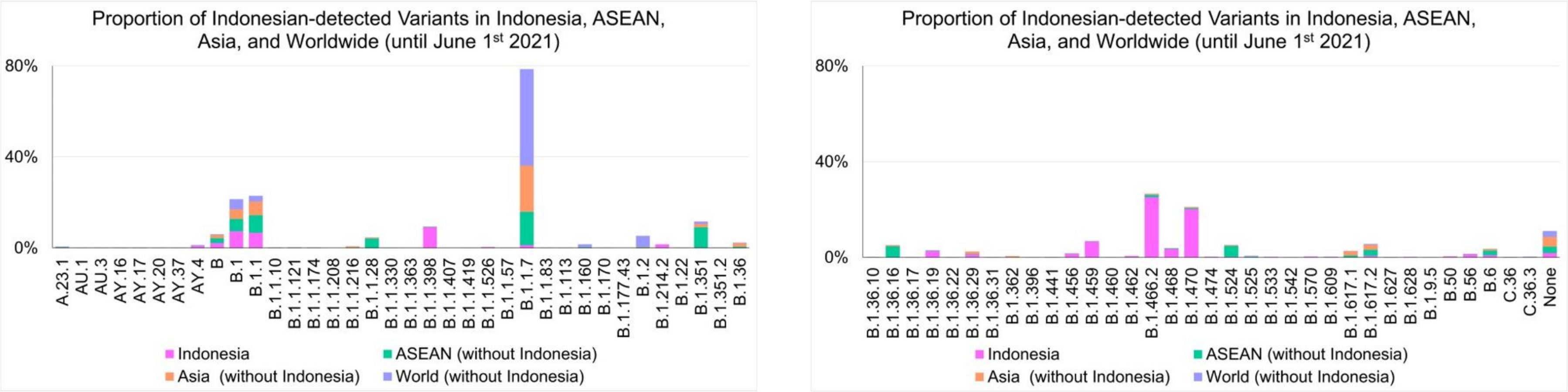

b. Delta Outbreak

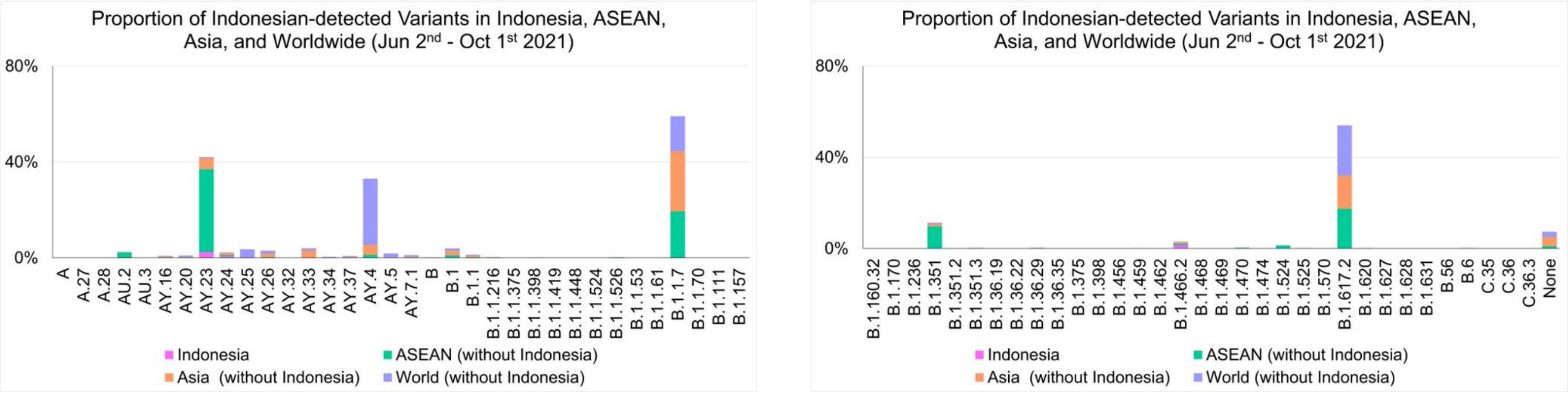

Figure S6

a.

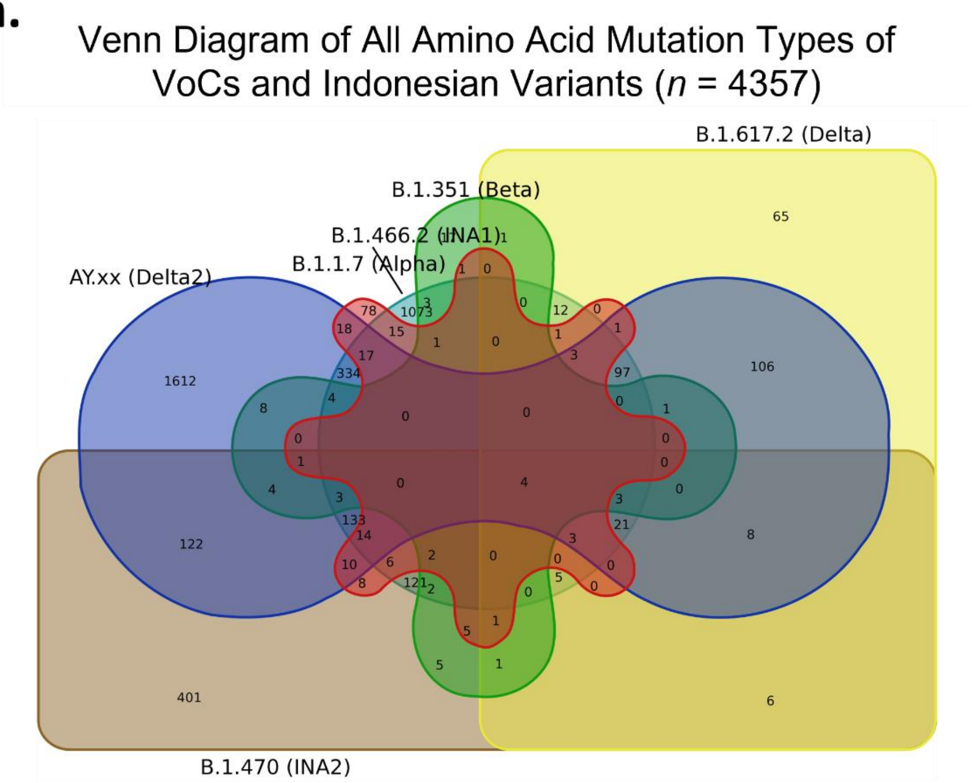
Venn Diagram of All Amino Acid Mutation Types of
VoCs and Indonesian Variants (n = 4357)
B.1.617.2 (Delta)
B.1.351 (Beta)
B.1.466.2 (INA1)
B.1.1.7 (Alpha)
AY.xx (Delta2)
B.1.470 (INA2)

b.

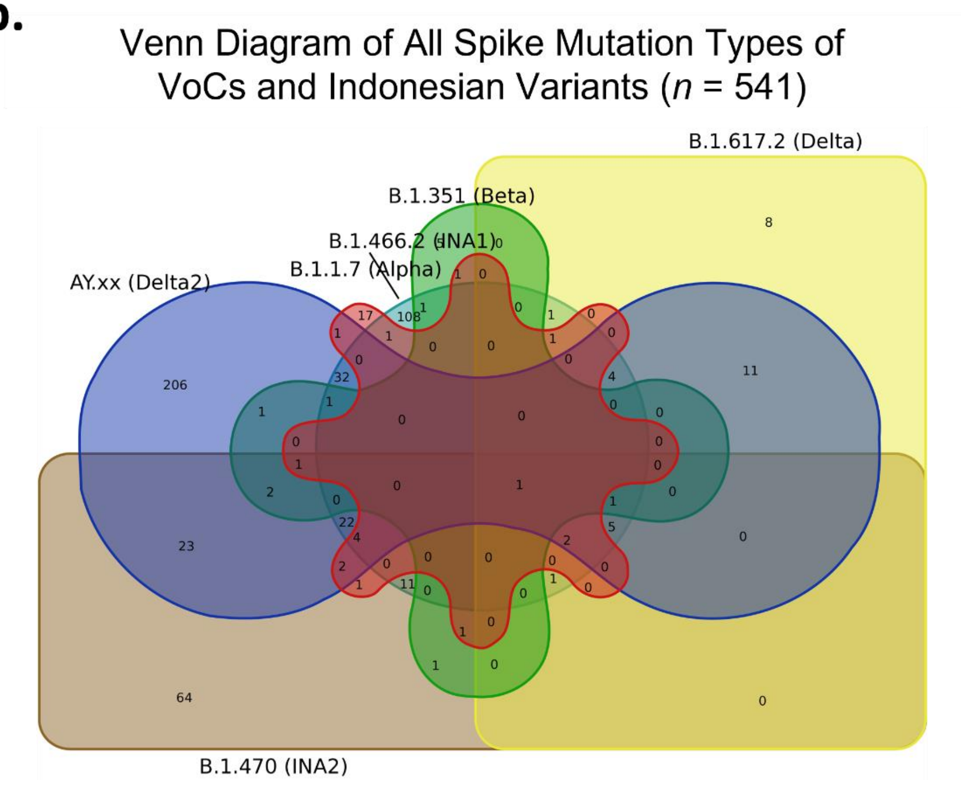
Venn Diagram of All Spike Mutation Types of
VoCs and Indonesian Variants (n = 541)
B.1.617.2 (Delta)
B.1.351 (Beta)
B.1.466.2 (INA1)
B.1.1.7 (Alpha)
AY.xx (Delta2)
B.1.470 (INA2)

Figure S7

a

Originating country of imported genomes

Sample ID	Collection Date	Imported Location
EPI_ISL_576387	20/08/2020	Saudi Arabia
EPI_ISL_1239222	31/12/2020	Non-Specific
EPI_ISL_1239223	4/01/2021	Non-Specific
EPI_ISL_1122422	10/01/2021	United Kingdom
EPI_ISL_1239143	8/02/2021	Non-Specific
EPI_ISL_2886178	13/03/2021	Malaysia
EPI_ISL_2886179	13/03/2021	Malaysia
EPI_ISL_2886170	13/03/2021	Malaysia
EPI_ISL_2886171	13/03/2021	Malaysia
EPI_ISL_2886174	13/03/2021	Malaysia
EPI_ISL_2886177	13/03/2021	Malaysia
EPI_ISL_2226647	21/04/2021	Non-Specific
EPI_ISL_2429146	27/04/2021	Non-Specific
EPI_ISL_2364675	29/04/2021	India
EPI_ISL_2226648	5/05/2021	Non-Specific
EPI_ISL_4056005	12/05/2021	Malaysia
EPI_ISL_4056006	12/05/2021	Malaysia
EPI_ISL_4056007	12/05/2021	Malaysia
EPI_ISL_4056008	12/05/2021	Malaysia
EPI_ISL_4742352	12/05/2021	Netherlands
EPI_ISL_2692993	19/05/2021	France
EPI_ISL_2692994	24/05/2021	Bosnia-Herzegovina

b

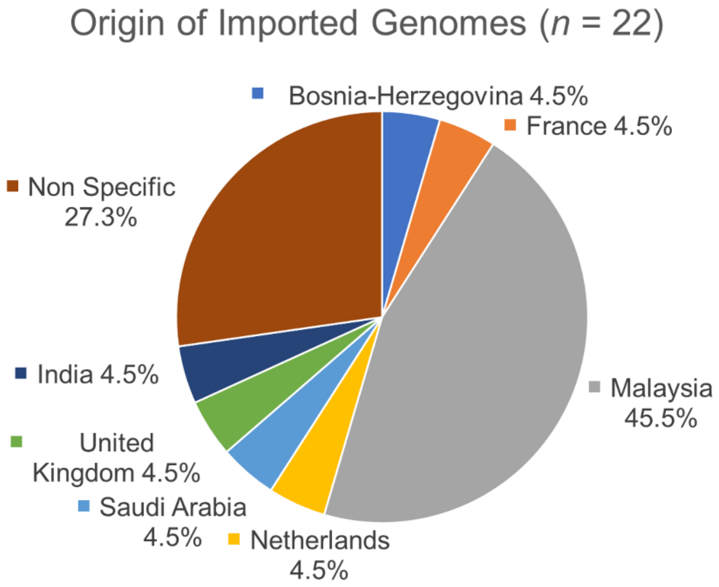

Figure S8

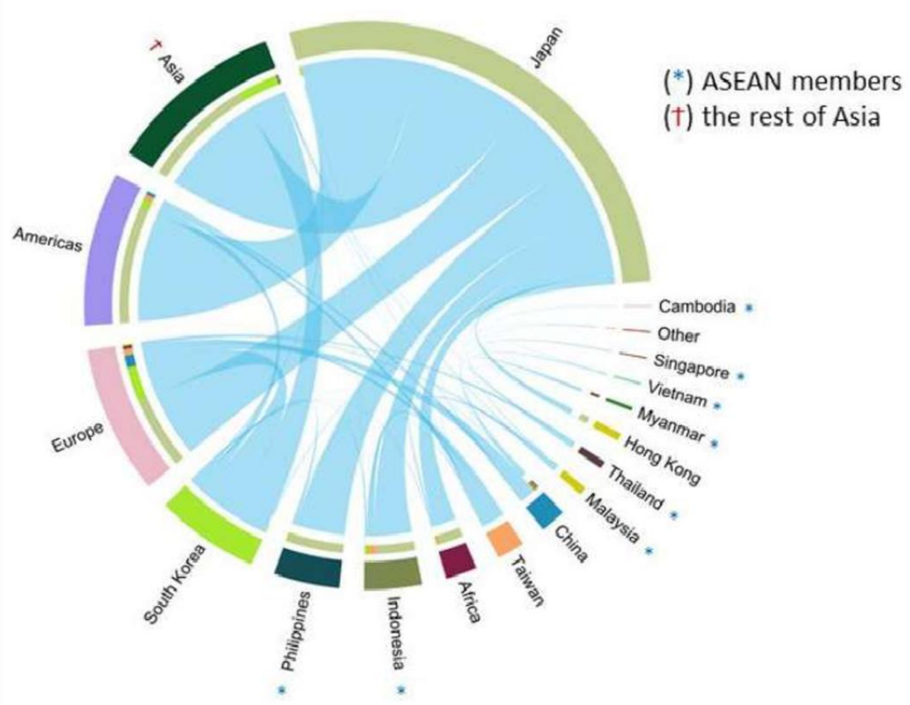

Supplement: Supplementary file 1 [file viruses-14-00778-s001.zip › viruses-1641476-supplementary/cahyani_etal_supplementary_figures.pdf]
