# Supplementary material for: Genome Profiling of SARS-CoV-2 in Indonesia, ASEAN and the Neighbouring East Asian Countries: Features, Challenges and Achievements
Source: Viruses. 2022 Apr 8;14(4):778. doi: 10.3390/v14040778 (PMC9027902; doi:10.3390/v14040778)
Supplement: Supplementary file 1 [file viruses-14-00778-s001.zip › viruses-1641476-supplementary/supplementary_tables.pdf]

Table S1. Genomes submitted by NICCRAT-BRIN team.

Accession ID	Collection Date	Patient Age	Gender	Pango Lineage	Pangolin Version	AA Substitutions	Is High Coverage?	Patient Status
EPI_ISL_1020188	13/01/2021	61	Male	B.1.459	16/09/2021	Spike_L18F,N_D377Y,NSP12_I539T,Spike_S689R,NSP3_P822L,N_A119S,NSP2_V284I,N_T205I,NS3_Q57H,NSP3_F53L,NS8_S67F,NS6_E54stop,NSP12_P323L,NSP16_I68V,Spike_D614G,NSP3_I483V,NSP3_N844S,NSP3_E37D	NO	Unknown
EPI_ISL_1117592	7/12/2020	67	Male	B.1.459	16/09/2021	NSP14_T173A,NSP3_P822L,N_S193I,NS3_Q57H,NSP12_P323L,Spike_D614G,NSP12_G596S,NSP2_P91L	YES	Hospitalized
EPI_ISL_1118280	6/12/2020	12	Male	B.1.470	16/09/2021	Spike_T719I,NSP3_L368F,NSP14_A323S,N_S194L,NSP6_H11Y,NSP3_T656I,NS3_Q57H,NS3_D27H,NSP12_P323L,Spike_D614G,NSP12_P227L	YES	Symptomatic
EPI_ISL_1020197	1/12/2020	22	Female	B.1.1.398	16/09/2021	Spike_P812Q,N_R203K,N_G204R,NSP12_V299F,NSP12_P323L,Spike_D614G,Spike_Q613H	YES	Asymptomatic
EPI_ISL_1117591	1/12/2020	20	Female	B.1.459	16/09/2021	NSP2_L271F,Spike_E654Q,NSP3_P822L,N_S193I,NSP12_K91R,NS3_Q57H,NSP12_P323L,N_P365L,Spike_D614G,NSP12_G596S,N_Q289H	NO	Symptomatic
EPI_ISL_1117590	30/11/2020	45	Female	B.1.1.398	16/09/2021	NS3_A99V,N_R203K,N_G204R,NSP3_S1285F,NSP12_P323L,Spike_D614G,NSP3_F814L	YES	Symptomatic

Accession ID	Collection Date	Patient Age	Gender	Pango Lineage	Pangolin Version	AA Substitutions	Is High Coverage?	Patient Status
EPI_ISL_1117451	9/11/2020	33	Female	B.1.1.398	16/09/2021	NSP6_L33M,NSP2_V447I,N_R203K,NSP6_F34V,NSP6_W31Y,N_G204R,NSP5_L220F,NSP6_Q30E,NSP12_P323L,Spike_D614G,NSP6_ins35VL,NSP6_L37F,NS3_S177T,NSP6_T29P	YES	Unknown
EPI_ISL_1137601	30/10/2020	53	Female	B.1.1.398	16/09/2021	N_R203K,NS3_T89I,N_G204R,NSP12_P323L,Spike_D614G,NSP13_E341D	NO	Symptomatic
EPI_ISL_1020192	13/10/2020	32	Female	B.1.1.398	16/09/2021	N_R203K,Spike_T719S,N_G204R,NSP3_M1436I,NSP12_P323L,Spike_D614G,Spike_Q613H	YES	Asymptomatic
EPI_ISL_1020203	18/09/2020	38	Male	B.1.1	16/09/2021	N_P365S,N_R203K,NSP3_L1328F,N_G204R,NSP12_P323L,Spike_D614G	YES	Symptomatic
EPI_ISL_1117456	7/09/2020	55	Male	B.1.459	16/09/2021	NS8_L118V,NSP3_P822L,NSP12_M666I,N_S193I,NS3_Q57H,NSP12_P323L,Spike_D614G,NSP12_G596S	YES	Unknown
EPI_ISL_1117457	7/09/2020	30	Male	B.1.459	16/09/2021	NSP3_P822L,N_S193I,NS3_Q57H,Spike_A348S,NSP12_P323L,Spike_D614G,NSP12_G596S,NSP16_A34V	YES	Asymptomatic

Blue and yellow highlights: mutations shared by all genomes

Table S2. List of Indonesian institutions that carried out SARS-CoV-2 WGS per 1 October 2021.

**Institution**	**Sequencing Platform**				
	**Illumina & Sanger**	**Illumina only**	**ONT**	**Sanger Only**	**Sub Total**
BRIN	-	-	11	-	11
BRIN-WJHL	-	-	55	-	55
EIJK	38	792	-	-	830
EIJK-NIHRD	-	383	-	-	383
EIJK-RSRM	-	437	-	-	437
EIJK-UNSRI	-	54	-	-	54
EIJK-UNWAR	-	353	-	-	353
EIJK-USK	-	6	-	-	6
EIJK-WJHL	-	85	-	-	85
GSILab	-	-	1323	1	1324
IRCVS	-	-	2	-	2
ITD-UNAIR	-	161	10	-	171
LIPI	-	-	223	-	223
**LIPI-NICCRAT**	**-**	**-**	**12**	**-**	**12**
LIPI-NIHRD	-	-	139	-	139
LIPI-UNSOED	-	-	15	-	15
UNHAS	-	-	44	-	44
MRINUPH	-	-	-	17	17
MRINUPH-EIJK	-	92	-	-	92
NIHRD	7	2021	12	17	2057
SHSIU	-	-	14	-	14
TFRIC	-	14	2	-	16
TUH	-	11	11	-	22
UA	-	70	-	-	70
UGM	-	161	-	-	161
UI	-	37	55	-	92

**Institution**	**Sequencing Platform**				
	**Illumina & Sanger**	**Illumina only**	**ONT**	**Sanger Only**	**Sub Total**
UI-NIHRD	-	46	-	-	46
UNS	-	10	-	-	10
UPNVJ	-	9	-	-	9
USU	-	23	-	-	23
WJHL-ITB	-	100	-	-	100
**Total**	**45**	**4865**	**1928**	**35**	**6873**

BRIN: National Research and Innovation Agency; EIJK: Eijkman Research Center for Molecular Biology; GSILab: Laboratory of Genomik Solidaritas Indonesia ; IRCVS: Indonesian Research Center for Veterinary Science; ITB: Bandung Institute of technology; ITD-UNAIR: Research Center for Vaccine Technology and Development, Institute of Tropical Disease, Airlangga University; LIPI: Indonesian Institute of Sciences; MRINUPH: Mochtar Riady Institute for Nanotechnology-Universitas Pelita Harapan; NICCRAT: Nottingham Indonesia Collaboration for Clinical Research and Training; NIHRD: National Institute of Health Research and Development; RSRM: Raden Mattaher Regional General Hospital ; SHSIU: Syarif Hidayatullah State Islamic University Jakarta; TFRIC: Padjadjaran University; Bandung Insitute of Technology; West Java Health Laboratory; TUH: Tanjungpura University Hospital; UA: Institute of Tropical Disease-Airlangga University; UGM: Gadjah Mada University; UHAS: Hasanuddin University; UI: University of Indonesia; UNS: Sebelas Maret University; UNSOED: Jenderal Soedirman University; UNSRI: Sriwijaya University; UNWAR: Warmadewa University; UPNVJ: University of Pembangunan Nasional Veteran Jakarta; USK: Syiah Kuala University; USU: Sumatera Utara University; WJHL: West Java Health Laboratory.

Table S3. Clinical metadata of genomes in Indonesia, Southeast and East Asia Pre-Delta Outbreak per 1 June 2021.

**Country**	**Live**	**Deceased**	**Not Hospitalized/ Asymptomatic**	**Hospitalized/ Symptomatic**	**No. Sequenced Genomes**	**Recorded Metadata (% Sequenced Genome)**
Japan	114	16	62	9,114	50,241	18.5%
South Korea	25	9	0	0	6216	0.5%
Philippines	3,593	73	0	11	4295	85.6%
Hong Kong	601	26	0	323	2837	33.5%
Singapore	0	0	0	553	2576	21.5%
**Indonesia**	**811**	**25**	**26**	**477**	**1806**	**74.1%**
Thailand	8	0	0	3	1404	0.8%
China	239	1	2	153	1220	32.4%
Malaysia	476	7	3	64	745	73.8%
Cambodia	278	0	0	0	281	98.9%
Taiwan	25	0	0	74	193	51.3%
Vietnam	79	0	0	3	158	51.9%
Myanmar	2	0	0	0	41	4.9%
Timor-Leste	0	0	0	0	19	0.0%
Brunei	0	0	0	0	10	0.0%

Table S4. Age and Sex Distribution of Countries.

**Country**	**% Ages of Total Population**			**% Sex of Total Population**	
	**0-14 Years** [1]	**15-64 Years** [2]	**>65 Years** [3]	**Female** [4]	**Male** [5]
China	13	71	11	48.7	51
Hong Kong	12	70	17	54.0	46
Indonesia	26	68	6	49.6	50
Japan	13	59	28	51.2	49
South Korea	13	72	15	49.9	50
United Kingdom	18	64	19	50.6	49

Table S5. Functions of genes and their mutation effects.

**Mutation**	**Variant**	**Gene Function**	**Mutation Effect**
NSP12_P323L	All shared	RNA-dependent RNA polymerase (RdRp) [6]	Correlate with increase mortality rate in the East Mediterranean Region when mutation is in single incidence [6]
NSP4_K35R	All shared	Transmembrane proteins involved in regulating the host immunity, modifying host cell organelles for viral replication and escape [7]	Currently no data
NSP6_L37F	All shared	Transmembrane protein involved in regulation of the host autophagy [8]	Correlate with asymptomatic infection; reduced abilities to activate inflammasomes and pyroptosis [8]
Spike_D614G	All shared	Trimeric spike (S) protein [9]	More stabilisation of ACE2 binding [9]
Spike_N439K	B.1.466.2	Receptor binding motif (RBM) of the trimeric (S) protein [10]	Enhanced binding affinity to the hACE2 receptor and confers resistance against several neutralizing monoclonal antibodies [10]
NS3_S220I	B.1.4770	Accessory protein to mask virus from class I major histocompatibility factors (MHC) [11]	Currently no data
NSP2_L217V	B.1.4770	Transmembrane protein [12]	Currently no data

References

1. Population ages 0-14 (% of total population) | Data, (n.d.). https://data.worldbank.org/indicator/SP.POP.0014.TO.ZS?name_desc=false&view=chart (accessed June 13, 2021).
2. Population ages 15-64 (% of total population) | Data, (n.d.). https://data.worldbank.org/indicator/SP.POP.1564.TO.ZS?name_desc=false&view=chart (accessed June 13, 2021).
3. Population ages 65 and above (% of total population) | Data, (n.d.). https://data.worldbank.org/indicator/SP.POP.65UP.TO.ZS?name_desc=false&view=chart (accessed June 13, 2021).
4. Population, female (% of total population) | Data, (n.d.). https://data.worldbank.org/indicator/SP.POP.TOTL.FE.ZS?name_desc=false (accessed June 13, 2021).
5. Population, male (% of total population) | Data, (n.d.). https://data.worldbank.org/indicator/SP.POP.TOTL.MA.ZS?name_desc=false (accessed June 13, 2021).
6. S. Omais, S. Kharroubi, H. Zaraket, No association between the SARS-CoV-2 variants and mortality rates in the Eastern Mediterranean Region, Gene. 801 (2021) 145843. https://doi.org/10.1016/J.GENE.2021.145843.
7. S. Thomas, Mapping the Nonstructural Transmembrane Proteins of Severe Acute Respiratory Syndrome Coronavirus 2, J. Comput. Biol. 28 (2021) 909–921. https://doi.org/10.1089/CMB.2020.0627/ASSET/IMAGES/LARGE/CMB.2020.0627_FIGURE5B.JPEG.
8. X. Sun, Y. Liu, Z. Huang, W. Xu, W. Hu, L. Yi, Z. Liu, H. Chan, J. Zeng, X. Liu, H. Chen, J. Yu, F.K.L. Chan, S.C. Ng, S.H. Wong, M.H. Wang, T. Gin, G.M. Joynt, D.S.C. Hui, X. Zou, Y. Shu, C.H.K. Cheng, S. Fang, H. Luo, J. Lu, M.T.V. Chan, L. Zhang, W.K.K. Wu, SARS-CoV-2 non-structural protein 6 triggers NLRP3-dependent pyroptosis by targeting ATP6AP1, Cell Death Differ. 2021. (2022) 1–15. https://doi.org/10.1038/s41418-021-00916-7.
9. J. Zhang, Y. Cai, T. Xiao, J. Lu, H. Peng, S.M. Sterling, R.M. Walsh, S. Rits-Volloch, H. Zhu, A.N. Woosley, W. Yang, P. Sliz, B. Chen, Structural impact on SARS-CoV-2 spike protein by D614G substitution, Science (80-.). 372 (2021) 525–530. https://doi.org/10.1126/SCIENCE.ABF2303/SUPPL_FILE/ABF2303_ZHANG_SM.PDF.
10. E.C. Thomson, L.E. Rosen, J.G. Shepherd, R. Spreafico, A. da Silva Filipe, J.A. Wojcechowskyj, C. Davis, L. Piccoli, D.J. Pascall, J. Dillen, S. Lytras, N. Czudnochowski, R. Shah, M. Meury, N. Jesudason, A. De Marco, K. Li, J. Bassi, A. O'Toole, D. Pinto, R.M. Colquhoun, K. Culap, B. Jackson, F. Zatta, A. Rambaut, S. Jaconi, V.B. Sreenu, J. Nix, I. Zhang, R.F. Jarrett, W.G. Glass, M. Beltramello, K. Nomikou, M. Pizzuto, L. Tong, E. Cameroni, T.I. Croll, N. Johnson, J. Di Iulio, A. Wickenhagen, A. Ceschi, A.M. Harbison, D. Mair, P. Ferrari, K. Smollett, F. Sallusto, S. Carmichael, C. Garzoni, J. Nichols, M. Galli, J. Hughes, A. Riva, A. Ho, M. Schiuma, M.G. Semple, P.J.M. Openshaw, E. Fadda, J.K. Baillie, J.D. Chodera, S.J. Rihn, S.J. Lycett, H.W. Virgin, A. Telenti, D. Corti, D.L. Robertson, G. Snell, Circulating SARS-CoV-2 spike N439K variants maintain fitness while evading antibody-mediated immunity, Cell. 184 (2021) 1171-1187.e20. https://doi.org/10.1016/J.CELL.2021.01.037.
11. S. Mudgal, R. Yadav, H.R. Haokip, A. Pandit, Y.S. Mary, Genomic variation and point mutations analysis of Indian COVID-19 patient samples submitted in GISAID database, J. Indian Chem. Soc. 98 (2021) 100156. https://doi.org/10.1016/J.JICS.2021.100156.
12. S. Angeletti, D. Benvenuto, M. Bianchi, M. Giovanetti, S. Pascarella, M. Ciccozzi, COVID-2019: The role of the nsp2 and nsp3 in its pathogenesis, J. Med. Virol. 92 (2020) 584–588. https://doi.org/10.1002/JMV.25719.
