# Supplementary material for: Genome Profiling of SARS-CoV-2 in Indonesia, ASEAN and the Neighbouring East Asian Countries: Features, Challenges and Achievements
Source: Viruses. 2022 Apr 8;14(4):778. doi: 10.3390/v14040778 (PMC9027902; doi:10.3390/v14040778)
Supplement: Supplementary file 1 [file viruses-14-00778-s001.zip › viruses-1641476-supplementary/TableS6_acknowledgement__all.pdf]

We gratefully acknowledge the following Authors from the Originating laboratories responsible for obtaining the specimens, as well as the Submitting laboratories where the genome data were generated and shared via GISAID, on which this research is based.

All Submitters of data may be contacted directly via www.gisaid.org

Authors are sorted alphabetically.

Accession ID	Originating Laboratory	Submitting Laboratory	Authors
EPI_ISL_1660412, EPI_ISL_1660418	AMC Cileunyi Hospital	West Java Health Laboratory; School of Life Sciences and Technology, Institut Teknologi Bandung	Aulia Saraswati Wicaksono; Azzania Fibriani; Cut Nur Cinthia Alamanda; Ema Rahmawati; Karimatu Khoirunnisa; Miftahul Faridl; Rifky Waluyajati Rachman; Rini Robiani; Ryan Bayusantika Ristandi
EPI_ISL_890185, EPI_ISL_890186, EPI_ISL_890187, EPI_ISL_891151, EPI_ISL_905731, EPI_ISL_911707, EPI_ISL_911709			
see above	Academic Hospital of Gadjah Mada University (RSA UGM)	Genetics Working Group (Pokja Genetik) Faculty of Medicine, Public Health and Nursing Universitas Gadjah Mada (FK-KMK UGM); Disease Investigation Center Wates Ministry of Agriculture Indonesia; Department of Microbiology FK-KMK UGM; Laboratorium Diagnostik Yayasan Tahija World Mosquito Program (WMP) Yogyakarta Center for Tropical Medicine FK-KMK UGM; Integrated Research center FK-KMK UGM; Department of Computer Science and Electronics FMIPA UGM	Aditya Rifqi Fauzi; Afiahayati; Alvin Santoso Kalim; Audric Kenny Tedja; Dwi AA Nugrahaningsih; Dwiki afandy; Dyah Ayu Puspitarani; Edwin W. Daniwijaya; Eggi Arguni; Endah Supriyati; Fadil Fahri; Gunadi; Hendra Wibawa; Kemala Athollah; Kristy Iskandar; Ludhang P. Rizki; Marcellus; Maria Patricia Inggriani; Mohamad S. Hakim; Nungki Anggorowati; Siswanto; Susan Simanjaya; Titik Nuryastuti; Tri Wibawa; Untung Riawan; William Widitjiarso
EPI_ISL_877129	Academic Hospital of Gajdah Mada University (RSA UGM)	Genetics Working Group (Pokja Genetik) Faculty of Medicine, Public Health and Nursing Universitas Gadjah Mada (FK-KMK UGM); Disease Investigation Center Wates Ministry of Agriculture Indonesia; Department of Microbiology FK-KMK UGM; Laboratorium Diagnostik Yayasan Tahija World Mosquito Program (WMP) Yogyakarta Center for Tropical Medicine FK-KMK UGM; Integrated Research center FK-KMK UGM; Department of Computer Science and Electronics FMIPA UGM	Afiahayati; Dwi AA Nugrahaningsih; Dwiki afandy; Dyah Ayu Puspitarani; Edwin W. Daniwijaya; Eggi Arguni; Endah Supriyati; Gunadi; Hendra Wibawa; Kristy Iskandar; Ludhang P. Rizki; Marcellus; Mohamad S. Hakim; Nungki Anggorowati; Siswanto; Titik Nuryastuti; Tri Wibawa
EPI_ISL_877126, EPI_ISL_877127, EPI_ISL_877128, EPI_ISL_877130, EPI_ISL_877131	Academic Hospital of Gajdah Mada University (RSA UGM)	Genetics Working Group (Pokja Genetik) Faculty of Medicine, Public Health and Nursing Universitas Gadjah Mada (FK-KMK UGM); Disease Investigation Center Wates Ministry of Agriculture Indonesia; Department of Microbiology FK-KMK UGM; Laboratorium Diagnostik Yayasan Tahija World Mosquito Program (WMP) Yogyakarta Center for Tropical Medicine FK-KMK UGM; Integrated Research center FK-KMK UGM; Department of Computer Science and Electronics FMIPA UGM	Aditya Rifqi Fauzi; Afiahayati; Alvin Santoso Kalim; Audric Kenny Tedja; Desyifa Mursalin; Dwi AA Nugrahaningsih; Edwin W. Daniwijaya; Eggi Arguni; Endah Supriyati; Fadil Fahri; Gunadi; Hendra Wibawa; Kemala Athollah; Kristy Iskandar; Ludhang P. Rizki; Marcellus; Maria Patricia Inggriani; Mohamad S. Hakim; Nungki Anggorowati; Siswanto; Susan Simanjaya; Titik Nuryastuti; Tri Wibawa; Untung Riawan; William Widitjiarso
EPI_ISL_1159382, EPI_ISL_1159387, EPI_ISL_2274438	Adi Husada Kapasari Hospital	Institute of Tropical Disease, Universitas Airlangga	Aldise M Nastri; Gatot Soegiarto; Hermanto Wijaya; Jezzy R Dewantari; Kazufumi Shimizu; Krisnoadi Rahardjo; Laksmi Wulandari; Maria I Lusida; Resti Yudhawati; Rima R Prasetya; Soetjipto; Yasuko Mori
EPI_ISL_458083, EPI_ISL_956271	Adi Husada Undaan Hospital	Institute of Tropical Disease, Universitas Airlangga	Aldise M Nastri; Gatot Soegiarto; Irawati Marga; Jezzy R Dewantari; Kazufumi Shimizu; Krisnoadi Rahardjo; Laksmi Wulandari; Maria I Lusida; Mitsuhiro Nishimura; Resti Yudhawati; Retno A Setyoningrum; Rima R Prasetya; Soetjipto; Yasuko Mori; Yohko K Shimizu
EPI_ISL_1335889, EPI_ISL_1335891	Advent Hospital Medan	Faculty of Medicine, Universitas Sumatera Utara; Institute of Tropical Disease, Universitas Airlangga	Aldise M Nastri; Franciscus Ginting; Inke N D Lubis; Irbah R Nainggolan; Jezzy R Dewantari; Kazufumi Shimizu; Krisnoadi Rahardjo; Maria I Lusida; Meliani; Mirzan Hasibuan; Muhammad Ichwan; R Andika D Cahyadi; R Lia Kusumawati; Ramadhan Bestari; Rima R Prasetya; Soetjipto; Yasuko Mori
EPI_ISL_747240	Al Islam Hospital	West Java Health Laboratory; School of Life Sciences and Technology, Institut Teknologi Bandung	Azzania Fibriani; Cut Nur Cinthia Alamanda; Ema Rahmawati; Isak Solihin; Karimatu Khoirunnisa; Miftahul Faridl; Rifky Waluyajati Rachman; Rini Robiani; Ryan Bayusantika Ristandi
EPI_ISL_2617439	Al-Irsyad Hospital	Institute of Tropical Disease, Universitas Airlangga	Aldise M Nastri; Febria Rachmanita; Gatot Soegiarto; Herlin Ferliana; Jezzy R Dewantari; Kazufumi Shimizu; Krisnoadi Rahardjo; Laksmi Wulandari; Maria I Lusida; Resti Yudhawati; Rima R Prasetya; Soetjipto; Yasuko Mori
EPI_ISL_877458, EPI_ISL_1165068, EPI_ISL_1165071, EPI_ISL_1789116, EPI_ISL_1789117, EPI_ISL_1795053, EPI_ISL_2274436			
see above	Anwar Medika General Hospital	Institute of Tropical Disease, Universitas Airlangga	Aldise M Nastri; Gatot Soegiarto; Jezzy R Dewantari; Kazufumi Shimizu; Krisnoadi Rahardjo; Laksmi Wulandari; Maria I Lusida; Muhammad Hamdan; Nungky Taniasari; Resti Yudhawati; Rima R Prasetya; Soetjipto; Yasuko Mori
EPI_ISL_1969249, EPI_ISL_1969250, EPI_ISL_2047551, EPI_ISL_2047552, EPI_ISL_2047553, EPI_ISL_2047554	BBLK Palembang	National Institute of Health Research and Development	Arie Ardiansyah Nugraha; Hana Apsari Pawestri; Hartanti Dian Ikawati; Kartika Dewi Puspa; Krisna Pangesti; Nelly Puspandari; Subangkit; Triyani Soekarso; Vivi Setiawaty
EPI_ISL_1534704, EPI_ISL_1534705	BBLK Surabaya	National Institute of Health Research and Development	Arie Ardiansyah Nugraha; Hana Apsari Pawestri; Hartanti Dian Ikawati; Kartika Dewi Puspa; Krisna Pangesti; Nelly Puspandari; Subangkit; Vivi Setiawaty
EPI_ISL_1915562, EPI_ISL_1915563, EPI_ISL_3138866, EPI_ISL_3138867, EPI_ISL_3138868, EPI_ISL_3138869, EPI_ISL_3138870, EPI_ISL_3138871, EPI_ISL_3138872, EPI_ISL_3138873, EPI_ISL_3138874, EPI_ISL_3138928, EPI_ISL_3138929, EPI_ISL_3138930, EPI_ISL_3138931, EPI_ISL_3138932, EPI_ISL_3138933, EPI_ISL_3138934, EPI_ISL_3138935, EPI_ISL_3138936, EPI_ISL_5107717, EPI_ISL_5107718, EPI_ISL_5107719, EPI_ISL_5107720, EPI_ISL_5107721, EPI_ISL_5107722, EPI_ISL_5107723, EPI_ISL_5107724, EPI_ISL_5107725, EPI_ISL_5107726, EPI_ISL_5107727, EPI_ISL_5107728, EPI_ISL_5107729, EPI_ISL_5107730, EPI_ISL_5107731, EPI_ISL_5107732, EPI_ISL_5107733, EPI_ISL_5107734, EPI_ISL_5107735, EPI_ISL_5107736, EPI_ISL_5107737, EPI_ISL_5107738, EPI_ISL_5107739, EPI_ISL_5107740, EPI_ISL_5107741, EPI_ISL_5107742, EPI_ISL_5107743, EPI_ISL_5107744, EPI_ISL_5107745, EPI_ISL_5107746, EPI_ISL_5107835, EPI_ISL_5107836, EPI_ISL_5107837, EPI_ISL_5107838, EPI_ISL_5107839, EPI_ISL_5107840, EPI_ISL_5107841, EPI_ISL_5107842, EPI_ISL_5107843, EPI_ISL_5107844			
see above	BBTKL PP Banjarbaru	National Institute of Health Research and Development	; Arie Ardiansyah Nugraha; Fajar Nur; Fajar Nur Sulistiyohadi; Hana Apsari Pawestri; Hartanti Dian Ikawati; Kartika Dewi Puspa; Krisna Pangesti; Nelly Puspandari; Subangkit; Sulistiyohadi; Triyani Soekarso; Vivi Setiawaty
EPI_ISL_1469239	BBTKL PP Jakarta	National Institute of Health Research and Development	Arie Ardiansyah Nugraha; Hana Apsari Pawestri; Hartanti Dian Ikawati; Kartika Dewi Puspa; Krisna Pangesti; Nelly Puspandari; Subangkit; Vivi Setiawaty
EPI_ISL_5107829	BBTKL PP Kelas 1 Makassar (Dinkes Polman/PKM Katumbangan)	National Institute of Health Research and Development	Arie Ardiansyah Nugraha; Fajar Nur Sulistiyohadi; Hana Apsari Pawestri; Hartanti Dian Ikawati; Kartika Dewi Puspa; Krisna Pangesti; Nelly Puspandari; Subangkit; Triyani Soekarso; Vivi Setiawaty
EPI_ISL_4254920	BBTKL PP Kelas 1 Makassar (Dinkes Polman/PKM Mapili)	National Institute of Health Research and Development	Arie Ardiansyah Nugraha; Fajar Nur Sulistiyohadi; Hana Apsari Pawestri; Hartanti Dian Ikawati; Kartika Dewi Puspa; Krisna Pangesti; Nelly Puspandari; Subangkit; Triyani Soekarso; Vivi Setiawaty
EPI_ISL_5107826, EPI_ISL_5107827	BBTKL PP Kelas 1 Makassar (Dinkes Polman/PKM Mapilli)	National Institute of Health Research and Development	Arie Ardiansyah Nugraha; Fajar Nur; Hana Apsari Pawestri; Hartanti Dian Ikawati; Kartika Dewi Puspa; Krisna Pangesti; Nelly Puspandari; Subangkit; Sulistiyohadi; Triyani Soekarso; Vivi Setiawaty
EPI_ISL_5107830, EPI_ISL_5107831, EPI_ISL_5107832	BBTKL PP Kelas 1 Makassar (Dinkes Polman/PKM Matangnga)	National Institute of Health Research and Development	Arie Ardiansyah Nugraha; Fajar Nur Sulistiyohadi; Hana Apsari Pawestri; Hartanti Dian Ikawati; Kartika Dewi Puspa; Krisna Pangesti; Nelly Puspandari; Subangkit; Triyani Soekarso; Vivi Setiawaty
EPI_ISL_5107833	BBTKL PP Kelas 1 Makassar (Dinkes Polman/PKM Pambusuang)	National Institute of Health Research and Development	Arie Ardiansyah Nugraha; Fajar Nur Sulistiyohadi; Hana Apsari Pawestri; Hartanti Dian Ikawati; Kartika Dewi Puspa; Krisna Pangesti; Nelly Puspandari; Subangkit; Triyani Soekarso; Vivi Setiawaty
EPI_ISL_4254921, EPI_ISL_4254922, EPI_ISL_4254923, EPI_ISL_5107819, EPI_ISL_5107820, EPI_ISL_5107821, EPI_ISL_5107822, EPI_ISL_5107823, EPI_ISL_5107824, EPI_ISL_5107825			
see above	BBTKL PP Kelas 1 Makassar (Dinkes Polman/PKM Pekkabata)	National Institute of Health Research and Development	Arie Ardiansyah Nugraha; Fajar Nur; Fajar Nur Sulistiyohadi; Hana Apsari Pawestri; Hartanti Dian Ikawati; Kartika Dewi Puspa; Krisna Pangesti; Nelly Puspandari; Subangkit; Sulistiyohadi; Triyani Soekarso; Vivi Setiawaty
EPI_ISL_5107828	BBTKL PP Kelas 1 Makassar (Dinkes Polman/PKM Wonomulyo)	National Institute of Health Research and Development	Arie Ardiansyah Nugraha; Fajar Nur; Hana Apsari Pawestri; Hartanti Dian Ikawati; Kartika Dewi Puspa; Krisna Pangesti; Nelly Puspandari; Subangkit; Sulistiyohadi; Triyani Soekarso; Vivi Setiawaty
EPI_ISL_4254919	BBTKL PP Kelas 1 Makassar (Dinkes Polman/RS Pratama Wonomulyo)	National Institute of Health Research and Development	Arie Ardiansyah Nugraha; Fajar Nur Sulistiyohadi; Hana Apsari Pawestri; Hartanti Dian Ikawati; Kartika Dewi Puspa; Krisna Pangesti; Nelly Puspandari; Subangkit; Triyani Soekarso; Vivi Setiawaty
EPI_ISL_5796035, EPI_ISL_5796036, EPI_ISL_5796037, EPI_ISL_5796038, EPI_ISL_5796039, EPI_ISL_5796040, EPI_ISL_5796041, EPI_ISL_5796042, EPI_ISL_5796043, EPI_ISL_5796044, EPI_ISL_5796045, EPI_ISL_5796046, EPI_ISL_5796047, EPI_ISL_5796048, EPI_ISL_5796049, EPI_ISL_5796050, EPI_ISL_5796051, EPI_ISL_5796052, EPI_ISL_5796053, EPI_ISL_5796054, EPI_ISL_5796055, EPI_ISL_5796056, EPI_ISL_5796057, EPI_ISL_5796058, EPI_ISL_5796059, EPI_ISL_5796060, EPI_ISL_5796061, EPI_ISL_5796062, EPI_ISL_5796063, EPI_ISL_5796064, EPI_ISL_5796065, EPI_ISL_5796083, EPI_ISL_5796084, EPI_ISL_5796087			
see above	BBTKL PP Kelas I Manado	National Institute of Health Research and Development	Arie Ardiansyah Nugraha; Fajar Nur Sulistiyohadi; Hana Apsari Pawestri; Hartanti Dian Ikawati; Kartika Dewi Puspa; Krisna Pangesti; Nelly Puspandari; Subangkit; Triyani Soekarso; Vivi Setiawaty
EPI_ISL_1257851, EPI_ISL_1257852, EPI_ISL_1257853, EPI_ISL_1257854	BBTKLPP Banjarbaru	National Institute of Health Research and Development	Arie Ardiansyah Nugraha; Dewi Hermawati; Hana Apsari Pawestri; Hartanti Dian Ikawati; Kartika Dewi Puspa; Krisna Pangesti; Mutia Raudah; Nelly Puspandari; Subangkit; Vivi Setiawaty
EPI_ISL_1169048	BBTKLPP Banjarbaru, Kalimantan Selatan	National Institute of Health Research and Development	Arie Ardiansyah Nugraha; Hana Apsari Pawestri; Hartanti Dian Ikawati; Kartika Dewi Puspa; Nelly Puspandari; Subangkit; Vivi Setiawaty
EPI_ISL_2620714, EPI_ISL_2868878, EPI_ISL_2868920, EPI_ISL_2868921	BBTKLPP Jakarta	National Institute of Health Research and Development	Arie Ardiansyah Nugraha; Hana Apsari Pawestri; Hartanti Dian Ikawati; Kartika Dewi Puspa; Krisna Pangesti; Nelly Puspandari; Subangkit; Triyani Soekarso; Vivi Setiawaty
EPI_ISL_2233088	BBTKLPP Kelas I Batam	National Institute of Health Research and Development	Arie Ardiansyah Nugraha; Budi Santosa; Hana Apsari Pawestri; Hartanti Dian Ikawati; Ida Neni Haryanti; Ismail; Kartika Dewi Puspa; Krisna Pangesti; Nelly Puspandari; Subangkit; Triyani Soekarso; Vivi Setiawaty
EPI_ISL_2226648	BBTKLPP Surabaya	Institute of Tropical Disease, Universitas Airlangga	Acub Zaenal; Aldise M Nastri; Gatot Soegiarto; Jezzy R Dewantari; Kazufumi Shimizu; Krisnoadi Rahardjo; Laksmi Wulandari; Maria I Lusida; Resti Yudhawati; Rima R Prasetya; Rosidi Roslan; Soetjipto; Yasuko Mori; Zahrotunnisa
EPI_ISL_2943235	BBTKLPP Yogyakarta	Genetics Working Group (Pokja Genetik) Faculty of Medicine, Public Health and Nursing Universitas Gadjah Mada (FK-KMK UGM); Disease Investigation Center Wates Ministry of Agriculture Indonesia; Department of Microbiology FK-KMK UGM; Laboratorium Diagnostik Yayasan Tahija World Mosquito Program (WMP) Yogyakarta Center for Tropical Medicine FK-KMK UGM; Integrated Research center FK-KMK UGM; Department of Computer Science and Electronics	Afiahayati; Dwi AA Nugrahaningsih; Dwi Indaryati; Edwin W. Daniwijaya; Eggi Arguni; Endah Supriyati; Gunadi; Hana F Hanifin; Hendra Wibawa; Indarto Sulistiyono; Irene Tania; Kristy Iskandar; Ludhang P. Rizki; Marcellus; Mohamad S. Hakim; Nungki Anggorowati; Pramesti G Dewi; Siswanto; Titik Nuryastuti; Tri Wibawa

		FMIPA UGM; RSUP Dr. Sardjito	
EPI_ISL_1257856, EPI_ISL_1257857, EPI_ISL_1257858	BBTKLPP Yogyakarta	National Institute of Health Research and Development	Arie Ardiansyah Nugraha; Hana Apsari Pawestri; Hartanti Dian Ikawati; Havid Setyawan; Indaryati; Irene; Kartika Dewi Puspa; Krisna Pangesti; Nelly Puspandari; Subangkit; Vivi Setiawaty
EPI_ISL_2617537, EPI_ISL_2617538, EPI_ISL_2617539, EPI_ISL_2617540, EPI_ISL_2617541, EPI_ISL_2617542	BLK Dan Pengujian Alkes Semarang Jateng	National Institute of Health Research and Development	Arie Ardiansyah Nugraha; Hana Apsari Pawestri; Hartanti Dian Ikawati; Kartika Dewi Puspa; Krisna Pangesti; Nelly Puspandari; Subangkit; Triyani Soekarso; Vivi Setiawaty
EPI_ISL_4104499, EPI_ISL_4104500, EPI_ISL_4104501, EPI_ISL_4104502, EPI_ISL_4104503, EPI_ISL_4104504, EPI_ISL_4104505, EPI_ISL_4104506, EPI_ISL_4104507, EPI_ISL_4104508, EPI_ISL_4104509, EPI_ISL_4104510, EPI_ISL_4104511, EPI_ISL_4104512, EPI_ISL_5022948			
see above	BLKPK Bangka Belitung (RSUD Depati Hamzah)	National Institute of Health Research and Development	Arie Ardiansyah Nugraha; Fajar Nur Sulistiyohadi; Hana Apsari Pawestri; Hartanti Dian Ikawati; Kartika Dewi Puspa; Krisna Pangesti; Nelly Puspandari; Subangkit; Triyani Soekarso; Vivi Setiawaty
EPI_ISL_2811837	BMC Mayapada Hospital	Eijkman Institute for Molecular Biology, National Research and Innovation Agency	Amin Soebandrio; Edison Johar; Frilasita A Yudhaputri; Hidayat Trimarsanto; Iskandar Adnan; Khin Saw Myint; Lidwina Priliani; Lydia V. Panggalo; Muhammad Rezki Rasyak; Safarina G Malik; Sukma Oktavianthi; Willy Agustine
EPI_ISL_1915579, EPI_ISL_2106975, EPI_ISL_5107784, EPI_ISL_5107785, EPI_ISL_5107786, EPI_ISL_5107787, EPI_ISL_5107788, EPI_ISL_5107789, EPI_ISL_5107790, EPI_ISL_5107791, EPI_ISL_5107792, EPI_ISL_5107793, EPI_ISL_5107794, EPI_ISL_5107795, EPI_ISL_5107796, EPI_ISL_5107797, EPI_ISL_5107799, EPI_ISL_5107800, EPI_ISL_5107801, EPI_ISL_5107802, EPI_ISL_5107803, EPI_ISL_5107804, EPI_ISL_5107805, EPI_ISL_5107806, EPI_ISL_5107807, EPI_ISL_5107808, EPI_ISL_5107809, EPI_ISL_5107810, EPI_ISL_5107811, EPI_ISL_5107812			
see above	BPOM Gorontalo	National Institute of Health Research and Development	Arie Ardiansyah Nugraha; Fajar Nur Sulistiyohadi; Hana Apsari Pawestri; Hartanti Dian Ikawati; Kartika Dewi Puspa; Krisna Pangesti; Nelly Puspandari; Subangkit; Triyani Soekarso; Vivi Setiawaty
EPI_ISL_3151609, EPI_ISL_3151616, EPI_ISL_3151623, EPI_ISL_3151630, EPI_ISL_3151636, EPI_ISL_3151643, EPI_ISL_3151676			
see above	BPOM Makassar	National Institute of Health Research and Development	Arie Ardiansyah Nugraha; Fajar Nur Sulistiyohadi; Hana Apsari Pawestri; Hartanti Dian Ikawati; Kartika Dewi Puspa; Krisna Pangesti; Nelly Puspandari; Subangkit; Triyani Soekarso; Vivi Setiawaty
EPI_ISL_1257855, EPI_ISL_5441860, EPI_ISL_5441861, EPI_ISL_5441862	BPOM Mamuju	National Institute of Health Research and Development	Arie Ardiansyah Nugraha; Fajar Nur Sulistiyohadi; Hana Apsari Pawestri; Hartanti Dian Ikawati; Kartika Dewi Puspa; Krisna Pangesti; Nelly Puspandari; Subangkit; Triyani Soekarso; Vivi Setiawaty
EPI_ISL_2854753, EPI_ISL_2854754, EPI_ISL_2854755, EPI_ISL_2854756, EPI_ISL_2868929, EPI_ISL_2987580, EPI_ISL_2987581, EPI_ISL_2987582, EPI_ISL_2987583, EPI_ISL_3019943			
see above	BPPK Aceh	National Institute of Health Research and Development	Arie Ardiansyah Nugraha; Fajar Nur Sulistiyohadi; Hana Apsari Pawestri; Hartanti Dian Ikawati; Kartika Dewi Puspa; Krisna Pangesti; Nelly Puspandari; Subangkit; Triyani Soekarso; Vivi Setiawaty
EPI_ISL_2101216, EPI_ISL_2101217, EPI_ISL_2101218, EPI_ISL_2101219, EPI_ISL_2101220, EPI_ISL_2101221, EPI_ISL_2101222, EPI_ISL_2101223, EPI_ISL_2101224, EPI_ISL_2101225, EPI_ISL_2101226, EPI_ISL_2101227, EPI_ISL_2101228			
see above	BRSU Tabanan	Eijkman Institute for Molecular Biology, National Agency for Research and Innovation; Molecular Biology Laboratory, Faculty Medicine and Health Sciences, Warmadewa University	Amin Soebandrio; Edison Johar; Erly Sintya; Frilasita A Yudhaputri; Hidayat Trimarsanto; I Wayan Duta Krisna; Iskandar Adnan; Khin Saw Myint; Lidwina Priliani; Lydia V. Panggalo; Muhammad Rezki Rasyak; Safarina G Malik; Sri Masyeni; Sukma Oktavianthi; Willy Agustine
EPI_ISL_1020186, EPI_ISL_1137600	BSL3 Laboratory, Bogor, West Java	Biosafety Level-3 Laboratory, Indonesian Institute of Sciences (LIPI)	Anggia Prasetyoputri; Eko Wahyu Putro; Gita Syahputra; Isa Nuryana; Ratih Asmana Ningrum; Sri Swasthikawati; Sugiyono Saputra; Syam Budi Iryanto
EPI_ISL_3138875, EPI_ISL_3138876, EPI_ISL_3138877, EPI_ISL_3138878, EPI_ISL_3138879, EPI_ISL_3138880, EPI_ISL_3138881, EPI_ISL_3138882, EPI_ISL_3138883, EPI_ISL_3138884			
see above	BTKL PP Ambon	National Institute of Health Research and Development	Arie Ardiansyah Nugraha; Fajar Nur Sulistiyohadi; Hana Apsari Pawestri; Hartanti Dian Ikawati; Kartika Dewi Puspa; Krisna Pangesti; Nelly Puspandari; Subangkit; Triyani Soekarso; Vivi Setiawaty
EPI_ISL_3138886, EPI_ISL_3138887, EPI_ISL_3138888, EPI_ISL_3138889, EPI_ISL_3138890, EPI_ISL_3138891, EPI_ISL_3138892, EPI_ISL_3138893, EPI_ISL_4254900, EPI_ISL_4254901, EPI_ISL_4254902, EPI_ISL_4254903, EPI_ISL_4254904, EPI_ISL_4254905, EPI_ISL_4254906, EPI_ISL_4254907, EPI_ISL_4254908, EPI_ISL_4254909, EPI_ISL_4254910, EPI_ISL_4254911, EPI_ISL_4254912, EPI_ISL_4254913, EPI_ISL_4254914, EPI_ISL_4254915, EPI_ISL_4254916, EPI_ISL_4254917, EPI_ISL_4254918, EPI_ISL_4730599, EPI_ISL_4730600, EPI_ISL_4730601, EPI_ISL_4730602, EPI_ISL_4730603, EPI_ISL_4730604, EPI_ISL_4730605, EPI_ISL_4730606, EPI_ISL_4730607, EPI_ISL_5022721, EPI_ISL_5022722, EPI_ISL_5022723, EPI_ISL_5022724, EPI_ISL_5022725, EPI_ISL_5022726, EPI_ISL_5022727, EPI_ISL_5022728, EPI_ISL_5022729, EPI_ISL_5022730, EPI_ISL_5022731, EPI_ISL_5022732, EPI_ISL_5022733, EPI_ISL_5022734, EPI_ISL_5022735, EPI_ISL_5022736, EPI_ISL_5022737, EPI_ISL_5022738, EPI_ISL_5022872, EPI_ISL_5022873, EPI_ISL_5022874, EPI_ISL_5022875, EPI_ISL_5022876, EPI_ISL_5022877, EPI_ISL_5022878, EPI_ISL_5022879, EPI_ISL_5022953, EPI_ISL_5107747, EPI_ISL_5107748, EPI_ISL_5107749, EPI_ISL_5107750, EPI_ISL_5107751, EPI_ISL_5107752, EPI_ISL_5107753, EPI_ISL_5107754, EPI_ISL_5107755, EPI_ISL_5107756, EPI_ISL_5107757, EPI_ISL_5107758, EPI_ISL_5107759, EPI_ISL_5107760, EPI_ISL_5107761, EPI_ISL_5107762, EPI_ISL_5107763, EPI_ISL_5107764, EPI_ISL_5107765, EPI_ISL_5107766, EPI_ISL_5107767, EPI_ISL_5107768, EPI_ISL_5107769, EPI_ISL_5107770, EPI_ISL_5107771, EPI_ISL_5107772, EPI_ISL_5107773, EPI_ISL_5107774, EPI_ISL_5107775, EPI_ISL_5107776, EPI_ISL_5107777, EPI_ISL_5107778, EPI_ISL_5107779, EPI_ISL_5107780, EPI_ISL_5107781, EPI_ISL_5107782, EPI_ISL_5107783, EPI_ISL_5441805, EPI_ISL_5441806, EPI_ISL_5441807, EPI_ISL_5441808, EPI_ISL_5441809, EPI_ISL_5441810, EPI_ISL_5441811, EPI_ISL_5441812, EPI_ISL_5441813, EPI_ISL_5441814, EPI_ISL_5441815, EPI_ISL_5441816, EPI_ISL_5441817, EPI_ISL_5441818, EPI_ISL_5441819, EPI_ISL_5441820, EPI_ISL_5441821, EPI_ISL_5441822, EPI_ISL_5441823, EPI_ISL_5441824, EPI_ISL_5441825, EPI_ISL_5441826, EPI_ISL_5441827, EPI_ISL_5441828, EPI_ISL_5441829, EPI_ISL_5441830, EPI_ISL_5441831, EPI_ISL_5441832, EPI_ISL_5441833, EPI_ISL_5441834, EPI_ISL_5441835, EPI_ISL_5441836, EPI_ISL_5441837, EPI_ISL_5441838, EPI_ISL_5441839, EPI_ISL_5441840, EPI_ISL_5441841, EPI_ISL_5441842, EPI_ISL_5441843, EPI_ISL_5441844, EPI_ISL_5441845, EPI_ISL_5441846, EPI_ISL_5441847, EPI_ISL_5441848, EPI_ISL_5441849, EPI_ISL_5441850, EPI_ISL_5441851, EPI_ISL_5441852, EPI_ISL_5441853			
see above	BTKL PP Kelas 1 Manado	National Institute of Health Research and Development	; Arie Ardiansyah Nugraha; Fajar Nur; Fajar Nur Sulistiyohadi; Hana Apsari Pawestri; Hartanti Dian Ikawati; Kartika Dewi Puspa; Krisna Pangesti; Nelly Puspandari; Subangkit; Sulistiyohadi; Triyani Soekarso; Vivi Setiawaty
EPI_ISL_3277504, EPI_ISL_3277505, EPI_ISL_3277506, EPI_ISL_3277507, EPI_ISL_3277508, EPI_ISL_3277509, EPI_ISL_3277510, EPI_ISL_3277511, EPI_ISL_3277512, EPI_ISL_3277513, EPI_ISL_3277514, EPI_ISL_3277515, EPI_ISL_3277516, EPI_ISL_3277517, EPI_ISL_3277518, EPI_ISL_3277519, EPI_ISL_3277520, EPI_ISL_3277521, EPI_ISL_3277522, EPI_ISL_3277523, EPI_ISL_3277524, EPI_ISL_3277525, EPI_ISL_3277526, EPI_ISL_3277527, EPI_ISL_3277530, EPI_ISL_3277533, EPI_ISL_3277534, EPI_ISL_3277536, EPI_ISL_3277537, EPI_ISL_3277538, EPI_ISL_3277539, EPI_ISL_3277540, EPI_ISL_3277541, EPI_ISL_3277542, EPI_ISL_3277543, EPI_ISL_3303967, EPI_ISL_3303968, EPI_ISL_3303969, EPI_ISL_3303970, EPI_ISL_3303971, EPI_ISL_3303972, EPI_ISL_3303973, EPI_ISL_3303974, EPI_ISL_3303975, EPI_ISL_3303976, EPI_ISL_3303977, EPI_ISL_3303978, EPI_ISL_3303979, EPI_ISL_3303980			
see above	BTKL PP Kelas I Batam	Indonesian Institute of Sciences (LIPI)/National Agency of Research and Innovation (BRIN) and National Institute of Health Research and Development (NIHRD)	Ade Andriani; Alfi T. Fathurahman; Andri Wardiana; Anggia Prasetyoputri; Anik B. Dharmayanthi; Dharmayanthi; Erwin F. Hasrianda; Hana Apsari Pawestri; Hariyatun; Indriawati; Isa Nuryana; Listiana Oktavia; Masrukhin; Puspita Lisdiyanti; Ratih A. Ningrum; Subangkit; Sugiyono Saputra; Syam B. Iryanto; Vivi Setiawaty; Yuliawati
EPI_ISL_2931726, EPI_ISL_2931735, EPI_ISL_2931743, EPI_ISL_2931753, EPI_ISL_2931754, EPI_ISL_2931762, EPI_ISL_2931773, EPI_ISL_2931788, EPI_ISL_2987645, EPI_ISL_2987646, EPI_ISL_2987647, EPI_ISL_2987648, EPI_ISL_2987649, EPI_ISL_2987650, EPI_ISL_2987651, EPI_ISL_2987652, EPI_ISL_2987653, EPI_ISL_3138917			
see above	BTKL PP Kelas I Batam	National Institute of Health Research and Development	; Arie Ardiansyah Nugraha; Fajar Nur Sulistiyohadi; Hana Apsari Pawestri; Hartanti Dian Ikawati; Kartika Dewi Puspa; Krisna Pangesti; Nelly Puspandari; Subangkit; Triyani Soekarso; Vivi Setiawaty
EPI_ISL_4004719, EPI_ISL_4004720, EPI_ISL_4004721, EPI_ISL_4004722, EPI_ISL_4004723, EPI_ISL_4004724, EPI_ISL_4004725, EPI_ISL_4004726, EPI_ISL_4004727, EPI_ISL_4004728, EPI_ISL_4004729, EPI_ISL_4004730, EPI_ISL_4004731, EPI_ISL_4004732, EPI_ISL_4004733, EPI_ISL_4004734, EPI_ISL_4004735			
see above	BTKL PP Kelas I Batam	National Research and Innovation Agency (BRIN-Indonesia, VenomCoV Project Team); National Institute of Health Research and Development (NIHRD)	Andri Wardiana; Anggia Prasetyoputri; Hana Apsari Pawestri; Indriawati; Isa Nuryana; Masrukhin; Puspita Lisdiyanti; Ratih A. Ningrum; Subangkit; Sugiyono Saputra; Syam B. Iryanto; Vivi Setiawaty
EPI_ISL_1257832, EPI_ISL_1257864	BTKL-PP Ambon	National Institute of Health Research and Development	Arie Ardiansyah Nugraha; Hana Apsari Pawestri; Hartanti Dian Ikawati; Kartika Dewi Puspa; Krisna Pangesti; Nelly Puspandari; Subangkit; Vivi Setiawaty
EPI_ISL_1263459	BTKLPP Ambon	National Institute of Health Research and Development	Arie Ardiansyah Nugraha; Hana Apsari Pawestri; Hartanti Dian Ikawati; Kartika Dewi Puspa; Krisna Pangesti; Nelly Puspandari; Subangkit; Vivi Setiawaty
EPI_ISL_3275038	BTKLPP Kelas 1 Manado	National Institute of Health Research and Development	Arie Ardiansyah Nugraha; Hana Apsari Pawestri; Hartanti Dian Ikawati; Kartika Dewi Puspa; Krisna Nur Andriana Pangesti; Nelly Puspandari; Subangkit; Triyani Soekarso; Vivi Setiawaty
EPI_ISL_3070872, EPI_ISL_3070873, EPI_ISL_3070874, EPI_ISL_3070875, EPI_ISL_3070876, EPI_ISL_3070877, EPI_ISL_3070878, EPI_ISL_3070879, EPI_ISL_3070880, EPI_ISL_3070881, EPI_ISL_3070882, EPI_ISL_3070883, EPI_ISL_3070884, EPI_ISL_3070885, EPI_ISL_3070886, EPI_ISL_3070887, EPI_ISL_3070888, EPI_ISL_3070889, EPI_ISL_3070890, EPI_ISL_3070891, EPI_ISL_3070892, EPI_ISL_3070893, EPI_ISL_3070894, EPI_ISL_3070895, EPI_ISL_3070896, EPI_ISL_3070897, EPI_ISL_3070898, EPI_ISL_3070899, EPI_ISL_3070900, EPI_ISL_3070901, EPI_ISL_3070902, EPI_ISL_3070903, EPI_ISL_3070904, EPI_ISL_3070905, EPI_ISL_3070906, EPI_ISL_3070907, EPI_ISL_3070908, EPI_ISL_3070909, EPI_ISL_3070910, EPI_ISL_3070911, EPI_ISL_3070912, EPI_ISL_3070913, EPI_ISL_3070914, EPI_ISL_3070915, EPI_ISL_3070916, EPI_ISL_3070917, EPI_ISL_3070918, EPI_ISL_3070919, EPI_ISL_3070920, EPI_ISL_3070921, EPI_ISL_3070922, EPI_ISL_3070923, EPI_ISL_3070924, EPI_ISL_3070925, EPI_ISL_3070926, EPI_ISL_3070927, EPI_ISL_3070928, EPI_ISL_3070929, EPI_ISL_3070930, EPI_ISL_3070931, EPI_ISL_3070932, EPI_ISL_3070933, EPI_ISL_3070934, EPI_ISL_3070935, EPI_ISL_3070936, EPI_ISL_3070937, EPI_ISL_3070938, EPI_ISL_3070939, EPI_ISL_3070940, EPI_ISL_3070941, EPI_ISL_3070942, EPI_ISL_3070943, EPI_ISL_3070944, EPI_ISL_3070945, EPI_ISL_3070946, EPI_ISL_3070947, EPI_ISL_3070948, EPI_ISL_3070949, EPI_ISL_3070950, EPI_ISL_3070951, EPI_ISL_3070952, EPI_ISL_3070953, EPI_ISL_3070954, EPI_ISL_3070955, EPI_ISL_3070956, EPI_ISL_3070957, EPI_ISL_3070958, EPI_ISL_3070959, EPI_ISL_3070960, EPI_ISL_3070961, EPI_ISL_3070962, EPI_ISL_3159699, EPI_ISL_3159702, EPI_ISL_3159705			
see above	BTKLPP Kelas I Batam	Eijkman Institute for Molecular Biology, National Research and Innovation Agency; National Institute of Health Research and Development	Amin Soebandrio; Edison Johar; Frilasita A Yudhaputri; Hana Apsari Pawestri; Hidayat Trimarsanto; Iskandar Adnan; Khin Saw Myint; Lidwina Priliani; Lydia V. Panggalo; Muhammad Rezki Rasyak; Safarina G Malik; Subangkit; Sukma Oktavianthi; Vivi Setiawaty; Willy Agustine
EPI_ISL_3138841, EPI_ISL_3138842, EPI_ISL_3138909, EPI_ISL_3138910, EPI_ISL_3138911, EPI_ISL_3138912, EPI_ISL_3138913, EPI_ISL_3138914, EPI_ISL_3138915, EPI_ISL_3138916			
see above	BTKLPP Kelas I Batam	National Institute of Health Research and Development	Arie Ardiansyah Nugraha; Fajar Nur Sulistiyohadi; Hana Apsari Pawestri; Hartanti Dian Ikawati; Kartika Dewi Puspa; Krisna Pangesti; Nelly Puspandari; Subangkit; Triyani Soekarso; Vivi Setiawaty
EPI_ISL_1265441, EPI_ISL_1265442, EPI_ISL_1265443, EPI_ISL_1265444, EPI_ISL_1265445, EPI_ISL_1265446, EPI_ISL_1265447, EPI_ISL_1265448, EPI_ISL_1265449, EPI_ISL_1265450, EPI_ISL_1265451, EPI_ISL_1265452, EPI_ISL_1265453, EPI_ISL_1265454, EPI_ISL_1265455, EPI_ISL_1265456, EPI_ISL_1265457, EPI_ISL_1265458, EPI_ISL_1265459, EPI_ISL_1265460, EPI_ISL_1265461, EPI_ISL_1265462			
see above	BTKLPP Kelas I Makassar	Eijkman Institute for Molecular Biology, Ministry of Research and Technology/National Agency for Research and Innovation; National Institute of Health Research and Development	Amin Soebandrio; Edison Johar; Frilasita A Yudhaputri; Hana Apsari Pawestri; Hidayat Trimarsanto; Iskandar Adnan; Khin Saw Myint; Lydia V. Panggalo; Safarina G Malik; Slamet; Sukma Oktavianthi; Vivi Setiawaty; Willy Agustine
EPI_ISL_2868875, EPI_ISL_2868887, EPI_ISL_2868889, EPI_ISL_2868891, EPI_ISL_2868892, EPI_ISL_2868894, EPI_ISL_2868896, EPI_ISL_2868897, EPI_ISL_2868899, EPI_ISL_2868901			
see above	BTKLPP Kelas I Makassar	National Institute of Health Research and Development	Arie Ardiansyah Nugraha; Hana Apsari Pawestri; Hartanti Dian Ikawati; Kartika Dewi Puspa; Krisna Pangesti; Nelly Puspandari; Subangkit; Triyani Soekarso; Vivi Setiawaty
EPI_ISL_574619, EPI_ISL_574620, EPI_ISL_574621, EPI_ISL_574622, EPI_ISL_574623	BTKLPP Kelas I Manado	Eijkman Institute for Molecular Biology, Ministry of Research and Technology/National Agency for Research and Innovation	Amin Soebandrio; David H Muljono; Edison Johar; Frilasita A Yudhaputri; Herawati Sudoyo; Hidayat Trimarsanto; Iskandar A Adnan; Khin Saw Myint; Safarina G Malik; Willy Agustine
EPI_ISL_2868923, EPI_ISL_2868925, EPI_ISL_2868926, EPI_ISL_2868928	BTKLPP Kelas I Manado	National Institute of Health Research and Development	Arie Ardiansyah Nugraha; Hana Apsari Pawestri; Hartanti Dian Ikawati; Kartika Dewi Puspa; Krisna Pangesti; Nelly Puspandari; Subangkit; Triyani Soekarso; Vivi Setiawaty
EPI_ISL_1534690, EPI_ISL_1534691, EPI_ISL_1534692, EPI_ISL_1534693, EPI_ISL_1534694, EPI_ISL_1534695, EPI_ISL_1534696			
see above	BTKLPP kelas 1 Batam	National Institute of Health Research and Development	Arie Ardiansyah Nugraha; Budi Santosa; Hana Apsari Pawestri; Hartanti Dian Ikawati; Ida Neni Haryanti; Ismail; Kartika Dewi Puspa; Krisna Pangesti; Nelly Puspandari; Subangkit; Vivi Setiawaty
EPI_ISL_2860278, EPI_ISL_2860279, EPI_ISL_2860280	Badan Besar Rehabilitasi BNN	Biosafety Level-3 Laboratory, Indonesian Institute of Sciences (LIPI)	Andri Wardiana; Anik B. Dharmayanthi; Idris; Indriawati; Listiana Oktavia; Masrukhin; Ratih A. Ningrum; Yuliawati
EPI_ISL_3691597, EPI_ISL_3691650, EPI_ISL_3691651, EPI_ISL_3691652, EPI_ISL_3691653, EPI_ISL_3691654, EPI_ISL_3691655, EPI_ISL_3691656, EPI_ISL_3691657, EPI_ISL_3691658, EPI_ISL_3691659, EPI_ISL_3691660, EPI_ISL_3691661, EPI_ISL_3691662, EPI_ISL_3691663, EPI_ISL_3691664, EPI_ISL_3691665, EPI_ISL_3691666, EPI_ISL_3691667, EPI_ISL_3691668			
see above	Badan Layanan Umum Daerah RS Konawe	National Institute of Health Research and Development	Arie Ardiansyah Nugraha; Fajar Nur Sulistiyohadi; Hana Apsari Pawestri; Hartanti Dian Ikawati; Kartika Dewi Puspa; Krisna Pangesti; Nelly Puspandari; Subangkit; Triyani Soekarso; Vivi Setiawaty
EPI_ISL_4104461, EPI_ISL_4104462, EPI_ISL_4104463, EPI_ISL_4104464, EPI_ISL_4104465, EPI_ISL_4104466, EPI_ISL_4104467, EPI_ISL_4104468, EPI_ISL_4104469, EPI_ISL_4104470, EPI_ISL_4104471, EPI_ISL_4104472, EPI_ISL_4104473, EPI_ISL_4104474, EPI_ISL_4104475, EPI_ISL_4104476, EPI_ISL_4104477, EPI_ISL_4104478, EPI_ISL_4104479, EPI_ISL_4104480, EPI_ISL_4104481, EPI_ISL_4104482, EPI_ISL_4104483, EPI_ISL_4104484, EPI_ISL_4104485, EPI_ISL_4104486, EPI_ISL_4104487, EPI_ISL_4104488, EPI_ISL_4104489, EPI_ISL_4104490, EPI_ISL_4104491, EPI_ISL_4104492, EPI_ISL_4104493, EPI_ISL_4104494, EPI_ISL_4104495, EPI_ISL_4104496, EPI_ISL_4104497, EPI_ISL_4104498			
see above	Badan Litbangkes Aceh	National Institute of Health Research and Development	Arie Ardiansyah Nugraha; Fajar Nur; Fajar Nur Sulistiyohadi; Hana Apsari Pawestri; Hartanti Dian Ikawati; Kartika Dewi Puspa; Krisna Pangesti; Nelly Puspandari; Subangkit; Sulistiyohadi; Triyani Soekarso; Vivi Setiawaty
EPI_ISL_5022947	Badan Litbangkes BPPK Aceh	National Institute of Health Research and Development	Arie Ardiansyah Nugraha; Fajar Nur Sulistiyohadi; Hana Apsari Pawestri; Hartanti Dian Ikawati; Kartika Dewi Puspa; Krisna Pangesti; Nelly Puspandari; Subangkit; Triyani Soekarso; Vivi Setiawaty

EPI_ISL_3267186, EPI_ISL_4056009, EPI_ISL_4056011, EPI_ISL_4056012	Balai Besar Laboratorium Kesehatan - Center for Health Laboratory Surabaya	Institute of Tropical Disease, Universitas Airlangga	Adi Pramono Hendrata; Aldise M Nastri; Gatot Soegiarto; Jezzy R Dewantari; Kazufumi Shimizu; Kohar Hari Santoso; Krisnoadi Rahardjo; Laksmi Wulandari; Maria I Lusida; Resti Yudhawati; Rima R Prasetya; Soetjipto; Yasuko Mori
EPI_ISL_791988	Balai Besar POM Semarang	National Institute of Health Research and Development	AA; Aryanti; HA; HD; Ikawati; KD; KNA; N; Nugraha; Pangesti; Pawestri; Puspa; Puspandari; Setiawaty; Soekarso; Subangkit; T; V
EPI_ISL_2537486, EPI_ISL_2537488, EPI_ISL_2537489, EPI_ISL_2537491, EPI_ISL_2537492, EPI_ISL_2538126, EPI_ISL_2543614, EPI_ISL_2543668, EPI_ISL_2544800, EPI_ISL_2544801, EPI_ISL_2558705, EPI_ISL_2558706, EPI_ISL_2558707, EPI_ISL_2558708, EPI_ISL_2558709, EPI_ISL_2558710, EPI_ISL_2558711, EPI_ISL_2558712, EPI_ISL_2558714, EPI_ISL_2558716, EPI_ISL_2558717, EPI_ISL_2558721, EPI_ISL_2558722, EPI_ISL_2558723, EPI_ISL_2558724, EPI_ISL_2558725, EPI_ISL_2558726, EPI_ISL_2558727, EPI_ISL_2558728, EPI_ISL_2558729, EPI_ISL_2558730, EPI_ISL_2558731, EPI_ISL_2558736, EPI_ISL_2558737			
see above	Balai Besar Penelitian dan Pengembangan dan Reservoir Penyakit, Salatiga	Genomik Solidaritas Indonesia Laboratorium	Alfin Mohammad Abdillah; Anindya Pradipta Susanto; Annisa Muthiah Sukirman; Anuraj Shankar; Ariel Pradipta; Budi Wiweko; Carissa Sintca Wijaya; Dhahlia Agustina Cahyono; Gracia Felias Enos Korompis; Joko Waluyo; Louisa Markus; Meutia Ayuputeri Kumaheri; Normastuti Adhini Tantyo; Pratiwi Sudarmono; Vania Gavrila Wikasa; Yudistira Aditya Rahmat
EPI_ISL_1020199	Balai Besar Rehabilitasi (BNN)	Biosafety Level-3 Laboratory, Indonesian Institute of Sciences (LIPI)	Alfi T. Fathurahman; Idris; Linda Sukmarini; Masrukhin; Ratih Asmana Ningrum; Ruby Setiawan; Sri Swasthikawati; Sugiyono Saputra
EPI_ISL_775599	Balai Besar Veteriner Maros	National Institute of Health Research and Development	AA; HA; HD; Ikawati; KD; KNA; Mangidi; Muflihanah; N; Nugraha; Pangesti; Pawestri; Puspa; Puspandari; R; Setiawaty; Soekarso; Subangkit; T; V
EPI_ISL_753698	Balai Besar Veteriner Maros, Sulawesi Selatan	National Institute of Health Research and Development	AA; HA; HD; Ikawati; KD; KNA; Mangidi; Muflihanah; N; Nugraha; Pangesti; Pawestri; Puspa; Puspandari; R; Setiawaty; Soekarso; Subangkit; T; V
EPI_ISL_4847177	Balai Kesehatan Masyarakat AHD Klaten	Balai Laboratorium Kesehatan Dan Pengujian Alat Kesehatan Provinsi Jawa Tengah	Alia Wijayanti; Dwi Nursetyaasih; Dyah Ayu; Endah Wahyu Y; Ismi Farhati; Purnomo; Sekti Handayani; Wiwik Peni; Yekti Hediningsih
EPI_ISL_4756054, EPI_ISL_4756056	Balai Kesehatan Masyarakat Klaten	Balai Laboratorium Kesehatan Dan Pengujian Alat Kesehatan Provinsi Jawa Tengah	Alia Wijayanti; Dwi Nursetyaasih; Dyah Ayu; Endah Wahyu Y; Ismi Farhati; Purnomo; Sekti Handayani; Wiwik Peni; Yekti Hediningsih
EPI_ISL_5616256, EPI_ISL_5616267, EPI_ISL_5616292, EPI_ISL_5616301, EPI_ISL_5616334, EPI_ISL_5616344, EPI_ISL_5616363, EPI_ISL_5616472, EPI_ISL_5616512			
see above	Balai Kesehatan Masyarakat Klaten	Balai Laboratorium Kesehatan dan Pengujian Alat Kesehatan Provinsi Jawa Tengah	Alia Wijayanti; Dwi Nursetyaasih; Dyah Ayu; Endah Wahyu Y; Ismi Farhati; Purnomo; Sekti Handayani; Wiwik Peni; Yekti Hediningsih
EPI_ISL_4826927, EPI_ISL_4829453, EPI_ISL_4847261, EPI_ISL_4847415	Balai Kesehatan Masyarakat Klaten AHD	Balai Laboratorium Kesehatan Dan Pengujian Alat Kesehatan Provinsi Jawa Tengah	Alia Wijayanti; Dwi Nursetyaasih; Dyah Ayu; Endah Wahyu Y; Ismi Farhati; Purnomo; Sekti Handayani; Wiwik Peni; Yekti Hediningsih
EPI_ISL_5616484	Balai Kesehatan Masyarakat Semarang	Balai Laboratorium Kesehatan dan Pengujian Alat Kesehatan Provinsi Jawa Tengah	Alia Wijayanti; Dwi Nursetyaasih; Dyah Ayu; Endah Wahyu Y; Ismi Farhati; Purnomo; Sekti Handayani; Wiwik Peni; Yekti Hediningsih
EPI_ISL_4824126	Balai Kesehatan Masyarakat Semarang BPSDMD	Balai Laboratorium Kesehatan Dan Pengujian Alat Kesehatan Provinsi Jawa Tengah	Alia Wijayanti; Dwi Nursetyaasih; Dyah Ayu; Endah Wahyu Y; Ismi Farhati; Purnomo; Sekti Handayani; Wiwik Peni; Yekti Hediningsih
EPI_ISL_4826621	Balai Kkesehatan Masyarakat Klaten AHD	Balai Laboratorium Kesehatan Dan Pengujian Alat Kesehatan Provinsi Jawa Tengah	Alia Wijayanti; Dwi Nursetyaasih; Dyah Ayu; Endah Wahyu Y; Ismi Farhati; Purnomo; Sekti Handayani; Wiwik Peni; Yekti Hediningsih
EPI_ISL_791978	Balai Labkes Lampung	National Institute of Health Research and Development	AA; HA; HD; Ikawati; KD; KNA; L; N; Nugraha; Pangesti; Pawestri; Puspa; Puspandari; Setiawaty; Soekarso; Subangkit; T; V; Yurina
EPI_ISL_791981	Balai Litbang Aceh	National Institute of Health Research and Development	AA; E; F; Fitria; HA; HD; Ichwansyah; Ikawati; KD; KNA; N; Nugraha; Pangesti; Pawestri; Puspa; Puspandari; Setiawaty; Soekarso; Subangkit; T; V
EPI_ISL_1257818, EPI_ISL_1257819, EPI_ISL_1257820, EPI_ISL_1257821, EPI_ISL_1257822	Balai Litbangkes Papua	National Institute of Health Research and Development	Antonius Octavian; Arie Ardiansyah Nugraha; Hana Apsari Pawestri; Hartanti Dian Ikawati; Hotma Sitompul; Kartika Dewi Puspa; Krisna Pangesti; Nelly Puspandari; Subangkit; Vivi Setiawaty
EPI_ISL_1257830, EPI_ISL_1257831, EPI_ISL_2047561, EPI_ISL_2047562, EPI_ISL_2047563, EPI_ISL_2047564, EPI_ISL_2047565, EPI_ISL_2047566, EPI_ISL_2047567, EPI_ISL_2047568, EPI_ISL_2047569			
see above	Balai Litbangkes Tanah Bumbu	National Institute of Health Research and Development	Arie Ardiansyah Nugraha; Hana Apsari Pawestri; Hartanti Dian Ikawati; Juhairiyah; Kartika Dewi Puspa; Krisna Pangesti; Nelly Puspandari; Subangkit; Syarif Hidayat; Triyani Soekarso; Vivi Setiawaty
EPI_ISL_4090578, EPI_ISL_4090579, EPI_ISL_4090580, EPI_ISL_4090581, EPI_ISL_4090582	Balai Penelitian Dan Pengembangan kesehatan Tanah Bumbu	National Institute of Health Research and Development	Arie Ardiansyah Nugraha; Fajar Nur Sulistiyohadi; Hana Apsari Pawestri; Hartanti Dian Ikawati; Kartika Dewi Puspa; Krisna Pangesti; Nelly Puspandari; Subangkit; Triyani Soekarso; Vivi Setiawaty
EPI_ISL_3394979, EPI_ISL_3394980, EPI_ISL_3394981, EPI_ISL_3394982, EPI_ISL_3394983, EPI_ISL_3394984, EPI_ISL_3394985, EPI_ISL_3394986, EPI_ISL_3394987, EPI_ISL_3394988, EPI_ISL_3394989, EPI_ISL_3394990, EPI_ISL_3394991, EPI_ISL_3394992, EPI_ISL_3394993, EPI_ISL_3394994, EPI_ISL_3394995, EPI_ISL_3394996, EPI_ISL_3394997, EPI_ISL_3394998, EPI_ISL_3394999, EPI_ISL_3395000, EPI_ISL_3395001, EPI_ISL_3395002, EPI_ISL_3395003, EPI_ISL_3395004, EPI_ISL_3395005, EPI_ISL_3395006, EPI_ISL_3395007			
see above	Balai Penelitian dan Pengembangan Aceh	National Institute of Health Research and Development	Arie Ardiansyah Nugraha; Fahmi Ichwansyah; Hana Apsari Pawestri; Hartanti Dian Ikawati; Kartika Dewi Puspa; Krisna Pangesti; Nelly Puspandari; Subangkit; Triyani Soekarso; Vivi Setiawaty
EPI_ISL_538506, EPI_ISL_538507, EPI_ISL_538512, EPI_ISL_1048406	Balai Penelitian dan Pengembangan Biomedis Papua	National Institute of Health Research and Development	A; AA; Anton Oktavianus; Arie Ardiansyah Nugraha; HA; HD; HML; Hana Apsari Pawestri; Hartanti Dian Ikawati; Hotma Sitompul; Hutapea; Ikawati; KD; KNA; Kartika Dewi Puspa; Krisna Nur Andriana Pangesti; M; Nelly Puspandari; Nugraha; Oktavian; Paisal; Pangesti; Pasaribu; Pawestri; Puspa; Setiawaty, V.; Soekarso; Subangkit; T; Vivi Setiawaty.
EPI_ISL_5107675, EPI_ISL_5107676, EPI_ISL_5107677, EPI_ISL_5107678, EPI_ISL_5107679, EPI_ISL_5107680, EPI_ISL_5107681, EPI_ISL_5107682, EPI_ISL_5107683, EPI_ISL_5107684, EPI_ISL_5107685, EPI_ISL_5107686, EPI_ISL_5107687, EPI_ISL_5107688, EPI_ISL_5107689, EPI_ISL_5107690, EPI_ISL_5107691, EPI_ISL_5107692, EPI_ISL_5107693, EPI_ISL_5107694, EPI_ISL_5107695, EPI_ISL_5107696, EPI_ISL_5107697, EPI_ISL_5107698, EPI_ISL_5107699, EPI_ISL_5107700, EPI_ISL_5107701, EPI_ISL_5107702, EPI_ISL_5107703, EPI_ISL_5107704, EPI_ISL_5107705, EPI_ISL_5107706, EPI_ISL_5107707, EPI_ISL_5107708, EPI_ISL_5107709, EPI_ISL_5107710, EPI_ISL_5107711, EPI_ISL_5107712, EPI_ISL_5107713, EPI_ISL_5107714, EPI_ISL_5107715			
see above	Balai Penelitian dan Pengembangan Kesehatan Papua	National Institute of Health Research and Development	Antonius Oktavian; Choirul Huda; Clara Imaniar; Hana Apsari Pawestri; Hana Krismawati; Hartanti Dian Ikawati; Hotma Hutapea; Moh.Fadjri Rokhmad; Ratna Tanjung; Sophia M.Yansip; Tri wahyuni; Triyani Soekarso; Vivi Setiawaty; Yuli Arisanti; Yustinus Maladan
EPI_ISL_4730586, EPI_ISL_4730587, EPI_ISL_4730588, EPI_ISL_4730592, EPI_ISL_4730593, EPI_ISL_4730594, EPI_ISL_4730595, EPI_ISL_4730596, EPI_ISL_4730597, EPI_ISL_4730598, EPI_ISL_5022694, EPI_ISL_5022695, EPI_ISL_5022696, EPI_ISL_5022697, EPI_ISL_5022698, EPI_ISL_5022699, EPI_ISL_5022700, EPI_ISL_5022701, EPI_ISL_5022702, EPI_ISL_5022703, EPI_ISL_5022704, EPI_ISL_5022705, EPI_ISL_5022706, EPI_ISL_5022707, EPI_ISL_5107858, EPI_ISL_5107859, EPI_ISL_5107860, EPI_ISL_5107861, EPI_ISL_5107862, EPI_ISL_5107863, EPI_ISL_5107864, EPI_ISL_5107865, EPI_ISL_5107866, EPI_ISL_5107867			
see above	Balai Pengawas Obat Dan Makanan Ambon	National Institute of Health Research and Development	Arie Ardiansyah Nugraha; Fajar Nur; Fajar Nur Sulistiyohadi; Hana Apsari Pawestri; Hartanti Dian Ikawati; Kartika Dewi Puspa; Krisna Pangesti; Nelly Puspandari; Subangkit; Sulistiyohadi; Triyani Soekarso; Vivi Setiawaty
EPI_ISL_1915572, EPI_ISL_1915573, EPI_ISL_1915574, EPI_ISL_1915575	Balitbang Tanah Bumbu	National Institute of Health Research and Development	Arie Ardiansyah Nugraha; Hana Apsari Pawestri; Hartanti Dian Ikawati; Kartika Dewi Puspa; Krisna Pangesti; Nelly Puspandari; Subangkit; Vivi Setiawaty
EPI_ISL_775594	Balitvet Lampung	National Institute of Health Research and Development	AA; E; EA; HA; HD; Ikawati; KD; KNA; N; Nugraha; Pangesti; Pawestri; Puspa; Puspandari; Saswiyanti; Setiawaty; Soekarso; Srihanto; Subangkit; T; V
EPI_ISL_4885303, EPI_ISL_4885304, EPI_ISL_4885305, EPI_ISL_4885306, EPI_ISL_4885307, EPI_ISL_4885308, EPI_ISL_4885309, EPI_ISL_4885310, EPI_ISL_4885311, EPI_ISL_4885312, EPI_ISL_4885313, EPI_ISL_4885314, EPI_ISL_4885315, EPI_ISL_4885316, EPI_ISL_4885317, EPI_ISL_4885318, EPI_ISL_4885319, EPI_ISL_4885320, EPI_ISL_4885321, EPI_ISL_4885322			
see above	Bandung Health Laboratory	West Java Health Laboratory	Azzania Fibriani; Cut Nur Cinthia Alamanda; Ema Rahmawati; Isak Solihin; Karimatu Khoirunnisa; Miftahul Faridl; Rifky Waluyajati Rachman; Rini Robiani; Ryan Bayusantika Ristandi
EPI_ISL_4885323, EPI_ISL_4885324, EPI_ISL_4885325, EPI_ISL_4885326, EPI_ISL_4885327, EPI_ISL_4885328, EPI_ISL_4885329, EPI_ISL_4885330, EPI_ISL_4885331, EPI_ISL_4885332, EPI_ISL_4885333			
see above	Bandung Health Polytechnic	West Java Health Laboratory	Azzania Fibriani; Cut Nur Cinthia Alamanda; Ema Rahmawati; Isak Solihin; Karimatu Khoirunnisa; Miftahul Faridl; Rifky Waluyajati Rachman; Rini Robiani; Ryan Bayusantika Ristandi
EPI_ISL_5328474, EPI_ISL_5328475, EPI_ISL_5328476, EPI_ISL_5328477, EPI_ISL_5328478, EPI_ISL_5328479, EPI_ISL_5328480, EPI_ISL_5328481, EPI_ISL_5328482, EPI_ISL_5328483, EPI_ISL_5328484, EPI_ISL_5328485, EPI_ISL_5328486, EPI_ISL_5328487, EPI_ISL_5328488, EPI_ISL_5328489, EPI_ISL_5328490, EPI_ISL_5328491, EPI_ISL_5328492			
see above	Bandung Health Polytechnic	West Java Health Laboratory; School of Life Sciences and Technology, Institut Teknologi Bandung	Azzania Fibriani; Cut Nur Cinthia Alamanda; Ema Rahmawati; Karimatu Khoirunnisa; Miftahul Faridl; Rifky Waluyajati Rachman; Rini Robiani; Ryan Bayusantika Ristandi
EPI_ISL_1615587, EPI_ISL_1615656, EPI_ISL_1633339, EPI_ISL_1634465, EPI_ISL_1660472	Bandung Public Health	West Java Health Laboratory; School of Life Sciences and Technology, Institut Teknologi Bandung	Aulia Saraswati Wicaksono; Azzania Fibriani; Cut Nur Cinthia Alamanda; Ema Rahmawati; Karimatu Khoirunnisa; Miftahul Faridl; Rifky Waluyajati Rachman; Rini Robiani; Ryan Bayusantika Ristandi
EPI_ISL_1072964	Bhayangkara Hospital Medan	Institute of Tropical Disease, Universitas Airlangga; Faculty of Medicine, Universitas Sumatera Utara	Aldise M Nastri; Franciscus Ginting; Inke N D Lubis; Irbah R Nainggolan; Jezzy R Dewantari; Kazufumi Shimizu; Krisnoadi Rahardjo; Maria I Lusida; Meliani; Mirzan Hasibuan; Muhammad Ichwan; R Andika D Cahyadi; R Lia Kusumawati; Ramadhan Bestari; Rima R Prasetya; Soetjipto; Yasuko Mori
EPI_ISL_1069153	Binjai Health Office	Institute of Tropical Disease, Universitas Airlangga; Fakultas Kedokteran, Universitas Sumatra Utara	Aldise M Nastri; Franciscus Ginting; Inke N D Lubis; Irbah R Nainggolan; Jezzy R Dewantari; Kazufumi Shimizu; Krisnoadi Rahardjo; Maria I Lusida; Meliani; Mirzan Hasibuan; Muhammad Ichwan; R Andika D Cahyadi; R Lia Kusumawati; Ramadhan Bestari; Rima R Prasetya; Soetjipto; Yasuko Mori
EPI_ISL_4004656, EPI_ISL_4004657, EPI_ISL_4004658	Biosafety Level 3 Laboratory BRIN	National Research and Innovation Agency (BRIN-Indonesia)	Ahmad Randy; Akhirta Atikana; Aliyatur Rosyidah; Beti E. Dewi; Dian F. Agustiyani; Hariyatun; Herjuno A. Nugroho; Lucia D. Antika; Marissa Angelina; Masrukhin; Popi H. Wisnuwardhani; Ratih A. Ningrum; Sugiyono Saputra; Syaiful Rizal; Syam B. Iryanto
EPI_ISL_747238	Bogor Public Health	West Java Health Laboratory; School of Life Sciences and Technology, Institut Teknologi Bandung	Azzania Fibriani; Cut Nur Cinthia Alamanda; Ema Rahmawati; Isak Solihin; Karimatu Khoirunnisa; Miftahul Faridl; Rifky Waluyajati Rachman; Rini Robiani; Ryan Bayusantika Ristandi
EPI_ISL_5328534, EPI_ISL_5328535, EPI_ISL_5328536	Bogor Regional Hospital	West Java Health Laboratory; School of Life Sciences and Technology, Institut Teknologi Bandung	Azzania Fibriani; Cut Nur Cinthia Alamanda; Ema Rahmawati; Karimatu Khoirunnisa; Miftahul Faridl; Rifky Waluyajati Rachman; Rini Robiani; Ryan Bayusantika Ristandi
EPI_ISL_3188923, EPI_ISL_3188924, EPI_ISL_3188925, EPI_ISL_3188926, EPI_ISL_3188927, EPI_ISL_3188928, EPI_ISL_3188929, EPI_ISL_3188930, EPI_ISL_3188931, EPI_ISL_3188932, EPI_ISL_3188933, EPI_ISL_3188934, EPI_ISL_3188935, EPI_ISL_3188936, EPI_ISL_3188937, EPI_ISL_3188938, EPI_ISL_3188939, EPI_ISL_3188940, EPI_ISL_3188941, EPI_ISL_3188942, EPI_ISL_3188943, EPI_ISL_3188944, EPI_ISL_3200768, EPI_ISL_3200769, EPI_ISL_3200770, EPI_ISL_3200771, EPI_ISL_3200772, EPI_ISL_3200773, EPI_ISL_3200774, EPI_ISL_3200775, EPI_ISL_3200776, EPI_ISL_3200777, EPI_ISL_3200778, EPI_ISL_3200779, EPI_ISL_3200780, EPI_ISL_3200781, EPI_ISL_3200782, EPI_ISL_3200783, EPI_ISL_3200784, EPI_ISL_3200785, EPI_ISL_3200786, EPI_ISL_3200787, EPI_ISL_3200788, EPI_ISL_3200789, EPI_ISL_3200790, EPI_ISL_3200791, EPI_ISL_3200792, EPI_ISL_3200793, EPI_ISL_3200794, EPI_ISL_3200795, EPI_ISL_3200796, EPI_ISL_3200797, EPI_ISL_3200798, EPI_ISL_3200799, EPI_ISL_3200800, EPI_ISL_3200801, EPI_ISL_3200802, EPI_ISL_3200803, EPI_ISL_3200804, EPI_ISL_3200805, EPI_ISL_3200806, EPI_ISL_3200807, EPI_ISL_3200808, EPI_ISL_3200809, EPI_ISL_3200810, EPI_ISL_3200811,			

EPI_ISL_3200812, EPI_ISL_3200813, EPI_ISL_3200814, EPI_ISL_3200815, EPI_ISL_3200816, EPI_ISL_3200817, EPI_ISL_3200818, EPI_ISL_3200819			
see above	Bunda Thamrin Hospital / EIJKMAN OXFORD CLINICAL RESEARCH UNIT	Genomik Solidaritas Indonesia Laboratorium	Anna Christina Brazia; Annisa Muthiah Sukirman; Anuraj Shankar; Ariel Pradipta; Ayodhia Pitaloka Pasaribu; Beni Satria; Carissa Sintca; Carissa Sintca Wijaya; Decy Subekti; Eka Airlangga; Gracia Felias Enos Korompis; Hendri Wijaya; Louisa Markus; Meutia Ayuputeri Kumaheri; Mutiara; Raph Hamers; Suwarti; Vania Gavrila Wikasa; Vania Gavrilla Wikasa
EPI_ISL_2931797	CIPAKU KOTA BOGOR	National Institute of Health Research and Development	; Arie Ardiansyah Nugraha; Hana Apsari Pawestri; Hartanti Dian Ikawati; Kartika Dewi Puspa; Krisna Pangesti; Nelly Puspandari; Subangkit; Triyani Soekarso; Vivi Setiawaty
EPI_ISL_5328539, EPI_ISL_5328540, EPI_ISL_5328541	Ciamis Public Health	West Java Health Laboratory; School of Life Sciences and Technology, Institut Teknologi Bandung	Azzania Fibriani; Cut Nur Cinthia Alamanda; Ema Rahmawati; Karimatu Khoirunnisa; Miftahul Faridl; Rifky Waluyajati Rachman; Rini Robiani; Ryan Bayusantika Ristandi
EPI_ISL_1346485, EPI_ISL_1398367, EPI_ISL_1398565, EPI_ISL_1398932, EPI_ISL_1398939, EPI_ISL_1398942, EPI_ISL_1398955, EPI_ISL_1398983			
see above	Cianjur Public Health	West Java Health Laboratory; School of Life Sciences and Technology, Institut Teknologi Bandung	Azzania Fibriani; Cut Nur Cinthia Alamanda; Ema Rahmawati; Isak Solihin; Kamila Tania; Karimatu Khoirunnisa; Miftahul Faridl; Rifky Waluyajati Rachman; Rini Robiani; Ryan Bayusantika Ristandi
EPI_ISL_1634474	Cimahi Public Health	West Java Health Laboratory; School of Life Sciences and Technology, Institut Teknologi Bandung	Aulia Saraswati Wicaksono; Azzania Fibriani; Cut Nur Cinthia Alamanda; Ema Rahmawati; Karimatu Khoirunnisa; Miftahul Faridl; Rifky Waluyajati Rachman; Rini Robiani; Ryan Bayusantika Ristandi
EPI_ISL_1534697, EPI_ISL_1534698, EPI_ISL_1534699, EPI_ISL_1534700, EPI_ISL_2106982	Ciputra Hospital Citra Garden City	National Institute of Health Research and Development	Arie Ardiansyah Nugraha; Hana Apsari Pawestri; Hartanti Dian Ikawati; Kartika Dewi Puspa; Krisna Pangesti; Nelly Puspandari; Subangkit; Vivi Setiawaty
EPI_ISL_2364584	Ciputra Hospital Citra Raya Tangerang	Eijkman Institute for Molecular Biology, National Agency for Research and Innovation	Amin Soebandrio; Edison Johar; Frilasita A Yudhaputri; Hidayat Trimarsanto; Iskandar Adnan; Khin Saw Myint; Lidwina Priliani; Lydia V. Panggalo; Muhammad Rezki Rasyak; Safarina G Malik; Sukma Oktavianthi; Willy Agustine
EPI_ISL_5417758, EPI_ISL_5417759, EPI_ISL_5417760, EPI_ISL_5417761, EPI_ISL_5417762, EPI_ISL_5417763	Cirebon Gunung Jati Regional Hospital	West Java Health Laboratory; School of Life Sciences and Technology, Institut Teknologi Bandung	Azzania Fibriani; Cut Nur Cinthia Alamanda; Ema Rahmawati; Karimatu Khoirunnisa; Miftahul Faridl; Rifky Waluyajati Rachman; Rini Robiani; Ryan Bayusantika Ristandi
EPI_ISL_877422, EPI_ISL_1795059	Citra Medika Hospital	Institute of Tropical Disease, Universitas Airlangga	Aldise M Nastri; Gatot Soegiarto; Jezzy R Dewantari; Kazufumi Shimizu; Krisnoadi Rahardjo; Laksmi Wulandari; Maria I Lusida; Resti Yudhawati; Rima R Prasetya; Soetjipto; Wahidah Rachmaniyah; Yasuko Mori
EPI_ISL_1248934, EPI_ISL_1249610, EPI_ISL_1250222, EPI_ISL_1258423, EPI_ISL_1258424, EPI_ISL_1258463, EPI_ISL_1258464, EPI_ISL_1258465, EPI_ISL_1263319, EPI_ISL_1263320, EPI_ISL_1263321, EPI_ISL_1263322, EPI_ISL_1263323, EPI_ISL_1630278, EPI_ISL_1630279, EPI_ISL_1630280, EPI_ISL_1630281, EPI_ISL_2691912, EPI_ISL_2692142, EPI_ISL_2692143, EPI_ISL_2692144, EPI_ISL_2692169, EPI_ISL_2692170, EPI_ISL_2692171, EPI_ISL_2692179, EPI_ISL_2692205, EPI_ISL_2692206, EPI_ISL_2692531, EPI_ISL_2692533, EPI_ISL_2692706, EPI_ISL_2692880, EPI_ISL_2692882, EPI_ISL_2692886, EPI_ISL_2692887, EPI_ISL_2692889, EPI_ISL_2692891, EPI_ISL_2692892, EPI_ISL_2692896, EPI_ISL_2692897, EPI_ISL_2692899, EPI_ISL_2692900, EPI_ISL_2692902, EPI_ISL_2692903, EPI_ISL_2692905			
see above	Clinical Microbiology Lab RSPTN Universitas Hasanuddin	Clinical Microbiology Lab RSPTN Universitas Hasanuddin	Gita Vita Soraya; Handayani; Handayani Halik; Muhammad Nasrum Massi; Muhammad Yusuf Usman; Rizalinda Sjahril; Rufika Shari; Triwibawa
EPI_ISL_747194, EPI_ISL_1122422, EPI_ISL_1122423, EPI_ISL_1122424, EPI_ISL_2692207, EPI_ISL_2692909, EPI_ISL_2692981, EPI_ISL_2692983, EPI_ISL_2692986, EPI_ISL_2692987, EPI_ISL_2692989, EPI_ISL_2692990, EPI_ISL_2692991, EPI_ISL_2692992, EPI_ISL_2692993, EPI_ISL_2692994, EPI_ISL_2692995, EPI_ISL_2993340, EPI_ISL_2993341, EPI_ISL_2993432, EPI_ISL_2993433, EPI_ISL_2993434, EPI_ISL_2993539, EPI_ISL_2993632, EPI_ISL_2993633, EPI_ISL_2993658, EPI_ISL_2993659, EPI_ISL_3000155, EPI_ISL_3456060, EPI_ISL_4847411, EPI_ISL_4847413, EPI_ISL_5054899, EPI_ISL_5054915, EPI_ISL_5054918			
see above	Clinical Microbiology Laboratory, Faculty of Medicine, Universitas Indonesia	Clinical Microbiology Laboratory, Faculty of Medicine, Universitas Indonesia	Andi Yasmon; Anis Karuniawati; Augustine Natasha; Dewi LF; Erni J. Nelwan; Faculty of Medicine; Febriani R; Fera Ibrahim; Fithriyah; Fitrhiyah; Ibrahim F; Karuniawati A; Makhtaromi A; Natasha A; Nurzahra I; Pratiwi Sudarmono; Rahmawaty RG; Saharman YR; Sudarmono P.; Syarurachman A; Universitas Indonesia; Yasmon A; Yulia Rosa Saharman; linical Microbiology Laboratory
EPI_ISL_1196774, EPI_ISL_1196792, EPI_ISL_1196793, EPI_ISL_1196795, EPI_ISL_1196798, EPI_ISL_2835609, EPI_ISL_2835610, EPI_ISL_2835643, EPI_ISL_2835644, EPI_ISL_2835645, EPI_ISL_2835646, EPI_ISL_2835647, EPI_ISL_2835648, EPI_ISL_2835649, EPI_ISL_2835650, EPI_ISL_2835651, EPI_ISL_2835652, EPI_ISL_2835653, EPI_ISL_2835654, EPI_ISL_2835655, EPI_ISL_2835656, EPI_ISL_2835684, EPI_ISL_2835685, EPI_ISL_3503134, EPI_ISL_3503135, EPI_ISL_3534497, EPI_ISL_3534498, EPI_ISL_3534499, EPI_ISL_3534500, EPI_ISL_3534501, EPI_ISL_3534502, EPI_ISL_3534505, EPI_ISL_3534508, EPI_ISL_3534510, EPI_ISL_3534513, EPI_ISL_3534514, EPI_ISL_3534515, EPI_ISL_3534516, EPI_ISL_3534517, EPI_ISL_3534518, EPI_ISL_3534519, EPI_ISL_3534520			
see above	Clinical Microbiology Laboratory, Faculty of Medicine, Universitas Indonesia	Faculty of Medicine, Universitas Indonesia	Andi Yasmon; Anis Karuniawati; Ari Fahrial Syam; Augustine Natasha; Badriul Hegar; Beti Ernawati; Beti Ernawati dewi; Budi Wiweko; Dwiana Ocviyanti; Fadilah; Fera Ibrahim; Fithriyah; Hana Apsari Pawestri; Khaerunissa Anbar Istiadi; Linda Erlina; Pratiwi Sudarmono; Rafika Indah Paramita; Vivi Setiawaty; Yulia Rosa
EPI_ISL_4392617	Clinical Pathology Laboratory, RSUP Dr. Hasan Sadikin	Eijkman Institute for Molecular Biology, National Agency for Research and Innovation	Amin Soebandrio; Edison Johar; Frilasita A Yudhaputri; Hidayat Trimarsanto; Iskandar Adnan; Jessica R Saragih; Khin Saw Myint; Lidwina Priliani; Lydia V Panggalo; Muhammad Rezki Rasyak; Safarina G Malik; Sukma Oktavianthi; Willy Agustine
EPI_ISL_3466962, EPI_ISL_3466968, EPI_ISL_3466974, EPI_ISL_3466979, EPI_ISL_3466986, EPI_ISL_3550572, EPI_ISL_3550574, EPI_ISL_3550575, EPI_ISL_3550576, EPI_ISL_3550577, EPI_ISL_3550578, EPI_ISL_3550579, EPI_ISL_3550580, EPI_ISL_3550581, EPI_ISL_3550582, EPI_ISL_3550583, EPI_ISL_3550584, EPI_ISL_3550585, EPI_ISL_3550586, EPI_ISL_3550587, EPI_ISL_3550588, EPI_ISL_3550589, EPI_ISL_3550590, EPI_ISL_3550591, EPI_ISL_3550592, EPI_ISL_3550593, EPI_ISL_3550594, EPI_ISL_3550595, EPI_ISL_3550596, EPI_ISL_3550597, EPI_ISL_3550598, EPI_ISL_3550599, EPI_ISL_3550600, EPI_ISL_3550601, EPI_ISL_3550602, EPI_ISL_3550603, EPI_ISL_3550604, EPI_ISL_3550605			
see above	Clinical Pathology Laboratory, RSUP Dr. Hasan Sadikin	Eijkman Institute for Molecular Biology, National Research and Innovation Agency	Amin Soebandrio; Edison Johar; Frilasita A Yudhaputri; Hidayat Trimarsanto; Iskandar Adnan; Jessica R. Saragih; Khin Saw Myint; Lidwina Priliani; Lydia V. Panggalo; Muhammad Rezki Rasyak; Safarina G Malik; Sukma Oktavianthi; Willy Agustine
EPI_ISL_1247019	Clinical microbiology lab, RSPTN Universitas Hasanuddin	Clinical microbiology lab, RSPTN Universitas Hasanuddin	Gita Vita Soraya; Handayani; Muhammad Nasrum Massi; Muhammad Yusuf Usman; Rizalinda Sjahril; Rufika Shari; Triwibawa
EPI_ISL_1335896	Columbia Asia Hospital Medan	Faculty of Medicine, Universitas Sumatera Utara; Institute of Tropical Disease, Universitas Airlangga	Aldise M Nastri; Franciscus Ginting; Inke N D Lubis; Irbah R Nainggolan; Jezzy R Dewantari; Kazufumi Shimizu; Krisnoadi Rahardjo; Maria I Lusida; Meliani; Mirzan Hasibuan; Muhammad Ichwan; R Andika D Cahyadi; R Lia Kusumawati; Ramadhan Bestari; Rima R Prasetya; Soetjipto; Yasuko Mori
EPI_ISL_1020204	DD Klinik Ciputat, South Tangerang, Banten	Biosafety Level-3 Laboratory, Indonesian Institute of Sciences (LIPI)	Anggia Prasetyoputri; Eko Wahyu Putro; Fahrurrozi; Isa Nuryana; Ratih Asmana Ningrum; Sri Swasthikawati; Sugiyono Saputra; Syam Budi Iryanto
EPI_ISL_3092457, EPI_ISL_3092464, EPI_ISL_3098445, EPI_ISL_3098759	DINAS KESEHATAN DKI JAKARTA	Genomik Solidaritas Indonesia Laboratorium	Alfin Mohammad Abdillah; Annisa Muthiah Sukirman; Anuraj Shankar; Ariel Pradipta; Carissa Sintca Wijaya; Dhahlia Agustina Cahyono; Dwi Oktavia; Gracia Felias Enos Korompis; Meutia Ayuputeri Kumaheri; Mohammad Anang Syarifudin; Ngabila Salama; Normastuti Adhini Tantyo; Tiranti Vindha; Vania Gavrila Wikasa; Widyastuti
EPI_ISL_2964704, EPI_ISL_2964706	DINKES KAB. GROBOGAN	Genetics Working Group (Pokja Genetik) Faculty of Medicine, Public Health and Nursing Universitas Gadjah Mada (FK-KMK UGM); Disease Investigation Center Wates Ministry of Agriculture Indonesia; Department of Microbiology FK-KMK UGM; Laboratorium Diagnostik Yayasan Tahija World Mosquito Program (WMP) Yogyakarta Center for Tropical Medicine FK-KMK UGM; Integrated Research Center FK-KMK UGM; Department of Computer Science and Electronics FMIPA UGM; RSUP Dr. Sardjito	Afiahayati; Dwi AA Nugrahaningsih; Dwi Indaryati; Dyah A Puspitarani; Edwin W. Daniwijaya; Eggi Arguni; Endah Supriyati; Gunadi; Hana F Hanifin; Hendra Wibawa; Indarto Sulistiyono; Irene Tania; Kristy Iskandar; Ludhang P. Rizki; Marcellus; Mohamad S. Hakim; Nungki Anggorowati; Pramesti G Dewi; Siswanto; Susan Simanjaya; Titik Nuryastuti; Tri Wibawa
EPI_ISL_2964697, EPI_ISL_2964700	DINKES KAB. JEPARA	Genetics Working Group (Pokja Genetik) Faculty of Medicine, Public Health and Nursing Universitas Gadjah Mada (FK-KMK UGM); Disease Investigation Center Wates Ministry of Agriculture Indonesia; Department of Microbiology FK-KMK UGM; Laboratorium Diagnostik Yayasan Tahija World Mosquito Program (WMP) Yogyakarta Center for Tropical Medicine FK-KMK UGM; Integrated Research Center FK-KMK UGM; Department of Computer Science and Electronics FMIPA UGM; RSUP Dr. Sardjito	Afiahayati; Alvina A Setiawan; Cita S Amalia; Dwi AA Nugrahaningsih; Dwi Indaryati; Edwin W. Daniwijaya; Eggi Arguni; Endah Supriyati; Gunadi; Hendra Wibawa; Indarto Sulistiyono; Khanza a Vujira; Kristy Iskandar; Ludhang P. Rizki; Marcellus; Mohamad S. Hakim; Nungki Anggorowati; Pramesti G Dewi; Siswanto; Titik Nuryastuti; Tri Wibawa
EPI_ISL_2931771, EPI_ISL_2931780, EPI_ISL_2931787	DINKES KOTA BOGOR	National Institute of Health Research and Development	; Arie Ardiansyah Nugraha; Hana Apsari Pawestri; Hartanti Dian Ikawati; Kartika Dewi Puspa; Krisna Pangesti; Nelly Puspandari; Subangkit; Triyani Soekarso; Vivi Setiawaty
EPI_ISL_2868916, EPI_ISL_2868918	DINKES Kab Bogor	National Institute of Health Research and Development	Arie Ardiansyah Nugraha; Hana Apsari Pawestri; Hartanti Dian Ikawati; Kartika Dewi Puspa; Krisna Pangesti; Nelly Puspandari; Subangkit; Triyani Soekarso; Vivi Setiawaty
EPI_ISL_2617550, EPI_ISL_2617551, EPI_ISL_2617552, EPI_ISL_2617553, EPI_ISL_2617554	DINKES Kab TAPIN KalSel	National Institute of Health Research and Development	Arie Ardiansyah Nugraha; Hana Apsari Pawestri; Hartanti Dian Ikawati; Kartika Dewi Puspa; Krisna Pangesti; Nelly Puspandari; Subangkit; Triyani Soekarso; Vivi Setiawaty
EPI_ISL_2932712	DINKES Kota Waringin Barat Kalteng	National Institute of Health Research and Development	; Arie Ardiansyah Nugraha; Hana Apsari Pawestri; Hartanti Dian Ikawati; Kartika Dewi Puspa; Krisna Pangesti; Nelly Puspandari; Subangkit; Triyani Soekarso; Vivi Setiawaty
EPI_ISL_2854762, EPI_ISL_2868937, EPI_ISL_2868939, EPI_ISL_2868941, EPI_ISL_2868942, EPI_ISL_2868944, EPI_ISL_2896196			
see above	DINKES Prov Kalimantan Timur	National Institute of Health Research and Development	Arie Ardiansyah Nugraha; Hana Apsari Pawestri; Hartanti Dian Ikawati; Kartika Dewi Puspa; Krisna Pangesti; Nelly Puspandari; Subangkit; Triyani Soekarso; Vivi Setiawaty
EPI_ISL_4730546, EPI_ISL_4730547, EPI_ISL_4730548, EPI_ISL_4730549, EPI_ISL_4730550, EPI_ISL_4730551, EPI_ISL_4730552, EPI_ISL_4730553, EPI_ISL_4730554			
see above	DINKES Provinsi Jambi	National Institute of Health Research and Development	Arie Ardiansyah Nugraha; Fajar Nur; Fajar Nur Sulistiyohadi; Hana Apsari Pawestri; Hartanti Dian Ikawati; Kartika Dewi Puspa; Krisna Pangesti; Nelly Puspandari; Subangkit; Sulistiyohadi; Triyani Soekarso; Vivi Setiawaty
EPI_ISL_4730536, EPI_ISL_4730537, EPI_ISL_4730538, EPI_ISL_4730539, EPI_ISL_4730540, EPI_ISL_4730541, EPI_ISL_4730542, EPI_ISL_4730543, EPI_ISL_4730544, EPI_ISL_4730545			
see above	DINKES Rejang Lebong Bengkulu	National Institute of Health Research and Development	Arie Ardiansyah Nugraha; Fajar Nur; Hana Apsari Pawestri; Hartanti Dian Ikawati; Kartika Dewi Puspa; Krisna Pangesti; Nelly Puspandari; Subangkit; Sulistiyohadi; Triyani Soekarso; Vivi Setiawaty
EPI_ISL_2954684	DKK Gunungkidul/ Puskesmas Girisubo	Genetics Working Group (Pokja Genetik) Faculty of Medicine, Public Health and Nursing Universitas Gadjah Mada (FK-KMK UGM); Disease Investigation Center Wates Ministry of Agriculture Indonesia; Department of Microbiology FK-KMK UGM; Laboratorium Diagnostik Yayasan Tahija World Mosquito Program (WMP) Yogyakarta Center for Tropical Medicine FK-KMK UGM; Integrated Research Center FK-KMK UGM; Department of Computer Science and Electronics FMIPA UGM; RSUP Dr. Sardjito	Afiahayati; Alvina A Setiawan; Cita S Amalia; Dwi AA Nugrahaningsih; Dwi Indaryati; Edwin W. Daniwijaya; Eggi Arguni; Endah Supriyati; Gunadi; Hendra Wibawa; Indarto Sulistiyono; Kristy Iskandar; Ludhang P. Rizki; Marcellus; Mohamad S. Hakim; Nungki Anggorowati; Pramesti G Dewi; Siswanto; Titik Nuryastuti; Tri Wibawa
EPI_ISL_2955335, EPI_ISL_2955586, EPI_ISL_2955597, EPI_ISL_2955598	DKK Gunungkidul/ Puskesmas Karangmojo I	Genetics Working Group (Pokja Genetik) Faculty of Medicine, Public Health and Nursing Universitas Gadjah Mada (FK-KMK UGM); Disease Investigation Center Wates Ministry of Agriculture Indonesia; Department of Microbiology FK-KMK UGM; Laboratorium Diagnostik Yayasan Tahija World Mosquito Program (WMP) Yogyakarta Center for Tropical Medicine FK-KMK UGM; Integrated Research Center FK-KMK UGM; Department of Computer Science and Electronics	Afiahayati; Alvina A Setiawan; Dwi AA Nugrahaningsih; Dwi Indaryati; Dyah A Puspitarani; Edwin W. Daniwijaya; Eggi Arguni; Endah Supriyati; Gunadi; Hana F Hanifin; Hendra Wibawa; Indarto Sulistiyono; Irene Tania; Khanza a Vujira; Kristy Iskandar; Ludhang P. Rizki; Marcellus; Mohamad S. Hakim; Nungki Anggorowati; Pramesti G Dewi; Siswanto; Susan Simanjaya; Titik Nuryastuti; Tri Wibawa

		FMIPA UGM; RSUP Dr. Sardjito	
EPI_ISL_2933110	DKK Sleman/Puskesmas Depok 1	Genetics Working Group (Pokja Genetik) Faculty of Medicine, Public Health and Nursing Universitas Gadjah Mada (FK-KMK UGM); Disease Investigation Center Wates Ministry of Agriculture Indonesia; Department of Microbiology FK-KMK UGM; Laboratorium Diagnostik Yayasan Tahija World Mosquito Program (WMP) Yogyakarta Center for Tropical Medicine FK-KMK UGM; Integrated Research Center FK-KMK UGM; Department of Computer Science and	Afiahayati; Alvina A Setiawan; Dwi AA Nugrahaningsih; Dwi Indaryati; Edwin W. Daniwijaya; Eggi Arguni; Endah Supriyati; Gunadi; Hendra Wibawa; Indarto Sulistiyono; Khanza a Vujira; Kristy Iskandar; Ludhang P. Rizki; Marcellus; Mohamad S. Hakim; Nungki Anggorowati; Pramesti G Dewi; Siswanto; Titik Nuryastuti; Tri Wibawa
EPI_ISL_2951626	DKK Sleman/Puskesmas Depok 1	Genetics Working Group (Pokja Genetik) Faculty of Medicine, Public Health and Nursing Universitas Gadjah Mada (FK-KMK UGM); Disease Investigation Center Wates Ministry of Agriculture Indonesia; Department of Microbiology FK-KMK UGM; Laboratorium Diagnostik Yayasan Tahija World Mosquito Program (WMP) Yogyakarta Center for Tropical Medicine FK-KMK UGM; Integrated Research Center FK-KMK UGM; Department of Computer Science and Electronics FMIPA UGM; RSUP Dr.	Afiahayati; Dwi AA Nugrahaningsih; Dwi Indaryati; Edwin W. Daniwijaya; Eggi Arguni; Endah Supriyati; Gunadi; Hana F Hanifin; Hendra Wibawa; Indarto Sulistiyono; Irene Tania; Kristy Iskandar; Ludhang P. Rizki; Marcellus; Mohamad S. Hakim; Nungki Anggorowati; Pramesti G Dewi; Siswanto; Titik Nuryastuti; Tri Wibawa
EPI_ISL_2932613, EPI_ISL_2933109, EPI_ISL_2943237, EPI_ISL_2952510, EPI_ISL_2953393, EPI_ISL_2955609	DKK Sleman/Puskesmas Depok 1	Genetics Working Group (Pokja Genetik) Faculty of Medicine, Public Health and Nursing Universitas Gadjah Mada (FK-KMK UGM); Disease Investigation Center Wates Ministry of Agriculture Indonesia; Department of Microbiology FK-KMK UGM; Laboratorium Diagnostik Yayasan Tahija World Mosquito Program (WMP) Yogyakarta Center for Tropical Medicine FK-KMK UGM; Integrated Research Center FK-KMK UGM; Department of Computer Science and Electronics FMIPA UGM; RSUP Dr. Sardjito	Afiahayati; Alvina A Setiawan; Cita S Amalia; Dwi AA Nugrahaningsih; Dwi Indaryati; Dyah A Puspitarani; Edwin W. Daniwijaya; Eggi Arguni; Endah Supriyati; Gunadi; Hana F Hanifin; Hendra Wibawa; Indarto Sulistiyono; Irene Tania; Khanza a Vujira; Kristy Iskandar; Ludhang P. Rizki; Marcellus; Mohamad S. Hakim; Nungki Anggorowati; Pramesti G Dewi; Siswanto; Susan Simanjaya; Titik Nuryastuti; Tri Wibawa
EPI_ISL_2955627, EPI_ISL_2955628	DKK Sleman/Puskesmas Ngemplak 2	Genetics Working Group (Pokja Genetik) Faculty of Medicine, Public Health and Nursing Universitas Gadjah Mada (FK-KMK UGM); Disease Investigation Center Wates Ministry of Agriculture Indonesia; Department of Microbiology FK-KMK UGM; Laboratorium Diagnostik Yayasan Tahija World Mosquito Program (WMP) Yogyakarta Center for Tropical Medicine FK-KMK UGM; Integrated Research Center FK-KMK UGM; Department of Computer Science and Electronics FMIPA UGM; RSUP Dr. Sardjito	Afiahayati; Dwi AA Nugrahaningsih; Dwi Indaryati; Dyah A Puspitarani; Edwin W. Daniwijaya; Eggi Arguni; Endah Supriyati; Gunadi; Hana F Hanifin; Hendra Wibawa; Indarto Sulistiyono; Irene Tania; Kristy Iskandar; Ludhang P. Rizki; Marcellus; Mohamad S. Hakim; Nungki Anggorowati; Pramesti G Dewi; Siswanto; Susan Simanjaya; Titik Nuryastuti; Tri Wibawa
EPI_ISL_1020205	DMC Dompet Dhuafa, South Tangerang, Banten	Biosafety Level-3 Laboratory, Indonesian Institute of Sciences (LIPI)	Anggia Prasetyoputri; Anik B. Dharmayanthi; Isa Nuryana; Nuruliawaty Utami; Ratih Asmana Ningrum; Sri Swasthikawati; Sugiyono Saputra; Syam Budi Iryanto
EPI_ISL_877459, EPI_ISL_1159389	Darmo Hospital	Institute of Tropical Disease, Universitas Airlangga	Aldise M Nastri; Gatot Soegiarto; Jezzy R Dewantari; Kazufumi Shimizu; Krisnoadi Rahardjo; Laksmi Wulandari; Maria I Lusida; Resti Yudhawati; Rima R Prasetya; Soetjipto; Sulung Budianto; Yasuko Mori
EPI_ISL_877420, EPI_ISL_882770, EPI_ISL_1165073, EPI_ISL_1795054, EPI_ISL_2284875	Darus Syifa' Islamic Hospital	Institute of Tropical Disease, Universitas Airlangga	Aldise M Nastri; Gatot Soegiarto; HM Faiz; Jezzy R Dewantari; Kazufumi Shimizu; Krisnoadi Rahardjo; Laksmi Wulandari; Maria I Lusida; Resti Yudhawati; Rima R Prasetya; Soetjipto; Yasuko Mori
EPI_ISL_3533117, EPI_ISL_3533118, EPI_ISL_3533119, EPI_ISL_3533120, EPI_ISL_3533121, EPI_ISL_3533122, EPI_ISL_3533123, EPI_ISL_3533124, EPI_ISL_3533125			
see above	Department of Internal Medicine, Cipto Mangunkusumo National General Hospital Indonesia Jakarta	Faculty of Medicine, Universitas Indonesia	Anis Karuniawati; Ari Fahrial Syam; Badriul Hegar; Budi Wiweko; Fadilah; Muhammad Firhad Idrus; Murdani Abdullah; Virly Nanda Muzelina
EPI_ISL_1335884, EPI_ISL_1335892, EPI_ISL_1335893, EPI_ISL_1335894, EPI_ISL_1335898, EPI_ISL_1335899	Department of Microbiology, Faculty of Medicine, Universitas Sumatera Utara	Faculty of Medicine, Universitas Sumatera Utara; Institute of Tropical Disease, Universitas Airlangga	Aldise M Nastri; Franciscus Ginting; Inke N D Lubis; Irbah R Nainggolan; Jezzy R Dewantari; Kazufumi Shimizu; Krisnoadi Rahardjo; Maria I Lusida; Meliani; Mirzan Hasibuan; Muhammad Ichwan; R Andika D Cahyadi; R Lia Kusumawati; Ramadhan Bestari; Rima R Prasetya; Soetjipto; Yasuko Mori
EPI_ISL_1072965, EPI_ISL_1072986	Department of Microbiology, Faculty of Medicine, Universitas Sumatera Utara	Institute of Tropical Disease, Universitas Airlangga; Faculty of Medicine, Universitas Sumatera Utara	Aldise M Nastri; Franciscus Ginting; Inke N D Lubis; Irbah R Nainggolan; Jezzy R Dewantari; Kazufumi Shimizu; Krisnoadi Rahardjo; Maria I Lusida; Meliani; Mirzan Hasibuan; Muhammad Ichwan; R Andika D Cahyadi; R Lia Kusumawati; Ramadhan Bestari; Rima R Prasetya; Soetjipto; Yasuko Mori
EPI_ISL_1341798	Depok Public Health	West Java Health Laboratory; School of Life Sciences and Technology, Institut Teknologi Bandung	Azzania Fibriani; Cut Nur Cinthia Alamanda; Ema Rahmawati; Isak Solihin; Karimatu Khoirunnisa; Miftahul Faridl; Rifky Waluyajati Rachman; Rini Robiani; Ryan Bayusantika Ristandi
EPI_ISL_1447329, EPI_ISL_1447330, EPI_ISL_1447331, EPI_ISL_1447332, EPI_ISL_1447333, EPI_ISL_1447334, EPI_ISL_1447335, EPI_ISL_1447336, EPI_ISL_1447337, EPI_ISL_1447338, EPI_ISL_1447339, EPI_ISL_1447340, EPI_ISL_1447341, EPI_ISL_1447342, EPI_ISL_1447343, EPI_ISL_1447344, EPI_ISL_1447345, EPI_ISL_1447346, EPI_ISL_1447347, EPI_ISL_1447348, EPI_ISL_1447349, EPI_ISL_1447350, EPI_ISL_1447351, EPI_ISL_1447352, EPI_ISL_1447353, EPI_ISL_1447354, EPI_ISL_1447355, EPI_ISL_1447356, EPI_ISL_1447357, EPI_ISL_1447358, EPI_ISL_1447359, EPI_ISL_1447360, EPI_ISL_1447361, EPI_ISL_1447362, EPI_ISL_1469232, EPI_ISL_1469233, EPI_ISL_1469234, EPI_ISL_1469235, EPI_ISL_1469236, EPI_ISL_1469237, EPI_ISL_1533034, EPI_ISL_1533035, EPI_ISL_1533036, EPI_ISL_1533037, EPI_ISL_1533038, EPI_ISL_1533039, EPI_ISL_1533040, EPI_ISL_1533041, EPI_ISL_1533042, EPI_ISL_1533043, EPI_ISL_1533044, EPI_ISL_1533045, EPI_ISL_1533046, EPI_ISL_1533047, EPI_ISL_1533048, EPI_ISL_1533049, EPI_ISL_1533050, EPI_ISL_1533051, EPI_ISL_1533052, EPI_ISL_1533053, EPI_ISL_1533054, EPI_ISL_1533055, EPI_ISL_1533056, EPI_ISL_1533057, EPI_ISL_1533058, EPI_ISL_1533059, EPI_ISL_1533060, EPI_ISL_1533061, EPI_ISL_1533062, EPI_ISL_1533063, EPI_ISL_1533064, EPI_ISL_1533065, EPI_ISL_1533066, EPI_ISL_1533067, EPI_ISL_1533068, EPI_ISL_1533069, EPI_ISL_1533070, EPI_ISL_1533071, EPI_ISL_1533072, EPI_ISL_1533073, EPI_ISL_1533074, EPI_ISL_1533075, EPI_ISL_1533076, EPI_ISL_1533077, EPI_ISL_1533078, EPI_ISL_1533079, EPI_ISL_1533080, EPI_ISL_1533081, EPI_ISL_1533082, EPI_ISL_1533083, EPI_ISL_1533084, EPI_ISL_1533085, EPI_ISL_1533086, EPI_ISL_1533087, EPI_ISL_1533088, EPI_ISL_1533089, EPI_ISL_1533090, EPI_ISL_1533091, EPI_ISL_1533092, EPI_ISL_1533093, EPI_ISL_1533094, EPI_ISL_1533095, EPI_ISL_1533096, EPI_ISL_1533097, EPI_ISL_1533098, EPI_ISL_1533099, EPI_ISL_1533100, EPI_ISL_1533101			
see above	Diagnostic and Research Center of Infectious Diseases, Medical Faculty, Andalas University	Diagnostic and Research Center of Infectious Diseases, Medical Faculty, Andalas University	Andani Eka Putra; Ayu Novita Trisnawati; Dede Rahman Agustian; Desmawati; Dessy Arisanty; Fauzul Azhim; Gestina Aliska; Ikhwan R. Sudji; Juane Plantika Menra; Linosefa; Mutia Lailani; Nia Ayuni Putri; Nita Afriani; SM Rezvi; Sekar Asri Tresnaningtyas; Siskalil Fahma; Syafrizayanti; Syandrez Prima Putra; Yolani Syaputri
EPI_ISL_3314974, EPI_ISL_3314975, EPI_ISL_3314976, EPI_ISL_3314977, EPI_ISL_3314978, EPI_ISL_3314979, EPI_ISL_3314980, EPI_ISL_3314981, EPI_ISL_3314982, EPI_ISL_3314983, EPI_ISL_3314984, EPI_ISL_3314985, EPI_ISL_3314986, EPI_ISL_3314987, EPI_ISL_3314988, EPI_ISL_3314989, EPI_ISL_3314990, EPI_ISL_3314991, EPI_ISL_3314992, EPI_ISL_3314993, EPI_ISL_3314994, EPI_ISL_3314995, EPI_ISL_3314996, EPI_ISL_3314997, EPI_ISL_3314998, EPI_ISL_3314999, EPI_ISL_3315000, EPI_ISL_3315001, EPI_ISL_3315002, EPI_ISL_3315003, EPI_ISL_3315004, EPI_ISL_3315005, EPI_ISL_3315006, EPI_ISL_3315007, EPI_ISL_3315008, EPI_ISL_3315009, EPI_ISL_3315010, EPI_ISL_3315011, EPI_ISL_3315012, EPI_ISL_3315013, EPI_ISL_3315014, EPI_ISL_3315015, EPI_ISL_3315016, EPI_ISL_3315017, EPI_ISL_3315018, EPI_ISL_3315019, EPI_ISL_3315020, EPI_ISL_3315021, EPI_ISL_3315022, EPI_ISL_3315023, EPI_ISL_3315024, EPI_ISL_3315025, EPI_ISL_3315026, EPI_ISL_3315027, EPI_ISL_3315028, EPI_ISL_3315029, EPI_ISL_3315030, EPI_ISL_3315031, EPI_ISL_3315032, EPI_ISL_3315033, EPI_ISL_3315034, EPI_ISL_3315035, EPI_ISL_3315036, EPI_ISL_3315037, EPI_ISL_3315038, EPI_ISL_3315039, EPI_ISL_3315040, EPI_ISL_3315041, EPI_ISL_3315042, EPI_ISL_3315043, EPI_ISL_3315044, EPI_ISL_3315045, EPI_ISL_3315046, EPI_ISL_3315047, EPI_ISL_3315048, EPI_ISL_3315049, EPI_ISL_3374525, EPI_ISL_3374526, EPI_ISL_3374527, EPI_ISL_3374528, EPI_ISL_3374529, EPI_ISL_3374530, EPI_ISL_3374676, EPI_ISL_3374677, EPI_ISL_3374678, EPI_ISL_3374679, EPI_ISL_3374680, EPI_ISL_3374681, EPI_ISL_3374682, EPI_ISL_3374683, EPI_ISL_3374684, EPI_ISL_3374685, EPI_ISL_3374686, EPI_ISL_3374687, EPI_ISL_3374688, EPI_ISL_3374689, EPI_ISL_3374690, EPI_ISL_3374691, EPI_ISL_3374692, EPI_ISL_3374693, EPI_ISL_3374694, EPI_ISL_3374695, EPI_ISL_3374696, EPI_ISL_3374697, EPI_ISL_3374698, EPI_ISL_3374699, EPI_ISL_3374700, EPI_ISL_3374701, EPI_ISL_3383407, EPI_ISL_3383408, EPI_ISL_3383409, EPI_ISL_3383410, EPI_ISL_3383411, EPI_ISL_3383412, EPI_ISL_3383413, EPI_ISL_3383414, EPI_ISL_3383415, EPI_ISL_3383416, EPI_ISL_3383417, EPI_ISL_3383418, EPI_ISL_3383419, EPI_ISL_3383420, EPI_ISL_3383421, EPI_ISL_3383422, EPI_ISL_3383423, EPI_ISL_3383424, EPI_ISL_3383425, EPI_ISL_3383426, EPI_ISL_3383427, EPI_ISL_3383428, EPI_ISL_3383429, EPI_ISL_3383430, EPI_ISL_3383431, EPI_ISL_3383432, EPI_ISL_3383433, EPI_ISL_3383434, EPI_ISL_3383435, EPI_ISL_3383436, EPI_ISL_3383437, EPI_ISL_3383438, EPI_ISL_3383439, EPI_ISL_3383440			
see above	Diagnostic and Research Center of Infectious Diseases, Medical Faculty, Andalas University	Genomik Solidaritas Indonesia Laboratorium / Diagnostic and Research Center of Infectious Diseases, Medical Faculty, Andalas University	Andani Eka Putra; Annisa Muthiah Sukirman; Anuraj Shankar; Ariel Pradipta; Carissa Sintca Wijaya; Dede Rahman Agustian; Dhahlia Agustina Cahyono; Gracia Felias Enos Korompis; Himawan Masyhuri; Linosefa; Louisa Markus; Meutia Ayuputeri Kumaheri; Reinhart Gabriel; Vania Gavrila Wikasa
EPI_ISL_1469250	Dinas Kesehatan Blitar	National Institute of Health Research and Development	Arie Ardiansyah Nugraha; Hana Apsari Pawestri; Hartanti Dian Ikawati; Kartika Dewi Puspa; Krisna Pangesti; Nelly Puspandari; Subangkit; Vivi Setiawaty
EPI_ISL_2047570, EPI_ISL_2047571	Dinas Kesehatan Kab Blitar	National Institute of Health Research and Development	Arie Ardiansyah Nugraha; Hana Apsari Pawestri; Hartanti Dian Ikawati; Kartika Dewi Puspa; Krisna Pangesti; Nelly Puspandari; Subangkit; Triyani Soekarso; Vivi Setiawaty
EPI_ISL_2918634	Dinas Kesehatan Kabupaten Indramayu	Eijkman Institute for Molecular Biology, National Research and Innovation Agency; West Java Health Laboratory	Amin Soebandrio; Azzania Fibriani; Cut Nur Cinthia Alamanda; Edison Johar; Ema Rahmawati; Frilasita A Yudhaputri; Hidayat Trimarsanto; Iskandar Adnan; Khin Saw Myint; Lidwina Priliani; Lydia V. Panggalo; Muhammad Rezki Rasyak; Rifky Waluyajati Rachman; Ryan Bayusantika Ristandi; Safarina G Malik; Sukma Oktavianthi; Willy Agustine
EPI_ISL_5616244, EPI_ISL_5616279, EPI_ISL_5616374, EPI_ISL_5616436, EPI_ISL_5616453, EPI_ISL_5616500, EPI_ISL_5616547, EPI_ISL_5616572, EPI_ISL_5616585, EPI_ISL_5616599			
see above	Dinas Kesehatan Kabupaten Pemalang	Balai Laboratorium Kesehatan dan Pengujian Alat Kesehatan Provinsi Jawa Tengah	Alia Wijayanti; Dwi Nursetyaasih; Dyah Ayu; Endah Wahyu Y; Ismi Farhati; Purnomo; Sekti Handayani; Wiwik Peni; Yekti Hediningsih
EPI_ISL_1469240, EPI_ISL_1469246, EPI_ISL_1469271, EPI_ISL_1469272, EPI_ISL_1469273, EPI_ISL_1469274, EPI_ISL_1469275, EPI_ISL_1469284			
see above	Dinas Kesehatan Kabupaten Ponorogo	National Institute of Health Research and Development	Arie Ardiansyah Nugraha; Hana Apsari Pawestri; Hartanti Dian Ikawati; Kartika Dewi Puspa; Krisna Pangesti; Nelly Puspandari; Subangkit; Vivi Setiawaty
EPI_ISL_2918633	Dinas Kesehatan Kabupaten Sumedang	Eijkman Institute for Molecular Biology, National Research and Innovation Agency; West Java Health Laboratory	Amin Soebandrio; Azzania Fibriani; Cut Nur Cinthia Alamanda; Edison Johar; Ema Rahmawati; Frilasita A Yudhaputri; Hidayat Trimarsanto; Iskandar Adnan; Khin Saw Myint; Lidwina Priliani; Lydia V. Panggalo; Muhammad Rezki Rasyak; Rifky Waluyajati Rachman; Ryan Bayusantika Ristandi; Safarina G Malik; Sukma Oktavianthi; Willy Agustine
EPI_ISL_2620713	Dinas Kesehatan Kabupaten Tapin Kalimantan Selatan	National Institute of Health Research and Development	Arie Ardiansyah Nugraha; Hana Apsari Pawestri; Hartanti Dian Ikawati; Kartika Dewi Puspa; Krisna Pangesti; Nelly Puspandari; Subangkit; Triyani Soekarso; Vivi Setiawaty
EPI_ISL_3138855, EPI_ISL_3138856, EPI_ISL_3138857, EPI_ISL_3138858, EPI_ISL_3138859	Dinas Kesehatan Klungkung	National Institute of Health Research and Development	; Arie Ardiansyah Nugraha; Fajar Nur Sulistiyohadi; Hana Apsari Pawestri; Hartanti Dian Ikawati; Kartika Dewi Puspa; Krisna Pangesti; Nelly Puspandari; Subangkit; Triyani Soekarso; Vivi Setiawaty
EPI_ISL_1855939	Dinas Kesehatan Kota Bogor	Indonesian Research Center for Veterinary Science / BBalitvet	Diana Nurjanah; Fadjry Djufry; Harimurti Nuradji; Ilham Chaidir; Ni Luh Putu Indi Dharmayanti; Risa Indriani; Sri Nowo Retno
EPI_ISL_2047531	Dinas Kesehatan Kota Denpasar	National Institute of Health Research and Development	Arie Ardiansyah Nugraha; Hana Apsari Pawestri; Hartanti Dian Ikawati; Kartika Dewi Puspa; Krisna Pangesti; Nelly Puspandari; Subangkit; Triyani Soekarso; Vivi Setiawaty
EPI_ISL_3159708, EPI_ISL_3159711, EPI_ISL_3159714, EPI_ISL_3159717, EPI_ISL_3159720, EPI_ISL_3159723, EPI_ISL_3159726, EPI_ISL_3159729, EPI_ISL_3159732, EPI_ISL_3159735, EPI_ISL_3159738, EPI_ISL_3159741, EPI_ISL_3159744, EPI_ISL_3159747, EPI_ISL_3159750, EPI_ISL_3159753, EPI_ISL_3159756, EPI_ISL_3159759, EPI_ISL_3159762, EPI_ISL_3159765, EPI_ISL_3159768, EPI_ISL_3159771, EPI_ISL_3159774, EPI_ISL_3159777, EPI_ISL_3159780, EPI_ISL_3159784, EPI_ISL_3159786, EPI_ISL_3159788, EPI_ISL_3159791, EPI_ISL_3159794, EPI_ISL_3159797, EPI_ISL_3159800, EPI_ISL_3159802, EPI_ISL_3159805, EPI_ISL_3159808, EPI_ISL_3159811, EPI_ISL_3159814, EPI_ISL_3159817, EPI_ISL_3159820, EPI_ISL_3159823, EPI_ISL_3159826, EPI_ISL_3159829, EPI_ISL_3159833, EPI_ISL_3159835, EPI_ISL_3159838, EPI_ISL_3159841, EPI_ISL_3159844, EPI_ISL_3159846, EPI_ISL_3159849, EPI_ISL_3159852, EPI_ISL_3159855, EPI_ISL_3159858, EPI_ISL_3159861, EPI_ISL_3159864, EPI_ISL_3159867, EPI_ISL_3159870, EPI_ISL_3159873, EPI_ISL_3159876, EPI_ISL_3159879, EPI_ISL_3159882, EPI_ISL_3159885, EPI_ISL_3159889, EPI_ISL_3159892, EPI_ISL_3159895, EPI_ISL_3159897, EPI_ISL_3159901, EPI_ISL_3159903, EPI_ISL_3159906, EPI_ISL_3159909, EPI_ISL_3159912, EPI_ISL_3159915, EPI_ISL_3159918, EPI_ISL_3159921, EPI_ISL_3159924, EPI_ISL_3159927, EPI_ISL_3159930, EPI_ISL_3159934, EPI_ISL_3159937, EPI_ISL_3159940, EPI_ISL_3159943, EPI_ISL_3159946, EPI_ISL_3159950, EPI_ISL_3159953, EPI_ISL_3159956, EPI_ISL_3159959, EPI_ISL_3159963, EPI_ISL_3159966, EPI_ISL_3159969, EPI_ISL_3159972, EPI_ISL_3159976, EPI_ISL_3159979, EPI_ISL_3159982, EPI_ISL_3159985, EPI_ISL_3159989, EPI_ISL_3159992, EPI_ISL_3159995, EPI_ISL_3159998, EPI_ISL_3160001, EPI_ISL_3160004, EPI_ISL_3160007, EPI_ISL_3160010, EPI_ISL_3160013, EPI_ISL_3160016, EPI_ISL_3160019, EPI_ISL_3160022, EPI_ISL_3160025, EPI_ISL_3160028, EPI_ISL_3160031, EPI_ISL_3160034, EPI_ISL_3160037, EPI_ISL_3160040, EPI_ISL_3160043, EPI_ISL_3160046, EPI_ISL_3160049, EPI_ISL_3160053, EPI_ISL_3160055, EPI_ISL_3160059, EPI_ISL_3160062, EPI_ISL_3160065, EPI_ISL_3160068, EPI_ISL_3160071, EPI_ISL_3160074, EPI_ISL_3160077, EPI_ISL_3160080, EPI_ISL_3160082, EPI_ISL_3160085, EPI_ISL_3160088, EPI_ISL_3160091, EPI_ISL_3160094, EPI_ISL_3160097, EPI_ISL_3160100, EPI_ISL_3160103, EPI_ISL_3160106, EPI_ISL_3160109, EPI_ISL_3160112, EPI_ISL_3160115, EPI_ISL_3160118, EPI_ISL_3160121, EPI_ISL_3160124, EPI_ISL_3160126, EPI_ISL_3160129, EPI_ISL_3160132, EPI_ISL_3160135, EPI_ISL_3160138, EPI_ISL_3160141, EPI_ISL_3160144, EPI_ISL_3160147, EPI_ISL_3160150, EPI_ISL_3160153, EPI_ISL_3160156, EPI_ISL_3160159, EPI_ISL_3160162, EPI_ISL_3160165, EPI_ISL_3160168, EPI_ISL_3160169, EPI_ISL_3160170, EPI_ISL_3160171, EPI_ISL_3160172, EPI_ISL_3160173, EPI_ISL_3160174, EPI_ISL_3160175, EPI_ISL_3160176, EPI_ISL_3160177, EPI_ISL_3160178, EPI_ISL_3160179, EPI_ISL_3160180, EPI_ISL_3160181, EPI_ISL_3160182, EPI_ISL_3160183, EPI_ISL_3160184, EPI_ISL_3160187, EPI_ISL_3160190, EPI_ISL_3160194, EPI_ISL_3160197, EPI_ISL_3160200, EPI_ISL_3160203, EPI_ISL_3550459, EPI_ISL_3550460, EPI_ISL_3550461, EPI_ISL_3550462, EPI_ISL_3550463, EPI_ISL_3550464, EPI_ISL_3550465, EPI_ISL_3550466, EPI_ISL_3550467, EPI_ISL_3550468, EPI_ISL_3550469, EPI_ISL_3550470, EPI_ISL_3550471, EPI_ISL_3550472, EPI_ISL_3550473, EPI_ISL_3550474, EPI_ISL_3550475, EPI_ISL_3550476, EPI_ISL_3550477, EPI_ISL_3550478, EPI_ISL_3550479, EPI_ISL_3550480,			

EPI_ISL_3550481, EPI_ISL_3550482, EPI_ISL_3550483, EPI_ISL_3550484, EPI_ISL_3550485, EPI_ISL_3550486, EPI_ISL_3550487, EPI_ISL_3550488, EPI_ISL_3550489, EPI_ISL_3550490, EPI_ISL_3550491, EPI_ISL_3550492, EPI_ISL_3550494, EPI_ISL_3550495, EPI_ISL_3550496, EPI_ISL_3550497, EPI_ISL_3550499, EPI_ISL_3550500, EPI_ISL_3550501, EPI_ISL_3550502, EPI_ISL_3550503, EPI_ISL_3550504, EPI_ISL_3550505, EPI_ISL_3550507, EPI_ISL_3550508, EPI_ISL_3550509, EPI_ISL_3550510, EPI_ISL_3550511, EPI_ISL_3550512, EPI_ISL_3550513, EPI_ISL_3550515, EPI_ISL_3550516, EPI_ISL_3550517, EPI_ISL_3550518, EPI_ISL_3550519, EPI_ISL_3550520, EPI_ISL_3550521, EPI_ISL_3550522, EPI_ISL_3550523, EPI_ISL_3550524, EPI_ISL_3550525, EPI_ISL_3550526, EPI_ISL_3550527, EPI_ISL_3550528, EPI_ISL_3550530, EPI_ISL_3550531, EPI_ISL_3550532, EPI_ISL_3550533, EPI_ISL_3550534, EPI_ISL_3550535, EPI_ISL_3550536, EPI_ISL_3550537, EPI_ISL_3550538, EPI_ISL_3550539, EPI_ISL_3550540, EPI_ISL_3550541, EPI_ISL_3550542, EPI_ISL_3550543, EPI_ISL_3550544, EPI_ISL_3550545, EPI_ISL_3550546, EPI_ISL_3550547, EPI_ISL_3550548, EPI_ISL_3550549, EPI_ISL_3550550, EPI_ISL_3550551, EPI_ISL_3550552, EPI_ISL_3550553, EPI_ISL_3550554			
see above	Dinas Kesehatan Provinsi Kalimantan Timur	Eijkman Institute for Molecular Biology, National Research and Innovation Agency; National Institute of Health Research and Development	Amin Soebandrio; Edison Johar; Frilasita A Yudhaputri; Hana Apsari Pawestri; Hidayat Trimarsanto; Iskandar Adnan; Jessica R. Saragih; Khin Saw Myint; Lidwina Priliani; Lydia V. Panggalo; Muhammad Rezki Rasyak; Safarina G Malik; Subangkit; Sukma Oktavianthi; Vivi Setiawaty; Willy Agustine
EPI_ISL_3541189, EPI_ISL_3541190, EPI_ISL_3541191	Dinas Kesehatan Provinsi Lampung	National Institute of Health Research and Development	Arie Ardiansyah Nugraha; Fajar Nur Sulistiyohadi; Hana Apsari Pawestri; Hartanti Dian Ikawati; Kartika Dewi Puspa; Krisna Pangesti; Nelly Puspandari; Subangkit; Triyani Soekarso; Vivi Setiawaty
EPI_ISL_3029943, EPI_ISL_3029944, EPI_ISL_3029946, EPI_ISL_3029947, EPI_ISL_3029948, EPI_ISL_3029950, EPI_ISL_3029951, EPI_ISL_3029952, EPI_ISL_3029953, EPI_ISL_3029954, EPI_ISL_3029955, EPI_ISL_3029956, EPI_ISL_3029957, EPI_ISL_3029958, EPI_ISL_3029960, EPI_ISL_3029961, EPI_ISL_3029962, EPI_ISL_3029963, EPI_ISL_3029964, EPI_ISL_3029965, EPI_ISL_3029966, EPI_ISL_3029967, EPI_ISL_3029968, EPI_ISL_3029969, EPI_ISL_3029970, EPI_ISL_3029971, EPI_ISL_3029972, EPI_ISL_3029973, EPI_ISL_3029974, EPI_ISL_3029975, EPI_ISL_3029976, EPI_ISL_3029977, EPI_ISL_3029978, EPI_ISL_3029979, EPI_ISL_3029980, EPI_ISL_3029981, EPI_ISL_3029982, EPI_ISL_3029983, EPI_ISL_3029984, EPI_ISL_3029985, EPI_ISL_3029986, EPI_ISL_3029987, EPI_ISL_3029988, EPI_ISL_3029989, EPI_ISL_3029990, EPI_ISL_3029991, EPI_ISL_3029992, EPI_ISL_3029993, EPI_ISL_3070860			
see above	Dinas Kesehatan Provinsi Nusa Tenggara Timur	Eijkman Institute for Molecular Biology, National Research and Innovation Agency	Amin Soebandrio; Edison Johar; Frilasita A Yudhaputri; Hidayat Trimarsanto; Iskandar Adnan; Khin Saw Myint; Lidwina Priliani; Lydia V. Panggalo; Muhammad Rezki Rasyak; Safarina G Malik; Sukma Oktavianthi; Willy Agustine
EPI_ISL_3151603, EPI_ISL_3151608, EPI_ISL_3151615, EPI_ISL_3151622, EPI_ISL_3151629, EPI_ISL_3151635, EPI_ISL_3151655, EPI_ISL_3151661, EPI_ISL_3278295, EPI_ISL_5022911, EPI_ISL_5022912, EPI_ISL_5022913, EPI_ISL_5022914, EPI_ISL_5022915			
see above	Dinas Kesehatan Provinsi Papua Barat	National Institute of Health Research and Development	Arie Ardiansyah Nugraha; Fajar Nur Sulistiyohadi; Hana Apsari Pawestri; Hartanti Dian Ikawati; Kartika Dewi Puspa; Krisna Pangesti; Nelly Puspandari; Subangkit; Triyani Soekarso; Vivi Setiawaty
EPI_ISL_5107886, EPI_ISL_5107887, EPI_ISL_5107888, EPI_ISL_5107889	Dinas Kesehatan Rejang Lebong Bengkulu	National Institute of Health Research and Development	Arie Ardiansyah Nugraha; Fajar Nur Sulistiyohadi; Hana Apsari Pawestri; Hartanti Dian Ikawati; Kartika Dewi Puspa; Krisna Pangesti; Nelly Puspandari; Subangkit; Triyani Soekarso; Vivi Setiawaty
EPI_ISL_2262280	Dinkes Kab Kota Waringin Barat	National Institute of Health Research and Development	Arie Ardiansyah Nugraha; Hana Apsari Pawestri; Hartanti Dian Ikawati; Kartika Dewi Puspa; Krisna Pangesti; Nelly Puspandari; Subangkit; Triyani Soekarso; Vivi Setiawaty
EPI_ISL_2262281	Dinkes Kab Kota Waringin Timur	National Institute of Health Research and Development	Arie Ardiansyah Nugraha; Hana Apsari Pawestri; Hartanti Dian Ikawati; Kartika Dewi Puspa; Krisna Pangesti; Nelly Puspandari; Subangkit; Triyani Soekarso; Vivi Setiawaty
EPI_ISL_3138809	Dinkes Kab. Kediri	National Institute of Health Research and Development	Arie Ardiansyah Nugraha; Fajar Nur Sulistiyohadi; Hana Apsari Pawestri; Hartanti Dian Ikawati; Kartika Dewi Puspa; Krisna Pangesti; Nelly Puspandari; Subangkit; Triyani Soekarso; Vivi Setiawaty
EPI_ISL_4090552, EPI_ISL_4090553, EPI_ISL_4090554, EPI_ISL_4090555	Dinkes Kabupaten Magetan (RSUD dr Sayidiman)	National Institute of Health Research and Development	Arie Ardiansyah Nugraha; Fajar Nur; Hana Apsari Pawestri; Hartanti Dian Ikawati; Kartika Dewi Puspa; Krisna Pangesti; Nelly Puspandari; Subangkit; Sulistiyohadi; Triyani Soekarso; Vivi Setiawaty
EPI_ISL_2965760, EPI_ISL_2965761, EPI_ISL_2965762, EPI_ISL_2965763, EPI_ISL_2965764, EPI_ISL_2965765, EPI_ISL_2965766, EPI_ISL_2965767, EPI_ISL_2965768, EPI_ISL_2965769, EPI_ISL_2987662, EPI_ISL_2987663			
see above	Dinkes Kabupaten Merauke	National Institute of Health Research and Development	Arie Ardiansyah Nugraha; Fajar Nur Sulistiyohadi; Hana Apsari Pawestri; Hartanti Dian Ikawati; Kartika Dewi Puspa; Krisna Pangesti; Nelly Puspandari; Subangkit; Triyani Soekarso; Vivi Setiawaty
EPI_ISL_4090619, EPI_ISL_4090620, EPI_ISL_4090621, EPI_ISL_4090622, EPI_ISL_4090623, EPI_ISL_4090624, EPI_ISL_4090625, EPI_ISL_4090626, EPI_ISL_4090627, EPI_ISL_4090628, EPI_ISL_4090629, EPI_ISL_4090630, EPI_ISL_4090631, EPI_ISL_4090632, EPI_ISL_4090633, EPI_ISL_4090634, EPI_ISL_4090635, EPI_ISL_4090636, EPI_ISL_4090637, EPI_ISL_4090638, EPI_ISL_4090639			
see above	Dinkes Kabupaten Morowali	National Institute of Health Research and Development	Arie Ardiansyah Nugraha; Fajar Nur Sulistiyohadi; Hana Apsari Pawestri; Hartanti Dian Ikawati; Kartika Dewi Puspa; Krisna Pangesti; Nelly Puspandari; Subangkit; Triyani Soekarso; Vivi Setiawaty
EPI_ISL_2987637, EPI_ISL_2987638, EPI_ISL_2987639, EPI_ISL_2987640, EPI_ISL_3019944	Dinkes Kalimantan Timur	National Institute of Health Research and Development	Arie Ardiansyah Nugraha; Fajar Nur Sulistiyohadi; Hana Apsari Pawestri; Hartanti Dian Ikawati; Kartika Dewi Puspa; Krisna Pangesti; Nelly Puspandari; Subangkit; Triyani Soekarso; Vivi Setiawaty
EPI_ISL_2262267	Dinkes Kepulauan Seribu	National Institute of Health Research and Development	Arie Ardiansyah Nugraha; Hana Apsari Pawestri; Hartanti Dian Ikawati; Kartika Dewi Puspa; Krisna Pangesti; Nelly Puspandari; Subangkit; Triyani Soekarso; Vivi Setiawaty
EPI_ISL_528753	Dinkes Kota Bogor	School of Life Sciences and Technology & School of Pharmacy-Institut Teknologi Bandung; Molecular Genetics Laboratory-Faculty of Medicine-Universitas Padjadjaran; Laboratorium Kesehatan Provinsi Jawa Barat	Agung Eru Wibowo; Azzania Fibriani; Catur Riani; Cut Nur Cinthia Alamanda; Davin H. E. Setiamarga; Ema Rahmawati; Hammam Riza; Hesti Lina Wiraswati; Husna Nugrahapraja; Irvan Faizal; Lia Faridah; Marselina Irasonia Tan; Rifky Waluyajati Rachman; Rizki Mardian; Ryan Bayusantika Ristandi; Savira Ekawardhani; Sony Solistia Wirawan; Tarwadi; Yunia Sribudiani
EPI_ISL_2617495	Dinkes Kota Tangerang	National Institute of Health Research and Development	Arie Ardiansyah Nugraha; Hana Apsari Pawestri; Hartanti Dian Ikawati; Kartika Dewi Puspa; Krisna Pangesti; Nelly Puspandari; Subangkit; Triyani Soekarso; Vivi Setiawaty
EPI_ISL_2854694, EPI_ISL_2854695, EPI_ISL_2854696	Dinkes Mojokerto	National Institute of Health Research and Development	Arie Ardiansyah Nugraha; Hana Apsari Pawestri; Hartanti Dian Ikawati; Kartika Dewi Puspa; Krisna Pangesti; Nelly Puspandari; Subangkit; Triyani Soekarso; Vivi Setiawaty
EPI_ISL_2931728, EPI_ISL_2931736, EPI_ISL_2931744, EPI_ISL_2931745, EPI_ISL_2931755, EPI_ISL_2931764, EPI_ISL_2931775, EPI_ISL_2931782, EPI_ISL_2931790			
see above	Dinkes Musi Banyu Asin	National Institute of Health Research and Development	; Arie Ardiansyah Nugraha; Hana Apsari Pawestri; Hartanti Dian Ikawati; Kartika Dewi Puspa; Krisna Pangesti; Nelly Puspandari; Subangkit; Triyani Soekarso; Vivi Setiawaty
EPI_ISL_4104518, EPI_ISL_4104519, EPI_ISL_4104520, EPI_ISL_4104521, EPI_ISL_4104522, EPI_ISL_4104523, EPI_ISL_4104524, EPI_ISL_4104525, EPI_ISL_4104526, EPI_ISL_4104527, EPI_ISL_4104528, EPI_ISL_4104529, EPI_ISL_4104530, EPI_ISL_4104531			
see above	Dinkes Provinsi Jambi	National Institute of Health Research and Development	Arie Ardiansyah Nugraha; Fajar Nur Sulistiyohadi; Hana Apsari Pawestri; Hartanti Dian Ikawati; Kartika Dewi Puspa; Krisna Pangesti; Nelly Puspandari; Subangkit; Triyani Soekarso; Vivi Setiawaty
EPI_ISL_528748	Dinkes Provinsi Jawa Barat	School of Life Sciences and Technology & School of Pharmacy-Institut Teknologi Bandung; Molecular Genetics Laboratory-Faculty of Medicine-Universitas Padjadjaran; Laboratorium Kesehatan Provinsi Jawa Barat	Adelina Khristiani Rahayu; Agung Eru Wibowo; Azzania Fibriani; Catur Riani; Cut Nur Cinthia Alamanda; Ema Rahmawati; Gusti Ayu Prani Pradani; Hammam Riza; Hesti Lina Wiraswati; Husna Nugrahapraja; Irvan Faizal; Lia Faridah; Marselina Irasonia Tan; Rifky Waluyajati Rachman; Ryan Bayusantika Ristandi; Savira Ekawardhani; Sony Solistia Wirawan; Tarwadi; Yunia Sribudiani
EPI_ISL_5743655, EPI_ISL_5743659, EPI_ISL_5743781, EPI_ISL_5743783, EPI_ISL_5743784, EPI_ISL_5743785, EPI_ISL_5743790, EPI_ISL_5743796			
see above	Dinkes Provinsi Kalimanta Utara	National Institute of Health Research and Development	Arie Ardiansyah Nugraha; Fajar Nur Sulistiyohadi; Hana Apsari Pawestri; Hartanti Dian Ikawati; Kartika Dewi Puspa; Krisna Pangesti; Nelly Puspandari; Subangkit; Triyani Soekarso; Vivi Setiawaty
EPI_ISL_2965778, EPI_ISL_2965779, EPI_ISL_2965780, EPI_ISL_2965781, EPI_ISL_2965782, EPI_ISL_2965783, EPI_ISL_2965784, EPI_ISL_2965785, EPI_ISL_2965786, EPI_ISL_2987635, EPI_ISL_2987636, EPI_ISL_3138940, EPI_ISL_3151604, EPI_ISL_3151610, EPI_ISL_3151611, EPI_ISL_3151617, EPI_ISL_3151618, EPI_ISL_3151624, EPI_ISL_3151625, EPI_ISL_3151637, EPI_ISL_3151638, EPI_ISL_3151644, EPI_ISL_3151645, EPI_ISL_3151649, EPI_ISL_3151650, EPI_ISL_3151656, EPI_ISL_3151657, EPI_ISL_3151658, EPI_ISL_3151662, EPI_ISL_3151663, EPI_ISL_3151664, EPI_ISL_3151669, EPI_ISL_3151670, EPI_ISL_3151671, EPI_ISL_3151677, EPI_ISL_3151678, EPI_ISL_3151679, EPI_ISL_3216574, EPI_ISL_3216575, EPI_ISL_3216576, EPI_ISL_3216577, EPI_ISL_3216578, EPI_ISL_3216579, EPI_ISL_3216580, EPI_ISL_3216581, EPI_ISL_3216582, EPI_ISL_3216583, EPI_ISL_3216584, EPI_ISL_3216585, EPI_ISL_3216586			
see above	Dinkes Provinsi Kalimantan Timur	National Institute of Health Research and Development	Arie Ardiansyah Nugraha; Fajar Nur Sulistiyohadi; Hana Apsari Pawestri; Hartanti Dian Ikawati; Kartika Dewi Puspa; Krisna Pangesti; Nelly Puspandari; Subangkit; Triyani Soekarso; Vivi Setiawaty
EPI_ISL_3691578, EPI_ISL_3691579	Dinkes Provinsi Kalimantan Timur (Labkesda Bontang)	National Institute of Health Research and Development	Arie Ardiansyah Nugraha; Hana Apsari Pawestri; Hartanti Dian Ikawati; Kartika Dewi Puspa; Krisna Pangesti; Nelly Puspandari; Subangkit; Triyani Soekarso; Vivi Setiawaty
EPI_ISL_3691577	Dinkes Provinsi Kalimantan Timur (Labkesprov Kaltim)	National Institute of Health Research and Development	Arie Ardiansyah Nugraha; Hana Apsari Pawestri; Hartanti Dian Ikawati; Kartika Dewi Puspa; Krisna Pangesti; Nelly Puspandari; Subangkit; Triyani Soekarso; Vivi Setiawaty
EPI_ISL_4520492, EPI_ISL_4520493, EPI_ISL_4520494, EPI_ISL_4520495, EPI_ISL_4520496, EPI_ISL_4520497, EPI_ISL_4520498, EPI_ISL_4520499, EPI_ISL_4520500, EPI_ISL_4520501, EPI_ISL_4520502, EPI_ISL_4520503, EPI_ISL_4520504, EPI_ISL_4520505, EPI_ISL_4520506, EPI_ISL_4520507, EPI_ISL_4520508, EPI_ISL_4520509, EPI_ISL_4520510, EPI_ISL_4520511, EPI_ISL_4520512, EPI_ISL_4520513, EPI_ISL_4520514, EPI_ISL_4520515, EPI_ISL_4520516, EPI_ISL_4520517, EPI_ISL_4520518, EPI_ISL_4520519, EPI_ISL_4520520, EPI_ISL_4520521, EPI_ISL_4520522, EPI_ISL_4520523, EPI_ISL_4520524, EPI_ISL_4520525, EPI_ISL_4520526, EPI_ISL_4520527, EPI_ISL_4520528, EPI_ISL_4520529, EPI_ISL_4520530, EPI_ISL_4520531, EPI_ISL_4520532, EPI_ISL_4520533, EPI_ISL_4520534, EPI_ISL_4520535, EPI_ISL_4520536, EPI_ISL_4520537, EPI_ISL_4997457, EPI_ISL_4997463, EPI_ISL_4997472, EPI_ISL_4997479, EPI_ISL_4997487, EPI_ISL_4997494, EPI_ISL_4997495, EPI_ISL_4997496, EPI_ISL_4997497, EPI_ISL_4997501, EPI_ISL_4997504, EPI_ISL_4997509, EPI_ISL_4997511, EPI_ISL_4997515, EPI_ISL_4997521, EPI_ISL_4997528, EPI_ISL_4997537, EPI_ISL_4997544, EPI_ISL_4997550, EPI_ISL_4997557, EPI_ISL_4997563, EPI_ISL_4997572, EPI_ISL_4997573, EPI_ISL_4997583, EPI_ISL_4997588, EPI_ISL_4997590, EPI_ISL_4997596, EPI_ISL_4997599, EPI_ISL_4997606, EPI_ISL_4997607, EPI_ISL_4997612, EPI_ISL_4997619, EPI_ISL_4997624, EPI_ISL_4997627, EPI_ISL_4997632, EPI_ISL_4997635, EPI_ISL_4997636, EPI_ISL_4997639, EPI_ISL_4997645, EPI_ISL_4997648, EPI_ISL_4997654, EPI_ISL_4997657, EPI_ISL_4997666, EPI_ISL_4997667, EPI_ISL_4997670, EPI_ISL_4997677, EPI_ISL_4997684, EPI_ISL_4997693			
see above	Dinkes Provinsi Kalimantan Timur (RSKD Balikpapan)	Faculty of Medicine, Universitas Indonesia	Ajeng Megawati Fajrin; Anis Karuniawati; Ari Fahrial Syam; Badriul Hegar; Beti Ernawati; Budi Wiweko; Dimas Ramadhian Noor; Fadilah; Fera Ibrahim; Hana Apsari Pawestri; Khaerunissa Anbar Istiadi; Linda Erlina; Rafika Indah Paramita; Subangkit; Vivi Setiawaty
EPI_ISL_2965770, EPI_ISL_2965771, EPI_ISL_2965772, EPI_ISL_2965773, EPI_ISL_2965774, EPI_ISL_2965775, EPI_ISL_2965776, EPI_ISL_2965777, EPI_ISL_2987641, EPI_ISL_2987642, EPI_ISL_2987643, EPI_ISL_2987644, EPI_ISL_3138897, EPI_ISL_3138898, EPI_ISL_3138899, EPI_ISL_3138900, EPI_ISL_3138901, EPI_ISL_3138902, EPI_ISL_3138903, EPI_ISL_3138904, EPI_ISL_3138905, EPI_ISL_3138906, EPI_ISL_5796024, EPI_ISL_5796025			
see above	Dinkes Provinsi Kalimantan Utara	National Institute of Health Research and Development	Arie Ardiansyah Nugraha; Fajar Nur; Fajar Nur Sulistiyohadi; Hana Apsari Pawestri; Hartanti Dian Ikawati; Kartika Dewi Puspa; Krisna Pangesti; Nelly Puspandari; Subangkit; Sulistiyohadi; Triyani Soekarso; Vivi Setiawaty
EPI_ISL_4090609, EPI_ISL_4090610, EPI_ISL_4090611, EPI_ISL_4090612, EPI_ISL_4090613, EPI_ISL_4254843, EPI_ISL_4254844, EPI_ISL_4254845, EPI_ISL_4254846, EPI_ISL_4254847, EPI_ISL_4254848, EPI_ISL_4254849, EPI_ISL_4254850			
see above	Dinkes Provinsi Kalimantan Utara (RS dr Soemarno Sostromojo)	National Institute of Health Research and Development	Arie Ardiansyah Nugraha; Fajar Nur; Fajar Nur Sulistiyohadi; Hana Apsari Pawestri; Hartanti Dian Ikawati; Kartika Dewi Puspa; Krisna Pangesti; Nelly Puspandari; Subangkit; Sulistiyohadi; Triyani Soekarso; Vivi Setiawaty
EPI_ISL_4090600, EPI_ISL_4090601, EPI_ISL_4090602, EPI_ISL_4090603, EPI_ISL_4254836, EPI_ISL_4254837, EPI_ISL_4254853, EPI_ISL_5022950			
see above	Dinkes Provinsi Kalimantan Utara (RSUD Akhmad Berahim)	National Institute of Health Research and Development	Arie Ardiansyah Nugraha; Fajar Nur; Fajar Nur Sulistiyohadi; Hana Apsari Pawestri; Hartanti Dian Ikawati; Kartika Dewi Puspa; Krisna Pangesti; Nelly Puspandari; Subangkit; Sulistiyohadi; Triyani Soekarso; Vivi Setiawaty
EPI_ISL_4090604, EPI_ISL_4090605, EPI_ISL_4090606, EPI_ISL_4090607, EPI_ISL_4090608, EPI_ISL_4254838, EPI_ISL_4254839, EPI_ISL_4254840, EPI_ISL_4254841, EPI_ISL_4254842			
see above	Dinkes Provinsi Kalimantan Utara (RSUD Malinau)	National Institute of Health Research and Development	Arie Ardiansyah Nugraha; Fajar Nur; Fajar Nur Sulistiyohadi; Hana Apsari Pawestri; Hartanti Dian Ikawati; Kartika Dewi Puspa; Krisna Pangesti; Nelly Puspandari; Subangkit; Sulistiyohadi; Triyani Soekarso; Vivi Setiawaty
EPI_ISL_4090595, EPI_ISL_4090596, EPI_ISL_4090597, EPI_ISL_4090598, EPI_ISL_4090599, EPI_ISL_4254831, EPI_ISL_4254832, EPI_ISL_4254833, EPI_ISL_4254834, EPI_ISL_4254835			
see above	Dinkes Provinsi Kalimantan Utara (RSUD Nunukan)	National Institute of Health Research and Development	Arie Ardiansyah Nugraha; Fajar Nur; Fajar Nur Sulistiyohadi; Hana Apsari Pawestri; Hartanti Dian Ikawati; Kartika Dewi Puspa; Krisna Pangesti; Nelly Puspandari; Subangkit; Sulistiyohadi; Triyani Soekarso; Vivi Setiawaty
EPI_ISL_4090614, EPI_ISL_4090615, EPI_ISL_4090616, EPI_ISL_4090617, EPI_ISL_4090618, EPI_ISL_4254851, EPI_ISL_4254852			
see above	Dinkes Provinsi Kalimantan	National Institute of Health Research and Development	Arie Ardiansyah Nugraha; Fajar Nur Sulistiyohadi; Hana Apsari Pawestri; Hartanti Dian Ikawati; Kartika Dewi Puspa; Krisna Pangesti; Nelly Puspandari; Subangkit; Triyani Soekarso; Vivi Setiawaty

	Utara (RSUD Tarakan)		
EPI_ISL_2931733, EPI_ISL_2931741, EPI_ISL_2931751, EPI_ISL_2931760	Dinkes Provinsi Lampung	National Institute of Health Research and Development	Arie Ardiansyah Nugraha; Hana Apsari Pawestri; Hartanti Dian Ikawati; Kartika Dewi Puspa; Krisna Pangesti; Nelly Puspandari; Subangkit; Triyani Soekarso; Vivi Setiawaty
EPI_ISL_3375297, EPI_ISL_3375298, EPI_ISL_3375299, EPI_ISL_3375300, EPI_ISL_3375301, EPI_ISL_3375302, EPI_ISL_3375303, EPI_ISL_3375304, EPI_ISL_3375305, EPI_ISL_3375306, EPI_ISL_3375307, EPI_ISL_3375308, EPI_ISL_3375309, EPI_ISL_3375310, EPI_ISL_3375311, EPI_ISL_3375312, EPI_ISL_3375313, EPI_ISL_3375314, EPI_ISL_3375315, EPI_ISL_3375316, EPI_ISL_3375317, EPI_ISL_3375318, EPI_ISL_3375319, EPI_ISL_3375320, EPI_ISL_3375321, EPI_ISL_3375322, EPI_ISL_3375323, EPI_ISL_3375324, EPI_ISL_3375325, EPI_ISL_3375326			
see above	Dinkes Provinsi Sulawesi Tengah	National Institute of Health Research and Development	Arie Ardiansyah Nugraha; Fajar Nur Sulistiyohadi; Hana Apsari Pawestri; Hartanti Dian Ikawati; Kartika Dewi Puspa; Krisna Pangesti; Nelly Puspandari; Subangkit; Triyani Soekarso; Vivi Setiawaty
EPI_ISL_4090584	Dinkes Provinsi Sulawesi Tengah RSUD Mokopido	National Institute of Health Research and Development	Arie Ardiansyah Nugraha; Fajar Nur Sulistiyohadi; Hana Apsari Pawestri; Hartanti Dian Ikawati; Kartika Dewi Puspa; Krisna Pangesti; Nelly Puspandari; Subangkit; Triyani Soekarso; Vivi Setiawaty
EPI_ISL_467374	Dinkes Samarinda	Eijkman Institute for Molecular Biology, Ministry of Research and Technology/National Agency for Research and Innovation	Amin Soebandrio; David H Muljono; Edison Johar; Frilasita A Yudhaputri; Hidayat Trimarsanto; Khin Saw Myint; Safarina G Malik
EPI_ISL_2233090	Dinkes Samarinda	National Institute of Health Research and Development	Arie Ardiansyah Nugraha; Hana Apsari Pawestri; Hartanti Dian Ikawati; Kartika Dewi Puspa; Krisna Pangesti; Nelly Puspandari; Subangkit; Triyani Soekarso; Vivi Setiawaty
EPI_ISL_754241	Dinkes Tasikmalaya	"School of Life Sciences and Technology & School of Pharmacy-Institut Teknologi Bandung; Molecular Genetics Laboratory-Faculty of Medicine-Universitas Padjadjaran; Laboratorium Kesehatan Provinsi Jawa Barat"	Agung Eru Wibowo; Azzania Fibriani; Catur Riani; Cut Nur Cinthia Alamanda; Ema Rahmawati; Hammam Riza; Hesti Lina Wiraswati; Husna Nugrahapraja; Irvan Faizal; Karimatu Khoirunnisa; Lia Faridah; Marselina Irasonia Tan; Miftahul Faridl; Rifky Waluyajati Rachman; Ryan Bayusantika Ristandi; Savira Ekawardhani; Soni Solistia Wirawan; Tarwadi; Yunia Sribudiani
EPI_ISL_1020197, EPI_ISL_1117591, EPI_ISL_2840871, EPI_ISL_2840872, EPI_ISL_2840873, EPI_ISL_2840874, EPI_ISL_2840875, EPI_ISL_2840876, EPI_ISL_2840877, EPI_ISL_2840879, EPI_ISL_2840880, EPI_ISL_2840886, EPI_ISL_2840887, EPI_ISL_2840888, EPI_ISL_2840890, EPI_ISL_2840891, EPI_ISL_2840892			
see above	Dinkes, Bogor, West Java	Biosafety Level-3 Laboratory, Indonesian Institute of Sciences (LIPI)	Ade Andriani; Andri Wardiana; Anggia Prasetyoputri; Anik Budhi Dharmayanthi; Asep M. Ridwanulloh; Asep M. Ridwanuloh; Eko Wahyu Putro; Erwin F. Hasrianda; Fahrurrozi; Gita Syahputra; Hariyatun; Herjuno A. Nugroho; Inswasti Cahyani; Listiana Oktavia; Matthew W. Loose; Mohammad Ilyas; Nova D. Yanthi; Puspita Lisdiyanti; Ratih A. Ningrum; Sugiyono Saputra; Susanti; Syam B. Iryanto; Syam Budi Iryanto; Wien Kusharyoto; Yuliawati
EPI_ISL_1041030, EPI_ISL_2991995	Dr. Cipto Mangunkusumo (RSCM) Hospital	Faculty of Medicine, Universitas Indonesia	Aliya Fatimah; Ari Fahrial Syam; Mei Riasanti; Rani Dwi Ningtias; Rina La Distia Nora
EPI_ISL_528752	Dr. H. A. Rotinsulu Lung Hospital	School of Pharmacy & School of Life Sciences and Technology - Institut Teknologi Bandung; Molecular Genetics Laboratory-Faculty of Medicine-Universitas Padjadjaran; Laboratorium Kesehatan Provinsi Jawa Barat	Adelina Khristiani Rahayu; Agung Eru Wibowo; Azzania Fibriani; Catur Riani; Cut Nur Cinthia Alamanda; Ema Rahmawati; Gusti Ayu Prani Pradani; Hammam Riza; Hesti Lina Wiraswati; Husna Nugrahapraja; Irvan Faizal; Lia Faridah; Marselina Irasonia Tan; Rifky Waluyajati Rachman; Ryan Bayusantika Ristandi; Savira Ekawardhani; Sony Solistia Wirawan; Tarwadi; Yunia Sribudiani
EPI_ISL_4885279, EPI_ISL_4885280, EPI_ISL_4885281, EPI_ISL_4885282, EPI_ISL_4885283, EPI_ISL_4885284	Dr. Slamet Garut Public Hospital	West Java Health Laboratory	Azzania Fibriani; Cut Nur Cinthia Alamanda; Ema Rahmawati; Isak Solihin; Karimatu Khoirunnisa; Miftahul Faridl; Rifky Waluyajati Rachman; Rini Robiani; Ryan Bayusantika Ristandi
EPI_ISL_4885266, EPI_ISL_4885267, EPI_ISL_4885268, EPI_ISL_4885269, EPI_ISL_4885270, EPI_ISL_4885271, EPI_ISL_4885272, EPI_ISL_4885273, EPI_ISL_4885274, EPI_ISL_4885275, EPI_ISL_4885276, EPI_ISL_4885277, EPI_ISL_4885278			
see above	Dr. Slamet Garut Public Hospital	West Java Health Laboratory; School of Life Sciences and Technology, Institut Teknologi Bandung	Azzania Fibriani; Cut Nur Cinthia Alamanda; Ema Rahmawati; Isak Solihin; Karimatu Khoirunnisa; Miftahul Faridl; Rifky Waluyajati Rachman; Rini Robiani; Ryan Bayusantika Ristandi
EPI_ISL_5328538	Dr. Slamet Garut Public Regional Hospital	West Java Health Laboratory; School of Life Sciences and Technology, Institut Teknologi Bandung	Azzania Fibriani; Cut Nur Cinthia Alamanda; Ema Rahmawati; Karimatu Khoirunnisa; Miftahul Faridl; Rifky Waluyajati Rachman; Rini Robiani; Ryan Bayusantika Ristandi
EPI_ISL_1364466, EPI_ISL_1364467, EPI_ISL_1364468, EPI_ISL_1366083, EPI_ISL_1366238, EPI_ISL_1366269, EPI_ISL_1366271			
see above	Dr. Soetomo Teaching Hospital	Research Center for Vaccine Technology and Development, Institute of Tropical Disease, Airlangga University	Andi Yasmin Wijaya; Aryati; Cita Rosita Sigit Prakoeswa; Damayanti Tinduh; Dominicus Husada; Dwiyanti Puspitasari; Fedik Abdul Rantam; Helen Susilowati; Jusak Nugraha; Neneng Dewi Kurniati; Ni Nyoman Tri Puspaningsih
EPI_ISL_4417852, EPI_ISL_4417853, EPI_ISL_4417854, EPI_ISL_4417855, EPI_ISL_4417856, EPI_ISL_4417857, EPI_ISL_4417858, EPI_ISL_4417861, EPI_ISL_4417862, EPI_ISL_4417863, EPI_ISL_4417864, EPI_ISL_4417865, EPI_ISL_4417866, EPI_ISL_4417867, EPI_ISL_4417868, EPI_ISL_4417869, EPI_ISL_4417870, EPI_ISL_4417871, EPI_ISL_4417872, EPI_ISL_4417873, EPI_ISL_4417874, EPI_ISL_4417875, EPI_ISL_4417876, EPI_ISL_4417885, EPI_ISL_4417886, EPI_ISL_4417887, EPI_ISL_4417888, EPI_ISL_4417889, EPI_ISL_4417890, EPI_ISL_4417891, EPI_ISL_4417892, EPI_ISL_4417893			
see above	EIJKMAN OXFORD CLINICAL RESEARCH UNIT / RS Fatmawati	Genomik Solidaritas Indonesia Laboratorium	Anis Karuniawati; Annisa Muthiah Sukirman; Anuraj Shankar; Ariel Pradipta; Carissa Sintca Wijaya; Decy Subekti; Dhahlia Agustina Cahyono; Dian Tri Wahyudi; Gracia Felias Enos Korompis; Himawan Masyhuri; Louisa Markus; Meutia Ayuputeri Kumaheri; Raph Hamers; Stefanus Bernard; Suwarti; Vera Irawany
EPI_ISL_4417877, EPI_ISL_4417878, EPI_ISL_4417879, EPI_ISL_4417880, EPI_ISL_4417881, EPI_ISL_4417882, EPI_ISL_4417883, EPI_ISL_4417884, EPI_ISL_4417915, EPI_ISL_4417916, EPI_ISL_4468051, EPI_ISL_4468052, EPI_ISL_4468053, EPI_ISL_4468054, EPI_ISL_4468055, EPI_ISL_4468056, EPI_ISL_4468057, EPI_ISL_4468534, EPI_ISL_4468535, EPI_ISL_4468536, EPI_ISL_4468537, EPI_ISL_4468541, EPI_ISL_5098163			
see above	EIJKMAN OXFORD CLINICAL RESEARCH UNIT / RS Pelni	Genomik Solidaritas Indonesia Laboratorium	Anis Karuniawati; Annisa Muthiah Sukirman; Anuraj Shankar; Ariel Pradipta; Carissa Sintca Wijaya; Decy Subekti; Dhahlia Agustina Cahyono; Dian Tri Wahyudi; Gracia Felias Enos Korompis; Himawan Masyhuri; Louisa Markus; Meutia Ayuputeri Kumaheri; Raph Hamers; Robert Sinto; Stefanus Bernard; Suwarti
EPI_ISL_4417894, EPI_ISL_4417896, EPI_ISL_4417897, EPI_ISL_4417898, EPI_ISL_4417899, EPI_ISL_4417900, EPI_ISL_4417905, EPI_ISL_4417906, EPI_ISL_4417907, EPI_ISL_4417909, EPI_ISL_4417910, EPI_ISL_4417911, EPI_ISL_4417912, EPI_ISL_4417913, EPI_ISL_4417914, EPI_ISL_4468538, EPI_ISL_5098157			
see above	EIJKMAN OXFORD CLINICAL RESEARCH UNIT / RSUD Pasar Minggu	Genomik Solidaritas Indonesia Laboratorium	; Anis Karuniawati; Annisa Muthiah Sukirman; Anuraj Shankar; Ariel Pradipta; Carissa Sintca Wijaya; Decy Subekti; Dhahlia Agustina Cahyono; Dian Tri Wahyudi; Dwi Utomo Nusantara; Gracia Felias Enos Korompis; Himawan Masyhuri; Louisa Markus; Meutia Ayuputeri Kumaheri; Raph Hamers; Stefanus Bernard; Suwarti
EPI_ISL_1622411	EKA Hospital BSD	Eijkman Institute for Molecular Biology, Ministry of Research and Technology/National Agency for Research and Innovation	Amin Soebandrio; Edison Johar; Frilasita A Yudhaputri; Hidayat Trimarsanto; Iskandar Adnan; Khin Saw Myint; Lydia V. Panggalo; Muhammad Rezki Rasyak; Safarina G Malik; Sukma Oktavianthi; Willy Agustine
EPI_ISL_2614730	EKA Hospital BSD	Eijkman Institute for Molecular Biology, National Research and Innovation Agency	Amin Soebandrio; Edison Johar; Frilasita A Yudhaputri; Hidayat Trimarsanto; Iskandar Adnan; Khin Saw Myint; Lidwina Priliani; Lydia V. Panggalo; Muhammad Rezki Rasyak; Safarina G Malik; Sukma Oktavianthi; Willy Agustine
EPI_ISL_889005, EPI_ISL_947293	Eijkman Institute for Molecular Biology, Ministry of Research and Technology/National Agency for Research and Innovation	Eijkman Institute for Molecular Biology, Ministry of Research and Technology/National Agency for Research and Innovation	Amin Soebandrio; Edison Johar; Frilasita A Yudhaputri; Hidayat Trimarsanto; Iskandar Adnan; Khin Saw Myint; Lydia V. Panggalo; Safarina G Malik; Sukma Oktavianthi; Willy Agustine
EPI_ISL_3159657, EPI_ISL_3159660, EPI_ISL_3159663, EPI_ISL_3159666, EPI_ISL_3159669, EPI_ISL_3159673, EPI_ISL_3159676, EPI_ISL_3159679, EPI_ISL_3159682, EPI_ISL_3159685, EPI_ISL_3159688, EPI_ISL_3159690			
see above	Eijkman Institute for Molecular Biology, National Research and Innovation Agency	Eijkman Institute for Molecular Biology, National Research and Innovation Agency	Amin Soebandrio; Edison Johar; Frilasita A Yudhaputri; Hidayat Trimarsanto; Iskandar Adnan; Khin Saw Myint; Lidwina Priliani; Lydia V. Panggalo; Muhammad Rezki Rasyak; Safarina G Malik; Sukma Oktavianthi; Willy Agustine
EPI_ISL_1335890	Elisabeth Hospital Medan	Faculty of Medicine, Universitas Sumatera Utara; Institute of Tropical Disease, Universitas Airlangga	Aldise M Nastri; Franciscus Ginting; Inke N D Lubis; Irbah R Nainggolan; Jezzy R Dewantari; Kazufumi Shimizu; Krisnoadi Rahardjo; Maria I Lusida; Meliani; Mirzan Hasibuan; Muhammad Ichwan; R Andika D Cahyadi; R Lia Kusumawati; Ramadhan Bestari; Rima R Prasetya; Soetjipto; Yasuko Mori
EPI_ISL_2614724	Excellent Beneficial Diagnostic Center	Eijkman Institute for Molecular Biology, National Research and Innovation Agency	Amin Soebandrio; Edison Johar; Frilasita A Yudhaputri; Hidayat Trimarsanto; Iskandar Adnan; Khin Saw Myint; Lidwina Priliani; Lydia V. Panggalo; Muhammad Rezki Rasyak; Safarina G Malik; Sukma Oktavianthi; Willy Agustine
EPI_ISL_4004701	Express Test Depok	National Research and Innovation Agency (BRIN-Indonesia)	Ahmad Fathoni; Anik B. Dharmayanthi; Asep M. Ridwanulloh; Hariyatun; Indriawati; Rifki Sadikin; Sugiyono Saputra; Suvia Widyaningrum; Syam B. Iryanto
EPI_ISL_5200479, EPI_ISL_5200480, EPI_ISL_5200481, EPI_ISL_5200482, EPI_ISL_5200483, EPI_ISL_5200484, EPI_ISL_5200485, EPI_ISL_5200486			
see above	FKIK UNIKA ATMA JAYA	Genomik Solidaritas Indonesia Laboratorium	Annisa Muthiah Sukirman; Anuraj Shankar; Ariel Pradipta; Carissa Sintca Wijaya; Dian Tri Wahyudi; Gracia Felias Enos Korompis; Louisa Markus; Maria Mardalena Martini Kaisar; Meutia Ayuputeri Kumaheri; Reinhart Gabriel; Sem Samuel Surja; Sheila Jonnatan; Soegianto Ali; Stefanus Bernard; Tria Asri Widowati; Vania Gavrila Wikasa
EPI_ISL_1416191	Fakultas Kedokteran Universitas Sumatera Utara	National Institute of Health Research and Development	Arie Ardiansyah Nugraha; Hana Apsari Pawestri; Hartanti Dian Ikawati; Kartika Dewi Puspa; Krisna Nur Andriana Pangesti; Nelly Puspandari; Subangkit; Vivi Setiawaty
EPI_ISL_1534701, EPI_ISL_1534702, EPI_ISL_1534703	Fakultas kedokteran Universitas Sumatra Utara	National Institute of Health Research and Development	Arie Ardiansyah Nugraha; Hana Apsari Pawestri; Hartanti Dian Ikawati; Kartika Dewi Puspa; Krisna Pangesti; Nelly Puspandari; Subangkit; Vivi Setiawaty
EPI_ISL_2382411, EPI_ISL_2382412	FarmaLab Indoutama (Wisma Atlet)	National Institute of Health Research and Development	Arie Ardiansyah Nugraha; Hana Apsari Pawestri; Hartanti Dian Ikawati; Kartika Dewi Puspa; Krisna Pangesti; Nelly Puspandari; Subangkit; Triyani Soekarso; Vivi Setiawaty
EPI_ISL_2382402, EPI_ISL_2382403, EPI_ISL_2382404, EPI_ISL_2382405, EPI_ISL_2382406, EPI_ISL_2382407, EPI_ISL_2429146			
see above	FarmaLab Indoutama Halim	National Institute of Health Research and Development	Arie Ardiansyah Nugraha; Hana Apsari Pawestri; Hartanti Dian Ikawati; Kartika Dewi Puspa; Krisna Pangesti; Nelly Puspandari; Subangkit; Triyani Soekarso; Vivi Setiawaty
EPI_ISL_2896195	Farmalab Halim Perdana Kusuma	National Institute of Health Research and Development	; Arie Ardiansyah Nugraha; Hana Apsari Pawestri; Hartanti Dian Ikawati; Kartika Dewi Puspa; Krisna Pangesti; Nelly Puspandari; Subangkit; Triyani Soekarso; Vivi Setiawaty
EPI_ISL_1257860, EPI_ISL_1257861, EPI_ISL_1257862	Farmalab Indoutama	National Institute of Health Research and Development	Arie Ardiansyah Nugraha; Hana Apsari Pawestri; Hartanti Dian Ikawati; Kartika Dewi Puspa; Krisna Pangesti; Nelly Puspandari; Subangkit; Vivi Setiawaty
EPI_ISL_1048372, EPI_ISL_1048375, EPI_ISL_1048381	Farmalab Indoutama Jakarta	National Institute of Health Research and Development	Arie Ardiansyah Nugraha; Hana Apsari Pawestri; Hartanti Dian Ikawati; Kartika Dewi Puspa; Krisna Nur Andriana Pangesti; Nelly Puspandari; Subangkit; Vivi Setiawaty.
EPI_ISL_3230249	GOOD DOCTOR / DINAS KESEHATAN DKI JAKARTA	Genomik Solidaritas Indonesia Laboratorium	Annisa Muthiah Sukirman; Anuraj Shankar; Ariel Pradipta; Carissa Sintca Wijaya; Gracia Felias Enos Korompis; Meutia Ayuputeri Kumaheri; Reinhart Gabriel; Vania Gavrila Wikasa; Venny Beauty
EPI_ISL_3548699	GSI INDY BINTARO	Genomik Solidaritas Indonesia Laboratorium	Annisa Muthiah Sukirman; Anuraj Shankar; Ariel Pradipta; Carissa Sintca Wijaya; Dhahlia Agustina Cahyono; Gracia Felias Enos Korompis; Louisa Markus; Meutia Ayuputeri Kumaheri; Neng Hilma Hamidah; Vania Gavrila Wikasa
EPI_ISL_3138823, EPI_ISL_3138824, EPI_ISL_3138825, EPI_ISL_3138826, EPI_ISL_3138827, EPI_ISL_3138828, EPI_ISL_3138829, EPI_ISL_3138830, EPI_ISL_3138831, EPI_ISL_3138832, EPI_ISL_4508316, EPI_ISL_4508317, EPI_ISL_4508318, EPI_ISL_4508319, EPI_ISL_4508320, EPI_ISL_4508321, EPI_ISL_4508322, EPI_ISL_4508323, EPI_ISL_5022813, EPI_ISL_5022814, EPI_ISL_5022815, EPI_ISL_5022816, EPI_ISL_5022817, EPI_ISL_5022818, EPI_ISL_5022819, EPI_ISL_5022820, EPI_ISL_5022821, EPI_ISL_5022822, EPI_ISL_5022823, EPI_ISL_5022824, EPI_ISL_5022825, EPI_ISL_5022826, EPI_ISL_5022827			

see above	GSI LAB	National Institute of Health Research and Development	Arie Ardiansyah Nugraha; Fajar Nur Sulistiyohadi; Hana Apsari Pawestri; Hartanti Dian Ikawati; Kartika Dewi Puspa; Krisna Pangesti; Meutia Kumaheri; Nelly Puspandari; Subangkit; Triyani Soekarso; Vivi Setiawaty
EPI_ISL_3548627, EPI_ISL_3548628, EPI_ISL_3548635, EPI_ISL_3548674, EPI_ISL_3548698, EPI_ISL_3548700, EPI_ISL_3548701, EPI_ISL_3548702, EPI_ISL_3695857			
see above	GSI MEDIKA PLAZA CILANDAK	Genomik Solidaritas Indonesia Laboratorium	Annisa Muthiah Sukirman; Anuraj Shankar; Ariel Pradipta; Carissa Sintca Wijaya; Dhahlia Agustina Cahyono; Dwi Oktavia; Gracia Felias Enos Korompis; Louisa Markus; Meutia Ayuputeri Kumaheri; Neng Hilma Hamidah; Ngabila Salama; Tiranti Vindha; Vania Gavrila Wikasa
EPI_ISL_2296526, EPI_ISL_2434061, EPI_ISL_2434123, EPI_ISL_2434157, EPI_ISL_2434161, EPI_ISL_2434168, EPI_ISL_2434173, EPI_ISL_2434175, EPI_ISL_2434973, EPI_ISL_2434978, EPI_ISL_2434988, EPI_ISL_2434991, EPI_ISL_2437093, EPI_ISL_2499962, EPI_ISL_2499963, EPI_ISL_2499964, EPI_ISL_2499965, EPI_ISL_2500217, EPI_ISL_2500218, EPI_ISL_2500362, EPI_ISL_2500464, EPI_ISL_2500465, EPI_ISL_2500466, EPI_ISL_2500467, EPI_ISL_2500468, EPI_ISL_2500469, EPI_ISL_2500470, EPI_ISL_2500471, EPI_ISL_2500488, EPI_ISL_2500489, EPI_ISL_2500490, EPI_ISL_2500491, EPI_ISL_2500492, EPI_ISL_2500493, EPI_ISL_2500494, EPI_ISL_2537490, EPI_ISL_2543760, EPI_ISL_2547285, EPI_ISL_2558713, EPI_ISL_2558715, EPI_ISL_2558718, EPI_ISL_2558719, EPI_ISL_2558720, EPI_ISL_2558732, EPI_ISL_2558733, EPI_ISL_2558734, EPI_ISL_2558735, EPI_ISL_2620719, EPI_ISL_2620720, EPI_ISL_2620721, EPI_ISL_2620722, EPI_ISL_2620723, EPI_ISL_2620724, EPI_ISL_2620725, EPI_ISL_2620726, EPI_ISL_2620727, EPI_ISL_2626594, EPI_ISL_2626595, EPI_ISL_2626596, EPI_ISL_2626597, EPI_ISL_2626598, EPI_ISL_2626599, EPI_ISL_2626600, EPI_ISL_2626601, EPI_ISL_2626602, EPI_ISL_2626603, EPI_ISL_2626604, EPI_ISL_2626605, EPI_ISL_2626606, EPI_ISL_2626607, EPI_ISL_2626608, EPI_ISL_2626609, EPI_ISL_2626610, EPI_ISL_2626611, EPI_ISL_2626612, EPI_ISL_2626613, EPI_ISL_2626614, EPI_ISL_2626615, EPI_ISL_2626616, EPI_ISL_2626617, EPI_ISL_2626618, EPI_ISL_2626619, EPI_ISL_2626620, EPI_ISL_2626839, EPI_ISL_2626840, EPI_ISL_2626841, EPI_ISL_2626842, EPI_ISL_2626843, EPI_ISL_2626844, EPI_ISL_2626845, EPI_ISL_2626846, EPI_ISL_2626847, EPI_ISL_2626848, EPI_ISL_2626849, EPI_ISL_2626850, EPI_ISL_2626851, EPI_ISL_2832726, EPI_ISL_2832727, EPI_ISL_2832728, EPI_ISL_2832729, EPI_ISL_2832730, EPI_ISL_2832731, EPI_ISL_2832732, EPI_ISL_2832733, EPI_ISL_2832734, EPI_ISL_2832735, EPI_ISL_2832736, EPI_ISL_2832737, EPI_ISL_2832738, EPI_ISL_2832739, EPI_ISL_2832740, EPI_ISL_2832741, EPI_ISL_2832742, EPI_ISL_2832743, EPI_ISL_2832744, EPI_ISL_2832745, EPI_ISL_3052638, EPI_ISL_3102025, EPI_ISL_3102030, EPI_ISL_3102034, EPI_ISL_3102044, EPI_ISL_3102136, EPI_ISL_3149971, EPI_ISL_3208080, EPI_ISL_3208084, EPI_ISL_3208085, EPI_ISL_3208087, EPI_ISL_3208088, EPI_ISL_3208089, EPI_ISL_3208090, EPI_ISL_3208091, EPI_ISL_3208092, EPI_ISL_3208093, EPI_ISL_3208094, EPI_ISL_3208095, EPI_ISL_3208096, EPI_ISL_3208097, EPI_ISL_3208098, EPI_ISL_3208099, EPI_ISL_3208100, EPI_ISL_3208101, EPI_ISL_3208102, EPI_ISL_3208103, EPI_ISL_3208104, EPI_ISL_3208105, EPI_ISL_3208106, EPI_ISL_3208107, EPI_ISL_3208108, EPI_ISL_3208109, EPI_ISL_3208110, EPI_ISL_3208111, EPI_ISL_3208112, EPI_ISL_3208113, EPI_ISL_3216522, EPI_ISL_3216523, EPI_ISL_3230178, EPI_ISL_3230179, EPI_ISL_3230180, EPI_ISL_3230181, EPI_ISL_3230182, EPI_ISL_3230183, EPI_ISL_3230184, EPI_ISL_3230185, EPI_ISL_3230186, EPI_ISL_3230187, EPI_ISL_3230188, EPI_ISL_3230189, EPI_ISL_3230190, EPI_ISL_3230191, EPI_ISL_3230192, EPI_ISL_3230193, EPI_ISL_3230194, EPI_ISL_3230195, EPI_ISL_3230196, EPI_ISL_3230197, EPI_ISL_3230198, EPI_ISL_3230233, EPI_ISL_3230244, EPI_ISL_3230245, EPI_ISL_3230246, EPI_ISL_3230248, EPI_ISL_3299023, EPI_ISL_3299210, EPI_ISL_3333239, EPI_ISL_3333249, EPI_ISL_3333250, EPI_ISL_3333251, EPI_ISL_3385442, EPI_ISL_3385443, EPI_ISL_3385467, EPI_ISL_3385468, EPI_ISL_3385469, EPI_ISL_3385470, EPI_ISL_3385471, EPI_ISL_3385472, EPI_ISL_3385473, EPI_ISL_3385474, EPI_ISL_3385475, EPI_ISL_3385476, EPI_ISL_3385477, EPI_ISL_3385478, EPI_ISL_3385479, EPI_ISL_3385480, EPI_ISL_3385481, EPI_ISL_3385482, EPI_ISL_3385483, EPI_ISL_3385484, EPI_ISL_3385485, EPI_ISL_3385486, EPI_ISL_3385487, EPI_ISL_3401850, EPI_ISL_3401854, EPI_ISL_3401856, EPI_ISL_3401857, EPI_ISL_3401860, EPI_ISL_3401862, EPI_ISL_3401864, EPI_ISL_3401865, EPI_ISL_3401866, EPI_ISL_3401896, EPI_ISL_3401897, EPI_ISL_3401904, EPI_ISL_3401905, EPI_ISL_3401906, EPI_ISL_3401907, EPI_ISL_3401908, EPI_ISL_3401909, EPI_ISL_3401910, EPI_ISL_3401911, EPI_ISL_3401912, EPI_ISL_3401913, EPI_ISL_3401914, EPI_ISL_3401915, EPI_ISL_3401916, EPI_ISL_3401917, EPI_ISL_3401918, EPI_ISL_3401919, EPI_ISL_3401920, EPI_ISL_3401921, EPI_ISL_3401922, EPI_ISL_3401923, EPI_ISL_3401924, EPI_ISL_3401925, EPI_ISL_3401926, EPI_ISL_3401927, EPI_ISL_3401928, EPI_ISL_3401929, EPI_ISL_3401930, EPI_ISL_3404373, EPI_ISL_3404374, EPI_ISL_3404375, EPI_ISL_3404376, EPI_ISL_3404377, EPI_ISL_3404378, EPI_ISL_3404379, EPI_ISL_3404380, EPI_ISL_3404381, EPI_ISL_3404382, EPI_ISL_3549704, EPI_ISL_3664304, EPI_ISL_3664312, EPI_ISL_3664313, EPI_ISL_3730775, EPI_ISL_3730778, EPI_ISL_3730783, EPI_ISL_3730788, EPI_ISL_3730789, EPI_ISL_3730790, EPI_ISL_3730791, EPI_ISL_3730799, EPI_ISL_3730805, EPI_ISL_3730806, EPI_ISL_3799747, EPI_ISL_3836269, EPI_ISL_3836270, EPI_ISL_3922120, EPI_ISL_3922121, EPI_ISL_3922122, EPI_ISL_3922123, EPI_ISL_3922124, EPI_ISL_3922125, EPI_ISL_3922126, EPI_ISL_3922127, EPI_ISL_3945477, EPI_ISL_3945478, EPI_ISL_3945479, EPI_ISL_3945480, EPI_ISL_3945481, EPI_ISL_3945482, EPI_ISL_3946312, EPI_ISL_3946313, EPI_ISL_3946314, EPI_ISL_3946315, EPI_ISL_4030490, EPI_ISL_4030491, EPI_ISL_4030492, EPI_ISL_4115724, EPI_ISL_4115831, EPI_ISL_4115842, EPI_ISL_4115873, EPI_ISL_4115881, EPI_ISL_4115884, EPI_ISL_4115895, EPI_ISL_4115898, EPI_ISL_4417848, EPI_ISL_4417859, EPI_ISL_4417860, EPI_ISL_4417901, EPI_ISL_4417902, EPI_ISL_4468539, EPI_ISL_4468540, EPI_ISL_4468545, EPI_ISL_4468546, EPI_ISL_4475424, EPI_ISL_4475425, EPI_ISL_4475426, EPI_ISL_4481528, EPI_ISL_4481532, EPI_ISL_4481536, EPI_ISL_4481540, EPI_ISL_4481544, EPI_ISL_4481553, EPI_ISL_4481562, EPI_ISL_4481566, EPI_ISL_4491521, EPI_ISL_4491522, EPI_ISL_4491523, EPI_ISL_4491524, EPI_ISL_4491525, EPI_ISL_4503095, EPI_ISL_4503096, EPI_ISL_4503097, EPI_ISL_4503098, EPI_ISL_4503099, EPI_ISL_4503100, EPI_ISL_4503101, EPI_ISL_4503102, EPI_ISL_4503103, EPI_ISL_4503104, EPI_ISL_4503105, EPI_ISL_4520546, EPI_ISL_4520547, EPI_ISL_4520548, EPI_ISL_4520549, EPI_ISL_4520550, EPI_ISL_4520551, EPI_ISL_4520552, EPI_ISL_4520553, EPI_ISL_4520554, EPI_ISL_4520555, EPI_ISL_4520556, EPI_ISL_4520557, EPI_ISL_4730631, EPI_ISL_4731098, EPI_ISL_4740189, EPI_ISL_4740192, EPI_ISL_4783729, EPI_ISL_4847070, EPI_ISL_5200492, EPI_ISL_5200497, EPI_ISL_5200498, EPI_ISL_5200503			
see above	Genomik Solidaritas Indonesia Laboratorium	Genomik Solidaritas Indonesia Laboratorium	Ahmad Zoebad Foeady; Alfin Mohammad Abdillah; Alfin Muhammad Abdillah; Andani Eka Putra; Andi Utama; Anindya Pradipta Susanto; Anna Christina Brazia; Annisa Muthiah Sukirman; Anuraj Shankar; Ariel Pradipta; Asma Mujahidah; Budi Wiweko; Carissa Sintca Wijaya; David Hendra Haryanto; Dede Rahman Agustian; Dhahlia Agustina Cahyono; Dian Tri Wahyudi; Dwi Oktavia; Esti Rahayuning Tias; Gracia Felias Enos Korompis; Himawan Masyhuri; Laely Indrayanti; Linosefa; Louisa Markus; Lulu Nursyifa; Meutia Ayuputeri Kumaheri; Neng Hilma Hamidah; Ngabila Salama; Normastuti Adhini Tantyo; Pratiwi Srikandi Yudhastari; Pratiwi Sudarmono; Reinhart Gabriel; Ridho Assidicky; Stefanus Bernard; Tiranti Vindha; Tryna Tania; Vania Gavrila Wikasa; Volta Widjaja; Yudistira Aditya Rahmat
EPI_ISL_1159385, EPI_ISL_2284877, EPI_ISL_2284881	Graha Medika Maternity Hospital	Institute of Tropical Disease, Universitas Airlangga	Aldise M Nastri; Diah R Kusumawati; Gatot Soegiarto; Jezzy R Dewantari; Kazufumi Shimizu; Krisnoadi Rahardjo; Laksmi Wulandari; Maria I Lusida; Resti Yudhawati; Rima R Prasetya; Soetjipto; Yasuko Mori
EPI_ISL_1346625, EPI_ISL_1346627	Hasan Sadikin Hospital	West Java Health Laboratory; School of Life Sciences and Technology, Institut Teknologi Bandung	Anggraini Alam; Azzania Fibriani; Basti Andriyoko; Cut Nur Cinthia Alamanda; Djatnika Setiabudi; Ema Rahmawati; Ida Parwati; Isak Solihin; Karimatu Khoirunnisa; Miftahul Faridl; Nina Susana Dewi; Rifky Waluyajati Rachman; Rini Robiani; Ryan Bayusantika Ristandi
EPI_ISL_1969239, EPI_ISL_1969240	Hotel Grand Sahid Jaya	National Institute of Health Research and Development	Arie Ardiansyah Nugraha; Hana Apsari Pawestri; Hartanti Dian Ikawati; Kartika Dewi Puspa; Krisna Pangesti; Nelly Puspandari; Subangkit; Triyani Soekarso; Vivi Setiawaty
EPI_ISL_1969241	Hotel Grand Tjokro	National Institute of Health Research and Development	Arie Ardiansyah Nugraha; Hana Apsari Pawestri; Hartanti Dian Ikawati; Kartika Dewi Puspa; Krisna Pangesti; Nelly Puspandari; Subangkit; Triyani Soekarso; Vivi Setiawaty
EPI_ISL_2233087, EPI_ISL_2233095	Hotel Horison	National Institute of Health Research and Development	Arie Ardiansyah Nugraha; Hana Apsari Pawestri; Hartanti Dian Ikawati; Kartika Dewi Puspa; Krisna Pangesti; Nelly Puspandari; Subangkit; Triyani Soekarso; Vivi Setiawaty
EPI_ISL_529961, EPI_ISL_759956	Husada Utama Hospital	Institute of Tropical Disease, Universitas Airlangga	Aldise M Nastri; Didi Dewanto; Gatot Soegiarto; Jezzy R Dewantari; Kazufumi Shimizu; Krisnoadi Rahardjo; Laksmi Wulandari; Maria I Lusida; Mitsuhiro Nishimura; Resti Yudhawati; Retno A Setyoningrum; Rima R Prasetya; Soetjipto; Yasuko Mori; Yohko K Shimizu
EPI_ISL_2617520, EPI_ISL_2617521	IMC LAB Jakarta Timur	National Institute of Health Research and Development	Arie Ardiansyah Nugraha; Hana Apsari Pawestri; Hartanti Dian Ikawati; Kartika Dewi Puspa; Krisna Pangesti; Nelly Puspandari; Subangkit; Triyani Soekarso; Vivi Setiawaty
EPI_ISL_5200493, EPI_ISL_5200502	INTIBIOS LAB	Genomik Solidaritas Indonesia Laboratorium	Agustine Agnes; Annisa Muthiah Sukirman; Anuraj Shankar; Ariel Pradipta; Carissa Sintca Wijaya; Gracia Felias Enos Korompis; Louisa Markus; Meutia Ayuputeri Kumaheri; Reinhart Gabriel; Stefanus Bernard; Vania Gavrila Wikasa; Yuni Suci Lestari
EPI_ISL_2886158	IVRCRD	Institute for Vector and Reservoir Control, Research and Development (IVRCRD)- National Institute of Health Research and Development (NIHRD), Indonesia	Aprilia Safitri; Arief Mulyono; Arif Suryo Prasetyo; Aryo Ardanto; Ayu Pradipta Pratiwi; Dimas Bagus Wicaksono Putro; Dwi Susilo; Esti Rahardianingtyas; Evi sulistyorini; Hana Apsari Pawestri; Joko Waluyo; Kusumaningtyas Sekar Negari; Lulus Susanti; Marjianto; Mega Tyas Prihatin; Muhidin; Mujiyanto; Nur Hidayati; Restu Choirul Saban; Ristiyanto; Sidiq Setyo Nugroho; Siti Alfiah; Sri Wahyuni Handayani; Subangkit; Tika Fiona Sari; Triwibowo Ambar Garjito; Vivi Setiawaty; Wimbi Kartika Ratnasari; Yusnita Mirna Anggraeni
EPI_ISL_2886166	IVRCRD Puskesmas Cebongan	Institute for Vector and Reservoir Control, Research and Development (IVRCRD)- National Institute of Health Research and Development (NIHRD), Indonesia	Aprilia Safitri; Arief Mulyono; Arif Suryo Prasetyo; Aryo Ardanto; Ayu Pradipta Pratiwi; Dimas Bagus Wicaksono Putro; Dwi Susilo; Esti Rahardianingtyas; Evi sulistyorini; Hana Apsari Pawestri; Joko Waluyo; Kusumaningtyas Sekar Negari; Lulus Susanti; Marjianto; Mega Tyas Prihatin; Muhidin; Mujiyanto; Nur Hidayati; Restu Choirul Saban; Ristiyanto; Sidiq Setyo Nugroho; Siti Alfiah; Sri Wahyuni Handayani; Subangkit; Tika Fiona Sari; Triwibowo Ambar Garjito; Vivi Setiawaty; Wimbi Kartika Ratnasari; Yusnita Mirna Anggraeni
EPI_ISL_2886160, EPI_ISL_2886161	IVRCRD Puskesmas Muntilan 1	Institute for Vector and Reservoir Control, Research and Development (IVRCRD)- National Institute of Health Research and Development (NIHRD), Indonesia	Aprilia Safitri; Arief Mulyono; Arif Suryo Prasetyo; Aryo Ardanto; Ayu Pradipta Pratiwi; Dimas Bagus Wicaksono Putro; Dwi Susilo; Esti Rahardianingtyas; Evi sulistyorini; Hana Apsari Pawestri; Joko Waluyo; Kusumaningtyas Sekar Negari; Lulus Susanti; Marjianto; Mega Tyas Prihatin; Muhidin; Mujiyanto; Nur Hidayati; Restu Choirul Saban; Ristiyanto; Sidiq Setyo Nugroho; Siti Alfiah; Sri Wahyuni Handayani; Subangkit; Tika Fiona Sari; Triwibowo Ambar Garjito; Vivi Setiawaty; Wimbi Kartika Ratnasari; Yusnita Mirna Anggraeni
EPI_ISL_2886167	IVRCRD Puskesmas Sidorejo Lor	Institute for Vector and Reservoir Control, Research and Development (IVRCRD)- National Institute of Health Research and Development (NIHRD), Indonesia	Aprilia Safitri; Arief Mulyono; Arif Suryo Prasetyo; Aryo Ardanto; Ayu Pradipta Pratiwi; Dimas Bagus Wicaksono Putro; Dwi Susilo; Esti Rahardianingtyas; Evi sulistyorini; Hana Apsari Pawestri; Joko Waluyo; Kusumaningtyas Sekar Negari; Lulus Susanti; Marjianto; Mega Tyas Prihatin; Muhidin; Mujiyanto; Nur Hidayati; Restu Choirul Saban; Ristiyanto; Sidiq Setyo Nugroho; Siti Alfiah; Sri Wahyuni Handayani; Subangkit; Tika Fiona Sari; Triwibowo Ambar Garjito; Vivi Setiawaty; Wimbi Kartika Ratnasari; Yusnita Mirna Anggraeni
EPI_ISL_2886164	IVRCRD Puskesmas Welahan 1	Institute for Vector and Reservoir Control, Research and Development (IVRCRD)- National Institute of Health Research and Development (NIHRD), Indonesia	Aprilia Safitri; Arief Mulyono; Arif Suryo Prasetyo; Aryo Ardanto; Ayu Pradipta Pratiwi; Dimas Bagus Wicaksono Putro; Dwi Susilo; Esti Rahardianingtyas; Evi sulistyorini; Hana Apsari Pawestri; Joko Waluyo; Kusumaningtyas Sekar Negari; Lulus Susanti; Marjianto; Mega Tyas Prihatin; Muhidin; Mujiyanto; Nur Hidayati; Restu Choirul Saban; Ristiyanto; Sidiq Setyo Nugroho; Siti Alfiah; Sri Wahyuni Handayani; Subangkit; Tika Fiona Sari; Triwibowo Ambar Garjito; Vivi Setiawaty; Wimbi Kartika Ratnasari; Yusnita Mirna Anggraeni
EPI_ISL_2886157, EPI_ISL_2886159	IVRCRD RSPAW	Institute for Vector and Reservoir Control, Research and Development (IVRCRD)- National Institute of Health Research and Development (NIHRD), Indonesia	Aprilia Safitri; Arief Mulyono; Arif Suryo Prasetyo; Aryo Ardanto; Ayu Pradipta Pratiwi; Dimas Bagus Wicaksono Putro; Dwi Susilo; Esti Rahardianingtyas; Evi sulistyorini; Hana Apsari Pawestri; Joko Waluyo; Kusumaningtyas Sekar Negari; Lulus Susanti; Marjianto; Mega Tyas Prihatin; Muhidin; Mujiyanto; Nur Hidayati; Restu Choirul Saban; Ristiyanto; Sidiq Setyo Nugroho; Siti Alfiah; Sri Wahyuni Handayani; Subangkit; Tika Fiona Sari; Triwibowo Ambar Garjito; Vivi Setiawaty; Wimbi Kartika Ratnasari; Yusnita Mirna Anggraeni
EPI_ISL_2886162, EPI_ISL_2886163, EPI_ISL_2886165	IVRCRD RSUD Budi Rahayu	Institute for Vector and Reservoir Control, Research and Development (IVRCRD)- National Institute of Health Research and Development (NIHRD), Indonesia	Aprilia Safitri; Arief Mulyono; Arif Suryo Prasetyo; Aryo Ardanto; Ayu Pradipta Pratiwi; Dimas Bagus Wicaksono Putro; Dwi Susilo; Esti Rahardianingtyas; Evi sulistyorini; Hana Apsari Pawestri; Joko Waluyo; Kusumaningtyas Sekar Negari; Lulus Susanti; Marjianto; Mega Tyas Prihatin; Muhidin; Mujiyanto; Nur Hidayati; Restu Choirul Saban; Ristiyanto; Sidiq Setyo Nugroho; Siti Alfiah; Sri Wahyuni Handayani; Subangkit; Tika Fiona Sari; Triwibowo Ambar Garjito; Vivi Setiawaty; Wimbi Kartika Ratnasari; Yusnita Mirna Anggraeni
EPI_ISL_528746	Immanuel Hospital	Molecular Genetics Laboratory-Faculty of Medicine-Universitas Padjadjaran; School of Life Sciences and Technology & School of Pharmacy-Institut Teknologi Bandung; Laboratorium Kesehatan Provinsi Jawa Barat	Agung Eru Wibowo; Azzania Fibriani; Catur Riani; Cut Nur Cinthia Alamanda; Ema Rahmawati; Fensi Amalina; Hammam Riza; Hesti Lina Wiraswati; Husna Nugrahapraja; Irvan Faizal; Lia Faridah; Marselina Irasonia Tan; Mas Rizky A.A. Syamsunarno; Rifky Waluyajati Rachman; Ryan Bayusantika Ristandi; Savira Ekawardhani; Sony Solistia Wirawan; Tarwadi; Tri Hanggono Achmad; Yunia Sribudiani
EPI_ISL_1660474	Immanuel Hospital	West Java Health Laboratory; School of Life Sciences and Technology, Institut Teknologi Bandung	Aulia Saraswati Wicaksono; Azzania Fibriani; Cut Nur Cinthia Alamanda; Ema Rahmawati; Karimatu Khoirunnisa; Miftahul Faridl; Rifky Waluyajati Rachman; Rini Robiani; Ryan Bayusantika Ristandi
EPI_ISL_1855390	Indonesian Research Center for Veterinary Science/BBalitvet	Indonesian Research Center for Veterinary Science/BBalitvet	Diana Nurjanah; Fadjry Djufry; Harimurti Nuradji; Ilham Chaidir; Ni Luh Putu Indi Dharmayanti; Risa Indriani; Sri Nowo Retno
EPI_ISL_1366503, EPI_ISL_1366505, EPI_ISL_1366509	Indrapura Field Hospital (part of Dr. Soetomo Teaching Hospital)	Research Center for Vaccine Technology and Development, Institute of Tropical Disease, Airlangga University	Andi Yasmin Wijaya; Aryati; Christijogo Soemartono Waloejo; Cita Rosita Sigit Prakoeswa; Damayanti Tinduh; Dominicus Husada; Dwiyanti Puspitasari; Fedik Abdul Rantam; Helen Susilowati; Jusak Nugraha; Neneng Dewi Kurniati; Ni Nyoman Tri Puspaningsih
EPI_ISL_2570805, EPI_ISL_2570806, EPI_ISL_4056005, EPI_ISL_4056006, EPI_ISL_4056007, EPI_ISL_4056008, EPI_ISL_4742352			
see above	Indrapura Field Hospital Surabaya	Institute of Tropical Disease, Universitas Airlangga	Aldise M Nastri; Febria Rachmanita; Gatot Soegiarto; Herlin Ferliana; Jezzy R Dewantari; Kazufumi Shimizu; Kohar Hari Santoso; Krisnoadi Rahardjo; Laksmi Wulandari; Maria I Lusida; Resti Yudhawati; Rima R Prasetya; Soetjipto; Yasuko Mori
EPI_ISL_2886168	Institute for Vector and Reservoir Control, Research and Development (IVRCRD)- National Institute of Health Research and Development (NIHRD), Indonesia	Institute for Vector and Reservoir Control, Research and Development (IVRCRD)- National Institute of Health Research and Development (NIHRD), Indonesia	Aprilia Safitri; Arief Mulyono; Arif Suryo Prasetyo; Aryo Ardanto; Ayu Pradipta Pratiwi; Dimas Bagus Wicaksono Putro; Dwi Susilo; Esti Rahardianingtyas; Evi sulistyorini; Hana Apsari Pawestri; Joko Waluyo; Kusumaningtyas Sekar Negari; Lulus Susanti; Marjianto; Mega Tyas Prihatin; Muhidin; Mujiyanto; Nur Hidayati; Restu Choirul Saban; Ristiyanto; Sidiq Setyo Nugroho; Siti Alfiah; Sri Wahyuni Handayani; Subangkit; Tika Fiona Sari; Triwibowo Ambar Garjito; Vivi Setiawaty; Wimbi Kartika Ratnasari; Yusnita Mirna Anggraeni
EPI_ISL_877421, EPI_ISL_882769, EPI_ISL_956280, EPI_ISL_956312, EPI_ISL_956316, EPI_ISL_1159384, EPI_ISL_1165072, EPI_ISL_1165074			
see above	Institute of Tropical Disease	Institute of Tropical Disease, Universitas Airlangga	Aldise M Nastri; Gatot Soegiarto; Jezzy R Dewantari; Kazufumi Shimizu; Krisnoadi Rahardjo; Laksmi Wulandari; Maria I Lusida; Resti Yudhawati; Rima R Prasetya; Soetjipto; Yasuko Mori
EPI_ISL_1789118, EPI_ISL_1789119, EPI_ISL_1789172, EPI_ISL_1795058, EPI_ISL_1795060, EPI_ISL_2284876, EPI_ISL_2284878, EPI_ISL_2284880, EPI_ISL_4056016, EPI_ISL_4056017			
see above	Institute of Tropical Disease, Universitas Airlangga	Institute of Tropical Disease, Universitas Airlangga	Aldise M Nastri; Gatot Soegiarto; Jezzy R Dewantari; Kazufumi Shimizu; Kohar Hari Santoso; Krisnoadi Rahardjo; Laksmi Wulandari; Maria I Lusida; Mitsuhiro Nishimura; Resti Yudhawati; Retno A Setyoningrum; Rima R Prasetya; Soetjipto; Yasuko Mori; Yohko K Shimizu
EPI_ISL_2631487, EPI_ISL_2631488, EPI_ISL_2631489, EPI_ISL_2631490, EPI_ISL_2631491, EPI_ISL_2631492, EPI_ISL_2631493, EPI_ISL_2631494, EPI_ISL_2631495, EPI_ISL_2631496, EPI_ISL_2631497, EPI_ISL_2722318, EPI_ISL_2722320, EPI_ISL_2722322, EPI_ISL_2722324, EPI_ISL_2722326, EPI_ISL_2722328, EPI_ISL_2722329, EPI_ISL_2722331, EPI_ISL_2722333, EPI_ISL_2722335, EPI_ISL_2722337, EPI_ISL_2722339, EPI_ISL_2722341, EPI_ISL_2722342, EPI_ISL_2722344, EPI_ISL_2722346			
see above	Intibios Karawang	Indonesian Institute of Sciences (LIPI)/National Agency of Research and Innovation (BRIN) and National Institute of Health Research and Development (NIHRD)	Ade Andriani; Alfi T. Fathurahman; Andri Wardiana; Anggia Prasetyoputri; Anik B. Dharmayanthi; Anik. B. Dharmayanthi; Asep M. Ridwanulloh; Erwin F. Hasrianda; Erwin Fajar Hasrianda; Gita Syahputra; Hariyatun; Idris; Indriawati; Isa Nuryana; Masrukhin; Puspita Lisdiyanti; Ratih A. Ningrum; Ruby Setiawan; Subangkit; Sugiyono Saputra; Syaiful Rizal; Syam B. Iryanto; Vivi Setiawaty; Wien Kusharyoto; Yayuk Sri Rahayu
EPI_ISL_4106135	Intibios Karawang	National Research and Innovation Agency (BRIN-Indonesia, VenomCoV Project Team)	Anggia Prasetyoputri; Erwin Fajar Hasrianda; Gita Syahputra; Idris; Indriawati; Ratih A. Ningrum; Subangkit; Sugiyono Saputra; Syaiful Rizal; Syam B. Iryanto; Vivi Setiawaty; Wien Kusharyoto; Yayuk Sri Rahayu
EPI_ISL_3946305	Intibios Lab & Klinik Ciputat	Genomik Solidaritas Indonesia Laboratorium	Annisa Muthiah Sukirman; Anuraj Shankar; Ariel Pradipta; Carissa Sintca Wijaya; Dhahlia Agustina Cahyono; Dwi Oktavia; Gracia Felias Enos Korompis; Himawan Masyhuri; Louisa Markus; Meutia Ayuputeri Kumaheri; Mouleidi Dwi Putri; Neng Hilma Hamidah; Ngabila Salama; Pratiwi Srikandi Yudhastari; Tiranti Vindha; Umar Soh
EPI_ISL_4475421, EPI_ISL_4847072	Intibios Lab Mangga Besar	Genomik Solidaritas Indonesia Laboratorium	Agustin Agnes; Annisa Muthiah Sukirman; Anuraj Shankar; Ariel Pradipta; Esti Rahayuning Tias; Gracia Felias Enos Korompis; Louisa Markus; Meutia Ayuputeri Kumaheri; Reinhart Gabriel; Stefanus Bernard; Vania Gavrila Wikasa; Yuni Suci Lestari
EPI_ISL_4475422,	Intibioslab	Genomik Solidaritas Indonesia Laboratorium	Agustine Agnes; Anna Christina Brazia; Annisa Muthiah Sukirman; Anuraj Shankar; Ariel Pradipta; Dian Tri Wahyudi; Esti Rahayuning Tias; Gracia Felias Enos Korompis; Louisa Markus; Meutia Ayuputeri Kumaheri; Mouleidi Dwi

EPI_ISL_4503094			Putri; Reinhart Gabriel; Stefanus Bernard; Umar Soh; Vania Gavrila Wikasa; Yuni Suci Lestari
EPI_ISL_956311	Islamic Hospital	Institute of Tropical Disease, Universitas Airlangga	Aldise M Nastri; Dodo Anondo; Gatot Soegiarto; Jezzy R Dewantari; Kazufumi Shimizu; Krisnoadi Rahardjo; Laksmi Wulandari; Maria I Lusida; Resti Yudhawati; Rima R Prasetya; Soetjipto; Yasuko Mori
EPI_ISL_3730781, EPI_ISL_3730784, EPI_ISL_3730792, EPI_ISL_3730793, EPI_ISL_3730794, EPI_ISL_3730795, EPI_ISL_3730796, EPI_ISL_3730800, EPI_ISL_3730801, EPI_ISL_3730803, EPI_ISL_3730804, EPI_ISL_3730807, EPI_ISL_3799748, EPI_ISL_3922114, EPI_ISL_3922115, EPI_ISL_3922116, EPI_ISL_3922117, EPI_ISL_3922118, EPI_ISL_3922119, EPI_ISL_4030482, EPI_ISL_4030483, EPI_ISL_4030484, EPI_ISL_4030485, EPI_ISL_4468050, EPI_ISL_4475423, EPI_ISL_4520545, EPI_ISL_5031091, EPI_ISL_5031092, EPI_ISL_5031093, EPI_ISL_5031094, EPI_ISL_5462141			
see above	KALGEN INNOLAB	Genomik Solidaritas Indonesia Laboratorium	Andi Utama; Anna Christina Brazia; Annisa Muthiah Sukirman; Anuraj Shankar; Ariel Pradipta; Arih Daimah; Asma Mujahidah; Carissa Sintca Wijaya; David Hendra Haryanto; Dhahlia Agustina Cahyono; Dian Tri Wahyudi; Dwi Oktavia; Esti Rahayuning Tias; Gracia Felias Enos Korompis; Himawan Masyhuri; Jedidiah Abel Mehola; Louisa Markus; Meutia Ayuputeri Kumaheri; Neng Hilma Hamidah; Ngabila Salama; Reinhart Gabriel; Ridho Assidicky; Stefanus Bernard; Tiranti Vindha; Vania Gavrila Wikasa; Vania Gavrila Wikasa Dhahlia Agustina Cahyono
EPI_ISL_3208078, EPI_ISL_3208079, EPI_ISL_3208081	KIMIA FARMA / DINAS KESEHATAN DKI JAKARTA	Genomik Solidaritas Indonesia Laboratorium	Annisa Muthiah Sukirman; Anuraj Shankar; Ariel Pradipta; Carissa Sintca Wijaya; Gracia Felias Enos Korompis; Latifah Anandari; Meutia Ayuputeri Kumaheri; Reinhart Gabriel; Vania Gavrila Wikasa
EPI_ISL_2854779	KKP Kelas II Cilacap	National Institute of Health Research and Development	Arie Ardiansyah Nugraha; Hana Apsari Pawestri; Hartanti Dian Ikawati; Kartika Dewi Puspa; Krisna Pangesti; Nelly Puspandari; Subangkit; Triyani Soekarso; Vivi Setiawaty
EPI_ISL_1257859	KKP Soekarno Hatta	National Institute of Health Research and Development	Arie Ardiansyah Nugraha; Hana Apsari Pawestri; Hartanti Dian Ikawati; Kartika Dewi Puspa; Krisna Pangesti; Nelly Puspandari; Subangkit; Vivi Setiawaty
EPI_ISL_2932610	KOTA Yogya DKK Gondokusuman II	Genetics Working Group (Pokja Genetik) Faculty of Medicine, Public Health and Nursing Universitas Gadjah Mada (FK-KMK UGM); Disease Investigation Center Wates Ministry of Agriculture Indonesia; Department of Microbiology FK-KMK UGM; Laboratorium Diagnostik Yayasan Tahija World Mosquito Program (WMP) Yogyakarta Center for	Afiahayati; Alvina A Setiawan; Dwi AA Nugrahaningsih; Dwi Indaryati; Edwin W. Daniwijaya; Eggi Arguni; Endah Supriyati; Gunadi; Hendra Wibawa; Indarto Sulistiyono; Khanza a Vujira; Kristy Iskandar; Ludhang P. Rizki; Marcellus; Mohamad S. Hakim; Nungki Anggorowati; Pramesti G Dewi; Siswanto; Titik Nuryastuti; Tri Wibawa
EPI_ISL_4885262, EPI_ISL_4885263, EPI_ISL_4951281	Kabupaten Karawang Lung Speciality Hospital	West Java Health Laboratory; School of Life Sciences and Technology, Institut Teknologi Bandung	Azzania Fibriani; Cut Nur Cinthia Alamanda; Ema Rahmawati; Isak Solihin; Karimatu Khoirunnisa; Miftahul Faridl; Rifky Waluyajati Rachman; Rini Robiani; Ryan Bayusantika Ristandi
EPI_ISL_3691649	Kantor Kesehatan Pelabuhan Bangka Belitung	National Institute of Health Research and Development	Arie Ardiansyah Nugraha; Fajar Nur Sulistiyohadi; Hana Apsari Pawestri; Hartanti Dian Ikawati; Kartika Dewi Puspa; Krisna Pangesti; Nelly Puspandari; Subangkit; Triyani Soekarso; Vivi Setiawaty
EPI_ISL_4730569, EPI_ISL_4730570, EPI_ISL_4730571, EPI_ISL_4730572, EPI_ISL_5022954, EPI_ISL_5107815, EPI_ISL_5107816, EPI_ISL_5107817, EPI_ISL_5107818			
see above	Kantor Kesehatan Pelabuhan Kelas 2 Cilacap	National Institute of Health Research and Development	Arie Ardiansyah Nugraha; Fajar Nur; Fajar Nur Sulistiyohadi; Hana Apsari Pawestri; Hartanti Dian Ikawati; Kartika Dewi Puspa; Krisna Pangesti; Nelly Puspandari; Subangkit; Sulistiyohadi; Triyani Soekarso; Vivi Setiawaty
EPI_ISL_2854781, EPI_ISL_2854782, EPI_ISL_2854783, EPI_ISL_2854784, EPI_ISL_2854785, EPI_ISL_2854786, EPI_ISL_2854787, EPI_ISL_2854788, EPI_ISL_2854789, EPI_ISL_2854790, EPI_ISL_2854791, EPI_ISL_2854792, EPI_ISL_2854793, EPI_ISL_2854794, EPI_ISL_2854795, EPI_ISL_2854796, EPI_ISL_2896199, EPI_ISL_2896201			
see above	Kantor Kesehatan Pelabuhan Kelas I Medan	National Institute of Health Research and Development	Arie Ardiansyah Nugraha; Hana Apsari Pawestri; Hartanti Dian Ikawati; Kartika Dewi Puspa; Krisna Pangesti; Nelly Puspandari; Subangkit; Triyani Soekarso; Vivi Setiawaty
EPI_ISL_2233091, EPI_ISL_2233092, EPI_ISL_2233093, EPI_ISL_2233096, EPI_ISL_2233097, EPI_ISL_2233098, EPI_ISL_2233099, EPI_ISL_2233100, EPI_ISL_2233101, EPI_ISL_2233102, EPI_ISL_2233103, EPI_ISL_2233104, EPI_ISL_2233105			
see above	Kantor Kesehatan Pelabuhan Kelas II Cilacap	National Institute of Health Research and Development	Arie Ardiansyah Nugraha; Hana Apsari Pawestri; Hartanti Dian Ikawati; Kartika Dewi Puspa; Krisna Pangesti; Nelly Puspandari; Subangkit; Triyani Soekarso; Vivi Setiawaty
EPI_ISL_3138838	Kantor Kesehatan Pelabuhan Kelas II Tarakan	National Institute of Health Research and Development	Arie Ardiansyah Nugraha; Fajar Nur Sulistiyohadi; Hana Apsari Pawestri; Hartanti Dian Ikawati; Kartika Dewi Puspa; Krisna Pangesti; Nelly Puspandari; Subangkit; Triyani Soekarso; Vivi Setiawaty
EPI_ISL_2854757, EPI_ISL_2854758, EPI_ISL_2854759	Kantor Kesehatan Pelabuhan Kelas III Bengkulu	National Institute of Health Research and Development	Arie Ardiansyah Nugraha; Hana Apsari Pawestri; Hartanti Dian Ikawati; Kartika Dewi Puspa; Krisna Pangesti; Nelly Puspandari; Subangkit; Triyani Soekarso; Vivi Setiawaty
EPI_ISL_1915564, EPI_ISL_1915565, EPI_ISL_2047555, EPI_ISL_2047556, EPI_ISL_2047557, EPI_ISL_2047558, EPI_ISL_2047559			
see above	Kantor Pengelola Sumbawa Technopark	National Institute of Health Research and Development	Arie Ardiansyah Nugraha; Arief Budi Witarto; Hana Apsari Pawestri; Hartanti Dian Ikawati; Kartika Dewi Puspa; Krisna Pangesti; Laily Indrayanti; Lalu Hamzi Fikri; Nelly Puspandari; Nurhandini Eka Dewi; Rian Adha Ardinata; Subangkit; Triyani Soekarso; Vivi Setiawaty
EPI_ISL_833498	Kartika Pulomas Hospital, Jakarta, Indonesia	Biosafety Level-3 Laboratory, Indonesian Institute of Sciences (LIPI)	Ade Andriani; Ahmad Fathoni; Andri Wardiana; Anggia Prasetyoputri; Anik Budhi Dharmayanthi; Isa Nuryana; Pinka Alisa Diena Shafa; Ratih Asmana Ningrum; Syam Budi Iryanto; Winda Tasia
EPI_ISL_1398357	Kasih Bunda Hospital	West Java Health Laboratory; School of Life Sciences and Technology, Institut Teknologi Bandung	Azzania Fibriani; Cut Nur Cinthia Alamanda; Ema Rahmawati; Isak Solihin; Kamila Tania; Karimatu Khoirunnisa; Miftahul Faridl; Rifky Waluyajati Rachman; Rini Robiani; Ryan Bayusantika Ristandi
EPI_ISL_518820, EPI_ISL_833388, EPI_ISL_833389, EPI_ISL_833390, EPI_ISL_833391, EPI_ISL_833495, EPI_ISL_833497, EPI_ISL_833500, EPI_ISL_833508			
see above	Klinik Apotek Dein, Jakarta, Indonesia	Biosafety Level-3 Laboratory, Indonesian Institute of Sciences (LIPI)	Ade Andriani; Ahmad Fathoni; Akhirta Atikana; Alfi Taufik Fathurahman; Andri Wardiana; Anggia Prasetyoputri; Anik Budhi Dharmayanthi; Ario Betha Juanssilfero; Asep M Ridwanuloh; Asrul M. Fuad; Dadang Supriatna; Dian Fitria Agustiyanti; Fahrurrozy; Hariyatun; Herman Irawan; Isa Nuryana; Pamella Apriliana; Popi Hadi Wisnuwardhani; Puspita Lisdiyanti; Ratih Asmana Ningrum; Rifki Sadikin; Saiful anwar; Sri Swasthikawati; Syam Budi Iryanto; Wien Kusharyoto
EPI_ISL_1020203, EPI_ISL_1117457, EPI_ISL_2840889	Klinik Apotik Dien, Jakarta	Biosafety Level-3 Laboratory, Indonesian Institute of Sciences (LIPI)	Ade Andriani; Anik Budhi Dharmayanthi; Asep M. Ridwanulloh; Asep M. Ridwanuloh; Eko Wahyu Putro; Eko Wahyu Putro; Gita Syahputra; Hariyatun; Inswasti Cahyani; Listiana Oktavia; Matthew W. Loose; Mohammad Ilyas; Sugiyono Saputra; Susanti; Syam Budi Iryanto; Wien Kusharyoto; Yuliawati
EPI_ISL_1284204	Klinik Ciputra Medical Center	Eijkman Institute for Molecular Biology, Ministry of Research and Technology/National Agency for Research and Innovation	Amin Soebandrio; Edison Johar; Frilasita A Yudhaputri; Hidayat Trimarsanto; Iskandar Adnan; Khin Saw Myint; Lydia V. Panggalo; Safarina G Malik; Sukma Oktavianthi; Willy Agustine
EPI_ISL_2860294, EPI_ISL_2860295, EPI_ISL_2860347	Klinik Dr Nurdin	Biosafety Level-3 Laboratory, Indonesian Institute of Sciences (LIPI)	Andri Wardiana; Anggia Prasetyoputri; Anik B. Dharmayanthi; Asep M. Ridwanulloh; Asep M.Ridwanulloh; Fahrurrozi; Indriawati; Linda Sukmarini; Listiana Oktavia; Nova D. Yanthi; Ruby Setiawan; Syam B. Iryanto; Yuliawati
EPI_ISL_2854713, EPI_ISL_2854714, EPI_ISL_2854715, EPI_ISL_2854716, EPI_ISL_2854717, EPI_ISL_2854718	Klinik Kimia Farma Garuda	National Institute of Health Research and Development	; Arie Ardiansyah Nugraha; Hana Apsari Pawestri; Hartanti Dian Ikawati; Kartika Dewi Puspa; Krisna Pangesti; Nelly Puspandari; Subangkit; Triyani Soekarso; Vivi Setiawaty
EPI_ISL_2614679	Klinik Lamina	Eijkman Institute for Molecular Biology, National Research and Innovation Agency	Amin Soebandrio; Edison Johar; Frilasita A Yudhaputri; Hidayat Trimarsanto; Iskandar Adnan; Khin Saw Myint; Lidwina Priliani; Lydia V. Panggalo; Muhammad Rezki Rasyak; Safarina G Malik; Sukma Oktavianthi; Willy Agustine
EPI_ISL_2860281, EPI_ISL_2860282, EPI_ISL_2860283, EPI_ISL_2860284, EPI_ISL_2860285, EPI_ISL_2860286, EPI_ISL_2860287, EPI_ISL_2860288, EPI_ISL_2860289, EPI_ISL_2860348, EPI_ISL_2860349, EPI_ISL_2860368			
see above	Klinik Primera Clinica	Biosafety Level-3 Laboratory, Indonesian Institute of Sciences (LIPI)	Andri Wardiana; Anggia Prasetyoputri; Anik B. Dharmayanthi; Annisa D. Septiani; Asep M. Ridwanulloh; Asep M.Ridwanulloh; Fahrurrozi; Idris; Indriawati; Linda Sukmarini; Listiana Oktavia; Lusiana; Masrukhin; Maya Komalasari; Nova D. Yanthi; Ratih A. Ningrum; Ruby Setiawan; Sri Swasthikawati; Sugiyono Saputra; Syam B. Iryanto; Yuliawati
EPI_ISL_4004697, EPI_ISL_4004704	Klinik Primera Clinica	National Research and Innovation Agency (BRIN-Indonesia)	Ahmad Fathoni; Anggia Prasetyoputri; Anik B. Dharmayanthi; Asep M. Ridwanulloh; Hariyatun; Indriawati; Rifki Sadikin; Sugiyono Saputra; Suvia Widyaningrum; Syam B. Iryanto
EPI_ISL_2844826	Klinik Primera Clinica, Jakarta	Biosafety Level-3 Laboratory, Indonesian Institute of Sciences (LIPI)	Anggia Prasetyoputri; Erwin F. Hasrianda; Fahrurrozi; Herjuno A. Nugroho; Nova D. Yanthi; Wien Kusharyoto
EPI_ISL_4106128	Klinik Primera Clinica, Jakarta	National Research and Innovation Agency (BRIN-Indonesia, VenomCoV Project Team)	Andri Wardiana; Anggia Prasetyoputri; Erwin F. Hasrianda; Fahrurrozi; Herjuno A. Nugroho; Nova D. Yanthi; Sugiyono Saputra; Syam B. Iryanto
EPI_ISL_1789112	Klinik Tanjung Laboratory	Institute of Tropical Disease, Universitas Airlangga	Aldise M Nastri; Gatot Soegiarto; Jezzy R Dewantari; Kazufumi Shimizu; Krisnoadi Rahardjo; Laksmi Wulandari; Maria I Lusida; Resti Yudhawati; Rima R Prasetya; Soetjipto; Yasuko Mori
EPI_ISL_1072962	Kota Nopan Primary Health Centre, Mandailing Natal District	Institute of Tropical Disease, Universitas Airlangga; Faculty of Medicine, Universitas Sumatera Utara	Aldise M Nastri; Franciscus Ginting; Inke N D Lubis; Irbah R Nainggolan; Jezzy R Dewantari; Kazufumi Shimizu; Krisnoadi Rahardjo; Maria I Lusida; Meliani; Mirzan Hasibuan; Muhammad Ichwan; R Andika D Cahyadi; R Lia Kusumawati; Ramadhan Bestari; Rima R Prasetya; Soetjipto; Yasuko Mori
EPI_ISL_1398353, EPI_ISL_1398369, EPI_ISL_1615617	Kuningan Public Health	West Java Health Laboratory; School of Life Sciences and Technology, Institut Teknologi Bandung	Aulia Saraswati Wicaksono; Azzania Fibriani; Cut Nur Cinthia Alamanda; Ema Rahmawati; Isak Solihin; Kamila Tania; Karimatu Khoirunnisa; Miftahul Faridl; Rifky Waluyajati Rachman; Rini Robiani; Ryan Bayusantika Ristandi
EPI_ISL_747239	Kuningan Publlic Health Office	West Java Health Laboratory; School of Life Sciences and Technology, Institut Teknologi Bandung	Azzania Fibriani; Cut Nur Cinthia Alamanda; Ema Rahmawati; Isak Solihin; Karimatu Khoirunnisa; Miftahul Faridl; Rifky Waluyajati Rachman; Rini Robiani; Ryan Bayusantika Ristandi
EPI_ISL_4090576	LAB Bina Husada Mitra Keluarga	National Institute of Health Research and Development	Arie Ardiansyah Nugraha; Fajar Nur Sulistiyohadi; Hana Apsari Pawestri; Hartanti Dian Ikawati; Kartika Dewi Puspa; Krisna Pangesti; Nelly Puspandari; Subangkit; Triyani Soekarso; Vivi Setiawaty
EPI_ISL_4508301, EPI_ISL_4508302, EPI_ISL_4508303, EPI_ISL_4508304, EPI_ISL_4508305, EPI_ISL_4508306, EPI_ISL_4508307, EPI_ISL_4508308, EPI_ISL_4508309, EPI_ISL_4508310, EPI_ISL_4508311, EPI_ISL_4508312, EPI_ISL_4508313, EPI_ISL_4508314, EPI_ISL_4508315			
see above	LAB Hamera Sarana Indonesia	National Institute of Health Research and Development	Arie Ardiansyah Nugraha; Fajar Nur Sulistiyohadi; Hana Apsari Pawestri; Hartanti Dian Ikawati; Kartika Dewi Puspa; Krisna Pangesti; Nelly Puspandari; Subangkit; Triyani Soekarso; Vivi Setiawaty
EPI_ISL_2790716, EPI_ISL_2790717, EPI_ISL_2790718, EPI_ISL_2790719, EPI_ISL_2790720, EPI_ISL_3418685	LAB MIKROBIOLOGI FKUI / DINAS KESEHATAN DKI JAKARTA	Genomik Solidaritas Indonesia Laboratorium	Alfin Mohammad Abdillah; Anna Christina Brazia; Annisa Muthiah Sukirman; Anuraj Shankar; Ariel Pradipta; Carissa Sintca Wijaya; Dhahlia Agustina Cahyono; Dwi Oktavia; Fera Ibrahim; Gracia Felias Enos Korompis; Meutia Ayuputeri Kumaheri; Ngabila Salama; Normastuti Adhini Tantyo; Pratiwi Sudarmono; Reinhart Gabriel; Tiranti Vindha; Vania Gavrila Wikasa; Widyastuti; Yulia Rosa
EPI_ISL_2617496, EPI_ISL_2617497, EPI_ISL_2617498	LAB Mitra Keluarga Pratama	National Institute of Health Research and Development	Arie Ardiansyah Nugraha; Hana Apsari Pawestri; Hartanti Dian Ikawati; Kartika Dewi Puspa; Krisna Pangesti; Nelly Puspandari; Subangkit; Triyani Soekarso; Vivi Setiawaty
EPI_ISL_2617500	LAB RS Hermina Depok	National Institute of Health Research and Development	Arie Ardiansyah Nugraha; Hana Apsari Pawestri; Hartanti Dian Ikawati; Kartika Dewi Puspa; Krisna Pangesti; Nelly Puspandari; Subangkit; Triyani Soekarso; Vivi Setiawaty
EPI_ISL_3138787,	LAB RS Kartika Pulomas	National Institute of Health Research and Development	Arie Ardiansyah Nugraha; Fajar Nur Sulistiyohadi; Hana Apsari Pawestri; Hartanti Dian Ikawati; Kartika Dewi Puspa; Krisna Pangesti; Nelly Puspandari; Subangkit; Triyani Soekarso; Vivi Setiawaty

EPI_ISL_3138788			
EPI_ISL_2854729	LAB RS Mitra Kemayoran	National Institute of Health Research and Development	; Arie Ardiansyah Nugraha; Hana Apsari Pawestri; Hartanti Dian Ikawati; Kartika Dewi Puspa; Krisna Pangesti; Nelly Puspandari; Subangkit; Triyani Soekarso; Vivi Setiawaty
EPI_ISL_2987599	LAB RSUD Koja Jakut	National Institute of Health Research and Development	Arie Ardiansyah Nugraha; Fajar Nur Sulistiyohadi; Hana Apsari Pawestri; Hartanti Dian Ikawati; Kartika Dewi Puspa; Krisna Pangesti; Nelly Puspandari; Subangkit; Triyani Soekarso; Vivi Setiawaty
EPI_ISL_3208075, EPI_ISL_3208076	LABKESDA / DINAS KESEHATAN DKI JAKARTA	Genomik Solidaritas Indonesia Laboratorium	Annisa Muthiah Sukirman; Anuraj Shankar; Ariel Pradipta; Carissa Sintca Wijaya; Gracia Felias Enos Korompis; Meutia Ayuputeri Kumaheri; Reinhart Gabriel; Tryna Tania; Vania Gavrila Wikasa
EPI_ISL_2723620, EPI_ISL_2723621, EPI_ISL_2723622, EPI_ISL_2723623, EPI_ISL_2723624, EPI_ISL_2723625, EPI_ISL_2723626, EPI_ISL_2723627, EPI_ISL_2723628, EPI_ISL_2723629, EPI_ISL_2723630, EPI_ISL_2723631, EPI_ISL_2723632, EPI_ISL_2723633, EPI_ISL_2723634, EPI_ISL_2723635, EPI_ISL_2723636, EPI_ISL_2723637, EPI_ISL_2723638, EPI_ISL_2723639, EPI_ISL_2723640, EPI_ISL_2723641, EPI_ISL_2723642, EPI_ISL_2723643, EPI_ISL_2723644, EPI_ISL_2723645, EPI_ISL_2723646, EPI_ISL_2723647, EPI_ISL_2723648, EPI_ISL_3374705			
see above	LABKESDA DKI JAKARTA	Genomik Solidaritas Indonesia Laboratorium	Alfin Mohammad Abdillah; Annisa Muthiah Sukirman; Anuraj Shankar; Ariel Pradipta; Carissa Sintca Wijaya; Dhahlia Agustina Cahyono; Dwi Oktavia; Gracia Felias Enos Korompis; Himawan Masyhuri; Louisa Markus; Meutia Ayuputeri Kumaheri; Mohammad Anang Syarifudin; Ngabila Salama; Normastuti Adhini Tantyo; Tiranti Vindha; Vania Gavrila Wikasa; Widyastuti
EPI_ISL_3836261	LABORATOIUM KLINIK MEDILAB	Genomik Solidaritas Indonesia Laboratorium	Anna Christina Brazia; Annisa Muthiah Sukirman; Anuraj Shankar; Ariel Pradipta; David Hendra Haryanto; Dhahlia Agustina Cahyono; Dwi Oktavia; Dwi Setiyani Wulandari; Fitri Winita; Gracia Felias Enos Korompis; Louisa Markus; Meutia Ayuputeri Kumaheri; Ngabila Salama; Pratiwi Srikandi Yudhastari; Reinhart Gabriel; Tiranti Vindha; Vania Gavrila Wikasa; Willy Setiawan
EPI_ISL_4783441, EPI_ISL_4783452, EPI_ISL_4783459, EPI_ISL_4783489, EPI_ISL_4783513, EPI_ISL_4783531, EPI_ISL_4783536, EPI_ISL_4783549, EPI_ISL_4783552, EPI_ISL_4783563, EPI_ISL_4783570, EPI_ISL_4783580, EPI_ISL_4783589, EPI_ISL_4783597, EPI_ISL_4783601, EPI_ISL_4783619, EPI_ISL_4783629, EPI_ISL_4783643, EPI_ISL_4783676, EPI_ISL_4783688, EPI_ISL_4783703, EPI_ISL_4849718			
see above	LABORATORIUM BIOMEDIK LONTAR FK UNRI	Genomik Solidaritas Indonesia Laboratorium	Annisa Muthiah Sukirman; Anuraj Shankar; Arfianti; Ariel Pradipta; Carissa Sintca Wijaya; Esti Rahayuning Tias; Gracia Felias Enos Korompis; Himawan Masyhuri; Louisa Markus; Maya Savira; Meutia Ayuputeri Kumaheri; Rahmat Azhari Kemal; Stefanus Bernard
EPI_ISL_3836260, EPI_ISL_3836262	LABORATORIUM KLINIK MEDILAB	Genomik Solidaritas Indonesia Laboratorium	Anna Christina Brazia; Annisa Muthiah Sukirman; Anuraj Shankar; Ariel Pradipta; David Hendra Haryanto; Dhahlia Agustina Cahyono; Dwi Oktavia; Dwi Setiyani Wulandari; Fitri Winita; Gracia Felias Enos Korompis; Louisa Markus; Meutia Ayuputeri Kumaheri; Ngabila Salama; Pratiwi Srikandi Yudhastari; Reinhart Gabriel; Tiranti Vindha; Vania Gavrila Wikasa; Willy Setiawan
EPI_ISL_3385417, EPI_ISL_3385418, EPI_ISL_3385419, EPI_ISL_3385420, EPI_ISL_3385421, EPI_ISL_3385422, EPI_ISL_3385423, EPI_ISL_3385424, EPI_ISL_3385425, EPI_ISL_3385426, EPI_ISL_3385427, EPI_ISL_3385428, EPI_ISL_3385429, EPI_ISL_3385430, EPI_ISL_3385431, EPI_ISL_3385432, EPI_ISL_3385433, EPI_ISL_3385434, EPI_ISL_3385435, EPI_ISL_3385436, EPI_ISL_3385437, EPI_ISL_3385438, EPI_ISL_3385439, EPI_ISL_3385440, EPI_ISL_3385441, EPI_ISL_3385444, EPI_ISL_3385445, EPI_ISL_3385446, EPI_ISL_3385447, EPI_ISL_3385448, EPI_ISL_3385449, EPI_ISL_3385450, EPI_ISL_3385451, EPI_ISL_3385452, EPI_ISL_3385453, EPI_ISL_3385454, EPI_ISL_3385455, EPI_ISL_3385456, EPI_ISL_3385457, EPI_ISL_3385458, EPI_ISL_3385459, EPI_ISL_3385460, EPI_ISL_3385461, EPI_ISL_3385462, EPI_ISL_3385463, EPI_ISL_3385464, EPI_ISL_3385465, EPI_ISL_3385466, EPI_ISL_3386094, EPI_ISL_3386095			
see above	LABORATORIUM RSUD PROF DR. W. Z. JOHANNES KUPANG	Genomik Solidaritas Indonesia Laboratorium	Annisa Muthiah Sukirman; Anuraj Shankar; Ariel Pradipta; Carissa Sintca Wijaya; Chandra Apriadi Panduwal; Dhahlia Agustina Cahyono; Don Bosko Joni Dumbaris; Gracia Felias Enos Korompis; Hermi Indita Malewa; Himawan Masyhuri; Louisa Markus; Meutia Ayuputeri Kumaheri; Vania Gavrila Wikasa
EPI_ISL_3548634, EPI_ISL_3548685, EPI_ISL_3548686, EPI_ISL_3548687	LABORATORIUM RSUP FATMAWATI	Genomik Solidaritas Indonesia Laboratorium	Annisa Muthiah Sukirman; Anuraj Shankar; Ariel Pradipta; Carissa Sintca Wijaya; Dhahlia Agustina Cahyono; Dwi Oktavia; Gracia Felias Enos Korompis; Jerry; Lidya Utami; Loli Simajuntak; Louisa Markus; Meutia Ayuputeri Kumaheri; Neng Hilma Hamidah; Ngabila Salama; Tiranti Vindha; Vania Gavrila Wikasa
EPI_ISL_2920084, EPI_ISL_2920099, EPI_ISL_2920100, EPI_ISL_2920101	LABORATORY OF CLINICAL MICROBIOLOGY, FACULTY OF MEDICINE, UNIVERSITY OF INDONESIA	LABORATORY OF CLINICAL MICROBIOLOGY, FACULTY OF MEDICINE, UNIVERSITY OF INDONESIA	RAHMAWATY RG; SUDARMONO PP
EPI_ISL_5022904	Lab GSI	National Institute of Health Research and Development	Arie Ardiansyah Nugraha; Fajar Nur Sulistiyohadi; Hana Apsari Pawestri; Hartanti Dian Ikawati; Kartika Dewi Puspa; Krisna Pangesti; Nelly Puspandari; Subangkit; Triyani Soekarso; Vivi Setiawaty
EPI_ISL_2987654, EPI_ISL_2987655, EPI_ISL_2987656, EPI_ISL_2987657, EPI_ISL_4254881, EPI_ISL_4254882, EPI_ISL_4254883, EPI_ISL_4254884, EPI_ISL_4254885, EPI_ISL_4254886, EPI_ISL_4254887, EPI_ISL_4254888, EPI_ISL_4254889, EPI_ISL_4254890, EPI_ISL_4254891, EPI_ISL_4254892, EPI_ISL_4254893, EPI_ISL_4254894, EPI_ISL_4254895, EPI_ISL_4254896, EPI_ISL_4254897, EPI_ISL_4254898, EPI_ISL_4254899, EPI_ISL_5022951, EPI_ISL_5022952			
see above	Lab Genetik Sumbawa	National Institute of Health Research and Development	Arie Ardiansyah Nugraha; Arief Budi Witarto; Fajar Nur Sulistiyohadi; Hana Apsari Pawestri; Hartanti Dian Ikawati; Kartika Dewi Puspa; Krisna Pangesti; Laily Indrayanti; Lalu Hamzi Fikri; Nelly Puspandari; Nurhandini Eka Dewi; Rastu Karyana; Rian Adha Ardinata; Subangkit; Triyani Soekarso; Vivi Setiawaty
EPI_ISL_3216557, EPI_ISL_3216558, EPI_ISL_3216559, EPI_ISL_3216560, EPI_ISL_3216561, EPI_ISL_3278294, EPI_ISL_3691575			
see above	Lab Genetik Sumbawa Technopark	National Institute of Health Research and Development	Arie Ardiansyah Nugraha; Arief Budi Witarto; Fajar Nur Sulistiyohadi; Hana Apsari Pawestri; Hartanti Dian Ikawati; Kartika Dewi Puspa; Krisna Pangesti; Laily Indrayanti; Lalu Hamzi Fikri; Nelly Puspandari; Nurhandini Eka Dewi; Rian Ardha Ardinata; Subangkit; Triyani Soekarso; Vivi Setiawaty
EPI_ISL_2617524, EPI_ISL_2617525, EPI_ISL_2617526, EPI_ISL_2617527, EPI_ISL_2617528, EPI_ISL_2617529, EPI_ISL_2617530, EPI_ISL_2617531, EPI_ISL_2617532, EPI_ISL_2617533, EPI_ISL_2617534, EPI_ISL_2617535			
see above	Lab Genomik Solidaritas Indonesia	National Institute of Health Research and Development	Arie Ardiansyah Nugraha; Hana Apsari Pawestri; Hartanti Dian Ikawati; Kartika Dewi Puspa; Krisna Pangesti; Meutia Kumaheri; Nelly Puspandari; Subangkit; Triyani Soekarso; Vivi Setiawaty
EPI_ISL_5022920, EPI_ISL_5022921, EPI_ISL_5441707, EPI_ISL_5441708, EPI_ISL_5441709, EPI_ISL_5441710, EPI_ISL_5441711, EPI_ISL_5441712, EPI_ISL_5441713, EPI_ISL_5441714, EPI_ISL_5441715, EPI_ISL_5441716, EPI_ISL_5441717, EPI_ISL_5441718, EPI_ISL_5441719, EPI_ISL_5441720, EPI_ISL_5441721, EPI_ISL_5441722, EPI_ISL_5441723, EPI_ISL_5441724, EPI_ISL_5441725, EPI_ISL_5441726, EPI_ISL_5441727, EPI_ISL_5441728, EPI_ISL_5441729, EPI_ISL_5441730, EPI_ISL_5441731, EPI_ISL_5441732, EPI_ISL_5441733, EPI_ISL_5441734, EPI_ISL_5441735, EPI_ISL_5441788, EPI_ISL_5441789, EPI_ISL_5441790, EPI_ISL_5441791, EPI_ISL_5441792, EPI_ISL_5441793, EPI_ISL_5441794, EPI_ISL_5441795, EPI_ISL_5441796, EPI_ISL_5441797, EPI_ISL_5441798, EPI_ISL_5441799, EPI_ISL_5441800, EPI_ISL_5441801, EPI_ISL_5441802, EPI_ISL_5441803, EPI_ISL_5441804, EPI_ISL_5441854, EPI_ISL_5441855, EPI_ISL_5441856, EPI_ISL_5441857, EPI_ISL_5441858, EPI_ISL_5441859, EPI_ISL_5743636, EPI_ISL_5743637, EPI_ISL_5743644, EPI_ISL_5743667, EPI_ISL_5743668, EPI_ISL_5743673, EPI_ISL_5743674, EPI_ISL_5743687, EPI_ISL_5743693, EPI_ISL_5743694, EPI_ISL_5743700, EPI_ISL_5743701, EPI_ISL_5743702, EPI_ISL_5743709, EPI_ISL_5743710, EPI_ISL_5743714, EPI_ISL_5743715, EPI_ISL_5743791, EPI_ISL_5743795, EPI_ISL_5743803			
see above	Lab Hamera Sarana Indonesia	National Institute of Health Research and Development	Arie Ardiansyah Nugraha; Fajar Nur; Fajar Nur Sulistiyohadi; Hana Apsari Pawestri; Hartanti Dian Ikawati; Kartika Dewi Puspa; Krisna Pangesti; Nelly Puspandari; Subangkit; Sulistiyohadi; Triyani Soekarso; Vivi Setiawaty
EPI_ISL_4508268, EPI_ISL_4508269, EPI_ISL_4508270, EPI_ISL_4508271, EPI_ISL_4508272, EPI_ISL_4508273, EPI_ISL_4508274, EPI_ISL_4508275, EPI_ISL_4508276, EPI_ISL_5022759, EPI_ISL_5022760, EPI_ISL_5022761, EPI_ISL_5022762, EPI_ISL_5022763, EPI_ISL_5022764, EPI_ISL_5022765, EPI_ISL_5022766			
see above	Lab Helix Depok	National Institute of Health Research and Development	; Arie Ardiansyah Nugraha; Fajar Nur; Fajar Nur Sulistiyohadi; Hana Apsari Pawestri; Hartanti Dian Ikawati; Kartika Dewi Puspa; Krisna Pangesti; Nelly Puspandari; Subangkit; Sulistiyohadi; Triyani Soekarso; Vivi Setiawaty
EPI_ISL_1469249, EPI_ISL_1469251	Lab Intibios Karawang	National Institute of Health Research and Development	Arie Ardiansyah Nugraha; Hana Apsari Pawestri; Hartanti Dian Ikawati; Kartika Dewi Puspa; Krisna Pangesti; Nelly Puspandari; Subangkit; Vivi Setiawaty
EPI_ISL_1969244, EPI_ISL_1969245, EPI_ISL_2047507, EPI_ISL_2047508, EPI_ISL_2047509, EPI_ISL_2047510, EPI_ISL_2047511, EPI_ISL_2047512			
see above	Lab RSUP DR Mohammad Hoesin Palembang	National Institute of Health Research and Development	Arie Ardiansyah Nugraha; Eny Rahmawati; Hana Apsari Pawestri; Hartanti Dian Ikawati; Kartika Dewi Puspa; Krisna Pangesti; Nelly Puspandari; Rogaya; Subangkit; Triyani Soekarso; Vivi Setiawaty
EPI_ISL_5022757, EPI_ISL_5022758, EPI_ISL_5022829, EPI_ISL_5022830, EPI_ISL_5022831, EPI_ISL_5022832, EPI_ISL_5022833, EPI_ISL_5022834, EPI_ISL_5022835, EPI_ISL_5022858, EPI_ISL_5022859, EPI_ISL_5022860, EPI_ISL_5022861, EPI_ISL_5022862, EPI_ISL_5022863, EPI_ISL_5022939, EPI_ISL_5022940, EPI_ISL_5022941, EPI_ISL_5022942			
see above	Lab Smartco	National Institute of Health Research and Development	; Arie Ardiansyah Nugraha; Fajar Nur Sulistiyohadi; Hana Apsari Pawestri; Hartanti Dian Ikawati; Kartika Dewi Puspa; Krisna Pangesti; Nelly Puspandari; Subangkit; Triyani Soekarso; Vivi Setiawaty
EPI_ISL_775596	Lab. Biologi Molekular FKIK Unwar, Bali	National Institute of Health Research and Development	AA; Dewi; HA; HD; Ikawati; KD; KNA; N; Nugraha; Pangesti; Pawestri; Puspa; Puspandari; SR; Setiawaty; Soekarso; Subangkit; T; V
EPI_ISL_5446153, EPI_ISL_5446158, EPI_ISL_5446159, EPI_ISL_5446163, EPI_ISL_5446165, EPI_ISL_5446167	Labkes Kota Bandung	Laboratorium Genetik Molekuler, Biomedik Lanjut, FK UNPAD	Chrysanti Murad; Dwi Agustian; Fensi Amalia; Husna Nugrahapraja; Ida Parwati; Jessi Annisa; Lidya Chaidir; Nurul Setia Rahayu; Rima Destya Triatin; Savira Ekawardhani; Tri Hanggono Ahmad; Yeni Rendieni; Yunia Sribudiani
EPI_ISL_3138843, EPI_ISL_3138844, EPI_ISL_3138845, EPI_ISL_3138846, EPI_ISL_3138847, EPI_ISL_3138848, EPI_ISL_3138849, EPI_ISL_3138850, EPI_ISL_3138852			
see above	Labkes Provinsi Bali	National Institute of Health Research and Development	Arie Ardiansyah Nugraha; Fajar Nur Sulistiyohadi; Hana Apsari Pawestri; Hartanti Dian Ikawati; Kartika Dewi Puspa; Krisna Pangesti; Nelly Puspandari; Subangkit; Triyani Soekarso; Vivi Setiawaty
EPI_ISL_3401852, EPI_ISL_3401853, EPI_ISL_3401858, EPI_ISL_3401861	Labkesda	Genomik Solidaritas Indonesia Laboratorium	Annisa Muthiah Sukirman; Anuraj Shankar; Ariel Pradipta; Carissa Sintca Wijaya; Dhahlia Agustina Cahyono; Dwi Oktavia; Gracia Felias Enos Korompis; Himawan Masyhuri; Louisa Markus; Meutia Ayuputeri Kumaheri; Ngabila Salama; Tiranti Vindha; Vania Gavrila Wikasa
EPI_ISL_2955330	Labkesda Bantul	Genetics Working Group (Pokja Genetik) Faculty of Medicine, Public Health and Nursing Universitas Gadjah Mada (FK-KMK UGM); Disease Investigation Center Wates Ministry of Agriculture Indonesia; Department of Microbiology FK-KMK UGM; Laboratorium Diagnostik Yayasan Tahija World Mosquito Program (WMP) Yogyakarta Center for Tropical Medicine FK-KMK UGM; Integrated Research Center FK-KMK UGM; Department of Computer Science and Electronics FMIPA UGM; RSUP Dr. Sardjito	Afiahayati; Alvina A Setiawan; Cita S Amalia; Dwi AA Nugrahaningsih; Dwi Indaryati; Edwin W. Daniwijaya; Eggi Arguni; Endah Supriyati; Gunadi; Hendra Wibawa; Indarto Sulistiyono; Kristy Iskandar; Ludhang P. Rizki; Marcellus; Mohamad S. Hakim; Nungki Anggorowati; Pramesti G Dewi; Siswanto; Titik Nuryastuti; Tri Wibawa
EPI_ISL_4969011	Labkesda Bantul DIY	Genetics Working Group (Pokja Genetik) Faculty of Medicine, Public Health and Nursing Universitas Gadjah Mada (FK-KMK UGM); Disease Investigation Center Wates Ministry of Agriculture Indonesia; Department of Microbiology FK-KMK UGM; Laboratorium Diagnostik Yayasan Tahija World Mosquito Program (WMP) Yogyakarta Center for Tropical Medicine FK-KMK UGM; Integrated Research Center FK-KMK UGM; Department of Computer Science and Electronics FMIPA UGM; RSUP Dr. Sardjito	Afiahayati; Alvina A Setiawan; Cita S Amalia; Dwi AA Nugrahaningsih; Dwi Indaryati; Edwin W. Daniwijaya; Eggi Arguni; Endah Supriyati; Gunadi; Hendra Wibawa; Indarto Sulistiyono; Kristy Iskandar; Ludhang P. Rizki; Marcellus; Mohamad S. Hakim; Nungki Anggorowati; Pramesti G Dewi; Siswanto; Titik Nuryastuti; Tri Wibawa
EPI_ISL_3946307	Labkesda DKI Jakarta	Genomik Solidaritas Indonesia Laboratorium	Annisa Muthiah Sukirman; Anuraj Shankar; Ariel Pradipta; Carissa Sintca Wijaya; Dhahlia Agustina Cahyono; Dwi Oktavia; Gracia Felias Enos Korompis; Himawan Masyhuri; Inten; Louisa Markus; Meutia Ayuputeri Kumaheri; Neng Hilma Hamidah; Ngabila Salama; Pratiwi Srikandi Yudhastari; Suhartiningsih; Tiranti Vindha
EPI_ISL_2987597, EPI_ISL_2987598, EPI_ISL_2987601, EPI_ISL_2987605, EPI_ISL_2987606, EPI_ISL_3138894, EPI_ISL_3138895, EPI_ISL_3138896, EPI_ISL_3216571, EPI_ISL_3216572, EPI_ISL_3216573			
see above	Labkesda DKI Jakarta	National Institute of Health Research and Development	Arie Ardiansyah Nugraha; Fajar Nur Sulistiyohadi; Hana Apsari Pawestri; Hartanti Dian Ikawati; Kartika Dewi Puspa; Krisna Pangesti; Nelly Puspandari; Subangkit; Triyani Soekarso; Vivi Setiawaty
EPI_ISL_2860350, EPI_ISL_2860351, EPI_ISL_2860352, EPI_ISL_2860353, EPI_ISL_2860354, EPI_ISL_2860355, EPI_ISL_2860356, EPI_ISL_2860357, EPI_ISL_2860358, EPI_ISL_2860359			
see above	Labkesda Depok	Biosafety Level-3 Laboratory, Indonesian Institute of Sciences (LIPI)	Andri Wardiana; Anggia Prasetyoputri; Anik B. Dharmayanthi; Elin Herliana; Harto W. Tamara; Herman Irawan; Kania R. Astuti; Kurniyawati; Masrukhin; Puspita Lisdiyanti; Ratih A. Ningrum; Ruby Setiawan; Sri Swasthikawati; Sugeng Suhadi; Sugiyono Saputra; Syaiful Rizal; Syam B. Iryanto
EPI_ISL_2931725, EPI_ISL_2931734, EPI_ISL_2931742, EPI_ISL_2931752, EPI_ISL_2931761	Labkesda Depok	National Institute of Health Research and Development	Arie Ardiansyah Nugraha; Hana Apsari Pawestri; Hartanti Dian Ikawati; Kartika Dewi Puspa; Krisna Pangesti; Nelly Puspandari; Subangkit; Triyani Soekarso; Vivi Setiawaty
EPI_ISL_1915578	Labkesda Jakarta	National Institute of Health Research and Development	Arie Ardiansyah Nugraha; Hana Apsari Pawestri; Hartanti Dian Ikawati; Kartika Dewi Puspa; Krisna Pangesti; Nelly Puspandari; Subangkit; Vivi Setiawaty

EPI_ISL_1469288, EPI_ISL_1469289, EPI_ISL_1534708, EPI_ISL_2106981	Labkesda Karawang	National Institute of Health Research and Development	Arie Ardiansyah Nugraha; Hana Apsari Pawestri; Hartanti Dian Ikawati; Kartika Dewi Puspa; Krisna Pangesti; Nelly Puspandari; Subangkit; Vivi Setiawaty
EPI_ISL_2854726, EPI_ISL_2854727, EPI_ISL_2854728, EPI_ISL_3216541, EPI_ISL_3216542, EPI_ISL_3216543, EPI_ISL_3216544, EPI_ISL_3216545			
see above	Labkesda Kota Tangerang	National Institute of Health Research and Development	; Arie Ardiansyah Nugraha; Fajar Nur Sulistiyohadi; Hana Apsari Pawestri; Hartanti Dian Ikawati; Kartika Dewi Puspa; Krisna Pangesti; Nelly Puspandari; Subangkit; Triyani Soekarso; Vivi Setiawaty
EPI_ISL_3691587, EPI_ISL_3691588, EPI_ISL_3691589, EPI_ISL_3691591, EPI_ISL_3691644, EPI_ISL_3691645, EPI_ISL_3691646, EPI_ISL_3691647, EPI_ISL_3691648, EPI_ISL_3691669, EPI_ISL_3691670, EPI_ISL_3691671, EPI_ISL_3691673, EPI_ISL_3691674, EPI_ISL_3691675, EPI_ISL_3691676, EPI_ISL_3691677			
see above	Labkesda Pangkalpinang	National Institute of Health Research and Development	Arie Ardiansyah Nugraha; Fajar Nur Sulistiyohadi; Hana Apsari Pawestri; Hartanti Dian Ikawati; Kartika Dewi Puspa; Krisna Pangesti; Nelly Puspandari; Subangkit; Triyani Soekarso; Vivi Setiawaty
EPI_ISL_2854731, EPI_ISL_2854732, EPI_ISL_2854733, EPI_ISL_2854734, EPI_ISL_2854735, EPI_ISL_2854736, EPI_ISL_3138810, EPI_ISL_3138811, EPI_ISL_3138812			
see above	Labkesda Prov DKI Jakarta	National Institute of Health Research and Development	; Arie Ardiansyah Nugraha; Fajar Nur Sulistiyohadi; Hana Apsari Pawestri; Hartanti Dian Ikawati; Kartika Dewi Puspa; Krisna Pangesti; Nelly Puspandari; Subangkit; Triyani Soekarso; Vivi Setiawaty
EPI_ISL_2233094	Labkesda Prov Kal Tim	National Institute of Health Research and Development	Arie Ardiansyah Nugraha; Hana Apsari Pawestri; Hartanti Dian Ikawati; Kartika Dewi Puspa; Krisna Pangesti; Nelly Puspandari; Subangkit; Triyani Soekarso; Vivi Setiawaty
EPI_ISL_3946306	Labkesda Prov. DKI Jakarta	Genomik Solidaritas Indonesia Laboratorium	Annisa Muthiah Sukirman; Anuraj Shankar; Ariel Pradipta; Carissa Sintca Wijaya; Dhahlia Agustina Cahyono; Dwi Oktavia; Gracia Felias Enos Korompis; Himawan Masyhuri; Louisa Markus; Meutia Ayuputeri Kumaheri; Neng Hilma Hamidah; Ngabila Salama; Pratiwi Srikandi Yudhastari; Suhartiningsih; Syifa Khairunnisa; Tiranti Vindha
EPI_ISL_3548629	Laboearorium RSPP	Genomik Solidaritas Indonesia Laboratorium	Annisa Muthiah Sukirman; Anuraj Shankar; Ariel Pradipta; Carissa Sintca Wijaya; Dhahlia Agustina Cahyono; Dian Prastiti; Dwi Oktavia; Gracia Felias Enos Korompis; Louisa Markus; Meutia Ayuputeri Kumaheri; Neng Hilma Hamidah; Ngabila Salama; Tiranti Vindha; Vania Gavrila Wikasa
EPI_ISL_3401859, EPI_ISL_3401868, EPI_ISL_3401872, EPI_ISL_3401873	Laboratorium RSUP Fatmawati	Genomik Solidaritas Indonesia Laboratorium	Annisa Muthiah Sukirman; Anuraj Shankar; Ariel Pradipta; Carissa Sintca Wijaya; Devi; Dhahlia Agustina Cahyono; Dwi Oktavia; Gracia Felias Enos Korompis; Himawan Masyhuri; Jerry; Lidya Utami; Loli Simajuntak; Louisa Markus; Meutia Ayuputeri Kumaheri; Ngabila Salama; Siska Situmorang; Tiranti Vindha; Triana Puspita Dewi; Vania Gavrila Wikasa
EPI_ISL_5103932, EPI_ISL_5103934, EPI_ISL_5103935, EPI_ISL_5103936, EPI_ISL_5103937, EPI_ISL_5103938, EPI_ISL_5103939, EPI_ISL_5103940, EPI_ISL_5103941, EPI_ISL_5103942, EPI_ISL_5103943, EPI_ISL_5103944, EPI_ISL_5103945, EPI_ISL_5103946, EPI_ISL_5103947, EPI_ISL_5103948, EPI_ISL_5103950, EPI_ISL_5103951, EPI_ISL_5103952, EPI_ISL_5103953, EPI_ISL_5103954, EPI_ISL_5103955, EPI_ISL_5103956, EPI_ISL_5103957, EPI_ISL_5103958, EPI_ISL_5103959, EPI_ISL_5103960			
see above	Laboratorium Biologi Molekuler, Patologi Klinik RS Hasan Sadikin	Laboratorium Genetik Molekuler, Biomedik Lanjut, FK UNPAD	Chrysanti Murad; Delita Prihatni; Dwi Agustian; Fensi Amalia; Ida Parwati; Jessi Annisa; Lidya Chaidir; Nuni Haeruni; Nurul Setia Rahayu; Savira Ekawardhani; Shinta; Tri Hanggono Ahmad; Yanu; Yeni Rendieni; Yunia Sribudiani
EPI_ISL_5103933, EPI_ISL_5103949	Laboratorium Biomolekuler RSUD Al Ihsan	Laboratorium Genetik Molekuler, Biomedik Lanjut, FK UNPAD	Chrysanti Murad; Dewi Rinakanti; Dwi Agustian; Fensi Amalia; Ida Parwati; Jessi Annisa; Lidya Chaidir; Nuni Haeruni; Nurul Setia Rahayu; Savira Ekawardhani; Shinta; Tri Hanggono Ahmad; Yanu; Yeni Rendieni; Yunia Sribudiani
EPI_ISL_5022784, EPI_ISL_5022785, EPI_ISL_5022786, EPI_ISL_5022787, EPI_ISL_5022788, EPI_ISL_5022789, EPI_ISL_5022790, EPI_ISL_5022791, EPI_ISL_5022792, EPI_ISL_5022793, EPI_ISL_5022794, EPI_ISL_5022795, EPI_ISL_5022796, EPI_ISL_5022797, EPI_ISL_5022798, EPI_ISL_5022799, EPI_ISL_5022803, EPI_ISL_5022804, EPI_ISL_5022805, EPI_ISL_5022806, EPI_ISL_5022807, EPI_ISL_5022808, EPI_ISL_5022809, EPI_ISL_5022810, EPI_ISL_5022811, EPI_ISL_5022812			
see above	Laboratorium Hamera Sarana Indonesia	National Institute of Health Research and Development	Arie Ardiansyah Nugraha; Fajar Nur Sulistiyohadi; Hana Apsari Pawestri; Hartanti Dian Ikawati; Kartika Dewi Puspa; Krisna Pangesti; Nelly Puspandari; Subangkit; Triyani Soekarso; Vivi Setiawaty
EPI_ISL_1415898	Laboratorium Intibios Karawang	National Institute of Health Research and Development	Arie Ardiansyah Nugraha; Hana Apsari Pawestri; Hartanti Dian Ikawati; Kartika Dewi Puspa; Krisna Nur Andriana Pangesti; Nelly Puspandari; Subangkit; Vivi Setiawaty
EPI_ISL_3030055	Laboratorium Kesehatan Daerah Kota Bandung, Dinas Kesehatan Kota Bandung	Eijkman Institute for Molecular Biology, National Research and Innovation Agency; West Java Health Laboratory	Amin Soebandrio; Azzania Fibriani; Cut Nur Cinthia Alamanda; Edison Johar; Ema Rahmawati; Frilasita A Yudhaputri; Hidayat Trimarsanto; Husna Nugrahapraja; Iskandar Adnan; Khin Saw Myint; Lidwina Priliani; Lydia V. Panggalo; Muhammad Rezki Rasyak; Rifky Waluyajati Rachman; Ryan Bayusantika Ristandi; Safarina G Malik; Sheila Widia Trisakti; Sukma Oktavianthi; Tati Sutarti Sastrowiyoto; Usman Abdul Jabar; Willy Agustine
EPI_ISL_4056004	Laboratorium Kesehatan Daerah Surabaya	Institute of Tropical Disease, Universitas Airlangga	Aldise M Nastri; Gatot Soegiarto; Jezzy R Dewantari; Kazufumi Shimizu; Kohar Hari Santoso; Krisnoadi Rahardjo; Laksmi Wulandari; Maria I Lusida; Resti Yudhawati; Rima R Prasetya; Soetjipto; Yasuko Mori
EPI_ISL_5536496, EPI_ISL_5536511, EPI_ISL_5536512, EPI_ISL_5536518, EPI_ISL_5536522, EPI_ISL_5536526, EPI_ISL_5536528, EPI_ISL_5536529, EPI_ISL_5536553, EPI_ISL_5536558, EPI_ISL_5536559, EPI_ISL_5536560, EPI_ISL_5536561, EPI_ISL_5536568, EPI_ISL_5536571, EPI_ISL_5536574, EPI_ISL_5536575			
see above	Laboratorium Kesehatan Kab. Bogor	National Research and Innovation Agency (BRIN-Indonesia, VenomCoV Project Team); Dinas Kesehatan Kab. Bogor	Ahmad R. Al Fiqri; Alvira R. Sativa; Anggia Prasetyoputri; Anik B. Dharmayanthi; Dimas Prapanca; Gustomo; Indriawati; Isnaini Z. Asyifa; Muhammad M. Adzdzakiy; Ratih A. Ningrum; Sugiyono Saputra; Syaiful Rizal; Syam B. Iryanto
EPI_ISL_5796080, EPI_ISL_5796081	Laboratorium Kesehatan Provinsi Bali	National Institute of Health Research and Development	Arie Ardiansyah Nugraha; Fajar Nur Sulistiyohadi; Hana Apsari Pawestri; Hartanti Dian Ikawati; Kartika Dewi Puspa; Krisna Pangesti; Nelly Puspandari; Subangkit; Triyani Soekarso; Vivi Setiawaty
EPI_ISL_511879	Laboratorium Kesehatan Provinsi Jawa Barat	Molecular Genetics Laboratory-Faculty of Medicine-Universitas Padjadjaran; School of Life Sciences and Technology & School of Pharmacy-Institut Teknologi Bandung; Laboratorium Kesehatan Provinsi Jawa Barat	Agung Eru Wibowo; Azzania Fibriani; Catur Riani; Cut Nur Cinthia Alamanda; Ema Rahmawati; Hammam Riza; Hesti Lina Wiraswati; Husna Nugrahapraja; Lia Faridah; Marselina Irasonia Tan; Rifky Waluyajati Rachman; Ryan Bayusantika Ristandi; Savira Ekawardhani; Soni Solistia Wirawan; Tarwadi; Yunia Sribudiani
EPI_ISL_528745	Laboratorium Kesehatan Provinsi Jawa Barat	School of Life Sciences and Technology & School of Pharmacy-Institut Teknologi Bandung; Molecular Genetics Laboratory-Faculty of Medicine-Universitas Padjadjaran; Laboratorium Kesehatan Provinsi Jawa Barat	Agung Eru Wibowo; Azzania Fibriani; Catur Riani; Cut Nur Cinthia Alamanda; Ema Rahmawati; Fensi Amalina; Hammam Riza; Hesti Lina Wiraswati; Husna Nugrahapraja; Irvan Faizal; Lia Faridah; Marselina Irasonia Tan; Mas Rizky A.A. Syamsunarno; Rifky Waluyajati Rachman; Ryan Bayusantika Ristandi; Savira Ekawardhani; Sony Solistia Wirawan; Tarwadi; Tri Hanggono Achmad; Yunia Sribudiani
EPI_ISL_3385402, EPI_ISL_3385403, EPI_ISL_3385404, EPI_ISL_3385405, EPI_ISL_3385406, EPI_ISL_3385407, EPI_ISL_3385408, EPI_ISL_3385409, EPI_ISL_3385410, EPI_ISL_3385411, EPI_ISL_3385412, EPI_ISL_3385413, EPI_ISL_3385414, EPI_ISL_3385415, EPI_ISL_3385416, EPI_ISL_3386093, EPI_ISL_3404356, EPI_ISL_3404357, EPI_ISL_3404358, EPI_ISL_3404359, EPI_ISL_3404360, EPI_ISL_3404361, EPI_ISL_3404362, EPI_ISL_3404363, EPI_ISL_3404364, EPI_ISL_3404365, EPI_ISL_3404366, EPI_ISL_3404367			
see above	Laboratorium Khusus Speedlab Jakarta	Genomik Solidaritas Indonesia Laboratorium	Annisa Muthiah Sukirman; Anuraj Shankar; Ariel Pradipta; Carissa Sintca Wijaya; Dhahlia Agustina Cahyono; Gracia Felias Enos Korompis; Himawan Masyhuri; Louisa Markus; Meutia Ayuputeri Kumaheri; Neng Hilma Hamidah; Vania Gavrila Wikasa
EPI_ISL_791977	Laboratorium Mikrobiologi Samarinda	National Institute of Health Research and Development	AA; HA; HD; Ikawati; KD; KNA; N; Nugraha; Pangesti; Pawestri; Puspa; Puspandari; Setiawaty; Soekarso; Subangkit; T; Tandirogang; V; Yadi
EPI_ISL_3691592, EPI_ISL_3691625, EPI_ISL_3691626, EPI_ISL_3691627, EPI_ISL_3691628, EPI_ISL_3691629, EPI_ISL_3691630, EPI_ISL_3691631, EPI_ISL_3691632, EPI_ISL_3691633, EPI_ISL_3691634, EPI_ISL_3691635, EPI_ISL_3691636, EPI_ISL_3691637, EPI_ISL_3691638, EPI_ISL_3691639, EPI_ISL_3691640, EPI_ISL_3691641, EPI_ISL_3691642, EPI_ISL_3691643			
see above	Laboratorium PCR PT Halmahera Persada Lygend	National Institute of Health Research and Development	Arie Ardiansyah Nugraha; Fajar Nur Sulistiyohadi; Hana Apsari Pawestri; Hartanti Dian Ikawati; Kartika Dewi Puspa; Krisna Pangesti; Nelly Puspandari; Subangkit; Triyani Soekarso; Vivi Setiawaty
EPI_ISL_3691594, EPI_ISL_3691596, EPI_ISL_3691617, EPI_ISL_3691618, EPI_ISL_3691619, EPI_ISL_3691620, EPI_ISL_3691621, EPI_ISL_3691622, EPI_ISL_3691623, EPI_ISL_3691624			
see above	Laboratorium PCR PT Nusa Halmahera Minerals	National Institute of Health Research and Development	Arie Ardiansyah Nugraha; Fajar Nur Sulistiyohadi; Hana Apsari Pawestri; Hartanti Dian Ikawati; Kartika Dewi Puspa; Krisna Pangesti; Nelly Puspandari; Subangkit; Triyani Soekarso; Vivi Setiawaty
EPI_ISL_5307520	Laboratorium RS Al Islam	Laboratorium Genetik Molekuler, Biomedik Lanjut, FK UNPAD	Chrysanti Murad; Dwi Agustian; Fensi Amalia; Ida Parwati; Jessi Annisa; Lidya Chaidir; Nuni Haeruni; Nurul Setia Rahayu; Rita Herawati; Savira Ekawardhani; Shinta; Tri Hanggono Ahmad; Yanu; Yeni Rendieni; Yunia Sribudiani
EPI_ISL_4468049	Laboratorium RSPAD Gatot Soebroto	Genomik Solidaritas Indonesia Laboratorium	Annisa Muthiah Sukirman; Anuraj Shankar; Ariel Pradipta; Carissa Sintca Wijaya; Dhahlia Agustina Cahyono; Dian Tri Wahyudi; Gracia Felias Enos Korompis; Himawan Masyhuri; Louisa Markus; Martina Lily Yana; Meutia Ayuputeri Kumaheri; Stefanus Bernard
EPI_ISL_3548640, EPI_ISL_3548689, EPI_ISL_3548690, EPI_ISL_3548691, EPI_ISL_3548692	Laboratorium RSPP	Genomik Solidaritas Indonesia Laboratorium	Annisa Muthiah Sukirman; Anuraj Shankar; Ariel Pradipta; Carissa Sintca Wijaya; Dhahlia Agustina Cahyono; Dwi Oktavia; Gracia Felias Enos Korompis; Louisa Markus; Meutia Ayuputeri Kumaheri; Neng Hilma Hamidah; Ngabila Salama; Tiranti Vindha; Vania Gavrila Wikasa; Wahyuningsih
EPI_ISL_4090575	Laboratorium RSUD Poso	National Institute of Health Research and Development	Arie Ardiansyah Nugraha; Fajar Nur Sulistiyohadi; Hana Apsari Pawestri; Hartanti Dian Ikawati; Kartika Dewi Puspa; Krisna Pangesti; Nelly Puspandari; Subangkit; Triyani Soekarso; Vivi Setiawaty
EPI_ISL_3086886, EPI_ISL_3086887, EPI_ISL_3086888, EPI_ISL_3086889, EPI_ISL_3086890, EPI_ISL_3086891, EPI_ISL_3086892, EPI_ISL_3086893, EPI_ISL_3086894, EPI_ISL_3086895, EPI_ISL_3086896			
see above	Laboratorium Riset Unsoed	Indonesian Institute of Sciences (LIPI) and Laboratorium Riset Universitas Jenderal Soedirman	Anggia Prasetyoputri; Anik B. Dharmayanthi; Dhadhang W. Kurniawan; Dody Novrial; Endang S. Kusmintarsih; Erwin F. Hasrianda; Herjuno A. Nugroho; Idris; Indriawati; Isa Nuryana; Listiana Oktavia; M. M. Rudi Prihatno; M. Z. Syamsul Hidayat; Masrukhin; Norman A. Prayogi; Puspita Lisdiyanti; Ratih A. Ningrum; Ruby Setiawan; Sugiyono Saputra; Suprayogi; Syaiful Rizal; Syam B. Iryanto; Wahyu Siswandari; Yuliawati
EPI_ISL_4106136, EPI_ISL_4106137, EPI_ISL_4106138, EPI_ISL_4106139	Laboratorium Riset Unsoed	National Research and Innovation Agency (BRIN-Indonesia, VenomCoV Project Team); Laboratorium Riset Unsoed	Dhadhang W. Kurniawan; Dody Novrial; Endang S. Kusmintarsih; Idris; Indriawati; Isa Nuryana; M. M. Rudi Prihatno; M. Z. Syamsul Hidayat; Norman A. Prayogi; Puspita Lisdiyanti; Ratih A. Ningrum; Sugiyono Saputra; Suprayogi; Syaiful Rizal; Syam B. Iryanto; Wahyu Siswandari
EPI_ISL_3466838, EPI_ISL_3466844, EPI_ISL_3466856, EPI_ISL_3466862, EPI_ISL_3466869, EPI_ISL_3466875, EPI_ISL_3466881, EPI_ISL_3466888, EPI_ISL_3466894, EPI_ISL_3466900, EPI_ISL_3466905, EPI_ISL_3466912, EPI_ISL_3466918, EPI_ISL_3466924, EPI_ISL_3466936, EPI_ISL_3466942, EPI_ISL_3466949, EPI_ISL_3466956, EPI_ISL_3550555, EPI_ISL_3550556, EPI_ISL_3550557, EPI_ISL_3550558, EPI_ISL_3550559, EPI_ISL_3550560, EPI_ISL_3550561, EPI_ISL_3550562, EPI_ISL_3550563, EPI_ISL_3550566, EPI_ISL_3550567, EPI_ISL_3550568, EPI_ISL_3550569, EPI_ISL_3550570, EPI_ISL_3550571			
see above	Laboratory of Clinical Microbiology, Faculty of Medicine, University of Indonesia	Eijkman Institute for Molecular Biology, National Research and Innovation Agency	Amin Soebandrio; Edison Johar; Frilasita A Yudhaputri; Hidayat Trimarsanto; Iskandar Adnan; Jessica R. Saragih; Khin Saw Myint; Lidwina Priliani; Lydia V. Panggalo; Muhammad Rezki Rasyak; Safarina G Malik; Sukma Oktavianthi; Willy Agustine
EPI_ISL_4056010	Malang Health Office	Institute of Tropical Disease, Universitas Airlangga	Aldise M Nastri; Gatot Soegiarto; Jezzy R Dewantari; Kazufumi Shimizu; Kohar Hari Santoso; Krisnoadi Rahardjo; Laksmi Wulandari; Maria I Lusida; Resti Yudhawati; Rima R Prasetya; Soetjipto; Yasuko Mori
EPI_ISL_1335888, EPI_ISL_1335895	Martha Friska Multatuli Hospital Medan	Faculty of Medicine, Universitas Sumatera Utara; Institute of Tropical Disease, Universitas Airlangga	Aldise M Nastri; Franciscus Ginting; Inke N D Lubis; Irbah R Nainggolan; Jezzy R Dewantari; Kazufumi Shimizu; Krisnoadi Rahardjo; Maria I Lusida; Meliani; Mirzan Hasibuan; Muhammad Ichwan; R Andika D Cahyadi; R Lia Kusumawati; Ramadhan Bestari; Rima R Prasetya; Soetjipto; Yasuko Mori
EPI_ISL_707692, EPI_ISL_707696, EPI_ISL_707778, EPI_ISL_707890, EPI_ISL_708652, EPI_ISL_882645, EPI_ISL_882646, EPI_ISL_882656, EPI_ISL_883957, EPI_ISL_933575, EPI_ISL_933576, EPI_ISL_1523640, EPI_ISL_1523641, EPI_ISL_1523642			
see above	Medical Research Center, Faculty of Medicine, Syarif Hidayatullah State Islamic University Jakarta	Medical Research Center, Faculty of Medicine, Syarif Hidayatullah State Islamic University Jakarta	Chris Adhiyanto; Dennis Nurjadi; Endah Wulandari; Erike A Suwarsono; Erike Suwarsono; Ferania Mela; Fika Ekayanti; Flori R Sari; Flori Ratna Sari; Hari Hendarto; Laifa Hendarmin; Rini Puspitaningrum; Zeti Harriyati
EPI_ISL_435281	Medistra Hospital Jakarta	Eijkman Institute for Molecular Biology, Ministry of Research and Technology/National Agency for Research and Innovation	Amin Soebandrio; David H Muljono; Edison Johar; Frilasita A Yudhaputri; Hidayat Trimarsanto; Khin Saw Myint; Safarina G Malik
EPI_ISL_1117592	Melania Hospital, Bagor , West	Biosafety Level-3 Laboratory, Indonesian Institute of Sciences (LIPI)	Ade Andriani; Anik Budhi Dharmayanthi; Asep M. Ridwanuloh; Eko Wahyu Putro; Gita Syahputra; Hariyatun; Inswasti Cahyani; Matthew W. Loose; Mohammad Ilyas; Susanti; Syam Budi Iryanto; Wien Kusharyoto

	Java		
EPI_ISL_1020192, EPI_ISL_1117450	Melania Hospital, Bagor , west Java	Biosafety Level-3 Laboratory, Indonesian Institute of Sciences (LIPI)	Ade Andriani; Anggia Prasetyoputri; Anik Budhi Dharmayanthi; Ari Sulistyo Wulandari; Asep M. Ridwanuloh; Eko Wahyu Putro; Gita Syahputra; Hariyatun; Inswasti Cahyani; Isa Nuryana; Matthew W. Loose; Mohammad Ilyas; Ratih Asmana Ningrum; Sri Swasthikawati; Sugiyono Saputra; Susanti; Syam Budi Iryanto; Wien Kusharyoto
EPI_ISL_1020195	Melania Hospital, Bogor , West Java	Biosafety Level-3 Laboratory, Indonesian Institute of Sciences (LIPI)	Anggia Prasetyoputri; Erwin Hasrianda; Fahrurrozi; Gita Syahputra; Herjuno A. Nugroho; Nova Dhilla Yanthi; Ratih Asmana Ningrum; Syam Budi Iryanto
EPI_ISL_1118280	Merial Health Hospital, Jakarta	Biosafety Level-3 Laboratory, Indonesian Institute of Sciences (LIPI)	Ade Andriani; Anik Budhi Dharmayanthi; Asep M. Ridwanuloh; Eko Wahyu Putro; Gita Syahputra; Hariyatun; Inswasti Cahyani; Matthew W. Loose; Mohammad Ilyas; Susanti; Syam Budi Iryanto; Wien Kusharyoto
EPI_ISL_1284054	Metro Hospitals Cikarang	Eijkman Institute for Molecular Biology, Ministry of Research and Technology/National Agency for Research and Innovation	Amin Soebandrio; Edison Johar; Frilasita A Yudhaputri; Hidayat Trimarsanto; Iskandar Adnan; Khin Saw Myint; Lydia V. Panggalo; Safarina G Malik; Sukma Oktavianthi; Willy Agustine
EPI_ISL_3550606, EPI_ISL_3550607, EPI_ISL_3550608, EPI_ISL_3550609, EPI_ISL_3550610, EPI_ISL_3550611, EPI_ISL_3550612, EPI_ISL_3550613, EPI_ISL_3550614, EPI_ISL_3550615, EPI_ISL_3550616, EPI_ISL_3550617, EPI_ISL_3550618, EPI_ISL_3550619, EPI_ISL_3550620, EPI_ISL_3550621, EPI_ISL_3550622, EPI_ISL_3550623, EPI_ISL_3550624, EPI_ISL_3550625, EPI_ISL_3550626, EPI_ISL_3550627, EPI_ISL_3550628, EPI_ISL_3550629, EPI_ISL_3550630, EPI_ISL_3550631, EPI_ISL_3550632, EPI_ISL_3550633, EPI_ISL_3550634, EPI_ISL_3550635, EPI_ISL_3550636, EPI_ISL_3550637, EPI_ISL_3550638, EPI_ISL_3550639, EPI_ISL_3550640, EPI_ISL_3550641, EPI_ISL_3550642, EPI_ISL_3550643, EPI_ISL_3550644, EPI_ISL_3550645, EPI_ISL_3550646, EPI_ISL_3550647, EPI_ISL_3550648, EPI_ISL_3550649, EPI_ISL_3550650, EPI_ISL_3550651, EPI_ISL_3550652			
see above	Microbiology Laboratory, Faculty of Medicine, University of Sumatera Utara	Eijkman Institute for Molecular Biology, National Research and Innovation Agency	Amin Soebandrio; Edison Johar; Frilasita A Yudhaputri; Hidayat Trimarsanto; Iskandar Adnan; Jessica R. Saragih; Khin Saw Myint; Lidwina Priliani; Lydia V. Panggalo; Muhammad Rezki Rasyak; Safarina G Malik; Sukma Oktavianthi; Willy Agustine
EPI_ISL_5021327, EPI_ISL_5021382, EPI_ISL_5021508	Microbiology Laboratory, Faculty of Medicine, University of Sumatera Utara	Eijkman Research Center for Molecular Biology, National Research and Innovation Agency; Microbiology Laboratory, Faculty of Medicine, University of Sumatera Utara	Amin Soebandrio; Edison Johar; Frilasita A Yudhaputri; Hidayat Trimarsanto; Inke Nadia Diniyanti Lubis; Irbah Rea Alvieda Nainggolan; Iskandar Adnan; Jessica R Saragih; Khin Saw Myint; Lidwina Priliani; Lydia V Panggalo; Mirzan Hasibuan; Muhammad Ichwan; Muhammad Rezki Rasyak; R Andika Dwi Cahyadi; R Lia Kusumawati; Ramadhan Bestari; Ranti Permatasari; Safarina G Malik; Sukma Oktavianthi; Tryna Tania; Willy Agustine
EPI_ISL_1615588, EPI_ISL_1634442, EPI_ISL_1660414	Mitra Kasih Hospital	West Java Health Laboratory; School of Life Sciences and Technology, Institut Teknologi Bandung	Aulia Saraswati Wicaksono; Azzania Fibriani; Cut Nur Cinthia Alamanda; Ema Rahmawati; Karimatu Khoirunnisa; Miftahul Faridl; Rifky Waluyajati Rachman; Rini Robiani; Ryan Bayusantika Ristandi
EPI_ISL_458079	Mitra Keluarga Hospital Kenjeran	Institute of Tropical Disease, Universitas Airlangga	Aldise M Nastri; Anastasia W Jefuna; Gatot Soegiarto; Jezzy R Dewantari; Kazufumi Shimizu; Krisnoadi Rahardjo; Laksmi Wulandari; Maria I Lusida; Mitsuhiro Nishimura; Resti Yudhawati; Retno A Setyoningrum; Rima R Prasetya; Soetjipto; Yasuko Mori; Yohko K Shimizu
EPI_ISL_759964, EPI_ISL_956276, EPI_ISL_2693671	Mitra Keluarga Hospital Waru	Institute of Tropical Disease, Universitas Airlangga	Aldise M Nastri; Christina Dian Anggraeni; Febria Rachmanita; Gatot Soegiarto; Herlin Ferliana; Jezzy R Dewantari; Kazufumi Shimizu; Krisnoadi Rahardjo; Laksmi Wulandari; Maria I Lusida; Resti Yudhawati; Rima R Prasetya; Soetjipto; Yasuko Mori
EPI_ISL_437192	Mitra Keluarga Kelapa Gading Hospital	Eijkman Institute for Molecular Biology, Ministry of Research and Technology/National Agency for Research and Innovation	Amin Soebandrio; David H Muljono; Edison Johar; Frilasita A Yudhaputri; Hidayat Trimarsanto; Khin Saw Myint; Safarina G Malik
EPI_ISL_2101022, EPI_ISL_2101023, EPI_ISL_2101024, EPI_ISL_2101025, EPI_ISL_2101026, EPI_ISL_2101027, EPI_ISL_2101028, EPI_ISL_2101029, EPI_ISL_2101030, EPI_ISL_2101031, EPI_ISL_2101032, EPI_ISL_2101034, EPI_ISL_2101035, EPI_ISL_2101036, EPI_ISL_2101037, EPI_ISL_2101038, EPI_ISL_2101039, EPI_ISL_2101040, EPI_ISL_2101041, EPI_ISL_2101042, EPI_ISL_2101043, EPI_ISL_2101044, EPI_ISL_2101045, EPI_ISL_2101046, EPI_ISL_2101047, EPI_ISL_2101048, EPI_ISL_2101049, EPI_ISL_2101050, EPI_ISL_2101051, EPI_ISL_2101052, EPI_ISL_2101053, EPI_ISL_2101054, EPI_ISL_2101055, EPI_ISL_2101056, EPI_ISL_2101057, EPI_ISL_2101058, EPI_ISL_2101059, EPI_ISL_2101060, EPI_ISL_2101061, EPI_ISL_2101062, EPI_ISL_2101063, EPI_ISL_2101064, EPI_ISL_2101065, EPI_ISL_2101066, EPI_ISL_2101067, EPI_ISL_2101068, EPI_ISL_2101069, EPI_ISL_2101070, EPI_ISL_2101071, EPI_ISL_2101072, EPI_ISL_2101073, EPI_ISL_2101074, EPI_ISL_2101075, EPI_ISL_2101076, EPI_ISL_2101077, EPI_ISL_2101078, EPI_ISL_2101079, EPI_ISL_2101080, EPI_ISL_2101081, EPI_ISL_2101082, EPI_ISL_2101083, EPI_ISL_2101084, EPI_ISL_2101085, EPI_ISL_2101086, EPI_ISL_2101087, EPI_ISL_2101088, EPI_ISL_2101089, EPI_ISL_2101090, EPI_ISL_2101091, EPI_ISL_2101092, EPI_ISL_2101093, EPI_ISL_2101094, EPI_ISL_2101095, EPI_ISL_2101096, EPI_ISL_2101097, EPI_ISL_2101098, EPI_ISL_2101099, EPI_ISL_2101100, EPI_ISL_2101102, EPI_ISL_2101103, EPI_ISL_2101104, EPI_ISL_2101105, EPI_ISL_2101106, EPI_ISL_2101107, EPI_ISL_2101108, EPI_ISL_2101109, EPI_ISL_2101110, EPI_ISL_2101111, EPI_ISL_2101113, EPI_ISL_2101114, EPI_ISL_2101115, EPI_ISL_2101116, EPI_ISL_2101117, EPI_ISL_2101118, EPI_ISL_2101119, EPI_ISL_2101120, EPI_ISL_2101121, EPI_ISL_2101122, EPI_ISL_2101123, EPI_ISL_2101125, EPI_ISL_2101126, EPI_ISL_2101127, EPI_ISL_2101128, EPI_ISL_2101129, EPI_ISL_2101130, EPI_ISL_2101131, EPI_ISL_2101132, EPI_ISL_2101133, EPI_ISL_2101134, EPI_ISL_2101135, EPI_ISL_2101136, EPI_ISL_2101137, EPI_ISL_2101138, EPI_ISL_2101139, EPI_ISL_2101140, EPI_ISL_2101141, EPI_ISL_2101142, EPI_ISL_2101143, EPI_ISL_2101144, EPI_ISL_2101145, EPI_ISL_2101146, EPI_ISL_2101147, EPI_ISL_2101148, EPI_ISL_2101149, EPI_ISL_2101150, EPI_ISL_2101151, EPI_ISL_2101152, EPI_ISL_2101153, EPI_ISL_2101154, EPI_ISL_2101155, EPI_ISL_2101156, EPI_ISL_2101157, EPI_ISL_2101158, EPI_ISL_2101159, EPI_ISL_2101160, EPI_ISL_2101161, EPI_ISL_2101162, EPI_ISL_2101163, EPI_ISL_2101164, EPI_ISL_2101165, EPI_ISL_2101166, EPI_ISL_2101167, EPI_ISL_2101168, EPI_ISL_2101169, EPI_ISL_2101170, EPI_ISL_2101171, EPI_ISL_2101172, EPI_ISL_2101173, EPI_ISL_2101174, EPI_ISL_2101175, EPI_ISL_2101176, EPI_ISL_2101177, EPI_ISL_2101178, EPI_ISL_2101179, EPI_ISL_2101180, EPI_ISL_2101181, EPI_ISL_2101182, EPI_ISL_2101183, EPI_ISL_2101184, EPI_ISL_2101185, EPI_ISL_2101186, EPI_ISL_2101187, EPI_ISL_2101188, EPI_ISL_2101189, EPI_ISL_2101190, EPI_ISL_2101191, EPI_ISL_2101192, EPI_ISL_2101193, EPI_ISL_2101194, EPI_ISL_2101195, EPI_ISL_2101196, EPI_ISL_2101197, EPI_ISL_2101198, EPI_ISL_2101199, EPI_ISL_2101200, EPI_ISL_2101202, EPI_ISL_2101203, EPI_ISL_2101204, EPI_ISL_2101205, EPI_ISL_2101206, EPI_ISL_2101207, EPI_ISL_2101208, EPI_ISL_2101209, EPI_ISL_2101210, EPI_ISL_2101211, EPI_ISL_2101212, EPI_ISL_2101213, EPI_ISL_2101214, EPI_ISL_2101215			
see above	Molecular Biology Laboratory, Faculty Medicine and Health Sciences, Warmadewa University	Eijkman Institute for Molecular Biology, National Agency for Research and Innovation; Molecular Biology Laboratory, Faculty Medicine and Health Sciences, Warmadewa University	Amin Soebandrio; Edison Johar; Erly Sintya; Frilasita A Yudhaputri; Hidayat Trimarsanto; Iskandar Adnan; Khin Saw Myint; Lidwina Priliani; Lydia V. Panggalo; Muhammad Rezki Rasyak; Safarina G Malik; Sri Masyeni; Sukma Oktavianthi; Willy Agustine
EPI_ISL_3030002	Molecular Biology Laboratory, Faculty Medicine and Health Sciences, Warmadewa University	Eijkman Institute for Molecular Biology, National Research and Innovation Agency; Faculty Medicine and Health Sciences, Warmadewa University	Amin Soebandrio; Edison Johar; Erly Sintya; Frilasita A Yudhaputri; Hidayat Trimarsanto; Iskandar Adnan; Khin Saw Myint; Lidwina Priliani; Lydia V. Panggalo; Muhammad Rezki Rasyak; Safarina G Malik; Sri Masyeni; Sukma Oktavianthi; Willy Agustine
EPI_ISL_1795055, EPI_ISL_1795057	Muhammadiyah Hospital	Institute of Tropical Disease, Universitas Airlangga	Aldise M Nastri; Gatot Soegiarto; Imam Suyuthi; Jezzy R Dewantari; Kazufumi Shimizu; Krisnoadi Rahardjo; Laksmi Wulandari; Maria I Lusida; Resti Yudhawati; Rima R Prasetya; Soetjipto; Yasuko Mori
EPI_ISL_1117456	Murni Teguh Hospital, Jakarta	Biosafety Level-3 Laboratory, Indonesian Institute of Sciences (LIPI)	Ade Andriani; Anik Budhi Dharmayanthi; Asep M. Ridwanuloh; Eko Wahyu Putro; Gita Syahputra; Hariyatun; Inswasti Cahyani; Matthew W. Loose; Mohammad Ilyas; Susanti; Syam Budi Iryanto; Wien Kusharyoto
EPI_ISL_5021389, EPI_ISL_5021397, EPI_ISL_5021401, EPI_ISL_5021403, EPI_ISL_5021446, EPI_ISL_5021455, EPI_ISL_5021481, EPI_ISL_5021502, EPI_ISL_5021516, EPI_ISL_5021519, EPI_ISL_5021531, EPI_ISL_5021542, EPI_ISL_5021545, EPI_ISL_5021559, EPI_ISL_5021588, EPI_ISL_5021591			
see above	Murni Teguh Memorial Hospital	Eijkman Research Center for Molecular Biology, National Research and Innovation Agency; Microbiology Laboratory, Faculty of Medicine, University of Sumatera Utara	Amin Soebandrio; Edison Johar; Frilasita A Yudhaputri; Hidayat Trimarsanto; Inke Nadia Diniyanti Lubis; Iskandar Adnan; Jessica R Saragih; Khin Saw Myint; Lidwina Priliani; Lydia V Panggalo; Muhammad Rezki Rasyak; Mutiara; R Lia Kusumawati; Safarina G Malik; Sahat Halim; Sukma Oktavianthi; Tryna Tania; Willy Agustine
EPI_ISL_3208058, EPI_ISL_3208059, EPI_ISL_3208060, EPI_ISL_3208061, EPI_ISL_3208062, EPI_ISL_3208063, EPI_ISL_3208064, EPI_ISL_3208065, EPI_ISL_3208066, EPI_ISL_3208067, EPI_ISL_3208068, EPI_ISL_3208069, EPI_ISL_3208070, EPI_ISL_3208071, EPI_ISL_3208072, EPI_ISL_3208073, EPI_ISL_3208074, EPI_ISL_3208086			
see above	Murni Teguh Memorial Hospital	Genomik Solidaritas Indonesia Laboratorium	Annisa Muthiah Sukirman; Anuraj Shankar; Ariel Pradipta; Carissa Sintca Wijaya; Gracia Felias Enos Korompis; Inke Nadia Diniyanti Lubis; Lia Kusumawati; Meutia Ayuputeri Kumaheri; Mutiara; Reinhart Gabriel; Ririe Fachrina Malisie; Tryna Tania; Vania Gavrila Wikasa
EPI_ISL_3200739, EPI_ISL_3200740, EPI_ISL_3200741, EPI_ISL_3200742, EPI_ISL_3200743, EPI_ISL_3200744, EPI_ISL_3200745, EPI_ISL_3200746, EPI_ISL_3200747, EPI_ISL_3200748, EPI_ISL_3200749, EPI_ISL_3200750, EPI_ISL_3200751, EPI_ISL_3200752, EPI_ISL_3200753, EPI_ISL_3200754, EPI_ISL_3200755, EPI_ISL_3200756, EPI_ISL_3200757, EPI_ISL_3200758, EPI_ISL_3200759, EPI_ISL_3200760, EPI_ISL_3200761, EPI_ISL_3200762, EPI_ISL_3200763, EPI_ISL_3200764, EPI_ISL_3200765, EPI_ISL_3200766, EPI_ISL_3200767			
see above	Murni Teguh Memorial Hospital / EIJKMAN OXFORD CLINICAL RESEARCH UNIT	Genomik Solidaritas Indonesia Laboratorium	Anna Christina Brazia; Annisa Muthiah Sukirman; Anuraj Shankar; Ariel Pradipta; Ayodhia Pitaloka Pasaribu; Carissa Sintca; Decy Subekti; Gracia Felias Enos Korompis; Hendri Wijaya; Louisa Markus; Meutia Ayuputeri Kumaheri; Mutiara; Raph Hamers; Suwarti; Vania Gavrila Wikasa
EPI_ISL_5022828	Naraya Medical Center	National Institute of Health Research and Development	Arie Ardiansyah Nugraha; Fajar Nur Sulistiyohadi; Hana Apsari Pawestri; Hartanti Dian Ikawati; Kartika Dewi Puspa; Krisna Pangesti; Nelly Puspandari; Subangkit; Triyani Soekarso; Vivi Setiawaty
EPI_ISL_4508259, EPI_ISL_4508260, EPI_ISL_4508261, EPI_ISL_4508262, EPI_ISL_4508263, EPI_ISL_4508264, EPI_ISL_4508265, EPI_ISL_4508266, EPI_ISL_4508267, EPI_ISL_5022769, EPI_ISL_5022770, EPI_ISL_5022771, EPI_ISL_5022772, EPI_ISL_5022773, EPI_ISL_5022774, EPI_ISL_5022775, EPI_ISL_5022776, EPI_ISL_5022777, EPI_ISL_5022778, EPI_ISL_5022779, EPI_ISL_5022780, EPI_ISL_5022781, EPI_ISL_5022782, EPI_ISL_5022783, EPI_ISL_5022937, EPI_ISL_5022938, EPI_ISL_5107845			
see above	Naraya Medical Center, Bekasi	National Institute of Health Research and Development	Arie Ardiansyah Nugraha; Fajar Nur; Fajar Nur Sulistiyohadi; Hana Apsari Pawestri; Hartanti Dian Ikawati; Kartika Dewi Puspa; Krisna Pangesti; Nelly Puspandari; Subangkit; Sulistiyohadi; Triyani Soekarso; Vivi Setiawaty
EPI_ISL_2284873	National Hospital	Institute of Tropical Disease, Universitas Airlangga	Aldise M Nastri; Azwan H Lubis; Gatot Soegiarto; Jezzy R Dewantari; Kazufumi Shimizu; Krisnoadi Rahardjo; Laksmi Wulandari; Maria I Lusida; Resti Yudhawati; Rima R Prasetya; Soetjipto; Yasuko Mori
EPI_ISL_1469238, EPI_ISL_1469241, EPI_ISL_1469242, EPI_ISL_1469243, EPI_ISL_1469244, EPI_ISL_1469245, EPI_ISL_1469252, EPI_ISL_1469255, EPI_ISL_1469259, EPI_ISL_1469260, EPI_ISL_1469261, EPI_ISL_1469262, EPI_ISL_1469263, EPI_ISL_1469264, EPI_ISL_1469265, EPI_ISL_1469266, EPI_ISL_1469267, EPI_ISL_1469268, EPI_ISL_1469269, EPI_ISL_1469270, EPI_ISL_1469276, EPI_ISL_1469277, EPI_ISL_1469278, EPI_ISL_1469290, EPI_ISL_1469291, EPI_ISL_1534706, EPI_ISL_1534707			
see above	National Institute Health Research and Development	National Institute of Health Research and Development	Agustiningsih; Arie Ardiansyah Nugraha; Fauzul Muna; Hana Apsari Pawestri; Hartanti Dian Ikawati; Herna; Holy Arif Wibowo; Irene Lorinda Indalao; Kartika Dewi Puspa; Kindi Adam; Krisna Nur Andriana Pangesti; Krisna Pangesti; Natalie Laurencia Kipuw; Nelly Puspandari; Ni Ketut Susilarini; Nike Susanti; Nur Ika Hariastuti; Nurika Hariastuti; Reni Herman; Ririn Ramadhany; Subangkit; Tati Febriyanti; Triyani Soekarso; Ully Alfi Nikmah; Vivi Setiawaty; Vivi Setiawaty.; Yuni Rukminiati
EPI_ISL_413219, EPI_ISL_414375, EPI_ISL_414376, EPI_ISL_414377, EPI_ISL_428207, EPI_ISL_428208, EPI_ISL_538504, EPI_ISL_538505, EPI_ISL_538508, EPI_ISL_538509, EPI_ISL_574433, EPI_ISL_576386, EPI_ISL_576387, EPI_ISL_747233, EPI_ISL_747234, EPI_ISL_747237, EPI_ISL_756376, EPI_ISL_756401, EPI_ISL_757287, EPI_ISL_833130, EPI_ISL_909772, EPI_ISL_910014, EPI_ISL_942960, EPI_ISL_942971, EPI_ISL_943548, EPI_ISL_949183, EPI_ISL_949192, EPI_ISL_953427, EPI_ISL_984276, EPI_ISL_984277, EPI_ISL_984309, EPI_ISL_984313, EPI_ISL_1118206, EPI_ISL_1118207, EPI_ISL_1118208, EPI_ISL_1118209, EPI_ISL_1118210, EPI_ISL_1118211, EPI_ISL_1118212, EPI_ISL_1118213, EPI_ISL_1118214, EPI_ISL_1118215, EPI_ISL_1118216, EPI_ISL_1118931, EPI_ISL_1118933, EPI_ISL_1169046, EPI_ISL_1169050, EPI_ISL_1169051, EPI_ISL_1169052, EPI_ISL_1169053, EPI_ISL_1169054, EPI_ISL_1169055, EPI_ISL_1169056, EPI_ISL_1169057, EPI_ISL_1169058, EPI_ISL_1169059, EPI_ISL_1169060, EPI_ISL_1170948, EPI_ISL_1170955, EPI_ISL_1239143, EPI_ISL_1239144, EPI_ISL_1239145, EPI_ISL_1239216, EPI_ISL_1239217, EPI_ISL_1239218, EPI_ISL_1239219, EPI_ISL_1239221, EPI_ISL_1239222, EPI_ISL_1239223, EPI_ISL_1257816, EPI_ISL_1257817, EPI_ISL_1391009, EPI_ISL_1391010, EPI_ISL_1391011, EPI_ISL_1391012, EPI_ISL_1391013, EPI_ISL_1391014, EPI_ISL_1391015, EPI_ISL_1391016, EPI_ISL_1391017, EPI_ISL_1391018, EPI_ISL_1411159, EPI_ISL_1413427, EPI_ISL_1415427, EPI_ISL_1824601, EPI_ISL_1824602, EPI_ISL_1824603, EPI_ISL_1824608, EPI_ISL_1824609, EPI_ISL_1824610, EPI_ISL_1824611, EPI_ISL_1824612, EPI_ISL_1824613, EPI_ISL_1824614, EPI_ISL_1824719, EPI_ISL_1915511, EPI_ISL_1915512, EPI_ISL_1915513, EPI_ISL_1915514, EPI_ISL_1915515, EPI_ISL_1915516, EPI_ISL_1915517, EPI_ISL_1915518, EPI_ISL_1915519, EPI_ISL_1915520, EPI_ISL_1915521, EPI_ISL_1915522, EPI_ISL_1915523, EPI_ISL_1915524, EPI_ISL_1915525, EPI_ISL_1915526, EPI_ISL_1915527, EPI_ISL_1915528, EPI_ISL_1915529, EPI_ISL_1915530, EPI_ISL_1915531, EPI_ISL_1915532, EPI_ISL_1915533, EPI_ISL_1915534, EPI_ISL_1915535, EPI_ISL_1915536, EPI_ISL_1915537, EPI_ISL_1915538, EPI_ISL_1915539, EPI_ISL_1915540, EPI_ISL_1915541, EPI_ISL_1915542, EPI_ISL_1915543, EPI_ISL_1915544, EPI_ISL_1915545, EPI_ISL_1915546, EPI_ISL_1915547, EPI_ISL_1915548, EPI_ISL_1915549, EPI_ISL_2106988, EPI_ISL_2429144			
see above	National Institute of Health Research and Development	National Institute of Health Research and Development	; A; AA; Adam; Agustiningsih; Arie Ardiansyah Nugraha; Asri Febriyani; Aulia Rizki; D; E; Estaria; F; Fauzul Muna; Febriyani; Febriyanti; HA; HD; Hana A Pawestri; Hana Apsari Pawestri; Hariastuti; Hartanti Dian Ikawati; Herman; Herna; Holy Arif Wibowo; IL; Ikawati; Indalao; Indalau; Indrasari; Irene Lorinda Indalao; J; Juwita Kurniawati; K; KD; KNA; Kartika Dewi Puspa; Kindi Adam; Kipuw; Krisna Nur Andriana Pangesti; Krisna Pangesti; Kurniawati; M; Monika; Muhammad Nurhadi; Muna; Mursinah; N; ND; NI; NK; NL; Nafiandi; Natalie Laurencia Kipuw; Natalie Laurentia Kipuw; Nelly Puspandari; Ni Ketut Susilarini; Nike Susanti; Nikmah; Nugraha; Nur Ika Hariastuti; Nurhadi; Nurika Hariastuti; Paisal; Pangesti; Pawestri; Pratiwi; Puspa; Puspandari; Puspandary; R; Ramadhany; Reni Herman; Ririn Ramadhany; Rizki; Rukminiati; Setiawaty; Setiawaty, V.; Siswanto; Soekarso; Subangkit; Susanti; Susilarini; T; Tati Febriyanti; Triyani Soekarso; UA; Uli Alfi Nikmah; Ully Alfi Nikmah; Uly Alfi Nikmah; V; Vivi; Vivi Setiawaty; Vivi Setiawaty.; Wibowo; Wulandari; Y; Yuni Rukminiati; risna Nur Andriana Pangesti; vivi Setiawaty
EPI_ISL_1335883, EPI_ISL_1335885, EPI_ISL_1335887, EPI_ISL_1335897	North Sumatera Health Office	Faculty of Medicine, Universitas Sumatera Utara; Institute of Tropical Disease, Universitas Airlangga	Aldise M Nastri; Franciscus Ginting; Inke N D Lubis; Irbah R Nainggolan; Jezzy R Dewantari; Kazufumi Shimizu; Krisnoadi Rahardjo; Maria I Lusida; Meliani; Mirzan Hasibuan; Muhammad Ichwan; R Andika D Cahyadi; R Lia Kusumawati; Ramadhan Bestari; Rima R Prasetya; Soetjipto; Yasuko Mori
EPI_ISL_1072963	North Sumatera Health Office	Institute of Tropical Disease, Universitas Airlangga; Faculty of Medicine, Universitas Sumatera Utara	Aldise M Nastri; Franciscus Ginting; Inke N D Lubis; Irbah R Nainggolan; Jezzy R Dewantari; Kazufumi Shimizu; Krisnoadi Rahardjo; Maria I Lusida; Meliani; Mirzan Hasibuan; Muhammad Ichwan; R Andika D Cahyadi; R Lia Kusumawati; Ramadhan Bestari; Rima R Prasetya; Soetjipto; Yasuko Mori
EPI_ISL_3138840	OMNI Hospitals Pekayon	National Institute of Health Research and Development	Arie Ardiansyah Nugraha; Fajar Nur Sulistiyohadi; Hana Apsari Pawestri; Hartanti Dian Ikawati; Kartika Dewi Puspa; Krisna Pangesti; Nelly Puspandari; Subangkit; Triyani Soekarso; Vivi Setiawaty
EPI_ISL_1915571	PKC Cilincing	National Institute of Health Research and Development	Arie Ardiansyah Nugraha; Hana Apsari Pawestri; Hartanti Dian Ikawati; Kartika Dewi Puspa; Krisna Pangesti; Nelly Puspandari; Subangkit; Vivi Setiawaty
EPI_ISL_1824607	PKC Jagakarsa	National Institute of Health Research and Development	Arie Ardiansyah Nugraha; Hana Apsari Pawestri; Hartanti Dian Ikawati; Kartika Dewi Puspa; Krisna Pangesti; Nelly Puspandari; Subangkit; Vivi Setiawaty
EPI_ISL_2106983, EPI_ISL_3138789, EPI_ISL_3138790, EPI_ISL_3138791	PKC Kramat Jati	National Institute of Health Research and Development	Arie Ardiansyah Nugraha; Fajar Nur Sulistiyohadi; Hana Apsari Pawestri; Hartanti Dian Ikawati; Kartika Dewi Puspa; Krisna Pangesti; Nelly Puspandari; Subangkit; Triyani Soekarso; Vivi Setiawaty
EPI_ISL_2931730, EPI_ISL_2931738, EPI_ISL_2931747, EPI_ISL_2931767, EPI_ISL_2931792, EPI_ISL_3019941, EPI_ISL_3019942, EPI_ISL_3138927			

see above	PKM Abiansemal III, Bali	National Institute of Health Research and Development	; Arie Ardiansyah Nugraha; Fajar Nur Sulistiyohadi; Hana Apsari Pawestri; Hartanti Dian Ikawati; Kartika Dewi Puspa; Krisna Pangesti; Nelly Puspandari; Subangkit; Triyani Soekarso; Vivi Setiawaty
EPI_ISL_3138839	PKM Kec Kramat Jati	National Institute of Health Research and Development	Arie Ardiansyah Nugraha; Fajar Nur Sulistiyohadi; Hana Apsari Pawestri; Hartanti Dian Ikawati; Kartika Dewi Puspa; Krisna Pangesti; Nelly Puspandari; Subangkit; Triyani Soekarso; Vivi Setiawaty
EPI_ISL_3401891, EPI_ISL_3401892, EPI_ISL_3401894	PKM Kelapa Gading	Genomik Solidaritas Indonesia Laboratorium	Annisa Muthiah Sukirman; Anuraj Shankar; Ariel Pradipta; Carissa Sintca Wijaya; Dhahlia Agustina Cahyono; Dwi Oktavia; Gracia Felias Enos Korompis; Himawan Masyhuri; Louisa Markus; Meutia Ayuputeri Kumaheri; Ngabila Salama; Tiranti Vindha; Vania Gavrila Wikasa
EPI_ISL_2617466, EPI_ISL_2617467, EPI_ISL_2617468, EPI_ISL_2617469, EPI_ISL_2617470, EPI_ISL_2617471, EPI_ISL_2617472, EPI_ISL_2617473, EPI_ISL_2617474, EPI_ISL_2617475, EPI_ISL_2617476, EPI_ISL_2617477, EPI_ISL_2617478, EPI_ISL_2617479, EPI_ISL_2617480, EPI_ISL_2617481, EPI_ISL_2617482, EPI_ISL_2617483, EPI_ISL_2617484, EPI_ISL_2617485, EPI_ISL_2617486, EPI_ISL_2617487, EPI_ISL_2617488, EPI_ISL_2617489, EPI_ISL_2617490, EPI_ISL_2617491, EPI_ISL_2617492			
see above	PKM SINDANG BARANG BOGOR	National Institute of Health Research and Development	Agustiningsih; Arie Ardiansyah Nugraha; Fauzul Muna; Hana Apsari Pawestri; Hartanti Dian Ikawati; Herna; Holy Arif Wibowo; Irene Lorinda Indalao; Kartika Dewi Puspa; Kindi Adam; Krisna Nur Andriana Pangesti; Natalie Laurencia Kipuw; Nelly Puspandari; Ni Ketut Susilarini; Nike Susanti; Nurika Hariastuti; Reni Herman; Ririn Ramadhany; Subangkit; Tati Febriyanti; Triyani Soekarso; Ully Alfi Nikmah; Vivi Setiawaty.; Yuni Rukminiati
EPI_ISL_1469258	PKM TEMPURAN KARAWANG	National Institute of Health Research and Development	Agustiningsih; Arie Ardiansyah Nugraha; Fauzul Muna; Hana Apsari Pawestri; Hartanti Dian Ikawati; Herna; Holy Arif Wibowo; Irene Lorinda Indalao; Kartika Dewi Puspa; Kindi Adam; Krisna Nur Andriana Pangesti; Natalie Laurencia Kipuw; Nelly Puspandari; Ni Ketut Susilarini; Nike Susanti; Nurika Hariastuti; Reni Herman; Ririn Ramadhany; Subangkit; Tati Febriyanti; Triyani Soekarso; Ully Alfi Nikmah; Vivi Setiawaty.; Yuni Rukminiati
EPI_ISL_3401893	PKM Tanjung Priok	Genomik Solidaritas Indonesia Laboratorium	Annisa Muthiah Sukirman; Anuraj Shankar; Ariel Pradipta; Carissa Sintca Wijaya; Dhahlia Agustina Cahyono; Dwi Oktavia; Gracia Felias Enos Korompis; Himawan Masyhuri; Louisa Markus; Meutia Ayuputeri Kumaheri; Ngabila Salama; Tiranti Vindha; Vania Gavrila Wikasa; Wahyudi
EPI_ISL_1824604, EPI_ISL_1915551, EPI_ISL_1915552, EPI_ISL_1915553	PRVKP FK UI	National Institute of Health Research and Development	Arie Ardiansyah Nugraha; Hana Apsari Pawestri; Hartanti Dian Ikawati; Kartika Dewi Puspa; Krisna Pangesti; Nelly Puspandari; Subangkit; Triyani Soekarso; Vivi Setiawaty
EPI_ISL_4508324, EPI_ISL_4508325, EPI_ISL_4508326, EPI_ISL_4508327, EPI_ISL_4508328, EPI_ISL_4508329, EPI_ISL_4508330, EPI_ISL_4508331, EPI_ISL_4508332, EPI_ISL_4508333, EPI_ISL_4730555, EPI_ISL_4730556, EPI_ISL_4730557, EPI_ISL_4730558, EPI_ISL_4730559, EPI_ISL_4730560, EPI_ISL_4730561, EPI_ISL_4730562, EPI_ISL_4730563, EPI_ISL_5022836, EPI_ISL_5022837, EPI_ISL_5022838, EPI_ISL_5022839, EPI_ISL_5022840, EPI_ISL_5022841, EPI_ISL_5022842, EPI_ISL_5022843, EPI_ISL_5022844, EPI_ISL_5022852, EPI_ISL_5022853, EPI_ISL_5022854, EPI_ISL_5022855, EPI_ISL_5022856, EPI_ISL_5022857, EPI_ISL_5022957, EPI_ISL_5107846, EPI_ISL_5107847, EPI_ISL_5107848, EPI_ISL_5107849, EPI_ISL_5107850, EPI_ISL_5107851, EPI_ISL_5107852, EPI_ISL_5107853, EPI_ISL_5107854, EPI_ISL_5107855, EPI_ISL_5441736, EPI_ISL_5441737, EPI_ISL_5441738, EPI_ISL_5441739, EPI_ISL_5441740, EPI_ISL_5441741, EPI_ISL_5441742, EPI_ISL_5441743, EPI_ISL_5441748, EPI_ISL_5441749, EPI_ISL_5441750, EPI_ISL_5441773, EPI_ISL_5441774, EPI_ISL_5441775, EPI_ISL_5441776, EPI_ISL_5441777, EPI_ISL_5441778, EPI_ISL_5441779, EPI_ISL_5441780, EPI_ISL_5441781, EPI_ISL_5441782, EPI_ISL_5441783, EPI_ISL_5743645, EPI_ISL_5743662, EPI_ISL_5743672, EPI_ISL_5743681, EPI_ISL_5743686, EPI_ISL_5743716, EPI_ISL_5743725, EPI_ISL_5743726, EPI_ISL_5743736, EPI_ISL_5743737, EPI_ISL_5743742, EPI_ISL_5743743, EPI_ISL_5743744, EPI_ISL_5743751, EPI_ISL_5743752, EPI_ISL_5743760, EPI_ISL_5743761, EPI_ISL_5743769, EPI_ISL_5743770, EPI_ISL_5743771, EPI_ISL_5743776			
see above	PRVKP FKUI	National Institute of Health Research and Development	Arie Ardiansyah Nugraha; Fajar Nur Sulistiyohadi; Hana Apsari Pawestri; Hartanti Dian Ikawati; Kartika Dewi Puspa; Krisna Pangesti; Nelly Puspandari; Subangkit; Triyani Soekarso; Vivi Setiawaty
EPI_ISL_5022916, EPI_ISL_5022917, EPI_ISL_5022918, EPI_ISL_5022919, EPI_ISL_5441744, EPI_ISL_5441745, EPI_ISL_5441746, EPI_ISL_5441747			
see above	PRVKP FKUI (Lab Hamera Sarana Indonesia)	National Institute of Health Research and Development	Arie Ardiansyah Nugraha; Fajar Nur Sulistiyohadi; Hana Apsari Pawestri; Hartanti Dian Ikawati; Kartika Dewi Puspa; Krisna Pangesti; Nelly Puspandari; Subangkit; Triyani Soekarso; Vivi Setiawaty
EPI_ISL_3730787	PT Kartika Bina Medikatama (Medika Plaza)	Genomik Solidaritas Indonesia Laboratorium	Anna Christina Brazia; Annisa Muthiah Sukirman; Anuraj Shankar; Ariel Pradipta; Dhahlia Agustina Cahyono; Dwi Oktavia; Gracia Felias Enos Korompis; Louisa Markus; Meutia Ayuputeri Kumaheri; Ngabila Salama; Rafikana Desi; Reinhart Gabriel; Tiranti Vindha; Vania Gavrila Wikasa
EPI_ISL_2987613, EPI_ISL_2987617, EPI_ISL_2987621, EPI_ISL_2987622, EPI_ISL_2987623, EPI_ISL_2987624	PUSKESMAS BOJONGSARI	National Institute of Health Research and Development	Arie Ardiansyah Nugraha; Fajar Nur Sulistiyohadi; Hana Apsari Pawestri; Hartanti Dian Ikawati; Kartika Dewi Puspa; Krisna Pangesti; Nelly Puspandari; Subangkit; Triyani Soekarso; Vivi Setiawaty
EPI_ISL_2337255	PUSKESMAS JEPARA	Genetics Working Group (Pokja Genetik) Faculty of Medicine, Public Health and Nursing Universitas Gadjah Mada (FK-KMK UGM); Disease Investigation Center Wates Ministry of Agriculture Indonesia; Department of Microbiology FK-KMK UGM; Laboratorium Diagnostik Yayasan Tahija World Mosquito Program (WMP) Yogyakarta Center for Tropical Medicine FK-KMK UGM; Integrated Research Center FK-KMK UGM; Department of Computer Science and Electronics FMIPA UGM; RSUP Dr. Sardjito	Afiahayati; Alvina A Setiawan; Cita S Amalia; Dwi AA Nugrahaningsih; Edwin W. Daniwijaya; Eggi Arguni; Endah Supriyati; Gunadi; Hendra Wibawa; Kristy Iskandar; Ludhang P. Rizki; Marcellus; Mohamad S. Hakim; Nungki Anggorowati; Siswanto; Titik Nuryastuti; Tri Wibawa
EPI_ISL_5200494, EPI_ISL_5200495, EPI_ISL_5200496, EPI_ISL_5200501	PUSKESMAS KECAMATAN CAKUNG	Genomik Solidaritas Indonesia Laboratorium	Annisa Muthiah Sukirman; Anuraj Shankar; Ariel Pradipta; Carissa Sintca Wijaya; Gracia Felias Enos Korompis; Junaidah; Louisa Markus; Meutia Ayuputeri Kumaheri; Rahmaniwati Sulaiman; Reinhart Gabriel; Santi Rosamarlia; Stefanus Bernard; Vania Gavrila Wikasa
EPI_ISL_3730786, EPI_ISL_3730798	PUSKESMAS KECAMATAN PANCORAN	Genomik Solidaritas Indonesia Laboratorium	Aisyah Rachmadini; Anna Christina Brazia; Annisa Muthiah Sukirman; Anuraj Shankar; Ariel Pradipta; Dhahlia Agustina Cahyono; Dwi Oktavia; Gracia Felias Enos Korompis; Louisa Markus; Meutia Ayuputeri Kumaheri; Ngabila Salama; Reinhart Gabriel; Tiranti Vindha; Vania Gavrila Wikasa
EPI_ISL_3541163, EPI_ISL_3541164	PUSKESMAS KECAMATAN PASAR MINGGU	National Institute of Health Research and Development	Arie Ardiansyah Nugraha; Fajar Nur Sulistiyohadi; Hana Apsari Pawestri; Hartanti Dian Ikawati; Kartika Dewi Puspa; Krisna Pangesti; Nelly Puspandari; Subangkit; Triyani Soekarso; Vivi Setiawaty
EPI_ISL_2987612, EPI_ISL_2987620	PUSKESMAS SINDANG BARANG	National Institute of Health Research and Development	Arie Ardiansyah Nugraha; Fajar Nur Sulistiyohadi; Hana Apsari Pawestri; Hartanti Dian Ikawati; Kartika Dewi Puspa; Krisna Pangesti; Nelly Puspandari; Subangkit; Triyani Soekarso; Vivi Setiawaty
EPI_ISL_2987614	PUSKESMAS TANA SEREAL	National Institute of Health Research and Development	Arie Ardiansyah Nugraha; Fajar Nur Sulistiyohadi; Hana Apsari Pawestri; Hartanti Dian Ikawati; Kartika Dewi Puspa; Krisna Pangesti; Nelly Puspandari; Subangkit; Triyani Soekarso; Vivi Setiawaty
EPI_ISL_2987616	PUSKESMAS TANAH SEREAL BOGOR	National Institute of Health Research and Development	Arie Ardiansyah Nugraha; Fajar Nur Sulistiyohadi; Hana Apsari Pawestri; Hartanti Dian Ikawati; Kartika Dewi Puspa; Krisna Pangesti; Nelly Puspandari; Subangkit; Triyani Soekarso; Vivi Setiawaty
EPI_ISL_956277, EPI_ISL_956314, EPI_ISL_1159388, EPI_ISL_1165075, EPI_ISL_1789202	Paru Hospital	Institute of Tropical Disease, Universitas Airlangga	Aldise M Nastri; Dyah Retno; Gatot Soegiarto; Jezzy R Dewantari; Kazufumi Shimizu; Krisnoadi Rahardjo; Laksmi Wulandari; Maria I Lusida; Resti Yudhawati; Rima R Prasetya; Soetjipto; Yasuko Mori
EPI_ISL_5328537	Pelabuhan Ratu Sukabumi Regional Hospital	West Java Health Laboratory; School of Life Sciences and Technology, Institut Teknologi Bandung	Azzania Fibriani; Cut Nur Cinthia Alamanda; Ema Rahmawati; Karimatu Khoirunnisa; Miftahul Faridl; Rifky Waluyajati Rachman; Rini Robiani; Ryan Bayusantika Ristandi
EPI_ISL_4885264, EPI_ISL_4885265	Pelabuhan Ratu Sukabumi Regional Public Hospital	West Java Health Laboratory; School of Life Sciences and Technology, Institut Teknologi Bandung	Azzania Fibriani; Cut Nur Cinthia Alamanda; Ema Rahmawati; Isak Solihin; Karimatu Khoirunnisa; Miftahul Faridl; Rifky Waluyajati Rachman; Rini Robiani; Ryan Bayusantika Ristandi
EPI_ISL_5616413	Politeknik Kesehatan Kemenkes Semarang	Balai Laboratorium Kesehatan dan Pengujian Alat Kesehatan Provinsi Jawa Tengah	Alia Wijayanti; Dwi Nursetyaasih; Dyah Ayu; Endah Wahyu Y; Ismi Farhati; Purnomo; Sekti Handayani; Wiwik Peni; Yekti Hediningsih
EPI_ISL_759959	Premier Hospital Surabaya	Institute of Tropical Disease, Universitas Airlangga	Aldise M Nastri; Gatot Soegiarto; Hartono Tanto; Jezzy R Dewantari; Kazufumi Shimizu; Krisnoadi Rahardjo; Laksmi Wulandari; Maria I Lusida; Resti Yudhawati; Rima R Prasetya; Soetjipto; Yasuko Mori
EPI_ISL_759955, EPI_ISL_877423, EPI_ISL_877456, EPI_ISL_877457, EPI_ISL_956307, EPI_ISL_2284868, EPI_ISL_2284882, EPI_ISL_4056014			
see above	Primasatya Husada Citra Hospital	Institute of Tropical Disease, Universitas Airlangga	Aldise M Nastri; Gatot Soegiarto; Jezzy R Dewantari; Kazufumi Shimizu; Kohar Hari Santoso; Krisnoadi Rahardjo; Laksmi Wulandari; Maria I Lusida; Pudji Djanuartono; Resti Yudhawati; Rima R Prasetya; Soetjipto; Yasuko Mori
EPI_ISL_1020179, EPI_ISL_1020181, EPI_ISL_1020191, EPI_ISL_2840868	Primera Clinica, Jakarta	Biosafety Level-3 Laboratory, Indonesian Institute of Sciences (LIPI)	Andri Wardiana; Anggia Prasetyoputri; Anik B. Dharmayanthi; Ari S. Wulandari; Erwin F. Hasrianda; Fahrurrozi; Herjuno A. Nugroho; Indriawati; Isa Nuryana; Nova D. Yanthi; Ratih Asmana Ningrum; Sugiyono Saputra; Syaiful Rizal; Syam B. Iryanto; Syam Budi Iryanto
EPI_ISL_2631478, EPI_ISL_2631479, EPI_ISL_2631480	Prodia Karawang	Indonesian Institute of Sciences (LIPI)/National Agency of Research and Innovation (BRIN) and National Institute of Health Research and Development (NIHRD)	Alfi T. Fathurahman; Andri Wardiana; Anik B. Dharmayanthi; Asep M. Ridwanulloh; Erwin F. Hasrianda; Hariyatun; Ratih A. Ningrum; Ruby Setiawan; Subangkit; Sugiyono Saputra; Syam B. Iryanto; Vivi Setiawaty; Yayuk Sri Rahayu
EPI_ISL_538513	Provincial Health Laboratory Bekasi West Java	National Institute of Health Research and Development	AA; HA; HD; Ikawati; KD; KNA; Nugraha; Paisal; Pangesti; Pawestri; Puspa; Setiawaty, V.; Soekarso; Subangkit; T
EPI_ISL_1398936	Purwakarta Public Health	West Java Health Laboratory	Azzania Fibriani; Cut Nur Cinthia Alamanda; Ema Rahmawati; Isak Solihin; Kamila Tania; Karimatu Khoirunnisa; Miftahul Faridl; Rifky Waluyajati Rachman; Rini Robiani; Ryan Bayusantika Ristandi
EPI_ISL_1634467	Purwakarta Public Health	West Java Health Laboratory; School of Life Sciences and Technology, Institut Teknologi Bandung	Aulia Saraswati Wicaksono; Azzania Fibriani; Cut Nur Cinthia Alamanda; Ema Rahmawati; Karimatu Khoirunnisa; Miftahul Faridl; Rifky Waluyajati Rachman; Rini Robiani; Ryan Bayusantika Ristandi
EPI_ISL_437189	Pusat Pertamina Hospital	Eijkman Institute for Molecular Biology, Ministry of Research and Technology/National Agency for Research and Innovation	Amin Soebandrio; David H Muljono; Edison Johar; Frilasita A Yudhaputri; Hidayat Trimarsanto; Khin Saw Myint; Safarina G Malik
EPI_ISL_3030049	Puskesmas Arcamanik, Dinas Kesehatan Kota Bandung	Eijkman Institute for Molecular Biology, National Research and Innovation Agency; West Java Health Laboratory	Amin Soebandrio; Azzania Fibriani; Cut Nur Cinthia Alamanda; Edison Johar; Ema Rahmawati; Frilasita A Yudhaputri; Hidayat Trimarsanto; Husna Nugrahapraja; Iskandar Adnan; Khin Saw Myint; Lidwina Priliani; Lydia V. Panggalo; Muhammad Rezki Rasyak; Rifky Waluyajati Rachman; Ryan Bayusantika Ristandi; Safarina G Malik; Sheila Widia Trisakti; Sukma Oktavianthi; Tati Sutarti Sastrowiyoto; Usman Abdul Jabar; Willy Agustine
EPI_ISL_2918646, EPI_ISL_2918647, EPI_ISL_2918648, EPI_ISL_2918649	Puskesmas Baleendah	Eijkman Institute for Molecular Biology, National Research and Innovation Agency; West Java Health Laboratory	Amin Soebandrio; Azzania Fibriani; Cut Nur Cinthia Alamanda; Edison Johar; Ema Rahmawati; Frilasita A Yudhaputri; Hidayat Trimarsanto; Iskandar Adnan; Khin Saw Myint; Lidwina Priliani; Lydia V. Panggalo; Muhammad Rezki Rasyak; Rifky Waluyajati Rachman; Ryan Bayusantika Ristandi; Safarina G Malik; Sukma Oktavianthi; Willy Agustine
EPI_ISL_2918639	Puskesmas Banjaran Kota	Eijkman Institute for Molecular Biology, National Research and Innovation Agency; West Java Health Laboratory	Amin Soebandrio; Azzania Fibriani; Cut Nur Cinthia Alamanda; Edison Johar; Ema Rahmawati; Frilasita A Yudhaputri; Hidayat Trimarsanto; Iskandar Adnan; Khin Saw Myint; Lidwina Priliani; Lydia V. Panggalo; Muhammad Rezki Rasyak; Rifky Waluyajati Rachman; Ryan Bayusantika Ristandi; Safarina G Malik; Sukma Oktavianthi; Willy Agustine
EPI_ISL_610155	Puskesmas Cangkrep	Genetics Working Group (Pokja Genetik) Faculty of Medicine, Public Health and Nursing Universitas Gadjah Mada (FK-KMK UGM); Disease Investigation Center Wates Ministry of Agriculture Indonesia; Department of Microbiology FK-KMK UGM; Laboratorium Diagnostik Yayasan Tahija World Mosquito Program (WMP) Yogyakarta Center for Tropical Medicine FK-KMK UGM; Integrated Research Center FK-KMK UGM; Department of Computer Science and Electronics FMIPA UGM; Balai Besar Teknik Kesehatan Lingkungan dan Pengendalian Penyakit (BBTKLPP) Yogyakarta	Afiahayati; Dwi AA Nugrahaningsih; Edwin W. Daniwijaya; Eggi Arguni; Endah Supriyati; Fadil Fahri; Gunadi; Havid Setyawan; Hendra Wibawa; Indaryati; Irene; Kristy Iskandar; Ludhang P. Rizki; Marcellus; Mohamad S. Hakim; Nawang Sukestiningsih; Nungki Anggorowati; Ririn Enggy Yuliyanti; Siswanto; Titik Nuryastuti; Tri Wibawa
EPI_ISL_3030044	Puskesmas Ciawigebang	Eijkman Institute for Molecular Biology, National Research and Innovation Agency; West Java Health Laboratory	Amin Soebandrio; Azzania Fibriani; Cut Nur Cinthia Alamanda; Edison Johar; Ema Rahmawati; Frilasita A Yudhaputri; Hidayat Trimarsanto; Iskandar Adnan; Khin Saw Myint; Lidwina Priliani; Lydia V. Panggalo; Muhammad Rezki

			Rasyak; Rifky Waluyajati Rachman; Ryan Bayusantika Ristandi; Safarina G Malik; Sukma Oktavianthi; Willy Agustine
EPI_ISL_2964681	Puskesmas Colomadu 1 DKK Karanganyar	Genetics Working Group (Pokja Genetik) Faculty of Medicine, Public Health and Nursing Universitas Gadjah Mada (FK-KMK UGM); Disease Investigation Center Wates Ministry of Agriculture Indonesia; Department of Microbiology FK-KMK UGM; Laboratorium Diagnostik Yayasan Tahija World Mosquito Program (WMP) Yogyakarta Center for Tropical Medicine FK-KMK UGM; Integrated Research Center FK-KMK UGM; Department of Computer Science and Electronics FMIPA UGM; RSUP Dr. Sardjito	Afiahayati; Alvina A Setiawan; Dwi AA Nugrahaningsih; Dwi Indaryati; Edwin W. Daniwijaya; Eggi Arguni; Endah Supriyati; Gunadi; Hendra Wibawa; Indarto Sulistiyono; Khanza a Vujira; Kristy Iskandar; Ludhang P. Rizki; Marcellus; Mohamad S. Hakim; Nungki Anggorowati; Pramesti G Dewi; Siswanto; Titik Nuryastuti; Tri Wibawa
EPI_ISL_4828221	Puskesmas Doplang Blora	Balai Laboratorium Kesehatan Dan Pengujian Alat Kesehatan Provinsi Jawa Tengah	Alia Wijayanti; Dwi Nursetyaasih; Dyah Ayu; Endah Wahyu Y; Ismi Farhati; Purnomo; Sekti Handayani; Wiwik Peni; Yekti Hediningsih
EPI_ISL_4969122, EPI_ISL_4969127, EPI_ISL_4969136, EPI_ISL_4969298, EPI_ISL_4969302, EPI_ISL_5010083, EPI_ISL_5010110, EPI_ISL_5010321			
see above	Puskesmas Galur I	Genetics Working Group (Pokja Genetik) Faculty of Medicine, Public Health and Nursing Universitas Gadjah Mada (FK-KMK UGM); Disease Investigation Center Wates Ministry of Agriculture Indonesia; Department of Microbiology FK-KMK UGM; Laboratorium Diagnostik Yayasan Tahija World Mosquito Program (WMP) Yogyakarta Center for Tropical Medicine FK-KMK UGM; Integrated Research Center FK-KMK UGM; Department of Computer Science and Electronics FMIPA UGM; RSUP Dr. Sardjito	Afiahayati; Alvina A Setiawan; Cita S Amalia; Dwi AA Nugrahaningsih; Dwi Indaryati; Dyah A Puspitarani; Edwin W. Daniwijaya; Eggi Arguni; Endah Supriyati; Gunadi; Hana F Hanifin; Hendra Wibawa; Indarto Sulistiyono; Irene Tania; Khanza a Vujira; Kristy Iskandar; Ludhang P. Rizki; Marcellus; Mohamad S. Hakim; Nungki Anggorowati; Pramesti G Dewi; Siswanto; Susan Simanjaya; Titik Nuryastuti; Tri Wibawa
EPI_ISL_4982801	Puskesmas Grimulyo I	Genetics Working Group (Pokja Genetik) Faculty of Medicine, Public Health and Nursing Universitas Gadjah Mada (FK-KMK UGM); Disease Investigation Center Wates Ministry of Agriculture Indonesia; Department of Microbiology FK-KMK UGM; Laboratorium Diagnostik Yayasan Tahija World Mosquito Program (WMP) Yogyakarta Center for Tropical Medicine FK-KMK UGM; Integrated Research Center FK-KMK UGM; Department of Computer Science and Electronics FMIPA UGM; RSUP Dr. Sardjito	Afiahayati; Dwi AA Nugrahaningsih; Dwi Indaryati; Edwin W. Daniwijaya; Eggi Arguni; Endah Supriyati; Gunadi; Hana F Hanifin; Hendra Wibawa; Indarto Sulistiyono; Irene Tania; Kristy Iskandar; Ludhang P. Rizki; Marcellus; Mohamad S. Hakim; Nungki Anggorowati; Pramesti G Dewi; Siswanto; Titik Nuryastuti; Tri Wibawa
EPI_ISL_763066	Puskesmas Grogol, Sukoharjo	Universitas Sebelas Maret (UNS); Rumah Sakit UNS (RS-UNS); National Institute of Health Research and Development, Indonesian Ministry of Health.	Afif A Ghufron; Arie A Nugraha; Betty Suryawati; Dewi Puspa; Hana A Pawestri; Hartanti Dian Ika; Hartono; Kartika; Maryani; Revi G H Novika; Vivi Setiawaty.; Yulia Sari
EPI_ISL_3030043	Puskesmas Hantara	Eijkman Institute for Molecular Biology, National Research and Innovation Agency; West Java Health Laboratory	Amin Soebandrio; Azzania Fibriani; Cut Nur Cinthia Alamanda; Edison Johar; Ema Rahmawati; Frilasita A Yudhaputri; Hidayat Trimarsanto; Iskandar Adnan; Khin Saw Myint; Lidwina Priliani; Lydia V. Panggalo; Muhammad Rezki Rasyak; Rifky Waluyajati Rachman; Ryan Bayusantika Ristandi; Safarina G Malik; Sukma Oktavianthi; Willy Agustine
EPI_ISL_763070	Puskesmas Jambu Kulon, Klaten	Universitas Sebelas Maret (UNS); Rumah Sakit UNS (RS-UNS); National Institute of Health Research and Development, Indonesian Ministry of Health.	Afif A Ghufron; Arie A Nugraha; Betty Suryawati.; Dewi Puspa; Hana A Pawestri; Hartanti Dian Ika; Hartono; Kartika; Maryani; Vivi Setiawaty; Yulia Sari
EPI_ISL_3030045, EPI_ISL_3030046, EPI_ISL_3030058	Puskesmas Jatihandap, Dinas Kesehatan Kota Bandung	Eijkman Institute for Molecular Biology, National Research and Innovation Agency; West Java Health Laboratory	Ade Fany Safitri; Amin Soebandrio; Azzania Fibriani; Cut Nur Cinthia Alamanda; Edison Johar; Ema Rahmawati; Frilasita A Yudhaputri; Hidayat Trimarsanto; Husna Nugrahapraja; Iskandar Adnan; Khin Saw Myint; Lidwina Priliani; Lydia V. Panggalo; Muhammad Rezki Rasyak; Ranza Fadhlil Pratama; Rifky Waluyajati Rachman; Ryan Bayusantika Ristandi; Safarina G Malik; Sukma Oktavianthi; Tati Sutarti Sastrowiyoto; Willy Agustine
EPI_ISL_3030041	Puskesmas Jelekong	Eijkman Institute for Molecular Biology, National Research and Innovation Agency; West Java Health Laboratory	Amin Soebandrio; Azzania Fibriani; Cut Nur Cinthia Alamanda; Edison Johar; Ema Rahmawati; Frilasita A Yudhaputri; Hidayat Trimarsanto; Iskandar Adnan; Khin Saw Myint; Lidwina Priliani; Lydia V. Panggalo; Muhammad Rezki Rasyak; Rifky Waluyajati Rachman; Ryan Bayusantika Ristandi; Safarina G Malik; Sukma Oktavianthi; Willy Agustine
EPI_ISL_5010106	Puskesmas Kalibawang	Genetics Working Group (Pokja Genetik) Faculty of Medicine, Public Health and Nursing Universitas Gadjah Mada (FK-KMK UGM); Disease Investigation Center Wates Ministry of Agriculture Indonesia; Department of Microbiology FK-KMK UGM; Laboratorium Diagnostik Yayasan Tahija World Mosquito Program (WMP) Yogyakarta Center for Tropical Medicine FK-KMK UGM; Integrated Research Center FK-KMK UGM; Department of Computer Science and Electronics FMIPA UGM; RSUP Dr. Sardjito	Afiahayati; Alvina A Setiawan; Cita S Amalia; Dwi AA Nugrahaningsih; Dwi Indaryati; Edwin W. Daniwijaya; Eggi Arguni; Endah Supriyati; Gunadi; Hendra Wibawa; Indarto Sulistiyono; Kristy Iskandar; Ludhang P. Rizki; Marcellus; Mohamad S. Hakim; Nungki Anggorowati; Pramesti G Dewi; Siswanto; Titik Nuryastuti; Tri Wibawa
EPI_ISL_4828937	Puskesmas Karangrayung II – Kab Grobogan	Balai Laboratorium Kesehatan Dan Pengujian Alat Kesehatan Provinsi Jawa Tengah	Alia Wijayanti; Dwi Nursetyaasih; Dyah Ayu; Endah Wahyu Y; Ismi Farhati; Purnomo; Sekti Handayani; Wiwik Peni; Yekti Hediningsih
EPI_ISL_3401900, EPI_ISL_3401901, EPI_ISL_3401902, EPI_ISL_3401903	Puskesmas Kecamatan Pandemangan	Genomik Solidaritas Indonesia Laboratorium	Annisa Muthiah Sukirman; Anuraj Shankar; Ariel Pradipta; Carissa Sintca Wijaya; Dhahlia Agustina Cahyono; Dwi Oktavia; Gracia Felias Enos Korompis; Himawan Masyhuri; Louisa Markus; Meutia Ayuputeri Kumaheri; Ngabila Salama; Tiranti Vindha; Vania Gavrila Wikasa
EPI_ISL_3401855, EPI_ISL_3401879, EPI_ISL_3401880, EPI_ISL_3401881, EPI_ISL_3401882, EPI_ISL_3401883, EPI_ISL_3401884, EPI_ISL_3401885, EPI_ISL_3401886, EPI_ISL_3401887, EPI_ISL_3401888, EPI_ISL_3401889, EPI_ISL_3401890, EPI_ISL_3664305, EPI_ISL_3664306, EPI_ISL_3664307, EPI_ISL_3664308, EPI_ISL_3664309, EPI_ISL_3664310, EPI_ISL_3664311			
see above	Puskesmas Kecamatan Tanjung Priok	Genomik Solidaritas Indonesia Laboratorium	Ahrahayati Wildany; Annisa Muthiah Sukirman; Anuraj Shankar; Ariel Pradipta; Carissa Sintca Wijaya; Dhahlia Agustina Cahyono; Dwi Oktavia; Gracia Felias Enos Korompis; Himawan Masyhuri; Louisa Markus; Meutia Ayuputeri Kumaheri; Ngabila Salama; Tiranti Vindha; Vania Gavrila Wikasa
EPI_ISL_2918640	Puskesmas Kiangroke	Eijkman Institute for Molecular Biology, National Research and Innovation Agency; West Java Health Laboratory	Amin Soebandrio; Azzania Fibriani; Cut Nur Cinthia Alamanda; Edison Johar; Ema Rahmawati; Frilasita A Yudhaputri; Hidayat Trimarsanto; Iskandar Adnan; Khin Saw Myint; Lidwina Priliani; Lydia V. Panggalo; Muhammad Rezki Rasyak; Rifky Waluyajati Rachman; Ryan Bayusantika Ristandi; Safarina G Malik; Sukma Oktavianthi; Willy Agustine
EPI_ISL_4983057, EPI_ISL_5010024, EPI_ISL_5010104, EPI_ISL_5010355	Puskesmas Kokap II	Genetics Working Group (Pokja Genetik) Faculty of Medicine, Public Health and Nursing Universitas Gadjah Mada (FK-KMK UGM); Disease Investigation Center Wates Ministry of Agriculture Indonesia; Department of Microbiology FK-KMK UGM; Laboratorium Diagnostik Yayasan Tahija World Mosquito Program (WMP) Yogyakarta Center for Tropical Medicine FK-KMK UGM; Integrated Research Center FK-KMK UGM; Department of Computer Science and Electronics FMIPA UGM; RSUP Dr. Sardjito	Afiahayati; Alvina A Setiawan; Cita S Amalia; Dwi AA Nugrahaningsih; Dwi Indaryati; Dyah A Puspitarani; Edwin W. Daniwijaya; Eggi Arguni; Endah Supriyati; Gunadi; Hana F Hanifin; Hendra Wibawa; Indarto Sulistiyono; Irene Tania; Kristy Iskandar; Ludhang P. Rizki; Marcellus; Mohamad S. Hakim; Nungki Anggorowati; Pramesti G Dewi; Siswanto; Susan Simanjaya; Titik Nuryastuti; Tri Wibawa
EPI_ISL_2918631	Puskesmas Kramatmulya	Eijkman Institute for Molecular Biology, National Research and Innovation Agency; West Java Health Laboratory	Amin Soebandrio; Azzania Fibriani; Cut Nur Cinthia Alamanda; Edison Johar; Ema Rahmawati; Frilasita A Yudhaputri; Hidayat Trimarsanto; Iskandar Adnan; Khin Saw Myint; Lidwina Priliani; Lydia V. Panggalo; Muhammad Rezki Rasyak; Rifky Waluyajati Rachman; Ryan Bayusantika Ristandi; Safarina G Malik; Sukma Oktavianthi; Willy Agustine
EPI_ISL_5010073	Puskesmas Lendah I	Genetics Working Group (Pokja Genetik) Faculty of Medicine, Public Health and Nursing Universitas Gadjah Mada (FK-KMK UGM); Disease Investigation Center Wates Ministry of Agriculture Indonesia; Department of Microbiology FK-KMK UGM; Laboratorium Diagnostik Yayasan Tahija World Mosquito Program (WMP) Yogyakarta Center for Tropical Medicine FK-KMK UGM; Integrated Research Center FK-KMK UGM; Department of Computer Science and Electronics FMIPA UGM; RSUP Dr. Sardjito	Afiahayati; Alvina A Setiawan; Cita S Amalia; Dwi AA Nugrahaningsih; Dwi Indaryati; Edwin W. Daniwijaya; Eggi Arguni; Endah Supriyati; Gunadi; Hendra Wibawa; Indarto Sulistiyono; Kristy Iskandar; Ludhang P. Rizki; Marcellus; Mohamad S. Hakim; Nungki Anggorowati; Pramesti G Dewi; Siswanto; Titik Nuryastuti; Tri Wibawa
EPI_ISL_3030042	Puskesmas Luragung	Eijkman Institute for Molecular Biology, National Research and Innovation Agency; West Java Health Laboratory	Amin Soebandrio; Azzania Fibriani; Cut Nur Cinthia Alamanda; Edison Johar; Ema Rahmawati; Frilasita A Yudhaputri; Hidayat Trimarsanto; Iskandar Adnan; Khin Saw Myint; Lidwina Priliani; Lydia V. Panggalo; Muhammad Rezki Rasyak; Rifky Waluyajati Rachman; Ryan Bayusantika Ristandi; Safarina G Malik; Sukma Oktavianthi; Willy Agustine
EPI_ISL_2918632, EPI_ISL_2918636	Puskesmas Margajaya	Eijkman Institute for Molecular Biology, National Research and Innovation Agency; West Java Health Laboratory	Amin Soebandrio; Azzania Fibriani; Cut Nur Cinthia Alamanda; Edison Johar; Ema Rahmawati; Frilasita A Yudhaputri; Hidayat Trimarsanto; Iskandar Adnan; Khin Saw Myint; Lidwina Priliani; Lydia V. Panggalo; Muhammad Rezki Rasyak; Rifky Waluyajati Rachman; Ryan Bayusantika Ristandi; Safarina G Malik; Sukma Oktavianthi; Willy Agustine
EPI_ISL_632936	Puskesmas Mlati 1 Sleman	Genetics Working Group (Pokja Genetik) Faculty of Medicine, Public Health and Nursing Universitas Gadjah Mada (FK-KMK UGM); Disease Investigation Center Wates Ministry of Agriculture Indonesia; Department of Microbiology FK-KMK UGM; Laboratorium Diagnostik Yayasan Tahija World Mosquito Program (WMP) Yogyakarta Center for Tropical Medicine FK-KMK UGM; Integrated Research Center FK-KMK UGM; Department of Computer Science and Electronics FMIPA UGM; RSUP Dr. Sardjito	Afiahayati; Bambang Sigit Riyanto; Dwi AA Nugrahaningsih; Edwin W. Daniwijaya; Eggi Arguni; Eko Budiono; Endah Supriyati; Ernawati; Gunadi; Hendra Wibawa; Heni Retnowulan; Ika Trisnawati; Ira Puspitawati; Kristy Iskandar; Ludhang P. Rizki; Marcellus; Mohamad S. Hakim; Munawar Gani; Nungki Anggorowati; Nur Imma Fatimah Harahap; Nur Rahmi Ananda; Osman Sianipar; Riat El Khair; Satria Maulana; Siswanto; Sumardi; Titik Nuryastuti; Tri Wibawa; Umi Solekhah Intansari; Untung Wirawan; William Widitjiarso; Yunika Puspadewi; Elizabeth Henny Herningtiyas
EPI_ISL_5010492	Puskesmas PLERET	Genetics Working Group (Pokja Genetik) Faculty of Medicine, Public Health and Nursing Universitas Gadjah Mada (FK-KMK UGM); Disease Investigation Center Wates Ministry of Agriculture Indonesia; Department of Microbiology FK-KMK UGM; Laboratorium Diagnostik Yayasan Tahija World Mosquito Program (WMP) Yogyakarta Center for Tropical Medicine FK-KMK UGM; Integrated Research Center FK-KMK UGM; Department of Computer Science and Electronics FMIPA UGM; RSUP Dr. Sardjito	Afiahayati; Alvina A Setiawan; Dwi AA Nugrahaningsih; Dwi Indaryati; Edwin W. Daniwijaya; Eggi Arguni; Endah Supriyati; Gunadi; Hendra Wibawa; Indarto Sulistiyono; Khanza a Vujira; Kristy Iskandar; Ludhang P. Rizki; Marcellus; Mohamad S. Hakim; Nungki Anggorowati; Pramesti G Dewi; Siswanto; Titik Nuryastuti; Tri Wibawa
EPI_ISL_2918638, EPI_ISL_3030040	Puskesmas Pakutandang	Eijkman Institute for Molecular Biology, National Research and Innovation Agency; West Java Health Laboratory	Amin Soebandrio; Azzania Fibriani; Cut Nur Cinthia Alamanda; Edison Johar; Ema Rahmawati; Frilasita A Yudhaputri; Hidayat Trimarsanto; Iskandar Adnan; Khin Saw Myint; Lidwina Priliani; Lydia V. Panggalo; Muhammad Rezki Rasyak; Rifky Waluyajati Rachman; Ryan Bayusantika Ristandi; Safarina G Malik; Sukma Oktavianthi; Willy Agustine
EPI_ISL_766037	Puskesmas Palmerah	Eijkman Institute for Molecular Biology, Ministry of Research and Technology/National Agency for Research and Innovation	Amin Soebandrio; David H Muljono; Edison Johar; Frilasita A Yudhaputri; Hidayat Trimarsanto; Khin Saw Myint; Safarina G Malik
EPI_ISL_2918628	Puskesmas Pameungpeuk	Eijkman Institute for Molecular Biology, National Research and Innovation Agency; West Java Health Laboratory	Amin Soebandrio; Azzania Fibriani; Cut Nur Cinthia Alamanda; Edison Johar; Ema Rahmawati; Frilasita A Yudhaputri; Hidayat Trimarsanto; Iskandar Adnan; Khin Saw Myint; Lidwina Priliani; Lydia V. Panggalo; Muhammad Rezki Rasyak; Rifky Waluyajati Rachman; Ryan Bayusantika Ristandi; Safarina G Malik; Sukma Oktavianthi; Willy Agustine
EPI_ISL_2918630, EPI_ISL_2918637	Puskesmas Pangalengan	Eijkman Institute for Molecular Biology, National Research and Innovation Agency; West Java Health Laboratory	Amin Soebandrio; Azzania Fibriani; Cut Nur Cinthia Alamanda; Edison Johar; Ema Rahmawati; Frilasita A Yudhaputri; Hidayat Trimarsanto; Iskandar Adnan; Khin Saw Myint; Lidwina Priliani; Lydia V. Panggalo; Muhammad Rezki Rasyak; Rifky Waluyajati Rachman; Ryan Bayusantika Ristandi; Safarina G Malik; Sukma Oktavianthi; Willy Agustine
EPI_ISL_4980593	Puskesmas Pengasih I	Genetics Working Group (Pokja Genetik) Faculty of Medicine, Public Health and Nursing Universitas Gadjah Mada (FK-KMK UGM); Disease Investigation Center Wates Ministry of Agriculture Indonesia; Department of Microbiology FK-KMK UGM; Laboratorium Diagnostik Yayasan Tahija World Mosquito Program (WMP) Yogyakarta Center for Tropical Medicine FK-KMK UGM; Integrated Research Center FK-KMK UGM; Department of Computer Science and Electronics FMIPA UGM; RSUP Dr. Sardjito	Afiahayati; Alvina A Setiawan; Dwi AA Nugrahaningsih; Dwi Indaryati; Edwin W. Daniwijaya; Eggi Arguni; Endah Supriyati; Gunadi; Hendra Wibawa; Indarto Sulistiyono; Khanza a Vujira; Kristy Iskandar; Ludhang P. Rizki; Marcellus; Mohamad S. Hakim; Nungki Anggorowati; Pramesti G Dewi; Siswanto; Titik Nuryastuti; Tri Wibawa
EPI_ISL_4980431, EPI_ISL_4983391	Puskesmas Pengasih II	Genetics Working Group (Pokja Genetik) Faculty of Medicine, Public Health and Nursing Universitas Gadjah Mada (FK-KMK UGM); Disease Investigation Center Wates Ministry of Agriculture Indonesia; Department of Microbiology FK-KMK UGM; Laboratorium Diagnostik Yayasan Tahija World Mosquito Program (WMP) Yogyakarta Center for Tropical Medicine FK-KMK UGM; Integrated Research Center FK-KMK UGM; Department of Computer Science and Electronics FMIPA UGM; RSUP Dr. Sardjito	Afiahayati; Alvina A Setiawan; Cita S Amalia; Dwi AA Nugrahaningsih; Dwi Indaryati; Edwin W. Daniwijaya; Eggi Arguni; Endah Supriyati; Gunadi; Hendra Wibawa; Indarto Sulistiyono; Khanza a Vujira; Kristy Iskandar; Ludhang P. Rizki; Marcellus; Mohamad S. Hakim; Nungki Anggorowati; Pramesti G Dewi; Siswanto; Titik Nuryastuti; Tri Wibawa
EPI_ISL_5010416	Puskesmas Pleret	Genetics Working Group (Pokja Genetik) Faculty of Medicine, Public Health and Nursing Universitas Gadjah Mada (FK-KMK UGM); Disease Investigation Center Wates Ministry of Agriculture Indonesia; Department of Microbiology FK-KMK UGM; Laboratorium Diagnostik Yayasan Tahija World Mosquito Program (WMP) Yogyakarta Center for Tropical Medicine FK-KMK UGM; Integrated Research Center FK-KMK UGM; Department of Computer Science and Electronics FMIPA UGM; RSUP Dr. Sardjito	Afiahayati; Dwi AA Nugrahaningsih; Dwi Indaryati; Dyah A Puspitarani; Edwin W. Daniwijaya; Eggi Arguni; Endah Supriyati; Gunadi; Hendra Wibawa; Indarto Sulistiyono; Kristy Iskandar; Ludhang P. Rizki; Marcellus; Mohamad S. Hakim; Nungki Anggorowati; Pramesti G Dewi; Siswanto; Susan Simanjaya; Titik Nuryastuti; Tri Wibawa
EPI_ISL_2918635	Puskesmas Rancakalong	Eijkman Institute for Molecular Biology, National Research and Innovation Agency; West Java Health Laboratory	Amin Soebandrio; Azzania Fibriani; Cut Nur Cinthia Alamanda; Edison Johar; Ema Rahmawati; Frilasita A Yudhaputri; Hidayat Trimarsanto; Iskandar Adnan; Khin Saw Myint; Lidwina Priliani; Lydia V. Panggalo; Muhammad Rezki Rasyak; Rifky Waluyajati Rachman; Ryan Bayusantika Ristandi; Safarina G Malik; Sukma Oktavianthi; Willy Agustine

EPI_ISL_2918641, EPI_ISL_2918642, EPI_ISL_2918643, EPI_ISL_2918644, EPI_ISL_2918645	Puskesmas Rancamanyar	Eijkman Institute for Molecular Biology, National Research and Innovation Agency; West Java Health Laboratory	Amin Soebandrio; Azzania Fibriani; Cut Nur Cinthia Alamanda; Edison Johar; Ema Rahmawati; Frilasita A Yudhaputri; Hidayat Trimarsanto; Iskandar Adnan; Khin Saw Myint; Lidwina Priliani; Lydia V. Panggalo; Muhammad Rezki Rasyak; Rifky Waluyajati Rachman; Ryan Bayusantika Ristandi; Safarina G Malik; Sukma Oktavianthi; Willy Agustine
EPI_ISL_5010063	Puskesmas Samigaluh I	Genetics Working Group (Pokja Genetik) Faculty of Medicine, Public Health and Nursing Universitas Gadjah Mada (FK-KMK UGM); Disease Investigation Center Wates Ministry of Agriculture Indonesia; Department of Microbiology FK-KMK UGM; Laboratorium Diagnostik Yayasan Tahija World Mosquito Program (WMP) Yogyakarta Center for Tropical Medicine FK-KMK UGM; Integrated Research Center FK-KMK UGM; Department of Computer Science and Electronics FMIPA UGM; RSUP Dr. Sardjito	Afiahayati; Dwi AA Nugrahaningsih; Dwi Indaryati; Dyah A Puspitarani; Edwin W. Daniwijaya; Eggi Arguni; Endah Supriyati; Gunadi; Hendra Wibawa; Indarto Sulistiyono; Kristy Iskandar; Ludhang P. Rizki; Marcellus; Mohamad S. Hakim; Nungki Anggorowati; Pramesti G Dewi; Siswanto; Susan Simanjaya; Titik Nuryastuti; Tri Wibawa
EPI_ISL_2918629	Puskesmas Sangkanhurip	Eijkman Institute for Molecular Biology, National Research and Innovation Agency; West Java Health Laboratory	Amin Soebandrio; Azzania Fibriani; Cut Nur Cinthia Alamanda; Edison Johar; Ema Rahmawati; Frilasita A Yudhaputri; Hidayat Trimarsanto; Iskandar Adnan; Khin Saw Myint; Lidwina Priliani; Lydia V. Panggalo; Muhammad Rezki Rasyak; Rifky Waluyajati Rachman; Ryan Bayusantika Ristandi; Safarina G Malik; Sukma Oktavianthi; Willy Agustine
EPI_ISL_3030053, EPI_ISL_3030059	Puskesmas Sarijadi, Dinas Kesehatan Kota Bandung	Eijkman Institute for Molecular Biology, National Research and Innovation Agency; West Java Health Laboratory	Ade Fany Safitri; Amin Soebandrio; Azzania Fibriani; Cut Nur Cinthia Alamanda; Edison Johar; Ema Rahmawati; Frilasita A Yudhaputri; Hidayat Trimarsanto; Husna Nugrahapraja; Iskandar Adnan; Khin Saw Myint; Lidwina Priliani; Lydia V. Panggalo; Muhammad Rezki Rasyak; Ranza Fadhlil Pratama; Rifky Waluyajati Rachman; Ryan Bayusantika Ristandi; Safarina G Malik; Sukma Oktavianthi; Tati Sutarti Sastrowiyoto; Willy Agustine
EPI_ISL_2918627	Puskesmas Sugihmukti	Eijkman Institute for Molecular Biology, National Research and Innovation Agency; West Java Health Laboratory	Amin Soebandrio; Azzania Fibriani; Cut Nur Cinthia Alamanda; Edison Johar; Ema Rahmawati; Frilasita A Yudhaputri; Hidayat Trimarsanto; Iskandar Adnan; Khin Saw Myint; Lidwina Priliani; Lydia V. Panggalo; Muhammad Rezki Rasyak; Rifky Waluyajati Rachman; Ryan Bayusantika Ristandi; Safarina G Malik; Sukma Oktavianthi; Willy Agustine
EPI_ISL_3030048	Puskesmas Sukawarna, Dinas Kesehatan Kota Bandung	Eijkman Institute for Molecular Biology, National Research and Innovation Agency; West Java Health Laboratory	Ade Fany Safitri; Amin Soebandrio; Azzania Fibriani; Cut Nur Cinthia Alamanda; Edison Johar; Ema Rahmawati; Frilasita A Yudhaputri; Hidayat Trimarsanto; Husna Nugrahapraja; Iskandar Adnan; Khin Saw Myint; Lidwina Priliani; Lydia V. Panggalo; Muhammad Rezki Rasyak; Ranza Fadhlil Pratama; Rifky Waluyajati Rachman; Ryan Bayusantika Ristandi; Safarina G Malik; Sukma Oktavianthi; Tati Sutarti Sastrowiyoto; Willy Agustine
EPI_ISL_574610	Puskesmas Tambora	Eijkman Institute for Molecular Biology, Ministry of Research and Technology/National Agency for Research and Innovation	Amin Soebandrio; David H Muljono; Edison Johar; Frilasita A Yudhaputri; Herawati Sudoyo; Hidayat Trimarsanto; Iskandar A Adnan; Khin Saw Myint; Safarina G Malik; Willy Agustine
EPI_ISL_4969291	Puskesmas Temon 2	Genetics Working Group (Pokja Genetik) Faculty of Medicine, Public Health and Nursing Universitas Gadjah Mada (FK-KMK UGM); Disease Investigation Center Wates Ministry of Agriculture Indonesia; Department of Microbiology FK-KMK UGM; Laboratorium Diagnostik Yayasan Tahija World Mosquito Program (WMP) Yogyakarta Center for Tropical Medicine FK-KMK UGM; Integrated Research Center FK-KMK UGM; Department of Computer Science and Electronics FMIPA UGM; RSUP Dr. Sardjito	Afiahayati; Alvina A Setiawan; Dwi AA Nugrahaningsih; Dwi Indaryati; Edwin W. Daniwijaya; Eggi Arguni; Endah Supriyati; Gunadi; Hendra Wibawa; Indarto Sulistiyono; Khanza a Vujira; Kristy Iskandar; Ludhang P. Rizki; Marcellus; Mohamad S. Hakim; Nungki Anggorowati; Pramesti G Dewi; Siswanto; Titik Nuryastuti; Tri Wibawa
EPI_ISL_2854780	Puskesmas kec Menteng	National Institute of Health Research and Development	Arie Ardiansyah Nugraha; Hana Apsari Pawestri; Hartanti Dian Ikawati; Kartika Dewi Puspa; Krisna Pangesti; Nelly Puspandari; Subangkit; Triyani Soekarso; Vivi Setiawaty
EPI_ISL_4004699, EPI_ISL_4004700, EPI_ISL_4004702	QR Lab	National Research and Innovation Agency (BRIN-Indonesia)	Ahmad Fathoni; Anggia Prasetyoputri; Anik B. Dharmayanthi; Asep M. Ridwanulloh; Hariyatun; Indriawati; Rifki Sadikin; Sugiyono Saputra; Suvia Widyaningrum; Syam B. Iryanto
EPI_ISL_518819, EPI_ISL_1020206	Qadr Hospital, Tangerang, Banten	Biosafety Level-3 Laboratory, Indonesian Institute of Sciences (LIPI)	Ade Andriani; Ahmad Fathoni; Andri Wardiana; Anggia Prasetyoputri; Anik Budhi Dharmayanthi; Ari Sulistyo Wulandari; Ario Betha Juanssilfero; Asep M Ridwanuloh; Eko Wahyu Putro; Gita Syahputra; Isa Nuryana; Puspita Lisdiyanti; Ratih Asmana Ningrum; Rifki Sadikin; Sri Swasthikawati; Sugiyono Saputra; Syam Budi Iryanto; Wien Kusharyoto
EPI_ISL_2854775, EPI_ISL_2931796, EPI_ISL_2965759	RS Advent Bandar Lampung	National Institute of Health Research and Development	; Arie Ardiansyah Nugraha; Fajar Nur Sulistiyohadi; Hana Apsari Pawestri; Hartanti Dian Ikawati; Kartika Dewi Puspa; Krisna Pangesti; Nelly Puspandari; Subangkit; Triyani Soekarso; Vivi Setiawaty
EPI_ISL_2364593	RS An-Nisa Tangerang	Eijkman Institute for Molecular Biology, National Agency for Research and Innovation	Amin Soebandrio; Edison Johar; Frilasita A Yudhaputri; Hidayat Trimarsanto; Iskandar Adnan; Khin Saw Myint; Lidwina Priliani; Lydia V. Panggalo; Muhammad Rezki Rasyak; Safarina G Malik; Sukma Oktavianthi; Willy Agustine
EPI_ISL_888980, EPI_ISL_889015, EPI_ISL_889022	RS Anna	Eijkman Institute for Molecular Biology, Ministry of Research and Technology/National Agency for Research and Innovation	Amin Soebandrio; Edison Johar; Frilasita A Yudhaputri; Hidayat Trimarsanto; Iskandar Adnan; Khin Saw Myint; Lydia V. Panggalo; Safarina G Malik; Sukma Oktavianthi; Willy Agustine
EPI_ISL_1283960	RS Asy-Syifaa Leuwiliang	Eijkman Institute for Molecular Biology, Ministry of Research and Technology/National Agency for Research and Innovation	Amin Soebandrio; Edison Johar; Frilasita A Yudhaputri; Hidayat Trimarsanto; Iskandar Adnan; Khin Saw Myint; Lydia V. Panggalo; Safarina G Malik; Sukma Oktavianthi; Willy Agustine
EPI_ISL_2364578	RS Asy-Syifaa Leuwiliang	Eijkman Institute for Molecular Biology, National Agency for Research and Innovation	Amin Soebandrio; Edison Johar; Frilasita A Yudhaputri; Hidayat Trimarsanto; Iskandar Adnan; Khin Saw Myint; Lidwina Priliani; Lydia V. Panggalo; Muhammad Rezki Rasyak; Safarina G Malik; Sukma Oktavianthi; Willy Agustine
EPI_ISL_3401867, EPI_ISL_3401895, EPI_ISL_3401898, EPI_ISL_3401899	RS Atma Jaya	Genomik Solidaritas Indonesia Laboratorium	Annisa Muthiah Sukirman; Anuraj Shankar; Ariel Pradipta; Carissa Sintca Wijaya; Dhahlia Agustina Cahyono; Dwi Oktavia; Gracia Felias Enos Korompis; Himawan Masyhuri; Louisa Markus; Meutia Ayuputeri Kumaheri; Ngabila Salama; Tiranti Vindha; Vania Gavrila Wikasa
EPI_ISL_3138851	RS Bali Jimbaran	National Institute of Health Research and Development	Arie Ardiansyah Nugraha; Fajar Nur Sulistiyohadi; Hana Apsari Pawestri; Hartanti Dian Ikawati; Kartika Dewi Puspa; Krisna Pangesti; Nelly Puspandari; Subangkit; Triyani Soekarso; Vivi Setiawaty
EPI_ISL_3138853, EPI_ISL_3138854	RS Balimed Karangasem	National Institute of Health Research and Development	; Arie Ardiansyah Nugraha; Fajar Nur Sulistiyohadi; Hana Apsari Pawestri; Hartanti Dian Ikawati; Kartika Dewi Puspa; Krisna Pangesti; Nelly Puspandari; Subangkit; Triyani Soekarso; Vivi Setiawaty
EPI_ISL_2631464, EPI_ISL_2631465, EPI_ISL_2631466, EPI_ISL_2631467, EPI_ISL_2631468	RS Bayukarta	Indonesian Institute of Sciences (LIPI)/National Agency of Research and Innovation (BRIN) and National Institute of Health Research and Development (NIHRD)	Anggia Prasetyoputri; Eko Wahyu Putro; Fahrurrozi; Herjuno A. Nugroho; Idris; Indriawati; Isa Nuryana; Linda Sukmarini; Listiana Oktavia; Nova D. Yanthi; Puspita Lisdiyanti; Sri Swasthikawati; Subangkit; Sugiyono Saputra; Syam B. Iryanto; Vivi Setiawaty; Yayuk Sri Rahayu; Yuliawati
EPI_ISL_1284044, EPI_ISL_1284048, EPI_ISL_1284201, EPI_ISL_1284310, EPI_ISL_1622365, EPI_ISL_1622381, EPI_ISL_1622403, EPI_ISL_1622404			
see above	RS Bhakti Husada Cikarang	Eijkman Institute for Molecular Biology, Ministry of Research and Technology/National Agency for Research and Innovation	Amin Soebandrio; Edison Johar; Frilasita A Yudhaputri; Hidayat Trimarsanto; Iskandar Adnan; Khin Saw Myint; Lydia V. Panggalo; Muhammad Rezki Rasyak; Safarina G Malik; Sukma Oktavianthi; Willy Agustine
EPI_ISL_2364573, EPI_ISL_2364574, EPI_ISL_2364590, EPI_ISL_2364604, EPI_ISL_2364629	RS Bhakti Husada Cikarang	Eijkman Institute for Molecular Biology, National Agency for Research and Innovation	Amin Soebandrio; Edison Johar; Frilasita A Yudhaputri; Hidayat Trimarsanto; Iskandar Adnan; Khin Saw Myint; Lidwina Priliani; Lydia V. Panggalo; Muhammad Rezki Rasyak; Safarina G Malik; Sukma Oktavianthi; Willy Agustine
EPI_ISL_2614708	RS Bhakti Husada Cikarang	Eijkman Institute for Molecular Biology, National Research and Innovation Agency	Amin Soebandrio; Edison Johar; Frilasita A Yudhaputri; Hidayat Trimarsanto; Iskandar Adnan; Khin Saw Myint; Lidwina Priliani; Lydia V. Panggalo; Muhammad Rezki Rasyak; Safarina G Malik; Sukma Oktavianthi; Willy Agustine
EPI_ISL_888979, EPI_ISL_1283991, EPI_ISL_1284121, EPI_ISL_1284125, EPI_ISL_1622414	RS Bhakti Mulia	Eijkman Institute for Molecular Biology, Ministry of Research and Technology/National Agency for Research and Innovation	Amin Soebandrio; Edison Johar; Frilasita A Yudhaputri; Hidayat Trimarsanto; Iskandar Adnan; Khin Saw Myint; Lydia V. Panggalo; Muhammad Rezki Rasyak; Safarina G Malik; Sukma Oktavianthi; Willy Agustine
EPI_ISL_2364595	RS Bhakti Mulia	Eijkman Institute for Molecular Biology, National Agency for Research and Innovation	Amin Soebandrio; Edison Johar; Frilasita A Yudhaputri; Hidayat Trimarsanto; Iskandar Adnan; Khin Saw Myint; Lidwina Priliani; Lydia V. Panggalo; Muhammad Rezki Rasyak; Safarina G Malik; Sukma Oktavianthi; Willy Agustine
EPI_ISL_2614652, EPI_ISL_2614653, EPI_ISL_2614654, EPI_ISL_2614665, EPI_ISL_2614704, EPI_ISL_2614722	RS Bhakti Mulia	Eijkman Institute for Molecular Biology, National Research and Innovation Agency	Amin Soebandrio; Edison Johar; Frilasita A Yudhaputri; Hidayat Trimarsanto; Iskandar Adnan; Khin Saw Myint; Lidwina Priliani; Lydia V. Panggalo; Muhammad Rezki Rasyak; Safarina G Malik; Sukma Oktavianthi; Willy Agustine
EPI_ISL_766035	RS Bhayangkara Brimob Kelapa Dua	Eijkman Institute for Molecular Biology, Ministry of Research and Technology/National Agency for Research and Innovation	Amin Soebandrio; David H Muljono; Edison Johar; Frilasita A Yudhaputri; Hidayat Trimarsanto; Khin Saw Myint; Safarina G Malik
EPI_ISL_5022922, EPI_ISL_5022923, EPI_ISL_5022924, EPI_ISL_5022925, EPI_ISL_5022926	RS Bhayangkara Jambi	National Institute of Health Research and Development	Arie Ardiansyah Nugraha; Fajar Nur Sulistiyohadi; Hana Apsari Pawestri; Hartanti Dian Ikawati; Kartika Dewi Puspa; Krisna Pangesti; Nelly Puspandari; Subangkit; Triyani Soekarso; Vivi Setiawaty
EPI_ISL_4730608, EPI_ISL_4730609, EPI_ISL_4730610, EPI_ISL_4730611, EPI_ISL_4730612	RS Bhayangkara TK II Makassar	National Institute of Health Research and Development	Arie Ardiansyah Nugraha; Fajar Nur Sulistiyohadi; Hana Apsari Pawestri; Hartanti Dian Ikawati; Kartika Dewi Puspa; Krisna Pangesti; Nelly Puspandari; Subangkit; Triyani Soekarso; Vivi Setiawaty
EPI_ISL_5796066, EPI_ISL_5796067, EPI_ISL_5796068, EPI_ISL_5796069, EPI_ISL_5796070, EPI_ISL_5796071, EPI_ISL_5796072, EPI_ISL_5796073, EPI_ISL_5796074, EPI_ISL_5796075			
see above	RS Bhayangkara TK III Banjarmasin	National Institute of Health Research and Development	Arie Ardiansyah Nugraha; Fajar Nur Sulistiyohadi; Hana Apsari Pawestri; Hartanti Dian Ikawati; Kartika Dewi Puspa; Krisna Pangesti; Nelly Puspandari; Subangkit; Triyani Soekarso; Vivi Setiawaty
EPI_ISL_766039, EPI_ISL_766048	RS Bhayangkara Tk. III	Eijkman Institute for Molecular Biology, Ministry of Research and Technology/National Agency for Research and Innovation	Amin Soebandrio; David H Muljono; Edison Johar; Frilasita A Yudhaputri; Hidayat Trimarsanto; Khin Saw Myint; Safarina G Malik
EPI_ISL_1283967	RS Bina Husada Bogor	Eijkman Institute for Molecular Biology, Ministry of Research and Technology/National Agency for Research and Innovation	Amin Soebandrio; Edison Johar; Frilasita A Yudhaputri; Hidayat Trimarsanto; Iskandar Adnan; Khin Saw Myint; Lydia V. Panggalo; Safarina G Malik; Sukma Oktavianthi; Willy Agustine
EPI_ISL_947249,	RS Bina Husada Cibinong	Eijkman Institute for Molecular Biology, Ministry of Research and Technology/National Agency for Research and	Amin Soebandrio; Edison Johar; Frilasita A Yudhaputri; Hidayat Trimarsanto; Iskandar Adnan; Khin Saw Myint; Lydia V. Panggalo; Safarina G Malik; Sukma Oktavianthi; Willy Agustine

EPI_ISL_947252, EPI_ISL_947257		Innovation	
EPI_ISL_1284069, EPI_ISL_1284072, EPI_ISL_1622383	RS Bina Sehat Mandiri	Eijkman Institute for Molecular Biology, Ministry of Research and Technology/National Agency for Research and Innovation	Amin Soebandrio; Edison Johar; Frilasita A Yudhaputri; Hidayat Trimarsanto; Iskandar Adnan; Khin Saw Myint; Lydia V. Panggalo; Muhammad Rezki Rasyak; Safarina G Malik; Sukma Oktavianthi; Willy Agustine
EPI_ISL_3070858	RS Bogor Senior	Eijkman Institute for Molecular Biology, National Research and Innovation Agency	Amin Soebandrio; Edison Johar; Frilasita A Yudhaputri; Hidayat Trimarsanto; Iskandar Adnan; Khin Saw Myint; Lidwina Priliani; Lydia V. Panggalo; Muhammad Rezki Rasyak; Safarina G Malik; Sukma Oktavianthi; Willy Agustine
EPI_ISL_1915577	RS Bunda	National Institute of Health Research and Development	Arie Ardiansyah Nugraha; Hana Apsari Pawestri; Hartanti Dian Ikawati; Kartika Dewi Puspa; Krisna Pangesti; Nelly Puspandari; Subangkit; Vivi Setiawaty
EPI_ISL_4491502, EPI_ISL_4491503, EPI_ISL_4491504, EPI_ISL_4491505, EPI_ISL_4491506, EPI_ISL_4491507, EPI_ISL_4491508, EPI_ISL_4491509, EPI_ISL_4491510, EPI_ISL_4491511, EPI_ISL_4491512, EPI_ISL_4491513, EPI_ISL_4491515, EPI_ISL_4491516, EPI_ISL_4491517, EPI_ISL_4491518, EPI_ISL_4491519, EPI_ISL_4491520			
see above	RS Bunda Thamrin / EIJKMAN OXFORD CLINICAL RESEARCH UNIT	Genomik Solidaritas Indonesia Laboratorium	Annisa Muthiah Sukirman; Anuraj Shankar; Ariel Pradipta; Ayodhia Pitaloka Pasaribu; Decy Subekti; Dhahlia Agustina Cahyono; Eka Airlangga; Esti Rahayuning Tias; Gracia Felias Enos Korompis; Hasanul Arifin; Hendri Wijaya; Louisa Markus; Meutia Ayuputeri Kumaheri; Raph Hamers; Reinhart Gabriel; Stefanus Bernard; Suwarti; Vania Gavrila Wikasa
EPI_ISL_1283979, EPI_ISL_1284058, EPI_ISL_1284186, EPI_ISL_1622410	RS Cendana	Eijkman Institute for Molecular Biology, Ministry of Research and Technology/National Agency for Research and Innovation	Amin Soebandrio; Edison Johar; Frilasita A Yudhaputri; Hidayat Trimarsanto; Iskandar Adnan; Khin Saw Myint; Lydia V. Panggalo; Muhammad Rezki Rasyak; Safarina G Malik; Sukma Oktavianthi; Willy Agustine
EPI_ISL_2868902, EPI_ISL_2868907, EPI_ISL_2868909, EPI_ISL_2868910, EPI_ISL_2987596	RS Cinta Kasih Tzu Chi	National Institute of Health Research and Development	Arie Ardiansyah Nugraha; Fajar Nur Sulistiyohadi; Hana Apsari Pawestri; Hartanti Dian Ikawati; Kartika Dewi Puspa; Krisna Pangesti; Nelly Puspandari; Subangkit; Triyani Soekarso; Vivi Setiawaty
EPI_ISL_3138767	RS Cinta Kasih Tzuchi	National Institute of Health Research and Development	Arie Ardiansyah Nugraha; Fajar Nur Sulistiyohadi; Hana Apsari Pawestri; Hartanti Dian Ikawati; Kartika Dewi Puspa; Krisna Pangesti; Nelly Puspandari; Subangkit; Triyani Soekarso; Vivi Setiawaty
EPI_ISL_3086880	RS Citra Medika	Indonesian Institute of Sciences (LIPI)	Annisa D. Septiani; Asep M. Ridwanulloh; Indriawati; Isa Nuryana; Lusiana; Maya Komalasari; Michael Saut Parulian; Ocha P. Meitha; Sugiyono Saputra; Syaiful Rizal; Willery Yeo; Yuhoniz A. Maryam
EPI_ISL_888987	RS Citra Medika Depok	Eijkman Institute for Molecular Biology, Ministry of Research and Technology/National Agency for Research and Innovation	Amin Soebandrio; Edison Johar; Frilasita A Yudhaputri; Hidayat Trimarsanto; Iskandar Adnan; Khin Saw Myint; Lydia V. Panggalo; Safarina G Malik; Sukma Oktavianthi; Willy Agustine
EPI_ISL_5021361, EPI_ISL_5021369, EPI_ISL_5021489, EPI_ISL_5021497, EPI_ISL_5021536, EPI_ISL_5021552, EPI_ISL_5021569, EPI_ISL_5021577, EPI_ISL_5021587, EPI_ISL_5021590			
see above	RS Columbia Asia Medan	Eijkman Research Center for Molecular Biology, National Research and Innovation Agency; Microbiology Laboratory, Faculty of Medicine, University of Sumatera Utara	Amin Soebandrio; Ananda Wibawanta Ginting; Dian Dwi Wahyuni; Edison Johar; Frilasita A Yudhaputri; Hidayat Trimarsanto; Inke Nadia Diniyanti Lubis; Iskandar Adnan; Jessica R Saragih; Khin Saw Myint; Lidwina Priliani; Lydia V Panggalo; Muhammad Rezki Rasyak; R Lia Kusumawati; Safarina G Malik; Sukma Oktavianthi; Tryna Tania; Willy Agustine
EPI_ISL_4760490	RS Darurat Covid Wonokerto Kab Pekalongan	Balai Laboratorium Kesehatan Dan Pengujian Alat Kesehatan Provinsi Jawa Tengah	Alia Wijayanti; Dwi Nursetyaasih; Dyah Ayu; Endah Wahyu Y; Ismi Farhati; Purnomo; Sekti Handayani; Wiwik Peni; Yekti Hediningsih
EPI_ISL_4730564, EPI_ISL_4730565, EPI_ISL_4730566, EPI_ISL_4730567, EPI_ISL_4730568, EPI_ISL_5022958, EPI_ISL_5022959			
see above	RS Darurat Penanganan Covid-19 Wisma Atlet Kemayoran	National Institute of Health Research and Development	Arie Ardiansyah Nugraha; Fajar Nur Sulistiyohadi; Hana Apsari Pawestri; Hartanti Dian Ikawati; Kartika Dewi Puspa; Krisna Pangesti; Nelly Puspandari; Subangkit; Triyani Soekarso; Vivi Setiawaty
EPI_ISL_2337254	RS Dr Oen Solo Baru	Genetics Working Group (Pokja Genetik) Faculty of Medicine, Public Health and Nursing Universitas Gadjah Mada (FK-KMK UGM); Disease Investigation Center Wates Ministry of Agriculture Indonesia; Department of Microbiology FK-KMK UGM; Laboratorium Diagnostik Yayasan Tahija World Mosquito Program (WMP) Yogyakarta Center for Tropical Medicine FK-KMK UGM; Integrated Research Center FK-KMK UGM; Department of Computer Science and Electronics FMIPA UGM; RSUP Dr. Sardjito	Afiahayati; Dwi AA Nugrahaningsih; Edwin W. Daniwijaya; Eggi Arguni; Endah Supriyati; Gunadi; Hana F Hanifin; Hendra Wibawa; Irene Tania; Kristy Iskandar; Ludhang P. Rizki; Marcellus; Mohamad S. Hakim; Nungki Anggorowati; Siswanto; Titik Nuryastuti; Tri Wibawa
EPI_ISL_2964917, EPI_ISL_2964918, EPI_ISL_2964919, EPI_ISL_2964920, EPI_ISL_2964921	RS Dr. OEN KANDANG SAPI SOLO	Genetics Working Group (Pokja Genetik) Faculty of Medicine, Public Health and Nursing Universitas Gadjah Mada (FK-KMK UGM); Disease Investigation Center Wates Ministry of Agriculture Indonesia; Department of Microbiology FK-KMK UGM; Laboratorium Diagnostik Yayasan Tahija World Mosquito Program (WMP) Yogyakarta Center for Tropical Medicine FK-KMK UGM; Integrated Research Center FK-KMK UGM; Department of Computer Science and Electronics FMIPA UGM; RSUP Dr. Sardjito	Afiahayati; Alvina A Setiawan; Cita S Amalia; Dwi AA Nugrahaningsih; Dwi Indaryati; Dyah A Puspitarani; Edwin W. Daniwijaya; Eggi Arguni; Endah Supriyati; Gunadi; Hana F Hanifin; Hendra Wibawa; Indarto Sulistiyono; Irene Tania; Khanza a Vujira; Kristy Iskandar; Ludhang P. Rizki; Marcellus; Mohamad S. Hakim; Nungki Anggorowati; Pramesti G Dewi; Siswanto; Susan Simanjaya; Titik Nuryastuti; Tri Wibawa
EPI_ISL_2964707, EPI_ISL_2964872	RS Dr. Oen Kandang Sapi Solo	Genetics Working Group (Pokja Genetik) Faculty of Medicine, Public Health and Nursing Universitas Gadjah Mada (FK-KMK UGM); Disease Investigation Center Wates Ministry of Agriculture Indonesia; Department of Microbiology FK-KMK UGM; Laboratorium Diagnostik Yayasan Tahija World Mosquito Program (WMP) Yogyakarta Center for Tropical Medicine FK-KMK UGM; Integrated Research Center FK-KMK UGM; Department of Computer Science and Electronics FMIPA UGM; RSUP Dr. Sardjito	Afiahayati; Alvina A Setiawan; Cita S Amalia; Dwi AA Nugrahaningsih; Dwi Indaryati; Dyah A Puspitarani; Edwin W. Daniwijaya; Eggi Arguni; Endah Supriyati; Gunadi; Hendra Wibawa; Indarto Sulistiyono; Kristy Iskandar; Ludhang P. Rizki; Marcellus; Mohamad S. Hakim; Nungki Anggorowati; Pramesti G Dewi; Siswanto; Susan Simanjaya; Titik Nuryastuti; Tri Wibawa
EPI_ISL_2844824	RS Family Medical center, Bogor, West Java	Biosafety Level-3 Laboratory, Indonesian Institute of Sciences (LIPI)	Anggia Prasetyoputri; Erwin F. Hasrianda; Fahrurrozi; Herjuno A. Nugroho; Nova D. Yanthi; Ratih A. Ningrum
EPI_ISL_568693, EPI_ISL_574603	RS Freeport Tembagapura	Eijkman Institute for Molecular Biology, Ministry of Research and Technology/National Agency for Research and Innovation	Amin Soebandrio; David H Muljono; Edison Johar; Frilasita A Yudhaputri; Herawati Sudoyo; Hidayat Trimarsanto; Iskandar A Adnan; Khin Saw Myint; Safarina G Malik; Willy Agustine
EPI_ISL_3836265	RS GADING PLUIT	Genomik Solidaritas Indonesia Laboratorium	Anna Christina Brazia; Annisa Muthiah Sukirman; Anuraj Shankar; Ariel Pradipta; David Hendra Haryanto; Dhahlia Agustina Cahyono; Dwi Oktavia; Eko Susanti; Gracia Felias Enos Korompis; Louisa Markus; Meutia Ayuputeri Kumaheri; Ngabila Salama; Pratiwi Srikandi Yudhastari; Reinhart Gabriel; Tiranti Vindha; Vania Gavrila Wikasa
EPI_ISL_2868915	RS Gading Pluit	National Institute of Health Research and Development	Arie Ardiansyah Nugraha; Hana Apsari Pawestri; Hartanti Dian Ikawati; Kartika Dewi Puspa; Krisna Pangesti; Nelly Puspandari; Subangkit; Triyani Soekarso; Vivi Setiawaty
EPI_ISL_2364586, EPI_ISL_2364591	RS Graha Juanda	Eijkman Institute for Molecular Biology, National Agency for Research and Innovation	Amin Soebandrio; Edison Johar; Frilasita A Yudhaputri; Hidayat Trimarsanto; Iskandar Adnan; Khin Saw Myint; Lidwina Priliani; Lydia V. Panggalo; Muhammad Rezki Rasyak; Safarina G Malik; Sukma Oktavianthi; Willy Agustine
EPI_ISL_1284303	RS Grha Kedoya	Eijkman Institute for Molecular Biology, Ministry of Research and Technology/National Agency for Research and Innovation	Amin Soebandrio; Edison Johar; Frilasita A Yudhaputri; Hidayat Trimarsanto; Iskandar Adnan; Khin Saw Myint; Lydia V. Panggalo; Safarina G Malik; Sukma Oktavianthi; Willy Agustine
EPI_ISL_2614728, EPI_ISL_2614729	RS Grha Kedoya	Eijkman Institute for Molecular Biology, National Research and Innovation Agency	Amin Soebandrio; Edison Johar; Frilasita A Yudhaputri; Hidayat Trimarsanto; Iskandar Adnan; Khin Saw Myint; Lidwina Priliani; Lydia V. Panggalo; Muhammad Rezki Rasyak; Safarina G Malik; Sukma Oktavianthi; Willy Agustine
EPI_ISL_3030000	RS Grha MM2100	Eijkman Institute for Molecular Biology, National Research and Innovation Agency	Amin Soebandrio; Edison Johar; Frilasita A Yudhaputri; Hidayat Trimarsanto; Iskandar Adnan; Khin Saw Myint; Lidwina Priliani; Lydia V. Panggalo; Muhammad Rezki Rasyak; Safarina G Malik; Sukma Oktavianthi; Willy Agustine
EPI_ISL_4115720, EPI_ISL_4115728, EPI_ISL_4115731, EPI_ISL_4115734, EPI_ISL_4115738, EPI_ISL_4115742, EPI_ISL_4115745, EPI_ISL_4115748, EPI_ISL_4115751, EPI_ISL_4115755, EPI_ISL_4115758, EPI_ISL_4115762, EPI_ISL_4115765, EPI_ISL_4115768, EPI_ISL_4115771, EPI_ISL_4115774, EPI_ISL_4115778, EPI_ISL_4115782, EPI_ISL_4115786, EPI_ISL_4115789, EPI_ISL_4115793, EPI_ISL_4115796, EPI_ISL_4115799, EPI_ISL_4115802, EPI_ISL_4115806, EPI_ISL_4115810, EPI_ISL_4115813, EPI_ISL_4115817, EPI_ISL_4115820, EPI_ISL_4115823, EPI_ISL_4115827, EPI_ISL_4115834, EPI_ISL_4115839, EPI_ISL_4115845, EPI_ISL_4115850, EPI_ISL_4115853, EPI_ISL_4115856, EPI_ISL_4115859, EPI_ISL_4115864, EPI_ISL_4115867, EPI_ISL_4115870, EPI_ISL_4115878, EPI_ISL_4115888, EPI_ISL_4115891, EPI_ISL_4115901, EPI_ISL_4115904, EPI_ISL_4115908, EPI_ISL_4740190, EPI_ISL_4740191, EPI_ISL_4740193			
see above	RS HASAN SADIKIN BANDUNG	Genomik Solidaritas Indonesia Laboratorium	Annisa Muthiah Sukirman; Anuraj Shankar; Ariel Pradipta; Basti Andriyoko; Carissa Sintca Wijaya; Delita Prihatni; Dewi Kartika Turbawati; Dhahlia Agustina Cahyono; Gracia Felias Enos Korompis; Himawan Masyhuri; Louisa Markus; Meutia Ayuputeri Kumaheri; Neng Hilma Hamidah; Pratiwi Srikandi Yudhastari; Vania Gavrila Wikasa; Vivi Setyawati
EPI_ISL_574613, EPI_ISL_574615, EPI_ISL_1622367, EPI_ISL_1622384, EPI_ISL_1622385	RS Harapan Bunda	Eijkman Institute for Molecular Biology, Ministry of Research and Technology/National Agency for Research and Innovation	Amin Soebandrio; David H Muljono; Edison Johar; Frilasita A Yudhaputri; Herawati Sudoyo; Hidayat Trimarsanto; Iskandar A Adnan; Iskandar Adnan; Khin Saw Myint; Lydia V. Panggalo; Muhammad Rezki Rasyak; Safarina G Malik; Sukma Oktavianthi; Willy Agustine
EPI_ISL_2013509, EPI_ISL_2364576, EPI_ISL_2364608, EPI_ISL_2364609, EPI_ISL_2364635	RS Harapan Bunda	Eijkman Institute for Molecular Biology, National Agency for Research and Innovation	Amin Soebandrio; Edison Johar; Frilasita A Yudhaputri; Hidayat Trimarsanto; Iskandar Adnan; Khin Saw Myint; Lidwina Priliani; Lydia V. Panggalo; Muhammad Rezki Rasyak; Safarina G Malik; Sukma Oktavianthi; Willy Agustine
EPI_ISL_2614655, EPI_ISL_2614690, EPI_ISL_2614697, EPI_ISL_2614698, EPI_ISL_2614714	RS Harapan Bunda	Eijkman Institute for Molecular Biology, National Research and Innovation Agency	Amin Soebandrio; Edison Johar; Frilasita A Yudhaputri; Hidayat Trimarsanto; Iskandar Adnan; Khin Saw Myint; Lidwina Priliani; Lydia V. Panggalo; Muhammad Rezki Rasyak; Safarina G Malik; Sukma Oktavianthi; Willy Agustine
EPI_ISL_1284176, EPI_ISL_1284240	RS Harapan Mulia Cibarusah	Eijkman Institute for Molecular Biology, Ministry of Research and Technology/National Agency for Research and Innovation	Amin Soebandrio; Edison Johar; Frilasita A Yudhaputri; Hidayat Trimarsanto; Iskandar Adnan; Khin Saw Myint; Lydia V. Panggalo; Safarina G Malik; Sukma Oktavianthi; Willy Agustine
EPI_ISL_2614699	RS Harapan Mulia Cibarusah	Eijkman Institute for Molecular Biology, National Research and Innovation Agency	Amin Soebandrio; Edison Johar; Frilasita A Yudhaputri; Hidayat Trimarsanto; Iskandar Adnan; Khin Saw Myint; Lidwina Priliani; Lydia V. Panggalo; Muhammad Rezki Rasyak; Safarina G Malik; Sukma Oktavianthi; Willy Agustine
EPI_ISL_947290	RS Hermina Bitung	Eijkman Institute for Molecular Biology, Ministry of Research and Technology/National Agency for Research and Innovation	Amin Soebandrio; Edison Johar; Frilasita A Yudhaputri; Hidayat Trimarsanto; Iskandar Adnan; Khin Saw Myint; Lydia V. Panggalo; Safarina G Malik; Sukma Oktavianthi; Willy Agustine
EPI_ISL_888983	RS Hermina Daan Mogot	Eijkman Institute for Molecular Biology, Ministry of Research and Technology/National Agency for Research and Innovation	Amin Soebandrio; Edison Johar; Frilasita A Yudhaputri; Hidayat Trimarsanto; Iskandar Adnan; Khin Saw Myint; Lydia V. Panggalo; Safarina G Malik; Sukma Oktavianthi; Willy Agustine
EPI_ISL_888984	RS Hermina Depok	Eijkman Institute for Molecular Biology, Ministry of Research and Technology/National Agency for Research and Innovation	Amin Soebandrio; Edison Johar; Frilasita A Yudhaputri; Hidayat Trimarsanto; Iskandar Adnan; Khin Saw Myint; Lydia V. Panggalo; Safarina G Malik; Sukma Oktavianthi; Willy Agustine
EPI_ISL_2614736, EPI_ISL_3029934, EPI_ISL_3029935	RS Hermina Depok	Eijkman Institute for Molecular Biology, National Research and Innovation Agency	Amin Soebandrio; Edison Johar; Frilasita A Yudhaputri; Hidayat Trimarsanto; Iskandar Adnan; Khin Saw Myint; Lidwina Priliani; Lydia V. Panggalo; Muhammad Rezki Rasyak; Safarina G Malik; Sukma Oktavianthi; Willy Agustine

EPI_ISL_2854712, EPI_ISL_2854723, EPI_ISL_2854724	RS Hermina Depok	National Institute of Health Research and Development	; Arie Ardiansyah Nugraha; Hana Apsari Pawestri; Hartanti Dian Ikawati; Kartika Dewi Puspa; Krisna Pangesti; Nelly Puspandari; Subangkit; Triyani Soekarso; Vivi Setiawaty
EPI_ISL_2854725	RS Hermina Galaxy	National Institute of Health Research and Development	; Arie Ardiansyah Nugraha; Hana Apsari Pawestri; Hartanti Dian Ikawati; Kartika Dewi Puspa; Krisna Pangesti; Nelly Puspandari; Subangkit; Triyani Soekarso; Vivi Setiawaty
EPI_ISL_2617518	RS Hermina Kemayoran	National Institute of Health Research and Development	Arie Ardiansyah Nugraha; Hana Apsari Pawestri; Hartanti Dian Ikawati; Kartika Dewi Puspa; Krisna Pangesti; Nelly Puspandari; Subangkit; Triyani Soekarso; Vivi Setiawaty
EPI_ISL_574612	RS Hermina Mekarsari	Eijkman Institute for Molecular Biology, Ministry of Research and Technology/National Agency for Research and Innovation	Amin Soebandrio; David H Muljono; Edison Johar; Frilasita A Yudhaputri; Herawati Sudoyo; Hidayat Trimarsanto; Iskandar A Adnan; Khin Saw Myint; Safarina G Malik; Willy Agustine
EPI_ISL_3029937	RS Hermina Periuk	Eijkman Institute for Molecular Biology, National Research and Innovation Agency	Amin Soebandrio; Edison Johar; Frilasita A Yudhaputri; Hidayat Trimarsanto; Iskandar Adnan; Khin Saw Myint; Lidwina Priliani; Lydia V. Panggalo; Muhammad Rezki Rasyak; Safarina G Malik; Sukma Oktavianthi; Willy Agustine
EPI_ISL_888985	RS Hermina Serpong	Eijkman Institute for Molecular Biology, Ministry of Research and Technology/National Agency for Research and Innovation	Amin Soebandrio; Edison Johar; Frilasita A Yudhaputri; Hidayat Trimarsanto; Iskandar Adnan; Khin Saw Myint; Lydia V. Panggalo; Safarina G Malik; Sukma Oktavianthi; Willy Agustine
EPI_ISL_889017	RS Hermina Tangerang	Eijkman Institute for Molecular Biology, Ministry of Research and Technology/National Agency for Research and Innovation	Amin Soebandrio; Edison Johar; Frilasita A Yudhaputri; Hidayat Trimarsanto; Iskandar Adnan; Khin Saw Myint; Lydia V. Panggalo; Safarina G Malik; Sukma Oktavianthi; Willy Agustine
EPI_ISL_574607	RS Husada	Eijkman Institute for Molecular Biology, Ministry of Research and Technology/National Agency for Research and Innovation	Amin Soebandrio; David H Muljono; Edison Johar; Frilasita A Yudhaputri; Herawati Sudoyo; Hidayat Trimarsanto; Iskandar A Adnan; Khin Saw Myint; Safarina G Malik; Willy Agustine
EPI_ISL_2617519, EPI_ISL_2617522, EPI_ISL_2617523, EPI_ISL_3138837	RS IMC Bintaro	National Institute of Health Research and Development	Arie Ardiansyah Nugraha; Fajar Nur Sulistiyohadi; Hana Apsari Pawestri; Hartanti Dian Ikawati; Kartika Dewi Puspa; Krisna Pangesti; Nelly Puspandari; Subangkit; Triyani Soekarso; Vivi Setiawaty
EPI_ISL_4004698, EPI_ISL_4004703	RS Ibu dan Anak Permata Pertiwi	National Research and Innovation Agency (BRIN-Indonesia)	Ahmad Fathoni; Anggia Prasetyoputri; Anik B. Dharmayanthi; Asep M. Ridwanulloh; Hariyatun; Indriawati; Rifki Sadikin; Sugiyono Saputra; Suvia Widyaningrum; Syam B. Iryanto
EPI_ISL_2854748	RS Ichsan Medical Centre Bintaro Tangsel	National Institute of Health Research and Development	; Arie Ardiansyah Nugraha; Hana Apsari Pawestri; Hartanti Dian Ikawati; Kartika Dewi Puspa; Krisna Pangesti; Nelly Puspandari; Subangkit; Triyani Soekarso; Vivi Setiawaty
EPI_ISL_4778322	RS Islam Muhammadiyah Rodliyah Achid Moga Pemalang	Balai Laboratorium Kesehatan Dan Pengujian Alat Kesehatan Provinsi Jawa Tengah	Alia Wijayanti; Dwi Nursetyaasih; Dyah Ayu; Endah Wahyu Y; Ismi Farhati; Purnomo; Sekti Handayani; Wiwik Peni; Yekti Hediningsih
EPI_ISL_4757248, EPI_ISL_4760364	RS Islam Purwodadi Grobogan	Balai Laboratorium Kesehatan Dan Pengujian Alat Kesehatan Provinsi Jawa Tengah	Alia Wijayanti; Dwi Nursetyaasih; Dyah Ayu; Endah Wahyu Y; Ismi Farhati; Purnomo; Sekti Handayani; Wiwik Peni; Yekti Hediningsih
EPI_ISL_1283987, EPI_ISL_1284322	RS Islam Sari Asih Ar-Rahmah	Eijkman Institute for Molecular Biology, Ministry of Research and Technology/National Agency for Research and Innovation	Amin Soebandrio; Edison Johar; Frilasita A Yudhaputri; Hidayat Trimarsanto; Iskandar Adnan; Khin Saw Myint; Lydia V. Panggalo; Safarina G Malik; Sukma Oktavianthi; Willy Agustine
EPI_ISL_2364605	RS Islam Sari Asih Ar-Rahmah	Eijkman Institute for Molecular Biology, National Agency for Research and Innovation	Amin Soebandrio; Edison Johar; Frilasita A Yudhaputri; Hidayat Trimarsanto; Iskandar Adnan; Khin Saw Myint; Lidwina Priliani; Lydia V. Panggalo; Muhammad Rezki Rasyak; Safarina G Malik; Sukma Oktavianthi; Willy Agustine
EPI_ISL_4968087, EPI_ISL_4969010, EPI_ISL_4969082, EPI_ISL_4969086	RS JIH	Genetics Working Group (Pokja Genetik) Faculty of Medicine, Public Health and Nursing Universitas Gadjah Mada (FK-KMK UGM); Disease Investigation Center Wates Ministry of Agriculture Indonesia; Department of Microbiology FK-KMK UGM; Laboratorium Diagnostik Yayasan Tahija World Mosquito Program (WMP) Yogyakarta Center for Tropical Medicine FK-KMK UGM; Integrated Research Center FK-KMK UGM; Department of Computer Science and Electronics FMIPA UGM; RSUP Dr. Sardjito	Afiahayati; Alvina A Setiawan; Cita S Amalia; Dwi AA Nugrahaningsih; Dwi Indaryati; Dyah A Puspitarani; Edwin W. Daniwijaya; Eggi Arguni; Endah Supriyati; Gunadi; Hana F Hanifin; Hendra Wibawa; Indarto Sulistiyono; Irene Tania; Khanza a Vujira; Kristy Iskandar; Ludhang P. Rizki; Marcellus; Mohamad S. Hakim; Nungki Anggorowati; Pramesti G Dewi; Siswanto; Susan Simanjaya; Titik Nuryastuti; Tri Wibawa
EPI_ISL_3138833, EPI_ISL_3138834, EPI_ISL_3138835, EPI_ISL_3138836	RS Jantung Dan Pembuluh Darah Harapan Kita	National Institute of Health Research and Development	Arie Ardiansyah Nugraha; Fajar Nur Sulistiyohadi; Hana Apsari Pawestri; Hartanti Dian Ikawati; Kartika Dewi Puspa; Krisna Pangesti; Nelly Puspandari; Subangkit; Triyani Soekarso; Vivi Setiawaty
EPI_ISL_3138860, EPI_ISL_3138861, EPI_ISL_3138862	RS Jantung dan Pembulu Darah Harapan Kita	National Institute of Health Research and Development	; Arie Ardiansyah Nugraha; Fajar Nur Sulistiyohadi; Hana Apsari Pawestri; Hartanti Dian Ikawati; Kartika Dewi Puspa; Krisna Pangesti; Nelly Puspandari; Subangkit; Triyani Soekarso; Vivi Setiawaty
EPI_ISL_3216546, EPI_ISL_3216547	RS Jantung dan Pembuluh Darah Harapan Kita	National Institute of Health Research and Development	Arie Ardiansyah Nugraha; Fajar Nur Sulistiyohadi; Hana Apsari Pawestri; Hartanti Dian Ikawati; Kartika Dewi Puspa; Krisna Pangesti; Nelly Puspandari; Subangkit; Triyani Soekarso; Vivi Setiawaty
EPI_ISL_4090556, EPI_ISL_4090557, EPI_ISL_4090558, EPI_ISL_4090559, EPI_ISL_4090560	RS Karakau Medika	National Institute of Health Research and Development	Arie Ardiansyah Nugraha; Fajar Nur; Hana Apsari Pawestri; Hartanti Dian Ikawati; Kartika Dewi Puspa; Krisna Pangesti; Nelly Puspandari; Subangkit; Sulistiyohadi; Triyani Soekarso; Vivi Setiawaty
EPI_ISL_4417826, EPI_ISL_4417827, EPI_ISL_4417828, EPI_ISL_4417829, EPI_ISL_4417830, EPI_ISL_4417831, EPI_ISL_4417832, EPI_ISL_4417833, EPI_ISL_4417834, EPI_ISL_4417835, EPI_ISL_4417836, EPI_ISL_4417837, EPI_ISL_4417838, EPI_ISL_4417839, EPI_ISL_4417840, EPI_ISL_4417841, EPI_ISL_4417842, EPI_ISL_4417843, EPI_ISL_4417844, EPI_ISL_4417845, EPI_ISL_4417846, EPI_ISL_4417847, EPI_ISL_4417849, EPI_ISL_4417850, EPI_ISL_4417851, EPI_ISL_4468542, EPI_ISL_4491514, EPI_ISL_5098156			
see above	RS Karitas / EIJKMAN OXFORD CLINICAL RESEARCH UNIT	Genomik Solidaritas Indonesia Laboratorium	; Anis Karuniawati; Annisa Muthiah Sukirman; Anuraj Shankar; Ariel Pradipta; Bonifacius; Carissa Sintca Wijaya; Claus Bough; Decy Subekti; Dhahlia Agustina Cahyono; Dian Tri Wahyudi; Dwi Utomo Nusantara; Esti Rahayuning Tias; Gracia Felias Enos Korompis; Himawan Masyhuri; Kevin Baird; Louisa Markus; Meutia Ayuputeri Kumaheri; Raph Hamers; Reinhart Gabriel; Stefanus Bernard; Suwarti; Vania Gavrila Wikasa
EPI_ISL_2854749, EPI_ISL_2868905, EPI_ISL_3138820, EPI_ISL_3138821, EPI_ISL_3138822, EPI_ISL_3278297	RS Kartika Pulomas	National Institute of Health Research and Development	Arie Ardiansyah Nugraha; Fajar Nur Sulistiyohadi; Hana Apsari Pawestri; Hartanti Dian Ikawati; Kartika Dewi Puspa; Krisna Pangesti; Nelly Puspandari; Subangkit; Triyani Soekarso; Vivi Setiawaty
EPI_ISL_763064	RS Kasih Ibu, Surakarta	Universitas Sebelas Maret (UNS); Rumah Sakit UNS (RS-UNS); National Institute of Health Research and Development, Indonesian Ministry of Health.	Afif A Ghufron; Arie A Nugraha; Betty Suryawati; Dewi Puspa; Hana A Pawestri; Hartanti Dian Ika; Hartono; Kartika; Maryani; Revi G H Novika; Vivi Setiawaty.; Yulia Sari
EPI_ISL_4825226, EPI_ISL_4825512	RS Ken Saras Ungaran	Balai Laboratorium Kesehatan Dan Pengujian Alat Kesehatan Provinsi Jawa Tengah	Alia Wijayanti; Dwi Nursetyaasih; Dyah Ayu; Endah Wahyu Y; Ismi Farhati; Purnomo; Sekti Handayani; Wiwik Peni; Yekti Hediningsih
EPI_ISL_4847179	RS Ken Saras Ungaran Semarang	Balai Laboratorium Kesehatan Dan Pengujian Alat Kesehatan Provinsi Jawa Tengah	Alia Wijayanti; Dwi Nursetyaasih; Dyah Ayu; Endah Wahyu Y; Ismi Farhati; Purnomo; Sekti Handayani; Wiwik Peni; Yekti Hediningsih
EPI_ISL_3030052, EPI_ISL_3030056, EPI_ISL_3030057	RS Khusus Gigi dan Mulut Kota Bandung, Dinas Kesehatan Kota Bandung	Eijkman Institute for Molecular Biology, National Research and Innovation Agency; West Java Health Laboratory	Ade Fany Safitri; Amin Soebandrio; Azzania Fibriani; Cut Nur Cinthia Alamanda; Edison Johar; Ema Rahmawati; Frilasita A Yudhaputri; Hidayat Trimarsanto; Husna Nugrahapraja; Iskandar Adnan; Khin Saw Myint; Lidwina Priliani; Lydia V. Panggalo; Muhammad Rezki Rasyak; Ranza Fadhlil Pratama; Rifky Waluyajati Rachman; Ryan Bayusantika Ristandi; Safarina G Malik; Sukma Oktavianthi; Tati Sutarti Sastrowiyoto; Willy Agustine
EPI_ISL_2987604	RS Khusus Kanker MRCCC Siloam Semanggi	National Institute of Health Research and Development	Arie Ardiansyah Nugraha; Fajar Nur Sulistiyohadi; Hana Apsari Pawestri; Hartanti Dian Ikawati; Kartika Dewi Puspa; Krisna Pangesti; Nelly Puspandari; Subangkit; Triyani Soekarso; Vivi Setiawaty
EPI_ISL_4004705	RS Khusus Paru Kabupaten Karawang	National Research and Innovation Agency (BRIN-Indonesia, VenomCoV Project Team); West Java Health Laboratory	Ahmad Fathoni; Anggia Prasetyoputri; Anik B. Dharmayanthi; Asep M. Ridwanulloh; Azzania Fibriani; Cut Nur Cinthia Alamanda; Ema Rahmawati; Hariyatun; Indriawati; Karimatu Khoirunnisa; Miftahul Faridl; Ratih A. Ningrum; Rifki Sadikin; Rifky Waluyajati Rachman; Rini Robiani; Ryan Bayusantika Ristandi; Sugiyono Saputra; Syam B. Iryanto
EPI_ISL_1469247, EPI_ISL_1469256, EPI_ISL_1469257, EPI_ISL_1469285, EPI_ISL_1469286	RS Khusus Paru Karawang	National Institute of Health Research and Development	Arie Ardiansyah Nugraha; Hana Apsari Pawestri; Hartanti Dian Ikawati; Kartika Dewi Puspa; Krisna Pangesti; Nelly Puspandari; Subangkit; Vivi Setiawaty
EPI_ISL_2868931	RS Kogabwilhan II Surabaya	National Institute of Health Research and Development	Arie Ardiansyah Nugraha; Hana Apsari Pawestri; Hartanti Dian Ikawati; Kartika Dewi Puspa; Krisna Pangesti; Nelly Puspandari; Subangkit; Triyani Soekarso; Vivi Setiawaty
EPI_ISL_2854778	RS Krakatau Medika, Cilegon	National Institute of Health Research and Development	Arie Ardiansyah Nugraha; Hana Apsari Pawestri; Hartanti Dian Ikawati; Kartika Dewi Puspa; Krisna Pangesti; Nelly Puspandari; Subangkit; Triyani Soekarso; Vivi Setiawaty
EPI_ISL_568692, EPI_ISL_574614, EPI_ISL_574616, EPI_ISL_574617, EPI_ISL_574618, EPI_ISL_766047	RS Kramat 128	Eijkman Institute for Molecular Biology, Ministry of Research and Technology/National Agency for Research and Innovation	Amin Soebandrio; David H Muljono; Edison Johar; Frilasita A Yudhaputri; Herawati Sudoyo; Hidayat Trimarsanto; Iskandar A Adnan; Khin Saw Myint; Safarina G Malik; Willy Agustine
EPI_ISL_2854699, EPI_ISL_2854700	RS Larangan Kogabwilhan II Indrapura Sby	National Institute of Health Research and Development	Arie Ardiansyah Nugraha; Hana Apsari Pawestri; Hartanti Dian Ikawati; Kartika Dewi Puspa; Krisna Pangesti; Nelly Puspandari; Subangkit; Triyani Soekarso; Vivi Setiawaty
EPI_ISL_538499	RS Lavallete Malang East Java	National Institute of Health Research and Development	AA; HA; HD; Ikawati; KD; KNA; Nugraha; Paisal; Pangesti; Pawestri; Puspa; Setiawaty, V.; Soekarso; Subangkit; T
EPI_ISL_1469254	RS Lira Medika Karawang	National Institute of Health Research and Development	Arie Ardiansyah Nugraha; Hana Apsari Pawestri; Hartanti Dian Ikawati; Kartika Dewi Puspa; Krisna Pangesti; Nelly Puspandari; Subangkit; Vivi Setiawaty
EPI_ISL_833393, EPI_ISL_833492	RS MMC Jakarta, Indonesia	Biosafety Level-3 Laboratory, Indonesian Institute of Sciences (LIPI)	Ade Andriani; Ahmad Fathoni; Andri Wardiana; Anggia Prasetyoputri; Anik Budhi Dharmayanthi; Eva Erdayani; Isa Nuryana; Linda Sukmarini; Nova Dilla Yanthi; Ratih Asmana Ningrum; Syam Budi Iryanto; Yuliawati
EPI_ISL_833503, EPI_ISL_833504, EPI_ISL_833505, EPI_ISL_833507	RS MMC, Jakarta, Indonesia	Biosafety Level-3 Laboratory, Indonesian Institute of Sciences (LIPI)	Ade Andriani; Ahmad Fathoni; Ahmad Randy; Andri Wardiana; Anggia Prasetyoputri; Anik Budhi Dharmayanthi; Idris; Isa Nuryana; Listiana Oktavia; Muhammad Ridwan; Pangda Sopha; Ratih Asmana Ningrum; Ruby Setiawan; Syaiful Rizal; Syam Budi Iryanto

EPI_ISL_2790724, EPI_ISL_2790725, EPI_ISL_2790726, EPI_ISL_2790727, EPI_ISL_2790728, EPI_ISL_2790729	RS MRCCC SILOAM SEMANGGI / DINAS KESEHATAN DKI JAKARTA	Genomik Solidaritas Indonesia Laboratorium	Alfin Mohammad Abdillah; Anna Christina Brazia; Annisa Muthiah Sukirman; Anuraj Shankar; Ariel Pradipta; Carissa Sintca Wijaya; Dhahlia Agustina Cahyono; Dwi Oktavia; Gracia Felias Enos Korompis; Meutia Ayuputeri Kumaheri; Ngabila Salama; Normastuti Adhini Tantyo; Reinhart Gabriel; Tiranti Vindha; Vania Gavrila Wikasa; Widyastuti
EPI_ISL_1469248, EPI_ISL_1469253, EPI_ISL_1469287, EPI_ISL_2106980	RS Mandaya Karawang	National Institute of Health Research and Development	Arie Ardiansyah Nugraha; Hana Apsari Pawestri; Hartanti Dian Ikawati; Kartika Dewi Puspa; Krisna Pangesti; Nelly Puspandari; Subangkit; Vivi Setiawaty
EPI_ISL_1622373, EPI_ISL_1622426	RS Mary Cileungsi Hijau	Eijkman Institute for Molecular Biology, Ministry of Research and Technology/National Agency for Research and Innovation	Amin Soebandrio; Edison Johar; Frilasita A Yudhaputri; Hidayat Trimarsanto; Iskandar Adnan; Khin Saw Myint; Lydia V. Panggalo; Muhammad Rezki Rasyak; Safarina G Malik; Sukma Oktavianthi; Willy Agustine
EPI_ISL_2364601, EPI_ISL_2364614, EPI_ISL_2364638, EPI_ISL_2364641	RS Mary Cileungsi Hijau	Eijkman Institute for Molecular Biology, National Agency for Research and Innovation	Amin Soebandrio; Edison Johar; Frilasita A Yudhaputri; Hidayat Trimarsanto; Iskandar Adnan; Khin Saw Myint; Lidwina Priliani; Lydia V. Panggalo; Muhammad Rezki Rasyak; Safarina G Malik; Sukma Oktavianthi; Willy Agustine
EPI_ISL_2614693	RS Mary Cileungsi Hijau	Eijkman Institute for Molecular Biology, National Research and Innovation Agency	Amin Soebandrio; Edison Johar; Frilasita A Yudhaputri; Hidayat Trimarsanto; Iskandar Adnan; Khin Saw Myint; Lidwina Priliani; Lydia V. Panggalo; Muhammad Rezki Rasyak; Safarina G Malik; Sukma Oktavianthi; Willy Agustine
EPI_ISL_1284244, EPI_ISL_1284247, EPI_ISL_1622389	RS Masmitra	Eijkman Institute for Molecular Biology, Ministry of Research and Technology/National Agency for Research and Innovation	Amin Soebandrio; Edison Johar; Frilasita A Yudhaputri; Hidayat Trimarsanto; Iskandar Adnan; Khin Saw Myint; Lydia V. Panggalo; Muhammad Rezki Rasyak; Safarina G Malik; Sukma Oktavianthi; Willy Agustine
EPI_ISL_2364575	RS Masmitra	Eijkman Institute for Molecular Biology, National Agency for Research and Innovation	Amin Soebandrio; Edison Johar; Frilasita A Yudhaputri; Hidayat Trimarsanto; Iskandar Adnan; Khin Saw Myint; Lidwina Priliani; Lydia V. Panggalo; Muhammad Rezki Rasyak; Safarina G Malik; Sukma Oktavianthi; Willy Agustine
EPI_ISL_2987615, EPI_ISL_2987618, EPI_ISL_2987619	RS Mata Cicendo Bandung	National Institute of Health Research and Development	Arie Ardiansyah Nugraha; Fajar Nur Sulistiyohadi; Hana Apsari Pawestri; Hartanti Dian Ikawati; Kartika Dewi Puspa; Krisna Pangesti; Nelly Puspandari; Subangkit; Triyani Soekarso; Vivi Setiawaty
EPI_ISL_4783718	RS Mayapada Jakarta Selatan	Genomik Solidaritas Indonesia Laboratorium	Annisa Muthiah Sukirman; Anuraj Shankar; Ariel Pradipta; Benny Hosiana Tumbelaka; Carissa Sintca Wijaya; Esti Rahayuning Tias; Gracia Felias Enos Korompis; Himawan Masyhuri; Louisa Markus; Marliana; Meutia Ayuputeri Kumaheri; Shinta Vr Hutajulu; Stefanus Bernard
EPI_ISL_3466023	RS Mayapada Tangerang	Eijkman Institute for Molecular Biology, National Research and Innovation Agency	Amin Soebandrio; Edison Johar; Frilasita A Yudhaputri; Hidayat Trimarsanto; Iskandar Adnan; Jessica R. Saragih; Khin Saw Myint; Lidwina Priliani; Lydia V. Panggalo; Muhammad Rezki Rasyak; Safarina G Malik; Sukma Oktavianthi; Willy Agustine
EPI_ISL_1622382	RS Medika Dramaga	Eijkman Institute for Molecular Biology, Ministry of Research and Technology/National Agency for Research and Innovation	Amin Soebandrio; Edison Johar; Frilasita A Yudhaputri; Hidayat Trimarsanto; Iskandar Adnan; Khin Saw Myint; Lydia V. Panggalo; Muhammad Rezki Rasyak; Safarina G Malik; Sukma Oktavianthi; Willy Agustine
EPI_ISL_2364589, EPI_ISL_2364606, EPI_ISL_2364607	RS Medika Dramaga	Eijkman Institute for Molecular Biology, National Agency for Research and Innovation	Amin Soebandrio; Edison Johar; Frilasita A Yudhaputri; Hidayat Trimarsanto; Iskandar Adnan; Khin Saw Myint; Lidwina Priliani; Lydia V. Panggalo; Muhammad Rezki Rasyak; Safarina G Malik; Sukma Oktavianthi; Willy Agustine
EPI_ISL_1284129	RS Medika Permata Hijau	Eijkman Institute for Molecular Biology, Ministry of Research and Technology/National Agency for Research and Innovation	Amin Soebandrio; Edison Johar; Frilasita A Yudhaputri; Hidayat Trimarsanto; Iskandar Adnan; Khin Saw Myint; Lydia V. Panggalo; Safarina G Malik; Sukma Oktavianthi; Willy Agustine
EPI_ISL_2614648	RS Medika Permata Hijau	Eijkman Institute for Molecular Biology, National Research and Innovation Agency	Amin Soebandrio; Edison Johar; Frilasita A Yudhaputri; Hidayat Trimarsanto; Iskandar Adnan; Khin Saw Myint; Lidwina Priliani; Lydia V. Panggalo; Muhammad Rezki Rasyak; Safarina G Malik; Sukma Oktavianthi; Willy Agustine
EPI_ISL_1284215, EPI_ISL_1284273, EPI_ISL_1284277, EPI_ISL_1622391, EPI_ISL_1622408, EPI_ISL_1622412, EPI_ISL_1622421, EPI_ISL_1622422			
see above	RS Medirossa Cikarang	Eijkman Institute for Molecular Biology, Ministry of Research and Technology/National Agency for Research and Innovation	Amin Soebandrio; Edison Johar; Frilasita A Yudhaputri; Hidayat Trimarsanto; Iskandar Adnan; Khin Saw Myint; Lydia V. Panggalo; Muhammad Rezki Rasyak; Safarina G Malik; Sukma Oktavianthi; Willy Agustine
EPI_ISL_2364568, EPI_ISL_2364659, EPI_ISL_2364663	RS Medirossa Cikarang	Eijkman Institute for Molecular Biology, National Agency for Research and Innovation	Amin Soebandrio; Edison Johar; Frilasita A Yudhaputri; Hidayat Trimarsanto; Iskandar Adnan; Khin Saw Myint; Lidwina Priliani; Lydia V. Panggalo; Muhammad Rezki Rasyak; Safarina G Malik; Sukma Oktavianthi; Willy Agustine
EPI_ISL_2614667, EPI_ISL_2614671, EPI_ISL_2614688, EPI_ISL_2614689, EPI_ISL_2614711	RS Medirossa Cikarang	Eijkman Institute for Molecular Biology, National Research and Innovation Agency	Amin Soebandrio; Edison Johar; Frilasita A Yudhaputri; Hidayat Trimarsanto; Iskandar Adnan; Khin Saw Myint; Lidwina Priliani; Lydia V. Panggalo; Muhammad Rezki Rasyak; Safarina G Malik; Sukma Oktavianthi; Willy Agustine
EPI_ISL_2918613	RS Medistra	Eijkman Institute for Molecular Biology, National Research and Innovation Agency	Amin Soebandrio; Edison Johar; Frilasita A Yudhaputri; Hidayat Trimarsanto; Iskandar Adnan; Khin Saw Myint; Lidwina Priliani; Lydia V. Panggalo; Muhammad Rezki Rasyak; Safarina G Malik; Sukma Oktavianthi; Willy Agustine
EPI_ISL_833509, EPI_ISL_833510	RS Melania, Bogor, Indonesia	Biosafety Level-3 Laboratory, Indonesian Institute of Sciences (LIPI)	Ade Andriani; Ahmad Fathoni; Andri Wardiana; Anggia Prasetyoputri; Anik Budhi Dharmayanthi; Isa Nuryana; Ratih Asmana Ningrum; Syam Budi Iryanto
EPI_ISL_5022905, EPI_ISL_5022906	RS Metta Medika II Sibolga	National Institute of Health Research and Development	Arie Ardiansyah Nugraha; Fajar Nur Sulistiyohadi; Hana Apsari Pawestri; Hartanti Dian Ikawati; Kartika Dewi Puspa; Krisna Pangesti; Nelly Puspandari; Subangkit; Triyani Soekarso; Vivi Setiawaty
EPI_ISL_889016	RS Mitra Husada	Eijkman Institute for Molecular Biology, Ministry of Research and Technology/National Agency for Research and Innovation	Amin Soebandrio; Edison Johar; Frilasita A Yudhaputri; Hidayat Trimarsanto; Iskandar Adnan; Khin Saw Myint; Lydia V. Panggalo; Safarina G Malik; Sukma Oktavianthi; Willy Agustine
EPI_ISL_1284210	RS Mitra Husada Pringsewu	Eijkman Institute for Molecular Biology, Ministry of Research and Technology/National Agency for Research and Innovation	Amin Soebandrio; Edison Johar; Frilasita A Yudhaputri; Hidayat Trimarsanto; Iskandar Adnan; Khin Saw Myint; Lydia V. Panggalo; Safarina G Malik; Sukma Oktavianthi; Willy Agustine
EPI_ISL_1284105	RS Mitra Husada Tangerang	Eijkman Institute for Molecular Biology, Ministry of Research and Technology/National Agency for Research and Innovation	Amin Soebandrio; Edison Johar; Frilasita A Yudhaputri; Hidayat Trimarsanto; Iskandar Adnan; Khin Saw Myint; Lydia V. Panggalo; Safarina G Malik; Sukma Oktavianthi; Willy Agustine
EPI_ISL_2614710	RS Mitra Husada Tangerang	Eijkman Institute for Molecular Biology, National Research and Innovation Agency	Amin Soebandrio; Edison Johar; Frilasita A Yudhaputri; Hidayat Trimarsanto; Iskandar Adnan; Khin Saw Myint; Lidwina Priliani; Lydia V. Panggalo; Muhammad Rezki Rasyak; Safarina G Malik; Sukma Oktavianthi; Willy Agustine
EPI_ISL_3070963	RS Mitra Kasih	Eijkman Institute for Molecular Biology, National Research and Innovation Agency; West Java Health Laboratory	Amin Soebandrio; Azzania Fibriani; Cut Nur Cinthia Alamanda; Edison Johar; Ema Rahmawati; Frilasita A Yudhaputri; Hidayat Trimarsanto; Iskandar Adnan; Khin Saw Myint; Lidwina Priliani; Lydia V. Panggalo; Muhammad Rezki Rasyak; Rifky Waluyajati Rachman; Ryan Bayusantika Ristandi; Safarina G Malik; Sukma Oktavianthi; Willy Agustine
EPI_ISL_2987600, EPI_ISL_2987602	RS Mitra Keluarga	National Institute of Health Research and Development	Arie Ardiansyah Nugraha; Fajar Nur Sulistiyohadi; Hana Apsari Pawestri; Hartanti Dian Ikawati; Kartika Dewi Puspa; Krisna Pangesti; Nelly Puspandari; Subangkit; Triyani Soekarso; Vivi Setiawaty
EPI_ISL_1284269	RS Mitra Keluarga Bekasi Barat	Eijkman Institute for Molecular Biology, Ministry of Research and Technology/National Agency for Research and Innovation	Amin Soebandrio; Edison Johar; Frilasita A Yudhaputri; Hidayat Trimarsanto; Iskandar Adnan; Khin Saw Myint; Lydia V. Panggalo; Safarina G Malik; Sukma Oktavianthi; Willy Agustine
EPI_ISL_2364632	RS Mitra Keluarga Bekasi Barat	Eijkman Institute for Molecular Biology, National Agency for Research and Innovation	Amin Soebandrio; Edison Johar; Frilasita A Yudhaputri; Hidayat Trimarsanto; Iskandar Adnan; Khin Saw Myint; Lidwina Priliani; Lydia V. Panggalo; Muhammad Rezki Rasyak; Safarina G Malik; Sukma Oktavianthi; Willy Agustine
EPI_ISL_766034, EPI_ISL_766036, EPI_ISL_889009	RS Mitra Keluarga Bintaro	Eijkman Institute for Molecular Biology, Ministry of Research and Technology/National Agency for Research and Innovation	Amin Soebandrio; David H Muljono; Edison Johar; Frilasita A Yudhaputri; Hidayat Trimarsanto; Iskandar Adnan; Khin Saw Myint; Lydia V. Panggalo; Safarina G Malik; Sukma Oktavianthi; Willy Agustine
EPI_ISL_2614721	RS Mitra Keluarga Bintaro	Eijkman Institute for Molecular Biology, National Research and Innovation Agency	Amin Soebandrio; Edison Johar; Frilasita A Yudhaputri; Hidayat Trimarsanto; Iskandar Adnan; Khin Saw Myint; Lidwina Priliani; Lydia V. Panggalo; Muhammad Rezki Rasyak; Safarina G Malik; Sukma Oktavianthi; Willy Agustine
EPI_ISL_888999, EPI_ISL_947301, EPI_ISL_1284207	RS Mitra Keluarga Cibubur	Eijkman Institute for Molecular Biology, Ministry of Research and Technology/National Agency for Research and Innovation	Amin Soebandrio; Edison Johar; Frilasita A Yudhaputri; Hidayat Trimarsanto; Iskandar Adnan; Khin Saw Myint; Lydia V. Panggalo; Safarina G Malik; Sukma Oktavianthi; Willy Agustine
EPI_ISL_2364579, EPI_ISL_2364600	RS Mitra Keluarga Cibubur	Eijkman Institute for Molecular Biology, National Agency for Research and Innovation	Amin Soebandrio; Edison Johar; Frilasita A Yudhaputri; Hidayat Trimarsanto; Iskandar Adnan; Khin Saw Myint; Lidwina Priliani; Lydia V. Panggalo; Muhammad Rezki Rasyak; Safarina G Malik; Sukma Oktavianthi; Willy Agustine
EPI_ISL_3029941, EPI_ISL_3029942, EPI_ISL_3029995, EPI_ISL_3029999	RS Mitra Keluarga Cibubur	Eijkman Institute for Molecular Biology, National Research and Innovation Agency	Amin Soebandrio; Edison Johar; Frilasita A Yudhaputri; Hidayat Trimarsanto; Iskandar Adnan; Khin Saw Myint; Lidwina Priliani; Lydia V. Panggalo; Muhammad Rezki Rasyak; Safarina G Malik; Sukma Oktavianthi; Willy Agustine
EPI_ISL_2013045	RS Mitra Keluarga Cikarang	Eijkman Institute for Molecular Biology, National Agency for Research and Innovation	Amin Soebandrio; Edison Johar; Frilasita A Yudhaputri; Hidayat Trimarsanto; Iskandar Adnan; Khin Saw Myint; Lydia V. Panggalo; Muhammad Rezki Rasyak; Safarina G Malik; Sukma Oktavianthi; Willy Agustine
EPI_ISL_947328	RS Mitra Keluarga Depok	Eijkman Institute for Molecular Biology, Ministry of Research and Technology/National Agency for Research and Innovation	Amin Soebandrio; Edison Johar; Frilasita A Yudhaputri; Hidayat Trimarsanto; Iskandar Adnan; Khin Saw Myint; Lydia V. Panggalo; Safarina G Malik; Sukma Oktavianthi; Willy Agustine
EPI_ISL_888991, EPI_ISL_888992, EPI_ISL_889003, EPI_ISL_889004, EPI_ISL_947270, EPI_ISL_947326, EPI_ISL_947327, EPI_ISL_1283933, EPI_ISL_1283941, EPI_ISL_1283945, EPI_ISL_1283949, EPI_ISL_1283953, EPI_ISL_1283964, EPI_ISL_1283982, EPI_ISL_1283984, EPI_ISL_1284007, EPI_ISL_1284008, EPI_ISL_1284021, EPI_ISL_1284025, EPI_ISL_1284063, EPI_ISL_1284133, EPI_ISL_1284136, EPI_ISL_1284251, EPI_ISL_1284254, EPI_ISL_1284307, EPI_ISL_1622360, EPI_ISL_1622361, EPI_ISL_1622371, EPI_ISL_1622372, EPI_ISL_1622379, EPI_ISL_1622380, EPI_ISL_1622388, EPI_ISL_1622413, EPI_ISL_1622428, EPI_ISL_1622429, EPI_ISL_1622430			
see above	RS Mitra Keluarga Gading Serpong	Eijkman Institute for Molecular Biology, Ministry of Research and Technology/National Agency for Research and Innovation	Amin Soebandrio; Edison Johar; Frilasita A Yudhaputri; Hidayat Trimarsanto; Iskandar Adnan; Khin Saw Myint; Lydia V. Panggalo; Muhammad Rezki Rasyak; Safarina G Malik; Sukma Oktavianthi; Willy Agustine
EPI_ISL_2364587, EPI_ISL_2364588, EPI_ISL_2364592, EPI_ISL_2364603, EPI_ISL_2364616, EPI_ISL_2364617	RS Mitra Keluarga Gading Serpong	Eijkman Institute for Molecular Biology, National Agency for Research and Innovation	Amin Soebandrio; Edison Johar; Frilasita A Yudhaputri; Hidayat Trimarsanto; Iskandar Adnan; Khin Saw Myint; Lidwina Priliani; Lydia V. Panggalo; Muhammad Rezki Rasyak; Safarina G Malik; Sukma Oktavianthi; Willy Agustine

EPI_ISL_2614650, EPI_ISL_2614651, EPI_ISL_2614661, EPI_ISL_2614662, EPI_ISL_2614668, EPI_ISL_2614675, EPI_ISL_2614701, EPI_ISL_2614702, EPI_ISL_2614703, EPI_ISL_2614712, EPI_ISL_2614717, EPI_ISL_2614718, EPI_ISL_3070859, EPI_ISL_3465996			
see above	RS Mitra Keluarga Gading Serpong	Eijkman Institute for Molecular Biology, National Research and Innovation Agency	Amin Soebandrio; Edison Johar; Frilasita A Yudhaputri; Hidayat Trimarsanto; Iskandar Adnan; Jessica R. Saragih; Khin Saw Myint; Lidwina Priliani; Lydia V. Panggalo; Muhammad Rezki Rasyak; Safarina G Malik; Sukma Oktavianthi; Willy Agustine
EPI_ISL_1283998, EPI_ISL_1284017, EPI_ISL_1284101, EPI_ISL_1284163, EPI_ISL_1284165, EPI_ISL_1284219, EPI_ISL_1284223, EPI_ISL_1284284, EPI_ISL_1284288, EPI_ISL_1284292, EPI_ISL_1284314, EPI_ISL_1284327, EPI_ISL_1284331, EPI_ISL_1284335, EPI_ISL_1622409			
see above	RS Mitra Keluarga Kalideres	Eijkman Institute for Molecular Biology, Ministry of Research and Technology/National Agency for Research and Innovation	Amin Soebandrio; Edison Johar; Frilasita A Yudhaputri; Hidayat Trimarsanto; Iskandar Adnan; Khin Saw Myint; Lydia V. Panggalo; Muhammad Rezki Rasyak; Safarina G Malik; Sukma Oktavianthi; Willy Agustine
EPI_ISL_2364610	RS Mitra Keluarga Kalideres	Eijkman Institute for Molecular Biology, National Agency for Research and Innovation	Amin Soebandrio; Edison Johar; Frilasita A Yudhaputri; Hidayat Trimarsanto; Iskandar Adnan; Khin Saw Myint; Lidwina Priliani; Lydia V. Panggalo; Muhammad Rezki Rasyak; Safarina G Malik; Sukma Oktavianthi; Willy Agustine
EPI_ISL_2617493	RS Mitra Keluarga Kalideres	National Institute of Health Research and Development	Arie Ardiansyah Nugraha; Hana Apsari Pawestri; Hartanti Dian Ikawati; Kartika Dewi Puspa; Krisna Pangesti; Nelly Puspandari; Subangkit; Triyani Soekarso; Vivi Setiawaty
EPI_ISL_574608, EPI_ISL_947322, EPI_ISL_1283929, EPI_ISL_1283994, EPI_ISL_1622425	RS Mitra Keluarga Kelapa Gading	Eijkman Institute for Molecular Biology, Ministry of Research and Technology/National Agency for Research and Innovation	Amin Soebandrio; David H Muljono; Edison Johar; Frilasita A Yudhaputri; Herawati Sudoyo; Hidayat Trimarsanto; Iskandar A Adnan; Iskandar Adnan; Khin Saw Myint; Lydia V. Panggalo; Muhammad Rezki Rasyak; Safarina G Malik; Sukma Oktavianthi; Willy Agustine
EPI_ISL_2013047, EPI_ISL_2364596, EPI_ISL_2364618, EPI_ISL_2364619	RS Mitra Keluarga Kelapa Gading	Eijkman Institute for Molecular Biology, National Agency for Research and Innovation	Amin Soebandrio; Edison Johar; Frilasita A Yudhaputri; Hidayat Trimarsanto; Iskandar Adnan; Khin Saw Myint; Lidwina Priliani; Lydia V. Panggalo; Muhammad Rezki Rasyak; Safarina G Malik; Sukma Oktavianthi; Willy Agustine
EPI_ISL_2614656, EPI_ISL_2614657, EPI_ISL_2614666, EPI_ISL_2614676	RS Mitra Keluarga Kelapa Gading	Eijkman Institute for Molecular Biology, National Research and Innovation Agency	Amin Soebandrio; Edison Johar; Frilasita A Yudhaputri; Hidayat Trimarsanto; Iskandar Adnan; Khin Saw Myint; Lidwina Priliani; Lydia V. Panggalo; Muhammad Rezki Rasyak; Safarina G Malik; Sukma Oktavianthi; Willy Agustine
EPI_ISL_3030001	RS Mitra Keluarga Kemayoran	Eijkman Institute for Molecular Biology, National Research and Innovation Agency	Amin Soebandrio; Edison Johar; Frilasita A Yudhaputri; Hidayat Trimarsanto; Iskandar Adnan; Khin Saw Myint; Lidwina Priliani; Lydia V. Panggalo; Muhammad Rezki Rasyak; Safarina G Malik; Sukma Oktavianthi; Willy Agustine
EPI_ISL_889012, EPI_ISL_1284067, EPI_ISL_1284197	RS Mitra Keluarga Pratama Jatiasih	Eijkman Institute for Molecular Biology, Ministry of Research and Technology/National Agency for Research and Innovation	Amin Soebandrio; Edison Johar; Frilasita A Yudhaputri; Hidayat Trimarsanto; Iskandar Adnan; Khin Saw Myint; Lydia V. Panggalo; Safarina G Malik; Sukma Oktavianthi; Willy Agustine
EPI_ISL_2617512	RS Mitra Kemayoran	National Institute of Health Research and Development	Arie Ardiansyah Nugraha; Hana Apsari Pawestri; Hartanti Dian Ikawati; Kartika Dewi Puspa; Krisna Pangesti; Nelly Puspandari; Subangkit; Triyani Soekarso; Vivi Setiawaty
EPI_ISL_3030050, EPI_ISL_3030051	RS Muhammadiyah Bandung, Dinas Kesehatan Kota Bandung	Eijkman Institute for Molecular Biology, National Research and Innovation Agency; West Java Health Laboratory	Ade Fany Safitri; Amin Soebandrio; Azzania Fibriani; Cut Nur Cinthia Alamanda; Edison Johar; Ema Rahmawati; Frilasita A Yudhaputri; Hidayat Trimarsanto; Husna Nugrahapraja; Iskandar Adnan; Khin Saw Myint; Lidwina Priliani; Lydia V. Panggalo; Muhammad Rezki Rasyak; Ranza Fadhlil Pratama; Rifky Waluyajati Rachman; Ryan Bayusantika Ristandi; Safarina G Malik; Sukma Oktavianthi; Tati Sutarti Sastrowiyoto; Willy Agustine
EPI_ISL_1284258	RS Mulia Pajajaran	Eijkman Institute for Molecular Biology, Ministry of Research and Technology/National Agency for Research and Innovation	Amin Soebandrio; Edison Johar; Frilasita A Yudhaputri; Hidayat Trimarsanto; Iskandar Adnan; Khin Saw Myint; Lydia V. Panggalo; Safarina G Malik; Sukma Oktavianthi; Willy Agustine
EPI_ISL_1283932, EPI_ISL_1284266	RS Mulya Tangerang	Eijkman Institute for Molecular Biology, Ministry of Research and Technology/National Agency for Research and Innovation	Amin Soebandrio; Edison Johar; Frilasita A Yudhaputri; Hidayat Trimarsanto; Iskandar Adnan; Khin Saw Myint; Lydia V. Panggalo; Safarina G Malik; Sukma Oktavianthi; Willy Agustine
EPI_ISL_1284031, EPI_ISL_1284262	RS OMNI Alam Sutera	Eijkman Institute for Molecular Biology, Ministry of Research and Technology/National Agency for Research and Innovation	Amin Soebandrio; Edison Johar; Frilasita A Yudhaputri; Hidayat Trimarsanto; Iskandar Adnan; Khin Saw Myint; Lydia V. Panggalo; Safarina G Malik; Sukma Oktavianthi; Willy Agustine
EPI_ISL_1622427	RS OMNI Pulomas	Eijkman Institute for Molecular Biology, Ministry of Research and Technology/National Agency for Research and Innovation	Amin Soebandrio; Edison Johar; Frilasita A Yudhaputri; Hidayat Trimarsanto; Iskandar Adnan; Khin Saw Myint; Lydia V. Panggalo; Muhammad Rezki Rasyak; Safarina G Malik; Sukma Oktavianthi; Willy Agustine
EPI_ISL_2614719, EPI_ISL_2709359, EPI_ISL_2709360, EPI_ISL_2709378, EPI_ISL_2709383, EPI_ISL_2709389, EPI_ISL_2709390, EPI_ISL_2709395, EPI_ISL_2811830, EPI_ISL_2811831, EPI_ISL_2811832			
see above	RS OMNI Pulomas	Eijkman Institute for Molecular Biology, National Research and Innovation Agency	Amin Soebandrio; Edison Johar; Frilasita A Yudhaputri; Hidayat Trimarsanto; Iskandar Adnan; Khin Saw Myint; Lidwina Priliani; Lydia V. Panggalo; Muhammad Rezki Rasyak; Safarina G Malik; Sukma Oktavianthi; Willy Agustine
EPI_ISL_889020	RS Omni Cikarang	Eijkman Institute for Molecular Biology, Ministry of Research and Technology/National Agency for Research and Innovation	Amin Soebandrio; Edison Johar; Frilasita A Yudhaputri; Hidayat Trimarsanto; Iskandar Adnan; Khin Saw Myint; Lydia V. Panggalo; Safarina G Malik; Sukma Oktavianthi; Willy Agustine
EPI_ISL_888981, EPI_ISL_1284002	RS Omni Pulomas	Eijkman Institute for Molecular Biology, Ministry of Research and Technology/National Agency for Research and Innovation	Amin Soebandrio; Edison Johar; Frilasita A Yudhaputri; Hidayat Trimarsanto; Iskandar Adnan; Khin Saw Myint; Lydia V. Panggalo; Safarina G Malik; Sukma Oktavianthi; Willy Agustine
EPI_ISL_2723600, EPI_ISL_2723601, EPI_ISL_2723602, EPI_ISL_2723603, EPI_ISL_2723604	RS PELNI	Genomik Solidaritas Indonesia Laboratorium	Alfin Mohammad Abdillah; Annisa Muthiah Sukirman; Anuraj Shankar; Ariel Pradipta; Carissa Sintca Wijaya; Dhahlia Agustina Cahyono; Dwi Oktavia; Gracia Felias Enos Korompis; Meutia Ayuputeri Kumaheri; Mohammad Anang Syarifudin; Ngabila Salama; Normastuti Adhini Tantyo; Tiranti Vindha; Vania Gavrila Wikasa; Widyastuti
EPI_ISL_3836266	RS PLUIT	Genomik Solidaritas Indonesia Laboratorium	Anna Christina Brazia; Annisa Muthiah Sukirman; Anuraj Shankar; Ariel Pradipta; David Hendra Haryanto; Dhahlia Agustina Cahyono; Dwi Oktavia; Eko Susanti; Gracia Felias Enos Korompis; Louisa Markus; Meutia Ayuputeri Kumaheri; Ngabila Salama; Pratiwi Srikandi Yudhastari; Reinhart Gabriel; Tiranti Vindha; Vania Gavrila Wikasa
EPI_ISL_833493, EPI_ISL_833494, EPI_ISL_833496	RS PMI Bogor, Indonesia	Biosafety Level-3 Laboratory, Indonesian Institute of Sciences (LIPI)	Ade Andriani; Ahmad Fathoni; Andri Wardiana; Anggia Prasetyoputri; Anik Budhi Dharmayanthi; Anky Zannati; Arie Wulandari; Indriawati; Isa Nuryana; Maritsa Nurfatwa; Nurulliawaty; Ratih Asmana Ningrum; Syam Budi Iryanto; Yana Rubiyana
EPI_ISL_833506	RS PMI, Bogor, Indonesia	Biosafety Level-3 Laboratory, Indonesian Institute of Sciences (LIPI)	Ade Andriani; Ahmad Fathoni; Andri Wardiana; Anggia Prasetyoputri; Anik Budhi Dharmayanthi; Gilang Akbariani; Isa Nuryana; Ratih Asmana Ningrum; Satrio Haryo Benowo; Syam Budi Iryanto
EPI_ISL_1020175, EPI_ISL_1020180, EPI_ISL_1020184, EPI_ISL_1020193, EPI_ISL_1020196, EPI_ISL_1117451, EPI_ISL_1117589, EPI_ISL_1137601, EPI_ISL_2840894, EPI_ISL_2844815, EPI_ISL_2844822, EPI_ISL_2844825, EPI_ISL_2844827, EPI_ISL_2844828, EPI_ISL_2844831			
see above	RS PMI, Bogor, West Java	Biosafety Level-3 Laboratory, Indonesian Institute of Sciences (LIPI)	Ade Andriani; Alfi T. Fathurahman; Andri Wardiana; Anggia Prasetyoputri; Anik B. Dharmayanthi; Anik Budhi Dharmayanthi; Ari S. Wulandari; Asep M. Ridwanulloh; Asep M. Ridwanuloh; Eko Wahyu Putro; Eko Wahyu Putro; Erwin F. Hasrianda; Fahrurrozi; Gita Syahputra; Hariyatun; Herjuno A. Nugroho; Idris; Indriawati; Inswasti Cahyani; Isa Nuryana; Linda Sukmarini; Listiana Oktavia; Matthew W. Loose; Mohammad Ilyas; Nova D. Yanthi; Puspita Lisdiyanti; Ratih A. Ningrum; Ratih Asmana Ningrum; Sri Swasthikawati; Sugiyono Saputra; Sugiyono Saputra; Susanti; Syaiful Rizal; Syam B. Iryanto; Syam Budi Iryanto; Wien Kusharyoto; Yuliawati
EPI_ISL_4106123, EPI_ISL_4106127	RS PMI, Bogor, West Java	National Research and Innovation Agency (BRIN-Indonesia, VenomCoV Project Team)	Andri Wardiana; Anggia Prasetyoputri; Erwin F. Hasrianda; Fahrurrozi; Herjuno A. Nugroho; Nova D. Yanthi; Sugiyono Saputra; Syam B. Iryanto
EPI_ISL_2943230	RS Panti Rapih	Genetics Working Group (Pokja Genetik) Faculty of Medicine, Public Health and Nursing Universitas Gadjah Mada (FK-KMK UGM); Disease Investigation Center Wates Ministry of Agriculture Indonesia; Department of Microbiology FK-KMK UGM; Laboratorium Diagnostik Yayasan Tahija World Mosquito Program (WMP) Yogyakarta Center for Tropical Medicine FK-KMK UGM; Integrated Research Center FK-KMK UGM; Department of Computer Science and Electronics	Afiahayati; Alvina A Setiawan; Dwi AA Nugrahaningsih; Dwi Indaryati; Edwin W. Daniwijaya; Eggi Arguni; Endah Supriyati; Gunadi; Hendra Wibawa; Indarto Sulistiyono; Khanza a Vujira; Kristy Iskandar; Ludhang P. Rizki; Marcellus; Mohamad S. Hakim; Nungki Anggorowati; Pramesti G Dewi; Siswanto; Titik Nuryastuti; Tri Wibawa
EPI_ISL_2943232	RS Panti Rapih	Genetics Working Group (Pokja Genetik) Faculty of Medicine, Public Health and Nursing Universitas Gadjah Mada (FK-KMK UGM); Disease Investigation Center Wates Ministry of Agriculture Indonesia; Department of Microbiology FK-KMK UGM; Laboratorium Diagnostik Yayasan Tahija World Mosquito Program (WMP) Yogyakarta Center for Tropical Medicine FK-KMK UGM; Integrated Research Center FK-KMK UGM; Department of Computer Science and Electronics FMIPA UGM; RSUP Dr. Sardjito	Afiahayati; Alvina A Setiawan; Cita S Amalia; Dwi AA Nugrahaningsih; Dwi Indaryati; Edwin W. Daniwijaya; Eggi Arguni; Endah Supriyati; Gunadi; Hendra Wibawa; Indarto Sulistiyono; Kristy Iskandar; Ludhang P. Rizki; Marcellus; Mohamad S. Hakim; Nungki Anggorowati; Pramesti G Dewi; Siswanto; Titik Nuryastuti; Tri Wibawa
EPI_ISL_610158	RS Panti Rini	Genetics Working Group (Pokja Genetik) Faculty of Medicine, Public Health and Nursing Universitas Gadjah Mada (FK-KMK UGM); Disease Investigation Center Wates Ministry of Agriculture Indonesia; Department of Microbiology FK-KMK UGM; Laboratorium Diagnostik Yayasan Tahija World Mosquito Program (WMP) Yogyakarta Center for Tropical Medicine FK-KMK UGM; Integrated Research Center FK-KMK UGM; Department of Computer Science and Electronics FMIPA UGM	Afiahayati; Dwi AA Nugrahaningsih; Dwiki Afandy; Dyah Ayu Puspitarani; Edwin W. Daniwijaya; Eggi Arguni; Endah Supriyati; Gunadi; Hendra Wibawa; Kristy Iskandar; Ludhang P. Rizki; Marcellus; Mohamad S. Hakim; Nungki Anggorowati; Siswanto; Titik Nuryastuti; Tri Wibawa; V. Noegroho Isti Donodjati; Wawan Triharjanto
EPI_ISL_763063	RS Panti Waluyo, Surakarta	Universitas Sebelas Maret (UNS); Rumah Sakit UNS (RS-UNS); National Institute of Health Research and Development, Indonesian Ministry of Health.	Afif A Ghufron; Arie A Nugraha; Betty Suryawati; Dewi Puspa; Hana A Pawestri; Hartanti Dian Ika; Hartono; Kartika; Maryani; Revi G H Novika; Vivi Setiawaty.; Yulia Sari
EPI_ISL_568688, EPI_ISL_766043, EPI_ISL_766045	RS Pelni	Eijkman Institute for Molecular Biology, Ministry of Research and Technology/National Agency for Research and Innovation	Amin Soebandrio; David H Muljono; Edison Johar; Frilasita A Yudhaputri; Herawati Sudoyo; Hidayat Trimarsanto; Iskandar A Adnan; Khin Saw Myint; Safarina G Malik; Willy Agustine
EPI_ISL_2614725, EPI_ISL_2614726, EPI_ISL_2614727, EPI_ISL_2614731, EPI_ISL_2709366, EPI_ISL_2709367, EPI_ISL_2709368, EPI_ISL_2709382, EPI_ISL_3029940			
see above	RS Pelni	Eijkman Institute for Molecular Biology, National Research and Innovation Agency	Amin Soebandrio; Edison Johar; Frilasita A Yudhaputri; Hidayat Trimarsanto; Iskandar Adnan; Khin Saw Myint; Lidwina Priliani; Lydia V. Panggalo; Muhammad Rezki Rasyak; Safarina G Malik; Sukma Oktavianthi; Willy Agustine
EPI_ISL_2364577	RS Permata Bekasi	Eijkman Institute for Molecular Biology, National Agency for Research and Innovation	Amin Soebandrio; Edison Johar; Frilasita A Yudhaputri; Hidayat Trimarsanto; Iskandar Adnan; Khin Saw Myint; Lidwina Priliani; Lydia V. Panggalo; Muhammad Rezki Rasyak; Safarina G Malik; Sukma Oktavianthi; Willy Agustine
EPI_ISL_1284190, EPI_ISL_1284193, EPI_ISL_1622416, EPI_ISL_1622417, EPI_ISL_1622434	RS Permata Cibubur	Eijkman Institute for Molecular Biology, Ministry of Research and Technology/National Agency for Research and Innovation	Amin Soebandrio; Edison Johar; Frilasita A Yudhaputri; Hidayat Trimarsanto; Iskandar Adnan; Khin Saw Myint; Lydia V. Panggalo; Muhammad Rezki Rasyak; Safarina G Malik; Sukma Oktavianthi; Willy Agustine
EPI_ISL_2364597, EPI_ISL_2364646, EPI_ISL_2364650	RS Permata Cibubur	Eijkman Institute for Molecular Biology, National Agency for Research and Innovation	Amin Soebandrio; Edison Johar; Frilasita A Yudhaputri; Hidayat Trimarsanto; Iskandar Adnan; Khin Saw Myint; Lidwina Priliani; Lydia V. Panggalo; Muhammad Rezki Rasyak; Safarina G Malik; Sukma Oktavianthi; Willy Agustine
EPI_ISL_2614681, EPI_ISL_2614682, EPI_ISL_2614694,	RS Permata Cibubur	Eijkman Institute for Molecular Biology, National Research and Innovation Agency	Amin Soebandrio; Edison Johar; Frilasita A Yudhaputri; Hidayat Trimarsanto; Iskandar Adnan; Khin Saw Myint; Lidwina Priliani; Lydia V. Panggalo; Muhammad Rezki Rasyak; Safarina G Malik; Sukma Oktavianthi; Willy Agustine

EPI_ISL_2709394			
EPI_ISL_775597	RS Pertamina Sorong	National Institute of Health Research and Development	AA; HA; HD; Ikawati; KD; KNA; N; Nugraha; Pangesti; Pawestri; Puspa; Puspandari; Setiawaty; Soekarso; Subangkit; T; V; Yosepin
EPI_ISL_766041	RS Polri	Eijkman Institute for Molecular Biology, Ministry of Research and Technology/National Agency for Research and Innovation	Amin Soebandrio; David H Muljono; Edison Johar; Frilasita A Yudhaputri; Hidayat Trimarsanto; Khin Saw Myint; Safarina G Malik
EPI_ISL_888989, EPI_ISL_888990	RS Pondok Indah Bintaro Jaya	Eijkman Institute for Molecular Biology, Ministry of Research and Technology/National Agency for Research and Innovation	Amin Soebandrio; Edison Johar; Frilasita A Yudhaputri; Hidayat Trimarsanto; Iskandar Adnan; Khin Saw Myint; Lydia V. Panggalo; Safarina G Malik; Sukma Oktavianthi; Willy Agustine
EPI_ISL_435282, EPI_ISL_435283, EPI_ISL_437190, EPI_ISL_437191, EPI_ISL_888982	RS Pondok Indah Hospital – Pondok Indah	Eijkman Institute for Molecular Biology, Ministry of Research and Technology/National Agency for Research and Innovation	Amin Soebandrio; David H Muljono; Edison Johar; Frilasita A Yudhaputri; Hidayat Trimarsanto; Iskandar Adnan; Khin Saw Myint; Lydia V. Panggalo; Safarina G Malik; Sukma Oktavianthi; Willy Agustine
EPI_ISL_2614735, EPI_ISL_2614737, EPI_ISL_2614738, EPI_ISL_2709358, EPI_ISL_2709369, EPI_ISL_2709373, EPI_ISL_2709374, EPI_ISL_2709375, EPI_ISL_2709376, EPI_ISL_2709377, EPI_ISL_2709381, EPI_ISL_2709387, EPI_ISL_2709391, EPI_ISL_2709401, EPI_ISL_2709402, EPI_ISL_2811834, EPI_ISL_2811835, EPI_ISL_2811836, EPI_ISL_2918604, EPI_ISL_2918605, EPI_ISL_2918606, EPI_ISL_2918607, EPI_ISL_2918609, EPI_ISL_2918610, EPI_ISL_2918611, EPI_ISL_2918612, EPI_ISL_3029931, EPI_ISL_3029932, EPI_ISL_3029997, EPI_ISL_3029998			
see above	RS Pondok Indah Pondok Indah	Eijkman Institute for Molecular Biology, National Research and Innovation Agency	Amin Soebandrio; Edison Johar; Frilasita A Yudhaputri; Hidayat Trimarsanto; Iskandar Adnan; Khin Saw Myint; Lidwina Priliani; Lydia V. Panggalo; Muhammad Rezki Rasyak; Safarina G Malik; Sukma Oktavianthi; Willy Agustine
EPI_ISL_5021156	RS Pondok Indah Pondok Indah	Eijkman Research Center for Molecular Biology, National Research and Innovation Agency	Amin Soebandrio; Edison Johar; Frilasita A Yudhaputri; Hidayat Trimarsanto; Iskandar Adnan; Jessica R Saragih; Khin Saw Myint; Lidwina Priliani; Lydia V Panggalo; Muhammad Rezki Rasyak; Safarina G Malik; Sukma Oktavianthi; Willy Agustine
EPI_ISL_568689	RS Pondok Indah Puri Indah	Eijkman Institute for Molecular Biology, Ministry of Research and Technology/National Agency for Research and Innovation	Amin Soebandrio; David H Muljono; Edison Johar; Frilasita A Yudhaputri; Herawati Sudoyo; Hidayat Trimarsanto; Iskandar A Adnan; Khin Saw Myint; Safarina G Malik; Willy Agustine
EPI_ISL_3159693, EPI_ISL_3466003, EPI_ISL_3466009, EPI_ISL_3550458	RS Pondok Indah Puri Indah	Eijkman Institute for Molecular Biology, National Research and Innovation Agency	Amin Soebandrio; Edison Johar; Frilasita A Yudhaputri; Hidayat Trimarsanto; Iskandar Adnan; Jessica R. Saragih; Khin Saw Myint; Lidwina Priliani; Lydia V. Panggalo; Muhammad Rezki Rasyak; Safarina G Malik; Sukma Oktavianthi; Willy Agustine
EPI_ISL_574611	RS Premier Bintaro	Eijkman Institute for Molecular Biology, Ministry of Research and Technology/National Agency for Research and Innovation	Amin Soebandrio; David H Muljono; Edison Johar; Frilasita A Yudhaputri; Herawati Sudoyo; Hidayat Trimarsanto; Iskandar A Adnan; Khin Saw Myint; Safarina G Malik; Willy Agustine
EPI_ISL_2709403, EPI_ISL_3029938	RS Premier Bintaro	Eijkman Institute for Molecular Biology, National Research and Innovation Agency	Amin Soebandrio; Edison Johar; Frilasita A Yudhaputri; Hidayat Trimarsanto; Iskandar Adnan; Khin Saw Myint; Lidwina Priliani; Lydia V. Panggalo; Muhammad Rezki Rasyak; Safarina G Malik; Sukma Oktavianthi; Willy Agustine
EPI_ISL_2987634	RS Premier Surabaya	National Institute of Health Research and Development	Arie Ardiansyah Nugraha; Fajar Nur Sulistiyohadi; Hana Apsari Pawestri; Hartanti Dian Ikawati; Kartika Dewi Puspa; Krisna Pangesti; Nelly Puspandari; Subangkit; Triyani Soekarso; Vivi Setiawaty
EPI_ISL_2965787, EPI_ISL_2965788	RS Prima Husada Cipta	National Institute of Health Research and Development	Arie Ardiansyah Nugraha; Fajar Nur Sulistiyohadi; Hana Apsari Pawestri; Hartanti Dian Ikawati; Kartika Dewi Puspa; Krisna Pangesti; Nelly Puspandari; Subangkit; Triyani Soekarso; Vivi Setiawaty
EPI_ISL_538500, EPI_ISL_538501	RS Prima Husada Cipta Medan North Sumatra	National Institute of Health Research and Development	AA; HA; HD; Ikawati; KD; KNA; Nugraha; Paisal; Pangesti; Pawestri; Puspa; Setiawaty, V.; Soekarso; Subangkit; T
EPI_ISL_2631481, EPI_ISL_2631482, EPI_ISL_2631483, EPI_ISL_2631484, EPI_ISL_2631485, EPI_ISL_2631486	RS Primaya	Indonesian Institute of Sciences (LIPI)/National Agency of Research and Innovation (BRIN) and National Institute of Health Research and Development (NIHRD)	Alfi T. Fathurahman; Andri Wardiana; Anggia Prasetyoputri; Anik B. Dharmayanthi; Asep M. Ridwanulloh; Erwin F. Hasrianda; Hariyatun; Idris; Ratih A. Ningrum; Ruby Setiawan; Subangkit; Sugiyono Saputra; Syam B. Iryanto; Vivi Setiawaty; Yayuk Sri Rahayu
EPI_ISL_1284057	RS Primaya Tangerang	Eijkman Institute for Molecular Biology, Ministry of Research and Technology/National Agency for Research and Innovation	Amin Soebandrio; Edison Johar; Frilasita A Yudhaputri; Hidayat Trimarsanto; Iskandar Adnan; Khin Saw Myint; Lydia V. Panggalo; Safarina G Malik; Sukma Oktavianthi; Willy Agustine
EPI_ISL_2896198	RS Pusat Otak Nasional Prof dr. Mahar Mardjono	National Institute of Health Research and Development	Arie Ardiansyah Nugraha; Hana Apsari Pawestri; Hartanti Dian Ikawati; Kartika Dewi Puspa; Krisna Pangesti; Nelly Puspandari; Subangkit; Triyani Soekarso; Vivi Setiawaty
EPI_ISL_2614733, EPI_ISL_2614734, EPI_ISL_2709362, EPI_ISL_2709363, EPI_ISL_2709364, EPI_ISL_2709365, EPI_ISL_2709370, EPI_ISL_2709371, EPI_ISL_2709372, EPI_ISL_2709379, EPI_ISL_2709380			
see above	RS Pusat Pertamina	Eijkman Institute for Molecular Biology, National Research and Innovation Agency	Amin Soebandrio; Edison Johar; Frilasita A Yudhaputri; Hidayat Trimarsanto; Iskandar Adnan; Khin Saw Myint; Lidwina Priliani; Lydia V. Panggalo; Muhammad Rezki Rasyak; Safarina G Malik; Sukma Oktavianthi; Willy Agustine
EPI_ISL_888988, EPI_ISL_947262, EPI_ISL_1622418	RS Qadr	Eijkman Institute for Molecular Biology, Ministry of Research and Technology/National Agency for Research and Innovation	Amin Soebandrio; Edison Johar; Frilasita A Yudhaputri; Hidayat Trimarsanto; Iskandar Adnan; Khin Saw Myint; Lydia V. Panggalo; Muhammad Rezki Rasyak; Safarina G Malik; Sukma Oktavianthi; Willy Agustine
EPI_ISL_833499, EPI_ISL_833571	RS Qadr, Tangerang, Indonesia	Biosafety Level-3 Laboratory, Indonesian Institute of Sciences (LIPI)	Ade Andriani; Ahmad Fathoni; Andri Wardiana; Anggia Prasetyoputri; Anik Budhi Dharmayanthi; Erwin Fajar Hasrianda; Isa Nuryana; Ratih Asmana Ningrum; Ratna Dwi Ramadani; Ryan Haryo Setiawan; Sugiyono Saputra; Syam Budi Iryanto
EPI_ISL_5010527	RS Rajawali Citra	Genetics Working Group (Pokja Genetik) Faculty of Medicine, Public Health and Nursing Universitas Gadjah Mada (FK-KMK UGM); Disease Investigation Center Wates Ministry of Agriculture Indonesia; Department of Microbiology FK-KMK UGM; Laboratorium Diagnostik Yayasan Tahija World Mosquito Program (WMP) Yogyakarta Center for Tropical Medicine FK-KMK UGM; Integrated Research Center FK-KMK UGM; Department of Computer Science and Electronics FMIPA UGM; RSUP Dr. Sardjito	Afiahayati; Alvina A Setiawan; Cita S Amalia; Dwi AA Nugrahaningsih; Dwi Indaryati; Edwin W. Daniwijaya; Eggi Arguni; Endah Supriyati; Gunadi; Hendra Wibawa; Indarto Sulistiyono; Kristy Iskandar; Ludhang P. Rizki; Marcellus; Mohamad S. Hakim; Nungki Anggorowati; Pramesti G Dewi; Siswanto; Titik Nuryastuti; Tri Wibawa
EPI_ISL_889013	RS Rawa Lumbu	Eijkman Institute for Molecular Biology, Ministry of Research and Technology/National Agency for Research and Innovation	Amin Soebandrio; Edison Johar; Frilasita A Yudhaputri; Hidayat Trimarsanto; Iskandar Adnan; Khin Saw Myint; Lydia V. Panggalo; Safarina G Malik; Sukma Oktavianthi; Willy Agustine
EPI_ISL_3070862	RS Rawa Lumbu	Eijkman Institute for Molecular Biology, National Research and Innovation Agency	Amin Soebandrio; Edison Johar; Frilasita A Yudhaputri; Hidayat Trimarsanto; Iskandar Adnan; Khin Saw Myint; Lidwina Priliani; Lydia V. Panggalo; Muhammad Rezki Rasyak; Safarina G Malik; Sukma Oktavianthi; Willy Agustine
EPI_ISL_4741896	RS Roemani Muhammadiyah	Balai Laboratorium Kesehatan Dan Pengujian Alat Kesehatan Provinsi Jawa Tengah	Alia Wijayanti; Dwi Nursetyaasih; Dyah Ayu; Endah Wahyu Y; Ismi Farhati; Purnomo; Sekti Handayani; Wiwik Peni; Yekti Hediningsih
EPI_ISL_2868912, EPI_ISL_2868913	RS Royal Progress	National Institute of Health Research and Development	Arie Ardiansyah Nugraha; Hana Apsari Pawestri; Hartanti Dian Ikawati; Kartika Dewi Puspa; Krisna Pangesti; Nelly Puspandari; Subangkit; Triyani Soekarso; Vivi Setiawaty
EPI_ISL_1284281	RS Royal Taruma	Eijkman Institute for Molecular Biology, Ministry of Research and Technology/National Agency for Research and Innovation	Amin Soebandrio; Edison Johar; Frilasita A Yudhaputri; Hidayat Trimarsanto; Iskandar Adnan; Khin Saw Myint; Lydia V. Panggalo; Safarina G Malik; Sukma Oktavianthi; Willy Agustine
EPI_ISL_1284052, EPI_ISL_1284078	RS Rumah Indonesia Sehat	Eijkman Institute for Molecular Biology, Ministry of Research and Technology/National Agency for Research and Innovation	Amin Soebandrio; Edison Johar; Frilasita A Yudhaputri; Hidayat Trimarsanto; Iskandar Adnan; Khin Saw Myint; Lydia V. Panggalo; Safarina G Malik; Sukma Oktavianthi; Willy Agustine
EPI_ISL_791985	RS Santa Maria Pekanbaru	National Institute of Health Research and Development	AA; Fridayenti; HA; HD; Ikawati; KD; KNA; N; Nugraha; Pangesti; Pawestri; Puspa; Puspandari; Setiawaty; Soekarso; Subangkit; T; V
EPI_ISL_766042, EPI_ISL_947265, EPI_ISL_947320, EPI_ISL_1622363, EPI_ISL_1622364	RS Sari Asih Ciledug	Eijkman Institute for Molecular Biology, Ministry of Research and Technology/National Agency for Research and Innovation	Amin Soebandrio; David H Muljono; Edison Johar; Frilasita A Yudhaputri; Hidayat Trimarsanto; Iskandar Adnan; Khin Saw Myint; Lydia V. Panggalo; Muhammad Rezki Rasyak; Safarina G Malik; Sukma Oktavianthi; Willy Agustine
EPI_ISL_2364599	RS Sari Asih Ciledug	Eijkman Institute for Molecular Biology, National Agency for Research and Innovation	Amin Soebandrio; Edison Johar; Frilasita A Yudhaputri; Hidayat Trimarsanto; Iskandar Adnan; Khin Saw Myint; Lidwina Priliani; Lydia V. Panggalo; Muhammad Rezki Rasyak; Safarina G Malik; Sukma Oktavianthi; Willy Agustine
EPI_ISL_2709385, EPI_ISL_2709386	RS Sari Asih Cipondoh	Eijkman Institute for Molecular Biology, National Research and Innovation Agency	Amin Soebandrio; Edison Johar; Frilasita A Yudhaputri; Hidayat Trimarsanto; Iskandar Adnan; Khin Saw Myint; Lidwina Priliani; Lydia V. Panggalo; Muhammad Rezki Rasyak; Safarina G Malik; Sukma Oktavianthi; Willy Agustine
EPI_ISL_833501, EPI_ISL_833502	RS Sayang Bunda, Makasar, Indonesia	Biosafety Level-3 Laboratory, Indonesian Institute of Sciences (LIPI)	Ade Andriani; Ahmad Fathoni; Andri Wardiana; Anggia Prasetyoputri; Anik Budhi Dharmayanthi; Fajarrudin Ahmad; Herjuno Ari Eko Nugroho; I sa Nuryana; Isa Nuryana; Masrukhin; Onny Nurrahman Marwayana; Ratih Asmana Ningrum; Syam Budi Iryanto
EPI_ISL_2617464, EPI_ISL_2617465	RS Semen Gresik	National Institute of Health Research and Development	Agustiningsih; Arie Ardiansyah Nugraha; Fauzul Muna; Hana Apsari Pawestri; Hartanti Dian Ikawati; Herna; Holy Arif Wibowo; Irene Lorinda Indalao; Kartika Dewi Puspa; Kindi Adam; Krisna Nur Andriana Pangesti; Natalie Laurencia Kipuw; Nelly Puspandari; Ni Ketut Susilarini; Nike Susanti; Nurika Hariastuti; Reni Herman; Ririn Ramadhany; Subangkit; Tati Febriyanti; Triyani Soekarso; Ully Alfi Nikmah; Vivi Setiawaty.; Yuni Rukminiati
EPI_ISL_889008	RS Sentra Medika Cibinong	Eijkman Institute for Molecular Biology, Ministry of Research and Technology/National Agency for Research and Innovation	Amin Soebandrio; Edison Johar; Frilasita A Yudhaputri; Hidayat Trimarsanto; Iskandar Adnan; Khin Saw Myint; Lydia V. Panggalo; Safarina G Malik; Sukma Oktavianthi; Willy Agustine
EPI_ISL_2364615	RS Sentra Medika Cibinong	Eijkman Institute for Molecular Biology, National Agency for Research and Innovation	Amin Soebandrio; Edison Johar; Frilasita A Yudhaputri; Hidayat Trimarsanto; Iskandar Adnan; Khin Saw Myint; Lidwina Priliani; Lydia V. Panggalo; Muhammad Rezki Rasyak; Safarina G Malik; Sukma Oktavianthi; Willy Agustine
EPI_ISL_2709392, EPI_ISL_3029994	RS Sentra Medika Cibinong	Eijkman Institute for Molecular Biology, National Research and Innovation Agency	Amin Soebandrio; Edison Johar; Frilasita A Yudhaputri; Hidayat Trimarsanto; Iskandar Adnan; Khin Saw Myint; Lidwina Priliani; Lydia V. Panggalo; Muhammad Rezki Rasyak; Safarina G Malik; Sukma Oktavianthi; Willy Agustine
EPI_ISL_947307, EPI_ISL_947309, EPI_ISL_1283936, EPI_ISL_1284027, EPI_ISL_1284029, EPI_ISL_1284156, EPI_ISL_1284160, EPI_ISL_1284318, EPI_ISL_1284340, EPI_ISL_1622362, EPI_ISL_1622374, EPI_ISL_1622390, EPI_ISL_1622419, EPI_ISL_1622420, EPI_ISL_1622431, EPI_ISL_1622432			
see above	RS Sentra Medika Cikarang	Eijkman Institute for Molecular Biology, Ministry of Research and Technology/National Agency for Research and Innovation	Amin Soebandrio; Edison Johar; Frilasita A Yudhaputri; Hidayat Trimarsanto; Iskandar Adnan; Khin Saw Myint; Lydia V. Panggalo; Muhammad Rezki Rasyak; Safarina G Malik; Sukma Oktavianthi; Willy Agustine
EPI_ISL_2013043, EPI_ISL_2013044, EPI_ISL_2364569, EPI_ISL_2364570, EPI_ISL_2364571, EPI_ISL_2364572, EPI_ISL_2364581, EPI_ISL_2364582, EPI_ISL_2364621, EPI_ISL_2364624, EPI_ISL_2364626			
see above	RS Sentra Medika Cikarang	Eijkman Institute for Molecular Biology, National Agency for Research and Innovation	Amin Soebandrio; Edison Johar; Frilasita A Yudhaputri; Hidayat Trimarsanto; Iskandar Adnan; Khin Saw Myint; Lidwina Priliani; Lydia V. Panggalo; Muhammad Rezki Rasyak; Safarina G Malik; Sukma Oktavianthi; Willy Agustine
EPI_ISL_2614649, EPI_ISL_2614660, EPI_ISL_2614672, EPI_ISL_2614673, EPI_ISL_2614674, EPI_ISL_2614683, EPI_ISL_2614684, EPI_ISL_2614685, EPI_ISL_2614686, EPI_ISL_2614687, EPI_ISL_2614695, EPI_ISL_2614696, EPI_ISL_2614706, EPI_ISL_2614707, EPI_ISL_2614713, EPI_ISL_2614720			
see above	RS Sentra Medika Cikarang	Eijkman Institute for Molecular Biology, National Research and Innovation Agency	Amin Soebandrio; Edison Johar; Frilasita A Yudhaputri; Hidayat Trimarsanto; Iskandar Adnan; Khin Saw Myint; Lidwina Priliani; Lydia V. Panggalo; Muhammad Rezki Rasyak; Safarina G Malik; Sukma Oktavianthi; Willy Agustine
EPI_ISL_947323	RS Siloam Kebon Jeruk	Eijkman Institute for Molecular Biology, Ministry of Research and Technology/National Agency for Research and Innovation	Amin Soebandrio; Edison Johar; Frilasita A Yudhaputri; Hidayat Trimarsanto; Iskandar Adnan; Khin Saw Myint; Lydia V. Panggalo; Safarina G Malik; Sukma Oktavianthi; Willy Agustine

EPI_ISL_574606	RS Siloam Purwakarta	Eijkman Institute for Molecular Biology, Ministry of Research and Technology/National Agency for Research and Innovation	Amin Soebandrio; David H Muljono; Edison Johar; Frilasita A Yudhaputri; Herawati Sudoyo; Hidayat Trimarsanto; Iskandar A Adnan; Khin Saw Myint; Safarina G Malik; Willy Agustine
EPI_ISL_1622369, EPI_ISL_1622424	RS Sumber Waras	Eijkman Institute for Molecular Biology, Ministry of Research and Technology/National Agency for Research and Innovation	Amin Soebandrio; Edison Johar; Frilasita A Yudhaputri; Hidayat Trimarsanto; Iskandar Adnan; Khin Saw Myint; Lydia V. Panggalo; Muhammad Rezki Rasyak; Safarina G Malik; Sukma Oktavianthi; Willy Agustine
EPI_ISL_2364602	RS Sumber Waras	Eijkman Institute for Molecular Biology, National Agency for Research and Innovation	Amin Soebandrio; Edison Johar; Frilasita A Yudhaputri; Hidayat Trimarsanto; Iskandar Adnan; Khin Saw Myint; Lidwina Priliani; Lydia V. Panggalo; Muhammad Rezki Rasyak; Safarina G Malik; Sukma Oktavianthi; Willy Agustine
EPI_ISL_2614646, EPI_ISL_2614647, EPI_ISL_2614669, EPI_ISL_2614670, EPI_ISL_2614715, EPI_ISL_2614732	RS Sumber Waras	Eijkman Institute for Molecular Biology, National Research and Innovation Agency	Amin Soebandrio; Edison Johar; Frilasita A Yudhaputri; Hidayat Trimarsanto; Iskandar Adnan; Khin Saw Myint; Lidwina Priliani; Lydia V. Panggalo; Muhammad Rezki Rasyak; Safarina G Malik; Sukma Oktavianthi; Willy Agustine
EPI_ISL_833039	RS TK II 02.05.01 dr. AK Gani Palembang	National Institute of Health Research and Development	A; AA; HA; HD; Ikawati; KD; KNA; N; Nugraha; Pangesti; Pawestri; Puspa; Puspandari; RP; Setiawaty; Soekarso; Subangkit; T; Tantula; V; Wiradharma
EPI_ISL_2854698, EPI_ISL_2896197	RS TK II M Ridwan Meuraksa	National Institute of Health Research and Development	Arie Ardiansyah Nugraha; Hana Apsari Pawestri; Hartanti Dian Ikawati; Kartika Dewi Puspa; Krisna Pangesti; Nelly Puspandari; Subangkit; Triyani Soekarso; Vivi Setiawaty
EPI_ISL_1284034, EPI_ISL_1284035, EPI_ISL_1622375	RS Taman Harapan Baru	Eijkman Institute for Molecular Biology, Ministry of Research and Technology/National Agency for Research and Innovation	Amin Soebandrio; Edison Johar; Frilasita A Yudhaputri; Hidayat Trimarsanto; Iskandar Adnan; Khin Saw Myint; Lydia V. Panggalo; Muhammad Rezki Rasyak; Safarina G Malik; Sukma Oktavianthi; Willy Agustine
EPI_ISL_766040	RS Tingkat II Moh. Ridwan Meuraksa	Eijkman Institute for Molecular Biology, Ministry of Research and Technology/National Agency for Research and Innovation	Amin Soebandrio; David H Muljono; Edison Johar; Frilasita A Yudhaputri; Hidayat Trimarsanto; Khin Saw Myint; Safarina G Malik
EPI_ISL_1020176, EPI_ISL_1020177, EPI_ISL_1020178, EPI_ISL_1020182, EPI_ISL_1020183, EPI_ISL_1020185, EPI_ISL_1020187, EPI_ISL_1020188, EPI_ISL_1020189, EPI_ISL_1020190, EPI_ISL_1020198, EPI_ISL_1020200, EPI_ISL_1020201, EPI_ISL_1020202, EPI_ISL_1117452, EPI_ISL_1117453, EPI_ISL_1117454, EPI_ISL_1117455, EPI_ISL_1117590, EPI_ISL_1137602, EPI_ISL_1137603, EPI_ISL_2840869, EPI_ISL_2840870, EPI_ISL_2840878, EPI_ISL_2840881, EPI_ISL_2840882, EPI_ISL_2840883, EPI_ISL_2840884, EPI_ISL_2840885, EPI_ISL_2840893			
see above	RS Trimitra, Bogor, West java	Biosafety Level-3 Laboratory, Indonesian Institute of Sciences (LIPI)	Ade Andriani; Alfi T. Fathurahman; Andri Wardiana; Anggia Prasetyoputri; Anik B. Dharmayanthi; Anik Budhi Dharmayanthi; Ari S. Wulandari; Asep M. Ridwanulloh; Asep M. Ridwanuloh; Eko Wahyu Putro; Erwin F. Hasrianda; Erwin Hasrianda; Fahrurrozi; Gita Syahputra; Hariyatun; Herjuno A. Nugroho; Idris; Indriawati; Inswasti Cahyani; Isa Nuryana; Linda Sukmarini; Listiana Oktavia; Masrukhin; Matthew W. Loose; Mohammad Ilyas; Nova D. Yanthi; Nova Dhilla Yanthi; Puspita Lisdiyanti; Ratih A. Ningrum; Ratih Asmana Ningrum; Ruby Setiawan; Sri Swasthikawati; Sugiyono Saputra; Sugiyono Saputra; Susanti; Syaiful Rizal; Syam B. Iryanto; Syam Budi Iryanto; Wien Kusharyoto; Yuliawati
EPI_ISL_759702	RS UNS Surakarta	Universitas Sebelas Maret (UNS), Rumah sakit Universitas Sebelas Maret (RS-UNS) Surakarta, National Institute of Health Research and Development, Indonesian Ministry of Health	Afif Avicena Gufron; Arie Ardiansyah Nugraha; Betty Suryawati; Dewi Puspa; Hana Apsari Pawestri; Hartanti Dian Ika; Kartika; Vivi Setiawaty; Yulia sari; hartono; maryani
EPI_ISL_759701	RS UNS, Surakarta	UNS/RS-UNS, Faculty of Medicine	Afif Avicenna Gufron; Arie Ardiansyah Nugraha; Betty Suryawati; HanaHana Apsari Pawestri; Hartanti Dian Ika; Hartono; Kartika Dewi Puspa; Maryani; Vivi Setiawaty.; Yulia Sari
EPI_ISL_947278	RS Umum Ciputra Hospital Citragarden City	Eijkman Institute for Molecular Biology, Ministry of Research and Technology/National Agency for Research and Innovation	Amin Soebandrio; Edison Johar; Frilasita A Yudhaputri; Hidayat Trimarsanto; Iskandar Adnan; Khin Saw Myint; Lydia V. Panggalo; Safarina G Malik; Sukma Oktavianthi; Willy Agustine
EPI_ISL_4104513, EPI_ISL_4104514, EPI_ISL_4104515, EPI_ISL_4104516, EPI_ISL_4104517	RS Umum Daerah Dr. M. Yunus Bengkulu	National Institute of Health Research and Development	Arie Ardiansyah Nugraha; Fajar Nur Sulistiyohadi; Hana Apsari Pawestri; Hartanti Dian Ikawati; Kartika Dewi Puspa; Krisna Pangesti; Nelly Puspandari; Subangkit; Triyani Soekarso; Vivi Setiawaty
EPI_ISL_1284083	RS Umum Harapan Bunda	Eijkman Institute for Molecular Biology, Ministry of Research and Technology/National Agency for Research and Innovation	Amin Soebandrio; Edison Johar; Frilasita A Yudhaputri; Hidayat Trimarsanto; Iskandar Adnan; Khin Saw Myint; Lydia V. Panggalo; Safarina G Malik; Sukma Oktavianthi; Willy Agustine
EPI_ISL_2262254, EPI_ISL_2262255, EPI_ISL_2262257, EPI_ISL_2262260, EPI_ISL_2262261, EPI_ISL_2262262, EPI_ISL_2262263, EPI_ISL_2262265, EPI_ISL_2854693, EPI_ISL_2854701, EPI_ISL_2854702, EPI_ISL_2854703, EPI_ISL_2854704			
see above	RS Umum Kartika Pulomas	National Institute of Health Research and Development	Arie Ardiansyah Nugraha; Hana Apsari Pawestri; Hartanti Dian Ikawati; Kartika Dewi Puspa; Krisna Pangesti; Nelly Puspandari; Subangkit; Triyani Soekarso; Vivi Setiawaty
EPI_ISL_2844832	RS Umum Medika Dramaga, Bogor, West java	Biosafety Level-3 Laboratory, Indonesian Institute of Sciences (LIPI)	Andri Wardiana; Anggia Prasetyoputri; Erwin F. Hasrianda; Fahrurrozi; Herjuno A. Nugroho; Nova D. Yanthi
EPI_ISL_2617494, EPI_ISL_2617499, EPI_ISL_2617501, EPI_ISL_2617513, EPI_ISL_2617516, EPI_ISL_2617517	RS Umum Medistra	National Institute of Health Research and Development	Arie Ardiansyah Nugraha; Hana Apsari Pawestri; Hartanti Dian Ikawati; Kartika Dewi Puspa; Krisna Pangesti; Nelly Puspandari; Subangkit; Triyani Soekarso; Vivi Setiawaty
EPI_ISL_947275	RS Umum Mulia Pajajaran	Eijkman Institute for Molecular Biology, Ministry of Research and Technology/National Agency for Research and Innovation	Amin Soebandrio; Edison Johar; Frilasita A Yudhaputri; Hidayat Trimarsanto; Iskandar Adnan; Khin Saw Myint; Lydia V. Panggalo; Safarina G Malik; Sukma Oktavianthi; Willy Agustine
EPI_ISL_2860290, EPI_ISL_2860291, EPI_ISL_2860292, EPI_ISL_2860293, EPI_ISL_2860332, EPI_ISL_2860333, EPI_ISL_2860334, EPI_ISL_2860335, EPI_ISL_2860336, EPI_ISL_2860337, EPI_ISL_2860338, EPI_ISL_2860339, EPI_ISL_2860340, EPI_ISL_2860341, EPI_ISL_2860342, EPI_ISL_2860343, EPI_ISL_2860344, EPI_ISL_2860345, EPI_ISL_2860346, EPI_ISL_2860360, EPI_ISL_2860361, EPI_ISL_2860362, EPI_ISL_2860363, EPI_ISL_2860364, EPI_ISL_2860365, EPI_ISL_2860366, EPI_ISL_2860367			
see above	RS Umum Trimitra	Biosafety Level-3 Laboratory, Indonesian Institute of Sciences (LIPI)	Ade Andriani; Andri Wardiana; Anggia Prasetyoputri; Anik B. Dharmayanthi; Annisa D. Septiani; Asep M. Ridwanulloh; Asep M.Ridwanulloh; Fahrurrozi; Idris; Indriawati; Linda Sukmarini; Listiana Oktavia; Lusiana; Masrukhin; Maya Komalasari; Michael S. Parulian; Michael Saut Parulian; Nova D. Yanthi; Ocha P. Meitha; Puspita Lisdiyanti; Ratih A. Ningrum; Ruby Setiawan; Sri Swasthikawati; Sugiyono Saputra; Syam B. Iryanto; Wien Kusharyoto; Willery Yeo; Yuhoniz A. Maryam; Yuliawati
EPI_ISL_3086881, EPI_ISL_3086882, EPI_ISL_3086883, EPI_ISL_3086884, EPI_ISL_3086885	RS Umum Trimitra	Indonesian Institute of Sciences (LIPI)	Andri Wardiana; Anggia Prasetyoputri; Anik B. Dharmayanthi; Annisa D. Septiani; Indriawati; Isa Nuryana; Lusiana; Maya Komalasari; Michael Saut Parulian; Ocha P. Meitha; Ratih A. Ningrum; Sugiyono Saputra; Syaiful Rizal; Syam B. Iryanto; Willery Yeo; Yuhoniz A. Maryam
EPI_ISL_2844812, EPI_ISL_2844813, EPI_ISL_2844814, EPI_ISL_2844816, EPI_ISL_2844817, EPI_ISL_2844818, EPI_ISL_2844819, EPI_ISL_2844820, EPI_ISL_2844821, EPI_ISL_2844823, EPI_ISL_2844829, EPI_ISL_2844830			
see above	RS Umum Trimitra, Bogor, West java	Biosafety Level-3 Laboratory, Indonesian Institute of Sciences (LIPI)	Alfi T. Fathurahman; Andri Wardiana; Anggia Prasetyoputri; Anik B. Dharmayanthi; Erwin F. Hasrianda; Fahrurrozi; Herjuno A. Nugroho; Idris; Linda Sukmarini; Nova D. Yanthi; Puspita Lisdiyanti; Ratih A. Ningrum; Sri Swasthikawati; Sugiyono Saputra; Syam B. Iryanto
EPI_ISL_4106122, EPI_ISL_4106124, EPI_ISL_4106125, EPI_ISL_4106126	RS Umum Trimitra, Bogor, West java	National Research and Innovation Agency (BRIN-Indonesia, VenomCoV Project Team)	Andri Wardiana; Anggia Prasetyoputri; Erwin F. Hasrianda; Fahrurrozi; Herjuno A. Nugroho; Nova D. Yanthi; Sugiyono Saputra; Syam B. Iryanto
EPI_ISL_3279414	RS Universitas Brawijaya	Institute of Tropical Disease, Universitas Airlangga	Aldise M Nastri; Gatot Soegiarto; Jezzy R Dewantari; Kazufumi Shimizu; Krisnoadi Rahardjo; Laksmi Wulandari; Maria I Lusida; Resti Yudhawati; Rima R Prasetya; Soetjipto; Sri Andarini; Yasuko Mori
EPI_ISL_3138863, EPI_ISL_3138864, EPI_ISL_3138865	RS Universitas Brawijaya	National Institute of Health Research and Development	; Arie Ardiansyah Nugraha; Fajar Nur Sulistiyohadi; Hana Apsari Pawestri; Hartanti Dian Ikawati; Kartika Dewi Puspa; Krisna Pangesti; Nelly Puspandari; Subangkit; Triyani Soekarso; Vivi Setiawaty
EPI_ISL_2854697	RS Universitas Brawijaya Malang	National Institute of Health Research and Development	Arie Ardiansyah Nugraha; Hana Apsari Pawestri; Hartanti Dian Ikawati; Kartika Dewi Puspa; Krisna Pangesti; Nelly Puspandari; Subangkit; Triyani Soekarso; Vivi Setiawaty
EPI_ISL_5021323, EPI_ISL_5021333, EPI_ISL_5021344, EPI_ISL_5021349, EPI_ISL_5021357, EPI_ISL_5021375, EPI_ISL_5021410, EPI_ISL_5021416, EPI_ISL_5021420, EPI_ISL_5021424, EPI_ISL_5021430, EPI_ISL_5021433, EPI_ISL_5021438, EPI_ISL_5021461, EPI_ISL_5021471, EPI_ISL_5021527, EPI_ISL_5021581, EPI_ISL_5021589			
see above	RS Universitas Sumatera Utara	Eijkman Research Center for Molecular Biology, National Research and Innovation Agency; Microbiology Laboratory, Faculty of Medicine, University of Sumatera Utara	Amin Soebandrio; Edison Johar; Frilasita A Yudhaputri; Hidayat Trimarsanto; Inke Nadia Diniyanti Lubis; Irbah Rea Alvieda Nainggolan; Iskandar Adnan; Jessica R Saragih; Khin Saw Myint; Lidwina Priliani; Lydia V Panggalo; Mirzan Hasibuan; Muhammad Ichwan; Muhammad Rezki Rasyak; R Andika Dwi Cahyadi; R Lia Kusumawati; Ramadhan Bestari; Ranti Permatasari; Safarina G Malik; Sukma Oktavianthi; Tryna Tania; Willy Agustine
EPI_ISL_5107868, EPI_ISL_5107869, EPI_ISL_5107870, EPI_ISL_5107871, EPI_ISL_5107872, EPI_ISL_5107873, EPI_ISL_5107874, EPI_ISL_5107875, EPI_ISL_5107876, EPI_ISL_5107877, EPI_ISL_5107878, EPI_ISL_5107879, EPI_ISL_5107880, EPI_ISL_5107881, EPI_ISL_5107882, EPI_ISL_5107883, EPI_ISL_5107884, EPI_ISL_5107885			
see above	RS Universitas Tanjung Pura	National Institute of Health Research and Development	Andriani; Arie Ardiansyah Nugraha; Delima Fajar Liana; Fajar Nur Sulistiyohadi; Hana Apsari Pawestri; Hartanti Dian Ikawati; Kartika Dewi Puspa; Krisna Pangesti; Nelly Puspandari; Puji Astuti; Sofi Siti Shofiyah; Subangkit; Triyani Soekarso; Virhan Novianry; Vivi Setiawaty
EPI_ISL_4849719, EPI_ISL_5200499, EPI_ISL_5462140	RS YARSI	Genomik Solidaritas Indonesia Laboratorium	Annisa Muthiah Sukirman; Anuraj Shankar; Ariel Pradipta; Carissa Sintca Wijaya; Dian Tri Wahyudi; Esti Rahayuning Tias; Gracia Felias Enos Korompis; Himawan Masyhuri; Intan Razari; Louisa Markus; Meutia Ayuputeri Kumaheri; Nida; Nurhayani Fatimah; Rana Zara Athaya; Reinhart Gabriel; Sayang Rahmadini; Stefanus Bernard; Syukrini Bahri; Vania Gavrila Wikasa
EPI_ISL_2364675	RS Yarsi	Eijkman Institute for Molecular Biology, National Agency for Research and Innovation	Amin Soebandrio; Edison Johar; Frilasita A Yudhaputri; Hidayat Trimarsanto; Iskandar Adnan; Khin Saw Myint; Lidwina Priliani; Lydia V. Panggalo; Muhammad Rezki Rasyak; Safarina G Malik; Sukma Oktavianthi; Willy Agustine
EPI_ISL_3278299	RS. Moh Ridwan Meuraksa	National Institute of Health Research and Development	Arie Ardiansyah Nugraha; Hana Apsari Pawestri; Hartanti Dian Ikawati; Kartika Dewi Puspa; Krisna Pangesti; Nelly Puspandari; Subangkit; Triyani Soekarso; Vivi Setiawaty
EPI_ISL_3138785	RS. Moh Ridwan Meuraksa.	National Institute of Health Research and Development	Arie Ardiansyah Nugraha; Fajar Nur Sulistiyohadi; Hana Apsari Pawestri; Hartanti Dian Ikawati; Kartika Dewi Puspa; Krisna Pangesti; Nelly Puspandari; Subangkit; Triyani Soekarso; Vivi Setiawaty
EPI_ISL_4779276	RS. Muhammadiyah Rodliyah Achid Moga Pemalang	Balai Laboratorium Kesehatan Dan Pengujian Alat Kesehatan Provinsi Jawa Tengah	Alia Wijayanti; Dwi Nursetyaasih; Dyah Ayu; Endah Wahyu Y; Ismi Farhati; Purnomo; Sekti Handayani; Wiwik Peni; Yekti Hediningsih
EPI_ISL_5616563, EPI_ISL_5616620	RS. Panti Rahayu Yakkum Purwodadi Grobongan	Balai Laboratorium Kesehatan dan Pengujian Alat Kesehatan Provinsi Jawa Tengah	Alia Wijayanti; Dwi Nursetyaasih; Dyah Ayu; Endah Wahyu Y; Ismi Farhati; Purnomo; Sekti Handayani; Wiwik Peni; Yekti Hediningsih
EPI_ISL_2955608	RSA UGM	Genetics Working Group (Pokja Genetik) Faculty of Medicine, Public Health and Nursing Universitas Gadjah Mada (FK-KMK UGM); Disease Investigation Center Wates Ministry of Agriculture Indonesia; Department of Microbiology FK-KMK UGM; Laboratorium Diagnostik Yayasan Tahija World Mosquito Program (WMP) Yogyakarta Center for Tropical Medicine FK-KMK UGM; Integrated Research Center FK-KMK UGM; Department of Computer Science and Electronics	Afiahayati; Dwi AA Nugrahaningsih; Dwi Indaryati; Dyah A Puspitarani; Edwin W. Daniwijaya; Eggi Arguni; Endah Supriyati; Gunadi; Hendra Wibawa; Indarto Sulistiyono; Kristy Iskandar; Ludhang P. Rizki; Marcellus; Mohamad S. Hakim; Nungki Anggorowati; Pramesti G Dewi; Siswanto; Susan Simanjaya; Titik Nuryastuti; Tri Wibawa

		FMIPA UGM; RSUP Dr. Sardjito	
EPI_ISL_902737, EPI_ISL_902749, EPI_ISL_906050, EPI_ISL_906051, EPI_ISL_906052, EPI_ISL_985398	RSA UGM	Genetics Working Group (Pokja Genetik) Faculty of Medicine, Public Health and Nursing Universitas Gadjah Mada (FK-KMK UGM); Disease Investigation Center Wates Ministry of Agriculture Indonesia; Department of Microbiology FK-KMK UGM; Laboratorium Diagnostik Yayasan Tahija World Mosquito Program (WMP) Yogyakarta Center for Tropical Medicine FK-KMK UGM; Integrated Research center FK-KMK UGM; Department of Computer Science and Electronics FMIPA UGM	Aditya Rifqi Fauzi; Afiahayati; Alvin Santoso Kalim; Dwi AA Nugrahaningsih; Dwiki Afandy; Dwiki afandy; Dyah Ayu Puspitarani; Edwin W. Daniwijaya; Eggi Arguni; Endah Supriyati; Fadil Fahri; Gunadi; Hendra Wibawa; Kemala Athollah; Kristy Iskandar; Ludhang P. Rizki; Marcellus; Maria Patricia Inggriani; Mohamad S. Hakim; Nungki Anggorowati; Siswanto; Susan Simanjaya; Titik Nuryastuti; Tri Wibawa; Untung Riawan; William Widitjiarso
EPI_ISL_576145	RSA Universitas Gadjah Mada	Genetics Working Group (Pokja Genetik) Faculty of Medicine, Public Health and Nursing Universitas Gadjah Mada (FK-KMK UGM); Disease Investigation Center Wates Ministry of Agriculture Indonesia; Department of Microbiology FK-KMK UGM; Laboratorium Diagnostik Yayasan Tahija World Mosquito Program (WMP) Yogyakarta Center for Tropical Medicine FK-KMK UGM; Integrated Research Center FK-KMK UGM; Department of Computer Science and Electronics FMIPA UGM	Afiahayati; Dwi AA Nugrahaningsih; Edwin W. Daniwijaya; Eggi Arguni; Endah Supriyati; Fadil Fahri; Gunadi; Hendra Wibawa; Kristy Iskandar; Ludhang P. Rizki; Marcellus; Mohamad S. Hakim; Nungki Anggorowati; Siswanto; Titik Nuryastuti; Tri Wibawa; William Widitjiarso
EPI_ISL_3548630, EPI_ISL_3548631, EPI_ISL_3548632, EPI_ISL_3548637, EPI_ISL_3548638, EPI_ISL_3548639, EPI_ISL_3548641, EPI_ISL_3548642, EPI_ISL_3548643, EPI_ISL_3548644, EPI_ISL_3548645, EPI_ISL_3548646, EPI_ISL_3548647, EPI_ISL_3548648, EPI_ISL_3548649, EPI_ISL_3548650, EPI_ISL_3548651, EPI_ISL_3548652, EPI_ISL_3548653, EPI_ISL_3548654, EPI_ISL_3548655, EPI_ISL_3548656, EPI_ISL_3548657, EPI_ISL_3548658, EPI_ISL_3548659, EPI_ISL_3548660, EPI_ISL_3548661, EPI_ISL_3548662, EPI_ISL_3548663, EPI_ISL_3548664, EPI_ISL_3548665, EPI_ISL_3548666, EPI_ISL_3548667, EPI_ISL_3548668, EPI_ISL_3548669, EPI_ISL_3548670, EPI_ISL_3548671, EPI_ISL_3548672, EPI_ISL_3548673, EPI_ISL_3548675, EPI_ISL_3548676, EPI_ISL_3548677, EPI_ISL_3548678, EPI_ISL_3548679, EPI_ISL_3548680, EPI_ISL_3548681, EPI_ISL_3548682, EPI_ISL_3548683, EPI_ISL_3548684, EPI_ISL_3730776, EPI_ISL_3730777, EPI_ISL_3730779, EPI_ISL_3730780, EPI_ISL_3730782, EPI_ISL_3730802, EPI_ISL_5462135, EPI_ISL_5462136, EPI_ISL_5462137, EPI_ISL_5462138			
see above	RSAB HARAPAN KITA	Genomik Solidaritas Indonesia Laboratorium	Anna Christina Brazia; Annisa Muthiah Sukirman; Anuraj Shankar; Ariel Pradipta; Carissa Sintca Wijaya; Dhahlia Agustina Cahyono; Dwi Oktavia; Esti Rahayuning Tias; Gracia Felias Enos Korompis; Himawan Masyhuri; Louisa Markus; Meutia Ayuputeri Kumaheri; Neng Hilma Hamidah; Ngabila Salama; Ni Sayu Dewi Budhiyani; Reinhart Gabriel; Stefanus Bernard; Tiranti Vindha; Vania Gavrila Wikasa
EPI_ISL_1284140, EPI_ISL_1284145, EPI_ISL_1622376, EPI_ISL_1622377, EPI_ISL_1622397, EPI_ISL_1622398, EPI_ISL_1622399			
see above	RSAB Harapan Kita	Eijkman Institute for Molecular Biology, Ministry of Research and Technology/National Agency for Research and Innovation	Amin Soebandrio; Edison Johar; Frilasita A Yudhaputri; Hidayat Trimarsanto; Iskandar Adnan; Khin Saw Myint; Lydia V. Panggalo; Muhammad Rezki Rasyak; Safarina G Malik; Sukma Oktavianthi; Willy Agustine
EPI_ISL_2614678	RSAB Harapan Kita	Eijkman Institute for Molecular Biology, National Research and Innovation Agency	Amin Soebandrio; Edison Johar; Frilasita A Yudhaputri; Hidayat Trimarsanto; Iskandar Adnan; Khin Saw Myint; Lidwina Priliani; Lydia V. Panggalo; Muhammad Rezki Rasyak; Safarina G Malik; Sukma Oktavianthi; Willy Agustine
EPI_ISL_5462139	RSAB Harapan Kita	Genomik Solidaritas Indonesia Laboratorium	Annisa Muthiah Sukirman; Anuraj Shankar; Ariel Pradipta; Esti Rahayuning Tias; Gracia Felias Enos Korompis; Himawan Masyhuri; Louisa Markus; Meutia Ayuputeri Kumaheri; Ni Sayu Dewi Budhiyani; Stefanus Bernard; Vania Gavrila Wikasa
EPI_ISL_529138	RSAL Dr. Ramelan Surabaya	Institute of Tropical Disease, Universitas Airlangga	Aldise M Nastri; Gatot Soegiarto; Jezzy R Dewantari; Kazufumi Shimizu; Krisnoadi Rahardjo; Laksmi Wulandari; Maria I Lusida; Mitsuhiro Nishimura; Radito Soesanto; Resti Yudhawati; Retno A Setyoningrum; Rima R Prasetya; Soetjipto; Yasuko Mori; Yohko K Shimizu
EPI_ISL_3138792, EPI_ISL_3138793, EPI_ISL_3138794	RSAU Esnawan Antariksa	National Institute of Health Research and Development	Arie Ardiansyah Nugraha; Fajar Nur Sulistiyohadi; Hana Apsari Pawestri; Hartanti Dian Ikawati; Kartika Dewi Puspa; Krisna Pangesti; Nelly Puspandari; Subangkit; Triyani Soekarso; Vivi Setiawaty
EPI_ISL_5446152, EPI_ISL_5446156	RSAU dr.M. Salamun	Laboratorium Genetik Molekuler, Biomedik Lanjut, FK UNPAD	Any Yuliani; Chrysanti Murad; Dwi Agustian; Fensi Amalia; Jessi Annisa; Lidya Chaidir; Nuni Haeruni; Nurul Setia Rahayu; Rima Destya Triatin; Savira Ekawardhani; Shinta; Tri Hanggono Ahmad; Yanu; Yeni Rendieni; Yunia Sribudiani
EPI_ISL_2709384, EPI_ISL_2709388, EPI_ISL_2918608, EPI_ISL_3029996	RSCM	Eijkman Institute for Molecular Biology, National Research and Innovation Agency	Amin Soebandrio; Edison Johar; Frilasita A Yudhaputri; Hidayat Trimarsanto; Iskandar Adnan; Khin Saw Myint; Lidwina Priliani; Lydia V. Panggalo; Muhammad Rezki Rasyak; Safarina G Malik; Sukma Oktavianthi; Willy Agustine
EPI_ISL_2987603, EPI_ISL_2987607, EPI_ISL_2987609, EPI_ISL_3278300, EPI_ISL_4508295, EPI_ISL_4508296, EPI_ISL_4508297, EPI_ISL_4508298, EPI_ISL_4508299, EPI_ISL_4508300, EPI_ISL_4508334, EPI_ISL_4508335, EPI_ISL_4508336, EPI_ISL_4508337, EPI_ISL_5022800, EPI_ISL_5022801, EPI_ISL_5022802, EPI_ISL_5022845, EPI_ISL_5022846, EPI_ISL_5022847, EPI_ISL_5022848, EPI_ISL_5022849, EPI_ISL_5022850, EPI_ISL_5022851, EPI_ISL_5022894, EPI_ISL_5022895, EPI_ISL_5022896, EPI_ISL_5022897, EPI_ISL_5022898, EPI_ISL_5022899, EPI_ISL_5022900, EPI_ISL_5022901, EPI_ISL_5022902, EPI_ISL_5022903, EPI_ISL_5022943, EPI_ISL_5022944, EPI_ISL_5022945, EPI_ISL_5107834, EPI_ISL_5107856, EPI_ISL_5107857, EPI_ISL_5441751, EPI_ISL_5441752, EPI_ISL_5441753, EPI_ISL_5441754, EPI_ISL_5441755, EPI_ISL_5441756, EPI_ISL_5441757, EPI_ISL_5441758, EPI_ISL_5441759, EPI_ISL_5441760, EPI_ISL_5441761, EPI_ISL_5441762, EPI_ISL_5441763, EPI_ISL_5441764, EPI_ISL_5441769, EPI_ISL_5441770, EPI_ISL_5441771, EPI_ISL_5441772, EPI_ISL_5743648, EPI_ISL_5743649, EPI_ISL_5743650, EPI_ISL_5743654, EPI_ISL_5743663, EPI_ISL_5743682, EPI_ISL_5743778, EPI_ISL_5743780			
see above	RSDC Wisma Atlet Kemayoran	National Institute of Health Research and Development	Arie Ardiansyah Nugraha; Fajar Nur Sulistiyohadi; Hana Apsari Pawestri; Hartanti Dian Ikawati; Kartika Dewi Puspa; Krisna Pangesti; Nelly Puspandari; Subangkit; Triyani Soekarso; Vivi Setiawaty
EPI_ISL_759968	RSI Darus Syifa'	Institute of Tropical Disease, Universitas Airlangga	Aldise M Nastri; Gatot Soegiarto; HM Faiz; Jezzy R Dewantari; Kazufumi Shimizu; Krisnoadi Rahardjo; Laksmi Wulandari; Maria I Lusida; Resti Yudhawati; Rima R Prasetya; Soetjipto; Yasuko Mori
EPI_ISL_4969084	RSI NU Demak	Genetics Working Group (Pokja Genetik) Faculty of Medicine, Public Health and Nursing Universitas Gadjah Mada (FK-KMK UGM); Disease Investigation Center Wates Ministry of Agriculture Indonesia; Department of Microbiology FK-KMK UGM; Laboratorium Diagnostik Yayasan Tahija World Mosquito Program (WMP) Yogyakarta Center for Tropical Medicine FK-KMK UGM; Integrated Research Center FK-KMK UGM; Department of Computer Science and Electronics FMIPA UGM; RSUP Dr. Sardjito	Afiahayati; Alvina A Setiawan; Dwi AA Nugrahaningsih; Dwi Indaryati; Edwin W. Daniwijaya; Eggi Arguni; Endah Supriyati; Gunadi; Hendra Wibawa; Indarto Sulistiyono; Khanza a Vujira; Kristy Iskandar; Ludhang P. Rizki; Marcellus; Mohamad S. Hakim; Nungki Anggorowati; Pramesti G Dewi; Siswanto; Titik Nuryastuti; Tri Wibawa
EPI_ISL_2364656	RSIA Assyifa	Eijkman Institute for Molecular Biology, National Agency for Research and Innovation	Amin Soebandrio; Edison Johar; Frilasita A Yudhaputri; Hidayat Trimarsanto; Iskandar Adnan; Khin Saw Myint; Lidwina Priliani; Lydia V. Panggalo; Muhammad Rezki Rasyak; Safarina G Malik; Sukma Oktavianthi; Willy Agustine
EPI_ISL_3730785, EPI_ISL_3730797, EPI_ISL_3946309, EPI_ISL_3946310, EPI_ISL_3946311	RSIA Budhi Jaya	Genomik Solidaritas Indonesia Laboratorium	Anna Christina Brazia; Annisa Muthiah Sukirman; Anuraj Shankar; Ariel Pradipta; Carissa Sintca Wijaya; Dhahlia Agustina Cahyono; Dwi Oktavia; Gracia Felias Enos Korompis; Himawan Masyhuri; Louisa Markus; Lulu Nursyifa; Meutia Ayuputeri Kumaheri; Neng Hilma Hamidah; Ngabila Salama; Pratiwi Srikandi Yudhastari; Reinhart Gabriel; Tiranti Vindha; Vania Gavrila Wikasa
EPI_ISL_1622378	RSIA Bun	Eijkman Institute for Molecular Biology, Ministry of Research and Technology/National Agency for Research and Innovation	Amin Soebandrio; Edison Johar; Frilasita A Yudhaputri; Hidayat Trimarsanto; Iskandar Adnan; Khin Saw Myint; Lydia V. Panggalo; Muhammad Rezki Rasyak; Safarina G Malik; Sukma Oktavianthi; Willy Agustine
EPI_ISL_1283996, EPI_ISL_1284149, EPI_ISL_1284152	RSIA Bunda Sejahtera	Eijkman Institute for Molecular Biology, Ministry of Research and Technology/National Agency for Research and Innovation	Amin Soebandrio; Edison Johar; Frilasita A Yudhaputri; Hidayat Trimarsanto; Iskandar Adnan; Khin Saw Myint; Lydia V. Panggalo; Safarina G Malik; Sukma Oktavianthi; Willy Agustine
EPI_ISL_2364598	RSIA Bunda Sejahtera	Eijkman Institute for Molecular Biology, National Agency for Research and Innovation	Amin Soebandrio; Edison Johar; Frilasita A Yudhaputri; Hidayat Trimarsanto; Iskandar Adnan; Khin Saw Myint; Lidwina Priliani; Lydia V. Panggalo; Muhammad Rezki Rasyak; Safarina G Malik; Sukma Oktavianthi; Willy Agustine
EPI_ISL_947284	RSIA Bunda Sejati	Eijkman Institute for Molecular Biology, Ministry of Research and Technology/National Agency for Research and Innovation	Amin Soebandrio; Edison Johar; Frilasita A Yudhaputri; Hidayat Trimarsanto; Iskandar Adnan; Khin Saw Myint; Lydia V. Panggalo; Safarina G Malik; Sukma Oktavianthi; Willy Agustine
EPI_ISL_2614716	RSIA Bunda Sejati	Eijkman Institute for Molecular Biology, National Research and Innovation Agency	Amin Soebandrio; Edison Johar; Frilasita A Yudhaputri; Hidayat Trimarsanto; Iskandar Adnan; Khin Saw Myint; Lidwina Priliani; Lydia V. Panggalo; Muhammad Rezki Rasyak; Safarina G Malik; Sukma Oktavianthi; Willy Agustine
EPI_ISL_3030054	RSIA Grha Bunda, Dinas Kesehatan Kota Bandung	Eijkman Institute for Molecular Biology, National Research and Innovation Agency; West Java Health Laboratory	Ade Fany Safitri; Amin Soebandrio; Azzania Fibriani; Cut Nur Cinthia Alamanda; Edison Johar; Ema Rahmawati; Frilasita A Yudhaputri; Hidayat Trimarsanto; Husna Nugrahapraja; Iskandar Adnan; Khin Saw Myint; Lidwina Priliani; Lydia V. Panggalo; Muhammad Rezki Rasyak; Ranza Fadhlil Pratama; Rifky Waluyajati Rachman; Ryan Bayusantika Ristandi; Safarina G Malik; Sukma Oktavianthi; Tati Sutarti Sastrowiyoto; Willy Agustine
EPI_ISL_889021, EPI_ISL_1622392	RSIA Kenari Graha Medika	Eijkman Institute for Molecular Biology, Ministry of Research and Technology/National Agency for Research and Innovation	Amin Soebandrio; Edison Johar; Frilasita A Yudhaputri; Hidayat Trimarsanto; Iskandar Adnan; Khin Saw Myint; Lydia V. Panggalo; Muhammad Rezki Rasyak; Safarina G Malik; Sukma Oktavianthi; Willy Agustine
EPI_ISL_947287, EPI_ISL_947325, EPI_ISL_1284074, EPI_ISL_1284076	RSIA PKU Muhammadiyah	Eijkman Institute for Molecular Biology, Ministry of Research and Technology/National Agency for Research and Innovation	Amin Soebandrio; Edison Johar; Frilasita A Yudhaputri; Hidayat Trimarsanto; Iskandar Adnan; Khin Saw Myint; Lydia V. Panggalo; Safarina G Malik; Sukma Oktavianthi; Willy Agustine
EPI_ISL_2364666, EPI_ISL_2364669	RSIA PKU Muhammadiyah	Eijkman Institute for Molecular Biology, National Agency for Research and Innovation	Amin Soebandrio; Edison Johar; Frilasita A Yudhaputri; Hidayat Trimarsanto; Iskandar Adnan; Khin Saw Myint; Lidwina Priliani; Lydia V. Panggalo; Muhammad Rezki Rasyak; Safarina G Malik; Sukma Oktavianthi; Willy Agustine
EPI_ISL_889019, EPI_ISL_947273, EPI_ISL_1283923, EPI_ISL_1283924	RSIA Sayyidah	Eijkman Institute for Molecular Biology, Ministry of Research and Technology/National Agency for Research and Innovation	Amin Soebandrio; Edison Johar; Frilasita A Yudhaputri; Hidayat Trimarsanto; Iskandar Adnan; Khin Saw Myint; Lydia V. Panggalo; Safarina G Malik; Sukma Oktavianthi; Willy Agustine
EPI_ISL_1283957, EPI_ISL_1283970, EPI_ISL_1284178, EPI_ISL_1622370, EPI_ISL_1622435	RSIA Selasih Medika	Eijkman Institute for Molecular Biology, Ministry of Research and Technology/National Agency for Research and Innovation	Amin Soebandrio; Edison Johar; Frilasita A Yudhaputri; Hidayat Trimarsanto; Iskandar Adnan; Khin Saw Myint; Lydia V. Panggalo; Muhammad Rezki Rasyak; Safarina G Malik; Sukma Oktavianthi; Willy Agustine
EPI_ISL_2614680	RSIA Selasih Medika	Eijkman Institute for Molecular Biology, National Research and Innovation Agency	Amin Soebandrio; Edison Johar; Frilasita A Yudhaputri; Hidayat Trimarsanto; Iskandar Adnan; Khin Saw Myint; Lidwina Priliani; Lydia V. Panggalo; Muhammad Rezki Rasyak; Safarina G Malik; Sukma Oktavianthi; Willy Agustine
EPI_ISL_1284087, EPI_ISL_1284182, EPI_ISL_1284296, EPI_ISL_1622368, EPI_ISL_1622386, EPI_ISL_1622393, EPI_ISL_1622423, EPI_ISL_1622433			
see above	RSJ Dr. Soeharto Heerdjan	Eijkman Institute for Molecular Biology, Ministry of Research and Technology/National Agency for Research and Innovation	Amin Soebandrio; Edison Johar; Frilasita A Yudhaputri; Hidayat Trimarsanto; Iskandar Adnan; Khin Saw Myint; Lydia V. Panggalo; Muhammad Rezki Rasyak; Safarina G Malik; Sukma Oktavianthi; Willy Agustine
EPI_ISL_2364583, EPI_ISL_2364672	RSJ Dr. Soeharto Heerdjan	Eijkman Institute for Molecular Biology, National Agency for Research and Innovation	Amin Soebandrio; Edison Johar; Frilasita A Yudhaputri; Hidayat Trimarsanto; Iskandar Adnan; Khin Saw Myint; Lidwina Priliani; Lydia V. Panggalo; Muhammad Rezki Rasyak; Safarina G Malik; Sukma Oktavianthi; Willy Agustine
EPI_ISL_2614658, EPI_ISL_2614659, EPI_ISL_2614663, EPI_ISL_2614664, EPI_ISL_2614677, EPI_ISL_2614691, EPI_ISL_2614705, EPI_ISL_2614709			
see above	RSJ Dr. Soeharto Heerdjan	Eijkman Institute for Molecular Biology, National Research and Innovation Agency	Amin Soebandrio; Edison Johar; Frilasita A Yudhaputri; Hidayat Trimarsanto; Iskandar Adnan; Khin Saw Myint; Lidwina Priliani; Lydia V. Panggalo; Muhammad Rezki Rasyak; Safarina G Malik; Sukma Oktavianthi; Willy Agustine
EPI_ISL_5021103, EPI_ISL_5021107, EPI_ISL_5021113, EPI_ISL_5021118, EPI_ISL_5021127, EPI_ISL_5021132, EPI_ISL_5021137, EPI_ISL_5021145, EPI_ISL_5021147			
see above	RSJ Dr. Soeharto Heerdjan	Eijkman Research Center for Molecular Biology, National Research and Innovation Agency	Amin Soebandrio; Edison Johar; Frilasita A Yudhaputri; Hidayat Trimarsanto; Iskandar Adnan; Jessica R Saragih; Khin Saw Myint; Lidwina Priliani; Lydia V Panggalo; Muhammad Rezki Rasyak; Safarina G Malik; Sukma Oktavianthi; Willy Agustine
EPI_ISL_4741237	RSJD Dr Amino Gondohutomo	Balai Laboratorium Kesehatan Dan Pengujian Alat Kesehatan Provinsi Jawa Tengah	Dwi Nursetyaasih; Dyah Ayu; Endah Wahyu Y; Ismi Farhati; Purnomo; Sekti Handayani; Wiwik Peni; Yekti Hediningsih
EPI_ISL_574609, EPI_ISL_766038	RSJPD Harapan Kita	Eijkman Institute for Molecular Biology, Ministry of Research and Technology/National Agency for Research and Innovation	Amin Soebandrio; David H Muljono; Edison Johar; Frilasita A Yudhaputri; Herawati Sudoyo; Hidayat Trimarsanto; Iskandar A Adnan; Khin Saw Myint; Safarina G Malik; Willy Agustine
EPI_ISL_2631454, EPI_ISL_2631455, EPI_ISL_2631456, EPI_ISL_2631457, EPI_ISL_2631458, EPI_ISL_2631459, EPI_ISL_2631460, EPI_ISL_2631461, EPI_ISL_2631462, EPI_ISL_2631463			

see above	RSK Paru Karawang	Indonesian Institute of Sciences (LIPI)/National Agency of Research and Innovation (BRIN) and National Institute of Health Research and Development (NIHRD)	; Anggia Prasetyoputri; Anik B. Dharmayanthi; Eko Wahyu Putro; Fahrurrozi; Herjuno A. Nugroho; Idris; Indriawati; Isa Nuryana; Linda Sukmarini; Listiana Oktavia; Nova D. Yanthi; Puspita Lisdiyanti; Ratih A. Ningrum; Ruby Setiawan; Sri Swasthikawati; Subangkit; Syam B. Iryanto; Vivi Setiawaty; Vivi Setiawaty Puspita Lisdiyanti; Yayuk Sri Rahayu; Yuliawati
EPI_ISL_2931727, EPI_ISL_2931763, EPI_ISL_2931774, EPI_ISL_2931781, EPI_ISL_2931789	RSKIA Kota Bandung	National Institute of Health Research and Development	; Arie Ardiansyah Nugraha; Hana Apsari Pawestri; Hartanti Dian Ikawati; Kartika Dewi Puspa; Krisna Pangesti; Nelly Puspandari; Subangkit; Triyani Soekarso; Vivi Setiawaty
EPI_ISL_3308870, EPI_ISL_3308963	RSPAW	Institute for Vector and Reservoir Control, Research and Development (IVRCRD)- National Institute of Health Research and Development (NIHRD), Indonesia	Aprilia Safitri; Arief Mulyono; Arif Suryo Prasetyo; Aryo Ardanto; Ayu Pradipta Pratiwi; Dimas Bagus Wicaksono Putro; Dwi Susilo; Esti Rahardianingtyas; Evi sulistyorini; Hana Apsari Pawestri; Joko Waluyo; Kusumaningtyas Sekar Negari; Lulus Susanti; Marjianto; Mega Tyas Prihatin; Muhidin; Mujiyanto; Nur Hidayati; Restu Choirul Saban; Ristiyanto; Sidiq Setyo Nugroho; Siti Alfiah; Sri Wahyuni Handayani; Subangkit; Tika Fiona Sari; Triwibowo Ambar Garjito; Vivi Setiawaty; Wimbi Kartika Ratnasari; Yusnita Mirna Anggraeni
EPI_ISL_2858700, EPI_ISL_4481519, EPI_ISL_4481523, EPI_ISL_4481548, EPI_ISL_4481557, EPI_ISL_5200491, EPI_ISL_5200500			
see above	RSPI PROF DR. SULIANTI SAROSO	Genomik Solidaritas Indonesia Laboratorium	Ahmad Zoebad Foeady; Alfin Muhammad Abdillah; Anindya Pradipta Susanto; Anna Christina Brazia; Annisa Muthiah Sukirman; Anuraj Shankar; Ariel Pradipta; Carissa Sintca Wijaya; Dhahlia Agustina Cahyono; Dian Tri Wahyudi; Esti Rahayuning Tias; Gracia Felias Enos Korompis; Himawan Masyhuri; Louisa Markus; Meutia Ayuputeri Kumaheri; Normastuti Adhini Tantyo; Pompini Agustina Sitompul; Reinhart Gabriel; Stefanus Bernard; Teguh Sarry Hartono; Vania Gavrila Wikasa
EPI_ISL_3548636, EPI_ISL_3548693, EPI_ISL_3548694, EPI_ISL_3548695, EPI_ISL_3548696, EPI_ISL_3548697, EPI_ISL_3945456, EPI_ISL_3945457, EPI_ISL_3945458, EPI_ISL_3945459, EPI_ISL_3945460, EPI_ISL_3945461, EPI_ISL_3945462, EPI_ISL_3945463, EPI_ISL_3945464, EPI_ISL_3945465, EPI_ISL_3945466, EPI_ISL_3945467, EPI_ISL_3945468, EPI_ISL_3945469, EPI_ISL_3945470, EPI_ISL_3945471, EPI_ISL_3945472, EPI_ISL_3945473, EPI_ISL_3945474, EPI_ISL_3945475, EPI_ISL_3945476, EPI_ISL_4730630			
see above	RSPI PROF DR. SULIANTI SAROSO, RS	Genomik Solidaritas Indonesia Laboratorium	Anna Christina Brazia; Annisa Muthiah Sukirman; Anuraj Shankar; Ariel Pradipta; Carissa Sintca Wijaya; David Hendra Haryanto; Dhahlia Agustina Cahyono; Dwi Oktavia; Gracia Felias Enos Korompis; Louisa Markus; Meutia Ayuputeri Kumaheri; Neng Hilma Hamidah; Ngabila Salama; Pratiwi Srikandi Yudhastari; Reinhart Gabriel; Teguh Sarry Hartono; Tiranti Vindha; Vania Gavrila Wikasa
EPI_ISL_1915566, EPI_ISL_1915567, EPI_ISL_1915568	RSPI Prof DR Sulianti Saroso Jakarta Utara	National Institute of Health Research and Development	Arie Ardiansyah Nugraha; Hana Apsari Pawestri; Hartanti Dian Ikawati; Kartika Dewi Puspa; Krisna Pangesti; Nelly Puspandari; Subangkit; Triyani Soekarso; Vivi Setiawaty
EPI_ISL_3401851, EPI_ISL_3401863, EPI_ISL_3401874, EPI_ISL_3401875, EPI_ISL_3401876, EPI_ISL_3401877, EPI_ISL_3401878, EPI_ISL_3664303			
see above	RSPI Prof DR. Sulianti Saroso, Rs	Genomik Solidaritas Indonesia Laboratorium	Annisa Muthiah Sukirman; Anuraj Shankar; Ariel Pradipta; Carissa Sintca Wijaya; Dhahlia Agustina Cahyono; Dwi Oktavia; Gracia Felias Enos Korompis; Himawan Masyhuri; Louisa Markus; Meutia Ayuputeri Kumaheri; Ngabila Salama; Tiranti Vindha; Vania Gavrila Wikasa
EPI_ISL_2987592, EPI_ISL_2987593, EPI_ISL_2987594, EPI_ISL_2987595	RSPI Prof dr Sulianti Saroso	National Institute of Health Research and Development	Arie Ardiansyah Nugraha; Fajar Nur Sulistiyohadi; Hana Apsari Pawestri; Hartanti Dian Ikawati; Kartika Dewi Puspa; Krisna Pangesti; Nelly Puspandari; Subangkit; Triyani Soekarso; Vivi Setiawaty
EPI_ISL_2854770, EPI_ISL_2854771, EPI_ISL_2854772, EPI_ISL_2854773, EPI_ISL_2854774	RSPI Prof. DR. Sulianti Saroso	National Institute of Health Research and Development	Arie Ardiansyah Nugraha; Hana Apsari Pawestri; Hartanti Dian Ikawati; Kartika Dewi Puspa; Krisna Pangesti; Nelly Puspandari; Subangkit; Triyani Soekarso; Vivi Setiawaty
EPI_ISL_2047574, EPI_ISL_2047575, EPI_ISL_2047576, EPI_ISL_2106984, EPI_ISL_2106985, EPI_ISL_2106986, EPI_ISL_2262269, EPI_ISL_2262271, EPI_ISL_2262273, EPI_ISL_2262283, EPI_ISL_2262285, EPI_ISL_2262288, EPI_ISL_2262289, EPI_ISL_2262291, EPI_ISL_2262295, EPI_ISL_2382416, EPI_ISL_3138813, EPI_ISL_3138814, EPI_ISL_3138815, EPI_ISL_3138816, EPI_ISL_3138817, EPI_ISL_3138818, EPI_ISL_3138819			
see above	RSPI Prof. Dr. Sulianti Saroso	National Institute of Health Research and Development	Arie Ardiansyah Nugraha; Fajar Nur Sulistiyohadi; Hana Apsari Pawestri; Hartanti Dian Ikawati; Kartika Dewi Puspa; Krisna Pangesti; Nelly Puspandari; Subangkit; Triyani Soekarso; Vivi Setiawaty
EPI_ISL_1257847, EPI_ISL_1257848, EPI_ISL_1257849, EPI_ISL_1257850	RSPI Prof.Dr.Sulianti Saroso	National Institute of Health Research and Development	Arie Ardiansyah Nugraha; Hana Apsari Pawestri; Hartanti Dian Ikawati; Kartika Dewi Puspa; Krisna Pangesti; Nelly Puspandari; Subangkit; Vivi Setiawaty
EPI_ISL_3333238, EPI_ISL_3333240, EPI_ISL_3333241, EPI_ISL_3333242, EPI_ISL_3333243, EPI_ISL_3333244, EPI_ISL_3333245, EPI_ISL_3333246, EPI_ISL_3333247, EPI_ISL_3333248			
see above	RSPI SULIANTI SAROSO	Genomik Solidaritas Indonesia Laboratorium	Annisa Muthiah Sukirman; Anuraj Shankar; Ariel Pradipta; Carissa Sintca Wijaya; Dhahlia Agustina Cahyono; Gracia Felias Enos Korompis; Louisa Markus; Meutia Ayuputeri Kumaheri; Reinhart Gabriel; Vania Gavrila Wikasa
EPI_ISL_2868873, EPI_ISL_2868876, EPI_ISL_2868879, EPI_ISL_2868881, EPI_ISL_2868883, EPI_ISL_2868884, EPI_ISL_2868886, EPI_ISL_3138804, EPI_ISL_3138805, EPI_ISL_3138806, EPI_ISL_3138807, EPI_ISL_3138808			
see above	RSPI Sulianti Saroso	National Institute of Health Research and Development	Arie Ardiansyah Nugraha; Fajar Nur Sulistiyohadi; Hana Apsari Pawestri; Hartanti Dian Ikawati; Kartika Dewi Puspa; Krisna Pangesti; Nelly Puspandari; Subangkit; Triyani Soekarso; Vivi Setiawaty
EPI_ISL_1915560, EPI_ISL_1915561	RSPI Sulianti Saroso Jakarta Utara	National Institute of Health Research and Development	Arie Ardiansyah Nugraha; Hana Apsari Pawestri; Hartanti Dian Ikawati; Kartika Dewi Puspa; Krisna Pangesti; Nelly Puspandari; Subangkit; Triyani Soekarso; Vivi Setiawaty
EPI_ISL_3374704	RSU ADHYAKSA	Genomik Solidaritas Indonesia Laboratorium	Annisa Muthiah Sukirman; Anuraj Shankar; Ariel Pradipta; Carissa Sintca Wijaya; Dhahlia Agustina Cahyono; Gracia Felias Enos Korompis; Himawan Masyhuri; Louisa Markus; Meutia Ayuputeri Kumaheri; Vania Gavrila Wikasa
EPI_ISL_2709393, EPI_ISL_2709398, EPI_ISL_2709399	RSU Adhyaksa	Eijkman Institute for Molecular Biology, National Research and Innovation Agency	Amin Soebandrio; Edison Johar; Frilasita A Yudhaputri; Hidayat Trimarsanto; Iskandar Adnan; Khin Saw Myint; Lidwina Priliani; Lydia V. Panggalo; Muhammad Rezki Rasyak; Safarina G Malik; Sukma Oktavianthi; Willy Agustine
EPI_ISL_947268	RSU Anggrek Mas	Eijkman Institute for Molecular Biology, Ministry of Research and Technology/National Agency for Research and Innovation	Amin Soebandrio; Edison Johar; Frilasita A Yudhaputri; Hidayat Trimarsanto; Iskandar Adnan; Khin Saw Myint; Lydia V. Panggalo; Safarina G Malik; Sukma Oktavianthi; Willy Agustine
EPI_ISL_888997	RSU Asysyifaa	Eijkman Institute for Molecular Biology, Ministry of Research and Technology/National Agency for Research and Innovation	Amin Soebandrio; Edison Johar; Frilasita A Yudhaputri; Hidayat Trimarsanto; Iskandar Adnan; Khin Saw Myint; Lydia V. Panggalo; Safarina G Malik; Sukma Oktavianthi; Willy Agustine
EPI_ISL_889001, EPI_ISL_1284040, EPI_ISL_1284169, EPI_ISL_1284172	RSU Azra	Eijkman Institute for Molecular Biology, Ministry of Research and Technology/National Agency for Research and Innovation	Amin Soebandrio; Edison Johar; Frilasita A Yudhaputri; Hidayat Trimarsanto; Iskandar Adnan; Khin Saw Myint; Lydia V. Panggalo; Safarina G Malik; Sukma Oktavianthi; Willy Agustine
EPI_ISL_888986	RSU Bhakti Kartini	Eijkman Institute for Molecular Biology, Ministry of Research and Technology/National Agency for Research and Innovation	Amin Soebandrio; Edison Johar; Frilasita A Yudhaputri; Hidayat Trimarsanto; Iskandar Adnan; Khin Saw Myint; Lydia V. Panggalo; Safarina G Malik; Sukma Oktavianthi; Willy Agustine
EPI_ISL_947247, EPI_ISL_947260, EPI_ISL_947312, EPI_ISL_947329, EPI_ISL_947330	RSU Bunda Mulia	Eijkman Institute for Molecular Biology, Ministry of Research and Technology/National Agency for Research and Innovation	Amin Soebandrio; Edison Johar; Frilasita A Yudhaputri; Hidayat Trimarsanto; Iskandar Adnan; Khin Saw Myint; Lydia V. Panggalo; Safarina G Malik; Sukma Oktavianthi; Willy Agustine
EPI_ISL_1283973	RSU Bunda Mulia Cikarang	Eijkman Institute for Molecular Biology, Ministry of Research and Technology/National Agency for Research and Innovation	Amin Soebandrio; Edison Johar; Frilasita A Yudhaputri; Hidayat Trimarsanto; Iskandar Adnan; Khin Saw Myint; Lydia V. Panggalo; Safarina G Malik; Sukma Oktavianthi; Willy Agustine
EPI_ISL_3029933, EPI_ISL_3070863, EPI_ISL_3070864, EPI_ISL_3070865, EPI_ISL_3070866	RSU Dinda Tangerang	Eijkman Institute for Molecular Biology, National Research and Innovation Agency	Amin Soebandrio; Edison Johar; Frilasita A Yudhaputri; Hidayat Trimarsanto; Iskandar Adnan; Khin Saw Myint; Lidwina Priliani; Lydia V. Panggalo; Muhammad Rezki Rasyak; Safarina G Malik; Sukma Oktavianthi; Willy Agustine
EPI_ISL_888996	RSU Graha Juanda	Eijkman Institute for Molecular Biology, Ministry of Research and Technology/National Agency for Research and Innovation	Amin Soebandrio; Edison Johar; Frilasita A Yudhaputri; Hidayat Trimarsanto; Iskandar Adnan; Khin Saw Myint; Lydia V. Panggalo; Safarina G Malik; Sukma Oktavianthi; Willy Agustine
EPI_ISL_1284000, EPI_ISL_1284064, EPI_ISL_1284109, EPI_ISL_1284114, EPI_ISL_1284118, EPI_ISL_1284299, EPI_ISL_1622387, EPI_ISL_1622400, EPI_ISL_1622415			
see above	RSU HGA Depok	Eijkman Institute for Molecular Biology, Ministry of Research and Technology/National Agency for Research and Innovation	Amin Soebandrio; Edison Johar; Frilasita A Yudhaputri; Hidayat Trimarsanto; Iskandar Adnan; Khin Saw Myint; Lydia V. Panggalo; Muhammad Rezki Rasyak; Safarina G Malik; Sukma Oktavianthi; Willy Agustine
EPI_ISL_2364653	RSU HGA Depok	Eijkman Institute for Molecular Biology, National Agency for Research and Innovation	Amin Soebandrio; Edison Johar; Frilasita A Yudhaputri; Hidayat Trimarsanto; Iskandar Adnan; Khin Saw Myint; Lidwina Priliani; Lydia V. Panggalo; Muhammad Rezki Rasyak; Safarina G Malik; Sukma Oktavianthi; Willy Agustine
EPI_ISL_2614692, EPI_ISL_2614700, EPI_ISL_2709396, EPI_ISL_2709397, EPI_ISL_3029939	RSU HGA Depok	Eijkman Institute for Molecular Biology, National Research and Innovation Agency	Amin Soebandrio; Edison Johar; Frilasita A Yudhaputri; Hidayat Trimarsanto; Iskandar Adnan; Khin Saw Myint; Lidwina Priliani; Lydia V. Panggalo; Muhammad Rezki Rasyak; Safarina G Malik; Sukma Oktavianthi; Willy Agustine
EPI_ISL_889006, EPI_ISL_947304, EPI_ISL_947315, EPI_ISL_1283927, EPI_ISL_1284049, EPI_ISL_1284168, EPI_ISL_1284226, EPI_ISL_1284230, EPI_ISL_1284234, EPI_ISL_1284237			
see above	RSU Harapan Bunda	Eijkman Institute for Molecular Biology, Ministry of Research and Technology/National Agency for Research and Innovation	Amin Soebandrio; Edison Johar; Frilasita A Yudhaputri; Hidayat Trimarsanto; Iskandar Adnan; Khin Saw Myint; Lydia V. Panggalo; Safarina G Malik; Sukma Oktavianthi; Willy Agustine
EPI_ISL_2987610, EPI_ISL_2987611	RSU Hermina Kemayoran	National Institute of Health Research and Development	Arie Ardiansyah Nugraha; Fajar Nur Sulistiyohadi; Hana Apsari Pawestri; Hartanti Dian Ikawati; Kartika Dewi Puspa; Krisna Pangesti; Nelly Puspandari; Subangkit; Triyani Soekarso; Vivi Setiawaty
EPI_ISL_947296	RSU Kartika Husada	Eijkman Institute for Molecular Biology, Ministry of Research and Technology/National Agency for Research and Innovation	Amin Soebandrio; Edison Johar; Frilasita A Yudhaputri; Hidayat Trimarsanto; Iskandar Adnan; Khin Saw Myint; Lydia V. Panggalo; Safarina G Malik; Sukma Oktavianthi; Willy Agustine
EPI_ISL_2854740, EPI_ISL_2854741, EPI_ISL_2854742, EPI_ISL_2854743, EPI_ISL_2854744, EPI_ISL_3138786, EPI_ISL_3138796, EPI_ISL_3138797, EPI_ISL_3138798, EPI_ISL_3138799, EPI_ISL_3138800, EPI_ISL_3138801			
see above	RSU Kartika Pulomas	National Institute of Health Research and Development	; Arie Ardiansyah Nugraha; Fajar Nur Sulistiyohadi; Hana Apsari Pawestri; Hartanti Dian Ikawati; Kartika Dewi Puspa; Krisna Pangesti; Nelly Puspandari; Subangkit; Triyani Soekarso; Vivi Setiawaty

EPI_ISL_888994	RSU MMC	Eijkman Institute for Molecular Biology, Ministry of Research and Technology/National Agency for Research and Innovation	Amin Soebandrio; Edison Johar; Frilasita A Yudhaputri; Hidayat Trimarsanto; Iskandar Adnan; Khin Saw Myint; Lydia V. Panggalo; Safarina G Malik; Sukma Oktavianthi; Willy Agustine
EPI_ISL_889010, EPI_ISL_889014, EPI_ISL_889018, EPI_ISL_947281	RSU Medika Dramaga	Eijkman Institute for Molecular Biology, Ministry of Research and Technology/National Agency for Research and Innovation	Amin Soebandrio; Edison Johar; Frilasita A Yudhaputri; Hidayat Trimarsanto; Iskandar Adnan; Khin Saw Myint; Lydia V. Panggalo; Safarina G Malik; Sukma Oktavianthi; Willy Agustine
EPI_ISL_888993, EPI_ISL_947298, EPI_ISL_947317, EPI_ISL_947324, EPI_ISL_1284212	RSU Medirossa	Eijkman Institute for Molecular Biology, Ministry of Research and Technology/National Agency for Research and Innovation	Amin Soebandrio; Edison Johar; Frilasita A Yudhaputri; Hidayat Trimarsanto; Iskandar Adnan; Khin Saw Myint; Lydia V. Panggalo; Safarina G Malik; Sukma Oktavianthi; Willy Agustine
EPI_ISL_947255	RSU Meilia	Eijkman Institute for Molecular Biology, Ministry of Research and Technology/National Agency for Research and Innovation	Amin Soebandrio; Edison Johar; Frilasita A Yudhaputri; Hidayat Trimarsanto; Iskandar Adnan; Khin Saw Myint; Lydia V. Panggalo; Safarina G Malik; Sukma Oktavianthi; Willy Agustine
EPI_ISL_4090566, EPI_ISL_4090567, EPI_ISL_4090568, EPI_ISL_4090569, EPI_ISL_4090570	RSU Melinda 2	National Institute of Health Research and Development	Arie Ardiansyah Nugraha; Fajar Nur Sulistiyohadi; Hana Apsari Pawestri; Hartanti Dian Ikawati; Kartika Dewi Puspa; Krisna Pangesti; Nelly Puspandari; Subangkit; Triyani Soekarso; Vivi Setiawaty
EPI_ISL_2987608	RSU Mitra Keluarga Kalideres	National Institute of Health Research and Development	Arie Ardiansyah Nugraha; Fajar Nur Sulistiyohadi; Hana Apsari Pawestri; Hartanti Dian Ikawati; Kartika Dewi Puspa; Krisna Pangesti; Nelly Puspandari; Subangkit; Triyani Soekarso; Vivi Setiawaty
EPI_ISL_4741904	RSU PKU Muhammadiyah GUBUG	Balai Laboratorium Kesehatan Dan Pengujian Alat Kesehatan Provinsi Jawa Tengah	Alia Wijayanti; Dwi Nursetyaasih; Dyah Ayu; Endah Wahyu Y; Ismi Farhati; Purnomo; Sekti Handayani; Wiwik Peni; Yekti Hediningsih
EPI_ISL_889011	RSU Permata Cibubur	Eijkman Institute for Molecular Biology, Ministry of Research and Technology/National Agency for Research and Innovation	Amin Soebandrio; Edison Johar; Frilasita A Yudhaputri; Hidayat Trimarsanto; Iskandar Adnan; Khin Saw Myint; Lydia V. Panggalo; Safarina G Malik; Sukma Oktavianthi; Willy Agustine
EPI_ISL_2854777	RSU Permata Depok	National Institute of Health Research and Development	Arie Ardiansyah Nugraha; Hana Apsari Pawestri; Hartanti Dian Ikawati; Kartika Dewi Puspa; Krisna Pangesti; Nelly Puspandari; Subangkit; Triyani Soekarso; Vivi Setiawaty
EPI_ISL_1622394, EPI_ISL_1622401, EPI_ISL_1622402	RSU Proklamasi	Eijkman Institute for Molecular Biology, Ministry of Research and Technology/National Agency for Research and Innovation	Amin Soebandrio; Edison Johar; Frilasita A Yudhaputri; Hidayat Trimarsanto; Iskandar Adnan; Khin Saw Myint; Lydia V. Panggalo; Muhammad Rezki Rasyak; Safarina G Malik; Sukma Oktavianthi; Willy Agustine
EPI_ISL_833392	RSU Sayang Bunda, Makasar,Indonesia	Biosafety Level-3 Laboratory, Indonesian Institute of Sciences (LIPI)	Ade Andriani; Ahmad Fathoni; Andri Wardiana; Anggia Prasetyoputri; Anik Budhi Dharmayanthi; Dini Nurdiani; Isa Nuryana; Ratih Asmana Ningrum; Syam Budi Iryanto; Wahyuni
EPI_ISL_4741903	RSU Siaga Medika Pemalang	Balai Laboratorium Kesehatan Dan Pengujian Alat Kesehatan Provinsi Jawa Tengah	Alia Wijayanti; Dwi Nursetyaasih; Dyah Ayu; Endah Wahyu Y; Ismi Farhati; Purnomo; Sekti Handayani; Wiwik Peni; Yekti Hediningsih
EPI_ISL_3216555, EPI_ISL_3216556, EPI_ISL_3691574	RSU Siloam, Bekasi	National Institute of Health Research and Development	Arie Ardiansyah Nugraha; Fajar Nur Sulistiyohadi; Hana Apsari Pawestri; Hartanti Dian Ikawati; Kartika Dewi Puspa; Krisna Pangesti; Nelly Puspandari; Subangkit; Triyani Soekarso; Vivi Setiawaty
EPI_ISL_2854776	RSU St Carolus	National Institute of Health Research and Development	Arie Ardiansyah Nugraha; Hana Apsari Pawestri; Hartanti Dian Ikawati; Kartika Dewi Puspa; Krisna Pangesti; Nelly Puspandari; Subangkit; Triyani Soekarso; Vivi Setiawaty
EPI_ISL_888995, EPI_ISL_889002, EPI_ISL_1284004, EPI_ISL_1284090, EPI_ISL_1284093, EPI_ISL_1284096, EPI_ISL_1284174, EPI_ISL_1284344			
see above	RSU Sumber Waras	Eijkman Institute for Molecular Biology, Ministry of Research and Technology/National Agency for Research and Innovation	Amin Soebandrio; Edison Johar; Frilasita A Yudhaputri; Hidayat Trimarsanto; Iskandar Adnan; Khin Saw Myint; Lydia V. Panggalo; Safarina G Malik; Sukma Oktavianthi; Willy Agustine
EPI_ISL_2932713	RSU dr Abdul Radjak	National Institute of Health Research and Development	Arie Ardiansyah Nugraha; Hana Apsari Pawestri; Hartanti Dian Ikawati; Kartika Dewi Puspa; Krisna Pangesti; Nelly Puspandari; Subangkit; Triyani Soekarso; Vivi Setiawaty
EPI_ISL_4783428, EPI_ISL_4783469, EPI_ISL_4783478, EPI_ISL_4783499, EPI_ISL_4783523, EPI_ISL_4783608, EPI_ISL_4783611, EPI_ISL_4783654, EPI_ISL_4783668			
see above	RSUD ARIFIN ACHMAD PROVINSI RIAU	Genomik Solidaritas Indonesia Laboratorium	Annisa Muthiah Sukirman; Anuraj Shankar; Ariel Pradipta; Carissa Sintca Wijaya; Dewi Anggraini; Esti Rahayuning Tias; Fajri Marindra Siregar; Gracia Felias Enos Korompis; Himawan Masyhuri; Louisa Markus; Meutia Ayuputeri Kumaheri; Rahmat Azhari Kemal; Stefanus Bernard
EPI_ISL_2233089	RSUD Abdul Moeis	National Institute of Health Research and Development	Arie Ardiansyah Nugraha; Hana Apsari Pawestri; Hartanti Dian Ikawati; Kartika Dewi Puspa; Krisna Pangesti; Nelly Puspandari; Subangkit; Triyani Soekarso; Vivi Setiawaty
EPI_ISL_5446154, EPI_ISL_5446155, EPI_ISL_5446157, EPI_ISL_5446160, EPI_ISL_5446161, EPI_ISL_5446162, EPI_ISL_5446164, EPI_ISL_5446166, EPI_ISL_5446168, EPI_ISL_5446169, EPI_ISL_5446170, EPI_ISL_5446171			
see above	RSUD Al Ihsan	Laboratorium Genetik Molekuler, Biomedik Lanjut, FK UNPAD	Chrysanti Murad; Dewi Rinakanti; Dwi Agustian; Fensi Amalia; Jessi Annisa; Lidya Chaidir; Nurul Setia Rahayu; Rima Destya Triatin; Savira Ekawardhani; Tri Hanggono Ahmad; Yeni Rendieni; Yunia Sribudiani
EPI_ISL_5796026, EPI_ISL_5796027, EPI_ISL_5796028, EPI_ISL_5796029, EPI_ISL_5796030, EPI_ISL_5796031, EPI_ISL_5796032, EPI_ISL_5796033, EPI_ISL_5796034, EPI_ISL_5796082, EPI_ISL_5796086, EPI_ISL_5796088			
see above	RSUD Anugerah Tomohon	National Institute of Health Research and Development	Arie Ardiansyah Nugraha; Fajar Nur; Fajar Nur Sulistiyohadi; Hana Apsari Pawestri; Hartanti Dian Ikawati; Kartika Dewi Puspa; Krisna Pangesti; Nelly Puspandari; Subangkit; Sulistiyohadi; Triyani Soekarso; Vivi Setiawaty
EPI_ISL_3138937, EPI_ISL_3138938, EPI_ISL_3138939, EPI_ISL_3138941, EPI_ISL_3216532, EPI_ISL_3216533, EPI_ISL_3216534, EPI_ISL_3216535, EPI_ISL_3216536, EPI_ISL_3216537, EPI_ISL_3216538, EPI_ISL_3216539, EPI_ISL_3216540, EPI_ISL_3541192, EPI_ISL_3541193, EPI_ISL_3541194, EPI_ISL_3541195, EPI_ISL_3541196, EPI_ISL_3541197, EPI_ISL_3541198, EPI_ISL_3541199, EPI_ISL_3541200, EPI_ISL_3541201, EPI_ISL_3541202, EPI_ISL_3541203, EPI_ISL_3541204, EPI_ISL_3541205, EPI_ISL_3541206, EPI_ISL_3541207, EPI_ISL_3541208, EPI_ISL_3541209, EPI_ISL_3541210, EPI_ISL_3541211, EPI_ISL_3541212, EPI_ISL_3541213, EPI_ISL_3541214, EPI_ISL_3541215, EPI_ISL_3541216, EPI_ISL_3541217, EPI_ISL_3541218, EPI_ISL_3541219, EPI_ISL_3541220, EPI_ISL_3541221, EPI_ISL_3691572, EPI_ISL_3691585, EPI_ISL_3691586			
see above	RSUD Arifin Achmad	National Institute of Health Research and Development	Arie Ardiansyah Nugraha; Fajar Nur Sulistiyohadi; Fajri Marindra; Hana Apsari Pawestri; Hartanti Dian Ikawati; Kartika Dewi Puspa; Krisna Pangesti; Nelly Puspandari; Subangkit; Triyani Soekarso; Vivi Setiawaty
EPI_ISL_2233106, EPI_ISL_2233107, EPI_ISL_2233108, EPI_ISL_2233109, EPI_ISL_2258213, EPI_ISL_2258218	RSUD Arifin Ahmad Riau	National Institute of Health Research and Development	Arie Ardiansyah Nugraha; Fajri Marinda; Hana Apsari Pawestri; Hartanti Dian Ikawati; Kartika Dewi Puspa; Krisna Pangesti; Nelly Puspandari; Subangkit; Triyani Soekarso; Vivi Setiawaty
EPI_ISL_1824616, EPI_ISL_1824617, EPI_ISL_1824618	RSUD Bahteramas Kendari	National Institute of Health Research and Development	Arie Ardiansyah Nugraha; Hana Apsari Pawestri; Hartanti Dian Ikawati; Kartika Dewi Puspa; Krisna Pangesti; Nelly Puspandari; Subangkit; Vivi Setiawaty
EPI_ISL_2047532, EPI_ISL_2106979, EPI_ISL_2617543, EPI_ISL_2617544, EPI_ISL_2617545, EPI_ISL_2617546, EPI_ISL_2617547, EPI_ISL_2617548, EPI_ISL_2617549, EPI_ISL_2854763, EPI_ISL_2854764, EPI_ISL_2854765, EPI_ISL_2854766, EPI_ISL_2854767, EPI_ISL_2854768, EPI_ISL_2854769, EPI_ISL_2896200			
see above	RSUD Bali Mandara	National Institute of Health Research and Development	Arie Ardiansyah Nugraha; Hana Apsari Pawestri; Hartanti Dian Ikawati; I Wayan Agus Gede Manik Saputra; Kartika Dewi Puspa; Krisna Pangesti; Nelly Puspandari; Subangkit; Triyani Soekarso; Vivi Setiawaty
EPI_ISL_538503	RSUD Bali Mandara Denpasar Bali	National Institute of Health Research and Development	AA; HA; HD; Ikawati; KD; KNA; Nugraha; Paisal; Pangesti; Pawestri; Puspa; Setiawaty, V.; Soekarso; Subangkit; T
EPI_ISL_458081, EPI_ISL_529964	RSUD Bangil Pasuruan	Institute of Tropical Disease, Universitas Airlangga	Aldise M Nastri; Arma Roosalina; Gatot Soegiarto; Jezzy R Dewantari; Kazufumi Shimizu; Krisnoadi Rahardjo; Laksmi Wulandari; Maria I Lusida; Mitsuhiro Nishimura; Resti Yudhawati; Retno A Setyoningrum; Rima R Prasetya; Soetjipto; Yasuko Mori; Yohko K Shimizu
EPI_ISL_2600952, EPI_ISL_2600982, EPI_ISL_2601030, EPI_ISL_2601031, EPI_ISL_2601032	RSUD Banyumas	Genetics Working Group (Pokja Genetik) Faculty of Medicine, Public Health and Nursing Universitas Gadjah Mada (FK-KMK UGM); Disease Investigation Center Wates Ministry of Agriculture Indonesia; Department of Microbiology FK-KMK UGM; Laboratorium Diagnostik Yayasan Tahija World Mosquito Program (WMP) Yogyakarta Center for Tropical Medicine FK-KMK UGM; Integrated Research Center FK-KMK UGM; Department of Computer Science and Electronics FMIPA UGM; RSUP Dr. Sardjito	Afiahayati; Alvina A Setiawan; Cita S Amalia; Dwi AA Nugrahaningsih; Dwi Indaryati; Dyah A Puspitarani; Edwin W. Daniwijaya; Eggi Arguni; Endah Supriyati; Gunadi; Hana F Hanifin; Hendra Wibawa; Indarto Sulistiyono; Irene Tania; Khanza a Vujira; Kristy Iskandar; Ludhang P. Rizki; Marcellus; Mohamad S. Hakim; Nungki Anggorowati; Pramesti G Dewi; Siswanto; Susan Simanjaya; Titik Nuryastuti; Tri Wibawa
EPI_ISL_791980	RSUD Blambangan Banyuwangi	National Institute of Health Research and Development	AA; HA; HD; Ikawati; KD; KNA; N; Noor; Nugraha; Pangesti; Pawestri; Puspa; Puspandari; RI; Setiawaty; Soekarso; Subangkit; T; V
EPI_ISL_2262294, EPI_ISL_3216562, EPI_ISL_3216563, EPI_ISL_3216564, EPI_ISL_3216565, EPI_ISL_3216566, EPI_ISL_3216567, EPI_ISL_3216568, EPI_ISL_3216569, EPI_ISL_3216570, EPI_ISL_3691576			
see above	RSUD Brebes	National Institute of Health Research and Development	Arie Ardiansyah Nugraha; Fajar Nur Sulistiyohadi; Hana Apsari Pawestri; Hartanti Dian Ikawati; Kartika Dewi Puspa; Krisna Pangesti; Nelly Puspandari; Subangkit; Triyani Soekarso; Vivi Setiawaty
EPI_ISL_576383	RSUD Budi Rahayu Kota Magelang	Genetics Working Group (Pokja Genetik) Faculty of Medicine, Public Health and Nursing Universitas Gadjah Mada (FK-KMK UGM); Disease Investigation Center Wates Ministry of Agriculture Indonesia; Department of Microbiology FK-KMK UGM; Laboratorium Diagnostik Yayasan Tahija World Mosquito Program (WMP) Yogyakarta Center for Tropical Medicine FK-KMK UGM; Integrated Research Center FK-KMK UGM; Department of Computer Science and Electronics FMIPA UGM; Balai Besar Teknik Kesehatan Lingkungan dan Pengendalian Penyakit (BBTKLPP) Yogyakarta	Aditya Rifqi Fauzi; Afiahayati; Ari Meliyanti; Audric Kenny Tedja; Dwi AA Nugrahaningsih; Edwin W. Daniwijaya; Eggi Arguni; Endah Supriyati; Gunadi; Havid Setyawan; Hendra Wibawa; Indaryati; Irene; Kristy Iskandar; Ludhang P. Rizki; Marcellus; Merliana Sari Situmeang; Mohamad S. Hakim; Nungki Anggorowati; Siswanto; Titik Nuryastuti; Tri Wibawa
EPI_ISL_759703	RSUD Bung Karno	Universitas Sebelas Maret (UNS), Rumah sakit Universitas Sebelas Maret (RS-UNS) Surakarta, National Institute of Health Research and Development, Indonesian Ministry of Health	Afif Avicena Gufron; Arie Ardiansyah Nugraha; Betty Suryawati; Dewi Puspa; Hana Apsari Pawestri; Hartanti Dian Ika; Kartika; Vivi Setiawaty; Yulia sari; hartono; maryani
EPI_ISL_759705	RSUD Bung Karno, Surakarta	Universitas Sebelas Maret (UNS); Rumah Sakit Universitas Sebelas Maret (RS-UNS), Surakarta; National Institute of Health Research and Development, Indonesian Ministry of Health , Jakarta).	Afif Avicena Gufron; Arie Ardiansyah Nugraha; Betty Suryawati; Dewi Puspa; Hana Apsari Pawestri; Hartanti Dian Ika; Hartono; Kartika; Maryani; Vivi Setiawaty.; Yulia Sari
EPI_ISL_3216520	RSUD CEMPAKA PUTIH / DINAS KESEHATAN DKI JAKARTA	Genomik Solidaritas Indonesia Laboratorium	Alfin Mohammad Abdillah; Anna Christina Brazia; Annisa Muthiah Sukirman; Anuraj Shankar; Ariel Pradipta; Carissa Sintca Wijaya; Dhahlia Agustina Cahyono; Gracia Felias Enos Korompis; Himawan Masyhuri; Meutia Ayuputeri Kumaheri; Neng Hilma Hamidah; Normastuti Adhini Tantyo; Reinhart Gabriel; Vania Gavrila Wikasa
EPI_ISL_2335090, EPI_ISL_2335146, EPI_ISL_2335456, EPI_ISL_2335872, EPI_ISL_2336149	RSUD CILACAP	Genetics Working Group (Pokja Genetik) Faculty of Medicine, Public Health and Nursing Universitas Gadjah Mada (FK-KMK UGM); Disease Investigation Center Wates Ministry of Agriculture Indonesia; Department of Microbiology FK-KMK UGM; Laboratorium Diagnostik Yayasan Tahija World Mosquito Program (WMP) Yogyakarta Center for Tropical Medicine FK-KMK UGM; Integrated Research Center FK-KMK UGM; Department of Computer Science and Electronics FMIPA UGM; RSUP Dr. Sardjito	Afiahayati; Alvina A Setiawan; Cita S Amalia; Dwi AA Nugrahaningsih; Dwi Indaryati; Dyah A Puspitarani; Edwin W. Daniwijaya; Eggi Arguni; Endah Supriyati; Gunadi; Hana F Hanifin; Hendra Wibawa; I Putu Aditio A; Indarto Sulistiyono; Irene Tania; Kristy Iskandar; Ludhang P. Rizki; Marcellus; Maria P Inggriani; Mohamad S. Hakim; Nungki Anggorowati; Pramesti G Dewi; Siswanto; Susan Simanjaya; Titik Nuryastuti; Tri Wibawa; Untung Riawan
EPI_ISL_2854745, EPI_ISL_2854746,	RSUD Ciawi	National Institute of Health Research and Development	; Arie Ardiansyah Nugraha; Hana Apsari Pawestri; Hartanti Dian Ikawati; Kartika Dewi Puspa; Krisna Pangesti; Nelly Puspandari; Subangkit; Triyani Soekarso; Vivi Setiawaty

EPI_ISL_2854747			
EPI_ISL_2333654, EPI_ISL_2333810, EPI_ISL_2334058, EPI_ISL_2334094, EPI_ISL_2334474, EPI_ISL_2334587, EPI_ISL_2335021, EPI_ISL_2600582, EPI_ISL_2600951, EPI_ISL_2601033			
see above	RSUD Cilacap	Genetics Working Group (Pokja Genetik) Faculty of Medicine, Public Health and Nursing Universitas Gadjah Mada (FK-KMK UGM); Disease Investigation Center Wates Ministry of Agriculture Indonesia; Department of Microbiology FK-KMK UGM; Laboratorium Diagnostik Yayasan Tahija World Mosquito Program (WMP) Yogyakarta Center for Tropical Medicine FK-KMK UGM; Integrated Research Center FK-KMK UGM; Department of Computer Science and Electronics FMIPA UGM; RSUP Dr. Sardjito	Afiahayati; Alvina A Setiawan; Cita S Amalia; Dwi AA Nugrahaningsih; Dwi Indaryati; Dyah A Puspitarani; Edwin W. Daniwijaya; Eggi Arguni; Endah Supriyati; Gunadi; Hana F Hanifin; Hendra Wibawa; I Putu Aditio A; Indarto Sulistiyono; Irene Tania; Kristy Iskandar; Ludhang P. Rizki; Marcellus; Maria P Inggriani; Mohamad S. Hakim; Nungki Anggorowati; Pramesti G Dewi; Siswanto; Susan Simanjaya; Titik Nuryastuti; Tri Wibawa; Untung Riawan
EPI_ISL_888998, EPI_ISL_889000, EPI_ISL_889007, EPI_ISL_1283938, EPI_ISL_1283988, EPI_ISL_1284014, EPI_ISL_1284061, EPI_ISL_1622366, EPI_ISL_1622395, EPI_ISL_1622396, EPI_ISL_1622405, EPI_ISL_1622406, EPI_ISL_1622407			
see above	RSUD Cileungsi	Eijkman Institute for Molecular Biology, Ministry of Research and Technology/National Agency for Research and Innovation	Amin Soebandrio; Edison Johar; Frilasita A Yudhaputri; Hidayat Trimarsanto; Iskandar Adnan; Khin Saw Myint; Lydia V. Panggalo; Muhammad Rezki Rasyak; Safarina G Malik; Sukma Oktavianthi; Willy Agustine
EPI_ISL_2013046, EPI_ISL_2364580, EPI_ISL_2364585, EPI_ISL_2364594, EPI_ISL_2364611, EPI_ISL_2364612, EPI_ISL_2364613, EPI_ISL_2364644			
see above	RSUD Cileungsi	Eijkman Institute for Molecular Biology, National Agency for Research and Innovation	Amin Soebandrio; Edison Johar; Frilasita A Yudhaputri; Hidayat Trimarsanto; Iskandar Adnan; Khin Saw Myint; Lidwina Priliani; Lydia V. Panggalo; Muhammad Rezki Rasyak; Safarina G Malik; Sukma Oktavianthi; Willy Agustine
EPI_ISL_2709400	RSUD Cileungsi	Eijkman Institute for Molecular Biology, National Research and Innovation Agency	Amin Soebandrio; Edison Johar; Frilasita A Yudhaputri; Hidayat Trimarsanto; Iskandar Adnan; Khin Saw Myint; Lidwina Priliani; Lydia V. Panggalo; Muhammad Rezki Rasyak; Safarina G Malik; Sukma Oktavianthi; Willy Agustine
EPI_ISL_791979	RSUD Curup Bengkulu	National Institute of Health Research and Development	AA; HA; HD; Ikawati; KD; KNA; Mardiani; N; Nugraha; Pangesti; Pawestri; Puspa; Puspandari; Setiawaty; Soekarso; Subangkit; T; V
EPI_ISL_2854690, EPI_ISL_2854691, EPI_ISL_2854692	RSUD DR Moewardi Surakarta	National Institute of Health Research and Development	Arie Ardiansyah Nugraha; Hana Apsari Pawestri; Hartanti Dian Ikawati; Kartika Dewi Puspa; Krisna Pangesti; Nelly Puspandari; Subangkit; Triyani Soekarso; Vivi Setiawaty
EPI_ISL_2964684, EPI_ISL_2964688, EPI_ISL_2964692, EPI_ISL_2964941, EPI_ISL_2964942, EPI_ISL_2964943, EPI_ISL_2964944, EPI_ISL_2964956, EPI_ISL_2964957			
see above	RSUD DR. MOEWARDI	Genetics Working Group (Pokja Genetik) Faculty of Medicine, Public Health and Nursing Universitas Gadjah Mada (FK-KMK UGM); Disease Investigation Center Wates Ministry of Agriculture Indonesia; Department of Microbiology FK-KMK UGM; Laboratorium Diagnostik Yayasan Tahija World Mosquito Program (WMP) Yogyakarta Center for Tropical Medicine FK-KMK UGM; Integrated Research Center FK-KMK UGM; Department of Computer Science and Electronics FMIPA UGM	Afiahayati; Alvina A Setiawan; Cita S Amalia; Dwi AA Nugrahaningsih; Dwi Indaryati; Dyah A Puspitarani; Edwin W. Daniwijaya; Eggi Arguni; Endah Supriyati; Gunadi; Hana F Hanifin; Hendra Wibawa; I Putu Aditio A; Indarto Sulistiyono; Irene Tania; Kristy Iskandar; Ludhang P. Rizki; Marcellus; Mohamad S. Hakim; Nungki Anggorowati; Pramesti G Dewi; Siswanto; Susan Simanjaya; Titik Nuryastuti; Tri Wibawa
EPI_ISL_2964677	RSUD DR. MOEWARDI Surakarta	Genetics Working Group (Pokja Genetik) Faculty of Medicine, Public Health and Nursing Universitas Gadjah Mada (FK-KMK UGM); Disease Investigation Center Wates Ministry of Agriculture Indonesia; Department of Microbiology FK-KMK UGM; Laboratorium Diagnostik Yayasan Tahija World Mosquito Program (WMP) Yogyakarta Center for Tropical Medicine FK-KMK UGM; Integrated Research Center FK-KMK UGM; Department of Computer Science and Electronics FMIPA UGM; RSUP Dr. Sardjito	Afiahayati; Alvina A Setiawan; Dwi AA Nugrahaningsih; Dwi Indaryati; Edwin W. Daniwijaya; Eggi Arguni; Endah Supriyati; Gunadi; Hendra Wibawa; Indarto Sulistiyono; Khanza a Vujira; Kristy Iskandar; Ludhang P. Rizki; Marcellus; Mohamad S. Hakim; Nungki Anggorowati; Pramesti G Dewi; Siswanto; Titik Nuryastuti; Tri Wibawa
EPI_ISL_2854737, EPI_ISL_2854738, EPI_ISL_2854739	RSUD Dr Doris Sylvanus Kalteng	National Institute of Health Research and Development	; Arie Ardiansyah Nugraha; Hana Apsari Pawestri; Hartanti Dian Ikawati; Kartika Dewi Puspa; Krisna Pangesti; Nelly Puspandari; Subangkit; Triyani Soekarso; Vivi Setiawaty
EPI_ISL_833040	RSUD Dr. Abdul Aziz Singkawang	National Institute of Health Research and Development	AA; HA; HD; HN; Ikawati; Junus; KD; KNA; N; Nugraha; Pangesti; Pawestri; Puspa; Puspandari; Setiawaty; Soekarso; Subangkit; T; V
EPI_ISL_759967	RSUD Dr. Iskak Tulungagung	Institute of Tropical Disease, Universitas Airlangga	Aldise M Nastri; Gatot Soegiarto; Jezzy R Dewantari; Kazufumi Shimizu; Krisnoadi Rahardjo; Laksmi Wulandari; Maria I Lusida; Resti Yudhawati; Rima R Prasetya; Soetjipto; Supriyanto Dharmoredjo; Yasuko Mori
EPI_ISL_4741899, EPI_ISL_4756064	RSUD Dr. M. Ashari Kabupaten Pemalang	Balai Laboratorium Kesehatan Dan Pengujian Alat Kesehatan Provinsi Jawa Tengah	Alia Wijayanti; Dwi Nursetyaasih; Dyah Ayu; Endah Wahyu Y; Ismi Farhati; Purnomo; Sekti Handayani; Wiwik Peni; Yekti Hediningsih
EPI_ISL_956308, EPI_ISL_956310	RSUD Dr. M. Soewandhie	Institute of Tropical Disease, Universitas Airlangga	Aldise M Nastri; Febria Rachmanita; Gatot Soegiarto; Jezzy R Dewantari; Kazufumi Shimizu; Krisnoadi Rahardjo; Laksmi Wulandari; Maria I Lusida; Resti Yudhawati; Rima R Prasetya; Soetjipto; Yasuko Mori
EPI_ISL_4254872, EPI_ISL_4254873, EPI_ISL_4254874, EPI_ISL_4254875, EPI_ISL_4254876, EPI_ISL_4254877, EPI_ISL_4254878, EPI_ISL_4254879, EPI_ISL_4254880			
see above	RSUD Dr. Saiful Anwar Jawa Timur	National Institute of Health Research and Development	Arie Ardiansyah Nugraha; Fajar Nur Sulistiyohadi; Hana Apsari Pawestri; Hartanti Dian Ikawati; Kartika Dewi Puspa; Krisna Pangesti; Nelly Puspandari; Subangkit; Triyani Soekarso; Vivi Setiawaty
EPI_ISL_4004659, EPI_ISL_4004660, EPI_ISL_4004661, EPI_ISL_4004662, EPI_ISL_4004663, EPI_ISL_4004664, EPI_ISL_4004665, EPI_ISL_4004666, EPI_ISL_4004667, EPI_ISL_4004668, EPI_ISL_4004669, EPI_ISL_4004670, EPI_ISL_4004671, EPI_ISL_4106117, EPI_ISL_4106118			
see above	RSUD Dr. Slamet Garut	National Research and Innovation Agency (BRIN-Indonesia, VenomCoV Project Team); West Java Health Laboratory	Anik B. Dharmayanthi; Azzania Fibriani; Cut Nur Cinthia Alamanda; Ema Rahmawati; Indriawati; Karimatu Khoirunnisa; Listiana Oktavia; Masrukhin; Miftahul Faridl; Ratih A. Ningrum; Rifky Waluyajati Rachman; Rini Robiani; Ryan Bayusantika Ristandi; Sugiyono Saputra; Syaiful Rizal; Syam B. Iryanto
EPI_ISL_1005695	RSUD Dr. Soedirman	Genetics Working Group (Pokja Genetik) Faculty of Medicine, Public Health and Nursing Universitas Gadjah Mada (FK-KMK UGM); Disease Investigation Center Wates Ministry of Agriculture Indonesia; Department of Microbiology FK-KMK UGM; Laboratorium Diagnostik Yayasan Tahija World Mosquito Program (WMP) Yogyakarta Center for Tropical Medicine FK-KMK UGM; Integrated Research Center FK-KMK UGM; Department of Computer Science and Electronics FMIPA UGM; Balai Besar Teknik Kesehatan Lingkungan dan Pengendalian Penyakit (BBTKLPP) Yogyakarta	Afiahayati; Arry Setyowati; Audric Kenny Tedja; Chusni Mubarakh; Dwi AA Nugrahaningsih; Edwin W. Daniwijaya; Eggi Arguni; Endah Supriyati; Gunadi; Havid Setyawan; Hendra Wibawa; Indaryati; Irene; Kristy Iskandar; Ludhang P. Rizki; Marcellus; Maria Patricia Inggriani; Mohamad S. Hakim; Nungki Anggorowati; Siswanto; Titik Nuryastuti; Tri Wibawa; Try Nirmala Sari
EPI_ISL_437188, EPI_ISL_529965, EPI_ISL_560991, EPI_ISL_759962, EPI_ISL_759965, EPI_ISL_2226647, EPI_ISL_2274437, EPI_ISL_2284869, EPI_ISL_2284870, EPI_ISL_2284871, EPI_ISL_2284872, EPI_ISL_2284884			
see above	RSUD Dr. Soetomo	Institute of Tropical Disease, Universitas Airlangga	Acub Zaenal; Aldise M Nastri; Gatot Soegiarto; Jezzy R Dewantari; Joni Wahyuhadi; Kazufumi Shimizu; Krisnoadi Rahardjo; Laksmi Wulandari; Maria I Lusida; Mitsuhiro Nishimura; Neneng D Kurniati; Resti Y Meliana; Resti Yudhawati; Retno A Setyoningrum; Rima R Prasetya; Soetjipto; Yasuko Mori; Yohko K Shimizu
EPI_ISL_610161, EPI_ISL_610162, EPI_ISL_985396, EPI_ISL_985397, EPI_ISL_1005696, EPI_ISL_1005697, EPI_ISL_1005698			
see above	RSUD Dr. Tjitrowardojo	Genetics Working Group (Pokja Genetik) Faculty of Medicine, Public Health and Nursing Universitas Gadjah Mada (FK-KMK UGM); Disease Investigation Center Wates Ministry of Agriculture Indonesia; Department of Microbiology FK-KMK UGM; Laboratorium Diagnostik Yayasan Tahija World Mosquito Program (WMP) Yogyakarta Center for Tropical Medicine FK-KMK UGM; Integrated Research Center FK-KMK UGM; Department of Computer Science and Electronics FMIPA UGM; Balai Besar Teknik Kesehatan Lingkungan dan Pengendalian Penyakit (BBTKLPP) Yogyakarta	Aditya Rifqi Fauzi; Afiahayati; Alvin Santoso Kalim; Arry Setyowati; Chusni Mubarakh; Desyifa Mursalin; Dwi AA Nugrahaningsih; Dwiki afandy; Dyah Ayu Puspitarani; Edwin W. Daniwijaya; Eggi Arguni; Endah Supriyati; Fadil Fahri; Gunadi; Havid Setyawan; Hendra Wibawa; Indaryati; Irene; Kemala Athollah; Kristy Iskandar; Ludhang P. Rizki; Marcellus; Mohamad S. Hakim; Nungki Anggorowati; Siswanto; Susan Simanjaya; Titik Nuryastuti; Tri Wibawa; Try Nirmala Sari; Untung Riawan; William Widitjiarso; Wuryanto
EPI_ISL_2854750, EPI_ISL_2854751, EPI_ISL_2854752, EPI_ISL_2932710, EPI_ISL_2987658, EPI_ISL_2987659, EPI_ISL_2987660, EPI_ISL_2987661, EPI_ISL_3019945, EPI_ISL_3151601, EPI_ISL_3151605, EPI_ISL_3151606, EPI_ISL_3151612, EPI_ISL_3151613, EPI_ISL_3151619, EPI_ISL_3151620, EPI_ISL_3151626, EPI_ISL_3151627, EPI_ISL_3151631, EPI_ISL_3151632, EPI_ISL_3151633, EPI_ISL_3151639, EPI_ISL_3151640, EPI_ISL_3151646, EPI_ISL_3151651, EPI_ISL_3151652, EPI_ISL_3151665, EPI_ISL_3151666, EPI_ISL_3151672, EPI_ISL_3151673, EPI_ISL_3278296			
see above	RSUD Dr. W.Z. Johanes kupang	National Institute of Health Research and Development	Arie Ardiansyah Nugraha; Fajar Nur Sulistiyohadi; Hana Apsari Pawestri; Hartanti Dian Ikawati; Kartika Dewi Puspa; Krisna Pangesti; Nelly Puspandari; Subangkit; Triyani Soekarso; Vivi Setiawaty
EPI_ISL_4056015	RSUD Dr. Wahidin Sudirohusodo	Institute of Tropical Disease, Universitas Airlangga	Aldise M Nastri; Gatot Soegiarto; Jezzy R Dewantari; Kazufumi Shimizu; Kohar Hari Santoso; Krisnoadi Rahardjo; Laksmi Wulandari; Maria I Lusida; Resti Yudhawati; Rima R Prasetya; Soetjipto; Yasuko Mori
EPI_ISL_3230176, EPI_ISL_3230177, EPI_ISL_3374702, EPI_ISL_3374703, EPI_ISL_3548633, EPI_ISL_3548688, EPI_ISL_3836263, EPI_ISL_3836264, EPI_ISL_3946308, EPI_ISL_4030489, EPI_ISL_4475419, EPI_ISL_4475420			
see above	RSUD KEBAYORAN BARU	Genomik Solidaritas Indonesia Laboratorium	Anna Christina Brazia; Annisa Muthiah Sukirman; Anuraj Shankar; Ariel Pradipta; Carissa Sintca Wijaya; David Hendra Haryanto; Dewi Isnawati Q.I; Dhahlia Agustina Cahyono; Dwi Oktavia; Esti Rahayuning Tias; Gracia Felias Enos Korompis; Heru; Himawan Masyhuri; Juniar Dwi Jayanti; Louisa Markus; Melisa; Melly Ariyanti; Meutia Ayuputeri Kumaheri; Neng Hilma Hamidah; Ngabila Salama; Ollien Kartika; Pratiwi Srikandi Yudhastari; Reinhart Gabriel; Stefanus; Stefanus Bernard; Tiranti Vindha; Vania Gavrila Wikasa
EPI_ISL_2790721, EPI_ISL_2790722, EPI_ISL_2790723, EPI_ISL_2790730, EPI_ISL_3418696	RSUD KEBAYORAN BARU / DINAS KESEHATAN DKI JAKARTA	Genomik Solidaritas Indonesia Laboratorium	Alfin Mohammad Abdillah; Anna Christina Brazia; Annisa Muthiah Sukirman; Anuraj Shankar; Ariel Pradipta; Ariel Pradipta Submitter information; Carissa Sintca Wijaya; Dhahlia Agustina Cahyono; Dwi Oktavia; Gracia Felias Enos Korompis; Meutia Ayuputeri Kumaheri; Ngabila Salama; Normastuti Adhini Tantyo; Reinhart Gabriel; Tiranti Vindha; Vania Gavrila Wikasa; Widyastuti
EPI_ISL_3208082, EPI_ISL_3208083	RSUD KOJA / DINAS KESEHATAN DKI JAKARTA	Genomik Solidaritas Indonesia Laboratorium	Annisa Muthiah Sukirman; Anuraj Shankar; Ariel Pradipta; Carissa Sintca Wijaya; Gracia Felias Enos Korompis; Meutia Ayuputeri Kumaheri; Reinhart Gabriel; Tryna Tania; Vania Gavrila Wikasa
EPI_ISL_2987630, EPI_ISL_2987631, EPI_ISL_2987632, EPI_ISL_2987633, EPI_ISL_3216587, EPI_ISL_3216588, EPI_ISL_3216589, EPI_ISL_3216590, EPI_ISL_3216591, EPI_ISL_3216592, EPI_ISL_3216593, EPI_ISL_3216594, EPI_ISL_3216595			
see above	RSUD KRMT Wongsonegoro	National Institute of Health Research and Development	Arie Ardiansyah Nugraha; Fajar Nur Sulistiyohadi; Hana Apsari Pawestri; Hartanti Dian Ikawati; Kartika Dewi Puspa; Krisna Pangesti; Nelly Puspandari; Subangkit; Triyani Soekarso; Vivi Setiawaty
EPI_ISL_4969091, EPI_ISL_4969096, EPI_ISL_4969115, EPI_ISL_4969119	RSUD KRT. Setjonegoro Wonosobo	Genetics Working Group (Pokja Genetik) Faculty of Medicine, Public Health and Nursing Universitas Gadjah Mada (FK-KMK UGM); Disease Investigation Center Wates Ministry of Agriculture Indonesia; Department of Microbiology FK-KMK UGM; Laboratorium Diagnostik Yayasan Tahija World Mosquito Program (WMP) Yogyakarta Center for Tropical Medicine FK-KMK UGM; Integrated Research Center FK-KMK UGM; Department of Computer Science and Electronics FMIPA UGM; RSUP Dr. Sardjito	. Marcellus; Afiahayati; Alvina A Setiawan; Bambang Sigit Riyanto; Dwi AA Nugrahaningsih; Dwi Indaryati; Dyah A Puspitarani; Edwin W. Daniwijaya; Eggi Arguni; Eko Budiono; Endah Supriyati; Gunadi; Hana F Hanifin; Hendra Wibawa; Heni Retnowulan; Ika Trisnawati; Indarto Sulistiyono; Ira Puspitawati; Irene Tania; Khanza a Vujira; Kristy Iskandar; Ludhang P. Rizki; Marcellus; Mohamad S. Hakim; Munawar Gani; Nungki Anggorowati; Nur Imma Fatimah Harahap; Nur Rahmi Ananda; Osman Sianipar; Pramesti G Dewi; Riat El Khair; Satria Maulana; Siswanto; Sumardi; Susan Simanjaya; Titik Nuryastuti; Tri Wibawa; Umi Solekhah Intansari; Yunika Puspadewi; Elizabeth Henny Herningtiyas
EPI_ISL_2047529	RSUD Kab Buleleng	National Institute of Health Research and Development	Arie Ardiansyah Nugraha; Hana Apsari Pawestri; Hartanti Dian Ikawati; Kartika Dewi Puspa; Krisna Pangesti; Nelly Puspandari; Subangkit; Triyani Soekarso; Vivi Setiawaty
EPI_ISL_2931750, EPI_ISL_2931759, EPI_ISL_2931769, EPI_ISL_2931770, EPI_ISL_2931777, EPI_ISL_2931779, EPI_ISL_2931786, EPI_ISL_2931795, EPI_ISL_2987591, EPI_ISL_5796078, EPI_ISL_5796079			
see above	RSUD Kabupaten Buleleng	National Institute of Health Research and Development	; Arie Ardiansyah Nugraha; Fajar Nur Sulistiyohadi; Hana Apsari Pawestri; Hartanti Dian Ikawati; Kartika Dewi Puspa; Krisna Pangesti; Nelly Puspandari; Subangkit; Triyani Soekarso; Vivi Setiawaty
EPI_ISL_5796076, EPI_ISL_5796077, EPI_ISL_5796085	RSUD Kabupaten Tabanan	National Institute of Health Research and Development	Arie Ardiansyah Nugraha; Fajar Nur Sulistiyohadi; Hana Apsari Pawestri; Hartanti Dian Ikawati; Kartika Dewi Puspa; Krisna Pangesti; Nelly Puspandari; Subangkit; Triyani Soekarso; Vivi Setiawaty
EPI_ISL_5022907, EPI_ISL_5022908, EPI_ISL_5022909, EPI_ISL_5022910	RSUD Kabupaten Teluk Bintuni Papua Barat	National Institute of Health Research and Development	Arie Ardiansyah Nugraha; Fajar Nur Sulistiyohadi; Hana Apsari Pawestri; Hartanti Dian Ikawati; Kartika Dewi Puspa; Krisna Pangesti; Nelly Puspandari; Subangkit; Triyani Soekarso; Vivi Setiawaty

EPI_ISL_2631469, EPI_ISL_2631470, EPI_ISL_2631471, EPI_ISL_2631472, EPI_ISL_2631473, EPI_ISL_2631474, EPI_ISL_2631475, EPI_ISL_2631476, EPI_ISL_2631477			
see above	RSUD Karawang	Indonesian Institute of Sciences (LIPI)/National Agency of Research and Innovation (BRIN) and National Institute of Health Research and Development (NIHRD)	; Andri Wardiana; Anggia Prasetyoputri; Anik B. Dharmayanthi; Eko Wahyu Putro; Fahrurrozi; Gita Syahputra; Herjuno A. Nugroho; Idris; Indriawati; Isa Nuryana; Linda Sukmarini; Listiana Oktavia; Nova D. Yanthi; Puspita Lisdiyanti; Ratih A. Ningrum; Sri Swasthikawati; Subangkit; Sugiyono Saputra; Syam B. Iryanto; Vivi Setiawaty; Vivi Setiawaty Puspita Lisdiyanti; Yayuk Sri Rahayu; Yuliawati
EPI_ISL_2758647	RSUD Kebayoran Baru	Genomik Solidaritas Indonesia Laboratorium	Ahmad Zoebad Foeady; Alfin Mohammad Abdillah; Annisa Muthiah Sukirman; Anuraj Shankar; Ariel Pradipta; Carissa Sintca Wijaya; Dhahlia Agustina Cahyono; Gracia Felias Enos Korompis; Meutia Ayuputeri Kumaheri; Normastuti Adhini Tantyo; Vania Gavrila Wikasa
EPI_ISL_2617536	RSUD Kebayoran Baru	National Institute of Health Research and Development	Arie Ardiansyah Nugraha; Hana Apsari Pawestri; Hartanti Dian Ikawati; Kartika Dewi Puspa; Krisna Pangesti; Nelly Puspandari; Subangkit; Triyani Soekarso; Vivi Setiawaty
EPI_ISL_2101229, EPI_ISL_2101230, EPI_ISL_2101231, EPI_ISL_2101232, EPI_ISL_2101233, EPI_ISL_2101234, EPI_ISL_2101235, EPI_ISL_2101236, EPI_ISL_2101237, EPI_ISL_2101238, EPI_ISL_2101239, EPI_ISL_2101240, EPI_ISL_2101241, EPI_ISL_2101242, EPI_ISL_2101243, EPI_ISL_2101244, EPI_ISL_2101245, EPI_ISL_2101246, EPI_ISL_2101247, EPI_ISL_2101248, EPI_ISL_2101249, EPI_ISL_2101250, EPI_ISL_2101251, EPI_ISL_2101252, EPI_ISL_2101253, EPI_ISL_2101254, EPI_ISL_2101255			
see above	RSUD Klungkung	Eijkman Institute for Molecular Biology, National Agency for Research and Innovation; Molecular Biology Laboratory, Faculty Medicine and Health Sciences, Warmadewa University	Amin Soebandrio; Edison Johar; Erly Sintya; Frilasita A Yudhaputri; Hidayat Trimarsanto; I Komang Parwata; Iskandar Adnan; Khin Saw Myint; Lidwina Priliani; Lydia V. Panggalo; Muhammad Rezki Rasyak; Safarina G Malik; Sri Masyeni; Sukma Oktavianthi; Willy Agustine
EPI_ISL_775598	RSUD Kota Mataram	National Institute of Health Research and Development	AA; HA; HD; Ikawati; KD; KNA; N; Nugraha; Pangesti; Pawestri; Puspa; Puspandari; SK; Sari; Setiawaty; Soekarso; Subangkit; T; V
EPI_ISL_4090561, EPI_ISL_4090562, EPI_ISL_4090563, EPI_ISL_4090564, EPI_ISL_4090565	RSUD Kota Surakarta	National Institute of Health Research and Development	Arie Ardiansyah Nugraha; Fajar Nur; Fajar Nur Sulistiyohadi; Hana Apsari Pawestri; Hartanti Dian Ikawati; Kartika Dewi Puspa; Krisna Pangesti; Nelly Puspandari; Subangkit; Sulistiyohadi; Triyani Soekarso; Vivi Setiawaty
EPI_ISL_763068	RSUD Kota Surakarta	Universitas Sebelas Maret (UNS); Rumah Sakit UNS (RS-UNS); National Institute of Health Research and Development, Indonesian Ministry of Health.	Afif A Ghufron; Arie A Nugraha; Betty Suryawati.; Dewi Puspa; Hana A Pawestri; Hartanti Dian Ika; Hartono; Kartika; Maryani; Revi G H Novika; Vivi Setiawaty; Yulia Sari
EPI_ISL_2854719	RSUD Kota Tangerang	National Institute of Health Research and Development	; Arie Ardiansyah Nugraha; Hana Apsari Pawestri; Hartanti Dian Ikawati; Kartika Dewi Puspa; Krisna Pangesti; Nelly Puspandari; Subangkit; Triyani Soekarso; Vivi Setiawaty
EPI_ISL_3836267, EPI_ISL_3836268	RSUD MAMPANG PRAPATAN	Genomik Solidaritas Indonesia Laboratorium	Anna Christina Brazia; Annisa Muthiah Sukirman; Anuraj Shankar; Ariel Pradipta; David Hendra Haryanto; Dhahlia Agustina Cahyono; Didiet Adi Permana; Dwi Oktavia; Gracia Felias Enos Korompis; Louisa Markus; Meutia Ayuputeri Kumaheri; Ngabila Salama; Pratiwi Srikandi Yudhastari; Reinhart Gabriel; Tiranti Vindha; Vania Gavrila Wikasa
EPI_ISL_3208077	RSUD MATRAMAN / DINAS KESEHATAN DKI JAKARTA	Genomik Solidaritas Indonesia Laboratorium	Annisa Muthiah Sukirman; Anuraj Shankar; Ariel Pradipta; Carissa Sintca Wijaya; Gracia Felias Enos Korompis; Meutia Ayuputeri Kumaheri; Reinhart Gabriel; Tryna Tania; Vania Gavrila Wikasa
EPI_ISL_3138795	RSUD Mampang	National Institute of Health Research and Development	Arie Ardiansyah Nugraha; Fajar Nur Sulistiyohadi; Hana Apsari Pawestri; Hartanti Dian Ikawati; Kartika Dewi Puspa; Krisna Pangesti; Nelly Puspandari; Subangkit; Triyani Soekarso; Vivi Setiawaty
EPI_ISL_2101256, EPI_ISL_2101257, EPI_ISL_2101258, EPI_ISL_2101259, EPI_ISL_2101260, EPI_ISL_2101261, EPI_ISL_2101262, EPI_ISL_2101263, EPI_ISL_2101264, EPI_ISL_2101265, EPI_ISL_2101266, EPI_ISL_2101267, EPI_ISL_2101268, EPI_ISL_2101269, EPI_ISL_2101270, EPI_ISL_2101271, EPI_ISL_2101272, EPI_ISL_2101273, EPI_ISL_2101274, EPI_ISL_2101275, EPI_ISL_2101276, EPI_ISL_2101277, EPI_ISL_2101278, EPI_ISL_2101279, EPI_ISL_2101280, EPI_ISL_2101281, EPI_ISL_2101282, EPI_ISL_2101283, EPI_ISL_2101284, EPI_ISL_2101285, EPI_ISL_2101286, EPI_ISL_2101287, EPI_ISL_2101288, EPI_ISL_2101290, EPI_ISL_2101291, EPI_ISL_2101292, EPI_ISL_2101293, EPI_ISL_2101294, EPI_ISL_2101295, EPI_ISL_2101296, EPI_ISL_2101297, EPI_ISL_2101298, EPI_ISL_2101299, EPI_ISL_2101300, EPI_ISL_2101301, EPI_ISL_2101302, EPI_ISL_2101303, EPI_ISL_2101304, EPI_ISL_2101305, EPI_ISL_2101306, EPI_ISL_2101307, EPI_ISL_2101308, EPI_ISL_2101309, EPI_ISL_2101310, EPI_ISL_2101311, EPI_ISL_2101312, EPI_ISL_2101313, EPI_ISL_2101314, EPI_ISL_2101315, EPI_ISL_2101316, EPI_ISL_2101317, EPI_ISL_2101318, EPI_ISL_2101319, EPI_ISL_2101320, EPI_ISL_2101321, EPI_ISL_2101322, EPI_ISL_2101323, EPI_ISL_2101324, EPI_ISL_2101325, EPI_ISL_2101326, EPI_ISL_2101327, EPI_ISL_2101328, EPI_ISL_2101329, EPI_ISL_2101330, EPI_ISL_2101331, EPI_ISL_2101332, EPI_ISL_2101333, EPI_ISL_2101334, EPI_ISL_2101335, EPI_ISL_2101336, EPI_ISL_2101337, EPI_ISL_2101338, EPI_ISL_2101339, EPI_ISL_2101340, EPI_ISL_2101341, EPI_ISL_2101342			
see above	RSUD Mangusada	Eijkman Institute for Molecular Biology, National Agency for Research and Innovation; Molecular Biology Laboratory, Faculty Medicine and Health Sciences, Warmadewa University	Amin Soebandrio; Edison Johar; Erly Sintya; Frilasita A Yudhaputri; Hidayat Trimarsanto; Ida Ayu Wayan Mahayani; Iskandar Adnan; Khin Saw Myint; Lidwina Priliani; Lydia V. Panggalo; Muhammad Rezki Rasyak; Safarina G Malik; Sri Masyeni; Sukma Oktavianthi; Willy Agustine
EPI_ISL_3030003, EPI_ISL_3030004, EPI_ISL_3030005, EPI_ISL_3030006, EPI_ISL_3030007, EPI_ISL_3030008, EPI_ISL_3030009, EPI_ISL_3030010, EPI_ISL_3030011, EPI_ISL_3030012, EPI_ISL_3030013, EPI_ISL_3030014, EPI_ISL_3030015, EPI_ISL_3030016, EPI_ISL_3030017, EPI_ISL_3030018, EPI_ISL_3030019, EPI_ISL_3030020, EPI_ISL_3030021, EPI_ISL_3030022, EPI_ISL_3030023, EPI_ISL_3030024, EPI_ISL_3030025, EPI_ISL_3030026, EPI_ISL_3030027, EPI_ISL_3030028, EPI_ISL_3030029, EPI_ISL_3030030, EPI_ISL_3030031, EPI_ISL_3030032, EPI_ISL_3030033, EPI_ISL_3030034, EPI_ISL_3030035, EPI_ISL_3030036, EPI_ISL_3030037, EPI_ISL_3030038, EPI_ISL_3030039			
see above	RSUD Mangusada	Eijkman Institute for Molecular Biology, National Research and Innovation Agency; Faculty Medicine and Health Sciences, Warmadewa University	Amin Soebandrio; Edison Johar; Erly Sintya; Frilasita A Yudhaputri; Hidayat Trimarsanto; Ida Ayu Wayan Mahayani; Iskandar Adnan; Khin Saw Myint; Lidwina Priliani; Lydia V. Panggalo; Muhammad Rezki Rasyak; Safarina G Malik; Sri Masyeni; Sukma Oktavianthi; Willy Agustine
EPI_ISL_3138885	RSUD Mangusada Badung	National Institute of Health Research and Development	Arie Ardiansyah Nugraha; Fajar Nur Sulistiyohadi; Hana Apsari Pawestri; Hartanti Dian Ikawati; Kartika Dewi Puspa; Krisna Pangesti; Nelly Puspandari; Subangkit; Triyani Soekarso; Vivi Setiawaty
EPI_ISL_2617514, EPI_ISL_2617515, EPI_ISL_2620712	RSUD Mataram Prov Nusa Tenggara Barat	National Institute of Health Research and Development	Arie Ardiansyah Nugraha; Hana Apsari Pawestri; Hartanti Dian Ikawati; Kartika Dewi Puspa; Krisna Pangesti; Laily Indrayanti; Nelly Puspandari; Subangkit; Triyani Soekarso; Vivi Setiawaty
EPI_ISL_791986	RSUD Morotai Maluku Utara	National Institute of Health Research and Development	AA; Denggo; HA; HD; Ikawati; KD; KNA; N; Nugraha; Pangesti; Pawestri; Puspa; Puspandari; SD; Setiawaty; Soekarso; Subangkit; T; V
EPI_ISL_2854730, EPI_ISL_4104532, EPI_ISL_4104533, EPI_ISL_4104534, EPI_ISL_4104535, EPI_ISL_4104536, EPI_ISL_4104537			
see above	RSUD Nganjuk	National Institute of Health Research and Development	; Arie Ardiansyah Nugraha; Fajar Nur Sulistiyohadi; Hana Apsari Pawestri; Hartanti Dian Ikawati; Kartika Dewi Puspa; Krisna Pangesti; Nelly Puspandari; Subangkit; Triyani Soekarso; Vivi Setiawaty
EPI_ISL_516829	RSUD Nyi Ageng Serang	Genetics Working Group (Pokja Genetik) Faculty of Medicine, Public Health and Nursing Universitas Gadjah Mada (FK-KMK UGM), Disease Investigation Center Wates Ministry of Agriculture Indonesia, Department of Microbiology FK-KMK UGM, Laboratorium Diagnostik Yayasan Tahija World Mosquito Program (WMP) Yogyakarta Center for Tropical Medicine FK-KMK UGM, Integrated Research Center FK-KMK UGM, Department of Computer Science and Electronics FMIPA UGM	. Marcellus; . Siswanto; Afiahayati; Beby Dewi Sartika; Dwi AA Nugrahaningsih; Dyah Ayu Puspitarani; Edwin W. Daniwijaya; Eggi Arguni; Endah Supriyati; Gunadi; Hendra Wibawa; Ludhang P. Rizki; Mohamad S. Hakim; Titik Nuryastuti; Tri Wibawa
EPI_ISL_4978455, EPI_ISL_4980931, EPI_ISL_4981198, EPI_ISL_4981563, EPI_ISL_5010016, EPI_ISL_5010036, EPI_ISL_5010038, EPI_ISL_5010046, EPI_ISL_5010054, EPI_ISL_5010412			
see above	RSUD Nyi Ageng Serang	Genetics Working Group (Pokja Genetik) Faculty of Medicine, Public Health and Nursing Universitas Gadjah Mada (FK-KMK UGM); Disease Investigation Center Wates Ministry of Agriculture Indonesia; Department of Microbiology FK-KMK UGM; Laboratorium Diagnostik Yayasan Tahija World Mosquito Program (WMP) Yogyakarta Center for Tropical Medicine FK-KMK UGM; Integrated Research Center FK-KMK UGM; Department of Computer Science and Electronics FMIPA UGM; RSUP Dr. Sardjito	. Marcellus; Afiahayati; Alvina A Setiawan; Bambang Sigit Riyanto; Cita S Amalia; Dwi AA Nugrahaningsih; Dwi Indaryati; Dyah A Puspitarani; Edwin W. Daniwijaya; Eggi Arguni; Eko Budiono; Endah Supriyati; Gunadi; Hana F Hanifin; Hendra Wibawa; Heni Retnowulan; I Putu Aditio A; Ika Trisnawati; Indarto Sulistiyono; Ira Puspitawati; Irene Tania; Khanza a Vujira; Kristy Iskandar; Ludhang P. Rizki; Marcellus; Mohamad S. Hakim; Munawar Gani; Nungki Anggorowati; Nur Imma Fatimah Harahap; Nur Rahmi Ananda; Osman Sianipar; Pramesti G Dewi; Riat El Khair; Satria Maulana; Siswanto; Sumardi; Susan Simanjaya; Titik Nuryastuti; Tri Wibawa; Umi Solekhah Intansari; Yunika Puspadewi; Elizabeth Henny Herningtiyas
EPI_ISL_2790731	RSUD PASAR MINGGU / DINAS KESEHATAN DKI JAKARTA	Genomik Solidaritas Indonesia Laboratorium	Alfin Mohammad Abdillah; Anna Christina Brazia; Annisa Muthiah Sukirman; Anuraj Shankar; Ariel Pradipta; Carissa Sintca Wijaya; Dhahlia Agustina Cahyono; Dwi Oktavia; Gracia Felias Enos Korompis; Meutia Ayuputeri Kumaheri; Ngabila Salama; Normastuti Adhini Tantyo; Reinhart Gabriel; Tiranti Vindha; Vania Gavrila Wikasa; Widyastuti
EPI_ISL_3384770, EPI_ISL_3384771, EPI_ISL_3384772, EPI_ISL_3384773, EPI_ISL_3384774, EPI_ISL_3384775, EPI_ISL_3384776, EPI_ISL_3384777, EPI_ISL_3384778, EPI_ISL_3384779, EPI_ISL_3384780, EPI_ISL_3384781, EPI_ISL_3384782, EPI_ISL_3384783, EPI_ISL_3385398, EPI_ISL_3385399, EPI_ISL_3385400, EPI_ISL_3385401, EPI_ISL_3664279			
see above	RSUD PROVINSI NTB	Genomik Solidaritas Indonesia Laboratorium	Annisa Muthiah Sukirman; Anuraj Shankar; Ariel Pradipta; Carissa Sintca Wijaya; Dhahlia Agustina Cahyono; Gracia Felias Enos Korompis; Himawan Masyhuri; Louisa Markus; Meutia Ayuputeri Kumaheri; Vania Gavrila Wikasa
EPI_ISL_2868904	RSUD Pademangan	National Institute of Health Research and Development	Arie Ardiansyah Nugraha; Hana Apsari Pawestri; Hartanti Dian Ikawati; Kartika Dewi Puspa; Krisna Pangesti; Nelly Puspandari; Subangkit; Triyani Soekarso; Vivi Setiawaty
EPI_ISL_2854683, EPI_ISL_2854684, EPI_ISL_2854685, EPI_ISL_2854686, EPI_ISL_2854687, EPI_ISL_2854688, EPI_ISL_2854689, EPI_ISL_2896202, EPI_ISL_2932711, EPI_ISL_3138949			
see above	RSUD Palangkaraya	National Institute of Health Research and Development	Arie Ardiansyah Nugraha; Fajar Nur Sulistiyohadi; Hana Apsari Pawestri; Hartanti Dian Ikawati; Kartika Dewi Puspa; Krisna Pangesti; Nelly Puspandari; Subangkit; Triyani Soekarso; Vivi Setiawaty
EPI_ISL_2954064	RSUD Panembahan Senopati, Bantul	Genetics Working Group (Pokja Genetik) Faculty of Medicine, Public Health and Nursing Universitas Gadjah Mada (FK-KMK UGM); Disease Investigation Center Wates Ministry of Agriculture Indonesia; Department of Microbiology FK-KMK UGM; Laboratorium Diagnostik Yayasan Tahija World Mosquito Program (WMP) Yogyakarta Center for Tropical Medicine FK-KMK UGM; Integrated Research Center FK-KMK UGM; Department of Computer	Afiahayati; Dwi AA Nugrahaningsih; Dwi Indaryati; Edwin W. Daniwijaya; Eggi Arguni; Endah Supriyati; Gunadi; Hana F Hanifin; Hendra Wibawa; Indarto Sulistiyono; Irene Tania; Kristy Iskandar; Ludhang P. Rizki; Marcellus; Mohamad S. Hakim; Nungki Anggorowati; Pramesti G Dewi; Siswanto; Titik Nuryastuti; Tri Wibawa
EPI_ISL_4279162, EPI_ISL_4279163, EPI_ISL_4279164, EPI_ISL_4279165, EPI_ISL_4279166, EPI_ISL_4279167, EPI_ISL_4279168, EPI_ISL_4279169, EPI_ISL_4279170, EPI_ISL_4279171, EPI_ISL_4279172, EPI_ISL_4279173, EPI_ISL_4279174, EPI_ISL_4279175, EPI_ISL_4279176, EPI_ISL_4279177, EPI_ISL_4279178			
see above	RSUD Pasangkayu Sulawesi Barat	National Institute of Health Research and Development	Arie Ardiansyah Nugraha; Fajar Nur; Fajar Nur Sulistiyohadi; Hana Apsari Pawestri; Hartanti Dian Ikawati; Kartika Dewi Puspa; Krisna Pangesti; Nelly Puspandari; Subangkit; Sulistiyohadi; Triyani Soekarso; Vivi Setiawaty
EPI_ISL_4417895, EPI_ISL_4417903, EPI_ISL_4417904, EPI_ISL_4417908	RSUD Pasar Minggu / EIJKMAN OXFORD CLINICAL RESEARCH UNIT	Genomik Solidaritas Indonesia Laboratorium	Anis Karuniawati; Annisa Muthiah Sukirman; Anuraj Shankar; Ariel Pradipta; Carissa Sintca Wijaya; Decy Subekti; Dhahlia Agustina Cahyono; Dian Tri Wahyudi; Dwi Utomo Nusantara; Gracia Felias Enos Korompis; Himawan Masyhuri; Louisa Markus; Meutia Ayuputeri Kumaheri; Raph Hamers; Robert Sinto; Stefanus Bernard; Suwarti
EPI_ISL_1300521	RSUD Pasarminggu	Stem Cell Lab, Universitas Pembangunan Nasional Veteran Jakarta	Andri Pramesyanti Pramono; Megadianty Mokoginta; Ria Maria Theresa; Yudhi Nugraha; Yudi Amiarno
EPI_ISL_1300496, EPI_ISL_1300522, EPI_ISL_1300535, EPI_ISL_1300540, EPI_ISL_1300644, EPI_ISL_1300660, EPI_ISL_1300661			
see above	RSUD Pasarminggu	Stem Cell Lab, Universitas Pembangunan Nasional Veteran Jakarta (UPNVJ)	Andri Pramesyanti Pramono; Megadianty Mokoginta; Ria Maria Theresa; Yudhi Nugraha; Yudi Amiarno
EPI_ISL_1297194	RSUD Pasarminggu	Stem Cell Lab. Universitas Pembangunan Nasional Veteran Jakarta (UPNVJ)	Andri Pramesyanti Pramono; Megadianty Mokoginta; Ria Maria Theresa; Yudhi Nugraha; Yudi Amiarno
EPI_ISL_4004706, EPI_ISL_4004707, EPI_ISL_4004708, EPI_ISL_4004709, EPI_ISL_4004710, EPI_ISL_4004711, EPI_ISL_4004712, EPI_ISL_4004713, EPI_ISL_4004714, EPI_ISL_4004715, EPI_ISL_4004716, EPI_ISL_4004717, EPI_ISL_4004718, EPI_ISL_4106119			
see above	RSUD Pelabuhan Ratu Sukabumi	National Research and Innovation Agency (BRIN-Indonesia, VenomCoV Project Team); West Java Health Laboratory	Ahmad Fathoni; Anggia Prasetyoputri; Anik B. Dharmayanthi; Asep M. Ridwanulloh; Azzania Fibriani; Cut Nur Cinthia Alamanda; Ema Rahmawati; Hariyatun; Herjuno Ari Nugroho; Indriawati; Isa Nuryana; Karimatu Khoirunnisa; Karimatu Khoirunnisa Rifki Sadikin; Masrukhin; Miftahul Faridl; Puspita Lisdiyanti; Ratih A. Ningrum; Rifki Sadikin; Rifky Waluyajati Rachman; Rini Robiani; Ryan Bayusantika Ristandi; Sugiyono Saputra; Suvia Widyaningrum; Syaiful Rizal; Syam B. Iryanto; ratih A. Ningrum
EPI_ISL_568686	RSUD Prof. DR. Margono Soekarjo	Eijkman Institute for Molecular Biology, Ministry of Research and Technology/National Agency for Research and Innovation	Amin Soebandrio; David H Muljono; Edison Johar; Frilasita A Yudhaputri; Herawati Sudoyo; Hidayat Trimarsanto; Iskandar A Adnan; Khin Saw Myint; Safarina G Malik; Willy Agustine
EPI_ISL_766033	RSUD Prof. DR. W. Z. Johannes Kupang	Eijkman Institute for Molecular Biology, Ministry of Research and Technology/National Agency for Research and Innovation	Amin Soebandrio; David H Muljono; Edison Johar; Frilasita A Yudhaputri; Hidayat Trimarsanto; Khin Saw Myint; Safarina G Malik
EPI_ISL_3216548, EPI_ISL_3216549, EPI_ISL_3216550, EPI_ISL_3216551, EPI_ISL_3216552, EPI_ISL_3216553, EPI_ISL_3216554, EPI_ISL_3691573			
see above	RSUD Provinsi Nusa Tenggara Barat	National Institute of Health Research and Development	Arie Ardiansyah Nugraha; Fajar Nur Sulistiyohadi; Hana Apsari Pawestri; Hartanti Dian Ikawati; Kartika Dewi Puspa; Krisna Pangesti; Laily Indrayanti; Nelly Puspandari; Subangkit; Triyani Soekarso; Vivi Setiawaty
EPI_ISL_3151602, EPI_ISL_3151607, EPI_ISL_3151614, EPI_ISL_3151621, EPI_ISL_3151628, EPI_ISL_3151634, EPI_ISL_3151641, EPI_ISL_3151642, EPI_ISL_3151647, EPI_ISL_3151648, EPI_ISL_3151653, EPI_ISL_3151654, EPI_ISL_3151659, EPI_ISL_3151660, EPI_ISL_3151667, EPI_ISL_3151668, EPI_ISL_3151674, EPI_ISL_3151675			
see above	RSUD Provinsi Papua Barat	National Institute of Health Research and Development	Arie Ardiansyah Nugraha; Fajar Nur Sulistiyohadi; Hana Apsari Pawestri; Hartanti Dian Ikawati; Kartika Dewi Puspa; Krisna Pangesti; Nelly Puspandari; Subangkit; Triyani Soekarso; Vivi Setiawaty
EPI_ISL_3070964, EPI_ISL_3070965, EPI_ISL_3070966, EPI_ISL_3070967, EPI_ISL_3070968, EPI_ISL_3070969, EPI_ISL_3070970, EPI_ISL_3070971, EPI_ISL_3070972, EPI_ISL_3070973, EPI_ISL_3070974, EPI_ISL_3070975, EPI_ISL_3070976, EPI_ISL_3070977, EPI_ISL_3070978, EPI_ISL_3070979, EPI_ISL_3070980, EPI_ISL_3070981, EPI_ISL_3070982, EPI_ISL_3070983, EPI_ISL_3070984, EPI_ISL_3070985, EPI_ISL_3070986, EPI_ISL_3070987, EPI_ISL_3070988, EPI_ISL_3070989, EPI_ISL_3070990, EPI_ISL_3070991, EPI_ISL_3070992, EPI_ISL_3070993, EPI_ISL_3070994, EPI_ISL_3070995, EPI_ISL_3070996, EPI_ISL_3070997, EPI_ISL_3070998, EPI_ISL_3070999, EPI_ISL_3071000, EPI_ISL_3071001, EPI_ISL_3071002, EPI_ISL_3071003, EPI_ISL_3071004, EPI_ISL_3071005, EPI_ISL_3071006, EPI_ISL_3071007,			

EPI_ISL_3071008, EPI_ISL_3071009, EPI_ISL_3071010, EPI_ISL_3071011, EPI_ISL_3071012, EPI_ISL_3071013, EPI_ISL_3071014, EPI_ISL_3071015, EPI_ISL_3071016, EPI_ISL_3071017, EPI_ISL_3071018, EPI_ISL_3071020, EPI_ISL_3071021, EPI_ISL_3071022, EPI_ISL_3071023, EPI_ISL_3071024, EPI_ISL_3071025, EPI_ISL_3071026, EPI_ISL_3071027, EPI_ISL_3071028, EPI_ISL_3071029, EPI_ISL_3071030, EPI_ISL_3071031, EPI_ISL_3071032, EPI_ISL_3071033, EPI_ISL_3071034, EPI_ISL_3071035, EPI_ISL_3071036, EPI_ISL_3071037, EPI_ISL_3071038, EPI_ISL_3071039, EPI_ISL_3071040, EPI_ISL_3071041, EPI_ISL_3071042, EPI_ISL_3071043, EPI_ISL_3071044, EPI_ISL_3071045, EPI_ISL_3071046, EPI_ISL_3071047, EPI_ISL_3071048, EPI_ISL_3071049, EPI_ISL_3071050, EPI_ISL_3071051, EPI_ISL_3071052, EPI_ISL_3071053, EPI_ISL_3071054, EPI_ISL_3071055, EPI_ISL_3071056, EPI_ISL_3071057, EPI_ISL_3071058, EPI_ISL_3071059, EPI_ISL_3071060, EPI_ISL_3071061, EPI_ISL_3071062, EPI_ISL_3071063, EPI_ISL_3071064, EPI_ISL_3071065, EPI_ISL_3071066, EPI_ISL_3160206, EPI_ISL_3160209, EPI_ISL_3160212, EPI_ISL_3160214, EPI_ISL_3160215, EPI_ISL_3160216, EPI_ISL_3160217, EPI_ISL_3160218, EPI_ISL_3160219, EPI_ISL_3160220, EPI_ISL_3160221, EPI_ISL_3160222, EPI_ISL_3160223, EPI_ISL_3160224, EPI_ISL_3160225, EPI_ISL_3160226, EPI_ISL_3160227, EPI_ISL_3160228, EPI_ISL_3160229, EPI_ISL_3160230, EPI_ISL_3160231, EPI_ISL_3160232, EPI_ISL_3160234, EPI_ISL_3160235, EPI_ISL_3160236, EPI_ISL_3160237, EPI_ISL_3160238, EPI_ISL_3160239, EPI_ISL_3160240, EPI_ISL_3160241, EPI_ISL_3160242, EPI_ISL_3160243, EPI_ISL_3160244, EPI_ISL_3160245, EPI_ISL_3160246, EPI_ISL_3160247, EPI_ISL_3160248, EPI_ISL_3160249, EPI_ISL_3160250, EPI_ISL_3160251, EPI_ISL_3160252, EPI_ISL_3160253, EPI_ISL_3160255, EPI_ISL_3160259, EPI_ISL_3160263, EPI_ISL_3160267, EPI_ISL_3160270, EPI_ISL_3160274, EPI_ISL_3160277, EPI_ISL_3160284, EPI_ISL_3160291, EPI_ISL_3160294, EPI_ISL_3160297, EPI_ISL_3160300, EPI_ISL_3160303, EPI_ISL_3160307, EPI_ISL_3160310, EPI_ISL_3160314, EPI_ISL_3160317, EPI_ISL_3160320, EPI_ISL_3160324, EPI_ISL_3160327, EPI_ISL_3160330, EPI_ISL_3160333, EPI_ISL_3160336, EPI_ISL_3160339, EPI_ISL_3160343, EPI_ISL_3160346, EPI_ISL_3160349, EPI_ISL_3160353, EPI_ISL_3160356, EPI_ISL_3160359, EPI_ISL_3160362, EPI_ISL_3160365, EPI_ISL_3160366, EPI_ISL_3160367, EPI_ISL_3160368, EPI_ISL_3160369, EPI_ISL_3160370, EPI_ISL_3160371, EPI_ISL_3160373, EPI_ISL_3160375, EPI_ISL_3160376, EPI_ISL_3160377, EPI_ISL_3160378, EPI_ISL_3160379, EPI_ISL_3160380, EPI_ISL_3160381, EPI_ISL_3160382, EPI_ISL_3160383, EPI_ISL_3160384, EPI_ISL_3160385, EPI_ISL_3160386, EPI_ISL_3160387, EPI_ISL_3160388, EPI_ISL_3160389, EPI_ISL_3160390, EPI_ISL_3160391, EPI_ISL_3160392, EPI_ISL_3160393, EPI_ISL_3160394, EPI_ISL_3160395, EPI_ISL_3160396, EPI_ISL_3160398, EPI_ISL_3466028, EPI_ISL_3466034, EPI_ISL_3466040, EPI_ISL_3466046, EPI_ISL_3466053, EPI_ISL_3466060, EPI_ISL_3466067, EPI_ISL_3466072, EPI_ISL_3466079, EPI_ISL_3466085, EPI_ISL_3466091, EPI_ISL_3466097, EPI_ISL_3466104, EPI_ISL_3466110, EPI_ISL_3466117, EPI_ISL_3466123, EPI_ISL_3466129, EPI_ISL_3466136, EPI_ISL_3466142, EPI_ISL_3466149, EPI_ISL_3466155, EPI_ISL_3466162, EPI_ISL_3466168, EPI_ISL_3466173, EPI_ISL_3466179, EPI_ISL_3466185, EPI_ISL_3466191, EPI_ISL_3466197, EPI_ISL_3466203, EPI_ISL_3466210, EPI_ISL_3466217, EPI_ISL_3466229, EPI_ISL_3466234, EPI_ISL_3466241, EPI_ISL_3466247, EPI_ISL_3466253, EPI_ISL_3466260, EPI_ISL_3466266, EPI_ISL_3466272, EPI_ISL_3466278, EPI_ISL_3466284, EPI_ISL_3466291, EPI_ISL_3466297, EPI_ISL_3466303, EPI_ISL_3466310, EPI_ISL_3466316, EPI_ISL_3466322, EPI_ISL_3466328, EPI_ISL_3466335, EPI_ISL_3466341, EPI_ISL_3466347, EPI_ISL_3466353, EPI_ISL_3466359, EPI_ISL_3466366, EPI_ISL_3466372, EPI_ISL_3466379, EPI_ISL_3466385, EPI_ISL_3466392, EPI_ISL_3466398, EPI_ISL_3466405, EPI_ISL_3466412, EPI_ISL_3466418, EPI_ISL_3466424, EPI_ISL_3466431, EPI_ISL_3466438, EPI_ISL_3466444, EPI_ISL_3466450, EPI_ISL_3466456, EPI_ISL_3466462, EPI_ISL_3466468, EPI_ISL_3466475, EPI_ISL_3466478, EPI_ISL_3466479, EPI_ISL_3466480, EPI_ISL_3466481, EPI_ISL_3466482, EPI_ISL_3466483, EPI_ISL_3466484, EPI_ISL_3466485, EPI_ISL_3466486, EPI_ISL_3466487, EPI_ISL_3466488, EPI_ISL_3466489, EPI_ISL_3466490, EPI_ISL_3466491, EPI_ISL_3466492, EPI_ISL_3466493, EPI_ISL_3466494, EPI_ISL_3466495, EPI_ISL_3466496, EPI_ISL_3466497, EPI_ISL_3466498, EPI_ISL_3466499, EPI_ISL_3466500, EPI_ISL_3466501, EPI_ISL_3466503, EPI_ISL_3466509, EPI_ISL_3466514, EPI_ISL_3466519, EPI_ISL_3466526, EPI_ISL_3466531, EPI_ISL_3466537, EPI_ISL_3466544, EPI_ISL_3466550, EPI_ISL_3466556, EPI_ISL_3466562, EPI_ISL_3466569, EPI_ISL_3466575, EPI_ISL_3466581, EPI_ISL_3466587, EPI_ISL_3466593, EPI_ISL_3466598, EPI_ISL_3466605, EPI_ISL_3466610, EPI_ISL_3466617, EPI_ISL_3466624, EPI_ISL_3466629, EPI_ISL_3466636, EPI_ISL_3466640, EPI_ISL_3466646, EPI_ISL_3466652, EPI_ISL_3466659, EPI_ISL_3466666, EPI_ISL_3466670, EPI_ISL_3466676, EPI_ISL_3466681, EPI_ISL_3466687, EPI_ISL_3466693, EPI_ISL_3466699, EPI_ISL_3466704, EPI_ISL_3466711, EPI_ISL_3466718, EPI_ISL_3466724, EPI_ISL_3466731, EPI_ISL_3466737, EPI_ISL_3466743, EPI_ISL_3466755, EPI_ISL_3466761, EPI_ISL_3466767, EPI_ISL_3466773, EPI_ISL_3466779, EPI_ISL_3466785, EPI_ISL_3466791, EPI_ISL_3466797, EPI_ISL_3466804, EPI_ISL_3466811, EPI_ISL_3466817, EPI_ISL_3466824, EPI_ISL_3466830			
see above	RSUD Raden Mattaher	Eijkman Institute for Molecular Biology, National Research and Innovation Agency; Raden Mattaher Regional General Hospital	Amin Soebandrio; Edison Johar; Fery Kusnadi; Frilasita A Yudhaputri; Hidayat Trimarsanto; Iskandar Adnan; Jessica R. Saragih; Khin Saw Myint; Lidwina Priliani; Lydia V. Panggalo; Muhammad Rezki Rasyak; Safarina G Malik; Sotianingsih; Sukma Oktavianthi; Willy Agustine; Yusnidar
EPI_ISL_4761488	RSUD SUNAN KALIJAGA DEMAK	Balai Laboratorium Kesehatan Dan Pengujian Alat Kesehatan Provinsi Jawa Tengah	Alia Wijayanti; Dwi Nursetyaasih; Dyah Ayu; Endah Wahyu Y; Ismi Farhati; Purnomo; Sekti Handayani; Wiwik Peni; Yekti Hediningsih
EPI_ISL_632937	RSUD Saptosari Gunung Kidul	Genetics Working Group (Pokja Genetik) Faculty of Medicine, Public Health and Nursing Universitas Gadjah Mada (FK-KMK UGM); Disease Investigation Center Wates Ministry of Agriculture Indonesia; Department of Microbiology FK-KMK UGM; Laboratorium Diagnostik Yayasan Tahija World Mosquito Program (WMP) Yogyakarta Center for Tropical Medicine FK-KMK UGM; Integrated Research Center FK-KMK UGM; Department of Computer Science and Electronics FMIPA UGM; Balai Besar Teknik Kesehatan Lingkungan dan Pengendalian Penyakit (BBTKLPP) Yogyakarta	Afiahayati; Audric Kenny Tedja; Dwi AA Nugrahaningsih; Edwin W. Daniwijaya; Eggi Arguni; Eko Darmawan; Endah Supriyati; Gunadi; Havid Setyawan; Hendra Wibawa; Indaryati; Irene; Kristy Iskandar; Ludhang P. Rizki; Marcellus; Maria Patricia Inggriani; Mohamad S. Hakim; Nungki Anggorowati; Siswanto; Titik Nuryastuti; Tri Wibawa
EPI_ISL_529966, EPI_ISL_529967, EPI_ISL_759963, EPI_ISL_759966, EPI_ISL_956274, EPI_ISL_956309	RSUD Sidoarjo	Institute of Tropical Disease, Universitas Airlangga	Aldise M Nastri; Atok Irawan; Gatot Soegiarto; Jezzy R Dewantari; Kazufumi Shimizu; Krisnoadi Rahardjo; Laksmi Wulandari; Maria I Lusida; Mitsuhiro Nishimura; Resti Yudhawati; Retno A Setyoningrum; Rima R Prasetya; Soetjipto; Yasuko Mori; Yohko K Shimizu
EPI_ISL_4004672, EPI_ISL_4004673, EPI_ISL_4004674, EPI_ISL_4004675, EPI_ISL_4004676, EPI_ISL_4004677, EPI_ISL_4004678, EPI_ISL_4004679, EPI_ISL_4004680, EPI_ISL_4004681, EPI_ISL_4004682, EPI_ISL_4004683, EPI_ISL_4004684, EPI_ISL_4004685, EPI_ISL_4004686, EPI_ISL_4004687, EPI_ISL_4004688, EPI_ISL_4004689, EPI_ISL_4004690, EPI_ISL_4004691, EPI_ISL_4004692, EPI_ISL_4004693, EPI_ISL_4004694, EPI_ISL_4004695, EPI_ISL_4004696, EPI_ISL_5536497, EPI_ISL_5536498, EPI_ISL_5536499, EPI_ISL_5536500, EPI_ISL_5536501, EPI_ISL_5536502, EPI_ISL_5536503, EPI_ISL_5536504, EPI_ISL_5536505, EPI_ISL_5536506, EPI_ISL_5536507, EPI_ISL_5536508, EPI_ISL_5536509, EPI_ISL_5536510, EPI_ISL_5536513, EPI_ISL_5536514, EPI_ISL_5536515, EPI_ISL_5536516, EPI_ISL_5536517, EPI_ISL_5536519, EPI_ISL_5536520, EPI_ISL_5536521, EPI_ISL_5536523, EPI_ISL_5536524, EPI_ISL_5536525, EPI_ISL_5536527, EPI_ISL_5536530, EPI_ISL_5536531, EPI_ISL_5536532, EPI_ISL_5536533, EPI_ISL_5536534, EPI_ISL_5536535, EPI_ISL_5536536, EPI_ISL_5536537, EPI_ISL_5536538, EPI_ISL_5536539, EPI_ISL_5536540, EPI_ISL_5536541, EPI_ISL_5536542, EPI_ISL_5536543, EPI_ISL_5536544, EPI_ISL_5536545, EPI_ISL_5536547, EPI_ISL_5536548, EPI_ISL_5536549, EPI_ISL_5536550, EPI_ISL_5536551, EPI_ISL_5536552, EPI_ISL_5536554, EPI_ISL_5536555, EPI_ISL_5536556, EPI_ISL_5536557, EPI_ISL_5536565, EPI_ISL_5536566, EPI_ISL_5536567, EPI_ISL_5536569, EPI_ISL_5536570, EPI_ISL_5536572, EPI_ISL_5536573			
see above	RSUD Singaparna Medika Citrautama	National Research and Innovation Agency (BRIN-Indonesia, VenomCoV Project Team); West Java Health Laboratory	Ahmad Fathoni; Ahmad R. Al Fiqri; Alvira R. Sativa; Anggia Prasetyoputri; Anik B. Dharmayanthi; Azzania Fibriani; Cut Nur Cinthia Alamanda; Dian F. Agustiyani; Ema Rahmawati; Erwin Hasrianda; Hariyatun; Herjuno A. Nugroho; Indriawati; Isa Nuryana; Isnaini Z. Asyifa; Karimatu Khoirunnisa; Listiana Oktavia; M. Mumtaz Adzdzakiy; Masrukhin; Miftahul Faridl; Muhammad M. Adzdzakiy; Popi H. Wisnuwardhani; Ratih A. Ningrum; Rifki Sadikin; Rifky W. Rachman; Rifky Waluyajati Rachman; Rini Robiani; Ryan B. Ristandi; Ryan Bayusantika Ristandi; Sugiyono Saputra; Syaiful Rizal; Syam B. Iryanto
EPI_ISL_1915576, EPI_ISL_2047572, EPI_ISL_2047573	RSUD Siti Fatimah Prov Sumatera Selatan	National Institute of Health Research and Development	Arie Ardiansyah Nugraha; Hana Apsari Pawestri; Hartanti Dian Ikawati; Kartika Dewi Puspa; Krisna Pangesti; Nelly Puspandari; Subangkit; Triyani Soekarso; Vivi Setiawaty
EPI_ISL_538498	RSUD Soediono Madiun East Java	National Institute of Health Research and Development	AA; HA; HD; Ikawati; KD; KNA; Nugraha; Paisal; Pangesti; Pawestri; Puspa; Setiawaty, V.; Soekarso; Subangkit; T
EPI_ISL_538502	RSUD Sultan Imanudin Pangkalan Bun Center Kalimantan	National Institute of Health Research and Development	AA; HA; HD; Ikawati; KD; KNA; Nugraha; Paisal; Pangesti; Pawestri; Puspa; Setiawaty, V.; Soekarso; Subangkit; T
EPI_ISL_4756069, EPI_ISL_4824657	RSUD Sunan Kalijaga Demak	Balai Laboratorium Kesehatan Dan Pengujian Alat Kesehatan Provinsi Jawa Tengah	Alia Wijayanti; Dwi Nursetyaasih; Dyah Ayu; Endah Wahyu Y; Ismi Farhati; Purnomo; Sekti Handayani; Wiwik Peni; Yekti Hediningsih
EPI_ISL_5616384	RSUD Sunan Kalijaga Demak	Balai Laboratorium Kesehatan dan Pengujian Alat Kesehatan Provinsi Jawa Tengah	Alia Wijayanti; Dwi Nursetyaasih; Dyah Ayu; Endah Wahyu Y; Ismi Farhati; Purnomo; Sekti Handayani; Wiwik Peni; Yekti Hediningsih
EPI_ISL_4969177, EPI_ISL_4969179	RSUD Sunan Kalijaga Demak	Genetics Working Group (Pokja Genetik) Faculty of Medicine, Public Health and Nursing Universitas Gadjah Mada (FK-KMK UGM); Disease Investigation Center Wates Ministry of Agriculture Indonesia; Department of Microbiology FK-KMK UGM; Laboratorium Diagnostik Yayasan Tahija World Mosquito Program (WMP) Yogyakarta Center for Tropical Medicine FK-KMK UGM; Integrated Research Center FK-KMK UGM; Department of Computer Science and Electronics FMIPA UGM; RSUP Dr. Sardjito	Afiahayati; Alvina A Setiawan; Dwi AA Nugrahaningsih; Dwi Indaryati; Dyah A Puspitarani; Edwin W. Daniwijaya; Eggi Arguni; Endah Supriyati; Gunadi; Hendra Wibawa; Indarto Sulistiyono; Khanza a Vujira; Kristy Iskandar; Ludhang P. Rizki; Marcellus; Mohamad S. Hakim; Nungki Anggorowati; Pramesti G Dewi; Siswanto; Susan Simanjaya; Titik Nuryastuti; Tri Wibawa
EPI_ISL_1469279, EPI_ISL_1469280, EPI_ISL_1469281, EPI_ISL_1469282, EPI_ISL_1469283	RSUD Surakarta	National Institute of Health Research and Development	Arie Ardiansyah Nugraha; Hana Apsari Pawestri; Hartanti Dian Ikawati; Kartika Dewi Puspa; Krisna Pangesti; Nelly Puspandari; Subangkit; Vivi Setiawaty
EPI_ISL_3138942, EPI_ISL_3138943	RSUD Tarakan	National Institute of Health Research and Development	Arie Ardiansyah Nugraha; Fajar Nur Sulistiyohadi; Hana Apsari Pawestri; Hartanti Dian Ikawati; Kartika Dewi Puspa; Krisna Pangesti; Nelly Puspandari; Subangkit; Triyani Soekarso; Vivi Setiawaty
EPI_ISL_2047560, EPI_ISL_3216596, EPI_ISL_3216597, EPI_ISL_3216598	RSUD Tarakan Jakarta	National Institute of Health Research and Development	Arie Ardiansyah Nugraha; Fajar Nur Sulistiyohadi; Hana Apsari Pawestri; Hartanti Dian Ikawati; Juhairiyah; Kartika Dewi Puspa; Krisna Pangesti; Nelly Puspandari; Subangkit; Syarif Hidayat; Triyani Soekarso; Vivi Setiawaty
EPI_ISL_803876	RSUD Tarakan Kalimantan Utara	National Institute of Health Research and Development	AA; HA; HD; Ikawati; K; KD; KNA; N; Nugraha; Pangesti; Pawestri; Puspa; Puspandari; R; Setiawaty; Sindunata; Soekarso; Sriwedari; Subangkit; T; V
EPI_ISL_3138907, EPI_ISL_3138908	RSUD Tarutung	National Institute of Health Research and Development	Arie Ardiansyah Nugraha; Fajar Nur Sulistiyohadi; Hana Apsari Pawestri; Hartanti Dian Ikawati; Kartika Dewi Puspa; Krisna Pangesti; Nelly Puspandari; Subangkit; Triyani Soekarso; Vivi Setiawaty
EPI_ISL_4090640, EPI_ISL_4090641, EPI_ISL_4090642	RSUD Teluk Wondama Papua Barat	National Institute of Health Research and Development	Arie Ardiansyah Nugraha; Fajar Nur Sulistiyohadi; Hana Apsari Pawestri; Hartanti Dian Ikawati; Kartika Dewi Puspa; Krisna Pangesti; Nelly Puspandari; Subangkit; Triyani Soekarso; Vivi Setiawaty
EPI_ISL_1915580, EPI_ISL_1915581, EPI_ISL_1915582	RSUD UNDATA Palu	National Institute of Health Research and Development	Arie Ardiansyah Nugraha; Hana Apsari Pawestri; Hartanti Dian Ikawati; Kartika Dewi Puspa; Krisna Pangesti; Nelly Puspandari; Rosa Dwi Wahyuni; Subangkit; Vivi Setiawaty
EPI_ISL_538510	RSUD Ulin Banjarmasin South Kalimantan	National Institute of Health Research and Development	AA; HA; HD; Ikawati; KD; KNA; M; Nugraha; Paisal; Pangesti; Pasaribu; Pawestri; Puspa; Setiawaty, V.; Soekarso; Subangkit; T
EPI_ISL_753699	RSUD Ulin Banjarmasin Kalimantan Selatan	National Institute of Health Research and Development	AA; HA; HD; Ikawati; KD; KNA; M; N; Nugraha; Pangesti; Pasaribu; Pawestri; Puspa; Puspandari; Setiawaty; Soekarso; Subangkit; T; V
EPI_ISL_538511	RSUD Wahidin Sudirohusodo Mojokerto East Java	National Institute of Health Research and Development	AA; HA; HD; Ikawati; KD; KNA; Nugraha; Paisal; Pangesti; Pawestri; Puspa; Setiawaty, V.; Soekarso; Subangkit; T
EPI_ISL_791989	RSUD Waluyo Jati Kota Kraksaan, Jawa Timur	National Institute of Health Research and Development	AA; HA; HD; I; Ikawati; KD; KNA; Muhashonah; N; Nugraha; Pangesti; Pawestri; Puspa; Puspandari; Setiawaty; Soekarso; Subangkit; T; V
EPI_ISL_575331	RSUD Wates	Genetics Working Group (Pokja Genetik) Faculty of Medicine, Public Health and Nursing Universitas Gadjah Mada (FK-KMK UGM); Disease Investigation Center Wates Ministry of Agriculture Indonesia; Department of Microbiology FK-KMK UGM; Laboratorium Diagnostik Yayasan Tahija World Mosquito Program (WMP) Yogyakarta Center for Tropical Medicine FK-KMK UGM; Integrated Research Center FK-KMK UGM; Department of Computer Science and Electronics FMIPA UGM	. Marcellus; Afiahayati; Alvin S. Kalim; Bastianto Kusumajaya; Dwi AA Nugrahaningsih; Edwin W. Daniwijaya; Eggi Arguni; Endah Supriyati; Gunadi; Hendra Wibawa; Kristy Iskandar; Ludhang P. Rizki; Mohamad S. Hakim; Nungki Anggorowati; Siswanto; Susan Simanjaya; Titik Nuryastuti; Tri Wibawa; Zumrati Ahmad
EPI_ISL_3691590, EPI_ISL_3691598, EPI_ISL_3691599, EPI_ISL_3691600, EPI_ISL_3691601, EPI_ISL_3691602, EPI_ISL_3691603, EPI_ISL_3691604, EPI_ISL_3691605, EPI_ISL_3691606, EPI_ISL_3691607, EPI_ISL_3691608, EPI_ISL_3691609, EPI_ISL_3691610, EPI_ISL_3691611, EPI_ISL_3691612, EPI_ISL_3691613, EPI_ISL_3691614, EPI_ISL_3691615, EPI_ISL_3691616			
see above	RSUD dr Abdul Rivai Berau	National Institute of Health Research and Development	Arie Ardiansyah Nugraha; Fajar Nur; Fajar Nur Sulistiyohadi; Hana Apsari Pawestri; Hartanti Dian Ikawati; Kartika Dewi Puspa; Krisna Pangesti; Nelly Puspandari; Subangkit; Sulistiyohadi; Triyani Soekarso; Vivi Setiawaty
EPI_ISL_5022864, EPI_ISL_5022865, EPI_ISL_5022866, EPI_ISL_5022867, EPI_ISL_5022868, EPI_ISL_5022869, EPI_ISL_5022870, EPI_ISL_5022871, EPI_ISL_5107890, EPI_ISL_5107891, EPI_ISL_5107892, EPI_ISL_5107893, EPI_ISL_5107894, EPI_ISL_5107895, EPI_ISL_5107896, EPI_ISL_5107897, EPI_ISL_5107898, EPI_ISL_5107899			
see above	RSUD dr H Ansari Saleh Banjarmasin	National Institute of Health Research and Development	Arie Ardiansyah Nugraha; Fajar Nur; Fajar Nur Sulistiyohadi; Hana Apsari Pawestri; Hartanti Dian Ikawati; Kartika Dewi Puspa; Krisna Pangesti; Nelly Puspandari; Subangkit; Sulistiyohadi; Triyani Soekarso; Vivi Setiawaty
EPI_ISL_4104538, EPI_ISL_4104539, EPI_ISL_4104540, EPI_ISL_4104541, EPI_ISL_4104542, EPI_ISL_4104543, EPI_ISL_4104544, EPI_ISL_4104545, EPI_ISL_4104546, EPI_ISL_4104547, EPI_ISL_4104548, EPI_ISL_4104549, EPI_ISL_4104550, EPI_ISL_4104551, EPI_ISL_4104552, EPI_ISL_5022946			
see above	RSUD dr H Chasan Boesoirie	National Institute of Health Research and Development	Arie Ardiansyah Nugraha; Fajar Nur Sulistiyohadi; Hana Apsari Pawestri; Hartanti Dian Ikawati; Kartika Dewi Puspa; Krisna Pangesti; Nelly Puspandari; Subangkit; Triyani Soekarso; Vivi Setiawaty
EPI_ISL_4254867, EPI_ISL_4254868, EPI_ISL_4254869, EPI_ISL_4254870,	RSUD dr H Koesnadi Bondowoso	National Institute of Health Research and Development	Arie Ardiansyah Nugraha; Fajar Nur Sulistiyohadi; Hana Apsari Pawestri; Hartanti Dian Ikawati; Kartika Dewi Puspa; Krisna Pangesti; Nelly Puspandari; Subangkit; Triyani Soekarso; Vivi Setiawaty

EPI_ISL_4254871			
EPI_ISL_3691672, EPI_ISL_3691678, EPI_ISL_3691679, EPI_ISL_3691680	RSUD dr H Marsidi Judono	National Institute of Health Research and Development	Arie Ardiansyah Nugraha; Fajar Nur Sulistiyohadi; Hana Apsari Pawestri; Hartanti Dian Ikawati; Kartika Dewi Puspa; Krisna Pangesti; Nelly Puspandari; Subangkit; Triyani Soekarso; Vivi Setiawaty
EPI_ISL_2502615	RSUD dr Loekmono Hadi	Genetics Working Group (Pokja Genetik) Faculty of Medicine, Public Health and Nursing Universitas Gadjah Mada (FK-KMK UGM); Disease Investigation Center Wates Ministry of Agriculture Indonesia; Department of Microbiology FK-KMK UGM; Laboratorium Diagnostik Yayasan Tahija World Mosquito Program (WMP) Yogyakarta Center for Tropical Medicine FK-KMK UGM; Integrated Research Center FK-KMK UGM; Department of Computer Science and Electronics FMIPA UGM; RSUP Dr. Sardjito	Afiahayati; Dwi AA Nugrahaningsih; Dwi Indaryati; Dyah A Puspitarani; Edwin W. Daniwijaya; Eggi Arguni; Endah Supriyati; Gunadi; Hendra Wibawa; Indarto Sulistiyono; Kristy Iskandar; Ludhang P. Rizki; Marcellus; Mohamad S. Hakim; Nungki Anggorowati; Pramesti G Dewi; Siswanto; Susan Simanjaya; Titik Nuryastuti; Tri Wibawa
EPI_ISL_4090583	RSUD dr Sayidiman Magetan	National Institute of Health Research and Development	Arie Ardiansyah Nugraha; Fajar Nur Sulistiyohadi; Hana Apsari Pawestri; Hartanti Dian Ikawati; Kartika Dewi Puspa; Krisna Pangesti; Nelly Puspandari; Subangkit; Triyani Soekarso; Vivi Setiawaty
EPI_ISL_2987625, EPI_ISL_2987626, EPI_ISL_2987627, EPI_ISL_2987628, EPI_ISL_2987629, EPI_ISL_3019946	RSUD dr Slamet Garut	National Institute of Health Research and Development	Arie Ardiansyah Nugraha; Fajar Nur Sulistiyohadi; Hana Apsari Pawestri; Hartanti Dian Ikawati; Kartika Dewi Puspa; Krisna Pangesti; Nelly Puspandari; Subangkit; Triyani Soekarso; Vivi Setiawaty
EPI_ISL_2931729, EPI_ISL_2931737, EPI_ISL_2931746, EPI_ISL_2931756, EPI_ISL_2931757, EPI_ISL_2931765, EPI_ISL_2931766, EPI_ISL_2931776, EPI_ISL_2931783, EPI_ISL_2931791			
see above	RSUD dr Soediran Mangun Sumarso, Wonogiri	National Institute of Health Research and Development	; Arie Ardiansyah Nugraha; Hana Apsari Pawestri; Hartanti Dian Ikawati; Kartika Dewi Puspa; Krisna Pangesti; Nelly Puspandari; Subangkit; Triyani Soekarso; Vivi Setiawaty
EPI_ISL_3216524, EPI_ISL_3216525, EPI_ISL_3216526, EPI_ISL_3216527, EPI_ISL_3216528, EPI_ISL_3216529, EPI_ISL_3216530, EPI_ISL_3216531, EPI_ISL_3278293			
see above	RSUD dr Soehadi Prijonegoro Sragen	National Institute of Health Research and Development	Arie Ardiansyah Nugraha; Fajar Nur Sulistiyohadi; Hana Apsari Pawestri; Hartanti Dian Ikawati; Kartika Dewi Puspa; Krisna Pangesti; Nelly Puspandari; Subangkit; Triyani Soekarso; Vivi Setiawaty
EPI_ISL_2258207, EPI_ISL_2262276, EPI_ISL_2262277	RSUD dr Soetomo Surabaya	National Institute of Health Research and Development	Arie Ardiansyah Nugraha; Hana Apsari Pawestri; Hartanti Dian Ikawati; Kartika Dewi Puspa; Krisna Pangesti; Nelly Puspandari; Subangkit; Triyani Soekarso; Vivi Setiawaty
EPI_ISL_4730582, EPI_ISL_4730583, EPI_ISL_4730584, EPI_ISL_4730585, EPI_ISL_4736609, EPI_ISL_5022708, EPI_ISL_5022709, EPI_ISL_5022710, EPI_ISL_5022711, EPI_ISL_5022712, EPI_ISL_5022713, EPI_ISL_5022714, EPI_ISL_5022715, EPI_ISL_5022716, EPI_ISL_5022717, EPI_ISL_5022718, EPI_ISL_5022719, EPI_ISL_5022720			
see above	RSUD dr. H. Ansari Saleh Banjarmasin	National Institute of Health Research and Development	Arie Ardiansyah Nugraha; Fajar Nur Sulistiyohadi; Hana Apsari Pawestri; Hartanti Dian Ikawati; Kartika Dewi Puspa; Krisna Pangesti; Nelly Puspandari; Subangkit; Triyani Soekarso; Vivi Setiawaty
EPI_ISL_3404368, EPI_ISL_3404369, EPI_ISL_3404371, EPI_ISL_3404372	RSUD dr. H. Marsidi Judono Belitung	Genomik Solidaritas Indonesia Laboratorium	Annisa Muthiah Sukirman; Anuraj Shankar; Ariel Pradipta; Carissa Sintca Wijaya; Dhahlia Agustina Cahyono; Gracia Felias Enos Korompis; Louisa Markus; Meutia Ayuputeri Kumaheri; Neng Hilma Hamidah; Teguh A.Maulana Gultom; Titik Handayani Tanujaya; Vania Gavrila Wikasa
EPI_ISL_3404370	RSUD dr. H. Marsidi Judono, Belitung	Genomik Solidaritas Indonesia Laboratorium	Annisa Muthiah Sukirman; Anuraj Shankar; Ariel Pradipta; Carissa Sintca Wijaya; Dhahlia Agustina Cahyono; Gracia Felias Enos Korompis; Louisa Markus; Meutia Ayuputeri Kumaheri; Neng Hilma Hamidah; Teguh A.Maulana Gultom; Titik Handayani Tanujaya; Vania Gavrila Wikasa
EPI_ISL_4030488	RSUD dr. H.Marsidi Judono	Genomik Solidaritas Indonesia Laboratorium	Annisa Muthiah Sukirman; Anuraj Shankar; Ariel Pradipta; Carissa Sintca Wijaya; David Hendra Haryanto; Dhahlia Agustina Cahyono; Dwi Oktavia; Gracia Felias Enos Korompis; Hendra; Himawan Masyhuri; Louisa Markus; Meutia Ayuputeri Kumaheri; Neng Hilma Hamidah; Ngabila Salama; R. Anggi Wahyu Nugroho; Tiranti Vindha; Titik Handayani Tanujaya
EPI_ISL_1824615	RSUD dr. Hasri Ainun Habibie Gorontalo	National Institute of Health Research and Development	Arie Ardiansyah Nugraha; Hana Apsari Pawestri; Hartanti Dian Ikawati; Kartika Dewi Puspa; Krisna Pangesti; Nelly Puspandari; Subangkit; Vivi Setiawaty
EPI_ISL_791983	RSUD dr. Kanujoso Djatiwibowo Balikpapan	National Institute of Health Research and Development	AA; HA; HD; Ikawati; Ivanna; KD; KNA; N; Nugraha; Pangesti; Pawestri; Puspa; Puspandari; Setiawaty; Soekarso; Subangkit; T; V
EPI_ISL_2502616, EPI_ISL_2502627, EPI_ISL_2502628, EPI_ISL_2502629, EPI_ISL_2502667, EPI_ISL_2502687, EPI_ISL_2502717, EPI_ISL_2502763, EPI_ISL_2502764, EPI_ISL_2502765, EPI_ISL_2503134, EPI_ISL_2506037, EPI_ISL_2506038, EPI_ISL_2506039, EPI_ISL_2506040, EPI_ISL_2506041, EPI_ISL_2506042, EPI_ISL_2534078, EPI_ISL_2534079, EPI_ISL_2534082, EPI_ISL_2534289, EPI_ISL_2534338, EPI_ISL_2534339, EPI_ISL_2534346, EPI_ISL_2534376, EPI_ISL_2534377, EPI_ISL_2534378, EPI_ISL_2534379, EPI_ISL_2534454, EPI_ISL_2534455, EPI_ISL_2534456, EPI_ISL_2534457, EPI_ISL_2534458, EPI_ISL_2534489, EPI_ISL_2534490, EPI_ISL_2534518			
see above	RSUD dr. LOEKMONO HADI	Genetics Working Group (Pokja Genetik) Faculty of Medicine, Public Health and Nursing Universitas Gadjah Mada (FK-KMK UGM); Disease Investigation Center Wates Ministry of Agriculture Indonesia; Department of Microbiology FK-KMK UGM; Laboratorium Diagnostik Yayasan Tahija World Mosquito Program (WMP) Yogyakarta Center for Tropical Medicine FK-KMK UGM; Integrated Research Center FK-KMK UGM; Department of Computer Science and Electronics FMIPA UGM; RSUP Dr. Sardjito	Afiahayati; Alvina A Setiawan; Cita S Amalia; Dwi AA Nugrahaningsih; Dwi Indaryati; Dyah A Puspitarani; Edwin W. Daniwijaya; Eggi Arguni; Endah Supriyati; Gunadi; Hana F Hanifin; Hendra Wibawa; Indarto Sulistiyono; Irene Tania; Khanza a Vujira; Kristy Iskandar; Ludhang P. Rizki; Marcellus; Mohamad S. Hakim; Nungki Anggorowati; Pramesti G Dewi; Siswanto; Susan Simanjaya; Titik Nuryastuti; Tri Wibawa
EPI_ISL_791987	RSUD dr. Loekmono Hadi Kudus	National Institute of Health Research and Development	AA; HA; HD; Ikawati; KD; KNA; Khoiriyah; N; Nugraha; Pangesti; Pawestri; Puspa; Puspandari; S; Setiawaty; Soekarso; Subangkit; T; V
EPI_ISL_3279412	RSUD dr. Mohammad Zyn Sampang	Institute of Tropical Disease, Universitas Airlangga	Agus Akhmadi; Aldise M Nastri; Gatot Soegiarto; Jezzy R Dewantari; Kazufumi Shimizu; Krisnoadi Rahardjo; Laksmi Wulandari; Maria I Lusida; Resti Yudhawati; Rima R Prasetya; Soetjipto; Yasuko Mori
EPI_ISL_3279413	RSUD dr. Soedarsono	Institute of Tropical Disease, Universitas Airlangga	Aldise M Nastri; Gatot Soegiarto; Jezzy R Dewantari; Kazufumi Shimizu; Krisnoadi Rahardjo; Laksmi Wulandari; M Burhan; Maria I Lusida; Resti Yudhawati; Rima R Prasetya; Soetjipto; Yasuko Mori
EPI_ISL_3267187	RSUD dr. Soegiri Lamongan	Institute of Tropical Disease, Universitas Airlangga	Aldise M Nastri; Gatot Soegiarto; Jezzy R Dewantari; Kazufumi Shimizu; Krisnoadi Rahardjo; Laksmi Wulandari; M Chaidir Annas; Maria I Lusida; Resti Yudhawati; Rima R Prasetya; Soetjipto; Yasuko Mori
EPI_ISL_2854711	RSUD dr. Zaenoel Abidin	National Institute of Health Research and Development	; Arie Ardiansyah Nugraha; Hana Apsari Pawestri; Hartanti Dian Ikawati; Kartika Dewi Puspa; Krisna Pangesti; Nelly Puspandari; Subangkit; Triyani Soekarso; Vivi Setiawaty
EPI_ISL_4030486, EPI_ISL_4030487	RSUD dr.H.Marsidi Judono	Genomik Solidaritas Indonesia Laboratorium	Annisa Muthiah Sukirman; Anuraj Shankar; Ariel Pradipta; Carissa Sintca Wijaya; David Hendra Haryanto; Dhahlia Agustina Cahyono; Dwi Oktavia; Gracia Felias Enos Korompis; Hendra; Himawan Masyhuri; Louisa Markus; Meutia Ayuputeri Kumaheri; Neng Hilma Hamidah; Ngabila Salama; R. Anggi Wahyu Nugroho; Tiranti Vindha; Titik Handayani Tanujaya
EPI_ISL_4761496	RSUD. Dr. R Soedjati Purwodadi Grobogan	Balai Laboratorium Kesehatan Dan Pengujian Alat Kesehatan Provinsi Jawa Tengah	Alia Wijayanti; Dwi Nursetyaasih; Dyah Ayu; Endah Wahyu Y; Ismi Farhati; Purnomo; Sekti Handayani; Wiwik Peni; Yekti Hediningsih
EPI_ISL_5616355, EPI_ISL_5616421	RSUD. Dr. R Soedjati Purwodadi Grobogan	Balai Laboratorium Kesehatan dan Pengujian Alat Kesehatan Provinsi Jawa Tengah	Alia Wijayanti; Dwi Nursetyaasih; Dyah Ayu; Endah Wahyu Y; Ismi Farhati; Purnomo; Sekti Handayani; Wiwik Peni; Yekti Hediningsih
EPI_ISL_1257865, EPI_ISL_1257866	RSUP Adam Malik	National Institute of Health Research and Development	Ade Rahmaini; Arie Ardiansyah Nugraha; Hana Apsari Pawestri; Hartanti Dian Ikawati; Kartika Dewi Puspa; Krisna Pangesti; Maliana; Nelly Elfrida Samosir; Nelly Puspandari; Rina Yunita; Subangkit; Vivi Setiawaty
EPI_ISL_1169049	RSUP Adam Malik Medan Sumatera Utara	National Institute of Health Research and Development	Arie Ardiansyah Nugraha; Hana Apsari Pawestri; Hartanti Dian Ikawati; Kartika Dewi Puspa; Nelly Puspandari; Subangkit; Vivi Setiawaty
EPI_ISL_791982	RSUP DR KARIADI Semarang	National Institute of Health Research and Development	AA; HA; HD; I; Ikawati; KD; KNA; N; Nugraha; Pangesti; Pawestri; Puspa; Puspandari; Puspitasari; Setiawaty; Soekarso; Subangkit; T; V
EPI_ISL_1257823, EPI_ISL_1257824, EPI_ISL_1257825, EPI_ISL_1257826, EPI_ISL_2854667, EPI_ISL_2854668, EPI_ISL_2854669, EPI_ISL_2854670, EPI_ISL_2854671, EPI_ISL_2854672			
see above	RSUP DR Mohammad Hoesin	National Institute of Health Research and Development	Arie Ardiansyah Nugraha; Eny Rahmawati; Hana Apsari Pawestri; Hartanti Dian Ikawati; Kartika Dewi Puspa; Krisna Pangesti; Nelly Puspandari; Rogaya; Subangkit; Triyani Soekarso; Vivi Setiawaty
EPI_ISL_2338534	RSUP DR SARDJITO	Genetics Working Group (Pokja Genetik) Faculty of Medicine, Public Health and Nursing Universitas Gadjah Mada (FK-KMK UGM); Disease Investigation Center Wates Ministry of Agriculture Indonesia; Department of Microbiology FK-KMK UGM; Laboratorium Diagnostik Yayasan Tahija World Mosquito Program (WMP) Yogyakarta Center for Tropical Medicine FK-KMK UGM; Integrated Research Center FK-KMK UGM; Department of Computer Science and Electronics FMIPA UGM; RSUP Dr. Sardjito	. Marcellus; Afiahayati; Bambang Sigit Riyanto; Dwi AA Nugrahaningsih; Dyah A Puspitarani; Edwin W. Daniwijaya; Eggi Arguni; Eko Budiono; Endah Supriyati; Gunadi; Hendra Wibawa; Heni Retnowulan; Ika Trisnawati; Ira Puspitawati; Kristy Iskandar; Ludhang P. Rizki; Mohamad S. Hakim; Munawar Gani; Nungki Anggorowati; Nur Imma Fatimah Harahap; Nur Rahmi Ananda; Osman Sianipar; Riat El Khair; Satria Maulana; Siswanto; Sumardi; Titik Nuryastuti; Tri Wibawa; Umi Solekhah Intansari; Untung Riawan; Yunika Puspadewi; Elizabeth Henny Herningtiyas
EPI_ISL_3138918, EPI_ISL_3138919, EPI_ISL_3138920, EPI_ISL_3138921, EPI_ISL_3138922, EPI_ISL_3138923, EPI_ISL_3138924, EPI_ISL_3138925, EPI_ISL_3138926			
see above	RSUP Dr Kariadi Semarang	National Institute of Health Research and Development	Arie Ardiansyah Nugraha; Fajar Nur Sulistiyohadi; Hana Apsari Pawestri; Hartanti Dian Ikawati; Kartika Dewi Puspa; Krisna Pangesti; Nelly Puspandari; Subangkit; Triyani Soekarso; Vivi Setiawaty
EPI_ISL_576115	RSUP Dr Sardjito	Genetics Working Group (Pokja Genetik) Faculty of Medicine, Public Health and Nursing Universitas Gadjah Mada (FK-KMK UGM); Disease Investigation Center Wates Ministry of Agriculture Indonesia; Department of Microbiology FK-KMK UGM; Laboratorium Diagnostik Yayasan Tahija World Mosquito Program (WMP) Yogyakarta Center for Tropical Medicine FK-KMK UGM; Integrated Research Center FK-KMK UGM; Department of Computer Science and Electronics FMIPA UGM	. Marcellus; Afiahayati; Dwi AA Nugrahaningsih; Edwin W. Daniwijaya; Eggi Arguni; Endah Supriyati; Gunadi; Hendra Wibawa; Ika Trisnawati; Ira Puspitawati; Kristy Iskandar; Ludhang P. Rizki; Maria Patricia Inggriani; Mohamad S. Hakim; Nungki Anggorowati; Nur Imma Fatimah Harahap; Osman Sianipar; Riat El Khair; Siswanto; Titik Nuryastuti; Tri Wibawa; Umi Solekhah Intansari; Untung Wirawan; Yunika Puspadewi; Elizabeth Henny Herningtiyas
EPI_ISL_2955607, EPI_ISL_3417523, EPI_ISL_3417524, EPI_ISL_4969112	RSUP Dr Sardjito	Genetics Working Group (Pokja Genetik) Faculty of Medicine, Public Health and Nursing Universitas Gadjah Mada (FK-KMK UGM); Disease Investigation Center Wates Ministry of Agriculture Indonesia; Department of Microbiology FK-KMK UGM; Laboratorium Diagnostik Yayasan Tahija World Mosquito Program (WMP) Yogyakarta Center for Tropical Medicine FK-KMK UGM; Integrated Research Center FK-KMK UGM; Department of Computer Science and Electronics FMIPA UGM; RSUP Dr. Sardjito	. Marcellus; Afiahayati; Alvina A Setiawan; Bambang Sigit Riyanto; Dwi AA Nugrahaningsih; Dwi Indaryati; Edwin W. Daniwijaya; Eggi Arguni; Eko Budiono; Endah Supriyati; Gunadi; Hana F Hanifin; Hendra Wibawa; Heni Retnowulan; Ika Trisnawati; Indarto Sulistiyono; Ira Puspitawati; Irene Tania; Khanza a Vujira; Kristy Iskandar; Ludhang P. Rizki; Marcellus; Mohamad S. Hakim; Munawar Gani; Nungki Anggorowati; Nur Imma Fatimah Harahap; Nur Rahmi Ananda; Osman Sianipar; Pramesti G Dewi; Riat El Khair; Satria Maulana; Siswanto; Sumardi; Titik Nuryastuti; Tri Wibawa; Umi Solekhah Intansari; Yunika Puspadewi; Elizabeth Henny Herningtiyas
EPI_ISL_1169047	RSUP Dr. Mohammad Hoesin Palembang Sumatera Selatan	National Institute of Health Research and Development	Arie Ardiansyah Nugraha; Hana Apsari Pawestri; Hartanti Dian Ikawati; Kartika Dewi Puspa; Nelly Puspandari; Subangkit; Vivi Setiawaty
EPI_ISL_2824988, EPI_ISL_2824989, EPI_ISL_2824990, EPI_ISL_2824991, EPI_ISL_2824992, EPI_ISL_2824993, EPI_ISL_2824994, EPI_ISL_2824995, EPI_ISL_2824996, EPI_ISL_2824997, EPI_ISL_2824998, EPI_ISL_2824999, EPI_ISL_2825000, EPI_ISL_2825001, EPI_ISL_2825002, EPI_ISL_2825003, EPI_ISL_2825004, EPI_ISL_2825005, EPI_ISL_2825006, EPI_ISL_2825007, EPI_ISL_2825008, EPI_ISL_2825009, EPI_ISL_2825010, EPI_ISL_2825011, EPI_ISL_2825012, EPI_ISL_2825013, EPI_ISL_2825014, EPI_ISL_2825015, EPI_ISL_2825016, EPI_ISL_2825017, EPI_ISL_2825018, EPI_ISL_2825019, EPI_ISL_2825020, EPI_ISL_2825021, EPI_ISL_2825022, EPI_ISL_3418699, EPI_ISL_3418866, EPI_ISL_3419363			
see above	RSUP Dr. Hasan Sadikin Bandung	Genomik Solidaritas Indonesia Laboratorium	Alfin Mohammad Abdillah; Anna Christina Brazia; Annisa Muthiah Sukirman; Anuraj Shankar; Ariel Pradipta; Basti Andriyoko; Carissa Sintca Wijaya; Delita Prihatni; Dewi Kartika Turbawati; Dhahlia Agustina Cahyono; Gracia Felias Enos Korompis; Louisa Markus; Meutia Ayuputeri Kumaheri; Normastuti Adhini Tantyo; Reinhart Gabriel; Vania Gavrila Wikasa; Vivi Setyawati

EPI_ISL_2854720, EPI_ISL_2854721, EPI_ISL_2854722	RSUP Dr. Hasan Sadikin Bandung	National Institute of Health Research and Development	; Arie Ardiansyah Nugraha; Hana Apsari Pawestri; Hartanti Dian Ikawati; Kartika Dewi Puspa; Krisna Pangesti; Nelly Puspandari; Subangkit; Triyani Soekarso; Vivi Setiawaty
EPI_ISL_2918614, EPI_ISL_2918615, EPI_ISL_2918616, EPI_ISL_2918617, EPI_ISL_2918618, EPI_ISL_2918619, EPI_ISL_2918620, EPI_ISL_2918621, EPI_ISL_2918622, EPI_ISL_2918623, EPI_ISL_2918626, EPI_ISL_3029905, EPI_ISL_3029906, EPI_ISL_3029907, EPI_ISL_3029908, EPI_ISL_3029909, EPI_ISL_3029910, EPI_ISL_3029911, EPI_ISL_3029912, EPI_ISL_3029913, EPI_ISL_3029914, EPI_ISL_3029915, EPI_ISL_3029916, EPI_ISL_3029917, EPI_ISL_3029918, EPI_ISL_3029919, EPI_ISL_3029920, EPI_ISL_3029921, EPI_ISL_3029922, EPI_ISL_3029923, EPI_ISL_3029924, EPI_ISL_3029925, EPI_ISL_3029926, EPI_ISL_3029927, EPI_ISL_3029928, EPI_ISL_3029929, EPI_ISL_3029930, EPI_ISL_3070856, EPI_ISL_3070857			
see above	RSUP Dr. Kariadi	Eijkman Institute for Molecular Biology, National Research and Innovation Agency	Amin Soebandrio; Edison Johar; Frilasita A Yudhaputri; Hidayat Trimarsanto; Iskandar Adnan; Khin Saw Myint; Lidwina Priliani; Lydia V. Panggalo; Muhammad Rezki Rasyak; Safarina G Malik; Sukma Oktavianthi; Willy Agustine
EPI_ISL_766032	RSUP Dr. Sardjito	Eijkman Institute for Molecular Biology, Ministry of Research and Technology/National Agency for Research and Innovation	Amin Soebandrio; David H Muljono; Edison Johar; Frilasita A Yudhaputri; Hidayat Trimarsanto; Khin Saw Myint; Safarina G Malik
EPI_ISL_2943173	RSUP Dr. Sardjito	Genetics Working Group (Pokja Genetik) Faculty of Medicine, Public Health and Nursing Universitas Gadjah Mada (FK-KMK UGM); Disease Investigation Center Wates Ministry of Agriculture Indonesia; Department of Microbiology FK-KMK UGM; Laboratorium Diagnostik Yayasan Tahija World Mosquito Program	. Marcellus; Afiahayati; Bambang Sigit Riyanto; Dwi AA Nugrahaningsih; Edwin W. Daniwijaya; Eggi Arguni; Eko Budiono; Endah Supriyati; Gunadi; Hana F Hanifin; Hendra Wibawa; Heni Retnowulan; Ika Trisnawati; Ira Puspitawati; Irene Tania; Kristy Iskandar; Ludhang P. Rizki; Mohamad S. Hakim; Munawar Gani; Nungki Anggorowati; Nur Imma Fatimah Harahap; Nur Rahmi Ananda; Osman Sianipar; Riat El Khair; Satria Maulana; Siswanto; Sumardi; Titik Nuryastuti; Tri Wibawa; Umi Solekhah Intansari; Yunika Puspadewi; Elizabeth Henny Herningtiyas
EPI_ISL_576113, EPI_ISL_576114, EPI_ISL_576116, EPI_ISL_576128, EPI_ISL_576130	RSUP Dr. Sardjito	Genetics Working Group (Pokja Genetik) Faculty of Medicine, Public Health and Nursing Universitas Gadjah Mada (FK-KMK UGM); Disease Investigation Center Wates Ministry of Agriculture Indonesia; Department of Microbiology FK-KMK UGM; Laboratorium Diagnostik Yayasan Tahija World Mosquito Program (WMP) Yogyakarta Center for Tropical Medicine FK-KMK UGM; Integrated Research Center FK-KMK UGM; Department of Computer Science and Electronics FMIPA UGM	. Marcellus; Aditya Rifqi Fauzi; Afiahayati; Alvin S. Kalim; Alvin Santoso Kalim; Amalia Setyati; Audric Kenny Tedja; Bambang Sigit Riyanto; Cahya Dewi Satria; Desyifa Mursalin; Dwi AA Nugrahaningsih; Dwikisworo Setyowireni; Dyah Ayu Puspitarani; Edwin W. Daniwijaya; Eggi Arguni; Eko Budiono; Elisabeth Siti Herini; Endah Supriyati; Fadil Fahri; Gunadi; Hendra Wibawa; Heni Retnowulan; Ida Safitri Laksanawati; Ika Trisnawati; Ira Puspitawati; Kemala Athollah; Kristy Iskandar; Ludhang P. Rizki; Marcellus; Mohamad S. Hakim; Munawar Gani; Nungki Anggorowati; Nur Imma Fatimah Harahap; Nur Rahmi Ananda; Osman Sianipar; Riat El Khair; Rina Triasih; Ririn Enggy Yuliyanti; Satria Maulana; Siswanto; Sumardi; Susan Simanjaya; Titik Nuryastuti; Titis Widowati; Tri Wibawa; Umi Solekhah Intansari; William Widitjiarso; Yunika Puspadewi; Elizabeth Henny Herningtiyas
EPI_ISL_862039, EPI_ISL_862040, EPI_ISL_862041, EPI_ISL_872188, EPI_ISL_872189, EPI_ISL_872190, EPI_ISL_885142, EPI_ISL_2601039			
see above	RSUP Dr. Sardjito	Genetics Working Group (Pokja Genetik) Faculty of Medicine, Public Health and Nursing Universitas Gadjah Mada (FK-KMK UGM); Disease Investigation Center Wates Ministry of Agriculture Indonesia; Department of Microbiology FK-KMK UGM; Laboratorium Diagnostik Yayasan Tahija World Mosquito Program (WMP) Yogyakarta Center for Tropical Medicine FK-KMK UGM; Integrated Research Center FK-KMK UGM; Department of Computer Science and Electronics FMIPA UGM; RSUP Dr. Sardjito	. Marcellus; Aditya Rifqi Fauzi; Afiahayati; Alvin S. Kalim; Alvin Santoso Kalim; Audric Kenny Tedja; Bambang Sigit Riyanto; Dwi AA Nugrahaningsih; Dwiki afandy; Dyah Ayu Puspitarani; Edwin W. Daniwijaya; Eggi Arguni; Eko Budiono; Endah Supriyati; Fadil Fahri; Gunadi; Hana F Hanifin; Hendra Wibawa; Heni Retnowulan; Ika Trisnawati; Ira Puspitawati; Irene Tania; Kemala Athollah; Kristy Iskandar; Ludhang P. Rizki; Maria Patricia Inggriani; Mohamad S. Hakim; Munawar Gani; Nungki Anggorowati; Nur Imma Fatimah Harahap; Nur Rahmi Ananda; Osman Sianipar; Riat El Khair; Satria Maulana; Siswanto; Sumardi; Susan Simanjaya; Titik Nuryastuti; Tri Wibawa; Umi Solekhah Intansari; Untung Riawan; William Widitjiarso; Yunika Puspadewi; Elizabeth Henny Herningtiyas
EPI_ISL_1257827, EPI_ISL_1257828, EPI_ISL_1257829, EPI_ISL_1257863	RSUP Dr. Sardjito	National Institute of Health Research and Development	Arie Ardiansyah Nugraha; Hana Apsari Pawestri; Hartanti Dian Ikawati; Kartika Dewi Puspa; Krisna Pangesti; Nelly Puspandari; Subangkit; Vivi Setiawaty
EPI_ISL_3216515, EPI_ISL_3216516, EPI_ISL_3216517, EPI_ISL_3216518, EPI_ISL_3216519, EPI_ISL_3216521, EPI_ISL_3230238, EPI_ISL_3230239, EPI_ISL_3230240, EPI_ISL_3230241, EPI_ISL_3230242, EPI_ISL_3230243, EPI_ISL_3230247			
see above	RSUP FATMAWATI / DINAS KESEHATAN DKI JAKARTA	Genomik Solidaritas Indonesia Laboratorium	Alfin Mohammad Abdillah; Anna Christina Brazia; Annisa Muthiah Sukirman; Anuraj Shankar; Ariel Pradipta; Carissa Sintca Wijaya; Dhahlia Agustina Cahyono; Gracia Felias Enos Korompis; Hartini Cahyadi; Himawan Masyhuri; Irwan Idris; Jerry; Leonardo H Simanjuntak; Lidya Utami; Loli Simajuntak; Meutia Ayuputeri Kumaheri; Neng Hilma Hamidah; Normastuti Adhini Tantyo; Reinhart Gabriel; Vania Gavrila Wikasa
EPI_ISL_467376, EPI_ISL_568687, EPI_ISL_568690, EPI_ISL_568691, EPI_ISL_766044, EPI_ISL_766046	RSUP Fatmawati	Eijkman Institute for Molecular Biology, Ministry of Research and Technology/National Agency for Research and Innovation	Amin Soebandrio; David H Muljono; Edison Johar; Frilasita A Yudhaputri; Herawati Sudoyo; Hidayat Trimarsanto; Iskandar A Adnan; Khin Saw Myint; Safarina G Malik; Willy Agustine
EPI_ISL_1257842, EPI_ISL_1257843, EPI_ISL_1257844, EPI_ISL_1257845, EPI_ISL_1257846, EPI_ISL_2262252	RSUP Fatmawati	National Institute of Health Research and Development	Arie Ardiansyah Nugraha; Hana Apsari Pawestri; Hartanti Dian Ikawati; Kartika Dewi Puspa; Krisna Pangesti; Lidya Utami; Luci Liana; Lusiya Ningsih; Nelly Puspandari; Subangkit; Triyani Soekarso; Vivi Setiawaty
EPI_ISL_2854673, EPI_ISL_2854674, EPI_ISL_2854675, EPI_ISL_2854676, EPI_ISL_2854677, EPI_ISL_2854678, EPI_ISL_2854679, EPI_ISL_2854680, EPI_ISL_2854681, EPI_ISL_2854682, EPI_ISL_2931731, EPI_ISL_2931732, EPI_ISL_2931739, EPI_ISL_2931740, EPI_ISL_2931748, EPI_ISL_2931749, EPI_ISL_2931758, EPI_ISL_2931768, EPI_ISL_2931778, EPI_ISL_2931784, EPI_ISL_2931785, EPI_ISL_2931793, EPI_ISL_2931794, EPI_ISL_2932709, EPI_ISL_2987664, EPI_ISL_2987665, EPI_ISL_4254854, EPI_ISL_4254855, EPI_ISL_4254856, EPI_ISL_4254857, EPI_ISL_4254858, EPI_ISL_4254859, EPI_ISL_4254860, EPI_ISL_4254861, EPI_ISL_4254862, EPI_ISL_4254863, EPI_ISL_4254864, EPI_ISL_4254865, EPI_ISL_4254866			
see above	RSUP H Adam Malik	National Institute of Health Research and Development	; Arie Ardiansyah Nugraha; Fajar Nur Sulistiyohadi; Hana Apsari Pawestri; Hartanti Dian Ikawati; Kartika Dewi Puspa; Krisna Pangesti; Nelly Puspandari; Subangkit; Triyani Soekarso; Vivi Setiawaty
EPI_ISL_2047513, EPI_ISL_2047514, EPI_ISL_2047515, EPI_ISL_2047516, EPI_ISL_2047517, EPI_ISL_2047518, EPI_ISL_2047519, EPI_ISL_2047520, EPI_ISL_2047521, EPI_ISL_2047522, EPI_ISL_2047523, EPI_ISL_2047524, EPI_ISL_2047525, EPI_ISL_2047526, EPI_ISL_2047527, EPI_ISL_2047528, EPI_ISL_2106987			
see above	RSUP H Adam Malik Medan	National Institute of Health Research and Development	Ade Rahmaini; Arie Ardiansyah Nugraha; Hana Apsari Pawestri; Hartanti Dian Ikawati; Kartika Dewi Puspa; Krisna Pangesti; Maliana; Nelly Elfrida Samosir; Nelly Puspandari; Rina Yunita; Subangkit; Triyani Soekarso; Vivi Setiawaty
EPI_ISL_3138944, EPI_ISL_3138945, EPI_ISL_3138946, EPI_ISL_3138947, EPI_ISL_3138948	RSUP Nasional Dr. Cipto Mangunkusumo	National Institute of Health Research and Development	Arie Ardiansyah Nugraha; Fajar Nur Sulistiyohadi; Hana Apsari Pawestri; Hartanti Dian Ikawati; Kartika Dewi Puspa; Krisna Pangesti; Nelly Puspandari; Subangkit; Triyani Soekarso; Vivi Setiawaty
EPI_ISL_3186060, EPI_ISL_3186061, EPI_ISL_3186064, EPI_ISL_3186065, EPI_ISL_3186066, EPI_ISL_3186067, EPI_ISL_3186068, EPI_ISL_3186069, EPI_ISL_3186070, EPI_ISL_3186071, EPI_ISL_3186072, EPI_ISL_3186073, EPI_ISL_3186074, EPI_ISL_3194636, EPI_ISL_3374423, EPI_ISL_3374424, EPI_ISL_3374425			
see above	RSUP Persahabatan, Jakarta	Biosafety Level-3 Laboratory, Indonesian Institute of Sciences (LIPI)	Ade Andriani; Ahmad R. H. Utomo; Alfi T. Fathurahman; Andhika Candra Putra; Andri Wardiana; Anggia Prasetyoputri; Anik B. Dharmayanthi; Asep M. Ridwanulloh; Asep M. Ridwanuloh; Budi Haryanto; Hariyatun; Idris; Indriawati; Isa Nuryana; Jamal Zaini; Linda Sukmarini; Ratih A. Ningrum; Sri Swasthikawati; Sugiyono Saputra; Syaiful Rizal; Syam B. Iryanto; Zahrah Saniyyah
EPI_ISL_4106129, EPI_ISL_4106130, EPI_ISL_4106131, EPI_ISL_4106132, EPI_ISL_4106133, EPI_ISL_4106134	RSUP Persahabatan, Jakarta	National Research and Innovation Agency (BRIN-Indonesia, VenomCoV Project Team)	Ade Andriani; Ahmad R. H. Utomo; Andhika Candra Putra; Andri Wardiana; Anggia Prasetyoputri; Asep M. Ridwanuloh; Budi Haryanto; Hariyatun; Jamal Zaini; Ratih A. Ningrum; Sugiyono Saputra; Zahrah Saniyyah
EPI_ISL_467375, EPI_ISL_568694, EPI_ISL_574604	RSUP Prof. Dr. R. Kandou Manado	Eijkman Institute for Molecular Biology, Ministry of Research and Technology/National Agency for Research and Innovation	Amin Soebandrio; David H Muljono; Edison Johar; Frilasita A Yudhaputri; Herawati Sudoyo; Hidayat Trimarsanto; Iskandar A Adnan; Khin Saw Myint; Safarina G Malik; Willy Agustine
EPI_ISL_1257833, EPI_ISL_1257834, EPI_ISL_1257835, EPI_ISL_1257836, EPI_ISL_1257837, EPI_ISL_1257838, EPI_ISL_1257839, EPI_ISL_1257840, EPI_ISL_1257841, EPI_ISL_1824605, EPI_ISL_1824606, EPI_ISL_1915550, EPI_ISL_1915554, EPI_ISL_1915555, EPI_ISL_1915556, EPI_ISL_1915557, EPI_ISL_1915558, EPI_ISL_1915559, EPI_ISL_1915569, EPI_ISL_1915570, EPI_ISL_2106976, EPI_ISL_2106977, EPI_ISL_2106978, EPI_ISL_2987584, EPI_ISL_2987585, EPI_ISL_2987586, EPI_ISL_2987587, EPI_ISL_2987588, EPI_ISL_2987589, EPI_ISL_2987590, EPI_ISL_3138768, EPI_ISL_3138769, EPI_ISL_3138770, EPI_ISL_3138771, EPI_ISL_3138772, EPI_ISL_3138773, EPI_ISL_3138774, EPI_ISL_3138775, EPI_ISL_3138776, EPI_ISL_3138777, EPI_ISL_3138778, EPI_ISL_3138779, EPI_ISL_3138780, EPI_ISL_3138781, EPI_ISL_3138782, EPI_ISL_3138783, EPI_ISL_3138784, EPI_ISL_3278298, EPI_ISL_3691593, EPI_ISL_4090585, EPI_ISL_4090586, EPI_ISL_4090587, EPI_ISL_4090588, EPI_ISL_4090589			
see above	RSUP Sanglah	National Institute of Health Research and Development	Arie Ardiansyah Nugraha; Fajar Nur Sulistiyohadi; Hana Apsari Pawestri; Hartanti Dian Ikawati; I Gede Ketut Sajinadiyasa; I Nengah Tony Rustawan; Kartika Dewi Puspa; Krisna Pangesti; Nelly Puspandari; Subangkit; Triyani Soekarso; Vivi Setiawaty
EPI_ISL_5022739, EPI_ISL_5022740, EPI_ISL_5022741, EPI_ISL_5022742, EPI_ISL_5022743, EPI_ISL_5022744, EPI_ISL_5022745, EPI_ISL_5022746, EPI_ISL_5022747, EPI_ISL_5022748, EPI_ISL_5022749, EPI_ISL_5022750, EPI_ISL_5022751, EPI_ISL_5022752, EPI_ISL_5022753, EPI_ISL_5022754, EPI_ISL_5022755, EPI_ISL_5022756			
see above	RSUP Sanglah Bali	National Institute of Health Research and Development	; Arie Ardiansyah Nugraha; Fajar Nur Sulistiyohadi; Hana Apsari Pawestri; Hartanti Dian Ikawati; Kartika Dewi Puspa; Krisna Pangesti; Nelly Puspandari; Subangkit; Triyani Soekarso; Vivi Setiawaty
EPI_ISL_2955606	RSUP Sardjito	Genetics Working Group (Pokja Genetik) Faculty of Medicine, Public Health and Nursing Universitas Gadjah Mada (FK-KMK UGM); Disease Investigation Center Wates Ministry of Agriculture Indonesia; Department of Microbiology FK-KMK UGM; Laboratorium Diagnostik Yayasan Tahija World Mosquito Program (WMP) Yogyakarta Center for Tropical Medicine FK-KMK UGM; Integrated Research Center FK-KMK UGM; Department of Computer Science and Electronics FMIPA UGM; RSUP Dr. Sardjito	. Marcellus; Afiahayati; Alvina A Setiawan; Bambang Sigit Riyanto; Dwi AA Nugrahaningsih; Edwin W. Daniwijaya; Eggi Arguni; Eko Budiono; Endah Supriyati; Gunadi; Hendra Wibawa; Heni Retnowulan; Ika Trisnawati; Ira Puspitawati; Khanza a Vujira; Kristy Iskandar; Ludhang P. Rizki; Mohamad S. Hakim; Munawar Gani; Nungki Anggorowati; Nur Imma Fatimah Harahap; Nur Rahmi Ananda; Osman Sianipar; Riat El Khair; Satria Maulana; Siswanto; Sumardi; Titik Nuryastuti; Tri Wibawa; Umi Solekhah Intansari; Yunika Puspadewi; Elizabeth Henny Herningtiyas
EPI_ISL_3375327, EPI_ISL_3375328, EPI_ISL_3375329, EPI_ISL_3375330, EPI_ISL_3375331, EPI_ISL_3375332, EPI_ISL_3375333, EPI_ISL_3375334, EPI_ISL_3375335, EPI_ISL_3375336, EPI_ISL_3375337, EPI_ISL_3375338, EPI_ISL_3375339, EPI_ISL_3375340, EPI_ISL_3375341, EPI_ISL_3375342, EPI_ISL_3375343, EPI_ISL_3375344, EPI_ISL_3375345, EPI_ISL_3375346, EPI_ISL_3375347, EPI_ISL_3375348, EPI_ISL_3375349, EPI_ISL_3375350, EPI_ISL_3375351, EPI_ISL_3375352, EPI_ISL_3375353, EPI_ISL_3691580			
see above	RSUP dr. M Djamil Padang	National Institute of Health Research and Development	Arie Ardiansyah Nugraha; Deswita Sari; Fajar Nur Sulistiyohadi; Hana Apsari Pawestri; Hartanti Dian Ikawati; Kartika Dewi Puspa; Krisna Pangesti; Nelly Puspandari; Subangkit; Triyani Soekarso; Vivi Setiawaty
EPI_ISL_4392618, EPI_ISL_4392619, EPI_ISL_4392620, EPI_ISL_4392621, EPI_ISL_4392622, EPI_ISL_4392623, EPI_ISL_4392624, EPI_ISL_4392625, EPI_ISL_4392626, EPI_ISL_4392627, EPI_ISL_4392628, EPI_ISL_4392629, EPI_ISL_4392630, EPI_ISL_4392631, EPI_ISL_4392632, EPI_ISL_4392633, EPI_ISL_4392634, EPI_ISL_4392635, EPI_ISL_4392636, EPI_ISL_4392637, EPI_ISL_4392638, EPI_ISL_4392639, EPI_ISL_4392640, EPI_ISL_4392641, EPI_ISL_4392642, EPI_ISL_4392643, EPI_ISL_4392644, EPI_ISL_4392646, EPI_ISL_4392647, EPI_ISL_4392648, EPI_ISL_4392649, EPI_ISL_4392650, EPI_ISL_4392651, EPI_ISL_4392652, EPI_ISL_4392653, EPI_ISL_4392654, EPI_ISL_4392655, EPI_ISL_4392656, EPI_ISL_4392657, EPI_ISL_4392658, EPI_ISL_4392659, EPI_ISL_4392660, EPI_ISL_4392662			
see above	RSUP dr. Mohammad Hoesin/Fakultas Kedokteran Universitas Sriwijaya	Eijkman Institute for Molecular Biology, National Agency for Research and Innovation; RSUP dr. Mohammad Hoesin/Fakultas Kedokteran Universitas Sriwijaya	Ahmad Ghiffari; Amin Soebandrio; Chairil Anwar; Edison Johar; Eny Rahmawati; Frilasita A Yudhaputri; Hidayat Trimarsanto; Iche Andriani Liberty; Iskandar Adnan; Jessica R Saragih; Khin Saw Myint; Lidwina Priliani; Lydia V Panggalo; Muhammad Rezki Rasyak; Nelda Aprilia Salim; Phey Liana; Safarina G Malik; Sukma Oktavianthi; Willy Agustine; Yulia Iriani
EPI_ISL_3070867, EPI_ISL_3070868, EPI_ISL_3070869, EPI_ISL_3070870, EPI_ISL_3070871	RSUP dr. Mohammad Hoesin/Fakultas Kedokteran Universitas Sriwijaya	Eijkman Institute for Molecular Biology, National Research and Innovation Agency	Ahmad Ghiffari; Amin Soebandrio; Chairil Anwar; Edison Johar; Eny Rahmawati; Frilasita A Yudhaputri; Hidayat Trimarsanto; Iche Andriani Liberty; Iskandar Adnan; Khin Saw Myint; Lidwina Priliani; Lydia V. Panggalo; Muhammad Rezki Rasyak; Nelda Aprilia Salim; Phey Liana; Safarina G Malik; Sukma Oktavianthi; Willy Agustine; Yulia Iriani
EPI_ISL_4549379, EPI_ISL_4549380, EPI_ISL_4549381, EPI_ISL_4549382, EPI_ISL_4549383, EPI_ISL_4549384	RSUP dr. Mohammad Hoesin/Fakultas Kedokteran Universitas Sriwijaya	Eijkman Research Center for Molecular Biology, National Research and Innovation Agency; RSUP dr. Mohammad Hoesin/Fakultas Kedokteran Universitas Sriwijaya	Ahmad Ghiffari; Amin Soebandrio; Chairil Anwar; Edison Johar; Eny Rahmawati; Frilasita A Yudhaputri; Hidayat Trimarsanto; Iche Andriani Liberty; Iskandar Adnan; Jessica R Saragih; Khin Saw Myint; Lidwina Priliani; Lydia V Panggalo; Muhammad Rezki Rasyak; Nelda Aprilia Salim; Phey Liana; Safarina G Malik; Sukma Oktavianthi; Willy Agustine; Yulia Iriani
EPI_ISL_525492	RSUP dr. SOERADJI TIRTONEGORO	Genetics Working Group (Pokja Genetik) Faculty of Medicine, Public Health and Nursing Universitas Gadjah Mada (FK-KMK UGM), Disease Investigation Center Wates Ministry of Agriculture Indonesia, Department of Microbiology FK-	. Marcellus; . Siswanto; Afiahayati; Alvin S. Kalim; Dwi AA Nugrahaningsih; Dwiki Afandy; Edwin W. Daniwijaya; Eggi Arguni; Endah Supriyati; Gunadi; Hendra Wibawa; Indah Juliana; Kurniyanto; Ludhang P. Rizki; Mohamad S. Hakim; Titik Nuryastuti; Tri Wibawa

		KMK UGM, Laboratorium Diagnostik Yayasan Tahija World Mosquito Program (WMP) Yogyakarta Center for Tropical Medicine FK-KMK UGM, Integrated Research Center FK-KMK UGM, Department of Computer Science and Electronics FMIPA UGM	
EPI_ISL_574605	RSUP dr. Sardjito	Eijkman Institute for Molecular Biology, Ministry of Research and Technology/National Agency for Research and Innovation	Amin Soebandrio; David H Muljono; Edison Johar; Frilasita A Yudhaputri; Herawati Sudoyo; Hidayat Trimarsanto; Iskandar A Adnan; Khin Saw Myint; Safarina G Malik; Willy Agustine
EPI_ISL_4090590, EPI_ISL_4090591, EPI_ISL_4090592, EPI_ISL_4090593, EPI_ISL_4090594	RSUP dr. Wahidin Sudirohusodo	National Institute of Health Research and Development	Arie Ardiansyah Nugraha; Fajar Nur Sulistiyohadi; Hana Apsari Pawestri; Hartanti Dian Ikawati; Kartika Dewi Puspa; Krisna Pangesti; Nelly Puspandari; Subangkit; Triyani Soekarso; Vivi Setiawaty
EPI_ISL_2601661, EPI_ISL_2601662, EPI_ISL_2601663, EPI_ISL_2601664, EPI_ISL_2601665, EPI_ISL_2601666, EPI_ISL_2601667, EPI_ISL_2601668, EPI_ISL_2601669, EPI_ISL_2601670			
see above	RSUPN Cipto Mangunkusumo	Genomik Solidaritas Indonesia Laboratorium	Ahmad Zoebad Foeady; Alfin Mohammad Abdillah; Anindya Pradipta Susanto; Annisa Muthiah Sukirman; Anuraj Shankar; Ariel Pradipta; Budi Wiweko; Carissa Sintca Wijaya; Dhahlia Agustina Cahyono; Eka Ginanjar; Eric Tenda; Gracia Felias Enos Korompis; Louisa Markus; Meutia Ayuputeri Kumaheri; Normastuti Adhini Tantyo; Nuri Dyah Indrasari; Pratiwi Sudarmono; Vania Gavrila Wikasa
EPI_ISL_3160399	RSUPN Dr. Cipto Mangunkusumo	Eijkman Institute for Molecular Biology, National Research and Innovation Agency	Amin Soebandrio; Edison Johar; Frilasita A Yudhaputri; Hidayat Trimarsanto; Iskandar Adnan; Khin Saw Myint; Lidwina Priliani; Lydia V. Panggalo; Muhammad Rezki Rasyak; Safarina G Malik; Sukma Oktavianthi; Willy Agustine
EPI_ISL_984264, EPI_ISL_1239220	RSUPN Dr. Cipto Mangunkusumo	National Institute of Health Research and Development	Agustiningsih; Arie Ardiansyah Nugraha; Dewi Wulandari; Fauzul Muna; Hana Apsari Pawestri; Hartanti Dian Ikawati; Herna; Holy Arif Wibowo; Irene Lorinda Indalao; Kartika Dewi Puspa; Kindi Adam; Krisna Nur Andriana Pangesti; Natalie Laurencia Kipuw; Nelly Puspandari; Ni Ketut Susilarini; Nike Susanti; Nuri Dyah Indrasari; Nurika Hariastuti; Reni Herman; Ririn Ramadhany; Subangkit; Tati Febriyanti; Triyani Soekarso; Ully Alfi Nikmah; Vivi Setiawaty; Vivi Setiawaty.; Yuni Rukminiati
EPI_ISL_2617502, EPI_ISL_2617503, EPI_ISL_2617504, EPI_ISL_2617505, EPI_ISL_2617506, EPI_ISL_2617507, EPI_ISL_2617508, EPI_ISL_2617509, EPI_ISL_2617510, EPI_ISL_2617511			
see above	RSUPP Persahabatan Jakarta	National Institute of Health Research and Development	Arie Ardiansyah Nugraha; Hana Apsari Pawestri; Hartanti Dian Ikawati; Kartika Dewi Puspa; Krisna Pangesti; Nelly Puspandari; Subangkit; Triyani Soekarso; Vivi Setiawaty
EPI_ISL_3755956, EPI_ISL_3755957, EPI_ISL_3755958, EPI_ISL_3755959, EPI_ISL_3755960, EPI_ISL_3755961, EPI_ISL_3755962, EPI_ISL_3755963, EPI_ISL_3755964, EPI_ISL_3755965, EPI_ISL_3755966, EPI_ISL_4503235, EPI_ISL_4503236, EPI_ISL_4503237			
see above	RUMAH SAKIT DAERAH IDAMAN KOTA BANJARBARU	Genomik Solidaritas Indonesia Laboratorium	Anna Christina Brazia; Annisa Muthiah Sukirman; Anuraj Shankar; Ariel Pradipta; David Hendra Haryanto; Dhahlia Agustina Cahyono; Gracia Felias Enos Korompis; Louisa Markus; Meutia Ayuputeri Kumaheri; Pratiwi Srikandi Yudhastari; Rahimul Yakin; Reinhart Gabriel; Vania Gavrila Wikasa
EPI_ISL_4549295, EPI_ISL_4549296, EPI_ISL_4549297, EPI_ISL_4549298, EPI_ISL_4549299, EPI_ISL_4549300, EPI_ISL_4549301, EPI_ISL_4549302, EPI_ISL_4549303, EPI_ISL_4549304, EPI_ISL_4549305, EPI_ISL_4549306, EPI_ISL_4549307, EPI_ISL_4549308, EPI_ISL_4549309, EPI_ISL_4549310, EPI_ISL_4549311, EPI_ISL_4549312, EPI_ISL_4549313, EPI_ISL_4549314, EPI_ISL_4549315, EPI_ISL_4549316, EPI_ISL_4549317, EPI_ISL_4549318, EPI_ISL_4549319, EPI_ISL_4549320, EPI_ISL_4549321, EPI_ISL_4549322, EPI_ISL_4549323, EPI_ISL_4549324, EPI_ISL_4549325, EPI_ISL_4549326, EPI_ISL_4549327, EPI_ISL_4549328, EPI_ISL_4549329, EPI_ISL_4549330, EPI_ISL_4549331, EPI_ISL_4549332, EPI_ISL_4549333, EPI_ISL_4549334, EPI_ISL_4549335, EPI_ISL_4549336, EPI_ISL_4549337, EPI_ISL_4549338, EPI_ISL_4549339, EPI_ISL_4549340, EPI_ISL_4549341, EPI_ISL_4549342, EPI_ISL_4549343, EPI_ISL_4549344, EPI_ISL_4549345, EPI_ISL_4549346, EPI_ISL_4549348, EPI_ISL_4549349, EPI_ISL_4549350, EPI_ISL_4549351, EPI_ISL_4549353, EPI_ISL_4549354, EPI_ISL_4549355, EPI_ISL_4549356, EPI_ISL_4549357, EPI_ISL_4549358, EPI_ISL_4549359, EPI_ISL_4549360, EPI_ISL_4549361, EPI_ISL_4549362, EPI_ISL_4549363, EPI_ISL_4549364, EPI_ISL_4549365, EPI_ISL_4549366, EPI_ISL_4549367, EPI_ISL_4549368, EPI_ISL_4549369, EPI_ISL_4549370, EPI_ISL_4549371, EPI_ISL_4549372, EPI_ISL_4549373, EPI_ISL_4549374, EPI_ISL_4549375, EPI_ISL_4549376, EPI_ISL_4549377, EPI_ISL_4549378, EPI_ISL_5021163, EPI_ISL_5021167, EPI_ISL_5021172, EPI_ISL_5021178, EPI_ISL_5021185, EPI_ISL_5021188, EPI_ISL_5021197, EPI_ISL_5021199, EPI_ISL_5021205, EPI_ISL_5021208, EPI_ISL_5021211, EPI_ISL_5021217, EPI_ISL_5021224, EPI_ISL_5021233, EPI_ISL_5021241, EPI_ISL_5021245, EPI_ISL_5021249, EPI_ISL_5021257, EPI_ISL_5021262, EPI_ISL_5021268, EPI_ISL_5021272, EPI_ISL_5021278, EPI_ISL_5021286, EPI_ISL_5021294, EPI_ISL_5021300, EPI_ISL_5021303, EPI_ISL_5021308, EPI_ISL_5021316, EPI_ISL_5395721, EPI_ISL_5395722, EPI_ISL_5395723, EPI_ISL_5395724, EPI_ISL_5395725, EPI_ISL_5395726, EPI_ISL_5395727, EPI_ISL_5395728, EPI_ISL_5395729, EPI_ISL_5395730, EPI_ISL_5395731, EPI_ISL_5395732, EPI_ISL_5395733, EPI_ISL_5395734, EPI_ISL_5395735, EPI_ISL_5395736, EPI_ISL_5395737, EPI_ISL_5395738, EPI_ISL_5395739, EPI_ISL_5395740, EPI_ISL_5395741, EPI_ISL_5395742, EPI_ISL_5395744, EPI_ISL_5395745, EPI_ISL_5395746, EPI_ISL_5395747, EPI_ISL_5395748, EPI_ISL_5395749, EPI_ISL_5395750, EPI_ISL_5395751, EPI_ISL_5395752, EPI_ISL_5395753, EPI_ISL_5395754, EPI_ISL_5395755, EPI_ISL_5395756, EPI_ISL_5395757, EPI_ISL_5395758, EPI_ISL_5395759, EPI_ISL_5395760, EPI_ISL_5395761, EPI_ISL_5395762, EPI_ISL_5395763, EPI_ISL_5395764, EPI_ISL_5395765, EPI_ISL_5395766, EPI_ISL_5395767, EPI_ISL_5395768, EPI_ISL_5395769, EPI_ISL_5395771, EPI_ISL_5395772, EPI_ISL_5395773, EPI_ISL_5395774, EPI_ISL_5395775, EPI_ISL_5395776, EPI_ISL_5395777, EPI_ISL_5395778, EPI_ISL_5395779, EPI_ISL_5395783, EPI_ISL_5395784, EPI_ISL_5395785, EPI_ISL_5395786, EPI_ISL_5395787, EPI_ISL_5395788, EPI_ISL_5395789, EPI_ISL_5395790, EPI_ISL_5395791, EPI_ISL_5395792, EPI_ISL_5395793, EPI_ISL_5395794, EPI_ISL_5395795, EPI_ISL_5395796, EPI_ISL_5395797, EPI_ISL_5395798, EPI_ISL_5395799, EPI_ISL_5395800, EPI_ISL_5395801, EPI_ISL_5395802			
see above	Raden Mattaher Regional General Hospital (RSUD)	Eijkman Research Center for Molecular Biology, National Research and Innovation Agency; Raden Mattaher Regional General Hospital (RSUD)	Amin Soebandrio; Edison Johar; Fery Kusnadi; Frilasita A Yudhaputri; Hidayat Trimarsanto; Iskandar Adnan; Jessica R Saragih; Khin Saw Myint; Lidwina Priliani; Lydia V Panggalo; Muhammad Rezki Rasyak; Safarina G Malik; Sotianingsih; Sukma Oktavianthi; Willy Agustine; Yusnidar
EPI_ISL_1165076	Rahman Rahim Hospital	Institute of Tropical Disease, Universitas Airlangga	Aldise M Nastri; Gatot Soegiarto; Jezzy R Dewantari; Kazufumi Shimizu; Krisnoadi Rahardjo; Laksmi Wulandari; Maria I Lusida; Rendra E Febrianto; Resti Yudhawati; Rima R Prasetya; Soetjipto; Yasuko Mori
EPI_ISL_1159391	Royal Hospital	Institute of Tropical Disease, Universitas Airlangga	Aldise M Nastri; Gatot Soegiarto; Henny P Margastuti; Jezzy R Dewantari; Kazufumi Shimizu; Krisnoadi Rahardjo; Laksmi Wulandari; Maria I Lusida; Resti Yudhawati; Rima R Prasetya; Soetjipto; Yasuko Mori
EPI_ISL_3401869, EPI_ISL_3401870	Rsia Budhi Jaya	Genomik Solidaritas Indonesia Laboratorium	Annisa Muthiah Sukirman; Anuraj Shankar; Ariel Pradipta; Carissa Sintca Wijaya; Dhahlia Agustina Cahyono; Dwi Oktavia; Gracia Felias Enos Korompis; Himawan Masyhuri; Louisa Markus; Lulu Nursyifa; Meutia Ayuputeri Kumaheri; Ngabila Salama; Tiranti Vindha; Vania Gavrila Wikasa
EPI_ISL_3401871	Rsud Kebayoran Baru	Genomik Solidaritas Indonesia Laboratorium	Annisa Muthiah Sukirman; Anuraj Shankar; Ariel Pradipta; Carissa Sintca Wijaya; Dhahlia Agustina Cahyono; Dwi Oktavia; Gracia Felias Enos Korompis; Himawan Masyhuri; Louisa Markus; Meutia Ayuputeri Kumaheri; Ngabila Salama; Ridho Aulia Rahman; Tiranti Vindha; Vania Gavrila Wikasa
EPI_ISL_4090643, EPI_ISL_4090644	Rumah Sakit Advent Bandar Lampung	National Institute of Health Research and Development	Arie Ardiansyah Nugraha; Fajar Nur Sulistiyohadi; Hana Apsari Pawestri; Hartanti Dian Ikawati; Kartika Dewi Puspa; Krisna Pangesti; Nelly Puspandari; Subangkit; Triyani Soekarso; Vivi Setiawaty
EPI_ISL_516800	Rumah Sakit Akademik Universitas Gadjah Mada	Genetics Working Group (Pokja Genetik) Faculty of Medicine, Public Health and Nursing Universitas Gadjah Mada (FK-KMK UGM); Disease Investigation Center Wates Ministry of Agriculture Indonesia; Department of Microbiology FK-KMK UGM; Laboratorium Diagnostik Yayasan Tahija World Mosquito Program (WMP) Yogyakarta Center for Tropical Medicine FK-KMK UGM; Integrated Research center FK-KMK UGM; Department of Computer Science and Electronics FMIPA UGM	. Marcellus; Afiahayati; Alvin S. Kalim; Desyifa Mursalin; Dwi AA Nugrahaningsih; Edwin W. Daniwijaya; Eggi Arguni; Endah Supriyati; Gunadi; Hendra Wibawa; Ludhang P. Rizki; Mohamad S. Hakim; Siswanto; Titik Nuryastuti; Tri Wibawa
EPI_ISL_4968082	Rumah Sakit Bhayangkara Polda DIY	Genetics Working Group (Pokja Genetik) Faculty of Medicine, Public Health and Nursing Universitas Gadjah Mada (FK-KMK UGM); Disease Investigation Center Wates Ministry of Agriculture Indonesia; Department of Microbiology FK-KMK UGM; Laboratorium Diagnostik Yayasan Tahija World Mosquito Program (WMP) Yogyakarta Center for Tropical Medicine FK-KMK UGM; Integrated Research Center FK-KMK UGM; Department of Computer Science and Electronics FMIPA UGM; RSUP Dr. Sardjito	Afiahayati; Dwi AA Nugrahaningsih; Dwi Indaryati; Edwin W. Daniwijaya; Eggi Arguni; Endah Supriyati; Gunadi; Hana F Hanifin; Hendra Wibawa; Indarto Sulistiyono; Irene Tania; Kristy Iskandar; Ludhang P. Rizki; Marcellus; Mohamad S. Hakim; Nungki Anggorowati; Pramesti G Dewi; Siswanto; Titik Nuryastuti; Tri Wibawa
EPI_ISL_4768047	Rumah Sakit Islam Al-Ikhlas Pemalang	Balai Laboratorium Kesehatan Dan Pengujian Alat Kesehatan Provinsi Jawa Tengah	Alia Wijayanti; Dwi Nursetyaasih; Dyah Ayu; Endah Wahyu Y; Ismi Farhati; Purnomo; Sekti Handayani; Wiwik Peni; Yekti Hediningsih
EPI_ISL_5616317, EPI_ISL_5616399	Rumah Sakit Islam Sultan Agung Semarang	Balai Laboratorium Kesehatan dan Pengujian Alat Kesehatan Provinsi Jawa Tengah	Alia Wijayanti; Dwi Nursetyaasih; Dyah Ayu; Endah Wahyu Y; Ismi Farhati; Purnomo; Sekti Handayani; Wiwik Peni; Yekti Hediningsih
EPI_ISL_5616630, EPI_ISL_5616635	Rumah Sakit Jiwa Daerah Dr. RM. Soedjarwadi Klaten	Balai Laboratorium Kesehatan dan Pengujian Alat Kesehatan Provinsi Jawa Tengah	Alia Wijayanti; Dwi Nursetyaasih; Dyah Ayu; Endah Wahyu Y; Ismi Farhati; Purnomo; Sekti Handayani; Wiwik Peni; Yekti Hediningsih
EPI_ISL_516806	Rumah Sakit PKU Gamping	Genetics Working Group (Pokja Genetik) Faculty of Medicine, Public Health and Nursing Universitas Gadjah Mada (FK-KMK UGM); Disease Investigation Center Wates Ministry of Agriculture Indonesia; Department of Microbiology FK-KMK UGM; Laboratorium Diagnostik Yayasan Tahija World Mosquito Program (WMP) Yogyakarta Center for Tropical Medicine FK-KMK UGM; Integrated Research center FK-KMK UGM; Department of Computer Science and Electronics FMIPA UGM	. Afiahayati; . Marcellus; . Siswanto; Ardorisye Saptaty Fornia; Dwi AA Nugrahaningsih; Edwin W. Daniwijaya; Eggi Arguni; Endah Supriyati; Gunadi; Hendra Wibawa; Kemala Athollah; Ludhang P. Rizki; Mohamad S. Hakim; Titik Nuryastuti; Tri Wibawa
EPI_ISL_4761491	Rumah Sakit PKU Muhammadiyah Cepu	Balai Laboratorium Kesehatan Dan Pengujian Alat Kesehatan Provinsi Jawa Tengah	Alia Wijayanti; Dwi Nursetyaasih; Dyah Ayu; Endah Wahyu Y; Ismi Farhati; Purnomo; Sekti Handayani; Wiwik Peni; Yekti Hediningsih
EPI_ISL_3230236	Rumah Sakit Pantai Indah Kapuk / DINAS KESEHATAN DKI JAKARTA	Genomik Solidaritas Indonesia Laboratorium	Annisa Muthiah Sukirman; Anuraj Shankar; Ariel Pradipta; Carissa Sintca Wijaya; Gracia Felias Enos Korompis; Hartini Cahyadi; Irwan Idris; Leonardo H Simanjuntak; Meutia Ayuputeri Kumaheri; Reinhart Gabriel; Vania Gavrila Wikasa
EPI_ISL_4756061	Rumah Sakit Pelita Anugerah Demak	Balai Laboratorium Kesehatan Dan Pengujian Alat Kesehatan Provinsi Jawa Tengah	Alia Wijayanti; Dwi Nursetyaasih; Dyah Ayu; Endah Wahyu Y; Ismi Farhati; Purnomo; Sekti Handayani; Wiwik Peni; Yekti Hediningsih
EPI_ISL_5616539	Rumah Sakit Permata Medika Semarang	Balai Laboratorium Kesehatan dan Pengujian Alat Kesehatan Provinsi Jawa Tengah	Alia Wijayanti; Dwi Nursetyaasih; Dyah Ayu; Endah Wahyu Y; Ismi Farhati; Purnomo; Sekti Handayani; Wiwik Peni; Yekti Hediningsih
EPI_ISL_5616612	Rumah Sakit Roemani Semarang	Balai Laboratorium Kesehatan dan Pengujian Alat Kesehatan Provinsi Jawa Tengah	Alia Wijayanti; Dwi Nursetyaasih; Dyah Ayu; Endah Wahyu Y; Ismi Farhati; Purnomo; Sekti Handayani; Wiwik Peni; Yekti Hediningsih
EPI_ISL_2875769, EPI_ISL_2875770, EPI_ISL_2875772, EPI_ISL_2875773, EPI_ISL_2875775, EPI_ISL_2875776, EPI_ISL_2875778, EPI_ISL_2875780, EPI_ISL_2875781, EPI_ISL_2875783, EPI_ISL_2875784, EPI_ISL_2875786, EPI_ISL_2875787			
see above	Rumah Sakit Umum Daerah Kota Mataram	Genomik Solidaritas Indonesia Laboratorium	Ahmad Zoebad Foeady; Alfin Muhammad Abdillah; Anindya Pradipta Susanto; Anna Christina Brazia; Annisa Muthiah Sukirman; Anuraj Shankar; Ariel Pradipta; Dhahlia Agustina Cahyono; Gracia Felias Enos Korompis; Himawan Masyhuri; Laely Indrayanti; Louisa Markus; Meutia Ayuputeri Kumaheri; Normastuti Adhini Tantyo
EPI_ISL_1969246, EPI_ISL_1969247, EPI_ISL_1969248, EPI_ISL_2047533, EPI_ISL_2047534, EPI_ISL_2047535, EPI_ISL_2047536, EPI_ISL_2047537, EPI_ISL_2047538, EPI_ISL_2047539, EPI_ISL_2047540, EPI_ISL_2047541, EPI_ISL_2047542, EPI_ISL_2047543, EPI_ISL_2047544, EPI_ISL_2047545, EPI_ISL_2047546, EPI_ISL_2047547, EPI_ISL_2047548, EPI_ISL_2047549, EPI_ISL_2047550			
see above	Rumah Sakit Umum Daerah Palangkaraya	National Institute of Health Research and Development	Alia Yunita; Arie Ardiansyah Nugraha; Fransiska JLA; Hana Apsari Pawestri; Hartanti Dian Ikawati; Kartika Dewi Puspa; Krisna Pangesti; Mayawati Mewo; Nelly Puspandari; Subangkit; Triyani Soekarso; Vivi Setiawaty
EPI_ISL_5616230, EPI_ISL_5616392, EPI_ISL_5616525	Rumah Sakit Umum Daerah Tugurejo	Balai Laboratorium Kesehatan dan Pengujian Alat Kesehatan Provinsi Jawa Tengah	Alia Wijayanti; Dwi Nursetyaasih; Dyah Ayu; Endah Wahyu Y; Ismi Farhati; Purnomo; Sekti Handayani; Wiwik Peni; Yekti Hediningsih
EPI_ISL_3541165, EPI_ISL_3541166, EPI_ISL_3541167, EPI_ISL_3541168, EPI_ISL_3541169, EPI_ISL_3541170, EPI_ISL_3541171, EPI_ISL_3541172, EPI_ISL_3541173, EPI_ISL_3541174, EPI_ISL_3541175, EPI_ISL_3541176, EPI_ISL_3541177, EPI_ISL_3541178, EPI_ISL_3541179, EPI_ISL_3541180, EPI_ISL_3541181, EPI_ISL_3541182, EPI_ISL_3541183, EPI_ISL_3541184, EPI_ISL_3541185, EPI_ISL_3541186, EPI_ISL_3541187, EPI_ISL_3541188, EPI_ISL_3691582, EPI_ISL_3691583, EPI_ISL_3691584			
see above	Rumah Sakit Umum Daerah Ulin Banjarmasin	National Institute of Health Research and Development	Arie Ardiansyah Nugraha; Fajar Nur Sulistiyohadi; Hana Apsari Pawestri; Hartanti Dian Ikawati; Kartika Dewi Puspa; Krisna Pangesti; Munaroh Pasaribu; Nelly Puspandari; Subangkit; Triyani Soekarso; Vivi Setiawaty
EPI_ISL_2860296, EPI_ISL_2860297, EPI_ISL_2860298, EPI_ISL_2860299	Rumah Sakit Umum Medika Dramaga	Biosafety Level-3 Laboratory, Indonesian Institute of Sciences (LIPI)	Andri Wardiana; Anik B. Dharmayanthi; Asep M.Ridwanulloh; Indriawati; Listiana Oktavia; Sugiyono Saputra; Syam B. Iryanto; Yuliawati
EPI_ISL_1048373	Rumah Sakit Umum Pusat Dr.	National Institute of Health Research and Development	Arie Ardiansyah Nugraha; Hana Apsari Pawestri; Hartanti Dian Ikawati; Iva Puspitasari; Kartika Dewi Puspa; Krisna Nur Andriana Pangesti; Nelly Puspandari; Subangkit; Vivi Setiawaty.

	Kariadi Semarang		
EPI_ISL_1265171, EPI_ISL_4090577	Rumah Sakit Umum Pusat Fatmawati	National Institute of Health Research and Development	Arie Ardiansyah Nugraha; Fajar Nur Sulistiyohadi; Hana Apsari Pawestri; Hartanti Dian Ikawati; Kartika Dewi Puspa; Krisna Pangesti; Lidya Utami; Luci Liana; Lusiya Ningsih; Nelly Puspandari; Subangkit; Triyani Soekarso; Vivi Setiawaty
EPI_ISL_1265273	Rumah Sakit Umum Pusat H. Adam Malik	National Institute of Health Research and Development	Ade Rahmaini; Arie Ardiansyah Nugraha; Hana Apsari Pawestri; Hartanti Dian Ikawati; Kartika Dewi Puspa; Krisna Pangesti; Maliana; Nelly Elfrida Samosir; Nelly Puspandari; Rina Yunita; Subangkit; Vivi Setiawaty
EPI_ISL_1265168	Rumah Sakit Umum Pusat Sanglah	National Institute of Health Research and Development	Arie Ardiansyah Nugraha; Hana Apsari Pawestri; Hartanti Dian Ikawati; I Gede Ketut Sajinadiyasa; I Nengah Tony Rustawan; Kartika Dewi Puspa; Krisna Pangesti; Nelly Puspandari; Subangkit; Vivi Setiawaty
EPI_ISL_3957749	Rumah Sakit Umum Siloam (RSUS)	Mochtar Riady Institute for Nanotechnology-Universitas Pelita Harapan; Eijkman Institute for Molecular Biology, National Research and Innovation Agency	Aksar C Lages; Amin Soebandrio; David Rustandi; Edison Johar; Febi Andriani; Frilasita A Yudhaputri; Hidayat Trimarsanto; Irawan Yusuf; Iskandar Adnan; Ivet M Suriapranata; Juandy Jo; Khin Saw Myint; Lidwina Priliani; Lydia V. Panggalo; Muhammad Rezki Rasyak; Rinaldy Kusuma; Riska N Taufik; Rury M Wahyuni; Safarina G Malik; Sukma Oktavianthi; Tandry Meriyanti; Willy Agustine; Young O Larasati
EPI_ISL_759842	Rumah Sakit Universitas Sebelas Maret (RS-UNS)	Universitas Sebelas Maret (UNS); Rumah Sakit Universitas Sebelas Maret (RS-UNS), Surakarta; National Institute of Health Research and Development, Indonesian Ministry of Health, Jakarta.	Afif Avicena Ghufron; Arie Ardiansyah Nugraha; Betty Suryawati; Dewi Puspa; Hana Apsari Pawestri; Hartanti Dian Ika; Hartono; Kartika; Maryani; Vivi Setiawaty.; Yulia Sari
EPI_ISL_4730573, EPI_ISL_4730574, EPI_ISL_4730575, EPI_ISL_4730576, EPI_ISL_4730577, EPI_ISL_4730578, EPI_ISL_4730579, EPI_ISL_4730580, EPI_ISL_4730581, EPI_ISL_5022955			
see above	Rumah Sakit Universitas Tanjung Pura	National Institute of Health Research and Development	Arie Ardiansyah Nugraha; Fajar Nur Sulistiyohadi; Hana Apsari Pawestri; Hartanti Dian Ikawati; Kartika Dewi Puspa; Krisna Pangesti; Nelly Puspandari; Subangkit; Triyani Soekarso; Vivi Setiawaty
EPI_ISL_3375354, EPI_ISL_3375355, EPI_ISL_3375356, EPI_ISL_3375357, EPI_ISL_3375358, EPI_ISL_3375359, EPI_ISL_3375360, EPI_ISL_3375361, EPI_ISL_3375362, EPI_ISL_3375363, EPI_ISL_3375364, EPI_ISL_3375365, EPI_ISL_3375366, EPI_ISL_3375367, EPI_ISL_3375368, EPI_ISL_3375369, EPI_ISL_3375370, EPI_ISL_3375371, EPI_ISL_3375372, EPI_ISL_3375373, EPI_ISL_3375374, EPI_ISL_3375375, EPI_ISL_3375376, EPI_ISL_3375377, EPI_ISL_3375378, EPI_ISL_3375379, EPI_ISL_3375380, EPI_ISL_3375381, EPI_ISL_3375382, EPI_ISL_3375383, EPI_ISL_3375384, EPI_ISL_3375385, EPI_ISL_3375386, EPI_ISL_3375387, EPI_ISL_3691581			
see above	Rumah Sakit Universitas Tanjungpura	National Institute of Health Research and Development	Andriani; Arie Ardiansyah Nugraha; Delima Fajar Liana; Fajar Nur Sulistiyohadi; Hana Apsari Pawestri; Hartanti Dian Ikawati; Kartika Dewi Puspa; Krisna Pangesti; Nelly Puspandari; Puji Astuti; Puji Astuti Vivi Setiawaty; Sofi Siti Shofiyah; Subangkit; Triyani Soekarso; Virhan Novianry; Vivi Setiawaty
EPI_ISL_4468543, EPI_ISL_4468544	SMARTCO LABORATORY & RESEARCH	Genomik Solidaritas Indonesia Laboratorium	Annisa Muthiah Sukirman; Anuraj Shankar; Ariel Pradipta; Carissa Sintca Wijaya; Dhahlia Agustina Cahyono; Dian Tri Wahyudi; Gracia Felias Enos Korompis; Himawan Masyhuri; Louisa Markus; Maria Mardalena Martini Kaisar; Meutia Ayuputeri Kumaheri; Sem Samuel Surja; Stefanus Bernard; Suratno Lulut Ratnoglik
EPI_ISL_2723605, EPI_ISL_2723606, EPI_ISL_2723607, EPI_ISL_2723608, EPI_ISL_2723609, EPI_ISL_2723610, EPI_ISL_2723611, EPI_ISL_2723612, EPI_ISL_2723613, EPI_ISL_2723614, EPI_ISL_2723615, EPI_ISL_2723616, EPI_ISL_2723617, EPI_ISL_2723618, EPI_ISL_2723619			
see above	SPEEDLAB	Genomik Solidaritas Indonesia Laboratorium	Alfin Mohammad Abdillah; Annisa Muthiah Sukirman; Anuraj Shankar; Ariel Pradipta; Carissa Sintca Wijaya; Dhahlia Agustina Cahyono; Dwi Oktavia; Gracia Felias Enos Korompis; Meutia Ayuputeri Kumaheri; Mohammad Anang Syarifudin; Ngabila Salama; Normastuti Adhini Tantyo; Tiranti Vindha; Vania Gavrila Wikasa; Widyastuti
EPI_ISL_529718	Santo Borromeus Hospital	Laboratorium Kesehatan Provinsi Jawa Barat; School of Life Sciences and Technology & School of Pharmacy-Institut Teknologi Bandung; Molecular Genetics Laboratory-Faculty of Medicine-Universitas Padjadjaran	Adelina Khristiani Rahayu; Agung Eru Wibowo; Azzania Fibriani; Catur Riani; Cut Nur Cinthia Alamanda; Ema Rahmawati; Gusti Ayu Prani Pradani; Hammam Riza; Hesti Lina Wiraswati; Husna Nugrahapraja; Irvan Faizal; Lia Faridah; Marselina Irasonia Tan; Rifky Waluyajati Rachman; Ryan Bayusantika Ristandi; Savira Ekawardhani; Sony Solistia Wirawan; Tarwadi; Yunia Sribudiani
EPI_ISL_528751	Santo Borromeus Hospital	Molecular Genetics Laboratory-Faculty of Medicine-Universitas Padjadjaran; School of Life Sciences and Technology & School of Pharmacy-Institut Teknologi Bandung; Laboratorium Kesehatan Provinsi Jawa Barat	Agung Eru Wibowo; Azzania Fibriani; Catur Riani; Cut Nur Cinthia Alamanda; Ema Rahmawati; Fensi Amalina; Hammam Riza; Hesti Lina Wiraswati; Husna Nugrahapraja; Irvan Faizal; Lia Faridah; Marselina Irasonia Tan; Mas Rizky A.A. Syamsunarno; Rifky Waluyajati Rachman; Ryan Bayusantika Ristandi; Savira Ekawardhani; Sony Solistia Wirawan; Tarwadi; Tri Hanggono Achmad; Yunia Sribudiani
EPI_ISL_528750, EPI_ISL_528759	Santo Borromeus Hospital	School of Life Sciences and Technology & School of Pharmacy-Institut Teknologi Bandung; Molecular Genetics Laboratory-Faculty of Medicine-Universitas Padjadjaran; Laboratorium Kesehatan Provinsi Jawa Barat	Agung Eru Wibowo; Azzania Fibriani; Catur Riani; Cut Nur Cinthia Alamanda; Ema Rahmawati; Fensi Amalina; Hammam Riza; Hesti Lina Wiraswati; Husna Nugrahapraja; Irvan Faizal; Karimatu Khoirunnisa; Lia Faridah; Marselina Irasonia Tan; Mas Rizky A.A. Syamsunarno; Miftahul Faridl; Rifky Waluyajati Rachman; Ryan Bayusantika Ristandi; Savira Ekawardhani; Sony Solistia Wirawan; Tarwadi; Tri Hanggono Achmad; Yunia Sribudiani
EPI_ISL_528747	Santo Borromeus Hospital	School of Pharmacy & School of Life Sciences and Technology - Institut Teknologi Bandung; Molecular Genetics Laboratory-Faculty of Medicine-Universitas Padjadjaran; Laboratorium Kesehatan Provinsi Jawa Barat	Agung Eru Wibowo; Azzania Fibriani; Catur Riani; Cut Nur Cinthia Alamanda; Ema Rahmawati; Hammam Riza; Hesti Lina Wiraswati; Husna Nugrahapraja; Irvan Faizal; Karimatu Khoirunnisa; Lia Faridah; Marselina Irasonia Tan; Miftahul Faridl; Rifky Waluyajati Rachman; Ryan Bayusantika Ristandi; Savira Ekawardhani; Sony Solistia Wirawan; Tarwadi; Yunia Sribudiani
EPI_ISL_1615658	Santo Borromeus Hospital	West Java Health Laboratory; School of Life Sciences and Technology, Institut Teknologi Bandung	Aulia Saraswati Wicaksono; Azzania Fibriani; Cut Nur Cinthia Alamanda; Ema Rahmawati; Karimatu Khoirunnisa; Miftahul Faridl; Rifky Waluyajati Rachman; Rini Robiani; Ryan Bayusantika Ristandi
EPI_ISL_3691595	Santos Hospital Bandung	National Institute of Health Research and Development	Arie Ardiansyah Nugraha; Fajar Nur Sulistiyohadi; Hana Apsari Pawestri; Hartanti Dian Ikawati; Kartika Dewi Puspa; Krisna Pangesti; Nelly Puspandari; Subangkit; Triyani Soekarso; Vivi Setiawaty
EPI_ISL_754239	Santosa Hospital Bandung Central	"School of Life Sciences and Technology & School of Pharmacy-Institut Teknologi Bandung; Molecular Genetics Laboratory-Faculty of Medicine-Universitas Padjadjaran; Laboratorium Kesehatan Provinsi Jawa Barat"	Agung Eru Wibowo; Azzania Fibriani; Catur Riani; Cut Nur Cinthia Alamanda; Ema Rahmawati; Hammam Riza; Hesti Lina Wiraswati; Husna Nugrahapraja; Irvan Faizal; Karimatu Khoirunnisa; Lia Faridah; Marselina Irasonia Tan; Miftahul Faridl; Rifky Waluyajati Rachman; Ryan Bayusantika Ristandi; Savira Ekawardhani; Soni Solistia Wirawan; Tarwadi; Yunia Sribudiani
EPI_ISL_528749	Santosa Hospital Bandung Central	School of Life Sciences and Technology & School of Pharmacy-Institut Teknologi Bandung; Molecular Genetics Laboratory-Faculty of Medicine-Universitas Padjadjaran; Laboratorium Kesehatan Provinsi Jawa Barat	Agung Eru Wibowo; Azzania Fibriani; Catur Riani; Cut Nur Cinthia Alamanda; Davin H. E. Setiamarga; Ema Rahmawati; Hammam Riza; Hesti Lina Wiraswati; Husna Nugrahapraja; Irvan Faizal; Lia Faridah; Marselina Irasonia Tan; Rifky Waluyajati Rachman; Rizki Mardian; Ryan Bayusantika Ristandi; Savira Ekawardhani; Sony Solistia Wirawan; Tarwadi; Yunia Sribudiani
EPI_ISL_511878	Santosa Hospital Bandung Kopo	Laboratorium Kesehatan Provinsi Jawa Barat; Molecular Genetics Laboratory-Faculty of Medicine-Universitas Padjadjaran; School of Life Sciences and Technology & School of Pharmacy-Institut Teknologi Bandung	Agung Eru Wibowo; Azzania Fibriani; Catur Riani; Cut Nur Cinthia Alamanda; Ema Rahmawati; Hammam Riza; Hesti Lina Wiraswati; Husna Nugrahapraja; Lia Faridah; Marselina Irasonia Tan; Rifky Waluyajati Rachman; Ryan Bayusantika Ristandi; Savira Ekawardhani; Soni Solistia Wirawan; Tarwadi; Yunia Sribudiani
EPI_ISL_1615586, EPI_ISL_1634471, EPI_ISL_1634475, EPI_ISL_1660419	Santosa Kopo Hospital	West Java Health Laboratory; School of Life Sciences and Technology, Institut Teknologi Bandung	Aulia Saraswati Wicaksono; Azzania Fibriani; Cut Nur Cinthia Alamanda; Ema Rahmawati; Karimatu Khoirunnisa; Miftahul Faridl; Rifky Waluyajati Rachman; Rini Robiani; Ryan Bayusantika Ristandi
EPI_ISL_1159390	Semen Gresik Hospital	Institute of Tropical Disease, Universitas Airlangga	Aldise M Nastri; Gatot Soegiarto; Jezzy R Dewantari; Kazufumi Shimizu; Krisnoadi Rahardjo; Laksmi Wulandari; Maria I Lusida; Muchdor; Resti Yudhawati; Rima R Prasetya; Soetjipto; Yasuko Mori
EPI_ISL_1795056	Siloam Hospital	Institute of Tropical Disease, Universitas Airlangga	Aldise M Nastri; Gatot Soegiarto; Jezzy R Dewantari; Kazufumi Shimizu; Krisnoadi Rahardjo; Laksmi Wulandari; Maria I Lusida; Maria M Padmidewi; Mitsuhiro Nishimura; Resti Yudhawati; Retno A Setyoningrum; Rima R Prasetya; Soetjipto; Yasuko Mori; Yohko K Shimizu
EPI_ISL_4885349, EPI_ISL_4885350	Siloam Hospital	West Java Health Laboratory	Azzania Fibriani; Cut Nur Cinthia Alamanda; Ema Rahmawati; Isak Solihin; Karimatu Khoirunnisa; Miftahul Faridl; Rifky Waluyajati Rachman; Rini Robiani; Ryan Bayusantika Ristandi
EPI_ISL_3957707, EPI_ISL_3957708, EPI_ISL_3957726, EPI_ISL_3957734, EPI_ISL_3957763, EPI_ISL_3957764	Siloam Hospital Bangka Belitung (SHBB)	Mochtar Riady Institute for Nanotechnology-Universitas Pelita Harapan; Eijkman Institute for Molecular Biology, National Research and Innovation Agency	Aksar C Lages; Amin Soebandrio; David Rustandi; Edison Johar; Febi Andriani; Frilasita A Yudhaputri; Hidayat Trimarsanto; Irawan Yusuf; Iskandar Adnan; Ivet M Suriapranata; Juandy Jo; Khin Saw Myint; Lidwina Priliani; Lydia V. Panggalo; Muhammad Rezki Rasyak; Rinaldy Kusuma; Riska N Taufik; Rury M Wahyuni; Safarina G Malik; Sukma Oktavianthi; Tandry Meriyanti; Tri Shinta Kurniasih; Willy Agustine; Young O Larasati
EPI_ISL_3957691, EPI_ISL_3957692, EPI_ISL_3957693, EPI_ISL_3957697, EPI_ISL_3957698, EPI_ISL_3957717, EPI_ISL_3957718, EPI_ISL_3957720, EPI_ISL_3957721			
see above	Siloam Hospital Jambi (SHJB)	Mochtar Riady Institute for Nanotechnology-Universitas Pelita Harapan; Eijkman Institute for Molecular Biology, National Research and Innovation Agency	Aksar C Lages; Amin Soebandrio; David Rustandi; Edison Johar; Febi Andriani; Frilasita A Yudhaputri; Hidayat Trimarsanto; Irawan Yusuf; Iskandar Adnan; Ivet M Suriapranata; Juandy Jo; Khin Saw Myint; Lidwina Priliani; Lydia V. Panggalo; Muhammad Rezki Rasyak; Rinaldy Kusuma; Riska N Taufik; Rury M Wahyuni; Safarina G Malik; Sukma Oktavianthi; Tandry Meriyanti; Tri Shinta Kurniasih; Willy Agustine; Young O Larasati
EPI_ISL_3957744	Siloam Hospital Kelapa Dua (SHKD)	Mochtar Riady Institute for Nanotechnology-Universitas Pelita Harapan; Eijkman Institute for Molecular Biology, National Research and Innovation Agency	Aksar C Lages; Amin Soebandrio; David Rustandi; Edison Johar; Febi Andriani; Frilasita A Yudhaputri; Hidayat Trimarsanto; Irawan Yusuf; Iskandar Adnan; Ivet M Suriapranata; Juandy Jo; Khin Saw Myint; Lidwina Priliani; Lydia V. Panggalo; Muhammad Rezki Rasyak; Rinaldy Kusuma; Riska N Taufik; Rury M Wahyuni; Safarina G Malik; Sukma Oktavianthi; Tandry Meriyanti; Tri Shinta Kurniasih; Willy Agustine; Young O Larasati
EPI_ISL_3957694, EPI_ISL_3957704, EPI_ISL_3957705, EPI_ISL_3957706, EPI_ISL_3957709, EPI_ISL_3957710, EPI_ISL_3957719, EPI_ISL_3957722, EPI_ISL_3957723, EPI_ISL_3957724, EPI_ISL_3957725, EPI_ISL_3957731, EPI_ISL_3957732, EPI_ISL_3957733, EPI_ISL_3957752, EPI_ISL_3957753, EPI_ISL_3957754, EPI_ISL_3957756, EPI_ISL_3957757, EPI_ISL_3957758, EPI_ISL_3957759, EPI_ISL_3957761, EPI_ISL_3957768			
see above	Siloam Hospital Labuan Bajo (SHLB)	Mochtar Riady Institute for Nanotechnology-Universitas Pelita Harapan; Eijkman Institute for Molecular Biology, National Research and Innovation Agency	Aksar C Lages; Amin Soebandrio; David Rustandi; Edison Johar; Febi Andriani; Frilasita A Yudhaputri; Hidayat Trimarsanto; Irawan Yusuf; Iskandar Adnan; Ivet M Suriapranata; Juandy Jo; Khin Saw Myint; Lidwina Priliani; Lydia V. Panggalo; Muhammad Rezki Rasyak; Rinaldy Kusuma; Riska N Taufik; Rury M Wahyuni; Safarina G Malik; Sukma Oktavianthi; Tandry Meriyanti; Tri Shinta Kurniasih; Willy Agustine; Young O Larasati
EPI_ISL_438546, EPI_ISL_438547, EPI_ISL_438548, EPI_ISL_438549	Siloam Hospital Lippo Village	Mochtar Riady Institute for Nanotechnology, Universitas Pelita Harapan	Aksar C Lages; David Rustandi; Febi Andriani; Irawan Yusuf; Ivet M Suriapranata; Riska N Taufik; Tri Shinta Kurniasih
EPI_ISL_779383, EPI_ISL_779384, EPI_ISL_779385, EPI_ISL_779386, EPI_ISL_779387, EPI_ISL_779388, EPI_ISL_779389, EPI_ISL_779390, EPI_ISL_779391, EPI_ISL_779392, EPI_ISL_779393, EPI_ISL_779394, EPI_ISL_779395			
see above	Siloam Hospital Lippo Village	Mochtar Riady Institute for Nanotechnology-Universitas Pelita Harapan	Aksar C Lages; David Rustandi; Febi Andriani; Irawan Yusuf; Ivet M Suriapranata; Rinaldy Kusuma; Riska N Taufik; Rury M Wahyuni; Tri Shinta Kurniasih; Young O Larasati
EPI_ISL_3957684, EPI_ISL_3957736, EPI_ISL_3957742, EPI_ISL_3957746, EPI_ISL_3957747, EPI_ISL_3957750, EPI_ISL_3957755, EPI_ISL_3957772, EPI_ISL_3957773, EPI_ISL_4037168, EPI_ISL_4037172			
see above	Siloam Hospital Lippo Village (SHLV)	Mochtar Riady Institute for Nanotechnology-Universitas Pelita Harapan; Eijkman Institute for Molecular Biology, National Research and Innovation Agency	Aksar C Lages; Amin Soebandrio; David Rustandi; Edison Johar; Febi Andriani; Frilasita A Yudhaputri; Hidayat Trimarsanto; Irawan Yusuf; Iskandar Adnan; Ivet M Suriapranata; Juandy Jo; Khin Saw Myint; Lidwina Priliani; Lydia V. Panggalo; Muhammad Rezki Rasyak; Rinaldy Kusuma; Riska N Taufik; Rury M Wahyuni; Safarina G Malik; Sukma Oktavianthi; Tandry Meriyanti; Tri Shinta Kurniasih; Willy Agustine; Young O Larasati
EPI_ISL_3957685, EPI_ISL_3957686	Siloam Hospital Mampang (SHMA)	Mochtar Riady Institute for Nanotechnology-Universitas Pelita Harapan; Eijkman Institute for Molecular Biology, National Research and Innovation Agency	Aksar C Lages; Amin Soebandrio; David Rustandi; Edison Johar; Febi Andriani; Frilasita A Yudhaputri; Hidayat Trimarsanto; Irawan Yusuf; Iskandar Adnan; Ivet M Suriapranata; Juandy Jo; Khin Saw Myint; Lidwina Priliani; Lydia V. Panggalo; Muhammad Rezki Rasyak; Rinaldy Kusuma; Riska N Taufik; Rury M Wahyuni; Safarina G Malik; Sukma Oktavianthi; Tandry Meriyanti; Tri Shinta Kurniasih; Willy Agustine; Young O Larasati
EPI_ISL_3957681, EPI_ISL_3957682, EPI_ISL_3957689, EPI_ISL_3957690	Siloam Hospital Manado (SHMN)	Mochtar Riady Institute for Nanotechnology-Universitas Pelita Harapan; Eijkman Institute for Molecular Biology, National Research and Innovation Agency	Aksar C Lages; Amin Soebandrio; David Rustandi; Edison Johar; Febi Andriani; Frilasita A Yudhaputri; Hidayat Trimarsanto; Irawan Yusuf; Iskandar Adnan; Ivet M Suriapranata; Juandy Jo; Khin Saw Myint; Lidwina Priliani; Lydia V. Panggalo; Muhammad Rezki Rasyak; Rinaldy Kusuma; Riska N Taufik; Rury M Wahyuni; Safarina G Malik; Sukma Oktavianthi; Tandry Meriyanti; Tri Shinta Kurniasih; Willy Agustine; Young O Larasati
EPI_ISL_3957687, EPI_ISL_3957688, EPI_ISL_3957712, EPI_ISL_4037167	Siloam Hospital Paal Dua (SHPD)	Mochtar Riady Institute for Nanotechnology-Universitas Pelita Harapan; Eijkman Institute for Molecular Biology, National Research and Innovation Agency	Aksar C Lages; Amin Soebandrio; David Rustandi; Edison Johar; Febi Andriani; Frilasita A Yudhaputri; Hidayat Trimarsanto; Irawan Yusuf; Iskandar Adnan; Ivet M Suriapranata; Juandy Jo; Khin Saw Myint; Lidwina Priliani; Lydia V. Panggalo; Muhammad Rezki Rasyak; Rinaldy Kusuma; Riska N Taufik; Rury M Wahyuni; Safarina G Malik; Sukma Oktavianthi; Tandry Meriyanti; Tri Shinta Kurniasih; Willy Agustine; Young O Larasati
EPI_ISL_3957683, EPI_ISL_3957700, EPI_ISL_3957701, EPI_ISL_3957702, EPI_ISL_3957711, EPI_ISL_3957714, EPI_ISL_3957715, EPI_ISL_3957727, EPI_ISL_3957728, EPI_ISL_3957729, EPI_ISL_3957735, EPI_ISL_3957738, EPI_ISL_3957739, EPI_ISL_3957745, EPI_ISL_3957765, EPI_ISL_3957767, EPI_ISL_3957771, EPI_ISL_4037169, EPI_ISL_4037170, EPI_ISL_4037171, EPI_ISL_4037173			
see above	Siloam Hospital Palangkaraya (SHPR)	Mochtar Riady Institute for Nanotechnology-Universitas Pelita Harapan; Eijkman Institute for Molecular Biology, National Research and Innovation Agency	Aksar C Lages; Amin Soebandrio; David Rustandi; Edison Johar; Febi Andriani; Frilasita A Yudhaputri; Hidayat Trimarsanto; Irawan Yusuf; Iskandar Adnan; Ivet M Suriapranata; Juandy Jo; Khin Saw Myint; Lidwina Priliani; Lydia V. Panggalo; Muhammad Rezki Rasyak; Rinaldy Kusuma; Riska N Taufik; Rury M Wahyuni; Safarina G Malik; Sukma Oktavianthi; Tandry Meriyanti; Tri Shinta Kurniasih; Willy Agustine; Young O Larasati
EPI_ISL_3957695, EPI_ISL_3957696, EPI_ISL_3957699, EPI_ISL_3957703, EPI_ISL_3957713, EPI_ISL_3957716, EPI_ISL_3957730, EPI_ISL_3957737, EPI_ISL_3957740, EPI_ISL_3957741, EPI_ISL_3957743, EPI_ISL_3957748, EPI_ISL_3957751, EPI_ISL_3957760, EPI_ISL_3957762, EPI_ISL_3957766, EPI_ISL_3957769, EPI_ISL_3957770			
see above	Siloam Hospital Purwakarta (SHPW)	Mochtar Riady Institute for Nanotechnology-Universitas Pelita Harapan; Eijkman Institute for Molecular Biology, National Research and Innovation Agency	Aksar C Lages; Amin Soebandrio; David Rustandi; Edison Johar; Febi Andriani; Frilasita A Yudhaputri; Hidayat Trimarsanto; Irawan Yusuf; Iskandar Adnan; Ivet M Suriapranata; Juandy Jo; Khin Saw Myint; Lidwina Priliani; Lydia V. Panggalo; Muhammad Rezki Rasyak; Rinaldy Kusuma; Riska N Taufik; Rury M Wahyuni; Safarina G Malik; Sukma Oktavianthi; Tandry Meriyanti; Tri Shinta Kurniasih; Willy Agustine; Young O Larasati
EPI_ISL_437187	Siloam Hospitals	Institute of Tropical Disease, Universitas Airlangga	Aldise M Nastri; Gatot Soegiarto; Jezzy R Dewantari; Kazufumi Shimizu; Krisnoadi Rahardjo; Laksmi Wulandari; Maria I Lusida; Maria M Padmidewi; Mitsuhiro Nishimura; Resti Y Meliana; Retno A Setyoningrum; Rima R Prasetya; Soetjipto; Yasuko Mori; Yohko K Shimizu

EPI_ISL_1265173	Siloam Hospitals Yogyakarta	National Institute of Health Research and Development	Arie Ardiansyah Nugraha; Hana Apsari Pawestri; Hartanti Dian Ikawati; Kartika Dewi Puspa; Krisna Pangesti; Nelly Puspandari; Subangkit; Vivi Setiawaty
EPI_ISL_759957, EPI_ISL_877419, EPI_ISL_877460, EPI_ISL_877538, EPI_ISL_882771, EPI_ISL_888591, EPI_ISL_888593, EPI_ISL_956278, EPI_ISL_956279, EPI_ISL_956281, EPI_ISL_956313, EPI_ISL_956315, EPI_ISL_1159383, EPI_ISL_1159386, EPI_ISL_1165067, EPI_ISL_1165069, EPI_ISL_1165070, EPI_ISL_1789113, EPI_ISL_1789114, EPI_ISL_1789115, EPI_ISL_2284874, EPI_ISL_2284879, EPI_ISL_2284883, EPI_ISL_4056018, EPI_ISL_4742355			
see above	Siti Khodijah Hospital	Institute of Tropical Disease, Universitas Airlangga	Aldise M Nastri; Gatot Soegiarto; Jezzy R Dewantari; Kazufumi Shimizu; Kohar Hari Santoso; Krisnoadi Rahardjo; Laksmi Wulandari; Maria I Lusida; Muhammad Hamdan; Resti Yudhawati; Rima R Prasetya; Soetjipto; Yasuko Mori
EPI_ISL_2931772	SmartCO Lab	National Institute of Health Research and Development	; Arie Ardiansyah Nugraha; Hana Apsari Pawestri; Hartanti Dian Ikawati; Kartika Dewi Puspa; Krisna Pangesti; Nelly Puspandari; Subangkit; Triyani Soekarso; Vivi Setiawaty
EPI_ISL_2382413, EPI_ISL_2382414, EPI_ISL_2382415, EPI_ISL_3138802, EPI_ISL_3138803	Smartco Lab	National Institute of Health Research and Development	Arie Ardiansyah Nugraha; Fajar Nur Sulistiyohadi; Hana Apsari Pawestri; Hartanti Dian Ikawati; Kartika Dewi Puspa; Krisna Pangesti; Nelly Puspandari; Subangkit; Triyani Soekarso; Vivi Setiawaty
EPI_ISL_872897	Stem Cell Research and Development Center	Stem Cell Research and Development Center	Aristika Dinaryanti; Deya Karsari; Eryk Hendrianto; Igo Syaiful Ihsan; Naimah Putri; Nora Ertanti; Purwati
EPI_ISL_1341802, EPI_ISL_1341907, EPI_ISL_1343818, EPI_ISL_1398267, EPI_ISL_1398355, EPI_ISL_1398359, EPI_ISL_1398375, EPI_ISL_1398561, EPI_ISL_1398924, EPI_ISL_1398927, EPI_ISL_1398930, EPI_ISL_1398981, EPI_ISL_1615590, EPI_ISL_1615620, EPI_ISL_1634477, EPI_ISL_1660417, EPI_ISL_1660420			
see above	Subang Public Health	West Java Health Laboratory; School of Life Sciences and Technology, Institut Teknologi Bandung	Aulia Saraswati Wicaksono; Azzania Fibriani; Cut Nur Cinthia Alamanda; Ema Rahmawati; Isak Solihin; Kamila Tania; Karimatu Khoirunnisa; Miftahul Faridl; Rifky Waluyajati Rachman; Rini Robiani; Ryan Bayusantika Ristandi
EPI_ISL_747241	Subang Public Health Office	West Java Health Laboratory; School of Life Sciences and Technology, Institut Teknologi Bandung	Azzania Fibriani; Cut Nur Cinthia Alamanda; Ema Rahmawati; Isak Solihin; Karimatu Khoirunnisa; Miftahul Faridl; Rifky Waluyajati Rachman; Rini Robiani; Ryan Bayusantika Ristandi
EPI_ISL_747235, EPI_ISL_1341801	Sukabumi Health Laboratory	West Java Health Laboratory; School of Life Sciences and Technology, Institut Teknologi Bandung	Azzania Fibriani; Cut Nur Cinthia Alamanda; Ema Rahmawati; Isak Solihin; Karimatu Khoirunnisa; Miftahul Faridl; Rifky Waluyajati Rachman; Rini Robiani; Ryan Bayusantika Ristandi
EPI_ISL_747236, EPI_ISL_1398374	Sukabumi Public Health	West Java Health Laboratory; School of Life Sciences and Technology, Institut Teknologi Bandung	Azzania Fibriani; Cut Nur Cinthia Alamanda; Ema Rahmawati; Isak Solihin; Kamila Tania; Karimatu Khoirunnisa; Miftahul Faridl; Rifky Waluyajati Rachman; Rini Robiani; Ryan Bayusantika Ristandi
EPI_ISL_1398365, EPI_ISL_1398980, EPI_ISL_1615589, EPI_ISL_1615621	Sumedang Public Health	West Java Health Laboratory; School of Life Sciences and Technology, Institut Teknologi Bandung	Aulia Saraswati Wicaksono; Azzania Fibriani; Cut Nur Cinthia Alamanda; Ema Rahmawati; Isak Solihin; Kamila Tania; Karimatu Khoirunnisa; Miftahul Faridl; Rifky Waluyajati Rachman; Rini Robiani; Ryan Bayusantika Ristandi
EPI_ISL_529719	Sumedang Regional General Hospital	Laboratorium Kesehatan Provinsi Jawa Barat; School of Life Sciences and Technology & School of Pharmacy-Institut Teknologi Bandung; Molecular Genetics Laboratory-Faculty of Medicine-Universitas Padjadjaran	Adelina Khristiani Rahayu; Agung Eru Wibowo; Azzania Fibriani; Catur Riani; Cut Nur Cinthia Alamanda; Ema Rahmawati; Gusti Ayu Prani Pradani; Hammam Riza; Hesti Lina Wiraswati; Husna Nugrahapraja; Irvan Faizal; Lia Faridah; Marselina Irasonia Tan; Rifky Waluyajati Rachman; Ryan Bayusantika Ristandi; Savira Ekawardhani; Sony Solistia Wirawan; Tarwadi; Yunia Sribudiani
EPI_ISL_1335886	Sundari Hospital Medan	Faculty of Medicine, Universitas Sumatera Utara; Institute of Tropical Disease, Universitas Airlangga	Aldise M Nastri; Franciscus Ginting; Inke N D Lubis; Irbah R Nainggolan; Jezzy R Dewantari; Kazufumi Shimizu; Krisnoadi Rahardjo; Maria I Lusida; Meliani; Mirzan Hasibuan; Muhammad Ichwan; R Andika D Cahyadi; R Lia Kusumawati; Ramadhan Bestari; Rima R Prasetya; Soetjipto; Yasuko Mori
EPI_ISL_2510687, EPI_ISL_2570808, EPI_ISL_2570809, EPI_ISL_2570811, EPI_ISL_2617435, EPI_ISL_2617436, EPI_ISL_2617437, EPI_ISL_2617438, EPI_ISL_2693669, EPI_ISL_2693670			
see above	Surabaya Health Office	Institute of Tropical Disease, Universitas Airlangga	Aldise M Nastri; Febria Rachmanita; Gatot Soegiarto; Herlin Ferliana; Jezzy R Dewantari; Kazufumi Shimizu; Krisnoadi Rahardjo; Laksmi Wulandari; Maria I Lusida; Resti Yudhawati; Rima R Prasetya; Soetjipto; Yasuko Mori
EPI_ISL_759960, EPI_ISL_1159381	Surabaya Public Health Office	Institute of Tropical Disease, Universitas Airlangga	Aldise M Nastri; Febria Rachmanita; Gatot Soegiarto; Jezzy R Dewantari; Kazufumi Shimizu; Krisnoadi Rahardjo; Laksmi Wulandari; Maria I Lusida; Resti Yudhawati; Rima R Prasetya; Soetjipto; Yasuko Mori
EPI_ISL_1969242, EPI_ISL_1969243	Swissbel Hotel Airport	National Institute of Health Research and Development	Arie Ardiansyah Nugraha; Hana Apsari Pawestri; Hartanti Dian Ikawati; Kartika Dewi Puspa; Krisna Pangesti; Nelly Puspandari; Subangkit; Triyani Soekarso; Vivi Setiawaty
EPI_ISL_3466992, EPI_ISL_3467002, EPI_ISL_3467007, EPI_ISL_3467014, EPI_ISL_3550653, EPI_ISL_3550654	Syiah Kuala University	Eijkman Institute for Molecular Biology, National Research and Innovation Agency; Syiah Kuala University	Agung Pranata; Amin Soebandrio; Edison Johar; Frilasita A Yudhaputri; Hamdani; Harapan Harapan; Hidayat Trimarsanto; Ichsan Ichsan; Irwansyah; Iskandar Adnan; Jessica R. Saragih; Khin Saw Myint; Kurnia Fitri Jamil; Lidwina Priliani; Lydia V. Panggalo; Maimun Syukri; Mudatsir Mudatsir; Muhammad Rezki Rasyak; Razali Thalib; Rudi Kurniawan; Safarina G Malik; Samsul Rizal; Sarwo Edhy S; Sukma Oktavianthi; Willy Agustine
EPI_ISL_902917, EPI_ISL_902918, EPI_ISL_902919, EPI_ISL_902921, EPI_ISL_902922, EPI_ISL_902924, EPI_ISL_911633, EPI_ISL_911675, EPI_ISL_911708, EPI_ISL_911732, EPI_ISL_911750, EPI_ISL_1250844, EPI_ISL_1250845, EPI_ISL_1250846, EPI_ISL_1250851, EPI_ISL_1250859, EPI_ISL_2886170, EPI_ISL_2886171, EPI_ISL_2886174, EPI_ISL_2886177, EPI_ISL_2886178, EPI_ISL_2886179			
see above	Tanjungpura University Hospital	Tanjungpura University Hospital	Ambar Rialita; Andriani; Arie Ardiansyah Nugraha; Arif Wicaksono; Delima Fajar Liana; Eka Ardiani Putri; Eka Ardiani Putri EA; Hana Apsari Pawestri; Hartanti Dian Ikawati; Helmi Sastriawan; Kahtan MI; Kartika Dewi Puspa; Liana DF; Mahyarudin; Muhammad Ibnu Kahtan; Muhammad Kahtan; Novianry V; Puji Astuti; Puji Astuti P; Putri EA; Rialita A; Rini Andriani; Sastriawan W; Shofiyah SS Astuti P; Sofi Siti Shofiyah; Virhan Novianry; Vivi Setiawaty; Willy Handoko; Windarti W; Wiwik Windarti
EPI_ISL_4885285, EPI_ISL_4885286, EPI_ISL_4885287, EPI_ISL_4885288, EPI_ISL_4885289, EPI_ISL_4885290, EPI_ISL_4885291, EPI_ISL_4885292, EPI_ISL_4885293, EPI_ISL_4885294, EPI_ISL_4885295, EPI_ISL_4885296, EPI_ISL_4885297, EPI_ISL_4885298, EPI_ISL_4885299, EPI_ISL_4885300, EPI_ISL_4885301, EPI_ISL_4885302			
see above	Tasikmalaya Public Health	West Java Health Laboratory	Azzania Fibriani; Cut Nur Cinthia Alamanda; Ema Rahmawati; Isak Solihin; Karimatu Khoirunnisa; Miftahul Faridl; Rifky Waluyajati Rachman; Rini Robiani; Ryan Bayusantika Ristandi
EPI_ISL_1341799, EPI_ISL_1341800, EPI_ISL_1634470	Tasikmalaya Public Health	West Java Health Laboratory; School of Life Sciences and Technology, Institut Teknologi Bandung	Aulia Saraswati Wicaksono; Azzania Fibriani; Cut Nur Cinthia Alamanda; Ema Rahmawati; Isak Solihin; Karimatu Khoirunnisa; Miftahul Faridl; Rifky Waluyajati Rachman; Rini Robiani; Ryan Bayusantika Ristandi
EPI_ISL_576385	Tirta Medical Center Angsana, Banjarmasin Kalsel	National Institute of Health Research and Development	AA; HA; HD; Ikawati; KD; KNA; Nugraha; Paisal; Pangesti; Pawestri; Puspa; Setiawaty; Soekarso; Subangkit; T; V
EPI_ISL_1020194	UMMI Hospital, Bogor, West Java	Biosafety Level-3 Laboratory, Indonesian Institute of Sciences (LIPI)	Anggia Prasetyoputri; Gita Syahputra; Isa Nuryana; Ratih Asmana Ningrum; Sri Swasthikawati; Sugiyono Saputra; Syam Budi Iryanto
EPI_ISL_4730613, EPI_ISL_4730614, EPI_ISL_4730615, EPI_ISL_4730616, EPI_ISL_4730617, EPI_ISL_4730618, EPI_ISL_4730619, EPI_ISL_4730620, EPI_ISL_4730621, EPI_ISL_5022880, EPI_ISL_5022881, EPI_ISL_5022882, EPI_ISL_5022883, EPI_ISL_5022884, EPI_ISL_5022885, EPI_ISL_5022886, EPI_ISL_5022887, EPI_ISL_5022888, EPI_ISL_5022889, EPI_ISL_5022890, EPI_ISL_5022891, EPI_ISL_5022892, EPI_ISL_5022893, EPI_ISL_5022956			
see above	UPT LABKES DINKES SULTENG	National Institute of Health Research and Development	Arie Ardiansyah Nugraha; Fajar Nur Sulistiyohadi; Hana Apsari Pawestri; Hartanti Dian Ikawati; Kartika Dewi Puspa; Krisna Pangesti; Nelly Puspandari; Subangkit; Triyani Soekarso; Vivi Setiawaty
EPI_ISL_2047530	UPT LabKes Sulawesi Tengah	National Institute of Health Research and Development	Arie Ardiansyah Nugraha; Hana Apsari Pawestri; Hartanti Dian Ikawati; Kartika Dewi Puspa; Krisna Pangesti; Nelly Puspandari; Subangkit; Triyani Soekarso; Vivi Setiawaty
EPI_ISL_2854760, EPI_ISL_2854761, EPI_ISL_2868933, EPI_ISL_2868934, EPI_ISL_2868936	UPTD Balai LABKES Kep. Bangka Belitung	National Institute of Health Research and Development	Arie Ardiansyah Nugraha; Hana Apsari Pawestri; Hartanti Dian Ikawati; Kartika Dewi Puspa; Krisna Pangesti; Nelly Puspandari; Subangkit; Triyani Soekarso; Vivi Setiawaty
EPI_ISL_5022927, EPI_ISL_5022928, EPI_ISL_5022929, EPI_ISL_5022930, EPI_ISL_5022931, EPI_ISL_5022932, EPI_ISL_5022933, EPI_ISL_5022934, EPI_ISL_5022935, EPI_ISL_5022936			
see above	UPTD LABKESDA Kota Baru Jambi	National Institute of Health Research and Development	Arie Ardiansyah Nugraha; Fajar Nur Sulistiyohadi; Hana Apsari Pawestri; Hartanti Dian Ikawati; Kartika Dewi Puspa; Krisna Pangesti; Nelly Puspandari; Subangkit; Triyani Soekarso; Vivi Setiawaty
EPI_ISL_2854705, EPI_ISL_2854706, EPI_ISL_2854707	UPTD LABKESDA Kota Samarinda	National Institute of Health Research and Development	Arie Ardiansyah Nugraha; Hana Apsari Pawestri; Hartanti Dian Ikawati; Kartika Dewi Puspa; Krisna Pangesti; Nelly Puspandari; Subangkit; Triyani Soekarso; Vivi Setiawaty
EPI_ISL_3086878	UPTD Labkesda Depok	Indonesian Institute of Sciences (LIPI) and Laboratorium Kesehatan Daerah (Labkesda) Kota Depok	Anik B. Dharmayanthi; Elin Herliana; Erwin F. Hasrianda; Harto W. Tamara; Herjuno A. Nugroho; Idris; Kania R. Astuti; Kurniyawati; Listiana Oktavia; Masrukhin; Ratih A. Ningrum; Ruby Setiawan; Sugeng Suhadi; Syaiful Rizal; Syam B. Iryanto; Yuliawati
EPI_ISL_2631442, EPI_ISL_2631443, EPI_ISL_2631444, EPI_ISL_2631445, EPI_ISL_2631446, EPI_ISL_2631447, EPI_ISL_2631448, EPI_ISL_2631449, EPI_ISL_2631450, EPI_ISL_2631451, EPI_ISL_2631452, EPI_ISL_2631453			
see above	UPTD Labkesda Karawang	Indonesian Institute of Sciences (LIPI)/National Agency of Research and Innovation (BRIN) and National Institute of Health Research and Development (NIHRD)	Andri Wardiana; Anggia Prasetyoputri; Anik B. Dharmayanthi; Gita Syahputra; Herjuno A. Nugroho; Idris; Indriawati; Isa Nuryana; Listiana Oktavia; Puspita Lisdiyanti; Ratih A. Ningrum; Sri Swasthikawati; Subangkit; Sugiyono Saputra; Syam B. Iryanto; Vivi Setiawaty; Vivi Setiawaty Puspita; Vivi Setiawaty Puspita Lisdiyanti; Yayuk Sri Rahayu; Yuliawati
EPI_ISL_3086869, EPI_ISL_3086876, EPI_ISL_3086877	UPTD Puskesmas Cimanggis	Indonesian Institute of Sciences (LIPI) and Laboratorium Kesehatan Daerah (Labkesda) Kota Depok	Andri Wardiana; Elin Herliana; Erwin F. Hasrianda; Harto W. Tamara; Herjuno A. Nugroho; Idris; Kania R. Astuti; Kurniyawati; Listiana Oktavia; Masrukhin; Ratih A. Ningrum; Ruby Setiawan; Sugeng Suhadi; Sugiyono Saputra; Syaiful Rizal; Syam B. Iryanto; Yuliawati
EPI_ISL_4090571, EPI_ISL_4090572, EPI_ISL_4090573, EPI_ISL_4090574, EPI_ISL_5022949	UPTD Puskesmas Kab Tabanan	National Institute of Health Research and Development	Arie Ardiansyah Nugraha; Fajar Nur Sulistiyohadi; Hana Apsari Pawestri; Hartanti Dian Ikawati; Kartika Dewi Puspa; Krisna Pangesti; Nelly Puspandari; Subangkit; Triyani Soekarso; Vivi Setiawaty
EPI_ISL_3086865, EPI_ISL_3086867, EPI_ISL_3086873, EPI_ISL_3086879	UPTD Puskesmas Limo	Indonesian Institute of Sciences (LIPI) and Laboratorium Kesehatan Daerah (Labkesda) Kota Depok	Anggia Prasetyoputri; Anik B. Dharmayanthi; Elin Herliana; Erwin F. Hasrianda; Harto W. Tamara; Herjuno A. Nugroho; Idris; Kania R. Astuti; Kurniyawati; Listiana Oktavia; Masrukhin; Puspita Lisdiyanti; Ratih A. Ningrum; Ruby Setiawan; Sugeng Suhadi; Syaiful Rizal; Syam B. Iryanto; Yuliawati
EPI_ISL_3086866, EPI_ISL_3086871, EPI_ISL_3086872, EPI_ISL_3086874	UPTD Puskesmas Mampang	Indonesian Institute of Sciences (LIPI) and Laboratorium Kesehatan Daerah (Labkesda) Kota Depok	Anggia Prasetyoputri; Elin Herliana; Erwin F. Hasrianda; Harto W. Tamara; Herjuno A. Nugroho; Idris; Kania R. Astuti; Kurniyawati; Listiana Oktavia; Masrukhin; Puspita Lisdiyanti; Ratih A. Ningrum; Ruby Setiawan; Sugeng Suhadi; Sugiyono Saputra; Syaiful Rizal; Syam B. Iryanto; Yuliawati
EPI_ISL_3086868	UPTD Puskesmas Pondok Sukmajaya	Indonesian Institute of Sciences (LIPI) and Laboratorium Kesehatan Daerah (Labkesda) Kota Depok	Andri Wardiana; Elin Herliana; Erwin F. Hasrianda; Harto W. Tamara; Herjuno A. Nugroho; Idris; Kania R. Astuti; Kurniyawati; Listiana Oktavia; Masrukhin; Ratih A. Ningrum; Ruby Setiawan; Sugeng Suhadi; Syaiful Rizal; Syam B. Iryanto; Yuliawati
EPI_ISL_3086870	UPTD Puskesmas Sawangan	Indonesian Institute of Sciences (LIPI) and Laboratorium Kesehatan Daerah (Labkesda) Kota Depok	Andri Wardiana; Elin Herliana; Erwin F. Hasrianda; Harto W. Tamara; Herjuno A. Nugroho; Idris; Kania R. Astuti; Kurniyawati; Listiana Oktavia; Masrukhin; Ratih A. Ningrum; Ruby Setiawan; Sugeng Suhadi; Syaiful Rizal; Syam B. Iryanto; Yuliawati

EPI_ISL_3086875	UPTD Puskesmas Tanah Baru	Indonesian Institute of Sciences (LIPI) and Laboratorium Kesehatan Daerah (Labkesda) Kota Depok	Elin Herliana; Erwin F. Hasrianda; Harto W. Tamara; Herjuno A. Nugroho; Idris; Kania R. Astuti; Kurniyawati; Listiana Oktavia; Masrukhin; Ratih A. Ningrum; Ruby Setiawan; Sugeng Suhadi; Sugiyono Saputra; Syaiful Rizal; Syam B. Iryanto; Yuliawati
EPI_ISL_2854708, EPI_ISL_2854709, EPI_ISL_2854710	UPTDLABKESDA Kota Depok	National Institute of Health Research and Development	Arie Ardiansyah Nugraha; Hana Apsari Pawestri; Hartanti Dian Ikawati; Kartika Dewi Puspa; Krisna Pangesti; Nelly Puspandari; Subangkit; Triyani Soekarso; Vivi Setiawaty
EPI_ISL_2614723	US Embassy Health Unit	Eijkman Institute for Molecular Biology, National Research and Innovation Agency	Amin Soebandrio; Edison Johar; Frilasita A Yudhaputri; Hidayat Trimarsanto; Iskandar Adnan; Khin Saw Myint; Lidwina Priliani; Lydia V. Panggalo; Muhammad Rezki Rasyak; Safarina G Malik; Sukma Oktavianthi; Willy Agustine
EPI_ISL_458082, EPI_ISL_529962, EPI_ISL_529963, EPI_ISL_759958, EPI_ISL_759961, EPI_ISL_956270, EPI_ISL_956273, EPI_ISL_4056013, EPI_ISL_4056019			
see above	Universitas Airlangga Hospital	Institute of Tropical Disease, Universitas Airlangga	Aldise M Nastri; Gatot Soegiarto; Jezzy R Dewantari; Kazufumi Shimizu; Kohar Hari Santoso; Krisnoadi Rahardjo; Laksmi Wulandari; Maria I Lusida; Mitsuhiro Nishimura; Nasronudin; Resti Yudhawati; Retno A Setyoningrum; Rima R Prasetya; Soetjipto; Yasuko Mori; Yohko K Shimizu
EPI_ISL_1196279, EPI_ISL_1196280, EPI_ISL_1196281, EPI_ISL_1196303, EPI_ISL_1196304, EPI_ISL_3374593	Universitas Indonesia Hospital	Faculty of Medicine, Universitas Indonesia	Anis Karuniawati; Ardiana Kusumaningrum; Ari Fahrial Syam; Astuti Giantini; Aulia Reski Widyaningrum; Badriul Hegar; Budi Wiweko; Dimas Seto; Doni Usman; Dwiana Ocviyanti; Fadilah; Ferdi Anda Sitepu; Irandi Putra Pratomo; Khaerunissa Anbar Istiadi; Linda Erlina; Putra Mahanaim Tampubolon; Rafika Indah Paramita
EPI_ISL_4508277, EPI_ISL_4508278, EPI_ISL_4508279, EPI_ISL_4508280, EPI_ISL_4508281, EPI_ISL_4508282, EPI_ISL_4508283, EPI_ISL_4508284, EPI_ISL_4508285, EPI_ISL_4508286, EPI_ISL_4508287, EPI_ISL_4508288, EPI_ISL_4508289, EPI_ISL_4508290, EPI_ISL_4508291, EPI_ISL_4508292, EPI_ISL_4508293, EPI_ISL_4508294			
see above	WISMA ATLET	National Institute of Health Research and Development	Arie Ardiansyah Nugraha; Fajar Nur Sulistiyohadi; Hana Apsari Pawestri; Hartanti Dian Ikawati; Kartika Dewi Puspa; Krisna Pangesti; Nelly Puspandari; Subangkit; Triyani Soekarso; Vivi Setiawaty
EPI_ISL_5328472, EPI_ISL_5328473	Waled Regional Public Hospital	West Java Health Laboratory; School of Life Sciences and Technology, Institut Teknologi Bandung	Azzania Fibriani; Cut Nur Cinthia Alamanda; Ema Rahmawati; Karimatu Khoirunnisa; Miftahul Faridl; Rifky Waluyajati Rachman; Rini Robiani; Ryan Bayusantika Ristandi
EPI_ISL_1398934	West Bandung Public Health	West Java Health Laboratory	Azzania Fibriani; Cut Nur Cinthia Alamanda; Ema Rahmawati; Isak Solihin; Kamila Tania; Karimatu Khoirunnisa; Miftahul Faridl; Rifky Waluyajati Rachman; Rini Robiani; Ryan Bayusantika Ristandi
EPI_ISL_1340862, EPI_ISL_1340863, EPI_ISL_1340864, EPI_ISL_1340865, EPI_ISL_1340866, EPI_ISL_1340867, EPI_ISL_1341792, EPI_ISL_1341793, EPI_ISL_1341794, EPI_ISL_1341795, EPI_ISL_1341796, EPI_ISL_1341797, EPI_ISL_1345232, EPI_ISL_1346484, EPI_ISL_1398361, EPI_ISL_1398563, EPI_ISL_1398568, EPI_ISL_1398929, EPI_ISL_1398984, EPI_ISL_1660415, EPI_ISL_1660473			
see above	West Bandung Public Health	West Java Health Laboratory; School of Life Sciences and Technology, Institut Teknologi Bandung	Aulia Saraswati Wicaksono; Azzania Fibriani; Cut Nur Cinthia Alamanda; Ema Rahmawati; Isak Solihin; Kamila Tania; Karimatu Khoirunnisa; Miftahul Faridl; Rifky Waluyajati Rachman; Rini Robiani; Ryan Bayusantika Ristandi
EPI_ISL_2709404, EPI_ISL_2709405, EPI_ISL_2709406, EPI_ISL_2709407, EPI_ISL_2709408, EPI_ISL_2709409, EPI_ISL_2709410, EPI_ISL_2709411, EPI_ISL_2709412, EPI_ISL_2709413, EPI_ISL_2709414, EPI_ISL_2709415, EPI_ISL_2709416, EPI_ISL_2709418, EPI_ISL_2709419, EPI_ISL_2709420, EPI_ISL_2709421, EPI_ISL_2709422, EPI_ISL_2709423, EPI_ISL_2709424, EPI_ISL_2709425, EPI_ISL_2709426, EPI_ISL_2709427, EPI_ISL_2709428, EPI_ISL_2709429, EPI_ISL_2709430, EPI_ISL_2709431, EPI_ISL_2709432, EPI_ISL_2709433, EPI_ISL_2709434, EPI_ISL_2709435, EPI_ISL_2709436, EPI_ISL_2709437, EPI_ISL_2709438, EPI_ISL_2709439, EPI_ISL_2709440, EPI_ISL_2709441, EPI_ISL_2709442, EPI_ISL_2709443, EPI_ISL_2709444, EPI_ISL_2709445, EPI_ISL_2709446			
see above	West Java Health Laboratory	Eijkman Institute for Molecular Biology, National Research and Innovation Agency; West Java Health Laboratory	Amin Soebandrio; Azzania Fibriani; Cut Nur Cinthia Alamanda; Edison Johar; Ema Rahmawati; Frilasita A Yudhaputri; Hidayat Trimarsanto; Iskandar Adnan; Khin Saw Myint; Lidwina Priliani; Lydia V. Panggalo; Muhammad Rezki Rasyak; Rifky Waluyajati Rachman; Ryan Bayusantika Ristandi; Safarina G Malik; Sukma Oktavianthi; Willy Agustine
EPI_ISL_745033, EPI_ISL_4885334, EPI_ISL_4885335, EPI_ISL_4885336, EPI_ISL_4885337, EPI_ISL_4885338, EPI_ISL_4885339, EPI_ISL_4885340, EPI_ISL_4885341, EPI_ISL_4885342, EPI_ISL_4885343, EPI_ISL_4885344, EPI_ISL_4885345, EPI_ISL_4885346, EPI_ISL_4885347, EPI_ISL_4885348			
see above	West Java Health Laboratory	West Java Health Laboratory	Azzania Fibriani; Cut Nur Cinthia Alamanda; Ema Rahmawati; Isak Solihin; Karimatu Khoirunnisa; Miftahul Faridl; Rifky Waluyajati Rachman; Rini Robiani; Ryan Bayusantika Ristandi
EPI_ISL_1343218, EPI_ISL_1344587, EPI_ISL_1346047, EPI_ISL_1398363, EPI_ISL_1398371, EPI_ISL_1398562, EPI_ISL_1398567, EPI_ISL_1615600, EPI_ISL_1615660, EPI_ISL_1660413, EPI_ISL_1660416, EPI_ISL_5328493, EPI_ISL_5328494, EPI_ISL_5328495, EPI_ISL_5328496, EPI_ISL_5328497, EPI_ISL_5328498, EPI_ISL_5328499, EPI_ISL_5328500, EPI_ISL_5328501, EPI_ISL_5328502, EPI_ISL_5328503, EPI_ISL_5328504, EPI_ISL_5328505, EPI_ISL_5328506, EPI_ISL_5328507, EPI_ISL_5328508, EPI_ISL_5328509, EPI_ISL_5328510, EPI_ISL_5328511, EPI_ISL_5328512, EPI_ISL_5328513, EPI_ISL_5328514, EPI_ISL_5328515, EPI_ISL_5328516, EPI_ISL_5328517, EPI_ISL_5328518, EPI_ISL_5328519, EPI_ISL_5328520, EPI_ISL_5328521, EPI_ISL_5328522, EPI_ISL_5328523, EPI_ISL_5328524, EPI_ISL_5328525, EPI_ISL_5328526, EPI_ISL_5328527, EPI_ISL_5328528, EPI_ISL_5328529, EPI_ISL_5328530, EPI_ISL_5328531, EPI_ISL_5328532, EPI_ISL_5328533			
see above	West Java Health Laboratory	West Java Health Laboratory; School of Life Sciences and Technology, Institut Teknologi Bandung	Aulia Saraswati Wicaksono; Azzania Fibriani; Cut Nur Cinthia Alamanda; Ema Rahmawati; Isak Solihin; Kamila Tania; Karimatu Khoirunnisa; Miftahul Faridl; Rifky Waluyajati Rachman; Rini Robiani; Ryan Bayusantika Ristandi
EPI_ISL_2382408, EPI_ISL_2382409, EPI_ISL_2382410	Wisma Atlet	National Institute of Health Research and Development	Arie Ardiansyah Nugraha; Hana Apsari Pawestri; Hartanti Dian Ikawati; Kartika Dewi Puspa; Krisna Pangesti; Nelly Puspandari; Subangkit; Triyani Soekarso; Vivi Setiawaty

We gratefully acknowledge the following Authors from the Originating laboratories responsible for obtaining the specimens, as well as the Submitting laboratories where the genome data were generated and shared via GISAID, on which this research is based.

All Submitters of data may be contacted directly via www.gisaid.org

Authors are sorted alphabetically.

Accession ID	Originating Laboratory	Submitting Laboratory	Authors
EPI_ISL_1122419, EPI_ISL_1122420, EPI_ISL_1122421	National Public Health Laboratory, National Centre for Infectious Diseases	National Virology Reference Laboratory	Lin Cui; Raymond Tzer Pin Lin; Surita Taib; Tze Minn Mak; Zainun Zaini; Zhenyang Zhou
EPI_ISL_435674, EPI_ISL_435675, EPI_ISL_435676, EPI_ISL_435677, EPI_ISL_443187, EPI_ISL_962877, EPI_ISL_962878, EPI_ISL_3602079, EPI_ISL_3602082, EPI_ISL_3602083, EPI_ISL_3602085, EPI_ISL_3602088, EPI_ISL_3602090, EPI_ISL_3602091, EPI_ISL_3602093, EPI_ISL_3602096, EPI_ISL_3602100, EPI_ISL_3602101, EPI_ISL_3602103, EPI_ISL_3602104, EPI_ISL_3602107, EPI_ISL_3602110, EPI_ISL_3602112, EPI_ISL_3681492, EPI_ISL_3681496, EPI_ISL_3681499, EPI_ISL_3681501, EPI_ISL_3681504, EPI_ISL_3681508, EPI_ISL_3681511, EPI_ISL_3681514, EPI_ISL_3681516, EPI_ISL_3681519, EPI_ISL_3681523, EPI_ISL_3681525			
see above	National Virology Reference Laboratory	National Public Health Laboratory, National Centre for Infectious Diseases	Chavatte Jean-Marc; Cui Lin; Katherine Ching; Lin Cui; Lin Raymond Tzer Pin; Mak Tze Minn; Octavia Sophie; Raymond Tzer Pin Lin; Royce Ang; Taib Surita; Tze Minn Mak; Zaini Zainun; Zhenyang Zhou

We gratefully acknowledge the following Authors from the Originating laboratories responsible for obtaining the specimens, as well as the Submitting laboratories where the genome data were generated and shared via GISAID, on which this research is based.

All Submitters of data may be contacted directly via www.gisaid.org

Authors are sorted alphabetically.

Accession ID	Originating Laboratory	Submitting Laboratory	Authors
EPI_ISL_480601, EPI_ISL_480602	National Health Laboratory, Timor-Leste	MDU-PHL	Antonia da Costa, E.; Baird, R.; Barreto, I.; Caly, L.; Canisia, D.; Dakh, F.; Dolores de Jesus da Costa, M.; Douglas, N.; Francis, J.; Freeman, K.; Jayanti Pereira Tilman, A.; Marr, I.; Sait, M.; Salles de Sousa, A.; Schultz, M.; Seemann, T.; Sherry, N.; Soares da Silva, E.; Wapling, J.; Ximenes, J.
EPI_ISL_456596, EPI_ISL_456597, EPI_ISL_456598, EPI_ISL_456599, EPI_ISL_456600, EPI_ISL_456601, EPI_ISL_456602, EPI_ISL_456603, EPI_ISL_456604, EPI_ISL_456605, EPI_ISL_456606, EPI_ISL_456607, EPI_ISL_456608, EPI_ISL_456609, EPI_ISL_456610, EPI_ISL_456611, EPI_ISL_456612			
see above	National Health Laboratory, Timor-Leste	Microbiological Diagnostic Unit Public Health Laboratory, The Peter Doherty Institute for Infection and Immunity	Antonia da Costa, E.; Baird, R.; Barreto, I.; Caly, L.; Canisia, D.; Dakh, F.; Dolores de Jesus da Costa, M.; Douglas, N.; Francis, J.; Freeman, K.; Jayanti Pereira Tilman, A.; Marr, I.; Sait, M.; Salles de Sousa, A.; Schultz, M.; Seemann, T.; Sherry, N.; Soares da Silva, E.; Wapling, J.; Ximenes, J.

We gratefully acknowledge the following Authors from the Originating laboratories responsible for obtaining the specimens, as well as the Submitting laboratories where the genome data were generated and shared via GISAID, on which this research is based.

All Submitters of data may be contacted directly via www.gisaid.org

Authors are sorted alphabetically.

Accession ID	Originating Laboratory	Submitting Laboratory	Authors
EPI_ISL_1495992, EPI_ISL_1495993, EPI_ISL_1495994, EPI_ISL_1495996, EPI_ISL_1495997	ATGenes Co. Ltd.	National Institute of Health, Department of Medical Sciences, Ministry of Public Health, Thailand	Hathaikan Tanchai; Natchaya Khiadsang; Nuttida Thongpramul; Pakorn Piromtong; Pilailuk Okada; Ratana Tacharoenmuang; Siripaporn Phuygun; Sittiporn Parnmen; Sunthareeya Waicharoen.; Thanutsapa Thanadachakull
EPI_ISL_3827430	Atsamat Hospital	Division of Genomic Medicine and Innovation support,Department of Medical Sciences, Ministry of Public Health, Thailand	Archawin Rojanawiwat; Jirapha Pakdee; Natthakul Bunneang; Nuanjun Wichukchinda; Penpitcha Thawong; Pilailuk Akkapaiboon Okada; Pundharika Piboonsiri; Surakameth Mahasirimongkol; Waritta Sawaengdee
EPI_ISL_403962, EPI_ISL_403963	Bamrasnaradura Hospital	1. Department of Medical Sciences, Ministry of Public Health, Thailand 2. Thai Red Cross Emerging Infectious Diseases - Health Science Centre 3. Department of Disease Control, Ministry of Public Health, Thailand	Buathong; Chittaganpitch; Malinee; Mekha; Nanthawan; Okada; Parnmen; Phuygun; Pilailuk; Rome; Siripaporn; Sittiporn; Sunthareeya; Supaporn; Thanadachakul; Thanutsapa; Wacharapluesadee; Waicharoen; Warawan; Wongboot
EPI_ISL_635199	Bamrasnaradura Infectious Disease Institute	Molecular HIV and Emerging Infectious Diseases Laboratory	Sumonmal UTTAYAMAKUL
EPI_ISL_434692, EPI_ISL_434693, EPI_ISL_434694, EPI_ISL_434696, EPI_ISL_515469	Bamrasnaradura hospital	National Institute of Health. Department of medical Sciences, Ministry of Public Health, Thailand	Chittaganpitch; Malinee; Okada; Parnmen; Phuygun; Pilailuk; Siripaporn; Sittiporn; Sunthareeya; Thanadachakul; Thanutsapa; Waicharoen; Warawan; Wongboot
EPI_ISL_3827388, EPI_ISL_3827433	Ban Dung Crown Prince Hospital	Division of Genomic Medicine and Innovation support,Department of Medical Sciences, Ministry of Public Health, Thailand	Archawin Rojanawiwat; Jirapha Pakdee; Natthakul Bunneang; Nuanjun Wichukchinda; Penpitcha Thawong; Pilailuk Akkapaiboon Okada; Pundharika Piboonsiri; Surakameth Mahasirimongkol; Waritta Sawaengdee
EPI_ISL_3827317, EPI_ISL_3827321, EPI_ISL_3827322, EPI_ISL_3827323, EPI_ISL_3827332, EPI_ISL_3827333, EPI_ISL_3827447			
see above	Ban hong Hospital	Division of Genomic Medicine and Innovation support,Department of Medical Sciences, Ministry of Public Health, Thailand	Archawin Rojanawiwat; Jirapha Pakdee; Natthakul Bunneang; Nuanjun Wichukchinda; Penpitcha Thawong; Pilailuk Akkapaiboon Okada; Pundharika Piboonsiri; Surakameth Mahasirimongkol; Waritta Sawaengdee
EPI_ISL_3827176, EPI_ISL_3827399, EPI_ISL_3827400, EPI_ISL_3827401, EPI_ISL_3827402, EPI_ISL_3827403, EPI_ISL_3827404, EPI_ISL_3827405, EPI_ISL_3827406, EPI_ISL_3827407, EPI_ISL_3827408, EPI_ISL_3827409, EPI_ISL_3827410, EPI_ISL_3827411, EPI_ISL_3827412, EPI_ISL_3827413, EPI_ISL_3827414, EPI_ISL_3827415, EPI_ISL_3827416, EPI_ISL_3827417, EPI_ISL_3827418, EPI_ISL_3827419, EPI_ISL_3827420, EPI_ISL_3827421, EPI_ISL_3827422, EPI_ISL_3827423, EPI_ISL_3827424, EPI_ISL_3827435, EPI_ISL_3827436, EPI_ISL_3827437, EPI_ISL_3827438, EPI_ISL_3827439, EPI_ISL_3827440, EPI_ISL_3827441, EPI_ISL_3827449, EPI_ISL_3827450, EPI_ISL_3827451, EPI_ISL_3827452, EPI_ISL_3827454, EPI_ISL_3827456, EPI_ISL_3827459, EPI_ISL_3827463, EPI_ISL_3827464, EPI_ISL_3827466, EPI_ISL_3827467, EPI_ISL_3827468, EPI_ISL_3827470, EPI_ISL_3827471, EPI_ISL_3827472, EPI_ISL_3827475, EPI_ISL_3827476, EPI_ISL_3827477, EPI_ISL_3827480, EPI_ISL_3827483, EPI_ISL_3827484, EPI_ISL_3827485, EPI_ISL_3827486			
see above	Bangkok Health Centre	Division of Genomic Medicine and Innovation support,Department of Medical Sciences, Ministry of Public Health, Thailand	Archawin Rojanawiwat; Jirapha Pakdee; Natthakul Bunneang; Nuanjun Wichukchinda; Penpitcha Thawong; Pilailuk Akkapaiboon Okada; Pundharika Piboonsiri; Surakameth Mahasirimongkol; Waritta Sawaengdee
EPI_ISL_2433317	Bangkok Hospital Phuket	Division of Genomic Medicine and Innovation support,Department of Medical Sciences, Ministry of Public Health, Thailand	Archawin Rojanawiwat; Jirapha Pakdee; Natthakul Bunneang; Nuanjun Wichukchinda; Penpitcha Thawong; Pilailuk Akkapaiboon Okada; Pundharika Piboonsiri; Surakameth Mahasirimongkol; Waritta Sawaengdee
EPI_ISL_4235358, EPI_ISL_4235366, EPI_ISL_4235373, EPI_ISL_4235433	Bangkok Metropolition Administration	Division of Genomic Medicine and Innovation support,Department of Medical Sciences, Ministry of Public Health, Thailand	Archawin Rojanawiwat; Jirapha Pakdee; Natthakul Bunneang; Nuanjun Wichukchinda; Penpitcha Thawong; Pilailuk Akkapaiboon Okada; Pundharika Piboonsiri; Surakameth Mahasirimongkol; Waritta Sawaengdee
EPI_ISL_3827194, EPI_ISL_3827195, EPI_ISL_3827196	Bangkok hospital Pattaya	Division of Genomic Medicine and Innovation support,Department of Medical Sciences, Ministry of Public Health, Thailand	Archawin Rojanawiwat; Jirapha Pakdee; Natthakul Bunneang; Nuanjun Wichukchinda; Penpitcha Thawong; Pilailuk Akkapaiboon Okada; Pundharika Piboonsiri; Surakameth Mahasirimongkol; Waritta Sawaengdee
EPI_ISL_1497602, EPI_ISL_1497603	Bangpakok 9 International Hospital	National Institute of Health, Department of Medical Sciences, Ministry of Public Health, Thailand	Hathaikan Tanchai; Natchaya Khiadsang; Nuttida Thongpramul; Pakorn Piromtong; Pilailuk Okada; Ratana Tacharoenmuang; Siripaporn Phuygun; Sittiporn Parnmen; Sunthareeya Waicharoen.; Thanutsapa Thanadachakull
EPI_ISL_699484, EPI_ISL_708721, EPI_ISL_708722, EPI_ISL_708736	Bangpakok 9 international hospital	National Institute of Health, Department of Medical Sciences, Ministry of Public Health, Thailand	Malinee Chittaganpitch; Pilailuk Okada; Siripaporn Phuygun; Sittiporn Parnmen; Sunthareeya Waicharoen; Thanutsapa Thanadachakul; Warawan Wongboot
EPI_ISL_3827336, EPI_ISL_3827337	Bangplee Hospital	Division of Genomic Medicine and Innovation support,Department of Medical Sciences, Ministry of Public Health, Thailand	Archawin Rojanawiwat; Jirapha Pakdee; Natthakul Bunneang; Nuanjun Wichukchinda; Penpitcha Thawong; Pilailuk Akkapaiboon Okada; Pundharika Piboonsiri; Surakameth Mahasirimongkol; Waritta Sawaengdee
EPI_ISL_493137, EPI_ISL_493139	Center for Research and Innovation, Faculty of Medical Technology, Mahidol University	Center for Research and Innovation, Faculty of Medical Technology, Mahidol University	Anek Mungaomklang; Hatairat Lerdsamran; Jarunee Prasertsopon; Kamolthip Atsawawaranunt; Kantima Sangsiriwut; Nattakan Thinpan; Pilaipan Puthavathana; Prabda Praphasiri; Somrak Sirikhetkon; Tipsuda Chanmanee
EPI_ISL_450875, EPI_ISL_450876, EPI_ISL_450877, EPI_ISL_450878, EPI_ISL_450879, EPI_ISL_450880, EPI_ISL_450881, EPI_ISL_450882, EPI_ISL_450883, EPI_ISL_450884, EPI_ISL_450885, EPI_ISL_450886, EPI_ISL_450887, EPI_ISL_450888, EPI_ISL_450889, EPI_ISL_450890, EPI_ISL_450891, EPI_ISL_450892, EPI_ISL_450893, EPI_ISL_450894, EPI_ISL_450895, EPI_ISL_450896, EPI_ISL_450897, EPI_ISL_450898, EPI_ISL_450899, EPI_ISL_450900, EPI_ISL_450901, EPI_ISL_450902, EPI_ISL_450903, EPI_ISL_450904, EPI_ISL_450905, EPI_ISL_450906, EPI_ISL_450907, EPI_ISL_450908, EPI_ISL_450909, EPI_ISL_450910, EPI_ISL_450911, EPI_ISL_450912, EPI_ISL_450913, EPI_ISL_450914, EPI_ISL_450915, EPI_ISL_450916, EPI_ISL_450917, EPI_ISL_450918, EPI_ISL_450919, EPI_ISL_450920, EPI_ISL_450921, EPI_ISL_450922, EPI_ISL_450923, EPI_ISL_450924, EPI_ISL_450925, EPI_ISL_450926, EPI_ISL_450927, EPI_ISL_450928, EPI_ISL_450929, EPI_ISL_450930, EPI_ISL_450931, EPI_ISL_450932, EPI_ISL_450933, EPI_ISL_450934, EPI_ISL_450935, EPI_ISL_450936, EPI_ISL_450937, EPI_ISL_450938, EPI_ISL_450939, EPI_ISL_450940, EPI_ISL_450941, EPI_ISL_450942, EPI_ISL_450943, EPI_ISL_450944, EPI_ISL_450945, EPI_ISL_450946, EPI_ISL_450947, EPI_ISL_450948, EPI_ISL_450949, EPI_ISL_450950, EPI_ISL_450951, EPI_ISL_450952, EPI_ISL_450953, EPI_ISL_450954, EPI_ISL_450955, EPI_ISL_450956, EPI_ISL_450957, EPI_ISL_450958, EPI_ISL_450959, EPI_ISL_450960, EPI_ISL_450961, EPI_ISL_450962, EPI_ISL_450963, EPI_ISL_450964, EPI_ISL_450965, EPI_ISL_450966, EPI_ISL_450967, EPI_ISL_450968, EPI_ISL_450969, EPI_ISL_450970, EPI_ISL_450971, EPI_ISL_450972, EPI_ISL_450973, EPI_ISL_450974, EPI_ISL_450975, EPI_ISL_450976, EPI_ISL_450977, EPI_ISL_450978, EPI_ISL_450979, EPI_ISL_450980, EPI_ISL_450981, EPI_ISL_450982, EPI_ISL_450983, EPI_ISL_450984, EPI_ISL_450985, EPI_ISL_450986, EPI_ISL_450987, EPI_ISL_450988, EPI_ISL_450989, EPI_ISL_450990, EPI_ISL_450991, EPI_ISL_450992, EPI_ISL_450993, EPI_ISL_450994, EPI_ISL_450995, EPI_ISL_450996, EPI_ISL_450997, EPI_ISL_450998, EPI_ISL_450999, EPI_ISL_451000, EPI_ISL_451001, EPI_ISL_451002, EPI_ISL_451003, EPI_ISL_451004, EPI_ISL_451005, EPI_ISL_451006, EPI_ISL_451007, EPI_ISL_451008, EPI_ISL_451009, EPI_ISL_451010, EPI_ISL_451011, EPI_ISL_451012, EPI_ISL_451013, EPI_ISL_451014, EPI_ISL_451015, EPI_ISL_451016, EPI_ISL_451017, EPI_ISL_451018, EPI_ISL_451019, EPI_ISL_451020, EPI_ISL_451021, EPI_ISL_451022, EPI_ISL_451023, EPI_ISL_451024, EPI_ISL_451025, EPI_ISL_451026, EPI_ISL_451027, EPI_ISL_451028, EPI_ISL_451029, EPI_ISL_451030, EPI_ISL_451031, EPI_ISL_451032, EPI_ISL_451033, EPI_ISL_451034, EPI_ISL_451035, EPI_ISL_451036, EPI_ISL_451037, EPI_ISL_451038, EPI_ISL_451039, EPI_ISL_451040, EPI_ISL_451041, EPI_ISL_451042, EPI_ISL_451043, EPI_ISL_451044, EPI_ISL_451045, EPI_ISL_451046, EPI_ISL_451047, EPI_ISL_451048, EPI_ISL_451049, EPI_ISL_451050, EPI_ISL_451051, EPI_ISL_451052, EPI_ISL_451053, EPI_ISL_451054, EPI_ISL_451055, EPI_ISL_451056, EPI_ISL_451057, EPI_ISL_451058, EPI_ISL_451059, EPI_ISL_451060, EPI_ISL_451061, EPI_ISL_451062, EPI_ISL_451063, EPI_ISL_451064, EPI_ISL_451065, EPI_ISL_451066, EPI_ISL_451067, EPI_ISL_451068, EPI_ISL_451069, EPI_ISL_451070, EPI_ISL_451071, EPI_ISL_451072, EPI_ISL_451073, EPI_ISL_451074			
see above	Center of Excellence in Clinical Virology	Center of Excellence in Clinical Virology	Auphimai, C.; Chansaenroj, J.; Nilyanimit, P.; Poovorawan, Y.; Puenpa, J.; Suwannakarn, K.; Yorsaeng, R.
EPI_ISL_1346626, EPI_ISL_1346628, EPI_ISL_1346630, EPI_ISL_1346631, EPI_ISL_1346634, EPI_ISL_1346636, EPI_ISL_4254666, EPI_ISL_4254674, EPI_ISL_4254675, EPI_ISL_4254677, EPI_ISL_4254678, EPI_ISL_4254726, EPI_ISL_4254828, EPI_ISL_4254829, EPI_ISL_4254830, EPI_ISL_4255004, EPI_ISL_4255005, EPI_ISL_4255166, EPI_ISL_4255167, EPI_ISL_4255168, EPI_ISL_4255169, EPI_ISL_4255170, EPI_ISL_4255171, EPI_ISL_4255179, EPI_ISL_4255418, EPI_ISL_4255541, EPI_ISL_4255542, EPI_ISL_4255543			
see above	Center of Excellence in Clinical Virology	Research Unit of Systems Microbiology	Amornmas Kongkliang; Amornmas Kongklieng; Anek Mungaomklang; Jira Chansaenroj; Jiratchaya Puenpa; Kamolthip Atsawawaranunt; Nungruthai Suntronwong; Nutsada Saengdao; Oraphan Mayuramart; Pakkaporn Panwijitkul; Pattaraporn Nimsamer; Patthaya Rattanakomol; Ritthideach Yorsaeng; Rujipat Wasitthankasem; Sunchai Payungporn; Suwanna Petto; Vichan Pawan; Vichan Pawun; Vorthon Sawaswong; Yong Poovorawan; Yong Poovowan
EPI_ISL_455583, EPI_ISL_455594	Central Chest Institute of Thailand	National Institute of Health. Department of medical Sciences, Ministry of Public Health, Thailand	Chittaganpitch; Malinee; Okada; Parnmen; Phuygun; Pilailuk; Siripaporn; Sittiporn; Sunthareeya; Thanadachakul; Thanutsapa; Waicharoen; Warawan; Wongboot
EPI_ISL_430842	Central chest Institute of Thailand	National Institute of Health. Department of medical Sciences, Ministry of Public Health, Thailand	Chittaganpitch; Malinee; Okada; Parnmen; Phuygun; Pilailuk; Siripaporn; Sittiporn; Sunthareeya; Thanadachakul; Thanutsapa; Waicharoen; Warawan; Wongboot
EPI_ISL_4647505, EPI_ISL_4647506, EPI_ISL_4647511, EPI_ISL_4647512, EPI_ISL_4647522, EPI_ISL_4647523, EPI_ISL_4647528			
see above	Chainat Narenthon Hospital	Division of Genomic Medicine and Innovation support,Department of Medical Sciences, Ministry of Public Health, Thailand	Archawin Rojanawiwat; Jirapha Pakdee; Natthakul Bunneang; Nuanjun Wichukchinda; Penpitcha Thawong; Pilailuk Akkapaiboon Okada; Pundharika Piboonsiri; Surakameth Mahasirimongkol; Waritta Sawaengdee
EPI_ISL_3827386	Chaiwan Hospiral	Division of Genomic Medicine and Innovation support,Department of Medical Sciences, Ministry of Public Health, Thailand	Archawin Rojanawiwat; Jirapha Pakdee; Natthakul Bunneang; Nuanjun Wichukchinda; Penpitcha Thawong; Pilailuk Akkapaiboon Okada; Pundharika Piboonsiri; Surakameth Mahasirimongkol; Waritta Sawaengdee
EPI_ISL_3407820, EPI_ISL_3419393	Chaiyaphum Hospital	National Institute of Health, Department of Medical Sciences, Ministry of Public Health, Thailand	; Natchaya Khiadsang; Nuttida Thongpramul; Pakorn Piromtong; Pilailuk Okada; Ratana Tacharoenmuang; Siripaporn Phuygun; Sittiporn Parnmen; Sunthareeya Waicharoen; Thanutsapa Thanadachakul; Warawan Wongboot; sirikanda wimol
EPI_ISL_2433327, EPI_ISL_2433328, EPI_ISL_2433329, EPI_ISL_2433330, EPI_ISL_2433342, EPI_ISL_2433343, EPI_ISL_2433344, EPI_ISL_2433345			
see above	Chana Hospital	Division of Genomic Medicine and Innovation support,Department of Medical Sciences, Ministry of Public Health, Thailand	Archawin Rojanawiwat; Jirapha Pakdee; Natthakul Bunneang; Nuanjun Wichukchinda; Penpitcha Thawong; Pilailuk Akkapaiboon Okada; Pundharika Piboonsiri; Surakameth Mahasirimongkol; Waritta Sawaengdee
EPI_ISL_3407001	Chaophya Hospital	National Institute of Health, Department of Medical Sciences, Ministry of Public Health, Thailand	; Natchaya Khiadsang; Nuttida Thongpramul; Pakorn Piromtong; Pilailuk Okada; Ratana Tacharoenmuang; Siripaporn Phuygun; Sittiporn Parnmen; Sunthareeya Waicharoen; Thanutsapa Thanadachakul; Warawan Wongboot; sirikanda wimol
EPI_ISL_4235111, EPI_ISL_4235114, EPI_ISL_4235116, EPI_ISL_4235125, EPI_ISL_4235126, EPI_ISL_4235127, EPI_ISL_4235132, EPI_ISL_4235133, EPI_ISL_4235134, EPI_ISL_4235135, EPI_ISL_4235139, EPI_ISL_4235140, EPI_ISL_4235142, EPI_ISL_4235146, EPI_ISL_4235147, EPI_ISL_4235148, EPI_ISL_4235149, EPI_ISL_4235154, EPI_ISL_4235155, EPI_ISL_4235156, EPI_ISL_4235157, EPI_ISL_4235162, EPI_ISL_4235163, EPI_ISL_4235164, EPI_ISL_4235165, EPI_ISL_4235170, EPI_ISL_4235171, EPI_ISL_4235172, EPI_ISL_4235173, EPI_ISL_4235178, EPI_ISL_4235179, EPI_ISL_4235180, EPI_ISL_4235184, EPI_ISL_4235185, EPI_ISL_4235186, EPI_ISL_4235187, EPI_ISL_4235191, EPI_ISL_4235193, EPI_ISL_4235198, EPI_ISL_4235200, EPI_ISL_4235201, EPI_ISL_4235204, EPI_ISL_4235205, EPI_ISL_4235208, EPI_ISL_4235209, EPI_ISL_4235210, EPI_ISL_4235211, EPI_ISL_4235213, EPI_ISL_4235215, EPI_ISL_4235216, EPI_ISL_4235217, EPI_ISL_4235218, EPI_ISL_4235219, EPI_ISL_4235222, EPI_ISL_4235223, EPI_ISL_4235224, EPI_ISL_4235225, EPI_ISL_4235226, EPI_ISL_4235230, EPI_ISL_4235231, EPI_ISL_4235232, EPI_ISL_4235233, EPI_ISL_4235234, EPI_ISL_4235237, EPI_ISL_4235238, EPI_ISL_4235239, EPI_ISL_4235240, EPI_ISL_4235241, EPI_ISL_4235245, EPI_ISL_4235246, EPI_ISL_4235247, EPI_ISL_4235248, EPI_ISL_4235249, EPI_ISL_4235253, EPI_ISL_4235254, EPI_ISL_4235255, EPI_ISL_4235256, EPI_ISL_4235260, EPI_ISL_4235261, EPI_ISL_4235262, EPI_ISL_4235263, EPI_ISL_4235264, EPI_ISL_4235268, EPI_ISL_4235269, EPI_ISL_4235270, EPI_ISL_4235271, EPI_ISL_4235277, EPI_ISL_4235278, EPI_ISL_4235282, EPI_ISL_4235283, EPI_ISL_4235284, EPI_ISL_4235285, EPI_ISL_4235315, EPI_ISL_4235322, EPI_ISL_4647033, EPI_ISL_4647039, EPI_ISL_4647047, EPI_ISL_4647057, EPI_ISL_4647062, EPI_ISL_4647063, EPI_ISL_4647067, EPI_ISL_4647068, EPI_ISL_4647070, EPI_ISL_4647211, EPI_ISL_4647219, EPI_ISL_4647235, EPI_ISL_4647243, EPI_ISL_4647258, EPI_ISL_4647273, EPI_ISL_4647283, EPI_ISL_4647284, EPI_ISL_4647293			
see above	Charoenkrung pracharak hospital	Division of Genomic Medicine and Innovation support,Department of Medical Sciences, Ministry of Public Health, Thailand	Archawin Rojanawiwat; Jirapha Pakdee; Natthakul Bunneang; Nuanjun Wichukchinda; Penpitcha Thawong; Pilailuk Akkapaiboon Okada; Pundharika Piboonsiri; Surakameth Mahasirimongkol; Waritta Sawaengdee

EPI_ISL_2433287, EPI_ISL_2433290, EPI_ISL_2433305, EPI_ISL_2433533, EPI_ISL_2433534, EPI_ISL_2433535, EPI_ISL_2433536, EPI_ISL_2433537, EPI_ISL_2433538, EPI_ISL_2433539, EPI_ISL_2433540, EPI_ISL_2433541, EPI_ISL_2433542, EPI_ISL_2433543, EPI_ISL_2433544, EPI_ISL_2433545, EPI_ISL_2433546, EPI_ISL_2433547, EPI_ISL_2433548, EPI_ISL_2433549, EPI_ISL_2433550, EPI_ISL_2433551, EPI_ISL_2433619, EPI_ISL_2433620, EPI_ISL_2433621, EPI_ISL_2433622, EPI_ISL_2433623, EPI_ISL_2433624, EPI_ISL_2433625, EPI_ISL_2433626, EPI_ISL_2433627, EPI_ISL_2433628, EPI_ISL_2433629, EPI_ISL_2433630, EPI_ISL_2433631, EPI_ISL_2433632, EPI_ISL_2433633, EPI_ISL_2433634, EPI_ISL_2433635, EPI_ISL_2433636, EPI_ISL_2433637, EPI_ISL_2433638, EPI_ISL_2433639, EPI_ISL_2433640, EPI_ISL_2433641, EPI_ISL_2433642, EPI_ISL_2433643, EPI_ISL_2433644, EPI_ISL_2433645, EPI_ISL_2433650, EPI_ISL_2433651			
see above	Chulabhorn Hospital	Division of Genomic Medicine and Innovation support,Department of Medical Sciences, Ministry of Public Health, Thailand	Archawin Rojanawiwat; Jirapha Pakdee; Natthakul Bunneang; Nuanjun Wichukchinda; Penpitcha Thawong; Pilailuk Akkapaiboon Okada; Pundharika Piboonsiri; Surakameth Mahasirimongkol; Waritta Sawaengdee
EPI_ISL_2840598, EPI_ISL_2840599, EPI_ISL_2840600, EPI_ISL_2877030, EPI_ISL_2877031, EPI_ISL_3038908, EPI_ISL_3038909, EPI_ISL_3407843			
see above	Chulabhorn Hospital	National Institute of Health, Department of Medical Sciences, Ministry of Public Health, Thailand	; Natchaya Khiadsang; Natchaya Khiadsang; Nuttida Thongpramul; Pakorn Piromtong; Pilailuk Okada; Ratana Tacharoenmuang; Siripaporn Phuygun; Sittiporn Parnmen; Sunthareeya Waicharoen; Thanutsapa Thanadachakul; Warawan Wongboot; sirikanda wimol
EPI_ISL_4647340, EPI_ISL_4647355, EPI_ISL_4647368, EPI_ISL_4647384	Department of Disease Control	Division of Genomic Medicine and Innovation support,Department of Medical Sciences, Ministry of Public Health, Thailand	Archawin Rojanawiwat; Jirapha Pakdee; Natthakul Bunneang; Nuanjun Wichukchinda; Penpitcha Thawong; Pilailuk Akkapaiboon Okada; Pundharika Piboonsiri; Surakameth Mahasirimongkol; Waritta Sawaengdee
EPI_ISL_3407855, EPI_ISL_3407856, EPI_ISL_3407867, EPI_ISL_3407868, EPI_ISL_3471401, EPI_ISL_3531662, EPI_ISL_3665935, EPI_ISL_3665936, EPI_ISL_3665937, EPI_ISL_3665938, EPI_ISL_3665939, EPI_ISL_3665940, EPI_ISL_3665941, EPI_ISL_3665942, EPI_ISL_3665943, EPI_ISL_3665944, EPI_ISL_3665945, EPI_ISL_3665946, EPI_ISL_3665947, EPI_ISL_3665948, EPI_ISL_3665949, EPI_ISL_3665950, EPI_ISL_3665959, EPI_ISL_3665960			
see above	Department of Disease Control	National Institute of Health, Department of Medical Sciences, Ministry of Public Health, Thailand	; Natchaya Khiadsang; Nuttida Thongpramul; Pakorn Piromtong; Pilailuk Okada; Ratana Tacharoenmuang; Siripaporn Phuygun; Sittiporn Parnmen; Sunthareeya Waicharoen; Thanutsapa Thanadachakul; Warawan Wongboot; sirikanda wimol
EPI_ISL_2970356, EPI_ISL_2970357	Department of Microbiology, Mahidol University	Department of Microbiology, Mahidol University	Arunee Thitithanyanont
EPI_ISL_2628995, EPI_ISL_2663240	Department of Veterinary Public Health, Faculty of Veterinary Science, Chulalongkorn University	Department of Veterinary Public Health, Faculty of Veterinary Science, Chulalongkorn University	Boonyapisitsopa, S.; Bunpapong, N.; Chaiyawong, S.; Chamsai, E.; Charoenkul, K.; Jairak, W.; Poovorawan; Surachetpong, S.; Suwannakarn, K.; Tangwangvivat, R.; Tantilertcharoen, R.; Techakriengkrai, N.; Udom, K.; Y. and Amonsin, A.
EPI_ISL_2628963	Department of Veterinary Public Health, Faculty of Veterinary Science, Chulalongkorn University, Bangkok, Thailand, Center of Excellence for Emerging and Re-emerging Infectious Diseases in Animals, and One Health Research Cluster, Faculty of Veterinary Science, Chulalongkorn University	Department of Veterinary Public Health, Faculty of Veterinary Science, Chulalongkorn University, Bangkok, Thailand, Center of Excellence for Emerging and Re-emerging Infectious Diseases in Animals, and One Health Research Cluster, Faculty of Veterinary Science, Chulalongkorn University	Boonyapisitsopa, S.; Bunpapong, N.; Chaiyawong, S.; Chamsai, E.; Charoenkul, K.; Jairak, W.; Poovorawan; Surachetpong, S.; Suwannakarn, K.; Tangwangvivat, R.; Tantilertcharoen, R.; Techakriengkrai, N.; Udom, K.; Y. and Amonsin, A.
EPI_ISL_1491561, EPI_ISL_1491562, EPI_ISL_1491563, EPI_ISL_1491564, EPI_ISL_1491565, EPI_ISL_1491566, EPI_ISL_1491567, EPI_ISL_1491568, EPI_ISL_1491569, EPI_ISL_1491572, EPI_ISL_2104744, EPI_ISL_2104745, EPI_ISL_2104755, EPI_ISL_2104756, EPI_ISL_2348800, EPI_ISL_2348801, EPI_ISL_2348802, EPI_ISL_2348803, EPI_ISL_2348825, EPI_ISL_2348826, EPI_ISL_2348827, EPI_ISL_3716304, EPI_ISL_3716305, EPI_ISL_3716306, EPI_ISL_3716311, EPI_ISL_3716314, EPI_ISL_3716317, EPI_ISL_3716318, EPI_ISL_3716320, EPI_ISL_3716321, EPI_ISL_3716324, EPI_ISL_3716325, EPI_ISL_3716326, EPI_ISL_3716328, EPI_ISL_3716329, EPI_ISL_3716330, EPI_ISL_3716334, EPI_ISL_3716335, EPI_ISL_3716336, EPI_ISL_3716337, EPI_ISL_3716339, EPI_ISL_3716340, EPI_ISL_3716342, EPI_ISL_3716345			
see above	Division of Epidemiology	COVID-19 Network Investigations (CONI) Alliance	Angkana Huang; Anthony R. Jones; Araya Keskesorn; Arporn Wangwiwatsin; Arthicha Wongkumma; Atika Awae; Bhakbhoom Panthan; Chattiwutti Tanabat; Chodmanee Sukunpun; Chonticha Klungtong; Duangkamon Loesbanluechai; Ekawat Pasomsub; Elizabeth Batty; Insee Sensorn; Janjira Thaipadungpanit; Juraiwan Ungsathaphornphon; Kamonchanok Chuayjan; Kawinna Kerdsalung; Khajohn Joonlasak; Kingkan Rakmanee; Krittikorn Kumpornsin; Namfon Kotanan; Pantila Taweewigyakarn; Prangsiri Nalaem; Prapai Kreerat; Rattanapong Kompao; Rusmanira Khwankerd; Saraiya Chete; Sawanya Chantutanon; Sawvanit Lamun; Sitipatimoh Mina; Stefan Fernandez; Thanat Chookajorn; Theerarat Kochakarn; Treewat Watthanachockchai; Wachirapun Chainontee; Wasun Chantratita; Wudtichai Manasatienkij
EPI_ISL_4235395	Division of Genomic Medicine and Innovation support,Department of Medical Sciences, Ministry of Public Health, Thailand	Division of Genomic Medicine and Innovation support,Department of Medical Sciences, Ministry of Public Health, Thailand	Archawin Rojanawiwat; Jirapha Pakdee; Natthakul Bunneang; Nuanjun Wichukchinda; Penpitcha Thawong; Pilailuk Akkapaiboon Okada; Pundharika Piboonsiri; Surakameth Mahasirimongkol; Waritta Sawaengdee
EPI_ISL_2351158	Division of Genomic medicine and Innonation support,Department of medical Sciences, Ministry of Public Health, Thailand	Division of Genomic Medicine and Innovation support,Department of Medical Sciences, Ministry of Public Health, Thailand	Archawin Rojanawiwat; Jirapha Pakdee; Natthakul Bunneang; Nuanjun Wichukchinda; Penpitcha Thawong; Pilailuk Akkapaiboon Okada; Pundharika Piboonsiri; Surakameth Mahasirimongkol; Waritta Sawaengdee
EPI_ISL_2348491, EPI_ISL_2348495, EPI_ISL_2350937, EPI_ISL_2350938, EPI_ISL_2350939, EPI_ISL_2350940, EPI_ISL_2350941, EPI_ISL_2350942, EPI_ISL_2350943, EPI_ISL_2350944, EPI_ISL_2350945, EPI_ISL_2350946, EPI_ISL_2350947, EPI_ISL_2350948, EPI_ISL_2350949, EPI_ISL_2350950, EPI_ISL_2350951, EPI_ISL_2350952, EPI_ISL_2350953, EPI_ISL_2350954, EPI_ISL_2350955, EPI_ISL_2350956, EPI_ISL_2350957, EPI_ISL_2350958, EPI_ISL_2350959			
see above	Ekachai Hospital	Division of Genomic Medicine and Innovation support,Department of Medical Sciences, Ministry of Public Health, Thailand	Archawin Rojanawiwat; Jirapha Pakdee; Natthakul Bunneang; Nuanjun Wichukchinda; Penpitcha Thawong; Pilailuk Akkapaiboon Okada; Pundharika Piboonsiri; Surakameth Mahasirimongkol; Waritta Sawaengdee
EPI_ISL_2433380, EPI_ISL_2433381	Emergency Operation Center	Division of Genomic Medicine and Innovation support,Department of Medical Sciences, Ministry of Public Health, Thailand	Archawin Rojanawiwat; Jirapha Pakdee; Natthakul Bunneang; Nuanjun Wichukchinda; Penpitcha Thawong; Pilailuk Akkapaiboon Okada; Pundharika Piboonsiri; Surakameth Mahasirimongkol; Waritta Sawaengdee
EPI_ISL_708822, EPI_ISL_708824, EPI_ISL_708825, EPI_ISL_708826, EPI_ISL_768612, EPI_ISL_768613	Emergency Operation Center, (EOC)	National Institute of Health, Department of Medical Sciences, Ministry of Public Health, Thailand	; Malinee Chittaganpitch; Natchaya Khiadsang; Pakorn Piromtong; Pilailuk Okada; Ratana Tacharoenmuang; Siripaporn Phuygun; Sittiporn Parnmen; Sunthareeya Waicharoen; Thanutsapa Thanadachakul; Warawan Wongboot; sirikanda wimol
EPI_ISL_1491576, EPI_ISL_1491577, EPI_ISL_1491578, EPI_ISL_1491579, EPI_ISL_1491580, EPI_ISL_1491581, EPI_ISL_1491582, EPI_ISL_1491583, EPI_ISL_1538420, EPI_ISL_1538429, EPI_ISL_1538430, EPI_ISL_1538431, EPI_ISL_1545668, EPI_ISL_2802857			
see above	Faculty of Medicine Vajira Hospital	COVID-19 Network Investigations (CONI) Alliance	Ampan Vimonvattana; Anan Manomaipiboon; Angkana Huang; Anthony R. Jones; Arporn Wangwiwatsin; Bhakbhoom Panthan; Chayanit Phutthanu; Chonticha Klungtong; Duangkamon Loesbanluechai; Ekawat Pasomsub; Elizabeth Batty; Insee Sensorn; Jakravoot Maneerit; Janjira Thaipadungpanit; Khajohn Joonlasak; Kingkan Rakmanee; Krittikorn Kumpornsin; Namfon Kotanan; Stefan Fernandez; Sunisa Dongphooyao; Thanat Chookajorn; Theerarat Kochakarn; Tonsan Hansirisathit; Treewat Watthanachockchai; Uraporn Phumisantiphong; Wasun Chantratita; Wipawee Thongsopa; Wudtichai Manasatienkij
EPI_ISL_2348808, EPI_ISL_2348809, EPI_ISL_2348810, EPI_ISL_2678114, EPI_ISL_2678115, EPI_ISL_2678116, EPI_ISL_2678117, EPI_ISL_2678118, EPI_ISL_2678119, EPI_ISL_2678120, EPI_ISL_2678121, EPI_ISL_2678122, EPI_ISL_2678123, EPI_ISL_2678124, EPI_ISL_2678125			
see above	Faculty of Medicine, Prince of Songkla University	COVID-19 Network Investigations (CONI) Alliance	Angkana Huang; Anthony R. Jones; Arporn Wangwiwatsin; Bhakbhoom Panthan; Chonticha Klungtong; Duangkamon Loesbanluechai; Ekawat Pasomsub; Elizabeth Batty; Insee Sensorn; Janjira Thaipadungpanit; Khajohn Joonlasak; Kingkan Rakmanee; Krittikorn Kumpornsin; Namfon Kotanan; Smonrapat Surasombatpattana; Stefan Fernandez; Thanat Chookajorn; Theerarat Kochakarn; Treewat Watthanachockchai; Wasun Chantratita; Wudtichai Manasatienkij
EPI_ISL_455587	H.R.H. Maha Chakri Sirindhorn Medical Center	National Institute of Health. Department of medical Sciences, Ministry of Public Health, Thailand	Chittaganpitch; Malinee; Okada; Parnmen; Phuygun; Pilailuk; Siripaporn; Sittiporn; Sunthareeya; Thanadachakul; Thanutsapa; Waicharoen; Warawan; Wongboot
EPI_ISL_2433320, EPI_ISL_2433321	Hatyai District Public Health Office	Division of Genomic Medicine and Innovation support,Department of Medical Sciences, Ministry of Public Health, Thailand	Archawin Rojanawiwat; Jirapha Pakdee; Natthakul Bunneang; Nuanjun Wichukchinda; Penpitcha Thawong; Pilailuk Akkapaiboon Okada; Pundharika Piboonsiri; Surakameth Mahasirimongkol; Waritta Sawaengdee
EPI_ISL_4235401	Health Service Center 1 Sapan Mon (Mon Bridge)	Division of Genomic Medicine and Innovation support,Department of Medical Sciences, Ministry of Public Health, Thailand	Archawin Rojanawiwat; Jirapha Pakdee; Natthakul Bunneang; Nuanjun Wichukchinda; Penpitcha Thawong; Pilailuk Akkapaiboon Okada; Pundharika Piboonsiri; Surakameth Mahasirimongkol; Waritta Sawaengdee
EPI_ISL_458023, EPI_ISL_458024, EPI_ISL_458025, EPI_ISL_458026, EPI_ISL_458027, EPI_ISL_458028, EPI_ISL_498255, EPI_ISL_498256, EPI_ISL_498257, EPI_ISL_498258, EPI_ISL_498259, EPI_ISL_498260, EPI_ISL_498261, EPI_ISL_707709, EPI_ISL_707710			
see above	Hospital for Tropical Diseases	COVID-19 Network Investigations (CONI) Alliance	Akanitt Jittmittraphap; Angkana Huang; Anthony R. Jones; Arporn Wangwiwatsin; Bhakbhoom Panthan; Chonticha Klungtong; Ekawat Pasomsub; Elizabeth Batty; Insee Sensorn; Janjira Thaipadungpanit; Khajohn Joonsalak; Kingkan Rakmanee; Krittikorn Kumpornsin; Namfon Kotanan; Nantarat Chantawat; Pornsawan Leaungwutiwong; Stefan Fernandez; Thanat Chookajorn; Theerarat Kochakarn; Treewat Watthanachockchai; Viravarn Luvira; Wasun Chantratita; Weena Janwitthayanan; Wudtichai Manasatienkij
EPI_ISL_3826498, EPI_ISL_3826499, EPI_ISL_3826500	Institute Of Dermatology	Division of Genomic Medicine and Innovation support,Department of Medical Sciences, Ministry of Public Health, Thailand	Archawin Rojanawiwat; Jirapha Pakdee; Natthakul Bunneang; Nuanjun Wichukchinda; Penpitcha Thawong; Pilailuk Akkapaiboon Okada; Pundharika Piboonsiri; Surakameth Mahasirimongkol; Waritta Sawaengdee
EPI_ISL_707707, EPI_ISL_707708, EPI_ISL_728557, EPI_ISL_728558, EPI_ISL_728559, EPI_ISL_728560, EPI_ISL_728561, EPI_ISL_728562, EPI_ISL_728563, EPI_ISL_733528, EPI_ISL_733529, EPI_ISL_733530, EPI_ISL_733531, EPI_ISL_733532, EPI_ISL_733533, EPI_ISL_733534, EPI_ISL_733535, EPI_ISL_733536, EPI_ISL_733537, EPI_ISL_733538, EPI_ISL_733539, EPI_ISL_733540, EPI_ISL_733541, EPI_ISL_733542, EPI_ISL_733543, EPI_ISL_733544, EPI_ISL_733545, EPI_ISL_733546, EPI_ISL_733547, EPI_ISL_733548, EPI_ISL_733549, EPI_ISL_733550, EPI_ISL_733551, EPI_ISL_733552, EPI_ISL_733553, EPI_ISL_733554, EPI_ISL_733555, EPI_ISL_733556, EPI_ISL_733557, EPI_ISL_733558, EPI_ISL_733559, EPI_ISL_733560, EPI_ISL_733561, EPI_ISL_733562, EPI_ISL_733563, EPI_ISL_733564, EPI_ISL_733565, EPI_ISL_733566, EPI_ISL_733567, EPI_ISL_812929, EPI_ISL_812930, EPI_ISL_812931, EPI_ISL_812932, EPI_ISL_812933, EPI_ISL_812934, EPI_ISL_812935, EPI_ISL_812936, EPI_ISL_812937, EPI_ISL_812938, EPI_ISL_812939, EPI_ISL_812940, EPI_ISL_812941, EPI_ISL_812942, EPI_ISL_812943, EPI_ISL_812944, EPI_ISL_812945, EPI_ISL_812946, EPI_ISL_812947, EPI_ISL_812948, EPI_ISL_812949, EPI_ISL_812950, EPI_ISL_812951, EPI_ISL_812952, EPI_ISL_812953, EPI_ISL_812954, EPI_ISL_812955, EPI_ISL_882773, EPI_ISL_882774, EPI_ISL_882775, EPI_ISL_882776, EPI_ISL_882777, EPI_ISL_882778, EPI_ISL_882779, EPI_ISL_882781, EPI_ISL_882782, EPI_ISL_882783, EPI_ISL_882784, EPI_ISL_882785, EPI_ISL_882786, EPI_ISL_882787, EPI_ISL_882788, EPI_ISL_882789, EPI_ISL_882790, EPI_ISL_882791, EPI_ISL_882792, EPI_ISL_882793, EPI_ISL_882794, EPI_ISL_882800, EPI_ISL_882801, EPI_ISL_882802, EPI_ISL_882803, EPI_ISL_882804, EPI_ISL_887105, EPI_ISL_1138828, EPI_ISL_1138829, EPI_ISL_1491531, EPI_ISL_1491532, EPI_ISL_1491533, EPI_ISL_1491534, EPI_ISL_1491535, EPI_ISL_1491536, EPI_ISL_1491537, EPI_ISL_1491538, EPI_ISL_1491539, EPI_ISL_1491540, EPI_ISL_1491541, EPI_ISL_1491542, EPI_ISL_1491543, EPI_ISL_1491544, EPI_ISL_1491545, EPI_ISL_1491546, EPI_ISL_1491547, EPI_ISL_1491548, EPI_ISL_1491549, EPI_ISL_1491550, EPI_ISL_1491551, EPI_ISL_1491552, EPI_ISL_1491553, EPI_ISL_1491554, EPI_ISL_1491570, EPI_ISL_1491571, EPI_ISL_1491574, EPI_ISL_1511124, EPI_ISL_1511125, EPI_ISL_1511126, EPI_ISL_1511127, EPI_ISL_1511128, EPI_ISL_1511129, EPI_ISL_1511130, EPI_ISL_1511131, EPI_ISL_1511132, EPI_ISL_1511133, EPI_ISL_1511134, EPI_ISL_1511135, EPI_ISL_1511136, EPI_ISL_1511137, EPI_ISL_1511138, EPI_ISL_1538418, EPI_ISL_1538419, EPI_ISL_1538421, EPI_ISL_1538422, EPI_ISL_1538423, EPI_ISL_1538424, EPI_ISL_1538425, EPI_ISL_1538426, EPI_ISL_1538427, EPI_ISL_1538428, EPI_ISL_2104730, EPI_ISL_2348793, EPI_ISL_2348794, EPI_ISL_2348795, EPI_ISL_2348796, EPI_ISL_2348797, EPI_ISL_2348798, EPI_ISL_2348799, EPI_ISL_2348806, EPI_ISL_2348807, EPI_ISL_2348811, EPI_ISL_2348812, EPI_ISL_2348813, EPI_ISL_2348814, EPI_ISL_2348815, EPI_ISL_2348816, EPI_ISL_2348817, EPI_ISL_2348818, EPI_ISL_2348824, EPI_ISL_2348828, EPI_ISL_2348829, EPI_ISL_2678092, EPI_ISL_2678093, EPI_ISL_2678094, EPI_ISL_2678095, EPI_ISL_2678096, EPI_ISL_2678097, EPI_ISL_2678098, EPI_ISL_2678099, EPI_ISL_2678100, EPI_ISL_2723695, EPI_ISL_2928541, EPI_ISL_2928542, EPI_ISL_2928543, EPI_ISL_2928544, EPI_ISL_2928545, EPI_ISL_2928546, EPI_ISL_2928547, EPI_ISL_2928548, EPI_ISL_2928549, EPI_ISL_2928550			
see above	Institute for Urban Disease Control and Prevention	COVID-19 Network Investigations (CONI) Alliance	Amornmas Kongklieng; Anek Mungaomklang; Angkana Huang; Anthony R. Jones; Arporn Wangwiwatsin; Bhakbhoom Panthan; Chonticha Klungtong; Duangkamon Loesbanluechai; Ekawat Pasomsub; Elizabeth Batty; Insee Sensorn; Janjira Thaipadungpanit; Kamolthip Atsawawaranunt; Khajohn Joonlasak; Khajohn Joonsalak; Kingkan Rakmanee; Krittikorn Kumpornsin; Namfon Kotanan; Prayuth Kaewmalang; Pukkaporn Parnwijitkul; Stefan Fernandez; Thanat Chookajorn; Theerarat Kochakarn; Treewat Watthanachockchai; Vichan Pawun; Wasun Chantratita; Wudtichai Manasatienkij
EPI_ISL_3419372, EPI_ISL_3419373, EPI_ISL_3419374, EPI_ISL_3419375, EPI_ISL_3419376, EPI_ISL_3419377, EPI_ISL_3419378, EPI_ISL_3419379, EPI_ISL_3419380, EPI_ISL_3419381, EPI_ISL_3419382, EPI_ISL_3419383, EPI_ISL_3419384, EPI_ISL_3419385, EPI_ISL_3419386, EPI_ISL_3419387, EPI_ISL_3419388, EPI_ISL_3419389			
see above	Institute for Urban Disease Control and Prevention	Division of Genomic Medicine and Innovation support,Department of Medical Sciences, Ministry of Public Health, Thailand	Archawin Rojanawiwat; Jirapha Pakdee; Natthakul Bunneang; Nuanjun Wichukchinda; Penpitcha Thawong; Pilailuk Akkapaiboon Okada; Pundharika Piboonsiri; Surakameth Mahasirimongkol; Waritta Sawaengdee
EPI_ISL_4235359, EPI_ISL_4235367, EPI_ISL_4235374, EPI_ISL_4235427, EPI_ISL_4235434, EPI_ISL_4235440	Institute of dermatology	Division of Genomic Medicine and Innovation support,Department of Medical Sciences, Ministry of Public Health, Thailand	Archawin Rojanawiwat; Jirapha Pakdee; Natthakul Bunneang; Nuanjun Wichukchinda; Penpitcha Thawong; Pilailuk Akkapaiboon Okada; Pundharika Piboonsiri; Surakameth Mahasirimongkol; Waritta Sawaengdee
EPI_ISL_3402019	Kanchanadit Hospital	National Institute of Health, Department of Medical Sciences, Ministry of Public Health, Thailand	; Natchaya Khiadsang; Nuttida Thongpramul; Pakorn Piromtong; Pilailuk Okada; Ratana Tacharoenmuang; Siripaporn Phuygun; Sittiporn Parnmen; Sunthareeya Waicharoen; Thanutsapa Thanadachakul; Warawan Wongboot; sirikanda wimol
EPI_ISL_3038905, EPI_ISL_3038906, EPI_ISL_3038907	Karunvej Pathumthani Hospital	National Institute of Health, Department of Medical Sciences, Ministry of Public Health, Thailand	; Natchaya Khiadsang; Nuttida Thongpramul; Pakorn Piromtong; Pilailuk Okada; Ratana Tacharoenmuang; Siripaporn Phuygun; Sittiporn Parnmen; Sunthareeya Waicharoen; Thanutsapa Thanadachakul; Warawan Wongboot; sirikanda wimol
EPI_ISL_4235141	Kasemrad Bangkae Hospital	Division of Genomic Medicine and Innovation support,Department of	Archawin Rojanawiwat; Jirapha Pakdee; Natthakul Bunneang; Nuanjun Wichukchinda; Penpitcha Thawong; Pilailuk Akkapaiboon Okada; Pundharika Piboonsiri; Surakameth Mahasirimongkol; Waritta Sawaengdee

		Medical Sciences, Ministry of Public Health, Thailand	
EPI_ISL_4106144	Kasemrad Hospital Rattanathibet	National Institute of Health, Department of Medical Sciences, Ministry of Public Health, Thailand	Archawin Rojanawiwat; Natchaya Khiadsang; Nuttida Thongpramul; Pakorn Piromtong; Pilailuk Okada; Ratana Tacharoenmuang; Siripaporn Phuygun; Sittiporn Parnmen; Sunthareeya Waicharoen; Thanutsapa Thanadachakul; Warawan Wongboot; sirikanda wimol
EPI_ISL_3827181, EPI_ISL_3827182, EPI_ISL_3827183, EPI_ISL_3827184, EPI_ISL_3827185, EPI_ISL_3827469	Kasemrad hospital	Division of Genomic Medicine and Innovation support,Department of Medical Sciences, Ministry of Public Health, Thailand	Archawin Rojanawiwat; Jirapha Pakdee; Natthakul Bunneang; Nuanjun Wichukchinda; Penpitcha Thawong; Pilailuk Akkapaiboon Okada; Pundharika Piboonsiri; Surakameth Mahasirimongkol; Waritta Sawaengdee
EPI_ISL_2433295, EPI_ISL_2433307, EPI_ISL_2433313, EPI_ISL_2433315, EPI_ISL_2433364, EPI_ISL_2433365, EPI_ISL_2433366, EPI_ISL_2433367, EPI_ISL_2433368, EPI_ISL_2433369, EPI_ISL_2433370, EPI_ISL_2433371, EPI_ISL_2433372, EPI_ISL_2433373, EPI_ISL_2433374, EPI_ISL_2433375, EPI_ISL_2433376, EPI_ISL_2433377, EPI_ISL_2433378, EPI_ISL_2433379, EPI_ISL_2433572, EPI_ISL_2433573, EPI_ISL_2433574, EPI_ISL_2433575, EPI_ISL_2433576, EPI_ISL_2433577, EPI_ISL_2433578, EPI_ISL_2433579, EPI_ISL_2433580, EPI_ISL_2433581, EPI_ISL_2433582, EPI_ISL_2433583, EPI_ISL_2433584, EPI_ISL_2433585, EPI_ISL_2433586, EPI_ISL_2433587, EPI_ISL_2433588, EPI_ISL_2433589, EPI_ISL_2433590, EPI_ISL_2433591, EPI_ISL_2433592, EPI_ISL_2433593, EPI_ISL_2433594, EPI_ISL_2433595, EPI_ISL_2433596, EPI_ISL_2433597, EPI_ISL_2433598, EPI_ISL_2433599, EPI_ISL_2433600, EPI_ISL_2433601, EPI_ISL_2433602, EPI_ISL_2433603, EPI_ISL_2433604, EPI_ISL_2433605, EPI_ISL_2433606, EPI_ISL_2433607, EPI_ISL_2433608, EPI_ISL_2433609, EPI_ISL_2433610, EPI_ISL_2433611, EPI_ISL_2433612, EPI_ISL_2433613, EPI_ISL_2433614, EPI_ISL_2433615, EPI_ISL_2433616, EPI_ISL_2433617			
see above	Kasemrad international hospital	Division of Genomic Medicine and Innovation support,Department of Medical Sciences, Ministry of Public Health, Thailand	Archawin Rojanawiwat; Jirapha Pakdee; Natthakul Bunneang; Nuanjun Wichukchinda; Penpitcha Thawong; Pilailuk Akkapaiboon Okada; Pundharika Piboonsiri; Surakameth Mahasirimongkol; Waritta Sawaengdee
EPI_ISL_2840605	Kasemrad international hospital ratthanatibeth	National Institute of Health, Department of Medical Sciences, Ministry of Public Health, Thailand	; Natchaya Khiadsang; Nuttida Thongpramul; Pakorn Piromtong; Pilailuk Okada; Ratana Tacharoenmuang; Siripaporn Phuygun; Sittiporn Parnmen; Sunthareeya Waicharoen; Thanutsapa Thanadachakul; Warawan Wongboot; sirikanda wimol
EPI_ISL_2510689, EPI_ISL_4488503, EPI_ISL_4488505, EPI_ISL_4488507, EPI_ISL_4488511, EPI_ISL_4488513, EPI_ISL_4488515, EPI_ISL_4488517, EPI_ISL_4488521, EPI_ISL_4488523, EPI_ISL_4488525, EPI_ISL_4488527, EPI_ISL_4488528, EPI_ISL_4488529, EPI_ISL_4488530, EPI_ISL_4488531, EPI_ISL_4488533, EPI_ISL_4488535, EPI_ISL_4488537, EPI_ISL_4488539, EPI_ISL_4488541, EPI_ISL_4488542, EPI_ISL_4488544, EPI_ISL_4488546, EPI_ISL_4488549, EPI_ISL_4488550, EPI_ISL_4488552, EPI_ISL_4488554, EPI_ISL_4488556, EPI_ISL_4488558, EPI_ISL_4488560, EPI_ISL_4488561, EPI_ISL_4488563, EPI_ISL_4488568, EPI_ISL_4488570, EPI_ISL_4488571, EPI_ISL_4488573, EPI_ISL_4488575, EPI_ISL_4488577, EPI_ISL_4488579, EPI_ISL_4488581, EPI_ISL_4488585, EPI_ISL_4488587, EPI_ISL_4488589, EPI_ISL_4488591, EPI_ISL_4488592, EPI_ISL_4488594, EPI_ISL_4488596, EPI_ISL_4488598, EPI_ISL_4488600, EPI_ISL_4488602, EPI_ISL_4488605, EPI_ISL_4488606, EPI_ISL_4488608, EPI_ISL_4488610, EPI_ISL_4488614, EPI_ISL_4488617, EPI_ISL_4488619, EPI_ISL_4488621, EPI_ISL_4488623, EPI_ISL_4488625, EPI_ISL_4488626, EPI_ISL_4488628, EPI_ISL_4488630, EPI_ISL_4488632, EPI_ISL_4488636, EPI_ISL_4488640, EPI_ISL_4488642, EPI_ISL_4488644, EPI_ISL_4488646, EPI_ISL_4488647, EPI_ISL_4488649			
see above	King Chulalongkorn Memorial Hospital	Thai Red Cross Emerging Infectious Diseases Clinical Center and Faculty of Medicine, Chulalongkorn University	Anthony R. Jones; Chonticha Klungthong; Gompol Suwanpimolkul; Khajohn Joonlasak; Leilani Paitoonpong; Opass Putcharoen; Pattama Torvorapanit; Piyawan Chinnawirotpisan; Stefan Fernandez; Supaporn Wacharapluesadee; Torvorapanit Pattama; Watsamon Jantarabenjakul; Wudtichai Manasatienkij
EPI_ISL_4647535, EPI_ISL_4647538, EPI_ISL_4647543, EPI_ISL_4647546, EPI_ISL_4647553, EPI_ISL_4647560, EPI_ISL_4647566			
see above	King Prajadhipok Memorial Hospital	Division of Genomic Medicine and Innovation support,Department of Medical Sciences, Ministry of Public Health, Thailand	Archawin Rojanawiwat; Jirapha Pakdee; Natthakul Bunneang; Nuanjun Wichukchinda; Penpitcha Thawong; Pilailuk Akkapaiboon Okada; Pundharika Piboonsiri; Surakameth Mahasirimongkol; Waritta Sawaengdee
EPI_ISL_2433318, EPI_ISL_3826483, EPI_ISL_3826484, EPI_ISL_3826485, EPI_ISL_3826486, EPI_ISL_3826487, EPI_ISL_3826488, EPI_ISL_3826490, EPI_ISL_3826491, EPI_ISL_3826492, EPI_ISL_3826493, EPI_ISL_3826494, EPI_ISL_3826495, EPI_ISL_3826496, EPI_ISL_3826497, EPI_ISL_3826606, EPI_ISL_3826607, EPI_ISL_3826608, EPI_ISL_3826609, EPI_ISL_3826610, EPI_ISL_3826611, EPI_ISL_3826612, EPI_ISL_3826613, EPI_ISL_3826614, EPI_ISL_3826615, EPI_ISL_3826616, EPI_ISL_3826617, EPI_ISL_3826618, EPI_ISL_3826619, EPI_ISL_3826620, EPI_ISL_3826621, EPI_ISL_3826622, EPI_ISL_3826623, EPI_ISL_3826624, EPI_ISL_3826625, EPI_ISL_3826626, EPI_ISL_3826627, EPI_ISL_3826628, EPI_ISL_3826629, EPI_ISL_3826630, EPI_ISL_3826631, EPI_ISL_3826632, EPI_ISL_3826633, EPI_ISL_3826634, EPI_ISL_3826635, EPI_ISL_3826636, EPI_ISL_3826637, EPI_ISL_3826638, EPI_ISL_3826639, EPI_ISL_3826640, EPI_ISL_3826641, EPI_ISL_3826642, EPI_ISL_3826643, EPI_ISL_3826644, EPI_ISL_3826645, EPI_ISL_3826646, EPI_ISL_3826647, EPI_ISL_3826648, EPI_ISL_3826649, EPI_ISL_3826650, EPI_ISL_3826651, EPI_ISL_3826652, EPI_ISL_3826653, EPI_ISL_3826654, EPI_ISL_3826655, EPI_ISL_3826656, EPI_ISL_3826657, EPI_ISL_3826658, EPI_ISL_3826659, EPI_ISL_3826660, EPI_ISL_3826661, EPI_ISL_3826662, EPI_ISL_3826663, EPI_ISL_3826664, EPI_ISL_3826665, EPI_ISL_3826666, EPI_ISL_3826667, EPI_ISL_3826668, EPI_ISL_3826669, EPI_ISL_3826670, EPI_ISL_3826671, EPI_ISL_3826672, EPI_ISL_3826673, EPI_ISL_3826674, EPI_ISL_3826675, EPI_ISL_3826676, EPI_ISL_3826677, EPI_ISL_3826678, EPI_ISL_3826679, EPI_ISL_3826680, EPI_ISL_3826681, EPI_ISL_3826682, EPI_ISL_3826683, EPI_ISL_3826684, EPI_ISL_3826685, EPI_ISL_3826686, EPI_ISL_3826687, EPI_ISL_3826688, EPI_ISL_3826689, EPI_ISL_3826690, EPI_ISL_3826691, EPI_ISL_3826692, EPI_ISL_3826693, EPI_ISL_3826694, EPI_ISL_3826695, EPI_ISL_3826696, EPI_ISL_3826697, EPI_ISL_3826698, EPI_ISL_3826699, EPI_ISL_3826700, EPI_ISL_3826701, EPI_ISL_3826702, EPI_ISL_3826703, EPI_ISL_3826704, EPI_ISL_3826705, EPI_ISL_3826706, EPI_ISL_3826707, EPI_ISL_3826708, EPI_ISL_3826709, EPI_ISL_3826710, EPI_ISL_3826711, EPI_ISL_3826712, EPI_ISL_3826713, EPI_ISL_3826714, EPI_ISL_3826715, EPI_ISL_3826716, EPI_ISL_3826717, EPI_ISL_3826718, EPI_ISL_3826719, EPI_ISL_3826720, EPI_ISL_3826721, EPI_ISL_3826722, EPI_ISL_3826723, EPI_ISL_3826724, EPI_ISL_3826725, EPI_ISL_3826726, EPI_ISL_3826727, EPI_ISL_3826728, EPI_ISL_3826729, EPI_ISL_3826730, EPI_ISL_3826731, EPI_ISL_3826732, EPI_ISL_3826733, EPI_ISL_3826734, EPI_ISL_3826735, EPI_ISL_3826736, EPI_ISL_3826737, EPI_ISL_3826738, EPI_ISL_3826739, EPI_ISL_3826740, EPI_ISL_3826741, EPI_ISL_3826742, EPI_ISL_3826743, EPI_ISL_3826744, EPI_ISL_3826745, EPI_ISL_3826746, EPI_ISL_3826747, EPI_ISL_3826760, EPI_ISL_3826761, EPI_ISL_3826765, EPI_ISL_3826783, EPI_ISL_3826787, EPI_ISL_3827173, EPI_ISL_3827178, EPI_ISL_3827179, EPI_ISL_3827180, EPI_ISL_3827201, EPI_ISL_3827202, EPI_ISL_3827203, EPI_ISL_3827204, EPI_ISL_3827205, EPI_ISL_3827206, EPI_ISL_3827207, EPI_ISL_3827208, EPI_ISL_3827209, EPI_ISL_3827210, EPI_ISL_3827211, EPI_ISL_3827212, EPI_ISL_3827213, EPI_ISL_3827214, EPI_ISL_3827215, EPI_ISL_3827216, EPI_ISL_3827217, EPI_ISL_3827218, EPI_ISL_3827219, EPI_ISL_3827220, EPI_ISL_3827221, EPI_ISL_3827222, EPI_ISL_3827223, EPI_ISL_3827224, EPI_ISL_3827225, EPI_ISL_3827226, EPI_ISL_3827227, EPI_ISL_3827228, EPI_ISL_3827229, EPI_ISL_3827230, EPI_ISL_3827231, EPI_ISL_3827232, EPI_ISL_3827233, EPI_ISL_3827234, EPI_ISL_3827235, EPI_ISL_3827236, EPI_ISL_3827237, EPI_ISL_3827238, EPI_ISL_3827239, EPI_ISL_3827240, EPI_ISL_3827241, EPI_ISL_3827242, EPI_ISL_3827243, EPI_ISL_3827244, EPI_ISL_3827245, EPI_ISL_3827246, EPI_ISL_3827247, EPI_ISL_3827248, EPI_ISL_3827249, EPI_ISL_3827250, EPI_ISL_3827251, EPI_ISL_3827252, EPI_ISL_3827253, EPI_ISL_3827254, EPI_ISL_3827255, EPI_ISL_3827256, EPI_ISL_3827257, EPI_ISL_3827258, EPI_ISL_3827259, EPI_ISL_3827260, EPI_ISL_3827261, EPI_ISL_3827262, EPI_ISL_3827263, EPI_ISL_3827264, EPI_ISL_3827265, EPI_ISL_3827425, EPI_ISL_3827426, EPI_ISL_3827427, EPI_ISL_3827428, EPI_ISL_3827443, EPI_ISL_3827460, EPI_ISL_3827462, EPI_ISL_3827487, EPI_ISL_4235107, EPI_ISL_4235123, EPI_ISL_4235131, EPI_ISL_4235138, EPI_ISL_4235145, EPI_ISL_4235152, EPI_ISL_4235160, EPI_ISL_4235168, EPI_ISL_4235176, EPI_ISL_4235189, EPI_ISL_4235196, EPI_ISL_4235203, EPI_ISL_4235244, EPI_ISL_4235252, EPI_ISL_4235259, EPI_ISL_4235267, EPI_ISL_4235275, EPI_ISL_4235276, EPI_ISL_4235281, EPI_ISL_4235288, EPI_ISL_4235289, EPI_ISL_4235293, EPI_ISL_4235294, EPI_ISL_4235299, EPI_ISL_4235300, EPI_ISL_4235305, EPI_ISL_4235311, EPI_ISL_4235318, EPI_ISL_4235324, EPI_ISL_4235325, EPI_ISL_4235330, EPI_ISL_4235331, EPI_ISL_4235336, EPI_ISL_4235337, EPI_ISL_4235341, EPI_ISL_4235342, EPI_ISL_4235347, EPI_ISL_4235348, EPI_ISL_4235352, EPI_ISL_4235353, EPI_ISL_4647188, EPI_ISL_4647196, EPI_ISL_4647228, EPI_ISL_4647238, EPI_ISL_4647250, EPI_ISL_4647266, EPI_ISL_4647272, EPI_ISL_4647279, EPI_ISL_4647297			
see above	Klang Hospital	Division of Genomic Medicine and Innovation support,Department of Medical Sciences, Ministry of Public Health, Thailand	Archawin Rojanawiwat; Jirapha Pakdee; Natthakul Bunneang; Nuanjun Wichukchinda; Penpitcha Thawong; Pilailuk Akkapaiboon Okada; Pundharika Piboonsiri; Surakameth Mahasirimongkol; Waritta Sawaengdee
EPI_ISL_3827434	Kumpawapi Hospital	Division of Genomic Medicine and Innovation support,Department of Medical Sciences, Ministry of Public Health, Thailand	Archawin Rojanawiwat; Jirapha Pakdee; Natthakul Bunneang; Nuanjun Wichukchinda; Penpitcha Thawong; Pilailuk Akkapaiboon Okada; Pundharika Piboonsiri; Surakameth Mahasirimongkol; Waritta Sawaengdee
EPI_ISL_3827390, EPI_ISL_3827432	Kutchap Hospital Udonthani	Division of Genomic Medicine and Innovation support,Department of Medical Sciences, Ministry of Public Health, Thailand	Archawin Rojanawiwat; Jirapha Pakdee; Natthakul Bunneang; Nuanjun Wichukchinda; Penpitcha Thawong; Pilailuk Akkapaiboon Okada; Pundharika Piboonsiri; Surakameth Mahasirimongkol; Waritta Sawaengdee
EPI_ISL_3827316, EPI_ISL_3827318, EPI_ISL_3827319, EPI_ISL_3827320, EPI_ISL_3827324, EPI_ISL_3827325, EPI_ISL_3827326, EPI_ISL_3827327, EPI_ISL_3827328, EPI_ISL_3827488			
see above	Lamphun Hospital	Division of Genomic Medicine and Innovation support,Department of Medical Sciences, Ministry of Public Health, Thailand	Archawin Rojanawiwat; Jirapha Pakdee; Natthakul Bunneang; Nuanjun Wichukchinda; Penpitcha Thawong; Pilailuk Akkapaiboon Okada; Pundharika Piboonsiri; Surakameth Mahasirimongkol; Waritta Sawaengdee
EPI_ISL_3827306, EPI_ISL_3827307	Maesai hospital	Division of Genomic Medicine and Innovation support,Department of Medical Sciences, Ministry of Public Health, Thailand	Archawin Rojanawiwat; Jirapha Pakdee; Natthakul Bunneang; Nuanjun Wichukchinda; Penpitcha Thawong; Pilailuk Akkapaiboon Okada; Pundharika Piboonsiri; Surakameth Mahasirimongkol; Waritta Sawaengdee
EPI_ISL_3827364	Mahasarakham Hospital	Division of Genomic Medicine and Innovation support,Department of Medical Sciences, Ministry of Public Health, Thailand	Archawin Rojanawiwat; Jirapha Pakdee; Natthakul Bunneang; Nuanjun Wichukchinda; Penpitcha Thawong; Pilailuk Akkapaiboon Okada; Pundharika Piboonsiri; Surakameth Mahasirimongkol; Waritta Sawaengdee
EPI_ISL_3665964	Mahasarakham University	National Institute of Health, Department of Medical Sciences, Ministry of Public Health, Thailand	; Natchaya Khiadsang; Nuttida Thongpramul; Pakorn Piromtong; Pilailuk Okada; Ratana Tacharoenmuang; Siripaporn Phuygun; Sittiporn Parnmen; Sunthareeya Waicharoen; Thanutsapa Thanadachakul; Warawan Wongboot; sirikanda wimol
EPI_ISL_3827199, EPI_ISL_3827200	Makarak Hospital	Division of Genomic Medicine and Innovation support,Department of Medical Sciences, Ministry of Public Health, Thailand	Archawin Rojanawiwat; Jirapha Pakdee; Natthakul Bunneang; Nuanjun Wichukchinda; Penpitcha Thawong; Pilailuk Akkapaiboon Okada; Pundharika Piboonsiri; Surakameth Mahasirimongkol; Waritta Sawaengdee
EPI_ISL_2433316, EPI_ISL_2433382, EPI_ISL_2433383, EPI_ISL_2433384, EPI_ISL_2433385, EPI_ISL_2433386, EPI_ISL_2433387, EPI_ISL_2433388, EPI_ISL_2433389, EPI_ISL_2433390, EPI_ISL_2433391, EPI_ISL_2433392, EPI_ISL_2433393, EPI_ISL_2433394, EPI_ISL_2433395, EPI_ISL_2433396, EPI_ISL_2433397, EPI_ISL_2433398, EPI_ISL_2433399, EPI_ISL_2433400, EPI_ISL_2433401, EPI_ISL_2433402, EPI_ISL_2433403, EPI_ISL_2433404, EPI_ISL_2433405, EPI_ISL_2433406, EPI_ISL_2433407, EPI_ISL_2433408, EPI_ISL_2433409, EPI_ISL_2433410, EPI_ISL_2433411, EPI_ISL_2433412, EPI_ISL_2433413, EPI_ISL_2433414, EPI_ISL_2433415, EPI_ISL_2433416, EPI_ISL_2433417, EPI_ISL_2433418, EPI_ISL_2433419, EPI_ISL_2433420, EPI_ISL_2433421, EPI_ISL_2433422, EPI_ISL_2433423, EPI_ISL_2433424, EPI_ISL_2433425, EPI_ISL_2433426, EPI_ISL_2433427, EPI_ISL_2433428, EPI_ISL_2433429, EPI_ISL_2433430, EPI_ISL_2433431, EPI_ISL_2433478, EPI_ISL_2433479, EPI_ISL_2433480, EPI_ISL_2433481, EPI_ISL_2433482, EPI_ISL_2433483, EPI_ISL_2433484, EPI_ISL_2433485, EPI_ISL_2433486, EPI_ISL_2433487, EPI_ISL_2433488, EPI_ISL_2433489, EPI_ISL_2433490, EPI_ISL_2433491, EPI_ISL_2433492, EPI_ISL_2433493, EPI_ISL_2433494, EPI_ISL_2433495, EPI_ISL_2433496, EPI_ISL_4235297, EPI_ISL_4235303, EPI_ISL_4235309, EPI_ISL_4235316, EPI_ISL_4235323, EPI_ISL_4235329, EPI_ISL_4235334, EPI_ISL_4235340, EPI_ISL_4235345, EPI_ISL_4235351, EPI_ISL_4235356			
see above	Medical Correctional Hospital	Division of Genomic Medicine and Innovation support,Department of Medical Sciences, Ministry of Public Health, Thailand	Archawin Rojanawiwat; Jirapha Pakdee; Natthakul Bunneang; Nuanjun Wichukchinda; Penpitcha Thawong; Pilailuk Akkapaiboon Okada; Pundharika Piboonsiri; Surakameth Mahasirimongkol; Waritta Sawaengdee
EPI_ISL_4635443, EPI_ISL_4636441	Mettapracharak Watraikhing Hospital	National Institute of Health, Department of Medical Sciences, Ministry of Public Health, Thailand	Archawin Rojanawiwat; Natchaya Khiadsang; Nuttida Thongpramul; Pakorn Piromtong; Pilailuk Okada; Ratana Tacharoenmuang; Siripaporn Phuygun; Sittiporn Parnmen; Sunthareeya Waicharoen; Thanutsapa Thanadachakul; Warawan Wongboot; sirikanda wimol
EPI_ISL_2350960, EPI_ISL_2350961, EPI_ISL_2350962, EPI_ISL_2350963, EPI_ISL_2350964, EPI_ISL_2350965, EPI_ISL_2350966, EPI_ISL_2350967, EPI_ISL_2350968, EPI_ISL_2350969, EPI_ISL_2350970, EPI_ISL_2350971, EPI_ISL_2350972, EPI_ISL_2350973, EPI_ISL_2350974, EPI_ISL_2350975, EPI_ISL_2350976, EPI_ISL_2350977, EPI_ISL_2350978, EPI_ISL_2350979, EPI_ISL_2350980, EPI_ISL_2350981, EPI_ISL_2350982, EPI_ISL_2350983, EPI_ISL_2350984, EPI_ISL_2350985, EPI_ISL_2350986, EPI_ISL_2350987, EPI_ISL_2350988, EPI_ISL_2350989, EPI_ISL_2350990, EPI_ISL_2350991, EPI_ISL_2350992, EPI_ISL_2350993, EPI_ISL_2350994, EPI_ISL_2350995, EPI_ISL_2350996, EPI_ISL_2350997, EPI_ISL_2350998, EPI_ISL_2350999, EPI_ISL_2351000, EPI_ISL_2351001, EPI_ISL_2351002, EPI_ISL_2351003, EPI_ISL_2351004, EPI_ISL_2351005, EPI_ISL_2351006, EPI_ISL_2351007, EPI_ISL_2351008, EPI_ISL_2351009, EPI_ISL_2351010, EPI_ISL_2351011, EPI_ISL_2351012, EPI_ISL_2351013, EPI_ISL_2351014, EPI_ISL_2351015, EPI_ISL_2351016, EPI_ISL_2351017, EPI_ISL_2351018, EPI_ISL_2351019, EPI_ISL_2351020, EPI_ISL_2351021, EPI_ISL_2351022, EPI_ISL_2351023, EPI_ISL_2351024, EPI_ISL_2351025, EPI_ISL_2351026, EPI_ISL_2351027, EPI_ISL_2351028, EPI_ISL_2351029, EPI_ISL_2351030, EPI_ISL_2351031, EPI_ISL_2351032, EPI_ISL_2351033, EPI_ISL_2351034, EPI_ISL_2351035, EPI_ISL_2351036, EPI_ISL_2351037, EPI_ISL_2351038, EPI_ISL_2351039, EPI_ISL_2351040, EPI_ISL_2351041, EPI_ISL_2351042, EPI_ISL_2351043, EPI_ISL_2351044, EPI_ISL_2351045, EPI_ISL_2351046, EPI_ISL_2351047, EPI_ISL_2351048, EPI_ISL_2351049, EPI_ISL_2351050, EPI_ISL_2351051			
see above	Mongkutwattana General Hospital	Division of Genomic Medicine and Innovation support,Department of Medical Sciences, Ministry of Public Health, Thailand	Archawin Rojanawiwat; Jirapha Pakdee; Natthakul Bunneang; Nuanjun Wichukchinda; Penpitcha Thawong; Pilailuk Akkapaiboon Okada; Pundharika Piboonsiri; Surakameth Mahasirimongkol; Waritta Sawaengdee
EPI_ISL_3827366, EPI_ISL_3827367, EPI_ISL_3827368, EPI_ISL_3827370, EPI_ISL_3827371, EPI_ISL_3827372, EPI_ISL_3827373, EPI_ISL_3827374			
see above	Nakhonphanom Provincial Health Office	Division of Genomic Medicine and Innovation support,Department of Medical Sciences, Ministry of Public Health, Thailand	Archawin Rojanawiwat; Jirapha Pakdee; Natthakul Bunneang; Nuanjun Wichukchinda; Penpitcha Thawong; Pilailuk Akkapaiboon Okada; Pundharika Piboonsiri; Surakameth Mahasirimongkol; Waritta Sawaengdee
EPI_ISL_3827334, EPI_ISL_3827335	Nakornping Hospital	Division of Genomic Medicine and Innovation support,Department of Medical Sciences, Ministry of Public Health, Thailand	Archawin Rojanawiwat; Jirapha Pakdee; Natthakul Bunneang; Nuanjun Wichukchinda; Penpitcha Thawong; Pilailuk Akkapaiboon Okada; Pundharika Piboonsiri; Surakameth Mahasirimongkol; Waritta Sawaengdee
EPI_ISL_3827398	Namsom hospital	Division of Genomic Medicine and Innovation support,Department of Medical Sciences, Ministry of Public Health, Thailand	Archawin Rojanawiwat; Jirapha Pakdee; Natthakul Bunneang; Nuanjun Wichukchinda; Penpitcha Thawong; Pilailuk Akkapaiboon Okada; Pundharika Piboonsiri; Surakameth Mahasirimongkol; Waritta Sawaengdee
EPI_ISL_2433296, EPI_ISL_2433301, EPI_ISL_2433303, EPI_ISL_2433304, EPI_ISL_2433308, EPI_ISL_2433310, EPI_ISL_2433432, EPI_ISL_2433433, EPI_ISL_2433434, EPI_ISL_2433435, EPI_ISL_2433436, EPI_ISL_2433437, EPI_ISL_2433438, EPI_ISL_2433439, EPI_ISL_2433440, EPI_ISL_2433441, EPI_ISL_2433556, EPI_ISL_2433559, EPI_ISL_2433560, EPI_ISL_2433561, EPI_ISL_2433562, EPI_ISL_2433563, EPI_ISL_2433564, EPI_ISL_2433565, EPI_ISL_2433566, EPI_ISL_2433567, EPI_ISL_2433568, EPI_ISL_2433569, EPI_ISL_2433570, EPI_ISL_2433571, EPI_ISL_2433647, EPI_ISL_2433648, EPI_ISL_2433649, EPI_ISL_4557927, EPI_ISL_4647084, EPI_ISL_4647085, EPI_ISL_4647091, EPI_ISL_4647100, EPI_ISL_4647106, EPI_ISL_4647111, EPI_ISL_4647117, EPI_ISL_4647126, EPI_ISL_4647129			
see above	Naradhiwas Rajanagarindra Hospital	Division of Genomic Medicine and Innovation support,Department of Medical Sciences, Ministry of Public Health, Thailand	Archawin Rojanawiwat; Jirapha Pakdee; Natthakul Bunneang; Nuanjun Wichukchinda; Penpitcha Thawong; Pilailuk Akkapaiboon Okada; Pundharika Piboonsiri; Surakameth Mahasirimongkol; Waritta Sawaengdee
EPI_ISL_2433447, EPI_ISL_2433448, EPI_ISL_2433449, EPI_ISL_2433450, EPI_ISL_2433451, EPI_ISL_2433452, EPI_ISL_2433552			
see above	Naresuan University Hospital	Division of Genomic Medicine and Innovation support,Department of Medical Sciences, Ministry of Public Health, Thailand	Archawin Rojanawiwat; Jirapha Pakdee; Natthakul Bunneang; Nuanjun Wichukchinda; Penpitcha Thawong; Pilailuk Akkapaiboon Okada; Pundharika Piboonsiri; Surakameth Mahasirimongkol; Waritta Sawaengdee
EPI_ISL_3827186, EPI_ISL_3827188, EPI_ISL_3827189, EPI_ISL_3827190, EPI_ISL_3827191, EPI_ISL_3827192	National Healthcare System,co	Division of Genomic Medicine and Innovation support,Department of Medical Sciences, Ministry of Public Health, Thailand	Archawin Rojanawiwat; Jirapha Pakdee; Natthakul Bunneang; Nuanjun Wichukchinda; Penpitcha Thawong; Pilailuk Akkapaiboon Okada; Pundharika Piboonsiri; Surakameth Mahasirimongkol; Waritta Sawaengdee
EPI_ISL_489991, EPI_ISL_515471, EPI_ISL_515472, EPI_ISL_515473, EPI_ISL_515474, EPI_ISL_515475, EPI_ISL_515476, EPI_ISL_515477, EPI_ISL_2020164, EPI_ISL_3403345, EPI_ISL_3665951			
see above	National Institute of Health, Department of Medical Sciences, Ministry of Public Health, Thailand	National Institute of Health, Department of Medical Sciences, Ministry of Public Health, Thailand	; Chittaganpitch; Hathaikan Tanchai; Malinee; Malinee Chittaganpitch; Natchaya Khiadsang; Nuttida Thongpramul; Okada; Pakorn Piromtong; Parnmen; Phuygun; Pilailuk; Pilailuk Okada; Ratana Tacharoenmuang; Siripaporn; Siripaporn Phuygun; Sittiporn; Sittiporn Parnmen; Sunthareeya; Sunthareeya Waicharoen; Sunthareeya Waicharoen.; Thanadachakul; Thanutsapa; Thanutsapa Thanadachakul; Thanutsapa Thanadachakull; Waicharoen; Warawan; Warawan Wongboot; Wongboot; sirikanda wimol

EPI_ISL_450197, EPI_ISL_455584, EPI_ISL_455589, EPI_ISL_455590, EPI_ISL_455591, EPI_ISL_455592, EPI_ISL_455593, EPI_ISL_455624, EPI_ISL_515287, EPI_ISL_515288, EPI_ISL_515289, EPI_ISL_515290, EPI_ISL_515291, EPI_ISL_515463, EPI_ISL_515470			
see above	National Institute of Health. Department of medical Sciences, Ministry of Public Health, Thailand	National Institute of Health. Department of medical Sciences, Ministry of Public Health, Thailand	Chittaganpitch; Malinee; Okada; Parnmen; Phuygun; Pilailuk; Siripaporn; Sittiporn; Sunthareeya; Thanadachakul; Thanutsapa; Waicharoen; Warawan; Wongboot
EPI_ISL_3791305, EPI_ISL_3791308, EPI_ISL_4279185	Navavej Hospital	COVID-19 Network Investigations (CONI) Alliance	Angkana Huang; Anthony R. Jones; Arporn Wangwiwatsin; Bhakbhoom Panthan; Chonticha Klungtong; Duangkamon Loesbanluechai; Ekawat Pasomsub; Elizabeth Batty; Insee Sensorn; Janjira Thaipadungpanit; Khajohn Joonlasak; Kingkan Rakmanee; Krittikorn Kumpornsin; Namfon Kotanan; Stefan Fernandez; Thanat Chookajorn; Theerarat Kochakarn; Treewat Watthanachockchai; Wasun Chantratita; Wudtichai Manasatienkij
EPI_ISL_4262470	Neurological Institute of Thailand	National Institute of Health, Department of Medical Sciences, Ministry of Public Health, Thailand	Archawin Rojanawiwat; Natchaya Khiadsang; Nuttida Thongpramul; Pakorn Piromtong; Pilailuk Okada; Ratana Tacharoenmuang; Siripaporn Phuygun; Sittiporn Parnmen; Sunthareeya Waicharoen; Thanutsapa Thanadachakul; Warawan Wongboot; sirikanda wimol
EPI_ISL_1495998	NewGen Laboratory Medical Technology Clinic	National Institute of Health, Department of Medical Sciences, Ministry of Public Health, Thailand	Hathaikan Tanchai; Natchaya Khiadsang; Nuttida Thongpramul; Pakorn Piromtong; Pilailuk Okada; Ratana Tacharoenmuang; Siripaporn Phuygun; Sittiporn Parnmen; Sunthareeya Waicharoen.; Thanutsapa Thanadachakull
EPI_ISL_3827387, EPI_ISL_3827389	Nong Han Hospital	Division of Genomic Medicine and Innovation support,Department of Medical Sciences, Ministry of Public Health, Thailand	Archawin Rojanawiwat; Jirapha Pakdee; Natthakul Bunneang; Nuanjun Wichukchinda; Penpitcha Thawong; Pilailuk Akkapaiboon Okada; Pundharika Piboonsiri; Surakameth Mahasirimongkol; Waritta Sawaengdee
EPI_ISL_3827381, EPI_ISL_3827382, EPI_ISL_3827383, EPI_ISL_3827384	Nonghin Hospital	Division of Genomic Medicine and Innovation support,Department of Medical Sciences, Ministry of Public Health, Thailand	Archawin Rojanawiwat; Jirapha Pakdee; Natthakul Bunneang; Nuanjun Wichukchinda; Penpitcha Thawong; Pilailuk Akkapaiboon Okada; Pundharika Piboonsiri; Surakameth Mahasirimongkol; Waritta Sawaengdee
EPI_ISL_3827369	Nongkhai Hospital	Division of Genomic Medicine and Innovation support,Department of Medical Sciences, Ministry of Public Health, Thailand	Archawin Rojanawiwat; Jirapha Pakdee; Natthakul Bunneang; Nuanjun Wichukchinda; Penpitcha Thawong; Pilailuk Akkapaiboon Okada; Pundharika Piboonsiri; Surakameth Mahasirimongkol; Waritta Sawaengdee
EPI_ISL_3827431	Nonsaad Hospital	Division of Genomic Medicine and Innovation support,Department of Medical Sciences, Ministry of Public Health, Thailand	Archawin Rojanawiwat; Jirapha Pakdee; Natthakul Bunneang; Nuanjun Wichukchinda; Penpitcha Thawong; Pilailuk Akkapaiboon Okada; Pundharika Piboonsiri; Surakameth Mahasirimongkol; Waritta Sawaengdee
EPI_ISL_3403346, EPI_ISL_3403347, EPI_ISL_3403348, EPI_ISL_3403349, EPI_ISL_3407817, EPI_ISL_3407826, EPI_ISL_3407838			
see above	Nonthaburi Public Health Office	National Institute of Health, Department of Medical Sciences, Ministry of Public Health, Thailand	; Natchaya Khiadsang; Nuttida Thongpramul; Pakorn Piromtong; Pilailuk Okada; Ratana Tacharoenmuang; Siripaporn Phuygun; Sittiporn Parnmen; Sunthareeya Waicharoen; Thanutsapa Thanadachakul; Warawan Wongboot; sirikanda wimol
EPI_ISL_3827346, EPI_ISL_3827347, EPI_ISL_3827348, EPI_ISL_3827349, EPI_ISL_3827350, EPI_ISL_3827351, EPI_ISL_3827352, EPI_ISL_3827353, EPI_ISL_3827354, EPI_ISL_3827355			
see above	Occicare Medical Center	Division of Genomic Medicine and Innovation support,Department of Medical Sciences, Ministry of Public Health, Thailand	Archawin Rojanawiwat; Jirapha Pakdee; Natthakul Bunneang; Nuanjun Wichukchinda; Penpitcha Thawong; Pilailuk Akkapaiboon Okada; Pundharika Piboonsiri; Surakameth Mahasirimongkol; Waritta Sawaengdee
EPI_ISL_4647659	Office of Disease Prevention and Control Region 11 Nakhon Si Thammarat	Division of Genomic Medicine and Innovation support,Department of Medical Sciences, Ministry of Public Health, Thailand	Archawin Rojanawiwat; Jirapha Pakdee; Natthakul Bunneang; Nuanjun Wichukchinda; Penpitcha Thawong; Pilailuk Akkapaiboon Okada; Pundharika Piboonsiri; Surakameth Mahasirimongkol; Waritta Sawaengdee
EPI_ISL_2233453	Office of Disease Prevention and Control Region 12 Songkhla	COVID-19 Network Investigations (CONI) Alliance	Angkana Huang; Anthony R. Jones; Arporn Wangwiwatsin; Bhakbhoom Panthan; Chonticha Klungtong; Duangkamon Loesbanluechai; Ekawat Pasomsub; Elizabeth Batty; Insee Sensorn; Janjira Thaipadungpanit; Khajohn Joonlasak; Kingkan Rakmanee; Krittikorn Kumpornsin; Namfon Kotanan; Sawanya Chantutanon; Stefan Fernandez; Thanat Chookajorn; Theerarat Kochakarn; Treewat Watthanachockchai; Wasun Chantratita; Wiphaporn Thabthimthong; Wudtichai Manasatienkij
EPI_ISL_2348804, EPI_ISL_2348805, EPI_ISL_2348819, EPI_ISL_2348820, EPI_ISL_2348821, EPI_ISL_2348822, EPI_ISL_2348835, EPI_ISL_2348836, EPI_ISL_2678111, EPI_ISL_2678112, EPI_ISL_2678113			
see above	Office of Diseases Prevention and Control Region 12 Songkhla	COVID-19 Network Investigations (CONI) Alliance	Angkana Huang; Anthony R. Jones; Arporn Wangwiwatsin; Bhakbhoom Panthan; Chonticha Klungtong; Duangkamon Loesbanluechai; Ekawat Pasomsub; Elizabeth Batty; Insee Sensorn; Janjira Thaipadungpanit; Khajohn Joonlasak; Kingkan Rakmanee; Krittikorn Kumpornsin; Namfon Kotanan; Sawanya Chantutanon; Stefan Fernandez; Thanat Chookajorn; Theerarat Kochakarn; Treewat Watthanachockchai; Wasun Chantratita; Wiphaporn Thabthimthong; Wudtichai Manasatienkij
EPI_ISL_882772, EPI_ISL_882795, EPI_ISL_882796, EPI_ISL_882797, EPI_ISL_882798, EPI_ISL_882799, EPI_ISL_1073956, EPI_ISL_1073957, EPI_ISL_1073959, EPI_ISL_1073961, EPI_ISL_1073962, EPI_ISL_1073963, EPI_ISL_1073965, EPI_ISL_1073981, EPI_ISL_1073982, EPI_ISL_1073983, EPI_ISL_1073984, EPI_ISL_1073985, EPI_ISL_1073986, EPI_ISL_1073987, EPI_ISL_1073988, EPI_ISL_1073989, EPI_ISL_1073990, EPI_ISL_1073991, EPI_ISL_1073992, EPI_ISL_1073993, EPI_ISL_1073994, EPI_ISL_1073995, EPI_ISL_1073996, EPI_ISL_1073997, EPI_ISL_1073998, EPI_ISL_1073999, EPI_ISL_1074000, EPI_ISL_1074001, EPI_ISL_1074002, EPI_ISL_1074003, EPI_ISL_1074004, EPI_ISL_1074005, EPI_ISL_1074006, EPI_ISL_1074007, EPI_ISL_1074008, EPI_ISL_1074009, EPI_ISL_1074010, EPI_ISL_1074011, EPI_ISL_1074012, EPI_ISL_1074013, EPI_ISL_1074014, EPI_ISL_1074015, EPI_ISL_1074016, EPI_ISL_1074017, EPI_ISL_1074018, EPI_ISL_1074019, EPI_ISL_1074020, EPI_ISL_1074021, EPI_ISL_1074022, EPI_ISL_1074023, EPI_ISL_1074024, EPI_ISL_1138830, EPI_ISL_1138831, EPI_ISL_1138832, EPI_ISL_1138833, EPI_ISL_1138834, EPI_ISL_1138835, EPI_ISL_1138836, EPI_ISL_1138837, EPI_ISL_1138838, EPI_ISL_1138839, EPI_ISL_1138840, EPI_ISL_1138841, EPI_ISL_1138842, EPI_ISL_1138843, EPI_ISL_1138848, EPI_ISL_1138849, EPI_ISL_1138850, EPI_ISL_1138851, EPI_ISL_1138852, EPI_ISL_1138853, EPI_ISL_1138854, EPI_ISL_1138855, EPI_ISL_1138856, EPI_ISL_1138857, EPI_ISL_1138858, EPI_ISL_1138859, EPI_ISL_1138860, EPI_ISL_1138861, EPI_ISL_1138862, EPI_ISL_1138863, EPI_ISL_1138864, EPI_ISL_1138865, EPI_ISL_1138866, EPI_ISL_1138867, EPI_ISL_1138868, EPI_ISL_1138869, EPI_ISL_1138870, EPI_ISL_1138871, EPI_ISL_1138872, EPI_ISL_1138873, EPI_ISL_1138874, EPI_ISL_1138875, EPI_ISL_1138876, EPI_ISL_1138877, EPI_ISL_1491575, EPI_ISL_2104721, EPI_ISL_2104722, EPI_ISL_2104723, EPI_ISL_2104724, EPI_ISL_2104725, EPI_ISL_2104726, EPI_ISL_2104727, EPI_ISL_2104728, EPI_ISL_2104729			
see above	Office of Diseases Prevention and Control Region 4 Saraburi	COVID-19 Network Investigations (CONI) Alliance	Amornmas Kongklieng; Anek Mungaomklang; Angkana Huang; Anthony R. Jones; Arporn Wangwiwatsin; Bhakbhoom Panthan; Chonticha Klungtong; Duangkamon Loesbanluechai; Ekawat Pasomsub; Elizabeth Batty; Insee Sensorn; Janjira Thaipadungpanit; Jutikul Kaewmalakul; Kamolthip Atsawawaranunt; Khajohn Joonlasak; Khajohn Joonsalak; Kingkan Rakmanee; Krittikorn Kumpornsin; Namfon Kotanan; Nathamon Runnachot; Pakjira Rimdusit; Payon Pengyo; Praima Moonmuang; Prayuth Kaewmalang; Pukkaporn Parnwijitkul; Sataporn Hatsadichart; Sirinapa Singthong; Siriporn Lakesukthom; Siriwan Yaemnimnual; Stefan Fernandez; Suttiruk Changchawai; Thanat Chookajorn; Theerarat Kochakarn; Treewat Watthanachockchai; Vichan Pawun; Wasun Chantratita; Wonvimol Lemprasert; Wudtichai Manasatienkij
EPI_ISL_4647408	PUBLIC HEALTH SERVICE CENTER 39, RAT BURANA	Division of Genomic Medicine and Innovation support,Department of Medical Sciences, Ministry of Public Health, Thailand	Archawin Rojanawiwat; Jirapha Pakdee; Natthakul Bunneang; Nuanjun Wichukchinda; Penpitcha Thawong; Pilailuk Akkapaiboon Okada; Pundharika Piboonsiri; Surakameth Mahasirimongkol; Waritta Sawaengdee
EPI_ISL_4647318, EPI_ISL_4647426	PUBLIC HEALTH SERVICE CENTER 46, KANTARAT UTHIT	Division of Genomic Medicine and Innovation support,Department of Medical Sciences, Ministry of Public Health, Thailand	Archawin Rojanawiwat; Jirapha Pakdee; Natthakul Bunneang; Nuanjun Wichukchinda; Penpitcha Thawong; Pilailuk Akkapaiboon Okada; Pundharika Piboonsiri; Surakameth Mahasirimongkol; Waritta Sawaengdee
EPI_ISL_4647298, EPI_ISL_4647308	PUBLIC HEALTH SERVICE CENTER 53, THUNG SONG HONG	Division of Genomic Medicine and Innovation support,Department of Medical Sciences, Ministry of Public Health, Thailand	Archawin Rojanawiwat; Jirapha Pakdee; Natthakul Bunneang; Nuanjun Wichukchinda; Penpitcha Thawong; Pilailuk Akkapaiboon Okada; Pundharika Piboonsiri; Surakameth Mahasirimongkol; Waritta Sawaengdee
EPI_ISL_4647392	PUBLIC HEALTH SERVICE CENTER 54 (THAT IAM)	Division of Genomic Medicine and Innovation support,Department of Medical Sciences, Ministry of Public Health, Thailand	Archawin Rojanawiwat; Jirapha Pakdee; Natthakul Bunneang; Nuanjun Wichukchinda; Penpitcha Thawong; Pilailuk Akkapaiboon Okada; Pundharika Piboonsiri; Surakameth Mahasirimongkol; Waritta Sawaengdee
EPI_ISL_4647350, EPI_ISL_4647374, EPI_ISL_4647433, EPI_ISL_4647438, EPI_ISL_4647444	PUBLIC HEALTH SERVICE CENTER 59, THUNG KHRU 2	Division of Genomic Medicine and Innovation support,Department of Medical Sciences, Ministry of Public Health, Thailand	Archawin Rojanawiwat; Jirapha Pakdee; Natthakul Bunneang; Nuanjun Wichukchinda; Penpitcha Thawong; Pilailuk Akkapaiboon Okada; Pundharika Piboonsiri; Surakameth Mahasirimongkol; Waritta Sawaengdee
EPI_ISL_455605	Panyananthaphikkhu Chonprathan Medical Center	National Institute of Health. Department of medical Sciences, Ministry of Public Health, Thailand	Chittaganpitch; Malinee; Okada; Parnmen; Phuygun; Pilailuk; Siripaporn; Sittiporn; Sunthareeya; Thanadachakul; Thanutsapa; Waicharoen; Warawan; Wongboot
EPI_ISL_434701	Panyanunthaphikkhu Chonprathan Medical Center (PCMC)	National Institute of Health. Department of medical Sciences, Ministry of Public Health, Thailand	Chittaganpitch; Malinee; Okada; Parnmen; Phuygun; Pilailuk; Siripaporn; Sittiporn; Sunthareeya; Thanadachakul; Thanutsapa; Waicharoen; Warawan; Wongboot
EPI_ISL_1251227	Paolo Hospital Rangsit	National Institute of Health, Department of Medical Sciences, Ministry of Public Health, Thailand	Hathaikan Tanchai; Natchaya Khiadsang; Nuttida Thongpramul; Pakorn Piromtong; Pilailuk Okada; Ratana Tacharoenmuang; Siripaporn Phuygun; Sittiporn Parnmen; Sunthareeya Waicharoen.; Thanutsapa Thanadachakull
EPI_ISL_3827174, EPI_ISL_3827330, EPI_ISL_3827331	Pasang Hospital	Division of Genomic Medicine and Innovation support,Department of Medical Sciences, Ministry of Public Health, Thailand	Archawin Rojanawiwat; Jirapha Pakdee; Natthakul Bunneang; Nuanjun Wichukchinda; Penpitcha Thawong; Pilailuk Akkapaiboon Okada; Pundharika Piboonsiri; Surakameth Mahasirimongkol; Waritta Sawaengdee
EPI_ISL_2433291, EPI_ISL_2433319, EPI_ISL_2433555, EPI_ISL_2433646	Pattani Hoapital	Division of Genomic Medicine and Innovation support,Department of Medical Sciences, Ministry of Public Health, Thailand	Archawin Rojanawiwat; Jirapha Pakdee; Natthakul Bunneang; Nuanjun Wichukchinda; Penpitcha Thawong; Pilailuk Akkapaiboon Okada; Pundharika Piboonsiri; Surakameth Mahasirimongkol; Waritta Sawaengdee
EPI_ISL_3827299, EPI_ISL_3827300, EPI_ISL_3827301, EPI_ISL_3827302, EPI_ISL_3827303, EPI_ISL_3827397, EPI_ISL_3827442, EPI_ISL_3827444, EPI_ISL_3827445, EPI_ISL_3827448, EPI_ISL_3827473			
see above	Phayao Hospital	Division of Genomic Medicine and Innovation support,Department of Medical Sciences, Ministry of Public Health, Thailand	Archawin Rojanawiwat; Jirapha Pakdee; Natthakul Bunneang; Nuanjun Wichukchinda; Penpitcha Thawong; Pilailuk Akkapaiboon Okada; Pundharika Piboonsiri; Surakameth Mahasirimongkol; Waritta Sawaengdee
EPI_ISL_3827308, EPI_ISL_3827309, EPI_ISL_3827310, EPI_ISL_3827311, EPI_ISL_3827312, EPI_ISL_3827313, EPI_ISL_3827314, EPI_ISL_3827315			
see above	Phayao Ram Hospital	Division of Genomic Medicine and Innovation support,Department of Medical Sciences, Ministry of Public Health, Thailand	Archawin Rojanawiwat; Jirapha Pakdee; Natthakul Bunneang; Nuanjun Wichukchinda; Penpitcha Thawong; Pilailuk Akkapaiboon Okada; Pundharika Piboonsiri; Surakameth Mahasirimongkol; Waritta Sawaengdee
EPI_ISL_2346471, EPI_ISL_2348489, EPI_ISL_2348490	Phra Chom Klao Hospital	Division of Genomic medicine and Innonation support,Department of medical Sciences, Ministry of Public Health, Thailand	Archawin Rojanawiwat; Jirapha Pakdee; Natthakul Bunneang; Nuanjun Wichukchinda; Penpitcha Thawong; Pilailuk Akkapaiboon Okada; Pundharika Piboonsiri; Surakameth Mahasirimongkol; Waritta Sawaengdee
EPI_ISL_3665961	Phra Chom Klao Hospital	National Institute of Health, Department of Medical Sciences, Ministry of Public Health, Thailand	; Natchaya Khiadsang; Nuttida Thongpramul; Pakorn Piromtong; Pilailuk Okada; Ratana Tacharoenmuang; Siripaporn Phuygun; Sittiporn Parnmen; Sunthareeya Waicharoen; Thanutsapa Thanadachakul; Warawan Wongboot; sirikanda wimol
EPI_ISL_3406997, EPI_ISL_3406998, EPI_ISL_4635437	Phra Nang Klao Hospital	National Institute of Health, Department of Medical Sciences, Ministry of Public Health, Thailand	; Archawin Rojanawiwat; Natchaya Khiadsang; Nuttida Thongpramul; Pakorn Piromtong; Pilailuk Okada; Ratana Tacharoenmuang; Siripaporn Phuygun; Sittiporn Parnmen; Sunthareeya Waicharoen; Thanutsapa Thanadachakul; Warawan Wongboot; sirikanda wimol
EPI_ISL_3827304	Phrae Hospital	Division of Genomic Medicine and Innovation support,Department of Medical Sciences, Ministry of Public Health, Thailand	Archawin Rojanawiwat; Jirapha Pakdee; Natthakul Bunneang; Nuanjun Wichukchinda; Penpitcha Thawong; Pilailuk Akkapaiboon Okada; Pundharika Piboonsiri; Surakameth Mahasirimongkol; Waritta Sawaengdee
EPI_ISL_455585, EPI_ISL_455608	Phramongkutklao Hospital	National Institute of Health. Department of medical Sciences, Ministry of Public Health, Thailand	Chittaganpitch; Malinee; Okada; Parnmen; Phuygun; Pilailuk; Siripaporn; Sittiporn; Sunthareeya; Thanadachakul; Thanutsapa; Waicharoen; Warawan; Wongboot
EPI_ISL_3827171	Phu Kradueng Hospital	Division of Genomic Medicine and Innovation support,Department of Medical Sciences, Ministry of Public Health, Thailand	Archawin Rojanawiwat; Jirapha Pakdee; Natthakul Bunneang; Nuanjun Wichukchinda; Penpitcha Thawong; Pilailuk Akkapaiboon Okada; Pundharika Piboonsiri; Surakameth Mahasirimongkol; Waritta Sawaengdee
EPI_ISL_2433288, EPI_ISL_2433557	Phuket Provisional Hospital	Division of Genomic Medicine and Innovation support,Department of Medical Sciences, Ministry of Public Health, Thailand	Archawin Rojanawiwat; Jirapha Pakdee; Natthakul Bunneang; Nuanjun Wichukchinda; Penpitcha Thawong; Pilailuk Akkapaiboon Okada; Pundharika Piboonsiri; Surakameth Mahasirimongkol; Waritta Sawaengdee

EPI_ISL_3407004	Phyathai 3 Hospital	National Institute of Health, Department of Medical Sciences, Ministry of Public Health, Thailand	; Natchaya Khiadsang; Nuttida Thongpramul; Pakorn Piromtong; Pilailuk Okada; Ratana Tacharoenmuang; Siripaporn Phuygun; Sittiporn Parnmen; Sunthareeya Waicharoen; Thanutsapa Thanadachakul; Warawan Wongboot; sirikanda wimol
EPI_ISL_3827187, EPI_ISL_3827193, EPI_ISL_3827197, EPI_ISL_3827198, EPI_ISL_3827429	Phyathai Hospital	Division of Genomic Medicine and Innovation support,Department of Medical Sciences, Ministry of Public Health, Thailand	Archawin Rojanawiwat; Jirapha Pakdee; Natthakul Bunneang; Nuanjun Wichukchinda; Penpitcha Thawong; Pilailuk Akkapaiboon Okada; Pundharika Piboonsiri; Surakameth Mahasirimongkol; Waritta Sawaengdee
EPI_ISL_1173247, EPI_ISL_1495988, EPI_ISL_1495989, EPI_ISL_1495990, EPI_ISL_1495991, EPI_ISL_1497598, EPI_ISL_3665952, EPI_ISL_3665953, EPI_ISL_3665954, EPI_ISL_3665962, EPI_ISL_3665963			
see above	Piyavate Hospital	National Institute of Health, Department of Medical Sciences, Ministry of Public Health, Thailand	; Hathaikan Tanchai; Natchaya Khiadsang; Natchaya Khiadsang; Nuttida Thongpramul; Pakorn Piromtong; Pilailuk Okada; Ratana Tacharoenmuang; Siripaporn Phuygun; Sittiporn Parnmen; Sunthareeya Waicharoen; Sunthareeya Waicharoen.; Thanutsapa Thanadachakul; Thanutsapa Thanadachakull; Warawan Wongboot; sirikanda wimol
EPI_ISL_3827377, EPI_ISL_3827378, EPI_ISL_3827380, EPI_ISL_3827453	Pla pak Hospital	Division of Genomic Medicine and Innovation support,Department of Medical Sciences, Ministry of Public Health, Thailand	Archawin Rojanawiwat; Jirapha Pakdee; Natthakul Bunneang; Nuanjun Wichukchinda; Penpitcha Thawong; Pilailuk Akkapaiboon Okada; Pundharika Piboonsiri; Surakameth Mahasirimongkol; Waritta Sawaengdee
EPI_ISL_3892044, EPI_ISL_3892046, EPI_ISL_3892048, EPI_ISL_3892049, EPI_ISL_3892051, EPI_ISL_3892052, EPI_ISL_3892055, EPI_ISL_3892057, EPI_ISL_3892058, EPI_ISL_3892061, EPI_ISL_3892062, EPI_ISL_3892065, EPI_ISL_3892067, EPI_ISL_3892069, EPI_ISL_3892075, EPI_ISL_3892079, EPI_ISL_3892085, EPI_ISL_3892088, EPI_ISL_3892089, EPI_ISL_3892092, EPI_ISL_3892094, EPI_ISL_3892095, EPI_ISL_3892097, EPI_ISL_3892098, EPI_ISL_3892100, EPI_ISL_3892107, EPI_ISL_3892109, EPI_ISL_3892111, EPI_ISL_3892113, EPI_ISL_3892115, EPI_ISL_3892119, EPI_ISL_3892124, EPI_ISL_3892130, EPI_ISL_3892135, EPI_ISL_3892138, EPI_ISL_3892140, EPI_ISL_3892141, EPI_ISL_3892144, EPI_ISL_3892146, EPI_ISL_3892148, EPI_ISL_3892150, EPI_ISL_3892153, EPI_ISL_3892154, EPI_ISL_3892155, EPI_ISL_3892157, EPI_ISL_3892158, EPI_ISL_3892160, EPI_ISL_3892162, EPI_ISL_3892165, EPI_ISL_3892167, EPI_ISL_3892168, EPI_ISL_3892171, EPI_ISL_3892173, EPI_ISL_3892176, EPI_ISL_3892178, EPI_ISL_3892180			
see above	Port Health and Quarantine, Suvarnabhumi Airport	Thai Red Cross Emerging Infectious Diseases Clinical Center and Faculty of Medicine, Chulalongkorn University	Anutsara Jitsatja; Jiratchaya Nuanpirom; Nattakarn Thippamom; Opass Putcharoen; Rawinan Soma; Rome Buathong; Sasiprapa Ninwattana; Sininat Petcharat; Sirirak Thanasakulprasert; Sopon Iamsirithaworn; Supaporn Wacharapluesadee
EPI_ISL_430841, EPI_ISL_434698, EPI_ISL_434699, EPI_ISL_434702, EPI_ISL_434703, EPI_ISL_434704, EPI_ISL_434705, EPI_ISL_434706, EPI_ISL_455606, EPI_ISL_455607			
see above	Praram 9 Hospital	National Institute of Health. Department of medical Sciences, Ministry of Public Health, Thailand	Chittaganpitch; Malinee; Okada; Parnmen; Phuygun; Pilailuk; Siripaporn; Sittiporn; Sunthareeya; Thanadachakul; Thanutsapa; Waicharoen; Warawan; Wongboot
EPI_ISL_2351054, EPI_ISL_2351055, EPI_ISL_2351056, EPI_ISL_2351057, EPI_ISL_2351058, EPI_ISL_2351059, EPI_ISL_2351060, EPI_ISL_4235383, EPI_ISL_4235391, EPI_ISL_4235398, EPI_ISL_4235413			
see above	Public Health Center 23 Siphraya	Division of Genomic Medicine and Innovation support,Department of Medical Sciences, Ministry of Public Health, Thailand	Archawin Rojanawiwat; Jirapha Pakdee; Natthakul Bunneang; Nuanjun Wichukchinda; Penpitcha Thawong; Pilailuk Akkapaiboon Okada; Pundharika Piboonsiri; Surakameth Mahasirimongkol; Waritta Sawaengdee
EPI_ISL_4235375, EPI_ISL_4235399, EPI_ISL_4235407, EPI_ISL_4235430, EPI_ISL_4235436	Public Health Center 28 Krung Thon Buri,	Division of Genomic Medicine and Innovation support,Department of Medical Sciences, Ministry of Public Health, Thailand	Archawin Rojanawiwat; Jirapha Pakdee; Natthakul Bunneang; Nuanjun Wichukchinda; Penpitcha Thawong; Pilailuk Akkapaiboon Okada; Pundharika Piboonsiri; Surakameth Mahasirimongkol; Waritta Sawaengdee
EPI_ISL_4235431, EPI_ISL_4235437	Public Health Center 29	Division of Genomic Medicine and Innovation support,Department of Medical Sciences, Ministry of Public Health, Thailand	Archawin Rojanawiwat; Jirapha Pakdee; Natthakul Bunneang; Nuanjun Wichukchinda; Penpitcha Thawong; Pilailuk Akkapaiboon Okada; Pundharika Piboonsiri; Surakameth Mahasirimongkol; Waritta Sawaengdee
EPI_ISL_4235363, EPI_ISL_4235370, EPI_ISL_4235378, EPI_ISL_4235386	Public Health Center 31	Division of Genomic Medicine and Innovation support,Department of Medical Sciences, Ministry of Public Health, Thailand	Archawin Rojanawiwat; Jirapha Pakdee; Natthakul Bunneang; Nuanjun Wichukchinda; Penpitcha Thawong; Pilailuk Akkapaiboon Okada; Pundharika Piboonsiri; Surakameth Mahasirimongkol; Waritta Sawaengdee
EPI_ISL_3826508, EPI_ISL_3826509, EPI_ISL_3826510, EPI_ISL_3826511, EPI_ISL_3826512, EPI_ISL_3826513, EPI_ISL_3826514, EPI_ISL_3826515, EPI_ISL_3826766			
see above	Public Health Center 32 Maris Tintamusik	Division of Genomic Medicine and Innovation support,Department of Medical Sciences, Ministry of Public Health, Thailand	Archawin Rojanawiwat; Jirapha Pakdee; Natthakul Bunneang; Nuanjun Wichukchinda; Penpitcha Thawong; Pilailuk Akkapaiboon Okada; Pundharika Piboonsiri; Surakameth Mahasirimongkol; Waritta Sawaengdee
EPI_ISL_4235357	Public Health Center 4 Dindang	Division of Genomic Medicine and Innovation support,Department of Medical Sciences, Ministry of Public Health, Thailand	Archawin Rojanawiwat; Jirapha Pakdee; Natthakul Bunneang; Nuanjun Wichukchinda; Penpitcha Thawong; Pilailuk Akkapaiboon Okada; Pundharika Piboonsiri; Surakameth Mahasirimongkol; Waritta Sawaengdee
EPI_ISL_4235408, EPI_ISL_4235424, EPI_ISL_4235438	Public Health Center 44	Division of Genomic Medicine and Innovation support,Department of Medical Sciences, Ministry of Public Health, Thailand	Archawin Rojanawiwat; Jirapha Pakdee; Natthakul Bunneang; Nuanjun Wichukchinda; Penpitcha Thawong; Pilailuk Akkapaiboon Okada; Pundharika Piboonsiri; Surakameth Mahasirimongkol; Waritta Sawaengdee
EPI_ISL_4235396, EPI_ISL_4235404, EPI_ISL_4235412, EPI_ISL_4235417	Public Health Center 46	Division of Genomic Medicine and Innovation support,Department of Medical Sciences, Ministry of Public Health, Thailand	Archawin Rojanawiwat; Jirapha Pakdee; Natthakul Bunneang; Nuanjun Wichukchinda; Penpitcha Thawong; Pilailuk Akkapaiboon Okada; Pundharika Piboonsiri; Surakameth Mahasirimongkol; Waritta Sawaengdee
EPI_ISL_4235362, EPI_ISL_4235422, EPI_ISL_4235443	Public Health Center 48	Division of Genomic Medicine and Innovation support,Department of Medical Sciences, Ministry of Public Health, Thailand	Archawin Rojanawiwat; Jirapha Pakdee; Natthakul Bunneang; Nuanjun Wichukchinda; Penpitcha Thawong; Pilailuk Akkapaiboon Okada; Pundharika Piboonsiri; Surakameth Mahasirimongkol; Waritta Sawaengdee
EPI_ISL_2351103, EPI_ISL_2351104, EPI_ISL_2351105, EPI_ISL_2351106, EPI_ISL_2351107, EPI_ISL_2351108, EPI_ISL_2351109, EPI_ISL_2351110, EPI_ISL_2351111, EPI_ISL_2351112, EPI_ISL_2351113, EPI_ISL_2351114, EPI_ISL_2351115, EPI_ISL_2351116, EPI_ISL_2351117, EPI_ISL_2351118, EPI_ISL_2351119, EPI_ISL_2351120, EPI_ISL_2351121, EPI_ISL_2351122, EPI_ISL_2351123, EPI_ISL_2351124, EPI_ISL_2351125, EPI_ISL_2351126, EPI_ISL_2351127, EPI_ISL_2351128, EPI_ISL_2351129, EPI_ISL_2351130, EPI_ISL_2351131, EPI_ISL_2351132, EPI_ISL_2351133, EPI_ISL_2351134, EPI_ISL_2351135, EPI_ISL_2351136, EPI_ISL_2351137, EPI_ISL_2351138, EPI_ISL_2351139, EPI_ISL_2351140, EPI_ISL_2351141, EPI_ISL_2351142, EPI_ISL_2351143, EPI_ISL_2351144, EPI_ISL_2351145, EPI_ISL_2351146, EPI_ISL_2351147, EPI_ISL_2351148, EPI_ISL_2351149, EPI_ISL_2351150, EPI_ISL_2351151, EPI_ISL_3826778			
see above	Public Health Center 52 Samsennok	Division of Genomic Medicine and Innovation support,Department of Medical Sciences, Ministry of Public Health, Thailand	Archawin Rojanawiwat; Jirapha Pakdee; Natthakul Bunneang; Nuanjun Wichukchinda; Penpitcha Thawong; Pilailuk Akkapaiboon Okada; Pundharika Piboonsiri; Surakameth Mahasirimongkol; Waritta Sawaengdee
EPI_ISL_4235381, EPI_ISL_4235387, EPI_ISL_4235389, EPI_ISL_4235393, EPI_ISL_4235411, EPI_ISL_4235415	Public Health Center 53	Division of Genomic Medicine and Innovation support,Department of Medical Sciences, Ministry of Public Health, Thailand	Archawin Rojanawiwat; Jirapha Pakdee; Natthakul Bunneang; Nuanjun Wichukchinda; Penpitcha Thawong; Pilailuk Akkapaiboon Okada; Pundharika Piboonsiri; Surakameth Mahasirimongkol; Waritta Sawaengdee
EPI_ISL_4235418, EPI_ISL_4235445	Public Health Center 61	Division of Genomic Medicine and Innovation support,Department of Medical Sciences, Ministry of Public Health, Thailand	Archawin Rojanawiwat; Jirapha Pakdee; Natthakul Bunneang; Nuanjun Wichukchinda; Penpitcha Thawong; Pilailuk Akkapaiboon Okada; Pundharika Piboonsiri; Surakameth Mahasirimongkol; Waritta Sawaengdee
EPI_ISL_4235419, EPI_ISL_4235420	Public Health Center 63	Division of Genomic Medicine and Innovation support,Department of Medical Sciences, Ministry of Public Health, Thailand	Archawin Rojanawiwat; Jirapha Pakdee; Natthakul Bunneang; Nuanjun Wichukchinda; Penpitcha Thawong; Pilailuk Akkapaiboon Okada; Pundharika Piboonsiri; Surakameth Mahasirimongkol; Waritta Sawaengdee
EPI_ISL_4647324	Public Health Center 64 khong Sam Wa	Division of Genomic Medicine and Innovation support,Department of Medical Sciences, Ministry of Public Health, Thailand	Archawin Rojanawiwat; Jirapha Pakdee; Natthakul Bunneang; Nuanjun Wichukchinda; Penpitcha Thawong; Pilailuk Akkapaiboon Okada; Pundharika Piboonsiri; Surakameth Mahasirimongkol; Waritta Sawaengdee
EPI_ISL_2351080	Public Health Center 65	Division of Genomic Medicine and Innovation support,Department of Medical Sciences, Ministry of Public Health, Thailand	Archawin Rojanawiwat; Jirapha Pakdee; Natthakul Bunneang; Nuanjun Wichukchinda; Penpitcha Thawong; Pilailuk Akkapaiboon Okada; Pundharika Piboonsiri; Surakameth Mahasirimongkol; Waritta Sawaengdee
EPI_ISL_4235364	Public Health Center 67 Taweewattana	Division of Genomic Medicine and Innovation support,Department of Medical Sciences, Ministry of Public Health, Thailand	Archawin Rojanawiwat; Jirapha Pakdee; Natthakul Bunneang; Nuanjun Wichukchinda; Penpitcha Thawong; Pilailuk Akkapaiboon Okada; Pundharika Piboonsiri; Surakameth Mahasirimongkol; Waritta Sawaengdee
EPI_ISL_4235390, EPI_ISL_4235392, EPI_ISL_4235406	Public Health Center 69	Division of Genomic Medicine and Innovation support,Department of Medical Sciences, Ministry of Public Health, Thailand	Archawin Rojanawiwat; Jirapha Pakdee; Natthakul Bunneang; Nuanjun Wichukchinda; Penpitcha Thawong; Pilailuk Akkapaiboon Okada; Pundharika Piboonsiri; Surakameth Mahasirimongkol; Waritta Sawaengdee
EPI_ISL_4647349	Public Health Center 7 Boonmee Bururatchrangsan	Division of Genomic Medicine and Innovation support,Department of Medical Sciences, Ministry of Public Health, Thailand	Archawin Rojanawiwat; Jirapha Pakdee; Natthakul Bunneang; Nuanjun Wichukchinda; Penpitcha Thawong; Pilailuk Akkapaiboon Okada; Pundharika Piboonsiri; Surakameth Mahasirimongkol; Waritta Sawaengdee
EPI_ISL_2351200, EPI_ISL_2351201, EPI_ISL_2351202	Public Health Centre 29	Division of Genomic Medicine and Innovation support,Department of Medical Sciences, Ministry of Public Health, Thailand	Archawin Rojanawiwat; Jirapha Pakdee; Natthakul Bunneang; Nuanjun Wichukchinda; Penpitcha Thawong; Pilailuk Akkapaiboon Okada; Pundharika Piboonsiri; Surakameth Mahasirimongkol; Waritta Sawaengdee
EPI_ISL_2351203, EPI_ISL_2351204	Public Health Centre 33	Division of Genomic Medicine and Innovation support,Department of Medical Sciences, Ministry of Public Health, Thailand	Archawin Rojanawiwat; Jirapha Pakdee; Natthakul Bunneang; Nuanjun Wichukchinda; Penpitcha Thawong; Pilailuk Akkapaiboon Okada; Pundharika Piboonsiri; Surakameth Mahasirimongkol; Waritta Sawaengdee
EPI_ISL_2351199, EPI_ISL_2351205, EPI_ISL_2351206, EPI_ISL_2351207, EPI_ISL_2351208, EPI_ISL_2351209, EPI_ISL_2351210, EPI_ISL_2351211, EPI_ISL_2351212, EPI_ISL_2351213, EPI_ISL_2351214, EPI_ISL_2351215			
see above	Public Health Centre 36	Division of Genomic Medicine and Innovation support,Department of Medical Sciences, Ministry of Public Health, Thailand	Archawin Rojanawiwat; Jirapha Pakdee; Natthakul Bunneang; Nuanjun Wichukchinda; Penpitcha Thawong; Pilailuk Akkapaiboon Okada; Pundharika Piboonsiri; Surakameth Mahasirimongkol; Waritta Sawaengdee
EPI_ISL_2351052, EPI_ISL_2351053, EPI_ISL_2351152, EPI_ISL_2351153, EPI_ISL_2351196, EPI_ISL_2351197, EPI_ISL_2351198			
see above	Public Health Centre 40	Division of Genomic Medicine and Innovation support,Department of Medical Sciences, Ministry of Public Health, Thailand	Archawin Rojanawiwat; Jirapha Pakdee; Natthakul Bunneang; Nuanjun Wichukchinda; Penpitcha Thawong; Pilailuk Akkapaiboon Okada; Pundharika Piboonsiri; Surakameth Mahasirimongkol; Waritta Sawaengdee
EPI_ISL_3826506, EPI_ISL_3826507	Public Health Centre 58 Lom-Phimsen Fak Udom	Division of Genomic Medicine and Innovation support,Department of Medical Sciences, Ministry of Public Health, Thailand	Archawin Rojanawiwat; Jirapha Pakdee; Natthakul Bunneang; Nuanjun Wichukchinda; Penpitcha Thawong; Pilailuk Akkapaiboon Okada; Pundharika Piboonsiri; Surakameth Mahasirimongkol; Waritta Sawaengdee
EPI_ISL_2351081, EPI_ISL_2351082, EPI_ISL_2351083, EPI_ISL_2351084, EPI_ISL_2351085, EPI_ISL_2351086, EPI_ISL_2351087, EPI_ISL_2351088, EPI_ISL_2351089, EPI_ISL_2351090, EPI_ISL_2351091, EPI_ISL_2351092, EPI_ISL_2351093, EPI_ISL_2351094, EPI_ISL_2351095, EPI_ISL_2351096, EPI_ISL_2351097, EPI_ISL_2351098, EPI_ISL_2351099, EPI_ISL_2351100, EPI_ISL_2351101, EPI_ISL_2351102			
see above	Public Health Centre 6	Division of Genomic Medicine and Innovation support,Department of Medical Sciences, Ministry of Public Health, Thailand	Archawin Rojanawiwat; Jirapha Pakdee; Natthakul Bunneang; Nuanjun Wichukchinda; Penpitcha Thawong; Pilailuk Akkapaiboon Okada; Pundharika Piboonsiri; Surakameth Mahasirimongkol; Waritta Sawaengdee

EPI_ISL_3826517, EPI_ISL_3826518, EPI_ISL_3826519, EPI_ISL_3826520	Public Health Centre 6 Female Culture Authority	Division of Genomic Medicine and Innovation support,Department of Medical Sciences, Ministry of Public Health, Thailand	Archawin Rojanawiwat; Jirapha Pakdee; Natthakul Bunneang; Nuanjun Wichukchinda; Penpitcha Thawong; Pilailuk Akkapaiboon Okada; Pundharika Piboonsiri; Surakameth Mahasirimongkol; Waritta Sawaengdee
EPI_ISL_2351061, EPI_ISL_2351062, EPI_ISL_2351063, EPI_ISL_2351064, EPI_ISL_2351065, EPI_ISL_2351066, EPI_ISL_2351067, EPI_ISL_2351068			
see above	Public Health Centre 67	Division of Genomic Medicine and Innovation support,Department of Medical Sciences, Ministry of Public Health, Thailand	Archawin Rojanawiwat; Jirapha Pakdee; Natthakul Bunneang; Nuanjun Wichukchinda; Penpitcha Thawong; Pilailuk Akkapaiboon Okada; Pundharika Piboonsiri; Surakameth Mahasirimongkol; Waritta Sawaengdee
EPI_ISL_4647359	Public Health Service Center 1 Sapan Mon (Mon Bridge)	Division of Genomic Medicine and Innovation support,Department of Medical Sciences, Ministry of Public Health, Thailand	Archawin Rojanawiwat; Jirapha Pakdee; Natthakul Bunneang; Nuanjun Wichukchinda; Penpitcha Thawong; Pilailuk Akkapaiboon Okada; Pundharika Piboonsiri; Surakameth Mahasirimongkol; Waritta Sawaengdee
EPI_ISL_2351069, EPI_ISL_2351070, EPI_ISL_2351071, EPI_ISL_2351072, EPI_ISL_2351073, EPI_ISL_2351074, EPI_ISL_2351075, EPI_ISL_2351076, EPI_ISL_2351077, EPI_ISL_2351078, EPI_ISL_2351079, EPI_ISL_2351159, EPI_ISL_2351160, EPI_ISL_2351161, EPI_ISL_2351162, EPI_ISL_2351163, EPI_ISL_2351164, EPI_ISL_2351165, EPI_ISL_2351166, EPI_ISL_2351167, EPI_ISL_2351168, EPI_ISL_2351169, EPI_ISL_2351170, EPI_ISL_2351171, EPI_ISL_2351172, EPI_ISL_2351173, EPI_ISL_2351174, EPI_ISL_2351175, EPI_ISL_2351176, EPI_ISL_2351177, EPI_ISL_2351178, EPI_ISL_2351179, EPI_ISL_2351180, EPI_ISL_2351181, EPI_ISL_2351182, EPI_ISL_2351183, EPI_ISL_2351184, EPI_ISL_2351185, EPI_ISL_2351186, EPI_ISL_2351187, EPI_ISL_2351188, EPI_ISL_2351189, EPI_ISL_2351190, EPI_ISL_2351191, EPI_ISL_2351192, EPI_ISL_2351193, EPI_ISL_2351194, EPI_ISL_2351195			
see above	Public Health Service Center 1 Saphan Mon	Division of Genomic Medicine and Innovation support,Department of Medical Sciences, Ministry of Public Health, Thailand	Archawin Rojanawiwat; Jirapha Pakdee; Natthakul Bunneang; Nuanjun Wichukchinda; Penpitcha Thawong; Pilailuk Akkapaiboon Okada; Pundharika Piboonsiri; Surakameth Mahasirimongkol; Waritta Sawaengdee
EPI_ISL_3826755, EPI_ISL_3826756, EPI_ISL_3826757, EPI_ISL_3826758	Public Health Service Center 13 Maitree Vanich	Division of Genomic Medicine and Innovation support,Department of Medical Sciences, Ministry of Public Health, Thailand	Archawin Rojanawiwat; Jirapha Pakdee; Natthakul Bunneang; Nuanjun Wichukchinda; Penpitcha Thawong; Pilailuk Akkapaiboon Okada; Pundharika Piboonsiri; Surakameth Mahasirimongkol; Waritta Sawaengdee
EPI_ISL_3826504, EPI_ISL_3826752, EPI_ISL_3826753, EPI_ISL_3826754	Public Health Service Center 15 Lat Phrao	Division of Genomic Medicine and Innovation support,Department of Medical Sciences, Ministry of Public Health, Thailand	Archawin Rojanawiwat; Jirapha Pakdee; Natthakul Bunneang; Nuanjun Wichukchinda; Penpitcha Thawong; Pilailuk Akkapaiboon Okada; Pundharika Piboonsiri; Surakameth Mahasirimongkol; Waritta Sawaengdee
EPI_ISL_4235400	Public Health Service Center 17 Pracha Niwet	Division of Genomic Medicine and Innovation support,Department of Medical Sciences, Ministry of Public Health, Thailand	Archawin Rojanawiwat; Jirapha Pakdee; Natthakul Bunneang; Nuanjun Wichukchinda; Penpitcha Thawong; Pilailuk Akkapaiboon Okada; Pundharika Piboonsiri; Surakameth Mahasirimongkol; Waritta Sawaengdee
EPI_ISL_4235382	Public Health Service Center 18	Division of Genomic Medicine and Innovation support,Department of Medical Sciences, Ministry of Public Health, Thailand	Archawin Rojanawiwat; Jirapha Pakdee; Natthakul Bunneang; Nuanjun Wichukchinda; Penpitcha Thawong; Pilailuk Akkapaiboon Okada; Pundharika Piboonsiri; Surakameth Mahasirimongkol; Waritta Sawaengdee
EPI_ISL_4647367	Public Health Service Center 18 Mongkol Wonwangtan	Division of Genomic Medicine and Innovation support,Department of Medical Sciences, Ministry of Public Health, Thailand	Archawin Rojanawiwat; Jirapha Pakdee; Natthakul Bunneang; Nuanjun Wichukchinda; Penpitcha Thawong; Pilailuk Akkapaiboon Okada; Pundharika Piboonsiri; Surakameth Mahasirimongkol; Waritta Sawaengdee
EPI_ISL_3826501, EPI_ISL_3826502, EPI_ISL_3826503	Public Health Service Center 20	Division of Genomic Medicine and Innovation support,Department of Medical Sciences, Ministry of Public Health, Thailand	Archawin Rojanawiwat; Jirapha Pakdee; Natthakul Bunneang; Nuanjun Wichukchinda; Penpitcha Thawong; Pilailuk Akkapaiboon Okada; Pundharika Piboonsiri; Surakameth Mahasirimongkol; Waritta Sawaengdee
EPI_ISL_2351154, EPI_ISL_2351155, EPI_ISL_2351156, EPI_ISL_2351157, EPI_ISL_4235360, EPI_ISL_4235361, EPI_ISL_4235365, EPI_ISL_4235371, EPI_ISL_4235377, EPI_ISL_4235379, EPI_ISL_4235385, EPI_ISL_4235388, EPI_ISL_4235405, EPI_ISL_4235428, EPI_ISL_4235435, EPI_ISL_4235441, EPI_ISL_4235442, EPI_ISL_4235444			
see above	Public Health Service Center 24 Bang Khen	Division of Genomic Medicine and Innovation support,Department of Medical Sciences, Ministry of Public Health, Thailand	Archawin Rojanawiwat; Jirapha Pakdee; Natthakul Bunneang; Nuanjun Wichukchinda; Penpitcha Thawong; Pilailuk Akkapaiboon Okada; Pundharika Piboonsiri; Surakameth Mahasirimongkol; Waritta Sawaengdee
EPI_ISL_4235369	Public Health Service Center 25 Huai Khwang	Division of Genomic Medicine and Innovation support,Department of Medical Sciences, Ministry of Public Health, Thailand	Archawin Rojanawiwat; Jirapha Pakdee; Natthakul Bunneang; Nuanjun Wichukchinda; Penpitcha Thawong; Pilailuk Akkapaiboon Okada; Pundharika Piboonsiri; Surakameth Mahasirimongkol; Waritta Sawaengdee
EPI_ISL_4647416	Public Health Service Center 27, Chan Chimphaibun	Division of Genomic Medicine and Innovation support,Department of Medical Sciences, Ministry of Public Health, Thailand	Archawin Rojanawiwat; Jirapha Pakdee; Natthakul Bunneang; Nuanjun Wichukchinda; Penpitcha Thawong; Pilailuk Akkapaiboon Okada; Pundharika Piboonsiri; Surakameth Mahasirimongkol; Waritta Sawaengdee
EPI_ISL_3826516, EPI_ISL_3826785	Public Health Service Center 30 Wat Chao Arm	Division of Genomic Medicine and Innovation support,Department of Medical Sciences, Ministry of Public Health, Thailand	Archawin Rojanawiwat; Jirapha Pakdee; Natthakul Bunneang; Nuanjun Wichukchinda; Penpitcha Thawong; Pilailuk Akkapaiboon Okada; Pundharika Piboonsiri; Surakameth Mahasirimongkol; Waritta Sawaengdee
EPI_ISL_4557923	Public Health Service Center 32 Maris Tintamusik	Division of Genomic Medicine and Innovation support,Department of Medical Sciences, Ministry of Public Health, Thailand	Archawin Rojanawiwat; Jirapha Pakdee; Natthakul Bunneang; Nuanjun Wichukchinda; Penpitcha Thawong; Pilailuk Akkapaiboon Okada; Pundharika Piboonsiri; Surakameth Mahasirimongkol; Waritta Sawaengdee
EPI_ISL_3826505, EPI_ISL_3826521, EPI_ISL_3826522, EPI_ISL_3826523, EPI_ISL_3826773, EPI_ISL_3826790	Public Health Service Center 34:Po Sri	Division of Genomic Medicine and Innovation support,Department of Medical Sciences, Ministry of Public Health, Thailand	Archawin Rojanawiwat; Jirapha Pakdee; Natthakul Bunneang; Nuanjun Wichukchinda; Penpitcha Thawong; Pilailuk Akkapaiboon Okada; Pundharika Piboonsiri; Surakameth Mahasirimongkol; Waritta Sawaengdee
EPI_ISL_3826489	Public Health Service Center 46 Kantarat Uthit	Division of Genomic Medicine and Innovation support,Department of Medical Sciences, Ministry of Public Health, Thailand	Archawin Rojanawiwat; Jirapha Pakdee; Natthakul Bunneang; Nuanjun Wichukchinda; Penpitcha Thawong; Pilailuk Akkapaiboon Okada; Pundharika Piboonsiri; Surakameth Mahasirimongkol; Waritta Sawaengdee
EPI_ISL_4235372, EPI_ISL_4235380, EPI_ISL_4235402, EPI_ISL_4235403, EPI_ISL_4235410, EPI_ISL_4235416, EPI_ISL_4235423, EPI_ISL_4235425, EPI_ISL_4235429, EPI_ISL_4235432			
see above	Public Health Service Center 59 Thung Khru	Division of Genomic Medicine and Innovation support,Department of Medical Sciences, Ministry of Public Health, Thailand	Archawin Rojanawiwat; Jirapha Pakdee; Natthakul Bunneang; Nuanjun Wichukchinda; Penpitcha Thawong; Pilailuk Akkapaiboon Okada; Pundharika Piboonsiri; Surakameth Mahasirimongkol; Waritta Sawaengdee
EPI_ISL_4647336, EPI_ISL_4647378	Public Health Service Center 61	Division of Genomic Medicine and Innovation support,Department of Medical Sciences, Ministry of Public Health, Thailand	Archawin Rojanawiwat; Jirapha Pakdee; Natthakul Bunneang; Nuanjun Wichukchinda; Penpitcha Thawong; Pilailuk Akkapaiboon Okada; Pundharika Piboonsiri; Surakameth Mahasirimongkol; Waritta Sawaengdee
EPI_ISL_4235397, EPI_ISL_4235439, EPI_ISL_4557924, EPI_ISL_4647331, EPI_ISL_4647332, EPI_ISL_4647398	Public Health Service Center 62 Tuangrat Sasanawin	Division of Genomic Medicine and Innovation support,Department of Medical Sciences, Ministry of Public Health, Thailand	Archawin Rojanawiwat; Jirapha Pakdee; Natthakul Bunneang; Nuanjun Wichukchinda; Penpitcha Thawong; Pilailuk Akkapaiboon Okada; Pundharika Piboonsiri; Surakameth Mahasirimongkol; Waritta Sawaengdee
EPI_ISL_4235368, EPI_ISL_4235376, EPI_ISL_4235384, EPI_ISL_4235414, EPI_ISL_4235421, EPI_ISL_4235426, EPI_ISL_4647076, EPI_ISL_4647307			
see above	Public Health Service Center 65 Ruksasuk Bang Bon	Division of Genomic Medicine and Innovation support,Department of Medical Sciences, Ministry of Public Health, Thailand	Archawin Rojanawiwat; Jirapha Pakdee; Natthakul Bunneang; Nuanjun Wichukchinda; Penpitcha Thawong; Pilailuk Akkapaiboon Okada; Pundharika Piboonsiri; Surakameth Mahasirimongkol; Waritta Sawaengdee
EPI_ISL_4647415	Public Health Service Center 68 Saphan Sung	Division of Genomic Medicine and Innovation support,Department of Medical Sciences, Ministry of Public Health, Thailand	Archawin Rojanawiwat; Jirapha Pakdee; Natthakul Bunneang; Nuanjun Wichukchinda; Penpitcha Thawong; Pilailuk Akkapaiboon Okada; Pundharika Piboonsiri; Surakameth Mahasirimongkol; Waritta Sawaengdee
EPI_ISL_4235409	Public Health Service Center 7 Bunmee Pururachrungson	Division of Genomic Medicine and Innovation support,Department of Medical Sciences, Ministry of Public Health, Thailand	Archawin Rojanawiwat; Jirapha Pakdee; Natthakul Bunneang; Nuanjun Wichukchinda; Penpitcha Thawong; Pilailuk Akkapaiboon Okada; Pundharika Piboonsiri; Surakameth Mahasirimongkol; Waritta Sawaengdee
EPI_ISL_4235394	Public Health Service Center 9	Division of Genomic Medicine and Innovation support,Department of Medical Sciences, Ministry of Public Health, Thailand	Archawin Rojanawiwat; Jirapha Pakdee; Natthakul Bunneang; Nuanjun Wichukchinda; Penpitcha Thawong; Pilailuk Akkapaiboon Okada; Pundharika Piboonsiri; Surakameth Mahasirimongkol; Waritta Sawaengdee
EPI_ISL_768616, EPI_ISL_1497608	Rajavithi Hospital	National Institute of Health, Department of Medical Sciences, Ministry of Public Health, Thailand	; Hathaikan Tanchai; Natchaya Khiadsang; Nuttida Thongpramul; Pakorn Piromtong; Pilailuk Okada; Ratana Tacharoenmuang; Siripaporn Phuygun; Sittiporn Parnmen; Sunthareeya Waicharoen; Sunthareeya Waicharoen.; Thanutsapa Thanadachakul; Thanutsapa Thanadachakull; Warawan Wongboot; sirikanda wimol
EPI_ISL_2433346, EPI_ISL_2433347	Rajyindee Hospital	Division of Genomic Medicine and Innovation support,Department of Medical Sciences, Ministry of Public Health, Thailand	Archawin Rojanawiwat; Jirapha Pakdee; Natthakul Bunneang; Nuanjun Wichukchinda; Penpitcha Thawong; Pilailuk Akkapaiboon Okada; Pundharika Piboonsiri; Surakameth Mahasirimongkol; Waritta Sawaengdee
EPI_ISL_882780, EPI_ISL_1138847, EPI_ISL_1491573, EPI_ISL_2104733, EPI_ISL_2104734, EPI_ISL_2104735, EPI_ISL_2104743, EPI_ISL_2104747, EPI_ISL_2104748, EPI_ISL_2348823, EPI_ISL_2678109, EPI_ISL_2928531, EPI_ISL_2928537			
see above	Ramathibodi Chakri Naruebodindra Hospital	COVID-19 Network Investigations (CONI) Alliance	Angkana Huang; Anthony R. Jones; Arporn Wangwiwatsin; Bhakbhoom Panthan; Chonticha Klungtong; Duangkamon Loesbanluechai; Ekawat Pasomsub; Elizabeth Batty; Insee Sensorn; Janjira Thaipadungpanit; Khajohn Joonlasak; Khajohn Joonsalak; Kingkan Rakmanee; Krittikorn Kumpornsin; Namfon Kotanan; Stefan Fernandez; Thanat Chookajorn; Theerarat Kochakarn; Treewat Watthanachockchai; Wasun Chantratita; Wudtichai Manasatienkij
EPI_ISL_423039, EPI_ISL_423040, EPI_ISL_423041, EPI_ISL_423042, EPI_ISL_423043, EPI_ISL_429164, EPI_ISL_429165, EPI_ISL_429166, EPI_ISL_429167, EPI_ISL_429168, EPI_ISL_429169, EPI_ISL_429170, EPI_ISL_429171, EPI_ISL_429172, EPI_ISL_429173, EPI_ISL_429174, EPI_ISL_429175, EPI_ISL_429176, EPI_ISL_429177, EPI_ISL_429178, EPI_ISL_429179, EPI_ISL_429180, EPI_ISL_429181, EPI_ISL_429182, EPI_ISL_429183, EPI_ISL_429184, EPI_ISL_445380, EPI_ISL_446996, EPI_ISL_446997, EPI_ISL_446998, EPI_ISL_446999, EPI_ISL_447000, EPI_ISL_447001, EPI_ISL_447002, EPI_ISL_447003, EPI_ISL_447004, EPI_ISL_447005, EPI_ISL_447006, EPI_ISL_447007, EPI_ISL_447008, EPI_ISL_447009, EPI_ISL_447010, EPI_ISL_447011, EPI_ISL_447012, EPI_ISL_447013, EPI_ISL_447014, EPI_ISL_447015, EPI_ISL_447016, EPI_ISL_447017, EPI_ISL_447018, EPI_ISL_447019, EPI_ISL_447020, EPI_ISL_447021, EPI_ISL_447022, EPI_ISL_447023, EPI_ISL_447024, EPI_ISL_447025, EPI_ISL_447026, EPI_ISL_447027, EPI_ISL_447028, EPI_ISL_447029, EPI_ISL_450723, EPI_ISL_455908, EPI_ISL_455909, EPI_ISL_455910, EPI_ISL_455911, EPI_ISL_455912, EPI_ISL_455913, EPI_ISL_455914, EPI_ISL_455915, EPI_ISL_455916, EPI_ISL_455917, EPI_ISL_455918, EPI_ISL_455919, EPI_ISL_455920, EPI_ISL_455921, EPI_ISL_455922, EPI_ISL_455923, EPI_ISL_455924, EPI_ISL_455925, EPI_ISL_455926, EPI_ISL_455927, EPI_ISL_455928, EPI_ISL_455929, EPI_ISL_455930, EPI_ISL_455931, EPI_ISL_455932, EPI_ISL_455933, EPI_ISL_455934, EPI_ISL_455935, EPI_ISL_455936, EPI_ISL_455937, EPI_ISL_455938, EPI_ISL_455939, EPI_ISL_455940, EPI_ISL_455941, EPI_ISL_455942, EPI_ISL_455943, EPI_ISL_455944, EPI_ISL_455945, EPI_ISL_455946, EPI_ISL_455947, EPI_ISL_455948, EPI_ISL_498262, EPI_ISL_498263, EPI_ISL_498264, EPI_ISL_498265, EPI_ISL_498266, EPI_ISL_512847, EPI_ISL_512848, EPI_ISL_512849, EPI_ISL_512850, EPI_ISL_512851, EPI_ISL_512852, EPI_ISL_512853, EPI_ISL_512854, EPI_ISL_512855, EPI_ISL_512856, EPI_ISL_512857, EPI_ISL_512858, EPI_ISL_512859, EPI_ISL_512860, EPI_ISL_512861, EPI_ISL_512862, EPI_ISL_512863, EPI_ISL_512864, EPI_ISL_512865, EPI_ISL_512866, EPI_ISL_512867, EPI_ISL_512868, EPI_ISL_512869, EPI_ISL_512870, EPI_ISL_512871, EPI_ISL_512872, EPI_ISL_568580, EPI_ISL_568581, EPI_ISL_568582, EPI_ISL_568583, EPI_ISL_568584, EPI_ISL_568585, EPI_ISL_568586, EPI_ISL_693334, EPI_ISL_693335, EPI_ISL_693336, EPI_ISL_693337, EPI_ISL_693338, EPI_ISL_693339, EPI_ISL_693340, EPI_ISL_693341, EPI_ISL_693342, EPI_ISL_693343, EPI_ISL_693344, EPI_ISL_693345, EPI_ISL_693346, EPI_ISL_693347, EPI_ISL_693348, EPI_ISL_693349, EPI_ISL_693350, EPI_ISL_693351, EPI_ISL_693352, EPI_ISL_693353, EPI_ISL_693354, EPI_ISL_693355, EPI_ISL_693356, EPI_ISL_693357, EPI_ISL_693358, EPI_ISL_693359, EPI_ISL_693360, EPI_ISL_693361, EPI_ISL_693362, EPI_ISL_693363, EPI_ISL_693364, EPI_ISL_693365, EPI_ISL_693366, EPI_ISL_693367, EPI_ISL_693368, EPI_ISL_693369, EPI_ISL_693370, EPI_ISL_693371, EPI_ISL_693372, EPI_ISL_693373, EPI_ISL_693374, EPI_ISL_693375, EPI_ISL_693376, EPI_ISL_693377, EPI_ISL_693378, EPI_ISL_693379, EPI_ISL_693380, EPI_ISL_693381, EPI_ISL_693382, EPI_ISL_693383, EPI_ISL_693384, EPI_ISL_693385, EPI_ISL_693386, EPI_ISL_812923, EPI_ISL_812924, EPI_ISL_812925, EPI_ISL_812926, EPI_ISL_812927, EPI_ISL_812928, EPI_ISL_1073953, EPI_ISL_1073954, EPI_ISL_1073955, EPI_ISL_1073958, EPI_ISL_1073960, EPI_ISL_1073964, EPI_ISL_1073966, EPI_ISL_1073967, EPI_ISL_1073968, EPI_ISL_1073969, EPI_ISL_1073970, EPI_ISL_1073971, EPI_ISL_1073972, EPI_ISL_1073973, EPI_ISL_1073974, EPI_ISL_1073975, EPI_ISL_1073976, EPI_ISL_1073977, EPI_ISL_1073978, EPI_ISL_1073979, EPI_ISL_1073980, EPI_ISL_1138844, EPI_ISL_1138845, EPI_ISL_1138846, EPI_ISL_1491555, EPI_ISL_1491556, EPI_ISL_1491557, EPI_ISL_1491558, EPI_ISL_1491559, EPI_ISL_1491560, EPI_ISL_2104731, EPI_ISL_2104732, EPI_ISL_2104736, EPI_ISL_2104737, EPI_ISL_2104738, EPI_ISL_2104739, EPI_ISL_2104740, EPI_ISL_2104741, EPI_ISL_2104742, EPI_ISL_2104746, EPI_ISL_2104749, EPI_ISL_2104750, EPI_ISL_2104751, EPI_ISL_2104752, EPI_ISL_2104753, EPI_ISL_2104754, EPI_ISL_2348830, EPI_ISL_2348831, EPI_ISL_2348832, EPI_ISL_2348833, EPI_ISL_2348834, EPI_ISL_2678101, EPI_ISL_2678102, EPI_ISL_2678103, EPI_ISL_2678104, EPI_ISL_2678105, EPI_ISL_2678106, EPI_ISL_2678107, EPI_ISL_2678108, EPI_ISL_2678110, EPI_ISL_2802831, EPI_ISL_2802832, EPI_ISL_2802833, EPI_ISL_2802834, EPI_ISL_2802835, EPI_ISL_2802836, EPI_ISL_2802837, EPI_ISL_2802838, EPI_ISL_2802839, EPI_ISL_2802840, EPI_ISL_2802841, EPI_ISL_2802842, EPI_ISL_2802843, EPI_ISL_2802844, EPI_ISL_2802845, EPI_ISL_2802846, EPI_ISL_2802847, EPI_ISL_2802848, EPI_ISL_2802849, EPI_ISL_2802850, EPI_ISL_2802851, EPI_ISL_2802852, EPI_ISL_2802853, EPI_ISL_2802854, EPI_ISL_2802855, EPI_ISL_2802856, EPI_ISL_2840479, EPI_ISL_2928523, EPI_ISL_2928524, EPI_ISL_2928525, EPI_ISL_2928526, EPI_ISL_2928527, EPI_ISL_2928528, EPI_ISL_2928529, EPI_ISL_2928530, EPI_ISL_2928532, EPI_ISL_2928533, EPI_ISL_2928534, EPI_ISL_2928535, EPI_ISL_2928536, EPI_ISL_2928538, EPI_ISL_2928539, EPI_ISL_2928540, EPI_ISL_3152903, EPI_ISL_3152904, EPI_ISL_3152905, EPI_ISL_3152906, EPI_ISL_3152907, EPI_ISL_3152908, EPI_ISL_3152909, EPI_ISL_3152910, EPI_ISL_3152911, EPI_ISL_3152912, EPI_ISL_3152913, EPI_ISL_3152914, EPI_ISL_3152915, EPI_ISL_3152916, EPI_ISL_3152917, EPI_ISL_3152918, EPI_ISL_3152919, EPI_ISL_3152920, EPI_ISL_3152921, EPI_ISL_3152922, EPI_ISL_3152923, EPI_ISL_3152924, EPI_ISL_3152925, EPI_ISL_3152926, EPI_ISL_3152927, EPI_ISL_3152928, EPI_ISL_3152929, EPI_ISL_3152930, EPI_ISL_3152931, EPI_ISL_3152932, EPI_ISL_3152933, EPI_ISL_3152934, EPI_ISL_3152935, EPI_ISL_3152936, EPI_ISL_3152937, EPI_ISL_3157503, EPI_ISL_3157941, EPI_ISL_3158259, EPI_ISL_3716308, EPI_ISL_3716309, EPI_ISL_3716310, EPI_ISL_3716312, EPI_ISL_3716313, EPI_ISL_3716315, EPI_ISL_3716322, EPI_ISL_3716323, EPI_ISL_3716338, EPI_ISL_3716341, EPI_ISL_4279186, EPI_ISL_4279187, EPI_ISL_4279188, EPI_ISL_4279189, EPI_ISL_4279190, EPI_ISL_4554543, EPI_ISL_4554545, EPI_ISL_4554546, EPI_ISL_4554548, EPI_ISL_4554550, EPI_ISL_4554552, EPI_ISL_4554553, EPI_ISL_4554555, EPI_ISL_4554557, EPI_ISL_4554559, EPI_ISL_4554560			
see above	Ramathibodi Hospital	COVID-19 Network Investigations (CONI) Alliance	Angkana Huang; Anthony R. Jones; Arporn Wangwiwatsin; Bhakbhoom Panthan; Chonticha Klungtong; Duangkamon Loesbanluechai; Ekawat Pasomsub; Elizabeth Batty; Insee Sensorn; Janjira Thaipadungpanit; Khajohn

			Joonlasak; Khajohn Joonsalak; Kingkan Rakmanee; Krittikorn Kumpornsin; Namfon Kotanan; Poramate Jiaranai; Stefan Fernandez; Thanat Chookajorn; Theerarat Kochakarn; Treewat Watthanachockchai; Wasun Chantratita; Wudtichai Manasatienkij
EPI_ISL_515465, EPI_ISL_515467, EPI_ISL_515468	Ramathibodi Hospital	National Institute of Health. Department of medical Sciences, Ministry of Public Health, Thailand	Chittaganpitch; Malinee; Okada; Parnmen; Phuygun; Pilailuk; Siripaporn; Sittiporn; Sunthareeya; Thanadachakul; Thanutsapa; Waicharoen; Warawan; Wongboot
EPI_ISL_1497606	Ramkhamhaeng Hospital	National Institute of Health, Department of Medical Sciences, Ministry of Public Health, Thailand	Hathaikan Tanchai; Natchaya Khiadsang; Nuttida Thongpramul; Pakorn Piromtong; Pilailuk Okada; Ratana Tacharoenmuang; Siripaporn Phuygun; Sittiporn Parnmen; Sunthareeya Waicharoen.; Thanutsapa Thanadachakull
EPI_ISL_434700, EPI_ISL_455604, EPI_ISL_515292	Ramkhamhaeng Hospital	National Institute of Health. Department of medical Sciences, Ministry of Public Health, Thailand	Chittaganpitch; Malinee; Okada; Parnmen; Phuygun; Pilailuk; Siripaporn; Sittiporn; Sunthareeya; Thanadachakul; Thanutsapa; Waicharoen; Warawan; Wongboot
EPI_ISL_2348492, EPI_ISL_2348493, EPI_ISL_2348494, EPI_ISL_2350930, EPI_ISL_2350931, EPI_ISL_2350932, EPI_ISL_2350933, EPI_ISL_2350934, EPI_ISL_2350935, EPI_ISL_2350936			
see above	Ratchaburi Hospital	Division of Genomic Medicine and Innovation support,Department of Medical Sciences, Ministry of Public Health, Thailand	Archawin Rojanawiwat; Jirapha Pakdee; Natthakul Bunneang; Nuanjun Wichukchinda; Penpitcha Thawong; Pilailuk Akkapaiboon Okada; Pundharika Piboonsiri; Surakameth Mahasirimongkol; Waritta Sawaengdee
EPI_ISL_2433326, EPI_ISL_2433337	Rattaphum Hospital	Division of Genomic Medicine and Innovation support,Department of Medical Sciences, Ministry of Public Health, Thailand	Archawin Rojanawiwat; Jirapha Pakdee; Natthakul Bunneang; Nuanjun Wichukchinda; Penpitcha Thawong; Pilailuk Akkapaiboon Okada; Pundharika Piboonsiri; Surakameth Mahasirimongkol; Waritta Sawaengdee
EPI_ISL_2433292, EPI_ISL_2433312, EPI_ISL_2433314, EPI_ISL_2433442, EPI_ISL_2433443, EPI_ISL_2433444, EPI_ISL_2433445, EPI_ISL_2433446, EPI_ISL_2433618			
see above	Regional Medical Science Center 2 Phitsanulok	Division of Genomic Medicine and Innovation support,Department of Medical Sciences, Ministry of Public Health, Thailand	Archawin Rojanawiwat; Jirapha Pakdee; Natthakul Bunneang; Nuanjun Wichukchinda; Penpitcha Thawong; Pilailuk Akkapaiboon Okada; Pundharika Piboonsiri; Surakameth Mahasirimongkol; Waritta Sawaengdee
EPI_ISL_3038912	Regional Medical Science Center 2 Phitsanulok	National Institute of Health, Department of Medical Sciences, Ministry of Public Health, Thailand	; Natchaya Khiadsang; Nuttida Thongpramul; Pakorn Piromtong; Pilailuk Okada; Ratana Tacharoenmuang; Siripaporn Phuygun; Sittiporn Parnmen; Sunthareeya Waicharoen; Thanutsapa Thanadachakul; Warawan Wongboot; sirikanda wimol
EPI_ISL_4647568, EPI_ISL_4647573, EPI_ISL_4647582	Regional Medical Sciences Center 1 Chiang Mai	Division of Genomic Medicine and Innovation support,Department of Medical Sciences, Ministry of Public Health, Thailand	Archawin Rojanawiwat; Jirapha Pakdee; Natthakul Bunneang; Nuanjun Wichukchinda; Penpitcha Thawong; Pilailuk Akkapaiboon Okada; Pundharika Piboonsiri; Surakameth Mahasirimongkol; Waritta Sawaengdee
EPI_ISL_3402015, EPI_ISL_3402016, EPI_ISL_3407823, EPI_ISL_3407824, EPI_ISL_3407825, EPI_ISL_3407827, EPI_ISL_3407854, EPI_ISL_3534190, EPI_ISL_3534191, EPI_ISL_3534192, EPI_ISL_3534193, EPI_ISL_4117756, EPI_ISL_4117767, EPI_ISL_4634614, EPI_ISL_4635444, EPI_ISL_4635445, EPI_ISL_4635446, EPI_ISL_4636445, EPI_ISL_4636452			
see above	Regional Medical Sciences Center 1 Chiang Mai	National Institute of Health, Department of Medical Sciences, Ministry of Public Health, Thailand	; Archawin Rojanawiwat; Natchaya Khiadsang; Nuttida Thongpramul; Pakorn Piromtong; Pilailuk Okada; Ratana Tacharoenmuang; Siripaporn Phuygun; Sittiporn Parnmen; Sunthareeya Waicharoen; Thanutsapa Thanadachakul; Warawan Wongboot; sirikanda wimol
EPI_ISL_768524, EPI_ISL_768528, EPI_ISL_768529, EPI_ISL_768609, EPI_ISL_768614, EPI_ISL_1251232, EPI_ISL_1251233, EPI_ISL_1251234, EPI_ISL_1251235, EPI_ISL_1251236, EPI_ISL_3407815, EPI_ISL_3471407, EPI_ISL_3471408, EPI_ISL_3471417, EPI_ISL_3534200, EPI_ISL_3590625, EPI_ISL_3590626, EPI_ISL_3797059, EPI_ISL_3797060, EPI_ISL_3797061, EPI_ISL_4117771, EPI_ISL_4117775, EPI_ISL_4636420, EPI_ISL_4636449, EPI_ISL_4636450			
see above	Regional Medical Sciences Center 1/1 Chiang Rai	National Institute of Health, Department of Medical Sciences, Ministry of Public Health, Thailand	; Archawin Rojanawiwat; Hathaikan Tanchai; Natchaya Khiadsang; Nuttida Thongpramul; Pakorn Piromtong; Pilailuk Okada; Ratana Tacharoenmuang; Siripaporn Phuygun; Sittiporn Parnmen; Sunthareeya Waicharoen; Sunthareeya Waicharoen.; Thanutsapa Thanadachakul; Thanutsapa Thanadachakull; Warawan Wongboot; sirikanda wimol
EPI_ISL_2433285, EPI_ISL_2433300, EPI_ISL_2433306, EPI_ISL_2433516, EPI_ISL_2433517, EPI_ISL_2433518, EPI_ISL_2433519, EPI_ISL_2433520, EPI_ISL_2433521, EPI_ISL_2433522, EPI_ISL_2433523, EPI_ISL_2433524, EPI_ISL_2433525, EPI_ISL_2433526, EPI_ISL_2433527, EPI_ISL_2433528, EPI_ISL_2433529, EPI_ISL_2433530, EPI_ISL_2433531, EPI_ISL_2433532, EPI_ISL_2433554, EPI_ISL_4647586, EPI_ISL_4647591, EPI_ISL_4647596, EPI_ISL_4647601, EPI_ISL_4647608, EPI_ISL_4647617, EPI_ISL_4647666, EPI_ISL_4647674, EPI_ISL_4647680, EPI_ISL_4647686, EPI_ISL_4647687, EPI_ISL_4647692, EPI_ISL_4647693, EPI_ISL_4647699			
see above	Regional Medical Sciences Center 1/1 Chiangrai	Division of Genomic Medicine and Innovation support,Department of Medical Sciences, Ministry of Public Health, Thailand	Archawin Rojanawiwat; Jirapha Pakdee; Natthakul Bunneang; Nuanjun Wichukchinda; Penpitcha Thawong; Pilailuk Akkapaiboon Okada; Pundharika Piboonsiri; Surakameth Mahasirimongkol; Waritta Sawaengdee
EPI_ISL_2877032, EPI_ISL_2877033	Regional Medical Sciences Center 1/1 Chiangrai	National Institute of Health, Department of Medical Sciences, Ministry of Public Health, Thailand	; Natchaya Khiadsang; Nuttida Thongpramul; Pakorn Piromtong; Pilailuk Okada; Ratana Tacharoenmuang; Siripaporn Phuygun; Sittiporn Parnmen; Sunthareeya Waicharoen; Thanutsapa Thanadachakul; Warawan Wongboot; sirikanda wimol
EPI_ISL_3038911, EPI_ISL_3407862	Regional Medical Sciences Center 10 Ubonratchathani	National Institute of Health, Department of Medical Sciences, Ministry of Public Health, Thailand	; Natchaya Khiadsang; Natchaya Khiadsang; Nuttida Thongpramul; Pakorn Piromtong; Pilailuk Okada; Ratana Tacharoenmuang; Siripaporn Phuygun; Sittiporn Parnmen; Sunthareeya Waicharoen; Thanutsapa Thanadachakul; Warawan Wongboot; sirikanda wimol
EPI_ISL_3407828, EPI_ISL_3407829, EPI_ISL_3407837, EPI_ISL_3407839, EPI_ISL_3407840, EPI_ISL_3407849, EPI_ISL_3407858, EPI_ISL_3407859, EPI_ISL_3419391, EPI_ISL_3419392, EPI_ISL_3471400, EPI_ISL_3534199, EPI_ISL_4050576, EPI_ISL_4636439, EPI_ISL_4646106			
see above	Regional Medical Sciences Center 11 Surat Thani	National Institute of Health, Department of Medical Sciences, Ministry of Public Health, Thailand	; Archawin Rojanawiwat; Natchaya Khiadsang; Nuttida Thongpramul; Pakorn Piromtong; Pilailuk Okada; Ratana Tacharoenmuang; Siripaporn Phuygun; Sittiporn Parnmen; Sunthareeya Waicharoen; Thanutsapa Thanadachakul; Warawan Wongboot; sirikanda wimol
EPI_ISL_4647621	Regional Medical Sciences Center 11/1 Phuket	Division of Genomic Medicine and Innovation support,Department of Medical Sciences, Ministry of Public Health, Thailand	Archawin Rojanawiwat; Jirapha Pakdee; Natthakul Bunneang; Nuanjun Wichukchinda; Penpitcha Thawong; Pilailuk Akkapaiboon Okada; Pundharika Piboonsiri; Surakameth Mahasirimongkol; Waritta Sawaengdee
EPI_ISL_3402017, EPI_ISL_3403344, EPI_ISL_3407821, EPI_ISL_3407852, EPI_ISL_3407861, EPI_ISL_3419394, EPI_ISL_3471402, EPI_ISL_3471403, EPI_ISL_3471411, EPI_ISL_3471412, EPI_ISL_3471413, EPI_ISL_3531670, EPI_ISL_3531674, EPI_ISL_4050582, EPI_ISL_4050583, EPI_ISL_4106143, EPI_ISL_4117778, EPI_ISL_4117783, EPI_ISL_4170070, EPI_ISL_4635423, EPI_ISL_4635424, EPI_ISL_4635425, EPI_ISL_4635426, EPI_ISL_4635427, EPI_ISL_4636446, EPI_ISL_4636454			
see above	Regional Medical Sciences Center 11/1 Phuket	National Institute of Health, Department of Medical Sciences, Ministry of Public Health, Thailand	; Archawin Rojanawiwat; Natchaya Khiadsang; Nuttida Thongpramul; Pakorn Piromtong; Pilailuk Okada; Ratana Tacharoenmuang; Siripaporn Phuygun; Sittiporn Parnmen; Sunthareeya Waicharoen; Thanutsapa Thanadachakul; Warawan Wongboot; sirikanda wimol
EPI_ISL_699464, EPI_ISL_708723, EPI_ISL_708735	Regional Medical Sciences Center 11/1 phuket	National Institute of Health, Department of Medical Sciences, Ministry of Public Health, Thailand	Malinee Chittaganpitch; Pilailuk Okada; Siripaporn Phuygun; Sittiporn Parnmen; Sunthareeya Waicharoen; Thanutsapa Thanadachakul; Warawan Wongboot
EPI_ISL_3826474, EPI_ISL_3826475, EPI_ISL_3826481, EPI_ISL_3826764, EPI_ISL_3826769, EPI_ISL_3826772, EPI_ISL_3826774, EPI_ISL_3826777, EPI_ISL_3826781			
see above	Regional Medical Sciences Center 12 Songkhla	Division of Genomic Medicine and Innovation support,Department of Medical Sciences, Ministry of Public Health, Thailand	Archawin Rojanawiwat; Jirapha Pakdee; Natthakul Bunneang; Nuanjun Wichukchinda; Penpitcha Thawong; Pilailuk Akkapaiboon Okada; Pundharika Piboonsiri; Surakameth Mahasirimongkol; Waritta Sawaengdee
EPI_ISL_2840595, EPI_ISL_2840596, EPI_ISL_2840597, EPI_ISL_3342067, EPI_ISL_3342068, EPI_ISL_3403351, EPI_ISL_3403352, EPI_ISL_3403353, EPI_ISL_3403354, EPI_ISL_3403355, EPI_ISL_3407844, EPI_ISL_3407848, EPI_ISL_3407853, EPI_ISL_3407860, EPI_ISL_3797054, EPI_ISL_3797055, EPI_ISL_3797056, EPI_ISL_3797057, EPI_ISL_3797058, EPI_ISL_3801323, EPI_ISL_4050574, EPI_ISL_4050579, EPI_ISL_4050581, EPI_ISL_4050584, EPI_ISL_4106140, EPI_ISL_4106146, EPI_ISL_4117786, EPI_ISL_4117789, EPI_ISL_4117794, EPI_ISL_4200230, EPI_ISL_4200236, EPI_ISL_4200238, EPI_ISL_4200240, EPI_ISL_4200241, EPI_ISL_4200243, EPI_ISL_4200245, EPI_ISL_4200247, EPI_ISL_4200249, EPI_ISL_4200251, EPI_ISL_4254660, EPI_ISL_4254662, EPI_ISL_4254663, EPI_ISL_4254664, EPI_ISL_4254667, EPI_ISL_4254668, EPI_ISL_4254669, EPI_ISL_4254670, EPI_ISL_4254671, EPI_ISL_4254672, EPI_ISL_4262471, EPI_ISL_4262472, EPI_ISL_4262473, EPI_ISL_4348476, EPI_ISL_4348478, EPI_ISL_4348479, EPI_ISL_4634615, EPI_ISL_4635432, EPI_ISL_4635433, EPI_ISL_4635434, EPI_ISL_4635435, EPI_ISL_4635436, EPI_ISL_4636421, EPI_ISL_4636422, EPI_ISL_4636435, EPI_ISL_4636436, EPI_ISL_4636437, EPI_ISL_4636438, EPI_ISL_4636457, EPI_ISL_4646126			
see above	Regional Medical Sciences Center 12 Songkhla	National Institute of Health, Department of Medical Sciences, Ministry of Public Health, Thailand	; Archawin Rojanawiwat; Natchaya Khiadsang; Natchaya Khiadsang; Nuttida Thongpramul; Pakorn Piromtong; Pilailuk Okada; Ratana Tacharoenmuang; Siripaporn Phuygun; Sittiporn Parnmen; Sunthareeya Waicharoen; Thanutsapa Thanadachakul; Warawan Wongboot; sirikanda wimol
EPI_ISL_3403350, EPI_ISL_3407816, EPI_ISL_3407845, EPI_ISL_3407847, EPI_ISL_3407850, EPI_ISL_3471414, EPI_ISL_3471415, EPI_ISL_3471416, EPI_ISL_3531679, EPI_ISL_3534185, EPI_ISL_3534186, EPI_ISL_3534194, EPI_ISL_3534195, EPI_ISL_3534196, EPI_ISL_3534197, EPI_ISL_3534198, EPI_ISL_3590629, EPI_ISL_4050575, EPI_ISL_4200232, EPI_ISL_4200234, EPI_ISL_4254661, EPI_ISL_4634616, EPI_ISL_4635429, EPI_ISL_4635430, EPI_ISL_4635431, EPI_ISL_4636424, EPI_ISL_4636425			
see above	Regional Medical Sciences Center 12/1 Trang	National Institute of Health, Department of Medical Sciences, Ministry of Public Health, Thailand	; Archawin Rojanawiwat; Natchaya Khiadsang; Nuttida Thongpramul; Pakorn Piromtong; Pilailuk Okada; Ratana Tacharoenmuang; Siripaporn Phuygun; Sittiporn Parnmen; Sunthareeya Waicharoen; Thanutsapa Thanadachakul; Warawan Wongboot; sirikanda wimol
EPI_ISL_3826572, EPI_ISL_3826573, EPI_ISL_3826574, EPI_ISL_3826575, EPI_ISL_3826576, EPI_ISL_3826577, EPI_ISL_3826578, EPI_ISL_3826579, EPI_ISL_3826580, EPI_ISL_3826581, EPI_ISL_3826582, EPI_ISL_3826583, EPI_ISL_3826584, EPI_ISL_3826585, EPI_ISL_3826586, EPI_ISL_3826587, EPI_ISL_3826588, EPI_ISL_3826589, EPI_ISL_3826590, EPI_ISL_3826591, EPI_ISL_3826592, EPI_ISL_3826593, EPI_ISL_3826594, EPI_ISL_3826595, EPI_ISL_3826596, EPI_ISL_3826597, EPI_ISL_3826598, EPI_ISL_3826599, EPI_ISL_3826600, EPI_ISL_3826601, EPI_ISL_3826602, EPI_ISL_3826603, EPI_ISL_3826604, EPI_ISL_3826605, EPI_ISL_3826791, EPI_ISL_3826800, EPI_ISL_3826801			
see above	Regional Medical Sciences Center 2 Phitsanulok	Division of Genomic Medicine and Innovation support,Department of Medical Sciences, Ministry of Public Health, Thailand	Archawin Rojanawiwat; Jirapha Pakdee; Natthakul Bunneang; Nuanjun Wichukchinda; Penpitcha Thawong; Pilailuk Akkapaiboon Okada; Pundharika Piboonsiri; Surakameth Mahasirimongkol; Waritta Sawaengdee
EPI_ISL_3406995, EPI_ISL_3407818, EPI_ISL_3407832, EPI_ISL_3407835, EPI_ISL_3407836, EPI_ISL_3407871, EPI_ISL_3407874, EPI_ISL_4170071, EPI_ISL_4348477, EPI_ISL_4636453			
see above	Regional Medical Sciences Center 2 Phitsanulok	National Institute of Health, Department of Medical Sciences, Ministry of Public Health, Thailand	; Archawin Rojanawiwat; Natchaya Khiadsang; Nuttida Thongpramul; Pakorn Piromtong; Pilailuk Okada; Ratana Tacharoenmuang; Siripaporn Phuygun; Sittiporn Parnmen; Sunthareeya Waicharoen; Thanutsapa Thanadachakul; Warawan Wongboot; sirikanda wimol
EPI_ISL_3407819, EPI_ISL_3407822, EPI_ISL_3407831, EPI_ISL_3407863, EPI_ISL_3407865, EPI_ISL_3471405, EPI_ISL_4050578, EPI_ISL_4262467, EPI_ISL_4262468, EPI_ISL_4262474, EPI_ISL_4262475, EPI_ISL_4348469, EPI_ISL_4348471, EPI_ISL_4348472, EPI_ISL_4348473, EPI_ISL_4348474, EPI_ISL_4348475, EPI_ISL_4634612, EPI_ISL_4636428, EPI_ISL_4636429, EPI_ISL_4636442, EPI_ISL_4636443, EPI_ISL_4636444, EPI_ISL_4636458			
see above	Regional Medical Sciences Center 3 Nakhon Sawan	National Institute of Health, Department of Medical Sciences, Ministry of Public Health, Thailand	; Archawin Rojanawiwat; Natchaya Khiadsang; Nuttida Thongpramul; Pakorn Piromtong; Pilailuk Okada; Ratana Tacharoenmuang; Siripaporn Phuygun; Sittiporn Parnmen; Sunthareeya Waicharoen; Thanutsapa Thanadachakul; Warawan Wongboot; sirikanda wimol
EPI_ISL_3342062, EPI_ISL_3342063	Regional Medical Sciences Center 3 Nakhonsawan	National Institute of Health, Department of Medical Sciences, Ministry of Public Health, Thailand	; Natchaya Khiadsang; Nuttida Thongpramul; Pakorn Piromtong; Pilailuk Okada; Ratana Tacharoenmuang; Siripaporn Phuygun; Sittiporn Parnmen; Sunthareeya Waicharoen; Thanutsapa Thanadachakul; Warawan Wongboot; sirikanda wimol
EPI_ISL_3471406, EPI_ISL_4262469	Regional Medical Sciences Center 4 Saraburi	National Institute of Health, Department of Medical Sciences, Ministry of Public Health, Thailand	; Archawin Rojanawiwat; Natchaya Khiadsang; Nuttida Thongpramul; Pakorn Piromtong; Pilailuk Okada; Ratana Tacharoenmuang; Siripaporn Phuygun; Sittiporn Parnmen; Sunthareeya Waicharoen; Thanutsapa Thanadachakul; Warawan Wongboot; sirikanda wimol
EPI_ISL_768530, EPI_ISL_768531, EPI_ISL_768532, EPI_ISL_768533, EPI_ISL_768534, EPI_ISL_768535, EPI_ISL_768536, EPI_ISL_768537, EPI_ISL_768538, EPI_ISL_768539, EPI_ISL_768540, EPI_ISL_768541, EPI_ISL_768542, EPI_ISL_768543, EPI_ISL_768544, EPI_ISL_768615, EPI_ISL_768617, EPI_ISL_3342061, EPI_ISL_3406999, EPI_ISL_3407000, EPI_ISL_3407833, EPI_ISL_3407872, EPI_ISL_3419318			
see above	Regional Medical Sciences Center 5 Samut Songkhram	National Institute of Health, Department of Medical Sciences, Ministry of Public Health, Thailand	; Natchaya Khiadsang; Nuttida Thongpramul; Pakorn Piromtong; Pilailuk Okada; Ratana Tacharoenmuang; Siripaporn Phuygun; Sittiporn Parnmen; Sunthareeya Waicharoen; Thanutsapa Thanadachakul; Warawan Wongboot; sirikanda wimol
EPI_ISL_2348496, EPI_ISL_2348497, EPI_ISL_2350921, EPI_ISL_2350922, EPI_ISL_2350923, EPI_ISL_2350924, EPI_ISL_2350925, EPI_ISL_2350926, EPI_ISL_2350927, EPI_ISL_2350928, EPI_ISL_2350929, EPI_ISL_3826473, EPI_ISL_3826477, EPI_ISL_3826478, EPI_ISL_3826479, EPI_ISL_3826480, EPI_ISL_3826482, EPI_ISL_3826524, EPI_ISL_3826528, EPI_ISL_3826529, EPI_ISL_3826530, EPI_ISL_3826531, EPI_ISL_3826532, EPI_ISL_3826533, EPI_ISL_3826534, EPI_ISL_3826535, EPI_ISL_3826536, EPI_ISL_3826537, EPI_ISL_3826538, EPI_ISL_3826539, EPI_ISL_3826540, EPI_ISL_3826541, EPI_ISL_3826542, EPI_ISL_3826543, EPI_ISL_3826544, EPI_ISL_3826545, EPI_ISL_3826546, EPI_ISL_3826547, EPI_ISL_3826548, EPI_ISL_3826549, EPI_ISL_3826550, EPI_ISL_3826551, EPI_ISL_3826552, EPI_ISL_3826553, EPI_ISL_3826554, EPI_ISL_3826555, EPI_ISL_3826556, EPI_ISL_3826557, EPI_ISL_3826558, EPI_ISL_3826559, EPI_ISL_3826560, EPI_ISL_3826561, EPI_ISL_3826562, EPI_ISL_3826563, EPI_ISL_3826564, EPI_ISL_3826565, EPI_ISL_3826566, EPI_ISL_3826567, EPI_ISL_3826568, EPI_ISL_3826569, EPI_ISL_3826570, EPI_ISL_3826571, EPI_ISL_3826748, EPI_ISL_3826749, EPI_ISL_3826750, EPI_ISL_3826751, EPI_ISL_3826759, EPI_ISL_3826762, EPI_ISL_3826763, EPI_ISL_3826767, EPI_ISL_3826771, EPI_ISL_3826775, EPI_ISL_3826776, EPI_ISL_3826779, EPI_ISL_3826780, EPI_ISL_3826784, EPI_ISL_3826786, EPI_ISL_3826788, EPI_ISL_3826789, EPI_ISL_3826794, EPI_ISL_3826795, EPI_ISL_3826796, EPI_ISL_3826797, EPI_ISL_3826798, EPI_ISL_3826799, EPI_ISL_4557925, EPI_ISL_4647622, EPI_ISL_4647626, EPI_ISL_4647635, EPI_ISL_4647640, EPI_ISL_4647641, EPI_ISL_4647647, EPI_ISL_4647650, EPI_ISL_4647655, EPI_ISL_4647658			
see above	Regional Medical Sciences Center 6 Chonburi	Division of Genomic Medicine and Innovation support,Department of Medical Sciences, Ministry of Public Health, Thailand	Archawin Rojanawiwat; Jirapha Pakdee; Natthakul Bunneang; Nuanjun Wichukchinda; Penpitcha Thawong; Pilailuk Akkapaiboon Okada; Pundharika Piboonsiri; Surakameth Mahasirimongkol; Waritta Sawaengdee
EPI_ISL_3402018, EPI_ISL_3406994, EPI_ISL_3407830, EPI_ISL_3407834, EPI_ISL_3407841, EPI_ISL_3419316, EPI_ISL_3419395, EPI_ISL_3419397, EPI_ISL_3471409, EPI_ISL_3471410, EPI_ISL_3531666, EPI_ISL_3534187, EPI_ISL_3534188, EPI_ISL_3534189, EPI_ISL_3590617, EPI_ISL_3590618, EPI_ISL_3590619, EPI_ISL_3590620, EPI_ISL_3590621, EPI_ISL_3590622, EPI_ISL_3631655, EPI_ISL_3631656, EPI_ISL_3631657, EPI_ISL_3631658, EPI_ISL_3636271, EPI_ISL_3636272, EPI_ISL_3797062, EPI_ISL_4050577, EPI_ISL_4050580, EPI_ISL_4106141, EPI_ISL_4106142, EPI_ISL_4106145, EPI_ISL_4106147, EPI_ISL_4117797, EPI_ISL_4117802, EPI_ISL_4117805, EPI_ISL_4170072, EPI_ISL_4170073, EPI_ISL_4170074, EPI_ISL_4201551, EPI_ISL_4254659, EPI_ISL_4254665, EPI_ISL_4634613, EPI_ISL_4634617, EPI_ISL_4635438, EPI_ISL_4635439, EPI_ISL_4635440, EPI_ISL_4635441, EPI_ISL_4635442, EPI_ISL_4636418, EPI_ISL_4636419, EPI_ISL_4636426, EPI_ISL_4636427, EPI_ISL_4636440, EPI_ISL_4636448, EPI_ISL_4636451, EPI_ISL_4636455, EPI_ISL_4636456, EPI_ISL_4646118			

see above	Regional Medical Sciences Center 6 Chonburi	National Institute of Health, Department of Medical Sciences, Ministry of Public Health, Thailand	; Archawin Rojanawiwat; Natchaya Khiadsang; Nuttida Thongpramul; Pakorn Piromtong; Pilailuk Okada; Ratana Tacharoenmuang; Siripaporn Phuygun; Sittiporn Parnmen; Sunthareeya Waicharoen; Thanutsapa Thanadachakul; Warawan Wongboot; sirikanda wimol
EPI_ISL_3406993, EPI_ISL_3406996, EPI_ISL_3407002, EPI_ISL_3407003, EPI_ISL_3407842, EPI_ISL_3407846, EPI_ISL_3407866, EPI_ISL_3407869, EPI_ISL_3407873, EPI_ISL_3665955, EPI_ISL_4348470, EPI_ISL_4636430, EPI_ISL_4636431, EPI_ISL_4636432, EPI_ISL_4636433, EPI_ISL_4636434, EPI_ISL_4646091, EPI_ISL_4646092, EPI_ISL_4646099, EPI_ISL_4646105, EPI_ISL_4646127			
see above	Regional Medical Sciences Center 7 Khon Kaen	National Institute of Health, Department of Medical Sciences, Ministry of Public Health, Thailand	; Archawin Rojanawiwat; Natchaya Khiadsang; Nuttida Thongpramul; Pakorn Piromtong; Pilailuk Okada; Ratana Tacharoenmuang; Siripaporn Phuygun; Sittiporn Parnmen; Sunthareeya Waicharoen; Thanutsapa Thanadachakul; Warawan Wongboot; sirikanda wimol
EPI_ISL_1251229, EPI_ISL_1251230, EPI_ISL_1251231	Regional Medical Sciences Center 7 Khon Kean	National Institute of Health, Department of Medical Sciences, Ministry of Public Health, Thailand	Hathaikan Tanchai; Natchaya Khiadsang; Nuttida Thongpramul; Pakorn Piromtong; Pilailuk Okada; Ratana Tacharoenmuang; Siripaporn Phuygun; Sittiporn Parnmen; Sunthareeya Waicharoen.; Thanutsapa Thanadachakull
EPI_ISL_2877034, EPI_ISL_2877035, EPI_ISL_2877036, EPI_ISL_2877037, EPI_ISL_3038903, EPI_ISL_3038910, EPI_ISL_3342069, EPI_ISL_3402020, EPI_ISL_3402021, EPI_ISL_3402022, EPI_ISL_3407851, EPI_ISL_3407870, EPI_ISL_3419396, EPI_ISL_3590627, EPI_ISL_3590628, EPI_ISL_3801324, EPI_ISL_4636423			
see above	Regional Medical Sciences Center 8 Udonthani	National Institute of Health, Department of Medical Sciences, Ministry of Public Health, Thailand	; Archawin Rojanawiwat; Natchaya Khiadsang; Natchaya Khiadsang; Nuttida Thongpramul; Pakorn Piromtong; Pilailuk Okada; Ratana Tacharoenmuang; Siripaporn Phuygun; Sittiporn Parnmen; Sunthareeya Waicharoen; Thanutsapa Thanadachakul; Warawan Wongboot; sirikanda wimol
EPI_ISL_3342066, EPI_ISL_3407857, EPI_ISL_3407864, EPI_ISL_3419317, EPI_ISL_3471404, EPI_ISL_3590623, EPI_ISL_3590624, EPI_ISL_3631659, EPI_ISL_4635428, EPI_ISL_4636447, EPI_ISL_4646112			
see above	Regional Medical Sciences Center 9 Nakhon Ratchasima	National Institute of Health, Department of Medical Sciences, Ministry of Public Health, Thailand	; Archawin Rojanawiwat; Natchaya Khiadsang; Nuttida Thongpramul; Pakorn Piromtong; Pilailuk Okada; Ratana Tacharoenmuang; Siripaporn Phuygun; Sittiporn Parnmen; Sunthareeya Waicharoen; Thanutsapa Thanadachakul; Warawan Wongboot; sirikanda wimol
EPI_ISL_3827175, EPI_ISL_3827266, EPI_ISL_3827267, EPI_ISL_3827268, EPI_ISL_3827269, EPI_ISL_3827270, EPI_ISL_3827271, EPI_ISL_3827272, EPI_ISL_3827273, EPI_ISL_3827274, EPI_ISL_3827275, EPI_ISL_3827276, EPI_ISL_3827277, EPI_ISL_3827278, EPI_ISL_3827279, EPI_ISL_3827280, EPI_ISL_3827281, EPI_ISL_3827282, EPI_ISL_3827283, EPI_ISL_3827284, EPI_ISL_3827285, EPI_ISL_3827286, EPI_ISL_3827287, EPI_ISL_3827288, EPI_ISL_3827289, EPI_ISL_3827290, EPI_ISL_3827291, EPI_ISL_3827292, EPI_ISL_3827293, EPI_ISL_3827294, EPI_ISL_3827295, EPI_ISL_3827296, EPI_ISL_3827297, EPI_ISL_3827298, EPI_ISL_3827392, EPI_ISL_3827393, EPI_ISL_3827394, EPI_ISL_3827395, EPI_ISL_3827396, EPI_ISL_3827457, EPI_ISL_3827478, EPI_ISL_3827481, EPI_ISL_3827482			
see above	Regional Medical Sciences Centre 12/1 Trang	Division of Genomic Medicine and Innovation support,Department of Medical Sciences, Ministry of Public Health, Thailand	Archawin Rojanawiwat; Jirapha Pakdee; Natthakul Bunneang; Nuanjun Wichukchinda; Penpitcha Thawong; Pilailuk Akkapaiboon Okada; Pundharika Piboonsiri; Surakameth Mahasirimongkol; Waritta Sawaengdee
EPI_ISL_708810, EPI_ISL_708811	Regional medical sciences center 2 Phitsanulok	National Institute of Health, Department of Medical Sciences, Ministry of Public Health, Thailand	Malinee Chittaganpitch; Pakorn Piromtong; Pilailuk Okada; Siripaporn Phuygun; Sittiporn Parnmen; Sunthareeya Waicharoen; Thanutsapa Thanadachakul; Warawan Wongboot
EPI_ISL_755588, EPI_ISL_768521, EPI_ISL_768610, EPI_ISL_768611	Regional medical sciences center 6 Chonburi	National Institute of Health, Department of Medical Sciences, Ministry of Public Health, Thailand	; Malinee Chittaganpitch; Natchaya Khiadsang; Pakorn Piromtong; Pilailuk Okada; Ratana Tacharoenmuang; Siripaporn Phuygun; Sittiporn Parnmen; Sunthareeya Waicharoen; Thanutsapa Thanadachakul; Warawan Wongboot; sirikanda wimol
EPI_ISL_708737, EPI_ISL_708797, EPI_ISL_708800, EPI_ISL_708801, EPI_ISL_708802, EPI_ISL_708804, EPI_ISL_708806, EPI_ISL_708807, EPI_ISL_708808			
see above	Regional medical sciences center 6 chonburi	National Institute of Health, Department of Medical Sciences, Ministry of Public Health, Thailand	Malinee Chittaganpitch; Pakorn Piromtong; Pilailuk Okada; Siripaporn Phuygun; Sittiporn Parnmen; Sunthareeya Waicharoen; Thanutsapa Thanadachakul; Warawan Wongboot
EPI_ISL_3827357, EPI_ISL_3827358, EPI_ISL_3827359, EPI_ISL_3827363, EPI_ISL_3827455, EPI_ISL_3827479	Roi-Et Hospital	Division of Genomic Medicine and Innovation support,Department of Medical Sciences, Ministry of Public Health, Thailand	Archawin Rojanawiwat; Jirapha Pakdee; Natthakul Bunneang; Nuanjun Wichukchinda; Penpitcha Thawong; Pilailuk Akkapaiboon Okada; Pundharika Piboonsiri; Surakameth Mahasirimongkol; Waritta Sawaengdee
EPI_ISL_1495995	Sai Noi Hospital	National Institute of Health, Department of Medical Sciences, Ministry of Public Health, Thailand	Hathaikan Tanchai; Natchaya Khiadsang; Nuttida Thongpramul; Pakorn Piromtong; Pilailuk Okada; Ratana Tacharoenmuang; Siripaporn Phuygun; Sittiporn Parnmen; Sunthareeya Waicharoen.; Thanutsapa Thanadachakull
EPI_ISL_1497599, EPI_ISL_1497600, EPI_ISL_1497601, EPI_ISL_1497604, EPI_ISL_1497605, EPI_ISL_1497607, EPI_ISL_1497609			
see above	Samutsakhon Provincial Public Health Office	National Institute of Health, Department of Medical Sciences, Ministry of Public Health, Thailand	Hathaikan Tanchai; Natchaya Khiadsang; Nuttida Thongpramul; Pakorn Piromtong; Pilailuk Okada; Ratana Tacharoenmuang; Siripaporn Phuygun; Sittiporn Parnmen; Sunthareeya Waicharoen.; Thanutsapa Thanadachakull
EPI_ISL_2802818, EPI_ISL_2802819, EPI_ISL_2802820, EPI_ISL_2802821, EPI_ISL_2802822, EPI_ISL_2802823, EPI_ISL_2802824, EPI_ISL_2802825, EPI_ISL_2802826, EPI_ISL_2802827, EPI_ISL_2802828, EPI_ISL_2802829, EPI_ISL_2802830, EPI_ISL_2928512, EPI_ISL_2928513, EPI_ISL_2928514, EPI_ISL_2928515, EPI_ISL_2928516, EPI_ISL_2928517, EPI_ISL_2928518, EPI_ISL_2928519, EPI_ISL_2928520, EPI_ISL_2928521, EPI_ISL_2928522, EPI_ISL_3716307, EPI_ISL_3716316, EPI_ISL_3716319, EPI_ISL_3716327, EPI_ISL_3716331, EPI_ISL_3716332, EPI_ISL_3716333, EPI_ISL_3716343, EPI_ISL_3716344, EPI_ISL_3716346, EPI_ISL_3791311, EPI_ISL_3791314, EPI_ISL_3791318, EPI_ISL_3791322, EPI_ISL_3791325, EPI_ISL_3791338, EPI_ISL_3791349, EPI_ISL_4279182, EPI_ISL_4279183, EPI_ISL_4279184			
see above	Shoklo Malaria Research Unit	COVID-19 Network Investigations (CONI) Alliance	Angkana Huang; Anthony R. Jones; Arporn Wangwiwatsin; Bhakbhoom Panthan; Chonticha Klungtong; Duangkamon Loesbanluechai; Ekawat Pasomsub; Elizabeth Batty; Francois Nosten; Insee Sensorn; Janjira Thaipadungpanit; Khajohn Joonlasak; Kingkan Rakmanee; Krittikorn Kumpornsin; Namfon Kotanan; Stefan Fernandez; Thanat Chookajorn; Theerarat Kochakarn; Treewat Watthanachockchai; Wanitda Watthanaworawit; Wasun Chantratita; Wudtichai Manasatienkij
EPI_ISL_3827172	Si Bun Rueang Hospital	Division of Genomic Medicine and Innovation support,Department of Medical Sciences, Ministry of Public Health, Thailand	Archawin Rojanawiwat; Jirapha Pakdee; Natthakul Bunneang; Nuanjun Wichukchinda; Penpitcha Thawong; Pilailuk Akkapaiboon Okada; Pundharika Piboonsiri; Surakameth Mahasirimongkol; Waritta Sawaengdee
EPI_ISL_768523	Singburi Hospital	National Institute of Health, Department of Medical Sciences, Ministry of Public Health, Thailand	; Natchaya Khiadsang; Pakorn Piromtong; Pilailuk Okada; Ratana Tacharoenmuang; Siripaporn Phuygun; Sittiporn Parnmen; Sunthareeya Waicharoen; Thanutsapa Thanadachakul; Warawan Wongboot; sirikanda wimol
EPI_ISL_4235100, EPI_ISL_4235102, EPI_ISL_4235118, EPI_ISL_4235120, EPI_ISL_4235128, EPI_ISL_4235129, EPI_ISL_4235136, EPI_ISL_4235137, EPI_ISL_4235143, EPI_ISL_4235144, EPI_ISL_4235150, EPI_ISL_4235151, EPI_ISL_4235158, EPI_ISL_4235159, EPI_ISL_4235166, EPI_ISL_4235167, EPI_ISL_4235174, EPI_ISL_4235175, EPI_ISL_4235181, EPI_ISL_4235182, EPI_ISL_4235188, EPI_ISL_4235194, EPI_ISL_4235202, EPI_ISL_4235206, EPI_ISL_4235214, EPI_ISL_4235228, EPI_ISL_4235243, EPI_ISL_4235251, EPI_ISL_4235258, EPI_ISL_4235266, EPI_ISL_4235274, EPI_ISL_4235280, EPI_ISL_4235290, EPI_ISL_4235291, EPI_ISL_4235295, EPI_ISL_4235296, EPI_ISL_4235301, EPI_ISL_4235302, EPI_ISL_4235306, EPI_ISL_4235307, EPI_ISL_4235308, EPI_ISL_4235312, EPI_ISL_4235313, EPI_ISL_4235314, EPI_ISL_4235319, EPI_ISL_4235320, EPI_ISL_4235326, EPI_ISL_4235327, EPI_ISL_4235328, EPI_ISL_4235332, EPI_ISL_4235333, EPI_ISL_4235338, EPI_ISL_4235339, EPI_ISL_4235343, EPI_ISL_4235344, EPI_ISL_4235349, EPI_ISL_4235350, EPI_ISL_4235354, EPI_ISL_4235355			
see above	Sirindhorn Hospital	Division of Genomic Medicine and Innovation support,Department of Medical Sciences, Ministry of Public Health, Thailand	Archawin Rojanawiwat; Jirapha Pakdee; Natthakul Bunneang; Nuanjun Wichukchinda; Penpitcha Thawong; Pilailuk Akkapaiboon Okada; Pundharika Piboonsiri; Surakameth Mahasirimongkol; Waritta Sawaengdee
EPI_ISL_3038904, EPI_ISL_3665956, EPI_ISL_3665957	Siriraj Hospital	National Institute of Health, Department of Medical Sciences, Ministry of Public Health, Thailand	; Natchaya Khiadsang; Natchaya Khiadsang; Nuttida Thongpramul; Pakorn Piromtong; Pilailuk Okada; Ratana Tacharoenmuang; Siripaporn Phuygun; Sittiporn Parnmen; Sunthareeya Waicharoen; Thanutsapa Thanadachakul; Warawan Wongboot; sirikanda wimol
EPI_ISL_515464	Siriraj Hospital	National Institute of Health. Department of medical Sciences, Ministry of Public Health, Thailand	Chittaganpitch; Malinee; Okada; Parnmen; Phuygun; Pilailuk; Siripaporn; Sittiporn; Sunthareeya; Thanadachakul; Thanutsapa; Waicharoen; Warawan; Wongboot
EPI_ISL_756375	Siriraj Hospital, Mahidol University	National Institute of Health, Department of Medical Sciences, Ministry of Public Health, Thailand	Archiraya Pattama; Chutikarn Chaimayo; Natchaya Khiadsang; Navin Horthongkham; Niracha Athipanyasilp; Pakorn Piromtong; Pilailuk Okada; Ratana Tacharoenmuang; Ruengpung Sutthent; Sirikanda wimol; Siripaporn Phuygun; Sittiporn Parnmen; Sunthareeya Waicharoen; Thanutsapa Thanadachakul; Wannee Kantakamalakul.; Warawan Wongboot
EPI_ISL_447906, EPI_ISL_447907, EPI_ISL_447908, EPI_ISL_455586	Siriraj hospital	National Institute of Health. Department of medical Sciences, Ministry of Public Health, Thailand	Chittaganpitch; Malinee; Navin Horthongkham; Okada; Parnmen; Phuygun; Pilailuk; Siripaporn; Sittiporn; Sunthareeya; Thanadachakul; Thanutsapa; Waicharoen; Warawan; Wongboot
EPI_ISL_3827465	Sirivej Hospital Chanthaburi	Division of Genomic Medicine and Innovation support,Department of Medical Sciences, Ministry of Public Health, Thailand	Archawin Rojanawiwat; Jirapha Pakdee; Natthakul Bunneang; Nuanjun Wichukchinda; Penpitcha Thawong; Pilailuk Akkapaiboon Okada; Pundharika Piboonsiri; Surakameth Mahasirimongkol; Waritta Sawaengdee
EPI_ISL_3827375, EPI_ISL_3827376, EPI_ISL_3827379, EPI_ISL_3827385	Song Dao Hospital	Division of Genomic Medicine and Innovation support,Department of Medical Sciences, Ministry of Public Health, Thailand	Archawin Rojanawiwat; Jirapha Pakdee; Natthakul Bunneang; Nuanjun Wichukchinda; Penpitcha Thawong; Pilailuk Akkapaiboon Okada; Pundharika Piboonsiri; Surakameth Mahasirimongkol; Waritta Sawaengdee
EPI_ISL_2433286, EPI_ISL_2433289, EPI_ISL_2433297, EPI_ISL_2433299, EPI_ISL_2433311, EPI_ISL_2433322, EPI_ISL_2433323, EPI_ISL_2433324, EPI_ISL_2433325, EPI_ISL_2433331, EPI_ISL_2433332, EPI_ISL_2433333, EPI_ISL_2433334, EPI_ISL_2433335, EPI_ISL_2433336, EPI_ISL_2433338, EPI_ISL_2433339, EPI_ISL_2433340, EPI_ISL_2433341, EPI_ISL_2433348, EPI_ISL_2433349, EPI_ISL_2433350			
see above	Songkhla Hospital	Division of Genomic Medicine and Innovation support,Department of Medical Sciences, Ministry of Public Health, Thailand	Archawin Rojanawiwat; Jirapha Pakdee; Natthakul Bunneang; Nuanjun Wichukchinda; Penpitcha Thawong; Pilailuk Akkapaiboon Okada; Pundharika Piboonsiri; Surakameth Mahasirimongkol; Waritta Sawaengdee
EPI_ISL_2433298, EPI_ISL_2433351, EPI_ISL_2433352, EPI_ISL_2433353, EPI_ISL_2433354, EPI_ISL_2433355, EPI_ISL_2433356, EPI_ISL_2433357, EPI_ISL_2433358, EPI_ISL_2433359, EPI_ISL_2433360, EPI_ISL_2433361, EPI_ISL_2433362, EPI_ISL_2433363			
see above	Songkhla Provincial Public Health Office	Division of Genomic Medicine and Innovation support,Department of Medical Sciences, Ministry of Public Health, Thailand	Archawin Rojanawiwat; Jirapha Pakdee; Natthakul Bunneang; Nuanjun Wichukchinda; Penpitcha Thawong; Pilailuk Akkapaiboon Okada; Pundharika Piboonsiri; Surakameth Mahasirimongkol; Waritta Sawaengdee
EPI_ISL_3827356, EPI_ISL_3827458, EPI_ISL_3827474	Suddhavej Hospital	Division of Genomic Medicine and Innovation support,Department of Medical Sciences, Ministry of Public Health, Thailand	Archawin Rojanawiwat; Jirapha Pakdee; Natthakul Bunneang; Nuanjun Wichukchinda; Penpitcha Thawong; Pilailuk Akkapaiboon Okada; Pundharika Piboonsiri; Surakameth Mahasirimongkol; Waritta Sawaengdee
EPI_ISL_3827365	Suwankhuha Hospital	Division of Genomic Medicine and Innovation support,Department of Medical Sciences, Ministry of Public Health, Thailand	Archawin Rojanawiwat; Jirapha Pakdee; Natthakul Bunneang; Nuanjun Wichukchinda; Penpitcha Thawong; Pilailuk Akkapaiboon Okada; Pundharika Piboonsiri; Surakameth Mahasirimongkol; Waritta Sawaengdee
EPI_ISL_3827360, EPI_ISL_3827361, EPI_ISL_3827362	Suwannaphum Hospital	Division of Genomic Medicine and Innovation support,Department of Medical Sciences, Ministry of Public Health, Thailand	Archawin Rojanawiwat; Jirapha Pakdee; Natthakul Bunneang; Nuanjun Wichukchinda; Penpitcha Thawong; Pilailuk Akkapaiboon Okada; Pundharika Piboonsiri; Surakameth Mahasirimongkol; Waritta Sawaengdee
EPI_ISL_708821, EPI_ISL_768522	Synphaet Hospital	National Institute of Health, Department of Medical Sciences, Ministry of Public Health, Thailand	; Malinee Chittaganpitch; Natchaya Khiadsang; Pakorn Piromtong; Pilailuk Okada; Ratana Tacharoenmuang; Siripaporn Phuygun; Sittiporn Parnmen; Sunthareeya Waicharoen; Thanutsapa Thanadachakul; Warawan Wongboot; sirikanda wimol
EPI_ISL_2433294, EPI_ISL_2433309, EPI_ISL_2433453, EPI_ISL_2433454, EPI_ISL_2433455, EPI_ISL_2433456, EPI_ISL_2433457, EPI_ISL_2433458, EPI_ISL_2433459, EPI_ISL_2433460, EPI_ISL_2433461, EPI_ISL_2433462, EPI_ISL_2433463, EPI_ISL_2433464, EPI_ISL_2433465, EPI_ISL_2433466, EPI_ISL_2433467, EPI_ISL_2433468, EPI_ISL_2433469, EPI_ISL_2433470, EPI_ISL_2433471, EPI_ISL_2433472, EPI_ISL_2433553, EPI_ISL_2433558			
see above	Taksin King the Great Hospital	Division of Genomic Medicine and Innovation support,Department of Medical Sciences, Ministry of Public Health, Thailand	Archawin Rojanawiwat; Jirapha Pakdee; Natthakul Bunneang; Nuanjun Wichukchinda; Penpitcha Thawong; Pilailuk Akkapaiboon Okada; Pundharika Piboonsiri; Surakameth Mahasirimongkol; Waritta Sawaengdee
EPI_ISL_4235109, EPI_ISL_4235124, EPI_ISL_4235153, EPI_ISL_4235161, EPI_ISL_4235169, EPI_ISL_4235177, EPI_ISL_4235183, EPI_ISL_4235190, EPI_ISL_4235197, EPI_ISL_4235212, EPI_ISL_4235220, EPI_ISL_4235221, EPI_ISL_4235227, EPI_ISL_4235229, EPI_ISL_4235235, EPI_ISL_4235236, EPI_ISL_4235242, EPI_ISL_4235250, EPI_ISL_4235257, EPI_ISL_4235265, EPI_ISL_4235272, EPI_ISL_4235273, EPI_ISL_4235279, EPI_ISL_4235286, EPI_ISL_4235287, EPI_ISL_4235292, EPI_ISL_4235298, EPI_ISL_4235304, EPI_ISL_4235310, EPI_ISL_4235317, EPI_ISL_4235335, EPI_ISL_4235346, EPI_ISL_4557921, EPI_ISL_4557922, EPI_ISL_4647029, EPI_ISL_4647032, EPI_ISL_4647137, EPI_ISL_4647142, EPI_ISL_4647147, EPI_ISL_4647149, EPI_ISL_4647150, EPI_ISL_4647154, EPI_ISL_4647160, EPI_ISL_4647167, EPI_ISL_4647174, EPI_ISL_4647180			

see above	Taksin hospital	Division of Genomic Medicine and Innovation support,Department of Medical Sciences, Ministry of Public Health, Thailand	Archawin Rojanawiwat; Jirapha Pakdee; Natthakul Bunneang; Nuanjun Wichukchinda; Penpitcha Thawong; Pilailuk Akkapaiboon Okada; Pundharika Piboonsiri; Surakameth Mahasirimongkol; Waritta Sawaengdee
EPI_ISL_4647445, EPI_ISL_4647451, EPI_ISL_4647456, EPI_ISL_4647462, EPI_ISL_4647463, EPI_ISL_4647470, EPI_ISL_4647471, EPI_ISL_4647478			
see above	Takuapa Hospital	Division of Genomic Medicine and Innovation support,Department of Medical Sciences, Ministry of Public Health, Thailand	Archawin Rojanawiwat; Jirapha Pakdee; Natthakul Bunneang; Nuanjun Wichukchinda; Penpitcha Thawong; Pilailuk Akkapaiboon Okada; Pundharika Piboonsiri; Surakameth Mahasirimongkol; Waritta Sawaengdee
EPI_ISL_2426254, EPI_ISL_2426255, EPI_ISL_2426256, EPI_ISL_2426257, EPI_ISL_2426258, EPI_ISL_2426259	Thai Red Cross Emerging Infectious Diseases Clinical Center and Faculty of Medicine, Chulalongkorn University	Thai Red Cross Emerging Infectious Diseases Clinical Center and Faculty of Medicine, Chulalongkorn University	Anthony R. Jones; Chonticha Klungthong; Gompol Suwanpimolkul; Khajohn Joonlasak; Leilani Paitoonpong; Opass Putcharoen; Piyawan Chinnawirotpisan; Stefan Fernandez; Supaporn Wacharapluesadee; Torvorapanit Pattama; Watsamon Jantarabenjakul; Wudtichai Manasatienkij
EPI_ISL_877227, EPI_ISL_877766, EPI_ISL_918160, EPI_ISL_918161, EPI_ISL_918162, EPI_ISL_918163, EPI_ISL_918166, EPI_ISL_934971, EPI_ISL_934972, EPI_ISL_937531, EPI_ISL_937532, EPI_ISL_937533, EPI_ISL_937534, EPI_ISL_937535, EPI_ISL_937536, EPI_ISL_937537, EPI_ISL_937538, EPI_ISL_937539, EPI_ISL_937540, EPI_ISL_937541, EPI_ISL_937542, EPI_ISL_937543, EPI_ISL_937544, EPI_ISL_956272, EPI_ISL_956275, EPI_ISL_976893, EPI_ISL_976894, EPI_ISL_976895, EPI_ISL_976896, EPI_ISL_976897, EPI_ISL_976898, EPI_ISL_976899, EPI_ISL_976900, EPI_ISL_976901, EPI_ISL_976902, EPI_ISL_976903, EPI_ISL_976904, EPI_ISL_976905, EPI_ISL_976906, EPI_ISL_976907, EPI_ISL_976908, EPI_ISL_976909, EPI_ISL_981004, EPI_ISL_984303, EPI_ISL_984304, EPI_ISL_1007658, EPI_ISL_1007659, EPI_ISL_1117527, EPI_ISL_1117528, EPI_ISL_1278368, EPI_ISL_1278369, EPI_ISL_1278370, EPI_ISL_1278371, EPI_ISL_1278372, EPI_ISL_1296442, EPI_ISL_1296443, EPI_ISL_1296444, EPI_ISL_1296445, EPI_ISL_1296446, EPI_ISL_1296447, EPI_ISL_1296448, EPI_ISL_1296449, EPI_ISL_1296450, EPI_ISL_1296451, EPI_ISL_1296452, EPI_ISL_1296453, EPI_ISL_1296454, EPI_ISL_1296455, EPI_ISL_1296456, EPI_ISL_1296457, EPI_ISL_1296458, EPI_ISL_1296459, EPI_ISL_1296460, EPI_ISL_1296461, EPI_ISL_1296462, EPI_ISL_1708575, EPI_ISL_1969991, EPI_ISL_2566380, EPI_ISL_2566381, EPI_ISL_2566382, EPI_ISL_2566383, EPI_ISL_2566384, EPI_ISL_2566385, EPI_ISL_2566386, EPI_ISL_2566387, EPI_ISL_2566388, EPI_ISL_2566389, EPI_ISL_2566390, EPI_ISL_2566391, EPI_ISL_2566392, EPI_ISL_2566393, EPI_ISL_2566394, EPI_ISL_2566395, EPI_ISL_2566396, EPI_ISL_2566397, EPI_ISL_2566398, EPI_ISL_2566399, EPI_ISL_2566400, EPI_ISL_2566401, EPI_ISL_2566402, EPI_ISL_2566403, EPI_ISL_2566404, EPI_ISL_2566405, EPI_ISL_2566406, EPI_ISL_2566407, EPI_ISL_2566408, EPI_ISL_2566409, EPI_ISL_2566410, EPI_ISL_2566411, EPI_ISL_2566412, EPI_ISL_2566413, EPI_ISL_2566414, EPI_ISL_2566415, EPI_ISL_2566416, EPI_ISL_2566417, EPI_ISL_2566418, EPI_ISL_2566419, EPI_ISL_2566420, EPI_ISL_2566421, EPI_ISL_2566422, EPI_ISL_2566423, EPI_ISL_2566424, EPI_ISL_2566425, EPI_ISL_2566426, EPI_ISL_2566427, EPI_ISL_2566428, EPI_ISL_2566429, EPI_ISL_2566430, EPI_ISL_2566431, EPI_ISL_2566432, EPI_ISL_2566433, EPI_ISL_2566434, EPI_ISL_2566435, EPI_ISL_2566436, EPI_ISL_2566437, EPI_ISL_2566438, EPI_ISL_2566439, EPI_ISL_2566440, EPI_ISL_2566441, EPI_ISL_2566442, EPI_ISL_2566443			
see above	Thai Red Cross Emerging Infectious Diseases Health Science Centre, Chulalongkorn Hospital, Faculty of Medicine, Chulalongkorn University	Thai Red Cross Emerging Infectious Diseases Center and Faculty of Medicine, Chulalongkorn University	Anthony R. Jones; Apaporn Rodpan; Chanida Ruchisrisarod; Chaniya Jamsaim; Chonticha Klungthong; Duangtip Singhakun; Gompol Suwanpimolkul; Kamolthip Atsawawaranunt; Kanthita Worachotsueptrakun; Khajohn Joonlasak; Krirk Asavametha; Leilani Paitoonpong; M.D.; Non Chindavech; Opass Putcharoen; Panpit Suwangool; Panpit Suwangool; Pasin Hemachudha; Pattama Torvorapanit; Piyapan Prueksapanich; Piyawan Chinnawirotpisan; Pornvimol Leethong; Ratchada Kitsommart; Ratima samorn; Rome Buathong; Sininat Petcharat; Sininat Petcharat; Sopon Iamsirithaworn; Stefan Fernandez; Supaporn Wacharapluesadee; Teerada Ponpinit; Thiravat Hemachudha; Thirawat Supharatpariyakorn; Vichan Pawun; Watsamon Jantarabenjakul; Weenassarin Ampoot; Wichai Thanasopon; Wudtichai Manasatienkij; Yutthana Joyjinda; Yutthana Joyjinda
EPI_ISL_3665958	Thainakarin Hospital	National Institute of Health, Department of Medical Sciences, Ministry of Public Health, Thailand	; Natchaya Khiadsang; Nuttida Thongpramul; Pakorn Piromtong; Pilailuk Okada; Ratana Tacharoenmuang; Siripaporn Phuygun; Sittiporn Parnmen; Sunthareeya Waicharoen; Thanutsapa Thanadachakul; Warawan Wongboot; sirikanda wimol
EPI_ISL_434707	Thammasat University Hospital	National Institute of Health. Department of medical Sciences, Ministry of Public Health, Thailand	Chittaganpitch; Malinee; Okada; Parnmen; Phuygun; Pilailuk; Siripaporn; Sittiporn; Sunthareeya; Thanadachakul; Thanutsapa; Waicharoen; Warawan; Wongboot
EPI_ISL_3826472, EPI_ISL_3826476, EPI_ISL_3826525, EPI_ISL_3826526, EPI_ISL_3826527, EPI_ISL_3826768, EPI_ISL_3826770, EPI_ISL_3826782, EPI_ISL_3826792, EPI_ISL_3826793, EPI_ISL_3827177, EPI_ISL_3827338, EPI_ISL_3827339, EPI_ISL_3827340, EPI_ISL_3827341, EPI_ISL_3827342, EPI_ISL_3827343, EPI_ISL_3827344, EPI_ISL_3827345, EPI_ISL_3827446, EPI_ISL_3827461			
see above	The Office of Disease Prevention and Control 6	Division of Genomic Medicine and Innovation support,Department of Medical Sciences, Ministry of Public Health, Thailand	Archawin Rojanawiwat; Jirapha Pakdee; Natthakul Bunneang; Nuanjun Wichukchinda; Penpitcha Thawong; Pilailuk Akkapaiboon Okada; Pundharika Piboonsiri; Surakameth Mahasirimongkol; Waritta Sawaengdee
EPI_ISL_1497597, EPI_ISL_2877029	The office of Disease Prevention and Control 6, Chonburi	National Institute of Health, Department of Medical Sciences, Ministry of Public Health, Thailand	; Hathaikan Tanchai; Natchaya Khiadsang; Nuttida Thongpramul; Pakorn Piromtong; Pilailuk Okada; Ratana Tacharoenmuang; Siripaporn Phuygun; Sittiporn Parnmen; Sunthareeya Waicharoen; Sunthareeya Waicharoen.; Thanutsapa Thanadachakul; Thanutsapa Thanadachakull; Warawan Wongboot; sirikanda wimol
EPI_ISL_2433302, EPI_ISL_2433473, EPI_ISL_2433474, EPI_ISL_2433475, EPI_ISL_2433476, EPI_ISL_2433477, EPI_ISL_2433497, EPI_ISL_2433498, EPI_ISL_2433499, EPI_ISL_2433500, EPI_ISL_2433501, EPI_ISL_2433502, EPI_ISL_2433503, EPI_ISL_2433504, EPI_ISL_2433505, EPI_ISL_2433506, EPI_ISL_2433507, EPI_ISL_2433508, EPI_ISL_2433509, EPI_ISL_2433510, EPI_ISL_2433511, EPI_ISL_2433512, EPI_ISL_2433513, EPI_ISL_2433514, EPI_ISL_2433515			
see above	Trang Hospital	Division of Genomic Medicine and Innovation support,Department of Medical Sciences, Ministry of Public Health, Thailand	Archawin Rojanawiwat; Jirapha Pakdee; Natthakul Bunneang; Nuanjun Wichukchinda; Penpitcha Thawong; Pilailuk Akkapaiboon Okada; Pundharika Piboonsiri; Surakameth Mahasirimongkol; Waritta Sawaengdee
EPI_ISL_455588	Trang Hospital	National Institute of Health. Department of medical Sciences, Ministry of Public Health, Thailand	Chittaganpitch; Malinee; Okada; Parnmen; Phuygun; Pilailuk; Siripaporn; Sittiporn; Sunthareeya; Thanadachakul; Thanutsapa; Waicharoen; Warawan; Wongboot
EPI_ISL_4557926	Trat Hospital	Division of Genomic Medicine and Innovation support,Department of Medical Sciences, Ministry of Public Health, Thailand	Archawin Rojanawiwat; Jirapha Pakdee; Natthakul Bunneang; Nuanjun Wichukchinda; Penpitcha Thawong; Pilailuk Akkapaiboon Okada; Pundharika Piboonsiri; Surakameth Mahasirimongkol; Waritta Sawaengdee
EPI_ISL_3827391	Udon Thani Provincial Health Office	Division of Genomic Medicine and Innovation support,Department of Medical Sciences, Ministry of Public Health, Thailand	Archawin Rojanawiwat; Jirapha Pakdee; Natthakul Bunneang; Nuanjun Wichukchinda; Penpitcha Thawong; Pilailuk Akkapaiboon Okada; Pundharika Piboonsiri; Surakameth Mahasirimongkol; Waritta Sawaengdee
EPI_ISL_708815, EPI_ISL_708816, EPI_ISL_708817, EPI_ISL_708818, EPI_ISL_708819, EPI_ISL_2840601, EPI_ISL_2840602, EPI_ISL_2840603, EPI_ISL_2840604, EPI_ISL_3342064, EPI_ISL_3342065			
see above	Urban Institute for Disease Prevention and Control	National Institute of Health, Department of Medical Sciences, Ministry of Public Health, Thailand	; Malinee Chittaganpitch; Natchaya Khiadsang; Natchaya Khiadsang; Nuttida Thongpramul; Pakorn Piromtong; Pilailuk Okada; Ratana Tacharoenmuang; Siripaporn Phuygun; Sittiporn Parnmen; Sunthareeya Waicharoen; Thanutsapa Thanadachakul; Warawan Wongboot; sirikanda wimol
EPI_ISL_4647479, EPI_ISL_4647483, EPI_ISL_4647484, EPI_ISL_4647489, EPI_ISL_4647490, EPI_ISL_4647494, EPI_ISL_4647500			
see above	Vachira Phuket Hospital	Division of Genomic Medicine and Innovation support,Department of Medical Sciences, Ministry of Public Health, Thailand	Archawin Rojanawiwat; Jirapha Pakdee; Natthakul Bunneang; Nuanjun Wichukchinda; Penpitcha Thawong; Pilailuk Akkapaiboon Okada; Pundharika Piboonsiri; Surakameth Mahasirimongkol; Waritta Sawaengdee
EPI_ISL_708820	Vajira Hospital	National Institute of Health, Department of Medical Sciences, Ministry of Public Health, Thailand	Malinee Chittaganpitch; Pakorn Piromtong; Pilailuk Okada; Siripaporn Phuygun; Sittiporn Parnmen; Sunthareeya Waicharoen; Thanutsapa Thanadachakul; Warawan Wongboot
EPI_ISL_4235105, EPI_ISL_4235122, EPI_ISL_4235130, EPI_ISL_4235192, EPI_ISL_4235195, EPI_ISL_4235199, EPI_ISL_4235207, EPI_ISL_4235321, EPI_ISL_4647202, EPI_ISL_4647251			
see above	Vibharam Hospital	Division of Genomic Medicine and Innovation support,Department of Medical Sciences, Ministry of Public Health, Thailand	Archawin Rojanawiwat; Jirapha Pakdee; Natthakul Bunneang; Nuanjun Wichukchinda; Penpitcha Thawong; Pilailuk Akkapaiboon Okada; Pundharika Piboonsiri; Surakameth Mahasirimongkol; Waritta Sawaengdee
EPI_ISL_708823	Vibharam Hospital	National Institute of Health, Department of Medical Sciences, Ministry of Public Health, Thailand	Malinee Chittaganpitch; Pakorn Piromtong; Pilailuk Okada; Siripaporn Phuygun; Sittiporn Parnmen; Sunthareeya Waicharoen; Thanutsapa Thanadachakul; Warawan Wongboot
EPI_ISL_3827329	Wiang Nong Long Hospital	Division of Genomic Medicine and Innovation support,Department of Medical Sciences, Ministry of Public Health, Thailand	Archawin Rojanawiwat; Jirapha Pakdee; Natthakul Bunneang; Nuanjun Wichukchinda; Penpitcha Thawong; Pilailuk Akkapaiboon Okada; Pundharika Piboonsiri; Surakameth Mahasirimongkol; Waritta Sawaengdee
EPI_ISL_3827305	Wiang Pa Pao Hospital	Division of Genomic Medicine and Innovation support,Department of Medical Sciences, Ministry of Public Health, Thailand	Archawin Rojanawiwat; Jirapha Pakdee; Natthakul Bunneang; Nuanjun Wichukchinda; Penpitcha Thawong; Pilailuk Akkapaiboon Okada; Pundharika Piboonsiri; Surakameth Mahasirimongkol; Waritta Sawaengdee
EPI_ISL_1251228	Wiset Chai Chan Hospital	National Institute of Health, Department of Medical Sciences, Ministry of Public Health, Thailand	Hathaikan Tanchai; Natchaya Khiadsang; Nuttida Thongpramul; Pakorn Piromtong; Pilailuk Okada; Ratana Tacharoenmuang; Siripaporn Phuygun; Sittiporn Parnmen; Sunthareeya Waicharoen.; Thanutsapa Thanadachakull
EPI_ISL_708812, EPI_ISL_708813, EPI_ISL_708814	World Medical Hospital	National Institute of Health, Department of Medical Sciences, Ministry of Public Health, Thailand	Malinee Chittaganpitch; Pakorn Piromtong; Pilailuk Okada; Siripaporn Phuygun; Sittiporn Parnmen; Sunthareeya Waicharoen; Thanutsapa Thanadachakul; Warawan Wongboot
EPI_ISL_2433293	Yala Hospital	Division of Genomic Medicine and Innovation support,Department of Medical Sciences, Ministry of Public Health, Thailand	Archawin Rojanawiwat; Jirapha Pakdee; Natthakul Bunneang; Nuanjun Wichukchinda; Penpitcha Thawong; Pilailuk Akkapaiboon Okada; Pundharika Piboonsiri; Surakameth Mahasirimongkol; Waritta Sawaengdee
EPI_ISL_450196	bumrungrad international hospital	National Institute of Health. Department of medical Sciences, Ministry of Public Health, Thailand	Chittaganpitch; Malinee; Okada; Parnmen; Phuygun; Pilailuk; Siripaporn; Sittiporn; Sunthareeya; Thanadachakul; Thanutsapa; Waicharoen; Warawan; Wongboot
EPI_ISL_447909, EPI_ISL_447910, EPI_ISL_447911, EPI_ISL_447912, EPI_ISL_447913, EPI_ISL_447914, EPI_ISL_447915, EPI_ISL_447916, EPI_ISL_447917, EPI_ISL_447918, EPI_ISL_447919, EPI_ISL_447920, EPI_ISL_447921			
see above	n/a	National Institute of Health. Department of medical Sciences, Ministry of Public Health, Thailand	Chittaganpitch; Malinee; Okada; Parnmen; Phuygun; Pilailuk; Siripaporn; Sittiporn; Sunthareeya; Thanadachakul; Thanutsapa; Waicharoen; Warawan; Wongboot
EPI_ISL_430837	n/a	Thai National Influenza Center, Department of medical Science, Ministry of Public Health, Thailand	Chittaganpitch; Malinee; Okada; Parnmen; Phuygun; Pilailuk; Siripaporn; Sittiporn; Sunthareeya; Thanadachakul; Thanutsapa; Waicharoen; Warawan; Wongboot
EPI_ISL_437602, EPI_ISL_437603, EPI_ISL_437604, EPI_ISL_437605, EPI_ISL_437606, EPI_ISL_437607, EPI_ISL_437608, EPI_ISL_437609, EPI_ISL_437610, EPI_ISL_437611, EPI_ISL_437612, EPI_ISL_437613, EPI_ISL_437614, EPI_ISL_437615, EPI_ISL_437616, EPI_ISL_437617, EPI_ISL_437618, EPI_ISL_437619, EPI_ISL_437620, EPI_ISL_437621, EPI_ISL_437622, EPI_ISL_437623, EPI_ISL_437624			
see above	unknown	Faculty of Medicine	Buathong, R.; Bunprakob, S.; Ghai, S.; Joyjinda, Y.; Mungaomklang, A.; Petcharat, S.; Plipat; Prasithsirikul, W.; Rodpan, A.; Sirichan, N.; T. and Hemachudha, T.; Wacharapluesadee, S.
EPI_ISL_434695, EPI_ISL_434697, EPI_ISL_434708	unknown	National Institute of Health. Department of medical Sciences, Ministry of Public Health, Thailand	Chittaganpitch; Malinee; Okada; Parnmen; Phuygun; Pilailuk; Siripaporn; Sittiporn; Sunthareeya; Thanadachakul; Thanutsapa; Waicharoen; Warawan; Wongboot

We gratefully acknowledge the following Authors from the Originating laboratories responsible for obtaining the specimens, as well as the Submitting laboratories where the genome data were generated and shared via GISAID, on which this research is based.

All Submitters of data may be contacted directly via www.gisaid.org

Authors are sorted alphabetically.

Accession ID	Originating Laboratory	Submitting Laboratory	Authors
EPI_ISL_406031, EPI_ISL_420082, EPI_ISL_420083, EPI_ISL_420084, EPI_ISL_420085, EPI_ISL_421641, EPI_ISL_421651, EPI_ISL_428488, EPI_ISL_428489, EPI_ISL_428490, EPI_ISL_428491, EPI_ISL_429882, EPI_ISL_429883, EPI_ISL_429884, EPI_ISL_857314, EPI_ISL_1381386, EPI_ISL_2455264, EPI_ISL_2455327, EPI_ISL_2455329, EPI_ISL_2455330, EPI_ISL_2705575, EPI_ISL_3000157, EPI_ISL_3000160, EPI_ISL_3000409, EPI_ISL_3000790, EPI_ISL_3001055, EPI_ISL_3001368, EPI_ISL_3001841, EPI_ISL_3002178, EPI_ISL_3040140, EPI_ISL_3040141, EPI_ISL_3040142, EPI_ISL_3040143, EPI_ISL_3040144, EPI_ISL_3040145, EPI_ISL_3040146, EPI_ISL_3040147, EPI_ISL_3040148, EPI_ISL_3040149, EPI_ISL_3040151, EPI_ISL_3040152, EPI_ISL_3987576			
see above	Centers for Disease Control, R.O.C. (Taiwan)	Centers for Disease Control, R.O.C. (Taiwan)	Ji-Rong Yang; Jung-Jung Mu; Jung-Jung-Mu; Ming-Tsan Liu; Ming-Tsan-Liu; Shu-Ying Li; Yu-Chi Lin; Yu-Chi-Lin
EPI_ISL_408489, EPI_ISL_410218, EPI_ISL_413592, EPI_ISL_422407, EPI_ISL_422408, EPI_ISL_422409, EPI_ISL_422410, EPI_ISL_422411, EPI_ISL_422412, EPI_ISL_422413, EPI_ISL_422414, EPI_ISL_422415, EPI_ISL_422416, EPI_ISL_422417, EPI_ISL_422418, EPI_ISL_422419, EPI_ISL_422420, EPI_ISL_422421, EPI_ISL_422422, EPI_ISL_447614, EPI_ISL_447615, EPI_ISL_447616, EPI_ISL_447617, EPI_ISL_447618, EPI_ISL_447619, EPI_ISL_447620, EPI_ISL_447621, EPI_ISL_447622, EPI_ISL_463007, EPI_ISL_534336, EPI_ISL_693302, EPI_ISL_693303, EPI_ISL_693304, EPI_ISL_693305, EPI_ISL_693306, EPI_ISL_738064, EPI_ISL_738065, EPI_ISL_740547, EPI_ISL_872591, EPI_ISL_872592, EPI_ISL_872593, EPI_ISL_872594, EPI_ISL_872595, EPI_ISL_872596, EPI_ISL_872597, EPI_ISL_872598, EPI_ISL_872599, EPI_ISL_1010728, EPI_ISL_1010729, EPI_ISL_1010730, EPI_ISL_1010731, EPI_ISL_1020315, EPI_ISL_1020316, EPI_ISL_1039160, EPI_ISL_1041957, EPI_ISL_1041958, EPI_ISL_1667469, EPI_ISL_1667471, EPI_ISL_1667472, EPI_ISL_1667474, EPI_ISL_1667475, EPI_ISL_2307350, EPI_ISL_3979387			
see above	Department of Laboratory Medicine, National Taiwan University Hospital	Microbial Genomics Core Lab, National Taiwan University Centers of Genomic and Precision Medicine	Chiao-Ling Li; Hurng-Yi Wang; Pei-Jer Chen; Shan-Chwen Chang; Shiou-Hwei Yeh; Sui-Yuan Chang; Tzi-Yuan Wang; Ya-Yun Lai; You-Yu Lin
EPI_ISL_4169856	Dr.Chiaho Shih	Dr.Chiaho Shih	An-Ting Liou; Chih-Hsu Chang; Ching-Yi Tsai; Inn-Wen Chong; Jeng-Yi Li; Jer-Ming Chang; Jih-Jin Tsai; Mei-Yeh Jade Lu; Ming-Feng Hou; Ping-Chang Lin; Shang-Jyh Hwang; Wen-Chang Lin; Yu-Chen Cheng; Yuh-Jyh Jong; and Chiaho Shih
EPI_ISL_415741, EPI_ISL_415742, EPI_ISL_415743, EPI_ISL_417518, EPI_ISL_417519, EPI_ISL_417520, EPI_ISL_417521, EPI_ISL_417522, EPI_ISL_417523, EPI_ISL_417524, EPI_ISL_417525, EPI_ISL_424969, EPI_ISL_424970, EPI_ISL_424971, EPI_ISL_424972, EPI_ISL_424973, EPI_ISL_424974, EPI_ISL_424975, EPI_ISL_424978, EPI_ISL_444274, EPI_ISL_444275, EPI_ISL_444276, EPI_ISL_444277, EPI_ISL_444278, EPI_ISL_452178, EPI_ISL_452179, EPI_ISL_464092, EPI_ISL_464093, EPI_ISL_464094, EPI_ISL_660543, EPI_ISL_660544, EPI_ISL_660545, EPI_ISL_667807, EPI_ISL_667808, EPI_ISL_667809, EPI_ISL_956317, EPI_ISL_956318, EPI_ISL_956320, EPI_ISL_956321, EPI_ISL_956322, EPI_ISL_956323, EPI_ISL_956324, EPI_ISL_956325, EPI_ISL_956326, EPI_ISL_956327, EPI_ISL_956328, EPI_ISL_956329, EPI_ISL_956330, EPI_ISL_956331, EPI_ISL_2249472, EPI_ISL_2249473, EPI_ISL_2249499, EPI_ISL_2249500, EPI_ISL_2249515, EPI_ISL_2249516, EPI_ISL_2249517, EPI_ISL_2249597, EPI_ISL_2249653, EPI_ISL_2249836, EPI_ISL_2250151, EPI_ISL_2250152, EPI_ISL_2250184			
see above	Laboratory Medicine	Department of Laboratory Medicine, Lin-Kou Chang Gung Memorial Hospital, Taoyuan, Taiwan	Cheng-Hsun Chiu; Cheng-Ta Yang; Chi-Hsien Huang; Chung-Guei Huang; Guang-Wu Chen; Kuang-Tso Le; Kuo-Chien Tsao; Kuo-Ming Lee; Mei-Jen Hsiao; Peng-Nien Huang; Po-Wei Huang; Shin-Ru Shih; Shu-Li Yang; Shu-Min Lin; Yi-Chun Liu; Yu-Nong Gong
EPI_ISL_411915	Laboratory Medicine	Department of Laboratory Medicine, Lin-Kou Chang Gung Memorial Hospital, Taoyuan, Taiwan.	Chung-Guei Huang; Kuo-Chien Tsao; Shin-Ru Shih; Shu-Li Yang; Yhu-Chering Huang; Yi-Chun Li; Yu-Nong Gong
EPI_ISL_2544700, EPI_ISL_2544701, EPI_ISL_2544702, EPI_ISL_2544703, EPI_ISL_2544704, EPI_ISL_2544705, EPI_ISL_2544706, EPI_ISL_2544707, EPI_ISL_2544708, EPI_ISL_2544709, EPI_ISL_2544710, EPI_ISL_2544711, EPI_ISL_2544712, EPI_ISL_2544713, EPI_ISL_2544714			
see above	Research Center for Emerging Viral Infections, Chang Gung University, Taiwan	Research Center for Emerging Viral Infections, Chang Gung University, Taiwan	Carol Wang; Chung-Guei Huang; Hsiao-Chen Tu; Hui-Ying Weng; Hung-Yu Shu; Jason Su; Jora Lin; Kuo-Ming Lee; Po-Wei Huang; Pocky Lai; Shih-Feng Tsai; Shin-Ru Shih; Shu-Li Yang; Tsu-Lan Wu; Yu-Nong Gong; Yung-Feng Lin
EPI_ISL_426629, EPI_ISL_426630, EPI_ISL_426631, EPI_ISL_426632, EPI_ISL_427392, EPI_ISL_427393, EPI_ISL_427394, EPI_ISL_427395, EPI_ISL_427396, EPI_ISL_427397, EPI_ISL_427398, EPI_ISL_428229, EPI_ISL_428230, EPI_ISL_428231, EPI_ISL_436099, EPI_ISL_436100, EPI_ISL_436101, EPI_ISL_436102, EPI_ISL_436103, EPI_ISL_436104, EPI_ISL_436105, EPI_ISL_436106, EPI_ISL_436107, EPI_ISL_436108, EPI_ISL_447252, EPI_ISL_447253, EPI_ISL_447254, EPI_ISL_447255, EPI_ISL_447256, EPI_ISL_447257, EPI_ISL_447591, EPI_ISL_447592, EPI_ISL_447593, EPI_ISL_457726, EPI_ISL_457730, EPI_ISL_457733, EPI_ISL_458029, EPI_ISL_2692999, EPI_ISL_2693000, EPI_ISL_2693004, EPI_ISL_2693005, EPI_ISL_2693006, EPI_ISL_4096803, EPI_ISL_4096805, EPI_ISL_4096807, EPI_ISL_4578347			
see above	TSGH-CP molecular lab	TSGH-CP molecular lab	Cherng-Lih Perng; Chien-Wen Chen; Chih-Kai Chang; Feng-Yee Chang; Hsing-Yi Chung; Hung-Sheng Shang; Jung-Chung Lin; Kuo-Ming Yeh; Kuo-Sheng Hung; Ming-Jr JIAN; Ming-Jr Jian; Sheng-Kang Chiu; Shih-Hung Tsai; Tien-Yao Chang
EPI_ISL_437336	TSGH-CP molecular lab, Division of Clinical Pathology, Department of Pathology	TSGH-CP molecular lab, Division of Clinical Pathology, Department of Pathology	Cherng-Lih Perng; Chien-Wen Chen; Chih-Kai Chang; Feng-Yee Chang; Hsing-Yi Chung; Hung-Sheng Shang; Jung-Chung Lin; Kuo-Ming Yeh; Kuo-Sheng Hung; Ming-Jr JIAN; Sheng-Kang Chiu; Shih-Hung Tsai; Tien-Yao Chang
EPI_ISL_411926, EPI_ISL_411927	Taiwan Centers for Disease Control	Taiwan Centers for Disease Control	Ji-Rong Yang; Jung-Jung Mu; Ming-Tsan-Liu; Yu-Chi-Lin
EPI_ISL_493198, EPI_ISL_493199, EPI_ISL_493200, EPI_ISL_493201, EPI_ISL_493202, EPI_ISL_493203, EPI_ISL_493204, EPI_ISL_493205, EPI_ISL_493206, EPI_ISL_493207, EPI_ISL_493208, EPI_ISL_494747			
see above	Virology Lab,Department of Pathology, National Cheng Kung University Hospital	Virology Lab,Department of Pathology, National Cheng Kung University Hospital	Huey-Pin Tsai; Huey-Pin Tsai et al; et al

We gratefully acknowledge the following Authors from the Originating laboratories responsible for obtaining the specimens, as well as the Submitting laboratories where the genome data were generated and shared via GISAID, on which this research is based.

All Submitters of data may be contacted directly via www.gisaid.org

Authors are sorted alphabetically.

Accession ID	Originating Laboratory	Submitting Laboratory	Authors
EPI_ISL_620406	Biomedical Science and Technology, Konkuk University, Laboratory of Cytokine Immunology (Room 601-1) IBST,Konkuk University	Biomedical Science and Technology, Konkuk University, Laboratory of Cytokine Immunology (Room 601-1) IBST,Konkuk University	Kim, S.
EPI_ISL_420799, EPI_ISL_420800, EPI_ISL_420801	Brian D. Allgood Army Community Hospital	Pathogen Discovery, Respiratory Viruses Branch, Division of Viral Diseases, Centers for Disease Control and Prevention	Alison S. Laufer Halpin; Anne Uehara; Christopher A. Elkins; Clinton R. Paden; Haibin Wang; Jasmine Padilla; Jing Zhang; Justin Lee; Krista Queen; Mary S. Keckler; Rachel Marine; Suxiang Tong; Yan Li; Ying Tao
EPI_ISL_516783, EPI_ISL_516784, EPI_ISL_516785, EPI_ISL_522439, EPI_ISL_522440, EPI_ISL_522441, EPI_ISL_522442, EPI_ISL_522443, EPI_ISL_522444, EPI_ISL_522445, EPI_ISL_522446, EPI_ISL_522447, EPI_ISL_522448, EPI_ISL_522449, EPI_ISL_522450, EPI_ISL_522451, EPI_ISL_522452, EPI_ISL_522453, EPI_ISL_522455, EPI_ISL_522456, EPI_ISL_522457, EPI_ISL_522458, EPI_ISL_522459, EPI_ISL_522460, EPI_ISL_522461, EPI_ISL_522462, EPI_ISL_522463, EPI_ISL_522464, EPI_ISL_522467, EPI_ISL_522468, EPI_ISL_522469, EPI_ISL_522470, EPI_ISL_522471, EPI_ISL_522472, EPI_ISL_522473, EPI_ISL_522476, EPI_ISL_522479, EPI_ISL_522480, EPI_ISL_522481, EPI_ISL_522482, EPI_ISL_522483, EPI_ISL_522485, EPI_ISL_522491, EPI_ISL_522492, EPI_ISL_522493, EPI_ISL_522494, EPI_ISL_522495, EPI_ISL_522496, EPI_ISL_522497, EPI_ISL_522502, EPI_ISL_522505, EPI_ISL_522508, EPI_ISL_522510, EPI_ISL_522515, EPI_ISL_522516, EPI_ISL_522518, EPI_ISL_526730, EPI_ISL_526731, EPI_ISL_526734, EPI_ISL_526735, EPI_ISL_526736, EPI_ISL_526737, EPI_ISL_526738, EPI_ISL_526739, EPI_ISL_526740, EPI_ISL_526741, EPI_ISL_526742, EPI_ISL_526743, EPI_ISL_526744, EPI_ISL_526745, EPI_ISL_526746			
see above	Center for Laboratory Control of Infectious Diseases, Korea Centers for Diseases Control and Prevention	Center for Laboratory Control of Infectious Diseases, Korea Centers for Diseases Control and Prevention	Ae Kyung Park; Eunkyung Shin; Heui Man Kim; Jeong-Min Kim; Jin Sun No; Junyoung Kim; Myung Guk Han; Yoon-Seok Chung
EPI_ISL_479662, EPI_ISL_591070	College of Veterinary Medicine, Chungnam National University	College of Veterinary Medicine, Chungnam National University	S.H. and Jang, Y.; Seo; Seo, S.
EPI_ISL_411929	Department of Clinical Diagnostics	Department of Clinical Diagnostics	C.K.; Choe; Choi; G.-W.; J.-S. and Oh; J.Y.; Kang; Kim; Kim, N.; Kwon; Lee; M.-D.; M.-W.; N.-J.; P.G.; Park; S.-J.; Seo; Seong; W.B.; Yun, J.
EPI_ISL_485398, EPI_ISL_985394, EPI_ISL_1007662, EPI_ISL_1007663, EPI_ISL_1007664, EPI_ISL_1007665, EPI_ISL_1007666, EPI_ISL_1007667, EPI_ISL_1007668, EPI_ISL_1007669, EPI_ISL_1007670, EPI_ISL_1007671, EPI_ISL_1007672, EPI_ISL_1007673, EPI_ISL_1007674, EPI_ISL_1007675, EPI_ISL_1007676, EPI_ISL_1007677, EPI_ISL_1007678, EPI_ISL_1007679, EPI_ISL_2226216, EPI_ISL_2226217, EPI_ISL_2226218, EPI_ISL_2226219, EPI_ISL_2226220, EPI_ISL_2226221, EPI_ISL_2226222, EPI_ISL_2226223, EPI_ISL_2226224, EPI_ISL_2226225, EPI_ISL_2226226, EPI_ISL_2226227, EPI_ISL_2226228			
see above	Department of Internal Medicine, College of Medicine, Chosun University	Department of Internal Medicine, College of Medicine, Chosun University	D.-M.; Dong-Min Kim; Kim
EPI_ISL_507039	Department of Microbiology, College of Medicine and Medical Research Institute Chungbuk National University	Department of Microbiology, College of Medicine and Medical Research Institute Chungbuk National University	Eun-Ha Kim; Hye Won Jeong; Mark Anthony B. Casel; Se-Mi Kim; Seong-Gyu Kim; Su-Jin Park; Young Ki Choi; Young-Il Kim
EPI_ISL_1069194	Department of Microbiology, College of Medicine and Medical Research Institute Chungbuk National University	Department of Microbiology, College of Medicine and Medical Research Institute Chungbuk National University	Bum Sik Chin; Eun Ji Kim; Eun-Ha Kim; Haryoung Poo; Hye Won Jeong; In-Pil Mo; Jae U. Jung; Jae-Hyung Chang; Jihye Um; Jun-Sun Park; Ki-Hyun Chung; Kwang-Min Yu; Mark Anthony B. Casel; Min-Suk Song; Ok-Jun Lee; Richard J. Webby; Se-Mi Kim; Seong-Gyu Kim; Seunghun Lee; Su-Jin Park; Suan-Sin Foo; Van Dam Lai; Yeonjae Kim; Young Ki Choi; Young-Il Kim
EPI_ISL_413017, EPI_ISL_413018, EPI_ISL_413514, EPI_ISL_413516	Department of Microbiology, Institute for Viral Diseases, College of Medicine, Korea University	Department of Microbiology, Institute for Viral Diseases, College of Medicine, Korea University	Changmin Kang; Cui Chunguang; Dong Min Kim; Gee Eun Lee; Gee eun Lee; Hee Jin Cheong; Heedo Park; Jeonghun Kim; Ji Yun Noh; Jin Gu Yoon; Jin Il Kim; Joon Young Song; Joon-Yong Bae; Jungmin Lee; Juyoung Cho; Kyeong-ryeol Shin; Man-Seong Park; Woo Joo Kim
EPI_ISL_738139, EPI_ISL_738141, EPI_ISL_738142, EPI_ISL_747245, EPI_ISL_747246, EPI_ISL_747247, EPI_ISL_747248, EPI_ISL_747249, EPI_ISL_747250, EPI_ISL_747251, EPI_ISL_747252, EPI_ISL_747253, EPI_ISL_747254, EPI_ISL_747255, EPI_ISL_747256, EPI_ISL_747257, EPI_ISL_747258, EPI_ISL_747259, EPI_ISL_747260, EPI_ISL_747261, EPI_ISL_747262, EPI_ISL_747263, EPI_ISL_747264, EPI_ISL_747265, EPI_ISL_747266, EPI_ISL_747267, EPI_ISL_747268, EPI_ISL_747269, EPI_ISL_747270, EPI_ISL_747271, EPI_ISL_747272, EPI_ISL_747273, EPI_ISL_747274, EPI_ISL_747275, EPI_ISL_747276, EPI_ISL_747277, EPI_ISL_747278, EPI_ISL_747279, EPI_ISL_747280, EPI_ISL_747281, EPI_ISL_747284, EPI_ISL_747285, EPI_ISL_747286, EPI_ISL_747287, EPI_ISL_747288, EPI_ISL_747289, EPI_ISL_747290, EPI_ISL_747291, EPI_ISL_747292, EPI_ISL_747293, EPI_ISL_747294, EPI_ISL_747295, EPI_ISL_747296, EPI_ISL_747297, EPI_ISL_747298, EPI_ISL_747299, EPI_ISL_747300, EPI_ISL_747301, EPI_ISL_747302, EPI_ISL_747303, EPI_ISL_747304, EPI_ISL_747305, EPI_ISL_747306, EPI_ISL_747307, EPI_ISL_747308, EPI_ISL_747309, EPI_ISL_747310, EPI_ISL_747311, EPI_ISL_747312, EPI_ISL_747313, EPI_ISL_747314, EPI_ISL_747315, EPI_ISL_747316, EPI_ISL_747317, EPI_ISL_747318, EPI_ISL_747319, EPI_ISL_747320, EPI_ISL_747321, EPI_ISL_747322, EPI_ISL_747323, EPI_ISL_747324, EPI_ISL_747325, EPI_ISL_747326, EPI_ISL_747327, EPI_ISL_747328, EPI_ISL_747329, EPI_ISL_747330, EPI_ISL_747331, EPI_ISL_747332, EPI_ISL_747333, EPI_ISL_747334, EPI_ISL_747335, EPI_ISL_747336, EPI_ISL_747337, EPI_ISL_747338, EPI_ISL_747339, EPI_ISL_747340, EPI_ISL_747341, EPI_ISL_747342, EPI_ISL_747343, EPI_ISL_747344, EPI_ISL_747345, EPI_ISL_747346, EPI_ISL_747347, EPI_ISL_747348, EPI_ISL_747349, EPI_ISL_747350, EPI_ISL_747351, EPI_ISL_747352, EPI_ISL_747353, EPI_ISL_747354, EPI_ISL_747355, EPI_ISL_747356, EPI_ISL_747357, EPI_ISL_747358, EPI_ISL_747359, EPI_ISL_747360, EPI_ISL_747361, EPI_ISL_747362, EPI_ISL_747363, EPI_ISL_747364, EPI_ISL_747365, EPI_ISL_747366, EPI_ISL_747367, EPI_ISL_747368, EPI_ISL_747369, EPI_ISL_747370, EPI_ISL_747371, EPI_ISL_747372, EPI_ISL_747373, EPI_ISL_747374, EPI_ISL_747375, EPI_ISL_747376, EPI_ISL_747377, EPI_ISL_747378, EPI_ISL_747379, EPI_ISL_747380, EPI_ISL_747381, EPI_ISL_747382, EPI_ISL_747383, EPI_ISL_747384, EPI_ISL_747385, EPI_ISL_747386, EPI_ISL_747387, EPI_ISL_747388, EPI_ISL_747389, EPI_ISL_747390, EPI_ISL_747391, EPI_ISL_747392, EPI_ISL_747393, EPI_ISL_747394, EPI_ISL_747395, EPI_ISL_747396, EPI_ISL_747397, EPI_ISL_747398, EPI_ISL_747399, EPI_ISL_747400, EPI_ISL_747401, EPI_ISL_747402, EPI_ISL_747403, EPI_ISL_747404, EPI_ISL_747405, EPI_ISL_747406, EPI_ISL_747407, EPI_ISL_747408, EPI_ISL_747409, EPI_ISL_747410, EPI_ISL_747411, EPI_ISL_747412, EPI_ISL_747413, EPI_ISL_747414, EPI_ISL_747415, EPI_ISL_747416, EPI_ISL_747417, EPI_ISL_747418, EPI_ISL_747419, EPI_ISL_747420, EPI_ISL_747421, EPI_ISL_747425, EPI_ISL_747426, EPI_ISL_747427, EPI_ISL_747428, EPI_ISL_747429, EPI_ISL_747430, EPI_ISL_747431, EPI_ISL_747432, EPI_ISL_747433, EPI_ISL_747434, EPI_ISL_747435, EPI_ISL_747436, EPI_ISL_747437, EPI_ISL_747438, EPI_ISL_747439, EPI_ISL_747440, EPI_ISL_747441, EPI_ISL_747442, EPI_ISL_747443, EPI_ISL_747444, EPI_ISL_747445, EPI_ISL_747446, EPI_ISL_747447, EPI_ISL_747448, EPI_ISL_747449, EPI_ISL_747450, EPI_ISL_747451, EPI_ISL_747452, EPI_ISL_747453, EPI_ISL_747454, EPI_ISL_747455, EPI_ISL_747456, EPI_ISL_747457, EPI_ISL_747458, EPI_ISL_759991, EPI_ISL_759992, EPI_ISL_759993, EPI_ISL_759994, EPI_ISL_759995, EPI_ISL_759996, EPI_ISL_759997, EPI_ISL_759998, EPI_ISL_759999, EPI_ISL_760000, EPI_ISL_760001, EPI_ISL_760002, EPI_ISL_760003, EPI_ISL_760004, EPI_ISL_760005, EPI_ISL_760006, EPI_ISL_760007, EPI_ISL_760008, EPI_ISL_760009, EPI_ISL_760010, EPI_ISL_760011, EPI_ISL_760012, EPI_ISL_760013, EPI_ISL_760014, EPI_ISL_760015, EPI_ISL_760016, EPI_ISL_760017, EPI_ISL_760018, EPI_ISL_760019, EPI_ISL_760020, EPI_ISL_760021, EPI_ISL_760022, EPI_ISL_760023, EPI_ISL_760024, EPI_ISL_760025, EPI_ISL_760026, EPI_ISL_760027, EPI_ISL_760028, EPI_ISL_760029, EPI_ISL_760030, EPI_ISL_760060, EPI_ISL_760061, EPI_ISL_760062, EPI_ISL_760063, EPI_ISL_760064, EPI_ISL_760065, EPI_ISL_760066, EPI_ISL_760067, EPI_ISL_760068, EPI_ISL_760069, EPI_ISL_760070, EPI_ISL_760071, EPI_ISL_760072, EPI_ISL_760073, EPI_ISL_760074, EPI_ISL_760075, EPI_ISL_760076, EPI_ISL_760077, EPI_ISL_760078, EPI_ISL_760079, EPI_ISL_760080, EPI_ISL_760081, EPI_ISL_760082, EPI_ISL_760083, EPI_ISL_760084, EPI_ISL_760085, EPI_ISL_760086, EPI_ISL_760087, EPI_ISL_760088, EPI_ISL_760089, EPI_ISL_760090, EPI_ISL_760091, EPI_ISL_760092, EPI_ISL_760093, EPI_ISL_760094, EPI_ISL_760095, EPI_ISL_760096, EPI_ISL_760097, EPI_ISL_760098, EPI_ISL_760099, EPI_ISL_760100, EPI_ISL_760101, EPI_ISL_760102, EPI_ISL_760103, EPI_ISL_760104, EPI_ISL_760105, EPI_ISL_760106, EPI_ISL_760107, EPI_ISL_760108, EPI_ISL_760109, EPI_ISL_760110, EPI_ISL_760111, EPI_ISL_760112, EPI_ISL_760113, EPI_ISL_760114, EPI_ISL_760115, EPI_ISL_760116, EPI_ISL_760117, EPI_ISL_760118, EPI_ISL_760119, EPI_ISL_760120, EPI_ISL_760121, EPI_ISL_760122, EPI_ISL_760123, EPI_ISL_760124, EPI_ISL_760125, EPI_ISL_760126, EPI_ISL_760127, EPI_ISL_760128, EPI_ISL_760129, EPI_ISL_760130, EPI_ISL_760131, EPI_ISL_760132, EPI_ISL_760133, EPI_ISL_760134, EPI_ISL_760135, EPI_ISL_760136, EPI_ISL_760137, EPI_ISL_760138, EPI_ISL_760139, EPI_ISL_760140, EPI_ISL_760141, EPI_ISL_760142, EPI_ISL_760143, EPI_ISL_760144, EPI_ISL_760145, EPI_ISL_760146, EPI_ISL_760147, EPI_ISL_760148, EPI_ISL_760149, EPI_ISL_760150, EPI_ISL_760151, EPI_ISL_760152, EPI_ISL_760153, EPI_ISL_760154, EPI_ISL_760155, EPI_ISL_760156, EPI_ISL_760157, EPI_ISL_760158, EPI_ISL_760159, EPI_ISL_760160, EPI_ISL_760161, EPI_ISL_760162, EPI_ISL_760163, EPI_ISL_760164, EPI_ISL_760165, EPI_ISL_760166, EPI_ISL_760167, EPI_ISL_760168, EPI_ISL_760169, EPI_ISL_760170, EPI_ISL_760171, EPI_ISL_760172, EPI_ISL_760173, EPI_ISL_760174, EPI_ISL_760175, EPI_ISL_760176, EPI_ISL_760177, EPI_ISL_760178, EPI_ISL_760179, EPI_ISL_760180, EPI_ISL_760181, EPI_ISL_760182, EPI_ISL_760183, EPI_ISL_760184, EPI_ISL_760185, EPI_ISL_760186, EPI_ISL_760187, EPI_ISL_760188, EPI_ISL_760189, EPI_ISL_760190, EPI_ISL_760191, EPI_ISL_760192, EPI_ISL_760193, EPI_ISL_760194, EPI_ISL_760195, EPI_ISL_760196, EPI_ISL_760197, EPI_ISL_760198, EPI_ISL_760199, EPI_ISL_760200, EPI_ISL_760201, EPI_ISL_760202, EPI_ISL_760203, EPI_ISL_760204, EPI_ISL_760205, EPI_ISL_760206, EPI_ISL_760207, EPI_ISL_760208, EPI_ISL_760209, EPI_ISL_760210, EPI_ISL_760211, EPI_ISL_760212, EPI_ISL_760213, EPI_ISL_760214, EPI_ISL_760215, EPI_ISL_760216, EPI_ISL_760217, EPI_ISL_760218, EPI_ISL_760219, EPI_ISL_760220, EPI_ISL_760221, EPI_ISL_760222, EPI_ISL_760223, EPI_ISL_760224, EPI_ISL_760225, EPI_ISL_760226, EPI_ISL_760227, EPI_ISL_760228, EPI_ISL_760229, EPI_ISL_760230, EPI_ISL_760231, EPI_ISL_760232, EPI_ISL_760233, EPI_ISL_760234, EPI_ISL_760235, EPI_ISL_760236, EPI_ISL_760237, EPI_ISL_760238, EPI_ISL_760239, EPI_ISL_760240, EPI_ISL_760241, EPI_ISL_760242, EPI_ISL_760243, EPI_ISL_760244, EPI_ISL_760245, EPI_ISL_762992, EPI_ISL_833249, EPI_ISL_850181, EPI_ISL_850182, EPI_ISL_850183, EPI_ISL_850184, EPI_ISL_850185, EPI_ISL_850186, EPI_ISL_850187, EPI_ISL_850188, EPI_ISL_850189, EPI_ISL_850190, EPI_ISL_850191, EPI_ISL_850192, EPI_ISL_850193, EPI_ISL_850194, EPI_ISL_850195, EPI_ISL_850196, EPI_ISL_850197, EPI_ISL_850198, EPI_ISL_850199, EPI_ISL_850200, EPI_ISL_850201, EPI_ISL_850202, EPI_ISL_850203, EPI_ISL_850204, EPI_ISL_850205, EPI_ISL_850206, EPI_ISL_850207, EPI_ISL_850208, EPI_ISL_850209, EPI_ISL_850210, EPI_ISL_850211, EPI_ISL_850212, EPI_ISL_850213, EPI_ISL_850214, EPI_ISL_850215, EPI_ISL_850216, EPI_ISL_850217, EPI_ISL_850218, EPI_ISL_850219, EPI_ISL_850220, EPI_ISL_850221, EPI_ISL_850222, EPI_ISL_850223, EPI_ISL_850224, EPI_ISL_850225, EPI_ISL_850226, EPI_ISL_850227, EPI_ISL_850228, EPI_ISL_850229, EPI_ISL_850230, EPI_ISL_850231, EPI_ISL_850232, EPI_ISL_850233, EPI_ISL_850234, EPI_ISL_850235, EPI_ISL_850236, EPI_ISL_850237, EPI_ISL_850238, EPI_ISL_850239, EPI_ISL_850240, EPI_ISL_850241, EPI_ISL_850242, EPI_ISL_850243, EPI_ISL_850244, EPI_ISL_850245, EPI_ISL_850246, EPI_ISL_850247, EPI_ISL_850248, EPI_ISL_850249, EPI_ISL_850250, EPI_ISL_850251, EPI_ISL_850252, EPI_ISL_850253, EPI_ISL_850254, EPI_ISL_850255, EPI_ISL_850256, EPI_ISL_850257, EPI_ISL_850258, EPI_ISL_850259, EPI_ISL_850260, EPI_ISL_850261, EPI_ISL_850262, EPI_ISL_850263, EPI_ISL_850264, EPI_ISL_850265, EPI_ISL_850266, EPI_ISL_850267, EPI_ISL_850268, EPI_ISL_850269, EPI_ISL_850270, EPI_ISL_850271, EPI_ISL_850272, EPI_ISL_850273, EPI_ISL_850274, EPI_ISL_850275, EPI_ISL_850276, EPI_ISL_850277, EPI_ISL_850278, EPI_ISL_850279, EPI_ISL_850280, EPI_ISL_850281, EPI_ISL_850282, EPI_ISL_850283, EPI_ISL_850284, EPI_ISL_850285, EPI_ISL_850286, EPI_ISL_850287, EPI_ISL_850288, EPI_ISL_850289, EPI_ISL_850290, EPI_ISL_850291, EPI_ISL_850292, EPI_ISL_850293, EPI_ISL_850294, EPI_ISL_850295, EPI_ISL_850296, EPI_ISL_850297, EPI_ISL_850298, EPI_ISL_850299, EPI_ISL_850300, EPI_ISL_850301, EPI_ISL_850302, EPI_ISL_850303, EPI_ISL_850304, EPI_ISL_850305, EPI_ISL_850306, EPI_ISL_850307, EPI_ISL_850308, EPI_ISL_850309, EPI_ISL_850310, EPI_ISL_850311, EPI_ISL_850312, EPI_ISL_850313, EPI_ISL_850314, EPI_ISL_850315, EPI_ISL_850316, EPI_ISL_850317, EPI_ISL_850318, EPI_ISL_850319, EPI_ISL_850320, EPI_ISL_850321, EPI_ISL_850322, EPI_ISL_850323, EPI_ISL_850324, EPI_ISL_850325, EPI_ISL_850326, EPI_ISL_850327, EPI_ISL_850328, EPI_ISL_850329, EPI_ISL_850330, EPI_ISL_850331, EPI_ISL_850332, EPI_ISL_850333, EPI_ISL_850334, EPI_ISL_850335, EPI_ISL_850336, EPI_ISL_850337, EPI_ISL_850338, EPI_ISL_850339, EPI_ISL_850340, EPI_ISL_850341, EPI_ISL_850342, EPI_ISL_850343, EPI_ISL_850344, EPI_ISL_850345, EPI_ISL_850346, EPI_ISL_850347, EPI_ISL_850348, EPI_ISL_850349, EPI_ISL_850350, EPI_ISL_850351, EPI_ISL_850352, EPI_ISL_850353, EPI_ISL_850354, EPI_ISL_850355, EPI_ISL_850356, EPI_ISL_850357, EPI_ISL_850358, EPI_ISL_850359, EPI_ISL_850360, EPI_ISL_850361, EPI_ISL_850362, EPI_ISL_850363, EPI_ISL_850364, EPI_ISL_850365, EPI_ISL_850366, EPI_ISL_850367, EPI_ISL_850368, EPI_ISL_850369, EPI_ISL_850370, EPI_ISL_850371, EPI_ISL_850372, EPI_ISL_850373, EPI_ISL_850374, EPI_ISL_850375, EPI_ISL_850376, EPI_ISL_850377, EPI_ISL_850378, EPI_ISL_850379, EPI_ISL_850380, EPI_ISL_850381, EPI_ISL_850382, EPI_ISL_850383, EPI_ISL_850384, EPI_ISL_850385, EPI_ISL_850386, EPI_ISL_850387, EPI_ISL_850388, EPI_ISL_850389, EPI_ISL_850390, EPI_ISL_850391, EPI_ISL_850392, EPI_ISL_850393, EPI_ISL_850394, EPI_ISL_850395, EPI_ISL_850396, EPI_ISL_850397, EPI_ISL_850398, EPI_ISL_850399, EPI_ISL_850400, EPI_ISL_850401, EPI_ISL_850402, EPI_ISL_850403, EPI_ISL_850404, EPI_ISL_850405, EPI_ISL_850406, EPI_ISL_850407, EPI_ISL_850408, EPI_ISL_850409, EPI_ISL_850410, EPI_ISL_850411, EPI_ISL_850412, EPI_ISL_850413, EPI_ISL_850414, EPI_ISL_850415, EPI_ISL_850416, EPI_ISL_850417, EPI_ISL_850418, EPI_ISL_850419, EPI_ISL_850420, EPI_ISL_850421, EPI_ISL_850422, EPI_ISL_850423, EPI_ISL_850424, EPI_ISL_850425, EPI_ISL_850426, EPI_ISL_850427, EPI_ISL_850428, EPI_ISL_850429, EPI_ISL_850430, EPI_ISL_850431, EPI_ISL_850432, EPI_ISL_850433, EPI_ISL_850434, EPI_ISL_850435, EPI_ISL_850436, EPI_ISL_850437, EPI_ISL_850438, EPI_ISL_850439, EPI_ISL_850440, EPI_ISL_850441, EPI_ISL_850442, EPI_ISL_850443, EPI_ISL_850444, EPI_ISL_850445, EPI_ISL_850446, EPI_ISL_850447, EPI_ISL_850448, EPI_ISL_850449, EPI_ISL_850450, EPI_ISL_850451, EPI_ISL_850452, EPI_ISL_850453, EPI_ISL_850454, EPI_ISL_850455, EPI_ISL_850456, EPI_ISL_850457, EPI_ISL_850458, EPI_ISL_850459, EPI_ISL_850460, EPI_ISL_850461, EPI_ISL_850462, EPI_ISL_850463, EPI_ISL_850464, EPI_ISL_850465, EPI_ISL_850466, EPI_ISL_850467, EPI_ISL_850468, EPI_ISL_850469, EPI_ISL_850470, EPI_ISL_850471, EPI_ISL_850472, EPI_ISL_850473, EPI_ISL_850474, EPI_ISL_850475, EPI_ISL_850476, EPI_ISL_850477, EPI_ISL_850478, EPI_ISL_850479, EPI_ISL_850480, EPI_ISL_850481, EPI_ISL_850482, EPI_ISL_850483, EPI_ISL_850484, EPI_ISL_850485, EPI_ISL_850486, EPI_ISL_850487, EPI_ISL_850488, EPI_ISL_850489, EPI_ISL_850490, EPI_ISL_850491, EPI_ISL_850492, EPI_ISL_850493, EPI_ISL_850494, EPI_ISL_850495, EPI_ISL_850496, EPI_ISL_850497, EPI_ISL_850498, EPI_ISL_850499, EPI_ISL_850500, EPI_ISL_850501, EPI_ISL_850502, EPI_ISL_850503, EPI_ISL_850504, EPI_ISL_850505, EPI_ISL_850653, EPI_ISL_850654, EPI_ISL_850655, EPI_ISL_850656, EPI_ISL_850657, EPI_ISL_850658, EPI_ISL_850659, EPI_ISL_850660, EPI_ISL_850661, EPI_ISL_850662, EPI_ISL_955939, EPI_ISL_955940, EPI_ISL_955941, EPI_ISL_955942, EPI_ISL_955943, EPI_ISL_955944, EPI_ISL_955945, EPI_ISL_955946, EPI_ISL_955947, EPI_ISL_955948, EPI_ISL_955949, EPI_ISL_955950, EPI_ISL_955951, EPI_ISL_955952, EPI_ISL_955953, EPI_ISL_955954, EPI_ISL_955955, EPI_ISL_955956, EPI_ISL_955957, EPI_ISL_955958, EPI_ISL_955959, EPI_ISL_955960, EPI_ISL_955961, EPI_ISL_955962, EPI_ISL_955963, EPI_ISL_955964, EPI_ISL_955965, EPI_ISL_955966, EPI_ISL_955967, EPI_ISL_955968, EPI_ISL_955969, EPI_ISL_955970, EPI_ISL_955971, EPI_ISL_955972, EPI_ISL_955973, EPI_ISL_955974, EPI_ISL_955975, EPI_ISL_955976, EPI_ISL_955977, EPI_ISL_955978, EPI_ISL_955979, EPI_ISL_955980, EPI_ISL_955981, EPI_ISL_959476, EPI_ISL_959477, EPI_ISL_959478, EPI_ISL_959479, EPI_ISL_959480, EPI_ISL_959481, EPI_ISL_959482, EPI_ISL_959483, EPI_ISL_959484, EPI_ISL_959485, EPI_ISL_959486, EPI_ISL_959487, EPI_ISL_959488, EPI_ISL_959489, EPI_ISL_959490, EPI_ISL_959491, EPI_ISL_959492, EPI_ISL_959493, EPI_ISL_959494, EPI_ISL_959495, EPI_ISL_959496, EPI_ISL_959497, EPI_ISL_959498, EPI_ISL_959499, EPI_ISL_959500, EPI_ISL_959501, EPI_ISL_959502, EPI_ISL_959503, EPI_ISL_959504, EPI_ISL_959505, EPI_ISL_959506, EPI_ISL_959507, EPI_ISL_959508, EPI_ISL_959509, EPI_ISL_959510, EPI_ISL_959511, EPI_ISL_959512, EPI_ISL_959513, EPI_ISL_959514, EPI_ISL_959515, EPI_ISL_959516, EPI_ISL_959517, EPI_ISL_959518, EPI_ISL_959519, EPI_ISL_959520, EPI_ISL_959521, EPI_ISL_959522, EPI_ISL_959523, EPI_ISL_959524, EPI_ISL_959525, EPI_ISL_959526, EPI_ISL_959527, EPI_ISL_959528, EPI_ISL_959529, EPI_ISL_959530, EPI_ISL_959531, EPI_ISL_959532, EPI_ISL_959533, EPI_ISL_959534, EPI_ISL_959535, EPI_ISL_959536, EPI_ISL_959537, EPI_ISL_994668, EPI_ISL_994669, EPI_ISL_994670, EPI_ISL_994671, EPI_ISL_994672, EPI_ISL_994673, EPI_ISL_994674, EPI_ISL_994675, EPI_ISL_994676, EPI_ISL_994677, EPI_ISL_994678, EPI_ISL_994679, EPI_ISL_994680, EPI_ISL_994681, EPI_ISL_994682, EPI_ISL_994683, EPI_ISL_994684, EPI_ISL_994685, EPI_ISL_994686, EPI_ISL_994687, EPI_ISL_994688, EPI_ISL_994689, EPI_ISL_994690, EPI_ISL_994691, EPI_ISL_994692, EPI_ISL_994693, EPI_ISL_994694, EPI_ISL_994695, EPI_ISL_994696, EPI_ISL_994697, EPI_ISL_994698, EPI_ISL_994699, EPI_ISL_994700, EPI_ISL_994701, EPI_ISL_994702, EPI_ISL_994703, EPI_ISL_994704, EPI_ISL_994705, EPI_ISL_994706, EPI_ISL_994707, EPI_ISL_994708, EPI_ISL_994709, EPI_ISL_994710, EPI_ISL_994711, EPI_ISL_994712, EPI_ISL_994713, EPI_ISL_994714, EPI_ISL_994715, EPI_ISL_994716, EPI_ISL_994717, EPI_ISL_994718, EPI_ISL_994719, EPI_ISL_994720, EPI_ISL_994721, EPI_ISL_994722, EPI_ISL_994723, EPI_ISL_994724, EPI_ISL_994725, EPI_ISL_994726, EPI_ISL_994727, EPI_ISL_994728, EPI_ISL_994729, EPI_ISL_994730, EPI_ISL_994731, EPI_ISL_994732, EPI_ISL_994733, EPI_ISL_994734, EPI_ISL_994735, EPI_ISL_994736, EPI_ISL_994737, EPI_ISL_994738, EPI_ISL_994741, EPI_ISL_994742, EPI_ISL_994743, EPI_ISL_994744, EPI_ISL_994745, EPI_ISL_994746, EPI_ISL_994747, EPI_ISL_994748, EPI_ISL_994749, EPI_ISL_994750, EPI_ISL_994751, EPI_ISL_994752, EPI_ISL_994753, EPI_ISL_994754, EPI_ISL_994755, EPI_ISL_994756, EPI_ISL_994757, EPI_ISL_994758, EPI_ISL_994759, EPI_ISL_994760, EPI_ISL_994761, EPI_ISL_994762, EPI_ISL_994763, EPI_ISL_994764, EPI_ISL_994765, EPI_ISL_994766, EPI_ISL_994767, EPI_ISL_994768, EPI_ISL_994769, EPI_ISL_994770, EPI_ISL_994771, EPI_ISL_994772, EPI_ISL_994773, EPI_ISL_994774, EPI_ISL_994775, EPI_ISL_994776, EPI_ISL_994777, EPI_ISL_994778, EPI_ISL_994779, EPI_ISL_994780, EPI_ISL_994781, EPI_ISL_994782, EPI_ISL_994783, EPI_ISL_994784, EPI_ISL_994785, EPI_ISL_994786, EPI_ISL_994787, EPI_ISL_994788, EPI_ISL_994789, EPI_ISL_994790, EPI_ISL_994791, EPI_ISL_994792, EPI_ISL_994793, EPI_ISL_994794, EPI_ISL_994795, EPI_ISL_994796, EPI_ISL_994797, EPI_ISL_994798, EPI_ISL_994799, EPI_ISL_994800, EPI_ISL_994801, EPI_ISL_995192, EPI_ISL_995193, EPI_ISL_995194, EPI_ISL_995195, EPI_ISL_995196, EPI_ISL_995197, EPI_ISL_995198, EPI_ISL_995199, EPI_ISL_995200, EPI_ISL_995201, EPI_ISL_995202, EPI_ISL_995203, EPI_ISL_995204, EPI_ISL_995205, EPI_ISL_995206, EPI_ISL_995207, EPI_ISL_995208, EPI_ISL_995209, EPI_ISL_995210, EPI_ISL_995211, EPI_ISL_995212, EPI_ISL_995213, EPI_ISL_995214, EPI_ISL_995215, EPI_ISL_995216, EPI_ISL_995217, EPI_ISL_995218, EPI_ISL_995219, EPI_ISL_995220, EPI_ISL_995221, EPI_ISL_995222, EPI_ISL_995223, EPI_ISL_995224, EPI_ISL_995225, EPI_ISL_995226, EPI_ISL_995227, EPI_ISL_995228, EPI_ISL_995229, EPI_ISL_995230, EPI_ISL_995231, EPI_ISL_995232, EPI_ISL_995233, EPI_ISL_995234, EPI_ISL_995235, EPI_ISL_995236, EPI_ISL_995237, EPI_ISL_995238, EPI_ISL_995239, EPI_ISL_995240, EPI_ISL_995241, EPI_ISL_995242, EPI_ISL_995243, EPI_ISL_995244, EPI_ISL_995245, EPI_ISL_995246, EPI_ISL_995247, EPI_ISL_995248, EPI_ISL_995249, EPI_ISL_995250, EPI_ISL_995251, EPI_ISL_995252, EPI_ISL_995253, EPI_ISL_995254, EPI_ISL_995255, EPI_ISL_995256, EPI_ISL_995257, EPI_ISL_995258, EPI_ISL_995259, EPI_ISL_995260, EPI_ISL_995261, EPI_ISL_995262, EPI_ISL_995263, EPI_ISL_995264, EPI_ISL_995265, EPI_ISL_995266, EPI_ISL_995267, EPI_ISL_995268, EPI_ISL_995269, EPI_ISL_995270, EPI_ISL_995271, EPI_ISL_995272, EPI_ISL_995273, EPI_ISL_995274, EPI_ISL_995275, EPI_ISL_995276, EPI_ISL_995277, EPI_ISL_995278, EPI_ISL_995279, EPI_ISL_995280, EPI_ISL_995281, EPI_ISL_995282, EPI_ISL_995283, EPI_ISL_995284, EPI_ISL_995285, EPI_ISL_995286, EPI_ISL_995287, EPI_ISL_995288, EPI_ISL_995289, EPI_ISL_995290, EPI_ISL_995291, EPI_ISL_995292, EPI_ISL_995293, EPI_ISL_995308, EPI_ISL_995309, EPI_ISL_995310, EPI_ISL_995311, EPI_ISL_995312, EPI_ISL_995313, EPI_ISL_995314, EPI_ISL_995315, EPI_ISL_995316, EPI_ISL_995317, EPI_ISL_995318, EPI_ISL_995319, EPI_ISL_995320, EPI_ISL_995321, EPI_ISL_995322, EPI_ISL_995323, EPI_ISL_995324, EPI_ISL_995325, EPI_ISL_995326, EPI_ISL_995327, EPI_ISL_995328, EPI_ISL_995329, EPI_ISL_995330, EPI_ISL_995765, EPI_ISL_995766, EPI_ISL_995767, EPI_ISL_995768, EPI_ISL_995769, EPI_ISL_995770, EPI_ISL_995771, EPI_ISL_995772, EPI_ISL_995773, EPI_ISL_995774, EPI_ISL_995775, EPI_ISL_995776, EPI_ISL_995777, EPI_ISL_995778, EPI_ISL_995779, EPI_ISL_995780, EPI_ISL_995781, EPI_ISL_995782, EPI_ISL_995783, EPI_ISL_995784, EPI_ISL_995785, EPI_ISL_995786, EPI_ISL_995787, EPI_ISL_995788, EPI_ISL_995789, EPI_ISL_995790, EPI_ISL_995791, EPI_ISL_995792, EPI_ISL_995793, EPI_ISL_995794, EPI_ISL_995795, EPI_ISL_995796, EPI_ISL_995797, EPI_ISL_995798, EPI_ISL_995799, EPI_ISL_995800, EPI_ISL_995801, EPI_ISL_995802, EPI_ISL_995803, EPI_ISL_995804, EPI_ISL_995805, EPI_ISL_995806, EPI_ISL_995807, EPI_ISL_995808, EPI_ISL_995809, EPI_ISL_995810, EPI_ISL_995811, EPI_ISL_995812, EPI_ISL_995813, EPI_ISL_995814, EPI_ISL_995815, EPI_ISL_995816, EPI_ISL_995817, EPI_ISL_995818, EPI_ISL_995819, EPI_ISL_995820, EPI_ISL_995821, EPI_ISL_995822, EPI_ISL_995823, EPI_ISL_995824, EPI_ISL_995825, EPI_ISL_995826, EPI_ISL_995827, EPI_ISL_995828, EPI_ISL_995829, EPI_ISL_995830, EPI_ISL_995831, EPI_ISL_995832, EPI_ISL_995833, EPI_ISL_995834, EPI_ISL_995835, EPI_ISL_995836, EPI_ISL_995837, EPI_ISL_995838, EPI_ISL_1007643, EPI_ISL_1007644, EPI_ISL_1007645, EPI_ISL_1007646, EPI_ISL_1007647, EPI_ISL_1007648, EPI_ISL_1007649, EPI_ISL_1007650, EPI_ISL_1007651, EPI_ISL_1007652, EPI_ISL_1007653, EPI_ISL_1007654, EPI_ISL_1007655, EPI_ISL_1007656, EPI_ISL_1007657, EPI_ISL_1063537, EPI_ISL_1063538, EPI_ISL_1063539, EPI_ISL_1063540, EPI_ISL_1063541, EPI_ISL_1063542, EPI_ISL_1063543, EPI_ISL_1063544, EPI_ISL_1063545, EPI_ISL_1063546, EPI_ISL_1063547, EPI_ISL_1063548, EPI_ISL_1063549, EPI_ISL_1063550, EPI_ISL_1063551, EPI_ISL_1063552, EPI_ISL_1063553, EPI_ISL_1063554, EPI_ISL_1063555, EPI_ISL_1063556, EPI_ISL_1063557, EPI_ISL_1063558, EPI_ISL_1063559, EPI_ISL_1063560, EPI_ISL_1063561, EPI_ISL_1063562, EPI_ISL_1063563, EPI_ISL_1063564, EPI_ISL_1063565, EPI_ISL_1063566, EPI_ISL_1063567, EPI_ISL_1063568, EPI_ISL_1063569, EPI_ISL_1063570, EPI_ISL_1063571, EPI_ISL_1063572, EPI_ISL_1063573, EPI_ISL_1063574, EPI_ISL_1063575, EPI_ISL_1063576, EPI_ISL_1063577, EPI_ISL_1063578, EPI_ISL_1063579, EPI_ISL_1063580, EPI_ISL_1063581, EPI_ISL_1063582, EPI_ISL_1063583, EPI_ISL_1063584, EPI_ISL_1063585, EPI_ISL_1063586, EPI_ISL_1063587, EPI_ISL_1063588, EPI_ISL_1063589, EPI_ISL_1063590, EPI_ISL_1063591, EPI_ISL_1063592, EPI_ISL_1063593, EPI_ISL_1063594, EPI_ISL_1063595, EPI_ISL_1063596, EPI_ISL_1063597, EPI_ISL_1063598, EPI_ISL_1063599, EPI_ISL_1063600, EPI_ISL_1063601, EPI_ISL_1063602, EPI_ISL_1063603, EPI_ISL_1063604, EPI_ISL_1063605, EPI_ISL_1063606, EPI_ISL_1063607, EPI_ISL_1063608, EPI_ISL_1063609, EPI_ISL_1063610, EPI_ISL_1063611, EPI_ISL_1063612, EPI_ISL_1063613, EPI_ISL_1063614, EPI_ISL_1063615, EPI_ISL_1063616, EPI_ISL_1063617, EPI_ISL_1063618, EPI_ISL_1063619, EPI_ISL_1063620, EPI_ISL_1063621, EPI_ISL_1063622, EPI_ISL_1063623, EPI_ISL_1063624, EPI_ISL_1063625, EPI_ISL_1063626, EPI_ISL_1063628, EPI_ISL_1063629, EPI_ISL_1063630, EPI_ISL_1063631, EPI_ISL_1063632, EPI_ISL_1063633, EPI_ISL_1063634, EPI_ISL_1063635, EPI_ISL_1063636, EPI_ISL_1063637, EPI_ISL_1063638, EPI_ISL_1063639, EPI_ISL_1063640, EPI_ISL_1063641, EPI_ISL_1063642, EPI_ISL_1063643, EPI_ISL_1063644, EPI_ISL_1063645, EPI_ISL_1063646, EPI_ISL_1063647, EPI_ISL_1063648, EPI_ISL_1063649, EPI_ISL_1063650, EPI_ISL_1063651, EPI_ISL_1063652, EPI_ISL_1063653, EPI_ISL_1063654, EPI_ISL_1063655, EPI_ISL_1063656, EPI_ISL_1063657, EPI_ISL_1063658, EPI_ISL_1063659, EPI_ISL_1063660, EPI_ISL_1063661, EPI_ISL_1063662, EPI_ISL_1063663, EPI_ISL_1063664, EPI_ISL_1063665, EPI_ISL_1063666, EPI_ISL_1063667, EPI_ISL_1063668, EPI_ISL_1063669, EPI_ISL_1063670, EPI_ISL_1063671, EPI_ISL_1063672, EPI_ISL_1063673, EPI_ISL_1063674, EPI_ISL_1063675, EPI_ISL_1063676, EPI_ISL_1063677, EPI_ISL_1063678, EPI_ISL_1063679, EPI_ISL_1063680, EPI_ISL_1063681, EPI_ISL_1063682, EPI_ISL_1063683, EPI_ISL_1063684, EPI_ISL_1063685, EPI_ISL_1063686, EPI_ISL_1063687, EPI_ISL_1063688, EPI_ISL_1063689, EPI_ISL_1063690, EPI_ISL_1063691, EPI_ISL_1063692, EPI_ISL_1063693, EPI_ISL_1063694, EPI_ISL_1063695, EPI_ISL_1063696, EPI_ISL_1063697, EPI_ISL_1063698, EPI_ISL_1063699, EPI_ISL_1063700, EPI_ISL_1063701, EPI_ISL_1063702, EPI_ISL_1063703, EPI_ISL_1063704, EPI_ISL_1063705, EPI_ISL_1063706, EPI_ISL_1063707, EPI_ISL_1063708, EPI_ISL_1063709, EPI_ISL_1063710, EPI_ISL_1063711, EPI_ISL_1063712, EPI_ISL_1063713, EPI_ISL_1063714, EPI_ISL_1063715, EPI_ISL_1063716, EPI_ISL_1063717, EPI_ISL_1063718, EPI_ISL_1063719, EPI_ISL_1063720, EPI_ISL_1063721, EPI_ISL_1063722, EPI_ISL_1063723, EPI_ISL_1063724, EPI_ISL_1063725, EPI_ISL_1063726, EPI_ISL_1063727, EPI_ISL_1063728, EPI_ISL_1063729, EPI_ISL_1063730, EPI_ISL_1063731, EPI_ISL_1063732, EPI_ISL_1063733, EPI_ISL_1063734, EPI_ISL_1063735, EPI_ISL_1063736, EPI_ISL_1063737, EPI_ISL_1063738, EPI_ISL_1063739, EPI_ISL_1063740, EPI_ISL_1063741, EPI_ISL_1063742, EPI_ISL_1063743, EPI_ISL_1063744, EPI_ISL_1063745, EPI_ISL_1063746, EPI_ISL_1063747, EPI_ISL_1063748, EPI_ISL_1063749, EPI_ISL_1063750, EPI_ISL_1063751, EPI_ISL_1063752, EPI_ISL_1063753, EPI_ISL_1063754, EPI_ISL_1063755, EPI_ISL_1063756, EPI_ISL_1063757, EPI_ISL_1063758, EPI_ISL_1063759, EPI_ISL_1063760, EPI_ISL_1063761, EPI_ISL_1063762, EPI_ISL_1063763, EPI_ISL_1063764, EPI_ISL_1063765, EPI_ISL_1063766, EPI_ISL_1063767, EPI_ISL_1063768, EPI_ISL_1063769, EPI_ISL_1063770, EPI_ISL_1063771, EPI_ISL_1063772, EPI_ISL_1063773, EPI_ISL_1138900, EPI_ISL_1138901, EPI_ISL_1138902, EPI_ISL_1138903, EPI_ISL_1138904, EPI_ISL_1138905, EPI_ISL_1138906, EPI_ISL_1138907, EPI_ISL_1138908, EPI_ISL_1138909, EPI_ISL_1138910, EPI_ISL_1138911, EPI_ISL_1138912, EPI_ISL_1138913, EPI_ISL_1138914, EPI_ISL_1138915, EPI_ISL_1138916, EPI_ISL_1138917, EPI_ISL_1138918, EPI_ISL_1138919, EPI_ISL_1138920, EPI_ISL_1138921, EPI_ISL_1138922, EPI_ISL_1138923, EPI_ISL_1138924, EPI_ISL_1138925, EPI_ISL_1138926, EPI_ISL_1138927, EPI_ISL_1138928, EPI_ISL_1138929, EPI_ISL_1138930, EPI_ISL_1138931, EPI_ISL_1138932, EPI_ISL_1138933, EPI_ISL_1138934, EPI_ISL_1138935, EPI_ISL_1138936, EPI_ISL_1138937, EPI_ISL_1138938, EPI_ISL_1138939, EPI_ISL_1138940, EPI_ISL_1138941, EPI_ISL_1138942, EPI_ISL_1138943, EPI_ISL_1138944, EPI_ISL_1138945, EPI_ISL_1138946, EPI_ISL_1138947, EPI_ISL_1138948, EPI_ISL_1138949, EPI_ISL_1138950, EPI_ISL_1138951, EPI_ISL_1138952, EPI_ISL_1138953, EPI_ISL_1138954, EPI_ISL_1138955, EPI_ISL_1138956, EPI_ISL_1138957, EPI_ISL_1138958, EPI_ISL_1138959, EPI_ISL_1138960, EPI_ISL_1138961, EPI_ISL_1138962, EPI_ISL_1138963, EPI_ISL_1138964, EPI_ISL_1138965, EPI_ISL_1138966, EPI_ISL_1138967, EPI_ISL_1138968, EPI_ISL_1138970, EPI_ISL_1138971, EPI_ISL_1138972, EPI_ISL_1138973, EPI_ISL_1138974, EPI_ISL_1138975, EPI_ISL_1138976, EPI_ISL_1138977, EPI_ISL_1138978, EPI_ISL_1138979, EPI_ISL_1138980, EPI_ISL_1138981, EPI_ISL_1138982, EPI_ISL_1165001, EPI_ISL_1165002, EPI_ISL_1165003, EPI_ISL_1165004, EPI_ISL_1165005, EPI_ISL_1165006, EPI_ISL_1165007, EPI_ISL_1165008, EPI_ISL_1165009, EPI_ISL_1165010, EPI_ISL_1165011, EPI_ISL_1165012, EPI_ISL_1165013, EPI_ISL_1165014, EPI_ISL_1165015, EPI_ISL_1165016, EPI_ISL_1165017, EPI_ISL_1165018, EPI_ISL_1165019, EPI_ISL_1165020, EPI_ISL_1165021, EPI_ISL_1165022, EPI_ISL_1165023, EPI_ISL_1165024, EPI_ISL_1165025, EPI_ISL_1165026, EPI_ISL_1165027, EPI_ISL_1165028, EPI_ISL_1165029, EPI_ISL_1165030, EPI_ISL_1165031, EPI_ISL_1165032, EPI_ISL_1165033, EPI_ISL_1165034, EPI_ISL_1165035, EPI_ISL_1165036, EPI_ISL_1165037, EPI_ISL_1165038, EPI_ISL_1165039, EPI_ISL_1165040, EPI_ISL_1165041, EPI_ISL_1165042, EPI_ISL_1165043, EPI_ISL_1165044, EPI_ISL_1165045, EPI_ISL_1165046, EPI_ISL_1165047, EPI_ISL_1165048, EPI_ISL_1165049, EPI_ISL_1165050, EPI_ISL_1165051, EPI_ISL_1165052, EPI_ISL_1165053, EPI_ISL_1165054, EPI_ISL_1165055, EPI_ISL_1165056, EPI_ISL_1165057, EPI_ISL_1165058, EPI_ISL_1165059, EPI_ISL_1165060, EPI_ISL_1165061, EPI_ISL_1165062, EPI_ISL_1165063, EPI_ISL_1165064, EPI_ISL_1165065, EPI_ISL_1209021, EPI_ISL_1209408, EPI_ISL_1209409, EPI_ISL_1209410, EPI_ISL_1209411, EPI_ISL_1209412, EPI_ISL_1209413, EPI_ISL_1209414, EPI_ISL_1209415, EPI_ISL_1209416, EPI_ISL_1209417, EPI_ISL_1209418, EPI_ISL_1209419, EPI_ISL_1209420, EPI_ISL_1209421, EPI_ISL_1209422, EPI_ISL_1209423, EPI_ISL_1209424, EPI_ISL_1209425, EPI_ISL_1209426, EPI_ISL_1209427, EPI_ISL_1209428, EPI_ISL_1209429, EPI_ISL_1209430, EPI_ISL_1209431, EPI_ISL_1209432, EPI_ISL_1209433, EPI_ISL_1209434, EPI_ISL_1209435, EPI_ISL_1209436, EPI_ISL_1209437, EPI_ISL_1209438, EPI_ISL_1209439, EPI_ISL_1209440, EPI_ISL_1209441, EPI_ISL_1209442, EPI_ISL_1209443, EPI_ISL_1209444, EPI_ISL_1209445, EPI_ISL_1209446, EPI_ISL_1209447, EPI_ISL_1209448, EPI_ISL_1209449, EPI_ISL_1209450, EPI_ISL_1209451, EPI_ISL_1209452, EPI_ISL_1228588, EPI_ISL_1228589, EPI_ISL_1228590, EPI_ISL_1228591, EPI_ISL_1228592, EPI_ISL_1228593, EPI_ISL_1228594, EPI_ISL_1228595, EPI_ISL_1228596, EPI_ISL_1228597, EPI_ISL_1228598, EPI_ISL_1228599, EPI_ISL_1228600, EPI_ISL_1228601, EPI_ISL_1228602, EPI_ISL_1228603, EPI_ISL_1228604, EPI_ISL_1228605, EPI_ISL_1228606, EPI_ISL_1228607, EPI_ISL_1228608, EPI_ISL_1228609, EPI_ISL_1228610, EPI_ISL_1228611, EPI_ISL_1228612, EPI_ISL_1228613, EPI_ISL_1228614, EPI_ISL_1228615, EPI_ISL_1228616, EPI_ISL_1228617, EPI_ISL_1228618, EPI_ISL_1228619, EPI_ISL_1228620, EPI_ISL_1228621, EPI_ISL_1228622, EPI_ISL_1228623, EPI_ISL_1228624, EPI_ISL_1228625, EPI_ISL_1228626, EPI_ISL_1228627, EPI_ISL_1228628, EPI_ISL_1228629, EPI_ISL_1228630, EPI_ISL_1228631, EPI_ISL_1228632, EPI_ISL_1228633, EPI_ISL_1228634, EPI_ISL_1228635, EPI_ISL_1228636, EPI_ISL_1228637, EPI_ISL_1228638, EPI_ISL_1228639, EPI_ISL_1228640, EPI_ISL_1228641, EPI_ISL_1228642, EPI_ISL_1228643, EPI_ISL_1228644, EPI_ISL_1228645, EPI_ISL_1228646, EPI_ISL_1228647, EPI_ISL_1228648, EPI_ISL_1228649, EPI_ISL_1228650, EPI_ISL_1228651, EPI_ISL_1228652, EPI_ISL_1228653, EPI_ISL_1228654, EPI_ISL_1228655, EPI_ISL_1228656, EPI_ISL_1228657, EPI_ISL_1228658, EPI_ISL_1228659, EPI_ISL_1228660, EPI_ISL_1228661,			

EPI_ISL_1228662, EPI_ISL_1228663, EPI_ISL_1228664, EPI_ISL_1252366, EPI_ISL_1252367, EPI_ISL_1252368, EPI_ISL_1252369, EPI_ISL_1252370, EPI_ISL_1252371, EPI_ISL_1252372, EPI_ISL_1252373, EPI_ISL_1252374, EPI_ISL_1252375, EPI_ISL_1252376, EPI_ISL_1252377, EPI_ISL_1252378, EPI_ISL_1252379, EPI_ISL_1252380, EPI_ISL_1252381, EPI_ISL_1252382, EPI_ISL_1252383, EPI_ISL_1252384,
EPI_ISL_1252385, EPI_ISL_1252386, EPI_ISL_1252387, EPI_ISL_1252388, EPI_ISL_1252389, EPI_ISL_1252390, EPI_ISL_1252391, EPI_ISL_1252392, EPI_ISL_1252393, EPI_ISL_1252394, EPI_ISL_1252395, EPI_ISL_1252396, EPI_ISL_1252397, EPI_ISL_1252398, EPI_ISL_1252399, EPI_ISL_1252400, EPI_ISL_1252401, EPI_ISL_1252402, EPI_ISL_1252403, EPI_ISL_1252404, EPI_ISL_1252405, EPI_ISL_1252406,
EPI_ISL_1252407, EPI_ISL_1252408, EPI_ISL_1252409, EPI_ISL_1252410, EPI_ISL_1252411, EPI_ISL_1252412, EPI_ISL_1252413, EPI_ISL_1252414, EPI_ISL_1252415, EPI_ISL_1252416, EPI_ISL_1252417, EPI_ISL_1252418, EPI_ISL_1252419, EPI_ISL_1252420, EPI_ISL_1252421, EPI_ISL_1252422, EPI_ISL_1252423, EPI_ISL_1252424, EPI_ISL_1252425, EPI_ISL_1252426, EPI_ISL_1252427, EPI_ISL_1252428,
EPI_ISL_1252429, EPI_ISL_1252430, EPI_ISL_1252431, EPI_ISL_1252432, EPI_ISL_1252433, EPI_ISL_1252434, EPI_ISL_1252435, EPI_ISL_1252436, EPI_ISL_1252437, EPI_ISL_1252438, EPI_ISL_1252439, EPI_ISL_1252440, EPI_ISL_1252441, EPI_ISL_1252442, EPI_ISL_1252443, EPI_ISL_1252444, EPI_ISL_1252445, EPI_ISL_1315328, EPI_ISL_1315329, EPI_ISL_1315330, EPI_ISL_1315331, EPI_ISL_1315332,
EPI_ISL_1315333, EPI_ISL_1315334, EPI_ISL_1315335, EPI_ISL_1315336, EPI_ISL_1315337, EPI_ISL_1315338, EPI_ISL_1315339, EPI_ISL_1315340, EPI_ISL_1315341, EPI_ISL_1315342, EPI_ISL_1315343, EPI_ISL_1315344, EPI_ISL_1315345, EPI_ISL_1315346, EPI_ISL_1315347, EPI_ISL_1315348, EPI_ISL_1315349, EPI_ISL_1315350, EPI_ISL_1315351, EPI_ISL_1315352, EPI_ISL_1315353, EPI_ISL_1315354,
EPI_ISL_1315355, EPI_ISL_1315356, EPI_ISL_1315357, EPI_ISL_1315358, EPI_ISL_1315359, EPI_ISL_1315360, EPI_ISL_1315361, EPI_ISL_1315362, EPI_ISL_1315363, EPI_ISL_1315364, EPI_ISL_1315365, EPI_ISL_1315366, EPI_ISL_1315367, EPI_ISL_1315368, EPI_ISL_1315369, EPI_ISL_1315370, EPI_ISL_1315371, EPI_ISL_1315372, EPI_ISL_1315373, EPI_ISL_1315374, EPI_ISL_1315375, EPI_ISL_1315376,
EPI_ISL_1315377, EPI_ISL_1315378, EPI_ISL_1315379, EPI_ISL_1315380, EPI_ISL_1315381, EPI_ISL_1315382, EPI_ISL_1315383, EPI_ISL_1315384, EPI_ISL_1315385, EPI_ISL_1315386, EPI_ISL_1315387, EPI_ISL_1315388, EPI_ISL_1315389, EPI_ISL_1315390, EPI_ISL_1315391, EPI_ISL_1315392, EPI_ISL_1315393, EPI_ISL_1315394, EPI_ISL_1315395, EPI_ISL_1315396, EPI_ISL_1315397, EPI_ISL_1315398,
EPI_ISL_1315399, EPI_ISL_1315400, EPI_ISL_1315401, EPI_ISL_1315402, EPI_ISL_1315403, EPI_ISL_1315404, EPI_ISL_1315405, EPI_ISL_1315406, EPI_ISL_1315407, EPI_ISL_1315408, EPI_ISL_1315409, EPI_ISL_1315410, EPI_ISL_1315411, EPI_ISL_1315412, EPI_ISL_1315413, EPI_ISL_1315414, EPI_ISL_1315415, EPI_ISL_1315416, EPI_ISL_1315417, EPI_ISL_1315418, EPI_ISL_1315419, EPI_ISL_1315420,
EPI_ISL_1318989, EPI_ISL_1318990, EPI_ISL_1318991, EPI_ISL_1318992, EPI_ISL_1318993, EPI_ISL_1318994, EPI_ISL_1318995, EPI_ISL_1318996, EPI_ISL_1318997, EPI_ISL_1318998, EPI_ISL_1318999, EPI_ISL_1319000, EPI_ISL_1319001, EPI_ISL_1319002, EPI_ISL_1319003, EPI_ISL_1319004, EPI_ISL_1319005, EPI_ISL_1319006, EPI_ISL_1319007, EPI_ISL_1319008, EPI_ISL_1319009, EPI_ISL_1319010,
EPI_ISL_1319011, EPI_ISL_1319012, EPI_ISL_1319013, EPI_ISL_1319014, EPI_ISL_1319015, EPI_ISL_1319016, EPI_ISL_1319017, EPI_ISL_1319018, EPI_ISL_1319019, EPI_ISL_1319020, EPI_ISL_1319021, EPI_ISL_1319022, EPI_ISL_1319023, EPI_ISL_1319024, EPI_ISL_1319025, EPI_ISL_1319026, EPI_ISL_1319027, EPI_ISL_1319028, EPI_ISL_1319029, EPI_ISL_1319030, EPI_ISL_1319031, EPI_ISL_1319032,
EPI_ISL_1319033, EPI_ISL_1319034, EPI_ISL_1319035, EPI_ISL_1319036, EPI_ISL_1319037, EPI_ISL_1319038, EPI_ISL_1319039, EPI_ISL_1319040, EPI_ISL_1319041, EPI_ISL_1489527, EPI_ISL_1489529, EPI_ISL_1489530, EPI_ISL_1489531, EPI_ISL_1489532, EPI_ISL_1489533, EPI_ISL_1489534, EPI_ISL_1489535, EPI_ISL_1489536, EPI_ISL_1489537, EPI_ISL_1489538, EPI_ISL_1489539, EPI_ISL_1489540,
EPI_ISL_1489541, EPI_ISL_1489542, EPI_ISL_1489543, EPI_ISL_1489544, EPI_ISL_1489545, EPI_ISL_1489546, EPI_ISL_1489547, EPI_ISL_1489548, EPI_ISL_1489549, EPI_ISL_1489550, EPI_ISL_1489551, EPI_ISL_1489552, EPI_ISL_1489553, EPI_ISL_1489554, EPI_ISL_1489555, EPI_ISL_1489556, EPI_ISL_1489557, EPI_ISL_1489558, EPI_ISL_1489559, EPI_ISL_1489560, EPI_ISL_1489561, EPI_ISL_1489562,
EPI_ISL_1489563, EPI_ISL_1489564, EPI_ISL_1489565, EPI_ISL_1489566, EPI_ISL_1489567, EPI_ISL_1489568, EPI_ISL_1489569, EPI_ISL_1489570, EPI_ISL_1489571, EPI_ISL_1489572, EPI_ISL_1489573, EPI_ISL_1489574, EPI_ISL_1489575, EPI_ISL_1489576, EPI_ISL_1489577, EPI_ISL_1489578, EPI_ISL_1489579, EPI_ISL_1489580, EPI_ISL_1489581, EPI_ISL_1489582, EPI_ISL_1489583, EPI_ISL_1489584,
EPI_ISL_1489585, EPI_ISL_1489586, EPI_ISL_1489587, EPI_ISL_1489588, EPI_ISL_1489589, EPI_ISL_1489590, EPI_ISL_1489591, EPI_ISL_1489592, EPI_ISL_1489593, EPI_ISL_1489594, EPI_ISL_1489595, EPI_ISL_1489596, EPI_ISL_1489597, EPI_ISL_1489598, EPI_ISL_1489599, EPI_ISL_1489600, EPI_ISL_1489601, EPI_ISL_1489602, EPI_ISL_1489603, EPI_ISL_1489604, EPI_ISL_1489605, EPI_ISL_1489606,
EPI_ISL_1489607, EPI_ISL_1489608, EPI_ISL_1489609, EPI_ISL_1489610, EPI_ISL_1489611, EPI_ISL_1489612, EPI_ISL_1489613, EPI_ISL_1489614, EPI_ISL_1489615, EPI_ISL_1489616, EPI_ISL_1489617, EPI_ISL_1489618, EPI_ISL_1489619, EPI_ISL_1489620, EPI_ISL_1489621, EPI_ISL_1489622, EPI_ISL_1489623, EPI_ISL_1489624, EPI_ISL_1489625, EPI_ISL_1489626, EPI_ISL_1489627, EPI_ISL_1489628,
EPI_ISL_1489629, EPI_ISL_1489630, EPI_ISL_1489631, EPI_ISL_1489632, EPI_ISL_1489633, EPI_ISL_1489634, EPI_ISL_1489635, EPI_ISL_1489636, EPI_ISL_1489637, EPI_ISL_1489638, EPI_ISL_1489639, EPI_ISL_1489640, EPI_ISL_1489641, EPI_ISL_1489642, EPI_ISL_1489643, EPI_ISL_1489644, EPI_ISL_1489645, EPI_ISL_1489646, EPI_ISL_1489647, EPI_ISL_1489648, EPI_ISL_1489649, EPI_ISL_1489650,
EPI_ISL_1489651, EPI_ISL_1489652, EPI_ISL_1489653, EPI_ISL_1489654, EPI_ISL_1489655, EPI_ISL_1489656, EPI_ISL_1489942, EPI_ISL_1489943, EPI_ISL_1489944, EPI_ISL_1489945, EPI_ISL_1489946, EPI_ISL_1489947, EPI_ISL_1489948, EPI_ISL_1489949, EPI_ISL_1489950, EPI_ISL_1489951, EPI_ISL_1489952, EPI_ISL_1489953, EPI_ISL_1489954, EPI_ISL_1489955, EPI_ISL_1489956, EPI_ISL_1489957,
EPI_ISL_1489958, EPI_ISL_1489959, EPI_ISL_1489960, EPI_ISL_1489961, EPI_ISL_1489962, EPI_ISL_1489963, EPI_ISL_1489964, EPI_ISL_1489965, EPI_ISL_1489966, EPI_ISL_1489967, EPI_ISL_1489968, EPI_ISL_1489969, EPI_ISL_1489970, EPI_ISL_1489971, EPI_ISL_1489972, EPI_ISL_1489973, EPI_ISL_1489974, EPI_ISL_1489975, EPI_ISL_1489976, EPI_ISL_1489977, EPI_ISL_1489978, EPI_ISL_1489979,
EPI_ISL_1489980, EPI_ISL_1489981, EPI_ISL_1489982, EPI_ISL_1489983, EPI_ISL_1489984, EPI_ISL_1489985, EPI_ISL_1489986, EPI_ISL_1489987, EPI_ISL_1489988, EPI_ISL_1489989, EPI_ISL_1489990, EPI_ISL_1489991, EPI_ISL_1489992, EPI_ISL_1489993, EPI_ISL_1489994, EPI_ISL_1489995, EPI_ISL_1489996, EPI_ISL_1489997, EPI_ISL_1489998, EPI_ISL_1489999, EPI_ISL_1490000, EPI_ISL_1490001,
EPI_ISL_1490002, EPI_ISL_1490003, EPI_ISL_1490004, EPI_ISL_1490005, EPI_ISL_1490006, EPI_ISL_1490007, EPI_ISL_1490008, EPI_ISL_1490009, EPI_ISL_1490010, EPI_ISL_1490011, EPI_ISL_1490012, EPI_ISL_1490013, EPI_ISL_1490014, EPI_ISL_1490015, EPI_ISL_1490016, EPI_ISL_1490017, EPI_ISL_1490018, EPI_ISL_1490019, EPI_ISL_1490020, EPI_ISL_1490021, EPI_ISL_1490022, EPI_ISL_1490023,
EPI_ISL_1490024, EPI_ISL_1490025, EPI_ISL_1490026, EPI_ISL_1490027, EPI_ISL_1490028, EPI_ISL_1490029, EPI_ISL_1490030, EPI_ISL_1490031, EPI_ISL_1490032, EPI_ISL_1490033, EPI_ISL_1490034, EPI_ISL_1490035, EPI_ISL_1490036, EPI_ISL_1490037, EPI_ISL_1490038, EPI_ISL_1490039, EPI_ISL_1490040, EPI_ISL_1490041, EPI_ISL_1490042, EPI_ISL_1490043, EPI_ISL_1490044, EPI_ISL_1490045,
EPI_ISL_1490046, EPI_ISL_1490047, EPI_ISL_1490048, EPI_ISL_1490049, EPI_ISL_1490050, EPI_ISL_1490051, EPI_ISL_1490052, EPI_ISL_1490053, EPI_ISL_1490054, EPI_ISL_1490055, EPI_ISL_1490056, EPI_ISL_1490057, EPI_ISL_1490058, EPI_ISL_1490059, EPI_ISL_1490060, EPI_ISL_1490061, EPI_ISL_1490062, EPI_ISL_1490063, EPI_ISL_1490064, EPI_ISL_1490065, EPI_ISL_1490066, EPI_ISL_1490067,
EPI_ISL_1490068, EPI_ISL_1490069, EPI_ISL_1490070, EPI_ISL_1490071, EPI_ISL_1490072, EPI_ISL_1490073, EPI_ISL_1490074, EPI_ISL_1490075, EPI_ISL_1490076, EPI_ISL_1490077, EPI_ISL_1490078, EPI_ISL_1490079, EPI_ISL_1490080, EPI_ISL_1490081, EPI_ISL_1490082, EPI_ISL_1490083, EPI_ISL_1490084, EPI_ISL_1490085, EPI_ISL_1490086, EPI_ISL_1490087, EPI_ISL_1490088, EPI_ISL_1490089,
EPI_ISL_1490090, EPI_ISL_1490091, EPI_ISL_1490092, EPI_ISL_1490093, EPI_ISL_1490094, EPI_ISL_1490095, EPI_ISL_1490096, EPI_ISL_1490097, EPI_ISL_1490098, EPI_ISL_1490099, EPI_ISL_1490100, EPI_ISL_1490101, EPI_ISL_1490102, EPI_ISL_1490103, EPI_ISL_1490104, EPI_ISL_1490105, EPI_ISL_1490106, EPI_ISL_1490107, EPI_ISL_1490108, EPI_ISL_1490109, EPI_ISL_1490110, EPI_ISL_1490111,
EPI_ISL_1490112, EPI_ISL_1490113, EPI_ISL_1490114, EPI_ISL_1490115, EPI_ISL_1490116, EPI_ISL_1490117, EPI_ISL_1490118, EPI_ISL_1490119, EPI_ISL_1490120, EPI_ISL_1490121, EPI_ISL_1490122, EPI_ISL_1490123, EPI_ISL_1490124, EPI_ISL_1490125, EPI_ISL_1490126, EPI_ISL_1490127, EPI_ISL_1490128, EPI_ISL_1490129, EPI_ISL_1490130, EPI_ISL_1490131, EPI_ISL_1490132, EPI_ISL_1490133,
EPI_ISL_1490134, EPI_ISL_1490135, EPI_ISL_1490136, EPI_ISL_1490137, EPI_ISL_1490138, EPI_ISL_1490139, EPI_ISL_1490140, EPI_ISL_1490141, EPI_ISL_1490142, EPI_ISL_1490143, EPI_ISL_1490144, EPI_ISL_1490145, EPI_ISL_1490146, EPI_ISL_1490147, EPI_ISL_1490148, EPI_ISL_1490149, EPI_ISL_1490150, EPI_ISL_1490151, EPI_ISL_1490152, EPI_ISL_1490153, EPI_ISL_1490154, EPI_ISL_1490155,
EPI_ISL_1490156, EPI_ISL_1490157, EPI_ISL_1490158, EPI_ISL_1490159, EPI_ISL_1490160, EPI_ISL_1490161, EPI_ISL_1490162, EPI_ISL_1490163, EPI_ISL_1490164, EPI_ISL_1490167, EPI_ISL_1490168, EPI_ISL_1490169, EPI_ISL_1490170, EPI_ISL_1490171, EPI_ISL_1490172, EPI_ISL_1490173, EPI_ISL_1490174, EPI_ISL_1490175, EPI_ISL_1490176, EPI_ISL_1490177, EPI_ISL_1490178, EPI_ISL_1490179,
EPI_ISL_1490180, EPI_ISL_1490181, EPI_ISL_1490182, EPI_ISL_1490183, EPI_ISL_1490184, EPI_ISL_1587452, EPI_ISL_1587453, EPI_ISL_1587454, EPI_ISL_1587455, EPI_ISL_1587456, EPI_ISL_1587457, EPI_ISL_1587458, EPI_ISL_1587459, EPI_ISL_1587460, EPI_ISL_1587461, EPI_ISL_1587462, EPI_ISL_1587463, EPI_ISL_1587464, EPI_ISL_1587465, EPI_ISL_1587466, EPI_ISL_1587467, EPI_ISL_1587468,
EPI_ISL_1587469, EPI_ISL_1587470, EPI_ISL_1587471, EPI_ISL_1587472, EPI_ISL_1587473, EPI_ISL_1587474, EPI_ISL_1587475, EPI_ISL_1587476, EPI_ISL_1587477, EPI_ISL_1587478, EPI_ISL_1587479, EPI_ISL_1587480, EPI_ISL_1587481, EPI_ISL_1587482, EPI_ISL_1587483, EPI_ISL_1587484, EPI_ISL_1587485, EPI_ISL_1587486, EPI_ISL_1587487, EPI_ISL_1587488, EPI_ISL_1587489, EPI_ISL_1587490,
EPI_ISL_1587491, EPI_ISL_1587492, EPI_ISL_1587493, EPI_ISL_1587494, EPI_ISL_1587495, EPI_ISL_1587496, EPI_ISL_1587497, EPI_ISL_1587498, EPI_ISL_1587499, EPI_ISL_1587500, EPI_ISL_1587501, EPI_ISL_1587502, EPI_ISL_1587503, EPI_ISL_1587504, EPI_ISL_1587505, EPI_ISL_1587506, EPI_ISL_1587507, EPI_ISL_1587508, EPI_ISL_1587509, EPI_ISL_1587510, EPI_ISL_1587511, EPI_ISL_1587512,
EPI_ISL_1587513, EPI_ISL_1587514, EPI_ISL_1587515, EPI_ISL_1587516, EPI_ISL_1587517, EPI_ISL_1587518, EPI_ISL_1587519, EPI_ISL_1587520, EPI_ISL_1587521, EPI_ISL_1587522, EPI_ISL_1587523, EPI_ISL_1587524, EPI_ISL_1587525, EPI_ISL_1587526, EPI_ISL_1587527, EPI_ISL_1587528, EPI_ISL_1587529, EPI_ISL_1587530, EPI_ISL_1587531, EPI_ISL_1587532, EPI_ISL_1587533, EPI_ISL_1587534,
EPI_ISL_1587535, EPI_ISL_1587536, EPI_ISL_1587537, EPI_ISL_1587538, EPI_ISL_1587539, EPI_ISL_1587540, EPI_ISL_1587541, EPI_ISL_1587542, EPI_ISL_1587543, EPI_ISL_1587544, EPI_ISL_1587545, EPI_ISL_1587546, EPI_ISL_1587547, EPI_ISL_1587548, EPI_ISL_1587549, EPI_ISL_1587550, EPI_ISL_1622336, EPI_ISL_1622337, EPI_ISL_1622338, EPI_ISL_1622339, EPI_ISL_1622340, EPI_ISL_1622341,
EPI_ISL_1622342, EPI_ISL_1622343, EPI_ISL_1622344, EPI_ISL_1622345, EPI_ISL_1622346, EPI_ISL_1622347, EPI_ISL_1622348, EPI_ISL_1622349, EPI_ISL_1622350, EPI_ISL_1622351, EPI_ISL_1622352, EPI_ISL_1622353, EPI_ISL_1622354, EPI_ISL_1622355, EPI_ISL_1622356, EPI_ISL_1622357, EPI_ISL_1622358, EPI_ISL_1622436, EPI_ISL_1622437, EPI_ISL_1622438, EPI_ISL_1622439, EPI_ISL_1622440,
EPI_ISL_1622441, EPI_ISL_1622442, EPI_ISL_1622443, EPI_ISL_1622444, EPI_ISL_1622445, EPI_ISL_1622446, EPI_ISL_1622447, EPI_ISL_1622448, EPI_ISL_1622449, EPI_ISL_1622450, EPI_ISL_1622451, EPI_ISL_1622452, EPI_ISL_1622453, EPI_ISL_1622454, EPI_ISL_1622455, EPI_ISL_1622456, EPI_ISL_1622457, EPI_ISL_1622458, EPI_ISL_1622459, EPI_ISL_1622460, EPI_ISL_1622461, EPI_ISL_1622462,
EPI_ISL_1622463, EPI_ISL_1622464, EPI_ISL_1622465, EPI_ISL_1622466, EPI_ISL_1622467, EPI_ISL_1622468, EPI_ISL_1622469, EPI_ISL_1622470, EPI_ISL_1622471, EPI_ISL_1622472, EPI_ISL_1622473, EPI_ISL_1622474, EPI_ISL_1622475, EPI_ISL_1622476, EPI_ISL_1622477, EPI_ISL_1622478, EPI_ISL_1622479, EPI_ISL_1622480, EPI_ISL_1622481, EPI_ISL_1622482, EPI_ISL_1622483, EPI_ISL_1622484,
EPI_ISL_1622485, EPI_ISL_1622486, EPI_ISL_1622487, EPI_ISL_1622488, EPI_ISL_1622489, EPI_ISL_1622490, EPI_ISL_1622491, EPI_ISL_1622492, EPI_ISL_1622493, EPI_ISL_1622494, EPI_ISL_1622495, EPI_ISL_1622496, EPI_ISL_1622497, EPI_ISL_1622498, EPI_ISL_1622499, EPI_ISL_1645676, EPI_ISL_1645683, EPI_ISL_1645685, EPI_ISL_1645687, EPI_ISL_1645689, EPI_ISL_1645691, EPI_ISL_1645693,
EPI_ISL_1645695, EPI_ISL_1645697, EPI_ISL_1645699, EPI_ISL_1645701, EPI_ISL_1645702, EPI_ISL_1645704, EPI_ISL_1645706, EPI_ISL_1645712, EPI_ISL_1645714, EPI_ISL_1645716, EPI_ISL_1645718, EPI_ISL_1645720, EPI_ISL_1645722, EPI_ISL_1645724, EPI_ISL_1645726, EPI_ISL_1645728, EPI_ISL_1645730, EPI_ISL_1645732, EPI_ISL_1645734, EPI_ISL_1645735, EPI_ISL_1645737, EPI_ISL_1645739,
EPI_ISL_1645741, EPI_ISL_1645743, EPI_ISL_1645745, EPI_ISL_1645747, EPI_ISL_1645754, EPI_ISL_1645756, EPI_ISL_1645758, EPI_ISL_1645760, EPI_ISL_1645761, EPI_ISL_1645763, EPI_ISL_1645765, EPI_ISL_1645766, EPI_ISL_1645768, EPI_ISL_1645770, EPI_ISL_1645771, EPI_ISL_1645773, EPI_ISL_1645775, EPI_ISL_1645777, EPI_ISL_1645779, EPI_ISL_1645781, EPI_ISL_1645783, EPI_ISL_1645785,
EPI_ISL_1645787, EPI_ISL_1645795, EPI_ISL_1645797, EPI_ISL_1645798, EPI_ISL_1645800, EPI_ISL_1645802, EPI_ISL_1645804, EPI_ISL_1645806, EPI_ISL_1645808, EPI_ISL_1645810, EPI_ISL_1645812, EPI_ISL_1645814, EPI_ISL_1645816, EPI_ISL_1645818, EPI_ISL_1645820, EPI_ISL_1645822, EPI_ISL_1645824, EPI_ISL_1645826, EPI_ISL_1645828, EPI_ISL_1645830, EPI_ISL_1645832, EPI_ISL_1645833,
EPI_ISL_1645835, EPI_ISL_1645837, EPI_ISL_1645839, EPI_ISL_1645841, EPI_ISL_1645843, EPI_ISL_1645845, EPI_ISL_1645847, EPI_ISL_1645849, EPI_ISL_1645851, EPI_ISL_1645852, EPI_ISL_1645855, EPI_ISL_1645857, EPI_ISL_1645864, EPI_ISL_1645866, EPI_ISL_1645867, EPI_ISL_1645868, EPI_ISL_1645869, EPI_ISL_1645870, EPI_ISL_1645872, EPI_ISL_1645874, EPI_ISL_1645881, EPI_ISL_1645883,
EPI_ISL_1645885, EPI_ISL_1645887, EPI_ISL_1645889, EPI_ISL_1645891, EPI_ISL_1645893, EPI_ISL_1645895, EPI_ISL_1645897, EPI_ISL_1645898, EPI_ISL_1645900, EPI_ISL_1645903, EPI_ISL_1645905, EPI_ISL_1645906, EPI_ISL_1645908, EPI_ISL_1645910, EPI_ISL_1645912, EPI_ISL_1645914, EPI_ISL_1645916, EPI_ISL_1645918, EPI_ISL_1645920, EPI_ISL_1645923, EPI_ISL_1645925, EPI_ISL_1645927,
EPI_ISL_1645928, EPI_ISL_1645930, EPI_ISL_1645932, EPI_ISL_1645934, EPI_ISL_1645936, EPI_ISL_1645938, EPI_ISL_1645940, EPI_ISL_1645942, EPI_ISL_1645943, EPI_ISL_1645945, EPI_ISL_1645947, EPI_ISL_1645949, EPI_ISL_1645951, EPI_ISL_1645953, EPI_ISL_1645955, EPI_ISL_1645957, EPI_ISL_1645959, EPI_ISL_1645961, EPI_ISL_1645962, EPI_ISL_1645964, EPI_ISL_1645966, EPI_ISL_1645968,
EPI_ISL_1645970, EPI_ISL_1645972, EPI_ISL_1645974, EPI_ISL_1645976, EPI_ISL_1645978, EPI_ISL_1645980, EPI_ISL_1645982, EPI_ISL_1645983, EPI_ISL_1645985, EPI_ISL_1645987, EPI_ISL_1645989, EPI_ISL_1645991, EPI_ISL_1645993, EPI_ISL_1645996, EPI_ISL_1646002, EPI_ISL_1646004, EPI_ISL_1646006, EPI_ISL_1646008, EPI_ISL_1646010, EPI_ISL_1646012, EPI_ISL_1646014, EPI_ISL_1646016,
EPI_ISL_1646018, EPI_ISL_1646020, EPI_ISL_1646022, EPI_ISL_1646023, EPI_ISL_1646026, EPI_ISL_1646028, EPI_ISL_1646029, EPI_ISL_1646032, EPI_ISL_1646033, EPI_ISL_1646035, EPI_ISL_1646037, EPI_ISL_1646038, EPI_ISL_1646041, EPI_ISL_1646042, EPI_ISL_1646044, EPI_ISL_1646046, EPI_ISL_1646048, EPI_ISL_1646050, EPI_ISL_1646052, EPI_ISL_1646054, EPI_ISL_1646056, EPI_ISL_1646058,
EPI_ISL_1646060, EPI_ISL_1646062, EPI_ISL_1646064, EPI_ISL_1646065, EPI_ISL_1646067, EPI_ISL_1646069, EPI_ISL_1646071, EPI_ISL_1646073, EPI_ISL_1646079, EPI_ISL_1646081, EPI_ISL_1646083, EPI_ISL_1646085, EPI_ISL_1646087, EPI_ISL_1646089, EPI_ISL_1646090, EPI_ISL_1646097, EPI_ISL_1646098, EPI_ISL_1646100, EPI_ISL_1646102, EPI_ISL_1646104, EPI_ISL_1646106, EPI_ISL_1646108,
EPI_ISL_1646110, EPI_ISL_1646112, EPI_ISL_1646114, EPI_ISL_1646116, EPI_ISL_1646118, EPI_ISL_1646119, EPI_ISL_1646121, EPI_ISL_1646123, EPI_ISL_1646125, EPI_ISL_1646127, EPI_ISL_1646129, EPI_ISL_1646131, EPI_ISL_1646133, EPI_ISL_1646135, EPI_ISL_1646137, EPI_ISL_1646139, EPI_ISL_1646141, EPI_ISL_1646143, EPI_ISL_1646145, EPI_ISL_1646147, EPI_ISL_1646149, EPI_ISL_1646151,
EPI_ISL_1646153, EPI_ISL_1646154, EPI_ISL_1646157, EPI_ISL_1646158, EPI_ISL_1646160, EPI_ISL_1646162, EPI_ISL_1646164, EPI_ISL_1646166, EPI_ISL_1646168, EPI_ISL_1646170, EPI_ISL_1646172, EPI_ISL_1646174, EPI_ISL_1646176, EPI_ISL_1646178, EPI_ISL_1646179, EPI_ISL_1646182, EPI_ISL_1646184, EPI_ISL_1646186, EPI_ISL_1646187, EPI_ISL_1646189, EPI_ISL_1646191, EPI_ISL_1646193,
EPI_ISL_1646195, EPI_ISL_1646197, EPI_ISL_1646199, EPI_ISL_1646201, EPI_ISL_1646203, EPI_ISL_1646204, EPI_ISL_1646207, EPI_ISL_1646208, EPI_ISL_1646210, EPI_ISL_1646212, EPI_ISL_1646214, EPI_ISL_1646216, EPI_ISL_1646218, EPI_ISL_1646220, EPI_ISL_1646222, EPI_ISL_1646224, EPI_ISL_1646226, EPI_ISL_1646228, EPI_ISL_1646230, EPI_ISL_1646232, EPI_ISL_1646234, EPI_ISL_1646236,
EPI_ISL_1646238, EPI_ISL_1646240, EPI_ISL_1646242, EPI_ISL_1646244, EPI_ISL_1646246, EPI_ISL_1646248, EPI_ISL_1646250, EPI_ISL_1646251, EPI_ISL_1646253, EPI_ISL_1646256, EPI_ISL_1646258, EPI_ISL_1646259, EPI_ISL_1646261, EPI_ISL_1646263, EPI_ISL_1646265, EPI_ISL_1646267, EPI_ISL_1646269, EPI_ISL_1646271, EPI_ISL_1646273, EPI_ISL_1646275, EPI_ISL_1646277, EPI_ISL_1646279,
EPI_ISL_1646281, EPI_ISL_1646283, EPI_ISL_1646285, EPI_ISL_1646287, EPI_ISL_1646289, EPI_ISL_1646291, EPI_ISL_1646293, EPI_ISL_1646294, EPI_ISL_1646296, EPI_ISL_1646299, EPI_ISL_1646300, EPI_ISL_1646302, EPI_ISL_1646304, EPI_ISL_1646306, EPI_ISL_1646308, EPI_ISL_1646310, EPI_ISL_1646311, EPI_ISL_1646313, EPI_ISL_1646315, EPI_ISL_1646317, EPI_ISL_1646319, EPI_ISL_1646321,
EPI_ISL_1646323, EPI_ISL_1646325, EPI_ISL_1646327, EPI_ISL_1646329, EPI_ISL_1646331, EPI_ISL_1646332, EPI_ISL_1646334, EPI_ISL_1646336, EPI_ISL_1646338, EPI_ISL_1646340, EPI_ISL_1646342, EPI_ISL_1646343, EPI_ISL_1646345, EPI_ISL_1646347, EPI_ISL_1646349, EPI_ISL_1646351, EPI_ISL_1646353, EPI_ISL_1646354, EPI_ISL_1646357, EPI_ISL_1646358, EPI_ISL_1646360, EPI_ISL_1646362,
EPI_ISL_1646363, EPI_ISL_1646365, EPI_ISL_1646367, EPI_ISL_1646369, EPI_ISL_1646371, EPI_ISL_1646373, EPI_ISL_1646375, EPI_ISL_1646377, EPI_ISL_1646378, EPI_ISL_1646380, EPI_ISL_1646381, EPI_ISL_1646383, EPI_ISL_1646385, EPI_ISL_1646387, EPI_ISL_1646388, EPI_ISL_1646390, EPI_ISL_1646392, EPI_ISL_1646394, EPI_ISL_1646396, EPI_ISL_1646398, EPI_ISL_1646400, EPI_ISL_1646401,
EPI_ISL_1646404, EPI_ISL_1646406, EPI_ISL_1646408, EPI_ISL_1646409, EPI_ISL_1646411, EPI_ISL_1646414, EPI_ISL_1646415, EPI_ISL_1646417, EPI_ISL_1646419, EPI_ISL_1646421, EPI_ISL_1646423, EPI_ISL_1646425, EPI_ISL_1646427, EPI_ISL_1646429, EPI_ISL_1646431, EPI_ISL_1646432, EPI_ISL_1646434, EPI_ISL_1646436, EPI_ISL_1646438, EPI_ISL_1646439, EPI_ISL_1646442, EPI_ISL_1646443,
EPI_ISL_1646445, EPI_ISL_1646447, EPI_ISL_1646448, EPI_ISL_1646449, EPI_ISL_1646450, EPI_ISL_1646452, EPI_ISL_1646454, EPI_ISL_1646456, EPI_ISL_1646458, EPI_ISL_1646460, EPI_ISL_1646462, EPI_ISL_1646464, EPI_ISL_1646466, EPI_ISL_1646468, EPI_ISL_1646469, EPI_ISL_1646471, EPI_ISL_1646473, EPI_ISL_1646475, EPI_ISL_1646477, EPI_ISL_1646479, EPI_ISL_1646481, EPI_ISL_1646483,
EPI_ISL_1646485, EPI_ISL_1646486, EPI_ISL_1646488, EPI_ISL_1646490, EPI_ISL_1646492, EPI_ISL_1646494, EPI_ISL_1646496, EPI_ISL_1646498, EPI_ISL_1646500, EPI_ISL_1646503, EPI_ISL_1646505, EPI_ISL_1646507, EPI_ISL_1646509, EPI_ISL_1646511, EPI_ISL_1646512, EPI_ISL_1646514, EPI_ISL_1646516, EPI_ISL_1646518, EPI_ISL_1646520, EPI_ISL_1646521, EPI_ISL_1646523, EPI_ISL_1646525,
EPI_ISL_1646527, EPI_ISL_1646529, EPI_ISL_1646531, EPI_ISL_1646532, EPI_ISL_1646534, EPI_ISL_1646536, EPI_ISL_1646537, EPI_ISL_1646539, EPI_ISL_1646541, EPI_ISL_1646543, EPI_ISL_1646545, EPI_ISL_1646547, EPI_ISL_1646549, EPI_ISL_1646551, EPI_ISL_1646553, EPI_ISL_1646555, EPI_ISL_1646557, EPI_ISL_1646559, EPI_ISL_1646561, EPI_ISL_1646563, EPI_ISL_1646565, EPI_ISL_1646566,
EPI_ISL_1646568, EPI_ISL_1646570, EPI_ISL_1646572, EPI_ISL_1646574, EPI_ISL_1646576, EPI_ISL_1646578, EPI_ISL_1646580, EPI_ISL_1646582, EPI_ISL_1646584, EPI_ISL_1646586, EPI_ISL_1646588, EPI_ISL_1646589, EPI_ISL_1646592, EPI_ISL_1646594, EPI_ISL_1646596, EPI_ISL_1646598, EPI_ISL_1646600, EPI_ISL_1646602, EPI_ISL_1646604, EPI_ISL_1646606, EPI_ISL_1646608, EPI_ISL_1646610,
EPI_ISL_1646611, EPI_ISL_1646613, EPI_ISL_1646615, EPI_ISL_1646617, EPI_ISL_1646619, EPI_ISL_1646621, EPI_ISL_1646623, EPI_ISL_1646625, EPI_ISL_1646627, EPI_ISL_1646629, EPI_ISL_1646631, EPI_ISL_1646633, EPI_ISL_1646634, EPI_ISL_1646636, EPI_ISL_1646638, EPI_ISL_1646640, EPI_ISL_1646642, EPI_ISL_1646644, EPI_ISL_1646646, EPI_ISL_1646648, EPI_ISL_1646650, EPI_ISL_1646652,
EPI_ISL_1646654, EPI_ISL_1646656, EPI_ISL_1646658, EPI_ISL_1646660, EPI_ISL_1646662, EPI_ISL_1646663, EPI_ISL_1646665, EPI_ISL_1646667, EPI_ISL_1646669, EPI_ISL_1646671, EPI_ISL_1646673, EPI_ISL_1646675, EPI_ISL_1646677, EPI_ISL_1646679, EPI_ISL_1646681, EPI_ISL_1646682, EPI_ISL_1646686, EPI_ISL_1646688, EPI_ISL_1646690, EPI_ISL_1646692, EPI_ISL_1646693, EPI_ISL_1646695,
EPI_ISL_1646697, EPI_ISL_1646700, EPI_ISL_1646701, EPI_ISL_1646703, EPI_ISL_1646705, EPI_ISL_1646707, EPI_ISL_1646709, EPI_ISL_1646711, EPI_ISL_1646713, EPI_ISL_1646715, EPI_ISL_1646717, EPI_ISL_1646719, EPI_ISL_1646721, EPI_ISL_1646723, EPI_ISL_1646725, EPI_ISL_1646727, EPI_ISL_1646728, EPI_ISL_1646731, EPI_ISL_1646732, EPI_ISL_1646734, EPI_ISL_1646736, EPI_ISL_1646738,
EPI_ISL_1646740, EPI_ISL_1646742, EPI_ISL_1646744, EPI_ISL_1646746, EPI_ISL_1646748, EPI_ISL_1646750, EPI_ISL_1646752, EPI_ISL_1646753, EPI_ISL_1646755, EPI_ISL_1646757, EPI_ISL_1646759, EPI_ISL_1646760, EPI_ISL_1646762, EPI_ISL_1646764, EPI_ISL_1646766, EPI_ISL_1646768, EPI_ISL_1646769, EPI_ISL_1646771, EPI_ISL_1646773, EPI_ISL_1646775, EPI_ISL_1646777, EPI_ISL_1646780,
EPI_ISL_1646781, EPI_ISL_1646783, EPI_ISL_1646785, EPI_ISL_1646787, EPI_ISL_1646789, EPI_ISL_1646791, EPI_ISL_1646793, EPI_ISL_1646795, EPI_ISL_1646797, EPI_ISL_1646798, EPI_ISL_1646800, EPI_ISL_1646802, EPI_ISL_1646804, EPI_ISL_1646806, EPI_ISL_1646808, EPI_ISL_1646810, EPI_ISL_1646812, EPI_ISL_1646814, EPI_ISL_1646816, EPI_ISL_1646817, EPI_ISL_1646819, EPI_ISL_1646821,
EPI_ISL_1646823, EPI_ISL_1646825, EPI_ISL_1646827, EPI_ISL_1646828, EPI_ISL_1646830, EPI_ISL_1646832, EPI_ISL_1646834, EPI_ISL_1646836, EPI_ISL_1646838, EPI_ISL_1646840, EPI_ISL_1646842, EPI_ISL_1646844, EPI_ISL_1646846, EPI_ISL_1646848, EPI_ISL_1646850, EPI_ISL_1646852, EPI_ISL_1646854, EPI_ISL_1646856, EPI_ISL_1646858, EPI_ISL_1646860, EPI_ISL_1646862, EPI_ISL_1646864,
EPI_ISL_1646866, EPI_ISL_1646868, EPI_ISL_1646870, EPI_ISL_1646871, EPI_ISL_1646873, EPI_ISL_1646875, EPI_ISL_1646877, EPI_ISL_1646879, EPI_ISL_1646881, EPI_ISL_1646883, EPI_ISL_1646885, EPI_ISL_1646887, EPI_ISL_1646889, EPI_ISL_1646891, EPI_ISL_1646892, EPI_ISL_1646893, EPI_ISL_1646894, EPI_ISL_1646895, EPI_ISL_1646896, EPI_ISL_1646897, EPI_ISL_1646898, EPI_ISL_1646899,
EPI_ISL_1646900, EPI_ISL_1646901, EPI_ISL_1646902, EPI_ISL_1646903, EPI_ISL_1646904, EPI_ISL_1646905, EPI_ISL_1646906, EPI_ISL_1646907, EPI_ISL_1646908, EPI_ISL_1646909, EPI_ISL_1646910, EPI_ISL_1646911, EPI_ISL_1646912, EPI_ISL_1646913, EPI_ISL_1646914, EPI_ISL_1646915, EPI_ISL_1646916, EPI_ISL_1646917, EPI_ISL_1646918, EPI_ISL_1646919, EPI_ISL_1646920, EPI_ISL_1646921,
EPI_ISL_1646922, EPI_ISL_1646923, EPI_ISL_1646924, EPI_ISL_1646925, EPI_ISL_1646926, EPI_ISL_1646927, EPI_ISL_1646928, EPI_ISL_1646929, EPI_ISL_1646930, EPI_ISL_1646931, EPI_ISL_1646932, EPI_ISL_1646933, EPI_ISL_1646934, EPI_ISL_1646935, EPI_ISL_1646936, EPI_ISL_1646937, EPI_ISL_1646938, EPI_ISL_1646939, EPI_ISL_1646940, EPI_ISL_1646941, EPI_ISL_1646942, EPI_ISL_1646943,
EPI_ISL_1646944, EPI_ISL_1646945, EPI_ISL_1646946, EPI_ISL_1646947, EPI_ISL_1646948, EPI_ISL_1646949, EPI_ISL_1646950, EPI_ISL_1646951, EPI_ISL_1646952, EPI_ISL_1646953, EPI_ISL_1646954, EPI_ISL_1646955, EPI_ISL_1646956, EPI_ISL_1646957, EPI_ISL_1646958, EPI_ISL_1646959, EPI_ISL_1646960, EPI_ISL_1646961, EPI_ISL_1646962, EPI_ISL_1646963, EPI_ISL_1646964, EPI_ISL_1646965,
EPI_ISL_1646966, EPI_ISL_1646967, EPI_ISL_1646968, EPI_ISL_1646969, EPI_ISL_1646970, EPI_ISL_1646971, EPI_ISL_1646972, EPI_ISL_1646973, EPI_ISL_1646974, EPI_ISL_1646975, EPI_ISL_1646976, EPI_ISL_1646977, EPI_ISL_1646978, EPI_ISL_1646979, EPI_ISL_1646980, EPI_ISL_1646981, EPI_ISL_1646982, EPI_ISL_1646983, EPI_ISL_1646984, EPI_ISL_1646985, EPI_ISL_1646986, EPI_ISL_1646987,
EPI_ISL_1646988, EPI_ISL_1646989, EPI_ISL_1646990, EPI_ISL_1646991, EPI_ISL_1646992, EPI_ISL_1646993, EPI_ISL_1646994, EPI_ISL_1646995, EPI_ISL_1646996, EPI_ISL_1646997, EPI_ISL_1646998, EPI_ISL_1646999, EPI_ISL_1647000, EPI_ISL_1647001, EPI_ISL_1647002, EPI_ISL_1647003, EPI_ISL_1647004, EPI_ISL_1647005, EPI_ISL_1647006, EPI_ISL_1647007, EPI_ISL_1647008, EPI_ISL_1647009,
EPI_ISL_1647010, EPI_ISL_1647011, EPI_ISL_1647012, EPI_ISL_1647013, EPI_ISL_1647014, EPI_ISL_1647015, EPI_ISL_1647016, EPI_ISL_1647017, EPI_ISL_1647018, EPI_ISL_1647019, EPI_ISL_1647020, EPI_ISL_1647021, EPI_ISL_1647022, EPI_ISL_1647023, EPI_ISL_1647024, EPI_ISL_1647025, EPI_ISL_1647026, EPI_ISL_1647347, EPI_ISL_1647348, EPI_ISL_1647349, EPI_ISL_1647350, EPI_ISL_1647351,
EPI_ISL_1647352, EPI_ISL_1647353, EPI_ISL_1675253, EPI_ISL_1675254, EPI_ISL_1675255, EPI_ISL_1675256, EPI_ISL_1675257, EPI_ISL_1675258, EPI_ISL_1675259, EPI_ISL_1675260, EPI_ISL_1675261, EPI_ISL_1675262, EPI_ISL_1675263, EPI_ISL_1675264, EPI_ISL_1675265, EPI_ISL_1675266, EPI_ISL_1675267, EPI_ISL_1675268, EPI_ISL_1675269, EPI_ISL_1675270, EPI_ISL_1675271, EPI_ISL_1675272,
EPI_ISL_1675273, EPI_ISL_1675274, EPI_ISL_1675275, EPI_ISL_1675276, EPI_ISL_1675277, EPI_ISL_1675278, EPI_ISL_1675279, EPI_ISL_1675280, EPI_ISL_1675281, EPI_ISL_1675282, EPI_ISL_1675283, EPI_ISL_1675284, EPI_ISL_1675285, EPI_ISL_1675286, EPI_ISL_1675287, EPI_ISL_1675288, EPI_ISL_1675289, EPI_ISL_1675290, EPI_ISL_1675291, EPI_ISL_1675292, EPI_ISL_1744537, EPI_ISL_1744538,
EPI_ISL_1744539, EPI_ISL_1744540, EPI_ISL_1744541, EPI_ISL_1744542, EPI_ISL_1744543, EPI_ISL_1744544, EPI_ISL_1744545, EPI_ISL_1744546, EPI_ISL_1744547, EPI_ISL_1744548, EPI_ISL_1744549, EPI_ISL_1744550, EPI_ISL_1744551, EPI_ISL_1744552, EPI_ISL_1744553, EPI_ISL_1744554, EPI_ISL_1744555, EPI_ISL_1744556, EPI_ISL_1744557, EPI_ISL_1744558, EPI_ISL_1744559, EPI_ISL_1744560,
EPI_ISL_1744561, EPI_ISL_1744562, EPI_ISL_1744563, EPI_ISL_1744564, EPI_ISL_1744565, EPI_ISL_1744566, EPI_ISL_1744567, EPI_ISL_1744568, EPI_ISL_1744569, EPI_ISL_1744570, EPI_ISL_1744571, EPI_ISL_1744572, EPI_ISL_1744573, EPI_ISL_1744574, EPI_ISL_1744575, EPI_ISL_1744576, EPI_ISL_1744577, EPI_ISL_1744578, EPI_ISL_1744579, EPI_ISL_1744580, EPI_ISL_1744581, EPI_ISL_1744582,
EPI_ISL_1744583, EPI_ISL_1744584, EPI_ISL_1744585, EPI_ISL_1744586, EPI_ISL_1744587, EPI_ISL_1744588, EPI_ISL_1744589, EPI_ISL_1744590, EPI_ISL_1744591, EPI_ISL_1744592, EPI_ISL_1744593, EPI_ISL_1744594, EPI_ISL_1744595, EPI_ISL_1744596, EPI_ISL_1744597, EPI_ISL_1744598, EPI_ISL_1744599, EPI_ISL_1744600, EPI_ISL_1744601, EPI_ISL_1744602, EPI_ISL_1744603, EPI_ISL_1744604,
EPI_ISL_1744605, EPI_ISL_1744606, EPI_ISL_1744607, EPI_ISL_1744608, EPI_ISL_1744609, EPI_ISL_1744610, EPI_ISL_1744611, EPI_ISL_1744612, EPI_ISL_1744613, EPI_ISL_1744614, EPI_ISL_1744615, EPI_ISL_1744616, EPI_ISL_1744617, EPI_ISL_1744618, EPI_ISL_1744619, EPI_ISL_1744620, EPI_ISL_1744621, EPI_ISL_1744622, EPI_ISL_1744623, EPI_ISL_1744624, EPI_ISL_1744625, EPI_ISL_1744626,
EPI_ISL_1744627, EPI_ISL_1744628, EPI_ISL_1744629, EPI_ISL_1744630, EPI_ISL_1744631, EPI_ISL_1744632, EPI_ISL_1744633, EPI_ISL_1744634, EPI_ISL_1744635, EPI_ISL_1744636, EPI_ISL_1744637, EPI_ISL_1744638, EPI_ISL_1744639, EPI_ISL_1744640, EPI_ISL_1744641, EPI_ISL_1744642, EPI_ISL_1744643, EPI_ISL_1744644, EPI_ISL_1744645, EPI_ISL_1744646, EPI_ISL_1744647, EPI_ISL_1744648,
EPI_ISL_1744649, EPI_ISL_1744650, EPI_ISL_1744651, EPI_ISL_1744652, EPI_ISL_1744653, EPI_ISL_1744654, EPI_ISL_1744655, EPI_ISL_1744656, EPI_ISL_1744657, EPI_ISL_1744658, EPI_ISL_1744659, EPI_ISL_1744660, EPI_ISL_1744661, EPI_ISL_1744662, EPI_ISL_1744663, EPI_ISL_1744664, EPI_ISL_1744665, EPI_ISL_1744666, EPI_ISL_1744667, EPI_ISL_1744668, EPI_ISL_1744669, EPI_ISL_1744670,
EPI_ISL_1744671, EPI_ISL_1744672, EPI_ISL_1744673, EPI_ISL_1744674, EPI_ISL_1744675, EPI_ISL_1744676, EPI_ISL_1744677, EPI_ISL_1744678, EPI_ISL_1744679, EPI_ISL_1744680, EPI_ISL_1744681, EPI_ISL_1744682, EPI_ISL_1744683, EPI_ISL_1744684, EPI_ISL_1744685, EPI_ISL_1744686, EPI_ISL_1744687, EPI_ISL_1744688, EPI_ISL_1744689, EPI_ISL_1744690, EPI_ISL_1744691, EPI_ISL_1744692,
EPI_ISL_1744693, EPI_ISL_1744694, EPI_ISL_1744695, EPI_ISL_1744696, EPI_ISL_1744697, EPI_ISL_1744698, EPI_ISL_1744699, EPI_ISL_1744700, EPI_ISL_1744701, EPI_ISL_1744702, EPI_ISL_1744703, EPI_ISL_1744704, EPI_ISL_1744705, EPI_ISL_1744706, EPI_ISL_1744707, EPI_ISL_1744708, EPI_ISL_1744709, EPI_ISL_1744710, EPI_ISL_1744711, EPI_ISL_1744712, EPI_ISL_1744713, EPI_ISL_1744714,
EPI_ISL_1744715, EPI_ISL_1744716, EPI_ISL_1744717, EPI_ISL_1744718, EPI_ISL_1744719, EPI_ISL_1744720, EPI_ISL_1744721, EPI_ISL_1744722, EPI_ISL_1744723, EPI_ISL_1744724, EPI_ISL_1744725, EPI_ISL_1744726, EPI_ISL_1744727, EPI_ISL_1744728, EPI_ISL_1744729, EPI_ISL_1744730, EPI_ISL_1744731, EPI_ISL_1744732, EPI_ISL_1744733, EPI_ISL_1744734, EPI_ISL_1744735, EPI_ISL_1744736,
EPI_ISL_1744737, EPI_ISL_1744738, EPI_ISL_1744739, EPI_ISL_1744740, EPI_ISL_1744741, EPI_ISL_1744742, EPI_ISL_1744743, EPI_ISL_1744744, EPI_ISL_1744745, EPI_ISL_1744746, EPI_ISL_1744747, EPI_ISL_1744748, EPI_ISL_1744749, EPI_ISL_1744750, EPI_ISL_1744751, EPI_ISL_1744752, EPI_ISL_1744753, EPI_ISL_1744754, EPI_ISL_1744755, EPI_ISL_1744756, EPI_ISL_1744757, EPI_ISL_1744758,
EPI_ISL_1744759, EPI_ISL_1744760, EPI_ISL_1744761, EPI_ISL_1744762, EPI_ISL_1744763, EPI_ISL_1744764, EPI_ISL_1744765, EPI_ISL_1744766, EPI_ISL_1744767, EPI_ISL_1744768, EPI_ISL_1744769, EPI_ISL_1744770, EPI_ISL_1744771, EPI_ISL_1744772, EPI_ISL_1744773, EPI_ISL_1744774, EPI_ISL_1744775, EPI_ISL_1744776, EPI_ISL_1744777, EPI_ISL_1744778, EPI_ISL_1744779, EPI_ISL_1744780,
EPI_ISL_1744781, EPI_ISL_1744782, EPI_ISL_1744783, EPI_ISL_1744784, EPI_ISL_1744785, EPI_ISL_1744786, EPI_ISL_1744787, EPI_ISL_1744788, EPI_ISL_1744789, EPI_ISL_1744790, EPI_ISL_1744791, EPI_ISL_1744792, EPI_ISL_1744793, EPI_ISL_1744794, EPI_ISL_1744795, EPI_ISL_1744796, EPI_ISL_1744797, EPI_ISL_1744798, EPI_ISL_1744799, EPI_ISL_1744800, EPI_ISL_1744801, EPI_ISL_1744802,
EPI_ISL_1744803, EPI_ISL_1744804, EPI_ISL_1744805, EPI_ISL_1744806, EPI_ISL_1744807, EPI_ISL_1744808, EPI_ISL_1744809, EPI_ISL_1744810, EPI_ISL_1744811, EPI_ISL_1744812, EPI_ISL_1744813, EPI_ISL_1744814, EPI_ISL_1744815, EPI_ISL_1744816, EPI_ISL_1744817, EPI_ISL_1744818, EPI_ISL_1744819, EPI_ISL_1744820, EPI_ISL_1744821, EPI_ISL_1744822, EPI_ISL_1744823, EPI_ISL_1744824,
EPI_ISL_1744825, EPI_ISL_1744826, EPI_ISL_1744827, EPI_ISL_1744828, EPI_ISL_1744829, EPI_ISL_1744830, EPI_ISL_1744831, EPI_ISL_1744832, EPI_ISL_1744833, EPI_ISL_1744834, EPI_ISL_1744835, EPI_ISL_1744836, EPI_ISL_1744837, EPI_ISL_1744838, EPI_ISL_1744839, EPI_ISL_1744840, EPI_ISL_1744841, EPI_ISL_1744842, EPI_ISL_1744843, EPI_ISL_1744844, EPI_ISL_1744845, EPI_ISL_1744846,
EPI_ISL_1744847, EPI_ISL_1744848, EPI_ISL_1744849, EPI_ISL_1744850, EPI_ISL_1744851, EPI_ISL_1744852, EPI_ISL_1744853, EPI_ISL_1744854, EPI_ISL_1744855, EPI_ISL_1744856, EPI_ISL_1744857, EPI_ISL_1744858, EPI_ISL_1744859, EPI_ISL_1744860, EPI_ISL_1744861, EPI_ISL_1744862, EPI_ISL_1744863, EPI_ISL_1744864, EPI_ISL_1744865, EPI_ISL_1744866, EPI_ISL_1744867, EPI_ISL_1744868,
EPI_ISL_1744869, EPI_ISL_1744870, EPI_ISL_1744871, EPI_ISL_1744872, EPI_ISL_1744873, EPI_ISL_1744874, EPI_ISL_1744875, EPI_ISL_1744876, EPI_ISL_1744877, EPI_ISL_1744878, EPI_ISL_1744879, EPI_ISL_1744880, EPI_ISL_1744881, EPI_ISL_1744882, EPI_ISL_1934642, EPI_ISL_1934643, EPI_ISL_1934644, EPI_ISL_1934645, EPI_ISL_1934646, EPI_ISL_1934647, EPI_ISL_1934648, EPI_ISL_1934649,
EPI_ISL_1934650, EPI_ISL_1934651, EPI_ISL_1934652, EPI_ISL_1934653, EPI_ISL_1934654, EPI_ISL_1934655, EPI_ISL_1934656, EPI_ISL_1934657, EPI_ISL_1934658, EPI_ISL_1934659, EPI_ISL_1934660, EPI_ISL_1934661, EPI_ISL_1934662, EPI_ISL_1934663, EPI_ISL_1934664, EPI_ISL_1934665, EPI_ISL_1934666, EPI_ISL_1934667, EPI_ISL_1934668, EPI_ISL_1934669, EPI_ISL_1934670, EPI_ISL_1934671,
EPI_ISL_1934672, EPI_ISL_1934673, EPI_ISL_1934674, EPI_ISL_1934675, EPI_ISL_1934676, EPI_ISL_1934677, EPI_ISL_1934678, EPI_ISL_1934679, EPI_ISL_1934680, EPI_ISL_1934681, EPI_ISL_1934682, EPI_ISL_1934683, EPI_ISL_1934684, EPI_ISL_1934685, EPI_ISL_1934686, EPI_ISL_1934687, EPI_ISL_1934688, EPI_ISL_1934689, EPI_ISL_1934690, EPI_ISL_1934691, EPI_ISL_1934692, EPI_ISL_1934693,
EPI_ISL_1934694, EPI_ISL_1934695, EPI_ISL_1934696, EPI_ISL_1934697, EPI_ISL_1934698, EPI_ISL_1934699, EPI_ISL_1934700, EPI_ISL_1934701, EPI_ISL_1934702, EPI_ISL_1934703, EPI_ISL_1934704, EPI_ISL_1934705, EPI_ISL_1934706, EPI_ISL_1934707, EPI_ISL_1934708, EPI_ISL_1934709, EPI_ISL_1934710, EPI_ISL_1934711, EPI_ISL_1934712, EPI_ISL_1934713, EPI_ISL_1934714, EPI_ISL_1934715,
EPI_ISL_1934716, EPI_ISL_1934717, EPI_ISL_1934718, EPI_ISL_1934719, EPI_ISL_1934720, EPI_ISL_1934721, EPI_ISL_1934722, EPI_ISL_1934723, EPI_ISL_1934724, EPI_ISL_1934725, EPI_ISL_1934726, EPI_ISL_1934727, EPI_ISL_1934728, EPI_ISL_1934729, EPI_ISL_1934730, EPI_ISL_1934731, EPI_ISL_1934732, EPI_ISL_1934733, EPI_ISL_1934734, EPI_ISL_1934735, EPI_ISL_1934736, EPI_ISL_1934737,
EPI_ISL_1934738, EPI_ISL_1934739, EPI_ISL_1934740, EPI_ISL_1934741, EPI_ISL_1934742, EPI_ISL_1934743, EPI_ISL_1934744, EPI_ISL_1934745, EPI_ISL_1934746, EPI_ISL_1934747, EPI_ISL_1934748, EPI_ISL_1934749, EPI_ISL_1934750, EPI_ISL_1934751, EPI_ISL_1934752, EPI_ISL_1934753, EPI_ISL_1934754, EPI_ISL_1934755, EPI_ISL_1934756, EPI_ISL_1934757, EPI_ISL_1934758, EPI_ISL_1934759,
EPI_ISL_1934760, EPI_ISL_1934761, EPI_ISL_1934762, EPI_ISL_1934763, EPI_ISL_1934764, EPI_ISL_1934765, EPI_ISL_1934766, EPI_ISL_1934767, EPI_ISL_1934768, EPI_ISL_1934769, EPI_ISL_1934770, EPI_ISL_1934771, EPI_ISL_1934772, EPI_ISL_1934773, EPI_ISL_1934774, EPI_ISL_1934775, EPI_ISL_1934776, EPI_ISL_1934777, EPI_ISL_1934778, EPI_ISL_1934779, EPI_ISL_1934780, EPI_ISL_1934781,
EPI_ISL_1934782, EPI_ISL_1934783, EPI_ISL_1934784, EPI_ISL_1934785, EPI_ISL_1934786, EPI_ISL_1934787, EPI_ISL_1934788, EPI_ISL_1934789, EPI_ISL_1934790, EPI_ISL_1934791, EPI_ISL_1934792, EPI_ISL_1934793, EPI_ISL_1934794, EPI_ISL_1934795, EPI_ISL_1934796, EPI_ISL_1934797, EPI_ISL_1934798, EPI_ISL_1934799, EPI_ISL_1934800, EPI_ISL_1934801, EPI_ISL_1934802, EPI_ISL_1934803,
EPI_ISL_1934804, EPI_ISL_1934805, EPI_ISL_1934806, EPI_ISL_1934807, EPI_ISL_1934808, EPI_ISL_1934809, EPI_ISL_1934810, EPI_ISL_1934811, EPI_ISL_1934812, EPI_ISL_1934813, EPI_ISL_1934814, EPI_ISL_1934815, EPI_ISL_1934816, EPI_ISL_1934817, EPI_ISL_1934818, EPI_ISL_1934819, EPI_ISL_1934820, EPI_ISL_1934821, EPI_ISL_1934822, EPI_ISL_1934823, EPI_ISL_1934824, EPI_ISL_1934825,
EPI_ISL_1934826, EPI_ISL_1934827, EPI_ISL_1934828, EPI_ISL_1934829, EPI_ISL_1934830, EPI_ISL_1934831, EPI_ISL_1934832, EPI_ISL_1934833, EPI_ISL_1934834, EPI_ISL_1934835, EPI_ISL_1934836, EPI_ISL_1934837, EPI_ISL_1934838, EPI_ISL_1934839, EPI_ISL_1934840, EPI_ISL_1934841, EPI_ISL_1934842, EPI_ISL_1934843, EPI_ISL_1934844, EPI_ISL_1934845, EPI_ISL_1934846, EPI_ISL_1934847,
EPI_ISL_1934848, EPI_ISL_1934849, EPI_ISL_1934850, EPI_ISL_1934851, EPI_ISL_1934852, EPI_ISL_1934853, EPI_ISL_1935653, EPI_ISL_1935654, EPI_ISL_1935655, EPI_ISL_1935656, EPI_ISL_1935657, EPI_ISL_1935658, EPI_ISL_1935659, EPI_ISL_1935660, EPI_ISL_1935661, EPI_ISL_1935662, EPI_ISL_1935663, EPI_ISL_1935664, EPI_ISL_1935665, EPI_ISL_1935666, EPI_ISL_1935667, EPI_ISL_1935668,
EPI_ISL_1935669, EPI_ISL_1935670, EPI_ISL_1935671, EPI_ISL_1935672, EPI_ISL_1935673, EPI_ISL_1935674, EPI_ISL_1935675, EPI_ISL_1935676, EPI_ISL_1935677, EPI_ISL_1935678, EPI_ISL_1935679, EPI_ISL_1935680, EPI_ISL_1935681, EPI_ISL_1935682, EPI_ISL_1935683, EPI_ISL_1935684, EPI_ISL_1935685, EPI_ISL_1935686, EPI_ISL_1935687, EPI_ISL_1935688, EPI_ISL_1935689, EPI_ISL_1935690,
EPI_ISL_1935691, EPI_ISL_1935692, EPI_ISL_1935693, EPI_ISL_1935694, EPI_ISL_1935695, EPI_ISL_1935696, EPI_ISL_1935697, EPI_ISL_1935698, EPI_ISL_1935699, EPI_ISL_1935700, EPI_ISL_1935701, EPI_ISL_1935702, EPI_ISL_1935703, EPI_ISL_1935704, EPI_ISL_1935705, EPI_ISL_1935706, EPI_ISL_1935707, EPI_ISL_1935708, EPI_ISL_1935709, EPI_ISL_1935710, EPI_ISL_1935711, EPI_ISL_1935712,
EPI_ISL_1935713, EPI_ISL_1935714, EPI_ISL_1935715, EPI_ISL_1935716, EPI_ISL_1935717, EPI_ISL_1935718, EPI_ISL_1935719, EPI_ISL_1935720, EPI_ISL_1935721, EPI_ISL_1935722, EPI_ISL_1935723, EPI_ISL_1935724, EPI_ISL_1935725, EPI_ISL_1935726, EPI_ISL_1935727, EPI_ISL_1935728, EPI_ISL_1935729, EPI_ISL_1935730, EPI_ISL_1935731, EPI_ISL_1935732, EPI_ISL_1935733, EPI_ISL_1935734,
EPI_ISL_1935735, EPI_ISL_1935736, EPI_ISL_1935737, EPI_ISL_1935738, EPI_ISL_1935739, EPI_ISL_1935740, EPI_ISL_1935741, EPI_ISL_1935742, EPI_ISL_1935743, EPI_ISL_1935744, EPI_ISL_1935745, EPI_ISL_1935746, EPI_ISL_1935747, EPI_ISL_1935748, EPI_ISL_1935749, EPI_ISL_1935750, EPI_ISL_1935751, EPI_ISL_1935752, EPI_ISL_1935753, EPI_ISL_1935754, EPI_ISL_1935755, EPI_ISL_1935756,
EPI_ISL_1935757, EPI_ISL_1935758, EPI_ISL_1935759, EPI_ISL_1935760, EPI_ISL_1935761, EPI_ISL_1935762, EPI_ISL_1935763, EPI_ISL_1935764, EPI_ISL_1935765, EPI_ISL_1935766, EPI_ISL_1935767, EPI_ISL_1935768, EPI_ISL_1935769, EPI_ISL_1935770, EPI_ISL_1935771, EPI_ISL_1935772, EPI_ISL_1935773, EPI_ISL_1935774, EPI_ISL_1935775, EPI_ISL_1935776, EPI_ISL_1935777, EPI_ISL_1935778,
EPI_ISL_1935779, EPI_ISL_1935780, EPI_ISL_1935781, EPI_ISL_1935782, EPI_ISL_1935783, EPI_ISL_1935784, EPI_ISL_1935785, EPI_ISL_1935786, EPI_ISL_1935787, EPI_ISL_1935788, EPI_ISL_1935789, EPI_ISL_1935790, EPI_ISL_1935791, EPI_ISL_1935792, EPI_ISL_1935793, EPI_ISL_1935794, EPI_ISL_1935795, EPI_ISL_1935796, EPI_ISL_1935797, EPI_ISL_1935798, EPI_ISL_1935799, EPI_ISL_1935800,
EPI_ISL_1935801, EPI_ISL_1935802, EPI_ISL_1935803, EPI_ISL_1935804, EPI_ISL_1935805, EPI_ISL_1935806, EPI_ISL_1935807, EPI_ISL_1935808, EPI_ISL_1935809, EPI_ISL_1935810, EPI_ISL_1935811, EPI_ISL_1935812, EPI_ISL_1935813, EPI_ISL_1935814, EPI_ISL_1935815, EPI_ISL_1935816, EPI_ISL_1935817, EPI_ISL_1935818, EPI_ISL_1935819, EPI_ISL_1935820, EPI_ISL_1935821, EPI_ISL_1935822,
EPI_ISL_1935823, EPI_ISL_1935824, EPI_ISL_1935825, EPI_ISL_1935826, EPI_ISL_1935827, EPI_ISL_1935828, EPI_ISL_1935829, EPI_ISL_1935830, EPI_ISL_1935831, EPI_ISL_1935832, EPI_ISL_1935833, EPI_ISL_1935834, EPI_ISL_1935835, EPI_ISL_1935836, EPI_ISL_1935837, EPI_ISL_1935838, EPI_ISL_1935839, EPI_ISL_1935840, EPI_ISL_1935841, EPI_ISL_1935842, EPI_ISL_1935843, EPI_ISL_1935844,
EPI_ISL_1935845, EPI_ISL_1935846, EPI_ISL_1935847, EPI_ISL_1935848, EPI_ISL_1935849, EPI_ISL_1935850, EPI_ISL_1935851, EPI_ISL_1935852, EPI_ISL_1935853, EPI_ISL_1935854, EPI_ISL_1935855, EPI_ISL_1935856, EPI_ISL_1935857, EPI_ISL_1935858, EPI_ISL_1935859, EPI_ISL_1935860, EPI_ISL_1935861, EPI_ISL_1935862, EPI_ISL_1935863, EPI_ISL_1935864, EPI_ISL_1935865, EPI_ISL_1935866,
EPI_ISL_1935867, EPI_ISL_1935868, EPI_ISL_1935869, EPI_ISL_1935870, EPI_ISL_1935871, EPI_ISL_1935872, EPI_ISL_1935873, EPI_ISL_1935874, EPI_ISL_1935875, EPI_ISL_1935876, EPI_ISL_1935877, EPI_ISL_1935878, EPI_ISL_1935879, EPI_ISL_1935880, EPI_ISL_1935881, EPI_ISL_1935882, EPI_ISL_1935883, EPI_ISL_1935884, EPI_ISL_1935885, EPI_ISL_1935886, EPI_ISL_1935887, EPI_ISL_1935888,
EPI_ISL_1935889, EPI_ISL_1935890, EPI_ISL_1935891, EPI_ISL_1935892, EPI_ISL_1935893, EPI_ISL_1935894, EPI_ISL_1935895, EPI_ISL_1935896, EPI_ISL_1935897, EPI_ISL_1935898, EPI_ISL_1935899, EPI_ISL_1935900, EPI_ISL_1935901, EPI_ISL_1935902, EPI_ISL_1935903, EPI_ISL_1935904, EPI_ISL_1935905, EPI_ISL_1935906, EPI_ISL_1935907, EPI_ISL_1935908, EPI_ISL_1935909, EPI_ISL_1935910,
EPI_ISL_1935911, EPI_ISL_1935912, EPI_ISL_1935913, EPI_ISL_1935914, EPI_ISL_1935915, EPI_ISL_1935916, EPI_ISL_1935917, EPI_ISL_1935918, EPI_ISL_1935919, EPI_ISL_1935920, EPI_ISL_1935921, EPI_ISL_1935922, EPI_ISL_1935923, EPI_ISL_1935924, EPI_ISL_1935925, EPI_ISL_1935926, EPI_ISL_1935927, EPI_ISL_1935928, EPI_ISL_1935929, EPI_ISL_1935930, EPI_ISL_1935931, EPI_ISL_1935932,

EPI_ISL_1935933, EPI_ISL_1935934, EPI_ISL_1935935, EPI_ISL_1935936, EPI_ISL_1935937, EPI_ISL_1935938, EPI_ISL_1935939, EPI_ISL_1935940, EPI_ISL_1935941, EPI_ISL_1935942, EPI_ISL_1935943, EPI_ISL_1935944, EPI_ISL_1935945, EPI_ISL_1935946, EPI_ISL_1935947, EPI_ISL_1935948, EPI_ISL_1935949, EPI_ISL_1935950, EPI_ISL_1935951, EPI_ISL_1935952, EPI_ISL_1935953, EPI_ISL_1935954,
EPI_ISL_1935955, EPI_ISL_1935956, EPI_ISL_1935957, EPI_ISL_1935958, EPI_ISL_1935959, EPI_ISL_1935960, EPI_ISL_1935961, EPI_ISL_1935962, EPI_ISL_1935963, EPI_ISL_1935964, EPI_ISL_1935965, EPI_ISL_1935966, EPI_ISL_1935967, EPI_ISL_1935968, EPI_ISL_1935969, EPI_ISL_1935970, EPI_ISL_1935971, EPI_ISL_1935972, EPI_ISL_1935973, EPI_ISL_1935974, EPI_ISL_1935975, EPI_ISL_1935976,
EPI_ISL_1935977, EPI_ISL_1935978, EPI_ISL_1935979, EPI_ISL_1935980, EPI_ISL_1935981, EPI_ISL_1935982, EPI_ISL_1935983, EPI_ISL_1935984, EPI_ISL_1935985, EPI_ISL_1935986, EPI_ISL_1935987, EPI_ISL_1935988, EPI_ISL_1935989, EPI_ISL_1935990, EPI_ISL_1935991, EPI_ISL_1935992, EPI_ISL_1935993, EPI_ISL_1935994, EPI_ISL_1935995, EPI_ISL_1935996, EPI_ISL_1935997, EPI_ISL_1935998,
EPI_ISL_1935999, EPI_ISL_1936000, EPI_ISL_1936001, EPI_ISL_1936002, EPI_ISL_1936003, EPI_ISL_1936004, EPI_ISL_1936005, EPI_ISL_1936006, EPI_ISL_1936007, EPI_ISL_1936008, EPI_ISL_1936009, EPI_ISL_1936010, EPI_ISL_1936011, EPI_ISL_1936012, EPI_ISL_1936013, EPI_ISL_1936014, EPI_ISL_1936015, EPI_ISL_1936016, EPI_ISL_1936017, EPI_ISL_1936018, EPI_ISL_1936019, EPI_ISL_1936020,
EPI_ISL_1936021, EPI_ISL_1936022, EPI_ISL_1936023, EPI_ISL_1936024, EPI_ISL_1936025, EPI_ISL_1936026, EPI_ISL_1936027, EPI_ISL_1936028, EPI_ISL_1936029, EPI_ISL_1936030, EPI_ISL_1936031, EPI_ISL_1936032, EPI_ISL_1936033, EPI_ISL_1936034, EPI_ISL_1936035, EPI_ISL_1936036, EPI_ISL_1936037, EPI_ISL_1936038, EPI_ISL_1936039, EPI_ISL_1936040, EPI_ISL_1936041, EPI_ISL_1936042,
EPI_ISL_1936043, EPI_ISL_1936044, EPI_ISL_1936045, EPI_ISL_1936046, EPI_ISL_1936047, EPI_ISL_1936048, EPI_ISL_1936049, EPI_ISL_1936050, EPI_ISL_1936051, EPI_ISL_1936052, EPI_ISL_1936053, EPI_ISL_1936054, EPI_ISL_1936055, EPI_ISL_1936056, EPI_ISL_1936057, EPI_ISL_1936058, EPI_ISL_1936059, EPI_ISL_1936060, EPI_ISL_1936061, EPI_ISL_1936062, EPI_ISL_1936063, EPI_ISL_1936064,
EPI_ISL_1936065, EPI_ISL_1936066, EPI_ISL_1936067, EPI_ISL_1936068, EPI_ISL_1936069, EPI_ISL_1936070, EPI_ISL_1936071, EPI_ISL_1936072, EPI_ISL_1936073, EPI_ISL_1936074, EPI_ISL_1936075, EPI_ISL_1936076, EPI_ISL_1936077, EPI_ISL_1936078, EPI_ISL_1936079, EPI_ISL_1936080, EPI_ISL_1936081, EPI_ISL_1936082, EPI_ISL_1936083, EPI_ISL_1936084, EPI_ISL_1936085, EPI_ISL_1936086,
EPI_ISL_1936087, EPI_ISL_1936088, EPI_ISL_1936089, EPI_ISL_1936090, EPI_ISL_1936091, EPI_ISL_1936092, EPI_ISL_1936093, EPI_ISL_1936094, EPI_ISL_1936095, EPI_ISL_1936096, EPI_ISL_1936097, EPI_ISL_1936098, EPI_ISL_1936099, EPI_ISL_1936100, EPI_ISL_1936101, EPI_ISL_1936465, EPI_ISL_1936466, EPI_ISL_1936467, EPI_ISL_1936468, EPI_ISL_1936469, EPI_ISL_1936470, EPI_ISL_1936471,
EPI_ISL_1936472, EPI_ISL_1936473, EPI_ISL_1936474, EPI_ISL_1936475, EPI_ISL_1936476, EPI_ISL_1936477, EPI_ISL_1936478, EPI_ISL_1936479, EPI_ISL_1936480, EPI_ISL_1936481, EPI_ISL_1936482, EPI_ISL_1936483, EPI_ISL_1936484, EPI_ISL_1936485, EPI_ISL_1936486, EPI_ISL_1936487, EPI_ISL_1936488, EPI_ISL_1936489, EPI_ISL_1936490, EPI_ISL_1936491, EPI_ISL_1936492, EPI_ISL_1936493,
EPI_ISL_1936494, EPI_ISL_1936495, EPI_ISL_1936496, EPI_ISL_1936497, EPI_ISL_1936498, EPI_ISL_1936499, EPI_ISL_1936500, EPI_ISL_1936501, EPI_ISL_1936502, EPI_ISL_1936503, EPI_ISL_1936504, EPI_ISL_1936505, EPI_ISL_1936506, EPI_ISL_1936507, EPI_ISL_1936508, EPI_ISL_1936509, EPI_ISL_1936510, EPI_ISL_1936511, EPI_ISL_1936512, EPI_ISL_1936513, EPI_ISL_1936514, EPI_ISL_1936515,
EPI_ISL_1936516, EPI_ISL_1936517, EPI_ISL_1936518, EPI_ISL_1936519, EPI_ISL_1936520, EPI_ISL_1936521, EPI_ISL_1936522, EPI_ISL_1936523, EPI_ISL_1936524, EPI_ISL_1936525, EPI_ISL_1936526, EPI_ISL_1936527, EPI_ISL_1936528, EPI_ISL_1936529, EPI_ISL_1936530, EPI_ISL_1936531, EPI_ISL_1936532, EPI_ISL_1936533, EPI_ISL_1936534, EPI_ISL_1936535, EPI_ISL_1936536, EPI_ISL_1936537,
EPI_ISL_1936538, EPI_ISL_1936539, EPI_ISL_1936540, EPI_ISL_1936541, EPI_ISL_1936542, EPI_ISL_1936543, EPI_ISL_1936544, EPI_ISL_1936545, EPI_ISL_1936546, EPI_ISL_1936547, EPI_ISL_1936548, EPI_ISL_1936549, EPI_ISL_1936550, EPI_ISL_1936551, EPI_ISL_1936552, EPI_ISL_1936553, EPI_ISL_1936554, EPI_ISL_1936555, EPI_ISL_1936556, EPI_ISL_1936557, EPI_ISL_1936558, EPI_ISL_1936559,
EPI_ISL_1936560, EPI_ISL_1936561, EPI_ISL_1936562, EPI_ISL_1936563, EPI_ISL_1936564, EPI_ISL_1936565, EPI_ISL_1936566, EPI_ISL_1936567, EPI_ISL_1936568, EPI_ISL_1936569, EPI_ISL_1936570, EPI_ISL_1936571, EPI_ISL_1936572, EPI_ISL_1936573, EPI_ISL_1936574, EPI_ISL_1936575, EPI_ISL_1936576, EPI_ISL_1936577, EPI_ISL_1936578, EPI_ISL_1936579, EPI_ISL_1936580, EPI_ISL_1936581,
EPI_ISL_1936582, EPI_ISL_1936583, EPI_ISL_1936584, EPI_ISL_1936585, EPI_ISL_1936586, EPI_ISL_1936587, EPI_ISL_1936588, EPI_ISL_1936589, EPI_ISL_1936590, EPI_ISL_1936591, EPI_ISL_1936592, EPI_ISL_1936593, EPI_ISL_1936594, EPI_ISL_1936595, EPI_ISL_1936596, EPI_ISL_1936597, EPI_ISL_1936598, EPI_ISL_1936599, EPI_ISL_1936600, EPI_ISL_1936601, EPI_ISL_1936602, EPI_ISL_1936603,
EPI_ISL_1936604, EPI_ISL_1936605, EPI_ISL_1936606, EPI_ISL_1936607, EPI_ISL_1936608, EPI_ISL_1936609, EPI_ISL_1936610, EPI_ISL_1936611, EPI_ISL_1936612, EPI_ISL_1936613, EPI_ISL_1936614, EPI_ISL_1936615, EPI_ISL_1936616, EPI_ISL_1936617, EPI_ISL_1936618, EPI_ISL_1936619, EPI_ISL_1936620, EPI_ISL_1936621, EPI_ISL_1936622, EPI_ISL_1936623, EPI_ISL_1936624, EPI_ISL_1936625,
EPI_ISL_1936626, EPI_ISL_1936627, EPI_ISL_1936628, EPI_ISL_1936629, EPI_ISL_1936630, EPI_ISL_1936631, EPI_ISL_1936632, EPI_ISL_1936633, EPI_ISL_1936634, EPI_ISL_1936635, EPI_ISL_1936636, EPI_ISL_1936637, EPI_ISL_1936638, EPI_ISL_1936639, EPI_ISL_1936640, EPI_ISL_1936641, EPI_ISL_1936642, EPI_ISL_1936643, EPI_ISL_1936644, EPI_ISL_1936645, EPI_ISL_1936646, EPI_ISL_1936647,
EPI_ISL_1936648, EPI_ISL_1936649, EPI_ISL_1936650, EPI_ISL_1936651, EPI_ISL_1936652, EPI_ISL_1936653, EPI_ISL_1936654, EPI_ISL_1936655, EPI_ISL_1936656, EPI_ISL_1936657, EPI_ISL_1936658, EPI_ISL_1936659, EPI_ISL_1936660, EPI_ISL_1936661, EPI_ISL_1936662, EPI_ISL_1936663, EPI_ISL_1936664, EPI_ISL_1936665, EPI_ISL_1936666, EPI_ISL_1936667, EPI_ISL_1936668, EPI_ISL_1936669,
EPI_ISL_1936670, EPI_ISL_1936671, EPI_ISL_1936672, EPI_ISL_1936673, EPI_ISL_1936674, EPI_ISL_1936675, EPI_ISL_1936676, EPI_ISL_1936677, EPI_ISL_1936678, EPI_ISL_1936679, EPI_ISL_2161033, EPI_ISL_2161034, EPI_ISL_2161035, EPI_ISL_2161036, EPI_ISL_2161037, EPI_ISL_2161038, EPI_ISL_2161039, EPI_ISL_2161040, EPI_ISL_2161041, EPI_ISL_2161042, EPI_ISL_2161043, EPI_ISL_2161044,
EPI_ISL_2161045, EPI_ISL_2161046, EPI_ISL_2161047, EPI_ISL_2161048, EPI_ISL_2161049, EPI_ISL_2161050, EPI_ISL_2161051, EPI_ISL_2161052, EPI_ISL_2161053, EPI_ISL_2161054, EPI_ISL_2161055, EPI_ISL_2161056, EPI_ISL_2161057, EPI_ISL_2161058, EPI_ISL_2161059, EPI_ISL_2161060, EPI_ISL_2161061, EPI_ISL_2161062, EPI_ISL_2161063, EPI_ISL_2161064, EPI_ISL_2161065, EPI_ISL_2161066,
EPI_ISL_2161067, EPI_ISL_2161068, EPI_ISL_2161069, EPI_ISL_2161070, EPI_ISL_2161071, EPI_ISL_2161072, EPI_ISL_2161073, EPI_ISL_2161074, EPI_ISL_2161075, EPI_ISL_2161076, EPI_ISL_2161077, EPI_ISL_2161078, EPI_ISL_2161079, EPI_ISL_2161080, EPI_ISL_2161081, EPI_ISL_2161082, EPI_ISL_2161083, EPI_ISL_2161084, EPI_ISL_2161085, EPI_ISL_2161086, EPI_ISL_2161087, EPI_ISL_2161088,
EPI_ISL_2161089, EPI_ISL_2161090, EPI_ISL_2161091, EPI_ISL_2161092, EPI_ISL_2161093, EPI_ISL_2161094, EPI_ISL_2161095, EPI_ISL_2161096, EPI_ISL_2161097, EPI_ISL_2161098, EPI_ISL_2161099, EPI_ISL_2161100, EPI_ISL_2161101, EPI_ISL_2161102, EPI_ISL_2161103, EPI_ISL_2161104, EPI_ISL_2161105, EPI_ISL_2161106, EPI_ISL_2161107, EPI_ISL_2161108, EPI_ISL_2161109, EPI_ISL_2161110,
EPI_ISL_2161111, EPI_ISL_2161112, EPI_ISL_2161113, EPI_ISL_2161114, EPI_ISL_2161115, EPI_ISL_2161116, EPI_ISL_2161117, EPI_ISL_2161118, EPI_ISL_2161119, EPI_ISL_2161120, EPI_ISL_2161121, EPI_ISL_2161122, EPI_ISL_2161123, EPI_ISL_2161124, EPI_ISL_2161125, EPI_ISL_2161126, EPI_ISL_2161127, EPI_ISL_2161128, EPI_ISL_2161129, EPI_ISL_2161130, EPI_ISL_2161131, EPI_ISL_2161132,
EPI_ISL_2161133, EPI_ISL_2161134, EPI_ISL_2161135, EPI_ISL_2161136, EPI_ISL_2161137, EPI_ISL_2161138, EPI_ISL_2161139, EPI_ISL_2161140, EPI_ISL_2161141, EPI_ISL_2161142, EPI_ISL_2161143, EPI_ISL_2161144, EPI_ISL_2161145, EPI_ISL_2161146, EPI_ISL_2161147, EPI_ISL_2161148, EPI_ISL_2161149, EPI_ISL_2161150, EPI_ISL_2161151, EPI_ISL_2161152, EPI_ISL_2161153, EPI_ISL_2161154,
EPI_ISL_2161155, EPI_ISL_2161156, EPI_ISL_2161157, EPI_ISL_2161158, EPI_ISL_2161159, EPI_ISL_2161160, EPI_ISL_2161161, EPI_ISL_2161162, EPI_ISL_2161163, EPI_ISL_2161164, EPI_ISL_2161165, EPI_ISL_2161166, EPI_ISL_2284527, EPI_ISL_2284528, EPI_ISL_2284529, EPI_ISL_2284530, EPI_ISL_2284531, EPI_ISL_2284532, EPI_ISL_2284533, EPI_ISL_2284534, EPI_ISL_2284535, EPI_ISL_2284536,
EPI_ISL_2284537, EPI_ISL_2284538, EPI_ISL_2284539, EPI_ISL_2284540, EPI_ISL_2284541, EPI_ISL_2284542, EPI_ISL_2284543, EPI_ISL_2284544, EPI_ISL_2284545, EPI_ISL_2284546, EPI_ISL_2284547, EPI_ISL_2284548, EPI_ISL_2284549, EPI_ISL_2284550, EPI_ISL_2284551, EPI_ISL_2284552, EPI_ISL_2284553, EPI_ISL_2284554, EPI_ISL_2284555, EPI_ISL_2284556, EPI_ISL_2284557, EPI_ISL_2284558,
EPI_ISL_2284559, EPI_ISL_2284560, EPI_ISL_2284561, EPI_ISL_2284562, EPI_ISL_2284563, EPI_ISL_2284564, EPI_ISL_2284565, EPI_ISL_2284566, EPI_ISL_2284567, EPI_ISL_2284568, EPI_ISL_2284569, EPI_ISL_2284570, EPI_ISL_2284571, EPI_ISL_2284572, EPI_ISL_2284573, EPI_ISL_2284574, EPI_ISL_2284575, EPI_ISL_2284576, EPI_ISL_2284577, EPI_ISL_2284578, EPI_ISL_2284579, EPI_ISL_2284580,
EPI_ISL_2284581, EPI_ISL_2284582, EPI_ISL_2284583, EPI_ISL_2284584, EPI_ISL_2284585, EPI_ISL_2284586, EPI_ISL_2284587, EPI_ISL_2284588, EPI_ISL_2284589, EPI_ISL_2284590, EPI_ISL_2284591, EPI_ISL_2284592, EPI_ISL_2284593, EPI_ISL_2284594, EPI_ISL_2284595, EPI_ISL_2284596, EPI_ISL_2284597, EPI_ISL_2284598, EPI_ISL_2284599, EPI_ISL_2284600, EPI_ISL_2284601, EPI_ISL_2284602,
EPI_ISL_2284603, EPI_ISL_2284604, EPI_ISL_2284605, EPI_ISL_2284606, EPI_ISL_2284607, EPI_ISL_2284608, EPI_ISL_2284609, EPI_ISL_2284610, EPI_ISL_2284611, EPI_ISL_2284612, EPI_ISL_2284613, EPI_ISL_2284614, EPI_ISL_2284615, EPI_ISL_2284616, EPI_ISL_2284617, EPI_ISL_2284618, EPI_ISL_2284619, EPI_ISL_2284620, EPI_ISL_2284621, EPI_ISL_2284622, EPI_ISL_2284623, EPI_ISL_2284624,
EPI_ISL_2284625, EPI_ISL_2284626, EPI_ISL_2284627, EPI_ISL_2284628, EPI_ISL_2284629, EPI_ISL_2284630, EPI_ISL_2284631, EPI_ISL_2284632, EPI_ISL_2284633, EPI_ISL_2284634, EPI_ISL_2284635, EPI_ISL_2284636, EPI_ISL_2284637, EPI_ISL_2284638, EPI_ISL_2284639, EPI_ISL_2284640, EPI_ISL_2284641, EPI_ISL_2284642, EPI_ISL_2284643, EPI_ISL_2284644, EPI_ISL_2284645, EPI_ISL_2284646,
EPI_ISL_2284647, EPI_ISL_2284648, EPI_ISL_2284649, EPI_ISL_2284650, EPI_ISL_2284651, EPI_ISL_2284652, EPI_ISL_2284653, EPI_ISL_2284654, EPI_ISL_2284655, EPI_ISL_2284656, EPI_ISL_2284657, EPI_ISL_2284658, EPI_ISL_2284659, EPI_ISL_2284660, EPI_ISL_2284661, EPI_ISL_2284662, EPI_ISL_2284663, EPI_ISL_2284664, EPI_ISL_2284665, EPI_ISL_2284666, EPI_ISL_2284667, EPI_ISL_2284668,
EPI_ISL_2284669, EPI_ISL_2284670, EPI_ISL_2284671, EPI_ISL_2332243, EPI_ISL_2332244, EPI_ISL_2332245, EPI_ISL_2332246, EPI_ISL_2332247, EPI_ISL_2332248, EPI_ISL_2332249, EPI_ISL_2332250, EPI_ISL_2332251, EPI_ISL_2332252, EPI_ISL_2332253, EPI_ISL_2332254, EPI_ISL_2332255, EPI_ISL_2332256, EPI_ISL_2332257, EPI_ISL_2332258, EPI_ISL_2332259, EPI_ISL_2332260, EPI_ISL_2332261,
EPI_ISL_2332262, EPI_ISL_2332263, EPI_ISL_2332264, EPI_ISL_2332265, EPI_ISL_2332266, EPI_ISL_2332267, EPI_ISL_2332268, EPI_ISL_2332269, EPI_ISL_2332270, EPI_ISL_2332271, EPI_ISL_2332272, EPI_ISL_2332273, EPI_ISL_2332274, EPI_ISL_2332275, EPI_ISL_2332276, EPI_ISL_2332277, EPI_ISL_2332278, EPI_ISL_2332279, EPI_ISL_2332280, EPI_ISL_2332281, EPI_ISL_2332282, EPI_ISL_2332283,
EPI_ISL_2332284, EPI_ISL_2332285, EPI_ISL_2332286, EPI_ISL_2332287, EPI_ISL_2332288, EPI_ISL_2332289, EPI_ISL_2332290, EPI_ISL_2332291, EPI_ISL_2332292, EPI_ISL_2332293, EPI_ISL_2332294, EPI_ISL_2332295, EPI_ISL_2332296, EPI_ISL_2332297, EPI_ISL_2332298, EPI_ISL_2332299, EPI_ISL_2332300, EPI_ISL_2332301, EPI_ISL_2332302, EPI_ISL_2332303, EPI_ISL_2332304, EPI_ISL_2332305,
EPI_ISL_2332306, EPI_ISL_2332307, EPI_ISL_2332308, EPI_ISL_2332309, EPI_ISL_2332310, EPI_ISL_2332311, EPI_ISL_2332312, EPI_ISL_2332313, EPI_ISL_2332314, EPI_ISL_2332315, EPI_ISL_2332316, EPI_ISL_2332317, EPI_ISL_2332318, EPI_ISL_2332319, EPI_ISL_2332320, EPI_ISL_2332321, EPI_ISL_2332322, EPI_ISL_2332323, EPI_ISL_2332324, EPI_ISL_2332325, EPI_ISL_2332326, EPI_ISL_2332327,
EPI_ISL_2332328, EPI_ISL_2332329, EPI_ISL_2332330, EPI_ISL_2332331, EPI_ISL_2332332, EPI_ISL_2332333, EPI_ISL_2332334, EPI_ISL_2332335, EPI_ISL_2332336, EPI_ISL_2332337, EPI_ISL_2332338, EPI_ISL_2332339, EPI_ISL_2332340, EPI_ISL_2332341, EPI_ISL_2332342, EPI_ISL_2332343, EPI_ISL_2332344, EPI_ISL_2332345, EPI_ISL_2332346, EPI_ISL_2332347, EPI_ISL_2332348, EPI_ISL_2332349,
EPI_ISL_2332350, EPI_ISL_2332351, EPI_ISL_2332352, EPI_ISL_2332353, EPI_ISL_2332354, EPI_ISL_2332355, EPI_ISL_2332356, EPI_ISL_2332357, EPI_ISL_2332358, EPI_ISL_2332359, EPI_ISL_2332360, EPI_ISL_2332361, EPI_ISL_2332362, EPI_ISL_2332363, EPI_ISL_2332364, EPI_ISL_2332365, EPI_ISL_2332366, EPI_ISL_2332367, EPI_ISL_2332368, EPI_ISL_2332369, EPI_ISL_2332370, EPI_ISL_2332371,
EPI_ISL_2332372, EPI_ISL_2332373, EPI_ISL_2332374, EPI_ISL_2332375, EPI_ISL_2332376, EPI_ISL_2332377, EPI_ISL_2332378, EPI_ISL_2332379, EPI_ISL_2332380, EPI_ISL_2332381, EPI_ISL_2332382, EPI_ISL_2332383, EPI_ISL_2332384, EPI_ISL_2332385, EPI_ISL_2332386, EPI_ISL_2332387, EPI_ISL_2332388, EPI_ISL_2332389, EPI_ISL_2332390, EPI_ISL_2332391, EPI_ISL_2332392, EPI_ISL_2332393,
EPI_ISL_2332394, EPI_ISL_2332395, EPI_ISL_2332396, EPI_ISL_2332397, EPI_ISL_2332398, EPI_ISL_2332399, EPI_ISL_2332400, EPI_ISL_2332401, EPI_ISL_2332402, EPI_ISL_2332403, EPI_ISL_2332404, EPI_ISL_2332405, EPI_ISL_2332406, EPI_ISL_2332407, EPI_ISL_2332408, EPI_ISL_2332409, EPI_ISL_2332410, EPI_ISL_2332411, EPI_ISL_2332412, EPI_ISL_2332413, EPI_ISL_2332414, EPI_ISL_2332415,
EPI_ISL_2332416, EPI_ISL_2332417, EPI_ISL_2332418, EPI_ISL_2332419, EPI_ISL_2332420, EPI_ISL_2332421, EPI_ISL_2332422, EPI_ISL_2332423, EPI_ISL_2332424, EPI_ISL_2332425, EPI_ISL_2332426, EPI_ISL_2332427, EPI_ISL_2332428, EPI_ISL_2332429, EPI_ISL_2332430, EPI_ISL_2332431, EPI_ISL_2332432, EPI_ISL_2332433, EPI_ISL_2332434, EPI_ISL_2332435, EPI_ISL_2332436, EPI_ISL_2332437,
EPI_ISL_2332438, EPI_ISL_2332439, EPI_ISL_2332440, EPI_ISL_2332441, EPI_ISL_2332442, EPI_ISL_2332443, EPI_ISL_2332444, EPI_ISL_2332445, EPI_ISL_2332446, EPI_ISL_2332447, EPI_ISL_2332448, EPI_ISL_2332449, EPI_ISL_2332450, EPI_ISL_2332451, EPI_ISL_2332452, EPI_ISL_2332453, EPI_ISL_2332454, EPI_ISL_2332455, EPI_ISL_2332456, EPI_ISL_2332457, EPI_ISL_2332458, EPI_ISL_2332459,
EPI_ISL_2332460, EPI_ISL_2332461, EPI_ISL_2332462, EPI_ISL_2332463, EPI_ISL_2332464, EPI_ISL_2332465, EPI_ISL_2332466, EPI_ISL_2332467, EPI_ISL_2332468, EPI_ISL_2332469, EPI_ISL_2332470, EPI_ISL_2332471, EPI_ISL_2332472, EPI_ISL_2332473, EPI_ISL_2332474, EPI_ISL_2332475, EPI_ISL_2332476, EPI_ISL_2332477, EPI_ISL_2332478, EPI_ISL_2332479, EPI_ISL_2332480, EPI_ISL_2332481,
EPI_ISL_2332482, EPI_ISL_2332483, EPI_ISL_2332484, EPI_ISL_2332485, EPI_ISL_2332486, EPI_ISL_2332487, EPI_ISL_2332488, EPI_ISL_2332489, EPI_ISL_2332490, EPI_ISL_2332491, EPI_ISL_2332492, EPI_ISL_2332493, EPI_ISL_2332494, EPI_ISL_2332495, EPI_ISL_2332496, EPI_ISL_2332497, EPI_ISL_2332498, EPI_ISL_2332499, EPI_ISL_2332500, EPI_ISL_2332501, EPI_ISL_2332502, EPI_ISL_2332503,
EPI_ISL_2332504, EPI_ISL_2332505, EPI_ISL_2332506, EPI_ISL_2332507, EPI_ISL_2332508, EPI_ISL_2332509, EPI_ISL_2332510, EPI_ISL_2332511, EPI_ISL_2332512, EPI_ISL_2332513, EPI_ISL_2332514, EPI_ISL_2332515, EPI_ISL_2332516, EPI_ISL_2332517, EPI_ISL_2332518, EPI_ISL_2332519, EPI_ISL_2332520, EPI_ISL_2332521, EPI_ISL_2332522, EPI_ISL_2332523, EPI_ISL_2332524, EPI_ISL_2332525,
EPI_ISL_2332526, EPI_ISL_2332527, EPI_ISL_2332528, EPI_ISL_2332529, EPI_ISL_2332530, EPI_ISL_2332531, EPI_ISL_2332532, EPI_ISL_2332533, EPI_ISL_2332534, EPI_ISL_2332535, EPI_ISL_2332536, EPI_ISL_2332537, EPI_ISL_2332538, EPI_ISL_2332539, EPI_ISL_2332540, EPI_ISL_2332541, EPI_ISL_2332542, EPI_ISL_2332543, EPI_ISL_2332544, EPI_ISL_2332545, EPI_ISL_2332546, EPI_ISL_2332547,
EPI_ISL_2332548, EPI_ISL_2332549, EPI_ISL_2332550, EPI_ISL_2332551, EPI_ISL_2332552, EPI_ISL_2332553, EPI_ISL_2332554, EPI_ISL_2332555, EPI_ISL_2332556, EPI_ISL_2332557, EPI_ISL_2332558, EPI_ISL_2332559, EPI_ISL_2332560, EPI_ISL_2332561, EPI_ISL_2332562, EPI_ISL_2332563, EPI_ISL_2332564, EPI_ISL_2332565, EPI_ISL_2332566, EPI_ISL_2332567, EPI_ISL_2332568, EPI_ISL_2332569,
EPI_ISL_2332570, EPI_ISL_2332571, EPI_ISL_2332572, EPI_ISL_2332573, EPI_ISL_2332574, EPI_ISL_2332575, EPI_ISL_2332576, EPI_ISL_2332577, EPI_ISL_2332578, EPI_ISL_2332579, EPI_ISL_2361092, EPI_ISL_2361093, EPI_ISL_2361094, EPI_ISL_2361095, EPI_ISL_2361096, EPI_ISL_2361097, EPI_ISL_2361098, EPI_ISL_2361099, EPI_ISL_2361100, EPI_ISL_2361101, EPI_ISL_2361102, EPI_ISL_2361103,
EPI_ISL_2361104, EPI_ISL_2361105, EPI_ISL_2361106, EPI_ISL_2361107, EPI_ISL_2361108, EPI_ISL_2361109, EPI_ISL_2361110, EPI_ISL_2361111, EPI_ISL_2361112, EPI_ISL_2361113, EPI_ISL_2361114, EPI_ISL_2361115, EPI_ISL_2361116, EPI_ISL_2361117, EPI_ISL_2361118, EPI_ISL_2361119, EPI_ISL_2361120, EPI_ISL_2361121, EPI_ISL_2361122, EPI_ISL_2361123, EPI_ISL_2361124, EPI_ISL_2361125,
EPI_ISL_2361126, EPI_ISL_2361127, EPI_ISL_2361128, EPI_ISL_2361129, EPI_ISL_2361130, EPI_ISL_2361131, EPI_ISL_2361132, EPI_ISL_2361133, EPI_ISL_2361134, EPI_ISL_2361135, EPI_ISL_2361136, EPI_ISL_2361137, EPI_ISL_2361138, EPI_ISL_2361139, EPI_ISL_2361140, EPI_ISL_2361141, EPI_ISL_2361142, EPI_ISL_2361143, EPI_ISL_2361144, EPI_ISL_2361145, EPI_ISL_2361146, EPI_ISL_2361147,
EPI_ISL_2361148, EPI_ISL_2361149, EPI_ISL_2361150, EPI_ISL_2361151, EPI_ISL_2361152, EPI_ISL_2361153, EPI_ISL_2361154, EPI_ISL_2361155, EPI_ISL_2361156, EPI_ISL_2361157, EPI_ISL_2361158, EPI_ISL_2361159, EPI_ISL_2361160, EPI_ISL_2361161, EPI_ISL_2361162, EPI_ISL_2361163, EPI_ISL_2361164, EPI_ISL_2361165, EPI_ISL_2361166, EPI_ISL_2361167, EPI_ISL_2361168, EPI_ISL_2361169,
EPI_ISL_2361170, EPI_ISL_2361171, EPI_ISL_2361172, EPI_ISL_2361173, EPI_ISL_2361174, EPI_ISL_2361175, EPI_ISL_2361176, EPI_ISL_2361177, EPI_ISL_2361178, EPI_ISL_2361179, EPI_ISL_2361180, EPI_ISL_2361181, EPI_ISL_2361182, EPI_ISL_2361183, EPI_ISL_2361184, EPI_ISL_2361185, EPI_ISL_2361186, EPI_ISL_2361187, EPI_ISL_2361188, EPI_ISL_2361189, EPI_ISL_2361190, EPI_ISL_2361191,
EPI_ISL_2361192, EPI_ISL_2361193, EPI_ISL_2361194, EPI_ISL_2361195, EPI_ISL_2361196, EPI_ISL_2361197, EPI_ISL_2361198, EPI_ISL_2361199, EPI_ISL_2361200, EPI_ISL_2361201, EPI_ISL_2361202, EPI_ISL_2361203, EPI_ISL_2361204, EPI_ISL_2361205, EPI_ISL_2361206, EPI_ISL_2361207, EPI_ISL_2361208, EPI_ISL_2361209, EPI_ISL_2361210, EPI_ISL_2361211, EPI_ISL_2361212, EPI_ISL_2361213,
EPI_ISL_2361214, EPI_ISL_2361215, EPI_ISL_2361216, EPI_ISL_2361217, EPI_ISL_2361218, EPI_ISL_2361219, EPI_ISL_2361220, EPI_ISL_2361221, EPI_ISL_2361222, EPI_ISL_2361223, EPI_ISL_2361224, EPI_ISL_2361225, EPI_ISL_2361226, EPI_ISL_2361227, EPI_ISL_2361228, EPI_ISL_2361229, EPI_ISL_2361230, EPI_ISL_2361231, EPI_ISL_2361232, EPI_ISL_2361233, EPI_ISL_2361234, EPI_ISL_2361235,
EPI_ISL_2361236, EPI_ISL_2361237, EPI_ISL_2361238, EPI_ISL_2361239, EPI_ISL_2361240, EPI_ISL_2361241, EPI_ISL_2361242, EPI_ISL_2361243, EPI_ISL_2361244, EPI_ISL_2361245, EPI_ISL_2361246, EPI_ISL_2361247, EPI_ISL_2361248, EPI_ISL_2361249, EPI_ISL_2361250, EPI_ISL_2361251, EPI_ISL_2361252, EPI_ISL_2361253, EPI_ISL_2361254, EPI_ISL_2361255, EPI_ISL_2361256, EPI_ISL_2361257,
EPI_ISL_2361258, EPI_ISL_2361259, EPI_ISL_2361260, EPI_ISL_2361261, EPI_ISL_2361262, EPI_ISL_2361263, EPI_ISL_2361264, EPI_ISL_2361265, EPI_ISL_2464281, EPI_ISL_2464282, EPI_ISL_2464283, EPI_ISL_2464284, EPI_ISL_2464285, EPI_ISL_2464286, EPI_ISL_2464287, EPI_ISL_2464288, EPI_ISL_2464289, EPI_ISL_2464290, EPI_ISL_2464291, EPI_ISL_2464292, EPI_ISL_2464293, EPI_ISL_2464294,
EPI_ISL_2464295, EPI_ISL_2464296, EPI_ISL_2464297, EPI_ISL_2464298, EPI_ISL_2464299, EPI_ISL_2464300, EPI_ISL_2464301, EPI_ISL_2464302, EPI_ISL_2464303, EPI_ISL_2464304, EPI_ISL_2464305, EPI_ISL_2464306, EPI_ISL_2464307, EPI_ISL_2464308, EPI_ISL_2464309, EPI_ISL_2464310, EPI_ISL_2464311, EPI_ISL_2464312, EPI_ISL_2464313, EPI_ISL_2464314, EPI_ISL_2464315, EPI_ISL_2464316,
EPI_ISL_2464317, EPI_ISL_2464318, EPI_ISL_2464319, EPI_ISL_2464320, EPI_ISL_2464321, EPI_ISL_2464322, EPI_ISL_2464323, EPI_ISL_2464324, EPI_ISL_2464325, EPI_ISL_2464326, EPI_ISL_2464327, EPI_ISL_2464328, EPI_ISL_2464329, EPI_ISL_2464330, EPI_ISL_2464331, EPI_ISL_2464332, EPI_ISL_2464333, EPI_ISL_2464334, EPI_ISL_2464335, EPI_ISL_2464336, EPI_ISL_2464337, EPI_ISL_2464338,
EPI_ISL_2464339, EPI_ISL_2464340, EPI_ISL_2464341, EPI_ISL_2464342, EPI_ISL_2464343, EPI_ISL_2464344, EPI_ISL_2464345, EPI_ISL_2464346, EPI_ISL_2464347, EPI_ISL_2464348, EPI_ISL_2464349, EPI_ISL_2464350, EPI_ISL_2464351, EPI_ISL_2464352, EPI_ISL_2464353, EPI_ISL_2464354, EPI_ISL_2464355, EPI_ISL_2464356, EPI_ISL_2464357, EPI_ISL_2464358, EPI_ISL_2464359, EPI_ISL_2464360,
EPI_ISL_2464361, EPI_ISL_2464362, EPI_ISL_2464363, EPI_ISL_2464364, EPI_ISL_2464365, EPI_ISL_2464366, EPI_ISL_2464367, EPI_ISL_2464368, EPI_ISL_2464369, EPI_ISL_2464370, EPI_ISL_2464371, EPI_ISL_2464372, EPI_ISL_2464373, EPI_ISL_2464374, EPI_ISL_2464375, EPI_ISL_2710384, EPI_ISL_2710385, EPI_ISL_2710386, EPI_ISL_2710387, EPI_ISL_2710388, EPI_ISL_2710389, EPI_ISL_2710390,
EPI_ISL_2710391, EPI_ISL_2710392, EPI_ISL_2710393, EPI_ISL_2710394, EPI_ISL_2710395, EPI_ISL_2710396, EPI_ISL_2710397, EPI_ISL_2710398, EPI_ISL_2710399, EPI_ISL_2710400, EPI_ISL_2710401, EPI_ISL_2710402, EPI_ISL_2710403, EPI_ISL_2710404, EPI_ISL_2710405, EPI_ISL_2710406, EPI_ISL_2710407, EPI_ISL_2710408, EPI_ISL_2710409, EPI_ISL_2710410, EPI_ISL_2710411, EPI_ISL_2710412,
EPI_ISL_2710413, EPI_ISL_2710414, EPI_ISL_2710415, EPI_ISL_2710416, EPI_ISL_2710417, EPI_ISL_2710418, EPI_ISL_2710419, EPI_ISL_2710420, EPI_ISL_2710421, EPI_ISL_2710422, EPI_ISL_2710423, EPI_ISL_2710424, EPI_ISL_2710425, EPI_ISL_2710426, EPI_ISL_2710427, EPI_ISL_2710428, EPI_ISL_2710429, EPI_ISL_2710430, EPI_ISL_2710431, EPI_ISL_2710432, EPI_ISL_2710433, EPI_ISL_2710434,
EPI_ISL_2710435, EPI_ISL_2710436, EPI_ISL_2710437, EPI_ISL_2710438, EPI_ISL_2710439, EPI_ISL_2710440, EPI_ISL_2710441, EPI_ISL_2710442, EPI_ISL_2710443, EPI_ISL_2710444, EPI_ISL_2710445, EPI_ISL_2710446, EPI_ISL_2710447, EPI_ISL_2710448, EPI_ISL_2710449, EPI_ISL_2710450, EPI_ISL_2710451, EPI_ISL_2710452, EPI_ISL_2710453, EPI_ISL_2710454, EPI_ISL_2710455, EPI_ISL_2710456,
EPI_ISL_2710457, EPI_ISL_2710458, EPI_ISL_2710459, EPI_ISL_2710460, EPI_ISL_2710461, EPI_ISL_2710462, EPI_ISL_2710463, EPI_ISL_2710464, EPI_ISL_2710465, EPI_ISL_2710466, EPI_ISL_2710467, EPI_ISL_2710468, EPI_ISL_2710469, EPI_ISL_2710470, EPI_ISL_2710471, EPI_ISL_2710472, EPI_ISL_2710473, EPI_ISL_2710474, EPI_ISL_2710475, EPI_ISL_2710476, EPI_ISL_2710477, EPI_ISL_2710478,
EPI_ISL_2710479, EPI_ISL_2710480, EPI_ISL_2710481, EPI_ISL_2710482, EPI_ISL_2710483, EPI_ISL_2710484, EPI_ISL_2710485, EPI_ISL_2710486, EPI_ISL_2710487, EPI_ISL_2710488, EPI_ISL_2710489, EPI_ISL_2710490, EPI_ISL_2710491, EPI_ISL_2710492, EPI_ISL_2710493, EPI_ISL_2710494, EPI_ISL_2710495, EPI_ISL_2710496, EPI_ISL_2710497, EPI_ISL_2710498, EPI_ISL_2710499, EPI_ISL_2710500,
EPI_ISL_2710501, EPI_ISL_2710502, EPI_ISL_2710503, EPI_ISL_2710504, EPI_ISL_2710505, EPI_ISL_2710506, EPI_ISL_2710507, EPI_ISL_2710508, EPI_ISL_2710509, EPI_ISL_2710510, EPI_ISL_2710511, EPI_ISL_2710512, EPI_ISL_2710513, EPI_ISL_2710514, EPI_ISL_2710515, EPI_ISL_2710516, EPI_ISL_2710517, EPI_ISL_2710518, EPI_ISL_2710519, EPI_ISL_2710520, EPI_ISL_2710521, EPI_ISL_2710522,
EPI_ISL_2710523, EPI_ISL_2710524, EPI_ISL_2710525, EPI_ISL_2710526, EPI_ISL_2710527, EPI_ISL_2710528, EPI_ISL_2710529, EPI_ISL_2710530, EPI_ISL_2710531, EPI_ISL_2710532, EPI_ISL_2710533, EPI_ISL_2710534, EPI_ISL_2710535, EPI_ISL_2710536, EPI_ISL_2710537, EPI_ISL_2710538, EPI_ISL_2710539, EPI_ISL_2710540, EPI_ISL_2710541, EPI_ISL_2710542, EPI_ISL_2710543, EPI_ISL_2710544,
EPI_ISL_2710545, EPI_ISL_2710546, EPI_ISL_2710547, EPI_ISL_2710548, EPI_ISL_2710549, EPI_ISL_2710884, EPI_ISL_2710885, EPI_ISL_2710886, EPI_ISL_2710887, EPI_ISL_2710888, EPI_ISL_2710889, EPI_ISL_2710890, EPI_ISL_2710891, EPI_ISL_2710892, EPI_ISL_2710893, EPI_ISL_2710894, EPI_ISL_2710895, EPI_ISL_2710896, EPI_ISL_2710897, EPI_ISL_2710898, EPI_ISL_2710899, EPI_ISL_2710900,
EPI_ISL_2710901, EPI_ISL_2710902, EPI_ISL_2710903, EPI_ISL_2710904, EPI_ISL_2710905, EPI_ISL_2710906, EPI_ISL_2710907, EPI_ISL_2710908, EPI_ISL_2710909, EPI_ISL_2710910, EPI_ISL_2710911, EPI_ISL_2710912, EPI_ISL_2710913, EPI_ISL_2710914, EPI_ISL_2710915, EPI_ISL_2710916, EPI_ISL_2710917, EPI_ISL_2710918, EPI_ISL_2710919, EPI_ISL_2710920, EPI_ISL_2710921, EPI_ISL_2710922,
EPI_ISL_2710923, EPI_ISL_2710924, EPI_ISL_2710925, EPI_ISL_2710926, EPI_ISL_2710927, EPI_ISL_2710928, EPI_ISL_2710929, EPI_ISL_2710930, EPI_ISL_2710931, EPI_ISL_2710932, EPI_ISL_2710933, EPI_ISL_2710934, EPI_ISL_2710935, EPI_ISL_2710936, EPI_ISL_2710937, EPI_ISL_2710938, EPI_ISL_2710939, EPI_ISL_2710940, EPI_ISL_2710941, EPI_ISL_2710942, EPI_ISL_2710943, EPI_ISL_2710944,
EPI_ISL_2710945, EPI_ISL_2710946, EPI_ISL_2710947, EPI_ISL_2710948, EPI_ISL_2710949, EPI_ISL_2710950, EPI_ISL_2710951, EPI_ISL_2710952, EPI_ISL_2710953, EPI_ISL_2710954, EPI_ISL_2710955, EPI_ISL_2710956, EPI_ISL_2710957, EPI_ISL_2710958, EPI_ISL_2710959, EPI_ISL_2710960, EPI_ISL_2710961, EPI_ISL_2710962, EPI_ISL_2710963, EPI_ISL_2710964, EPI_ISL_2710965, EPI_ISL_2710966,
EPI_ISL_2710967, EPI_ISL_2710968, EPI_ISL_2710969, EPI_ISL_2710970, EPI_ISL_2710971, EPI_ISL_2710972, EPI_ISL_2710973, EPI_ISL_2710974, EPI_ISL_2710975, EPI_ISL_2710976, EPI_ISL_2710977, EPI_ISL_2710978, EPI_ISL_2710979, EPI_ISL_2710980, EPI_ISL_2710981, EPI_ISL_2710982, EPI_ISL_2710983, EPI_ISL_2710984, EPI_ISL_2710985, EPI_ISL_2710986, EPI_ISL_2710987, EPI_ISL_2710988,
EPI_ISL_2710989, EPI_ISL_2710990, EPI_ISL_2710991, EPI_ISL_2710992, EPI_ISL_2710993, EPI_ISL_2710994, EPI_ISL_2710995, EPI_ISL_2710996, EPI_ISL_2710997, EPI_ISL_2710998, EPI_ISL_2710999, EPI_ISL_2711000, EPI_ISL_2711001, EPI_ISL_2711002, EPI_ISL_2711003, EPI_ISL_2711004, EPI_ISL_2711005, EPI_ISL_2711006, EPI_ISL_2711007, EPI_ISL_2711008, EPI_ISL_2711009, EPI_ISL_2711010,
EPI_ISL_2711011, EPI_ISL_2711012, EPI_ISL_2711013, EPI_ISL_2711014, EPI_ISL_2711015, EPI_ISL_2711016, EPI_ISL_2711017, EPI_ISL_2711018, EPI_ISL_2711019, EPI_ISL_2711020, EPI_ISL_2711021, EPI_ISL_2711022, EPI_ISL_2711023, EPI_ISL_2711024, EPI_ISL_2711025, EPI_ISL_2711026, EPI_ISL_2711027, EPI_ISL_2711028, EPI_ISL_2711029, EPI_ISL_2711030, EPI_ISL_2711031, EPI_ISL_2711032,
EPI_ISL_2711033, EPI_ISL_2711034, EPI_ISL_2711035, EPI_ISL_2711036, EPI_ISL_2711037, EPI_ISL_2711038, EPI_ISL_2711039, EPI_ISL_2711040, EPI_ISL_2711041, EPI_ISL_2711042, EPI_ISL_2711043, EPI_ISL_2711044, EPI_ISL_2711045, EPI_ISL_2711046, EPI_ISL_2711047, EPI_ISL_2711048, EPI_ISL_2711049, EPI_ISL_2711050, EPI_ISL_2711051, EPI_ISL_2711052, EPI_ISL_2711053, EPI_ISL_2711054,
EPI_ISL_2711055, EPI_ISL_2711056, EPI_ISL_2711057, EPI_ISL_2711058, EPI_ISL_2711059, EPI_ISL_2711060, EPI_ISL_2711061, EPI_ISL_2711062, EPI_ISL_2711063, EPI_ISL_2711064, EPI_ISL_2711065, EPI_ISL_2711066, EPI_ISL_2711067, EPI_ISL_2711068, EPI_ISL_2711069, EPI_ISL_2711070, EPI_ISL_2711071, EPI_ISL_2711072, EPI_ISL_2711073, EPI_ISL_2711074, EPI_ISL_2711075, EPI_ISL_2711076,
EPI_ISL_2711077, EPI_ISL_2711078, EPI_ISL_2711079, EPI_ISL_2711080, EPI_ISL_2711081, EPI_ISL_2711082, EPI_ISL_2711083, EPI_ISL_2711084, EPI_ISL_2711085, EPI_ISL_2711086, EPI_ISL_2711087, EPI_ISL_2711088, EPI_ISL_2711089, EPI_ISL_2711090, EPI_ISL_2711091, EPI_ISL_2711092, EPI_ISL_2711093, EPI_ISL_2711094, EPI_ISL_2711095, EPI_ISL_2711096, EPI_ISL_2711097, EPI_ISL_2711098,
EPI_ISL_2711099, EPI_ISL_2711100, EPI_ISL_2711101, EPI_ISL_2711102, EPI_ISL_2711103, EPI_ISL_2711104, EPI_ISL_2711105, EPI_ISL_2711106, EPI_ISL_2711107, EPI_ISL_2711108, EPI_ISL_2711109, EPI_ISL_2711110, EPI_ISL_2711111, EPI_ISL_2711112, EPI_ISL_2711113, EPI_ISL_2711114, EPI_ISL_2711115, EPI_ISL_2711116, EPI_ISL_2711117, EPI_ISL_2711118, EPI_ISL_2711119, EPI_ISL_2711120,
EPI_ISL_2711121, EPI_ISL_2711122, EPI_ISL_2711123, EPI_ISL_2711124, EPI_ISL_2711125, EPI_ISL_2711126, EPI_ISL_2711127, EPI_ISL_2711128, EPI_ISL_2711129, EPI_ISL_2711130, EPI_ISL_2711131, EPI_ISL_2711132, EPI_ISL_2711133, EPI_ISL_2711134, EPI_ISL_2711135, EPI_ISL_2711136, EPI_ISL_2711137, EPI_ISL_2711138, EPI_ISL_2711139, EPI_ISL_2711140, EPI_ISL_2711141, EPI_ISL_2711142,
EPI_ISL_2711143, EPI_ISL_2711144, EPI_ISL_2711145, EPI_ISL_2711146, EPI_ISL_2711147, EPI_ISL_2711148, EPI_ISL_2711149, EPI_ISL_2711150, EPI_ISL_2711151, EPI_ISL_2711152, EPI_ISL_2711153, EPI_ISL_2711154, EPI_ISL_2711155, EPI_ISL_2711156, EPI_ISL_2711157, EPI_ISL_2711158, EPI_ISL_2711159, EPI_ISL_2711160, EPI_ISL_2711161, EPI_ISL_2711162, EPI_ISL_2711163, EPI_ISL_2711164,
EPI_ISL_2711165, EPI_ISL_2711166, EPI_ISL_2711167, EPI_ISL_2711168, EPI_ISL_2711169, EPI_ISL_2711170, EPI_ISL_2711171, EPI_ISL_2711172, EPI_ISL_2711173, EPI_ISL_2711174, EPI_ISL_2711175, EPI_ISL_2711176, EPI_ISL_2711177, EPI_ISL_2711178, EPI_ISL_2711179, EPI_ISL_2711180, EPI_ISL_2711181, EPI_ISL_2711182, EPI_ISL_2711183, EPI_ISL_2711184, EPI_ISL_2711185, EPI_ISL_2711186,
EPI_ISL_2711187, EPI_ISL_2711188, EPI_ISL_2711189, EPI_ISL_2711190, EPI_ISL_2711191, EPI_ISL_2711192, EPI_ISL_2711193, EPI_ISL_2711194, EPI_ISL_2711195, EPI_ISL_2711196, EPI_ISL_2711197, EPI_ISL_2711198, EPI_ISL_2711199, EPI_ISL_2711200, EPI_ISL_2711201, EPI_ISL_2711202, EPI_ISL_2711203, EPI_ISL_2711204, EPI_ISL_2711205, EPI_ISL_2711206, EPI_ISL_2711207, EPI_ISL_2711208,
EPI_ISL_2711209, EPI_ISL_2711210, EPI_ISL_2711211, EPI_ISL_2711212, EPI_ISL_2711213, EPI_ISL_2711214, EPI_ISL_2711215, EPI_ISL_2711216, EPI_ISL_2711217, EPI_ISL_2711218, EPI_ISL_2711219, EPI_ISL_2711220, EPI_ISL_2711221, EPI_ISL_2711222, EPI_ISL_2711223, EPI_ISL_2711224, EPI_ISL_2711225, EPI_ISL_2711226, EPI_ISL_2711227, EPI_ISL_2711228, EPI_ISL_2711229, EPI_ISL_2711230,
EPI_ISL_2711231, EPI_ISL_2711232, EPI_ISL_2711233, EPI_ISL_2711234, EPI_ISL_2711235, EPI_ISL_2711236, EPI_ISL_2711237, EPI_ISL_2711238, EPI_ISL_2711239, EPI_ISL_2711240, EPI_ISL_2711241, EPI_ISL_2711242, EPI_ISL_2711243, EPI_ISL_2711244, EPI_ISL_2711245, EPI_ISL_2711246, EPI_ISL_2711247, EPI_ISL_2711248, EPI_ISL_2711249, EPI_ISL_2711250, EPI_ISL_2711251, EPI_ISL_2711252,
EPI_ISL_2711253, EPI_ISL_2711254, EPI_ISL_2711255, EPI_ISL_2711256, EPI_ISL_2711257, EPI_ISL_2711258, EPI_ISL_2711259, EPI_ISL_2711260, EPI_ISL_2711261, EPI_ISL_2711262, EPI_ISL_2711263, EPI_ISL_2711264, EPI_ISL_2711265, EPI_ISL_2711266, EPI_ISL_2711267, EPI_ISL_2711268, EPI_ISL_2711269, EPI_ISL_2711270, EPI_ISL_2711271, EPI_ISL_2711272, EPI_ISL_2711273, EPI_ISL_2711274,
EPI_ISL_2711275, EPI_ISL_2711276, EPI_ISL_2711277, EPI_ISL_2711278, EPI_ISL_2711279, EPI_ISL_2711280, EPI_ISL_2711281, EPI_ISL_2711282, EPI_ISL_2711283, EPI_ISL_2711284, EPI_ISL_2711285, EPI_ISL_2711286, EPI_ISL_2711287, EPI_ISL_2711288, EPI_ISL_2711289, EPI_ISL_2711290, EPI_ISL_2711291, EPI_ISL_2711292, EPI_ISL_2711293, EPI_ISL_2711294, EPI_ISL_2711295, EPI_ISL_2711296,
EPI_ISL_2711297, EPI_ISL_2711298, EPI_ISL_2711299, EPI_ISL_2711300, EPI_ISL_2711301, EPI_ISL_2711302, EPI_ISL_2711303, EPI_ISL_2711304, EPI_ISL_2711305, EPI_ISL_2711306, EPI_ISL_2711307, EPI_ISL_2711308, EPI_ISL_2711309, EPI_ISL_2711310, EPI_ISL_2711311, EPI_ISL_2711312, EPI_ISL_2711313, EPI_ISL_2711314, EPI_ISL_2711315, EPI_ISL_2711316, EPI_ISL_2711317, EPI_ISL_2711318,
EPI_ISL_2711319, EPI_ISL_2711320, EPI_ISL_2711321, EPI_ISL_2711322, EPI_ISL_2711323, EPI_ISL_2711324, EPI_ISL_2711325, EPI_ISL_2711326, EPI_ISL_2711327, EPI_ISL_2711328, EPI_ISL_2711329, EPI_ISL_2711330, EPI_ISL_2711331, EPI_ISL_2711332, EPI_ISL_2711333, EPI_ISL_2711334, EPI_ISL_2711335, EPI_ISL_2711336, EPI_ISL_2711337, EPI_ISL_2711338, EPI_ISL_2711339, EPI_ISL_2711340,
EPI_ISL_2711341, EPI_ISL_2711342, EPI_ISL_2711343, EPI_ISL_2711344, EPI_ISL_2711345, EPI_ISL_2711346, EPI_ISL_2711347, EPI_ISL_2711348, EPI_ISL_2711349, EPI_ISL_2711350, EPI_ISL_2711351, EPI_ISL_2711352, EPI_ISL_2711353, EPI_ISL_2711354, EPI_ISL_2711355, EPI_ISL_2711356, EPI_ISL_2711357, EPI_ISL_2711358, EPI_ISL_2711359, EPI_ISL_2711360, EPI_ISL_2711361, EPI_ISL_2711362,
EPI_ISL_2711363, EPI_ISL_2711364, EPI_ISL_2711365, EPI_ISL_2711366, EPI_ISL_2711367, EPI_ISL_2711368, EPI_ISL_2711369, EPI_ISL_2711370, EPI_ISL_2711371, EPI_ISL_2711372, EPI_ISL_2711373, EPI_ISL_2711374, EPI_ISL_2711375, EPI_ISL_2711376, EPI_ISL_2711377, EPI_ISL_2711378, EPI_ISL_2711379, EPI_ISL_2711380, EPI_ISL_2711381, EPI_ISL_2711382, EPI_ISL_2711383, EPI_ISL_2711384,
EPI_ISL_2711385, EPI_ISL_2711386, EPI_ISL_2711387, EPI_ISL_2711388, EPI_ISL_2711389, EPI_ISL_2711390, EPI_ISL_2711391, EPI_ISL_2711392, EPI_ISL_2711393, EPI_ISL_2711394, EPI_ISL_2711395, EPI_ISL_2711396, EPI_ISL_2711397, EPI_ISL_2711398, EPI_ISL_2711399, EPI_ISL_2711400, EPI_ISL_2711401, EPI_ISL_2711402, EPI_ISL_2711403, EPI_ISL_2711404, EPI_ISL_2711405, EPI_ISL_2711406,
EPI_ISL_2711407, EPI_ISL_2711408, EPI_ISL_2711409, EPI_ISL_2711410, EPI_ISL_2711411, EPI_ISL_2711412, EPI_ISL_2711413, EPI_ISL_2711414, EPI_ISL_2711415, EPI_ISL_2711416, EPI_ISL_2711417, EPI_ISL_2711418, EPI_ISL_2711419, EPI_ISL_2711420, EPI_ISL_2711421, EPI_ISL_2711422, EPI_ISL_2711423, EPI_ISL_2711424, EPI_ISL_2711425, EPI_ISL_2711426, EPI_ISL_2711427, EPI_ISL_2711428,
EPI_ISL_2711429, EPI_ISL_2711430, EPI_ISL_2711431, EPI_ISL_2711432, EPI_ISL_2711433, EPI_ISL_2711434, EPI_ISL_2711435, EPI_ISL_2711436, EPI_ISL_2711437, EPI_ISL_2711438, EPI_ISL_2711439, EPI_ISL_2711440, EPI_ISL_2711441, EPI_ISL_2711442, EPI_ISL_2711443, EPI_ISL_2711444, EPI_ISL_2711445, EPI_ISL_2711446, EPI_ISL_2711447, EPI_ISL_2711448, EPI_ISL_2711449, EPI_ISL_2711450,
EPI_ISL_2711451, EPI_ISL_2711452, EPI_ISL_2711453, EPI_ISL_2711454, EPI_ISL_2711455, EPI_ISL_2711456, EPI_ISL_2711457, EPI_ISL_2711458, EPI_ISL_2711459, EPI_ISL_2711460, EPI_ISL_2711461, EPI_ISL_2711462, EPI_ISL_2711463, EPI_ISL_2711464, EPI_ISL_2711465, EPI_ISL_2711466, EPI_ISL_2711467, EPI_ISL_2711468, EPI_ISL_2711469, EPI_ISL_2711470, EPI_ISL_2711471, EPI_ISL_2711472,
EPI_ISL_2711473, EPI_ISL_2711474, EPI_ISL_2711475, EPI_ISL_2711476, EPI_ISL_2711477, EPI_ISL_2711478, EPI_ISL_2711479, EPI_ISL_2711480, EPI_ISL_2711481, EPI_ISL_2711482, EPI_ISL_2711483, EPI_ISL_2711484, EPI_ISL_2711485, EPI_ISL_2711486, EPI_ISL_2711487, EPI_ISL_2711488, EPI_ISL_2711489, EPI_ISL_2711490, EPI_ISL_2711491, EPI_ISL_2711492, EPI_ISL_2711493, EPI_ISL_2711494,
EPI_ISL_2711495, EPI_ISL_2711496, EPI_ISL_2711497, EPI_ISL_2711498, EPI_ISL_2711499, EPI_ISL_2711500, EPI_ISL_2711501, EPI_ISL_2711502, EPI_ISL_2711503, EPI_ISL_2711504, EPI_ISL_2711505, EPI_ISL_2711506, EPI_ISL_2711507, EPI_ISL_2711508, EPI_ISL_2711509, EPI_ISL_2711510, EPI_ISL_2711511, EPI_ISL_2711512, EPI_ISL_2711513, EPI_ISL_2711514, EPI_ISL_2711515, EPI_ISL_2711516,
EPI_ISL_2711517, EPI_ISL_2711518, EPI_ISL_2711519, EPI_ISL_2711520, EPI_ISL_2711521, EPI_ISL_2711522, EPI_ISL_2711523, EPI_ISL_2711524, EPI_ISL_2711525, EPI_ISL_2711526, EPI_ISL_2711527, EPI_ISL_2711528, EPI_ISL_2711529, EPI_ISL_2711530, EPI_ISL_2711531, EPI_ISL_2711532, EPI_ISL_2711533, EPI_ISL_2711534, EPI_ISL_2711535, EPI_ISL_2711536, EPI_ISL_2711537, EPI_ISL_2711538,
EPI_ISL_2711539, EPI_ISL_2711540, EPI_ISL_2711541, EPI_ISL_2711542, EPI_ISL_2711543, EPI_ISL_2711544, EPI_ISL_2711545, EPI_ISL_2711546, EPI_ISL_2711547, EPI_ISL_2711548, EPI_ISL_2711549, EPI_ISL_2711550, EPI_ISL_2711551, EPI_ISL_2711552, EPI_ISL_2711553, EPI_ISL_2711554, EPI_ISL_2711555, EPI_ISL_2711556, EPI_ISL_2711557, EPI_ISL_2711558, EPI_ISL_2711559, EPI_ISL_2711560,
EPI_ISL_2711561, EPI_ISL_2711562, EPI_ISL_2711563, EPI_ISL_2711564, EPI_ISL_2711565, EPI_ISL_2711566, EPI_ISL_2711567, EPI_ISL_2711568, EPI_ISL_2711569, EPI_ISL_2711570, EPI_ISL_2711571, EPI_ISL_2711572, EPI_ISL_2711573, EPI_ISL_2711574, EPI_ISL_2711575, EPI_ISL_2711576, EPI_ISL_2711577, EPI_ISL_2711578, EPI_ISL_2711579, EPI_ISL_2711580, EPI_ISL_2711581, EPI_ISL_2711582,
EPI_ISL_2711583, EPI_ISL_2711584, EPI_ISL_2711585, EPI_ISL_2711586, EPI_ISL_2711587, EPI_ISL_2711588, EPI_ISL_2711589, EPI_ISL_2711590, EPI_ISL_2711591, EPI_ISL_2711592, EPI_ISL_2711593, EPI_ISL_2711594, EPI_ISL_2711595, EPI_ISL_2711596, EPI_ISL_2711597, EPI_ISL_2711598, EPI_ISL_2711599, EPI_ISL_2711600, EPI_ISL_2711601, EPI_ISL_2711602, EPI_ISL_2711603, EPI_ISL_2711604,
EPI_ISL_2711605, EPI_ISL_2711606, EPI_ISL_2711607, EPI_ISL_2711608, EPI_ISL_2711609, EPI_ISL_2711610, EPI_ISL_2711611, EPI_ISL_2711612, EPI_ISL_2711613, EPI_ISL_2711614, EPI_ISL_2711615, EPI_ISL_2711616, EPI_ISL_2711617, EPI_ISL_2711618, EPI_ISL_2711619, EPI_ISL_2711620, EPI_ISL_2711621, EPI_ISL_2711622, EPI_ISL_2711623, EPI_ISL_2711624, EPI_ISL_2711625, EPI_ISL_2711626,
EPI_ISL_2711627, EPI_ISL_2711628, EPI_ISL_2711629, EPI_ISL_2711630, EPI_ISL_2711631, EPI_ISL_2711632, EPI_ISL_2711633, EPI_ISL_2711634, EPI_ISL_2711635, EPI_ISL_2711636, EPI_ISL_2711637, EPI_ISL_2711638, EPI_ISL_2711639, EPI_ISL_2711640, EPI_ISL_2711641, EPI_ISL_2711642, EPI_ISL_2711643, EPI_ISL_2711644, EPI_ISL_2711645, EPI_ISL_2711646, EPI_ISL_2711647, EPI_ISL_2711648,
EPI_ISL_2711649, EPI_ISL_2711650, EPI_ISL_2711651, EPI_ISL_2711652, EPI_ISL_2711653, EPI_ISL_2711654, EPI_ISL_2711655, EPI_ISL_2711656, EPI_ISL_2711657, EPI_ISL_2711658, EPI_ISL_2711659, EPI_ISL_2711660, EPI_ISL_2711661, EPI_ISL_2711662, EPI_ISL_2711663, EPI_ISL_2711664, EPI_ISL_2711665, EPI_ISL_2711666, EPI_ISL_2711667, EPI_ISL_2711668, EPI_ISL_2711669, EPI_ISL_2711670,
EPI_ISL_2711671, EPI_ISL_2711672, EPI_ISL_2711673, EPI_ISL_2711674, EPI_ISL_2711675, EPI_ISL_2711676, EPI_ISL_2711677, EPI_ISL_2711678, EPI_ISL_2711679, EPI_ISL_2711680, EPI_ISL_2711681, EPI_ISL_2711682, EPI_ISL_2711683, EPI_ISL_2711684, EPI_ISL_2711685, EPI_ISL_2711686, EPI_ISL_2711687, EPI_ISL_2711688, EPI_ISL_2711689, EPI_ISL_2711690, EPI_ISL_2711691, EPI_ISL_2711692,
EPI_ISL_2711693, EPI_ISL_2711694, EPI_ISL_2711695, EPI_ISL_2711696, EPI_ISL_2711697, EPI_ISL_2711698, EPI_ISL_2711699, EPI_ISL_2711700, EPI_ISL_2711701, EPI_ISL_2711702, EPI_ISL_2711703, EPI_ISL_2711704, EPI_ISL_2711705, EPI_ISL_2711706, EPI_ISL_2711707, EPI_ISL_2711708, EPI_ISL_2711709, EPI_ISL_2711710, EPI_ISL_2711711, EPI_ISL_2711712, EPI_ISL_2711713, EPI_ISL_2711714,
EPI_ISL_2711715, EPI_ISL_2711716, EPI_ISL_2711717, EPI_ISL_2711718, EPI_ISL_2711719, EPI_ISL_2711720, EPI_ISL_2711721, EPI_ISL_2711722, EPI_ISL_2711723, EPI_ISL_2711724, EPI_ISL_2711725, EPI_ISL_2711726, EPI_ISL_2711727, EPI_ISL_2711728, EPI_ISL_2711729, EPI_ISL_2711730, EPI_ISL_2711731, EPI_ISL_2711732, EPI_ISL_2711733, EPI_ISL_2711734, EPI_ISL_2711735, EPI_ISL_2711736,
EPI_ISL_2711737, EPI_ISL_2711738, EPI_ISL_2711739, EPI_ISL_2711740, EPI_ISL_2711741, EPI_ISL_2711742, EPI_ISL_2711743, EPI_ISL_2711744, EPI_ISL_2711745, EPI_ISL_2711746, EPI_ISL_2711747, EPI_ISL_2711748, EPI_ISL_2711749, EPI_ISL_2711750, EPI_ISL_2711751, EPI_ISL_2711752, EPI_ISL_2711753, EPI_ISL_2711754, EPI_ISL_2711755, EPI_ISL_2711756, EPI_ISL_2711757, EPI_ISL_2711758,
EPI_ISL_2711759, EPI_ISL_2711760, EPI_ISL_2711761, EPI_ISL_2711762, EPI_ISL_2711763, EPI_ISL_2711764, EPI_ISL_2711765, EPI_ISL_2711766, EPI_ISL_2711767, EPI_ISL_2711768, EPI_ISL_2711769, EPI_ISL_2711770, EPI_ISL_2711771, EPI_ISL_2711772, EPI_ISL_2711773, EPI_ISL_2711774, EPI_ISL_2711775, EPI_ISL_2711776, EPI_ISL_2711777, EPI_ISL_2711778, EPI_ISL_2711779, EPI_ISL_2711780,
EPI_ISL_2711781, EPI_ISL_2711782, EPI_ISL_2711783, EPI_ISL_2711784, EPI_ISL_2711785, EPI_ISL_2711786, EPI_ISL_2711787, EPI_ISL_2711788, EPI_ISL_2711789, EPI_ISL_2711790, EPI_ISL_2711791, EPI_ISL_2711792, EPI_ISL_2711793, EPI_ISL_2711794, EPI_ISL_2711795, EPI_ISL_2711796, EPI_ISL_2711797, EPI_ISL_2711798, EPI_ISL_2711799, EPI_ISL_2711800, EPI_ISL_2711801, EPI_ISL_2711802,
EPI_ISL_2711803, EPI_ISL_2711804, EPI_ISL_2711805, EPI_ISL_2711806, EPI_ISL_2711807, EPI_ISL_2711808, EPI_ISL_2711809, EPI_ISL_2711810, EPI_ISL_2711811, EPI_ISL_2711812, EPI_ISL_2711813, EPI_ISL_2711814, EPI_ISL_2711815, EPI_ISL_2711816, EPI_ISL_2711817, EPI_ISL_2711818, EPI_ISL_2711819, EPI_ISL_2711820, EPI_ISL_2711821, EPI_ISL_2711822, EPI_ISL_2711823, EPI_ISL_2711824,
EPI_ISL_2711825, EPI_ISL_2711826, EPI_ISL_2711827, EPI_ISL_2711828, EPI_ISL_2711829, EPI_ISL_2711830, EPI_ISL_2711831, EPI_ISL_2711832, EPI_ISL_2711833, EPI_ISL_2711834, EPI_ISL_2711835, EPI_ISL_2711836, EPI_ISL_2711837, EPI_ISL_2711838, EPI_ISL_2711839, EPI_ISL_2711840, EPI_ISL_2711841, EPI_ISL_2711842, EPI_ISL_2711843, EPI_ISL_2711844, EPI_ISL_2711845, EPI_ISL_2711846,
EPI_ISL_2711847, EPI_ISL_2711848, EPI_ISL_2711849, EPI_ISL_2711850, EPI_ISL_2711851, EPI_ISL_2711852, EPI_ISL_2711853, EPI_ISL_2711854, EPI_ISL_2711855, EPI_ISL_2711856, EPI_ISL_2711857, EPI_ISL_2711858, EPI_ISL_2711859, EPI_ISL_2711860, EPI_ISL_2711861, EPI_ISL_2711862, EPI_ISL_2711863, EPI_ISL_2711864, EPI_ISL_2711865, EPI_ISL_2711866, EPI_ISL_2711867, EPI_ISL_2711868,

EPI_ISL_2711869, EPI_ISL_2711870, EPI_ISL_2711871, EPI_ISL_2711872, EPI_ISL_2711873, EPI_ISL_2711874, EPI_ISL_2711875, EPI_ISL_2711876, EPI_ISL_2711877, EPI_ISL_2711878, EPI_ISL_2711879, EPI_ISL_2711880, EPI_ISL_2711881, EPI_ISL_2711882, EPI_ISL_2711883, EPI_ISL_2711884, EPI_ISL_2711885, EPI_ISL_2711886, EPI_ISL_2711887, EPI_ISL_2711888, EPI_ISL_2711889, EPI_ISL_2711890,
EPI_ISL_2711891, EPI_ISL_2711892, EPI_ISL_2711893, EPI_ISL_2711894, EPI_ISL_2711895, EPI_ISL_2711896, EPI_ISL_2711897, EPI_ISL_2711898, EPI_ISL_2711899, EPI_ISL_2711900, EPI_ISL_2711901, EPI_ISL_2711902, EPI_ISL_2711903, EPI_ISL_2711904, EPI_ISL_2711905, EPI_ISL_2711906, EPI_ISL_2711907, EPI_ISL_2711908, EPI_ISL_2711909, EPI_ISL_2711910, EPI_ISL_2711911, EPI_ISL_2711912,
EPI_ISL_2711913, EPI_ISL_2711914, EPI_ISL_2711915, EPI_ISL_2711916, EPI_ISL_2711917, EPI_ISL_2711918, EPI_ISL_2711919, EPI_ISL_2711920, EPI_ISL_2711921, EPI_ISL_2711922, EPI_ISL_2711923, EPI_ISL_2711924, EPI_ISL_2711925, EPI_ISL_2711926, EPI_ISL_2711927, EPI_ISL_2711928, EPI_ISL_2711929, EPI_ISL_2711930, EPI_ISL_2711931, EPI_ISL_2711932, EPI_ISL_2711933, EPI_ISL_2711934,
EPI_ISL_2711935, EPI_ISL_2711936, EPI_ISL_2711937, EPI_ISL_2711938, EPI_ISL_2711939, EPI_ISL_2711940, EPI_ISL_2711941, EPI_ISL_2711942, EPI_ISL_2711943, EPI_ISL_2711944, EPI_ISL_2711945, EPI_ISL_2711946, EPI_ISL_2711947, EPI_ISL_2711948, EPI_ISL_2711949, EPI_ISL_2711950, EPI_ISL_2711951, EPI_ISL_2711952, EPI_ISL_2711953, EPI_ISL_2711954, EPI_ISL_2711955, EPI_ISL_2711956,
EPI_ISL_2711957, EPI_ISL_2711958, EPI_ISL_2711959, EPI_ISL_2711960, EPI_ISL_2711961, EPI_ISL_2711962, EPI_ISL_2711963, EPI_ISL_2711964, EPI_ISL_2711965, EPI_ISL_2711966, EPI_ISL_2711967, EPI_ISL_2711968, EPI_ISL_2711969, EPI_ISL_2711970, EPI_ISL_2711971, EPI_ISL_2711972, EPI_ISL_2711973, EPI_ISL_2711974, EPI_ISL_2711975, EPI_ISL_2711976, EPI_ISL_2711977, EPI_ISL_2711978,
EPI_ISL_2711979, EPI_ISL_2711980, EPI_ISL_2711981, EPI_ISL_2711982, EPI_ISL_2711983, EPI_ISL_2711984, EPI_ISL_2711985, EPI_ISL_2711986, EPI_ISL_2711987, EPI_ISL_2711988, EPI_ISL_2711989, EPI_ISL_2711990, EPI_ISL_2711991, EPI_ISL_2711992, EPI_ISL_2711993, EPI_ISL_2711994, EPI_ISL_2711995, EPI_ISL_2711996, EPI_ISL_2711997, EPI_ISL_2711998, EPI_ISL_2711999, EPI_ISL_2712000,
EPI_ISL_2712001, EPI_ISL_2712002, EPI_ISL_2712003, EPI_ISL_2712004, EPI_ISL_2712005, EPI_ISL_2712006, EPI_ISL_2712007, EPI_ISL_2712008, EPI_ISL_2712009, EPI_ISL_2712010, EPI_ISL_2712011, EPI_ISL_2712012, EPI_ISL_2712013, EPI_ISL_2712014, EPI_ISL_2712015, EPI_ISL_2712016, EPI_ISL_2712017, EPI_ISL_2712018, EPI_ISL_2712019, EPI_ISL_2712020, EPI_ISL_2712021, EPI_ISL_2712022,
EPI_ISL_2712023, EPI_ISL_2712024, EPI_ISL_2712025, EPI_ISL_2712026, EPI_ISL_2712027, EPI_ISL_2712028, EPI_ISL_2712029, EPI_ISL_2712030, EPI_ISL_2712031, EPI_ISL_2712032, EPI_ISL_2712033, EPI_ISL_2712034, EPI_ISL_2712035, EPI_ISL_2712036, EPI_ISL_2712037, EPI_ISL_2712038, EPI_ISL_2712039, EPI_ISL_2712040, EPI_ISL_2712041, EPI_ISL_2712042, EPI_ISL_2712043, EPI_ISL_2712044,
EPI_ISL_2712045, EPI_ISL_2712046, EPI_ISL_2712047, EPI_ISL_2712048, EPI_ISL_2712049, EPI_ISL_2712050, EPI_ISL_2712051, EPI_ISL_2712052, EPI_ISL_2712053, EPI_ISL_2712054, EPI_ISL_2712055, EPI_ISL_2712056, EPI_ISL_2712057, EPI_ISL_2712058, EPI_ISL_2712059, EPI_ISL_2712060, EPI_ISL_2712061, EPI_ISL_2712062, EPI_ISL_2712063, EPI_ISL_2712064, EPI_ISL_2712065, EPI_ISL_2712066,
EPI_ISL_2712067, EPI_ISL_2712068, EPI_ISL_2712069, EPI_ISL_2712070, EPI_ISL_2712071, EPI_ISL_2712072, EPI_ISL_2712073, EPI_ISL_2712074, EPI_ISL_2712075, EPI_ISL_2712076, EPI_ISL_2712077, EPI_ISL_2712078, EPI_ISL_2712079, EPI_ISL_2712080, EPI_ISL_2712081, EPI_ISL_2712082, EPI_ISL_2712083, EPI_ISL_2712084, EPI_ISL_2712085, EPI_ISL_2712086, EPI_ISL_2712087, EPI_ISL_2712088,
EPI_ISL_2712089, EPI_ISL_2712090, EPI_ISL_2712091, EPI_ISL_2712092, EPI_ISL_2712093, EPI_ISL_2712094, EPI_ISL_2712095, EPI_ISL_2712096, EPI_ISL_2712097, EPI_ISL_2712098, EPI_ISL_2712099, EPI_ISL_2712100, EPI_ISL_2712101, EPI_ISL_2712102, EPI_ISL_2712103, EPI_ISL_2712104, EPI_ISL_2712105, EPI_ISL_2712106, EPI_ISL_2712107, EPI_ISL_2712108, EPI_ISL_2712109, EPI_ISL_2712110,
EPI_ISL_2712111, EPI_ISL_2712112, EPI_ISL_2712113, EPI_ISL_2712114, EPI_ISL_2712115, EPI_ISL_2712116, EPI_ISL_2712117, EPI_ISL_2712118, EPI_ISL_2712119, EPI_ISL_2712120, EPI_ISL_2712121, EPI_ISL_2712122, EPI_ISL_2712123, EPI_ISL_2712124, EPI_ISL_2712125, EPI_ISL_2712126, EPI_ISL_2712127, EPI_ISL_2712128, EPI_ISL_2712129, EPI_ISL_2712130, EPI_ISL_2712131, EPI_ISL_2712132,
EPI_ISL_2712133, EPI_ISL_2712134, EPI_ISL_2712135, EPI_ISL_2712136, EPI_ISL_2712137, EPI_ISL_2712138, EPI_ISL_2712139, EPI_ISL_2712140, EPI_ISL_2712141, EPI_ISL_2712142, EPI_ISL_2712143, EPI_ISL_2712144, EPI_ISL_2712145, EPI_ISL_2712146, EPI_ISL_2712147, EPI_ISL_2712148, EPI_ISL_2712149, EPI_ISL_2712150, EPI_ISL_2712151, EPI_ISL_2712152, EPI_ISL_2712153, EPI_ISL_2712154,
EPI_ISL_2712155, EPI_ISL_2712156, EPI_ISL_2712157, EPI_ISL_2712158, EPI_ISL_2712159, EPI_ISL_2712160, EPI_ISL_2712161, EPI_ISL_2712162, EPI_ISL_2712163, EPI_ISL_2712164, EPI_ISL_2712165, EPI_ISL_2712166, EPI_ISL_2712167, EPI_ISL_2712168, EPI_ISL_2712169, EPI_ISL_2712170, EPI_ISL_2712171, EPI_ISL_2712172, EPI_ISL_2712173, EPI_ISL_2712174, EPI_ISL_2712175, EPI_ISL_2712176,
EPI_ISL_2712177, EPI_ISL_2712178, EPI_ISL_2712179, EPI_ISL_2712180, EPI_ISL_2712181, EPI_ISL_2712182, EPI_ISL_2712183, EPI_ISL_2712184, EPI_ISL_2712185, EPI_ISL_2712186, EPI_ISL_2712187, EPI_ISL_2712188, EPI_ISL_2712189, EPI_ISL_2712190, EPI_ISL_2712191, EPI_ISL_2712192, EPI_ISL_2712193, EPI_ISL_2712194, EPI_ISL_2712195, EPI_ISL_2712196, EPI_ISL_2712197, EPI_ISL_2712198,
EPI_ISL_2712199, EPI_ISL_2712200, EPI_ISL_2712201, EPI_ISL_2712202, EPI_ISL_2712203, EPI_ISL_2712204, EPI_ISL_2712205, EPI_ISL_2712206, EPI_ISL_2712207, EPI_ISL_2712208, EPI_ISL_2712209, EPI_ISL_2712210, EPI_ISL_2712211, EPI_ISL_2712212, EPI_ISL_2712213, EPI_ISL_2712214, EPI_ISL_2712215, EPI_ISL_2712216, EPI_ISL_2712217, EPI_ISL_2712218, EPI_ISL_2712219, EPI_ISL_2712220,
EPI_ISL_2712221, EPI_ISL_2712222, EPI_ISL_2712223, EPI_ISL_2712224, EPI_ISL_2712225, EPI_ISL_2712226, EPI_ISL_2712227, EPI_ISL_2712228, EPI_ISL_2712229, EPI_ISL_2712230, EPI_ISL_2712231, EPI_ISL_2712232, EPI_ISL_2712233, EPI_ISL_2712234, EPI_ISL_2712235, EPI_ISL_2712236, EPI_ISL_2712237, EPI_ISL_2712238, EPI_ISL_2712239, EPI_ISL_2712240, EPI_ISL_2712241, EPI_ISL_2712242,
EPI_ISL_2712243, EPI_ISL_2712244, EPI_ISL_2712245, EPI_ISL_2712246, EPI_ISL_2712247, EPI_ISL_2712248, EPI_ISL_2712249, EPI_ISL_2712250, EPI_ISL_2712251, EPI_ISL_2712252, EPI_ISL_2712253, EPI_ISL_2712254, EPI_ISL_2712255, EPI_ISL_2712256, EPI_ISL_2712257, EPI_ISL_2712258, EPI_ISL_2712259, EPI_ISL_2712260, EPI_ISL_2712261, EPI_ISL_2712262, EPI_ISL_2712263, EPI_ISL_2712264,
EPI_ISL_2712265, EPI_ISL_2712266, EPI_ISL_2712267, EPI_ISL_2712268, EPI_ISL_2712269, EPI_ISL_2712270, EPI_ISL_2712271, EPI_ISL_2712272, EPI_ISL_2712273, EPI_ISL_2712274, EPI_ISL_2712275, EPI_ISL_2712276, EPI_ISL_2712277, EPI_ISL_2712278, EPI_ISL_2712279, EPI_ISL_2712280, EPI_ISL_2712281, EPI_ISL_2712282, EPI_ISL_2712283, EPI_ISL_2712284, EPI_ISL_2712285, EPI_ISL_2712286,
EPI_ISL_2712287, EPI_ISL_2712288, EPI_ISL_2712289, EPI_ISL_2712290, EPI_ISL_2712291, EPI_ISL_2712292, EPI_ISL_2712293, EPI_ISL_2712294, EPI_ISL_2712295, EPI_ISL_2712296, EPI_ISL_2712297, EPI_ISL_2712298, EPI_ISL_2712299, EPI_ISL_2712300, EPI_ISL_2712301, EPI_ISL_2712302, EPI_ISL_2712303, EPI_ISL_2712304, EPI_ISL_2712305, EPI_ISL_2712306, EPI_ISL_2712307, EPI_ISL_2712308,
EPI_ISL_2712309, EPI_ISL_2712310, EPI_ISL_2712311, EPI_ISL_2712312, EPI_ISL_2712313, EPI_ISL_2712314, EPI_ISL_2712315, EPI_ISL_2712316, EPI_ISL_2712317, EPI_ISL_2712318, EPI_ISL_2712319, EPI_ISL_2712320, EPI_ISL_2712321, EPI_ISL_2712322, EPI_ISL_2712323, EPI_ISL_2712324, EPI_ISL_2712325, EPI_ISL_2712326, EPI_ISL_2712327, EPI_ISL_2712328, EPI_ISL_2712329, EPI_ISL_2712330,
EPI_ISL_2712331, EPI_ISL_2712332, EPI_ISL_2712333, EPI_ISL_2712334, EPI_ISL_2712335, EPI_ISL_2712336, EPI_ISL_2712337, EPI_ISL_2712338, EPI_ISL_2712339, EPI_ISL_2712340, EPI_ISL_2712341, EPI_ISL_2712342, EPI_ISL_2712343, EPI_ISL_2712344, EPI_ISL_2712345, EPI_ISL_2712346, EPI_ISL_2712347, EPI_ISL_2712348, EPI_ISL_2712349, EPI_ISL_2712350, EPI_ISL_2712351, EPI_ISL_2712352,
EPI_ISL_2712353, EPI_ISL_2712354, EPI_ISL_2712355, EPI_ISL_2712356, EPI_ISL_2712357, EPI_ISL_2712358, EPI_ISL_2712359, EPI_ISL_2712360, EPI_ISL_2712361, EPI_ISL_2712362, EPI_ISL_2712363, EPI_ISL_2712364, EPI_ISL_2712365, EPI_ISL_2712366, EPI_ISL_2712367, EPI_ISL_2712368, EPI_ISL_2712369, EPI_ISL_2712370, EPI_ISL_2712371, EPI_ISL_2712372, EPI_ISL_2712373, EPI_ISL_2712374,
EPI_ISL_2712375, EPI_ISL_2712376, EPI_ISL_2712377, EPI_ISL_2712378, EPI_ISL_2712379, EPI_ISL_2712380, EPI_ISL_2712381, EPI_ISL_2712382, EPI_ISL_2712383, EPI_ISL_2712384, EPI_ISL_2712385, EPI_ISL_2712386, EPI_ISL_2712387, EPI_ISL_2712388, EPI_ISL_2712389, EPI_ISL_2712390, EPI_ISL_2712391, EPI_ISL_2712392, EPI_ISL_2712393, EPI_ISL_2712394, EPI_ISL_2712395, EPI_ISL_2712396,
EPI_ISL_2712397, EPI_ISL_2712398, EPI_ISL_2712399, EPI_ISL_2712400, EPI_ISL_2712401, EPI_ISL_2712402, EPI_ISL_2712403, EPI_ISL_2712404, EPI_ISL_2712405, EPI_ISL_2712406, EPI_ISL_2712407, EPI_ISL_2712408, EPI_ISL_2712409, EPI_ISL_2712410, EPI_ISL_2712411, EPI_ISL_2712412, EPI_ISL_2712413, EPI_ISL_2712414, EPI_ISL_2712415, EPI_ISL_2712416, EPI_ISL_2712417, EPI_ISL_2712418,
EPI_ISL_2712419, EPI_ISL_2712420, EPI_ISL_2712421, EPI_ISL_2712422, EPI_ISL_2712423, EPI_ISL_2712424, EPI_ISL_2712425, EPI_ISL_2712426, EPI_ISL_2712427, EPI_ISL_2712428, EPI_ISL_2712429, EPI_ISL_2712430, EPI_ISL_2712431, EPI_ISL_2712432, EPI_ISL_2712433, EPI_ISL_2712434, EPI_ISL_2712435, EPI_ISL_2712436, EPI_ISL_2712437, EPI_ISL_2712438, EPI_ISL_2712439, EPI_ISL_2712440,
EPI_ISL_2712441, EPI_ISL_2712442, EPI_ISL_2712443, EPI_ISL_2712444, EPI_ISL_2712445, EPI_ISL_2712446, EPI_ISL_2712447, EPI_ISL_2712448, EPI_ISL_2712449, EPI_ISL_2712450, EPI_ISL_2754319, EPI_ISL_2754320, EPI_ISL_2754321, EPI_ISL_2754322, EPI_ISL_2754323, EPI_ISL_2754324, EPI_ISL_2754325, EPI_ISL_2754326, EPI_ISL_2754327, EPI_ISL_2754328, EPI_ISL_2754329, EPI_ISL_2754330,
EPI_ISL_2754331, EPI_ISL_2754332, EPI_ISL_2754333, EPI_ISL_2754334, EPI_ISL_2754335, EPI_ISL_2754336, EPI_ISL_2754337, EPI_ISL_2754338, EPI_ISL_2754339, EPI_ISL_2754340, EPI_ISL_2754341, EPI_ISL_2754342, EPI_ISL_2754343, EPI_ISL_2754344, EPI_ISL_2754345, EPI_ISL_2754346, EPI_ISL_2754347, EPI_ISL_2754348, EPI_ISL_2754349, EPI_ISL_2754350, EPI_ISL_2754351, EPI_ISL_2754352,
EPI_ISL_2754353, EPI_ISL_2754354, EPI_ISL_2754355, EPI_ISL_2754356, EPI_ISL_2754357, EPI_ISL_2754358, EPI_ISL_2754359, EPI_ISL_2754360, EPI_ISL_2754361, EPI_ISL_2754362, EPI_ISL_2754363, EPI_ISL_2754364, EPI_ISL_2754365, EPI_ISL_2754366, EPI_ISL_2754367, EPI_ISL_2754368, EPI_ISL_2754369, EPI_ISL_2754371, EPI_ISL_2754372, EPI_ISL_2754373, EPI_ISL_2754374, EPI_ISL_2754375,
EPI_ISL_2754376, EPI_ISL_2754377, EPI_ISL_2754378, EPI_ISL_2754379, EPI_ISL_2754380, EPI_ISL_2754381, EPI_ISL_2754382, EPI_ISL_2754383, EPI_ISL_2754384, EPI_ISL_2754385, EPI_ISL_2754386, EPI_ISL_2754387, EPI_ISL_2754388, EPI_ISL_2754389, EPI_ISL_2754390, EPI_ISL_2754391, EPI_ISL_2754392, EPI_ISL_2754393, EPI_ISL_2754394, EPI_ISL_2754395, EPI_ISL_2754396, EPI_ISL_2754397,
EPI_ISL_2754399, EPI_ISL_2754400, EPI_ISL_2754401, EPI_ISL_2754402, EPI_ISL_2754403, EPI_ISL_2754404, EPI_ISL_2754405, EPI_ISL_2754406, EPI_ISL_2754407, EPI_ISL_2754408, EPI_ISL_2754409, EPI_ISL_2754410, EPI_ISL_2754411, EPI_ISL_2754412, EPI_ISL_2754413, EPI_ISL_2754414, EPI_ISL_2754415, EPI_ISL_2754416, EPI_ISL_2754417, EPI_ISL_2754418, EPI_ISL_2754419, EPI_ISL_2754420,
EPI_ISL_2754421, EPI_ISL_2754422, EPI_ISL_2754423, EPI_ISL_2754424, EPI_ISL_2754425, EPI_ISL_2754426, EPI_ISL_2754427, EPI_ISL_2754428, EPI_ISL_2754429, EPI_ISL_2754430, EPI_ISL_2754431, EPI_ISL_2754432, EPI_ISL_2754433, EPI_ISL_2754434, EPI_ISL_2754435, EPI_ISL_2754436, EPI_ISL_2754437, EPI_ISL_2754438, EPI_ISL_2754439, EPI_ISL_2754440, EPI_ISL_2754441, EPI_ISL_2754442,
EPI_ISL_2754443, EPI_ISL_2754444, EPI_ISL_2754445, EPI_ISL_2754446, EPI_ISL_2754447, EPI_ISL_2754448, EPI_ISL_2754449, EPI_ISL_2754450, EPI_ISL_2754451, EPI_ISL_2754452, EPI_ISL_2754453, EPI_ISL_2754454, EPI_ISL_2754455, EPI_ISL_2754456, EPI_ISL_2754457, EPI_ISL_2754458, EPI_ISL_2754459, EPI_ISL_2754460, EPI_ISL_2754461, EPI_ISL_2754462, EPI_ISL_2754463, EPI_ISL_2754464,
EPI_ISL_2754465, EPI_ISL_2754466, EPI_ISL_2754467, EPI_ISL_2754469, EPI_ISL_2754470, EPI_ISL_2754471, EPI_ISL_2754472, EPI_ISL_2754473, EPI_ISL_2754474, EPI_ISL_2754475, EPI_ISL_2754476, EPI_ISL_2754477, EPI_ISL_2754478, EPI_ISL_2754479, EPI_ISL_2754480, EPI_ISL_2754481, EPI_ISL_2754482, EPI_ISL_2754483, EPI_ISL_2754484, EPI_ISL_2754485, EPI_ISL_2754486, EPI_ISL_2754487,
EPI_ISL_2754488, EPI_ISL_2754489, EPI_ISL_2754490, EPI_ISL_2754491, EPI_ISL_2754492, EPI_ISL_2754493, EPI_ISL_2754494, EPI_ISL_2754495, EPI_ISL_2754496, EPI_ISL_2754497, EPI_ISL_2754498, EPI_ISL_2754499, EPI_ISL_2754500, EPI_ISL_2754501, EPI_ISL_2754502, EPI_ISL_2754503, EPI_ISL_2754504, EPI_ISL_2754505, EPI_ISL_2754506, EPI_ISL_2754507, EPI_ISL_2754508, EPI_ISL_2754509,
EPI_ISL_2754510, EPI_ISL_2754511, EPI_ISL_2754512, EPI_ISL_2754513, EPI_ISL_2754514, EPI_ISL_2754515, EPI_ISL_2754516, EPI_ISL_2754518, EPI_ISL_2754519, EPI_ISL_2754520, EPI_ISL_2754521, EPI_ISL_2754522, EPI_ISL_2754523, EPI_ISL_2754524, EPI_ISL_2754525, EPI_ISL_2754526, EPI_ISL_2754527, EPI_ISL_2754528, EPI_ISL_2754529, EPI_ISL_2754530, EPI_ISL_2754531, EPI_ISL_2754532,
EPI_ISL_2754533, EPI_ISL_2754534, EPI_ISL_2754535, EPI_ISL_2754536, EPI_ISL_2754537, EPI_ISL_2754538, EPI_ISL_2754539, EPI_ISL_2754540, EPI_ISL_2754541, EPI_ISL_2754542, EPI_ISL_2754543, EPI_ISL_2754544, EPI_ISL_2754545, EPI_ISL_2754546, EPI_ISL_2754547, EPI_ISL_2754548, EPI_ISL_2754550, EPI_ISL_2754551, EPI_ISL_2754552, EPI_ISL_2754553, EPI_ISL_2754554, EPI_ISL_2754555,
EPI_ISL_2754556, EPI_ISL_2754557, EPI_ISL_2754558, EPI_ISL_2754559, EPI_ISL_2754560, EPI_ISL_2754561, EPI_ISL_2754562, EPI_ISL_2754563, EPI_ISL_2754564, EPI_ISL_2754565, EPI_ISL_2754566, EPI_ISL_2754567, EPI_ISL_2754568, EPI_ISL_2754569, EPI_ISL_2754570, EPI_ISL_2754571, EPI_ISL_2754572, EPI_ISL_2754573, EPI_ISL_2754574, EPI_ISL_2754575, EPI_ISL_2754576, EPI_ISL_2754577,
EPI_ISL_2754578, EPI_ISL_2754579, EPI_ISL_2754580, EPI_ISL_2754582, EPI_ISL_2754583, EPI_ISL_2754584, EPI_ISL_2754585, EPI_ISL_2754586, EPI_ISL_2754587, EPI_ISL_2754588, EPI_ISL_2754589, EPI_ISL_2754590, EPI_ISL_2754591, EPI_ISL_2754592, EPI_ISL_2754593, EPI_ISL_2754594, EPI_ISL_2754595, EPI_ISL_2754596, EPI_ISL_2754597, EPI_ISL_2754598, EPI_ISL_2754599, EPI_ISL_2754600,
EPI_ISL_2754601, EPI_ISL_2754602, EPI_ISL_2754603, EPI_ISL_2754604, EPI_ISL_2754605, EPI_ISL_2754606, EPI_ISL_2754607, EPI_ISL_2754609, EPI_ISL_2754610, EPI_ISL_2754611, EPI_ISL_2754612, EPI_ISL_2754613, EPI_ISL_2754614, EPI_ISL_2754615, EPI_ISL_2754616, EPI_ISL_2754617, EPI_ISL_2754618, EPI_ISL_2754619, EPI_ISL_2754620, EPI_ISL_2754621, EPI_ISL_2754622, EPI_ISL_2754623,
EPI_ISL_2754624, EPI_ISL_2754625, EPI_ISL_2754626, EPI_ISL_2754627, EPI_ISL_2754629, EPI_ISL_2754630, EPI_ISL_2754631, EPI_ISL_2754632, EPI_ISL_2754633, EPI_ISL_2754634, EPI_ISL_2754635, EPI_ISL_2754636, EPI_ISL_2754637, EPI_ISL_2754638, EPI_ISL_2754639, EPI_ISL_2754640, EPI_ISL_2754641, EPI_ISL_2754642, EPI_ISL_2754643, EPI_ISL_2754644, EPI_ISL_2754645, EPI_ISL_2754646,
EPI_ISL_2754647, EPI_ISL_2754648, EPI_ISL_2754649, EPI_ISL_2754650, EPI_ISL_2754651, EPI_ISL_2754652, EPI_ISL_2754653, EPI_ISL_2754654, EPI_ISL_2754655, EPI_ISL_2754656, EPI_ISL_2754657, EPI_ISL_2754658, EPI_ISL_2754659, EPI_ISL_2754660, EPI_ISL_2754661, EPI_ISL_2754662, EPI_ISL_2754663, EPI_ISL_2754664, EPI_ISL_2754666, EPI_ISL_2754667, EPI_ISL_2754668, EPI_ISL_2754669,
EPI_ISL_2754670, EPI_ISL_2754671, EPI_ISL_2754672, EPI_ISL_2754673, EPI_ISL_2754674, EPI_ISL_2754675, EPI_ISL_2754676, EPI_ISL_2754677, EPI_ISL_2754678, EPI_ISL_2754679, EPI_ISL_2754680, EPI_ISL_2754681, EPI_ISL_2754682, EPI_ISL_2754683, EPI_ISL_2754684, EPI_ISL_2754685, EPI_ISL_2754686, EPI_ISL_2754687, EPI_ISL_2754688, EPI_ISL_2754689, EPI_ISL_2754690, EPI_ISL_2754691,
EPI_ISL_2754692, EPI_ISL_2754693, EPI_ISL_2754694, EPI_ISL_2754695, EPI_ISL_2754696, EPI_ISL_2754697, EPI_ISL_2754698, EPI_ISL_2754699, EPI_ISL_2754700, EPI_ISL_2754701, EPI_ISL_2754702, EPI_ISL_2754703, EPI_ISL_2754704, EPI_ISL_2754705, EPI_ISL_2754706, EPI_ISL_2754707, EPI_ISL_2754708, EPI_ISL_2754709, EPI_ISL_2754710, EPI_ISL_2754711, EPI_ISL_2754712, EPI_ISL_2754713,
EPI_ISL_2754714, EPI_ISL_2754715, EPI_ISL_2754716, EPI_ISL_2754717, EPI_ISL_2754718, EPI_ISL_2754719, EPI_ISL_2754720, EPI_ISL_2754721, EPI_ISL_2754722, EPI_ISL_2754723, EPI_ISL_2754724, EPI_ISL_2754725, EPI_ISL_2754726, EPI_ISL_2754727, EPI_ISL_2754728, EPI_ISL_2754729, EPI_ISL_2754730, EPI_ISL_2754731, EPI_ISL_2754732, EPI_ISL_2754733, EPI_ISL_2754734, EPI_ISL_2754735,
EPI_ISL_2754736, EPI_ISL_2754737, EPI_ISL_2754738, EPI_ISL_2754739, EPI_ISL_2754740, EPI_ISL_2754741, EPI_ISL_2754742, EPI_ISL_2754743, EPI_ISL_2754744, EPI_ISL_2754745, EPI_ISL_2754746, EPI_ISL_2754747, EPI_ISL_2754748, EPI_ISL_2754749, EPI_ISL_2754750, EPI_ISL_2754751, EPI_ISL_2754752, EPI_ISL_2754753, EPI_ISL_2754754, EPI_ISL_2754755, EPI_ISL_2754756, EPI_ISL_2754757,
EPI_ISL_2754758, EPI_ISL_2754759, EPI_ISL_2754760, EPI_ISL_2754761, EPI_ISL_2754762, EPI_ISL_2754763, EPI_ISL_2754764, EPI_ISL_2754765, EPI_ISL_2754766, EPI_ISL_2754767, EPI_ISL_2754768, EPI_ISL_2754769, EPI_ISL_2754770, EPI_ISL_2754771, EPI_ISL_2754772, EPI_ISL_2754773, EPI_ISL_2754774, EPI_ISL_2754775, EPI_ISL_2754776, EPI_ISL_2754777, EPI_ISL_2754778, EPI_ISL_2754779,
EPI_ISL_2754780, EPI_ISL_2754781, EPI_ISL_2754782, EPI_ISL_2754783, EPI_ISL_2754784, EPI_ISL_2754785, EPI_ISL_2754786, EPI_ISL_2754787, EPI_ISL_2754788, EPI_ISL_2754789, EPI_ISL_2754790, EPI_ISL_2754791, EPI_ISL_2754792, EPI_ISL_2754793, EPI_ISL_2754794, EPI_ISL_2754795, EPI_ISL_2754796, EPI_ISL_2754797, EPI_ISL_2754798, EPI_ISL_2754799, EPI_ISL_2754800, EPI_ISL_2754801,
EPI_ISL_2754802, EPI_ISL_2754804, EPI_ISL_2754805, EPI_ISL_2754807, EPI_ISL_2754808, EPI_ISL_2754809, EPI_ISL_2754810, EPI_ISL_2754811, EPI_ISL_2754812, EPI_ISL_2754813, EPI_ISL_2754814, EPI_ISL_2754815, EPI_ISL_2754816, EPI_ISL_2754817, EPI_ISL_2754818, EPI_ISL_2754819, EPI_ISL_2754820, EPI_ISL_2754821, EPI_ISL_2754822, EPI_ISL_2754823, EPI_ISL_2754824, EPI_ISL_2754826,
EPI_ISL_2754827, EPI_ISL_2754828, EPI_ISL_2754829, EPI_ISL_2754830, EPI_ISL_2754831, EPI_ISL_2754832, EPI_ISL_2754833, EPI_ISL_2754834, EPI_ISL_2754835, EPI_ISL_2754836, EPI_ISL_2754837, EPI_ISL_2754838, EPI_ISL_2754839, EPI_ISL_2754840, EPI_ISL_2754841, EPI_ISL_2754842, EPI_ISL_2754844, EPI_ISL_2754845, EPI_ISL_2754846, EPI_ISL_2754847, EPI_ISL_2754848, EPI_ISL_2754849,
EPI_ISL_2754850, EPI_ISL_2754851, EPI_ISL_2754852, EPI_ISL_2754853, EPI_ISL_2863957, EPI_ISL_2863958, EPI_ISL_2863959, EPI_ISL_2863960, EPI_ISL_2863961, EPI_ISL_2863962, EPI_ISL_2863963, EPI_ISL_2863964, EPI_ISL_2863965, EPI_ISL_2863966, EPI_ISL_2863967, EPI_ISL_2863968, EPI_ISL_2863969, EPI_ISL_2863970, EPI_ISL_2863971, EPI_ISL_2863972, EPI_ISL_2863973, EPI_ISL_2863974,
EPI_ISL_2863975, EPI_ISL_2863976, EPI_ISL_2863977, EPI_ISL_2863978, EPI_ISL_2863979, EPI_ISL_2863980, EPI_ISL_2863981, EPI_ISL_2863982, EPI_ISL_2863983, EPI_ISL_2863984, EPI_ISL_2863985, EPI_ISL_2863986, EPI_ISL_2863987, EPI_ISL_2863988, EPI_ISL_2863989, EPI_ISL_2863990, EPI_ISL_2863991, EPI_ISL_2863992, EPI_ISL_2863993, EPI_ISL_2863994, EPI_ISL_2863995, EPI_ISL_2863996,
EPI_ISL_2863997, EPI_ISL_2863998, EPI_ISL_2863999, EPI_ISL_2864000, EPI_ISL_2864001, EPI_ISL_2864002, EPI_ISL_2864003, EPI_ISL_2864004, EPI_ISL_2864005, EPI_ISL_2864006, EPI_ISL_2864007, EPI_ISL_2864008, EPI_ISL_2864009, EPI_ISL_2864010, EPI_ISL_2864011, EPI_ISL_2864012, EPI_ISL_2864013, EPI_ISL_2864014, EPI_ISL_2864015, EPI_ISL_2864016, EPI_ISL_2864017, EPI_ISL_2864018,
EPI_ISL_2864019, EPI_ISL_2864020, EPI_ISL_2864021, EPI_ISL_2864022, EPI_ISL_2864023, EPI_ISL_2864024, EPI_ISL_2864025, EPI_ISL_2864026, EPI_ISL_2864027, EPI_ISL_2864028, EPI_ISL_2864029, EPI_ISL_2864030, EPI_ISL_2864031, EPI_ISL_2864032, EPI_ISL_2864033, EPI_ISL_2864034, EPI_ISL_2864035, EPI_ISL_2864036, EPI_ISL_2864037, EPI_ISL_2864038, EPI_ISL_2864039, EPI_ISL_2864040,
EPI_ISL_2864041, EPI_ISL_2864042, EPI_ISL_2864043, EPI_ISL_2864044, EPI_ISL_2864045, EPI_ISL_2864046, EPI_ISL_2864047, EPI_ISL_2864048, EPI_ISL_2864049, EPI_ISL_2864050, EPI_ISL_2864051, EPI_ISL_2864052, EPI_ISL_2864053, EPI_ISL_2864054, EPI_ISL_2864055, EPI_ISL_2864056, EPI_ISL_2864057, EPI_ISL_2864058, EPI_ISL_2864059, EPI_ISL_2864060, EPI_ISL_2864061, EPI_ISL_2864062,
EPI_ISL_2864063, EPI_ISL_2864064, EPI_ISL_2864065, EPI_ISL_2864066, EPI_ISL_2864067, EPI_ISL_2864068, EPI_ISL_2864069, EPI_ISL_2864070, EPI_ISL_2864071, EPI_ISL_2864072, EPI_ISL_2864073, EPI_ISL_2864074, EPI_ISL_2864075, EPI_ISL_2864076, EPI_ISL_2864077, EPI_ISL_2864078, EPI_ISL_2864079, EPI_ISL_2864080, EPI_ISL_2864081, EPI_ISL_2864082, EPI_ISL_2864083, EPI_ISL_2864084,
EPI_ISL_2864085, EPI_ISL_2864086, EPI_ISL_2887349, EPI_ISL_2887350, EPI_ISL_2887351, EPI_ISL_2887352, EPI_ISL_2887353, EPI_ISL_2887354, EPI_ISL_2887355, EPI_ISL_2887356, EPI_ISL_2887357, EPI_ISL_2887358, EPI_ISL_2887359, EPI_ISL_2887360, EPI_ISL_2887361, EPI_ISL_2887362, EPI_ISL_2887363, EPI_ISL_2887364, EPI_ISL_2887365, EPI_ISL_2887366, EPI_ISL_2887367, EPI_ISL_2887368,
EPI_ISL_2887369, EPI_ISL_2887370, EPI_ISL_2887371, EPI_ISL_2887372, EPI_ISL_2887373, EPI_ISL_2887374, EPI_ISL_2887375, EPI_ISL_2887376, EPI_ISL_2887377, EPI_ISL_2887378, EPI_ISL_2887379, EPI_ISL_2887380, EPI_ISL_2887381, EPI_ISL_2887382, EPI_ISL_2887383, EPI_ISL_2887384, EPI_ISL_2887385, EPI_ISL_2887386, EPI_ISL_2887387, EPI_ISL_2887388, EPI_ISL_2887389, EPI_ISL_2887390,
EPI_ISL_2887391, EPI_ISL_2887392, EPI_ISL_2887393, EPI_ISL_2887394, EPI_ISL_2887395, EPI_ISL_2887396, EPI_ISL_2887397, EPI_ISL_2887398, EPI_ISL_2887399, EPI_ISL_2887400, EPI_ISL_2887401, EPI_ISL_2887402, EPI_ISL_2887403, EPI_ISL_2887404, EPI_ISL_2887405, EPI_ISL_2887406, EPI_ISL_2887407, EPI_ISL_2887408, EPI_ISL_2887409, EPI_ISL_2887410, EPI_ISL_2887411, EPI_ISL_2887412,
EPI_ISL_2887413, EPI_ISL_2887414, EPI_ISL_2887415, EPI_ISL_2887416, EPI_ISL_2967250, EPI_ISL_2967251, EPI_ISL_2967252, EPI_ISL_2967253, EPI_ISL_2967254, EPI_ISL_2967255, EPI_ISL_2967256, EPI_ISL_2967257, EPI_ISL_2967258, EPI_ISL_2967259, EPI_ISL_2967260, EPI_ISL_2967261, EPI_ISL_2967262, EPI_ISL_2967263, EPI_ISL_2967264, EPI_ISL_2967265, EPI_ISL_2967266, EPI_ISL_2967267,
EPI_ISL_2967268, EPI_ISL_2967269, EPI_ISL_2967270, EPI_ISL_2967271, EPI_ISL_2967272, EPI_ISL_2967273, EPI_ISL_2967274, EPI_ISL_2967275, EPI_ISL_2967276, EPI_ISL_2967277, EPI_ISL_2967278, EPI_ISL_2967279, EPI_ISL_2967280, EPI_ISL_2967281, EPI_ISL_2967282, EPI_ISL_2967283, EPI_ISL_2967284, EPI_ISL_2967285, EPI_ISL_2967286, EPI_ISL_2967287, EPI_ISL_2967288, EPI_ISL_2967289,
EPI_ISL_2967290, EPI_ISL_2967291, EPI_ISL_2967292, EPI_ISL_2967293, EPI_ISL_2967294, EPI_ISL_2967295, EPI_ISL_2967296, EPI_ISL_2967297, EPI_ISL_2967298, EPI_ISL_2967299, EPI_ISL_2967300, EPI_ISL_2967301, EPI_ISL_2967302, EPI_ISL_2967303, EPI_ISL_2967304, EPI_ISL_2967305, EPI_ISL_2967306, EPI_ISL_2967307, EPI_ISL_2967308, EPI_ISL_2967309, EPI_ISL_2967310, EPI_ISL_2967311,
EPI_ISL_2967312, EPI_ISL_2967313, EPI_ISL_2967314, EPI_ISL_2967315, EPI_ISL_2967316, EPI_ISL_2967317, EPI_ISL_2967318, EPI_ISL_2967319, EPI_ISL_2967320, EPI_ISL_2967321, EPI_ISL_2967322, EPI_ISL_2967323, EPI_ISL_2967324, EPI_ISL_2967325, EPI_ISL_2967326, EPI_ISL_2967327, EPI_ISL_2967328, EPI_ISL_2967329, EPI_ISL_2967330, EPI_ISL_2967331, EPI_ISL_2967332, EPI_ISL_2967333,
EPI_ISL_2967334, EPI_ISL_2967335, EPI_ISL_2967336, EPI_ISL_2967337, EPI_ISL_2967338, EPI_ISL_2967339, EPI_ISL_2967340, EPI_ISL_2967341, EPI_ISL_2967342, EPI_ISL_2967343, EPI_ISL_2967344, EPI_ISL_2967345, EPI_ISL_2967346, EPI_ISL_2967347, EPI_ISL_2967348, EPI_ISL_2967349, EPI_ISL_2967350, EPI_ISL_2967351, EPI_ISL_2967352, EPI_ISL_2967353, EPI_ISL_2967354, EPI_ISL_2967355,
EPI_ISL_2967356, EPI_ISL_2967357, EPI_ISL_2967358, EPI_ISL_2967359, EPI_ISL_2967360, EPI_ISL_2967361, EPI_ISL_2967362, EPI_ISL_2967363, EPI_ISL_2967364, EPI_ISL_2967365, EPI_ISL_2967366, EPI_ISL_2967367, EPI_ISL_2967368, EPI_ISL_2967369, EPI_ISL_2967370, EPI_ISL_2967371, EPI_ISL_2967372, EPI_ISL_2967373, EPI_ISL_2967374, EPI_ISL_2967375, EPI_ISL_2967376, EPI_ISL_2967377,
EPI_ISL_2967378, EPI_ISL_2967379, EPI_ISL_2967380, EPI_ISL_2967381, EPI_ISL_2967382, EPI_ISL_2967383, EPI_ISL_2967384, EPI_ISL_2967385, EPI_ISL_2967386, EPI_ISL_2967387, EPI_ISL_2967388, EPI_ISL_2967389, EPI_ISL_2967390, EPI_ISL_2967391, EPI_ISL_2967392, EPI_ISL_2967393, EPI_ISL_2967394, EPI_ISL_2967395, EPI_ISL_2967396, EPI_ISL_2967397, EPI_ISL_2967398, EPI_ISL_2967399,
EPI_ISL_2967400, EPI_ISL_2967401, EPI_ISL_2967402, EPI_ISL_2967403, EPI_ISL_2967404, EPI_ISL_2967405, EPI_ISL_2967406, EPI_ISL_2967407, EPI_ISL_2967408, EPI_ISL_2967409, EPI_ISL_2967410, EPI_ISL_2967411, EPI_ISL_2967412, EPI_ISL_2967413, EPI_ISL_2967414, EPI_ISL_2967415, EPI_ISL_2967416, EPI_ISL_2967417, EPI_ISL_2967418, EPI_ISL_2967419, EPI_ISL_2967420, EPI_ISL_2967421,
EPI_ISL_2967422, EPI_ISL_2967423, EPI_ISL_2967424, EPI_ISL_2967425, EPI_ISL_2967426, EPI_ISL_2967427, EPI_ISL_2967428, EPI_ISL_2967429, EPI_ISL_2967430, EPI_ISL_2967431, EPI_ISL_2967432, EPI_ISL_2967433, EPI_ISL_2967434, EPI_ISL_2967435, EPI_ISL_2967436, EPI_ISL_2967437, EPI_ISL_2967438, EPI_ISL_2967439, EPI_ISL_2967440, EPI_ISL_2967441, EPI_ISL_2967442, EPI_ISL_2967443,
EPI_ISL_2967444, EPI_ISL_2967445, EPI_ISL_2967446, EPI_ISL_2967447, EPI_ISL_2967448, EPI_ISL_2967449, EPI_ISL_2967450, EPI_ISL_2967451, EPI_ISL_2967452, EPI_ISL_2967453, EPI_ISL_2967454, EPI_ISL_2967455, EPI_ISL_2967456, EPI_ISL_2967457, EPI_ISL_2967458, EPI_ISL_2967459, EPI_ISL_2967460, EPI_ISL_2967461, EPI_ISL_2967462, EPI_ISL_2967463, EPI_ISL_2967464, EPI_ISL_2967465,
EPI_ISL_2967466, EPI_ISL_2967467, EPI_ISL_2967468, EPI_ISL_2967469, EPI_ISL_2967470, EPI_ISL_2967471, EPI_ISL_2967472, EPI_ISL_2967473, EPI_ISL_2967474, EPI_ISL_2967475, EPI_ISL_2967476, EPI_ISL_2967477, EPI_ISL_2967478, EPI_ISL_2967479, EPI_ISL_2967480, EPI_ISL_2967481, EPI_ISL_2967482, EPI_ISL_2967483, EPI_ISL_2967484, EPI_ISL_2967485, EPI_ISL_2967486, EPI_ISL_2967487,
EPI_ISL_2967488, EPI_ISL_2967489, EPI_ISL_2967490, EPI_ISL_2967491, EPI_ISL_2967492, EPI_ISL_2967493, EPI_ISL_2967494, EPI_ISL_2967495, EPI_ISL_2967496, EPI_ISL_2967497, EPI_ISL_2967498, EPI_ISL_2967499, EPI_ISL_2967500, EPI_ISL_2967501, EPI_ISL_2967502, EPI_ISL_2967503, EPI_ISL_2967504, EPI_ISL_2967505, EPI_ISL_2967506, EPI_ISL_2967507, EPI_ISL_2967508, EPI_ISL_2967509,
EPI_ISL_2967510, EPI_ISL_2967511, EPI_ISL_2967512, EPI_ISL_2967513, EPI_ISL_2967514, EPI_ISL_2967515, EPI_ISL_2967516, EPI_ISL_2967517, EPI_ISL_2967518, EPI_ISL_2967519, EPI_ISL_2967520, EPI_ISL_2967521, EPI_ISL_2967522, EPI_ISL_2967523, EPI_ISL_2967524, EPI_ISL_2967525, EPI_ISL_2967526, EPI_ISL_2967527, EPI_ISL_2967528, EPI_ISL_2967529, EPI_ISL_2967530, EPI_ISL_2967531,
EPI_ISL_2967532, EPI_ISL_2967533, EPI_ISL_2967534, EPI_ISL_2967535, EPI_ISL_2967536, EPI_ISL_2967537, EPI_ISL_2967538, EPI_ISL_2967539, EPI_ISL_2967540, EPI_ISL_2967541, EPI_ISL_2967542, EPI_ISL_2967543, EPI_ISL_2967544, EPI_ISL_2967545, EPI_ISL_2967546, EPI_ISL_2967547, EPI_ISL_2967548, EPI_ISL_2967549, EPI_ISL_2967550, EPI_ISL_2967551, EPI_ISL_2967552, EPI_ISL_2967553,
EPI_ISL_2967554, EPI_ISL_2967555, EPI_ISL_2967556, EPI_ISL_2967557, EPI_ISL_2967558, EPI_ISL_2967559, EPI_ISL_2967560, EPI_ISL_2967561, EPI_ISL_2967562, EPI_ISL_2967563, EPI_ISL_2967564, EPI_ISL_2967565, EPI_ISL_2967566, EPI_ISL_2967567, EPI_ISL_2967568, EPI_ISL_2967569, EPI_ISL_2967570, EPI_ISL_2967571, EPI_ISL_2967572, EPI_ISL_2967573, EPI_ISL_2967574, EPI_ISL_2967575,
EPI_ISL_2967576, EPI_ISL_2967577, EPI_ISL_2967578, EPI_ISL_2967579, EPI_ISL_2967580, EPI_ISL_2967581

see above	Division of Emerging Infectious Diseases, Bureau of Infectious Diseases Diagnosis Control, Korea Disease Control and Prevention Agency	Division of Emerging Infectious Diseases, Bureau of Infectious Diseases Diagnosis Control, Korea Disease Control and Prevention Agency	Ae Kyung Park; Chae Young Lee; Chaeyoung Lee; Eun-Jin Kim; Heui Man Kim; Hyeokjin Lee; Il-Hwan Kim; Jaehee Lee; Jeong-Ah Kim; Jeong-Ah Kimg; Jeong-Min Kim; Jin Sun No; Namjoo Lee; Sang Hee Woo
EPI_ISL_413513, EPI_ISL_413515	Division of Infectious Diseases, Department of Internal Medicine, Korea University College of Medicine	Department of Microbiology, Institute for Viral Diseases, College of Medicine, Korea University	Changmin Kang; Cui Chunguang; Gee Eun Lee; Hee Jin Cheong; Heedo Park; Jeonghun Kim; Ji Yun Noh; Jin Gu Yoon; Jin Il Kim; Joon Young Song; Joon-Yong Bae; Jungmin Lee; Juyoung Cho; Kyeong-ryeol Shin; Man-Seong Park; Woo Joo Kim
EPI_ISL_660109, EPI_ISL_660110	Division of Infectious Diseases, Department of Internal Medicine, Yonsei University College of Medicine	Department of biotechnology, Yonsei University	Jae sun Yoon; Jin Young Ahn; Jun Yong Choi*; Jung Ho Kim; Minwoo Kim; Youn-Jung Lee; and Jong-Won Oh*

EPI_ISL_2631329, EPI_ISL_2631330, EPI_ISL_2631331, EPI_ISL_2631332, EPI_ISL_2631333, EPI_ISL_2631334, EPI_ISL_2631335, EPI_ISL_2631336, EPI_ISL_2631337, EPI_ISL_2631544, EPI_ISL_2649888, EPI_ISL_2649889, EPI_ISL_2649890

see above	Division of Infectious Diseases, Sejong Institute of Health and Environment	Division of Infectious Diseases, Sejong Institute of Health and Environment	Chaehyeon Sim; Ho-Eun Park; Hyejin Jang; Jae Hyeon Seo; Ji Won Kim; Ji Yeon Yang; Ji-Yun Kim; Mi-Sook Eom; Mihee Yun; Nina Yoo; Sang-Gu Yeo

EPI_ISL_812956, EPI_ISL_812957, EPI_ISL_812958, EPI_ISL_812959, EPI_ISL_812960, EPI_ISL_812961, EPI_ISL_812962, EPI_ISL_812963, EPI_ISL_812964, EPI_ISL_812965

see above	Division of Pathogen Resource Management, Korea National Institute of Health, Korea Disease Control and Prevention Agency	Division of Pathogen Resource Management, Korea National Institute of Health, Korea Disease Control and Prevention Agency	Choi; Choi, C.; E.-J.; J.-M.; Kim; M.-r.; S.T.; S.Y.; Y.S.; Yun

EPI_ISL_412869, EPI_ISL_412870, EPI_ISL_412871, EPI_ISL_412872, EPI_ISL_412873, EPI_ISL_425117, EPI_ISL_425118, EPI_ISL_426163, EPI_ISL_426164, EPI_ISL_426166, EPI_ISL_426167, EPI_ISL_426168, EPI_ISL_426169, EPI_ISL_426171, EPI_ISL_426173, EPI_ISL_426180, EPI_ISL_426181, EPI_ISL_426182, EPI_ISL_426183, EPI_ISL_426187, EPI_ISL_427809, EPI_ISL_427810, EPI_ISL_427811, EPI_ISL_427812,
EPI_ISL_427813, EPI_ISL_471425, EPI_ISL_471426, EPI_ISL_471438, EPI_ISL_471439, EPI_ISL_471440, EPI_ISL_471441, EPI_ISL_471442, EPI_ISL_471443, EPI_ISL_471444, EPI_ISL_471445, EPI_ISL_471446, EPI_ISL_471447, EPI_ISL_471448, EPI_ISL_471449, EPI_ISL_471450, EPI_ISL_471451, EPI_ISL_471452, EPI_ISL_471453, EPI_ISL_471454, EPI_ISL_471455, EPI_ISL_481370, EPI_ISL_481371, EPI_ISL_481372,
EPI_ISL_481373, EPI_ISL_481374, EPI_ISL_481375, EPI_ISL_481376, EPI_ISL_481377, EPI_ISL_481378, EPI_ISL_481379, EPI_ISL_497951, EPI_ISL_497953, EPI_ISL_497954, EPI_ISL_497955, EPI_ISL_497956, EPI_ISL_497957, EPI_ISL_497958, EPI_ISL_497959, EPI_ISL_497960, EPI_ISL_497961, EPI_ISL_497962, EPI_ISL_497963, EPI_ISL_497964, EPI_ISL_497965, EPI_ISL_497966, EPI_ISL_497967, EPI_ISL_497968,
EPI_ISL_497969, EPI_ISL_497970, EPI_ISL_497971, EPI_ISL_497972, EPI_ISL_497973, EPI_ISL_497974, EPI_ISL_497975, EPI_ISL_497976, EPI_ISL_497977, EPI_ISL_497978, EPI_ISL_497979, EPI_ISL_497980, EPI_ISL_497981, EPI_ISL_497982, EPI_ISL_497983, EPI_ISL_497984, EPI_ISL_497985, EPI_ISL_497986, EPI_ISL_497987, EPI_ISL_497988, EPI_ISL_497989, EPI_ISL_497990, EPI_ISL_497991, EPI_ISL_497992,
EPI_ISL_497993, EPI_ISL_497994, EPI_ISL_497995, EPI_ISL_497996, EPI_ISL_497997, EPI_ISL_497998, EPI_ISL_497999, EPI_ISL_498000, EPI_ISL_498001, EPI_ISL_498002, EPI_ISL_498003, EPI_ISL_498004, EPI_ISL_498005, EPI_ISL_498006, EPI_ISL_498007, EPI_ISL_498008, EPI_ISL_498009, EPI_ISL_498010, EPI_ISL_498011, EPI_ISL_498012, EPI_ISL_498013, EPI_ISL_498014, EPI_ISL_498015, EPI_ISL_498016,
EPI_ISL_498017, EPI_ISL_498018, EPI_ISL_498019, EPI_ISL_498020, EPI_ISL_498021, EPI_ISL_498022, EPI_ISL_498023, EPI_ISL_498024, EPI_ISL_498025, EPI_ISL_498026, EPI_ISL_498027, EPI_ISL_498028, EPI_ISL_498029, EPI_ISL_498030, EPI_ISL_498031, EPI_ISL_498032, EPI_ISL_498033, EPI_ISL_498034, EPI_ISL_498035, EPI_ISL_498036, EPI_ISL_498037, EPI_ISL_498038, EPI_ISL_498039, EPI_ISL_498040,
EPI_ISL_498041, EPI_ISL_498042, EPI_ISL_498043, EPI_ISL_498044, EPI_ISL_498045, EPI_ISL_498046, EPI_ISL_498047, EPI_ISL_498048, EPI_ISL_498049, EPI_ISL_498050, EPI_ISL_498051, EPI_ISL_498052, EPI_ISL_498053, EPI_ISL_506947, EPI_ISL_506948, EPI_ISL_506949, EPI_ISL_506950, EPI_ISL_506951, EPI_ISL_506952, EPI_ISL_506953, EPI_ISL_506954, EPI_ISL_506955, EPI_ISL_506956, EPI_ISL_506957,
EPI_ISL_506958, EPI_ISL_506959, EPI_ISL_506960, EPI_ISL_506961, EPI_ISL_506962, EPI_ISL_506963, EPI_ISL_506964, EPI_ISL_506965, EPI_ISL_506966, EPI_ISL_506967, EPI_ISL_506968, EPI_ISL_506969, EPI_ISL_506970, EPI_ISL_506971, EPI_ISL_506972, EPI_ISL_506973, EPI_ISL_506974, EPI_ISL_506975, EPI_ISL_506976, EPI_ISL_506977, EPI_ISL_506978, EPI_ISL_506979, EPI_ISL_506980, EPI_ISL_506981,
EPI_ISL_506982, EPI_ISL_506983, EPI_ISL_506984, EPI_ISL_506985, EPI_ISL_506986, EPI_ISL_506987, EPI_ISL_506988, EPI_ISL_506989, EPI_ISL_506990, EPI_ISL_506991, EPI_ISL_506992, EPI_ISL_506993, EPI_ISL_506994, EPI_ISL_506995, EPI_ISL_510547, EPI_ISL_510548, EPI_ISL_510549, EPI_ISL_510550, EPI_ISL_510551, EPI_ISL_510552, EPI_ISL_510553, EPI_ISL_510554, EPI_ISL_510555, EPI_ISL_510556,
EPI_ISL_510557, EPI_ISL_510558, EPI_ISL_510559, EPI_ISL_510560, EPI_ISL_510561, EPI_ISL_510562, EPI_ISL_510563, EPI_ISL_510564, EPI_ISL_510565, EPI_ISL_510566, EPI_ISL_510567, EPI_ISL_510568, EPI_ISL_510569, EPI_ISL_510570, EPI_ISL_510571, EPI_ISL_510572, EPI_ISL_510573, EPI_ISL_510574, EPI_ISL_510575, EPI_ISL_510576, EPI_ISL_510577, EPI_ISL_510578, EPI_ISL_510579, EPI_ISL_510580,
EPI_ISL_510581, EPI_ISL_510582, EPI_ISL_510583, EPI_ISL_510584, EPI_ISL_510585, EPI_ISL_510586, EPI_ISL_510587, EPI_ISL_510588, EPI_ISL_510589, EPI_ISL_510590, EPI_ISL_510591, EPI_ISL_510592, EPI_ISL_510593, EPI_ISL_510594, EPI_ISL_510595, EPI_ISL_510596, EPI_ISL_510597, EPI_ISL_510598, EPI_ISL_510599, EPI_ISL_510600, EPI_ISL_510601, EPI_ISL_510602, EPI_ISL_510603, EPI_ISL_510604,
EPI_ISL_510605, EPI_ISL_510606, EPI_ISL_510607, EPI_ISL_510608, EPI_ISL_510609, EPI_ISL_510610, EPI_ISL_510611, EPI_ISL_510612, EPI_ISL_510613, EPI_ISL_510614, EPI_ISL_510615, EPI_ISL_510616, EPI_ISL_510617, EPI_ISL_510618, EPI_ISL_510619, EPI_ISL_510620, EPI_ISL_510621, EPI_ISL_510622, EPI_ISL_510623, EPI_ISL_510624, EPI_ISL_510625, EPI_ISL_510626, EPI_ISL_510627, EPI_ISL_510628,
EPI_ISL_510629, EPI_ISL_510630, EPI_ISL_510631, EPI_ISL_510632, EPI_ISL_510633, EPI_ISL_510634, EPI_ISL_510635, EPI_ISL_510636, EPI_ISL_510637, EPI_ISL_510638, EPI_ISL_510639, EPI_ISL_510640, EPI_ISL_510641, EPI_ISL_510642, EPI_ISL_510643, EPI_ISL_510644, EPI_ISL_510645, EPI_ISL_510646, EPI_ISL_510647, EPI_ISL_510648, EPI_ISL_510649, EPI_ISL_510650, EPI_ISL_510651, EPI_ISL_510652,
EPI_ISL_510653, EPI_ISL_510654, EPI_ISL_510655, EPI_ISL_510656, EPI_ISL_510657, EPI_ISL_510658, EPI_ISL_510659, EPI_ISL_510660, EPI_ISL_510661, EPI_ISL_510662, EPI_ISL_510663, EPI_ISL_510664, EPI_ISL_510665, EPI_ISL_510666, EPI_ISL_510667, EPI_ISL_510668, EPI_ISL_510669, EPI_ISL_510670, EPI_ISL_510671, EPI_ISL_510672, EPI_ISL_510673, EPI_ISL_510674, EPI_ISL_510675, EPI_ISL_510676,
EPI_ISL_510677, EPI_ISL_510678, EPI_ISL_510679, EPI_ISL_510680, EPI_ISL_510681, EPI_ISL_510682, EPI_ISL_510683, EPI_ISL_510684, EPI_ISL_510685, EPI_ISL_510686, EPI_ISL_510687, EPI_ISL_510688, EPI_ISL_514756, EPI_ISL_514757, EPI_ISL_514758, EPI_ISL_514759, EPI_ISL_514760, EPI_ISL_514761, EPI_ISL_514762, EPI_ISL_514763, EPI_ISL_514764, EPI_ISL_514765, EPI_ISL_514766, EPI_ISL_514767,
EPI_ISL_514768, EPI_ISL_514769, EPI_ISL_514770, EPI_ISL_514771, EPI_ISL_514772, EPI_ISL_514773, EPI_ISL_514774, EPI_ISL_514775, EPI_ISL_514776, EPI_ISL_514777, EPI_ISL_514778, EPI_ISL_514779, EPI_ISL_514780, EPI_ISL_514781, EPI_ISL_514782, EPI_ISL_514783, EPI_ISL_514784, EPI_ISL_514785, EPI_ISL_514786, EPI_ISL_514787, EPI_ISL_514788, EPI_ISL_514789, EPI_ISL_514790, EPI_ISL_514791,
EPI_ISL_514792, EPI_ISL_514793, EPI_ISL_514794, EPI_ISL_514795, EPI_ISL_514796, EPI_ISL_514797, EPI_ISL_514798, EPI_ISL_514799, EPI_ISL_514800, EPI_ISL_514801, EPI_ISL_514802, EPI_ISL_514803, EPI_ISL_514804, EPI_ISL_514805, EPI_ISL_514806, EPI_ISL_514807, EPI_ISL_514808, EPI_ISL_514809, EPI_ISL_514810, EPI_ISL_514811, EPI_ISL_514812, EPI_ISL_514813, EPI_ISL_514814, EPI_ISL_514815,
EPI_ISL_514816, EPI_ISL_514817, EPI_ISL_514818, EPI_ISL_514819, EPI_ISL_514820, EPI_ISL_514821, EPI_ISL_514822, EPI_ISL_514823, EPI_ISL_514824, EPI_ISL_514825, EPI_ISL_514826, EPI_ISL_514827, EPI_ISL_514828, EPI_ISL_514829, EPI_ISL_514830, EPI_ISL_514831, EPI_ISL_514832, EPI_ISL_514833, EPI_ISL_514834, EPI_ISL_514835, EPI_ISL_514836, EPI_ISL_514837, EPI_ISL_514838, EPI_ISL_514839,
EPI_ISL_514840, EPI_ISL_514841, EPI_ISL_514842, EPI_ISL_514843, EPI_ISL_514844, EPI_ISL_514845, EPI_ISL_514846, EPI_ISL_514847, EPI_ISL_514848, EPI_ISL_514849, EPI_ISL_514850, EPI_ISL_514851, EPI_ISL_514852, EPI_ISL_514853, EPI_ISL_514854, EPI_ISL_514855, EPI_ISL_514856, EPI_ISL_514857, EPI_ISL_514858, EPI_ISL_514859, EPI_ISL_514860, EPI_ISL_514861, EPI_ISL_514862, EPI_ISL_514863,
EPI_ISL_514864, EPI_ISL_514865, EPI_ISL_514866, EPI_ISL_514867, EPI_ISL_514868, EPI_ISL_514869, EPI_ISL_514870, EPI_ISL_514871, EPI_ISL_514872, EPI_ISL_514873, EPI_ISL_514874, EPI_ISL_514875, EPI_ISL_514876, EPI_ISL_514877, EPI_ISL_514878, EPI_ISL_514879, EPI_ISL_514880, EPI_ISL_514881, EPI_ISL_514882, EPI_ISL_514883, EPI_ISL_514884, EPI_ISL_514885, EPI_ISL_514886, EPI_ISL_514887,

EPI_ISL_514888, EPI_ISL_514889, EPI_ISL_514890, EPI_ISL_514891, EPI_ISL_514892, EPI_ISL_514893, EPI_ISL_514894, EPI_ISL_514895, EPI_ISL_514896, EPI_ISL_514897, EPI_ISL_514898, EPI_ISL_514899, EPI_ISL_514900, EPI_ISL_514901, EPI_ISL_514902, EPI_ISL_514903, EPI_ISL_514904, EPI_ISL_514905, EPI_ISL_514906, EPI_ISL_514907, EPI_ISL_514908, EPI_ISL_514909, EPI_ISL_514910, EPI_ISL_514911, EPI_ISL_514912, EPI_ISL_514913, EPI_ISL_514914, EPI_ISL_514915, EPI_ISL_514916, EPI_ISL_514917, EPI_ISL_514918, EPI_ISL_514919, EPI_ISL_514920, EPI_ISL_514921, EPI_ISL_514922, EPI_ISL_514923, EPI_ISL_514924, EPI_ISL_514925, EPI_ISL_514926, EPI_ISL_514927, EPI_ISL_514928, EPI_ISL_514929, EPI_ISL_514930, EPI_ISL_514931, EPI_ISL_514932, EPI_ISL_514933, EPI_ISL_514934, EPI_ISL_514935, EPI_ISL_514936, EPI_ISL_514937, EPI_ISL_514938, EPI_ISL_514939, EPI_ISL_514940, EPI_ISL_514941, EPI_ISL_514942, EPI_ISL_514943, EPI_ISL_514944, EPI_ISL_514945, EPI_ISL_514946, EPI_ISL_514947, EPI_ISL_514948, EPI_ISL_514949, EPI_ISL_514950, EPI_ISL_514951, EPI_ISL_514952, EPI_ISL_514953, EPI_ISL_514954, EPI_ISL_514955, EPI_ISL_514956, EPI_ISL_514957, EPI_ISL_514958, EPI_ISL_514959, EPI_ISL_514960, EPI_ISL_514961, EPI_ISL_514962, EPI_ISL_514963, EPI_ISL_514964, EPI_ISL_514965, EPI_ISL_514966, EPI_ISL_514967, EPI_ISL_514968, EPI_ISL_514969, EPI_ISL_514970, EPI_ISL_514971, EPI_ISL_514972, EPI_ISL_514973, EPI_ISL_514974, EPI_ISL_514975, EPI_ISL_514976, EPI_ISL_514977, EPI_ISL_514978, EPI_ISL_514979, EPI_ISL_514980, EPI_ISL_514981, EPI_ISL_514982, EPI_ISL_514983, EPI_ISL_514984, EPI_ISL_514985, EPI_ISL_514986, EPI_ISL_514987, EPI_ISL_514988, EPI_ISL_514989, EPI_ISL_514990, EPI_ISL_514991, EPI_ISL_514992, EPI_ISL_514993, EPI_ISL_514994, EPI_ISL_514995, EPI_ISL_514996, EPI_ISL_514997, EPI_ISL_514998, EPI_ISL_514999, EPI_ISL_515000, EPI_ISL_515001, EPI_ISL_515002, EPI_ISL_515003, EPI_ISL_515004, EPI_ISL_515005, EPI_ISL_515006, EPI_ISL_515007, EPI_ISL_515008, EPI_ISL_515009, EPI_ISL_515010, EPI_ISL_515011, EPI_ISL_515012, EPI_ISL_515013, EPI_ISL_515014, EPI_ISL_515015, EPI_ISL_515016, EPI_ISL_515017, EPI_ISL_515018, EPI_ISL_515019, EPI_ISL_515020, EPI_ISL_515021, EPI_ISL_515022, EPI_ISL_515023, EPI_ISL_515024, EPI_ISL_515025, EPI_ISL_515026, EPI_ISL_515027, EPI_ISL_515028, EPI_ISL_515029, EPI_ISL_515030, EPI_ISL_515031, EPI_ISL_515032, EPI_ISL_515033, EPI_ISL_515034, EPI_ISL_515035, EPI_ISL_515036, EPI_ISL_515037, EPI_ISL_515038, EPI_ISL_515039, EPI_ISL_515040, EPI_ISL_515041, EPI_ISL_515042, EPI_ISL_515043, EPI_ISL_515044, EPI_ISL_515045, EPI_ISL_515046, EPI_ISL_515047, EPI_ISL_515048, EPI_ISL_515049, EPI_ISL_515050, EPI_ISL_515051, EPI_ISL_515052, EPI_ISL_515053, EPI_ISL_515054, EPI_ISL_522454, EPI_ISL_522465, EPI_ISL_522466, EPI_ISL_522474, EPI_ISL_522475, EPI_ISL_522477, EPI_ISL_522478, EPI_ISL_522484, EPI_ISL_522486, EPI_ISL_522487, EPI_ISL_522488, EPI_ISL_522489, EPI_ISL_522490, EPI_ISL_522498, EPI_ISL_522499, EPI_ISL_522500, EPI_ISL_522501, EPI_ISL_522503, EPI_ISL_522504, EPI_ISL_522506, EPI_ISL_522507, EPI_ISL_522509, EPI_ISL_522511, EPI_ISL_522512, EPI_ISL_522513, EPI_ISL_522514, EPI_ISL_522517, EPI_ISL_522519, EPI_ISL_522520, EPI_ISL_522521, EPI_ISL_522522, EPI_ISL_522523, EPI_ISL_522524, EPI_ISL_522525, EPI_ISL_522526, EPI_ISL_522527, EPI_ISL_522528, EPI_ISL_522529, EPI_ISL_522530, EPI_ISL_522531, EPI_ISL_522532, EPI_ISL_522533, EPI_ISL_522534, EPI_ISL_522535, EPI_ISL_522536, EPI_ISL_522537, EPI_ISL_522538, EPI_ISL_522539, EPI_ISL_522540, EPI_ISL_522541, EPI_ISL_522542, EPI_ISL_522543, EPI_ISL_522544, EPI_ISL_526700, EPI_ISL_526701, EPI_ISL_526702, EPI_ISL_526703, EPI_ISL_526704, EPI_ISL_526705, EPI_ISL_526706, EPI_ISL_526707, EPI_ISL_526708, EPI_ISL_526709, EPI_ISL_526710, EPI_ISL_526711, EPI_ISL_526712, EPI_ISL_526713, EPI_ISL_526714, EPI_ISL_526715, EPI_ISL_526716, EPI_ISL_526717, EPI_ISL_526718, EPI_ISL_526719, EPI_ISL_526720, EPI_ISL_526721, EPI_ISL_526722, EPI_ISL_526723, EPI_ISL_526724, EPI_ISL_526725, EPI_ISL_526726, EPI_ISL_526727, EPI_ISL_526728, EPI_ISL_526729, EPI_ISL_526732, EPI_ISL_526733			
see above	Division of Viral Diseases, Center for Laboratory Control of Infectious Diseases, Korea Centers for Diseases Control and Prevention	Division of Viral Diseases, Center for Laboratory Control of Infectious Diseases, Korea Centers for Diseases Control and Prevention	Daesang Lee; Dong Hyun Song; Heui Man Kim; Hye-Jun Jo; Jeong-Min Kim; Jun-Sub Kim; Junhyeong Jang; Mi-Seon Kim; Myung Guk Han; Namjoo Lee; Sang Hee Woo; Sehee Park; Seong Tae Jeong; Yoon-Seok Chung
EPI_ISL_541942	Health and Environmental Research Institute of Gwangju	Health & Environment Institute of Gwangju	Ji-eun Lee; Min Ji Kim
EPI_ISL_540993, EPI_ISL_540994, EPI_ISL_540995, EPI_ISL_540996, EPI_ISL_540997, EPI_ISL_540998, EPI_ISL_540999, EPI_ISL_541000, EPI_ISL_541001, EPI_ISL_541002, EPI_ISL_541003, EPI_ISL_541004, EPI_ISL_541005, EPI_ISL_541006, EPI_ISL_541007			
see above	Health and Environmental Research Institute of Gwangju Metropolitan city	Health and Environmental Research Institute of Gwangju Metropolitan city	Ji-eun Lee; Min Ji Kim
EPI_ISL_407193	Korea Centers for Disease Control & Prevention (KCDC) Center for Laboratory Control of Infectious Diseases Division of Viral Diseases	Korea Centers for Disease Control & Prevention (KCDC) Center for Laboratory Control of Infectious Diseases Division of Viral Diseases	Heui Man Kim; Hye-Joon Jo; Jeong-Min Kim; Mi-Seon Kim; Myung Guk Han; Namjoo Lee; SangHee Woo; Sehee Park; Yoon-Seok Chung
EPI_ISL_3026044	Laboratory Medicine, Hallym University College of Medicine	Laboratory Medicine, Hallym University College of Medicine	J.-S.; Kim
EPI_ISL_1038791, EPI_ISL_1038792	US Air Force School of Aerospace Medicine	US Air Force School of Aerospace Medicine	Amanda Javorina; Anthony Fries; Clarise Starr; Elizabeth Macias; Jennifer Meyer; Sarah Purves; William Buggele; William Gruner
EPI_ISL_485001, EPI_ISL_485002, EPI_ISL_485388, EPI_ISL_485389, EPI_ISL_485390, EPI_ISL_485391, EPI_ISL_485392, EPI_ISL_485393, EPI_ISL_485394, EPI_ISL_485395, EPI_ISL_485396			
see above	University of Ulsan College of Medicine and Asan Medical Center	University of Ulsan College of Medicine and Asan Medical Center	Heungsup Sung; Jaewoong Lee; Jina Lee; Jiwon Jung; Jongsik Chun; Kihyun Lee; Kuenyoul Park; Kyu-Hwa Hur; Mauricio Chalita; Mi-Na Kim; Seok-Hwan Yoon; Sung-Han Kim; and Hae Kyung Lee

We gratefully acknowledge the following Authors from the Originating laboratories responsible for obtaining the specimens, as well as the Submitting laboratories where the genome data were generated and shared via GISAID, on which this research is based.

All Submitters of data may be contacted directly via www.gisaid.org

Authors are sorted alphabetically.

Accession ID	Originating Laboratory	Submitting Laboratory	Authors
EPI_ISL_3118360, EPI_ISL_3118361, EPI_ISL_3118362, EPI_ISL_3118363, EPI_ISL_3118364	Department of Internal Medicine, Jeonbuk National University Medical School	Microbiological Analysis Team, Biometrology Group, Division of Chemical and Biological Metrology, Korea Research Institute of Standards and Science (KRISS)	Changseop Lee; Changwoo Park; Dongju Park; Edmond Changkyun Park; Hana Yi and Seil Kim; Hee Min Yoo; Kwan Woo Kim; Zohaib ul Hassan

EPI_ISL_3024809, EPI_ISL_3024810, EPI_ISL_3024811, EPI_ISL_3024812, EPI_ISL_3024813, EPI_ISL_3024814, EPI_ISL_3024815, EPI_ISL_3024816, EPI_ISL_3024817, EPI_ISL_3024818, EPI_ISL_3024819, EPI_ISL_3024820, EPI_ISL_3024821, EPI_ISL_3024822, EPI_ISL_3024823, EPI_ISL_3024824, EPI_ISL_3024825, EPI_ISL_3024826, EPI_ISL_3024827, EPI_ISL_3024828, EPI_ISL_3024829, EPI_ISL_3024830, EPI_ISL_3024831, EPI_ISL_3024832, EPI_ISL_3024833, EPI_ISL_3024834, EPI_ISL_3024835, EPI_ISL_3024836, EPI_ISL_3024837, EPI_ISL_3024838, EPI_ISL_3024839, EPI_ISL_3024840, EPI_ISL_3024841, EPI_ISL_3024842, EPI_ISL_3024843, EPI_ISL_3024844, EPI_ISL_3024845, EPI_ISL_3024846, EPI_ISL_3024847, EPI_ISL_3024848, EPI_ISL_3024849, EPI_ISL_3024850, EPI_ISL_3024851, EPI_ISL_3026045, EPI_ISL_3026046, EPI_ISL_3026047, EPI_ISL_3026048, EPI_ISL_3026049, EPI_ISL_3026050, EPI_ISL_3026051, EPI_ISL_3026052, EPI_ISL_3026053, EPI_ISL_3026054, EPI_ISL_3026055, EPI_ISL_3026056, EPI_ISL_3026057, EPI_ISL_3026058, EPI_ISL_3026059, EPI_ISL_3026060, EPI_ISL_3026061, EPI_ISL_3026062, EPI_ISL_3026063, EPI_ISL_3026064, EPI_ISL_3026065, EPI_ISL_3026066, EPI_ISL_3026067, EPI_ISL_3026068, EPI_ISL_3026069, EPI_ISL_3026070, EPI_ISL_3026071, EPI_ISL_3026072, EPI_ISL_3026073, EPI_ISL_3026074, EPI_ISL_3026075, EPI_ISL_3026076, EPI_ISL_3026077, EPI_ISL_3026078, EPI_ISL_3026079, EPI_ISL_3026080, EPI_ISL_3026081, EPI_ISL_3026082, EPI_ISL_3026083, EPI_ISL_3026084, EPI_ISL_3026085, EPI_ISL_3026086, EPI_ISL_3026087, EPI_ISL_3026088, EPI_ISL_3026089, EPI_ISL_3026090, EPI_ISL_3026091, EPI_ISL_3026092, EPI_ISL_3026093, EPI_ISL_3026094, EPI_ISL_3026095, EPI_ISL_3026096, EPI_ISL_3026097, EPI_ISL_3026098, EPI_ISL_3026099, EPI_ISL_3026100, EPI_ISL_3026101, EPI_ISL_3026102, EPI_ISL_3026103, EPI_ISL_3026104, EPI_ISL_3026105, EPI_ISL_3026106, EPI_ISL_3026107, EPI_ISL_3026108, EPI_ISL_3026109, EPI_ISL_3026110, EPI_ISL_3026111, EPI_ISL_3026112, EPI_ISL_3026113, EPI_ISL_3026114, EPI_ISL_3026115, EPI_ISL_3026116, EPI_ISL_3026117, EPI_ISL_3026118, EPI_ISL_3026119, EPI_ISL_3026120, EPI_ISL_3026121, EPI_ISL_3026122, EPI_ISL_3026123, EPI_ISL_3026124, EPI_ISL_3026125, EPI_ISL_3026126, EPI_ISL_3026127, EPI_ISL_3026128, EPI_ISL_3026129, EPI_ISL_3026130, EPI_ISL_3026131, EPI_ISL_3026132, EPI_ISL_3026133, EPI_ISL_3026134, EPI_ISL_3026135, EPI_ISL_3026136, EPI_ISL_3026137, EPI_ISL_3026138, EPI_ISL_3026139, EPI_ISL_3026140, EPI_ISL_3026141, EPI_ISL_3026142, EPI_ISL_3026143, EPI_ISL_3026144, EPI_ISL_3026145, EPI_ISL_3026146, EPI_ISL_3026147, EPI_ISL_3026148, EPI_ISL_3026149, EPI_ISL_3026150, EPI_ISL_3026151, EPI_ISL_3026152, EPI_ISL_3026153, EPI_ISL_3026154, EPI_ISL_3026155, EPI_ISL_3026156, EPI_ISL_3026157, EPI_ISL_3026158, EPI_ISL_3026159, EPI_ISL_3026160, EPI_ISL_3026161, EPI_ISL_3026162, EPI_ISL_3026163, EPI_ISL_3026164, EPI_ISL_3026165, EPI_ISL_3026166, EPI_ISL_3026167, EPI_ISL_3026168, EPI_ISL_3026169, EPI_ISL_3026170, EPI_ISL_3026171, EPI_ISL_3026172, EPI_ISL_3026173, EPI_ISL_3026174, EPI_ISL_3026175, EPI_ISL_3026176, EPI_ISL_3026177, EPI_ISL_3026178, EPI_ISL_3026179, EPI_ISL_3026180, EPI_ISL_3026181, EPI_ISL_3026182, EPI_ISL_3026183, EPI_ISL_3026184, EPI_ISL_3026185, EPI_ISL_3026186, EPI_ISL_3026187, EPI_ISL_3026188, EPI_ISL_3026189, EPI_ISL_3026190, EPI_ISL_3026191, EPI_ISL_3026192, EPI_ISL_3026193, EPI_ISL_3026194, EPI_ISL_3026195, EPI_ISL_3026196, EPI_ISL_3026197, EPI_ISL_3026198, EPI_ISL_3026199, EPI_ISL_3026200, EPI_ISL_3026201, EPI_ISL_3026202, EPI_ISL_3026203, EPI_ISL_3026204, EPI_ISL_3026205, EPI_ISL_3026206, EPI_ISL_3026207, EPI_ISL_3026208, EPI_ISL_3026209, EPI_ISL_3026210, EPI_ISL_3026211, EPI_ISL_3026212, EPI_ISL_3026213, EPI_ISL_3026214, EPI_ISL_3026215, EPI_ISL_3026216, EPI_ISL_3026217, EPI_ISL_3026218, EPI_ISL_3026219, EPI_ISL_3026220, EPI_ISL_3026221, EPI_ISL_3026222, EPI_ISL_3026223, EPI_ISL_3026224, EPI_ISL_3026225, EPI_ISL_3026226, EPI_ISL_3026227, EPI_ISL_3026228, EPI_ISL_3026229, EPI_ISL_3026230, EPI_ISL_3026231, EPI_ISL_3026232, EPI_ISL_3026233, EPI_ISL_3026234, EPI_ISL_3026235, EPI_ISL_3026236, EPI_ISL_3026237, EPI_ISL_3026238, EPI_ISL_3026239, EPI_ISL_3026240, EPI_ISL_3026241, EPI_ISL_3026242, EPI_ISL_3026243, EPI_ISL_3026244, EPI_ISL_3026245, EPI_ISL_3026246, EPI_ISL_3026247, EPI_ISL_3026248, EPI_ISL_3026249, EPI_ISL_3026250, EPI_ISL_3026251, EPI_ISL_3026252, EPI_ISL_3026253, EPI_ISL_3026254, EPI_ISL_3026255, EPI_ISL_3026256, EPI_ISL_3026257, EPI_ISL_3026258, EPI_ISL_3026259, EPI_ISL_3026260, EPI_ISL_3026261, EPI_ISL_3026262, EPI_ISL_3026263, EPI_ISL_3026264, EPI_ISL_3026265, EPI_ISL_3026266, EPI_ISL_3026267, EPI_ISL_3026268, EPI_ISL_3026269, EPI_ISL_3026270, EPI_ISL_3026271, EPI_ISL_3026272, EPI_ISL_3026273, EPI_ISL_3026274, EPI_ISL_3026275, EPI_ISL_3026276, EPI_ISL_3026277, EPI_ISL_3026278, EPI_ISL_3026279, EPI_ISL_3026280, EPI_ISL_3026281, EPI_ISL_3026282, EPI_ISL_3026283, EPI_ISL_3026284, EPI_ISL_3026285, EPI_ISL_3026286, EPI_ISL_3026287, EPI_ISL_3026288, EPI_ISL_3026289, EPI_ISL_3026290, EPI_ISL_3026291, EPI_ISL_3026292, EPI_ISL_3026293, EPI_ISL_3026294, EPI_ISL_3026295, EPI_ISL_3026296, EPI_ISL_3026297, EPI_ISL_3026298, EPI_ISL_3026299, EPI_ISL_3026300, EPI_ISL_3026301, EPI_ISL_3026302, EPI_ISL_3026303, EPI_ISL_3026304, EPI_ISL_3026305, EPI_ISL_3026306, EPI_ISL_3026307, EPI_ISL_3026308, EPI_ISL_3026309, EPI_ISL_3026310, EPI_ISL_3026311, EPI_ISL_3026312, EPI_ISL_3026313, EPI_ISL_3026314, EPI_ISL_3026315, EPI_ISL_3026316, EPI_ISL_3026317, EPI_ISL_3026318, EPI_ISL_3026319, EPI_ISL_3026320, EPI_ISL_3026321, EPI_ISL_3026322, EPI_ISL_3026323, EPI_ISL_3026324, EPI_ISL_3026325, EPI_ISL_3026326, EPI_ISL_3026327, EPI_ISL_3026328, EPI_ISL_3026329, EPI_ISL_3026330, EPI_ISL_3026331, EPI_ISL_3026332, EPI_ISL_3026333, EPI_ISL_3026334, EPI_ISL_3026335, EPI_ISL_3026336, EPI_ISL_3026337, EPI_ISL_3026338, EPI_ISL_3026339, EPI_ISL_3026340, EPI_ISL_3026341, EPI_ISL_3026342, EPI_ISL_3026343, EPI_ISL_3026344, EPI_ISL_3026345, EPI_ISL_3026346, EPI_ISL_3026347, EPI_ISL_3026348, EPI_ISL_3026349, EPI_ISL_3026350, EPI_ISL_3026351, EPI_ISL_3026352, EPI_ISL_3026353, EPI_ISL_3026354, EPI_ISL_3026355, EPI_ISL_3026356, EPI_ISL_3026357, EPI_ISL_3026358, EPI_ISL_3026359, EPI_ISL_3026360, EPI_ISL_3026361, EPI_ISL_3026362, EPI_ISL_3026363, EPI_ISL_3026364, EPI_ISL_3026365, EPI_ISL_3026366, EPI_ISL_3026367, EPI_ISL_3026368, EPI_ISL_3026369, EPI_ISL_3026370, EPI_ISL_3026371, EPI_ISL_3026372, EPI_ISL_3026373, EPI_ISL_3026374, EPI_ISL_3026375, EPI_ISL_3026376, EPI_ISL_3026377, EPI_ISL_3026378, EPI_ISL_3026379, EPI_ISL_3026380, EPI_ISL_3026381, EPI_ISL_3026382, EPI_ISL_3026383, EPI_ISL_3026384, EPI_ISL_3026385, EPI_ISL_3026386, EPI_ISL_3026387, EPI_ISL_3026388, EPI_ISL_3026389, EPI_ISL_3026390, EPI_ISL_3026391, EPI_ISL_3026392, EPI_ISL_3026393, EPI_ISL_3026394, EPI_ISL_3026395, EPI_ISL_3026396, EPI_ISL_3026397, EPI_ISL_3026398, EPI_ISL_3026399, EPI_ISL_3026400, EPI_ISL_3026401, EPI_ISL_3026402, EPI_ISL_3026403, EPI_ISL_3026404, EPI_ISL_3026405, EPI_ISL_3026406, EPI_ISL_3026407, EPI_ISL_3026408, EPI_ISL_3026409, EPI_ISL_3026410, EPI_ISL_3026411, EPI_ISL_3026412, EPI_ISL_3026413, EPI_ISL_3026414, EPI_ISL_3026415, EPI_ISL_3026416, EPI_ISL_3026417, EPI_ISL_3026418, EPI_ISL_3026419, EPI_ISL_3026420, EPI_ISL_3026421, EPI_ISL_3026422, EPI_ISL_3026423, EPI_ISL_3026424, EPI_ISL_3026425, EPI_ISL_3026426, EPI_ISL_3026427, EPI_ISL_3026428, EPI_ISL_3026429, EPI_ISL_3026430, EPI_ISL_3026431, EPI_ISL_3026432, EPI_ISL_3026433, EPI_ISL_3026434, EPI_ISL_3026435, EPI_ISL_3026436, EPI_ISL_3026437, EPI_ISL_3026438, EPI_ISL_3026439, EPI_ISL_3026440, EPI_ISL_3026441, EPI_ISL_3026442, EPI_ISL_3026443, EPI_ISL_3026444, EPI_ISL_3026445, EPI_ISL_3026446, EPI_ISL_3026447, EPI_ISL_3026448, EPI_ISL_3026449, EPI_ISL_3026450, EPI_ISL_3026451, EPI_ISL_3026452, EPI_ISL_3026453, EPI_ISL_3026454, EPI_ISL_3026455, EPI_ISL_3026456, EPI_ISL_3026457, EPI_ISL_3026458, EPI_ISL_3026459, EPI_ISL_3026460, EPI_ISL_3026461, EPI_ISL_3026462, EPI_ISL_3026463, EPI_ISL_3026464, EPI_ISL_3026465, EPI_ISL_3026466, EPI_ISL_3026467, EPI_ISL_3026468, EPI_ISL_3026469, EPI_ISL_3026470, EPI_ISL_3026471, EPI_ISL_3026472, EPI_ISL_3026473, EPI_ISL_3026474, EPI_ISL_3026475, EPI_ISL_3026476, EPI_ISL_3026477, EPI_ISL_3026478, EPI_ISL_3026479, EPI_ISL_3026480, EPI_ISL_3026481, EPI_ISL_3026482, EPI_ISL_3026483, EPI_ISL_3026484, EPI_ISL_3026485, EPI_ISL_3026486, EPI_ISL_3026487, EPI_ISL_3026488, EPI_ISL_3026489, EPI_ISL_3026490, EPI_ISL_3026491, EPI_ISL_3026492, EPI_ISL_3026493, EPI_ISL_3026494, EPI_ISL_3026495, EPI_ISL_3026496, EPI_ISL_3026497, EPI_ISL_3026498, EPI_ISL_3026499, EPI_ISL_3026500, EPI_ISL_3026501, EPI_ISL_3026502, EPI_ISL_3026503, EPI_ISL_3026504, EPI_ISL_3026505, EPI_ISL_3026506, EPI_ISL_3026507, EPI_ISL_3026508, EPI_ISL_3026509, EPI_ISL_3026510, EPI_ISL_3026511, EPI_ISL_3026512, EPI_ISL_3026513, EPI_ISL_3026514, EPI_ISL_3026515, EPI_ISL_3026516, EPI_ISL_3026517, EPI_ISL_3026518, EPI_ISL_3026519, EPI_ISL_3026520, EPI_ISL_3026521, EPI_ISL_3026522, EPI_ISL_3026523, EPI_ISL_3026524, EPI_ISL_3026525, EPI_ISL_3026526, EPI_ISL_3026527, EPI_ISL_3026528, EPI_ISL_3026529, EPI_ISL_3026530, EPI_ISL_3026531, EPI_ISL_3026532, EPI_ISL_3026533, EPI_ISL_3026534, EPI_ISL_3026535, EPI_ISL_3026536, EPI_ISL_3026537, EPI_ISL_3026538, EPI_ISL_3026539, EPI_ISL_3026540, EPI_ISL_3026541, EPI_ISL_3026542, EPI_ISL_3026543, EPI_ISL_3026544, EPI_ISL_3026545, EPI_ISL_3026546, EPI_ISL_3026547, EPI_ISL_3026548, EPI_ISL_3026549, EPI_ISL_3026550, EPI_ISL_3026551, EPI_ISL_3026552, EPI_ISL_3026553, EPI_ISL_3026554, EPI_ISL_3026555, EPI_ISL_3026556, EPI_ISL_3026557, EPI_ISL_3026558, EPI_ISL_3026559, EPI_ISL_3026560, EPI_ISL_3026561, EPI_ISL_3026562, EPI_ISL_3026563, EPI_ISL_3026564, EPI_ISL_3026565, EPI_ISL_3026566, EPI_ISL_3026567, EPI_ISL_3026568, EPI_ISL_3026569, EPI_ISL_3026570, EPI_ISL_3026571, EPI_ISL_3026572, EPI_ISL_3026573, EPI_ISL_3026574, EPI_ISL_3026575, EPI_ISL_3026576, EPI_ISL_3026577, EPI_ISL_3026578, EPI_ISL_3026579, EPI_ISL_3026580, EPI_ISL_3026581, EPI_ISL_3026582, EPI_ISL_3026583, EPI_ISL_3026584, EPI_ISL_3026585, EPI_ISL_3026586, EPI_ISL_3026587, EPI_ISL_3026588, EPI_ISL_3026589, EPI_ISL_3026590, EPI_ISL_3026591, EPI_ISL_3026592, EPI_ISL_3026593, EPI_ISL_3026594, EPI_ISL_3026595, EPI_ISL_3026596, EPI_ISL_3026597, EPI_ISL_3026598, EPI_ISL_3026599, EPI_ISL_3026600, EPI_ISL_3026601, EPI_ISL_3026602, EPI_ISL_3026603, EPI_ISL_3026604, EPI_ISL_3026605, EPI_ISL_3026606, EPI_ISL_3026607, EPI_ISL_3026608, EPI_ISL_3026609, EPI_ISL_3026610, EPI_ISL_3026611, EPI_ISL_3026612, EPI_ISL_3026613, EPI_ISL_3026614, EPI_ISL_3026615, EPI_ISL_3026616, EPI_ISL_3026617, EPI_ISL_3026618, EPI_ISL_3026619, EPI_ISL_3026620, EPI_ISL_3026621, EPI_ISL_3026622, EPI_ISL_3026623, EPI_ISL_3026624, EPI_ISL_3026625, EPI_ISL_3026626, EPI_ISL_3026627, EPI_ISL_3026628, EPI_ISL_3026629, EPI_ISL_3026630, EPI_ISL_3026631, EPI_ISL_3026632, EPI_ISL_3026633, EPI_ISL_3026634, EPI_ISL_3026635, EPI_ISL_3026636, EPI_ISL_3026637, EPI_ISL_3026638, EPI_ISL_3026639, EPI_ISL_3026640, EPI_ISL_3026641, EPI_ISL_3026642, EPI_ISL_3026643, EPI_ISL_3026644, EPI_ISL_3026645, EPI_ISL_3026646, EPI_ISL_3026647, EPI_ISL_3026648, EPI_ISL_3026649, EPI_ISL_3026650, EPI_ISL_3026651, EPI_ISL_3026652, EPI_ISL_3026653, EPI_ISL_3026654, EPI_ISL_3026655, EPI_ISL_3026656, EPI_ISL_3026657, EPI_ISL_3026658, EPI_ISL_3026659, EPI_ISL_3026660, EPI_ISL_3026661, EPI_ISL_3026662, EPI_ISL_3026663, EPI_ISL_3026664, EPI_ISL_3026665, EPI_ISL_3026666, EPI_ISL_3026667, EPI_ISL_3026668, EPI_ISL_3026669, EPI_ISL_3026670, EPI_ISL_3026671, EPI_ISL_3026672, EPI_ISL_3026673, EPI_ISL_3026674, EPI_ISL_3026675, EPI_ISL_3026676, EPI_ISL_3026677, EPI_ISL_3026678, EPI_ISL_3026679, EPI_ISL_3026680, EPI_ISL_3026681, EPI_ISL_3026682, EPI_ISL_3026683, EPI_ISL_3026684, EPI_ISL_3026685, EPI_ISL_3026686, EPI_ISL_3026687, EPI_ISL_3026688, EPI_ISL_3026689, EPI_ISL_3026690, EPI_ISL_3026691, EPI_ISL_3026692, EPI_ISL_3026693, EPI_ISL_3026694, EPI_ISL_3026695, EPI_ISL_3026696, EPI_ISL_3026697, EPI_ISL_3026698, EPI_ISL_3026700, EPI_ISL_3026701, EPI_ISL_3026702, EPI_ISL_3026703, EPI_ISL_3026704, EPI_ISL_3026705, EPI_ISL_3026706, EPI_ISL_3026707, EPI_ISL_3026708, EPI_ISL_3026709, EPI_ISL_3026710, EPI_ISL_3026711, EPI_ISL_3026712, EPI_ISL_3026713, EPI_ISL_3026714, EPI_ISL_3026715, EPI_ISL_3026716, EPI_ISL_3026717, EPI_ISL_3026718, EPI_ISL_3026719, EPI_ISL_3026720, EPI_ISL_3026721, EPI_ISL_3026722, EPI_ISL_3026723, EPI_ISL_3026724, EPI_ISL_3026725, EPI_ISL_3026726, EPI_ISL_3026727, EPI_ISL_3026728, EPI_ISL_3026729, EPI_ISL_3026730, EPI_ISL_3026731, EPI_ISL_3026732, EPI_ISL_3026733, EPI_ISL_3026734, EPI_ISL_3026735, EPI_ISL_3026736, EPI_ISL_3026737, EPI_ISL_3026738, EPI_ISL_3026739, EPI_ISL_3026740, EPI_ISL_3026741, EPI_ISL_3026742, EPI_ISL_3026743, EPI_ISL_3026744, EPI_ISL_3026745, EPI_ISL_3026746, EPI_ISL_3026747, EPI_ISL_3026748, EPI_ISL_3026749, EPI_ISL_3026750, EPI_ISL_3026751, EPI_ISL_3026752, EPI_ISL_3026753, EPI_ISL_3026754, EPI_ISL_3026755, EPI_ISL_3026756, EPI_ISL_3026757, EPI_ISL_3026758, EPI_ISL_3026759, EPI_ISL_3026760, EPI_ISL_3026761, EPI_ISL_3026762, EPI_ISL_3026763, EPI_ISL_3026764, EPI_ISL_3026765, EPI_ISL_3026766, EPI_ISL_3026767, EPI_ISL_3026768, EPI_ISL_3026769, EPI_ISL_3026770, EPI_ISL_3026771, EPI_ISL_3026772, EPI_ISL_3026773, EPI_ISL_3026774, EPI_ISL_3026775, EPI_ISL_3026776, EPI_ISL_3026777, EPI_ISL_3026778, EPI_ISL_3026779, EPI_ISL_3026780, EPI_ISL_3026781, EPI_ISL_3026782, EPI_ISL_3026783, EPI_ISL_3026784, EPI_ISL_3026785, EPI_ISL_3026786, EPI_ISL_3026787, EPI_ISL_3026788, EPI_ISL_3026789, EPI_ISL_3026790, EPI_ISL_3026791, EPI_ISL_3026792, EPI_ISL_3026793, EPI_ISL_3026794, EPI_ISL_3026795, EPI_ISL_3026796, EPI_ISL_3026797, EPI_ISL_3026798, EPI_ISL_3026799, EPI_ISL_3026800, EPI_ISL_3026801, EPI_ISL_3026802, EPI_ISL_3026803, EPI_ISL_3026804, EPI_ISL_3026805, EPI_ISL_3026806, EPI_ISL_3026807, EPI_ISL_3026808, EPI_ISL_3026809, EPI_ISL_3026810, EPI_ISL_3026811, EPI_ISL_3026812, EPI_ISL_3026813, EPI_ISL_3026814, EPI_ISL_3026815, EPI_ISL_3026816, EPI_ISL_3026817, EPI_ISL_3026818, EPI_ISL_3026819, EPI_ISL_3026820, EPI_ISL_3026821, EPI_ISL_3026822, EPI_ISL_3026823, EPI_ISL_3026824, EPI_ISL_3026825, EPI_ISL_3026826, EPI_ISL_3026827, EPI_ISL_3026828, EPI_ISL_3026829, EPI_ISL_3026830, EPI_ISL_3026831, EPI_ISL_3026832, EPI_ISL_3026833, EPI_ISL_3026834, EPI_ISL_3026835, EPI_ISL_3026836, EPI_ISL_3026837, EPI_ISL_3026838, EPI_ISL_3026839, EPI_ISL_3026840, EPI_ISL_3026841, EPI_ISL_3026842, EPI_ISL_3026843, EPI_ISL_3026844, EPI_ISL_3026845, EPI_ISL_3026846, EPI_ISL_3026847, EPI_ISL_3026848, EPI_ISL_3026849, EPI_ISL_3026850, EPI_ISL_3026851, EPI_ISL_3026852, EPI_ISL_3026853, EPI_ISL_3026854, EPI_ISL_3026855, EPI_ISL_3026856, EPI_ISL_3026857, EPI_ISL_3026858, EPI_ISL_3026859, EPI_ISL_3026860, EPI_ISL_3118428, EPI_ISL_3118429, EPI_ISL_3118430, EPI_ISL_3118431, EPI_ISL_3118432, EPI_ISL_3146637, EPI_ISL_3146638, EPI_ISL_3146639, EPI_ISL_3146640, EPI_ISL_3146641, EPI_ISL_3146642, EPI_ISL_3146643, EPI_ISL_3146644, EPI_ISL_3146645, EPI_ISL_3146646, EPI_ISL_3146647, EPI_ISL_3146648, EPI_ISL_3146649, EPI_ISL_3146650, EPI_ISL_3146651, EPI_ISL_3146652, EPI_ISL_3146653, EPI_ISL_3146654, EPI_ISL_3146655, EPI_ISL_3146656, EPI_ISL_3146657, EPI_ISL_3146658, EPI_ISL_3146659, EPI_ISL_3146660, EPI_ISL_3146661, EPI_ISL_3146662, EPI_ISL_3146663, EPI_ISL_3146664, EPI_ISL_3146665, EPI_ISL_3146666, EPI_ISL_3146667, EPI_ISL_3146668, EPI_ISL_3146669, EPI_ISL_3146670, EPI_ISL_3146671, EPI_ISL_3146672, EPI_ISL_3146673, EPI_ISL_3146674, EPI_ISL_3146675, EPI_ISL_3146676, EPI_ISL_3146677, EPI_ISL_3146678, EPI_ISL_3146679, EPI_ISL_3146680, EPI_ISL_3146681, EPI_ISL_3146682, EPI_ISL_3146683, EPI_ISL_3146684, EPI_ISL_3146685, EPI_ISL_3146686, EPI_ISL_3146687, EPI_ISL_3146688, EPI_ISL_3146689, EPI_ISL_3146690, EPI_ISL_3146691, EPI_ISL_3146692, EPI_ISL_3146693, EPI_ISL_3146694, EPI_ISL_3146695, EPI_ISL_3146696, EPI_ISL_3146697, EPI_ISL_3146698, EPI_ISL_3146699, EPI_ISL_3146700, EPI_ISL_3146701, EPI_ISL_3146702, EPI_ISL_3146703, EPI_ISL_3146704, EPI_ISL_3146705, EPI_ISL_3146706, EPI_ISL_3146707, EPI_ISL_3146708, EPI_ISL_3146709, EPI_ISL_3146710, EPI_ISL_3146711, EPI_ISL_3146712, EPI_ISL_3146713, EPI_ISL_3146714, EPI_ISL_3146715, EPI_ISL_3146716, EPI_ISL_3146717, EPI_ISL_3146718, EPI_ISL_3146719, EPI_ISL_3146720, EPI_ISL_3146721, EPI_ISL_3146722, EPI_ISL_3146723, EPI_ISL_3146724, EPI_ISL_3146725, EPI_ISL_3146726, EPI_ISL_3146727, EPI_ISL_3146728, EPI_ISL_3146729, EPI_ISL_3146730, EPI_ISL_3146731, EPI_ISL_3146732, EPI_ISL_3146733, EPI_ISL_3146734, EPI_ISL_3146735, EPI_ISL_3146736, EPI_ISL_3146737, EPI_ISL_3146739, EPI_ISL_3146741, EPI_ISL_3146742, EPI_ISL_3146744, EPI_ISL_3146746, EPI_ISL_3146748, EPI_ISL_3146750, EPI_ISL_3146751, EPI_ISL_3146753, EPI_ISL_3146755, EPI_ISL_3146757, EPI_ISL_3146759, EPI_ISL_3146760, EPI_ISL_3146762, EPI_ISL_3146764, EPI_ISL_3146765, EPI_ISL_3146767, EPI_ISL_3146769, EPI_ISL_3146771, EPI_ISL_3146772, EPI_ISL_3146774, EPI_ISL_3146776, EPI_ISL_3146777, EPI_ISL_3146778, EPI_ISL_3146780, EPI_ISL_3146782, EPI_ISL_3146783, EPI_ISL_3146785, EPI_ISL_3146787, EPI_ISL_3146788, EPI_ISL_3146790, EPI_ISL_3146792, EPI_ISL_3146793, EPI_ISL_3146795, EPI_ISL_3146797, EPI_ISL_3146798, EPI_ISL_3146800, EPI_ISL_3146802, EPI_ISL_3146803, EPI_ISL_3146804, EPI_ISL_3146805, EPI_ISL_3146806, EPI_ISL_3146807, EPI_ISL_3146808, EPI_ISL_3146809, EPI_ISL_3146810, EPI_ISL_3146811, EPI_ISL_3146812, EPI_ISL_3146813, EPI_ISL_3146814, EPI_ISL_3146815, EPI_ISL_3146816, EPI_ISL_3146817, EPI_ISL_3146818, EPI_ISL_3146819, EPI_ISL_3146820, EPI_ISL_3146821, EPI_ISL_3146822, EPI_ISL_3146823, EPI_ISL_3146824, EPI_ISL_3146825, EPI_ISL_3146826, EPI_ISL_3146827, EPI_ISL_3146828, EPI_ISL_3146829, EPI_ISL_3146830, EPI_ISL_3146831, EPI_ISL_3146832, EPI_ISL_3146833, EPI_ISL_3146834, EPI_ISL_3146835, EPI_ISL_3146836, EPI_ISL_3146837, EPI_ISL_3146838, EPI_ISL_3146839, EPI_ISL_3146840, EPI_ISL_3146841, EPI_ISL_3146842, EPI_ISL_3146843, EPI_ISL_3146844, EPI_ISL_3146845, EPI_ISL_3146846, EPI_ISL_3146847, EPI_ISL_3146848, EPI_ISL_3146849, EPI_ISL_3146850, EPI_ISL_3146851, EPI_ISL_3146852, EPI_ISL_3146853, EPI_ISL_3146854, EPI_ISL_3146855, EPI_ISL_3146856, EPI_ISL_3146857, EPI_ISL_3146858, EPI_ISL_3146859, EPI_ISL_3146860, EPI_ISL_3146861, EPI_ISL_3146862, EPI_ISL_3146863, EPI_ISL_3146864, EPI_ISL_3146865, EPI_ISL_3146866, EPI_ISL_3146867, EPI_ISL_3146868, EPI_ISL_3146869, EPI_ISL_3146870, EPI_ISL_3146871, EPI_ISL_3146872, EPI_ISL_3146873, EPI_ISL_3146874, EPI_ISL_3146875, EPI_ISL_3146876, EPI_ISL_3146877, EPI_ISL_3146878, EPI_ISL_3146879, EPI_ISL_3146880, EPI_ISL_3146881, EPI_ISL_3146882, EPI_ISL_3146883, EPI_ISL_3146884, EPI_ISL_3146885, EPI_ISL_3146886, EPI_ISL_3146887, EPI_ISL_3146888, EPI_ISL_3146889, EPI_ISL_3146890, EPI_ISL_3146891, EPI_ISL_3146892, EPI_ISL_3146893, EPI_ISL_3146894, EPI_ISL_3146895, EPI_ISL_3146896, EPI_ISL_3146897, EPI_ISL_3146898, EPI_ISL_3146899, EPI_ISL_3146900, EPI_ISL_3146901, EPI_ISL_3146902, EPI_ISL_3146903, EPI_ISL_3146904, EPI_ISL_3146905, EPI_ISL_3146906, EPI_ISL_3146907, EPI_ISL_3146908, EPI_ISL_3146909, EPI_ISL_3146910, EPI_ISL_3146911, EPI_ISL_3146912, EPI_ISL_3146913, EPI_ISL_3146914, EPI_ISL_3146915, EPI_ISL_3146916, EPI_ISL_3146917, EPI_ISL_3146918, EPI_ISL_3146919, EPI_ISL_3146920, EPI_ISL_3146921, EPI_ISL_3146922, EPI_ISL_3146923, EPI_ISL_3146924, EPI_ISL_3146925, EPI_ISL_3146926, EPI_ISL_3146927, EPI_ISL_3146928, EPI_ISL_3146929, EPI_ISL_3146930, EPI_ISL_3146931, EPI_ISL_3146932, EPI_ISL_3146933, EPI_ISL_3146934, EPI_ISL_3146935, EPI_ISL_3146936, EPI_ISL_3146937, EPI_ISL_3146938, EPI_ISL_3146939, EPI_ISL_3146940, EPI_ISL_3146941, EPI_ISL_3146942, EPI_ISL_3146943, EPI_ISL_3146944, EPI_ISL_3146945, EPI_ISL_3146946, EPI_ISL_3146947, EPI_ISL_3146948, EPI_ISL_3146949, EPI_ISL_3146950, EPI_ISL_3146951, EPI_ISL_3146952, EPI_ISL_3146953, EPI_ISL_3146954, EPI_ISL_3146955, EPI_ISL_3146956, EPI_ISL_3146957, EPI_ISL_3146958, EPI_ISL_3146959, EPI_ISL_3146960, EPI_ISL_3146961, EPI_ISL_3146962, EPI_ISL_3146963, EPI_ISL_3146964, EPI_ISL_3146965, EPI_ISL_3146966, EPI_ISL_3146967, EPI_ISL_3146968, EPI_ISL_3146969, EPI_ISL_3146970, EPI_ISL_3146971, EPI_ISL_3146972, EPI_ISL_3146973, EPI_ISL_3146974, EPI_ISL_3146975, EPI_ISL_3146976, EPI_ISL_3146977, EPI_ISL_3146978, EPI_ISL_3146979, EPI_ISL_3146980, EPI_ISL_3146981, EPI_ISL_3146982, EPI_ISL_3146983, EPI_ISL_3146984, EPI_ISL_3146985, EPI_ISL_3146986, EPI_ISL_3146987, EPI_ISL_3146988, EPI_ISL_3146989, EPI_ISL_3146990, EPI_ISL_3146991, EPI_ISL_3146992, EPI_ISL_3146993, EPI_ISL_3146994, EPI_ISL_3146995, EPI_ISL_3146996, EPI_ISL_3146997, EPI_ISL_3146998, EPI_ISL_3146999, EPI_ISL_3147000, EPI_ISL_3147001, EPI_ISL_3147002, EPI_ISL_3147003, EPI_ISL_3147004, EPI_ISL_3147005, EPI_ISL_3147006, EPI_ISL_3147007, EPI_ISL_3147008, EPI_ISL_3147009, EPI_ISL_3147010, EPI_ISL_3147011, EPI_ISL_3147012, EPI_ISL_3147013, EPI_ISL_3147014, EPI_ISL_3147015, EPI_ISL_3147016, EPI_ISL_3147017, EPI_ISL_3147018, EPI_ISL_3147019, EPI_ISL_3147020, EPI_ISL_3147021, EPI_ISL_3147022, EPI_ISL_3147023, EPI_ISL_3147024, EPI_ISL_3147025, EPI_ISL_3147026, EPI_ISL_3147027, EPI_ISL_3147028, EPI_ISL_3147029, EPI_ISL_3147030, EPI_ISL_3147031, EPI_ISL_3147032, EPI_ISL_3147033, EPI_ISL_3147034, EPI_ISL_3147035, EPI_ISL_3147036, EPI_ISL_3147037, EPI_ISL_3147038, EPI_ISL_3147039, EPI_ISL_3147040, EPI_ISL_3147041, EPI_ISL_3147042, EPI_ISL_3147043, EPI_ISL_3147044, EPI_ISL_3147045, EPI_ISL_3147046, EPI_ISL_3147047, EPI_ISL_3147048, EPI_ISL_3147049, EPI_ISL_3147050, EPI_ISL_3147051, EPI_ISL_3147052, EPI_ISL_3147053, EPI_ISL_3147054, EPI_ISL_3147055, EPI_ISL_3147056, EPI_ISL_3147057, EPI_ISL_3147058, EPI_ISL_3147059, EPI_ISL_3147060, EPI_ISL_3147061, EPI_ISL_3147062, EPI_ISL_3147063, EPI_ISL_3147064, EPI_ISL_3147065, EPI_ISL_3147066, EPI_ISL_3147067, EPI_ISL_3147068, EPI_ISL_3147069, EPI_ISL_3147070, EPI_ISL_3147071, EPI_ISL_3147072, EPI_ISL_3147073, EPI_ISL_3147074, EPI_ISL_3147075, EPI_ISL_3147076, EPI_ISL_3147077, EPI_ISL_3147078, EPI_ISL_3147079, EPI_ISL_3147080, EPI_ISL_3147081, EPI_ISL_3147082, EPI_ISL_3147083, EPI_ISL_3147084, EPI_ISL_3147085, EPI_ISL_3147086, EPI_ISL_3147087, EPI_ISL_3147088, EPI_ISL_3147089, EPI_ISL_3147090, EPI_ISL_3147091, EPI_ISL_3147092, EPI_ISL_3147093, EPI_ISL_3147094, EPI_ISL_3147095, EPI_ISL_3147096, EPI_ISL_3147097, EPI_ISL_3147098, EPI_ISL_3147099, EPI_ISL_3147100, EPI_ISL_3147101, EPI_ISL_3147102, EPI_ISL_3147103, EPI_ISL_3147104, EPI_ISL_3147105, EPI_ISL_3147106, EPI_ISL_3147107, EPI_ISL_3147108, EPI_ISL_3147109, EPI_ISL_3147110, EPI_ISL_3147111, EPI_ISL_3147112, EPI_ISL_3147113, EPI_ISL_3147114, EPI_ISL_3147115, EPI_ISL_3147116, EPI_ISL_3147117, EPI_ISL_3147118, EPI_ISL_3147119, EPI_ISL_3147120, EPI_ISL_3147121, EPI_ISL_3147122, EPI_ISL_3147123, EPI_ISL_3147124, EPI_ISL_3147125, EPI_ISL_3147126, EPI_ISL_3147127, EPI_ISL_3147128, EPI_ISL_3147129, EPI_ISL_3147130, EPI_ISL_3147131, EPI_ISL_3147132, EPI_ISL_3147133, EPI_ISL_3147134, EPI_ISL_3147135, EPI_ISL_3147136, EPI_ISL_3147137, EPI_ISL_3147138, EPI_ISL_3147139, EPI_ISL_3147140, EPI_ISL_3147141, EPI_ISL_3147142, EPI_ISL_3147143, EPI_ISL_3147144, EPI_ISL_3147145, EPI_ISL_3147146, EPI_ISL_3147147, EPI_ISL_3147148, EPI_ISL_3147149, EPI_ISL_3147150, EPI_ISL_3147151, EPI_ISL_3147152, EPI_ISL_3147153, EPI_ISL_3147154, EPI_ISL_3147155, EPI_ISL_3147156, EPI_ISL_3147157, EPI_ISL_3147158, EPI_ISL_3147159, EPI_ISL_3147160, EPI_ISL_3147161, EPI_ISL_3147162, EPI_ISL_3147163, EPI_ISL_3147164, EPI_ISL_3147165, EPI_ISL_3147166, EPI_ISL_3147167, EPI_ISL_3147168, EPI_ISL_3147169, EPI_ISL_3147170, EPI_ISL_3147171, EPI_ISL_3147172, EPI_ISL_3147173, EPI_ISL_3147174, EPI_ISL_3147175, EPI_ISL_3147176, EPI_ISL_3147177, EPI_ISL_3147178, EPI_ISL_3368912, EPI_ISL_3368913, EPI_ISL_3368914, EPI_ISL_3368915, EPI_ISL_3368916, EPI_ISL_3368917, EPI_ISL_3368918, EPI_ISL_3368919, EPI_ISL_3368920, EPI_ISL_3368921, EPI_ISL_3368922, EPI_ISL_3368923, EPI_ISL_3368924, EPI_ISL_3368925, EPI_ISL_3368926, EPI_ISL_3368927, EPI_ISL_3368928, EPI_ISL_3368929, EPI_ISL_3368930, EPI_ISL_3368931, EPI_ISL_3368932, EPI_ISL_3368933, EPI_ISL_3368934, EPI_ISL_3368935, EPI_ISL_3368936, EPI_ISL_3368937, EPI_ISL_3368938, EPI_ISL_3368939, EPI_ISL_3368940, EPI_ISL_3368941, EPI_ISL_3368942, EPI_ISL_3368943, EPI_ISL_3368944, EPI_ISL_3368945, EPI_ISL_3368946, EPI_ISL_3368947, EPI_ISL_3368948, EPI_ISL_3368949, EPI_ISL_3368950, EPI_ISL_3368951, EPI_ISL_3368952, EPI_ISL_3368953, EPI_ISL_3368954, EPI_ISL_3368955, EPI_ISL_3368956, EPI_ISL_3368957, EPI_ISL_3368958, EPI_ISL_3368959, EPI_ISL_3368960, EPI_ISL_3368961, EPI_ISL_3368962, EPI_ISL_3368963, EPI_ISL_3368964, EPI_ISL_3368965, EPI_ISL_3368966, EPI_ISL_3368967, EPI_ISL_3368968, EPI_ISL_3368969, EPI_ISL_3368970, EPI_ISL_3368971, EPI_ISL_3368972, EPI_ISL_3368973, EPI_ISL_3368974, EPI_ISL_3368975, EPI_ISL_3368976, EPI_ISL_3368977, EPI_ISL_3368978, EPI_ISL_3368979, EPI_ISL_3368980, EPI_ISL_3368981, EPI_ISL_3368982, EPI_ISL_3368983, EPI_ISL_3368984, EPI_ISL_3368985, EPI_ISL_3368986, EPI_ISL_3368987, EPI_ISL_3368988, EPI_ISL_3368989, EPI_ISL_3368990, EPI_ISL_3368991, EPI_ISL_3368992, EPI_ISL_3368993, EPI_ISL_3368994, EPI_ISL_3368995, EPI_ISL_3368996, EPI_ISL_3368997, EPI_ISL_3368998, EPI_ISL_3368999, EPI_ISL_3369000, EPI_ISL_3369001, EPI_ISL_3369002, EPI_ISL_3369003, EPI_ISL_3369004, EPI_ISL_3369005, EPI_ISL_3369006, EPI_ISL_3369007, EPI_ISL_3369008, EPI_ISL_3369009, EPI_ISL_3369010, EPI_ISL_3369011, EPI_ISL_3369012, EPI_ISL_3369013, EPI_ISL_3369014, EPI_ISL_3369015, EPI_ISL_3369016, EPI_ISL_3369017, EPI_ISL_3369018, EPI_ISL_3369019, EPI_ISL_3369020, EPI_ISL_3369021, EPI_ISL_3369022, EPI_ISL_3369023, EPI_ISL_3369024, EPI_ISL_3369025, EPI_ISL_3369026, EPI_ISL_3369027, EPI_ISL_3369028, EPI_ISL_3369029, EPI_ISL_3369030, EPI_ISL_3369031, EPI_ISL_3369032, EPI_ISL_3369033, EPI_ISL_3369034, EPI_ISL_3369035, EPI_ISL_3369036, EPI_ISL_3369037, EPI_ISL_3369038, EPI_ISL_3369039, EPI_ISL_3369040, EPI_ISL_3369041, EPI_ISL_3369042, EPI_ISL_3369043, EPI_ISL_3369044, EPI_ISL_3369045, EPI_ISL_3369046, EPI_ISL_3369047, EPI_ISL_3369048, EPI_ISL_3369049, EPI_ISL_3369050, EPI_ISL_3369051, EPI_ISL_3369052, EPI_ISL_3369053, EPI_ISL_3369054, EPI_ISL_3369055, EPI_ISL_3369056, EPI_ISL_3369057, EPI_ISL_3369058, EPI_ISL_3369059, EPI_ISL_3369060, EPI_ISL_3369061, EPI_ISL_3369062, EPI_ISL_3369063, EPI_ISL_3369064, EPI_ISL_3369065, EPI_ISL_3369066, EPI_ISL_3369067, EPI_ISL_3369068, EPI_ISL_3369069, EPI_ISL_3369070, EPI_ISL_3369071, EPI_ISL_3369072, EPI_ISL_3369073, EPI_ISL_3369074, EPI_ISL_3369075, EPI_ISL_3369076, EPI_ISL_3369077, EPI_ISL_3369078, EPI_ISL_3369079, EPI_ISL_3369080, EPI_ISL_3369081, EPI_ISL_3369082, EPI_ISL_3369083, EPI_ISL_3369084, EPI_ISL_3369085, EPI_ISL_3369086, EPI_ISL_3369087, EPI_ISL_3369088, EPI_ISL_3369089, EPI_ISL_3369090, EPI_ISL_3369091, EPI_ISL_3369092, EPI_ISL_3369093, EPI_ISL_3369094, EPI_ISL_3369095, EPI_ISL_3369096, EPI_ISL_3369097, EPI_ISL_3369098, EPI_ISL_3369099, EPI_ISL_3369100, EPI_ISL_3369101, EPI_ISL_3369102, EPI_ISL_3369103, EPI_ISL_3369104, EPI_ISL_3369105, EPI_ISL_3369106, EPI_ISL_3369107, EPI_ISL_3369108, EPI_ISL_3369109, EPI_ISL_3369110, EPI_ISL_3369111, EPI_ISL_3369112, EPI_ISL_3369113, EPI_ISL_3369114, EPI_ISL_3369115, EPI_ISL_3369116, EPI_ISL_3369117, EPI_ISL_3369118, EPI_ISL_3369119, EPI_ISL_3369120, EPI_ISL_3369121, EPI_ISL_3369122, EPI_ISL_3369123, EPI_ISL_3369124, EPI_ISL_3369125, EPI_ISL_3369126, EPI_ISL_3369127, EPI_ISL_3369128, EPI_ISL_3369129, EPI_ISL_3369130, EPI_ISL_3369131, EPI_ISL_3369132, EPI_ISL_3369133, EPI_ISL_3369134, EPI_ISL_3369135, EPI_ISL_3369136, EPI_ISL_3369137, EPI_ISL_3369138, EPI_ISL_3369139, EPI_ISL_3369140, EPI_ISL_3369141, EPI_ISL_3369142, EPI_ISL_3369143, EPI_ISL_3369144, EPI_ISL_3369145, EPI_ISL_3369146, EPI_ISL_3369147, EPI_ISL_3369148, EPI_ISL_3369149, EPI_ISL_3369150, EPI_ISL_3369151, EPI_ISL_3369152, EPI_ISL_3369153, EPI_ISL_3369154, EPI_ISL_3369155, EPI_ISL_3369156, EPI_ISL_3369157, EPI_ISL_3369158, EPI_ISL_3369159, EPI_ISL_3369160, EPI_ISL_3369161, EPI_ISL_3369162, EPI_ISL_3369163, EPI_ISL_3369164, EPI_ISL_3369165, EPI_ISL_3369166, EPI_ISL_3369167, EPI_ISL_3369168, EPI_ISL_3369169, EPI_ISL_3369170, EPI_ISL_3369171, EPI_ISL_3369172, EPI_ISL_3369173, EPI_ISL_3369174, EPI_ISL_3369175, EPI_ISL_3369176, EPI_ISL_3369177, EPI_ISL_3369178, EPI_ISL_3369179, EPI_ISL_3369180, EPI_ISL_3369181, EPI_ISL_3369182, EPI_ISL_3369183, EPI_ISL_3369184, EPI_ISL_3369185, EPI_ISL_3369186, EPI_ISL_3369187, EPI_ISL_3369188, EPI_ISL_3369189, EPI_ISL_3369190, EPI_ISL_3369191, EPI_ISL_3369192, EPI_ISL_3369193, EPI_ISL_3369194, EPI_ISL_3369195, EPI_ISL_3369196, EPI_ISL_3369197, EPI_ISL_3369198, EPI_ISL_3369199, EPI_ISL_3369200, EPI_ISL_3369201, EPI_ISL_3369202, EPI_ISL_3369203, EPI_ISL_3369204, EPI_ISL_3369205, EPI_ISL_3369206, EPI_ISL_3369207, EPI_ISL_3369208, EPI_ISL_3369209, EPI_ISL_3369210, EPI_ISL_3369211, EPI_ISL_3369212, EPI_ISL_3369213, EPI_ISL_3369214, EPI_ISL_3369215, EPI_ISL_3369216, EPI_ISL_3369217, EPI_ISL_3369218, EPI_ISL_3369219, EPI_ISL_3369220, EPI_ISL_3369221, EPI_ISL_3369222, EPI_ISL_3369223, EPI_ISL_3369224, EPI_ISL_3369225, EPI_ISL_3369226, EPI_ISL_3369227, EPI_ISL_3369228, EPI_ISL_3369229, EPI_ISL_3369230, EPI_ISL_3369231, EPI_ISL_3369232, EPI_ISL_3369233, EPI_ISL_3369234, EPI_ISL_3369235, EPI_ISL_3369236, EPI_ISL_3369237, EPI_ISL_3369238, EPI_ISL_3369239, EPI_ISL_3369240, EPI_ISL_3369241, EPI_ISL_3369242, EPI_ISL_3369243, EPI_ISL_3369244, EPI_ISL_3369245, EPI_ISL_3369246, EPI_ISL_3369247, EPI_ISL_3369248, EPI_ISL_3369249, EPI_ISL_3369250, EPI_ISL_3369251, EPI_ISL_3369252, EPI_ISL_3369253, EPI_ISL_3369254, EPI_ISL_3369255, EPI_ISL_3369256, EPI_ISL_3369257, EPI_ISL_3369258, EPI_ISL_3369259, EPI_ISL_3369260, EPI_ISL_3369261, EPI_ISL_3369262, EPI_ISL_3369263, EPI_ISL_3369264, EPI_ISL_3369265, EPI_ISL_3369266, EPI_ISL_3369267, EPI_ISL_3369268, EPI_ISL_3369269, EPI_ISL_3369270, EPI_ISL_3369271, EPI_ISL_3369272, EPI_ISL_3369273, EPI_ISL_3369274, EPI_ISL_3448953, EPI_ISL_3448954, EPI_ISL_3448955, EPI_ISL_3451875, EPI_ISL_3451876, EPI_ISL_3451877, EPI_ISL_3451878, EPI_ISL_3451879, EPI_ISL_3451880, EPI_ISL_3451881, EPI_ISL_3451882, EPI_ISL_3451883, EPI_ISL_3451884, EPI_ISL_3451885, EPI_ISL_3451886, EPI_ISL_3451887, EPI_ISL_3451888, EPI_ISL_3451889, EPI_ISL_3451890, EPI_ISL_3451891, EPI_ISL_3451892, EPI_ISL_3451893, EPI_ISL_3451894, EPI_ISL_3451895, EPI_ISL_3451896, EPI_ISL_3451897, EPI_ISL_3451898, EPI_ISL_3451899, EPI_ISL_3451900, EPI_ISL_3451901, EPI_ISL_3451902, EPI_ISL_3451903, EPI_ISL_3451904, EPI_ISL_3451905, EPI_ISL_3451906, EPI_ISL_3451907, EPI_ISL_3451908, EPI_ISL_3451909, EPI_ISL_3451910, EPI_ISL_3451911, EPI_ISL_3451912, EPI_ISL_3451913, EPI_ISL_3451914, EPI_ISL_3451915, EPI_ISL_3451916, EPI_ISL_3451917, EPI_ISL_3451918, EPI_ISL_3451919, EPI_ISL_3451920, EPI_ISL_3451921, EPI_ISL_3451922, EPI_ISL_3451923, EPI_ISL_3451924, EPI_ISL_3451925, EPI_ISL_3451926, EPI_ISL_3451927, EPI_ISL_3451928, EPI_ISL_3451929, EPI_ISL_3451930, EPI_ISL_3451931, EPI_ISL_3451932, EPI_ISL_3451933, EPI_ISL_3451934, EPI_ISL_3451935, EPI_ISL_3451936, EPI_ISL_3451937, EPI_ISL_3451938, EPI_ISL_3451939, EPI_ISL_3451940, EPI_ISL_3451941, EPI_ISL_3451942, EPI_ISL_3451943, EPI_ISL_3451944, EPI_ISL_3451945, EPI_ISL_3451946, EPI_ISL_3451947, EPI_ISL_3451948, EPI_ISL_3451949, EPI_ISL_3451950, EPI_ISL_3451951, EPI_ISL_3451952, EPI_ISL_3451953, EPI_ISL_3451954, EPI_ISL_3451955, EPI_ISL_3451956, EPI_ISL_3451957, EPI_ISL_3451958, EPI_ISL_3451959, EPI_ISL_3451960, EPI_ISL_3451961, EPI_ISL_3451962, EPI_ISL_3451963, EPI_ISL_3451964, EPI_ISL_3451965, EPI_ISL_3451966, EPI_ISL_3451967, EPI_ISL_3451968, EPI_ISL_3451969, EPI_ISL_3451970, EPI_ISL_3451971, EPI_ISL_3451972, EPI_ISL_3451973, EPI_ISL_3451974, EPI_ISL_3451975, EPI_ISL_3451976, EPI_ISL_3451977, EPI_ISL_3451978, EPI_ISL_3451979, EPI_ISL_3451980, EPI_ISL_3451981, EPI_ISL_3451982, EPI_ISL_3451983, EPI_ISL_3451984, EPI_ISL_3451985, EPI_ISL_3451986, EPI_ISL_3451987, EPI_ISL_3451988, EPI_ISL_3451989, EPI_ISL_3451990, EPI_ISL_3451991, EPI_ISL_3451992, EPI_ISL_3451993, EPI_ISL_3451994, EPI_ISL_3451995, EPI_ISL_3451996, EPI_ISL_3451997, EPI_ISL_3451998, EPI_ISL_3451999, EPI_ISL_3452000, EPI_ISL_3452001, EPI_ISL_3452002, EPI_ISL_3452003, EPI_ISL_3452004, EPI_ISL_3452005, EPI_ISL_3452006, EPI_ISL_3452007, EPI_ISL_3452008, EPI_ISL_3452009, EPI_ISL_3452010, EPI_ISL_3452011, EPI_ISL_3452012, EPI_ISL_3452013, EPI_ISL_3452014, EPI_ISL_3452015, EPI_ISL_3452016, EPI_ISL_3452017, EPI_ISL_3452018, EPI_ISL_3452019, EPI_ISL_3452020, EPI_ISL_3452021, EPI_ISL_3452022, EPI_ISL_3452023, EPI_ISL_3452024, EPI_ISL_3452025, EPI_ISL_3452026, EPI_ISL_3452027, EPI_ISL_3452028, EPI_ISL_3452029, EPI_ISL_3452030, EPI_ISL_3452031, EPI_ISL_3452032, EPI_ISL_3452033, EPI_ISL_3452034, EPI_ISL_3452035, EPI_ISL_3452036, EPI_ISL_3452037, EPI_ISL_3452038, EPI_ISL_3452039, EPI_ISL_3452040, EPI_ISL_3452041, EPI_ISL_3452042, EPI_ISL_3452043, EPI_ISL_3452044, EPI_ISL_3452045, EPI_ISL_3452046, EPI_ISL_3452047, EPI_ISL_3452048, EPI_ISL_3452049, EPI_ISL_3452050, EPI_ISL_3452051, EPI_ISL_3452052, EPI_ISL_3452053, EPI_ISL_3452054, EPI_ISL_3452055, EPI_ISL_3452056, EPI_ISL_3452057, EPI_ISL_3452058, EPI_ISL_3452059, EPI_ISL_3452060, EPI_ISL_3452061, EPI_ISL_3452062, EPI_ISL_3452063, EPI_ISL_3452064, EPI_ISL_3452065, EPI_ISL_3452066, EPI_ISL_3452067, EPI_ISL_3452068, EPI_ISL_3452069, EPI_ISL_3452070, EPI_ISL_3452071, EPI_ISL_3452072, EPI_ISL_3452073, EPI_ISL_3452074, EPI_ISL_3452075, EPI_ISL_3452076, EPI_ISL_3452077, EPI_ISL_3452078, EPI_ISL_3452079, EPI_ISL_3452080, EPI_ISL_3452081, EPI_ISL_3452082, EPI_ISL_3452083, EPI_ISL_3452084, EPI_ISL_3452085, EPI_ISL_3452086, EPI_ISL_3452087, EPI_ISL_3452088, EPI_ISL_3452089, EPI_ISL_3452090, EPI_ISL_3452092, EPI_ISL_3452093, EPI_ISL_3452095, EPI_ISL_3452097, EPI_ISL_3452098, EPI_ISL_3452100, EPI_ISL_3452102, EPI_ISL_3452103, EPI_ISL_3452105, EPI_ISL_3452106, EPI_ISL_3452108, EPI_ISL_3452109, EPI_ISL_3452111, EPI_ISL_3452113, EPI_ISL_3452114, EPI_ISL_3452116, EPI_ISL_3452117, EPI_ISL_3452119, EPI_ISL_3452120, EPI_ISL_3452122, EPI_ISL_3452123, EPI_ISL_3452125, EPI_ISL_3452126, EPI_ISL_3452127, EPI_ISL_3452128, EPI_ISL_3452130, EPI_ISL_3452131, EPI_ISL_3452133, EPI_ISL_3452134, EPI_ISL_3452136, EPI_ISL_3452137, EPI_ISL_3452139, EPI_ISL_3452140, EPI_ISL_3452142, EPI_ISL_3452143, EPI_ISL_3452145, EPI_ISL_3452146, EPI_ISL_3452148, EPI_ISL_3452149, EPI_ISL_3452151, EPI_ISL_3452152, EPI_ISL_3452154, EPI_ISL_3452155, EPI_ISL_3452157, EPI_ISL_3452158, EPI_ISL_3452160, EPI_ISL_3452162, EPI_ISL_3452163, EPI_ISL_3452165, EPI_ISL_3452166, EPI_ISL_3452168, EPI_ISL_3452169, EPI_ISL_3452171, EPI_ISL_3452172, EPI_ISL_3452174, EPI_ISL_3452176, EPI_ISL_3452177, EPI_ISL_3452179, EPI_ISL_3452180, EPI_ISL_3452182, EPI_ISL_3452183, EPI_ISL_3452185, EPI_ISL_3452187, EPI_ISL_3452188, EPI_ISL_3452189, EPI_ISL_3452191, EPI_ISL_3452193, EPI_ISL_3452194, EPI_ISL_3452196, EPI_ISL_3452197, EPI_ISL_3452199, EPI_ISL_3452200, EPI_ISL_3452202, EPI_ISL_3452203, EPI_ISL_3452205, EPI_ISL_3452206, EPI_ISL_3452208, EPI_ISL_3452210, EPI_ISL_3452211, EPI_ISL_3452213, EPI_ISL_3452214, EPI_ISL_3452216, EPI_ISL_3452218, EPI_ISL_3452219, EPI_ISL_3452221, EPI_ISL_3452222, EPI_ISL_3452224, EPI_ISL_3452225, EPI_ISL_3452227, EPI_ISL_3452228, EPI_ISL_3452230, EPI_ISL_3452231, EPI_ISL_3452233, EPI_ISL_3452234, EPI_ISL_3452236, EPI_ISL_3452237, EPI_ISL_3452239, EPI_ISL_3452241, EPI_ISL_3452242, EPI_ISL_3452244, EPI_ISL_3452245, EPI_ISL_3452247, EPI_ISL_3452248, EPI_ISL_3452250, EPI_ISL_3452251, EPI_ISL_3452253, EPI_ISL_3452254, EPI_ISL_3452256, EPI_ISL_3452257, EPI_ISL_3452259, EPI_ISL_3452260, EPI_ISL_3452262, EPI_ISL_3452263, EPI_ISL_3452265, EPI_ISL_3452266, EPI_ISL_3452268, EPI_ISL_3452270, EPI_ISL_3452271, EPI_ISL_3452273, EPI_ISL_3452275, EPI_ISL_3452276, EPI_ISL_3452278, EPI_ISL_3452279, EPI_ISL_3452281, EPI_ISL_3452283, EPI_ISL_3452284, EPI_ISL_3452286, EPI_ISL_3452287, EPI_ISL_3452289, EPI_ISL_3452290, EPI_ISL_3452291, EPI_ISL_3452293, EPI_ISL_3452295, EPI_ISL_3452296, EPI_ISL_3452298, EPI_ISL_3452299, EPI_ISL_3452301, EPI_ISL_3452303, EPI_ISL_3452304, EPI_ISL_3452306, EPI_ISL_3452307, EPI_ISL_3452309, EPI_ISL_3452311, EPI_ISL_3452312, EPI_ISL_3452314, EPI_ISL_3452316, EPI_ISL_3452318, EPI_ISL_3452319, EPI_ISL_3452321, EPI_ISL_3452322, EPI_ISL_3452324, EPI_ISL_3452325,

EPI_ISL_3452327, EPI_ISL_3452329, EPI_ISL_3452331, EPI_ISL_3452332, EPI_ISL_3452334, EPI_ISL_3452335, EPI_ISL_3452337, EPI_ISL_3452339, EPI_ISL_3452340, EPI_ISL_3452342, EPI_ISL_3452343, EPI_ISL_3452345, EPI_ISL_3452346, EPI_ISL_3452348, EPI_ISL_3452349, EPI_ISL_3452351, EPI_ISL_3452353, EPI_ISL_3452354, EPI_ISL_3452356, EPI_ISL_3452357, EPI_ISL_3452359, EPI_ISL_3452360,
EPI_ISL_3452362, EPI_ISL_3452364, EPI_ISL_3452365, EPI_ISL_3452367, EPI_ISL_3452368, EPI_ISL_3452370, EPI_ISL_3452371, EPI_ISL_3452373, EPI_ISL_3452374, EPI_ISL_3452376, EPI_ISL_3452378, EPI_ISL_3452379, EPI_ISL_3452381, EPI_ISL_3452382, EPI_ISL_3452384, EPI_ISL_3452385, EPI_ISL_3452387, EPI_ISL_3452389, EPI_ISL_3452390, EPI_ISL_3452392, EPI_ISL_3452393, EPI_ISL_3452395,
EPI_ISL_3452396, EPI_ISL_3452398, EPI_ISL_3452399, EPI_ISL_3452401, EPI_ISL_3452402, EPI_ISL_3452404, EPI_ISL_3452405, EPI_ISL_3452407, EPI_ISL_3452408, EPI_ISL_3452410, EPI_ISL_3452411, EPI_ISL_3452413, EPI_ISL_3452414, EPI_ISL_3452416, EPI_ISL_3452417, EPI_ISL_3452419, EPI_ISL_3452421, EPI_ISL_3452422, EPI_ISL_3452424, EPI_ISL_3452425, EPI_ISL_3452427, EPI_ISL_3452429,
EPI_ISL_3452430, EPI_ISL_3452432, EPI_ISL_3452433, EPI_ISL_3452435, EPI_ISL_3452436, EPI_ISL_3452438, EPI_ISL_3452439, EPI_ISL_3452440, EPI_ISL_3452441, EPI_ISL_3452442, EPI_ISL_3452443, EPI_ISL_3452444, EPI_ISL_3452445, EPI_ISL_3452446, EPI_ISL_3452447, EPI_ISL_3452448, EPI_ISL_3452449, EPI_ISL_3452450, EPI_ISL_3452451, EPI_ISL_3452452, EPI_ISL_3452453, EPI_ISL_3452454,
EPI_ISL_3452455, EPI_ISL_3452456, EPI_ISL_3452457, EPI_ISL_3452458, EPI_ISL_3452459, EPI_ISL_3452460, EPI_ISL_3452461, EPI_ISL_3452462, EPI_ISL_3452463, EPI_ISL_3452464, EPI_ISL_3452465, EPI_ISL_3452466, EPI_ISL_3452467, EPI_ISL_3452468, EPI_ISL_3452469, EPI_ISL_3452470, EPI_ISL_3452471, EPI_ISL_3452472, EPI_ISL_3452473, EPI_ISL_3452474, EPI_ISL_3452475, EPI_ISL_3452476,
EPI_ISL_3452477, EPI_ISL_3452478, EPI_ISL_3452479, EPI_ISL_3452480, EPI_ISL_3452481, EPI_ISL_3452482, EPI_ISL_3452483, EPI_ISL_3452484, EPI_ISL_3452485, EPI_ISL_3452486, EPI_ISL_3452487, EPI_ISL_3452488, EPI_ISL_3452489, EPI_ISL_3452490, EPI_ISL_3452491, EPI_ISL_3452492, EPI_ISL_3452493, EPI_ISL_3452494, EPI_ISL_3452495, EPI_ISL_3452496, EPI_ISL_3544612, EPI_ISL_3544613,
EPI_ISL_3544614, EPI_ISL_3544615, EPI_ISL_3544616, EPI_ISL_3544617, EPI_ISL_3544618, EPI_ISL_3544619, EPI_ISL_3544620, EPI_ISL_3544621, EPI_ISL_3544622, EPI_ISL_3544623, EPI_ISL_3544624, EPI_ISL_3544625, EPI_ISL_3544626, EPI_ISL_3544627, EPI_ISL_3544628, EPI_ISL_3544629, EPI_ISL_3544630, EPI_ISL_3544631, EPI_ISL_3544632, EPI_ISL_3544633, EPI_ISL_3544634, EPI_ISL_3544635,
EPI_ISL_3544636, EPI_ISL_3544637, EPI_ISL_3544638, EPI_ISL_3544639, EPI_ISL_3544640, EPI_ISL_3544641, EPI_ISL_3544642, EPI_ISL_3544643, EPI_ISL_3544644, EPI_ISL_3544645, EPI_ISL_3544646, EPI_ISL_3544647, EPI_ISL_3544648, EPI_ISL_3544649, EPI_ISL_3544650, EPI_ISL_3544651, EPI_ISL_3544652, EPI_ISL_3544653, EPI_ISL_3544654, EPI_ISL_3544655, EPI_ISL_3544656, EPI_ISL_3544657,
EPI_ISL_3544658, EPI_ISL_3544659, EPI_ISL_3544660, EPI_ISL_3544661, EPI_ISL_3544662, EPI_ISL_3544663, EPI_ISL_3544664, EPI_ISL_3544665, EPI_ISL_3544666, EPI_ISL_3544667, EPI_ISL_3544668, EPI_ISL_3544669, EPI_ISL_3544670, EPI_ISL_3544671, EPI_ISL_3544672, EPI_ISL_3544673, EPI_ISL_3544674, EPI_ISL_3544675, EPI_ISL_3544676, EPI_ISL_3544677, EPI_ISL_3544678, EPI_ISL_3544679,
EPI_ISL_3544680, EPI_ISL_3544681, EPI_ISL_3544682, EPI_ISL_3544683, EPI_ISL_3544684, EPI_ISL_3544685, EPI_ISL_3544686, EPI_ISL_3544687, EPI_ISL_3544688, EPI_ISL_3544689, EPI_ISL_3544690, EPI_ISL_3544691, EPI_ISL_3544692, EPI_ISL_3544693, EPI_ISL_3544694, EPI_ISL_3544695, EPI_ISL_3544696, EPI_ISL_3544697, EPI_ISL_3544698, EPI_ISL_3544699, EPI_ISL_3544700, EPI_ISL_3544701,
EPI_ISL_3544702, EPI_ISL_3544703, EPI_ISL_3544704, EPI_ISL_3544705, EPI_ISL_3544706, EPI_ISL_3544707, EPI_ISL_3544708, EPI_ISL_3544709, EPI_ISL_3544710, EPI_ISL_3544711, EPI_ISL_3544712, EPI_ISL_3544713, EPI_ISL_3544714, EPI_ISL_3544715, EPI_ISL_3544716, EPI_ISL_3544717, EPI_ISL_3544718, EPI_ISL_3544719, EPI_ISL_3544720, EPI_ISL_3544721, EPI_ISL_3544722, EPI_ISL_3544723,
EPI_ISL_3544724, EPI_ISL_3544725, EPI_ISL_3544726, EPI_ISL_3544727, EPI_ISL_3544728, EPI_ISL_3544729, EPI_ISL_3544730, EPI_ISL_3544731, EPI_ISL_3544732, EPI_ISL_3544733, EPI_ISL_3544734, EPI_ISL_3544735, EPI_ISL_3544736, EPI_ISL_3544737, EPI_ISL_3544738, EPI_ISL_3544739, EPI_ISL_3544740, EPI_ISL_3544741, EPI_ISL_3544742, EPI_ISL_3544743, EPI_ISL_3544744, EPI_ISL_3544745,
EPI_ISL_3544746, EPI_ISL_3544747, EPI_ISL_3544748, EPI_ISL_3544749, EPI_ISL_3544750, EPI_ISL_3544751, EPI_ISL_3544752, EPI_ISL_3544753, EPI_ISL_3544754, EPI_ISL_3544755, EPI_ISL_3544756, EPI_ISL_3544757, EPI_ISL_3544758, EPI_ISL_3544759, EPI_ISL_3544760, EPI_ISL_3544761, EPI_ISL_3544762, EPI_ISL_3544763, EPI_ISL_3544764, EPI_ISL_3544765, EPI_ISL_3544766, EPI_ISL_3544767,
EPI_ISL_3544768, EPI_ISL_3544769, EPI_ISL_3544770, EPI_ISL_3544771, EPI_ISL_3544772, EPI_ISL_3544773, EPI_ISL_3544774, EPI_ISL_3544775, EPI_ISL_3544776, EPI_ISL_3544777, EPI_ISL_3544778, EPI_ISL_3544779, EPI_ISL_3544780, EPI_ISL_3544781, EPI_ISL_3544782, EPI_ISL_3544783, EPI_ISL_3544784, EPI_ISL_3544785, EPI_ISL_3544786, EPI_ISL_3544787, EPI_ISL_3544788, EPI_ISL_3544789,
EPI_ISL_3544790, EPI_ISL_3544791, EPI_ISL_3544792, EPI_ISL_3544793, EPI_ISL_3544794, EPI_ISL_3544795, EPI_ISL_3544796, EPI_ISL_3544797, EPI_ISL_3544798, EPI_ISL_3544799, EPI_ISL_3544800, EPI_ISL_3544801, EPI_ISL_3544802, EPI_ISL_3544803, EPI_ISL_3544804, EPI_ISL_3544805, EPI_ISL_3544806, EPI_ISL_3544807, EPI_ISL_3544808, EPI_ISL_3544809, EPI_ISL_3544810, EPI_ISL_3544811,
EPI_ISL_3544812, EPI_ISL_3544813, EPI_ISL_3544814, EPI_ISL_3544815, EPI_ISL_3544816, EPI_ISL_3544817, EPI_ISL_3544818, EPI_ISL_3544819, EPI_ISL_3544820, EPI_ISL_3544821, EPI_ISL_3544822, EPI_ISL_3544823, EPI_ISL_3544824, EPI_ISL_3544825, EPI_ISL_3544826, EPI_ISL_3544827, EPI_ISL_3544828, EPI_ISL_3544829, EPI_ISL_3544830, EPI_ISL_3544831, EPI_ISL_3544832, EPI_ISL_3544833,
EPI_ISL_3544834, EPI_ISL_3544835, EPI_ISL_3544836, EPI_ISL_3544837, EPI_ISL_3544838, EPI_ISL_3544839, EPI_ISL_3544840, EPI_ISL_3544841, EPI_ISL_3544842, EPI_ISL_3544843, EPI_ISL_3544844, EPI_ISL_3544845, EPI_ISL_3544846, EPI_ISL_3544847, EPI_ISL_3544848, EPI_ISL_3544849, EPI_ISL_3544850, EPI_ISL_3544851, EPI_ISL_3544852, EPI_ISL_3544853, EPI_ISL_3544854, EPI_ISL_3544855,
EPI_ISL_3544856, EPI_ISL_3544857, EPI_ISL_3544858, EPI_ISL_3544859, EPI_ISL_3544860, EPI_ISL_3544861, EPI_ISL_3544862, EPI_ISL_3544863, EPI_ISL_3544864, EPI_ISL_3544865, EPI_ISL_3544866, EPI_ISL_3544867, EPI_ISL_3544868, EPI_ISL_3544869, EPI_ISL_3544870, EPI_ISL_3544871, EPI_ISL_3544872, EPI_ISL_3544873, EPI_ISL_3544874, EPI_ISL_3544875, EPI_ISL_3544876, EPI_ISL_3544877,
EPI_ISL_3544878, EPI_ISL_3544879, EPI_ISL_3544880, EPI_ISL_3544881, EPI_ISL_3544882, EPI_ISL_3544883, EPI_ISL_3544884, EPI_ISL_3544885, EPI_ISL_3544886, EPI_ISL_3544887, EPI_ISL_3544888, EPI_ISL_3544889, EPI_ISL_3544890, EPI_ISL_3544891, EPI_ISL_3544892, EPI_ISL_3544893, EPI_ISL_3544894, EPI_ISL_3544895, EPI_ISL_3544896, EPI_ISL_3544897, EPI_ISL_3544898, EPI_ISL_3544899,
EPI_ISL_3544900, EPI_ISL_3544901, EPI_ISL_3544902, EPI_ISL_3544903, EPI_ISL_3544904, EPI_ISL_3544905, EPI_ISL_3544906, EPI_ISL_3544907, EPI_ISL_3544908, EPI_ISL_3544909, EPI_ISL_3544910, EPI_ISL_3544911, EPI_ISL_3544912, EPI_ISL_3544913, EPI_ISL_3544914, EPI_ISL_3544915, EPI_ISL_3544916, EPI_ISL_3544917, EPI_ISL_3544918, EPI_ISL_3544919, EPI_ISL_3544920, EPI_ISL_3544921,
EPI_ISL_3544922, EPI_ISL_3544923, EPI_ISL_3544924, EPI_ISL_3544925, EPI_ISL_3544926, EPI_ISL_3544927, EPI_ISL_3544928, EPI_ISL_3544929, EPI_ISL_3544930, EPI_ISL_3544931, EPI_ISL_3544932, EPI_ISL_3544933, EPI_ISL_3544934, EPI_ISL_3544935, EPI_ISL_3544936, EPI_ISL_3544937, EPI_ISL_3544938, EPI_ISL_3544939, EPI_ISL_3544940, EPI_ISL_3544941, EPI_ISL_3544942, EPI_ISL_3544943,
EPI_ISL_3544944, EPI_ISL_3544945, EPI_ISL_3544946, EPI_ISL_3544947, EPI_ISL_3544948, EPI_ISL_3544949, EPI_ISL_3544950, EPI_ISL_3544951, EPI_ISL_3544952, EPI_ISL_3544953, EPI_ISL_3544954, EPI_ISL_3544955, EPI_ISL_3544956, EPI_ISL_3544957, EPI_ISL_3544958, EPI_ISL_3544959, EPI_ISL_3544960, EPI_ISL_3544961, EPI_ISL_3544962, EPI_ISL_3544963, EPI_ISL_3544964, EPI_ISL_3544965,
EPI_ISL_3544966, EPI_ISL_3544967, EPI_ISL_3544968, EPI_ISL_3544969, EPI_ISL_3544970, EPI_ISL_3544971, EPI_ISL_3544972, EPI_ISL_3544973, EPI_ISL_3544974, EPI_ISL_3544975, EPI_ISL_3544976, EPI_ISL_3544977, EPI_ISL_3544978, EPI_ISL_3544979, EPI_ISL_3544980, EPI_ISL_3544981, EPI_ISL_3544982, EPI_ISL_3544983, EPI_ISL_3544984, EPI_ISL_3544985, EPI_ISL_3544986, EPI_ISL_3544987,
EPI_ISL_3544988, EPI_ISL_3544989, EPI_ISL_3544990, EPI_ISL_3544991, EPI_ISL_3544992, EPI_ISL_3544993, EPI_ISL_3544994, EPI_ISL_3544995, EPI_ISL_3544996, EPI_ISL_3544997, EPI_ISL_3544998, EPI_ISL_3544999, EPI_ISL_3545000, EPI_ISL_3545001, EPI_ISL_3545002, EPI_ISL_3545003, EPI_ISL_3545004, EPI_ISL_3545005, EPI_ISL_3545006, EPI_ISL_3545007, EPI_ISL_3545008, EPI_ISL_3545009,
EPI_ISL_3545010, EPI_ISL_3545011, EPI_ISL_3545012, EPI_ISL_3545013, EPI_ISL_3545014, EPI_ISL_3545015, EPI_ISL_3545016, EPI_ISL_3545017, EPI_ISL_3545018, EPI_ISL_3545019, EPI_ISL_3545020, EPI_ISL_3545021, EPI_ISL_3545022, EPI_ISL_3545023, EPI_ISL_3545024, EPI_ISL_3545025, EPI_ISL_3545026, EPI_ISL_3545027, EPI_ISL_3545028, EPI_ISL_3545029, EPI_ISL_3545030, EPI_ISL_3545031,
EPI_ISL_3545032, EPI_ISL_3545033, EPI_ISL_3545034, EPI_ISL_3545035, EPI_ISL_3545036, EPI_ISL_3545037, EPI_ISL_3545038, EPI_ISL_3545039, EPI_ISL_3545040, EPI_ISL_3545041, EPI_ISL_3545042, EPI_ISL_3545043, EPI_ISL_3545044, EPI_ISL_3545045, EPI_ISL_3545046, EPI_ISL_3545047, EPI_ISL_3545048, EPI_ISL_3545049, EPI_ISL_3545050, EPI_ISL_3545051, EPI_ISL_3545052, EPI_ISL_3545053,
EPI_ISL_3545054, EPI_ISL_3545055, EPI_ISL_3545056, EPI_ISL_3545057, EPI_ISL_3545058, EPI_ISL_3545059, EPI_ISL_3545060, EPI_ISL_3545061, EPI_ISL_3545062, EPI_ISL_3545063, EPI_ISL_3545064, EPI_ISL_3545065, EPI_ISL_3545066, EPI_ISL_3545067, EPI_ISL_3545068, EPI_ISL_3545069, EPI_ISL_3545070, EPI_ISL_3545071, EPI_ISL_3545072, EPI_ISL_3545073, EPI_ISL_3545074, EPI_ISL_3545075,
EPI_ISL_3545076, EPI_ISL_3545077, EPI_ISL_3545078, EPI_ISL_3545079, EPI_ISL_3545080, EPI_ISL_3545081, EPI_ISL_3545082, EPI_ISL_3545083, EPI_ISL_3545084, EPI_ISL_3545085, EPI_ISL_3545086, EPI_ISL_3545087, EPI_ISL_3545088, EPI_ISL_3545089, EPI_ISL_3545090, EPI_ISL_3545091, EPI_ISL_3545092, EPI_ISL_3545093, EPI_ISL_3545094, EPI_ISL_3545095, EPI_ISL_3545096, EPI_ISL_3545097,
EPI_ISL_3545098, EPI_ISL_3545099, EPI_ISL_3545100, EPI_ISL_3545101, EPI_ISL_3545102, EPI_ISL_3545103, EPI_ISL_3545104, EPI_ISL_3545105, EPI_ISL_3545106, EPI_ISL_3545107, EPI_ISL_3545108, EPI_ISL_3545109, EPI_ISL_3545110, EPI_ISL_3545111, EPI_ISL_3545112, EPI_ISL_3545113, EPI_ISL_3545114, EPI_ISL_3545115, EPI_ISL_3545116, EPI_ISL_3545117, EPI_ISL_3545118, EPI_ISL_3545119,
EPI_ISL_3545120, EPI_ISL_3545121, EPI_ISL_3545122, EPI_ISL_3545123, EPI_ISL_3545124, EPI_ISL_3545125, EPI_ISL_3545126, EPI_ISL_3545127, EPI_ISL_3545128, EPI_ISL_3545129, EPI_ISL_3545130, EPI_ISL_3545131, EPI_ISL_3545132, EPI_ISL_3545133, EPI_ISL_3545134, EPI_ISL_3545135, EPI_ISL_3545136, EPI_ISL_3545137, EPI_ISL_3545138, EPI_ISL_3545139, EPI_ISL_3545140, EPI_ISL_3545141,
EPI_ISL_3545142, EPI_ISL_3545143, EPI_ISL_3545144, EPI_ISL_3545145, EPI_ISL_3545146, EPI_ISL_3545147, EPI_ISL_3545148, EPI_ISL_3545149, EPI_ISL_3545150, EPI_ISL_3545151, EPI_ISL_3545152, EPI_ISL_3545153, EPI_ISL_3545154, EPI_ISL_3545155, EPI_ISL_3545156, EPI_ISL_3545157, EPI_ISL_3545158, EPI_ISL_3545159, EPI_ISL_3545160, EPI_ISL_3545161, EPI_ISL_3545162, EPI_ISL_3545163,
EPI_ISL_3545164, EPI_ISL_3545165, EPI_ISL_3545166, EPI_ISL_3545167, EPI_ISL_3545168, EPI_ISL_3545169, EPI_ISL_3545170, EPI_ISL_3545171, EPI_ISL_3545172, EPI_ISL_3545173, EPI_ISL_3545174, EPI_ISL_3545175, EPI_ISL_3545176, EPI_ISL_3545177, EPI_ISL_3545178, EPI_ISL_3545179, EPI_ISL_3545180, EPI_ISL_3545181, EPI_ISL_3545182, EPI_ISL_3545183, EPI_ISL_3545184, EPI_ISL_3545185,
EPI_ISL_3545186, EPI_ISL_3545187, EPI_ISL_3545188, EPI_ISL_3545189, EPI_ISL_3545190, EPI_ISL_3545191, EPI_ISL_3545192, EPI_ISL_3545193, EPI_ISL_3545194, EPI_ISL_3545195, EPI_ISL_3545196, EPI_ISL_3545197, EPI_ISL_3545198, EPI_ISL_3545199, EPI_ISL_3545200, EPI_ISL_3545201, EPI_ISL_3545202, EPI_ISL_3545203, EPI_ISL_3545204, EPI_ISL_3545205, EPI_ISL_3545206, EPI_ISL_3545207,
EPI_ISL_3545208, EPI_ISL_3545209, EPI_ISL_3545210, EPI_ISL_3545211, EPI_ISL_3545212, EPI_ISL_3545213, EPI_ISL_3545214, EPI_ISL_3545215, EPI_ISL_3545216, EPI_ISL_3545217, EPI_ISL_3545218, EPI_ISL_3545219, EPI_ISL_3545220, EPI_ISL_3545221, EPI_ISL_3545222, EPI_ISL_3545223, EPI_ISL_3545224, EPI_ISL_3545225, EPI_ISL_3545226, EPI_ISL_3545227, EPI_ISL_3545228, EPI_ISL_3545229,
EPI_ISL_3674045, EPI_ISL_3674047, EPI_ISL_3674050, EPI_ISL_3674054, EPI_ISL_3674056, EPI_ISL_3674061, EPI_ISL_3674064, EPI_ISL_3674066, EPI_ISL_3674069, EPI_ISL_3674073, EPI_ISL_3674075, EPI_ISL_3674078, EPI_ISL_3674081, EPI_ISL_3674084, EPI_ISL_3674087, EPI_ISL_3674089, EPI_ISL_3674092, EPI_ISL_3674094, EPI_ISL_3674097, EPI_ISL_3674101, EPI_ISL_3674103, EPI_ISL_3674106,
EPI_ISL_3674110, EPI_ISL_3674113, EPI_ISL_3674116, EPI_ISL_3674119, EPI_ISL_3674121, EPI_ISL_3674124, EPI_ISL_3674128, EPI_ISL_3674130, EPI_ISL_3674133, EPI_ISL_3674136, EPI_ISL_3674138, EPI_ISL_3674142, EPI_ISL_3674145, EPI_ISL_3674147, EPI_ISL_3674150, EPI_ISL_3674153, EPI_ISL_3674156, EPI_ISL_3674159, EPI_ISL_3674162, EPI_ISL_3674165, EPI_ISL_3674168, EPI_ISL_3674171,
EPI_ISL_3674173, EPI_ISL_3674176, EPI_ISL_3674180, EPI_ISL_3674182, EPI_ISL_3674185, EPI_ISL_3674189, EPI_ISL_3674192, EPI_ISL_3674194, EPI_ISL_3674197, EPI_ISL_3674201, EPI_ISL_3674204, EPI_ISL_3674206, EPI_ISL_3674209, EPI_ISL_3674213, EPI_ISL_3674215, EPI_ISL_3674218, EPI_ISL_3674222, EPI_ISL_3674224, EPI_ISL_3674229, EPI_ISL_3674231, EPI_ISL_3674235, EPI_ISL_3674238,
EPI_ISL_3674240, EPI_ISL_3674244, EPI_ISL_3674247, EPI_ISL_3674249, EPI_ISL_3674252, EPI_ISL_3674255, EPI_ISL_3674258, EPI_ISL_3674261, EPI_ISL_3674264, EPI_ISL_3674267, EPI_ISL_3674270, EPI_ISL_3674298, EPI_ISL_3674301, EPI_ISL_3674303, EPI_ISL_3674307, EPI_ISL_3674310, EPI_ISL_3674312, EPI_ISL_3674315, EPI_ISL_3674318, EPI_ISL_3674319, EPI_ISL_3674323, EPI_ISL_3674326,
EPI_ISL_3674328, EPI_ISL_3674331, EPI_ISL_3674333, EPI_ISL_3674338, EPI_ISL_3674341, EPI_ISL_3674344, EPI_ISL_3674346, EPI_ISL_3674349, EPI_ISL_3674353, EPI_ISL_3674355, EPI_ISL_3674359, EPI_ISL_3674362, EPI_ISL_3674365, EPI_ISL_3674368, EPI_ISL_3674371, EPI_ISL_3674374, EPI_ISL_3674378, EPI_ISL_3674380, EPI_ISL_3674383, EPI_ISL_3674387, EPI_ISL_3674389, EPI_ISL_3674392,
EPI_ISL_3674396, EPI_ISL_3674398, EPI_ISL_3674401, EPI_ISL_3674404, EPI_ISL_3674406, EPI_ISL_3674409, EPI_ISL_3674412, EPI_ISL_3674415, EPI_ISL_3674418, EPI_ISL_3674421, EPI_ISL_3674424, EPI_ISL_3674427, EPI_ISL_3674430, EPI_ISL_3674433, EPI_ISL_3674437, EPI_ISL_3674439, EPI_ISL_3674442, EPI_ISL_3674445, EPI_ISL_3674449, EPI_ISL_3674451, EPI_ISL_3674454, EPI_ISL_3674458,
EPI_ISL_3674460, EPI_ISL_3674463, EPI_ISL_3674467, EPI_ISL_3674469, EPI_ISL_3674472, EPI_ISL_3674475, EPI_ISL_3674477, EPI_ISL_3674481, EPI_ISL_3674484, EPI_ISL_3674486, EPI_ISL_3674490, EPI_ISL_3674493, EPI_ISL_3674495, EPI_ISL_3674498, EPI_ISL_3674501, EPI_ISL_3674503, EPI_ISL_3674506, EPI_ISL_3674510, EPI_ISL_3674513, EPI_ISL_3674515, EPI_ISL_3674519, EPI_ISL_3674522,
EPI_ISL_3674524, EPI_ISL_3674527, EPI_ISL_3674531, EPI_ISL_3674533, EPI_ISL_3674536, EPI_ISL_3674540, EPI_ISL_3674542, EPI_ISL_3674545, EPI_ISL_3674548, EPI_ISL_3674552, EPI_ISL_3674554, EPI_ISL_3674557, EPI_ISL_3674561, EPI_ISL_3674563, EPI_ISL_3674566, EPI_ISL_3674570, EPI_ISL_3674572, EPI_ISL_3674576, EPI_ISL_3674579, EPI_ISL_3674582, EPI_ISL_3674584, EPI_ISL_3674587,
EPI_ISL_3674591, EPI_ISL_3674594, EPI_ISL_3674597, EPI_ISL_3674600, EPI_ISL_3674603, EPI_ISL_3674607, EPI_ISL_3674609, EPI_ISL_3674613, EPI_ISL_3674616, EPI_ISL_3674620, EPI_ISL_3674622, EPI_ISL_3674625, EPI_ISL_3674628, EPI_ISL_3674632, EPI_ISL_3674634, EPI_ISL_3674637, EPI_ISL_3674640, EPI_ISL_3674643, EPI_ISL_3674646, EPI_ISL_3674649, EPI_ISL_3674652, EPI_ISL_3674655,
EPI_ISL_3674659, EPI_ISL_3674661, EPI_ISL_3674664, EPI_ISL_3674667, EPI_ISL_3674669, EPI_ISL_3674673, EPI_ISL_3674676, EPI_ISL_3674678, EPI_ISL_3674682, EPI_ISL_3674685, EPI_ISL_3674687, EPI_ISL_3674690, EPI_ISL_3674694, EPI_ISL_3674697, EPI_ISL_3674699, EPI_ISL_3674703, EPI_ISL_3674706, EPI_ISL_3674709, EPI_ISL_3674711, EPI_ISL_3674714, EPI_ISL_3674717, EPI_ISL_3674720,
EPI_ISL_3674723, EPI_ISL_3674726, EPI_ISL_3674729, EPI_ISL_3674732, EPI_ISL_3674734, EPI_ISL_3674737, EPI_ISL_3674740, EPI_ISL_3674743, EPI_ISL_3674746, EPI_ISL_3674748, EPI_ISL_3674750, EPI_ISL_3674754, EPI_ISL_3674758, EPI_ISL_3674761, EPI_ISL_3674763, EPI_ISL_3674766, EPI_ISL_3674770, EPI_ISL_3674772, EPI_ISL_3674775, EPI_ISL_3674779, EPI_ISL_3674783, EPI_ISL_3674785,
EPI_ISL_3674788, EPI_ISL_3772616, EPI_ISL_3772619, EPI_ISL_3772621, EPI_ISL_3772623, EPI_ISL_3772625, EPI_ISL_3772627, EPI_ISL_3772629, EPI_ISL_3772631, EPI_ISL_3772633, EPI_ISL_3772635, EPI_ISL_3772637, EPI_ISL_3772639, EPI_ISL_3772641, EPI_ISL_3772644, EPI_ISL_3772646, EPI_ISL_3772648, EPI_ISL_3772650, EPI_ISL_3772652, EPI_ISL_3772653, EPI_ISL_3772655, EPI_ISL_3772657,
EPI_ISL_3772659, EPI_ISL_3772661, EPI_ISL_3772664, EPI_ISL_3772666, EPI_ISL_3772668, EPI_ISL_3772670, EPI_ISL_3772672, EPI_ISL_3772674, EPI_ISL_3772676, EPI_ISL_3772678, EPI_ISL_3772680, EPI_ISL_3772682, EPI_ISL_3772684, EPI_ISL_3772686, EPI_ISL_3772689, EPI_ISL_3772691, EPI_ISL_3772693, EPI_ISL_3772695, EPI_ISL_3772697, EPI_ISL_3772699, EPI_ISL_3772701, EPI_ISL_3772703,
EPI_ISL_3772705, EPI_ISL_3772707, EPI_ISL_3772709, EPI_ISL_3772712, EPI_ISL_3772714, EPI_ISL_3772716, EPI_ISL_3772718, EPI_ISL_3772720, EPI_ISL_3772722, EPI_ISL_3772724, EPI_ISL_3772726, EPI_ISL_3772728, EPI_ISL_3772730, EPI_ISL_3772732, EPI_ISL_3772734, EPI_ISL_3772736, EPI_ISL_3772738, EPI_ISL_3772740, EPI_ISL_3772742, EPI_ISL_3772744, EPI_ISL_3772746, EPI_ISL_3772748,
EPI_ISL_3772750, EPI_ISL_3772752, EPI_ISL_3772754, EPI_ISL_3772756, EPI_ISL_3772758, EPI_ISL_3772760, EPI_ISL_3772762, EPI_ISL_3772764, EPI_ISL_3772766, EPI_ISL_3772768, EPI_ISL_3772770, EPI_ISL_3772772, EPI_ISL_3772774, EPI_ISL_3772776, EPI_ISL_3772778, EPI_ISL_3772780, EPI_ISL_3772782, EPI_ISL_3772784, EPI_ISL_3772786, EPI_ISL_3772788, EPI_ISL_3772790, EPI_ISL_3772792,
EPI_ISL_3772794, EPI_ISL_3772796, EPI_ISL_3772798, EPI_ISL_3772800, EPI_ISL_3772802, EPI_ISL_3772804, EPI_ISL_3772806, EPI_ISL_3772808, EPI_ISL_3772810, EPI_ISL_3772812, EPI_ISL_3772815, EPI_ISL_3772817, EPI_ISL_3772819, EPI_ISL_3772821, EPI_ISL_3772823, EPI_ISL_3772826, EPI_ISL_3772828, EPI_ISL_3772830, EPI_ISL_3772832, EPI_ISL_3772834, EPI_ISL_3772836, EPI_ISL_3772838,
EPI_ISL_3772840, EPI_ISL_3772842, EPI_ISL_3772844, EPI_ISL_3772847, EPI_ISL_3772849, EPI_ISL_3772851, EPI_ISL_3772853, EPI_ISL_3772855, EPI_ISL_3772857, EPI_ISL_3772859, EPI_ISL_3772861, EPI_ISL_3772863, EPI_ISL_3772865, EPI_ISL_3772867, EPI_ISL_3772869, EPI_ISL_3772871, EPI_ISL_3772873, EPI_ISL_3772875, EPI_ISL_3772877, EPI_ISL_3772879, EPI_ISL_3772881, EPI_ISL_3772883,
EPI_ISL_3772885, EPI_ISL_3772887, EPI_ISL_3772889, EPI_ISL_3772891, EPI_ISL_3772893, EPI_ISL_3772895, EPI_ISL_3772897, EPI_ISL_3772899, EPI_ISL_3772902, EPI_ISL_3772907, EPI_ISL_3772909, EPI_ISL_3772911, EPI_ISL_3772913, EPI_ISL_3772915, EPI_ISL_3772917, EPI_ISL_3772919, EPI_ISL_3772921, EPI_ISL_3772923, EPI_ISL_3772925, EPI_ISL_3772927, EPI_ISL_3772929, EPI_ISL_3772931,
EPI_ISL_3772933, EPI_ISL_3772935, EPI_ISL_3772937, EPI_ISL_3772939, EPI_ISL_3772941, EPI_ISL_3772943, EPI_ISL_3772945, EPI_ISL_3772948, EPI_ISL_3772950, EPI_ISL_3772952, EPI_ISL_3772954, EPI_ISL_3772956, EPI_ISL_3772958, EPI_ISL_3772960, EPI_ISL_3772962, EPI_ISL_3772964, EPI_ISL_3772966, EPI_ISL_3772968, EPI_ISL_3772970, EPI_ISL_3772973, EPI_ISL_3772975, EPI_ISL_3772977,
EPI_ISL_3772978, EPI_ISL_3772980, EPI_ISL_3772983, EPI_ISL_3772985, EPI_ISL_3772987, EPI_ISL_3772989, EPI_ISL_3772991, EPI_ISL_3772993, EPI_ISL_3772995, EPI_ISL_3772997, EPI_ISL_3772999, EPI_ISL_3773001, EPI_ISL_3773004, EPI_ISL_3773006, EPI_ISL_3773008, EPI_ISL_3773009, EPI_ISL_3773012, EPI_ISL_3773014, EPI_ISL_3773016, EPI_ISL_3773018, EPI_ISL_3773020, EPI_ISL_3773023,
EPI_ISL_3773025, EPI_ISL_3773027, EPI_ISL_3773029, EPI_ISL_3773032, EPI_ISL_3773034, EPI_ISL_3773036, EPI_ISL_3773037, EPI_ISL_3773039, EPI_ISL_3773041, EPI_ISL_3773044, EPI_ISL_3773046, EPI_ISL_3773048, EPI_ISL_3773050, EPI_ISL_3869567, EPI_ISL_3869568, EPI_ISL_3869569, EPI_ISL_3869570, EPI_ISL_3869571, EPI_ISL_3869572, EPI_ISL_3869573, EPI_ISL_3869574, EPI_ISL_3869575,
EPI_ISL_3869576, EPI_ISL_3869577, EPI_ISL_3869578, EPI_ISL_3869579, EPI_ISL_3869580, EPI_ISL_3869581, EPI_ISL_3869582, EPI_ISL_3869583, EPI_ISL_3869584, EPI_ISL_3869585, EPI_ISL_3869586, EPI_ISL_3869587, EPI_ISL_3869588, EPI_ISL_3869589, EPI_ISL_3869590, EPI_ISL_3869591, EPI_ISL_3869592, EPI_ISL_3869593, EPI_ISL_3869594, EPI_ISL_3869595, EPI_ISL_3869596, EPI_ISL_3869597,
EPI_ISL_3869598, EPI_ISL_3869599, EPI_ISL_3869600, EPI_ISL_3869601, EPI_ISL_3869602, EPI_ISL_3869603, EPI_ISL_3869604, EPI_ISL_3869605, EPI_ISL_3869606, EPI_ISL_3869607, EPI_ISL_3869608, EPI_ISL_3869609, EPI_ISL_3869610, EPI_ISL_3869611, EPI_ISL_3869612, EPI_ISL_3869613, EPI_ISL_3869614, EPI_ISL_3869615, EPI_ISL_3869616, EPI_ISL_3869617, EPI_ISL_3869618, EPI_ISL_3869619,
EPI_ISL_3869620, EPI_ISL_3869621, EPI_ISL_3869622, EPI_ISL_3869623, EPI_ISL_3869624, EPI_ISL_3869625, EPI_ISL_3869626, EPI_ISL_3869627, EPI_ISL_3869628, EPI_ISL_3869629, EPI_ISL_3869630, EPI_ISL_3869631, EPI_ISL_3869632, EPI_ISL_3869633, EPI_ISL_3869634, EPI_ISL_3869635, EPI_ISL_3869636, EPI_ISL_3869637, EPI_ISL_3869638, EPI_ISL_3869639, EPI_ISL_3869640, EPI_ISL_3869641,
EPI_ISL_3869642, EPI_ISL_3869643, EPI_ISL_3869644, EPI_ISL_3869645, EPI_ISL_3869646, EPI_ISL_3869647, EPI_ISL_3869648, EPI_ISL_3869649, EPI_ISL_3869650, EPI_ISL_3869651, EPI_ISL_3869652, EPI_ISL_3869653, EPI_ISL_3869654, EPI_ISL_3869655, EPI_ISL_3869656, EPI_ISL_3869657, EPI_ISL_3869658, EPI_ISL_3869659, EPI_ISL_3869660, EPI_ISL_3869661, EPI_ISL_3869662, EPI_ISL_3869663,
EPI_ISL_3869664, EPI_ISL_3869665, EPI_ISL_3869666, EPI_ISL_3869667, EPI_ISL_3869668, EPI_ISL_3869669, EPI_ISL_3869670, EPI_ISL_3869671, EPI_ISL_3869672, EPI_ISL_3869673, EPI_ISL_3869674, EPI_ISL_3869675, EPI_ISL_3869676, EPI_ISL_3869677, EPI_ISL_3869678, EPI_ISL_3869679, EPI_ISL_3869680, EPI_ISL_3869681, EPI_ISL_3869682, EPI_ISL_3869683, EPI_ISL_3869684, EPI_ISL_3869685,
EPI_ISL_3869686, EPI_ISL_3869687, EPI_ISL_3869688, EPI_ISL_3869689, EPI_ISL_3869690, EPI_ISL_3869691, EPI_ISL_3869692, EPI_ISL_3869693, EPI_ISL_3869694, EPI_ISL_3869695, EPI_ISL_3869696, EPI_ISL_3869697, EPI_ISL_3869698, EPI_ISL_3869699, EPI_ISL_3869700, EPI_ISL_3869701, EPI_ISL_3869702, EPI_ISL_3869703, EPI_ISL_3869704, EPI_ISL_3869705, EPI_ISL_3869706, EPI_ISL_3869707,
EPI_ISL_3869708, EPI_ISL_3869709, EPI_ISL_3869710, EPI_ISL_3869711, EPI_ISL_3869712, EPI_ISL_3869713, EPI_ISL_3869714, EPI_ISL_3869715, EPI_ISL_3869716, EPI_ISL_3869717, EPI_ISL_3869718, EPI_ISL_3869719, EPI_ISL_3869720, EPI_ISL_3869721, EPI_ISL_3869722, EPI_ISL_3869723, EPI_ISL_3869724, EPI_ISL_3869725, EPI_ISL_3869726, EPI_ISL_3869727, EPI_ISL_3869728, EPI_ISL_3869729,
EPI_ISL_3869730, EPI_ISL_3869731, EPI_ISL_3869732, EPI_ISL_3869733, EPI_ISL_3869734, EPI_ISL_3869735, EPI_ISL_3869736, EPI_ISL_3869737, EPI_ISL_3869738, EPI_ISL_3869739, EPI_ISL_3869740, EPI_ISL_3869741, EPI_ISL_3869742, EPI_ISL_3869743, EPI_ISL_3869744, EPI_ISL_3869745, EPI_ISL_3869746, EPI_ISL_3869747, EPI_ISL_3869748, EPI_ISL_3869749, EPI_ISL_3869750, EPI_ISL_3869751,
EPI_ISL_3869752, EPI_ISL_3869753, EPI_ISL_3869754, EPI_ISL_3869755, EPI_ISL_3869756, EPI_ISL_3869757, EPI_ISL_3869758, EPI_ISL_3869759, EPI_ISL_3869760, EPI_ISL_3869761, EPI_ISL_3869762, EPI_ISL_3869763, EPI_ISL_3869764, EPI_ISL_3869765, EPI_ISL_3869766, EPI_ISL_3869767, EPI_ISL_3869768, EPI_ISL_3869769, EPI_ISL_3869770, EPI_ISL_3869771, EPI_ISL_3869772, EPI_ISL_3869773,
EPI_ISL_3869774, EPI_ISL_3869775, EPI_ISL_3869776, EPI_ISL_3869777, EPI_ISL_3869778, EPI_ISL_3869779, EPI_ISL_3869780, EPI_ISL_3869781, EPI_ISL_3869782, EPI_ISL_3869783, EPI_ISL_3869784, EPI_ISL_3869785, EPI_ISL_3869786, EPI_ISL_3869787, EPI_ISL_3869788, EPI_ISL_3869789, EPI_ISL_3869790, EPI_ISL_3869791, EPI_ISL_3869792, EPI_ISL_3869793, EPI_ISL_3869794, EPI_ISL_3869795,
EPI_ISL_3869796, EPI_ISL_3869797, EPI_ISL_3869798, EPI_ISL_3869799, EPI_ISL_3869800, EPI_ISL_3869801, EPI_ISL_3869802, EPI_ISL_3869803, EPI_ISL_3869804, EPI_ISL_3869805, EPI_ISL_3869806, EPI_ISL_3869807, EPI_ISL_3869808, EPI_ISL_3869809, EPI_ISL_3869810, EPI_ISL_3869811, EPI_ISL_3869812, EPI_ISL_3869813, EPI_ISL_3869814, EPI_ISL_3869815, EPI_ISL_3869816, EPI_ISL_3869817,
EPI_ISL_3869818, EPI_ISL_3869819, EPI_ISL_3869820, EPI_ISL_3869821, EPI_ISL_3869822, EPI_ISL_3869823, EPI_ISL_3869824, EPI_ISL_3869825, EPI_ISL_3869826, EPI_ISL_3869827, EPI_ISL_3869828, EPI_ISL_3869829, EPI_ISL_3869830, EPI_ISL_3869831, EPI_ISL_3869832, EPI_ISL_3869833, EPI_ISL_3869834, EPI_ISL_3869835, EPI_ISL_3869836, EPI_ISL_3869837, EPI_ISL_3869838, EPI_ISL_3869839,
EPI_ISL_3869840, EPI_ISL_3869841, EPI_ISL_3869842, EPI_ISL_3869843, EPI_ISL_3869844, EPI_ISL_3869845, EPI_ISL_3869846, EPI_ISL_3869847, EPI_ISL_3869848, EPI_ISL_3869849, EPI_ISL_3869850, EPI_ISL_3869851, EPI_ISL_3869852, EPI_ISL_3869853, EPI_ISL_3869854, EPI_ISL_3869855, EPI_ISL_3869856, EPI_ISL_3869857, EPI_ISL_3869858, EPI_ISL_3869859, EPI_ISL_3869860, EPI_ISL_3869861,
EPI_ISL_3869862, EPI_ISL_3869863, EPI_ISL_3869864, EPI_ISL_3869865, EPI_ISL_3869866, EPI_ISL_3869867, EPI_ISL_3869868, EPI_ISL_3869869, EPI_ISL_3869870, EPI_ISL_3869871, EPI_ISL_3869872, EPI_ISL_3869873, EPI_ISL_3869874, EPI_ISL_3869875, EPI_ISL_3869876, EPI_ISL_3869877, EPI_ISL_3869878, EPI_ISL_3869879, EPI_ISL_3869880, EPI_ISL_3869881, EPI_ISL_3869882, EPI_ISL_3869883,
EPI_ISL_3869884, EPI_ISL_3869885, EPI_ISL_3869886, EPI_ISL_3869887, EPI_ISL_3869888, EPI_ISL_3869889, EPI_ISL_3869890, EPI_ISL_3869891, EPI_ISL_3869892, EPI_ISL_3869893, EPI_ISL_3869894, EPI_ISL_3869895, EPI_ISL_3869896, EPI_ISL_3869897, EPI_ISL_3869898, EPI_ISL_3869899, EPI_ISL_3869900, EPI_ISL_3869901, EPI_ISL_3869902, EPI_ISL_3869903, EPI_ISL_3869904, EPI_ISL_3869905,
EPI_ISL_3869906, EPI_ISL_3869907, EPI_ISL_3869908, EPI_ISL_3869909, EPI_ISL_3869910, EPI_ISL_3869911, EPI_ISL_3869912, EPI_ISL_3869913, EPI_ISL_3869914, EPI_ISL_3869915, EPI_ISL_3869916, EPI_ISL_3869917, EPI_ISL_3869918, EPI_ISL_3869919, EPI_ISL_3869920, EPI_ISL_3869921, EPI_ISL_3869922, EPI_ISL_3869923, EPI_ISL_3869924, EPI_ISL_3869925, EPI_ISL_3869926, EPI_ISL_3869927,
EPI_ISL_3869928, EPI_ISL_3869929, EPI_ISL_3869930, EPI_ISL_3869931, EPI_ISL_3869932, EPI_ISL_3869933, EPI_ISL_3869934, EPI_ISL_3869935, EPI_ISL_3869936, EPI_ISL_3869937, EPI_ISL_3869938, EPI_ISL_3869939, EPI_ISL_3869940, EPI_ISL_3869941, EPI_ISL_3869942, EPI_ISL_3869943, EPI_ISL_3869944, EPI_ISL_3869945, EPI_ISL_3869946, EPI_ISL_3869947, EPI_ISL_3869948, EPI_ISL_3869949,
EPI_ISL_3869950, EPI_ISL_3869951, EPI_ISL_3869952, EPI_ISL_3869953, EPI_ISL_3869954, EPI_ISL_3869955, EPI_ISL_3869956, EPI_ISL_3869957, EPI_ISL_3869958, EPI_ISL_3869959, EPI_ISL_3869960, EPI_ISL_3869961, EPI_ISL_3869962, EPI_ISL_3869963, EPI_ISL_3869964, EPI_ISL_3869965, EPI_ISL_3869966, EPI_ISL_3869967, EPI_ISL_3869968, EPI_ISL_3869969, EPI_ISL_3869970, EPI_ISL_3869971,
EPI_ISL_3869972, EPI_ISL_3869973, EPI_ISL_3869974, EPI_ISL_3869975, EPI_ISL_3869976, EPI_ISL_3869977, EPI_ISL_3869978, EPI_ISL_3869979, EPI_ISL_3869980, EPI_ISL_3869981, EPI_ISL_3869982, EPI_ISL_3869983, EPI_ISL_3869984, EPI_ISL_3869985, EPI_ISL_3869986, EPI_ISL_3869987, EPI_ISL_3869988, EPI_ISL_3869989, EPI_ISL_3869990, EPI_ISL_3869991, EPI_ISL_3869992, EPI_ISL_3869993,
EPI_ISL_3869994, EPI_ISL_3869995, EPI_ISL_3869996, EPI_ISL_3869997, EPI_ISL_3869998, EPI_ISL_3869999, EPI_ISL_3870000, EPI_ISL_3870001, EPI_ISL_3870002, EPI_ISL_3870003, EPI_ISL_3870004, EPI_ISL_3870005, EPI_ISL_3870006, EPI_ISL_3870007, EPI_ISL_3870008, EPI_ISL_3870009, EPI_ISL_3870010, EPI_ISL_3870011, EPI_ISL_3870012, EPI_ISL_3870013, EPI_ISL_3870014, EPI_ISL_3870015,
EPI_ISL_3870016, EPI_ISL_3870017, EPI_ISL_3870018, EPI_ISL_3870019, EPI_ISL_3870020, EPI_ISL_3870021, EPI_ISL_3870022, EPI_ISL_3870023, EPI_ISL_3870024, EPI_ISL_3870025, EPI_ISL_3870026, EPI_ISL_3870027, EPI_ISL_3870028, EPI_ISL_3870029, EPI_ISL_3870030, EPI_ISL_3870031, EPI_ISL_3870032, EPI_ISL_3870033, EPI_ISL_3870034, EPI_ISL_3870035, EPI_ISL_3870036, EPI_ISL_3870037,
EPI_ISL_3870038, EPI_ISL_3870039, EPI_ISL_3870040, EPI_ISL_3870041, EPI_ISL_3870042, EPI_ISL_3870043, EPI_ISL_3870044, EPI_ISL_3870045, EPI_ISL_3870046, EPI_ISL_3870047, EPI_ISL_3870048, EPI_ISL_3870049, EPI_ISL_3870050, EPI_ISL_3870051, EPI_ISL_3870052, EPI_ISL_3870053, EPI_ISL_3870054, EPI_ISL_3870055, EPI_ISL_3870056, EPI_ISL_3870057, EPI_ISL_3870058, EPI_ISL_3870059,
EPI_ISL_3870060, EPI_ISL_3870061, EPI_ISL_3870062, EPI_ISL_3870063, EPI_ISL_3870064, EPI_ISL_3870065, EPI_ISL_3870066, EPI_ISL_3870067, EPI_ISL_3870068, EPI_ISL_3870069, EPI_ISL_3933495, EPI_ISL_3933496, EPI_ISL_3933497, EPI_ISL_3933498, EPI_ISL_3933499, EPI_ISL_3933500, EPI_ISL_3933501, EPI_ISL_3933502, EPI_ISL_3933503, EPI_ISL_3933504, EPI_ISL_3933505, EPI_ISL_3933506,
EPI_ISL_3933507, EPI_ISL_3933508, EPI_ISL_3933509, EPI_ISL_3933510, EPI_ISL_3933511, EPI_ISL_3933512, EPI_ISL_3933513, EPI_ISL_3933514, EPI_ISL_3933515, EPI_ISL_3933516, EPI_ISL_3933517, EPI_ISL_3933518, EPI_ISL_3933519, EPI_ISL_3933520, EPI_ISL_3933521, EPI_ISL_3933522, EPI_ISL_3933523, EPI_ISL_3933524, EPI_ISL_3933525, EPI_ISL_3933526, EPI_ISL_3933527, EPI_ISL_3933528,
EPI_ISL_3933529, EPI_ISL_3933530, EPI_ISL_3933531, EPI_ISL_3933532, EPI_ISL_3933533, EPI_ISL_3933534, EPI_ISL_3933535, EPI_ISL_3933536, EPI_ISL_3933537, EPI_ISL_3933538, EPI_ISL_3933539, EPI_ISL_3933540, EPI_ISL_3933541, EPI_ISL_3933542, EPI_ISL_3933543, EPI_ISL_3933544, EPI_ISL_3933545, EPI_ISL_3933546, EPI_ISL_3933547, EPI_ISL_3933548, EPI_ISL_3933549, EPI_ISL_4203818,
EPI_ISL_4203820, EPI_ISL_4203822, EPI_ISL_4203824, EPI_ISL_4203825, EPI_ISL_4203827, EPI_ISL_4203829, EPI_ISL_4203831, EPI_ISL_4203832, EPI_ISL_4203834, EPI_ISL_4203836, EPI_ISL_4203838, EPI_ISL_4203839, EPI_ISL_4203841, EPI_ISL_4203843, EPI_ISL_4203844, EPI_ISL_4203847, EPI_ISL_4203849, EPI_ISL_4203851, EPI_ISL_4203853, EPI_ISL_4203854, EPI_ISL_4203856, EPI_ISL_4203858,
EPI_ISL_4203859, EPI_ISL_4203861, EPI_ISL_4203863, EPI_ISL_4203864, EPI_ISL_4203866, EPI_ISL_4203868, EPI_ISL_4203869, EPI_ISL_4203871, EPI_ISL_4203873, EPI_ISL_4203874, EPI_ISL_4203876, EPI_ISL_4203878, EPI_ISL_4203880, EPI_ISL_4203882, EPI_ISL_4203883, EPI_ISL_4203885, EPI_ISL_4203887, EPI_ISL_4203888, EPI_ISL_4203890, EPI_ISL_4203892, EPI_ISL_4203894, EPI_ISL_4203896,
EPI_ISL_4203897, EPI_ISL_4203899, EPI_ISL_4203901, EPI_ISL_4203903, EPI_ISL_4203904, EPI_ISL_4203906, EPI_ISL_4203908, EPI_ISL_4203910, EPI_ISL_4203911, EPI_ISL_4203913, EPI_ISL_4203915, EPI_ISL_4203917, EPI_ISL_4203918, EPI_ISL_4203920, EPI_ISL_4203922, EPI_ISL_4203924, EPI_ISL_4203925, EPI_ISL_4203927, EPI_ISL_4203929, EPI_ISL_4203930, EPI_ISL_4203932, EPI_ISL_4203934,
EPI_ISL_4203936, EPI_ISL_4203937, EPI_ISL_4203939, EPI_ISL_4203941, EPI_ISL_4203943, EPI_ISL_4203944, EPI_ISL_4203946, EPI_ISL_4203948, EPI_ISL_4203950, EPI_ISL_4203951, EPI_ISL_4203953, EPI_ISL_4203955, EPI_ISL_4203957, EPI_ISL_4203958, EPI_ISL_4203960, EPI_ISL_4203961, EPI_ISL_4203963, EPI_ISL_4203965, EPI_ISL_4203966, EPI_ISL_4203968, EPI_ISL_4203970, EPI_ISL_4203972,
EPI_ISL_4203974, EPI_ISL_4203976, EPI_ISL_4203978, EPI_ISL_4203980, EPI_ISL_4203982, EPI_ISL_4203983, EPI_ISL_4203985, EPI_ISL_4203987, EPI_ISL_4203989, EPI_ISL_4203990, EPI_ISL_4203992, EPI_ISL_4203994, EPI_ISL_4203996, EPI_ISL_4203997, EPI_ISL_4203999, EPI_ISL_4204001, EPI_ISL_4204003, EPI_ISL_4204004, EPI_ISL_4204006, EPI_ISL_4204008, EPI_ISL_4204009, EPI_ISL_4204011,
EPI_ISL_4204013, EPI_ISL_4204015, EPI_ISL_4204016, EPI_ISL_4204018, EPI_ISL_4204020, EPI_ISL_4204022, EPI_ISL_4204023, EPI_ISL_4204025, EPI_ISL_4204027, EPI_ISL_4204028, EPI_ISL_4204030, EPI_ISL_4204032, EPI_ISL_4204034, EPI_ISL_4204035, EPI_ISL_4204037, EPI_ISL_4204039, EPI_ISL_4204041, EPI_ISL_4204042, EPI_ISL_4204044, EPI_ISL_4204046, EPI_ISL_4204047, EPI_ISL_4204049,
EPI_ISL_4204051, EPI_ISL_4204052, EPI_ISL_4204054, EPI_ISL_4204056, EPI_ISL_4204058, EPI_ISL_4204059, EPI_ISL_4204061, EPI_ISL_4204063, EPI_ISL_4204065, EPI_ISL_4204067, EPI_ISL_4204068, EPI_ISL_4204070, EPI_ISL_4204072, EPI_ISL_4204073, EPI_ISL_4204075, EPI_ISL_4204077, EPI_ISL_4204078, EPI_ISL_4204079, EPI_ISL_4204080, EPI_ISL_4204081, EPI_ISL_4204082, EPI_ISL_4204083,
EPI_ISL_4204084, EPI_ISL_4204085, EPI_ISL_4204086, EPI_ISL_4204087, EPI_ISL_4204088, EPI_ISL_4204089, EPI_ISL_4204090, EPI_ISL_4204091, EPI_ISL_4204092, EPI_ISL_4204093, EPI_ISL_4204094, EPI_ISL_4204095, EPI_ISL_4204096, EPI_ISL_4204097, EPI_ISL_4204098, EPI_ISL_4204099, EPI_ISL_4204100, EPI_ISL_4204101, EPI_ISL_4204102, EPI_ISL_4204103, EPI_ISL_4204104, EPI_ISL_4204105,
EPI_ISL_4204106, EPI_ISL_4204107, EPI_ISL_4204108, EPI_ISL_4204109, EPI_ISL_4204110, EPI_ISL_4204111, EPI_ISL_4204112, EPI_ISL_4204113, EPI_ISL_4204114, EPI_ISL_4204115, EPI_ISL_4204116, EPI_ISL_4204117, EPI_ISL_4204118, EPI_ISL_4204119, EPI_ISL_4204120, EPI_ISL_4204121, EPI_ISL_4204122, EPI_ISL_4204123, EPI_ISL_4204124, EPI_ISL_4204125, EPI_ISL_4204126, EPI_ISL_4204127,
EPI_ISL_4204128, EPI_ISL_4204129, EPI_ISL_4204130, EPI_ISL_4204131, EPI_ISL_4204132, EPI_ISL_4204133, EPI_ISL_4204134, EPI_ISL_4204135, EPI_ISL_4204136, EPI_ISL_4204137, EPI_ISL_4204138, EPI_ISL_4204139, EPI_ISL_4204140, EPI_ISL_4204141, EPI_ISL_4204142, EPI_ISL_4204143, EPI_ISL_4204144, EPI_ISL_4204145, EPI_ISL_4204146, EPI_ISL_4204147, EPI_ISL_4204148, EPI_ISL_4204150,
EPI_ISL_4204151, EPI_ISL_4204152, EPI_ISL_4204153, EPI_ISL_4204154, EPI_ISL_4204155, EPI_ISL_4204156, EPI_ISL_4204157, EPI_ISL_4204158, EPI_ISL_4204159, EPI_ISL_4204160, EPI_ISL_4204161, EPI_ISL_4204162, EPI_ISL_4204163, EPI_ISL_4204164, EPI_ISL_4204165, EPI_ISL_4204166, EPI_ISL_4204167, EPI_ISL_4204168, EPI_ISL_4204169, EPI_ISL_4204170, EPI_ISL_4204171, EPI_ISL_4204172,
EPI_ISL_4204173, EPI_ISL_4204174, EPI_ISL_4204175, EPI_ISL_4204176, EPI_ISL_4204177, EPI_ISL_4204178, EPI_ISL_4204179, EPI_ISL_4204180, EPI_ISL_4204181, EPI_ISL_4204182, EPI_ISL_4204183, EPI_ISL_4204184, EPI_ISL_4204185, EPI_ISL_4204186, EPI_ISL_4204187, EPI_ISL_4204188, EPI_ISL_4204189, EPI_ISL_4204190, EPI_ISL_4204191, EPI_ISL_4204192, EPI_ISL_4204193, EPI_ISL_4204194,
EPI_ISL_4204195, EPI_ISL_4204196, EPI_ISL_4204197, EPI_ISL_4204198, EPI_ISL_4204199, EPI_ISL_4204200, EPI_ISL_4204201, EPI_ISL_4204202, EPI_ISL_4204203, EPI_ISL_4204204, EPI_ISL_4204205, EPI_ISL_4204206, EPI_ISL_4204207, EPI_ISL_4204208, EPI_ISL_4204209, EPI_ISL_4204210, EPI_ISL_4204211, EPI_ISL_4204212, EPI_ISL_4204213, EPI_ISL_4204214, EPI_ISL_4204215, EPI_ISL_4204216,
EPI_ISL_4204217, EPI_ISL_4204218, EPI_ISL_4204219, EPI_ISL_4204220, EPI_ISL_4204221, EPI_ISL_4204222, EPI_ISL_4204223, EPI_ISL_4204224, EPI_ISL_4204225, EPI_ISL_4204226, EPI_ISL_4204227, EPI_ISL_4204228, EPI_ISL_4204229, EPI_ISL_4204230, EPI_ISL_4204231, EPI_ISL_4204232, EPI_ISL_4204233, EPI_ISL_4204234, EPI_ISL_4204235, EPI_ISL_4204236, EPI_ISL_4204237, EPI_ISL_4204238,
EPI_ISL_4204239, EPI_ISL_4204240, EPI_ISL_4204241, EPI_ISL_4204242, EPI_ISL_4204243, EPI_ISL_4204244, EPI_ISL_4204245, EPI_ISL_4204246, EPI_ISL_4204247, EPI_ISL_4204248, EPI_ISL_4204249, EPI_ISL_4204250, EPI_ISL_4204251, EPI_ISL_4204252, EPI_ISL_4204253, EPI_ISL_4204254, EPI_ISL_4204255, EPI_ISL_4204256, EPI_ISL_4204257, EPI_ISL_4204258, EPI_ISL_4204259, EPI_ISL_4204260,
EPI_ISL_4204261, EPI_ISL_4204262, EPI_ISL_4204263, EPI_ISL_4204264, EPI_ISL_4204265, EPI_ISL_4204266, EPI_ISL_4204267, EPI_ISL_4204268, EPI_ISL_4204269, EPI_ISL_4204270, EPI_ISL_4204271, EPI_ISL_4204272, EPI_ISL_4204273, EPI_ISL_4204274, EPI_ISL_4204275, EPI_ISL_4204276, EPI_ISL_4204277, EPI_ISL_4204278, EPI_ISL_4204279, EPI_ISL_4204280, EPI_ISL_4204281, EPI_ISL_4204282,
EPI_ISL_4204283, EPI_ISL_4204284, EPI_ISL_4204285, EPI_ISL_4204286, EPI_ISL_4204287, EPI_ISL_4204288, EPI_ISL_4204289, EPI_ISL_4204290, EPI_ISL_4204291, EPI_ISL_4204292, EPI_ISL_4204293, EPI_ISL_4204294, EPI_ISL_4204295, EPI_ISL_4204296, EPI_ISL_4204297, EPI_ISL_4204298, EPI_ISL_4204299, EPI_ISL_4204300, EPI_ISL_4204301, EPI_ISL_4204302, EPI_ISL_4204303, EPI_ISL_4204304,
EPI_ISL_4204305, EPI_ISL_4204306, EPI_ISL_4204307, EPI_ISL_4204308, EPI_ISL_4204309, EPI_ISL_4204310, EPI_ISL_4204311, EPI_ISL_4204312, EPI_ISL_4204313, EPI_ISL_4204314, EPI_ISL_4204315, EPI_ISL_4204316, EPI_ISL_4204317, EPI_ISL_4204318, EPI_ISL_4204319, EPI_ISL_4204320, EPI_ISL_4204321, EPI_ISL_4204322, EPI_ISL_4204323, EPI_ISL_4204324, EPI_ISL_4204325, EPI_ISL_4204326,
EPI_ISL_4204327, EPI_ISL_4204328, EPI_ISL_4204329, EPI_ISL_4204330, EPI_ISL_4204331, EPI_ISL_4204332, EPI_ISL_4204333, EPI_ISL_4204334, EPI_ISL_4204335, EPI_ISL_4204336, EPI_ISL_4204337, EPI_ISL_4204338, EPI_ISL_4204339, EPI_ISL_4204340, EPI_ISL_4204341, EPI_ISL_4204342, EPI_ISL_4204343, EPI_ISL_4204344, EPI_ISL_4204346, EPI_ISL_4204347, EPI_ISL_4204348, EPI_ISL_4204349,
EPI_ISL_4204350, EPI_ISL_4204351, EPI_ISL_4204352, EPI_ISL_4204353, EPI_ISL_4204354, EPI_ISL_4204355, EPI_ISL_4204356, EPI_ISL_4204357, EPI_ISL_4204358, EPI_ISL_4204359, EPI_ISL_4204360, EPI_ISL_4204361, EPI_ISL_4204362, EPI_ISL_4204363, EPI_ISL_4204364, EPI_ISL_4204365, EPI_ISL_4204366, EPI_ISL_4204367, EPI_ISL_4204368, EPI_ISL_4204369, EPI_ISL_4204370, EPI_ISL_4204371,
EPI_ISL_4204372, EPI_ISL_4204373, EPI_ISL_4204374, EPI_ISL_4204375, EPI_ISL_4204376, EPI_ISL_4204377, EPI_ISL_4204378, EPI_ISL_4204379, EPI_ISL_4204380, EPI_ISL_4204381, EPI_ISL_4204382, EPI_ISL_4204383, EPI_ISL_4204384, EPI_ISL_4204385, EPI_ISL_4204386, EPI_ISL_4204387, EPI_ISL_4204388, EPI_ISL_4204389, EPI_ISL_4204390, EPI_ISL_4204391, EPI_ISL_4204392, EPI_ISL_4204393,
EPI_ISL_4204394, EPI_ISL_4204395, EPI_ISL_4204396, EPI_ISL_4204397, EPI_ISL_4204398, EPI_ISL_4204399, EPI_ISL_4204400, EPI_ISL_4204401, EPI_ISL_4204402, EPI_ISL_4204403, EPI_ISL_4204404, EPI_ISL_4204405, EPI_ISL_4204406, EPI_ISL_4204407, EPI_ISL_4204408, EPI_ISL_4204409, EPI_ISL_4204410, EPI_ISL_4204411, EPI_ISL_4204412, EPI_ISL_4204413, EPI_ISL_4204414, EPI_ISL_4204415,
EPI_ISL_4204416, EPI_ISL_4204417, EPI_ISL_4204418, EPI_ISL_4204419, EPI_ISL_4204420, EPI_ISL_4204421, EPI_ISL_4204422, EPI_ISL_4204423, EPI_ISL_4204424, EPI_ISL_4204425, EPI_ISL_4204426, EPI_ISL_4204427, EPI_ISL_4204428, EPI_ISL_4204429, EPI_ISL_4204430, EPI_ISL_4204431, EPI_ISL_4204432, EPI_ISL_4204433, EPI_ISL_4204434, EPI_ISL_4204435, EPI_ISL_4204436, EPI_ISL_4204437,
EPI_ISL_4204438, EPI_ISL_4204439, EPI_ISL_4204440, EPI_ISL_4204441, EPI_ISL_4204442, EPI_ISL_4204443, EPI_ISL_4204444, EPI_ISL_4204445, EPI_ISL_4204446, EPI_ISL_4204447, EPI_ISL_4204448, EPI_ISL_4204449, EPI_ISL_4204450, EPI_ISL_4204451, EPI_ISL_4475591, EPI_ISL_4475592, EPI_ISL_4475593, EPI_ISL_4475594, EPI_ISL_4475595, EPI_ISL_4475596, EPI_ISL_4475597, EPI_ISL_4475598,
EPI_ISL_4475599, EPI_ISL_4475600, EPI_ISL_4475601, EPI_ISL_4475602, EPI_ISL_4475603, EPI_ISL_4475604, EPI_ISL_4475605, EPI_ISL_4475606, EPI_ISL_4475607, EPI_ISL_4475608, EPI_ISL_4475609, EPI_ISL_4475610, EPI_ISL_4475611, EPI_ISL_4475612, EPI_ISL_4475613, EPI_ISL_4475614, EPI_ISL_4475615, EPI_ISL_4475616, EPI_ISL_4475617, EPI_ISL_4475618, EPI_ISL_4475619, EPI_ISL_4475620,
EPI_ISL_4475621, EPI_ISL_4475622, EPI_ISL_4475623, EPI_ISL_4475624, EPI_ISL_4475625, EPI_ISL_4475626, EPI_ISL_4475627, EPI_ISL_4475628, EPI_ISL_4475629, EPI_ISL_4475630, EPI_ISL_4475631, EPI_ISL_4475632, EPI_ISL_4475633, EPI_ISL_4475634, EPI_ISL_4475635, EPI_ISL_4475636, EPI_ISL_4475637, EPI_ISL_4475638, EPI_ISL_4475639, EPI_ISL_4475640, EPI_ISL_4475641, EPI_ISL_4475642,
EPI_ISL_4475643, EPI_ISL_4475644, EPI_ISL_4475645, EPI_ISL_4475646, EPI_ISL_4475647, EPI_ISL_4475648, EPI_ISL_4475649, EPI_ISL_4475650, EPI_ISL_4475651, EPI_ISL_4475652, EPI_ISL_4475653, EPI_ISL_4475654, EPI_ISL_4475655, EPI_ISL_4475656, EPI_ISL_4475657, EPI_ISL_4475658, EPI_ISL_4475659, EPI_ISL_4475660, EPI_ISL_4475661, EPI_ISL_4475662, EPI_ISL_4475663, EPI_ISL_4475664,
EPI_ISL_4475665, EPI_ISL_4475666, EPI_ISL_4475667, EPI_ISL_4475668, EPI_ISL_4475669, EPI_ISL_4475670, EPI_ISL_4475671, EPI_ISL_4475672, EPI_ISL_4475673, EPI_ISL_4475674, EPI_ISL_4475675, EPI_ISL_4475676, EPI_ISL_4475677, EPI_ISL_4475678, EPI_ISL_4475679, EPI_ISL_4475680, EPI_ISL_4475681, EPI_ISL_4475682, EPI_ISL_4475683, EPI_ISL_4475684, EPI_ISL_4475685, EPI_ISL_4475686,
EPI_ISL_4475687, EPI_ISL_4475688, EPI_ISL_4475689, EPI_ISL_4475690, EPI_ISL_4475691, EPI_ISL_4475692, EPI_ISL_4475693, EPI_ISL_4475694, EPI_ISL_4475695, EPI_ISL_4475696, EPI_ISL_4475697, EPI_ISL_4475698, EPI_ISL_4475699, EPI_ISL_4475700, EPI_ISL_4475701, EPI_ISL_4475702, EPI_ISL_4475703, EPI_ISL_4475704, EPI_ISL_4475705, EPI_ISL_4475706, EPI_ISL_4475707, EPI_ISL_4475708,
EPI_ISL_4475709, EPI_ISL_4475710, EPI_ISL_4475711, EPI_ISL_4570332, EPI_ISL_4570342, EPI_ISL_4570343, EPI_ISL_4570344, EPI_ISL_4633310, EPI_ISL_4633311, EPI_ISL_4633312, EPI_ISL_4633313, EPI_ISL_4633314, EPI_ISL_4633315, EPI_ISL_4633316, EPI_ISL_4633317, EPI_ISL_4633318, EPI_ISL_4633319, EPI_ISL_4633320, EPI_ISL_4633321, EPI_ISL_4633322, EPI_ISL_4633323, EPI_ISL_4633324,

EPI_ISL_4633325, EPI_ISL_4633326, EPI_ISL_4633327, EPI_ISL_4633328, EPI_ISL_4633329, EPI_ISL_4633330, EPI_ISL_4633331, EPI_ISL_4633332, EPI_ISL_4633333, EPI_ISL_4633334, EPI_ISL_4633335, EPI_ISL_4633336, EPI_ISL_4633337, EPI_ISL_4633338, EPI_ISL_4633339, EPI_ISL_4633340, EPI_ISL_4633341, EPI_ISL_4633342, EPI_ISL_4633343, EPI_ISL_4633344, EPI_ISL_4633345, EPI_ISL_4633346, EPI_ISL_4633347, EPI_ISL_4633348, EPI_ISL_4633349, EPI_ISL_4633350, EPI_ISL_4633351, EPI_ISL_4633352, EPI_ISL_4633353, EPI_ISL_4633354, EPI_ISL_4633355, EPI_ISL_4633356, EPI_ISL_4633357, EPI_ISL_4633358, EPI_ISL_4633359, EPI_ISL_4633360, EPI_ISL_4633361, EPI_ISL_4633362, EPI_ISL_4633363, EPI_ISL_4633364, EPI_ISL_4633365, EPI_ISL_4633366, EPI_ISL_4633367, EPI_ISL_4633368, EPI_ISL_4633369, EPI_ISL_4633370, EPI_ISL_4633371, EPI_ISL_4633372, EPI_ISL_4633373, EPI_ISL_4633374, EPI_ISL_4633375, EPI_ISL_4633376, EPI_ISL_4633377, EPI_ISL_4633378, EPI_ISL_4633379, EPI_ISL_4633380, EPI_ISL_4633381, EPI_ISL_4633382, EPI_ISL_4633383, EPI_ISL_4633384, EPI_ISL_4633385, EPI_ISL_4633386, EPI_ISL_4633387, EPI_ISL_4633388, EPI_ISL_4633389, EPI_ISL_4633390, EPI_ISL_4633391, EPI_ISL_4633392, EPI_ISL_4633393, EPI_ISL_4633394, EPI_ISL_4633395, EPI_ISL_4633396, EPI_ISL_4633397, EPI_ISL_4633398, EPI_ISL_4633399, EPI_ISL_4633400, EPI_ISL_4633401, EPI_ISL_4633402, EPI_ISL_4633403, EPI_ISL_4633404, EPI_ISL_4633405, EPI_ISL_4633406, EPI_ISL_4633407, EPI_ISL_4633408, EPI_ISL_4633409, EPI_ISL_4633413, EPI_ISL_4633414, EPI_ISL_4633415, EPI_ISL_4633416, EPI_ISL_4633417, EPI_ISL_4633418, EPI_ISL_4633419, EPI_ISL_4633420, EPI_ISL_4633421, EPI_ISL_4633422, EPI_ISL_4633423, EPI_ISL_4633424, EPI_ISL_4633425, EPI_ISL_4633426, EPI_ISL_4633427, EPI_ISL_4633428, EPI_ISL_4633429, EPI_ISL_4633430, EPI_ISL_4633431, EPI_ISL_4633432, EPI_ISL_4633433, EPI_ISL_4633434, EPI_ISL_4633435, EPI_ISL_4633436, EPI_ISL_4633437, EPI_ISL_4633438, EPI_ISL_4633439, EPI_ISL_4633440, EPI_ISL_4633441, EPI_ISL_4633442, EPI_ISL_4633443, EPI_ISL_4633444, EPI_ISL_4633445, EPI_ISL_4633446, EPI_ISL_4633447, EPI_ISL_4633448, EPI_ISL_4633449, EPI_ISL_4633450, EPI_ISL_4633451, EPI_ISL_4633452, EPI_ISL_4633453, EPI_ISL_4633454, EPI_ISL_4633455, EPI_ISL_4633456, EPI_ISL_4633457, EPI_ISL_4633458, EPI_ISL_4633459, EPI_ISL_4633460, EPI_ISL_4633461, EPI_ISL_4633462, EPI_ISL_4633463, EPI_ISL_4633464, EPI_ISL_4633465, EPI_ISL_4633466, EPI_ISL_4633467, EPI_ISL_4633468, EPI_ISL_4633469, EPI_ISL_4633470, EPI_ISL_4633471, EPI_ISL_4633472, EPI_ISL_4633473, EPI_ISL_4633474, EPI_ISL_4633475, EPI_ISL_4633476, EPI_ISL_4633477, EPI_ISL_4633478, EPI_ISL_4633479, EPI_ISL_4633480, EPI_ISL_4633481, EPI_ISL_4633482, EPI_ISL_4633483, EPI_ISL_4633484, EPI_ISL_4633485, EPI_ISL_4633486, EPI_ISL_4633487, EPI_ISL_4633488, EPI_ISL_4633489, EPI_ISL_4633490, EPI_ISL_4633491, EPI_ISL_4633492, EPI_ISL_4633493, EPI_ISL_4633494, EPI_ISL_4633495, EPI_ISL_4633496, EPI_ISL_4633497, EPI_ISL_4633498, EPI_ISL_4633499, EPI_ISL_4633500, EPI_ISL_4633501, EPI_ISL_4633502, EPI_ISL_4633503, EPI_ISL_4633504, EPI_ISL_4633505, EPI_ISL_4633506, EPI_ISL_4633507, EPI_ISL_4633511, EPI_ISL_4633512, EPI_ISL_4633513, EPI_ISL_4633514, EPI_ISL_4633515, EPI_ISL_4633516, EPI_ISL_4633517, EPI_ISL_4633518, EPI_ISL_4633519, EPI_ISL_4633520, EPI_ISL_4633521, EPI_ISL_4633522, EPI_ISL_4633523, EPI_ISL_4633524, EPI_ISL_4633525, EPI_ISL_4633526, EPI_ISL_4633527, EPI_ISL_4633528, EPI_ISL_4633529, EPI_ISL_4633530, EPI_ISL_4633531, EPI_ISL_4633532, EPI_ISL_4633533, EPI_ISL_4633534, EPI_ISL_4633535, EPI_ISL_4633536, EPI_ISL_4633537, EPI_ISL_4633538, EPI_ISL_4633539, EPI_ISL_4633540, EPI_ISL_4633541, EPI_ISL_4633542, EPI_ISL_4633543, EPI_ISL_4633544, EPI_ISL_4633545, EPI_ISL_4633546, EPI_ISL_4633547, EPI_ISL_4633548, EPI_ISL_4633549, EPI_ISL_4633550, EPI_ISL_4633551, EPI_ISL_4633552, EPI_ISL_4633553, EPI_ISL_4633554, EPI_ISL_4633555, EPI_ISL_4633556, EPI_ISL_4633557, EPI_ISL_4633558, EPI_ISL_4633559, EPI_ISL_4633560, EPI_ISL_4633561, EPI_ISL_4633562, EPI_ISL_4633563, EPI_ISL_4633564, EPI_ISL_4633565, EPI_ISL_4633566, EPI_ISL_4633567, EPI_ISL_4633568, EPI_ISL_4633569, EPI_ISL_4633570, EPI_ISL_4633571, EPI_ISL_4633572, EPI_ISL_4633573, EPI_ISL_4633574, EPI_ISL_4633575, EPI_ISL_4633576, EPI_ISL_4633577, EPI_ISL_4633578, EPI_ISL_4633579, EPI_ISL_4633580, EPI_ISL_4633581, EPI_ISL_4633582, EPI_ISL_4633583, EPI_ISL_4633584, EPI_ISL_4633585, EPI_ISL_4633586, EPI_ISL_4633587, EPI_ISL_4633588, EPI_ISL_4633589, EPI_ISL_4633590, EPI_ISL_4633591, EPI_ISL_4633592, EPI_ISL_4633593, EPI_ISL_4633594, EPI_ISL_4633595, EPI_ISL_4633596, EPI_ISL_4633597, EPI_ISL_4633598, EPI_ISL_4633599, EPI_ISL_4633600, EPI_ISL_4633601, EPI_ISL_4633602, EPI_ISL_4633603, EPI_ISL_4633604, EPI_ISL_4633605, EPI_ISL_4633606, EPI_ISL_4633607, EPI_ISL_4633608, EPI_ISL_4633609, EPI_ISL_4633610, EPI_ISL_4633611, EPI_ISL_4633612, EPI_ISL_4633613, EPI_ISL_4633614, EPI_ISL_4633615, EPI_ISL_4633616, EPI_ISL_4633617, EPI_ISL_4633618, EPI_ISL_4633619, EPI_ISL_4633620, EPI_ISL_4633621, EPI_ISL_4633622, EPI_ISL_4633623, EPI_ISL_4633624, EPI_ISL_4633625, EPI_ISL_4633626, EPI_ISL_4633627, EPI_ISL_4633628, EPI_ISL_4633629, EPI_ISL_4633630, EPI_ISL_4633631, EPI_ISL_4633632, EPI_ISL_4633633, EPI_ISL_4633634, EPI_ISL_4633635, EPI_ISL_4633636, EPI_ISL_4633637, EPI_ISL_4633638, EPI_ISL_4633639, EPI_ISL_4633640, EPI_ISL_4633641, EPI_ISL_4633642, EPI_ISL_4633643, EPI_ISL_4633644, EPI_ISL_4633645, EPI_ISL_4633646, EPI_ISL_4633647, EPI_ISL_4633648, EPI_ISL_4633649, EPI_ISL_4633650, EPI_ISL_4633651, EPI_ISL_4633652, EPI_ISL_4633653, EPI_ISL_4633654, EPI_ISL_4633655, EPI_ISL_4633656, EPI_ISL_4633657, EPI_ISL_4633658, EPI_ISL_4633659, EPI_ISL_4633660, EPI_ISL_4633661, EPI_ISL_4633662, EPI_ISL_4633663, EPI_ISL_4633664, EPI_ISL_4633665, EPI_ISL_4633666, EPI_ISL_4633667, EPI_ISL_4633668, EPI_ISL_4633669, EPI_ISL_4633670, EPI_ISL_4633671, EPI_ISL_4633672, EPI_ISL_4633673, EPI_ISL_4633674, EPI_ISL_4633675, EPI_ISL_4633676, EPI_ISL_4633677, EPI_ISL_4633678, EPI_ISL_4633679, EPI_ISL_4633680, EPI_ISL_4633681, EPI_ISL_4633682, EPI_ISL_4633683, EPI_ISL_4633684, EPI_ISL_4633685, EPI_ISL_4633686, EPI_ISL_4633687, EPI_ISL_4633688, EPI_ISL_4633689, EPI_ISL_4633690, EPI_ISL_4633691, EPI_ISL_4633692, EPI_ISL_4633693, EPI_ISL_4633694, EPI_ISL_4633695, EPI_ISL_4633696, EPI_ISL_4633697, EPI_ISL_4633698, EPI_ISL_4633699, EPI_ISL_4633700, EPI_ISL_4633701, EPI_ISL_4633702, EPI_ISL_4633703, EPI_ISL_4633704, EPI_ISL_4633705, EPI_ISL_4633706, EPI_ISL_4633707, EPI_ISL_4633708, EPI_ISL_4633709, EPI_ISL_4633710, EPI_ISL_4633711, EPI_ISL_4633712, EPI_ISL_4633713, EPI_ISL_4633714, EPI_ISL_4633715, EPI_ISL_4633716, EPI_ISL_4633717, EPI_ISL_4633718, EPI_ISL_4633719, EPI_ISL_4633720, EPI_ISL_4633721, EPI_ISL_4633722, EPI_ISL_4633723, EPI_ISL_4633724, EPI_ISL_4633725, EPI_ISL_4633726, EPI_ISL_4633727, EPI_ISL_4633728, EPI_ISL_4633729, EPI_ISL_4633730, EPI_ISL_4633731, EPI_ISL_4633732, EPI_ISL_4633733, EPI_ISL_4633734, EPI_ISL_4633735, EPI_ISL_4633736, EPI_ISL_4633737, EPI_ISL_4633738, EPI_ISL_4633739, EPI_ISL_4633740, EPI_ISL_4633741, EPI_ISL_4633742, EPI_ISL_4633743, EPI_ISL_4633744, EPI_ISL_4633745, EPI_ISL_4633746, EPI_ISL_4633747, EPI_ISL_4633748, EPI_ISL_4633749, EPI_ISL_4633750, EPI_ISL_4633751, EPI_ISL_4633752, EPI_ISL_4633753, EPI_ISL_4633754, EPI_ISL_4633755, EPI_ISL_4633756, EPI_ISL_4633757, EPI_ISL_4633758, EPI_ISL_4633759, EPI_ISL_4633760, EPI_ISL_4633761, EPI_ISL_4633762, EPI_ISL_4633763, EPI_ISL_4633764, EPI_ISL_4633765, EPI_ISL_4633766, EPI_ISL_4633767, EPI_ISL_4633768, EPI_ISL_4633769, EPI_ISL_4633770, EPI_ISL_4633771, EPI_ISL_4633772, EPI_ISL_4633773, EPI_ISL_4633774, EPI_ISL_4633775, EPI_ISL_4633776, EPI_ISL_4633777, EPI_ISL_4633778, EPI_ISL_4633779, EPI_ISL_4633780, EPI_ISL_4633781, EPI_ISL_4633782, EPI_ISL_4633783, EPI_ISL_4633784, EPI_ISL_4633785, EPI_ISL_4633786, EPI_ISL_4633787, EPI_ISL_4633788, EPI_ISL_4633793, EPI_ISL_4633794, EPI_ISL_4633795, EPI_ISL_4633796, EPI_ISL_4633797, EPI_ISL_4633798, EPI_ISL_4633799, EPI_ISL_4633800, EPI_ISL_4633801, EPI_ISL_4633802			
see above	Division of Emerging Infectious Diseases, Bureau of Infectious Diseases Diagnosis Control, Korea Disease Control and Prevention Agency	Division of Emerging Infectious Diseases, Bureau of Infectious Diseases Diagnosis Control, Korea Disease Control and Prevention Agency	Ae Kyung Park; Chae Young Lee; Eun-Jin Kim; Heui Man Kim; Il-Hwan Kim; Jaehee Lee; Jeong-Ah Kim; Jeong-Min Kim; Namjoo Lee; Sang Hee Woo
EPI_ISL_3411836	Division of Infectious Diseases, Department of Internal Medicine, Yonsei University College of Medicine	Department of Biotechnology, Yonsei University	Jun Yong Choi*; Jung Ho Kim; Minwoo Kim; Youn-Jung Lee; and Jong-Won Oh*

We gratefully acknowledge the following Authors from the Originating laboratories responsible for obtaining the specimens, as well as the Submitting laboratories where the genome data were generated and shared via GISAID, on which this research is based.

All Submitters of data may be contacted directly via www.gisaid.org

Authors are sorted alphabetically.

Accession ID	Originating Laboratory	Submitting Laboratory	Authors
EPI_ISL_476805, EPI_ISL_476806, EPI_ISL_476807, EPI_ISL_476808, EPI_ISL_476809, EPI_ISL_476810, EPI_ISL_476811, EPI_ISL_476812, EPI_ISL_476815			
see above	Department of Laboratory Medicine Tan Tock Seng Hospital	Department of Laboratory Medicine Tan Tock Seng Hospital	Barkham TMS; Chen YYC; Li C; Maurer-Stroh S; Nagarajan N; Sessions OM; Tang WY; Zair X
EPI_ISL_477172, EPI_ISL_477174, EPI_ISL_477175, EPI_ISL_477177, EPI_ISL_477178, EPI_ISL_477180, EPI_ISL_477182, EPI_ISL_477184, EPI_ISL_477187, EPI_ISL_477188, EPI_ISL_477189, EPI_ISL_477190, EPI_ISL_477191, EPI_ISL_477192, EPI_ISL_479482, EPI_ISL_479483, EPI_ISL_479484, EPI_ISL_479485, EPI_ISL_479486, EPI_ISL_479487, EPI_ISL_479488, EPI_ISL_479489, EPI_ISL_479490, EPI_ISL_479491, EPI_ISL_479492, EPI_ISL_492978, EPI_ISL_492979			
see above	Department of Laboratory Medicine Tan Tock Seng Hospital	Department of Laboratory Medicine Tan Tock Seng Hospital	Barkham TMS; Chen YYC; Li C; Maurer-Stroh S; Nagarajan N; Sessions OM; Tang WY; Zair X
EPI_ISL_516801, EPI_ISL_516802, EPI_ISL_516803, EPI_ISL_516804, EPI_ISL_516805	Department of Laboratory Medicine, Tan Tock Seng Hospital	Department of Laboratory Medicine, Tan Tock Seng Hospital	Barkham TMS; Chen YYC; Li C; Maurer-Stroh S; Nagarajan N; Sessions OM; Tang WY; Zair X
EPI_ISL_476795, EPI_ISL_476796, EPI_ISL_476797, EPI_ISL_476813, EPI_ISL_476814, EPI_ISL_476816, EPI_ISL_476817, EPI_ISL_476818, EPI_ISL_476819, EPI_ISL_476820, EPI_ISL_476821, EPI_ISL_507002, EPI_ISL_507003, EPI_ISL_507004, EPI_ISL_507005, EPI_ISL_507006, EPI_ISL_538435, EPI_ISL_538436, EPI_ISL_538437, EPI_ISL_538438, EPI_ISL_538439, EPI_ISL_538440, EPI_ISL_538441, EPI_ISL_538442, EPI_ISL_538443, EPI_ISL_538444, EPI_ISL_538445, EPI_ISL_538446, EPI_ISL_538447, EPI_ISL_538448, EPI_ISL_538449, EPI_ISL_538450, EPI_ISL_538451, EPI_ISL_538452, EPI_ISL_538453, EPI_ISL_538454, EPI_ISL_538455, EPI_ISL_538456, EPI_ISL_538457, EPI_ISL_538458, EPI_ISL_538459, EPI_ISL_538460, EPI_ISL_538461, EPI_ISL_538462, EPI_ISL_538463, EPI_ISL_538464, EPI_ISL_538465, EPI_ISL_538466, EPI_ISL_538467, EPI_ISL_538468, EPI_ISL_538469, EPI_ISL_538470, EPI_ISL_538471, EPI_ISL_538472, EPI_ISL_538473, EPI_ISL_538474, EPI_ISL_538475, EPI_ISL_538476, EPI_ISL_538477, EPI_ISL_538478, EPI_ISL_538479, EPI_ISL_538480, EPI_ISL_538481, EPI_ISL_538482, EPI_ISL_538483, EPI_ISL_538484, EPI_ISL_538485, EPI_ISL_538486, EPI_ISL_538487, EPI_ISL_538488, EPI_ISL_538489, EPI_ISL_538490, EPI_ISL_538491, EPI_ISL_538492, EPI_ISL_538493, EPI_ISL_538494, EPI_ISL_538495, EPI_ISL_538496, EPI_ISL_538497, EPI_ISL_648043, EPI_ISL_648044, EPI_ISL_648045, EPI_ISL_648046, EPI_ISL_648047, EPI_ISL_648048, EPI_ISL_648049, EPI_ISL_648050, EPI_ISL_648051, EPI_ISL_648052, EPI_ISL_648053, EPI_ISL_648054, EPI_ISL_648055, EPI_ISL_648056, EPI_ISL_648057, EPI_ISL_648058, EPI_ISL_648059, EPI_ISL_648060, EPI_ISL_648061, EPI_ISL_648062, EPI_ISL_648063, EPI_ISL_648064, EPI_ISL_648065, EPI_ISL_648066, EPI_ISL_648067, EPI_ISL_648068, EPI_ISL_648069, EPI_ISL_648070, EPI_ISL_648071, EPI_ISL_648072, EPI_ISL_648073, EPI_ISL_648074, EPI_ISL_648075, EPI_ISL_648076, EPI_ISL_648077, EPI_ISL_648078, EPI_ISL_648079, EPI_ISL_648080, EPI_ISL_648081, EPI_ISL_648082, EPI_ISL_648083, EPI_ISL_648084, EPI_ISL_648085, EPI_ISL_648086, EPI_ISL_648087, EPI_ISL_648088, EPI_ISL_648089, EPI_ISL_648090, EPI_ISL_648091, EPI_ISL_648092, EPI_ISL_648093, EPI_ISL_648094, EPI_ISL_648095, EPI_ISL_648096, EPI_ISL_648097, EPI_ISL_648098, EPI_ISL_648099, EPI_ISL_648100, EPI_ISL_648101, EPI_ISL_648102, EPI_ISL_648103, EPI_ISL_648104, EPI_ISL_648105, EPI_ISL_648106, EPI_ISL_648107, EPI_ISL_648108, EPI_ISL_648109, EPI_ISL_648110, EPI_ISL_648111, EPI_ISL_648112, EPI_ISL_648113, EPI_ISL_648114, EPI_ISL_648115, EPI_ISL_648116, EPI_ISL_648117, EPI_ISL_648118, EPI_ISL_648119, EPI_ISL_648120, EPI_ISL_648121, EPI_ISL_648610, EPI_ISL_648611, EPI_ISL_648612, EPI_ISL_648613, EPI_ISL_648614, EPI_ISL_648615, EPI_ISL_648616, EPI_ISL_648617, EPI_ISL_648618, EPI_ISL_648619, EPI_ISL_648620, EPI_ISL_648621, EPI_ISL_648622, EPI_ISL_648623, EPI_ISL_648624, EPI_ISL_648625, EPI_ISL_648626, EPI_ISL_648627, EPI_ISL_648628, EPI_ISL_648629, EPI_ISL_648630, EPI_ISL_648631, EPI_ISL_648632, EPI_ISL_648633, EPI_ISL_648634, EPI_ISL_648635, EPI_ISL_648636, EPI_ISL_648637, EPI_ISL_648638, EPI_ISL_648639, EPI_ISL_648640, EPI_ISL_648641, EPI_ISL_648642, EPI_ISL_648643, EPI_ISL_648644, EPI_ISL_648645, EPI_ISL_648646, EPI_ISL_648647, EPI_ISL_648648, EPI_ISL_648649, EPI_ISL_648650, EPI_ISL_648651, EPI_ISL_648652, EPI_ISL_648653, EPI_ISL_648654, EPI_ISL_648655, EPI_ISL_648656, EPI_ISL_648657, EPI_ISL_648658, EPI_ISL_648659, EPI_ISL_648660, EPI_ISL_648661, EPI_ISL_648662, EPI_ISL_648663, EPI_ISL_648664, EPI_ISL_648665, EPI_ISL_648666, EPI_ISL_648667, EPI_ISL_648668, EPI_ISL_648669, EPI_ISL_648670, EPI_ISL_648671, EPI_ISL_648672, EPI_ISL_648673, EPI_ISL_648674, EPI_ISL_648675, EPI_ISL_648676, EPI_ISL_648678, EPI_ISL_648679, EPI_ISL_648680, EPI_ISL_648681, EPI_ISL_648682, EPI_ISL_648683, EPI_ISL_648684, EPI_ISL_648685, EPI_ISL_648686, EPI_ISL_648687, EPI_ISL_648688, EPI_ISL_648689, EPI_ISL_648690, EPI_ISL_648691, EPI_ISL_648692, EPI_ISL_648693, EPI_ISL_648694, EPI_ISL_648695, EPI_ISL_648696, EPI_ISL_648697, EPI_ISL_648698, EPI_ISL_648699, EPI_ISL_648700, EPI_ISL_648701, EPI_ISL_648702, EPI_ISL_648703, EPI_ISL_648704, EPI_ISL_648705, EPI_ISL_648706, EPI_ISL_648707, EPI_ISL_648708, EPI_ISL_648709, EPI_ISL_648710, EPI_ISL_648711, EPI_ISL_648712, EPI_ISL_648713, EPI_ISL_648714, EPI_ISL_648715, EPI_ISL_648716, EPI_ISL_648717, EPI_ISL_648718, EPI_ISL_648719, EPI_ISL_648720, EPI_ISL_648721, EPI_ISL_648722, EPI_ISL_648723, EPI_ISL_648724, EPI_ISL_648725, EPI_ISL_648726, EPI_ISL_648727, EPI_ISL_648728, EPI_ISL_648729, EPI_ISL_648730, EPI_ISL_648731, EPI_ISL_648732, EPI_ISL_648733, EPI_ISL_648734, EPI_ISL_648735, EPI_ISL_648736, EPI_ISL_648737, EPI_ISL_648738, EPI_ISL_648739, EPI_ISL_648740, EPI_ISL_648741, EPI_ISL_648742, EPI_ISL_648743, EPI_ISL_648744, EPI_ISL_648745, EPI_ISL_648746, EPI_ISL_648747, EPI_ISL_648748, EPI_ISL_648749, EPI_ISL_648750, EPI_ISL_648751, EPI_ISL_648752, EPI_ISL_648753, EPI_ISL_648754, EPI_ISL_648755, EPI_ISL_648756, EPI_ISL_648757, EPI_ISL_648758, EPI_ISL_648759, EPI_ISL_648760, EPI_ISL_648761, EPI_ISL_648762, EPI_ISL_648763, EPI_ISL_648764, EPI_ISL_648765, EPI_ISL_648766, EPI_ISL_648767, EPI_ISL_648768, EPI_ISL_648769, EPI_ISL_648770, EPI_ISL_648771, EPI_ISL_648772, EPI_ISL_648773, EPI_ISL_648774, EPI_ISL_648775, EPI_ISL_648776, EPI_ISL_648777, EPI_ISL_648778, EPI_ISL_648779, EPI_ISL_648780, EPI_ISL_648781, EPI_ISL_648782, EPI_ISL_648783, EPI_ISL_648784, EPI_ISL_648785, EPI_ISL_648786, EPI_ISL_648787, EPI_ISL_648788, EPI_ISL_648789, EPI_ISL_648790, EPI_ISL_648791, EPI_ISL_648792, EPI_ISL_648793, EPI_ISL_648794, EPI_ISL_648795, EPI_ISL_648796, EPI_ISL_648797, EPI_ISL_648798, EPI_ISL_648799, EPI_ISL_648800, EPI_ISL_648801, EPI_ISL_648802, EPI_ISL_648803, EPI_ISL_648804, EPI_ISL_648805, EPI_ISL_648806, EPI_ISL_648807, EPI_ISL_648808, EPI_ISL_648809, EPI_ISL_648810, EPI_ISL_648811, EPI_ISL_648812, EPI_ISL_648813, EPI_ISL_648814, EPI_ISL_648815, EPI_ISL_648816, EPI_ISL_648817, EPI_ISL_648818, EPI_ISL_648819, EPI_ISL_648820			
see above	Department of Laboratory Medicine, Tan Tock Seng Hospital	Department of Laboratory Medicine, Tan Tock Seng Hospital	Barkham TMS; Chen YYC; Li C; Lim JX; Maurer-Stroh S; Nagarajan N; Sessions OM; Tang WY; Zair X
EPI_ISL_477170	Department of Laboratory, Medicine Tan Tock Seng Hospital	Department of Laboratory Medicine Tan Tock Seng Hospital	Barkham TMS; Chen YYC; Li C; Maurer-Stroh S; Nagarajan N; Sessions OM; Tang WY; Zair X
EPI_ISL_477171	Department of Laboratory, Medicine Tan Tock Seng Hospital	Department of Laboratory, Medicine Tan Tock Seng Hospital	Barkham TMS; Chen YYC; Li C; Maurer-Stroh S; Nagarajan N; Sessions OM; Tang WY; Zair X
EPI_ISL_407988, EPI_ISL_410535, EPI_ISL_414378, EPI_ISL_414379, EPI_ISL_414380, EPI_ISL_420099, EPI_ISL_420100, EPI_ISL_420101, EPI_ISL_420102, EPI_ISL_420103, EPI_ISL_420104, EPI_ISL_420105, EPI_ISL_420106, EPI_ISL_420107, EPI_ISL_420108, EPI_ISL_420109, EPI_ISL_420110, EPI_ISL_420111			
see above	National Centre for Infectious Diseases	Programme in Emerging Infectious Diseases, Duke-NUS Medical School	Barnaby E Young; Danielle E Anderson; David CB Lye; Gavin JD Smith; Jayanthi Jayakumar; Linfa Wang; Louisa Sun; Martin Linster; Yan Zhuang; Yee Sin Leo; Yvonne CF Su
EPI_ISL_410719	National Public Health Laboratory	National Public Health Laboratory	Cui L; Lin RTP; Mak TM; Octavia S
EPI_ISL_410716	National Public Health Laboratory, National Centre for Infectious Diseases	National Centre for Infectious Diseases, National Centre for Infectious Diseases	Cui L; Lin RTP; Mak TM; Octavia S
EPI_ISL_410713, EPI_ISL_410714, EPI_ISL_410715, EPI_ISL_418992, EPI_ISL_418993, EPI_ISL_418994, EPI_ISL_418995, EPI_ISL_418996, EPI_ISL_418997, EPI_ISL_418998, EPI_ISL_418999, EPI_ISL_419000, EPI_ISL_419001, EPI_ISL_422428, EPI_ISL_422429, EPI_ISL_422430, EPI_ISL_422431, EPI_ISL_422432, EPI_ISL_422433, EPI_ISL_422434, EPI_ISL_422435, EPI_ISL_428822, EPI_ISL_428823, EPI_ISL_428824, EPI_ISL_428825, EPI_ISL_428826, EPI_ISL_428827, EPI_ISL_428828, EPI_ISL_428829, EPI_ISL_428830, EPI_ISL_428831, EPI_ISL_428832, EPI_ISL_428833, EPI_ISL_428834, EPI_ISL_428835, EPI_ISL_428836, EPI_ISL_428837, EPI_ISL_428838, EPI_ISL_428839, EPI_ISL_428840, EPI_ISL_428841, EPI_ISL_428842, EPI_ISL_428843, EPI_ISL_428844, EPI_ISL_428845, EPI_ISL_428846, EPI_ISL_428847, EPI_ISL_428848, EPI_ISL_428849, EPI_ISL_428850, EPI_ISL_435678, EPI_ISL_435679, EPI_ISL_435680, EPI_ISL_435681, EPI_ISL_435682, EPI_ISL_435683, EPI_ISL_435684, EPI_ISL_435685, EPI_ISL_435686, EPI_ISL_435687, EPI_ISL_435688, EPI_ISL_435689, EPI_ISL_435690, EPI_ISL_435691, EPI_ISL_435692, EPI_ISL_435693, EPI_ISL_435694, EPI_ISL_435695, EPI_ISL_435696, EPI_ISL_435697, EPI_ISL_435698, EPI_ISL_435699, EPI_ISL_435700, EPI_ISL_443188, EPI_ISL_443189, EPI_ISL_443190, EPI_ISL_443191, EPI_ISL_443192, EPI_ISL_443193, EPI_ISL_443194, EPI_ISL_443195, EPI_ISL_443196, EPI_ISL_443197, EPI_ISL_443198, EPI_ISL_443199, EPI_ISL_443200, EPI_ISL_443201, EPI_ISL_443202, EPI_ISL_443203, EPI_ISL_443204, EPI_ISL_443205, EPI_ISL_443206, EPI_ISL_443207, EPI_ISL_443208, EPI_ISL_443209, EPI_ISL_443210, EPI_ISL_443211, EPI_ISL_443212, EPI_ISL_443213, EPI_ISL_443214, EPI_ISL_443215, EPI_ISL_443216, EPI_ISL_443217, EPI_ISL_443218, EPI_ISL_443219, EPI_ISL_443220, EPI_ISL_443221, EPI_ISL_443222, EPI_ISL_443223, EPI_ISL_443224, EPI_ISL_443225, EPI_ISL_443226, EPI_ISL_443227, EPI_ISL_443228, EPI_ISL_443229, EPI_ISL_443230, EPI_ISL_443231, EPI_ISL_443232, EPI_ISL_443233, EPI_ISL_443234, EPI_ISL_443235, EPI_ISL_443236, EPI_ISL_443237, EPI_ISL_443238, EPI_ISL_443239, EPI_ISL_443240, EPI_ISL_443241, EPI_ISL_443242, EPI_ISL_443243, EPI_ISL_443244, EPI_ISL_443245, EPI_ISL_443246, EPI_ISL_443247, EPI_ISL_443248, EPI_ISL_443249, EPI_ISL_462276, EPI_ISL_462277, EPI_ISL_462278, EPI_ISL_462279, EPI_ISL_462280, EPI_ISL_462281, EPI_ISL_462282, EPI_ISL_462283, EPI_ISL_462284, EPI_ISL_462285, EPI_ISL_462286, EPI_ISL_462287, EPI_ISL_462288, EPI_ISL_462289, EPI_ISL_462290, EPI_ISL_462291, EPI_ISL_462292, EPI_ISL_462293, EPI_ISL_462294, EPI_ISL_462295, EPI_ISL_462296, EPI_ISL_462297, EPI_ISL_462298, EPI_ISL_462299, EPI_ISL_462300, EPI_ISL_462301, EPI_ISL_462302, EPI_ISL_462303, EPI_ISL_462304, EPI_ISL_462305, EPI_ISL_462306, EPI_ISL_462307, EPI_ISL_462308, EPI_ISL_462309, EPI_ISL_462310, EPI_ISL_462311, EPI_ISL_462312, EPI_ISL_462313, EPI_ISL_462314, EPI_ISL_462315, EPI_ISL_462316, EPI_ISL_462317, EPI_ISL_462318, EPI_ISL_462319, EPI_ISL_462320, EPI_ISL_462321, EPI_ISL_462322, EPI_ISL_462323, EPI_ISL_462324, EPI_ISL_462325, EPI_ISL_462326, EPI_ISL_462327, EPI_ISL_462328, EPI_ISL_462329, EPI_ISL_462330, EPI_ISL_462331, EPI_ISL_462332, EPI_ISL_462333, EPI_ISL_462334, EPI_ISL_462335, EPI_ISL_462336, EPI_ISL_462337, EPI_ISL_462338, EPI_ISL_462339, EPI_ISL_462340, EPI_ISL_462341, EPI_ISL_462342, EPI_ISL_462343, EPI_ISL_462344, EPI_ISL_462345, EPI_ISL_462346, EPI_ISL_462347, EPI_ISL_462348, EPI_ISL_462349, EPI_ISL_462350, EPI_ISL_462351, EPI_ISL_462352, EPI_ISL_462353, EPI_ISL_462354, EPI_ISL_462355, EPI_ISL_462356, EPI_ISL_462357, EPI_ISL_462358, EPI_ISL_462359, EPI_ISL_462360, EPI_ISL_462361, EPI_ISL_462362, EPI_ISL_462363, EPI_ISL_462364, EPI_ISL_462365, EPI_ISL_462366, EPI_ISL_462367, EPI_ISL_462368, EPI_ISL_462369, EPI_ISL_462370, EPI_ISL_462371, EPI_ISL_462372, EPI_ISL_462373, EPI_ISL_462374, EPI_ISL_462375, EPI_ISL_462376, EPI_ISL_462377, EPI_ISL_462378, EPI_ISL_462379, EPI_ISL_462380, EPI_ISL_462381, EPI_ISL_462382, EPI_ISL_462383, EPI_ISL_462384, EPI_ISL_462385, EPI_ISL_462386, EPI_ISL_462387, EPI_ISL_462388, EPI_ISL_462389, EPI_ISL_462390, EPI_ISL_462391, EPI_ISL_462392, EPI_ISL_462393, EPI_ISL_462394, EPI_ISL_462395, EPI_ISL_462396, EPI_ISL_462397, EPI_ISL_462398, EPI_ISL_462399, EPI_ISL_462400, EPI_ISL_462401, EPI_ISL_462402, EPI_ISL_462403, EPI_ISL_462404, EPI_ISL_462405, EPI_ISL_462406, EPI_ISL_462407, EPI_ISL_462408, EPI_ISL_462409, EPI_ISL_462410, EPI_ISL_462411, EPI_ISL_462412, EPI_ISL_462413, EPI_ISL_462414, EPI_ISL_462415, EPI_ISL_462416, EPI_ISL_462417, EPI_ISL_462418, EPI_ISL_462419, EPI_ISL_462420, EPI_ISL_462421, EPI_ISL_462422, EPI_ISL_462423, EPI_ISL_462424, EPI_ISL_462425, EPI_ISL_462426, EPI_ISL_462427, EPI_ISL_462428, EPI_ISL_462429, EPI_ISL_462430, EPI_ISL_462431, EPI_ISL_462432, EPI_ISL_462433, EPI_ISL_469080, EPI_ISL_469081, EPI_ISL_469082, EPI_ISL_469083, EPI_ISL_469084, EPI_ISL_469085, EPI_ISL_469086, EPI_ISL_469087, EPI_ISL_469088, EPI_ISL_469089, EPI_ISL_469090, EPI_ISL_469091, EPI_ISL_469092, EPI_ISL_469093, EPI_ISL_469094, EPI_ISL_469095, EPI_ISL_469096, EPI_ISL_469097, EPI_ISL_469098, EPI_ISL_469099, EPI_ISL_469100, EPI_ISL_469101, EPI_ISL_469102, EPI_ISL_469103, EPI_ISL_469104, EPI_ISL_469105, EPI_ISL_469106, EPI_ISL_469107, EPI_ISL_469108, EPI_ISL_469109, EPI_ISL_469110, EPI_ISL_469111, EPI_ISL_469112, EPI_ISL_469113, EPI_ISL_469114, EPI_ISL_469115, EPI_ISL_469116, EPI_ISL_469117, EPI_ISL_469118, EPI_ISL_469119, EPI_ISL_469120, EPI_ISL_469121, EPI_ISL_469122, EPI_ISL_469123, EPI_ISL_469124, EPI_ISL_469125, EPI_ISL_469126, EPI_ISL_469127, EPI_ISL_469128, EPI_ISL_469129, EPI_ISL_469130, EPI_ISL_469131, EPI_ISL_469132, EPI_ISL_469133, EPI_ISL_469134, EPI_ISL_469135, EPI_ISL_469136, EPI_ISL_469137, EPI_ISL_469138, EPI_ISL_469139, EPI_ISL_469140, EPI_ISL_469141, EPI_ISL_469142, EPI_ISL_469143, EPI_ISL_469144, EPI_ISL_469145, EPI_ISL_469146, EPI_ISL_469147, EPI_ISL_469148, EPI_ISL_469149, EPI_ISL_469150, EPI_ISL_469151, EPI_ISL_469152, EPI_ISL_469153, EPI_ISL_469154, EPI_ISL_469155, EPI_ISL_469274, EPI_ISL_475937, EPI_ISL_475938, EPI_ISL_475939, EPI_ISL_475940, EPI_ISL_475941, EPI_ISL_475942, EPI_ISL_475943, EPI_ISL_475944, EPI_ISL_475945, EPI_ISL_475946, EPI_ISL_475947, EPI_ISL_475948, EPI_ISL_475949, EPI_ISL_475950, EPI_ISL_475951, EPI_ISL_475952, EPI_ISL_475953, EPI_ISL_475954, EPI_ISL_475955, EPI_ISL_475956, EPI_ISL_475957, EPI_ISL_475958, EPI_ISL_475959, EPI_ISL_475960, EPI_ISL_475961, EPI_ISL_475962, EPI_ISL_475963, EPI_ISL_475964, EPI_ISL_475965, EPI_ISL_475966, EPI_ISL_475967, EPI_ISL_475968, EPI_ISL_475969, EPI_ISL_475970, EPI_ISL_475971, EPI_ISL_475972, EPI_ISL_475973, EPI_ISL_475974, EPI_ISL_475975, EPI_ISL_475976, EPI_ISL_475977, EPI_ISL_475978, EPI_ISL_475979, EPI_ISL_475980, EPI_ISL_475981, EPI_ISL_475982, EPI_ISL_475983, EPI_ISL_475984, EPI_ISL_475985, EPI_ISL_475986, EPI_ISL_475987, EPI_ISL_475988, EPI_ISL_475989, EPI_ISL_475990, EPI_ISL_475991, EPI_ISL_475992, EPI_ISL_475993, EPI_ISL_475994, EPI_ISL_475995, EPI_ISL_475996, EPI_ISL_475997, EPI_ISL_475998, EPI_ISL_475999, EPI_ISL_479574, EPI_ISL_479575, EPI_ISL_479576, EPI_ISL_479577, EPI_ISL_479578, EPI_ISL_479579, EPI_ISL_479580, EPI_ISL_479581, EPI_ISL_479582, EPI_ISL_479583, EPI_ISL_479584, EPI_ISL_479585, EPI_ISL_479586, EPI_ISL_479587, EPI_ISL_479588, EPI_ISL_479589, EPI_ISL_479590, EPI_ISL_479591, EPI_ISL_479592, EPI_ISL_479593, EPI_ISL_479594, EPI_ISL_479595, EPI_ISL_479596, EPI_ISL_479597, EPI_ISL_479598, EPI_ISL_479599, EPI_ISL_479600, EPI_ISL_479601, EPI_ISL_479602, EPI_ISL_479603, EPI_ISL_483576, EPI_ISL_483577, EPI_ISL_483578, EPI_ISL_483579, EPI_ISL_483580, EPI_ISL_483581, EPI_ISL_483582, EPI_ISL_483583, EPI_ISL_483584, EPI_ISL_483585, EPI_ISL_483586, EPI_ISL_483587, EPI_ISL_483588, EPI_ISL_483589, EPI_ISL_483590, EPI_ISL_483591, EPI_ISL_483592, EPI_ISL_483593, EPI_ISL_483594, EPI_ISL_483595, EPI_ISL_483596, EPI_ISL_483597, EPI_ISL_483598, EPI_ISL_483599, EPI_ISL_483600, EPI_ISL_483601, EPI_ISL_483602, EPI_ISL_483603, EPI_ISL_483604, EPI_ISL_483605, EPI_ISL_483606, EPI_ISL_483607, EPI_ISL_483608, EPI_ISL_483609, EPI_ISL_483610, EPI_ISL_483611, EPI_ISL_483612, EPI_ISL_483613, EPI_ISL_483614, EPI_ISL_483615, EPI_ISL_483616, EPI_ISL_483617, EPI_ISL_483618, EPI_ISL_483619, EPI_ISL_483620, EPI_ISL_483621, EPI_ISL_490049, EPI_ISL_490050, EPI_ISL_490051, EPI_ISL_490052, EPI_ISL_490053, EPI_ISL_490054, EPI_ISL_490055, EPI_ISL_490056, EPI_ISL_490057, EPI_ISL_490058, EPI_ISL_490059, EPI_ISL_490060, EPI_ISL_490061, EPI_ISL_490062, EPI_ISL_490063, EPI_ISL_490064, EPI_ISL_490065, EPI_ISL_490066, EPI_ISL_490067, EPI_ISL_490068, EPI_ISL_490069, EPI_ISL_490070, EPI_ISL_490071, EPI_ISL_490072, EPI_ISL_490073, EPI_ISL_490074, EPI_ISL_490075, EPI_ISL_490076, EPI_ISL_490077, EPI_ISL_490078, EPI_ISL_490079, EPI_ISL_493390, EPI_ISL_493391, EPI_ISL_493392, EPI_ISL_493393, EPI_ISL_493394, EPI_ISL_493395, EPI_ISL_493396, EPI_ISL_493397, EPI_ISL_493398, EPI_ISL_493399, EPI_ISL_493400, EPI_ISL_493401, EPI_ISL_493402, EPI_ISL_493403, EPI_ISL_493404, EPI_ISL_493405, EPI_ISL_493406, EPI_ISL_493407, EPI_ISL_493408, EPI_ISL_493409, EPI_ISL_493410, EPI_ISL_493411, EPI_ISL_493412, EPI_ISL_493413, EPI_ISL_493414, EPI_ISL_493415, EPI_ISL_493416, EPI_ISL_493417, EPI_ISL_493418, EPI_ISL_493419, EPI_ISL_493420, EPI_ISL_493421, EPI_ISL_493422, EPI_ISL_493423, EPI_ISL_493424, EPI_ISL_493425, EPI_ISL_498564, EPI_ISL_498565, EPI_ISL_498566, EPI_ISL_498567, EPI_ISL_498568, EPI_ISL_498569, EPI_ISL_498570, EPI_ISL_498571, EPI_ISL_498572, EPI_ISL_498573, EPI_ISL_498574, EPI_ISL_498575, EPI_ISL_498576, EPI_ISL_498577, EPI_ISL_498578, EPI_ISL_498579, EPI_ISL_498580, EPI_ISL_498581, EPI_ISL_498582, EPI_ISL_498583, EPI_ISL_498584, EPI_ISL_498585, EPI_ISL_498586, EPI_ISL_498587, EPI_ISL_498588, EPI_ISL_498589, EPI_ISL_498590, EPI_ISL_498591, EPI_ISL_498592, EPI_ISL_498593, EPI_ISL_498594, EPI_ISL_498595, EPI_ISL_498596, EPI_ISL_498597, EPI_ISL_498598, EPI_ISL_498599, EPI_ISL_498600, EPI_ISL_498601, EPI_ISL_498602, EPI_ISL_498603, EPI_ISL_498604, EPI_ISL_498605, EPI_ISL_498606, EPI_ISL_498607, EPI_ISL_498608, EPI_ISL_498609, EPI_ISL_498610, EPI_ISL_498611, EPI_ISL_498612, EPI_ISL_498613, EPI_ISL_498614, EPI_ISL_498615, EPI_ISL_498616, EPI_ISL_498617, EPI_ISL_498618, EPI_ISL_512822, EPI_ISL_512823, EPI_ISL_512824, EPI_ISL_512825, EPI_ISL_512826, EPI_ISL_512827, EPI_ISL_512828, EPI_ISL_512829, EPI_ISL_512830, EPI_ISL_512831, EPI_ISL_512832, EPI_ISL_512833, EPI_ISL_512834, EPI_ISL_512835, EPI_ISL_512836, EPI_ISL_512837, EPI_ISL_512838, EPI_ISL_512839, EPI_ISL_512840, EPI_ISL_512841, EPI_ISL_512842, EPI_ISL_516807, EPI_ISL_516808, EPI_ISL_516809, EPI_ISL_516810, EPI_ISL_516811, EPI_ISL_516812, EPI_ISL_516813, EPI_ISL_516814, EPI_ISL_516815, EPI_ISL_516816, EPI_ISL_516817, EPI_ISL_516818, EPI_ISL_516819, EPI_ISL_516820, EPI_ISL_516821, EPI_ISL_516822, EPI_ISL_516823, EPI_ISL_516824, EPI_ISL_516825, EPI_ISL_516826, EPI_ISL_516827, EPI_ISL_516828, EPI_ISL_527359, EPI_ISL_527360, EPI_ISL_527361, EPI_ISL_527362, EPI_ISL_527363, EPI_ISL_527364, EPI_ISL_527365, EPI_ISL_527366, EPI_ISL_527367, EPI_ISL_527368, EPI_ISL_527369, EPI_ISL_527370, EPI_ISL_527371, EPI_ISL_527372, EPI_ISL_527373, EPI_ISL_527374, EPI_ISL_527375, EPI_ISL_527376, EPI_ISL_527377, EPI_ISL_527378, EPI_ISL_527379, EPI_ISL_536412, EPI_ISL_536413, EPI_ISL_536414, EPI_ISL_536415, EPI_ISL_536416, EPI_ISL_536417, EPI_ISL_536418, EPI_ISL_536419, EPI_ISL_536420, EPI_ISL_536421, EPI_ISL_536422, EPI_ISL_536423, EPI_ISL_536424, EPI_ISL_536425, EPI_ISL_536426, EPI_ISL_536427, EPI_ISL_536428, EPI_ISL_536429, EPI_ISL_536430, EPI_ISL_536431, EPI_ISL_536432, EPI_ISL_536433, EPI_ISL_536434, EPI_ISL_536435, EPI_ISL_536436, EPI_ISL_536437, EPI_ISL_536438, EPI_ISL_536439, EPI_ISL_536440, EPI_ISL_536441, EPI_ISL_536442, EPI_ISL_536443, EPI_ISL_536444, EPI_ISL_536445, EPI_ISL_536446, EPI_ISL_536447, EPI_ISL_536448, EPI_ISL_536449, EPI_ISL_536450, EPI_ISL_536451, EPI_ISL_536452, EPI_ISL_536453, EPI_ISL_536454, EPI_ISL_548972, EPI_ISL_548973, EPI_ISL_548974, EPI_ISL_548975, EPI_ISL_548976, EPI_ISL_548977, EPI_ISL_548978, EPI_ISL_548979, EPI_ISL_548980, EPI_ISL_548981, EPI_ISL_548982, EPI_ISL_548983, EPI_ISL_548984, EPI_ISL_548985, EPI_ISL_548986, EPI_ISL_548987, EPI_ISL_548988, EPI_ISL_548989, EPI_ISL_548990, EPI_ISL_548991, EPI_ISL_548992, EPI_ISL_548993, EPI_ISL_548994, EPI_ISL_548995, EPI_ISL_548996, EPI_ISL_548997, EPI_ISL_548998, EPI_ISL_548999, EPI_ISL_549000, EPI_ISL_549001, EPI_ISL_549002, EPI_ISL_549003, EPI_ISL_549004, EPI_ISL_549005, EPI_ISL_574493, EPI_ISL_574494, EPI_ISL_574495, EPI_ISL_574496, EPI_ISL_574497, EPI_ISL_574498, EPI_ISL_574499, EPI_ISL_574500, EPI_ISL_574501, EPI_ISL_574502, EPI_ISL_574503, EPI_ISL_574504, EPI_ISL_574505, EPI_ISL_574506, EPI_ISL_574507, EPI_ISL_574508, EPI_ISL_574509, EPI_ISL_574510, EPI_ISL_574511, EPI_ISL_574512, EPI_ISL_574513, EPI_ISL_574514, EPI_ISL_574515, EPI_ISL_574516, EPI_ISL_574517, EPI_ISL_574518, EPI_ISL_574519, EPI_ISL_574520, EPI_ISL_574521, EPI_ISL_574522, EPI_ISL_574523, EPI_ISL_574524, EPI_ISL_574525, EPI_ISL_574526, EPI_ISL_574527, EPI_ISL_574528, EPI_ISL_574529, EPI_ISL_574530, EPI_ISL_574531, EPI_ISL_574532, EPI_ISL_574533, EPI_ISL_574534, EPI_ISL_574535, EPI_ISL_574536, EPI_ISL_574537, EPI_ISL_574538, EPI_ISL_574539, EPI_ISL_574540, EPI_ISL_574541, EPI_ISL_574542, EPI_ISL_596456, EPI_ISL_596457, EPI_ISL_596458, EPI_ISL_596459, EPI_ISL_596460, EPI_ISL_596461, EPI_ISL_596462, EPI_ISL_596463, EPI_ISL_596464, EPI_ISL_596465, EPI_ISL_596466, EPI_ISL_596467, EPI_ISL_596468, EPI_ISL_596469, EPI_ISL_596470, EPI_ISL_596471, EPI_ISL_596472, EPI_ISL_596473, EPI_ISL_596474, EPI_ISL_596475, EPI_ISL_596476, EPI_ISL_596477, EPI_ISL_596478, EPI_ISL_596479, EPI_ISL_596480, EPI_ISL_596481, EPI_ISL_596482, EPI_ISL_596483, EPI_ISL_596484, EPI_ISL_596485, EPI_ISL_596486, EPI_ISL_596487, EPI_ISL_596488, EPI_ISL_596489, EPI_ISL_596490, EPI_ISL_596491, EPI_ISL_596492, EPI_ISL_596493, EPI_ISL_596494, EPI_ISL_596495, EPI_ISL_596496, EPI_ISL_596497, EPI_ISL_605817, EPI_ISL_605818, EPI_ISL_605819, EPI_ISL_605820, EPI_ISL_605821, EPI_ISL_605822, EPI_ISL_605823, EPI_ISL_605824, EPI_ISL_626628, EPI_ISL_626629, EPI_ISL_626630, EPI_ISL_626631, EPI_ISL_626632, EPI_ISL_626633, EPI_ISL_626634, EPI_ISL_626635, EPI_ISL_626636, EPI_ISL_626637, EPI_ISL_626638, EPI_ISL_626639, EPI_ISL_626640, EPI_ISL_626641, EPI_ISL_626642, EPI_ISL_626643, EPI_ISL_626644, EPI_ISL_626645, EPI_ISL_626646, EPI_ISL_645115, EPI_ISL_645116, EPI_ISL_645117, EPI_ISL_645118, EPI_ISL_645119, EPI_ISL_645120, EPI_ISL_645121, EPI_ISL_645122, EPI_ISL_645123, EPI_ISL_645124, EPI_ISL_645125, EPI_ISL_645126, EPI_ISL_645127, EPI_ISL_645128, EPI_ISL_645129, EPI_ISL_645130, EPI_ISL_645131, EPI_ISL_645132, EPI_ISL_654810, EPI_ISL_654811, EPI_ISL_654812, EPI_ISL_654813, EPI_ISL_654814, EPI_ISL_654815, EPI_ISL_654816, EPI_ISL_654817, EPI_ISL_693307, EPI_ISL_693308, EPI_ISL_693309, EPI_ISL_693310, EPI_ISL_693311, EPI_ISL_693312, EPI_ISL_693313, EPI_ISL_693314, EPI_ISL_693315, EPI_ISL_693316, EPI_ISL_693317, EPI_ISL_693318, EPI_ISL_693319, EPI_ISL_693320, EPI_ISL_693321, EPI_ISL_693322, EPI_ISL_693323, EPI_ISL_693324, EPI_ISL_693325, EPI_ISL_693326, EPI_ISL_693327, EPI_ISL_693328, EPI_ISL_693329, EPI_ISL_693330, EPI_ISL_693331, EPI_ISL_728179, EPI_ISL_728180, EPI_ISL_728181, EPI_ISL_728182, EPI_ISL_728183, EPI_ISL_728184, EPI_ISL_728185, EPI_ISL_728186, EPI_ISL_728187, EPI_ISL_728188, EPI_ISL_728189, EPI_ISL_728190, EPI_ISL_728191, EPI_ISL_728192, EPI_ISL_728193, EPI_ISL_728194, EPI_ISL_728195, EPI_ISL_728196, EPI_ISL_728197, EPI_ISL_728198, EPI_ISL_728199, EPI_ISL_728200, EPI_ISL_728201, EPI_ISL_754074, EPI_ISL_754075, EPI_ISL_754076, EPI_ISL_754077, EPI_ISL_754078, EPI_ISL_754079, EPI_ISL_754080, EPI_ISL_754081, EPI_ISL_754082, EPI_ISL_754083, EPI_ISL_754084, EPI_ISL_754085, EPI_ISL_754086, EPI_ISL_754087, EPI_ISL_754088, EPI_ISL_754089, EPI_ISL_754090, EPI_ISL_754091, EPI_ISL_754092, EPI_ISL_754093, EPI_ISL_754094, EPI_ISL_754095, EPI_ISL_754096, EPI_ISL_754097, EPI_ISL_754098, EPI_ISL_754099, EPI_ISL_754100, EPI_ISL_754101, EPI_ISL_754102, EPI_ISL_754103, EPI_ISL_754104, EPI_ISL_754105, EPI_ISL_754106, EPI_ISL_754107, EPI_ISL_754108, EPI_ISL_768618, EPI_ISL_768619, EPI_ISL_768620, EPI_ISL_768621, EPI_ISL_768622, EPI_ISL_768623, EPI_ISL_768624, EPI_ISL_768625, EPI_ISL_768626, EPI_ISL_768627, EPI_ISL_768628, EPI_ISL_768629, EPI_ISL_768630, EPI_ISL_768631, EPI_ISL_768632, EPI_ISL_803958, EPI_ISL_803959, EPI_ISL_803960, EPI_ISL_803961, EPI_ISL_803962, EPI_ISL_803963, EPI_ISL_803964, EPI_ISL_803965, EPI_ISL_803966, EPI_ISL_803967, EPI_ISL_803968, EPI_ISL_803969, EPI_ISL_803970, EPI_ISL_803971, EPI_ISL_803972, EPI_ISL_803973, EPI_ISL_803974, EPI_ISL_803975, EPI_ISL_803976, EPI_ISL_803977, EPI_ISL_803978, EPI_ISL_803979, EPI_ISL_803980, EPI_ISL_803981, EPI_ISL_803982, EPI_ISL_803983, EPI_ISL_803984, EPI_ISL_803985, EPI_ISL_803986, EPI_ISL_803987, EPI_ISL_803988, EPI_ISL_803989, EPI_ISL_803990, EPI_ISL_803991, EPI_ISL_803992, EPI_ISL_803993, EPI_ISL_803994, EPI_ISL_803995, EPI_ISL_803996, EPI_ISL_803997, EPI_ISL_803998, EPI_ISL_803999, EPI_ISL_804000, EPI_ISL_804001, EPI_ISL_804002, EPI_ISL_804003, EPI_ISL_804004, EPI_ISL_804005, EPI_ISL_804006, EPI_ISL_825064, EPI_ISL_825065, EPI_ISL_825066, EPI_ISL_825067, EPI_ISL_825068, EPI_ISL_825069, EPI_ISL_825070, EPI_ISL_825071, EPI_ISL_825072, EPI_ISL_825073, EPI_ISL_825074, EPI_ISL_825075, EPI_ISL_825076, EPI_ISL_825077, EPI_ISL_825078, EPI_ISL_825079, EPI_ISL_825080, EPI_ISL_825081, EPI_ISL_825082, EPI_ISL_825083, EPI_ISL_833373, EPI_ISL_833374, EPI_ISL_833375, EPI_ISL_833376, EPI_ISL_833377, EPI_ISL_833378, EPI_ISL_833379, EPI_ISL_833380, EPI_ISL_833381, EPI_ISL_833382, EPI_ISL_833383, EPI_ISL_833384, EPI_ISL_833385, EPI_ISL_833386, EPI_ISL_833387, EPI_ISL_857469, EPI_ISL_857470, EPI_ISL_857471, EPI_ISL_857472, EPI_ISL_857473, EPI_ISL_857474, EPI_ISL_857475, EPI_ISL_857476, EPI_ISL_857477, EPI_ISL_857478, EPI_ISL_857479, EPI_ISL_857480, EPI_ISL_857481, EPI_ISL_857482, EPI_ISL_857483, EPI_ISL_857484, EPI_ISL_857485, EPI_ISL_862816, EPI_ISL_862817, EPI_ISL_862818, EPI_ISL_862819, EPI_ISL_862820, EPI_ISL_862821, EPI_ISL_862822, EPI_ISL_862823, EPI_ISL_862824, EPI_ISL_862825, EPI_ISL_862826, EPI_ISL_862827, EPI_ISL_862828, EPI_ISL_862829, EPI_ISL_862830, EPI_ISL_891231, EPI_ISL_891232, EPI_ISL_891233, EPI_ISL_891234, EPI_ISL_891235, EPI_ISL_891236, EPI_ISL_891237, EPI_ISL_891238, EPI_ISL_891239, EPI_ISL_891240, EPI_ISL_891241, EPI_ISL_891242, EPI_ISL_891243, EPI_ISL_891244, EPI_ISL_891245, EPI_ISL_891246, EPI_ISL_891247, EPI_ISL_891248, EPI_ISL_906833, EPI_ISL_906834, EPI_ISL_906835, EPI_ISL_906836, EPI_ISL_906837, EPI_ISL_906838, EPI_ISL_906839, EPI_ISL_906840, EPI_ISL_906841, EPI_ISL_906842, EPI_ISL_906843, EPI_ISL_906844, EPI_ISL_906845, EPI_ISL_906846, EPI_ISL_937515, EPI_ISL_937516, EPI_ISL_937517, EPI_ISL_937518, EPI_ISL_937519, EPI_ISL_937520, EPI_ISL_937521, EPI_ISL_937522, EPI_ISL_937523, EPI_ISL_937524, EPI_ISL_937525, EPI_ISL_937526, EPI_ISL_937527, EPI_ISL_937528, EPI_ISL_937529, EPI_ISL_937530, EPI_ISL_953385, EPI_ISL_953386, EPI_ISL_953387, EPI_ISL_953388, EPI_ISL_953389, EPI_ISL_953390, EPI_ISL_953391, EPI_ISL_953392, EPI_ISL_953393, EPI_ISL_953394, EPI_ISL_953395, EPI_ISL_953396, EPI_ISL_953397, EPI_ISL_953398, EPI_ISL_953399, EPI_ISL_953400, EPI_ISL_953401, EPI_ISL_981007, EPI_ISL_981008, EPI_ISL_981009, EPI_ISL_981010, EPI_ISL_981011, EPI_ISL_981012, EPI_ISL_981013, EPI_ISL_981014, EPI_ISL_981015, EPI_ISL_981016, EPI_ISL_981017, EPI_ISL_981018, EPI_ISL_981019, EPI_ISL_981020, EPI_ISL_981021, EPI_ISL_981022, EPI_ISL_981023, EPI_ISL_981024, EPI_ISL_981025, EPI_ISL_981026, EPI_ISL_981027, EPI_ISL_981028, EPI_ISL_981029, EPI_ISL_995294, EPI_ISL_995295, EPI_ISL_995297, EPI_ISL_995298, EPI_ISL_995299, EPI_ISL_995300, EPI_ISL_995301, EPI_ISL_995302, EPI_ISL_995303, EPI_ISL_995305, EPI_ISL_995306, EPI_ISL_995307, EPI_ISL_1034260, EPI_ISL_1034261, EPI_ISL_1034262, EPI_ISL_1034263, EPI_ISL_1034264, EPI_ISL_1034265, EPI_ISL_1034266, EPI_ISL_1034267, EPI_ISL_1034268, EPI_ISL_1034269, EPI_ISL_1034270, EPI_ISL_1034271, EPI_ISL_1034272, EPI_ISL_1034273, EPI_ISL_1081922, EPI_ISL_1081924, EPI_ISL_1081926, EPI_ISL_1081928, EPI_ISL_1081930, EPI_ISL_1081932, EPI_ISL_1081934, EPI_ISL_1081935, EPI_ISL_1081937, EPI_ISL_1081939, EPI_ISL_1081941, EPI_ISL_1081943, EPI_ISL_1081945, EPI_ISL_1081946, EPI_ISL_1081947, EPI_ISL_1081948, EPI_ISL_1081949, EPI_ISL_1081950, EPI_ISL_1081951, EPI_ISL_1081952, EPI_ISL_1098833, EPI_ISL_1098834, EPI_ISL_1098835, EPI_ISL_1098836, EPI_ISL_1098837, EPI_ISL_1164353, EPI_ISL_1164354, EPI_ISL_1164355, EPI_ISL_1164356, EPI_ISL_1164357, EPI_ISL_1164358, EPI_ISL_1164359, EPI_ISL_1173248, EPI_ISL_1173249, EPI_ISL_1173250, EPI_ISL_1173251, EPI_ISL_1173252, EPI_ISL_1173253, EPI_ISL_1173254, EPI_ISL_1173255, EPI_ISL_1173256, EPI_ISL_1229162, EPI_ISL_1229163, EPI_ISL_1229164, EPI_ISL_1229165, EPI_ISL_1229166, EPI_ISL_1229167, EPI_ISL_1229168, EPI_ISL_1252446, EPI_ISL_1252447, EPI_ISL_1252448, EPI_ISL_1252449, EPI_ISL_1252450, EPI_ISL_1252451, EPI_ISL_1252452, EPI_ISL_1252453, EPI_ISL_1252454, EPI_ISL_1252455, EPI_ISL_1295938, EPI_ISL_1295939, EPI_ISL_1295940, EPI_ISL_1295941, EPI_ISL_1295942, EPI_ISL_1312382, EPI_ISL_1312383, EPI_ISL_1312384, EPI_ISL_1312385, EPI_ISL_1312386, EPI_ISL_1312387, EPI_ISL_1312388, EPI_ISL_1315626, EPI_ISL_1315627, EPI_ISL_1315628, EPI_ISL_1367545, EPI_ISL_1367546, EPI_ISL_1367547, EPI_ISL_1367548, EPI_ISL_1367549, EPI_ISL_1367550, EPI_ISL_1367551, EPI_ISL_1367552, EPI_ISL_1367553, EPI_ISL_1367554, EPI_ISL_1367555, EPI_ISL_1367556, EPI_ISL_1367557, EPI_ISL_1367558, EPI_ISL_1367559, EPI_ISL_1367560,			

EPI_ISL_1367561, EPI_ISL_1367562, EPI_ISL_1367563, EPI_ISL_1367564, EPI_ISL_1367565, EPI_ISL_1442942, EPI_ISL_1442943, EPI_ISL_1442944, EPI_ISL_1442945, EPI_ISL_1442946, EPI_ISL_1442947, EPI_ISL_1442948, EPI_ISL_1442949, EPI_ISL_1442950, EPI_ISL_1442951, EPI_ISL_1442952, EPI_ISL_1442953, EPI_ISL_1442954, EPI_ISL_1442955, EPI_ISL_1476987, EPI_ISL_1476988, EPI_ISL_1476989,
EPI_ISL_1476990, EPI_ISL_1476991, EPI_ISL_1476992, EPI_ISL_1476993, EPI_ISL_1476994, EPI_ISL_1476995, EPI_ISL_1476996, EPI_ISL_1476997, EPI_ISL_1476998, EPI_ISL_1476999, EPI_ISL_1477000, EPI_ISL_1477001, EPI_ISL_1477002, EPI_ISL_1477003, EPI_ISL_1477004, EPI_ISL_1477005, EPI_ISL_1477006, EPI_ISL_1477007, EPI_ISL_1477008, EPI_ISL_1477009, EPI_ISL_1477010, EPI_ISL_1477011,
EPI_ISL_1477012, EPI_ISL_1477013, EPI_ISL_1477014, EPI_ISL_1477015, EPI_ISL_1477016, EPI_ISL_1477017, EPI_ISL_1477018, EPI_ISL_1477019, EPI_ISL_1477020, EPI_ISL_1477021, EPI_ISL_1477022, EPI_ISL_1477023, EPI_ISL_1477024, EPI_ISL_1477025, EPI_ISL_1477026, EPI_ISL_1477027, EPI_ISL_1477028, EPI_ISL_1477029, EPI_ISL_1477030, EPI_ISL_1477031, EPI_ISL_1477032, EPI_ISL_1477033,
EPI_ISL_1477034, EPI_ISL_1477035, EPI_ISL_1477036, EPI_ISL_1477037, EPI_ISL_1477038, EPI_ISL_1477039, EPI_ISL_1477040, EPI_ISL_1477041, EPI_ISL_1477042, EPI_ISL_1489717, EPI_ISL_1489718, EPI_ISL_1489719, EPI_ISL_1489720, EPI_ISL_1489721, EPI_ISL_1489722, EPI_ISL_1489723, EPI_ISL_1489724, EPI_ISL_1489725, EPI_ISL_1489726, EPI_ISL_1489727, EPI_ISL_1489728, EPI_ISL_1519355,
EPI_ISL_1519356, EPI_ISL_1519357, EPI_ISL_1519358, EPI_ISL_1519360, EPI_ISL_1519361, EPI_ISL_1519362, EPI_ISL_1519363, EPI_ISL_1519364, EPI_ISL_1519365, EPI_ISL_1519366, EPI_ISL_1519368, EPI_ISL_1519369, EPI_ISL_1519370, EPI_ISL_1519371, EPI_ISL_1519372, EPI_ISL_1519373, EPI_ISL_1519374, EPI_ISL_1519375, EPI_ISL_1519377, EPI_ISL_1519378, EPI_ISL_1519379, EPI_ISL_1519380,
EPI_ISL_1519381, EPI_ISL_1519382, EPI_ISL_1519383, EPI_ISL_1519384, EPI_ISL_1519386, EPI_ISL_1519388, EPI_ISL_1519391, EPI_ISL_1519393, EPI_ISL_1519396, EPI_ISL_1519398, EPI_ISL_1519401, EPI_ISL_1519405, EPI_ISL_1519407, EPI_ISL_1519410, EPI_ISL_1519413, EPI_ISL_1519416, EPI_ISL_1519419, EPI_ISL_1524777, EPI_ISL_1524778, EPI_ISL_1524779, EPI_ISL_1524780, EPI_ISL_1524781,
EPI_ISL_1524782, EPI_ISL_1524783, EPI_ISL_1524784, EPI_ISL_1524785, EPI_ISL_1524786, EPI_ISL_1524787, EPI_ISL_1524788, EPI_ISL_1524789, EPI_ISL_1524790, EPI_ISL_1524791, EPI_ISL_1524792, EPI_ISL_1524793, EPI_ISL_1524794, EPI_ISL_1524795, EPI_ISL_1524796, EPI_ISL_1524797, EPI_ISL_1524798, EPI_ISL_1524799, EPI_ISL_1524800, EPI_ISL_1524801, EPI_ISL_1543939, EPI_ISL_1543940,
EPI_ISL_1543941, EPI_ISL_1543942, EPI_ISL_1543943, EPI_ISL_1543944, EPI_ISL_1543945, EPI_ISL_1543946, EPI_ISL_1543947, EPI_ISL_1543948, EPI_ISL_1543949, EPI_ISL_1543950, EPI_ISL_1543951, EPI_ISL_1543952, EPI_ISL_1543953, EPI_ISL_1543954, EPI_ISL_1543955, EPI_ISL_1543956, EPI_ISL_1543957, EPI_ISL_1543958, EPI_ISL_1543959, EPI_ISL_1543960, EPI_ISL_1543961, EPI_ISL_1543962,
EPI_ISL_1543963, EPI_ISL_1543964, EPI_ISL_1543965, EPI_ISL_1543966, EPI_ISL_1543967, EPI_ISL_1543968, EPI_ISL_1543969, EPI_ISL_1543970, EPI_ISL_1543971, EPI_ISL_1543972, EPI_ISL_1543973, EPI_ISL_1543974, EPI_ISL_1543975, EPI_ISL_1543976, EPI_ISL_1543977, EPI_ISL_1543978, EPI_ISL_1543979, EPI_ISL_1543980, EPI_ISL_1543981, EPI_ISL_1543982, EPI_ISL_1545779, EPI_ISL_1568430,
EPI_ISL_1568432, EPI_ISL_1620131, EPI_ISL_1620132, EPI_ISL_1620133, EPI_ISL_1620134, EPI_ISL_1620135, EPI_ISL_1620136, EPI_ISL_1620137, EPI_ISL_1620138, EPI_ISL_1620139, EPI_ISL_1620140, EPI_ISL_1620141, EPI_ISL_1620142, EPI_ISL_1620143, EPI_ISL_1620144, EPI_ISL_1620145, EPI_ISL_1620146, EPI_ISL_1620147, EPI_ISL_1620148, EPI_ISL_1620149, EPI_ISL_1620150, EPI_ISL_1620151,
EPI_ISL_1620152, EPI_ISL_1620153, EPI_ISL_1620154, EPI_ISL_1620155, EPI_ISL_1620156, EPI_ISL_1620157, EPI_ISL_1620158, EPI_ISL_1620159, EPI_ISL_1620160, EPI_ISL_1620161, EPI_ISL_1620162, EPI_ISL_1620163, EPI_ISL_1620164, EPI_ISL_1620165, EPI_ISL_1620166, EPI_ISL_1620167, EPI_ISL_1620168, EPI_ISL_1620169, EPI_ISL_1620170, EPI_ISL_1634423, EPI_ISL_1634424, EPI_ISL_1634425,
EPI_ISL_1634426, EPI_ISL_1634427, EPI_ISL_1634428, EPI_ISL_1634430, EPI_ISL_1634431, EPI_ISL_1634432, EPI_ISL_1634433, EPI_ISL_1652091, EPI_ISL_1652092, EPI_ISL_1652093, EPI_ISL_1652094, EPI_ISL_1652095, EPI_ISL_1652096, EPI_ISL_1652097, EPI_ISL_1652098, EPI_ISL_1652099, EPI_ISL_1652100, EPI_ISL_1652101, EPI_ISL_1652102, EPI_ISL_1652103, EPI_ISL_1652104, EPI_ISL_1652105,
EPI_ISL_1652106, EPI_ISL_1652108, EPI_ISL_1652109, EPI_ISL_1652110, EPI_ISL_1652111, EPI_ISL_1652112, EPI_ISL_1652113, EPI_ISL_1652114, EPI_ISL_1652115, EPI_ISL_1652116, EPI_ISL_1652117, EPI_ISL_1652118, EPI_ISL_1652119, EPI_ISL_1652120, EPI_ISL_1652122, EPI_ISL_1652123, EPI_ISL_1652198, EPI_ISL_1652199, EPI_ISL_1652200, EPI_ISL_1652531, EPI_ISL_1652532, EPI_ISL_1668967,
EPI_ISL_1704809, EPI_ISL_1704810, EPI_ISL_1704811, EPI_ISL_1704812, EPI_ISL_1704813, EPI_ISL_1704814, EPI_ISL_1704815, EPI_ISL_1704816, EPI_ISL_1704817, EPI_ISL_1704818, EPI_ISL_1704819, EPI_ISL_1704820, EPI_ISL_1704821, EPI_ISL_1704822, EPI_ISL_1704823, EPI_ISL_1704824, EPI_ISL_1704825, EPI_ISL_1704826, EPI_ISL_1704827, EPI_ISL_1704828, EPI_ISL_1704829, EPI_ISL_1704830,
EPI_ISL_1704831, EPI_ISL_1704832, EPI_ISL_1704833, EPI_ISL_1704834, EPI_ISL_1704835, EPI_ISL_1704836, EPI_ISL_1704837, EPI_ISL_1704838, EPI_ISL_1704839, EPI_ISL_1704840, EPI_ISL_1704841, EPI_ISL_1704842, EPI_ISL_1704843, EPI_ISL_1704844, EPI_ISL_1704845, EPI_ISL_1704846, EPI_ISL_1704847, EPI_ISL_1704848, EPI_ISL_1704849, EPI_ISL_1704850, EPI_ISL_1704851, EPI_ISL_1719868,
EPI_ISL_1719869, EPI_ISL_1719870, EPI_ISL_1719871, EPI_ISL_1719872, EPI_ISL_1719873, EPI_ISL_1719874, EPI_ISL_1719875, EPI_ISL_1719876, EPI_ISL_1719877, EPI_ISL_1719878, EPI_ISL_1719879, EPI_ISL_1719880, EPI_ISL_1719881, EPI_ISL_1719882, EPI_ISL_1719883, EPI_ISL_1719884, EPI_ISL_1719885, EPI_ISL_1719886, EPI_ISL_1719887, EPI_ISL_1719888, EPI_ISL_1719889, EPI_ISL_1719890,
EPI_ISL_1719891, EPI_ISL_1719892, EPI_ISL_1719893, EPI_ISL_1719894, EPI_ISL_1719895, EPI_ISL_1719896, EPI_ISL_1719897, EPI_ISL_1719898, EPI_ISL_1719899, EPI_ISL_1719900, EPI_ISL_1719901, EPI_ISL_1719902, EPI_ISL_1719903, EPI_ISL_1719904, EPI_ISL_1752677, EPI_ISL_1816925, EPI_ISL_1816926, EPI_ISL_1816927, EPI_ISL_1816928, EPI_ISL_1816929, EPI_ISL_1816930, EPI_ISL_1816931,
EPI_ISL_1816932, EPI_ISL_1816933, EPI_ISL_1816934, EPI_ISL_1816935, EPI_ISL_1816936, EPI_ISL_1816937, EPI_ISL_1816938, EPI_ISL_1816939, EPI_ISL_1816940, EPI_ISL_1816941, EPI_ISL_1816942, EPI_ISL_1816943, EPI_ISL_1816944, EPI_ISL_1816945, EPI_ISL_1816946, EPI_ISL_1816947, EPI_ISL_1816948, EPI_ISL_1816949, EPI_ISL_1816950, EPI_ISL_1816951, EPI_ISL_1816952, EPI_ISL_1816953,
EPI_ISL_1816954, EPI_ISL_1816955, EPI_ISL_1816956, EPI_ISL_1816957, EPI_ISL_1816958, EPI_ISL_1816959, EPI_ISL_1816960, EPI_ISL_1816961, EPI_ISL_1816962, EPI_ISL_1816963, EPI_ISL_1816964, EPI_ISL_1816965, EPI_ISL_1816966, EPI_ISL_1816967, EPI_ISL_1816968, EPI_ISL_1816969, EPI_ISL_1816970, EPI_ISL_1816971, EPI_ISL_1816972, EPI_ISL_1816973, EPI_ISL_1897601, EPI_ISL_1897602,
EPI_ISL_1897603, EPI_ISL_1897604, EPI_ISL_1897605, EPI_ISL_1897606, EPI_ISL_1897607, EPI_ISL_1897608, EPI_ISL_1897609, EPI_ISL_1897610, EPI_ISL_1897611, EPI_ISL_1897612, EPI_ISL_1897613, EPI_ISL_1897614, EPI_ISL_1897615, EPI_ISL_1897616, EPI_ISL_1897617, EPI_ISL_1897618, EPI_ISL_1897619, EPI_ISL_1897620, EPI_ISL_1897621, EPI_ISL_1897622, EPI_ISL_1897623, EPI_ISL_1897624,
EPI_ISL_1897625, EPI_ISL_1897626, EPI_ISL_1897627, EPI_ISL_1897628, EPI_ISL_1897629, EPI_ISL_1897630, EPI_ISL_1897631, EPI_ISL_1897632, EPI_ISL_1897633, EPI_ISL_1897634, EPI_ISL_1897635, EPI_ISL_1897636, EPI_ISL_1897637, EPI_ISL_1897638, EPI_ISL_1897639, EPI_ISL_1897640, EPI_ISL_1897641, EPI_ISL_1914639, EPI_ISL_1914640, EPI_ISL_1914641, EPI_ISL_1914642, EPI_ISL_1914643,
EPI_ISL_1914644, EPI_ISL_1914645, EPI_ISL_1914646, EPI_ISL_1914647, EPI_ISL_1914648, EPI_ISL_1914649, EPI_ISL_1914650, EPI_ISL_1914651, EPI_ISL_1914652, EPI_ISL_1914653, EPI_ISL_1914654, EPI_ISL_1914655, EPI_ISL_1914656, EPI_ISL_1914657, EPI_ISL_1914658, EPI_ISL_1914659, EPI_ISL_1914660, EPI_ISL_1914661, EPI_ISL_1914662, EPI_ISL_2349709, EPI_ISL_2349710, EPI_ISL_2349711,
EPI_ISL_2349712, EPI_ISL_2349713, EPI_ISL_2349714, EPI_ISL_2349715, EPI_ISL_2349716, EPI_ISL_2349717, EPI_ISL_2349718, EPI_ISL_2349719, EPI_ISL_2349720, EPI_ISL_2349721, EPI_ISL_2349722, EPI_ISL_2349723, EPI_ISL_2349724, EPI_ISL_2349725, EPI_ISL_2349726, EPI_ISL_2349727, EPI_ISL_2349728, EPI_ISL_2349729, EPI_ISL_2349730, EPI_ISL_2349731, EPI_ISL_2349732, EPI_ISL_2349733,
EPI_ISL_2349734, EPI_ISL_2349735, EPI_ISL_2349736, EPI_ISL_2349737, EPI_ISL_2349738, EPI_ISL_2349739, EPI_ISL_2349740, EPI_ISL_2349741, EPI_ISL_2349742, EPI_ISL_2349743, EPI_ISL_2349744, EPI_ISL_2349745, EPI_ISL_2349746, EPI_ISL_2349747, EPI_ISL_2349748, EPI_ISL_2349749, EPI_ISL_2349750, EPI_ISL_2349751, EPI_ISL_2349752, EPI_ISL_2349753, EPI_ISL_2349754, EPI_ISL_2349755,
EPI_ISL_2349756, EPI_ISL_2349757, EPI_ISL_2349758, EPI_ISL_2349759, EPI_ISL_2349760, EPI_ISL_2349761, EPI_ISL_2349762, EPI_ISL_2349763, EPI_ISL_2349764, EPI_ISL_2349765, EPI_ISL_2349766, EPI_ISL_2349767, EPI_ISL_2349768, EPI_ISL_2349769, EPI_ISL_2349770, EPI_ISL_2349771, EPI_ISL_2349772, EPI_ISL_2349773, EPI_ISL_2349774, EPI_ISL_2349775, EPI_ISL_2349776, EPI_ISL_2349777,
EPI_ISL_2349778, EPI_ISL_2349779, EPI_ISL_2349780, EPI_ISL_2349781, EPI_ISL_2349782, EPI_ISL_2349783, EPI_ISL_2349784, EPI_ISL_2349785, EPI_ISL_2349786, EPI_ISL_2349787, EPI_ISL_2349788, EPI_ISL_2349789, EPI_ISL_2349790, EPI_ISL_2349791, EPI_ISL_2349792, EPI_ISL_2349793, EPI_ISL_2349794, EPI_ISL_2349795, EPI_ISL_2349796, EPI_ISL_2349797, EPI_ISL_2349798, EPI_ISL_2349799,
EPI_ISL_2349800, EPI_ISL_2349801, EPI_ISL_2349802, EPI_ISL_2349803, EPI_ISL_2349804, EPI_ISL_2349805, EPI_ISL_2349806, EPI_ISL_2349807, EPI_ISL_2349808, EPI_ISL_2349809, EPI_ISL_2349810, EPI_ISL_2349811, EPI_ISL_2349812, EPI_ISL_2349813, EPI_ISL_2349814, EPI_ISL_2349815, EPI_ISL_2349816, EPI_ISL_2349817, EPI_ISL_2349818, EPI_ISL_2349819, EPI_ISL_2349820, EPI_ISL_2349821,
EPI_ISL_2349822, EPI_ISL_2349823, EPI_ISL_2349824, EPI_ISL_2349825, EPI_ISL_2349826, EPI_ISL_2349827, EPI_ISL_2349828, EPI_ISL_2349829, EPI_ISL_2349830, EPI_ISL_2349831, EPI_ISL_2349832, EPI_ISL_2349833, EPI_ISL_2349834, EPI_ISL_2349835, EPI_ISL_2349836, EPI_ISL_2349837, EPI_ISL_2349838, EPI_ISL_2349839, EPI_ISL_2349840, EPI_ISL_2349841, EPI_ISL_2349842, EPI_ISL_2349843,
EPI_ISL_2349844, EPI_ISL_2349845, EPI_ISL_2349846, EPI_ISL_2349847, EPI_ISL_2349848, EPI_ISL_2349849, EPI_ISL_2349850, EPI_ISL_2349851, EPI_ISL_2349852, EPI_ISL_2349853, EPI_ISL_2349854, EPI_ISL_2349855, EPI_ISL_2349856, EPI_ISL_2349857, EPI_ISL_2349858, EPI_ISL_2349859, EPI_ISL_2349860, EPI_ISL_2349861, EPI_ISL_2349862, EPI_ISL_2349863, EPI_ISL_2349864, EPI_ISL_2349865,
EPI_ISL_2349866, EPI_ISL_2349867, EPI_ISL_2349868, EPI_ISL_2349869, EPI_ISL_2349870, EPI_ISL_2349871, EPI_ISL_2349872, EPI_ISL_2349873, EPI_ISL_2349874, EPI_ISL_2349875, EPI_ISL_2349876, EPI_ISL_2349877, EPI_ISL_2349878, EPI_ISL_2508617, EPI_ISL_2508618, EPI_ISL_2508619, EPI_ISL_2508620, EPI_ISL_2508621, EPI_ISL_2508622, EPI_ISL_2508623, EPI_ISL_2508624, EPI_ISL_2508625,
EPI_ISL_2508626, EPI_ISL_2508627, EPI_ISL_2508628, EPI_ISL_2508629, EPI_ISL_2508630, EPI_ISL_2508631, EPI_ISL_2508632, EPI_ISL_2508633, EPI_ISL_2508634, EPI_ISL_2508635, EPI_ISL_2508636, EPI_ISL_2508637, EPI_ISL_2508638, EPI_ISL_2508639, EPI_ISL_2508640, EPI_ISL_2508641, EPI_ISL_2508642, EPI_ISL_2508643, EPI_ISL_2508644, EPI_ISL_2508645, EPI_ISL_2508646, EPI_ISL_2508647,
EPI_ISL_2508648, EPI_ISL_2508649, EPI_ISL_2508650, EPI_ISL_2508651, EPI_ISL_2508652, EPI_ISL_2508653, EPI_ISL_2508654, EPI_ISL_2508655, EPI_ISL_2508656, EPI_ISL_2508657, EPI_ISL_2508658, EPI_ISL_2508659, EPI_ISL_2508660, EPI_ISL_2508661, EPI_ISL_2508662, EPI_ISL_2508663, EPI_ISL_2508664, EPI_ISL_2508665, EPI_ISL_2508666, EPI_ISL_2508667, EPI_ISL_2508668, EPI_ISL_2508669,
EPI_ISL_2508670, EPI_ISL_2508671, EPI_ISL_2508672, EPI_ISL_2508673, EPI_ISL_2508674, EPI_ISL_2508675, EPI_ISL_2508676, EPI_ISL_2508677, EPI_ISL_2508678, EPI_ISL_2508679, EPI_ISL_2508680, EPI_ISL_2508681, EPI_ISL_2508682, EPI_ISL_2508683, EPI_ISL_2508684, EPI_ISL_2508685, EPI_ISL_2508686, EPI_ISL_2508687, EPI_ISL_2508688, EPI_ISL_2508689, EPI_ISL_2508690, EPI_ISL_2508691,
EPI_ISL_2508692, EPI_ISL_2508693, EPI_ISL_2508694, EPI_ISL_2508695, EPI_ISL_2508696, EPI_ISL_2508697, EPI_ISL_2508698, EPI_ISL_2508699, EPI_ISL_2508700, EPI_ISL_2508701, EPI_ISL_2508702, EPI_ISL_2508703, EPI_ISL_2508704, EPI_ISL_2508705, EPI_ISL_2508706, EPI_ISL_2508707, EPI_ISL_2508708, EPI_ISL_2508709, EPI_ISL_2508710, EPI_ISL_2508711, EPI_ISL_2508712, EPI_ISL_2508713,
EPI_ISL_2508714, EPI_ISL_2508715, EPI_ISL_2508716, EPI_ISL_2508717, EPI_ISL_2508718, EPI_ISL_2508719, EPI_ISL_2508720, EPI_ISL_2508721, EPI_ISL_2508722, EPI_ISL_2508723, EPI_ISL_2508724, EPI_ISL_2508725, EPI_ISL_2508726, EPI_ISL_2508727, EPI_ISL_2508728, EPI_ISL_2508729, EPI_ISL_2508730, EPI_ISL_2508731, EPI_ISL_2508732, EPI_ISL_2508733, EPI_ISL_2508734, EPI_ISL_2508735,
EPI_ISL_2508736, EPI_ISL_2508737, EPI_ISL_2508738, EPI_ISL_2508739, EPI_ISL_2508740, EPI_ISL_2508741, EPI_ISL_2508742, EPI_ISL_2508743, EPI_ISL_2508744, EPI_ISL_2508745, EPI_ISL_2508746, EPI_ISL_2508747, EPI_ISL_2508748, EPI_ISL_2508749, EPI_ISL_2508750, EPI_ISL_2508751, EPI_ISL_2508752, EPI_ISL_2508753, EPI_ISL_2508754, EPI_ISL_2508755, EPI_ISL_2508756, EPI_ISL_2508757,
EPI_ISL_2508758, EPI_ISL_2508759, EPI_ISL_2508760, EPI_ISL_2508761, EPI_ISL_2508762, EPI_ISL_2508763, EPI_ISL_2508764, EPI_ISL_2508765, EPI_ISL_2508766, EPI_ISL_2508767, EPI_ISL_2508768, EPI_ISL_2508769, EPI_ISL_2508770, EPI_ISL_2508771, EPI_ISL_2508772, EPI_ISL_2508773, EPI_ISL_2508774, EPI_ISL_2508775, EPI_ISL_2508776, EPI_ISL_2508777, EPI_ISL_2508778, EPI_ISL_2508779,
EPI_ISL_2508780, EPI_ISL_2508781, EPI_ISL_2508782, EPI_ISL_2508783, EPI_ISL_2508784, EPI_ISL_2508785, EPI_ISL_2508786, EPI_ISL_2508787, EPI_ISL_2508788, EPI_ISL_2508789, EPI_ISL_2508790, EPI_ISL_2508791, EPI_ISL_2508792, EPI_ISL_2508793, EPI_ISL_2508794, EPI_ISL_2508795, EPI_ISL_2508796, EPI_ISL_2508797, EPI_ISL_2508798, EPI_ISL_2508799, EPI_ISL_2508800, EPI_ISL_2508801,
EPI_ISL_2508802, EPI_ISL_2508803, EPI_ISL_2508804, EPI_ISL_2508805, EPI_ISL_2508806, EPI_ISL_2508807, EPI_ISL_2508808, EPI_ISL_2508809, EPI_ISL_2508810, EPI_ISL_2508811, EPI_ISL_2508812, EPI_ISL_2508813, EPI_ISL_2508814, EPI_ISL_2508815, EPI_ISL_2508816, EPI_ISL_2508817, EPI_ISL_2508818, EPI_ISL_2508819, EPI_ISL_2508820, EPI_ISL_2508821, EPI_ISL_2508822, EPI_ISL_2508823,
EPI_ISL_2508824, EPI_ISL_2508825, EPI_ISL_2508826, EPI_ISL_2508827, EPI_ISL_2508828, EPI_ISL_2508829, EPI_ISL_2508830, EPI_ISL_2508831, EPI_ISL_2508832, EPI_ISL_2508833, EPI_ISL_2508834, EPI_ISL_2508835, EPI_ISL_2508836, EPI_ISL_2508837, EPI_ISL_2508838, EPI_ISL_2508839, EPI_ISL_2508840, EPI_ISL_2508841, EPI_ISL_2508842, EPI_ISL_2508843, EPI_ISL_2508844, EPI_ISL_2508845,
EPI_ISL_2508846, EPI_ISL_2508847, EPI_ISL_2508848, EPI_ISL_2508849, EPI_ISL_2508850, EPI_ISL_2508851, EPI_ISL_2508852, EPI_ISL_2508853, EPI_ISL_2508854, EPI_ISL_2508855, EPI_ISL_2508856, EPI_ISL_2508857, EPI_ISL_2508858, EPI_ISL_2508859, EPI_ISL_2508860, EPI_ISL_2508861, EPI_ISL_2508862, EPI_ISL_2508863, EPI_ISL_2508864, EPI_ISL_2508865, EPI_ISL_2508866, EPI_ISL_2508867,
EPI_ISL_2508868, EPI_ISL_2508869, EPI_ISL_2508870, EPI_ISL_2508871, EPI_ISL_2508872, EPI_ISL_2508873, EPI_ISL_2508874, EPI_ISL_2508875, EPI_ISL_2508876, EPI_ISL_2508877, EPI_ISL_2508878, EPI_ISL_2508879, EPI_ISL_2508880, EPI_ISL_2508881, EPI_ISL_2508882, EPI_ISL_2508883, EPI_ISL_2508884, EPI_ISL_2508885, EPI_ISL_2508886, EPI_ISL_2508887, EPI_ISL_2508888, EPI_ISL_2508889,
EPI_ISL_2508890, EPI_ISL_2508891, EPI_ISL_2508892, EPI_ISL_2508893, EPI_ISL_2508894, EPI_ISL_2508895, EPI_ISL_2508896, EPI_ISL_2508897, EPI_ISL_2508898, EPI_ISL_2508899, EPI_ISL_2508900, EPI_ISL_2508901, EPI_ISL_2508902, EPI_ISL_2508903, EPI_ISL_2508904, EPI_ISL_2508905, EPI_ISL_2508906, EPI_ISL_2508907, EPI_ISL_2508908, EPI_ISL_2508909, EPI_ISL_2508910, EPI_ISL_2508911,
EPI_ISL_2508912, EPI_ISL_2508913, EPI_ISL_2508914, EPI_ISL_2508915, EPI_ISL_2508916, EPI_ISL_2508917, EPI_ISL_2508918, EPI_ISL_2508919, EPI_ISL_2508920, EPI_ISL_2508921, EPI_ISL_2508922, EPI_ISL_2508923, EPI_ISL_2508924, EPI_ISL_2508925, EPI_ISL_2508926, EPI_ISL_2508927, EPI_ISL_2508928, EPI_ISL_2508929, EPI_ISL_2508930, EPI_ISL_2508931, EPI_ISL_2508932, EPI_ISL_2508933,
EPI_ISL_2508934, EPI_ISL_2508935, EPI_ISL_2508936, EPI_ISL_2508937, EPI_ISL_2508938, EPI_ISL_2508939, EPI_ISL_2508940, EPI_ISL_2508941, EPI_ISL_2508942, EPI_ISL_2508943, EPI_ISL_2508944, EPI_ISL_2508945, EPI_ISL_2508946, EPI_ISL_2508947, EPI_ISL_2508948, EPI_ISL_2508949, EPI_ISL_2508950, EPI_ISL_2508951, EPI_ISL_2508952, EPI_ISL_2508953, EPI_ISL_2508954, EPI_ISL_2508955,
EPI_ISL_2508956, EPI_ISL_2508957, EPI_ISL_2508958, EPI_ISL_2508959, EPI_ISL_2508960, EPI_ISL_2508961, EPI_ISL_2508962, EPI_ISL_2508963, EPI_ISL_2508964, EPI_ISL_2508965, EPI_ISL_2508966, EPI_ISL_2508967, EPI_ISL_2508968, EPI_ISL_2508969, EPI_ISL_2508970, EPI_ISL_2508971, EPI_ISL_2508972, EPI_ISL_2508973, EPI_ISL_2508974, EPI_ISL_2508975, EPI_ISL_2508976, EPI_ISL_2508977,
EPI_ISL_2508978, EPI_ISL_2508979, EPI_ISL_2508980, EPI_ISL_2508981, EPI_ISL_2508982, EPI_ISL_2508983, EPI_ISL_2508984, EPI_ISL_2508985, EPI_ISL_2508986, EPI_ISL_2508987, EPI_ISL_2508988, EPI_ISL_2508989, EPI_ISL_2508990, EPI_ISL_2508991, EPI_ISL_2508992, EPI_ISL_2508993, EPI_ISL_2508994, EPI_ISL_2508995, EPI_ISL_2508996, EPI_ISL_2508997, EPI_ISL_2508998, EPI_ISL_2508999,
EPI_ISL_2509000, EPI_ISL_2509001, EPI_ISL_2509002, EPI_ISL_2509003, EPI_ISL_2509004, EPI_ISL_2509005, EPI_ISL_2509006, EPI_ISL_2509007, EPI_ISL_2509008, EPI_ISL_2509009, EPI_ISL_2509010, EPI_ISL_2509011, EPI_ISL_2509012, EPI_ISL_2509013, EPI_ISL_2509014, EPI_ISL_2509015, EPI_ISL_2509016, EPI_ISL_2509017, EPI_ISL_2509018, EPI_ISL_2509019, EPI_ISL_2509020, EPI_ISL_2509021,
EPI_ISL_2509022, EPI_ISL_2509023, EPI_ISL_2509024, EPI_ISL_2509025, EPI_ISL_2509026, EPI_ISL_2509027, EPI_ISL_2509028, EPI_ISL_2509029, EPI_ISL_2509030, EPI_ISL_2509031, EPI_ISL_2509032, EPI_ISL_2509033, EPI_ISL_2509034, EPI_ISL_2509035, EPI_ISL_2509036, EPI_ISL_2509037, EPI_ISL_2509038, EPI_ISL_2509039, EPI_ISL_2509040, EPI_ISL_2509041, EPI_ISL_2509042, EPI_ISL_2509043,
EPI_ISL_2509044, EPI_ISL_2509045, EPI_ISL_2509046, EPI_ISL_2509047, EPI_ISL_2509048, EPI_ISL_2509049, EPI_ISL_2509050, EPI_ISL_2509051, EPI_ISL_2509052, EPI_ISL_2509053, EPI_ISL_2509054, EPI_ISL_2509055, EPI_ISL_2509056, EPI_ISL_2509057, EPI_ISL_2509058, EPI_ISL_2509059, EPI_ISL_2509060, EPI_ISL_2509061, EPI_ISL_2509062, EPI_ISL_2509063, EPI_ISL_2509064, EPI_ISL_2509065,
EPI_ISL_2509066, EPI_ISL_2509067, EPI_ISL_2509068, EPI_ISL_2509069, EPI_ISL_2509070, EPI_ISL_2509071, EPI_ISL_2509072, EPI_ISL_2509073, EPI_ISL_2509074, EPI_ISL_2509075, EPI_ISL_2509076, EPI_ISL_2509077, EPI_ISL_2509078, EPI_ISL_2509079, EPI_ISL_2509080, EPI_ISL_2509081, EPI_ISL_2509082, EPI_ISL_2509083, EPI_ISL_2509084, EPI_ISL_2509085, EPI_ISL_2509086, EPI_ISL_2509087,
EPI_ISL_2509088, EPI_ISL_2509089, EPI_ISL_2509090, EPI_ISL_2509091, EPI_ISL_2509092, EPI_ISL_2509093, EPI_ISL_2509094, EPI_ISL_2509095, EPI_ISL_2509096, EPI_ISL_2509097, EPI_ISL_2509098, EPI_ISL_2509099, EPI_ISL_2509100, EPI_ISL_2509101, EPI_ISL_2509102, EPI_ISL_2509103, EPI_ISL_2509104, EPI_ISL_2509105, EPI_ISL_2509106, EPI_ISL_2509107, EPI_ISL_2509108, EPI_ISL_2509109,
EPI_ISL_2509110, EPI_ISL_2509111, EPI_ISL_2621885, EPI_ISL_2621886, EPI_ISL_2621887, EPI_ISL_2621888, EPI_ISL_2621889, EPI_ISL_2621890, EPI_ISL_2621891, EPI_ISL_2621892, EPI_ISL_2621893, EPI_ISL_2621894, EPI_ISL_2621895, EPI_ISL_2621896, EPI_ISL_2621897, EPI_ISL_2621898, EPI_ISL_2621899, EPI_ISL_2621900, EPI_ISL_2621901, EPI_ISL_2621902, EPI_ISL_2621903, EPI_ISL_2621904,
EPI_ISL_2621905, EPI_ISL_2621906, EPI_ISL_2621907, EPI_ISL_2621908, EPI_ISL_2621909, EPI_ISL_2621910, EPI_ISL_2621911, EPI_ISL_2621912, EPI_ISL_2621913, EPI_ISL_2621914, EPI_ISL_2621915, EPI_ISL_2621916, EPI_ISL_2621917, EPI_ISL_2621918, EPI_ISL_2621919, EPI_ISL_2621920, EPI_ISL_2621921, EPI_ISL_2621922, EPI_ISL_2621923, EPI_ISL_2621924, EPI_ISL_2621925, EPI_ISL_2621926,
EPI_ISL_2621927, EPI_ISL_2621928, EPI_ISL_2621929, EPI_ISL_2621930, EPI_ISL_2621931, EPI_ISL_2621932, EPI_ISL_2621933, EPI_ISL_2621934, EPI_ISL_2621935, EPI_ISL_2621936, EPI_ISL_2621937, EPI_ISL_2621938, EPI_ISL_2621939, EPI_ISL_2621940, EPI_ISL_2621941, EPI_ISL_2621942, EPI_ISL_2621943, EPI_ISL_2621944, EPI_ISL_2621945, EPI_ISL_2621946, EPI_ISL_2621947, EPI_ISL_2621948,
EPI_ISL_2621949, EPI_ISL_2621950, EPI_ISL_2621951, EPI_ISL_2621952, EPI_ISL_2621953, EPI_ISL_2621954, EPI_ISL_2621955, EPI_ISL_2621956, EPI_ISL_2621957, EPI_ISL_2621958, EPI_ISL_2621959, EPI_ISL_2621960, EPI_ISL_2621961, EPI_ISL_2621962, EPI_ISL_2621963, EPI_ISL_2621964, EPI_ISL_2627533, EPI_ISL_2627534, EPI_ISL_2627535, EPI_ISL_2627536, EPI_ISL_2627537, EPI_ISL_2627538,
EPI_ISL_2627539, EPI_ISL_2627540, EPI_ISL_2627541, EPI_ISL_2627542, EPI_ISL_2627543, EPI_ISL_2627544, EPI_ISL_2627545, EPI_ISL_2627546, EPI_ISL_2627547, EPI_ISL_2627548, EPI_ISL_2627549, EPI_ISL_2627550, EPI_ISL_2627551, EPI_ISL_2627552, EPI_ISL_2627553, EPI_ISL_2627554, EPI_ISL_2627555, EPI_ISL_2627556, EPI_ISL_2627557, EPI_ISL_2627558, EPI_ISL_2627559, EPI_ISL_2627560,
EPI_ISL_2627561, EPI_ISL_2627562, EPI_ISL_2627563, EPI_ISL_2627564, EPI_ISL_2627565, EPI_ISL_2627566, EPI_ISL_2627567, EPI_ISL_2627568, EPI_ISL_2627569, EPI_ISL_2627570, EPI_ISL_2627571, EPI_ISL_2627572, EPI_ISL_2627573, EPI_ISL_2627574, EPI_ISL_2627575, EPI_ISL_2627576, EPI_ISL_2627577, EPI_ISL_2627578, EPI_ISL_2627579, EPI_ISL_2627580, EPI_ISL_2710081, EPI_ISL_2710082,
EPI_ISL_2710083, EPI_ISL_2710084, EPI_ISL_2710085, EPI_ISL_2710086, EPI_ISL_2710087, EPI_ISL_2710088, EPI_ISL_2710089, EPI_ISL_2710090, EPI_ISL_2710091, EPI_ISL_2710092, EPI_ISL_2710093, EPI_ISL_2710094, EPI_ISL_2710095, EPI_ISL_2710096, EPI_ISL_2710097, EPI_ISL_2710098, EPI_ISL_2710099, EPI_ISL_2710100, EPI_ISL_2710101, EPI_ISL_2710102, EPI_ISL_2710103, EPI_ISL_2710104,
EPI_ISL_2710105, EPI_ISL_2710106, EPI_ISL_2710107, EPI_ISL_2710108, EPI_ISL_2710109, EPI_ISL_2710110, EPI_ISL_2710111, EPI_ISL_2710112, EPI_ISL_2710113, EPI_ISL_2710114, EPI_ISL_2710115, EPI_ISL_2710116, EPI_ISL_2710117, EPI_ISL_2710118, EPI_ISL_2710119, EPI_ISL_2710120, EPI_ISL_2710121, EPI_ISL_2710122, EPI_ISL_2710123, EPI_ISL_2710124, EPI_ISL_2710125, EPI_ISL_2710126,
EPI_ISL_2710127, EPI_ISL_2710128, EPI_ISL_2710129, EPI_ISL_2710130, EPI_ISL_2710131, EPI_ISL_2710132, EPI_ISL_2710133, EPI_ISL_2710134, EPI_ISL_2710135, EPI_ISL_2710136, EPI_ISL_2710137, EPI_ISL_2710138, EPI_ISL_2710139, EPI_ISL_2710140, EPI_ISL_2710141, EPI_ISL_2710142, EPI_ISL_2710143, EPI_ISL_2710144, EPI_ISL_2710145, EPI_ISL_2710146, EPI_ISL_2710147, EPI_ISL_2710148,
EPI_ISL_2710149, EPI_ISL_2710150, EPI_ISL_2710151, EPI_ISL_2710152, EPI_ISL_2710153, EPI_ISL_2710154, EPI_ISL_2710155, EPI_ISL_2710156, EPI_ISL_2710157, EPI_ISL_2710158, EPI_ISL_2710159, EPI_ISL_2710160, EPI_ISL_2710161, EPI_ISL_2710162, EPI_ISL_2710163, EPI_ISL_2710164, EPI_ISL_2710165, EPI_ISL_2710166, EPI_ISL_2710167, EPI_ISL_2710168, EPI_ISL_2710169, EPI_ISL_2710170,
EPI_ISL_2710171, EPI_ISL_2790295, EPI_ISL_2790296, EPI_ISL_2790297, EPI_ISL_2790298, EPI_ISL_2790299, EPI_ISL_2790300, EPI_ISL_2790301, EPI_ISL_2790302, EPI_ISL_2790303, EPI_ISL_2790304, EPI_ISL_2790305, EPI_ISL_2790332, EPI_ISL_2790333, EPI_ISL_2790334, EPI_ISL_2790335, EPI_ISL_2790336, EPI_ISL_2790337, EPI_ISL_2790338, EPI_ISL_2790339, EPI_ISL_2790340, EPI_ISL_2790341,
EPI_ISL_2790342, EPI_ISL_2790343, EPI_ISL_2790344, EPI_ISL_2790345, EPI_ISL_2790346, EPI_ISL_2790347, EPI_ISL_2790348, EPI_ISL_2790349, EPI_ISL_2790350, EPI_ISL_2790351, EPI_ISL_2790352, EPI_ISL_2790353, EPI_ISL_2790354, EPI_ISL_2790355, EPI_ISL_2790356, EPI_ISL_2790357, EPI_ISL_2790358, EPI_ISL_2790359, EPI_ISL_2790360, EPI_ISL_2790361, EPI_ISL_2790362, EPI_ISL_2790363,
EPI_ISL_2790364, EPI_ISL_2790365, EPI_ISL_2790366, EPI_ISL_2790367, EPI_ISL_2790368, EPI_ISL_2790369, EPI_ISL_2790370, EPI_ISL_2790371, EPI_ISL_2790372, EPI_ISL_2790373, EPI_ISL_2790374, EPI_ISL_2790375, EPI_ISL_2790376, EPI_ISL_2790377, EPI_ISL_2790378, EPI_ISL_2790379, EPI_ISL_2790380, EPI_ISL_2790381, EPI_ISL_2790382, EPI_ISL_2790383, EPI_ISL_2790384, EPI_ISL_2790385,
EPI_ISL_2790386, EPI_ISL_2790387, EPI_ISL_2820263, EPI_ISL_2820264, EPI_ISL_2820265, EPI_ISL_2820266, EPI_ISL_2820267, EPI_ISL_2820268, EPI_ISL_2820269, EPI_ISL_2820270, EPI_ISL_2820271, EPI_ISL_2820272, EPI_ISL_2820273, EPI_ISL_2820274, EPI_ISL_2820275, EPI_ISL_2820276, EPI_ISL_2820277, EPI_ISL_2820278, EPI_ISL_2820279, EPI_ISL_2820280, EPI_ISL_2820281, EPI_ISL_2820282,
EPI_ISL_2820283, EPI_ISL_2820284, EPI_ISL_2820285, EPI_ISL_2820286, EPI_ISL_2820287, EPI_ISL_2887781, EPI_ISL_2887782, EPI_ISL_2887783, EPI_ISL_2887784, EPI_ISL_2887785, EPI_ISL_2887786, EPI_ISL_2887787, EPI_ISL_2887788, EPI_ISL_2887789, EPI_ISL_2887790, EPI_ISL_2887791, EPI_ISL_2887792, EPI_ISL_2887793, EPI_ISL_2887794, EPI_ISL_2887795, EPI_ISL_2887796, EPI_ISL_2978680,
EPI_ISL_2978681, EPI_ISL_2982227, EPI_ISL_2982228, EPI_ISL_2982229, EPI_ISL_2982230, EPI_ISL_2982231, EPI_ISL_2982232, EPI_ISL_2982233, EPI_ISL_2982234, EPI_ISL_2982235, EPI_ISL_2982236, EPI_ISL_2982237, EPI_ISL_2982238, EPI_ISL_2982239, EPI_ISL_2982240, EPI_ISL_2982241, EPI_ISL_2982242, EPI_ISL_2982243, EPI_ISL_2982244, EPI_ISL_2982245, EPI_ISL_2982246, EPI_ISL_2982247,
EPI_ISL_2982248, EPI_ISL_2982249, EPI_ISL_2982250, EPI_ISL_2982251, EPI_ISL_2982252, EPI_ISL_2982253, EPI_ISL_2982254, EPI_ISL_2982255, EPI_ISL_2982256, EPI_ISL_2982257, EPI_ISL_2982258, EPI_ISL_2982259, EPI_ISL_3007538, EPI_ISL_3007540, EPI_ISL_3007542, EPI_ISL_3007544, EPI_ISL_3007546, EPI_ISL_3007548, EPI_ISL_3007550, EPI_ISL_3007552, EPI_ISL_3007554, EPI_ISL_3007556,
EPI_ISL_3007558, EPI_ISL_3007560, EPI_ISL_3007562, EPI_ISL_3007564, EPI_ISL_3007566, EPI_ISL_3007568, EPI_ISL_3007570, EPI_ISL_3007572, EPI_ISL_3007574, EPI_ISL_3007576, EPI_ISL_3007578, EPI_ISL_3007580, EPI_ISL_3007582, EPI_ISL_3007585, EPI_ISL_3007587, EPI_ISL_3007589, EPI_ISL_3008927, EPI_ISL_3008928, EPI_ISL_3008929, EPI_ISL_3008930, EPI_ISL_3008931, EPI_ISL_3008932,
EPI_ISL_3008933, EPI_ISL_3008934, EPI_ISL_3008935, EPI_ISL_3008936, EPI_ISL_3008937, EPI_ISL_3008938, EPI_ISL_3008939, EPI_ISL_3008940, EPI_ISL_3008941, EPI_ISL_3008942, EPI_ISL_3008943, EPI_ISL_3008944, EPI_ISL_3008945, EPI_ISL_3008946, EPI_ISL_3008947, EPI_ISL_3008948, EPI_ISL_3008949, EPI_ISL_3008950, EPI_ISL_3008951, EPI_ISL_3008952, EPI_ISL_3008953, EPI_ISL_3008954,
EPI_ISL_3008955, EPI_ISL_3008956, EPI_ISL_3008957, EPI_ISL_3008958, EPI_ISL_3008959, EPI_ISL_3008960, EPI_ISL_3008961, EPI_ISL_3008962, EPI_ISL_3008963, EPI_ISL_3008964, EPI_ISL_3008965, EPI_ISL_3008966, EPI_ISL_3008967, EPI_ISL_3008968, EPI_ISL_3008969, EPI_ISL_3008970, EPI_ISL_3008971, EPI_ISL_3008972, EPI_ISL_3008973, EPI_ISL_3008974, EPI_ISL_3008975, EPI_ISL_3008976,
EPI_ISL_3008977, EPI_ISL_3008978, EPI_ISL_3008979, EPI_ISL_3008980, EPI_ISL_3008981, EPI_ISL_3008982, EPI_ISL_3008983, EPI_ISL_3008984, EPI_ISL_3008985, EPI_ISL_3008986, EPI_ISL_3008987, EPI_ISL_3008988, EPI_ISL_3008989, EPI_ISL_3008990, EPI_ISL_3008991, EPI_ISL_3008992, EPI_ISL_3008993, EPI_ISL_3008994, EPI_ISL_3008995, EPI_ISL_3008996, EPI_ISL_3008997, EPI_ISL_3008998,
EPI_ISL_3008999, EPI_ISL_3009000, EPI_ISL_3009001, EPI_ISL_3009002, EPI_ISL_3009003, EPI_ISL_3009004, EPI_ISL_3009005, EPI_ISL_3009006, EPI_ISL_3009007, EPI_ISL_3009008, EPI_ISL_3009009, EPI_ISL_3009010, EPI_ISL_3009011, EPI_ISL_3009012, EPI_ISL_3009013, EPI_ISL_3009014, EPI_ISL_3009015, EPI_ISL_3009016, EPI_ISL_3009017, EPI_ISL_3009018, EPI_ISL_3009019, EPI_ISL_3009020,
EPI_ISL_3009021, EPI_ISL_3009022, EPI_ISL_3009023, EPI_ISL_3009024, EPI_ISL_3009025, EPI_ISL_3009026, EPI_ISL_3009027, EPI_ISL_3009028, EPI_ISL_3009029, EPI_ISL_3009030, EPI_ISL_3009031, EPI_ISL_3009032, EPI_ISL_3009033, EPI_ISL_3009034, EPI_ISL_3009035, EPI_ISL_3009036, EPI_ISL_3009037, EPI_ISL_3009038, EPI_ISL_3009039, EPI_ISL_3009040, EPI_ISL_3009041, EPI_ISL_3009042,
EPI_ISL_3009043, EPI_ISL_3009044, EPI_ISL_3009045, EPI_ISL_3009046, EPI_ISL_3009047, EPI_ISL_3026034, EPI_ISL_3026035, EPI_ISL_3026036, EPI_ISL_3026037, EPI_ISL_3026038, EPI_ISL_3026039, EPI_ISL_3052685, EPI_ISL_3052686, EPI_ISL_3052687, EPI_ISL_3052688, EPI_ISL_3052689, EPI_ISL_3052690, EPI_ISL_3052691, EPI_ISL_3052692, EPI_ISL_3052693, EPI_ISL_3052694, EPI_ISL_3052695,
EPI_ISL_3052696, EPI_ISL_3052697, EPI_ISL_3052698, EPI_ISL_3052699, EPI_ISL_3052700, EPI_ISL_3052701, EPI_ISL_3052702, EPI_ISL_3052704, EPI_ISL_3052705, EPI_ISL_3052706, EPI_ISL_3052707, EPI_ISL_3052708, EPI_ISL_3052709, EPI_ISL_3052710, EPI_ISL_3052711, EPI_ISL_3052712, EPI_ISL_3052713, EPI_ISL_3052714, EPI_ISL_3052715, EPI_ISL_3052716, EPI_ISL_3052717, EPI_ISL_3052718,
EPI_ISL_3052719, EPI_ISL_3052720, EPI_ISL_3052721, EPI_ISL_3052722, EPI_ISL_3052723, EPI_ISL_3052724, EPI_ISL_3052725, EPI_ISL_3052726, EPI_ISL_3052727, EPI_ISL_3052728, EPI_ISL_3052729, EPI_ISL_3052730, EPI_ISL_3052731, EPI_ISL_3052732, EPI_ISL_3052733, EPI_ISL_3052734, EPI_ISL_3052735, EPI_ISL_3052736, EPI_ISL_3052737, EPI_ISL_3052738, EPI_ISL_3052739, EPI_ISL_3052740,
EPI_ISL_3052741, EPI_ISL_3052742, EPI_ISL_3052743, EPI_ISL_3052744, EPI_ISL_3052745, EPI_ISL_3052746, EPI_ISL_3052747, EPI_ISL_3052748, EPI_ISL_3052749, EPI_ISL_3052750, EPI_ISL_3052751, EPI_ISL_3052752, EPI_ISL_3052753, EPI_ISL_3052754, EPI_ISL_3052755, EPI_ISL_3052756, EPI_ISL_3052757, EPI_ISL_3052758, EPI_ISL_3052759, EPI_ISL_3052760, EPI_ISL_3052761, EPI_ISL_3052762,
EPI_ISL_3052763, EPI_ISL_3052764, EPI_ISL_3052765, EPI_ISL_3052766, EPI_ISL_3052767, EPI_ISL_3052768, EPI_ISL_3052769, EPI_ISL_3052770, EPI_ISL_3052771, EPI_ISL_3052772, EPI_ISL_3052773, EPI_ISL_3052774, EPI_ISL_3052775, EPI_ISL_3052776, EPI_ISL_3052777, EPI_ISL_3052778, EPI_ISL_3052779, EPI_ISL_3052780, EPI_ISL_3052781, EPI_ISL_3052782, EPI_ISL_3052783, EPI_ISL_3052784,
EPI_ISL_3052785, EPI_ISL_3052786, EPI_ISL_3052787, EPI_ISL_3052788, EPI_ISL_3052789, EPI_ISL_3052790, EPI_ISL_3052791, EPI_ISL_3052792, EPI_ISL_3052793, EPI_ISL_3052794, EPI_ISL_3052795, EPI_ISL_3052796, EPI_ISL_3052797, EPI_ISL_3052798, EPI_ISL_3052799, EPI_ISL_3052800, EPI_ISL_3052801, EPI_ISL_3052802, EPI_ISL_3052803, EPI_ISL_3052804, EPI_ISL_3052805, EPI_ISL_3052806,
EPI_ISL_3052807, EPI_ISL_3052808, EPI_ISL_3052809, EPI_ISL_3052810, EPI_ISL_3052811, EPI_ISL_3052812, EPI_ISL_3052813, EPI_ISL_3052814, EPI_ISL_3052815, EPI_ISL_3052816, EPI_ISL_3052817, EPI_ISL_3052818, EPI_ISL_3052819, EPI_ISL_3052820, EPI_ISL_3052821, EPI_ISL_3052822, EPI_ISL_3052823, EPI_ISL_3052824, EPI_ISL_3052825, EPI_ISL_3052826, EPI_ISL_3052827, EPI_ISL_3052828,
EPI_ISL_3052829, EPI_ISL_3052830, EPI_ISL_3052831, EPI_ISL_3052832, EPI_ISL_3052833, EPI_ISL_3052834, EPI_ISL_3052835, EPI_ISL_3052836, EPI_ISL_3052837, EPI_ISL_3052838, EPI_ISL_3052839, EPI_ISL_3052840, EPI_ISL_3052841, EPI_ISL_3052842, EPI_ISL_3052843, EPI_ISL_3052844, EPI_ISL_3052845, EPI_ISL_3052846, EPI_ISL_3052847, EPI_ISL_3052848, EPI_ISL_3052849, EPI_ISL_3052850,
EPI_ISL_3052851, EPI_ISL_3052852, EPI_ISL_3052853, EPI_ISL_3052854, EPI_ISL_3052855, EPI_ISL_3052856, EPI_ISL_3052857, EPI_ISL_3052858, EPI_ISL_3052859, EPI_ISL_3052860, EPI_ISL_3052861, EPI_ISL_3052862, EPI_ISL_3052863, EPI_ISL_3052864, EPI_ISL_3052865, EPI_ISL_3052866, EPI_ISL_3052867, EPI_ISL_3052868, EPI_ISL_3052869, EPI_ISL_3052870, EPI_ISL_3052871, EPI_ISL_3052872,
EPI_ISL_3052873, EPI_ISL_3052874, EPI_ISL_3052875, EPI_ISL_3052876, EPI_ISL_3052877, EPI_ISL_3052878, EPI_ISL_3052879, EPI_ISL_3052880, EPI_ISL_3052881, EPI_ISL_3052882, EPI_ISL_3052883, EPI_ISL_3052884, EPI_ISL_3052885, EPI_ISL_3052886, EPI_ISL_3052887, EPI_ISL_3052888, EPI_ISL_3052889, EPI_ISL_3052890, EPI_ISL_3052891, EPI_ISL_3052892, EPI_ISL_3052893, EPI_ISL_3052894,
EPI_ISL_3052895, EPI_ISL_3052896, EPI_ISL_3052897, EPI_ISL_3052898, EPI_ISL_3052899, EPI_ISL_3052900, EPI_ISL_3052901, EPI_ISL_3052902, EPI_ISL_3052903, EPI_ISL_3052904, EPI_ISL_3087335, EPI_ISL_3087336, EPI_ISL_3087337, EPI_ISL_3087338, EPI_ISL_3087339, EPI_ISL_3087340, EPI_ISL_3087341, EPI_ISL_3087342, EPI_ISL_3087343, EPI_ISL_3087344, EPI_ISL_3087345, EPI_ISL_3087346,
EPI_ISL_3087347, EPI_ISL_3087348, EPI_ISL_3087349, EPI_ISL_3087350, EPI_ISL_3087351, EPI_ISL_3087352, EPI_ISL_3087353, EPI_ISL_3087354, EPI_ISL_3087355, EPI_ISL_3087356, EPI_ISL_3087357, EPI_ISL_3087358, EPI_ISL_3087359, EPI_ISL_3087360, EPI_ISL_3087361, EPI_ISL_3087362, EPI_ISL_3087363, EPI_ISL_3087364, EPI_ISL_3087365, EPI_ISL_3087366, EPI_ISL_3087367, EPI_ISL_3087368,
EPI_ISL_3087369, EPI_ISL_3087370, EPI_ISL_3087371, EPI_ISL_3087372, EPI_ISL_3087373, EPI_ISL_3087374, EPI_ISL_3087375, EPI_ISL_3087376, EPI_ISL_3087377, EPI_ISL_3087378, EPI_ISL_3087379, EPI_ISL_3087380, EPI_ISL_3087381, EPI_ISL_3087382, EPI_ISL_3087383, EPI_ISL_3087384, EPI_ISL_3087385, EPI_ISL_3087386, EPI_ISL_3087387, EPI_ISL_3087388, EPI_ISL_3087389, EPI_ISL_3087390,
EPI_ISL_3087391, EPI_ISL_3087392, EPI_ISL_3087393, EPI_ISL_3087394, EPI_ISL_3087395, EPI_ISL_3087396, EPI_ISL_3087397, EPI_ISL_3087398, EPI_ISL_3087399, EPI_ISL_3087400, EPI_ISL_3087401, EPI_ISL_3087402, EPI_ISL_3087403, EPI_ISL_3087404, EPI_ISL_3087405, EPI_ISL_3087406, EPI_ISL_3087407, EPI_ISL_3087408, EPI_ISL_3087409, EPI_ISL_3087410, EPI_ISL_3087411, EPI_ISL_3087412,
EPI_ISL_3087413, EPI_ISL_3087414, EPI_ISL_3087415, EPI_ISL_3087416, EPI_ISL_3087417, EPI_ISL_3087418, EPI_ISL_3087419, EPI_ISL_3087420, EPI_ISL_3087421, EPI_ISL_3087422, EPI_ISL_3087423, EPI_ISL_3087424, EPI_ISL_3087425, EPI_ISL_3087426, EPI_ISL_3087427, EPI_ISL_3087428, EPI_ISL_3087429, EPI_ISL_3087430, EPI_ISL_3087431, EPI_ISL_3087432, EPI_ISL_3087433, EPI_ISL_3087434,
EPI_ISL_3087435, EPI_ISL_3087436, EPI_ISL_3087437, EPI_ISL_3087438, EPI_ISL_3087439, EPI_ISL_3087440, EPI_ISL_3087441, EPI_ISL_3087442, EPI_ISL_3087443, EPI_ISL_3087444, EPI_ISL_3087445, EPI_ISL_3087446, EPI_ISL_3087447, EPI_ISL_3087448, EPI_ISL_3087449, EPI_ISL_3087450, EPI_ISL_3087451, EPI_ISL_3087452, EPI_ISL_3087453, EPI_ISL_3087454, EPI_ISL_3087455, EPI_ISL_3087456,
EPI_ISL_3087457, EPI_ISL_3087458, EPI_ISL_3087459, EPI_ISL_3087460, EPI_ISL_3087461, EPI_ISL_3087462, EPI_ISL_3087463, EPI_ISL_3087464, EPI_ISL_3087465, EPI_ISL_3087466, EPI_ISL_3087467, EPI_ISL_3087468, EPI_ISL_3087469, EPI_ISL_3087470, EPI_ISL_3087471, EPI_ISL_3087472, EPI_ISL_3087473, EPI_ISL_3087474, EPI_ISL_3087475, EPI_ISL_3087476, EPI_ISL_3087477, EPI_ISL_3087478,
EPI_ISL_3087479, EPI_ISL_3087480, EPI_ISL_3087481, EPI_ISL_3087482, EPI_ISL_3087483, EPI_ISL_3087484, EPI_ISL_3087485, EPI_ISL_3087486, EPI_ISL_3087487, EPI_ISL_3087488, EPI_ISL_3087489, EPI_ISL_3087490, EPI_ISL_3087491, EPI_ISL_3087492, EPI_ISL_3087493, EPI_ISL_3087494, EPI_ISL_3087495, EPI_ISL_3087496, EPI_ISL_3087497, EPI_ISL_3087498, EPI_ISL_3087499, EPI_ISL_3087500,
EPI_ISL_3087501, EPI_ISL_3087502, EPI_ISL_3087503, EPI_ISL_3087504, EPI_ISL_3087505, EPI_ISL_3087506, EPI_ISL_3087507, EPI_ISL_3087508, EPI_ISL_3087509, EPI_ISL_3087510, EPI_ISL_3087511, EPI_ISL_3087512, EPI_ISL_3087513, EPI_ISL_3087514, EPI_ISL_3087515, EPI_ISL_3087516, EPI_ISL_3087517, EPI_ISL_3087518, EPI_ISL_3087519, EPI_ISL_3087520, EPI_ISL_3087521, EPI_ISL_3087522,
EPI_ISL_3087523, EPI_ISL_3087524, EPI_ISL_3087525, EPI_ISL_3087526, EPI_ISL_3087527, EPI_ISL_3087528, EPI_ISL_3087529, EPI_ISL_3087530, EPI_ISL_3087531, EPI_ISL_3087532, EPI_ISL_3087533, EPI_ISL_3087534, EPI_ISL_3087535, EPI_ISL_3087536, EPI_ISL_3087537, EPI_ISL_3087538, EPI_ISL_3087539, EPI_ISL_3087540, EPI_ISL_3087541, EPI_ISL_3087542, EPI_ISL_3087543, EPI_ISL_3087544,
EPI_ISL_3087545, EPI_ISL_3087546, EPI_ISL_3087547, EPI_ISL_3087548, EPI_ISL_3087549, EPI_ISL_3087550, EPI_ISL_3087551, EPI_ISL_3087552, EPI_ISL_3087553, EPI_ISL_3087554, EPI_ISL_3087555, EPI_ISL_3087556, EPI_ISL_3087557, EPI_ISL_3087558, EPI_ISL_3087559, EPI_ISL_3087560, EPI_ISL_3087561, EPI_ISL_3151827, EPI_ISL_3151828, EPI_ISL_3151829, EPI_ISL_3151830, EPI_ISL_3151831,
EPI_ISL_3151832, EPI_ISL_3151833, EPI_ISL_3151834, EPI_ISL_3151835, EPI_ISL_3151836, EPI_ISL_3151837, EPI_ISL_3151838, EPI_ISL_3151839, EPI_ISL_3151840, EPI_ISL_3151841, EPI_ISL_3151842, EPI_ISL_3151843, EPI_ISL_3151844, EPI_ISL_3151845, EPI_ISL_3151846, EPI_ISL_3151847, EPI_ISL_3151848, EPI_ISL_3151849, EPI_ISL_3151850, EPI_ISL_3151851, EPI_ISL_3151852, EPI_ISL_3151853,
EPI_ISL_3151854, EPI_ISL_3151855, EPI_ISL_3151856, EPI_ISL_3151857, EPI_ISL_3151858, EPI_ISL_3151859, EPI_ISL_3151860, EPI_ISL_3151861, EPI_ISL_3151862, EPI_ISL_3151863, EPI_ISL_3151864, EPI_ISL_3151865, EPI_ISL_3151866, EPI_ISL_3151867, EPI_ISL_3151868, EPI_ISL_3151869, EPI_ISL_3151870, EPI_ISL_3151871, EPI_ISL_3151872, EPI_ISL_3151873, EPI_ISL_3151874, EPI_ISL_3151875,
EPI_ISL_3151876, EPI_ISL_3151877, EPI_ISL_3151878, EPI_ISL_3151879, EPI_ISL_3151880, EPI_ISL_3151881, EPI_ISL_3151882, EPI_ISL_3151883, EPI_ISL_3151884, EPI_ISL_3151885, EPI_ISL_3151886, EPI_ISL_3151887, EPI_ISL_3151888, EPI_ISL_3151889, EPI_ISL_3151890, EPI_ISL_3151891, EPI_ISL_3151892, EPI_ISL_3151893, EPI_ISL_3151894, EPI_ISL_3151895, EPI_ISL_3151896, EPI_ISL_3151897,
EPI_ISL_3151898, EPI_ISL_3151899, EPI_ISL_3151900, EPI_ISL_3151901, EPI_ISL_3151902, EPI_ISL_3151903, EPI_ISL_3151904, EPI_ISL_3151905, EPI_ISL_3151906, EPI_ISL_3151907, EPI_ISL_3151908, EPI_ISL_3151909, EPI_ISL_3151910, EPI_ISL_3151911, EPI_ISL_3151912, EPI_ISL_3151913, EPI_ISL_3151914, EPI_ISL_3151915, EPI_ISL_3151916, EPI_ISL_3151917, EPI_ISL_3151918, EPI_ISL_3151919,
EPI_ISL_3151920, EPI_ISL_3151921, EPI_ISL_3151922, EPI_ISL_3151923, EPI_ISL_3151924, EPI_ISL_3151925, EPI_ISL_3151926, EPI_ISL_3151927, EPI_ISL_3151928, EPI_ISL_3151929, EPI_ISL_3151930, EPI_ISL_3151931, EPI_ISL_3151932, EPI_ISL_3151933, EPI_ISL_3151934, EPI_ISL_3151935, EPI_ISL_3151936, EPI_ISL_3151937, EPI_ISL_3151938, EPI_ISL_3151939, EPI_ISL_3151940, EPI_ISL_3151941,
EPI_ISL_3151942, EPI_ISL_3151943, EPI_ISL_3151944, EPI_ISL_3152444, EPI_ISL_3152445, EPI_ISL_3152446, EPI_ISL_3152447, EPI_ISL_3152448, EPI_ISL_3152449, EPI_ISL_3152450, EPI_ISL_3152451, EPI_ISL_3152452, EPI_ISL_3152453, EPI_ISL_3152454, EPI_ISL_3152455, EPI_ISL_3152456, EPI_ISL_3152457, EPI_ISL_3152458, EPI_ISL_3152459, EPI_ISL_3152460, EPI_ISL_3152461, EPI_ISL_3152462,
EPI_ISL_3152463, EPI_ISL_3152464, EPI_ISL_3152465, EPI_ISL_3152466, EPI_ISL_3152467, EPI_ISL_3152468, EPI_ISL_3152469, EPI_ISL_3152470, EPI_ISL_3152471, EPI_ISL_3152472, EPI_ISL_3152473, EPI_ISL_3152474, EPI_ISL_3152475, EPI_ISL_3152476, EPI_ISL_3152477, EPI_ISL_3152478, EPI_ISL_3152479, EPI_ISL_3152480, EPI_ISL_3152481, EPI_ISL_3152482, EPI_ISL_3152483, EPI_ISL_3152484,
EPI_ISL_3152485, EPI_ISL_3152486, EPI_ISL_3152487, EPI_ISL_3152488, EPI_ISL_3152489, EPI_ISL_3152490, EPI_ISL_3152491, EPI_ISL_3152492, EPI_ISL_3152493, EPI_ISL_3152494, EPI_ISL_3152495, EPI_ISL_3152496, EPI_ISL_3152497, EPI_ISL_3152498, EPI_ISL_3152499, EPI_ISL_3152500, EPI_ISL_3152501, EPI_ISL_3152502, EPI_ISL_3152503, EPI_ISL_3152504, EPI_ISL_3152505, EPI_ISL_3152506,
EPI_ISL_3152507, EPI_ISL_3152508, EPI_ISL_3152509, EPI_ISL_3152510, EPI_ISL_3152511, EPI_ISL_3152512, EPI_ISL_3152513, EPI_ISL_3152514, EPI_ISL_3152515, EPI_ISL_3152516, EPI_ISL_3152517, EPI_ISL_3152518, EPI_ISL_3152519, EPI_ISL_3152520, EPI_ISL_3152521, EPI_ISL_3152522, EPI_ISL_3152523, EPI_ISL_3152524, EPI_ISL_3152525, EPI_ISL_3152526, EPI_ISL_3152527, EPI_ISL_3152528,
EPI_ISL_3152529, EPI_ISL_3152530, EPI_ISL_3152531, EPI_ISL_3152532, EPI_ISL_3152533, EPI_ISL_3152534, EPI_ISL_3152535, EPI_ISL_3152536, EPI_ISL_3152537, EPI_ISL_3152538, EPI_ISL_3152539, EPI_ISL_3152540, EPI_ISL_3152541, EPI_ISL_3152542, EPI_ISL_3152543, EPI_ISL_3152544, EPI_ISL_3152545, EPI_ISL_3152546, EPI_ISL_3152547, EPI_ISL_3152548, EPI_ISL_3152549, EPI_ISL_3152550,
EPI_ISL_3152551, EPI_ISL_3152552, EPI_ISL_3152553, EPI_ISL_3152554, EPI_ISL_3152555, EPI_ISL_3152556, EPI_ISL_3152557, EPI_ISL_3152558, EPI_ISL_3185969, EPI_ISL_3185970, EPI_ISL_3185971, EPI_ISL_3185972, EPI_ISL_3185973, EPI_ISL_3185974, EPI_ISL_3185975, EPI_ISL_3185976, EPI_ISL_3185977, EPI_ISL_3185978, EPI_ISL_3185979, EPI_ISL_3185980, EPI_ISL_3185981, EPI_ISL_3185982,
EPI_ISL_3185983, EPI_ISL_3185984, EPI_ISL_3185985, EPI_ISL_3185986, EPI_ISL_3185987, EPI_ISL_3185988, EPI_ISL_3185989, EPI_ISL_3185990, EPI_ISL_3185991, EPI_ISL_3185992, EPI_ISL_3185993, EPI_ISL_3185994, EPI_ISL_3185995, EPI_ISL_3185996, EPI_ISL_3185997, EPI_ISL_3185998, EPI_ISL_3185999, EPI_ISL_3186000, EPI_ISL_3186001, EPI_ISL_3186002, EPI_ISL_3186003, EPI_ISL_3186004,
EPI_ISL_3186005, EPI_ISL_3186006, EPI_ISL_3186007, EPI_ISL_3186008, EPI_ISL_3186009, EPI_ISL_3186010, EPI_ISL_3186011, EPI_ISL_3186012, EPI_ISL_3186013, EPI_ISL_3186014, EPI_ISL_3186015, EPI_ISL_3186016, EPI_ISL_3186017, EPI_ISL_3186018, EPI_ISL_3186019, EPI_ISL_3186020, EPI_ISL_3186021, EPI_ISL_3186022, EPI_ISL_3186023, EPI_ISL_3186024, EPI_ISL_3186025, EPI_ISL_3186026,
EPI_ISL_3186027, EPI_ISL_3186028, EPI_ISL_3186029, EPI_ISL_3186030, EPI_ISL_3186031, EPI_ISL_3186032, EPI_ISL_3186033, EPI_ISL_3186034, EPI_ISL_3186035, EPI_ISL_3186036, EPI_ISL_3186037, EPI_ISL_3186038, EPI_ISL_3186039, EPI_ISL_3186040, EPI_ISL_3186041, EPI_ISL_3186042, EPI_ISL_3186043, EPI_ISL_3186044, EPI_ISL_3186045, EPI_ISL_3186046, EPI_ISL_3188672, EPI_ISL_3188673,

EPI_ISL_3188674, EPI_ISL_3188675, EPI_ISL_3188676, EPI_ISL_3188677, EPI_ISL_3188678, EPI_ISL_3188679, EPI_ISL_3188680, EPI_ISL_3188681, EPI_ISL_3188682, EPI_ISL_3188683, EPI_ISL_3188684, EPI_ISL_3188685, EPI_ISL_3188686, EPI_ISL_3188687, EPI_ISL_3188688, EPI_ISL_3188689, EPI_ISL_3188690, EPI_ISL_3188691, EPI_ISL_3188692, EPI_ISL_3188693, EPI_ISL_3188694, EPI_ISL_3188695,
EPI_ISL_3188696, EPI_ISL_3188697, EPI_ISL_3188698, EPI_ISL_3188699, EPI_ISL_3188700, EPI_ISL_3188701, EPI_ISL_3188702, EPI_ISL_3188703, EPI_ISL_3188704, EPI_ISL_3188705, EPI_ISL_3188706, EPI_ISL_3188707, EPI_ISL_3188708, EPI_ISL_3188709, EPI_ISL_3188710, EPI_ISL_3188711, EPI_ISL_3188712, EPI_ISL_3188713, EPI_ISL_3188714, EPI_ISL_3188715, EPI_ISL_3188716, EPI_ISL_3188717,
EPI_ISL_3188718, EPI_ISL_3188719, EPI_ISL_3188720, EPI_ISL_3188721, EPI_ISL_3188722, EPI_ISL_3188723, EPI_ISL_3188724, EPI_ISL_3188725, EPI_ISL_3188726, EPI_ISL_3188727, EPI_ISL_3188728, EPI_ISL_3188729, EPI_ISL_3188730, EPI_ISL_3188731, EPI_ISL_3188732, EPI_ISL_3188733, EPI_ISL_3188734, EPI_ISL_3188735, EPI_ISL_3188736, EPI_ISL_3188737, EPI_ISL_3188738, EPI_ISL_3188739,
EPI_ISL_3188740, EPI_ISL_3188741, EPI_ISL_3188742, EPI_ISL_3188743, EPI_ISL_3188744, EPI_ISL_3188745, EPI_ISL_3188746, EPI_ISL_3188747, EPI_ISL_3188748, EPI_ISL_3188749, EPI_ISL_3188750, EPI_ISL_3188751, EPI_ISL_3188752, EPI_ISL_3188753, EPI_ISL_3188754, EPI_ISL_3188755, EPI_ISL_3188756, EPI_ISL_3188757, EPI_ISL_3188758, EPI_ISL_3188759, EPI_ISL_3188760, EPI_ISL_3188761,
EPI_ISL_3188762, EPI_ISL_3188763, EPI_ISL_3188764, EPI_ISL_3188765, EPI_ISL_3188766, EPI_ISL_3188767, EPI_ISL_3188768, EPI_ISL_3188769, EPI_ISL_3188770, EPI_ISL_3188771, EPI_ISL_3188772, EPI_ISL_3188773, EPI_ISL_3188774, EPI_ISL_3188775, EPI_ISL_3188776, EPI_ISL_3188777, EPI_ISL_3188778, EPI_ISL_3188779, EPI_ISL_3188780, EPI_ISL_3188781, EPI_ISL_3188782, EPI_ISL_3188783,
EPI_ISL_3188784, EPI_ISL_3188785, EPI_ISL_3188786, EPI_ISL_3188787, EPI_ISL_3188788, EPI_ISL_3188789, EPI_ISL_3188790, EPI_ISL_3188791, EPI_ISL_3188792, EPI_ISL_3188793, EPI_ISL_3188794, EPI_ISL_3188795, EPI_ISL_3188796, EPI_ISL_3188797, EPI_ISL_3188798, EPI_ISL_3188799, EPI_ISL_3188800, EPI_ISL_3188801, EPI_ISL_3188802, EPI_ISL_3188803, EPI_ISL_3188804, EPI_ISL_3188805,
EPI_ISL_3188806, EPI_ISL_3188807, EPI_ISL_3188808, EPI_ISL_3188809, EPI_ISL_3188810, EPI_ISL_3188811, EPI_ISL_3188812, EPI_ISL_3261984, EPI_ISL_3261985, EPI_ISL_3261986, EPI_ISL_3261987, EPI_ISL_3261988, EPI_ISL_3261989, EPI_ISL_3261990, EPI_ISL_3261991, EPI_ISL_3261992, EPI_ISL_3261993, EPI_ISL_3261994, EPI_ISL_3261995, EPI_ISL_3261996, EPI_ISL_3261997, EPI_ISL_3261998,
EPI_ISL_3261999, EPI_ISL_3262000, EPI_ISL_3262001, EPI_ISL_3262002, EPI_ISL_3262003, EPI_ISL_3262004, EPI_ISL_3262005, EPI_ISL_3262006, EPI_ISL_3262007, EPI_ISL_3262008, EPI_ISL_3262009, EPI_ISL_3262010, EPI_ISL_3262011, EPI_ISL_3262012, EPI_ISL_3262013, EPI_ISL_3262014, EPI_ISL_3262015, EPI_ISL_3262016, EPI_ISL_3262017, EPI_ISL_3262018, EPI_ISL_3262019, EPI_ISL_3262020,
EPI_ISL_3262021, EPI_ISL_3262022, EPI_ISL_3262023, EPI_ISL_3262024, EPI_ISL_3262025, EPI_ISL_3262026, EPI_ISL_3262027, EPI_ISL_3262028, EPI_ISL_3262029, EPI_ISL_3262030, EPI_ISL_3262031, EPI_ISL_3262032, EPI_ISL_3262033, EPI_ISL_3262034, EPI_ISL_3262035, EPI_ISL_3262036, EPI_ISL_3262037, EPI_ISL_3262038, EPI_ISL_3262039, EPI_ISL_3262040, EPI_ISL_3262041, EPI_ISL_3262042,
EPI_ISL_3262043, EPI_ISL_3262044, EPI_ISL_3262045, EPI_ISL_3262046, EPI_ISL_3262047, EPI_ISL_3262048, EPI_ISL_3262049, EPI_ISL_3262050, EPI_ISL_3262051, EPI_ISL_3262052, EPI_ISL_3262053, EPI_ISL_3262054, EPI_ISL_3262055, EPI_ISL_3262056, EPI_ISL_3262057, EPI_ISL_3262058, EPI_ISL_3262059, EPI_ISL_3262060, EPI_ISL_3262061, EPI_ISL_3262062, EPI_ISL_3262063, EPI_ISL_3262064,
EPI_ISL_3262065, EPI_ISL_3262066, EPI_ISL_3262067, EPI_ISL_3262068, EPI_ISL_3262069, EPI_ISL_3262070, EPI_ISL_3262071, EPI_ISL_3262072, EPI_ISL_3262073, EPI_ISL_3262074, EPI_ISL_3262075, EPI_ISL_3262076, EPI_ISL_3262077, EPI_ISL_3262078, EPI_ISL_3262079, EPI_ISL_3262080, EPI_ISL_3262081, EPI_ISL_3262082, EPI_ISL_3262083, EPI_ISL_3262084, EPI_ISL_3262085, EPI_ISL_3262086,
EPI_ISL_3262087, EPI_ISL_3262088, EPI_ISL_3262089, EPI_ISL_3262090, EPI_ISL_3262091, EPI_ISL_3262092, EPI_ISL_3262093, EPI_ISL_3262094, EPI_ISL_3262095, EPI_ISL_3262096, EPI_ISL_3262097, EPI_ISL_3262098, EPI_ISL_3262099, EPI_ISL_3262100, EPI_ISL_3262101, EPI_ISL_3262102, EPI_ISL_3262103, EPI_ISL_3262104, EPI_ISL_3262105, EPI_ISL_3262106, EPI_ISL_3262107, EPI_ISL_3262108,
EPI_ISL_3262109, EPI_ISL_3262110, EPI_ISL_3262111, EPI_ISL_3262112, EPI_ISL_3262113, EPI_ISL_3262114, EPI_ISL_3262115, EPI_ISL_3262116, EPI_ISL_3262117, EPI_ISL_3266126, EPI_ISL_3266127, EPI_ISL_3266128, EPI_ISL_3266129, EPI_ISL_3266130, EPI_ISL_3266131, EPI_ISL_3266132, EPI_ISL_3266133, EPI_ISL_3266134, EPI_ISL_3266135, EPI_ISL_3266136, EPI_ISL_3266137, EPI_ISL_3266138,
EPI_ISL_3266139, EPI_ISL_3266140, EPI_ISL_3266141, EPI_ISL_3266142, EPI_ISL_3266143, EPI_ISL_3266144, EPI_ISL_3266145, EPI_ISL_3266146, EPI_ISL_3266147, EPI_ISL_3266148, EPI_ISL_3266149, EPI_ISL_3266150, EPI_ISL_3266151, EPI_ISL_3266152, EPI_ISL_3266153, EPI_ISL_3266154, EPI_ISL_3266155, EPI_ISL_3266156, EPI_ISL_3266157, EPI_ISL_3266158, EPI_ISL_3266159, EPI_ISL_3266160,
EPI_ISL_3266161, EPI_ISL_3266162, EPI_ISL_3266163, EPI_ISL_3266164, EPI_ISL_3266165, EPI_ISL_3266166, EPI_ISL_3266167, EPI_ISL_3266168, EPI_ISL_3266169, EPI_ISL_3266170, EPI_ISL_3266171, EPI_ISL_3266172, EPI_ISL_3266173, EPI_ISL_3266174, EPI_ISL_3266175, EPI_ISL_3266176, EPI_ISL_3266177, EPI_ISL_3266178, EPI_ISL_3266179, EPI_ISL_3266180, EPI_ISL_3266181, EPI_ISL_3266182,
EPI_ISL_3266183, EPI_ISL_3266184, EPI_ISL_3266185, EPI_ISL_3266186, EPI_ISL_3266187, EPI_ISL_3266188, EPI_ISL_3266189, EPI_ISL_3266190, EPI_ISL_3266191, EPI_ISL_3266192, EPI_ISL_3266193, EPI_ISL_3266194, EPI_ISL_3266195, EPI_ISL_3266196, EPI_ISL_3266197, EPI_ISL_3266198, EPI_ISL_3266199, EPI_ISL_3266200, EPI_ISL_3266201, EPI_ISL_3266202, EPI_ISL_3266203, EPI_ISL_3266204,
EPI_ISL_3266205, EPI_ISL_3266206, EPI_ISL_3266207, EPI_ISL_3266208, EPI_ISL_3266209, EPI_ISL_3307964, EPI_ISL_3307965, EPI_ISL_3307966, EPI_ISL_3307967, EPI_ISL_3307968, EPI_ISL_3307969, EPI_ISL_3307970, EPI_ISL_3307971, EPI_ISL_3307972, EPI_ISL_3307973, EPI_ISL_3307974, EPI_ISL_3307975, EPI_ISL_3307976, EPI_ISL_3307977, EPI_ISL_3307978, EPI_ISL_3307979, EPI_ISL_3307980,
EPI_ISL_3307981, EPI_ISL_3307982, EPI_ISL_3307983, EPI_ISL_3307984, EPI_ISL_3307985, EPI_ISL_3307986, EPI_ISL_3307987, EPI_ISL_3307988, EPI_ISL_3307989, EPI_ISL_3307990, EPI_ISL_3307991, EPI_ISL_3307992, EPI_ISL_3307993, EPI_ISL_3307994, EPI_ISL_3307995, EPI_ISL_3307996, EPI_ISL_3307997, EPI_ISL_3307998, EPI_ISL_3307999, EPI_ISL_3308000, EPI_ISL_3308001, EPI_ISL_3308002,
EPI_ISL_3308003, EPI_ISL_3308004, EPI_ISL_3308005, EPI_ISL_3308006, EPI_ISL_3308007, EPI_ISL_3308008, EPI_ISL_3308009, EPI_ISL_3308010, EPI_ISL_3308011, EPI_ISL_3308012, EPI_ISL_3308013, EPI_ISL_3308014, EPI_ISL_3308015, EPI_ISL_3308016, EPI_ISL_3308017, EPI_ISL_3308018, EPI_ISL_3308019, EPI_ISL_3308020, EPI_ISL_3308021, EPI_ISL_3308022, EPI_ISL_3308023, EPI_ISL_3308024,
EPI_ISL_3308025, EPI_ISL_3308026, EPI_ISL_3308027, EPI_ISL_3308028, EPI_ISL_3308029, EPI_ISL_3308030, EPI_ISL_3308031, EPI_ISL_3308032, EPI_ISL_3308033, EPI_ISL_3308034, EPI_ISL_3308035, EPI_ISL_3308036, EPI_ISL_3308037, EPI_ISL_3308038, EPI_ISL_3308039, EPI_ISL_3308040, EPI_ISL_3308041, EPI_ISL_3308042, EPI_ISL_3308043, EPI_ISL_3308044, EPI_ISL_3308045, EPI_ISL_3308046,
EPI_ISL_3308047, EPI_ISL_3308048, EPI_ISL_3308049, EPI_ISL_3308050, EPI_ISL_3308051, EPI_ISL_3308052, EPI_ISL_3308053, EPI_ISL_3308054, EPI_ISL_3308055, EPI_ISL_3308056, EPI_ISL_3308057, EPI_ISL_3308058, EPI_ISL_3308059, EPI_ISL_3308060, EPI_ISL_3308061, EPI_ISL_3308062, EPI_ISL_3308063, EPI_ISL_3308064, EPI_ISL_3308065, EPI_ISL_3308066, EPI_ISL_3308067, EPI_ISL_3308068,
EPI_ISL_3308069, EPI_ISL_3308070, EPI_ISL_3308071, EPI_ISL_3308072, EPI_ISL_3308073, EPI_ISL_3308074, EPI_ISL_3308075, EPI_ISL_3308076, EPI_ISL_3308077, EPI_ISL_3308078, EPI_ISL_3308079, EPI_ISL_3308080, EPI_ISL_3308081, EPI_ISL_3308082, EPI_ISL_3308083, EPI_ISL_3308084, EPI_ISL_3308085, EPI_ISL_3308086, EPI_ISL_3308087, EPI_ISL_3308088, EPI_ISL_3308089, EPI_ISL_3308090,
EPI_ISL_3308091, EPI_ISL_3308092, EPI_ISL_3308093, EPI_ISL_3308094, EPI_ISL_3308095, EPI_ISL_3308096, EPI_ISL_3308097, EPI_ISL_3308098, EPI_ISL_3308099, EPI_ISL_3308100, EPI_ISL_3308101, EPI_ISL_3308102, EPI_ISL_3308103, EPI_ISL_3308104, EPI_ISL_3308105, EPI_ISL_3308106, EPI_ISL_3308107, EPI_ISL_3308108, EPI_ISL_3308109, EPI_ISL_3308110, EPI_ISL_3308111, EPI_ISL_3308112,
EPI_ISL_3308113, EPI_ISL_3308114, EPI_ISL_3308115, EPI_ISL_3308116, EPI_ISL_3308117, EPI_ISL_3308118, EPI_ISL_3308119, EPI_ISL_3308120, EPI_ISL_3308121, EPI_ISL_3308122, EPI_ISL_3308123, EPI_ISL_3308124, EPI_ISL_3308125, EPI_ISL_3308126, EPI_ISL_3308127, EPI_ISL_3308128, EPI_ISL_3308129, EPI_ISL_3308130, EPI_ISL_3308131, EPI_ISL_3308132, EPI_ISL_3308133, EPI_ISL_3308134,
EPI_ISL_3308135, EPI_ISL_3308136, EPI_ISL_3308137, EPI_ISL_3308138, EPI_ISL_3308139, EPI_ISL_3308140, EPI_ISL_3308141, EPI_ISL_3308142, EPI_ISL_3308143, EPI_ISL_3308144, EPI_ISL_3308145, EPI_ISL_3308146, EPI_ISL_3308147, EPI_ISL_3308148, EPI_ISL_3308150, EPI_ISL_3308151, EPI_ISL_3308152, EPI_ISL_3308153, EPI_ISL_3308154, EPI_ISL_3308155, EPI_ISL_3308156, EPI_ISL_3308157,
EPI_ISL_3308158, EPI_ISL_3308159, EPI_ISL_3308160, EPI_ISL_3308161, EPI_ISL_3308162, EPI_ISL_3308163, EPI_ISL_3308164, EPI_ISL_3308165, EPI_ISL_3308166, EPI_ISL_3308167, EPI_ISL_3387152, EPI_ISL_3387153, EPI_ISL_3387154, EPI_ISL_3387155, EPI_ISL_3387156, EPI_ISL_3387157, EPI_ISL_3387158, EPI_ISL_3387159, EPI_ISL_3387160, EPI_ISL_3387161, EPI_ISL_3387162, EPI_ISL_3387163,
EPI_ISL_3387164, EPI_ISL_3387165, EPI_ISL_3387166, EPI_ISL_3387167, EPI_ISL_3387168, EPI_ISL_3387169, EPI_ISL_3387170, EPI_ISL_3387171, EPI_ISL_3387172, EPI_ISL_3387173, EPI_ISL_3387174, EPI_ISL_3387175, EPI_ISL_3387176, EPI_ISL_3387177, EPI_ISL_3387178, EPI_ISL_3387179, EPI_ISL_3387180, EPI_ISL_3387181, EPI_ISL_3387182, EPI_ISL_3387183, EPI_ISL_3387184, EPI_ISL_3387185,
EPI_ISL_3387186, EPI_ISL_3387187, EPI_ISL_3387188, EPI_ISL_3387189, EPI_ISL_3387190, EPI_ISL_3387191, EPI_ISL_3387192, EPI_ISL_3387193, EPI_ISL_3387194, EPI_ISL_3387195, EPI_ISL_3387196, EPI_ISL_3387197, EPI_ISL_3387198, EPI_ISL_3387199, EPI_ISL_3387200, EPI_ISL_3387201, EPI_ISL_3387202, EPI_ISL_3387203, EPI_ISL_3387204, EPI_ISL_3387205, EPI_ISL_3387206, EPI_ISL_3387207,
EPI_ISL_3387208, EPI_ISL_3387209, EPI_ISL_3387210, EPI_ISL_3387211, EPI_ISL_3387212, EPI_ISL_3387213, EPI_ISL_3387214, EPI_ISL_3387215, EPI_ISL_3387216, EPI_ISL_3387217, EPI_ISL_3387218, EPI_ISL_3387219, EPI_ISL_3387220, EPI_ISL_3387221, EPI_ISL_3387222, EPI_ISL_3387223, EPI_ISL_3387224, EPI_ISL_3387225, EPI_ISL_3387226, EPI_ISL_3387227, EPI_ISL_3387228, EPI_ISL_3387229,
EPI_ISL_3387230, EPI_ISL_3393544, EPI_ISL_3393545, EPI_ISL_3393546, EPI_ISL_3393547, EPI_ISL_3393548, EPI_ISL_3393549, EPI_ISL_3393550, EPI_ISL_3393551, EPI_ISL_3393552, EPI_ISL_3393553, EPI_ISL_3393554, EPI_ISL_3393555, EPI_ISL_3393556, EPI_ISL_3393557, EPI_ISL_3393558, EPI_ISL_3393559, EPI_ISL_3393560, EPI_ISL_3393561, EPI_ISL_3393562, EPI_ISL_3393563, EPI_ISL_3393564,
EPI_ISL_3393565, EPI_ISL_3393566, EPI_ISL_3393567, EPI_ISL_3393568, EPI_ISL_3393569, EPI_ISL_3393570, EPI_ISL_3393571, EPI_ISL_3393572, EPI_ISL_3393573, EPI_ISL_3393574, EPI_ISL_3393575, EPI_ISL_3393576, EPI_ISL_3393577, EPI_ISL_3393578, EPI_ISL_3393579, EPI_ISL_3393580, EPI_ISL_3393581, EPI_ISL_3393582, EPI_ISL_3393583, EPI_ISL_3393584, EPI_ISL_3393585, EPI_ISL_3393586,
EPI_ISL_3393587, EPI_ISL_3393588, EPI_ISL_3393589, EPI_ISL_3393590, EPI_ISL_3393591, EPI_ISL_3393592, EPI_ISL_3393593, EPI_ISL_3393594, EPI_ISL_3393595, EPI_ISL_3393596, EPI_ISL_3393597, EPI_ISL_3393598, EPI_ISL_3393599, EPI_ISL_3393600, EPI_ISL_3393601, EPI_ISL_3393602, EPI_ISL_3393603, EPI_ISL_3393604, EPI_ISL_3393605, EPI_ISL_3393606, EPI_ISL_3393607, EPI_ISL_3393608,
EPI_ISL_3393609, EPI_ISL_3393610, EPI_ISL_3393611, EPI_ISL_3393612, EPI_ISL_3393613, EPI_ISL_3393614, EPI_ISL_3393615, EPI_ISL_3393616, EPI_ISL_3393617, EPI_ISL_3393618, EPI_ISL_3393619, EPI_ISL_3393620, EPI_ISL_3393621, EPI_ISL_3393622, EPI_ISL_3393623, EPI_ISL_3393624, EPI_ISL_3393625, EPI_ISL_3393626, EPI_ISL_3393627, EPI_ISL_3393628, EPI_ISL_3393629, EPI_ISL_3393630,
EPI_ISL_3393631, EPI_ISL_3393632, EPI_ISL_3393633, EPI_ISL_3393634, EPI_ISL_3393635, EPI_ISL_3393636, EPI_ISL_3393637, EPI_ISL_3393638, EPI_ISL_3393639, EPI_ISL_3393640, EPI_ISL_3393641, EPI_ISL_3393642, EPI_ISL_3393643, EPI_ISL_3393644, EPI_ISL_3393645, EPI_ISL_3393646, EPI_ISL_3393647, EPI_ISL_3393648, EPI_ISL_3393649, EPI_ISL_3393650, EPI_ISL_3393651, EPI_ISL_3393652,
EPI_ISL_3393653, EPI_ISL_3393654, EPI_ISL_3426927, EPI_ISL_3426928, EPI_ISL_3426929, EPI_ISL_3426930, EPI_ISL_3426931, EPI_ISL_3426932, EPI_ISL_3426933, EPI_ISL_3426934, EPI_ISL_3426935, EPI_ISL_3426936, EPI_ISL_3426937, EPI_ISL_3426938, EPI_ISL_3426939, EPI_ISL_3426940, EPI_ISL_3426941, EPI_ISL_3426942, EPI_ISL_3426943, EPI_ISL_3426944, EPI_ISL_3426945, EPI_ISL_3426946,
EPI_ISL_3426947, EPI_ISL_3426948, EPI_ISL_3426949, EPI_ISL_3426950, EPI_ISL_3426951, EPI_ISL_3426952, EPI_ISL_3426953, EPI_ISL_3426954, EPI_ISL_3426955, EPI_ISL_3426956, EPI_ISL_3426957, EPI_ISL_3426958, EPI_ISL_3426959, EPI_ISL_3426960, EPI_ISL_3426961, EPI_ISL_3426962, EPI_ISL_3426963, EPI_ISL_3426964, EPI_ISL_3426965, EPI_ISL_3426966, EPI_ISL_3426967, EPI_ISL_3426968,
EPI_ISL_3426969, EPI_ISL_3426970, EPI_ISL_3426971, EPI_ISL_3426972, EPI_ISL_3426973, EPI_ISL_3426974, EPI_ISL_3426975, EPI_ISL_3426976, EPI_ISL_3426977, EPI_ISL_3426978, EPI_ISL_3426979, EPI_ISL_3426980, EPI_ISL_3426981, EPI_ISL_3426982, EPI_ISL_3426983, EPI_ISL_3426984, EPI_ISL_3426985, EPI_ISL_3426986, EPI_ISL_3426987, EPI_ISL_3426988, EPI_ISL_3426989, EPI_ISL_3426990,
EPI_ISL_3426991, EPI_ISL_3426992, EPI_ISL_3426993, EPI_ISL_3426994, EPI_ISL_3426995, EPI_ISL_3426996, EPI_ISL_3426997, EPI_ISL_3426998, EPI_ISL_3426999, EPI_ISL_3427000, EPI_ISL_3427001, EPI_ISL_3427002, EPI_ISL_3427003, EPI_ISL_3427004, EPI_ISL_3427005, EPI_ISL_3427006, EPI_ISL_3427007, EPI_ISL_3427008, EPI_ISL_3427009, EPI_ISL_3427010, EPI_ISL_3427011, EPI_ISL_3427012,
EPI_ISL_3427013, EPI_ISL_3427014, EPI_ISL_3427015, EPI_ISL_3427016, EPI_ISL_3427017, EPI_ISL_3427018, EPI_ISL_3427019, EPI_ISL_3427020, EPI_ISL_3427021, EPI_ISL_3427022, EPI_ISL_3427023, EPI_ISL_3427024, EPI_ISL_3427025, EPI_ISL_3427026, EPI_ISL_3427027, EPI_ISL_3427028, EPI_ISL_3427029, EPI_ISL_3427030, EPI_ISL_3427031, EPI_ISL_3427032, EPI_ISL_3427033, EPI_ISL_3427034,
EPI_ISL_3427035, EPI_ISL_3427036, EPI_ISL_3427037, EPI_ISL_3427038, EPI_ISL_3427039, EPI_ISL_3427040, EPI_ISL_3427041, EPI_ISL_3427042, EPI_ISL_3427043, EPI_ISL_3427044, EPI_ISL_3427045, EPI_ISL_3427046, EPI_ISL_3427047, EPI_ISL_3427048, EPI_ISL_3427049, EPI_ISL_3427050, EPI_ISL_3427051, EPI_ISL_3427052, EPI_ISL_3427053, EPI_ISL_3427054, EPI_ISL_3427055, EPI_ISL_3427056,
EPI_ISL_3427057, EPI_ISL_3427058, EPI_ISL_3427059, EPI_ISL_3427060, EPI_ISL_3427061, EPI_ISL_3427062, EPI_ISL_3427063, EPI_ISL_3427064, EPI_ISL_3427065, EPI_ISL_3427066, EPI_ISL_3427067, EPI_ISL_3427068, EPI_ISL_3427069, EPI_ISL_3427070, EPI_ISL_3427071, EPI_ISL_3427072, EPI_ISL_3427073, EPI_ISL_3427074, EPI_ISL_3427075, EPI_ISL_3427076, EPI_ISL_3427077, EPI_ISL_3427078,
EPI_ISL_3427079, EPI_ISL_3427080, EPI_ISL_3427081, EPI_ISL_3427082, EPI_ISL_3427083, EPI_ISL_3427084, EPI_ISL_3427085, EPI_ISL_3427086, EPI_ISL_3427087, EPI_ISL_3427088, EPI_ISL_3427089, EPI_ISL_3427090, EPI_ISL_3427091, EPI_ISL_3427092, EPI_ISL_3427093, EPI_ISL_3427094, EPI_ISL_3427095, EPI_ISL_3427096, EPI_ISL_3427097, EPI_ISL_3427098, EPI_ISL_3427099, EPI_ISL_3427100,
EPI_ISL_3427101, EPI_ISL_3427102, EPI_ISL_3427103, EPI_ISL_3427104, EPI_ISL_3427105, EPI_ISL_3427106, EPI_ISL_3427107, EPI_ISL_3427108, EPI_ISL_3427109, EPI_ISL_3427110, EPI_ISL_3427111, EPI_ISL_3427112, EPI_ISL_3543183, EPI_ISL_3543184, EPI_ISL_3543185, EPI_ISL_3543186, EPI_ISL_3543187, EPI_ISL_3543188, EPI_ISL_3543189, EPI_ISL_3543190, EPI_ISL_3543191, EPI_ISL_3543192,
EPI_ISL_3543193, EPI_ISL_3543194, EPI_ISL_3543195, EPI_ISL_3543196, EPI_ISL_3543197, EPI_ISL_3543198, EPI_ISL_3543199, EPI_ISL_3543200, EPI_ISL_3543201, EPI_ISL_3543202, EPI_ISL_3543203, EPI_ISL_3543204, EPI_ISL_3543205, EPI_ISL_3543206, EPI_ISL_3543207, EPI_ISL_3543208, EPI_ISL_3543209, EPI_ISL_3543210, EPI_ISL_3543211, EPI_ISL_3543212, EPI_ISL_3543213, EPI_ISL_3543214,
EPI_ISL_3543215, EPI_ISL_3543216, EPI_ISL_3543217, EPI_ISL_3543218, EPI_ISL_3543219, EPI_ISL_3543220, EPI_ISL_3543221, EPI_ISL_3543222, EPI_ISL_3543223, EPI_ISL_3543224, EPI_ISL_3543225, EPI_ISL_3543226, EPI_ISL_3543227, EPI_ISL_3543228, EPI_ISL_3543229, EPI_ISL_3543230, EPI_ISL_3543231, EPI_ISL_3543232, EPI_ISL_3543233, EPI_ISL_3543234, EPI_ISL_3543235, EPI_ISL_3543236,
EPI_ISL_3543237, EPI_ISL_3543238, EPI_ISL_3543239, EPI_ISL_3543240, EPI_ISL_3543241, EPI_ISL_3543242, EPI_ISL_3543243, EPI_ISL_3543244, EPI_ISL_3543245, EPI_ISL_3543246, EPI_ISL_3543247, EPI_ISL_3543248, EPI_ISL_3543249, EPI_ISL_3543250, EPI_ISL_3543251, EPI_ISL_3543252, EPI_ISL_3543253, EPI_ISL_3543254, EPI_ISL_3543255, EPI_ISL_3543256, EPI_ISL_3543257, EPI_ISL_3543258,
EPI_ISL_3543259, EPI_ISL_3543260, EPI_ISL_3543261, EPI_ISL_3543262, EPI_ISL_3543263, EPI_ISL_3543264, EPI_ISL_3543265, EPI_ISL_3543266, EPI_ISL_3543267, EPI_ISL_3543268, EPI_ISL_3543269, EPI_ISL_3543270, EPI_ISL_3543271, EPI_ISL_3543272, EPI_ISL_3543273, EPI_ISL_3543274, EPI_ISL_3543275, EPI_ISL_3543276, EPI_ISL_3543277, EPI_ISL_3543278, EPI_ISL_3543279, EPI_ISL_3543280,
EPI_ISL_3543281, EPI_ISL_3543282, EPI_ISL_3543283, EPI_ISL_3543284, EPI_ISL_3572883, EPI_ISL_3572886, EPI_ISL_3572888, EPI_ISL_3572890, EPI_ISL_3572892, EPI_ISL_3572894, EPI_ISL_3572897, EPI_ISL_3572899, EPI_ISL_3572901, EPI_ISL_3572903, EPI_ISL_3572905, EPI_ISL_3572907, EPI_ISL_3572909, EPI_ISL_3572911, EPI_ISL_3572913, EPI_ISL_3572916, EPI_ISL_3572918, EPI_ISL_3572920,
EPI_ISL_3572922, EPI_ISL_3572924, EPI_ISL_3572926, EPI_ISL_3572929, EPI_ISL_3572931, EPI_ISL_3572933, EPI_ISL_3572935, EPI_ISL_3572937, EPI_ISL_3572939, EPI_ISL_3572941, EPI_ISL_3572943, EPI_ISL_3572945, EPI_ISL_3572947, EPI_ISL_3572949, EPI_ISL_3572951, EPI_ISL_3572953, EPI_ISL_3572956, EPI_ISL_3572958, EPI_ISL_3572960, EPI_ISL_3572962, EPI_ISL_3572964, EPI_ISL_3572966,
EPI_ISL_3572968, EPI_ISL_3572971, EPI_ISL_3572973, EPI_ISL_3572975, EPI_ISL_3572977, EPI_ISL_3572979, EPI_ISL_3572981, EPI_ISL_3572984, EPI_ISL_3572986, EPI_ISL_3572988, EPI_ISL_3572990, EPI_ISL_3572992, EPI_ISL_3572995, EPI_ISL_3572997, EPI_ISL_3572999, EPI_ISL_3573001, EPI_ISL_3573004, EPI_ISL_3573006, EPI_ISL_3573008, EPI_ISL_3573011, EPI_ISL_3573013, EPI_ISL_3573015,
EPI_ISL_3573017, EPI_ISL_3588264, EPI_ISL_3588265, EPI_ISL_3588267, EPI_ISL_3588268, EPI_ISL_3588269, EPI_ISL_3588270, EPI_ISL_3588271, EPI_ISL_3588272, EPI_ISL_3588273, EPI_ISL_3588274, EPI_ISL_3588275, EPI_ISL_3588276, EPI_ISL_3588277, EPI_ISL_3588278, EPI_ISL_3588279, EPI_ISL_3588280, EPI_ISL_3588281, EPI_ISL_3588283, EPI_ISL_3588284, EPI_ISL_3588285, EPI_ISL_3588286,
EPI_ISL_3588289, EPI_ISL_3588290, EPI_ISL_3588291, EPI_ISL_3588294, EPI_ISL_3588295, EPI_ISL_3588298, EPI_ISL_3588299, EPI_ISL_3588300, EPI_ISL_3588301, EPI_ISL_3588302, EPI_ISL_3588303, EPI_ISL_3588304, EPI_ISL_3588305, EPI_ISL_3588306, EPI_ISL_3588307, EPI_ISL_3588308, EPI_ISL_3588309, EPI_ISL_3588310, EPI_ISL_3588311, EPI_ISL_3588312, EPI_ISL_3588313, EPI_ISL_3588314,
EPI_ISL_3588315, EPI_ISL_3588316, EPI_ISL_3588317, EPI_ISL_3588318, EPI_ISL_3588319, EPI_ISL_3588320, EPI_ISL_3588321, EPI_ISL_3588322, EPI_ISL_3588323, EPI_ISL_3588324, EPI_ISL_3588325, EPI_ISL_3588326, EPI_ISL_3588327, EPI_ISL_3588328, EPI_ISL_3588329, EPI_ISL_3588330, EPI_ISL_3588331, EPI_ISL_3595937, EPI_ISL_3595938, EPI_ISL_3595939, EPI_ISL_3595940, EPI_ISL_3595941,
EPI_ISL_3595942, EPI_ISL_3595943, EPI_ISL_3595944, EPI_ISL_3595945, EPI_ISL_3595946, EPI_ISL_3595947, EPI_ISL_3595948, EPI_ISL_3595949, EPI_ISL_3595950, EPI_ISL_3595951, EPI_ISL_3595952, EPI_ISL_3595953, EPI_ISL_3595954, EPI_ISL_3595955, EPI_ISL_3595956, EPI_ISL_3595957, EPI_ISL_3595958, EPI_ISL_3595959, EPI_ISL_3595960, EPI_ISL_3595961, EPI_ISL_3595962, EPI_ISL_3595963,
EPI_ISL_3595964, EPI_ISL_3595965, EPI_ISL_3595966, EPI_ISL_3595967, EPI_ISL_3595968, EPI_ISL_3595969, EPI_ISL_3595970, EPI_ISL_3595971, EPI_ISL_3595972, EPI_ISL_3595973, EPI_ISL_3595974, EPI_ISL_3595975, EPI_ISL_3595976, EPI_ISL_3595977, EPI_ISL_3595978, EPI_ISL_3595979, EPI_ISL_3595980, EPI_ISL_3595981, EPI_ISL_3595982, EPI_ISL_3595983, EPI_ISL_3595984, EPI_ISL_3595985,
EPI_ISL_3595986, EPI_ISL_3595987, EPI_ISL_3595988, EPI_ISL_3595989, EPI_ISL_3595990, EPI_ISL_3595991, EPI_ISL_3595992, EPI_ISL_3595993, EPI_ISL_3595994, EPI_ISL_3595995, EPI_ISL_3595996, EPI_ISL_3595997, EPI_ISL_3595998, EPI_ISL_3595999, EPI_ISL_3596000, EPI_ISL_3596001, EPI_ISL_3596003, EPI_ISL_3596004, EPI_ISL_3596005, EPI_ISL_3596006, EPI_ISL_3596007, EPI_ISL_3596008,
EPI_ISL_3596009, EPI_ISL_3596010, EPI_ISL_3596011, EPI_ISL_3596012, EPI_ISL_3596013, EPI_ISL_3596014, EPI_ISL_3596015, EPI_ISL_3596016, EPI_ISL_3596017, EPI_ISL_3596018, EPI_ISL_3596019, EPI_ISL_3596020, EPI_ISL_3596021, EPI_ISL_3596022, EPI_ISL_3596023, EPI_ISL_3596024, EPI_ISL_3596025, EPI_ISL_3596026, EPI_ISL_3596027, EPI_ISL_3596028, EPI_ISL_3596029, EPI_ISL_3596030,
EPI_ISL_3596031, EPI_ISL_3596032, EPI_ISL_3596033, EPI_ISL_3596034, EPI_ISL_3596036, EPI_ISL_3596037, EPI_ISL_3596039, EPI_ISL_3596040, EPI_ISL_3596041, EPI_ISL_3596042, EPI_ISL_3596043, EPI_ISL_3596044, EPI_ISL_3596045, EPI_ISL_3596046, EPI_ISL_3596047, EPI_ISL_3596048, EPI_ISL_3596050, EPI_ISL_3596051, EPI_ISL_3596052, EPI_ISL_3596053, EPI_ISL_3631638, EPI_ISL_3730708,
EPI_ISL_3730709, EPI_ISL_3730710, EPI_ISL_3730711, EPI_ISL_3730712, EPI_ISL_3730713, EPI_ISL_3730714, EPI_ISL_3730715, EPI_ISL_3730716, EPI_ISL_3730717, EPI_ISL_3730718, EPI_ISL_3730719, EPI_ISL_3730720, EPI_ISL_3730721, EPI_ISL_3730722, EPI_ISL_3730723, EPI_ISL_3730724, EPI_ISL_3730725, EPI_ISL_3730726, EPI_ISL_3730727, EPI_ISL_3730728, EPI_ISL_3730729, EPI_ISL_3730730,
EPI_ISL_3730731, EPI_ISL_3730732, EPI_ISL_3730733, EPI_ISL_3730734, EPI_ISL_3730735, EPI_ISL_3730736, EPI_ISL_3730737, EPI_ISL_3730738, EPI_ISL_3730739, EPI_ISL_3730740, EPI_ISL_3730741, EPI_ISL_3730742, EPI_ISL_3730743, EPI_ISL_3730744, EPI_ISL_3730745, EPI_ISL_3730746, EPI_ISL_3730747, EPI_ISL_3730748, EPI_ISL_3730749, EPI_ISL_3730750, EPI_ISL_3730751, EPI_ISL_3730752,
EPI_ISL_3730753, EPI_ISL_3730754, EPI_ISL_3730755, EPI_ISL_3730756, EPI_ISL_3730757, EPI_ISL_3730758, EPI_ISL_3730759, EPI_ISL_3730760, EPI_ISL_3730761, EPI_ISL_3730762, EPI_ISL_3730763, EPI_ISL_3730764, EPI_ISL_3730765, EPI_ISL_3730766, EPI_ISL_3730767, EPI_ISL_3730768, EPI_ISL_3730769, EPI_ISL_3730770, EPI_ISL_3730771, EPI_ISL_3730772, EPI_ISL_3730773, EPI_ISL_3730774,
EPI_ISL_3737326, EPI_ISL_3737327, EPI_ISL_3737328, EPI_ISL_3737329, EPI_ISL_3737330, EPI_ISL_3737331, EPI_ISL_3737332, EPI_ISL_3737333, EPI_ISL_3737334, EPI_ISL_3737335, EPI_ISL_3737336, EPI_ISL_3737337, EPI_ISL_3737338, EPI_ISL_3737339, EPI_ISL_3737340, EPI_ISL_3737341, EPI_ISL_3737342, EPI_ISL_3737343, EPI_ISL_3737344, EPI_ISL_3737345, EPI_ISL_3737346, EPI_ISL_3737347,
EPI_ISL_3737348, EPI_ISL_3737349, EPI_ISL_3737350, EPI_ISL_3737351, EPI_ISL_3737352, EPI_ISL_3737353, EPI_ISL_3737354, EPI_ISL_3737355, EPI_ISL_3737356, EPI_ISL_3737357, EPI_ISL_3737358, EPI_ISL_3737359, EPI_ISL_3737360, EPI_ISL_3737361, EPI_ISL_3737362, EPI_ISL_3737363, EPI_ISL_3737364, EPI_ISL_3737365, EPI_ISL_3737366, EPI_ISL_3737367, EPI_ISL_3737368, EPI_ISL_3737369,
EPI_ISL_3737370, EPI_ISL_3737371, EPI_ISL_3737372, EPI_ISL_3737373, EPI_ISL_3737374, EPI_ISL_3737375, EPI_ISL_3737376, EPI_ISL_3737377, EPI_ISL_3737378, EPI_ISL_3737379, EPI_ISL_3737380, EPI_ISL_3737381, EPI_ISL_3737382, EPI_ISL_3737383, EPI_ISL_3737384, EPI_ISL_3737385, EPI_ISL_3737386, EPI_ISL_3737387, EPI_ISL_3737388, EPI_ISL_3737389, EPI_ISL_3737390, EPI_ISL_3737391,
EPI_ISL_3737392, EPI_ISL_3737393, EPI_ISL_3737394, EPI_ISL_3737395, EPI_ISL_3737396, EPI_ISL_3737397, EPI_ISL_3737398, EPI_ISL_3737399, EPI_ISL_3737400, EPI_ISL_3737401, EPI_ISL_3737402, EPI_ISL_3737403, EPI_ISL_3737404, EPI_ISL_3737405, EPI_ISL_3737406, EPI_ISL_3737407, EPI_ISL_3737408, EPI_ISL_3737409, EPI_ISL_3737410, EPI_ISL_3737411, EPI_ISL_3737412, EPI_ISL_3737413,
EPI_ISL_3737414, EPI_ISL_3737415, EPI_ISL_3737416, EPI_ISL_3737417, EPI_ISL_3737418, EPI_ISL_3737419, EPI_ISL_3737420, EPI_ISL_3737421, EPI_ISL_3737422, EPI_ISL_3737423, EPI_ISL_3737424, EPI_ISL_3737425, EPI_ISL_3737426, EPI_ISL_3737427, EPI_ISL_3737428, EPI_ISL_3737429, EPI_ISL_3737430, EPI_ISL_3783893, EPI_ISL_3783918, EPI_ISL_3783927, EPI_ISL_3783935, EPI_ISL_3783941,
EPI_ISL_3783966, EPI_ISL_3783986, EPI_ISL_3784009, EPI_ISL_3784032, EPI_ISL_3784050, EPI_ISL_3784056, EPI_ISL_3784065, EPI_ISL_3784072, EPI_ISL_3784078, EPI_ISL_3784084, EPI_ISL_3784091, EPI_ISL_3784099, EPI_ISL_3784124, EPI_ISL_3784142, EPI_ISL_3784165, EPI_ISL_3784190, EPI_ISL_3784216, EPI_ISL_3784239, EPI_ISL_3784263, EPI_ISL_3784288, EPI_ISL_3784314, EPI_ISL_3784333,
EPI_ISL_3784355, EPI_ISL_3784378, EPI_ISL_3784400, EPI_ISL_3784423, EPI_ISL_3784441, EPI_ISL_3784464, EPI_ISL_3784483, EPI_ISL_3784505, EPI_ISL_3784528, EPI_ISL_3784550, EPI_ISL_3784572, EPI_ISL_3784597, EPI_ISL_3784621, EPI_ISL_3784640, EPI_ISL_3784665, EPI_ISL_3784689, EPI_ISL_3784712, EPI_ISL_3784737, EPI_ISL_3784757, EPI_ISL_3784765, EPI_ISL_3784771, EPI_ISL_3784779,
EPI_ISL_3784786, EPI_ISL_3784793, EPI_ISL_3784801, EPI_ISL_3784808, EPI_ISL_3784816, EPI_ISL_3784823, EPI_ISL_3784830, EPI_ISL_3784837, EPI_ISL_3784845, EPI_ISL_3784853, EPI_ISL_3784862, EPI_ISL_3784869, EPI_ISL_3784877, EPI_ISL_3784885, EPI_ISL_3784908, EPI_ISL_3784916, EPI_ISL_3784921, EPI_ISL_3784930, EPI_ISL_3784958, EPI_ISL_3784980, EPI_ISL_3784988, EPI_ISL_3784995,
EPI_ISL_3785004, EPI_ISL_3785029, EPI_ISL_3785036, EPI_ISL_3785042, EPI_ISL_3785050, EPI_ISL_3785073, EPI_ISL_3785099, EPI_ISL_3785121, EPI_ISL_3785128, EPI_ISL_3785134, EPI_ISL_3785142, EPI_ISL_3785164, EPI_ISL_3785184, EPI_ISL_3785208, EPI_ISL_3785229, EPI_ISL_3785250, EPI_ISL_3785256, EPI_ISL_3785267, EPI_ISL_3785273, EPI_ISL_3785296, EPI_ISL_3785318, EPI_ISL_3785339,
EPI_ISL_3785382, EPI_ISL_3785407, EPI_ISL_3785413, EPI_ISL_3785420, EPI_ISL_3785430, EPI_ISL_3785436, EPI_ISL_3785443, EPI_ISL_3785449, EPI_ISL_3785455, EPI_ISL_3785484, EPI_ISL_3785506, EPI_ISL_3785524, EPI_ISL_3785530, EPI_ISL_3785538, EPI_ISL_3785543, EPI_ISL_3785550, EPI_ISL_3785555, EPI_ISL_3785560, EPI_ISL_3785566, EPI_ISL_3785574, EPI_ISL_3785595, EPI_ISL_3785632,
EPI_ISL_3785638, EPI_ISL_3785644, EPI_ISL_3785652, EPI_ISL_3785674, EPI_ISL_3785691, EPI_ISL_3785712, EPI_ISL_3785730, EPI_ISL_3785750, EPI_ISL_3785770, EPI_ISL_3785794, EPI_ISL_3785801, EPI_ISL_3785808, EPI_ISL_3785819, EPI_ISL_3785840, EPI_ISL_3785870, EPI_ISL_3785893, EPI_ISL_3785915, EPI_ISL_3785941, EPI_ISL_3785964, EPI_ISL_3785988, EPI_ISL_3786008, EPI_ISL_3786032,
EPI_ISL_3786079, EPI_ISL_3786105, EPI_ISL_3786129, EPI_ISL_3786137, EPI_ISL_3789757, EPI_ISL_3789763, EPI_ISL_3789767, EPI_ISL_3789798, EPI_ISL_3789814, EPI_ISL_3789830, EPI_ISL_3789846, EPI_ISL_3789862, EPI_ISL_3789878, EPI_ISL_3789892, EPI_ISL_3789910, EPI_ISL_3789922, EPI_ISL_3789934, EPI_ISL_3789950, EPI_ISL_3789954, EPI_ISL_3789960, EPI_ISL_3789965, EPI_ISL_3789968,
EPI_ISL_3789978, EPI_ISL_3789994, EPI_ISL_3790008, EPI_ISL_3790024, EPI_ISL_3790040, EPI_ISL_3790057, EPI_ISL_3790074, EPI_ISL_3790093, EPI_ISL_3790107, EPI_ISL_3790119, EPI_ISL_3790136, EPI_ISL_3790151, EPI_ISL_3790168, EPI_ISL_3790185, EPI_ISL_3790200, EPI_ISL_3790216, EPI_ISL_3790233, EPI_ISL_3790237, EPI_ISL_3790241, EPI_ISL_3790245, EPI_ISL_3790250, EPI_ISL_3790255,
EPI_ISL_3790260, EPI_ISL_3790265, EPI_ISL_3790270, EPI_ISL_3790274, EPI_ISL_3790278, EPI_ISL_3790284, EPI_ISL_3790289, EPI_ISL_3790294, EPI_ISL_3790310, EPI_ISL_3790326, EPI_ISL_3790340, EPI_ISL_3790355, EPI_ISL_3790371, EPI_ISL_3790388, EPI_ISL_3790393, EPI_ISL_3790398, EPI_ISL_3790403, EPI_ISL_3790408, EPI_ISL_3790419, EPI_ISL_3790434, EPI_ISL_3790449, EPI_ISL_3790466,
EPI_ISL_3790484, EPI_ISL_3790500, EPI_ISL_3790515, EPI_ISL_3790531, EPI_ISL_3790549, EPI_ISL_3790565, EPI_ISL_3790584, EPI_ISL_3790597, EPI_ISL_3790611, EPI_ISL_3790621, EPI_ISL_3790631, EPI_ISL_3790638, EPI_ISL_3790647, EPI_ISL_3790658, EPI_ISL_3790668, EPI_ISL_3790678, EPI_ISL_3790689, EPI_ISL_3790700, EPI_ISL_3790711, EPI_ISL_3790719, EPI_ISL_3790722, EPI_ISL_3790725,
EPI_ISL_3790729, EPI_ISL_3790732, EPI_ISL_3790737, EPI_ISL_3790740, EPI_ISL_3790743, EPI_ISL_3790747, EPI_ISL_3790751, EPI_ISL_3790754, EPI_ISL_3790758, EPI_ISL_3790761, EPI_ISL_3790765, EPI_ISL_3924145, EPI_ISL_3924146, EPI_ISL_3924147, EPI_ISL_3924148, EPI_ISL_3924149, EPI_ISL_3924150, EPI_ISL_3924151, EPI_ISL_3924152, EPI_ISL_3924153, EPI_ISL_3924154, EPI_ISL_3924155,
EPI_ISL_3924156, EPI_ISL_3924157, EPI_ISL_3924158, EPI_ISL_3924159, EPI_ISL_3924160, EPI_ISL_3924161, EPI_ISL_3924162, EPI_ISL_3924163, EPI_ISL_3924164, EPI_ISL_3924165, EPI_ISL_3924166, EPI_ISL_3924167, EPI_ISL_3924168, EPI_ISL_3924169, EPI_ISL_3924170, EPI_ISL_3924171, EPI_ISL_3924172, EPI_ISL_3924173, EPI_ISL_3924174, EPI_ISL_3924175, EPI_ISL_3924176, EPI_ISL_3924177,
EPI_ISL_3924178, EPI_ISL_3924179, EPI_ISL_3924180, EPI_ISL_3924181, EPI_ISL_3924182, EPI_ISL_3924183, EPI_ISL_3924184, EPI_ISL_3924185, EPI_ISL_3924186, EPI_ISL_3924187, EPI_ISL_3924188, EPI_ISL_3924189, EPI_ISL_3924190, EPI_ISL_3924191, EPI_ISL_3924192, EPI_ISL_3924193, EPI_ISL_3924194, EPI_ISL_3924195, EPI_ISL_3924196, EPI_ISL_3924197, EPI_ISL_3924198, EPI_ISL_3924199,
EPI_ISL_3924200, EPI_ISL_3924201, EPI_ISL_3924202, EPI_ISL_3924203, EPI_ISL_3924204, EPI_ISL_3924205, EPI_ISL_3924206, EPI_ISL_3924207, EPI_ISL_3924208, EPI_ISL_3924209, EPI_ISL_3924210, EPI_ISL_3924211, EPI_ISL_3924212, EPI_ISL_3924213, EPI_ISL_3924214, EPI_ISL_3924215, EPI_ISL_3924216, EPI_ISL_3924217, EPI_ISL_3924218, EPI_ISL_3924219, EPI_ISL_3924220, EPI_ISL_3924221,
EPI_ISL_3924222, EPI_ISL_3924223, EPI_ISL_3924224, EPI_ISL_3924225, EPI_ISL_3924226, EPI_ISL_3924227, EPI_ISL_3924228, EPI_ISL_3924229, EPI_ISL_3924230, EPI_ISL_3924231, EPI_ISL_3924232, EPI_ISL_3924233, EPI_ISL_3924234, EPI_ISL_3924235, EPI_ISL_3924236, EPI_ISL_3924237, EPI_ISL_3924238, EPI_ISL_3924239, EPI_ISL_3924240, EPI_ISL_3924241, EPI_ISL_3924242, EPI_ISL_3924243,
EPI_ISL_3924244, EPI_ISL_3924245, EPI_ISL_3924384, EPI_ISL_3924385, EPI_ISL_3924386, EPI_ISL_3924387, EPI_ISL_3924388, EPI_ISL_3924389, EPI_ISL_3924390, EPI_ISL_3924391, EPI_ISL_3924392, EPI_ISL_3924393, EPI_ISL_3924394, EPI_ISL_3924395, EPI_ISL_3924396, EPI_ISL_3924397, EPI_ISL_3924398, EPI_ISL_3924399, EPI_ISL_3924400, EPI_ISL_3924401, EPI_ISL_3924402, EPI_ISL_3924403,
EPI_ISL_3924404, EPI_ISL_3924405, EPI_ISL_3924406, EPI_ISL_3924407, EPI_ISL_3924408, EPI_ISL_3924409, EPI_ISL_3924410, EPI_ISL_3924411, EPI_ISL_3924412, EPI_ISL_3924413, EPI_ISL_3924414, EPI_ISL_3924415, EPI_ISL_3924416, EPI_ISL_3924417, EPI_ISL_3924418, EPI_ISL_3924419, EPI_ISL_3924420, EPI_ISL_3924421, EPI_ISL_3924422, EPI_ISL_3924423, EPI_ISL_3924424, EPI_ISL_3924425,
EPI_ISL_3924426, EPI_ISL_3924427, EPI_ISL_3924428, EPI_ISL_3924429, EPI_ISL_3924430, EPI_ISL_3924431, EPI_ISL_3924432, EPI_ISL_3924433, EPI_ISL_3924434, EPI_ISL_3924435, EPI_ISL_3924436, EPI_ISL_3924437, EPI_ISL_3924438, EPI_ISL_3924439, EPI_ISL_3924440, EPI_ISL_3924441, EPI_ISL_3924442, EPI_ISL_3924443, EPI_ISL_3924444, EPI_ISL_3924445, EPI_ISL_3924446, EPI_ISL_3924447,
EPI_ISL_3924448, EPI_ISL_3924449, EPI_ISL_3924450, EPI_ISL_3924451, EPI_ISL_3924452, EPI_ISL_3924453, EPI_ISL_3924454, EPI_ISL_3924455, EPI_ISL_3924456, EPI_ISL_3924457, EPI_ISL_3924458, EPI_ISL_3924459, EPI_ISL_3924460, EPI_ISL_3924461, EPI_ISL_3924462, EPI_ISL_3924463, EPI_ISL_3924464, EPI_ISL_3924465, EPI_ISL_3924466, EPI_ISL_3924467, EPI_ISL_3924468, EPI_ISL_3924469,
EPI_ISL_3924470, EPI_ISL_3924471, EPI_ISL_3924472, EPI_ISL_3924473, EPI_ISL_3924474, EPI_ISL_3924475, EPI_ISL_3924476, EPI_ISL_3924477, EPI_ISL_3924478, EPI_ISL_3924479, EPI_ISL_3924480, EPI_ISL_3924481, EPI_ISL_3924482, EPI_ISL_3924483, EPI_ISL_3924484, EPI_ISL_3924485, EPI_ISL_3924486, EPI_ISL_3924487, EPI_ISL_3924488, EPI_ISL_3924489, EPI_ISL_3924490, EPI_ISL_3924491,
EPI_ISL_3924492, EPI_ISL_3924493, EPI_ISL_3924494, EPI_ISL_3924495, EPI_ISL_3924496, EPI_ISL_3924497, EPI_ISL_3924498, EPI_ISL_3924499, EPI_ISL_3924500, EPI_ISL_3924501, EPI_ISL_3924502, EPI_ISL_3924503, EPI_ISL_3924504, EPI_ISL_3924505, EPI_ISL_3924506, EPI_ISL_3924507, EPI_ISL_3924508, EPI_ISL_3924509, EPI_ISL_3924510, EPI_ISL_3924511, EPI_ISL_3924512, EPI_ISL_3924513,
EPI_ISL_3924514, EPI_ISL_3924515, EPI_ISL_3924516, EPI_ISL_3924517, EPI_ISL_3924518, EPI_ISL_3924519, EPI_ISL_3924520, EPI_ISL_3924521, EPI_ISL_3924522, EPI_ISL_3924523, EPI_ISL_3924524, EPI_ISL_3924525, EPI_ISL_3924526, EPI_ISL_3924527, EPI_ISL_3924528, EPI_ISL_3924529, EPI_ISL_3924530, EPI_ISL_3924531, EPI_ISL_3924532, EPI_ISL_3924533, EPI_ISL_3924534, EPI_ISL_3924535,
EPI_ISL_3949671, EPI_ISL_3949672, EPI_ISL_3949673, EPI_ISL_3949674, EPI_ISL_3949675, EPI_ISL_3949676, EPI_ISL_3949677, EPI_ISL_3949678, EPI_ISL_3949679, EPI_ISL_3949680, EPI_ISL_3949681, EPI_ISL_3949682, EPI_ISL_3949683, EPI_ISL_3949684, EPI_ISL_3949685, EPI_ISL_3949686, EPI_ISL_3949687, EPI_ISL_3949688, EPI_ISL_3949689, EPI_ISL_3949690, EPI_ISL_3949691, EPI_ISL_3949692,
EPI_ISL_3949693, EPI_ISL_3949694, EPI_ISL_3949695, EPI_ISL_3949696, EPI_ISL_3949697, EPI_ISL_3949698, EPI_ISL_3949699, EPI_ISL_3949700, EPI_ISL_3949701, EPI_ISL_3949702, EPI_ISL_3949703, EPI_ISL_3949704, EPI_ISL_3949705, EPI_ISL_3949706, EPI_ISL_3949707, EPI_ISL_3949708, EPI_ISL_3949709, EPI_ISL_3949710, EPI_ISL_3949711, EPI_ISL_3949712, EPI_ISL_3949713, EPI_ISL_3949714,
EPI_ISL_3949715, EPI_ISL_3949716, EPI_ISL_3949717, EPI_ISL_3949718, EPI_ISL_3949719, EPI_ISL_3949720, EPI_ISL_3949721, EPI_ISL_3949722, EPI_ISL_3949723, EPI_ISL_3949724, EPI_ISL_3949725, EPI_ISL_3949726, EPI_ISL_3949727, EPI_ISL_3949728, EPI_ISL_3949729, EPI_ISL_3949730, EPI_ISL_3949731, EPI_ISL_3949732, EPI_ISL_3949733, EPI_ISL_3949734, EPI_ISL_3949735, EPI_ISL_3949736,
EPI_ISL_3949737, EPI_ISL_3949738, EPI_ISL_3949739, EPI_ISL_3949740, EPI_ISL_3949741, EPI_ISL_3949742, EPI_ISL_3949743, EPI_ISL_3949744, EPI_ISL_3949745, EPI_ISL_3949746, EPI_ISL_3949747, EPI_ISL_3949748, EPI_ISL_3949749, EPI_ISL_3949750, EPI_ISL_3949751, EPI_ISL_3949752, EPI_ISL_3949753, EPI_ISL_3949754, EPI_ISL_3949755, EPI_ISL_3949756, EPI_ISL_3949757, EPI_ISL_3949758,
EPI_ISL_3949759, EPI_ISL_3949760, EPI_ISL_3949761, EPI_ISL_3949762, EPI_ISL_3949763, EPI_ISL_3949764, EPI_ISL_3949765, EPI_ISL_3949766, EPI_ISL_3949767, EPI_ISL_3949768, EPI_ISL_3949769, EPI_ISL_3949770, EPI_ISL_3949771, EPI_ISL_3949772, EPI_ISL_3949773, EPI_ISL_3949774, EPI_ISL_3949775, EPI_ISL_3949776, EPI_ISL_3949777, EPI_ISL_3949778, EPI_ISL_3949779, EPI_ISL_3949780,
EPI_ISL_3949781, EPI_ISL_3949782, EPI_ISL_3949783, EPI_ISL_3949784, EPI_ISL_3949785, EPI_ISL_3949786, EPI_ISL_3949787, EPI_ISL_3949788, EPI_ISL_3949789, EPI_ISL_3949790, EPI_ISL_3949791, EPI_ISL_3949792, EPI_ISL_3949793, EPI_ISL_3949794, EPI_ISL_3949795, EPI_ISL_3949796, EPI_ISL_3949797, EPI_ISL_3949798, EPI_ISL_3949799, EPI_ISL_3949800, EPI_ISL_3949801, EPI_ISL_3949802,
EPI_ISL_3949803, EPI_ISL_3949804, EPI_ISL_3949805, EPI_ISL_3949806, EPI_ISL_3949807, EPI_ISL_3949808, EPI_ISL_3949809, EPI_ISL_3949810, EPI_ISL_3949811, EPI_ISL_3949812, EPI_ISL_3949813, EPI_ISL_3949814, EPI_ISL_3949815, EPI_ISL_3949816, EPI_ISL_3949817, EPI_ISL_3949818, EPI_ISL_3949819, EPI_ISL_3949820, EPI_ISL_3949821, EPI_ISL_3949822, EPI_ISL_3949823, EPI_ISL_3949824,
EPI_ISL_3949825, EPI_ISL_3949826, EPI_ISL_3949827, EPI_ISL_3949828, EPI_ISL_3949829, EPI_ISL_3949830, EPI_ISL_3949831, EPI_ISL_3949832, EPI_ISL_3949833, EPI_ISL_3949834, EPI_ISL_3949835, EPI_ISL_3949836, EPI_ISL_3949837, EPI_ISL_3949838, EPI_ISL_3949839, EPI_ISL_3949840, EPI_ISL_3949841, EPI_ISL_3949842, EPI_ISL_3949843, EPI_ISL_3949844, EPI_ISL_3949845, EPI_ISL_3949846,
EPI_ISL_3949847, EPI_ISL_3949848, EPI_ISL_3949849, EPI_ISL_3949850, EPI_ISL_3949851, EPI_ISL_3949852, EPI_ISL_3949853, EPI_ISL_3949854, EPI_ISL_3949855, EPI_ISL_3949856, EPI_ISL_3949857, EPI_ISL_3949858, EPI_ISL_3949859, EPI_ISL_3949860, EPI_ISL_3949861, EPI_ISL_3949862, EPI_ISL_3949863, EPI_ISL_3949864, EPI_ISL_3949865, EPI_ISL_3949866, EPI_ISL_3949867, EPI_ISL_3949868,
EPI_ISL_3949869, EPI_ISL_3949870, EPI_ISL_3949871, EPI_ISL_3949872, EPI_ISL_3949873, EPI_ISL_3949874, EPI_ISL_3949875, EPI_ISL_3949876, EPI_ISL_3949877, EPI_ISL_3949878, EPI_ISL_3949879, EPI_ISL_3949880, EPI_ISL_3949881, EPI_ISL_3949882, EPI_ISL_3949883, EPI_ISL_3949884, EPI_ISL_3949885, EPI_ISL_3949886, EPI_ISL_3949887, EPI_ISL_3949888, EPI_ISL_3949889, EPI_ISL_3949890,
EPI_ISL_3949891, EPI_ISL_3949892, EPI_ISL_3949893, EPI_ISL_3949894, EPI_ISL_3949895, EPI_ISL_3949896, EPI_ISL_3949897, EPI_ISL_3949898, EPI_ISL_3949899, EPI_ISL_3949900, EPI_ISL_3949901, EPI_ISL_3949902, EPI_ISL_3949903, EPI_ISL_3949904, EPI_ISL_3949905, EPI_ISL_3949906, EPI_ISL_3949907, EPI_ISL_3949908, EPI_ISL_3949909, EPI_ISL_3949910, EPI_ISL_3949911, EPI_ISL_3949912,
EPI_ISL_3949913, EPI_ISL_3949914, EPI_ISL_3949915, EPI_ISL_4073318, EPI_ISL_4073319, EPI_ISL_4073320, EPI_ISL_4073321, EPI_ISL_4073322, EPI_ISL_4073323, EPI_ISL_4073324, EPI_ISL_4073325, EPI_ISL_4073326, EPI_ISL_4073327, EPI_ISL_4073328, EPI_ISL_4073329, EPI_ISL_4073330, EPI_ISL_4073331, EPI_ISL_4073332, EPI_ISL_4073333, EPI_ISL_4073334, EPI_ISL_4073335, EPI_ISL_4073336,
EPI_ISL_4073337, EPI_ISL_4073338, EPI_ISL_4073339, EPI_ISL_4073340, EPI_ISL_4073341, EPI_ISL_4073342, EPI_ISL_4073343, EPI_ISL_4073344, EPI_ISL_4073345, EPI_ISL_4073346, EPI_ISL_4073347, EPI_ISL_4073348, EPI_ISL_4073349, EPI_ISL_4073350, EPI_ISL_4073351, EPI_ISL_4073352, EPI_ISL_4073353, EPI_ISL_4073354, EPI_ISL_4073355, EPI_ISL_4073356, EPI_ISL_4073357, EPI_ISL_4073358,
EPI_ISL_4073359, EPI_ISL_4073360, EPI_ISL_4073361, EPI_ISL_4073362, EPI_ISL_4073363, EPI_ISL_4073364, EPI_ISL_4073365, EPI_ISL_4073366, EPI_ISL_4073367, EPI_ISL_4073368, EPI_ISL_4073369, EPI_ISL_4073370, EPI_ISL_4073371, EPI_ISL_4073372, EPI_ISL_4073373, EPI_ISL_4073374, EPI_ISL_4073375, EPI_ISL_4073376, EPI_ISL_4073377, EPI_ISL_4073378, EPI_ISL_4073379, EPI_ISL_4073380,
EPI_ISL_4073381, EPI_ISL_4073382, EPI_ISL_4073383, EPI_ISL_4073384, EPI_ISL_4073385, EPI_ISL_4073386, EPI_ISL_4073387, EPI_ISL_4073388, EPI_ISL_4073389, EPI_ISL_4073390, EPI_ISL_4073391, EPI_ISL_4073392, EPI_ISL_4073393, EPI_ISL_4073394, EPI_ISL_4073395, EPI_ISL_4073396, EPI_ISL_4073397, EPI_ISL_4073398, EPI_ISL_4073400, EPI_ISL_4073401, EPI_ISL_4073402, EPI_ISL_4073403,
EPI_ISL_4073404, EPI_ISL_4073405, EPI_ISL_4073406, EPI_ISL_4073407, EPI_ISL_4073408, EPI_ISL_4073409, EPI_ISL_4073410, EPI_ISL_4073411, EPI_ISL_4073412, EPI_ISL_4073413, EPI_ISL_4073414, EPI_ISL_4073415, EPI_ISL_4073416, EPI_ISL_4073417, EPI_ISL_4079207, EPI_ISL_4079208, EPI_ISL_4079209, EPI_ISL_4079210, EPI_ISL_4079211, EPI_ISL_4079212, EPI_ISL_4079213, EPI_ISL_4079214,
EPI_ISL_4079215, EPI_ISL_4079216, EPI_ISL_4079217, EPI_ISL_4079218, EPI_ISL_4079219, EPI_ISL_4079220, EPI_ISL_4079221, EPI_ISL_4079222, EPI_ISL_4079223, EPI_ISL_4079224, EPI_ISL_4079225, EPI_ISL_4079226, EPI_ISL_4079227, EPI_ISL_4079228, EPI_ISL_4079229, EPI_ISL_4079230, EPI_ISL_4079231, EPI_ISL_4079232, EPI_ISL_4079233, EPI_ISL_4079234, EPI_ISL_4079235, EPI_ISL_4079236,
EPI_ISL_4079237, EPI_ISL_4079238, EPI_ISL_4079239, EPI_ISL_4079240, EPI_ISL_4079241, EPI_ISL_4079242, EPI_ISL_4079243, EPI_ISL_4079244, EPI_ISL_4079245, EPI_ISL_4079246, EPI_ISL_4079247, EPI_ISL_4079248, EPI_ISL_4079249, EPI_ISL_4079250, EPI_ISL_4079251, EPI_ISL_4079252, EPI_ISL_4079253, EPI_ISL_4079254, EPI_ISL_4079255, EPI_ISL_4079256, EPI_ISL_4079257, EPI_ISL_4079258,
EPI_ISL_4079259, EPI_ISL_4079260, EPI_ISL_4079261, EPI_ISL_4079262, EPI_ISL_4079263, EPI_ISL_4079264, EPI_ISL_4079265, EPI_ISL_4079266, EPI_ISL_4079267, EPI_ISL_4079268, EPI_ISL_4079269, EPI_ISL_4079270, EPI_ISL_4079271, EPI_ISL_4079272, EPI_ISL_4079273, EPI_ISL_4079274, EPI_ISL_4079275, EPI_ISL_4079276, EPI_ISL_4079277, EPI_ISL_4079278, EPI_ISL_4079279, EPI_ISL_4079280,
EPI_ISL_4079281, EPI_ISL_4079282, EPI_ISL_4079283, EPI_ISL_4079284, EPI_ISL_4079285, EPI_ISL_4079286, EPI_ISL_4079287, EPI_ISL_4079288, EPI_ISL_4079289, EPI_ISL_4079290, EPI_ISL_4079291, EPI_ISL_4079292, EPI_ISL_4079293, EPI_ISL_4079294, EPI_ISL_4079295, EPI_ISL_4079296, EPI_ISL_4079297, EPI_ISL_4079298, EPI_ISL_4079299, EPI_ISL_4079300, EPI_ISL_4079301, EPI_ISL_4079302,
EPI_ISL_4079303, EPI_ISL_4079304, EPI_ISL_4079305, EPI_ISL_4079306, EPI_ISL_4079307, EPI_ISL_4079308, EPI_ISL_4079309, EPI_ISL_4079310, EPI_ISL_4079311, EPI_ISL_4079312, EPI_ISL_4079313, EPI_ISL_4079314, EPI_ISL_4079315, EPI_ISL_4079316, EPI_ISL_4079317, EPI_ISL_4079318, EPI_ISL_4079319, EPI_ISL_4079320, EPI_ISL_4079321, EPI_ISL_4079322, EPI_ISL_4079323, EPI_ISL_4079324,
EPI_ISL_4079325, EPI_ISL_4079326, EPI_ISL_4079327, EPI_ISL_4079328, EPI_ISL_4079329, EPI_ISL_4079330, EPI_ISL_4079331, EPI_ISL_4079332, EPI_ISL_4079333, EPI_ISL_4079334, EPI_ISL_4079335, EPI_ISL_4079336, EPI_ISL_4079337, EPI_ISL_4079338, EPI_ISL_4079339, EPI_ISL_4079340, EPI_ISL_4079341, EPI_ISL_4079342, EPI_ISL_4079343, EPI_ISL_4079344, EPI_ISL_4079345, EPI_ISL_4079346,

EPI_ISL_4079347, EPI_ISL_4079348, EPI_ISL_4079349, EPI_ISL_4079350, EPI_ISL_4079351, EPI_ISL_4079352, EPI_ISL_4079353, EPI_ISL_4079354, EPI_ISL_4079355, EPI_ISL_4079356, EPI_ISL_4079357, EPI_ISL_4137236, EPI_ISL_4137237, EPI_ISL_4137238, EPI_ISL_4137239, EPI_ISL_4137240, EPI_ISL_4137241, EPI_ISL_4137242, EPI_ISL_4137243, EPI_ISL_4137244, EPI_ISL_4137245, EPI_ISL_4137246, EPI_ISL_4137247, EPI_ISL_4137248, EPI_ISL_4137249, EPI_ISL_4137250, EPI_ISL_4137251, EPI_ISL_4137252, EPI_ISL_4137253, EPI_ISL_4137254, EPI_ISL_4137255, EPI_ISL_4137256, EPI_ISL_4137257, EPI_ISL_4137258, EPI_ISL_4137259, EPI_ISL_4137260, EPI_ISL_4137261, EPI_ISL_4137262, EPI_ISL_4137263, EPI_ISL_4137264, EPI_ISL_4137265, EPI_ISL_4137266, EPI_ISL_4137267, EPI_ISL_4137268, EPI_ISL_4137269, EPI_ISL_4137270, EPI_ISL_4137271, EPI_ISL_4137272, EPI_ISL_4137273, EPI_ISL_4137274, EPI_ISL_4137275, EPI_ISL_4137276, EPI_ISL_4137277, EPI_ISL_4137278, EPI_ISL_4137279, EPI_ISL_4137280, EPI_ISL_4137281, EPI_ISL_4137282, EPI_ISL_4137283, EPI_ISL_4137284, EPI_ISL_4137285, EPI_ISL_4137286, EPI_ISL_4137287, EPI_ISL_4137288, EPI_ISL_4137289, EPI_ISL_4137290, EPI_ISL_4137291, EPI_ISL_4137292, EPI_ISL_4137293, EPI_ISL_4137294, EPI_ISL_4137295, EPI_ISL_4137296, EPI_ISL_4137297, EPI_ISL_4137298, EPI_ISL_4137299, EPI_ISL_4137300, EPI_ISL_4137301, EPI_ISL_4137302, EPI_ISL_4137303, EPI_ISL_4137304, EPI_ISL_4137305, EPI_ISL_4137306, EPI_ISL_4137307, EPI_ISL_4137308, EPI_ISL_4137309, EPI_ISL_4137310, EPI_ISL_4137311, EPI_ISL_4137312, EPI_ISL_4137313, EPI_ISL_4137314, EPI_ISL_4137315, EPI_ISL_4137316, EPI_ISL_4137317, EPI_ISL_4137318, EPI_ISL_4137319, EPI_ISL_4137320, EPI_ISL_4137321, EPI_ISL_4137322, EPI_ISL_4137323, EPI_ISL_4137324, EPI_ISL_4137325, EPI_ISL_4137326, EPI_ISL_4137327, EPI_ISL_4137328, EPI_ISL_4137329, EPI_ISL_4137330, EPI_ISL_4137331, EPI_ISL_4137332, EPI_ISL_4137333, EPI_ISL_4137334, EPI_ISL_4137335, EPI_ISL_4137336, EPI_ISL_4137337, EPI_ISL_4137338, EPI_ISL_4137339, EPI_ISL_4137340, EPI_ISL_4137341, EPI_ISL_4137342, EPI_ISL_4137343, EPI_ISL_4137344, EPI_ISL_4137345, EPI_ISL_4137346, EPI_ISL_4137347, EPI_ISL_4137348, EPI_ISL_4137349, EPI_ISL_4137350, EPI_ISL_4137351, EPI_ISL_4137352, EPI_ISL_4137353, EPI_ISL_4137354, EPI_ISL_4137355, EPI_ISL_4137356, EPI_ISL_4137357, EPI_ISL_4137358, EPI_ISL_4137359, EPI_ISL_4137360, EPI_ISL_4137361, EPI_ISL_4137363, EPI_ISL_4137364, EPI_ISL_4137365, EPI_ISL_4137366, EPI_ISL_4137367, EPI_ISL_4137368, EPI_ISL_4137369, EPI_ISL_4137370, EPI_ISL_4137371, EPI_ISL_4137372, EPI_ISL_4137373, EPI_ISL_4137374, EPI_ISL_4137375, EPI_ISL_4137376, EPI_ISL_4137377, EPI_ISL_4137378, EPI_ISL_4137379, EPI_ISL_4137380, EPI_ISL_4137381, EPI_ISL_4137382, EPI_ISL_4137383, EPI_ISL_4137384, EPI_ISL_4137385, EPI_ISL_4137386, EPI_ISL_4137387, EPI_ISL_4137388, EPI_ISL_4137389, EPI_ISL_4137390, EPI_ISL_4137391, EPI_ISL_4137392, EPI_ISL_4137393, EPI_ISL_4137394, EPI_ISL_4137395, EPI_ISL_4137396, EPI_ISL_4137397, EPI_ISL_4137398, EPI_ISL_4137399, EPI_ISL_4137400, EPI_ISL_4137401, EPI_ISL_4137402, EPI_ISL_4169825, EPI_ISL_4169826, EPI_ISL_4271662, EPI_ISL_4271663, EPI_ISL_4271664, EPI_ISL_4271665, EPI_ISL_4271666, EPI_ISL_4271667, EPI_ISL_4271668, EPI_ISL_4271669, EPI_ISL_4271670, EPI_ISL_4271671, EPI_ISL_4271672, EPI_ISL_4271673, EPI_ISL_4271674, EPI_ISL_4271675, EPI_ISL_4271676, EPI_ISL_4271677, EPI_ISL_4271678, EPI_ISL_4271679, EPI_ISL_4271680, EPI_ISL_4271681, EPI_ISL_4271682, EPI_ISL_4271683, EPI_ISL_4271684, EPI_ISL_4271685, EPI_ISL_4271686, EPI_ISL_4271687, EPI_ISL_4271688, EPI_ISL_4271689, EPI_ISL_4271690, EPI_ISL_4271691, EPI_ISL_4271692, EPI_ISL_4271693, EPI_ISL_4271694, EPI_ISL_4271695, EPI_ISL_4271696, EPI_ISL_4271697, EPI_ISL_4271698, EPI_ISL_4271699, EPI_ISL_4271700, EPI_ISL_4271701, EPI_ISL_4271702, EPI_ISL_4271703, EPI_ISL_4271704, EPI_ISL_4271705, EPI_ISL_4271706, EPI_ISL_4271707, EPI_ISL_4271708, EPI_ISL_4271709, EPI_ISL_4271710, EPI_ISL_4271711, EPI_ISL_4271712, EPI_ISL_4271713, EPI_ISL_4271714, EPI_ISL_4271715, EPI_ISL_4271716, EPI_ISL_4271717, EPI_ISL_4271718, EPI_ISL_4271719, EPI_ISL_4271720, EPI_ISL_4271721, EPI_ISL_4271722, EPI_ISL_4271723, EPI_ISL_4271724, EPI_ISL_4271725, EPI_ISL_4271726, EPI_ISL_4271727, EPI_ISL_4271728, EPI_ISL_4271729, EPI_ISL_4271730, EPI_ISL_4271731, EPI_ISL_4271732, EPI_ISL_4271733, EPI_ISL_4271734, EPI_ISL_4271735, EPI_ISL_4271736, EPI_ISL_4271737, EPI_ISL_4271738, EPI_ISL_4271739, EPI_ISL_4271740, EPI_ISL_4271741, EPI_ISL_4271742, EPI_ISL_4271743, EPI_ISL_4271744, EPI_ISL_4271745, EPI_ISL_4271746, EPI_ISL_4271747, EPI_ISL_4271748, EPI_ISL_4271749, EPI_ISL_4271750, EPI_ISL_4271751, EPI_ISL_4271752, EPI_ISL_4271753, EPI_ISL_4271754, EPI_ISL_4271755, EPI_ISL_4271756, EPI_ISL_4271757, EPI_ISL_4271758, EPI_ISL_4271759, EPI_ISL_4271760, EPI_ISL_4271761, EPI_ISL_4271762, EPI_ISL_4271763, EPI_ISL_4271764, EPI_ISL_4271765, EPI_ISL_4271766, EPI_ISL_4271767, EPI_ISL_4271768, EPI_ISL_4271769, EPI_ISL_4271770, EPI_ISL_4271771, EPI_ISL_4271772, EPI_ISL_4271773, EPI_ISL_4271774, EPI_ISL_4271775, EPI_ISL_4271776, EPI_ISL_4271777, EPI_ISL_4271778, EPI_ISL_4271779, EPI_ISL_4271780, EPI_ISL_4271781, EPI_ISL_4271782, EPI_ISL_4271783, EPI_ISL_4271784, EPI_ISL_4271785, EPI_ISL_4271786, EPI_ISL_4271787, EPI_ISL_4271788, EPI_ISL_4271789, EPI_ISL_4271790, EPI_ISL_4271791, EPI_ISL_4271792, EPI_ISL_4271793, EPI_ISL_4271794, EPI_ISL_4271795, EPI_ISL_4271796, EPI_ISL_4271797, EPI_ISL_4271798, EPI_ISL_4271799, EPI_ISL_4271800, EPI_ISL_4271801, EPI_ISL_4271802, EPI_ISL_4271803, EPI_ISL_4271804, EPI_ISL_4271805, EPI_ISL_4271806, EPI_ISL_4271807, EPI_ISL_4271808, EPI_ISL_4271809, EPI_ISL_4271810, EPI_ISL_4271811, EPI_ISL_4271812, EPI_ISL_4271813, EPI_ISL_4271814, EPI_ISL_4271815, EPI_ISL_4271816, EPI_ISL_4271817, EPI_ISL_4271818, EPI_ISL_4271819, EPI_ISL_4271820, EPI_ISL_4271821, EPI_ISL_4271822, EPI_ISL_4271823, EPI_ISL_4271824, EPI_ISL_4271825, EPI_ISL_4271826, EPI_ISL_4271827, EPI_ISL_4341672, EPI_ISL_4341673, EPI_ISL_4341674, EPI_ISL_4341675, EPI_ISL_4341676, EPI_ISL_4341677, EPI_ISL_4341678, EPI_ISL_4341679, EPI_ISL_4341680, EPI_ISL_4341681, EPI_ISL_4341682, EPI_ISL_4341683, EPI_ISL_4341684, EPI_ISL_4341685, EPI_ISL_4341686, EPI_ISL_4341687, EPI_ISL_4341688, EPI_ISL_4341689, EPI_ISL_4341690, EPI_ISL_4341691, EPI_ISL_4341692, EPI_ISL_4341693, EPI_ISL_4341695, EPI_ISL_4341696, EPI_ISL_4341698, EPI_ISL_4341700, EPI_ISL_4341702, EPI_ISL_4341704, EPI_ISL_4341706, EPI_ISL_4341707, EPI_ISL_4341713, EPI_ISL_4341719, EPI_ISL_4341721, EPI_ISL_4341722, EPI_ISL_4341725, EPI_ISL_4341726, EPI_ISL_4341728, EPI_ISL_4341730, EPI_ISL_4341732, EPI_ISL_4341733, EPI_ISL_4341735, EPI_ISL_4341737, EPI_ISL_4341739, EPI_ISL_4341745, EPI_ISL_4341747, EPI_ISL_4341749, EPI_ISL_4341751, EPI_ISL_4341753, EPI_ISL_4341754, EPI_ISL_4341756, EPI_ISL_4341763, EPI_ISL_4341765, EPI_ISL_4341766, EPI_ISL_4341768, EPI_ISL_4341770, EPI_ISL_4341772, EPI_ISL_4341774, EPI_ISL_4341780, EPI_ISL_4341786, EPI_ISL_4341788, EPI_ISL_4341790, EPI_ISL_4341791, EPI_ISL_4341793, EPI_ISL_4341795, EPI_ISL_4341797, EPI_ISL_4341799, EPI_ISL_4341800, EPI_ISL_4341802, EPI_ISL_4341804, EPI_ISL_4341806, EPI_ISL_4341807, EPI_ISL_4341809, EPI_ISL_4341815, EPI_ISL_4341821, EPI_ISL_4341827, EPI_ISL_4341829, EPI_ISL_4341831, EPI_ISL_4341832, EPI_ISL_4341834, EPI_ISL_4341836, EPI_ISL_4341838, EPI_ISL_4341840, EPI_ISL_4341842, EPI_ISL_4341844, EPI_ISL_4341846, EPI_ISL_4341847, EPI_ISL_4341849, EPI_ISL_4341851, EPI_ISL_4341853, EPI_ISL_4341855, EPI_ISL_4341856, EPI_ISL_4341858, EPI_ISL_4341860, EPI_ISL_4341862, EPI_ISL_4341864, EPI_ISL_4341869, EPI_ISL_4341871, EPI_ISL_4341873, EPI_ISL_4341874, EPI_ISL_4341876, EPI_ISL_4341878, EPI_ISL_4341884, EPI_ISL_4341886, EPI_ISL_4341888, EPI_ISL_4341890, EPI_ISL_4341892, EPI_ISL_4341894, EPI_ISL_4341896, EPI_ISL_4341897, EPI_ISL_4341899, EPI_ISL_4341901, EPI_ISL_4341903, EPI_ISL_4341905, EPI_ISL_4341907, EPI_ISL_4341909, EPI_ISL_4341910, EPI_ISL_4341912, EPI_ISL_4341914, EPI_ISL_4341916, EPI_ISL_4341918, EPI_ISL_4341925, EPI_ISL_4341930, EPI_ISL_4341935, EPI_ISL_4341942, EPI_ISL_4341944, EPI_ISL_4341946, EPI_ISL_4341948, EPI_ISL_4341949, EPI_ISL_4341951, EPI_ISL_4341953, EPI_ISL_4341959, EPI_ISL_4341961, EPI_ISL_4341962, EPI_ISL_4341964, EPI_ISL_4341966, EPI_ISL_4341968, EPI_ISL_4341970, EPI_ISL_4341976, EPI_ISL_4341978, EPI_ISL_4341980, EPI_ISL_4341981, EPI_ISL_4341983, EPI_ISL_4341985, EPI_ISL_4341987, EPI_ISL_4341989, EPI_ISL_4341991, EPI_ISL_4341993, EPI_ISL_4341994, EPI_ISL_4341996, EPI_ISL_4341998, EPI_ISL_4342003, EPI_ISL_4342009, EPI_ISL_4342015, EPI_ISL_4342020, EPI_ISL_4342022, EPI_ISL_4342024, EPI_ISL_4342026, EPI_ISL_4342028, EPI_ISL_4342029, EPI_ISL_4342031, EPI_ISL_4342033, EPI_ISL_4342035, EPI_ISL_4342036, EPI_ISL_4342038, EPI_ISL_4342040, EPI_ISL_4342042, EPI_ISL_4342044, EPI_ISL_4342046, EPI_ISL_4342047, EPI_ISL_4342049, EPI_ISL_4342051, EPI_ISL_4504029, EPI_ISL_4504030, EPI_ISL_4504031, EPI_ISL_4504032, EPI_ISL_4504033, EPI_ISL_4504034, EPI_ISL_4504035, EPI_ISL_4504036, EPI_ISL_4504037, EPI_ISL_4504038, EPI_ISL_4504039, EPI_ISL_4504040, EPI_ISL_4504041, EPI_ISL_4504042, EPI_ISL_4504043, EPI_ISL_4504044, EPI_ISL_4504045, EPI_ISL_4504046, EPI_ISL_4504047, EPI_ISL_4504048, EPI_ISL_4504049, EPI_ISL_4504050, EPI_ISL_4504051, EPI_ISL_4504052, EPI_ISL_4504053, EPI_ISL_4504054, EPI_ISL_4504055, EPI_ISL_4504056, EPI_ISL_4504057, EPI_ISL_4504058, EPI_ISL_4504059, EPI_ISL_4504060, EPI_ISL_4504061, EPI_ISL_4504062, EPI_ISL_4504063, EPI_ISL_4504064, EPI_ISL_4504065, EPI_ISL_4504066, EPI_ISL_4504067, EPI_ISL_4504068, EPI_ISL_4504069, EPI_ISL_4504070, EPI_ISL_4504071, EPI_ISL_4504072, EPI_ISL_4504073, EPI_ISL_4504074, EPI_ISL_4504075, EPI_ISL_4504076, EPI_ISL_4504077, EPI_ISL_4504078, EPI_ISL_4504079, EPI_ISL_4504080, EPI_ISL_4504081, EPI_ISL_4504082, EPI_ISL_4504083, EPI_ISL_4504084, EPI_ISL_4504085, EPI_ISL_4504086, EPI_ISL_4504087, EPI_ISL_4504088, EPI_ISL_4504089, EPI_ISL_4504090, EPI_ISL_4504091, EPI_ISL_4504092, EPI_ISL_4504093, EPI_ISL_4504094, EPI_ISL_4504095, EPI_ISL_4504096, EPI_ISL_4504097, EPI_ISL_4504098, EPI_ISL_4504099, EPI_ISL_4504100, EPI_ISL_4504101, EPI_ISL_4504102, EPI_ISL_4504103, EPI_ISL_4504104, EPI_ISL_4504105, EPI_ISL_4504106, EPI_ISL_4504107, EPI_ISL_4504108, EPI_ISL_4504109, EPI_ISL_4504110, EPI_ISL_4504111, EPI_ISL_4504112, EPI_ISL_4504113, EPI_ISL_4504114, EPI_ISL_4504115, EPI_ISL_4504116, EPI_ISL_4504117, EPI_ISL_4504118, EPI_ISL_4504119, EPI_ISL_4504120, EPI_ISL_4504121, EPI_ISL_4504122, EPI_ISL_4504123, EPI_ISL_4504124, EPI_ISL_4504125, EPI_ISL_4504126, EPI_ISL_4504127, EPI_ISL_4504128, EPI_ISL_4504129, EPI_ISL_4504130, EPI_ISL_4504131, EPI_ISL_4504132, EPI_ISL_4504133, EPI_ISL_4504134, EPI_ISL_4504135, EPI_ISL_4504136, EPI_ISL_4504137, EPI_ISL_4504138, EPI_ISL_4504139, EPI_ISL_4504140, EPI_ISL_4504141, EPI_ISL_4504142, EPI_ISL_4504143, EPI_ISL_4504144, EPI_ISL_4504145, EPI_ISL_4504146, EPI_ISL_4504147, EPI_ISL_4504148, EPI_ISL_4504149, EPI_ISL_4504150, EPI_ISL_4504151, EPI_ISL_4504152, EPI_ISL_4504153, EPI_ISL_4504154, EPI_ISL_4504155, EPI_ISL_4504156, EPI_ISL_4504157, EPI_ISL_4504158, EPI_ISL_4504159, EPI_ISL_4504160, EPI_ISL_4504161, EPI_ISL_4504162, EPI_ISL_4504163, EPI_ISL_4504164, EPI_ISL_4504165, EPI_ISL_4504166, EPI_ISL_4504167, EPI_ISL_4504168, EPI_ISL_4504169, EPI_ISL_4504170, EPI_ISL_4504171, EPI_ISL_4504172, EPI_ISL_4504173, EPI_ISL_4504174, EPI_ISL_4504175, EPI_ISL_4504176, EPI_ISL_4504177, EPI_ISL_4504178, EPI_ISL_4504179, EPI_ISL_4504180, EPI_ISL_4504181, EPI_ISL_4504182, EPI_ISL_4504183, EPI_ISL_4504184, EPI_ISL_4504185, EPI_ISL_4504186, EPI_ISL_4504187, EPI_ISL_4504188, EPI_ISL_4504189, EPI_ISL_4504190, EPI_ISL_4504191, EPI_ISL_4504192, EPI_ISL_4557756, EPI_ISL_4557757, EPI_ISL_4557758, EPI_ISL_4557759, EPI_ISL_4557760, EPI_ISL_4557761, EPI_ISL_4557762, EPI_ISL_4557763, EPI_ISL_4557764, EPI_ISL_4557765, EPI_ISL_4557766, EPI_ISL_4557767, EPI_ISL_4557768, EPI_ISL_4557769, EPI_ISL_4557770, EPI_ISL_4557771, EPI_ISL_4557772, EPI_ISL_4557773, EPI_ISL_4557774, EPI_ISL_4557775, EPI_ISL_4557776, EPI_ISL_4557777, EPI_ISL_4557778, EPI_ISL_4557779, EPI_ISL_4557780, EPI_ISL_4557781, EPI_ISL_4557782, EPI_ISL_4557783, EPI_ISL_4557784, EPI_ISL_4557785, EPI_ISL_4557786, EPI_ISL_4557787, EPI_ISL_4557788, EPI_ISL_4557789, EPI_ISL_4557790, EPI_ISL_4557791, EPI_ISL_4557792, EPI_ISL_4557793, EPI_ISL_4557794, EPI_ISL_4557795, EPI_ISL_4557796, EPI_ISL_4557797, EPI_ISL_4557798, EPI_ISL_4557799, EPI_ISL_4557800, EPI_ISL_4557801, EPI_ISL_4557802, EPI_ISL_4557803, EPI_ISL_4557804, EPI_ISL_4557805, EPI_ISL_4557806, EPI_ISL_4557807, EPI_ISL_4557808, EPI_ISL_4557809, EPI_ISL_4557810, EPI_ISL_4557811, EPI_ISL_4557812, EPI_ISL_4557813, EPI_ISL_4557814, EPI_ISL_4557815, EPI_ISL_4557816, EPI_ISL_4557817, EPI_ISL_4557818, EPI_ISL_4557819, EPI_ISL_4557820, EPI_ISL_4557821, EPI_ISL_4557822, EPI_ISL_4557823, EPI_ISL_4557824, EPI_ISL_4557825, EPI_ISL_4557826, EPI_ISL_4557827, EPI_ISL_4557828, EPI_ISL_4557829, EPI_ISL_4557830, EPI_ISL_4557831, EPI_ISL_4557832, EPI_ISL_4557833, EPI_ISL_4557834, EPI_ISL_4557835, EPI_ISL_4557836, EPI_ISL_4557837, EPI_ISL_4557838, EPI_ISL_4557839, EPI_ISL_4557840, EPI_ISL_4557841, EPI_ISL_4557842, EPI_ISL_4557843, EPI_ISL_4557844, EPI_ISL_4557845, EPI_ISL_4557846, EPI_ISL_4557847, EPI_ISL_4557848, EPI_ISL_4557849, EPI_ISL_4557850, EPI_ISL_4557851, EPI_ISL_4557852, EPI_ISL_4557853, EPI_ISL_4557854, EPI_ISL_4557855, EPI_ISL_4557856, EPI_ISL_4557857, EPI_ISL_4557858, EPI_ISL_4557859, EPI_ISL_4557860, EPI_ISL_4557861, EPI_ISL_4557862, EPI_ISL_4557863, EPI_ISL_4557864, EPI_ISL_4557865, EPI_ISL_4557866, EPI_ISL_4557867, EPI_ISL_4557868, EPI_ISL_4557869, EPI_ISL_4557870, EPI_ISL_4557871, EPI_ISL_4557872, EPI_ISL_4557873, EPI_ISL_4557874, EPI_ISL_4557875, EPI_ISL_4557876, EPI_ISL_4557877, EPI_ISL_4557878, EPI_ISL_4557879, EPI_ISL_4557880, EPI_ISL_4557881, EPI_ISL_4557882, EPI_ISL_4557883, EPI_ISL_4557884, EPI_ISL_4557885, EPI_ISL_4557886, EPI_ISL_4557887, EPI_ISL_4557888, EPI_ISL_4557889, EPI_ISL_4557890, EPI_ISL_4557891, EPI_ISL_4557892, EPI_ISL_4557893, EPI_ISL_4557894, EPI_ISL_4557895, EPI_ISL_4557896, EPI_ISL_4557897, EPI_ISL_4557898, EPI_ISL_4557899, EPI_ISL_4557900, EPI_ISL_4557901, EPI_ISL_4557902, EPI_ISL_4557903, EPI_ISL_4557904, EPI_ISL_4557905, EPI_ISL_4557906, EPI_ISL_4557907, EPI_ISL_4557908, EPI_ISL_4557909, EPI_ISL_4557910, EPI_ISL_4557911, EPI_ISL_4557912, EPI_ISL_4557913, EPI_ISL_4557914, EPI_ISL_4557915, EPI_ISL_4557916, EPI_ISL_4557917, EPI_ISL_4557918, EPI_ISL_4557919, EPI_ISL_4557920			
see above	National Public Health Laboratory, National Centre for Infectious Diseases	National Public Health Laboratory, National Centre for Infectious Diseases	Chavatte JM; Chavatte Jean-Marc; Cui L; Cui Lin; Grace Jie Yin Ngan; Grace Ngan; Grace Ngan Jie Yin; Katherine Ching; Lin Cui; Lin RTP; Lin Raymond Tzer Pin; Mak TM; Mak Tze Minn; Octavia S; Octavia Sophie; Raymond Tzer Pin Lin; Royce Ang; Samuel Loo; Sophie Octavia; Tze Minn Mak; Zhenyang Zhou; Zhou Z
EPI_ISL_574479, EPI_ISL_574480, EPI_ISL_574481, EPI_ISL_574482, EPI_ISL_574483, EPI_ISL_574484, EPI_ISL_574485, EPI_ISL_574486, EPI_ISL_574487, EPI_ISL_574488, EPI_ISL_574489, EPI_ISL_574490, EPI_ISL_574491, EPI_ISL_574492			
see above	Programme in Emerging Infectious Diseases, Duke-NUS Medical School	National Public Health Laboratory, National Centre for Infectious Diseases	Adrian Eng Zheng Kang; Danielle E Anderson; Lin Cui; Raymond Tzer Pin Lin; Sophie Octavia; Tze Minn Mak; Zhenyang Zhou
EPI_ISL_462085, EPI_ISL_462086, EPI_ISL_462087, EPI_ISL_462088, EPI_ISL_462089, EPI_ISL_482672, EPI_ISL_482673, EPI_ISL_482674, EPI_ISL_482675, EPI_ISL_482676, EPI_ISL_482677, EPI_ISL_482678, EPI_ISL_482679, EPI_ISL_482680, EPI_ISL_482681, EPI_ISL_482682, EPI_ISL_482683, EPI_ISL_482684, EPI_ISL_482685, EPI_ISL_482686, EPI_ISL_482687, EPI_ISL_482688, EPI_ISL_482689, EPI_ISL_482690, EPI_ISL_482691, EPI_ISL_482692, EPI_ISL_482693, EPI_ISL_482694, EPI_ISL_482695, EPI_ISL_482696, EPI_ISL_482697, EPI_ISL_482698, EPI_ISL_482699, EPI_ISL_500539, EPI_ISL_500540, EPI_ISL_500541, EPI_ISL_500542, EPI_ISL_500543, EPI_ISL_500544, EPI_ISL_500545, EPI_ISL_500546, EPI_ISL_500547, EPI_ISL_500548, EPI_ISL_500549, EPI_ISL_500550, EPI_ISL_500551, EPI_ISL_500552, EPI_ISL_500553, EPI_ISL_500554, EPI_ISL_500555, EPI_ISL_500556, EPI_ISL_500557, EPI_ISL_500558, EPI_ISL_500559, EPI_ISL_500560, EPI_ISL_500561, EPI_ISL_500562, EPI_ISL_500563, EPI_ISL_500564, EPI_ISL_500565, EPI_ISL_500566, EPI_ISL_500567, EPI_ISL_500568, EPI_ISL_500569, EPI_ISL_500570, EPI_ISL_500571, EPI_ISL_500572, EPI_ISL_509372, EPI_ISL_509373, EPI_ISL_509374, EPI_ISL_509375, EPI_ISL_509376, EPI_ISL_509377, EPI_ISL_509378, EPI_ISL_509379, EPI_ISL_509380, EPI_ISL_509381, EPI_ISL_509382, EPI_ISL_509383, EPI_ISL_509384, EPI_ISL_509385, EPI_ISL_509386, EPI_ISL_509387, EPI_ISL_509388, EPI_ISL_509389, EPI_ISL_509390, EPI_ISL_509391, EPI_ISL_509392, EPI_ISL_509393, EPI_ISL_509394, EPI_ISL_509395, EPI_ISL_517996, EPI_ISL_517997, EPI_ISL_517998, EPI_ISL_517999, EPI_ISL_518000, EPI_ISL_518001, EPI_ISL_518002, EPI_ISL_518003, EPI_ISL_518004, EPI_ISL_518005, EPI_ISL_518006, EPI_ISL_518007, EPI_ISL_518008, EPI_ISL_518009, EPI_ISL_518010, EPI_ISL_518011, EPI_ISL_518012, EPI_ISL_518013, EPI_ISL_518014, EPI_ISL_518015, EPI_ISL_518016, EPI_ISL_518017, EPI_ISL_524445, EPI_ISL_524446, EPI_ISL_524447, EPI_ISL_524448, EPI_ISL_524449, EPI_ISL_583893, EPI_ISL_583894, EPI_ISL_583895, EPI_ISL_583896, EPI_ISL_583897, EPI_ISL_583898, EPI_ISL_583899, EPI_ISL_583900, EPI_ISL_610152, EPI_ISL_610153, EPI_ISL_610154, EPI_ISL_768324, EPI_ISL_891251, EPI_ISL_891252, EPI_ISL_891253, EPI_ISL_1749430, EPI_ISL_1749431, EPI_ISL_1749432, EPI_ISL_1749433, EPI_ISL_1749434, EPI_ISL_1749435, EPI_ISL_1749436, EPI_ISL_1749437, EPI_ISL_1969690, EPI_ISL_2464555, EPI_ISL_2464556, EPI_ISL_2464557, EPI_ISL_2464558, EPI_ISL_2464559, EPI_ISL_2566444, EPI_ISL_2566445, EPI_ISL_2812146, EPI_ISL_2812147, EPI_ISL_2812148, EPI_ISL_2812149, EPI_ISL_2812150, EPI_ISL_2812151, EPI_ISL_2812152, EPI_ISL_2812153, EPI_ISL_2812154, EPI_ISL_2812155, EPI_ISL_2812156, EPI_ISL_3062664, EPI_ISL_3062665, EPI_ISL_3062666, EPI_ISL_3062667, EPI_ISL_3062668, EPI_ISL_3062669, EPI_ISL_3062670, EPI_ISL_3062671, EPI_ISL_3062672			
see above	Singapore General Hospital	Department of Microbiology	Chayaporn Suphavilai; Chenhao Li; Chenhao Li; James Sim Heng Chiak; Karrie Ko; Karrie Ko; Kenneth Xin Long Chan; Kern Rei Chng; Kern Rei Chng; Kian Sing Chan; Kian Sing Chan; Kun Lee Lim; Kun Lee Lim; Lynette Oon; Lynette Oon; Niranjan Nagarajan; Niranjan Nagarajan; Nurdyana Abdul Rahman; Nurdyana Abdul Rahman; Sui Sin Goh; Suphavilai Chayaporn
EPI_ISL_406973	Singapore General Hospital	National Public Health Laboratory	Chavatte JM; Cui; L; Lin; Mak; Octavia S; RTP; TM; ZY; Zhou
EPI_ISL_407987	Singapore General Hospital	Programme in Emerging Infectious Diseases, Duke-NUS Medical School	Danielle E Anderson; Gavin JD Smith; Jayanthi Jayakumar; Jenny GH Low; Kian Sing Chan; Linfa Wang; Lynette LE Oon; Martin Linster; Yan Zhuang; Yvonne CF Su
EPI_ISL_2464560, EPI_ISL_2464561, EPI_ISL_2464562, EPI_ISL_2464563, EPI_ISL_2464564	Singapore General Hospital	Singapore General Hospital	Chayaporn Suphavilai; James Sim Heng Chiak; Karrie Ko; Kenneth Xin Long Chan; Kern Rei Chng; Kian Sing Chan; Kun Lee Lim; Lynette Oon; Niranjan Nagarajan; Nurdyana Abdul Rahman; Sui Sin Goh
EPI_ISL_410536, EPI_ISL_410537	Singapore General Hospital, Molecular Laboratory, Division of Pathology	Programme in Emerging Infectious Diseases, Duke-NUS Medical School	Danielle E Anderson; Gavin JD Smith; Jayanthi Jayakumar; Jenny GH Low; Kian Sing Chan; Lynette LE Oon; Martin Linster; Shirin Kalimuddin; Yan Zhuang; Yvonne CF Su

We gratefully acknowledge the following Authors from the Originating laboratories responsible for obtaining the specimens, as well as the Submitting laboratories where the genome data were generated and shared via GISAID, on which this research is based.

All Submitters of data may be contacted directly via www.gisaid.org

Authors are sorted alphabetically.

Accession ID	Originating Laboratory	Submitting Laboratory	Authors
EPI_ISL_4459417	ALABANG MEDICAL CENTER	Research Institute for Tropical Medicine/Tohoku University	Allen Anthony Laraño; Ardiane Ysabelle Dolor; Catalino Demetria; Clyde Dapat; Emmanuel Kagning Tsinda; Francisco Gerardo Polotan; Hitoshi Oshitani; Inez Andrea Medado; Jefferson Earl Halog; Lei Lanna Dancel; Leilanie Nacion; Ma. Angelica Tujan; Machiko Okamoto; Maria Carmen Corpuz; Mariko Siato-Obata; Mayuko Saito
EPI_ISL_4459353	APACIBLE MEMORIAL DISTRICT HOSPITAL	Research Institute for Tropical Medicine/Tohoku University	Allen Anthony Laraño; Ardiane Ysabelle Dolor; Catalino Demetria; Clyde Dapat; Emmanuel Kagning Tsinda; Francisco Gerardo Polotan; Hitoshi Oshitani; Inez Andrea Medado; Jefferson Earl Halog; Lei Lanna Dancel; Leilanie Nacion; Ma. Angelica Tujan; Machiko Okamoto; Maria Carmen Corpuz; Mariko Siato-Obata; Mayuko Saito
EPI_ISL_2859131, EPI_ISL_2859134, EPI_ISL_2859136, EPI_ISL_2859140, EPI_ISL_2859150, EPI_ISL_2859151, EPI_ISL_2859152, EPI_ISL_2859154, EPI_ISL_2859163, EPI_ISL_2859167, EPI_ISL_2859245, EPI_ISL_2859259, EPI_ISL_2859266, EPI_ISL_2859280, EPI_ISL_2859288, EPI_ISL_2859321, EPI_ISL_2859333, EPI_ISL_2859339, EPI_ISL_2859526, EPI_ISL_2859895, EPI_ISL_2859916, EPI_ISL_2859958			
see above	Adela Serra TY Memorial Center	Philippine Genome Center	Alethea R. de Guzman; Anna Ong-Lim; Arianne A. Zamora; Benedict A. Maralit; Carlo M. Lapid; Celia Carlos; Devon Ray Pacial; Diomedes A. Carino; Edsel Maurice Salvaña; El King D. Morado; Elcid Aaron R. Pangilinan; Eva Maria Cutiongco-de la Paz; Francis A. Tablizo; Henrietta Marie Rodriguez; Jaime C. Montoya; Jan Michael C. Yap; Jarvin E. Nipales; Jo-Hannah S. Llames; John Q. Wong; Joshua Gregor A. Dizon; Juan Antonio R. Magalang; Karol Sophia Agape R. Padilla; Kenneth M. Kim; Kris P. Punayan; Krisitna Patriz Dela Cruz; Lindsay Claire D.L. Carandang; Ma. Exanil Plantig; Marc Edsel C. Ayes; Maria Rosario Singh-Vergeire and Cynthia P. Saloma; Maria Sofia L. Yangzon; Marielle M Gamboa; Marissa Alejandria; Nina Francesca Bustamante; Razel Nikka M. Hao; Renato Jacinto Q. Mantaring; Rianna Patricia S. Cruz; Sheila Mae M. Araiza; Yvonne Valerie Austria; Zipporah Mariebelle R. Enriquez; Zyrel V. Mollejon
EPI_ISL_2153828	Alfonso Rural Health Unit	Philippine Genome Center	Alethea R. de Guzman; Anna Ong-Lim; Arianne A. Zamora; Asia Louisa U. Chong; Benedict A. Maralit; Candice Francheska B. Tambaoan; Carlo M. Lapid; Celia Carlos; Devon Ray Pacial; Edsel Maurice Salvaña; El King D. Morado; Eva Maria Cutiongco-de la Paz; Francis A. Tablizo; Irish Coleen A. Asin; Jaime C. Montoya; Jan Michael C. Yap; Jo-Hannah S. Llames; John Q. Wong; Joshua Gregor A. Dizon; Juan Antonio R. Magalang; Karol Sophia Agape R. Padilla; Kenneth M. Kim; Kris P. Punayan; Marc Edsel C. Ayes; Marc Jerrone R. Castro; Maria Rosario Singh-Vergeire and Cynthia P. Saloma; Maria Sofia L. Yangzon; Marissa Alejandria; Razel Nikka M. Hao; Rianna Patricia S. Cruz; Sheila Mae M. Araiza
EPI_ISL_410302	Amalea Dulcene Nicolasora Research Institute for Tropical Medicine, Molecular Biology Laboratory	Amalea Dulcene Nicolasora Research Institute for Tropical Medicine, Molecular Biology Laboratory	A.D.; E.S.; F.M.; I.P.; J.G.; K.M.; M.A.; Manalo; Medado; Mercado; Nicolasora; O.T. and Cruz; Onza; Polotan; Tujan
EPI_ISL_2188084, EPI_ISL_2188273, EPI_ISL_2188882, EPI_ISL_2188942, EPI_ISL_2188944, EPI_ISL_2189005, EPI_ISL_2189011, EPI_ISL_2189026, EPI_ISL_2189074, EPI_ISL_2189076, EPI_ISL_2189198, EPI_ISL_2189456, EPI_ISL_2189461, EPI_ISL_2189502, EPI_ISL_2189509, EPI_ISL_4715245			
see above	Amang Rodriguez Memorial Medical Center	Philippine Genome Center	Alethea R. de Guzman; Anna Ong-Lim; Arianne A. Zamora; Asia Louisa U. Chong; Benedict A. Maralit; Candice Francheska B. Tambaoan; Carlo M. Lapid; Celia Carlos; Cynthia P. Saloma; Devon Ray Pacial; Diomedes A. Cariño; Edsel Maurice Salvana; Edsel Maurice Salvaña; El King D. Morado; Elcid Aaron R. Pangilinan; Eva Maria Cutiongco-de la Paz; Francis A. Tablizo; Henrietta Marie Rodriguez; Irish Coleen A. Asin; Jaime C. Montoya; Jan Michael C. Yap; Jarvin E. Nipales; Jo-Hannah S. Llames; John Q. Wong; Joshua Gregor A. Dizon; Juan Antonio R. Magalang; Karol Sophia Agape R. Padilla; Kenneth M. Kim; Kris P. Punayan; Kristina Patriz Dela Cruz; Lindsay Clare D.L. Carandang; Ma. Exanil Plantig; Marc Edsel C. Ayes; Marc Jerrone R. Castro; Maria Rosario Singh-Vergeire; Maria Rosario Singh-Vergeire and Cynthia P. Saloma; Maria Sofia L. Yangzon; Marielle M. Gamboa; Marissa Alejandria; Niña Francesca Bustamante; Razel Nikka M. Hao; Renato Jacinto Q. Mantaring; Rianna Patricia S. Cruz; Sheila Mae M. Araiza; Shiela Mae M. Araiza; Yvonne Valerie Austria; Zipporah Mariebelle R. Enriquez; Zyrel V. Mollejon
EPI_ISL_2155067, EPI_ISL_2155087, EPI_ISL_2155090, EPI_ISL_2155115, EPI_ISL_2155117, EPI_ISL_2155170, EPI_ISL_2155174, EPI_ISL_2155196, EPI_ISL_2155222, EPI_ISL_2155230, EPI_ISL_2155265, EPI_ISL_2155279, EPI_ISL_2155333, EPI_ISL_2155336, EPI_ISL_2156551, EPI_ISL_2156552			
see above	Amosup Seamen's Hospital	Philippine Genome Center	Alethea R. de Guzman; Anna Ong-Lim; Arianne A. Zamora; Asia Louisa U. Chong; Benedict A. Maralit; Candice Francheska B. Tambaoan; Carlo M. Lapid; Celia Carlos; Devon Ray Pacial; Edsel Maurice Salvaña; El King D. Morado; Elcid Aaron R. Pangilinan; Eva Maria Cutiongco-de la Paz; Francis A. Tablizo; Irish Coleen A. Asin; Jaime C. Montoya; Jan Michael C. Yap; Jo-Hannah S. Llames; John Q. Wong; Joshua Gregor A. Dizon; Juan Antonio R. Magalang; Karol Sophia Agape R. Padilla; Kenneth M. Kim; Kris P. Punayan; Marc Edsel C. Ayes; Marc Jerrone R. Castro; Maria Rosario Singh-Vergeire and Cynthia P. Saloma; Maria Sofia L. Yangzon; Marissa Alejandria; Razel Nikka M. Hao; Rianna Patricia S. Cruz; Sheila Mae M. Araiza
EPI_ISL_2105510, EPI_ISL_2105513, EPI_ISL_2105518, EPI_ISL_2105519, EPI_ISL_2105520, EPI_ISL_2153903, EPI_ISL_2153909, EPI_ISL_2153915, EPI_ISL_2153924, EPI_ISL_2153987, EPI_ISL_2153997, EPI_ISL_2153998, EPI_ISL_2154031, EPI_ISL_2154034, EPI_ISL_2154041, EPI_ISL_2154047			
see above	Army General Hospital Molecular Laboratory	Philippine Genome Center	Alethea R. de Guzman; Anna Ong-Lim; Arianne A. Zamora; Asia Louisa U. Chong; Benedict A. Maralit; Candice Francheska B. Tambaoan; Carlo M. Lapid; Celia Carlos; Devon Ray Pacial; Edsel Maurice Salvaña; El King D. Morado; Eva Maria Cutiongco-de la Paz; Francis A. Tablizo; Irish Coleen A. Asin; Jaime C. Montoya; Jan Michael C. Yap; Jo-Hannah S. Llames; John Q. Wong; Joshua Gregor A. Dizon; Juan Antonio R. Magalang; Karol Sophia Agape R. Padilla; Kenneth M. Kim; Kris P. Punayan; Marc Edsel C. Ayes; Marc Jerrone R. Castro; Maria Rosario Singh-Vergeire and Cynthia P. Saloma; Maria Sofia L. Yangzon; Marissa Alejandria; Razel Nikka M. Hao; Rianna Patricia S. Cruz; Sheila Mae M. Araiza
EPI_ISL_3105864	Asian Hospital and Medical Center	Research Institute for Tropical Medicine	Clyde Dapat; Deana Mae Ocampo; Emmanuel Kagning Tsinda; Francisco Gerardo Polotan; Hitoshi Oshitani; Inez Andrea Medado; Jefferson Earl Halog; Joana Ina Manalo; Lei Lanna Dancel; Ma Angelica Tujan; Mariko Saito-Obata; Mayuko Saito; Michiko Okamoto; Samantha Louise Bado
EPI_ISL_4459391, EPI_ISL_4459401, EPI_ISL_4459411	BIÑAN CITY HEALTH OFFICE II	Research Institute for Tropical Medicine/Tohoku University	Allen Anthony Laraño; Ardiane Ysabelle Dolor; Catalino Demetria; Clyde Dapat; Emmanuel Kagning Tsinda; Francisco Gerardo Polotan; Hitoshi Oshitani; Inez Andrea Medado; Jefferson Earl Halog; Lei Lanna Dancel; Leilanie Nacion; Ma. Angelica Tujan; Machiko Okamoto; Maria Carmen Corpuz; Mariko Siato-Obata; Mayuko Saito
EPI_ISL_4459404, EPI_ISL_4459407	BIÑAN DOCTORS' HOSPITAL, INC.	Research Institute for Tropical Medicine/Tohoku University	Allen Anthony Laraño; Ardiane Ysabelle Dolor; Catalino Demetria; Clyde Dapat; Emmanuel Kagning Tsinda; Francisco Gerardo Polotan; Hitoshi Oshitani; Inez Andrea Medado; Jefferson Earl Halog; Lei Lanna Dancel; Leilanie Nacion; Ma. Angelica Tujan; Machiko Okamoto; Maria Carmen Corpuz; Mariko Siato-Obata; Mayuko Saito
EPI_ISL_2156318, EPI_ISL_2156319	BULACAN MEDICAL CENTER	Philippine Genome Center	Alethea R. de Guzman; Anna Ong-Lim; Arianne A. Zamora; Asia Louisa U. Chong; Benedict A. Maralit; Candice Francheska B. Tambaoan; Carlo M. Lapid; Celia Carlos; Devon Ray Pacial; Edsel Maurice Salvaña; El King D. Morado; Elcid Aaron R. Pangilinan; Eva Maria Cutiongco-de la Paz; Francis A. Tablizo; Irish Coleen A. Asin; Jaime C. Montoya; Jan Michael C. Yap; Jo-Hannah S. Llames; John Q. Wong; Joshua Gregor A. Dizon; Juan Antonio R. Magalang; Karol Sophia Agape R. Padilla; Kenneth M. Kim; Kris P. Punayan; Marc Edsel C. Ayes; Marc Jerrone R. Castro; Maria Rosario Singh-Vergeire and Cynthia P. Saloma; Maria Sofia L. Yangzon; Marissa Alejandria; Razel Nikka M. Hao; Rianna Patricia S. Cruz; Sheila Mae M. Araiza
EPI_ISL_4459474	BULALACAO MUNICIPAL HEALTH OFFICE	Research Institute for Tropical Medicine/Tohoku University	Allen Anthony Laraño; Ardiane Ysabelle Dolor; Catalino Demetria; Clyde Dapat; Emmanuel Kagning Tsinda; Francisco Gerardo Polotan; Hitoshi Oshitani; Inez Andrea Medado; Jefferson Earl Halog; Lei Lanna Dancel; Leilanie Nacion; Ma. Angelica Tujan; Machiko Okamoto; Maria Carmen Corpuz; Mariko Siato-Obata; Mayuko Saito
EPI_ISL_2155396	Bagong Cainta Municipal Hospital (PGC)	Philippine Genome Center	Alethea R. de Guzman; Anna Ong-Lim; Arianne A. Zamora; Asia Louisa U. Chong; Benedict A. Maralit; Candice Francheska B. Tambaoan; Carlo M. Lapid; Celia Carlos; Devon Ray Pacial; Edsel Maurice Salvaña; El King D. Morado; Eva Maria Cutiongco-de la Paz; Francis A. Tablizo; Irish Coleen A. Asin; Jaime C. Montoya; Jan Michael C. Yap; Jo-Hannah S. Llames; John Q. Wong; Joshua Gregor A. Dizon; Juan Antonio R. Magalang; Karol Sophia Agape R. Padilla; Kenneth M. Kim; Kris P. Punayan; Marc Edsel C. Ayes; Marc Jerrone R. Castro; Maria Rosario Singh-Vergeire and Cynthia P. Saloma; Maria Sofia L. Yangzon; Marissa Alejandria; Razel Nikka M. Hao; Rianna Patricia S. Cruz; Sheila Mae M. Araiza
EPI_ISL_2105283, EPI_ISL_2105284, EPI_ISL_2105421, EPI_ISL_2153809, EPI_ISL_2153833, EPI_ISL_2153837, EPI_ISL_2153849, EPI_ISL_2153858, EPI_ISL_2153865, EPI_ISL_2153873, EPI_ISL_2153878, EPI_ISL_2153881, EPI_ISL_2153882, EPI_ISL_2153888, EPI_ISL_2153890, EPI_ISL_2153893, EPI_ISL_2153894, EPI_ISL_2153896, EPI_ISL_2153897, EPI_ISL_2153899, EPI_ISL_2153901, EPI_ISL_2153907, EPI_ISL_2153910, EPI_ISL_2153911, EPI_ISL_2153912, EPI_ISL_2153913, EPI_ISL_2153917, EPI_ISL_2153919, EPI_ISL_2153921, EPI_ISL_2153922, EPI_ISL_2153923, EPI_ISL_2153926, EPI_ISL_2153929, EPI_ISL_2153933, EPI_ISL_2153938, EPI_ISL_2153945, EPI_ISL_2153947, EPI_ISL_2153949, EPI_ISL_2153950, EPI_ISL_2153955, EPI_ISL_2153960, EPI_ISL_2153974, EPI_ISL_2153979, EPI_ISL_2153993, EPI_ISL_2153995, EPI_ISL_2154001, EPI_ISL_2154007, EPI_ISL_2154009, EPI_ISL_2154011, EPI_ISL_2154012, EPI_ISL_2154017, EPI_ISL_2154021, EPI_ISL_2154023, EPI_ISL_2154029, EPI_ISL_2154030, EPI_ISL_2154032, EPI_ISL_2154033, EPI_ISL_2154035, EPI_ISL_2154036, EPI_ISL_2154037, EPI_ISL_2154039, EPI_ISL_2154040, EPI_ISL_2154042, EPI_ISL_2154043, EPI_ISL_2154388, EPI_ISL_2154391, EPI_ISL_2154392, EPI_ISL_2154393, EPI_ISL_2154396, EPI_ISL_2154397, EPI_ISL_2154398, EPI_ISL_2154400, EPI_ISL_2154405, EPI_ISL_2154409, EPI_ISL_2154428, EPI_ISL_2154429, EPI_ISL_2154434, EPI_ISL_2154435, EPI_ISL_2154437, EPI_ISL_2154438, EPI_ISL_2154439, EPI_ISL_2154441, EPI_ISL_2154445, EPI_ISL_2154447, EPI_ISL_2154451, EPI_ISL_2154453, EPI_ISL_2154461, EPI_ISL_2154462, EPI_ISL_2154464, EPI_ISL_2154465, EPI_ISL_2154466, EPI_ISL_2154470, EPI_ISL_2154471, EPI_ISL_2154477, EPI_ISL_2154481, EPI_ISL_2154482, EPI_ISL_2154484, EPI_ISL_2154485, EPI_ISL_2154486, EPI_ISL_2154489, EPI_ISL_2154492, EPI_ISL_2154494, EPI_ISL_2154500, EPI_ISL_2154502, EPI_ISL_2154503, EPI_ISL_2154508, EPI_ISL_2154511, EPI_ISL_2154512, EPI_ISL_2154514, EPI_ISL_2154515, EPI_ISL_2154517, EPI_ISL_2154520, EPI_ISL_2154522, EPI_ISL_2154524, EPI_ISL_2154530, EPI_ISL_2154531, EPI_ISL_2154535, EPI_ISL_2154536, EPI_ISL_2154539, EPI_ISL_2154542, EPI_ISL_2154543, EPI_ISL_2154544, EPI_ISL_2154549, EPI_ISL_2154552, EPI_ISL_2154562, EPI_ISL_2154567, EPI_ISL_2154569, EPI_ISL_2154573, EPI_ISL_2154577, EPI_ISL_2154578, EPI_ISL_2154579, EPI_ISL_2154583, EPI_ISL_2154586, EPI_ISL_2154589, EPI_ISL_2154591, EPI_ISL_2154599, EPI_ISL_2154601, EPI_ISL_2154604, EPI_ISL_2154605, EPI_ISL_2154606, EPI_ISL_2154608, EPI_ISL_2154609, EPI_ISL_2154615, EPI_ISL_2154617, EPI_ISL_2154624, EPI_ISL_2154625, EPI_ISL_2154626, EPI_ISL_2154627, EPI_ISL_2154628, EPI_ISL_2154635, EPI_ISL_2154636, EPI_ISL_2154639, EPI_ISL_2154642, EPI_ISL_2154644, EPI_ISL_2154647, EPI_ISL_2154649, EPI_ISL_2154652, EPI_ISL_2154653, EPI_ISL_2154656, EPI_ISL_2154657, EPI_ISL_2154658, EPI_ISL_2154659, EPI_ISL_2154661, EPI_ISL_2154663, EPI_ISL_2154667, EPI_ISL_2154672, EPI_ISL_2154674, EPI_ISL_2154675, EPI_ISL_2154677, EPI_ISL_2154680, EPI_ISL_2154681, EPI_ISL_2154682, EPI_ISL_2154692, EPI_ISL_2154694, EPI_ISL_2154695, EPI_ISL_2154705, EPI_ISL_2154711, EPI_ISL_2154713, EPI_ISL_2154715, EPI_ISL_2154716, EPI_ISL_2154717, EPI_ISL_2154718, EPI_ISL_2154722, EPI_ISL_2154728, EPI_ISL_2154731, EPI_ISL_2154741, EPI_ISL_2154743, EPI_ISL_2154746, EPI_ISL_2154747, EPI_ISL_2154748, EPI_ISL_2154750, EPI_ISL_2154752, EPI_ISL_2154753, EPI_ISL_2154759, EPI_ISL_2154760, EPI_ISL_2154762, EPI_ISL_2154764, EPI_ISL_2154765, EPI_ISL_2154767, EPI_ISL_2154772, EPI_ISL_2154774, EPI_ISL_2154775, EPI_ISL_2154778, EPI_ISL_2154779, EPI_ISL_2154785, EPI_ISL_2154790, EPI_ISL_2154791, EPI_ISL_2154798, EPI_ISL_2154799, EPI_ISL_2154803, EPI_ISL_2154809, EPI_ISL_2154811, EPI_ISL_2154812, EPI_ISL_2154815, EPI_ISL_2154818, EPI_ISL_2154820, EPI_ISL_2154822, EPI_ISL_2154826, EPI_ISL_2154827, EPI_ISL_2154829, EPI_ISL_2154832, EPI_ISL_2154833, EPI_ISL_2154834, EPI_ISL_2154836, EPI_ISL_2154837, EPI_ISL_2154842, EPI_ISL_2154843, EPI_ISL_2154845, EPI_ISL_2154846, EPI_ISL_2154847, EPI_ISL_2154850, EPI_ISL_2154852, EPI_ISL_2154853, EPI_ISL_2154858, EPI_ISL_2154859, EPI_ISL_2154862, EPI_ISL_2154865, EPI_ISL_2154867, EPI_ISL_2154870, EPI_ISL_2154871, EPI_ISL_2154879, EPI_ISL_2154881, EPI_ISL_2154882, EPI_ISL_2154883, EPI_ISL_2154885, EPI_ISL_2154889, EPI_ISL_2154893, EPI_ISL_2154897, EPI_ISL_2154898, EPI_ISL_2154899, EPI_ISL_2154900, EPI_ISL_2154903, EPI_ISL_2154904, EPI_ISL_2154905, EPI_ISL_2154908, EPI_ISL_2154910, EPI_ISL_2154913, EPI_ISL_2154914, EPI_ISL_2154916, EPI_ISL_2154919, EPI_ISL_2154921, EPI_ISL_2154927, EPI_ISL_2154935, EPI_ISL_2154939, EPI_ISL_2154940, EPI_ISL_2154946, EPI_ISL_2154952, EPI_ISL_2154954, EPI_ISL_2154966, EPI_ISL_2155002, EPI_ISL_2155003, EPI_ISL_2155004, EPI_ISL_2155005, EPI_ISL_2155008, EPI_ISL_2155011, EPI_ISL_2155012, EPI_ISL_2155013, EPI_ISL_2155014, EPI_ISL_2155053, EPI_ISL_2155081, EPI_ISL_2155121, EPI_ISL_2155139, EPI_ISL_2155150, EPI_ISL_2155151, EPI_ISL_2155154, EPI_ISL_2155175, EPI_ISL_2155198, EPI_ISL_2155204, EPI_ISL_2155209, EPI_ISL_2155235, EPI_ISL_2155249, EPI_ISL_2155263, EPI_ISL_2155264, EPI_ISL_2155293, EPI_ISL_2155339, EPI_ISL_2155354, EPI_ISL_2156129, EPI_ISL_2156130, EPI_ISL_2156131, EPI_ISL_2156132, EPI_ISL_2156133, EPI_ISL_2156134, EPI_ISL_2156135, EPI_ISL_2156136, EPI_ISL_2156137, EPI_ISL_2156138, EPI_ISL_2156139, EPI_ISL_2156140, EPI_ISL_2156329, EPI_ISL_2156330, EPI_ISL_2156344, EPI_ISL_2156345, EPI_ISL_2156346, EPI_ISL_2156382, EPI_ISL_2156383, EPI_ISL_2156384, EPI_ISL_2156385, EPI_ISL_2156386, EPI_ISL_2156387, EPI_ISL_2156408, EPI_ISL_2156409, EPI_ISL_2156423, EPI_ISL_2156424, EPI_ISL_2156425, EPI_ISL_2156426, EPI_ISL_2156449, EPI_ISL_2156450, EPI_ISL_2156451, EPI_ISL_2156469, EPI_ISL_2156470, EPI_ISL_2156472, EPI_ISL_2156473, EPI_ISL_2156529, EPI_ISL_2156546, EPI_ISL_2156554, EPI_ISL_2156565, EPI_ISL_2156571, EPI_ISL_2156572, EPI_ISL_2156573, EPI_ISL_2156587, EPI_ISL_2156588, EPI_ISL_2156594, EPI_ISL_2156595, EPI_ISL_2156598, EPI_ISL_2156599, EPI_ISL_2156601, EPI_ISL_2156602, EPI_ISL_2156603, EPI_ISL_2156604, EPI_ISL_2156610, EPI_ISL_2156611, EPI_ISL_2156615, EPI_ISL_2156616, EPI_ISL_2156618, EPI_ISL_2156619, EPI_ISL_2156620, EPI_ISL_2156621, EPI_ISL_2156622, EPI_ISL_2156623, EPI_ISL_2156627, EPI_ISL_2156628, EPI_ISL_2156629, EPI_ISL_2171197, EPI_ISL_2188050, EPI_ISL_2188057, EPI_ISL_2188070, EPI_ISL_2188142, EPI_ISL_2188185, EPI_ISL_2188197, EPI_ISL_2188204, EPI_ISL_2188211, EPI_ISL_2188224, EPI_ISL_2188231, EPI_ISL_2188683, EPI_ISL_2188693, EPI_ISL_2188702, EPI_ISL_2188724, EPI_ISL_2188812, EPI_ISL_2188823, EPI_ISL_2188839, EPI_ISL_2188867, EPI_ISL_2189145, EPI_ISL_2859141, EPI_ISL_2859174, EPI_ISL_2859193, EPI_ISL_2859194, EPI_ISL_2859195, EPI_ISL_2859196, EPI_ISL_2859197, EPI_ISL_2859198, EPI_ISL_2859199, EPI_ISL_2859200, EPI_ISL_2859203, EPI_ISL_2859204, EPI_ISL_2859205, EPI_ISL_2859206, EPI_ISL_2859208, EPI_ISL_2859209, EPI_ISL_2859210, EPI_ISL_2859211, EPI_ISL_2859212, EPI_ISL_2859213, EPI_ISL_2859214, EPI_ISL_2859215, EPI_ISL_2859216, EPI_ISL_2859217, EPI_ISL_2859228, EPI_ISL_2859233, EPI_ISL_2859235, EPI_ISL_2859271, EPI_ISL_2859273, EPI_ISL_2859275, EPI_ISL_2859291, EPI_ISL_2859298, EPI_ISL_2859299, EPI_ISL_2859308, EPI_ISL_2859319, EPI_ISL_2859336, EPI_ISL_2859340, EPI_ISL_2859507, EPI_ISL_2859672, EPI_ISL_2859674, EPI_ISL_2859688, EPI_ISL_2859693, EPI_ISL_2859698, EPI_ISL_2859706, EPI_ISL_2859712, EPI_ISL_2859713, EPI_ISL_2859749, EPI_ISL_2859758, EPI_ISL_2859764, EPI_ISL_2859771, EPI_ISL_2859773, EPI_ISL_2859776, EPI_ISL_2859799, EPI_ISL_2859819, EPI_ISL_2859833, EPI_ISL_2859836, EPI_ISL_2859848, EPI_ISL_2859857, EPI_ISL_2859859, EPI_ISL_2859930, EPI_ISL_2859942, EPI_ISL_2859953, EPI_ISL_2860094, EPI_ISL_4712308, EPI_ISL_4712330, EPI_ISL_4712378, EPI_ISL_4712448, EPI_ISL_4712508, EPI_ISL_4712538, EPI_ISL_4712550, EPI_ISL_4712583, EPI_ISL_4712671, EPI_ISL_4712682, EPI_ISL_4712710, EPI_ISL_4712740, EPI_ISL_4712759, EPI_ISL_4712807, EPI_ISL_4712910, EPI_ISL_4712963, EPI_ISL_4713103, EPI_ISL_4713170, EPI_ISL_4713864, EPI_ISL_4713919, EPI_ISL_4713991, EPI_ISL_4714040, EPI_ISL_4714190, EPI_ISL_4714195, EPI_ISL_4714210, EPI_ISL_4714358, EPI_ISL_4714438, EPI_ISL_4714466, EPI_ISL_4714538, EPI_ISL_4714648, EPI_ISL_4714691, EPI_ISL_4714723, EPI_ISL_4714868, EPI_ISL_4714915, EPI_ISL_4714923			
see above	Baguio General Hospital Medical Center (BGHMC)	Philippine Genome Center	Alethea R. de Guzman; Anna Ong-Lim; Arianne A. Zamora; Asia Louisa U. Chong; Benedict A. Maralit; Candice Francheska B. Tambaoan; Carlo M. Lapid; Celia Carlos; Cynthia P. Saloma; Devon Ray Pacial; Diomedes A. Carino; Diomedes A. Cariño; Edsel Maurice Salvana; Edsel Maurice Salvaña; El King D. Morado; Elcid Aaron R. Pangilinan; Eva Maria Cutiongco-de la Paz; Francis A. Tablizo; Henrietta Marie Rodriguez; Irish Coleen A. Asin; Jaime C. Montoya; Jan Michael C. Yap; Jarvin E. Nipales; Jo-Hannah S. Llames; John Q. Wong; Joshua Gregor A. Dizon; Juan Antonio R. Magalang; Karol Sophia Agape R. Padilla; Kenneth M. Kim; Kris P. Punayan; Krisitna Patriz Dela Cruz; Kristina Patriz Dela Cruz; Lindsay Claire D.L. Carandang; Lindsay Clare D.L. Carandang; Ma. Exanil Plantig; Marc Edsel C. Ayes; Marc Jerrone R. Castro; Maria Rosario Singh-Vergeire; Maria Rosario Singh-Vergeire and Cynthia P. Saloma; Maria Sofia L. Yangzon; Marielle M Gamboa; Marielle M. Gamboa; Marissa Alejandria; Nina Francesca Bustamante; Niña Francesca Bustamante; Razel Nikka M. Hao; Renato Jacinto Q. Mantaring; Rianna Patricia S. Cruz; Sheila Mae M. Araiza; Shiela Mae M. Araiza; Yvonne Valerie Austria; Zipporah Mariebelle R. Enriquez; Zyrel V. Mollejon
EPI_ISL_1081797, EPI_ISL_1081798, EPI_ISL_1081799, EPI_ISL_1081800, EPI_ISL_1081801, EPI_ISL_1081802, EPI_ISL_1081803, EPI_ISL_1081804, EPI_ISL_1081805, EPI_ISL_1081806, EPI_ISL_1081807, EPI_ISL_1081808, EPI_ISL_1081809, EPI_ISL_1081823, EPI_ISL_1081824, EPI_ISL_1081825, EPI_ISL_1081826			
see above	Baguio General Hospital and Medical Center	Philippine Genome Center	Alethea R. de Guzman; Anna Ong-Lim; Arianne A. Zamora; Asia Louisa U. Chong; Benedict A. Maralit; Candice Francheska B. Tambaoan; Carlo M. Lapid; Celia Carlos; Cynthia P. Saloma; Devon Ray Pacial; Edsel Maurice Salvaña; El King D. Morado; Eva Maria Cutiongco-de la Paz; Francis A. Tablizo; Irish Coleen A. Asin; Jaime C. Montoya; Jan Michael C. Yap; Jo-Hannah S. Llames; John Q. Wong; Joshua Gregor A. Dizon; Juan Antonio R. Magalang; Karol Sophia Agape R. Padilla; Kenneth M. Kim; Kris P. Punayan; Marc Edsel C. Ayes; Marc Jerrone R. Castro; Maria Sofia L. Yangzon; Marissa Alejandria; Razel Nikka M. Hao; Rianna Patricia S. Cruz; Sheila Mae M. Araiza; and Maria Rosario Singh-Vergeire
EPI_ISL_2156418, EPI_ISL_2156434	Bataan General Hospital	Philippine Genome Center	Alethea R. de Guzman; Anna Ong-Lim; Arianne A. Zamora; Asia Louisa U. Chong; Benedict A. Maralit; Candice Francheska B. Tambaoan; Carlo M. Lapid; Celia Carlos; Devon Ray Pacial; Edsel Maurice Salvaña; El King D. Morado; Elcid Aaron R. Pangilinan; Eva Maria Cutiongco-de la Paz; Francis A. Tablizo; Irish Coleen A. Asin; Jaime C. Montoya; Jan Michael C. Yap; Jo-Hannah S. Llames; John Q. Wong; Joshua Gregor A. Dizon; Juan Antonio R. Magalang; Karol Sophia Agape R. Padilla; Kenneth M. Kim; Kris P. Punayan; Marc Edsel C. Ayes; Marc Jerrone R. Castro; Maria Rosario Singh-Vergeire and Cynthia P. Saloma; Maria Sofia L. Yangzon; Marissa Alejandria; Razel Nikka M. Hao; Rianna Patricia S. Cruz; Sheila Mae M. Araiza
EPI_ISL_2155070, EPI_ISL_2155126, EPI_ISL_2155251, EPI_ISL_2155252, EPI_ISL_2155253, EPI_ISL_2155286, EPI_ISL_2155295, EPI_ISL_2155328, EPI_ISL_2155591, EPI_ISL_2155592, EPI_ISL_2155593, EPI_ISL_2155594, EPI_ISL_2155595, EPI_ISL_2155596, EPI_ISL_2155597, EPI_ISL_2155598, EPI_ISL_2155599, EPI_ISL_2156141, EPI_ISL_2156142, EPI_ISL_2156143, EPI_ISL_2156144, EPI_ISL_2156145, EPI_ISL_2156146, EPI_ISL_2156147, EPI_ISL_2156577, EPI_ISL_2156597, EPI_ISL_2156609, EPI_ISL_2188757, EPI_ISL_2188783, EPI_ISL_2859139, EPI_ISL_2859185, EPI_ISL_2859202, EPI_ISL_2859221, EPI_ISL_2859226, EPI_ISL_2859230, EPI_ISL_2859232, EPI_ISL_2859234, EPI_ISL_2859240, EPI_ISL_2859241, EPI_ISL_2859253, EPI_ISL_2859256, EPI_ISL_2859257, EPI_ISL_2859263, EPI_ISL_2859290,			

EPI_ISL_2859328, EPI_ISL_2859343, EPI_ISL_2859369, EPI_ISL_2859655, EPI_ISL_2859810, EPI_ISL_2859820, EPI_ISL_2859864, EPI_ISL_2859888, EPI_ISL_2859899, EPI_ISL_2859901, EPI_ISL_2859912, EPI_ISL_2859931, EPI_ISL_2859935, EPI_ISL_4716123			
see above	Bataan General Hospital and Medical Center	Philippine Genome Center	Alethea R. de Guzman; Anna Ong-Lim; Arianne A. Zamora; Asia Louisa U. Chong; Benedict A. Maralit; Candice Francheska B. Tambaoan; Carlo M. Lapid; Celia Carlos; Cynthia P. Saloma; Devon Ray Pacial; Diomedes A. Carino; Diomedes A. Cariño; Edsel Maurice Salvana; Edsel Maurice Salvaña; El King D. Morado; Elcid Aaron R. Pangilinan; Eva Maria Cutiongco-de la Paz; Francis A. Tablizo; Henrietta Marie Rodriguez; Irish Coleen A. Asin; Jaime C. Montoya; Jan Michael C. Yap; Jarvin E. Nipales; Jo-Hannah S. Llames; John Q. Wong; Joshua Gregor A. Dizon; Juan Antonio R. Magalang; Karol Sophia Agape R. Padilla; Kenneth M. Kim; Kris P. Punayan; Krisitna Patriz Dela Cruz; Kristina Patriz Dela Cruz; Lindsay Claire D.L. Carandang; Lindsay Clare D.L. Carandang; Ma. Exanil Plantig; Marc Edsel C. Ayes; Marc Jerrone R. Castro; Maria Rosario Singh-Vergeire; Maria Rosario Singh-Vergeire and Cynthia P. Saloma; Maria Sofia L. Yangzon; Marielle M Gamboa; Marielle M. Gamboa; Marissa Alejandria; Nina Francesca Bustamante; Niña Francesca Bustamante; Razel Nikka M. Hao; Renato Jacinto Q. Mantaring; Rianna Patricia S. Cruz; Sheila Mae M. Araiza; Shiela Mae M. Araiza; Yvonne Valerie Austria; Zipporah Mariebelle R. Enriquez; Zyrel V. Mollejon
EPI_ISL_833333, EPI_ISL_833334, EPI_ISL_833340, EPI_ISL_833341	Batangas City Health Office	Research Institute for Tropical Medicine	Catalino Demetria; Daria Manalo; Edelwisa Mercado; Francisco Gerardo Polotan; Hannah Leah Morito; Inez Andrea Medado; John Leonard Chan; Kirstyn Brunker; Ma Angelica Tujan; Othoniel Jan Onza
EPI_ISL_1069187	Batangas Provincial Health Office	Research Institute for Tropical Medicine	Catalino Demetria; Daria Manalo; Edelwisa Mercado; Francisco Gerardo Polotan; Hannah Leah Morito; Inez Andrea Medado; John Leonard Chan; Joseph Hughes; Kirstyn Brunker; Ma Angelica Tujan; Othoniel Jan Onza
EPI_ISL_2155518, EPI_ISL_2155519	Bernandino General Hospital I (PGC)	Philippine Genome Center	Alethea R. de Guzman; Anna Ong-Lim; Arianne A. Zamora; Asia Louisa U. Chong; Benedict A. Maralit; Candice Francheska B. Tambaoan; Carlo M. Lapid; Celia Carlos; Devon Ray Pacial; Edsel Maurice Salvaña; El King D. Morado; Eva Maria Cutiongco-de la Paz; Francis A. Tablizo; Irish Coleen A. Asin; Jaime C. Montoya; Jan Michael C. Yap; Jo-Hannah S. Llames; John Q. Wong; Joshua Gregor A. Dizon; Juan Antonio R. Magalang; Karol Sophia Agape R. Padilla; Kenneth M. Kim; Kris P. Punayan; Marc Edsel C. Ayes; Marc Jerrone R. Castro; Maria Rosario Singh-Vergeire and Cynthia P. Saloma; Maria Sofia L. Yangzon; Marissa Alejandria; Razel Nikka M. Hao; Rianna Patricia S. Cruz; Sheila Mae M. Araiza
EPI_ISL_430843	Bethany Hospital	Research Institute for Tropical Medicine	Bautista; Brunker, K.; C.S.; C.T.; D.L.; Demetria; E.S.; F.G.M.; I.A.P.; Manalo; Medado; Mercado; O.J.T.; Onza; Polotan
EPI_ISL_4715887, EPI_ISL_4715902, EPI_ISL_4715979, EPI_ISL_4716008, EPI_ISL_4716022, EPI_ISL_4716230, EPI_ISL_4716272, EPI_ISL_4716320, EPI_ISL_4716346, EPI_ISL_4716389, EPI_ISL_4716415, EPI_ISL_4716505, EPI_ISL_4716514, EPI_ISL_4716596, EPI_ISL_4716657, EPI_ISL_4716679, EPI_ISL_4716689, EPI_ISL_4716697, EPI_ISL_4716806, EPI_ISL_4716827, EPI_ISL_4716829, EPI_ISL_4716849, EPI_ISL_4716914, EPI_ISL_4716949, EPI_ISL_4716986, EPI_ISL_4716992, EPI_ISL_4717019, EPI_ISL_4717036, EPI_ISL_4717105, EPI_ISL_4717154, EPI_ISL_4717212, EPI_ISL_4717303, EPI_ISL_4717373			
see above	Bicol Regional Diagnostic and Reference Laboratory	Philippine Genome Center	Alethea R. de Guzman; Anna Ong-Lim; Arianne A. Zamora; Benedict A. Maralit; Carlo M. Lapid; Celia Carlos; Cynthia P. Saloma; Devon Ray Pacial; Diomedes A. Cariño; Edsel Maurice Salvana; El King D. Morado; Elcid Aaron R. Pangilinan; Eva Maria Cutiongco-de la Paz; Francis A. Tablizo; Henrietta Marie Rodriguez; Jaime C. Montoya; Jan Michael C. Yap; Jarvin E. Nipales; Jo-Hannah S. Llames; John Q. Wong; Joshua Gregor A. Dizon; Juan Antonio R. Magalang; Karol Sophia Agape R. Padilla; Kenneth M. Kim; Kris P. Punayan; Kristina Patriz Dela Cruz; Lindsay Clare D.L. Carandang; Ma. Exanil Plantig; Marc Edsel C. Ayes; Maria Rosario Singh-Vergeire; Maria Sofia L. Yangzon; Marielle M. Gamboa; Marissa Alejandria; Niña Francesca Bustamante; Razel Nikka M. Hao; Renato Jacinto Q. Mantaring; Rianna Patricia S. Cruz; Shiela Mae M. Araiza; Yvonne Valerie Austria; Zipporah Mariebelle R. Enriquez; Zyrel V. Mollejon
EPI_ISL_1213504, EPI_ISL_1213506, EPI_ISL_1213508, EPI_ISL_1213509, EPI_ISL_1213511, EPI_ISL_1213513, EPI_ISL_1213514, EPI_ISL_1213516, EPI_ISL_1213518, EPI_ISL_1213519, EPI_ISL_1213521, EPI_ISL_1213523, EPI_ISL_1213524, EPI_ISL_1213526, EPI_ISL_1213528, EPI_ISL_1213530, EPI_ISL_1213531, EPI_ISL_2091460, EPI_ISL_2105463, EPI_ISL_2105464, EPI_ISL_2105465, EPI_ISL_2105467, EPI_ISL_2105480, EPI_ISL_2105486, EPI_ISL_2105494, EPI_ISL_2105498, EPI_ISL_2105507, EPI_ISL_2156022, EPI_ISL_2156475, EPI_ISL_2156481			
see above	Bohol Containerized PCR Laboratory	Philippine Genome Center	Alethea R. de Guzman; Anna Ong-Lim; Arianne A. Zamora; Asia Louisa U. Chong; Benedict A. Maralit; Candice Francheska B. Tambaoan; Carlo M. Lapid; Celia Carlos; Devon Ray Pacial; Edsel Maurice Salvaña; El King D. Morado; Elcid Aaron R. Pangilinan; Eva Maria Cutiongco-de la Paz; Francis A. Tablizo; Irish Coleen A. Asin; Jaime C. Montoya; Jan Michael C. Yap; Jo-Hannah S. Llames; John Q. Wong; Joshua Gregor A. Dizon; Juan Antonio R. Magalang; Karol Sophia Agape R. Padilla; Kenneth M. Kim; Kris P. Punayan; Marc Edsel C. Ayes; Marc Jerrone R. Castro; Maria Rosario Singh-Vergeire and Cynthia P. Saloma; Maria Sofia L. Yangzon; Marissa Alejandria; Razel Nikka M. Hao; Rianna Patricia S. Cruz; Sheila Mae M. Araiza
EPI_ISL_833343	Buenavista Rural Health Unit	Research Institute for Tropical Medicine	Catalino Demetria; Daria Manalo; Edelwisa Mercado; Francisco Gerardo Polotan; Hannah Leah Morito; Inez Andrea Medado; John Leonard Chan; Kirstyn Brunker; Ma Angelica Tujan; Othoniel Jan Onza
EPI_ISL_2105378, EPI_ISL_2154933, EPI_ISL_2154934, EPI_ISL_2154936, EPI_ISL_2154937, EPI_ISL_2154944, EPI_ISL_2154945, EPI_ISL_2154951, EPI_ISL_2154953, EPI_ISL_2154960, EPI_ISL_2154962, EPI_ISL_2154963, EPI_ISL_2155601, EPI_ISL_2155602, EPI_ISL_2171196			
see above	Bulacan Medical Center	Philippine Genome Center	Alethea R. de Guzman; Anna Ong-Lim; Arianne A. Zamora; Asia Louisa U. Chong; Benedict A. Maralit; Candice Francheska B. Tambaoan; Carlo M. Lapid; Celia Carlos; Devon Ray Pacial; Edsel Maurice Salvaña; El King D. Morado; Elcid Aaron R. Pangilinan; Eva Maria Cutiongco-de la Paz; Francis A. Tablizo; Irish Coleen A. Asin; Jaime C. Montoya; Jan Michael C. Yap; Jo-Hannah S. Llames; John Q. Wong; Joshua Gregor A. Dizon; Juan Antonio R. Magalang; Karol Sophia Agape R. Padilla; Kenneth M. Kim; Kris P. Punayan; Marc Edsel C. Ayes; Marc Jerrone R. Castro; Maria Rosario Singh-Vergeire and Cynthia P. Saloma; Maria Sofia L. Yangzon; Marissa Alejandria; Razel Nikka M. Hao; Rianna Patricia S. Cruz; Sheila Mae M. Araiza
EPI_ISL_2558054, EPI_ISL_2558056, EPI_ISL_2558058, EPI_ISL_2558059, EPI_ISL_2558061, EPI_ISL_2558062, EPI_ISL_2558063			
see above	Bureau Of Quarantine (BOQ)	Philippine Genome Center	Alethea R. de Guzman; Anna Ong-Lim; Arianne A. Zamora; Asia Louisa U. Chong; Benedict A. Maralit; Candice Francheska B. Tambaoan; Carlo M. Lapid; Celia Carlos; Devon Ray Pacial; Edsel Maurice Salvaña; El King D. Morado; Elcid Aaron R. Pangilinan; Eva Maria Cutiongco-de la Paz; Francis A. Tablizo; Irish Coleen A. Asin; Jaime C. Montoya; Jan Michael C. Yap; Jo-Hannah S. Llames; John Q. Wong; Joshua Gregor A. Dizon; Juan Antonio R. Magalang; Karol Sophia Agape R. Padilla; Kenneth M. Kim; Kris P. Punayan; Marc Edsel C. Ayes; Maria Rosario Singh-Vergeire and Cynthia P. Saloma; Maria Sofia L. Yangzon; Marissa Alejandria; Razel Nikka M. Hao; Renato Jacinto Q. Mantaring; Rianna Patricia S. Cruz; Sheila Mae M. Araiza
EPI_ISL_833336	Bureau of Quarantine	Research Institute for Tropical Medicine	Catalino Demetria; Daria Manalo; Edelwisa Mercado; Francisco Gerardo Polotan; Hannah Leah Morito; Inez Andrea Medado; John Leonard Chan; Kirstyn Brunker; Ma Angelica Tujan; Othoniel Jan Onza
EPI_ISL_2105414, EPI_ISL_2105436, EPI_ISL_2105456, EPI_ISL_2105522, EPI_ISL_2105524, EPI_ISL_2105525, EPI_ISL_2105528, EPI_ISL_2105531, EPI_ISL_2105534, EPI_ISL_2105536, EPI_ISL_2105537, EPI_ISL_2105542, EPI_ISL_2105544, EPI_ISL_2105548, EPI_ISL_2105552, EPI_ISL_2105559, EPI_ISL_2105563, EPI_ISL_2105564, EPI_ISL_2155226, EPI_ISL_2155267, EPI_ISL_2156148, EPI_ISL_2156149, EPI_ISL_2156150, EPI_ISL_2156151, EPI_ISL_2156153, EPI_ISL_2156154, EPI_ISL_2156340, EPI_ISL_2156341, EPI_ISL_2156342, EPI_ISL_2156343, EPI_ISL_2156362, EPI_ISL_2156363, EPI_ISL_2156379, EPI_ISL_2156380, EPI_ISL_2156381, EPI_ISL_2156397, EPI_ISL_2156398, EPI_ISL_2156399, EPI_ISL_2156400, EPI_ISL_2156421, EPI_ISL_2156422, EPI_ISL_2156444, EPI_ISL_2156445, EPI_ISL_2156446, EPI_ISL_2156447, EPI_ISL_2156448, EPI_ISL_2156463, EPI_ISL_2156464, EPI_ISL_2156465, EPI_ISL_2156466, EPI_ISL_2156467, EPI_ISL_2156468, EPI_ISL_2188097, EPI_ISL_2188870, EPI_ISL_2188906, EPI_ISL_2188913, EPI_ISL_2188936, EPI_ISL_2188994, EPI_ISL_2189046, EPI_ISL_2189115, EPI_ISL_2189127, EPI_ISL_2189157, EPI_ISL_2189233, EPI_ISL_2189252, EPI_ISL_2189273, EPI_ISL_2189274, EPI_ISL_2189296, EPI_ISL_2189358, EPI_ISL_2189362, EPI_ISL_2189377, EPI_ISL_2189380, EPI_ISL_2189401, EPI_ISL_2189486, EPI_ISL_2189493, EPI_ISL_2859207, EPI_ISL_2860202, EPI_ISL_2860216, EPI_ISL_2860238, EPI_ISL_2860239, EPI_ISL_2860243, EPI_ISL_2860246, EPI_ISL_2860251, EPI_ISL_2860253, EPI_ISL_4713266, EPI_ISL_4715869, EPI_ISL_4716508, EPI_ISL_4716550, EPI_ISL_4716606, EPI_ISL_4716652, EPI_ISL_4716748, EPI_ISL_4716768, EPI_ISL_4716774, EPI_ISL_4716894, EPI_ISL_4716984, EPI_ISL_4717188, EPI_ISL_4717220, EPI_ISL_4717253, EPI_ISL_4717319			
see above	Butuan Medical Center	Philippine Genome Center	Alethea R. de Guzman; Anna Ong-Lim; Arianne A. Zamora; Asia Louisa U. Chong; Benedict A. Maralit; Candice Francheska B. Tambaoan; Carlo M. Lapid; Celia Carlos; Cynthia P. Saloma; Devon Ray Pacial; Diomedes A. Carino; Diomedes A. Cariño; Edsel Maurice Salvana; Edsel Maurice Salvaña; El King D. Morado; Elcid Aaron R. Pangilinan; Eva Maria Cutiongco-de la Paz; Francis A. Tablizo; Henrietta Marie Rodriguez; Irish Coleen A. Asin; Jaime C. Montoya; Jan Michael C. Yap; Jarvin E. Nipales; Jo-Hannah S. Llames; John Q. Wong; Joshua Gregor A. Dizon; Juan Antonio R. Magalang; Karol Sophia Agape R. Padilla; Kenneth M. Kim; Kris P. Punayan; Krisitna Patriz Dela Cruz; Kristina Patriz Dela Cruz; Lindsay Claire D.L. Carandang; Lindsay Clare D.L. Carandang; Ma. Exanil Plantig; Marc Edsel C. Ayes; Marc Jerrone R. Castro; Maria Rosario Singh-Vergeire; Maria Rosario Singh-Vergeire and Cynthia P. Saloma; Maria Sofia L. Yangzon; Marielle M Gamboa; Marielle M. Gamboa; Marissa Alejandria; Nina Francesca Bustamante; Niña Francesca Bustamante; Razel Nikka M. Hao; Renato Jacinto Q. Mantaring; Rianna Patricia S. Cruz; Sheila Mae M. Araiza; Shiela Mae M. Araiza; Yvonne Valerie Austria; Zipporah Mariebelle R. Enriquez; Zyrel V. Mollejon
EPI_ISL_4459368	CALAMBA DOCTORS' HOSPITAL	Research Institute for Tropical Medicine/Tohoku University	Allen Anthony Laraño; Ardiane Ysabelle Dolor; Catalino Demetria; Clyde Dapat; Emmanuel Kagning Tsinda; Francisco Gerardo Polotan; Hitoshi Oshitani; Inez Andrea Medado; Jefferson Earl Halog; Lei Lanna Dancel; Leilanie Nacion; Ma. Angelica Tujan; Machiko Okamoto; Maria Carmen Corpuz; Mariko Siato-Obata; Mayuko Saito
EPI_ISL_2156578	Cagayan Valley CHD TB Reference Laboratory (GeneXpert)	Philippine Genome Center	Alethea R. de Guzman; Anna Ong-Lim; Arianne A. Zamora; Asia Louisa U. Chong; Benedict A. Maralit; Candice Francheska B. Tambaoan; Carlo M. Lapid; Celia Carlos; Devon Ray Pacial; Edsel Maurice Salvaña; El King D. Morado; Elcid Aaron R. Pangilinan; Eva Maria Cutiongco-de la Paz; Francis A. Tablizo; Irish Coleen A. Asin; Jaime C. Montoya; Jan Michael C. Yap; Jo-Hannah S. Llames; John Q. Wong; Joshua Gregor A. Dizon; Juan Antonio R. Magalang; Karol Sophia Agape R. Padilla; Kenneth M. Kim; Kris P. Punayan; Marc Edsel C. Ayes; Marc Jerrone R. Castro; Maria Rosario Singh-Vergeire and Cynthia P. Saloma; Maria Sofia L. Yangzon; Marissa Alejandria; Razel Nikka M. Hao; Rianna Patricia S. Cruz; Sheila Mae M. Araiza
EPI_ISL_2105218, EPI_ISL_2105219, EPI_ISL_2105220, EPI_ISL_2105221, EPI_ISL_2105222, EPI_ISL_2105223, EPI_ISL_2105224, EPI_ISL_2105225, EPI_ISL_2105226, EPI_ISL_2105230, EPI_ISL_2105231, EPI_ISL_2105233, EPI_ISL_2105234, EPI_ISL_2105235, EPI_ISL_2105236, EPI_ISL_2105237, EPI_ISL_2105239, EPI_ISL_2105242, EPI_ISL_2105243, EPI_ISL_2105244, EPI_ISL_2105245, EPI_ISL_2105246, EPI_ISL_2105247, EPI_ISL_2105248, EPI_ISL_2105249, EPI_ISL_2105250, EPI_ISL_2105251, EPI_ISL_2105253, EPI_ISL_2105254, EPI_ISL_2105255, EPI_ISL_2105257, EPI_ISL_2105258, EPI_ISL_2105259, EPI_ISL_2105260, EPI_ISL_2105261, EPI_ISL_2105262, EPI_ISL_2105263, EPI_ISL_2105265, EPI_ISL_2105268, EPI_ISL_2105269, EPI_ISL_2105270, EPI_ISL_2105271, EPI_ISL_2105273, EPI_ISL_2105276, EPI_ISL_2105277, EPI_ISL_2105278, EPI_ISL_2105279, EPI_ISL_2105281, EPI_ISL_2105282, EPI_ISL_2105285, EPI_ISL_2105286, EPI_ISL_2105291, EPI_ISL_2105299, EPI_ISL_2105322, EPI_ISL_2105363, EPI_ISL_2105392, EPI_ISL_2105406, EPI_ISL_2105409, EPI_ISL_2105448, EPI_ISL_2105449, EPI_ISL_2105453, EPI_ISL_2105529, EPI_ISL_2105532, EPI_ISL_2105549, EPI_ISL_2105550, EPI_ISL_2105551, EPI_ISL_2154394, EPI_ISL_2154399, EPI_ISL_2154404, EPI_ISL_2154407, EPI_ISL_2154408, EPI_ISL_2154410, EPI_ISL_2154411, EPI_ISL_2154412, EPI_ISL_2154414, EPI_ISL_2154415, EPI_ISL_2154419, EPI_ISL_2154422, EPI_ISL_2154424, EPI_ISL_2154425, EPI_ISL_2154426, EPI_ISL_2154427, EPI_ISL_2154430, EPI_ISL_2154431, EPI_ISL_2154432, EPI_ISL_2154446, EPI_ISL_2154455, EPI_ISL_2154459, EPI_ISL_2154460, EPI_ISL_2154472, EPI_ISL_2154476, EPI_ISL_2154478, EPI_ISL_2154479, EPI_ISL_2154487, EPI_ISL_2154488, EPI_ISL_2154491, EPI_ISL_2154495, EPI_ISL_2154497, EPI_ISL_2154498, EPI_ISL_2154499, EPI_ISL_2154501, EPI_ISL_2154505, EPI_ISL_2154507, EPI_ISL_2154513, EPI_ISL_2154516, EPI_ISL_2154523, EPI_ISL_2154526, EPI_ISL_2154529, EPI_ISL_2154532, EPI_ISL_2154533, EPI_ISL_2154534, EPI_ISL_2154537, EPI_ISL_2154540, EPI_ISL_2154545, EPI_ISL_2154546, EPI_ISL_2154560, EPI_ISL_2154563, EPI_ISL_2154570, EPI_ISL_2154572, EPI_ISL_2154581, EPI_ISL_2154584, EPI_ISL_2154588, EPI_ISL_2154596, EPI_ISL_2154600, EPI_ISL_2154602, EPI_ISL_2154603, EPI_ISL_2154607, EPI_ISL_2154610, EPI_ISL_2154611, EPI_ISL_2154613, EPI_ISL_2154614, EPI_ISL_2154633, EPI_ISL_2154634, EPI_ISL_2154637, EPI_ISL_2154638, EPI_ISL_2154640, EPI_ISL_2154641, EPI_ISL_2154666, EPI_ISL_2154669, EPI_ISL_2154671, EPI_ISL_2154678, EPI_ISL_2154684, EPI_ISL_2154686, EPI_ISL_2154687, EPI_ISL_2154688, EPI_ISL_2154689, EPI_ISL_2154690, EPI_ISL_2154691, EPI_ISL_2154704, EPI_ISL_2154706, EPI_ISL_2154709, EPI_ISL_2154710, EPI_ISL_2154723, EPI_ISL_2154730, EPI_ISL_2154732, EPI_ISL_2154733, EPI_ISL_2154738, EPI_ISL_2154739, EPI_ISL_2154744, EPI_ISL_2154745, EPI_ISL_2154751, EPI_ISL_2154757, EPI_ISL_2154758, EPI_ISL_2154768, EPI_ISL_2154771, EPI_ISL_2154773, EPI_ISL_2154776, EPI_ISL_2154777, EPI_ISL_2154781, EPI_ISL_2154782, EPI_ISL_2154783, EPI_ISL_2154786, EPI_ISL_2154787, EPI_ISL_2154804, EPI_ISL_2154814, EPI_ISL_2154823, EPI_ISL_2154824, EPI_ISL_2154825, EPI_ISL_2154838, EPI_ISL_2154839, EPI_ISL_2154841, EPI_ISL_2154844, EPI_ISL_2154849, EPI_ISL_2154851, EPI_ISL_2154854, EPI_ISL_2154856, EPI_ISL_2154861, EPI_ISL_2154866, EPI_ISL_2154868, EPI_ISL_2154873, EPI_ISL_2154874, EPI_ISL_2154877, EPI_ISL_2154878, EPI_ISL_2154880, EPI_ISL_2154886, EPI_ISL_2154887, EPI_ISL_2154888, EPI_ISL_2154895, EPI_ISL_2154896, EPI_ISL_2154902, EPI_ISL_2154911, EPI_ISL_2154912, EPI_ISL_2154915, EPI_ISL_2154918, EPI_ISL_2154920, EPI_ISL_2154922, EPI_ISL_2154924, EPI_ISL_2154925, EPI_ISL_2154928, EPI_ISL_2154930, EPI_ISL_2154938, EPI_ISL_2154948, EPI_ISL_2154955, EPI_ISL_2154957, EPI_ISL_2154959, EPI_ISL_2154961, EPI_ISL_2154964, EPI_ISL_2154965, EPI_ISL_2154967, EPI_ISL_2155108, EPI_ISL_2155123, EPI_ISL_2155130, EPI_ISL_2155137, EPI_ISL_2155144, EPI_ISL_2155145, EPI_ISL_2155161, EPI_ISL_2155191, EPI_ISL_2155194, EPI_ISL_2155238, EPI_ISL_2155283, EPI_ISL_2155310, EPI_ISL_2155337, EPI_ISL_2155346, EPI_ISL_2155351, EPI_ISL_2155357, EPI_ISL_2155703, EPI_ISL_2155704, EPI_ISL_2155705, EPI_ISL_2155706, EPI_ISL_2155707, EPI_ISL_2155708, EPI_ISL_2155709, EPI_ISL_2155710, EPI_ISL_2155711, EPI_ISL_2155712, EPI_ISL_2155713, EPI_ISL_2155714, EPI_ISL_2155715, EPI_ISL_2155716, EPI_ISL_2155717, EPI_ISL_2155718, EPI_ISL_2155719, EPI_ISL_2155720, EPI_ISL_2155721, EPI_ISL_2155722, EPI_ISL_2155723, EPI_ISL_2155724, EPI_ISL_2155725, EPI_ISL_2155726, EPI_ISL_2155727, EPI_ISL_2155728, EPI_ISL_2156206, EPI_ISL_2156207, EPI_ISL_2156208, EPI_ISL_2156209, EPI_ISL_2156210, EPI_ISL_2156211, EPI_ISL_2156212, EPI_ISL_2156213, EPI_ISL_2156214, EPI_ISL_2156215, EPI_ISL_2156216, EPI_ISL_2156217, EPI_ISL_2156218, EPI_ISL_2156219, EPI_ISL_2156367, EPI_ISL_2156415, EPI_ISL_2156427, EPI_ISL_2156428, EPI_ISL_2156429, EPI_ISL_2156430, EPI_ISL_2156431, EPI_ISL_2156432, EPI_ISL_2156433, EPI_ISL_2156460, EPI_ISL_2156506, EPI_ISL_2156515, EPI_ISL_2156553, EPI_ISL_2156579, EPI_ISL_2156580, EPI_ISL_2156581, EPI_ISL_2156582, EPI_ISL_2156625, EPI_ISL_2156630, EPI_ISL_2156650, EPI_ISL_2156652, EPI_ISL_2156660, EPI_ISL_2156663, EPI_ISL_2156679, EPI_ISL_2156682, EPI_ISL_2156683, EPI_ISL_2156700, EPI_ISL_2156705, EPI_ISL_2156712, EPI_ISL_2156714, EPI_ISL_2156720, EPI_ISL_2156729, EPI_ISL_2156732, EPI_ISL_2156733, EPI_ISL_2156734, EPI_ISL_2156754, EPI_ISL_2156757, EPI_ISL_2156762, EPI_ISL_2156763, EPI_ISL_2156765, EPI_ISL_2188061, EPI_ISL_2188720, EPI_ISL_2859201, EPI_ISL_2859239, EPI_ISL_2859255, EPI_ISL_2859261, EPI_ISL_2859694, EPI_ISL_2860197, EPI_ISL_2860201, EPI_ISL_2860203, EPI_ISL_2860206, EPI_ISL_2860207, EPI_ISL_2860208, EPI_ISL_2860209, EPI_ISL_2860210, EPI_ISL_2860211, EPI_ISL_4712255, EPI_ISL_4712267, EPI_ISL_4712271, EPI_ISL_4712280, EPI_ISL_4712285, EPI_ISL_4712289, EPI_ISL_4712320, EPI_ISL_4712321, EPI_ISL_4712517, EPI_ISL_4712602, EPI_ISL_4712621, EPI_ISL_4712700, EPI_ISL_4712726, EPI_ISL_4712856, EPI_ISL_4713834, EPI_ISL_4713842, EPI_ISL_4713915, EPI_ISL_4713939, EPI_ISL_4713963, EPI_ISL_4713979, EPI_ISL_4713983, EPI_ISL_4713984, EPI_ISL_4714001, EPI_ISL_4714017, EPI_ISL_4714044, EPI_ISL_4714053, EPI_ISL_4714067, EPI_ISL_4714079, EPI_ISL_4714096, EPI_ISL_4714111, EPI_ISL_4714144, EPI_ISL_4714148, EPI_ISL_4714203, EPI_ISL_4714235, EPI_ISL_4714247, EPI_ISL_4714281, EPI_ISL_4714294, EPI_ISL_4714314, EPI_ISL_4714316, EPI_ISL_4714323, EPI_ISL_4714350, EPI_ISL_4714354, EPI_ISL_4714380, EPI_ISL_4714388, EPI_ISL_4714393, EPI_ISL_4714422, EPI_ISL_4714428, EPI_ISL_4714435, EPI_ISL_4714444, EPI_ISL_4714460, EPI_ISL_4714472, EPI_ISL_4714501, EPI_ISL_4714509, EPI_ISL_4714558, EPI_ISL_4714565, EPI_ISL_4714578, EPI_ISL_4714603, EPI_ISL_4714618, EPI_ISL_4714709, EPI_ISL_4714793, EPI_ISL_4714801, EPI_ISL_4714878, EPI_ISL_4714956, EPI_ISL_4714970, EPI_ISL_4715014, EPI_ISL_4715022, EPI_ISL_4715042, EPI_ISL_4715120, EPI_ISL_4715176, EPI_ISL_4715460			
see above	Cagayan Valley Medical Center Molecular Laboratory	Philippine Genome Center	Alethea R. de Guzman; Anna Ong-Lim; Arianne A. Zamora; Asia Louisa U. Chong; Benedict A. Maralit; Candice Francheska B. Tambaoan; Carlo M. Lapid; Celia Carlos; Cynthia P. Saloma; Devon Ray Pacial; Diomedes A. Carino; Diomedes A. Cariño; Edsel Maurice Salvana; Edsel Maurice Salvaña; El King D. Morado; Elcid Aaron R. Pangilinan; Eva Maria Cutiongco-de la Paz; Francis A. Tablizo; Henrietta Marie Rodriguez; Irish Coleen A. Asin; Jaime C. Montoya; Jan Michael C. Yap; Jarvin E. Nipales; Jo-Hannah S. Llames; John Q. Wong; Joshua Gregor A. Dizon; Juan Antonio R. Magalang; Karol Sophia Agape R. Padilla; Kenneth M. Kim; Kris P. Punayan; Krisitna Patriz Dela Cruz; Kristina Patriz Dela Cruz; Lindsay Claire D.L. Carandang; Lindsay Clare D.L. Carandang; Ma. Exanil Plantig; Marc Edsel C. Ayes; Marc Jerrone R. Castro; Maria Rosario Singh-Vergeire; Maria Rosario Singh-Vergeire and Cynthia P. Saloma; Maria Sofia L. Yangzon; Marielle M Gamboa; Marielle M. Gamboa; Marissa Alejandria; Nina Francesca Bustamante; Niña Francesca Bustamante; Razel Nikka M. Hao; Renato Jacinto Q. Mantaring; Rianna Patricia S. Cruz; Sheila Mae M. Araiza; Shiela Mae M. Araiza; Yvonne Valerie Austria; Zipporah Mariebelle R. Enriquez; Zyrel V. Mollejon
EPI_ISL_2155521, EPI_ISL_2155522, EPI_ISL_2155523, EPI_ISL_2155524	Cainta Health Office (PGC)	Philippine Genome Center	Alethea R. de Guzman; Anna Ong-Lim; Arianne A. Zamora; Asia Louisa U. Chong; Benedict A. Maralit; Candice Francheska B. Tambaoan; Carlo M. Lapid; Celia Carlos; Devon Ray Pacial; Edsel Maurice Salvaña; El King D. Morado; Eva Maria Cutiongco-de la Paz; Francis A. Tablizo; Irish Coleen A. Asin; Jaime C. Montoya; Jan Michael C. Yap; Jo-Hannah S. Llames; John Q. Wong; Joshua Gregor A. Dizon; Juan Antonio R. Magalang; Karol Sophia Agape R. Padilla; Kenneth M. Kim; Kris P. Punayan; Marc Edsel C. Ayes; Marc Jerrone R. Castro; Maria Rosario Singh-Vergeire and Cynthia P. Saloma; Maria Sofia L. Yangzon; Marissa Alejandria; Razel Nikka M. Hao; Rianna Patricia S. Cruz; Sheila Mae M. Araiza
EPI_ISL_1069176	Calamba Doctors Hospital	Research Institute for Tropical Medicine	Catalino Demetria; Daria Manalo; Edelwisa Mercado; Francisco Gerardo Polotan; Hannah Leah Morito; Inez Andrea Medado; John Leonard Chan; Joseph Hughes; Kirstyn Brunker; Ma Angelica Tujan; Othoniel Jan Onza
EPI_ISL_3105869	Candelaria Rural Health Unit	Research Institute for Tropical Medicine	Clyde Dapat; Deana Mae Ocampo; Emmanuel Kagning Tsinda; Francisco Gerardo Polotan; Hitoshi Oshitani; Inez Andrea Medado; Jefferson Earl Halog; Joana Ina Manalo; Lei Lanna Dancel; Ma Angelica Tujan; Mariko Saito-Obata; Mayuko Saito; Michiko Okamoto; Samantha Louise Bado
EPI_ISL_2154490, EPI_ISL_2154819	Cardinal Santos Medical Center	Philippine Genome Center	Alethea R. de Guzman; Anna Ong-Lim; Arianne A. Zamora; Asia Louisa U. Chong; Benedict A. Maralit; Candice Francheska B. Tambaoan; Carlo M. Lapid; Celia Carlos; Devon Ray Pacial; Edsel Maurice Salvaña; El King D. Morado; Eva Maria Cutiongco-de la Paz; Francis A. Tablizo; Irish Coleen A. Asin; Jaime C. Montoya; Jan Michael C. Yap; Jo-Hannah S. Llames; John Q. Wong; Joshua Gregor A. Dizon; Juan Antonio R. Magalang; Karol Sophia Agape R. Padilla; Kenneth M. Kim; Kris P. Punayan; Marc Edsel C. Ayes; Marc Jerrone R. Castro; Maria Rosario Singh-Vergeire and Cynthia P. Saloma; Maria Sofia L. Yangzon; Marissa Alejandria; Razel Nikka M. Hao; Rianna Patricia S. Cruz; Sheila Mae M. Araiza
EPI_ISL_491472	Cardinal Santos Medical Center	Research Institute for Tropical Medicine	Catalino Demetria; Criselda Bautista; Daria Manalo; Edelwisa Mercado; Francisco Gerardo Polotan; Inez Andrea Medado; Kirstyn Brunker; Ma. Angelica Tujan; Othoniel Jan Onza
EPI_ISL_1122426, EPI_ISL_1122427, EPI_ISL_1122428, EPI_ISL_1122429, EPI_ISL_1122430, EPI_ISL_1122431, EPI_ISL_1122432, EPI_ISL_1122433, EPI_ISL_1122434, EPI_ISL_1122435, EPI_ISL_1122436, EPI_ISL_1122437, EPI_ISL_1122438, EPI_ISL_1122439, EPI_ISL_1122440, EPI_ISL_1122441, EPI_ISL_1122442, EPI_ISL_1122443, EPI_ISL_1122444, EPI_ISL_1122445, EPI_ISL_1122446, EPI_ISL_1122447, EPI_ISL_1122448, EPI_ISL_1122449, EPI_ISL_1122450, EPI_ISL_1122451, EPI_ISL_1122452, EPI_ISL_1122453, EPI_ISL_1122454, EPI_ISL_1213533, EPI_ISL_1213535, EPI_ISL_1213537, EPI_ISL_1213539, EPI_ISL_1213540, EPI_ISL_1213542, EPI_ISL_1213544, EPI_ISL_1213546, EPI_ISL_1213547, EPI_ISL_1213549, EPI_ISL_1213551, EPI_ISL_1213552, EPI_ISL_1213554, EPI_ISL_1213556, EPI_ISL_1213558, EPI_ISL_1213559, EPI_ISL_1213561, EPI_ISL_1219702, EPI_ISL_2091458, EPI_ISL_2091461, EPI_ISL_2105296, EPI_ISL_2105320, EPI_ISL_2105327, EPI_ISL_2105329, EPI_ISL_2105332, EPI_ISL_2105379, EPI_ISL_2105383, EPI_ISL_2105468, EPI_ISL_2105469, EPI_ISL_2105476, EPI_ISL_2105477, EPI_ISL_2105479, EPI_ISL_2105485, EPI_ISL_2105491, EPI_ISL_2105492, EPI_ISL_2105495, EPI_ISL_2105496, EPI_ISL_2105499, EPI_ISL_2105500, EPI_ISL_2105501, EPI_ISL_2105503, EPI_ISL_2105505, EPI_ISL_2105526, EPI_ISL_2105527, EPI_ISL_2105560, EPI_ISL_2155587, EPI_ISL_2155588, EPI_ISL_2155589, EPI_ISL_2155590, EPI_ISL_2155919, EPI_ISL_2155920, EPI_ISL_2155921, EPI_ISL_2155922, EPI_ISL_2155923, EPI_ISL_2155924, EPI_ISL_2155925, EPI_ISL_2155926, EPI_ISL_2155927, EPI_ISL_2155928, EPI_ISL_2155929, EPI_ISL_2155930, EPI_ISL_2155931, EPI_ISL_2155932, EPI_ISL_2155933, EPI_ISL_2155934, EPI_ISL_2155935, EPI_ISL_2155936, EPI_ISL_2155937, EPI_ISL_2155938, EPI_ISL_2155939, EPI_ISL_2155940, EPI_ISL_2156124, EPI_ISL_2156125, EPI_ISL_2156126, EPI_ISL_2156127, EPI_ISL_2156128, EPI_ISL_4712088, EPI_ISL_4712137, EPI_ISL_4712238, EPI_ISL_4712247, EPI_ISL_4712417, EPI_ISL_4712615, EPI_ISL_4712793, EPI_ISL_4712950, EPI_ISL_4713399, EPI_ISL_4713556, EPI_ISL_4713727, EPI_ISL_4713733, EPI_ISL_4713803, EPI_ISL_4713804, EPI_ISL_4713901, EPI_ISL_4713959, EPI_ISL_4714105, EPI_ISL_4714676, EPI_ISL_4714823, EPI_ISL_4715283, EPI_ISL_4715388, EPI_ISL_4715399, EPI_ISL_4715404, EPI_ISL_4715415, EPI_ISL_4715430, EPI_ISL_4715441, EPI_ISL_4715445, EPI_ISL_4715451, EPI_ISL_4715465, EPI_ISL_4715473, EPI_ISL_4715486, EPI_ISL_4715488, EPI_ISL_4715501, EPI_ISL_4715508, EPI_ISL_4715523, EPI_ISL_4715531, EPI_ISL_4715536, EPI_ISL_4715545, EPI_ISL_4715549, EPI_ISL_4715553, EPI_ISL_4715563, EPI_ISL_4715569, EPI_ISL_4715578, EPI_ISL_4715583, EPI_ISL_4715585, EPI_ISL_4715587, EPI_ISL_4717390			
see above	Cebu TB Reference	Philippine Genome	Alethea R. de Guzman; Anna Ong-Lim; Arianne A. Zamora; Asia Louisa U. Chong; Benedict A. Maralit; Candice Francheska B. Tambaoan; Carlo M. Lapid; Celia Carlos; Cynthia P. Saloma; Devon Ray Pacial; Diomedes A. Cariño; Edsel Maurice Salvana; Edsel Maurice Salvaña; El King D. Morado; Elcid Aaron R. Pangilinan; Eva

	Laboratory	Center	Maria Cutiongco-de la Paz; Francis A. Tablizo; Henrietta Marie Rodriguez; Irish Coleen A. Asin; Jaime C. Montoya; Jan Michael C. Yap; Jarvin E. Nipales; Jo-Hannah S. Llames; John Q. Wong; Joshua Gregor A. Dizon; Juan Antonio R. Magalang; Karol Sophia Agape R. Padilla; Kenneth M. Kim; Kris P. Punayan; Kristina Patriz Dela Cruz; Lindsay Clare D.L. Carandang; Ma. Exanil Plantig; Marc Edsel C. Ayes; Marc Jerrone R. Castro; Maria Rosario Singh-Vergeire; Maria Rosario Singh-Vergeire and Cynthia P. Saloma; Maria Sofia L. Yangzon; Marielle M. Gamboa; Marissa Alejandria; Niña Francesca Bustamante; Razel Nikka M. Hao; Renato Jacinto Q. Mantaring; Rianna Patricia S. Cruz; Sheila Mae M. Araiza; Shiela Mae M. Araiza; Yvonne Valerie Austria; Zipporah Mariebelle R. Enriquez; Zyrel V. Mollejon
EPI_ISL_2105547, EPI_ISL_2156494, EPI_ISL_4714975	Chinese General Hospital	Philippine Genome Center	Alethea R. de Guzman; Anna Ong-Lim; Arianne A. Zamora; Asia Louisa U. Chong; Benedict A. Maralit; Candice Francheska B. Tambaoan; Carlo M. Lapid; Celia Carlos; Cynthia P. Saloma; Devon Ray Pacial; Diomedes A. Cariño; Edsel Maurice Salvana; Edsel Maurice Salvaña; El King D. Morado; Elcid Aaron R. Pangilinan; Eva Maria Cutiongco-de la Paz; Francis A. Tablizo; Henrietta Marie Rodriguez; Irish Coleen A. Asin; Jaime C. Montoya; Jan Michael C. Yap; Jarvin E. Nipales; Jo-Hannah S. Llames; John Q. Wong; Joshua Gregor A. Dizon; Juan Antonio R. Magalang; Karol Sophia Agape R. Padilla; Kenneth M. Kim; Kris P. Punayan; Kristina Patriz Dela Cruz; Lindsay Clare D.L. Carandang; Ma. Exanil Plantig; Marc Edsel C. Ayes; Marc Jerrone R. Castro; Maria Rosario Singh-Vergeire; Maria Rosario Singh-Vergeire and Cynthia P. Saloma; Maria Sofia L. Yangzon; Marielle M. Gamboa; Marissa Alejandria; Niña Francesca Bustamante; Razel Nikka M. Hao; Renato Jacinto Q. Mantaring; Rianna Patricia S. Cruz; Sheila Mae M. Araiza; Shiela Mae M. Araiza; Yvonne Valerie Austria; Zipporah Mariebelle R. Enriquez; Zyrel V. Mollejon
EPI_ISL_833337	Christ the Saviour General Hospital - Batangas	Research Institute for Tropical Medicine	Catalino Demetria; Daria Manalo; Edelwisa Mercado; Francisco Gerardo Polotan; Hannah Leah Morito; Inez Andrea Medado; John Leonard Chan; Kirstyn Brunker; Ma Angelica Tujan; Othoniel Jan Onza
EPI_ISL_3105871	City Health Office - San Juan	Research Institute for Tropical Medicine	Clyde Dapat; Deana Mae Ocampo; Emmanuel Kagning Tsinda; Francisco Gerardo Polotan; Hitoshi Oshitani; Inez Andrea Medado; Jefferson Earl Halog; Joana Ina Manalo; Lei Lanna Dancel; Ma Angelica Tujan; Mariko Saito-Obata; Mayuko Saito; Michiko Okamoto; Samantha Louise Bado
EPI_ISL_2859300	Ciudad Medical Zamboanga	Philippine Genome Center	Alethea R. de Guzman; Anna Ong-Lim; Arianne A. Zamora; Benedict A. Maralit; Carlo M. Lapid; Celia Carlos; Devon Ray Pacial; Diomedes A. Carino; Edsel Maurice Salvaña; El King D. Morado; Elcid Aaron R. Pangilinan; Eva Maria Cutiongco-de la Paz; Francis A. Tablizo; Henrietta Marie Rodriguez; Jaime C. Montoya; Jan Michael C. Yap; Jarvin E. Nipales; Jo-Hannah S. Llames; John Q. Wong; Joshua Gregor A. Dizon; Juan Antonio R. Magalang; Karol Sophia Agape R. Padilla; Kenneth M. Kim; Kris P. Punayan; Krisitna Patriz Dela Cruz; Lindsay Claire D.L. Carandang; Ma. Exanil Plantig; Marc Edsel C. Ayes; Maria Rosario Singh-Vergeire and Cynthia P. Saloma; Maria Sofia L. Yangzon; Marielle M Gamboa; Marissa Alejandria; Nina Francesca Bustamante; Razel Nikka M. Hao; Renato Jacinto Q. Mantaring; Rianna Patricia S. Cruz; Sheila Mae M. Araiza; Yvonne Valerie Austria; Zipporah Mariebelle R. Enriquez; Zyrel V. Mollejon
EPI_ISL_1213563, EPI_ISL_2105538, EPI_ISL_2105543, EPI_ISL_2156166, EPI_ISL_2156281, EPI_ISL_2156282, EPI_ISL_2156283, EPI_ISL_2156295, EPI_ISL_2156296, EPI_ISL_2156297, EPI_ISL_2156298, EPI_ISL_2156299, EPI_ISL_2156327, EPI_ISL_2171163, EPI_ISL_2171229, EPI_ISL_2188677, EPI_ISL_2188728, EPI_ISL_2188775, EPI_ISL_2189064, EPI_ISL_2189421, EPI_ISL_2189445, EPI_ISL_2859243, EPI_ISL_2859254, EPI_ISL_2859264, EPI_ISL_2859302, EPI_ISL_2859372, EPI_ISL_2860215			
see above	Cotabato Regional and Medical Center	Philippine Genome Center	Alethea R. de Guzman; Anna Ong-Lim; Arianne A. Zamora; Asia Louisa U. Chong; Benedict A. Maralit; Candice Francheska B. Tambaoan; Carlo M. Lapid; Celia Carlos; Devon Ray Pacial; Diomedes A. Carino; Edsel Maurice Salvaña; El King D. Morado; Elcid Aaron R. Pangilinan; Eva Maria Cutiongco-de la Paz; Francis A. Tablizo; Henrietta Marie Rodriguez; Irish Coleen A. Asin; Jaime C. Montoya; Jan Michael C. Yap; Jarvin E. Nipales; Jo-Hannah S. Llames; John Q. Wong; Joshua Gregor A. Dizon; Juan Antonio R. Magalang; Karol Sophia Agape R. Padilla; Kenneth M. Kim; Kris P. Punayan; Krisitna Patriz Dela Cruz; Lindsay Claire D.L. Carandang; Ma. Exanil Plantig; Marc Edsel C. Ayes; Marc Jerrone R. Castro; Maria Rosario Singh-Vergeire and Cynthia P. Saloma; Maria Sofia L. Yangzon; Marielle M Gamboa; Marissa Alejandria; Nina Francesca Bustamante; Razel Nikka M. Hao; Renato Jacinto Q. Mantaring; Rianna Patricia S. Cruz; Sheila Mae M. Araiza; Yvonne Valerie Austria; Zipporah Mariebelle R. Enriquez; Zyrel V. Mollejon
EPI_ISL_3105882	Cruz-Rabe Maternity and General Hospital	Research Institute for Tropical Medicine	Clyde Dapat; Deana Mae Ocampo; Emmanuel Kagning Tsinda; Francisco Gerardo Polotan; Hitoshi Oshitani; Inez Andrea Medado; Jefferson Earl Halog; Joana Ina Manalo; Lei Lanna Dancel; Ma Angelica Tujan; Mariko Saito-Obata; Mayuko Saito; Michiko Okamoto; Samantha Louise Bado
EPI_ISL_4459365, EPI_ISL_4459527	D.T. PROTACIO HOSPITAL	Research Institute for Tropical Medicine/Tohoku University	Allen Anthony Laraño; Ardiane Ysabelle Dolor; Catalino Demetria; Clyde Dapat; Emmanuel Kagning Tsinda; Francisco Gerardo Polotan; Hitoshi Oshitani; Inez Andrea Medado; Jefferson Earl Halog; Lei Lanna Dancel; Leilanie Nacion; Ma. Angelica Tujan; Machiko Okamoto; Maria Carmen Corpuz; Mariko Siato-Obata; Mayuko Saito
EPI_ISL_1069186	D.T. Protacio Hospital	Research Institute for Tropical Medicine	Catalino Demetria; Daria Manalo; Edelwisa Mercado; Francisco Gerardo Polotan; Hannah Leah Morito; Inez Andrea Medado; John Leonard Chan; Joseph Hughes; Kirstyn Brunker; Ma Angelica Tujan; Othoniel Jan Onza
EPI_ISL_4459296, EPI_ISL_4459299, EPI_ISL_4459303, EPI_ISL_4459359	DON BOSCO HEALTH CENTER	Research Institute for Tropical Medicine/Tohoku University	Allen Anthony Laraño; Ardiane Ysabelle Dolor; Catalino Demetria; Clyde Dapat; Emmanuel Kagning Tsinda; Francisco Gerardo Polotan; Hitoshi Oshitani; Inez Andrea Medado; Jefferson Earl Halog; Lei Lanna Dancel; Leilanie Nacion; Ma. Angelica Tujan; Machiko Okamoto; Maria Carmen Corpuz; Mariko Siato-Obata; Mayuko Saito
EPI_ISL_4459477	DOÑA MARTA HEALTH CENTER	Research Institute for Tropical Medicine/Tohoku University	Allen Anthony Laraño; Ardiane Ysabelle Dolor; Catalino Demetria; Clyde Dapat; Emmanuel Kagning Tsinda; Francisco Gerardo Polotan; Hitoshi Oshitani; Inez Andrea Medado; Jefferson Earl Halog; Lei Lanna Dancel; Leilanie Nacion; Ma. Angelica Tujan; Machiko Okamoto; Maria Carmen Corpuz; Mariko Siato-Obata; Mayuko Saito
EPI_ISL_2156564	Daniel O. Mercado Medical Center	Philippine Genome Center	Alethea R. de Guzman; Anna Ong-Lim; Arianne A. Zamora; Asia Louisa U. Chong; Benedict A. Maralit; Candice Francheska B. Tambaoan; Carlo M. Lapid; Celia Carlos; Devon Ray Pacial; Edsel Maurice Salvaña; El King D. Morado; Elcid Aaron R. Pangilinan; Eva Maria Cutiongco-de la Paz; Francis A. Tablizo; Irish Coleen A. Asin; Jaime C. Montoya; Jan Michael C. Yap; Jo-Hannah S. Llames; John Q. Wong; Joshua Gregor A. Dizon; Juan Antonio R. Magalang; Karol Sophia Agape R. Padilla; Kenneth M. Kim; Kris P. Punayan; Marc Edsel C. Ayes; Marc Jerrone R. Castro; Maria Rosario Singh-Vergeire and Cynthia P. Saloma; Maria Sofia L. Yangzon; Marissa Alejandria; Razel Nikka M. Hao; Rianna Patricia S. Cruz; Sheila Mae M. Araiza
EPI_ISL_1081828, EPI_ISL_1081840	Davao One World	Philippine Genome Center	Alethea R. de Guzman; Anna Ong-Lim; Arianne A. Zamora; Asia Louisa U. Chong; Benedict A. Maralit; Candice Francheska B. Tambaoan; Carlo M. Lapid; Celia Carlos; Cynthia P. Saloma; Devon Ray Pacial; Edsel Maurice Salvaña; El King D. Morado; Eva Maria Cutiongco-de la Paz; Francis A. Tablizo; Irish Coleen A. Asin; Jaime C. Montoya; Jan Michael C. Yap; Jo-Hannah S. Llames; John Q. Wong; Joshua Gregor A. Dizon; Juan Antonio R. Magalang; Karol Sophia Agape R. Padilla; Kenneth M. Kim; Kris P. Punayan; Marc Edsel C. Ayes; Marc Jerrone R. Castro; Maria Sofia L. Yangzon; Marissa Alejandria; Razel Nikka M. Hao; Rianna Patricia S. Cruz; Sheila Mae M. Araiza; and Maria Rosario Singh-Vergeire
EPI_ISL_2105288, EPI_ISL_2105311, EPI_ISL_2105313, EPI_ISL_2105318, EPI_ISL_2105326, EPI_ISL_2105328, EPI_ISL_2105331, EPI_ISL_2105339, EPI_ISL_2105352, EPI_ISL_2105361, EPI_ISL_2105395, EPI_ISL_2105396, EPI_ISL_2105397, EPI_ISL_2105429, EPI_ISL_2105440, EPI_ISL_2105455, EPI_ISL_2105530, EPI_ISL_2105533, EPI_ISL_2105545, EPI_ISL_2155224, EPI_ISL_2155641, EPI_ISL_2155642, EPI_ISL_2155643, EPI_ISL_2156371, EPI_ISL_2156403, EPI_ISL_2156404, EPI_ISL_2156405, EPI_ISL_2156406, EPI_ISL_2156419, EPI_ISL_2156498, EPI_ISL_2156499, EPI_ISL_2156507, EPI_ISL_2156508, EPI_ISL_4712015, EPI_ISL_4712023, EPI_ISL_4712031, EPI_ISL_4712068, EPI_ISL_4712077, EPI_ISL_4712165, EPI_ISL_4712183			
see above	Davao One World Diagnostic Center Incorporated	Philippine Genome Center	Alethea R. de Guzman; Anna Ong-Lim; Arianne A. Zamora; Asia Louisa U. Chong; Benedict A. Maralit; Candice Francheska B. Tambaoan; Carlo M. Lapid; Celia Carlos; Cynthia P. Saloma; Devon Ray Pacial; Diomedes A. Cariño; Edsel Maurice Salvana; Edsel Maurice Salvaña; El King D. Morado; Elcid Aaron R. Pangilinan; Eva Maria Cutiongco-de la Paz; Francis A. Tablizo; Henrietta Marie Rodriguez; Irish Coleen A. Asin; Jaime C. Montoya; Jan Michael C. Yap; Jarvin E. Nipales; Jo-Hannah S. Llames; John Q. Wong; Joshua Gregor A. Dizon; Juan Antonio R. Magalang; Karol Sophia Agape R. Padilla; Kenneth M. Kim; Kris P. Punayan; Kristina Patriz Dela Cruz; Lindsay Clare D.L. Carandang; Ma. Exanil Plantig; Marc Edsel C. Ayes; Marc Jerrone R. Castro; Maria Rosario Singh-Vergeire; Maria Rosario Singh-Vergeire and Cynthia P. Saloma; Maria Sofia L. Yangzon; Marielle M. Gamboa; Marissa Alejandria; Niña Francesca Bustamante; Razel Nikka M. Hao; Renato Jacinto Q. Mantaring; Rianna Patricia S. Cruz; Sheila Mae M. Araiza; Shiela Mae M. Araiza; Yvonne Valerie Austria; Zipporah Mariebelle R. Enriquez; Zyrel V. Mollejon
EPI_ISL_4630042, EPI_ISL_4712045, EPI_ISL_4712050, EPI_ISL_4712089, EPI_ISL_4712115, EPI_ISL_4712156, EPI_ISL_4712175, EPI_ISL_4712436, EPI_ISL_4712514, EPI_ISL_4712922, EPI_ISL_4712929, EPI_ISL_4712953, EPI_ISL_4713002, EPI_ISL_4713056, EPI_ISL_4713115, EPI_ISL_4713185, EPI_ISL_4713302, EPI_ISL_4713358, EPI_ISL_4713411, EPI_ISL_4713472, EPI_ISL_4713495, EPI_ISL_4713571, EPI_ISL_4713588, EPI_ISL_4713607, EPI_ISL_4713631, EPI_ISL_4713673, EPI_ISL_4713813, EPI_ISL_4713815, EPI_ISL_4713851, EPI_ISL_4713928, EPI_ISL_4713942, EPI_ISL_4713972, EPI_ISL_4714008, EPI_ISL_4714091, EPI_ISL_4714172, EPI_ISL_4714185, EPI_ISL_4714288, EPI_ISL_4714295, EPI_ISL_4714386, EPI_ISL_4714477, EPI_ISL_4714499, EPI_ISL_4714553, EPI_ISL_4714572, EPI_ISL_4714609, EPI_ISL_4714633, EPI_ISL_4714693, EPI_ISL_4714716, EPI_ISL_4714745, EPI_ISL_4714757, EPI_ISL_4714810, EPI_ISL_4714840, EPI_ISL_4714896, EPI_ISL_4714918, EPI_ISL_4714991, EPI_ISL_4715039, EPI_ISL_4715098, EPI_ISL_4715102, EPI_ISL_4715150, EPI_ISL_4715154, EPI_ISL_4715188, EPI_ISL_4715262, EPI_ISL_4715289, EPI_ISL_4715295, EPI_ISL_4715300, EPI_ISL_4715543			
see above	Davao Regional Medical Center	Philippine Genome Center	Alethea R. de Guzman; Alyssa Joyce E. Telles; Anna Ong-Lim; Arianne A. Zamora; Benedict A. Maralit; Carlo M. Lapid; Celia Carlos; Cynthia P. Saloma; Devon Ray Pacial; Diomedes A. Cariño; Edsel Maurice Salvaña; El King D. Morado; Elcid Aaron R. Pangilinan; Eva Maria Cutiongco-de la Paz; Francis A. Tablizo; Henrietta Marie Rodriguez; Jaime C. Montoya; Jan Michael C. Yap; Jarvin E. Nipales; Jo-Hannah S. Llames; John Michael Egana; John Q. Wong; Joshua Gregor A. Dizon; Joshua Jose Endozo; Juan Antonio R. Magalang; Karol Sophia Agape R. Padilla; Kenneth M. Kim; Kris P. Punayan; Kristina Patriz Dela Cruz; Lindsay Clare D.L. Carandang; Ma. Exanil Plantig; Marc Edsel C. Ayes; Maria Rosario Singh-Vergeire; Maria Sofia L. Yangzon; Marielle M. Gamboa; Marissa Alejandria; Niña Francesca Bustamante; Razel Nikka M. Hao; Renato Jacinto Q. Mantaring; Rianna Patricia S. Cruz; Shiela Mae M. Araiza; Yvonne Valerie Austria; Zipporah Mariebelle R. Enriquez; Zyrel V. Mollejon
EPI_ISL_2859231, EPI_ISL_2860186, EPI_ISL_2860190, EPI_ISL_2860192	Democrito O. Plaza Memorial Hospital	Philippine Genome Center	Alethea R. de Guzman; Anna Ong-Lim; Arianne A. Zamora; Benedict A. Maralit; Carlo M. Lapid; Celia Carlos; Devon Ray Pacial; Diomedes A. Carino; Edsel Maurice Salvaña; El King D. Morado; Elcid Aaron R. Pangilinan; Eva Maria Cutiongco-de la Paz; Francis A. Tablizo; Henrietta Marie Rodriguez; Jaime C. Montoya; Jan Michael C. Yap; Jarvin E. Nipales; Jo-Hannah S. Llames; John Q. Wong; Joshua Gregor A. Dizon; Juan Antonio R. Magalang; Karol Sophia Agape R. Padilla; Kenneth M. Kim; Kris P. Punayan; Krisitna Patriz Dela Cruz; Lindsay Claire D.L. Carandang; Ma. Exanil Plantig; Marc Edsel C. Ayes; Maria Rosario Singh-Vergeire and Cynthia P. Saloma; Maria Sofia L. Yangzon; Marielle M Gamboa; Marissa Alejandria; Nina Francesca Bustamante; Razel Nikka M. Hao; Renato Jacinto Q. Mantaring; Rianna Patricia S. Cruz; Sheila Mae M. Araiza; Yvonne Valerie Austria; Zipporah Mariebelle R. Enriquez; Zyrel V. Mollejon
EPI_ISL_1081796	Detoxicare	Philippine Genome Center	Alethea R. de Guzman; Anna Ong-Lim; Arianne A. Zamora; Asia Louisa U. Chong; Benedict A. Maralit; Candice Francheska B. Tambaoan; Carlo M. Lapid; Celia Carlos; Cynthia P. Saloma; Devon Ray Pacial; Edsel Maurice Salvaña; El King D. Morado; Eva Maria Cutiongco-de la Paz; Francis A. Tablizo; Irish Coleen A. Asin; Jaime C. Montoya; Jan Michael C. Yap; Jo-Hannah S. Llames; John Q. Wong; Joshua Gregor A. Dizon; Juan Antonio R. Magalang; Karol Sophia Agape R. Padilla; Kenneth M. Kim; Kris P. Punayan; Marc Edsel C. Ayes; Marc Jerrone R. Castro; Maria Sofia L. Yangzon; Marissa Alejandria; Razel Nikka M. Hao; Rianna Patricia S. Cruz; Sheila Mae M. Araiza; and Maria Rosario Singh-Vergeire
EPI_ISL_2105252, EPI_ISL_2154442, EPI_ISL_2154468, EPI_ISL_2154480, EPI_ISL_2154558, EPI_ISL_2154612, EPI_ISL_2154698, EPI_ISL_2154700, EPI_ISL_2154806, EPI_ISL_2154872, EPI_ISL_2154891, EPI_ISL_2154894, EPI_ISL_2156113, EPI_ISL_2156114, EPI_ISL_2156115, EPI_ISL_2156500, EPI_ISL_2188698, EPI_ISL_2188729, EPI_ISL_2188778, EPI_ISL_2188822, EPI_ISL_2188838, EPI_ISL_2860252, EPI_ISL_4713435			
see above	Detoxicare Molecular Diagnostics Laboratory	Philippine Genome Center	Alethea R. de Guzman; Anna Ong-Lim; Arianne A. Zamora; Asia Louisa U. Chong; Benedict A. Maralit; Candice Francheska B. Tambaoan; Carlo M. Lapid; Celia Carlos; Cynthia P. Saloma; Devon Ray Pacial; Diomedes A. Carino; Diomedes A. Cariño; Edsel Maurice Salvana; Edsel Maurice Salvaña; El King D. Morado; Elcid Aaron R. Pangilinan; Eva Maria Cutiongco-de la Paz; Francis A. Tablizo; Henrietta Marie Rodriguez; Irish Coleen A. Asin; Jaime C. Montoya; Jan Michael C. Yap; Jarvin E. Nipales; Jo-Hannah S. Llames; John Q. Wong; Joshua Gregor A. Dizon; Juan Antonio R. Magalang; Karol Sophia Agape R. Padilla; Kenneth M. Kim; Kris P. Punayan; Krisitna Patriz Dela Cruz; Kristina Patriz Dela Cruz; Lindsay Claire D.L. Carandang; Lindsay Clare D.L. Carandang; Ma. Exanil Plantig; Marc Edsel C. Ayes; Marc Jerrone R. Castro; Maria Rosario Singh-Vergeire; Maria Rosario Singh-Vergeire and Cynthia P. Saloma; Maria Sofia L. Yangzon; Marielle M Gamboa; Marielle M. Gamboa; Marissa Alejandria; Nina Francesca Bustamante; Niña Francesca Bustamante; Razel Nikka M. Hao; Renato Jacinto Q. Mantaring; Rianna Patricia S. Cruz; Sheila Mae M. Araiza; Shiela Mae M. Araiza; Yvonne Valerie Austria; Zipporah Mariebelle R. Enriquez; Zyrel V. Mollejon
EPI_ISL_2859225	Divine Word Hospital Viralogy Laboratory	Philippine Genome Center	Alethea R. de Guzman; Anna Ong-Lim; Arianne A. Zamora; Benedict A. Maralit; Carlo M. Lapid; Celia Carlos; Devon Ray Pacial; Diomedes A. Carino; Edsel Maurice Salvaña; El King D. Morado; Elcid Aaron R. Pangilinan; Eva Maria Cutiongco-de la Paz; Francis A. Tablizo; Henrietta Marie Rodriguez; Jaime C. Montoya; Jan Michael C. Yap; Jarvin E. Nipales; Jo-Hannah S. Llames; John Q. Wong; Joshua Gregor A. Dizon; Juan Antonio R. Magalang; Karol Sophia Agape R. Padilla; Kenneth M. Kim; Kris P. Punayan; Krisitna Patriz Dela Cruz; Lindsay Claire D.L. Carandang; Ma. Exanil Plantig; Marc Edsel C. Ayes; Maria Rosario Singh-Vergeire and Cynthia P. Saloma; Maria Sofia L. Yangzon; Marielle M Gamboa; Marissa Alejandria; Nina Francesca Bustamante; Razel Nikka M. Hao; Renato Jacinto Q. Mantaring; Rianna Patricia S. Cruz; Sheila Mae M. Araiza; Yvonne Valerie Austria; Zipporah Mariebelle R. Enriquez; Zyrel V. Mollejon
EPI_ISL_2189042	Dr. Arturo Pingoy Medical Center	Philippine Genome Center	Alethea R. de Guzman; Anna Ong-Lim; Arianne A. Zamora; Asia Louisa U. Chong; Benedict A. Maralit; Candice Francheska B. Tambaoan; Carlo M. Lapid; Celia Carlos; Devon Ray Pacial; Edsel Maurice Salvaña; El King D. Morado; Elcid Aaron R. Pangilinan; Eva Maria Cutiongco-de la Paz; Francis A. Tablizo; Irish Coleen A. Asin; Jaime C. Montoya; Jan Michael C. Yap; Jo-Hannah S. Llames; John Q. Wong; Joshua Gregor A. Dizon; Juan Antonio R. Magalang; Karol Sophia Agape R. Padilla; Kenneth M. Kim; Kris P. Punayan; Marc Edsel C. Ayes; Maria Rosario Singh-Vergeire and Cynthia P. Saloma; Maria Sofia L. Yangzon; Marissa Alejandria; Razel Nikka M. Hao; Renato Jacinto Q. Mantaring; Rianna Patricia S. Cruz; Sheila Mae M. Araiza
EPI_ISL_2105306, EPI_ISL_2105360, EPI_ISL_2105365, EPI_ISL_2105433, EPI_ISL_2105541, EPI_ISL_2154027, EPI_ISL_2156167, EPI_ISL_2156168, EPI_ISL_2156169, EPI_ISL_2188121, EPI_ISL_2188278, EPI_ISL_2188921, EPI_ISL_2189291, EPI_ISL_2189420, EPI_ISL_4712334			
see above	Dr. Jorge P. Royeca Hospital	Philippine Genome Center	Alethea R. de Guzman; Anna Ong-Lim; Arianne A. Zamora; Asia Louisa U. Chong; Benedict A. Maralit; Candice Francheska B. Tambaoan; Carlo M. Lapid; Celia Carlos; Cynthia P. Saloma; Devon Ray Pacial; Diomedes A. Cariño; Edsel Maurice Salvana; Edsel Maurice Salvaña; El King D. Morado; Elcid Aaron R. Pangilinan; Eva Maria Cutiongco-de la Paz; Francis A. Tablizo; Henrietta Marie Rodriguez; Irish Coleen A. Asin; Jaime C. Montoya; Jan Michael C. Yap; Jarvin E. Nipales; Jo-Hannah S. Llames; John Q. Wong; Joshua Gregor A. Dizon; Juan Antonio R. Magalang; Karol Sophia Agape R. Padilla; Kenneth M. Kim; Kris P. Punayan; Kristina Patriz Dela Cruz; Lindsay Clare D.L. Carandang; Ma. Exanil Plantig; Marc Edsel C. Ayes; Marc Jerrone R. Castro; Maria Rosario Singh-Vergeire; Maria Rosario Singh-Vergeire and Cynthia P. Saloma; Maria Sofia L. Yangzon; Marielle M. Gamboa; Marissa Alejandria; Niña Francesca Bustamante; Razel Nikka M. Hao; Renato Jacinto Q. Mantaring; Rianna Patricia S. Cruz; Sheila Mae M. Araiza; Shiela Mae M. Araiza; Yvonne Valerie Austria; Zipporah Mariebelle R. Enriquez; Zyrel V. Mollejon
EPI_ISL_3105876	Dr. Jose P. Rizal Memorial District Hospital	Research Institute for Tropical Medicine	Clyde Dapat; Deana Mae Ocampo; Emmanuel Kagning Tsinda; Francisco Gerardo Polotan; Hitoshi Oshitani; Inez Andrea Medado; Jefferson Earl Halog; Joana Ina Manalo; Lei Lanna Dancel; Ma Angelica Tujan; Mariko Saito-Obata; Mayuko Saito; Michiko Okamoto; Samantha Louise Bado
EPI_ISL_4716619, EPI_ISL_4716876, EPI_ISL_4717420, EPI_ISL_4717439	Dr. Rafael S. Tumbokon Memorial Hospital	Philippine Genome Center	Alethea R. de Guzman; Anna Ong-Lim; Arianne A. Zamora; Benedict A. Maralit; Carlo M. Lapid; Celia Carlos; Cynthia P. Saloma; Devon Ray Pacial; Diomedes A. Cariño; Edsel Maurice Salvana; El King D. Morado; Elcid Aaron R. Pangilinan; Eva Maria Cutiongco-de la Paz; Francis A. Tablizo; Henrietta Marie Rodriguez; Jaime C. Montoya; Jan Michael C. Yap; Jarvin E. Nipales; Jo-Hannah S. Llames; John Q. Wong; Joshua Gregor A. Dizon; Juan Antonio R. Magalang; Karol Sophia Agape R. Padilla; Kenneth M. Kim; Kris P. Punayan; Kristina Patriz Dela Cruz; Lindsay Clare D.L. Carandang; Ma. Exanil Plantig; Marc Edsel C. Ayes; Maria Rosario Singh-Vergeire; Maria Sofia L. Yangzon; Marielle M. Gamboa; Marissa Alejandria; Niña Francesca Bustamante; Razel Nikka M. Hao; Renato Jacinto Q. Mantaring; Rianna Patricia S. Cruz; Shiela Mae M. Araiza; Yvonne Valerie Austria; Zipporah Mariebelle R. Enriquez; Zyrel V. Mollejon
EPI_ISL_2859168, EPI_ISL_2859229, EPI_ISL_2859247, EPI_ISL_2859373, EPI_ISL_2859374, EPI_ISL_2859375, EPI_ISL_2859376, EPI_ISL_2859378, EPI_ISL_2859379			
see above	Eastern Visayas Regional Covid-19 Testing Center	Philippine Genome Center	Alethea R. de Guzman; Anna Ong-Lim; Arianne A. Zamora; Benedict A. Maralit; Carlo M. Lapid; Celia Carlos; Devon Ray Pacial; Diomedes A. Carino; Edsel Maurice Salvaña; El King D. Morado; Elcid Aaron R. Pangilinan; Eva Maria Cutiongco-de la Paz; Francis A. Tablizo; Henrietta Marie Rodriguez; Jaime C. Montoya; Jan Michael C. Yap; Jarvin E. Nipales; Jo-Hannah S. Llames; John Q. Wong; Joshua Gregor A. Dizon; Juan Antonio R. Magalang; Karol Sophia Agape R. Padilla; Kenneth M. Kim; Kris P. Punayan; Krisitna Patriz Dela Cruz; Lindsay Claire D.L. Carandang; Ma. Exanil Plantig; Marc Edsel C. Ayes; Maria Rosario Singh-Vergeire and Cynthia P. Saloma; Maria Sofia L. Yangzon; Marielle M Gamboa; Marissa Alejandria; Nina Francesca Bustamante; Razel Nikka M. Hao; Renato Jacinto Q. Mantaring; Rianna Patricia S. Cruz; Sheila Mae M. Araiza; Yvonne Valerie Austria; Zipporah Mariebelle R. Enriquez; Zyrel V. Mollejon
EPI_ISL_2155605	F.Y. Manalo Medical Foundation Inc.- New Era General Hospital (PGC)	Philippine Genome Center	Alethea R. de Guzman; Anna Ong-Lim; Arianne A. Zamora; Asia Louisa U. Chong; Benedict A. Maralit; Candice Francheska B. Tambaoan; Carlo M. Lapid; Celia Carlos; Devon Ray Pacial; Edsel Maurice Salvaña; El King D. Morado; Eva Maria Cutiongco-de la Paz; Francis A. Tablizo; Irish Coleen A. Asin; Jaime C. Montoya; Jan Michael C. Yap; Jo-Hannah S. Llames; John Q. Wong; Joshua Gregor A. Dizon; Juan Antonio R. Magalang; Karol Sophia Agape R. Padilla; Kenneth M. Kim; Kris P. Punayan; Marc Edsel C. Ayes; Marc Jerrone R. Castro; Maria Rosario Singh-Vergeire and Cynthia P. Saloma; Maria Sofia L. Yangzon; Marissa Alejandria; Razel Nikka M. Hao; Rianna Patricia S. Cruz; Sheila Mae M. Araiza
EPI_ISL_4459531	FOUNDATION OF OUR LADY OF PEACE	Research Institute for Tropical	Allen Anthony Laraño; Ardiane Ysabelle Dolor; Catalino Demetria; Clyde Dapat; Emmanuel Kagning Tsinda; Francisco Gerardo Polotan; Hitoshi Oshitani; Inez Andrea Medado; Jefferson Earl Halog; Lei Lanna Dancel; Leilanie Nacion; Ma. Angelica Tujan; Machiko Okamoto; Maria Carmen Corpuz; Mariko Siato-Obata; Mayuko Saito

	MISSION, INC.-OUR LADY OF PEACE HOSPITAL	Medicine/Tohoku University	
EPI_ISL_2155412, EPI_ISL_2155413, EPI_ISL_2155414	Fatima University Medical Center Antipolo Corp.	Philippine Genome Center	Alethea R. de Guzman; Anna Ong-Lim; Arianne A. Zamora; Asia Louisa U. Chong; Benedict A. Maralit; Candice Francheska B. Tambaoan; Carlo M. Lapid; Celia Carlos; Devon Ray Pacial; Edsel Maurice Salvaña; El King D. Morado; Eva Maria Cutiongco-de la Paz; Francis A. Tablizo; Irish Coleen A. Asin; Jaime C. Montoya; Jan Michael C. Yap; Jo-Hannah S. Llames; John Q. Wong; Joshua Gregor A. Dizon; Juan Antonio R. Magalang; Karol Sophia Agape R. Padilla; Kenneth M. Kim; Kris P. Punayan; Marc Edsel C. Ayes; Marc Jerrone R. Castro; Maria Rosario Singh-Vergeire and Cynthia P. Saloma; Maria Sofia L. Yangzon; Marissa Alejandria; Razel Nikka M. Hao; Rianna Patricia S. Cruz; Sheila Mae M. Araiza
EPI_ISL_1069189	Filinvest Tent Covid Quarantine Facility	Research Institute for Tropical Medicine	Catalino Demetria; Daria Manalo; Edelwisa Mercado; Francisco Gerardo Polotan; Hannah Leah Morito; Inez Andrea Medado; John Leonard Chan; Joseph Hughes; Kirstyn Brunker; Ma Angelica Tujan; Othoniel Jan Onza
EPI_ISL_1081812, EPI_ISL_2105393, EPI_ISL_2105553, EPI_ISL_2154421, EPI_ISL_2154458, EPI_ISL_2154554, EPI_ISL_2154907, EPI_ISL_2155007, EPI_ISL_2155066, EPI_ISL_2155078, EPI_ISL_2155756, EPI_ISL_2155757, EPI_ISL_2155758, EPI_ISL_2155759, EPI_ISL_2156221, EPI_ISL_2156222, EPI_ISL_2156293, EPI_ISL_2156322, EPI_ISL_2156335, EPI_ISL_2156336, EPI_ISL_2156401, EPI_ISL_2156458, EPI_ISL_2156483, EPI_ISL_2171137, EPI_ISL_2188684, EPI_ISL_2188704, EPI_ISL_2188761, EPI_ISL_2188769, EPI_ISL_2188772, EPI_ISL_2188779, EPI_ISL_2188852, EPI_ISL_2189057, EPI_ISL_2189065, EPI_ISL_2189174, EPI_ISL_2189206, EPI_ISL_2189284, EPI_ISL_2189303, EPI_ISL_2189348, EPI_ISL_2189369, EPI_ISL_2189399, EPI_ISL_2189460, EPI_ISL_2558053, EPI_ISL_2558057, EPI_ISL_2859502, EPI_ISL_2859513, EPI_ISL_2859531, EPI_ISL_2859561, EPI_ISL_2859587, EPI_ISL_2859594, EPI_ISL_2859625, EPI_ISL_2859628, EPI_ISL_2859629, EPI_ISL_2859634, EPI_ISL_2859720, EPI_ISL_2859742, EPI_ISL_2859925, EPI_ISL_2859970, EPI_ISL_2859979, EPI_ISL_2860007, EPI_ISL_2860050, EPI_ISL_2860097, EPI_ISL_2860098, EPI_ISL_2860138, EPI_ISL_2860146, EPI_ISL_2860150, EPI_ISL_2860182, EPI_ISL_2860187, EPI_ISL_2860193, EPI_ISL_2860195, EPI_ISL_2860198, EPI_ISL_2860199, EPI_ISL_2860200, EPI_ISL_2860204, EPI_ISL_2860213, EPI_ISL_2860217, EPI_ISL_2860228, EPI_ISL_2860229, EPI_ISL_2860230, EPI_ISL_2860231, EPI_ISL_2860232, EPI_ISL_2860233, EPI_ISL_2860234, EPI_ISL_2860235, EPI_ISL_2860237, EPI_ISL_4717388			
see above	First Aide Diagnostic Center	Philippine Genome Center	Alethea R. de Guzman; Anna Ong-Lim; Arianne A. Zamora; Asia Louisa U. Chong; Benedict A. Maralit; Candice Francheska B. Tambaoan; Carlo M. Lapid; Celia Carlos; Cynthia P. Saloma; Devon Ray Pacial; Diomedes A. Carino; Diomedes A. Cariño; Edsel Maurice Salvana; Edsel Maurice Salvaña; El King D. Morado; Elcid Aaron R. Pangilinan; Eva Maria Cutiongco-de la Paz; Francis A. Tablizo; Henrietta Marie Rodriguez; Irish Coleen A. Asin; Jaime C. Montoya; Jan Michael C. Yap; Jarvin E. Nipales; Jo-Hannah S. Llames; John Q. Wong; Joshua Gregor A. Dizon; Juan Antonio R. Magalang; Karol Sophia Agape R. Padilla; Kenneth M. Kim; Kris P. Punayan; Krisitna Patriz Dela Cruz; Kristina Patriz Dela Cruz; Lindsay Claire D.L. Carandang; Lindsay Clare D.L. Carandang; Ma. Exanil Plantig; Marc Edsel C. Ayes; Marc Jerrone R. Castro; Maria Rosario Singh-Vergeire; Maria Rosario Singh-Vergeire and Cynthia P. Saloma; Maria Sofia L. Yangzon; Marielle M Gamboa; Marielle M. Gamboa; Marissa Alejandria; Nina Francesca Bustamante; Niña Francesca Bustamante; Razel Nikka M. Hao; Renato Jacinto Q. Mantaring; Rianna Patricia S. Cruz; Sheila Mae M. Araiza; Shiela Mae M. Araiza; Yvonne Valerie Austria; Zipporah Mariebelle R. Enriquez; Zyrel V. Mollejon; and Maria Rosario Singh-Vergeire
EPI_ISL_2105466, EPI_ISL_2105471, EPI_ISL_2105473, EPI_ISL_2105481, EPI_ISL_2105482, EPI_ISL_2105483, EPI_ISL_2105484, EPI_ISL_2105488, EPI_ISL_2105490, EPI_ISL_2105493, EPI_ISL_2105497, EPI_ISL_2105502, EPI_ISL_2105504, EPI_ISL_2105523, EPI_ISL_2105555, EPI_ISL_2155973, EPI_ISL_2155974, EPI_ISL_2155975, EPI_ISL_2155976, EPI_ISL_2156184			
see above	Governor Celestino Gallares Memorial Hospital	Philippine Genome Center	Alethea R. de Guzman; Anna Ong-Lim; Arianne A. Zamora; Asia Louisa U. Chong; Benedict A. Maralit; Candice Francheska B. Tambaoan; Carlo M. Lapid; Celia Carlos; Devon Ray Pacial; Edsel Maurice Salvaña; El King D. Morado; Eva Maria Cutiongco-de la Paz; Francis A. Tablizo; Irish Coleen A. Asin; Jaime C. Montoya; Jan Michael C. Yap; Jo-Hannah S. Llames; John Q. Wong; Joshua Gregor A. Dizon; Juan Antonio R. Magalang; Karol Sophia Agape R. Padilla; Kenneth M. Kim; Kris P. Punayan; Marc Edsel C. Ayes; Marc Jerrone R. Castro; Maria Rosario Singh-Vergeire and Cynthia P. Saloma; Maria Sofia L. Yangzon; Marissa Alejandria; Razel Nikka M. Hao; Rianna Patricia S. Cruz; Sheila Mae M. Araiza
EPI_ISL_2156188, EPI_ISL_2156189, EPI_ISL_2156567	Graman Medical & Maternity Hospital Inc Molecular Laboratory	Philippine Genome Center	Alethea R. de Guzman; Anna Ong-Lim; Arianne A. Zamora; Asia Louisa U. Chong; Benedict A. Maralit; Candice Francheska B. Tambaoan; Carlo M. Lapid; Celia Carlos; Devon Ray Pacial; Edsel Maurice Salvaña; El King D. Morado; Elcid Aaron R. Pangilinan; Eva Maria Cutiongco-de la Paz; Francis A. Tablizo; Irish Coleen A. Asin; Jaime C. Montoya; Jan Michael C. Yap; Jo-Hannah S. Llames; John Q. Wong; Joshua Gregor A. Dizon; Juan Antonio R. Magalang; Karol Sophia Agape R. Padilla; Kenneth M. Kim; Kris P. Punayan; Marc Edsel C. Ayes; Marc Jerrone R. Castro; Maria Rosario Singh-Vergeire and Cynthia P. Saloma; Maria Sofia L. Yangzon; Marissa Alejandria; Razel Nikka M. Hao; Rianna Patricia S. Cruz; Sheila Mae M. Araiza
EPI_ISL_4459326, EPI_ISL_4459350, EPI_ISL_4459397, EPI_ISL_4459420, EPI_ISL_4459468	H.M. CORACHEA GENERAL HOSPITAL	Research Institute for Tropical Medicine/Tohoku University	Allen Anthony Laraño; Ardiane Ysabelle Dolor; Catalino Demetria; Clyde Dapat; Emmanuel Kagning Tsinda; Francisco Gerardo Polotan; Hitoshi Oshitani; Inez Andrea Medado; Jefferson Earl Halog; Lei Lanna Dancel; Leilanie Nacion; Ma. Angelica Tujan; Machiko Okamoto; Maria Carmen Corpuz; Mariko Siato-Obata; Mayuko Saito
EPI_ISL_1069185	H.M. Corachea General Hospital	Research Institute for Tropical Medicine	Catalino Demetria; Daria Manalo; Edelwisa Mercado; Francisco Gerardo Polotan; Hannah Leah Morito; Inez Andrea Medado; John Leonard Chan; Joseph Hughes; Kirstyn Brunker; Ma Angelica Tujan; Othoniel Jan Onza
EPI_ISL_2154406, EPI_ISL_2154457, EPI_ISL_2154483, EPI_ISL_2154571, EPI_ISL_2154702, EPI_ISL_2154755, EPI_ISL_2154793, EPI_ISL_2154816, EPI_ISL_2154817, EPI_ISL_2154830			
see above	Health Delivery Systems, Inc.	Philippine Genome Center	Alethea R. de Guzman; Anna Ong-Lim; Arianne A. Zamora; Asia Louisa U. Chong; Benedict A. Maralit; Candice Francheska B. Tambaoan; Carlo M. Lapid; Celia Carlos; Devon Ray Pacial; Edsel Maurice Salvaña; El King D. Morado; Eva Maria Cutiongco-de la Paz; Francis A. Tablizo; Irish Coleen A. Asin; Jaime C. Montoya; Jan Michael C. Yap; Jo-Hannah S. Llames; John Q. Wong; Joshua Gregor A. Dizon; Juan Antonio R. Magalang; Karol Sophia Agape R. Padilla; Kenneth M. Kim; Kris P. Punayan; Marc Edsel C. Ayes; Marc Jerrone R. Castro; Maria Rosario Singh-Vergeire and Cynthia P. Saloma; Maria Sofia L. Yangzon; Marissa Alejandria; Razel Nikka M. Hao; Rianna Patricia S. Cruz; Sheila Mae M. Araiza
EPI_ISL_2155845, EPI_ISL_2155846, EPI_ISL_2155848, EPI_ISL_2155849, EPI_ISL_2156291	Hi-Precision Diagnostic Center	Philippine Genome Center	Alethea R. de Guzman; Anna Ong-Lim; Arianne A. Zamora; Asia Louisa U. Chong; Benedict A. Maralit; Candice Francheska B. Tambaoan; Carlo M. Lapid; Celia Carlos; Devon Ray Pacial; Edsel Maurice Salvaña; El King D. Morado; Elcid Aaron R. Pangilinan; Eva Maria Cutiongco-de la Paz; Francis A. Tablizo; Irish Coleen A. Asin; Jaime C. Montoya; Jan Michael C. Yap; Jo-Hannah S. Llames; John Q. Wong; Joshua Gregor A. Dizon; Juan Antonio R. Magalang; Karol Sophia Agape R. Padilla; Kenneth M. Kim; Kris P. Punayan; Marc Edsel C. Ayes; Marc Jerrone R. Castro; Maria Rosario Singh-Vergeire and Cynthia P. Saloma; Maria Sofia L. Yangzon; Marissa Alejandria; Razel Nikka M. Hao; Rianna Patricia S. Cruz; Sheila Mae M. Araiza
EPI_ISL_1213565, EPI_ISL_1213566, EPI_ISL_2091459, EPI_ISL_2155184, EPI_ISL_2155215, EPI_ISL_2155461, EPI_ISL_2155462, EPI_ISL_2155463, EPI_ISL_2155464, EPI_ISL_2155465, EPI_ISL_2155466, EPI_ISL_2155467, EPI_ISL_2155468, EPI_ISL_2155469, EPI_ISL_2155470, EPI_ISL_2155471, EPI_ISL_2155472, EPI_ISL_2155473, EPI_ISL_2155474, EPI_ISL_2155475, EPI_ISL_2155476, EPI_ISL_2156116, EPI_ISL_2156410, EPI_ISL_2156411, EPI_ISL_2156478, EPI_ISL_2188929, EPI_ISL_2188972, EPI_ISL_2189008, EPI_ISL_2189103, EPI_ISL_2189171, EPI_ISL_2189523, EPI_ISL_2859981, EPI_ISL_2860008, EPI_ISL_2860028, EPI_ISL_2860105, EPI_ISL_2860164, EPI_ISL_4713560, EPI_ISL_4713618, EPI_ISL_4716336, EPI_ISL_4716525			
see above	Hi-Precision Diagnostic Center (QC)	Philippine Genome Center	Alethea R. de Guzman; Anna Ong-Lim; Arianne A. Zamora; Asia Louisa U. Chong; Benedict A. Maralit; Candice Francheska B. Tambaoan; Carlo M. Lapid; Celia Carlos; Cynthia P. Saloma; Devon Ray Pacial; Diomedes A. Carino; Diomedes A. Cariño; Edsel Maurice Salvana; Edsel Maurice Salvaña; El King D. Morado; Elcid Aaron R. Pangilinan; Eva Maria Cutiongco-de la Paz; Francis A. Tablizo; Henrietta Marie Rodriguez; Irish Coleen A. Asin; Jaime C. Montoya; Jan Michael C. Yap; Jarvin E. Nipales; Jo-Hannah S. Llames; John Q. Wong; Joshua Gregor A. Dizon; Juan Antonio R. Magalang; Karol Sophia Agape R. Padilla; Kenneth M. Kim; Kris P. Punayan; Krisitna Patriz Dela Cruz; Kristina Patriz Dela Cruz; Lindsay Claire D.L. Carandang; Lindsay Clare D.L. Carandang; Ma. Exanil Plantig; Marc Edsel C. Ayes; Marc Jerrone R. Castro; Maria Rosario Singh-Vergeire; Maria Rosario Singh-Vergeire and Cynthia P. Saloma; Maria Sofia L. Yangzon; Marielle M Gamboa; Marielle M. Gamboa; Marissa Alejandria; Nina Francesca Bustamante; Niña Francesca Bustamante; Razel Nikka M. Hao; Renato Jacinto Q. Mantaring; Rianna Patricia S. Cruz; Sheila Mae M. Araiza; Shiela Mae M. Araiza; Yvonne Valerie Austria; Zipporah Mariebelle R. Enriquez; Zyrel V. Mollejon
EPI_ISL_1122457	Hi-Precision Diagnostics	Philippine Genome Center	Alethea R. de Guzman; Anna Ong-Lim; Arianne A. Zamora; Asia Louisa U. Chong; Benedict A. Maralit; Candice Francheska B. Tambaoan; Carlo M. Lapid; Celia Carlos; Devon Ray Pacial; Edsel Maurice Salvaña; El King D. Morado; Eva Maria Cutiongco-de la Paz; Francis A. Tablizo; Irish Coleen A. Asin; Jaime C. Montoya; Jan Michael C. Yap; Jo-Hannah S. Llames; John Q. Wong; Joshua Gregor A. Dizon; Juan Antonio R. Magalang; Karol Sophia Agape R. Padilla; Kenneth M. Kim; Kris P. Punayan; Marc Edsel C. Ayes; Marc Jerrone R. Castro; Maria Rosario Singh-Vergeire and Cynthia P. Saloma; Maria Sofia L. Yangzon; Marissa Alejandria; Razel Nikka M. Hao; Rianna Patricia S. Cruz; Sheila Mae M. Araiza
EPI_ISL_2105506, EPI_ISL_2105508, EPI_ISL_2189410, EPI_ISL_2859581, EPI_ISL_2859738, EPI_ISL_2859789, EPI_ISL_2859892, EPI_ISL_2860027, EPI_ISL_2860061, EPI_ISL_2860067, EPI_ISL_2860133			
see above	IOM- Manila Health Centre Laboratory	Philippine Genome Center	Alethea R. de Guzman; Anna Ong-Lim; Arianne A. Zamora; Asia Louisa U. Chong; Benedict A. Maralit; Candice Francheska B. Tambaoan; Carlo M. Lapid; Celia Carlos; Devon Ray Pacial; Diomedes A. Carino; Edsel Maurice Salvaña; El King D. Morado; Elcid Aaron R. Pangilinan; Eva Maria Cutiongco-de la Paz; Francis A. Tablizo; Henrietta Marie Rodriguez; Irish Coleen A. Asin; Jaime C. Montoya; Jan Michael C. Yap; Jarvin E. Nipales; Jo-Hannah S. Llames; John Q. Wong; Joshua Gregor A. Dizon; Juan Antonio R. Magalang; Karol Sophia Agape R. Padilla; Kenneth M. Kim; Kris P. Punayan; Krisitna Patriz Dela Cruz; Lindsay Claire D.L. Carandang; Ma. Exanil Plantig; Marc Edsel C. Ayes; Marc Jerrone R. Castro; Maria Rosario Singh-Vergeire and Cynthia P. Saloma; Maria Sofia L. Yangzon; Marielle M Gamboa; Marissa Alejandria; Nina Francesca Bustamante; Razel Nikka M. Hao; Renato Jacinto Q. Mantaring; Rianna Patricia S. Cruz; Sheila Mae M. Araiza; Yvonne Valerie Austria; Zipporah Mariebelle R. Enriquez; Zyrel V. Mollejon
EPI_ISL_2155001, EPI_ISL_2155323, EPI_ISL_2155644, EPI_ISL_2155645, EPI_ISL_2155646, EPI_ISL_2155647, EPI_ISL_2155648, EPI_ISL_2155649, EPI_ISL_2155650, EPI_ISL_2155651, EPI_ISL_2155652, EPI_ISL_2155653, EPI_ISL_2155654, EPI_ISL_2155655, EPI_ISL_2155656, EPI_ISL_2155657, EPI_ISL_2156190, EPI_ISL_2156191, EPI_ISL_2156192, EPI_ISL_2156193, EPI_ISL_2156194, EPI_ISL_2156195, EPI_ISL_2156196, EPI_ISL_2188177, EPI_ISL_2188239, EPI_ISL_2188745, EPI_ISL_4713882, EPI_ISL_4715880, EPI_ISL_4715899, EPI_ISL_4715986, EPI_ISL_4716224, EPI_ISL_4716258, EPI_ISL_4716331, EPI_ISL_4716613, EPI_ISL_4716980			
see above	Ilocos Training and Regional Medical Center	Philippine Genome Center	Alethea R. de Guzman; Anna Ong-Lim; Arianne A. Zamora; Asia Louisa U. Chong; Benedict A. Maralit; Candice Francheska B. Tambaoan; Carlo M. Lapid; Celia Carlos; Cynthia P. Saloma; Devon Ray Pacial; Diomedes A. Cariño; Edsel Maurice Salvana; Edsel Maurice Salvaña; El King D. Morado; Elcid Aaron R. Pangilinan; Eva Maria Cutiongco-de la Paz; Francis A. Tablizo; Henrietta Marie Rodriguez; Irish Coleen A. Asin; Jaime C. Montoya; Jan Michael C. Yap; Jarvin E. Nipales; Jo-Hannah S. Llames; John Q. Wong; Joshua Gregor A. Dizon; Juan Antonio R. Magalang; Karol Sophia Agape R. Padilla; Kenneth M. Kim; Kris P. Punayan; Kristina Patriz Dela Cruz; Lindsay Clare D.L. Carandang; Ma. Exanil Plantig; Marc Edsel C. Ayes; Marc Jerrone R. Castro; Maria Rosario Singh-Vergeire; Maria Rosario Singh-Vergeire and Cynthia P. Saloma; Maria Sofia L. Yangzon; Marielle M. Gamboa; Marissa Alejandria; Niña Francesca Bustamante; Razel Nikka M. Hao; Renato Jacinto Q. Mantaring; Rianna Patricia S. Cruz; Sheila Mae M. Araiza; Shiela Mae M. Araiza; Yvonne Valerie Austria; Zipporah Mariebelle R. Enriquez; Zyrel V. Mollejon
EPI_ISL_634976	Institute of Molecular Biology and Biotechnology, National Institutes of Health	Institute of Molecular Biology and Biotechnology, National Institutes of Health	Arevalo, G.; Dungca; E.M.; N.T. and Salvana; Schwem, B.
EPI_ISL_4459456	JABEZ MEDICAL CENTER	Research Institute for Tropical Medicine/Tohoku University	Allen Anthony Laraño; Ardiane Ysabelle Dolor; Catalino Demetria; Clyde Dapat; Emmanuel Kagning Tsinda; Francisco Gerardo Polotan; Hitoshi Oshitani; Inez Andrea Medado; Jefferson Earl Halog; Lei Lanna Dancel; Leilanie Nacion; Ma. Angelica Tujan; Machiko Okamoto; Maria Carmen Corpuz; Mariko Siato-Obata; Mayuko Saito
EPI_ISL_3105867	Jecsons Medical Center	Research Institute for Tropical Medicine	Clyde Dapat; Deana Mae Ocampo; Emmanuel Kagning Tsinda; Francisco Gerardo Polotan; Hitoshi Oshitani; Inez Andrea Medado; Jefferson Earl Halog; Joana Ina Manalo; Lei Lanna Dancel; Ma Angelica Tujan; Mariko Saito-Obata; Mayuko Saito; Michiko Okamoto; Samantha Louise Bado
EPI_ISL_410314, EPI_ISL_410344, EPI_ISL_410345	Joanna Ina Manalo Research Institute for Tropical Medicine, Molecular Biology Laboratory	Joanna Ina Manalo Research Institute for Tropical Medicine, Molecular Biology Laboratory	A.D.; Cruz; E.S.; F.M.; I.P.; J.I.; J.I. and Nicolasora; K.M. and Polotan; M.A.; Manalo; Medado; Mercado; Nicolasora; O.T.; Onza; Tujan
EPI_ISL_2188983, EPI_ISL_2189465, EPI_ISL_4713689	Jose B. Lingad Memorial General Hospital	Philippine Genome Center	Alethea R. de Guzman; Anna Ong-Lim; Arianne A. Zamora; Asia Louisa U. Chong; Benedict A. Maralit; Candice Francheska B. Tambaoan; Carlo M. Lapid; Celia Carlos; Cynthia P. Saloma; Devon Ray Pacial; Diomedes A. Cariño; Edsel Maurice Salvana; Edsel Maurice Salvaña; El King D. Morado; Elcid Aaron R. Pangilinan; Eva Maria Cutiongco-de la Paz; Francis A. Tablizo; Henrietta Marie Rodriguez; Irish Coleen A. Asin; Jaime C. Montoya; Jan Michael C. Yap; Jarvin E. Nipales; Jo-Hannah S. Llames; John Q. Wong; Joshua Gregor A. Dizon; Juan Antonio R. Magalang; Karol Sophia Agape R. Padilla; Kenneth M. Kim; Kris P. Punayan; Kristina Patriz Dela Cruz; Lindsay Clare D.L. Carandang; Ma. Exanil Plantig; Marc Edsel C. Ayes; Maria Rosario Singh-Vergeire; Maria Rosario Singh-Vergeire and Cynthia P. Saloma; Maria Sofia L. Yangzon; Marielle M. Gamboa; Marissa Alejandria; Niña Francesca Bustamante; Razel Nikka M. Hao; Renato Jacinto Q. Mantaring; Rianna Patricia S. Cruz; Sheila Mae M. Araiza; Shiela Mae M. Araiza; Yvonne Valerie Austria; Zipporah Mariebelle R. Enriquez; Zyrel V. Mollejon
EPI_ISL_4714031, EPI_ISL_4714666, EPI_ISL_4715057, EPI_ISL_4715171	Kairos Diagnostics Laboratory	Philippine Genome Center	Alethea R. de Guzman; Anna Ong-Lim; Arianne A. Zamora; Benedict A. Maralit; Carlo M. Lapid; Celia Carlos; Cynthia P. Saloma; Devon Ray Pacial; Diomedes A. Cariño; Edsel Maurice Salvana; El King D. Morado; Elcid Aaron R. Pangilinan; Eva Maria Cutiongco-de la Paz; Francis A. Tablizo; Henrietta Marie Rodriguez; Jaime C. Montoya; Jan Michael C. Yap; Jarvin E. Nipales; Jo-Hannah S. Llames; John Q. Wong; Joshua Gregor A. Dizon; Juan Antonio R. Magalang; Karol Sophia Agape R. Padilla; Kenneth M. Kim; Kris P. Punayan; Kristina Patriz Dela Cruz; Lindsay Clare D.L. Carandang; Ma. Exanil Plantig; Marc Edsel C. Ayes; Maria Rosario Singh-Vergeire; Maria Sofia L. Yangzon; Marielle M. Gamboa; Marissa Alejandria; Niña Francesca Bustamante; Razel Nikka M. Hao; Renato Jacinto Q. Mantaring; Rianna Patricia S. Cruz; Shiela Mae M. Araiza; Yvonne Valerie Austria; Zipporah Mariebelle R. Enriquez; Zyrel V. Mollejon
EPI_ISL_4459525	LEVERIZA HEALTH CENTER	Research Institute for Tropical Medicine/Tohoku University	Allen Anthony Laraño; Ardiane Ysabelle Dolor; Catalino Demetria; Clyde Dapat; Emmanuel Kagning Tsinda; Francisco Gerardo Polotan; Hitoshi Oshitani; Inez Andrea Medado; Jefferson Earl Halog; Lei Lanna Dancel; Leilanie Nacion; Ma. Angelica Tujan; Machiko Okamoto; Maria Carmen Corpuz; Mariko Siato-Obata; Mayuko Saito
EPI_ISL_4459387	LOBO MUNICIPAL HEALTH OFFICE	Research Institute for Tropical Medicine/Tohoku University	Allen Anthony Laraño; Ardiane Ysabelle Dolor; Catalino Demetria; Clyde Dapat; Emmanuel Kagning Tsinda; Francisco Gerardo Polotan; Hitoshi Oshitani; Inez Andrea Medado; Jefferson Earl Halog; Lei Lanna Dancel; Leilanie Nacion; Ma. Angelica Tujan; Machiko Okamoto; Maria Carmen Corpuz; Mariko Siato-Obata; Mayuko Saito
EPI_ISL_4459362	LUMBAN RURAL HEALTH UNIT	Research Institute for Tropical Medicine/Tohoku University	Allen Anthony Laraño; Ardiane Ysabelle Dolor; Catalino Demetria; Clyde Dapat; Emmanuel Kagning Tsinda; Francisco Gerardo Polotan; Hitoshi Oshitani; Inez Andrea Medado; Jefferson Earl Halog; Lei Lanna Dancel; Leilanie Nacion; Ma. Angelica Tujan; Machiko Okamoto; Maria Carmen Corpuz; Mariko Siato-Obata; Mayuko Saito
EPI_ISL_3105875	La Huerta Health Center	Research Institute for Tropical Medicine	Clyde Dapat; Deana Mae Ocampo; Emmanuel Kagning Tsinda; Francisco Gerardo Polotan; Hitoshi Oshitani; Inez Andrea Medado; Jefferson Earl Halog; Joana Ina Manalo; Lei Lanna Dancel; Ma Angelica Tujan; Mariko Saito-Obata; Mayuko Saito; Michiko Okamoto; Samantha Louise Bado
EPI_ISL_2155729, EPI_ISL_2155730	Laguna Holy Family Hospital, Inc.	Philippine Genome Center	Alethea R. de Guzman; Anna Ong-Lim; Arianne A. Zamora; Asia Louisa U. Chong; Benedict A. Maralit; Candice Francheska B. Tambaoan; Carlo M. Lapid; Celia Carlos; Devon Ray Pacial; Edsel Maurice Salvaña; El King D. Morado; Eva Maria Cutiongco-de la Paz; Francis A. Tablizo; Irish Coleen A. Asin; Jaime C. Montoya; Jan Michael C. Yap; Jo-Hannah S. Llames; John Q. Wong; Joshua Gregor A. Dizon; Juan Antonio R. Magalang; Karol Sophia Agape R. Padilla; Kenneth M. Kim; Kris P. Punayan; Marc Edsel C. Ayes; Marc Jerrone R. Castro; Maria Rosario Singh-Vergeire and Cynthia P. Saloma; Maria Sofia L. Yangzon; Marissa Alejandria; Razel Nikka M.

			Hao; Rianna Patricia S. Cruz; Sheila Mae M. Araiza
EPI_ISL_1069174	Laguna Medical Center, Inc	Research Institute for Tropical Medicine	Catalino Demetria; Daria Manalo; Edelwisa Mercado; Francisco Gerardo Polotan; Hannah Leah Morito; Inez Andrea Medado; John Leonard Chan; Joseph Hughes; Kirstyn Brunker; Ma Angelica Tujan; Othoniel Jan Onza
EPI_ISL_2155069, EPI_ISL_2155106, EPI_ISL_2155158, EPI_ISL_2155160, EPI_ISL_2155188, EPI_ISL_2155199, EPI_ISL_2155212, EPI_ISL_2155271, EPI_ISL_2155345, EPI_ISL_2155640, EPI_ISL_2155972, EPI_ISL_2156407, EPI_ISL_2156420, EPI_ISL_2156489, EPI_ISL_2156524, EPI_ISL_2156637, EPI_ISL_2188046, EPI_ISL_2188205, EPI_ISL_2188206, EPI_ISL_2188909, EPI_ISL_2188999, EPI_ISL_2189321, EPI_ISL_2189333, EPI_ISL_2189339, EPI_ISL_2189353, EPI_ISL_2189359, EPI_ISL_2189398, EPI_ISL_2189413, EPI_ISL_2859610, EPI_ISL_2859619, EPI_ISL_2859650, EPI_ISL_2859660, EPI_ISL_2859747, EPI_ISL_2859783, EPI_ISL_2859803, EPI_ISL_2859804, EPI_ISL_2859809, EPI_ISL_2859821, EPI_ISL_2859827, EPI_ISL_2859886, EPI_ISL_2859924, EPI_ISL_2860012, EPI_ISL_2860034, EPI_ISL_2860054, EPI_ISL_2860085, EPI_ISL_2860115, EPI_ISL_2860116, EPI_ISL_2860152, EPI_ISL_2860171, EPI_ISL_4712489, EPI_ISL_4712903, EPI_ISL_4713005, EPI_ISL_4713129, EPI_ISL_4713172, EPI_ISL_4713237, EPI_ISL_4713280, EPI_ISL_4713329, EPI_ISL_4713441, EPI_ISL_4713490, EPI_ISL_4713568			
see above	Laguna Provincial Hospital - San Pablo District Hospital	Philippine Genome Center	Alethea R. de Guzman; Anna Ong-Lim; Arianne A. Zamora; Asia Louisa U. Chong; Benedict A. Maralit; Candice Francheska B. Tambaoan; Carlo M. Lapid; Celia Carlos; Cynthia P. Saloma; Devon Ray Pacial; Diomedes A. Carino; Diomedes A. Cariño; Edsel Maurice Salvana; Edsel Maurice Salvaña; El King D. Morado; Elcid Aaron R. Pangilinan; Eva Maria Cutiongco-de la Paz; Francis A. Tablizo; Henrietta Marie Rodriguez; Irish Coleen A. Asin; Jaime C. Montoya; Jan Michael C. Yap; Jarvin E. Nipales; Jo-Hannah S. Llames; John Q. Wong; Joshua Gregor A. Dizon; Juan Antonio R. Magalang; Karol Sophia Agape R. Padilla; Kenneth M. Kim; Kris P. Punayan; Krisitna Patriz Dela Cruz; Kristina Patriz Dela Cruz; Lindsay Claire D.L. Carandang; Lindsay Clare D.L. Carandang; Ma. Exanil Plantig; Marc Edsel C. Ayes; Marc Jerrone R. Castro; Maria Rosario Singh-Vergeire; Maria Rosario Singh-Vergeire and Cynthia P. Saloma; Maria Sofia L. Yangzon; Marielle M Gamboa; Marielle M. Gamboa; Marissa Alejandria; Nina Francesca Bustamante; Niña Francesca Bustamante; Razel Nikka M. Hao; Renato Jacinto Q. Mantaring; Rianna Patricia S. Cruz; Sheila Mae M. Araiza; Shiela Mae M. Araiza; Yvonne Valerie Austria; Zipporah Mariebelle R. Enriquez; Zyrel V. Mollejon
EPI_ISL_2188182, EPI_ISL_2188878, EPI_ISL_2189318, EPI_ISL_2189343	Las PiÃ±as General Hospital and Salellite Trauma Center	Philippine Genome Center	Alethea R. de Guzman; Anna Ong-Lim; Arianne A. Zamora; Asia Louisa U. Chong; Benedict A. Maralit; Candice Francheska B. Tambaoan; Carlo M. Lapid; Celia Carlos; Devon Ray Pacial; Edsel Maurice Salvaña; El King D. Morado; Elcid Aaron R. Pangilinan; Eva Maria Cutiongco-de la Paz; Francis A. Tablizo; Irish Coleen A. Asin; Jaime C. Montoya; Jan Michael C. Yap; Jo-Hannah S. Llames; John Q. Wong; Joshua Gregor A. Dizon; Juan Antonio R. Magalang; Karol Sophia Agape R. Padilla; Kenneth M. Kim; Kris P. Punayan; Marc Edsel C. Ayes; Marc Jerrone R. Castro; Maria Rosario Singh-Vergeire and Cynthia P. Saloma; Maria Sofia L. Yangzon; Marissa Alejandria; Razel Nikka M. Hao; Renato Jacinto Q. Mantaring; Rianna Patricia S. Cruz; Sheila Mae M. Araiza
EPI_ISL_3105877, EPI_ISL_3105881, EPI_ISL_3105885	Las Piñas General Hospital and Satellite Trauma Center	Research Institute for Tropical Medicine	Clyde Dapat; Deana Mae Ocampo; Emmanuel Kagning Tsinda; Francisco Gerardo Polotan; Hitoshi Oshitani; Inez Andrea Medado; Jefferson Earl Halog; Joana Ina Manalo; Lei Lanna Dancel; Ma Angelica Tujan; Mariko Saito-Obata; Mayuko Saito; Michiko Okamoto; Samantha Louise Bado
EPI_ISL_1069188	Lumban Rural Health Unit	Research Institute for Tropical Medicine	Catalino Demetria; Daria Manalo; Edelwisa Mercado; Francisco Gerardo Polotan; Hannah Leah Morito; Inez Andrea Medado; John Leonard Chan; Joseph Hughes; Kirstyn Brunker; Ma Angelica Tujan; Othoniel Jan Onza
EPI_ISL_430844	Lung Center of the Philippines	Research Institute for Tropical Medicine	Bautista; Brunker, K.; C.S.; C.T.; D.L.; Demetria; E.S.; F.G.M.; I.A.P.; Manalo; Medado; Mercado; O.J.T.; Onza; Polotan
EPI_ISL_1213568, EPI_ISL_2155762, EPI_ISL_2155763, EPI_ISL_2155764, EPI_ISL_2155765, EPI_ISL_2155766, EPI_ISL_2156223, EPI_ISL_2156224, EPI_ISL_2156225, EPI_ISL_2156321, EPI_ISL_2156513, EPI_ISL_2156514, EPI_ISL_2156523, EPI_ISL_2156527, EPI_ISL_2859188, EPI_ISL_2859315, EPI_ISL_4712850			
see above	Lung Center of the Philippines (LCP)	Philippine Genome Center	Alethea R. de Guzman; Anna Ong-Lim; Arianne A. Zamora; Asia Louisa U. Chong; Benedict A. Maralit; Candice Francheska B. Tambaoan; Carlo M. Lapid; Celia Carlos; Cynthia P. Saloma; Devon Ray Pacial; Diomedes A. Carino; Diomedes A. Cariño; Edsel Maurice Salvana; Edsel Maurice Salvaña; El King D. Morado; Elcid Aaron R. Pangilinan; Eva Maria Cutiongco-de la Paz; Francis A. Tablizo; Henrietta Marie Rodriguez; Irish Coleen A. Asin; Jaime C. Montoya; Jan Michael C. Yap; Jarvin E. Nipales; Jo-Hannah S. Llames; John Q. Wong; Joshua Gregor A. Dizon; Juan Antonio R. Magalang; Karol Sophia Agape R. Padilla; Kenneth M. Kim; Kris P. Punayan; Krisitna Patriz Dela Cruz; Kristina Patriz Dela Cruz; Lindsay Claire D.L. Carandang; Lindsay Clare D.L. Carandang; Ma. Exanil Plantig; Marc Edsel C. Ayes; Marc Jerrone R. Castro; Maria Rosario Singh-Vergeire; Maria Rosario Singh-Vergeire and Cynthia P. Saloma; Maria Sofia L. Yangzon; Marielle M Gamboa; Marielle M. Gamboa; Marissa Alejandria; Nina Francesca Bustamante; Niña Francesca Bustamante; Razel Nikka M. Hao; Renato Jacinto Q. Mantaring; Rianna Patricia S. Cruz; Sheila Mae M. Araiza; Shiela Mae M. Araiza; Yvonne Valerie Austria; Zipporah Mariebelle R. Enriquez; Zyrel V. Mollejon
EPI_ISL_4459480	MA. ESTRELLA GENERAL HOSPITAL	Research Institute for Tropical Medicine/Tohoku University	Allen Anthony Laraño; Ardiane Ysabelle Dolor; Catalino Demetria; Clyde Dapat; Emmanuel Kagning Tsinda; Francisco Gerardo Polotan; Hitoshi Oshitani; Inez Andrea Medado; Jefferson Earl Halog; Lei Lanna Dancel; Leilanie Nacion; Ma. Angelica Tujan; Machiko Okamoto; Maria Carmen Corpuz; Mariko Siato-Obata; Mayuko Saito
EPI_ISL_4459430, EPI_ISL_4459471	MATAAS NA KAHOY RURAL HEALTH UNIT	Research Institute for Tropical Medicine/Tohoku University	Allen Anthony Laraño; Ardiane Ysabelle Dolor; Catalino Demetria; Clyde Dapat; Emmanuel Kagning Tsinda; Francisco Gerardo Polotan; Hitoshi Oshitani; Inez Andrea Medado; Jefferson Earl Halog; Lei Lanna Dancel; Leilanie Nacion; Ma. Angelica Tujan; Machiko Okamoto; Maria Carmen Corpuz; Mariko Siato-Obata; Mayuko Saito
EPI_ISL_4459292	MEDICAL CENTER MUNTINLUPA, INC	Research Institute for Tropical Medicine/Tohoku University	Allen Anthony Laraño; Ardiane Ysabelle Dolor; Catalino Demetria; Clyde Dapat; Emmanuel Kagning Tsinda; Francisco Gerardo Polotan; Hitoshi Oshitani; Inez Andrea Medado; Jefferson Earl Halog; Lei Lanna Dancel; Leilanie Nacion; Ma. Angelica Tujan; Machiko Okamoto; Maria Carmen Corpuz; Mariko Siato-Obata; Mayuko Saito
EPI_ISL_4459427	METRO LEMERY MEDICAL CENTER	Research Institute for Tropical Medicine/Tohoku University	Allen Anthony Laraño; Ardiane Ysabelle Dolor; Catalino Demetria; Clyde Dapat; Emmanuel Kagning Tsinda; Francisco Gerardo Polotan; Hitoshi Oshitani; Inez Andrea Medado; Jefferson Earl Halog; Lei Lanna Dancel; Leilanie Nacion; Ma. Angelica Tujan; Machiko Okamoto; Maria Carmen Corpuz; Mariko Siato-Obata; Mayuko Saito
EPI_ISL_4459356	METRO SAN JOSE MEDICAL CENTER	Research Institute for Tropical Medicine/Tohoku University	Allen Anthony Laraño; Ardiane Ysabelle Dolor; Catalino Demetria; Clyde Dapat; Emmanuel Kagning Tsinda; Francisco Gerardo Polotan; Hitoshi Oshitani; Inez Andrea Medado; Jefferson Earl Halog; Lei Lanna Dancel; Leilanie Nacion; Ma. Angelica Tujan; Machiko Okamoto; Maria Carmen Corpuz; Mariko Siato-Obata; Mayuko Saito
EPI_ISL_4459450, EPI_ISL_4459453, EPI_ISL_4459483	MOGPOG RURAL HEALTH UNIT	Research Institute for Tropical Medicine/Tohoku University	Allen Anthony Laraño; Ardiane Ysabelle Dolor; Catalino Demetria; Clyde Dapat; Emmanuel Kagning Tsinda; Francisco Gerardo Polotan; Hitoshi Oshitani; Inez Andrea Medado; Jefferson Earl Halog; Lei Lanna Dancel; Leilanie Nacion; Ma. Angelica Tujan; Machiko Okamoto; Maria Carmen Corpuz; Mariko Siato-Obata; Mayuko Saito
EPI_ISL_2154931, EPI_ISL_2155406, EPI_ISL_2155407, EPI_ISL_2155408, EPI_ISL_2155409, EPI_ISL_2155410, EPI_ISL_2155411, EPI_ISL_4716025			
see above	Makati Medical Center	Philippine Genome Center	Alethea R. de Guzman; Anna Ong-Lim; Arianne A. Zamora; Asia Louisa U. Chong; Benedict A. Maralit; Candice Francheska B. Tambaoan; Carlo M. Lapid; Celia Carlos; Cynthia P. Saloma; Devon Ray Pacial; Diomedes A. Cariño; Edsel Maurice Salvana; Edsel Maurice Salvaña; El King D. Morado; Elcid Aaron R. Pangilinan; Eva Maria Cutiongco-de la Paz; Francis A. Tablizo; Henrietta Marie Rodriguez; Irish Coleen A. Asin; Jaime C. Montoya; Jan Michael C. Yap; Jarvin E. Nipales; Jo-Hannah S. Llames; John Q. Wong; Joshua Gregor A. Dizon; Juan Antonio R. Magalang; Karol Sophia Agape R. Padilla; Kenneth M. Kim; Kris P. Punayan; Kristina Patriz Dela Cruz; Lindsay Clare D.L. Carandang; Ma. Exanil Plantig; Marc Edsel C. Ayes; Marc Jerrone R. Castro; Maria Rosario Singh-Vergeire; Maria Rosario Singh-Vergeire and Cynthia P. Saloma; Maria Sofia L. Yangzon; Marielle M. Gamboa; Marissa Alejandria; Niña Francesca Bustamante; Razel Nikka M. Hao; Renato Jacinto Q. Mantaring; Rianna Patricia S. Cruz; Sheila Mae M. Araiza; Shiela Mae M. Araiza; Yvonne Valerie Austria; Zipporah Mariebelle R. Enriquez; Zyrel V. Mollejon
EPI_ISL_430838	Makati Medical Center	Research Institute for Tropical Medicine	Bautista; Brunker, K.; C.S.; C.T.; D.L.; Demetria; E.S.; F.G.M.; I.A.P.; Manalo; Medado; Mercado; O.J.T.; Onza; Polotan
EPI_ISL_2154004	Mandaluyong City Medical Center	Philippine Genome Center	Alethea R. de Guzman; Anna Ong-Lim; Arianne A. Zamora; Asia Louisa U. Chong; Benedict A. Maralit; Candice Francheska B. Tambaoan; Carlo M. Lapid; Celia Carlos; Devon Ray Pacial; Edsel Maurice Salvaña; El King D. Morado; Eva Maria Cutiongco-de la Paz; Francis A. Tablizo; Irish Coleen A. Asin; Jaime C. Montoya; Jan Michael C. Yap; Jo-Hannah S. Llames; John Q. Wong; Joshua Gregor A. Dizon; Juan Antonio R. Magalang; Karol Sophia Agape R. Padilla; Kenneth M. Kim; Kris P. Punayan; Marc Edsel C. Ayes; Marc Jerrone R. Castro; Maria Rosario Singh-Vergeire and Cynthia P. Saloma; Maria Sofia L. Yangzon; Marissa Alejandria; Razel Nikka M. Hao; Rianna Patricia S. Cruz; Sheila Mae M. Araiza
EPI_ISL_1122456, EPI_ISL_1213569, EPI_ISL_2105475, EPI_ISL_2105558, EPI_ISL_2155010, EPI_ISL_2155096, EPI_ISL_2155107, EPI_ISL_2155297, EPI_ISL_2155487, EPI_ISL_2155488, EPI_ISL_2155489, EPI_ISL_2155490, EPI_ISL_2155491, EPI_ISL_2155492, EPI_ISL_2155493, EPI_ISL_2155494, EPI_ISL_2155495, EPI_ISL_2155496, EPI_ISL_2155497, EPI_ISL_2155498, EPI_ISL_2155499, EPI_ISL_2155500, EPI_ISL_2155501, EPI_ISL_2155502, EPI_ISL_2155503, EPI_ISL_2155504, EPI_ISL_2155505, EPI_ISL_2155506, EPI_ISL_2155507, EPI_ISL_2155508, EPI_ISL_2155509, EPI_ISL_2155510, EPI_ISL_2155511, EPI_ISL_2155512, EPI_ISL_2155513, EPI_ISL_2155514, EPI_ISL_2155515, EPI_ISL_2155516, EPI_ISL_2155517, EPI_ISL_2156118, EPI_ISL_2156119, EPI_ISL_2156120, EPI_ISL_2156121, EPI_ISL_2156122, EPI_ISL_2156123, EPI_ISL_2156452, EPI_ISL_2156453, EPI_ISL_2156596, EPI_ISL_2156613, EPI_ISL_2156662, EPI_ISL_2156667, EPI_ISL_2156697, EPI_ISL_2156730, EPI_ISL_2156731, EPI_ISL_4715305			
see above	Manila Doctors Hospital	Philippine Genome Center	Alethea R. de Guzman; Anna Ong-Lim; Arianne A. Zamora; Asia Louisa U. Chong; Benedict A. Maralit; Candice Francheska B. Tambaoan; Carlo M. Lapid; Celia Carlos; Cynthia P. Saloma; Devon Ray Pacial; Diomedes A. Cariño; Edsel Maurice Salvana; Edsel Maurice Salvaña; El King D. Morado; Elcid Aaron R. Pangilinan; Eva Maria Cutiongco-de la Paz; Francis A. Tablizo; Henrietta Marie Rodriguez; Irish Coleen A. Asin; Jaime C. Montoya; Jan Michael C. Yap; Jarvin E. Nipales; Jo-Hannah S. Llames; John Q. Wong; Joshua Gregor A. Dizon; Juan Antonio R. Magalang; Karol Sophia Agape R. Padilla; Kenneth M. Kim; Kris P. Punayan; Kristina Patriz Dela Cruz; Lindsay Clare D.L. Carandang; Ma. Exanil Plantig; Marc Edsel C. Ayes; Marc Jerrone R. Castro; Maria Rosario Singh-Vergeire; Maria Rosario Singh-Vergeire and Cynthia P. Saloma; Maria Sofia L. Yangzon; Marielle M. Gamboa; Marissa Alejandria; Niña Francesca Bustamante; Razel Nikka M. Hao; Renato Jacinto Q. Mantaring; Rianna Patricia S. Cruz; Sheila Mae M. Araiza; Shiela Mae M. Araiza; Yvonne Valerie Austria; Zipporah Mariebelle R. Enriquez; Zyrel V. Mollejon
EPI_ISL_2859236, EPI_ISL_4714241, EPI_ISL_4714252, EPI_ISL_4714571, EPI_ISL_4714657, EPI_ISL_4714729, EPI_ISL_4714833, EPI_ISL_4715269, EPI_ISL_4715431, EPI_ISL_4715437, EPI_ISL_4715480, EPI_ISL_4715494, EPI_ISL_4715513, EPI_ISL_4715519, EPI_ISL_4715554, EPI_ISL_4715597, EPI_ISL_4715603			
see above	Manuel J. Santos Hospital	Philippine Genome Center	Alethea R. de Guzman; Anna Ong-Lim; Arianne A. Zamora; Benedict A. Maralit; Carlo M. Lapid; Celia Carlos; Cynthia P. Saloma; Devon Ray Pacial; Diomedes A. Carino; Diomedes A. Cariño; Edsel Maurice Salvana; Edsel Maurice Salvaña; El King D. Morado; Elcid Aaron R. Pangilinan; Eva Maria Cutiongco-de la Paz; Francis A. Tablizo; Henrietta Marie Rodriguez; Jaime C. Montoya; Jan Michael C. Yap; Jarvin E. Nipales; Jo-Hannah S. Llames; John Q. Wong; Joshua Gregor A. Dizon; Juan Antonio R. Magalang; Karol Sophia Agape R. Padilla; Kenneth M. Kim; Kris P. Punayan; Krisitna Patriz Dela Cruz; Kristina Patriz Dela Cruz; Lindsay Claire D.L. Carandang; Lindsay Clare D.L. Carandang; Ma. Exanil Plantig; Marc Edsel C. Ayes; Maria Rosario Singh-Vergeire; Maria Rosario Singh-Vergeire and Cynthia P. Saloma; Maria Sofia L. Yangzon; Marielle M Gamboa; Marielle M. Gamboa; Marissa Alejandria; Nina Francesca Bustamante; Niña Francesca Bustamante; Razel Nikka M. Hao; Renato Jacinto Q. Mantaring; Rianna Patricia S. Cruz; Sheila Mae M. Araiza; Shiela Mae M. Araiza; Yvonne Valerie Austria; Zipporah Mariebelle R. Enriquez; Zyrel V. Mollejon
EPI_ISL_2105535, EPI_ISL_2107799, EPI_ISL_2156278, EPI_ISL_2156279, EPI_ISL_2156280, EPI_ISL_2156487, EPI_ISL_2156488, EPI_ISL_2189326, EPI_ISL_4712342, EPI_ISL_4712450, EPI_ISL_4712563, EPI_ISL_4712768, EPI_ISL_4712778, EPI_ISL_4712800, EPI_ISL_4712943, EPI_ISL_4713012, EPI_ISL_4713039, EPI_ISL_4713066, EPI_ISL_4713260, EPI_ISL_4713419, EPI_ISL_4713613, EPI_ISL_4713638, EPI_ISL_4713662, EPI_ISL_4714084, EPI_ISL_4714685, EPI_ISL_4714783, EPI_ISL_4715232, EPI_ISL_4715310, EPI_ISL_4715322, EPI_ISL_4715334, EPI_ISL_4715351, EPI_ISL_4715358			
see above	Maria Reyna Xavier University Hospital	Philippine Genome Center	Alethea R. de Guzman; Anna Ong-Lim; Arianne A. Zamora; Asia Louisa U. Chong; Benedict A. Maralit; Candice Francheska B. Tambaoan; Carlo M. Lapid; Celia Carlos; Cynthia P. Saloma; Devon Ray Pacial; Diomedes A. Cariño; Edsel Maurice Salvana; Edsel Maurice Salvaña; El King D. Morado; Elcid Aaron R. Pangilinan; Eva Maria Cutiongco-de la Paz; Francis A. Tablizo; Henrietta Marie Rodriguez; Irish Coleen A. Asin; Jaime C. Montoya; Jan Michael C. Yap; Jarvin E. Nipales; Jo-Hannah S. Llames; John Q. Wong; Joshua Gregor A. Dizon; Juan Antonio R. Magalang; Karol Sophia Agape R. Padilla; Kenneth M. Kim; Kris P. Punayan; Kristina Patriz Dela Cruz; Lindsay Clare D.L. Carandang; Ma. Exanil Plantig; Marc Edsel C. Ayes; Marc Jerrone R. Castro; Maria Rosario Singh-Vergeire; Maria Rosario Singh-Vergeire and Cynthia P. Saloma; Maria Sofia L. Yangzon; Marielle M. Gamboa; Marissa Alejandria; Niña Francesca Bustamante; Razel Nikka M. Hao; Renato Jacinto Q. Mantaring; Rianna Patricia S. Cruz; Sheila Mae M. Araiza; Shiela Mae M. Araiza; Yvonne Valerie Austria; Zipporah Mariebelle R. Enriquez; Zyrel V. Mollejon
EPI_ISL_2155378, EPI_ISL_2155379, EPI_ISL_2155380, EPI_ISL_2155381, EPI_ISL_2155382, EPI_ISL_2155383, EPI_ISL_2155384, EPI_ISL_2155385, EPI_ISL_2156097, EPI_ISL_2156098, EPI_ISL_2188112, EPI_ISL_2188712, EPI_ISL_2188716, EPI_ISL_2188771, EPI_ISL_2189203, EPI_ISL_2189207, EPI_ISL_2189302, EPI_ISL_2189342, EPI_ISL_2189475, EPI_ISL_2189522, EPI_ISL_2859603, EPI_ISL_2859679, EPI_ISL_2859727, EPI_ISL_2859734, EPI_ISL_2859933, EPI_ISL_4713893, EPI_ISL_4714586, EPI_ISL_4715049, EPI_ISL_4715241, EPI_ISL_4715874, EPI_ISL_4716137, EPI_ISL_4716203, EPI_ISL_4716453, EPI_ISL_4716520, EPI_ISL_4716734, EPI_ISL_4717075, EPI_ISL_4717204, EPI_ISL_4717370			
see above	Mariano Marcos Memorial Hospital Medical Center	Philippine Genome Center	Alethea R. de Guzman; Anna Ong-Lim; Arianne A. Zamora; Asia Louisa U. Chong; Benedict A. Maralit; Candice Francheska B. Tambaoan; Carlo M. Lapid; Celia Carlos; Cynthia P. Saloma; Devon Ray Pacial; Diomedes A. Carino; Diomedes A. Cariño; Edsel Maurice Salvana; Edsel Maurice Salvaña; El King D. Morado; Elcid Aaron R. Pangilinan; Eva Maria Cutiongco-de la Paz; Francis A. Tablizo; Henrietta Marie Rodriguez; Irish Coleen A. Asin; Jaime C. Montoya; Jan Michael C. Yap; Jarvin E. Nipales; Jo-Hannah S. Llames; John Q. Wong; Joshua Gregor A. Dizon; Juan Antonio R. Magalang; Karol Sophia Agape R. Padilla; Kenneth M. Kim; Kris P. Punayan; Krisitna Patriz Dela Cruz; Kristina Patriz Dela Cruz; Lindsay Claire D.L. Carandang; Lindsay Clare D.L. Carandang; Ma. Exanil Plantig; Marc Edsel C. Ayes; Marc Jerrone R. Castro; Maria Rosario Singh-Vergeire; Maria Rosario Singh-Vergeire and Cynthia P. Saloma; Maria Sofia L. Yangzon; Marielle M Gamboa; Marielle M. Gamboa; Marissa Alejandria; Nina Francesca Bustamante; Niña Francesca Bustamante; Razel Nikka M. Hao; Renato Jacinto Q. Mantaring; Rianna Patricia S. Cruz; Sheila Mae M. Araiza; Shiela Mae M. Araiza; Yvonne Valerie Austria; Zipporah Mariebelle R. Enriquez; Zyrel V. Mollejon
EPI_ISL_2155659, EPI_ISL_2155660, EPI_ISL_2155661, EPI_ISL_2155662, EPI_ISL_2156417, EPI_ISL_2188109, EPI_ISL_2188180, EPI_ISL_2188240, EPI_ISL_2188241, EPI_ISL_2188718, EPI_ISL_2188735, EPI_ISL_2188764, EPI_ISL_2188789, EPI_ISL_2188800, EPI_ISL_2188813, EPI_ISL_2188847, EPI_ISL_2188850, EPI_ISL_2188888, EPI_ISL_2188910, EPI_ISL_2188918, EPI_ISL_2188926, EPI_ISL_2188979, EPI_ISL_2188996, EPI_ISL_2189002, EPI_ISL_2189013, EPI_ISL_2189025, EPI_ISL_2189063, EPI_ISL_2189077, EPI_ISL_2189089, EPI_ISL_2189109, EPI_ISL_2189141, EPI_ISL_2189165, EPI_ISL_2189170, EPI_ISL_2189188, EPI_ISL_2189192, EPI_ISL_2189205, EPI_ISL_2189223, EPI_ISL_2189280, EPI_ISL_2189309, EPI_ISL_2189310, EPI_ISL_2189364, EPI_ISL_2189405, EPI_ISL_2189419, EPI_ISL_2189441, EPI_ISL_2189477, EPI_ISL_2859573, EPI_ISL_2859585, EPI_ISL_2859588, EPI_ISL_2859642, EPI_ISL_2859665, EPI_ISL_2859670, EPI_ISL_2859675, EPI_ISL_2859682, EPI_ISL_2859685, EPI_ISL_2859690, EPI_ISL_2859691, EPI_ISL_2859692, EPI_ISL_2859699, EPI_ISL_2859707, EPI_ISL_2859710, EPI_ISL_2859726, EPI_ISL_2859735, EPI_ISL_2859741, EPI_ISL_2859744, EPI_ISL_2859761, EPI_ISL_2859990, EPI_ISL_2860005, EPI_ISL_2860021, EPI_ISL_2860029, EPI_ISL_2860125, EPI_ISL_2860156, EPI_ISL_2860158, EPI_ISL_2860166, EPI_ISL_2860167, EPI_ISL_2860180, EPI_ISL_4714594			
see above	Mary Mediatrix Medical Center	Philippine Genome Center	Alethea R. de Guzman; Anna Ong-Lim; Arianne A. Zamora; Asia Louisa U. Chong; Benedict A. Maralit; Candice Francheska B. Tambaoan; Carlo M. Lapid; Celia Carlos; Cynthia P. Saloma; Devon Ray Pacial; Diomedes A. Carino; Diomedes A. Cariño; Edsel Maurice Salvana; Edsel Maurice Salvaña; El King D. Morado; Elcid Aaron R. Pangilinan; Eva Maria Cutiongco-de la Paz; Francis A. Tablizo; Henrietta Marie Rodriguez; Irish Coleen A. Asin; Jaime C. Montoya; Jan Michael C. Yap; Jarvin E. Nipales; Jo-Hannah S. Llames; John Q. Wong; Joshua Gregor A. Dizon; Juan Antonio R. Magalang; Karol Sophia Agape R. Padilla; Kenneth M. Kim; Kris P. Punayan; Krisitna Patriz Dela Cruz; Kristina Patriz Dela Cruz; Lindsay Claire D.L. Carandang; Lindsay Clare D.L. Carandang; Ma. Exanil Plantig; Marc Edsel C. Ayes; Marc Jerrone R. Castro; Maria Rosario Singh-Vergeire; Maria Rosario Singh-Vergeire and Cynthia P. Saloma; Maria Sofia L. Yangzon; Marielle M Gamboa; Marielle M. Gamboa; Marissa Alejandria; Nina Francesca Bustamante; Niña Francesca Bustamante; Razel Nikka M. Hao; Renato Jacinto Q. Mantaring; Rianna Patricia S. Cruz; Sheila Mae M. Araiza; Shiela Mae M. Araiza; Yvonne Valerie Austria; Zipporah Mariebelle R. Enriquez; Zyrel V. Mollejon
EPI_ISL_1069182, EPI_ISL_1069183	Metro San Jose Medical Center	Research Institute for Tropical Medicine	Catalino Demetria; Daria Manalo; Edelwisa Mercado; Francisco Gerardo Polotan; Hannah Leah Morito; Inez Andrea Medado; John Leonard Chan; Joseph Hughes; Kirstyn Brunker; Ma Angelica Tujan; Othoniel Jan Onza
EPI_ISL_2155658	Morong Rural Health Unit (PGC)	Philippine Genome Center	Alethea R. de Guzman; Anna Ong-Lim; Arianne A. Zamora; Asia Louisa U. Chong; Benedict A. Maralit; Candice Francheska B. Tambaoan; Carlo M. Lapid; Celia Carlos; Devon Ray Pacial; Edsel Maurice Salvaña; El King D. Morado; Eva Maria Cutiongco-de la Paz; Francis A. Tablizo; Irish Coleen A. Asin; Jaime C. Montoya; Jan Michael C. Yap; Jo-Hannah S. Llames; John Q. Wong; Joshua Gregor A. Dizon; Juan Antonio R. Magalang; Karol Sophia Agape R. Padilla; Kenneth M. Kim; Kris P. Punayan; Marc Edsel C. Ayes; Marc Jerrone R. Castro; Maria Rosario Singh-Vergeire and Cynthia P. Saloma; Maria Sofia L. Yangzon; Marissa Alejandria; Razel Nikka M. Hao; Rianna Patricia S. Cruz; Sheila Mae M. Araiza

EPI_ISL_2155678	National Center for Mental Health (PGC)	Philippine Genome Center	Alethea R. de Guzman; Anna Ong-Lim; Arianne A. Zamora; Asia Louisa U. Chong; Benedict A. Maralit; Candice Francheska B. Tambaoan; Carlo M. Lapid; Celia Carlos; Devon Ray Pacial; Edsel Maurice Salvaña; El King D. Morado; Eva Maria Cutiongco-de la Paz; Francis A. Tablizo; Irish Coleen A. Asin; Jaime C. Montoya; Jan Michael C. Yap; Jo-Hannah S. Llames; John Q. Wong; Joshua Gregor A. Dizon; Juan Antonio R. Magalang; Karol Sophia Agape R. Padilla; Kenneth M. Kim; Kris P. Punayan; Marc Edsel C. Ayes; Marc Jerrone R. Castro; Maria Rosario Singh-Vergeire and Cynthia P. Saloma; Maria Sofia L. Yangzon; Marissa Alejandria; Razel Nikka M. Hao; Rianna Patricia S. Cruz; Sheila Mae M. Araiza
EPI_ISL_434554, EPI_ISL_434555, EPI_ISL_434556, EPI_ISL_434557, EPI_ISL_434558	National Institutes of Health, University of the Philippines Manila	Philippine Genome Center	Alessandra C. Sanchez; Benedict A. Maralit; Bernard Demot; Carlo M. Lapid; Christina Tan; El King D. Morado; Eva Maria Cutiongco-de la Paz; Francis A. Tablizo; Haifa L.Gaza; Jan Michael C. Yap; Jarvin E. Nipales; Jo-Hannah S. Llames; John Mark Velasco; Joshua Gregor A. Dizon; Joy Ann Petronio-Santos; Julius Aaron Mejia; Kris P. Punayan; Kristianne Arielle D. Gabriel; Maribell Dollete; Marissa M. Alejandria; Raul V. Destura; Shana F. Genavia; Shebna Rose D. Fabilloren; Shiela Mae M. Araiza; Sonia Salamat; and Cynthia P. Saloma
EPI_ISL_431833	National Institutes of Health, University of the Philippines Manila	Philippine Genome Center, University of the Philippines System	Alessandra C. Sanchez; Benedict A. Maralit; Bernard Demot; Carlo M. Lapid; Christina Tan; El King D. Morado; Eva Maria Cutiongco-de la Paz; Francis A. Tablizo; Haifa L.Gaza; Jan Michael C. Yap; Jarvin E. Nipales; Jo-Hannah S. Llames; John Mark Velasco; Joshua Gregor A. Dizon; Joy Ann Petronio-Santos; Julius Aaron Mejia; Kris P. Punayan; Kristianne Arielle D. Gabriel; Maribell Dollete; Marissa M. Alejandria; Raul V. Destura; Shana F. Genavia; Shebna Rose D. Fabilloren; Shiela Mae M. Araiza; Sonia Salamat; and Cynthia P. Saloma
EPI_ISL_833332	National Jail Management and Penology Training Institute	Research Institute for Tropical Medicine	Catalino Demetria; Daria Manalo; Edelwisa Mercado; Francisco Gerardo Polotan; Hannah Leah Morito; Inez Andrea Medado; John Leonard Chan; Kirstyn Brunker; Ma Angelica Tujan; Othoniel Jan Onza
EPI_ISL_2155663, EPI_ISL_2156517, EPI_ISL_2156518, EPI_ISL_2156606, EPI_ISL_2171098, EPI_ISL_2171208, EPI_ISL_2188077, EPI_ISL_2188159, EPI_ISL_2188753, EPI_ISL_2188845, EPI_ISL_2188851, EPI_ISL_2188879, EPI_ISL_2188922, EPI_ISL_2189131, EPI_ISL_2189297, EPI_ISL_2189403, EPI_ISL_2189458, EPI_ISL_2859514, EPI_ISL_2859560, EPI_ISL_2859618			
see above	National Kidney and Transplant Institute (NKTI)	Philippine Genome Center	Alethea R. de Guzman; Anna Ong-Lim; Arianne A. Zamora; Asia Louisa U. Chong; Benedict A. Maralit; Candice Francheska B. Tambaoan; Carlo M. Lapid; Celia Carlos; Devon Ray Pacial; Diomedes A. Carino; Edsel Maurice Salvaña; El King D. Morado; Elcid Aaron R. Pangilinan; Eva Maria Cutiongco-de la Paz; Francis A. Tablizo; Henrietta Marie Rodriguez; Irish Coleen A. Asin; Jaime C. Montoya; Jan Michael C. Yap; Jarvin E. Nipales; Jo-Hannah S. Llames; John Q. Wong; Joshua Gregor A. Dizon; Juan Antonio R. Magalang; Karol Sophia Agape R. Padilla; Kenneth M. Kim; Kris P. Punayan; Krisitna Patriz Dela Cruz; Lindsay Claire D.L. Carandang; Ma. Exanil Plantig; Marc Edsel C. Ayes; Marc Jerrone R. Castro; Maria Rosario Singh-Vergeire and Cynthia P. Saloma; Maria Sofia L. Yangzon; Marielle M Gamboa; Marissa Alejandria; Nina Francesca Bustamante; Razel Nikka M. Hao; Renato Jacinto Q. Mantaring; Rianna Patricia S. Cruz; Sheila Mae M. Araiza; Yvonne Valerie Austria; Zipporah Mariebelle R. Enriquez; Zyrel V. Mollejon
EPI_ISL_1213571, EPI_ISL_1213573, EPI_ISL_2105398, EPI_ISL_2105399, EPI_ISL_2105400, EPI_ISL_2105403, EPI_ISL_2105404, EPI_ISL_2105407, EPI_ISL_2105408, EPI_ISL_2105410, EPI_ISL_2105412, EPI_ISL_2105413, EPI_ISL_2105415, EPI_ISL_2105416, EPI_ISL_2105417, EPI_ISL_2105419, EPI_ISL_2105422, EPI_ISL_2105423, EPI_ISL_2105426, EPI_ISL_2105427, EPI_ISL_2105430, EPI_ISL_2105431, EPI_ISL_2105435, EPI_ISL_2105437, EPI_ISL_2105438, EPI_ISL_2105439, EPI_ISL_2105441, EPI_ISL_2105443, EPI_ISL_2105444, EPI_ISL_2105445, EPI_ISL_2105447, EPI_ISL_2105450, EPI_ISL_2105451, EPI_ISL_2105452, EPI_ISL_2105454, EPI_ISL_2105457, EPI_ISL_2105458, EPI_ISL_2105460, EPI_ISL_2107794, EPI_ISL_2107796, EPI_ISL_2107797, EPI_ISL_2155195, EPI_ISL_2155270, EPI_ISL_2155327			
see above	Negros Oriental Provincial Hospital	Philippine Genome Center	Alethea R. de Guzman; Anna Ong-Lim; Arianne A. Zamora; Asia Louisa U. Chong; Benedict A. Maralit; Candice Francheska B. Tambaoan; Carlo M. Lapid; Celia Carlos; Devon Ray Pacial; Edsel Maurice Salvaña; El King D. Morado; Eva Maria Cutiongco-de la Paz; Francis A. Tablizo; Irish Coleen A. Asin; Jaime C. Montoya; Jan Michael C. Yap; Jo-Hannah S. Llames; John Q. Wong; Joshua Gregor A. Dizon; Juan Antonio R. Magalang; Karol Sophia Agape R. Padilla; Kenneth M. Kim; Kris P. Punayan; Marc Edsel C. Ayes; Marc Jerrone R. Castro; Maria Rosario Singh-Vergeire and Cynthia P. Saloma; Maria Sofia L. Yangzon; Marissa Alejandria; Razel Nikka M. Hao; Rianna Patricia S. Cruz; Sheila Mae M. Araiza
EPI_ISL_2105401, EPI_ISL_2105539, EPI_ISL_2105554, EPI_ISL_2105556, EPI_ISL_2105561, EPI_ISL_2156185, EPI_ISL_2156186, EPI_ISL_2156187, EPI_ISL_2156402, EPI_ISL_2156485, EPI_ISL_2156486, EPI_ISL_2156584, EPI_ISL_2156585, EPI_ISL_2156591, EPI_ISL_2156592, EPI_ISL_2156593, EPI_ISL_2189113, EPI_ISL_2189152, EPI_ISL_2189187, EPI_ISL_2189255, EPI_ISL_2189279, EPI_ISL_2189350, EPI_ISL_4712038, EPI_ISL_4712177, EPI_ISL_4713956, EPI_ISL_4714619, EPI_ISL_4714771, EPI_ISL_4714952, EPI_ISL_4715078, EPI_ISL_4715093, EPI_ISL_4715113, EPI_ISL_4715132, EPI_ISL_4715318			
see above	Northern Mindanao TB Regional Center	Philippine Genome Center	Alethea R. de Guzman; Anna Ong-Lim; Arianne A. Zamora; Asia Louisa U. Chong; Benedict A. Maralit; Candice Francheska B. Tambaoan; Carlo M. Lapid; Celia Carlos; Cynthia P. Saloma; Devon Ray Pacial; Diomedes A. Cariño; Edsel Maurice Salvana; Edsel Maurice Salvaña; El King D. Morado; Elcid Aaron R. Pangilinan; Eva Maria Cutiongco-de la Paz; Francis A. Tablizo; Henrietta Marie Rodriguez; Irish Coleen A. Asin; Jaime C. Montoya; Jan Michael C. Yap; Jarvin E. Nipales; Jo-Hannah S. Llames; John Q. Wong; Joshua Gregor A. Dizon; Juan Antonio R. Magalang; Karol Sophia Agape R. Padilla; Kenneth M. Kim; Kris P. Punayan; Kristina Patriz Dela Cruz; Lindsay Clare D.L. Carandang; Ma. Exanil Plantig; Marc Edsel C. Ayes; Marc Jerrone R. Castro; Maria Rosario Singh-Vergeire; Maria Rosario Singh-Vergeire and Cynthia P. Saloma; Maria Sofia L. Yangzon; Marielle M. Gamboa; Marissa Alejandria; Niña Francesca Bustamante; Razel Nikka M. Hao; Renato Jacinto Q. Mantaring; Rianna Patricia S. Cruz; Sheila Mae M. Araiza; Shiela Mae M. Araiza; Yvonne Valerie Austria; Zipporah Mariebelle R. Enriquez; Zyrel V. Mollejon
EPI_ISL_4459433, EPI_ISL_4459486	ODIONGAN RURAL HEALTH UNIT	Research Institute for Tropical Medicine/Tohoku University	Allen Anthony Laraño; Ardiane Ysabelle Dolor; Catalino Demetria; Clyde Dapat; Emmanuel Kagning Tsinda; Francisco Gerardo Polotan; Hitoshi Oshitani; Inez Andrea Medado; Jefferson Earl Halog; Lei Lanna Dancel; Leilanie Nacion; Ma. Angelica Tujan; Machiko Okamoto; Maria Carmen Corpuz; Mariko Siato-Obata; Mayuko Saito
EPI_ISL_4459306, EPI_ISL_4459436, EPI_ISL_4459522	OLIVAREZ GENERAL HOSPITAL	Research Institute for Tropical Medicine/Tohoku University	Allen Anthony Laraño; Ardiane Ysabelle Dolor; Catalino Demetria; Clyde Dapat; Emmanuel Kagning Tsinda; Francisco Gerardo Polotan; Hitoshi Oshitani; Inez Andrea Medado; Jefferson Earl Halog; Lei Lanna Dancel; Leilanie Nacion; Ma. Angelica Tujan; Machiko Okamoto; Maria Carmen Corpuz; Mariko Siato-Obata; Mayuko Saito
EPI_ISL_4459499	ORIENTAL MINDORO SOUTHERN DISTRICT HOSPITAL	Research Institute for Tropical Medicine/Tohoku University	Allen Anthony Laraño; Ardiane Ysabelle Dolor; Catalino Demetria; Clyde Dapat; Emmanuel Kagning Tsinda; Francisco Gerardo Polotan; Hitoshi Oshitani; Inez Andrea Medado; Jefferson Earl Halog; Lei Lanna Dancel; Leilanie Nacion; Ma. Angelica Tujan; Machiko Okamoto; Maria Carmen Corpuz; Mariko Siato-Obata; Mayuko Saito
EPI_ISL_4459465	OSPITAL NG LIPA	Research Institute for Tropical Medicine/Tohoku University	Allen Anthony Laraño; Ardiane Ysabelle Dolor; Catalino Demetria; Clyde Dapat; Emmanuel Kagning Tsinda; Francisco Gerardo Polotan; Hitoshi Oshitani; Inez Andrea Medado; Jefferson Earl Halog; Lei Lanna Dancel; Leilanie Nacion; Ma. Angelica Tujan; Machiko Okamoto; Maria Carmen Corpuz; Mariko Siato-Obata; Mayuko Saito
EPI_ISL_2188053, EPI_ISL_2188249	OSPITAL NG MUNTINLUPA	Philippine Genome Center	Alethea R. de Guzman; Anna Ong-Lim; Arianne A. Zamora; Asia Louisa U. Chong; Benedict A. Maralit; Candice Francheska B. Tambaoan; Carlo M. Lapid; Celia Carlos; Devon Ray Pacial; Edsel Maurice Salvaña; El King D. Morado; Elcid Aaron R. Pangilinan; Eva Maria Cutiongco-de la Paz; Francis A. Tablizo; Irish Coleen A. Asin; Jaime C. Montoya; Jan Michael C. Yap; Jo-Hannah S. Llames; John Q. Wong; Joshua Gregor A. Dizon; Juan Antonio R. Magalang; Karol Sophia Agape R. Padilla; Kenneth M. Kim; Kris P. Punayan; Marc Edsel C. Ayes; Marc Jerrone R. Castro; Maria Rosario Singh-Vergeire and Cynthia P. Saloma; Maria Sofia L. Yangzon; Marissa Alejandria; Razel Nikka M. Hao; Rianna Patricia S. Cruz; Sheila Mae M. Araiza
EPI_ISL_4459384, EPI_ISL_4459394, EPI_ISL_4459501	OSPITAL NG PARAÑAQUE	Research Institute for Tropical Medicine/Tohoku University	Allen Anthony Laraño; Ardiane Ysabelle Dolor; Catalino Demetria; Clyde Dapat; Emmanuel Kagning Tsinda; Francisco Gerardo Polotan; Hitoshi Oshitani; Inez Andrea Medado; Jefferson Earl Halog; Lei Lanna Dancel; Leilanie Nacion; Ma. Angelica Tujan; Machiko Okamoto; Maria Carmen Corpuz; Mariko Siato-Obata; Mayuko Saito
EPI_ISL_4459423	OTHERS - BALAYAN RURAL HEALTH UNIT	Research Institute for Tropical Medicine/Tohoku University	Allen Anthony Laraño; Ardiane Ysabelle Dolor; Catalino Demetria; Clyde Dapat; Emmanuel Kagning Tsinda; Francisco Gerardo Polotan; Hitoshi Oshitani; Inez Andrea Medado; Jefferson Earl Halog; Lei Lanna Dancel; Leilanie Nacion; Ma. Angelica Tujan; Machiko Okamoto; Maria Carmen Corpuz; Mariko Siato-Obata; Mayuko Saito
EPI_ISL_4459495	OTHERS - CHD REGION 4B	Research Institute for Tropical Medicine/Tohoku University	Allen Anthony Laraño; Ardiane Ysabelle Dolor; Catalino Demetria; Clyde Dapat; Emmanuel Kagning Tsinda; Francisco Gerardo Polotan; Hitoshi Oshitani; Inez Andrea Medado; Jefferson Earl Halog; Lei Lanna Dancel; Leilanie Nacion; Ma. Angelica Tujan; Machiko Okamoto; Maria Carmen Corpuz; Mariko Siato-Obata; Mayuko Saito
EPI_ISL_4459381	OTHERS - FILINVEST TENT COVID QUARANTINE FACILITY	Research Institute for Tropical Medicine/Tohoku University	Allen Anthony Laraño; Ardiane Ysabelle Dolor; Catalino Demetria; Clyde Dapat; Emmanuel Kagning Tsinda; Francisco Gerardo Polotan; Hitoshi Oshitani; Inez Andrea Medado; Jefferson Earl Halog; Lei Lanna Dancel; Leilanie Nacion; Ma. Angelica Tujan; Machiko Okamoto; Maria Carmen Corpuz; Mariko Siato-Obata; Mayuko Saito
EPI_ISL_4459339	OTHERS - MARTIN MARASIGAN MEMORIAL DISTRICT HOSPITAL	Research Institute for Tropical Medicine/Tohoku University	Allen Anthony Laraño; Ardiane Ysabelle Dolor; Catalino Demetria; Clyde Dapat; Emmanuel Kagning Tsinda; Francisco Gerardo Polotan; Hitoshi Oshitani; Inez Andrea Medado; Jefferson Earl Halog; Lei Lanna Dancel; Leilanie Nacion; Ma. Angelica Tujan; Machiko Okamoto; Maria Carmen Corpuz; Mariko Siato-Obata; Mayuko Saito
EPI_ISL_4459289	OTHERS - OSPITAL NG PARAÑAQUE DISTRICT II	Research Institute for Tropical Medicine/Tohoku University	Allen Anthony Laraño; Ardiane Ysabelle Dolor; Catalino Demetria; Clyde Dapat; Emmanuel Kagning Tsinda; Francisco Gerardo Polotan; Hitoshi Oshitani; Inez Andrea Medado; Jefferson Earl Halog; Lei Lanna Dancel; Leilanie Nacion; Ma. Angelica Tujan; Machiko Okamoto; Maria Carmen Corpuz; Mariko Siato-Obata; Mayuko Saito
EPI_ISL_4459462	OTHERS - TANAUAN CITY HEALTH OFFICE	Research Institute for Tropical Medicine/Tohoku University	Allen Anthony Laraño; Ardiane Ysabelle Dolor; Catalino Demetria; Clyde Dapat; Emmanuel Kagning Tsinda; Francisco Gerardo Polotan; Hitoshi Oshitani; Inez Andrea Medado; Jefferson Earl Halog; Lei Lanna Dancel; Leilanie Nacion; Ma. Angelica Tujan; Machiko Okamoto; Maria Carmen Corpuz; Mariko Siato-Obata; Mayuko Saito
EPI_ISL_2188997	Occidental Mindoro Provincial Hospital	Philippine Genome Center	Alethea R. de Guzman; Anna Ong-Lim; Arianne A. Zamora; Asia Louisa U. Chong; Benedict A. Maralit; Candice Francheska B. Tambaoan; Carlo M. Lapid; Celia Carlos; Devon Ray Pacial; Edsel Maurice Salvaña; El King D. Morado; Elcid Aaron R. Pangilinan; Eva Maria Cutiongco-de la Paz; Francis A. Tablizo; Irish Coleen A. Asin; Jaime C. Montoya; Jan Michael C. Yap; Jo-Hannah S. Llames; John Q. Wong; Joshua Gregor A. Dizon; Juan Antonio R. Magalang; Karol Sophia Agape R. Padilla; Kenneth M. Kim; Kris P. Punayan; Marc Edsel C. Ayes; Maria Rosario Singh-Vergeire and Cynthia P. Saloma; Maria Sofia L. Yangzon; Marissa Alejandria; Razel Nikka M. Hao; Renato Jacinto Q. Mantaring; Rianna Patricia S. Cruz; Sheila Mae M. Araiza
EPI_ISL_3105865	Olivarez General Hospital	Research Institute for Tropical Medicine	Clyde Dapat; Deana Mae Ocampo; Emmanuel Kagning Tsinda; Francisco Gerardo Polotan; Hitoshi Oshitani; Inez Andrea Medado; Jefferson Earl Halog; Joana Ina Manalo; Lei Lanna Dancel; Ma Angelica Tujan; Mariko Saito-Obata; Mayuko Saito; Michiko Okamoto; Samantha Louise Bado
EPI_ISL_2859615, EPI_ISL_2859703, EPI_ISL_2859840, EPI_ISL_2859881	Oriental Mindoro Provincial Hospital	Philippine Genome Center	Alethea R. de Guzman; Anna Ong-Lim; Arianne A. Zamora; Benedict A. Maralit; Carlo M. Lapid; Celia Carlos; Devon Ray Pacial; Diomedes A. Carino; Edsel Maurice Salvaña; El King D. Morado; Elcid Aaron R. Pangilinan; Eva Maria Cutiongco-de la Paz; Francis A. Tablizo; Henrietta Marie Rodriguez; Jaime C. Montoya; Jan Michael C. Yap; Jarvin E. Nipales; Jo-Hannah S. Llames; John Q. Wong; Joshua Gregor A. Dizon; Juan Antonio R. Magalang; Karol Sophia Agape R. Padilla; Kenneth M. Kim; Kris P. Punayan; Krisitna Patriz Dela Cruz; Lindsay Claire D.L. Carandang; Ma. Exanil Plantig; Marc Edsel C. Ayes; Maria Rosario Singh-Vergeire and Cynthia P. Saloma; Maria Sofia L. Yangzon; Marielle M Gamboa; Marissa Alejandria; Nina Francesca Bustamante; Razel Nikka M. Hao; Renato Jacinto Q. Mantaring; Rianna Patricia S. Cruz; Sheila Mae M. Araiza; Yvonne Valerie Austria; Zipporah Mariebelle R. Enriquez; Zyrel V. Mollejon
EPI_ISL_3105878	Ospital ng Biñan	Research Institute for Tropical Medicine	Clyde Dapat; Deana Mae Ocampo; Emmanuel Kagning Tsinda; Francisco Gerardo Polotan; Hitoshi Oshitani; Inez Andrea Medado; Jefferson Earl Halog; Joana Ina Manalo; Lei Lanna Dancel; Ma Angelica Tujan; Mariko Saito-Obata; Mayuko Saito; Michiko Okamoto; Samantha Louise Bado
EPI_ISL_2155701, EPI_ISL_2155702, EPI_ISL_2156368, EPI_ISL_2156394, EPI_ISL_2171224, EPI_ISL_2188038, EPI_ISL_2188042, EPI_ISL_2188076, EPI_ISL_2188093, EPI_ISL_2188101, EPI_ISL_2188148, EPI_ISL_2188219, EPI_ISL_2188675, EPI_ISL_2188676, EPI_ISL_2188690, EPI_ISL_2188748, EPI_ISL_2188815, EPI_ISL_2859500, EPI_ISL_2859506, EPI_ISL_2859681, EPI_ISL_2859732, EPI_ISL_2859740, EPI_ISL_2859756, EPI_ISL_2859906, EPI_ISL_2859971, EPI_ISL_2860040, EPI_ISL_2860106, EPI_ISL_2860159, EPI_ISL_2860161, EPI_ISL_2860194, EPI_ISL_2860205, EPI_ISL_2860241, EPI_ISL_4714336, EPI_ISL_4714651, EPI_ISL_4715252, EPI_ISL_4715331, EPI_ISL_4715344			
see above	Ospital ng Imus	Philippine Genome Center	Alethea R. de Guzman; Anna Ong-Lim; Arianne A. Zamora; Asia Louisa U. Chong; Benedict A. Maralit; Candice Francheska B. Tambaoan; Carlo M. Lapid; Celia Carlos; Cynthia P. Saloma; Devon Ray Pacial; Diomedes A. Carino; Diomedes A. Cariño; Edsel Maurice Salvana; Edsel Maurice Salvaña; El King D. Morado; Elcid Aaron R. Pangilinan; Eva Maria Cutiongco-de la Paz; Francis A. Tablizo; Henrietta Marie Rodriguez; Irish Coleen A. Asin; Jaime C. Montoya; Jan Michael C. Yap; Jarvin E. Nipales; Jo-Hannah S. Llames; John Q. Wong; Joshua Gregor A. Dizon; Juan Antonio R. Magalang; Karol Sophia Agape R. Padilla; Kenneth M. Kim; Kris P. Punayan; Krisitna Patriz Dela Cruz; Kristina Patriz Dela Cruz; Lindsay Claire D.L. Carandang; Lindsay Clare D.L. Carandang; Ma. Exanil Plantig; Marc Edsel C. Ayes; Marc Jerrone R. Castro; Maria Rosario Singh-Vergeire; Maria Rosario Singh-Vergeire and Cynthia P. Saloma; Maria Sofia L. Yangzon; Marielle M Gamboa; Marielle M. Gamboa; Marissa Alejandria; Nina Francesca Bustamante; Niña Francesca Bustamante; Razel Nikka M. Hao; Renato Jacinto Q. Mantaring; Rianna Patricia S. Cruz; Sheila Mae M. Araiza; Shiela Mae M. Araiza; Yvonne Valerie Austria; Zipporah Mariebelle R. Enriquez; Zyrel V. Mollejon
EPI_ISL_1069184	Ospital ng Lipa	Research Institute for Tropical Medicine	Catalino Demetria; Daria Manalo; Edelwisa Mercado; Francisco Gerardo Polotan; Hannah Leah Morito; Inez Andrea Medado; John Leonard Chan; Joseph Hughes; Kirstyn Brunker; Ma Angelica Tujan; Othoniel Jan Onza

EPI_ISL_3105873, EPI_ISL_3105874, EPI_ISL_3105884	Ospital ng Makati	Research Institute for Tropical Medicine	Clyde Dapat; Deana Mae Ocampo; Emmanuel Kagning Tsinda; Francisco Gerardo Polotan; Hitoshi Oshitani; Inez Andrea Medado; Jefferson Earl Halog; Joana Ina Manalo; Lei Lanna Dancel; Ma Angelica Tujan; Mariko Saito-Obata; Mayuko Saito; Michiko Okamoto; Samantha Louise Bado
EPI_ISL_2171150, EPI_ISL_2171162, EPI_ISL_2171170, EPI_ISL_2171183, EPI_ISL_2188067, EPI_ISL_2188106, EPI_ISL_2188117, EPI_ISL_2188191, EPI_ISL_2188707, EPI_ISL_2189058, EPI_ISL_2189295			
see above	Ospital ng ParaÃ±aque II	Philippine Genome Center	Alethea R. de Guzman; Anna Ong-Lim; Arianne A. Zamora; Asia Louisa U. Chong; Benedict A. Maralit; Candice Francheska B. Tambaoan; Carlo M. Lapid; Celia Carlos; Devon Ray Pacial; Edsel Maurice Salvaña; El King D. Morado; Elcid Aaron R. Pangilinan; Eva Maria Cutiongco-de la Paz; Francis A. Tablizo; Irish Coleen A. Asin; Jaime C. Montoya; Jan Michael C. Yap; Jo-Hannah S. Llames; John Q. Wong; Joshua Gregor A. Dizon; Juan Antonio R. Magalang; Karol Sophia Agape R. Padilla; Kenneth M. Kim; Kris P. Punayan; Marc Edsel C. Ayes; Marc Jerrone R. Castro; Maria Rosario Singh-Vergeire and Cynthia P. Saloma; Maria Sofia L. Yangzon; Marissa Alejandria; Razel Nikka M. Hao; Renato Jacinto Q. Mantaring; Rianna Patricia S. Cruz; Sheila Mae M. Araiza
EPI_ISL_4459440	PALUAN COMMUNITY HOSPITAL	Research Institute for Tropical Medicine/Tohoku University	Allen Anthony Laraño; Ardiane Ysabelle Dolor; Catalino Demetria; Clyde Dapat; Emmanuel Kagning Tsinda; Francisco Gerardo Polotan; Hitoshi Oshitani; Inez Andrea Medado; Jefferson Earl Halog; Lei Lanna Dancel; Leilanie Nacion; Ma. Angelica Tujan; Machiko Okamoto; Maria Carmen Corpuz; Mariko Siato-Obata; Mayuko Saito
EPI_ISL_2171219	PARKWAY MEDICAL AND DIAGNOSTIC CENTER	Philippine Genome Center	Alethea R. de Guzman; Anna Ong-Lim; Arianne A. Zamora; Asia Louisa U. Chong; Benedict A. Maralit; Candice Francheska B. Tambaoan; Carlo M. Lapid; Celia Carlos; Devon Ray Pacial; Edsel Maurice Salvaña; El King D. Morado; Elcid Aaron R. Pangilinan; Eva Maria Cutiongco-de la Paz; Francis A. Tablizo; Irish Coleen A. Asin; Jaime C. Montoya; Jan Michael C. Yap; Jo-Hannah S. Llames; John Q. Wong; Joshua Gregor A. Dizon; Juan Antonio R. Magalang; Karol Sophia Agape R. Padilla; Kenneth M. Kim; Kris P. Punayan; Marc Edsel C. Ayes; Marc Jerrone R. Castro; Maria Rosario Singh-Vergeire and Cynthia P. Saloma; Maria Sofia L. Yangzon; Marissa Alejandria; Razel Nikka M. Hao; Rianna Patricia S. Cruz; Sheila Mae M. Araiza
EPI_ISL_2188100, EPI_ISL_2188124, EPI_ISL_2188140, EPI_ISL_2188158, EPI_ISL_2188220, EPI_ISL_2188703, EPI_ISL_2188776, EPI_ISL_2188796, EPI_ISL_2188804			
see above	PASIG CITY CHILDREN'S HOSPITAL	Philippine Genome Center	Alethea R. de Guzman; Anna Ong-Lim; Arianne A. Zamora; Asia Louisa U. Chong; Benedict A. Maralit; Candice Francheska B. Tambaoan; Carlo M. Lapid; Celia Carlos; Devon Ray Pacial; Edsel Maurice Salvaña; El King D. Morado; Elcid Aaron R. Pangilinan; Eva Maria Cutiongco-de la Paz; Francis A. Tablizo; Irish Coleen A. Asin; Jaime C. Montoya; Jan Michael C. Yap; Jo-Hannah S. Llames; John Q. Wong; Joshua Gregor A. Dizon; Juan Antonio R. Magalang; Karol Sophia Agape R. Padilla; Kenneth M. Kim; Kris P. Punayan; Marc Edsel C. Ayes; Marc Jerrone R. Castro; Maria Rosario Singh-Vergeire and Cynthia P. Saloma; Maria Sofia L. Yangzon; Marissa Alejandria; Razel Nikka M. Hao; Renato Jacinto Q. Mantaring; Rianna Patricia S. Cruz; Sheila Mae M. Araiza
EPI_ISL_2171124	PCR MED Molecular Laboratory	Philippine Genome Center	Alethea R. de Guzman; Anna Ong-Lim; Arianne A. Zamora; Asia Louisa U. Chong; Benedict A. Maralit; Candice Francheska B. Tambaoan; Carlo M. Lapid; Celia Carlos; Devon Ray Pacial; Edsel Maurice Salvaña; El King D. Morado; Elcid Aaron R. Pangilinan; Eva Maria Cutiongco-de la Paz; Francis A. Tablizo; Irish Coleen A. Asin; Jaime C. Montoya; Jan Michael C. Yap; Jo-Hannah S. Llames; John Q. Wong; Joshua Gregor A. Dizon; Juan Antonio R. Magalang; Karol Sophia Agape R. Padilla; Kenneth M. Kim; Kris P. Punayan; Marc Edsel C. Ayes; Marc Jerrone R. Castro; Maria Rosario Singh-Vergeire and Cynthia P. Saloma; Maria Sofia L. Yangzon; Marissa Alejandria; Razel Nikka M. Hao; Rianna Patricia S. Cruz; Sheila Mae M. Araiza
EPI_ISL_1213574, EPI_ISL_1213576, EPI_ISL_2105474, EPI_ISL_2105478, EPI_ISL_2155941, EPI_ISL_2155942, EPI_ISL_2155943, EPI_ISL_2155944, EPI_ISL_2155945, EPI_ISL_2155946, EPI_ISL_2155947, EPI_ISL_2155948, EPI_ISL_2155949, EPI_ISL_2155950, EPI_ISL_2155951, EPI_ISL_2155952, EPI_ISL_2155953, EPI_ISL_2155954, EPI_ISL_2155955, EPI_ISL_2155956, EPI_ISL_2155957, EPI_ISL_2155958, EPI_ISL_2155959, EPI_ISL_2155960, EPI_ISL_2155961, EPI_ISL_2155962, EPI_ISL_2155963, EPI_ISL_2156284, EPI_ISL_2156300, EPI_ISL_2156316, EPI_ISL_2156317, EPI_ISL_2156328			
see above	PHILIPPINE AIRPORT DIAGNOSTIC LABORATORY	Philippine Genome Center	Alethea R. de Guzman; Anna Ong-Lim; Arianne A. Zamora; Asia Louisa U. Chong; Benedict A. Maralit; Candice Francheska B. Tambaoan; Carlo M. Lapid; Celia Carlos; Devon Ray Pacial; Edsel Maurice Salvaña; El King D. Morado; Elcid Aaron R. Pangilinan; Eva Maria Cutiongco-de la Paz; Francis A. Tablizo; Irish Coleen A. Asin; Jaime C. Montoya; Jan Michael C. Yap; Jo-Hannah S. Llames; John Q. Wong; Joshua Gregor A. Dizon; Juan Antonio R. Magalang; Karol Sophia Agape R. Padilla; Kenneth M. Kim; Kris P. Punayan; Marc Edsel C. Ayes; Marc Jerrone R. Castro; Maria Rosario Singh-Vergeire and Cynthia P. Saloma; Maria Sofia L. Yangzon; Marissa Alejandria; Razel Nikka M. Hao; Rianna Patricia S. Cruz; Sheila Mae M. Araiza
EPI_ISL_2188129	PNP General Hospital	Philippine Genome Center	Alethea R. de Guzman; Anna Ong-Lim; Arianne A. Zamora; Asia Louisa U. Chong; Benedict A. Maralit; Candice Francheska B. Tambaoan; Carlo M. Lapid; Celia Carlos; Devon Ray Pacial; Edsel Maurice Salvaña; El King D. Morado; Elcid Aaron R. Pangilinan; Eva Maria Cutiongco-de la Paz; Francis A. Tablizo; Irish Coleen A. Asin; Jaime C. Montoya; Jan Michael C. Yap; Jo-Hannah S. Llames; John Q. Wong; Joshua Gregor A. Dizon; Juan Antonio R. Magalang; Karol Sophia Agape R. Padilla; Kenneth M. Kim; Kris P. Punayan; Marc Edsel C. Ayes; Marc Jerrone R. Castro; Maria Rosario Singh-Vergeire and Cynthia P. Saloma; Maria Sofia L. Yangzon; Marissa Alejandria; Razel Nikka M. Hao; Rianna Patricia S. Cruz; Sheila Mae M. Araiza
EPI_ISL_4712048, EPI_ISL_4712127, EPI_ISL_4712153, EPI_ISL_4712186, EPI_ISL_4712200, EPI_ISL_4712391, EPI_ISL_4712572, EPI_ISL_4712640, EPI_ISL_4712766, EPI_ISL_4712875			
see above	PRC SUBIC MOLECULAR LABORATORY	Philippine Genome Center	Alethea R. de Guzman; Arianne A. Zamora; Benedict A. Maralit; Carlo M. Lapid; Celia Carlos; Cynthia P. Saloma; Devon Ray Pacial; Diomedes A. Cariño; Edsel Maurice Salvana; El King D. Morado; Elcid Aaron R. Pangilinan; Eva Maria Cutiongco-de la Paz; Francis A. Tablizo; Henrietta Marie Rodriguez; Jaime C. Montoya; Jan Michael C. Yap; Jarvin E. Nipales; Jo-Hannah S. Llames; John Q. Wong; Joshua Gregor A. Dizon; Juan Antonio R. Magalang; Karol Sophia Agape R. Padilla; Kenneth M. Kim; Kris P. Punayan; Kristina Patriz Dela Cruz; Lindsay Clare D.L. Carandang; Ma. Exanil Plantig; Marc Edsel C. Ayes; Maria Rosario Singh-Vergeire; Maria Sofia L. Yangzon; Marielle M. Gamboa; Marissa Alejandria; Niña Francesca Bustamante; Razel Nikka M. Hao; Renato Jacinto Q. Mantaring; Rianna Patricia S. Cruz; Shiela Mae M. Araiza; Yvonne Valerie Austria; Zipporah Mariebelle R. Enriquez; Zyrel V. Mollejon
EPI_ISL_1081821	PRIME CARE ALPHA COVID-19 TESTING LABORATORY	Philippine Genome Center	Alethea R. de Guzman; Anna Ong-Lim; Arianne A. Zamora; Asia Louisa U. Chong; Benedict A. Maralit; Candice Francheska B. Tambaoan; Carlo M. Lapid; Celia Carlos; Cynthia P. Saloma; Devon Ray Pacial; Edsel Maurice Salvaña; El King D. Morado; Eva Maria Cutiongco-de la Paz; Francis A. Tablizo; Irish Coleen A. Asin; Jaime C. Montoya; Jan Michael C. Yap; Jo-Hannah S. Llames; John Q. Wong; Joshua Gregor A. Dizon; Juan Antonio R. Magalang; Karol Sophia Agape R. Padilla; Kenneth M. Kim; Kris P. Punayan; Marc Edsel C. Ayes; Marc Jerrone R. Castro; Maria Sofia L. Yangzon; Marissa Alejandria; Razel Nikka M. Hao; Rianna Patricia S. Cruz; Sheila Mae M. Araiza; and Maria Rosario Singh-Vergeire
EPI_ISL_2105557	PROVIDERS MULTI-PURPOSE COOPERATIVE MEDICAL CENTER	Philippine Genome Center	Alethea R. de Guzman; Anna Ong-Lim; Arianne A. Zamora; Asia Louisa U. Chong; Benedict A. Maralit; Candice Francheska B. Tambaoan; Carlo M. Lapid; Celia Carlos; Devon Ray Pacial; Edsel Maurice Salvaña; El King D. Morado; Eva Maria Cutiongco-de la Paz; Francis A. Tablizo; Irish Coleen A. Asin; Jaime C. Montoya; Jan Michael C. Yap; Jo-Hannah S. Llames; John Q. Wong; Joshua Gregor A. Dizon; Juan Antonio R. Magalang; Karol Sophia Agape R. Padilla; Kenneth M. Kim; Kris P. Punayan; Marc Edsel C. Ayes; Marc Jerrone R. Castro; Maria Rosario Singh-Vergeire and Cynthia P. Saloma; Maria Sofia L. Yangzon; Marissa Alejandria; Razel Nikka M. Hao; Rianna Patricia S. Cruz; Sheila Mae M. Araiza
EPI_ISL_1069181	Pagbilao Rural Health Unit	Research Institute for Tropical Medicine	Catalino Demetria; Daria Manalo; Edelwisa Mercado; Francisco Gerardo Polotan; Hannah Leah Morito; Inez Andrea Medado; John Leonard Chan; Joseph Hughes; Kirstyn Brunker; Ma Angelica Tujan; Othoniel Jan Onza
EPI_ISL_1069175	Pangil Rural Health Unit	Research Institute for Tropical Medicine	Catalino Demetria; Daria Manalo; Edelwisa Mercado; Francisco Gerardo Polotan; Hannah Leah Morito; Inez Andrea Medado; John Leonard Chan; Joseph Hughes; Kirstyn Brunker; Ma Angelica Tujan; Othoniel Jan Onza
EPI_ISL_2154984, EPI_ISL_2154995, EPI_ISL_2155009, EPI_ISL_2155211, EPI_ISL_2155269, EPI_ISL_2155330, EPI_ISL_2155415, EPI_ISL_2155416, EPI_ISL_2155417, EPI_ISL_2155418, EPI_ISL_2155419, EPI_ISL_2155420, EPI_ISL_2155421, EPI_ISL_2155422, EPI_ISL_2155423, EPI_ISL_2156110, EPI_ISL_2156111, EPI_ISL_2156112, EPI_ISL_2156331, EPI_ISL_2156388, EPI_ISL_2156456, EPI_ISL_2156526, EPI_ISL_2156659, EPI_ISL_2188237, EPI_ISL_2188715, EPI_ISL_2188749, EPI_ISL_2188768, EPI_ISL_2188780, EPI_ISL_2188786, EPI_ISL_2188820, EPI_ISL_2188962, EPI_ISL_2188967, EPI_ISL_2189021, EPI_ISL_2189082, EPI_ISL_2189328, EPI_ISL_2189382, EPI_ISL_2189484, EPI_ISL_2189492, EPI_ISL_2859166, EPI_ISL_2859516, EPI_ISL_2859551, EPI_ISL_2859556, EPI_ISL_2859595, EPI_ISL_2859676, EPI_ISL_2860001, EPI_ISL_4712411, EPI_ISL_4712472, EPI_ISL_4712588, EPI_ISL_4712653, EPI_ISL_4712773, EPI_ISL_4712864, EPI_ISL_4712997, EPI_ISL_4713818, EPI_ISL_4713854, EPI_ISL_4713905, EPI_ISL_4714153, EPI_ISL_4714226, EPI_ISL_4714700, EPI_ISL_4714879, EPI_ISL_4715066, EPI_ISL_4715141, EPI_ISL_4715958, EPI_ISL_4716211			
see above	Parkway Medical and Diagnostic Center	Philippine Genome Center	Alethea R. de Guzman; Anna Ong-Lim; Arianne A. Zamora; Asia Louisa U. Chong; Benedict A. Maralit; Candice Francheska B. Tambaoan; Carlo M. Lapid; Celia Carlos; Cynthia P. Saloma; Devon Ray Pacial; Diomedes A. Carino; Diomedes A. Cariño; Edsel Maurice Salvana; Edsel Maurice Salvaña; El King D. Morado; Elcid Aaron R. Pangilinan; Eva Maria Cutiongco-de la Paz; Francis A. Tablizo; Henrietta Marie Rodriguez; Irish Coleen A. Asin; Jaime C. Montoya; Jan Michael C. Yap; Jarvin E. Nipales; Jo-Hannah S. Llames; John Q. Wong; Joshua Gregor A. Dizon; Juan Antonio R. Magalang; Karol Sophia Agape R. Padilla; Kenneth M. Kim; Kris P. Punayan; Krisitna Patriz Dela Cruz; Kristina Patriz Dela Cruz; Lindsay Claire D.L. Carandang; Lindsay Clare D.L. Carandang; Ma. Exanil Plantig; Marc Edsel C. Ayes; Marc Jerrone R. Castro; Maria Rosario Singh-Vergeire; Maria Rosario Singh-Vergeire and Cynthia P. Saloma; Maria Sofia L. Yangzon; Marielle M Gamboa; Marielle M. Gamboa; Marissa Alejandria; Nina Francesca Bustamante; Niña Francesca Bustamante; Razel Nikka M. Hao; Renato Jacinto Q. Mantaring; Rianna Patricia S. Cruz; Sheila Mae M. Araiza; Shiela Mae M. Araiza; Yvonne Valerie Austria; Zipporah Mariebelle R. Enriquez; Zyrel V. Mollejon
EPI_ISL_1081822	Parkway Medical and Diagnotic Center	Philippine Genome Center	Alethea R. de Guzman; Anna Ong-Lim; Arianne A. Zamora; Asia Louisa U. Chong; Benedict A. Maralit; Candice Francheska B. Tambaoan; Carlo M. Lapid; Celia Carlos; Cynthia P. Saloma; Devon Ray Pacial; Edsel Maurice Salvaña; El King D. Morado; Eva Maria Cutiongco-de la Paz; Francis A. Tablizo; Irish Coleen A. Asin; Jaime C. Montoya; Jan Michael C. Yap; Jo-Hannah S. Llames; John Q. Wong; Joshua Gregor A. Dizon; Juan Antonio R. Magalang; Karol Sophia Agape R. Padilla; Kenneth M. Kim; Kris P. Punayan; Marc Edsel C. Ayes; Marc Jerrone R. Castro; Maria Sofia L. Yangzon; Marissa Alejandria; Razel Nikka M. Hao; Rianna Patricia S. Cruz; Sheila Mae M. Araiza; and Maria Rosario Singh-Vergeire
EPI_ISL_2155058, EPI_ISL_2155169, EPI_ISL_2155187, EPI_ISL_2155308, EPI_ISL_2155397, EPI_ISL_2155398, EPI_ISL_2155399, EPI_ISL_2155400, EPI_ISL_2155401, EPI_ISL_2155402, EPI_ISL_2155403, EPI_ISL_2155404, EPI_ISL_2155883, EPI_ISL_2155884, EPI_ISL_2155885, EPI_ISL_2155886, EPI_ISL_2155887, EPI_ISL_2155888, EPI_ISL_2156109, EPI_ISL_2156364, EPI_ISL_2156412, EPI_ISL_2171094, EPI_ISL_2171095, EPI_ISL_2171106, EPI_ISL_2171131, EPI_ISL_2171145, EPI_ISL_2171158, EPI_ISL_2171165, EPI_ISL_2171168, EPI_ISL_2171180, EPI_ISL_2188874, EPI_ISL_2188877, EPI_ISL_2188907, EPI_ISL_2188925, EPI_ISL_2188928, EPI_ISL_2188931, EPI_ISL_2189015, EPI_ISL_2189017, EPI_ISL_2189034, EPI_ISL_2189050, EPI_ISL_2189080, EPI_ISL_2189084, EPI_ISL_2189104, EPI_ISL_2189148, EPI_ISL_2189180, EPI_ISL_2189184, EPI_ISL_2189197, EPI_ISL_2189220, EPI_ISL_2189224, EPI_ISL_2189225, EPI_ISL_2189232, EPI_ISL_2189234, EPI_ISL_2189245, EPI_ISL_2189251, EPI_ISL_2189263, EPI_ISL_2189264, EPI_ISL_2189286, EPI_ISL_2189288, EPI_ISL_2189290, EPI_ISL_2189337, EPI_ISL_2189344, EPI_ISL_2189370, EPI_ISL_2189381, EPI_ISL_2189383, EPI_ISL_2189385, EPI_ISL_2189416, EPI_ISL_2189432, EPI_ISL_2189437, EPI_ISL_2189451, EPI_ISL_2189452, EPI_ISL_2189466, EPI_ISL_2189468, EPI_ISL_2189499, EPI_ISL_2189505, EPI_ISL_2189512, EPI_ISL_2189515, EPI_ISL_2189517, EPI_ISL_2189526, EPI_ISL_4712229, EPI_ISL_4712295, EPI_ISL_4712360, EPI_ISL_4712384, EPI_ISL_4712451, EPI_ISL_4712464, EPI_ISL_4712480, EPI_ISL_4712494, EPI_ISL_4712513, EPI_ISL_4712597, EPI_ISL_4712610, EPI_ISL_4712631, EPI_ISL_4712656, EPI_ISL_4712695, EPI_ISL_4712717, EPI_ISL_4712761, EPI_ISL_4712783, EPI_ISL_4712813, EPI_ISL_4712829, EPI_ISL_4712952, EPI_ISL_4712971, EPI_ISL_4712977, EPI_ISL_4712981, EPI_ISL_4713009, EPI_ISL_4713021, EPI_ISL_4713027, EPI_ISL_4713044, EPI_ISL_4713077, EPI_ISL_4713107, EPI_ISL_4713146, EPI_ISL_4713179, EPI_ISL_4713193, EPI_ISL_4713210, EPI_ISL_4713216, EPI_ISL_4713243, EPI_ISL_4713255, EPI_ISL_4713290, EPI_ISL_4713292, EPI_ISL_4713338, EPI_ISL_4713380, EPI_ISL_4713395, EPI_ISL_4713448, EPI_ISL_4713455, EPI_ISL_4713523, EPI_ISL_4713539, EPI_ISL_4713541, EPI_ISL_4713561, EPI_ISL_4713601, EPI_ISL_4713656, EPI_ISL_4713658, EPI_ISL_4713668, EPI_ISL_4713679			
see above	Pasig City Children's Hospital - Child's Hope	Philippine Genome Center	Alethea R. de Guzman; Anna Ong-Lim; Arianne A. Zamora; Asia Louisa U. Chong; Benedict A. Maralit; Candice Francheska B. Tambaoan; Carlo M. Lapid; Celia Carlos; Cynthia P. Saloma; Devon Ray Pacial; Diomedes A. Cariño; Edsel Maurice Salvana; Edsel Maurice Salvaña; El King D. Morado; Elcid Aaron R. Pangilinan; Eva Maria Cutiongco-de la Paz; Francis A. Tablizo; Henrietta Marie Rodriguez; Irish Coleen A. Asin; Jaime C. Montoya; Jan Michael C. Yap; Jarvin E. Nipales; Jo-Hannah S. Llames; John Q. Wong; Joshua Gregor A. Dizon; Juan Antonio R. Magalang; Karol Sophia Agape R. Padilla; Kenneth M. Kim; Kris P. Punayan; Kristina Patriz Dela Cruz; Lindsay Clare D.L. Carandang; Ma. Exanil Plantig; Marc Edsel C. Ayes; Marc Jerrone R. Castro; Maria Rosario Singh-Vergeire; Maria Rosario Singh-Vergeire and Cynthia P. Saloma; Maria Sofia L. Yangzon; Marielle M. Gamboa; Marissa Alejandria; Niña Francesca Bustamante; Razel Nikka M. Hao; Renato Jacinto Q. Mantaring; Rianna Patricia S. Cruz; Sheila Mae M. Araiza; Shiela Mae M. Araiza; Yvonne Valerie Austria; Zipporah Mariebelle R. Enriquez; Zyrel V. Mollejon
EPI_ISL_430845	Pasig City General Hospital	Research Institute for Tropical Medicine	Bautista; Brunker, K.; C.S.; C.T.; D.L.; Demetria; E.S.; F.G.M.; I.A.P.; Manalo; Medado; Mercado; O.J.T.; Onza; Polotan
EPI_ISL_1081820, EPI_ISL_2105229, EPI_ISL_2105562, EPI_ISL_2156156, EPI_ISL_2156157, EPI_ISL_2156158, EPI_ISL_2156159, EPI_ISL_2156160, EPI_ISL_2156161, EPI_ISL_2156162, EPI_ISL_2156163, EPI_ISL_2156164, EPI_ISL_2156165, EPI_ISL_2156490, EPI_ISL_2156532, EPI_ISL_2156536, EPI_ISL_2156541			
see above	Philippine Airport Diagnostic Laboratory	Philippine Genome Center	Alethea R. de Guzman; Anna Ong-Lim; Arianne A. Zamora; Asia Louisa U. Chong; Benedict A. Maralit; Candice Francheska B. Tambaoan; Carlo M. Lapid; Celia Carlos; Cynthia P. Saloma; Devon Ray Pacial; Edsel Maurice Salvaña; El King D. Morado; Elcid Aaron R. Pangilinan; Eva Maria Cutiongco-de la Paz; Francis A. Tablizo; Irish Coleen A. Asin; Jaime C. Montoya; Jan Michael C. Yap; Jo-Hannah S. Llames; John Q. Wong; Joshua Gregor A. Dizon; Juan Antonio R. Magalang; Karol Sophia Agape R. Padilla; Kenneth M. Kim; Kris P. Punayan; Marc Edsel C. Ayes; Marc Jerrone R. Castro; Maria Rosario Singh-Vergeire and Cynthia P. Saloma; Maria Sofia L. Yangzon; Marissa Alejandria; Razel Nikka M. Hao; Rianna Patricia S. Cruz; Sheila Mae M. Araiza; and Maria Rosario Singh-Vergeire
EPI_ISL_2107795, EPI_ISL_2155091, EPI_ISL_2155155, EPI_ISL_2155213, EPI_ISL_2155259, EPI_ISL_2155262, EPI_ISL_2155292, EPI_ISL_2155302, EPI_ISL_2155325, EPI_ISL_2155329, EPI_ISL_2155348, EPI_ISL_2156749			
see above	Philippine Children's Medical Center	Philippine Genome Center	Alethea R. de Guzman; Anna Ong-Lim; Arianne A. Zamora; Asia Louisa U. Chong; Benedict A. Maralit; Candice Francheska B. Tambaoan; Carlo M. Lapid; Celia Carlos; Devon Ray Pacial; Edsel Maurice Salvaña; El King D. Morado; Elcid Aaron R. Pangilinan; Eva Maria Cutiongco-de la Paz; Francis A. Tablizo; Irish Coleen A. Asin; Jaime C. Montoya; Jan Michael C. Yap; Jo-Hannah S. Llames; John Q. Wong; Joshua Gregor A. Dizon; Juan Antonio R. Magalang; Karol Sophia Agape R. Padilla; Kenneth M. Kim; Kris P. Punayan; Marc Edsel C. Ayes; Marc Jerrone R. Castro; Maria Rosario Singh-Vergeire and Cynthia P. Saloma; Maria Sofia L. Yangzon; Marissa Alejandria; Razel Nikka M. Hao; Rianna Patricia S. Cruz; Sheila Mae M. Araiza
EPI_ISL_2155790, EPI_ISL_2155791, EPI_ISL_2155792, EPI_ISL_2155793, EPI_ISL_2155794, EPI_ISL_2155795, EPI_ISL_2155796, EPI_ISL_2156021, EPI_ISL_2156248, EPI_ISL_2156496, EPI_ISL_2156503, EPI_ISL_2171243, EPI_ISL_2171244, EPI_ISL_2171245, EPI_ISL_2171246, EPI_ISL_2171247, EPI_ISL_2171248, EPI_ISL_2171249, EPI_ISL_2171250, EPI_ISL_2171251, EPI_ISL_2171252, EPI_ISL_2171253, EPI_ISL_2171255, EPI_ISL_2171256, EPI_ISL_2171257, EPI_ISL_2171258, EPI_ISL_2171259, EPI_ISL_2171260, EPI_ISL_2171261, EPI_ISL_2171262, EPI_ISL_2171263, EPI_ISL_2171264, EPI_ISL_2188887, EPI_ISL_2188939, EPI_ISL_2188950, EPI_ISL_2188984, EPI_ISL_2189022, EPI_ISL_2189092, EPI_ISL_2189116, EPI_ISL_2189268, EPI_ISL_2189347, EPI_ISL_2189357, EPI_ISL_2189361, EPI_ISL_2189363, EPI_ISL_2189376, EPI_ISL_2189395, EPI_ISL_2189430, EPI_ISL_2189519, EPI_ISL_2859142, EPI_ISL_2859160, EPI_ISL_2859161, EPI_ISL_2859170, EPI_ISL_2859172, EPI_ISL_2859179, EPI_ISL_2859182, EPI_ISL_2859191, EPI_ISL_2859219, EPI_ISL_2859222, EPI_ISL_2859237, EPI_ISL_2859238, EPI_ISL_2859249, EPI_ISL_2859250, EPI_ISL_2859258, EPI_ISL_2859262, EPI_ISL_2859267, EPI_ISL_2859277, EPI_ISL_2859284, EPI_ISL_2859303, EPI_ISL_2859316, EPI_ISL_2859320, EPI_ISL_2859329, EPI_ISL_2859337, EPI_ISL_2859338, EPI_ISL_2859342, EPI_ISL_2859354, EPI_ISL_2859360, EPI_ISL_2859695, EPI_ISL_2859779, EPI_ISL_2859891, EPI_ISL_2859898, EPI_ISL_2860036, EPI_ISL_2860045, EPI_ISL_2860227, EPI_ISL_2860247, EPI_ISL_4712822, EPI_ISL_4713720, EPI_ISL_4715244, EPI_ISL_4716632, EPI_ISL_4717276			
see above	Philippine General Hospital (PGH)	Philippine Genome Center	Alethea R. de Guzman; Anna Ong-Lim; Arianne A. Zamora; Asia Louisa U. Chong; Benedict A. Maralit; Candice Francheska B. Tambaoan; Carlo M. Lapid; Celia Carlos; Cynthia P. Saloma; Devon Ray Pacial; Diomedes A. Carino; Diomedes A. Cariño; Edsel Maurice Salvana; Edsel Maurice Salvaña; El King D. Morado; Elcid Aaron R. Pangilinan; Eva Maria Cutiongco-de la Paz; Francis A. Tablizo; Henrietta Marie Rodriguez; Irish Coleen A. Asin; Jaime C. Montoya; Jan Michael C. Yap; Jarvin E. Nipales; Jo-Hannah S. Llames; John Q. Wong; Joshua Gregor A. Dizon; Juan Antonio R. Magalang; Karol Sophia Agape R. Padilla; Kenneth M. Kim; Kris P. Punayan; Krisitna Patriz Dela Cruz; Kristina Patriz Dela Cruz; Lindsay Claire D.L. Carandang; Lindsay Clare D.L. Carandang; Ma. Exanil Plantig; Marc Edsel C. Ayes; Marc Jerrone R. Castro; Maria Rosario Singh-Vergeire; Maria Rosario Singh-Vergeire and Cynthia P. Saloma; Maria Sofia L. Yangzon; Marielle M Gamboa; Marielle M. Gamboa; Marissa Alejandria; Nina Francesca Bustamante; Niña Francesca Bustamante; Razel Nikka M. Hao; Renato Jacinto Q. Mantaring; Rianna Patricia S. Cruz; Sheila Mae M. Araiza; Shiela Mae M. Araiza; Yvonne Valerie Austria; Zipporah Mariebelle R. Enriquez; Zyrel V. Mollejon
EPI_ISL_2105514, EPI_ISL_2105515, EPI_ISL_2153812, EPI_ISL_2153818, EPI_ISL_2153821, EPI_ISL_2153822, EPI_ISL_2153823, EPI_ISL_2153827, EPI_ISL_2153829, EPI_ISL_2153830, EPI_ISL_2153832, EPI_ISL_2153834, EPI_ISL_2153841, EPI_ISL_2153843, EPI_ISL_2153844, EPI_ISL_2153845, EPI_ISL_2153848, EPI_ISL_2153852, EPI_ISL_2153853, EPI_ISL_2153854, EPI_ISL_2153859, EPI_ISL_2153861, EPI_ISL_2153862, EPI_ISL_2153866, EPI_ISL_2153867, EPI_ISL_2153868, EPI_ISL_2153870, EPI_ISL_2153872, EPI_ISL_2153874, EPI_ISL_2153879, EPI_ISL_2153880, EPI_ISL_2153884, EPI_ISL_2153886, EPI_ISL_2153889, EPI_ISL_2153898, EPI_ISL_2153900, EPI_ISL_2153904, EPI_ISL_2153905, EPI_ISL_2153906, EPI_ISL_2153916, EPI_ISL_2153918, EPI_ISL_2153920, EPI_ISL_2153925, EPI_ISL_2153931, EPI_ISL_2153935, EPI_ISL_2153936, EPI_ISL_2153940, EPI_ISL_2153942, EPI_ISL_2153943, EPI_ISL_2153948, EPI_ISL_2153952, EPI_ISL_2153953, EPI_ISL_2153957, EPI_ISL_2153963, EPI_ISL_2153965, EPI_ISL_2153966, EPI_ISL_2153972, EPI_ISL_2153975, EPI_ISL_2153977, EPI_ISL_2153978, EPI_ISL_2153981, EPI_ISL_2153983, EPI_ISL_2153984, EPI_ISL_2153988, EPI_ISL_2153994, EPI_ISL_2154000, EPI_ISL_2154002, EPI_ISL_2154006, EPI_ISL_2154008, EPI_ISL_2154010, EPI_ISL_2154016, EPI_ISL_2154018, EPI_ISL_2154019, EPI_ISL_2154022, EPI_ISL_2154024, EPI_ISL_2154026, EPI_ISL_2154044, EPI_ISL_2154046, EPI_ISL_2154048, EPI_ISL_2154050			
see above	Philippine Genome Center - Biobank (NCR)	Philippine Genome Center	Alethea R. de Guzman; Anna Ong-Lim; Arianne A. Zamora; Asia Louisa U. Chong; Benedict A. Maralit; Candice Francheska B. Tambaoan; Carlo M. Lapid; Celia Carlos; Devon Ray Pacial; Edsel Maurice Salvaña; El King D. Morado; Eva Maria Cutiongco-de la Paz; Francis A. Tablizo; Irish Coleen A. Asin; Jaime C. Montoya; Jan Michael C. Yap; Jo-Hannah S. Llames; John Q. Wong; Joshua Gregor A. Dizon; Juan Antonio R. Magalang; Karol Sophia Agape R. Padilla; Kenneth M. Kim; Kris P. Punayan; Marc Edsel C. Ayes; Marc Jerrone R. Castro; Maria Rosario Singh-Vergeire and Cynthia P. Saloma; Maria Sofia L. Yangzon; Marissa Alejandria; Razel Nikka M. Hao; Rianna Patricia S. Cruz; Sheila Mae M. Araiza
EPI_ISL_2105509, EPI_ISL_2105511, EPI_ISL_2105512, EPI_ISL_2105516, EPI_ISL_2105517, EPI_ISL_2105521, EPI_ISL_2153808, EPI_ISL_2153810, EPI_ISL_2153811, EPI_ISL_2153813, EPI_ISL_2153814, EPI_ISL_2153815, EPI_ISL_2153816, EPI_ISL_2153817, EPI_ISL_2153819, EPI_ISL_2153820, EPI_ISL_2153824, EPI_ISL_2153825, EPI_ISL_2153826, EPI_ISL_2153831, EPI_ISL_2153835, EPI_ISL_2153836, EPI_ISL_2153838, EPI_ISL_2153839, EPI_ISL_2153840, EPI_ISL_2153842, EPI_ISL_2153846, EPI_ISL_2153847, EPI_ISL_2153850, EPI_ISL_2153851, EPI_ISL_2153855, EPI_ISL_2153856, EPI_ISL_2153857, EPI_ISL_2153863, EPI_ISL_2153864, EPI_ISL_2153869, EPI_ISL_2153871, EPI_ISL_2153875, EPI_ISL_2153876, EPI_ISL_2153877, EPI_ISL_2153885, EPI_ISL_2153887, EPI_ISL_2153891, EPI_ISL_2153892, EPI_ISL_2153895, EPI_ISL_2153902, EPI_ISL_2153908, EPI_ISL_2153914, EPI_ISL_2153927, EPI_ISL_2153928, EPI_ISL_2153930, EPI_ISL_2153932, EPI_ISL_2153934, EPI_ISL_2153937, EPI_ISL_2153939, EPI_ISL_2153941, EPI_ISL_2153944, EPI_ISL_2153946, EPI_ISL_2153951, EPI_ISL_2153954, EPI_ISL_2153956, EPI_ISL_2153958, EPI_ISL_2153959, EPI_ISL_2153961, EPI_ISL_2153962, EPI_ISL_2153964, EPI_ISL_2153967, EPI_ISL_2153968, EPI_ISL_2153969, EPI_ISL_2153971, EPI_ISL_2153973, EPI_ISL_2153976, EPI_ISL_2153980, EPI_ISL_2153982, EPI_ISL_2153985, EPI_ISL_2153986, EPI_ISL_2153989, EPI_ISL_2153990, EPI_ISL_2153991, EPI_ISL_2153992, EPI_ISL_2153996, EPI_ISL_2153999, EPI_ISL_2154003, EPI_ISL_2154005, EPI_ISL_2154014, EPI_ISL_2154015, EPI_ISL_2154020, EPI_ISL_2154025, EPI_ISL_2154045, EPI_ISL_2154049, EPI_ISL_2154051			
see above	Philippine Genome Center - Biobank (Region	Philippine Genome Center	Alethea R. de Guzman; Anna Ong-Lim; Arianne A. Zamora; Asia Louisa U. Chong; Benedict A. Maralit; Candice Francheska B. Tambaoan; Carlo M. Lapid; Celia Carlos; Devon Ray Pacial; Edsel Maurice Salvaña; El King D. Morado; Eva Maria Cutiongco-de la Paz; Francis A. Tablizo; Irish Coleen A. Asin; Jaime C. Montoya; Jan Michael C. Yap; Jo-Hannah S. Llames; John Q. Wong; Joshua Gregor A. Dizon; Juan Antonio R. Magalang; Karol Sophia Agape R. Padilla; Kenneth M. Kim; Kris P. Punayan; Marc Edsel C. Ayes; Marc Jerrone R. Castro; Maria Rosario Singh-Vergeire and Cynthia P. Saloma; Maria Sofia L. Yangzon; Marissa Alejandria; Razel Nikka M.

	IV-A)		Hao; Rianna Patricia S. Cruz; Sheila Mae M. Araiza
EPI_ISL_2171212	Philippine Genome Center UP-Diliman (NHB)	Philippine Genome Center	Alethea R. de Guzman; Anna Ong-Lim; Arianne A. Zamora; Asia Louisa U. Chong; Benedict A. Maralit; Candice Francheska B. Tambaoan; Carlo M. Lapid; Celia Carlos; Devon Ray Pacial; Edsel Maurice Salvaña; El King D. Morado; Elcid Aaron R. Pangilinan; Eva Maria Cutiongco-de la Paz; Francis A. Tablizo; Irish Coleen A. Asin; Jaime C. Montoya; Jan Michael C. Yap; Jo-Hannah S. Llames; John Q. Wong; Joshua Gregor A. Dizon; Juan Antonio R. Magalang; Karol Sophia Agape R. Padilla; Kenneth M. Kim; Kris P. Punayan; Marc Edsel C. Ayes; Marc Jerrone R. Castro; Maria Rosario Singh-Vergeire and Cynthia P. Saloma; Maria Sofia L. Yangzon; Marissa Alejandria; Razel Nikka M. Hao; Rianna Patricia S. Cruz; Sheila Mae M. Araiza
EPI_ISL_2156256, EPI_ISL_2156257	Philippine Genome Center UP-DilimanÂ¬â€ (QC)	Philippine Genome Center	Alethea R. de Guzman; Anna Ong-Lim; Arianne A. Zamora; Asia Louisa U. Chong; Benedict A. Maralit; Candice Francheska B. Tambaoan; Carlo M. Lapid; Celia Carlos; Devon Ray Pacial; Edsel Maurice Salvaña; El King D. Morado; Elcid Aaron R. Pangilinan; Eva Maria Cutiongco-de la Paz; Francis A. Tablizo; Irish Coleen A. Asin; Jaime C. Montoya; Jan Michael C. Yap; Jo-Hannah S. Llames; John Q. Wong; Joshua Gregor A. Dizon; Juan Antonio R. Magalang; Karol Sophia Agape R. Padilla; Kenneth M. Kim; Kris P. Punayan; Marc Edsel C. Ayes; Marc Jerrone R. Castro; Maria Rosario Singh-Vergeire and Cynthia P. Saloma; Maria Sofia L. Yangzon; Marissa Alejandria; Razel Nikka M. Hao; Rianna Patricia S. Cruz; Sheila Mae M. Araiza
EPI_ISL_2155405	Philippine Heart Center (PGC)	Philippine Genome Center	Alethea R. de Guzman; Anna Ong-Lim; Arianne A. Zamora; Asia Louisa U. Chong; Benedict A. Maralit; Candice Francheska B. Tambaoan; Carlo M. Lapid; Celia Carlos; Devon Ray Pacial; Edsel Maurice Salvaña; El King D. Morado; Eva Maria Cutiongco-de la Paz; Francis A. Tablizo; Irish Coleen A. Asin; Jaime C. Montoya; Jan Michael C. Yap; Jo-Hannah S. Llames; John Q. Wong; Joshua Gregor A. Dizon; Juan Antonio R. Magalang; Karol Sophia Agape R. Padilla; Kenneth M. Kim; Kris P. Punayan; Marc Edsel C. Ayes; Marc Jerrone R. Castro; Maria Rosario Singh-Vergeire and Cynthia P. Saloma; Maria Sofia L. Yangzon; Marissa Alejandria; Razel Nikka M. Hao; Rianna Patricia S. Cruz; Sheila Mae M. Araiza
EPI_ISL_2189117, EPI_ISL_2189440, EPI_ISL_2189454, EPI_ISL_4715852, EPI_ISL_4715930, EPI_ISL_4716551, EPI_ISL_4717128			
see above	Philippine National Police Crime Laboratory (PNP Crime Lab.)	Philippine Genome Center	Alethea R. de Guzman; Anna Ong-Lim; Arianne A. Zamora; Asia Louisa U. Chong; Benedict A. Maralit; Candice Francheska B. Tambaoan; Carlo M. Lapid; Celia Carlos; Cynthia P. Saloma; Devon Ray Pacial; Diomedes A. Cariño; Edsel Maurice Salvana; Edsel Maurice Salvaña; El King D. Morado; Elcid Aaron R. Pangilinan; Eva Maria Cutiongco-de la Paz; Francis A. Tablizo; Henrietta Marie Rodriguez; Irish Coleen A. Asin; Jaime C. Montoya; Jan Michael C. Yap; Jarvin E. Nipales; Jo-Hannah S. Llames; John Q. Wong; Joshua Gregor A. Dizon; Juan Antonio R. Magalang; Karol Sophia Agape R. Padilla; Kenneth M. Kim; Kris P. Punayan; Kristina Patriz Dela Cruz; Lindsay Clare D.L. Carandang; Ma. Exanil Plantig; Marc Edsel C. Ayes; Maria Rosario Singh-Vergeire; Maria Rosario Singh-Vergeire and Cynthia P. Saloma; Maria Sofia L. Yangzon; Marielle M. Gamboa; Marissa Alejandria; Niña Francesca Bustamante; Razel Nikka M. Hao; Renato Jacinto Q. Mantaring; Rianna Patricia S. Cruz; Sheila Mae M. Araiza; Shiela Mae M. Araiza; Yvonne Valerie Austria; Zipporah Mariebelle R. Enriquez; Zyrel V. Mollejon
EPI_ISL_1081793, EPI_ISL_1081794	Philippine Red Cross	Philippine Genome Center	Alethea R. de Guzman; Anna Ong-Lim; Arianne A. Zamora; Asia Louisa U. Chong; Benedict A. Maralit; Candice Francheska B. Tambaoan; Carlo M. Lapid; Celia Carlos; Cynthia P. Saloma; Devon Ray Pacial; Edsel Maurice Salvaña; El King D. Morado; Eva Maria Cutiongco-de la Paz; Francis A. Tablizo; Irish Coleen A. Asin; Jaime C. Montoya; Jan Michael C. Yap; Jo-Hannah S. Llames; John Q. Wong; Joshua Gregor A. Dizon; Juan Antonio R. Magalang; Karol Sophia Agape R. Padilla; Kenneth M. Kim; Kris P. Punayan; Marc Edsel C. Ayes; Marc Jerrone R. Castro; Maria Sofia L. Yangzon; Marissa Alejandria; Razel Nikka M. Hao; Rianna Patricia S. Cruz; Sheila Mae M. Araiza; and Maria Rosario Singh-Vergeire
EPI_ISL_1081831	Philippine Red Cross - Clark	Philippine Genome Center	Alethea R. de Guzman; Anna Ong-Lim; Arianne A. Zamora; Asia Louisa U. Chong; Benedict A. Maralit; Candice Francheska B. Tambaoan; Carlo M. Lapid; Celia Carlos; Cynthia P. Saloma; Devon Ray Pacial; Edsel Maurice Salvaña; El King D. Morado; Eva Maria Cutiongco-de la Paz; Francis A. Tablizo; Irish Coleen A. Asin; Jaime C. Montoya; Jan Michael C. Yap; Jo-Hannah S. Llames; John Q. Wong; Joshua Gregor A. Dizon; Juan Antonio R. Magalang; Karol Sophia Agape R. Padilla; Kenneth M. Kim; Kris P. Punayan; Marc Edsel C. Ayes; Marc Jerrone R. Castro; Maria Sofia L. Yangzon; Marissa Alejandria; Razel Nikka M. Hao; Rianna Patricia S. Cruz; Sheila Mae M. Araiza; and Maria Rosario Singh-Vergeire
EPI_ISL_2154450, EPI_ISL_2155231, EPI_ISL_2155676, EPI_ISL_2155677, EPI_ISL_2155979, EPI_ISL_2155980, EPI_ISL_2155981, EPI_ISL_2156201, EPI_ISL_2156325, EPI_ISL_2156338, EPI_ISL_2156339, EPI_ISL_2156350, EPI_ISL_2171198, EPI_ISL_2188902, EPI_ISL_2188961, EPI_ISL_2189059, EPI_ISL_2189091, EPI_ISL_2189135, EPI_ISL_2189250, EPI_ISL_2189277, EPI_ISL_2189366, EPI_ISL_2189464, EPI_ISL_2189476, EPI_ISL_2189490, EPI_ISL_2558052, EPI_ISL_2859187, EPI_ISL_2859701, EPI_ISL_2859730, EPI_ISL_2859733, EPI_ISL_2859965, EPI_ISL_2860018, EPI_ISL_2860038, EPI_ISL_2860185, EPI_ISL_2860191, EPI_ISL_4712637, EPI_ISL_4713798, EPI_ISL_4714272, EPI_ISL_4716189, EPI_ISL_4716283, EPI_ISL_4716704, EPI_ISL_4717361			
see above	Philippine Red Cross - Clark Molecular Laboratory	Philippine Genome Center	Alethea R. de Guzman; Anna Ong-Lim; Arianne A. Zamora; Asia Louisa U. Chong; Benedict A. Maralit; Candice Francheska B. Tambaoan; Carlo M. Lapid; Celia Carlos; Cynthia P. Saloma; Devon Ray Pacial; Diomedes A. Carino; Diomedes A. Cariño; Edsel Maurice Salvana; Edsel Maurice Salvaña; El King D. Morado; Elcid Aaron R. Pangilinan; Eva Maria Cutiongco-de la Paz; Francis A. Tablizo; Henrietta Marie Rodriguez; Irish Coleen A. Asin; Jaime C. Montoya; Jan Michael C. Yap; Jarvin E. Nipales; Jo-Hannah S. Llames; John Q. Wong; Joshua Gregor A. Dizon; Juan Antonio R. Magalang; Karol Sophia Agape R. Padilla; Kenneth M. Kim; Kris P. Punayan; Krisitna Patriz Dela Cruz; Kristina Patriz Dela Cruz; Lindsay Claire D.L. Carandang; Lindsay Clare D.L. Carandang; Ma. Exanil Plantig; Marc Edsel C. Ayes; Marc Jerrone R. Castro; Maria Rosario Singh-Vergeire; Maria Rosario Singh-Vergeire and Cynthia P. Saloma; Maria Sofia L. Yangzon; Marielle M Gamboa; Marielle M. Gamboa; Marissa Alejandria; Nina Francesca Bustamante; Niña Francesca Bustamante; Razel Nikka M. Hao; Renato Jacinto Q. Mantaring; Rianna Patricia S. Cruz; Sheila Mae M. Araiza; Shiela Mae M. Araiza; Yvonne Valerie Austria; Zipporah Mariebelle R. Enriquez; Zyrel V. Mollejon
EPI_ISL_2105405, EPI_ISL_2105420, EPI_ISL_2105432, EPI_ISL_2105446, EPI_ISL_2105459	Philippine Red Cross - Misamis Oriental	Philippine Genome Center	Alethea R. de Guzman; Anna Ong-Lim; Arianne A. Zamora; Asia Louisa U. Chong; Benedict A. Maralit; Candice Francheska B. Tambaoan; Carlo M. Lapid; Celia Carlos; Devon Ray Pacial; Edsel Maurice Salvaña; El King D. Morado; Eva Maria Cutiongco-de la Paz; Francis A. Tablizo; Irish Coleen A. Asin; Jaime C. Montoya; Jan Michael C. Yap; Jo-Hannah S. Llames; John Q. Wong; Joshua Gregor A. Dizon; Juan Antonio R. Magalang; Karol Sophia Agape R. Padilla; Kenneth M. Kim; Kris P. Punayan; Marc Edsel C. Ayes; Marc Jerrone R. Castro; Maria Rosario Singh-Vergeire and Cynthia P. Saloma; Maria Sofia L. Yangzon; Marissa Alejandria; Razel Nikka M. Hao; Rianna Patricia S. Cruz; Sheila Mae M. Araiza
EPI_ISL_2105472, EPI_ISL_2107793, EPI_ISL_2107798, EPI_ISL_2154968, EPI_ISL_2154970, EPI_ISL_2154971, EPI_ISL_2154979, EPI_ISL_2154980, EPI_ISL_2154981, EPI_ISL_2154983, EPI_ISL_2154987, EPI_ISL_2154996, EPI_ISL_2154997, EPI_ISL_2155062, EPI_ISL_2155065, EPI_ISL_2155125, EPI_ISL_2155132, EPI_ISL_2155138, EPI_ISL_2155173, EPI_ISL_2155182, EPI_ISL_2155200, EPI_ISL_2155205, EPI_ISL_2155217, EPI_ISL_2155228, EPI_ISL_2155239, EPI_ISL_2155242, EPI_ISL_2155272, EPI_ISL_2155274, EPI_ISL_2155277, EPI_ISL_2155278, EPI_ISL_2155291, EPI_ISL_2155303, EPI_ISL_2155306, EPI_ISL_2155315, EPI_ISL_2155435, EPI_ISL_2155436, EPI_ISL_2155437, EPI_ISL_2155438, EPI_ISL_2155439, EPI_ISL_2155440, EPI_ISL_2155441, EPI_ISL_2155442, EPI_ISL_2155443, EPI_ISL_2155444, EPI_ISL_2155445, EPI_ISL_2155446, EPI_ISL_2155447, EPI_ISL_2155448, EPI_ISL_2155449, EPI_ISL_2155450, EPI_ISL_2155451, EPI_ISL_2155452, EPI_ISL_2155453, EPI_ISL_2155454, EPI_ISL_2155455, EPI_ISL_2155456, EPI_ISL_2155457, EPI_ISL_2155458, EPI_ISL_2155459, EPI_ISL_2155460, EPI_ISL_2155889, EPI_ISL_2155890, EPI_ISL_2155891, EPI_ISL_2155892, EPI_ISL_2155893, EPI_ISL_2155894, EPI_ISL_2155895, EPI_ISL_2155896, EPI_ISL_2155897, EPI_ISL_2155898, EPI_ISL_2155899, EPI_ISL_2155900, EPI_ISL_2155901, EPI_ISL_2155902, EPI_ISL_2155903, EPI_ISL_2155904, EPI_ISL_2155905, EPI_ISL_2155906, EPI_ISL_2155907, EPI_ISL_2155908, EPI_ISL_2155909, EPI_ISL_2155910, EPI_ISL_2155911, EPI_ISL_2155912, EPI_ISL_2155913, EPI_ISL_2155914, EPI_ISL_2155915, EPI_ISL_2155916, EPI_ISL_2155917, EPI_ISL_2155918, EPI_ISL_2156285, EPI_ISL_2156320, EPI_ISL_2156454, EPI_ISL_2156455, EPI_ISL_2156474, EPI_ISL_2156543, EPI_ISL_2156547, EPI_ISL_2156548, EPI_ISL_2156556, EPI_ISL_2156605, EPI_ISL_2171090, EPI_ISL_2171103, EPI_ISL_2171147, EPI_ISL_2171178, EPI_ISL_2171182, EPI_ISL_2171186, EPI_ISL_2171201, EPI_ISL_2171211, EPI_ISL_2188110, EPI_ISL_2188154, EPI_ISL_2188160, EPI_ISL_2188221, EPI_ISL_2188678, EPI_ISL_2188686, EPI_ISL_2188689, EPI_ISL_2188719, EPI_ISL_2188744, EPI_ISL_2188751, EPI_ISL_2188788, EPI_ISL_2188805, EPI_ISL_2188824, EPI_ISL_2188859, EPI_ISL_2188896, EPI_ISL_2188916, EPI_ISL_2188927, EPI_ISL_2188969, EPI_ISL_2188973, EPI_ISL_2189029, EPI_ISL_2189070, EPI_ISL_2189095, EPI_ISL_2189105, EPI_ISL_2189126, EPI_ISL_2189146, EPI_ISL_2189154, EPI_ISL_2189159, EPI_ISL_2189172, EPI_ISL_2189175, EPI_ISL_2189185, EPI_ISL_2189196, EPI_ISL_2189199, EPI_ISL_2189242, EPI_ISL_2189248, EPI_ISL_2189259, EPI_ISL_2189262, EPI_ISL_2189287, EPI_ISL_2189304, EPI_ISL_2189320, EPI_ISL_2189330, EPI_ISL_2189345, EPI_ISL_2189352, EPI_ISL_2189393, EPI_ISL_2189407, EPI_ISL_2189478, EPI_ISL_2189496, EPI_ISL_2189525, EPI_ISL_2859515, EPI_ISL_2859598, EPI_ISL_2859791, EPI_ISL_2859829, EPI_ISL_2859852, EPI_ISL_2859870, EPI_ISL_2859909, EPI_ISL_2859928, EPI_ISL_2859980, EPI_ISL_2860096, EPI_ISL_2860140			
see above	Philippine Red Cross - National Blood Center	Philippine Genome Center	Alethea R. de Guzman; Anna Ong-Lim; Arianne A. Zamora; Asia Louisa U. Chong; Benedict A. Maralit; Candice Francheska B. Tambaoan; Carlo M. Lapid; Celia Carlos; Devon Ray Pacial; Diomedes A. Carino; Edsel Maurice Salvaña; El King D. Morado; Elcid Aaron R. Pangilinan; Eva Maria Cutiongco-de la Paz; Francis A. Tablizo; Henrietta Marie Rodriguez; Irish Coleen A. Asin; Jaime C. Montoya; Jan Michael C. Yap; Jarvin E. Nipales; Jo-Hannah S. Llames; John Q. Wong; Joshua Gregor A. Dizon; Juan Antonio R. Magalang; Karol Sophia Agape R. Padilla; Kenneth M. Kim; Kris P. Punayan; Krisitna Patriz Dela Cruz; Lindsay Claire D.L. Carandang; Ma. Exanil Plantig; Marc Edsel C. Ayes; Marc Jerrone R. Castro; Maria Rosario Singh-Vergeire and Cynthia P. Saloma; Maria Sofia L. Yangzon; Marielle M Gamboa; Marissa Alejandria; Nina Francesca Bustamante; Razel Nikka M. Hao; Renato Jacinto Q. Mantaring; Rianna Patricia S. Cruz; Sheila Mae M. Araiza; Yvonne Valerie Austria; Zipporah Mariebelle R. Enriquez; Zyrel V. Mollejon
EPI_ISL_1081810, EPI_ISL_1081811, EPI_ISL_1081813, EPI_ISL_1081814, EPI_ISL_1081815, EPI_ISL_1081816, EPI_ISL_1081817, EPI_ISL_1081818, EPI_ISL_1081830, EPI_ISL_1213578, EPI_ISL_2154974, EPI_ISL_2154975, EPI_ISL_2154976, EPI_ISL_2154998, EPI_ISL_2155006, EPI_ISL_2155015, EPI_ISL_2155052, EPI_ISL_2155057, EPI_ISL_2155059, EPI_ISL_2155063, EPI_ISL_2155071, EPI_ISL_2155082, EPI_ISL_2155110, EPI_ISL_2155113, EPI_ISL_2155114, EPI_ISL_2155127, EPI_ISL_2155133, EPI_ISL_2155147, EPI_ISL_2155156, EPI_ISL_2155157, EPI_ISL_2155171, EPI_ISL_2155179, EPI_ISL_2155203, EPI_ISL_2155220, EPI_ISL_2155225, EPI_ISL_2155229, EPI_ISL_2155237, EPI_ISL_2155254, EPI_ISL_2155258, EPI_ISL_2155260, EPI_ISL_2155285, EPI_ISL_2155290, EPI_ISL_2155294, EPI_ISL_2155309, EPI_ISL_2155324, EPI_ISL_2155338, EPI_ISL_2155349, EPI_ISL_2155767, EPI_ISL_2155768, EPI_ISL_2155769, EPI_ISL_2155770, EPI_ISL_2155771, EPI_ISL_2155772, EPI_ISL_2155773, EPI_ISL_2155774, EPI_ISL_2155775, EPI_ISL_2155776, EPI_ISL_2155777, EPI_ISL_2155778, EPI_ISL_2155779, EPI_ISL_2155780, EPI_ISL_2155781, EPI_ISL_2155782, EPI_ISL_2155783, EPI_ISL_2155784, EPI_ISL_2155785, EPI_ISL_2156011, EPI_ISL_2156012, EPI_ISL_2156013, EPI_ISL_2156014, EPI_ISL_2156015, EPI_ISL_2156016, EPI_ISL_2156017, EPI_ISL_2156018, EPI_ISL_2156019, EPI_ISL_2156020, EPI_ISL_2156228, EPI_ISL_2156229, EPI_ISL_2156230, EPI_ISL_2156231, EPI_ISL_2156232, EPI_ISL_2156233, EPI_ISL_2156234, EPI_ISL_2156235, EPI_ISL_2156236, EPI_ISL_2156237, EPI_ISL_2156238, EPI_ISL_2156239, EPI_ISL_2156240, EPI_ISL_2156241, EPI_ISL_2156242, EPI_ISL_2156243, EPI_ISL_2156244, EPI_ISL_2156245, EPI_ISL_2156246, EPI_ISL_2156247, EPI_ISL_2156292, EPI_ISL_2156303, EPI_ISL_2156304, EPI_ISL_2156305, EPI_ISL_2156306, EPI_ISL_2156307, EPI_ISL_2156308, EPI_ISL_2156309, EPI_ISL_2156310, EPI_ISL_2156311, EPI_ISL_2156312, EPI_ISL_2156315, EPI_ISL_2156333, EPI_ISL_2156334, EPI_ISL_2156347, EPI_ISL_2156348, EPI_ISL_2156365, EPI_ISL_2156366, EPI_ISL_2156391, EPI_ISL_2156392, EPI_ISL_2156393, EPI_ISL_2156535, EPI_ISL_2156566, EPI_ISL_2156575, EPI_ISL_2156614, EPI_ISL_2171091, EPI_ISL_2171092, EPI_ISL_2171093, EPI_ISL_2171097, EPI_ISL_2171099, EPI_ISL_2171100, EPI_ISL_2171101, EPI_ISL_2171102, EPI_ISL_2171105, EPI_ISL_2171108, EPI_ISL_2171109, EPI_ISL_2171110, EPI_ISL_2171112, EPI_ISL_2171113, EPI_ISL_2171114, EPI_ISL_2171119, EPI_ISL_2171122, EPI_ISL_2171127, EPI_ISL_2171128, EPI_ISL_2171134, EPI_ISL_2171136, EPI_ISL_2171140, EPI_ISL_2171141, EPI_ISL_2171142, EPI_ISL_2171146, EPI_ISL_2171148, EPI_ISL_2171151, EPI_ISL_2171152, EPI_ISL_2171155, EPI_ISL_2171156, EPI_ISL_2171157, EPI_ISL_2171160, EPI_ISL_2171164, EPI_ISL_2171166, EPI_ISL_2171167, EPI_ISL_2171169, EPI_ISL_2171174, EPI_ISL_2171184, EPI_ISL_2171189, EPI_ISL_2171191, EPI_ISL_2171192, EPI_ISL_2171194, EPI_ISL_2171195, EPI_ISL_2171200, EPI_ISL_2171202, EPI_ISL_2171205, EPI_ISL_2171207, EPI_ISL_2171209, EPI_ISL_2171214, EPI_ISL_2171217, EPI_ISL_2171220, EPI_ISL_2171222, EPI_ISL_2171223, EPI_ISL_2171225, EPI_ISL_2171226, EPI_ISL_2171228, EPI_ISL_2171231, EPI_ISL_2171232, EPI_ISL_2171235, EPI_ISL_2171236, EPI_ISL_2171237, EPI_ISL_2171238, EPI_ISL_2171239, EPI_ISL_2188045, EPI_ISL_2188048, EPI_ISL_2188071, EPI_ISL_2188075, EPI_ISL_2188080, EPI_ISL_2188089, EPI_ISL_2188099, EPI_ISL_2188113, EPI_ISL_2188120, EPI_ISL_2188147, EPI_ISL_2188178, EPI_ISL_2188212, EPI_ISL_2188225, EPI_ISL_2188228, EPI_ISL_2188234, EPI_ISL_2188247, EPI_ISL_2188250, EPI_ISL_2188260, EPI_ISL_2188266, EPI_ISL_2188269, EPI_ISL_2188271, EPI_ISL_2188685, EPI_ISL_2188694, EPI_ISL_2188699, EPI_ISL_2188710, EPI_ISL_2188711, EPI_ISL_2188717, EPI_ISL_2188723, EPI_ISL_2188736, EPI_ISL_2188740, EPI_ISL_2188741, EPI_ISL_2188743, EPI_ISL_2188747, EPI_ISL_2188752, EPI_ISL_2188758, EPI_ISL_2188763, EPI_ISL_2188767, EPI_ISL_2188770, EPI_ISL_2188785, EPI_ISL_2188787, EPI_ISL_2188793, EPI_ISL_2188798, EPI_ISL_2188801, EPI_ISL_2188803, EPI_ISL_2188811, EPI_ISL_2188817, EPI_ISL_2188826, EPI_ISL_2188842, EPI_ISL_2188843, EPI_ISL_2188849, EPI_ISL_2188853, EPI_ISL_2188854, EPI_ISL_2188856, EPI_ISL_2188858, EPI_ISL_2188860, EPI_ISL_2188865, EPI_ISL_2188868, EPI_ISL_2188871, EPI_ISL_2188873, EPI_ISL_2188880, EPI_ISL_2188890, EPI_ISL_2188892, EPI_ISL_2188898, EPI_ISL_2188914, EPI_ISL_2188933, EPI_ISL_2188935, EPI_ISL_2188938, EPI_ISL_2188941, EPI_ISL_2188943, EPI_ISL_2188949, EPI_ISL_2188952, EPI_ISL_2188977, EPI_ISL_2188981, EPI_ISL_2189003, EPI_ISL_2189014, EPI_ISL_2189016, EPI_ISL_2189027, EPI_ISL_2189035, EPI_ISL_2189038, EPI_ISL_2189047, EPI_ISL_2189052, EPI_ISL_2189055, EPI_ISL_2189056, EPI_ISL_2189066, EPI_ISL_2189081, EPI_ISL_2189124, EPI_ISL_2189136, EPI_ISL_2189139, EPI_ISL_2189149, EPI_ISL_2189163, EPI_ISL_2189164, EPI_ISL_2189173, EPI_ISL_2189178, EPI_ISL_2189183, EPI_ISL_2189194, EPI_ISL_2189204, EPI_ISL_2189210, EPI_ISL_2189213, EPI_ISL_2189218, EPI_ISL_2189241, EPI_ISL_2189243, EPI_ISL_2189253, EPI_ISL_2189267, EPI_ISL_2189281, EPI_ISL_2189301, EPI_ISL_2189329, EPI_ISL_2189334, EPI_ISL_2189346, EPI_ISL_2189349, EPI_ISL_2189365, EPI_ISL_2189450, EPI_ISL_2189455, EPI_ISL_2189457, EPI_ISL_2189459, EPI_ISL_2189510, EPI_ISL_2189511, EPI_ISL_2189513, EPI_ISL_2189520, EPI_ISL_2859497, EPI_ISL_2859498, EPI_ISL_2859499, EPI_ISL_2859501, EPI_ISL_2859504, EPI_ISL_2859505, EPI_ISL_2859508, EPI_ISL_2859509, EPI_ISL_2859510, EPI_ISL_2859511, EPI_ISL_2859512, EPI_ISL_2859518, EPI_ISL_2859519, EPI_ISL_2859521, EPI_ISL_2859522, EPI_ISL_2859523, EPI_ISL_2859524, EPI_ISL_2859525, EPI_ISL_2859528, EPI_ISL_2859529, EPI_ISL_2859530, EPI_ISL_2859532, EPI_ISL_2859533, EPI_ISL_2859534, EPI_ISL_2859535, EPI_ISL_2859537, EPI_ISL_2859538, EPI_ISL_2859540, EPI_ISL_2859541, EPI_ISL_2859543, EPI_ISL_2859544, EPI_ISL_2859546, EPI_ISL_2859548, EPI_ISL_2859549, EPI_ISL_2859550, EPI_ISL_2859552, EPI_ISL_2859553, EPI_ISL_2859554, EPI_ISL_2859555, EPI_ISL_2859557, EPI_ISL_2859558, EPI_ISL_2859559, EPI_ISL_2859562, EPI_ISL_2859563, EPI_ISL_2859564, EPI_ISL_2859565, EPI_ISL_2859566, EPI_ISL_2859567, EPI_ISL_2859568, EPI_ISL_2859569, EPI_ISL_2859570, EPI_ISL_2859571, EPI_ISL_2859572, EPI_ISL_2859574, EPI_ISL_2859575, EPI_ISL_2859577, EPI_ISL_2859579, EPI_ISL_2859580, EPI_ISL_2859582, EPI_ISL_2859583, EPI_ISL_2859584, EPI_ISL_2859586, EPI_ISL_2859589, EPI_ISL_2859590, EPI_ISL_2859591, EPI_ISL_2859592, EPI_ISL_2859593, EPI_ISL_2859596, EPI_ISL_2859597, EPI_ISL_2859599, EPI_ISL_2859600, EPI_ISL_2859601, EPI_ISL_2859604, EPI_ISL_2859605, EPI_ISL_2859607, EPI_ISL_2859608, EPI_ISL_2859609, EPI_ISL_2859611, EPI_ISL_2859612, EPI_ISL_2859614, EPI_ISL_2859616, EPI_ISL_2859617, EPI_ISL_2859621, EPI_ISL_2859622, EPI_ISL_2859623, EPI_ISL_2859624, EPI_ISL_2859630, EPI_ISL_2859633, EPI_ISL_2859636, EPI_ISL_2859637, EPI_ISL_2859638, EPI_ISL_2859639, EPI_ISL_2859640, EPI_ISL_2859643, EPI_ISL_2859647, EPI_ISL_2859648, EPI_ISL_2859651, EPI_ISL_2859652, EPI_ISL_2859653, EPI_ISL_2859654, EPI_ISL_2859656, EPI_ISL_2859657, EPI_ISL_2859661, EPI_ISL_2859662, EPI_ISL_2859663, EPI_ISL_2859664, EPI_ISL_2859666, EPI_ISL_2859667, EPI_ISL_2859677, EPI_ISL_2859687, EPI_ISL_2859700, EPI_ISL_2859704, EPI_ISL_2859709, EPI_ISL_2859717, EPI_ISL_2859722, EPI_ISL_2859725, EPI_ISL_2859731, EPI_ISL_2859736, EPI_ISL_2859750, EPI_ISL_2859753, EPI_ISL_2859757, EPI_ISL_2859763, EPI_ISL_2859770, EPI_ISL_2859775, EPI_ISL_2859777, EPI_ISL_2859781, EPI_ISL_2859784, EPI_ISL_2859786, EPI_ISL_2859787, EPI_ISL_2859790, EPI_ISL_2859792, EPI_ISL_2859793, EPI_ISL_2859795, EPI_ISL_2859796, EPI_ISL_2859797, EPI_ISL_2859798, EPI_ISL_2859800, EPI_ISL_2859801, EPI_ISL_2859802, EPI_ISL_2859805, EPI_ISL_2859807, EPI_ISL_2859808, EPI_ISL_2859814, EPI_ISL_2859818, EPI_ISL_2859822, EPI_ISL_2859823, EPI_ISL_2859826, EPI_ISL_2859831, EPI_ISL_2859834, EPI_ISL_2859835, EPI_ISL_2859837, EPI_ISL_2859838, EPI_ISL_2859839, EPI_ISL_2859842, EPI_ISL_2859843, EPI_ISL_2859846, EPI_ISL_2859847, EPI_ISL_2859849, EPI_ISL_2859851, EPI_ISL_2859854, EPI_ISL_2859855, EPI_ISL_2859856, EPI_ISL_2859858, EPI_ISL_2859860, EPI_ISL_2859863, EPI_ISL_2859865, EPI_ISL_2859866, EPI_ISL_2859867, EPI_ISL_2859871, EPI_ISL_2859872, EPI_ISL_2859873, EPI_ISL_2859874, EPI_ISL_2859876, EPI_ISL_2859877, EPI_ISL_2859878, EPI_ISL_2859879, EPI_ISL_2859880, EPI_ISL_2859882, EPI_ISL_2859883, EPI_ISL_2859884, EPI_ISL_2859885, EPI_ISL_2859887, EPI_ISL_2859889, EPI_ISL_2859890, EPI_ISL_2859893, EPI_ISL_2859894, EPI_ISL_2859896, EPI_ISL_2859897, EPI_ISL_2859902, EPI_ISL_2859903, EPI_ISL_2859904, EPI_ISL_2859905, EPI_ISL_2859907, EPI_ISL_2859908, EPI_ISL_2859911, EPI_ISL_2859914, EPI_ISL_2859915, EPI_ISL_2859917, EPI_ISL_2859918, EPI_ISL_2859920, EPI_ISL_2859922, EPI_ISL_2859923, EPI_ISL_2859926, EPI_ISL_2859927, EPI_ISL_2859929, EPI_ISL_2859932, EPI_ISL_2859934, EPI_ISL_2859936, EPI_ISL_2859937, EPI_ISL_2859939, EPI_ISL_2859940, EPI_ISL_2859941, EPI_ISL_2859943, EPI_ISL_2859945, EPI_ISL_2859946, EPI_ISL_2859947, EPI_ISL_2859948, EPI_ISL_2859949, EPI_ISL_2859950, EPI_ISL_2859952, EPI_ISL_2859954, EPI_ISL_2859955, EPI_ISL_2859962, EPI_ISL_2859964, EPI_ISL_2859966, EPI_ISL_2859968, EPI_ISL_2859969, EPI_ISL_2859974, EPI_ISL_2859975, EPI_ISL_2859977, EPI_ISL_2859984, EPI_ISL_2859986, EPI_ISL_2859988, EPI_ISL_2859992, EPI_ISL_2859994, EPI_ISL_2859995, EPI_ISL_2859996, EPI_ISL_2860002, EPI_ISL_2860006, EPI_ISL_2860023, EPI_ISL_2860024, EPI_ISL_2860025, EPI_ISL_2860026, EPI_ISL_2860032, EPI_ISL_2860037, EPI_ISL_2860039, EPI_ISL_2860041, EPI_ISL_2860042, EPI_ISL_2860043, EPI_ISL_2860044, EPI_ISL_2860046, EPI_ISL_2860048, EPI_ISL_2860049, EPI_ISL_2860052, EPI_ISL_2860055, EPI_ISL_2860057, EPI_ISL_2860058, EPI_ISL_2860060, EPI_ISL_2860062, EPI_ISL_2860064, EPI_ISL_2860069, EPI_ISL_2860070, EPI_ISL_2860071, EPI_ISL_2860072, EPI_ISL_2860073, EPI_ISL_2860074, EPI_ISL_2860075, EPI_ISL_2860076, EPI_ISL_2860078, EPI_ISL_2860082, EPI_ISL_2860086, EPI_ISL_2860087, EPI_ISL_2860090, EPI_ISL_2860093, EPI_ISL_2860100, EPI_ISL_2860103, EPI_ISL_2860104, EPI_ISL_2860107, EPI_ISL_2860110, EPI_ISL_2860111, EPI_ISL_2860113, EPI_ISL_2860124, EPI_ISL_2860127, EPI_ISL_2860128, EPI_ISL_2860131, EPI_ISL_2860132, EPI_ISL_2860134, EPI_ISL_2860139, EPI_ISL_2860142, EPI_ISL_2860145, EPI_ISL_2860148, EPI_ISL_2860154, EPI_ISL_2860157, EPI_ISL_2860160, EPI_ISL_2860163, EPI_ISL_2860165, EPI_ISL_2860174, EPI_ISL_2860178, EPI_ISL_2860179, EPI_ISL_2860181, EPI_ISL_2860184, EPI_ISL_2860212, EPI_ISL_2860214, EPI_ISL_2860236, EPI_ISL_4712058, EPI_ISL_4712094, EPI_ISL_4712109, EPI_ISL_4712121, EPI_ISL_4712140, EPI_ISL_4712630, EPI_ISL_4712647, EPI_ISL_4712658, EPI_ISL_4712689, EPI_ISL_4712753, EPI_ISL_4712835, EPI_ISL_4712868, EPI_ISL_4712914, EPI_ISL_4713061, EPI_ISL_4713128, EPI_ISL_4713155, EPI_ISL_4713277, EPI_ISL_4713287, EPI_ISL_4713322, EPI_ISL_4713407, EPI_ISL_4713425, EPI_ISL_4713467, EPI_ISL_4713582, EPI_ISL_4713857, EPI_ISL_4714123, EPI_ISL_4714625, EPI_ISL_4714639, EPI_ISL_4714752, EPI_ISL_4714765, EPI_ISL_4714903, EPI_ISL_4715067, EPI_ISL_4715216, EPI_ISL_4715925, EPI_ISL_4716062, EPI_ISL_4716095, EPI_ISL_4716183, EPI_ISL_4716233, EPI_ISL_4716243, EPI_ISL_4716248, EPI_ISL_4716420, EPI_ISL_4716546, EPI_ISL_4716563, EPI_ISL_4716574, EPI_ISL_4716650, EPI_ISL_4716758, EPI_ISL_4716781, EPI_ISL_4716802, EPI_ISL_4716840, EPI_ISL_4716934, EPI_ISL_4716969, EPI_ISL_4717042, EPI_ISL_4717051, EPI_ISL_4717056, EPI_ISL_4717062, EPI_ISL_4717108, EPI_ISL_4717114, EPI_ISL_4717196, EPI_ISL_4717219, EPI_ISL_4717228, EPI_ISL_4717233, EPI_ISL_4717238, EPI_ISL_4717248, EPI_ISL_4717263, EPI_ISL_4717270, EPI_ISL_4717280, EPI_ISL_4717288, EPI_ISL_4717337			
see above	Philippine Red Cross - Port Area	Philippine Genome Center	Alethea R. de Guzman; Anna Ong-Lim; Arianne A. Zamora; Asia Louisa U. Chong; Benedict A. Maralit; Candice Francheska B. Tambaoan; Carlo M. Lapid; Celia Carlos; Cynthia P. Saloma; Devon Ray Pacial; Diomedes A. Carino; Diomedes A. Cariño; Edsel Maurice Salvana; Edsel Maurice Salvaña; El King D. Morado; Elcid Aaron R. Pangilinan; Eva Maria Cutiongco-de la Paz; Francis A. Tablizo; Henrietta Marie Rodriguez; Irish Coleen A. Asin; Jaime C. Montoya; Jan Michael C. Yap; Jarvin E. Nipales; Jo-Hannah S. Llames; John Q. Wong; Joshua Gregor A. Dizon; Juan Antonio R. Magalang; Karol Sophia Agape R. Padilla; Kenneth M. Kim; Kris P. Punayan; Krisitna Patriz Dela Cruz; Kristina Patriz Dela Cruz; Lindsay Claire D.L. Carandang; Lindsay Clare D.L. Carandang; Ma. Exanil Plantig; Marc Edsel C. Ayes; Marc Jerrone R. Castro; Maria Rosario Singh-Vergeire; Maria Rosario Singh-Vergeire and Cynthia P. Saloma; Maria Sofia L. Yangzon; Marielle M Gamboa; Marielle M. Gamboa; Marissa Alejandria; Nina Francesca Bustamante; Niña Francesca Bustamante; Razel Nikka M. Hao; Renato Jacinto Q. Mantaring; Rianna Patricia S. Cruz; Sheila Mae M. Araiza; Shiela Mae M. Araiza; Yvonne Valerie Austria; Zipporah Mariebelle R. Enriquez; Zyrel V. Mollejon; and Maria Rosario Singh-Vergeire
EPI_ISL_4712314, EPI_ISL_4712351, EPI_ISL_4712374, EPI_ISL_4712377, EPI_ISL_4712402, EPI_ISL_4712458, EPI_ISL_4712525, EPI_ISL_4712530, EPI_ISL_4712548, EPI_ISL_4712582, EPI_ISL_4712592, EPI_ISL_4712606, EPI_ISL_4712670, EPI_ISL_4712676, EPI_ISL_4712716, EPI_ISL_4712734, EPI_ISL_4712745, EPI_ISL_4712782, EPI_ISL_4712840, EPI_ISL_4712869, EPI_ISL_4712881, EPI_ISL_4712897, EPI_ISL_4712938, EPI_ISL_4712958, EPI_ISL_4712965, EPI_ISL_4713139, EPI_ISL_4713202, EPI_ISL_4713223, EPI_ISL_4713249, EPI_ISL_4713271, EPI_ISL_4713350, EPI_ISL_4713394, EPI_ISL_4713479, EPI_ISL_4713484, EPI_ISL_4713574, EPI_ISL_4713825, EPI_ISL_4713949, EPI_ISL_4714196, EPI_ISL_4714268, EPI_ISL_4714332, EPI_ISL_4714374, EPI_ISL_4714454, EPI_ISL_4714495, EPI_ISL_4714527, EPI_ISL_4714751, EPI_ISL_4714816, EPI_ISL_4714852, EPI_ISL_4715159, EPI_ISL_4715276, EPI_ISL_4716466			
see above	Philippine Red Cross - Subic Molecular Laboratory	Philippine Genome Center	Alethea R. de Guzman; Anna Ong-Lim; Arianne A. Zamora; Benedict A. Maralit; Carlo M. Lapid; Celia Carlos; Cynthia P. Saloma; Devon Ray Pacial; Diomedes A. Cariño; Edsel Maurice Salvana; El King D. Morado; Elcid Aaron R. Pangilinan; Eva Maria Cutiongco-de la Paz; Francis A. Tablizo; Henrietta Marie Rodriguez; Jaime C. Montoya; Jan Michael C. Yap; Jarvin E. Nipales; Jo-Hannah S. Llames; John Q. Wong; Joshua Gregor A. Dizon; Juan Antonio R. Magalang; Karol Sophia Agape R. Padilla; Kenneth M. Kim; Kris P. Punayan; Kristina Patriz Dela Cruz; Lindsay Clare D.L. Carandang; Ma. Exanil Plantig; Marc Edsel C. Ayes; Maria Rosario Singh-Vergeire; Maria Sofia L. Yangzon; Marielle M. Gamboa; Marissa Alejandria; Niña Francesca Bustamante; Razel Nikka M. Hao; Renato Jacinto Q. Mantaring; Rianna Patricia S. Cruz; Shiela Mae M. Araiza; Yvonne Valerie Austria; Zipporah Mariebelle R. Enriquez; Zyrel V. Mollejon
EPI_ISL_2859164, EPI_ISL_2859312, EPI_ISL_2859318, EPI_ISL_2859325, EPI_ISL_2859332, EPI_ISL_2859353, EPI_ISL_2859361, EPI_ISL_2859365, EPI_ISL_2859370, EPI_ISL_4714027, EPI_ISL_4714449, EPI_ISL_4715184			
see above	Philippine Red Cross - Zamboanga Molecular Laboratory	Philippine Genome Center	Alethea R. de Guzman; Anna Ong-Lim; Arianne A. Zamora; Benedict A. Maralit; Carlo M. Lapid; Celia Carlos; Cynthia P. Saloma; Devon Ray Pacial; Diomedes A. Carino; Diomedes A. Cariño; Edsel Maurice Salvana; Edsel Maurice Salvaña; El King D. Morado; Elcid Aaron R. Pangilinan; Eva Maria Cutiongco-de la Paz; Francis A. Tablizo; Henrietta Marie Rodriguez; Jaime C. Montoya; Jan Michael C. Yap; Jarvin E. Nipales; Jo-Hannah S. Llames; John Q. Wong; Joshua Gregor A. Dizon; Juan Antonio R. Magalang; Karol Sophia Agape R. Padilla; Kenneth M. Kim; Kris P. Punayan; Krisitna Patriz Dela Cruz; Kristina Patriz Dela Cruz; Lindsay Claire D.L. Carandang; Lindsay Clare D.L. Carandang; Ma. Exanil Plantig; Marc Edsel C. Ayes; Maria Rosario Singh-Vergeire; Maria Rosario Singh-Vergeire and Cynthia P. Saloma; Maria Sofia L. Yangzon; Marielle M Gamboa; Marielle M. Gamboa; Marissa Alejandria; Nina Francesca Bustamante; Niña Francesca Bustamante; Razel Nikka M. Hao; Renato Jacinto Q. Mantaring; Rianna Patricia S. Cruz; Sheila Mae M. Araiza; Shiela Mae M. Araiza; Yvonne Valerie Austria; Zipporah Mariebelle R. Enriquez; Zyrel V. Mollejon
EPI_ISL_2154403, EPI_ISL_2154629, EPI_ISL_4714179	Philippine Red Cross Isabela Molecular Laboratory	Philippine Genome Center	Alethea R. de Guzman; Anna Ong-Lim; Arianne A. Zamora; Asia Louisa U. Chong; Benedict A. Maralit; Candice Francheska B. Tambaoan; Carlo M. Lapid; Celia Carlos; Cynthia P. Saloma; Devon Ray Pacial; Diomedes A. Cariño; Edsel Maurice Salvana; Edsel Maurice Salvaña; El King D. Morado; Elcid Aaron R. Pangilinan; Eva Maria Cutiongco-de la Paz; Francis A. Tablizo; Henrietta Marie Rodriguez; Irish Coleen A. Asin; Jaime C. Montoya; Jan Michael C. Yap; Jarvin E. Nipales; Jo-Hannah S. Llames; John Q. Wong; Joshua Gregor A. Dizon; Juan Antonio R. Magalang; Karol Sophia Agape R. Padilla; Kenneth M. Kim; Kris P. Punayan; Kristina Patriz Dela Cruz; Lindsay Clare D.L. Carandang; Ma. Exanil Plantig; Marc Edsel C. Ayes; Marc Jerrone R. Castro; Maria Rosario Singh-Vergeire; Maria Rosario Singh-Vergeire and Cynthia P. Saloma; Maria Sofia L. Yangzon; Marielle M. Gamboa; Marissa Alejandria; Niña Francesca Bustamante; Razel Nikka M. Hao; Renato Jacinto Q. Mantaring; Rianna Patricia S. Cruz; Sheila Mae M. Araiza; Shiela Mae M. Araiza; Yvonne Valerie Austria; Zipporah Mariebelle R. Enriquez; Zyrel V. Mollejon
EPI_ISL_1213580, EPI_ISL_2105381, EPI_ISL_2105424, EPI_ISL_2105434, EPI_ISL_2105487, EPI_ISL_2154977, EPI_ISL_2154985, EPI_ISL_2154992, EPI_ISL_2154993, EPI_ISL_2154999, EPI_ISL_2155061, EPI_ISL_2155165, EPI_ISL_2155331, EPI_ISL_2155731, EPI_ISL_2155732, EPI_ISL_2155733, EPI_ISL_2155734, EPI_ISL_2155735, EPI_ISL_2155736, EPI_ISL_2155737, EPI_ISL_2155738, EPI_ISL_2155739, EPI_ISL_2155740, EPI_ISL_2155741, EPI_ISL_2155742, EPI_ISL_2155743, EPI_ISL_2155744, EPI_ISL_2155745, EPI_ISL_2155746, EPI_ISL_2155747, EPI_ISL_2155748, EPI_ISL_2155749, EPI_ISL_2155750, EPI_ISL_2155751, EPI_ISL_2155752, EPI_ISL_2155753, EPI_ISL_2155754, EPI_ISL_2155755, EPI_ISL_2156001, EPI_ISL_2156002, EPI_ISL_2156003, EPI_ISL_2156004, EPI_ISL_2156005, EPI_ISL_2156006, EPI_ISL_2156007, EPI_ISL_2156008, EPI_ISL_2156009, EPI_ISL_2156220, EPI_ISL_2156294, EPI_ISL_2156323, EPI_ISL_2156324, EPI_ISL_2156337, EPI_ISL_2156414, EPI_ISL_2156459, EPI_ISL_2156476, EPI_ISL_2156484, EPI_ISL_2156504, EPI_ISL_2156505, EPI_ISL_2171138, EPI_ISL_2171143, EPI_ISL_2171149, EPI_ISL_2171159, EPI_ISL_2171161, EPI_ISL_2171203, EPI_ISL_2188078, EPI_ISL_2188122, EPI_ISL_2188128, EPI_ISL_2188133, EPI_ISL_2188138, EPI_ISL_2188199, EPI_ISL_2188226, EPI_ISL_2188680, EPI_ISL_2188697, EPI_ISL_2188708, EPI_ISL_2188726, EPI_ISL_2188759, EPI_ISL_2188762, EPI_ISL_2188794, EPI_ISL_2188797, EPI_ISL_2188802, EPI_ISL_2188816, EPI_ISL_2188855, EPI_ISL_2188863, EPI_ISL_2859812, EPI_ISL_2859832, EPI_ISL_2859845, EPI_ISL_2859985, EPI_ISL_2860031, EPI_ISL_2860059, EPI_ISL_2860083, EPI_ISL_2860117, EPI_ISL_2860153, EPI_ISL_4715126			
see above	Philippine Red Cross Logistics and	Philippine Genome Center	Alethea R. de Guzman; Anna Ong-Lim; Arianne A. Zamora; Asia Louisa U. Chong; Benedict A. Maralit; Candice Francheska B. Tambaoan; Carlo M. Lapid; Celia Carlos; Cynthia P. Saloma; Devon Ray Pacial; Diomedes A. Carino; Diomedes A. Cariño; Edsel Maurice Salvana; Edsel Maurice Salvaña; El King D. Morado; Elcid Aaron R. Pangilinan; Eva Maria Cutiongco-de la Paz; Francis A. Tablizo; Henrietta Marie Rodriguez; Irish Coleen A. Asin; Jaime C. Montoya; Jan Michael C. Yap; Jarvin E. Nipales; Jo-Hannah S. Llames; John Q. Wong; Joshua Gregor A. Dizon; Juan Antonio R. Magalang; Karol Sophia Agape R. Padilla; Kenneth M. Kim; Kris P. Punayan;

	Multipurpose Center		Krisitna Patriz Dela Cruz; Kristina Patriz Dela Cruz; Lindsay Claire D.L. Carandang; Lindsay Clare D.L. Carandang; Ma. Exanil Plantig; Marc Edsel C. Ayes; Marc Jerrone R. Castro; Maria Rosario Singh-Vergeire; Maria Rosario Singh-Vergeire and Cynthia P. Saloma; Maria Sofia L. Yangzon; Marielle M Gamboa; Marielle M. Gamboa; Marissa Alejandria; Nina Francesca Bustamante; Niña Francesca Bustamante; Razel Nikka M. Hao; Renato Jacinto Q. Mantaring; Rianna Patricia S. Cruz; Sheila Mae M. Araiza; Shiela Mae M. Araiza; Yvonne Valerie Austria; Zipporah Mariebelle R. Enriquez; Zyrel V. Mollejon
EPI_ISL_2153860, EPI_ISL_2153883, EPI_ISL_2153970, EPI_ISL_2154013, EPI_ISL_2154028, EPI_ISL_2154401, EPI_ISL_2154413, EPI_ISL_2154528, EPI_ISL_2154551, EPI_ISL_2154557, EPI_ISL_2154559, EPI_ISL_2154585, EPI_ISL_2154618, EPI_ISL_2154662, EPI_ISL_2154724, EPI_ISL_2154756, EPI_ISL_2154792, EPI_ISL_2154876			
see above	Philippine Red Cross Molecular Laboratory	Philippine Genome Center	Alethea R. de Guzman; Anna Ong-Lim; Arianne A. Zamora; Asia Louisa U. Chong; Benedict A. Maralit; Candice Francheska B. Tambaoan; Carlo M. Lapid; Celia Carlos; Devon Ray Pacial; Edsel Maurice Salvaña; El King D. Morado; Eva Maria Cutiongco-de la Paz; Francis A. Tablizo; Irish Coleen A. Asin; Jaime C. Montoya; Jan Michael C. Yap; Jo-Hannah S. Llames; John Q. Wong; Joshua Gregor A. Dizon; Juan Antonio R. Magalang; Karol Sophia Agape R. Padilla; Kenneth M. Kim; Kris P. Punayan; Marc Edsel C. Ayes; Marc Jerrone R. Castro; Maria Rosario Singh-Vergeire and Cynthia P. Saloma; Maria Sofia L. Yangzon; Marissa Alejandria; Razel Nikka M. Hao; Rianna Patricia S. Cruz; Sheila Mae M. Araiza
EPI_ISL_2155256, EPI_ISL_2155603, EPI_ISL_2155604, EPI_ISL_2156155, EPI_ISL_2156477, EPI_ISL_2156542, EPI_ISL_2156612, EPI_ISL_2188188, EPI_ISL_2188733, EPI_ISL_2188739, EPI_ISL_2188773, EPI_ISL_2188806, EPI_ISL_2188825, EPI_ISL_2188834, EPI_ISL_2189299, EPI_ISL_2189409, EPI_ISL_2860196, EPI_ISL_2860240, EPI_ISL_2860242, EPI_ISL_2860248, EPI_ISL_2860249, EPI_ISL_2860250, EPI_ISL_4713918, EPI_ISL_4713922, EPI_ISL_4713941, EPI_ISL_4714032, EPI_ISL_4714141, EPI_ISL_4714306, EPI_ISL_4714368, EPI_ISL_4714405, EPI_ISL_4714412, EPI_ISL_4714416, EPI_ISL_4714598, EPI_ISL_4715165, EPI_ISL_4715831, EPI_ISL_4715844, EPI_ISL_4715912, EPI_ISL_4715921, EPI_ISL_4715945, EPI_ISL_4715968, EPI_ISL_4715978, EPI_ISL_4715995, EPI_ISL_4716016, EPI_ISL_4716017, EPI_ISL_4716020, EPI_ISL_4716037, EPI_ISL_4716089, EPI_ISL_4716099, EPI_ISL_4716144, EPI_ISL_4716159, EPI_ISL_4716298, EPI_ISL_4716306, EPI_ISL_4716311, EPI_ISL_4716375, EPI_ISL_4716397, EPI_ISL_4716427, EPI_ISL_4716446, EPI_ISL_4716486, EPI_ISL_4716493, EPI_ISL_4716504, EPI_ISL_4716674, EPI_ISL_4716706, EPI_ISL_4716722, EPI_ISL_4716728, EPI_ISL_4716786, EPI_ISL_4716821, EPI_ISL_4716906, EPI_ISL_4716925, EPI_ISL_4717015, EPI_ISL_4717458			
see above	Philippine Red Cross-Batangas Chapter Molecular Laboratory	Philippine Genome Center	Alethea R. de Guzman; Anna Ong-Lim; Arianne A. Zamora; Asia Louisa U. Chong; Benedict A. Maralit; Candice Francheska B. Tambaoan; Carlo M. Lapid; Celia Carlos; Cynthia P. Saloma; Devon Ray Pacial; Diomedes A. Carino; Diomedes A. Cariño; Edsel Maurice Salvana; Edsel Maurice Salvaña; El King D. Morado; Elcid Aaron R. Pangilinan; Eva Maria Cutiongco-de la Paz; Francis A. Tablizo; Henrietta Marie Rodriguez; Irish Coleen A. Asin; Jaime C. Montoya; Jan Michael C. Yap; Jarvin E. Nipales; Jo-Hannah S. Llames; John Q. Wong; Joshua Gregor A. Dizon; Juan Antonio R. Magalang; Karol Sophia Agape R. Padilla; Kenneth M. Kim; Kris P. Punayan; Krisitna Patriz Dela Cruz; Kristina Patriz Dela Cruz; Lindsay Claire D.L. Carandang; Lindsay Clare D.L. Carandang; Ma. Exanil Plantig; Marc Edsel C. Ayes; Marc Jerrone R. Castro; Maria Rosario Singh-Vergeire; Maria Rosario Singh-Vergeire and Cynthia P. Saloma; Maria Sofia L. Yangzon; Marielle M Gamboa; Marielle M. Gamboa; Marissa Alejandria; Nina Francesca Bustamante; Niña Francesca Bustamante; Razel Nikka M. Hao; Renato Jacinto Q. Mantaring; Rianna Patricia S. Cruz; Sheila Mae M. Araiza; Shiela Mae M. Araiza; Yvonne Valerie Austria; Zipporah Mariebelle R. Enriquez; Zyrel V. Mollejon
EPI_ISL_1069179, EPI_ISL_1069180	Polillo Rural Health Unit	Research Institute for Tropical Medicine	Catalino Demetria; Daria Manalo; Edelwisa Mercado; Francisco Gerardo Polotan; Hannah Leah Morito; Inez Andrea Medado; John Leonard Chan; Joseph Hughes; Kirstyn Brunker; Ma Angelica Tujan; Othoniel Jan Onza
EPI_ISL_3105868	President Ramon Magsaysay Memorial Hospital	Research Institute for Tropical Medicine	Clyde Dapat; Deana Mae Ocampo; Emmanuel Kagning Tsinda; Francisco Gerardo Polotan; Hitoshi Oshitani; Inez Andrea Medado; Jefferson Earl Halog; Joana Ina Manalo; Lei Lanna Dancel; Ma Angelica Tujan; Mariko Saito-Obata; Mayuko Saito; Michiko Okamoto; Samantha Louise Bado
EPI_ISL_1213581, EPI_ISL_2155977, EPI_ISL_2155978, EPI_ISL_2156197, EPI_ISL_2156198, EPI_ISL_2156199, EPI_ISL_2156200, EPI_ISL_2171154, EPI_ISL_2171204, EPI_ISL_2188209, EPI_ISL_2188799, EPI_ISL_2188818, EPI_ISL_2188829, EPI_ISL_2188857, EPI_ISL_2188864, EPI_ISL_2188951, EPI_ISL_2189000, EPI_ISL_2189097, EPI_ISL_2189108, EPI_ISL_2189120, EPI_ISL_2189341, EPI_ISL_2859626, EPI_ISL_2859683, EPI_ISL_2859686, EPI_ISL_2859728, EPI_ISL_2859766, EPI_ISL_2859774, EPI_ISL_2859961, EPI_ISL_2859963, EPI_ISL_2859982, EPI_ISL_2859987, EPI_ISL_2860013, EPI_ISL_2860017, EPI_ISL_2860053, EPI_ISL_2860122, EPI_ISL_2860155			
see above	Prime Care Alpha Covid-19 Testing Laboratory	Philippine Genome Center	Alethea R. de Guzman; Anna Ong-Lim; Arianne A. Zamora; Asia Louisa U. Chong; Benedict A. Maralit; Candice Francheska B. Tambaoan; Carlo M. Lapid; Celia Carlos; Devon Ray Pacial; Diomedes A. Carino; Edsel Maurice Salvaña; El King D. Morado; Elcid Aaron R. Pangilinan; Eva Maria Cutiongco-de la Paz; Francis A. Tablizo; Henrietta Marie Rodriguez; Irish Coleen A. Asin; Jaime C. Montoya; Jan Michael C. Yap; Jarvin E. Nipales; Jo-Hannah S. Llames; John Q. Wong; Joshua Gregor A. Dizon; Juan Antonio R. Magalang; Karol Sophia Agape R. Padilla; Kenneth M. Kim; Kris P. Punayan; Krisitna Patriz Dela Cruz; Lindsay Claire D.L. Carandang; Ma. Exanil Plantig; Marc Edsel C. Ayes; Marc Jerrone R. Castro; Maria Rosario Singh-Vergeire and Cynthia P. Saloma; Maria Sofia L. Yangzon; Marielle M Gamboa; Marissa Alejandria; Nina Francesca Bustamante; Razel Nikka M. Hao; Renato Jacinto Q. Mantaring; Rianna Patricia S. Cruz; Sheila Mae M. Araiza; Yvonne Valerie Austria; Zipporah Mariebelle R. Enriquez; Zyrel V. Mollejon
EPI_ISL_491475	Protacio Hospital	Research Institute for Tropical Medicine	Catalino Demetria; Criselda Bautista; Daria Manalo; Edelwisa Mercado; Francisco Gerardo Polotan; Inez Andrea Medado; Kirstyn Brunker; Ma. Angelica Tujan; Othoniel Jan Onza
EPI_ISL_4459329	QUEZON MEDICAL CENTER	Research Institute for Tropical Medicine/Tohoku University	Allen Anthony Laraño; Ardiane Ysabelle Dolor; Catalino Demetria; Clyde Dapat; Emmanuel Kagning Tsinda; Francisco Gerardo Polotan; Hitoshi Oshitani; Inez Andrea Medado; Jefferson Earl Halog; Lei Lanna Dancel; Leilanie Nacion; Ma. Angelica Tujan; Machiko Okamoto; Maria Carmen Corpuz; Mariko Siato-Obata; Mayuko Saito
EPI_ISL_2154973, EPI_ISL_2154989, EPI_ISL_2155073, EPI_ISL_2155084, EPI_ISL_2155168, EPI_ISL_2155186, EPI_ISL_2155207, EPI_ISL_2155216, EPI_ISL_2155243, EPI_ISL_2155268, EPI_ISL_2155344, EPI_ISL_2155760, EPI_ISL_2155761, EPI_ISL_2156010, EPI_ISL_2156276, EPI_ISL_2156531, EPI_ISL_2156557, EPI_ISL_2188037, EPI_ISL_2188065, EPI_ISL_2188105, EPI_ISL_2188127, EPI_ISL_2188163, EPI_ISL_2188189, EPI_ISL_2188251, EPI_ISL_2188263, EPI_ISL_2188754, EPI_ISL_2188784, EPI_ISL_2188809, EPI_ISL_2188876, EPI_ISL_2188885, EPI_ISL_2189028, EPI_ISL_2189040, EPI_ISL_2189075, EPI_ISL_2189129, EPI_ISL_2189181, EPI_ISL_2189191, EPI_ISL_2189200, EPI_ISL_2189216, EPI_ISL_2189219, EPI_ISL_2189256, EPI_ISL_2189258, EPI_ISL_2189271, EPI_ISL_2189411, EPI_ISL_2189417, EPI_ISL_2189448, EPI_ISL_2189527, EPI_ISL_2859177, EPI_ISL_2859520, EPI_ISL_2859527, EPI_ISL_2859539, EPI_ISL_2859545, EPI_ISL_2859635, EPI_ISL_2859658, EPI_ISL_2859678, EPI_ISL_2859697, EPI_ISL_2859723, EPI_ISL_2859737, EPI_ISL_2859739, EPI_ISL_2859743, EPI_ISL_2859752, EPI_ISL_2859755, EPI_ISL_2859778, EPI_ISL_2859780, EPI_ISL_2859782, EPI_ISL_2859785, EPI_ISL_2859794, EPI_ISL_2859806, EPI_ISL_2859811, EPI_ISL_2859813, EPI_ISL_2859816, EPI_ISL_2859817, EPI_ISL_2859824, EPI_ISL_2859825, EPI_ISL_2859828, EPI_ISL_2859853, EPI_ISL_2859862, EPI_ISL_2859875, EPI_ISL_2859900, EPI_ISL_2859951, EPI_ISL_2859973, EPI_ISL_2859997, EPI_ISL_2859998, EPI_ISL_2860000, EPI_ISL_2860009, EPI_ISL_2860010, EPI_ISL_2860019, EPI_ISL_2860022, EPI_ISL_2860033, EPI_ISL_2860035, EPI_ISL_2860063, EPI_ISL_2860077, EPI_ISL_2860080, EPI_ISL_2860091, EPI_ISL_2860101, EPI_ISL_2860102, EPI_ISL_2860108, EPI_ISL_2860112, EPI_ISL_2860118, EPI_ISL_2860119, EPI_ISL_2860129, EPI_ISL_2860135, EPI_ISL_2860137, EPI_ISL_2860149, EPI_ISL_2860168, EPI_ISL_2860172, EPI_ISL_2860173, EPI_ISL_4712429, EPI_ISL_4712534, EPI_ISL_4712662, EPI_ISL_4712706, EPI_ISL_4712892, EPI_ISL_4713008, EPI_ISL_4713033, EPI_ISL_4713052, EPI_ISL_4713075, EPI_ISL_4713086, EPI_ISL_4713095, EPI_ISL_4713174, EPI_ISL_4713176, EPI_ISL_4713365, EPI_ISL_4713370, EPI_ISL_4713388, EPI_ISL_4713496, EPI_ISL_4713517, EPI_ISL_4713531, EPI_ISL_4713628, EPI_ISL_4713872, EPI_ISL_4714218, EPI_ISL_4715853, EPI_ISL_4716149, EPI_ISL_4716844, EPI_ISL_4716888, EPI_ISL_4717323			
see above	Qualimed Hospital Sta. Rosa	Philippine Genome Center	Alethea R. de Guzman; Anna Ong-Lim; Arianne A. Zamora; Asia Louisa U. Chong; Benedict A. Maralit; Candice Francheska B. Tambaoan; Carlo M. Lapid; Celia Carlos; Cynthia P. Saloma; Devon Ray Pacial; Diomedes A. Carino; Diomedes A. Cariño; Edsel Maurice Salvana; Edsel Maurice Salvaña; El King D. Morado; Elcid Aaron R. Pangilinan; Eva Maria Cutiongco-de la Paz; Francis A. Tablizo; Henrietta Marie Rodriguez; Irish Coleen A. Asin; Jaime C. Montoya; Jan Michael C. Yap; Jarvin E. Nipales; Jo-Hannah S. Llames; John Q. Wong; Joshua Gregor A. Dizon; Juan Antonio R. Magalang; Karol Sophia Agape R. Padilla; Kenneth M. Kim; Kris P. Punayan; Krisitna Patriz Dela Cruz; Kristina Patriz Dela Cruz; Lindsay Claire D.L. Carandang; Lindsay Clare D.L. Carandang; Ma. Exanil Plantig; Marc Edsel C. Ayes; Marc Jerrone R. Castro; Maria Rosario Singh-Vergeire; Maria Rosario Singh-Vergeire and Cynthia P. Saloma; Maria Sofia L. Yangzon; Marielle M Gamboa; Marielle M. Gamboa; Marissa Alejandria; Nina Francesca Bustamante; Niña Francesca Bustamante; Razel Nikka M. Hao; Renato Jacinto Q. Mantaring; Rianna Patricia S. Cruz; Sheila Mae M. Araiza; Shiela Mae M. Araiza; Yvonne Valerie Austria; Zipporah Mariebelle R. Enriquez; Zyrel V. Mollejon
EPI_ISL_2105310	Quezon City General Hospital (PGC)	Philippine Genome Center	Alethea R. de Guzman; Anna Ong-Lim; Arianne A. Zamora; Asia Louisa U. Chong; Benedict A. Maralit; Candice Francheska B. Tambaoan; Carlo M. Lapid; Celia Carlos; Devon Ray Pacial; Edsel Maurice Salvaña; El King D. Morado; Eva Maria Cutiongco-de la Paz; Francis A. Tablizo; Irish Coleen A. Asin; Jaime C. Montoya; Jan Michael C. Yap; Jo-Hannah S. Llames; John Q. Wong; Joshua Gregor A. Dizon; Juan Antonio R. Magalang; Karol Sophia Agape R. Padilla; Kenneth M. Kim; Kris P. Punayan; Marc Edsel C. Ayes; Marc Jerrone R. Castro; Maria Rosario Singh-Vergeire and Cynthia P. Saloma; Maria Sofia L. Yangzon; Marissa Alejandria; Razel Nikka M. Hao; Rianna Patricia S. Cruz; Sheila Mae M. Araiza
EPI_ISL_833342	Quezon Medical Center	Research Institute for Tropical Medicine	Catalino Demetria; Daria Manalo; Edelwisa Mercado; Francisco Gerardo Polotan; Hannah Leah Morito; Inez Andrea Medado; John Leonard Chan; Kirstyn Brunker; Ma Angelica Tujan; Othoniel Jan Onza
EPI_ISL_4459378, EPI_ISL_4459508, EPI_ISL_4459511	RESEARCH INSTITUTE FOR TROPICAL MEDICINE	Research Institute for Tropical Medicine/Tohoku University	Allen Anthony Laraño; Ardiane Ysabelle Dolor; Catalino Demetria; Clyde Dapat; Emmanuel Kagning Tsinda; Francisco Gerardo Polotan; Hitoshi Oshitani; Inez Andrea Medado; Jefferson Earl Halog; Lei Lanna Dancel; Leilanie Nacion; Ma. Angelica Tujan; Machiko Okamoto; Maria Carmen Corpuz; Mariko Siato-Obata; Mayuko Saito
EPI_ISL_1061412	RHS - PRO MIMAROPA	Research Institute for Tropical Medicine	Catalino Demetria; Daria Manalo; Edelwisa Mercado; Francisco Gerardo Polotan; Hannah Leah Morito; Inez Andrea Medado; John Leonard Chan; Joseph Hughes; Kirstyn Brunker; Ma Angelica Tujan; Othoniel Jan Onza
EPI_ISL_2155786, EPI_ISL_2155787	RURAL HEALTH UNIT - NAGCARLAN	Philippine Genome Center	Alethea R. de Guzman; Anna Ong-Lim; Arianne A. Zamora; Asia Louisa U. Chong; Benedict A. Maralit; Candice Francheska B. Tambaoan; Carlo M. Lapid; Celia Carlos; Devon Ray Pacial; Edsel Maurice Salvaña; El King D. Morado; Eva Maria Cutiongco-de la Paz; Francis A. Tablizo; Irish Coleen A. Asin; Jaime C. Montoya; Jan Michael C. Yap; Jo-Hannah S. Llames; John Q. Wong; Joshua Gregor A. Dizon; Juan Antonio R. Magalang; Karol Sophia Agape R. Padilla; Kenneth M. Kim; Kris P. Punayan; Marc Edsel C. Ayes; Marc Jerrone R. Castro; Maria Rosario Singh-Vergeire and Cynthia P. Saloma; Maria Sofia L. Yangzon; Marissa Alejandria; Razel Nikka M. Hao; Rianna Patricia S. Cruz; Sheila Mae M. Araiza
EPI_ISL_2155319	RURAL HEALTH UNIT - RIZAL	Philippine Genome Center	Alethea R. de Guzman; Anna Ong-Lim; Arianne A. Zamora; Asia Louisa U. Chong; Benedict A. Maralit; Candice Francheska B. Tambaoan; Carlo M. Lapid; Celia Carlos; Devon Ray Pacial; Edsel Maurice Salvaña; El King D. Morado; Eva Maria Cutiongco-de la Paz; Francis A. Tablizo; Irish Coleen A. Asin; Jaime C. Montoya; Jan Michael C. Yap; Jo-Hannah S. Llames; John Q. Wong; Joshua Gregor A. Dizon; Juan Antonio R. Magalang; Karol Sophia Agape R. Padilla; Kenneth M. Kim; Kris P. Punayan; Marc Edsel C. Ayes; Marc Jerrone R. Castro; Maria Rosario Singh-Vergeire and Cynthia P. Saloma; Maria Sofia L. Yangzon; Marissa Alejandria; Razel Nikka M. Hao; Rianna Patricia S. Cruz; Sheila Mae M. Araiza
EPI_ISL_1069190, EPI_ISL_1069191	Research Institite for Tropical Medicine	Research Institute for Tropical Medicine	Catalino Demetria; Daria Manalo; Edelwisa Mercado; Francisco Gerardo Polotan; Hannah Leah Morito; Inez Andrea Medado; John Leonard Chan; Joseph Hughes; Kirstyn Brunker; Ma Angelica Tujan; Othoniel Jan Onza
EPI_ISL_1081829	Research Institute for Tropical Medicine	Philippine Genome Center	Alethea R. de Guzman; Anna Ong-Lim; Arianne A. Zamora; Asia Louisa U. Chong; Benedict A. Maralit; Candice Francheska B. Tambaoan; Carlo M. Lapid; Celia Carlos; Cynthia P. Saloma; Devon Ray Pacial; Edsel Maurice Salvaña; El King D. Morado; Eva Maria Cutiongco-de la Paz; Francis A. Tablizo; Irish Coleen A. Asin; Jaime C. Montoya; Jan Michael C. Yap; Jo-Hannah S. Llames; John Q. Wong; Joshua Gregor A. Dizon; Juan Antonio R. Magalang; Karol Sophia Agape R. Padilla; Kenneth M. Kim; Kris P. Punayan; Marc Edsel C. Ayes; Marc Jerrone R. Castro; Maria Sofia L. Yangzon; Marissa Alejandria; Razel Nikka M. Hao; Rianna Patricia S. Cruz; Sheila Mae M. Araiza; and Maria Rosario Singh-Vergeire
EPI_ISL_430839, EPI_ISL_491467, EPI_ISL_491468, EPI_ISL_491474, EPI_ISL_3105866	Research Institute for Tropical Medicine	Research Institute for Tropical Medicine	Bautista; Brunker, K.; C.S.; C.T.; Catalino Demetria; Clyde Dapat; Criselda Bautista; D.L.; Daria Manalo; Deana Mae Ocampo; Demetria; E.S.; Edelwisa Mercado; Emmanuel Kagning Tsinda; F.G.M.; Francisco Gerardo Polotan; Hitoshi Oshitani; I.A.P.; Inez Andrea Medado; Jefferson Earl Halog; Joana Ina Manalo; Kirstyn Brunker; Lei Lanna Dancel; Ma Angelica Tujan; Ma. Angelica Tujan; Manalo; Mariko Saito-Obata; Mayuko Saito; Medado; Mercado; Michiko Okamoto; O.J.T.; Onza; Othoniel Jan Onza; Polotan; Samantha Louise Bado
EPI_ISL_2105289, EPI_ISL_2105304, EPI_ISL_2105337, EPI_ISL_2105418, EPI_ISL_2105442, EPI_ISL_2105461, EPI_ISL_2154387, EPI_ISL_2154390, EPI_ISL_2154395, EPI_ISL_2154418, EPI_ISL_2154423, EPI_ISL_2154443, EPI_ISL_2154444, EPI_ISL_2154449, EPI_ISL_2154454, EPI_ISL_2154463, EPI_ISL_2154469, EPI_ISL_2154473, EPI_ISL_2154474, EPI_ISL_2154475, EPI_ISL_2154509, EPI_ISL_2154510, EPI_ISL_2154518, EPI_ISL_2154521, EPI_ISL_2154527, EPI_ISL_2154541, EPI_ISL_2154548, EPI_ISL_2154550, EPI_ISL_2154553, EPI_ISL_2154556, EPI_ISL_2154561, EPI_ISL_2154564, EPI_ISL_2154574, EPI_ISL_2154576, EPI_ISL_2154580, EPI_ISL_2154587, EPI_ISL_2154592, EPI_ISL_2154594, EPI_ISL_2154597, EPI_ISL_2154598, EPI_ISL_2154622, EPI_ISL_2154630, EPI_ISL_2154643, EPI_ISL_2154645, EPI_ISL_2154646, EPI_ISL_2154650, EPI_ISL_2154654, EPI_ISL_2154660, EPI_ISL_2154665, EPI_ISL_2154668, EPI_ISL_2154673, EPI_ISL_2154676, EPI_ISL_2154679, EPI_ISL_2154683, EPI_ISL_2154693, EPI_ISL_2154697, EPI_ISL_2154714, EPI_ISL_2154719, EPI_ISL_2154725, EPI_ISL_2154726, EPI_ISL_2154727, EPI_ISL_2154729, EPI_ISL_2154734, EPI_ISL_2154736, EPI_ISL_2154737, EPI_ISL_2154740, EPI_ISL_2154742, EPI_ISL_2154749, EPI_ISL_2154763, EPI_ISL_2154766, EPI_ISL_2154769, EPI_ISL_2154770, EPI_ISL_2154794, EPI_ISL_2154795, EPI_ISL_2154800, EPI_ISL_2154801, EPI_ISL_2154802, EPI_ISL_2154807, EPI_ISL_2154821, EPI_ISL_2154835, EPI_ISL_2154855, EPI_ISL_2154857, EPI_ISL_2154860, EPI_ISL_2154869, EPI_ISL_2154884, EPI_ISL_2154890, EPI_ISL_2154892, EPI_ISL_2154909, EPI_ISL_2154950, EPI_ISL_2154988, EPI_ISL_2155051, EPI_ISL_2155054, EPI_ISL_2155055, EPI_ISL_2155056, EPI_ISL_2155060, EPI_ISL_2155072, EPI_ISL_2155074, EPI_ISL_2155075, EPI_ISL_2155076, EPI_ISL_2155077, EPI_ISL_2155079, EPI_ISL_2155083, EPI_ISL_2155086, EPI_ISL_2155092, EPI_ISL_2155093, EPI_ISL_2155094, EPI_ISL_2155095, EPI_ISL_2155097, EPI_ISL_2155099, EPI_ISL_2155100, EPI_ISL_2155101, EPI_ISL_2155102, EPI_ISL_2155103, EPI_ISL_2155105, EPI_ISL_2155109, EPI_ISL_2155111, EPI_ISL_2155112, EPI_ISL_2155116, EPI_ISL_2155119, EPI_ISL_2155120, EPI_ISL_2155122, EPI_ISL_2155124, EPI_ISL_2155129, EPI_ISL_2155131, EPI_ISL_2155134, EPI_ISL_2155135, EPI_ISL_2155136, EPI_ISL_2155143, EPI_ISL_2155149, EPI_ISL_2155152, EPI_ISL_2155153, EPI_ISL_2155159, EPI_ISL_2155162, EPI_ISL_2155163, EPI_ISL_2155164, EPI_ISL_2155166, EPI_ISL_2155167, EPI_ISL_2155172, EPI_ISL_2155176, EPI_ISL_2155177, EPI_ISL_2155178, EPI_ISL_2155180, EPI_ISL_2155181, EPI_ISL_2155183, EPI_ISL_2155185, EPI_ISL_2155189, EPI_ISL_2155190, EPI_ISL_2155192, EPI_ISL_2155193, EPI_ISL_2155197, EPI_ISL_2155201, EPI_ISL_2155206, EPI_ISL_2155208, EPI_ISL_2155210, EPI_ISL_2155214, EPI_ISL_2155219, EPI_ISL_2155221, EPI_ISL_2155223, EPI_ISL_2155227, EPI_ISL_2155232, EPI_ISL_2155233, EPI_ISL_2155234, EPI_ISL_2155236, EPI_ISL_2155240, EPI_ISL_2155241, EPI_ISL_2155244, EPI_ISL_2155245, EPI_ISL_2155247, EPI_ISL_2155248, EPI_ISL_2155250, EPI_ISL_2155255, EPI_ISL_2155261, EPI_ISL_2155266, EPI_ISL_2155273, EPI_ISL_2155275, EPI_ISL_2155280, EPI_ISL_2155281, EPI_ISL_2155282, EPI_ISL_2155284, EPI_ISL_2155287, EPI_ISL_2155288, EPI_ISL_2155289, EPI_ISL_2155296, EPI_ISL_2155298, EPI_ISL_2155299, EPI_ISL_2155300, EPI_ISL_2155301, EPI_ISL_2155305, EPI_ISL_2155307, EPI_ISL_2155311, EPI_ISL_2155312, EPI_ISL_2155314, EPI_ISL_2155316, EPI_ISL_2155320, EPI_ISL_2155321, EPI_ISL_2155322, EPI_ISL_2155326, EPI_ISL_2155332, EPI_ISL_2155334, EPI_ISL_2155335, EPI_ISL_2155340, EPI_ISL_2155342, EPI_ISL_2155343, EPI_ISL_2155347, EPI_ISL_2155350, EPI_ISL_2155353, EPI_ISL_2155355, EPI_ISL_2155358, EPI_ISL_2155606, EPI_ISL_2155607, EPI_ISL_2155608, EPI_ISL_2155609, EPI_ISL_2155610, EPI_ISL_2155611, EPI_ISL_2155612, EPI_ISL_2155613, EPI_ISL_2155614, EPI_ISL_2155615, EPI_ISL_2155616, EPI_ISL_2155617, EPI_ISL_2155618, EPI_ISL_2155619, EPI_ISL_2155620, EPI_ISL_2155621, EPI_ISL_2155622, EPI_ISL_2155623, EPI_ISL_2155624, EPI_ISL_2155625, EPI_ISL_2155626, EPI_ISL_2155627, EPI_ISL_2155628, EPI_ISL_2155629, EPI_ISL_2155630, EPI_ISL_2155631, EPI_ISL_2155632, EPI_ISL_2155633, EPI_ISL_2155634, EPI_ISL_2155635, EPI_ISL_2155636, EPI_ISL_2155637, EPI_ISL_2155638, EPI_ISL_2155639, EPI_ISL_2155964, EPI_ISL_2155965, EPI_ISL_2155966, EPI_ISL_2155967, EPI_ISL_2155968, EPI_ISL_2155969, EPI_ISL_2155970, EPI_ISL_2155971, EPI_ISL_2156170, EPI_ISL_2156171, EPI_ISL_2156172, EPI_ISL_2156173, EPI_ISL_2156174, EPI_ISL_2156175, EPI_ISL_2156176, EPI_ISL_2156177, EPI_ISL_2156178, EPI_ISL_2156179, EPI_ISL_2156180, EPI_ISL_2156181, EPI_ISL_2156182, EPI_ISL_2156183, EPI_ISL_2156326, EPI_ISL_2156351, EPI_ISL_2156352, EPI_ISL_2156353, EPI_ISL_2156354, EPI_ISL_2156355, EPI_ISL_2156356, EPI_ISL_2156357, EPI_ISL_2156358, EPI_ISL_2156359, EPI_ISL_2156360, EPI_ISL_2156361, EPI_ISL_2156372, EPI_ISL_2156373, EPI_ISL_2156374, EPI_ISL_2156375, EPI_ISL_2156376, EPI_ISL_2156377, EPI_ISL_2156378, EPI_ISL_2156396, EPI_ISL_2156435, EPI_ISL_2156436, EPI_ISL_2156437, EPI_ISL_2156438, EPI_ISL_2156439, EPI_ISL_2156440, EPI_ISL_2156441, EPI_ISL_2156442, EPI_ISL_2156443, EPI_ISL_2156493, EPI_ISL_2156519, EPI_ISL_2156520, EPI_ISL_2156521, EPI_ISL_2156525, EPI_ISL_2156528, EPI_ISL_2156539, EPI_ISL_2156540, EPI_ISL_2156544, EPI_ISL_2156545, EPI_ISL_2156558, EPI_ISL_2156559, EPI_ISL_2156560, EPI_ISL_2156561, EPI_ISL_2156562, EPI_ISL_2156563, EPI_ISL_2156568, EPI_ISL_2156569, EPI_ISL_2156570, EPI_ISL_2156574, EPI_ISL_2156600, EPI_ISL_2156607, EPI_ISL_2156608, EPI_ISL_2156617, EPI_ISL_2156626, EPI_ISL_2156631, EPI_ISL_2156632, EPI_ISL_2156633, EPI_ISL_2156634, EPI_ISL_2156635, EPI_ISL_2156638, EPI_ISL_2156639, EPI_ISL_2156640, EPI_ISL_2156641, EPI_ISL_2156642, EPI_ISL_2156643, EPI_ISL_2156644, EPI_ISL_2156645, EPI_ISL_2156646, EPI_ISL_2156647, EPI_ISL_2156648, EPI_ISL_2156649, EPI_ISL_2156651, EPI_ISL_2156653, EPI_ISL_2156654, EPI_ISL_2156655, EPI_ISL_2156656, EPI_ISL_2156657, EPI_ISL_2156661, EPI_ISL_2156664, EPI_ISL_2156665, EPI_ISL_2156669, EPI_ISL_2156670, EPI_ISL_2156671, EPI_ISL_2156674, EPI_ISL_2156675, EPI_ISL_2156676, EPI_ISL_2156680, EPI_ISL_2156681, EPI_ISL_2156684, EPI_ISL_2156696, EPI_ISL_2156698, EPI_ISL_2156699, EPI_ISL_2156701, EPI_ISL_2156702, EPI_ISL_2156703, EPI_ISL_2156704, EPI_ISL_2156706, EPI_ISL_2156707, EPI_ISL_2156708, EPI_ISL_2156709, EPI_ISL_2156710, EPI_ISL_2156713, EPI_ISL_2156715, EPI_ISL_2156716, EPI_ISL_2156717, EPI_ISL_2156718, EPI_ISL_2156719, EPI_ISL_2156721, EPI_ISL_2156722, EPI_ISL_2156723, EPI_ISL_2156724, EPI_ISL_2156725, EPI_ISL_2156726, EPI_ISL_2156727, EPI_ISL_2156728, EPI_ISL_2156735, EPI_ISL_2156736, EPI_ISL_2156737, EPI_ISL_2156738, EPI_ISL_2156739, EPI_ISL_2156740, EPI_ISL_2156741, EPI_ISL_2156742, EPI_ISL_2156743, EPI_ISL_2156744, EPI_ISL_2156745, EPI_ISL_2156746, EPI_ISL_2156747, EPI_ISL_2156748, EPI_ISL_2156750, EPI_ISL_2156751, EPI_ISL_2156752, EPI_ISL_2156753, EPI_ISL_2156755, EPI_ISL_2156756, EPI_ISL_2156758, EPI_ISL_2156759, EPI_ISL_2156764, EPI_ISL_2171123, EPI_ISL_2171215, EPI_ISL_2188043, EPI_ISL_2188079, EPI_ISL_2188172, EPI_ISL_2188274, EPI_ISL_2188701, EPI_ISL_2188742, EPI_ISL_2188750, EPI_ISL_2188756, EPI_ISL_2188782, EPI_ISL_2188836, EPI_ISL_2188841, EPI_ISL_2188846, EPI_ISL_2188848, EPI_ISL_2859133, EPI_ISL_2859135, EPI_ISL_2859146, EPI_ISL_2859147, EPI_ISL_2859148, EPI_ISL_2859149, EPI_ISL_2859155, EPI_ISL_2859156, EPI_ISL_2859159, EPI_ISL_2859162, EPI_ISL_2859165, EPI_ISL_2859173, EPI_ISL_2859178, EPI_ISL_2859180, EPI_ISL_2859181, EPI_ISL_2859183, EPI_ISL_2859184, EPI_ISL_2859189, EPI_ISL_2859220, EPI_ISL_2859223, EPI_ISL_2859224, EPI_ISL_2859227, EPI_ISL_2859242, EPI_ISL_2859244, EPI_ISL_2859246, EPI_ISL_2859248, EPI_ISL_2859251, EPI_ISL_2859252, EPI_ISL_2859265, EPI_ISL_2859268, EPI_ISL_2859276, EPI_ISL_2859278, EPI_ISL_2859282, EPI_ISL_2859293, EPI_ISL_2859295, EPI_ISL_2859307, EPI_ISL_2859310, EPI_ISL_2859311, EPI_ISL_2859314, EPI_ISL_2859322, EPI_ISL_2859326, EPI_ISL_2859331, EPI_ISL_2859335, EPI_ISL_2859346, EPI_ISL_2859347, EPI_ISL_2859350, EPI_ISL_2859351, EPI_ISL_2859355, EPI_ISL_2859357, EPI_ISL_2859362, EPI_ISL_2859363, EPI_ISL_2859364, EPI_ISL_2859377, EPI_ISL_2859380, EPI_ISL_2859606, EPI_ISL_2859620, EPI_ISL_2859627, EPI_ISL_2859631, EPI_ISL_2859632, EPI_ISL_2859641, EPI_ISL_2859644, EPI_ISL_2859646, EPI_ISL_2859671, EPI_ISL_2859684, EPI_ISL_2859719, EPI_ISL_2859748, EPI_ISL_2859759, EPI_ISL_2859767, EPI_ISL_2859788, EPI_ISL_2859815, EPI_ISL_2859841, EPI_ISL_2859844, EPI_ISL_2859850, EPI_ISL_2859869, EPI_ISL_2859944, EPI_ISL_2859959, EPI_ISL_2860014, EPI_ISL_2860066, EPI_ISL_2860081, EPI_ISL_2860126, EPI_ISL_2860136, EPI_ISL_2860162, EPI_ISL_4713304, EPI_ISL_4714061, EPI_ISL_4714275, EPI_ISL_4714517, EPI_ISL_4714962, EPI_ISL_4715008, EPI_ISL_4715087, EPI_ISL_4715142, EPI_ISL_4715258, EPI_ISL_4715891, EPI_ISL_4716001, EPI_ISL_4716047, EPI_ISL_4716074, EPI_ISL_4716075, EPI_ISL_4716181, EPI_ISL_4716192, EPI_ISL_4716193, EPI_ISL_4716197, EPI_ISL_4716278, EPI_ISL_4716318, EPI_ISL_4716321, EPI_ISL_4716425, EPI_ISL_4716445, EPI_ISL_4716460, EPI_ISL_4716530, EPI_ISL_4716628, EPI_ISL_4716648, EPI_ISL_4716655, EPI_ISL_4716661, EPI_ISL_4716676, EPI_ISL_4716703, EPI_ISL_4716719, EPI_ISL_4716739, EPI_ISL_4716863, EPI_ISL_4717155			
see above	Research Institute for Tropical Medicine, Inc. (RITM)	Philippine Genome Center	Alethea R. de Guzman; Anna Ong-Lim; Arianne A. Zamora; Asia Louisa U. Chong; Benedict A. Maralit; Candice Francheska B. Tambaoan; Carlo M. Lapid; Celia Carlos; Cynthia P. Saloma; Devon Ray Pacial; Diomedes A. Carino; Diomedes A. Cariño; Edsel Maurice Salvana; Edsel Maurice Salvaña; El King D. Morado; Elcid Aaron R. Pangilinan; Eva Maria Cutiongco-de la Paz; Francis A. Tablizo; Henrietta Marie Rodriguez; Irish Coleen A. Asin; Jaime C. Montoya; Jan Michael C. Yap; Jarvin E. Nipales; Jo-Hannah S. Llames; John Q. Wong; Joshua Gregor A. Dizon; Juan Antonio R. Magalang; Karol Sophia Agape R. Padilla; Kenneth M. Kim; Kris P. Punayan; Krisitna Patriz Dela Cruz; Kristina Patriz Dela Cruz; Lindsay Claire D.L. Carandang; Lindsay Clare D.L. Carandang; Ma. Exanil Plantig; Marc Edsel C. Ayes; Marc Jerrone R. Castro; Maria Rosario Singh-Vergeire; Maria Rosario Singh-Vergeire and Cynthia P. Saloma; Maria Sofia L. Yangzon; Marielle M Gamboa; Marielle M. Gamboa; Marissa Alejandria; Nina Francesca Bustamante; Niña Francesca Bustamante; Razel Nikka M. Hao; Renato Jacinto Q. Mantaring; Rianna Patricia S. Cruz; Sheila Mae M. Araiza; Shiela Mae M. Araiza; Yvonne Valerie Austria; Zipporah Mariebelle R. Enriquez; Zyrel V. Mollejon
EPI_ISL_430456	Rizal Medical Center	Research Institute for	Bautista; Brunker, K.; C.S.; C.T.; D.L.; Demetria; E.S.; F.G.M.; I.A.P.; Manalo; Medado; Mercado; O.J.T.; Onza; Polotan

		Tropical Medicine	
EPI_ISL_3105870	Rizal Provincial Hospital System - Antipolo Annex 1	Research Institute for Tropical Medicine	Clyde Dapat; Deana Mae Ocampo; Emmanuel Kagning Tsinda; Francisco Gerardo Polotan; Hitoshi Oshitani; Inez Andrea Medado; Jefferson Earl Halog; Joana Ina Manalo; Lei Lanna Dancel; Ma Angelica Tujan; Mariko Saito-Obata; Mayuko Saito; Michiko Okamoto; Samantha Louise Bado
EPI_ISL_491298	Rural Health Unit - Calauan, Laguna	Research Institute for Tropical Medicine	Bautista; Brunker, K.; C.S.; C.T.; D.L.; Demetria; E.S.; F.G.M.; I.A.P.; M.A.A.; Manalo; Medado; Mercado; O.J.T.; Onza; Polotan; Tujan
EPI_ISL_4459524	SABLAYAN RURAL HEALTH UNIT	Research Institute for Tropical Medicine/Tohoku University	Allen Anthony Laraño; Ardiane Ysabelle Dolor; Catalino Demetria; Clyde Dapat; Emmanuel Kagning Tsinda; Francisco Gerardo Polotan; Hitoshi Oshitani; Inez Andrea Medado; Jefferson Earl Halog; Lei Lanna Dancel; Leilanie Nacion; Ma. Angelica Tujan; Machiko Okamoto; Maria Carmen Corpuz; Mariko Siato-Obata; Mayuko Saito
EPI_ISL_2188060, EPI_ISL_2188108, EPI_ISL_2188126, EPI_ISL_2188135, EPI_ISL_2188276, EPI_ISL_4712444, EPI_ISL_4712556			
see above	SAN ANTONIO CITY OF ILAGAN HOSPITAL	Philippine Genome Center	Alethea R. de Guzman; Anna Ong-Lim; Arianne A. Zamora; Asia Louisa U. Chong; Benedict A. Maralit; Candice Francheska B. Tambaoan; Carlo M. Lapid; Celia Carlos; Cynthia P. Saloma; Devon Ray Pacial; Diomedes A. Cariño; Edsel Maurice Salvana; Edsel Maurice Salvaña; El King D. Morado; Elcid Aaron R. Pangilinan; Eva Maria Cutiongco-de la Paz; Francis A. Tablizo; Henrietta Marie Rodriguez; Irish Coleen A. Asin; Jaime C. Montoya; Jan Michael C. Yap; Jarvin E. Nipales; Jo-Hannah S. Llames; John Q. Wong; Joshua Gregor A. Dizon; Juan Antonio R. Magalang; Karol Sophia Agape R. Padilla; Kenneth M. Kim; Kris P. Punayan; Kristina Patriz Dela Cruz; Lindsay Clare D.L. Carandang; Ma. Exanil Plantig; Marc Edsel C. Ayes; Marc Jerrone R. Castro; Maria Rosario Singh-Vergeire; Maria Rosario Singh-Vergeire and Cynthia P. Saloma; Maria Sofia L. Yangzon; Marielle M. Gamboa; Marissa Alejandria; Niña Francesca Bustamante; Razel Nikka M. Hao; Renato Jacinto Q. Mantaring; Rianna Patricia S. Cruz; Sheila Mae M. Araiza; Shiela Mae M. Araiza; Yvonne Valerie Austria; Zipporah Mariebelle R. Enriquez; Zyrel V. Mollejon
EPI_ISL_4459414	SAN JOSE MUNICIPAL HEALTH OFFICE	Research Institute for Tropical Medicine/Tohoku University	Allen Anthony Laraño; Ardiane Ysabelle Dolor; Catalino Demetria; Clyde Dapat; Emmanuel Kagning Tsinda; Francisco Gerardo Polotan; Hitoshi Oshitani; Inez Andrea Medado; Jefferson Earl Halog; Lei Lanna Dancel; Leilanie Nacion; Ma. Angelica Tujan; Machiko Okamoto; Maria Carmen Corpuz; Mariko Siato-Obata; Mayuko Saito
EPI_ISL_4459505	SAN JUAN DE DIOS EDUCATIONAL FOUNDATION INC.	Research Institute for Tropical Medicine/Tohoku University	Allen Anthony Laraño; Ardiane Ysabelle Dolor; Catalino Demetria; Clyde Dapat; Emmanuel Kagning Tsinda; Francisco Gerardo Polotan; Hitoshi Oshitani; Inez Andrea Medado; Jefferson Earl Halog; Lei Lanna Dancel; Leilanie Nacion; Ma. Angelica Tujan; Machiko Okamoto; Maria Carmen Corpuz; Mariko Siato-Obata; Mayuko Saito
EPI_ISL_4459333	SAN JUAN RURAL HEALTH UNIT	Research Institute for Tropical Medicine/Tohoku University	Allen Anthony Laraño; Ardiane Ysabelle Dolor; Catalino Demetria; Clyde Dapat; Emmanuel Kagning Tsinda; Francisco Gerardo Polotan; Hitoshi Oshitani; Inez Andrea Medado; Jefferson Earl Halog; Lei Lanna Dancel; Leilanie Nacion; Ma. Angelica Tujan; Machiko Okamoto; Maria Carmen Corpuz; Mariko Siato-Obata; Mayuko Saito
EPI_ISL_2155600	SAN PABLO CITY GENERAL HOSPITAL	Philippine Genome Center	Alethea R. de Guzman; Anna Ong-Lim; Arianne A. Zamora; Asia Louisa U. Chong; Benedict A. Maralit; Candice Francheska B. Tambaoan; Carlo M. Lapid; Celia Carlos; Devon Ray Pacial; Edsel Maurice Salvaña; El King D. Morado; Eva Maria Cutiongco-de la Paz; Francis A. Tablizo; Irish Coleen A. Asin; Jaime C. Montoya; Jan Michael C. Yap; Jo-Hannah S. Llames; John Q. Wong; Joshua Gregor A. Dizon; Juan Antonio R. Magalang; Karol Sophia Agape R. Padilla; Kenneth M. Kim; Kris P. Punayan; Marc Edsel C. Ayes; Marc Jerrone R. Castro; Maria Rosario Singh-Vergeire and Cynthia P. Saloma; Maria Sofia L. Yangzon; Marissa Alejandria; Razel Nikka M. Hao; Rianna Patricia S. Cruz; Sheila Mae M. Araiza
EPI_ISL_2155068, EPI_ISL_2155140, EPI_ISL_2155141	SAN PABLO CITY HEALTH OFFICE	Philippine Genome Center	Alethea R. de Guzman; Anna Ong-Lim; Arianne A. Zamora; Asia Louisa U. Chong; Benedict A. Maralit; Candice Francheska B. Tambaoan; Carlo M. Lapid; Celia Carlos; Devon Ray Pacial; Edsel Maurice Salvaña; El King D. Morado; Eva Maria Cutiongco-de la Paz; Francis A. Tablizo; Irish Coleen A. Asin; Jaime C. Montoya; Jan Michael C. Yap; Jo-Hannah S. Llames; John Q. Wong; Joshua Gregor A. Dizon; Juan Antonio R. Magalang; Karol Sophia Agape R. Padilla; Kenneth M. Kim; Kris P. Punayan; Marc Edsel C. Ayes; Marc Jerrone R. Castro; Maria Rosario Singh-Vergeire and Cynthia P. Saloma; Maria Sofia L. Yangzon; Marissa Alejandria; Razel Nikka M. Hao; Rianna Patricia S. Cruz; Sheila Mae M. Araiza
EPI_ISL_4459529	SAN PEDRO DOCTORS HOSPITAL, INC.	Research Institute for Tropical Medicine/Tohoku University	Allen Anthony Laraño; Ardiane Ysabelle Dolor; Catalino Demetria; Clyde Dapat; Emmanuel Kagning Tsinda; Francisco Gerardo Polotan; Hitoshi Oshitani; Inez Andrea Medado; Jefferson Earl Halog; Lei Lanna Dancel; Leilanie Nacion; Ma. Angelica Tujan; Machiko Okamoto; Maria Carmen Corpuz; Mariko Siato-Obata; Mayuko Saito
EPI_ISL_4459518	SAN PEDRO RURAL HEALTH UNIT I	Research Institute for Tropical Medicine/Tohoku University	Allen Anthony Laraño; Ardiane Ysabelle Dolor; Catalino Demetria; Clyde Dapat; Emmanuel Kagning Tsinda; Francisco Gerardo Polotan; Hitoshi Oshitani; Inez Andrea Medado; Jefferson Earl Halog; Lei Lanna Dancel; Leilanie Nacion; Ma. Angelica Tujan; Machiko Okamoto; Maria Carmen Corpuz; Mariko Siato-Obata; Mayuko Saito
EPI_ISL_4459310	SAN ROQUE HEALTH CENTER	Research Institute for Tropical Medicine/Tohoku University	Allen Anthony Laraño; Ardiane Ysabelle Dolor; Catalino Demetria; Clyde Dapat; Emmanuel Kagning Tsinda; Francisco Gerardo Polotan; Hitoshi Oshitani; Inez Andrea Medado; Jefferson Earl Halog; Lei Lanna Dancel; Leilanie Nacion; Ma. Angelica Tujan; Machiko Okamoto; Maria Carmen Corpuz; Mariko Siato-Obata; Mayuko Saito
EPI_ISL_4459342, EPI_ISL_4459446	SANTA CRUZ DISTRICT HOSPITAL	Research Institute for Tropical Medicine/Tohoku University	Allen Anthony Laraño; Ardiane Ysabelle Dolor; Catalino Demetria; Clyde Dapat; Emmanuel Kagning Tsinda; Francisco Gerardo Polotan; Hitoshi Oshitani; Inez Andrea Medado; Jefferson Earl Halog; Lei Lanna Dancel; Leilanie Nacion; Ma. Angelica Tujan; Machiko Okamoto; Maria Carmen Corpuz; Mariko Siato-Obata; Mayuko Saito
EPI_ISL_4459490	SANTA CRUZ RURAL HEALTH UNIT I	Research Institute for Tropical Medicine/Tohoku University	Allen Anthony Laraño; Ardiane Ysabelle Dolor; Catalino Demetria; Clyde Dapat; Emmanuel Kagning Tsinda; Francisco Gerardo Polotan; Hitoshi Oshitani; Inez Andrea Medado; Jefferson Earl Halog; Lei Lanna Dancel; Leilanie Nacion; Ma. Angelica Tujan; Machiko Okamoto; Maria Carmen Corpuz; Mariko Siato-Obata; Mayuko Saito
EPI_ISL_4459443	SANTA CRUZ RURAL HEALTH UNIT II	Research Institute for Tropical Medicine/Tohoku University	Allen Anthony Laraño; Ardiane Ysabelle Dolor; Catalino Demetria; Clyde Dapat; Emmanuel Kagning Tsinda; Francisco Gerardo Polotan; Hitoshi Oshitani; Inez Andrea Medado; Jefferson Earl Halog; Lei Lanna Dancel; Leilanie Nacion; Ma. Angelica Tujan; Machiko Okamoto; Maria Carmen Corpuz; Mariko Siato-Obata; Mayuko Saito
EPI_ISL_4459346	SANTA TERESITA RURAL HEALTH UNIT	Research Institute for Tropical Medicine/Tohoku University	Allen Anthony Laraño; Ardiane Ysabelle Dolor; Catalino Demetria; Clyde Dapat; Emmanuel Kagning Tsinda; Francisco Gerardo Polotan; Hitoshi Oshitani; Inez Andrea Medado; Jefferson Earl Halog; Lei Lanna Dancel; Leilanie Nacion; Ma. Angelica Tujan; Machiko Okamoto; Maria Carmen Corpuz; Mariko Siato-Obata; Mayuko Saito
EPI_ISL_2156586	SOLANA RURAL HEALTH UNIT	Philippine Genome Center	Alethea R. de Guzman; Anna Ong-Lim; Arianne A. Zamora; Asia Louisa U. Chong; Benedict A. Maralit; Candice Francheska B. Tambaoan; Carlo M. Lapid; Celia Carlos; Devon Ray Pacial; Edsel Maurice Salvaña; El King D. Morado; Elcid Aaron R. Pangilinan; Eva Maria Cutiongco-de la Paz; Francis A. Tablizo; Irish Coleen A. Asin; Jaime C. Montoya; Jan Michael C. Yap; Jo-Hannah S. Llames; John Q. Wong; Joshua Gregor A. Dizon; Juan Antonio R. Magalang; Karol Sophia Agape R. Padilla; Kenneth M. Kim; Kris P. Punayan; Marc Edsel C. Ayes; Marc Jerrone R. Castro; Maria Rosario Singh-Vergeire and Cynthia P. Saloma; Maria Sofia L. Yangzon; Marissa Alejandria; Razel Nikka M. Hao; Rianna Patricia S. Cruz; Sheila Mae M. Araiza
EPI_ISL_1081795	SOUTH SUPER HI-WAY	Philippine Genome Center	Alethea R. de Guzman; Anna Ong-Lim; Arianne A. Zamora; Asia Louisa U. Chong; Benedict A. Maralit; Candice Francheska B. Tambaoan; Carlo M. Lapid; Celia Carlos; Cynthia P. Saloma; Devon Ray Pacial; Edsel Maurice Salvaña; El King D. Morado; Eva Maria Cutiongco-de la Paz; Francis A. Tablizo; Irish Coleen A. Asin; Jaime C. Montoya; Jan Michael C. Yap; Jo-Hannah S. Llames; John Q. Wong; Joshua Gregor A. Dizon; Juan Antonio R. Magalang; Karol Sophia Agape R. Padilla; Kenneth M. Kim; Kris P. Punayan; Marc Edsel C. Ayes; Marc Jerrone R. Castro; Maria Sofia L. Yangzon; Marissa Alejandria; Razel Nikka M. Hao; Rianna Patricia S. Cruz; Sheila Mae M. Araiza; and Maria Rosario Singh-Vergeire
EPI_ISL_4459287	ST. CLARE'S MEDICAL CENTER, INC.	Research Institute for Tropical Medicine/Tohoku University	Allen Anthony Laraño; Ardiane Ysabelle Dolor; Catalino Demetria; Clyde Dapat; Emmanuel Kagning Tsinda; Francisco Gerardo Polotan; Hitoshi Oshitani; Inez Andrea Medado; Jefferson Earl Halog; Lei Lanna Dancel; Leilanie Nacion; Ma. Angelica Tujan; Machiko Okamoto; Maria Carmen Corpuz; Mariko Siato-Obata; Mayuko Saito
EPI_ISL_2171104	STA. ROSA CHO - GENEXPERT LABORATORY	Philippine Genome Center	Alethea R. de Guzman; Anna Ong-Lim; Arianne A. Zamora; Asia Louisa U. Chong; Benedict A. Maralit; Candice Francheska B. Tambaoan; Carlo M. Lapid; Celia Carlos; Devon Ray Pacial; Edsel Maurice Salvaña; El King D. Morado; Elcid Aaron R. Pangilinan; Eva Maria Cutiongco-de la Paz; Francis A. Tablizo; Irish Coleen A. Asin; Jaime C. Montoya; Jan Michael C. Yap; Jo-Hannah S. Llames; John Q. Wong; Joshua Gregor A. Dizon; Juan Antonio R. Magalang; Karol Sophia Agape R. Padilla; Kenneth M. Kim; Kris P. Punayan; Marc Edsel C. Ayes; Marc Jerrone R. Castro; Maria Rosario Singh-Vergeire and Cynthia P. Saloma; Maria Sofia L. Yangzon; Marissa Alejandria; Razel Nikka M. Hao; Rianna Patricia S. Cruz; Sheila Mae M. Araiza
EPI_ISL_2105340, EPI_ISL_2105377, EPI_ISL_2105394, EPI_ISL_2155797, EPI_ISL_2155798, EPI_ISL_2155799, EPI_ISL_2155800, EPI_ISL_2155801, EPI_ISL_2155802, EPI_ISL_2155803, EPI_ISL_2155804, EPI_ISL_2155805, EPI_ISL_2155806, EPI_ISL_2155807, EPI_ISL_2155808, EPI_ISL_2155809, EPI_ISL_2155810, EPI_ISL_2155811, EPI_ISL_2155812, EPI_ISL_2156250, EPI_ISL_2156251, EPI_ISL_2156390, EPI_ISL_2156413, EPI_ISL_2171139, EPI_ISL_2171190, EPI_ISL_2189088, EPI_ISL_2189147, EPI_ISL_2189397, EPI_ISL_4713592			
see above	Safeguard DNA Diagnostic, Inc.	Philippine Genome Center	Alethea R. de Guzman; Anna Ong-Lim; Arianne A. Zamora; Asia Louisa U. Chong; Benedict A. Maralit; Candice Francheska B. Tambaoan; Carlo M. Lapid; Celia Carlos; Cynthia P. Saloma; Devon Ray Pacial; Diomedes A. Cariño; Edsel Maurice Salvana; Edsel Maurice Salvaña; El King D. Morado; Elcid Aaron R. Pangilinan; Eva Maria Cutiongco-de la Paz; Francis A. Tablizo; Henrietta Marie Rodriguez; Irish Coleen A. Asin; Jaime C. Montoya; Jan Michael C. Yap; Jarvin E. Nipales; Jo-Hannah S. Llames; John Q. Wong; Joshua Gregor A. Dizon; Juan Antonio R. Magalang; Karol Sophia Agape R. Padilla; Kenneth M. Kim; Kris P. Punayan; Kristina Patriz Dela Cruz; Lindsay Clare D.L. Carandang; Ma. Exanil Plantig; Marc Edsel C. Ayes; Marc Jerrone R. Castro; Maria Rosario Singh-Vergeire; Maria Rosario Singh-Vergeire and Cynthia P. Saloma; Maria Sofia L. Yangzon; Marielle M. Gamboa; Marissa Alejandria; Niña Francesca Bustamante; Razel Nikka M. Hao; Renato Jacinto Q. Mantaring; Rianna Patricia S. Cruz; Sheila Mae M. Araiza; Shiela Mae M. Araiza; Yvonne Valerie Austria; Zipporah Mariebelle R. Enriquez; Zyrel V. Mollejon
EPI_ISL_2105287, EPI_ISL_2155664, EPI_ISL_2155665, EPI_ISL_2155666, EPI_ISL_2155667, EPI_ISL_2155668, EPI_ISL_2155669, EPI_ISL_2155670, EPI_ISL_2155671, EPI_ISL_2155672, EPI_ISL_2155673, EPI_ISL_2155674, EPI_ISL_2155675, EPI_ISL_2156497, EPI_ISL_2156583, EPI_ISL_2156590, EPI_ISL_2188960, EPI_ISL_2859702, EPI_ISL_2859714, EPI_ISL_2859921, EPI_ISL_4714022			
see above	San Antonio City of Ilagan Hospital	Philippine Genome Center	Alethea R. de Guzman; Anna Ong-Lim; Arianne A. Zamora; Asia Louisa U. Chong; Benedict A. Maralit; Candice Francheska B. Tambaoan; Carlo M. Lapid; Celia Carlos; Cynthia P. Saloma; Devon Ray Pacial; Diomedes A. Carino; Diomedes A. Cariño; Edsel Maurice Salvana; Edsel Maurice Salvaña; El King D. Morado; Elcid Aaron R. Pangilinan; Eva Maria Cutiongco-de la Paz; Francis A. Tablizo; Henrietta Marie Rodriguez; Irish Coleen A. Asin; Jaime C. Montoya; Jan Michael C. Yap; Jarvin E. Nipales; Jo-Hannah S. Llames; John Q. Wong; Joshua Gregor A. Dizon; Juan Antonio R. Magalang; Karol Sophia Agape R. Padilla; Kenneth M. Kim; Kris P. Punayan; Krisitna Patriz Dela Cruz; Kristina Patriz Dela Cruz; Lindsay Claire D.L. Carandang; Lindsay Clare D.L. Carandang; Ma. Exanil Plantig; Marc Edsel C. Ayes; Marc Jerrone R. Castro; Maria Rosario Singh-Vergeire; Maria Rosario Singh-Vergeire and Cynthia P. Saloma; Maria Sofia L. Yangzon; Marielle M Gamboa; Marielle M. Gamboa; Marissa Alejandria; Nina Francesca Bustamante; Niña Francesca Bustamante; Razel Nikka M. Hao; Renato Jacinto Q. Mantaring; Rianna Patricia S. Cruz; Sheila Mae M. Araiza; Shiela Mae M. Araiza; Yvonne Valerie Austria; Zipporah Mariebelle R. Enriquez; Zyrel V. Mollejon
EPI_ISL_2155842, EPI_ISL_2155843, EPI_ISL_2155844, EPI_ISL_2155847, EPI_ISL_2156314, EPI_ISL_2156389	San Lazaro Hospital	Philippine Genome Center	Alethea R. de Guzman; Anna Ong-Lim; Arianne A. Zamora; Asia Louisa U. Chong; Benedict A. Maralit; Candice Francheska B. Tambaoan; Carlo M. Lapid; Celia Carlos; Devon Ray Pacial; Edsel Maurice Salvaña; El King D. Morado; Elcid Aaron R. Pangilinan; Eva Maria Cutiongco-de la Paz; Francis A. Tablizo; Irish Coleen A. Asin; Jaime C. Montoya; Jan Michael C. Yap; Jo-Hannah S. Llames; John Q. Wong; Joshua Gregor A. Dizon; Juan Antonio R. Magalang; Karol Sophia Agape R. Padilla; Kenneth M. Kim; Kris P. Punayan; Marc Edsel C. Ayes; Marc Jerrone R. Castro; Maria Rosario Singh-Vergeire and Cynthia P. Saloma; Maria Sofia L. Yangzon; Marissa Alejandria; Razel Nikka M. Hao; Rianna Patricia S. Cruz; Sheila Mae M. Araiza
EPI_ISL_491465, EPI_ISL_491466, EPI_ISL_491471	San Lazaro Hospital	Research Institute for Tropical Medicine	Catalino Demetria; Criselda Bautista; Daria Manalo; Edelwisa Mercado; Francisco Gerardo Polotan; Inez Andrea Medado; Kirstyn Brunker; Ma. Angelica Tujan; Othoniel Jan Onza
EPI_ISL_2155098, EPI_ISL_2156226, EPI_ISL_2156227,	San Lazaro Hospital (SLH)	Philippine Genome Center	Alethea R. de Guzman; Anna Ong-Lim; Arianne A. Zamora; Asia Louisa U. Chong; Benedict A. Maralit; Candice Francheska B. Tambaoan; Carlo M. Lapid; Celia Carlos; Devon Ray Pacial; Edsel Maurice Salvaña; El King D. Morado; Elcid Aaron R. Pangilinan; Eva Maria Cutiongco-de la Paz; Francis A. Tablizo; Irish Coleen A. Asin; Jaime C. Montoya; Jan Michael C. Yap; Jo-Hannah S. Llames; John Q. Wong; Joshua Gregor A. Dizon; Juan Antonio R. Magalang; Karol Sophia Agape R. Padilla; Kenneth M. Kim; Kris P. Punayan; Marc Edsel C. Ayes; Marc Jerrone R. Castro; Maria Rosario Singh-Vergeire and Cynthia P. Saloma; Maria Sofia L. Yangzon; Marissa Alejandria; Razel Nikka M. Hao; Rianna Patricia S. Cruz; Sheila Mae M. Araiza

EPI_ISL_2156482			
EPI_ISL_3105880	San Pascual Rural Health Unit	Research Institute for Tropical Medicine	Clyde Dapat; Deana Mae Ocampo; Emmanuel Kagning Tsinda; Francisco Gerardo Polotan; Hitoshi Oshitani; Inez Andrea Medado; Jefferson Earl Halog; Joana Ina Manalo; Lei Lanna Dancel; Ma Angelica Tujan; Mariko Saito-Obata; Mayuko Saito; Michiko Okamoto; Samantha Louise Bado
EPI_ISL_1069177, EPI_ISL_1069178	Sariaya Rural Health Unit	Research Institute for Tropical Medicine	Catalino Demetria; Daria Manalo; Edelwisa Mercado; Francisco Gerardo Polotan; Hannah Leah Morito; Inez Andrea Medado; John Leonard Chan; Joseph Hughes; Kirstyn Brunker; Ma Angelica Tujan; Othoniel Jan Onza
EPI_ISL_833335, EPI_ISL_833338	Siniloan Rural Health Unit	Research Institute for Tropical Medicine	Catalino Demetria; Daria Manalo; Edelwisa Mercado; Francisco Gerardo Polotan; Hannah Leah Morito; Inez Andrea Medado; John Leonard Chan; Kirstyn Brunker; Ma Angelica Tujan; Othoniel Jan Onza
EPI_ISL_2105232, EPI_ISL_2105238, EPI_ISL_2105274, EPI_ISL_2105275, EPI_ISL_2154389, EPI_ISL_2154416, EPI_ISL_2154433, EPI_ISL_2154448, EPI_ISL_2154456, EPI_ISL_2154496, EPI_ISL_2154504, EPI_ISL_2154519, EPI_ISL_2154575, EPI_ISL_2154593, EPI_ISL_2154595, EPI_ISL_2154619, EPI_ISL_2154621, EPI_ISL_2154631, EPI_ISL_2154632, EPI_ISL_2154651, EPI_ISL_2154655, EPI_ISL_2154664, EPI_ISL_2154685, EPI_ISL_2154696, EPI_ISL_2154707, EPI_ISL_2154708, EPI_ISL_2154712, EPI_ISL_2154735, EPI_ISL_2154754, EPI_ISL_2154788, EPI_ISL_2154796, EPI_ISL_2154808, EPI_ISL_2154840, EPI_ISL_2154848, EPI_ISL_2154917, EPI_ISL_2154926, EPI_ISL_2154941, EPI_ISL_2154949, EPI_ISL_2154958, EPI_ISL_2156636, EPI_ISL_2156658, EPI_ISL_2171121, EPI_ISL_2171133, EPI_ISL_2171171, EPI_ISL_2171176, EPI_ISL_2171206, EPI_ISL_2171218, EPI_ISL_2188940, EPI_ISL_2189024, EPI_ISL_2189112, EPI_ISL_2189118, EPI_ISL_2189222, EPI_ISL_2189228, EPI_ISL_2189293, EPI_ISL_2189331, EPI_ISL_2189396, EPI_ISL_2189453, EPI_ISL_2189462, EPI_ISL_2189521, EPI_ISL_4713344, EPI_ISL_4713651, EPI_ISL_4713667			
see above	South Super Highway Molecular Diagnostic Laboratory	Philippine Genome Center	Alethea R. de Guzman; Anna Ong-Lim; Arianne A. Zamora; Asia Louisa U. Chong; Benedict A. Maralit; Candice Francheska B. Tambaoan; Carlo M. Lapid; Celia Carlos; Cynthia P. Saloma; Devon Ray Pacial; Diomedes A. Cariño; Edsel Maurice Salvana; Edsel Maurice Salvaña; El King D. Morado; Elcid Aaron R. Pangilinan; Eva Maria Cutiongco-de la Paz; Francis A. Tablizo; Henrietta Marie Rodriguez; Irish Coleen A. Asin; Jaime C. Montoya; Jan Michael C. Yap; Jarvin E. Nipales; Jo-Hannah S. Llames; John Q. Wong; Joshua Gregor A. Dizon; Juan Antonio R. Magalang; Karol Sophia Agape R. Padilla; Kenneth M. Kim; Kris P. Punayan; Kristina Patriz Dela Cruz; Lindsay Clare D.L. Carandang; Ma. Exanil Plantig; Marc Edsel C. Ayes; Marc Jerrone R. Castro; Maria Rosario Singh-Vergeire; Maria Rosario Singh-Vergeire and Cynthia P. Saloma; Maria Sofia L. Yangzon; Marielle M. Gamboa; Marissa Alejandria; Niña Francesca Bustamante; Razel Nikka M. Hao; Renato Jacinto Q. Mantaring; Rianna Patricia S. Cruz; Sheila Mae M. Araiza; Shiela Mae M. Araiza; Yvonne Valerie Austria; Zipporah Mariebelle R. Enriquez; Zyrel V. Mollejon
EPI_ISL_2154417, EPI_ISL_2154420, EPI_ISL_2154436, EPI_ISL_2154440, EPI_ISL_2154467, EPI_ISL_2154493, EPI_ISL_2154506, EPI_ISL_2154538, EPI_ISL_2154547, EPI_ISL_2154565, EPI_ISL_2154566, EPI_ISL_2154568, EPI_ISL_2154590, EPI_ISL_2154616, EPI_ISL_2154620, EPI_ISL_2154623, EPI_ISL_2154670, EPI_ISL_2154701, EPI_ISL_2154720, EPI_ISL_2154721, EPI_ISL_2154761, EPI_ISL_2154780, EPI_ISL_2154784, EPI_ISL_2154797, EPI_ISL_2154828, EPI_ISL_2154831, EPI_ISL_2154864, EPI_ISL_2154875, EPI_ISL_2154901, EPI_ISL_2154932, EPI_ISL_2154943, EPI_ISL_2155080, EPI_ISL_2155276, EPI_ISL_2155813, EPI_ISL_2155814, EPI_ISL_2155815, EPI_ISL_2155816, EPI_ISL_2155817, EPI_ISL_2155818, EPI_ISL_2155819, EPI_ISL_2155820, EPI_ISL_2155821, EPI_ISL_2156252, EPI_ISL_2156253, EPI_ISL_2156254, EPI_ISL_2156255, EPI_ISL_2156495, EPI_ISL_2156522, EPI_ISL_2156666, EPI_ISL_2156668, EPI_ISL_2156672, EPI_ISL_2156673, EPI_ISL_2156677, EPI_ISL_2156678, EPI_ISL_2156685, EPI_ISL_2156686, EPI_ISL_2156687, EPI_ISL_2156688, EPI_ISL_2156689, EPI_ISL_2156690, EPI_ISL_2156691, EPI_ISL_2156692, EPI_ISL_2156693, EPI_ISL_2156694, EPI_ISL_2156695, EPI_ISL_2859673, EPI_ISL_2859830, EPI_ISL_4712081, EPI_ISL_4712193, EPI_ISL_4712369, EPI_ISL_4712396, EPI_ISL_4712503, EPI_ISL_4712886, EPI_ISL_4713847, EPI_ISL_4713876, EPI_ISL_4714317			
see above	Southern Isabela Medical Center Molecular Diagnostic & Research Laboratory (SIMC - MDRL)	Philippine Genome Center	Alethea R. de Guzman; Anna Ong-Lim; Arianne A. Zamora; Asia Louisa U. Chong; Benedict A. Maralit; Candice Francheska B. Tambaoan; Carlo M. Lapid; Celia Carlos; Cynthia P. Saloma; Devon Ray Pacial; Diomedes A. Carino; Diomedes A. Cariño; Edsel Maurice Salvana; Edsel Maurice Salvaña; El King D. Morado; Elcid Aaron R. Pangilinan; Eva Maria Cutiongco-de la Paz; Francis A. Tablizo; Henrietta Marie Rodriguez; Irish Coleen A. Asin; Jaime C. Montoya; Jan Michael C. Yap; Jarvin E. Nipales; Jo-Hannah S. Llames; John Q. Wong; Joshua Gregor A. Dizon; Juan Antonio R. Magalang; Karol Sophia Agape R. Padilla; Kenneth M. Kim; Kris P. Punayan; Krisitna Patriz Dela Cruz; Kristina Patriz Dela Cruz; Lindsay Claire D.L. Carandang; Lindsay Clare D.L. Carandang; Ma. Exanil Plantig; Marc Edsel C. Ayes; Marc Jerrone R. Castro; Maria Rosario Singh-Vergeire; Maria Rosario Singh-Vergeire and Cynthia P. Saloma; Maria Sofia L. Yangzon; Marielle M Gamboa; Marielle M. Gamboa; Marissa Alejandria; Nina Francesca Bustamante; Niña Francesca Bustamante; Razel Nikka M. Hao; Renato Jacinto Q. Mantaring; Rianna Patricia S. Cruz; Sheila Mae M. Araiza; Shiela Mae M. Araiza; Yvonne Valerie Austria; Zipporah Mariebelle R. Enriquez; Zyrel V. Mollejon
EPI_ISL_1122455	Southern Philippines Medical Center	Philippine Genome Center	Alethea R. de Guzman; Anna Ong-Lim; Arianne A. Zamora; Asia Louisa U. Chong; Benedict A. Maralit; Candice Francheska B. Tambaoan; Carlo M. Lapid; Celia Carlos; Devon Ray Pacial; Edsel Maurice Salvaña; El King D. Morado; Eva Maria Cutiongco-de la Paz; Francis A. Tablizo; Irish Coleen A. Asin; Jaime C. Montoya; Jan Michael C. Yap; Jo-Hannah S. Llames; John Q. Wong; Joshua Gregor A. Dizon; Juan Antonio R. Magalang; Karol Sophia Agape R. Padilla; Kenneth M. Kim; Kris P. Punayan; Marc Edsel C. Ayes; Marc Jerrone R. Castro; Maria Rosario Singh-Vergeire and Cynthia P. Saloma; Maria Sofia L. Yangzon; Marissa Alejandria; Razel Nikka M. Hao; Rianna Patricia S. Cruz; Sheila Mae M. Araiza
EPI_ISL_1081827	Southern Philippines Medical Center (Davao)	Philippine Genome Center	Alethea R. de Guzman; Anna Ong-Lim; Arianne A. Zamora; Asia Louisa U. Chong; Benedict A. Maralit; Candice Francheska B. Tambaoan; Carlo M. Lapid; Celia Carlos; Cynthia P. Saloma; Devon Ray Pacial; Edsel Maurice Salvaña; El King D. Morado; Eva Maria Cutiongco-de la Paz; Francis A. Tablizo; Irish Coleen A. Asin; Jaime C. Montoya; Jan Michael C. Yap; Jo-Hannah S. Llames; John Q. Wong; Joshua Gregor A. Dizon; Juan Antonio R. Magalang; Karol Sophia Agape R. Padilla; Kenneth M. Kim; Kris P. Punayan; Marc Edsel C. Ayes; Marc Jerrone R. Castro; Maria Sofia L. Yangzon; Marissa Alejandria; Razel Nikka M. Hao; Rianna Patricia S. Cruz; Sheila Mae M. Araiza; and Maria Rosario Singh-Vergeire
EPI_ISL_2105240, EPI_ISL_2105241, EPI_ISL_2105256, EPI_ISL_2105264, EPI_ISL_2105266, EPI_ISL_2105267, EPI_ISL_2105272, EPI_ISL_2107792			
see above	Southern Philippines Medical Center (SPMC)	Philippine Genome Center	Alethea R. de Guzman; Anna Ong-Lim; Arianne A. Zamora; Asia Louisa U. Chong; Benedict A. Maralit; Candice Francheska B. Tambaoan; Carlo M. Lapid; Celia Carlos; Devon Ray Pacial; Edsel Maurice Salvaña; El King D. Morado; Eva Maria Cutiongco-de la Paz; Francis A. Tablizo; Irish Coleen A. Asin; Jaime C. Montoya; Jan Michael C. Yap; Jo-Hannah S. Llames; John Q. Wong; Joshua Gregor A. Dizon; Juan Antonio R. Magalang; Karol Sophia Agape R. Padilla; Kenneth M. Kim; Kris P. Punayan; Marc Edsel C. Ayes; Marc Jerrone R. Castro; Maria Rosario Singh-Vergeire and Cynthia P. Saloma; Maria Sofia L. Yangzon; Marissa Alejandria; Razel Nikka M. Hao; Rianna Patricia S. Cruz; Sheila Mae M. Araiza
EPI_ISL_2105290, EPI_ISL_2105293, EPI_ISL_2105294, EPI_ISL_2105295, EPI_ISL_2105297, EPI_ISL_2105298, EPI_ISL_2105300, EPI_ISL_2105301, EPI_ISL_2105302, EPI_ISL_2105303, EPI_ISL_2105305, EPI_ISL_2105307, EPI_ISL_2105308, EPI_ISL_2105309, EPI_ISL_2105312, EPI_ISL_2105314, EPI_ISL_2105315, EPI_ISL_2105316, EPI_ISL_2105317, EPI_ISL_2105319, EPI_ISL_2105321, EPI_ISL_2105324, EPI_ISL_2105330, EPI_ISL_2105333, EPI_ISL_2105334, EPI_ISL_2105336, EPI_ISL_2105338, EPI_ISL_2105342, EPI_ISL_2105343, EPI_ISL_2105344, EPI_ISL_2105345, EPI_ISL_2105346, EPI_ISL_2105347, EPI_ISL_2105348, EPI_ISL_2105349, EPI_ISL_2105350, EPI_ISL_2105351, EPI_ISL_2105353, EPI_ISL_2105354, EPI_ISL_2105355, EPI_ISL_2105356, EPI_ISL_2105357, EPI_ISL_2105358, EPI_ISL_2105359, EPI_ISL_2105362, EPI_ISL_2105364, EPI_ISL_2105366, EPI_ISL_2105367, EPI_ISL_2105368, EPI_ISL_2105370, EPI_ISL_2105371, EPI_ISL_2105372, EPI_ISL_2105373, EPI_ISL_2105374, EPI_ISL_2105375, EPI_ISL_2105376, EPI_ISL_2105380, EPI_ISL_2105382, EPI_ISL_2105384, EPI_ISL_2105385, EPI_ISL_2105386, EPI_ISL_2105387, EPI_ISL_2105388, EPI_ISL_2105389, EPI_ISL_2105390, EPI_ISL_2105402, EPI_ISL_2105425, EPI_ISL_2105428, EPI_ISL_2155788, EPI_ISL_2155789			
see above	Southern Philippines Medical Center (SPMC-SNL-MBL)	Philippine Genome Center	Alethea R. de Guzman; Anna Ong-Lim; Arianne A. Zamora; Asia Louisa U. Chong; Benedict A. Maralit; Candice Francheska B. Tambaoan; Carlo M. Lapid; Celia Carlos; Devon Ray Pacial; Edsel Maurice Salvaña; El King D. Morado; Eva Maria Cutiongco-de la Paz; Francis A. Tablizo; Irish Coleen A. Asin; Jaime C. Montoya; Jan Michael C. Yap; Jo-Hannah S. Llames; John Q. Wong; Joshua Gregor A. Dizon; Juan Antonio R. Magalang; Karol Sophia Agape R. Padilla; Kenneth M. Kim; Kris P. Punayan; Marc Edsel C. Ayes; Marc Jerrone R. Castro; Maria Rosario Singh-Vergeire and Cynthia P. Saloma; Maria Sofia L. Yangzon; Marissa Alejandria; Razel Nikka M. Hao; Rianna Patricia S. Cruz; Sheila Mae M. Araiza
EPI_ISL_2156457, EPI_ISL_4717318	St. Luke's Medical Center - BGC (HB)	Philippine Genome Center	Alethea R. de Guzman; Anna Ong-Lim; Arianne A. Zamora; Asia Louisa U. Chong; Benedict A. Maralit; Candice Francheska B. Tambaoan; Carlo M. Lapid; Celia Carlos; Cynthia P. Saloma; Devon Ray Pacial; Diomedes A. Cariño; Edsel Maurice Salvana; Edsel Maurice Salvaña; El King D. Morado; Elcid Aaron R. Pangilinan; Eva Maria Cutiongco-de la Paz; Francis A. Tablizo; Henrietta Marie Rodriguez; Irish Coleen A. Asin; Jaime C. Montoya; Jan Michael C. Yap; Jarvin E. Nipales; Jo-Hannah S. Llames; John Q. Wong; Joshua Gregor A. Dizon; Juan Antonio R. Magalang; Karol Sophia Agape R. Padilla; Kenneth M. Kim; Kris P. Punayan; Kristina Patriz Dela Cruz; Lindsay Clare D.L. Carandang; Ma. Exanil Plantig; Marc Edsel C. Ayes; Marc Jerrone R. Castro; Maria Rosario Singh-Vergeire; Maria Rosario Singh-Vergeire and Cynthia P. Saloma; Maria Sofia L. Yangzon; Marielle M. Gamboa; Marissa Alejandria; Niña Francesca Bustamante; Razel Nikka M. Hao; Renato Jacinto Q. Mantaring; Rianna Patricia S. Cruz; Sheila Mae M. Araiza; Shiela Mae M. Araiza; Yvonne Valerie Austria; Zipporah Mariebelle R. Enriquez; Zyrel V. Mollejon
EPI_ISL_2154038	St. Luke's Medical Center- QC	Philippine Genome Center	Alethea R. de Guzman; Anna Ong-Lim; Arianne A. Zamora; Asia Louisa U. Chong; Benedict A. Maralit; Candice Francheska B. Tambaoan; Carlo M. Lapid; Celia Carlos; Devon Ray Pacial; Edsel Maurice Salvaña; El King D. Morado; Eva Maria Cutiongco-de la Paz; Francis A. Tablizo; Irish Coleen A. Asin; Jaime C. Montoya; Jan Michael C. Yap; Jo-Hannah S. Llames; John Q. Wong; Joshua Gregor A. Dizon; Juan Antonio R. Magalang; Karol Sophia Agape R. Padilla; Kenneth M. Kim; Kris P. Punayan; Marc Edsel C. Ayes; Marc Jerrone R. Castro; Maria Rosario Singh-Vergeire and Cynthia P. Saloma; Maria Sofia L. Yangzon; Marissa Alejandria; Razel Nikka M. Hao; Rianna Patricia S. Cruz; Sheila Mae M. Araiza
EPI_ISL_833339	St. Pascual De Baylon Hospital	Research Institute for Tropical Medicine	Catalino Demetria; Daria Manalo; Edelwisa Mercado; Francisco Gerardo Polotan; Hannah Leah Morito; Inez Andrea Medado; John Leonard Chan; Kirstyn Brunker; Ma Angelica Tujan; Othoniel Jan Onza
EPI_ISL_2188201, EPI_ISL_2188920, EPI_ISL_2188968, EPI_ISL_2188990, EPI_ISL_2188993, EPI_ISL_2189079, EPI_ISL_2189107, EPI_ISL_2189138, EPI_ISL_2189156, EPI_ISL_2189162, EPI_ISL_2189201, EPI_ISL_2189247, EPI_ISL_2189257, EPI_ISL_2189306, EPI_ISL_2189422, EPI_ISL_2189438, EPI_ISL_2189449, EPI_ISL_2189498, EPI_ISL_2859978, EPI_ISL_2859999, EPI_ISL_4714887, EPI_ISL_4715007, EPI_ISL_4715109, EPI_ISL_4715190			
see above	Sta. Ana Hospital	Philippine Genome Center	Alethea R. de Guzman; Anna Ong-Lim; Arianne A. Zamora; Asia Louisa U. Chong; Benedict A. Maralit; Candice Francheska B. Tambaoan; Carlo M. Lapid; Celia Carlos; Cynthia P. Saloma; Devon Ray Pacial; Diomedes A. Carino; Diomedes A. Cariño; Edsel Maurice Salvana; Edsel Maurice Salvaña; El King D. Morado; Elcid Aaron R. Pangilinan; Eva Maria Cutiongco-de la Paz; Francis A. Tablizo; Henrietta Marie Rodriguez; Irish Coleen A. Asin; Jaime C. Montoya; Jan Michael C. Yap; Jarvin E. Nipales; Jo-Hannah S. Llames; John Q. Wong; Joshua Gregor A. Dizon; Juan Antonio R. Magalang; Karol Sophia Agape R. Padilla; Kenneth M. Kim; Kris P. Punayan; Krisitna Patriz Dela Cruz; Kristina Patriz Dela Cruz; Lindsay Claire D.L. Carandang; Lindsay Clare D.L. Carandang; Ma. Exanil Plantig; Marc Edsel C. Ayes; Marc Jerrone R. Castro; Maria Rosario Singh-Vergeire; Maria Rosario Singh-Vergeire and Cynthia P. Saloma; Maria Sofia L. Yangzon; Marielle M Gamboa; Marielle M. Gamboa; Marissa Alejandria; Nina Francesca Bustamante; Niña Francesca Bustamante; Razel Nikka M. Hao; Renato Jacinto Q. Mantaring; Rianna Patricia S. Cruz; Sheila Mae M. Araiza; Shiela Mae M. Araiza; Yvonne Valerie Austria; Zipporah Mariebelle R. Enriquez; Zyrel V. Mollejon
EPI_ISL_2188066, EPI_ISL_2188143, EPI_ISL_2188238, EPI_ISL_2189012, EPI_ISL_2189143, EPI_ISL_4713312	Sta. Rosa CHO	Philippine Genome Center	Alethea R. de Guzman; Anna Ong-Lim; Arianne A. Zamora; Asia Louisa U. Chong; Benedict A. Maralit; Candice Francheska B. Tambaoan; Carlo M. Lapid; Celia Carlos; Cynthia P. Saloma; Devon Ray Pacial; Diomedes A. Cariño; Edsel Maurice Salvana; Edsel Maurice Salvaña; El King D. Morado; Elcid Aaron R. Pangilinan; Eva Maria Cutiongco-de la Paz; Francis A. Tablizo; Henrietta Marie Rodriguez; Irish Coleen A. Asin; Jaime C. Montoya; Jan Michael C. Yap; Jarvin E. Nipales; Jo-Hannah S. Llames; John Q. Wong; Joshua Gregor A. Dizon; Juan Antonio R. Magalang; Karol Sophia Agape R. Padilla; Kenneth M. Kim; Kris P. Punayan; Kristina Patriz Dela Cruz; Lindsay Clare D.L. Carandang; Ma. Exanil Plantig; Marc Edsel C. Ayes; Marc Jerrone R. Castro; Maria Rosario Singh-Vergeire; Maria Rosario Singh-Vergeire and Cynthia P. Saloma; Maria Sofia L. Yangzon; Marielle M. Gamboa; Marissa Alejandria; Niña Francesca Bustamante; Razel Nikka M. Hao; Renato Jacinto Q. Mantaring; Rianna Patricia S. Cruz; Sheila Mae M. Araiza; Shiela Mae M. Araiza; Yvonne Valerie Austria; Zipporah Mariebelle R. Enriquez; Zyrel V. Mollejon
EPI_ISL_2188875, EPI_ISL_2188895, EPI_ISL_2188903, EPI_ISL_2188911, EPI_ISL_2188934, EPI_ISL_2188947, EPI_ISL_2188970, EPI_ISL_2189010, EPI_ISL_2189123, EPI_ISL_2189144, EPI_ISL_2189153, EPI_ISL_2189155, EPI_ISL_2189158, EPI_ISL_2189166, EPI_ISL_2189179, EPI_ISL_2189190, EPI_ISL_2189195, EPI_ISL_2189230, EPI_ISL_2189308, EPI_ISL_2189325, EPI_ISL_2189389, EPI_ISL_2189390, EPI_ISL_2189402, EPI_ISL_2189424, EPI_ISL_2189433, EPI_ISL_2189470, EPI_ISL_2189480, EPI_ISL_2189482, EPI_ISL_2189487			
see above	Sta. Rosa CHO-GENEXPERT LABORATORY	Philippine Genome Center	Alethea R. de Guzman; Anna Ong-Lim; Arianne A. Zamora; Asia Louisa U. Chong; Benedict A. Maralit; Candice Francheska B. Tambaoan; Carlo M. Lapid; Celia Carlos; Devon Ray Pacial; Edsel Maurice Salvaña; El King D. Morado; Elcid Aaron R. Pangilinan; Eva Maria Cutiongco-de la Paz; Francis A. Tablizo; Irish Coleen A. Asin; Jaime C. Montoya; Jan Michael C. Yap; Jo-Hannah S. Llames; John Q. Wong; Joshua Gregor A. Dizon; Juan Antonio R. Magalang; Karol Sophia Agape R. Padilla; Kenneth M. Kim; Kris P. Punayan; Marc Edsel C. Ayes; Maria Rosario Singh-Vergeire and Cynthia P. Saloma; Maria Sofia L. Yangzon; Marissa Alejandria; Razel Nikka M. Hao; Renato Jacinto Q. Mantaring; Rianna Patricia S. Cruz; Sheila Mae M. Araiza
EPI_ISL_4459319, EPI_ISL_4459323	TALISAY RURAL HEALTH UNIT	Research Institute for Tropical Medicine/Tohoku University	Allen Anthony Laraño; Ardiane Ysabelle Dolor; Catalino Demetria; Clyde Dapat; Emmanuel Kagning Tsinda; Francisco Gerardo Polotan; Hitoshi Oshitani; Inez Andrea Medado; Jefferson Earl Halog; Lei Lanna Dancel; Leilanie Nacion; Ma. Angelica Tujan; Machiko Okamoto; Maria Carmen Corpuz; Mariko Siato-Obata; Mayuko Saito
EPI_ISL_4459459	TAYSAN RURAL HEALTH UNIT	Research Institute for Tropical Medicine/Tohoku University	Allen Anthony Laraño; Ardiane Ysabelle Dolor; Catalino Demetria; Clyde Dapat; Emmanuel Kagning Tsinda; Francisco Gerardo Polotan; Hitoshi Oshitani; Inez Andrea Medado; Jefferson Earl Halog; Lei Lanna Dancel; Leilanie Nacion; Ma. Angelica Tujan; Machiko Okamoto; Maria Carmen Corpuz; Mariko Siato-Obata; Mayuko Saito
EPI_ISL_2105462, EPI_ISL_2105470, EPI_ISL_2105489, EPI_ISL_2155850, EPI_ISL_2155851, EPI_ISL_2155852, EPI_ISL_2155853, EPI_ISL_2155854, EPI_ISL_2155855, EPI_ISL_2155856, EPI_ISL_2155857, EPI_ISL_2155858, EPI_ISL_2155859, EPI_ISL_2155860, EPI_ISL_2155861, EPI_ISL_2155862, EPI_ISL_2155863, EPI_ISL_2155864, EPI_ISL_2155865, EPI_ISL_2155866, EPI_ISL_2155867, EPI_ISL_2155868, EPI_ISL_2155869, EPI_ISL_2155870, EPI_ISL_2155871, EPI_ISL_2155872, EPI_ISL_2155873, EPI_ISL_2155874, EPI_ISL_2155875, EPI_ISL_2155876, EPI_ISL_2155877, EPI_ISL_2155878, EPI_ISL_2155879, EPI_ISL_2155880, EPI_ISL_2155881, EPI_ISL_2155882, EPI_ISL_2156258, EPI_ISL_2156259, EPI_ISL_2156260, EPI_ISL_2156262, EPI_ISL_2156263, EPI_ISL_2156264, EPI_ISL_2156286, EPI_ISL_2156287, EPI_ISL_2156288, EPI_ISL_2156289, EPI_ISL_2156290, EPI_ISL_2156301, EPI_ISL_2156302, EPI_ISL_2156313, EPI_ISL_2156332, EPI_ISL_4713501			
see above	THE LORD'S GRACE MEDICAL AND INDUSTRIAL CLINIC	Philippine Genome Center	Alethea R. de Guzman; Anna Ong-Lim; Arianne A. Zamora; Asia Louisa U. Chong; Benedict A. Maralit; Candice Francheska B. Tambaoan; Carlo M. Lapid; Celia Carlos; Cynthia P. Saloma; Devon Ray Pacial; Diomedes A. Cariño; Edsel Maurice Salvana; Edsel Maurice Salvaña; El King D. Morado; Elcid Aaron R. Pangilinan; Eva Maria Cutiongco-de la Paz; Francis A. Tablizo; Henrietta Marie Rodriguez; Irish Coleen A. Asin; Jaime C. Montoya; Jan Michael C. Yap; Jarvin E. Nipales; Jo-Hannah S. Llames; John Q. Wong; Joshua Gregor A. Dizon; Juan Antonio R. Magalang; Karol Sophia Agape R. Padilla; Kenneth M. Kim; Kris P. Punayan; Kristina Patriz Dela Cruz; Lindsay Clare D.L. Carandang; Ma. Exanil Plantig; Marc Edsel C. Ayes; Marc Jerrone R. Castro; Maria Rosario Singh-Vergeire; Maria Rosario Singh-Vergeire and Cynthia P. Saloma; Maria Sofia L. Yangzon; Marielle M. Gamboa; Marissa Alejandria; Niña Francesca Bustamante; Razel Nikka M. Hao; Renato Jacinto Q. Mantaring; Rianna Patricia S. Cruz; Sheila Mae M. Araiza; Shiela Mae M. Araiza; Yvonne Valerie Austria; Zipporah Mariebelle R. Enriquez; Zyrel V. Mollejon
EPI_ISL_4712302, EPI_ISL_4712823, EPI_ISL_4713328, EPI_ISL_4713429, EPI_ISL_4713508, EPI_ISL_4713646, EPI_ISL_4713708			
see above	THE MEDICAL CITY	Philippine Genome Center	Alethea R. de Guzman; Anna Ong-Lim; Arianne A. Zamora; Benedict A. Maralit; Carlo M. Lapid; Celia Carlos; Cynthia P. Saloma; Devon Ray Pacial; Diomedes A. Cariño; Edsel Maurice Salvana; El King D. Morado; Elcid Aaron R. Pangilinan; Eva Maria Cutiongco-de la Paz; Francis A. Tablizo; Henrietta Marie Rodriguez; Jaime C. Montoya; Jan Michael C. Yap; Jarvin E. Nipales; Jo-Hannah S. Llames; John Q. Wong; Joshua Gregor A. Dizon; Juan Antonio R. Magalang; Karol Sophia Agape R. Padilla; Kenneth M. Kim; Kris P. Punayan; Kristina Patriz Dela Cruz; Lindsay Clare D.L. Carandang; Ma. Exanil Plantig; Marc Edsel C. Ayes; Maria Rosario Singh-Vergeire; Maria Sofia L. Yangzon; Marielle M. Gamboa; Marissa Alejandria; Niña Francesca Bustamante; Razel Nikka M. Hao; Renato Jacinto Q. Mantaring; Rianna Patricia S. Cruz; Sheila Mae M. Araiza; Yvonne Valerie Austria; Zipporah Mariebelle R. Enriquez; Zyrel V. Mollejon
EPI_ISL_4459375, EPI_ISL_4459492	THE PREMIER MEDICAL CENTER	Research Institute for Tropical Medicine/Tohoku University	Allen Anthony Laraño; Ardiane Ysabelle Dolor; Catalino Demetria; Clyde Dapat; Emmanuel Kagning Tsinda; Francisco Gerardo Polotan; Hitoshi Oshitani; Inez Andrea Medado; Jefferson Earl Halog; Lei Lanna Dancel; Leilanie Nacion; Ma. Angelica Tujan; Machiko Okamoto; Maria Carmen Corpuz; Mariko Siato-Obata; Mayuko Saito
EPI_ISL_4459335	TORRIJOS RURAL HEALTH UNIT	Research Institute for Tropical Medicine/Tohoku University	Allen Anthony Laraño; Ardiane Ysabelle Dolor; Catalino Demetria; Clyde Dapat; Emmanuel Kagning Tsinda; Francisco Gerardo Polotan; Hitoshi Oshitani; Inez Andrea Medado; Jefferson Earl Halog; Lei Lanna Dancel; Leilanie Nacion; Ma. Angelica Tujan; Machiko Okamoto; Maria Carmen Corpuz; Mariko Siato-Obata; Mayuko Saito
EPI_ISL_3105872	Taguig City Health Office	Research Institute for Tropical Medicine	Clyde Dapat; Deana Mae Ocampo; Emmanuel Kagning Tsinda; Francisco Gerardo Polotan; Hitoshi Oshitani; Inez Andrea Medado; Jefferson Earl Halog; Joana Ina Manalo; Lei Lanna Dancel; Ma Angelica Tujan; Mariko Saito-Obata; Mayuko Saito; Michiko Okamoto; Samantha Louise Bado

EPI_ISL_3105879	Taguig-Pateros District Hospital	Research Institute for Tropical Medicine	Clyde Dapat; Deana Mae Ocampo; Emmanuel Kagning Tsinda; Francisco Gerardo Polotan; Hitoshi Oshitani; Inez Andrea Medado; Jefferson Earl Halog; Joana Ina Manalo; Lei Lanna Dancel; Ma Angelica Tujan; Mariko Saito-Obata; Mayuko Saito; Michiko Okamoto; Samantha Louise Bado
EPI_ISL_3105883	Tanauan City Health Office	Research Institute for Tropical Medicine	Clyde Dapat; Deana Mae Ocampo; Emmanuel Kagning Tsinda; Francisco Gerardo Polotan; Hitoshi Oshitani; Inez Andrea Medado; Jefferson Earl Halog; Joana Ina Manalo; Lei Lanna Dancel; Ma Angelica Tujan; Mariko Saito-Obata; Mayuko Saito; Michiko Okamoto; Samantha Louise Bado
EPI_ISL_2156249, EPI_ISL_2156265, EPI_ISL_2156266, EPI_ISL_2156267, EPI_ISL_2156268, EPI_ISL_2156269, EPI_ISL_2156270, EPI_ISL_2156271, EPI_ISL_2156272, EPI_ISL_2156273, EPI_ISL_2156274, EPI_ISL_2156275, EPI_ISL_2189311, EPI_ISL_2189312			
see above	Tarlac Provincial Hospital - Molecular Pathology Laboratory	Philippine Genome Center	Alethea R. de Guzman; Anna Ong-Lim; Arianne A. Zamora; Asia Louisa U. Chong; Benedict A. Maralit; Candice Francheska B. Tambaoan; Carlo M. Lapid; Celia Carlos; Devon Ray Pacial; Edsel Maurice Salvaña; El King D. Morado; Elcid Aaron R. Pangilinan; Eva Maria Cutiongco-de la Paz; Francis A. Tablizo; Irish Coleen A. Asin; Jaime C. Montoya; Jan Michael C. Yap; Jo-Hannah S. Llames; John Q. Wong; Joshua Gregor A. Dizon; Juan Antonio R. Magalang; Karol Sophia Agape R. Padilla; Kenneth M. Kim; Kris P. Punayan; Marc Edsel C. Ayes; Marc Jerrone R. Castro; Maria Rosario Singh-Vergeire and Cynthia P. Saloma; Maria Sofia L. Yangzon; Marissa Alejandria; Razel Nikka M. Hao; Renato Jacinto Q. Mantaring; Rianna Patricia S. Cruz; Sheila Mae M. Araiza
EPI_ISL_2155520	Taytay Municipal Health Office (PGC)	Philippine Genome Center	Alethea R. de Guzman; Anna Ong-Lim; Arianne A. Zamora; Asia Louisa U. Chong; Benedict A. Maralit; Candice Francheska B. Tambaoan; Carlo M. Lapid; Celia Carlos; Devon Ray Pacial; Edsel Maurice Salvaña; El King D. Morado; Eva Maria Cutiongco-de la Paz; Francis A. Tablizo; Irish Coleen A. Asin; Jaime C. Montoya; Jan Michael C. Yap; Jo-Hannah S. Llames; John Q. Wong; Joshua Gregor A. Dizon; Juan Antonio R. Magalang; Karol Sophia Agape R. Padilla; Kenneth M. Kim; Kris P. Punayan; Marc Edsel C. Ayes; Marc Jerrone R. Castro; Maria Rosario Singh-Vergeire and Cynthia P. Saloma; Maria Sofia L. Yangzon; Marissa Alejandria; Razel Nikka M. Hao; Rianna Patricia S. Cruz; Sheila Mae M. Araiza
EPI_ISL_1213583, EPI_ISL_2155341, EPI_ISL_4716112, EPI_ISL_4717432, EPI_ISL_4717438, EPI_ISL_4717463	Teresita Jalandoni Provincial Hospital	Philippine Genome Center	Alethea R. de Guzman; Anna Ong-Lim; Arianne A. Zamora; Asia Louisa U. Chong; Benedict A. Maralit; Candice Francheska B. Tambaoan; Carlo M. Lapid; Celia Carlos; Cynthia P. Saloma; Devon Ray Pacial; Diomedes A. Cariño; Edsel Maurice Salvana; Edsel Maurice Salvaña; El King D. Morado; Elcid Aaron R. Pangilinan; Eva Maria Cutiongco-de la Paz; Francis A. Tablizo; Henrietta Marie Rodriguez; Irish Coleen A. Asin; Jaime C. Montoya; Jan Michael C. Yap; Jarvin E. Nipales; Jo-Hannah S. Llames; John Q. Wong; Joshua Gregor A. Dizon; Juan Antonio R. Magalang; Karol Sophia Agape R. Padilla; Kenneth M. Kim; Kris P. Punayan; Kristina Patriz Dela Cruz; Lindsay Clare D.L. Carandang; Ma. Exanil Plantig; Marc Edsel C. Ayes; Marc Jerrone R. Castro; Maria Rosario Singh-Vergeire; Maria Rosario Singh-Vergeire and Cynthia P. Saloma; Maria Sofia L. Yangzon; Marielle M. Gamboa; Marissa Alejandria; Niña Francesca Bustamante; Razel Nikka M. Hao; Renato Jacinto Q. Mantaring; Rianna Patricia S. Cruz; Sheila Mae M. Araiza; Shiela Mae M. Araiza; Yvonne Valerie Austria; Zipporah Mariebelle R. Enriquez; Zyrel V. Mollejon
EPI_ISL_1081832, EPI_ISL_1081833, EPI_ISL_1081839, EPI_ISL_1081841, EPI_ISL_1122458, EPI_ISL_1213585, EPI_ISL_1213586, EPI_ISL_1219701, EPI_ISL_2105227, EPI_ISL_2105280, EPI_ISL_2105540, EPI_ISL_2105546, EPI_ISL_2154402, EPI_ISL_2154525, EPI_ISL_2154555, EPI_ISL_2154699, EPI_ISL_2154863, EPI_ISL_2154906, EPI_ISL_2155064, EPI_ISL_2155085, EPI_ISL_2155118, EPI_ISL_2155317, EPI_ISL_2155352, EPI_ISL_2155356, EPI_ISL_2155386, EPI_ISL_2155387, EPI_ISL_2155388, EPI_ISL_2155389, EPI_ISL_2155390, EPI_ISL_2155391, EPI_ISL_2155392, EPI_ISL_2155393, EPI_ISL_2155394, EPI_ISL_2155395, EPI_ISL_2156099, EPI_ISL_2156100, EPI_ISL_2156101, EPI_ISL_2156102, EPI_ISL_2156103, EPI_ISL_2156104, EPI_ISL_2156105, EPI_ISL_2156106, EPI_ISL_2156107, EPI_ISL_2156108, EPI_ISL_2156479, EPI_ISL_2156480, EPI_ISL_2156491, EPI_ISL_2156502, EPI_ISL_2156511, EPI_ISL_2156512, EPI_ISL_2156516, EPI_ISL_2156534, EPI_ISL_2156550, EPI_ISL_2171096, EPI_ISL_2171107, EPI_ISL_2171111, EPI_ISL_2171115, EPI_ISL_2171116, EPI_ISL_2171118, EPI_ISL_2171120, EPI_ISL_2171125, EPI_ISL_2171126, EPI_ISL_2171129, EPI_ISL_2171130, EPI_ISL_2171132, EPI_ISL_2171135, EPI_ISL_2171153, EPI_ISL_2171172, EPI_ISL_2171173, EPI_ISL_2171181, EPI_ISL_2171185, EPI_ISL_2171187, EPI_ISL_2171188, EPI_ISL_2171193, EPI_ISL_2171210, EPI_ISL_2171213, EPI_ISL_2171216, EPI_ISL_2171221, EPI_ISL_2171227, EPI_ISL_2171230, EPI_ISL_2171233, EPI_ISL_2171234, EPI_ISL_2188039, EPI_ISL_2188041, EPI_ISL_2188054, EPI_ISL_2188059, EPI_ISL_2188069, EPI_ISL_2188073, EPI_ISL_2188074, EPI_ISL_2188094, EPI_ISL_2188096, EPI_ISL_2188102, EPI_ISL_2188104, EPI_ISL_2188107, EPI_ISL_2188111, EPI_ISL_2188114, EPI_ISL_2188118, EPI_ISL_2188139, EPI_ISL_2188155, EPI_ISL_2188168, EPI_ISL_2188169, EPI_ISL_2188174, EPI_ISL_2188179, EPI_ISL_2188184, EPI_ISL_2188190, EPI_ISL_2188193, EPI_ISL_2188195, EPI_ISL_2188213, EPI_ISL_2188216, EPI_ISL_2188217, EPI_ISL_2188218, EPI_ISL_2188243, EPI_ISL_2188245, EPI_ISL_2188246, EPI_ISL_2188248, EPI_ISL_2188252, EPI_ISL_2188253, EPI_ISL_2188267, EPI_ISL_2188275, EPI_ISL_2188679, EPI_ISL_2188681, EPI_ISL_2188688, EPI_ISL_2188692, EPI_ISL_2188695, EPI_ISL_2188696, EPI_ISL_2188700, EPI_ISL_2188706, EPI_ISL_2188721, EPI_ISL_2188722, EPI_ISL_2188725, EPI_ISL_2188732, EPI_ISL_2188734, EPI_ISL_2188737, EPI_ISL_2188766, EPI_ISL_2188774, EPI_ISL_2188781, EPI_ISL_2188791, EPI_ISL_2188807, EPI_ISL_2188808, EPI_ISL_2188819, EPI_ISL_2188827, EPI_ISL_2188828, EPI_ISL_2188830, EPI_ISL_2188833, EPI_ISL_2188840, EPI_ISL_2188869, EPI_ISL_2188872, EPI_ISL_2188881, EPI_ISL_2188883, EPI_ISL_2188889, EPI_ISL_2188893, EPI_ISL_2188897, EPI_ISL_2188901, EPI_ISL_2188915, EPI_ISL_2188917, EPI_ISL_2188919, EPI_ISL_2188923, EPI_ISL_2188924, EPI_ISL_2188930, EPI_ISL_2188932, EPI_ISL_2188945, EPI_ISL_2188946, EPI_ISL_2188953, EPI_ISL_2188955, EPI_ISL_2188957, EPI_ISL_2188959, EPI_ISL_2188965, EPI_ISL_2188966, EPI_ISL_2188971, EPI_ISL_2188974, EPI_ISL_2188976, EPI_ISL_2188978, EPI_ISL_2188980, EPI_ISL_2188982, EPI_ISL_2188985, EPI_ISL_2188987, EPI_ISL_2188992, EPI_ISL_2189001, EPI_ISL_2189004, EPI_ISL_2189006, EPI_ISL_2189009, EPI_ISL_2189019, EPI_ISL_2189020, EPI_ISL_2189023, EPI_ISL_2189032, EPI_ISL_2189033, EPI_ISL_2189036, EPI_ISL_2189037, EPI_ISL_2189043, EPI_ISL_2189044, EPI_ISL_2189045, EPI_ISL_2189048, EPI_ISL_2189049, EPI_ISL_2189054, EPI_ISL_2189060, EPI_ISL_2189061, EPI_ISL_2189078, EPI_ISL_2189083, EPI_ISL_2189085, EPI_ISL_2189086, EPI_ISL_2189087, EPI_ISL_2189090, EPI_ISL_2189093, EPI_ISL_2189094, EPI_ISL_2189098, EPI_ISL_2189099, EPI_ISL_2189100, EPI_ISL_2189106, EPI_ISL_2189111, EPI_ISL_2189121, EPI_ISL_2189122, EPI_ISL_2189125, EPI_ISL_2189140, EPI_ISL_2189151, EPI_ISL_2189168, EPI_ISL_2189177, EPI_ISL_2189182, EPI_ISL_2189189, EPI_ISL_2189202, EPI_ISL_2189208, EPI_ISL_2189209, EPI_ISL_2189211, EPI_ISL_2189212, EPI_ISL_2189214, EPI_ISL_2189221, EPI_ISL_2189229, EPI_ISL_2189235, EPI_ISL_2189236, EPI_ISL_2189237, EPI_ISL_2189238, EPI_ISL_2189239, EPI_ISL_2189240, EPI_ISL_2189244, EPI_ISL_2189246, EPI_ISL_2189254, EPI_ISL_2189260, EPI_ISL_2189266, EPI_ISL_2189272, EPI_ISL_2189276, EPI_ISL_2189278, EPI_ISL_2189282, EPI_ISL_2189283, EPI_ISL_2189285, EPI_ISL_2189289, EPI_ISL_2189292, EPI_ISL_2189294, EPI_ISL_2189305, EPI_ISL_2189307, EPI_ISL_2189314, EPI_ISL_2189315, EPI_ISL_2189316, EPI_ISL_2189317, EPI_ISL_2189323, EPI_ISL_2189324, EPI_ISL_2189332, EPI_ISL_2189335, EPI_ISL_2189340, EPI_ISL_2189351, EPI_ISL_2189360, EPI_ISL_2189371, EPI_ISL_2189373, EPI_ISL_2189387, EPI_ISL_2189388, EPI_ISL_2189391, EPI_ISL_2189392, EPI_ISL_2189400, EPI_ISL_2189406, EPI_ISL_2189412, EPI_ISL_2189414, EPI_ISL_2189415, EPI_ISL_2189426, EPI_ISL_2189427, EPI_ISL_2189428, EPI_ISL_2189429, EPI_ISL_2189434, EPI_ISL_2189436, EPI_ISL_2189442, EPI_ISL_2189443, EPI_ISL_2189444, EPI_ISL_2189447, EPI_ISL_2189463, EPI_ISL_2189469, EPI_ISL_2189472, EPI_ISL_2189474, EPI_ISL_2189479, EPI_ISL_2189481, EPI_ISL_2189483, EPI_ISL_2189485, EPI_ISL_2189489, EPI_ISL_2189491, EPI_ISL_2189494, EPI_ISL_2189495, EPI_ISL_2189503, EPI_ISL_2189504, EPI_ISL_2189506, EPI_ISL_2189507, EPI_ISL_2189508, EPI_ISL_2189514, EPI_ISL_2189516, EPI_ISL_2189518, EPI_ISL_2189524, EPI_ISL_4714939, EPI_ISL_4715074, EPI_ISL_4715839, EPI_ISL_4715908, EPI_ISL_4716040, EPI_ISL_4716638, EPI_ISL_4716666, EPI_ISL_4717091, EPI_ISL_4717149			
see above	The Lord's Grace Medical and Industrial Clinic	Philippine Genome Center	Alethea R. de Guzman; Anna Ong-Lim; Arianne A. Zamora; Asia Louisa U. Chong; Benedict A. Maralit; Candice Francheska B. Tambaoan; Carlo M. Lapid; Celia Carlos; Cynthia P. Saloma; Devon Ray Pacial; Diomedes A. Cariño; Edsel Maurice Salvana; Edsel Maurice Salvaña; El King D. Morado; Elcid Aaron R. Pangilinan; Eva Maria Cutiongco-de la Paz; Francis A. Tablizo; Henrietta Marie Rodriguez; Irish Coleen A. Asin; Jaime C. Montoya; Jan Michael C. Yap; Jarvin E. Nipales; Jo-Hannah S. Llames; John Q. Wong; Joshua Gregor A. Dizon; Juan Antonio R. Magalang; Karol Sophia Agape R. Padilla; Kenneth M. Kim; Kris P. Punayan; Kristina Patriz Dela Cruz; Lindsay Clare D.L. Carandang; Ma. Exanil Plantig; Marc Edsel C. Ayes; Marc Jerrone R. Castro; Maria Rosario Singh-Vergeire; Maria Rosario Singh-Vergeire and Cynthia P. Saloma; Maria Sofia L. Yangzon; Marielle M. Gamboa; Marissa Alejandria; Niña Francesca Bustamante; Razel Nikka M. Hao; Renato Jacinto Q. Mantaring; Rianna Patricia S. Cruz; Sheila Mae M. Araiza; Shiela Mae M. Araiza; Yvonne Valerie Austria; Zipporah Mariebelle R. Enriquez; Zyrel V. Mollejon; and Maria Rosario Singh-Vergeire
EPI_ISL_2156202, EPI_ISL_2156203, EPI_ISL_2156204, EPI_ISL_2156205, EPI_ISL_2188884, EPI_ISL_2188886, EPI_ISL_2188894, EPI_ISL_2188899, EPI_ISL_2188900, EPI_ISL_2188904, EPI_ISL_2188908, EPI_ISL_2188912, EPI_ISL_2188937, EPI_ISL_2188948, EPI_ISL_2188954, EPI_ISL_2188956, EPI_ISL_2188958, EPI_ISL_2188963, EPI_ISL_2188975, EPI_ISL_2188986, EPI_ISL_2188988, EPI_ISL_2188989, EPI_ISL_2188991, EPI_ISL_2188995, EPI_ISL_2188998, EPI_ISL_2189007, EPI_ISL_2189018, EPI_ISL_2189030, EPI_ISL_2189031, EPI_ISL_2189039, EPI_ISL_2189041, EPI_ISL_2189051, EPI_ISL_2189053, EPI_ISL_2189062, EPI_ISL_2189067, EPI_ISL_2189068, EPI_ISL_2189069, EPI_ISL_2189071, EPI_ISL_2189073, EPI_ISL_2189096, EPI_ISL_2189101, EPI_ISL_2189102, EPI_ISL_2189110, EPI_ISL_2189114, EPI_ISL_2189119, EPI_ISL_2189128, EPI_ISL_2189130, EPI_ISL_2189132, EPI_ISL_2189134, EPI_ISL_2189137, EPI_ISL_2189142, EPI_ISL_2189150, EPI_ISL_2189160, EPI_ISL_2189161, EPI_ISL_2189167, EPI_ISL_2189169, EPI_ISL_2189176, EPI_ISL_2189186, EPI_ISL_2189215, EPI_ISL_2189217, EPI_ISL_2189226, EPI_ISL_2189227, EPI_ISL_2189231, EPI_ISL_2189261, EPI_ISL_2189265, EPI_ISL_2189269, EPI_ISL_2189270, EPI_ISL_2189275, EPI_ISL_2189298, EPI_ISL_2189319, EPI_ISL_2189322, EPI_ISL_2189327, EPI_ISL_2189336, EPI_ISL_2189338, EPI_ISL_2189354, EPI_ISL_2189356, EPI_ISL_2189367, EPI_ISL_2189368, EPI_ISL_2189372, EPI_ISL_2189374, EPI_ISL_2189375, EPI_ISL_2189379, EPI_ISL_2189384, EPI_ISL_2189394, EPI_ISL_2189404, EPI_ISL_2189408, EPI_ISL_2189418, EPI_ISL_2189425, EPI_ISL_2189431, EPI_ISL_2189435, EPI_ISL_2189439, EPI_ISL_2189446, EPI_ISL_2189467, EPI_ISL_2189473, EPI_ISL_2189488, EPI_ISL_2189497, EPI_ISL_2189500, EPI_ISL_2189501, EPI_ISL_2859503, EPI_ISL_2859517, EPI_ISL_2859536, EPI_ISL_2859542, EPI_ISL_2859547, EPI_ISL_2859576, EPI_ISL_2859578, EPI_ISL_2859602, EPI_ISL_2859613, EPI_ISL_2859645, EPI_ISL_2859649, EPI_ISL_2859659, EPI_ISL_2859668, EPI_ISL_2859669, EPI_ISL_2859680, EPI_ISL_2859689, EPI_ISL_2859696, EPI_ISL_2859705, EPI_ISL_2859708, EPI_ISL_2859711, EPI_ISL_2859715, EPI_ISL_2859716, EPI_ISL_2859718, EPI_ISL_2859721, EPI_ISL_2859724, EPI_ISL_2859729, EPI_ISL_2859745, EPI_ISL_2859746, EPI_ISL_2859751, EPI_ISL_2859754, EPI_ISL_2859760, EPI_ISL_2859762, EPI_ISL_2859765, EPI_ISL_2859768, EPI_ISL_2859772, EPI_ISL_2859861, EPI_ISL_2859868, EPI_ISL_2859910, EPI_ISL_2859913, EPI_ISL_2859919, EPI_ISL_2859938, EPI_ISL_2859956, EPI_ISL_2859957, EPI_ISL_2859960, EPI_ISL_2859967, EPI_ISL_2859972, EPI_ISL_2859976, EPI_ISL_2859983, EPI_ISL_2859989, EPI_ISL_2859991, EPI_ISL_2859993, EPI_ISL_2860003, EPI_ISL_2860004, EPI_ISL_2860011, EPI_ISL_2860015, EPI_ISL_2860016, EPI_ISL_2860020, EPI_ISL_2860030, EPI_ISL_2860047, EPI_ISL_2860051, EPI_ISL_2860056, EPI_ISL_2860065, EPI_ISL_2860068, EPI_ISL_2860079, EPI_ISL_2860084, EPI_ISL_2860088, EPI_ISL_2860089, EPI_ISL_2860092, EPI_ISL_2860095, EPI_ISL_2860099, EPI_ISL_2860109, EPI_ISL_2860114, EPI_ISL_2860120, EPI_ISL_2860121, EPI_ISL_2860123, EPI_ISL_2860130, EPI_ISL_2860141, EPI_ISL_2860143, EPI_ISL_2860144, EPI_ISL_2860147, EPI_ISL_2860151, EPI_ISL_2860169, EPI_ISL_2860170, EPI_ISL_2860175, EPI_ISL_2860176, EPI_ISL_2860177, EPI_ISL_2860183, EPI_ISL_2860188, EPI_ISL_2860189, EPI_ISL_2860245, EPI_ISL_4713227, EPI_ISL_4715391, EPI_ISL_4715421, EPI_ISL_4715990, EPI_ISL_4716083, EPI_ISL_4717134			
see above	The Medical City	Philippine Genome Center	Alethea R. de Guzman; Anna Ong-Lim; Arianne A. Zamora; Asia Louisa U. Chong; Benedict A. Maralit; Candice Francheska B. Tambaoan; Carlo M. Lapid; Celia Carlos; Cynthia P. Saloma; Devon Ray Pacial; Diomedes A. Carino; Diomedes A. Cariño; Edsel Maurice Salvana; Edsel Maurice Salvaña; El King D. Morado; Elcid Aaron R. Pangilinan; Eva Maria Cutiongco-de la Paz; Francis A. Tablizo; Henrietta Marie Rodriguez; Irish Coleen A. Asin; Jaime C. Montoya; Jan Michael C. Yap; Jarvin E. Nipales; Jo-Hannah S. Llames; John Q. Wong; Joshua Gregor A. Dizon; Juan Antonio R. Magalang; Karol Sophia Agape R. Padilla; Kenneth M. Kim; Kris P. Punayan; Krisitna Patriz Dela Cruz; Kristina Patriz Dela Cruz; Lindsay Claire D.L. Carandang; Lindsay Clare D.L. Carandang; Ma. Exanil Plantig; Marc Edsel C. Ayes; Marc Jerrone R. Castro; Maria Rosario Singh-Vergeire; Maria Rosario Singh-Vergeire and Cynthia P. Saloma; Maria Sofia L. Yangzon; Marielle M Gamboa; Marielle M. Gamboa; Marissa Alejandria; Nina Francesca Bustamante; Niña Francesca Bustamante; Razel Nikka M. Hao; Renato Jacinto Q. Mantaring; Rianna Patricia S. Cruz; Sheila Mae M. Araiza; Shiela Mae M. Araiza; Yvonne Valerie Austria; Zipporah Mariebelle R. Enriquez; Zyrel V. Mollejon
EPI_ISL_491473	The Medical City	Research Institute for Tropical Medicine	Catalino Demetria; Criselda Bautista; Daria Manalo; Edelwisa Mercado; Francisco Gerardo Polotan; Inez Andrea Medado; Kirstyn Brunker; Ma. Angelica Tujan; Othoniel Jan Onza
EPI_ISL_1213588	The Medical City - Ortigas	Philippine Genome Center	Alethea R. de Guzman; Anna Ong-Lim; Arianne A. Zamora; Asia Louisa U. Chong; Benedict A. Maralit; Candice Francheska B. Tambaoan; Carlo M. Lapid; Celia Carlos; Devon Ray Pacial; Edsel Maurice Salvaña; El King D. Morado; Eva Maria Cutiongco-de la Paz; Francis A. Tablizo; Irish Coleen A. Asin; Jaime C. Montoya; Jan Michael C. Yap; Jo-Hannah S. Llames; John Q. Wong; Joshua Gregor A. Dizon; Juan Antonio R. Magalang; Karol Sophia Agape R. Padilla; Kenneth M. Kim; Kris P. Punayan; Marc Edsel C. Ayes; Marc Jerrone R. Castro; Maria Rosario Singh-Vergeire and Cynthia P. Saloma; Maria Sofia L. Yangzon; Marissa Alejandria; Razel Nikka M. Hao; Rianna Patricia S. Cruz; Sheila Mae M. Araiza
EPI_ISL_2105292, EPI_ISL_2105323, EPI_ISL_2105335, EPI_ISL_2105369, EPI_ISL_2105411, EPI_ISL_2155128, EPI_ISL_2155142, EPI_ISL_2155146, EPI_ISL_2155148, EPI_ISL_2155202, EPI_ISL_2155246, EPI_ISL_2155257, EPI_ISL_2155679, EPI_ISL_2155680, EPI_ISL_2155681, EPI_ISL_2155682, EPI_ISL_2155683, EPI_ISL_2155684, EPI_ISL_2155685, EPI_ISL_2155686, EPI_ISL_2155687, EPI_ISL_2155688, EPI_ISL_2155689, EPI_ISL_2155690, EPI_ISL_2155691, EPI_ISL_2155692, EPI_ISL_2155693, EPI_ISL_2155694, EPI_ISL_2155695, EPI_ISL_2155696, EPI_ISL_2155697, EPI_ISL_2155698, EPI_ISL_2155699, EPI_ISL_2155700, EPI_ISL_2155982, EPI_ISL_2155983, EPI_ISL_2155984, EPI_ISL_2155985, EPI_ISL_2155986, EPI_ISL_2155987, EPI_ISL_2155988, EPI_ISL_2155989, EPI_ISL_2155990, EPI_ISL_2155991, EPI_ISL_2155992, EPI_ISL_2155993, EPI_ISL_2155994, EPI_ISL_2155995, EPI_ISL_2155996, EPI_ISL_2155997, EPI_ISL_2155998, EPI_ISL_2155999, EPI_ISL_2156000, EPI_ISL_2156277, EPI_ISL_2156349, EPI_ISL_2156369, EPI_ISL_2156370, EPI_ISL_2156395, EPI_ISL_2156416, EPI_ISL_2156461, EPI_ISL_2156462, EPI_ISL_2156492, EPI_ISL_2156576, EPI_ISL_2156589, EPI_ISL_2188040, EPI_ISL_2188044, EPI_ISL_2188047, EPI_ISL_2188049, EPI_ISL_2188051, EPI_ISL_2188052, EPI_ISL_2188055, EPI_ISL_2188056, EPI_ISL_2188058, EPI_ISL_2188062, EPI_ISL_2188063, EPI_ISL_2188064, EPI_ISL_2188068, EPI_ISL_2188072, EPI_ISL_2188082, EPI_ISL_2188083, EPI_ISL_2188085, EPI_ISL_2188086, EPI_ISL_2188087, EPI_ISL_2188088, EPI_ISL_2188090, EPI_ISL_2188091, EPI_ISL_2188092, EPI_ISL_2188095, EPI_ISL_2188098, EPI_ISL_2188115, EPI_ISL_2188116, EPI_ISL_2188119, EPI_ISL_2188125, EPI_ISL_2188130, EPI_ISL_2188131, EPI_ISL_2188132, EPI_ISL_2188134, EPI_ISL_2188136, EPI_ISL_2188137, EPI_ISL_2188141, EPI_ISL_2188144, EPI_ISL_2188145, EPI_ISL_2188146, EPI_ISL_2188149, EPI_ISL_2188150, EPI_ISL_2188151, EPI_ISL_2188152, EPI_ISL_2188153, EPI_ISL_2188156, EPI_ISL_2188157, EPI_ISL_2188161, EPI_ISL_2188162, EPI_ISL_2188164, EPI_ISL_2188165, EPI_ISL_2188166, EPI_ISL_2188167, EPI_ISL_2188170, EPI_ISL_2188171, EPI_ISL_2188173, EPI_ISL_2188175, EPI_ISL_2188176, EPI_ISL_2188181, EPI_ISL_2188183, EPI_ISL_2188186, EPI_ISL_2188187, EPI_ISL_2188192, EPI_ISL_2188194, EPI_ISL_2188196, EPI_ISL_2188198, EPI_ISL_2188200, EPI_ISL_2188202, EPI_ISL_2188203, EPI_ISL_2188207, EPI_ISL_2188208, EPI_ISL_2188210, EPI_ISL_2188214, EPI_ISL_2188215, EPI_ISL_2188222, EPI_ISL_2188223, EPI_ISL_2188227, EPI_ISL_2188229, EPI_ISL_2188230, EPI_ISL_2188232, EPI_ISL_2188233, EPI_ISL_2188235, EPI_ISL_2188236, EPI_ISL_2188242, EPI_ISL_2188244, EPI_ISL_2188254, EPI_ISL_2188255, EPI_ISL_2188256, EPI_ISL_2188257, EPI_ISL_2188258, EPI_ISL_2188259, EPI_ISL_2188261, EPI_ISL_2188262, EPI_ISL_2188264, EPI_ISL_2188265, EPI_ISL_2188268, EPI_ISL_2188270, EPI_ISL_2188272, EPI_ISL_2188277, EPI_ISL_2188682, EPI_ISL_2188687, EPI_ISL_2188705, EPI_ISL_2188709, EPI_ISL_2188713, EPI_ISL_2188714, EPI_ISL_2188727, EPI_ISL_2188730, EPI_ISL_2188731, EPI_ISL_2188738, EPI_ISL_2188746, EPI_ISL_2188755, EPI_ISL_2188765, EPI_ISL_2188777, EPI_ISL_2188790, EPI_ISL_2188792, EPI_ISL_2188795, EPI_ISL_2188810, EPI_ISL_2188814, EPI_ISL_2188821, EPI_ISL_2188831, EPI_ISL_2188832, EPI_ISL_2188837, EPI_ISL_2188844, EPI_ISL_2188861, EPI_ISL_2188866			
see above	The Medical City -Â Ortigas	Philippine Genome Center	Alethea R. de Guzman; Anna Ong-Lim; Arianne A. Zamora; Asia Louisa U. Chong; Benedict A. Maralit; Candice Francheska B. Tambaoan; Carlo M. Lapid; Celia Carlos; Devon Ray Pacial; Edsel Maurice Salvaña; El King D. Morado; Elcid Aaron R. Pangilinan; Eva Maria Cutiongco-de la Paz; Francis A. Tablizo; Irish Coleen A. Asin; Jaime C. Montoya; Jan Michael C. Yap; Jo-Hannah S. Llames; John Q. Wong; Joshua Gregor A. Dizon; Juan Antonio R. Magalang; Karol Sophia Agape R. Padilla; Kenneth M. Kim; Kris P. Punayan; Marc Edsel C. Ayes; Marc Jerrone R. Castro; Maria Rosario Singh-Vergeire and Cynthia P. Saloma; Maria Sofia L. Yangzon; Marissa Alejandria; Razel Nikka M. Hao; Renato Jacinto Q. Mantaring; Rianna Patricia S. Cruz; Sheila Mae M. Araiza
EPI_ISL_4459371, EPI_ISL_4459515	UNIHEALTH PARAÑAQUE HOSPITAL AND MEDICAL CENTER, INC.	Research Institute for Tropical Medicine/Tohoku University	Allen Anthony Laraño; Ardiane Ysabelle Dolor; Catalino Demetria; Clyde Dapat; Emmanuel Kagning Tsinda; Francisco Gerardo Polotan; Hitoshi Oshitani; Inez Andrea Medado; Jefferson Earl Halog; Lei Lanna Dancel; Leilanie Nacion; Ma. Angelica Tujan; Machiko Okamoto; Maria Carmen Corpuz; Mariko Siato-Obata; Mayuko Saito
EPI_ISL_2105325	UP Health Services (PGC)	Philippine Genome Center	Alethea R. de Guzman; Anna Ong-Lim; Arianne A. Zamora; Asia Louisa U. Chong; Benedict A. Maralit; Candice Francheska B. Tambaoan; Carlo M. Lapid; Celia Carlos; Devon Ray Pacial; Edsel Maurice Salvaña; El King D. Morado; Eva Maria Cutiongco-de la Paz; Francis A. Tablizo; Irish Coleen A. Asin; Jaime C. Montoya; Jan Michael C. Yap; Jo-Hannah S. Llames; John Q. Wong; Joshua Gregor A. Dizon; Juan Antonio R. Magalang; Karol Sophia Agape R. Padilla; Kenneth M. Kim; Kris P. Punayan; Marc Edsel C. Ayes; Marc Jerrone R. Castro; Maria Rosario Singh-Vergeire and Cynthia P. Saloma; Maria Sofia L. Yangzon; Marissa Alejandria; Razel Nikka M. Hao; Rianna Patricia S. Cruz; Sheila Mae M. Araiza
EPI_ISL_2171175, EPI_ISL_2171177, EPI_ISL_2171179, EPI_ISL_2171199, EPI_ISL_2171254, EPI_ISL_2188691, EPI_ISL_2188760, EPI_ISL_2188835			
see above	UP-PGH Molecular Laboratory	Philippine Genome Center	Alethea R. de Guzman; Anna Ong-Lim; Arianne A. Zamora; Asia Louisa U. Chong; Benedict A. Maralit; Candice Francheska B. Tambaoan; Carlo M. Lapid; Celia Carlos; Devon Ray Pacial; Edsel Maurice Salvaña; El King D. Morado; Elcid Aaron R. Pangilinan; Eva Maria Cutiongco-de la Paz; Francis A. Tablizo; Irish Coleen A. Asin; Jaime C. Montoya; Jan Michael C. Yap; Jo-Hannah S. Llames; John Q. Wong; Joshua Gregor A. Dizon; Juan Antonio R. Magalang; Karol Sophia Agape R. Padilla; Kenneth M. Kim; Kris P. Punayan; Marc Edsel C. Ayes; Marc Jerrone R. Castro; Maria Rosario Singh-Vergeire and Cynthia P. Saloma; Maria Sofia L. Yangzon; Marissa Alejandria; Razel Nikka M. Hao; Renato Jacinto Q. Mantaring; Rianna Patricia S. Cruz; Sheila Mae M. Araiza
EPI_ISL_2154969, EPI_ISL_2154972, EPI_ISL_2154978, EPI_ISL_2154982, EPI_ISL_2154990, EPI_ISL_2154991, EPI_ISL_2154994, EPI_ISL_2155000, EPI_ISL_2155089, EPI_ISL_2155525, EPI_ISL_2155526, EPI_ISL_2155527, EPI_ISL_2155528, EPI_ISL_2155529, EPI_ISL_2155530, EPI_ISL_2155531, EPI_ISL_2155532, EPI_ISL_2155533, EPI_ISL_2155534, EPI_ISL_2155535, EPI_ISL_2155536, EPI_ISL_2155537, EPI_ISL_2155538, EPI_ISL_2155539, EPI_ISL_2155540, EPI_ISL_2155541, EPI_ISL_2155542, EPI_ISL_2155543, EPI_ISL_2155544, EPI_ISL_2155545, EPI_ISL_2155546, EPI_ISL_2155547, EPI_ISL_2155548, EPI_ISL_2155549, EPI_ISL_2155550, EPI_ISL_2155551, EPI_ISL_2155552, EPI_ISL_2155553, EPI_ISL_2155554, EPI_ISL_2155555, EPI_ISL_2155556, EPI_ISL_2155557, EPI_ISL_2155558, EPI_ISL_2155559, EPI_ISL_2155560, EPI_ISL_2155561, EPI_ISL_2155562, EPI_ISL_2155563, EPI_ISL_2155564, EPI_ISL_2155565, EPI_ISL_2155566, EPI_ISL_2155567, EPI_ISL_2155568, EPI_ISL_2155569, EPI_ISL_2155570, EPI_ISL_2155571, EPI_ISL_2155572, EPI_ISL_2155573, EPI_ISL_2155574, EPI_ISL_2155575, EPI_ISL_2155576, EPI_ISL_2155577, EPI_ISL_2155578, EPI_ISL_2155579, EPI_ISL_2155580, EPI_ISL_2155581, EPI_ISL_2155582, EPI_ISL_2155583, EPI_ISL_2155584, EPI_ISL_2155585, EPI_ISL_2155586, EPI_ISL_2156537, EPI_ISL_2156711			
see above	UPLB Covid-19 Molecular Diagnostic Laboratory	Philippine Genome Center	Alethea R. de Guzman; Anna Ong-Lim; Arianne A. Zamora; Asia Louisa U. Chong; Benedict A. Maralit; Candice Francheska B. Tambaoan; Carlo M. Lapid; Celia Carlos; Devon Ray Pacial; Edsel Maurice Salvaña; El King D. Morado; Elcid Aaron R. Pangilinan; Eva Maria Cutiongco-de la Paz; Francis A. Tablizo; Irish Coleen A. Asin; Jaime C. Montoya; Jan Michael C. Yap; Jo-Hannah S. Llames; John Q. Wong; Joshua Gregor A. Dizon; Juan Antonio R. Magalang; Karol Sophia Agape R. Padilla; Kenneth M. Kim; Kris P. Punayan; Marc Edsel C. Ayes; Marc Jerrone R. Castro; Maria Rosario Singh-Vergeire and Cynthia P. Saloma; Maria Sofia L. Yangzon; Marissa Alejandria; Razel Nikka M. Hao; Rianna Patricia S. Cruz; Sheila Mae M. Araiza
EPI_ISL_1081819	University of the East Ramon Magsaysay Memorial Medical Center	Philippine Genome Center	Alethea R. de Guzman; Anna Ong-Lim; Arianne A. Zamora; Asia Louisa U. Chong; Benedict A. Maralit; Candice Francheska B. Tambaoan; Carlo M. Lapid; Celia Carlos; Cynthia P. Saloma; Devon Ray Pacial; Edsel Maurice Salvaña; El King D. Morado; Eva Maria Cutiongco-de la Paz; Francis A. Tablizo; Irish Coleen A. Asin; Jaime C. Montoya; Jan Michael C. Yap; Jo-Hannah S. Llames; John Q. Wong; Joshua Gregor A. Dizon; Juan Antonio R. Magalang; Karol Sophia Agape R. Padilla; Kenneth M. Kim; Kris P. Punayan; Marc Edsel C. Ayes; Marc Jerrone R. Castro; Maria Sofia L. Yangzon; Marissa Alejandria; Razel Nikka M. Hao; Rianna Patricia S. Cruz; Sheila Mae M. Araiza; and Maria Rosario Singh-Vergeire
EPI_ISL_2154813, EPI_ISL_2154986, EPI_ISL_2155304, EPI_ISL_2155313, EPI_ISL_2155485, EPI_ISL_2155486, EPI_ISL_2156117, EPI_ISL_2156533, EPI_ISL_2156555, EPI_ISL_2156624, EPI_ISL_2188862, EPI_ISL_4714256			
see above	University of the East Ramon Magsaysay Memorial Medical Center (UERM)	Philippine Genome Center	Alethea R. de Guzman; Anna Ong-Lim; Arianne A. Zamora; Asia Louisa U. Chong; Benedict A. Maralit; Candice Francheska B. Tambaoan; Carlo M. Lapid; Celia Carlos; Cynthia P. Saloma; Devon Ray Pacial; Diomedes A. Cariño; Edsel Maurice Salvana; Edsel Maurice Salvaña; El King D. Morado; Elcid Aaron R. Pangilinan; Eva Maria Cutiongco-de la Paz; Francis A. Tablizo; Henrietta Marie Rodriguez; Irish Coleen A. Asin; Jaime C. Montoya; Jan Michael C. Yap; Jarvin E. Nipales; Jo-Hannah S. Llames; John Q. Wong; Joshua Gregor A. Dizon; Juan Antonio R. Magalang; Karol Sophia Agape R. Padilla; Kenneth M. Kim; Kris P. Punayan; Kristina Patriz Dela Cruz; Lindsay Clare D.L. Carandang; Ma. Exanil Plantig; Marc Edsel C. Ayes; Marc Jerrone R. Castro; Maria Rosario Singh-Vergeire; Maria Rosario Singh-Vergeire and Cynthia P. Saloma; Maria Sofia L. Yangzon; Marielle M. Gamboa; Marissa Alejandria; Niña Francesca Bustamante; Razel Nikka M. Hao; Renato Jacinto Q. Mantaring; Rianna Patricia S. Cruz; Sheila Mae M. Araiza; Shiela Mae M. Araiza; Yvonne Valerie Austria; Zipporah Mariebelle R. Enriquez; Zyrel V. Mollejon
EPI_ISL_4713121	University of the Philippines - PGH-MRL	Philippine Genome Center	Alethea R. de Guzman; Anna Ong-Lim; Arianne A. Zamora; Benedict A. Maralit; Carlo M. Lapid; Celia Carlos; Cynthia P. Saloma; Devon Ray Pacial; Diomedes A. Cariño; Edsel Maurice Salvana; El King D. Morado; Elcid Aaron R. Pangilinan; Eva Maria Cutiongco-de la Paz; Francis A. Tablizo; Henrietta Marie Rodriguez; Jaime C. Montoya; Jan Michael C. Yap; Jarvin E. Nipales; Jo-Hannah S. Llames; John Q. Wong; Joshua Gregor A. Dizon; Juan Antonio R. Magalang; Karol Sophia Agape R. Padilla; Kenneth M. Kim; Kris P. Punayan; Kristina Patriz Dela Cruz; Lindsay Clare D.L. Carandang; Ma. Exanil Plantig; Marc Edsel C. Ayes; Maria Rosario Singh-Vergeire; Maria Sofia L. Yangzon; Marielle M. Gamboa; Marissa Alejandria; Niña Francesca Bustamante; Razel Nikka M. Hao; Renato Jacinto Q. Mantaring; Rianna Patricia S. Cruz; Shiela Mae M. Araiza; Yvonne Valerie Austria; Zipporah Mariebelle R. Enriquez; Zyrel V. Mollejon
EPI_ISL_2105228, EPI_ISL_2105391, EPI_ISL_2154452, EPI_ISL_2154582, EPI_ISL_2154648, EPI_ISL_2154789, EPI_ISL_2154805, EPI_ISL_2154810, EPI_ISL_2154923, EPI_ISL_2154929, EPI_ISL_2154942, EPI_ISL_2154947, EPI_ISL_2155104, EPI_ISL_2155218, EPI_ISL_2155318, EPI_ISL_2155477, EPI_ISL_2155478, EPI_ISL_2155479, EPI_ISL_2155480, EPI_ISL_2155481, EPI_ISL_2155482, EPI_ISL_2155483, EPI_ISL_2155484, EPI_ISL_2156760, EPI_ISL_2156761, EPI_ISL_2156766, EPI_ISL_2171117, EPI_ISL_2188081, EPI_ISL_2188103, EPI_ISL_2188123, EPI_ISL_2188891, EPI_ISL_2188905, EPI_ISL_2188964, EPI_ISL_2189072, EPI_ISL_2189133, EPI_ISL_2189193, EPI_ISL_2189249, EPI_ISL_2189300, EPI_ISL_2189313, EPI_ISL_2189355, EPI_ISL_2189378, EPI_ISL_2189386, EPI_ISL_2189423, EPI_ISL_2189471, EPI_ISL_2558055, EPI_ISL_2558060, EPI_ISL_2859132, EPI_ISL_2859158, EPI_ISL_2859190, EPI_ISL_2859313, EPI_ISL_2859323, EPI_ISL_2860244			
see above	University of the	Philippine Genome	Alethea R. de Guzman; Anna Ong-Lim; Arianne A. Zamora; Asia Louisa U. Chong; Benedict A. Maralit; Candice Francheska B. Tambaoan; Carlo M. Lapid; Celia Carlos; Devon Ray Pacial; Diomedes A. Carino; Edsel Maurice Salvaña; El King D. Morado; Elcid Aaron R. Pangilinan; Eva Maria Cutiongco-de la Paz; Francis A. Tablizo;

	Philippines National Institutes of Health (UP NIH)	Center	Henrietta Marie Rodriguez; Irish Coleen A. Asin; Jaime C. Montoya; Jan Michael C. Yap; Jarvin E. Nipales; Jo-Hannah S. Llames; John Q. Wong; Joshua Gregor A. Dizon; Juan Antonio R. Magalang; Karol Sophia Agape R. Padilla; Kenneth M. Kim; Kris P. Punayan; Krisitna Patriz Dela Cruz; Lindsay Claire D.L. Carandang; Ma. Exanil Plantig; Marc Edsel C. Ayes; Marc Jerrone R. Castro; Maria Rosario Singh-Vergeire and Cynthia P. Saloma; Maria Sofia L. Yangzon; Marielle M Gamboa; Marissa Alejandria; Nina Francesca Bustamante; Razel Nikka M. Hao; Renato Jacinto Q. Mantaring; Rianna Patricia S. Cruz; Sheila Mae M. Araiza; Yvonne Valerie Austria; Zipporah Mariebelle R. Enriquez; Zyrel V. Mollejon
EPI_ISL_4459313, EPI_ISL_4459316	VILLAMOR HEALTH CENTER	Research Institute for Tropical Medicine/Tohoku University	Allen Anthony Laraño; Ardiane Ysabelle Dolor; Catalino Demetria; Clyde Dapat; Emmanuel Kagning Tsinda; Francisco Gerardo Polotan; Hitoshi Oshitani; Inez Andrea Medado; Jefferson Earl Halog; Lei Lanna Dancel; Leilanie Nacion; Ma. Angelica Tujan; Machiko Okamoto; Maria Carmen Corpuz; Mariko Siato-Obata; Mayuko Saito
EPI_ISL_2090606, EPI_ISL_2090607, EPI_ISL_2090608, EPI_ISL_2090609, EPI_ISL_2090610, EPI_ISL_2090611, EPI_ISL_2090612, EPI_ISL_2090613, EPI_ISL_2090614, EPI_ISL_2090615, EPI_ISL_2090616			
see above	VIROLOGY, AFRIMS	VIROLOGY, AFRIMS	A.T.; Chinnawirotpisan, P.; Chua; Claire Navarro, F.; Corazon Diones, P.; D. Jr.; Fernandez, S.; H. Jr.; Huang; J.M.; Jones, A.; Joonlasak, K.; Klungthong, C.; M.T.; Manasatienkij, W.; Tabinas; V. II; Valderama; Velasco; Villa
EPI_ISL_4714163, EPI_ISL_4714392, EPI_ISL_4714465, EPI_ISL_4714647, EPI_ISL_4714800, EPI_ISL_4715028, EPI_ISL_4715222, EPI_ISL_4715247			
see above	VITALAB Molecular Laboratory	Philippine Genome Center	Alethea R. de Guzman; Anna Ong-Lim; Arianne A. Zamora; Benedict A. Maralit; Carlo M. Lapid; Celia Carlos; Cynthia P. Saloma; Devon Ray Pacial; Diomedes A. Cariño; Edsel Maurice Salvana; El King D. Morado; Elcid Aaron R. Pangilinan; Eva Maria Cutiongco-de la Paz; Francis A. Tablizo; Henrietta Marie Rodriguez; Jaime C. Montoya; Jan Michael C. Yap; Jarvin E. Nipales; Jo-Hannah S. Llames; John Q. Wong; Joshua Gregor A. Dizon; Juan Antonio R. Magalang; Karol Sophia Agape R. Padilla; Kenneth M. Kim; Kris P. Punayan; Kristina Patriz Dela Cruz; Lindsay Clare D.L. Carandang; Ma. Exanil Plantig; Marc Edsel C. Ayes; Maria Rosario Singh-Vergeire; Maria Sofia L. Yangzon; Marielle M. Gamboa; Marissa Alejandria; Niña Francesca Bustamante; Razel Nikka M. Hao; Renato Jacinto Q. Mantaring; Rianna Patricia S. Cruz; Shiela Mae M. Araiza; Yvonne Valerie Austria; Zipporah Mariebelle R. Enriquez; Zyrel V. Mollejon
EPI_ISL_2105341, EPI_ISL_2155424, EPI_ISL_2155425, EPI_ISL_2155426, EPI_ISL_2155427, EPI_ISL_2155428, EPI_ISL_2155429, EPI_ISL_2155430, EPI_ISL_2155431, EPI_ISL_2155432, EPI_ISL_2155433, EPI_ISL_2155434, EPI_ISL_2156501, EPI_ISL_2156509, EPI_ISL_2156510, EPI_ISL_2156530, EPI_ISL_2156538, EPI_ISL_2156549, EPI_ISL_2156767			
see above	Valenzuela Hope Molecular Laboratory	Philippine Genome Center	Alethea R. de Guzman; Anna Ong-Lim; Arianne A. Zamora; Asia Louisa U. Chong; Benedict A. Maralit; Candice Francheska B. Tambaoan; Carlo M. Lapid; Celia Carlos; Devon Ray Pacial; Edsel Maurice Salvaña; El King D. Morado; Elcid Aaron R. Pangilinan; Eva Maria Cutiongco-de la Paz; Francis A. Tablizo; Irish Coleen A. Asin; Jaime C. Montoya; Jan Michael C. Yap; Jo-Hannah S. Llames; John Q. Wong; Joshua Gregor A. Dizon; Juan Antonio R. Magalang; Karol Sophia Agape R. Padilla; Kenneth M. Kim; Kris P. Punayan; Marc Edsel C. Ayes; Marc Jerrone R. Castro; Maria Rosario Singh-Vergeire and Cynthia P. Saloma; Maria Sofia L. Yangzon; Marissa Alejandria; Razel Nikka M. Hao; Rianna Patricia S. Cruz; Sheila Mae M. Araiza
EPI_ISL_430840	Veterans Memorial Medical Center	Research Institute for Tropical Medicine	Bautista; Brunker, K.; C.S.; C.T.; D.L.; Demetria; E.S.; F.G.M.; I.A.P.; Manalo; Medado; Mercado; O.J.T.; Onza; Polotan
EPI_ISL_2859769, EPI_ISL_4713699, EPI_ISL_4713715	Vicente Sotto Memorial Medical Center (VSMMC)	Philippine Genome Center	Alethea R. de Guzman; Anna Ong-Lim; Arianne A. Zamora; Benedict A. Maralit; Carlo M. Lapid; Celia Carlos; Cynthia P. Saloma; Devon Ray Pacial; Diomedes A. Carino; Diomedes A. Cariño; Edsel Maurice Salvana; Edsel Maurice Salvaña; El King D. Morado; Elcid Aaron R. Pangilinan; Eva Maria Cutiongco-de la Paz; Francis A. Tablizo; Henrietta Marie Rodriguez; Jaime C. Montoya; Jan Michael C. Yap; Jarvin E. Nipales; Jo-Hannah S. Llames; John Q. Wong; Joshua Gregor A. Dizon; Juan Antonio R. Magalang; Karol Sophia Agape R. Padilla; Kenneth M. Kim; Kris P. Punayan; Krisitna Patriz Dela Cruz; Kristina Patriz Dela Cruz; Lindsay Claire D.L. Carandang; Lindsay Clare D.L. Carandang; Ma. Exanil Plantig; Marc Edsel C. Ayes; Maria Rosario Singh-Vergeire; Maria Rosario Singh-Vergeire and Cynthia P. Saloma; Maria Sofia L. Yangzon; Marielle M Gamboa; Marielle M. Gamboa; Marissa Alejandria; Nina Francesca Bustamante; Niña Francesca Bustamante; Razel Nikka M. Hao; Renato Jacinto Q. Mantaring; Rianna Patricia S. Cruz; Sheila Mae M. Araiza; Shiela Mae M. Araiza; Yvonne Valerie Austria; Zipporah Mariebelle R. Enriquez; Zyrel V. Mollejon
EPI_ISL_2859143, EPI_ISL_2859153, EPI_ISL_2859157, EPI_ISL_2859283, EPI_ISL_2859286, EPI_ISL_2859292, EPI_ISL_2859296			
see above	West Metro Medical Center GeneXpert Laboratory	Philippine Genome Center	Alethea R. de Guzman; Anna Ong-Lim; Arianne A. Zamora; Benedict A. Maralit; Carlo M. Lapid; Celia Carlos; Devon Ray Pacial; Diomedes A. Carino; Edsel Maurice Salvaña; El King D. Morado; Elcid Aaron R. Pangilinan; Eva Maria Cutiongco-de la Paz; Francis A. Tablizo; Henrietta Marie Rodriguez; Jaime C. Montoya; Jan Michael C. Yap; Jarvin E. Nipales; Jo-Hannah S. Llames; John Q. Wong; Joshua Gregor A. Dizon; Juan Antonio R. Magalang; Karol Sophia Agape R. Padilla; Kenneth M. Kim; Kris P. Punayan; Krisitna Patriz Dela Cruz; Lindsay Claire D.L. Carandang; Ma. Exanil Plantig; Marc Edsel C. Ayes; Maria Rosario Singh-Vergeire and Cynthia P. Saloma; Maria Sofia L. Yangzon; Marielle M Gamboa; Marissa Alejandria; Nina Francesca Bustamante; Razel Nikka M. Hao; Renato Jacinto Q. Mantaring; Rianna Patricia S. Cruz; Sheila Mae M. Araiza; Yvonne Valerie Austria; Zipporah Mariebelle R. Enriquez; Zyrel V. Mollejon
EPI_ISL_4715895, EPI_ISL_4715939, EPI_ISL_4716030, EPI_ISL_4716051, EPI_ISL_4716056, EPI_ISL_4716065, EPI_ISL_4716101, EPI_ISL_4716104, EPI_ISL_4716111, EPI_ISL_4716117, EPI_ISL_4716130, EPI_ISL_4716164, EPI_ISL_4716174, EPI_ISL_4716215, EPI_ISL_4716288, EPI_ISL_4716326, EPI_ISL_4716342, EPI_ISL_4716354, EPI_ISL_4716361, EPI_ISL_4716368, EPI_ISL_4716396, EPI_ISL_4716408, EPI_ISL_4716479, EPI_ISL_4716539, EPI_ISL_4716578, EPI_ISL_4716584, EPI_ISL_4716712, EPI_ISL_4716755, EPI_ISL_4716759, EPI_ISL_4716788, EPI_ISL_4716830, EPI_ISL_4716910, EPI_ISL_4716918, EPI_ISL_4716976, EPI_ISL_4717068, EPI_ISL_4717080, EPI_ISL_4717116, EPI_ISL_4717125, EPI_ISL_4717172, EPI_ISL_4717240, EPI_ISL_4717251, EPI_ISL_4717271, EPI_ISL_4717347, EPI_ISL_4717397, EPI_ISL_4717398, EPI_ISL_4717407, EPI_ISL_4717444, EPI_ISL_4717452, EPI_ISL_4717467			
see above	Western Visayas Medical Center (WVMC)	Philippine Genome Center	Alethea R. de Guzman; Anna Ong-Lim; Arianne A. Zamora; Benedict A. Maralit; Carlo M. Lapid; Celia Carlos; Cynthia P. Saloma; Devon Ray Pacial; Diomedes A. Cariño; Edsel Maurice Salvana; El King D. Morado; Elcid Aaron R. Pangilinan; Eva Maria Cutiongco-de la Paz; Francis A. Tablizo; Henrietta Marie Rodriguez; Jaime C. Montoya; Jan Michael C. Yap; Jarvin E. Nipales; Jo-Hannah S. Llames; John Q. Wong; Joshua Gregor A. Dizon; Juan Antonio R. Magalang; Karol Sophia Agape R. Padilla; Kenneth M. Kim; Kris P. Punayan; Kristina Patriz Dela Cruz; Lindsay Clare D.L. Carandang; Ma. Exanil Plantig; Marc Edsel C. Ayes; Maria Rosario Singh-Vergeire; Maria Sofia L. Yangzon; Marielle M. Gamboa; Marissa Alejandria; Niña Francesca Bustamante; Razel Nikka M. Hao; Renato Jacinto Q. Mantaring; Rianna Patricia S. Cruz; Shiela Mae M. Araiza; Yvonne Valerie Austria; Zipporah Mariebelle R. Enriquez; Zyrel V. Mollejon
EPI_ISL_4712265	Western Visayas State University - Molecular Diagnostic Laboratory	Philippine Genome Center	Alethea R. de Guzman; Anna Ong-Lim; Arianne A. Zamora; Benedict A. Maralit; Carlo M. Lapid; Celia Carlos; Cynthia P. Saloma; Devon Ray Pacial; Diomedes A. Cariño; Edsel Maurice Salvana; El King D. Morado; Elcid Aaron R. Pangilinan; Eva Maria Cutiongco-de la Paz; Francis A. Tablizo; Henrietta Marie Rodriguez; Jaime C. Montoya; Jan Michael C. Yap; Jarvin E. Nipales; Jo-Hannah S. Llames; John Q. Wong; Joshua Gregor A. Dizon; Juan Antonio R. Magalang; Karol Sophia Agape R. Padilla; Kenneth M. Kim; Kris P. Punayan; Kristina Patriz Dela Cruz; Lindsay Clare D.L. Carandang; Ma. Exanil Plantig; Marc Edsel C. Ayes; Maria Rosario Singh-Vergeire; Maria Sofia L. Yangzon; Marielle M. Gamboa; Marissa Alejandria; Niña Francesca Bustamante; Razel Nikka M. Hao; Renato Jacinto Q. Mantaring; Rianna Patricia S. Cruz; Shiela Mae M. Araiza; Yvonne Valerie Austria; Zipporah Mariebelle R. Enriquez; Zyrel V. Mollejon
EPI_ISL_2859138, EPI_ISL_2859186, EPI_ISL_2859305, EPI_ISL_2859317, EPI_ISL_2859324, EPI_ISL_2859330, EPI_ISL_2859334, EPI_ISL_2859349, EPI_ISL_2859352, EPI_ISL_2859356, EPI_ISL_2859359, EPI_ISL_2859367			
see above	Zamboanga City Medical Center	Philippine Genome Center	Alethea R. de Guzman; Anna Ong-Lim; Arianne A. Zamora; Benedict A. Maralit; Carlo M. Lapid; Celia Carlos; Devon Ray Pacial; Diomedes A. Carino; Edsel Maurice Salvaña; El King D. Morado; Elcid Aaron R. Pangilinan; Eva Maria Cutiongco-de la Paz; Francis A. Tablizo; Henrietta Marie Rodriguez; Jaime C. Montoya; Jan Michael C. Yap; Jarvin E. Nipales; Jo-Hannah S. Llames; John Q. Wong; Joshua Gregor A. Dizon; Juan Antonio R. Magalang; Karol Sophia Agape R. Padilla; Kenneth M. Kim; Kris P. Punayan; Krisitna Patriz Dela Cruz; Lindsay Claire D.L. Carandang; Ma. Exanil Plantig; Marc Edsel C. Ayes; Maria Rosario Singh-Vergeire and Cynthia P. Saloma; Maria Sofia L. Yangzon; Marielle M Gamboa; Marissa Alejandria; Nina Francesca Bustamante; Razel Nikka M. Hao; Renato Jacinto Q. Mantaring; Rianna Patricia S. Cruz; Sheila Mae M. Araiza; Yvonne Valerie Austria; Zipporah Mariebelle R. Enriquez; Zyrel V. Mollejon
EPI_ISL_2859145, EPI_ISL_2859272, EPI_ISL_2859274, EPI_ISL_2859279, EPI_ISL_2859294, EPI_ISL_2859301, EPI_ISL_2859306, EPI_ISL_2859327, EPI_ISL_2859341, EPI_ISL_2859348, EPI_ISL_2859358, EPI_ISL_2859366, EPI_ISL_2859368, EPI_ISL_2859371, EPI_ISL_4712024, EPI_ISL_4712104, EPI_ISL_4712141, EPI_ISL_4712146, EPI_ISL_4712178, EPI_ISL_4712199, EPI_ISL_4714068, EPI_ISL_4714737, EPI_ISL_4714787, EPI_ISL_4714846, EPI_ISL_4714932, EPI_ISL_4715033, EPI_ISL_4715228, EPI_ISL_4716376, EPI_ISL_4717145			
see above	Zamboanga City Medical Center GeneXpert Laboratory	Philippine Genome Center	Alethea R. de Guzman; Anna Ong-Lim; Arianne A. Zamora; Benedict A. Maralit; Carlo M. Lapid; Celia Carlos; Cynthia P. Saloma; Devon Ray Pacial; Diomedes A. Carino; Diomedes A. Cariño; Edsel Maurice Salvana; Edsel Maurice Salvaña; El King D. Morado; Elcid Aaron R. Pangilinan; Eva Maria Cutiongco-de la Paz; Francis A. Tablizo; Henrietta Marie Rodriguez; Jaime C. Montoya; Jan Michael C. Yap; Jarvin E. Nipales; Jo-Hannah S. Llames; John Q. Wong; Joshua Gregor A. Dizon; Juan Antonio R. Magalang; Karol Sophia Agape R. Padilla; Kenneth M. Kim; Kris P. Punayan; Krisitna Patriz Dela Cruz; Kristina Patriz Dela Cruz; Lindsay Claire D.L. Carandang; Lindsay Clare D.L. Carandang; Ma. Exanil Plantig; Marc Edsel C. Ayes; Maria Rosario Singh-Vergeire; Maria Rosario Singh-Vergeire and Cynthia P. Saloma; Maria Sofia L. Yangzon; Marielle M Gamboa; Marielle M. Gamboa; Marissa Alejandria; Nina Francesca Bustamante; Niña Francesca Bustamante; Razel Nikka M. Hao; Renato Jacinto Q. Mantaring; Rianna Patricia S. Cruz; Sheila Mae M. Araiza; Shiela Mae M. Araiza; Yvonne Valerie Austria; Zipporah Mariebelle R. Enriquez; Zyrel V. Mollejon
EPI_ISL_2859137, EPI_ISL_2859144, EPI_ISL_2859169, EPI_ISL_2859171, EPI_ISL_2859175, EPI_ISL_2859176, EPI_ISL_2859192, EPI_ISL_2859218, EPI_ISL_2859260, EPI_ISL_2859269, EPI_ISL_2859270, EPI_ISL_2859304, EPI_ISL_2859309, EPI_ISL_2859344, EPI_ISL_2859345, EPI_ISL_2859381, EPI_ISL_2860218, EPI_ISL_4713630, EPI_ISL_4713933, EPI_ISL_4714047, EPI_ISL_4714136, EPI_ISL_4714399, EPI_ISL_4714861, EPI_ISL_4714907, EPI_ISL_4714947, EPI_ISL_4715198, EPI_ISL_4715205, EPI_ISL_4715833, EPI_ISL_4715859, EPI_ISL_4716170, EPI_ISL_4716242, EPI_ISL_4716469, EPI_ISL_4716498, EPI_ISL_4716557, EPI_ISL_4716570, EPI_ISL_4716593, EPI_ISL_4716633, EPI_ISL_4716685, EPI_ISL_4716764, EPI_ISL_4716797, EPI_ISL_4716812, EPI_ISL_4716854, EPI_ISL_4716867, EPI_ISL_4716869, EPI_ISL_4716870, EPI_ISL_4716882, EPI_ISL_4716899, EPI_ISL_4716938, EPI_ISL_4716941, EPI_ISL_4716947, EPI_ISL_4716959, EPI_ISL_4716964, EPI_ISL_4716987, EPI_ISL_4716997, EPI_ISL_4717007, EPI_ISL_4717026, EPI_ISL_4717032, EPI_ISL_4717038, EPI_ISL_4717039, EPI_ISL_4717045, EPI_ISL_4717087, EPI_ISL_4717096, EPI_ISL_4717112, EPI_ISL_4717118, EPI_ISL_4717162, EPI_ISL_4717179, EPI_ISL_4717200, EPI_ISL_4717274, EPI_ISL_4717293, EPI_ISL_4717300, EPI_ISL_4717307, EPI_ISL_4717314, EPI_ISL_4717329, EPI_ISL_4717351, EPI_ISL_4717352, EPI_ISL_4717379, EPI_ISL_4717383, EPI_ISL_4717402, EPI_ISL_4717411, EPI_ISL_4717426, EPI_ISL_4717448			
see above	Zamboanga City Medical Center-Department of Agriculture Satellite Laboratory for COVID-19 Testing	Philippine Genome Center	Alethea R. de Guzman; Anna Ong-Lim; Arianne A. Zamora; Benedict A. Maralit; Carlo M. Lapid; Celia Carlos; Cynthia P. Saloma; Devon Ray Pacial; Diomedes A. Carino; Diomedes A. Cariño; Edsel Maurice Salvana; Edsel Maurice Salvaña; El King D. Morado; Elcid Aaron R. Pangilinan; Eva Maria Cutiongco-de la Paz; Francis A. Tablizo; Henrietta Marie Rodriguez; Jaime C. Montoya; Jan Michael C. Yap; Jarvin E. Nipales; Jo-Hannah S. Llames; John Q. Wong; Joshua Gregor A. Dizon; Juan Antonio R. Magalang; Karol Sophia Agape R. Padilla; Kenneth M. Kim; Kris P. Punayan; Krisitna Patriz Dela Cruz; Kristina Patriz Dela Cruz; Lindsay Claire D.L. Carandang; Lindsay Clare D.L. Carandang; Ma. Exanil Plantig; Marc Edsel C. Ayes; Maria Rosario Singh-Vergeire; Maria Rosario Singh-Vergeire and Cynthia P. Saloma; Maria Sofia L. Yangzon; Marielle M Gamboa; Marielle M. Gamboa; Marissa Alejandria; Nina Francesca Bustamante; Niña Francesca Bustamante; Razel Nikka M. Hao; Renato Jacinto Q. Mantaring; Rianna Patricia S. Cruz; Sheila Mae M. Araiza; Shiela Mae M. Araiza; Yvonne Valerie Austria; Zipporah Mariebelle R. Enriquez; Zyrel V. Mollejon
EPI_ISL_2859281, EPI_ISL_2859285, EPI_ISL_2859287, EPI_ISL_2859289, EPI_ISL_2859297, EPI_ISL_4713887, EPI_ISL_4714117, EPI_ISL_4714140, EPI_ISL_4714158, EPI_ISL_4714951, EPI_ISL_4715482			
see above	Zamboanga Del Sur Medical Center PCR Laboratory	Philippine Genome Center	Alethea R. de Guzman; Anna Ong-Lim; Arianne A. Zamora; Benedict A. Maralit; Carlo M. Lapid; Celia Carlos; Cynthia P. Saloma; Devon Ray Pacial; Diomedes A. Carino; Diomedes A. Cariño; Edsel Maurice Salvana; Edsel Maurice Salvaña; El King D. Morado; Elcid Aaron R. Pangilinan; Eva Maria Cutiongco-de la Paz; Francis A. Tablizo; Henrietta Marie Rodriguez; Jaime C. Montoya; Jan Michael C. Yap; Jarvin E. Nipales; Jo-Hannah S. Llames; John Q. Wong; Joshua Gregor A. Dizon; Juan Antonio R. Magalang; Karol Sophia Agape R. Padilla; Kenneth M. Kim; Kris P. Punayan; Krisitna Patriz Dela Cruz; Kristina Patriz Dela Cruz; Lindsay Claire D.L. Carandang; Lindsay Clare D.L. Carandang; Ma. Exanil Plantig; Marc Edsel C. Ayes; Maria Rosario Singh-Vergeire; Maria Rosario Singh-Vergeire and Cynthia P. Saloma; Maria Sofia L. Yangzon; Marielle M Gamboa; Marielle M. Gamboa; Marissa Alejandria; Nina Francesca Bustamante; Niña Francesca Bustamante; Razel Nikka M. Hao; Renato Jacinto Q. Mantaring; Rianna Patricia S. Cruz; Sheila Mae M. Araiza; Shiela Mae M. Araiza; Yvonne Valerie Austria; Zipporah Mariebelle R. Enriquez; Zyrel V. Mollejon

We gratefully acknowledge the following Authors from the Originating laboratories responsible for obtaining the specimens, as well as the Submitting laboratories where the genome data were generated and shared via GISAID, on which this research is based.

All Submitters of data may be contacted directly via www.gisaid.org

Authors are sorted alphabetically.

Accession ID	Originating Laboratory	Submitting Laboratory	Authors
EPI_ISL_2592630, EPI_ISL_2593764, EPI_ISL_2595725, EPI_ISL_2595726, EPI_ISL_2595860, EPI_ISL_2596341, EPI_ISL_2596803, EPI_ISL_2597228, EPI_ISL_2597229, EPI_ISL_2597312, EPI_ISL_2612300			
see above	DSMRC	DSMRC	Khine Zaw Oo; Ko Ko Lwin; Ko Ko Win; Kyee Myint; Nay Myo Aung; Pai Khant Kyaw; Phyo Kyaw Aung; Thet Wai Oo; Zaw Win Htun
EPI_ISL_833041	Defence Services Medical Research Center, Biological Research Laboratory	Defence Services Medical Research Center, Biological Research Laboratory	Khine Zaw Oo; Ko Ko Lwin; Ko Ko Win; Kyaw Wanna; Kyee Myint; Nay Myo Aung; Phyo Kyaw Aung; Sat Paing Htoo; Thein Zaw; Zaw Win Htun
EPI_ISL_896572, EPI_ISL_896573, EPI_ISL_896574, EPI_ISL_896575, EPI_ISL_896576, EPI_ISL_896577, EPI_ISL_896578, EPI_ISL_896579, EPI_ISL_896580, EPI_ISL_896581, EPI_ISL_896582, EPI_ISL_896583, EPI_ISL_896584, EPI_ISL_896585, EPI_ISL_896586, EPI_ISL_896587, EPI_ISL_896588, EPI_ISL_896589, EPI_ISL_896590, EPI_ISL_896591, EPI_ISL_896592, EPI_ISL_896593, EPI_ISL_896594, EPI_ISL_896595, EPI_ISL_896596, EPI_ISL_896597, EPI_ISL_896598, EPI_ISL_896599, EPI_ISL_896600, EPI_ISL_896601			
see above	Department of Medical Research	DMR	Myat Htut Nyunt
EPI_ISL_512844	Department of Medical Research	DMR_Myanmar	Aung Kyaw Kyaw; Aung Zaw Latt; Hlaing Myat Thu; Hnin Ohnmar Soe; Htin Lin; Kay Thi Aye; Lai Lai San; Myat Htut Nyunt; Nan Aye Thida Oo; Ni Ni Zaw; Phyu Win Ei; Su Mon Win; Theingi Win Myat; Wah Wah Aung; Yi Yi Kyaw; Zaw Than Htun
EPI_ISL_3246035, EPI_ISL_3246036, EPI_ISL_3246037, EPI_ISL_3246038, EPI_ISL_3246039, EPI_ISL_3246040, EPI_ISL_3246041, EPI_ISL_3246042, EPI_ISL_3246043, EPI_ISL_3246044, EPI_ISL_3246045, EPI_ISL_3246046, EPI_ISL_3246047			
see above	Myanmar	National Institute of Health, Department of Medical Sciences, Ministry of Public Health, Thailand	; Natchaya Khiadsang; Nuttida Thongpramul; Pakorn Piromtong; Pilailuk Okada; Ratana Tacharoenmuang; Siripaporn Phuygun; Sittiporn Parnmen; Sunthareeya Waicharoen; Thanutsapa Thanadachakul; Warawan Wongboot; sirikanda wimol
EPI_ISL_3252972, EPI_ISL_3252973, EPI_ISL_3252974, EPI_ISL_3252975, EPI_ISL_3252976, EPI_ISL_3252977, EPI_ISL_3252978, EPI_ISL_3252979, EPI_ISL_3252980, EPI_ISL_3252981			
see above	National Health Laboratory, Ministry Of Health And Sports, The Republic Of The Union Of Myanmar	Pathogen Genomics Center, National Institute of Infectious Diseases	Eh Htoo Pe; Htay Htay Tin; Kentaro Itokawa; Makoto Kuroda; Masanori Hashino; Reiko Saito; Rina Tanaka; Tsuyoshi Sekizuka; Wint Wint Phyu
EPI_ISL_849736, EPI_ISL_849737, EPI_ISL_849738, EPI_ISL_849739, EPI_ISL_849740, EPI_ISL_849741, EPI_ISL_849742, EPI_ISL_849743			
see above	Special Operations Medical Research Division, Defence Services Medical Research Centre	Special Operations Medical Research Division, Defence Services Medical Research Centre	Aung; Htun; K.K.; K.Z.; Lwin; Myint, K.; N.M.; Oo; P.K.; Win; Z.W.; Zaw, T.
EPI_ISL_434709	unknown	National Institute of Health. Department of medical Sciences, Ministry of Public Health, Thailand	Chittaganpitch; Malinee; Okada; Parnmen; Phuygun; Pilailuk; Siripaporn; Sittiporn; Sunthareeya; Thanadachakul; Thanutsapa; Waicharoen; Warawan; Wongboot

We gratefully acknowledge the following Authors from the Originating laboratories responsible for obtaining the specimens, as well as the Submitting laboratories where the genome data were generated and shared via GISAID, on which this research is based.

All Submitters of data may be contacted directly via www.gisaid.org

Authors are sorted alphabetically.

Accession ID	Originating Laboratory	Submitting Laboratory	Authors
EPI_ISL_4601562, EPI_ISL_4601563, EPI_ISL_4601566, EPI_ISL_4601578	Akademi Latihan Ketenteraan	Malaysia Genome Institute	Azrin Ahmad; Enizza Kasim; Irni Suhayu Sapian; Mohd Faizal Abu Bakar; Mohd Ghows Mohd Azzam.; Mohd Noor Mat Isa; Nor Azfa Johari; Nurhezreen Md Iqbal; Shamsidar Sopie; Siti Noraini Othman; Yusuf Muhammad Noor
EPI_ISL_2854052, EPI_ISL_3425884, EPI_ISL_4096804, EPI_ISL_4096806, EPI_ISL_4096808, EPI_ISL_4096809, EPI_ISL_4096810			
see above	Ampang Hospital	Institute for Medical Research, Infectious Disease Research Centre, National Institutes of Health, Ministry of Health Malaysia	Ahmad FA; Ahmad Fazilah NA; Anasir MI; Azizan MA; Kamel K; Mohd Zawawi Z; Norhisham SN; Ramly N; Robert F; Suppiah J; Thayan R
EPI_ISL_3266062	Asia Muar Clinic	Institute for Medical Research, Infectious Disease Research Centre, National Institutes of Health, Ministry of Health Malaysia	Anasir MI; Azizan MA; Kamel K; Mohd Zawawi Z; Ramly N; Robert F; Suppiah J; Thayan R
EPI_ISL_4367711, EPI_ISL_4368189	BIOTROPIK, Universiti Malaysia Pahang, Malaysia	BIOTROPIK, Universiti Malaysia Pahang, Malaysia	Hajar Fauzan Bin Ahmad
EPI_ISL_2811998	Bandar Kuantan Health Clinic	Institute for Medical Research, Infectious Disease Research Centre, National Institutes of Health, Ministry of Health Malaysia	Azizan MA; Kamel K; Mohd Zawawi Z; Ramly N; Robert F; Suppiah J; Thayan R
EPI_ISL_4348280, EPI_ISL_4348281, EPI_ISL_4348298, EPI_ISL_4348299, EPI_ISL_4348300	Banting Hospital	Institute for Medical Research, Infectious Disease Research Centre, National Institutes of Health, Ministry of Health Malaysia	Ahmad FA; Ahmad Fazilah NA; Anasir MI; Azizan MA; Kamel K; Mohd Zawawi Z; Norhisyam SN; Ramly N; Robert F; Suppiah J; Thayan R
EPI_ISL_3446665, EPI_ISL_3446666, EPI_ISL_3446668	Batu Health Clinic	Institute for Medical Research, Infectious Disease Research Centre, National Institutes of Health, Ministry of Health Malaysia	Anasir MI; Azizan MA; Kamel K; Mohd Zawawi Z; Ramly N; Robert F; Suppiah J; Thayan R
EPI_ISL_2854069	Bayan Lepas Health Clinic	Institute for Medical Research, Infectious Disease Research Centre, National Institutes of Health, Ministry of Health Malaysia	Anasir MI; Azizan MA; Kamel K; Mohd Zawawi Z; Ramly N; Robert F; Suppiah J; Thayan R
EPI_ISL_4101546	Beaufort District Health Office	Institute for Medical Research, Infectious Disease Research Centre, National Institutes of Health, Ministry of Health Malaysia	Ahmad FA; Ahmad Fazilah NA; Anasir MI; Azizan MA; Kamel K; Mohd Zawawi Z; Norhisham SN; Ramly N; Robert F; Suppiah J; Thayan R
EPI_ISL_3769373	Bilik Gerakan Hospital Sungai Buloh	iPROMISE, UiTM	Ariza Adnan; Fadzilah Mohd Nor; Lim Wai Feng; Mohd Asif Mohd Sukri; Mohd Nur Fakhruzzaman Noorizhab; Mohd Zaki Salleh; Sazzli Shahlan Kassim; Siti Farah Alwani Mohd Nawi; Siti Hamimah Sheikh Abdul Kadir; Teh Lay Kek; Wang Seok Mui
EPI_ISL_1973325, EPI_ISL_1973326, EPI_ISL_1973327, EPI_ISL_1973328, EPI_ISL_2020238, EPI_ISL_2020239, EPI_ISL_2020240			
see above	Bintulu Hospital	Institute for Medical Research, Infectious Disease Research Centre, National Institutes of Health, Ministry of Health Malaysia	Azizan MA; Kamel K; Mohd Zawawi Z; Ramly N; Robert F; Suppiah J; Thayan R
EPI_ISL_4051657, EPI_ISL_4051658, EPI_ISL_4051659, EPI_ISL_4051660, EPI_ISL_4051726, EPI_ISL_4051727, EPI_ISL_4051728, EPI_ISL_4051729, EPI_ISL_4051819, EPI_ISL_4051820, EPI_ISL_4051821, EPI_ISL_4051822, EPI_ISL_4051823, EPI_ISL_4051824, EPI_ISL_4051825, EPI_ISL_4051826, EPI_ISL_4051846, EPI_ISL_4051848, EPI_ISL_4051860			
see above	Bintulu Hospital PCR Lab	Institute of Health and Community Medicine	Chan Chia Jui; Chua Hock Hin; David Perera; Ooi Mong How; Tan Lee See; Tonnii Sia Loong Loong; Wong Jyn Shan
EPI_ISL_4460067, EPI_ISL_4460068, EPI_ISL_4460069, EPI_ISL_4460070, EPI_ISL_4460071, EPI_ISL_4460072, EPI_ISL_4460073, EPI_ISL_4460074, EPI_ISL_4460075, EPI_ISL_4460076, EPI_ISL_4460077, EPI_ISL_4460078, EPI_ISL_4460079, EPI_ISL_4460080, EPI_ISL_4460081, EPI_ISL_4460082, EPI_ISL_4460083, EPI_ISL_4460084, EPI_ISL_4460085, EPI_ISL_4460086, EPI_ISL_4460087, EPI_ISL_4460088, EPI_ISL_4460089, EPI_ISL_4460090, EPI_ISL_4460091, EPI_ISL_4460092, EPI_ISL_4460093, EPI_ISL_4460094, EPI_ISL_4460095, EPI_ISL_4460096, EPI_ISL_4460156, EPI_ISL_4460157, EPI_ISL_4460158, EPI_ISL_4460159, EPI_ISL_4460160, EPI_ISL_4460161, EPI_ISL_4460162, EPI_ISL_4460163, EPI_ISL_4460164, EPI_ISL_4460165, EPI_ISL_4460166, EPI_ISL_4460167, EPI_ISL_4460170, EPI_ISL_4460171, EPI_ISL_4460172, EPI_ISL_4460173, EPI_ISL_4460174, EPI_ISL_4460175, EPI_ISL_4460176, EPI_ISL_4460177, EPI_ISL_4460178, EPI_ISL_4460179, EPI_ISL_4460180, EPI_ISL_4460181, EPI_ISL_4460182, EPI_ISL_4460183, EPI_ISL_4460184, EPI_ISL_4460185, EPI_ISL_4460186, EPI_ISL_4460187, EPI_ISL_4460188, EPI_ISL_4460189, EPI_ISL_4460190, EPI_ISL_4460191, EPI_ISL_4460192, EPI_ISL_4460193, EPI_ISL_4460194, EPI_ISL_4460195, EPI_ISL_4460196, EPI_ISL_4460197, EPI_ISL_4460198, EPI_ISL_4460199, EPI_ISL_4460200, EPI_ISL_4460201, EPI_ISL_4460202, EPI_ISL_4460203, EPI_ISL_4460204, EPI_ISL_4460205, EPI_ISL_4460206, EPI_ISL_4460315, EPI_ISL_4460316, EPI_ISL_4460317, EPI_ISL_4460318, EPI_ISL_4460319, EPI_ISL_4460320, EPI_ISL_4460321, EPI_ISL_4460322, EPI_ISL_4460323, EPI_ISL_4460324, EPI_ISL_4460325, EPI_ISL_4460326, EPI_ISL_4460327, EPI_ISL_4460328, EPI_ISL_4460329, EPI_ISL_4460330, EPI_ISL_4460331, EPI_ISL_4460332, EPI_ISL_4460333, EPI_ISL_4460334, EPI_ISL_4460335, EPI_ISL_4460336, EPI_ISL_4460337, EPI_ISL_4460338, EPI_ISL_4460339, EPI_ISL_4460340, EPI_ISL_4460341, EPI_ISL_4460342, EPI_ISL_4460343, EPI_ISL_4460344, EPI_ISL_4460345, EPI_ISL_4460346, EPI_ISL_4460347, EPI_ISL_4460348, EPI_ISL_4460349			
see above	Bintulu Hospital PCR Lab, Bintulu	Institute of Health and Community Medicine	Chan Chia Jui; Chua Hock Hin; David Perera; Ooi Mong How; Tan Lee See; Tonnii Sia Loong Loong; Wong Jyn Shan
EPI_ISL_718167, EPI_ISL_718174, EPI_ISL_718176, EPI_ISL_718178, EPI_ISL_718182, EPI_ISL_718185, EPI_ISL_718191, EPI_ISL_718192, EPI_ISL_718197, EPI_ISL_718198, EPI_ISL_718203, EPI_ISL_718204, EPI_ISL_718205, EPI_ISL_718216, EPI_ISL_718218, EPI_ISL_718222, EPI_ISL_1363119, EPI_ISL_1363120, EPI_ISL_1363121, EPI_ISL_1363122, EPI_ISL_1363123, EPI_ISL_2367474, EPI_ISL_2367476, EPI_ISL_2367482, EPI_ISL_2367483, EPI_ISL_2367484, EPI_ISL_2367485, EPI_ISL_2367486, EPI_ISL_2367487, EPI_ISL_2367488, EPI_ISL_2367534, EPI_ISL_2379984, EPI_ISL_2379985, EPI_ISL_2379986, EPI_ISL_2379987, EPI_ISL_2379988, EPI_ISL_2380011, EPI_ISL_2380012, EPI_ISL_2380013, EPI_ISL_2380014, EPI_ISL_2380015, EPI_ISL_2380016, EPI_ISL_2380017, EPI_ISL_2380018, EPI_ISL_2380019, EPI_ISL_2380020, EPI_ISL_2380021, EPI_ISL_2380022, EPI_ISL_2380023, EPI_ISL_2380024, EPI_ISL_2380025, EPI_ISL_2380026, EPI_ISL_2380027, EPI_ISL_2380028, EPI_ISL_2380029, EPI_ISL_2380030, EPI_ISL_2380031, EPI_ISL_2380032, EPI_ISL_2380033, EPI_ISL_2535604, EPI_ISL_2535607, EPI_ISL_2535608, EPI_ISL_2535619, EPI_ISL_2535620, EPI_ISL_2535630, EPI_ISL_2535631, EPI_ISL_2535632, EPI_ISL_2535641, EPI_ISL_2535642, EPI_ISL_2535652, EPI_ISL_2535653, EPI_ISL_2535660, EPI_ISL_2535661, EPI_ISL_2535672, EPI_ISL_2535673, EPI_ISL_2535683, EPI_ISL_2535695, EPI_ISL_2535696, EPI_ISL_2535697, EPI_ISL_2535698, EPI_ISL_2535699, EPI_ISL_2535700, EPI_ISL_2535701, EPI_ISL_2535702, EPI_ISL_2535731, EPI_ISL_2535732, EPI_ISL_2535733, EPI_ISL_2535734, EPI_ISL_2535735, EPI_ISL_2535736, EPI_ISL_2535737, EPI_ISL_2535738, EPI_ISL_2535739, EPI_ISL_2535740, EPI_ISL_2535741, EPI_ISL_2535742, EPI_ISL_2535743, EPI_ISL_2535744, EPI_ISL_2535745, EPI_ISL_2535746, EPI_ISL_2535747, EPI_ISL_2535748, EPI_ISL_2535749, EPI_ISL_2535750, EPI_ISL_2535751, EPI_ISL_2535752, EPI_ISL_2535753, EPI_ISL_2535754, EPI_ISL_2535755, EPI_ISL_2535756, EPI_ISL_2535757, EPI_ISL_2535758, EPI_ISL_2535759, EPI_ISL_2535760, EPI_ISL_2535761, EPI_ISL_2535762, EPI_ISL_2535763, EPI_ISL_2535764, EPI_ISL_2535765, EPI_ISL_2535766, EPI_ISL_2535767, EPI_ISL_2535768, EPI_ISL_2535769, EPI_ISL_2535770, EPI_ISL_2535771, EPI_ISL_2535772, EPI_ISL_2535780, EPI_ISL_2535781, EPI_ISL_2608379, EPI_ISL_2608380, EPI_ISL_2608381, EPI_ISL_2608382, EPI_ISL_2608383, EPI_ISL_2608384, EPI_ISL_2608385, EPI_ISL_2608386, EPI_ISL_2608387, EPI_ISL_2608388, EPI_ISL_2608389, EPI_ISL_2608390, EPI_ISL_2608391, EPI_ISL_2608392, EPI_ISL_2608393, EPI_ISL_2608394, EPI_ISL_2608395, EPI_ISL_2608396, EPI_ISL_2608397, EPI_ISL_2608398, EPI_ISL_2608399, EPI_ISL_2608400, EPI_ISL_2608401, EPI_ISL_2608402, EPI_ISL_2608403, EPI_ISL_2608404, EPI_ISL_2608405, EPI_ISL_4051645, EPI_ISL_4051674, EPI_ISL_4051675, EPI_ISL_4051696, EPI_ISL_4051697, EPI_ISL_4051698, EPI_ISL_4051699, EPI_ISL_4051700, EPI_ISL_4051701, EPI_ISL_4051702, EPI_ISL_4051827, EPI_ISL_4051855, EPI_ISL_4051856, EPI_ISL_4460102, EPI_ISL_4460103, EPI_ISL_4460104, EPI_ISL_4460105, EPI_ISL_4460106, EPI_ISL_4460107, EPI_ISL_4460108, EPI_ISL_4460109, EPI_ISL_4460110, EPI_ISL_4460111, EPI_ISL_4460112, EPI_ISL_4460113, EPI_ISL_4460114, EPI_ISL_4460115, EPI_ISL_4460116, EPI_ISL_4460117, EPI_ISL_4460118, EPI_ISL_4460119, EPI_ISL_4460120, EPI_ISL_4460121, EPI_ISL_4460122, EPI_ISL_4460123, EPI_ISL_4460124, EPI_ISL_4460125, EPI_ISL_4460126, EPI_ISL_4460127, EPI_ISL_4460221, EPI_ISL_4460222, EPI_ISL_4460223, EPI_ISL_4460224, EPI_ISL_4460225, EPI_ISL_4460226, EPI_ISL_4460227, EPI_ISL_4460231, EPI_ISL_4460232, EPI_ISL_4460233, EPI_ISL_4460234, EPI_ISL_4460235, EPI_ISL_4460236, EPI_ISL_4460237, EPI_ISL_4460238, EPI_ISL_4460239, EPI_ISL_4460240, EPI_ISL_4460241, EPI_ISL_4460242, EPI_ISL_4460243, EPI_ISL_4460244, EPI_ISL_4460245, EPI_ISL_4460246, EPI_ISL_4460247, EPI_ISL_4460248, EPI_ISL_4460249, EPI_ISL_4460250, EPI_ISL_4460251, EPI_ISL_4460252, EPI_ISL_4460253, EPI_ISL_4460254, EPI_ISL_4460255, EPI_ISL_4460256, EPI_ISL_4460257, EPI_ISL_4460258, EPI_ISL_4460259, EPI_ISL_4460260, EPI_ISL_4460261, EPI_ISL_4460262, EPI_ISL_4460263, EPI_ISL_4460264, EPI_ISL_4460265, EPI_ISL_4460266, EPI_ISL_4460267, EPI_ISL_4460268, EPI_ISL_4460269, EPI_ISL_4460270, EPI_ISL_4460271, EPI_ISL_4460272, EPI_ISL_4460273, EPI_ISL_4460274, EPI_ISL_4460275, EPI_ISL_4460276, EPI_ISL_4460277, EPI_ISL_4460278, EPI_ISL_4460279, EPI_ISL_4460280, EPI_ISL_4460281, EPI_ISL_4460282, EPI_ISL_4460283, EPI_ISL_4460284, EPI_ISL_4460285, EPI_ISL_4460286, EPI_ISL_4460287, EPI_ISL_4460288, EPI_ISL_4460289, EPI_ISL_4460290, EPI_ISL_4460291, EPI_ISL_4460292, EPI_ISL_4460293, EPI_ISL_4460294, EPI_ISL_4460295, EPI_ISL_4460296, EPI_ISL_4460297, EPI_ISL_4460298, EPI_ISL_4460299, EPI_ISL_4460300, EPI_ISL_4460301, EPI_ISL_4460302, EPI_ISL_4460303, EPI_ISL_4460304, EPI_ISL_4460305, EPI_ISL_4460306, EPI_ISL_4460307, EPI_ISL_4460308, EPI_ISL_4460309, EPI_ISL_4460310, EPI_ISL_4460311, EPI_ISL_4460312, EPI_ISL_4460313, EPI_ISL_4460314			
see above	Borneo Medical Centre	Institute of Health and Community Medicine	Chan Chia Jui; Chua Hock Hin; David Perera; Ooi Mong How; Tonnii Sia Loong Loong; Wong Jyn Shan; Wong Kiing Aik
EPI_ISL_4460350, EPI_ISL_4460351, EPI_ISL_4460352, EPI_ISL_4460353, EPI_ISL_4460354, EPI_ISL_4460355, EPI_ISL_4460356, EPI_ISL_4460357, EPI_ISL_4460358, EPI_ISL_4460359, EPI_ISL_4460360, EPI_ISL_4460361, EPI_ISL_4460362, EPI_ISL_4460363, EPI_ISL_4460364, EPI_ISL_4460365, EPI_ISL_4460366, EPI_ISL_4460367, EPI_ISL_4460368, EPI_ISL_4460369, EPI_ISL_4460370, EPI_ISL_4460371, EPI_ISL_4460372, EPI_ISL_4460373, EPI_ISL_4460374, EPI_ISL_4460375, EPI_ISL_4460376, EPI_ISL_4460377, EPI_ISL_4460378, EPI_ISL_4460379, EPI_ISL_4460380, EPI_ISL_4460381, EPI_ISL_4460382			
see above	Borneo Specialist Hospital, Miri	Institute of Health and Community Medicine	Chan Chia Jui; Chua Hock Hin; David Perera; Ooi Mong How; Tonnii Sia Loong Loong; Wong Jyn Shan
EPI_ISL_3446671, EPI_ISL_3446673	Bukit Changgang Health Clinic	Institute for Medical Research, Infectious Disease Research Centre, National Institutes of Health, Ministry of Health Malaysia	Anasir MI; Azizan MA; Kamel K; Mohd Zawawi Z; Ramly N; Robert F; Suppiah J; Thayan R
EPI_ISL_3446676	Bukit Kuda Health Clinic	Institute for Medical Research, Infectious Disease Research Centre, National Institutes of Health, Ministry of Health Malaysia	Anasir MI; Azizan MA; Kamel K; Mohd Zawawi Z; Ramly N; Robert F; Suppiah J; Thayan R
EPI_ISL_3446674	Bukit Kuda Heath Clinic	Institute for Medical Research, Infectious Disease Research Centre, National Institutes of Health, Ministry of Health Malaysia	Anasir MI; Azizan MA; Kamel K; Mohd Zawawi Z; Ramly N; Robert F; Suppiah J; Thayan R
EPI_ISL_4122269	CAC ILKKM	iPROMISE, UiTM	Ariza Adnan; Fadzilah Mohd Nor; Lim Wai Feng; Mohd Asif Mohd Sukri; Mohd Nur Fakhruzzaman Noorizhab; Mohd Zaki Salleh; Sazzli Shahlan Kassim; Siti Farah Alwani Mohd Nawi; Siti Hamimah Sheikh Abdul Kadir; Teh Lay Kek; Wang Seok Mui
EPI_ISL_4122272	CAC ILKKM Kangar Perlis	iPROMISE, UiTM	Ariza Adnan; Fadzilah Mohd Nor; Lim Wai Feng; Mohd Asif Mohd Sukri; Mohd Nur Fakhruzzaman Noorizhab; Mohd Zaki Salleh; Sazzli Shahlan Kassim; Siti Farah Alwani Mohd Nawi; Siti Hamimah Sheikh Abdul Kadir; Teh Lay Kek; Wang Seok Mui
EPI_ISL_4565023	CPRC HOSPITAL SG BULOH	Tropical Infectious Diseases Research & Education Centre (TIDREC), Universiti Malaya	AsmaAnati CheMatSeri; Che-Norainon Yaacob; Jia-Yi Tan; Jo-Ern Wong; Kim-Kee Tan; Mulya-Mustika-Sari Zulkifli; Noor Syahida Azizan; Nur-Hidayana Mahfodz; Sazaly AbuBakar; Siti-Sarah Nor’e
EPI_ISL_4565033	CPRC IPJKNT	Tropical Infectious Diseases Research & Education Centre (TIDREC), Universiti Malaya	AsmaAnati CheMatSeri; Che-Norainon Yaacob; Jia-Yi Tan; Jo-Ern Wong; Kim-Kee Tan; Mulya-Mustika-Sari Zulkifli; Noor Syahida Azizan; Nur-Hidayana Mahfodz; Sazaly AbuBakar; Siti-Sarah Nor’e
EPI_ISL_3980722, EPI_ISL_3980723, EPI_ISL_3980725, EPI_ISL_3980726, EPI_ISL_3980728, EPI_ISL_4564996, EPI_ISL_4564997, EPI_ISL_4564998, EPI_ISL_4564999, EPI_ISL_4565000, EPI_ISL_4565001, EPI_ISL_4565013, EPI_ISL_4565014, EPI_ISL_4565015, EPI_ISL_4565016, EPI_ISL_4565017, EPI_ISL_4565032, EPI_ISL_4565034, EPI_ISL_4565035, EPI_ISL_4565036, EPI_ISL_4565037, EPI_ISL_4565038, EPI_ISL_4565039, EPI_ISL_4565041, EPI_ISL_4565042			
see above	CPRC JKNT	Tropical Infectious Diseases Research & Education Centre (TIDREC), Universiti Malaya	AsmaAnati CheMatSeri; Che-Norainon Yaacob; Jia-Yi Tan; Jo-Ern Wong; Kim-Kee Tan; Mulya-Mustika-Sari Zulkifli; Noor Syahida Azizan; Nur-Hidayana Mahfodz; Sazaly AbuBakar; Siti-Sarah Nor’e
EPI_ISL_3980727	CPRC Jabatan kesihatan Negeri Terengganu	Tropical Infectious Diseases Research & Education Centre (TIDREC), Universiti Malaya	AsmaAnati CheMatSeri; Che-Norainon Yaacob; Jia-Yi Tan; Jo-Ern Wong; Kim-Kee Tan; Mulya-Mustika-Sari Zulkifli; Noor Syahida Azizan; Nur-Hidayana Mahfodz; Sazaly AbuBakar; Siti-Sarah Nor’e
EPI_ISL_4051646, EPI_ISL_4051647, EPI_ISL_4051648, EPI_ISL_4051649, EPI_ISL_4051650, EPI_ISL_4051651, EPI_ISL_4051652, EPI_ISL_4051664, EPI_ISL_4051665, EPI_ISL_4051666, EPI_ISL_4051667, EPI_ISL_4051668, EPI_ISL_4051669, EPI_ISL_4051670, EPI_ISL_4051671, EPI_ISL_4051672, EPI_ISL_4051673, EPI_ISL_4051676, EPI_ISL_4051678, EPI_ISL_4051679, EPI_ISL_4051703, EPI_ISL_4051704, EPI_ISL_4051772, EPI_ISL_4051773, EPI_ISL_4051774, EPI_ISL_4051775, EPI_ISL_4051776, EPI_ISL_4051777, EPI_ISL_4051778, EPI_ISL_4051779, EPI_ISL_4051780, EPI_ISL_4051781, EPI_ISL_4051782, EPI_ISL_4051783, EPI_ISL_4051784, EPI_ISL_4051785, EPI_ISL_4051786, EPI_ISL_4051787, EPI_ISL_4051788, EPI_ISL_4051789, EPI_ISL_4051818, EPI_ISL_4051833, EPI_ISL_4051834, EPI_ISL_4051835, EPI_ISL_4051836, EPI_ISL_4051839, EPI_ISL_4051847, EPI_ISL_4051859, EPI_ISL_4051863			
see above	CRC, Sibu Hospital	Institute of Health and Community Medicine	Chan Chia Jui; Chua Hock Hin; David Perera; Ooi Mong How; Tonnii Sia Loong Loong; Wong Jyn Shan; Wong Kiing Aik
EPI_ISL_3374151	Cameron Highlands District Health Office	Institute for Medical Research, Infectious Disease Research Centre, National Institutes of Health, Ministry of Health Malaysia	Anasir MI; Azizan MA; Kamel K; Mohd Zawawi Z; Ramly N; Robert F; Suppiah J; Thayan R
EPI_ISL_3374233, EPI_ISL_3374709	Cheras District Health Office	Institute for Medical Research, Infectious Disease Research Centre, National Institutes of Health, Ministry of Health Malaysia	Anasir MI; Azizan MA; Kamel K; Mohd Zawawi Z; Ramly N; Robert F; Suppiah J; Thayan R
EPI_ISL_4460207, EPI_ISL_4460208, EPI_ISL_4460209, EPI_ISL_4460210, EPI_ISL_4460211, EPI_ISL_4460217, EPI_ISL_4460218, EPI_ISL_4460219, EPI_ISL_4460220, EPI_ISL_4460228, EPI_ISL_4460229			
see above	Clinical Research Centre (CRC), Sibu Hospital, Sibu	Institute of Health and Community Medicine	Chan Chia Jui; Chua Hock Hin; David Perera; Ooi Mong How; Tonnii Sia Loong Loong; Wong Jyn Shan; Wong Kiing Aik
EPI_ISL_2367511, EPI_ISL_2367512, EPI_ISL_2367513, EPI_ISL_2367520, EPI_ISL_2367521, EPI_ISL_2367522, EPI_ISL_2367523, EPI_ISL_2367524, EPI_ISL_2367525, EPI_ISL_2367526, EPI_ISL_2367527, EPI_ISL_2367528, EPI_ISL_2367529, EPI_ISL_2367530, EPI_ISL_2367531, EPI_ISL_2367532, EPI_ISL_2367533, EPI_ISL_2367535, EPI_ISL_2367536, EPI_ISL_2367537, EPI_ISL_2367538, EPI_ISL_2379957, EPI_ISL_2379958, EPI_ISL_2379959, EPI_ISL_2379960, EPI_ISL_2535703, EPI_ISL_2535704, EPI_ISL_2535705, EPI_ISL_2535706, EPI_ISL_2535707, EPI_ISL_2535708, EPI_ISL_2535709, EPI_ISL_2535710, EPI_ISL_2535711, EPI_ISL_2535712, EPI_ISL_2535713, EPI_ISL_2535714, EPI_ISL_2535715, EPI_ISL_2535716, EPI_ISL_2535717, EPI_ISL_2535718, EPI_ISL_2535719, EPI_ISL_2535720, EPI_ISL_2535721, EPI_ISL_2535722, EPI_ISL_2535723, EPI_ISL_2535724, EPI_ISL_2535725, EPI_ISL_2535726, EPI_ISL_2535727, EPI_ISL_2535728, EPI_ISL_2535729, EPI_ISL_2535730			
see above	Clinical Research Centre Hospital Sibu	Institute of Health and Community Medicine	Chan Chia Jui; Chua Hock Hin; David Perera; Ooi Mong How; Tonnii Sia Loong Loong; Wong Jyn Shan; Wong Kiing Aik
EPI_ISL_2608406, EPI_ISL_2608407, EPI_ISL_2608408, EPI_ISL_2608409, EPI_ISL_2608410, EPI_ISL_2608411, EPI_ISL_2608412, EPI_ISL_2608413, EPI_ISL_2608414			

see above	Clinical Research Centre, Sarawak General Hospital	Institute of Health and Community Medicine	Chan Chia Jui; Chua Hock Hin; David Perera; Ooi Mong How; Tonnii Sia Loong Loong; Wong Jyn Shan; Wong Kiing Aik
EPI_ISL_2827788, EPI_ISL_2827789, EPI_ISL_2827790	DEPARTMENT OF MEDICAL MICROBIOLOGY & PARASITOLOGY, SCHOOL OF MEDICAL SCIENCES	DEPARTMENT OF MEDICAL MICROBIOLOGY & PARASITOLOGY, SCHOOL OF MEDICAL SCIENCES	A.H.; A.S.; Alexander Chong; Ayub, Q.; Azmi; Banga Singh; Chan; Chua; Deris; E.A.R.; Eng; Engku Nur Syafirah; Halim; Harun, A.; Hussin, Z.; K.I.; K.K.; Khalid; L.H.; Lau; Lee; Leela, A.; Lim; M.F.; M.N.F.; M.Z.H.; Mat Salleh; Md Zain, M.; Md Zoqratt; Muhammad; Musa; Musa, N.; N.H.; N.L.; N.M.; N.S.; Omar, S.; Rahman, S.; Rosline, H.; S.C.; S.Y.; Samsudin; W.C.; W.L.; W.M.; W.W.H.; Y.Y.; Yaacob; Z.Z.; Zahiruddin
EPI_ISL_2342546, EPI_ISL_2342547, EPI_ISL_2342548, EPI_ISL_2342549, EPI_ISL_2342550, EPI_ISL_2342551, EPI_ISL_2342552, EPI_ISL_2342553, EPI_ISL_2342554, EPI_ISL_2342555, EPI_ISL_2342556, EPI_ISL_2342557, EPI_ISL_2342558, EPI_ISL_2342559, EPI_ISL_2342560, EPI_ISL_2342561, EPI_ISL_2342562, EPI_ISL_2342563, EPI_ISL_2342564, EPI_ISL_2342565			
see above	DNA Laboratories Sdn Bhd	Malaysia Genome Institute	Azrin Ahmad; Enizza Kasim; Irni Suhayu Sapian; Mohd Faizal Abu Bakar; Mohd Noor Mat Isa; Nor Azfa Johari.; Nurhezreen Md Iqbal; Shamsidar Sopie; Siti Noraini Othman; Wong Yong Wee; Yusuf Muhammad Noor
EPI_ISL_1059902	DNA Laboratories Sdn. Bhd.	Malaysia Genome Institute	Azrin Ahmad; Enizza Kasim; Irni Suhayu Sapian; Mohd Faizal Abu Bakar; Mohd Noor Mat Isa; Nor Azfa Johari.; Nurhezreen Md Iqbal; Shamsidar Sopie; Siti Noraini Othman; Wong Yong Wee; Yusuf Muhammad Noor
EPI_ISL_1435783, EPI_ISL_1435785, EPI_ISL_1435787, EPI_ISL_1435789, EPI_ISL_1435791, EPI_ISL_1435792, EPI_ISL_1435794, EPI_ISL_1435796, EPI_ISL_1435798, EPI_ISL_1435800			
see above	DNALAB Sdn. Bhd.	Malaysia Genome Institute	Azrin Ahmad; Enizza Kasim; Irni Suhayu Sapian; Mohd Faizal Abu Bakar; Mohd Noor Mat Isa; Nor Azfa Johari.; Nurhezreen Md Iqbal; Shamsidar Sopie; Siti Noraini Othman; Wong Yong Wee; Yusuf Muhammad Noor
EPI_ISL_3446678	Dengkil Health Clinic	Institute for Medical Research, Infectious Disease Research Centre, National Institutes of Health, Ministry of Health Malaysia	Anasir MI; Azizan MA; Kamel K; Mohd Zawawi Z; Ramly N; Robert F; Suppiah J; Thayan R
EPI_ISL_2988859, EPI_ISL_2988860, EPI_ISL_2988861, EPI_ISL_2988862, EPI_ISL_2988863, EPI_ISL_2988864, EPI_ISL_2988865, EPI_ISL_2988866, EPI_ISL_2988867, EPI_ISL_2988868, EPI_ISL_2988869, EPI_ISL_2988870, EPI_ISL_2988875, EPI_ISL_2988876, EPI_ISL_2988877			
see above	Department of Medical Microbiology and Parasitology, Hospital Universiti Sains Malaysia	Molecular Research Laboratory	Abdul Haris bin Muhammad; Ahmad Sukari Bin Halim; Alexander Chong Shu Chien; Aswini Leela; Azian Harun; Chan Yean Yean; Chua Wei Chuan; Chua Wei Lian; Engku Nur Syafirah bt Engku Abd Rahman; Kamarul Imran Musa; Kirnpal Kaur Banga Singh; Lau Nyok Sean; Lee Lih Huey; Lee Yeong Yeh; Lim Shu Yong; Maizun binti Mohd Zain; Muhammad Fazli bin Khalid; Muhammad Nashrul Farhan Samsudin; Muhammad Zarul Hanifah Bin Md Zoqratt; Najib Majdi Bin Yaacob; Nazmi Liana Binti Azmi; Noor Hafizan binti Mat Salleh; Nurfadhlina Musa; Qasim Ayub; Rosline Hassan; Sadequr Rahman; Syahida binti Omar; Wan Mohd Zahiruddin Wan Mohammad; Wilhelm Eng Wei Han; Zaini bin Hussin; Zakuan Zainy Deris
EPI_ISL_2151509, EPI_ISL_2151510, EPI_ISL_2151541, EPI_ISL_2151584, EPI_ISL_2554730, EPI_ISL_2554731	Department of Medical Microbiology, Hospital Pengajar Universiti Putra Malaysia	Department of Medical Microbiology, Hospital Pengajar Universiti Putra Malaysia	Boo Sook Yee; Chin Siang Tean; Hui-Yee Chee; Jia-Yong Lam; Mohd Syis Zulkipli; Narcisse Joseph; Syafinaz Amin-Nordin; Yien-Ping Wong
EPI_ISL_861717, EPI_ISL_861718, EPI_ISL_861719, EPI_ISL_861720, EPI_ISL_861721, EPI_ISL_861722, EPI_ISL_861723, EPI_ISL_861724, EPI_ISL_861725, EPI_ISL_861726, EPI_ISL_861729, EPI_ISL_977585, EPI_ISL_977586, EPI_ISL_977587, EPI_ISL_977588, EPI_ISL_977589, EPI_ISL_977590, EPI_ISL_2342566, EPI_ISL_2342567, EPI_ISL_2342568, EPI_ISL_2342569, EPI_ISL_2342570, EPI_ISL_2342571, EPI_ISL_2342572, EPI_ISL_2342573, EPI_ISL_2342574, EPI_ISL_2342575, EPI_ISL_2342576, EPI_ISL_2342577, EPI_ISL_2342578, EPI_ISL_2342579, EPI_ISL_2342580, EPI_ISL_2342581, EPI_ISL_2342582, EPI_ISL_2342583, EPI_ISL_2342584, EPI_ISL_3162191, EPI_ISL_3162192, EPI_ISL_3162193, EPI_ISL_3162194, EPI_ISL_3162195, EPI_ISL_3162196, EPI_ISL_3162197, EPI_ISL_3162198, EPI_ISL_3162199, EPI_ISL_3162200, EPI_ISL_3162201, EPI_ISL_3162202, EPI_ISL_3162203, EPI_ISL_3162204, EPI_ISL_3162205, EPI_ISL_3162206, EPI_ISL_3162207, EPI_ISL_3162208, EPI_ISL_3162209, EPI_ISL_3162210, EPI_ISL_3162211, EPI_ISL_3162212, EPI_ISL_3162213, EPI_ISL_3162214, EPI_ISL_3162215, EPI_ISL_3162216, EPI_ISL_3162217, EPI_ISL_3162218, EPI_ISL_3162219, EPI_ISL_3162220, EPI_ISL_3162221, EPI_ISL_3162222, EPI_ISL_3162223			
see above	Department of Medical Microbiology, Hospital Pengajar Universiti Putra Malaysia	Malaysia Genome Institute	Avisha Richards; Azrin Ahmad; Enizza Kasim; Hui-Yee Chee; Irni Suhayu Sapian; Mohd Anuar Jonet; Mohd Faizal Abu Bakar; Mohd Noor Mat Isa; Muhammad MI; Narcisse Joseph; Nor Azfa Johari; Nor Zahrin Hasran; Nurhezreen Md Iqbal; Shamsidar Sopie; Siti Noraini Othman; Syafinaz Amin-Nordin; Yusuf Muhammad Noor
EPI_ISL_417917	Department of Medical Microbiology, University Malaya Medical Centre	Department of Medical Microbiology	Adeeba KAMARULZAMAN; Chee Kuan WONG; Cindy Shuan Ju TEH; I-Ching SAM; Sasheela PONNAMPALAVANAR; Sharifah Faridah SYED OMAR; University Malaya Medical Centre COVID Team; Vijayan MUNUSAMY; Yoke Fun Chan; Yoong Min CHONG
EPI_ISL_417918, EPI_ISL_417919, EPI_ISL_417920, EPI_ISL_501176, EPI_ISL_501177, EPI_ISL_501178, EPI_ISL_501179, EPI_ISL_501180, EPI_ISL_501181, EPI_ISL_501182, EPI_ISL_501183, EPI_ISL_501184, EPI_ISL_501185, EPI_ISL_501186, EPI_ISL_501187, EPI_ISL_501188, EPI_ISL_501189, EPI_ISL_501190, EPI_ISL_501191, EPI_ISL_501192, EPI_ISL_501193, EPI_ISL_501194, EPI_ISL_501195, EPI_ISL_501196, EPI_ISL_501197, EPI_ISL_501198, EPI_ISL_501199, EPI_ISL_501200, EPI_ISL_501201, EPI_ISL_501202, EPI_ISL_501203, EPI_ISL_501204, EPI_ISL_501205, EPI_ISL_501206, EPI_ISL_501207, EPI_ISL_501208, EPI_ISL_501209, EPI_ISL_501210, EPI_ISL_501211, EPI_ISL_501212, EPI_ISL_501213, EPI_ISL_501214, EPI_ISL_501215, EPI_ISL_501216, EPI_ISL_501217, EPI_ISL_501218, EPI_ISL_501219, EPI_ISL_501220, EPI_ISL_501221, EPI_ISL_501222, EPI_ISL_501223, EPI_ISL_501224, EPI_ISL_501225, EPI_ISL_501226, EPI_ISL_501227, EPI_ISL_501228, EPI_ISL_506996, EPI_ISL_506997, EPI_ISL_506998, EPI_ISL_506999, EPI_ISL_507000, EPI_ISL_2769381, EPI_ISL_2769382, EPI_ISL_2769383, EPI_ISL_2769384, EPI_ISL_2769385, EPI_ISL_2769386, EPI_ISL_2769387, EPI_ISL_2769388, EPI_ISL_2769389, EPI_ISL_2769390, EPI_ISL_2769391, EPI_ISL_2769392, EPI_ISL_2769393, EPI_ISL_2769394, EPI_ISL_2769395, EPI_ISL_2769396, EPI_ISL_2769397, EPI_ISL_2769398, EPI_ISL_2769399, EPI_ISL_2769400, EPI_ISL_2769401, EPI_ISL_2769402, EPI_ISL_2769403, EPI_ISL_2769404, EPI_ISL_2769405, EPI_ISL_2769406, EPI_ISL_2769407, EPI_ISL_2769408, EPI_ISL_2769409, EPI_ISL_2769410, EPI_ISL_2769411, EPI_ISL_2769412, EPI_ISL_2769413, EPI_ISL_2769414, EPI_ISL_2769415, EPI_ISL_2769416, EPI_ISL_2769417, EPI_ISL_2769418, EPI_ISL_2769419, EPI_ISL_2769420, EPI_ISL_2769421, EPI_ISL_2769422, EPI_ISL_2769423, EPI_ISL_2769424, EPI_ISL_2769425, EPI_ISL_2769426, EPI_ISL_2769427, EPI_ISL_2769428, EPI_ISL_2769429, EPI_ISL_2769430, EPI_ISL_2769431, EPI_ISL_2769432, EPI_ISL_2769433, EPI_ISL_2769434, EPI_ISL_2769435, EPI_ISL_2769436, EPI_ISL_2769437, EPI_ISL_2769438, EPI_ISL_2784328, EPI_ISL_2784329, EPI_ISL_3246345, EPI_ISL_3246346, EPI_ISL_3246347, EPI_ISL_3246348, EPI_ISL_3246349, EPI_ISL_3246350, EPI_ISL_3246351, EPI_ISL_3246352, EPI_ISL_3246353, EPI_ISL_3246354, EPI_ISL_3246355, EPI_ISL_3246356, EPI_ISL_3246357, EPI_ISL_3246358, EPI_ISL_3246359, EPI_ISL_3246360, EPI_ISL_3246361, EPI_ISL_3246362, EPI_ISL_3246363, EPI_ISL_3246364, EPI_ISL_3246365, EPI_ISL_3246366, EPI_ISL_3246367, EPI_ISL_3246368, EPI_ISL_3246369, EPI_ISL_3246370, EPI_ISL_3246371, EPI_ISL_3246372, EPI_ISL_3246373, EPI_ISL_3246374, EPI_ISL_3246375, EPI_ISL_3246376, EPI_ISL_3246377, EPI_ISL_3246378, EPI_ISL_3246379, EPI_ISL_3246380, EPI_ISL_3246381, EPI_ISL_3246382, EPI_ISL_3246383, EPI_ISL_3246384, EPI_ISL_3246385, EPI_ISL_3246386, EPI_ISL_3246387, EPI_ISL_3246388, EPI_ISL_3246389, EPI_ISL_3246390, EPI_ISL_3246391, EPI_ISL_3246392, EPI_ISL_3246393, EPI_ISL_3246394, EPI_ISL_3246395, EPI_ISL_3246396, EPI_ISL_3246397, EPI_ISL_3246398, EPI_ISL_3246399, EPI_ISL_3246400, EPI_ISL_3246401, EPI_ISL_3246402, EPI_ISL_3246403, EPI_ISL_3246404, EPI_ISL_3246405, EPI_ISL_3246406, EPI_ISL_3246407, EPI_ISL_3246408, EPI_ISL_3246409, EPI_ISL_3246410, EPI_ISL_3246411, EPI_ISL_3246412, EPI_ISL_3246413, EPI_ISL_3246414, EPI_ISL_3246415, EPI_ISL_3246416, EPI_ISL_3246417, EPI_ISL_3246418, EPI_ISL_3246419, EPI_ISL_3246420, EPI_ISL_3246421, EPI_ISL_3246422, EPI_ISL_3246423, EPI_ISL_3246424, EPI_ISL_3246425, EPI_ISL_3246426, EPI_ISL_3246427, EPI_ISL_3246428, EPI_ISL_3246429, EPI_ISL_3246430, EPI_ISL_3492535, EPI_ISL_4056087, EPI_ISL_4056088, EPI_ISL_4056089, EPI_ISL_4056090, EPI_ISL_4056092, EPI_ISL_4056093, EPI_ISL_4056094, EPI_ISL_4056095, EPI_ISL_4056096, EPI_ISL_4056097, EPI_ISL_4056098, EPI_ISL_4056099, EPI_ISL_4056100, EPI_ISL_4056101, EPI_ISL_4056102, EPI_ISL_4056103, EPI_ISL_4056104, EPI_ISL_4056105, EPI_ISL_4056106, EPI_ISL_4056107, EPI_ISL_4056108, EPI_ISL_4056109, EPI_ISL_4056110, EPI_ISL_4056111, EPI_ISL_4056112, EPI_ISL_4056113, EPI_ISL_4056114, EPI_ISL_4056115, EPI_ISL_4056116, EPI_ISL_4056117, EPI_ISL_4056118, EPI_ISL_4056119, EPI_ISL_4056120, EPI_ISL_4056121, EPI_ISL_4056122, EPI_ISL_4056123, EPI_ISL_4056124, EPI_ISL_4056125, EPI_ISL_4056127, EPI_ISL_4056128, EPI_ISL_4056129, EPI_ISL_4056130, EPI_ISL_4056131, EPI_ISL_4056132, EPI_ISL_4056133, EPI_ISL_4056134, EPI_ISL_4056135, EPI_ISL_4056136, EPI_ISL_4056137, EPI_ISL_4056138, EPI_ISL_4056139, EPI_ISL_4056140, EPI_ISL_4056141, EPI_ISL_4056142, EPI_ISL_4056143, EPI_ISL_4056144, EPI_ISL_4056145, EPI_ISL_4056146, EPI_ISL_4056147, EPI_ISL_4056148, EPI_ISL_4056149, EPI_ISL_4056150, EPI_ISL_4056151, EPI_ISL_4056152, EPI_ISL_4056153, EPI_ISL_4056154, EPI_ISL_4056155, EPI_ISL_4056156, EPI_ISL_4056157, EPI_ISL_4056158, EPI_ISL_4056159, EPI_ISL_4056160, EPI_ISL_4056161, EPI_ISL_4056162, EPI_ISL_4056163, EPI_ISL_4056165, EPI_ISL_4056166, EPI_ISL_4056167, EPI_ISL_4056168, EPI_ISL_4056169, EPI_ISL_4056170, EPI_ISL_4056171, EPI_ISL_4056172, EPI_ISL_4056173, EPI_ISL_4056174, EPI_ISL_4056175, EPI_ISL_4056176, EPI_ISL_4056177, EPI_ISL_4071805			
see above	Department of Medical Microbiology, University Malaya Medical Centre	Department of Medical Microbiology, Faculty of Medicine, University of Malaya	Adeeba KAMARULZAMAN; Chee Kuan WONG; Cindy Shuan Ju TEH; Fadhil Hadi JAMALUDDIN; Han Ming GAN; I-Ching SAM; Izzati Kausar; Jennifer Chong; Jolene Yin Ling FU; Sasheela PONNAMPALAVANAR; Sharifah Faridah SYED OMAR; University Malaya Medical Centre COVID Team; Vijayan MUNUSAMY; Yoke Fun CHAN; Yoke Fun Chan; Yoong Min CHONG
EPI_ISL_2621676, EPI_ISL_2621677, EPI_ISL_2622006, EPI_ISL_2622007, EPI_ISL_2622045, EPI_ISL_2622047, EPI_ISL_2622079			
see above	Department of Pathology	Centre for Research in Advanced Tropical Bioscience	Hajar Fauzan Bin Ahmad; Norhidayah Binti Kamarudin; Ummu Afeera Zainulabid
EPI_ISL_2622046, EPI_ISL_2622088, EPI_ISL_2622089	Department of Pathology	Centre for Research in Advanced Tropical Bioscience Universiti Malaysia Pahang, 26300 Gambang, Pahang, Malaysia	Hajar Fauzan Bin Ahmad; Norhidayah Binti Kamarudin; Ummu Afeera Zainulabid
EPI_ISL_3221325, EPI_ISL_3239916, EPI_ISL_3241561	Department of Pathology & Laboratory Medicine	Centre for Research in Advanced Tropical Bioscience (Biotropic Centre)	Hajar Fauzan Ahmad; How Soon Hin and Hajar Fauzan Ahmad; Mohd Nazli Kamarulzaman; Norhidayah Kamarudin; Ummu Afeera Zainulabid
EPI_ISL_3221322, EPI_ISL_3221323	Department of Pathology and Laboratory Medicine, Kulliyyah of Medicine, International Islamic University of Malaysia	Centre for Research in Advanced Tropical Bioscience (Biotropic Centre)	Hajar Fauzan Bin Ahmad; How Soon Hin and Hajar Fauzan Ahmad; Norhidayah Kamarudin; Ummu Afeera Zainulabid
EPI_ISL_4096801	Duchess of Kent Hospital	Institute for Medical Research, Infectious Disease Research Centre, National Institutes of Health, Ministry of Health Malaysia	Ahmad FA; Ahmad Fazilah NA; Anasir MI; Azizan MA; Kamel K; Mohd Zawawi Z; Norhisham SN; Ramly N; Robert F; Suppiah J; Thayan R
EPI_ISL_1919861	Duchess of Kent Hospital, Sandakan	Institute for Medical Research, Infectious Disease Research Centre, National Institutes of Health, Ministry of Health Malaysia	Kamel K; Mohd Zawawi Z; Suppiah J; Thayan R
EPI_ISL_4602782, EPI_ISL_4602785	Forensic Department of Queen Elizabeth Hospital	Institute for Medical Research, Infectious Disease Research Centre, National Institutes of Health, Ministry of Health Malaysia	Ahmad FA; Ahmad Fazilah NA; Anasir MI; Azizan MA; Kamel K; Mohd Zawawi Z; Norhisyam SN; Ramly N; Robert F; Suppiah J; Thayan R
EPI_ISL_3858139	GLENEAGLES HOSPITAL MEDINI JOHOR	UKM Medical Molecular Biology Institute (UMBI)	Mira Farzana binti Mohamad Mokhtar
EPI_ISL_3769343	GLENEAGLES HOSPITAL MEDINI JOHOR	iPROMISE, UiTM	Ariza Adnan; Fadzilah Mohd Nor; Lim Wai Feng; Mohd Asif Mohd Sukri; Mohd Nur Fakhruzzaman Noorizhab; Mohd Zaki Salleh; Sazzli Shahlan Kassim; Siti Farah Alwani Mohd Nawi; Siti Hamimah Sheikh Abdul Kadir; Teh Lay Kek; Wang Seok Mui
EPI_ISL_3334218	Gleneagles Hospital Medini Johor	Institute for Medical Research, Infectious Disease Research Centre, National Institutes of Health, Ministry of Health Malaysia	Anasir MI; Azizan MA; Kamel K; Mohd Zawawi Z; Ramly N; Robert F; Suppiah J; Thayan R
EPI_ISL_2549562, EPI_ISL_3333232	Gribbles Pathology	Institute for Medical Research, Infectious Disease Research Centre, National Institutes of Health, Ministry of Health Malaysia	Anasir MI; Azizan MA; Kamel K; Mohd Zawawi Z; Ramly N; Robert F; Suppiah J; Thayan R
EPI_ISL_3333233, EPI_ISL_3767019	Gribbles Pathology (M) Sdn Bhd	Institute for Medical Research, Infectious Disease Research Centre, National Institutes of Health, Ministry of Health Malaysia	Anasir MI; Azizan MA; Kamel K; Mohd Zawawi Z; Ramly N; Robert F; Suppiah J; Thayan R
EPI_ISL_2233383	Gribbles Pathology Malaysia	Institute for Medical Research, Infectious Disease Research Centre, National Institutes of Health, Ministry of Health Malaysia	Azizan MA; Kamel K; Mohd Zawawi Z; Ramly N; Robert F; Suppiah J; Thayan R
EPI_ISL_4051681, EPI_ISL_4051705, EPI_ISL_4051770, EPI_ISL_4051771, EPI_ISL_4051796, EPI_ISL_4051797, EPI_ISL_4051798, EPI_ISL_4051799, EPI_ISL_4051800, EPI_ISL_4051801, EPI_ISL_4051802, EPI_ISL_4051803, EPI_ISL_4051804, EPI_ISL_4051805, EPI_ISL_4051806, EPI_ISL_4051807, EPI_ISL_4051808, EPI_ISL_4051809, EPI_ISL_4051810, EPI_ISL_4051811, EPI_ISL_4051812, EPI_ISL_4051813, EPI_ISL_4051814, EPI_ISL_4051815, EPI_ISL_4051816, EPI_ISL_4051817, EPI_ISL_4051828, EPI_ISL_4051840, EPI_ISL_4051841, EPI_ISL_4051843, EPI_ISL_4460141, EPI_ISL_4460142, EPI_ISL_4460143, EPI_ISL_4460144, EPI_ISL_4460145, EPI_ISL_4460146, EPI_ISL_4460147, EPI_ISL_4460148, EPI_ISL_4460149, EPI_ISL_4460150, EPI_ISL_4460151, EPI_ISL_4460152, EPI_ISL_4460153, EPI_ISL_4460154, EPI_ISL_4460155, EPI_ISL_4460168, EPI_ISL_4460169			
see above	Gribbles Pathology, Kuching	Institute of Health and Community Medicine	Chan Chia Jui; Chua Hock Hin; David Perera; Ooi Mong How; Reagan Entigu Linton; Tonnii Sia Loong Loong; Wong Jyn Shan
EPI_ISL_4565025, EPI_ISL_4565027, EPI_ISL_4565029	H.Banting	Tropical Infectious Diseases Research & Education Centre (TIDREC), Universiti Malaya	AsmaAnati CheMatSeri; Che-Norainon Yaacob; Jia-Yi Tan; Jo-Ern Wong; Kim-Kee Tan; Mulya-Mustika-Sari Zulkifli; Noor Syahida Azizan; Nur-Hidayana Mahfodz; Sazaly AbuBakar; Siti-Sarah Nor’e
EPI_ISL_3980741, EPI_ISL_3980743, EPI_ISL_3980745, EPI_ISL_4565044, EPI_ISL_4565045	HOSPITAL AMPANG	Tropical Infectious Diseases Research & Education Centre (TIDREC), Universiti Malaya	AsmaAnati CheMatSeri; Che-Norainon Yaacob; Jia-Yi Tan; Jo-Ern Wong; Kim-Kee Tan; Mulya-Mustika-Sari Zulkifli; Noor Syahida Azizan; Nur-Hidayana Mahfodz; Sazaly AbuBakar; Siti-Sarah Nor’e
EPI_ISL_4565040	HOSPITAL ANGKATAN TENTERA TUANKU MIZAN	Tropical Infectious Diseases Research & Education Centre (TIDREC), Universiti Malaya	AsmaAnati CheMatSeri; Che-Norainon Yaacob; Jia-Yi Tan; Jo-Ern Wong; Kim-Kee Tan; Mulya-Mustika-Sari Zulkifli; Noor Syahida Azizan; Nur-Hidayana Mahfodz; Sazaly AbuBakar; Siti-Sarah Nor’e
EPI_ISL_4565043	HOSPITAL BAHAGIA ULU KINTA	Tropical Infectious Diseases Research & Education Centre (TIDREC), Universiti Malaya	AsmaAnati CheMatSeri; Che-Norainon Yaacob; Jia-Yi Tan; Jo-Ern Wong; Kim-Kee Tan; Mulya-Mustika-Sari Zulkifli; Noor Syahida Azizan; Nur-Hidayana Mahfodz; Sazaly AbuBakar; Siti-Sarah Nor’e
EPI_ISL_3945537	HOSPITAL PAKAR SULTANAH FATIMAH, MUAR	UKM Medical Molecular Biology Institute (UMBI)	Nur Alyaa Afifah Md Shahri
EPI_ISL_4122279, EPI_ISL_4122282, EPI_ISL_4122284, EPI_ISL_4122288, EPI_ISL_4122516, EPI_ISL_4122544, EPI_ISL_4122563, EPI_ISL_4435514, EPI_ISL_4435517, EPI_ISL_4435520, EPI_ISL_4435521, EPI_ISL_4435523, EPI_ISL_4435525, EPI_ISL_4435526, EPI_ISL_4435527			
see above	HOSPITAL PULAU PINANG	iPROMISE, UiTM	Ariza Adnan; Fadzilah Mohd Nor; Lim Wai Feng; Mohd Asif Mohd Sukri; Mohd Nur Fakhruzzaman Noorizhab; Mohd Zaki Salleh; Sazzli Shahlan Kassim; Siti Farah Alwani Mohd Nawi; Siti Hamimah Sheikh Abdul Kadir; Teh Lay Kek; Wang Seok Mui
EPI_ISL_4122489, EPI_ISL_4435524	HOSPITAL SG BAKAP	iPROMISE, UiTM	Ariza Adnan; Fadzilah Mohd Nor; Lim Wai Feng; Mohd Asif Mohd Sukri; Mohd Nur Fakhruzzaman Noorizhab; Mohd Zaki Salleh; Sazzli Shahlan Kassim; Siti Farah Alwani Mohd Nawi; Siti Hamimah Sheikh Abdul Kadir; Teh Lay Kek; Wang Seok Mui
EPI_ISL_3858163, EPI_ISL_3858184, EPI_ISL_3858185, EPI_ISL_3858186, EPI_ISL_3945538, EPI_ISL_3945539, EPI_ISL_3945540, EPI_ISL_3945541, EPI_ISL_3945542, EPI_ISL_3945543, EPI_ISL_3945544, EPI_ISL_4071989, EPI_ISL_4071990, EPI_ISL_4071991, EPI_ISL_4071992, EPI_ISL_4071994, EPI_ISL_4071995, EPI_ISL_4071997, EPI_ISL_4101591, EPI_ISL_4101599, EPI_ISL_4236993, EPI_ISL_4236996, EPI_ISL_4513507, EPI_ISL_4513508			
see above	HOSPITAL SULTANAH NORA ISMAIL	UKM Medical Molecular Biology Institute (UMBI)	Mira Farzana binti Mohamad Mokhtar; Nur Alyaa Afifah Md Shahri
EPI_ISL_4122292, EPI_ISL_4122540	HPP	iPROMISE, UiTM	Ariza Adnan; Fadzilah Mohd Nor; Lim Wai Feng; Mohd Asif Mohd Sukri; Mohd Nur Fakhruzzaman Noorizhab; Mohd Zaki Salleh; Sazzli Shahlan Kassim; Siti Farah Alwani Mohd Nawi; Siti Hamimah Sheikh Abdul Kadir; Teh Lay Kek; Wang Seok Mui
EPI_ISL_3858142, EPI_ISL_3858144, EPI_ISL_3858146	HPSF MUAR	UKM Medical Molecular Biology Institute (UMBI)	Mira Farzana binti Mohamad Mokhtar
EPI_ISL_4071993, EPI_ISL_4101595,	HPSF, MUAR	UKM Medical Molecular Biology Institute (UMBI)	Mira Farzana binti Mohamad Mokhtar; Nur Alyaa Afifah Md Shahri

EPI_ISL_4101596, EPI_ISL_4236995, EPI_ISL_4236997			
EPI_ISL_3980747, EPI_ISL_3980748, EPI_ISL_3980750, EPI_ISL_3980751, EPI_ISL_3980752, EPI_ISL_3980753, EPI_ISL_3980754, EPI_ISL_4565007, EPI_ISL_4565008, EPI_ISL_4565009, EPI_ISL_4565010, EPI_ISL_4565011, EPI_ISL_4565012			
see above	HRPB, Ipoh	Tropical Infectious Diseases Research & Education Centre (TIDREC), Universiti Malaya	AsmaAnati CheMatSeri; Che-Norainon Yaacob; Jia-Yi Tan; Jo-Ern Wong; Kim-Kee Tan; Mulya-Mustika-Sari Zulkifli; Noor Syahida Azizan; Nur-Hidayana Mahfodz; Sazaly AbuBakar; Siti-Sarah Nor'e
EPI_ISL_1196864	Hospital Alor Gajah	Institute for Medical Research, Infectious Disease Research Centre, National Institutes of Health, Ministry of Health Malaysia	Kamel K; Mohd Zawawi Z; Ramly N; Robert F; Suppiah J; Thayan R
EPI_ISL_4557932, EPI_ISL_4557933, EPI_ISL_4557934, EPI_ISL_4557942, EPI_ISL_4557943, EPI_ISL_4557944, EPI_ISL_4557945, EPI_ISL_4557946, EPI_ISL_4557947, EPI_ISL_4557948, EPI_ISL_4557949, EPI_ISL_4557950, EPI_ISL_4557951			
see above	Hospital Ampang	Malaysia Genome Institute	Azrin Ahmad; Enizza Kasim; Irni Suhayu Sapian; Mohd Faizal Abu Bakar; Mohd Ghows Mohd Azzam.; Mohd Noor Mat Isa; Nor Azfa Johari; Nurhezreen Md Iqbal; Shamsidar Sopie; Siti Noraini Othman; Yusuf Muhammad Noor
EPI_ISL_2727836	Hospital Angkatan Tentera Kota Kinabalu	Institute for Medical Research, Infectious Disease Research Centre, National Institutes of Health, Ministry of Health Malaysia	Azizan MA; Kamel K; Mohd Zawawi Z; Ramly N; Robert F; Suppiah J; Thayan R
EPI_ISL_2727837	Hospital Angkatan Tentera Wilayah Kota Kinabalu	Institute for Medical Research, Infectious Disease Research Centre, National Institutes of Health, Ministry of Health Malaysia	Azizan MA; Kamel K; Mohd Zawawi Z; Ramly N; Robert F; Suppiah J; Thayan R
EPI_ISL_3980749	Hospital Batu Gajah	Tropical Infectious Diseases Research & Education Centre (TIDREC), Universiti Malaya	AsmaAnati CheMatSeri; Che-Norainon Yaacob; Jia-Yi Tan; Jo-Ern Wong; Kim-Kee Tan; Mulya-Mustika-Sari Zulkifli; Noor Syahida Azizan; Nur-Hidayana Mahfodz; Sazaly AbuBakar; Siti-Sarah Nor'e
EPI_ISL_4513510	Hospital Canselor Tuanku Muhriz (HCTM)	UKM Medical Molecular Biology Institute (UMBI)	Mira Farzana binti Mohamad Mokhtar
EPI_ISL_4463193, EPI_ISL_4463194, EPI_ISL_4463195	Hospital Canselor Tuanku Muhriz UKM	UKM Medical Molecular Biology Institute (UMBI)	Nur Alyaa Afifah Md Shahri
EPI_ISL_936494	Hospital Enche Besar Hajjah Khalsom Kluang	Institute for Medical Research, Infectious Disease Research Centre, National Institutes of Health, Ministry of Health Malaysia	Azizan MA; Kamel K; Suppiah J; Thayan R
EPI_ISL_4122511, EPI_ISL_4435515, EPI_ISL_4435516, EPI_ISL_4435518, EPI_ISL_4435519	Hospital Kepala Batas	iPROMISE, UiTM	Ariza Adnan; Fadzilah Mohd Nor; Lim Wai Feng; Mohd Asif Mohd Sukri; Mohd Nur Fakhruzzaman Noorizhab; Mohd Zaki Salleh; Sazzli Shahlan Kassim; Siti Farah Alwani Mohd Nawi; Siti Hamimah Sheikh Abdul Kadir; Teh Lay Kek; Wang Seok Mui
EPI_ISL_4557935, EPI_ISL_4601559	Hospital Kuala Lumpur	Malaysia Genome Institute	Azrin Ahmad; Enizza Kasim; Irni Suhayu Sapian; Mohd Faizal Abu Bakar; Mohd Ghows Mohd Azzam.; Mohd Noor Mat Isa; Nor Azfa Johari; Nurhezreen Md Iqbal; Shamsidar Sopie; Siti Noraini Othman; Yusuf Muhammad Noor
EPI_ISL_2233382, EPI_ISL_2550733	Hospital Labuan	Institute for Medical Research, Infectious Disease Research Centre, National Institutes of Health, Ministry of Health Malaysia	Azizan MA; Kamel K; Mohd Zawawi Z; Ramly N; Robert F; Suppiah J; Thayan R
EPI_ISL_1673257	Hospital Lahad Datu	Institute for Medical Research, Infectious Disease Research Centre, National Institutes of Health, Ministry of Health Malaysia Jalan Setia Murni U13/52, Bandar Setia Alam, 40170 Selangor, Malaysia	Kamel K; Mohd Zawawi Z; Suppiah J; Thayan R
EPI_ISL_4601565, EPI_ISL_4601570, EPI_ISL_4601571, EPI_ISL_4601579	Hospital Lahad Datu	Malaysia Genome Institute	Azrin Ahmad; Enizza Kasim; Irni Suhayu Sapian; Mohd Faizal Abu Bakar; Mohd Ghows Mohd Azzam.; Mohd Noor Mat Isa; Nor Azfa Johari; Nurhezreen Md Iqbal; Shamsidar Sopie; Siti Noraini Othman; Yusuf Muhammad Noor
EPI_ISL_2379461	Hospital Melaka	Institute for Medical Research, Infectious Disease Research Centre, National Inst	Azizan MA; Kamel K; Mohd Zawawi Z; Ramly N; Robert F; Suppiah J; Thayan R
EPI_ISL_1196660, EPI_ISL_1196705, EPI_ISL_1196706, EPI_ISL_1196768, EPI_ISL_1196770, EPI_ISL_1196771	Hospital Melaka	Institute for Medical Research, Infectious Disease Research Centre, National Institutes of Health, Ministry of Health Malaysia	Kamel K; Mohd Zawawi Z; Ramly N; Robert F; Suppiah J; Thayan R
EPI_ISL_4513497	Hospital Melaka	UKM Medical Molecular Biology Institute (UMBI)	Mira Farzana binti Mohamad Mokhtar
EPI_ISL_4122275	Hospital P. Pinang	iPROMISE, UiTM	Ariza Adnan; Fadzilah Mohd Nor; Lim Wai Feng; Mohd Asif Mohd Sukri; Mohd Nur Fakhruzzaman Noorizhab; Mohd Zaki Salleh; Sazzli Shahlan Kassim; Siti Farah Alwani Mohd Nawi; Siti Hamimah Sheikh Abdul Kadir; Teh Lay Kek; Wang Seok Mui
EPI_ISL_1673337	Hospital Pakar Sultanah Fatimah	Institute for Medical Research, Infectious Disease Research Centre, National Institutes of Health, Ministry of Health Malaysia	Kamel K; Mohd Zawawi Z; Suppiah J; Thayan R
EPI_ISL_1196794, EPI_ISL_1196796, EPI_ISL_1196797	Hospital Pulau Pinang	Institute for Medical Research, Infectious Disease Research Centre, National Institutes of Health, Ministry of Health Malaysia	Kamel K; Mohd Zawawi Z; Ramly N; Robert F; Suppiah J; Thayan R
EPI_ISL_936488, EPI_ISL_1186114, EPI_ISL_1405917	Hospital Queen Elizabeth	Institute for Medical Research, Infectious Disease Research Centre, National Institutes of Health, Ministry of Health Malaysia	Azizan MA; Kamel K; Mohd Zawawi Z; Ramly N; Robert F; Suppiah J; Thayan R
EPI_ISL_4601560	Hospital Queen Elizabeth 2	Malaysia Genome Institute	Azrin Ahmad; Enizza Kasim; Irni Suhayu Sapian; Mohd Faizal Abu Bakar; Mohd Ghows Mohd Azzam.; Mohd Noor Mat Isa; Nor Azfa Johari; Nurhezreen Md Iqbal; Shamsidar Sopie; Siti Noraini Othman; Yusuf Muhammad Noor
EPI_ISL_936489	Hospital Raja Perempuan Zainab II	Institute for Medical Research, Infectious Disease Research Centre, National Institutes of Health, Ministry of Health Malaysia	Azizan MA; Kamel K; Suppiah J; Thayan R
EPI_ISL_2429132	Hospital Raja Permaisuri Bainun	Institute for Medical Research, Infectious Disease Research Centre, National Institutes of Health, Ministry of Health Malaysia	Azizan MA; Kamel K; Mohd Zawawi Z; Ramly N; Robert F; Suppiah J; Thayan R
EPI_ISL_1673288	Hospital Segamat Johor	Institute for Medical Research, Infectious Disease Research Centre, National Institutes of Health, Ministry of Health Malaysia	Kamel K; Mohd Zawawi Z; Suppiah J; Thayan R
EPI_ISL_4601580	Hospital Selayang	Malaysia Genome Institute	Azrin Ahmad; Enizza Kasim; Irni Suhayu Sapian; Mohd Faizal Abu Bakar; Mohd Ghows Mohd Azzam.; Mohd Noor Mat Isa; Nor Azfa Johari; Nurhezreen Md Iqbal; Shamsidar Sopie; Siti Noraini Othman; Yusuf Muhammad Noor
EPI_ISL_3769378	Hospital Serdang	iPROMISE, UiTM	Ariza Adnan; Fadzilah Mohd Nor; Lim Wai Feng; Mohd Asif Mohd Sukri; Mohd Nur Fakhruzzaman Noorizhab; Mohd Zaki Salleh; Sazzli Shahlan Kassim; Siti Farah Alwani Mohd Nawi; Siti Hamimah Sheikh Abdul Kadir; Teh Lay Kek; Wang Seok Mui
EPI_ISL_1673280	Hospital Sultan Ismail	Institute for Medical Research, Infectious Disease Research Centre, National Institutes of Health, Ministry of Health Malaysia	Kamel K; Mohd Zawawi Z; Suppiah J; Thayan R
EPI_ISL_1673283	Hospital Sultanah Aminah	Institute for Medical Research, Infectious Disease Research Centre, National Institutes of Health, Ministry of Health Malaysia	Kamel K; Mohd Zawawi Z; Suppiah J; Thayan R
EPI_ISL_1673284	Hospital Sultanah Nora Ismail	Institute for Medical Research, Infectious Disease Research Centre, National Institutes of Health, Ministry of Health Malaysia	Kamel K; Mohd Zawawi Z; Suppiah J; Thayan R
EPI_ISL_4101581	Hospital Sultanah Nora Ismail	UKM Medical Molecular Biology Institute (UMBI)	Nur Alyaa Afifah Md Shahri
EPI_ISL_2650001, EPI_ISL_2650002	Hospital Sultanah Nur Zahirah Kuala Terengganu	Institute for Medical Research, Infectious Disease Research Centre, National Institutes of Health, Ministry of Health Malaysia	Azizan MA; Kamel K; Mohd Zawawi Z; Ramly N; Robert F; Suppiah J; Thayan R
EPI_ISL_4557928, EPI_ISL_4557929, EPI_ISL_4557930, EPI_ISL_4557931, EPI_ISL_4557936, EPI_ISL_4557937, EPI_ISL_4557938, EPI_ISL_4557939, EPI_ISL_4557940, EPI_ISL_4557941, EPI_ISL_4557955, EPI_ISL_4558235, EPI_ISL_4558236, EPI_ISL_4601572, EPI_ISL_4601573, EPI_ISL_4601574, EPI_ISL_4601575, EPI_ISL_4601576, EPI_ISL_4601577			
see above	Hospital Sungai Buloh	Malaysia Genome Institute	Azrin Ahmad; Enizza Kasim; Irni Suhayu Sapian; Mohd Faizal Abu Bakar; Mohd Ghows Mohd Azzam.; Mohd Noor Mat Isa; Nor Azfa Johari; Nurhezreen Md Iqbal; Shamsidar Sopie; Siti Noraini Othman; Yusuf Muhammad Noor
EPI_ISL_1673309	Hospital Temenggong Seri Maharaja Tun Ibrahim	Institute for Medical Research, Infectious Disease Research Centre, National Institutes of Health, Ministry of Health Malaysia	Kamel K; Mohd Zawawi Z; Suppiah J; Thayan R
EPI_ISL_2549576	Hospital Tuanku Fauziah	Institute for Medical Research, Infectious Disease Research Centre, National Institutes of Health, Ministry of Health Malaysia	Azizan MA; Kamel K; Mohd Zawawi Z; Ramly N; Robert F; Suppiah J; Thayan R
EPI_ISL_2620716	Hospital Tuanku Fauziah, Kangar	Institute for Medical Research, Infectious Disease Research Centre, National Institutes of Health, Ministry of Health	Azizan MA; Kamel K; Mohd Zawawi Z; Ramly N; Robert F; Suppiah J; Thayan R
EPI_ISL_2620715	Hospital Tuanku Fauziah, Kangar	Institute for Medical Research, Infectious Disease Research Centre, National Institutes of Health, Ministry of Health Malaysia	Azizan MA; Kamel K; Mohd Zawawi Z; Ramly N; Robert F; Suppiah J; Thayan R
EPI_ISL_1672633	Hospital Tuanku Jaafar Seremban	Institute for Medical Research, Infectious Disease Research Centre, National Institutes of Health, Ministry of Health Malaysia	Kamel K; Mohd Zawawi Z; Suppiah J; Thayan R
EPI_ISL_4101584	Hospital Tuanku Jaafar Seremban	UKM Medical Molecular Biology Institute (UMBI)	Nur Alyaa Afifah Md Shahri
EPI_ISL_2650003	Hospital Wanita Dan Kanak-Kanak Sabah	Institute for Medical Research, Infectious Disease Research Centre, National Institutes of Health, Ministry of Health Malaysia	40170 Selangor; Bandar Setia Alam; Jalan Setia Murni U13/52; Malaysia
EPI_ISL_3762499	Hulu Perak District Health Office	Institute for Medical Research, Infectious Disease Research Centre, National Institutes of Health, Ministry of Health Malaysia	Anasir MI; Azizan MA; Kamel K; Mohd Zawawi Z; Ramly N; Robert F; Suppiah J; Thayan R
EPI_ISL_4101580	ICU Dahlia Hospital Melaka	UKM Medical Molecular Biology Institute (UMBI)	Nur Alyaa Afifah Md Shahri
EPI_ISL_3945545, EPI_ISL_3945546, EPI_ISL_3945547, EPI_ISL_3945548, EPI_ISL_3945549, EPI_ISL_4071977, EPI_ISL_4071978, EPI_ISL_4071979, EPI_ISL_4071980, EPI_ISL_4071981, EPI_ISL_4071983, EPI_ISL_4071984			
see above	Institut Biologi Molekul Perubatan UKM	UKM Medical Molecular Biology Institute (UMBI)	Mira Farzana binti Mohamad Mokhtar; Nur Alyaa Afifah Md Shahri
EPI_ISL_455790, EPI_ISL_455791, EPI_ISL_455792, EPI_ISL_455793	Institute for Medical Research, Infectious Disease Research Centre, National Institutes of Health, Ministry of Health Malaysia	Malaysia Genome Institute	Azrin Ahmad; Enizza Kasim; Irni Suhayu Sapian; Jeyanthi Suppiah; Mohd Faizal Abu Bakar; Mohd Noor Mat Isa; Norazah Ahmad; Nurhezreen Md Iqbal; Ravindran Thayan; Shahrul Hisham Zainal Ariffin; Shamsidar Sopie; Siti Noraini Othman; Tahir Aris; Yusuf Muhammad Noor; Zarina Mohd Zawawi
EPI_ISL_430439	Institute for Medical Research, Infectious Disease Research Centre, National Institutes of Health, Ministry of Health Malaysia	Institute for Medical Research Infectious Disease Research Centre, National Institutes of Health, Ministry of Health Malaysia	Abdul-Wahid.M-Z; Ahmad.N; Azizan.M-A; Hisham.H-A; Kalyanasundram.J; Mat-Sharani.S; Mohd-Zain.R; Mohd-Zawawi.Z; Suppiah.J; Tan.L-P; Thayan.R

EPI_ISL_459957	Institute for Medical Research, Infectious Disease Research Centre, National Institutes of Health, Minis	Institute for Medical Research, Infectious Disease Research Centre, National Institutes of Health, Minis	Ellan K; Kalyanasundram J; Kamel KA; Mohd-Zain R; Mohd-Zawawi Z; Suppiah J; Thayan R
EPI_ISL_459953	Institute for Medical Research, Infectious Disease Research Centre, National Institutes of Health, Ministry of Health Malaysia	Institute for Medical Research Infectious Disease Research Centre, National Institutes of Health, Ministry of Health Malaysia	Ellan K; Kalyanasundram J; Kamel KA; Mohd-Zain R; Mohd-Zawawi Z; Suppiah J; Thayan R
EPI_ISL_430440, EPI_ISL_430441, EPI_ISL_430442, EPI_ISL_430443, EPI_ISL_430444, EPI_ISL_459954, EPI_ISL_459955, EPI_ISL_459956, EPI_ISL_489992, EPI_ISL_489993, EPI_ISL_489994, EPI_ISL_490014, EPI_ISL_490015, EPI_ISL_490016, EPI_ISL_490047, EPI_ISL_490048, EPI_ISL_490089, EPI_ISL_490090, EPI_ISL_490091, EPI_ISL_490092, EPI_ISL_490093, EPI_ISL_490094, EPI_ISL_490095, EPI_ISL_490096, EPI_ISL_490097, EPI_ISL_490098, EPI_ISL_490099, EPI_ISL_490100, EPI_ISL_490101, EPI_ISL_490102, EPI_ISL_490103, EPI_ISL_596449, EPI_ISL_644671, EPI_ISL_644672, EPI_ISL_718267, EPI_ISL_718268, EPI_ISL_718269, EPI_ISL_718270, EPI_ISL_718271, EPI_ISL_718272, EPI_ISL_718274, EPI_ISL_718275, EPI_ISL_718276, EPI_ISL_718277, EPI_ISL_718278, EPI_ISL_718279, EPI_ISL_718280, EPI_ISL_718281, EPI_ISL_718282, EPI_ISL_718283, EPI_ISL_718284, EPI_ISL_718285, EPI_ISL_718286, EPI_ISL_718287, EPI_ISL_718288, EPI_ISL_718289, EPI_ISL_718290, EPI_ISL_718291, EPI_ISL_718292, EPI_ISL_718293, EPI_ISL_718294, EPI_ISL_718295, EPI_ISL_718296, EPI_ISL_718297, EPI_ISL_718298, EPI_ISL_718299, EPI_ISL_718300, EPI_ISL_718301, EPI_ISL_718302, EPI_ISL_718303, EPI_ISL_718304, EPI_ISL_718305, EPI_ISL_718306, EPI_ISL_718307, EPI_ISL_718308, EPI_ISL_718309, EPI_ISL_718310, EPI_ISL_718311, EPI_ISL_718312, EPI_ISL_718313, EPI_ISL_718314, EPI_ISL_718315, EPI_ISL_728157, EPI_ISL_728158, EPI_ISL_728160, EPI_ISL_728161, EPI_ISL_728162, EPI_ISL_728163, EPI_ISL_728164, EPI_ISL_728165, EPI_ISL_728166, EPI_ISL_728167, EPI_ISL_728169, EPI_ISL_728170, EPI_ISL_728178, EPI_ISL_728209, EPI_ISL_728210, EPI_ISL_728211, EPI_ISL_728212, EPI_ISL_728241, EPI_ISL_728242, EPI_ISL_728243, EPI_ISL_728244, EPI_ISL_728245, EPI_ISL_728246, EPI_ISL_728247, EPI_ISL_728248, EPI_ISL_728249, EPI_ISL_728250, EPI_ISL_728251, EPI_ISL_728252, EPI_ISL_728253, EPI_ISL_877228, EPI_ISL_912397, EPI_ISL_912399, EPI_ISL_912400, EPI_ISL_934424, EPI_ISL_936491, EPI_ISL_936496, EPI_ISL_944094, EPI_ISL_944095, EPI_ISL_944096, EPI_ISL_944099, EPI_ISL_944100, EPI_ISL_944101, EPI_ISL_944102, EPI_ISL_944103, EPI_ISL_944104, EPI_ISL_962203, EPI_ISL_962204, EPI_ISL_962205, EPI_ISL_962206, EPI_ISL_962207, EPI_ISL_962523, EPI_ISL_962524, EPI_ISL_962525, EPI_ISL_962526, EPI_ISL_962527, EPI_ISL_968089, EPI_ISL_1055037, EPI_ISL_1055261, EPI_ISL_1055262, EPI_ISL_1055263, EPI_ISL_1055264, EPI_ISL_1055361, EPI_ISL_1055362, EPI_ISL_1055363, EPI_ISL_1055364, EPI_ISL_1055365, EPI_ISL_1068849, EPI_ISL_1068850, EPI_ISL_1068851, EPI_ISL_1068927, EPI_ISL_1068928, EPI_ISL_1068929, EPI_ISL_1081327, EPI_ISL_1081333, EPI_ISL_1081350, EPI_ISL_1081351, EPI_ISL_1081352, EPI_ISL_1114717, EPI_ISL_1114718, EPI_ISL_1114719, EPI_ISL_1114720, EPI_ISL_1114721, EPI_ISL_1114722, EPI_ISL_1114723, EPI_ISL_1114724, EPI_ISL_1114726, EPI_ISL_1114734, EPI_ISL_1114744, EPI_ISL_1114765, EPI_ISL_1138598, EPI_ISL_1138750, EPI_ISL_1186028, EPI_ISL_1196866, EPI_ISL_1196867, EPI_ISL_1196930, EPI_ISL_1204503, EPI_ISL_1263458, EPI_ISL_1263460, EPI_ISL_1263540, EPI_ISL_1264762, EPI_ISL_1265161, EPI_ISL_1405966, EPI_ISL_1406078, EPI_ISL_1406146, EPI_ISL_1406148, EPI_ISL_1406153, EPI_ISL_1406178, EPI_ISL_1406185, EPI_ISL_1406187, EPI_ISL_1406192, EPI_ISL_1406194, EPI_ISL_1406197, EPI_ISL_1406199, EPI_ISL_1406201, EPI_ISL_1406204, EPI_ISL_1406242, EPI_ISL_1406244, EPI_ISL_1406247, EPI_ISL_1406250, EPI_ISL_1406274, EPI_ISL_1406277, EPI_ISL_1406281, EPI_ISL_1406286, EPI_ISL_1406292, EPI_ISL_1406294, EPI_ISL_1406295, EPI_ISL_1424064, EPI_ISL_1424065, EPI_ISL_1424130, EPI_ISL_1424143, EPI_ISL_1424466, EPI_ISL_1424467, EPI_ISL_1424468, EPI_ISL_1424470, EPI_ISL_1424471, EPI_ISL_1424510, EPI_ISL_1424513, EPI_ISL_1424518, EPI_ISL_1583006, EPI_ISL_1583007, EPI_ISL_1585242, EPI_ISL_1673287, EPI_ISL_1673293, EPI_ISL_1673296, EPI_ISL_1673299, EPI_ISL_1673304, EPI_ISL_1673307, EPI_ISL_1673332, EPI_ISL_1673336, EPI_ISL_1673338, EPI_ISL_1673340, EPI_ISL_1673341, EPI_ISL_1673342, EPI_ISL_1673344, EPI_ISL_1673345, EPI_ISL_1673420, EPI_ISL_1673683, EPI_ISL_1673735, EPI_ISL_1696746, EPI_ISL_1696747, EPI_ISL_1696748, EPI_ISL_1696749, EPI_ISL_1696750, EPI_ISL_1696751, EPI_ISL_1696752, EPI_ISL_1696753, EPI_ISL_1696754, EPI_ISL_1696755, EPI_ISL_1696756, EPI_ISL_1696757, EPI_ISL_1696758, EPI_ISL_1714796, EPI_ISL_1714797, EPI_ISL_1714798, EPI_ISL_1787254, EPI_ISL_1787317, EPI_ISL_1787318, EPI_ISL_1787319, EPI_ISL_1787320, EPI_ISL_1787321, EPI_ISL_1787322, EPI_ISL_1787323, EPI_ISL_1787324, EPI_ISL_1787689, EPI_ISL_1787983, EPI_ISL_1788079, EPI_ISL_1788080, EPI_ISL_1788081, EPI_ISL_1788082, EPI_ISL_1789203, EPI_ISL_1789204, EPI_ISL_1805505, EPI_ISL_1805508, EPI_ISL_1805509, EPI_ISL_1807185, EPI_ISL_1807186, EPI_ISL_1807188, EPI_ISL_1807206, EPI_ISL_1807207, EPI_ISL_1919650, EPI_ISL_1919651, EPI_ISL_1919652, EPI_ISL_1919653, EPI_ISL_1919654, EPI_ISL_1919860, EPI_ISL_1972534, EPI_ISL_1972535, EPI_ISL_1972536, EPI_ISL_1972537, EPI_ISL_2090886, EPI_ISL_2090887, EPI_ISL_2090889, EPI_ISL_2090890, EPI_ISL_2090891, EPI_ISL_2091000, EPI_ISL_2379433, EPI_ISL_2379443, EPI_ISL_2379650, EPI_ISL_2429134, EPI_ISL_2429136, EPI_ISL_2429137, EPI_ISL_2429138, EPI_ISL_2429141, EPI_ISL_2429142, EPI_ISL_2429143, EPI_ISL_2429145, EPI_ISL_2625633, EPI_ISL_2625635, EPI_ISL_2684231, EPI_ISL_2684232, EPI_ISL_2684233, EPI_ISL_2684545, EPI_ISL_2685359, EPI_ISL_2685766, EPI_ISL_2685835, EPI_ISL_2685836, EPI_ISL_2685837, EPI_ISL_2685838, EPI_ISL_2685839, EPI_ISL_2685840, EPI_ISL_2685841, EPI_ISL_2685843, EPI_ISL_2685844, EPI_ISL_2685845, EPI_ISL_2811884, EPI_ISL_2811887, EPI_ISL_2815375, EPI_ISL_2819024, EPI_ISL_2819571, EPI_ISL_2819919, EPI_ISL_2896287, EPI_ISL_3247527, EPI_ISL_3247528, EPI_ISL_3247529, EPI_ISL_3247700, EPI_ISL_3247768, EPI_ISL_3247769, EPI_ISL_3247770, EPI_ISL_3247771, EPI_ISL_3247772			
see above	Institute for Medical Research, Infectious Disease Research Centre, National Institutes of Health, Ministry of Health Malaysia	Institute for Medical Research, Infectious Disease Research Centre, National Institutes of Health, Ministry of Health Malaysia	Abdul-Wahid.M-Z; Ahmad.N; Anasir MI; Azizan MA; Azizan.M-A; Ellan K; Hisham.H-A; Kalyanasundram J; Kalyanasundram.J; Kamel K; Kamel KA; Mat-Sharani.S; Moh Zawawi Z; Mohd Zawawi Z; Mohd-Zain R; Mohd-Zain.R; Mohd-Zawawi Z; Mohd-Zawawi.Z; Ramly MN; Ramly N; Robert F; Suppiah J; Suppiah.J; Tan.L-P; Tengku-Abd-Rashid.T-R; Tengku-Rogayah.TAR; Thayan R; Thayan.R
EPI_ISL_2367539, EPI_ISL_2367540, EPI_ISL_2367541, EPI_ISL_2367542, EPI_ISL_2367543, EPI_ISL_2367544, EPI_ISL_2367545, EPI_ISL_2367546, EPI_ISL_2367547, EPI_ISL_2367548, EPI_ISL_2367549, EPI_ISL_2367550, EPI_ISL_2367551, EPI_ISL_2367552, EPI_ISL_2367553, EPI_ISL_2367554, EPI_ISL_2367555, EPI_ISL_2367556, EPI_ISL_2367557, EPI_ISL_2367558, EPI_ISL_2367559, EPI_ISL_2367560, EPI_ISL_2367561, EPI_ISL_2367562, EPI_ISL_2379894, EPI_ISL_2379895, EPI_ISL_2379896, EPI_ISL_2379897, EPI_ISL_2379898, EPI_ISL_2379899, EPI_ISL_2379900, EPI_ISL_2379901, EPI_ISL_2379902, EPI_ISL_2379903, EPI_ISL_2379904, EPI_ISL_2379905, EPI_ISL_2379906, EPI_ISL_2379907, EPI_ISL_2379908, EPI_ISL_2379909, EPI_ISL_2379910, EPI_ISL_2379911, EPI_ISL_2379912, EPI_ISL_2379913, EPI_ISL_2379914, EPI_ISL_2379915, EPI_ISL_2379916, EPI_ISL_2379917, EPI_ISL_2379918, EPI_ISL_2379919, EPI_ISL_2379920, EPI_ISL_2379921, EPI_ISL_2379922, EPI_ISL_2379923, EPI_ISL_2379924, EPI_ISL_2379925, EPI_ISL_2379926, EPI_ISL_2379927, EPI_ISL_2379929, EPI_ISL_2379930, EPI_ISL_2379931, EPI_ISL_2379932, EPI_ISL_2379933, EPI_ISL_2379934, EPI_ISL_2608374, EPI_ISL_2608375, EPI_ISL_2608376			
see above	Institute of Health and Community Medicine	Institute of Health and Community Medicine	Chan Chia Jui; Chua Hock Hin; David Perera; Ooi Mong How; Tonnii Sia Loong Loong; Wong Jyn Shan; Wong Kiing Aik
EPI_ISL_3071986, EPI_ISL_3071987, EPI_ISL_3100296, EPI_ISL_3100297, EPI_ISL_3100681, EPI_ISL_3100682, EPI_ISL_3100981			
see above	Ipoh Public Health Laboratory	Institute for Medical Research, Infectious Disease Research Centre, National Institutes of Health, Ministry of Health Malaysia	Anasir MI; Azizan MA; Kamel K; Mohd Zawawi Z; Ramly N; Robert F; Suppiah J; Thayan R
EPI_ISL_1919422, EPI_ISL_1919645, EPI_ISL_1919646	Ipoh Public Health Laboratory (MKAI), Ministry of Health Malaysia	Institute for Medical Research, Infectious Disease Research Centre, National Institutes of Health, Ministry	Kamel K; Mohd Zawawi Z; Suppiah J; Thayan R
EPI_ISL_1821883, EPI_ISL_1821884, EPI_ISL_1919272, EPI_ISL_1919273, EPI_ISL_1919647, EPI_ISL_1919648, EPI_ISL_1919649, EPI_ISL_1919858, EPI_ISL_1919859, EPI_ISL_1919944, EPI_ISL_1919945, EPI_ISL_1919946, EPI_ISL_1919947, EPI_ISL_1919949, EPI_ISL_1919950, EPI_ISL_1919951, EPI_ISL_1919952, EPI_ISL_1919986, EPI_ISL_1919987, EPI_ISL_1921767, EPI_ISL_1921769, EPI_ISL_1921770, EPI_ISL_2620718, EPI_ISL_2620728, EPI_ISL_2620744, EPI_ISL_2620745, EPI_ISL_2620901, EPI_ISL_2621621, EPI_ISL_2621622, EPI_ISL_2621623, EPI_ISL_2621624, EPI_ISL_2621625, EPI_ISL_2621626, EPI_ISL_2621627, EPI_ISL_2621628, EPI_ISL_2621629, EPI_ISL_2621630, EPI_ISL_2621631, EPI_ISL_2650007, EPI_ISL_2683498, EPI_ISL_2815329, EPI_ISL_2815330, EPI_ISL_2815377, EPI_ISL_2921400, EPI_ISL_3334021, EPI_ISL_3334182			
see above	Ipoh Public Health Laboratory (MKAI), Ministry of Health Malaysia	Institute for Medical Research, Infectious Disease Research Centre, National Institutes of Health, Ministry of Health Malaysia	Anasir MI; Azizan MA; Kamel K; Mohd Zawawi Z; Ramly N; Robert F; Suppiah J; Thayan R
EPI_ISL_4122508	JABATAN KESIHATAN NEGERI PERLIS	iPROMISE, UiTM	Ariza Adnan; Fadzilah Mohd Nor; Lim Wai Feng; Mohd Asif Mohd Sukri; Mohd Nur Fakhruzzaman Noorizhab; Mohd Zaki Salleh; Sazzli Shahlan Kassim; Siti Farah Alwani Mohd Nawi; Siti Hamimah Sheikh Abdul Kadir; Teh Lay Kek; Wang Seok Mui
EPI_ISL_4435528	JABATAN PATOLOGI, HOSPITAL PULAU PINANG	iPROMISE, UiTM	Ariza Adnan; Fadzilah Mohd Nor; Lim Wai Feng; Mohd Asif Mohd Sukri; Mohd Nur Fakhruzzaman Noorizhab; Mohd Zaki Salleh; Sazzli Shahlan Kassim; Siti Farah Alwani Mohd Nawi; Siti Hamimah Sheikh Abdul Kadir; Teh Lay Kek; Wang Seok Mui
EPI_ISL_3980708, EPI_ISL_3980710, EPI_ISL_3980717	JABATAN PERUBATAN FORENSIK HOSPITAL SULTANAH BAHIYAH	Tropical Infectious Diseases Research & Education Centre (TIDREC), Universiti Malaya	AsmaAnati CheMatSeri; Che-Norainon Yaacob; Jia-Yi Tan; Jo-Ern Wong; Kim-Kee Tan; Mulya-Mustika-Sari Zulkifli; Noor Syahida Azizan; Nur-Hidayana Mahfodz; Sazaly AbuBakar; Siti-Sarah Nor'e
EPI_ISL_4101590, EPI_ISL_4463190, EPI_ISL_4463191, EPI_ISL_4463192	JABATAN PERUBATAN FORENSIK, HOSPITAL TUANKU JA'AFAR SEREMBAN	UKM Medical Molecular Biology Institute (UMBI)	Nur Alyaa Afifah Md Shahri
EPI_ISL_4122483	JKN Komtar	iPROMISE, UiTM	Ariza Adnan; Fadzilah Mohd Nor; Lim Wai Feng; Mohd Asif Mohd Sukri; Mohd Nur Fakhruzzaman Noorizhab; Mohd Zaki Salleh; Sazzli Shahlan Kassim; Siti Farah Alwani Mohd Nawi; Siti Hamimah Sheikh Abdul Kadir; Teh Lay Kek; Wang Seok Mui
EPI_ISL_3769355, EPI_ISL_3769356, EPI_ISL_3769357, EPI_ISL_3769358, EPI_ISL_3769359, EPI_ISL_3769360, EPI_ISL_3769361, EPI_ISL_3769362, EPI_ISL_3769363, EPI_ISL_3769365, EPI_ISL_3769366, EPI_ISL_3769367, EPI_ISL_3769368, EPI_ISL_3769369, EPI_ISL_3769370, EPI_ISL_3769371, EPI_ISL_3769372, EPI_ISL_4122295, EPI_ISL_4122298, EPI_ISL_4122301, EPI_ISL_4122304, EPI_ISL_4122319, EPI_ISL_4122330, EPI_ISL_4122341, EPI_ISL_4122352, EPI_ISL_4122363, EPI_ISL_4122505, EPI_ISL_4122533, EPI_ISL_4122537, EPI_ISL_4435511, EPI_ISL_4435513, EPI_ISL_4435529, EPI_ISL_4435530, EPI_ISL_4435531, EPI_ISL_4435532, EPI_ISL_4435533, EPI_ISL_4435534, EPI_ISL_4435535, EPI_ISL_4435536, EPI_ISL_4435537, EPI_ISL_4435538, EPI_ISL_4435539, EPI_ISL_4435540, EPI_ISL_4435541, EPI_ISL_4435542, EPI_ISL_4435543, EPI_ISL_4435544, EPI_ISL_4435545, EPI_ISL_4435546, EPI_ISL_4435547, EPI_ISL_4435548, EPI_ISL_4435549			
see above	JKN PAHANG	iPROMISE, UiTM	Ariza Adnan; Fadzilah Mohd Nor; Lim Wai Feng; Mohd Asif Mohd Sukri; Mohd Nur Fakhruzzaman Noorizhab; Mohd Zaki Salleh; Sazzli Shahlan Kassim; Siti Farah Alwani Mohd Nawi; Siti Hamimah Sheikh Abdul Kadir; Teh Lay Kek; Wang Seok Mui
EPI_ISL_3769354, EPI_ISL_4122308, EPI_ISL_4122527, EPI_ISL_4122530, EPI_ISL_4435550	JKN Pahang	iPROMISE, UiTM	Ariza Adnan; Fadzilah Mohd Nor; Lim Wai Feng; Mohd Asif Mohd Sukri; Mohd Nur Fakhruzzaman Noorizhab; Mohd Zaki Salleh; Sazzli Shahlan Kassim; Siti Farah Alwani Mohd Nawi; Siti Hamimah Sheikh Abdul Kadir; Teh Lay Kek; Wang Seok Mui
EPI_ISL_3281367	JSS Medical Lab SDN BHD	Institute for Medical Research, Infectious Disease Research Centre, National Institutes of Health, Ministry of Health Malaysia	Anasir MI; Azizan MA; Kamel K; Mohd Zawawi Z; Ramly N; Robert F; Suppiah J; Thayan R
EPI_ISL_3980709, EPI_ISL_3980711, EPI_ISL_3980713, EPI_ISL_3980714, EPI_ISL_3980715, EPI_ISL_3980716, EPI_ISL_3980718, EPI_ISL_3980720			
see above	Jabatan Forensik Hospital Sultanah Bahiyah	Tropical Infectious Diseases Research & Education Centre (TIDREC), Universiti Malaya	AsmaAnati CheMatSeri; Che-Norainon Yaacob; Jia-Yi Tan; Jo-Ern Wong; Kim-Kee Tan; Mulya-Mustika-Sari Zulkifli; Noor Syahida Azizan; Nur-Hidayana Mahfodz; Sazaly AbuBakar; Siti-Sarah Nor'e
EPI_ISL_4565046	Jabatan Perubatan, Hospital Kuala Lumpur	Tropical Infectious Diseases Research & Education Centre (TIDREC), Universiti Malaya	AsmaAnati CheMatSeri; Che-Norainon Yaacob; Jia-Yi Tan; Jo-Ern Wong; Kim-Kee Tan; Mulya-Mustika-Sari Zulkifli; Noor Syahida Azizan; Nur-Hidayana Mahfodz; Sazaly AbuBakar; Siti-Sarah Nor'e
EPI_ISL_3446679	Jenjarom Health Clinic	Institute for Medical Research, Infectious Disease Research Centre, National Institutes of Health, Ministry of Health Malaysia	Anasir MI; Azizan MA; Kamel K; Mohd Zawawi Z; Ramly N; Robert F; Suppiah J; Thayan R
EPI_ISL_3446681, EPI_ISL_3446683	Jeram Health Clinic	Institute for Medical Research, Infectious Disease Research Centre, National Institutes of Health, Ministry of Health Malaysia	Anasir MI; Azizan MA; Kamel K; Mohd Zawawi Z; Ramly N; Robert F; Suppiah J; Thayan R
EPI_ISL_2091022, EPI_ISL_2233384, EPI_ISL_2550714, EPI_ISL_2550731, EPI_ISL_2625621, EPI_ISL_2625624, EPI_ISL_2625625, EPI_ISL_2625626, EPI_ISL_2625627, EPI_ISL_2625628, EPI_ISL_2625629, EPI_ISL_2625630, EPI_ISL_2625631, EPI_ISL_2649996, EPI_ISL_2650256, EPI_ISL_3100124, EPI_ISL_3100125, EPI_ISL_3100298, EPI_ISL_3100465, EPI_ISL_3100679, EPI_ISL_3100680, EPI_ISL_3374152, EPI_ISL_3419710			
see above	Johor Bahru Public Health Laboratory	Institute for Medical Research, Infectious Disease Research Centre, National Institutes of Health, Ministry of Health Malaysia	Anasir MI; Azizan MA; Kamel K; Mohd Zawawi Z; Ramly N; Robert F; Suppiah J; Thayan R
EPI_ISL_2757930, EPI_ISL_2757951, EPI_ISL_2812981	Johor State Health Department	Institute for Medical Research, Infectious Disease Research Centre, National Institutes of Health, Ministry of Health Malaysia	Azizan MA; Kamel K; Mohd Zawawi Z; Ramly N; Robert F; Suppiah J; Thayan R
EPI_ISL_4565050	KK PEROL	Tropical Infectious Diseases Research & Education Centre (TIDREC), Universiti Malaya	AsmaAnati CheMatSeri; Che-Norainon Yaacob; Jia-Yi Tan; Jo-Ern Wong; Kim-Kee Tan; Mulya-Mustika-Sari Zulkifli; Noor Syahida Azizan; Nur-Hidayana Mahfodz; Sazaly AbuBakar; Siti-Sarah Nor'e
EPI_ISL_4565049	KK Pos Brooke	Tropical Infectious Diseases Research & Education Centre (TIDREC), Universiti Malaya	AsmaAnati CheMatSeri; Che-Norainon Yaacob; Jia-Yi Tan; Jo-Ern Wong; Kim-Kee Tan; Mulya-Mustika-Sari Zulkifli; Noor Syahida Azizan; Nur-Hidayana Mahfodz; Sazaly AbuBakar; Siti-Sarah Nor'e
EPI_ISL_4565030	KLINIK KESIHATAN LOJING	Tropical Infectious Diseases Research & Education Centre (TIDREC), Universiti Malaya	AsmaAnati CheMatSeri; Che-Norainon Yaacob; Jia-Yi Tan; Jo-Ern Wong; Kim-Kee Tan; Mulya-Mustika-Sari Zulkifli; Noor Syahida Azizan; Nur-Hidayana Mahfodz; Sazaly AbuBakar; Siti-Sarah Nor'e
EPI_ISL_4122374, EPI_ISL_4435512	KLINIK KESIHATAN TRIANG	iPROMISE, UiTM	Ariza Adnan; Fadzilah Mohd Nor; Lim Wai Feng; Mohd Asif Mohd Sukri; Mohd Nur Fakhruzzaman Noorizhab; Mohd Zaki Salleh; Sazzli Shahlan Kassim; Siti Farah Alwani Mohd Nawi; Siti Hamimah Sheikh Abdul Kadir; Teh Lay Kek; Wang Seok Mui
EPI_ISL_3333234, EPI_ISL_4101560, EPI_ISL_4101561, EPI_ISL_4101562, EPI_ISL_4101563, EPI_ISL_4101564, EPI_ISL_4101565, EPI_ISL_4101566			
see above	Kajang Hospital	Institute for Medical Research, Infectious Disease Research Centre, National Institutes of Health, Ministry of Health Malaysia	Ahmad FA; Ahmad Fazilah NA; Anasir MI; Azizan MA; Kamel K; Mohd Zawawi Z; Norhisham SN; Ramly N; Robert F; Suppiah J; Thayan R
EPI_ISL_3446663, EPI_ISL_3446664	Kampung Bandar Health Clinic	Institute for Medical Research, Infectious Disease Research Centre, National Institutes of Health, Ministry of Health Malaysia	Anasir MI; Azizan MA; Kamel K; Mohd Zawawi Z; Ramly N; Robert F; Suppiah J; Thayan R
EPI_ISL_3050137	Kapit Hospital	Institute for Medical Research, Infectious Disease Research Centre, National Institutes of Health, Ministry of Health Malaysia	Anasir MI; Azizan MA; Kamel K; Mohd Zawawi Z; Ramly N; Robert F; Suppiah J; Thayan R
EPI_ISL_3446686, EPI_ISL_3446688	Kebun Baru Health Clinic	Institute for Medical Research, Infectious Disease Research Centre, National Institutes of Health, Ministry of Health Malaysia	Anasir MI; Azizan MA; Kamel K; Mohd Zawawi Z; Ramly N; Robert F; Suppiah J; Thayan R
EPI_ISL_2812576, EPI_ISL_2854053	Kemaman Hospital	Institute for Medical Research, Infectious Disease Research Centre, National Institutes of Health, Ministry of Health Malaysia	Anasir MI; Azizan MA; Kamel K; Mohd Zawawi Z; Ramly N; Robert F; Suppiah J; Thayan R
EPI_ISL_3723542	Kepala Batas Hospital	Institute for Medical Research, Infectious Disease Research Centre, National Institutes of Health, Ministry of Health Malaysia	Anasir MI; Azizan MA; Kamel K; Mohd Zawawi Z; Ramly N; Robert F; Suppiah J; Thayan R
EPI_ISL_4101548	Kinabatangan Area Health Office	Institute for Medical Research, Infectious Disease Research Centre, National Institutes of Health, Ministry of Health Malaysia	Ahmad FA; Ahmad Fazilah NA; Anasir MI; Azizan MA; Kamel K; Mohd Zawawi Z; Norhisham SN; Ramly N; Robert F; Suppiah J; Thayan R

EPI_ISL_4101558	Kinabatangan Center For Disease Control	Institute for Medical Research, Infectious Disease Research Centre, National Institutes of Health, Ministry of Health Malaysia	Ahmad FA; Ahmad Fazilah NA; Anasir MI; Azizan MA; Kamel K; Mohd Zawawi Z; Norhisham SN; Ramly N; Robert F; Suppiah J; Thayan R
EPI_ISL_4565047	Kk badang	Tropical Infectious Diseases Research & Education Centre (TIDREC), Universiti Malaya	AsmaAnati CheMatSeri; Che-Norainon Yaacob; Jia-Yi Tan; Jo-Ern Wong; Kim-Kee Tan; Mulya-Mustika-Sari Zulkifli; Noor Syahida Azizan; Nur-Hidayana Mahfodz; Sazaly AbuBakar; Siti-Sarah Nor'e
EPI_ISL_1972549, EPI_ISL_1972550	Klinik Kesihatan Bentong	Institute for Medical Research, Infectious Disease Research Centre, National Institutes of Health, Ministry of Health Malaysia	Azizan MA; Kamel K; Mohd Zawawi Z; Ramly N; Robert F; Suppiah J; Thayan R
EPI_ISL_1972553	Klinik Kesihatan Karak	Institute for Medical Research, Infectious Disease Research Centre, National Institutes of Health, Ministry of Health Malaysia	Azizan MA; Kamel K; Mohd Zawawi Z; Ramly N; Robert F; Suppiah J; Thayan R
EPI_ISL_3769383	Klinik Kesihatan Kuala Perlis	iPROMISE, UiTM	Ariza Adnan; Fadzilah Mohd Nor; Lim Wai Feng; Mohd Asif Mohd Sukri; Mohd Nur Fakhruzzaman Noorizhab; Mohd Zaki Salleh; Sazzli Shahlan Kassim; Siti Farah Alwani Mohd Nawi; Siti Hamimah Sheikh Abdul Kadir; Teh Lay Kek; Wang Seok Mui
EPI_ISL_1972548, EPI_ISL_1972551, EPI_ISL_1972552	Klinik Kesihatan Lurah Bilut (Felda)	Institute for Medical Research, Infectious Disease Research Centre, National Institutes of Health, Ministry of Health Malaysia	Azizan MA; Kamel K; Mohd Zawawi Z; Ramly N; Robert F; Suppiah J; Thayan R
EPI_ISL_3980724	Klinik Kesihatan Padang Rengas	Tropical Infectious Diseases Research & Education Centre (TIDREC), Universiti Malaya	AsmaAnati CheMatSeri; Che-Norainon Yaacob; Jia-Yi Tan; Jo-Ern Wong; Kim-Kee Tan; Mulya-Mustika-Sari Zulkifli; Noor Syahida Azizan; Nur-Hidayana Mahfodz; Sazaly AbuBakar; Siti-Sarah Nor'e
EPI_ISL_2811874	Klinik Kesihatan Pasir Akar	Institute for Medical Research, Infectious Disease Research Centre, National Institutes of Health, Ministry of Health Malaysia	Azizan MA; Kamel K; Mohd Zawawi Z; Ramly N; Robert F; Suppiah J; Thayan R
EPI_ISL_3762822	Kluang District Health Office	Institute for Medical Research, Infectious Disease Research Centre, National Institutes of Health, Ministry of Health Malaysia	Anasir MI; Azizan MA; Kamel K; Mohd Zawawi Z; Ramly N; Robert F; Suppiah J; Thayan R
EPI_ISL_1973324, EPI_ISL_2020075, EPI_ISL_2020076, EPI_ISL_2020077, EPI_ISL_2020078, EPI_ISL_2020165, EPI_ISL_2020166, EPI_ISL_2020167, EPI_ISL_3333236, EPI_ISL_3333581, EPI_ISL_3333593			
see above	Kota Bharu Public Health Laboratory	Institute for Medical Research, Infectious Disease Research Centre, National Institutes of Health, Ministry of Health Malaysia	Anasir MI; Azizan MA; Kamel K; Mohd Zawawi Z; Ramly N; Robert F; Suppiah J; Thayan R; uppiah J
EPI_ISL_2988871, EPI_ISL_2988872, EPI_ISL_2988873, EPI_ISL_2988874	Kota Bharu Public Health Laboratory	Molecular Research Laboratory	Abdul Haris bin Muhammad; Ahmad Sukari Bin Halim; Alexander Chong Shu Chien; Aswini Leela; Azian Harun; Chan Yean Yean; Chua Wei Chuan; Chua Wei Lian; Engku Nur Syafirah bt Engku Abd Rahman; Kamarul Imran Musa; Kirnpal Kaur Banga Singh; Lau Nyok Sean; Lee Lih Huey; Lee Yeong Yeh; Lim Shu Yong; Maizun binti Mohd Zain; Muhammad Fazli bin Khalid; Muhammad Nashrul Farhan Samsudin; Muhammad Zarul Hanifah Bin Md Zoqratt; Najib Majdi Bin Yaacob; Nazmi Liana Binti Azmi; Noor Hafizan binti Mat Salleh; Nurfadhlina Musa; Qasim Ayub; Rosline Hassan; Sadequr Rahman; Syahida binti Omar; Wan Mohd Zahiruddin Wan Mohammad; Wilhelm Eng Wei Han; Zaini bin Hussin; Zakuan Zainy Deris
EPI_ISL_4101547	Kota Marudu District Health Office	Institute for Medical Research, Infectious Disease Research Centre, National Institutes of Health, Ministry of Health Malaysia	Ahmad FA; Ahmad Fazilah NA; Anasir MI; Azizan MA; Kamel K; Mohd Zawawi Z; Norhisham SN; Ramly N; Robert F; Suppiah J; Thayan R
EPI_ISL_4096795	Kuala Kubu Baru Hospital	Institute for Medical Research, Infectious Disease Research Centre, National Institutes of Health, Ministry of Health Malaysia	Ahmad FA; Ahmad Fazilah NA; Anasir MI; Azizan MA; Kamel K; Mohd Zawawi Z; Norhisham SN; Ramly N; Robert F; Suppiah J; Thayan R
EPI_ISL_3446657	Kuala Kubu Bharu Hospital	Institute for Medical Research, Infectious Disease Research Centre, National Institutes of Health, Ministry of Health Malaysia	Anasir MI; Azizan MA; Kamel K; Mohd Zawawi Z; Ramly N; Robert F; Suppiah J; Thayan R
EPI_ISL_3446717, EPI_ISL_3446719, EPI_ISL_3446726, EPI_ISL_3446728	Kuala Langat District Health Office	Institute for Medical Research, Infectious Disease Research Centre, National Institutes of Health, Ministry of Health Malaysia	Anasir MI; Azizan MA; Kamel K; Mohd Zawawi Z; Ramly N; Robert F; Suppiah J; Thayan R
EPI_ISL_2815331, EPI_ISL_4348275, EPI_ISL_4348276	Kuala Lumpur General Hospital	Institute for Medical Research, Infectious Disease Research Centre, National Institutes of Health, Ministry of Health Malaysia	Ahmad FA; Ahmad Fazilah NA; Anasir MI; Azizan MA; Kamel K; Mohd Zawawi Z; Norhisyam SN; Ramly N; Robert F; Suppiah J; Thayan R
EPI_ISL_2649993, EPI_ISL_3071860, EPI_ISL_3101006	Kuala Lumpur Hospital	Institute for Medical Research, Infectious Disease Research Centre, National Institutes of Health, Ministry of Health Malaysia	Anasir MI; Azizan MA; Kamel K; Mohd Zawawi Z; Ramly N; Robert F; Suppiah J; Thayan R
EPI_ISL_3425312, EPI_ISL_3425462, EPI_ISL_3764515, EPI_ISL_3765760	Kuala Lumpur International Airport (KLIA) Health Office	Institute for Medical Research, Infectious Disease Research Centre, National Institutes of Health, Ministry of Health Malaysia	Anasir MI; Azizan MA; Kamel K; Mohd Zawawi Z; Ramly N; Robert F; Suppiah J; Thayan R
EPI_ISL_2811865, EPI_ISL_2811866, EPI_ISL_2811867, EPI_ISL_2811868, EPI_ISL_2811869, EPI_ISL_2811870	Kuala Lumpur International Healthcare Centre (KLIHC)	Institute for Medical Research, Infectious Disease Research Centre, National Institutes of Health, Ministry of Health Malaysia	Azizan MA; Kamel K; Mohd Zawawi Z; Ramly N; Robert F; Suppiah J; Thaya; Thayan R
EPI_ISL_3071988	Kuala Pilah District Health Office	Institute for Medical Research, Infectious Disease Research Centre, National Institutes of Health, Ministry of Health Malaysia	Anasir MI; Kamel K; Mohd Zawawi Z; Ramly N; Robert F; Suppiah J; Thayan R
EPI_ISL_3120236	Kuala Pilah Health District Office	Institute for Medical Research, Infectious Disease Research Centre, National Institutes of Health, Ministry of Health Malaysia	Anasir MI; Azizan MA; Kamel K; Mohd Zawawi Z; Ramly N; Robert F; Suppiah J; Thayan R
EPI_ISL_3446692, EPI_ISL_3446695	Kuala Selangor Health Clinic	Institute for Medical Research, Infectious Disease Research Centre, National Institutes of Health, Ministry of Health Malaysia	Anasir MI; Azizan MA; Kamel K; Mohd Zawawi Z; Ramly N; Robert F; Suppiah J; Thayan R
EPI_ISL_2854187	Kuala Terengganu District Health Office	Institute for Medical Research, Infectious Disease Research Centre, National Institutes of Health, Ministry of Health Malaysia	Anasir MI; Azizan MA; Kamel K; Mohd Zawawi Z; Ramly N; Robert F; Suppiah J; Thayan R
EPI_ISL_3446658	Kuala kubu Bharu Hospital	Institute for Medical Research, Infectious Disease Research Centre, National Institutes of Health, Ministry of Health Malaysia	Anasir MI; Azizan MA; Kamel K; Mohd Zawawi Z; Ramly N; Robert F; Suppiah J; Thayan R
EPI_ISL_4096812	Kudat District Health Office	Institute for Medical Research, Infectious Disease Research Centre, National Institutes of Health, Ministry of Health Malaysia	Ahmad FA; Ahmad Fazilah NA; Anasir MI; Azizan MA; Kamel K; Mohd Zawawi Z; Norhisham SN; Ramly N; Robert F; Suppiah J; Thayan R
EPI_ISL_2649991	Kudat Hospital	Institute for Medical Research, Infectious Disease Research Centre, National Institutes of Health, Ministry of Health Malaysia	Azizan MA; Kamel K; Mohd Zawawi Z; Ramly N; Robert F; Suppiah J; Thayan R
EPI_ISL_2621664	Kulim Hospital	Institute for Medical Research, Infectious Disease Research Centre, National Institutes of Health, Ministry of Health Malaysia	Azizan MA; Kamel K; Mohd Zawawi Z; Ramly N; Robert F; Suppiah J; Thayan R
EPI_ISL_3221324	Kulliyyah of Medicine, International Islamic University Malaysia, Bandar Indera Mahkota Campus, Jalan Sultan Ahmad Shah, 25200 Kuantan, Pahang Darul Makmur	Centre for Research in Advanced Tropical Bioscience (Biotropic Centre)	How Soon Hin and Hajar Fauzan Ahmad; Norhidayah Kamarudin; Ummu Afeera Zainulabid
EPI_ISL_3334843, EPI_ISL_3355465	Lablink (M) Sdn. Bhd	Institute for Medical Research, Infectious Disease Research Centre, National Institutes of Health, Ministry of Health Malaysia	Anasir MI; Azizan MA; Kamel K; Mohd Zawawi Z; Ramly N; Robert F; Suppiah J; Thayan R
EPI_ISL_3355464, EPI_ISL_3374523, EPI_ISL_3374524	Lablink (M) Sdn. Bhd.	Institute for Medical Research, Infectious Disease Research Centre, National Institutes of Health, Ministry of Health Malaysia	Anasir MI; Azizan MA; Kamel K; Mohd Zawawi Z; Ramly N; Robert F; Suppiah J; Thayan R
EPI_ISL_1585270, EPI_ISL_1585271, EPI_ISL_2549741, EPI_ISL_2757931, EPI_ISL_2812651, EPI_ISL_2812971, EPI_ISL_2854048, EPI_ISL_2921610, EPI_ISL_2921611, EPI_ISL_2924057, EPI_ISL_2931921			
see above	Labuan Hospital	Institute for Medical Research, Infectious Disease Research Centre, National Institutes of Health, Ministry of Health Malaysia	Anasir MI; Azizan MA; Kamel K; Mohd Zawawi Z; Ramly N; Robert F; Suppiah J; Thayan R
EPI_ISL_3762823, EPI_ISL_3762824	Lahad Datu Area Health Office	Institute for Medical Research, Infectious Disease Research Centre, National Institutes of Health, Ministry of Health Malaysia	Anasir MI; Azizan MA; Kamel K; Mohd Zawawi Z; Ramly N; Robert F; Suppiah J; Thayan R
EPI_ISL_2854068, EPI_ISL_4096793, EPI_ISL_4096794, EPI_ISL_4348240, EPI_ISL_4348270, EPI_ISL_4348271, EPI_ISL_4348272			
see above	Lahad Datu Hospital	Institute for Medical Research, Infectious Disease Research Centre, National Institutes of Health, Ministry of Health Malaysia	Ahmad FA; Ahmad Fazilah NA; Anasir MI; Azizan MA; Kamel K; Mohd Zawawi Z; Norhisham SN; Norhisyam SN; Ramly N; Robert F; Suppiah J; Thayan R
EPI_ISL_3120112, EPI_ISL_3120113, EPI_ISL_4602781	Lembah Pantai Health Office	Institute for Medical Research, Infectious Disease Research Centre, National Institutes of Health, Ministry of Health Malaysia	Ahmad FA; Ahmad Fazilah NA; Anasir MI; Azizan MA; Kamel K; Mohd Zawawi Z; Norhisyam SN; Ramly N; Robert F; Suppiah J; Thayan R
EPI_ISL_3266067	Likas Hospital	Institute for Medical Research, Infectious Disease Research Centre, National Institutes of Health, Ministry of Health Malaysia	Anasir MI; Azizan MA; Kamel K; Mohd Zawawi Z; Ramly N; Robert F; Suppiah J; Thayan R
EPI_ISL_807151, EPI_ISL_807152, EPI_ISL_807153	Makmal Kesihatan Awam Kebangsaan, Kementerian Kesihatan Malaysia	Integrative Pharmacogenomics Institute (iPROMISE)	Ariza Adnan; Fadzilah Mohd Nor; Hani Mat Hussin; Lim Wai Feng; Mohd Nur Fakhruzzaman Noorizhab; Mohd Zaki Salleh; Nor Zahrin Hasran; Sazzli Shahlan Kassim; Siti Farah Alwani Mohd Nawi; Siti Hamimah Sheikh Abdul Kadir; Teh Lay Kek; Wang Seok Mui
EPI_ISL_807150	Makmal Kesihatan Awam Kebangsaan, Kementerian Kesihatan Malaysia.	Integrative Pharmacogenomics Institute (iPROMISE)	Ariza Adnan; Fadzilah Mohd Nor; Hani Mat Hussin; Lim Wai Feng; Mohd Nur Fakhruzzaman Noorizhab; Mohd Zaki Salleh; Nor Zahrin Hasran; Sazzli Shahlan Kassim; Siti Farah Alwani Mohd Nawi; Siti Hamimah Sheikh Abdul Kadir; Teh Lay Kek; Wang Seok Mui
EPI_ISL_936490	Makmal Kesihatan Awam Kota Bharu	Institute for Medical Research, Infectious Disease Research Centre, National Institutes of Health, Ministry of Health Malaysia	Azizan MA; Kamel K; Suppiah J; Thayan R
EPI_ISL_528738, EPI_ISL_528739, EPI_ISL_528740, EPI_ISL_528741, EPI_ISL_528742, EPI_ISL_528743, EPI_ISL_528744, EPI_ISL_568873, EPI_ISL_568874, EPI_ISL_568875, EPI_ISL_582124, EPI_ISL_977591, EPI_ISL_977592, EPI_ISL_977593, EPI_ISL_1059900, EPI_ISL_1059901, EPI_ISL_1435802, EPI_ISL_1435804, EPI_ISL_1435806, EPI_ISL_1435808, EPI_ISL_1435810, EPI_ISL_1435812, EPI_ISL_1435814, EPI_ISL_1435816, EPI_ISL_1435817, EPI_ISL_1435820, EPI_ISL_2342585, EPI_ISL_2342586, EPI_ISL_2342587, EPI_ISL_2342588, EPI_ISL_2342589, EPI_ISL_2342590, EPI_ISL_2342591, EPI_ISL_2342592, EPI_ISL_2342593, EPI_ISL_2342594, EPI_ISL_2342595, EPI_ISL_2342596, EPI_ISL_2342597, EPI_ISL_2342598, EPI_ISL_2342599, EPI_ISL_2342600, EPI_ISL_3356252, EPI_ISL_3356253, EPI_ISL_3356254, EPI_ISL_3356255, EPI_ISL_3356256, EPI_ISL_3356257, EPI_ISL_3356258, EPI_ISL_3356259, EPI_ISL_3356260, EPI_ISL_3356261, EPI_ISL_3356262, EPI_ISL_3356263, EPI_ISL_3356264, EPI_ISL_3356265, EPI_ISL_3356266, EPI_ISL_3356267, EPI_ISL_3356268, EPI_ISL_3356269, EPI_ISL_3356270, EPI_ISL_3356271, EPI_ISL_3356272, EPI_ISL_3356273, EPI_ISL_3356274, EPI_ISL_3356275, EPI_ISL_3356276, EPI_ISL_3356277, EPI_ISL_3356278, EPI_ISL_3356279, EPI_ISL_3356280, EPI_ISL_3356281			
see above	Malaysia Genome Institute	Malaysia Genome Institute	Azrin Ahmad; Enizza Kasim; Gan Han Ming; Irni Suhayu Sapian; Mohd Faizal Abu Bakar; Mohd Noor Mat Isa; Nor Azfa Johari; Nor Azfa Johari.; Nurhezreen Md Iqbal; Shahrul Hisham Zainal Ariffin; Shamsidar Sopie; Siti Noraini Othman; Tan Ju Lin; Yusuf Muhammad Noor
EPI_ISL_2854188	Marang District Health Office	Institute for Medical Research, Infectious Disease Research Centre, National Institutes of Health, Ministry of Health Malaysia	Anasir MI; Azizan MA; Kamel K; Mohd Zawawi Z; Ramly N; Robert F; Suppiah J; Thayan R

EPI_ISL_4101543	Marudu District Health Office	Institute for Medical Research, Infectious Disease Research Centre, National Institutes of Health, Ministry of Health Malaysia	Ahmad FA; Ahmad Fazilah NA; Anasir MI; Azizan MA; Kamel K; Mohd Zawawi Z; Norhisham SN; Ramly N; Robert F; Suppiah J; Thayan R
EPI_ISL_4101544	Marudu District Health Ofiice	Institute for Medical Research, Infectious Disease Research Centre, National Institutes of Health, Ministry of Health Malaysia	Ahmad FA; Ahmad Fazilah NA; Anasir MI; Azizan MA; Kamel K; Mohd Zawawi Z; Norhisham SN; Ramly N; Robert F; Suppiah J; Thayan R
EPI_ISL_4602788, EPI_ISL_4603809, EPI_ISL_4604200	Medical Forensic Unit, Duchess of Kent Hospital	Institute for Medical Research, Infectious Disease Research Centre, National Institutes of Health, Ministry of Health Malaysia	Ahmad FA; Ahmad Fazilah NA; Anasir MI; Azizan MA; Kamel K; Mohd Zawawi Z; Norhisyam SN; Ramly N; Robert F; Suppiah J; Thayan R
EPI_ISL_2684234, EPI_ISL_2684235, EPI_ISL_2811876, EPI_ISL_2811877, EPI_ISL_2814916, EPI_ISL_2815327, EPI_ISL_2815328, EPI_ISL_2854049, EPI_ISL_2854050, EPI_ISL_2854051, EPI_ISL_2921996, EPI_ISL_3071855, EPI_ISL_3071856, EPI_ISL_3266063, EPI_ISL_3266064, EPI_ISL_3266065, EPI_ISL_3762497, EPI_ISL_3762498			
see above	Melaka Hospital	Institute for Medical Research, Infectious Disease Research Centre, National Institutes of Health, Ministry of Health Malaysia	Anasir MI; Azizan MA; Kamel K; Mohd Zawawi Z; Ramly N; Robert F; Suppiah J; Thayan R
EPI_ISL_455312, EPI_ISL_455313	Microbiology Unit, Department of Pathology & Laboratory Medicine, IIUM Medical Centre	SEA Microbiome Unit, Faculty of Industrial Sciences & Technology, Universiti Malaysia Pahang	Ahmad Hafiz Bin Zulkifly; Ahmad Hafiz Zulkifly; Ahmad Mahfuz Gazali; Hajar Fauzan Ahmad; IIUM Medical Centre Covid19 Taskforce; Mohd Fazli Farida Asras; Mohd Nazli Bin Kamarulzaman; Muhammad Adam Lee Abdullah; Norhidayah Binti Kamarudin; Norhidayah Kamarudin; UMP Covid19 Team
EPI_ISL_718132, EPI_ISL_718133, EPI_ISL_718134, EPI_ISL_718135, EPI_ISL_718136, EPI_ISL_718137, EPI_ISL_718138, EPI_ISL_718139, EPI_ISL_718140, EPI_ISL_718141, EPI_ISL_718142, EPI_ISL_718143, EPI_ISL_718144, EPI_ISL_718145, EPI_ISL_718146, EPI_ISL_718147, EPI_ISL_718148, EPI_ISL_718149, EPI_ISL_718150, EPI_ISL_718151, EPI_ISL_718152, EPI_ISL_718153, EPI_ISL_718154, EPI_ISL_718155, EPI_ISL_718156, EPI_ISL_718157, EPI_ISL_718158, EPI_ISL_718159, EPI_ISL_718160, EPI_ISL_718161, EPI_ISL_718162, EPI_ISL_718163, EPI_ISL_718164, EPI_ISL_718165, EPI_ISL_718166, EPI_ISL_718168, EPI_ISL_718169, EPI_ISL_718170, EPI_ISL_718171, EPI_ISL_718172, EPI_ISL_718173, EPI_ISL_718175, EPI_ISL_718177, EPI_ISL_718179, EPI_ISL_718180, EPI_ISL_718181, EPI_ISL_718183, EPI_ISL_718184, EPI_ISL_718186, EPI_ISL_718187, EPI_ISL_718188, EPI_ISL_718189, EPI_ISL_718190, EPI_ISL_718193, EPI_ISL_718194, EPI_ISL_718195, EPI_ISL_718196, EPI_ISL_718199, EPI_ISL_718200, EPI_ISL_718201, EPI_ISL_718202, EPI_ISL_718206, EPI_ISL_718207, EPI_ISL_718208, EPI_ISL_718209, EPI_ISL_718210, EPI_ISL_718211, EPI_ISL_718212, EPI_ISL_718213, EPI_ISL_718214, EPI_ISL_718215, EPI_ISL_718217, EPI_ISL_718219, EPI_ISL_718220, EPI_ISL_718221, EPI_ISL_1318203, EPI_ISL_1318204, EPI_ISL_1318205, EPI_ISL_1318206, EPI_ISL_1318207, EPI_ISL_1318208, EPI_ISL_1318209, EPI_ISL_1318210, EPI_ISL_1318211, EPI_ISL_1318212, EPI_ISL_1318213, EPI_ISL_1318214, EPI_ISL_1318215, EPI_ISL_1318216, EPI_ISL_1318217, EPI_ISL_1318218, EPI_ISL_1318219, EPI_ISL_1334365, EPI_ISL_2367469, EPI_ISL_2367470, EPI_ISL_2367471, EPI_ISL_2367472, EPI_ISL_2367473, EPI_ISL_2367475, EPI_ISL_2367477, EPI_ISL_2367478, EPI_ISL_2367479, EPI_ISL_2367480, EPI_ISL_2367481, EPI_ISL_2367489, EPI_ISL_2367490, EPI_ISL_2367491, EPI_ISL_2367492, EPI_ISL_2367493, EPI_ISL_2367494, EPI_ISL_2367495, EPI_ISL_2367496, EPI_ISL_2367497, EPI_ISL_2367498, EPI_ISL_2367499, EPI_ISL_2367500, EPI_ISL_2367501, EPI_ISL_2367502, EPI_ISL_2367503, EPI_ISL_2367504, EPI_ISL_2367505, EPI_ISL_2367506, EPI_ISL_2367507, EPI_ISL_2367508, EPI_ISL_2367509, EPI_ISL_2367510, EPI_ISL_2367514, EPI_ISL_2367515, EPI_ISL_2367516, EPI_ISL_2367517, EPI_ISL_2367518, EPI_ISL_2367519, EPI_ISL_2379928, EPI_ISL_2379935, EPI_ISL_2379936, EPI_ISL_2379937, EPI_ISL_2379938, EPI_ISL_2379939, EPI_ISL_2379940, EPI_ISL_2379941, EPI_ISL_2379942, EPI_ISL_2379943, EPI_ISL_2379944, EPI_ISL_2379945, EPI_ISL_2379946, EPI_ISL_2379947, EPI_ISL_2379948, EPI_ISL_2379949, EPI_ISL_2379950, EPI_ISL_2379951, EPI_ISL_2379952, EPI_ISL_2379953, EPI_ISL_2379954, EPI_ISL_2379955, EPI_ISL_2379956, EPI_ISL_2379961, EPI_ISL_2379962, EPI_ISL_2379963, EPI_ISL_2379964, EPI_ISL_2379965, EPI_ISL_2379966, EPI_ISL_2379967, EPI_ISL_2379968, EPI_ISL_2379969, EPI_ISL_2379970, EPI_ISL_2379971, EPI_ISL_2379972, EPI_ISL_2379973, EPI_ISL_2379974, EPI_ISL_2379975, EPI_ISL_2379976, EPI_ISL_2379977, EPI_ISL_2379978, EPI_ISL_2379979, EPI_ISL_2379980, EPI_ISL_2379981, EPI_ISL_2379982, EPI_ISL_2379983, EPI_ISL_2379989, EPI_ISL_2379990, EPI_ISL_2379991, EPI_ISL_2379992, EPI_ISL_2379993, EPI_ISL_2379994, EPI_ISL_2379995, EPI_ISL_2379996, EPI_ISL_2379997, EPI_ISL_2379998, EPI_ISL_2379999, EPI_ISL_2380000, EPI_ISL_2380001, EPI_ISL_2380002, EPI_ISL_2380003, EPI_ISL_2380004, EPI_ISL_2380005, EPI_ISL_2380006, EPI_ISL_2380007, EPI_ISL_2380008, EPI_ISL_2380009, EPI_ISL_2380010, EPI_ISL_2535602, EPI_ISL_2535603, EPI_ISL_2535605, EPI_ISL_2535606, EPI_ISL_2535609, EPI_ISL_2535610, EPI_ISL_2535611, EPI_ISL_2535612, EPI_ISL_2535613, EPI_ISL_2535614, EPI_ISL_2535615, EPI_ISL_2535616, EPI_ISL_2535617, EPI_ISL_2535618, EPI_ISL_2535621, EPI_ISL_2535622, EPI_ISL_2535623, EPI_ISL_2535624, EPI_ISL_2535625, EPI_ISL_2535626, EPI_ISL_2535627, EPI_ISL_2535628, EPI_ISL_2535629, EPI_ISL_2535633, EPI_ISL_2535634, EPI_ISL_2535635, EPI_ISL_2535636, EPI_ISL_2535637, EPI_ISL_2535638, EPI_ISL_2535639, EPI_ISL_2535640, EPI_ISL_2535643, EPI_ISL_2535644, EPI_ISL_2535645, EPI_ISL_2535646, EPI_ISL_2535647, EPI_ISL_2535648, EPI_ISL_2535649, EPI_ISL_2535650, EPI_ISL_2535651, EPI_ISL_2535654, EPI_ISL_2535655, EPI_ISL_2535656, EPI_ISL_2535657, EPI_ISL_2535658, EPI_ISL_2535659, EPI_ISL_2535662, EPI_ISL_2535663, EPI_ISL_2535664, EPI_ISL_2535665, EPI_ISL_2535666, EPI_ISL_2535667, EPI_ISL_2535668, EPI_ISL_2535669, EPI_ISL_2535670, EPI_ISL_2535671, EPI_ISL_2535674, EPI_ISL_2535675, EPI_ISL_2535676, EPI_ISL_2535677, EPI_ISL_2535678, EPI_ISL_2535679, EPI_ISL_2535680, EPI_ISL_2535681, EPI_ISL_2535682, EPI_ISL_2535684, EPI_ISL_2535685, EPI_ISL_2535686, EPI_ISL_2535687, EPI_ISL_2535688, EPI_ISL_2535689, EPI_ISL_2535690, EPI_ISL_2535691, EPI_ISL_2535692, EPI_ISL_2535693, EPI_ISL_2535694, EPI_ISL_2535773, EPI_ISL_2535774, EPI_ISL_2535775, EPI_ISL_2535776, EPI_ISL_2535777, EPI_ISL_2535778, EPI_ISL_2535779, EPI_ISL_2608377, EPI_ISL_2608378, EPI_ISL_4051653, EPI_ISL_4051661, EPI_ISL_4051680, EPI_ISL_4051682, EPI_ISL_4051683, EPI_ISL_4051684, EPI_ISL_4051685, EPI_ISL_4051686, EPI_ISL_4051687, EPI_ISL_4051688, EPI_ISL_4051689, EPI_ISL_4051690, EPI_ISL_4051691, EPI_ISL_4051692, EPI_ISL_4051693, EPI_ISL_4051694, EPI_ISL_4051695, EPI_ISL_4051706, EPI_ISL_4051707, EPI_ISL_4051708, EPI_ISL_4051709, EPI_ISL_4051710, EPI_ISL_4051711, EPI_ISL_4051712, EPI_ISL_4051713, EPI_ISL_4051714, EPI_ISL_4051715, EPI_ISL_4051716, EPI_ISL_4051717, EPI_ISL_4051718, EPI_ISL_4051719, EPI_ISL_4051720, EPI_ISL_4051737, EPI_ISL_4051738, EPI_ISL_4051748, EPI_ISL_4051764, EPI_ISL_4051765, EPI_ISL_4051766, EPI_ISL_4051767, EPI_ISL_4051768, EPI_ISL_4051769, EPI_ISL_4051790, EPI_ISL_4051791, EPI_ISL_4051792, EPI_ISL_4051793, EPI_ISL_4051794, EPI_ISL_4051795, EPI_ISL_4051829, EPI_ISL_4051831, EPI_ISL_4051832, EPI_ISL_4051837, EPI_ISL_4051838, EPI_ISL_4051844, EPI_ISL_4051845, EPI_ISL_4051850, EPI_ISL_4051851, EPI_ISL_4051852, EPI_ISL_4051853, EPI_ISL_4051854, EPI_ISL_4051858, EPI_ISL_4051861, EPI_ISL_4051862, EPI_ISL_4460128, EPI_ISL_4460129, EPI_ISL_4460130, EPI_ISL_4460131, EPI_ISL_4460132, EPI_ISL_4460133, EPI_ISL_4460134, EPI_ISL_4460135, EPI_ISL_4460136, EPI_ISL_4460137, EPI_ISL_4460138, EPI_ISL_4460139, EPI_ISL_4460140, EPI_ISL_4460230, EPI_ISL_4460393, EPI_ISL_4460394, EPI_ISL_4460395			
see above	Ministry of Health Hospitals	Institute of Health and Community Medicine	Chan Chia Jui; Chua Hock Hin; David Perera; Ooi Mong How; Tan Lee See; Tonnii Sia Loong Loong; Wong Jyn Shan; Wong Kiing Aik
EPI_ISL_2614740, EPI_ISL_2614741, EPI_ISL_2614742	Miri Hospital	Institute for Medical Research, Infectious Disease Research Centre, National Institutes of Health, Ministry of Health Malaysia	Azizan MA; Kamel K; Mohd Zawawi Z; Ramly N; Robert F; Suppiah J; Thayan R
EPI_ISL_4051654, EPI_ISL_4051655, EPI_ISL_4051656, EPI_ISL_4051721, EPI_ISL_4051722, EPI_ISL_4051723, EPI_ISL_4051724, EPI_ISL_4051725, EPI_ISL_4051857, EPI_ISL_4051864, EPI_ISL_4460097, EPI_ISL_4460098, EPI_ISL_4460099, EPI_ISL_4460100, EPI_ISL_4460101, EPI_ISL_4460212, EPI_ISL_4460213, EPI_ISL_4460214, EPI_ISL_4460215, EPI_ISL_4460216			
see above	Miri Hospital Molecular Diagnostic Lab	Institute of Health and Community Medicine	Chan Chia Jui; Chua Hock Hin; David Perera; Hanis Syazwani Mohd Hassan; Ooi Mong How; Tonnii Sia Loong Loong; Wong Jyn Shan
EPI_ISL_4460383, EPI_ISL_4460384, EPI_ISL_4460385, EPI_ISL_4460386, EPI_ISL_4460387	Miri Hospital Molecular Diagnostic Lab, Miri	Institute of Health and Community Medicine	Chan Chia Jui; Chua Hock Hin; David Perera; Hanis Syazwani Mohd Hassan; Ooi Mong How; Tonnii Sia Loong Loong; Wong Jyn Shan
EPI_ISL_3266032	Muar Clinic	Institute for Medical Research, Infectious Disease Research Centre, National Institutes of Health, Ministry of Health Malaysia	Anasir MI; Azizan MA; Kamel K; Mohd Zawawi Z; Ramly N; Robert F; Suppiah J; Thayan R
EPI_ISL_4101545	Nabawan District Health Ofiice	Institute for Medical Research, Infectious Disease Research Centre, National Institutes of Health, Ministry of Health Malaysia	Ahmad FA; Ahmad Fazilah NA; Anasir MI; Azizan MA; Kamel K; Mohd Zawawi Z; Norhisham SN; Ramly N; Robert F; Suppiah J; Thayan R
EPI_ISL_3281413	National Cancer Institute	Institute for Medical Research, Infectious Disease Research Centre, National Institutes of Health, Ministry of Health Malaysia	Anasir MI; Azizan MA; Kamel K; Mohd Zawawi Z; Ramly N; Robert F; Suppiah J; Thayan R
EPI_ISL_2379748	National Heart Institute	Institute for Medical Research, Infectious Disease Research Centre, National Institutes of Health, Ministry of Health Malaysia	Azizan MA; Kamel K; Mohd Zawawi Z; Ramly N; Robert F; Suppiah J; Thayan R
EPI_ISL_4347856	National Institute of Medical Forensic	Institute for Medical Research, Infectious Disease Research Centre, National Institutes of Health, Ministry of Health Malaysia	Ahmad FA; Ahmad Fazilah NA; Anasir MI; Azizan MA; Kamel K; Mohd Zawawi Z; Norhisyam SN; Ramly N; Robert F; Suppiah J; Thayan R
EPI_ISL_4347725	National Medical Forensic Institute	Institute for Medical Research, Infectious Disease Research Centre, National Institutes of Health, Ministry of Health Malaysia	Ahmad FA; Ahmad Fazilah NA; Anasir MI; Azizan MA; Kamel K; Mohd Zawawi Z; Norhisyam SN; Ramly N; Robert F; Suppiah J; Thayan R
EPI_ISL_738086	National Public Health Laboratory	Department of Medical Microbiology, Hospital Pengajar Universiti Putra Malaysia	Hui-Yee Chee; Jia-Yong Lam; Muhammad MI; Narcisse Joseph; Nor Zahrin Hasran; Syafinaz Amin-Nordin; Yien-Ping Wong
EPI_ISL_3281014, EPI_ISL_3281015, EPI_ISL_3281016, EPI_ISL_3769582	National Public Health Laboratory	Institute for Medical Research, Infectious Disease Research Centre, National Institutes of Health, Ministry of Health Malaysia	Anasir MI; Azizan MA; Kamel K; Mohd Zawawi Z; Ramly N; Robert F; Suppiah J; Thayan R
EPI_ISL_416829, EPI_ISL_416866, EPI_ISL_416884, EPI_ISL_416885, EPI_ISL_416886, EPI_ISL_416907	National Public Health Laboratory	Malaysia Genome Institute	Azrin Ahmad; Enizza Kasim; Hani Mat Hussin; Irni Suhayu Sapian; Mohd Faizal Abu Bakar; Mohd Noor Mat Isa; Noorliza Mohamad Noordin; Nor Azfa Johari; Norazimah Tajudin; Nurhezreen Md Iqbal; Rehan Shuhada Abu Bakar; Selvanesan Sengol; Shahrul Hisham Zainal Ariffin; Shamsidar Sopie; Siti Noraini Othman; W Afiza W Mohd Arifin; Yu Kie Chem; Yusuf Muhammad Noor
EPI_ISL_410487, EPI_ISL_410488, EPI_ISL_410489	National Public Health Laboratory	National Public Health Laboratory	A.B.; M.H.; M.N. and Hani; Noorliza; Norazimah, T.; Rehan Shuhada; Selvanesan, S.; Yu Kie, C.
EPI_ISL_1972889, EPI_ISL_1972890, EPI_ISL_1972891, EPI_ISL_1972892, EPI_ISL_1972893, EPI_ISL_1972894, EPI_ISL_1972895, EPI_ISL_2001114, EPI_ISL_2001115, EPI_ISL_2001213, EPI_ISL_2001429, EPI_ISL_2001430, EPI_ISL_2001481, EPI_ISL_2001482, EPI_ISL_2001643, EPI_ISL_2001644, EPI_ISL_2001645, EPI_ISL_2001654, EPI_ISL_2001655, EPI_ISL_2001656, EPI_ISL_2001659, EPI_ISL_2009116, EPI_ISL_2009193, EPI_ISL_2009232, EPI_ISL_2009233, EPI_ISL_2009234, EPI_ISL_2009305, EPI_ISL_2009529, EPI_ISL_2009530, EPI_ISL_2009595			
see above	National Public Health Laboratory Malaysia	National Public Health Laboratory Malaysia	Aziyati O; Chong CK; Hani MH; Hannah PYP; JL Tan; Kamal HKZ; Mohd AY; Noorliza MN; Norazimah T; Norazimah T Rehan SAB Selvanesan S; Norhayati R; NurulAina MCA; Nurulsyahida I; Rehan SAB; Selvanesan S; Ushananthiny R; W.NurAfiza WMA Zirwatul AA; YF Ngeow; Yukie C
EPI_ISL_3334190, EPI_ISL_3334217	National Public Health Laboratory, Sungai Buloh	Institute for Medical Research, Infectious Disease Research Centre, National Institutes of Health, Ministry of Health Malaysia	Anasir MI; Azizan MA; Kamel K; Mohd Zawawi Z; Ramly N; Robert F; Suppiah J; Thayan R
EPI_ISL_4122383, EPI_ISL_4122394, EPI_ISL_4122406, EPI_ISL_4122415, EPI_ISL_4122427, EPI_ISL_4122479, EPI_ISL_4122486, EPI_ISL_4122551			
see above	PEJ KESIHATAN BARAT DAYA	iPROMISE, UiTM	Ariza Adnan; Fadzilah Mohd Nor; Lim Wai Feng; Mohd Asif Mohd Sukri; Mohd Nur Fakhruzzaman Noorizhab; Mohd Zaki Salleh; Sazzli Shahlan Kassim; Siti Farah Alwani Mohd Nawi; Siti Hamimah Sheikh Abdul Kadir; Teh Lay Kek; Wang Seok Mui
EPI_ISL_4071986, EPI_ISL_4071987, EPI_ISL_4101588	PEJABAT KESIHATAN DAERAH BATU PAHAT	UKM Medical Molecular Biology Institute (UMBI)	Mira Farzana binti Mohamad Mokhtar; Nur Alyaa Afifah Md Shahri
EPI_ISL_4101592	PEJABAT KESIHATAN DAERAH JELEBU	UKM Medical Molecular Biology Institute (UMBI)	Nur Alyaa Afifah Md Shahri
EPI_ISL_4565051, EPI_ISL_4565052	PEJABAT KESIHATAN DAERAH MACHANG	Tropical Infectious Diseases Research & Education Centre (TIDREC), Universiti Malaya	AsmaAnati CheMatSeri; Che-Norainon Yaacob; Jia-Yi Tan; Jo-Ern Wong; Kim-Kee Tan; Mulya-Mustika-Sari Zulkifli; Noor Syahida Azizan; Nur-Hidayana Mahfodz; Sazaly AbuBakar; Siti-Sarah Nor'e
EPI_ISL_4565006, EPI_ISL_4565031	PEJABAT KESIHATAN DAERAH TUMPAT	Tropical Infectious Diseases Research & Education Centre (TIDREC), Universiti Malaya	AsmaAnati CheMatSeri; Che-Norainon Yaacob; Jia-Yi Tan; Jo-Ern Wong; Kim-Kee Tan; Mulya-Mustika-Sari Zulkifli; Noor Syahida Azizan; Nur-Hidayana Mahfodz; Sazaly AbuBakar; Siti-Sarah Nor'e
EPI_ISL_4236998	PKD ALOR GAJAH	UKM Medical Molecular Biology Institute (UMBI)	Mira Farzana binti Mohamad Mokhtar
EPI_ISL_3858171	PKD Alor Gajah	UKM Medical Molecular Biology Institute (UMBI)	Mira Farzana binti Mohamad Mokhtar
EPI_ISL_4565002, EPI_ISL_4565003, EPI_ISL_4565004, EPI_ISL_4565005	PKD BACHOK	Tropical Infectious Diseases Research & Education Centre (TIDREC), Universiti Malaya	AsmaAnati CheMatSeri; Che-Norainon Yaacob; Jia-Yi Tan; Jo-Ern Wong; Kim-Kee Tan; Mulya-Mustika-Sari Zulkifli; Noor Syahida Azizan; Nur-Hidayana Mahfodz; Sazaly AbuBakar; Siti-Sarah Nor'e
EPI_ISL_4071998, EPI_ISL_4101582, EPI_ISL_4101583	PKD JELEBU	UKM Medical Molecular Biology Institute (UMBI)	Mira Farzana binti Mohamad Mokhtar; Nur Alyaa Afifah Md Shahri
EPI_ISL_4071985	PKD JOHOR BAHRU	UKM Medical Molecular Biology Institute (UMBI)	Mira Farzana binti Mohamad Mokhtar
EPI_ISL_4101593, EPI_ISL_4463183, EPI_ISL_4463184, EPI_ISL_4463189	PKD Jempol	UKM Medical Molecular Biology Institute (UMBI)	Nur Alyaa Afifah Md Shahri
EPI_ISL_4513506	PKD KLUANG	UKM Medical Molecular Biology Institute (UMBI)	Mira Farzana binti Mohamad Mokhtar
EPI_ISL_3945553	PKD KOTA TINGGI	UKM Medical Molecular Biology Institute (UMBI)	Nur Alyaa Afifah Md Shahri
EPI_ISL_4513488, EPI_ISL_4513489, EPI_ISL_4513490, EPI_ISL_4513491, EPI_ISL_4513492, EPI_ISL_4513493	PKD KUALA PILAH	UKM Medical Molecular Biology Institute (UMBI)	Mira Farzana binti Mohamad Mokhtar
EPI_ISL_4236987	PKD KULAI	UKM Medical Molecular Biology Institute (UMBI)	Mira Farzana binti Mohamad Mokhtar
EPI_ISL_4101586, EPI_ISL_4101587, EPI_ISL_4101589	PKD MUAR	UKM Medical Molecular Biology Institute (UMBI)	Nur Alyaa Afifah Md Shahri
EPI_ISL_3945554, EPI_ISL_4463177, EPI_ISL_4463178	PKD SEREMBAN	UKM Medical Molecular Biology Institute (UMBI)	Nur Alyaa Afifah Md Shahri

EPI_ISL_3945555, EPI_ISL_3945556, EPI_ISL_4101597, EPI_ISL_4101598, EPI_ISL_4236999, EPI_ISL_4237000, EPI_ISL_4237001, EPI_ISL_4237002, EPI_ISL_4463176, EPI_ISL_4463181, EPI_ISL_4463182, EPI_ISL_4513494, EPI_ISL_4513495			
see above	PKD TAMPIN	UKM Medical Molecular Biology Institute (UMBI)	Mira Farzana binti Mohamad Mokhtar; Nur Alyaa Afifah Md Shahri
EPI_ISL_3980731	PKD Tanah Merah	Tropical Infectious Diseases Research & Education Centre (TIDREC), Universiti Malaya	AsmaAnati CheMatSeri; Che-Norainon Yaacob; Jia-Yi Tan; Jo-Ern Wong; Kim-Kee Tan; Mulya-Mustika-Sari Zulkifli; Noor Syahida Azizan; Nur-Hidayana Mahfodz; Sazaly AbuBakar; Siti-Sarah Nor'e
EPI_ISL_2921263, EPI_ISL_2921264, EPI_ISL_2921265, EPI_ISL_2921266	Pahang State Health Department	Institute for Medical Research, Infectious Disease Research Centre, National Institutes of Health, Ministry of Health Malaysia	Anasir MI; Azizan MA; Kamel K; Mohd Zawawi Z; Ramly N; Robert F; Suppiah J; Thayan R
EPI_ISL_3446696, EPI_ISL_3446697, EPI_ISL_3762835	Pandamaran Health Clinic	Institute for Medical Research, Infectious Disease Research Centre, National Institutes of Health, Ministry of Health Malaysia	Anasir MI; Azizan MA; Kamel K; Mohd Zawawi Z; Ramly N; Robert F; Suppiah J; Thayan R
EPI_ISL_2921262	Pantai Hospital Kuala Lumpur	Institute for Medical Research, Infectious Disease Research Centre, National Institutes of Health, Ministry of Health Malaysia	Anasir MI; Azizan MA; Kamel K; Mohd Zawawi Z; Ramly N; Robert F; Suppiah J; Thayan R
EPI_ISL_2811871, EPI_ISL_2811873	Pasir Mas Town Health Clinic	Institute for Medical Research, Infectious Disease Research Centre, National Institutes of Health, Ministry of Health Malaysia	Azizan MA; Kamel K; Mohd Zawawi Z; Ramly N; Robert F; Suppiah J; Thayan R
EPI_ISL_3945550, EPI_ISL_3945551, EPI_ISL_3945552	Pejabat Kesihatan Batu Pahat	UKM Medical Molecular Biology Institute (UMBI)	Nur Alyaa Afifah Md Shahri
EPI_ISL_4122468	Pejabat Kesihatan Daerah Kangar	iPROMISE, UiTM	Ariza Adnan; Fadzilah Mohd Nor; Lim Wai Feng; Mohd Asif Mohd Sukri; Mohd Nur Fakhruzzaman Noorizhab; Mohd Zaki Salleh; Sazzli Shahlan Kassim; Siti Farah Alwani Mohd Nawi; Siti Hamimah Sheikh Abdul Kadir; Teh Lay Kek; Wang Seok Mui
EPI_ISL_4557953, EPI_ISL_4558237, EPI_ISL_4558238	Pejabat Kesihatan Daerah Kota Marudu	Malaysia Genome Institute	Azrin Ahmad; Enizza Kasim; Irni Suhayu Sapian; Mohd Faizal Abu Bakar; Mohd Ghows Mohd Azzam.; Mohd Noor Mat Isa; Nor Azfa Johari; Nurhezreen Md Iqbal; Shamsidar Sopie; Siti Noraini Othman; Yusuf Muhammad Noor
EPI_ISL_4557954	Pejabat Kesihatan Daerah Sipitang	Malaysia Genome Institute	Azrin Ahmad; Enizza Kasim; Irni Suhayu Sapian; Mohd Faizal Abu Bakar; Mohd Ghows Mohd Azzam.; Mohd Noor Mat Isa; Nor Azfa Johari; Nurhezreen Md Iqbal; Shamsidar Sopie; Siti Noraini Othman; Yusuf Muhammad Noor
EPI_ISL_4557952, EPI_ISL_4601567, EPI_ISL_4601568, EPI_ISL_4601569	Pejabat Kesihatan Kawasan Tawau	Malaysia Genome Institute	Azrin Ahmad; Enizza Kasim; Irni Suhayu Sapian; Mohd Faizal Abu Bakar; Mohd Ghows Mohd Azzam.; Mohd Noor Mat Isa; Nor Azfa Johari; Nurhezreen Md Iqbal; Shamsidar Sopie; Siti Noraini Othman; Yusuf Muhammad Noor
EPI_ISL_3769345	Pejabat Kesihatan Pintu Antarabangsa	iPROMISE, UiTM	Ariza Adnan; Fadzilah Mohd Nor; Lim Wai Feng; Mohd Asif Mohd Sukri; Mohd Nur Fakhruzzaman Noorizhab; Mohd Zaki Salleh; Sazzli Shahlan Kassim; Siti Farah Alwani Mohd Nawi; Siti Hamimah Sheikh Abdul Kadir; Teh Lay Kek; Wang Seok Mui
EPI_ISL_3769339, EPI_ISL_3769344, EPI_ISL_3769349, EPI_ISL_3769350, EPI_ISL_3769351, EPI_ISL_3769352, EPI_ISL_3769353, EPI_ISL_4122437, EPI_ISL_4122547			
see above	Pejabat Kesihatan Pintu Masuk Antarabangsa	iPROMISE, UiTM	Ariza Adnan; Fadzilah Mohd Nor; Lim Wai Feng; Mohd Asif Mohd Sukri; Mohd Nur Fakhruzzaman Noorizhab; Mohd Zaki Salleh; Sazzli Shahlan Kassim; Siti Farah Alwani Mohd Nawi; Siti Hamimah Sheikh Abdul Kadir; Teh Lay Kek; Wang Seok Mui
EPI_ISL_3769336, EPI_ISL_3769337, EPI_ISL_3769338, EPI_ISL_3769340, EPI_ISL_3769341, EPI_ISL_3769342, EPI_ISL_3769346, EPI_ISL_3769347, EPI_ISL_3769348, EPI_ISL_4122447			
see above	Pejabat Kesihatan Pintu Masuk Antarabangsa Pulau Pinang	iPROMISE, UiTM	Ariza Adnan; Fadzilah Mohd Nor; Lim Wai Feng; Mohd Asif Mohd Sukri; Mohd Nur Fakhruzzaman Noorizhab; Mohd Zaki Salleh; Sazzli Shahlan Kassim; Siti Farah Alwani Mohd Nawi; Siti Hamimah Sheikh Abdul Kadir; Teh Lay Kek; Wang Seok Mui
EPI_ISL_4565048	Pejabat Kesihatan daerah Jeli	Tropical Infectious Diseases Research & Education Centre (TIDREC), Universiti Malaya	AsmaAnati CheMatSeri; Che-Norainon Yaacob; Jia-Yi Tan; Jo-Ern Wong; Kim-Kee Tan; Mulya-Mustika-Sari Zulkifli; Noor Syahida Azizan; Nur-Hidayana Mahfodz; Sazaly AbuBakar; Siti-Sarah Nor'e
EPI_ISL_2811984	Pekan Tajau Health Clinic	Institute for Medical Research, Infectious Disease Research Centre, National Institutes of Health, Ministry of Health Malaysia	Azizan MA; Kamel K; Mohd Zawawi Z; Ramly N; Robert F; Suppiah J; Thayan R
EPI_ISL_2649998, EPI_ISL_2649999, EPI_ISL_3723541, EPI_ISL_3723543, EPI_ISL_3723544, EPI_ISL_3723545, EPI_ISL_3762825, EPI_ISL_3762826, EPI_ISL_3762827, EPI_ISL_4348327			
see above	Penang General Hospital	Institute for Medical Research, Infectious Disease Research Centre, National Institutes of Health, Ministry of Health Malaysia	Ahmad FA; Ahmad Fazilah NA; Anasir MI; Azizan MA; Kamel K; Mohd Zawawi Z; Norhisyam SN; Ramly N; Robert F; Suppiah J; Thayan R
EPI_ISL_3762773, EPI_ISL_3762789, EPI_ISL_3762790, EPI_ISL_3762818	Penang State Health Department	Institute for Medical Research, Infectious Disease Research Centre, National Institutes of Health, Ministry of Health Malaysia	Anasir MI; Azizan MA; Kamel K; Mohd Zawawi Z; Ramly N; Robert F; Suppiah J; Thayan R
EPI_ISL_3247358, EPI_ISL_3247403	Perak State Health Department	Institute for Medical Research, Infectious Disease Research Centre, National Institutes of Health, Ministry of Health Malaysia	Anasir MI; Azizan MA; Kamel K; Mohd Zawawi Z; Ramly N; Robert F; Suppiah J; Thayan R
EPI_ISL_3769364	Pkd pekan	iPROMISE, UiTM	Ariza Adnan; Fadzilah Mohd Nor; Lim Wai Feng; Mohd Asif Mohd Sukri; Mohd Nur Fakhruzzaman Noorizhab; Mohd Zaki Salleh; Sazzli Shahlan Kassim; Siti Farah Alwani Mohd Nawi; Siti Hamimah Sheikh Abdul Kadir; Teh Lay Kek; Wang Seok Mui
EPI_ISL_3762828, EPI_ISL_3762829	Port Dickson District Health Office	Institute for Medical Research, Infectious Disease Research Centre, National Institutes of Health, Ministry of Health Malaysia	Anasir MI; Azizan MA; Kamel K; Mohd Zawawi Z; Ramly N; Robert F; Suppiah J; Thayan R
EPI_ISL_2550758, EPI_ISL_2550759	Port Dickson Hospital	Institute for Medical Research, Infectious Disease Research Centre, National Institutes of Health, Ministry of Health Malaysia	Azizan MA; Kamel K; Mohd Zawawi Z; Ramly N; Robert F; Suppiah J; Thayan R
EPI_ISL_3446656	Port Klang Health Clinic	Institute for Medical Research, Infectious Disease Research Centre, National Institutes of Health, Ministry of Health Malaysia	Anasir MI; Azizan MA; Kamel K; Mohd Zawawi Z; Ramly N; Robert F; Suppiah J; Thayan R
EPI_ISL_3446702	Pulau Ketam Health Clinic	Institute for Medical Research, Infectious Disease Research Centre, National Institutes of Health, Ministry of Health Malaysia	Anasir MI; Azizan MA; Kamel K; Mohd Zawawi Z; Ramly N; Robert F; Suppiah J; Thayan R
EPI_ISL_3374154, EPI_ISL_3374706, EPI_ISL_3374707, EPI_ISL_3374708, EPI_ISL_4638334	Putrajaya Health Office	Institute for Medical Research, Infectious Disease Research Centre, National Institutes of Health, Ministry of Health Malaysia	Ahmad FA; Ahmad Fazilah NA; Anasir MI; Azizan MA; Kamel K; Mohd Zawawi Z; Norhisyam SN; Ramly N; Robert F; Suppiah J; Thayan R
EPI_ISL_4348302	Putrajaya Hospital	Institute for Medical Research, Infectious Disease Research Centre, National Institutes of Health, Ministry of Health Malaysia	Ahmad FA; Ahmad Fazilah NA; Anasir MI; Azizan MA; Kamel K; Mohd Zawawi Z; Norhisyam SN; Ramly N; Robert F; Suppiah J; Thayan R
EPI_ISL_2650027, EPI_ISL_2650430, EPI_ISL_2650432, EPI_ISL_2650499, EPI_ISL_2650505, EPI_ISL_2854106, EPI_ISL_3762495, EPI_ISL_4346231			
see above	Queen Elizabeth Hospital	Institute for Medical Research, Infectious Disease Research Centre, National Institutes of Health, Ministry of Health Malaysia	Ahmad FA; Ahmad Fazilah NA; Anasir MI; Azizan MA; Kamel K; Mohd Zawawi Z; Norhisyam SN; Ramly N; Robert F; Suppiah J; Thayan R
EPI_ISL_2757941, EPI_ISL_2757942, EPI_ISL_2931924, EPI_ISL_3374470	Queen Elizabeth Hospital, Kota Kinabalu	Institute for Medical Research, Infectious Disease Research Centre, National Institutes of Health, Ministry of Health Malaysia	Anasir MI; Azizan MA; Kamel K; Mohd Zawawi Z; Ramly N; Robert F; Suppiah J; Thayan R
EPI_ISL_3120110	Raja Perempuan Zainab II Hospital	Institute for Medical Research, Infectious Disease Research Centre, National Institutes of Health, Ministry of Health Malaysia	Anasir MI; Azizan MA; Kamel K; Mohd Zawawi Z; Ramly N; Robert F; Suppiah J; Thayan R
EPI_ISL_2811886	Raja Perempuan Zainab II Hospital, Kota Bharu	Institute for Medical Research, Infectious Disease Research Centre, National Institutes of Health, Ministry of Health Malaysia	Azizan MA; Kamel K; Mohd Zawawi Z; Ramly N; Robert F; Suppiah J; Thayan R
EPI_ISL_3762496, EPI_ISL_3762830	Raja Permaisuri Bainun Hospital	Institute for Medical Research, Infectious Disease Research Centre, National Institutes of Health, Ministry of Health Malaysia	Anasir MI; Azizan MA; Kamel K; Mohd Zawawi Z; Ramly N; Robert F; Suppiah J; Thayan R
EPI_ISL_2812026, EPI_ISL_2812027, EPI_ISL_2812028	Rembau Health Clinic	Institute for Medical Research, Infectious Disease Research Centre, National Institutes of Health, Ministry of Health Malaysia	Azizan MA; Kamel K; Mohd Zawawi Z; Ramly N; Robert F; Suppiah J; Thayan R
EPI_ISL_4236988	SWAT TEAM, HPSF	UKM Medical Molecular Biology Institute (UMBI)	Mira Farzana binti Mohamad Mokhtar
EPI_ISL_4347923, EPI_ISL_4347983, EPI_ISL_4348047	Sabah State Health Department	Institute for Medical Research, Infectious Disease Research Centre, National Institutes of Health, Ministry of Health Malaysia	Ahmad FA; Ahmad Fazilah NA; Anasir MI; Azizan MA; Kamel K; Mohd Zawawi Z; Norhisyam SN; Ramly N; Robert F; Suppiah J; Thayan R
EPI_ISL_4638327	Salak Health Clinic	Institute for Medical Research, Infectious Disease Research Centre, National Institutes of Health, Ministry of Health Malaysia	Ahmad FA; Ahmad Fazilah NA; Anasir MI; Azizan MA; Kamel K; Mohd Zawawi Z; Norhisyam SN; Ramly N; Robert F; Suppiah J; Thayan R
EPI_ISL_936495, EPI_ISL_2614745	Sarawak General Hospital	Institute for Medical Research, Infectious Disease Research Centre, National Institutes of Health, Ministry of Health Malaysia	Azizan MA; Kamel K; Mohd Zawawi Z; Ramly N; Robert F; Suppiah J; Thayan R
EPI_ISL_4051662, EPI_ISL_4051663, EPI_ISL_4051677, EPI_ISL_4051730, EPI_ISL_4051731, EPI_ISL_4051732, EPI_ISL_4051733, EPI_ISL_4051734, EPI_ISL_4051735, EPI_ISL_4051736, EPI_ISL_4051739, EPI_ISL_4051740, EPI_ISL_4051741, EPI_ISL_4051742, EPI_ISL_4051743, EPI_ISL_4051744, EPI_ISL_4051745, EPI_ISL_4051746, EPI_ISL_4051747, EPI_ISL_4051749, EPI_ISL_4051750, EPI_ISL_4051751, EPI_ISL_4051752, EPI_ISL_4051753, EPI_ISL_4051754, EPI_ISL_4051755, EPI_ISL_4051756, EPI_ISL_4051757, EPI_ISL_4051758, EPI_ISL_4051759, EPI_ISL_4051760, EPI_ISL_4051761, EPI_ISL_4051762, EPI_ISL_4051763, EPI_ISL_4051830, EPI_ISL_4051842, EPI_ISL_4051849			
see above	Sarawak Heart Centre (SHC)	Institute of Health and Community Medicine	Chan Chia Jui; Chua Hock Hin; David Perera; Ooi Mong How; Tonnii Sia Loong Loong; Wong Jyn Shan; Wong Kiing Aik
EPI_ISL_4460388, EPI_ISL_4460389, EPI_ISL_4460390, EPI_ISL_4460391, EPI_ISL_4460392	Sarawak Heart Centre (SHC), Kota Samarahan	Institute of Health and Community Medicine	Chan Chia Jui; Chua Hock Hin; David Perera; Ooi Mong How; Tonnii Sia Loong Loong; Wong Jyn Shan
EPI_ISL_3762831, EPI_ISL_3762832	Seberang Perai Tengah District Health Office	Institute for Medical Research, Infectious Disease Research Centre, National Institutes of Health, Ministry of Health Malaysia	Anasir MI; Azizan MA; Kamel K; Mohd Zawawi Z; Ramly N; Robert F; Suppiah J; Thayan R
EPI_ISL_2649995	Segamat Hospital	Institute for Medical Research, Infectious Disease Research Centre, National Institutes of Health, Ministry of Health Malaysia	Azizan MA; Kamel K; Mohd Zawawi Z; Ramly N; Robert F; Suppiah J; Thayan R
EPI_ISL_3071859, EPI_ISL_3120111, EPI_ISL_4096802, EPI_ISL_4348303	Selayang Hospital	Institute for Medical Research, Infectious Disease Research Centre, National Institutes of Health, Ministry of Health Malaysia	Ahmad FA; Ahmad Fazilah NA; Anasir MI; Azizan MA; Kamel K; Mohd Zawawi Z; Norhisham SN; Norhisyam SN; Ramly N; Robert F; Suppiah J; Thayan R
EPI_ISL_2815376, EPI_ISL_3333235, EPI_ISL_3356282, EPI_ISL_3356283, EPI_ISL_3356352, EPI_ISL_4101567, EPI_ISL_4348277, EPI_ISL_4348304			
see above	Serdang Hospital	Institute for Medical Research, Infectious Disease Research Centre, National Institutes of Health, Ministry of Health Malaysia	Ahmad FA; Ahmad Fazilah NA; Anasir MI; Azizan MA; Kamel K; Mohd Zawawi Z; Norhisham SN; Norhisyam SN; Ramly N; Robert F; Suppiah J; Thayan R
EPI_ISL_3050258, EPI_ISL_3278292	Shah Alam Hospital	Institute for Medical Research, Infectious Disease Research Centre, National Institutes of Health, Ministry of Health Malaysia	Anasir MI; Azizan MA; Kamel K; Mohd Zawawi Z; Ramly N; Robert F; Suppiah J; Thayan R

EPI_ISL_3723540	Sibu Hospital	Institute for Medical Research, Infectious Disease Research Centre, National Institutes of Health, Ministry of Health Malaysia	Anasir MI; Azizan MA; Kamel K; Mohd Zawawi Z; Ramly N; Robert F; Suppiah J; Thayan R
EPI_ISL_3446703	Sijangkang Health Clinic	Institute for Medical Research, Infectious Disease Research Centre, National Institutes of Health, Ministry of Health Malaysia	Anasir MI; Azizan MA; Kamel K; Mohd Zawawi Z; Ramly N; Robert F; Suppiah J; Thayan R
EPI_ISL_4101551, EPI_ISL_4101552, EPI_ISL_4101554, EPI_ISL_4101556, EPI_ISL_4101557, EPI_ISL_4101559	Sipitang District Health Office	Institute for Medical Research, Infectious Disease Research Centre, National Institutes of Health, Ministry of Health Malaysia	Ahmad FA; Ahmad Fazilah NA; Anasir MI; Azizan MA; Kamel K; Mohd Zawawi Z; Norhisham SN; Ramly N; Robert F; Suppiah J; Thayan R
EPI_ISL_3333563	Slim River Hospital	Institute for Medical Research, Infectious Disease Research Centre, National Institutes of Health, Ministry of Health Malaysia	Anasir MI; Azizan MA; Kamel K; Mohd Zawawi Z; Ramly N; Robert F; Suppiah J; Thayan R
EPI_ISL_3120116	Sultan Abdul Halim Hospital	Institute for Medical Research, Infectious Disease Research Centre, National Institutes of Health, Ministry of Health Malaysia	Anasir MI; Azizan MA; Kamel K; Mohd Zawawi Z; Ramly N; Robert F; Suppiah J; Thayan R
EPI_ISL_2828708	Sultan Ahmad Shah Medical Centre @IIUM(SASMEC@IIUM)	Centre for Research in Advanced Tropical Bioscience (Biotropic Centre)	Hajar Fauzan Bin Ahmad
EPI_ISL_2812706, EPI_ISL_2921612, EPI_ISL_2924137, EPI_ISL_2924139	Sultan Haji Ahmad Shah Hospital	Institute for Medical Research, Infectious Disease Research Centre, National Institutes of Health, Ministry of Health Malaysia	Anasir MI; Azizan MA; Kamel K; Mohd Zawawi Z; Ramly N; Robert F; Suppiah J; Thayan R
EPI_ISL_2649997, EPI_ISL_2854107, EPI_ISL_3762737	Sultan Ismail Hospital	Institute for Medical Research, Infectious Disease Research Centre, National Institutes of Health, Ministry of Health Malaysia	Anasir MI; Azizan MA; Kamel K; Mohd Zawawi Z; Ramly N; Robert F; Suppiah J; Thayan R
EPI_ISL_2650028	Sultanah Aminah Hospital	Institute for Medical Research, Infectious Disease Research Centre, National Institutes of Health, Ministry of Health Malaysia	Azizan MA; Kamel K; Mohd Zawawi Z; Ramly N; Robert F; Suppiah J; Thayan R
EPI_ISL_2091024, EPI_ISL_2625637	Sultanah Aminah Hospital, Johor Bahru	Institute for Medical Research, Infectious Disease Research Centre, National Institutes of Health, Ministry of Health Malaysia	Azizan MA; Kamel K; Mohd Zawawi Z; Ramly N; Robert F; Suppiah J; Thayan R
EPI_ISL_2650014, EPI_ISL_2650015, EPI_ISL_3278301, EPI_ISL_3278302, EPI_ISL_4348321, EPI_ISL_4348322, EPI_ISL_4348323, EPI_ISL_4348325			
see above	Sultanah Bahiyah Hospital	Institute for Medical Research, Infectious Disease Research Centre, National Institutes of Health, Ministry of Health Malaysia	Ahmad FA; Ahmad Fazilah NA; Anasir MI; Azizan MA; Kamel K; Mohd Zawawi Z; Norhisyam SN; Ramly N; Robert F; Suppiah J; Thayan R
EPI_ISL_1972365, EPI_ISL_1972366, EPI_ISL_1972533, EPI_ISL_2614739, EPI_ISL_2683247, EPI_ISL_2683496, EPI_ISL_2684236, EPI_ISL_2811885, EPI_ISL_2812654, EPI_ISL_2812704, EPI_ISL_2815656, EPI_ISL_2815971, EPI_ISL_3100982, EPI_ISL_3120143, EPI_ISL_3120314, EPI_ISL_3333237, EPI_ISL_3333656, EPI_ISL_3333657, EPI_ISL_3333929, EPI_ISL_3333930, EPI_ISL_3333931, EPI_ISL_3374484			
see above	Sultanah Bahiyah Hospital, Alor Setar	Institute for Medical Research, Infectious Disease Research Centre, National Institutes of Health, Ministry of Health Malaysia	Anasir MI; Azizan MA; Kamel K; Mohd Zawawi Z; Ramly N; Robert F; Suppiah J; Thayan R
EPI_ISL_2931920, EPI_ISL_2931923	Sultanah Fatimah Specialist Hospital, Muar	Institute for Medical Research, Infectious Disease Research Centre, National Institutes of Health, Ministry of Health Malaysia	Anasir MI; Azizan MA; Kamel K; Mohd Zawawi Z; Ramly N; Robert F; Suppiah J; Thayan R
EPI_ISL_2811878, EPI_ISL_2811879, EPI_ISL_2811880, EPI_ISL_2811881, EPI_ISL_2811882, EPI_ISL_2811883	Sultanah Nora Ismail Hospital, Batu Pahat	Institute for Medical Research, Infectious Disease Research Centre, National Institutes of Health, Ministry of Health Malaysia	Azizan MA; Kamel K; Mohd Zawawi Z; Ramly N; Robert F; Suppiah J; Thayan R
EPI_ISL_2650000, EPI_ISL_3278291	Sultanah Nur Zahirah Hospital	Institute for Medical Research, Infectious Disease Research Centre, National Institutes of Health, Ministry of Health Malaysia	Anasir MI; Azizan MA; Kamel K; Mohd Zawawi Z; Ramly N; Robert F; Suppiah J; Thayan R
EPI_ISL_1972356, EPI_ISL_2091020, EPI_ISL_2614743, EPI_ISL_2625623, EPI_ISL_2650004, EPI_ISL_2650005, EPI_ISL_2650010, EPI_ISL_2683956, EPI_ISL_3120237, EPI_ISL_3425706, EPI_ISL_3425707, EPI_ISL_3425789, EPI_ISL_4096796, EPI_ISL_4096797, EPI_ISL_4096798, EPI_ISL_4096799, EPI_ISL_4348278, EPI_ISL_4348279, EPI_ISL_4348301			
see above	Sungai Buloh Hospital	Institute for Medical Research, Infectious Disease Research Centre, National Institutes of Health, Ministry of Health Malaysia	Ahmad FA; Ahmad Fazilah NA; Anasir MI; Azizan MA; Kamel K; Mohd Zawawi Z; Norhisham SN; Norhisyam SN; Ramly N; Robert F; Suppiah J; Thayan R
EPI_ISL_3762819	Tampin District Health	Institute for Medical Research, Infectious Disease Research Centre, National Institutes of Health, Ministry of Health Malaysia	Anasir MI; Azizan MA; Kamel K; Mohd Zawawi Z; Ramly N; Robert F; Suppiah J; Thayan R
EPI_ISL_3762738, EPI_ISL_3762758, EPI_ISL_3762820, EPI_ISL_3762821	Tampin District Health Office	Institute for Medical Research, Infectious Disease Research Centre, National Institutes of Health, Ministry of Health Malaysia	Anasir MI; Azizan MA; Kamel K; Mohd Zawawi Z; Ramly N; Robert F; Suppiah J; Thayan R
EPI_ISL_1972538, EPI_ISL_1972539, EPI_ISL_1972540, EPI_ISL_1972547	Tampin Hospital	Institute for Medical Research, Infectious Disease Research Centre, National Institutes of Health, Ministry of Health Malaysia	Azizan MA; Kamel K; Mohd Zawawi Z; Ramly N; Robert F; Suppiah J; Thayan R
EPI_ISL_3446704, EPI_ISL_3446706	Tanjung Sepat Health Clinic	Institute for Medical Research, Infectious Disease Research Centre, National Institutes of Health, Ministry of Health Malaysia	Anasir MI; Azizan MA; Kamel K; Mohd Zawawi Z; Ramly N; Robert F; Suppiah J; Thayan R
EPI_ISL_3762833, EPI_ISL_3762834, EPI_ISL_3762836, EPI_ISL_4346360, EPI_ISL_4346611, EPI_ISL_4347144, EPI_ISL_4348149			
see above	Tawau Area Health Office	Institute for Medical Research, Infectious Disease Research Centre, National Institutes of Health, Ministry of Health Malaysia	Ahmad FA; Ahmad Fazilah NA; Anasir MI; Azizan MA; Kamel K; Mohd Zawawi Z; Norhisyam SN; Ramly N; Robert F; Suppiah J; Thayan R
EPI_ISL_1919862, EPI_ISL_1919863, EPI_ISL_2379749, EPI_ISL_2650506, EPI_ISL_2854070, EPI_ISL_2854103, EPI_ISL_2854104, EPI_ISL_2854105, EPI_ISL_2854108, EPI_ISL_2854109, EPI_ISL_2854174, EPI_ISL_2854186, EPI_ISL_4348273, EPI_ISL_4348274			
see above	Tawau Hospital	Institute for Medical Research, Infectious Disease Research Centre, National Institutes of Health, Ministry of Health Malaysia	40170 Selangor; Ahmad FA; Ahmad Fazilah NA; Anasir MI; Azizan MA; Bandar Setia Alam; Jalan Setia Murni U13/52; Kamel K; Malaysia; Mohd Zawawi Z; Norhisyam SN; Ramly N; Robert F; Suppiah J; Thayan R
EPI_ISL_3446716	Telok Panglima Garang Health Clinic	Institute for Medical Research, Infectious Disease Research Centre, National Institutes of Health, Ministry of Health Malaysia	Anasir MI; Azizan MA; Kamel K; Mohd Zawawi Z; Ramly N; Robert F; Suppiah J; Thayan R
EPI_ISL_3446709, EPI_ISL_3446712	Teluk Datok Health Clinic	Institute for Medical Research, Infectious Disease Research Centre, National Institutes of Health, Ministry of Health Malaysia	Anasir MI; Azizan MA; Kamel K; Mohd Zawawi Z; Ramly N; Robert F; Suppiah J; Thayan R
EPI_ISL_3446714	Teluk Panglima Garang Health Clinic	Institute for Medical Research, Infectious Disease Research Centre, National Institutes of Health, Ministry of Health Malaysia	Anasir MI; Azizan MA; Kamel K; Mohd Zawawi Z; Ramly N; Robert F; Suppiah J; Thayan R
EPI_ISL_3100126, EPI_ISL_3100172, EPI_ISL_3100173, EPI_ISL_3100174	Tengku Ampuan Afzan Hospital	Institute for Medical Research, Infectious Disease Research Centre, National Institutes of Health, Ministry of Health Malaysia	Anasir MI; Azizan MA; Kamel K; Mohd Zawawi Z; Ramly N; Robert F; Suppiah J; Thayan R
EPI_ISL_2811959	Tengku Ampuan Afzan Hospital (HTAA)	Institute for Medical Research, Infectious Disease Research Centre, National Institutes of Health, Ministry of Health Malaysia	Azizan MA; Kamel K; Mohd Zawawi Z; Ramly N; Robert F; Suppiah J; Thayan R
EPI_ISL_2921997, EPI_ISL_3071857, EPI_ISL_3071858, EPI_ISL_3120114, EPI_ISL_3120115, EPI_ISL_3120138, EPI_ISL_3355519, EPI_ISL_3446659, EPI_ISL_3446660, EPI_ISL_3446661, EPI_ISL_4636748			
see above	Tengku Ampuan Rahimah Hospital	Institute for Medical Research, Infectious Disease Research Centre, National Institutes of Health, Ministry of Health Malaysia	Ahmad FA; Ahmad Fazilah NA; Anasir MI; Azizan MA; Kamel K; Mohd Zawawi Z; Norhisyam SN; Ramly N; Robert F; Suppiah J; Thayan R
EPI_ISL_4096811, EPI_ISL_4101549, EPI_ISL_4101550	Tenom District Health Office	Institute for Medical Research, Infectious Disease Research Centre, National Institutes of Health, Ministry of Health Malaysia	Ahmad FA; Ahmad Fazilah NA; Anasir MI; Azizan MA; Kamel K; Mohd Zawawi Z; Norhisham SN; Ramly N; Robert F; Suppiah J; Thayan R
EPI_ISL_2921260, EPI_ISL_2921261	Terengganu State Health Department	Institute for Medical Research, Infectious Disease Research Centre, National Institutes of Health, Ministry of Health Malaysia	Anasir MI; Azizan MA; Kamel K; Mohd Zawawi Z; Ramly N; Robert F; Suppiah J; Thayan R
EPI_ISL_4347590, EPI_ISL_4348365	Thomson Hospital Kota Damansara	Institute for Medical Research, Infectious Disease Research Centre, National Institutes of Health, Ministry of Health Malaysia	Ahmad FA; Ahmad Fazilah NA; Anasir MI; Azizan MA; Kamel K; Mohd Zawawi Z; Norhisyam SN; Ramly N; Robert F; Suppiah J; Thayan R
EPI_ISL_4601561, EPI_ISL_4601564	Thomson Hospital Kota Damansara	Malaysia Genome Institute	Azrin Ahmad; Enizza Kasim; Irni Suhayu Sapian; Mohd Faizal Abu Bakar; Mohd Ghows Mohd Azzam.; Mohd Noor Mat Isa; Nor Azfa Johari; Nurhezreen Md Iqbal; Shamsidar Sopie; Siti Noraini Othman; Yusuf Muhammad Noor
EPI_ISL_3429606	Tropical Infectious Diseases Research and Education Centre (TIDREC), Universiti Malaya, Kuala Lumpur, Malaysia	Tropical Infectious Diseases Research and Education Centre (TIDREC), Universiti Malaya, Kuala Lumpur, Malaysia	AsmaAnati CheMatSeri; Boon-Teong Teoh; Che-Norainon Yaacob; Chee-Sieng Khor; Jefree Johari; Jia-Yi Tan; Jo-Ern Wong; Juraina Abd-Jamil; Kim-Kee Tan; Mulya-Mustika-Sari Zulkifli; Noor Syahida Azizan; Nur-Hidayana Mahfodz; Sazaly AbuBakar; Siti-Sarah Nor'e; Vunjia Tiong
EPI_ISL_2614744, EPI_ISL_2649992	Tuanku Ampuan Najihah Hospital, Kuala Pilah	Institute for Medical Research, Infectious Disease Research Centre, National Institutes of Health, Ministry of Health Malaysia	Azizan MA; Kamel K; Mohd Zawawi Z; Ramly N; Robert F; Suppiah J; Thayan R
EPI_ISL_2649994	Tuanku Fauziah Hopsital	Institute for Medical Research, Infectious Disease Research Centre, National Institutes of Health, Ministry of Health Malaysia	Azizan MA; Kamel K; Mohd Zawawi Z; Ramly N; Robert F; Suppiah J; Thayan R
EPI_ISL_2931922, EPI_ISL_3333562	Tuanku Fauziah Hospital	Institute for Medical Research, Infectious Disease Research Centre, National Institutes of Health, Ministry of Health Malaysia	Anasir MI; Azizan MA; Kamel K; Mohd Zawawi Z; Ramly N; Robert F; Suppiah J; Thayan R
EPI_ISL_2650006, EPI_ISL_2811872	Tumpat Hospital	Institute for Medical Research, Infectious Disease Research Centre, National Institutes of Health, Ministry of Health Malaysia	Azizan MA; Kamel K; Mohd Zawawi Z; Ramly N; Robert F; Suppiah J; Thayan R
EPI_ISL_1068996, EPI_ISL_1068997, EPI_ISL_1068998, EPI_ISL_1068999, EPI_ISL_1069076, EPI_ISL_1069078	UCSI University	Institute for Medical Research, Infectious Disease Research Centre, National Institutes of Health, Ministry of Health Malaysia	Azizan MA; Kamel K; Sekaran SD; Suppiah J; Thayan R
EPI_ISL_3769374, EPI_ISL_3769376	UKA	iPROMISE, UiTM	Ariza Adnan; Fadzilah Mohd Nor; Lim Wai Feng; Mohd Asif Mohd Sukri; Mohd Nur Fakhruzzaman Noorizhab; Mohd Zaki Salleh; Sazzli Shahlan Kassim; Siti Farah Alwani Mohd Nawi; Siti Hamimah Sheikh Abdul Kadir; Teh Lay Kek; Wang Seok Mui
EPI_ISL_3858148	UKA HPSF	UKM Medical Molecular Biology Institute (UMBI)	Mira Farzana binti Mohamad Mokhtar
EPI_ISL_4236990	UKA HPSF MUAR	UKM Medical Molecular Biology Institute (UMBI)	Mira Farzana binti Mohamad Mokhtar
EPI_ISL_4236985, EPI_ISL_4236992	UKKP,HOSPITAL PERMAI	UKM Medical Molecular Biology Institute (UMBI)	Mira Farzana binti Mohamad Mokhtar

EPI_ISL_4435522	UNIT FORENSIK SUNGAI BAKAP	iPROMISE, UiTM	Ariza Adnan; Fadzilah Mohd Nor; Lim Wai Feng; Mohd Asif Mohd Sukri; Mohd Nur Fakhruzzaman Noorizhab; Mohd Zaki Salleh; Sazzli Shahlan Kassim; Siti Farah Alwani Mohd Nawi; Siti Hamimah Sheikh Abdul Kadir; Teh Lay Kek; Wang Seok Mui
EPI_ISL_3980698	UNIT KESELAMATAN DAN KESIHATAN HOSPITAL SULTANAH BAHIYAH	Tropical Infectious Diseases Research & Education Centre (TIDREC), Universiti Malaya	AsmaAnati CheMatSeri; Che-Norainon Yaacob; Jia-Yi Tan; Jo-Ern Wong; Kim-Kee Tan; Mulya-Mustika-Sari Zulkifli; Noor Syahida Azizan; Nur-Hidayana Mahfodz; Sazaly AbuBakar; Siti-Sarah Nor'e
EPI_ISL_3980712	UNIT KESELAMATAN DAN KESIHATAN PEKERJA	Tropical Infectious Diseases Research & Education Centre (TIDREC), Universiti Malaya	AsmaAnati CheMatSeri; Che-Norainon Yaacob; Jia-Yi Tan; Jo-Ern Wong; Kim-Kee Tan; Mulya-Mustika-Sari Zulkifli; Noor Syahida Azizan; Nur-Hidayana Mahfodz; Sazaly AbuBakar; Siti-Sarah Nor'e
EPI_ISL_3980696, EPI_ISL_3980697	UNIT KESELAMATAN DAN KESIHATAN PEKERJA HOSPITAL SULTANAH BAHIYAH	Tropical Infectious Diseases Research & Education Centre (TIDREC), Universiti Malaya	AsmaAnati CheMatSeri; Che-Norainon Yaacob; Jia-Yi Tan; Jo-Ern Wong; Kim-Kee Tan; Mulya-Mustika-Sari Zulkifli; Noor Syahida Azizan; Nur-Hidayana Mahfodz; Sazaly AbuBakar; Siti-Sarah Nor'e
EPI_ISL_4236986	UNIT KESIHATAN AWAM	UKM Medical Molecular Biology Institute (UMBI)	Mira Farzana binti Mohamad Mokhtar
EPI_ISL_3980730	UNIT KESIHATAN AWAM (UKA), HRPZ II	Tropical Infectious Diseases Research & Education Centre (TIDREC), Universiti Malaya	AsmaAnati CheMatSeri; Che-Norainon Yaacob; Jia-Yi Tan; Jo-Ern Wong; Kim-Kee Tan; Mulya-Mustika-Sari Zulkifli; Noor Syahida Azizan; Nur-Hidayana Mahfodz; Sazaly AbuBakar; Siti-Sarah Nor'e
EPI_ISL_3980732	UNIT KESIHATAN AWAM , HRPZII	Tropical Infectious Diseases Research & Education Centre (TIDREC), Universiti Malaya	AsmaAnati CheMatSeri; Che-Norainon Yaacob; Jia-Yi Tan; Jo-Ern Wong; Kim-Kee Tan; Mulya-Mustika-Sari Zulkifli; Noor Syahida Azizan; Nur-Hidayana Mahfodz; Sazaly AbuBakar; Siti-Sarah Nor'e
EPI_ISL_3980733	UNIT KESIHATAN AWAM ,UKA . HRPZ	Tropical Infectious Diseases Research & Education Centre (TIDREC), Universiti Malaya	AsmaAnati CheMatSeri; Che-Norainon Yaacob; Jia-Yi Tan; Jo-Ern Wong; Kim-Kee Tan; Mulya-Mustika-Sari Zulkifli; Noor Syahida Azizan; Nur-Hidayana Mahfodz; Sazaly AbuBakar; Siti-Sarah Nor'e
EPI_ISL_3858183, EPI_ISL_4071988, EPI_ISL_4071996, EPI_ISL_4101594, EPI_ISL_4236989, EPI_ISL_4236991, EPI_ISL_4236994, EPI_ISL_4513500, EPI_ISL_4513501, EPI_ISL_4513502, EPI_ISL_4513503, EPI_ISL_4513505, EPI_ISL_4513509			
see above	UNIT KESIHATAN AWAM HEBHK	UKM Medical Molecular Biology Institute (UMBI)	Mira Farzana binti Mohamad Mokhtar; Nur Alyaa Afifah Md Shahri
EPI_ISL_3980735	UNIT KESIHATAN AWAM HRPZII	Tropical Infectious Diseases Research & Education Centre (TIDREC), Universiti Malaya	AsmaAnati CheMatSeri; Che-Norainon Yaacob; Jia-Yi Tan; Jo-Ern Wong; Kim-Kee Tan; Mulya-Mustika-Sari Zulkifli; Noor Syahida Azizan; Nur-Hidayana Mahfodz; Sazaly AbuBakar; Siti-Sarah Nor'e
EPI_ISL_4513504	UNIT KESIHATAN AWAM UKA	UKM Medical Molecular Biology Institute (UMBI)	Mira Farzana binti Mohamad Mokhtar
EPI_ISL_3980734	UNIT KESIHATAN AWAM(UKA) HRPZ	Tropical Infectious Diseases Research & Education Centre (TIDREC), Universiti Malaya	AsmaAnati CheMatSeri; Che-Norainon Yaacob; Jia-Yi Tan; Jo-Ern Wong; Kim-Kee Tan; Mulya-Mustika-Sari Zulkifli; Noor Syahida Azizan; Nur-Hidayana Mahfodz; Sazaly AbuBakar; Siti-Sarah Nor'e
EPI_ISL_3980702	UNIT KESIHATAN DAN KESELAMATA PEKERJA HOSPITAL SULTANAH BAHIYAH	Tropical Infectious Diseases Research & Education Centre (TIDREC), Universiti Malaya	AsmaAnati CheMatSeri; Che-Norainon Yaacob; Jia-Yi Tan; Jo-Ern Wong; Kim-Kee Tan; Mulya-Mustika-Sari Zulkifli; Noor Syahida Azizan; Nur-Hidayana Mahfodz; Sazaly AbuBakar; Siti-Sarah Nor'e
EPI_ISL_3980701, EPI_ISL_3980703, EPI_ISL_3980706	UNIT KESIHATAN DAN KESELAMATAN HOSPITAL SULTANAH BAHIYAH	Tropical Infectious Diseases Research & Education Centre (TIDREC), Universiti Malaya	AsmaAnati CheMatSeri; Che-Norainon Yaacob; Jia-Yi Tan; Jo-Ern Wong; Kim-Kee Tan; Mulya-Mustika-Sari Zulkifli; Noor Syahida Azizan; Nur-Hidayana Mahfodz; Sazaly AbuBakar; Siti-Sarah Nor'e
EPI_ISL_3980700, EPI_ISL_3980704, EPI_ISL_3980705, EPI_ISL_3980707	UNIT KESIHATAN DAN KESELAMATAN PEKERJA HOSPITAL SULTANAH BAHIYAH	Tropical Infectious Diseases Research & Education Centre (TIDREC), Universiti Malaya	AsmaAnati CheMatSeri; Che-Norainon Yaacob; Jia-Yi Tan; Jo-Ern Wong; Kim-Kee Tan; Mulya-Mustika-Sari Zulkifli; Noor Syahida Azizan; Nur-Hidayana Mahfodz; Sazaly AbuBakar; Siti-Sarah Nor'e
EPI_ISL_3980699	UNIT KESIHATAN DAN KESIHATAN PEKERJA HOSPITAL SULTANAH BAHIYAH	Tropical Infectious Diseases Research & Education Centre (TIDREC), Universiti Malaya	AsmaAnati CheMatSeri; Che-Norainon Yaacob; Jia-Yi Tan; Jo-Ern Wong; Kim-Kee Tan; Mulya-Mustika-Sari Zulkifli; Noor Syahida Azizan; Nur-Hidayana Mahfodz; Sazaly AbuBakar; Siti-Sarah Nor'e
EPI_ISL_3858141, EPI_ISL_3858150, EPI_ISL_3858152, EPI_ISL_3858157, EPI_ISL_3858161, EPI_ISL_3858165, EPI_ISL_3858167, EPI_ISL_3858176, EPI_ISL_3858180, EPI_ISL_3858182, EPI_ISL_4101585, EPI_ISL_4463179, EPI_ISL_4463180, EPI_ISL_4463185, EPI_ISL_4463186, EPI_ISL_4463187, EPI_ISL_4463188, EPI_ISL_4513499			
see above	UNIT KPAS, PKD MELAKA TENGAH	UKM Medical Molecular Biology Institute (UMBI)	Mira Farzana binti Mohamad Mokhtar; Nur Alyaa Afifah Md Shahri
EPI_ISL_4122502	UNIT MIKROBIOLOGI, JABATAN PATOLOGI, HOSPITAL PULAU PINANG	iPROMISE, UiTM	Ariza Adnan; Fadzilah Mohd Nor; Lim Wai Feng; Mohd Asif Mohd Sukri; Mohd Nur Fakhruzzaman Noorizhab; Mohd Zaki Salleh; Sazzli Shahlan Kassim; Siti Farah Alwani Mohd Nawi; Siti Hamimah Sheikh Abdul Kadir; Teh Lay Kek; Wang Seok Mui
EPI_ISL_3980736, EPI_ISL_3980738, EPI_ISL_3980739, EPI_ISL_4565018, EPI_ISL_4565019, EPI_ISL_4565020, EPI_ISL_4565021, EPI_ISL_4565022, EPI_ISL_4565024, EPI_ISL_4565026, EPI_ISL_4565028			
see above	UNIT OSH, HOSPITAL SUNGAI BULOH	Tropical Infectious Diseases Research & Education Centre (TIDREC), Universiti Malaya	AsmaAnati CheMatSeri; Che-Norainon Yaacob; Jia-Yi Tan; Jo-Ern Wong; Kim-Kee Tan; Mulya-Mustika-Sari Zulkifli; Noor Syahida Azizan; Nur-Hidayana Mahfodz; Sazaly AbuBakar; Siti-Sarah Nor'e
EPI_ISL_3769375, EPI_ISL_3769377, EPI_ISL_3769379, EPI_ISL_3769380, EPI_ISL_3769381, EPI_ISL_3769382	UNIT OSH, HOSPITAL SUNGAI BULOH	iPROMISE, UiTM	Ariza Adnan; Fadzilah Mohd Nor; Lim Wai Feng; Mohd Asif Mohd Sukri; Mohd Nur Fakhruzzaman Noorizhab; Mohd Zaki Salleh; Sazzli Shahlan Kassim; Siti Farah Alwani Mohd Nawi; Siti Hamimah Sheikh Abdul Kadir; Teh Lay Kek; Wang Seok Mui
EPI_ISL_3374426	UiTM Sungai Buloh Hospital	Institute for Medical Research, Infectious Disease Research Centre, National Institutes of Health, Ministry of Health Malaysia	Anasir MI; Azizan MA; Kamel K; Mohd Zawawi Z; Ramly N; Robert F; Suppiah J; Thayan R
EPI_ISL_3980740	Uka HBTG	Tropical Infectious Diseases Research & Education Centre (TIDREC), Universiti Malaya	AsmaAnati CheMatSeri; Che-Norainon Yaacob; Jia-Yi Tan; Jo-Ern Wong; Kim-Kee Tan; Mulya-Mustika-Sari Zulkifli; Noor Syahida Azizan; Nur-Hidayana Mahfodz; Sazaly AbuBakar; Siti-Sarah Nor'e
EPI_ISL_3980742	Uka Hosp.Banting	Tropical Infectious Diseases Research & Education Centre (TIDREC), Universiti Malaya	AsmaAnati CheMatSeri; Che-Norainon Yaacob; Jia-Yi Tan; Jo-Ern Wong; Kim-Kee Tan; Mulya-Mustika-Sari Zulkifli; Noor Syahida Azizan; Nur-Hidayana Mahfodz; Sazaly AbuBakar; Siti-Sarah Nor'e
EPI_ISL_3980737, EPI_ISL_3980746	Uka Hospital Banting	Tropical Infectious Diseases Research & Education Centre (TIDREC), Universiti Malaya	AsmaAnati CheMatSeri; Che-Norainon Yaacob; Jia-Yi Tan; Jo-Ern Wong; Kim-Kee Tan; Mulya-Mustika-Sari Zulkifli; Noor Syahida Azizan; Nur-Hidayana Mahfodz; Sazaly AbuBakar; Siti-Sarah Nor'e
EPI_ISL_3980744	Uka Hospital Bnating	Tropical Infectious Diseases Research & Education Centre (TIDREC), Universiti Malaya	AsmaAnati CheMatSeri; Che-Norainon Yaacob; Jia-Yi Tan; Jo-Ern Wong; Kim-Kee Tan; Mulya-Mustika-Sari Zulkifli; Noor Syahida Azizan; Nur-Hidayana Mahfodz; Sazaly AbuBakar; Siti-Sarah Nor'e
EPI_ISL_4122458, EPI_ISL_4435551	Unit Kawalan Penyakit Berjangkit, Jabatan Kesihatan Negeri Perlis	iPROMISE, UiTM	Ariza Adnan; Fadzilah Mohd Nor; Lim Wai Feng; Mohd Asif Mohd Sukri; Mohd Nur Fakhruzzaman Noorizhab; Mohd Zaki Salleh; Sazzli Shahlan Kassim; Siti Farah Alwani Mohd Nawi; Siti Hamimah Sheikh Abdul Kadir; Teh Lay Kek; Wang Seok Mui
EPI_ISL_3980719, EPI_ISL_3980721	Unit Keselamatan dan Kesihatan Pekerja	Tropical Infectious Diseases Research & Education Centre (TIDREC), Universiti Malaya	AsmaAnati CheMatSeri; Che-Norainon Yaacob; Jia-Yi Tan; Jo-Ern Wong; Kim-Kee Tan; Mulya-Mustika-Sari Zulkifli; Noor Syahida Azizan; Nur-Hidayana Mahfodz; Sazaly AbuBakar; Siti-Sarah Nor'e
EPI_ISL_4513498	Unit keselamatan dan Kesihatan Pekerjaan	UKM Medical Molecular Biology Institute (UMBI)	Mira Farzana binti Mohamad Mokhtar
EPI_ISL_4513496	Unit keselamatan dan Kesihatan Pekerjaan Hospital Melaka	UKM Medical Molecular Biology Institute (UMBI)	Mira Farzana binti Mohamad Mokhtar
EPI_ISL_2811875	Universiti Sains Malaysia Hospital	Institute for Medical Research, Infectious Disease Research Centre, National Institutes of Health, Ministry of Health Malaysia	Azizan MA; Kamel K; Mohd Zawawi Z; Ramly N; Robert F; Suppiah J; Thayan R
EPI_ISL_877229, EPI_ISL_877230, EPI_ISL_877231, EPI_ISL_877232, EPI_ISL_877233, EPI_ISL_877234, EPI_ISL_877235, EPI_ISL_877236, EPI_ISL_877329, EPI_ISL_877330			
see above	University College Sedaya International (UCSI University)	Institute for Medical Research, Infectious Disease Research Centre, National Institutes of Health, Ministry of Health Malaysia	Azizan MA; Kamel K; Sekaran SD; Suppiah J; Thayan R
EPI_ISL_3980729	klinik kesihatan perol	Tropical Infectious Diseases Research & Education Centre (TIDREC), Universiti Malaya	AsmaAnati CheMatSeri; Che-Norainon Yaacob; Jia-Yi Tan; Jo-Ern Wong; Kim-Kee Tan; Mulya-Mustika-Sari Zulkifli; Noor Syahida Azizan; Nur-Hidayana Mahfodz; Sazaly AbuBakar; Siti-Sarah Nor'e

We gratefully acknowledge the following Authors from the Originating laboratories responsible for obtaining the specimens, as well as the Submitting laboratories where the genome data were generated and shared via GISAID, on which this research is based.

All Submitters of data may be contacted directly via www.gisaid.org

Authors are sorted alphabetically.

Accession ID	Originating Laboratory	Submitting Laboratory	Authors
EPI_ISL_3538928, EPI_ISL_3538933	IPL	Institut Pasteur du Laos	; Somphavan Somlor; Vincent Lacoste
EPI_ISL_3538929, EPI_ISL_3538930, EPI_ISL_3538931, EPI_ISL_3538932, EPI_ISL_3538934, EPI_ISL_3538935, EPI_ISL_3538936, EPI_ISL_3538937, EPI_ISL_3538938, EPI_ISL_3538939, EPI_ISL_3538940, EPI_ISL_3538941, EPI_ISL_3538942, EPI_ISL_3538943, EPI_ISL_3538944, EPI_ISL_3538945			
see above	NCLE	Institut Pasteur du Laos	; Somphavan Somlor; Vincent Lacoste
EPI_ISL_4302644, EPI_ISL_4302645, EPI_ISL_4302646, EPI_ISL_4302647, EPI_ISL_4302648	The Institut Pasteur du Laos	Pathogen Discovery Laboratory, Institut Pasteur	Baquero Salazar, E.; Bigot, T.; Bonomi, M.; Brey; Chretien, D.; Donati, F.; Douangboubpha, B.; Karami, Y.; Lacoste, V.; Lakeomany, K.; Munier, S.; Nilges, M.; P.T. and Eloit, M.; Paphaphanh, P.; Perot, P.; Phommavanh, N.; Regnault, B.; Rey, F.; Sanamxay, D.; Somlor, S.; Temmam, S.; Vongphayloth, K.; Xayaphet, V.; van der Werf, S.

We gratefully acknowledge the following Authors from the Originating laboratories responsible for obtaining the specimens, as well as the Submitting laboratories where the genome data were generated and shared via GISAID, on which this research is based.

All Submitters of data may be contacted directly via www.gisaid.org

Authors are sorted alphabetically.

Accession ID	Originating Laboratory	Submitting Laboratory	Authors
EPI_ISL_480204	Akita City Public Health Center	Pathogen Genomics Center, National Institute of Infectious Diseases	Hajime Kamiya; Kentaro Itokawa; Koichi Ito; Makoto Kuroda; Masanori Hashino; Motoi Suzuki; Rina Tanaka; Tsuyoshi Sekizuka
EPI_ISL_419297, EPI_ISL_419298, EPI_ISL_419309, EPI_ISL_419310, EPI_ISL_419311, EPI_ISL_479809, EPI_ISL_479810, EPI_ISL_479811, EPI_ISL_480004, EPI_ISL_480005, EPI_ISL_480006, EPI_ISL_480007, EPI_ISL_480008, EPI_ISL_480009, EPI_ISL_480010, EPI_ISL_480011, EPI_ISL_480012, EPI_ISL_480013, EPI_ISL_480014, EPI_ISL_684159, EPI_ISL_684160, EPI_ISL_684161, EPI_ISL_686659, EPI_ISL_686660, EPI_ISL_686661, EPI_ISL_686662, EPI_ISL_686663, EPI_ISL_686664, EPI_ISL_686665, EPI_ISL_686666, EPI_ISL_686667, EPI_ISL_686668, EPI_ISL_686669, EPI_ISL_686670, EPI_ISL_686671, EPI_ISL_686672, EPI_ISL_686673, EPI_ISL_686674, EPI_ISL_686675, EPI_ISL_686676, EPI_ISL_686677, EPI_ISL_686678, EPI_ISL_686679, EPI_ISL_686680, EPI_ISL_686681, EPI_ISL_686682, EPI_ISL_686683, EPI_ISL_686684, EPI_ISL_686685, EPI_ISL_686686, EPI_ISL_686687, EPI_ISL_686688, EPI_ISL_686689, EPI_ISL_686690, EPI_ISL_686691, EPI_ISL_686692, EPI_ISL_686693, EPI_ISL_686694, EPI_ISL_686695, EPI_ISL_686696, EPI_ISL_686697, EPI_ISL_686698, EPI_ISL_686699, EPI_ISL_686700, EPI_ISL_686701, EPI_ISL_686702, EPI_ISL_686703, EPI_ISL_686704, EPI_ISL_686705, EPI_ISL_686706, EPI_ISL_686707, EPI_ISL_686708, EPI_ISL_686709, EPI_ISL_686710, EPI_ISL_686711, EPI_ISL_686712, EPI_ISL_686713, EPI_ISL_686714, EPI_ISL_686715, EPI_ISL_686716, EPI_ISL_686717, EPI_ISL_686718, EPI_ISL_686719, EPI_ISL_686720, EPI_ISL_686721, EPI_ISL_686722, EPI_ISL_686723, EPI_ISL_686724, EPI_ISL_686725, EPI_ISL_686726, EPI_ISL_686727, EPI_ISL_686728, EPI_ISL_686729, EPI_ISL_686730, EPI_ISL_686731, EPI_ISL_686732, EPI_ISL_686733, EPI_ISL_686734, EPI_ISL_686735, EPI_ISL_686736, EPI_ISL_686737, EPI_ISL_686738, EPI_ISL_686739, EPI_ISL_686740, EPI_ISL_686741, EPI_ISL_686742, EPI_ISL_686743, EPI_ISL_686744, EPI_ISL_686745, EPI_ISL_686746, EPI_ISL_686747, EPI_ISL_686748, EPI_ISL_686749, EPI_ISL_686750, EPI_ISL_686751, EPI_ISL_686752, EPI_ISL_686753, EPI_ISL_686754, EPI_ISL_686755, EPI_ISL_686756, EPI_ISL_686757, EPI_ISL_686758, EPI_ISL_686759, EPI_ISL_686760, EPI_ISL_686761, EPI_ISL_686762, EPI_ISL_686763, EPI_ISL_686764, EPI_ISL_686765, EPI_ISL_686766, EPI_ISL_686767, EPI_ISL_686768, EPI_ISL_686769, EPI_ISL_686770, EPI_ISL_686771, EPI_ISL_686772, EPI_ISL_686773, EPI_ISL_686774, EPI_ISL_686775, EPI_ISL_686776, EPI_ISL_686777, EPI_ISL_686778, EPI_ISL_686779, EPI_ISL_686780, EPI_ISL_686781, EPI_ISL_686782, EPI_ISL_686783, EPI_ISL_686784, EPI_ISL_686785, EPI_ISL_686786, EPI_ISL_686787, EPI_ISL_686788, EPI_ISL_686789, EPI_ISL_686790, EPI_ISL_686791, EPI_ISL_686792, EPI_ISL_686793, EPI_ISL_686794, EPI_ISL_686795, EPI_ISL_686796, EPI_ISL_686797, EPI_ISL_686798, EPI_ISL_686799, EPI_ISL_686800, EPI_ISL_686801, EPI_ISL_686802, EPI_ISL_686803, EPI_ISL_686804, EPI_ISL_686805, EPI_ISL_686806, EPI_ISL_686807, EPI_ISL_686808, EPI_ISL_686809, EPI_ISL_686810, EPI_ISL_686811, EPI_ISL_686812, EPI_ISL_686813, EPI_ISL_686814, EPI_ISL_686815, EPI_ISL_686816, EPI_ISL_686817, EPI_ISL_686818, EPI_ISL_686819, EPI_ISL_686820, EPI_ISL_686821, EPI_ISL_686822, EPI_ISL_686823, EPI_ISL_686824, EPI_ISL_686825, EPI_ISL_686826, EPI_ISL_686827, EPI_ISL_686828, EPI_ISL_686829, EPI_ISL_686830, EPI_ISL_686831, EPI_ISL_686832, EPI_ISL_686833, EPI_ISL_686834, EPI_ISL_686835, EPI_ISL_686836, EPI_ISL_686837, EPI_ISL_686838, EPI_ISL_686839, EPI_ISL_686840, EPI_ISL_686841, EPI_ISL_686842, EPI_ISL_686843, EPI_ISL_686844, EPI_ISL_691284, EPI_ISL_691285, EPI_ISL_691286, EPI_ISL_691287, EPI_ISL_691288			
see above	Chiba Prefectural Institute of Public Health	Pathogen Genomics Center, National Institute of Infectious Diseases	Hajime Kamiya; Kentaro Itokawa; Makoto Kuroda; Masakatsu Taira; Masanori Hashino; Motoi Suzuki; Rina Tanaka; Tsuyoshi Sekizuka; Yushi Hachisu
EPI_ISL_481251, EPI_ISL_481252, EPI_ISL_481254, EPI_ISL_481255, EPI_ISL_481257, EPI_ISL_481258, EPI_ISL_481259, EPI_ISL_481260, EPI_ISL_481261, EPI_ISL_481263			
see above	Department of Emerging Infectious Diseases, Institute of Tropical Medicine, Nagasaki University	Department of Emerging Infectious Diseases, Institute of Tropical Medicine, Nagasaki University	Haruka Abe; Jiro Yasuda; Rokusuke Yoshikawa; Yuichiro Furusato
EPI_ISL_568556, EPI_ISL_568557, EPI_ISL_568558, EPI_ISL_568559, EPI_ISL_568560, EPI_ISL_568561, EPI_ISL_568562, EPI_ISL_568563, EPI_ISL_568564, EPI_ISL_568565, EPI_ISL_568566, EPI_ISL_568567, EPI_ISL_568568, EPI_ISL_568569, EPI_ISL_568570, EPI_ISL_568571, EPI_ISL_568572, EPI_ISL_568573, EPI_ISL_568574, EPI_ISL_568575, EPI_ISL_568576, EPI_ISL_636989, EPI_ISL_636990, EPI_ISL_636991, EPI_ISL_636992, EPI_ISL_636993, EPI_ISL_636994, EPI_ISL_636995, EPI_ISL_636996, EPI_ISL_636997, EPI_ISL_636998, EPI_ISL_636999, EPI_ISL_637000, EPI_ISL_637001, EPI_ISL_637002, EPI_ISL_637003, EPI_ISL_637004, EPI_ISL_637005, EPI_ISL_637006, EPI_ISL_637007, EPI_ISL_637008, EPI_ISL_637009, EPI_ISL_637010, EPI_ISL_637011, EPI_ISL_637012, EPI_ISL_637013, EPI_ISL_637014, EPI_ISL_637015, EPI_ISL_637016, EPI_ISL_637017, EPI_ISL_637018, EPI_ISL_637019, EPI_ISL_637020, EPI_ISL_637021, EPI_ISL_637075			
see above	Department of Infectious Diseases and Immunology, National Hospital Organization Nagoya Medical Center	Clinical Research Center, National Hospital Organization Nagoya Medical Center	Hirotaka Ode; Kazuhiro Matsuoka; Mai Kubota; Masakazu Matsuda; Mayumi Imahashi; Miho Nakasuji; Mikiko Mori; Nakasuji Miho; Yasumasa Iwatani; Yoshihiro Nakata; Yoshiyuki Yokomaku
EPI_ISL_644945, EPI_ISL_644946, EPI_ISL_644947, EPI_ISL_644948, EPI_ISL_644949, EPI_ISL_644950, EPI_ISL_644951, EPI_ISL_644952, EPI_ISL_644953, EPI_ISL_644954, EPI_ISL_644955, EPI_ISL_644956, EPI_ISL_644957, EPI_ISL_644958, EPI_ISL_644959, EPI_ISL_644960, EPI_ISL_644961, EPI_ISL_644962, EPI_ISL_644963, EPI_ISL_644964, EPI_ISL_644965, EPI_ISL_644966, EPI_ISL_644967, EPI_ISL_644968, EPI_ISL_644969, EPI_ISL_644970, EPI_ISL_644971, EPI_ISL_644972, EPI_ISL_644973, EPI_ISL_644974, EPI_ISL_644975, EPI_ISL_644976, EPI_ISL_644977, EPI_ISL_644978, EPI_ISL_644979, EPI_ISL_644980, EPI_ISL_644981, EPI_ISL_644982, EPI_ISL_644983, EPI_ISL_644984, EPI_ISL_644985, EPI_ISL_644986, EPI_ISL_644987, EPI_ISL_644988, EPI_ISL_644989, EPI_ISL_644990, EPI_ISL_644991, EPI_ISL_644992, EPI_ISL_644993, EPI_ISL_644994, EPI_ISL_644995, EPI_ISL_644996, EPI_ISL_644997, EPI_ISL_644999			
see above	Department of Infectious Diseases, Keio University School of Medicine, Tokyo, Japan	Center for Medical Genetics, Keio University School of Medicine, Tokyo, Japan	Haruhiko Siomi; Hirotsugu Ishizu; Kenjiro Kosaki; Kodai Abe; Yuka Iwasaki
EPI_ISL_479821, EPI_ISL_479822, EPI_ISL_479855, EPI_ISL_479856, EPI_ISL_479857, EPI_ISL_479858, EPI_ISL_479859, EPI_ISL_479860, EPI_ISL_479861, EPI_ISL_479868, EPI_ISL_479986, EPI_ISL_479987, EPI_ISL_479988, EPI_ISL_479989, EPI_ISL_480090, EPI_ISL_480091, EPI_ISL_480092, EPI_ISL_480093, EPI_ISL_480094, EPI_ISL_480095, EPI_ISL_480096, EPI_ISL_480097, EPI_ISL_480098, EPI_ISL_480099, EPI_ISL_480100, EPI_ISL_480101, EPI_ISL_480102, EPI_ISL_480205, EPI_ISL_480206, EPI_ISL_480207, EPI_ISL_480209, EPI_ISL_480210, EPI_ISL_480211, EPI_ISL_480212, EPI_ISL_480213, EPI_ISL_480214, EPI_ISL_480215, EPI_ISL_480216, EPI_ISL_480217, EPI_ISL_480218, EPI_ISL_480219, EPI_ISL_480220			
see above	Department of Infectious Diseases, Kobe Institute of Health	Pathogen Genomics Center, National Institute of Infectious Diseases	Hajime Kamiya; Kentaro Itokawa; Makoto Kuroda; Masanori Hashino; Motoi Suzuki; Rina Tanaka; Ryohei Nomoto; Tsuyoshi Sekizuka
EPI_ISL_693298, EPI_ISL_693299	Department of Microbiology, Yokohama City University School of Medicine	Department of Microbiology, Yokohama City University School of Medicine	Akihide Ryo; Ichiro Takeuchi; Kazuya Sakai; Kei Miyakawa; Mototsugu Nishii; Reo Matsumura; Ryo Saji
EPI_ISL_434561, EPI_ISL_434562	Department of Microbiology; Ryota Kumagai Tokyo Metropolitan Institute of Public Health	Department of Microbiology; Ryota Kumagai Tokyo Metropolitan Institute of Public Health	Asakura, H.; Chiba; Kumagai, R.; Nagashima, M.; T. and Sadamasu, K.; Yoshida, I.
EPI_ISL_414511	Department of Microbiology; Ryota Kumagai Tokyo Metropolitan Institute of Public Health	Tokyo Metoropolitan Institute of Public Health	Chiba; Kumagai, R.; Nagashima, M.; T. and Sadamasu, K.; Yoshida, I.
EPI_ISL_413459	Department of Pathology, Toshima Hospital	Pathogen Genomics Center, National Institute of Infectious Diseases	Haruka Nishioka; Harutaka Katano; Ippei Miyamoto; Ja-Mun Chong; Jun Yamazaki; Kentaro Itokawa; Makoto Kuroda; Masahiro Sano; Minoru Tobiume; Noriko Nakajima; Tadaki Suzuki; Takuya Adachi; Tsuyoshi Sekizuka; Yuko Sato
EPI_ISL_407084, EPI_ISL_529135	Department of Virology III, National Institute of Infectious Diseases	Pathogen Genomics Center, National Institute of Infectious Diseases	Kazuya Shirato; Kentaro Itokawa; Makoto Kuroda; Makoto Takeda; Masanori Hashino; Naganori Nao; Rina Tanaka; Shinji Watanabe; Shutoku Matsuyama; Takaji Wakita; Tsuyoshi Sekizuka
EPI_ISL_410531, EPI_ISL_410532	Dept. of Pathology, National Institute of Infectious Diseases	Pathogen Genomics Center, National Institute of Infectious Diseases	Harutaka Katano; Hideki Hasegawa; Kazuya Shirato; Makoto Kuroda; Makoto Takeda; Motoi Suzuki; Naganori Nao; Shutoku Matsuyama; Tadaki Suzuki; Takaji Wakita; Tsuyoshi Sekizuka
EPI_ISL_402126	Dept. of Virology III, National Institute of Infectious Diseases	Dept. of Virology III, National Institute of Infectious Diseases	Kazuya Shirato; Makoto Takeda; Naganori Nao; Shutoku Matsuyama
EPI_ISL_408665, EPI_ISL_408666, EPI_ISL_408667, EPI_ISL_408669	Dept. of Virology III, National Institute of Infectious Diseases	Pathogen Genomics Center, National Institute of Infectious Diseases	Kazuya Shirato; Makoto Kuroda; Makoto Takeda; Naganori Nao; Shutoku Matsuyama; Tsuyoshi Sekizuka
EPI_ISL_707928	Fujita Health University Hospital	Fujita Health University School of Medicine, Department of Microbiology	Aki Sakurai; Masahiro Suzuki; Yohei Doi
EPI_ISL_728000	Fujita Health University Okazaki Medical Center	Department of Virology and Parasitology, Fujita Health University School of Medicin	Aki Sakurai; Masahiro suzuki; Satoshi Komoto; Takayuki Murata; Takuma Ishihara; Tomihiko Ide; Yohei Doi
EPI_ISL_718273, EPI_ISL_727998, EPI_ISL_727999, EPI_ISL_728153, EPI_ISL_728154, EPI_ISL_728155, EPI_ISL_728156, EPI_ISL_728159			
see above	Fujita Health University Okazaki Medical Center	Department of Virology and Parasitology, Fujita Health University School of Medicine	Aki Sakurai; Masahiro suzuki; Satoshi Komoto; Takayuki Murata; Takuma Ishihara; Tomihiko Ide; Yohei Doi
EPI_ISL_728206, EPI_ISL_728207, EPI_ISL_728208	Fujita Health University Okazaki Medical Center	Fujita health University, Department of Microbiology	Aki Sakurai; Masahiro suzuki; Satoshi Komoto; Takayuki Murata; Takuma Ishihara; Tomihiko Ide; Yohei Doi
EPI_ISL_479967, EPI_ISL_479968, EPI_ISL_479969, EPI_ISL_479970, EPI_ISL_479971, EPI_ISL_479972, EPI_ISL_479973, EPI_ISL_479974, EPI_ISL_479975, EPI_ISL_479976, EPI_ISL_479977, EPI_ISL_479978, EPI_ISL_480120, EPI_ISL_480121, EPI_ISL_480122, EPI_ISL_480123, EPI_ISL_480124, EPI_ISL_480125, EPI_ISL_480126, EPI_ISL_480127, EPI_ISL_480128, EPI_ISL_480129, EPI_ISL_480130, EPI_ISL_480131, EPI_ISL_480132, EPI_ISL_480133, EPI_ISL_480134, EPI_ISL_480135, EPI_ISL_480136, EPI_ISL_480137, EPI_ISL_480138, EPI_ISL_480139, EPI_ISL_480140, EPI_ISL_480141, EPI_ISL_480142, EPI_ISL_480143, EPI_ISL_480144, EPI_ISL_480145, EPI_ISL_480146, EPI_ISL_480147, EPI_ISL_480148, EPI_ISL_480149, EPI_ISL_480150, EPI_ISL_480151, EPI_ISL_480152, EPI_ISL_480153, EPI_ISL_480154, EPI_ISL_480155, EPI_ISL_480156, EPI_ISL_480157, EPI_ISL_480158, EPI_ISL_480159, EPI_ISL_480160, EPI_ISL_480161, EPI_ISL_480162, EPI_ISL_480163, EPI_ISL_480164, EPI_ISL_480165, EPI_ISL_480166, EPI_ISL_480167, EPI_ISL_480168, EPI_ISL_480225			
see above	Fukui Prefectural Institute of Public Health and Environmental Science	Pathogen Genomics Center, National Institute of Infectious Diseases	Hajime Kamiya; Kentaro Itokawa; Makoto Kuroda; Masanori Hashino; Miho Toho; Motoi Suzuki; Rina Tanaka; Tsuyoshi Sekizuka
EPI_ISL_684408, EPI_ISL_684409, EPI_ISL_684410, EPI_ISL_684411, EPI_ISL_684412, EPI_ISL_684413, EPI_ISL_684414, EPI_ISL_684415, EPI_ISL_684416, EPI_ISL_684417, EPI_ISL_684418, EPI_ISL_684419, EPI_ISL_684420, EPI_ISL_684421, EPI_ISL_684422, EPI_ISL_684423, EPI_ISL_684424, EPI_ISL_684425, EPI_ISL_684426, EPI_ISL_684427, EPI_ISL_684428, EPI_ISL_684429, EPI_ISL_684430, EPI_ISL_684431, EPI_ISL_684432, EPI_ISL_684433, EPI_ISL_684434, EPI_ISL_684435, EPI_ISL_684436, EPI_ISL_684437, EPI_ISL_684438, EPI_ISL_684439, EPI_ISL_684440, EPI_ISL_684441, EPI_ISL_684442, EPI_ISL_684443, EPI_ISL_684444, EPI_ISL_684445, EPI_ISL_684446, EPI_ISL_684447, EPI_ISL_684448, EPI_ISL_684449, EPI_ISL_684450, EPI_ISL_684451, EPI_ISL_684452, EPI_ISL_684453, EPI_ISL_684454, EPI_ISL_684455, EPI_ISL_684456, EPI_ISL_684457, EPI_ISL_684458, EPI_ISL_684459, EPI_ISL_684460, EPI_ISL_684461, EPI_ISL_684462, EPI_ISL_684463, EPI_ISL_684464, EPI_ISL_684465, EPI_ISL_684466, EPI_ISL_684467, EPI_ISL_684468, EPI_ISL_684469, EPI_ISL_684470, EPI_ISL_684471, EPI_ISL_684472, EPI_ISL_684473, EPI_ISL_684474, EPI_ISL_684475, EPI_ISL_684476, EPI_ISL_684477, EPI_ISL_684478, EPI_ISL_684479, EPI_ISL_684480, EPI_ISL_684481, EPI_ISL_684482, EPI_ISL_684483, EPI_ISL_684634, EPI_ISL_684635, EPI_ISL_684636, EPI_ISL_684637, EPI_ISL_684638, EPI_ISL_684639, EPI_ISL_684640, EPI_ISL_684641, EPI_ISL_684642, EPI_ISL_684643, EPI_ISL_684644, EPI_ISL_684645, EPI_ISL_684646, EPI_ISL_684647, EPI_ISL_684648, EPI_ISL_684649, EPI_ISL_684650, EPI_ISL_684651, EPI_ISL_684652, EPI_ISL_684653, EPI_ISL_684654, EPI_ISL_684655, EPI_ISL_684656, EPI_ISL_684657, EPI_ISL_684658, EPI_ISL_684659, EPI_ISL_684660, EPI_ISL_684661, EPI_ISL_684662, EPI_ISL_684663, EPI_ISL_684664, EPI_ISL_684665, EPI_ISL_684666, EPI_ISL_684667, EPI_ISL_684668, EPI_ISL_684669, EPI_ISL_684670, EPI_ISL_684671, EPI_ISL_684672, EPI_ISL_684673, EPI_ISL_684674, EPI_ISL_684675, EPI_ISL_684676, EPI_ISL_684677, EPI_ISL_684678, EPI_ISL_684679, EPI_ISL_684680, EPI_ISL_684681, EPI_ISL_684682, EPI_ISL_684683, EPI_ISL_684684, EPI_ISL_684685, EPI_ISL_684686, EPI_ISL_684687, EPI_ISL_684688, EPI_ISL_684689, EPI_ISL_684690, EPI_ISL_684691, EPI_ISL_684692, EPI_ISL_684723, EPI_ISL_684724, EPI_ISL_684725, EPI_ISL_684726, EPI_ISL_684727, EPI_ISL_685918, EPI_ISL_685919, EPI_ISL_685920, EPI_ISL_685921, EPI_ISL_685922, EPI_ISL_685923, EPI_ISL_685924, EPI_ISL_687533, EPI_ISL_687534, EPI_ISL_687535, EPI_ISL_687536, EPI_ISL_687537, EPI_ISL_687538, EPI_ISL_687539, EPI_ISL_687540, EPI_ISL_687541, EPI_ISL_687542, EPI_ISL_687543, EPI_ISL_687544, EPI_ISL_687545, EPI_ISL_687546, EPI_ISL_687547, EPI_ISL_687548, EPI_ISL_687549, EPI_ISL_687550, EPI_ISL_687551, EPI_ISL_687552, EPI_ISL_687553, EPI_ISL_687554, EPI_ISL_687555, EPI_ISL_687556, EPI_ISL_687557, EPI_ISL_687558, EPI_ISL_687559, EPI_ISL_687560, EPI_ISL_688612, EPI_ISL_688619, EPI_ISL_688620, EPI_ISL_688621, EPI_ISL_688622, EPI_ISL_688623, EPI_ISL_688634, EPI_ISL_688897, EPI_ISL_688898, EPI_ISL_688899, EPI_ISL_688900, EPI_ISL_688901, EPI_ISL_688902, EPI_ISL_688903, EPI_ISL_688904, EPI_ISL_688905, EPI_ISL_688906, EPI_ISL_688907, EPI_ISL_688908, EPI_ISL_688909, EPI_ISL_688910, EPI_ISL_688911, EPI_ISL_688912, EPI_ISL_688913, EPI_ISL_688914, EPI_ISL_688915, EPI_ISL_688916, EPI_ISL_688917, EPI_ISL_688918, EPI_ISL_688919, EPI_ISL_688920, EPI_ISL_688921, EPI_ISL_688922, EPI_ISL_688923, EPI_ISL_688924, EPI_ISL_688925, EPI_ISL_688926, EPI_ISL_688927, EPI_ISL_688928, EPI_ISL_688929, EPI_ISL_688930, EPI_ISL_688931, EPI_ISL_688932, EPI_ISL_688933, EPI_ISL_688934, EPI_ISL_688935, EPI_ISL_688936, EPI_ISL_688937, EPI_ISL_688938, EPI_ISL_688939, EPI_ISL_688940, EPI_ISL_688941, EPI_ISL_688942, EPI_ISL_688943, EPI_ISL_688944, EPI_ISL_688945, EPI_ISL_688946, EPI_ISL_688947, EPI_ISL_688948, EPI_ISL_688949, EPI_ISL_688950, EPI_ISL_688951, EPI_ISL_688952, EPI_ISL_688953, EPI_ISL_688954, EPI_ISL_688955, EPI_ISL_688956, EPI_ISL_688957, EPI_ISL_688958, EPI_ISL_688959, EPI_ISL_688960, EPI_ISL_688961, EPI_ISL_688962, EPI_ISL_688963, EPI_ISL_688964, EPI_ISL_688965, EPI_ISL_688966, EPI_ISL_688967, EPI_ISL_688968, EPI_ISL_689460, EPI_ISL_689461, EPI_ISL_689462, EPI_ISL_689463, EPI_ISL_689464, EPI_ISL_689465, EPI_ISL_689466, EPI_ISL_689467, EPI_ISL_689468, EPI_ISL_689469, EPI_ISL_689470, EPI_ISL_689471, EPI_ISL_689472, EPI_ISL_689473, EPI_ISL_689474, EPI_ISL_689475, EPI_ISL_689476, EPI_ISL_689477, EPI_ISL_689478, EPI_ISL_689479, EPI_ISL_689480, EPI_ISL_689481, EPI_ISL_689482, EPI_ISL_689483, EPI_ISL_689484, EPI_ISL_689485, EPI_ISL_689486, EPI_ISL_689487, EPI_ISL_689488, EPI_ISL_689489, EPI_ISL_689490, EPI_ISL_689491, EPI_ISL_689492, EPI_ISL_689493, EPI_ISL_689494, EPI_ISL_689495, EPI_ISL_689496, EPI_ISL_689646, EPI_ISL_689692, EPI_ISL_689693, EPI_ISL_689694, EPI_ISL_689695, EPI_ISL_689696, EPI_ISL_689697, EPI_ISL_689759, EPI_ISL_689760, EPI_ISL_689761, EPI_ISL_689762, EPI_ISL_689763, EPI_ISL_689764, EPI_ISL_689765, EPI_ISL_689766, EPI_ISL_689767, EPI_ISL_689768, EPI_ISL_689769, EPI_ISL_689770, EPI_ISL_689771, EPI_ISL_689772, EPI_ISL_689773, EPI_ISL_689774, EPI_ISL_689775, EPI_ISL_689776, EPI_ISL_689777, EPI_ISL_689778, EPI_ISL_689779, EPI_ISL_689780, EPI_ISL_689781, EPI_ISL_689782, EPI_ISL_689783, EPI_ISL_689784, EPI_ISL_689785, EPI_ISL_689786, EPI_ISL_689787, EPI_ISL_689788, EPI_ISL_689789, EPI_ISL_689790, EPI_ISL_689791, EPI_ISL_689792, EPI_ISL_689793, EPI_ISL_689794, EPI_ISL_689795, EPI_ISL_689796, EPI_ISL_689797, EPI_ISL_689798, EPI_ISL_689799, EPI_ISL_689800, EPI_ISL_689801, EPI_ISL_689802, EPI_ISL_689803, EPI_ISL_689804, EPI_ISL_689805, EPI_ISL_689806, EPI_ISL_689807, EPI_ISL_689808, EPI_ISL_689809, EPI_ISL_689810, EPI_ISL_689811, EPI_ISL_689812, EPI_ISL_689813, EPI_ISL_689814, EPI_ISL_689815, EPI_ISL_689816, EPI_ISL_689817, EPI_ISL_689818, EPI_ISL_689819, EPI_ISL_689820, EPI_ISL_689821, EPI_ISL_689822, EPI_ISL_689823, EPI_ISL_689824, EPI_ISL_689825, EPI_ISL_690432, EPI_ISL_690433, EPI_ISL_690434, EPI_ISL_690435, EPI_ISL_690436, EPI_ISL_690437, EPI_ISL_690438, EPI_ISL_690439, EPI_ISL_690440, EPI_ISL_690441, EPI_ISL_690442, EPI_ISL_690443, EPI_ISL_690444, EPI_ISL_690445, EPI_ISL_690446, EPI_ISL_690447, EPI_ISL_690448, EPI_ISL_690449, EPI_ISL_690450, EPI_ISL_690451, EPI_ISL_690452, EPI_ISL_690453, EPI_ISL_690454, EPI_ISL_690455, EPI_ISL_690456, EPI_ISL_690457, EPI_ISL_690458, EPI_ISL_690459, EPI_ISL_690460, EPI_ISL_690461, EPI_ISL_690462, EPI_ISL_690463, EPI_ISL_690464, EPI_ISL_690465, EPI_ISL_690466, EPI_ISL_690467, EPI_ISL_690468, EPI_ISL_690469, EPI_ISL_690470, EPI_ISL_690471, EPI_ISL_690472, EPI_ISL_690473, EPI_ISL_690474, EPI_ISL_690475, EPI_ISL_690476, EPI_ISL_690477, EPI_ISL_690478, EPI_ISL_690479, EPI_ISL_690480, EPI_ISL_690481, EPI_ISL_690482, EPI_ISL_690483, EPI_ISL_690484, EPI_ISL_690485, EPI_ISL_690486, EPI_ISL_690487, EPI_ISL_690488, EPI_ISL_690489, EPI_ISL_690490, EPI_ISL_690491, EPI_ISL_690492, EPI_ISL_690493, EPI_ISL_690494, EPI_ISL_690495, EPI_ISL_690496, EPI_ISL_690497, EPI_ISL_690498, EPI_ISL_690499, EPI_ISL_690500, EPI_ISL_690501, EPI_ISL_690502, EPI_ISL_690503, EPI_ISL_690504, EPI_ISL_690505, EPI_ISL_690506, EPI_ISL_690507, EPI_ISL_690508, EPI_ISL_690509, EPI_ISL_690510, EPI_ISL_690511, EPI_ISL_690512, EPI_ISL_690513, EPI_ISL_690514, EPI_ISL_690515, EPI_ISL_690516, EPI_ISL_690517, EPI_ISL_690518, EPI_ISL_690519, EPI_ISL_690520, EPI_ISL_690521, EPI_ISL_690522, EPI_ISL_690523, EPI_ISL_690524, EPI_ISL_690525, EPI_ISL_690526, EPI_ISL_690982, EPI_ISL_690983, EPI_ISL_690984, EPI_ISL_690985, EPI_ISL_690986, EPI_ISL_691550, EPI_ISL_691551, EPI_ISL_691735, EPI_ISL_691756, EPI_ISL_691764, EPI_ISL_691765, EPI_ISL_692048, EPI_ISL_692049, EPI_ISL_692050, EPI_ISL_692219, EPI_ISL_692220, EPI_ISL_692221, EPI_ISL_692222, EPI_ISL_692223, EPI_ISL_692224, EPI_ISL_692225, EPI_ISL_692226, EPI_ISL_692227, EPI_ISL_692228, EPI_ISL_692229, EPI_ISL_692442, EPI_ISL_692443, EPI_ISL_692444, EPI_ISL_692445, EPI_ISL_692446, EPI_ISL_692447, EPI_ISL_692448, EPI_ISL_692449, EPI_ISL_692450, EPI_ISL_692451, EPI_ISL_692452, EPI_ISL_692453, EPI_ISL_692454, EPI_ISL_692455, EPI_ISL_692456, EPI_ISL_692457, EPI_ISL_692458, EPI_ISL_692459, EPI_ISL_692460, EPI_ISL_692461, EPI_ISL_692462, EPI_ISL_692463, EPI_ISL_692464, EPI_ISL_692465, EPI_ISL_692466, EPI_ISL_692467, EPI_ISL_692468, EPI_ISL_692469, EPI_ISL_692470, EPI_ISL_692471, EPI_ISL_692472, EPI_ISL_692473, EPI_ISL_692474, EPI_ISL_692475, EPI_ISL_692476, EPI_ISL_692477, EPI_ISL_692478, EPI_ISL_692479, EPI_ISL_692480, EPI_ISL_692481, EPI_ISL_692482, EPI_ISL_692483, EPI_ISL_692484, EPI_ISL_692485, EPI_ISL_692486, EPI_ISL_692487, EPI_ISL_692488, EPI_ISL_692489, EPI_ISL_692490, EPI_ISL_692491, EPI_ISL_692492, EPI_ISL_692493, EPI_ISL_692494, EPI_ISL_692495, EPI_ISL_692496, EPI_ISL_692497			
see above	Fukuoka Institute of Health and Environmental Sciences	Pathogen Genomics Center, National Institute of Infectious Diseases	Kentaro Itokawa; Makoto Kuroda; Masanori Hashino; Rina Tanaka; Tsuyoshi Sekizuka
EPI_ISL_480083, EPI_ISL_480084, EPI_ISL_480085, EPI_ISL_480086, EPI_ISL_480087, EPI_ISL_480088, EPI_ISL_480089			
see above	Gifu Prefectural Institute of Public Health and Environmental Sciences	Pathogen Genomics Center, National Institute of Infectious Diseases	Hajime Kamiya; Kentaro Itokawa; Makoto Kuroda; Masanori Hashino; Motoi Suzuki; Rina Tanaka; Tsuyoshi Sekizuka; Yoshihiko Kameyama
EPI_ISL_479850, EPI_ISL_479851, EPI_ISL_479852, EPI_ISL_479853, EPI_ISL_479854, EPI_ISL_479896, EPI_ISL_479897, EPI_ISL_479898, EPI_ISL_479899, EPI_ISL_479900, EPI_ISL_479901, EPI_ISL_480015, EPI_ISL_480016, EPI_ISL_480017, EPI_ISL_480018, EPI_ISL_480019, EPI_ISL_480020, EPI_ISL_480169, EPI_ISL_480170, EPI_ISL_480171, EPI_ISL_480172, EPI_ISL_480173, EPI_ISL_480174, EPI_ISL_480175, EPI_ISL_480176, EPI_ISL_480177, EPI_ISL_684127, EPI_ISL_684220, EPI_ISL_684221, EPI_ISL_684222, EPI_ISL_684223, EPI_ISL_684224, EPI_ISL_684225, EPI_ISL_684226, EPI_ISL_684227, EPI_ISL_684228, EPI_ISL_684229, EPI_ISL_684230, EPI_ISL_684231, EPI_ISL_684232, EPI_ISL_684233, EPI_ISL_684234, EPI_ISL_684235, EPI_ISL_684236, EPI_ISL_684237, EPI_ISL_684238, EPI_ISL_684239, EPI_ISL_684240, EPI_ISL_684241, EPI_ISL_684242, EPI_ISL_684243, EPI_ISL_684244, EPI_ISL_684245, EPI_ISL_684246, EPI_ISL_684247, EPI_ISL_684248, EPI_ISL_684249, EPI_ISL_684250, EPI_ISL_684251, EPI_ISL_684252, EPI_ISL_684253, EPI_ISL_684254, EPI_ISL_684255, EPI_ISL_684256, EPI_ISL_684257, EPI_ISL_684258, EPI_ISL_684259, EPI_ISL_684260, EPI_ISL_684261, EPI_ISL_684262, EPI_ISL_684263, EPI_ISL_684264, EPI_ISL_684265, EPI_ISL_684266, EPI_ISL_684267, EPI_ISL_684268, EPI_ISL_684269, EPI_ISL_684270, EPI_ISL_684271, EPI_ISL_684272, EPI_ISL_684273, EPI_ISL_684274, EPI_ISL_684275, EPI_ISL_684276, EPI_ISL_684277, EPI_ISL_684278, EPI_ISL_684279, EPI_ISL_684280, EPI_ISL_684281, EPI_ISL_684282, EPI_ISL_684283, EPI_ISL_684284, EPI_ISL_684285, EPI_ISL_684286, EPI_ISL_684287, EPI_ISL_684288, EPI_ISL_684325, EPI_ISL_684326, EPI_ISL_684327, EPI_ISL_684328, EPI_ISL_684329, EPI_ISL_684330, EPI_ISL_684331, EPI_ISL_684332, EPI_ISL_684333, EPI_ISL_684334, EPI_ISL_684335, EPI_ISL_684336, EPI_ISL_684337, EPI_ISL_684338, EPI_ISL_684566, EPI_ISL_684567, EPI_ISL_684568, EPI_ISL_684569, EPI_ISL_684570, EPI_ISL_684571, EPI_ISL_684572, EPI_ISL_684573, EPI_ISL_684574, EPI_ISL_684575, EPI_ISL_684576, EPI_ISL_684577, EPI_ISL_685279, EPI_ISL_685280, EPI_ISL_685281, EPI_ISL_689047, EPI_ISL_689048, EPI_ISL_689049, EPI_ISL_689050, EPI_ISL_689051, EPI_ISL_689052, EPI_ISL_689053, EPI_ISL_689054, EPI_ISL_689055, EPI_ISL_689056, EPI_ISL_689057, EPI_ISL_689058, EPI_ISL_689059, EPI_ISL_689060, EPI_ISL_689061, EPI_ISL_689062, EPI_ISL_689063, EPI_ISL_689064, EPI_ISL_689065, EPI_ISL_689066, EPI_ISL_689067, EPI_ISL_689068, EPI_ISL_689069, EPI_ISL_689070, EPI_ISL_689071, EPI_ISL_689072, EPI_ISL_689073, EPI_ISL_689074, EPI_ISL_689075, EPI_ISL_689076, EPI_ISL_689612, EPI_ISL_689617, EPI_ISL_689686, EPI_ISL_689687, EPI_ISL_689688, EPI_ISL_689689, EPI_ISL_689690, EPI_ISL_689691, EPI_ISL_690210, EPI_ISL_690211, EPI_ISL_690212, EPI_ISL_690213, EPI_ISL_690214, EPI_ISL_690215, EPI_ISL_690216, EPI_ISL_690217, EPI_ISL_690218, EPI_ISL_690219, EPI_ISL_690220, EPI_ISL_690221, EPI_ISL_690222, EPI_ISL_690223, EPI_ISL_690224, EPI_ISL_690225, EPI_ISL_690226, EPI_ISL_690227, EPI_ISL_690228, EPI_ISL_690229, EPI_ISL_690230, EPI_ISL_690231, EPI_ISL_690232, EPI_ISL_690233, EPI_ISL_690234, EPI_ISL_690235, EPI_ISL_690236, EPI_ISL_690237, EPI_ISL_690238,			

EPI_ISL_690239, EPI_ISL_690240, EPI_ISL_690241, EPI_ISL_690242, EPI_ISL_690243, EPI_ISL_690244, EPI_ISL_690245, EPI_ISL_690246, EPI_ISL_690247, EPI_ISL_690248, EPI_ISL_690249, EPI_ISL_690250, EPI_ISL_690251, EPI_ISL_690252, EPI_ISL_690253, EPI_ISL_692047, EPI_ISL_692316, EPI_ISL_692317, EPI_ISL_692318, EPI_ISL_692319, EPI_ISL_692320, EPI_ISL_692321, EPI_ISL_692322, EPI_ISL_692323, EPI_ISL_692324, EPI_ISL_692325, EPI_ISL_692326, EPI_ISL_692327			
see above	Gunma Prefectural Institute of Public Health and Environmental Sciences	Pathogen Genomics Center, National Institute of Infectious Diseases	Hajime Kamiya; Hiroyuki Tsukagoshi; Kentaro Itokawa; Makoto Kuroda; Masanori Hashino; Motoi Suzuki; Rina Tanaka; Tsuyoshi Sekizuka
EPI_ISL_479903, EPI_ISL_479904, EPI_ISL_479905, EPI_ISL_479906, EPI_ISL_479907, EPI_ISL_479908, EPI_ISL_479909, EPI_ISL_479910, EPI_ISL_479911, EPI_ISL_479912			
see above	Himeji City Institute of Environment and Health	Pathogen Genomics Center, National Institute of Infectious Diseases	Hajime Kamiya; Kentaro Itokawa; Makoto Kuroda; Masanori Hashino; Motoi Suzuki; Rina Tanaka; Tsuyoshi Sekizuka
EPI_ISL_480178, EPI_ISL_480179	Hiroshima City Institute of Public Health	Pathogen Genomics Center, National Institute of Infectious Diseases	Hajime Kamiya; Kentaro Itokawa; Kota Noritsune; Makoto Kuroda; Masanori Hashino; Motoi Suzuki; Rina Tanaka; Tsuyoshi Sekizuka
EPI_ISL_479792, EPI_ISL_479793, EPI_ISL_479794, EPI_ISL_479795, EPI_ISL_479801, EPI_ISL_479812, EPI_ISL_479813, EPI_ISL_479814, EPI_ISL_479815, EPI_ISL_479816, EPI_ISL_479817, EPI_ISL_479818, EPI_ISL_479819, EPI_ISL_479820			
see above	Hokkaido Institute of Public Health	Pathogen Genomics Center, National Institute of Infectious Diseases	Hajime Kamiya; Kentaro Itokawa; Makoto Kuroda; Masanori Hashino; Motoi Suzuki; Rika Komagome; Rina Tanaka; Tsuyoshi Sekizuka
EPI_ISL_479936, EPI_ISL_479937, EPI_ISL_479938, EPI_ISL_479939, EPI_ISL_479940, EPI_ISL_479941, EPI_ISL_479942, EPI_ISL_479943, EPI_ISL_479985, EPI_ISL_480021, EPI_ISL_480022, EPI_ISL_480023, EPI_ISL_480024, EPI_ISL_480025, EPI_ISL_480026, EPI_ISL_480027, EPI_ISL_480028, EPI_ISL_480029, EPI_ISL_480180, EPI_ISL_480181, EPI_ISL_480182, EPI_ISL_480183, EPI_ISL_480184, EPI_ISL_480185, EPI_ISL_480186, EPI_ISL_480187, EPI_ISL_480188, EPI_ISL_480189			
see above	Ibaraki Prefectural Institute of Public Health	Pathogen Genomics Center, National Institute of Infectious Diseases	Hajime Kamiya; Keiko Goto; Kentaro Itokawa; Makoto Kuroda; Masanori Hashino; Motoi Suzuki; Rina Tanaka; Tsuyoshi Sekizuka
EPI_ISL_419299, EPI_ISL_419300, EPI_ISL_479796	Ishikawa Prefectural Institute of Public Health and Environmental Science	Pathogen Genomics Center, National Institute of Infectious Diseases	Eri Nariai; Hajime Kamiya; Kentaro Itokawa; Makoto Kuroda; Masanori Hashino; Motoi Suzuki; Rina Tanaka; Sanae Kuramoto; Tsuyoshi Sekizuka
EPI_ISL_416565, EPI_ISL_416566, EPI_ISL_416567, EPI_ISL_416568, EPI_ISL_416569, EPI_ISL_416570, EPI_ISL_416571, EPI_ISL_416572, EPI_ISL_416573, EPI_ISL_416574, EPI_ISL_416575, EPI_ISL_416576, EPI_ISL_416577, EPI_ISL_416578, EPI_ISL_416579, EPI_ISL_416580, EPI_ISL_416581, EPI_ISL_416582, EPI_ISL_416583, EPI_ISL_416584, EPI_ISL_416585, EPI_ISL_416586, EPI_ISL_416587, EPI_ISL_416588, EPI_ISL_416589, EPI_ISL_416590, EPI_ISL_416591, EPI_ISL_416592, EPI_ISL_416593, EPI_ISL_416594, EPI_ISL_416595, EPI_ISL_416596, EPI_ISL_416597, EPI_ISL_416598, EPI_ISL_416599, EPI_ISL_416600, EPI_ISL_416601, EPI_ISL_416602, EPI_ISL_416603, EPI_ISL_416604, EPI_ISL_416605, EPI_ISL_416606, EPI_ISL_416607, EPI_ISL_416608, EPI_ISL_416609, EPI_ISL_416610, EPI_ISL_416611, EPI_ISL_416612, EPI_ISL_416613, EPI_ISL_416614, EPI_ISL_416615, EPI_ISL_416616, EPI_ISL_416617, EPI_ISL_416618, EPI_ISL_416619, EPI_ISL_416620, EPI_ISL_416621, EPI_ISL_416622, EPI_ISL_416623, EPI_ISL_416624, EPI_ISL_416625, EPI_ISL_416626, EPI_ISL_416627, EPI_ISL_416628, EPI_ISL_416629, EPI_ISL_416630, EPI_ISL_416631, EPI_ISL_416632, EPI_ISL_416633, EPI_ISL_416634, EPI_ISL_454749			
see above	Japanese Quarantine Stations	Pathogen Genomics Center, National Institute of Infectious Diseases	Hajime Kamiya; Hideki Hasegawa; Ikuyo Takayama; Kentaro Itokawa; Makoto Kuroda; Masanori Hashino; Motoi Suzuki; Rina Tanaka; Shinji Saito; Takaji Wakita; Takuri Takahashi; Takuya Yamagishi; Tsutomu Kageyama; Tsuyoshi Sekizuka
EPI_ISL_687043, EPI_ISL_687044, EPI_ISL_687045, EPI_ISL_687046, EPI_ISL_687047, EPI_ISL_687048, EPI_ISL_687049, EPI_ISL_687050, EPI_ISL_687051, EPI_ISL_687052, EPI_ISL_687053, EPI_ISL_687054, EPI_ISL_687055, EPI_ISL_687056, EPI_ISL_687057, EPI_ISL_687058, EPI_ISL_687059, EPI_ISL_687060, EPI_ISL_687061, EPI_ISL_687062, EPI_ISL_687063, EPI_ISL_687064, EPI_ISL_687065, EPI_ISL_687066, EPI_ISL_687067, EPI_ISL_687068, EPI_ISL_687069, EPI_ISL_687070, EPI_ISL_687071, EPI_ISL_687072, EPI_ISL_687073, EPI_ISL_687074, EPI_ISL_687075, EPI_ISL_687076, EPI_ISL_687077, EPI_ISL_687078, EPI_ISL_690818, EPI_ISL_690819, EPI_ISL_690820, EPI_ISL_690821, EPI_ISL_690822, EPI_ISL_690823, EPI_ISL_690824, EPI_ISL_690825, EPI_ISL_690826, EPI_ISL_690827, EPI_ISL_690828, EPI_ISL_690829, EPI_ISL_690830, EPI_ISL_690831, EPI_ISL_690832, EPI_ISL_690833, EPI_ISL_690834, EPI_ISL_690835, EPI_ISL_690836			
see above	Kanagawa Prefectural Institute of Public Health	Pathogen Genomics Center, National Institute of Infectious Diseases	Kentaro Itokawa; Makoto Kuroda; Masanori Hashino; Rina Tanaka; Tsuyoshi Sekizuka
EPI_ISL_469287, EPI_ISL_469288, EPI_ISL_469289, EPI_ISL_469290, EPI_ISL_469291, EPI_ISL_469292, EPI_ISL_469293, EPI_ISL_469294, EPI_ISL_469295, EPI_ISL_469296			
see above	Keio University Hospital	Keio University Hospital	Kenjiro Kosaki
EPI_ISL_437601, EPI_ISL_438947, EPI_ISL_438948, EPI_ISL_438949, EPI_ISL_438951, EPI_ISL_438952, EPI_ISL_438953, EPI_ISL_438954, EPI_ISL_438955, EPI_ISL_438956, EPI_ISL_438958, EPI_ISL_438959, EPI_ISL_438961, EPI_ISL_438962, EPI_ISL_438963, EPI_ISL_438964, EPI_ISL_438965, EPI_ISL_438966, EPI_ISL_438967, EPI_ISL_438968, EPI_ISL_438969, EPI_ISL_438970, EPI_ISL_438971, EPI_ISL_438972			
see above	Keio University School of Medicine	Keio University School of Medicine	; Haruhiko Siomi; Kenjiro Kosaki; Toshiki Takenouchi; Yuka Iwasaki
EPI_ISL_479990	Kitakyushu City Institute of Health and Environmental Sciences	Pathogen Genomics Center, National Institute of Infectious Diseases	Asuka Kikuchi Kentaro Itokawa; Hajime Kamiya; Katsuya Obata; Makoto Kuroda; Masanori Hashino; Motoi Suzuki; Rina Tanaka; Tsuyoshi Sekizuka
EPI_ISL_419296, EPI_ISL_479823	Kochi Prefectural Institute of Public Health	Pathogen Genomics Center, National Institute of Infectious Diseases	Akihiko Tokaji; Hajime Kamiya; Kentaro Itokawa; Makoto Kuroda; Masanori Hashino; Motoi Suzuki; Rina Tanaka; Tsuyoshi Sekizuka
EPI_ISL_480103, EPI_ISL_480104, EPI_ISL_480105, EPI_ISL_480106, EPI_ISL_480107, EPI_ISL_480108, EPI_ISL_480221, EPI_ISL_480222, EPI_ISL_480223			
see above	Koshigaya City Public Health Center	Pathogen Genomics Center, National Institute of Infectious Diseases	Aya Tamura; Hajime Kamiya; Kentaro Itokawa; Kyohei Sakata; Makoto Kuroda; Masanori Hashino; Motoi Suzuki; Rina Tanaka; Takumi Daimon; Tsuyoshi Sekizuka; Yoko Togawa; Yoshiko Hamada; Yuka Furui
EPI_ISL_479991, EPI_ISL_479992, EPI_ISL_479993, EPI_ISL_479994, EPI_ISL_479995, EPI_ISL_479996	Kumamoto City Public Health Research Institute	Pathogen Genomics Center, National Institute of Infectious Diseases	Hajime Kamiya; Kaori Tashiro; Kentaro Itokawa; Makoto Kuroda; Masanori Hashino; Motoi Suzuki; Rina Tanaka; Tsuyoshi Sekizuka
EPI_ISL_479824	Kumamoto PrefecturalInstituteof Public-Health and Environmental Science	Pathogen Genomics Center, National Institute of Infectious Diseases	Hajime Kamiya; Kentaro Itokawa; Makoto Kuroda; Masanori Hashino; Motoi Suzuki; Rina Tanaka; Shunsuke Yahiro; Tsuyoshi Sekizuka
EPI_ISL_479997, EPI_ISL_479998, EPI_ISL_479999, EPI_ISL_480000, EPI_ISL_480001	Nagano Environmental Conservation Research Institute	Pathogen Genomics Center, National Institute of Infectious Diseases	Hajime Kamiya; Kentaro Itokawa; Makoto Kuroda; Masanori Hashino; Motoi Suzuki; Naoko Shimodaira; Rina Tanaka; Tsuyoshi Sekizuka
EPI_ISL_480002, EPI_ISL_480003	Nagasaki Prefectural Institute for Environmental Research and Public Health	Pathogen Genomics Center, National Institute of Infectious Diseases	Fumiaki Matsumoto; Hajime Kamiya; Kentaro Itokawa; Makoto Kuroda; Masanori Hashino; Motoi Suzuki; Rina Tanaka; Tsuyoshi Sekizuka
EPI_ISL_480042, EPI_ISL_480043, EPI_ISL_480044, EPI_ISL_480045, EPI_ISL_480046, EPI_ISL_480047, EPI_ISL_480048, EPI_ISL_480049, EPI_ISL_480050, EPI_ISL_480051, EPI_ISL_480052, EPI_ISL_480053, EPI_ISL_480054, EPI_ISL_480055, EPI_ISL_480056, EPI_ISL_480057, EPI_ISL_480058, EPI_ISL_480059, EPI_ISL_480060, EPI_ISL_480061, EPI_ISL_480062, EPI_ISL_480063, EPI_ISL_480064			
see above	Nagoya City Public Health Research Institute	Pathogen Genomics Center, National Institute of Infectious Diseases	Hajime Kamiya; Kentaro Itokawa; Makoto Kuroda; Masanori Hashino; Motoi Suzuki; Rina Tanaka; Shinichiro Shibata; Takuya Miki; Tsuyoshi Sekizuka
EPI_ISL_479913, EPI_ISL_479914, EPI_ISL_479915, EPI_ISL_479916, EPI_ISL_479917, EPI_ISL_479918, EPI_ISL_479919, EPI_ISL_479920, EPI_ISL_479921, EPI_ISL_479922, EPI_ISL_479923, EPI_ISL_479924			
see above	Niigata City Public Health Research Institute	Pathogen Genomics Center, National Institute of Infectious Diseases	Hajime Kamiya; Kentaro Itokawa; Makoto Kuroda; Masanori Hashino; Motoi Suzuki; Rina Tanaka; Tsuyoshi Sekizuka; Yurie Takahashi
EPI_ISL_479869, EPI_ISL_479902, EPI_ISL_480226, EPI_ISL_685297, EPI_ISL_685298, EPI_ISL_685299, EPI_ISL_685300, EPI_ISL_685301, EPI_ISL_685302, EPI_ISL_685303, EPI_ISL_685304, EPI_ISL_685305, EPI_ISL_685306, EPI_ISL_685307, EPI_ISL_685308, EPI_ISL_685309, EPI_ISL_685310, EPI_ISL_687699, EPI_ISL_687700, EPI_ISL_687701, EPI_ISL_687702, EPI_ISL_687703, EPI_ISL_687704, EPI_ISL_687705, EPI_ISL_687706, EPI_ISL_687707, EPI_ISL_687708, EPI_ISL_687709, EPI_ISL_687710, EPI_ISL_687711, EPI_ISL_687712, EPI_ISL_687713, EPI_ISL_687714, EPI_ISL_687715, EPI_ISL_687716, EPI_ISL_687717, EPI_ISL_692051, EPI_ISL_692052			
see above	Niigata Prefectural Institute of Public Health and Environmental Sciences	Pathogen Genomics Center, National Institute of Infectious Diseases	Hajime Kamiya; Kentaro Itokawa; Makoto Kuroda; Masanori Hashino; Motoi Suzuki; Reiko Arai; Rina Tanaka; Tsuyoshi Sekizuka
EPI_ISL_479979, EPI_ISL_479980, EPI_ISL_479981, EPI_ISL_479982, EPI_ISL_479983, EPI_ISL_479984, EPI_ISL_480109, EPI_ISL_480110, EPI_ISL_480111, EPI_ISL_480112, EPI_ISL_480113, EPI_ISL_480114, EPI_ISL_480115, EPI_ISL_480116, EPI_ISL_480117, EPI_ISL_480118, EPI_ISL_480119			
see above	Oita Prefectural Institute of Public Health and Environmental Science	Pathogen Genomics Center, National Institute of Infectious Diseases	Hajime Kamiya; Kentaro Itokawa; Makoto Kuroda; Mari Sasaki; Masanori Hashino; Motoi Suzuki; Rina Tanaka; Tsuyoshi Sekizuka
EPI_ISL_479944, EPI_ISL_479945, EPI_ISL_479946, EPI_ISL_479947, EPI_ISL_479948, EPI_ISL_479949, EPI_ISL_479950, EPI_ISL_479951, EPI_ISL_479952, EPI_ISL_479953, EPI_ISL_479954, EPI_ISL_479955, EPI_ISL_479956, EPI_ISL_479957, EPI_ISL_479958, EPI_ISL_479966			
see above	Osaka Institute of Public Health	Pathogen Genomics Center, National Institute of Infectious Diseases	Hajime Kamiya; Kazushi Motomura; Kentaro Itokawa; Makoto Kuroda; Masanori Hashino; Motoi Suzuki; Rina Tanaka; Saeko Morikawa; Satoshi Hiroi; Tsuyoshi Sekizuka
EPI_ISL_480190, EPI_ISL_480191, EPI_ISL_480192, EPI_ISL_480193, EPI_ISL_480194, EPI_ISL_480195	Ota Health Center Welfare Section	Pathogen Genomics Center, National Institute of Infectious Diseases	Chika Takahashi; Hajime Kamiya; Kentaro Itokawa; Makoto Kuroda; Masanori Hashino; Motoi Suzuki; Rina Tanaka; Tsuyoshi Sekizuka
EPI_ISL_480208, EPI_ISL_591347, EPI_ISL_591348, EPI_ISL_591349, EPI_ISL_591350, EPI_ISL_591351, EPI_ISL_591352, EPI_ISL_591353, EPI_ISL_591354, EPI_ISL_591355, EPI_ISL_591356, EPI_ISL_591357, EPI_ISL_591358, EPI_ISL_591359, EPI_ISL_591360, EPI_ISL_591361, EPI_ISL_591362, EPI_ISL_591363, EPI_ISL_591364, EPI_ISL_591365, EPI_ISL_591366, EPI_ISL_591367, EPI_ISL_591368, EPI_ISL_591369, EPI_ISL_591370, EPI_ISL_591371, EPI_ISL_591372, EPI_ISL_591373, EPI_ISL_591374, EPI_ISL_591375, EPI_ISL_591376, EPI_ISL_591377, EPI_ISL_591378, EPI_ISL_591379, EPI_ISL_591380, EPI_ISL_591381, EPI_ISL_591382, EPI_ISL_591383, EPI_ISL_591384, EPI_ISL_591385, EPI_ISL_591386, EPI_ISL_591387, EPI_ISL_591388, EPI_ISL_591389, EPI_ISL_591390, EPI_ISL_591391, EPI_ISL_591392, EPI_ISL_591393, EPI_ISL_591394, EPI_ISL_591395, EPI_ISL_591396, EPI_ISL_591397, EPI_ISL_591398, EPI_ISL_591399, EPI_ISL_591400, EPI_ISL_591401, EPI_ISL_591402, EPI_ISL_591403, EPI_ISL_591404, EPI_ISL_591405, EPI_ISL_591406, EPI_ISL_591407, EPI_ISL_591408, EPI_ISL_591409, EPI_ISL_591410, EPI_ISL_591411, EPI_ISL_591412, EPI_ISL_591413, EPI_ISL_591414, EPI_ISL_591415, EPI_ISL_591416, EPI_ISL_591417, EPI_ISL_591418, EPI_ISL_591419, EPI_ISL_591420, EPI_ISL_591421, EPI_ISL_591422, EPI_ISL_591423, EPI_ISL_591424, EPI_ISL_591425, EPI_ISL_591426, EPI_ISL_591427, EPI_ISL_591428, EPI_ISL_591429, EPI_ISL_591430, EPI_ISL_591431, EPI_ISL_591432, EPI_ISL_591433, EPI_ISL_591434, EPI_ISL_591435, EPI_ISL_591436, EPI_ISL_591437, EPI_ISL_591438, EPI_ISL_591439, EPI_ISL_591440, EPI_ISL_591441, EPI_ISL_591442, EPI_ISL_591443, EPI_ISL_591444, EPI_ISL_591445, EPI_ISL_591446, EPI_ISL_591447, EPI_ISL_591448, EPI_ISL_591449, EPI_ISL_591450, EPI_ISL_591451, EPI_ISL_591452, EPI_ISL_591453, EPI_ISL_591454, EPI_ISL_591455, EPI_ISL_591456, EPI_ISL_591457, EPI_ISL_591458, EPI_ISL_591459, EPI_ISL_591460, EPI_ISL_591461, EPI_ISL_591462, EPI_ISL_591463, EPI_ISL_591464, EPI_ISL_591465, EPI_ISL_591466, EPI_ISL_591467, EPI_ISL_591468, EPI_ISL_591469, EPI_ISL_591470, EPI_ISL_591471, EPI_ISL_591472, EPI_ISL_591473, EPI_ISL_591474, EPI_ISL_591475, EPI_ISL_591476, EPI_ISL_591477, EPI_ISL_591478, EPI_ISL_591479, EPI_ISL_591480, EPI_ISL_591481, EPI_ISL_591482, EPI_ISL_591483, EPI_ISL_591535, EPI_ISL_591536, EPI_ISL_591537, EPI_ISL_591538, EPI_ISL_591539, EPI_ISL_667559, EPI_ISL_667560, EPI_ISL_667561, EPI_ISL_667562, EPI_ISL_667563, EPI_ISL_667564, EPI_ISL_667565, EPI_ISL_667566, EPI_ISL_667567, EPI_ISL_667568, EPI_ISL_667569, EPI_ISL_667570, EPI_ISL_667571, EPI_ISL_667572, EPI_ISL_667573, EPI_ISL_667574, EPI_ISL_667575, EPI_ISL_667576, EPI_ISL_667577, EPI_ISL_667578, EPI_ISL_667579, EPI_ISL_667580, EPI_ISL_667581, EPI_ISL_667582, EPI_ISL_667583, EPI_ISL_667584, EPI_ISL_667585, EPI_ISL_667586, EPI_ISL_667587, EPI_ISL_667588, EPI_ISL_667589, EPI_ISL_667590, EPI_ISL_667591, EPI_ISL_667592, EPI_ISL_667593, EPI_ISL_667594, EPI_ISL_667595, EPI_ISL_667596, EPI_ISL_667597, EPI_ISL_667598, EPI_ISL_667599, EPI_ISL_667600, EPI_ISL_667601, EPI_ISL_667602, EPI_ISL_667603, EPI_ISL_667604, EPI_ISL_667605, EPI_ISL_667606, EPI_ISL_667607, EPI_ISL_667608, EPI_ISL_667609, EPI_ISL_667610, EPI_ISL_667611, EPI_ISL_667612, EPI_ISL_667613, EPI_ISL_667614, EPI_ISL_667615, EPI_ISL_667616, EPI_ISL_667617, EPI_ISL_667618, EPI_ISL_667619, EPI_ISL_667620, EPI_ISL_667621, EPI_ISL_667622, EPI_ISL_667623, EPI_ISL_667624, EPI_ISL_667625, EPI_ISL_667626, EPI_ISL_667627, EPI_ISL_667628, EPI_ISL_667629, EPI_ISL_667630, EPI_ISL_667631, EPI_ISL_667632, EPI_ISL_667633, EPI_ISL_667634, EPI_ISL_667635, EPI_ISL_667636, EPI_ISL_667637, EPI_ISL_667638, EPI_ISL_667639, EPI_ISL_667640, EPI_ISL_667641, EPI_ISL_667642, EPI_ISL_667643, EPI_ISL_667644, EPI_ISL_667645, EPI_ISL_667646, EPI_ISL_667647, EPI_ISL_667648, EPI_ISL_667649, EPI_ISL_667650, EPI_ISL_667651, EPI_ISL_667652, EPI_ISL_667653, EPI_ISL_667654, EPI_ISL_667655, EPI_ISL_667656, EPI_ISL_667657, EPI_ISL_667658, EPI_ISL_667659, EPI_ISL_667660, EPI_ISL_667661, EPI_ISL_667662, EPI_ISL_667663, EPI_ISL_667664, EPI_ISL_667665, EPI_ISL_667666, EPI_ISL_667667, EPI_ISL_667668, EPI_ISL_667669, EPI_ISL_667670, EPI_ISL_667671, EPI_ISL_667672, EPI_ISL_667673, EPI_ISL_667674, EPI_ISL_667675, EPI_ISL_667676, EPI_ISL_667677, EPI_ISL_667678, EPI_ISL_667679, EPI_ISL_667680, EPI_ISL_667681, EPI_ISL_667682, EPI_ISL_667683, EPI_ISL_667684, EPI_ISL_667685, EPI_ISL_667686, EPI_ISL_667687, EPI_ISL_667688, EPI_ISL_667689, EPI_ISL_667690, EPI_ISL_667691, EPI_ISL_667692, EPI_ISL_667693, EPI_ISL_667694, EPI_ISL_667695, EPI_ISL_667696, EPI_ISL_667697, EPI_ISL_667698, EPI_ISL_667699, EPI_ISL_667700, EPI_ISL_667701, EPI_ISL_667702, EPI_ISL_667703, EPI_ISL_667704, EPI_ISL_667705, EPI_ISL_667706, EPI_ISL_667707, EPI_ISL_667708, EPI_ISL_667709, EPI_ISL_667710, EPI_ISL_667711, EPI_ISL_667712, EPI_ISL_667713, EPI_ISL_667714, EPI_ISL_667715, EPI_ISL_667716, EPI_ISL_667717, EPI_ISL_667718, EPI_ISL_667719, EPI_ISL_667720, EPI_ISL_667721, EPI_ISL_667722, EPI_ISL_667723, EPI_ISL_667724, EPI_ISL_667725, EPI_ISL_667726, EPI_ISL_667727, EPI_ISL_667728, EPI_ISL_667729, EPI_ISL_667730, EPI_ISL_667731, EPI_ISL_667732, EPI_ISL_667733, EPI_ISL_667734, EPI_ISL_667735, EPI_ISL_667736, EPI_ISL_667737, EPI_ISL_667738, EPI_ISL_667739, EPI_ISL_667740, EPI_ISL_667741, EPI_ISL_667742, EPI_ISL_667743, EPI_ISL_667744, EPI_ISL_667745, EPI_ISL_667746, EPI_ISL_667747, EPI_ISL_667748, EPI_ISL_667749, EPI_ISL_667750, EPI_ISL_667751, EPI_ISL_667752, EPI_ISL_667753, EPI_ISL_667754, EPI_ISL_667755, EPI_ISL_667756, EPI_ISL_667757, EPI_ISL_684110, EPI_ISL_684111, EPI_ISL_684112, EPI_ISL_684113, EPI_ISL_684114, EPI_ISL_684115, EPI_ISL_684116, EPI_ISL_684117, EPI_ISL_684118, EPI_ISL_684119, EPI_ISL_684120, EPI_ISL_684121, EPI_ISL_684122, EPI_ISL_684123, EPI_ISL_684124, EPI_ISL_684125, EPI_ISL_684126, EPI_ISL_684128, EPI_ISL_684129, EPI_ISL_684130, EPI_ISL_684131, EPI_ISL_684132, EPI_ISL_684133, EPI_ISL_684134, EPI_ISL_684135, EPI_ISL_684139, EPI_ISL_684140, EPI_ISL_684141, EPI_ISL_684142, EPI_ISL_684143, EPI_ISL_684144, EPI_ISL_684145, EPI_ISL_684146, EPI_ISL_684150, EPI_ISL_684151, EPI_ISL_684152, EPI_ISL_684153, EPI_ISL_684156, EPI_ISL_684157, EPI_ISL_684158, EPI_ISL_684162, EPI_ISL_684163, EPI_ISL_684164, EPI_ISL_684165, EPI_ISL_684166, EPI_ISL_684167, EPI_ISL_684168, EPI_ISL_684169, EPI_ISL_684170, EPI_ISL_684171, EPI_ISL_684172, EPI_ISL_684173, EPI_ISL_684174, EPI_ISL_684175, EPI_ISL_684176, EPI_ISL_684177, EPI_ISL_684178, EPI_ISL_684179, EPI_ISL_684180, EPI_ISL_684181, EPI_ISL_684182, EPI_ISL_684183, EPI_ISL_684184, EPI_ISL_684185, EPI_ISL_684186, EPI_ISL_684187, EPI_ISL_684188, EPI_ISL_684189, EPI_ISL_684190, EPI_ISL_684191, EPI_ISL_684192, EPI_ISL_684193, EPI_ISL_684194, EPI_ISL_684195, EPI_ISL_684196, EPI_ISL_684197, EPI_ISL_684198, EPI_ISL_684199, EPI_ISL_684200, EPI_ISL_684201, EPI_ISL_684202, EPI_ISL_684203, EPI_ISL_684204, EPI_ISL_684205, EPI_ISL_684206, EPI_ISL_684207, EPI_ISL_684208, EPI_ISL_684209, EPI_ISL_684210, EPI_ISL_684211, EPI_ISL_684212, EPI_ISL_684213, EPI_ISL_684214, EPI_ISL_684215, EPI_ISL_684216, EPI_ISL_684217, EPI_ISL_684218, EPI_ISL_684219, EPI_ISL_684289, EPI_ISL_684290, EPI_ISL_684291, EPI_ISL_684292, EPI_ISL_684293, EPI_ISL_684294, EPI_ISL_684295, EPI_ISL_684296, EPI_ISL_684297, EPI_ISL_684298, EPI_ISL_684299, EPI_ISL_684300, EPI_ISL_684301, EPI_ISL_684302, EPI_ISL_684303, EPI_ISL_684304, EPI_ISL_684305, EPI_ISL_684306, EPI_ISL_684307, EPI_ISL_684308, EPI_ISL_684309, EPI_ISL_684310, EPI_ISL_684311, EPI_ISL_684312, EPI_ISL_684313, EPI_ISL_684314, EPI_ISL_684315, EPI_ISL_684316, EPI_ISL_684317, EPI_ISL_684318, EPI_ISL_684319, EPI_ISL_684320, EPI_ISL_684321, EPI_ISL_684322, EPI_ISL_684323, EPI_ISL_684324, EPI_ISL_684339, EPI_ISL_684340, EPI_ISL_684341, EPI_ISL_684342, EPI_ISL_684343, EPI_ISL_684344, EPI_ISL_684345, EPI_ISL_684346, EPI_ISL_684347, EPI_ISL_684348, EPI_ISL_684349, EPI_ISL_684350, EPI_ISL_684351, EPI_ISL_684352, EPI_ISL_684353, EPI_ISL_684354, EPI_ISL_684355, EPI_ISL_684356, EPI_ISL_684357, EPI_ISL_684358, EPI_ISL_684359, EPI_ISL_684360, EPI_ISL_684361, EPI_ISL_684362, EPI_ISL_684363, EPI_ISL_684364, EPI_ISL_684365, EPI_ISL_684366, EPI_ISL_684367, EPI_ISL_684368, EPI_ISL_684369, EPI_ISL_684370, EPI_ISL_684371, EPI_ISL_684372, EPI_ISL_684373, EPI_ISL_684374, EPI_ISL_684375, EPI_ISL_684376, EPI_ISL_684377, EPI_ISL_684378, EPI_ISL_684379, EPI_ISL_684380, EPI_ISL_684381, EPI_ISL_684382, EPI_ISL_684383, EPI_ISL_684384, EPI_ISL_684385, EPI_ISL_684386, EPI_ISL_684387, EPI_ISL_684388, EPI_ISL_684389, EPI_ISL_684390, EPI_ISL_684391, EPI_ISL_684392, EPI_ISL_684393, EPI_ISL_684394, EPI_ISL_684395, EPI_ISL_684396, EPI_ISL_684397, EPI_ISL_684398, EPI_ISL_684399, EPI_ISL_684400, EPI_ISL_684401, EPI_ISL_684402, EPI_ISL_684403, EPI_ISL_684404, EPI_ISL_684405, EPI_ISL_684406, EPI_ISL_684407, EPI_ISL_684484, EPI_ISL_684485, EPI_ISL_684486, EPI_ISL_684487, EPI_ISL_684488, EPI_ISL_684489, EPI_ISL_684490, EPI_ISL_684491, EPI_ISL_684492, EPI_ISL_684493, EPI_ISL_684494, EPI_ISL_684495, EPI_ISL_684496, EPI_ISL_684497, EPI_ISL_684498, EPI_ISL_684499, EPI_ISL_684500, EPI_ISL_684501, EPI_ISL_684502, EPI_ISL_684503, EPI_ISL_684504, EPI_ISL_684505, EPI_ISL_684523, EPI_ISL_684524, EPI_ISL_684525, EPI_ISL_684526, EPI_ISL_684527, EPI_ISL_684528, EPI_ISL_684529, EPI_ISL_684530, EPI_ISL_684531, EPI_ISL_684532, EPI_ISL_684533, EPI_ISL_684534, EPI_ISL_684535, EPI_ISL_684536, EPI_ISL_684537, EPI_ISL_684538, EPI_ISL_684539, EPI_ISL_684540, EPI_ISL_684541, EPI_ISL_684542, EPI_ISL_684543, EPI_ISL_684544, EPI_ISL_684545, EPI_ISL_684546, EPI_ISL_684547, EPI_ISL_684548, EPI_ISL_684549, EPI_ISL_684550, EPI_ISL_684551, EPI_ISL_684552, EPI_ISL_684553, EPI_ISL_684554, EPI_ISL_684555, EPI_ISL_684558, EPI_ISL_684559, EPI_ISL_684560, EPI_ISL_684561, EPI_ISL_684562, EPI_ISL_684563, EPI_ISL_684564, EPI_ISL_684565, EPI_ISL_684578, EPI_ISL_684579, EPI_ISL_684580, EPI_ISL_684581, EPI_ISL_684582, EPI_ISL_684583, EPI_ISL_684584, EPI_ISL_684585, EPI_ISL_684586, EPI_ISL_684587, EPI_ISL_684588, EPI_ISL_684589, EPI_ISL_684590, EPI_ISL_684591, EPI_ISL_684592, EPI_ISL_684593, EPI_ISL_684594, EPI_ISL_684595, EPI_ISL_684596, EPI_ISL_684597, EPI_ISL_684598, EPI_ISL_684599, EPI_ISL_684600, EPI_ISL_684601, EPI_ISL_684602, EPI_ISL_684603, EPI_ISL_684604, EPI_ISL_684605, EPI_ISL_684606, EPI_ISL_684607, EPI_ISL_684608, EPI_ISL_684609, EPI_ISL_684610, EPI_ISL_684611, EPI_ISL_684612, EPI_ISL_684613, EPI_ISL_684614, EPI_ISL_684693, EPI_ISL_684694, EPI_ISL_684695, EPI_ISL_684696, EPI_ISL_684697, EPI_ISL_684698, EPI_ISL_684699, EPI_ISL_684700, EPI_ISL_684701, EPI_ISL_684702, EPI_ISL_684703, EPI_ISL_684704, EPI_ISL_684705, EPI_ISL_684706, EPI_ISL_684707, EPI_ISL_684708, EPI_ISL_684709, EPI_ISL_684710, EPI_ISL_684711, EPI_ISL_684712, EPI_ISL_684713, EPI_ISL_684714, EPI_ISL_684715, EPI_ISL_684716, EPI_ISL_684717, EPI_ISL_684718, EPI_ISL_684719, EPI_ISL_684720, EPI_ISL_684721, EPI_ISL_684722, EPI_ISL_684736, EPI_ISL_684737, EPI_ISL_684738, EPI_ISL_684739, EPI_ISL_684740, EPI_ISL_684741, EPI_ISL_684742, EPI_ISL_684743, EPI_ISL_684744, EPI_ISL_684745, EPI_ISL_684746, EPI_ISL_684747, EPI_ISL_684748, EPI_ISL_684749, EPI_ISL_684750, EPI_ISL_684751, EPI_ISL_684752, EPI_ISL_684753, EPI_ISL_684754, EPI_ISL_684755, EPI_ISL_684756, EPI_ISL_684757, EPI_ISL_684758, EPI_ISL_684759, EPI_ISL_684760, EPI_ISL_684761, EPI_ISL_684762, EPI_ISL_684763, EPI_ISL_684764, EPI_ISL_684765, EPI_ISL_684766, EPI_ISL_684767, EPI_ISL_684768, EPI_ISL_684769, EPI_ISL_684770, EPI_ISL_684771, EPI_ISL_684772, EPI_ISL_684773, EPI_ISL_684774, EPI_ISL_684775, EPI_ISL_684776, EPI_ISL_684777, EPI_ISL_684778, EPI_ISL_684779, EPI_ISL_684780, EPI_ISL_684781, EPI_ISL_684782, EPI_ISL_684783, EPI_ISL_684784, EPI_ISL_684785, EPI_ISL_684786, EPI_ISL_684787, EPI_ISL_684788, EPI_ISL_684789, EPI_ISL_684790, EPI_ISL_684791, EPI_ISL_684792, EPI_ISL_684793, EPI_ISL_684794, EPI_ISL_684795, EPI_ISL_684796, EPI_ISL_684797, EPI_ISL_684857, EPI_ISL_684858, EPI_ISL_684859, EPI_ISL_684860, EPI_ISL_684861, EPI_ISL_684862, EPI_ISL_684863, EPI_ISL_684864, EPI_ISL_684865, EPI_ISL_684866, EPI_ISL_684867, EPI_ISL_684868, EPI_ISL_684869, EPI_ISL_684870, EPI_ISL_684871, EPI_ISL_684872, EPI_ISL_684873, EPI_ISL_684874, EPI_ISL_684875, EPI_ISL_684876, EPI_ISL_684877, EPI_ISL_684878, EPI_ISL_684879, EPI_ISL_684880, EPI_ISL_685133, EPI_ISL_685134, EPI_ISL_685135, EPI_ISL_685136, EPI_ISL_685137, EPI_ISL_685138, EPI_ISL_685139, EPI_ISL_685140, EPI_ISL_685141, EPI_ISL_685142, EPI_ISL_685143, EPI_ISL_685144, EPI_ISL_685145, EPI_ISL_685146, EPI_ISL_685147, EPI_ISL_685148, EPI_ISL_685149, EPI_ISL_685150, EPI_ISL_685151, EPI_ISL_685152, EPI_ISL_685153, EPI_ISL_685154, EPI_ISL_685155, EPI_ISL_685156, EPI_ISL_685157, EPI_ISL_685158, EPI_ISL_685159, EPI_ISL_685160, EPI_ISL_685161, EPI_ISL_685162, EPI_ISL_685163, EPI_ISL_685164, EPI_ISL_685165, EPI_ISL_685166, EPI_ISL_685167, EPI_ISL_685168, EPI_ISL_685169, EPI_ISL_685170, EPI_ISL_685171, EPI_ISL_685172, EPI_ISL_685173, EPI_ISL_685174, EPI_ISL_685175, EPI_ISL_685176, EPI_ISL_685177, EPI_ISL_685178, EPI_ISL_685179, EPI_ISL_685180, EPI_ISL_685181, EPI_ISL_685182, EPI_ISL_685183, EPI_ISL_685184, EPI_ISL_685185, EPI_ISL_685186, EPI_ISL_685187, EPI_ISL_685188, EPI_ISL_685189, EPI_ISL_685190, EPI_ISL_685191, EPI_ISL_685192, EPI_ISL_685193, EPI_ISL_685194, EPI_ISL_685195, EPI_ISL_685196, EPI_ISL_685197, EPI_ISL_685198, EPI_ISL_685199, EPI_ISL_685200, EPI_ISL_685201, EPI_ISL_685202, EPI_ISL_685203, EPI_ISL_685204, EPI_ISL_685205, EPI_ISL_685206, EPI_ISL_685207, EPI_ISL_685208, EPI_ISL_685209, EPI_ISL_685210, EPI_ISL_685211, EPI_ISL_685212, EPI_ISL_685213, EPI_ISL_685214, EPI_ISL_685215, EPI_ISL_685216, EPI_ISL_685217, EPI_ISL_685218, EPI_ISL_685219, EPI_ISL_685238, EPI_ISL_685239, EPI_ISL_685240, EPI_ISL_685241, EPI_ISL_685242, EPI_ISL_685243, EPI_ISL_685244, EPI_ISL_685245, EPI_ISL_685246, EPI_ISL_685247, EPI_ISL_685248, EPI_ISL_685249, EPI_ISL_685250, EPI_ISL_685251, EPI_ISL_685252, EPI_ISL_685253, EPI_ISL_685254, EPI_ISL_685255, EPI_ISL_685256, EPI_ISL_685257, EPI_ISL_685258, EPI_ISL_685259, EPI_ISL_685260, EPI_ISL_685261, EPI_ISL_685262, EPI_ISL_685263, EPI_ISL_685264, EPI_ISL_685265, EPI_ISL_685266, EPI_ISL_685267, EPI_ISL_685268, EPI_ISL_685269, EPI_ISL_685270, EPI_ISL_685271, EPI_ISL_685272, EPI_ISL_685273, EPI_ISL_685274, EPI_ISL_685275, EPI_ISL_685276, EPI_ISL_685277, EPI_ISL_685278, EPI_ISL_685282, EPI_ISL_685283, EPI_ISL_685284, EPI_ISL_685285, EPI_ISL_685286, EPI_ISL_685287, EPI_ISL_685288, EPI_ISL_685289, EPI_ISL_685290, EPI_ISL_685291, EPI_ISL_685292, EPI_ISL_685293, EPI_ISL_685294, EPI_ISL_685295, EPI_ISL_685296, EPI_ISL_685311, EPI_ISL_685312, EPI_ISL_685313, EPI_ISL_685314, EPI_ISL_685315, EPI_ISL_685316, EPI_ISL_685317, EPI_ISL_685318, EPI_ISL_685319, EPI_ISL_685320, EPI_ISL_685321, EPI_ISL_685322, EPI_ISL_685323, EPI_ISL_685324, EPI_ISL_685325, EPI_ISL_685326, EPI_ISL_685327, EPI_ISL_685328, EPI_ISL_685329, EPI_ISL_685330, EPI_ISL_685331, EPI_ISL_685332, EPI_ISL_685333, EPI_ISL_685334, EPI_ISL_685335, EPI_ISL_685336, EPI_ISL_685337, EPI_ISL_685338, EPI_ISL_685339, EPI_ISL_685340, EPI_ISL_685341, EPI_ISL_685342, EPI_ISL_685343, EPI_ISL_685344, EPI_ISL_685345, EPI_ISL_685346, EPI_ISL_685347, EPI_ISL_685348, EPI_ISL_685349, EPI_ISL_685350, EPI_ISL_685351, EPI_ISL_685352, EPI_ISL_685353, EPI_ISL_685354, EPI_ISL_685355, EPI_ISL_685356, EPI_ISL_685357, EPI_ISL_685358, EPI_ISL_685359, EPI_ISL_685360, EPI_ISL_685361, EPI_ISL_685362, EPI_ISL_685363, EPI_ISL_685364, EPI_ISL_685365, EPI_ISL_685366, EPI_ISL_685367, EPI_ISL_685368, EPI_ISL_685369, EPI_ISL_685370, EPI_ISL_685371, EPI_ISL_685372, EPI_ISL_685373, EPI_ISL_685374, EPI_ISL_685375, EPI_ISL_685376, EPI_ISL_685377, EPI_ISL_685378, EPI_ISL_685379, EPI_ISL_685380, EPI_ISL_685381, EPI_ISL_685382, EPI_ISL_685383, EPI_ISL_685384, EPI_ISL_685385, EPI_ISL_685386, EPI_ISL_685387, EPI_ISL_685388, EPI_ISL_685389, EPI_ISL_685390, EPI_ISL_685391, EPI_ISL_685392, EPI_ISL_685393, EPI_ISL_685394, EPI_ISL_685395, EPI_ISL_685396, EPI_ISL_685397, EPI_ISL_685398, EPI_ISL_685399, EPI_ISL_685400, EPI_ISL_685401, EPI_ISL_685402, EPI_ISL_685403, EPI_ISL_685404, EPI_ISL_685405, EPI_ISL_685406, EPI_ISL_685505, EPI_ISL_685506, EPI_ISL_685507, EPI_ISL_685508, EPI_ISL_685509, EPI_ISL_685510, EPI_ISL_685511, EPI_ISL_685512, EPI_ISL_685513, EPI_ISL_685514, EPI_ISL_685515, EPI_ISL_685516, EPI_ISL_685517, EPI_ISL_685518, EPI_ISL_685519, EPI_ISL_685520, EPI_ISL_685521, EPI_ISL_685522, EPI_ISL_685523, EPI_ISL_685524, EPI_ISL_685525, EPI_ISL_685526, EPI_ISL_685527, EPI_ISL_685528, EPI_ISL_685529, EPI_ISL_685530, EPI_ISL_685531, EPI_ISL_685532, EPI_ISL_685533, EPI_ISL_685534, EPI_ISL_685535, EPI_ISL_685536, EPI_ISL_685537, EPI_ISL_685538, EPI_ISL_685539, EPI_ISL_685540, EPI_ISL_685541, EPI_ISL_685542, EPI_ISL_685543, EPI_ISL_685544, EPI_ISL_685545, EPI_ISL_685546, EPI_ISL_685547, EPI_ISL_685548, EPI_ISL_685549, EPI_ISL_685550, EPI_ISL_685551, EPI_ISL_685552, EPI_ISL_685553, EPI_ISL_685554, EPI_ISL_685555, EPI_ISL_685556, EPI_ISL_685557, EPI_ISL_685558, EPI_ISL_685559, EPI_ISL_685560, EPI_ISL_685561, EPI_ISL_685562, EPI_ISL_685563, EPI_ISL_685564, EPI_ISL_685565, EPI_ISL_685566, EPI_ISL_685567, EPI_ISL_685568, EPI_ISL_685569, EPI_ISL_685570, EPI_ISL_685571, EPI_ISL_685572, EPI_ISL_685573, EPI_ISL_685574, EPI_ISL_685575, EPI_ISL_685576, EPI_ISL_685577, EPI_ISL_685578, EPI_ISL_685579, EPI_ISL_685580, EPI_ISL_685581, EPI_ISL_685582, EPI_ISL_685583, EPI_ISL_685584,			

EPI_ISL_685585, EPI_ISL_685586, EPI_ISL_685587, EPI_ISL_685588, EPI_ISL_685589, EPI_ISL_685590, EPI_ISL_685591, EPI_ISL_685592, EPI_ISL_685593, EPI_ISL_685594, EPI_ISL_685595, EPI_ISL_685596, EPI_ISL_685597, EPI_ISL_685598, EPI_ISL_685599, EPI_ISL_685600, EPI_ISL_685601, EPI_ISL_685602, EPI_ISL_685603, EPI_ISL_685604, EPI_ISL_685605, EPI_ISL_685617, EPI_ISL_685618, EPI_ISL_685619,
EPI_ISL_685620, EPI_ISL_685621, EPI_ISL_685622, EPI_ISL_685623, EPI_ISL_685624, EPI_ISL_685625, EPI_ISL_685626, EPI_ISL_685627, EPI_ISL_685628, EPI_ISL_685629, EPI_ISL_685630, EPI_ISL_685631, EPI_ISL_685632, EPI_ISL_685633, EPI_ISL_685634, EPI_ISL_685635, EPI_ISL_685636, EPI_ISL_685637, EPI_ISL_685638, EPI_ISL_685639, EPI_ISL_685640, EPI_ISL_685641, EPI_ISL_685642, EPI_ISL_685643,
EPI_ISL_685644, EPI_ISL_685645, EPI_ISL_685646, EPI_ISL_685647, EPI_ISL_685648, EPI_ISL_685649, EPI_ISL_685650, EPI_ISL_685651, EPI_ISL_685652, EPI_ISL_685653, EPI_ISL_685654, EPI_ISL_685655, EPI_ISL_685656, EPI_ISL_685657, EPI_ISL_685658, EPI_ISL_685659, EPI_ISL_685660, EPI_ISL_685661, EPI_ISL_685662, EPI_ISL_685663, EPI_ISL_685664, EPI_ISL_685665, EPI_ISL_685695, EPI_ISL_685696,
EPI_ISL_685697, EPI_ISL_685698, EPI_ISL_685699, EPI_ISL_685700, EPI_ISL_685701, EPI_ISL_685702, EPI_ISL_685703, EPI_ISL_685704, EPI_ISL_685705, EPI_ISL_685706, EPI_ISL_685707, EPI_ISL_685708, EPI_ISL_685709, EPI_ISL_685710, EPI_ISL_685711, EPI_ISL_685712, EPI_ISL_685713, EPI_ISL_685714, EPI_ISL_685715, EPI_ISL_685716, EPI_ISL_685717, EPI_ISL_685718, EPI_ISL_685719, EPI_ISL_685720,
EPI_ISL_685721, EPI_ISL_685722, EPI_ISL_685723, EPI_ISL_685724, EPI_ISL_685725, EPI_ISL_685726, EPI_ISL_685727, EPI_ISL_685728, EPI_ISL_685729, EPI_ISL_685730, EPI_ISL_685731, EPI_ISL_685732, EPI_ISL_685733, EPI_ISL_685734, EPI_ISL_685735, EPI_ISL_685736, EPI_ISL_685737, EPI_ISL_685738, EPI_ISL_685739, EPI_ISL_685740, EPI_ISL_685741, EPI_ISL_685742, EPI_ISL_685743, EPI_ISL_685744,
EPI_ISL_685745, EPI_ISL_685746, EPI_ISL_685747, EPI_ISL_685748, EPI_ISL_685749, EPI_ISL_685750, EPI_ISL_685751, EPI_ISL_685752, EPI_ISL_685753, EPI_ISL_685754, EPI_ISL_685755, EPI_ISL_685756, EPI_ISL_685757, EPI_ISL_685758, EPI_ISL_685759, EPI_ISL_685760, EPI_ISL_685761, EPI_ISL_685762, EPI_ISL_685763, EPI_ISL_685764, EPI_ISL_685765, EPI_ISL_685766, EPI_ISL_685767, EPI_ISL_685768,
EPI_ISL_685769, EPI_ISL_685770, EPI_ISL_685771, EPI_ISL_685772, EPI_ISL_685773, EPI_ISL_685774, EPI_ISL_685775, EPI_ISL_685776, EPI_ISL_685777, EPI_ISL_685778, EPI_ISL_685779, EPI_ISL_685780, EPI_ISL_685781, EPI_ISL_685782, EPI_ISL_685783, EPI_ISL_685784, EPI_ISL_685785, EPI_ISL_685786, EPI_ISL_685787, EPI_ISL_685788, EPI_ISL_685789, EPI_ISL_685790, EPI_ISL_685791, EPI_ISL_685792,
EPI_ISL_685793, EPI_ISL_685794, EPI_ISL_685795, EPI_ISL_685796, EPI_ISL_685797, EPI_ISL_685798, EPI_ISL_685799, EPI_ISL_685800, EPI_ISL_685801, EPI_ISL_685802, EPI_ISL_685803, EPI_ISL_685804, EPI_ISL_685805, EPI_ISL_685806, EPI_ISL_685807, EPI_ISL_685808, EPI_ISL_685809, EPI_ISL_685810, EPI_ISL_685811, EPI_ISL_685812, EPI_ISL_685813, EPI_ISL_685814, EPI_ISL_685815, EPI_ISL_685816,
EPI_ISL_685817, EPI_ISL_685818, EPI_ISL_685819, EPI_ISL_685820, EPI_ISL_685821, EPI_ISL_685822, EPI_ISL_685823, EPI_ISL_685824, EPI_ISL_685825, EPI_ISL_685826, EPI_ISL_685827, EPI_ISL_685828, EPI_ISL_685829, EPI_ISL_685830, EPI_ISL_685831, EPI_ISL_685832, EPI_ISL_685833, EPI_ISL_685834, EPI_ISL_685835, EPI_ISL_685836, EPI_ISL_685837, EPI_ISL_685838, EPI_ISL_685839, EPI_ISL_685840,
EPI_ISL_685841, EPI_ISL_685842, EPI_ISL_685843, EPI_ISL_685844, EPI_ISL_685845, EPI_ISL_685846, EPI_ISL_685847, EPI_ISL_685848, EPI_ISL_685849, EPI_ISL_685850, EPI_ISL_685851, EPI_ISL_685852, EPI_ISL_685853, EPI_ISL_685854, EPI_ISL_685855, EPI_ISL_685856, EPI_ISL_685857, EPI_ISL_685858, EPI_ISL_685859, EPI_ISL_685860, EPI_ISL_685861, EPI_ISL_685862, EPI_ISL_685863, EPI_ISL_685864,
EPI_ISL_685865, EPI_ISL_685866, EPI_ISL_685867, EPI_ISL_685868, EPI_ISL_685869, EPI_ISL_685870, EPI_ISL_685871, EPI_ISL_685872, EPI_ISL_685873, EPI_ISL_685874, EPI_ISL_685875, EPI_ISL_685876, EPI_ISL_685877, EPI_ISL_685878, EPI_ISL_685879, EPI_ISL_685880, EPI_ISL_685881, EPI_ISL_685882, EPI_ISL_685883, EPI_ISL_685884, EPI_ISL_685885, EPI_ISL_685886, EPI_ISL_685887, EPI_ISL_685888,
EPI_ISL_685889, EPI_ISL_685890, EPI_ISL_685891, EPI_ISL_685892, EPI_ISL_685893, EPI_ISL_685894, EPI_ISL_685895, EPI_ISL_685896, EPI_ISL_685897, EPI_ISL_685898, EPI_ISL_685899, EPI_ISL_685900, EPI_ISL_685901, EPI_ISL_685902, EPI_ISL_685903, EPI_ISL_685904, EPI_ISL_685905, EPI_ISL_685906, EPI_ISL_685907, EPI_ISL_685908, EPI_ISL_685909, EPI_ISL_685910, EPI_ISL_685911, EPI_ISL_685912,
EPI_ISL_685913, EPI_ISL_685914, EPI_ISL_685915, EPI_ISL_685916, EPI_ISL_685917, EPI_ISL_685925, EPI_ISL_685926, EPI_ISL_685927, EPI_ISL_685928, EPI_ISL_685929, EPI_ISL_685930, EPI_ISL_685931, EPI_ISL_685932, EPI_ISL_685933, EPI_ISL_685934, EPI_ISL_685935, EPI_ISL_685936, EPI_ISL_685937, EPI_ISL_685938, EPI_ISL_685939, EPI_ISL_685940, EPI_ISL_685941, EPI_ISL_685942, EPI_ISL_685943,
EPI_ISL_685944, EPI_ISL_685945, EPI_ISL_685946, EPI_ISL_685947, EPI_ISL_685948, EPI_ISL_685949, EPI_ISL_685950, EPI_ISL_685951, EPI_ISL_685952, EPI_ISL_685953, EPI_ISL_685954, EPI_ISL_685955, EPI_ISL_685956, EPI_ISL_685957, EPI_ISL_685958, EPI_ISL_685959, EPI_ISL_685960, EPI_ISL_685961, EPI_ISL_685962, EPI_ISL_685988, EPI_ISL_685989, EPI_ISL_685990, EPI_ISL_685991, EPI_ISL_685992,
EPI_ISL_685993, EPI_ISL_685994, EPI_ISL_685995, EPI_ISL_685996, EPI_ISL_685997, EPI_ISL_685998, EPI_ISL_685999, EPI_ISL_686000, EPI_ISL_686001, EPI_ISL_686002, EPI_ISL_686003, EPI_ISL_686004, EPI_ISL_686005, EPI_ISL_686006, EPI_ISL_686007, EPI_ISL_686008, EPI_ISL_686009, EPI_ISL_686010, EPI_ISL_686011, EPI_ISL_686012, EPI_ISL_686013, EPI_ISL_686014, EPI_ISL_686015, EPI_ISL_686016,
EPI_ISL_686017, EPI_ISL_686018, EPI_ISL_686019, EPI_ISL_686020, EPI_ISL_686021, EPI_ISL_686022, EPI_ISL_686023, EPI_ISL_686024, EPI_ISL_686025, EPI_ISL_686026, EPI_ISL_686027, EPI_ISL_686028, EPI_ISL_686029, EPI_ISL_686030, EPI_ISL_686031, EPI_ISL_686032, EPI_ISL_686033, EPI_ISL_686034, EPI_ISL_686035, EPI_ISL_686036, EPI_ISL_686037, EPI_ISL_686038, EPI_ISL_686039, EPI_ISL_686040,
EPI_ISL_686041, EPI_ISL_686042, EPI_ISL_686043, EPI_ISL_686044, EPI_ISL_686045, EPI_ISL_686046, EPI_ISL_686047, EPI_ISL_686048, EPI_ISL_686049, EPI_ISL_686050, EPI_ISL_686051, EPI_ISL_686052, EPI_ISL_686053, EPI_ISL_686054, EPI_ISL_686055, EPI_ISL_686056, EPI_ISL_686057, EPI_ISL_686058, EPI_ISL_686059, EPI_ISL_686060, EPI_ISL_686061, EPI_ISL_686062, EPI_ISL_686063, EPI_ISL_686064,
EPI_ISL_686065, EPI_ISL_686066, EPI_ISL_686067, EPI_ISL_686068, EPI_ISL_686069, EPI_ISL_686070, EPI_ISL_686071, EPI_ISL_686072, EPI_ISL_686073, EPI_ISL_686074, EPI_ISL_686075, EPI_ISL_686076, EPI_ISL_686077, EPI_ISL_686078, EPI_ISL_686079, EPI_ISL_686080, EPI_ISL_686081, EPI_ISL_686082, EPI_ISL_686109, EPI_ISL_686110, EPI_ISL_686111, EPI_ISL_686112, EPI_ISL_686113, EPI_ISL_686114,
EPI_ISL_686115, EPI_ISL_686116, EPI_ISL_686117, EPI_ISL_686118, EPI_ISL_686119, EPI_ISL_686120, EPI_ISL_686121, EPI_ISL_686122, EPI_ISL_686123, EPI_ISL_686124, EPI_ISL_686125, EPI_ISL_686126, EPI_ISL_686127, EPI_ISL_686128, EPI_ISL_686129, EPI_ISL_686130, EPI_ISL_686131, EPI_ISL_686132, EPI_ISL_686133, EPI_ISL_686134, EPI_ISL_686135, EPI_ISL_686136, EPI_ISL_686137, EPI_ISL_686138,
EPI_ISL_686139, EPI_ISL_686140, EPI_ISL_686141, EPI_ISL_686142, EPI_ISL_686143, EPI_ISL_686144, EPI_ISL_686145, EPI_ISL_686146, EPI_ISL_686147, EPI_ISL_686148, EPI_ISL_686149, EPI_ISL_686150, EPI_ISL_686151, EPI_ISL_686152, EPI_ISL_686153, EPI_ISL_686154, EPI_ISL_686155, EPI_ISL_686156, EPI_ISL_686157, EPI_ISL_686158, EPI_ISL_686159, EPI_ISL_686160, EPI_ISL_686161, EPI_ISL_686162,
EPI_ISL_686163, EPI_ISL_686164, EPI_ISL_686165, EPI_ISL_686166, EPI_ISL_686167, EPI_ISL_686168, EPI_ISL_686169, EPI_ISL_686170, EPI_ISL_686171, EPI_ISL_686172, EPI_ISL_686173, EPI_ISL_686174, EPI_ISL_686175, EPI_ISL_686176, EPI_ISL_686177, EPI_ISL_686178, EPI_ISL_686179, EPI_ISL_686180, EPI_ISL_686181, EPI_ISL_686182, EPI_ISL_686183, EPI_ISL_686184, EPI_ISL_686185, EPI_ISL_686186,
EPI_ISL_686187, EPI_ISL_686188, EPI_ISL_686189, EPI_ISL_686190, EPI_ISL_686191, EPI_ISL_686192, EPI_ISL_686193, EPI_ISL_686194, EPI_ISL_686195, EPI_ISL_686196, EPI_ISL_686197, EPI_ISL_686198, EPI_ISL_686199, EPI_ISL_686200, EPI_ISL_686201, EPI_ISL_686202, EPI_ISL_686203, EPI_ISL_686204, EPI_ISL_686205, EPI_ISL_686206, EPI_ISL_686207, EPI_ISL_686208, EPI_ISL_686209, EPI_ISL_686210,
EPI_ISL_686211, EPI_ISL_686212, EPI_ISL_686213, EPI_ISL_686214, EPI_ISL_686215, EPI_ISL_686216, EPI_ISL_686217, EPI_ISL_686218, EPI_ISL_686219, EPI_ISL_686220, EPI_ISL_686221, EPI_ISL_686222, EPI_ISL_686223, EPI_ISL_686224, EPI_ISL_686225, EPI_ISL_686226, EPI_ISL_686227, EPI_ISL_686228, EPI_ISL_686229, EPI_ISL_686230, EPI_ISL_686231, EPI_ISL_686232, EPI_ISL_686233, EPI_ISL_686234,
EPI_ISL_686235, EPI_ISL_686236, EPI_ISL_686237, EPI_ISL_686238, EPI_ISL_686239, EPI_ISL_686240, EPI_ISL_686241, EPI_ISL_686242, EPI_ISL_686243, EPI_ISL_686244, EPI_ISL_686245, EPI_ISL_686246, EPI_ISL_686247, EPI_ISL_686248, EPI_ISL_686249, EPI_ISL_686250, EPI_ISL_686251, EPI_ISL_686252, EPI_ISL_686253, EPI_ISL_686254, EPI_ISL_686255, EPI_ISL_686256, EPI_ISL_686257, EPI_ISL_686258,
EPI_ISL_686259, EPI_ISL_686260, EPI_ISL_686261, EPI_ISL_686262, EPI_ISL_686263, EPI_ISL_686264, EPI_ISL_686265, EPI_ISL_686266, EPI_ISL_686267, EPI_ISL_686268, EPI_ISL_686269, EPI_ISL_686270, EPI_ISL_686271, EPI_ISL_686272, EPI_ISL_686273, EPI_ISL_686274, EPI_ISL_686275, EPI_ISL_686276, EPI_ISL_686277, EPI_ISL_686278, EPI_ISL_686279, EPI_ISL_686280, EPI_ISL_686281, EPI_ISL_686282,
EPI_ISL_686283, EPI_ISL_686284, EPI_ISL_686285, EPI_ISL_686286, EPI_ISL_686287, EPI_ISL_686288, EPI_ISL_686289, EPI_ISL_686290, EPI_ISL_686291, EPI_ISL_686292, EPI_ISL_686293, EPI_ISL_686294, EPI_ISL_686295, EPI_ISL_686296, EPI_ISL_686297, EPI_ISL_686298, EPI_ISL_686299, EPI_ISL_686300, EPI_ISL_686301, EPI_ISL_686302, EPI_ISL_686303, EPI_ISL_686304, EPI_ISL_686305, EPI_ISL_686306,
EPI_ISL_686307, EPI_ISL_686308, EPI_ISL_686309, EPI_ISL_686310, EPI_ISL_686311, EPI_ISL_686312, EPI_ISL_686313, EPI_ISL_686314, EPI_ISL_686315, EPI_ISL_686316, EPI_ISL_686317, EPI_ISL_686318, EPI_ISL_686319, EPI_ISL_686320, EPI_ISL_686321, EPI_ISL_686322, EPI_ISL_686323, EPI_ISL_686324, EPI_ISL_686325, EPI_ISL_686326, EPI_ISL_686327, EPI_ISL_686328, EPI_ISL_686329, EPI_ISL_686330,
EPI_ISL_686331, EPI_ISL_686332, EPI_ISL_686333, EPI_ISL_686334, EPI_ISL_686335, EPI_ISL_686336, EPI_ISL_686337, EPI_ISL_686338, EPI_ISL_686339, EPI_ISL_686340, EPI_ISL_686341, EPI_ISL_686342, EPI_ISL_686343, EPI_ISL_686344, EPI_ISL_686345, EPI_ISL_686346, EPI_ISL_686347, EPI_ISL_686348, EPI_ISL_686349, EPI_ISL_686350, EPI_ISL_686351, EPI_ISL_686352, EPI_ISL_686353, EPI_ISL_686354,
EPI_ISL_686355, EPI_ISL_686356, EPI_ISL_686357, EPI_ISL_686358, EPI_ISL_686359, EPI_ISL_686360, EPI_ISL_686361, EPI_ISL_686362, EPI_ISL_686363, EPI_ISL_686364, EPI_ISL_686365, EPI_ISL_686366, EPI_ISL_686367, EPI_ISL_686368, EPI_ISL_686369, EPI_ISL_686370, EPI_ISL_686371, EPI_ISL_686372, EPI_ISL_686373, EPI_ISL_686374, EPI_ISL_686375, EPI_ISL_686376, EPI_ISL_686377, EPI_ISL_686378,
EPI_ISL_686379, EPI_ISL_686380, EPI_ISL_686381, EPI_ISL_686382, EPI_ISL_686383, EPI_ISL_686384, EPI_ISL_686385, EPI_ISL_686386, EPI_ISL_686387, EPI_ISL_686388, EPI_ISL_686389, EPI_ISL_686390, EPI_ISL_686391, EPI_ISL_686392, EPI_ISL_686393, EPI_ISL_686394, EPI_ISL_686395, EPI_ISL_686396, EPI_ISL_686397, EPI_ISL_686398, EPI_ISL_686399, EPI_ISL_686400, EPI_ISL_686401, EPI_ISL_686402,
EPI_ISL_686403, EPI_ISL_686404, EPI_ISL_686405, EPI_ISL_686406, EPI_ISL_686407, EPI_ISL_686408, EPI_ISL_686409, EPI_ISL_686410, EPI_ISL_686411, EPI_ISL_686412, EPI_ISL_686413, EPI_ISL_686414, EPI_ISL_686415, EPI_ISL_686416, EPI_ISL_686417, EPI_ISL_686418, EPI_ISL_686419, EPI_ISL_686420, EPI_ISL_686421, EPI_ISL_686422, EPI_ISL_686423, EPI_ISL_686424, EPI_ISL_686425, EPI_ISL_686426,
EPI_ISL_686427, EPI_ISL_686428, EPI_ISL_686429, EPI_ISL_686430, EPI_ISL_686431, EPI_ISL_686432, EPI_ISL_686433, EPI_ISL_686434, EPI_ISL_686435, EPI_ISL_686436, EPI_ISL_686437, EPI_ISL_686438, EPI_ISL_686439, EPI_ISL_686440, EPI_ISL_686441, EPI_ISL_686442, EPI_ISL_686443, EPI_ISL_686444, EPI_ISL_686445, EPI_ISL_686446, EPI_ISL_686447, EPI_ISL_686448, EPI_ISL_686449, EPI_ISL_686450,
EPI_ISL_686451, EPI_ISL_686452, EPI_ISL_686453, EPI_ISL_686454, EPI_ISL_686455, EPI_ISL_686456, EPI_ISL_686457, EPI_ISL_686458, EPI_ISL_686459, EPI_ISL_686460, EPI_ISL_686461, EPI_ISL_686462, EPI_ISL_686463, EPI_ISL_686464, EPI_ISL_686465, EPI_ISL_686466, EPI_ISL_686467, EPI_ISL_686468, EPI_ISL_686469, EPI_ISL_686470, EPI_ISL_686471, EPI_ISL_686472, EPI_ISL_686473, EPI_ISL_686474,
EPI_ISL_686475, EPI_ISL_686476, EPI_ISL_686477, EPI_ISL_686478, EPI_ISL_686479, EPI_ISL_686480, EPI_ISL_686481, EPI_ISL_686482, EPI_ISL_686483, EPI_ISL_686484, EPI_ISL_686485, EPI_ISL_686486, EPI_ISL_686487, EPI_ISL_686488, EPI_ISL_686489, EPI_ISL_686490, EPI_ISL_686491, EPI_ISL_686492, EPI_ISL_686493, EPI_ISL_686494, EPI_ISL_686495, EPI_ISL_686496, EPI_ISL_686497, EPI_ISL_686498,
EPI_ISL_686499, EPI_ISL_686500, EPI_ISL_686501, EPI_ISL_686502, EPI_ISL_686503, EPI_ISL_686504, EPI_ISL_686505, EPI_ISL_686506, EPI_ISL_686507, EPI_ISL_686508, EPI_ISL_686509, EPI_ISL_686510, EPI_ISL_686511, EPI_ISL_686512, EPI_ISL_686513, EPI_ISL_686514, EPI_ISL_686515, EPI_ISL_686516, EPI_ISL_686517, EPI_ISL_686518, EPI_ISL_686519, EPI_ISL_686520, EPI_ISL_686612, EPI_ISL_686613,
EPI_ISL_686614, EPI_ISL_686615, EPI_ISL_686616, EPI_ISL_686617, EPI_ISL_686618, EPI_ISL_686619, EPI_ISL_686620, EPI_ISL_686621, EPI_ISL_686622, EPI_ISL_686623, EPI_ISL_686624, EPI_ISL_686625, EPI_ISL_686626, EPI_ISL_686627, EPI_ISL_686628, EPI_ISL_686629, EPI_ISL_686630, EPI_ISL_686631, EPI_ISL_686632, EPI_ISL_686633, EPI_ISL_686634, EPI_ISL_686635, EPI_ISL_686636, EPI_ISL_686637,
EPI_ISL_686638, EPI_ISL_686639, EPI_ISL_686640, EPI_ISL_686641, EPI_ISL_686642, EPI_ISL_686643, EPI_ISL_686644, EPI_ISL_686645, EPI_ISL_686646, EPI_ISL_686647, EPI_ISL_686648, EPI_ISL_686649, EPI_ISL_686650, EPI_ISL_686651, EPI_ISL_686652, EPI_ISL_686653, EPI_ISL_686654, EPI_ISL_686655, EPI_ISL_686656, EPI_ISL_686657, EPI_ISL_686658, EPI_ISL_686845, EPI_ISL_686846, EPI_ISL_686847,
EPI_ISL_686848, EPI_ISL_686849, EPI_ISL_686850, EPI_ISL_686851, EPI_ISL_686852, EPI_ISL_686853, EPI_ISL_686854, EPI_ISL_686855, EPI_ISL_686856, EPI_ISL_686857, EPI_ISL_686858, EPI_ISL_686859, EPI_ISL_686860, EPI_ISL_686861, EPI_ISL_686862, EPI_ISL_686863, EPI_ISL_686864, EPI_ISL_686865, EPI_ISL_686866, EPI_ISL_686867, EPI_ISL_686868, EPI_ISL_686869, EPI_ISL_686870, EPI_ISL_686871,
EPI_ISL_686872, EPI_ISL_686873, EPI_ISL_686874, EPI_ISL_686875, EPI_ISL_686876, EPI_ISL_686877, EPI_ISL_686878, EPI_ISL_686879, EPI_ISL_686880, EPI_ISL_686881, EPI_ISL_686882, EPI_ISL_686883, EPI_ISL_686884, EPI_ISL_686885, EPI_ISL_686886, EPI_ISL_686887, EPI_ISL_686888, EPI_ISL_686889, EPI_ISL_686890, EPI_ISL_686891, EPI_ISL_686892, EPI_ISL_686893, EPI_ISL_686894, EPI_ISL_686895,
EPI_ISL_686896, EPI_ISL_686897, EPI_ISL_686898, EPI_ISL_686899, EPI_ISL_686900, EPI_ISL_686901, EPI_ISL_686902, EPI_ISL_686903, EPI_ISL_686904, EPI_ISL_686905, EPI_ISL_686906, EPI_ISL_686907, EPI_ISL_686908, EPI_ISL_686909, EPI_ISL_686910, EPI_ISL_686911, EPI_ISL_686912, EPI_ISL_686913, EPI_ISL_686914, EPI_ISL_686915, EPI_ISL_686916, EPI_ISL_686917, EPI_ISL_686918, EPI_ISL_686919,
EPI_ISL_686920, EPI_ISL_686921, EPI_ISL_686922, EPI_ISL_686923, EPI_ISL_686924, EPI_ISL_686925, EPI_ISL_686926, EPI_ISL_686927, EPI_ISL_686928, EPI_ISL_686929, EPI_ISL_686930, EPI_ISL_686931, EPI_ISL_686932, EPI_ISL_686933, EPI_ISL_686934, EPI_ISL_686935, EPI_ISL_686936, EPI_ISL_686937, EPI_ISL_686938, EPI_ISL_686939, EPI_ISL_686940, EPI_ISL_686941, EPI_ISL_686942, EPI_ISL_686943,
EPI_ISL_686944, EPI_ISL_686945, EPI_ISL_686946, EPI_ISL_686947, EPI_ISL_686948, EPI_ISL_686949, EPI_ISL_686950, EPI_ISL_686951, EPI_ISL_686952, EPI_ISL_686953, EPI_ISL_686954, EPI_ISL_686955, EPI_ISL_686956, EPI_ISL_686957, EPI_ISL_686958, EPI_ISL_686959, EPI_ISL_686960, EPI_ISL_686961, EPI_ISL_686962, EPI_ISL_686963, EPI_ISL_686964, EPI_ISL_686965, EPI_ISL_686966, EPI_ISL_686967,
EPI_ISL_686968, EPI_ISL_686969, EPI_ISL_686970, EPI_ISL_686971, EPI_ISL_686972, EPI_ISL_686973, EPI_ISL_686974, EPI_ISL_686975, EPI_ISL_686976, EPI_ISL_686977, EPI_ISL_686978, EPI_ISL_686979, EPI_ISL_686980, EPI_ISL_686981, EPI_ISL_686982, EPI_ISL_686983, EPI_ISL_686984, EPI_ISL_686985, EPI_ISL_686986, EPI_ISL_686987, EPI_ISL_686988, EPI_ISL_686989, EPI_ISL_686990, EPI_ISL_686991,
EPI_ISL_686992, EPI_ISL_686993, EPI_ISL_686994, EPI_ISL_686995, EPI_ISL_686996, EPI_ISL_686997, EPI_ISL_686998, EPI_ISL_686999, EPI_ISL_687000, EPI_ISL_687001, EPI_ISL_687002, EPI_ISL_687003, EPI_ISL_687004, EPI_ISL_687005, EPI_ISL_687006, EPI_ISL_687007, EPI_ISL_687008, EPI_ISL_687009, EPI_ISL_687010, EPI_ISL_687011, EPI_ISL_687012, EPI_ISL_687013, EPI_ISL_687014, EPI_ISL_687015,
EPI_ISL_687016, EPI_ISL_687017, EPI_ISL_687018, EPI_ISL_687019, EPI_ISL_687020, EPI_ISL_687021, EPI_ISL_687022, EPI_ISL_687023, EPI_ISL_687024, EPI_ISL_687025, EPI_ISL_687026, EPI_ISL_687027, EPI_ISL_687028, EPI_ISL_687029, EPI_ISL_687030, EPI_ISL_687031, EPI_ISL_687032, EPI_ISL_687033, EPI_ISL_687034, EPI_ISL_687035, EPI_ISL_687036, EPI_ISL_687037, EPI_ISL_687038, EPI_ISL_687039,
EPI_ISL_687040, EPI_ISL_687041, EPI_ISL_687042, EPI_ISL_687079, EPI_ISL_687080, EPI_ISL_687081, EPI_ISL_687082, EPI_ISL_687083, EPI_ISL_687084, EPI_ISL_687085, EPI_ISL_687086, EPI_ISL_687087, EPI_ISL_687088, EPI_ISL_687089, EPI_ISL_687090, EPI_ISL_687091, EPI_ISL_687092, EPI_ISL_687093, EPI_ISL_687094, EPI_ISL_687095, EPI_ISL_687096, EPI_ISL_687097, EPI_ISL_687098, EPI_ISL_687099,
EPI_ISL_687100, EPI_ISL_687101, EPI_ISL_687102, EPI_ISL_687103, EPI_ISL_687104, EPI_ISL_687105, EPI_ISL_687106, EPI_ISL_687107, EPI_ISL_687108, EPI_ISL_687109, EPI_ISL_687110, EPI_ISL_687111, EPI_ISL_687112, EPI_ISL_687113, EPI_ISL_687114, EPI_ISL_687115, EPI_ISL_687116, EPI_ISL_687117, EPI_ISL_687118, EPI_ISL_687119, EPI_ISL_687120, EPI_ISL_687121, EPI_ISL_687122, EPI_ISL_687123,
EPI_ISL_687124, EPI_ISL_687125, EPI_ISL_687126, EPI_ISL_687127, EPI_ISL_687128, EPI_ISL_687129, EPI_ISL_687130, EPI_ISL_687131, EPI_ISL_687132, EPI_ISL_687133, EPI_ISL_687134, EPI_ISL_687135, EPI_ISL_687136, EPI_ISL_687137, EPI_ISL_687138, EPI_ISL_687139, EPI_ISL_687140, EPI_ISL_687141, EPI_ISL_687142, EPI_ISL_687143, EPI_ISL_687144, EPI_ISL_687145, EPI_ISL_687146, EPI_ISL_687147,
EPI_ISL_687148, EPI_ISL_687149, EPI_ISL_687150, EPI_ISL_687151, EPI_ISL_687152, EPI_ISL_687153, EPI_ISL_687154, EPI_ISL_687155, EPI_ISL_687156, EPI_ISL_687157, EPI_ISL_687158, EPI_ISL_687159, EPI_ISL_687160, EPI_ISL_687161, EPI_ISL_687162, EPI_ISL_687163, EPI_ISL_687164, EPI_ISL_687165, EPI_ISL_687166, EPI_ISL_687167, EPI_ISL_687168, EPI_ISL_687169, EPI_ISL_687170, EPI_ISL_687171,
EPI_ISL_687172, EPI_ISL_687173, EPI_ISL_687174, EPI_ISL_687175, EPI_ISL_687176, EPI_ISL_687177, EPI_ISL_687178, EPI_ISL_687179, EPI_ISL_687180, EPI_ISL_687181, EPI_ISL_687182, EPI_ISL_687183, EPI_ISL_687184, EPI_ISL_687185, EPI_ISL_687186, EPI_ISL_687187, EPI_ISL_687188, EPI_ISL_687189, EPI_ISL_687190, EPI_ISL_687191, EPI_ISL_687192, EPI_ISL_687193, EPI_ISL_687194, EPI_ISL_687195,
EPI_ISL_687196, EPI_ISL_687197, EPI_ISL_687198, EPI_ISL_687199, EPI_ISL_687200, EPI_ISL_687201, EPI_ISL_687202, EPI_ISL_687203, EPI_ISL_687204, EPI_ISL_687205, EPI_ISL_687206, EPI_ISL_687207, EPI_ISL_687208, EPI_ISL_687209, EPI_ISL_687210, EPI_ISL_687211, EPI_ISL_687212, EPI_ISL_687213, EPI_ISL_687214, EPI_ISL_687215, EPI_ISL_687216, EPI_ISL_687217, EPI_ISL_687218, EPI_ISL_687219,
EPI_ISL_687220, EPI_ISL_687221, EPI_ISL_687222, EPI_ISL_687223, EPI_ISL_687224, EPI_ISL_687225, EPI_ISL_687226, EPI_ISL_687227, EPI_ISL_687228, EPI_ISL_687229, EPI_ISL_687230, EPI_ISL_687231, EPI_ISL_687232, EPI_ISL_687233, EPI_ISL_687234, EPI_ISL_687235, EPI_ISL_687236, EPI_ISL_687237, EPI_ISL_687238, EPI_ISL_687239, EPI_ISL_687240, EPI_ISL_687241, EPI_ISL_687242, EPI_ISL_687243,
EPI_ISL_687244, EPI_ISL_687245, EPI_ISL_687246, EPI_ISL_687247, EPI_ISL_687248, EPI_ISL_687249, EPI_ISL_687250, EPI_ISL_687251, EPI_ISL_687252, EPI_ISL_687253, EPI_ISL_687254, EPI_ISL_687255, EPI_ISL_687256, EPI_ISL_687257, EPI_ISL_687258, EPI_ISL_687259, EPI_ISL_687260, EPI_ISL_687261, EPI_ISL_687262, EPI_ISL_687263, EPI_ISL_687264, EPI_ISL_687265, EPI_ISL_687266, EPI_ISL_687267,
EPI_ISL_687268, EPI_ISL_687269, EPI_ISL_687270, EPI_ISL_687271, EPI_ISL_687272, EPI_ISL_687273, EPI_ISL_687274, EPI_ISL_687275, EPI_ISL_687276, EPI_ISL_687277, EPI_ISL_687278, EPI_ISL_687279, EPI_ISL_687280, EPI_ISL_687281, EPI_ISL_687282, EPI_ISL_687283, EPI_ISL_687284, EPI_ISL_687285, EPI_ISL_687286, EPI_ISL_687287, EPI_ISL_687288, EPI_ISL_687289, EPI_ISL_687290, EPI_ISL_687291,
EPI_ISL_687292, EPI_ISL_687293, EPI_ISL_687294, EPI_ISL_687295, EPI_ISL_687296, EPI_ISL_687297, EPI_ISL_687298, EPI_ISL_687299, EPI_ISL_687300, EPI_ISL_687301, EPI_ISL_687302, EPI_ISL_687303, EPI_ISL_687304, EPI_ISL_687305, EPI_ISL_687306, EPI_ISL_687307, EPI_ISL_687308, EPI_ISL_687309, EPI_ISL_687310, EPI_ISL_687311, EPI_ISL_687312, EPI_ISL_687313, EPI_ISL_687314, EPI_ISL_687315,
EPI_ISL_687316, EPI_ISL_687317, EPI_ISL_687318, EPI_ISL_687319, EPI_ISL_687320, EPI_ISL_687321, EPI_ISL_687322, EPI_ISL_687323, EPI_ISL_687324, EPI_ISL_687325, EPI_ISL_687326, EPI_ISL_687327, EPI_ISL_687328, EPI_ISL_687329, EPI_ISL_687330, EPI_ISL_687331, EPI_ISL_687332, EPI_ISL_687333, EPI_ISL_687334, EPI_ISL_687335, EPI_ISL_687336, EPI_ISL_687337, EPI_ISL_687338, EPI_ISL_687339,
EPI_ISL_687340, EPI_ISL_687341, EPI_ISL_687342, EPI_ISL_687343, EPI_ISL_687344, EPI_ISL_687345, EPI_ISL_687346, EPI_ISL_687347, EPI_ISL_687348, EPI_ISL_687349, EPI_ISL_687350, EPI_ISL_687351, EPI_ISL_687352, EPI_ISL_687353, EPI_ISL_687354, EPI_ISL_687355, EPI_ISL_687356, EPI_ISL_687357, EPI_ISL_687358, EPI_ISL_687359, EPI_ISL_687360, EPI_ISL_687361, EPI_ISL_687362, EPI_ISL_687363,
EPI_ISL_687364, EPI_ISL_687365, EPI_ISL_687366, EPI_ISL_687367, EPI_ISL_687368, EPI_ISL_687369, EPI_ISL_687370, EPI_ISL_687371, EPI_ISL_687372, EPI_ISL_687373, EPI_ISL_687374, EPI_ISL_687375, EPI_ISL_687376, EPI_ISL_687377, EPI_ISL_687378, EPI_ISL_687379, EPI_ISL_687380, EPI_ISL_687381, EPI_ISL_687382, EPI_ISL_687383, EPI_ISL_687384, EPI_ISL_687385, EPI_ISL_687386, EPI_ISL_687387,
EPI_ISL_687388, EPI_ISL_687389, EPI_ISL_687390, EPI_ISL_687391, EPI_ISL_687392, EPI_ISL_687393, EPI_ISL_687394, EPI_ISL_687395, EPI_ISL_687396, EPI_ISL_687397, EPI_ISL_687398, EPI_ISL_687399, EPI_ISL_687400, EPI_ISL_687401, EPI_ISL_687402, EPI_ISL_687403, EPI_ISL_687404, EPI_ISL_687405, EPI_ISL_687406, EPI_ISL_687407, EPI_ISL_687408, EPI_ISL_687409, EPI_ISL_687410, EPI_ISL_687411,
EPI_ISL_687412, EPI_ISL_687413, EPI_ISL_687414, EPI_ISL_687415, EPI_ISL_687416, EPI_ISL_687417, EPI_ISL_687418, EPI_ISL_687419, EPI_ISL_687420, EPI_ISL_687421, EPI_ISL_687422, EPI_ISL_687423, EPI_ISL_687424, EPI_ISL_687425, EPI_ISL_687426, EPI_ISL_687427, EPI_ISL_687428, EPI_ISL_687429, EPI_ISL_687430, EPI_ISL_687431, EPI_ISL_687432, EPI_ISL_687433, EPI_ISL_687434, EPI_ISL_687435,
EPI_ISL_687436, EPI_ISL_687437, EPI_ISL_687438, EPI_ISL_687439, EPI_ISL_687440, EPI_ISL_687441, EPI_ISL_687442, EPI_ISL_687443, EPI_ISL_687444, EPI_ISL_687445, EPI_ISL_687446, EPI_ISL_687447, EPI_ISL_687448, EPI_ISL_687449, EPI_ISL_687450, EPI_ISL_687451, EPI_ISL_687452, EPI_ISL_687453, EPI_ISL_687454, EPI_ISL_687455, EPI_ISL_687456, EPI_ISL_687457, EPI_ISL_687458, EPI_ISL_687459,
EPI_ISL_687460, EPI_ISL_687461, EPI_ISL_687462, EPI_ISL_687463, EPI_ISL_687464, EPI_ISL_687465, EPI_ISL_687466, EPI_ISL_687467, EPI_ISL_687468, EPI_ISL_687469, EPI_ISL_687470, EPI_ISL_687471, EPI_ISL_687472, EPI_ISL_687473, EPI_ISL_687474, EPI_ISL_687475, EPI_ISL_687476, EPI_ISL_687477, EPI_ISL_687478, EPI_ISL_687479, EPI_ISL_687480, EPI_ISL_687481, EPI_ISL_687482, EPI_ISL_687483,
EPI_ISL_687484, EPI_ISL_687485, EPI_ISL_687486, EPI_ISL_687487, EPI_ISL_687488, EPI_ISL_687489, EPI_ISL_687490, EPI_ISL_687491, EPI_ISL_687492, EPI_ISL_687493, EPI_ISL_687494, EPI_ISL_687495, EPI_ISL_687496, EPI_ISL_687497, EPI_ISL_687498, EPI_ISL_687499, EPI_ISL_687500, EPI_ISL_687501, EPI_ISL_687502, EPI_ISL_687503, EPI_ISL_687504, EPI_ISL_687505, EPI_ISL_687506, EPI_ISL_687507,
EPI_ISL_687508, EPI_ISL_687509, EPI_ISL_687510, EPI_ISL_687511, EPI_ISL_687512, EPI_ISL_687513, EPI_ISL_687514, EPI_ISL_687515, EPI_ISL_687516, EPI_ISL_687517, EPI_ISL_687518, EPI_ISL_687519, EPI_ISL_687520, EPI_ISL_687521, EPI_ISL_687522, EPI_ISL_687523, EPI_ISL_687524, EPI_ISL_687525, EPI_ISL_687526, EPI_ISL_687527, EPI_ISL_687528, EPI_ISL_687529, EPI_ISL_687530, EPI_ISL_687531,
EPI_ISL_687532, EPI_ISL_687561, EPI_ISL_687562, EPI_ISL_687563, EPI_ISL_687564, EPI_ISL_687565, EPI_ISL_687566, EPI_ISL_687567, EPI_ISL_687568, EPI_ISL_687569, EPI_ISL_687570, EPI_ISL_687571, EPI_ISL_687572, EPI_ISL_687573, EPI_ISL_687574, EPI_ISL_687575, EPI_ISL_687576, EPI_ISL_687577, EPI_ISL_687578, EPI_ISL_687579, EPI_ISL_687580, EPI_ISL_687581, EPI_ISL_687582, EPI_ISL_687583,
EPI_ISL_687584, EPI_ISL_687585, EPI_ISL_687586, EPI_ISL_687587, EPI_ISL_687588, EPI_ISL_687589, EPI_ISL_687590, EPI_ISL_687591, EPI_ISL_687592, EPI_ISL_687593, EPI_ISL_687594, EPI_ISL_687595, EPI_ISL_687596, EPI_ISL_687597, EPI_ISL_687598, EPI_ISL_687599, EPI_ISL_687600, EPI_ISL_687601, EPI_ISL_687602, EPI_ISL_687603, EPI_ISL_687604, EPI_ISL_687605, EPI_ISL_687606, EPI_ISL_687607,
EPI_ISL_687608, EPI_ISL_687609, EPI_ISL_687610, EPI_ISL_687611, EPI_ISL_687612, EPI_ISL_687613, EPI_ISL_687614, EPI_ISL_687615, EPI_ISL_687616, EPI_ISL_687617, EPI_ISL_687618, EPI_ISL_687621, EPI_ISL_687622, EPI_ISL_687623, EPI_ISL_687624, EPI_ISL_687625, EPI_ISL_687626, EPI_ISL_687627, EPI_ISL_687628, EPI_ISL_687629, EPI_ISL_687630, EPI_ISL_687631, EPI_ISL_687632, EPI_ISL_687633,
EPI_ISL_687634, EPI_ISL_687635, EPI_ISL_687636, EPI_ISL_687637, EPI_ISL_687638, EPI_ISL_687639, EPI_ISL_687640, EPI_ISL_687641, EPI_ISL_687642, EPI_ISL_687643, EPI_ISL_687644, EPI_ISL_687645, EPI_ISL_687646, EPI_ISL_687647, EPI_ISL_687648, EPI_ISL_687649, EPI_ISL_687650, EPI_ISL_687651, EPI_ISL_687652, EPI_ISL_687653, EPI_ISL_687654, EPI_ISL_687655, EPI_ISL_687656, EPI_ISL_687657,
EPI_ISL_687658, EPI_ISL_687659, EPI_ISL_687660, EPI_ISL_687661, EPI_ISL_687662, EPI_ISL_687663, EPI_ISL_687664, EPI_ISL_687665, EPI_ISL_687666, EPI_ISL_687667, EPI_ISL_687668, EPI_ISL_687669, EPI_ISL_687670, EPI_ISL_687671, EPI_ISL_687672, EPI_ISL_687673, EPI_ISL_687674, EPI_ISL_687675, EPI_ISL_687676, EPI_ISL_687677, EPI_ISL_687678, EPI_ISL_687679, EPI_ISL_687680, EPI_ISL_687681,
EPI_ISL_687682, EPI_ISL_687683, EPI_ISL_687684, EPI_ISL_687685, EPI_ISL_687686, EPI_ISL_687687, EPI_ISL_687688, EPI_ISL_687689, EPI_ISL_687690, EPI_ISL_687691, EPI_ISL_687692, EPI_ISL_687693, EPI_ISL_687694, EPI_ISL_687695, EPI_ISL_687696, EPI_ISL_687697, EPI_ISL_687698, EPI_ISL_687718, EPI_ISL_687719, EPI_ISL_687720, EPI_ISL_687721, EPI_ISL_687722, EPI_ISL_687723, EPI_ISL_687724,
EPI_ISL_687725, EPI_ISL_687726, EPI_ISL_687727, EPI_ISL_687728, EPI_ISL_687729, EPI_ISL_687730, EPI_ISL_687731, EPI_ISL_687732, EPI_ISL_687733, EPI_ISL_687734, EPI_ISL_687735, EPI_ISL_687736, EPI_ISL_687737, EPI_ISL_687738, EPI_ISL_687739, EPI_ISL_687740, EPI_ISL_687741, EPI_ISL_687742, EPI_ISL_687743, EPI_ISL_687744, EPI_ISL_687745, EPI_ISL_687746, EPI_ISL_687747, EPI_ISL_687748,
EPI_ISL_687749, EPI_ISL_687750, EPI_ISL_687751, EPI_ISL_687752, EPI_ISL_687753, EPI_ISL_687754, EPI_ISL_687755, EPI_ISL_687756, EPI_ISL_687757, EPI_ISL_687758, EPI_ISL_687759, EPI_ISL_687760, EPI_ISL_687761, EPI_ISL_687762, EPI_ISL_687763, EPI_ISL_687764, EPI_ISL_687765, EPI_ISL_687766, EPI_ISL_687767, EPI_ISL_687768, EPI_ISL_687769, EPI_ISL_687770, EPI_ISL_687771, EPI_ISL_687772,
EPI_ISL_687773, EPI_ISL_687774, EPI_ISL_687775, EPI_ISL_687776, EPI_ISL_687777, EPI_ISL_687778, EPI_ISL_687779, EPI_ISL_687780, EPI_ISL_687781, EPI_ISL_687782, EPI_ISL_687783, EPI_ISL_687784, EPI_ISL_687785, EPI_ISL_687786, EPI_ISL_687787, EPI_ISL_687788, EPI_ISL_687789, EPI_ISL_687790, EPI_ISL_687791, EPI_ISL_687792, EPI_ISL_687793, EPI_ISL_687794, EPI_ISL_687795, EPI_ISL_687796,
EPI_ISL_687797, EPI_ISL_687798, EPI_ISL_687799, EPI_ISL_687800, EPI_ISL_687801, EPI_ISL_687802, EPI_ISL_687803, EPI_ISL_687804, EPI_ISL_687805, EPI_ISL_687806, EPI_ISL_687807, EPI_ISL_687808, EPI_ISL_687809, EPI_ISL_687810, EPI_ISL_687811, EPI_ISL_687812, EPI_ISL_687813, EPI_ISL_687814, EPI_ISL_687815, EPI_ISL_687816, EPI_ISL_687817, EPI_ISL_687818, EPI_ISL_687819, EPI_ISL_687820,
EPI_ISL_687821, EPI_ISL_687822, EPI_ISL_687823, EPI_ISL_687824, EPI_ISL_687825, EPI_ISL_687826, EPI_ISL_687827, EPI_ISL_687828, EPI_ISL_687829, EPI_ISL_687830, EPI_ISL_687831, EPI_ISL_687832, EPI_ISL_687833, EPI_ISL_687834, EPI_ISL_687835, EPI_ISL_687836, EPI_ISL_687837, EPI_ISL_687838, EPI_ISL_687839, EPI_ISL_687840, EPI_ISL_687841, EPI_ISL_687842, EPI_ISL_687843, EPI_ISL_687844,
EPI_ISL_687845, EPI_ISL_687846, EPI_ISL_687847, EPI_ISL_687848, EPI_ISL_687849, EPI_ISL_687850, EPI_ISL_687851, EPI_ISL_687852, EPI_ISL_687853, EPI_ISL_687854, EPI_ISL_687855, EPI_ISL_687856, EPI_ISL_687857, EPI_ISL_687858, EPI_ISL_687859, EPI_ISL_687860, EPI_ISL_687861, EPI_ISL_687862, EPI_ISL_687863, EPI_ISL_687864, EPI_ISL_687865, EPI_ISL_687866, EPI_ISL_687867, EPI_ISL_687868,
EPI_ISL_687869, EPI_ISL_687870, EPI_ISL_687871, EPI_ISL_687872, EPI_ISL_687873, EPI_ISL_687874, EPI_ISL_687875, EPI_ISL_687876, EPI_ISL_687877, EPI_ISL_687878, EPI_ISL_687879, EPI_ISL_687880, EPI_ISL_687881, EPI_ISL_687882, EPI_ISL_687883, EPI_ISL_687884, EPI_ISL_687885, EPI_ISL_687886, EPI_ISL_687887, EPI_ISL_687888, EPI_ISL_687889, EPI_ISL_687890, EPI_ISL_687891, EPI_ISL_687892,
EPI_ISL_687893, EPI_ISL_687894, EPI_ISL_687895, EPI_ISL_687896, EPI_ISL_687897, EPI_ISL_687898, EPI_ISL_687899, EPI_ISL_687900, EPI_ISL_687901, EPI_ISL_687902, EPI_ISL_687903, EPI_ISL_687904, EPI_ISL_687905, EPI_ISL_687906, EPI_ISL_687907, EPI_ISL_687908, EPI_ISL_687909, EPI_ISL_687910, EPI_ISL_687911, EPI_ISL_687912, EPI_ISL_687913, EPI_ISL_687914, EPI_ISL_687915, EPI_ISL_687916,
EPI_ISL_687917, EPI_ISL_687918, EPI_ISL_687919, EPI_ISL_687920, EPI_ISL_687921, EPI_ISL_687922, EPI_ISL_687923, EPI_ISL_687924, EPI_ISL_687925, EPI_ISL_687926, EPI_ISL_687927, EPI_ISL_687928, EPI_ISL_687929, EPI_ISL_687930, EPI_ISL_687931, EPI_ISL_687932, EPI_ISL_687933, EPI_ISL_687934, EPI_ISL_687935, EPI_ISL_687936, EPI_ISL_687937, EPI_ISL_687938, EPI_ISL_687939, EPI_ISL_687940,
EPI_ISL_687941, EPI_ISL_687942, EPI_ISL_687943, EPI_ISL_687944, EPI_ISL_687945, EPI_ISL_687946, EPI_ISL_687947, EPI_ISL_687948, EPI_ISL_687949, EPI_ISL_687950, EPI_ISL_687951, EPI_ISL_687952, EPI_ISL_687953, EPI_ISL_687954, EPI_ISL_687955, EPI_ISL_687956, EPI_ISL_687957, EPI_ISL_687958, EPI_ISL_687959, EPI_ISL_687960, EPI_ISL_687961, EPI_ISL_687962, EPI_ISL_687963, EPI_ISL_687964,
EPI_ISL_687965, EPI_ISL_687966, EPI_ISL_687967, EPI_ISL_687968, EPI_ISL_687969, EPI_ISL_687970, EPI_ISL_687971, EPI_ISL_687972, EPI_ISL_687973, EPI_ISL_687974, EPI_ISL_687975, EPI_ISL_687976, EPI_ISL_687977, EPI_ISL_687978, EPI_ISL_687979, EPI_ISL_687980, EPI_ISL_687981, EPI_ISL_687982, EPI_ISL_687983, EPI_ISL_687984, EPI_ISL_687985, EPI_ISL_687986, EPI_ISL_687987, EPI_ISL_687988,
EPI_ISL_687989, EPI_ISL_687990, EPI_ISL_687991, EPI_ISL_687992, EPI_ISL_687993, EPI_ISL_687994, EPI_ISL_687995, EPI_ISL_687996, EPI_ISL_687997, EPI_ISL_687998, EPI_ISL_687999, EPI_ISL_688000, EPI_ISL_688001, EPI_ISL_688002, EPI_ISL_688003, EPI_ISL_688004, EPI_ISL_688005, EPI_ISL_688006, EPI_ISL_688007, EPI_ISL_688008, EPI_ISL_688009, EPI_ISL_688010, EPI_ISL_688011, EPI_ISL_688012,
EPI_ISL_688013, EPI_ISL_688014, EPI_ISL_688015, EPI_ISL_688016, EPI_ISL_688017, EPI_ISL_688018, EPI_ISL_688019, EPI_ISL_688020, EPI_ISL_688021, EPI_ISL_688022, EPI_ISL_688023, EPI_ISL_688024, EPI_ISL_688025, EPI_ISL_688026, EPI_ISL_688027, EPI_ISL_688028, EPI_ISL_688029, EPI_ISL_688030, EPI_ISL_688031, EPI_ISL_688032, EPI_ISL_688033, EPI_ISL_688034, EPI_ISL_688035, EPI_ISL_688036,
EPI_ISL_688037, EPI_ISL_688038, EPI_ISL_688039, EPI_ISL_688040, EPI_ISL_688041, EPI_ISL_688042, EPI_ISL_688043, EPI_ISL_688044, EPI_ISL_688045, EPI_ISL_688046, EPI_ISL_688047, EPI_ISL_688048, EPI_ISL_688049, EPI_ISL_688050, EPI_ISL_688051, EPI_ISL_688052, EPI_ISL_688053, EPI_ISL_688054, EPI_ISL_688055, EPI_ISL_688056, EPI_ISL_688057, EPI_ISL_688058, EPI_ISL_688059, EPI_ISL_688060,
EPI_ISL_688061, EPI_ISL_688062, EPI_ISL_688063, EPI_ISL_688064, EPI_ISL_688065, EPI_ISL_688066, EPI_ISL_688067, EPI_ISL_688068, EPI_ISL_688069, EPI_ISL_688070, EPI_ISL_688071, EPI_ISL_688072, EPI_ISL_688073, EPI_ISL_688074, EPI_ISL_688075, EPI_ISL_688076, EPI_ISL_688077, EPI_ISL_688078, EPI_ISL_688079, EPI_ISL_688080, EPI_ISL_688081, EPI_ISL_688082, EPI_ISL_688083, EPI_ISL_688084,
EPI_ISL_688085, EPI_ISL_688086, EPI_ISL_688087, EPI_ISL_688088, EPI_ISL_688089, EPI_ISL_688090, EPI_ISL_688091, EPI_ISL_688092, EPI_ISL_688093, EPI_ISL_688094, EPI_ISL_688095, EPI_ISL_688096, EPI_ISL_688097, EPI_ISL_688098, EPI_ISL_688099, EPI_ISL_688100, EPI_ISL_688101, EPI_ISL_688102, EPI_ISL_688103, EPI_ISL_688104, EPI_ISL_688105, EPI_ISL_688106, EPI_ISL_688107, EPI_ISL_688108,
EPI_ISL_688109, EPI_ISL_688110, EPI_ISL_688111, EPI_ISL_688112, EPI_ISL_688113, EPI_ISL_688114, EPI_ISL_688115, EPI_ISL_688116, EPI_ISL_688117, EPI_ISL_688118, EPI_ISL_688119, EPI_ISL_688120, EPI_ISL_688121, EPI_ISL_688122, EPI_ISL_688123, EPI_ISL_688124, EPI_ISL_688125, EPI_ISL_688126, EPI_ISL_688127, EPI_ISL_688128, EPI_ISL_688129, EPI_ISL_688130, EPI_ISL_688131, EPI_ISL_688132,
EPI_ISL_688133, EPI_ISL_688134, EPI_ISL_688135, EPI_ISL_688136, EPI_ISL_688137, EPI_ISL_688138, EPI_ISL_688139, EPI_ISL_688140, EPI_ISL_688141, EPI_ISL_688142, EPI_ISL_688143, EPI_ISL_688144, EPI_ISL_688145, EPI_ISL_688146, EPI_ISL_688147, EPI_ISL_688148, EPI_ISL_688149, EPI_ISL_688150, EPI_ISL_688151, EPI_ISL_688152, EPI_ISL_688153, EPI_ISL_688154, EPI_ISL_688155, EPI_ISL_688156,
EPI_ISL_688157, EPI_ISL_688158, EPI_ISL_688159, EPI_ISL_688160, EPI_ISL_688161, EPI_ISL_688162, EPI_ISL_688163, EPI_ISL_688164, EPI_ISL_688165, EPI_ISL_688166, EPI_ISL_688167, EPI_ISL_688168, EPI_ISL_688169, EPI_ISL_688170, EPI_ISL_688171, EPI_ISL_688172, EPI_ISL_688173, EPI_ISL_688174, EPI_ISL_688175, EPI_ISL_688176, EPI_ISL_688177, EPI_ISL_688178, EPI_ISL_688179, EPI_ISL_688180,
EPI_ISL_688181, EPI_ISL_688182, EPI_ISL_688183, EPI_ISL_688184, EPI_ISL_688185, EPI_ISL_688186, EPI_ISL_688187, EPI_ISL_688188, EPI_ISL_688189, EPI_ISL_688190, EPI_ISL_688191, EPI_ISL_688192, EPI_ISL_688193, EPI_ISL_688194, EPI_ISL_688195, EPI_ISL_688196, EPI_ISL_688197, EPI_ISL_688198, EPI_ISL_688199, EPI_ISL_688200, EPI_ISL_688201, EPI_ISL_688202, EPI_ISL_688203, EPI_ISL_688204,
EPI_ISL_688205, EPI_ISL_688206, EPI_ISL_688207, EPI_ISL_688208, EPI_ISL_688209, EPI_ISL_688210, EPI_ISL_688211, EPI_ISL_688212, EPI_ISL_688213, EPI_ISL_688214, EPI_ISL_688215, EPI_ISL_688216, EPI_ISL_688217, EPI_ISL_688218, EPI_ISL_688219, EPI_ISL_688220, EPI_ISL_688221, EPI_ISL_688222, EPI_ISL_688223, EPI_ISL_688224, EPI_ISL_688225, EPI_ISL_688226, EPI_ISL_688227, EPI_ISL_688228,
EPI_ISL_688229, EPI_ISL_688230, EPI_ISL_688231, EPI_ISL_688232, EPI_ISL_688233, EPI_ISL_688234, EPI_ISL_688235, EPI_ISL_688236, EPI_ISL_688237, EPI_ISL_688238, EPI_ISL_688239, EPI_ISL_688240, EPI_ISL_688241, EPI_ISL_688242, EPI_ISL_688243, EPI_ISL_688244, EPI_ISL_688245, EPI_ISL_688246, EPI_ISL_688247, EPI_ISL_688248, EPI_ISL_688249, EPI_ISL_688250, EPI_ISL_688251, EPI_ISL_688252,
EPI_ISL_688253, EPI_ISL_688254, EPI_ISL_688255, EPI_ISL_688256, EPI_ISL_688257, EPI_ISL_688258, EPI_ISL_688259, EPI_ISL_688260, EPI_ISL_688261, EPI_ISL_688262, EPI_ISL_688263, EPI_ISL_688264, EPI_ISL_688265, EPI_ISL_688266, EPI_ISL_688267, EPI_ISL_688268, EPI_ISL_688269, EPI_ISL_688270, EPI_ISL_688271, EPI_ISL_688272, EPI_ISL_688273, EPI_ISL_688274, EPI_ISL_688275, EPI_ISL_688276,
EPI_ISL_688277, EPI_ISL_688278, EPI_ISL_688279, EPI_ISL_688280, EPI_ISL_688281, EPI_ISL_688282, EPI_ISL_688283, EPI_ISL_688402, EPI_ISL_688403, EPI_ISL_688404, EPI_ISL_688405, EPI_ISL_688406, EPI_ISL_688407, EPI_ISL_688408, EPI_ISL_688409, EPI_ISL_688410, EPI_ISL_688411, EPI_ISL_688412, EPI_ISL_688413, EPI_ISL_688414, EPI_ISL_688415, EPI_ISL_688416, EPI_ISL_688417, EPI_ISL_688418,
EPI_ISL_688419, EPI_ISL_688420, EPI_ISL_688421, EPI_ISL_688422, EPI_ISL_688423, EPI_ISL_688424, EPI_ISL_688425, EPI_ISL_688426, EPI_ISL_688427, EPI_ISL_688428, EPI_ISL_688429, EPI_ISL_688430, EPI_ISL_688431, EPI_ISL_688432, EPI_ISL_688433, EPI_ISL_688434, EPI_ISL_688435, EPI_ISL_688436, EPI_ISL_688437, EPI_ISL_688438, EPI_ISL_688439, EPI_ISL_688440, EPI_ISL_688441, EPI_ISL_688442,
EPI_ISL_688443, EPI_ISL_688444, EPI_ISL_688445, EPI_ISL_688446, EPI_ISL_688447, EPI_ISL_688448, EPI_ISL_688449, EPI_ISL_688450, EPI_ISL_688451, EPI_ISL_688452, EPI_ISL_688453, EPI_ISL_688454, EPI_ISL_688455, EPI_ISL_688456, EPI_ISL_688457, EPI_ISL_688458, EPI_ISL_688459, EPI_ISL_688460, EPI_ISL_688461, EPI_ISL_688462, EPI_ISL_688463, EPI_ISL_688464, EPI_ISL_688465, EPI_ISL_688466,
EPI_ISL_688467, EPI_ISL_688468, EPI_ISL_688469, EPI_ISL_688470, EPI_ISL_688471, EPI_ISL_688472, EPI_ISL_688473, EPI_ISL_688474, EPI_ISL_688475, EPI_ISL_688476, EPI_ISL_688477, EPI_ISL_688478, EPI_ISL_688479, EPI_ISL_688480, EPI_ISL_688481, EPI_ISL_688482, EPI_ISL_688483, EPI_ISL_688484, EPI_ISL_688485, EPI_ISL_688486, EPI_ISL_688487, EPI_ISL_688488, EPI_ISL_688489, EPI_ISL_688490,
EPI_ISL_688491, EPI_ISL_688492, EPI_ISL_688493, EPI_ISL_688494, EPI_ISL_688495, EPI_ISL_688496, EPI_ISL_688497, EPI_ISL_688498, EPI_ISL_688499, EPI_ISL_688500, EPI_ISL_688501, EPI_ISL_688502, EPI_ISL_688503, EPI_ISL_688504, EPI_ISL_688505, EPI_ISL_688506, EPI_ISL_688507, EPI_ISL_688508, EPI_ISL_688509, EPI_ISL_688510, EPI_ISL_688511, EPI_ISL_688512, EPI_ISL_688513, EPI_ISL_688514,
EPI_ISL_688515, EPI_ISL_688516, EPI_ISL_688517, EPI_ISL_688518, EPI_ISL_688519, EPI_ISL_688520, EPI_ISL_688521, EPI_ISL_688522, EPI_ISL_688523, EPI_ISL_688524, EPI_ISL_688525, EPI_ISL_688526, EPI_ISL_688527, EPI_ISL_688528, EPI_ISL_688529, EPI_ISL_688530, EPI_ISL_688531, EPI_ISL_688532, EPI_ISL_688533, EPI_ISL_688534, EPI_ISL_688535, EPI_ISL_688536, EPI_ISL_688537, EPI_ISL_688538,
EPI_ISL_688539, EPI_ISL_688540, EPI_ISL_688541, EPI_ISL_688542, EPI_ISL_688543, EPI_ISL_688544, EPI_ISL_688545, EPI_ISL_688546, EPI_ISL_688547, EPI_ISL_688548, EPI_ISL_688549, EPI_ISL_688550, EPI_ISL_688551, EPI_ISL_688552, EPI_ISL_688553, EPI_ISL_688554, EPI_ISL_688555, EPI_ISL_688556, EPI_ISL_688557, EPI_ISL_688558, EPI_ISL_688559, EPI_ISL_688560, EPI_ISL_688561, EPI_ISL_688562,
EPI_ISL_688563, EPI_ISL_688564, EPI_ISL_688565, EPI_ISL_688566, EPI_ISL_688567, EPI_ISL_688568, EPI_ISL_688569, EPI_ISL_688570, EPI_ISL_688571, EPI_ISL_688572, EPI_ISL_688573, EPI_ISL_688574, EPI_ISL_688575, EPI_ISL_688576, EPI_ISL_688577, EPI_ISL_688578, EPI_ISL_688579, EPI_ISL_688580, EPI_ISL_688581, EPI_ISL_688582, EPI_ISL_688605, EPI_ISL_688606, EPI_ISL_688607, EPI_ISL_688608,
EPI_ISL_688609, EPI_ISL_688610, EPI_ISL_688611, EPI_ISL_688613, EPI_ISL_688614, EPI_ISL_688615, EPI_ISL_688616, EPI_ISL_688617, EPI_ISL_688618, EPI_ISL_688624, EPI_ISL_688625, EPI_ISL_688626, EPI_ISL_688627, EPI_ISL_688628, EPI_ISL_688629, EPI_ISL_688630, EPI_ISL_688631, EPI_ISL_688632, EPI_ISL_688633, EPI_ISL_688635, EPI_ISL_688636, EPI_ISL_688637, EPI_ISL_688638, EPI_ISL_688639,
EPI_ISL_688640, EPI_ISL_688641, EPI_ISL_688642, EPI_ISL_688643, EPI_ISL_688644, EPI_ISL_688645, EPI_ISL_688646, EPI_ISL_688647, EPI_ISL_688648, EPI_ISL_688649, EPI_ISL_688650, EPI_ISL_688651, EPI_ISL_688652, EPI_ISL_688653, EPI_ISL_688654, EPI_ISL_688655, EPI_ISL_688656, EPI_ISL_688657, EPI_ISL_688658, EPI_ISL_688659, EPI_ISL_688660, EPI_ISL_688661, EPI_ISL_688662, EPI_ISL_688663,
EPI_ISL_688664, EPI_ISL_688665, EPI_ISL_688666, EPI_ISL_688667, EPI_ISL_688668, EPI_ISL_688669, EPI_ISL_688670, EPI_ISL_688671, EPI_ISL_688672, EPI_ISL_688673, EPI_ISL_688674, EPI_ISL_688675, EPI_ISL_688676, EPI_ISL_688677, EPI_ISL_688678, EPI_ISL_688679, EPI_ISL_688680, EPI_ISL_688681, EPI_ISL_688682, EPI_ISL_688683, EPI_ISL_688684, EPI_ISL_688685, EPI_ISL_688686, EPI_ISL_688687,
EPI_ISL_688688, EPI_ISL_688689, EPI_ISL_688690, EPI_ISL_688691, EPI_ISL_688692, EPI_ISL_688693, EPI_ISL_688694, EPI_ISL_688695, EPI_ISL_688696, EPI_ISL_688697, EPI_ISL_688698, EPI_ISL_688699, EPI_ISL_688710, EPI_ISL_688711, EPI_ISL_688712, EPI_ISL_688713, EPI_ISL_688714, EPI_ISL_688715, EPI_ISL_688716, EPI_ISL_688717, EPI_ISL_688718, EPI_ISL_688719, EPI_ISL_688720, EPI_ISL_688721,
EPI_ISL_688722, EPI_ISL_688723, EPI_ISL_688724, EPI_ISL_688725, EPI_ISL_688726, EPI_ISL_688727, EPI_ISL_688728, EPI_ISL_688729, EPI_ISL_688730, EPI_ISL_688731, EPI_ISL_688732, EPI_ISL_688733, EPI_ISL_688734, EPI_ISL_688735, EPI_ISL_688736, EPI_ISL_688737, EPI_ISL_688738, EPI_ISL_688739, EPI_ISL_688740, EPI_ISL_688741, EPI_ISL_688742, EPI_ISL_688743, EPI_ISL_688744, EPI_ISL_688745,
EPI_ISL_688746, EPI_ISL_688747, EPI_ISL_688748, EPI_ISL_688749, EPI_ISL_688750, EPI_ISL_688751, EPI_ISL_688752, EPI_ISL_688753, EPI_ISL_688754, EPI_ISL_688755, EPI_ISL_688756, EPI_ISL_688757, EPI_ISL_688758, EPI_ISL_688759, EPI_ISL_688760, EPI_ISL_688761, EPI_ISL_688762, EPI_ISL_688763, EPI_ISL_688764, EPI_ISL_688765, EPI_ISL_688766, EPI_ISL_688767, EPI_ISL_688768, EPI_ISL_688769,
EPI_ISL_688770, EPI_ISL_688771, EPI_ISL_688772, EPI_ISL_688773, EPI_ISL_688774, EPI_ISL_688775, EPI_ISL_688776, EPI_ISL_688777, EPI_ISL_688778, EPI_ISL_688779, EPI_ISL_688780, EPI_ISL_688781, EPI_ISL_688782, EPI_ISL_688783, EPI_ISL_688784, EPI_ISL_688785, EPI_ISL_688786, EPI_ISL_688787, EPI_ISL_688788, EPI_ISL_688789, EPI_ISL_688790, EPI_ISL_688791, EPI_ISL_688792, EPI_ISL_688793,
EPI_ISL_688794, EPI_ISL_688795, EPI_ISL_688796, EPI_ISL_688797, EPI_ISL_688798, EPI_ISL_688799, EPI_ISL_688800, EPI_ISL_688801, EPI_ISL_688802, EPI_ISL_688803, EPI_ISL_688804, EPI_ISL_688805, EPI_ISL_688806, EPI_ISL_688807, EPI_ISL_688808, EPI_ISL_688809, EPI_ISL_688810, EPI_ISL_688811, EPI_ISL_688812, EPI_ISL_688813, EPI_ISL_688814, EPI_ISL_688815, EPI_ISL_688816, EPI_ISL_688817,
EPI_ISL_688818, EPI_ISL_688819, EPI_ISL_688820, EPI_ISL_688821, EPI_ISL_688822, EPI_ISL_688823, EPI_ISL_688824, EPI_ISL_688825, EPI_ISL_688826, EPI_ISL_688827, EPI_ISL_688828, EPI_ISL_688829, EPI_ISL_688830, EPI_ISL_688831, EPI_ISL_688832, EPI_ISL_688833, EPI_ISL_688834, EPI_ISL_688835, EPI_ISL_688836, EPI_ISL_688837, EPI_ISL_688838, EPI_ISL_688839, EPI_ISL_688840, EPI_ISL_688841,

EPI_ISL_688842, EPI_ISL_688843, EPI_ISL_688844, EPI_ISL_688845, EPI_ISL_688846, EPI_ISL_688847, EPI_ISL_688848, EPI_ISL_688849, EPI_ISL_688850, EPI_ISL_688851, EPI_ISL_688852, EPI_ISL_688853, EPI_ISL_688854, EPI_ISL_688855, EPI_ISL_688856, EPI_ISL_688857, EPI_ISL_688858, EPI_ISL_688859, EPI_ISL_688860, EPI_ISL_688861, EPI_ISL_688862, EPI_ISL_688863, EPI_ISL_688864, EPI_ISL_688865,
EPI_ISL_688866, EPI_ISL_688867, EPI_ISL_688868, EPI_ISL_688869, EPI_ISL_688870, EPI_ISL_688871, EPI_ISL_688872, EPI_ISL_688873, EPI_ISL_688874, EPI_ISL_688875, EPI_ISL_688876, EPI_ISL_688877, EPI_ISL_688878, EPI_ISL_688879, EPI_ISL_688880, EPI_ISL_688881, EPI_ISL_688882, EPI_ISL_688883, EPI_ISL_688884, EPI_ISL_688885, EPI_ISL_688886, EPI_ISL_688887, EPI_ISL_688888, EPI_ISL_688889,
EPI_ISL_688890, EPI_ISL_688891, EPI_ISL_688892, EPI_ISL_688893, EPI_ISL_688894, EPI_ISL_688895, EPI_ISL_688896, EPI_ISL_688969, EPI_ISL_688970, EPI_ISL_688971, EPI_ISL_688972, EPI_ISL_688973, EPI_ISL_688974, EPI_ISL_688975, EPI_ISL_688976, EPI_ISL_688977, EPI_ISL_688978, EPI_ISL_688979, EPI_ISL_688980, EPI_ISL_688981, EPI_ISL_688982, EPI_ISL_688983, EPI_ISL_688984, EPI_ISL_688985,
EPI_ISL_688986, EPI_ISL_688987, EPI_ISL_688988, EPI_ISL_688989, EPI_ISL_688990, EPI_ISL_688991, EPI_ISL_688992, EPI_ISL_688993, EPI_ISL_688994, EPI_ISL_688995, EPI_ISL_688996, EPI_ISL_688997, EPI_ISL_688998, EPI_ISL_688999, EPI_ISL_689000, EPI_ISL_689001, EPI_ISL_689002, EPI_ISL_689003, EPI_ISL_689004, EPI_ISL_689005, EPI_ISL_689006, EPI_ISL_689007, EPI_ISL_689008, EPI_ISL_689009,
EPI_ISL_689010, EPI_ISL_689011, EPI_ISL_689012, EPI_ISL_689013, EPI_ISL_689014, EPI_ISL_689015, EPI_ISL_689016, EPI_ISL_689017, EPI_ISL_689018, EPI_ISL_689019, EPI_ISL_689020, EPI_ISL_689021, EPI_ISL_689022, EPI_ISL_689023, EPI_ISL_689024, EPI_ISL_689025, EPI_ISL_689026, EPI_ISL_689027, EPI_ISL_689028, EPI_ISL_689029, EPI_ISL_689030, EPI_ISL_689031, EPI_ISL_689032, EPI_ISL_689033,
EPI_ISL_689034, EPI_ISL_689035, EPI_ISL_689036, EPI_ISL_689037, EPI_ISL_689038, EPI_ISL_689039, EPI_ISL_689040, EPI_ISL_689041, EPI_ISL_689042, EPI_ISL_689043, EPI_ISL_689044, EPI_ISL_689045, EPI_ISL_689046, EPI_ISL_689089, EPI_ISL_689090, EPI_ISL_689091, EPI_ISL_689092, EPI_ISL_689093, EPI_ISL_689094, EPI_ISL_689095, EPI_ISL_689096, EPI_ISL_689097, EPI_ISL_689098, EPI_ISL_689099,
EPI_ISL_689100, EPI_ISL_689101, EPI_ISL_689102, EPI_ISL_689103, EPI_ISL_689104, EPI_ISL_689105, EPI_ISL_689106, EPI_ISL_689107, EPI_ISL_689108, EPI_ISL_689109, EPI_ISL_689110, EPI_ISL_689111, EPI_ISL_689112, EPI_ISL_689113, EPI_ISL_689114, EPI_ISL_689115, EPI_ISL_689116, EPI_ISL_689117, EPI_ISL_689118, EPI_ISL_689119, EPI_ISL_689120, EPI_ISL_689121, EPI_ISL_689122, EPI_ISL_689123,
EPI_ISL_689124, EPI_ISL_689125, EPI_ISL_689126, EPI_ISL_689127, EPI_ISL_689128, EPI_ISL_689129, EPI_ISL_689130, EPI_ISL_689131, EPI_ISL_689132, EPI_ISL_689133, EPI_ISL_689134, EPI_ISL_689135, EPI_ISL_689136, EPI_ISL_689137, EPI_ISL_689138, EPI_ISL_689139, EPI_ISL_689140, EPI_ISL_689141, EPI_ISL_689142, EPI_ISL_689143, EPI_ISL_689144, EPI_ISL_689145, EPI_ISL_689146, EPI_ISL_689147,
EPI_ISL_689148, EPI_ISL_689149, EPI_ISL_689150, EPI_ISL_689151, EPI_ISL_689152, EPI_ISL_689153, EPI_ISL_689154, EPI_ISL_689155, EPI_ISL_689156, EPI_ISL_689157, EPI_ISL_689158, EPI_ISL_689159, EPI_ISL_689160, EPI_ISL_689161, EPI_ISL_689162, EPI_ISL_689163, EPI_ISL_689164, EPI_ISL_689165, EPI_ISL_689166, EPI_ISL_689167, EPI_ISL_689168, EPI_ISL_689169, EPI_ISL_689170, EPI_ISL_689171,
EPI_ISL_689172, EPI_ISL_689173, EPI_ISL_689174, EPI_ISL_689175, EPI_ISL_689176, EPI_ISL_689177, EPI_ISL_689178, EPI_ISL_689179, EPI_ISL_689180, EPI_ISL_689181, EPI_ISL_689182, EPI_ISL_689183, EPI_ISL_689184, EPI_ISL_689185, EPI_ISL_689186, EPI_ISL_689187, EPI_ISL_689188, EPI_ISL_689189, EPI_ISL_689190, EPI_ISL_689191, EPI_ISL_689192, EPI_ISL_689193, EPI_ISL_689194, EPI_ISL_689195,
EPI_ISL_689196, EPI_ISL_689197, EPI_ISL_689198, EPI_ISL_689199, EPI_ISL_689200, EPI_ISL_689201, EPI_ISL_689202, EPI_ISL_689203, EPI_ISL_689204, EPI_ISL_689205, EPI_ISL_689206, EPI_ISL_689207, EPI_ISL_689208, EPI_ISL_689209, EPI_ISL_689210, EPI_ISL_689211, EPI_ISL_689212, EPI_ISL_689213, EPI_ISL_689214, EPI_ISL_689215, EPI_ISL_689216, EPI_ISL_689217, EPI_ISL_689218, EPI_ISL_689219,
EPI_ISL_689220, EPI_ISL_689221, EPI_ISL_689222, EPI_ISL_689223, EPI_ISL_689224, EPI_ISL_689225, EPI_ISL_689226, EPI_ISL_689227, EPI_ISL_689228, EPI_ISL_689229, EPI_ISL_689230, EPI_ISL_689231, EPI_ISL_689232, EPI_ISL_689233, EPI_ISL_689234, EPI_ISL_689235, EPI_ISL_689236, EPI_ISL_689237, EPI_ISL_689238, EPI_ISL_689239, EPI_ISL_689240, EPI_ISL_689241, EPI_ISL_689242, EPI_ISL_689243,
EPI_ISL_689244, EPI_ISL_689245, EPI_ISL_689246, EPI_ISL_689247, EPI_ISL_689248, EPI_ISL_689249, EPI_ISL_689250, EPI_ISL_689251, EPI_ISL_689252, EPI_ISL_689253, EPI_ISL_689254, EPI_ISL_689255, EPI_ISL_689256, EPI_ISL_689257, EPI_ISL_689258, EPI_ISL_689259, EPI_ISL_689260, EPI_ISL_689261, EPI_ISL_689262, EPI_ISL_689263, EPI_ISL_689264, EPI_ISL_689265, EPI_ISL_689266, EPI_ISL_689267,
EPI_ISL_689268, EPI_ISL_689269, EPI_ISL_689270, EPI_ISL_689271, EPI_ISL_689272, EPI_ISL_689273, EPI_ISL_689274, EPI_ISL_689275, EPI_ISL_689276, EPI_ISL_689277, EPI_ISL_689278, EPI_ISL_689279, EPI_ISL_689280, EPI_ISL_689281, EPI_ISL_689282, EPI_ISL_689283, EPI_ISL_689284, EPI_ISL_689285, EPI_ISL_689286, EPI_ISL_689287, EPI_ISL_689288, EPI_ISL_689289, EPI_ISL_689290, EPI_ISL_689291,
EPI_ISL_689292, EPI_ISL_689293, EPI_ISL_689294, EPI_ISL_689295, EPI_ISL_689296, EPI_ISL_689297, EPI_ISL_689298, EPI_ISL_689299, EPI_ISL_689300, EPI_ISL_689301, EPI_ISL_689302, EPI_ISL_689303, EPI_ISL_689304, EPI_ISL_689305, EPI_ISL_689306, EPI_ISL_689307, EPI_ISL_689308, EPI_ISL_689309, EPI_ISL_689310, EPI_ISL_689311, EPI_ISL_689312, EPI_ISL_689313, EPI_ISL_689314, EPI_ISL_689315,
EPI_ISL_689316, EPI_ISL_689317, EPI_ISL_689318, EPI_ISL_689319, EPI_ISL_689320, EPI_ISL_689321, EPI_ISL_689322, EPI_ISL_689323, EPI_ISL_689324, EPI_ISL_689325, EPI_ISL_689326, EPI_ISL_689327, EPI_ISL_689328, EPI_ISL_689329, EPI_ISL_689330, EPI_ISL_689331, EPI_ISL_689332, EPI_ISL_689333, EPI_ISL_689334, EPI_ISL_689335, EPI_ISL_689336, EPI_ISL_689337, EPI_ISL_689338, EPI_ISL_689339,
EPI_ISL_689340, EPI_ISL_689341, EPI_ISL_689342, EPI_ISL_689343, EPI_ISL_689344, EPI_ISL_689345, EPI_ISL_689346, EPI_ISL_689347, EPI_ISL_689348, EPI_ISL_689349, EPI_ISL_689350, EPI_ISL_689351, EPI_ISL_689352, EPI_ISL_689353, EPI_ISL_689354, EPI_ISL_689355, EPI_ISL_689356, EPI_ISL_689357, EPI_ISL_689358, EPI_ISL_689359, EPI_ISL_689360, EPI_ISL_689361, EPI_ISL_689362, EPI_ISL_689363,
EPI_ISL_689364, EPI_ISL_689365, EPI_ISL_689366, EPI_ISL_689367, EPI_ISL_689368, EPI_ISL_689369, EPI_ISL_689370, EPI_ISL_689371, EPI_ISL_689372, EPI_ISL_689373, EPI_ISL_689374, EPI_ISL_689375, EPI_ISL_689376, EPI_ISL_689377, EPI_ISL_689378, EPI_ISL_689379, EPI_ISL_689380, EPI_ISL_689381, EPI_ISL_689382, EPI_ISL_689383, EPI_ISL_689384, EPI_ISL_689385, EPI_ISL_689386, EPI_ISL_689387,
EPI_ISL_689388, EPI_ISL_689389, EPI_ISL_689390, EPI_ISL_689391, EPI_ISL_689392, EPI_ISL_689393, EPI_ISL_689394, EPI_ISL_689395, EPI_ISL_689396, EPI_ISL_689397, EPI_ISL_689398, EPI_ISL_689399, EPI_ISL_689400, EPI_ISL_689401, EPI_ISL_689402, EPI_ISL_689403, EPI_ISL_689404, EPI_ISL_689405, EPI_ISL_689406, EPI_ISL_689407, EPI_ISL_689408, EPI_ISL_689409, EPI_ISL_689410, EPI_ISL_689411,
EPI_ISL_689412, EPI_ISL_689413, EPI_ISL_689414, EPI_ISL_689415, EPI_ISL_689416, EPI_ISL_689417, EPI_ISL_689418, EPI_ISL_689419, EPI_ISL_689420, EPI_ISL_689421, EPI_ISL_689422, EPI_ISL_689423, EPI_ISL_689424, EPI_ISL_689425, EPI_ISL_689426, EPI_ISL_689427, EPI_ISL_689428, EPI_ISL_689429, EPI_ISL_689430, EPI_ISL_689431, EPI_ISL_689432, EPI_ISL_689433, EPI_ISL_689434, EPI_ISL_689435,
EPI_ISL_689436, EPI_ISL_689437, EPI_ISL_689438, EPI_ISL_689439, EPI_ISL_689440, EPI_ISL_689441, EPI_ISL_689442, EPI_ISL_689443, EPI_ISL_689444, EPI_ISL_689445, EPI_ISL_689446, EPI_ISL_689447, EPI_ISL_689448, EPI_ISL_689449, EPI_ISL_689450, EPI_ISL_689451, EPI_ISL_689452, EPI_ISL_689453, EPI_ISL_689454, EPI_ISL_689455, EPI_ISL_689456, EPI_ISL_689457, EPI_ISL_689458, EPI_ISL_689459,
EPI_ISL_689497, EPI_ISL_689498, EPI_ISL_689499, EPI_ISL_689500, EPI_ISL_689501, EPI_ISL_689502, EPI_ISL_689503, EPI_ISL_689504, EPI_ISL_689505, EPI_ISL_689506, EPI_ISL_689507, EPI_ISL_689508, EPI_ISL_689509, EPI_ISL_689510, EPI_ISL_689511, EPI_ISL_689512, EPI_ISL_689513, EPI_ISL_689514, EPI_ISL_689515, EPI_ISL_689516, EPI_ISL_689517, EPI_ISL_689518, EPI_ISL_689519, EPI_ISL_689520,
EPI_ISL_689521, EPI_ISL_689522, EPI_ISL_689523, EPI_ISL_689524, EPI_ISL_689525, EPI_ISL_689526, EPI_ISL_689527, EPI_ISL_689528, EPI_ISL_689529, EPI_ISL_689530, EPI_ISL_689531, EPI_ISL_689532, EPI_ISL_689533, EPI_ISL_689534, EPI_ISL_689535, EPI_ISL_689536, EPI_ISL_689537, EPI_ISL_689538, EPI_ISL_689539, EPI_ISL_689540, EPI_ISL_689541, EPI_ISL_689542, EPI_ISL_689543, EPI_ISL_689544,
EPI_ISL_689545, EPI_ISL_689546, EPI_ISL_689547, EPI_ISL_689548, EPI_ISL_689549, EPI_ISL_689550, EPI_ISL_689551, EPI_ISL_689552, EPI_ISL_689553, EPI_ISL_689554, EPI_ISL_689555, EPI_ISL_689556, EPI_ISL_689557, EPI_ISL_689558, EPI_ISL_689559, EPI_ISL_689560, EPI_ISL_689561, EPI_ISL_689562, EPI_ISL_689563, EPI_ISL_689564, EPI_ISL_689565, EPI_ISL_689566, EPI_ISL_689567, EPI_ISL_689568,
EPI_ISL_689569, EPI_ISL_689570, EPI_ISL_689571, EPI_ISL_689572, EPI_ISL_689573, EPI_ISL_689574, EPI_ISL_689575, EPI_ISL_689576, EPI_ISL_689577, EPI_ISL_689578, EPI_ISL_689579, EPI_ISL_689580, EPI_ISL_689581, EPI_ISL_689582, EPI_ISL_689583, EPI_ISL_689584, EPI_ISL_689585, EPI_ISL_689586, EPI_ISL_689587, EPI_ISL_689588, EPI_ISL_689589, EPI_ISL_689590, EPI_ISL_689591, EPI_ISL_689592,
EPI_ISL_689593, EPI_ISL_689594, EPI_ISL_689595, EPI_ISL_689596, EPI_ISL_689597, EPI_ISL_689598, EPI_ISL_689599, EPI_ISL_689600, EPI_ISL_689601, EPI_ISL_689602, EPI_ISL_689603, EPI_ISL_689604, EPI_ISL_689605, EPI_ISL_689606, EPI_ISL_689607, EPI_ISL_689608, EPI_ISL_689609, EPI_ISL_689610, EPI_ISL_689611, EPI_ISL_689613, EPI_ISL_689614, EPI_ISL_689615, EPI_ISL_689616, EPI_ISL_689618,
EPI_ISL_689619, EPI_ISL_689620, EPI_ISL_689621, EPI_ISL_689622, EPI_ISL_689623, EPI_ISL_689624, EPI_ISL_689625, EPI_ISL_689626, EPI_ISL_689627, EPI_ISL_689628, EPI_ISL_689629, EPI_ISL_689630, EPI_ISL_689631, EPI_ISL_689632, EPI_ISL_689633, EPI_ISL_689634, EPI_ISL_689635, EPI_ISL_689636, EPI_ISL_689637, EPI_ISL_689638, EPI_ISL_689639, EPI_ISL_689640, EPI_ISL_689641, EPI_ISL_689642,
EPI_ISL_689643, EPI_ISL_689644, EPI_ISL_689645, EPI_ISL_689647, EPI_ISL_689648, EPI_ISL_689649, EPI_ISL_689650, EPI_ISL_689651, EPI_ISL_689652, EPI_ISL_689653, EPI_ISL_689654, EPI_ISL_689655, EPI_ISL_689656, EPI_ISL_689657, EPI_ISL_689658, EPI_ISL_689659, EPI_ISL_689660, EPI_ISL_689661, EPI_ISL_689662, EPI_ISL_689663, EPI_ISL_689664, EPI_ISL_689665, EPI_ISL_689666, EPI_ISL_689667,
EPI_ISL_689668, EPI_ISL_689669, EPI_ISL_689670, EPI_ISL_689671, EPI_ISL_689672, EPI_ISL_689673, EPI_ISL_689674, EPI_ISL_689675, EPI_ISL_689676, EPI_ISL_689677, EPI_ISL_689678, EPI_ISL_689679, EPI_ISL_689680, EPI_ISL_689681, EPI_ISL_689682, EPI_ISL_689683, EPI_ISL_689684, EPI_ISL_689685, EPI_ISL_689698, EPI_ISL_689699, EPI_ISL_689700, EPI_ISL_689701, EPI_ISL_689702, EPI_ISL_689703,
EPI_ISL_689704, EPI_ISL_689705, EPI_ISL_689706, EPI_ISL_689707, EPI_ISL_689708, EPI_ISL_689709, EPI_ISL_689710, EPI_ISL_689711, EPI_ISL_689712, EPI_ISL_689713, EPI_ISL_689714, EPI_ISL_689715, EPI_ISL_689716, EPI_ISL_689717, EPI_ISL_689718, EPI_ISL_689719, EPI_ISL_689720, EPI_ISL_689721, EPI_ISL_689722, EPI_ISL_689723, EPI_ISL_689724, EPI_ISL_689725, EPI_ISL_689726, EPI_ISL_689727,
EPI_ISL_689728, EPI_ISL_689729, EPI_ISL_689730, EPI_ISL_689731, EPI_ISL_689732, EPI_ISL_689733, EPI_ISL_689734, EPI_ISL_689735, EPI_ISL_689736, EPI_ISL_689737, EPI_ISL_689738, EPI_ISL_689739, EPI_ISL_689740, EPI_ISL_689741, EPI_ISL_689742, EPI_ISL_689743, EPI_ISL_689744, EPI_ISL_689745, EPI_ISL_689746, EPI_ISL_689747, EPI_ISL_689748, EPI_ISL_689749, EPI_ISL_689750, EPI_ISL_689751,
EPI_ISL_689752, EPI_ISL_689753, EPI_ISL_689754, EPI_ISL_689755, EPI_ISL_689756, EPI_ISL_689757, EPI_ISL_689758, EPI_ISL_689826, EPI_ISL_689827, EPI_ISL_689828, EPI_ISL_689829, EPI_ISL_689830, EPI_ISL_689831, EPI_ISL_689832, EPI_ISL_689833, EPI_ISL_689834, EPI_ISL_689835, EPI_ISL_689836, EPI_ISL_689837, EPI_ISL_689838, EPI_ISL_689839, EPI_ISL_689840, EPI_ISL_689841, EPI_ISL_689842,
EPI_ISL_689843, EPI_ISL_689844, EPI_ISL_689845, EPI_ISL_689846, EPI_ISL_689847, EPI_ISL_689848, EPI_ISL_689849, EPI_ISL_689850, EPI_ISL_689851, EPI_ISL_689852, EPI_ISL_689853, EPI_ISL_689854, EPI_ISL_689855, EPI_ISL_689856, EPI_ISL_689857, EPI_ISL_689858, EPI_ISL_689859, EPI_ISL_689860, EPI_ISL_689861, EPI_ISL_689862, EPI_ISL_689863, EPI_ISL_689864, EPI_ISL_689865, EPI_ISL_689866,
EPI_ISL_689867, EPI_ISL_689868, EPI_ISL_689869, EPI_ISL_689870, EPI_ISL_689871, EPI_ISL_689872, EPI_ISL_689873, EPI_ISL_689874, EPI_ISL_689875, EPI_ISL_689876, EPI_ISL_689877, EPI_ISL_689878, EPI_ISL_689879, EPI_ISL_689880, EPI_ISL_689881, EPI_ISL_689882, EPI_ISL_689883, EPI_ISL_689884, EPI_ISL_689885, EPI_ISL_689886, EPI_ISL_689887, EPI_ISL_689888, EPI_ISL_689889, EPI_ISL_689890,
EPI_ISL_689891, EPI_ISL_689892, EPI_ISL_689893, EPI_ISL_689894, EPI_ISL_689895, EPI_ISL_689896, EPI_ISL_689897, EPI_ISL_689898, EPI_ISL_689899, EPI_ISL_689900, EPI_ISL_689901, EPI_ISL_689902, EPI_ISL_689903, EPI_ISL_689904, EPI_ISL_689905, EPI_ISL_689906, EPI_ISL_689907, EPI_ISL_689908, EPI_ISL_689909, EPI_ISL_689910, EPI_ISL_689911, EPI_ISL_689912, EPI_ISL_689913, EPI_ISL_689914,
EPI_ISL_689915, EPI_ISL_689916, EPI_ISL_689917, EPI_ISL_689918, EPI_ISL_689919, EPI_ISL_689920, EPI_ISL_689921, EPI_ISL_689922, EPI_ISL_689923, EPI_ISL_689924, EPI_ISL_689925, EPI_ISL_689926, EPI_ISL_689927, EPI_ISL_689928, EPI_ISL_689929, EPI_ISL_689930, EPI_ISL_689931, EPI_ISL_689932, EPI_ISL_689933, EPI_ISL_689934, EPI_ISL_689935, EPI_ISL_689936, EPI_ISL_689937, EPI_ISL_689938,
EPI_ISL_689939, EPI_ISL_689940, EPI_ISL_689941, EPI_ISL_689942, EPI_ISL_689943, EPI_ISL_689944, EPI_ISL_689945, EPI_ISL_689946, EPI_ISL_689947, EPI_ISL_689948, EPI_ISL_689949, EPI_ISL_689950, EPI_ISL_689951, EPI_ISL_689952, EPI_ISL_689953, EPI_ISL_689954, EPI_ISL_689955, EPI_ISL_689956, EPI_ISL_689957, EPI_ISL_689958, EPI_ISL_689959, EPI_ISL_689960, EPI_ISL_689961, EPI_ISL_689962,
EPI_ISL_689963, EPI_ISL_689964, EPI_ISL_689965, EPI_ISL_689966, EPI_ISL_689967, EPI_ISL_689968, EPI_ISL_689969, EPI_ISL_689970, EPI_ISL_689971, EPI_ISL_689972, EPI_ISL_689973, EPI_ISL_689974, EPI_ISL_689975, EPI_ISL_689976, EPI_ISL_689977, EPI_ISL_689978, EPI_ISL_689979, EPI_ISL_689980, EPI_ISL_689981, EPI_ISL_689982, EPI_ISL_689983, EPI_ISL_689984, EPI_ISL_689985, EPI_ISL_689986,
EPI_ISL_689987, EPI_ISL_689988, EPI_ISL_689989, EPI_ISL_689990, EPI_ISL_689991, EPI_ISL_689992, EPI_ISL_689993, EPI_ISL_689994, EPI_ISL_689995, EPI_ISL_689996, EPI_ISL_689997, EPI_ISL_689998, EPI_ISL_689999, EPI_ISL_690000, EPI_ISL_690001, EPI_ISL_690002, EPI_ISL_690003, EPI_ISL_690004, EPI_ISL_690005, EPI_ISL_690006, EPI_ISL_690007, EPI_ISL_690008, EPI_ISL_690009, EPI_ISL_690010,
EPI_ISL_690029, EPI_ISL_690030, EPI_ISL_690031, EPI_ISL_690032, EPI_ISL_690033, EPI_ISL_690034, EPI_ISL_690035, EPI_ISL_690036, EPI_ISL_690037, EPI_ISL_690038, EPI_ISL_690039, EPI_ISL_690040, EPI_ISL_690041, EPI_ISL_690042, EPI_ISL_690043, EPI_ISL_690044, EPI_ISL_690045, EPI_ISL_690046, EPI_ISL_690047, EPI_ISL_690048, EPI_ISL_690049, EPI_ISL_690050, EPI_ISL_690051, EPI_ISL_690052,
EPI_ISL_690053, EPI_ISL_690054, EPI_ISL_690055, EPI_ISL_690056, EPI_ISL_690057, EPI_ISL_690058, EPI_ISL_690059, EPI_ISL_690060, EPI_ISL_690061, EPI_ISL_690062, EPI_ISL_690063, EPI_ISL_690064, EPI_ISL_690065, EPI_ISL_690066, EPI_ISL_690067, EPI_ISL_690068, EPI_ISL_690069, EPI_ISL_690070, EPI_ISL_690071, EPI_ISL_690072, EPI_ISL_690073, EPI_ISL_690074, EPI_ISL_690075, EPI_ISL_690076,
EPI_ISL_690077, EPI_ISL_690078, EPI_ISL_690079, EPI_ISL_690080, EPI_ISL_690081, EPI_ISL_690082, EPI_ISL_690083, EPI_ISL_690084, EPI_ISL_690085, EPI_ISL_690086, EPI_ISL_690087, EPI_ISL_690088, EPI_ISL_690089, EPI_ISL_690090, EPI_ISL_690091, EPI_ISL_690092, EPI_ISL_690093, EPI_ISL_690094, EPI_ISL_690095, EPI_ISL_690096, EPI_ISL_690097, EPI_ISL_690098, EPI_ISL_690099, EPI_ISL_690100,
EPI_ISL_690101, EPI_ISL_690102, EPI_ISL_690103, EPI_ISL_690104, EPI_ISL_690105, EPI_ISL_690106, EPI_ISL_690107, EPI_ISL_690108, EPI_ISL_690109, EPI_ISL_690110, EPI_ISL_690111, EPI_ISL_690112, EPI_ISL_690113, EPI_ISL_690114, EPI_ISL_690115, EPI_ISL_690116, EPI_ISL_690117, EPI_ISL_690118, EPI_ISL_690119, EPI_ISL_690120, EPI_ISL_690121, EPI_ISL_690122, EPI_ISL_690123, EPI_ISL_690124,
EPI_ISL_690125, EPI_ISL_690126, EPI_ISL_690127, EPI_ISL_690128, EPI_ISL_690129, EPI_ISL_690130, EPI_ISL_690131, EPI_ISL_690132, EPI_ISL_690133, EPI_ISL_690134, EPI_ISL_690135, EPI_ISL_690136, EPI_ISL_690137, EPI_ISL_690138, EPI_ISL_690139, EPI_ISL_690140, EPI_ISL_690141, EPI_ISL_690142, EPI_ISL_690143, EPI_ISL_690144, EPI_ISL_690145, EPI_ISL_690146, EPI_ISL_690147, EPI_ISL_690148,
EPI_ISL_690149, EPI_ISL_690150, EPI_ISL_690151, EPI_ISL_690152, EPI_ISL_690153, EPI_ISL_690154, EPI_ISL_690155, EPI_ISL_690156, EPI_ISL_690157, EPI_ISL_690158, EPI_ISL_690159, EPI_ISL_690160, EPI_ISL_690161, EPI_ISL_690162, EPI_ISL_690163, EPI_ISL_690164, EPI_ISL_690165, EPI_ISL_690166, EPI_ISL_690167, EPI_ISL_690168, EPI_ISL_690169, EPI_ISL_690170, EPI_ISL_690171, EPI_ISL_690172,
EPI_ISL_690173, EPI_ISL_690174, EPI_ISL_690175, EPI_ISL_690176, EPI_ISL_690177, EPI_ISL_690178, EPI_ISL_690179, EPI_ISL_690180, EPI_ISL_690181, EPI_ISL_690182, EPI_ISL_690183, EPI_ISL_690184, EPI_ISL_690185, EPI_ISL_690186, EPI_ISL_690187, EPI_ISL_690188, EPI_ISL_690189, EPI_ISL_690190, EPI_ISL_690191, EPI_ISL_690192, EPI_ISL_690193, EPI_ISL_690194, EPI_ISL_690195, EPI_ISL_690196,
EPI_ISL_690197, EPI_ISL_690198, EPI_ISL_690199, EPI_ISL_690200, EPI_ISL_690201, EPI_ISL_690202, EPI_ISL_690203, EPI_ISL_690204, EPI_ISL_690205, EPI_ISL_690206, EPI_ISL_690207, EPI_ISL_690208, EPI_ISL_690209, EPI_ISL_690254, EPI_ISL_690255, EPI_ISL_690256, EPI_ISL_690257, EPI_ISL_690258, EPI_ISL_690259, EPI_ISL_690260, EPI_ISL_690261, EPI_ISL_690262, EPI_ISL_690263, EPI_ISL_690264,
EPI_ISL_690265, EPI_ISL_690266, EPI_ISL_690267, EPI_ISL_690268, EPI_ISL_690269, EPI_ISL_690270, EPI_ISL_690271, EPI_ISL_690272, EPI_ISL_690273, EPI_ISL_690274, EPI_ISL_690275, EPI_ISL_690276, EPI_ISL_690277, EPI_ISL_690278, EPI_ISL_690279, EPI_ISL_690280, EPI_ISL_690281, EPI_ISL_690282, EPI_ISL_690283, EPI_ISL_690284, EPI_ISL_690285, EPI_ISL_690286, EPI_ISL_690287, EPI_ISL_690288,
EPI_ISL_690289, EPI_ISL_690290, EPI_ISL_690291, EPI_ISL_690292, EPI_ISL_690293, EPI_ISL_690294, EPI_ISL_690295, EPI_ISL_690296, EPI_ISL_690297, EPI_ISL_690298, EPI_ISL_690299, EPI_ISL_690300, EPI_ISL_690301, EPI_ISL_690302, EPI_ISL_690303, EPI_ISL_690304, EPI_ISL_690305, EPI_ISL_690306, EPI_ISL_690307, EPI_ISL_690308, EPI_ISL_690309, EPI_ISL_690310, EPI_ISL_690311, EPI_ISL_690312,
EPI_ISL_690313, EPI_ISL_690314, EPI_ISL_690315, EPI_ISL_690316, EPI_ISL_690317, EPI_ISL_690318, EPI_ISL_690319, EPI_ISL_690320, EPI_ISL_690321, EPI_ISL_690322, EPI_ISL_690323, EPI_ISL_690324, EPI_ISL_690325, EPI_ISL_690326, EPI_ISL_690327, EPI_ISL_690328, EPI_ISL_690329, EPI_ISL_690330, EPI_ISL_690331, EPI_ISL_690332, EPI_ISL_690333, EPI_ISL_690334, EPI_ISL_690335, EPI_ISL_690336,
EPI_ISL_690337, EPI_ISL_690338, EPI_ISL_690339, EPI_ISL_690340, EPI_ISL_690341, EPI_ISL_690342, EPI_ISL_690343, EPI_ISL_690344, EPI_ISL_690345, EPI_ISL_690346, EPI_ISL_690347, EPI_ISL_690348, EPI_ISL_690349, EPI_ISL_690350, EPI_ISL_690351, EPI_ISL_690352, EPI_ISL_690353, EPI_ISL_690354, EPI_ISL_690355, EPI_ISL_690356, EPI_ISL_690357, EPI_ISL_690358, EPI_ISL_690359, EPI_ISL_690360,
EPI_ISL_690361, EPI_ISL_690362, EPI_ISL_690363, EPI_ISL_690364, EPI_ISL_690365, EPI_ISL_690366, EPI_ISL_690367, EPI_ISL_690368, EPI_ISL_690369, EPI_ISL_690370, EPI_ISL_690371, EPI_ISL_690372, EPI_ISL_690373, EPI_ISL_690374, EPI_ISL_690375, EPI_ISL_690376, EPI_ISL_690377, EPI_ISL_690378, EPI_ISL_690379, EPI_ISL_690380, EPI_ISL_690381, EPI_ISL_690382, EPI_ISL_690383, EPI_ISL_690384,
EPI_ISL_690385, EPI_ISL_690386, EPI_ISL_690387, EPI_ISL_690388, EPI_ISL_690389, EPI_ISL_690390, EPI_ISL_690391, EPI_ISL_690392, EPI_ISL_690393, EPI_ISL_690394, EPI_ISL_690395, EPI_ISL_690396, EPI_ISL_690397, EPI_ISL_690398, EPI_ISL_690399, EPI_ISL_690400, EPI_ISL_690401, EPI_ISL_690402, EPI_ISL_690403, EPI_ISL_690404, EPI_ISL_690405, EPI_ISL_690406, EPI_ISL_690407, EPI_ISL_690408,
EPI_ISL_690409, EPI_ISL_690410, EPI_ISL_690411, EPI_ISL_690412, EPI_ISL_690413, EPI_ISL_690414, EPI_ISL_690415, EPI_ISL_690416, EPI_ISL_690417, EPI_ISL_690418, EPI_ISL_690419, EPI_ISL_690420, EPI_ISL_690421, EPI_ISL_690422, EPI_ISL_690423, EPI_ISL_690424, EPI_ISL_690425, EPI_ISL_690426, EPI_ISL_690427, EPI_ISL_690428, EPI_ISL_690429, EPI_ISL_690430, EPI_ISL_690431, EPI_ISL_690527,
EPI_ISL_690528, EPI_ISL_690529, EPI_ISL_690530, EPI_ISL_690531, EPI_ISL_690532, EPI_ISL_690533, EPI_ISL_690534, EPI_ISL_690535, EPI_ISL_690536, EPI_ISL_690537, EPI_ISL_690538, EPI_ISL_690539, EPI_ISL_690540, EPI_ISL_690541, EPI_ISL_690542, EPI_ISL_690543, EPI_ISL_690544, EPI_ISL_690545, EPI_ISL_690546, EPI_ISL_690547, EPI_ISL_690548, EPI_ISL_690549, EPI_ISL_690550, EPI_ISL_690551,
EPI_ISL_690552, EPI_ISL_690553, EPI_ISL_690554, EPI_ISL_690555, EPI_ISL_690556, EPI_ISL_690557, EPI_ISL_690558, EPI_ISL_690559, EPI_ISL_690560, EPI_ISL_690561, EPI_ISL_690562, EPI_ISL_690563, EPI_ISL_690564, EPI_ISL_690565, EPI_ISL_690566, EPI_ISL_690567, EPI_ISL_690568, EPI_ISL_690569, EPI_ISL_690570, EPI_ISL_690571, EPI_ISL_690572, EPI_ISL_690573, EPI_ISL_690574, EPI_ISL_690575,
EPI_ISL_690576, EPI_ISL_690577, EPI_ISL_690578, EPI_ISL_690579, EPI_ISL_690580, EPI_ISL_690581, EPI_ISL_690582, EPI_ISL_690583, EPI_ISL_690584, EPI_ISL_690585, EPI_ISL_690586, EPI_ISL_690587, EPI_ISL_690588, EPI_ISL_690589, EPI_ISL_690590, EPI_ISL_690591, EPI_ISL_690592, EPI_ISL_690593, EPI_ISL_690594, EPI_ISL_690595, EPI_ISL_690596, EPI_ISL_690597, EPI_ISL_690598, EPI_ISL_690599,
EPI_ISL_690600, EPI_ISL_690601, EPI_ISL_690602, EPI_ISL_690603, EPI_ISL_690604, EPI_ISL_690605, EPI_ISL_690606, EPI_ISL_690607, EPI_ISL_690608, EPI_ISL_690609, EPI_ISL_690610, EPI_ISL_690611, EPI_ISL_690614, EPI_ISL_690615, EPI_ISL_690616, EPI_ISL_690617, EPI_ISL_690618, EPI_ISL_690620, EPI_ISL_690621, EPI_ISL_690622, EPI_ISL_690623, EPI_ISL_690624, EPI_ISL_690625, EPI_ISL_690629,
EPI_ISL_690632, EPI_ISL_690633, EPI_ISL_690634, EPI_ISL_690635, EPI_ISL_690636, EPI_ISL_690637, EPI_ISL_690684, EPI_ISL_690685, EPI_ISL_690686, EPI_ISL_690687, EPI_ISL_690688, EPI_ISL_690689, EPI_ISL_690690, EPI_ISL_690691, EPI_ISL_690692, EPI_ISL_690693, EPI_ISL_690694, EPI_ISL_690695, EPI_ISL_690696, EPI_ISL_690697, EPI_ISL_690698, EPI_ISL_690699, EPI_ISL_690700, EPI_ISL_690701,
EPI_ISL_690702, EPI_ISL_690703, EPI_ISL_690704, EPI_ISL_690705, EPI_ISL_690706, EPI_ISL_690707, EPI_ISL_690708, EPI_ISL_690709, EPI_ISL_690710, EPI_ISL_690711, EPI_ISL_690712, EPI_ISL_690713, EPI_ISL_690714, EPI_ISL_690715, EPI_ISL_690716, EPI_ISL_690717, EPI_ISL_690718, EPI_ISL_690719, EPI_ISL_690720, EPI_ISL_690721, EPI_ISL_690722, EPI_ISL_690723, EPI_ISL_690724, EPI_ISL_690725,
EPI_ISL_690726, EPI_ISL_690727, EPI_ISL_690728, EPI_ISL_690729, EPI_ISL_690730, EPI_ISL_690731, EPI_ISL_690732, EPI_ISL_690733, EPI_ISL_690734, EPI_ISL_690735, EPI_ISL_690736, EPI_ISL_690737, EPI_ISL_690738, EPI_ISL_690739, EPI_ISL_690740, EPI_ISL_690741, EPI_ISL_690742, EPI_ISL_690743, EPI_ISL_690744, EPI_ISL_690745, EPI_ISL_690746, EPI_ISL_690747, EPI_ISL_690748, EPI_ISL_690749,
EPI_ISL_690750, EPI_ISL_690751, EPI_ISL_690752, EPI_ISL_690753, EPI_ISL_690754, EPI_ISL_690755, EPI_ISL_690756, EPI_ISL_690757, EPI_ISL_690758, EPI_ISL_690759, EPI_ISL_690760, EPI_ISL_690761, EPI_ISL_690762, EPI_ISL_690763, EPI_ISL_690764, EPI_ISL_690765, EPI_ISL_690766, EPI_ISL_690767, EPI_ISL_690768, EPI_ISL_690769, EPI_ISL_690770, EPI_ISL_690771, EPI_ISL_690772, EPI_ISL_690773,
EPI_ISL_690774, EPI_ISL_690775, EPI_ISL_690776, EPI_ISL_690777, EPI_ISL_690778, EPI_ISL_690779, EPI_ISL_690780, EPI_ISL_690781, EPI_ISL_690782, EPI_ISL_690783, EPI_ISL_690784, EPI_ISL_690785, EPI_ISL_690786, EPI_ISL_690787, EPI_ISL_690788, EPI_ISL_690789, EPI_ISL_690790, EPI_ISL_690791, EPI_ISL_690845, EPI_ISL_690846, EPI_ISL_690847, EPI_ISL_690848, EPI_ISL_690849, EPI_ISL_690850,
EPI_ISL_690851, EPI_ISL_690852, EPI_ISL_690853, EPI_ISL_690854, EPI_ISL_690855, EPI_ISL_690856, EPI_ISL_690857, EPI_ISL_690858, EPI_ISL_690859, EPI_ISL_690860, EPI_ISL_690861, EPI_ISL_690862, EPI_ISL_690863, EPI_ISL_690864, EPI_ISL_690865, EPI_ISL_690866, EPI_ISL_690867, EPI_ISL_690868, EPI_ISL_690869, EPI_ISL_690870, EPI_ISL_690871, EPI_ISL_690872, EPI_ISL_690873, EPI_ISL_690874,
EPI_ISL_690875, EPI_ISL_690876, EPI_ISL_690877, EPI_ISL_690878, EPI_ISL_690879, EPI_ISL_690880, EPI_ISL_690881, EPI_ISL_690882, EPI_ISL_690883, EPI_ISL_690884, EPI_ISL_690885, EPI_ISL_690886, EPI_ISL_690887, EPI_ISL_690888, EPI_ISL_690889, EPI_ISL_690890, EPI_ISL_690891, EPI_ISL_690892, EPI_ISL_690893, EPI_ISL_690894, EPI_ISL_690895, EPI_ISL_690896, EPI_ISL_690897, EPI_ISL_690898,
EPI_ISL_690899, EPI_ISL_690900, EPI_ISL_690901, EPI_ISL_690909, EPI_ISL_690910, EPI_ISL_690911, EPI_ISL_690912, EPI_ISL_690913, EPI_ISL_690914, EPI_ISL_690915, EPI_ISL_690916, EPI_ISL_690917, EPI_ISL_690918, EPI_ISL_690919, EPI_ISL_690920, EPI_ISL_690921, EPI_ISL_690922, EPI_ISL_690923, EPI_ISL_690924, EPI_ISL_690925, EPI_ISL_690926, EPI_ISL_690927, EPI_ISL_690928, EPI_ISL_690929,
EPI_ISL_690930, EPI_ISL_690931, EPI_ISL_690932, EPI_ISL_690933, EPI_ISL_690934, EPI_ISL_690935, EPI_ISL_690936, EPI_ISL_690937, EPI_ISL_690938, EPI_ISL_690939, EPI_ISL_690940, EPI_ISL_690941, EPI_ISL_690942, EPI_ISL_690943, EPI_ISL_690944, EPI_ISL_690945, EPI_ISL_690946, EPI_ISL_690947, EPI_ISL_690948, EPI_ISL_690949, EPI_ISL_690950, EPI_ISL_690951, EPI_ISL_690952, EPI_ISL_690953,
EPI_ISL_690954, EPI_ISL_690955, EPI_ISL_690956, EPI_ISL_690957, EPI_ISL_690958, EPI_ISL_690959, EPI_ISL_690960, EPI_ISL_690961, EPI_ISL_690962, EPI_ISL_690963, EPI_ISL_690964, EPI_ISL_690965, EPI_ISL_690966, EPI_ISL_690967, EPI_ISL_690968, EPI_ISL_690969, EPI_ISL_690970, EPI_ISL_690971, EPI_ISL_690972, EPI_ISL_690973, EPI_ISL_690974, EPI_ISL_690975, EPI_ISL_690976, EPI_ISL_690977,
EPI_ISL_690978, EPI_ISL_690979, EPI_ISL_690980, EPI_ISL_690981, EPI_ISL_690987, EPI_ISL_690988, EPI_ISL_690989, EPI_ISL_690990, EPI_ISL_690991, EPI_ISL_690992, EPI_ISL_690993, EPI_ISL_690994, EPI_ISL_690995, EPI_ISL_690996, EPI_ISL_690997, EPI_ISL_690998, EPI_ISL_690999, EPI_ISL_691000, EPI_ISL_691001, EPI_ISL_691002, EPI_ISL_691003, EPI_ISL_691004, EPI_ISL_691005, EPI_ISL_691006,
EPI_ISL_691007, EPI_ISL_691008, EPI_ISL_691009, EPI_ISL_691010, EPI_ISL_691011, EPI_ISL_691012, EPI_ISL_691013, EPI_ISL_691014, EPI_ISL_691015, EPI_ISL_691016, EPI_ISL_691017, EPI_ISL_691018, EPI_ISL_691019, EPI_ISL_691020, EPI_ISL_691021, EPI_ISL_691022, EPI_ISL_691023, EPI_ISL_691024, EPI_ISL_691025, EPI_ISL_691026, EPI_ISL_691027, EPI_ISL_691028, EPI_ISL_691029, EPI_ISL_691030,
EPI_ISL_691031, EPI_ISL_691032, EPI_ISL_691033, EPI_ISL_691034, EPI_ISL_691035, EPI_ISL_691036, EPI_ISL_691037, EPI_ISL_691038, EPI_ISL_691039, EPI_ISL_691040, EPI_ISL_691041, EPI_ISL_691042, EPI_ISL_691043, EPI_ISL_691044, EPI_ISL_691045, EPI_ISL_691046, EPI_ISL_691047, EPI_ISL_691048, EPI_ISL_691049, EPI_ISL_691050, EPI_ISL_691051, EPI_ISL_691052, EPI_ISL_691053, EPI_ISL_691054,
EPI_ISL_691055, EPI_ISL_691056, EPI_ISL_691057, EPI_ISL_691058, EPI_ISL_691059, EPI_ISL_691060, EPI_ISL_691061, EPI_ISL_691062, EPI_ISL_691063, EPI_ISL_691064, EPI_ISL_691065, EPI_ISL_691066, EPI_ISL_691067, EPI_ISL_691068, EPI_ISL_691069, EPI_ISL_691070, EPI_ISL_691071, EPI_ISL_691072, EPI_ISL_691073, EPI_ISL_691074, EPI_ISL_691075, EPI_ISL_691076, EPI_ISL_691077, EPI_ISL_691078,
EPI_ISL_691079, EPI_ISL_691080, EPI_ISL_691081, EPI_ISL_691082, EPI_ISL_691083, EPI_ISL_691084, EPI_ISL_691085, EPI_ISL_691086, EPI_ISL_691087, EPI_ISL_691088, EPI_ISL_691089, EPI_ISL_691090, EPI_ISL_691091, EPI_ISL_691092, EPI_ISL_691093, EPI_ISL_691094, EPI_ISL_691095, EPI_ISL_691096, EPI_ISL_691097, EPI_ISL_691098, EPI_ISL_691099, EPI_ISL_691100, EPI_ISL_691101, EPI_ISL_691102,
EPI_ISL_691103, EPI_ISL_691104, EPI_ISL_691105, EPI_ISL_691106, EPI_ISL_691107, EPI_ISL_691108, EPI_ISL_691109, EPI_ISL_691110, EPI_ISL_691111, EPI_ISL_691112, EPI_ISL_691113, EPI_ISL_691114, EPI_ISL_691115, EPI_ISL_691116, EPI_ISL_691117, EPI_ISL_691118, EPI_ISL_691119, EPI_ISL_691120, EPI_ISL_691121, EPI_ISL_691122, EPI_ISL_691123, EPI_ISL_691124, EPI_ISL_691125, EPI_ISL_691126,
EPI_ISL_691127, EPI_ISL_691128, EPI_ISL_691129, EPI_ISL_691130, EPI_ISL_691131, EPI_ISL_691132, EPI_ISL_691133, EPI_ISL_691134, EPI_ISL_691135, EPI_ISL_691136, EPI_ISL_691137, EPI_ISL_691138, EPI_ISL_691139, EPI_ISL_691140, EPI_ISL_691141, EPI_ISL_691142, EPI_ISL_691143, EPI_ISL_691144, EPI_ISL_691145, EPI_ISL_691146, EPI_ISL_691147, EPI_ISL_691148, EPI_ISL_691149, EPI_ISL_691150,
EPI_ISL_691151, EPI_ISL_691152, EPI_ISL_691153, EPI_ISL_691154, EPI_ISL_691155, EPI_ISL_691156, EPI_ISL_691157, EPI_ISL_691158, EPI_ISL_691159, EPI_ISL_691160, EPI_ISL_691161, EPI_ISL_691162, EPI_ISL_691163, EPI_ISL_691164, EPI_ISL_691165, EPI_ISL_691166, EPI_ISL_691167, EPI_ISL_691168, EPI_ISL_691169, EPI_ISL_691170, EPI_ISL_691171, EPI_ISL_691172, EPI_ISL_691173, EPI_ISL_691174,
EPI_ISL_691175, EPI_ISL_691176, EPI_ISL_691177, EPI_ISL_691178, EPI_ISL_691179, EPI_ISL_691180, EPI_ISL_691181, EPI_ISL_691182, EPI_ISL_691183, EPI_ISL_691184, EPI_ISL_691185, EPI_ISL_691186, EPI_ISL_691187, EPI_ISL_691188, EPI_ISL_691189, EPI_ISL_691190, EPI_ISL_691191, EPI_ISL_691192, EPI_ISL_691193, EPI_ISL_691194, EPI_ISL_691195, EPI_ISL_691196, EPI_ISL_691197, EPI_ISL_691198,
EPI_ISL_691199, EPI_ISL_691200, EPI_ISL_691201, EPI_ISL_691202, EPI_ISL_691203, EPI_ISL_691204, EPI_ISL_691205, EPI_ISL_691206, EPI_ISL_691207, EPI_ISL_691208, EPI_ISL_691209, EPI_ISL_691210, EPI_ISL_691211, EPI_ISL_691212, EPI_ISL_691213, EPI_ISL_691214, EPI_ISL_691215, EPI_ISL_691216, EPI_ISL_691217, EPI_ISL_691218, EPI_ISL_691219, EPI_ISL_691220, EPI_ISL_691221, EPI_ISL_691222,
EPI_ISL_691223, EPI_ISL_691224, EPI_ISL_691225, EPI_ISL_691226, EPI_ISL_691227, EPI_ISL_691228, EPI_ISL_691229, EPI_ISL_691230, EPI_ISL_691231, EPI_ISL_691232, EPI_ISL_691233, EPI_ISL_691234, EPI_ISL_691235, EPI_ISL_691236, EPI_ISL_691237, EPI_ISL_691238, EPI_ISL_691239, EPI_ISL_691240, EPI_ISL_691241, EPI_ISL_691242, EPI_ISL_691243, EPI_ISL_691244, EPI_ISL_691245, EPI_ISL_691246,
EPI_ISL_691247, EPI_ISL_691248, EPI_ISL_691249, EPI_ISL_691250, EPI_ISL_691251, EPI_ISL_691252, EPI_ISL_691253, EPI_ISL_691254, EPI_ISL_691255, EPI_ISL_691256, EPI_ISL_691257, EPI_ISL_691258, EPI_ISL_691259, EPI_ISL_691260, EPI_ISL_691261, EPI_ISL_691262, EPI_ISL_691263, EPI_ISL_691264, EPI_ISL_691265, EPI_ISL_691266, EPI_ISL_691267, EPI_ISL_691268, EPI_ISL_691269, EPI_ISL_691270,
EPI_ISL_691271, EPI_ISL_691272, EPI_ISL_691273, EPI_ISL_691274, EPI_ISL_691275, EPI_ISL_691276, EPI_ISL_691277, EPI_ISL_691278, EPI_ISL_691279, EPI_ISL_691280, EPI_ISL_691281, EPI_ISL_691282, EPI_ISL_691283, EPI_ISL_691289, EPI_ISL_691290, EPI_ISL_691291, EPI_ISL_691292, EPI_ISL_691293, EPI_ISL_691294, EPI_ISL_691295, EPI_ISL_691296, EPI_ISL_691297, EPI_ISL_691298, EPI_ISL_691299,
EPI_ISL_691300, EPI_ISL_691301, EPI_ISL_691302, EPI_ISL_691303, EPI_ISL_691304, EPI_ISL_691305, EPI_ISL_691306, EPI_ISL_691307, EPI_ISL_691308, EPI_ISL_691309, EPI_ISL_691310, EPI_ISL_691311, EPI_ISL_691312, EPI_ISL_691313, EPI_ISL_691314, EPI_ISL_691315, EPI_ISL_691316, EPI_ISL_691317, EPI_ISL_691318, EPI_ISL_691319, EPI_ISL_691320, EPI_ISL_691321, EPI_ISL_691322, EPI_ISL_691323,
EPI_ISL_691324, EPI_ISL_691325, EPI_ISL_691326, EPI_ISL_691327, EPI_ISL_691328, EPI_ISL_691329, EPI_ISL_691330, EPI_ISL_691331, EPI_ISL_691332, EPI_ISL_691333, EPI_ISL_691334, EPI_ISL_691335, EPI_ISL_691336, EPI_ISL_691337, EPI_ISL_691338, EPI_ISL_691339, EPI_ISL_691340, EPI_ISL_691341, EPI_ISL_691342, EPI_ISL_691343, EPI_ISL_691344, EPI_ISL_691345, EPI_ISL_691346, EPI_ISL_691347,
EPI_ISL_691348, EPI_ISL_691349, EPI_ISL_691350, EPI_ISL_691351, EPI_ISL_691352, EPI_ISL_691353, EPI_ISL_691354, EPI_ISL_691355, EPI_ISL_691356, EPI_ISL_691357, EPI_ISL_691358, EPI_ISL_691359, EPI_ISL_691360, EPI_ISL_691361, EPI_ISL_691362, EPI_ISL_691363, EPI_ISL_691379, EPI_ISL_691380, EPI_ISL_691381, EPI_ISL_691382, EPI_ISL_691383, EPI_ISL_691384, EPI_ISL_691385, EPI_ISL_691386,
EPI_ISL_691387, EPI_ISL_691388, EPI_ISL_691389, EPI_ISL_691390, EPI_ISL_691391, EPI_ISL_691392, EPI_ISL_691393, EPI_ISL_691394, EPI_ISL_691395, EPI_ISL_691396, EPI_ISL_691397, EPI_ISL_691398, EPI_ISL_691399, EPI_ISL_691400, EPI_ISL_691401, EPI_ISL_691402, EPI_ISL_691403, EPI_ISL_691404, EPI_ISL_691405, EPI_ISL_691406, EPI_ISL_691407, EPI_ISL_691408, EPI_ISL_691409, EPI_ISL_691410,
EPI_ISL_691411, EPI_ISL_691412, EPI_ISL_691413, EPI_ISL_691414, EPI_ISL_691415, EPI_ISL_691416, EPI_ISL_691417, EPI_ISL_691418, EPI_ISL_691419, EPI_ISL_691420, EPI_ISL_691421, EPI_ISL_691422, EPI_ISL_691423, EPI_ISL_691424, EPI_ISL_691425, EPI_ISL_691426, EPI_ISL_691427, EPI_ISL_691428, EPI_ISL_691429, EPI_ISL_691430, EPI_ISL_691431, EPI_ISL_691432, EPI_ISL_691433, EPI_ISL_691434,
EPI_ISL_691435, EPI_ISL_691436, EPI_ISL_691437, EPI_ISL_691438, EPI_ISL_691439, EPI_ISL_691440, EPI_ISL_691441, EPI_ISL_691442, EPI_ISL_691443, EPI_ISL_691444, EPI_ISL_691445, EPI_ISL_691446, EPI_ISL_691447, EPI_ISL_691448, EPI_ISL_691449, EPI_ISL_691450, EPI_ISL_691451, EPI_ISL_691452, EPI_ISL_691453, EPI_ISL_691454, EPI_ISL_691455, EPI_ISL_691456, EPI_ISL_691457, EPI_ISL_691458,
EPI_ISL_691459, EPI_ISL_691460, EPI_ISL_691461, EPI_ISL_691462, EPI_ISL_691463, EPI_ISL_691464, EPI_ISL_691465, EPI_ISL_691466, EPI_ISL_691467, EPI_ISL_691468, EPI_ISL_691469, EPI_ISL_691470, EPI_ISL_691471, EPI_ISL_691472, EPI_ISL_691473, EPI_ISL_691474, EPI_ISL_691475, EPI_ISL_691476, EPI_ISL_691477, EPI_ISL_691478, EPI_ISL_691479, EPI_ISL_691480, EPI_ISL_691481, EPI_ISL_691482,
EPI_ISL_691483, EPI_ISL_691484, EPI_ISL_691485, EPI_ISL_691486, EPI_ISL_691487, EPI_ISL_691488, EPI_ISL_691489, EPI_ISL_691490, EPI_ISL_691491, EPI_ISL_691492, EPI_ISL_691493, EPI_ISL_691494, EPI_ISL_691495, EPI_ISL_691496, EPI_ISL_691497, EPI_ISL_691498, EPI_ISL_691499, EPI_ISL_691500, EPI_ISL_691501, EPI_ISL_691502, EPI_ISL_691503, EPI_ISL_691504, EPI_ISL_691505, EPI_ISL_691506,
EPI_ISL_691507, EPI_ISL_691508, EPI_ISL_691546, EPI_ISL_691547, EPI_ISL_691548, EPI_ISL_691549, EPI_ISL_691552, EPI_ISL_691553, EPI_ISL_691554, EPI_ISL_691555, EPI_ISL_691556, EPI_ISL_691557, EPI_ISL_691558, EPI_ISL_691559, EPI_ISL_691560, EPI_ISL_691561, EPI_ISL_691562, EPI_ISL_691563, EPI_ISL_691564, EPI_ISL_691565, EPI_ISL_691566, EPI_ISL_691567, EPI_ISL_691568, EPI_ISL_691569,
EPI_ISL_691570, EPI_ISL_691571, EPI_ISL_691572, EPI_ISL_691573, EPI_ISL_691574, EPI_ISL_691575, EPI_ISL_691576, EPI_ISL_691577, EPI_ISL_691578, EPI_ISL_691579, EPI_ISL_691580, EPI_ISL_691581, EPI_ISL_691582, EPI_ISL_691583, EPI_ISL_691584, EPI_ISL_691585, EPI_ISL_691586, EPI_ISL_691587, EPI_ISL_691588, EPI_ISL_691589, EPI_ISL_691590, EPI_ISL_691591, EPI_ISL_691592, EPI_ISL_691593,
EPI_ISL_691594, EPI_ISL_691595, EPI_ISL_691596, EPI_ISL_691597, EPI_ISL_691598, EPI_ISL_691599, EPI_ISL_691600, EPI_ISL_691601, EPI_ISL_691602, EPI_ISL_691603, EPI_ISL_691604, EPI_ISL_691605, EPI_ISL_691606, EPI_ISL_691607, EPI_ISL_691608, EPI_ISL_691609, EPI_ISL_691610, EPI_ISL_691611, EPI_ISL_691733, EPI_ISL_691734, EPI_ISL_691736, EPI_ISL_691737, EPI_ISL_691738, EPI_ISL_691739,
EPI_ISL_691740, EPI_ISL_691741, EPI_ISL_691742, EPI_ISL_691743, EPI_ISL_691744, EPI_ISL_691745, EPI_ISL_691746, EPI_ISL_691747, EPI_ISL_691748, EPI_ISL_691749, EPI_ISL_691750, EPI_ISL_691751, EPI_ISL_691752, EPI_ISL_691754, EPI_ISL_691755, EPI_ISL_691757, EPI_ISL_691758, EPI_ISL_691759, EPI_ISL_691760, EPI_ISL_691761, EPI_ISL_691762, EPI_ISL_691763, EPI_ISL_691767, EPI_ISL_691768,
EPI_ISL_691769, EPI_ISL_691770, EPI_ISL_691771, EPI_ISL_691772, EPI_ISL_691773, EPI_ISL_691774, EPI_ISL_691775, EPI_ISL_691776, EPI_ISL_691777, EPI_ISL_691778, EPI_ISL_691779, EPI_ISL_691780, EPI_ISL_691781, EPI_ISL_691782, EPI_ISL_691783, EPI_ISL_691784, EPI_ISL_691785, EPI_ISL_691786, EPI_ISL_691787, EPI_ISL_691788, EPI_ISL_691789, EPI_ISL_691790, EPI_ISL_691791, EPI_ISL_691792,
EPI_ISL_691793, EPI_ISL_691794, EPI_ISL_691795, EPI_ISL_691796, EPI_ISL_691797, EPI_ISL_691798, EPI_ISL_691799, EPI_ISL_691800, EPI_ISL_691801, EPI_ISL_691802, EPI_ISL_691803, EPI_ISL_691804, EPI_ISL_691805, EPI_ISL_691806, EPI_ISL_691807, EPI_ISL_691808, EPI_ISL_691809, EPI_ISL_691810, EPI_ISL_691811, EPI_ISL_691812, EPI_ISL_691813, EPI_ISL_691814, EPI_ISL_691815, EPI_ISL_691816,
EPI_ISL_691817, EPI_ISL_691818, EPI_ISL_691819, EPI_ISL_691820, EPI_ISL_691821, EPI_ISL_691822, EPI_ISL_691823, EPI_ISL_691824, EPI_ISL_691825, EPI_ISL_691826, EPI_ISL_691827, EPI_ISL_691828, EPI_ISL_691829, EPI_ISL_691830, EPI_ISL_691831, EPI_ISL_691832, EPI_ISL_691833, EPI_ISL_691834, EPI_ISL_691835, EPI_ISL_691836, EPI_ISL_691837, EPI_ISL_691838, EPI_ISL_691839, EPI_ISL_691840,
EPI_ISL_691841, EPI_ISL_691842, EPI_ISL_691843, EPI_ISL_691844, EPI_ISL_691845, EPI_ISL_691846, EPI_ISL_691847, EPI_ISL_691848, EPI_ISL_691849, EPI_ISL_691850, EPI_ISL_691851, EPI_ISL_691852, EPI_ISL_691853, EPI_ISL_691854, EPI_ISL_691855, EPI_ISL_691856, EPI_ISL_691857, EPI_ISL_691858, EPI_ISL_691859, EPI_ISL_691860, EPI_ISL_691861, EPI_ISL_691862, EPI_ISL_691863, EPI_ISL_691864,
EPI_ISL_691865, EPI_ISL_691866, EPI_ISL_691867, EPI_ISL_691868, EPI_ISL_691869, EPI_ISL_691870, EPI_ISL_691871, EPI_ISL_691872, EPI_ISL_691873, EPI_ISL_691874, EPI_ISL_691875, EPI_ISL_691876, EPI_ISL_691877, EPI_ISL_691878, EPI_ISL_691879, EPI_ISL_691880, EPI_ISL_691881, EPI_ISL_691882, EPI_ISL_691883, EPI_ISL_691884, EPI_ISL_691885, EPI_ISL_691886, EPI_ISL_691887, EPI_ISL_691888,
EPI_ISL_691889, EPI_ISL_691890, EPI_ISL_691891, EPI_ISL_691892, EPI_ISL_691893, EPI_ISL_691894, EPI_ISL_691895, EPI_ISL_691896, EPI_ISL_691897, EPI_ISL_691898, EPI_ISL_691899, EPI_ISL_691900, EPI_ISL_691901, EPI_ISL_691902, EPI_ISL_691903, EPI_ISL_691904, EPI_ISL_691905, EPI_ISL_691906, EPI_ISL_691907, EPI_ISL_691908, EPI_ISL_691909, EPI_ISL_691910, EPI_ISL_691911, EPI_ISL_691912,
EPI_ISL_691913, EPI_ISL_691914, EPI_ISL_691915, EPI_ISL_691916, EPI_ISL_691917, EPI_ISL_691918, EPI_ISL_691919, EPI_ISL_691920, EPI_ISL_691921, EPI_ISL_691922, EPI_ISL_691923, EPI_ISL_691924, EPI_ISL_691925, EPI_ISL_691926, EPI_ISL_691927, EPI_ISL_691928, EPI_ISL_691929, EPI_ISL_691930, EPI_ISL_691931, EPI_ISL_691932, EPI_ISL_691933, EPI_ISL_691934, EPI_ISL_691935, EPI_ISL_691936,
EPI_ISL_691937, EPI_ISL_691938, EPI_ISL_691939, EPI_ISL_691940, EPI_ISL_691941, EPI_ISL_691942, EPI_ISL_691943, EPI_ISL_691944, EPI_ISL_691945, EPI_ISL_691946, EPI_ISL_691947, EPI_ISL_691948, EPI_ISL_691949, EPI_ISL_691950, EPI_ISL_691951, EPI_ISL_691952, EPI_ISL_691953, EPI_ISL_691954, EPI_ISL_691955, EPI_ISL_691956, EPI_ISL_691957, EPI_ISL_691958, EPI_ISL_691959, EPI_ISL_691960,
EPI_ISL_691961, EPI_ISL_691962, EPI_ISL_691963, EPI_ISL_691964, EPI_ISL_691965, EPI_ISL_691966, EPI_ISL_691967, EPI_ISL_691968, EPI_ISL_691969, EPI_ISL_691970, EPI_ISL_691971, EPI_ISL_691972, EPI_ISL_691973, EPI_ISL_691974, EPI_ISL_691975, EPI_ISL_691976, EPI_ISL_691977, EPI_ISL_691978, EPI_ISL_691979, EPI_ISL_691980, EPI_ISL_691981, EPI_ISL_691982, EPI_ISL_691983, EPI_ISL_691984,
EPI_ISL_691985, EPI_ISL_691986, EPI_ISL_691987, EPI_ISL_691988, EPI_ISL_691989, EPI_ISL_691990, EPI_ISL_691991, EPI_ISL_691992, EPI_ISL_691993, EPI_ISL_691994, EPI_ISL_691995, EPI_ISL_691996, EPI_ISL_691997, EPI_ISL_691998, EPI_ISL_691999, EPI_ISL_692000, EPI_ISL_692001, EPI_ISL_692002, EPI_ISL_692003, EPI_ISL_692004, EPI_ISL_692005, EPI_ISL_692006, EPI_ISL_692007, EPI_ISL_692008,
EPI_ISL_692009, EPI_ISL_692010, EPI_ISL_692011, EPI_ISL_692012, EPI_ISL_692013, EPI_ISL_692014, EPI_ISL_692015, EPI_ISL_692016, EPI_ISL_692017, EPI_ISL_692018, EPI_ISL_692019, EPI_ISL_692020, EPI_ISL_692021, EPI_ISL_692022, EPI_ISL_692023, EPI_ISL_692024, EPI_ISL_692025, EPI_ISL_692026, EPI_ISL_692027, EPI_ISL_692028, EPI_ISL_692029, EPI_ISL_692030, EPI_ISL_692031, EPI_ISL_692032,
EPI_ISL_692033, EPI_ISL_692034, EPI_ISL_692035, EPI_ISL_692036, EPI_ISL_692037, EPI_ISL_692038, EPI_ISL_692039, EPI_ISL_692040, EPI_ISL_692041, EPI_ISL_692042, EPI_ISL_692043, EPI_ISL_692044, EPI_ISL_692045, EPI_ISL_692046, EPI_ISL_692059, EPI_ISL_692060, EPI_ISL_692061, EPI_ISL_692062, EPI_ISL_692063, EPI_ISL_692064, EPI_ISL_692065, EPI_ISL_692066, EPI_ISL_692067, EPI_ISL_692068,
EPI_ISL_692069, EPI_ISL_692070, EPI_ISL_692071, EPI_ISL_692072, EPI_ISL_692073, EPI_ISL_692074, EPI_ISL_692075, EPI_ISL_692076, EPI_ISL_692077, EPI_ISL_692078, EPI_ISL_692079, EPI_ISL_692080, EPI_ISL_692081, EPI_ISL_692082, EPI_ISL_692083, EPI_ISL_692084, EPI_ISL_692085, EPI_ISL_692086, EPI_ISL_692087, EPI_ISL_692088, EPI_ISL_692089, EPI_ISL_692090, EPI_ISL_692091, EPI_ISL_692092,
EPI_ISL_692093, EPI_ISL_692098, EPI_ISL_692099, EPI_ISL_692100, EPI_ISL_692101, EPI_ISL_692102, EPI_ISL_692103, EPI_ISL_692104, EPI_ISL_692105, EPI_ISL_692106, EPI_ISL_692107, EPI_ISL_692108, EPI_ISL_692109, EPI_ISL_692110, EPI_ISL_692111, EPI_ISL_692112, EPI_ISL_692113, EPI_ISL_692114, EPI_ISL_692115, EPI_ISL_692116, EPI_ISL_692117, EPI_ISL_692118, EPI_ISL_692129, EPI_ISL_692130,
EPI_ISL_692131, EPI_ISL_692132, EPI_ISL_692133, EPI_ISL_692134, EPI_ISL_692135, EPI_ISL_692136, EPI_ISL_692137, EPI_ISL_692138, EPI_ISL_692139, EPI_ISL_692140, EPI_ISL_692141, EPI_ISL_692142, EPI_ISL_692143, EPI_ISL_692144, EPI_ISL_692145, EPI_ISL_692146, EPI_ISL_692147, EPI_ISL_692148, EPI_ISL_692149, EPI_ISL_692150, EPI_ISL_692151, EPI_ISL_692152, EPI_ISL_692153, EPI_ISL_692154,
EPI_ISL_692155, EPI_ISL_692156, EPI_ISL_692157, EPI_ISL_692158, EPI_ISL_692159, EPI_ISL_692160, EPI_ISL_692161, EPI_ISL_692164, EPI_ISL_692165, EPI_ISL_692166, EPI_ISL_692167, EPI_ISL_692168, EPI_ISL_692169, EPI_ISL_692170, EPI_ISL_692171, EPI_ISL_692172, EPI_ISL_692173, EPI_ISL_692174, EPI_ISL_692175, EPI_ISL_692176, EPI_ISL_692177, EPI_ISL_692178, EPI_ISL_692179, EPI_ISL_692180,
EPI_ISL_692181, EPI_ISL_692182, EPI_ISL_692183, EPI_ISL_692184, EPI_ISL_692185, EPI_ISL_692186, EPI_ISL_692187, EPI_ISL_692188, EPI_ISL_692189, EPI_ISL_692190, EPI_ISL_692191, EPI_ISL_692192, EPI_ISL_692193, EPI_ISL_692194, EPI_ISL_692195, EPI_ISL_692196, EPI_ISL_692197, EPI_ISL_692198, EPI_ISL_692199, EPI_ISL_692200, EPI_ISL_692201, EPI_ISL_692202, EPI_ISL_692203, EPI_ISL_692204,

EPI_ISL_692205, EPI_ISL_692206, EPI_ISL_692207, EPI_ISL_692208, EPI_ISL_692209, EPI_ISL_692210, EPI_ISL_692211, EPI_ISL_692212, EPI_ISL_692213, EPI_ISL_692214, EPI_ISL_692215, EPI_ISL_692216, EPI_ISL_692217, EPI_ISL_692218, EPI_ISL_692230, EPI_ISL_692231, EPI_ISL_692232, EPI_ISL_692233, EPI_ISL_692234, EPI_ISL_692235, EPI_ISL_692236, EPI_ISL_692237, EPI_ISL_692238, EPI_ISL_692239, EPI_ISL_692240, EPI_ISL_692241, EPI_ISL_692242, EPI_ISL_692243, EPI_ISL_692244, EPI_ISL_692245, EPI_ISL_692246, EPI_ISL_692247, EPI_ISL_692248, EPI_ISL_692249, EPI_ISL_692250, EPI_ISL_692251, EPI_ISL_692252, EPI_ISL_692253, EPI_ISL_692254, EPI_ISL_692255, EPI_ISL_692256, EPI_ISL_692257, EPI_ISL_692258, EPI_ISL_692259, EPI_ISL_692260, EPI_ISL_692261, EPI_ISL_692262, EPI_ISL_692263, EPI_ISL_692264, EPI_ISL_692265, EPI_ISL_692266, EPI_ISL_692267, EPI_ISL_692268, EPI_ISL_692269, EPI_ISL_692270, EPI_ISL_692271, EPI_ISL_692272, EPI_ISL_692273, EPI_ISL_692274, EPI_ISL_692275, EPI_ISL_692276, EPI_ISL_692277, EPI_ISL_692278, EPI_ISL_692279, EPI_ISL_692280, EPI_ISL_692281, EPI_ISL_692282, EPI_ISL_692283, EPI_ISL_692328, EPI_ISL_692329, EPI_ISL_692330, EPI_ISL_692331, EPI_ISL_692332, EPI_ISL_692333, EPI_ISL_692334, EPI_ISL_692335, EPI_ISL_692336, EPI_ISL_692337, EPI_ISL_692338, EPI_ISL_692339, EPI_ISL_692340, EPI_ISL_692341, EPI_ISL_692342, EPI_ISL_692343, EPI_ISL_692344, EPI_ISL_692345, EPI_ISL_692346, EPI_ISL_692347, EPI_ISL_692348, EPI_ISL_692349, EPI_ISL_692350, EPI_ISL_692351, EPI_ISL_692352, EPI_ISL_692353, EPI_ISL_692354, EPI_ISL_692355, EPI_ISL_692356, EPI_ISL_692357, EPI_ISL_692358, EPI_ISL_692359, EPI_ISL_692360, EPI_ISL_692361, EPI_ISL_692362, EPI_ISL_692363, EPI_ISL_692364, EPI_ISL_692365, EPI_ISL_692366, EPI_ISL_692367, EPI_ISL_692368, EPI_ISL_692369, EPI_ISL_692370, EPI_ISL_692371, EPI_ISL_692372, EPI_ISL_692373, EPI_ISL_692374, EPI_ISL_692375, EPI_ISL_692376, EPI_ISL_692377, EPI_ISL_692378, EPI_ISL_692379, EPI_ISL_692380, EPI_ISL_692381, EPI_ISL_692382, EPI_ISL_692383, EPI_ISL_692384, EPI_ISL_692385, EPI_ISL_692386, EPI_ISL_692387, EPI_ISL_692388, EPI_ISL_692389, EPI_ISL_692390, EPI_ISL_692391, EPI_ISL_692392, EPI_ISL_692393, EPI_ISL_692394, EPI_ISL_692395, EPI_ISL_692396, EPI_ISL_692397, EPI_ISL_692398, EPI_ISL_692399, EPI_ISL_692400, EPI_ISL_692401, EPI_ISL_692402, EPI_ISL_692403, EPI_ISL_692404, EPI_ISL_692405, EPI_ISL_692406, EPI_ISL_692407, EPI_ISL_692408, EPI_ISL_692409, EPI_ISL_692410, EPI_ISL_692411, EPI_ISL_692412, EPI_ISL_692413, EPI_ISL_692414, EPI_ISL_692415, EPI_ISL_692416, EPI_ISL_692417, EPI_ISL_692418, EPI_ISL_692419, EPI_ISL_692420, EPI_ISL_692421, EPI_ISL_692422, EPI_ISL_692423, EPI_ISL_692424, EPI_ISL_692425, EPI_ISL_692426, EPI_ISL_692427, EPI_ISL_692428, EPI_ISL_692429, EPI_ISL_692430, EPI_ISL_692431, EPI_ISL_692432, EPI_ISL_692433, EPI_ISL_692434, EPI_ISL_692435, EPI_ISL_692436, EPI_ISL_692437, EPI_ISL_692438, EPI_ISL_692439, EPI_ISL_692440, EPI_ISL_692441, EPI_ISL_692498, EPI_ISL_692499, EPI_ISL_692500, EPI_ISL_692501, EPI_ISL_692502, EPI_ISL_692503, EPI_ISL_692504, EPI_ISL_692505, EPI_ISL_692506, EPI_ISL_692507, EPI_ISL_692508, EPI_ISL_692509, EPI_ISL_692510, EPI_ISL_692511, EPI_ISL_692512, EPI_ISL_692513, EPI_ISL_692514, EPI_ISL_692515, EPI_ISL_692516, EPI_ISL_692517, EPI_ISL_692518, EPI_ISL_692519, EPI_ISL_692520, EPI_ISL_692521, EPI_ISL_692522, EPI_ISL_692523, EPI_ISL_692524, EPI_ISL_692525, EPI_ISL_692526, EPI_ISL_692527, EPI_ISL_692528, EPI_ISL_692529, EPI_ISL_692530, EPI_ISL_692531, EPI_ISL_692532, EPI_ISL_692533, EPI_ISL_692534, EPI_ISL_692535, EPI_ISL_692536, EPI_ISL_692537, EPI_ISL_692538, EPI_ISL_692539, EPI_ISL_692540, EPI_ISL_692541, EPI_ISL_692542, EPI_ISL_692543, EPI_ISL_692544, EPI_ISL_692545, EPI_ISL_692546, EPI_ISL_692547, EPI_ISL_692548, EPI_ISL_692549, EPI_ISL_692550, EPI_ISL_692551, EPI_ISL_692552, EPI_ISL_692553, EPI_ISL_692554, EPI_ISL_692555, EPI_ISL_692556, EPI_ISL_692557, EPI_ISL_692558, EPI_ISL_692559, EPI_ISL_692560, EPI_ISL_692561, EPI_ISL_692562, EPI_ISL_692563, EPI_ISL_692564, EPI_ISL_692565, EPI_ISL_692566, EPI_ISL_692567, EPI_ISL_692568, EPI_ISL_692569, EPI_ISL_692570, EPI_ISL_692571, EPI_ISL_692572, EPI_ISL_692573, EPI_ISL_692574, EPI_ISL_692575, EPI_ISL_692576, EPI_ISL_692577, EPI_ISL_692578, EPI_ISL_692579, EPI_ISL_692580, EPI_ISL_692581, EPI_ISL_692582, EPI_ISL_692583, EPI_ISL_692584, EPI_ISL_692585, EPI_ISL_692586, EPI_ISL_692587, EPI_ISL_692588, EPI_ISL_692589, EPI_ISL_692590, EPI_ISL_692591, EPI_ISL_692592, EPI_ISL_692593, EPI_ISL_692594, EPI_ISL_692595, EPI_ISL_692596, EPI_ISL_692597, EPI_ISL_692598, EPI_ISL_692599, EPI_ISL_692600, EPI_ISL_692601, EPI_ISL_692602, EPI_ISL_692603, EPI_ISL_692604, EPI_ISL_692605, EPI_ISL_692606, EPI_ISL_692607, EPI_ISL_692608, EPI_ISL_692609, EPI_ISL_692610, EPI_ISL_692611, EPI_ISL_692612, EPI_ISL_692613, EPI_ISL_692614, EPI_ISL_692615, EPI_ISL_692616, EPI_ISL_692617, EPI_ISL_692618, EPI_ISL_692619, EPI_ISL_692620, EPI_ISL_692621, EPI_ISL_692622, EPI_ISL_692623, EPI_ISL_692624, EPI_ISL_692625, EPI_ISL_692626, EPI_ISL_692627, EPI_ISL_692628, EPI_ISL_692629, EPI_ISL_692630, EPI_ISL_692631, EPI_ISL_692632, EPI_ISL_692633, EPI_ISL_692634, EPI_ISL_692635, EPI_ISL_692636, EPI_ISL_692637, EPI_ISL_692638, EPI_ISL_692639, EPI_ISL_692640, EPI_ISL_692641, EPI_ISL_692642, EPI_ISL_692643, EPI_ISL_692644, EPI_ISL_692645, EPI_ISL_692646, EPI_ISL_692647, EPI_ISL_692648, EPI_ISL_692649, EPI_ISL_692650, EPI_ISL_692651, EPI_ISL_692652, EPI_ISL_692680, EPI_ISL_692681, EPI_ISL_692682, EPI_ISL_692683, EPI_ISL_692684, EPI_ISL_692685, EPI_ISL_692686, EPI_ISL_692687, EPI_ISL_692688, EPI_ISL_692689, EPI_ISL_692690, EPI_ISL_692691, EPI_ISL_692692, EPI_ISL_692693, EPI_ISL_692694, EPI_ISL_692695, EPI_ISL_692696, EPI_ISL_692697, EPI_ISL_692698, EPI_ISL_692699, EPI_ISL_692700, EPI_ISL_692701, EPI_ISL_692702, EPI_ISL_692703, EPI_ISL_692704, EPI_ISL_692705, EPI_ISL_692706, EPI_ISL_692707, EPI_ISL_692708, EPI_ISL_692709, EPI_ISL_692710, EPI_ISL_692711, EPI_ISL_692712, EPI_ISL_692713, EPI_ISL_692714, EPI_ISL_692715, EPI_ISL_692716, EPI_ISL_692717, EPI_ISL_692718, EPI_ISL_692719, EPI_ISL_692720, EPI_ISL_692721, EPI_ISL_692722, EPI_ISL_692723, EPI_ISL_692724, EPI_ISL_692725, EPI_ISL_692726, EPI_ISL_692727, EPI_ISL_692728, EPI_ISL_692729, EPI_ISL_692730, EPI_ISL_692731, EPI_ISL_692732, EPI_ISL_721568, EPI_ISL_721569, EPI_ISL_721570, EPI_ISL_721571, EPI_ISL_721572, EPI_ISL_721573, EPI_ISL_721574, EPI_ISL_721575, EPI_ISL_721576, EPI_ISL_721577, EPI_ISL_721578, EPI_ISL_721579, EPI_ISL_721580, EPI_ISL_721581, EPI_ISL_721582, EPI_ISL_721583, EPI_ISL_721584, EPI_ISL_721585, EPI_ISL_721586, EPI_ISL_721587, EPI_ISL_721588, EPI_ISL_721589, EPI_ISL_721590, EPI_ISL_721591, EPI_ISL_721592, EPI_ISL_721593, EPI_ISL_721594, EPI_ISL_721595, EPI_ISL_721596, EPI_ISL_721597, EPI_ISL_721598, EPI_ISL_721599, EPI_ISL_721600, EPI_ISL_721601, EPI_ISL_721602, EPI_ISL_721603, EPI_ISL_721604, EPI_ISL_721605, EPI_ISL_721606, EPI_ISL_721607, EPI_ISL_721608, EPI_ISL_721609, EPI_ISL_721610, EPI_ISL_721611, EPI_ISL_721612, EPI_ISL_721613, EPI_ISL_721614, EPI_ISL_721615, EPI_ISL_721616, EPI_ISL_721617, EPI_ISL_721618, EPI_ISL_721619, EPI_ISL_721620, EPI_ISL_721621, EPI_ISL_721622, EPI_ISL_721623, EPI_ISL_735439, EPI_ISL_735440, EPI_ISL_735441, EPI_ISL_735442, EPI_ISL_735443, EPI_ISL_736891, EPI_ISL_736892, EPI_ISL_736893, EPI_ISL_736894, EPI_ISL_736895, EPI_ISL_736896, EPI_ISL_736897, EPI_ISL_736898, EPI_ISL_736899, EPI_ISL_736900, EPI_ISL_736901, EPI_ISL_736902, EPI_ISL_736903, EPI_ISL_736904, EPI_ISL_736905, EPI_ISL_736906, EPI_ISL_736907, EPI_ISL_736908, EPI_ISL_736909, EPI_ISL_736910, EPI_ISL_736911, EPI_ISL_736912, EPI_ISL_736913, EPI_ISL_736914, EPI_ISL_736915, EPI_ISL_736916, EPI_ISL_736917, EPI_ISL_736918, EPI_ISL_736919, EPI_ISL_736920, EPI_ISL_736921, EPI_ISL_736922, EPI_ISL_736923, EPI_ISL_736924, EPI_ISL_737208			
see above	Pathogen Genomics Center, National Institute of Infectious Diseases	Pathogen Genomics Center, National Institute of Infectious Diseases	Hajime Kamiya; Kentaro Itokawa; Makoto Kuroda; Masanori Hashino; Motoi Suzuki; Rina Tanaka; Ryohei Nomoto; Tomoe Shimada; Tsuyoshi Sekizuka
EPI_ISL_479797, EPI_ISL_479798, EPI_ISL_479802, EPI_ISL_479803, EPI_ISL_479804, EPI_ISL_479870, EPI_ISL_479871			
see above	Sagamihara City Public Health Research Institute	Pathogen Genomics Center, National Institute of Infectious Diseases	Hajime Kamiya; Hiroshi Nakamura; Kentaro Itokawa; Makoto Kuroda; Masanori Hashino; Motoi Suzuki; Rina Tanaka; Tsuyoshi Sekizuka
EPI_ISL_416525	Saitama Medical University	Saitama Medical University	Kazuo Imai
EPI_ISL_416524	Saitama Medical University Hospital	Saitama Medical University	Kazuo Imai
EPI_ISL_419301, EPI_ISL_419302, EPI_ISL_419303, EPI_ISL_419304, EPI_ISL_419305, EPI_ISL_419306, EPI_ISL_419307, EPI_ISL_419308, EPI_ISL_479805, EPI_ISL_479806, EPI_ISL_479807, EPI_ISL_479808, EPI_ISL_479928, EPI_ISL_479929, EPI_ISL_479930, EPI_ISL_479931, EPI_ISL_479932, EPI_ISL_479933, EPI_ISL_479934, EPI_ISL_479935, EPI_ISL_684148, EPI_ISL_684149, EPI_ISL_684881, EPI_ISL_684882, EPI_ISL_684883, EPI_ISL_684884, EPI_ISL_684885, EPI_ISL_684886, EPI_ISL_684887, EPI_ISL_684888, EPI_ISL_684889, EPI_ISL_684890, EPI_ISL_684891, EPI_ISL_684892, EPI_ISL_684893, EPI_ISL_684894, EPI_ISL_684895, EPI_ISL_684896, EPI_ISL_684897, EPI_ISL_684898, EPI_ISL_684899, EPI_ISL_684900, EPI_ISL_684901, EPI_ISL_684902, EPI_ISL_684903, EPI_ISL_684904, EPI_ISL_684905, EPI_ISL_684906, EPI_ISL_684907, EPI_ISL_684908, EPI_ISL_684909, EPI_ISL_684910, EPI_ISL_684911, EPI_ISL_684912, EPI_ISL_684913, EPI_ISL_684914, EPI_ISL_684915, EPI_ISL_684916, EPI_ISL_684917, EPI_ISL_684918, EPI_ISL_684919, EPI_ISL_684920, EPI_ISL_684921, EPI_ISL_684922, EPI_ISL_684923, EPI_ISL_684924, EPI_ISL_684925, EPI_ISL_684926, EPI_ISL_684927, EPI_ISL_684928, EPI_ISL_684929, EPI_ISL_684930, EPI_ISL_684931, EPI_ISL_684932, EPI_ISL_684933, EPI_ISL_684934, EPI_ISL_684935, EPI_ISL_684936, EPI_ISL_684937, EPI_ISL_684938, EPI_ISL_684939, EPI_ISL_684940, EPI_ISL_684941, EPI_ISL_684942, EPI_ISL_684943, EPI_ISL_684944, EPI_ISL_684945, EPI_ISL_684946, EPI_ISL_684947, EPI_ISL_684948, EPI_ISL_684949, EPI_ISL_684950, EPI_ISL_684951, EPI_ISL_684952, EPI_ISL_684953, EPI_ISL_684954, EPI_ISL_684955, EPI_ISL_684956, EPI_ISL_684957, EPI_ISL_684958, EPI_ISL_684959, EPI_ISL_684960, EPI_ISL_684961, EPI_ISL_684962, EPI_ISL_684963, EPI_ISL_684964, EPI_ISL_684965, EPI_ISL_684966, EPI_ISL_684967, EPI_ISL_684968, EPI_ISL_684969, EPI_ISL_684970, EPI_ISL_684971, EPI_ISL_684972, EPI_ISL_684973, EPI_ISL_684974, EPI_ISL_684975, EPI_ISL_684976, EPI_ISL_684977, EPI_ISL_684978, EPI_ISL_684979, EPI_ISL_684980, EPI_ISL_684981, EPI_ISL_684982, EPI_ISL_684983, EPI_ISL_684984, EPI_ISL_684985, EPI_ISL_684986, EPI_ISL_684987, EPI_ISL_684988, EPI_ISL_684989, EPI_ISL_684990, EPI_ISL_684991, EPI_ISL_684992, EPI_ISL_684993, EPI_ISL_684994, EPI_ISL_684995, EPI_ISL_684996, EPI_ISL_684997, EPI_ISL_684998, EPI_ISL_684999, EPI_ISL_685000, EPI_ISL_685001, EPI_ISL_685002, EPI_ISL_685003, EPI_ISL_685004, EPI_ISL_685005, EPI_ISL_685006, EPI_ISL_685007, EPI_ISL_685008, EPI_ISL_685009, EPI_ISL_685010, EPI_ISL_685011, EPI_ISL_685012, EPI_ISL_685013, EPI_ISL_685014, EPI_ISL_685015, EPI_ISL_685016, EPI_ISL_685017, EPI_ISL_685018, EPI_ISL_685019, EPI_ISL_685020, EPI_ISL_685021, EPI_ISL_685022, EPI_ISL_685023, EPI_ISL_685024, EPI_ISL_685025, EPI_ISL_685026, EPI_ISL_685027, EPI_ISL_685028, EPI_ISL_685029, EPI_ISL_685030, EPI_ISL_685031, EPI_ISL_685032, EPI_ISL_685033, EPI_ISL_685034, EPI_ISL_685035, EPI_ISL_685036, EPI_ISL_685037, EPI_ISL_685038, EPI_ISL_685039, EPI_ISL_685040, EPI_ISL_685041, EPI_ISL_685042, EPI_ISL_685043, EPI_ISL_685044, EPI_ISL_685045, EPI_ISL_685046, EPI_ISL_685047, EPI_ISL_685048, EPI_ISL_685049, EPI_ISL_685050, EPI_ISL_685051, EPI_ISL_685052, EPI_ISL_685053, EPI_ISL_685054, EPI_ISL_685055, EPI_ISL_685056, EPI_ISL_685057, EPI_ISL_685058, EPI_ISL_685059, EPI_ISL_685060, EPI_ISL_685061, EPI_ISL_685062, EPI_ISL_685063, EPI_ISL_685064, EPI_ISL_685065, EPI_ISL_685066, EPI_ISL_685067, EPI_ISL_685068, EPI_ISL_685069, EPI_ISL_685070, EPI_ISL_685071, EPI_ISL_685072, EPI_ISL_685073, EPI_ISL_685074, EPI_ISL_685075, EPI_ISL_685076, EPI_ISL_685077, EPI_ISL_685078, EPI_ISL_685079, EPI_ISL_685080, EPI_ISL_685081, EPI_ISL_685082, EPI_ISL_685083, EPI_ISL_685084, EPI_ISL_685085, EPI_ISL_685086, EPI_ISL_685087, EPI_ISL_685088, EPI_ISL_685089, EPI_ISL_685090, EPI_ISL_685091, EPI_ISL_685092, EPI_ISL_685093, EPI_ISL_685094, EPI_ISL_685095, EPI_ISL_685096, EPI_ISL_685097, EPI_ISL_685098, EPI_ISL_685099, EPI_ISL_685100, EPI_ISL_685101, EPI_ISL_685102, EPI_ISL_685103, EPI_ISL_685104, EPI_ISL_685105, EPI_ISL_685106, EPI_ISL_685107, EPI_ISL_685108, EPI_ISL_685109, EPI_ISL_685110, EPI_ISL_685111, EPI_ISL_685112, EPI_ISL_685113, EPI_ISL_685114, EPI_ISL_685115, EPI_ISL_685116, EPI_ISL_685117, EPI_ISL_685118, EPI_ISL_685119, EPI_ISL_685120, EPI_ISL_685121, EPI_ISL_685122, EPI_ISL_685123, EPI_ISL_685124, EPI_ISL_685125, EPI_ISL_685126, EPI_ISL_685127, EPI_ISL_685128, EPI_ISL_685129, EPI_ISL_685130, EPI_ISL_685131, EPI_ISL_685132, EPI_ISL_686083, EPI_ISL_686084, EPI_ISL_686085, EPI_ISL_686086, EPI_ISL_686087, EPI_ISL_686088, EPI_ISL_686089, EPI_ISL_686090, EPI_ISL_686091, EPI_ISL_686092, EPI_ISL_686093, EPI_ISL_686094, EPI_ISL_686095, EPI_ISL_686096, EPI_ISL_686097, EPI_ISL_686098, EPI_ISL_686099, EPI_ISL_686100, EPI_ISL_686101, EPI_ISL_686102, EPI_ISL_686103, EPI_ISL_686104, EPI_ISL_686105, EPI_ISL_686106, EPI_ISL_686107, EPI_ISL_687619, EPI_ISL_687620, EPI_ISL_688284, EPI_ISL_688285, EPI_ISL_688286, EPI_ISL_688287, EPI_ISL_688288, EPI_ISL_688289, EPI_ISL_688290, EPI_ISL_688291, EPI_ISL_688292, EPI_ISL_688293, EPI_ISL_688294, EPI_ISL_688295, EPI_ISL_688296, EPI_ISL_688297, EPI_ISL_688298, EPI_ISL_688299, EPI_ISL_688300, EPI_ISL_688301, EPI_ISL_688302, EPI_ISL_688303, EPI_ISL_688304, EPI_ISL_688305, EPI_ISL_688306, EPI_ISL_688307, EPI_ISL_688308, EPI_ISL_688309, EPI_ISL_688310, EPI_ISL_688311, EPI_ISL_688312, EPI_ISL_688313, EPI_ISL_688314, EPI_ISL_688315, EPI_ISL_688316, EPI_ISL_688317, EPI_ISL_688318, EPI_ISL_688319, EPI_ISL_688320, EPI_ISL_688321, EPI_ISL_688322, EPI_ISL_688323, EPI_ISL_688324, EPI_ISL_688325, EPI_ISL_688326, EPI_ISL_688327, EPI_ISL_688328, EPI_ISL_688329, EPI_ISL_688330, EPI_ISL_688331, EPI_ISL_688332, EPI_ISL_688333, EPI_ISL_688334, EPI_ISL_688335, EPI_ISL_688336, EPI_ISL_688337, EPI_ISL_688338, EPI_ISL_688339, EPI_ISL_688340, EPI_ISL_688341, EPI_ISL_688342, EPI_ISL_688343, EPI_ISL_688344, EPI_ISL_688345, EPI_ISL_688346, EPI_ISL_688347, EPI_ISL_688348, EPI_ISL_688349, EPI_ISL_688350, EPI_ISL_688351, EPI_ISL_688352, EPI_ISL_688353, EPI_ISL_688354, EPI_ISL_688355, EPI_ISL_688356, EPI_ISL_688357, EPI_ISL_688358, EPI_ISL_688359, EPI_ISL_688360, EPI_ISL_688361, EPI_ISL_688362, EPI_ISL_688363, EPI_ISL_688364, EPI_ISL_688365, EPI_ISL_688366, EPI_ISL_688367, EPI_ISL_688368, EPI_ISL_688369, EPI_ISL_688370, EPI_ISL_688371, EPI_ISL_688372, EPI_ISL_688373, EPI_ISL_688374, EPI_ISL_688375, EPI_ISL_688376, EPI_ISL_688377, EPI_ISL_688378, EPI_ISL_688379, EPI_ISL_688380, EPI_ISL_688381, EPI_ISL_688382, EPI_ISL_688383, EPI_ISL_688384, EPI_ISL_688385, EPI_ISL_688386, EPI_ISL_688387, EPI_ISL_688388, EPI_ISL_688389, EPI_ISL_688390, EPI_ISL_688391, EPI_ISL_688392, EPI_ISL_688393, EPI_ISL_688394, EPI_ISL_688395, EPI_ISL_688396, EPI_ISL_688397, EPI_ISL_688398, EPI_ISL_688399, EPI_ISL_688400, EPI_ISL_688401, EPI_ISL_690619, EPI_ISL_690630, EPI_ISL_690631, EPI_ISL_690641, EPI_ISL_690642, EPI_ISL_690643, EPI_ISL_690644, EPI_ISL_690645, EPI_ISL_690646, EPI_ISL_690647, EPI_ISL_690648, EPI_ISL_690649, EPI_ISL_690650, EPI_ISL_690651, EPI_ISL_690652, EPI_ISL_690653, EPI_ISL_690654, EPI_ISL_690655, EPI_ISL_690656, EPI_ISL_690657, EPI_ISL_690658, EPI_ISL_690659, EPI_ISL_690660, EPI_ISL_690661, EPI_ISL_690662, EPI_ISL_690663, EPI_ISL_690664, EPI_ISL_690665, EPI_ISL_690666, EPI_ISL_690667, EPI_ISL_690668, EPI_ISL_690669, EPI_ISL_690670, EPI_ISL_690671, EPI_ISL_690672, EPI_ISL_690673, EPI_ISL_690674, EPI_ISL_690675, EPI_ISL_690676, EPI_ISL_690677, EPI_ISL_690678, EPI_ISL_690679, EPI_ISL_690680, EPI_ISL_690681, EPI_ISL_690682, EPI_ISL_690683, EPI_ISL_691364, EPI_ISL_691365, EPI_ISL_691366, EPI_ISL_691367, EPI_ISL_691368, EPI_ISL_691369, EPI_ISL_691370, EPI_ISL_691371, EPI_ISL_691372, EPI_ISL_691373, EPI_ISL_691374, EPI_ISL_691375, EPI_ISL_691376, EPI_ISL_691377, EPI_ISL_691378, EPI_ISL_691766			
see above	Saitama Prefectural Institute of Public Health	Pathogen Genomics Center, National Institute of Infectious Diseases	Hajime Kamiya; Hayato Ehara; Kentaro Itokawa; Makoto Kuroda; Masanori Hashino; Michiyo Shinohara; Motoi Suzuki; Rina Tanaka; Tsuyoshi Kishimoto; Tsuyoshi Sekizuka
EPI_ISL_479925, EPI_ISL_479926, EPI_ISL_479927, EPI_ISL_480065, EPI_ISL_480066, EPI_ISL_480067, EPI_ISL_480068, EPI_ISL_480069, EPI_ISL_480070, EPI_ISL_480071, EPI_ISL_480072, EPI_ISL_684147, EPI_ISL_686521, EPI_ISL_686522, EPI_ISL_686523, EPI_ISL_686524, EPI_ISL_686525, EPI_ISL_686526, EPI_ISL_686527, EPI_ISL_686528, EPI_ISL_686529, EPI_ISL_686530, EPI_ISL_686531, EPI_ISL_686532, EPI_ISL_686533, EPI_ISL_686534, EPI_ISL_688700, EPI_ISL_688701, EPI_ISL_688702, EPI_ISL_688703, EPI_ISL_688704, EPI_ISL_688705, EPI_ISL_688706, EPI_ISL_688707, EPI_ISL_688708, EPI_ISL_688709, EPI_ISL_690011, EPI_ISL_690012, EPI_ISL_690013, EPI_ISL_690014, EPI_ISL_690015, EPI_ISL_690016, EPI_ISL_690017, EPI_ISL_690018, EPI_ISL_690019, EPI_ISL_690020, EPI_ISL_690021, EPI_ISL_690022, EPI_ISL_690023, EPI_ISL_690024, EPI_ISL_690025, EPI_ISL_690026, EPI_ISL_690027, EPI_ISL_690028, EPI_ISL_690626, EPI_ISL_690627, EPI_ISL_690628, EPI_ISL_690837, EPI_ISL_690838, EPI_ISL_690839, EPI_ISL_690840, EPI_ISL_690841, EPI_ISL_690842, EPI_ISL_690843, EPI_ISL_690844, EPI_ISL_691509, EPI_ISL_691510, EPI_ISL_691511, EPI_ISL_691512, EPI_ISL_691513, EPI_ISL_691514, EPI_ISL_691515, EPI_ISL_691516, EPI_ISL_691517, EPI_ISL_691518, EPI_ISL_691519, EPI_ISL_691520, EPI_ISL_691521, EPI_ISL_691522, EPI_ISL_691523, EPI_ISL_691524, EPI_ISL_691525, EPI_ISL_691526, EPI_ISL_691527, EPI_ISL_691528, EPI_ISL_691529, EPI_ISL_691530, EPI_ISL_691531, EPI_ISL_691532, EPI_ISL_691533, EPI_ISL_691534, EPI_ISL_691535, EPI_ISL_691536, EPI_ISL_691537, EPI_ISL_691538, EPI_ISL_691539, EPI_ISL_691540, EPI_ISL_691541, EPI_ISL_691542, EPI_ISL_691543, EPI_ISL_691544, EPI_ISL_691545, EPI_ISL_691753, EPI_ISL_692053, EPI_ISL_692054, EPI_ISL_692055, EPI_ISL_692056, EPI_ISL_692057, EPI_ISL_692058, EPI_ISL_692284, EPI_ISL_692285, EPI_ISL_692286, EPI_ISL_692287, EPI_ISL_692288, EPI_ISL_692289, EPI_ISL_692290, EPI_ISL_692291, EPI_ISL_692292, EPI_ISL_692293, EPI_ISL_692294, EPI_ISL_692295, EPI_ISL_692296, EPI_ISL_692297, EPI_ISL_692298, EPI_ISL_692299, EPI_ISL_692300, EPI_ISL_692301, EPI_ISL_692302, EPI_ISL_692303, EPI_ISL_692304, EPI_ISL_692305, EPI_ISL_692306, EPI_ISL_692307, EPI_ISL_692308, EPI_ISL_692309, EPI_ISL_692310, EPI_ISL_692311, EPI_ISL_692312, EPI_ISL_692313, EPI_ISL_692314, EPI_ISL_692315, EPI_ISL_692653, EPI_ISL_692654, EPI_ISL_692655, EPI_ISL_692656, EPI_ISL_692657, EPI_ISL_692658, EPI_ISL_692659, EPI_ISL_692660, EPI_ISL_692661, EPI_ISL_692662, EPI_ISL_692663, EPI_ISL_692664, EPI_ISL_692665, EPI_ISL_692666, EPI_ISL_692667, EPI_ISL_692668, EPI_ISL_692669, EPI_ISL_692670, EPI_ISL_692671, EPI_ISL_692672, EPI_ISL_692673, EPI_ISL_692674, EPI_ISL_692675, EPI_ISL_692676, EPI_ISL_692677, EPI_ISL_692678, EPI_ISL_692679			
see above	Sakai City Institute of Public Health	Pathogen Genomics Center, National Institute of Infectious Diseases	Hajime Kamiya; Kentaro Itokawa; Makoto Kuroda; Masanori Hashino; Motoi Suzuki; Rina Tanaka; Tatsuya Miyoshi; Tsuyoshi Sekizuka
EPI_ISL_479799, EPI_ISL_479800, EPI_ISL_479826, EPI_ISL_479827, EPI_ISL_479828, EPI_ISL_479829, EPI_ISL_479830, EPI_ISL_479831, EPI_ISL_479832, EPI_ISL_479833, EPI_ISL_479834, EPI_ISL_479835, EPI_ISL_479836, EPI_ISL_479837, EPI_ISL_479838, EPI_ISL_479839, EPI_ISL_479840, EPI_ISL_479841, EPI_ISL_479842, EPI_ISL_479843, EPI_ISL_479844, EPI_ISL_479845, EPI_ISL_479846, EPI_ISL_479847, EPI_ISL_479848, EPI_ISL_479849, EPI_ISL_479872, EPI_ISL_479873, EPI_ISL_479874, EPI_ISL_479875, EPI_ISL_479876, EPI_ISL_479877, EPI_ISL_479878, EPI_ISL_479879, EPI_ISL_479880, EPI_ISL_479881, EPI_ISL_479882, EPI_ISL_479883, EPI_ISL_479884, EPI_ISL_479885			
see above	Sapporo City Institute of Public Health	Pathogen Genomics Center, National Institute of Infectious Diseases	Asami Ohnishi; Hajime Kamiya; Kentaro Itokawa; Makoto Kuroda; Masanori Hashino; Motoi Suzuki; Rina Tanaka; Tsuyoshi Sekizuka
EPI_ISL_480074, EPI_ISL_480075, EPI_ISL_480076, EPI_ISL_480077, EPI_ISL_480078, EPI_ISL_480079, EPI_ISL_480080, EPI_ISL_480081, EPI_ISL_480082			
see above	Shizuoka City Institute of Environmental Sciences and Public Health	Pathogen Genomics Center, National Institute of Infectious Diseases	Hajime Kamiya; Kenji Yagi; Kentaro Itokawa; Makoto Kuroda; Masanori Hashino; Motoi Suzuki; Rina Tanaka; Sou Okamura; Takaharu Maehata; Tsuyoshi Sekizuka; Yuji Kanazawa
EPI_ISL_412968, EPI_ISL_412969, EPI_ISL_420889, EPI_ISL_420890, EPI_ISL_445183, EPI_ISL_510545	Takayuki Hishiki Kanagawa Prefectural Institute of Public Health	Takayuki Hishiki Kanagawa Prefectural Institute of Public Health	An, T.; Hayashizaki; Hishiki, T.; Kawai, J.; Kogo, Y.; Matsuki, Y.; Sakuragi, J.; Shimizu, H.; Suzuki, R.; Tanaka, Y.; Usui, K.; Y. and Takasaki, T.
EPI_ISL_480030, EPI_ISL_480031, EPI_ISL_480032, EPI_ISL_480033, EPI_ISL_480034, EPI_ISL_480035, EPI_ISL_480036, EPI_ISL_480037, EPI_ISL_480038, EPI_ISL_480039, EPI_ISL_480040, EPI_ISL_480041, EPI_ISL_480073			
see above	Tochigi Prefectural Institute of Public Health and Environmental Science	Pathogen Genomics Center, National Institute of Infectious Diseases	Ako Nakajima; Hajime Kamiya; Kentaro Itokawa; Makoto Kuroda; Masanori Hashino; Motoi Suzuki; Rina Tanaka; Tsuyoshi Sekizuka
EPI_ISL_486890, EPI_ISL_486891, EPI_ISL_486892, EPI_ISL_486893, EPI_ISL_486894, EPI_ISL_486895, EPI_ISL_486896, EPI_ISL_486897, EPI_ISL_486898, EPI_ISL_486899, EPI_ISL_486900, EPI_ISL_486901, EPI_ISL_486902, EPI_ISL_486903, EPI_ISL_486904, EPI_ISL_486905, EPI_ISL_486906, EPI_ISL_486907, EPI_ISL_486908, EPI_ISL_486909, EPI_ISL_486910, EPI_ISL_486911			
see above	Tokyo Metoropolitan Institute of Public Health	Tokyo Metoropolitan Institute of Public Health	Asakura, H.; Chiba, T.; Kumagai, R.; Nagashima, M.; Sadamasu, K.; Yoshida, I.
EPI_ISL_479825, EPI_ISL_479886, EPI_ISL_479887, EPI_ISL_479888, EPI_ISL_479889, EPI_ISL_479890, EPI_ISL_479891, EPI_ISL_479892, EPI_ISL_479893, EPI_ISL_479894, EPI_ISL_479895, EPI_ISL_479959, EPI_ISL_479960, EPI_ISL_479961, EPI_ISL_479962, EPI_ISL_479963, EPI_ISL_479964, EPI_ISL_479965, EPI_ISL_480227, EPI_ISL_684108, EPI_ISL_684109, EPI_ISL_684136, EPI_ISL_684137, EPI_ISL_684138, EPI_ISL_684154, EPI_ISL_684155, EPI_ISL_684506, EPI_ISL_684507, EPI_ISL_684508, EPI_ISL_684509, EPI_ISL_684510, EPI_ISL_684511, EPI_ISL_684512, EPI_ISL_684513, EPI_ISL_684514, EPI_ISL_684515, EPI_ISL_684516, EPI_ISL_684517, EPI_ISL_684518, EPI_ISL_684519, EPI_ISL_684520, EPI_ISL_684521, EPI_ISL_684522, EPI_ISL_684556, EPI_ISL_684557, EPI_ISL_684615, EPI_ISL_684616, EPI_ISL_684617, EPI_ISL_684618, EPI_ISL_684619, EPI_ISL_684620, EPI_ISL_684621, EPI_ISL_684622, EPI_ISL_684623, EPI_ISL_684624, EPI_ISL_684625, EPI_ISL_684626, EPI_ISL_684627, EPI_ISL_684628, EPI_ISL_684629, EPI_ISL_684630, EPI_ISL_684631, EPI_ISL_684632, EPI_ISL_684633, EPI_ISL_684728, EPI_ISL_684729, EPI_ISL_684730, EPI_ISL_684731, EPI_ISL_684732, EPI_ISL_684733, EPI_ISL_684734, EPI_ISL_684735, EPI_ISL_685220, EPI_ISL_685221, EPI_ISL_685222, EPI_ISL_685223, EPI_ISL_685224, EPI_ISL_685225, EPI_ISL_685226, EPI_ISL_685227, EPI_ISL_685228, EPI_ISL_685229, EPI_ISL_685230, EPI_ISL_685231, EPI_ISL_685232, EPI_ISL_685233, EPI_ISL_685234, EPI_ISL_685235, EPI_ISL_685236, EPI_ISL_685237, EPI_ISL_685407, EPI_ISL_685408, EPI_ISL_685409, EPI_ISL_685410, EPI_ISL_685411, EPI_ISL_685412, EPI_ISL_685413, EPI_ISL_685414, EPI_ISL_685415, EPI_ISL_685416, EPI_ISL_685417, EPI_ISL_685418, EPI_ISL_685419, EPI_ISL_685420, EPI_ISL_685421, EPI_ISL_685422, EPI_ISL_685423, EPI_ISL_685424, EPI_ISL_685425, EPI_ISL_685426, EPI_ISL_685427, EPI_ISL_685428, EPI_ISL_685429, EPI_ISL_685430, EPI_ISL_685431, EPI_ISL_685432, EPI_ISL_685433, EPI_ISL_685434, EPI_ISL_685435, EPI_ISL_685436, EPI_ISL_685437, EPI_ISL_685438, EPI_ISL_685439, EPI_ISL_685440, EPI_ISL_685441, EPI_ISL_685442, EPI_ISL_685443, EPI_ISL_685444, EPI_ISL_685445, EPI_ISL_685446, EPI_ISL_685447, EPI_ISL_685448, EPI_ISL_685449, EPI_ISL_685450, EPI_ISL_685451, EPI_ISL_685452, EPI_ISL_685453, EPI_ISL_685454, EPI_ISL_685455, EPI_ISL_685456, EPI_ISL_685457, EPI_ISL_685458, EPI_ISL_685459, EPI_ISL_685460, EPI_ISL_685461, EPI_ISL_685462, EPI_ISL_685463, EPI_ISL_685464, EPI_ISL_685465, EPI_ISL_685466, EPI_ISL_685467, EPI_ISL_685468, EPI_ISL_685469, EPI_ISL_685470, EPI_ISL_685471, EPI_ISL_685472, EPI_ISL_685473, EPI_ISL_685474, EPI_ISL_685475, EPI_ISL_685476, EPI_ISL_685477, EPI_ISL_685478, EPI_ISL_685479, EPI_ISL_685480, EPI_ISL_685481, EPI_ISL_685482, EPI_ISL_685483, EPI_ISL_685484, EPI_ISL_685485, EPI_ISL_685486, EPI_ISL_685487, EPI_ISL_685488, EPI_ISL_685489, EPI_ISL_685490, EPI_ISL_685491, EPI_ISL_685492, EPI_ISL_685493, EPI_ISL_685494, EPI_ISL_685495, EPI_ISL_685496, EPI_ISL_685497, EPI_ISL_685606, EPI_ISL_685607, EPI_ISL_685608, EPI_ISL_685609, EPI_ISL_685610, EPI_ISL_685611, EPI_ISL_685612, EPI_ISL_685613, EPI_ISL_685614, EPI_ISL_685615, EPI_ISL_685616, EPI_ISL_685666, EPI_ISL_685667, EPI_ISL_685668, EPI_ISL_685669, EPI_ISL_685670, EPI_ISL_685671, EPI_ISL_685672, EPI_ISL_685673, EPI_ISL_685674, EPI_ISL_685675, EPI_ISL_685676, EPI_ISL_685677, EPI_ISL_685678, EPI_ISL_685679, EPI_ISL_685680, EPI_ISL_685681, EPI_ISL_685682, EPI_ISL_685683, EPI_ISL_685684, EPI_ISL_685685, EPI_ISL_685686, EPI_ISL_685687, EPI_ISL_685688, EPI_ISL_685689, EPI_ISL_685690, EPI_ISL_685691, EPI_ISL_685692, EPI_ISL_685693, EPI_ISL_685694, EPI_ISL_685963, EPI_ISL_685964, EPI_ISL_685965, EPI_ISL_685966, EPI_ISL_685967, EPI_ISL_685968, EPI_ISL_685969, EPI_ISL_685970, EPI_ISL_685971, EPI_ISL_685972, EPI_ISL_685973, EPI_ISL_685974, EPI_ISL_685975, EPI_ISL_685976, EPI_ISL_685977, EPI_ISL_685978, EPI_ISL_685979, EPI_ISL_685980, EPI_ISL_685981, EPI_ISL_685982, EPI_ISL_685983, EPI_ISL_685984, EPI_ISL_685985, EPI_ISL_685986, EPI_ISL_685987, EPI_ISL_688583, EPI_ISL_688584, EPI_ISL_688585, EPI_ISL_688586, EPI_ISL_688587, EPI_ISL_688588, EPI_ISL_688589, EPI_ISL_688590, EPI_ISL_688591, EPI_ISL_688592, EPI_ISL_688593, EPI_ISL_688594, EPI_ISL_688595, EPI_ISL_688596, EPI_ISL_688597, EPI_ISL_688598, EPI_ISL_688599, EPI_ISL_688600, EPI_ISL_688601, EPI_ISL_688602, EPI_ISL_688603, EPI_ISL_688604, EPI_ISL_689077, EPI_ISL_689078, EPI_ISL_689079, EPI_ISL_689080, EPI_ISL_689081, EPI_ISL_689082, EPI_ISL_689083, EPI_ISL_689084, EPI_ISL_689085, EPI_ISL_689086, EPI_ISL_689087, EPI_ISL_689088, EPI_ISL_690612, EPI_ISL_690613, EPI_ISL_690638, EPI_ISL_690639, EPI_ISL_690640, EPI_ISL_690792, EPI_ISL_690793, EPI_ISL_690794, EPI_ISL_690795, EPI_ISL_690796, EPI_ISL_690797, EPI_ISL_690798, EPI_ISL_690799, EPI_ISL_690800, EPI_ISL_690801, EPI_ISL_690802, EPI_ISL_690803, EPI_ISL_690804, EPI_ISL_690805, EPI_ISL_690806, EPI_ISL_690807, EPI_ISL_690808, EPI_ISL_690809, EPI_ISL_690810, EPI_ISL_690811, EPI_ISL_690812, EPI_ISL_690813, EPI_ISL_690814, EPI_ISL_690815, EPI_ISL_690816, EPI_ISL_690817, EPI_ISL_690902, EPI_ISL_690903, EPI_ISL_690904, EPI_ISL_690905, EPI_ISL_690906, EPI_ISL_690907, EPI_ISL_690908, EPI_ISL_692119, EPI_ISL_692120, EPI_ISL_692121, EPI_ISL_692122, EPI_ISL_692123, EPI_ISL_692124, EPI_ISL_692125, EPI_ISL_692126, EPI_ISL_692127, EPI_ISL_692128, EPI_ISL_692162, EPI_ISL_692163			
see above	Tokyo Metropolitan Institute of Public Health	Pathogen Genomics Center, National Institute of Infectious Diseases	Hajime Kamiya; Kenji Sadamasu; Kentaro Itokawa; Makoto Kuroda; Mami Nagashima; Masanori Hashino; Motoi Suzuki; Rina Tanaka; Takashi Chiba; Tsuyoshi Sekizuka
EPI_ISL_582135, EPI_ISL_582136, EPI_ISL_582137, EPI_ISL_582138, EPI_ISL_582139, EPI_ISL_582140, EPI_ISL_582141, EPI_ISL_582142, EPI_ISL_582143, EPI_ISL_582144, EPI_ISL_582145, EPI_ISL_582146, EPI_ISL_582147, EPI_ISL_649126, EPI_ISL_649127, EPI_ISL_649128, EPI_ISL_649129, EPI_ISL_649130, EPI_ISL_649131, EPI_ISL_649132, EPI_ISL_649133, EPI_ISL_649134, EPI_ISL_649135, EPI_ISL_649136, EPI_ISL_649137, EPI_ISL_649138, EPI_ISL_649139, EPI_ISL_649140, EPI_ISL_649141, EPI_ISL_649142, EPI_ISL_649143, EPI_ISL_649144			
see above	Tokyo Metropolitan Institute of Public Health, Department of Microbiology	Tokyo Metropolitan Institute of Public Health, Department of Microbiology	Asakura, H.; Chiba; Kumagai, R.; Nagashima, M.; T. and Sadamasu, K.; Yoshida, I.
EPI_ISL_480196, EPI_ISL_480197, EPI_ISL_480198, EPI_ISL_480199, EPI_ISL_480200, EPI_ISL_480201, EPI_ISL_480202, EPI_ISL_480203			
see above	Toyama Institute of Health	Pathogen Genomics Center, National Institute of Infectious Diseases	Hajime Kamiya; Kazunori Oishi; Kentaro Itokawa; Makoto Kuroda; Masae Itamochi; Masanori Hashino; Motoi Suzuki; Rina Tanaka; Tsuyoshi Sekizuka
EPI_ISL_418809	University of Wisconsin - Madison: Influenza Research Institute	University of Wisconsin Madison, AIDS Vaccine Research Laboratories	Gage Moreno; Katarina Braun; Peter Halfmann; et al.
EPI_ISL_479862, EPI_ISL_479863, EPI_ISL_479864, EPI_ISL_479865, EPI_ISL_479866, EPI_ISL_479867	Wakayama Prefectural Research Center of Environment and Public Health	Pathogen Genomics Center, National Institute of Infectious Diseases	Fumio Terasoma; Hajime Kamiya; Kentaro Itokawa; Makoto Kuroda; Masanori Hashino; Motoi Suzuki; Rina Tanaka; Tsuyoshi Sekizuka; Yosuke Hamajima
EPI_ISL_684798, EPI_ISL_684799, EPI_ISL_684800, EPI_ISL_684801, EPI_ISL_684802, EPI_ISL_684803, EPI_ISL_684804, EPI_ISL_684805, EPI_ISL_684806, EPI_ISL_684807, EPI_ISL_684808, EPI_ISL_684809, EPI_ISL_684810, EPI_ISL_684811, EPI_ISL_684812, EPI_ISL_684813, EPI_ISL_684814, EPI_ISL_684815, EPI_ISL_684816, EPI_ISL_684817, EPI_ISL_684818, EPI_ISL_684819, EPI_ISL_684820, EPI_ISL_684821, EPI_ISL_684822, EPI_ISL_684823, EPI_ISL_684824, EPI_ISL_684825, EPI_ISL_684826, EPI_ISL_684827, EPI_ISL_684828, EPI_ISL_684829, EPI_ISL_684830, EPI_ISL_684831, EPI_ISL_684832, EPI_ISL_684833, EPI_ISL_684834, EPI_ISL_684835, EPI_ISL_684836, EPI_ISL_684837, EPI_ISL_684838, EPI_ISL_684839, EPI_ISL_684840, EPI_ISL_684841, EPI_ISL_684842, EPI_ISL_684843, EPI_ISL_684844, EPI_ISL_684845, EPI_ISL_684846, EPI_ISL_684847, EPI_ISL_684848, EPI_ISL_684849, EPI_ISL_684850, EPI_ISL_684851, EPI_ISL_684852, EPI_ISL_684853, EPI_ISL_684854, EPI_ISL_684855, EPI_ISL_684856, EPI_ISL_685498, EPI_ISL_685499, EPI_ISL_685500, EPI_ISL_685501, EPI_ISL_685502, EPI_ISL_685503, EPI_ISL_685504, EPI_ISL_692094, EPI_ISL_692095, EPI_ISL_692096, EPI_ISL_692097			
see above	Yamagata Prefectural Institute of Public Health	Pathogen Genomics Center, National Institute of Infectious Diseases	Kentaro Itokawa; Makoto Kuroda; Masanori Hashino; Rina Tanaka; Tsuyoshi Sekizuka
EPI_ISL_479676, EPI_ISL_479677, EPI_ISL_479678, EPI_ISL_479679, EPI_ISL_479680, EPI_ISL_479681, EPI_ISL_479682, EPI_ISL_479683, EPI_ISL_479684, EPI_ISL_479685			

see above	unknown	Contact:Hiroyuki Asakura Tokyo Metropolitan Institute of Public Health, Department of Microbiology	Asakura, H.; Chiba, T.; Kumagai, R.; Nagashima, M.; Sadamasu, K.; Yoshida, I.
EPI_ISL_468724, EPI_ISL_468725	unknown	Contact:Ryota Kumagai Tokyo Metropolitan Institute of Public Health	Asakura, H.; Chiba, T.; Kumagai, R.; Nagashima, M.; Sadamasu, K.; Yoshida, I.
EPI_ISL_462439, EPI_ISL_462440, EPI_ISL_462441, EPI_ISL_462442, EPI_ISL_462443, EPI_ISL_462444, EPI_ISL_462445, EPI_ISL_462446			
see above	unknown	Ryota Kumagai Tokyo Metropolitan Institute of Public Health	Asakura, H.; Chiba, T.; Kumagai, R.; Nagashima, M.; Sadamasu, K.; Yoshida, I.

We gratefully acknowledge the following Authors from the Originating laboratories responsible for obtaining the specimens, as well as the Submitting laboratories where the genome data were generated and shared via GISAID, on which this research is based.

All Submitters of data may be contacted directly via www.gisaid.org

Authors are sorted alphabetically.

Accession ID	Originating Laboratory	Submitting Laboratory	Authors
EPI_ISL_2695884, EPI_ISL_2695962	Department of Developmental Medicine, Research Institute, Osaka Women's and Children's Hospital	Department of Developmental Medicine, Research Institute, Osaka Women's and Children's Hospital	Itaru Yanagihara; Yukiko Nakura
EPI_ISL_2693952, EPI_ISL_2693953, EPI_ISL_2693954, EPI_ISL_2693955, EPI_ISL_2693956, EPI_ISL_2693957	Department of Infectious Diseases and Immunology, National Hospital Organization Nagoya Medical Center	Clinical Research Center, National Hospital Organization Nagoya Medical Center	Hirotaka Ode; Kazuhiro Matsuoka; Mai Kubota; Masakazu Matsuda; Mayumi Imahashi; Miho Nakasuji; Mikiko Mori; Yasumasa Iwatani; Yoshihiro Nakata; Yoshiyuki Yokomaku
EPI_ISL_2358242, EPI_ISL_2358243, EPI_ISL_2358244, EPI_ISL_2358245, EPI_ISL_2358246, EPI_ISL_2358247, EPI_ISL_2358248, EPI_ISL_2358249, EPI_ISL_2358250, EPI_ISL_2358251, EPI_ISL_2358252, EPI_ISL_2358253, EPI_ISL_2358254, EPI_ISL_2358255, EPI_ISL_2358256, EPI_ISL_2358257, EPI_ISL_2358258, EPI_ISL_2358259, EPI_ISL_2358260, EPI_ISL_2358261, EPI_ISL_2358262, EPI_ISL_2358263, EPI_ISL_2358264, EPI_ISL_2358265, EPI_ISL_2358266, EPI_ISL_2358267, EPI_ISL_2358268, EPI_ISL_2358269, EPI_ISL_2358270, EPI_ISL_2358271, EPI_ISL_2358272, EPI_ISL_2358273, EPI_ISL_2358274, EPI_ISL_2358275, EPI_ISL_2358276, EPI_ISL_2358277, EPI_ISL_2358278, EPI_ISL_2358279, EPI_ISL_2358280, EPI_ISL_2358281, EPI_ISL_2358282, EPI_ISL_2358283, EPI_ISL_2358284, EPI_ISL_2358285, EPI_ISL_2358286, EPI_ISL_2358287, EPI_ISL_2358288, EPI_ISL_2358289, EPI_ISL_2358290, EPI_ISL_2358291, EPI_ISL_2358292, EPI_ISL_2358293, EPI_ISL_2358294, EPI_ISL_2358295, EPI_ISL_2358296, EPI_ISL_2358297, EPI_ISL_2358298, EPI_ISL_2358299, EPI_ISL_2358300, EPI_ISL_2358301, EPI_ISL_2358302, EPI_ISL_2358303, EPI_ISL_2358304, EPI_ISL_2358305, EPI_ISL_2358306, EPI_ISL_2358307, EPI_ISL_2358308, EPI_ISL_2358309, EPI_ISL_2358310, EPI_ISL_2358311, EPI_ISL_2358312, EPI_ISL_2358313, EPI_ISL_2358314, EPI_ISL_2358315, EPI_ISL_2358316, EPI_ISL_2358317, EPI_ISL_2358318, EPI_ISL_2358319, EPI_ISL_2358320, EPI_ISL_2358321, EPI_ISL_2358322, EPI_ISL_2358323, EPI_ISL_2358324, EPI_ISL_2358325, EPI_ISL_2358326, EPI_ISL_2358327, EPI_ISL_2358328, EPI_ISL_2358329, EPI_ISL_2358330, EPI_ISL_2358331, EPI_ISL_2358332, EPI_ISL_2358333, EPI_ISL_2358334, EPI_ISL_2358335, EPI_ISL_2358336, EPI_ISL_2358337, EPI_ISL_2358338, EPI_ISL_2358339, EPI_ISL_2358340, EPI_ISL_2358341, EPI_ISL_2358342, EPI_ISL_2358343, EPI_ISL_2358344, EPI_ISL_2358345, EPI_ISL_2358346, EPI_ISL_2358347, EPI_ISL_2358348, EPI_ISL_2358349, EPI_ISL_2358350, EPI_ISL_2358351, EPI_ISL_2358352, EPI_ISL_2358353, EPI_ISL_2358354, EPI_ISL_2358355, EPI_ISL_2358356, EPI_ISL_2358357, EPI_ISL_2358358, EPI_ISL_2358359, EPI_ISL_2358360, EPI_ISL_2358361, EPI_ISL_2358362, EPI_ISL_2358363, EPI_ISL_2358364, EPI_ISL_2358365, EPI_ISL_2358366, EPI_ISL_2358367, EPI_ISL_2358368, EPI_ISL_2358369, EPI_ISL_2358370, EPI_ISL_2358371, EPI_ISL_2358372, EPI_ISL_2358373, EPI_ISL_2358374, EPI_ISL_2358375, EPI_ISL_2358376, EPI_ISL_2358377, EPI_ISL_2358378, EPI_ISL_2358379, EPI_ISL_2358380, EPI_ISL_2358381, EPI_ISL_2358382, EPI_ISL_2358383, EPI_ISL_2358384, EPI_ISL_2358385, EPI_ISL_2358386, EPI_ISL_2358387, EPI_ISL_2358388, EPI_ISL_2358389, EPI_ISL_2358390, EPI_ISL_2358391, EPI_ISL_2358392, EPI_ISL_2358393, EPI_ISL_2358394, EPI_ISL_2358395, EPI_ISL_2358396, EPI_ISL_2358397, EPI_ISL_2358398, EPI_ISL_2358399, EPI_ISL_2358400, EPI_ISL_2358401, EPI_ISL_2358402, EPI_ISL_2358403, EPI_ISL_2358404, EPI_ISL_2358405, EPI_ISL_2358406, EPI_ISL_2358407, EPI_ISL_2358408, EPI_ISL_2358409, EPI_ISL_2358410, EPI_ISL_2358411, EPI_ISL_2358412, EPI_ISL_2358413, EPI_ISL_2358414, EPI_ISL_2358415, EPI_ISL_2358416, EPI_ISL_2358417, EPI_ISL_2358418, EPI_ISL_2358419, EPI_ISL_2358420, EPI_ISL_2358421, EPI_ISL_2358422, EPI_ISL_2358423, EPI_ISL_2358424, EPI_ISL_2358425, EPI_ISL_2358426, EPI_ISL_2358427, EPI_ISL_2358428, EPI_ISL_2358429, EPI_ISL_2358430, EPI_ISL_2358431, EPI_ISL_2358432, EPI_ISL_2358433, EPI_ISL_2358434, EPI_ISL_2358435, EPI_ISL_2358436, EPI_ISL_2358437, EPI_ISL_2358438, EPI_ISL_2358439, EPI_ISL_2358440, EPI_ISL_2358441, EPI_ISL_2358442, EPI_ISL_2358443, EPI_ISL_2358444, EPI_ISL_2358445, EPI_ISL_2358446, EPI_ISL_2358447, EPI_ISL_2358448, EPI_ISL_2358449, EPI_ISL_2358450, EPI_ISL_2358451, EPI_ISL_2358452, EPI_ISL_2358453, EPI_ISL_2358454, EPI_ISL_2358455, EPI_ISL_2358456, EPI_ISL_2358457, EPI_ISL_2358458, EPI_ISL_2358459, EPI_ISL_2358460, EPI_ISL_2358461, EPI_ISL_2358462, EPI_ISL_2358463, EPI_ISL_2358464, EPI_ISL_2358465, EPI_ISL_2358466, EPI_ISL_2358467, EPI_ISL_2358468, EPI_ISL_2358469, EPI_ISL_2358470, EPI_ISL_2358471, EPI_ISL_2358472, EPI_ISL_2358473, EPI_ISL_2358474, EPI_ISL_2358475, EPI_ISL_2358476, EPI_ISL_2358477, EPI_ISL_2358478, EPI_ISL_2358479, EPI_ISL_2358480, EPI_ISL_2358481, EPI_ISL_2358482, EPI_ISL_2358483, EPI_ISL_2358484, EPI_ISL_2358485, EPI_ISL_2358486, EPI_ISL_2358487, EPI_ISL_2358488, EPI_ISL_2358489, EPI_ISL_2358490, EPI_ISL_2358491, EPI_ISL_2358492, EPI_ISL_2358493, EPI_ISL_2358494, EPI_ISL_2358495, EPI_ISL_2358496, EPI_ISL_2358497, EPI_ISL_2358498, EPI_ISL_2358499, EPI_ISL_2358500, EPI_ISL_2358501, EPI_ISL_2358502, EPI_ISL_2358503, EPI_ISL_2358504, EPI_ISL_2358505, EPI_ISL_2358506, EPI_ISL_2358507, EPI_ISL_2358508, EPI_ISL_2358509, EPI_ISL_2358510, EPI_ISL_2358511, EPI_ISL_2358512, EPI_ISL_2358513, EPI_ISL_2358514, EPI_ISL_2358515, EPI_ISL_2358516, EPI_ISL_2358517, EPI_ISL_2358518, EPI_ISL_2358519, EPI_ISL_2358520, EPI_ISL_2358521, EPI_ISL_2358522, EPI_ISL_2358523, EPI_ISL_2358524, EPI_ISL_2358525, EPI_ISL_2358526, EPI_ISL_2358527, EPI_ISL_2358528, EPI_ISL_2358529, EPI_ISL_2358530, EPI_ISL_2358531, EPI_ISL_2358532, EPI_ISL_2358533, EPI_ISL_2358534, EPI_ISL_2358535, EPI_ISL_2358536, EPI_ISL_2358537, EPI_ISL_2358538, EPI_ISL_2358539, EPI_ISL_2358540, EPI_ISL_2358541, EPI_ISL_2358542, EPI_ISL_2358543, EPI_ISL_2358544, EPI_ISL_2358545, EPI_ISL_2358546, EPI_ISL_2358547, EPI_ISL_2358548, EPI_ISL_2358549, EPI_ISL_2358550, EPI_ISL_2358551, EPI_ISL_2358552, EPI_ISL_2358553, EPI_ISL_2358554, EPI_ISL_2358555, EPI_ISL_2358556, EPI_ISL_2358557, EPI_ISL_2358558, EPI_ISL_2358559, EPI_ISL_2358560, EPI_ISL_2358561, EPI_ISL_2358562, EPI_ISL_2358563, EPI_ISL_2358564, EPI_ISL_2358565, EPI_ISL_2358566, EPI_ISL_2358567, EPI_ISL_2358568, EPI_ISL_2358569, EPI_ISL_2358570, EPI_ISL_2358571, EPI_ISL_2358572, EPI_ISL_2358573, EPI_ISL_2358574, EPI_ISL_2358575, EPI_ISL_2358576, EPI_ISL_2358577, EPI_ISL_2358578, EPI_ISL_2358579, EPI_ISL_2358580, EPI_ISL_2358581, EPI_ISL_2358582, EPI_ISL_2358583, EPI_ISL_2358584, EPI_ISL_2358585, EPI_ISL_2358586, EPI_ISL_2358587, EPI_ISL_2358588, EPI_ISL_2358589, EPI_ISL_2358590, EPI_ISL_2358591, EPI_ISL_2358592, EPI_ISL_2358593, EPI_ISL_2358594, EPI_ISL_2358595, EPI_ISL_2358596, EPI_ISL_2358597, EPI_ISL_2358598, EPI_ISL_2358599, EPI_ISL_2358600, EPI_ISL_2358601, EPI_ISL_2358602, EPI_ISL_2358603, EPI_ISL_2358604, EPI_ISL_2358605, EPI_ISL_2358606, EPI_ISL_2358607, EPI_ISL_2358608, EPI_ISL_2358609, EPI_ISL_2358610, EPI_ISL_2358611, EPI_ISL_2358612, EPI_ISL_2358613, EPI_ISL_2358614, EPI_ISL_2358615, EPI_ISL_2358616, EPI_ISL_2358617, EPI_ISL_2358618, EPI_ISL_2358619, EPI_ISL_2358620, EPI_ISL_2358621, EPI_ISL_2358622, EPI_ISL_2358623, EPI_ISL_2358624, EPI_ISL_2358625, EPI_ISL_2358626, EPI_ISL_2358627, EPI_ISL_2358628, EPI_ISL_2358629, EPI_ISL_2358630, EPI_ISL_2358631, EPI_ISL_2358632, EPI_ISL_2358633, EPI_ISL_2358634, EPI_ISL_2358635, EPI_ISL_2358636, EPI_ISL_2358637, EPI_ISL_2358638, EPI_ISL_2358639, EPI_ISL_2358640, EPI_ISL_2358641, EPI_ISL_2358642, EPI_ISL_2358643, EPI_ISL_2358644, EPI_ISL_2358645, EPI_ISL_2358646, EPI_ISL_2358647, EPI_ISL_2358648, EPI_ISL_2358649, EPI_ISL_2358650, EPI_ISL_2358651, EPI_ISL_2358652, EPI_ISL_2358653, EPI_ISL_2358654, EPI_ISL_2358655, EPI_ISL_2358656, EPI_ISL_2358657, EPI_ISL_2358658, EPI_ISL_2358659, EPI_ISL_2358660, EPI_ISL_2358661, EPI_ISL_2358662, EPI_ISL_2358663, EPI_ISL_2358664, EPI_ISL_2358665, EPI_ISL_2358666, EPI_ISL_2358667, EPI_ISL_2358668, EPI_ISL_2358669, EPI_ISL_2358670, EPI_ISL_2358671, EPI_ISL_2358672, EPI_ISL_2358673, EPI_ISL_2358674, EPI_ISL_2358675, EPI_ISL_2358676, EPI_ISL_2358677, EPI_ISL_2358678, EPI_ISL_2358679, EPI_ISL_2358680, EPI_ISL_2358681, EPI_ISL_2358682, EPI_ISL_2358683, EPI_ISL_2358684, EPI_ISL_2358685, EPI_ISL_2358686, EPI_ISL_2358687, EPI_ISL_2358688, EPI_ISL_2358689, EPI_ISL_2358690, EPI_ISL_2358691, EPI_ISL_2358692, EPI_ISL_2358693, EPI_ISL_2358694, EPI_ISL_2358695, EPI_ISL_2358696, EPI_ISL_2358697, EPI_ISL_2358698, EPI_ISL_2358699, EPI_ISL_2358700, EPI_ISL_2358701, EPI_ISL_2358702, EPI_ISL_2358703, EPI_ISL_2358704, EPI_ISL_2358705, EPI_ISL_2358706, EPI_ISL_2358707, EPI_ISL_2358708, EPI_ISL_2358709, EPI_ISL_2358710, EPI_ISL_2358711, EPI_ISL_2358712, EPI_ISL_2358713, EPI_ISL_2358714, EPI_ISL_2358715, EPI_ISL_2358716, EPI_ISL_2358717, EPI_ISL_2358718, EPI_ISL_2358719, EPI_ISL_2358720, EPI_ISL_2358721, EPI_ISL_2358722, EPI_ISL_2358723, EPI_ISL_2358724, EPI_ISL_2358725, EPI_ISL_2358726, EPI_ISL_2358727, EPI_ISL_2358728, EPI_ISL_2358729, EPI_ISL_2358730, EPI_ISL_2358731, EPI_ISL_2358732, EPI_ISL_2358733, EPI_ISL_2358734, EPI_ISL_2358735, EPI_ISL_2358736, EPI_ISL_2358737, EPI_ISL_2358738, EPI_ISL_2358739, EPI_ISL_2358740, EPI_ISL_2358741, EPI_ISL_2358742, EPI_ISL_2358743, EPI_ISL_2358744, EPI_ISL_2358745, EPI_ISL_2358746, EPI_ISL_2358747, EPI_ISL_2358748, EPI_ISL_2358749, EPI_ISL_2358750, EPI_ISL_2358751, EPI_ISL_2358752, EPI_ISL_2358753, EPI_ISL_2358754, EPI_ISL_2358755, EPI_ISL_2358756, EPI_ISL_2358757, EPI_ISL_2358758, EPI_ISL_2358759, EPI_ISL_2358760, EPI_ISL_2358761, EPI_ISL_2358762, EPI_ISL_2358763, EPI_ISL_2358764, EPI_ISL_2358765, EPI_ISL_2358766, EPI_ISL_2358767, EPI_ISL_2358768, EPI_ISL_2358769, EPI_ISL_2358770, EPI_ISL_2358771, EPI_ISL_2358772, EPI_ISL_2358773, EPI_ISL_2358774, EPI_ISL_2358775, EPI_ISL_2358776, EPI_ISL_2358777, EPI_ISL_2358778, EPI_ISL_2358779, EPI_ISL_2358780, EPI_ISL_2358781, EPI_ISL_2358782, EPI_ISL_2358783, EPI_ISL_2358784, EPI_ISL_2358785, EPI_ISL_2358786, EPI_ISL_2358787, EPI_ISL_2358788, EPI_ISL_2358789, EPI_ISL_2358790, EPI_ISL_2358791, EPI_ISL_2358792, EPI_ISL_2358793, EPI_ISL_2358794, EPI_ISL_2358795, EPI_ISL_2358796, EPI_ISL_2358797, EPI_ISL_2358798, EPI_ISL_2358799, EPI_ISL_2358800, EPI_ISL_2358801, EPI_ISL_2358802, EPI_ISL_2358803, EPI_ISL_2358804, EPI_ISL_2358805, EPI_ISL_2358806, EPI_ISL_2358807, EPI_ISL_2358808, EPI_ISL_2358809, EPI_ISL_2358810, EPI_ISL_2358811, EPI_ISL_2358812, EPI_ISL_2358813, EPI_ISL_2358814, EPI_ISL_2358815, EPI_ISL_2358816, EPI_ISL_2358817, EPI_ISL_2358818, EPI_ISL_2358819, EPI_ISL_2358820, EPI_ISL_2358821, EPI_ISL_2358822, EPI_ISL_2358823, EPI_ISL_2358824, EPI_ISL_2358825, EPI_ISL_2358826, EPI_ISL_2358827, EPI_ISL_2358828, EPI_ISL_2358829, EPI_ISL_2358830, EPI_ISL_2358831, EPI_ISL_2358832, EPI_ISL_2358833, EPI_ISL_2358834, EPI_ISL_2358835, EPI_ISL_2358836, EPI_ISL_2358837, EPI_ISL_2358838, EPI_ISL_2358839, EPI_ISL_2358840, EPI_ISL_2358841, EPI_ISL_2358842, EPI_ISL_2358843, EPI_ISL_2358844, EPI_ISL_2358845, EPI_ISL_2358846, EPI_ISL_2358847, EPI_ISL_2358848, EPI_ISL_2358849, EPI_ISL_2358850, EPI_ISL_2358851, EPI_ISL_2358852, EPI_ISL_2358853, EPI_ISL_2358854, EPI_ISL_2358855, EPI_ISL_2358856, EPI_ISL_2358857, EPI_ISL_2358858, EPI_ISL_2358859, EPI_ISL_2358860, EPI_ISL_2358861, EPI_ISL_2358862, EPI_ISL_2358863, EPI_ISL_2358864, EPI_ISL_2358865, EPI_ISL_2358866, EPI_ISL_2358867, EPI_ISL_2358868, EPI_ISL_2358869, EPI_ISL_2358870, EPI_ISL_2358871, EPI_ISL_2358872, EPI_ISL_2358873, EPI_ISL_2358874, EPI_ISL_2358875, EPI_ISL_2358876, EPI_ISL_2358877, EPI_ISL_2358878, EPI_ISL_2358879, EPI_ISL_2358880, EPI_ISL_2358881, EPI_ISL_2358882, EPI_ISL_2358883, EPI_ISL_2358884, EPI_ISL_2358885, EPI_ISL_2358886, EPI_ISL_2358887, EPI_ISL_2358888, EPI_ISL_2358889, EPI_ISL_2358890, EPI_ISL_2358891, EPI_ISL_2358892, EPI_ISL_2358893, EPI_ISL_2358894, EPI_ISL_2358895, EPI_ISL_2358896, EPI_ISL_2358897, EPI_ISL_2358898, EPI_ISL_2358899, EPI_ISL_2358900, EPI_ISL_2358901, EPI_ISL_2358902, EPI_ISL_2358903, EPI_ISL_2358904, EPI_ISL_2358905, EPI_ISL_2358906, EPI_ISL_2358907, EPI_ISL_2358908, EPI_ISL_2358909, EPI_ISL_2358910, EPI_ISL_2358911, EPI_ISL_2358912, EPI_ISL_2358913, EPI_ISL_2358914, EPI_ISL_2358915, EPI_ISL_2358916, EPI_ISL_2358917, EPI_ISL_2358918, EPI_ISL_2358919, EPI_ISL_2358920, EPI_ISL_2358921, EPI_ISL_2358922, EPI_ISL_2358923, EPI_ISL_2358924, EPI_ISL_2358925, EPI_ISL_2358926, EPI_ISL_2358927, EPI_ISL_2358928, EPI_ISL_2358929, EPI_ISL_2358930, EPI_ISL_2358931, EPI_ISL_2358932, EPI_ISL_2358933, EPI_ISL_2358934, EPI_ISL_2358935, EPI_ISL_2358936, EPI_ISL_2358937, EPI_ISL_2358938, EPI_ISL_2358939, EPI_ISL_2358940, EPI_ISL_2358941, EPI_ISL_2358942, EPI_ISL_2358943, EPI_ISL_2358944, EPI_ISL_2358945, EPI_ISL_2358946, EPI_ISL_2358947, EPI_ISL_2358948, EPI_ISL_2358949, EPI_ISL_2358950, EPI_ISL_2358951, EPI_ISL_2358952, EPI_ISL_2358953, EPI_ISL_2358954, EPI_ISL_2358955, EPI_ISL_2358956, EPI_ISL_2358957, EPI_ISL_2358958, EPI_ISL_2358959, EPI_ISL_2358960, EPI_ISL_2358961, EPI_ISL_2358962, EPI_ISL_2358963, EPI_ISL_2358964, EPI_ISL_2358965, EPI_ISL_2358966, EPI_ISL_2358967, EPI_ISL_2358968, EPI_ISL_2358969, EPI_ISL_2358970, EPI_ISL_2358971, EPI_ISL_2358972, EPI_ISL_2358973, EPI_ISL_2358974, EPI_ISL_2358975, EPI_ISL_2358976, EPI_ISL_2358977, EPI_ISL_2358978, EPI_ISL_2358979, EPI_ISL_2358980, EPI_ISL_2358981, EPI_ISL_2358982, EPI_ISL_2358983, EPI_ISL_2358984, EPI_ISL_2358985, EPI_ISL_2358986, EPI_ISL_2358987, EPI_ISL_2358988, EPI_ISL_2358989, EPI_ISL_2358990, EPI_ISL_2358991, EPI_ISL_2358992, EPI_ISL_2358993, EPI_ISL_2358994, EPI_ISL_2358995, EPI_ISL_2358996, EPI_ISL_2358997, EPI_ISL_2358998, EPI_ISL_2358999, EPI_ISL_2359000, EPI_ISL_2359001, EPI_ISL_2359002, EPI_ISL_2359003, EPI_ISL_2359004, EPI_ISL_2359005, EPI_ISL_2359006, EPI_ISL_2359007, EPI_ISL_2359008, EPI_ISL_2359009, EPI_ISL_2359010, EPI_ISL_2359011, EPI_ISL_2359012, EPI_ISL_2359013, EPI_ISL_2359014, EPI_ISL_2359015, EPI_ISL_2359016, EPI_ISL_2359017, EPI_ISL_2359018, EPI_ISL_2359019, EPI_ISL_2359020, EPI_ISL_2359021, EPI_ISL_2359022, EPI_ISL_2359023, EPI_ISL_2359024, EPI_ISL_2359025, EPI_ISL_2359026, EPI_ISL_2359027, EPI_ISL_2359028, EPI_ISL_2359029, EPI_ISL_2359030, EPI_ISL_2359031, EPI_ISL_2359032, EPI_ISL_2359033, EPI_ISL_2359034, EPI_ISL_2359035, EPI_ISL_2359036, EPI_ISL_2359037, EPI_ISL_2359038, EPI_ISL_2359039, EPI_ISL_2359040, EPI_ISL_2359041, EPI_ISL_2359042, EPI_ISL_2359043, EPI_ISL_2359044, EPI_ISL_2359045, EPI_ISL_2359046, EPI_ISL_2359047, EPI_ISL_2359048, EPI_ISL_2359049, EPI_ISL_2359050, EPI_ISL_2359051, EPI_ISL_2359052, EPI_ISL_2359053, EPI_ISL_2359054, EPI_ISL_2359055, EPI_ISL_2359056, EPI_ISL_2359057, EPI_ISL_2359058, EPI_ISL_2359059, EPI_ISL_2359060, EPI_ISL_2359061, EPI_ISL_2359062, EPI_ISL_2359063, EPI_ISL_2359064, EPI_ISL_2359065, EPI_ISL_2359066, EPI_ISL_2359067, EPI_ISL_2359068, EPI_ISL_2359069, EPI_ISL_2359070, EPI_ISL_2359071, EPI_ISL_2359072, EPI_ISL_2359073, EPI_ISL_2359074, EPI_ISL_2359075, EPI_ISL_2359076, EPI_ISL_2359077, EPI_ISL_2359078, EPI_ISL_2359079, EPI_ISL_2359080, EPI_ISL_2359081, EPI_ISL_2359082, EPI_ISL_2359083, EPI_ISL_2359084, EPI_ISL_2359085, EPI_ISL_2359086, EPI_ISL_2359087, EPI_ISL_2359088, EPI_ISL_2359089, EPI_ISL_2359090, EPI_ISL_2359091, EPI_ISL_2359092, EPI_ISL_2359093, EPI_ISL_2359094, EPI_ISL_2359095, EPI_ISL_2359096, EPI_ISL_2359097, EPI_ISL_2359098, EPI_ISL_2359099, EPI_ISL_2359100, EPI_ISL_2359101, EPI_ISL_2359102, EPI_ISL_2359103, EPI_ISL_2359104, EPI_ISL_2359105, EPI_ISL_2359106, EPI_ISL_2359107, EPI_ISL_2359108, EPI_ISL_2359109, EPI_ISL_2359110, EPI_ISL_2359111, EPI_ISL_2359112, EPI_ISL_2359113, EPI_ISL_2359114, EPI_ISL_2359115, EPI_ISL_2359116, EPI_ISL_2359117, EPI_ISL_2359118, EPI_ISL_2359119, EPI_ISL_2359120, EPI_ISL_2359121, EPI_ISL_2359122, EPI_ISL_2359123, EPI_ISL_2359124, EPI_ISL_2359125, EPI_ISL_2359126, EPI_ISL_2359127, EPI_ISL_2359128, EPI_ISL_2359129, EPI_ISL_2359130, EPI_ISL_2359131, EPI_ISL_2359132, EPI_ISL_2359133, EPI_ISL_2359134, EPI_ISL_2359135, EPI_ISL_2359136, EPI_ISL_2359137, EPI_ISL_2359138, EPI_ISL_2359139, EPI_ISL_2359140, EPI_ISL_2359141, EPI_ISL_2359142, EPI_ISL_2359143, EPI_ISL_2359144, EPI_ISL_2359145, EPI_ISL_2359146, EPI_ISL_2359147, EPI_ISL_2359148, EPI_ISL_2359149, EPI_ISL_2359150, EPI_ISL_2359151, EPI_ISL_2359152, EPI_ISL_2359153, EPI_ISL_2359154, EPI_ISL_2359155, EPI_ISL_2359156, EPI_ISL_2359157, EPI_ISL_2359158, EPI_ISL_2359159, EPI_ISL_2359160, EPI_ISL_2359161, EPI_ISL_2359162, EPI_ISL_2359163, EPI_ISL_2359164, EPI_ISL_2359165, EPI_ISL_2359166, EPI_ISL_2359167, EPI_ISL_2359168, EPI_ISL_2359169, EPI_ISL_2359170, EPI_ISL_2359171, EPI_ISL_2359172, EPI_ISL_2359173, EPI_ISL_2359174, EPI_ISL_2359175, EPI_ISL_2359177, EPI_ISL_2359179, EPI_ISL_2359181, EPI_ISL_2359183, EPI_ISL_2359185, EPI_ISL_2359187, EPI_ISL_2359189, EPI_ISL_2359191, EPI_ISL_2359193, EPI_ISL_2359195, EPI_ISL_2359197, EPI_ISL_2359199, EPI_ISL_2359201, EPI_ISL_2359202, EPI_ISL_2359204, EPI_ISL_2359206, EPI_ISL_2359208, EPI_ISL_2359210, EPI_ISL_2359212, EPI_ISL_2359214, EPI_ISL_2359216, EPI_ISL_2359218, EPI_ISL_2359220, EPI_ISL_2359221, EPI_ISL_2359223, EPI_ISL_2359225, EPI_ISL_2359227, EPI_ISL_2359229, EPI_ISL_2359231, EPI_ISL_2359233, EPI_ISL_2359235, EPI_ISL_2359237, EPI_ISL_2359239, EPI_ISL_2359241, EPI_ISL_2359243, EPI_ISL_2359245, EPI_ISL_2359247, EPI_ISL_2359249, EPI_ISL_2359251, EPI_ISL_2359253, EPI_ISL_2359255, EPI_ISL_2359257, EPI_ISL_2359258, EPI_ISL_2359261, EPI_ISL_2359263, EPI_ISL_2359265, EPI_ISL_2359267, EPI_ISL_2359268, EPI_ISL_2359270, EPI_ISL_2359272, EPI_ISL_2359274, EPI_ISL_2359276, EPI_ISL_2359278, EPI_ISL_2359279, EPI_ISL_2359281, EPI_ISL_2359283, EPI_ISL_2359288, EPI_ISL_2359289, EPI_ISL_2359291, EPI_ISL_2359293, EPI_ISL_2359295, EPI_ISL_2359297, EPI_ISL_2359299, EPI_ISL_2359301, EPI_ISL_2359303, EPI_ISL_2359305, EPI_ISL_2359306, EPI_ISL_2359308, EPI_ISL_2359310, EPI_ISL_2359312, EPI_ISL_2359314, EPI_ISL_2359316, EPI_ISL_2359318, EPI_ISL_2359320, EPI_ISL_2359322, EPI_ISL_2359324, EPI_ISL_2359326, EPI_ISL_2359328, EPI_ISL_2359330, EPI_ISL_2359332, EPI_ISL_2359334, EPI_ISL_2359336, EPI_ISL_2359338, EPI_ISL_2359339, EPI_ISL_2359341, EPI_ISL_2359343, EPI_ISL_2359345, EPI_ISL_2359347, EPI_ISL_2359349, EPI_ISL_2359351, EPI_ISL_2359353, EPI_ISL_2359355, EPI_ISL_2359357, EPI_ISL_2359359, EPI_ISL_2359361, EPI_ISL_2359364, EPI_ISL_2359366, EPI_ISL_2359368, EPI_ISL_2359369, EPI_ISL_2359371, EPI_ISL_2359373, EPI_ISL_2359375, EPI_ISL_2359377, EPI_ISL_2359379, EPI_ISL_2359381, EPI_ISL_2359383, EPI_ISL_2359385, EPI_ISL_2359387, EPI_ISL_2359389, EPI_ISL_2359391, EPI_ISL_2359393, EPI_ISL_2359395, EPI_ISL_2359397, EPI_ISL_2359399, EPI_ISL_2359401, EPI_ISL_2359403, EPI_ISL_2359405, EPI_ISL_2359407, EPI_ISL_2359409, EPI_ISL_2359411, EPI_ISL_2359416, EPI_ISL_2359418, EPI_ISL_2359420, EPI_ISL_2359422, EPI_ISL_2359424, EPI_ISL_2359426, EPI_ISL_2359428, EPI_ISL_2359429, EPI_ISL_2359431, EPI_ISL_2359433, EPI_ISL_2359435, EPI_ISL_2359436, EPI_ISL_2359437, EPI_ISL_2359439, EPI_ISL_2359442, EPI_ISL_2359444, EPI_ISL_2359446, EPI_ISL_2359448, EPI_ISL_2359450, EPI_ISL_2359452, EPI_ISL_2359454, EPI_ISL_2359456, EPI_ISL_2359458, EPI_ISL_2359460, EPI_ISL_2359462, EPI_ISL_2359463, EPI_ISL_2359465, EPI_ISL_2359467, EPI_ISL_2359469, EPI_ISL_2359471, EPI_ISL_2359473, EPI_ISL_2359475, EPI_ISL_2359477, EPI_ISL_2359479, EPI_ISL_2359481, EPI_ISL_2359483, EPI_ISL_2359485, EPI_ISL_2359487, EPI_ISL_2359489, EPI_ISL_2359491, EPI_ISL_2359493, EPI_ISL_2359495, EPI_ISL_2359497, EPI_ISL_2359499, EPI_ISL_2359501, EPI_ISL_2359503, EPI_ISL_2359504, EPI_ISL_2359507, EPI_ISL_2359508, EPI_ISL_2359510, EPI_ISL_2359512, EPI_ISL_2359514, EPI_ISL_2359516, EPI_ISL_2359518, EPI_ISL_2359520, EPI_ISL_2359522, EPI_ISL_2359524, EPI_ISL_2359526, EPI_ISL_2359527, EPI_ISL_2359529, EPI_ISL_2359531, EPI_ISL_2359533, EPI_ISL_2359535, EPI_ISL_2359537, EPI_ISL_2359539, EPI_ISL_2359541, EPI_ISL_2359543, EPI_ISL_2359545, EPI_ISL_2359547, EPI_ISL_2359549, EPI_ISL_2359550, EPI_ISL_2359552, EPI_ISL_2359554, EPI_ISL_2359556, EPI_ISL_2359558, EPI_ISL_2359560, EPI_ISL_2359562, EPI_ISL_2359564, EPI_ISL_2359565, EPI_ISL_2359567, EPI_ISL_2359569, EPI_ISL_2359571, EPI_ISL_2359573, EPI_ISL_2359575, EPI_ISL_2359577, EPI_ISL_2359579, EPI_ISL_2359581, EPI_ISL_2359583, EPI_ISL_2359585, EPI_ISL_2359587, EPI_ISL_2359589, EPI_ISL_2359590, EPI_ISL_2359592, EPI_ISL_2359594, EPI_ISL_2359596, EPI_ISL_2359598, EPI_ISL_2359600, EPI_ISL_2359602, EPI_ISL_2359604, EPI_ISL_2359605, EPI_ISL_2359608, EPI_ISL_2359610, EPI_ISL_2359611, EPI_ISL_2359612, EPI_ISL_2359613, EPI_ISL_2359615, EPI_ISL_2359617, EPI_ISL_2359619, EPI_ISL_2359621, EPI_ISL_2359623, EPI_ISL_2359625, EPI_ISL_2359627, EPI_ISL_2359629, EPI_ISL_2359631, EPI_ISL_2359633, EPI_ISL_2359635, EPI_ISL_2359637, EPI_ISL_2359639, EPI_ISL_2359641, EPI_ISL_2359643, EPI_ISL_2359645, EPI_ISL_2359647, EPI_ISL_2359648, EPI_ISL_2359650, EPI_ISL_2359652, EPI_ISL_2359654, EPI_ISL_2359656, EPI_ISL_2359658, EPI_ISL_2359660, EPI_ISL_2359661, EPI_ISL_2359664, EPI_ISL_2359666, EPI_ISL_2359667, EPI_ISL_2359670, EPI_ISL_2359671, EPI_ISL_2359673, EPI_ISL_2359675, EPI_ISL_2359677, EPI_ISL_2359679, EPI_ISL_2359681, EPI_ISL_2359682, EPI_ISL_2359683, EPI_ISL_2359684, EPI_ISL_2359686, EPI_ISL_2359688, EPI_ISL_2359690, EPI_ISL_2359692, EPI_ISL_2359695, EPI_ISL_2359696, EPI_ISL_2359698, EPI_ISL_2359700, EPI_ISL_2359702, EPI_ISL_2359704, EPI_ISL_2359706, EPI_ISL_2359708, EPI_ISL_2359710, EPI_ISL_2359712, EPI_ISL_2359713, EPI_ISL_2359715, EPI_ISL_2359717, EPI_ISL_2359719, EPI_ISL_2359721, EPI_ISL_2359723, EPI_ISL_2359725, EPI_ISL_2359727, EPI_ISL_2359729, EPI_ISL_2359731, EPI_ISL_2359732, EPI_ISL_2359734, EPI_ISL_2359736, EPI_ISL_2359738, EPI_ISL_2359740, EPI_ISL_2359742, EPI_ISL_2359744, EPI_ISL_2359746, EPI_ISL_2359748, EPI_ISL_2359750, EPI_ISL_2359752, EPI_ISL_2359754, EPI_ISL_2359755, EPI_ISL_2359757, EPI_ISL_2359759, EPI_ISL_2359760, EPI_ISL_2359762, EPI_ISL_2359764, EPI_ISL_2359766, EPI_ISL_2359768, EPI_ISL_2359770, EPI_ISL_2359772, EPI_ISL_2359774, EPI_ISL_2359775, EPI_ISL_2359778, EPI_ISL_2359779, EPI_ISL_2359781, EPI_ISL_2359783, EPI_ISL_2359785, EPI_ISL_2359787, EPI_ISL_2359789, EPI_ISL_2359790, EPI_ISL_2359795, EPI_ISL_2359797, EPI_ISL_2359799, EPI_ISL_2359801, EPI_ISL_2359803, EPI_ISL_2359805, EPI_ISL_2359807, EPI_ISL_2359809, EPI_ISL_2359811, EPI_ISL_2359813, EPI_ISL_2359814, EPI_ISL_2359816, EPI_ISL_2359818, EPI_ISL_2359820, EPI_ISL_2359822, EPI_ISL_2359824, EPI_ISL_2359826, EPI_ISL_2359828, EPI_ISL_2359830, EPI_ISL_2359832, EPI_ISL_2359834, EPI_ISL_2359836, EPI_ISL_2359838, EPI_ISL_2359840, EPI_ISL_2359842, EPI_ISL_2359844, EPI_ISL_2359847, EPI_ISL_2359849, EPI_ISL_2359850, EPI_ISL_2359852, EPI_ISL_2359854, EPI_ISL_2359855, EPI_ISL_2359857, EPI_ISL_2359859, EPI_ISL_2359861, EPI_ISL_2359862, EPI_ISL_2359864, EPI_ISL_2359866, EPI_ISL_2359868, EPI_ISL_2359870, EPI_ISL_2359871, EPI_ISL_2359873, EPI_ISL_2359875, EPI_ISL_2359877, EPI_ISL_2359879, EPI_ISL_2359881, EPI_ISL_2359883, EPI_ISL_2359885, EPI_ISL_2359888, EPI_ISL_2359889, EPI_ISL_2359892, EPI_ISL_2359893, EPI_ISL_2359895, EPI_ISL_2359897, EPI_ISL_2359900, EPI_ISL_2359901, EPI_ISL_2359903, EPI_ISL_2359905, EPI_ISL_2359907, EPI_ISL_2359909, EPI_ISL_2359911, EPI_ISL_2359913, EPI_ISL_2359915, EPI_ISL_2359919, EPI_ISL_2359921, EPI_ISL_2359922, EPI_ISL_2359927, EPI_ISL_2359928, EPI_ISL_2359930, EPI_ISL_2359932, EPI_ISL_2359934, EPI_ISL_2359936, EPI_ISL_2359938, EPI_ISL_2359940, EPI_ISL_2359942, EPI_ISL_2359944, EPI_ISL_2359946, EPI_ISL_2359947, EPI_ISL_2359949, EPI_ISL_2359951, EPI_ISL_2359953, EPI_ISL_2359954, EPI_ISL_2359955, EPI_ISL_2359956, EPI_ISL_2359957, EPI_ISL_2359958, EPI_ISL_2359959, EPI_ISL_2359960, EPI_ISL_2359961, EPI_ISL_2359962, EPI_ISL_2359963, EPI_ISL_2359964, EPI_ISL_2359965, EPI_ISL_2359966, EPI_ISL_2359967, EPI_ISL_2359968, EPI_ISL_2359969, EPI_ISL_2359970, EPI_ISL_2359971, EPI_ISL_2359972, EPI_ISL_2359973, EPI_ISL_2359974, EPI_ISL_2359975, EPI_ISL_2359976, EPI_ISL_2359977, EPI_ISL_2359978, EPI_ISL_2359979, EPI_ISL_2359980, EPI_ISL_2359981, EPI_ISL_2359982, EPI_ISL_2359983, EPI_ISL_2359984, EPI_ISL_2359985, EPI_ISL_2359986, EPI_ISL_2359987, EPI_ISL_2359988, EPI_ISL_2359989, EPI_ISL_2359990, EPI_ISL_2359991, EPI_ISL_2359992, EPI_ISL_2359993, EPI_ISL_2359994, EPI_ISL_2359995, EPI_ISL_2359996, EPI_ISL_2359997, EPI_ISL_2359998, EPI_ISL_2359999, EPI_ISL_2360000, EPI_ISL_2360001, EPI_ISL_2360002, EPI_ISL_2360003, EPI_ISL_2360004, EPI_ISL_2360005, EPI_ISL_2360006, EPI_ISL_2360007, EPI_ISL_2360008, EPI_ISL_2360009, EPI_ISL_2360010, EPI_ISL_2360011, EPI_ISL_2360012, EPI_ISL_2360013, EPI_ISL_2360014, EPI_ISL_2360015, EPI_ISL_2360016, EPI_ISL_2360017, EPI_ISL_2360018, EPI_ISL_2360019, EPI_ISL_2360020, EPI_ISL_2360021, EPI_ISL_2360022, EPI_ISL_2360023, EPI_ISL_2360024, EPI_ISL_2360025, EPI_ISL_2360026, EPI_ISL_2360027, EPI_ISL_2360028, EPI_ISL_2360029, EPI_ISL_2360030, EPI_ISL_2360031, EPI_ISL_2360032, EPI_ISL_2360033, EPI_ISL_2360034, EPI_ISL_2360035, EPI_ISL_2360036, EPI_ISL_2360037, EPI_ISL_2360038, EPI_ISL_2360039, EPI_ISL_2360040, EPI_ISL_2360041, EPI_ISL_2360042, EPI_ISL_2360043, EPI_ISL_2360044, EPI_ISL_2360045, EPI_ISL_2360046, EPI_ISL_2360047, EPI_ISL_2360048, EPI_ISL_2360049, EPI_ISL_2360050, EPI_ISL_2360051, EPI_ISL_2360052, EPI_ISL_2360053, EPI_ISL_2360054, EPI_ISL_2360055, EPI_ISL_2360056, EPI_ISL_2360057, EPI_ISL_2360058, EPI_ISL_2360059, EPI_ISL_2360060, EPI_ISL_2360061, EPI_ISL_2360062, EPI_ISL_2360063, EPI_ISL_2360064, EPI_ISL_2360065, EPI_ISL_2360066, EPI_ISL_2360067, EPI_ISL_2360068, EPI_ISL_2360069, EPI_ISL_2360070, EPI_ISL_2360071, EPI_ISL_2360072, EPI_ISL_2360073, EPI_ISL_2360074, EPI_ISL_2360075, EPI_ISL_2360076, EPI_ISL_2360077, EPI_ISL_2360078, EPI_ISL_2360079, EPI_ISL_2360080, EPI_ISL_2360081, EPI_ISL_2360082, EPI_ISL_2360083, EPI_ISL_2360084, EPI_ISL_2360085, EPI_ISL_2360086, EPI_ISL_2360087, EPI_ISL_2360088, EPI_ISL_2360089, EPI_ISL_2360090, EPI_ISL_2360091, EPI_ISL_2360092, EPI_ISL_2360093, EPI_ISL_2360094, EPI_ISL_2360095, EPI_ISL_2360096, EPI_ISL_2360097, EPI_ISL_2360098, EPI_ISL_2360099, EPI_ISL_2360100, EPI_ISL_2360101, EPI_ISL_2360102, EPI_ISL_2360103, EPI_ISL_2360104, EPI_ISL_2360105, EPI_ISL_2360106, EPI_ISL_2360107, EPI_ISL_2360108, EPI_ISL_2360109, EPI_ISL_2360110, EPI_ISL_2360111, EPI_ISL_2360112, EPI_ISL_2360113, EPI_ISL_2360114, EPI_ISL_2360115, EPI_ISL_2360116, EPI_ISL_2360117, EPI_ISL_2360118, EPI_ISL_2360119, EPI_ISL_2360120, EPI_ISL_2360121, EPI_ISL_2360122, EPI_ISL_2360123, EPI_ISL_2360124, EPI_ISL_2360125, EPI_ISL_2360126, EPI_ISL_2360127, EPI_ISL_2360128, EPI_ISL_2360129, EPI_ISL_2360130, EPI_ISL_2360131, EPI_ISL_2360132, EPI_ISL_2360133, EPI_ISL_2360134, EPI_ISL_2360135, EPI_ISL_2360136, EPI_ISL_2360137, EPI_ISL_2360138, EPI_ISL_2360139, EPI_ISL_2360140, EPI_ISL_2360141, EPI_ISL_2360142, EPI_ISL_2360143, EPI_ISL_2360144, EPI_ISL_2360145, EPI_ISL_2360146, EPI_ISL_2360147, EPI_ISL_2360148, EPI_ISL_2360149, EPI_ISL_2360150, EPI_ISL_2360151, EPI_ISL_2360152, EPI_ISL_2360153, EPI_ISL_2360154, EPI_ISL_2360155, EPI_ISL_2360156, EPI_ISL_2360157, EPI_ISL_2360158, EPI_ISL_2360159, EPI_ISL_2360160, EPI_ISL_2360161, EPI_ISL_2360162, EPI_ISL_2360163, EPI_ISL_2360164, EPI_ISL_2360165, EPI_ISL_2360166, EPI_ISL_2360167, EPI_ISL_2360168, EPI_ISL_2360169, EPI_ISL_2360170, EPI_ISL_2360171, EPI_ISL_2360172, EPI_ISL_2360173, EPI_ISL_2360174, EPI_ISL_2360175, EPI_ISL_2360176, EPI_ISL_2360177, EPI_ISL_2360178, EPI_ISL_2360179, EPI_ISL_2360180, EPI_ISL_2360181, EPI_ISL_2360182, EPI_ISL_2360183, EPI_ISL_2360184, EPI_ISL_2360185, EPI_ISL_2360186, EPI_ISL_2360187, EPI_ISL_2360188, EPI_ISL_2360189, EPI_ISL_2360190, EPI_ISL_2360191, EPI_ISL_2360192, EPI_ISL_2360193, EPI_ISL_2360194, EPI_ISL_2360195, EPI_ISL_2360196, EPI_ISL_2360197, EPI_ISL_2360198, EPI_ISL_2360199, EPI_ISL_2360200, EPI_ISL_2360201, EPI_ISL_2360202, EPI_ISL_2360203, EPI_ISL_2360204, EPI_ISL_2360205, EPI_ISL_2360206, EPI_ISL_2360207, EPI_ISL_2360208, EPI_ISL_2360209, EPI_ISL_2360210, EPI_ISL_2360211, EPI_ISL_2360212, EPI_ISL_2360213, EPI_ISL_2360214, EPI_ISL_2360215, EPI_ISL_2360216			
see above	Department of Infectious Diseases, Kobe Institute of Health	Department of Infectious Diseases, Kobe Institute of Health	Kentaro Itokawa; Makoto Kuroda; Masanori Hashino; Noriko Nakanishi; Rina Tanaka; Ryohei Nomoto; Tomotada Iwamoto; Tsuyoshi Sekizuka
EPI_ISL_2535236	Department of Intensive Care Medicine, and clinical lab., Osaka Women's and Children's Hospital Dr. Muneyuki Takeuchi, and Dr Shinobu Ida	Department of Developmental Medicine, Research Institute, Osaka Women's and Children's Hospital	Itaru Yanagihara; Muneyuki Takeuchi; Shinobu Ida; Yukiko Nakura
EPI_ISL_2537481, EPI_ISL_2537482, EPI_ISL_2537483	Department of Intensive Care Medicine, and clinical lab., Osaka Women's and Children's Hospital Drs. Muneyuki Takeuchi, Shinobu Ida.	Department of Developmental Medicine, Research Institute, Osaka Women's and Children's Hospital	Itaru Yanagihara; Yukiko Nakura
EPI_ISL_2551183, EPI_ISL_2551519, EPI_ISL_2551520	Department of Microbiology, Tochigi Prefectural Institute of Public Health and Environmental Science	Genome Analysis Center, Kamma Memorial Hospital	Fuminori Mizukoshi; Hanako Yazawa; Hiroshi Kamma; Jun Ishii; Kaori Watanabe; Michiaki Masuda; Satoko Soma; Takuya Yazawa; Yoshiko Hitomi
EPI_ISL_2637224	Department of Microbiology, Yamanashi Institute for Public Health and Environment	Genome Analysis Center, Yamanashi Central Hospital	Yosuke Hirotsu
EPI_ISL_2476402, EPI_ISL_2476403, EPI_ISL_2476404, EPI_ISL_2476405, EPI_ISL_2476406, EPI_ISL_2476407, EPI_ISL_2476408			
see above	Department of Pathology, National Institute of Infectious Diseases	Department of Pathology, National Institute of Infectious Diseases	Harutaka Katano; Kentaro Itokawa; Makoto Kuroda; Shun Iida; Tadaki Suzuki; Tsuyoshi Sekizuka; Yuichiro Hirata
EPI_ISL_2716955, EPI_ISL_2716956, EPI_ISL_2716957, EPI_ISL_2716958, EPI_ISL_2716959, EPI_ISL_2716960, EPI_ISL_2716961, EPI_ISL_2716962, EPI_ISL_2716963, EPI_ISL_2716964, EPI_ISL_2716965, EPI_ISL_2716966, EPI_ISL_2716967, EPI_ISL_2716968, EPI_ISL_2716969, EPI_ISL_2716970, EPI_ISL_2716971, EPI_ISL_2716972, EPI_ISL_2716973			
see above	Department of Veterinary Science, Department of Virology I	Center for Influenza and Respiratory Virus Research	Akihiro Doi; Hideka Miura; Kaya Miyazaki; Seiichiro Fujisaki; Shinji Watanabe
EPI_ISL_2671849, EPI_ISL_2671850, EPI_ISL_2671851, EPI_ISL_2671852, EPI_ISL_2671853, EPI_ISL_2671854, EPI_ISL_2671855, EPI_ISL_2671856, EPI_ISL_2671857, EPI_ISL_2671858, EPI_ISL_2671859, EPI_ISL_2671860, EPI_ISL_2671861, EPI_ISL_2671862, EPI_ISL_2671863, EPI_ISL_2671864, EPI_ISL_2671865, EPI_ISL_2671888, EPI_ISL_2671889			
see above	Department of Veterinary Science, National Institute of Infectious Diseases	Department of Veterinary Science, National Institute of Infectious Diseases	Keita Ishijima; Ken Maeda; Shuetsu Fukushi; Soichi Yamada; Tsuguto Fujimoto; Tsukasa Yamamoto; Yudai Kuroda

EPI_ISL_2671866, EPI_ISL_2671867, EPI_ISL_2671868, EPI_ISL_2671869, EPI_ISL_2671870, EPI_ISL_2671871, EPI_ISL_2671872, EPI_ISL_2671873, EPI_ISL_2671874, EPI_ISL_2671875, EPI_ISL_2671876, EPI_ISL_2671877, EPI_ISL_2671878, EPI_ISL_2671879, EPI_ISL_2671880, EPI_ISL_2671881, EPI_ISL_2671882, EPI_ISL_2671883, EPI_ISL_2671884, EPI_ISL_2671885, EPI_ISL_2671886, EPI_ISL_2671887			
see above	Department of Virology I, National Institute of Infectious Diseases	Department of Veterinary Science, National Institute of Infectious Diseases	Keita Ishijima; Ken Maeda; Shuetsu Fukushi; Souichi Yamada; Tsuguto Fujimoto; Tsukasa Yamamoto; Yudai Kuroda
EPI_ISL_2509329, EPI_ISL_2509372, EPI_ISL_2509373, EPI_ISL_2509396, EPI_ISL_2509397, EPI_ISL_2509806, EPI_ISL_2509933, EPI_ISL_2509961, EPI_ISL_2510089, EPI_ISL_2510297, EPI_ISL_2510298, EPI_ISL_2510299, EPI_ISL_2510300, EPI_ISL_2614629, EPI_ISL_2614630, EPI_ISL_2614631, EPI_ISL_2614632, EPI_ISL_2614633, EPI_ISL_2695877			
see above	Dr. Yoshitaka Tamura Department of Clinical Laboratory, Osaka Habikino Medical Center	Department of Developmental Medicine, Research Institute, Osaka Women's and Children's Hospital	Itaru Yanagihara; Yukiko Nakur; Yukiko Nakura
EPI_ISL_2509337	Dr. Yoshitaka Tamura Department of Clinical Laboratory, Osaka Habikino Medical Center	Department of Developmental Medicine, Research Institute, Osaka Women's and Children's Hospital Department of Developmental Medicine, Research Institute, Osaka Women's and Children's Hospital	Itaru Yanagihara; Yukiko Nakura
EPI_ISL_2614634, EPI_ISL_2614635, EPI_ISL_2614636, EPI_ISL_2614637, EPI_ISL_2614638, EPI_ISL_2614641, EPI_ISL_2614642			
see above	Dr. Yuichiro Oba Department of General Medicine, Osaka General Medical Center	Department of Developmental Medicine, Research Institute, Osaka Women's and Children's Hospital	Itaru Yanagihara; Yukiko Nakura
EPI_ISL_2357237, EPI_ISL_2357238, EPI_ISL_2357239, EPI_ISL_2357240, EPI_ISL_2357241, EPI_ISL_2357242, EPI_ISL_2357243, EPI_ISL_2357244, EPI_ISL_2357245, EPI_ISL_2357246, EPI_ISL_2357247, EPI_ISL_2357248, EPI_ISL_2357249, EPI_ISL_2357250, EPI_ISL_2357251, EPI_ISL_2357252, EPI_ISL_2357253, EPI_ISL_2357254, EPI_ISL_2357255, EPI_ISL_2357256, EPI_ISL_2357257, EPI_ISL_2357258, EPI_ISL_2357259, EPI_ISL_2357260, EPI_ISL_2357261, EPI_ISL_2357262, EPI_ISL_2357263, EPI_ISL_2357264, EPI_ISL_2357265, EPI_ISL_2357266, EPI_ISL_2357267, EPI_ISL_2357268, EPI_ISL_2357269, EPI_ISL_2357270, EPI_ISL_2357271, EPI_ISL_2357272, EPI_ISL_2357273, EPI_ISL_2357274, EPI_ISL_2357275, EPI_ISL_2357276, EPI_ISL_2357277, EPI_ISL_2357278, EPI_ISL_2357279, EPI_ISL_2357280, EPI_ISL_2357281, EPI_ISL_2357282, EPI_ISL_2357283, EPI_ISL_2357284, EPI_ISL_2357285, EPI_ISL_2357286, EPI_ISL_2357287, EPI_ISL_2357288, EPI_ISL_2357289, EPI_ISL_2357290, EPI_ISL_2357291, EPI_ISL_2357292, EPI_ISL_2357293, EPI_ISL_2357294, EPI_ISL_2357295, EPI_ISL_2357296, EPI_ISL_2357297, EPI_ISL_2357298, EPI_ISL_2357299, EPI_ISL_2357300, EPI_ISL_2357301, EPI_ISL_2357302, EPI_ISL_2357303, EPI_ISL_2357304, EPI_ISL_2357305, EPI_ISL_2357306, EPI_ISL_2357307, EPI_ISL_2357308, EPI_ISL_2357309, EPI_ISL_2357310, EPI_ISL_2357311, EPI_ISL_2357312, EPI_ISL_2357313, EPI_ISL_2357314, EPI_ISL_2357315, EPI_ISL_2357316, EPI_ISL_2357317, EPI_ISL_2357318, EPI_ISL_2357319, EPI_ISL_2357320, EPI_ISL_2357321, EPI_ISL_2357322, EPI_ISL_2357323, EPI_ISL_2357324, EPI_ISL_2357325, EPI_ISL_2357326, EPI_ISL_2357327, EPI_ISL_2357328, EPI_ISL_2357329, EPI_ISL_2357330, EPI_ISL_2357331, EPI_ISL_2357332, EPI_ISL_2357333, EPI_ISL_2357334, EPI_ISL_2357335, EPI_ISL_2357336, EPI_ISL_2357337, EPI_ISL_2357338, EPI_ISL_2357339, EPI_ISL_2357340, EPI_ISL_2357341, EPI_ISL_2357342, EPI_ISL_2357343, EPI_ISL_2357344, EPI_ISL_2357345, EPI_ISL_2357346, EPI_ISL_2357347, EPI_ISL_2357348, EPI_ISL_2357349, EPI_ISL_2357350, EPI_ISL_2357351, EPI_ISL_2357352, EPI_ISL_2357353, EPI_ISL_2357354, EPI_ISL_2357355, EPI_ISL_2357356, EPI_ISL_2357357, EPI_ISL_2357358, EPI_ISL_2357359, EPI_ISL_2357360, EPI_ISL_2357361, EPI_ISL_2357362, EPI_ISL_2357363, EPI_ISL_2357364, EPI_ISL_2357365, EPI_ISL_2357366, EPI_ISL_2357367, EPI_ISL_2357368, EPI_ISL_2357369, EPI_ISL_2357370, EPI_ISL_2357371, EPI_ISL_2357372, EPI_ISL_2357373, EPI_ISL_2357374, EPI_ISL_2357375, EPI_ISL_2357376, EPI_ISL_2357377, EPI_ISL_2357378, EPI_ISL_2357379, EPI_ISL_2357380, EPI_ISL_2357381, EPI_ISL_2357382, EPI_ISL_2357383, EPI_ISL_2357384, EPI_ISL_2357385, EPI_ISL_2357386, EPI_ISL_2357387, EPI_ISL_2357388, EPI_ISL_2357389, EPI_ISL_2357390, EPI_ISL_2357391, EPI_ISL_2357392, EPI_ISL_2357393, EPI_ISL_2357394, EPI_ISL_2357395, EPI_ISL_2357396, EPI_ISL_2357397, EPI_ISL_2357398, EPI_ISL_2357399, EPI_ISL_2357400, EPI_ISL_2357401, EPI_ISL_2357402, EPI_ISL_2357403, EPI_ISL_2357404, EPI_ISL_2357405, EPI_ISL_2357406, EPI_ISL_2357407, EPI_ISL_2357408, EPI_ISL_2357409, EPI_ISL_2357410, EPI_ISL_2357411, EPI_ISL_2357412, EPI_ISL_2357413, EPI_ISL_2357414, EPI_ISL_2357415, EPI_ISL_2357416, EPI_ISL_2357417, EPI_ISL_2357418, EPI_ISL_2357419, EPI_ISL_2357420, EPI_ISL_2357421, EPI_ISL_2357422, EPI_ISL_2357423, EPI_ISL_2357424, EPI_ISL_2357425, EPI_ISL_2357426, EPI_ISL_2357427, EPI_ISL_2357428, EPI_ISL_2357429, EPI_ISL_2357430, EPI_ISL_2357431, EPI_ISL_2357432, EPI_ISL_2357433, EPI_ISL_2357434, EPI_ISL_2357435, EPI_ISL_2357436, EPI_ISL_2357437, EPI_ISL_2357438, EPI_ISL_2357439, EPI_ISL_2357440, EPI_ISL_2357441, EPI_ISL_2357442, EPI_ISL_2357443, EPI_ISL_2357444, EPI_ISL_2357445, EPI_ISL_2357446, EPI_ISL_2357447, EPI_ISL_2357448, EPI_ISL_2357449, EPI_ISL_2357450, EPI_ISL_2357451, EPI_ISL_2357452, EPI_ISL_2357453, EPI_ISL_2357454, EPI_ISL_2357455, EPI_ISL_2357456, EPI_ISL_2357457, EPI_ISL_2357458, EPI_ISL_2357459, EPI_ISL_2357460, EPI_ISL_2357461, EPI_ISL_2357462, EPI_ISL_2357463, EPI_ISL_2357464, EPI_ISL_2357465, EPI_ISL_2357466, EPI_ISL_2357467, EPI_ISL_2357468, EPI_ISL_2357469, EPI_ISL_2357470, EPI_ISL_2357471, EPI_ISL_2357472, EPI_ISL_2357473, EPI_ISL_2357474, EPI_ISL_2357475, EPI_ISL_2357476, EPI_ISL_2357477, EPI_ISL_2357478, EPI_ISL_2357479, EPI_ISL_2357480, EPI_ISL_2357481, EPI_ISL_2357482, EPI_ISL_2357483, EPI_ISL_2357484, EPI_ISL_2357485, EPI_ISL_2357486, EPI_ISL_2357487, EPI_ISL_2357488, EPI_ISL_2357489, EPI_ISL_2357490, EPI_ISL_2357491, EPI_ISL_2357492, EPI_ISL_2357493, EPI_ISL_2357494, EPI_ISL_2357495, EPI_ISL_2357496, EPI_ISL_2357497, EPI_ISL_2357498, EPI_ISL_2357499, EPI_ISL_2357500, EPI_ISL_2357501, EPI_ISL_2357502, EPI_ISL_2357503, EPI_ISL_2357504, EPI_ISL_2357505, EPI_ISL_2357506, EPI_ISL_2357507, EPI_ISL_2357508, EPI_ISL_2357509, EPI_ISL_2357510, EPI_ISL_2357511, EPI_ISL_2357512, EPI_ISL_2357513, EPI_ISL_2357514, EPI_ISL_2357515, EPI_ISL_2357516, EPI_ISL_2357517, EPI_ISL_2357518, EPI_ISL_2357519, EPI_ISL_2357520, EPI_ISL_2357521, EPI_ISL_2357522, EPI_ISL_2357523, EPI_ISL_2357524, EPI_ISL_2357525, EPI_ISL_2357526, EPI_ISL_2357527, EPI_ISL_2357528, EPI_ISL_2357529, EPI_ISL_2357530, EPI_ISL_2357531, EPI_ISL_2357532, EPI_ISL_2357533, EPI_ISL_2357534, EPI_ISL_2357535, EPI_ISL_2357536, EPI_ISL_2357537, EPI_ISL_2357538, EPI_ISL_2357539, EPI_ISL_2357540, EPI_ISL_2357541, EPI_ISL_2357542, EPI_ISL_2357543, EPI_ISL_2357544, EPI_ISL_2357545, EPI_ISL_2357546, EPI_ISL_2357547, EPI_ISL_2357548, EPI_ISL_2357549, EPI_ISL_2357550, EPI_ISL_2357551, EPI_ISL_2357552, EPI_ISL_2357553, EPI_ISL_2357554, EPI_ISL_2357555, EPI_ISL_2357556, EPI_ISL_2357557, EPI_ISL_2357558, EPI_ISL_2357559, EPI_ISL_2357560, EPI_ISL_2357561, EPI_ISL_2357562, EPI_ISL_2357563, EPI_ISL_2357564, EPI_ISL_2357565, EPI_ISL_2357566, EPI_ISL_2357567, EPI_ISL_2357568, EPI_ISL_2357569, EPI_ISL_2357570, EPI_ISL_2357571, EPI_ISL_2357572, EPI_ISL_2357573, EPI_ISL_2357574, EPI_ISL_2357575, EPI_ISL_2357576, EPI_ISL_2357577, EPI_ISL_2357578, EPI_ISL_2357579, EPI_ISL_2357580, EPI_ISL_2357581, EPI_ISL_2357582, EPI_ISL_2357583, EPI_ISL_2357584, EPI_ISL_2357585, EPI_ISL_2357586, EPI_ISL_2357587, EPI_ISL_2357588, EPI_ISL_2357589, EPI_ISL_2357590, EPI_ISL_2357591, EPI_ISL_2357592, EPI_ISL_2357593, EPI_ISL_2357594, EPI_ISL_2357595, EPI_ISL_2357596, EPI_ISL_2357597, EPI_ISL_2357598, EPI_ISL_2357599, EPI_ISL_2357600, EPI_ISL_2357601, EPI_ISL_2357602, EPI_ISL_2357603, EPI_ISL_2357604, EPI_ISL_2357605, EPI_ISL_2357606, EPI_ISL_2357607, EPI_ISL_2357608, EPI_ISL_2357609, EPI_ISL_2357610, EPI_ISL_2357611, EPI_ISL_2357612, EPI_ISL_2357613, EPI_ISL_2357614, EPI_ISL_2357615, EPI_ISL_2357617, EPI_ISL_2357618, EPI_ISL_2357619, EPI_ISL_2357620, EPI_ISL_2357621, EPI_ISL_2357622, EPI_ISL_2357623, EPI_ISL_2357624, EPI_ISL_2357625, EPI_ISL_2357626, EPI_ISL_2357627, EPI_ISL_2357628, EPI_ISL_2357629, EPI_ISL_2357630, EPI_ISL_2357631, EPI_ISL_2357632, EPI_ISL_2357633, EPI_ISL_2357634, EPI_ISL_2357635, EPI_ISL_2357636, EPI_ISL_2357637, EPI_ISL_2357638, EPI_ISL_2357639, EPI_ISL_2357640, EPI_ISL_2357641, EPI_ISL_2357642, EPI_ISL_2357643, EPI_ISL_2357644, EPI_ISL_2357645, EPI_ISL_2357646, EPI_ISL_2357647, EPI_ISL_2357648, EPI_ISL_2357649, EPI_ISL_2357650, EPI_ISL_2357651, EPI_ISL_2357652, EPI_ISL_2357653, EPI_ISL_2357654, EPI_ISL_2357655, EPI_ISL_2357656, EPI_ISL_2357657, EPI_ISL_2357658, EPI_ISL_2357659, EPI_ISL_2357660, EPI_ISL_2357661, EPI_ISL_2357662, EPI_ISL_2357663, EPI_ISL_2357664, EPI_ISL_2357665, EPI_ISL_2357666, EPI_ISL_2357667, EPI_ISL_2357668, EPI_ISL_2357669, EPI_ISL_2357670, EPI_ISL_2357671, EPI_ISL_2357672, EPI_ISL_2357673, EPI_ISL_2357674, EPI_ISL_2357675, EPI_ISL_2357676, EPI_ISL_2357677, EPI_ISL_2357678, EPI_ISL_2357679, EPI_ISL_2357680, EPI_ISL_2357681, EPI_ISL_2357682, EPI_ISL_2357683, EPI_ISL_2357684, EPI_ISL_2357685, EPI_ISL_2357686, EPI_ISL_2357687, EPI_ISL_2357688, EPI_ISL_2357689, EPI_ISL_2357690, EPI_ISL_2357691, EPI_ISL_2357692, EPI_ISL_2357693, EPI_ISL_2357694, EPI_ISL_2357695, EPI_ISL_2357696, EPI_ISL_2357697, EPI_ISL_2357698, EPI_ISL_2357699, EPI_ISL_2357700, EPI_ISL_2357701, EPI_ISL_2357702, EPI_ISL_2357703, EPI_ISL_2357704, EPI_ISL_2357705, EPI_ISL_2357706, EPI_ISL_2357707, EPI_ISL_2357708, EPI_ISL_2357709, EPI_ISL_2357710, EPI_ISL_2357711, EPI_ISL_2357712, EPI_ISL_2357713, EPI_ISL_2357714, EPI_ISL_2365421, EPI_ISL_2365422			
see above	Fukuoka Institute of Health and Environmental Sciences	Fukuoka Institute of Health and Environmental Sciences	Akira Ohishi; Asako Nakamura; Carl Yuki; Chiharu Katamune; Hiroaki Shigemura; Kentaro Itokawa; Makoto Kuroda; Masanori Hashino; Mitsuhiro Hamasaki; Rina Tanaka; Saori Ueda; Takayuki Kobayashi; Tsuyoshi Sekizuka; Yoshiki Etoh; Yuki Ashizuka
EPI_ISL_2391272, EPI_ISL_2391273, EPI_ISL_2391274, EPI_ISL_2391275, EPI_ISL_2391276, EPI_ISL_2391277, EPI_ISL_2391278, EPI_ISL_2391279, EPI_ISL_2391280, EPI_ISL_2391281, EPI_ISL_2391282, EPI_ISL_2391283, EPI_ISL_2391284, EPI_ISL_2391285, EPI_ISL_2391286, EPI_ISL_2391287			
see above	Fukushima Prefectural Institute of Public Health	Fukushima Prefectural Institute of Public Health	Kadzuhiro Kitakawa; Kazunori Suzuki; Kentaro Itokawa; Makoto Kuroda; Masanori Hashino; Naoko Kashiwabara; Rie Suzuki; Rina Tanaka; Shohei Fujita; Tsuyoshi Sekizuka
EPI_ISL_2566681	Genome Analysis Center, Kamma Memorial Hospital	Genome Analysis Center, Kamma Memorial Hospital	Hanako Yazawa; Hiroshi Kamma; Jun Ishii; Kaori Watanabe; Masaki Sato; Michiaki Masuda; Naruhisa Takano; Noriyuki Tannai; Satoko Soma; Takuya Yazawa
EPI_ISL_2349693, EPI_ISL_2349694, EPI_ISL_2349695, EPI_ISL_2349696, EPI_ISL_2349697, EPI_ISL_2349698, EPI_ISL_2349699, EPI_ISL_2349700, EPI_ISL_2349701, EPI_ISL_2349702, EPI_ISL_2349703, EPI_ISL_2349704, EPI_ISL_2349705, EPI_ISL_2349706, EPI_ISL_2349707, EPI_ISL_2349708, EPI_ISL_2364487, EPI_ISL_2364488, EPI_ISL_2364489, EPI_ISL_2364490, EPI_ISL_2364491, EPI_ISL_2364492, EPI_ISL_2364493, EPI_ISL_2364494, EPI_ISL_2364495, EPI_ISL_2364496, EPI_ISL_2364497, EPI_ISL_2364498, EPI_ISL_2364499, EPI_ISL_2364500, EPI_ISL_2364501, EPI_ISL_2364502, EPI_ISL_2364503, EPI_ISL_2364504, EPI_ISL_2364505, EPI_ISL_2364506, EPI_ISL_2364507, EPI_ISL_2364508, EPI_ISL_2364509, EPI_ISL_2364510, EPI_ISL_2364511, EPI_ISL_2364512, EPI_ISL_2364513, EPI_ISL_2364514, EPI_ISL_2364515, EPI_ISL_2364516, EPI_ISL_2364517, EPI_ISL_2364518, EPI_ISL_2364519, EPI_ISL_2364520, EPI_ISL_2364521, EPI_ISL_2364522, EPI_ISL_2365357, EPI_ISL_2462044, EPI_ISL_2462045, EPI_ISL_2462046, EPI_ISL_2462047, EPI_ISL_2462048, EPI_ISL_2462049, EPI_ISL_2462050, EPI_ISL_2462051, EPI_ISL_2462052, EPI_ISL_2462053, EPI_ISL_2462054, EPI_ISL_2462055, EPI_ISL_2462056, EPI_ISL_2462057, EPI_ISL_2462058, EPI_ISL_2497479, EPI_ISL_2497480, EPI_ISL_2497481, EPI_ISL_2497482, EPI_ISL_2497483, EPI_ISL_2497484, EPI_ISL_2497485, EPI_ISL_2497486, EPI_ISL_2497487, EPI_ISL_2497488, EPI_ISL_2497489, EPI_ISL_2497490, EPI_ISL_2497491, EPI_ISL_2497492, EPI_ISL_2497493, EPI_ISL_2497494, EPI_ISL_2497495, EPI_ISL_2565984, EPI_ISL_2565985, EPI_ISL_2565986, EPI_ISL_2565987, EPI_ISL_2565988, EPI_ISL_2565989, EPI_ISL_2565990, EPI_ISL_2565991, EPI_ISL_2565992, EPI_ISL_2565993, EPI_ISL_2565994, EPI_ISL_2565995, EPI_ISL_2565996, EPI_ISL_2565997, EPI_ISL_2621731, EPI_ISL_2621732, EPI_ISL_2621733, EPI_ISL_2621734, EPI_ISL_2621735, EPI_ISL_2621736, EPI_ISL_2621737, EPI_ISL_2637225, EPI_ISL_2637226, EPI_ISL_2637227, EPI_ISL_2637228, EPI_ISL_2637229, EPI_ISL_2637230, EPI_ISL_2637231, EPI_ISL_2644656, EPI_ISL_2644657, EPI_ISL_2644658, EPI_ISL_2644659, EPI_ISL_2644660, EPI_ISL_2644661, EPI_ISL_2644662, EPI_ISL_2644663, EPI_ISL_2644664, EPI_ISL_2644665, EPI_ISL_2644666, EPI_ISL_2644667, EPI_ISL_2644668, EPI_ISL_2644669, EPI_ISL_2644670, EPI_ISL_2644671, EPI_ISL_2657371, EPI_ISL_2657372, EPI_ISL_2657373, EPI_ISL_2657374, EPI_ISL_2696282, EPI_ISL_2696283, EPI_ISL_2696284, EPI_ISL_2696285, EPI_ISL_2696286, EPI_ISL_2696287, EPI_ISL_2696288, EPI_ISL_2696289, EPI_ISL_2696290, EPI_ISL_2696291, EPI_ISL_2696292, EPI_ISL_2696293, EPI_ISL_2696294, EPI_ISL_2696295, EPI_ISL_2696296, EPI_ISL_2696297, EPI_ISL_2754133, EPI_ISL_2754134, EPI_ISL_2754135, EPI_ISL_2754136, EPI_ISL_2754137, EPI_ISL_2754138			
see above	Genome Analysis Center, Yamanashi Central Hospital	Genome Analysis Center, Yamanashi Central Hospital	Yosuke Hirotsu
EPI_ISL_2565983, EPI_ISL_2727654	Genome Information Research Center, Research Institute for Microbial Diseases, Osaka University	Genome Information Research Center, Research Institute for Microbial Diseases, Osaka University	Daisuke Motooka; Hiroya Oki; Kentaro Tanaka; and Shota Nakamura
EPI_ISL_2349202, EPI_ISL_2349203, EPI_ISL_2349204, EPI_ISL_2349205, EPI_ISL_2349206, EPI_ISL_2349207, EPI_ISL_2349208, EPI_ISL_2349209, EPI_ISL_2349210, EPI_ISL_2349211, EPI_ISL_2349212, EPI_ISL_2349213, EPI_ISL_2349214, EPI_ISL_2349215, EPI_ISL_2349216, EPI_ISL_2349217, EPI_ISL_2349218, EPI_ISL_2349219, EPI_ISL_2349220, EPI_ISL_2349221, EPI_ISL_2349222, EPI_ISL_2349223, EPI_ISL_2349224, EPI_ISL_2349225, EPI_ISL_2349226, EPI_ISL_2349227, EPI_ISL_2349228, EPI_ISL_2349229, EPI_ISL_2349230, EPI_ISL_2349231, EPI_ISL_2349232, EPI_ISL_2349233, EPI_ISL_2349234, EPI_ISL_2349235, EPI_ISL_2349236, EPI_ISL_2349237, EPI_ISL_2349238, EPI_ISL_2349239, EPI_ISL_2349240, EPI_ISL_2349241, EPI_ISL_2349242, EPI_ISL_2349243, EPI_ISL_2349244, EPI_ISL_2349245, EPI_ISL_2349246, EPI_ISL_2349247, EPI_ISL_2349248, EPI_ISL_2349249, EPI_ISL_2349250, EPI_ISL_2349251, EPI_ISL_2349252, EPI_ISL_2349253, EPI_ISL_2349254, EPI_ISL_2349255, EPI_ISL_2349256, EPI_ISL_2349257, EPI_ISL_2349258, EPI_ISL_2349259, EPI_ISL_2349260, EPI_ISL_2349261, EPI_ISL_2349262, EPI_ISL_2349263, EPI_ISL_2349264, EPI_ISL_2349265, EPI_ISL_2349266, EPI_ISL_2349267, EPI_ISL_2349268			
see above	Gunma Prefectural Institute of Public Health and Environmental Sciences	Gunma Prefectural Institute of Public Health and Environmental Sciences	Daisuke Shinoda; Hiroyuki Tsukagoshi; Kentaro Itokawa; Makoto Kuroda; Mariko Saito; Masanori Hashino; Rina Tanaka; Tsuyoshi Sekizuka
EPI_ISL_2358038, EPI_ISL_2358039, EPI_ISL_2358040, EPI_ISL_2358041, EPI_ISL_2358042, EPI_ISL_2358043, EPI_ISL_2358044, EPI_ISL_2358045, EPI_ISL_2358046, EPI_ISL_2358047, EPI_ISL_2358048, EPI_ISL_2358049, EPI_ISL_2358050, EPI_ISL_2358051, EPI_ISL_2358052, EPI_ISL_2358053, EPI_ISL_2358054, EPI_ISL_2358055, EPI_ISL_2358056, EPI_ISL_2358057, EPI_ISL_2358058, EPI_ISL_2358059, EPI_ISL_2358060, EPI_ISL_2358061, EPI_ISL_2358062, EPI_ISL_2358063, EPI_ISL_2358064, EPI_ISL_2358065, EPI_ISL_2358066, EPI_ISL_2358067, EPI_ISL_2358068, EPI_ISL_2358069, EPI_ISL_2358070, EPI_ISL_2358071, EPI_ISL_2358072, EPI_ISL_2358073, EPI_ISL_2358074, EPI_ISL_2358075, EPI_ISL_2358076, EPI_ISL_2358077, EPI_ISL_2358078, EPI_ISL_2358079, EPI_ISL_2358080, EPI_ISL_2358081, EPI_ISL_2358082, EPI_ISL_2358083, EPI_ISL_2358084, EPI_ISL_2358085, EPI_ISL_2358086, EPI_ISL_2358087, EPI_ISL_2358088, EPI_ISL_2358089, EPI_ISL_2358090, EPI_ISL_2358091, EPI_ISL_2358092, EPI_ISL_2358093, EPI_ISL_2358094, EPI_ISL_2358095, EPI_ISL_2358096, EPI_ISL_2358097, EPI_ISL_2358098, EPI_ISL_2358099, EPI_ISL_2358100, EPI_ISL_2358101, EPI_ISL_2358102, EPI_ISL_2358103, EPI_ISL_2358104, EPI_ISL_2358105, EPI_ISL_2358106, EPI_ISL_2358107, EPI_ISL_2358108, EPI_ISL_2358109, EPI_ISL_2358110, EPI_ISL_2358111, EPI_ISL_2358112, EPI_ISL_2358113, EPI_ISL_2358114, EPI_ISL_2358115, EPI_ISL_2358116, EPI_ISL_2358117, EPI_ISL_2358118, EPI_ISL_2358119, EPI_ISL_2358120, EPI_ISL_2358121, EPI_ISL_2358122, EPI_ISL_2358123, EPI_ISL_2358124, EPI_ISL_2358125, EPI_ISL_2358126, EPI_ISL_2358127, EPI_ISL_2358128, EPI_ISL_2358129, EPI_ISL_2358130, EPI_ISL_2358131, EPI_ISL_2358132, EPI_ISL_2358133, EPI_ISL_2358134, EPI_ISL_2358135, EPI_ISL_2358136, EPI_ISL_2358137, EPI_ISL_2358138, EPI_ISL_2358139, EPI_ISL_2358140, EPI_ISL_2358141, EPI_ISL_2358142, EPI_ISL_2358143, EPI_ISL_2358144, EPI_ISL_2358145, EPI_ISL_2358146, EPI_ISL_2358147, EPI_ISL_2358148, EPI_ISL_2358149, EPI_ISL_2358150, EPI_ISL_2358151, EPI_ISL_2358152, EPI_ISL_2358153, EPI_ISL_2358154, EPI_ISL_2358155, EPI_ISL_2358156, EPI_ISL_2358157, EPI_ISL_2358158, EPI_ISL_2358159, EPI_ISL_2358160, EPI_ISL_2358161, EPI_ISL_2358162, EPI_ISL_2358163, EPI_ISL_2358164, EPI_ISL_2358165, EPI_ISL_2358166, EPI_ISL_2358167, EPI_ISL_2358168, EPI_ISL_2358169, EPI_ISL_2358170, EPI_ISL_2358171, EPI_ISL_2358172, EPI_ISL_2358173, EPI_ISL_2358174, EPI_ISL_2358175, EPI_ISL_2358176, EPI_ISL_2358177, EPI_ISL_2358178, EPI_ISL_2358179, EPI_ISL_2358180, EPI_ISL_2358181, EPI_ISL_2358182, EPI_ISL_2358183, EPI_ISL_2358184, EPI_ISL_2358185, EPI_ISL_2358186, EPI_ISL_2358187, EPI_ISL_2358188, EPI_ISL_2358189, EPI_ISL_2358190, EPI_ISL_2358191, EPI_ISL_2358192, EPI_ISL_2358193, EPI_ISL_2358194, EPI_ISL_2358195, EPI_ISL_2358196, EPI_ISL_2358197, EPI_ISL_2358198, EPI_ISL_2358199, EPI_ISL_2358200, EPI_ISL_2358201, EPI_ISL_2358202, EPI_ISL_2358203, EPI_ISL_2358204, EPI_ISL_2358205, EPI_ISL_2358206, EPI_ISL_2358207, EPI_ISL_2358208, EPI_ISL_2358209, EPI_ISL_2358210, EPI_ISL_2358211, EPI_ISL_2358212, EPI_ISL_2358213, EPI_ISL_2358214, EPI_ISL_2358215, EPI_ISL_2358216, EPI_ISL_2358217, EPI_ISL_2358218, EPI_ISL_2358219, EPI_ISL_2358220, EPI_ISL_2358221, EPI_ISL_2358222, EPI_ISL_2358223, EPI_ISL_2358224, EPI_ISL_2358225, EPI_ISL_2358226			
see above	Hokkaido Institute of Public Health	Hokkaido Institute of Public Health	Kazuhiro Okubo; Kentaro Itokawa; Makoto Kuroda; Masanori Hashino; Rina Tanaka; Tsuyoshi Sekizuka; Yuta Ohno
EPI_ISL_2364523, EPI_ISL_2364524, EPI_ISL_2364525, EPI_ISL_2364526, EPI_ISL_2364527, EPI_ISL_2364528, EPI_ISL_2364529, EPI_ISL_2364530, EPI_ISL_2364531, EPI_ISL_2364532, EPI_ISL_2364533, EPI_ISL_2364534, EPI_ISL_2364535, EPI_ISL_2364536, EPI_ISL_2364537, EPI_ISL_2364538, EPI_ISL_2364539, EPI_ISL_2364540, EPI_ISL_2364541, EPI_ISL_2364542, EPI_ISL_2364543, EPI_ISL_2364544, EPI_ISL_2364545, EPI_ISL_2364546, EPI_ISL_2364547, EPI_ISL_2364548, EPI_ISL_2364549, EPI_ISL_2364550, EPI_ISL_2364551, EPI_ISL_2364552, EPI_ISL_2364553, EPI_ISL_2364554, EPI_ISL_2364555, EPI_ISL_2364556, EPI_ISL_2364557, EPI_ISL_2364558, EPI_ISL_2364559, EPI_ISL_2364560, EPI_ISL_2364561, EPI_ISL_2364562, EPI_ISL_2364563, EPI_ISL_2364564, EPI_ISL_2364565, EPI_ISL_2364566			
see above	Hyogo Prefectural Institute of Public Health science	Pathogen Genomics Center, National Institute of Infectious Diseases	Denshi Takai; Etsuko Saito; Kenichi Ogita; Kentaro Itokawa; Kyoko Suzuki; Makoto Kuroda; Masanori Hashino; Masatsugu Chikahira; Miki Ogi; Rina Tanaka; Tomohiro Oshibe; Tsuyoshi Sekizuka; Yumi Akiyama
EPI_ISL_2350486, EPI_ISL_2350487, EPI_ISL_2350488, EPI_ISL_2350489, EPI_ISL_2350490, EPI_ISL_2350491, EPI_ISL_2350492, EPI_ISL_2350493, EPI_ISL_2350494, EPI_ISL_2350495, EPI_ISL_2350496, EPI_ISL_2350497, EPI_ISL_2350498, EPI_ISL_2350499, EPI_ISL_2350500, EPI_ISL_2350501, EPI_ISL_2350502, EPI_ISL_2350503, EPI_ISL_2350504, EPI_ISL_2350505, EPI_ISL_2350506, EPI_ISL_2350507, EPI_ISL_2350508, EPI_ISL_2350509, EPI_ISL_2350510, EPI_ISL_2350511, EPI_ISL_2350512, EPI_ISL_2350513, EPI_ISL_2350514, EPI_ISL_2350515, EPI_ISL_2350516, EPI_ISL_2350517, EPI_ISL_2350518, EPI_ISL_2350519, EPI_ISL_2350520, EPI_ISL_2350521, EPI_ISL_2350522, EPI_ISL_2350523, EPI_ISL_2350524, EPI_ISL_2350525, EPI_ISL_2350526, EPI_ISL_2350527, EPI_ISL_2350528, EPI_ISL_2350529, EPI_ISL_2350530, EPI_ISL_2350531, EPI_ISL_2350532, EPI_ISL_2350533, EPI_ISL_2350534, EPI_ISL_2350535, EPI_ISL_2350536, EPI_ISL_2350537, EPI_ISL_2350538, EPI_ISL_2350539, EPI_ISL_2350540, EPI_ISL_2350541, EPI_ISL_2350542, EPI_ISL_2350543, EPI_ISL_2350544, EPI_ISL_2350545, EPI_ISL_2350546, EPI_ISL_2350547, EPI_ISL_2350548, EPI_ISL_2350549, EPI_ISL_2350550, EPI_ISL_2350551, EPI_ISL_2350552, EPI_ISL_2350553, EPI_ISL_2350554, EPI_ISL_2350555, EPI_ISL_2350556, EPI_ISL_2350557, EPI_ISL_2350558, EPI_ISL_2350559, EPI_ISL_2350560, EPI_ISL_2350561, EPI_ISL_2350562, EPI_ISL_2350563, EPI_ISL_2350564, EPI_ISL_2350565, EPI_ISL_2350566, EPI_ISL_2350567, EPI_ISL_2350568, EPI_ISL_2350569, EPI_ISL_2350570, EPI_ISL_2350571, EPI_ISL_2350572, EPI_ISL_2350573, EPI_ISL_2350574, EPI_ISL_2350575, EPI_ISL_2350576, EPI_ISL_2350577, EPI_ISL_2350578, EPI_ISL_2350579, EPI_ISL_2350580, EPI_ISL_2350581, EPI_ISL_2350582, EPI_ISL_2350583, EPI_ISL_2350584, EPI_ISL_2350585, EPI_ISL_2350586, EPI_ISL_2350587, EPI_ISL_2350588, EPI_ISL_2350589, EPI_ISL_2350590, EPI_ISL_2350591, EPI_ISL_2350592, EPI_ISL_2350593, EPI_ISL_2350594, EPI_ISL_2350595, EPI_ISL_2350596, EPI_ISL_2350597, EPI_ISL_2350598, EPI_ISL_2350599, EPI_ISL_2350600, EPI_ISL_2350601, EPI_ISL_2350602, EPI_ISL_2350603, EPI_ISL_2350604, EPI_ISL_2350605, EPI_ISL_2350606, EPI_ISL_2350607, EPI_ISL_2350608, EPI_ISL_2350609, EPI_ISL_2350610, EPI_ISL_2350611, EPI_ISL_2350612, EPI_ISL_2350613, EPI_ISL_2350614, EPI_ISL_2350615, EPI_ISL_2350616, EPI_ISL_2350617, EPI_ISL_2350618, EPI_ISL_2350619, EPI_ISL_2350620, EPI_ISL_2350621, EPI_ISL_2350622, EPI_ISL_2350623, EPI_ISL_2350624, EPI_ISL_2350625, EPI_ISL_2350626, EPI_ISL_2350627, EPI_ISL_2350628, EPI_ISL_2350629, EPI_ISL_2350630, EPI_ISL_2350631, EPI_ISL_2350632, EPI_ISL_2350633, EPI_ISL_2350634, EPI_ISL_2350635, EPI_ISL_2350636, EPI_ISL_2350637, EPI_ISL_2350638, EPI_ISL_2350639, EPI_ISL_2350640, EPI_ISL_2350641, EPI_ISL_2350642, EPI_ISL_2350643, EPI_ISL_2350644, EPI_ISL_2350645, EPI_ISL_2350646, EPI_ISL_2350647, EPI_ISL_2350648, EPI_ISL_2350649, EPI_ISL_2350650, EPI_ISL_2350651, EPI_ISL_2350652, EPI_ISL_2350653, EPI_ISL_2350654, EPI_ISL_2350655, EPI_ISL_2350656, EPI_ISL_2350657, EPI_ISL_2350658, EPI_ISL_2350659, EPI_ISL_2350660, EPI_ISL_2350661, EPI_ISL_2350662, EPI_ISL_2350663, EPI_ISL_2350664, EPI_ISL_2350665, EPI_ISL_2350666, EPI_ISL_2350667, EPI_ISL_2350668, EPI_ISL_2350669, EPI_ISL_2350670, EPI_ISL_2350671, EPI_ISL_2350672, EPI_ISL_2350673, EPI_ISL_2350674, EPI_ISL_2350675, EPI_ISL_2350676, EPI_ISL_2350677, EPI_ISL_2350678, EPI_ISL_2350679, EPI_ISL_2350680, EPI_ISL_2350681, EPI_ISL_2350682, EPI_ISL_2350683, EPI_ISL_2350684, EPI_ISL_2350685, EPI_ISL_2350686, EPI_ISL_2350687, EPI_ISL_2350688, EPI_ISL_2350689, EPI_ISL_2350690, EPI_ISL_2350691, EPI_ISL_2350692, EPI_ISL_2350693, EPI_ISL_2350694, EPI_ISL_2350695, EPI_ISL_2350696, EPI_ISL_2350697, EPI_ISL_2350698, EPI_ISL_2350699, EPI_ISL_2350700, EPI_ISL_2350701, EPI_ISL_2350702, EPI_ISL_2350703, EPI_ISL_2350704, EPI_ISL_2350705, EPI_ISL_2350706, EPI_ISL_2350707, EPI_ISL_2350708, EPI_ISL_2350709, EPI_ISL_2350710, EPI_ISL_2350711, EPI_ISL_2350712, EPI_ISL_2350713, EPI_ISL_2350714, EPI_ISL_2350715, EPI_ISL_2350716, EPI_ISL_2350717, EPI_ISL_2350718, EPI_ISL_2350719, EPI_ISL_2350720, EPI_ISL_2350721, EPI_ISL_2350722, EPI_ISL_2350723, EPI_ISL_2350724, EPI_ISL_2350725, EPI_ISL_2350726, EPI_ISL_2350727, EPI_ISL_2350728, EPI_ISL_2350729, EPI_ISL_2350730, EPI_ISL_2350731, EPI_ISL_2350732, EPI_ISL_2350733, EPI_ISL_2350734, EPI_ISL_2350735, EPI_ISL_2350736, EPI_ISL_2350737, EPI_ISL_2350738, EPI_ISL_2350739, EPI_ISL_2350740, EPI_ISL_2350741, EPI_ISL_2350742, EPI_ISL_2350743, EPI_ISL_2350744, EPI_ISL_2350745, EPI_ISL_2350746, EPI_ISL_2350747, EPI_ISL_2350748, EPI_ISL_2350749, EPI_ISL_2350750, EPI_ISL_2350751, EPI_ISL_2350752, EPI_ISL_2350753, EPI_ISL_2350754, EPI_ISL_2350755, EPI_ISL_2350756, EPI_ISL_2350757, EPI_ISL_2350758, EPI_ISL_2350759, EPI_ISL_2350760, EPI_ISL_2350761, EPI_ISL_2350762, EPI_ISL_2350763, EPI_ISL_2350764, EPI_ISL_2350765, EPI_ISL_2350766, EPI_ISL_2350767, EPI_ISL_2350768, EPI_ISL_2350769, EPI_ISL_2350770, EPI_ISL_2350771, EPI_ISL_2350772, EPI_ISL_2350773, EPI_ISL_2350774, EPI_ISL_2350775, EPI_ISL_2350776, EPI_ISL_2350777, EPI_ISL_2350778, EPI_ISL_2350779, EPI_ISL_2350780, EPI_ISL_2350781, EPI_ISL_2350782, EPI_ISL_2350783, EPI_ISL_2462872, EPI_ISL_2462873, EPI_ISL_2462874, EPI_ISL_2462875, EPI_ISL_2462876, EPI_ISL_2462877, EPI_ISL_2462878			
see above	Ibaraki Prefectural Institute of Public Health	Ibaraki Prefectural Institute of Public Health	Keiko Goto; Kentaro Itokawa; Makoto Kuroda; Masanori Hashino; Rina Tanaka; Tsuyoshi Sekizuka
EPI_ISL_2671835, EPI_ISL_2671836, EPI_ISL_2671837, EPI_ISL_2671838, EPI_ISL_2671839, EPI_ISL_2671840, EPI_ISL_2671841, EPI_ISL_2671842, EPI_ISL_2671843, EPI_ISL_2671844, EPI_ISL_2671845, EPI_ISL_2671846, EPI_ISL_2671847, EPI_ISL_2671848, EPI_ISL_2671890, EPI_ISL_2671891, EPI_ISL_2671892, EPI_ISL_2671893, EPI_ISL_2671894, EPI_ISL_2671895, EPI_ISL_2671896, EPI_ISL_2671897, EPI_ISL_2671898, EPI_ISL_2671899			
see above	Juntendo University Hospital	Juntendo University Hospital	Koji Tuchiya; Yoko Tabe; Yoshie Hosaka
EPI_ISL_2361601, EPI_ISL_2361602, EPI_ISL_2361603, EPI_ISL_2361604, EPI_ISL_2361605, EPI_ISL_2361606, EPI_ISL_2361607, EPI_ISL_2361608, EPI_ISL_2361609, EPI_ISL_2361610, EPI_ISL_2361611, EPI_ISL_2361612, EPI_ISL_2361613, EPI_ISL_2361614, EPI_ISL_2361615, EPI_ISL_2361616, EPI_ISL_2361617, EPI_ISL_2544681, EPI_ISL_2544682, EPI_ISL_2544683, EPI_ISL_2544684, EPI_ISL_2544685, EPI_ISL_2544686, EPI_ISL_2544687, EPI_ISL_2544688, EPI_ISL_2544689, EPI_ISL_2544690, EPI_ISL_2544691, EPI_ISL_2544692, EPI_ISL_2544693, EPI_ISL_2544694, EPI_ISL_2544695, EPI_ISL_2544696, EPI_ISL_2544697, EPI_ISL_2544698			
see above	Kanagawa Prefectural Institute of Public Health	Kanagawa Prefectural Institute of Public Health	Itsumi Toyokura; Junichi Sakuragi; Kentaro Itokawa; Makiko Kondo; Makoto Kuroda; Masanori Hashino; Rieko Suzuki; Rina Tanaka; Sumi Watanabe; Takako Sano; Takayuki Hishiki; Tomohiko Takasaki; Tsuyoshi Sekizuka
EPI_ISL_2377697, EPI_ISL_2378699, EPI_ISL_2378709, EPI_ISL_2378710	Kawakita General Hospital	Tokyo Medical University Department of Microbiology	Hirofumi Miyake; Jun Suzuki; Kenji Sadamasu; Kenshirou Kroki; Mami Nagashima; Maya Isogai; Shigeki Nakamura; Takahiro Okai; Takako Yamazaki; Takushi Fujiwara; Tatsuya Inukai
EPI_ISL_2695878, EPI_ISL_2695879, EPI_ISL_2695880, EPI_ISL_2695881, EPI_ISL_2695883	Osaka General Medical Center	Department of Developmental Medicine, Research Institute, Osaka Women's and Children's Hospital	Itaru Yanagihara; Yukiko Nakura
EPI_ISL_2663782, EPI_ISL_2663783, EPI_ISL_2663784, EPI_ISL_2663785, EPI_ISL_2663786, EPI_ISL_2663787, EPI_ISL_2663788, EPI_ISL_2663789, EPI_ISL_2663790, EPI_ISL_2663791, EPI_ISL_2663792, EPI_ISL_2663793, EPI_ISL_2663794, EPI_ISL_2663795, EPI_ISL_2663796			

see above	Osaka University Hospital	Genome Information Research Center, Research Institute for Microbial Diseases, Osaka University	Daisuke Motooka; Haruhiko Hirata; Hiroshi Ogura; Hiroya Oki; Hisatake Matsumoto; Kentaro Tanaka; Shota Nakamura; Takeshi Ebihara; Yasuhiro Kato; and Atsushi Kumanogoh
EPI_ISL_2392147, EPI_ISL_2392148, EPI_ISL_2392149, EPI_ISL_2392150, EPI_ISL_2392151, EPI_ISL_2392152, EPI_ISL_2392153, EPI_ISL_2392154, EPI_ISL_2392155, EPI_ISL_2392156, EPI_ISL_2392157, EPI_ISL_2392158, EPI_ISL_2392159, EPI_ISL_2491330, EPI_ISL_2491331, EPI_ISL_2491332, EPI_ISL_2491333, EPI_ISL_2491334, EPI_ISL_2491335, EPI_ISL_2491336, EPI_ISL_2491337, EPI_ISL_2491338, EPI_ISL_2491339, EPI_ISL_2491340, EPI_ISL_2491341, EPI_ISL_2491342, EPI_ISL_2491343, EPI_ISL_2491344, EPI_ISL_2491345, EPI_ISL_2491346, EPI_ISL_2491347, EPI_ISL_2491348, EPI_ISL_2491349, EPI_ISL_2491350, EPI_ISL_2491351, EPI_ISL_2491352, EPI_ISL_2491353, EPI_ISL_2491354, EPI_ISL_2491355, EPI_ISL_2491356, EPI_ISL_2491357, EPI_ISL_2491358, EPI_ISL_2491359, EPI_ISL_2491360, EPI_ISL_2491361, EPI_ISL_2491362, EPI_ISL_2491363			
see above	Pathogen Genomics Center, National Institute of Infectious Diseases	Pathogen Genomics Center, National Institute of Infectious Diseases	Kentaro Itokawa; Makoto Kuroda; Masanori Hashino; Rina Tanaka; Tsuyoshi Sekizuka
EPI_ISL_2447604, EPI_ISL_2447605, EPI_ISL_2447606, EPI_ISL_2447607, EPI_ISL_2447608, EPI_ISL_2447609, EPI_ISL_2447610, EPI_ISL_2447611, EPI_ISL_2447612, EPI_ISL_2447613, EPI_ISL_2447615, EPI_ISL_2447617, EPI_ISL_2447620, EPI_ISL_2447623, EPI_ISL_2447625, EPI_ISL_2447628, EPI_ISL_2447631, EPI_ISL_2447634, EPI_ISL_2447636, EPI_ISL_2447639, EPI_ISL_2447642, EPI_ISL_2447644, EPI_ISL_2447648, EPI_ISL_2447650, EPI_ISL_2447653, EPI_ISL_2447656, EPI_ISL_2447659, EPI_ISL_2447662, EPI_ISL_2447664, EPI_ISL_2447667, EPI_ISL_2447670, EPI_ISL_2447673, EPI_ISL_2447676, EPI_ISL_2447678, EPI_ISL_2447680, EPI_ISL_2447683, EPI_ISL_2447686, EPI_ISL_2447689, EPI_ISL_2447692, EPI_ISL_2447694, EPI_ISL_2447697, EPI_ISL_2447700, EPI_ISL_2447988, EPI_ISL_2621738, EPI_ISL_2621739, EPI_ISL_2621740, EPI_ISL_2621741, EPI_ISL_2621742, EPI_ISL_2621743, EPI_ISL_2621744, EPI_ISL_2621745, EPI_ISL_2621746, EPI_ISL_2621747, EPI_ISL_2621748, EPI_ISL_2621749, EPI_ISL_2621750, EPI_ISL_2621751, EPI_ISL_2621752, EPI_ISL_2621753, EPI_ISL_2621754, EPI_ISL_2621755, EPI_ISL_2621756, EPI_ISL_2621757, EPI_ISL_2621758, EPI_ISL_2621759, EPI_ISL_2621760, EPI_ISL_2621761, EPI_ISL_2621762, EPI_ISL_2621763, EPI_ISL_2621764, EPI_ISL_2621765, EPI_ISL_2621766, EPI_ISL_2621767, EPI_ISL_2621768, EPI_ISL_2621769, EPI_ISL_2621770, EPI_ISL_2621771, EPI_ISL_2621772, EPI_ISL_2621773, EPI_ISL_2621774, EPI_ISL_2621775, EPI_ISL_2621776, EPI_ISL_2621777, EPI_ISL_2621778, EPI_ISL_2621779, EPI_ISL_2621780, EPI_ISL_2621781, EPI_ISL_2621782, EPI_ISL_2674806, EPI_ISL_2674807, EPI_ISL_2674808, EPI_ISL_2674809, EPI_ISL_2674810, EPI_ISL_2674811			
see above	SARS-CoV-2 testing team, National Institute of Infectious Diseases	Pathogen Genomics Center, National Institute of Infectious Diseases	Hazuka Y Furihata; Hiromizu Takahashi; Kentaro Itokawa; Makoto Kuroda; Masanori Hashino; Masumichi Saito; Naomi Nojiri; Nozomu Hanaoka; Rina Tanaka; Sana Uchikoba; Tsuguto Fujimoto; Tsuyoshi Sekizuka
EPI_ISL_2350403, EPI_ISL_2350404, EPI_ISL_2350405, EPI_ISL_2350406, EPI_ISL_2350407, EPI_ISL_2350408, EPI_ISL_2350409, EPI_ISL_2350410, EPI_ISL_2350411, EPI_ISL_2350412, EPI_ISL_2350413, EPI_ISL_2350414, EPI_ISL_2350415, EPI_ISL_2350416, EPI_ISL_2350417, EPI_ISL_2350418, EPI_ISL_2350419, EPI_ISL_2350420, EPI_ISL_2350421, EPI_ISL_2350422, EPI_ISL_2350423, EPI_ISL_2350424, EPI_ISL_2350425, EPI_ISL_2350426, EPI_ISL_2350427, EPI_ISL_2350428, EPI_ISL_2350429, EPI_ISL_2350430, EPI_ISL_2350431, EPI_ISL_2350432, EPI_ISL_2350433, EPI_ISL_2350434, EPI_ISL_2350435, EPI_ISL_2350436, EPI_ISL_2350437, EPI_ISL_2350438, EPI_ISL_2350439, EPI_ISL_2350440, EPI_ISL_2350441, EPI_ISL_2350442, EPI_ISL_2350443, EPI_ISL_2350444, EPI_ISL_2350445, EPI_ISL_2350446, EPI_ISL_2350447, EPI_ISL_2350448, EPI_ISL_2350449, EPI_ISL_2350450, EPI_ISL_2350451, EPI_ISL_2350452, EPI_ISL_2350453, EPI_ISL_2350454, EPI_ISL_2350455, EPI_ISL_2350456, EPI_ISL_2350457, EPI_ISL_2350458, EPI_ISL_2350459, EPI_ISL_2350460, EPI_ISL_2350461, EPI_ISL_2350462, EPI_ISL_2350463, EPI_ISL_2350464, EPI_ISL_2350465, EPI_ISL_2350466, EPI_ISL_2350467, EPI_ISL_2350468, EPI_ISL_2350469, EPI_ISL_2350470, EPI_ISL_2350471, EPI_ISL_2350472, EPI_ISL_2350473, EPI_ISL_2350474, EPI_ISL_2350475, EPI_ISL_2350476, EPI_ISL_2350477, EPI_ISL_2350478, EPI_ISL_2350479, EPI_ISL_2350480, EPI_ISL_2350481, EPI_ISL_2350482, EPI_ISL_2350483, EPI_ISL_2350484			
see above	Saitama Prefectural Institute of Public Health	Saitama Prefectural Institute of Public Health	Eri Aonuma; Hayato Ehara; Kentaro Itokawa; Makoto Kuroda; Masanori Hashino; Rina Tanaka; Satoshi Daisaki; Tsuyoshi Sekizuka
EPI_ISL_2357899, EPI_ISL_2357900, EPI_ISL_2357901, EPI_ISL_2357902, EPI_ISL_2357903, EPI_ISL_2357904, EPI_ISL_2357905, EPI_ISL_2357906, EPI_ISL_2357907, EPI_ISL_2357908, EPI_ISL_2357909, EPI_ISL_2357910, EPI_ISL_2357911, EPI_ISL_2357912, EPI_ISL_2357913, EPI_ISL_2357914, EPI_ISL_2357915, EPI_ISL_2357916, EPI_ISL_2357917, EPI_ISL_2357918, EPI_ISL_2357919, EPI_ISL_2357920, EPI_ISL_2357921, EPI_ISL_2357922, EPI_ISL_2357923, EPI_ISL_2357924, EPI_ISL_2357925, EPI_ISL_2357926, EPI_ISL_2357927, EPI_ISL_2357928, EPI_ISL_2357929, EPI_ISL_2357930, EPI_ISL_2357931, EPI_ISL_2357932, EPI_ISL_2357933, EPI_ISL_2357934, EPI_ISL_2357935, EPI_ISL_2357936, EPI_ISL_2357937, EPI_ISL_2357938, EPI_ISL_2357939, EPI_ISL_2357940, EPI_ISL_2357941, EPI_ISL_2357942, EPI_ISL_2357943, EPI_ISL_2357944, EPI_ISL_2357945, EPI_ISL_2357946, EPI_ISL_2357947, EPI_ISL_2357949, EPI_ISL_2357950, EPI_ISL_2357951, EPI_ISL_2357952, EPI_ISL_2357953, EPI_ISL_2357954, EPI_ISL_2357955, EPI_ISL_2357956, EPI_ISL_2357957, EPI_ISL_2357958			
see above	Sapporo City Institute of Public Health	Sapporo City Institute of Public Health	Asami Ohnishi; Kentaro Itokawa; Makoto Kuroda; Masanori Hashino; Masayuki Kikuchi; Rie Shimazaki; Rina Tanaka; Tsuyoshi Sekizuka; Yusuke Oguchi
EPI_ISL_2405217, EPI_ISL_2405218, EPI_ISL_2405219, EPI_ISL_2405220, EPI_ISL_2405221, EPI_ISL_2405222, EPI_ISL_2405223, EPI_ISL_2405224, EPI_ISL_2405225, EPI_ISL_2405226, EPI_ISL_2405227, EPI_ISL_2405228, EPI_ISL_2405229, EPI_ISL_2405230, EPI_ISL_2405231, EPI_ISL_2405232, EPI_ISL_2405233, EPI_ISL_2405234, EPI_ISL_2405235, EPI_ISL_2405236			
see above	Shimane Prefectural Institute of Public Health and Environmental Science	Shimane Prefectural Institute of Public Health and Environmental Science	Kentaro Itokawa; Makoto Kuroda; Masanori Hashino; Naoki Fujisawa; Rina Tanaka; Tsuyoshi Sekizuka
EPI_ISL_2551582	Tochigi Prefectural Institute of Environment and Health	Genome Analysis Center, Kamma Memorial Hospital	Fuminori Mizukoshi; Hanako Yazawa; Hiroshi Kamma; Jun Ishii; Kaori Watanabe; Michiaki Masuda; Satoko Soma; Takuya Yazawa; Yoshiko Hitomi
EPI_ISL_2438804, EPI_ISL_2438805, EPI_ISL_2438806, EPI_ISL_2438807, EPI_ISL_2438808, EPI_ISL_2438809, EPI_ISL_2438810, EPI_ISL_2438811, EPI_ISL_2438812, EPI_ISL_2438813, EPI_ISL_2438814, EPI_ISL_2438815, EPI_ISL_2438816, EPI_ISL_2438817, EPI_ISL_2438818, EPI_ISL_2438819, EPI_ISL_2438820, EPI_ISL_2438821, EPI_ISL_2438822, EPI_ISL_2438823, EPI_ISL_2438824, EPI_ISL_2438825, EPI_ISL_2438826, EPI_ISL_2438827, EPI_ISL_2438828, EPI_ISL_2438829, EPI_ISL_2438830, EPI_ISL_2438831, EPI_ISL_2438832, EPI_ISL_2438833, EPI_ISL_2438834, EPI_ISL_2438835, EPI_ISL_2438836, EPI_ISL_2438837, EPI_ISL_2438838, EPI_ISL_2438839, EPI_ISL_2438840, EPI_ISL_2438841, EPI_ISL_2438842, EPI_ISL_2438843, EPI_ISL_2438844, EPI_ISL_2438845, EPI_ISL_2438846, EPI_ISL_2438847, EPI_ISL_2438848, EPI_ISL_2438849, EPI_ISL_2438850, EPI_ISL_2438851, EPI_ISL_2438852, EPI_ISL_2438853, EPI_ISL_2461202, EPI_ISL_2461503, EPI_ISL_2461504, EPI_ISL_2461505, EPI_ISL_2461506, EPI_ISL_2461507, EPI_ISL_2461508, EPI_ISL_2462377, EPI_ISL_2462378, EPI_ISL_2462379, EPI_ISL_2462380, EPI_ISL_2462381, EPI_ISL_2462382, EPI_ISL_2462383, EPI_ISL_2462384, EPI_ISL_2462385, EPI_ISL_2462386, EPI_ISL_2462387, EPI_ISL_2462388, EPI_ISL_2462389, EPI_ISL_2462390, EPI_ISL_2462391, EPI_ISL_2462392, EPI_ISL_2462393, EPI_ISL_2462394, EPI_ISL_2462395, EPI_ISL_2462396, EPI_ISL_2462397, EPI_ISL_2462398, EPI_ISL_2462399, EPI_ISL_2462400, EPI_ISL_2562046, EPI_ISL_2562047, EPI_ISL_2562048, EPI_ISL_2562049, EPI_ISL_2562050, EPI_ISL_2562051, EPI_ISL_2562052, EPI_ISL_2562053, EPI_ISL_2562054, EPI_ISL_2562055, EPI_ISL_2562056, EPI_ISL_2562057, EPI_ISL_2562058, EPI_ISL_2651240, EPI_ISL_2651241, EPI_ISL_2651242, EPI_ISL_2651243, EPI_ISL_2651244, EPI_ISL_2651245, EPI_ISL_2651246, EPI_ISL_2651247, EPI_ISL_2651248, EPI_ISL_2651249, EPI_ISL_2709568, EPI_ISL_2709569, EPI_ISL_2709570, EPI_ISL_2709571, EPI_ISL_2709572, EPI_ISL_2709573, EPI_ISL_2709574, EPI_ISL_2709575, EPI_ISL_2709576, EPI_ISL_2709577, EPI_ISL_2709578, EPI_ISL_2709579, EPI_ISL_2709580, EPI_ISL_2709581, EPI_ISL_2709582, EPI_ISL_2709583, EPI_ISL_2709584, EPI_ISL_2709585, EPI_ISL_2709586, EPI_ISL_2709587, EPI_ISL_2709588, EPI_ISL_2709589, EPI_ISL_2709590, EPI_ISL_2709591, EPI_ISL_2709592, EPI_ISL_2709593, EPI_ISL_2709594, EPI_ISL_2709595, EPI_ISL_2709596, EPI_ISL_2709597, EPI_ISL_2709598, EPI_ISL_2709599, EPI_ISL_2709600, EPI_ISL_2709601, EPI_ISL_2709602, EPI_ISL_2723565, EPI_ISL_2723566, EPI_ISL_2723567, EPI_ISL_2723568, EPI_ISL_2723569			
see above	Tokyo Metropolitan Institute of Public Health	Tokyo Metropolitan Institute of Public Health	Akane Negishi; Hirofumi Miyake; Hiroshi Koike; Jun Suzuki; Kenji Sadamasu; Kenshirou Kuroki; Mami Nagashima; Masaki Hayashi; Maya Isogai; Takako Yamazaki; Takushi Fujiwara
EPI_ISL_2714903, EPI_ISL_2714904, EPI_ISL_2714905, EPI_ISL_2714906, EPI_ISL_2714907, EPI_ISL_2714908, EPI_ISL_2714909, EPI_ISL_2714910, EPI_ISL_2714911, EPI_ISL_2714912, EPI_ISL_2714913, EPI_ISL_2714914, EPI_ISL_2714915, EPI_ISL_2714916, EPI_ISL_2714917			
see above	Tokyo Metropolitan Institute of Public Health, Department of Microbiology	Tokyo Metropolitan Institute of Public Health, Department of Microbiology	Asakura, H.; Fujiwara, T.; J. and Sadamasu, K.; Kumagai, R.; Miyake, H.; Nagashima, M.; Suzuki; Yaoita, Y.; Yoshida, I.
EPI_ISL_2405201, EPI_ISL_2405237, EPI_ISL_2405360, EPI_ISL_2405361, EPI_ISL_2405362, EPI_ISL_2405590, EPI_ISL_2406026			
see above	Toyama Institute of Health	Toyama Institute of Health	Emiko Igarashi; Hideki Tani; Kazunori Oishi; Kentaro Itokawa; Makoto Kuroda; Masae Itamochi; Masanori Hashino; Noriko Inasaki; Rina Tanaka; Shunsuke Yazawa; Sumiyo Hasegawa; Tsuyoshi Sekizuka; Yumiko Saga

We gratefully acknowledge the following Authors from the Originating laboratories responsible for obtaining the specimens, as well as the Submitting laboratories where the genome data were generated and shared via GISAID, on which this research is based.

All Submitters of data may be contacted directly via www.gisaid.org

Authors are sorted alphabetically.

Accession ID	Originating Laboratory	Submitting Laboratory	Authors
EPI_ISL_2325628, EPI_ISL_2325629, EPI_ISL_2325630, EPI_ISL_2325631, EPI_ISL_2325632, EPI_ISL_2325633, EPI_ISL_2325634, EPI_ISL_2325635, EPI_ISL_2325636, EPI_ISL_2325637, EPI_ISL_2325638, EPI_ISL_2325639, EPI_ISL_2325640, EPI_ISL_2325641, EPI_ISL_2325642, EPI_ISL_2325643, EPI_ISL_2325644, EPI_ISL_2325645, EPI_ISL_2325646, EPI_ISL_2325647, EPI_ISL_2325648, EPI_ISL_2325649, EPI_ISL_2325650, EPI_ISL_2325651, EPI_ISL_2325652, EPI_ISL_2325653, EPI_ISL_2326211, EPI_ISL_2326212, EPI_ISL_2326213, EPI_ISL_2326648, EPI_ISL_2326649, EPI_ISL_2326650, EPI_ISL_2326651, EPI_ISL_2326652, EPI_ISL_2326653, EPI_ISL_2326654, EPI_ISL_2326655, EPI_ISL_2326656, EPI_ISL_2326657, EPI_ISL_2326658, EPI_ISL_2326659, EPI_ISL_2326660, EPI_ISL_2326661, EPI_ISL_2326662, EPI_ISL_2326663, EPI_ISL_2326664, EPI_ISL_2326665, EPI_ISL_2326666, EPI_ISL_2326667, EPI_ISL_2326668, EPI_ISL_2326669, EPI_ISL_2326670, EPI_ISL_2326671, EPI_ISL_2326672, EPI_ISL_2326673, EPI_ISL_2326674, EPI_ISL_2326675, EPI_ISL_2326676, EPI_ISL_2326677, EPI_ISL_2326678, EPI_ISL_2326679, EPI_ISL_2326680, EPI_ISL_2326681, EPI_ISL_2326682, EPI_ISL_2326683, EPI_ISL_2326684, EPI_ISL_2326685, EPI_ISL_2326686, EPI_ISL_2326687, EPI_ISL_2326688, EPI_ISL_2326689, EPI_ISL_2326690, EPI_ISL_2326691, EPI_ISL_2326692, EPI_ISL_2326693, EPI_ISL_2326694, EPI_ISL_2328818, EPI_ISL_2328819, EPI_ISL_2328820, EPI_ISL_2328821, EPI_ISL_2328822, EPI_ISL_2328823, EPI_ISL_2328824, EPI_ISL_2328825, EPI_ISL_2328826, EPI_ISL_2328827, EPI_ISL_2328828, EPI_ISL_2328829, EPI_ISL_2328830, EPI_ISL_2328831, EPI_ISL_2328832, EPI_ISL_2328833, EPI_ISL_2328834, EPI_ISL_2328835, EPI_ISL_2328836, EPI_ISL_2328837, EPI_ISL_2328838, EPI_ISL_2328839, EPI_ISL_2328840, EPI_ISL_2328841, EPI_ISL_2328842, EPI_ISL_2328843, EPI_ISL_2328844, EPI_ISL_2328845, EPI_ISL_2328846, EPI_ISL_2328847, EPI_ISL_2328848, EPI_ISL_2328849, EPI_ISL_2328850, EPI_ISL_2328851, EPI_ISL_2328852, EPI_ISL_2328853, EPI_ISL_2328854, EPI_ISL_2328855, EPI_ISL_2329678, EPI_ISL_2329679, EPI_ISL_2337239, EPI_ISL_2337240, EPI_ISL_2337241, EPI_ISL_2337242, EPI_ISL_2337243, EPI_ISL_2337244, EPI_ISL_2337245, EPI_ISL_2337246, EPI_ISL_2337247, EPI_ISL_2337248, EPI_ISL_2337249, EPI_ISL_2337250, EPI_ISL_2337251, EPI_ISL_2337252, EPI_ISL_2337253, EPI_ISL_2337256, EPI_ISL_2337257, EPI_ISL_2337258, EPI_ISL_2337259, EPI_ISL_2337260, EPI_ISL_2337261, EPI_ISL_2337262, EPI_ISL_2337263, EPI_ISL_2337264, EPI_ISL_2337265, EPI_ISL_2337266, EPI_ISL_2337267, EPI_ISL_2337268, EPI_ISL_2337269, EPI_ISL_2337270, EPI_ISL_2337271, EPI_ISL_2337272, EPI_ISL_2337273, EPI_ISL_2337274, EPI_ISL_2337275, EPI_ISL_2337276, EPI_ISL_2337277, EPI_ISL_2337278, EPI_ISL_2337279, EPI_ISL_2337280, EPI_ISL_2337281, EPI_ISL_2337282, EPI_ISL_2337283, EPI_ISL_2337284, EPI_ISL_2337285			
see above	Chiba Prefectural Institute of Public Health	Pathogen Genomics Center, National Institute of Infectious Diseases	Kentaro Itokawa; Makoto Kuroda; Masanori Hashino; Rina Tanaka; Tsuyoshi Sekizuka
EPI_ISL_2292081, EPI_ISL_2292082, EPI_ISL_2292083, EPI_ISL_2292084, EPI_ISL_2292085, EPI_ISL_2292086, EPI_ISL_2292087, EPI_ISL_2292088, EPI_ISL_2292089, EPI_ISL_2292090, EPI_ISL_2292091, EPI_ISL_2292092, EPI_ISL_2292093, EPI_ISL_2292094, EPI_ISL_2292095			
see above	Department of Infectious Diseases and Immunology, National Hospital Organization Nagoya Medical Center	Clinical Research Center, National Hospital Organization Nagoya Medical Center	Hirotaka Ode; Kazuhiro Matsuoka; Mai Kubota; Masakazu Matsuda; Mayumi Imahashi; Miho Nakasuji; Mikiko Mori; Yasumasa Iwatani; Yoshihiro Nakata; Yoshiyuki Yokomaku
EPI_ISL_2285723, EPI_ISL_2285724, EPI_ISL_2285725, EPI_ISL_2285726, EPI_ISL_2285727, EPI_ISL_2285728, EPI_ISL_2285729, EPI_ISL_2285730, EPI_ISL_2285731, EPI_ISL_2285732			
see above	Department of Pathology, National Institute of Infectious Diseases	Department of Pathology, National Institute of Infectious Diseases	Harutaka Katano; Tadaki Suzuki; Takayuki Kanno
EPI_ISL_2283768, EPI_ISL_2283769, EPI_ISL_2283770, EPI_ISL_2283771, EPI_ISL_2283772, EPI_ISL_2283773, EPI_ISL_2283774, EPI_ISL_2283775, EPI_ISL_2283776, EPI_ISL_2283777, EPI_ISL_2283778, EPI_ISL_2283779, EPI_ISL_2283780, EPI_ISL_2283781, EPI_ISL_2283782, EPI_ISL_2283783, EPI_ISL_2283784, EPI_ISL_2283785, EPI_ISL_2283786, EPI_ISL_2283787, EPI_ISL_2283788, EPI_ISL_2283789, EPI_ISL_2283790, EPI_ISL_2283791, EPI_ISL_2283792, EPI_ISL_2283793, EPI_ISL_2283794, EPI_ISL_2283795, EPI_ISL_2283796, EPI_ISL_2283797, EPI_ISL_2283798, EPI_ISL_2283799, EPI_ISL_2283800, EPI_ISL_2283801, EPI_ISL_2283802, EPI_ISL_2283803, EPI_ISL_2283804, EPI_ISL_2283805, EPI_ISL_2283806, EPI_ISL_2283807, EPI_ISL_2283808, EPI_ISL_2283809, EPI_ISL_2283810, EPI_ISL_2304298, EPI_ISL_2304299, EPI_ISL_2304300, EPI_ISL_2304301, EPI_ISL_2304302, EPI_ISL_2304303, EPI_ISL_2304304, EPI_ISL_2307325, EPI_ISL_2307326, EPI_ISL_2307327, EPI_ISL_2307328, EPI_ISL_2307329, EPI_ISL_2307330, EPI_ISL_2307331, EPI_ISL_2307332, EPI_ISL_2307333, EPI_ISL_2307334, EPI_ISL_2307335, EPI_ISL_2307336, EPI_ISL_2307337, EPI_ISL_2307338, EPI_ISL_2307339, EPI_ISL_2307340, EPI_ISL_2307341, EPI_ISL_2307342, EPI_ISL_2307343, EPI_ISL_2307344, EPI_ISL_2307345, EPI_ISL_2307346, EPI_ISL_2307347, EPI_ISL_2307348, EPI_ISL_2307349, EPI_ISL_2321226, EPI_ISL_2321227, EPI_ISL_2321228, EPI_ISL_2321229, EPI_ISL_2321230, EPI_ISL_2322453, EPI_ISL_2322454, EPI_ISL_2322455, EPI_ISL_2322456, EPI_ISL_2322457, EPI_ISL_2322458, EPI_ISL_2322459, EPI_ISL_2322460, EPI_ISL_2322461, EPI_ISL_2322462, EPI_ISL_2322463, EPI_ISL_2322464, EPI_ISL_2322465, EPI_ISL_2322466, EPI_ISL_2322467, EPI_ISL_2322468			
see above	Genome Analysis Center, Yamanashi Central Hospital	Genome Analysis Center, Yamanashi Central Hospital	Yosuke Hirotsu
EPI_ISL_2326393	Gunma Prefectural Institute of Public Health and Environmental Sciences	Pathogen Genomics Center, National Institute of Infectious Diseases	Kentaro Itokawa; Makoto Kuroda; Masanori Hashino; Rina Tanaka; Tsuyoshi Sekizuka
EPI_ISL_2326388	Ibaraki Prefectural Institute of Public Health	Pathogen Genomics Center, National Institute of Infectious Diseases	Kentaro Itokawa; Makoto Kuroda; Masanori Hashino; Rina Tanaka; Tsuyoshi Sekizuka
EPI_ISL_2337525, EPI_ISL_2337526, EPI_ISL_2337527, EPI_ISL_2337528, EPI_ISL_2337529, EPI_ISL_2337530, EPI_ISL_2337531, EPI_ISL_2337532, EPI_ISL_2337533, EPI_ISL_2337534, EPI_ISL_2337535, EPI_ISL_2337536, EPI_ISL_2337537, EPI_ISL_2337538, EPI_ISL_2337539, EPI_ISL_2337540, EPI_ISL_2337541, EPI_ISL_2337542, EPI_ISL_2337543, EPI_ISL_2337544, EPI_ISL_2337545, EPI_ISL_2337546, EPI_ISL_2337547, EPI_ISL_2337548, EPI_ISL_2337549, EPI_ISL_2337550, EPI_ISL_2337551, EPI_ISL_2337552, EPI_ISL_2337553, EPI_ISL_2337554, EPI_ISL_2337555, EPI_ISL_2337556, EPI_ISL_2337557			
see above	Niigata Prefectural Institute of Public Health and Environmental Sciences	Pathogen Genomics Center, National Institute of Infectious Diseases	Kentaro Itokawa; Makoto Kuroda; Masanori Hashino; Rina Tanaka; Tsuyoshi Sekizuka
EPI_ISL_2326185, EPI_ISL_2326394, EPI_ISL_2326395, EPI_ISL_2326396, EPI_ISL_2326397, EPI_ISL_2326398, EPI_ISL_2326399, EPI_ISL_2326400, EPI_ISL_2326401, EPI_ISL_2326402, EPI_ISL_2326403, EPI_ISL_2326404, EPI_ISL_2326857, EPI_ISL_2326858, EPI_ISL_2326859, EPI_ISL_2326860, EPI_ISL_2326861, EPI_ISL_2330519, EPI_ISL_2330520, EPI_ISL_2335458, EPI_ISL_2335459, EPI_ISL_2335460, EPI_ISL_2335461, EPI_ISL_2335462, EPI_ISL_2335463, EPI_ISL_2335464, EPI_ISL_2335465, EPI_ISL_2335466, EPI_ISL_2338397, EPI_ISL_2338398, EPI_ISL_2338399, EPI_ISL_2338400, EPI_ISL_2339144, EPI_ISL_2339145, EPI_ISL_2339146, EPI_ISL_2339147, EPI_ISL_2339148, EPI_ISL_2339149, EPI_ISL_2339150, EPI_ISL_2339151, EPI_ISL_2339152, EPI_ISL_2339153, EPI_ISL_2339154, EPI_ISL_2339155, EPI_ISL_2339156, EPI_ISL_2339157, EPI_ISL_2339158, EPI_ISL_2339159, EPI_ISL_2339160, EPI_ISL_2339161, EPI_ISL_2339162, EPI_ISL_2339163, EPI_ISL_2339164, EPI_ISL_2339165, EPI_ISL_2339166, EPI_ISL_2339167, EPI_ISL_2339168, EPI_ISL_2339169, EPI_ISL_2339170, EPI_ISL_2339171, EPI_ISL_2339172, EPI_ISL_2339173, EPI_ISL_2339174, EPI_ISL_2339175, EPI_ISL_2339176, EPI_ISL_2339177, EPI_ISL_2339178, EPI_ISL_2339179, EPI_ISL_2339180, EPI_ISL_2339181, EPI_ISL_2339182, EPI_ISL_2339183, EPI_ISL_2339184, EPI_ISL_2339185, EPI_ISL_2339186, EPI_ISL_2339187, EPI_ISL_2339188, EPI_ISL_2339189, EPI_ISL_2339190, EPI_ISL_2339191, EPI_ISL_2339192, EPI_ISL_2339193, EPI_ISL_2339194, EPI_ISL_2339195, EPI_ISL_2339196, EPI_ISL_2339197, EPI_ISL_2339198, EPI_ISL_2339199, EPI_ISL_2339200, EPI_ISL_2339201, EPI_ISL_2339202, EPI_ISL_2339203, EPI_ISL_2339204, EPI_ISL_2339205			
see above	Osaka Institute of Public Health, Morinomiya Center	Pathogen Genomics Center, National Institute of Infectious Diseases	Kentaro Itokawa; Makoto Kuroda; Masanori Hashino; Rina Tanaka; Tsuyoshi Sekizuka
EPI_ISL_2325618, EPI_ISL_2325619, EPI_ISL_2325620, EPI_ISL_2325621, EPI_ISL_2325622, EPI_ISL_2325623, EPI_ISL_2325624, EPI_ISL_2325625, EPI_ISL_2325626, EPI_ISL_2325627, EPI_ISL_2325654, EPI_ISL_2325655, EPI_ISL_2325656, EPI_ISL_2325657, EPI_ISL_2325658, EPI_ISL_2325659, EPI_ISL_2325660, EPI_ISL_2325661, EPI_ISL_2325662, EPI_ISL_2325663, EPI_ISL_2325664, EPI_ISL_2325665, EPI_ISL_2325666, EPI_ISL_2325667, EPI_ISL_2325668, EPI_ISL_2325669, EPI_ISL_2325670, EPI_ISL_2325671, EPI_ISL_2325672, EPI_ISL_2325673, EPI_ISL_2325674, EPI_ISL_2325675, EPI_ISL_2325676, EPI_ISL_2325677, EPI_ISL_2325678, EPI_ISL_2325679, EPI_ISL_2325680, EPI_ISL_2325681, EPI_ISL_2325682, EPI_ISL_2325683, EPI_ISL_2325684, EPI_ISL_2325685, EPI_ISL_2325686, EPI_ISL_2325687, EPI_ISL_2325688, EPI_ISL_2325689, EPI_ISL_2325690, EPI_ISL_2325691, EPI_ISL_2325692, EPI_ISL_2325693, EPI_ISL_2325694, EPI_ISL_2325695, EPI_ISL_2325696, EPI_ISL_2325697, EPI_ISL_2325698, EPI_ISL_2325699, EPI_ISL_2325700, EPI_ISL_2325701, EPI_ISL_2325702, EPI_ISL_2325703, EPI_ISL_2325704, EPI_ISL_2325705, EPI_ISL_2325706, EPI_ISL_2325707, EPI_ISL_2325708, EPI_ISL_2325709, EPI_ISL_2325710, EPI_ISL_2325711, EPI_ISL_2325712, EPI_ISL_2325713, EPI_ISL_2325714, EPI_ISL_2325715, EPI_ISL_2325716, EPI_ISL_2325717, EPI_ISL_2325718, EPI_ISL_2325719, EPI_ISL_2325720, EPI_ISL_2325721, EPI_ISL_2325722, EPI_ISL_2325723, EPI_ISL_2325724, EPI_ISL_2325725, EPI_ISL_2325726, EPI_ISL_2325727, EPI_ISL_2325728, EPI_ISL_2325729, EPI_ISL_2325730, EPI_ISL_2325731, EPI_ISL_2325732, EPI_ISL_2325733, EPI_ISL_2325734, EPI_ISL_2325735, EPI_ISL_2325736, EPI_ISL_2325737, EPI_ISL_2325738, EPI_ISL_2325739, EPI_ISL_2325740, EPI_ISL_2325741, EPI_ISL_2325742, EPI_ISL_2325743, EPI_ISL_2325744, EPI_ISL_2325745, EPI_ISL_2325746, EPI_ISL_2325747, EPI_ISL_2325748, EPI_ISL_2325749, EPI_ISL_2325750, EPI_ISL_2325751, EPI_ISL_2325752, EPI_ISL_2325753, EPI_ISL_2325754, EPI_ISL_2325755, EPI_ISL_2325756, EPI_ISL_2325757, EPI_ISL_2325758, EPI_ISL_2325759, EPI_ISL_2325760, EPI_ISL_2325761, EPI_ISL_2325762, EPI_ISL_2325763, EPI_ISL_2325764, EPI_ISL_2325765, EPI_ISL_2325766, EPI_ISL_2325767, EPI_ISL_2325768, EPI_ISL_2325769, EPI_ISL_2325770, EPI_ISL_2325771, EPI_ISL_2325772, EPI_ISL_2325773, EPI_ISL_2325774, EPI_ISL_2325775, EPI_ISL_2325776, EPI_ISL_2325777, EPI_ISL_2325778, EPI_ISL_2325779, EPI_ISL_2325780, EPI_ISL_2325781, EPI_ISL_2325782, EPI_ISL_2325783, EPI_ISL_2325784, EPI_ISL_2325785, EPI_ISL_2325786, EPI_ISL_2325787, EPI_ISL_2325788, EPI_ISL_2325789, EPI_ISL_2325790, EPI_ISL_2325791, EPI_ISL_2325792, EPI_ISL_2325793, EPI_ISL_2325794, EPI_ISL_2325795, EPI_ISL_2325796, EPI_ISL_2325797, EPI_ISL_2325798, EPI_ISL_2325799, EPI_ISL_2325800, EPI_ISL_2325801, EPI_ISL_2325802, EPI_ISL_2325803, EPI_ISL_2325804, EPI_ISL_2325805, EPI_ISL_2325806, EPI_ISL_2325807, EPI_ISL_2325808, EPI_ISL_2325809, EPI_ISL_2325810, EPI_ISL_2325811, EPI_ISL_2325812, EPI_ISL_2325813, EPI_ISL_2325814, EPI_ISL_2325815, EPI_ISL_2325816, EPI_ISL_2325817, EPI_ISL_2325818, EPI_ISL_2325819, EPI_ISL_2325820, EPI_ISL_2325821, EPI_ISL_2325822, EPI_ISL_2325823, EPI_ISL_2325824, EPI_ISL_2325825, EPI_ISL_2325826, EPI_ISL_2325827, EPI_ISL_2325828, EPI_ISL_2325829, EPI_ISL_2325830, EPI_ISL_2325831, EPI_ISL_2325832, EPI_ISL_2325833, EPI_ISL_2325834, EPI_ISL_2325835, EPI_ISL_2325836, EPI_ISL_2325837, EPI_ISL_2325838, EPI_ISL_2325839, EPI_ISL_2325840, EPI_ISL_2325841, EPI_ISL_2325842, EPI_ISL_2325843, EPI_ISL_2325844, EPI_ISL_2325845, EPI_ISL_2325846, EPI_ISL_2325847, EPI_ISL_2325848, EPI_ISL_2325849, EPI_ISL_2325850, EPI_ISL_2325851, EPI_ISL_2325852, EPI_ISL_2325853, EPI_ISL_2325854, EPI_ISL_2325855, EPI_ISL_2325856, EPI_ISL_2325857, EPI_ISL_2325858, EPI_ISL_2325859, EPI_ISL_2325860, EPI_ISL_2325861, EPI_ISL_2325862, EPI_ISL_2325863, EPI_ISL_2325864, EPI_ISL_2325865, EPI_ISL_2325866, EPI_ISL_2325867, EPI_ISL_2325868, EPI_ISL_2325869, EPI_ISL_2325870, EPI_ISL_2325871, EPI_ISL_2325872, EPI_ISL_2325873, EPI_ISL_2325874, EPI_ISL_2325875, EPI_ISL_2325876, EPI_ISL_2325877, EPI_ISL_2325878, EPI_ISL_2325879, EPI_ISL_2325880, EPI_ISL_2325881, EPI_ISL_2325882, EPI_ISL_2325883, EPI_ISL_2325884, EPI_ISL_2325885, EPI_ISL_2325886, EPI_ISL_2325887, EPI_ISL_2325888, EPI_ISL_2325889, EPI_ISL_2325890, EPI_ISL_2325891, EPI_ISL_2325892, EPI_ISL_2325893, EPI_ISL_2325894, EPI_ISL_2325895, EPI_ISL_2325896, EPI_ISL_2325897, EPI_ISL_2325898, EPI_ISL_2325899, EPI_ISL_2325900, EPI_ISL_2325901, EPI_ISL_2325902, EPI_ISL_2325903, EPI_ISL_2325904, EPI_ISL_2325905, EPI_ISL_2325906, EPI_ISL_2325907, EPI_ISL_2325908, EPI_ISL_2325909, EPI_ISL_2325910, EPI_ISL_2325911, EPI_ISL_2325912, EPI_ISL_2325913, EPI_ISL_2325914, EPI_ISL_2325915, EPI_ISL_2325916, EPI_ISL_2325917, EPI_ISL_2325918, EPI_ISL_2325919, EPI_ISL_2325920, EPI_ISL_2325921, EPI_ISL_2325922, EPI_ISL_2325923, EPI_ISL_2325924, EPI_ISL_2325925, EPI_ISL_2325926, EPI_ISL_2325927, EPI_ISL_2325928, EPI_ISL_2325929, EPI_ISL_2325930, EPI_ISL_2325931, EPI_ISL_2325932, EPI_ISL_2325933, EPI_ISL_2325934, EPI_ISL_2325935, EPI_ISL_2325936, EPI_ISL_2325937, EPI_ISL_2325938, EPI_ISL_2325939, EPI_ISL_2325940, EPI_ISL_2325941, EPI_ISL_2325942, EPI_ISL_2325943, EPI_ISL_2325944, EPI_ISL_2325945, EPI_ISL_2325946, EPI_ISL_2325947, EPI_ISL_2325948, EPI_ISL_2325949, EPI_ISL_2325950, EPI_ISL_2325951, EPI_ISL_2325952, EPI_ISL_2325953, EPI_ISL_2325954, EPI_ISL_2325955, EPI_ISL_2325956, EPI_ISL_2325957, EPI_ISL_2325958, EPI_ISL_2325959, EPI_ISL_2325960, EPI_ISL_2325961, EPI_ISL_2325962, EPI_ISL_2325963, EPI_ISL_2325964, EPI_ISL_2325965, EPI_ISL_2325966, EPI_ISL_2325967, EPI_ISL_2325968, EPI_ISL_2325969, EPI_ISL_2325970, EPI_ISL_2325971, EPI_ISL_2325972, EPI_ISL_2325973, EPI_ISL_2325974, EPI_ISL_2325975, EPI_ISL_2325976, EPI_ISL_2325977, EPI_ISL_2325978, EPI_ISL_2325979, EPI_ISL_2325980, EPI_ISL_2325981, EPI_ISL_2325982, EPI_ISL_2325983, EPI_ISL_2325984, EPI_ISL_2325985, EPI_ISL_2325986, EPI_ISL_2325987, EPI_ISL_2325988, EPI_ISL_2325989, EPI_ISL_2325990, EPI_ISL_2325991, EPI_ISL_2325992, EPI_ISL_2325993, EPI_ISL_2325994, EPI_ISL_2325995, EPI_ISL_2325996, EPI_ISL_2325997, EPI_ISL_2326210, EPI_ISL_2326214, EPI_ISL_2326215, EPI_ISL_2326332, EPI_ISL_2326333, EPI_ISL_2326334, EPI_ISL_2326335, EPI_ISL_2326336, EPI_ISL_2326337, EPI_ISL_2326338, EPI_ISL_2326339, EPI_ISL_2326340, EPI_ISL_2326364, EPI_ISL_2326365, EPI_ISL_2326366, EPI_ISL_2326367, EPI_ISL_2326368, EPI_ISL_2326369, EPI_ISL_2326370, EPI_ISL_2326371, EPI_ISL_2326372, EPI_ISL_2326373, EPI_ISL_2326374, EPI_ISL_2326375, EPI_ISL_2326376, EPI_ISL_2326377, EPI_ISL_2326378, EPI_ISL_2326379, EPI_ISL_2326380, EPI_ISL_2326381, EPI_ISL_2326382, EPI_ISL_2326383, EPI_ISL_2326384, EPI_ISL_2326385, EPI_ISL_2326386, EPI_ISL_2326387, EPI_ISL_2326389, EPI_ISL_2326390, EPI_ISL_2326391, EPI_ISL_2326392, EPI_ISL_2326405, EPI_ISL_2326406, EPI_ISL_2326407, EPI_ISL_2326408, EPI_ISL_2326409, EPI_ISL_2326410, EPI_ISL_2326411, EPI_ISL_2326412, EPI_ISL_2326413, EPI_ISL_2326414, EPI_ISL_2326415, EPI_ISL_2326416, EPI_ISL_2326417, EPI_ISL_2326418, EPI_ISL_2326419, EPI_ISL_2326420, EPI_ISL_2326421, EPI_ISL_2326422, EPI_ISL_2326423, EPI_ISL_2326424, EPI_ISL_2326425, EPI_ISL_2326426, EPI_ISL_2326427, EPI_ISL_2326428, EPI_ISL_2326429, EPI_ISL_2326430, EPI_ISL_2326431, EPI_ISL_2326432, EPI_ISL_2326433, EPI_ISL_2326434, EPI_ISL_2326435, EPI_ISL_2326436, EPI_ISL_2326437, EPI_ISL_2326438, EPI_ISL_2326439, EPI_ISL_2326440, EPI_ISL_2326441, EPI_ISL_2326442, EPI_ISL_2326443, EPI_ISL_2326444, EPI_ISL_2326445, EPI_ISL_2326446, EPI_ISL_2326447, EPI_ISL_2326448, EPI_ISL_2326449, EPI_ISL_2326450, EPI_ISL_2326451, EPI_ISL_2326452, EPI_ISL_2326453, EPI_ISL_2326454, EPI_ISL_2326455, EPI_ISL_2326456, EPI_ISL_2326457, EPI_ISL_2326458, EPI_ISL_2326459, EPI_ISL_2326460, EPI_ISL_2326461, EPI_ISL_2326462, EPI_ISL_2326463, EPI_ISL_2326695, EPI_ISL_2326696, EPI_ISL_2326697, EPI_ISL_2326698, EPI_ISL_2326699, EPI_ISL_2326700, EPI_ISL_2326701, EPI_ISL_2326702, EPI_ISL_2326703, EPI_ISL_2326704, EPI_ISL_2326705, EPI_ISL_2326706, EPI_ISL_2326707, EPI_ISL_2326708, EPI_ISL_2326709, EPI_ISL_2326710, EPI_ISL_2326711, EPI_ISL_2326712, EPI_ISL_2326713, EPI_ISL_2326714, EPI_ISL_2326715, EPI_ISL_2326716, EPI_ISL_2326717, EPI_ISL_2326718, EPI_ISL_2326719, EPI_ISL_2326720, EPI_ISL_2326721, EPI_ISL_2326722, EPI_ISL_2326723, EPI_ISL_2326724, EPI_ISL_2326725, EPI_ISL_2326726, EPI_ISL_2326727, EPI_ISL_2326728, EPI_ISL_2326729, EPI_ISL_2326730, EPI_ISL_2326731, EPI_ISL_2326732, EPI_ISL_2326733, EPI_ISL_2326734, EPI_ISL_2326735, EPI_ISL_2326736, EPI_ISL_2326737, EPI_ISL_2326738, EPI_ISL_2326739, EPI_ISL_2326740, EPI_ISL_2326741, EPI_ISL_2326742, EPI_ISL_2326743, EPI_ISL_2326744, EPI_ISL_2326745, EPI_ISL_2326746, EPI_ISL_2326747, EPI_ISL_2326748, EPI_ISL_2326749, EPI_ISL_2326750, EPI_ISL_2326751, EPI_ISL_2326752, EPI_ISL_2326753, EPI_ISL_2326754, EPI_ISL_2326755, EPI_ISL_2326756, EPI_ISL_2326757, EPI_ISL_2326758, EPI_ISL_2326759, EPI_ISL_2326760, EPI_ISL_2326761, EPI_ISL_2326762, EPI_ISL_2326763, EPI_ISL_2326764, EPI_ISL_2326765, EPI_ISL_2326766, EPI_ISL_2326767, EPI_ISL_2326768, EPI_ISL_2326769, EPI_ISL_2326770, EPI_ISL_2326771, EPI_ISL_2326772, EPI_ISL_2326773, EPI_ISL_2326774, EPI_ISL_2326775, EPI_ISL_2326776, EPI_ISL_2326777, EPI_ISL_2326778, EPI_ISL_2326779, EPI_ISL_2326780, EPI_ISL_2326781, EPI_ISL_2326782, EPI_ISL_2326783, EPI_ISL_2326784, EPI_ISL_2326785, EPI_ISL_2326786, EPI_ISL_2326787, EPI_ISL_2326788, EPI_ISL_2326789, EPI_ISL_2326790, EPI_ISL_2326791, EPI_ISL_2326792, EPI_ISL_2326793, EPI_ISL_2326794, EPI_ISL_2326795, EPI_ISL_2326796, EPI_ISL_2326797, EPI_ISL_2326798, EPI_ISL_2326799, EPI_ISL_2326800, EPI_ISL_2326801, EPI_ISL_2326802, EPI_ISL_2326803, EPI_ISL_2326804, EPI_ISL_2326805, EPI_ISL_2326806, EPI_ISL_2326807, EPI_ISL_2326808, EPI_ISL_2326809, EPI_ISL_2326810, EPI_ISL_2326811, EPI_ISL_2326812, EPI_ISL_2326813, EPI_ISL_2326814, EPI_ISL_2326815, EPI_ISL_2326816, EPI_ISL_2326817, EPI_ISL_2326818, EPI_ISL_2326819, EPI_ISL_2326820, EPI_ISL_2326821, EPI_ISL_2326822, EPI_ISL_2326823, EPI_ISL_2326824, EPI_ISL_2326825, EPI_ISL_2326826, EPI_ISL_2326827, EPI_ISL_2326828, EPI_ISL_2326829, EPI_ISL_2326830, EPI_ISL_2326831, EPI_ISL_2326832, EPI_ISL_2326833, EPI_ISL_2326834, EPI_ISL_2326835, EPI_ISL_2326836, EPI_ISL_2326837, EPI_ISL_2326838, EPI_ISL_2326839, EPI_ISL_2326840, EPI_ISL_2326841, EPI_ISL_2326842, EPI_ISL_2326843, EPI_ISL_2326844, EPI_ISL_2326845, EPI_ISL_2326846, EPI_ISL_2326847, EPI_ISL_2326848, EPI_ISL_2326849, EPI_ISL_2326850, EPI_ISL_2326851, EPI_ISL_2326852, EPI_ISL_2326853, EPI_ISL_2326854, EPI_ISL_2326855, EPI_ISL_2326856, EPI_ISL_2326862, EPI_ISL_2327088, EPI_ISL_2327089, EPI_ISL_2327090, EPI_ISL_2327091, EPI_ISL_2327092, EPI_ISL_2327093, EPI_ISL_2327094, EPI_ISL_2327095, EPI_ISL_2327096, EPI_ISL_2327097, EPI_ISL_2327098, EPI_ISL_2327099, EPI_ISL_2327100, EPI_ISL_2327101, EPI_ISL_2327102, EPI_ISL_2327103, EPI_ISL_2327104, EPI_ISL_2327105, EPI_ISL_2327106, EPI_ISL_2327107, EPI_ISL_2327108, EPI_ISL_2327109, EPI_ISL_2327110, EPI_ISL_2327111, EPI_ISL_2327112, EPI_ISL_2327113, EPI_ISL_2327114, EPI_ISL_2327115, EPI_ISL_2327116, EPI_ISL_2327117, EPI_ISL_2327118, EPI_ISL_2327119, EPI_ISL_2327120, EPI_ISL_2327121, EPI_ISL_2327122, EPI_ISL_2327123, EPI_ISL_2327124, EPI_ISL_2327125, EPI_ISL_2327126, EPI_ISL_2327127, EPI_ISL_2327128, EPI_ISL_2327129, EPI_ISL_2327130, EPI_ISL_2327131, EPI_ISL_2327132, EPI_ISL_2327133, EPI_ISL_2327134, EPI_ISL_2327135, EPI_ISL_2327136, EPI_ISL_2327137, EPI_ISL_2327138, EPI_ISL_2327139, EPI_ISL_2327140, EPI_ISL_2327141, EPI_ISL_2327142, EPI_ISL_2327143, EPI_ISL_2327144, EPI_ISL_2327145, EPI_ISL_2327146, EPI_ISL_2327147, EPI_ISL_2327148, EPI_ISL_2327149, EPI_ISL_2327150, EPI_ISL_2327151, EPI_ISL_2327152, EPI_ISL_2327153, EPI_ISL_2327154, EPI_ISL_2327155, EPI_ISL_2327156, EPI_ISL_2327157, EPI_ISL_2327158, EPI_ISL_2327159, EPI_ISL_2327160, EPI_ISL_2327161, EPI_ISL_2327162, EPI_ISL_2327163, EPI_ISL_2327164, EPI_ISL_2327165, EPI_ISL_2327166, EPI_ISL_2327167, EPI_ISL_2327168, EPI_ISL_2327169, EPI_ISL_2327170, EPI_ISL_2327171, EPI_ISL_2327172, EPI_ISL_2327173, EPI_ISL_2327174, EPI_ISL_2327175, EPI_ISL_2327176, EPI_ISL_2327177, EPI_ISL_2327178, EPI_ISL_2327179, EPI_ISL_2327180, EPI_ISL_2327476, EPI_ISL_2327477, EPI_ISL_2327478, EPI_ISL_2327479, EPI_ISL_2327480, EPI_ISL_2327481, EPI_ISL_2327482, EPI_ISL_2327483, EPI_ISL_2327484, EPI_ISL_2327485, EPI_ISL_2327486, EPI_ISL_2327487, EPI_ISL_2327488, EPI_ISL_2327489, EPI_ISL_2327490, EPI_ISL_2327491, EPI_ISL_2327492, EPI_ISL_2327493, EPI_ISL_2327494, EPI_ISL_2327495, EPI_ISL_2327496, EPI_ISL_2327497, EPI_ISL_2327498, EPI_ISL_2327499, EPI_ISL_2327500, EPI_ISL_2327501, EPI_ISL_2327502, EPI_ISL_2327503, EPI_ISL_2327504, EPI_ISL_2327505, EPI_ISL_2327506, EPI_ISL_2327507, EPI_ISL_2327508, EPI_ISL_2327509, EPI_ISL_2327510, EPI_ISL_2327511, EPI_ISL_2327512, EPI_ISL_2327513, EPI_ISL_2327514, EPI_ISL_2327515, EPI_ISL_2327516, EPI_ISL_2327517, EPI_ISL_2327518, EPI_ISL_2327519, EPI_ISL_2327520, EPI_ISL_2327521, EPI_ISL_2327522, EPI_ISL_2327523, EPI_ISL_2327524, EPI_ISL_2327525, EPI_ISL_2327526, EPI_ISL_2327527, EPI_ISL_2327528, EPI_ISL_2327529, EPI_ISL_2327530, EPI_ISL_2327531, EPI_ISL_2327532, EPI_ISL_2327533, EPI_ISL_2327534, EPI_ISL_2327535, EPI_ISL_2327536, EPI_ISL_2327537, EPI_ISL_2327538, EPI_ISL_2327539, EPI_ISL_2327540, EPI_ISL_2327541, EPI_ISL_2327542, EPI_ISL_2327543, EPI_ISL_2327544, EPI_ISL_2327545, EPI_ISL_2327546, EPI_ISL_2327547, EPI_ISL_2327548, EPI_ISL_2327549, EPI_ISL_2327550, EPI_ISL_2327551, EPI_ISL_2327552, EPI_ISL_2327553, EPI_ISL_2327554, EPI_ISL_2327555, EPI_ISL_2327556, EPI_ISL_2327557, EPI_ISL_2327558, EPI_ISL_2327559, EPI_ISL_2327560, EPI_ISL_2327561, EPI_ISL_2327562, EPI_ISL_2327563, EPI_ISL_2327564, EPI_ISL_2327565, EPI_ISL_2327566, EPI_ISL_2327567, EPI_ISL_2327568, EPI_ISL_2327569, EPI_ISL_2327570, EPI_ISL_2327571, EPI_ISL_2327572, EPI_ISL_2327573, EPI_ISL_2327574, EPI_ISL_2327575, EPI_ISL_2327576, EPI_ISL_2327577, EPI_ISL_2327578, EPI_ISL_2327579, EPI_ISL_2327580, EPI_ISL_2327581, EPI_ISL_2327582, EPI_ISL_2327583, EPI_ISL_2327584, EPI_ISL_2327585, EPI_ISL_2327586, EPI_ISL_2327587, EPI_ISL_2327588, EPI_ISL_2327589, EPI_ISL_2327590, EPI_ISL_2327591, EPI_ISL_2327592, EPI_ISL_2327593, EPI_ISL_2327594, EPI_ISL_2327595, EPI_ISL_2327596, EPI_ISL_2327597, EPI_ISL_2327598, EPI_ISL_2327599, EPI_ISL_2327600, EPI_ISL_2327601, EPI_ISL_2327602, EPI_ISL_2327603, EPI_ISL_2327604, EPI_ISL_2327605, EPI_ISL_2327606, EPI_ISL_2327607, EPI_ISL_2327608, EPI_ISL_2327609, EPI_ISL_2327610, EPI_ISL_2327611, EPI_ISL_2327612, EPI_ISL_2327613, EPI_ISL_2327614, EPI_ISL_2327615, EPI_ISL_2327616, EPI_ISL_2327617, EPI_ISL_2327618, EPI_ISL_2327619, EPI_ISL_2327620, EPI_ISL_2327621, EPI_ISL_2327622, EPI_ISL_2327623, EPI_ISL_2327624, EPI_ISL_2327625, EPI_ISL_2327626, EPI_ISL_2327627, EPI_ISL_2327628, EPI_ISL_2327629, EPI_ISL_2327630, EPI_ISL_2327631, EPI_ISL_2327632, EPI_ISL_2327633, EPI_ISL_2327634, EPI_ISL_2327635, EPI_ISL_2327636, EPI_ISL_2327637, EPI_ISL_2327638, EPI_ISL_2327639, EPI_ISL_2327640, EPI_ISL_2327641, EPI_ISL_2327642, EPI_ISL_2327643, EPI_ISL_2327644, EPI_ISL_2327645, EPI_ISL_2327646, EPI_ISL_2327647, EPI_ISL_2327648, EPI_ISL_2327649, EPI_ISL_2327650, EPI_ISL_2327651, EPI_ISL_2327652, EPI_ISL_2327653, EPI_ISL_2327654, EPI_ISL_2327655, EPI_ISL_2327656, EPI_ISL_2327657, EPI_ISL_2327658, EPI_ISL_2327659, EPI_ISL_2327660, EPI_ISL_2327661, EPI_ISL_2327662, EPI_ISL_2327663, EPI_ISL_2327664, EPI_ISL_2327665, EPI_ISL_2327666, EPI_ISL_2327667, EPI_ISL_2327668, EPI_ISL_2327669, EPI_ISL_2327670, EPI_ISL_2327671, EPI_ISL_2327672, EPI_ISL_2327673, EPI_ISL_2327674, EPI_ISL_2327675, EPI_ISL_2327676, EPI_ISL_2327677, EPI_ISL_2327678, EPI_ISL_2327679, EPI_ISL_2327680, EPI_ISL_2327681, EPI_ISL_2327682, EPI_ISL_2327683, EPI_ISL_2327684, EPI_ISL_2327685, EPI_ISL_2327686, EPI_ISL_2327687, EPI_ISL_2327688, EPI_ISL_2327689, EPI_ISL_2327690, EPI_ISL_2327691, EPI_ISL_2327692, EPI_ISL_2327693, EPI_ISL_2327694, EPI_ISL_2327695, EPI_ISL_2327696, EPI_ISL_2327697, EPI_ISL_2327698, EPI_ISL_2327699, EPI_ISL_2327700, EPI_ISL_2327701, EPI_ISL_2327702, EPI_ISL_2327703, EPI_ISL_2327704, EPI_ISL_2327705, EPI_ISL_2327706, EPI_ISL_2327707, EPI_ISL_2327708, EPI_ISL_2327709, EPI_ISL_2327710, EPI_ISL_2327711, EPI_ISL_2327712, EPI_ISL_2327713, EPI_ISL_2327714, EPI_ISL_2327715, EPI_ISL_2327716, EPI_ISL_2327717, EPI_ISL_2327718, EPI_ISL_2327719, EPI_ISL_2327720, EPI_ISL_2327721, EPI_ISL_2327722, EPI_ISL_2327723, EPI_ISL_2327724, EPI_ISL_2327725, EPI_ISL_2327726, EPI_ISL_2327727, EPI_ISL_2327728, EPI_ISL_2327729, EPI_ISL_2327730, EPI_ISL_2327731, EPI_ISL_2327732, EPI_ISL_2327733, EPI_ISL_2327734, EPI_ISL_2327735, EPI_ISL_2327736, EPI_ISL_2327737, EPI_ISL_2327738, EPI_ISL_2327739, EPI_ISL_2327740, EPI_ISL_2327741, EPI_ISL_2327742, EPI_ISL_2327743, EPI_ISL_2327744, EPI_ISL_2327745, EPI_ISL_2327746, EPI_ISL_2327747, EPI_ISL_2327748, EPI_ISL_2327749, EPI_ISL_2327750, EPI_ISL_2327751, EPI_ISL_2327752, EPI_ISL_2327753, EPI_ISL_2327754, EPI_ISL_2327755, EPI_ISL_2327756, EPI_ISL_2327757, EPI_ISL_2327758, EPI_ISL_2327759, EPI_ISL_2327760, EPI_ISL_2327761, EPI_ISL_2327762, EPI_ISL_2327763, EPI_ISL_2327764, EPI_ISL_2327765, EPI_ISL_2327766, EPI_ISL_2327767, EPI_ISL_2327768, EPI_ISL_2327769, EPI_ISL_2327770, EPI_ISL_2327771, EPI_ISL_2327772, EPI_ISL_2327773, EPI_ISL_2327774, EPI_ISL_2327775, EPI_ISL_2327776, EPI_ISL_2327777, EPI_ISL_2327778, EPI_ISL_2327779, EPI_ISL_2327780, EPI_ISL_2327781, EPI_ISL_2327782, EPI_ISL_2327783, EPI_ISL_2327784, EPI_ISL_2327785, EPI_ISL_2327786, EPI_ISL_2327787, EPI_ISL_2327788, EPI_ISL_2327789, EPI_ISL_2327790, EPI_ISL_2327791, EPI_ISL_2327792, EPI_ISL_2327793, EPI_ISL_2327794, EPI_ISL_2327795, EPI_ISL_2327796, EPI_ISL_2327797, EPI_ISL_2327798, EPI_ISL_2327799, EPI_ISL_2327800, EPI_ISL_2327801, EPI_ISL_2327802, EPI_ISL_2327803, EPI_ISL_2327804, EPI_ISL_2327805, EPI_ISL_2327806, EPI_ISL_2327807, EPI_ISL_2327808, EPI_ISL_2327809, EPI_ISL_2327810, EPI_ISL_2327811, EPI_ISL_2327812, EPI_ISL_2327813, EPI_ISL_2327814, EPI_ISL_2327815, EPI_ISL_2327816, EPI_ISL_2327817, EPI_ISL_2327818, EPI_ISL_2327819, EPI_ISL_2327820, EPI_ISL_2327821, EPI_ISL_2327822, EPI_ISL_2327823, EPI_ISL_2327824, EPI_ISL_2327825, EPI_ISL_2327826, EPI_ISL_2327827, EPI_ISL_2327828, EPI_ISL_2327829, EPI_ISL_2327830, EPI_ISL_2327831, EPI_ISL_2327832, EPI_ISL_2327833, EPI_ISL_2327834, EPI_ISL_2327835, EPI_ISL_2327836, EPI_ISL_2327837, EPI_ISL_2327838, EPI_ISL_2327839, EPI_ISL_2327840, EPI_ISL_2327841, EPI_ISL_2327842, EPI_ISL_2327843, EPI_ISL_2327844, EPI_ISL_2327845, EPI_ISL_2327846, EPI_ISL_2327847, EPI_ISL_2327848, EPI_ISL_2327849, EPI_ISL_2327850, EPI_ISL_2327851, EPI_ISL_2327852, EPI_ISL_2327853, EPI_ISL_2327854, EPI_ISL_2327855, EPI_ISL_2327856, EPI_ISL_2327857, EPI_ISL_2327858, EPI_ISL_2327859, EPI_ISL_2327860, EPI_ISL_2327861, EPI_ISL_2327862, EPI_ISL_2327863, EPI_ISL_2327864, EPI_ISL_2327865, EPI_ISL_2327866, EPI_ISL_2327867, EPI_ISL_2327868, EPI_ISL_2327869, EPI_ISL_2327870, EPI_ISL_2327871, EPI_ISL_2327872, EPI_ISL_2327873, EPI_ISL_2327874, EPI_ISL_2327875, EPI_ISL_2327876, EPI_ISL_2327877, EPI_ISL_2327878, EPI_ISL_2327879, EPI_ISL_2327880, EPI_ISL_2327881, EPI_ISL_2327882, EPI_ISL_2327883, EPI_ISL_2327884, EPI_ISL_2327885, EPI_ISL_2327886, EPI_ISL_2327887, EPI_ISL_2327888, EPI_ISL_2327889, EPI_ISL_2327890, EPI_ISL_2327891, EPI_ISL_2327892, EPI_ISL_2327893, EPI_ISL_2327894, EPI_ISL_2327895, EPI_ISL_2327896, EPI_ISL_2327897, EPI_ISL_2327898, EPI_ISL_2327899, EPI_ISL_2327900, EPI_ISL_2327901, EPI_ISL_2327902, EPI_ISL_2327903, EPI_ISL_2327904, EPI_ISL_2327905, EPI_ISL_2327906, EPI_ISL_2327907, EPI_ISL_2327908, EPI_ISL_2327909, EPI_ISL_2327910, EPI_ISL_2327911, EPI_ISL_2327912, EPI_ISL_2327913, EPI_ISL_2327914, EPI_ISL_2327915, EPI_ISL_2327916, EPI_ISL_2327917, EPI_ISL_2327918, EPI_ISL_2327919, EPI_ISL_2327920, EPI_ISL_2327921, EPI_ISL_2327922, EPI_ISL_2327923, EPI_ISL_2327924, EPI_ISL_2327925, EPI_ISL_2327926, EPI_ISL_2327927, EPI_ISL_2327928, EPI_ISL_2327929, EPI_ISL_2327930, EPI_ISL_2327931, EPI_ISL_2327932, EPI_ISL_2327933, EPI_ISL_2327934, EPI_ISL_2327935, EPI_ISL_2327936, EPI_ISL_2327937, EPI_ISL_2327938, EPI_ISL_2327939, EPI_ISL_2327940, EPI_ISL_2327941, EPI_ISL_2327942, EPI_ISL_2327943, EPI_ISL_2327944, EPI_ISL_2327945, EPI_ISL_2327946, EPI_ISL_2327947, EPI_ISL_2327948, EPI_ISL_2327949, EPI_ISL_2327950, EPI_ISL_2327951, EPI_ISL_2327952, EPI_ISL_2327953, EPI_ISL_2327954, EPI_ISL_2327955, EPI_ISL_2327956, EPI_ISL_2327957, EPI_ISL_2327958, EPI_ISL_2327959, EPI_ISL_2327960, EPI_ISL_2327961, EPI_ISL_2327962, EPI_ISL_2327963, EPI_ISL_2327964, EPI_ISL_2327965, EPI_ISL_2327966, EPI_ISL_2327967, EPI_ISL_2327968, EPI_ISL_2327969, EPI_ISL_2327970, EPI_ISL_2327971, EPI_ISL_2327972, EPI_ISL_2327973, EPI_ISL_2327974, EPI_ISL_2327975, EPI_ISL_2327976, EPI_ISL_2327977, EPI_ISL_2327978, EPI_ISL_2327979, EPI_ISL_2327980, EPI_ISL_2327981, EPI_ISL_2327982, EPI_ISL_2327983, EPI_ISL_2327984, EPI_ISL_2327985, EPI_ISL_2327986, EPI_ISL_2327987, EPI_ISL_2327988, EPI_ISL_2327989, EPI_ISL_2327990, EPI_ISL_2327991, EPI_ISL_2327992, EPI_ISL_2327993, EPI_ISL_2327994, EPI_ISL_2327995, EPI_ISL_2327996, EPI_ISL_2327997, EPI_ISL_2327998, EPI_ISL_2327999, EPI_ISL_2328000, EPI_ISL_2328001, EPI_ISL_2328002, EPI_ISL_2328003, EPI_ISL_2328004, EPI_ISL_2328005, EPI_ISL_2328006, EPI_ISL_2328007, EPI_ISL_2328008, EPI_ISL_2328009, EPI_ISL_2328010, EPI_ISL_2328011, EPI_ISL_2328012, EPI_ISL_2328013, EPI_ISL_2328014, EPI_ISL_2328015, EPI_ISL_2328016, EPI_ISL_2328017, EPI_ISL_2328018, EPI_ISL_2328019, EPI_ISL_2328020, EPI_ISL_2328021, EPI_ISL_2328022, EPI_ISL_2328023, EPI_ISL_2328024, EPI_ISL_2328025, EPI_ISL_2328026, EPI_ISL_2328027, EPI_ISL_2328028, EPI_ISL_2328029, EPI_ISL_2328030, EPI_ISL_2328031, EPI_ISL_2328032, EPI_ISL_2328033, EPI_ISL_2328034, EPI_ISL_2328035, EPI_ISL_2328036, EPI_ISL_2328037, EPI_ISL_2328038, EPI_ISL_2328039, EPI_ISL_2328040, EPI_ISL_2328041, EPI_ISL_2328042, EPI_ISL_2328043, EPI_ISL_2328044, EPI_ISL_2328045, EPI_ISL_2328046, EPI_ISL_2328047, EPI_ISL_2328048, EPI_ISL_2328049, EPI_ISL_2328050, EPI_ISL_2328051, EPI_ISL_2328052, EPI_ISL_2328053, EPI_ISL_2328054, EPI_ISL_2328055, EPI_ISL_2328056, EPI_ISL_2328057, EPI_ISL_2328058, EPI_ISL_2328059, EPI_ISL_2328060, EPI_ISL_2328061, EPI_ISL_2328062, EPI_ISL_2328063, EPI_ISL_2328064, EPI_ISL_2328065, EPI_ISL_2328066, EPI_ISL_2328067, EPI_ISL_2328068, EPI_ISL_2328069, EPI_ISL_2328070, EPI_ISL_2328071, EPI_ISL_2328072, EPI_ISL_2328073, EPI_ISL_2328074, EPI_ISL_2328075, EPI_ISL_2328076, EPI_ISL_2328077, EPI_ISL_2328078, EPI_ISL_2328079, EPI_ISL_2328080, EPI_ISL_2328081, EPI_ISL_2328082, EPI_ISL_2328083, EPI_ISL_2328084, EPI_ISL_2328085, EPI_ISL_2328086, EPI_ISL_2328087, EPI_ISL_2328088, EPI_ISL_2328089, EPI_ISL_2328090, EPI_ISL_2328091, EPI_ISL_2328092, EPI_ISL_2328093, EPI_ISL_2328094, EPI_ISL_2328095, EPI_ISL_2328096, EPI_ISL_2328097, EPI_ISL_2328098, EPI_ISL_2328099, EPI_ISL_2328100, EPI_ISL_2328101, EPI_ISL_2328102, EPI_ISL_2328103, EPI_ISL_2328104, EPI_ISL_2328105, EPI_ISL_2328106, EPI_ISL_2328107,			

EPI_ISL_2328108, EPI_ISL_2328109, EPI_ISL_2328110, EPI_ISL_2328111, EPI_ISL_2328112, EPI_ISL_2328113, EPI_ISL_2328114, EPI_ISL_2328115, EPI_ISL_2328116, EPI_ISL_2328117, EPI_ISL_2328118, EPI_ISL_2328119, EPI_ISL_2328120, EPI_ISL_2328121, EPI_ISL_2328122, EPI_ISL_2328123, EPI_ISL_2328124, EPI_ISL_2328125, EPI_ISL_2328126, EPI_ISL_2328127, EPI_ISL_2328128, EPI_ISL_2328129,
EPI_ISL_2328130, EPI_ISL_2328131, EPI_ISL_2328132, EPI_ISL_2328133, EPI_ISL_2328134, EPI_ISL_2328135, EPI_ISL_2328136, EPI_ISL_2328137, EPI_ISL_2328138, EPI_ISL_2328139, EPI_ISL_2328140, EPI_ISL_2328141, EPI_ISL_2328142, EPI_ISL_2328143, EPI_ISL_2328144, EPI_ISL_2328145, EPI_ISL_2328146, EPI_ISL_2328147, EPI_ISL_2328148, EPI_ISL_2328149, EPI_ISL_2328150, EPI_ISL_2328151,
EPI_ISL_2328152, EPI_ISL_2328153, EPI_ISL_2328154, EPI_ISL_2328155, EPI_ISL_2328156, EPI_ISL_2328157, EPI_ISL_2328158, EPI_ISL_2328159, EPI_ISL_2328160, EPI_ISL_2328161, EPI_ISL_2328162, EPI_ISL_2328163, EPI_ISL_2328164, EPI_ISL_2328165, EPI_ISL_2328166, EPI_ISL_2328167, EPI_ISL_2328168, EPI_ISL_2328169, EPI_ISL_2328170, EPI_ISL_2328171, EPI_ISL_2328172, EPI_ISL_2328173,
EPI_ISL_2328174, EPI_ISL_2328175, EPI_ISL_2328176, EPI_ISL_2328177, EPI_ISL_2328178, EPI_ISL_2328179, EPI_ISL_2328180, EPI_ISL_2328181, EPI_ISL_2328182, EPI_ISL_2328183, EPI_ISL_2328184, EPI_ISL_2328185, EPI_ISL_2328186, EPI_ISL_2328187, EPI_ISL_2328188, EPI_ISL_2328189, EPI_ISL_2328190, EPI_ISL_2328191, EPI_ISL_2328192, EPI_ISL_2328193, EPI_ISL_2328194, EPI_ISL_2328195,
EPI_ISL_2328196, EPI_ISL_2328197, EPI_ISL_2328198, EPI_ISL_2328199, EPI_ISL_2328200, EPI_ISL_2328201, EPI_ISL_2328202, EPI_ISL_2328203, EPI_ISL_2328204, EPI_ISL_2328205, EPI_ISL_2328206, EPI_ISL_2328207, EPI_ISL_2328208, EPI_ISL_2328209, EPI_ISL_2328210, EPI_ISL_2328211, EPI_ISL_2328212, EPI_ISL_2328213, EPI_ISL_2328214, EPI_ISL_2328215, EPI_ISL_2328216, EPI_ISL_2328217,
EPI_ISL_2328218, EPI_ISL_2328219, EPI_ISL_2328220, EPI_ISL_2328221, EPI_ISL_2328222, EPI_ISL_2328223, EPI_ISL_2328224, EPI_ISL_2328225, EPI_ISL_2328226, EPI_ISL_2328227, EPI_ISL_2328228, EPI_ISL_2328229, EPI_ISL_2328230, EPI_ISL_2328231, EPI_ISL_2328232, EPI_ISL_2328233, EPI_ISL_2328234, EPI_ISL_2328235, EPI_ISL_2328236, EPI_ISL_2328237, EPI_ISL_2328238, EPI_ISL_2328239,
EPI_ISL_2328240, EPI_ISL_2328241, EPI_ISL_2328242, EPI_ISL_2328243, EPI_ISL_2328244, EPI_ISL_2328245, EPI_ISL_2328246, EPI_ISL_2328247, EPI_ISL_2328248, EPI_ISL_2328249, EPI_ISL_2328250, EPI_ISL_2328251, EPI_ISL_2328252, EPI_ISL_2328253, EPI_ISL_2328254, EPI_ISL_2328255, EPI_ISL_2328256, EPI_ISL_2328257, EPI_ISL_2328258, EPI_ISL_2328259, EPI_ISL_2328260, EPI_ISL_2328261,
EPI_ISL_2328262, EPI_ISL_2328263, EPI_ISL_2328264, EPI_ISL_2328265, EPI_ISL_2328266, EPI_ISL_2328267, EPI_ISL_2328268, EPI_ISL_2328269, EPI_ISL_2328270, EPI_ISL_2328271, EPI_ISL_2328272, EPI_ISL_2328273, EPI_ISL_2328274, EPI_ISL_2328275, EPI_ISL_2328276, EPI_ISL_2328277, EPI_ISL_2328278, EPI_ISL_2328279, EPI_ISL_2328280, EPI_ISL_2328281, EPI_ISL_2328282, EPI_ISL_2328283,
EPI_ISL_2328284, EPI_ISL_2328285, EPI_ISL_2328286, EPI_ISL_2328287, EPI_ISL_2328288, EPI_ISL_2328289, EPI_ISL_2328290, EPI_ISL_2328291, EPI_ISL_2328292, EPI_ISL_2328293, EPI_ISL_2328294, EPI_ISL_2328295, EPI_ISL_2328296, EPI_ISL_2328297, EPI_ISL_2328298, EPI_ISL_2328299, EPI_ISL_2328300, EPI_ISL_2328301, EPI_ISL_2328302, EPI_ISL_2328303, EPI_ISL_2328304, EPI_ISL_2328305,
EPI_ISL_2328306, EPI_ISL_2328307, EPI_ISL_2328308, EPI_ISL_2328309, EPI_ISL_2328310, EPI_ISL_2328311, EPI_ISL_2328312, EPI_ISL_2328313, EPI_ISL_2328314, EPI_ISL_2328315, EPI_ISL_2328316, EPI_ISL_2328317, EPI_ISL_2328318, EPI_ISL_2328319, EPI_ISL_2328320, EPI_ISL_2328321, EPI_ISL_2328322, EPI_ISL_2328323, EPI_ISL_2328324, EPI_ISL_2328325, EPI_ISL_2328326, EPI_ISL_2328327,
EPI_ISL_2328328, EPI_ISL_2328329, EPI_ISL_2328330, EPI_ISL_2328331, EPI_ISL_2328332, EPI_ISL_2328333, EPI_ISL_2328334, EPI_ISL_2328335, EPI_ISL_2328336, EPI_ISL_2328337, EPI_ISL_2328338, EPI_ISL_2328339, EPI_ISL_2328340, EPI_ISL_2328341, EPI_ISL_2328342, EPI_ISL_2328343, EPI_ISL_2328344, EPI_ISL_2328345, EPI_ISL_2328346, EPI_ISL_2328347, EPI_ISL_2328348, EPI_ISL_2328349,
EPI_ISL_2328350, EPI_ISL_2328351, EPI_ISL_2328352, EPI_ISL_2328353, EPI_ISL_2328354, EPI_ISL_2328355, EPI_ISL_2328356, EPI_ISL_2328357, EPI_ISL_2328358, EPI_ISL_2328359, EPI_ISL_2328360, EPI_ISL_2328361, EPI_ISL_2328362, EPI_ISL_2328363, EPI_ISL_2328364, EPI_ISL_2328365, EPI_ISL_2328366, EPI_ISL_2328367, EPI_ISL_2328368, EPI_ISL_2328369, EPI_ISL_2328370, EPI_ISL_2328371,
EPI_ISL_2328372, EPI_ISL_2328373, EPI_ISL_2328374, EPI_ISL_2328375, EPI_ISL_2328376, EPI_ISL_2328377, EPI_ISL_2328378, EPI_ISL_2328379, EPI_ISL_2328380, EPI_ISL_2328381, EPI_ISL_2328382, EPI_ISL_2328383, EPI_ISL_2328384, EPI_ISL_2328385, EPI_ISL_2328386, EPI_ISL_2328387, EPI_ISL_2328388, EPI_ISL_2328389, EPI_ISL_2328390, EPI_ISL_2328391, EPI_ISL_2328392, EPI_ISL_2328393,
EPI_ISL_2328394, EPI_ISL_2328395, EPI_ISL_2328396, EPI_ISL_2328397, EPI_ISL_2328398, EPI_ISL_2328399, EPI_ISL_2328400, EPI_ISL_2328401, EPI_ISL_2328402, EPI_ISL_2328403, EPI_ISL_2328404, EPI_ISL_2328405, EPI_ISL_2328406, EPI_ISL_2328407, EPI_ISL_2328408, EPI_ISL_2328409, EPI_ISL_2328410, EPI_ISL_2328411, EPI_ISL_2328412, EPI_ISL_2328413, EPI_ISL_2328414, EPI_ISL_2328415,
EPI_ISL_2328416, EPI_ISL_2328417, EPI_ISL_2328418, EPI_ISL_2328419, EPI_ISL_2328420, EPI_ISL_2328421, EPI_ISL_2328422, EPI_ISL_2328423, EPI_ISL_2328424, EPI_ISL_2328425, EPI_ISL_2328426, EPI_ISL_2328427, EPI_ISL_2328428, EPI_ISL_2328429, EPI_ISL_2328430, EPI_ISL_2328431, EPI_ISL_2328432, EPI_ISL_2328433, EPI_ISL_2328434, EPI_ISL_2328435, EPI_ISL_2328436, EPI_ISL_2328437,
EPI_ISL_2328438, EPI_ISL_2328439, EPI_ISL_2328440, EPI_ISL_2328441, EPI_ISL_2328442, EPI_ISL_2328443, EPI_ISL_2328444, EPI_ISL_2328445, EPI_ISL_2328446, EPI_ISL_2328447, EPI_ISL_2328448, EPI_ISL_2328449, EPI_ISL_2328450, EPI_ISL_2328451, EPI_ISL_2328452, EPI_ISL_2328453, EPI_ISL_2328454, EPI_ISL_2328455, EPI_ISL_2328456, EPI_ISL_2328457, EPI_ISL_2328458, EPI_ISL_2328459,
EPI_ISL_2328460, EPI_ISL_2328461, EPI_ISL_2328462, EPI_ISL_2328463, EPI_ISL_2328464, EPI_ISL_2328465, EPI_ISL_2328466, EPI_ISL_2328467, EPI_ISL_2328468, EPI_ISL_2328469, EPI_ISL_2328470, EPI_ISL_2328471, EPI_ISL_2328472, EPI_ISL_2328473, EPI_ISL_2328474, EPI_ISL_2328475, EPI_ISL_2328476, EPI_ISL_2328477, EPI_ISL_2328478, EPI_ISL_2328479, EPI_ISL_2328480, EPI_ISL_2328481,
EPI_ISL_2328482, EPI_ISL_2328483, EPI_ISL_2328484, EPI_ISL_2328485, EPI_ISL_2328486, EPI_ISL_2328487, EPI_ISL_2328488, EPI_ISL_2328489, EPI_ISL_2328490, EPI_ISL_2328491, EPI_ISL_2328492, EPI_ISL_2328493, EPI_ISL_2328494, EPI_ISL_2328495, EPI_ISL_2328496, EPI_ISL_2328497, EPI_ISL_2328498, EPI_ISL_2328499, EPI_ISL_2328500, EPI_ISL_2328501, EPI_ISL_2328502, EPI_ISL_2328503,
EPI_ISL_2328504, EPI_ISL_2328505, EPI_ISL_2328506, EPI_ISL_2328507, EPI_ISL_2328508, EPI_ISL_2328509, EPI_ISL_2328510, EPI_ISL_2328511, EPI_ISL_2328512, EPI_ISL_2328513, EPI_ISL_2328514, EPI_ISL_2328515, EPI_ISL_2328516, EPI_ISL_2328517, EPI_ISL_2328518, EPI_ISL_2328519, EPI_ISL_2328520, EPI_ISL_2328521, EPI_ISL_2328522, EPI_ISL_2328523, EPI_ISL_2328524, EPI_ISL_2328525,
EPI_ISL_2328526, EPI_ISL_2328527, EPI_ISL_2328528, EPI_ISL_2328529, EPI_ISL_2328530, EPI_ISL_2328531, EPI_ISL_2328532, EPI_ISL_2328533, EPI_ISL_2328534, EPI_ISL_2328535, EPI_ISL_2328536, EPI_ISL_2328537, EPI_ISL_2328538, EPI_ISL_2328539, EPI_ISL_2328540, EPI_ISL_2328541, EPI_ISL_2328542, EPI_ISL_2328543, EPI_ISL_2328544, EPI_ISL_2328545, EPI_ISL_2328546, EPI_ISL_2328547,
EPI_ISL_2328548, EPI_ISL_2328549, EPI_ISL_2328550, EPI_ISL_2328551, EPI_ISL_2328552, EPI_ISL_2328553, EPI_ISL_2328554, EPI_ISL_2328555, EPI_ISL_2328556, EPI_ISL_2328557, EPI_ISL_2328558, EPI_ISL_2328559, EPI_ISL_2328560, EPI_ISL_2328561, EPI_ISL_2328562, EPI_ISL_2328563, EPI_ISL_2328564, EPI_ISL_2328565, EPI_ISL_2328566, EPI_ISL_2328567, EPI_ISL_2328568, EPI_ISL_2328569,
EPI_ISL_2328570, EPI_ISL_2328571, EPI_ISL_2328572, EPI_ISL_2328573, EPI_ISL_2328574, EPI_ISL_2328575, EPI_ISL_2328576, EPI_ISL_2328577, EPI_ISL_2328578, EPI_ISL_2328579, EPI_ISL_2328580, EPI_ISL_2328581, EPI_ISL_2328582, EPI_ISL_2328583, EPI_ISL_2328584, EPI_ISL_2328585, EPI_ISL_2328586, EPI_ISL_2328587, EPI_ISL_2328588, EPI_ISL_2328589, EPI_ISL_2328590, EPI_ISL_2328591,
EPI_ISL_2328592, EPI_ISL_2328593, EPI_ISL_2328594, EPI_ISL_2328595, EPI_ISL_2328596, EPI_ISL_2328597, EPI_ISL_2328598, EPI_ISL_2328599, EPI_ISL_2328600, EPI_ISL_2328601, EPI_ISL_2328602, EPI_ISL_2328856, EPI_ISL_2328857, EPI_ISL_2328858, EPI_ISL_2328859, EPI_ISL_2328860, EPI_ISL_2328861, EPI_ISL_2328862, EPI_ISL_2328863, EPI_ISL_2328864, EPI_ISL_2328865, EPI_ISL_2328866,
EPI_ISL_2328867, EPI_ISL_2328868, EPI_ISL_2328869, EPI_ISL_2328870, EPI_ISL_2328871, EPI_ISL_2328872, EPI_ISL_2328873, EPI_ISL_2328874, EPI_ISL_2328875, EPI_ISL_2328876, EPI_ISL_2328877, EPI_ISL_2328878, EPI_ISL_2328879, EPI_ISL_2328880, EPI_ISL_2328881, EPI_ISL_2328882, EPI_ISL_2328883, EPI_ISL_2328884, EPI_ISL_2328885, EPI_ISL_2328886, EPI_ISL_2328887, EPI_ISL_2328888,
EPI_ISL_2328889, EPI_ISL_2328890, EPI_ISL_2328891, EPI_ISL_2328892, EPI_ISL_2328893, EPI_ISL_2328894, EPI_ISL_2328895, EPI_ISL_2328896, EPI_ISL_2328897, EPI_ISL_2328898, EPI_ISL_2328899, EPI_ISL_2328900, EPI_ISL_2328901, EPI_ISL_2328902, EPI_ISL_2328903, EPI_ISL_2328904, EPI_ISL_2328905, EPI_ISL_2328906, EPI_ISL_2328907, EPI_ISL_2328908, EPI_ISL_2328909, EPI_ISL_2329610,
EPI_ISL_2329611, EPI_ISL_2329612, EPI_ISL_2329613, EPI_ISL_2329614, EPI_ISL_2329615, EPI_ISL_2329616, EPI_ISL_2329617, EPI_ISL_2329618, EPI_ISL_2329619, EPI_ISL_2329620, EPI_ISL_2329621, EPI_ISL_2329622, EPI_ISL_2329623, EPI_ISL_2329624, EPI_ISL_2329625, EPI_ISL_2329626, EPI_ISL_2329627, EPI_ISL_2329628, EPI_ISL_2329629, EPI_ISL_2329630, EPI_ISL_2329631, EPI_ISL_2329632,
EPI_ISL_2329633, EPI_ISL_2329634, EPI_ISL_2329635, EPI_ISL_2329636, EPI_ISL_2329637, EPI_ISL_2329638, EPI_ISL_2329639, EPI_ISL_2329640, EPI_ISL_2329641, EPI_ISL_2329642, EPI_ISL_2329643, EPI_ISL_2329644, EPI_ISL_2329645, EPI_ISL_2329646, EPI_ISL_2329647, EPI_ISL_2329648, EPI_ISL_2329649, EPI_ISL_2329650, EPI_ISL_2329651, EPI_ISL_2329652, EPI_ISL_2329653, EPI_ISL_2329654,
EPI_ISL_2329655, EPI_ISL_2329656, EPI_ISL_2329657, EPI_ISL_2329658, EPI_ISL_2329659, EPI_ISL_2329660, EPI_ISL_2329661, EPI_ISL_2329662, EPI_ISL_2329663, EPI_ISL_2329664, EPI_ISL_2329665, EPI_ISL_2329666, EPI_ISL_2329667, EPI_ISL_2329668, EPI_ISL_2329669, EPI_ISL_2329670, EPI_ISL_2329671, EPI_ISL_2329672, EPI_ISL_2329673, EPI_ISL_2329674, EPI_ISL_2329675, EPI_ISL_2329676,
EPI_ISL_2329677, EPI_ISL_2329680, EPI_ISL_2329681, EPI_ISL_2329682, EPI_ISL_2329683, EPI_ISL_2329684, EPI_ISL_2329685, EPI_ISL_2329686, EPI_ISL_2329687, EPI_ISL_2329688, EPI_ISL_2329689, EPI_ISL_2329690, EPI_ISL_2329691, EPI_ISL_2329692, EPI_ISL_2329693, EPI_ISL_2329694, EPI_ISL_2329695, EPI_ISL_2329696, EPI_ISL_2329697, EPI_ISL_2329698, EPI_ISL_2329699, EPI_ISL_2329700,
EPI_ISL_2329701, EPI_ISL_2329702, EPI_ISL_2329703, EPI_ISL_2329704, EPI_ISL_2329705, EPI_ISL_2329706, EPI_ISL_2329707, EPI_ISL_2329708, EPI_ISL_2329709, EPI_ISL_2329710, EPI_ISL_2329711, EPI_ISL_2329712, EPI_ISL_2329713, EPI_ISL_2329714, EPI_ISL_2329715, EPI_ISL_2329716, EPI_ISL_2329717, EPI_ISL_2329718, EPI_ISL_2329719, EPI_ISL_2329720, EPI_ISL_2329721, EPI_ISL_2329722,
EPI_ISL_2329723, EPI_ISL_2329724, EPI_ISL_2329725, EPI_ISL_2329726, EPI_ISL_2329727, EPI_ISL_2329728, EPI_ISL_2329729, EPI_ISL_2329730, EPI_ISL_2329731, EPI_ISL_2329732, EPI_ISL_2329733, EPI_ISL_2329734, EPI_ISL_2329735, EPI_ISL_2329736, EPI_ISL_2329737, EPI_ISL_2329738, EPI_ISL_2329739, EPI_ISL_2329740, EPI_ISL_2329741, EPI_ISL_2329742, EPI_ISL_2329743, EPI_ISL_2329744,
EPI_ISL_2329745, EPI_ISL_2329746, EPI_ISL_2329747, EPI_ISL_2329748, EPI_ISL_2329749, EPI_ISL_2329750, EPI_ISL_2329751, EPI_ISL_2329752, EPI_ISL_2329753, EPI_ISL_2329754, EPI_ISL_2329755, EPI_ISL_2329756, EPI_ISL_2329757, EPI_ISL_2329758, EPI_ISL_2329759, EPI_ISL_2329760, EPI_ISL_2329761, EPI_ISL_2329762, EPI_ISL_2329763, EPI_ISL_2329764, EPI_ISL_2329765, EPI_ISL_2329766,
EPI_ISL_2329767, EPI_ISL_2329768, EPI_ISL_2329769, EPI_ISL_2329770, EPI_ISL_2329771, EPI_ISL_2329772, EPI_ISL_2329773, EPI_ISL_2329774, EPI_ISL_2329775, EPI_ISL_2329776, EPI_ISL_2329777, EPI_ISL_2329778, EPI_ISL_2329779, EPI_ISL_2329780, EPI_ISL_2329781, EPI_ISL_2329782, EPI_ISL_2329783, EPI_ISL_2329784, EPI_ISL_2329785, EPI_ISL_2329786, EPI_ISL_2329787, EPI_ISL_2329788,
EPI_ISL_2329789, EPI_ISL_2329790, EPI_ISL_2329791, EPI_ISL_2329792, EPI_ISL_2329793, EPI_ISL_2329794, EPI_ISL_2329795, EPI_ISL_2329796, EPI_ISL_2329797, EPI_ISL_2329798, EPI_ISL_2329799, EPI_ISL_2329800, EPI_ISL_2329801, EPI_ISL_2329802, EPI_ISL_2329803, EPI_ISL_2329804, EPI_ISL_2329805, EPI_ISL_2329806, EPI_ISL_2329807, EPI_ISL_2329808, EPI_ISL_2329809, EPI_ISL_2329810,
EPI_ISL_2330328, EPI_ISL_2330329, EPI_ISL_2330330, EPI_ISL_2330331, EPI_ISL_2330332, EPI_ISL_2330333, EPI_ISL_2330334, EPI_ISL_2330335, EPI_ISL_2330336, EPI_ISL_2330337, EPI_ISL_2330338, EPI_ISL_2330339, EPI_ISL_2330340, EPI_ISL_2330341, EPI_ISL_2330342, EPI_ISL_2330343, EPI_ISL_2330344, EPI_ISL_2330345, EPI_ISL_2330346, EPI_ISL_2330347, EPI_ISL_2330348, EPI_ISL_2330349,
EPI_ISL_2330350, EPI_ISL_2330351, EPI_ISL_2330352, EPI_ISL_2330353, EPI_ISL_2330354, EPI_ISL_2330355, EPI_ISL_2330356, EPI_ISL_2330357, EPI_ISL_2330358, EPI_ISL_2330359, EPI_ISL_2330360, EPI_ISL_2330361, EPI_ISL_2330362, EPI_ISL_2330363, EPI_ISL_2330364, EPI_ISL_2330365, EPI_ISL_2330366, EPI_ISL_2330367, EPI_ISL_2330368, EPI_ISL_2330369, EPI_ISL_2330370, EPI_ISL_2330371,
EPI_ISL_2330372, EPI_ISL_2330373, EPI_ISL_2330374, EPI_ISL_2330375, EPI_ISL_2330376, EPI_ISL_2330377, EPI_ISL_2330378, EPI_ISL_2330379, EPI_ISL_2330380, EPI_ISL_2330381, EPI_ISL_2330382, EPI_ISL_2330383, EPI_ISL_2330384, EPI_ISL_2330385, EPI_ISL_2330386, EPI_ISL_2330387, EPI_ISL_2330388, EPI_ISL_2330389, EPI_ISL_2330390, EPI_ISL_2330391, EPI_ISL_2330392, EPI_ISL_2330393,
EPI_ISL_2330394, EPI_ISL_2330395, EPI_ISL_2330396, EPI_ISL_2330397, EPI_ISL_2330398, EPI_ISL_2330399, EPI_ISL_2330400, EPI_ISL_2330401, EPI_ISL_2330402, EPI_ISL_2330403, EPI_ISL_2330404, EPI_ISL_2330405, EPI_ISL_2330406, EPI_ISL_2330407, EPI_ISL_2330408, EPI_ISL_2330409, EPI_ISL_2330410, EPI_ISL_2330411, EPI_ISL_2330412, EPI_ISL_2330413, EPI_ISL_2330414, EPI_ISL_2330415,
EPI_ISL_2330416, EPI_ISL_2330417, EPI_ISL_2330418, EPI_ISL_2330419, EPI_ISL_2330420, EPI_ISL_2330421, EPI_ISL_2330422, EPI_ISL_2330423, EPI_ISL_2330424, EPI_ISL_2330425, EPI_ISL_2330426, EPI_ISL_2330427, EPI_ISL_2330428, EPI_ISL_2330429, EPI_ISL_2330430, EPI_ISL_2330431, EPI_ISL_2330432, EPI_ISL_2330433, EPI_ISL_2330434, EPI_ISL_2330435, EPI_ISL_2330436, EPI_ISL_2330437,
EPI_ISL_2330438, EPI_ISL_2330439, EPI_ISL_2330440, EPI_ISL_2330441, EPI_ISL_2330442, EPI_ISL_2330443, EPI_ISL_2330444, EPI_ISL_2330445, EPI_ISL_2330446, EPI_ISL_2330447, EPI_ISL_2330448, EPI_ISL_2330449, EPI_ISL_2330450, EPI_ISL_2330451, EPI_ISL_2330452, EPI_ISL_2330453, EPI_ISL_2330454, EPI_ISL_2330455, EPI_ISL_2330456, EPI_ISL_2330457, EPI_ISL_2330458, EPI_ISL_2330459,
EPI_ISL_2330460, EPI_ISL_2330461, EPI_ISL_2330462, EPI_ISL_2330463, EPI_ISL_2330464, EPI_ISL_2330465, EPI_ISL_2330466, EPI_ISL_2330467, EPI_ISL_2330468, EPI_ISL_2330469, EPI_ISL_2330470, EPI_ISL_2330471, EPI_ISL_2330472, EPI_ISL_2330473, EPI_ISL_2330474, EPI_ISL_2330475, EPI_ISL_2330476, EPI_ISL_2330477, EPI_ISL_2330478, EPI_ISL_2330479, EPI_ISL_2330480, EPI_ISL_2330481,
EPI_ISL_2330482, EPI_ISL_2330483, EPI_ISL_2330484, EPI_ISL_2330485, EPI_ISL_2330486, EPI_ISL_2330487, EPI_ISL_2330488, EPI_ISL_2330489, EPI_ISL_2330490, EPI_ISL_2330491, EPI_ISL_2330492, EPI_ISL_2330493, EPI_ISL_2330494, EPI_ISL_2330495, EPI_ISL_2330496, EPI_ISL_2330497, EPI_ISL_2330498, EPI_ISL_2330499, EPI_ISL_2330500, EPI_ISL_2330501, EPI_ISL_2330502, EPI_ISL_2330503,
EPI_ISL_2330504, EPI_ISL_2330505, EPI_ISL_2330506, EPI_ISL_2330507, EPI_ISL_2330508, EPI_ISL_2330509, EPI_ISL_2330510, EPI_ISL_2330511, EPI_ISL_2330512, EPI_ISL_2330513, EPI_ISL_2330514, EPI_ISL_2330515, EPI_ISL_2330516, EPI_ISL_2330517, EPI_ISL_2330518, EPI_ISL_2330521, EPI_ISL_2330522, EPI_ISL_2330523, EPI_ISL_2330524, EPI_ISL_2330525, EPI_ISL_2330526, EPI_ISL_2330527,
EPI_ISL_2330528, EPI_ISL_2330529, EPI_ISL_2330530, EPI_ISL_2330531, EPI_ISL_2330532, EPI_ISL_2330533, EPI_ISL_2330534, EPI_ISL_2330535, EPI_ISL_2330536, EPI_ISL_2330537, EPI_ISL_2330538, EPI_ISL_2330539, EPI_ISL_2330540, EPI_ISL_2330541, EPI_ISL_2330542, EPI_ISL_2330543, EPI_ISL_2330544, EPI_ISL_2330545, EPI_ISL_2330546, EPI_ISL_2330547, EPI_ISL_2330548, EPI_ISL_2330549,
EPI_ISL_2330550, EPI_ISL_2330551, EPI_ISL_2330552, EPI_ISL_2330553, EPI_ISL_2330554, EPI_ISL_2330555, EPI_ISL_2330556, EPI_ISL_2330557, EPI_ISL_2330558, EPI_ISL_2330559, EPI_ISL_2330560, EPI_ISL_2330561, EPI_ISL_2330562, EPI_ISL_2330563, EPI_ISL_2330564, EPI_ISL_2330565, EPI_ISL_2330566, EPI_ISL_2330567, EPI_ISL_2330568, EPI_ISL_2330569, EPI_ISL_2330570, EPI_ISL_2330571,
EPI_ISL_2330572, EPI_ISL_2330573, EPI_ISL_2330574, EPI_ISL_2330575, EPI_ISL_2330576, EPI_ISL_2330577, EPI_ISL_2330578, EPI_ISL_2330579, EPI_ISL_2330580, EPI_ISL_2330581, EPI_ISL_2330582, EPI_ISL_2330583, EPI_ISL_2330584, EPI_ISL_2330585, EPI_ISL_2330586, EPI_ISL_2330587, EPI_ISL_2330588, EPI_ISL_2330589, EPI_ISL_2330590, EPI_ISL_2330591, EPI_ISL_2330592, EPI_ISL_2330593,
EPI_ISL_2330594, EPI_ISL_2330595, EPI_ISL_2330596, EPI_ISL_2330597, EPI_ISL_2330598, EPI_ISL_2330599, EPI_ISL_2330600, EPI_ISL_2330601, EPI_ISL_2330602, EPI_ISL_2330603, EPI_ISL_2330604, EPI_ISL_2330605, EPI_ISL_2330606, EPI_ISL_2330607, EPI_ISL_2330608, EPI_ISL_2330609, EPI_ISL_2330610, EPI_ISL_2330611, EPI_ISL_2330612, EPI_ISL_2330613, EPI_ISL_2330614, EPI_ISL_2330615,
EPI_ISL_2330616, EPI_ISL_2330617, EPI_ISL_2330618, EPI_ISL_2330619, EPI_ISL_2330620, EPI_ISL_2330621, EPI_ISL_2330622, EPI_ISL_2330623, EPI_ISL_2330624, EPI_ISL_2330625, EPI_ISL_2330626, EPI_ISL_2330627, EPI_ISL_2330628, EPI_ISL_2331213, EPI_ISL_2331307, EPI_ISL_2331308, EPI_ISL_2331309, EPI_ISL_2331310, EPI_ISL_2331311, EPI_ISL_2331312, EPI_ISL_2331313, EPI_ISL_2331314,
EPI_ISL_2331315, EPI_ISL_2331316, EPI_ISL_2331317, EPI_ISL_2331318, EPI_ISL_2331319, EPI_ISL_2331320, EPI_ISL_2331321, EPI_ISL_2331322, EPI_ISL_2331323, EPI_ISL_2331324, EPI_ISL_2331325, EPI_ISL_2331326, EPI_ISL_2331327, EPI_ISL_2335233, EPI_ISL_2335234, EPI_ISL_2335235, EPI_ISL_2335236, EPI_ISL_2335237, EPI_ISL_2335238, EPI_ISL_2335239, EPI_ISL_2335240, EPI_ISL_2335241,
EPI_ISL_2335242, EPI_ISL_2335243, EPI_ISL_2335244, EPI_ISL_2335245, EPI_ISL_2335246, EPI_ISL_2335247, EPI_ISL_2335248, EPI_ISL_2335249, EPI_ISL_2335250, EPI_ISL_2335251, EPI_ISL_2335252, EPI_ISL_2335253, EPI_ISL_2335254, EPI_ISL_2335255, EPI_ISL_2335256, EPI_ISL_2335257, EPI_ISL_2335258, EPI_ISL_2335259, EPI_ISL_2335260, EPI_ISL_2335261, EPI_ISL_2335262, EPI_ISL_2335263,
EPI_ISL_2335264, EPI_ISL_2335265, EPI_ISL_2335266, EPI_ISL_2335267, EPI_ISL_2335268, EPI_ISL_2335269, EPI_ISL_2335270, EPI_ISL_2335271, EPI_ISL_2335272, EPI_ISL_2335273, EPI_ISL_2335274, EPI_ISL_2335275, EPI_ISL_2335276, EPI_ISL_2335277, EPI_ISL_2335278, EPI_ISL_2335279, EPI_ISL_2335280, EPI_ISL_2335281, EPI_ISL_2335282, EPI_ISL_2335283, EPI_ISL_2335284, EPI_ISL_2335285,
EPI_ISL_2335286, EPI_ISL_2335287, EPI_ISL_2335288, EPI_ISL_2335289, EPI_ISL_2335290, EPI_ISL_2335291, EPI_ISL_2335292, EPI_ISL_2335293, EPI_ISL_2335294, EPI_ISL_2335295, EPI_ISL_2335296, EPI_ISL_2335297, EPI_ISL_2335298, EPI_ISL_2335299, EPI_ISL_2335300, EPI_ISL_2335301, EPI_ISL_2335302, EPI_ISL_2335303, EPI_ISL_2335304, EPI_ISL_2335305, EPI_ISL_2335306, EPI_ISL_2335307,
EPI_ISL_2335308, EPI_ISL_2335309, EPI_ISL_2335310, EPI_ISL_2335311, EPI_ISL_2335312, EPI_ISL_2335313, EPI_ISL_2335314, EPI_ISL_2335315, EPI_ISL_2335316, EPI_ISL_2335317, EPI_ISL_2335318, EPI_ISL_2335319, EPI_ISL_2335320, EPI_ISL_2335321, EPI_ISL_2335322, EPI_ISL_2335323, EPI_ISL_2335324, EPI_ISL_2335325, EPI_ISL_2335326, EPI_ISL_2335327, EPI_ISL_2335328, EPI_ISL_2335329,
EPI_ISL_2335330, EPI_ISL_2335331, EPI_ISL_2335332, EPI_ISL_2335333, EPI_ISL_2335334, EPI_ISL_2335335, EPI_ISL_2335336, EPI_ISL_2335337, EPI_ISL_2335338, EPI_ISL_2335339, EPI_ISL_2335340, EPI_ISL_2335341, EPI_ISL_2335342, EPI_ISL_2335343, EPI_ISL_2335344, EPI_ISL_2335345, EPI_ISL_2335346, EPI_ISL_2335347, EPI_ISL_2335348, EPI_ISL_2335349, EPI_ISL_2335350, EPI_ISL_2335351,
EPI_ISL_2335352, EPI_ISL_2335353, EPI_ISL_2335354, EPI_ISL_2335355, EPI_ISL_2335356, EPI_ISL_2335357, EPI_ISL_2335358, EPI_ISL_2335359, EPI_ISL_2335360, EPI_ISL_2335361, EPI_ISL_2335362, EPI_ISL_2335363, EPI_ISL_2335364, EPI_ISL_2335365, EPI_ISL_2335366, EPI_ISL_2335367, EPI_ISL_2335368, EPI_ISL_2335369, EPI_ISL_2335370, EPI_ISL_2335371, EPI_ISL_2335372, EPI_ISL_2335373,
EPI_ISL_2335374, EPI_ISL_2335375, EPI_ISL_2335376, EPI_ISL_2335377, EPI_ISL_2335378, EPI_ISL_2335379, EPI_ISL_2335380, EPI_ISL_2335381, EPI_ISL_2335382, EPI_ISL_2335383, EPI_ISL_2335384, EPI_ISL_2335385, EPI_ISL_2335386, EPI_ISL_2335387, EPI_ISL_2335388, EPI_ISL_2335389, EPI_ISL_2335390, EPI_ISL_2335391, EPI_ISL_2335392, EPI_ISL_2335393, EPI_ISL_2335394, EPI_ISL_2335395,
EPI_ISL_2335396, EPI_ISL_2335397, EPI_ISL_2335398, EPI_ISL_2335399, EPI_ISL_2335400, EPI_ISL_2335401, EPI_ISL_2335402, EPI_ISL_2335403, EPI_ISL_2335404, EPI_ISL_2335405, EPI_ISL_2335406, EPI_ISL_2335407, EPI_ISL_2335408, EPI_ISL_2335409, EPI_ISL_2335410, EPI_ISL_2335411, EPI_ISL_2335412, EPI_ISL_2335413, EPI_ISL_2335414, EPI_ISL_2335415, EPI_ISL_2335416, EPI_ISL_2335417,
EPI_ISL_2335418, EPI_ISL_2335419, EPI_ISL_2335420, EPI_ISL_2335421, EPI_ISL_2335422, EPI_ISL_2335423, EPI_ISL_2335424, EPI_ISL_2335425, EPI_ISL_2335426, EPI_ISL_2335427, EPI_ISL_2335428, EPI_ISL_2335429, EPI_ISL_2335430, EPI_ISL_2335431, EPI_ISL_2335432, EPI_ISL_2335433, EPI_ISL_2335434, EPI_ISL_2335435, EPI_ISL_2335436, EPI_ISL_2335437, EPI_ISL_2335438, EPI_ISL_2335439,
EPI_ISL_2335440, EPI_ISL_2335441, EPI_ISL_2335442, EPI_ISL_2335443, EPI_ISL_2335444, EPI_ISL_2335445, EPI_ISL_2335446, EPI_ISL_2335447, EPI_ISL_2335448, EPI_ISL_2335449, EPI_ISL_2335450, EPI_ISL_2335451, EPI_ISL_2335452, EPI_ISL_2335453, EPI_ISL_2335454, EPI_ISL_2335455, EPI_ISL_2335457, EPI_ISL_2335467, EPI_ISL_2335468, EPI_ISL_2335469, EPI_ISL_2335470, EPI_ISL_2335471,
EPI_ISL_2335472, EPI_ISL_2335473, EPI_ISL_2335474, EPI_ISL_2335475, EPI_ISL_2335476, EPI_ISL_2335477, EPI_ISL_2335478, EPI_ISL_2335479, EPI_ISL_2335480, EPI_ISL_2335481, EPI_ISL_2335482, EPI_ISL_2335483, EPI_ISL_2335484, EPI_ISL_2335485, EPI_ISL_2335486, EPI_ISL_2335487, EPI_ISL_2335488, EPI_ISL_2335489, EPI_ISL_2335490, EPI_ISL_2335491, EPI_ISL_2335492, EPI_ISL_2335493,
EPI_ISL_2335494, EPI_ISL_2335495, EPI_ISL_2335496, EPI_ISL_2335497, EPI_ISL_2335498, EPI_ISL_2335499, EPI_ISL_2335500, EPI_ISL_2335501, EPI_ISL_2335502, EPI_ISL_2335503, EPI_ISL_2335504, EPI_ISL_2335505, EPI_ISL_2335506, EPI_ISL_2335507, EPI_ISL_2335508, EPI_ISL_2335509, EPI_ISL_2335510, EPI_ISL_2335511, EPI_ISL_2335512, EPI_ISL_2335513, EPI_ISL_2335514, EPI_ISL_2335515,
EPI_ISL_2335516, EPI_ISL_2335517, EPI_ISL_2335518, EPI_ISL_2335519, EPI_ISL_2335520, EPI_ISL_2335521, EPI_ISL_2335522, EPI_ISL_2335523, EPI_ISL_2335524, EPI_ISL_2335525, EPI_ISL_2335526, EPI_ISL_2335527, EPI_ISL_2335528, EPI_ISL_2335529, EPI_ISL_2335530, EPI_ISL_2335531, EPI_ISL_2335532, EPI_ISL_2335533, EPI_ISL_2335534, EPI_ISL_2335535, EPI_ISL_2335536, EPI_ISL_2335537,
EPI_ISL_2335538, EPI_ISL_2335539, EPI_ISL_2335540, EPI_ISL_2335541, EPI_ISL_2335542, EPI_ISL_2335543, EPI_ISL_2335544, EPI_ISL_2335545, EPI_ISL_2335546, EPI_ISL_2335547, EPI_ISL_2335548, EPI_ISL_2335549, EPI_ISL_2335550, EPI_ISL_2335551, EPI_ISL_2335552, EPI_ISL_2335553, EPI_ISL_2335554, EPI_ISL_2335555, EPI_ISL_2335556, EPI_ISL_2335557, EPI_ISL_2335558, EPI_ISL_2335559,
EPI_ISL_2335560, EPI_ISL_2335561, EPI_ISL_2335562, EPI_ISL_2335563, EPI_ISL_2335564, EPI_ISL_2335565, EPI_ISL_2335566, EPI_ISL_2335567, EPI_ISL_2335568, EPI_ISL_2335569, EPI_ISL_2335570, EPI_ISL_2335571, EPI_ISL_2335572, EPI_ISL_2335573, EPI_ISL_2335574, EPI_ISL_2335575, EPI_ISL_2335576, EPI_ISL_2335577, EPI_ISL_2335578, EPI_ISL_2335579, EPI_ISL_2335580, EPI_ISL_2335581,
EPI_ISL_2335582, EPI_ISL_2335583, EPI_ISL_2335584, EPI_ISL_2335585, EPI_ISL_2335586, EPI_ISL_2335587, EPI_ISL_2335588, EPI_ISL_2335589, EPI_ISL_2335590, EPI_ISL_2335591, EPI_ISL_2335592, EPI_ISL_2335593, EPI_ISL_2335594, EPI_ISL_2335595, EPI_ISL_2335596, EPI_ISL_2335597, EPI_ISL_2335598, EPI_ISL_2335599, EPI_ISL_2335600, EPI_ISL_2335601, EPI_ISL_2335602, EPI_ISL_2335603,
EPI_ISL_2335604, EPI_ISL_2335605, EPI_ISL_2335606, EPI_ISL_2335607, EPI_ISL_2335608, EPI_ISL_2335609, EPI_ISL_2335610, EPI_ISL_2335611, EPI_ISL_2335612, EPI_ISL_2335613, EPI_ISL_2335614, EPI_ISL_2335615, EPI_ISL_2335616, EPI_ISL_2335617, EPI_ISL_2335618, EPI_ISL_2335619, EPI_ISL_2335620, EPI_ISL_2335621, EPI_ISL_2335622, EPI_ISL_2335623, EPI_ISL_2335624, EPI_ISL_2335625,
EPI_ISL_2335626, EPI_ISL_2335627, EPI_ISL_2335628, EPI_ISL_2335629, EPI_ISL_2335630, EPI_ISL_2335631, EPI_ISL_2335632, EPI_ISL_2335633, EPI_ISL_2335634, EPI_ISL_2335635, EPI_ISL_2335636, EPI_ISL_2335637, EPI_ISL_2335638, EPI_ISL_2335639, EPI_ISL_2335640, EPI_ISL_2335641, EPI_ISL_2335642, EPI_ISL_2335643, EPI_ISL_2335644, EPI_ISL_2335645, EPI_ISL_2335646, EPI_ISL_2335647,
EPI_ISL_2335648, EPI_ISL_2335649, EPI_ISL_2335650, EPI_ISL_2335651, EPI_ISL_2335652, EPI_ISL_2335653, EPI_ISL_2335654, EPI_ISL_2335655, EPI_ISL_2335656, EPI_ISL_2335657, EPI_ISL_2335658, EPI_ISL_2335659, EPI_ISL_2335660, EPI_ISL_2335661, EPI_ISL_2335662, EPI_ISL_2335663, EPI_ISL_2335664, EPI_ISL_2335665, EPI_ISL_2335666, EPI_ISL_2335667, EPI_ISL_2335668, EPI_ISL_2335669,
EPI_ISL_2335670, EPI_ISL_2335671, EPI_ISL_2335672, EPI_ISL_2335673, EPI_ISL_2335674, EPI_ISL_2335675, EPI_ISL_2335676, EPI_ISL_2335677, EPI_ISL_2335678, EPI_ISL_2335679, EPI_ISL_2335680, EPI_ISL_2335681, EPI_ISL_2335682, EPI_ISL_2335683, EPI_ISL_2335684, EPI_ISL_2335685, EPI_ISL_2335686, EPI_ISL_2335687, EPI_ISL_2335688, EPI_ISL_2335689, EPI_ISL_2335690, EPI_ISL_2335691,
EPI_ISL_2335692, EPI_ISL_2335693, EPI_ISL_2335694, EPI_ISL_2335695, EPI_ISL_2335696, EPI_ISL_2335697, EPI_ISL_2335698, EPI_ISL_2335699, EPI_ISL_2335700, EPI_ISL_2335701, EPI_ISL_2335702, EPI_ISL_2335703, EPI_ISL_2335704, EPI_ISL_2335705, EPI_ISL_2335706, EPI_ISL_2335707, EPI_ISL_2335708, EPI_ISL_2335709, EPI_ISL_2335710, EPI_ISL_2335711, EPI_ISL_2335712, EPI_ISL_2335713,
EPI_ISL_2335714, EPI_ISL_2335715, EPI_ISL_2335716, EPI_ISL_2335717, EPI_ISL_2335718, EPI_ISL_2335719, EPI_ISL_2335720, EPI_ISL_2335721, EPI_ISL_2335722, EPI_ISL_2335723, EPI_ISL_2335724, EPI_ISL_2335725, EPI_ISL_2335726, EPI_ISL_2335727, EPI_ISL_2335728, EPI_ISL_2335729, EPI_ISL_2335730, EPI_ISL_2335731, EPI_ISL_2335732, EPI_ISL_2335733, EPI_ISL_2335734, EPI_ISL_2335735,
EPI_ISL_2335736, EPI_ISL_2335737, EPI_ISL_2335738, EPI_ISL_2335739, EPI_ISL_2335740, EPI_ISL_2335741, EPI_ISL_2335742, EPI_ISL_2335743, EPI_ISL_2335744, EPI_ISL_2335745, EPI_ISL_2335746, EPI_ISL_2335747, EPI_ISL_2335748, EPI_ISL_2335749, EPI_ISL_2335750, EPI_ISL_2335751, EPI_ISL_2335752, EPI_ISL_2335753, EPI_ISL_2335754, EPI_ISL_2335755, EPI_ISL_2335756, EPI_ISL_2335757,
EPI_ISL_2335758, EPI_ISL_2335759, EPI_ISL_2335760, EPI_ISL_2335761, EPI_ISL_2335762, EPI_ISL_2335763, EPI_ISL_2335764, EPI_ISL_2335765, EPI_ISL_2335766, EPI_ISL_2335767, EPI_ISL_2335768, EPI_ISL_2335769, EPI_ISL_2335770, EPI_ISL_2335771, EPI_ISL_2335772, EPI_ISL_2335773, EPI_ISL_2335774, EPI_ISL_2335775, EPI_ISL_2335776, EPI_ISL_2335777, EPI_ISL_2335778, EPI_ISL_2335779,
EPI_ISL_2335780, EPI_ISL_2335781, EPI_ISL_2335782, EPI_ISL_2335783, EPI_ISL_2335784, EPI_ISL_2335785, EPI_ISL_2335786, EPI_ISL_2335787, EPI_ISL_2335788, EPI_ISL_2335789, EPI_ISL_2335790, EPI_ISL_2335791, EPI_ISL_2335792, EPI_ISL_2335793, EPI_ISL_2335794, EPI_ISL_2335795, EPI_ISL_2335796, EPI_ISL_2335797, EPI_ISL_2335798, EPI_ISL_2335799, EPI_ISL_2335800, EPI_ISL_2335801,
EPI_ISL_2335802, EPI_ISL_2335803, EPI_ISL_2335804, EPI_ISL_2335805, EPI_ISL_2335806, EPI_ISL_2335807, EPI_ISL_2335808, EPI_ISL_2335809, EPI_ISL_2335810, EPI_ISL_2335811, EPI_ISL_2335812, EPI_ISL_2335813, EPI_ISL_2335814, EPI_ISL_2335815, EPI_ISL_2335816, EPI_ISL_2335817, EPI_ISL_2335818, EPI_ISL_2335819, EPI_ISL_2335820, EPI_ISL_2335821, EPI_ISL_2335822, EPI_ISL_2335823,
EPI_ISL_2335824, EPI_ISL_2335825, EPI_ISL_2335826, EPI_ISL_2335827, EPI_ISL_2335828, EPI_ISL_2335829, EPI_ISL_2335830, EPI_ISL_2335831, EPI_ISL_2335832, EPI_ISL_2335833, EPI_ISL_2335834, EPI_ISL_2335835, EPI_ISL_2335836, EPI_ISL_2335837, EPI_ISL_2335838, EPI_ISL_2335839, EPI_ISL_2335840, EPI_ISL_2335841, EPI_ISL_2335842, EPI_ISL_2335843, EPI_ISL_2335844, EPI_ISL_2335845,
EPI_ISL_2335846, EPI_ISL_2335847, EPI_ISL_2335848, EPI_ISL_2335849, EPI_ISL_2335850, EPI_ISL_2335851, EPI_ISL_2335852, EPI_ISL_2335853, EPI_ISL_2335854, EPI_ISL_2335855, EPI_ISL_2335856, EPI_ISL_2335857, EPI_ISL_2335858, EPI_ISL_2335859, EPI_ISL_2335860, EPI_ISL_2335861, EPI_ISL_2335862, EPI_ISL_2335863, EPI_ISL_2335864, EPI_ISL_2335865, EPI_ISL_2335866, EPI_ISL_2335867,
EPI_ISL_2335868, EPI_ISL_2335869, EPI_ISL_2335870, EPI_ISL_2335871, EPI_ISL_2335873, EPI_ISL_2335874, EPI_ISL_2335875, EPI_ISL_2335876, EPI_ISL_2335877, EPI_ISL_2335878, EPI_ISL_2335879, EPI_ISL_2335880, EPI_ISL_2335881, EPI_ISL_2335882, EPI_ISL_2335883, EPI_ISL_2335884, EPI_ISL_2335885, EPI_ISL_2335886, EPI_ISL_2335887, EPI_ISL_2335888, EPI_ISL_2335889, EPI_ISL_2335890,
EPI_ISL_2335891, EPI_ISL_2335892, EPI_ISL_2335893, EPI_ISL_2335894, EPI_ISL_2335895, EPI_ISL_2335896, EPI_ISL_2335897, EPI_ISL_2335898, EPI_ISL_2335899, EPI_ISL_2335900, EPI_ISL_2335901, EPI_ISL_2335902, EPI_ISL_2335903, EPI_ISL_2335904, EPI_ISL_2335905, EPI_ISL_2335906, EPI_ISL_2335907, EPI_ISL_2335908, EPI_ISL_2335909, EPI_ISL_2335910, EPI_ISL_2335911, EPI_ISL_2335912,
EPI_ISL_2335913, EPI_ISL_2335914, EPI_ISL_2335915, EPI_ISL_2335916, EPI_ISL_2335917, EPI_ISL_2335918, EPI_ISL_2335919, EPI_ISL_2335920, EPI_ISL_2335921, EPI_ISL_2335922, EPI_ISL_2335923, EPI_ISL_2335924, EPI_ISL_2335925, EPI_ISL_2335926, EPI_ISL_2335927, EPI_ISL_2335928, EPI_ISL_2335929, EPI_ISL_2335930, EPI_ISL_2335931, EPI_ISL_2335932, EPI_ISL_2335935, EPI_ISL_2335936,
EPI_ISL_2335937, EPI_ISL_2335938, EPI_ISL_2335939, EPI_ISL_2335940, EPI_ISL_2335941, EPI_ISL_2335942, EPI_ISL_2335943, EPI_ISL_2335944, EPI_ISL_2335945, EPI_ISL_2335946, EPI_ISL_2335947, EPI_ISL_2335948, EPI_ISL_2335949, EPI_ISL_2335950, EPI_ISL_2335951, EPI_ISL_2335952, EPI_ISL_2335953, EPI_ISL_2335954, EPI_ISL_2335955, EPI_ISL_2335956, EPI_ISL_2335957, EPI_ISL_2335958,
EPI_ISL_2335959, EPI_ISL_2335960, EPI_ISL_2335961, EPI_ISL_2335962, EPI_ISL_2335963, EPI_ISL_2335964, EPI_ISL_2335965, EPI_ISL_2335966, EPI_ISL_2335967, EPI_ISL_2335968, EPI_ISL_2335969, EPI_ISL_2335970, EPI_ISL_2335971, EPI_ISL_2335972, EPI_ISL_2335973, EPI_ISL_2335974, EPI_ISL_2335975, EPI_ISL_2335976, EPI_ISL_2335977, EPI_ISL_2335978, EPI_ISL_2335979, EPI_ISL_2335980,
EPI_ISL_2335981, EPI_ISL_2335982, EPI_ISL_2335983, EPI_ISL_2335984, EPI_ISL_2335985, EPI_ISL_2336355, EPI_ISL_2336356, EPI_ISL_2336357, EPI_ISL_2336358, EPI_ISL_2336359, EPI_ISL_2336360, EPI_ISL_2336361, EPI_ISL_2336362, EPI_ISL_2336363, EPI_ISL_2336364, EPI_ISL_2336365, EPI_ISL_2336366, EPI_ISL_2336367, EPI_ISL_2336368, EPI_ISL_2336369, EPI_ISL_2336370, EPI_ISL_2336371,
EPI_ISL_2336372, EPI_ISL_2336373, EPI_ISL_2336374, EPI_ISL_2336375, EPI_ISL_2336376, EPI_ISL_2336377, EPI_ISL_2336378, EPI_ISL_2336379, EPI_ISL_2336380, EPI_ISL_2336381, EPI_ISL_2336382, EPI_ISL_2336383, EPI_ISL_2336384, EPI_ISL_2336385, EPI_ISL_2336386, EPI_ISL_2336387, EPI_ISL_2336388, EPI_ISL_2336389, EPI_ISL_2336390, EPI_ISL_2336391, EPI_ISL_2336392, EPI_ISL_2336393,
EPI_ISL_2336394, EPI_ISL_2336395, EPI_ISL_2336396, EPI_ISL_2336397, EPI_ISL_2336398, EPI_ISL_2336399, EPI_ISL_2336400, EPI_ISL_2336401, EPI_ISL_2336402, EPI_ISL_2336403, EPI_ISL_2336404, EPI_ISL_2336405, EPI_ISL_2336406, EPI_ISL_2336407, EPI_ISL_2336408, EPI_ISL_2336409, EPI_ISL_2336410, EPI_ISL_2336411, EPI_ISL_2336412, EPI_ISL_2336413, EPI_ISL_2336414, EPI_ISL_2336415,
EPI_ISL_2336416, EPI_ISL_2336417, EPI_ISL_2336418, EPI_ISL_2336419, EPI_ISL_2336420, EPI_ISL_2336421, EPI_ISL_2336422, EPI_ISL_2336423, EPI_ISL_2336424, EPI_ISL_2336425, EPI_ISL_2336426, EPI_ISL_2336427, EPI_ISL_2336428, EPI_ISL_2336429, EPI_ISL_2336430, EPI_ISL_2336431, EPI_ISL_2336432, EPI_ISL_2336433, EPI_ISL_2336434, EPI_ISL_2336435, EPI_ISL_2336436, EPI_ISL_2336437,
EPI_ISL_2336438, EPI_ISL_2336439, EPI_ISL_2336440, EPI_ISL_2336441, EPI_ISL_2336442, EPI_ISL_2336443, EPI_ISL_2336444, EPI_ISL_2336445, EPI_ISL_2336446, EPI_ISL_2336447, EPI_ISL_2336448, EPI_ISL_2336449, EPI_ISL_2336450, EPI_ISL_2336451, EPI_ISL_2336452, EPI_ISL_2336453, EPI_ISL_2336454, EPI_ISL_2336455, EPI_ISL_2336456, EPI_ISL_2336457, EPI_ISL_2336458, EPI_ISL_2336459,
EPI_ISL_2336460, EPI_ISL_2336461, EPI_ISL_2336462, EPI_ISL_2336463, EPI_ISL_2336464, EPI_ISL_2336465, EPI_ISL_2336466, EPI_ISL_2336467, EPI_ISL_2336468, EPI_ISL_2336469, EPI_ISL_2336470, EPI_ISL_2336471, EPI_ISL_2336472, EPI_ISL_2336473, EPI_ISL_2336474, EPI_ISL_2336475, EPI_ISL_2336476, EPI_ISL_2336477, EPI_ISL_2336478, EPI_ISL_2336479, EPI_ISL_2336480, EPI_ISL_2336481,
EPI_ISL_2336482, EPI_ISL_2336749, EPI_ISL_2336750, EPI_ISL_2336751, EPI_ISL_2336752, EPI_ISL_2336753, EPI_ISL_2336754, EPI_ISL_2336755, EPI_ISL_2336756, EPI_ISL_2336757, EPI_ISL_2336758, EPI_ISL_2336759, EPI_ISL_2336760, EPI_ISL_2336761, EPI_ISL_2336762, EPI_ISL_2336763, EPI_ISL_2336764, EPI_ISL_2336765, EPI_ISL_2336766, EPI_ISL_2336767, EPI_ISL_2336768, EPI_ISL_2336769,
EPI_ISL_2336770, EPI_ISL_2336771, EPI_ISL_2336772, EPI_ISL_2336773, EPI_ISL_2336774, EPI_ISL_2336775, EPI_ISL_2336776, EPI_ISL_2336777, EPI_ISL_2336778, EPI_ISL_2336779, EPI_ISL_2336780, EPI_ISL_2336781, EPI_ISL_2336782, EPI_ISL_2336783, EPI_ISL_2336784, EPI_ISL_2336785, EPI_ISL_2336786, EPI_ISL_2336787, EPI_ISL_2336788, EPI_ISL_2336789, EPI_ISL_2336790, EPI_ISL_2336791,
EPI_ISL_2336792, EPI_ISL_2336793, EPI_ISL_2336794, EPI_ISL_2336795, EPI_ISL_2336796, EPI_ISL_2336797, EPI_ISL_2336798, EPI_ISL_2336799, EPI_ISL_2336800, EPI_ISL_2336801, EPI_ISL_2336802, EPI_ISL_2336803, EPI_ISL_2336804, EPI_ISL_2336805, EPI_ISL_2336806, EPI_ISL_2336807, EPI_ISL_2336808, EPI_ISL_2336809, EPI_ISL_2336810, EPI_ISL_2336811, EPI_ISL_2336812, EPI_ISL_2336813,
EPI_ISL_2336814, EPI_ISL_2336815, EPI_ISL_2336816, EPI_ISL_2336817, EPI_ISL_2336818, EPI_ISL_2336819, EPI_ISL_2336820, EPI_ISL_2336821, EPI_ISL_2336822, EPI_ISL_2336823, EPI_ISL_2336824, EPI_ISL_2336825, EPI_ISL_2336826, EPI_ISL_2336827, EPI_ISL_2336828, EPI_ISL_2336829, EPI_ISL_2336830, EPI_ISL_2336831, EPI_ISL_2336832, EPI_ISL_2336833, EPI_ISL_2336834, EPI_ISL_2336835,
EPI_ISL_2336836, EPI_ISL_2336837, EPI_ISL_2336838, EPI_ISL_2336839, EPI_ISL_2336840, EPI_ISL_2336841, EPI_ISL_2336842, EPI_ISL_2336843, EPI_ISL_2336844, EPI_ISL_2336845, EPI_ISL_2336846, EPI_ISL_2336847, EPI_ISL_2336848, EPI_ISL_2336849, EPI_ISL_2336850, EPI_ISL_2336851, EPI_ISL_2336852, EPI_ISL_2336853, EPI_ISL_2336854, EPI_ISL_2336855, EPI_ISL_2336856, EPI_ISL_2336857,
EPI_ISL_2336858, EPI_ISL_2336859, EPI_ISL_2336860, EPI_ISL_2336861, EPI_ISL_2336862, EPI_ISL_2336863, EPI_ISL_2336864, EPI_ISL_2336865, EPI_ISL_2336866, EPI_ISL_2336867, EPI_ISL_2336868, EPI_ISL_2336869, EPI_ISL_2336870, EPI_ISL_2336871, EPI_ISL_2336872, EPI_ISL_2336873, EPI_ISL_2336874, EPI_ISL_2336875, EPI_ISL_2336876, EPI_ISL_2336877, EPI_ISL_2336878, EPI_ISL_2336879,
EPI_ISL_2336880, EPI_ISL_2336881, EPI_ISL_2336882, EPI_ISL_2336883, EPI_ISL_2336884, EPI_ISL_2336885, EPI_ISL_2336886, EPI_ISL_2336887, EPI_ISL_2336888, EPI_ISL_2336889, EPI_ISL_2336890, EPI_ISL_2336891, EPI_ISL_2336892, EPI_ISL_2336893, EPI_ISL_2336894, EPI_ISL_2336895, EPI_ISL_2336896, EPI_ISL_2337286, EPI_ISL_2337287, EPI_ISL_2337288, EPI_ISL_2337289, EPI_ISL_2337290,
EPI_ISL_2337291, EPI_ISL_2337292, EPI_ISL_2337293, EPI_ISL_2337294, EPI_ISL_2337295, EPI_ISL_2337296, EPI_ISL_2337297, EPI_ISL_2337298, EPI_ISL_2337299, EPI_ISL_2337300, EPI_ISL_2337301, EPI_ISL_2337302, EPI_ISL_2337303, EPI_ISL_2337304, EPI_ISL_2337305, EPI_ISL_2337306, EPI_ISL_2337307, EPI_ISL_2337308, EPI_ISL_2337309, EPI_ISL_2337310, EPI_ISL_2337311, EPI_ISL_2337312,
EPI_ISL_2337313, EPI_ISL_2337314, EPI_ISL_2337315, EPI_ISL_2337316, EPI_ISL_2337317, EPI_ISL_2337318, EPI_ISL_2337319, EPI_ISL_2337320, EPI_ISL_2337321, EPI_ISL_2337322, EPI_ISL_2337323, EPI_ISL_2337324, EPI_ISL_2337325, EPI_ISL_2337326, EPI_ISL_2337327, EPI_ISL_2337328, EPI_ISL_2337329, EPI_ISL_2337330, EPI_ISL_2337331, EPI_ISL_2337332, EPI_ISL_2337333, EPI_ISL_2337334,
EPI_ISL_2337335, EPI_ISL_2337336, EPI_ISL_2337337, EPI_ISL_2337338, EPI_ISL_2337339, EPI_ISL_2337340, EPI_ISL_2337341, EPI_ISL_2337342, EPI_ISL_2337343, EPI_ISL_2337344, EPI_ISL_2337345, EPI_ISL_2337346, EPI_ISL_2337348, EPI_ISL_2337349, EPI_ISL_2337350, EPI_ISL_2337351, EPI_ISL_2337352, EPI_ISL_2337353, EPI_ISL_2337354, EPI_ISL_2337355, EPI_ISL_2337356, EPI_ISL_2337357,
EPI_ISL_2337358, EPI_ISL_2337359, EPI_ISL_2337360, EPI_ISL_2337361, EPI_ISL_2337362, EPI_ISL_2337363, EPI_ISL_2337364, EPI_ISL_2337365, EPI_ISL_2337366, EPI_ISL_2337367, EPI_ISL_2337368, EPI_ISL_2337369, EPI_ISL_2337370, EPI_ISL_2337371, EPI_ISL_2337372, EPI_ISL_2337373, EPI_ISL_2337374, EPI_ISL_2337375, EPI_ISL_2337376, EPI_ISL_2337377, EPI_ISL_2337378, EPI_ISL_2337379,
EPI_ISL_2337380, EPI_ISL_2337381, EPI_ISL_2337382, EPI_ISL_2337383, EPI_ISL_2337384, EPI_ISL_2337385, EPI_ISL_2337386, EPI_ISL_2337387, EPI_ISL_2337388, EPI_ISL_2337389, EPI_ISL_2337390, EPI_ISL_2337391, EPI_ISL_2337392, EPI_ISL_2337393, EPI_ISL_2337394, EPI_ISL_2337395, EPI_ISL_2337396, EPI_ISL_2337397, EPI_ISL_2337398, EPI_ISL_2337399, EPI_ISL_2337400, EPI_ISL_2337401,
EPI_ISL_2337402, EPI_ISL_2337403, EPI_ISL_2337404, EPI_ISL_2337405, EPI_ISL_2337406, EPI_ISL_2337407, EPI_ISL_2337408, EPI_ISL_2337409, EPI_ISL_2337410, EPI_ISL_2337411, EPI_ISL_2337412, EPI_ISL_2337413, EPI_ISL_2337414, EPI_ISL_2337415, EPI_ISL_2337416, EPI_ISL_2337417, EPI_ISL_2337418, EPI_ISL_2337419, EPI_ISL_2337420, EPI_ISL_2337421, EPI_ISL_2337422, EPI_ISL_2337423,
EPI_ISL_2337424, EPI_ISL_2337425, EPI_ISL_2337426, EPI_ISL_2337427, EPI_ISL_2337428, EPI_ISL_2337429, EPI_ISL_2337430, EPI_ISL_2337431, EPI_ISL_2337432, EPI_ISL_2337433, EPI_ISL_2337434, EPI_ISL_2337435, EPI_ISL_2337436, EPI_ISL_2337437, EPI_ISL_2337438, EPI_ISL_2337439, EPI_ISL_2337440, EPI_ISL_2337441, EPI_ISL_2337442, EPI_ISL_2337443, EPI_ISL_2337444, EPI_ISL_2337445,
EPI_ISL_2337446, EPI_ISL_2337447, EPI_ISL_2337448, EPI_ISL_2337449, EPI_ISL_2337450, EPI_ISL_2337451, EPI_ISL_2337452, EPI_ISL_2337453, EPI_ISL_2337454, EPI_ISL_2337455, EPI_ISL_2337456, EPI_ISL_2337457, EPI_ISL_2337458, EPI_ISL_2337459, EPI_ISL_2337460, EPI_ISL_2337461, EPI_ISL_2337462, EPI_ISL_2337463, EPI_ISL_2337464, EPI_ISL_2337465, EPI_ISL_2337466, EPI_ISL_2337467,
EPI_ISL_2337468, EPI_ISL_2337469, EPI_ISL_2337470, EPI_ISL_2337471, EPI_ISL_2337472, EPI_ISL_2337473, EPI_ISL_2337474, EPI_ISL_2337475, EPI_ISL_2337476, EPI_ISL_2337477, EPI_ISL_2337478, EPI_ISL_2337479, EPI_ISL_2337480, EPI_ISL_2337481, EPI_ISL_2337482, EPI_ISL_2337483, EPI_ISL_2337484, EPI_ISL_2337485, EPI_ISL_2337486, EPI_ISL_2337487, EPI_ISL_2337488, EPI_ISL_2337489,
EPI_ISL_2337490, EPI_ISL_2337491, EPI_ISL_2337492, EPI_ISL_2337493, EPI_ISL_2337494, EPI_ISL_2337495, EPI_ISL_2337496, EPI_ISL_2337497, EPI_ISL_2337498, EPI_ISL_2337499, EPI_ISL_2337500, EPI_ISL_2337501, EPI_ISL_2337502, EPI_ISL_2337503, EPI_ISL_2337504, EPI_ISL_2337505, EPI_ISL_2337506, EPI_ISL_2337507, EPI_ISL_2337508, EPI_ISL_2337509, EPI_ISL_2337510, EPI_ISL_2337511,
EPI_ISL_2337512, EPI_ISL_2337513, EPI_ISL_2337514, EPI_ISL_2337515, EPI_ISL_2337516, EPI_ISL_2337517, EPI_ISL_2337518, EPI_ISL_2337519, EPI_ISL_2337520, EPI_ISL_2337521, EPI_ISL_2337522, EPI_ISL_2337523, EPI_ISL_2337524, EPI_ISL_2337558, EPI_ISL_2337559, EPI_ISL_2337560, EPI_ISL_2337561, EPI_ISL_2337562, EPI_ISL_2337563, EPI_ISL_2337564, EPI_ISL_2337565, EPI_ISL_2337566,
EPI_ISL_2337567, EPI_ISL_2337568, EPI_ISL_2337569, EPI_ISL_2337570, EPI_ISL_2337571, EPI_ISL_2337572, EPI_ISL_2337573, EPI_ISL_2337574, EPI_ISL_2337575, EPI_ISL_2337576, EPI_ISL_2337577, EPI_ISL_2337578, EPI_ISL_2337579, EPI_ISL_2337580, EPI_ISL_2337581, EPI_ISL_2337582, EPI_ISL_2337583, EPI_ISL_2337584, EPI_ISL_2337585, EPI_ISL_2337586, EPI_ISL_2337587, EPI_ISL_2337588,
EPI_ISL_2337589, EPI_ISL_2337590, EPI_ISL_2337591, EPI_ISL_2337592, EPI_ISL_2337593, EPI_ISL_2337594, EPI_ISL_2337595, EPI_ISL_2337596, EPI_ISL_2337597, EPI_ISL_2337598, EPI_ISL_2337599, EPI_ISL_2337600, EPI_ISL_2337601, EPI_ISL_2337602, EPI_ISL_2337603, EPI_ISL_2337604, EPI_ISL_2337605, EPI_ISL_2337606, EPI_ISL_2337607, EPI_ISL_2337608, EPI_ISL_2337609, EPI_ISL_2337610,
EPI_ISL_2337611, EPI_ISL_2337612, EPI_ISL_2337613, EPI_ISL_2337614, EPI_ISL_2337615, EPI_ISL_2337616, EPI_ISL_2337617, EPI_ISL_2337618, EPI_ISL_2337619, EPI_ISL_2337620, EPI_ISL_2337621, EPI_ISL_2337622, EPI_ISL_2337623, EPI_ISL_2337624, EPI_ISL_2337625, EPI_ISL_2337626, EPI_ISL_2337627, EPI_ISL_2337628, EPI_ISL_2337629, EPI_ISL_2337630, EPI_ISL_2337631, EPI_ISL_2337632,
EPI_ISL_2338322, EPI_ISL_2338323, EPI_ISL_2338324, EPI_ISL_2338325, EPI_ISL_2338326, EPI_ISL_2338327, EPI_ISL_2338328, EPI_ISL_2338329, EPI_ISL_2338330, EPI_ISL_2338331, EPI_ISL_2338332, EPI_ISL_2338333, EPI_ISL_2338334, EPI_ISL_2338335, EPI_ISL_2338336, EPI_ISL_2338337, EPI_ISL_2338338, EPI_ISL_2338339, EPI_ISL_2338340, EPI_ISL_2338341, EPI_ISL_2338342, EPI_ISL_2338343,

EPI_ISL_2338344, EPI_ISL_2338345, EPI_ISL_2338346, EPI_ISL_2338347, EPI_ISL_2338348, EPI_ISL_2338349, EPI_ISL_2338350, EPI_ISL_2338351, EPI_ISL_2338352, EPI_ISL_2338353, EPI_ISL_2338354, EPI_ISL_2338355, EPI_ISL_2338356, EPI_ISL_2338357, EPI_ISL_2338358, EPI_ISL_2338359, EPI_ISL_2338360, EPI_ISL_2338361, EPI_ISL_2338362, EPI_ISL_2338363, EPI_ISL_2338364, EPI_ISL_2338365, EPI_ISL_2338366, EPI_ISL_2338367, EPI_ISL_2338368, EPI_ISL_2338369, EPI_ISL_2338370, EPI_ISL_2338371, EPI_ISL_2338372, EPI_ISL_2338373, EPI_ISL_2338374, EPI_ISL_2338375, EPI_ISL_2338376, EPI_ISL_2338377, EPI_ISL_2338378, EPI_ISL_2338379, EPI_ISL_2338380, EPI_ISL_2338381, EPI_ISL_2338382, EPI_ISL_2338383, EPI_ISL_2338384, EPI_ISL_2338385, EPI_ISL_2338386, EPI_ISL_2338387, EPI_ISL_2338388, EPI_ISL_2338389, EPI_ISL_2338390, EPI_ISL_2338391, EPI_ISL_2338392, EPI_ISL_2338393, EPI_ISL_2338394, EPI_ISL_2338395, EPI_ISL_2338396, EPI_ISL_2338401, EPI_ISL_2338402, EPI_ISL_2338403, EPI_ISL_2338404, EPI_ISL_2338405, EPI_ISL_2339035, EPI_ISL_2339036, EPI_ISL_2339037, EPI_ISL_2339038, EPI_ISL_2339039, EPI_ISL_2339040, EPI_ISL_2339041, EPI_ISL_2339042, EPI_ISL_2339043, EPI_ISL_2339044, EPI_ISL_2339045, EPI_ISL_2339046, EPI_ISL_2339047, EPI_ISL_2339048, EPI_ISL_2339049, EPI_ISL_2339050, EPI_ISL_2339051, EPI_ISL_2339052, EPI_ISL_2339053, EPI_ISL_2339054, EPI_ISL_2339055, EPI_ISL_2339056, EPI_ISL_2339057, EPI_ISL_2339058, EPI_ISL_2339059, EPI_ISL_2339060, EPI_ISL_2339061, EPI_ISL_2339062, EPI_ISL_2339063, EPI_ISL_2339064, EPI_ISL_2339065, EPI_ISL_2339066, EPI_ISL_2339067, EPI_ISL_2339068, EPI_ISL_2339069, EPI_ISL_2339070, EPI_ISL_2339071, EPI_ISL_2339072, EPI_ISL_2339073, EPI_ISL_2339074, EPI_ISL_2339075, EPI_ISL_2339076, EPI_ISL_2339077, EPI_ISL_2339078, EPI_ISL_2339079, EPI_ISL_2339080, EPI_ISL_2339081, EPI_ISL_2339082, EPI_ISL_2339083, EPI_ISL_2339084, EPI_ISL_2339085, EPI_ISL_2339086, EPI_ISL_2339087, EPI_ISL_2339088, EPI_ISL_2339089, EPI_ISL_2339090, EPI_ISL_2339091, EPI_ISL_2339092, EPI_ISL_2339093, EPI_ISL_2339094, EPI_ISL_2339095, EPI_ISL_2339096, EPI_ISL_2339097, EPI_ISL_2339098, EPI_ISL_2339099, EPI_ISL_2339100, EPI_ISL_2339101, EPI_ISL_2339102, EPI_ISL_2339103, EPI_ISL_2339104, EPI_ISL_2339105, EPI_ISL_2339106, EPI_ISL_2339107, EPI_ISL_2339108, EPI_ISL_2339109, EPI_ISL_2339110, EPI_ISL_2339111, EPI_ISL_2339112, EPI_ISL_2339113, EPI_ISL_2339114, EPI_ISL_2339115, EPI_ISL_2339116, EPI_ISL_2339117, EPI_ISL_2339118, EPI_ISL_2339119, EPI_ISL_2339120, EPI_ISL_2339121, EPI_ISL_2339122, EPI_ISL_2339123, EPI_ISL_2339124, EPI_ISL_2339125, EPI_ISL_2339126, EPI_ISL_2339127, EPI_ISL_2339128, EPI_ISL_2339129, EPI_ISL_2339130, EPI_ISL_2339131, EPI_ISL_2339132, EPI_ISL_2339133, EPI_ISL_2339134, EPI_ISL_2339135, EPI_ISL_2339136, EPI_ISL_2339137, EPI_ISL_2339138, EPI_ISL_2339139, EPI_ISL_2339140, EPI_ISL_2339141, EPI_ISL_2339142, EPI_ISL_2339143, EPI_ISL_2339206, EPI_ISL_2339207, EPI_ISL_2339208, EPI_ISL_2339209, EPI_ISL_2339210, EPI_ISL_2339211, EPI_ISL_2339212, EPI_ISL_2339213, EPI_ISL_2339214, EPI_ISL_2339215, EPI_ISL_2339216, EPI_ISL_2339217, EPI_ISL_2339218, EPI_ISL_2339219, EPI_ISL_2339220, EPI_ISL_2339221, EPI_ISL_2339222, EPI_ISL_2339223, EPI_ISL_2339224, EPI_ISL_2339225, EPI_ISL_2339226, EPI_ISL_2339227, EPI_ISL_2339228, EPI_ISL_2339229, EPI_ISL_2339230, EPI_ISL_2339231, EPI_ISL_2339232, EPI_ISL_2339233, EPI_ISL_2339234, EPI_ISL_2339235, EPI_ISL_2339236, EPI_ISL_2339237, EPI_ISL_2339238, EPI_ISL_2339239, EPI_ISL_2339240, EPI_ISL_2339241, EPI_ISL_2339242, EPI_ISL_2339243, EPI_ISL_2339244, EPI_ISL_2339245, EPI_ISL_2339246, EPI_ISL_2339247, EPI_ISL_2339248, EPI_ISL_2339249, EPI_ISL_2339250, EPI_ISL_2339251, EPI_ISL_2339252, EPI_ISL_2339253, EPI_ISL_2339254, EPI_ISL_2339255, EPI_ISL_2339256, EPI_ISL_2339257

see above	Pathogen Genomics Center, National Institute of Infectious Diseases	Pathogen Genomics Center, National Institute of Infectious Diseases	Kentaro Itokawa; Makoto Kuroda; Masanori Hashino; Rina Tanaka; Tsuyoshi Sekizuka

EPI_ISL_2325185, EPI_ISL_2325186, EPI_ISL_2325187, EPI_ISL_2325188, EPI_ISL_2325189, EPI_ISL_2325190, EPI_ISL_2325191, EPI_ISL_2325192, EPI_ISL_2325193, EPI_ISL_2325194, EPI_ISL_2325195, EPI_ISL_2325196, EPI_ISL_2325197, EPI_ISL_2325198, EPI_ISL_2325199, EPI_ISL_2325200, EPI_ISL_2325201, EPI_ISL_2325202, EPI_ISL_2325203, EPI_ISL_2325204, EPI_ISL_2325205, EPI_ISL_2325206, EPI_ISL_2325207, EPI_ISL_2325208, EPI_ISL_2325209, EPI_ISL_2325210, EPI_ISL_2325211, EPI_ISL_2325212, EPI_ISL_2325213, EPI_ISL_2325214, EPI_ISL_2325215, EPI_ISL_2325216, EPI_ISL_2325217, EPI_ISL_2325218, EPI_ISL_2325219, EPI_ISL_2325220, EPI_ISL_2325221, EPI_ISL_2325222, EPI_ISL_2325223, EPI_ISL_2325224, EPI_ISL_2325225, EPI_ISL_2325226, EPI_ISL_2325227, EPI_ISL_2325228, EPI_ISL_2325229, EPI_ISL_2325230, EPI_ISL_2325231, EPI_ISL_2325232, EPI_ISL_2325233, EPI_ISL_2325234, EPI_ISL_2325235, EPI_ISL_2325236, EPI_ISL_2325237, EPI_ISL_2325238, EPI_ISL_2325239, EPI_ISL_2325240, EPI_ISL_2325241, EPI_ISL_2325242, EPI_ISL_2325243, EPI_ISL_2325244, EPI_ISL_2325245, EPI_ISL_2325246, EPI_ISL_2325247, EPI_ISL_2325248, EPI_ISL_2325249, EPI_ISL_2325250, EPI_ISL_2325251, EPI_ISL_2325252, EPI_ISL_2325253, EPI_ISL_2325254, EPI_ISL_2325255, EPI_ISL_2325256, EPI_ISL_2325257, EPI_ISL_2325258, EPI_ISL_2325259, EPI_ISL_2325260, EPI_ISL_2325261, EPI_ISL_2325262, EPI_ISL_2325263, EPI_ISL_2325264, EPI_ISL_2325265, EPI_ISL_2325266, EPI_ISL_2325267, EPI_ISL_2325268, EPI_ISL_2325269, EPI_ISL_2325270, EPI_ISL_2325271, EPI_ISL_2325272, EPI_ISL_2325273, EPI_ISL_2325274, EPI_ISL_2325275, EPI_ISL_2325276, EPI_ISL_2325277, EPI_ISL_2325278, EPI_ISL_2325279, EPI_ISL_2325280, EPI_ISL_2325281, EPI_ISL_2325282, EPI_ISL_2325283, EPI_ISL_2325284, EPI_ISL_2325285, EPI_ISL_2325286, EPI_ISL_2325287, EPI_ISL_2325288, EPI_ISL_2325289, EPI_ISL_2325290, EPI_ISL_2325291, EPI_ISL_2325292, EPI_ISL_2325293, EPI_ISL_2325294, EPI_ISL_2325295, EPI_ISL_2325296, EPI_ISL_2325297, EPI_ISL_2325298, EPI_ISL_2325299, EPI_ISL_2325300, EPI_ISL_2325301, EPI_ISL_2325302, EPI_ISL_2325303, EPI_ISL_2325304, EPI_ISL_2325305, EPI_ISL_2325306, EPI_ISL_2325307, EPI_ISL_2325308, EPI_ISL_2325309, EPI_ISL_2325310, EPI_ISL_2325311, EPI_ISL_2325312, EPI_ISL_2325313, EPI_ISL_2325314, EPI_ISL_2325315, EPI_ISL_2325316, EPI_ISL_2325317, EPI_ISL_2325318, EPI_ISL_2325319, EPI_ISL_2325320, EPI_ISL_2325321, EPI_ISL_2325322, EPI_ISL_2325323, EPI_ISL_2325324, EPI_ISL_2325325, EPI_ISL_2325326, EPI_ISL_2325327, EPI_ISL_2325328, EPI_ISL_2325329, EPI_ISL_2325330, EPI_ISL_2325331, EPI_ISL_2325332, EPI_ISL_2325333, EPI_ISL_2325334, EPI_ISL_2325335, EPI_ISL_2325336, EPI_ISL_2325337, EPI_ISL_2325338, EPI_ISL_2325339, EPI_ISL_2325340, EPI_ISL_2325341, EPI_ISL_2325342, EPI_ISL_2325343, EPI_ISL_2325344, EPI_ISL_2325345, EPI_ISL_2325346, EPI_ISL_2325347, EPI_ISL_2325348, EPI_ISL_2325349, EPI_ISL_2325350, EPI_ISL_2325351, EPI_ISL_2325352, EPI_ISL_2325353, EPI_ISL_2325354, EPI_ISL_2325355, EPI_ISL_2325356, EPI_ISL_2325357, EPI_ISL_2325358, EPI_ISL_2325359, EPI_ISL_2325360, EPI_ISL_2325361, EPI_ISL_2325362, EPI_ISL_2325363, EPI_ISL_2325364, EPI_ISL_2325365, EPI_ISL_2325366, EPI_ISL_2325367, EPI_ISL_2325368, EPI_ISL_2325369, EPI_ISL_2325370, EPI_ISL_2325371, EPI_ISL_2325372, EPI_ISL_2325373, EPI_ISL_2325374, EPI_ISL_2325375, EPI_ISL_2325376, EPI_ISL_2325377, EPI_ISL_2325378, EPI_ISL_2325379, EPI_ISL_2325380, EPI_ISL_2325381, EPI_ISL_2325382, EPI_ISL_2325383, EPI_ISL_2325384, EPI_ISL_2325385, EPI_ISL_2325386, EPI_ISL_2325387, EPI_ISL_2325388, EPI_ISL_2325389, EPI_ISL_2325390, EPI_ISL_2325391, EPI_ISL_2325392, EPI_ISL_2325393, EPI_ISL_2325394, EPI_ISL_2325395, EPI_ISL_2325396, EPI_ISL_2325397, EPI_ISL_2325398, EPI_ISL_2325399, EPI_ISL_2325400, EPI_ISL_2325401, EPI_ISL_2325402, EPI_ISL_2325403, EPI_ISL_2325404, EPI_ISL_2325405, EPI_ISL_2325406, EPI_ISL_2325407, EPI_ISL_2325408, EPI_ISL_2325409, EPI_ISL_2325410, EPI_ISL_2325411, EPI_ISL_2325412, EPI_ISL_2325413, EPI_ISL_2325414, EPI_ISL_2325415, EPI_ISL_2325416, EPI_ISL_2325417, EPI_ISL_2325418, EPI_ISL_2325419, EPI_ISL_2325420, EPI_ISL_2325421, EPI_ISL_2325422, EPI_ISL_2325423, EPI_ISL_2325424, EPI_ISL_2325425, EPI_ISL_2325426, EPI_ISL_2325427, EPI_ISL_2325428, EPI_ISL_2325429, EPI_ISL_2325430, EPI_ISL_2325431, EPI_ISL_2325432, EPI_ISL_2325433, EPI_ISL_2325434, EPI_ISL_2325435, EPI_ISL_2325436, EPI_ISL_2325437, EPI_ISL_2325438, EPI_ISL_2325439, EPI_ISL_2325440, EPI_ISL_2325441, EPI_ISL_2325442, EPI_ISL_2325443, EPI_ISL_2325444, EPI_ISL_2325445, EPI_ISL_2325446, EPI_ISL_2325447, EPI_ISL_2325448, EPI_ISL_2325449, EPI_ISL_2325450, EPI_ISL_2325451, EPI_ISL_2325452, EPI_ISL_2325453, EPI_ISL_2325454, EPI_ISL_2325455, EPI_ISL_2325456, EPI_ISL_2325457, EPI_ISL_2325458, EPI_ISL_2325459, EPI_ISL_2325460, EPI_ISL_2325461, EPI_ISL_2325462, EPI_ISL_2325463, EPI_ISL_2325464, EPI_ISL_2325465, EPI_ISL_2325466, EPI_ISL_2325467, EPI_ISL_2325468, EPI_ISL_2325469, EPI_ISL_2325470, EPI_ISL_2325471, EPI_ISL_2325472, EPI_ISL_2325473, EPI_ISL_2325474, EPI_ISL_2325475, EPI_ISL_2325476, EPI_ISL_2325477, EPI_ISL_2325478, EPI_ISL_2325479, EPI_ISL_2325480, EPI_ISL_2325481, EPI_ISL_2325482, EPI_ISL_2325483, EPI_ISL_2325484, EPI_ISL_2325485, EPI_ISL_2325486, EPI_ISL_2325487, EPI_ISL_2325488, EPI_ISL_2325489, EPI_ISL_2325490, EPI_ISL_2325491, EPI_ISL_2325492, EPI_ISL_2325493, EPI_ISL_2325494, EPI_ISL_2325495, EPI_ISL_2325496, EPI_ISL_2325497, EPI_ISL_2325498, EPI_ISL_2325499, EPI_ISL_2325500, EPI_ISL_2325501, EPI_ISL_2325502, EPI_ISL_2325503, EPI_ISL_2325504, EPI_ISL_2325505, EPI_ISL_2325506, EPI_ISL_2325507, EPI_ISL_2325508, EPI_ISL_2325509, EPI_ISL_2325510, EPI_ISL_2325511, EPI_ISL_2325512, EPI_ISL_2325513, EPI_ISL_2325514, EPI_ISL_2325515, EPI_ISL_2325516, EPI_ISL_2325517, EPI_ISL_2325518, EPI_ISL_2325519, EPI_ISL_2325520, EPI_ISL_2325521, EPI_ISL_2325522, EPI_ISL_2325523, EPI_ISL_2325524, EPI_ISL_2325525, EPI_ISL_2325526, EPI_ISL_2325527, EPI_ISL_2325528, EPI_ISL_2325529, EPI_ISL_2325530, EPI_ISL_2325531, EPI_ISL_2325532, EPI_ISL_2325533, EPI_ISL_2325534, EPI_ISL_2325535, EPI_ISL_2325536, EPI_ISL_2325537, EPI_ISL_2325538, EPI_ISL_2325539, EPI_ISL_2325540, EPI_ISL_2325541, EPI_ISL_2325542, EPI_ISL_2325543, EPI_ISL_2325544, EPI_ISL_2325545, EPI_ISL_2325546, EPI_ISL_2325547, EPI_ISL_2325548, EPI_ISL_2325549, EPI_ISL_2325550, EPI_ISL_2325551, EPI_ISL_2325552, EPI_ISL_2325553, EPI_ISL_2325554, EPI_ISL_2325555, EPI_ISL_2325556, EPI_ISL_2325557, EPI_ISL_2325558, EPI_ISL_2325559, EPI_ISL_2325560, EPI_ISL_2325561, EPI_ISL_2325562, EPI_ISL_2325563, EPI_ISL_2325564, EPI_ISL_2325565, EPI_ISL_2325566, EPI_ISL_2325567, EPI_ISL_2325568, EPI_ISL_2325569, EPI_ISL_2325570, EPI_ISL_2325571, EPI_ISL_2325572, EPI_ISL_2325573, EPI_ISL_2325574, EPI_ISL_2325575, EPI_ISL_2325576, EPI_ISL_2325577, EPI_ISL_2325578, EPI_ISL_2325579, EPI_ISL_2325580, EPI_ISL_2325581, EPI_ISL_2325582, EPI_ISL_2325583, EPI_ISL_2325584, EPI_ISL_2325585, EPI_ISL_2325586, EPI_ISL_2325587, EPI_ISL_2325588, EPI_ISL_2325589, EPI_ISL_2325590, EPI_ISL_2325591, EPI_ISL_2325592, EPI_ISL_2325593, EPI_ISL_2325594, EPI_ISL_2325595, EPI_ISL_2325596, EPI_ISL_2325597, EPI_ISL_2325598, EPI_ISL_2325599, EPI_ISL_2325600, EPI_ISL_2325601, EPI_ISL_2325602, EPI_ISL_2325603, EPI_ISL_2325604, EPI_ISL_2325605, EPI_ISL_2325606, EPI_ISL_2325607, EPI_ISL_2325608, EPI_ISL_2325609, EPI_ISL_2325610, EPI_ISL_2325611, EPI_ISL_2325612, EPI_ISL_2325613, EPI_ISL_2325614, EPI_ISL_2325615, EPI_ISL_2325616, EPI_ISL_2325617, EPI_ISL_2325998, EPI_ISL_2325999, EPI_ISL_2326000, EPI_ISL_2326001, EPI_ISL_2326002, EPI_ISL_2326003, EPI_ISL_2326004, EPI_ISL_2326005, EPI_ISL_2326006, EPI_ISL_2326007, EPI_ISL_2326008, EPI_ISL_2326009, EPI_ISL_2326010, EPI_ISL_2326011, EPI_ISL_2326012, EPI_ISL_2326013, EPI_ISL_2326014, EPI_ISL_2326015, EPI_ISL_2326016, EPI_ISL_2326017, EPI_ISL_2326018, EPI_ISL_2326019, EPI_ISL_2326020, EPI_ISL_2326021, EPI_ISL_2326022, EPI_ISL_2326023, EPI_ISL_2326024, EPI_ISL_2326025, EPI_ISL_2326026, EPI_ISL_2326027, EPI_ISL_2326028, EPI_ISL_2326029, EPI_ISL_2326030, EPI_ISL_2326031, EPI_ISL_2326032, EPI_ISL_2326033, EPI_ISL_2326034, EPI_ISL_2326035, EPI_ISL_2326036, EPI_ISL_2326037, EPI_ISL_2326038, EPI_ISL_2326039, EPI_ISL_2326040, EPI_ISL_2326041, EPI_ISL_2326042, EPI_ISL_2326043, EPI_ISL_2326044, EPI_ISL_2326045, EPI_ISL_2326046, EPI_ISL_2326047, EPI_ISL_2326048, EPI_ISL_2326049, EPI_ISL_2326050, EPI_ISL_2326051, EPI_ISL_2326052, EPI_ISL_2326053, EPI_ISL_2326054, EPI_ISL_2326055, EPI_ISL_2326056, EPI_ISL_2326057, EPI_ISL_2326058, EPI_ISL_2326059, EPI_ISL_2326060, EPI_ISL_2326061, EPI_ISL_2326062, EPI_ISL_2326063, EPI_ISL_2326064, EPI_ISL_2326065, EPI_ISL_2326066, EPI_ISL_2326067, EPI_ISL_2326068, EPI_ISL_2326069, EPI_ISL_2326070, EPI_ISL_2326071, EPI_ISL_2326072, EPI_ISL_2326073, EPI_ISL_2326074, EPI_ISL_2326075, EPI_ISL_2326076, EPI_ISL_2326077, EPI_ISL_2326078, EPI_ISL_2326079, EPI_ISL_2326080, EPI_ISL_2326081, EPI_ISL_2326082, EPI_ISL_2326083, EPI_ISL_2326084, EPI_ISL_2326085, EPI_ISL_2326086, EPI_ISL_2326087, EPI_ISL_2326088, EPI_ISL_2326089, EPI_ISL_2326090, EPI_ISL_2326091, EPI_ISL_2326092, EPI_ISL_2326093, EPI_ISL_2326094, EPI_ISL_2326095, EPI_ISL_2326096, EPI_ISL_2326097, EPI_ISL_2326098, EPI_ISL_2326099, EPI_ISL_2326100, EPI_ISL_2326101, EPI_ISL_2326102, EPI_ISL_2326103, EPI_ISL_2326104, EPI_ISL_2326105, EPI_ISL_2326106, EPI_ISL_2326107, EPI_ISL_2326108, EPI_ISL_2326109, EPI_ISL_2326110, EPI_ISL_2326111, EPI_ISL_2326112, EPI_ISL_2326113, EPI_ISL_2326114, EPI_ISL_2326115, EPI_ISL_2326116, EPI_ISL_2326117, EPI_ISL_2326118, EPI_ISL_2326119, EPI_ISL_2326120, EPI_ISL_2326121, EPI_ISL_2326122, EPI_ISL_2326123, EPI_ISL_2326124, EPI_ISL_2326125, EPI_ISL_2326126, EPI_ISL_2326127, EPI_ISL_2326128, EPI_ISL_2326129, EPI_ISL_2326130, EPI_ISL_2326131, EPI_ISL_2326132, EPI_ISL_2326133, EPI_ISL_2326134, EPI_ISL_2326135, EPI_ISL_2326136, EPI_ISL_2326137, EPI_ISL_2326138, EPI_ISL_2326139, EPI_ISL_2326140, EPI_ISL_2326141, EPI_ISL_2326142, EPI_ISL_2326143, EPI_ISL_2326144, EPI_ISL_2326145, EPI_ISL_2326146, EPI_ISL_2326147, EPI_ISL_2326148, EPI_ISL_2326149, EPI_ISL_2326150, EPI_ISL_2326151, EPI_ISL_2326152, EPI_ISL_2326153, EPI_ISL_2326154, EPI_ISL_2326155, EPI_ISL_2326156, EPI_ISL_2326157, EPI_ISL_2326158, EPI_ISL_2326159, EPI_ISL_2326160, EPI_ISL_2326161, EPI_ISL_2326162, EPI_ISL_2326163, EPI_ISL_2326164, EPI_ISL_2326165, EPI_ISL_2326166, EPI_ISL_2326167, EPI_ISL_2326168, EPI_ISL_2326169, EPI_ISL_2326170, EPI_ISL_2326171, EPI_ISL_2326172, EPI_ISL_2326173, EPI_ISL_2326174, EPI_ISL_2326175, EPI_ISL_2326176, EPI_ISL_2326177, EPI_ISL_2326178, EPI_ISL_2326179, EPI_ISL_2326180, EPI_ISL_2326181, EPI_ISL_2326182, EPI_ISL_2326183, EPI_ISL_2326184, EPI_ISL_2326186, EPI_ISL_2326187, EPI_ISL_2326188, EPI_ISL_2326189, EPI_ISL_2326190, EPI_ISL_2326191, EPI_ISL_2326192, EPI_ISL_2326193, EPI_ISL_2326194, EPI_ISL_2326195, EPI_ISL_2326196, EPI_ISL_2326197, EPI_ISL_2326198, EPI_ISL_2326199, EPI_ISL_2326200, EPI_ISL_2326201, EPI_ISL_2326202, EPI_ISL_2326203, EPI_ISL_2326204, EPI_ISL_2326205, EPI_ISL_2326206, EPI_ISL_2326207, EPI_ISL_2326208, EPI_ISL_2326209, EPI_ISL_2326216, EPI_ISL_2326217, EPI_ISL_2326218, EPI_ISL_2326219, EPI_ISL_2326220, EPI_ISL_2326221, EPI_ISL_2326222, EPI_ISL_2326223, EPI_ISL_2326224, EPI_ISL_2326225, EPI_ISL_2326226, EPI_ISL_2326227, EPI_ISL_2326228, EPI_ISL_2326229, EPI_ISL_2326230, EPI_ISL_2326231, EPI_ISL_2326232, EPI_ISL_2326233, EPI_ISL_2326234, EPI_ISL_2326235, EPI_ISL_2326236, EPI_ISL_2326237, EPI_ISL_2326238, EPI_ISL_2326239, EPI_ISL_2326240, EPI_ISL_2326241, EPI_ISL_2326242, EPI_ISL_2326243, EPI_ISL_2326244, EPI_ISL_2326245, EPI_ISL_2326246, EPI_ISL_2326247, EPI_ISL_2326248, EPI_ISL_2326249, EPI_ISL_2326250, EPI_ISL_2326251, EPI_ISL_2326252, EPI_ISL_2326253, EPI_ISL_2326254, EPI_ISL_2326255, EPI_ISL_2326256, EPI_ISL_2326257, EPI_ISL_2326258, EPI_ISL_2326259, EPI_ISL_2326260, EPI_ISL_2326261, EPI_ISL_2326262, EPI_ISL_2326263, EPI_ISL_2326264, EPI_ISL_2326265, EPI_ISL_2326266, EPI_ISL_2326267, EPI_ISL_2326268, EPI_ISL_2326269, EPI_ISL_2326270, EPI_ISL_2326271, EPI_ISL_2326272, EPI_ISL_2326273, EPI_ISL_2326274, EPI_ISL_2326275, EPI_ISL_2326276, EPI_ISL_2326277, EPI_ISL_2326278, EPI_ISL_2326279, EPI_ISL_2326280, EPI_ISL_2326281, EPI_ISL_2326282, EPI_ISL_2326283, EPI_ISL_2326284, EPI_ISL_2326285, EPI_ISL_2326286, EPI_ISL_2326287, EPI_ISL_2326288, EPI_ISL_2326289, EPI_ISL_2326290, EPI_ISL_2326291, EPI_ISL_2326292, EPI_ISL_2326293, EPI_ISL_2326294, EPI_ISL_2326295, EPI_ISL_2326296, EPI_ISL_2326297, EPI_ISL_2326298, EPI_ISL_2326299, EPI_ISL_2326300, EPI_ISL_2326301, EPI_ISL_2326302, EPI_ISL_2326303, EPI_ISL_2326304, EPI_ISL_2326305, EPI_ISL_2326306, EPI_ISL_2326307, EPI_ISL_2326308, EPI_ISL_2326309, EPI_ISL_2326310, EPI_ISL_2326311, EPI_ISL_2326312, EPI_ISL_2326313, EPI_ISL_2326314, EPI_ISL_2326315, EPI_ISL_2326316, EPI_ISL_2326317, EPI_ISL_2326318, EPI_ISL_2326319, EPI_ISL_2326320, EPI_ISL_2326321, EPI_ISL_2326322, EPI_ISL_2326323, EPI_ISL_2326324, EPI_ISL_2326325, EPI_ISL_2326326, EPI_ISL_2326327, EPI_ISL_2326328, EPI_ISL_2326329, EPI_ISL_2326330, EPI_ISL_2326331, EPI_ISL_2326464, EPI_ISL_2326465, EPI_ISL_2326466, EPI_ISL_2326467, EPI_ISL_2326468, EPI_ISL_2326469, EPI_ISL_2326470, EPI_ISL_2326471, EPI_ISL_2326472, EPI_ISL_2326473, EPI_ISL_2326474, EPI_ISL_2326475, EPI_ISL_2326476, EPI_ISL_2326477, EPI_ISL_2326478, EPI_ISL_2326479, EPI_ISL_2326480, EPI_ISL_2326481, EPI_ISL_2326482, EPI_ISL_2326483, EPI_ISL_2326484, EPI_ISL_2326485, EPI_ISL_2326486, EPI_ISL_2326487, EPI_ISL_2326488, EPI_ISL_2326489, EPI_ISL_2326490, EPI_ISL_2326491, EPI_ISL_2326492, EPI_ISL_2326493, EPI_ISL_2326494, EPI_ISL_2326495, EPI_ISL_2326496, EPI_ISL_2326497, EPI_ISL_2326498, EPI_ISL_2326499, EPI_ISL_2326500, EPI_ISL_2326501, EPI_ISL_2326502, EPI_ISL_2326503, EPI_ISL_2326504, EPI_ISL_2326505, EPI_ISL_2326506, EPI_ISL_2326507, EPI_ISL_2326508, EPI_ISL_2326509, EPI_ISL_2326510, EPI_ISL_2326511, EPI_ISL_2326512, EPI_ISL_2326513, EPI_ISL_2326514, EPI_ISL_2326515, EPI_ISL_2326516, EPI_ISL_2326517, EPI_ISL_2326518, EPI_ISL_2326519, EPI_ISL_2326520, EPI_ISL_2326521, EPI_ISL_2326522, EPI_ISL_2326523, EPI_ISL_2326524, EPI_ISL_2326525, EPI_ISL_2326526, EPI_ISL_2326527, EPI_ISL_2326528, EPI_ISL_2326529, EPI_ISL_2326530, EPI_ISL_2326531, EPI_ISL_2326532, EPI_ISL_2326533, EPI_ISL_2326534, EPI_ISL_2326535, EPI_ISL_2326536, EPI_ISL_2326537, EPI_ISL_2326538, EPI_ISL_2326539, EPI_ISL_2326540, EPI_ISL_2326541, EPI_ISL_2326542, EPI_ISL_2326543, EPI_ISL_2326544, EPI_ISL_2326545, EPI_ISL_2326546, EPI_ISL_2326547, EPI_ISL_2326548, EPI_ISL_2326549, EPI_ISL_2326550, EPI_ISL_2326551, EPI_ISL_2326552, EPI_ISL_2326553, EPI_ISL_2326554, EPI_ISL_2326555, EPI_ISL_2326556, EPI_ISL_2326557, EPI_ISL_2326558, EPI_ISL_2326559, EPI_ISL_2326560, EPI_ISL_2326561, EPI_ISL_2326562, EPI_ISL_2326563, EPI_ISL_2326564, EPI_ISL_2326565, EPI_ISL_2326566, EPI_ISL_2326567, EPI_ISL_2326568, EPI_ISL_2326569, EPI_ISL_2326570, EPI_ISL_2326571, EPI_ISL_2326572, EPI_ISL_2326573, EPI_ISL_2326574, EPI_ISL_2326575, EPI_ISL_2326576, EPI_ISL_2326577, EPI_ISL_2326578, EPI_ISL_2326579, EPI_ISL_2326580, EPI_ISL_2326581, EPI_ISL_2326582, EPI_ISL_2326583, EPI_ISL_2326584, EPI_ISL_2326585, EPI_ISL_2326586, EPI_ISL_2326587, EPI_ISL_2326588, EPI_ISL_2326589, EPI_ISL_2326590, EPI_ISL_2326591, EPI_ISL_2326592, EPI_ISL_2326593, EPI_ISL_2326594, EPI_ISL_2326595, EPI_ISL_2326596, EPI_ISL_2326597, EPI_ISL_2326598, EPI_ISL_2326599, EPI_ISL_2326600, EPI_ISL_2326601, EPI_ISL_2326602, EPI_ISL_2326603, EPI_ISL_2326604, EPI_ISL_2326605, EPI_ISL_2326606, EPI_ISL_2326607, EPI_ISL_2326608, EPI_ISL_2326609, EPI_ISL_2326610, EPI_ISL_2326611, EPI_ISL_2326612, EPI_ISL_2326613, EPI_ISL_2326614, EPI_ISL_2326615, EPI_ISL_2326616, EPI_ISL_2326617, EPI_ISL_2326618, EPI_ISL_2326619, EPI_ISL_2326620, EPI_ISL_2326621, EPI_ISL_2326622, EPI_ISL_2326623, EPI_ISL_2326624, EPI_ISL_2326625, EPI_ISL_2326626, EPI_ISL_2326627, EPI_ISL_2326628, EPI_ISL_2326629, EPI_ISL_2326630, EPI_ISL_2326631, EPI_ISL_2326632, EPI_ISL_2326633, EPI_ISL_2326634, EPI_ISL_2326635, EPI_ISL_2326636, EPI_ISL_2326637, EPI_ISL_2326638, EPI_ISL_2326639, EPI_ISL_2326640, EPI_ISL_2326641, EPI_ISL_2326642, EPI_ISL_2326643, EPI_ISL_2326644, EPI_ISL_2326645, EPI_ISL_2326646, EPI_ISL_2326647, EPI_ISL_2326863, EPI_ISL_2326864, EPI_ISL_2326865, EPI_ISL_2326866, EPI_ISL_2326867, EPI_ISL_2326868, EPI_ISL_2326869, EPI_ISL_2326870, EPI_ISL_2326871, EPI_ISL_2326872, EPI_ISL_2326873, EPI_ISL_2326874, EPI_ISL_2326875, EPI_ISL_2326876, EPI_ISL_2326877, EPI_ISL_2326878, EPI_ISL_2326879, EPI_ISL_2326880, EPI_ISL_2326881, EPI_ISL_2326882, EPI_ISL_2326883, EPI_ISL_2326884, EPI_ISL_2326885, EPI_ISL_2326886, EPI_ISL_2326887, EPI_ISL_2326888, EPI_ISL_2326889, EPI_ISL_2326890, EPI_ISL_2326891, EPI_ISL_2326892, EPI_ISL_2326893, EPI_ISL_2326894, EPI_ISL_2326895, EPI_ISL_2326896, EPI_ISL_2326897, EPI_ISL_2326898, EPI_ISL_2326899, EPI_ISL_2326900, EPI_ISL_2326901, EPI_ISL_2326902, EPI_ISL_2326903, EPI_ISL_2326904, EPI_ISL_2326905, EPI_ISL_2326906, EPI_ISL_2326907, EPI_ISL_2326908, EPI_ISL_2326909, EPI_ISL_2326910, EPI_ISL_2326911, EPI_ISL_2326912, EPI_ISL_2326913, EPI_ISL_2326914, EPI_ISL_2326915, EPI_ISL_2326916, EPI_ISL_2326917, EPI_ISL_2326918, EPI_ISL_2326919, EPI_ISL_2326920, EPI_ISL_2326921, EPI_ISL_2326922, EPI_ISL_2326923, EPI_ISL_2326924, EPI_ISL_2326925, EPI_ISL_2326926, EPI_ISL_2326927, EPI_ISL_2326928, EPI_ISL_2326929, EPI_ISL_2326930, EPI_ISL_2326931, EPI_ISL_2326932, EPI_ISL_2326933, EPI_ISL_2326934, EPI_ISL_2326935, EPI_ISL_2326936, EPI_ISL_2326937, EPI_ISL_2326938, EPI_ISL_2326939, EPI_ISL_2326940, EPI_ISL_2326941, EPI_ISL_2326942, EPI_ISL_2326943, EPI_ISL_2326944, EPI_ISL_2326945, EPI_ISL_2326946, EPI_ISL_2326947, EPI_ISL_2326948, EPI_ISL_2326949, EPI_ISL_2326950, EPI_ISL_2326951, EPI_ISL_2326952, EPI_ISL_2326953, EPI_ISL_2326954, EPI_ISL_2326955, EPI_ISL_2326956, EPI_ISL_2326957, EPI_ISL_2326958, EPI_ISL_2326959, EPI_ISL_2326960, EPI_ISL_2326961, EPI_ISL_2326962, EPI_ISL_2326963, EPI_ISL_2326964, EPI_ISL_2326965, EPI_ISL_2326966, EPI_ISL_2326967, EPI_ISL_2326968, EPI_ISL_2326969, EPI_ISL_2326970, EPI_ISL_2326971, EPI_ISL_2326972, EPI_ISL_2326973, EPI_ISL_2326974, EPI_ISL_2326975, EPI_ISL_2326976, EPI_ISL_2326977, EPI_ISL_2326978, EPI_ISL_2326979, EPI_ISL_2326980, EPI_ISL_2326981, EPI_ISL_2326982, EPI_ISL_2326983, EPI_ISL_2326984, EPI_ISL_2326985, EPI_ISL_2326986, EPI_ISL_2326987, EPI_ISL_2326988, EPI_ISL_2326989, EPI_ISL_2326990, EPI_ISL_2326991, EPI_ISL_2326992, EPI_ISL_2326993, EPI_ISL_2326994, EPI_ISL_2326995, EPI_ISL_2326996, EPI_ISL_2326997, EPI_ISL_2326998, EPI_ISL_2326999, EPI_ISL_2327000, EPI_ISL_2327001, EPI_ISL_2327002, EPI_ISL_2327003, EPI_ISL_2327004, EPI_ISL_2327005, EPI_ISL_2327006, EPI_ISL_2327007, EPI_ISL_2327008, EPI_ISL_2327009, EPI_ISL_2327010, EPI_ISL_2327011, EPI_ISL_2327012, EPI_ISL_2327013, EPI_ISL_2327014, EPI_ISL_2327015, EPI_ISL_2327016, EPI_ISL_2327017, EPI_ISL_2327018, EPI_ISL_2327019, EPI_ISL_2327020, EPI_ISL_2327021, EPI_ISL_2327022, EPI_ISL_2327023, EPI_ISL_2327024, EPI_ISL_2327025, EPI_ISL_2327026, EPI_ISL_2327027, EPI_ISL_2327028, EPI_ISL_2327029, EPI_ISL_2327030, EPI_ISL_2327031, EPI_ISL_2327032, EPI_ISL_2327033, EPI_ISL_2327034, EPI_ISL_2327035, EPI_ISL_2327036, EPI_ISL_2327037, EPI_ISL_2327038, EPI_ISL_2327039, EPI_ISL_2327040, EPI_ISL_2327041, EPI_ISL_2327042, EPI_ISL_2327043, EPI_ISL_2327044, EPI_ISL_2327045, EPI_ISL_2327046, EPI_ISL_2327047, EPI_ISL_2327048, EPI_ISL_2327049, EPI_ISL_2327050, EPI_ISL_2327051, EPI_ISL_2327052, EPI_ISL_2327053, EPI_ISL_2327054, EPI_ISL_2327055, EPI_ISL_2327056, EPI_ISL_2327057, EPI_ISL_2327058, EPI_ISL_2327059, EPI_ISL_2327060, EPI_ISL_2327061, EPI_ISL_2327062, EPI_ISL_2327063, EPI_ISL_2327064, EPI_ISL_2327065, EPI_ISL_2327066, EPI_ISL_2327067, EPI_ISL_2327068, EPI_ISL_2327069, EPI_ISL_2327070, EPI_ISL_2327071, EPI_ISL_2327072, EPI_ISL_2327073, EPI_ISL_2327074, EPI_ISL_2327075, EPI_ISL_2327076, EPI_ISL_2327077, EPI_ISL_2327078, EPI_ISL_2327079, EPI_ISL_2327080, EPI_ISL_2327081, EPI_ISL_2327082, EPI_ISL_2327083, EPI_ISL_2327084, EPI_ISL_2327085, EPI_ISL_2327086, EPI_ISL_2327087, EPI_ISL_2327182, EPI_ISL_2327183, EPI_ISL_2327184, EPI_ISL_2327185, EPI_ISL_2327186, EPI_ISL_2327187, EPI_ISL_2327188, EPI_ISL_2327189, EPI_ISL_2327190, EPI_ISL_2327191, EPI_ISL_2327192, EPI_ISL_2327193, EPI_ISL_2327194, EPI_ISL_2327195, EPI_ISL_2327196, EPI_ISL_2327197, EPI_ISL_2327198, EPI_ISL_2327199, EPI_ISL_2327200, EPI_ISL_2327201, EPI_ISL_2327202, EPI_ISL_2327203, EPI_ISL_2327204, EPI_ISL_2327205, EPI_ISL_2327206, EPI_ISL_2327207, EPI_ISL_2327208, EPI_ISL_2327209, EPI_ISL_2327210, EPI_ISL_2327211, EPI_ISL_2327212, EPI_ISL_2327213, EPI_ISL_2327214, EPI_ISL_2327215, EPI_ISL_2327216, EPI_ISL_2327217, EPI_ISL_2327218, EPI_ISL_2327219, EPI_ISL_2327220, EPI_ISL_2327221, EPI_ISL_2327222, EPI_ISL_2327223, EPI_ISL_2327224, EPI_ISL_2327225, EPI_ISL_2327226, EPI_ISL_2327227, EPI_ISL_2327228, EPI_ISL_2327229, EPI_ISL_2327230, EPI_ISL_2327231, EPI_ISL_2327232, EPI_ISL_2327233, EPI_ISL_2327234, EPI_ISL_2327235, EPI_ISL_2327236, EPI_ISL_2327237, EPI_ISL_2327238, EPI_ISL_2327239, EPI_ISL_2327240, EPI_ISL_2327241, EPI_ISL_2327242, EPI_ISL_2327243, EPI_ISL_2327244, EPI_ISL_2327245, EPI_ISL_2327246, EPI_ISL_2327247, EPI_ISL_2327248, EPI_ISL_2327249, EPI_ISL_2327250, EPI_ISL_2327251, EPI_ISL_2327252, EPI_ISL_2327253, EPI_ISL_2327254, EPI_ISL_2327255, EPI_ISL_2327256, EPI_ISL_2327257, EPI_ISL_2327258, EPI_ISL_2327259, EPI_ISL_2327260, EPI_ISL_2327261, EPI_ISL_2327262, EPI_ISL_2327263, EPI_ISL_2327264, EPI_ISL_2327265, EPI_ISL_2327266, EPI_ISL_2327267, EPI_ISL_2327268, EPI_ISL_2327269, EPI_ISL_2327270, EPI_ISL_2327271, EPI_ISL_2327272, EPI_ISL_2327273, EPI_ISL_2327274, EPI_ISL_2327275, EPI_ISL_2327276, EPI_ISL_2327277, EPI_ISL_2327278, EPI_ISL_2327279, EPI_ISL_2327280, EPI_ISL_2327281, EPI_ISL_2327282, EPI_ISL_2327283, EPI_ISL_2327284, EPI_ISL_2327285, EPI_ISL_2327286, EPI_ISL_2327287, EPI_ISL_2327288, EPI_ISL_2327289, EPI_ISL_2327290, EPI_ISL_2327291, EPI_ISL_2327292, EPI_ISL_2327293, EPI_ISL_2327294, EPI_ISL_2327295, EPI_ISL_2327296, EPI_ISL_2327297, EPI_ISL_2327298, EPI_ISL_2327299, EPI_ISL_2327300, EPI_ISL_2327301, EPI_ISL_2327302, EPI_ISL_2327303, EPI_ISL_2327304, EPI_ISL_2327305, EPI_ISL_2327306, EPI_ISL_2327307, EPI_ISL_2327308, EPI_ISL_2327309, EPI_ISL_2327310, EPI_ISL_2327311, EPI_ISL_2327312, EPI_ISL_2327313, EPI_ISL_2327314, EPI_ISL_2327315, EPI_ISL_2327316, EPI_ISL_2327317, EPI_ISL_2327318, EPI_ISL_2327319, EPI_ISL_2327320, EPI_ISL_2327321, EPI_ISL_2327322, EPI_ISL_2327323, EPI_ISL_2327324, EPI_ISL_2327325, EPI_ISL_2327326, EPI_ISL_2327327, EPI_ISL_2327328, EPI_ISL_2327329, EPI_ISL_2327330, EPI_ISL_2327331, EPI_ISL_2327332, EPI_ISL_2327333, EPI_ISL_2327334, EPI_ISL_2327335, EPI_ISL_2327336, EPI_ISL_2327337, EPI_ISL_2327338, EPI_ISL_2327339, EPI_ISL_2327340, EPI_ISL_2327341, EPI_ISL_2327342, EPI_ISL_2327343, EPI_ISL_2327344, EPI_ISL_2327345, EPI_ISL_2327346, EPI_ISL_2327347, EPI_ISL_2327348, EPI_ISL_2327349, EPI_ISL_2327350, EPI_ISL_2327351, EPI_ISL_2327352, EPI_ISL_2327353, EPI_ISL_2327354, EPI_ISL_2327355, EPI_ISL_2327356, EPI_ISL_2327357, EPI_ISL_2327358, EPI_ISL_2327359, EPI_ISL_2327360, EPI_ISL_2327361, EPI_ISL_2327362, EPI_ISL_2327363, EPI_ISL_2327364, EPI_ISL_2327365, EPI_ISL_2327366, EPI_ISL_2327367, EPI_ISL_2327368, EPI_ISL_2327369, EPI_ISL_2327370, EPI_ISL_2327371, EPI_ISL_2327372, EPI_ISL_2327373, EPI_ISL_2327374, EPI_ISL_2327375, EPI_ISL_2327376, EPI_ISL_2327377, EPI_ISL_2327378, EPI_ISL_2327379, EPI_ISL_2327380, EPI_ISL_2327381, EPI_ISL_2327382, EPI_ISL_2327383, EPI_ISL_2327384, EPI_ISL_2327385, EPI_ISL_2327386, EPI_ISL_2327387, EPI_ISL_2327388, EPI_ISL_2327389, EPI_ISL_2327390, EPI_ISL_2327391, EPI_ISL_2327392, EPI_ISL_2327393, EPI_ISL_2327394, EPI_ISL_2327395, EPI_ISL_2327396, EPI_ISL_2327397, EPI_ISL_2327398, EPI_ISL_2327399, EPI_ISL_2327400, EPI_ISL_2327401, EPI_ISL_2327402, EPI_ISL_2327403, EPI_ISL_2327404, EPI_ISL_2327405, EPI_ISL_2327406, EPI_ISL_2327407, EPI_ISL_2327408, EPI_ISL_2327409, EPI_ISL_2327410, EPI_ISL_2327411, EPI_ISL_2327412, EPI_ISL_2327413, EPI_ISL_2327414, EPI_ISL_2327415, EPI_ISL_2327416, EPI_ISL_2327417, EPI_ISL_2327418, EPI_ISL_2327419, EPI_ISL_2327420, EPI_ISL_2327421, EPI_ISL_2327422, EPI_ISL_2327423, EPI_ISL_2327424, EPI_ISL_2327425, EPI_ISL_2327426, EPI_ISL_2327427, EPI_ISL_2327428, EPI_ISL_2327429, EPI_ISL_2327430, EPI_ISL_2327431, EPI_ISL_2327432, EPI_ISL_2327433, EPI_ISL_2327434, EPI_ISL_2327435, EPI_ISL_2327436, EPI_ISL_2327437, EPI_ISL_2327438, EPI_ISL_2327439, EPI_ISL_2327440, EPI_ISL_2327441, EPI_ISL_2327442, EPI_ISL_2327443, EPI_ISL_2327444, EPI_ISL_2327445, EPI_ISL_2327446, EPI_ISL_2327447, EPI_ISL_2327448, EPI_ISL_2327449, EPI_ISL_2327450, EPI_ISL_2327451, EPI_ISL_2327452, EPI_ISL_2327453, EPI_ISL_2327454, EPI_ISL_2327455, EPI_ISL_2327456, EPI_ISL_2327457, EPI_ISL_2327458, EPI_ISL_2327459, EPI_ISL_2327460, EPI_ISL_2327461, EPI_ISL_2327462, EPI_ISL_2327463, EPI_ISL_2327464, EPI_ISL_2327465, EPI_ISL_2327466, EPI_ISL_2327467, EPI_ISL_2327468, EPI_ISL_2327469, EPI_ISL_2327470, EPI_ISL_2327471, EPI_ISL_2327472, EPI_ISL_2327473, EPI_ISL_2327474, EPI_ISL_2327475, EPI_ISL_2328603, EPI_ISL_2328604, EPI_ISL_2328605, EPI_ISL_2328606, EPI_ISL_2328607, EPI_ISL_2328608, EPI_ISL_2328609, EPI_ISL_2328610, EPI_ISL_2328611, EPI_ISL_2328612, EPI_ISL_2328613, EPI_ISL_2328614, EPI_ISL_2328615, EPI_ISL_2328616, EPI_ISL_2328617, EPI_ISL_2328618, EPI_ISL_2328619, EPI_ISL_2328620, EPI_ISL_2328621, EPI_ISL_2328622, EPI_ISL_2328623, EPI_ISL_2328624, EPI_ISL_2328625, EPI_ISL_2328626, EPI_ISL_2328627, EPI_ISL_2328628, EPI_ISL_2328629, EPI_ISL_2328630, EPI_ISL_2328631, EPI_ISL_2328632, EPI_ISL_2328633, EPI_ISL_2328634, EPI_ISL_2328635, EPI_ISL_2328636, EPI_ISL_2328637, EPI_ISL_2328638, EPI_ISL_2328639, EPI_ISL_2328640, EPI_ISL_2328641, EPI_ISL_2328642, EPI_ISL_2328643, EPI_ISL_2328644, EPI_ISL_2328645, EPI_ISL_2328646, EPI_ISL_2328647, EPI_ISL_2328648, EPI_ISL_2328649, EPI_ISL_2328650, EPI_ISL_2328651, EPI_ISL_2328652, EPI_ISL_2328653, EPI_ISL_2328654, EPI_ISL_2328655, EPI_ISL_2328656, EPI_ISL_2328657, EPI_ISL_2328658, EPI_ISL_2328659, EPI_ISL_2328660, EPI_ISL_2328661, EPI_ISL_2328662, EPI_ISL_2328663, EPI_ISL_2328664, EPI_ISL_2328665, EPI_ISL_2328666, EPI_ISL_2328667, EPI_ISL_2328668, EPI_ISL_2328669, EPI_ISL_2328670, EPI_ISL_2328671, EPI_ISL_2328672, EPI_ISL_2328673, EPI_ISL_2328674, EPI_ISL_2328675, EPI_ISL_2328676, EPI_ISL_2328677, EPI_ISL_2328678, EPI_ISL_2328679, EPI_ISL_2328680, EPI_ISL_2328681, EPI_ISL_2328682, EPI_ISL_2328683, EPI_ISL_2328684, EPI_ISL_2328685, EPI_ISL_2328686, EPI_ISL_2328687, EPI_ISL_2328688, EPI_ISL_2328689, EPI_ISL_2328690, EPI_ISL_2328691, EPI_ISL_2328692, EPI_ISL_2328693, EPI_ISL_2328694, EPI_ISL_2328695, EPI_ISL_2328696, EPI_ISL_2328697, EPI_ISL_2328698, EPI_ISL_2328699, EPI_ISL_2328700, EPI_ISL_2328701, EPI_ISL_2328702, EPI_ISL_2328703, EPI_ISL_2328704, EPI_ISL_2328705, EPI_ISL_2328706, EPI_ISL_2328707, EPI_ISL_2328708, EPI_ISL_2328709, EPI_ISL_2328710, EPI_ISL_2328711, EPI_ISL_2328712, EPI_ISL_2328713, EPI_ISL_2328714, EPI_ISL_2328715, EPI_ISL_2328716, EPI_ISL_2328717, EPI_ISL_2328718, EPI_ISL_2328719, EPI_ISL_2328720, EPI_ISL_2328721, EPI_ISL_2328722, EPI_ISL_2328723, EPI_ISL_2328724, EPI_ISL_2328725, EPI_ISL_2328726, EPI_ISL_2328727, EPI_ISL_2328728, EPI_ISL_2328729, EPI_ISL_2328730, EPI_ISL_2328731, EPI_ISL_2328732, EPI_ISL_2328733, EPI_ISL_2328734, EPI_ISL_2328735, EPI_ISL_2328736, EPI_ISL_2328737, EPI_ISL_2328738, EPI_ISL_2328739, EPI_ISL_2328740, EPI_ISL_2328741, EPI_ISL_2328742, EPI_ISL_2328743, EPI_ISL_2328744, EPI_ISL_2328745, EPI_ISL_2328746, EPI_ISL_2328747, EPI_ISL_2328748, EPI_ISL_2328749, EPI_ISL_2328750, EPI_ISL_2328751, EPI_ISL_2328752, EPI_ISL_2328753, EPI_ISL_2328754, EPI_ISL_2328755, EPI_ISL_2328756, EPI_ISL_2328757, EPI_ISL_2328758, EPI_ISL_2328759, EPI_ISL_2328760, EPI_ISL_2328761, EPI_ISL_2328762, EPI_ISL_2328763, EPI_ISL_2328764, EPI_ISL_2328765, EPI_ISL_2328766, EPI_ISL_2328767, EPI_ISL_2328768, EPI_ISL_2328769, EPI_ISL_2328770, EPI_ISL_2328771, EPI_ISL_2328772, EPI_ISL_2328773, EPI_ISL_2328774, EPI_ISL_2328775, EPI_ISL_2328776, EPI_ISL_2328777, EPI_ISL_2328778, EPI_ISL_2328779, EPI_ISL_2328780, EPI_ISL_2328781, EPI_ISL_2328782, EPI_ISL_2328783, EPI_ISL_2328784, EPI_ISL_2328785, EPI_ISL_2328786, EPI_ISL_2328787, EPI_ISL_2328788, EPI_ISL_2328789, EPI_ISL_2328790, EPI_ISL_2328791, EPI_ISL_2328792, EPI_ISL_2328793, EPI_ISL_2328794, EPI_ISL_2328795, EPI_ISL_2328796, EPI_ISL_2328797, EPI_ISL_2328798, EPI_ISL_2328799, EPI_ISL_2328800, EPI_ISL_2328801, EPI_ISL_2328802, EPI_ISL_2328803, EPI_ISL_2328804, EPI_ISL_2328805, EPI_ISL_2328806, EPI_ISL_2328807, EPI_ISL_2328808, EPI_ISL_2328809, EPI_ISL_2328810, EPI_ISL_2328811, EPI_ISL_2328812, EPI_ISL_2328813, EPI_ISL_2328814, EPI_ISL_2328815, EPI_ISL_2328816, EPI_ISL_2328817, EPI_ISL_2328910, EPI_ISL_2328911, EPI_ISL_2328912, EPI_ISL_2328913, EPI_ISL_2328914, EPI_ISL_2328915, EPI_ISL_2328916, EPI_ISL_2328917, EPI_ISL_2328918, EPI_ISL_2328919, EPI_ISL_2328920, EPI_ISL_2328921, EPI_ISL_2328922, EPI_ISL_2328923, EPI_ISL_2328924, EPI_ISL_2328925, EPI_ISL_2328926, EPI_ISL_2328927, EPI_ISL_2328928, EPI_ISL_2328929, EPI_ISL_2328930, EPI_ISL_2328931, EPI_ISL_2328932, EPI_ISL_2328933, EPI_ISL_2328934, EPI_ISL_2328935, EPI_ISL_2328936, EPI_ISL_2328937, EPI_ISL_2328938, EPI_ISL_2328939, EPI_ISL_2328940, EPI_ISL_2328941, EPI_ISL_2328942, EPI_ISL_2328943, EPI_ISL_2328944, EPI_ISL_2328945, EPI_ISL_2328946, EPI_ISL_2328947, EPI_ISL_2328948, EPI_ISL_2328949, EPI_ISL_2328950, EPI_ISL_2328951, EPI_ISL_2328952, EPI_ISL_2328953, EPI_ISL_2328954, EPI_ISL_2328955, EPI_ISL_2328956, EPI_ISL_2328957, EPI_ISL_2328958, EPI_ISL_2328959, EPI_ISL_2328960, EPI_ISL_2328961, EPI_ISL_2328962, EPI_ISL_2328963, EPI_ISL_2328964, EPI_ISL_2328965, EPI_ISL_2328966, EPI_ISL_2328967, EPI_ISL_2328968, EPI_ISL_2328969, EPI_ISL_2328970, EPI_ISL_2328971, EPI_ISL_2328972, EPI_ISL_2328973, EPI_ISL_2328974, EPI_ISL_2328975, EPI_ISL_2328976, EPI_ISL_2328977, EPI_ISL_2328978, EPI_ISL_2328979, EPI_ISL_2328980, EPI_ISL_2328981, EPI_ISL_2328982, EPI_ISL_2328983, EPI_ISL_2328984, EPI_ISL_2328985, EPI_ISL_2328986, EPI_ISL_2328987, EPI_ISL_2328988, EPI_ISL_2328989, EPI_ISL_2328990, EPI_ISL_2328991, EPI_ISL_2328992, EPI_ISL_2328993, EPI_ISL_2328994, EPI_ISL_2328995, EPI_ISL_2328996, EPI_ISL_2328997, EPI_ISL_2328998, EPI_ISL_2328999, EPI_ISL_2329000, EPI_ISL_2329001, EPI_ISL_2329002, EPI_ISL_2329003, EPI_ISL_2329004, EPI_ISL_2329005, EPI_ISL_2329006, EPI_ISL_2329007, EPI_ISL_2329008, EPI_ISL_2329009, EPI_ISL_2329010, EPI_ISL_2329011, EPI_ISL_2329012, EPI_ISL_2329013, EPI_ISL_2329014, EPI_ISL_2329015, EPI_ISL_2329016, EPI_ISL_2329017, EPI_ISL_2329018, EPI_ISL_2329019, EPI_ISL_2329020, EPI_ISL_2329021, EPI_ISL_2329022, EPI_ISL_2329023, EPI_ISL_2329024, EPI_ISL_2329025, EPI_ISL_2329026, EPI_ISL_2329027, EPI_ISL_2329028, EPI_ISL_2329029, EPI_ISL_2329030, EPI_ISL_2329031, EPI_ISL_2329032, EPI_ISL_2329033, EPI_ISL_2329034, EPI_ISL_2329035, EPI_ISL_2329036, EPI_ISL_2329037, EPI_ISL_2329038, EPI_ISL_2329039, EPI_ISL_2329040, EPI_ISL_2329041, EPI_ISL_2329042, EPI_ISL_2329043, EPI_ISL_2329044, EPI_ISL_2329045, EPI_ISL_2329046, EPI_ISL_2329047, EPI_ISL_2329048, EPI_ISL_2329049, EPI_ISL_2329050, EPI_ISL_2329051, EPI_ISL_2329052, EPI_ISL_2329053, EPI_ISL_2329054, EPI_ISL_2329055, EPI_ISL_2329057, EPI_ISL_2329058, EPI_ISL_2329059, EPI_ISL_2329062, EPI_ISL_2329063, EPI_ISL_2329065, EPI_ISL_2329066, EPI_ISL_2329068, EPI_ISL_2329069, EPI_ISL_2329070, EPI_ISL_2329072, EPI_ISL_2329073, EPI_ISL_2329075, EPI_ISL_2329076, EPI_ISL_2329077, EPI_ISL_2329079, EPI_ISL_2329080, EPI_ISL_2329081, EPI_ISL_2329083, EPI_ISL_2329084, EPI_ISL_2329085, EPI_ISL_2329087, EPI_ISL_2329088, EPI_ISL_2329090, EPI_ISL_2329091, EPI_ISL_2329092, EPI_ISL_2329094, EPI_ISL_2329095, EPI_ISL_2329097, EPI_ISL_2329098, EPI_ISL_2329099, EPI_ISL_2329101, EPI_ISL_2329102, EPI_ISL_2329104, EPI_ISL_2329105, EPI_ISL_2329106, EPI_ISL_2329108, EPI_ISL_2329109, EPI_ISL_2329111, EPI_ISL_2329112, EPI_ISL_2329114, EPI_ISL_2329115, EPI_ISL_2329117, EPI_ISL_2329118, EPI_ISL_2329119, EPI_ISL_2329120, EPI_ISL_2329122, EPI_ISL_2329123, EPI_ISL_2329124, EPI_ISL_2329126, EPI_ISL_2329127, EPI_ISL_2329129, EPI_ISL_2329130, EPI_ISL_2329131, EPI_ISL_2329133, EPI_ISL_2329134, EPI_ISL_2329136, EPI_ISL_2329137, EPI_ISL_2329139, EPI_ISL_2329140, EPI_ISL_2329142, EPI_ISL_2329143, EPI_ISL_2329144, EPI_ISL_2329146, EPI_ISL_2329147, EPI_ISL_2329149, EPI_ISL_2329150, EPI_ISL_2329152, EPI_ISL_2329153, EPI_ISL_2329154, EPI_ISL_2329156, EPI_ISL_2329157, EPI_ISL_2329159, EPI_ISL_2329160, EPI_ISL_2329161, EPI_ISL_2329163, EPI_ISL_2329165, EPI_ISL_2329166, EPI_ISL_2329167, EPI_ISL_2329168, EPI_ISL_2329169, EPI_ISL_2329171, EPI_ISL_2329173, EPI_ISL_2329174, EPI_ISL_2329175, EPI_ISL_2329177, EPI_ISL_2329178, EPI_ISL_2329179, EPI_ISL_2329181, EPI_ISL_2329182, EPI_ISL_2329184, EPI_ISL_2329185, EPI_ISL_2329186, EPI_ISL_2329188, EPI_ISL_2329189, EPI_ISL_2329190, EPI_ISL_2329192, EPI_ISL_2329193, EPI_ISL_2329195, EPI_ISL_2329196, EPI_ISL_2329198, EPI_ISL_2329199, EPI_ISL_2329200, EPI_ISL_2329202, EPI_ISL_2329203, EPI_ISL_2329205, EPI_ISL_2329206, EPI_ISL_2329208, EPI_ISL_2329209, EPI_ISL_2329211, EPI_ISL_2329212, EPI_ISL_2329213, EPI_ISL_2329215, EPI_ISL_2329216, EPI_ISL_2329218, EPI_ISL_2329219, EPI_ISL_2329220, EPI_ISL_2329222, EPI_ISL_2329223, EPI_ISL_2329225, EPI_ISL_2329226, EPI_ISL_2329228, EPI_ISL_2329229, EPI_ISL_2329231, EPI_ISL_2329232, EPI_ISL_2329234, EPI_ISL_2329235, EPI_ISL_2329237, EPI_ISL_2329238, EPI_ISL_2329811, EPI_ISL_2329812, EPI_ISL_2329813, EPI_ISL_2329814, EPI_ISL_2329815, EPI_ISL_2329816, EPI_ISL_2329817, EPI_ISL_2329818, EPI_ISL_2329819, EPI_ISL_2329820, EPI_ISL_2329821, EPI_ISL_2329822, EPI_ISL_2329823, EPI_ISL_2329824, EPI_ISL_2329825, EPI_ISL_2329826, EPI_ISL_2329827, EPI_ISL_2329828, EPI_ISL_2329829, EPI_ISL_2329830, EPI_ISL_2329831, EPI_ISL_2329832, EPI_ISL_2329833, EPI_ISL_2329834, EPI_ISL_2329835, EPI_ISL_2329836, EPI_ISL_2329837, EPI_ISL_2329838, EPI_ISL_2329839, EPI_ISL_2329840, EPI_ISL_2329841, EPI_ISL_2329842, EPI_ISL_2329843, EPI_ISL_2329844, EPI_ISL_2329845, EPI_ISL_2329846, EPI_ISL_2329847, EPI_ISL_2329848, EPI_ISL_2329849, EPI_ISL_2329850, EPI_ISL_2329851, EPI_ISL_2329852, EPI_ISL_2329853, EPI_ISL_2329854, EPI_ISL_2329855, EPI_ISL_2329856, EPI_ISL_2329857, EPI_ISL_2329858, EPI_ISL_2329859, EPI_ISL_2329860, EPI_ISL_2329861, EPI_ISL_2329862, EPI_ISL_2329863, EPI_ISL_2329864, EPI_ISL_2329865, EPI_ISL_2329866, EPI_ISL_2329867, EPI_ISL_2329868, EPI_ISL_2329869, EPI_ISL_2329870, EPI_ISL_2329871, EPI_ISL_2329872, EPI_ISL_2329873, EPI_ISL_2329874, EPI_ISL_2329875, EPI_ISL_2329876, EPI_ISL_2329877, EPI_ISL_2329878, EPI_ISL_2329879, EPI_ISL_2329880, EPI_ISL_2329881, EPI_ISL_2329882, EPI_ISL_2329883, EPI_ISL_2329884, EPI_ISL_2329885, EPI_ISL_2329886, EPI_ISL_2329887, EPI_ISL_2329888, EPI_ISL_2329889, EPI_ISL_2329890, EPI_ISL_2329891, EPI_ISL_2329892, EPI_ISL_2329893, EPI_ISL_2329894, EPI_ISL_2329895, EPI_ISL_2329896, EPI_ISL_2329897, EPI_ISL_2329898, EPI_ISL_2329899, EPI_ISL_2329900, EPI_ISL_2329901, EPI_ISL_2329902, EPI_ISL_2329903, EPI_ISL_2329904, EPI_ISL_2329905, EPI_ISL_2329906, EPI_ISL_2329907, EPI_ISL_2329908, EPI_ISL_2329909, EPI_ISL_2329910, EPI_ISL_2329911, EPI_ISL_2329912, EPI_ISL_2329913, EPI_ISL_2329914, EPI_ISL_2329915, EPI_ISL_2329916, EPI_ISL_2329917, EPI_ISL_2329918, EPI_ISL_2329919, EPI_ISL_2329920, EPI_ISL_2329921, EPI_ISL_2329922, EPI_ISL_2329923, EPI_ISL_2329924, EPI_ISL_2329925, EPI_ISL_2329926, EPI_ISL_2329927, EPI_ISL_2329928, EPI_ISL_2329929, EPI_ISL_2329930, EPI_ISL_2329931, EPI_ISL_2329932, EPI_ISL_2329933, EPI_ISL_2329934, EPI_ISL_2329935, EPI_ISL_2329936, EPI_ISL_2329937, EPI_ISL_2329938, EPI_ISL_2329939, EPI_ISL_2329940, EPI_ISL_2329941, EPI_ISL_2329942, EPI_ISL_2329943, EPI_ISL_2329944, EPI_ISL_2329945, EPI_ISL_2329946, EPI_ISL_2329947, EPI_ISL_2329948, EPI_ISL_2329949, EPI_ISL_2329950, EPI_ISL_2329951, EPI_ISL_2329952, EPI_ISL_2329953, EPI_ISL_2329954, EPI_ISL_2329955, EPI_ISL_2329956, EPI_ISL_2329957, EPI_ISL_2329958, EPI_ISL_2329959, EPI_ISL_2329960, EPI_ISL_2329961, EPI_ISL_2329962, EPI_ISL_2329963, EPI_ISL_2329964, EPI_ISL_2329965, EPI_ISL_2329966, EPI_ISL_2329967, EPI_ISL_2329968, EPI_ISL_2329969, EPI_ISL_2329970, EPI_ISL_2329971, EPI_ISL_2329972, EPI_ISL_2329973, EPI_ISL_2329974, EPI_ISL_2329975, EPI_ISL_2329976, EPI_ISL_2329977, EPI_ISL_2329978, EPI_ISL_2329979, EPI_ISL_2329980, EPI_ISL_2329981, EPI_ISL_2329982, EPI_ISL_2329983, EPI_ISL_2329984, EPI_ISL_2329985, EPI_ISL_2329986, EPI_ISL_2329987, EPI_ISL_2329988, EPI_ISL_2329989, EPI_ISL_2329990, EPI_ISL_2329991, EPI_ISL_2329992, EPI_ISL_2329993, EPI_ISL_2329994, EPI_ISL_2329995, EPI_ISL_2329996, EPI_ISL_2329997, EPI_ISL_2329998, EPI_ISL_2329999, EPI_ISL_2330000, EPI_ISL_2330001, EPI_ISL_2330002, EPI_ISL_2330003, EPI_ISL_2330004, EPI_ISL_2330005, EPI_ISL_2330006, EPI_ISL_2330007, EPI_ISL_2330008, EPI_ISL_2330009, EPI_ISL_2330010, EPI_ISL_2330011, EPI_ISL_2330012, EPI_ISL_2330013,

EPI_ISL_2330014, EPI_ISL_2330015, EPI_ISL_2330016, EPI_ISL_2330017, EPI_ISL_2330018, EPI_ISL_2330019, EPI_ISL_2330020, EPI_ISL_2330021, EPI_ISL_2330022, EPI_ISL_2330023, EPI_ISL_2330024, EPI_ISL_2330025, EPI_ISL_2330026, EPI_ISL_2330027, EPI_ISL_2330028, EPI_ISL_2330029, EPI_ISL_2330030, EPI_ISL_2330031, EPI_ISL_2330032, EPI_ISL_2330033, EPI_ISL_2330034, EPI_ISL_2330035,
EPI_ISL_2330036, EPI_ISL_2330037, EPI_ISL_2330038, EPI_ISL_2330039, EPI_ISL_2330040, EPI_ISL_2330041, EPI_ISL_2330042, EPI_ISL_2330043, EPI_ISL_2330044, EPI_ISL_2330045, EPI_ISL_2330046, EPI_ISL_2330047, EPI_ISL_2330048, EPI_ISL_2330049, EPI_ISL_2330050, EPI_ISL_2330051, EPI_ISL_2330052, EPI_ISL_2330053, EPI_ISL_2330054, EPI_ISL_2330055, EPI_ISL_2330056, EPI_ISL_2330057,
EPI_ISL_2330058, EPI_ISL_2330059, EPI_ISL_2330060, EPI_ISL_2330061, EPI_ISL_2330062, EPI_ISL_2330063, EPI_ISL_2330064, EPI_ISL_2330065, EPI_ISL_2330066, EPI_ISL_2330067, EPI_ISL_2330068, EPI_ISL_2330069, EPI_ISL_2330070, EPI_ISL_2330071, EPI_ISL_2330072, EPI_ISL_2330073, EPI_ISL_2330074, EPI_ISL_2330075, EPI_ISL_2330076, EPI_ISL_2330077, EPI_ISL_2330078, EPI_ISL_2330079,
EPI_ISL_2330080, EPI_ISL_2330081, EPI_ISL_2330082, EPI_ISL_2330083, EPI_ISL_2330084, EPI_ISL_2330085, EPI_ISL_2330086, EPI_ISL_2330087, EPI_ISL_2330088, EPI_ISL_2330089, EPI_ISL_2330090, EPI_ISL_2330091, EPI_ISL_2330092, EPI_ISL_2330093, EPI_ISL_2330094, EPI_ISL_2330095, EPI_ISL_2330096, EPI_ISL_2330097, EPI_ISL_2330098, EPI_ISL_2330099, EPI_ISL_2330100, EPI_ISL_2330101,
EPI_ISL_2330102, EPI_ISL_2330103, EPI_ISL_2330104, EPI_ISL_2330105, EPI_ISL_2330106, EPI_ISL_2330107, EPI_ISL_2330108, EPI_ISL_2330109, EPI_ISL_2330110, EPI_ISL_2330111, EPI_ISL_2330112, EPI_ISL_2330113, EPI_ISL_2330114, EPI_ISL_2330115, EPI_ISL_2330116, EPI_ISL_2330117, EPI_ISL_2330118, EPI_ISL_2330119, EPI_ISL_2330120, EPI_ISL_2330121, EPI_ISL_2330122, EPI_ISL_2330123,
EPI_ISL_2330124, EPI_ISL_2330125, EPI_ISL_2330126, EPI_ISL_2330127, EPI_ISL_2330128, EPI_ISL_2330129, EPI_ISL_2330130, EPI_ISL_2330131, EPI_ISL_2330132, EPI_ISL_2330133, EPI_ISL_2330134, EPI_ISL_2330135, EPI_ISL_2330136, EPI_ISL_2330137, EPI_ISL_2330138, EPI_ISL_2330139, EPI_ISL_2330140, EPI_ISL_2330141, EPI_ISL_2330142, EPI_ISL_2330143, EPI_ISL_2330144, EPI_ISL_2330145,
EPI_ISL_2330146, EPI_ISL_2330147, EPI_ISL_2330148, EPI_ISL_2330149, EPI_ISL_2330150, EPI_ISL_2330151, EPI_ISL_2330152, EPI_ISL_2330153, EPI_ISL_2330154, EPI_ISL_2330155, EPI_ISL_2330156, EPI_ISL_2330157, EPI_ISL_2330158, EPI_ISL_2330159, EPI_ISL_2330160, EPI_ISL_2330161, EPI_ISL_2330162, EPI_ISL_2330163, EPI_ISL_2330164, EPI_ISL_2330165, EPI_ISL_2330166, EPI_ISL_2330167,
EPI_ISL_2330168, EPI_ISL_2330169, EPI_ISL_2330170, EPI_ISL_2330171, EPI_ISL_2330172, EPI_ISL_2330173, EPI_ISL_2330174, EPI_ISL_2330175, EPI_ISL_2330176, EPI_ISL_2330177, EPI_ISL_2330178, EPI_ISL_2330179, EPI_ISL_2330180, EPI_ISL_2330181, EPI_ISL_2330182, EPI_ISL_2330183, EPI_ISL_2330184, EPI_ISL_2330185, EPI_ISL_2330186, EPI_ISL_2330187, EPI_ISL_2330188, EPI_ISL_2330189,
EPI_ISL_2330190, EPI_ISL_2330191, EPI_ISL_2330192, EPI_ISL_2330193, EPI_ISL_2330194, EPI_ISL_2330195, EPI_ISL_2330196, EPI_ISL_2330197, EPI_ISL_2330198, EPI_ISL_2330199, EPI_ISL_2330200, EPI_ISL_2330201, EPI_ISL_2330202, EPI_ISL_2330203, EPI_ISL_2330204, EPI_ISL_2330205, EPI_ISL_2330206, EPI_ISL_2330207, EPI_ISL_2330208, EPI_ISL_2330209, EPI_ISL_2330210, EPI_ISL_2330211,
EPI_ISL_2330212, EPI_ISL_2330213, EPI_ISL_2330214, EPI_ISL_2330215, EPI_ISL_2330216, EPI_ISL_2330217, EPI_ISL_2330218, EPI_ISL_2330219, EPI_ISL_2330220, EPI_ISL_2330221, EPI_ISL_2330222, EPI_ISL_2330223, EPI_ISL_2330224, EPI_ISL_2330225, EPI_ISL_2330226, EPI_ISL_2330227, EPI_ISL_2330228, EPI_ISL_2330229, EPI_ISL_2330230, EPI_ISL_2330231, EPI_ISL_2330232, EPI_ISL_2330233,
EPI_ISL_2330234, EPI_ISL_2330235, EPI_ISL_2330236, EPI_ISL_2330237, EPI_ISL_2330238, EPI_ISL_2330239, EPI_ISL_2330240, EPI_ISL_2330241, EPI_ISL_2330242, EPI_ISL_2330243, EPI_ISL_2330244, EPI_ISL_2330245, EPI_ISL_2330246, EPI_ISL_2330247, EPI_ISL_2330248, EPI_ISL_2330249, EPI_ISL_2330250, EPI_ISL_2330251, EPI_ISL_2330252, EPI_ISL_2330253, EPI_ISL_2330254, EPI_ISL_2330255,
EPI_ISL_2330256, EPI_ISL_2330257, EPI_ISL_2330258, EPI_ISL_2330259, EPI_ISL_2330260, EPI_ISL_2330261, EPI_ISL_2330262, EPI_ISL_2330263, EPI_ISL_2330264, EPI_ISL_2330265, EPI_ISL_2330266, EPI_ISL_2330267, EPI_ISL_2330268, EPI_ISL_2330269, EPI_ISL_2330270, EPI_ISL_2330271, EPI_ISL_2330272, EPI_ISL_2330273, EPI_ISL_2330274, EPI_ISL_2330275, EPI_ISL_2330276, EPI_ISL_2330277,
EPI_ISL_2330278, EPI_ISL_2330279, EPI_ISL_2330280, EPI_ISL_2330281, EPI_ISL_2330282, EPI_ISL_2330283, EPI_ISL_2330284, EPI_ISL_2330285, EPI_ISL_2330286, EPI_ISL_2330287, EPI_ISL_2330288, EPI_ISL_2330289, EPI_ISL_2330290, EPI_ISL_2330291, EPI_ISL_2330292, EPI_ISL_2330293, EPI_ISL_2330294, EPI_ISL_2330295, EPI_ISL_2330296, EPI_ISL_2330297, EPI_ISL_2330298, EPI_ISL_2330299,
EPI_ISL_2330300, EPI_ISL_2330301, EPI_ISL_2330302, EPI_ISL_2330303, EPI_ISL_2330304, EPI_ISL_2330305, EPI_ISL_2330306, EPI_ISL_2330307, EPI_ISL_2330308, EPI_ISL_2330309, EPI_ISL_2330310, EPI_ISL_2330311, EPI_ISL_2330312, EPI_ISL_2330313, EPI_ISL_2330314, EPI_ISL_2330315, EPI_ISL_2330316, EPI_ISL_2330317, EPI_ISL_2330318, EPI_ISL_2330319, EPI_ISL_2330320, EPI_ISL_2330321,
EPI_ISL_2330322, EPI_ISL_2330323, EPI_ISL_2330324, EPI_ISL_2330325, EPI_ISL_2330326, EPI_ISL_2330327, EPI_ISL_2330629, EPI_ISL_2330630, EPI_ISL_2330631, EPI_ISL_2330632, EPI_ISL_2330633, EPI_ISL_2330634, EPI_ISL_2330635, EPI_ISL_2330636, EPI_ISL_2330637, EPI_ISL_2330638, EPI_ISL_2330639, EPI_ISL_2330640, EPI_ISL_2330641, EPI_ISL_2330642, EPI_ISL_2330643, EPI_ISL_2330644,
EPI_ISL_2330645, EPI_ISL_2330646, EPI_ISL_2330647, EPI_ISL_2330648, EPI_ISL_2330649, EPI_ISL_2330650, EPI_ISL_2330651, EPI_ISL_2330652, EPI_ISL_2330653, EPI_ISL_2330654, EPI_ISL_2330655, EPI_ISL_2330656, EPI_ISL_2330657, EPI_ISL_2330658, EPI_ISL_2330659, EPI_ISL_2330660, EPI_ISL_2330661, EPI_ISL_2330662, EPI_ISL_2330663, EPI_ISL_2330664, EPI_ISL_2330665, EPI_ISL_2330666,
EPI_ISL_2330667, EPI_ISL_2330668, EPI_ISL_2330669, EPI_ISL_2330670, EPI_ISL_2330671, EPI_ISL_2330672, EPI_ISL_2330673, EPI_ISL_2330674, EPI_ISL_2330675, EPI_ISL_2330676, EPI_ISL_2330677, EPI_ISL_2330678, EPI_ISL_2330679, EPI_ISL_2330680, EPI_ISL_2330681, EPI_ISL_2330682, EPI_ISL_2330683, EPI_ISL_2330684, EPI_ISL_2330685, EPI_ISL_2330686, EPI_ISL_2330687, EPI_ISL_2330688,
EPI_ISL_2330689, EPI_ISL_2330690, EPI_ISL_2330691, EPI_ISL_2330692, EPI_ISL_2330693, EPI_ISL_2330694, EPI_ISL_2330695, EPI_ISL_2330696, EPI_ISL_2330697, EPI_ISL_2330698, EPI_ISL_2330699, EPI_ISL_2330700, EPI_ISL_2330701, EPI_ISL_2330702, EPI_ISL_2330703, EPI_ISL_2330704, EPI_ISL_2330705, EPI_ISL_2330706, EPI_ISL_2330707, EPI_ISL_2330708, EPI_ISL_2330709, EPI_ISL_2330710,
EPI_ISL_2330711, EPI_ISL_2330712, EPI_ISL_2330713, EPI_ISL_2330714, EPI_ISL_2330715, EPI_ISL_2330716, EPI_ISL_2330717, EPI_ISL_2330718, EPI_ISL_2330719, EPI_ISL_2330720, EPI_ISL_2330721, EPI_ISL_2330722, EPI_ISL_2330723, EPI_ISL_2330724, EPI_ISL_2330725, EPI_ISL_2330726, EPI_ISL_2330727, EPI_ISL_2330728, EPI_ISL_2330729, EPI_ISL_2330730, EPI_ISL_2330731, EPI_ISL_2330732,
EPI_ISL_2330733, EPI_ISL_2330734, EPI_ISL_2330735, EPI_ISL_2330736, EPI_ISL_2330737, EPI_ISL_2330738, EPI_ISL_2330739, EPI_ISL_2330740, EPI_ISL_2330741, EPI_ISL_2330742, EPI_ISL_2330743, EPI_ISL_2330744, EPI_ISL_2330745, EPI_ISL_2330746, EPI_ISL_2330747, EPI_ISL_2330748, EPI_ISL_2330749, EPI_ISL_2330750, EPI_ISL_2330751, EPI_ISL_2330752, EPI_ISL_2330753, EPI_ISL_2330754,
EPI_ISL_2330755, EPI_ISL_2330756, EPI_ISL_2330757, EPI_ISL_2330758, EPI_ISL_2330759, EPI_ISL_2330760, EPI_ISL_2330761, EPI_ISL_2330762, EPI_ISL_2330763, EPI_ISL_2330764, EPI_ISL_2330765, EPI_ISL_2330766, EPI_ISL_2330767, EPI_ISL_2330768, EPI_ISL_2330769, EPI_ISL_2330770, EPI_ISL_2330771, EPI_ISL_2330772, EPI_ISL_2330773, EPI_ISL_2330774, EPI_ISL_2330775, EPI_ISL_2330776,
EPI_ISL_2330777, EPI_ISL_2330778, EPI_ISL_2330779, EPI_ISL_2330780, EPI_ISL_2330781, EPI_ISL_2330782, EPI_ISL_2330783, EPI_ISL_2330784, EPI_ISL_2330785, EPI_ISL_2330786, EPI_ISL_2330787, EPI_ISL_2330788, EPI_ISL_2330789, EPI_ISL_2330790, EPI_ISL_2330791, EPI_ISL_2330792, EPI_ISL_2330793, EPI_ISL_2330794, EPI_ISL_2330795, EPI_ISL_2330796, EPI_ISL_2330797, EPI_ISL_2330798,
EPI_ISL_2330799, EPI_ISL_2330800, EPI_ISL_2330801, EPI_ISL_2330802, EPI_ISL_2330803, EPI_ISL_2330804, EPI_ISL_2330805, EPI_ISL_2330806, EPI_ISL_2330807, EPI_ISL_2330808, EPI_ISL_2330809, EPI_ISL_2330810, EPI_ISL_2330811, EPI_ISL_2330812, EPI_ISL_2330813, EPI_ISL_2330814, EPI_ISL_2330815, EPI_ISL_2330816, EPI_ISL_2330817, EPI_ISL_2330818, EPI_ISL_2330819, EPI_ISL_2330820,
EPI_ISL_2330821, EPI_ISL_2330822, EPI_ISL_2330823, EPI_ISL_2330824, EPI_ISL_2330825, EPI_ISL_2330826, EPI_ISL_2330827, EPI_ISL_2330828, EPI_ISL_2330829, EPI_ISL_2330830, EPI_ISL_2330831, EPI_ISL_2330832, EPI_ISL_2330833, EPI_ISL_2330834, EPI_ISL_2330835, EPI_ISL_2330836, EPI_ISL_2330837, EPI_ISL_2330838, EPI_ISL_2330839, EPI_ISL_2330840, EPI_ISL_2330841, EPI_ISL_2330842,
EPI_ISL_2330843, EPI_ISL_2330844, EPI_ISL_2330845, EPI_ISL_2330846, EPI_ISL_2330847, EPI_ISL_2330848, EPI_ISL_2330849, EPI_ISL_2330850, EPI_ISL_2330851, EPI_ISL_2330852, EPI_ISL_2330853, EPI_ISL_2330854, EPI_ISL_2330855, EPI_ISL_2330856, EPI_ISL_2330857, EPI_ISL_2330858, EPI_ISL_2330859, EPI_ISL_2330860, EPI_ISL_2330861, EPI_ISL_2330862, EPI_ISL_2330863, EPI_ISL_2330864,
EPI_ISL_2330865, EPI_ISL_2330866, EPI_ISL_2330867, EPI_ISL_2330868, EPI_ISL_2330869, EPI_ISL_2330870, EPI_ISL_2330871, EPI_ISL_2330872, EPI_ISL_2330873, EPI_ISL_2330874, EPI_ISL_2330875, EPI_ISL_2330876, EPI_ISL_2330877, EPI_ISL_2330878, EPI_ISL_2330879, EPI_ISL_2330880, EPI_ISL_2330881, EPI_ISL_2330882, EPI_ISL_2330883, EPI_ISL_2330884, EPI_ISL_2330885, EPI_ISL_2330886,
EPI_ISL_2330887, EPI_ISL_2330888, EPI_ISL_2330889, EPI_ISL_2330890, EPI_ISL_2330891, EPI_ISL_2330892, EPI_ISL_2330893, EPI_ISL_2330894, EPI_ISL_2330895, EPI_ISL_2330896, EPI_ISL_2330897, EPI_ISL_2330898, EPI_ISL_2330899, EPI_ISL_2330900, EPI_ISL_2330901, EPI_ISL_2330902, EPI_ISL_2330903, EPI_ISL_2330904, EPI_ISL_2330905, EPI_ISL_2330906, EPI_ISL_2330907, EPI_ISL_2330908,
EPI_ISL_2330909, EPI_ISL_2330910, EPI_ISL_2330911, EPI_ISL_2330912, EPI_ISL_2330913, EPI_ISL_2330914, EPI_ISL_2330915, EPI_ISL_2330916, EPI_ISL_2330917, EPI_ISL_2330918, EPI_ISL_2330919, EPI_ISL_2330920, EPI_ISL_2330921, EPI_ISL_2330922, EPI_ISL_2330923, EPI_ISL_2330924, EPI_ISL_2330925, EPI_ISL_2330926, EPI_ISL_2330927, EPI_ISL_2330928, EPI_ISL_2330929, EPI_ISL_2330930,
EPI_ISL_2330931, EPI_ISL_2330932, EPI_ISL_2330933, EPI_ISL_2330934, EPI_ISL_2330935, EPI_ISL_2330936, EPI_ISL_2330937, EPI_ISL_2330938, EPI_ISL_2330939, EPI_ISL_2330940, EPI_ISL_2330941, EPI_ISL_2330942, EPI_ISL_2330943, EPI_ISL_2330944, EPI_ISL_2330945, EPI_ISL_2330946, EPI_ISL_2330947, EPI_ISL_2330948, EPI_ISL_2330949, EPI_ISL_2330950, EPI_ISL_2330951, EPI_ISL_2330952,
EPI_ISL_2330953, EPI_ISL_2330954, EPI_ISL_2330955, EPI_ISL_2330956, EPI_ISL_2330957, EPI_ISL_2330958, EPI_ISL_2330959, EPI_ISL_2330960, EPI_ISL_2330961, EPI_ISL_2330962, EPI_ISL_2330963, EPI_ISL_2330964, EPI_ISL_2330965, EPI_ISL_2330966, EPI_ISL_2330967, EPI_ISL_2330968, EPI_ISL_2330969, EPI_ISL_2330970, EPI_ISL_2330971, EPI_ISL_2330972, EPI_ISL_2330973, EPI_ISL_2330974,
EPI_ISL_2330975, EPI_ISL_2330976, EPI_ISL_2330977, EPI_ISL_2330978, EPI_ISL_2330979, EPI_ISL_2330980, EPI_ISL_2330981, EPI_ISL_2330982, EPI_ISL_2330983, EPI_ISL_2330984, EPI_ISL_2330985, EPI_ISL_2330986, EPI_ISL_2330987, EPI_ISL_2330988, EPI_ISL_2330989, EPI_ISL_2330990, EPI_ISL_2330991, EPI_ISL_2330992, EPI_ISL_2330993, EPI_ISL_2330994, EPI_ISL_2330995, EPI_ISL_2330996,
EPI_ISL_2330997, EPI_ISL_2330998, EPI_ISL_2330999, EPI_ISL_2331000, EPI_ISL_2331001, EPI_ISL_2331002, EPI_ISL_2331003, EPI_ISL_2331004, EPI_ISL_2331005, EPI_ISL_2331006, EPI_ISL_2331007, EPI_ISL_2331008, EPI_ISL_2331009, EPI_ISL_2331010, EPI_ISL_2331011, EPI_ISL_2331012, EPI_ISL_2331013, EPI_ISL_2331014, EPI_ISL_2331015, EPI_ISL_2331016, EPI_ISL_2331017, EPI_ISL_2331018,
EPI_ISL_2331019, EPI_ISL_2331020, EPI_ISL_2331021, EPI_ISL_2331022, EPI_ISL_2331023, EPI_ISL_2331024, EPI_ISL_2331025, EPI_ISL_2331026, EPI_ISL_2331027, EPI_ISL_2331028, EPI_ISL_2331029, EPI_ISL_2331030, EPI_ISL_2331031, EPI_ISL_2331032, EPI_ISL_2331033, EPI_ISL_2331034, EPI_ISL_2331035, EPI_ISL_2331036, EPI_ISL_2331037, EPI_ISL_2331038, EPI_ISL_2331039, EPI_ISL_2331040,
EPI_ISL_2331041, EPI_ISL_2331042, EPI_ISL_2331043, EPI_ISL_2331044, EPI_ISL_2331045, EPI_ISL_2331046, EPI_ISL_2331047, EPI_ISL_2331048, EPI_ISL_2331049, EPI_ISL_2331050, EPI_ISL_2331051, EPI_ISL_2331052, EPI_ISL_2331053, EPI_ISL_2331054, EPI_ISL_2331055, EPI_ISL_2331056, EPI_ISL_2331057, EPI_ISL_2331058, EPI_ISL_2331059, EPI_ISL_2331060, EPI_ISL_2331061, EPI_ISL_2331062,
EPI_ISL_2331063, EPI_ISL_2331064, EPI_ISL_2331065, EPI_ISL_2331066, EPI_ISL_2331067, EPI_ISL_2331068, EPI_ISL_2331069, EPI_ISL_2331070, EPI_ISL_2331071, EPI_ISL_2331072, EPI_ISL_2331073, EPI_ISL_2331074, EPI_ISL_2331075, EPI_ISL_2331076, EPI_ISL_2331077, EPI_ISL_2331078, EPI_ISL_2331079, EPI_ISL_2331080, EPI_ISL_2331081, EPI_ISL_2331082, EPI_ISL_2331083, EPI_ISL_2331084,
EPI_ISL_2331085, EPI_ISL_2331086, EPI_ISL_2331087, EPI_ISL_2331088, EPI_ISL_2331089, EPI_ISL_2331090, EPI_ISL_2331091, EPI_ISL_2331092, EPI_ISL_2331093, EPI_ISL_2331094, EPI_ISL_2331095, EPI_ISL_2331096, EPI_ISL_2331097, EPI_ISL_2331098, EPI_ISL_2331099, EPI_ISL_2331100, EPI_ISL_2331101, EPI_ISL_2331102, EPI_ISL_2331103, EPI_ISL_2331104, EPI_ISL_2331105, EPI_ISL_2331106,
EPI_ISL_2331107, EPI_ISL_2331108, EPI_ISL_2331109, EPI_ISL_2331110, EPI_ISL_2331111, EPI_ISL_2331112, EPI_ISL_2331113, EPI_ISL_2331114, EPI_ISL_2331115, EPI_ISL_2331116, EPI_ISL_2331117, EPI_ISL_2331118, EPI_ISL_2331119, EPI_ISL_2331120, EPI_ISL_2331121, EPI_ISL_2331122, EPI_ISL_2331123, EPI_ISL_2331124, EPI_ISL_2331125, EPI_ISL_2331126, EPI_ISL_2331127, EPI_ISL_2331128,
EPI_ISL_2331129, EPI_ISL_2331130, EPI_ISL_2331131, EPI_ISL_2331132, EPI_ISL_2331133, EPI_ISL_2331134, EPI_ISL_2331135, EPI_ISL_2331136, EPI_ISL_2331137, EPI_ISL_2331138, EPI_ISL_2331139, EPI_ISL_2331140, EPI_ISL_2331141, EPI_ISL_2331142, EPI_ISL_2331143, EPI_ISL_2331144, EPI_ISL_2331145, EPI_ISL_2331146, EPI_ISL_2331147, EPI_ISL_2331148, EPI_ISL_2331149, EPI_ISL_2331150,
EPI_ISL_2331151, EPI_ISL_2331152, EPI_ISL_2331153, EPI_ISL_2331154, EPI_ISL_2331155, EPI_ISL_2331156, EPI_ISL_2331157, EPI_ISL_2331158, EPI_ISL_2331159, EPI_ISL_2331160, EPI_ISL_2331161, EPI_ISL_2331162, EPI_ISL_2331163, EPI_ISL_2331164, EPI_ISL_2331165, EPI_ISL_2331166, EPI_ISL_2331167, EPI_ISL_2331168, EPI_ISL_2331169, EPI_ISL_2331170, EPI_ISL_2331171, EPI_ISL_2331172,
EPI_ISL_2331173, EPI_ISL_2331174, EPI_ISL_2331175, EPI_ISL_2331176, EPI_ISL_2331177, EPI_ISL_2331178, EPI_ISL_2331179, EPI_ISL_2331180, EPI_ISL_2331181, EPI_ISL_2331182, EPI_ISL_2331183, EPI_ISL_2331184, EPI_ISL_2331185, EPI_ISL_2331186, EPI_ISL_2331187, EPI_ISL_2331188, EPI_ISL_2331189, EPI_ISL_2331190, EPI_ISL_2331191, EPI_ISL_2331192, EPI_ISL_2331193, EPI_ISL_2331194,
EPI_ISL_2331195, EPI_ISL_2331196, EPI_ISL_2331197, EPI_ISL_2331198, EPI_ISL_2331199, EPI_ISL_2331200, EPI_ISL_2331201, EPI_ISL_2331202, EPI_ISL_2331203, EPI_ISL_2331204, EPI_ISL_2331205, EPI_ISL_2331206, EPI_ISL_2331207, EPI_ISL_2331208, EPI_ISL_2331209, EPI_ISL_2331210, EPI_ISL_2331211, EPI_ISL_2331212, EPI_ISL_2331214, EPI_ISL_2331215, EPI_ISL_2331216, EPI_ISL_2331217,
EPI_ISL_2331218, EPI_ISL_2331219, EPI_ISL_2331220, EPI_ISL_2331221, EPI_ISL_2331222, EPI_ISL_2331223, EPI_ISL_2331224, EPI_ISL_2331225, EPI_ISL_2331226, EPI_ISL_2331227, EPI_ISL_2331228, EPI_ISL_2331229, EPI_ISL_2331230, EPI_ISL_2331231, EPI_ISL_2331232, EPI_ISL_2331233, EPI_ISL_2331234, EPI_ISL_2331235, EPI_ISL_2331236, EPI_ISL_2331237, EPI_ISL_2331238, EPI_ISL_2331239,
EPI_ISL_2331240, EPI_ISL_2331241, EPI_ISL_2331242, EPI_ISL_2331243, EPI_ISL_2331244, EPI_ISL_2331245, EPI_ISL_2331246, EPI_ISL_2331247, EPI_ISL_2331248, EPI_ISL_2331249, EPI_ISL_2331250, EPI_ISL_2331251, EPI_ISL_2331252, EPI_ISL_2331253, EPI_ISL_2331254, EPI_ISL_2331255, EPI_ISL_2331256, EPI_ISL_2331257, EPI_ISL_2331258, EPI_ISL_2331259, EPI_ISL_2331260, EPI_ISL_2331261,
EPI_ISL_2331262, EPI_ISL_2331263, EPI_ISL_2331264, EPI_ISL_2331265, EPI_ISL_2331266, EPI_ISL_2331267, EPI_ISL_2331268, EPI_ISL_2331269, EPI_ISL_2331270, EPI_ISL_2331271, EPI_ISL_2331272, EPI_ISL_2331273, EPI_ISL_2331274, EPI_ISL_2331275, EPI_ISL_2331276, EPI_ISL_2331277, EPI_ISL_2331278, EPI_ISL_2331279, EPI_ISL_2331280, EPI_ISL_2331281, EPI_ISL_2331282, EPI_ISL_2331283,
EPI_ISL_2331284, EPI_ISL_2331285, EPI_ISL_2331286, EPI_ISL_2331287, EPI_ISL_2331288, EPI_ISL_2331289, EPI_ISL_2331290, EPI_ISL_2331291, EPI_ISL_2331292, EPI_ISL_2331293, EPI_ISL_2331294, EPI_ISL_2331295, EPI_ISL_2331296, EPI_ISL_2331297, EPI_ISL_2331298, EPI_ISL_2331299, EPI_ISL_2331300, EPI_ISL_2331301, EPI_ISL_2331302, EPI_ISL_2331303, EPI_ISL_2331304, EPI_ISL_2331305,
EPI_ISL_2331306, EPI_ISL_2335147, EPI_ISL_2335148, EPI_ISL_2335149, EPI_ISL_2335150, EPI_ISL_2335151, EPI_ISL_2335152, EPI_ISL_2335153, EPI_ISL_2335154, EPI_ISL_2335155, EPI_ISL_2335156, EPI_ISL_2335157, EPI_ISL_2335158, EPI_ISL_2335159, EPI_ISL_2335160, EPI_ISL_2335161, EPI_ISL_2335162, EPI_ISL_2335163, EPI_ISL_2335164, EPI_ISL_2335165, EPI_ISL_2335166, EPI_ISL_2335167,
EPI_ISL_2335168, EPI_ISL_2335169, EPI_ISL_2335170, EPI_ISL_2335171, EPI_ISL_2335172, EPI_ISL_2335173, EPI_ISL_2335174, EPI_ISL_2335175, EPI_ISL_2335176, EPI_ISL_2335177, EPI_ISL_2335178, EPI_ISL_2335179, EPI_ISL_2335180, EPI_ISL_2335181, EPI_ISL_2335182, EPI_ISL_2335183, EPI_ISL_2335184, EPI_ISL_2335185, EPI_ISL_2335186, EPI_ISL_2335187, EPI_ISL_2335188, EPI_ISL_2335189,
EPI_ISL_2335190, EPI_ISL_2335191, EPI_ISL_2335192, EPI_ISL_2335193, EPI_ISL_2335194, EPI_ISL_2335195, EPI_ISL_2335196, EPI_ISL_2335197, EPI_ISL_2335198, EPI_ISL_2335199, EPI_ISL_2335200, EPI_ISL_2335201, EPI_ISL_2335202, EPI_ISL_2335203, EPI_ISL_2335204, EPI_ISL_2335205, EPI_ISL_2335206, EPI_ISL_2335207, EPI_ISL_2335208, EPI_ISL_2335209, EPI_ISL_2335210, EPI_ISL_2335211,
EPI_ISL_2335212, EPI_ISL_2335213, EPI_ISL_2335214, EPI_ISL_2335215, EPI_ISL_2335216, EPI_ISL_2335217, EPI_ISL_2335218, EPI_ISL_2335219, EPI_ISL_2335220, EPI_ISL_2335221, EPI_ISL_2335222, EPI_ISL_2335223, EPI_ISL_2335224, EPI_ISL_2335225, EPI_ISL_2335226, EPI_ISL_2335227, EPI_ISL_2335228, EPI_ISL_2335229, EPI_ISL_2335230, EPI_ISL_2335231, EPI_ISL_2335232, EPI_ISL_2335986,
EPI_ISL_2335987, EPI_ISL_2335988, EPI_ISL_2335989, EPI_ISL_2335990, EPI_ISL_2335991, EPI_ISL_2335992, EPI_ISL_2335993, EPI_ISL_2335994, EPI_ISL_2335995, EPI_ISL_2335996, EPI_ISL_2335997, EPI_ISL_2335998, EPI_ISL_2335999, EPI_ISL_2336000, EPI_ISL_2336001, EPI_ISL_2336002, EPI_ISL_2336003, EPI_ISL_2336004, EPI_ISL_2336005, EPI_ISL_2336006, EPI_ISL_2336007, EPI_ISL_2336008,
EPI_ISL_2336009, EPI_ISL_2336010, EPI_ISL_2336011, EPI_ISL_2336012, EPI_ISL_2336013, EPI_ISL_2336014, EPI_ISL_2336015, EPI_ISL_2336016, EPI_ISL_2336017, EPI_ISL_2336018, EPI_ISL_2336019, EPI_ISL_2336020, EPI_ISL_2336021, EPI_ISL_2336022, EPI_ISL_2336023, EPI_ISL_2336024, EPI_ISL_2336025, EPI_ISL_2336026, EPI_ISL_2336027, EPI_ISL_2336028, EPI_ISL_2336029, EPI_ISL_2336030,
EPI_ISL_2336031, EPI_ISL_2336032, EPI_ISL_2336033, EPI_ISL_2336034, EPI_ISL_2336035, EPI_ISL_2336036, EPI_ISL_2336037, EPI_ISL_2336038, EPI_ISL_2336039, EPI_ISL_2336040, EPI_ISL_2336041, EPI_ISL_2336042, EPI_ISL_2336043, EPI_ISL_2336044, EPI_ISL_2336045, EPI_ISL_2336046, EPI_ISL_2336047, EPI_ISL_2336048, EPI_ISL_2336049, EPI_ISL_2336050, EPI_ISL_2336051, EPI_ISL_2336052,
EPI_ISL_2336053, EPI_ISL_2336054, EPI_ISL_2336055, EPI_ISL_2336056, EPI_ISL_2336057, EPI_ISL_2336058, EPI_ISL_2336059, EPI_ISL_2336060, EPI_ISL_2336061, EPI_ISL_2336062, EPI_ISL_2336063, EPI_ISL_2336064, EPI_ISL_2336065, EPI_ISL_2336066, EPI_ISL_2336067, EPI_ISL_2336068, EPI_ISL_2336069, EPI_ISL_2336070, EPI_ISL_2336071, EPI_ISL_2336072, EPI_ISL_2336073, EPI_ISL_2336074,
EPI_ISL_2336075, EPI_ISL_2336076, EPI_ISL_2336077, EPI_ISL_2336078, EPI_ISL_2336079, EPI_ISL_2336080, EPI_ISL_2336081, EPI_ISL_2336082, EPI_ISL_2336083, EPI_ISL_2336084, EPI_ISL_2336085, EPI_ISL_2336086, EPI_ISL_2336087, EPI_ISL_2336088, EPI_ISL_2336089, EPI_ISL_2336090, EPI_ISL_2336091, EPI_ISL_2336092, EPI_ISL_2336093, EPI_ISL_2336094, EPI_ISL_2336095, EPI_ISL_2336096,
EPI_ISL_2336097, EPI_ISL_2336098, EPI_ISL_2336099, EPI_ISL_2336100, EPI_ISL_2336101, EPI_ISL_2336102, EPI_ISL_2336103, EPI_ISL_2336104, EPI_ISL_2336105, EPI_ISL_2336106, EPI_ISL_2336107, EPI_ISL_2336108, EPI_ISL_2336109, EPI_ISL_2336110, EPI_ISL_2336111, EPI_ISL_2336112, EPI_ISL_2336113, EPI_ISL_2336114, EPI_ISL_2336115, EPI_ISL_2336116, EPI_ISL_2336117, EPI_ISL_2336118,
EPI_ISL_2336119, EPI_ISL_2336120, EPI_ISL_2336121, EPI_ISL_2336122, EPI_ISL_2336123, EPI_ISL_2336124, EPI_ISL_2336125, EPI_ISL_2336126, EPI_ISL_2336127, EPI_ISL_2336128, EPI_ISL_2336129, EPI_ISL_2336130, EPI_ISL_2336131, EPI_ISL_2336132, EPI_ISL_2336133, EPI_ISL_2336134, EPI_ISL_2336135, EPI_ISL_2336136, EPI_ISL_2336137, EPI_ISL_2336138, EPI_ISL_2336139, EPI_ISL_2336140,
EPI_ISL_2336141, EPI_ISL_2336142, EPI_ISL_2336143, EPI_ISL_2336144, EPI_ISL_2336145, EPI_ISL_2336146, EPI_ISL_2336147, EPI_ISL_2336148, EPI_ISL_2336254, EPI_ISL_2336255, EPI_ISL_2336256, EPI_ISL_2336257, EPI_ISL_2336258, EPI_ISL_2336259, EPI_ISL_2336260, EPI_ISL_2336261, EPI_ISL_2336262, EPI_ISL_2336263, EPI_ISL_2336264, EPI_ISL_2336265, EPI_ISL_2336266, EPI_ISL_2336267,
EPI_ISL_2336268, EPI_ISL_2336269, EPI_ISL_2336270, EPI_ISL_2336271, EPI_ISL_2336272, EPI_ISL_2336273, EPI_ISL_2336274, EPI_ISL_2336275, EPI_ISL_2336276, EPI_ISL_2336277, EPI_ISL_2336278, EPI_ISL_2336279, EPI_ISL_2336280, EPI_ISL_2336281, EPI_ISL_2336282, EPI_ISL_2336283, EPI_ISL_2336284, EPI_ISL_2336285, EPI_ISL_2336286, EPI_ISL_2336287, EPI_ISL_2336288, EPI_ISL_2336289,
EPI_ISL_2336290, EPI_ISL_2336291, EPI_ISL_2336292, EPI_ISL_2336293, EPI_ISL_2336294, EPI_ISL_2336295, EPI_ISL_2336296, EPI_ISL_2336297, EPI_ISL_2336298, EPI_ISL_2336299, EPI_ISL_2336300, EPI_ISL_2336301, EPI_ISL_2336302, EPI_ISL_2336303, EPI_ISL_2336304, EPI_ISL_2336305, EPI_ISL_2336306, EPI_ISL_2336307, EPI_ISL_2336308, EPI_ISL_2336309, EPI_ISL_2336310, EPI_ISL_2336311,
EPI_ISL_2336312, EPI_ISL_2336313, EPI_ISL_2336314, EPI_ISL_2336315, EPI_ISL_2336316, EPI_ISL_2336317, EPI_ISL_2336318, EPI_ISL_2336319, EPI_ISL_2336320, EPI_ISL_2336321, EPI_ISL_2336322, EPI_ISL_2336323, EPI_ISL_2336324, EPI_ISL_2336325, EPI_ISL_2336326, EPI_ISL_2336327, EPI_ISL_2336328, EPI_ISL_2336329, EPI_ISL_2336330, EPI_ISL_2336331, EPI_ISL_2336332, EPI_ISL_2336333,
EPI_ISL_2336334, EPI_ISL_2336335, EPI_ISL_2336336, EPI_ISL_2336337, EPI_ISL_2336338, EPI_ISL_2336339, EPI_ISL_2336340, EPI_ISL_2336341, EPI_ISL_2336342, EPI_ISL_2336343, EPI_ISL_2336344, EPI_ISL_2336345, EPI_ISL_2336346, EPI_ISL_2336347, EPI_ISL_2336348, EPI_ISL_2336349, EPI_ISL_2336350, EPI_ISL_2336351, EPI_ISL_2336352, EPI_ISL_2336353, EPI_ISL_2336354, EPI_ISL_2336483,
EPI_ISL_2336484, EPI_ISL_2336485, EPI_ISL_2336486, EPI_ISL_2336487, EPI_ISL_2336488, EPI_ISL_2336489, EPI_ISL_2336490, EPI_ISL_2336491, EPI_ISL_2336492, EPI_ISL_2336493, EPI_ISL_2336494, EPI_ISL_2336495, EPI_ISL_2336496, EPI_ISL_2336497, EPI_ISL_2336498, EPI_ISL_2336499, EPI_ISL_2336500, EPI_ISL_2336501, EPI_ISL_2336502, EPI_ISL_2336503, EPI_ISL_2336504, EPI_ISL_2336505,
EPI_ISL_2336506, EPI_ISL_2336507, EPI_ISL_2336508, EPI_ISL_2336509, EPI_ISL_2336510, EPI_ISL_2336511, EPI_ISL_2336512, EPI_ISL_2336513, EPI_ISL_2336514, EPI_ISL_2336515, EPI_ISL_2336516, EPI_ISL_2336517, EPI_ISL_2336518, EPI_ISL_2336519, EPI_ISL_2336520, EPI_ISL_2336521, EPI_ISL_2336522, EPI_ISL_2336523, EPI_ISL_2336524, EPI_ISL_2336525, EPI_ISL_2336526, EPI_ISL_2336527,
EPI_ISL_2336528, EPI_ISL_2336529, EPI_ISL_2336530, EPI_ISL_2336531, EPI_ISL_2336532, EPI_ISL_2336533, EPI_ISL_2336534, EPI_ISL_2336535, EPI_ISL_2336536, EPI_ISL_2336537, EPI_ISL_2336538, EPI_ISL_2336539, EPI_ISL_2336540, EPI_ISL_2336541, EPI_ISL_2336542, EPI_ISL_2336543, EPI_ISL_2336544, EPI_ISL_2336545, EPI_ISL_2336546, EPI_ISL_2336547, EPI_ISL_2336548, EPI_ISL_2336549,
EPI_ISL_2336550, EPI_ISL_2336551, EPI_ISL_2336552, EPI_ISL_2336553, EPI_ISL_2336554, EPI_ISL_2336555, EPI_ISL_2336556, EPI_ISL_2336557, EPI_ISL_2336558, EPI_ISL_2336559, EPI_ISL_2336560, EPI_ISL_2336561, EPI_ISL_2336562, EPI_ISL_2336563, EPI_ISL_2336564, EPI_ISL_2336565, EPI_ISL_2336566, EPI_ISL_2336567, EPI_ISL_2336568, EPI_ISL_2336569, EPI_ISL_2336570, EPI_ISL_2336571,
EPI_ISL_2336572, EPI_ISL_2336573, EPI_ISL_2336574, EPI_ISL_2336575, EPI_ISL_2336576, EPI_ISL_2336577, EPI_ISL_2336578, EPI_ISL_2336579, EPI_ISL_2336580, EPI_ISL_2336581, EPI_ISL_2336582, EPI_ISL_2336583, EPI_ISL_2336584, EPI_ISL_2336585, EPI_ISL_2336586, EPI_ISL_2336587, EPI_ISL_2336588, EPI_ISL_2336589, EPI_ISL_2336590, EPI_ISL_2336591, EPI_ISL_2336592, EPI_ISL_2336593,
EPI_ISL_2336594, EPI_ISL_2336595, EPI_ISL_2336596, EPI_ISL_2336597, EPI_ISL_2336598, EPI_ISL_2336599, EPI_ISL_2336600, EPI_ISL_2336601, EPI_ISL_2336602, EPI_ISL_2336603, EPI_ISL_2336604, EPI_ISL_2336605, EPI_ISL_2336606, EPI_ISL_2336607, EPI_ISL_2336608, EPI_ISL_2336609, EPI_ISL_2336610, EPI_ISL_2336611, EPI_ISL_2336612, EPI_ISL_2336613, EPI_ISL_2336614, EPI_ISL_2336615,
EPI_ISL_2336616, EPI_ISL_2336617, EPI_ISL_2336618, EPI_ISL_2336619, EPI_ISL_2336620, EPI_ISL_2336621, EPI_ISL_2336622, EPI_ISL_2336623, EPI_ISL_2336624, EPI_ISL_2336625, EPI_ISL_2336626, EPI_ISL_2336627, EPI_ISL_2336628, EPI_ISL_2336629, EPI_ISL_2336630, EPI_ISL_2336631, EPI_ISL_2336632, EPI_ISL_2336633, EPI_ISL_2336634, EPI_ISL_2336635, EPI_ISL_2336636, EPI_ISL_2336637,
EPI_ISL_2336638, EPI_ISL_2336639, EPI_ISL_2336640, EPI_ISL_2336641, EPI_ISL_2336642, EPI_ISL_2336643, EPI_ISL_2336644, EPI_ISL_2336645, EPI_ISL_2336646, EPI_ISL_2336647, EPI_ISL_2336648, EPI_ISL_2336649, EPI_ISL_2336650, EPI_ISL_2336651, EPI_ISL_2336652, EPI_ISL_2336653, EPI_ISL_2336654, EPI_ISL_2336655, EPI_ISL_2336656, EPI_ISL_2336657, EPI_ISL_2336658, EPI_ISL_2336659,
EPI_ISL_2336660, EPI_ISL_2336661, EPI_ISL_2336662, EPI_ISL_2336663, EPI_ISL_2336664, EPI_ISL_2336665, EPI_ISL_2336666, EPI_ISL_2336667, EPI_ISL_2336668, EPI_ISL_2336669, EPI_ISL_2336670, EPI_ISL_2336671, EPI_ISL_2336672, EPI_ISL_2336673, EPI_ISL_2336674, EPI_ISL_2336675, EPI_ISL_2336676, EPI_ISL_2336677, EPI_ISL_2336678, EPI_ISL_2336679, EPI_ISL_2336680, EPI_ISL_2336681,
EPI_ISL_2336682, EPI_ISL_2336683, EPI_ISL_2336684, EPI_ISL_2336685, EPI_ISL_2336686, EPI_ISL_2336687, EPI_ISL_2336688, EPI_ISL_2336689, EPI_ISL_2336690, EPI_ISL_2336691, EPI_ISL_2336692, EPI_ISL_2336693, EPI_ISL_2336694, EPI_ISL_2336695, EPI_ISL_2336696, EPI_ISL_2336697, EPI_ISL_2336698, EPI_ISL_2336699, EPI_ISL_2336700, EPI_ISL_2336701, EPI_ISL_2336702, EPI_ISL_2336703,
EPI_ISL_2336704, EPI_ISL_2336705, EPI_ISL_2336706, EPI_ISL_2336707, EPI_ISL_2336708, EPI_ISL_2336709, EPI_ISL_2336710, EPI_ISL_2336711, EPI_ISL_2336712, EPI_ISL_2336713, EPI_ISL_2336714, EPI_ISL_2336715, EPI_ISL_2336716, EPI_ISL_2336717, EPI_ISL_2336718, EPI_ISL_2336719, EPI_ISL_2336720, EPI_ISL_2336721, EPI_ISL_2336722, EPI_ISL_2336723, EPI_ISL_2336724, EPI_ISL_2336725,
EPI_ISL_2336726, EPI_ISL_2336727, EPI_ISL_2336728, EPI_ISL_2336729, EPI_ISL_2336730, EPI_ISL_2336731, EPI_ISL_2336732, EPI_ISL_2336733, EPI_ISL_2336734, EPI_ISL_2336735, EPI_ISL_2336736, EPI_ISL_2336737, EPI_ISL_2336738, EPI_ISL_2336739, EPI_ISL_2336740, EPI_ISL_2336741, EPI_ISL_2336742, EPI_ISL_2336743, EPI_ISL_2336744, EPI_ISL_2336745, EPI_ISL_2336746, EPI_ISL_2336747,
EPI_ISL_2336748, EPI_ISL_2336897, EPI_ISL_2336898, EPI_ISL_2336899, EPI_ISL_2336900, EPI_ISL_2336901, EPI_ISL_2336902, EPI_ISL_2336903, EPI_ISL_2336904, EPI_ISL_2336905, EPI_ISL_2336906, EPI_ISL_2336907, EPI_ISL_2336908, EPI_ISL_2336909, EPI_ISL_2336910, EPI_ISL_2336911, EPI_ISL_2336912, EPI_ISL_2336913, EPI_ISL_2336914, EPI_ISL_2336915, EPI_ISL_2336916, EPI_ISL_2336917,
EPI_ISL_2336918, EPI_ISL_2336919, EPI_ISL_2336920, EPI_ISL_2336921, EPI_ISL_2336922, EPI_ISL_2336923, EPI_ISL_2336924, EPI_ISL_2336925, EPI_ISL_2336926, EPI_ISL_2336927, EPI_ISL_2336928, EPI_ISL_2336929, EPI_ISL_2336930, EPI_ISL_2336931, EPI_ISL_2336932, EPI_ISL_2336933, EPI_ISL_2336934, EPI_ISL_2336935, EPI_ISL_2336936, EPI_ISL_2336937, EPI_ISL_2336938, EPI_ISL_2336939,
EPI_ISL_2336940, EPI_ISL_2336941, EPI_ISL_2336942, EPI_ISL_2336943, EPI_ISL_2336944, EPI_ISL_2336945, EPI_ISL_2336946, EPI_ISL_2336947, EPI_ISL_2336948, EPI_ISL_2336949, EPI_ISL_2336950, EPI_ISL_2336951, EPI_ISL_2336952, EPI_ISL_2336953, EPI_ISL_2336954, EPI_ISL_2336955, EPI_ISL_2336956, EPI_ISL_2336957, EPI_ISL_2336958, EPI_ISL_2336959, EPI_ISL_2336960, EPI_ISL_2336961,
EPI_ISL_2336962, EPI_ISL_2336963, EPI_ISL_2336964, EPI_ISL_2336965, EPI_ISL_2336966, EPI_ISL_2336967, EPI_ISL_2336968, EPI_ISL_2336969, EPI_ISL_2336970, EPI_ISL_2336971, EPI_ISL_2336972, EPI_ISL_2336973, EPI_ISL_2336974, EPI_ISL_2336975, EPI_ISL_2336976, EPI_ISL_2336977, EPI_ISL_2336978, EPI_ISL_2336979, EPI_ISL_2336980, EPI_ISL_2336981, EPI_ISL_2336982, EPI_ISL_2336983,
EPI_ISL_2336984, EPI_ISL_2336985, EPI_ISL_2336986, EPI_ISL_2336987, EPI_ISL_2336988, EPI_ISL_2336989, EPI_ISL_2336990, EPI_ISL_2336991, EPI_ISL_2336992, EPI_ISL_2336993, EPI_ISL_2336994, EPI_ISL_2336995, EPI_ISL_2336996, EPI_ISL_2336997, EPI_ISL_2336998, EPI_ISL_2336999, EPI_ISL_2337000, EPI_ISL_2337001, EPI_ISL_2337002, EPI_ISL_2337003, EPI_ISL_2337004, EPI_ISL_2337005,
EPI_ISL_2337006, EPI_ISL_2337007, EPI_ISL_2337008, EPI_ISL_2337009, EPI_ISL_2337010, EPI_ISL_2337011, EPI_ISL_2337012, EPI_ISL_2337013, EPI_ISL_2337014, EPI_ISL_2337015, EPI_ISL_2337016, EPI_ISL_2337017, EPI_ISL_2337018, EPI_ISL_2337019, EPI_ISL_2337020, EPI_ISL_2337021, EPI_ISL_2337022, EPI_ISL_2337023, EPI_ISL_2337024, EPI_ISL_2337025, EPI_ISL_2337026, EPI_ISL_2337027,
EPI_ISL_2337028, EPI_ISL_2337029, EPI_ISL_2337030, EPI_ISL_2337031, EPI_ISL_2337032, EPI_ISL_2337033, EPI_ISL_2337034, EPI_ISL_2337035, EPI_ISL_2337036, EPI_ISL_2337037, EPI_ISL_2337038, EPI_ISL_2337039, EPI_ISL_2337040, EPI_ISL_2337041, EPI_ISL_2337042, EPI_ISL_2337043, EPI_ISL_2337044, EPI_ISL_2337045, EPI_ISL_2337046, EPI_ISL_2337047, EPI_ISL_2337048, EPI_ISL_2337049,
EPI_ISL_2337050, EPI_ISL_2337051, EPI_ISL_2337052, EPI_ISL_2337053, EPI_ISL_2337054, EPI_ISL_2337055, EPI_ISL_2337056, EPI_ISL_2337057, EPI_ISL_2337058, EPI_ISL_2337059, EPI_ISL_2337060, EPI_ISL_2337061, EPI_ISL_2337062, EPI_ISL_2337063, EPI_ISL_2337064, EPI_ISL_2337065, EPI_ISL_2337066, EPI_ISL_2337067, EPI_ISL_2337068, EPI_ISL_2337069, EPI_ISL_2337070, EPI_ISL_2337071,
EPI_ISL_2337072, EPI_ISL_2337073, EPI_ISL_2337074, EPI_ISL_2337075, EPI_ISL_2337076, EPI_ISL_2337077, EPI_ISL_2337078, EPI_ISL_2337079, EPI_ISL_2337080, EPI_ISL_2337081, EPI_ISL_2337082, EPI_ISL_2337083, EPI_ISL_2337084, EPI_ISL_2337085, EPI_ISL_2337086, EPI_ISL_2337087, EPI_ISL_2337088, EPI_ISL_2337089, EPI_ISL_2337090, EPI_ISL_2337091, EPI_ISL_2337092, EPI_ISL_2337093,
EPI_ISL_2337094, EPI_ISL_2337095, EPI_ISL_2337096, EPI_ISL_2337097, EPI_ISL_2337098, EPI_ISL_2337099, EPI_ISL_2337100, EPI_ISL_2337101, EPI_ISL_2337102, EPI_ISL_2337103, EPI_ISL_2337104, EPI_ISL_2337105, EPI_ISL_2337106, EPI_ISL_2337107, EPI_ISL_2337108, EPI_ISL_2337109, EPI_ISL_2337110, EPI_ISL_2337111, EPI_ISL_2337112, EPI_ISL_2337113, EPI_ISL_2337114, EPI_ISL_2337115,
EPI_ISL_2337116, EPI_ISL_2337117, EPI_ISL_2337118, EPI_ISL_2337119, EPI_ISL_2337120, EPI_ISL_2337121, EPI_ISL_2337122, EPI_ISL_2337123, EPI_ISL_2337124, EPI_ISL_2337125, EPI_ISL_2337126, EPI_ISL_2337127, EPI_ISL_2337128, EPI_ISL_2337129, EPI_ISL_2337130, EPI_ISL_2337131, EPI_ISL_2337132, EPI_ISL_2337133, EPI_ISL_2337134, EPI_ISL_2337135, EPI_ISL_2337136, EPI_ISL_2337137,
EPI_ISL_2337138, EPI_ISL_2337139, EPI_ISL_2337140, EPI_ISL_2337141, EPI_ISL_2337142, EPI_ISL_2337143, EPI_ISL_2337144, EPI_ISL_2337145, EPI_ISL_2337146, EPI_ISL_2337147, EPI_ISL_2337148, EPI_ISL_2337149, EPI_ISL_2337150, EPI_ISL_2337151, EPI_ISL_2337152, EPI_ISL_2337153, EPI_ISL_2337154, EPI_ISL_2337155, EPI_ISL_2337156, EPI_ISL_2337157, EPI_ISL_2337158, EPI_ISL_2337159,
EPI_ISL_2337160, EPI_ISL_2337161, EPI_ISL_2337162, EPI_ISL_2337163, EPI_ISL_2337164, EPI_ISL_2337165, EPI_ISL_2337166, EPI_ISL_2337167, EPI_ISL_2337168, EPI_ISL_2337169, EPI_ISL_2337170, EPI_ISL_2337171, EPI_ISL_2337172, EPI_ISL_2337173, EPI_ISL_2337174, EPI_ISL_2337175, EPI_ISL_2337176, EPI_ISL_2337177, EPI_ISL_2337178, EPI_ISL_2337179, EPI_ISL_2337180, EPI_ISL_2337181,
EPI_ISL_2337182, EPI_ISL_2337183, EPI_ISL_2337184, EPI_ISL_2337185, EPI_ISL_2337186, EPI_ISL_2337187, EPI_ISL_2337188, EPI_ISL_2337189, EPI_ISL_2337190, EPI_ISL_2337191, EPI_ISL_2337192, EPI_ISL_2337193, EPI_ISL_2337194, EPI_ISL_2337195, EPI_ISL_2337196, EPI_ISL_2337197, EPI_ISL_2337198, EPI_ISL_2337199, EPI_ISL_2337200, EPI_ISL_2337201, EPI_ISL_2337202, EPI_ISL_2337203,
EPI_ISL_2337204, EPI_ISL_2337205, EPI_ISL_2337206, EPI_ISL_2337207, EPI_ISL_2337208, EPI_ISL_2337209, EPI_ISL_2337210, EPI_ISL_2337211, EPI_ISL_2337212, EPI_ISL_2337213, EPI_ISL_2337214, EPI_ISL_2337215, EPI_ISL_2337216, EPI_ISL_2337217, EPI_ISL_2337218, EPI_ISL_2337219, EPI_ISL_2337220, EPI_ISL_2337221, EPI_ISL_2337222, EPI_ISL_2337223, EPI_ISL_2337224, EPI_ISL_2337225,
EPI_ISL_2337226, EPI_ISL_2337227, EPI_ISL_2337228, EPI_ISL_2337229, EPI_ISL_2337230, EPI_ISL_2337231, EPI_ISL_2337232, EPI_ISL_2337233, EPI_ISL_2337234, EPI_ISL_2337235, EPI_ISL_2337236, EPI_ISL_2337237, EPI_ISL_2337238, EPI_ISL_2337633, EPI_ISL_2337634, EPI_ISL_2337635, EPI_ISL_2337636, EPI_ISL_2337637, EPI_ISL_2337638, EPI_ISL_2337639, EPI_ISL_2337640, EPI_ISL_2337641,
EPI_ISL_2337642, EPI_ISL_2337643, EPI_ISL_2337644, EPI_ISL_2337645, EPI_ISL_2337646, EPI_ISL_2337647, EPI_ISL_2337648, EPI_ISL_2337649, EPI_ISL_2337650, EPI_ISL_2337651, EPI_ISL_2337652, EPI_ISL_2337653, EPI_ISL_2337654, EPI_ISL_2337655, EPI_ISL_2337656, EPI_ISL_2337657, EPI_ISL_2337658, EPI_ISL_2337659, EPI_ISL_2337660, EPI_ISL_2337661, EPI_ISL_2337662, EPI_ISL_2337663,
EPI_ISL_2337664, EPI_ISL_2337665, EPI_ISL_2337666, EPI_ISL_2337667, EPI_ISL_2337668, EPI_ISL_2337669, EPI_ISL_2337670, EPI_ISL_2337671, EPI_ISL_2337672, EPI_ISL_2337673, EPI_ISL_2337674, EPI_ISL_2337675, EPI_ISL_2337676, EPI_ISL_2337677, EPI_ISL_2337678, EPI_ISL_2337679, EPI_ISL_2337680, EPI_ISL_2337681, EPI_ISL_2337682, EPI_ISL_2337683, EPI_ISL_2337684, EPI_ISL_2337685,
EPI_ISL_2337686, EPI_ISL_2337687, EPI_ISL_2337688, EPI_ISL_2337689, EPI_ISL_2337690, EPI_ISL_2337691, EPI_ISL_2337692, EPI_ISL_2337693, EPI_ISL_2337694, EPI_ISL_2337695, EPI_ISL_2337696, EPI_ISL_2337697, EPI_ISL_2337698, EPI_ISL_2337699, EPI_ISL_2337700, EPI_ISL_2337701, EPI_ISL_2337702, EPI_ISL_2337703, EPI_ISL_2337704, EPI_ISL_2337705, EPI_ISL_2337706, EPI_ISL_2337707,
EPI_ISL_2337708, EPI_ISL_2337709, EPI_ISL_2337710, EPI_ISL_2337711, EPI_ISL_2337712, EPI_ISL_2337713, EPI_ISL_2337714, EPI_ISL_2337715, EPI_ISL_2337716, EPI_ISL_2337717, EPI_ISL_2337718, EPI_ISL_2337719, EPI_ISL_2337720, EPI_ISL_2337721, EPI_ISL_2337722, EPI_ISL_2337723, EPI_ISL_2337724, EPI_ISL_2337725, EPI_ISL_2337726, EPI_ISL_2337727, EPI_ISL_2337728, EPI_ISL_2337729,
EPI_ISL_2337730, EPI_ISL_2337731, EPI_ISL_2337732, EPI_ISL_2337733, EPI_ISL_2337734, EPI_ISL_2337735, EPI_ISL_2337736, EPI_ISL_2337737, EPI_ISL_2337738, EPI_ISL_2337739, EPI_ISL_2337740, EPI_ISL_2337741, EPI_ISL_2337742, EPI_ISL_2337743, EPI_ISL_2337744, EPI_ISL_2337745, EPI_ISL_2337746, EPI_ISL_2337747, EPI_ISL_2337748, EPI_ISL_2337749, EPI_ISL_2337750, EPI_ISL_2337751,
EPI_ISL_2337752, EPI_ISL_2337753, EPI_ISL_2337754, EPI_ISL_2337755, EPI_ISL_2337756, EPI_ISL_2337757, EPI_ISL_2337758, EPI_ISL_2337759, EPI_ISL_2337760, EPI_ISL_2337761, EPI_ISL_2337762, EPI_ISL_2337763, EPI_ISL_2337764, EPI_ISL_2337765, EPI_ISL_2337766, EPI_ISL_2337767, EPI_ISL_2337768, EPI_ISL_2337769, EPI_ISL_2337770, EPI_ISL_2337771, EPI_ISL_2337772, EPI_ISL_2337773,
EPI_ISL_2337774, EPI_ISL_2337775, EPI_ISL_2337776, EPI_ISL_2337777, EPI_ISL_2337778, EPI_ISL_2337779, EPI_ISL_2337780, EPI_ISL_2337781, EPI_ISL_2337782, EPI_ISL_2337783, EPI_ISL_2337784, EPI_ISL_2337785, EPI_ISL_2337786, EPI_ISL_2337787, EPI_ISL_2337788, EPI_ISL_2337789, EPI_ISL_2337790, EPI_ISL_2337791, EPI_ISL_2337792, EPI_ISL_2337793, EPI_ISL_2337794, EPI_ISL_2337795,
EPI_ISL_2337796, EPI_ISL_2337797, EPI_ISL_2337798, EPI_ISL_2337799, EPI_ISL_2337800, EPI_ISL_2337801, EPI_ISL_2337802, EPI_ISL_2337803, EPI_ISL_2337804, EPI_ISL_2337805, EPI_ISL_2337806, EPI_ISL_2337807, EPI_ISL_2337808, EPI_ISL_2337809, EPI_ISL_2337810, EPI_ISL_2337811, EPI_ISL_2337812, EPI_ISL_2337813, EPI_ISL_2337814, EPI_ISL_2337815, EPI_ISL_2337816, EPI_ISL_2337817,
EPI_ISL_2337818, EPI_ISL_2337819, EPI_ISL_2337820, EPI_ISL_2337821, EPI_ISL_2337822, EPI_ISL_2337823, EPI_ISL_2337824, EPI_ISL_2337825, EPI_ISL_2337826, EPI_ISL_2337827, EPI_ISL_2337828, EPI_ISL_2337829, EPI_ISL_2337830, EPI_ISL_2337831, EPI_ISL_2337832, EPI_ISL_2337833, EPI_ISL_2337834, EPI_ISL_2337835, EPI_ISL_2337836, EPI_ISL_2337837, EPI_ISL_2337838, EPI_ISL_2337839,
EPI_ISL_2337840, EPI_ISL_2337841, EPI_ISL_2337842, EPI_ISL_2337843, EPI_ISL_2337844, EPI_ISL_2337845, EPI_ISL_2337846, EPI_ISL_2337847, EPI_ISL_2337848, EPI_ISL_2337849, EPI_ISL_2337850, EPI_ISL_2337851, EPI_ISL_2337852, EPI_ISL_2337853, EPI_ISL_2337854, EPI_ISL_2337855, EPI_ISL_2337856, EPI_ISL_2337857, EPI_ISL_2337858, EPI_ISL_2337859, EPI_ISL_2337860, EPI_ISL_2337861,
EPI_ISL_2337862, EPI_ISL_2337863, EPI_ISL_2337864, EPI_ISL_2337865, EPI_ISL_2337866, EPI_ISL_2337867, EPI_ISL_2337868, EPI_ISL_2337869, EPI_ISL_2337870, EPI_ISL_2337871, EPI_ISL_2337872, EPI_ISL_2337873, EPI_ISL_2337874, EPI_ISL_2337875, EPI_ISL_2337876, EPI_ISL_2337877, EPI_ISL_2337878, EPI_ISL_2337879, EPI_ISL_2337880, EPI_ISL_2337881, EPI_ISL_2337882, EPI_ISL_2337883,
EPI_ISL_2337884, EPI_ISL_2337885, EPI_ISL_2337886, EPI_ISL_2337887, EPI_ISL_2337888, EPI_ISL_2337889, EPI_ISL_2337890, EPI_ISL_2337891, EPI_ISL_2337892, EPI_ISL_2337893, EPI_ISL_2337894, EPI_ISL_2337895, EPI_ISL_2337896, EPI_ISL_2337897, EPI_ISL_2337898, EPI_ISL_2337899, EPI_ISL_2337900, EPI_ISL_2337901, EPI_ISL_2337902, EPI_ISL_2337903, EPI_ISL_2337904, EPI_ISL_2337905,
EPI_ISL_2337906, EPI_ISL_2337907, EPI_ISL_2337908, EPI_ISL_2337909, EPI_ISL_2337910, EPI_ISL_2337911, EPI_ISL_2337912, EPI_ISL_2337913, EPI_ISL_2337914, EPI_ISL_2337915, EPI_ISL_2337916, EPI_ISL_2337917, EPI_ISL_2337918, EPI_ISL_2337919, EPI_ISL_2337920, EPI_ISL_2337921, EPI_ISL_2337922, EPI_ISL_2337923, EPI_ISL_2337924, EPI_ISL_2337925, EPI_ISL_2337926, EPI_ISL_2337927,
EPI_ISL_2337928, EPI_ISL_2337929, EPI_ISL_2337930, EPI_ISL_2337931, EPI_ISL_2337932, EPI_ISL_2337933, EPI_ISL_2337934, EPI_ISL_2337935, EPI_ISL_2337936, EPI_ISL_2337937, EPI_ISL_2337938, EPI_ISL_2337939, EPI_ISL_2337940, EPI_ISL_2337941, EPI_ISL_2337942, EPI_ISL_2337943, EPI_ISL_2337944, EPI_ISL_2337945, EPI_ISL_2337946, EPI_ISL_2337947, EPI_ISL_2337948, EPI_ISL_2337949,
EPI_ISL_2337950, EPI_ISL_2337951, EPI_ISL_2337952, EPI_ISL_2337953, EPI_ISL_2337954, EPI_ISL_2337955, EPI_ISL_2337956, EPI_ISL_2337957, EPI_ISL_2337958, EPI_ISL_2337959, EPI_ISL_2337960, EPI_ISL_2337961, EPI_ISL_2337962, EPI_ISL_2337963, EPI_ISL_2337964, EPI_ISL_2337965, EPI_ISL_2337966, EPI_ISL_2337967, EPI_ISL_2337968, EPI_ISL_2337969, EPI_ISL_2337970, EPI_ISL_2337971,
EPI_ISL_2337972, EPI_ISL_2337973, EPI_ISL_2337974, EPI_ISL_2337975, EPI_ISL_2337976, EPI_ISL_2337977, EPI_ISL_2337978, EPI_ISL_2337979, EPI_ISL_2337980, EPI_ISL_2337981, EPI_ISL_2337982, EPI_ISL_2337983, EPI_ISL_2337984, EPI_ISL_2337985, EPI_ISL_2337986, EPI_ISL_2337987, EPI_ISL_2337988, EPI_ISL_2337989, EPI_ISL_2337990, EPI_ISL_2337991, EPI_ISL_2337992, EPI_ISL_2337993,
EPI_ISL_2337994, EPI_ISL_2337995, EPI_ISL_2337996, EPI_ISL_2337997, EPI_ISL_2337998, EPI_ISL_2337999, EPI_ISL_2338000, EPI_ISL_2338001, EPI_ISL_2338002, EPI_ISL_2338003, EPI_ISL_2338004, EPI_ISL_2338005, EPI_ISL_2338006, EPI_ISL_2338007, EPI_ISL_2338008, EPI_ISL_2338009, EPI_ISL_2338010, EPI_ISL_2338011, EPI_ISL_2338012, EPI_ISL_2338013, EPI_ISL_2338014, EPI_ISL_2338015,
EPI_ISL_2338016, EPI_ISL_2338017, EPI_ISL_2338018, EPI_ISL_2338019, EPI_ISL_2338020, EPI_ISL_2338021, EPI_ISL_2338022, EPI_ISL_2338023, EPI_ISL_2338024, EPI_ISL_2338025, EPI_ISL_2338026, EPI_ISL_2338027, EPI_ISL_2338028, EPI_ISL_2338029, EPI_ISL_2338030, EPI_ISL_2338031, EPI_ISL_2338032, EPI_ISL_2338033, EPI_ISL_2338034, EPI_ISL_2338035, EPI_ISL_2338036, EPI_ISL_2338037,
EPI_ISL_2338038, EPI_ISL_2338039, EPI_ISL_2338040, EPI_ISL_2338041, EPI_ISL_2338042, EPI_ISL_2338043, EPI_ISL_2338044, EPI_ISL_2338045, EPI_ISL_2338046, EPI_ISL_2338047, EPI_ISL_2338048, EPI_ISL_2338049, EPI_ISL_2338050, EPI_ISL_2338051, EPI_ISL_2338052, EPI_ISL_2338053, EPI_ISL_2338054, EPI_ISL_2338055, EPI_ISL_2338056, EPI_ISL_2338057, EPI_ISL_2338058, EPI_ISL_2338059,
EPI_ISL_2338060, EPI_ISL_2338061, EPI_ISL_2338062, EPI_ISL_2338063, EPI_ISL_2338064, EPI_ISL_2338065, EPI_ISL_2338066, EPI_ISL_2338067, EPI_ISL_2338068, EPI_ISL_2338069, EPI_ISL_2338070, EPI_ISL_2338071, EPI_ISL_2338072, EPI_ISL_2338073, EPI_ISL_2338074, EPI_ISL_2338075, EPI_ISL_2338076, EPI_ISL_2338077, EPI_ISL_2338078, EPI_ISL_2338079, EPI_ISL_2338080, EPI_ISL_2338081,
EPI_ISL_2338082, EPI_ISL_2338083, EPI_ISL_2338084, EPI_ISL_2338085, EPI_ISL_2338086, EPI_ISL_2338087, EPI_ISL_2338088, EPI_ISL_2338089, EPI_ISL_2338090, EPI_ISL_2338091, EPI_ISL_2338092, EPI_ISL_2338093, EPI_ISL_2338094, EPI_ISL_2338095, EPI_ISL_2338096, EPI_ISL_2338097, EPI_ISL_2338098, EPI_ISL_2338099, EPI_ISL_2338100, EPI_ISL_2338101, EPI_ISL_2338102, EPI_ISL_2338103,

EPI_ISL_2338104, EPI_ISL_2338105, EPI_ISL_2338106, EPI_ISL_2338107, EPI_ISL_2338108, EPI_ISL_2338109, EPI_ISL_2338110, EPI_ISL_2338111, EPI_ISL_2338112, EPI_ISL_2338113, EPI_ISL_2338114, EPI_ISL_2338115, EPI_ISL_2338116, EPI_ISL_2338117, EPI_ISL_2338118, EPI_ISL_2338119, EPI_ISL_2338120, EPI_ISL_2338121, EPI_ISL_2338122, EPI_ISL_2338123, EPI_ISL_2338124, EPI_ISL_2338125, EPI_ISL_2338126, EPI_ISL_2338127, EPI_ISL_2338128, EPI_ISL_2338129, EPI_ISL_2338130, EPI_ISL_2338131, EPI_ISL_2338132, EPI_ISL_2338133, EPI_ISL_2338134, EPI_ISL_2338135, EPI_ISL_2338136, EPI_ISL_2338137, EPI_ISL_2338138, EPI_ISL_2338139, EPI_ISL_2338140, EPI_ISL_2338141, EPI_ISL_2338142, EPI_ISL_2338143, EPI_ISL_2338144, EPI_ISL_2338145, EPI_ISL_2338146, EPI_ISL_2338147, EPI_ISL_2338148, EPI_ISL_2338149, EPI_ISL_2338150, EPI_ISL_2338151, EPI_ISL_2338152, EPI_ISL_2338153, EPI_ISL_2338154, EPI_ISL_2338155, EPI_ISL_2338156, EPI_ISL_2338157, EPI_ISL_2338158, EPI_ISL_2338159, EPI_ISL_2338160, EPI_ISL_2338161, EPI_ISL_2338162, EPI_ISL_2338163, EPI_ISL_2338164, EPI_ISL_2338165, EPI_ISL_2338166, EPI_ISL_2338167, EPI_ISL_2338168, EPI_ISL_2338169, EPI_ISL_2338170, EPI_ISL_2338171, EPI_ISL_2338172, EPI_ISL_2338173, EPI_ISL_2338174, EPI_ISL_2338175, EPI_ISL_2338176, EPI_ISL_2338177, EPI_ISL_2338178, EPI_ISL_2338179, EPI_ISL_2338180, EPI_ISL_2338181, EPI_ISL_2338182, EPI_ISL_2338183, EPI_ISL_2338184, EPI_ISL_2338185, EPI_ISL_2338186, EPI_ISL_2338187, EPI_ISL_2338188, EPI_ISL_2338189, EPI_ISL_2338190, EPI_ISL_2338191, EPI_ISL_2338192, EPI_ISL_2338193, EPI_ISL_2338194, EPI_ISL_2338195, EPI_ISL_2338196, EPI_ISL_2338197, EPI_ISL_2338198, EPI_ISL_2338199, EPI_ISL_2338200, EPI_ISL_2338201, EPI_ISL_2338202, EPI_ISL_2338203, EPI_ISL_2338204, EPI_ISL_2338205, EPI_ISL_2338206, EPI_ISL_2338207, EPI_ISL_2338208, EPI_ISL_2338209, EPI_ISL_2338210, EPI_ISL_2338211, EPI_ISL_2338212, EPI_ISL_2338213, EPI_ISL_2338214, EPI_ISL_2338215, EPI_ISL_2338216, EPI_ISL_2338217, EPI_ISL_2338218, EPI_ISL_2338219, EPI_ISL_2338220, EPI_ISL_2338221, EPI_ISL_2338222, EPI_ISL_2338223, EPI_ISL_2338224, EPI_ISL_2338225, EPI_ISL_2338226, EPI_ISL_2338227, EPI_ISL_2338228, EPI_ISL_2338229, EPI_ISL_2338230, EPI_ISL_2338231, EPI_ISL_2338232, EPI_ISL_2338233, EPI_ISL_2338234, EPI_ISL_2338235, EPI_ISL_2338236, EPI_ISL_2338237, EPI_ISL_2338238, EPI_ISL_2338239, EPI_ISL_2338240, EPI_ISL_2338241, EPI_ISL_2338242, EPI_ISL_2338243, EPI_ISL_2338244, EPI_ISL_2338245, EPI_ISL_2338246, EPI_ISL_2338247, EPI_ISL_2338248, EPI_ISL_2338249, EPI_ISL_2338250, EPI_ISL_2338251, EPI_ISL_2338252, EPI_ISL_2338253, EPI_ISL_2338254, EPI_ISL_2338255, EPI_ISL_2338256, EPI_ISL_2338257, EPI_ISL_2338258, EPI_ISL_2338259, EPI_ISL_2338260, EPI_ISL_2338261, EPI_ISL_2338262, EPI_ISL_2338263, EPI_ISL_2338264, EPI_ISL_2338265, EPI_ISL_2338266, EPI_ISL_2338267, EPI_ISL_2338268, EPI_ISL_2338269, EPI_ISL_2338270, EPI_ISL_2338271, EPI_ISL_2338272, EPI_ISL_2338273, EPI_ISL_2338274, EPI_ISL_2338275, EPI_ISL_2338276, EPI_ISL_2338277, EPI_ISL_2338278, EPI_ISL_2338279, EPI_ISL_2338280, EPI_ISL_2338281, EPI_ISL_2338282, EPI_ISL_2338283, EPI_ISL_2338284, EPI_ISL_2338285, EPI_ISL_2338286, EPI_ISL_2338287, EPI_ISL_2338288, EPI_ISL_2338289, EPI_ISL_2338290, EPI_ISL_2338291, EPI_ISL_2338292, EPI_ISL_2338293, EPI_ISL_2338294, EPI_ISL_2338295, EPI_ISL_2338296, EPI_ISL_2338297, EPI_ISL_2338298, EPI_ISL_2338299, EPI_ISL_2338300, EPI_ISL_2338301, EPI_ISL_2338302, EPI_ISL_2338303, EPI_ISL_2338304, EPI_ISL_2338305, EPI_ISL_2338306, EPI_ISL_2338307, EPI_ISL_2338308, EPI_ISL_2338309, EPI_ISL_2338310, EPI_ISL_2338311, EPI_ISL_2338312, EPI_ISL_2338313, EPI_ISL_2338314, EPI_ISL_2338315, EPI_ISL_2338316, EPI_ISL_2338317, EPI_ISL_2338318, EPI_ISL_2338319, EPI_ISL_2338320, EPI_ISL_2338321, EPI_ISL_2338406, EPI_ISL_2338407, EPI_ISL_2338408, EPI_ISL_2338409, EPI_ISL_2338410, EPI_ISL_2338411, EPI_ISL_2338412, EPI_ISL_2338413, EPI_ISL_2338414, EPI_ISL_2338415, EPI_ISL_2338416, EPI_ISL_2338417, EPI_ISL_2338418, EPI_ISL_2338419, EPI_ISL_2338420, EPI_ISL_2338421, EPI_ISL_2338422, EPI_ISL_2338423, EPI_ISL_2338424, EPI_ISL_2338425, EPI_ISL_2338426, EPI_ISL_2338427, EPI_ISL_2338428, EPI_ISL_2338429, EPI_ISL_2338430, EPI_ISL_2338431, EPI_ISL_2338432, EPI_ISL_2338433, EPI_ISL_2338434, EPI_ISL_2338435, EPI_ISL_2338436, EPI_ISL_2338437, EPI_ISL_2338438, EPI_ISL_2338439, EPI_ISL_2338440, EPI_ISL_2338441, EPI_ISL_2338442, EPI_ISL_2338443, EPI_ISL_2338444, EPI_ISL_2338445, EPI_ISL_2338446, EPI_ISL_2338447, EPI_ISL_2338448, EPI_ISL_2338449, EPI_ISL_2338450, EPI_ISL_2338451, EPI_ISL_2338452, EPI_ISL_2338453, EPI_ISL_2338454, EPI_ISL_2338455, EPI_ISL_2338456, EPI_ISL_2338457, EPI_ISL_2338458, EPI_ISL_2338459, EPI_ISL_2338460, EPI_ISL_2338461, EPI_ISL_2338462, EPI_ISL_2338463, EPI_ISL_2338464, EPI_ISL_2338465, EPI_ISL_2338466, EPI_ISL_2338467, EPI_ISL_2338468, EPI_ISL_2338469, EPI_ISL_2338470, EPI_ISL_2338471, EPI_ISL_2338472, EPI_ISL_2338473, EPI_ISL_2338474, EPI_ISL_2338475, EPI_ISL_2338476, EPI_ISL_2338477, EPI_ISL_2338478, EPI_ISL_2338479, EPI_ISL_2338480, EPI_ISL_2338481, EPI_ISL_2338482, EPI_ISL_2338483, EPI_ISL_2338484, EPI_ISL_2338485, EPI_ISL_2338486, EPI_ISL_2338487, EPI_ISL_2338488, EPI_ISL_2338489, EPI_ISL_2338490, EPI_ISL_2338491, EPI_ISL_2338492, EPI_ISL_2338493, EPI_ISL_2338494, EPI_ISL_2338495, EPI_ISL_2338496, EPI_ISL_2338497, EPI_ISL_2338498, EPI_ISL_2338499, EPI_ISL_2338500, EPI_ISL_2338501, EPI_ISL_2338502, EPI_ISL_2338503, EPI_ISL_2338504, EPI_ISL_2338505, EPI_ISL_2338506, EPI_ISL_2338507, EPI_ISL_2338508, EPI_ISL_2338509, EPI_ISL_2338510, EPI_ISL_2338511, EPI_ISL_2338512, EPI_ISL_2338513, EPI_ISL_2338514, EPI_ISL_2338515, EPI_ISL_2338516, EPI_ISL_2338517, EPI_ISL_2338518, EPI_ISL_2338519, EPI_ISL_2338520, EPI_ISL_2338521, EPI_ISL_2338522, EPI_ISL_2338523, EPI_ISL_2338524, EPI_ISL_2338525, EPI_ISL_2338526, EPI_ISL_2338527, EPI_ISL_2338528, EPI_ISL_2338529, EPI_ISL_2338530, EPI_ISL_2338531, EPI_ISL_2338532, EPI_ISL_2338533, EPI_ISL_2338535, EPI_ISL_2338536, EPI_ISL_2338537, EPI_ISL_2338538, EPI_ISL_2338539, EPI_ISL_2338540, EPI_ISL_2338541, EPI_ISL_2338542, EPI_ISL_2338543, EPI_ISL_2338544, EPI_ISL_2338545, EPI_ISL_2338546, EPI_ISL_2338547, EPI_ISL_2338548, EPI_ISL_2338549, EPI_ISL_2338550, EPI_ISL_2338551, EPI_ISL_2338552, EPI_ISL_2338553, EPI_ISL_2338554, EPI_ISL_2338555, EPI_ISL_2338556, EPI_ISL_2338557, EPI_ISL_2338558, EPI_ISL_2338559, EPI_ISL_2338560, EPI_ISL_2338561, EPI_ISL_2338562, EPI_ISL_2338563, EPI_ISL_2338564, EPI_ISL_2338565, EPI_ISL_2338566, EPI_ISL_2338567, EPI_ISL_2338568, EPI_ISL_2338569, EPI_ISL_2338570, EPI_ISL_2338571, EPI_ISL_2338572, EPI_ISL_2338573, EPI_ISL_2338574, EPI_ISL_2338575, EPI_ISL_2338576, EPI_ISL_2338577, EPI_ISL_2338578, EPI_ISL_2338579, EPI_ISL_2338580, EPI_ISL_2338581, EPI_ISL_2338582, EPI_ISL_2338583, EPI_ISL_2338584, EPI_ISL_2338585, EPI_ISL_2338586, EPI_ISL_2338587, EPI_ISL_2338588, EPI_ISL_2338589, EPI_ISL_2338590, EPI_ISL_2338591, EPI_ISL_2338592, EPI_ISL_2338593, EPI_ISL_2338594, EPI_ISL_2338595, EPI_ISL_2338596, EPI_ISL_2338597, EPI_ISL_2338598, EPI_ISL_2338599, EPI_ISL_2338600, EPI_ISL_2338601, EPI_ISL_2338602, EPI_ISL_2338603, EPI_ISL_2338604, EPI_ISL_2338605, EPI_ISL_2338606, EPI_ISL_2338607, EPI_ISL_2338608, EPI_ISL_2338609, EPI_ISL_2338610, EPI_ISL_2338611, EPI_ISL_2338612, EPI_ISL_2338613, EPI_ISL_2338614, EPI_ISL_2338615, EPI_ISL_2338616, EPI_ISL_2338617, EPI_ISL_2338618, EPI_ISL_2338619, EPI_ISL_2338620, EPI_ISL_2338621, EPI_ISL_2338622, EPI_ISL_2338623, EPI_ISL_2338624, EPI_ISL_2338625, EPI_ISL_2338626, EPI_ISL_2338627, EPI_ISL_2338628, EPI_ISL_2338629, EPI_ISL_2338630, EPI_ISL_2338631, EPI_ISL_2338632, EPI_ISL_2338633, EPI_ISL_2338634, EPI_ISL_2338635, EPI_ISL_2338636, EPI_ISL_2338637, EPI_ISL_2338638, EPI_ISL_2338639, EPI_ISL_2338640, EPI_ISL_2338641, EPI_ISL_2338642, EPI_ISL_2338643, EPI_ISL_2338644, EPI_ISL_2338645, EPI_ISL_2338646, EPI_ISL_2338647, EPI_ISL_2338648, EPI_ISL_2338649, EPI_ISL_2338650, EPI_ISL_2338651, EPI_ISL_2338652, EPI_ISL_2338653, EPI_ISL_2338654, EPI_ISL_2338655, EPI_ISL_2338656, EPI_ISL_2338657, EPI_ISL_2338658, EPI_ISL_2338659, EPI_ISL_2338660, EPI_ISL_2338661, EPI_ISL_2338662, EPI_ISL_2338663, EPI_ISL_2338664, EPI_ISL_2338665, EPI_ISL_2338666, EPI_ISL_2338667, EPI_ISL_2338668, EPI_ISL_2338669, EPI_ISL_2338670, EPI_ISL_2338671, EPI_ISL_2338672, EPI_ISL_2338673, EPI_ISL_2338674, EPI_ISL_2338675, EPI_ISL_2338676, EPI_ISL_2338677, EPI_ISL_2338678, EPI_ISL_2338679, EPI_ISL_2338680, EPI_ISL_2338681, EPI_ISL_2338682, EPI_ISL_2338683, EPI_ISL_2338684, EPI_ISL_2338685, EPI_ISL_2338686, EPI_ISL_2338687, EPI_ISL_2338688, EPI_ISL_2338689, EPI_ISL_2338690, EPI_ISL_2338691, EPI_ISL_2338692, EPI_ISL_2338693, EPI_ISL_2338694, EPI_ISL_2338695, EPI_ISL_2338696, EPI_ISL_2338697, EPI_ISL_2338698, EPI_ISL_2338699, EPI_ISL_2338700, EPI_ISL_2338701, EPI_ISL_2338702, EPI_ISL_2338703, EPI_ISL_2338704, EPI_ISL_2338705, EPI_ISL_2338706, EPI_ISL_2338707, EPI_ISL_2338708, EPI_ISL_2338709, EPI_ISL_2338710, EPI_ISL_2338711, EPI_ISL_2338712, EPI_ISL_2338713, EPI_ISL_2338714, EPI_ISL_2338715, EPI_ISL_2338716, EPI_ISL_2338717, EPI_ISL_2338718, EPI_ISL_2338719, EPI_ISL_2338720, EPI_ISL_2338721, EPI_ISL_2338722, EPI_ISL_2338723, EPI_ISL_2338724, EPI_ISL_2338725, EPI_ISL_2338726, EPI_ISL_2338727, EPI_ISL_2338728, EPI_ISL_2338729, EPI_ISL_2338730, EPI_ISL_2338731, EPI_ISL_2338732, EPI_ISL_2338733, EPI_ISL_2338734, EPI_ISL_2338735, EPI_ISL_2338736, EPI_ISL_2338737, EPI_ISL_2338738, EPI_ISL_2338739, EPI_ISL_2338740, EPI_ISL_2338741, EPI_ISL_2338742, EPI_ISL_2338743, EPI_ISL_2338744, EPI_ISL_2338745, EPI_ISL_2338746, EPI_ISL_2338747, EPI_ISL_2338748, EPI_ISL_2338749, EPI_ISL_2338750, EPI_ISL_2338751, EPI_ISL_2338752, EPI_ISL_2338753, EPI_ISL_2338754, EPI_ISL_2338755, EPI_ISL_2338756, EPI_ISL_2338757, EPI_ISL_2338758, EPI_ISL_2338759, EPI_ISL_2338760, EPI_ISL_2338761, EPI_ISL_2338762, EPI_ISL_2338763, EPI_ISL_2338764, EPI_ISL_2338765, EPI_ISL_2338766, EPI_ISL_2338767, EPI_ISL_2338768, EPI_ISL_2338769, EPI_ISL_2338770, EPI_ISL_2338771, EPI_ISL_2338772, EPI_ISL_2338773, EPI_ISL_2338774, EPI_ISL_2338775, EPI_ISL_2338776, EPI_ISL_2338777, EPI_ISL_2338778, EPI_ISL_2338779, EPI_ISL_2338780, EPI_ISL_2338781, EPI_ISL_2338782, EPI_ISL_2338783, EPI_ISL_2338784, EPI_ISL_2338785, EPI_ISL_2338786, EPI_ISL_2338787, EPI_ISL_2338788, EPI_ISL_2338789, EPI_ISL_2338790, EPI_ISL_2338791, EPI_ISL_2338792, EPI_ISL_2338793, EPI_ISL_2338794, EPI_ISL_2338795, EPI_ISL_2338796, EPI_ISL_2338797, EPI_ISL_2338798, EPI_ISL_2338799, EPI_ISL_2338800, EPI_ISL_2338801, EPI_ISL_2338802, EPI_ISL_2338803, EPI_ISL_2338804, EPI_ISL_2338805, EPI_ISL_2338806, EPI_ISL_2338807, EPI_ISL_2338808, EPI_ISL_2338809, EPI_ISL_2338810, EPI_ISL_2338811, EPI_ISL_2338812, EPI_ISL_2338813, EPI_ISL_2338814, EPI_ISL_2338815, EPI_ISL_2338816, EPI_ISL_2338817, EPI_ISL_2338818, EPI_ISL_2338819, EPI_ISL_2338820, EPI_ISL_2338821, EPI_ISL_2338822, EPI_ISL_2338823, EPI_ISL_2338824, EPI_ISL_2338825, EPI_ISL_2338826, EPI_ISL_2338827, EPI_ISL_2338828, EPI_ISL_2338829, EPI_ISL_2338830, EPI_ISL_2338831, EPI_ISL_2338832, EPI_ISL_2338833, EPI_ISL_2338834, EPI_ISL_2338835, EPI_ISL_2338836, EPI_ISL_2338837, EPI_ISL_2338838, EPI_ISL_2338839, EPI_ISL_2338840, EPI_ISL_2338841, EPI_ISL_2338842, EPI_ISL_2338843, EPI_ISL_2338844, EPI_ISL_2338845, EPI_ISL_2338846, EPI_ISL_2338847, EPI_ISL_2338848, EPI_ISL_2338849, EPI_ISL_2338850, EPI_ISL_2338851, EPI_ISL_2338852, EPI_ISL_2338853, EPI_ISL_2338854, EPI_ISL_2338855, EPI_ISL_2338856, EPI_ISL_2338857, EPI_ISL_2338858, EPI_ISL_2338859, EPI_ISL_2338860, EPI_ISL_2338861, EPI_ISL_2338862, EPI_ISL_2338863, EPI_ISL_2338864, EPI_ISL_2338865, EPI_ISL_2338866, EPI_ISL_2338867, EPI_ISL_2338868, EPI_ISL_2338869, EPI_ISL_2338870, EPI_ISL_2338871, EPI_ISL_2338872, EPI_ISL_2338873, EPI_ISL_2338874, EPI_ISL_2338875, EPI_ISL_2338876, EPI_ISL_2338877, EPI_ISL_2338878, EPI_ISL_2338879, EPI_ISL_2338880, EPI_ISL_2338881, EPI_ISL_2338882, EPI_ISL_2338883, EPI_ISL_2338884, EPI_ISL_2338885, EPI_ISL_2338886, EPI_ISL_2338887, EPI_ISL_2338888, EPI_ISL_2338889, EPI_ISL_2338890, EPI_ISL_2338891, EPI_ISL_2338892, EPI_ISL_2338893, EPI_ISL_2338894, EPI_ISL_2338895, EPI_ISL_2338896, EPI_ISL_2338897, EPI_ISL_2338898, EPI_ISL_2338899, EPI_ISL_2338900, EPI_ISL_2338901, EPI_ISL_2338902, EPI_ISL_2338903, EPI_ISL_2338904, EPI_ISL_2338905, EPI_ISL_2338906, EPI_ISL_2338907, EPI_ISL_2338908, EPI_ISL_2338909, EPI_ISL_2338910, EPI_ISL_2338911, EPI_ISL_2338912, EPI_ISL_2338913, EPI_ISL_2338914, EPI_ISL_2338915, EPI_ISL_2338916, EPI_ISL_2338917, EPI_ISL_2338918, EPI_ISL_2338919, EPI_ISL_2338920, EPI_ISL_2338921, EPI_ISL_2338922, EPI_ISL_2338923, EPI_ISL_2338924, EPI_ISL_2338925, EPI_ISL_2338926, EPI_ISL_2338927, EPI_ISL_2338928, EPI_ISL_2338929, EPI_ISL_2338930, EPI_ISL_2338931, EPI_ISL_2338932, EPI_ISL_2338933, EPI_ISL_2338934, EPI_ISL_2338935, EPI_ISL_2338936, EPI_ISL_2338937, EPI_ISL_2338938, EPI_ISL_2338939, EPI_ISL_2338940, EPI_ISL_2338941, EPI_ISL_2338942, EPI_ISL_2338943, EPI_ISL_2338944, EPI_ISL_2338945, EPI_ISL_2338946, EPI_ISL_2338947, EPI_ISL_2338948, EPI_ISL_2338949, EPI_ISL_2338950, EPI_ISL_2338951, EPI_ISL_2338952, EPI_ISL_2338953, EPI_ISL_2338954, EPI_ISL_2338955, EPI_ISL_2338956, EPI_ISL_2338957, EPI_ISL_2338958, EPI_ISL_2338959, EPI_ISL_2338960, EPI_ISL_2338961, EPI_ISL_2338962, EPI_ISL_2338963, EPI_ISL_2338964, EPI_ISL_2338965, EPI_ISL_2338966, EPI_ISL_2338967, EPI_ISL_2338968, EPI_ISL_2338969, EPI_ISL_2338970, EPI_ISL_2338971, EPI_ISL_2338972, EPI_ISL_2338973, EPI_ISL_2338974, EPI_ISL_2338975, EPI_ISL_2338976, EPI_ISL_2338977, EPI_ISL_2338978, EPI_ISL_2338979, EPI_ISL_2338980, EPI_ISL_2338981, EPI_ISL_2338982, EPI_ISL_2338983, EPI_ISL_2338984, EPI_ISL_2338985, EPI_ISL_2338986, EPI_ISL_2338987, EPI_ISL_2338988, EPI_ISL_2338989, EPI_ISL_2338990, EPI_ISL_2338991, EPI_ISL_2338992, EPI_ISL_2338993, EPI_ISL_2338994, EPI_ISL_2338995, EPI_ISL_2338996, EPI_ISL_2338997, EPI_ISL_2338998, EPI_ISL_2338999, EPI_ISL_2339000, EPI_ISL_2339001, EPI_ISL_2339002, EPI_ISL_2339003, EPI_ISL_2339004, EPI_ISL_2339005, EPI_ISL_2339006, EPI_ISL_2339007, EPI_ISL_2339008, EPI_ISL_2339009, EPI_ISL_2339010, EPI_ISL_2339011, EPI_ISL_2339012, EPI_ISL_2339013, EPI_ISL_2339014, EPI_ISL_2339015, EPI_ISL_2339016, EPI_ISL_2339017, EPI_ISL_2339018, EPI_ISL_2339019, EPI_ISL_2339020, EPI_ISL_2339021, EPI_ISL_2339022, EPI_ISL_2339023, EPI_ISL_2339024, EPI_ISL_2339025, EPI_ISL_2339026, EPI_ISL_2339027, EPI_ISL_2339028, EPI_ISL_2339029, EPI_ISL_2339030, EPI_ISL_2339031, EPI_ISL_2339032, EPI_ISL_2339033, EPI_ISL_2339034			
see above	SARS-CoV-2 testing team, National Institute of Infectious Diseases	Pathogen Genomics Center, National Institute of Infectious Diseases	Hazuka Y Furihata; Kentaro Itokawa; Makoto Kuroda; Masanori Hashino; Masumichi Saito; Naomi Nojiri; Nozomu Hanaoka; Rina Tanaka; Sana Uchikoba; Tsuguto Fujimoto; Tsuyoshi Sekizuka
EPI_ISL_2326341, EPI_ISL_2326342, EPI_ISL_2326343, EPI_ISL_2326344, EPI_ISL_2326345, EPI_ISL_2326346, EPI_ISL_2326347, EPI_ISL_2326348, EPI_ISL_2326349, EPI_ISL_2326350, EPI_ISL_2326351, EPI_ISL_2326352, EPI_ISL_2326353, EPI_ISL_2326354, EPI_ISL_2326355, EPI_ISL_2326356, EPI_ISL_2326357, EPI_ISL_2326358, EPI_ISL_2326359, EPI_ISL_2326360, EPI_ISL_2326361, EPI_ISL_2326362, EPI_ISL_2326363			
see above	Tokyo Metropolitan Institute of Public Health	Pathogen Genomics Center, National Institute of Infectious Diseases	Kentaro Itokawa; Makoto Kuroda; Masanori Hashino; Rina Tanaka; Tsuyoshi Sekizuka
EPI_ISL_2303990, EPI_ISL_2303991, EPI_ISL_2303992, EPI_ISL_2303993, EPI_ISL_2303994, EPI_ISL_2303995, EPI_ISL_2303996, EPI_ISL_2303997, EPI_ISL_2321182, EPI_ISL_2321183, EPI_ISL_2321184, EPI_ISL_2321185, EPI_ISL_2321186, EPI_ISL_2321187, EPI_ISL_2321188, EPI_ISL_2321189, EPI_ISL_2321190, EPI_ISL_2321191, EPI_ISL_2321192, EPI_ISL_2321193, EPI_ISL_2321194, EPI_ISL_2321195, EPI_ISL_2321196, EPI_ISL_2321197, EPI_ISL_2321198, EPI_ISL_2321199, EPI_ISL_2321200, EPI_ISL_2321201, EPI_ISL_2321202, EPI_ISL_2321203, EPI_ISL_2321204, EPI_ISL_2321205, EPI_ISL_2321206, EPI_ISL_2321207, EPI_ISL_2321208, EPI_ISL_2321209, EPI_ISL_2321210, EPI_ISL_2321211, EPI_ISL_2321212, EPI_ISL_2321213, EPI_ISL_2321214, EPI_ISL_2321215, EPI_ISL_2321216, EPI_ISL_2321217, EPI_ISL_2321218, EPI_ISL_2321219, EPI_ISL_2321220, EPI_ISL_2321221, EPI_ISL_2321222, EPI_ISL_2321223, EPI_ISL_2321224, EPI_ISL_2321225			
see above	Tokyo Metropolitan Institute of Public Health	Tokyo Metropolitan Institute of Public Health	Hirofumi_Miyake; Jun Suzuki; Kana Kimoto; Keiko_Iwakoshi; Kenji Sadamasu; Mami Nagashima; Masaki Hayashi; Mayu Takanashi; Sumiyo Yamamoto; Takako Yamazaki; Takashi Chiba; Takushi Fujiwara
EPI_ISL_2335933, EPI_ISL_2335934	Yamagata Prefectural Institute of Public Health	Pathogen Genomics Center, National Institute of Infectious Diseases	Kentaro Itokawa; Makoto Kuroda; Masanori Hashino; Rina Tanaka; Tsuyoshi Sekizuka

We gratefully acknowledge the following Authors from the Originating laboratories responsible for obtaining the specimens, as well as the Submitting laboratories where the genome data were generated and shared via GISAID, on which this research is based.

All Submitters of data may be contacted directly via www.gisaid.org

Authors are sorted alphabetically.

Accession ID	Originating Laboratory	Submitting Laboratory	Authors
EPI_ISL_1927241, EPI_ISL_1927242, EPI_ISL_1927244, EPI_ISL_1927245, EPI_ISL_1927247, EPI_ISL_1927248, EPI_ISL_1927249, EPI_ISL_1927251, EPI_ISL_1927252, EPI_ISL_1927254, EPI_ISL_1927255, EPI_ISL_1927257, EPI_ISL_1927258, EPI_ISL_1927260, EPI_ISL_1927261, EPI_ISL_1927263, EPI_ISL_1927264, EPI_ISL_1927266, EPI_ISL_1927267, EPI_ISL_1927269, EPI_ISL_1927270, EPI_ISL_1927271, EPI_ISL_1927273, EPI_ISL_1927275, EPI_ISL_1927460, EPI_ISL_1927462, EPI_ISL_1927463, EPI_ISL_1927849, EPI_ISL_1929239, EPI_ISL_1929399, EPI_ISL_1929401, EPI_ISL_1929402, EPI_ISL_1929404, EPI_ISL_1929405, EPI_ISL_1929424, EPI_ISL_1929426, EPI_ISL_1929427, EPI_ISL_1929435, EPI_ISL_1929528, EPI_ISL_1929530, EPI_ISL_1929531, EPI_ISL_1929533, EPI_ISL_1929534, EPI_ISL_1929535, EPI_ISL_1929537, EPI_ISL_1929539, EPI_ISL_1929540, EPI_ISL_1929541, EPI_ISL_1929543, EPI_ISL_1929545, EPI_ISL_1929546, EPI_ISL_1929548, EPI_ISL_1929549, EPI_ISL_1929551, EPI_ISL_1929552, EPI_ISL_1929554, EPI_ISL_1929555, EPI_ISL_1929557, EPI_ISL_1929558, EPI_ISL_1929560, EPI_ISL_1929561, EPI_ISL_1929563, EPI_ISL_1929564, EPI_ISL_1929566, EPI_ISL_1929567, EPI_ISL_1929569, EPI_ISL_1929570, EPI_ISL_1929572, EPI_ISL_1929573, EPI_ISL_1929575, EPI_ISL_1929576, EPI_ISL_1929578, EPI_ISL_1929579, EPI_ISL_1929581, EPI_ISL_1929582, EPI_ISL_1929584, EPI_ISL_1929585, EPI_ISL_1929587, EPI_ISL_1929588, EPI_ISL_1929590, EPI_ISL_1929591, EPI_ISL_1929593, EPI_ISL_1929594, EPI_ISL_1929596, EPI_ISL_1929597, EPI_ISL_1929599, EPI_ISL_1929600, EPI_ISL_1929602, EPI_ISL_1929603, EPI_ISL_1929605, EPI_ISL_1929606, EPI_ISL_1929608, EPI_ISL_1929609, EPI_ISL_1929611, EPI_ISL_1929612, EPI_ISL_1929614, EPI_ISL_1929615, EPI_ISL_1929617, EPI_ISL_1929618, EPI_ISL_1929620, EPI_ISL_1929621, EPI_ISL_1929623, EPI_ISL_1929624, EPI_ISL_1929626, EPI_ISL_1929627, EPI_ISL_1929629, EPI_ISL_1929630, EPI_ISL_1929632, EPI_ISL_1929633, EPI_ISL_1929635, EPI_ISL_1929636, EPI_ISL_1929638, EPI_ISL_1929639, EPI_ISL_1929641, EPI_ISL_1929642, EPI_ISL_1929643, EPI_ISL_1929645, EPI_ISL_1929646, EPI_ISL_1929648, EPI_ISL_1929649, EPI_ISL_1929651, EPI_ISL_1929652, EPI_ISL_1929654, EPI_ISL_1929655, EPI_ISL_1929656, EPI_ISL_1929658, EPI_ISL_1929659, EPI_ISL_1929661, EPI_ISL_1929662, EPI_ISL_1929664, EPI_ISL_1929665, EPI_ISL_1929667, EPI_ISL_1929668, EPI_ISL_1929670, EPI_ISL_1929671, EPI_ISL_1929673, EPI_ISL_1929674, EPI_ISL_1929675, EPI_ISL_1929677, EPI_ISL_1929678, EPI_ISL_1929680, EPI_ISL_1929681, EPI_ISL_1929683, EPI_ISL_1929684, EPI_ISL_1929686, EPI_ISL_1929687, EPI_ISL_1929689, EPI_ISL_1929690, EPI_ISL_1929692, EPI_ISL_1929693, EPI_ISL_1929695, EPI_ISL_1929696, EPI_ISL_1929698, EPI_ISL_1929699, EPI_ISL_1929701, EPI_ISL_1929702, EPI_ISL_1929704, EPI_ISL_1929705, EPI_ISL_1929707, EPI_ISL_1929708, EPI_ISL_1929710, EPI_ISL_1929711, EPI_ISL_1929713, EPI_ISL_1929714, EPI_ISL_1929716, EPI_ISL_1929717, EPI_ISL_1929719, EPI_ISL_1929720, EPI_ISL_1929722, EPI_ISL_1929723, EPI_ISL_1929725, EPI_ISL_1929727, EPI_ISL_1929728, EPI_ISL_1929730, EPI_ISL_1929731, EPI_ISL_1929732, EPI_ISL_1929734, EPI_ISL_1929735, EPI_ISL_1929737, EPI_ISL_1929739, EPI_ISL_1929740, EPI_ISL_1929742, EPI_ISL_1929743, EPI_ISL_1929745, EPI_ISL_1929746, EPI_ISL_1929748, EPI_ISL_1930241, EPI_ISL_1930243, EPI_ISL_1930245, EPI_ISL_1930246, EPI_ISL_1930248, EPI_ISL_1930249, EPI_ISL_1930251, EPI_ISL_1930252, EPI_ISL_1930254, EPI_ISL_1930256, EPI_ISL_1930257, EPI_ISL_1930259, EPI_ISL_1930260, EPI_ISL_1930262, EPI_ISL_1930263, EPI_ISL_1930265, EPI_ISL_1930266, EPI_ISL_1930268, EPI_ISL_1930269, EPI_ISL_1930271, EPI_ISL_1930272, EPI_ISL_1930274, EPI_ISL_1930275, EPI_ISL_1930277, EPI_ISL_1930279, EPI_ISL_1930280, EPI_ISL_1930282, EPI_ISL_1930283, EPI_ISL_1930285, EPI_ISL_1930286, EPI_ISL_1930288, EPI_ISL_1931618, EPI_ISL_1931970, EPI_ISL_1931971, EPI_ISL_1931972, EPI_ISL_1931974, EPI_ISL_1931976, EPI_ISL_1931977, EPI_ISL_1931980, EPI_ISL_1931984, EPI_ISL_1931988, EPI_ISL_1931992, EPI_ISL_1931995, EPI_ISL_1931998, EPI_ISL_1932002, EPI_ISL_1932004, EPI_ISL_1932005, EPI_ISL_1932007, EPI_ISL_1932008, EPI_ISL_1932010, EPI_ISL_1932011, EPI_ISL_1932013, EPI_ISL_1932014, EPI_ISL_1932016, EPI_ISL_1932017, EPI_ISL_1932019, EPI_ISL_1932020, EPI_ISL_1932022, EPI_ISL_1932023, EPI_ISL_1932025, EPI_ISL_1932027, EPI_ISL_1932028, EPI_ISL_1932030, EPI_ISL_1932032, EPI_ISL_1932033, EPI_ISL_1932034, EPI_ISL_1932036, EPI_ISL_1932038, EPI_ISL_1932039, EPI_ISL_1932041, EPI_ISL_1932308, EPI_ISL_1932309, EPI_ISL_1932310, EPI_ISL_1932311, EPI_ISL_1932312, EPI_ISL_1932313, EPI_ISL_1932725, EPI_ISL_1933148, EPI_ISL_1933149, EPI_ISL_1933150, EPI_ISL_1933151, EPI_ISL_1933152, EPI_ISL_1933153, EPI_ISL_1933154, EPI_ISL_1933155, EPI_ISL_1933156, EPI_ISL_1933157, EPI_ISL_1933158, EPI_ISL_1933159, EPI_ISL_1933160, EPI_ISL_1933161, EPI_ISL_1933162, EPI_ISL_1933163, EPI_ISL_1933164, EPI_ISL_1933165, EPI_ISL_1933166, EPI_ISL_1933167, EPI_ISL_1933168, EPI_ISL_1933169, EPI_ISL_1933170, EPI_ISL_1933171, EPI_ISL_1933172, EPI_ISL_1933173, EPI_ISL_1933174, EPI_ISL_1933175, EPI_ISL_1933176, EPI_ISL_1933177, EPI_ISL_1933178, EPI_ISL_1933179, EPI_ISL_1933180, EPI_ISL_1933181, EPI_ISL_1933182, EPI_ISL_1933183, EPI_ISL_1933184, EPI_ISL_1933185, EPI_ISL_1933186, EPI_ISL_1933187, EPI_ISL_1933188, EPI_ISL_1933189, EPI_ISL_1933190, EPI_ISL_1933191, EPI_ISL_1933192, EPI_ISL_1933193, EPI_ISL_1933194, EPI_ISL_1933195, EPI_ISL_1933196, EPI_ISL_1933197, EPI_ISL_1933198, EPI_ISL_1933199, EPI_ISL_1933200, EPI_ISL_1933201, EPI_ISL_1933202, EPI_ISL_1933203, EPI_ISL_1933204, EPI_ISL_1933205, EPI_ISL_1933206, EPI_ISL_1933207, EPI_ISL_1933208, EPI_ISL_1933209, EPI_ISL_1933210, EPI_ISL_1933211, EPI_ISL_1933212, EPI_ISL_1933213, EPI_ISL_1933214, EPI_ISL_1933215, EPI_ISL_1933382, EPI_ISL_1933383, EPI_ISL_1933384, EPI_ISL_1933385, EPI_ISL_1933437, EPI_ISL_1933438, EPI_ISL_1933439, EPI_ISL_1933440, EPI_ISL_1933441, EPI_ISL_1933442, EPI_ISL_1933443, EPI_ISL_1933444, EPI_ISL_1933445, EPI_ISL_1933446, EPI_ISL_1933638, EPI_ISL_1933725, EPI_ISL_1933726, EPI_ISL_1933727, EPI_ISL_1933728, EPI_ISL_1933729, EPI_ISL_1933730, EPI_ISL_1933731, EPI_ISL_1933732, EPI_ISL_1933733, EPI_ISL_1933734, EPI_ISL_1933735, EPI_ISL_1933749, EPI_ISL_1933933, EPI_ISL_1933934, EPI_ISL_1933935, EPI_ISL_1933936, EPI_ISL_1933937			
see above	Chiba Prefectural Institute of Public Health	Pathogen Genomics Center, National Institute of Infectious Diseases	Ai Kawana-Tachikawa; Chang-Kweng Lim; Eri Nakayama; Hazuka Y Furihata; Hidemasa Izumiya; Ken-ichi Lee; Kentaro Itokawa; Koichi Ishikawa; Makoto Kuroda; Masanori Hashino; Masatomo Morita; Masumichi Saito; Midori Nakamura-Hoshi; Motohiko Ogawa; Naomi Nojiri; Nobuo Koizumi; Nozomu Hanaoka; Rina Tanaka; Sana Uchikoba; Sayuri Seki; Shigeru Kusagawa; Shigeru Tajima; Shouji Yamamoto; Sunao Iyoda; Takahiro Maeki; Tsuguto Fujimoto; Tsuyoshi Sekizuka
EPI_ISL_1941590, EPI_ISL_1941592, EPI_ISL_1941593, EPI_ISL_1941596, EPI_ISL_1941598, EPI_ISL_1941600, EPI_ISL_1941601, EPI_ISL_1941603, EPI_ISL_1941606, EPI_ISL_1941607, EPI_ISL_1941610, EPI_ISL_1941611, EPI_ISL_1941614, EPI_ISL_1941615, EPI_ISL_1941616, EPI_ISL_1941617, EPI_ISL_1941621, EPI_ISL_1941623, EPI_ISL_1941627, EPI_ISL_1941630, EPI_ISL_1941632, EPI_ISL_1941634, EPI_ISL_1941635, EPI_ISL_1941638, EPI_ISL_1941640, EPI_ISL_1941641, EPI_ISL_1941644, EPI_ISL_1941645, EPI_ISL_1941647, EPI_ISL_1941649, EPI_ISL_1941650, EPI_ISL_1941651, EPI_ISL_1941652, EPI_ISL_1941653, EPI_ISL_1941654, EPI_ISL_1941655, EPI_ISL_1941656, EPI_ISL_1941657, EPI_ISL_1941658, EPI_ISL_1941659, EPI_ISL_1941662, EPI_ISL_1941663, EPI_ISL_1941665, EPI_ISL_1941667, EPI_ISL_1941669, EPI_ISL_1941671, EPI_ISL_1941673, EPI_ISL_1941676, EPI_ISL_1941678, EPI_ISL_1941680, EPI_ISL_1941682, EPI_ISL_1941683, EPI_ISL_1941686, EPI_ISL_1941687, EPI_ISL_1941689, EPI_ISL_1941692, EPI_ISL_1941694, EPI_ISL_1941696, EPI_ISL_1941697, EPI_ISL_1941699, EPI_ISL_1941701, EPI_ISL_1941703, EPI_ISL_1941705, EPI_ISL_1941707, EPI_ISL_1941709, EPI_ISL_1941711, EPI_ISL_1941713, EPI_ISL_1941716, EPI_ISL_1941718, EPI_ISL_1941721, EPI_ISL_1941725, EPI_ISL_1941728, EPI_ISL_1941730, EPI_ISL_1941733, EPI_ISL_1941735, EPI_ISL_1941737, EPI_ISL_1941740, EPI_ISL_1941741, EPI_ISL_1941743, EPI_ISL_1941745, EPI_ISL_1941746, EPI_ISL_1941748, EPI_ISL_1941750, EPI_ISL_1941752, EPI_ISL_1941753, EPI_ISL_1941756, EPI_ISL_1941758, EPI_ISL_1941760, EPI_ISL_1941762, EPI_ISL_1941764, EPI_ISL_1941766, EPI_ISL_1941767, EPI_ISL_1941769, EPI_ISL_1941771, EPI_ISL_1941772, EPI_ISL_1941774, EPI_ISL_1941775, EPI_ISL_1941777, EPI_ISL_1941780, EPI_ISL_1941781, EPI_ISL_1941783, EPI_ISL_1941784, EPI_ISL_1941785, EPI_ISL_1941786, EPI_ISL_1941788, EPI_ISL_1941789, EPI_ISL_1941791, EPI_ISL_1941794, EPI_ISL_1941795, EPI_ISL_1941797, EPI_ISL_1941799, EPI_ISL_1941802, EPI_ISL_1941803, EPI_ISL_1941806, EPI_ISL_1941807, EPI_ISL_1941808, EPI_ISL_1941810, EPI_ISL_1941811, EPI_ISL_1941814, EPI_ISL_1941815, EPI_ISL_1941817, EPI_ISL_1941819, EPI_ISL_1941822, EPI_ISL_1941823, EPI_ISL_1941826, EPI_ISL_1941827, EPI_ISL_1941830, EPI_ISL_1941832, EPI_ISL_1941833, EPI_ISL_1941835, EPI_ISL_1941838, EPI_ISL_1941839, EPI_ISL_1941842, EPI_ISL_1941843, EPI_ISL_1941846, EPI_ISL_1941848, EPI_ISL_1941849, EPI_ISL_1941851, EPI_ISL_1941852, EPI_ISL_1941853, EPI_ISL_1941854, EPI_ISL_1941855, EPI_ISL_1941856, EPI_ISL_1941857, EPI_ISL_1941858, EPI_ISL_1941859, EPI_ISL_1941860, EPI_ISL_1941861, EPI_ISL_1941862, EPI_ISL_1941863, EPI_ISL_1941864, EPI_ISL_1941865, EPI_ISL_1941866, EPI_ISL_1941867, EPI_ISL_1941868, EPI_ISL_1941869, EPI_ISL_1941870, EPI_ISL_1941871, EPI_ISL_1941872, EPI_ISL_1941873, EPI_ISL_1941874, EPI_ISL_1941876, EPI_ISL_1941877, EPI_ISL_1941878, EPI_ISL_1941879, EPI_ISL_1941880, EPI_ISL_1941881, EPI_ISL_1941882, EPI_ISL_1941883, EPI_ISL_1941884, EPI_ISL_1941885, EPI_ISL_1941886, EPI_ISL_1941887, EPI_ISL_1941888, EPI_ISL_1941889, EPI_ISL_1941890, EPI_ISL_1941891, EPI_ISL_1941892, EPI_ISL_1941893, EPI_ISL_1941894, EPI_ISL_1941895, EPI_ISL_1941896, EPI_ISL_1941897, EPI_ISL_1941898, EPI_ISL_1941899, EPI_ISL_1941900, EPI_ISL_1941901, EPI_ISL_1941902, EPI_ISL_1941903, EPI_ISL_1941904, EPI_ISL_1941905, EPI_ISL_1941906, EPI_ISL_1941907, EPI_ISL_1941908, EPI_ISL_1941909, EPI_ISL_1941910, EPI_ISL_1941911, EPI_ISL_1941912, EPI_ISL_1941913, EPI_ISL_1941914, EPI_ISL_1941915, EPI_ISL_1941916, EPI_ISL_1941917, EPI_ISL_1941918, EPI_ISL_1941919, EPI_ISL_1941920, EPI_ISL_1941922, EPI_ISL_1941923, EPI_ISL_1941924, EPI_ISL_1941925, EPI_ISL_1941926, EPI_ISL_1941927, EPI_ISL_1941928, EPI_ISL_1941929, EPI_ISL_1941930, EPI_ISL_1941931, EPI_ISL_1941932, EPI_ISL_1941933, EPI_ISL_1941934, EPI_ISL_1941935, EPI_ISL_1941936, EPI_ISL_1941937, EPI_ISL_1941938, EPI_ISL_1941939, EPI_ISL_1941940, EPI_ISL_1941941, EPI_ISL_1941942, EPI_ISL_1941943, EPI_ISL_1941944, EPI_ISL_1941945, EPI_ISL_1941946, EPI_ISL_1941947, EPI_ISL_1941948, EPI_ISL_1941949, EPI_ISL_1941950, EPI_ISL_1941951, EPI_ISL_1941952, EPI_ISL_1941953, EPI_ISL_1941954, EPI_ISL_1941955, EPI_ISL_1941956, EPI_ISL_1941957, EPI_ISL_1941958, EPI_ISL_1941959, EPI_ISL_1941960, EPI_ISL_1941961, EPI_ISL_1941962, EPI_ISL_1941963, EPI_ISL_1941964, EPI_ISL_1941965, EPI_ISL_1941966, EPI_ISL_1941967, EPI_ISL_1941968, EPI_ISL_1941969, EPI_ISL_1941970, EPI_ISL_1941971, EPI_ISL_1941972, EPI_ISL_1941973, EPI_ISL_1941974, EPI_ISL_1941975, EPI_ISL_1941976, EPI_ISL_1941977, EPI_ISL_1941978, EPI_ISL_1941979, EPI_ISL_1941980, EPI_ISL_1941981, EPI_ISL_1941982, EPI_ISL_1941983, EPI_ISL_1941984, EPI_ISL_1941985, EPI_ISL_1941986, EPI_ISL_1941987, EPI_ISL_1941988, EPI_ISL_1941989, EPI_ISL_1941990, EPI_ISL_1941991, EPI_ISL_1941992, EPI_ISL_1941993, EPI_ISL_1941994, EPI_ISL_1941995, EPI_ISL_1941996, EPI_ISL_1941997, EPI_ISL_1941998, EPI_ISL_1941999, EPI_ISL_1942000, EPI_ISL_1942001, EPI_ISL_1942002, EPI_ISL_1942003, EPI_ISL_1942004, EPI_ISL_1942005, EPI_ISL_1942006, EPI_ISL_1942007, EPI_ISL_1942008, EPI_ISL_1942009, EPI_ISL_1942010, EPI_ISL_1942011, EPI_ISL_1942012, EPI_ISL_1942013, EPI_ISL_1942014, EPI_ISL_1942015, EPI_ISL_1942016, EPI_ISL_1942017, EPI_ISL_1942018, EPI_ISL_1942019, EPI_ISL_1942020, EPI_ISL_1942021, EPI_ISL_1942022, EPI_ISL_1942023, EPI_ISL_1942024, EPI_ISL_1942025, EPI_ISL_1942026, EPI_ISL_1942027, EPI_ISL_1942028, EPI_ISL_1942029, EPI_ISL_1942030, EPI_ISL_1942031, EPI_ISL_1942032, EPI_ISL_1942033, EPI_ISL_1942034, EPI_ISL_1942035, EPI_ISL_1942036, EPI_ISL_1942037, EPI_ISL_1942038, EPI_ISL_1942039, EPI_ISL_1942040, EPI_ISL_1942041, EPI_ISL_1942042, EPI_ISL_1942043, EPI_ISL_1942044, EPI_ISL_1942045, EPI_ISL_1942046, EPI_ISL_1942047, EPI_ISL_1942048, EPI_ISL_1942049, EPI_ISL_1942050, EPI_ISL_1942051, EPI_ISL_1942052, EPI_ISL_1942053, EPI_ISL_1942054, EPI_ISL_1942055, EPI_ISL_1942056, EPI_ISL_1942057, EPI_ISL_1942058, EPI_ISL_1942059, EPI_ISL_1942060, EPI_ISL_1942061, EPI_ISL_1942062, EPI_ISL_1942063, EPI_ISL_1942064, EPI_ISL_1942065, EPI_ISL_1942066, EPI_ISL_1942067, EPI_ISL_1942068, EPI_ISL_1942069, EPI_ISL_1942070, EPI_ISL_1942071, EPI_ISL_1942072, EPI_ISL_1942073, EPI_ISL_1942074, EPI_ISL_1942075, EPI_ISL_1942076, EPI_ISL_1942077, EPI_ISL_1942078, EPI_ISL_1942079, EPI_ISL_1942080, EPI_ISL_1942081, EPI_ISL_1942082, EPI_ISL_1942083, EPI_ISL_1942084, EPI_ISL_1942085, EPI_ISL_1942086, EPI_ISL_1942087, EPI_ISL_1942088, EPI_ISL_1942089, EPI_ISL_1942090, EPI_ISL_1942091, EPI_ISL_1942092, EPI_ISL_1942093, EPI_ISL_1942094, EPI_ISL_1942095, EPI_ISL_1942096, EPI_ISL_1942097, EPI_ISL_1942098, EPI_ISL_1942099, EPI_ISL_1942100, EPI_ISL_1942101, EPI_ISL_1942102, EPI_ISL_1942103, EPI_ISL_1942104, EPI_ISL_1942105, EPI_ISL_1942106, EPI_ISL_1942107, EPI_ISL_1942108, EPI_ISL_1942109, EPI_ISL_1942110, EPI_ISL_1942111, EPI_ISL_1942112, EPI_ISL_1942113, EPI_ISL_1942114, EPI_ISL_1942115, EPI_ISL_1942116, EPI_ISL_1942117, EPI_ISL_1942118, EPI_ISL_1942119, EPI_ISL_1942120, EPI_ISL_1942121, EPI_ISL_1942122, EPI_ISL_1942123, EPI_ISL_1942124, EPI_ISL_1942125, EPI_ISL_1942126, EPI_ISL_1942127, EPI_ISL_1942128, EPI_ISL_1942129, EPI_ISL_1942130, EPI_ISL_1942131, EPI_ISL_1942132, EPI_ISL_1942133, EPI_ISL_1942134, EPI_ISL_1942135, EPI_ISL_1968981, EPI_ISL_1968982, EPI_ISL_1968983, EPI_ISL_1968984, EPI_ISL_1968985, EPI_ISL_1968986, EPI_ISL_1968987, EPI_ISL_1968988, EPI_ISL_1968989, EPI_ISL_1968990, EPI_ISL_1968991, EPI_ISL_1968992, EPI_ISL_1968993, EPI_ISL_1968994, EPI_ISL_1968995, EPI_ISL_1968996, EPI_ISL_1968997, EPI_ISL_1968998, EPI_ISL_1968999			
see above	Department of Infectious Diseases, Kobe Institute of Health	Department of Infectious Diseases, Kobe Institute of Health	Kentaro Itokawa; Makoto Kuroda; Masanori Hashino; Noriko Nakanishi; Rina Tanaka; Ryohei Nomoto; Tomotada Iwamoto; Tsuyoshi Sekizuka
EPI_ISL_2107432, EPI_ISL_2107433, EPI_ISL_2107434	Department of Intensive Care Medicine, and clinical lab., Osaka Women's and Children's Hospital	Department of Developmental Medicine, Research Institute, Osaka Women's and Children's Hospital	Itaru Yanagihara; Muneyuki Takeuchi; Shinobu Ida; Yukiko Nakura
EPI_ISL_2080355, EPI_ISL_2080356, EPI_ISL_2080479, EPI_ISL_2101018, EPI_ISL_2101020	Department of Microbiology, Tochigi Prefectural Institute of Public Health and Enviromental Science	Genome Analysis Center, Kamma Memorial Hospital	Fuminori Mizukoshi; Hanako Yazawa; Hiroshi Kamma; Jun Ishii; Kaori Watanabe; Michiaki Masuda; Satoko Soma; Takuya Yazawa; Yoshiko Hitomi
EPI_ISL_2080354, EPI_ISL_2080357, EPI_ISL_2080358, EPI_ISL_2080359, EPI_ISL_2101019	Department of Microbiology, Tochigi Prefectural Institute of Public Health and Environmental Science	Genome Analysis Center, Kamma Memorial Hospital	Fuminori Mizukoshi; Hanako Yazawa; Hiroshi Kamma; Jun Ishii; Kaori Watanabe; Michiaki Masuda; Satoko Soma; Takuya Yazawa; Yoshiko Hitomi
EPI_ISL_2158605, EPI_ISL_2158606, EPI_ISL_2158607, EPI_ISL_2158608, EPI_ISL_2158609, EPI_ISL_2158610, EPI_ISL_2158611, EPI_ISL_2158612, EPI_ISL_2158613, EPI_ISL_2158614, EPI_ISL_2158615, EPI_ISL_2158616, EPI_ISL_2158617			
see above	Department of Veterinary Science, National Institute of Infectious Diseases	Department of Veterinary Science, National Institute of Infectious Diseases	Keita Ishijima; Ken Maeda; Shuetsu Fukushi; Souichi Yamada; Tsukasa Yamamoto; Yudai Kuroda
EPI_ISL_2107438	Dr. Yoshitaka Tamura Department of Clinical Laboratory, Osaka Habikino Medical	Department of Developmental Medicine, Research Institute, Osaka Women's and Children's Hospital	Itaru Yanagihara; Yukiko Nakura
EPI_ISL_2107435, EPI_ISL_2107436, EPI_ISL_2107437, EPI_ISL_2107439, EPI_ISL_2107440, EPI_ISL_2107441, EPI_ISL_2107460, EPI_ISL_2107461, EPI_ISL_2107462, EPI_ISL_2107463, EPI_ISL_2107465, EPI_ISL_2107466, EPI_ISL_2107467, EPI_ISL_2107468			
see above	Dr. Yoshitaka Tamura Department of Clinical Laboratory, Osaka Habikino Medical Center	Department of Developmental Medicine, Research Institute, Osaka Women's and Children's Hospital	Itaru Yanagihara; Yukiko Nakura
EPI_ISL_2107464	Dr. Yoshitaka Tamura Department of Clinical Laboratory, Osaka Habikino Medical Center Dr. Yoshitaka Tamura Department of Clinical Laboratory, Osaka Habikino Medical Center	Department of Developmental Medicine, Research Institute, Osaka Women's and Children's Hospital	Itaru Yanagihara; Yukiko Nakura
EPI_ISL_2014854, EPI_ISL_2014855, EPI_ISL_2014856, EPI_ISL_2014857, EPI_ISL_2014858, EPI_ISL_2014859, EPI_ISL_2014860, EPI_ISL_2014861, EPI_ISL_2014862, EPI_ISL_2014863, EPI_ISL_2014864, EPI_ISL_2014865, EPI_ISL_2014866, EPI_ISL_2014867, EPI_ISL_2014868, EPI_ISL_2014869, EPI_ISL_2014870, EPI_ISL_2014871, EPI_ISL_2014872, EPI_ISL_2014873, EPI_ISL_2014874, EPI_ISL_2014875, EPI_ISL_2014876, EPI_ISL_2014877, EPI_ISL_2014878, EPI_ISL_2014879, EPI_ISL_2014880, EPI_ISL_2014881, EPI_ISL_2014882, EPI_ISL_2014883, EPI_ISL_2014884, EPI_ISL_2014885, EPI_ISL_2014886, EPI_ISL_2014887, EPI_ISL_2014888, EPI_ISL_2014889, EPI_ISL_2014890, EPI_ISL_2014891, EPI_ISL_2014892, EPI_ISL_2014893, EPI_ISL_2014894, EPI_ISL_2014895, EPI_ISL_2014896, EPI_ISL_2014897, EPI_ISL_2014898, EPI_ISL_2014899, EPI_ISL_2014900, EPI_ISL_2014901, EPI_ISL_2014902, EPI_ISL_2014903, EPI_ISL_2014904, EPI_ISL_2014905, EPI_ISL_2014906, EPI_ISL_2014907, EPI_ISL_2014908, EPI_ISL_2014909, EPI_ISL_2014910, EPI_ISL_2014911, EPI_ISL_2014912, EPI_ISL_2014913, EPI_ISL_2014914, EPI_ISL_2014915, EPI_ISL_2014916, EPI_ISL_2014917, EPI_ISL_2014918, EPI_ISL_2014919, EPI_ISL_2014920, EPI_ISL_2014921, EPI_ISL_2014922, EPI_ISL_2014923, EPI_ISL_2014924, EPI_ISL_2014925, EPI_ISL_2014926, EPI_ISL_2014927, EPI_ISL_2014928, EPI_ISL_2014929, EPI_ISL_2014930, EPI_ISL_2014931, EPI_ISL_2014932, EPI_ISL_2014933, EPI_ISL_2014934, EPI_ISL_2014935, EPI_ISL_2014936, EPI_ISL_2014937, EPI_ISL_2014938, EPI_ISL_2014939, EPI_ISL_2014940, EPI_ISL_2014941, EPI_ISL_2014942, EPI_ISL_2014943, EPI_ISL_2014944, EPI_ISL_2014945, EPI_ISL_2014946, EPI_ISL_2014947, EPI_ISL_2014948, EPI_ISL_2014949, EPI_ISL_2014950, EPI_ISL_2014951, EPI_ISL_2014952, EPI_ISL_2014953, EPI_ISL_2014954, EPI_ISL_2014955, EPI_ISL_2014956, EPI_ISL_2014957, EPI_ISL_2014958, EPI_ISL_2014959, EPI_ISL_2014960, EPI_ISL_2014961, EPI_ISL_2014962, EPI_ISL_2014963, EPI_ISL_2014964, EPI_ISL_2014965, EPI_ISL_2014966, EPI_ISL_2014967, EPI_ISL_2014968, EPI_ISL_2014969, EPI_ISL_2014970, EPI_ISL_2014971, EPI_ISL_2014972, EPI_ISL_2014973, EPI_ISL_2014974, EPI_ISL_2014975, EPI_ISL_2014976, EPI_ISL_2014977, EPI_ISL_2014978, EPI_ISL_2014979, EPI_ISL_2014980, EPI_ISL_2014981, EPI_ISL_2014982, EPI_ISL_2014983, EPI_ISL_2014984, EPI_ISL_2014985, EPI_ISL_2014986, EPI_ISL_2014987, EPI_ISL_2014988, EPI_ISL_2014989, EPI_ISL_2014990, EPI_ISL_2014991, EPI_ISL_2014992, EPI_ISL_2014993, EPI_ISL_2014994, EPI_ISL_2014995, EPI_ISL_2014996, EPI_ISL_2014997, EPI_ISL_2014998, EPI_ISL_2014999, EPI_ISL_2015000, EPI_ISL_2015001, EPI_ISL_2015002, EPI_ISL_2015003, EPI_ISL_2015004, EPI_ISL_2015005, EPI_ISL_2015006, EPI_ISL_2015007, EPI_ISL_2015008, EPI_ISL_2015009, EPI_ISL_2015010, EPI_ISL_2015011, EPI_ISL_2015012, EPI_ISL_2015013, EPI_ISL_2015014, EPI_ISL_2015015, EPI_ISL_2015016, EPI_ISL_2015017, EPI_ISL_2015018, EPI_ISL_2015019, EPI_ISL_2015020, EPI_ISL_2015021, EPI_ISL_2015022, EPI_ISL_2015023, EPI_ISL_2015024, EPI_ISL_2015025, EPI_ISL_2015026, EPI_ISL_2015027, EPI_ISL_2015028, EPI_ISL_2015029, EPI_ISL_2015030, EPI_ISL_2015031, EPI_ISL_2015032, EPI_ISL_2015033, EPI_ISL_2015034, EPI_ISL_2015035, EPI_ISL_2015036, EPI_ISL_2015037, EPI_ISL_2015038, EPI_ISL_2015039, EPI_ISL_2015040, EPI_ISL_2015041, EPI_ISL_2015042, EPI_ISL_2015043, EPI_ISL_2015044, EPI_ISL_2015045, EPI_ISL_2015046, EPI_ISL_2015047, EPI_ISL_2015048, EPI_ISL_2015049, EPI_ISL_2015050, EPI_ISL_2015051, EPI_ISL_2015052, EPI_ISL_2015053, EPI_ISL_2015054, EPI_ISL_2015055, EPI_ISL_2015056, EPI_ISL_2015057, EPI_ISL_2015058, EPI_ISL_2015059, EPI_ISL_2015060, EPI_ISL_2015061, EPI_ISL_2015062, EPI_ISL_2015063, EPI_ISL_2015064, EPI_ISL_2015065, EPI_ISL_2015066, EPI_ISL_2015067, EPI_ISL_2015068, EPI_ISL_2015069, EPI_ISL_2015070, EPI_ISL_2015071, EPI_ISL_2015072, EPI_ISL_2015073, EPI_ISL_2015074, EPI_ISL_2015075, EPI_ISL_2015076, EPI_ISL_2015077, EPI_ISL_2015078, EPI_ISL_2015079, EPI_ISL_2015080, EPI_ISL_2015081, EPI_ISL_2015082, EPI_ISL_2015083, EPI_ISL_2015084, EPI_ISL_2015085, EPI_ISL_2015086, EPI_ISL_2015087, EPI_ISL_2015088, EPI_ISL_2015089, EPI_ISL_2015090, EPI_ISL_2015091, EPI_ISL_2015092, EPI_ISL_2015093, EPI_ISL_2015094, EPI_ISL_2015095			
see above	Fukuoka Institute of Health and Environmental Sciences	Fukuoka Institute of Health and Environmental Sciences	Akira Ohishi; Asako Nakamura; Chiharu Katamune; Hiroaki Shigemura; Kentaro Itokawa; Makoto Kuroda; Masanori Hashino; Mitsuhiro Hamasaki; Rina Tanaka; Saori Ueda; Takayuki Kobayashi; Tsuyoshi Sekizuka; Yoshiki Etoh; Yuki Ashizuka
EPI_ISL_1929141, EPI_ISL_1929143	Fukuoka Institute of Health and Environmental Sciences	Pathogen Genomics Center, National Institute of Infectious Diseases	Hidemasa Izumiya; Ken Shimuta; Kentaro Itokawa; Makoto Kuroda; Masanori Hashino; Nobuo Koizumi; Rina Tanaka; Sunao Iyoda; Tsuyoshi Sekizuka
EPI_ISL_2080250, EPI_ISL_2080251, EPI_ISL_2080252, EPI_ISL_2080253, EPI_ISL_2080254, EPI_ISL_2080269, EPI_ISL_2080351			
see above	Genome Analysis Center, Kamma Memorial Hospital	Genome Analysis Center, Kamma Memorial Hospital	Hanako Yazawa; Hiroshi Kamma; Jun Ishii; Kaori Watanabe; Masaki Sato; Michiaki Masuda; Naruhisa Takano; Noriyuki Tannai; Satoko Soma; Takuya Yazawa
EPI_ISL_2235505, EPI_ISL_2235506, EPI_ISL_2235507, EPI_ISL_2235508, EPI_ISL_2235509, EPI_ISL_2235510, EPI_ISL_2235511, EPI_ISL_2235512, EPI_ISL_2235513, EPI_ISL_2235514, EPI_ISL_2235515, EPI_ISL_2235516, EPI_ISL_2235517, EPI_ISL_2235518, EPI_ISL_2235519, EPI_ISL_2235520			
see above	Genome Analysis Center, Yamanashi Central Hospital	Genome Analysis Center, Yamanashi Central Hospital	Yosuke Hirotsu
EPI_ISL_1922003, EPI_ISL_1922004, EPI_ISL_1922005, EPI_ISL_1922006, EPI_ISL_1922007, EPI_ISL_1922008, EPI_ISL_1922009, EPI_ISL_1922010, EPI_ISL_1922011, EPI_ISL_1922012, EPI_ISL_1922013, EPI_ISL_1922014, EPI_ISL_1922015, EPI_ISL_1922016, EPI_ISL_1922017, EPI_ISL_1922018, EPI_ISL_1922019, EPI_ISL_1922020, EPI_ISL_1922021, EPI_ISL_1922022, EPI_ISL_1922023, EPI_ISL_1922024, EPI_ISL_1922025, EPI_ISL_1922026, EPI_ISL_1922027, EPI_ISL_1922028, EPI_ISL_1922029, EPI_ISL_1922030, EPI_ISL_1922031, EPI_ISL_1922032, EPI_ISL_1922033, EPI_ISL_1922034, EPI_ISL_1922035, EPI_ISL_1922036, EPI_ISL_1922037, EPI_ISL_1922038, EPI_ISL_1922039, EPI_ISL_1922040, EPI_ISL_1922041			
see above	Gunma Prefectural Institute of Public Health and Environmental Sciences	Gunma Prefectural Institute of Public Health and Environmental Sciences	Daisuke Shinoda; Hiroyuki Tsukagoshi; Kentaro Itokawa; Makoto Kuroda; Mariko Saito; Masanori Hashino; Rina Tanaka; Tsuyoshi Sekizuka
EPI_ISL_1932420, EPI_ISL_1933570,	Gunma Prefectural Institute of Public Health and Environmental Sciences	Pathogen Genomics Center, National	Hazuka Y Furihata; Kentaro Itokawa; Makoto Kuroda; Masanori Hashino; Masumichi Saito; Naomi Nojiri; Nozomu Hanaoka; Rina Tanaka; Sana Uchikoba; Tsuguto Fujimoto; Tsuyoshi Sekizuka

EPI_ISL_1933571, EPI_ISL_1933572		Institute of Infectious Diseases	
EPI_ISL_2378724, EPI_ISL_2378725, EPI_ISL_2378726, EPI_ISL_2378727, EPI_ISL_2378728, EPI_ISL_2378729, EPI_ISL_2378730, EPI_ISL_2378731, EPI_ISL_2378732, EPI_ISL_2378733, EPI_ISL_2378734, EPI_ISL_2378735			
see above	Hiroyuki Asakura Tokyo Metropolitan Institute of Public Health Department of Microbiology	Hiroyuki Asakura Tokyo Metropolitan Institute of Public Health Department of Microbiology	Asakura, H.; Fujiwara, T.; J. and Sadamasu, K.; Kumagai, R.; Miyake, H.; Nagashima, M.; Suzuki; Yoshida, I.
EPI_ISL_2117076, EPI_ISL_2117078, EPI_ISL_2117081, EPI_ISL_2117083, EPI_ISL_2117086, EPI_ISL_2117089, EPI_ISL_2117092, EPI_ISL_2117095, EPI_ISL_2117098, EPI_ISL_2117100, EPI_ISL_2117102			
see above	Hiroyuki Asakura Tokyo Metropolitan Institute of Public Health Department of Microbiology	Hiroyuki Asakura Tokyo Metropolitan Institute of Public Health Department of Microbiology	Asakura, H.; Fujiwara, T.; J. and Sadamasu, K.; Miyake, H.; Nagashima, M.; Suzuki; Yoshida, I.
EPI_ISL_1967393, EPI_ISL_1967394, EPI_ISL_1967395, EPI_ISL_1967396, EPI_ISL_1967397, EPI_ISL_1967398, EPI_ISL_1967399, EPI_ISL_1967400, EPI_ISL_1967401, EPI_ISL_1967402, EPI_ISL_1967403, EPI_ISL_1967404, EPI_ISL_1967405, EPI_ISL_1967406, EPI_ISL_1967407, EPI_ISL_1967408, EPI_ISL_1967409, EPI_ISL_1967410, EPI_ISL_1967411, EPI_ISL_1967412, EPI_ISL_1967413, EPI_ISL_1967414, EPI_ISL_1967415, EPI_ISL_1967416, EPI_ISL_1967417, EPI_ISL_1967418, EPI_ISL_1967419, EPI_ISL_1967420, EPI_ISL_1967421, EPI_ISL_1967422, EPI_ISL_1967423, EPI_ISL_1967424, EPI_ISL_1967425, EPI_ISL_1967426, EPI_ISL_1967427, EPI_ISL_1967428, EPI_ISL_1967429, EPI_ISL_1967430, EPI_ISL_1967431, EPI_ISL_1967432, EPI_ISL_1967433, EPI_ISL_1967434, EPI_ISL_1967435, EPI_ISL_1967436, EPI_ISL_1967437, EPI_ISL_1967438, EPI_ISL_1967439, EPI_ISL_1967440, EPI_ISL_1967441, EPI_ISL_1967442, EPI_ISL_1967443, EPI_ISL_1967444, EPI_ISL_1967445, EPI_ISL_1967446, EPI_ISL_1967447, EPI_ISL_1967448, EPI_ISL_1967449, EPI_ISL_1967450, EPI_ISL_1967451, EPI_ISL_1967452, EPI_ISL_1967453, EPI_ISL_1967454, EPI_ISL_1967455, EPI_ISL_1967456, EPI_ISL_1967457, EPI_ISL_1967458, EPI_ISL_1967459, EPI_ISL_1967460, EPI_ISL_1967461, EPI_ISL_1967462, EPI_ISL_1967463, EPI_ISL_1967464, EPI_ISL_1967465, EPI_ISL_1967466, EPI_ISL_1967467, EPI_ISL_1967468, EPI_ISL_1967469, EPI_ISL_1967470, EPI_ISL_1967471, EPI_ISL_1967472, EPI_ISL_1967473			
see above	Hokkaido Institute of Public Health	Hokkaido Institute of Public Health	Kazuhiro Okubo; Kentaro Itokawa; Makoto Kuroda; Masanori Hashino; Rina Tanaka; Tsuyoshi Sekizuka; Yuta Ohno
EPI_ISL_2007394, EPI_ISL_2007395, EPI_ISL_2007396, EPI_ISL_2007397, EPI_ISL_2007398, EPI_ISL_2007399, EPI_ISL_2007400, EPI_ISL_2007401, EPI_ISL_2007402, EPI_ISL_2007403, EPI_ISL_2007404, EPI_ISL_2007405, EPI_ISL_2007406, EPI_ISL_2007407, EPI_ISL_2007408, EPI_ISL_2007409, EPI_ISL_2007410, EPI_ISL_2007411, EPI_ISL_2007412, EPI_ISL_2007413, EPI_ISL_2007414, EPI_ISL_2007415, EPI_ISL_2007416, EPI_ISL_2007417, EPI_ISL_2007418, EPI_ISL_2007419, EPI_ISL_2007420, EPI_ISL_2007421, EPI_ISL_2007422, EPI_ISL_2007423, EPI_ISL_2007424, EPI_ISL_2007425, EPI_ISL_2007426, EPI_ISL_2007427, EPI_ISL_2007428, EPI_ISL_2007429, EPI_ISL_2007430, EPI_ISL_2007431, EPI_ISL_2007432, EPI_ISL_2007433, EPI_ISL_2007434, EPI_ISL_2007435, EPI_ISL_2007436, EPI_ISL_2007437, EPI_ISL_2007438, EPI_ISL_2007439, EPI_ISL_2007440, EPI_ISL_2007441, EPI_ISL_2007442, EPI_ISL_2007443, EPI_ISL_2007444, EPI_ISL_2007445, EPI_ISL_2007446, EPI_ISL_2007447, EPI_ISL_2007448, EPI_ISL_2007449, EPI_ISL_2007450, EPI_ISL_2007451, EPI_ISL_2007452, EPI_ISL_2007453, EPI_ISL_2007454, EPI_ISL_2007455, EPI_ISL_2007456, EPI_ISL_2007457, EPI_ISL_2007458			
see above	Hyogo Prefectural Institute of Public Health science	Pathogen Genomics Center, National Institute of Infectious Diseases	Denshi Takai; Etsuko Saito; Kenichi Ogita; Kentaro Itokawa; Kyoko Suzuki; Makoto Kuroda; Masanori Hashino; Masatsugu Chikahira; Miki Ogi; Rina Tanaka; Tomohiro Oshibe; Tsuyoshi Sekizuka; Yumi Akiyama
EPI_ISL_1972916, EPI_ISL_1972917, EPI_ISL_1972918, EPI_ISL_1972919, EPI_ISL_1972920, EPI_ISL_1972921, EPI_ISL_1972922, EPI_ISL_1972923, EPI_ISL_1972924, EPI_ISL_1972925, EPI_ISL_1972926, EPI_ISL_1972927, EPI_ISL_1972928, EPI_ISL_1972929, EPI_ISL_1972930, EPI_ISL_1972931, EPI_ISL_1972932, EPI_ISL_1972933, EPI_ISL_1972934, EPI_ISL_1972935, EPI_ISL_1972936, EPI_ISL_1972937, EPI_ISL_1972938, EPI_ISL_1972939, EPI_ISL_1972940, EPI_ISL_1972941, EPI_ISL_1972942			
see above	Ibaraki Prefectural Institute of Public Health	Ibaraki Prefectural Institute of Public Health	Keiko Goto; Kentaro Itokawa; Makoto Kuroda; Masanori Hashino; Rina Tanaka; Tsuyoshi Sekizuka
EPI_ISL_1930966, EPI_ISL_1930968, EPI_ISL_1931499, EPI_ISL_1933479, EPI_ISL_1933480, EPI_ISL_1933537, EPI_ISL_1933538, EPI_ISL_1933539			
see above	Ibaraki Prefectural Institute of Public Health	Pathogen Genomics Center, National Institute of Infectious Diseases	Hazuka Y Furihata; Kentaro Itokawa; Makoto Kuroda; Masanori Hashino; Masumichi Saito; Naomi Nojiri; Nozomu Hanaoka; Rina Tanaka; Sana Uchikoba; Tsuguto Fujimoto; Tsuyoshi Sekizuka
EPI_ISL_1972571, EPI_ISL_1972572	Kanagawa Prefectural Institute of Public Health	Kanagawa Prefectural Institute of Public Health	Ichiro Furukawa; Junichi Sakuragi; Kentaro Itokawa; Makiko Kondo; Makoto Kuroda; Masanori Hashino; Rieko Suzuki; Rina Tanaka; Sumi Watanabe; Takako Sano; Takayuki Hishiki; Tomohiko Takasaki; Tsuyoshi Sekizuka
EPI_ISL_1930941, EPI_ISL_1930942, EPI_ISL_1931336	Kanagawa Prefectural Institute of Public Health	Pathogen Genomics Center, National Institute of Infectious Diseases	Hazuka Y Furihata; Hiromizu Takahashi; Kentaro Itokawa; Makoto Kuroda; Masanori Hashino; Masumichi Saito; Naomi Nojiri; Nozomu Hanaoka; Rina Tanaka; Tsuguto Fujimoto; Tsuyoshi Sekizuka
EPI_ISL_2107805, EPI_ISL_2107806, EPI_ISL_2107808, EPI_ISL_2107810, EPI_ISL_2107821	Kawakita General Hospital	Tokyo Medical University Department of Microbiology	Hirofumi Miyake; Jun Suzuki; Kenji Sadamasu; Mami Nagashima; Masaki Hayashi; Shigeki Nakamura; Takahiro Okai; Takako Yamazaki; Takushi Fujiwara; Tatsuya Inukai
EPI_ISL_2090605	Kei Haga Omura Satoshi Memorial Institute, Kitasato University, Infection Control and Immunology	Kei Haga Omura Satoshi Memorial Institute, Kitasato University, Infection Control and Immunology	Asanuma, H.; Ebisudani, T.; Fujii, M.; Fukunaga, K.; Haga, K.; Hamamoto, J.; Hishida, T.; K. and Sato, T.; Katayama; Mitsuishi, A.; Sugihara, K.; Sugimoto, S.; Todaka, R.; Yasuda, H.
EPI_ISL_1973091, EPI_ISL_1973092, EPI_ISL_1973093, EPI_ISL_1973094, EPI_ISL_1973095, EPI_ISL_1973096, EPI_ISL_1973097, EPI_ISL_1973098, EPI_ISL_1973099, EPI_ISL_1973100, EPI_ISL_1973101, EPI_ISL_1973102, EPI_ISL_1973103, EPI_ISL_1973104, EPI_ISL_1973105, EPI_ISL_1973106, EPI_ISL_1973107, EPI_ISL_1973108, EPI_ISL_1973109, EPI_ISL_1973110, EPI_ISL_1973111, EPI_ISL_1973112, EPI_ISL_1973113, EPI_ISL_1973114, EPI_ISL_1973115, EPI_ISL_1973116, EPI_ISL_1973117, EPI_ISL_1973118, EPI_ISL_1973119, EPI_ISL_1973120, EPI_ISL_1973121, EPI_ISL_1973122, EPI_ISL_1973123, EPI_ISL_1973124, EPI_ISL_1973125, EPI_ISL_1973126, EPI_ISL_1973127, EPI_ISL_1973128, EPI_ISL_1973129, EPI_ISL_1973130, EPI_ISL_1973131, EPI_ISL_1973132, EPI_ISL_1973133, EPI_ISL_1973134, EPI_ISL_1973135, EPI_ISL_1973136, EPI_ISL_1973137, EPI_ISL_1973138, EPI_ISL_1973139, EPI_ISL_1973140, EPI_ISL_1973141, EPI_ISL_1973142, EPI_ISL_1973143, EPI_ISL_1973144, EPI_ISL_1973145, EPI_ISL_1973146, EPI_ISL_1973147, EPI_ISL_1973148, EPI_ISL_1973149, EPI_ISL_1973150, EPI_ISL_1973151, EPI_ISL_1973152, EPI_ISL_1973153, EPI_ISL_1973154, EPI_ISL_1973155, EPI_ISL_1973156, EPI_ISL_1973157, EPI_ISL_1973158, EPI_ISL_1973159, EPI_ISL_1973160, EPI_ISL_1973161, EPI_ISL_1973162, EPI_ISL_1973163, EPI_ISL_1973164, EPI_ISL_1973165, EPI_ISL_1973166, EPI_ISL_1973167, EPI_ISL_1973168, EPI_ISL_1973169, EPI_ISL_1973170, EPI_ISL_1973171, EPI_ISL_1973172, EPI_ISL_1973173, EPI_ISL_1973174, EPI_ISL_1973175, EPI_ISL_1973176, EPI_ISL_1973177, EPI_ISL_1973178, EPI_ISL_1973179, EPI_ISL_1973180, EPI_ISL_1973181, EPI_ISL_1973182, EPI_ISL_1973183, EPI_ISL_1973184, EPI_ISL_1973185, EPI_ISL_1973186, EPI_ISL_1973187, EPI_ISL_1973188, EPI_ISL_1973189, EPI_ISL_1973190, EPI_ISL_1973191, EPI_ISL_1973192, EPI_ISL_1973193, EPI_ISL_1973194, EPI_ISL_1973195, EPI_ISL_1973196, EPI_ISL_1973197, EPI_ISL_1973198, EPI_ISL_1973199, EPI_ISL_1973200, EPI_ISL_1973201, EPI_ISL_1973202, EPI_ISL_1973203, EPI_ISL_1973204, EPI_ISL_1973205, EPI_ISL_1973206, EPI_ISL_1973207, EPI_ISL_1973208, EPI_ISL_1973209, EPI_ISL_1973210, EPI_ISL_1973211, EPI_ISL_1973212, EPI_ISL_1973213, EPI_ISL_1973214, EPI_ISL_1973215, EPI_ISL_1973216, EPI_ISL_1973217, EPI_ISL_1973218, EPI_ISL_1973219, EPI_ISL_1973220, EPI_ISL_1973221, EPI_ISL_1973222, EPI_ISL_1973223, EPI_ISL_1973224, EPI_ISL_1973225, EPI_ISL_1973226, EPI_ISL_1973227, EPI_ISL_1973228, EPI_ISL_1973229, EPI_ISL_1973230, EPI_ISL_1973231, EPI_ISL_1973232, EPI_ISL_1973233, EPI_ISL_1973234, EPI_ISL_1973235, EPI_ISL_1973236, EPI_ISL_1973237, EPI_ISL_1973238, EPI_ISL_1973239, EPI_ISL_1973240, EPI_ISL_1973241, EPI_ISL_1973242, EPI_ISL_1973243, EPI_ISL_1973244, EPI_ISL_1973245, EPI_ISL_1973246, EPI_ISL_1973247, EPI_ISL_1973248, EPI_ISL_1973249, EPI_ISL_1973250, EPI_ISL_1973251, EPI_ISL_1973252, EPI_ISL_1973253, EPI_ISL_1973254, EPI_ISL_1973255, EPI_ISL_1973256, EPI_ISL_1973257, EPI_ISL_1973258, EPI_ISL_1973259, EPI_ISL_1973260, EPI_ISL_1973261, EPI_ISL_1973262, EPI_ISL_1973263, EPI_ISL_1973264, EPI_ISL_1973265, EPI_ISL_1973266, EPI_ISL_1973267, EPI_ISL_1973268, EPI_ISL_1973269, EPI_ISL_1973270, EPI_ISL_1973271, EPI_ISL_1973272, EPI_ISL_1973273, EPI_ISL_1973274, EPI_ISL_1973275, EPI_ISL_1973276, EPI_ISL_1973277, EPI_ISL_1973278, EPI_ISL_1973279, EPI_ISL_1973280, EPI_ISL_1973281, EPI_ISL_1973282, EPI_ISL_1973283, EPI_ISL_1973284, EPI_ISL_1973285, EPI_ISL_1973286, EPI_ISL_1973287, EPI_ISL_1973288, EPI_ISL_1973289, EPI_ISL_1973290, EPI_ISL_1973291, EPI_ISL_1973292, EPI_ISL_1973293, EPI_ISL_1973294, EPI_ISL_1973295, EPI_ISL_1973296, EPI_ISL_1973297, EPI_ISL_1973298, EPI_ISL_1973299, EPI_ISL_1973300, EPI_ISL_1973301, EPI_ISL_1973302, EPI_ISL_1973303, EPI_ISL_1973304, EPI_ISL_1973305, EPI_ISL_1973306, EPI_ISL_1973307, EPI_ISL_1973308, EPI_ISL_1973309, EPI_ISL_1973310, EPI_ISL_1973311, EPI_ISL_1973312, EPI_ISL_1973313, EPI_ISL_1973314, EPI_ISL_1973315, EPI_ISL_1973316, EPI_ISL_1973317, EPI_ISL_1973318, EPI_ISL_1973319, EPI_ISL_1973320, EPI_ISL_1973321, EPI_ISL_1973322, EPI_ISL_1973323, EPI_ISL_1973329			
see above	Keio University School of Medicine	Keio University School of Medicine	Haruhiko Siomi; Hirotsugu Ishizu; Kenjiro Kosaki; Kodai Abe; Yuka Iwasaki
EPI_ISL_2020237, EPI_ISL_2020242	Kumamoto Prefectural Institute of Public-Health and Environmental Science	Kumamoto Prefectural Institute of Public-Health and Environmental Science	Kentaro Itokawa; Makoto Kuroda; Mami Sato; Masanori Hashino; Rina Tanaka; Shunsuke Yahiro; Tsuyoshi Sekizuka
EPI_ISL_1927447	Niigata Prefectural Institute of Public Health and Environmental Sciences	Pathogen Genomics Center, National Institute of Infectious Diseases	Kentaro Itokawa; Makoto Kuroda; Masanori Hashino; Rina Tanaka; Tsuyoshi Sekizuka
EPI_ISL_1926970, EPI_ISL_1926972, EPI_ISL_1926973, EPI_ISL_1926975, EPI_ISL_1926979, EPI_ISL_1926981, EPI_ISL_1926982, EPI_ISL_1926984, EPI_ISL_1926987, EPI_ISL_1926988, EPI_ISL_1927000, EPI_ISL_1927002, EPI_ISL_1927004, EPI_ISL_1927023, EPI_ISL_1927024, EPI_ISL_1927026, EPI_ISL_1927027, EPI_ISL_1927032, EPI_ISL_1927034, EPI_ISL_1927035, EPI_ISL_1927069, EPI_ISL_1927071, EPI_ISL_1927073, EPI_ISL_1927074, EPI_ISL_1927076, EPI_ISL_1927203, EPI_ISL_1927312, EPI_ISL_1927315, EPI_ISL_1927316, EPI_ISL_1927318, EPI_ISL_1927343, EPI_ISL_1927675, EPI_ISL_1927677, EPI_ISL_1927678, EPI_ISL_1927680, EPI_ISL_1927681, EPI_ISL_1927827, EPI_ISL_1928054, EPI_ISL_1928056, EPI_ISL_1928057, EPI_ISL_1928059, EPI_ISL_1928060, EPI_ISL_1928062, EPI_ISL_1928063, EPI_ISL_1928065, EPI_ISL_1928066, EPI_ISL_1928068, EPI_ISL_1928069, EPI_ISL_1928071, EPI_ISL_1928072, EPI_ISL_1928074, EPI_ISL_1928075, EPI_ISL_1928077, EPI_ISL_1928078, EPI_ISL_1928080, EPI_ISL_1928081, EPI_ISL_1928083, EPI_ISL_1928084, EPI_ISL_1928086, EPI_ISL_1928087, EPI_ISL_1928089, EPI_ISL_1928090, EPI_ISL_1928092, EPI_ISL_1928093, EPI_ISL_1928095, EPI_ISL_1928096, EPI_ISL_1928098, EPI_ISL_1928099, EPI_ISL_1928101, EPI_ISL_1928102, EPI_ISL_1928104, EPI_ISL_1928105, EPI_ISL_1928107, EPI_ISL_1928108, EPI_ISL_1928110, EPI_ISL_1928112, EPI_ISL_1929077, EPI_ISL_1929079, EPI_ISL_1929080, EPI_ISL_1929151, EPI_ISL_1929153, EPI_ISL_1929155, EPI_ISL_1929156, EPI_ISL_1929158, EPI_ISL_1929159, EPI_ISL_1929160, EPI_ISL_1929162, EPI_ISL_1929164, EPI_ISL_1929165, EPI_ISL_1929167, EPI_ISL_1929168, EPI_ISL_1929170, EPI_ISL_1929171, EPI_ISL_1929173, EPI_ISL_1929174, EPI_ISL_1929176, EPI_ISL_1929177, EPI_ISL_1929179, EPI_ISL_1929180, EPI_ISL_1929182, EPI_ISL_1929183, EPI_ISL_1929185, EPI_ISL_1929189, EPI_ISL_1929191, EPI_ISL_1929193, EPI_ISL_1929194, EPI_ISL_1929196, EPI_ISL_1929250, EPI_ISL_1929252, EPI_ISL_1929256, EPI_ISL_1929286, EPI_ISL_1929287, EPI_ISL_1929288, EPI_ISL_1929290, EPI_ISL_1929291, EPI_ISL_1929293, EPI_ISL_1929294, EPI_ISL_1929296, EPI_ISL_1929302, EPI_ISL_1929303, EPI_ISL_1929305, EPI_ISL_1929306, EPI_ISL_1929307, EPI_ISL_1929310, EPI_ISL_1929312, EPI_ISL_1929313, EPI_ISL_1929315, EPI_ISL_1929316, EPI_ISL_1929329, EPI_ISL_1929330, EPI_ISL_1929331, EPI_ISL_1929333, EPI_ISL_1929334, EPI_ISL_1929336, EPI_ISL_1929337, EPI_ISL_1929339, EPI_ISL_1929340, EPI_ISL_1929342, EPI_ISL_1929381, EPI_ISL_1929383, EPI_ISL_1929384, EPI_ISL_1929386, EPI_ISL_1929387, EPI_ISL_1929389, EPI_ISL_1930894, EPI_ISL_1930896, EPI_ISL_1930897, EPI_ISL_1930899, EPI_ISL_1930900, EPI_ISL_1930902, EPI_ISL_1930903, EPI_ISL_1930905, EPI_ISL_1930906, EPI_ISL_1930908, EPI_ISL_1930910, EPI_ISL_1930911, EPI_ISL_1930913, EPI_ISL_1930914, EPI_ISL_1930915, EPI_ISL_1930917, EPI_ISL_1930918, EPI_ISL_1930920, EPI_ISL_1930921, EPI_ISL_1930923, EPI_ISL_1930974, EPI_ISL_1930975, EPI_ISL_1930976, EPI_ISL_1930978, EPI_ISL_1930979, EPI_ISL_1930981, EPI_ISL_1930983, EPI_ISL_1930984, EPI_ISL_1930985, EPI_ISL_1930987, EPI_ISL_1930988, EPI_ISL_1930990, EPI_ISL_1930991, EPI_ISL_1930993, EPI_ISL_1930994, EPI_ISL_1930996, EPI_ISL_1930997, EPI_ISL_1930999, EPI_ISL_1931000, EPI_ISL_1931002, EPI_ISL_1931004, EPI_ISL_1931005, EPI_ISL_1931006, EPI_ISL_1931008, EPI_ISL_1931009, EPI_ISL_1931011, EPI_ISL_1931012, EPI_ISL_1931014, EPI_ISL_1931015, EPI_ISL_1931017, EPI_ISL_1931018, EPI_ISL_1931020, EPI_ISL_1931132, EPI_ISL_1931135, EPI_ISL_1931338, EPI_ISL_1931339, EPI_ISL_1931341, EPI_ISL_1931342, EPI_ISL_1931344, EPI_ISL_1931345, EPI_ISL_1931347, EPI_ISL_1931362, EPI_ISL_1931363, EPI_ISL_1931365, EPI_ISL_1931366, EPI_ISL_1931368, EPI_ISL_1931370, EPI_ISL_1931371, EPI_ISL_1931372, EPI_ISL_1931374, EPI_ISL_1931375, EPI_ISL_1931377, EPI_ISL_1931378, EPI_ISL_1931380, EPI_ISL_1931382, EPI_ISL_1931383, EPI_ISL_1931385, EPI_ISL_1931386, EPI_ISL_1931388, EPI_ISL_1931389, EPI_ISL_1931393, EPI_ISL_1931395, EPI_ISL_1931396, EPI_ISL_1931398, EPI_ISL_1931399, EPI_ISL_1931401, EPI_ISL_1931402, EPI_ISL_1931404, EPI_ISL_1931405, EPI_ISL_1931407, EPI_ISL_1931408, EPI_ISL_1931410, EPI_ISL_1931411, EPI_ISL_1931413, EPI_ISL_1931414, EPI_ISL_1931416, EPI_ISL_1931417, EPI_ISL_1931419, EPI_ISL_1931421, EPI_ISL_1931422, EPI_ISL_1931424, EPI_ISL_1931426, EPI_ISL_1931427, EPI_ISL_1931429, EPI_ISL_1931430, EPI_ISL_1931432, EPI_ISL_1931433, EPI_ISL_1931435, EPI_ISL_1931436, EPI_ISL_1931438, EPI_ISL_1931439, EPI_ISL_1931441, EPI_ISL_1931621, EPI_ISL_1931651, EPI_ISL_1931660, EPI_ISL_1931944, EPI_ISL_1931945, EPI_ISL_1931947, EPI_ISL_1931948, EPI_ISL_1932090, EPI_ISL_1932091, EPI_ISL_1932092, EPI_ISL_1932324, EPI_ISL_1932325, EPI_ISL_1932326, EPI_ISL_1932327, EPI_ISL_1932328, EPI_ISL_1932329, EPI_ISL_1932330, EPI_ISL_1932331, EPI_ISL_1932332, EPI_ISL_1932333, EPI_ISL_1932334, EPI_ISL_1932335, EPI_ISL_1932336, EPI_ISL_1932337, EPI_ISL_1932338, EPI_ISL_1932339, EPI_ISL_1932340, EPI_ISL_1932341, EPI_ISL_1932342, EPI_ISL_1932343, EPI_ISL_1932344, EPI_ISL_1932345, EPI_ISL_1932346, EPI_ISL_1932347, EPI_ISL_1932348, EPI_ISL_1932349, EPI_ISL_1932350, EPI_ISL_1932351, EPI_ISL_1932352, EPI_ISL_1932353, EPI_ISL_1932354, EPI_ISL_1932355, EPI_ISL_1932356, EPI_ISL_1932357, EPI_ISL_1932358, EPI_ISL_1932359, EPI_ISL_1932360, EPI_ISL_1932361, EPI_ISL_1932362, EPI_ISL_1932363, EPI_ISL_1932364, EPI_ISL_1932365, EPI_ISL_1932366, EPI_ISL_1932367, EPI_ISL_1932368, EPI_ISL_1932369, EPI_ISL_1932370, EPI_ISL_1932371, EPI_ISL_1932372, EPI_ISL_1932373, EPI_ISL_1932374, EPI_ISL_1932445, EPI_ISL_1932446, EPI_ISL_1932447, EPI_ISL_1932448, EPI_ISL_1932449, EPI_ISL_1932450, EPI_ISL_1932451, EPI_ISL_1932452, EPI_ISL_1932453, EPI_ISL_1932454, EPI_ISL_1932455, EPI_ISL_1932456, EPI_ISL_1932457, EPI_ISL_1932458, EPI_ISL_1932459, EPI_ISL_1932460, EPI_ISL_1932581, EPI_ISL_1932582, EPI_ISL_1932584, EPI_ISL_1932585, EPI_ISL_1932586, EPI_ISL_1932587, EPI_ISL_1932588, EPI_ISL_1932589, EPI_ISL_1932590, EPI_ISL_1932591, EPI_ISL_1932592, EPI_ISL_1932593, EPI_ISL_1932594, EPI_ISL_1932595, EPI_ISL_1932596, EPI_ISL_1932597, EPI_ISL_1932598, EPI_ISL_1932599, EPI_ISL_1932600, EPI_ISL_1932601, EPI_ISL_1932602, EPI_ISL_1932603, EPI_ISL_1932604, EPI_ISL_1932605, EPI_ISL_1932606, EPI_ISL_1932607, EPI_ISL_1932608, EPI_ISL_1932609, EPI_ISL_1932610, EPI_ISL_1932611, EPI_ISL_1932612, EPI_ISL_1932613, EPI_ISL_1932614, EPI_ISL_1932615, EPI_ISL_1932616, EPI_ISL_1932617, EPI_ISL_1932618, EPI_ISL_1932619, EPI_ISL_1932620, EPI_ISL_1932621, EPI_ISL_1932622, EPI_ISL_1932698, EPI_ISL_1932699, EPI_ISL_1932714, EPI_ISL_1932759, EPI_ISL_1932760, EPI_ISL_1932906, EPI_ISL_1932907, EPI_ISL_1932908, EPI_ISL_1932909, EPI_ISL_1933423, EPI_ISL_1933424, EPI_ISL_1933425, EPI_ISL_1933426, EPI_ISL_1933427, EPI_ISL_1933487, EPI_ISL_1933488, EPI_ISL_1933489, EPI_ISL_1933490, EPI_ISL_1933491, EPI_ISL_1933492, EPI_ISL_1933493, EPI_ISL_1933494, EPI_ISL_1933495, EPI_ISL_1933496, EPI_ISL_1933497, EPI_ISL_1933498, EPI_ISL_1933499, EPI_ISL_1933500, EPI_ISL_1933501, EPI_ISL_1933502, EPI_ISL_1933503, EPI_ISL_1933544, EPI_ISL_1933573, EPI_ISL_1933574, EPI_ISL_1933575, EPI_ISL_1933576, EPI_ISL_1933577, EPI_ISL_1933578, EPI_ISL_1933579, EPI_ISL_1933645, EPI_ISL_1933646, EPI_ISL_1933647, EPI_ISL_1933648, EPI_ISL_1933649, EPI_ISL_1933650, EPI_ISL_1933701, EPI_ISL_1933711, EPI_ISL_1933712, EPI_ISL_1933713, EPI_ISL_1933752, EPI_ISL_1933786, EPI_ISL_1933787, EPI_ISL_1933788, EPI_ISL_1933789, EPI_ISL_1933790, EPI_ISL_1933791, EPI_ISL_1933792, EPI_ISL_1933793, EPI_ISL_1933794, EPI_ISL_1933795, EPI_ISL_1933796, EPI_ISL_1933797, EPI_ISL_1933798, EPI_ISL_1933799, EPI_ISL_1933800, EPI_ISL_1933801, EPI_ISL_1933802, EPI_ISL_1933803, EPI_ISL_1933804, EPI_ISL_1933805, EPI_ISL_1933806, EPI_ISL_1933807, EPI_ISL_1933808, EPI_ISL_1933809, EPI_ISL_1933810, EPI_ISL_1933811, EPI_ISL_1933812, EPI_ISL_1933813, EPI_ISL_1933814, EPI_ISL_1933815, EPI_ISL_1933816, EPI_ISL_1933817, EPI_ISL_1933818, EPI_ISL_1933819, EPI_ISL_1933820, EPI_ISL_1933821, EPI_ISL_1933822, EPI_ISL_1933823, EPI_ISL_1933824, EPI_ISL_1933825, EPI_ISL_1933826, EPI_ISL_1933827, EPI_ISL_1933828, EPI_ISL_1933829, EPI_ISL_1933830, EPI_ISL_1933831, EPI_ISL_1933832, EPI_ISL_1933833, EPI_ISL_1933834, EPI_ISL_1933835, EPI_ISL_1933836, EPI_ISL_1934105, EPI_ISL_1934106, EPI_ISL_1934107, EPI_ISL_1934108, EPI_ISL_1934206, EPI_ISL_1934207, EPI_ISL_1934208, EPI_ISL_1934209, EPI_ISL_1934210, EPI_ISL_1934211, EPI_ISL_1934212, EPI_ISL_1934213, EPI_ISL_1934214, EPI_ISL_1934215, EPI_ISL_1934216, EPI_ISL_1934217, EPI_ISL_1934218, EPI_ISL_1934219, EPI_ISL_1934220, EPI_ISL_1934221, EPI_ISL_1934222, EPI_ISL_1934223, EPI_ISL_1934224, EPI_ISL_1934225, EPI_ISL_1934226, EPI_ISL_1934227, EPI_ISL_1934228, EPI_ISL_1934229, EPI_ISL_1934230, EPI_ISL_1934231, EPI_ISL_1934408, EPI_ISL_1934409			
see above	Osaka Institute of Public Health, Morinomiya Center	Pathogen Genomics Center, National Institute of Infectious Diseases	Ai Kawana-Tachikawa; Chang-Kweng Lim; Eri Nakayama; Hazuka Y Furihata; Hidemasa Izumiya; Hiromizu Takahashi; Ken Shimuta; Ken-ichi Lee; Kentaro Itokawa; Koichi Ishikawa; Makoto Kuroda; Masanori Hashino; Masatomo Morita; Masumichi Saito; Midori Nakamura-Hoshi; Motohiko Ogawa; Naomi Nojiri; Nobuo Koizumi; Nozomu Hanaoka; Rina Tanaka; Sana Uchikoba; Sayuri Seki; Shigeru Kusagawa; Shigeru Tajima; Shouji Yamamoto; Sunao Iyoda; Takahiro Maeki; Tsuguto Fujimoto; Tsuyoshi Sekizuka
EPI_ISL_1926969, EPI_ISL_1926976, EPI_ISL_1926978, EPI_ISL_1926985, EPI_ISL_1926990, EPI_ISL_1926992, EPI_ISL_1926993, EPI_ISL_1926994, EPI_ISL_1926996, EPI_ISL_1926997, EPI_ISL_1926999, EPI_ISL_1927005, EPI_ISL_1927007, EPI_ISL_1927008, EPI_ISL_1927010, EPI_ISL_1927011, EPI_ISL_1927013, EPI_ISL_1927014, EPI_ISL_1927016, EPI_ISL_1927017, EPI_ISL_1927019, EPI_ISL_1927020, EPI_ISL_1927022, EPI_ISL_1927029, EPI_ISL_1927030, EPI_ISL_1927044, EPI_ISL_1927045, EPI_ISL_1927047, EPI_ISL_1927048, EPI_ISL_1927050, EPI_ISL_1927051, EPI_ISL_1927053, EPI_ISL_1927054, EPI_ISL_1927056, EPI_ISL_1927058, EPI_ISL_1927059, EPI_ISL_1927061, EPI_ISL_1927062, EPI_ISL_1927064, EPI_ISL_1927065, EPI_ISL_1927067, EPI_ISL_1927068, EPI_ISL_1927077, EPI_ISL_1927079, EPI_ISL_1927080, EPI_ISL_1927082, EPI_ISL_1927083, EPI_ISL_1927084, EPI_ISL_1927086, EPI_ISL_1927087, EPI_ISL_1927089, EPI_ISL_1927090, EPI_ISL_1927092, EPI_ISL_1927093, EPI_ISL_1927095, EPI_ISL_1927096, EPI_ISL_1927098, EPI_ISL_1927099, EPI_ISL_1927101, EPI_ISL_1927102, EPI_ISL_1927190, EPI_ISL_1927192, EPI_ISL_1927193, EPI_ISL_1927195, EPI_ISL_1927196, EPI_ISL_1927197, EPI_ISL_1927199, EPI_ISL_1927200, EPI_ISL_1927202, EPI_ISL_1927207, EPI_ISL_1927208, EPI_ISL_1927209, EPI_ISL_1927211, EPI_ISL_1927212, EPI_ISL_1927214, EPI_ISL_1927215, EPI_ISL_1927217, EPI_ISL_1927221, EPI_ISL_1927223, EPI_ISL_1927229, EPI_ISL_1927236, EPI_ISL_1927286, EPI_ISL_1927288, EPI_ISL_1927293, EPI_ISL_1927294, EPI_ISL_1927296, EPI_ISL_1927309, EPI_ISL_1927311, EPI_ISL_1927314, EPI_ISL_1927319, EPI_ISL_1927321, EPI_ISL_1927322, EPI_ISL_1927324, EPI_ISL_1927327, EPI_ISL_1927328, EPI_ISL_1927330, EPI_ISL_1927331, EPI_ISL_1927333, EPI_ISL_1927334, EPI_ISL_1927336, EPI_ISL_1927337, EPI_ISL_1927339, EPI_ISL_1927341, EPI_ISL_1927342, EPI_ISL_1927349, EPI_ISL_1927351, EPI_ISL_1927352, EPI_ISL_1927354, EPI_ISL_1927355, EPI_ISL_1927357, EPI_ISL_1927395, EPI_ISL_1927398, EPI_ISL_1927430, EPI_ISL_1927448, EPI_ISL_1927450, EPI_ISL_1927451, EPI_ISL_1927453, EPI_ISL_1927454, EPI_ISL_1927456, EPI_ISL_1927457, EPI_ISL_1927459, EPI_ISL_1927465, EPI_ISL_1927466, EPI_ISL_1927468, EPI_ISL_1927469, EPI_ISL_1927471, EPI_ISL_1927472, EPI_ISL_1927473, EPI_ISL_1927475, EPI_ISL_1927476, EPI_ISL_1927478, EPI_ISL_1927479, EPI_ISL_1927481, EPI_ISL_1927482, EPI_ISL_1927484, EPI_ISL_1927485, EPI_ISL_1927487, EPI_ISL_1927488, EPI_ISL_1927490, EPI_ISL_1927491, EPI_ISL_1927492, EPI_ISL_1927494, EPI_ISL_1927496, EPI_ISL_1927497, EPI_ISL_1927498, EPI_ISL_1927500, EPI_ISL_1927501, EPI_ISL_1927503, EPI_ISL_1927505, EPI_ISL_1927506, EPI_ISL_1927508, EPI_ISL_1927509, EPI_ISL_1927511, EPI_ISL_1927512, EPI_ISL_1927514, EPI_ISL_1927515, EPI_ISL_1927517, EPI_ISL_1927518, EPI_ISL_1927520, EPI_ISL_1927521, EPI_ISL_1927523, EPI_ISL_1927524, EPI_ISL_1927525, EPI_ISL_1927527, EPI_ISL_1927528, EPI_ISL_1927530, EPI_ISL_1927531, EPI_ISL_1927533, EPI_ISL_1927534, EPI_ISL_1927536, EPI_ISL_1927537, EPI_ISL_1927539, EPI_ISL_1927540, EPI_ISL_1927542, EPI_ISL_1927543, EPI_ISL_1927545, EPI_ISL_1927546, EPI_ISL_1927547, EPI_ISL_1927549, EPI_ISL_1927550, EPI_ISL_1927552, EPI_ISL_1927553, EPI_ISL_1927555, EPI_ISL_1927556, EPI_ISL_1927558, EPI_ISL_1927559, EPI_ISL_1927561, EPI_ISL_1927563, EPI_ISL_1927564, EPI_ISL_1927566, EPI_ISL_1927567, EPI_ISL_1927569, EPI_ISL_1927570, EPI_ISL_1927571, EPI_ISL_1927573, EPI_ISL_1927575, EPI_ISL_1927576, EPI_ISL_1927578, EPI_ISL_1927579, EPI_ISL_1927581, EPI_ISL_1927582, EPI_ISL_1927583, EPI_ISL_1927585, EPI_ISL_1927586, EPI_ISL_1927588, EPI_ISL_1927589, EPI_ISL_1927591, EPI_ISL_1927592, EPI_ISL_1927594, EPI_ISL_1927595, EPI_ISL_1927596, EPI_ISL_1927598, EPI_ISL_1927599, EPI_ISL_1927601, EPI_ISL_1927602, EPI_ISL_1927604, EPI_ISL_1927605, EPI_ISL_1927606, EPI_ISL_1927608, EPI_ISL_1927609, EPI_ISL_1927611, EPI_ISL_1927612, EPI_ISL_1927614, EPI_ISL_1927615, EPI_ISL_1927617, EPI_ISL_1927618, EPI_ISL_1927620, EPI_ISL_1927621, EPI_ISL_1927623, EPI_ISL_1927624, EPI_ISL_1927626, EPI_ISL_1927627, EPI_ISL_1927629, EPI_ISL_1927630, EPI_ISL_1927632, EPI_ISL_1927633, EPI_ISL_1927635, EPI_ISL_1927637, EPI_ISL_1927638, EPI_ISL_1927640, EPI_ISL_1927641, EPI_ISL_1927643, EPI_ISL_1927644, EPI_ISL_1927645, EPI_ISL_1927647, EPI_ISL_1927649, EPI_ISL_1927650, EPI_ISL_1927652, EPI_ISL_1927653, EPI_ISL_1927655, EPI_ISL_1927656, EPI_ISL_1927658, EPI_ISL_1927659, EPI_ISL_1927660, EPI_ISL_1927662, EPI_ISL_1927663, EPI_ISL_1927665, EPI_ISL_1927667, EPI_ISL_1927668, EPI_ISL_1927670, EPI_ISL_1927671, EPI_ISL_1927673, EPI_ISL_1927674, EPI_ISL_1927683, EPI_ISL_1927684, EPI_ISL_1927686, EPI_ISL_1927687, EPI_ISL_1927689, EPI_ISL_1927690, EPI_ISL_1927692, EPI_ISL_1927693, EPI_ISL_1927695, EPI_ISL_1927696, EPI_ISL_1927698, EPI_ISL_1927699, EPI_ISL_1927701, EPI_ISL_1927702, EPI_ISL_1927704, EPI_ISL_1927705, EPI_ISL_1927707, EPI_ISL_1927708, EPI_ISL_1927710, EPI_ISL_1927711, EPI_ISL_1927713, EPI_ISL_1927714, EPI_ISL_1927716, EPI_ISL_1927717, EPI_ISL_1927719, EPI_ISL_1927720, EPI_ISL_1927722, EPI_ISL_1927723, EPI_ISL_1927725, EPI_ISL_1927726, EPI_ISL_1927727, EPI_ISL_1927729, EPI_ISL_1927730, EPI_ISL_1927732, EPI_ISL_1927733, EPI_ISL_1927735, EPI_ISL_1927736, EPI_ISL_1927738, EPI_ISL_1927739, EPI_ISL_1927741, EPI_ISL_1927742, EPI_ISL_1927744, EPI_ISL_1927745, EPI_ISL_1927746, EPI_ISL_1927748, EPI_ISL_1927749, EPI_ISL_1927751, EPI_ISL_1927752, EPI_ISL_1927753, EPI_ISL_1927755, EPI_ISL_1927756, EPI_ISL_1927758, EPI_ISL_1927759, EPI_ISL_1927761, EPI_ISL_1927762, EPI_ISL_1927764, EPI_ISL_1927765, EPI_ISL_1927767, EPI_ISL_1927768, EPI_ISL_1927770, EPI_ISL_1927771, EPI_ISL_1927773, EPI_ISL_1927774, EPI_ISL_1927776, EPI_ISL_1927777, EPI_ISL_1927779, EPI_ISL_1927780, EPI_ISL_1927782, EPI_ISL_1927783, EPI_ISL_1927785, EPI_ISL_1927786, EPI_ISL_1927788, EPI_ISL_1927789, EPI_ISL_1927791, EPI_ISL_1927792, EPI_ISL_1927794, EPI_ISL_1927795, EPI_ISL_1927797, EPI_ISL_1927798, EPI_ISL_1927800, EPI_ISL_1927801, EPI_ISL_1927803, EPI_ISL_1927805, EPI_ISL_1927806, EPI_ISL_1927808, EPI_ISL_1927809, EPI_ISL_1927813, EPI_ISL_1927816, EPI_ISL_1927818, EPI_ISL_1927819, EPI_ISL_1927821, EPI_ISL_1927822, EPI_ISL_1927824, EPI_ISL_1927825, EPI_ISL_1927851, EPI_ISL_1927852, EPI_ISL_1927854, EPI_ISL_1927855, EPI_ISL_1927857, EPI_ISL_1927858, EPI_ISL_1927860, EPI_ISL_1927861, EPI_ISL_1927863, EPI_ISL_1927864, EPI_ISL_1927866, EPI_ISL_1927867, EPI_ISL_1927869, EPI_ISL_1927870, EPI_ISL_1927872, EPI_ISL_1927873, EPI_ISL_1927875, EPI_ISL_1927876, EPI_ISL_1927878, EPI_ISL_1927879, EPI_ISL_1927881, EPI_ISL_1927882, EPI_ISL_1927884, EPI_ISL_1927885, EPI_ISL_1927887, EPI_ISL_1927888, EPI_ISL_1927890, EPI_ISL_1927891, EPI_ISL_1927893, EPI_ISL_1927894, EPI_ISL_1927896, EPI_ISL_1927897, EPI_ISL_1927898, EPI_ISL_1927900, EPI_ISL_1927901, EPI_ISL_1927903, EPI_ISL_1927904, EPI_ISL_1927906, EPI_ISL_1927907, EPI_ISL_1927909, EPI_ISL_1927910, EPI_ISL_1927912, EPI_ISL_1927913, EPI_ISL_1927915, EPI_ISL_1927916, EPI_ISL_1927918, EPI_ISL_1927919, EPI_ISL_1927921, EPI_ISL_1927922, EPI_ISL_1927924, EPI_ISL_1927925, EPI_ISL_1927927, EPI_ISL_1927928, EPI_ISL_1927930, EPI_ISL_1927931, EPI_ISL_1927933, EPI_ISL_1927935, EPI_ISL_1927936, EPI_ISL_1927937, EPI_ISL_1927939, EPI_ISL_1927940, EPI_ISL_1927942, EPI_ISL_1927944, EPI_ISL_1927945, EPI_ISL_1927946, EPI_ISL_1927948, EPI_ISL_1927949, EPI_ISL_1927951, EPI_ISL_1927952, EPI_ISL_1927954, EPI_ISL_1927955, EPI_ISL_1927957, EPI_ISL_1927958, EPI_ISL_1927960, EPI_ISL_1927961, EPI_ISL_1927963, EPI_ISL_1927964, EPI_ISL_1927966, EPI_ISL_1927967, EPI_ISL_1927969, EPI_ISL_1927970, EPI_ISL_1927972, EPI_ISL_1927973, EPI_ISL_1927975, EPI_ISL_1927976, EPI_ISL_1927978, EPI_ISL_1927979, EPI_ISL_1927981, EPI_ISL_1927982, EPI_ISL_1927984, EPI_ISL_1927985, EPI_ISL_1927987, EPI_ISL_1927988, EPI_ISL_1927990, EPI_ISL_1927991, EPI_ISL_1927993, EPI_ISL_1927994, EPI_ISL_1927996, EPI_ISL_1927997, EPI_ISL_1927999, EPI_ISL_1928000, EPI_ISL_1928002, EPI_ISL_1928003, EPI_ISL_1928005, EPI_ISL_1928006, EPI_ISL_1928008, EPI_ISL_1928009, EPI_ISL_1928011, EPI_ISL_1928012, EPI_ISL_1928014, EPI_ISL_1928015, EPI_ISL_1928017, EPI_ISL_1928018, EPI_ISL_1928020, EPI_ISL_1928021, EPI_ISL_1928023, EPI_ISL_1928024, EPI_ISL_1928026, EPI_ISL_1928027, EPI_ISL_1928029, EPI_ISL_1928030, EPI_ISL_1928032, EPI_ISL_1928033, EPI_ISL_1928035, EPI_ISL_1928036, EPI_ISL_1928038, EPI_ISL_1928039,			

EPI_ISL_1928041, EPI_ISL_1928042, EPI_ISL_1928044, EPI_ISL_1928045, EPI_ISL_1928047, EPI_ISL_1928048, EPI_ISL_1928050, EPI_ISL_1928051, EPI_ISL_1928053, EPI_ISL_1928113, EPI_ISL_1928114, EPI_ISL_1928116, EPI_ISL_1928117, EPI_ISL_1928119, EPI_ISL_1928120, EPI_ISL_1928122, EPI_ISL_1928123, EPI_ISL_1928125, EPI_ISL_1928126, EPI_ISL_1928128, EPI_ISL_1928129, EPI_ISL_1928131,
EPI_ISL_1928132, EPI_ISL_1928134, EPI_ISL_1928136, EPI_ISL_1928137, EPI_ISL_1928138, EPI_ISL_1928140, EPI_ISL_1928141, EPI_ISL_1928143, EPI_ISL_1928144, EPI_ISL_1928146, EPI_ISL_1928147, EPI_ISL_1928149, EPI_ISL_1928150, EPI_ISL_1928152, EPI_ISL_1928153, EPI_ISL_1928155, EPI_ISL_1928156, EPI_ISL_1928158, EPI_ISL_1928159, EPI_ISL_1928161, EPI_ISL_1928162, EPI_ISL_1928164,
EPI_ISL_1928165, EPI_ISL_1928167, EPI_ISL_1928168, EPI_ISL_1928169, EPI_ISL_1928171, EPI_ISL_1928173, EPI_ISL_1928174, EPI_ISL_1928175, EPI_ISL_1928177, EPI_ISL_1928178, EPI_ISL_1928180, EPI_ISL_1928181, EPI_ISL_1928183, EPI_ISL_1928184, EPI_ISL_1928186, EPI_ISL_1928187, EPI_ISL_1928189, EPI_ISL_1928190, EPI_ISL_1928192, EPI_ISL_1928193, EPI_ISL_1928195, EPI_ISL_1928196,
EPI_ISL_1928198, EPI_ISL_1928199, EPI_ISL_1928201, EPI_ISL_1928202, EPI_ISL_1928203, EPI_ISL_1928205, EPI_ISL_1928206, EPI_ISL_1928208, EPI_ISL_1928209, EPI_ISL_1928211, EPI_ISL_1928212, EPI_ISL_1928214, EPI_ISL_1928215, EPI_ISL_1928217, EPI_ISL_1928218, EPI_ISL_1928220, EPI_ISL_1928221, EPI_ISL_1928223, EPI_ISL_1928225, EPI_ISL_1928226, EPI_ISL_1928228, EPI_ISL_1928229,
EPI_ISL_1928230, EPI_ISL_1928232, EPI_ISL_1928233, EPI_ISL_1928235, EPI_ISL_1928236, EPI_ISL_1928238, EPI_ISL_1928239, EPI_ISL_1928241, EPI_ISL_1928242, EPI_ISL_1928244, EPI_ISL_1928245, EPI_ISL_1928247, EPI_ISL_1928248, EPI_ISL_1928250, EPI_ISL_1928251, EPI_ISL_1928253, EPI_ISL_1928254, EPI_ISL_1928256, EPI_ISL_1928257, EPI_ISL_1928259, EPI_ISL_1928260, EPI_ISL_1928261,
EPI_ISL_1928263, EPI_ISL_1928264, EPI_ISL_1928266, EPI_ISL_1928267, EPI_ISL_1928269, EPI_ISL_1928270, EPI_ISL_1928272, EPI_ISL_1928273, EPI_ISL_1928275, EPI_ISL_1928276, EPI_ISL_1928278, EPI_ISL_1928279, EPI_ISL_1928281, EPI_ISL_1928283, EPI_ISL_1928284, EPI_ISL_1928286, EPI_ISL_1928287, EPI_ISL_1928288, EPI_ISL_1928290, EPI_ISL_1928291, EPI_ISL_1928293, EPI_ISL_1928294,
EPI_ISL_1928296, EPI_ISL_1928297, EPI_ISL_1928299, EPI_ISL_1928300, EPI_ISL_1928302, EPI_ISL_1928303, EPI_ISL_1928305, EPI_ISL_1928306, EPI_ISL_1928308, EPI_ISL_1928309, EPI_ISL_1928311, EPI_ISL_1928312, EPI_ISL_1928313, EPI_ISL_1928315, EPI_ISL_1928316, EPI_ISL_1928318, EPI_ISL_1928320, EPI_ISL_1928321, EPI_ISL_1928323, EPI_ISL_1928324, EPI_ISL_1928325, EPI_ISL_1928327,
[truncated: 1,155,802 more chars]
