# Supplementary material for: Genome Profiling of SARS-CoV-2 in Indonesia, ASEAN and the Neighbouring East Asian Countries: Features, Challenges and Achievements
Source: Viruses. 2022 Apr 8;14(4):778. doi: 10.3390/v14040778 (PMC9027902; doi:10.3390/v14040778)
Supplement: Supplementary file 1 [file viruses-14-00778-s001.zip › viruses-1641476-supplementary/TableS7_acknowledgement__japan.pdf]

virus_name	epiID	submission_date
hCoV-19/Japan/OPHO-Hb7-47-2C/2021	EPI_ISL_3447067	2021-08-17
hCoV-19/Japan/OPHO-Hb7-49/2021	EPI_ISL_3446655	2021-08-17
hCoV-19/Japan/TKYK24329/2021	EPI_ISL_3447681	2021-08-17
hCoV-19/Japan/TKYK24382/2021	EPI_ISL_3447682	2021-08-17
hCoV-19/Japan/TKYK24555/2021	EPI_ISL_3457044	2021-08-17
hCoV-19/Japan/TKYK24656/2021	EPI_ISL_3447683	2021-08-17
hCoV-19/Japan/TKYK24693/2021	EPI_ISL_3457054	2021-08-17
hCoV-19/Japan/TKYK24775/2021	EPI_ISL_3447684	2021-08-17
hCoV-19/Japan/TKYK24800/2021	EPI_ISL_3457056	2021-08-17
hCoV-19/Japan/TKYK24909/2021	EPI_ISL_3447685	2021-08-17
hCoV-19/Japan/TKYK25032/2021	EPI_ISL_3447694	2021-08-17
hCoV-19/Japan/TKYK25048/2021	EPI_ISL_3457057	2021-08-17
hCoV-19/Japan/TKYK25073/2021	EPI_ISL_3457059	2021-08-17
hCoV-19/Japan/TKYK25078/2021	EPI_ISL_3457060	2021-08-17
hCoV-19/Japan/TKYK25079/2021	EPI_ISL_3457062	2021-08-17
hCoV-19/Japan/TKYK25364/2021	EPI_ISL_3447687	2021-08-17
hCoV-19/Japan/TKYK25376/2021	EPI_ISL_3447696	2021-08-17
hCoV-19/Japan/TKYK25619/2021	EPI_ISL_3447697	2021-08-17
hCoV-19/Japan/TKYK25625/2021	EPI_ISL_3447688	2021-08-17
hCoV-19/Japan/TKYK25639/2021	EPI_ISL_3447689	2021-08-17
hCoV-19/Japan/TKYK25641/2021	EPI_ISL_3447690	2021-08-17
hCoV-19/Japan/TKYK25645/2021	EPI_ISL_3447698	2021-08-17
hCoV-19/Japan/TKYK25735/2021	EPI_ISL_3447699	2021-08-17
hCoV-19/Japan/TKYK25778/2021	EPI_ISL_3447700	2021-08-17
hCoV-19/Japan/TKYK25787/2021	EPI_ISL_3447702	2021-08-17
hCoV-19/Japan/TKYK25789/2021	EPI_ISL_3447703	2021-08-17
hCoV-19/Japan/TKYK25797/2021	EPI_ISL_3447691	2021-08-17
hCoV-19/Japan/TKYK25806/2021	EPI_ISL_3447692	2021-08-17
hCoV-19/Japan/TKYK25808/2021	EPI_ISL_3447704	2021-08-17
hCoV-19/Japan/TKYK26257/2021	EPI_ISL_3447693	2021-08-17
hCoV-19/Japan/TKYK26259/2021	EPI_ISL_3447705	2021-08-17
hCoV-19/Japan/TKYS00473/2021	EPI_ISL_3447686	2021-08-17
hCoV-19/Japan/TKYS00474/2021	EPI_ISL_3447695	2021-08-17
hCoV-19/Japan/TKYS00484/2021	EPI_ISL_3447701	2021-08-17
hCoV-19/Japan/YCH0543/2021	EPI_ISL_3449308	2021-08-17
hCoV-19/Japan/YCH0544/2021	EPI_ISL_3449319	2021-08-17
hCoV-19/Japan/YCH0545/2021	EPI_ISL_3449309	2021-08-17
hCoV-19/Japan/YCH0546/2021	EPI_ISL_3449310	2021-08-17
hCoV-19/Japan/YCH0547/2021	EPI_ISL_3449304	2021-08-17
hCoV-19/Japan/YCH0548/2021	EPI_ISL_3449311	2021-08-17
hCoV-19/Japan/YCH0549/2021	EPI_ISL_3449305	2021-08-17
hCoV-19/Japan/YCH0550/2021	EPI_ISL_3449312	2021-08-17
hCoV-19/Japan/YCH0551/2021	EPI_ISL_3449313	2021-08-17
hCoV-19/Japan/YCH0552/2021	EPI_ISL_3449314	2021-08-17
hCoV-19/Japan/YCH0553/2021	EPI_ISL_3449315	2021-08-17
hCoV-19/Japan/YCH0554/2021	EPI_ISL_3449316	2021-08-17
hCoV-19/Japan/YCH0555/2021	EPI_ISL_3449306	2021-08-17
hCoV-19/Japan/YCH0556/2021	EPI_ISL_3449317	2021-08-17
hCoV-19/Japan/YCH0557/2021	EPI_ISL_3449318	2021-08-17
hCoV-19/Japan/YCH0558/2021	EPI_ISL_3449307	2021-08-17
hCoV-19/Japan/RIMD00206/2021	EPI_ISL_3477106	2021-08-18
hCoV-19/Japan/RIMD00207/2021	EPI_ISL_3477107	2021-08-18
hCoV-19/Japan/RIMD00208/2021	EPI_ISL_3477108	2021-08-18
hCoV-19/Japan/RIMD00209/2021	EPI_ISL_3477109	2021-08-18

virus_name	epiID	submission_date
hCoV-19/Japan/RIMD00210/2021	EPI_ISL_3477110	2021-08-18
hCoV-19/Japan/RIMD00211/2021	EPI_ISL_3477111	2021-08-18
hCoV-19/Japan/RIMD00212/2021	EPI_ISL_3477112	2021-08-18
hCoV-19/Japan/RIMD00213/2021	EPI_ISL_3477113	2021-08-18
hCoV-19/Japan/RIMD00214/2021	EPI_ISL_3477114	2021-08-18
hCoV-19/Japan/RIMD00215/2021	EPI_ISL_3477115	2021-08-18
hCoV-19/Japan/RIMD00216/2021	EPI_ISL_3477116	2021-08-18
hCoV-19/Japan/RIMD00218/2021	EPI_ISL_3477117	2021-08-18
hCoV-19/Japan/RIMD00219/2021	EPI_ISL_3477118	2021-08-18
hCoV-19/Japan/RIMD00220/2021	EPI_ISL_3477119	2021-08-18
hCoV-19/Japan/RIMD00221/2021	EPI_ISL_3477120	2021-08-18
hCoV-19/Japan/RIMD00222/2021	EPI_ISL_3477121	2021-08-18
hCoV-19/Japan/RIMD00223/2021	EPI_ISL_3477122	2021-08-18
hCoV-19/Japan/RIMD00224/2021	EPI_ISL_3477123	2021-08-18
hCoV-19/Japan/RIMD00225/2021	EPI_ISL_3477124	2021-08-18
hCoV-19/Japan/RIMD00226/2021	EPI_ISL_3477125	2021-08-18
hCoV-19/Japan/RIMD00227/2021	EPI_ISL_3477126	2021-08-18
hCoV-19/Japan/RIMD00228/2021	EPI_ISL_3477127	2021-08-18
hCoV-19/Japan/RIMD00229/2021	EPI_ISL_3477128	2021-08-18
hCoV-19/Japan/RIMD00230/2021	EPI_ISL_3477129	2021-08-18
hCoV-19/Japan/RIMD00231/2021	EPI_ISL_3477130	2021-08-18
hCoV-19/Japan/RIMD00232/2021	EPI_ISL_3477131	2021-08-18
hCoV-19/Japan/RIMD00233/2021	EPI_ISL_3477132	2021-08-18
hCoV-19/Japan/RIMD00234/2021	EPI_ISL_3477133	2021-08-18
hCoV-19/Japan/RIMD00235/2021	EPI_ISL_3477134	2021-08-18
hCoV-19/Japan/RIMD00236/2021	EPI_ISL_3477135	2021-08-18
hCoV-19/Japan/RIMD00237/2021	EPI_ISL_3477136	2021-08-18
hCoV-19/Japan/RIMD00238/2021	EPI_ISL_3477137	2021-08-18
hCoV-19/Japan/RIMD00239/2021	EPI_ISL_3477138	2021-08-18
hCoV-19/Japan/RIMD00240/2021	EPI_ISL_3477139	2021-08-18
hCoV-19/Japan/RIMD00241/2021	EPI_ISL_3477140	2021-08-18
hCoV-19/Japan/RIMD00242/2021	EPI_ISL_3477141	2021-08-18
hCoV-19/Japan/RIMD00243/2021	EPI_ISL_3477142	2021-08-18
hCoV-19/Japan/RIMD00244/2021	EPI_ISL_3477154	2021-08-18
hCoV-19/Japan/RIMD00245/2021	EPI_ISL_3477155	2021-08-18
hCoV-19/Japan/RIMD00246/2021	EPI_ISL_3477156	2021-08-18
hCoV-19/Japan/RIMD00247/2021	EPI_ISL_3477157	2021-08-18
hCoV-19/Japan/RIMD00248/2021	EPI_ISL_3477158	2021-08-18
hCoV-19/Japan/RIMD00249/2021	EPI_ISL_3477159	2021-08-18
hCoV-19/Japan/RIMD00250/2021	EPI_ISL_3477160	2021-08-18
hCoV-19/Japan/RIMD00251/2021	EPI_ISL_3477161	2021-08-18
hCoV-19/Japan/RIMD00252/2021	EPI_ISL_3477162	2021-08-18
hCoV-19/Japan/B178/2021	EPI_ISL_3494557	2021-08-19
hCoV-19/Japan/RIMD00253/2021	EPI_ISL_3501814	2021-08-19
hCoV-19/Japan/RIMD00254/2021	EPI_ISL_3501815	2021-08-19
hCoV-19/Japan/RIMD00255/2021	EPI_ISL_3502868	2021-08-19
hCoV-19/Japan/RIMD00256/2021	EPI_ISL_3502869	2021-08-19
hCoV-19/Japan/RIMD00257/2021	EPI_ISL_3502870	2021-08-19
hCoV-19/Japan/RIMD00258/2021	EPI_ISL_3502871	2021-08-19
hCoV-19/Japan/RIMD00259/2021	EPI_ISL_3502872	2021-08-19
hCoV-19/Japan/RIMD00260/2021	EPI_ISL_3502873	2021-08-19
hCoV-19/Japan/RIMD00261/2021	EPI_ISL_3502874	2021-08-19
hCoV-19/Japan/RIMD00262/2021	EPI_ISL_3502875	2021-08-19
hCoV-19/Japan/RIMD00263/2021	EPI_ISL_3502876	2021-08-19

virus_name	epiID	submission_date
hCoV-19/Japan/RIMD00264/2021	EPI_ISL_3502877	2021-08-19
hCoV-19/Japan/RIMD00265/2021	EPI_ISL_3502878	2021-08-19
hCoV-19/Japan/RIMD00266/2021	EPI_ISL_3502879	2021-08-19
hCoV-19/Japan/RIMD00267/2021	EPI_ISL_3502880	2021-08-19
hCoV-19/Japan/RIMD00268/2021	EPI_ISL_3502881	2021-08-19
hCoV-19/Japan/RIMD00269/2021	EPI_ISL_3502882	2021-08-19
hCoV-19/Japan/RIMD00270/2021	EPI_ISL_3502883	2021-08-19
hCoV-19/Japan/RIMD00271/2021	EPI_ISL_3502884	2021-08-19
hCoV-19/Japan/RIMD00272/2021	EPI_ISL_3502885	2021-08-19
hCoV-19/Japan/RIMD00273/2021	EPI_ISL_3502886	2021-08-19
hCoV-19/Japan/RIMD00274/2021	EPI_ISL_3503136	2021-08-19
hCoV-19/Japan/RIMD00275/2021	EPI_ISL_3503137	2021-08-19
hCoV-19/Japan/RIMD00276/2021	EPI_ISL_3503138	2021-08-19
hCoV-19/Japan/RIMD00277/2021	EPI_ISL_3503139	2021-08-19
hCoV-19/Japan/RIMD00278/2021	EPI_ISL_3503140	2021-08-19
hCoV-19/Japan/RIMD00279/2021	EPI_ISL_3503141	2021-08-19
hCoV-19/Japan/RIMD00280/2021	EPI_ISL_3503142	2021-08-19
hCoV-19/Japan/RIMD00281/2021	EPI_ISL_3503143	2021-08-19
hCoV-19/Japan/RIMD00282/2021	EPI_ISL_3503144	2021-08-19
hCoV-19/Japan/RIMD00283/2021	EPI_ISL_3503145	2021-08-19
hCoV-19/Japan/RIMD00284/2021	EPI_ISL_3503146	2021-08-19
hCoV-19/Japan/RIMD00285/2021	EPI_ISL_3503147	2021-08-19
hCoV-19/Japan/RIMD00286/2021	EPI_ISL_3503274	2021-08-19
hCoV-19/Japan/RIMD00287/2021	EPI_ISL_3503275	2021-08-19
hCoV-19/Japan/RIMD00288/2021	EPI_ISL_3503276	2021-08-19
hCoV-19/Japan/RIMD00289/2021	EPI_ISL_3503277	2021-08-19
hCoV-19/Japan/RIMD00290/2021	EPI_ISL_3503278	2021-08-19
hCoV-19/Japan/RIMD00291/2021	EPI_ISL_3503279	2021-08-19
hCoV-19/Japan/RIMD00292/2021	EPI_ISL_3503280	2021-08-19
hCoV-19/Japan/RIMD00293/2021	EPI_ISL_3503281	2021-08-19
hCoV-19/Japan/RIMD00294/2021	EPI_ISL_3503282	2021-08-19
hCoV-19/Japan/RIMD00295/2021	EPI_ISL_3503283	2021-08-19
hCoV-19/Japan/RIMD00296/2021	EPI_ISL_3503284	2021-08-19
hCoV-19/Japan/RIMD00297/2021	EPI_ISL_3503285	2021-08-19
hCoV-19/Japan/RIMD00298/2021	EPI_ISL_3503286	2021-08-19
hCoV-19/Japan/RIMD00299/2021	EPI_ISL_3503287	2021-08-19
hCoV-19/Japan/RIMD00300/2021	EPI_ISL_3503288	2021-08-19
hCoV-19/Japan/RIMD00301/2021	EPI_ISL_3503289	2021-08-19
hCoV-19/Japan/RIMD00302/2021	EPI_ISL_3503290	2021-08-19
hCoV-19/Japan/RIMD00303/2021	EPI_ISL_3503291	2021-08-19
hCoV-19/Japan/RIMD00304/2021	EPI_ISL_3502888	2021-08-19
hCoV-19/Japan/RIMD00305/2021	EPI_ISL_3502889	2021-08-19
hCoV-19/Japan/IC-1715/2021	EPI_ISL_3543168	2021-08-20
hCoV-19/Japan/IC-1716/2021	EPI_ISL_3543182	2021-08-20
hCoV-19/Japan/IC-1717/2021	EPI_ISL_3543178	2021-08-20
hCoV-19/Japan/IC-1718/2021	EPI_ISL_3543169	2021-08-20
hCoV-19/Japan/IC-1719/2021	EPI_ISL_3543160	2021-08-20
hCoV-19/Japan/IC-1720/2021	EPI_ISL_3543151	2021-08-20
hCoV-19/Japan/IC-1721/2021	EPI_ISL_3543141	2021-08-20
hCoV-19/Japan/IC-1722/2021	EPI_ISL_3543142	2021-08-20
hCoV-19/Japan/IC-1723/2021	EPI_ISL_3543179	2021-08-20
hCoV-19/Japan/IC-1724/2021	EPI_ISL_3543180	2021-08-20
hCoV-19/Japan/IC-1725/2021	EPI_ISL_3543181	2021-08-20
hCoV-19/Japan/IC-1726/2021	EPI_ISL_3543170	2021-08-20

virus_name	epiID	submission_date
hCoV-19/Japan/IC-1727/2021	EPI_ISL_3543171	2021-08-20
hCoV-19/Japan/IC-1728/2021	EPI_ISL_3543172	2021-08-20
hCoV-19/Japan/IC-1729/2021	EPI_ISL_3543161	2021-08-20
hCoV-19/Japan/IC-1730/2021	EPI_ISL_3543162	2021-08-20
hCoV-19/Japan/IC-1731/2021	EPI_ISL_3543152	2021-08-20
hCoV-19/Japan/IC-1732/2021	EPI_ISL_3543153	2021-08-20
hCoV-19/Japan/IC-1733/2021	EPI_ISL_3543154	2021-08-20
hCoV-19/Japan/IC-1734/2021	EPI_ISL_3543143	2021-08-20
hCoV-19/Japan/IC-1735/2021	EPI_ISL_3543144	2021-08-20
hCoV-19/Japan/IC-1736/2021	EPI_ISL_3543145	2021-08-20
hCoV-19/Japan/IC-1737/2021	EPI_ISL_3543132	2021-08-20
hCoV-19/Japan/IC-1738/2021	EPI_ISL_3543173	2021-08-20
hCoV-19/Japan/IC-1739/2021	EPI_ISL_3543163	2021-08-20
hCoV-19/Japan/IC-1740/2021	EPI_ISL_3543164	2021-08-20
hCoV-19/Japan/IC-1741/2021	EPI_ISL_3543165	2021-08-20
hCoV-19/Japan/IC-1742/2021	EPI_ISL_3543155	2021-08-20
hCoV-19/Japan/IC-1743/2021	EPI_ISL_3543156	2021-08-20
hCoV-19/Japan/IC-1744/2021	EPI_ISL_3543146	2021-08-20
hCoV-19/Japan/IC-1745/2021	EPI_ISL_3543133	2021-08-20
hCoV-19/Japan/IC-1746/2021	EPI_ISL_3543134	2021-08-20
hCoV-19/Japan/IC-1747/2021	EPI_ISL_3543135	2021-08-20
hCoV-19/Japan/IC-1748/2021	EPI_ISL_3543136	2021-08-20
hCoV-19/Japan/IC-1749/2021	EPI_ISL_3543137	2021-08-20
hCoV-19/Japan/IC-1750/2021	EPI_ISL_3543174	2021-08-20
hCoV-19/Japan/IC-1751/2021	EPI_ISL_3543175	2021-08-20
hCoV-19/Japan/IC-1752/2021	EPI_ISL_3543176	2021-08-20
hCoV-19/Japan/IC-1753/2021	EPI_ISL_3543177	2021-08-20
hCoV-19/Japan/IC-1754/2021	EPI_ISL_3543166	2021-08-20
hCoV-19/Japan/IC-1755/2021	EPI_ISL_3543167	2021-08-20
hCoV-19/Japan/IC-1756/2021	EPI_ISL_3543157	2021-08-20
hCoV-19/Japan/IC-1757/2021	EPI_ISL_3543158	2021-08-20
hCoV-19/Japan/IC-1758/2021	EPI_ISL_3543159	2021-08-20
hCoV-19/Japan/IC-1759/2021	EPI_ISL_3543147	2021-08-20
hCoV-19/Japan/IC-1760/2021	EPI_ISL_3543148	2021-08-20
hCoV-19/Japan/IC-1761/2021	EPI_ISL_3543149	2021-08-20
hCoV-19/Japan/IC-1762/2021	EPI_ISL_3543150	2021-08-20
hCoV-19/Japan/IC-1763/2021	EPI_ISL_3543138	2021-08-20
hCoV-19/Japan/IC-1764/2021	EPI_ISL_3543139	2021-08-20
hCoV-19/Japan/IC-1765/2021	EPI_ISL_3543140	2021-08-20
hCoV-19/Japan/IC-1766/2021	EPI_ISL_3543123	2021-08-20
hCoV-19/Japan/IC-1767/2021	EPI_ISL_3543124	2021-08-20
hCoV-19/Japan/IC-1768/2021	EPI_ISL_3543125	2021-08-20
hCoV-19/Japan/IC-1769/2021	EPI_ISL_3543126	2021-08-20
hCoV-19/Japan/IC-1770/2021	EPI_ISL_3543127	2021-08-20
hCoV-19/Japan/IC-1771/2021	EPI_ISL_3543128	2021-08-20
hCoV-19/Japan/IC-1772/2021	EPI_ISL_3543115	2021-08-20
hCoV-19/Japan/IC-1773/2021	EPI_ISL_3543116	2021-08-20
hCoV-19/Japan/IC-1774/2021	EPI_ISL_3543117	2021-08-20
hCoV-19/Japan/IC-1775/2021	EPI_ISL_3543118	2021-08-20
hCoV-19/Japan/IC-1776/2021	EPI_ISL_3543119	2021-08-20
hCoV-19/Japan/IC-1777/2021	EPI_ISL_3543129	2021-08-20
hCoV-19/Japan/IC-1778/2021	EPI_ISL_3543130	2021-08-20
hCoV-19/Japan/IC-1779/2021	EPI_ISL_3543131	2021-08-20
hCoV-19/Japan/IC-1780/2021	EPI_ISL_3543120	2021-08-20

virus_name	epiID	submission_date
hCoV-19/Japan/IC-1781/2021	EPI_ISL_3543121	2021-08-20
hCoV-19/Japan/IC-1782/2021	EPI_ISL_3543122	2021-08-20
hCoV-19/Japan/NIID-patho-20-247-1P0/2020	EPI_ISL_3541222	2021-08-20
hCoV-19/Japan/NIID-patho-20-312-36P0/2020	EPI_ISL_3541223	2021-08-20
hCoV-19/Japan/NIID-patho-20-312-38P0/2020	EPI_ISL_3541224	2021-08-20
hCoV-19/Japan/NIID-patho-20-312-39P0/2020	EPI_ISL_3541225	2021-08-20
hCoV-19/Japan/NIID-patho-21-144-18/2021	EPI_ISL_3541226	2021-08-20
hCoV-19/Japan/NIID-patho-21-144-35/2021	EPI_ISL_3541227	2021-08-20
hCoV-19/Japan/NIID-patho-21-144-41/2021	EPI_ISL_3541228	2021-08-20
hCoV-19/Japan/NIID-patho-21-144-48/2021	EPI_ISL_3541229	2021-08-20
hCoV-19/Japan/NIID-patho-21-180-8/2021	EPI_ISL_3541230	2021-08-20
hCoV-19/Japan/NIID-patho-21-189-2/2021	EPI_ISL_3541231	2021-08-20
hCoV-19/Japan/NIID-patho-CB21-009P0/2021	EPI_ISL_3541232	2021-08-20
hCoV-19/Japan/NIID-patho-CB21-033P0/2021	EPI_ISL_3541233	2021-08-20
hCoV-19/Japan/NIID-patho-CB21-059P0/2021	EPI_ISL_3541234	2021-08-20
hCoV-19/Japan/NIID-patho-CB21-062P0/2021	EPI_ISL_3541235	2021-08-20
hCoV-19/Japan/NIID-patho-CB21-075P0/2021	EPI_ISL_3541236	2021-08-20
hCoV-19/Japan/NIID-patho-CB21-077P0/2021	EPI_ISL_3541237	2021-08-20
hCoV-19/Japan/NIID-patho-CB21-078P0/2021	EPI_ISL_3541238	2021-08-20
hCoV-19/Japan/NIID-patho-CB21-094P0/2021	EPI_ISL_3541239	2021-08-20
hCoV-19/Japan/NIID-patho-CB21-095P0/2021	EPI_ISL_3541240	2021-08-20
hCoV-19/Japan/NIID-patho-CB21-102P0/2021	EPI_ISL_3541241	2021-08-20
hCoV-19/Japan/NIID-patho-CB21-103P0/2021	EPI_ISL_3541242	2021-08-20
hCoV-19/Japan/NIID-patho-CB21-108P0/2021	EPI_ISL_3541243	2021-08-20
hCoV-19/Japan/NIID-patho-CB21-110P0/2021	EPI_ISL_3541244	2021-08-20
hCoV-19/Japan/NIID-patho-CB21-111P0/2021	EPI_ISL_3541245	2021-08-20
hCoV-19/Japan/NIID-patho-CB21-113P0/2021	EPI_ISL_3541246	2021-08-20
hCoV-19/Japan/NIID-patho-CB21-115P0/2021	EPI_ISL_3541247	2021-08-20
hCoV-19/Japan/NIID-patho-CB21-125P0/2021	EPI_ISL_3541248	2021-08-20
hCoV-19/Japan/NIID-patho-CS4-15P0/2021	EPI_ISL_3541249	2021-08-20
hCoV-19/Japan/NIID-patho-CS4-18P0/2021	EPI_ISL_3541250	2021-08-20
hCoV-19/Japan/NIID-patho-CS4-20P0/2021	EPI_ISL_3541251	2021-08-20
hCoV-19/Japan/NIID-patho-CS4-21P0/2021	EPI_ISL_3541252	2021-08-20
hCoV-19/Japan/NIID-patho-CS4-26P0/2021	EPI_ISL_3541253	2021-08-20
hCoV-19/Japan/NIID-patho-CS4-28P0/2021	EPI_ISL_3541254	2021-08-20
hCoV-19/Japan/NIID-patho-CS4-9P0/2021	EPI_ISL_3541255	2021-08-20
hCoV-19/Japan/YCH0559/2021	EPI_ISL_3534412	2021-08-20
hCoV-19/Japan/YCH0560/2021	EPI_ISL_3534414	2021-08-20
hCoV-19/Japan/YCH0561/2021	EPI_ISL_3534416	2021-08-20
hCoV-19/Japan/YCH0562/2021	EPI_ISL_3534418	2021-08-20
hCoV-19/Japan/YCH0563/2021	EPI_ISL_3534420	2021-08-20
hCoV-19/Japan/YCH0564/2021	EPI_ISL_3534422	2021-08-20
hCoV-19/Japan/YCH0565/2021	EPI_ISL_3534424	2021-08-20
hCoV-19/Japan/YCH0566/2021	EPI_ISL_3534426	2021-08-20
hCoV-19/Japan/YCH0567/2021	EPI_ISL_3534428	2021-08-20
hCoV-19/Japan/YCH0568/2021	EPI_ISL_3534430	2021-08-20
hCoV-19/Japan/YCH0569/2021	EPI_ISL_3534432	2021-08-20
hCoV-19/Japan/YCH0570/2021	EPI_ISL_3534434	2021-08-20
hCoV-19/Japan/YCH0571/2021	EPI_ISL_3534436	2021-08-20
hCoV-19/Japan/YCH0572/2021	EPI_ISL_3534438	2021-08-20
hCoV-19/Japan/YCH0573/2021	EPI_ISL_3534440	2021-08-20
hCoV-19/Japan/YCH0574/2021	EPI_ISL_3534442	2021-08-20
hCoV-19/Japan/OPHO-ky-10-11/2021	EPI_ISL_3568731	2021-08-23
hCoV-19/Japan/OPHO-ky10-1/2021	EPI_ISL_3568625	2021-08-23

virus_name	epiID	submission_date
hCoV-19/Japan/OPHO-ky10-13/2021	EPI_ISL_3568732	2021-08-23
hCoV-19/Japan/OPHO-ky10-14/2021	EPI_ISL_3568733	2021-08-23
hCoV-19/Japan/OPHO-ky10-16/2021	EPI_ISL_3568734	2021-08-23
hCoV-19/Japan/OPHO-ky10-2/2021	EPI_ISL_3568844	2021-08-23
hCoV-19/Japan/OPHO-ky10-4/2021	EPI_ISL_3568845	2021-08-23
hCoV-19/Japan/OPHO-ky10-6/2021	EPI_ISL_3568627	2021-08-23
hCoV-19/Japan/OPHO-ky10-8/2021	EPI_ISL_3568867	2021-08-23
hCoV-19/Japan/OPHO-ky10-9/2021	EPI_ISL_3568639	2021-08-23
hCoV-19/Japan/OPHO-M23/2021	EPI_ISL_3568868	2021-08-23
hCoV-19/Japan/OPHO-M24/2021	EPI_ISL_3568870	2021-08-23
hCoV-19/Japan/CV00005609/2021	EPI_ISL_3655349	2021-08-24
hCoV-19/Japan/CV00005773/2021	EPI_ISL_3655365	2021-08-24
hCoV-19/Japan/CV00005925/2021	EPI_ISL_3655413	2021-08-24
hCoV-19/Japan/CV00006195/2021	EPI_ISL_3655366	2021-08-24
hCoV-19/Japan/CV00006641/2021	EPI_ISL_3655344	2021-08-24
hCoV-19/Japan/CV00038551/2021	EPI_ISL_3655460	2021-08-24
hCoV-19/Japan/CV00039051/2021	EPI_ISL_3655470	2021-08-24
hCoV-19/Japan/CV00040691/2021	EPI_ISL_3655360	2021-08-24
hCoV-19/Japan/CV00042126/2021	EPI_ISL_3655353	2021-08-24
hCoV-19/Japan/CV00043178/2021	EPI_ISL_3655409	2021-08-24
hCoV-19/Japan/CV00046376/2021	EPI_ISL_3655477	2021-08-24
hCoV-19/Japan/CV00046422/2021	EPI_ISL_3655421	2021-08-24
hCoV-19/Japan/CV00046512/2021	EPI_ISL_3655379	2021-08-24
hCoV-19/Japan/CV00046822/2021	EPI_ISL_3655414	2021-08-24
hCoV-19/Japan/CV00046880/2021	EPI_ISL_3655410	2021-08-24
hCoV-19/Japan/CV00047254/2021	EPI_ISL_3655357	2021-08-24
hCoV-19/Japan/CV00047276/2021	EPI_ISL_3655427	2021-08-24
hCoV-19/Japan/CV00047392/2021	EPI_ISL_3655397	2021-08-24
hCoV-19/Japan/CV00047565/2021	EPI_ISL_3655393	2021-08-24
hCoV-19/Japan/CV00047653/2021	EPI_ISL_3655474	2021-08-24
hCoV-19/Japan/CV00047773/2021	EPI_ISL_3655437	2021-08-24
hCoV-19/Japan/CV00047869/2021	EPI_ISL_3655411	2021-08-24
hCoV-19/Japan/CV00049531/2021	EPI_ISL_3655382	2021-08-24
hCoV-19/Japan/CV00049646/2021	EPI_ISL_3655428	2021-08-24
hCoV-19/Japan/CV00050921/2021	EPI_ISL_3655345	2021-08-24
hCoV-19/Japan/CV00052024/2021	EPI_ISL_3655415	2021-08-24
hCoV-19/Japan/CV00052220/2021	EPI_ISL_3655406	2021-08-24
hCoV-19/Japan/CV00052568/2021	EPI_ISL_3655383	2021-08-24
hCoV-19/Japan/CV00052647/2021	EPI_ISL_3655374	2021-08-24
hCoV-19/Japan/CV00052818/2021	EPI_ISL_3655372	2021-08-24
hCoV-19/Japan/CV00052882/2021	EPI_ISL_3655448	2021-08-24
hCoV-19/Japan/CV00054000/2021	EPI_ISL_3655358	2021-08-24
hCoV-19/Japan/CV00054001/2021	EPI_ISL_3655362	2021-08-24
hCoV-19/Japan/CV00054825/2021	EPI_ISL_3655375	2021-08-24
hCoV-19/Japan/CV00054827/2021	EPI_ISL_3655367	2021-08-24
hCoV-19/Japan/CV00055649/2021	EPI_ISL_3655398	2021-08-24
hCoV-19/Japan/CV00056017/2021	EPI_ISL_3655399	2021-08-24
hCoV-19/Japan/CV00056740/2021	EPI_ISL_3655438	2021-08-24
hCoV-19/Japan/CV00056788/2021	EPI_ISL_3655412	2021-08-24
hCoV-19/Japan/CV00059293/2021	EPI_ISL_3655407	2021-08-24
hCoV-19/Japan/CV00059711/2021	EPI_ISL_3655432	2021-08-24
hCoV-19/Japan/CV00059976/2021	EPI_ISL_3655451	2021-08-24
hCoV-19/Japan/CV00061124/2021	EPI_ISL_3655384	2021-08-24
hCoV-19/Japan/CV00062354/2021	EPI_ISL_3655385	2021-08-24

virus_name	epiID	submission_date
hCoV-19/Japan/CV00062389/2021	EPI_ISL_3655386	2021-08-24
hCoV-19/Japan/CV00062985/2021	EPI_ISL_3655394	2021-08-24
hCoV-19/Japan/CV00063007/2021	EPI_ISL_3655441	2021-08-24
hCoV-19/Japan/CV00063061/2021	EPI_ISL_3655452	2021-08-24
hCoV-19/Japan/CV00063583/2021	EPI_ISL_3655387	2021-08-24
hCoV-19/Japan/CV00063601/2021	EPI_ISL_3655449	2021-08-24
hCoV-19/Japan/CV00063678/2021	EPI_ISL_3655388	2021-08-24
hCoV-19/Japan/CV00063740/2021	EPI_ISL_3655454	2021-08-24
hCoV-19/Japan/CV00064150/2021	EPI_ISL_3655416	2021-08-24
hCoV-19/Japan/CV00064300/2021	EPI_ISL_3655370	2021-08-24
hCoV-19/Japan/CV00064517/2021	EPI_ISL_3655373	2021-08-24
hCoV-19/Japan/CV00064702/2021	EPI_ISL_3655377	2021-08-24
hCoV-19/Japan/CV00064896/2021	EPI_ISL_3655400	2021-08-24
hCoV-19/Japan/CV00065450/2021	EPI_ISL_3655389	2021-08-24
hCoV-19/Japan/CV00065823/2021	EPI_ISL_3655442	2021-08-24
hCoV-19/Japan/CV00065970/2021	EPI_ISL_3655433	2021-08-24
hCoV-19/Japan/CV00066058/2021	EPI_ISL_3655429	2021-08-24
hCoV-19/Japan/CV00066078/2021	EPI_ISL_3655434	2021-08-24
hCoV-19/Japan/CV00066380/2021	EPI_ISL_3655417	2021-08-24
hCoV-19/Japan/CV00066405/2021	EPI_ISL_3655418	2021-08-24
hCoV-19/Japan/CV00066448/2021	EPI_ISL_3655419	2021-08-24
hCoV-19/Japan/CV00066513/2021	EPI_ISL_3655443	2021-08-24
hCoV-19/Japan/CV00066646/2021	EPI_ISL_3655361	2021-08-24
hCoV-19/Japan/CV00066855/2021	EPI_ISL_3655395	2021-08-24
hCoV-19/Japan/CV00066905/2021	EPI_ISL_3655439	2021-08-24
hCoV-19/Japan/CV00067421/2021	EPI_ISL_3655455	2021-08-24
hCoV-19/Japan/CV00068854/2021	EPI_ISL_3655456	2021-08-24
hCoV-19/Japan/CV00069080/2021	EPI_ISL_3655390	2021-08-24
hCoV-19/Japan/CV00069347/2021	EPI_ISL_3655440	2021-08-24
hCoV-19/Japan/CV00071774/2021	EPI_ISL_3655401	2021-08-24
hCoV-19/Japan/CV00072007/2021	EPI_ISL_3655402	2021-08-24
hCoV-19/Japan/CV00072037/2021	EPI_ISL_3655403	2021-08-24
hCoV-19/Japan/CV00072809/2021	EPI_ISL_3655444	2021-08-24
hCoV-19/Japan/CV00075386/2021	EPI_ISL_3655364	2021-08-24
hCoV-19/Japan/CV00075987/2021	EPI_ISL_3655478	2021-08-24
hCoV-19/Japan/CV00077341/2021	EPI_ISL_3655469	2021-08-24
hCoV-19/Japan/CV00078222/2021	EPI_ISL_3655430	2021-08-24
hCoV-19/Japan/CV00078223/2021	EPI_ISL_3655431	2021-08-24
hCoV-19/Japan/CV00078406/2021	EPI_ISL_3655422	2021-08-24
hCoV-19/Japan/CV00079502/2021	EPI_ISL_3655435	2021-08-24
hCoV-19/Japan/CV00079782/2021	EPI_ISL_3655453	2021-08-24
hCoV-19/Japan/CV00080495/2021	EPI_ISL_3655354	2021-08-24
hCoV-19/Japan/CV00081721/2021	EPI_ISL_3655404	2021-08-24
hCoV-19/Japan/CV00083453/2021	EPI_ISL_3655350	2021-08-24
hCoV-19/Japan/CV00084587/2021	EPI_ISL_3655378	2021-08-24
hCoV-19/Japan/CV00084632/2021	EPI_ISL_3655371	2021-08-24
hCoV-19/Japan/CV00084911/2021	EPI_ISL_3655368	2021-08-24
hCoV-19/Japan/CV00085802/2021	EPI_ISL_3655376	2021-08-24
hCoV-19/Japan/CV00087463/2021	EPI_ISL_3655405	2021-08-24
hCoV-19/Japan/CV00087546/2021	EPI_ISL_3655380	2021-08-24
hCoV-19/Japan/CV00087692/2021	EPI_ISL_3655459	2021-08-24
hCoV-19/Japan/CV00087794/2021	EPI_ISL_3655381	2021-08-24
hCoV-19/Japan/CV00087930/2021	EPI_ISL_3655391	2021-08-24
hCoV-19/Japan/CV00089003/2021	EPI_ISL_3655462	2021-08-24

virus_name	epiID	submission_date
hCoV-19/Japan/CV00089005/2021	EPI_ISL_3655463	2021-08-24
hCoV-19/Japan/CV00089010/2021	EPI_ISL_3655464	2021-08-24
hCoV-19/Japan/CV00089011/2021	EPI_ISL_3655465	2021-08-24
hCoV-19/Japan/CV00106473/2021	EPI_ISL_3655458	2021-08-24
hCoV-19/Japan/MV00005281/2021	EPI_ISL_3655348	2021-08-24
hCoV-19/Japan/MV00010697/2021	EPI_ISL_3655346	2021-08-24
hCoV-19/Japan/MV00010818/2021	EPI_ISL_3655347	2021-08-24
hCoV-19/Japan/MV00010858/2021	EPI_ISL_3655363	2021-08-24
hCoV-19/Japan/MV00010886/2021	EPI_ISL_3655355	2021-08-24
hCoV-19/Japan/MV00011241/2021	EPI_ISL_3655392	2021-08-24
hCoV-19/Japan/MV00011276/2021	EPI_ISL_3655396	2021-08-24
hCoV-19/Japan/MV00011291/2021	EPI_ISL_3655408	2021-08-24
hCoV-19/Japan/MV00011320/2021	EPI_ISL_3655423	2021-08-24
hCoV-19/Japan/MV00011321/2021	EPI_ISL_3655424	2021-08-24
hCoV-19/Japan/MV00011323/2021	EPI_ISL_3655425	2021-08-24
hCoV-19/Japan/MV00011331/2021	EPI_ISL_3655426	2021-08-24
hCoV-19/Japan/MV00011502/2021	EPI_ISL_3655445	2021-08-24
hCoV-19/Japan/MV00011505/2021	EPI_ISL_3655446	2021-08-24
hCoV-19/Japan/MV00011515/2021	EPI_ISL_3655447	2021-08-24
hCoV-19/Japan/MV00011517/2021	EPI_ISL_3655450	2021-08-24
hCoV-19/Japan/MV00011535/2021	EPI_ISL_3655457	2021-08-24
hCoV-19/Japan/MV00011571/2021	EPI_ISL_3655461	2021-08-24
hCoV-19/Japan/MV00011581/2021	EPI_ISL_3655466	2021-08-24
hCoV-19/Japan/MV00011582/2021	EPI_ISL_3655467	2021-08-24
hCoV-19/Japan/MV00011599/2021	EPI_ISL_3655468	2021-08-24
hCoV-19/Japan/MV00011609/2021	EPI_ISL_3655471	2021-08-24
hCoV-19/Japan/MV00011630/2021	EPI_ISL_3655473	2021-08-24
hCoV-19/Japan/MV00011634/2021	EPI_ISL_3655475	2021-08-24
hCoV-19/Japan/MV00011641/2021	EPI_ISL_3655476	2021-08-24
hCoV-19/Japan/MV00014747/2021	EPI_ISL_3655351	2021-08-24
hCoV-19/Japan/MV00014751/2021	EPI_ISL_3655356	2021-08-24
hCoV-19/Japan/MV00014771/2021	EPI_ISL_3655359	2021-08-24
hCoV-19/Japan/MV00014782/2021	EPI_ISL_3655369	2021-08-24
hCoV-19/Japan/MV00015348/2021	EPI_ISL_3655352	2021-08-24
hCoV-19/Japan/MV00015496/2021	EPI_ISL_3655420	2021-08-24
hCoV-19/Japan/MV00016182/2021	EPI_ISL_3655436	2021-08-24
hCoV-19/Japan/MV00016413/2021	EPI_ISL_3655472	2021-08-24
hCoV-19/Japan/RIMD00306/2021	EPI_ISL_3689371	2021-08-25
hCoV-19/Japan/TKYK20623/2021	EPI_ISL_3673682	2021-08-25
hCoV-19/Japan/TKYK20785/2021	EPI_ISL_3673683	2021-08-25
hCoV-19/Japan/TKYK20786/2021	EPI_ISL_3673684	2021-08-25
hCoV-19/Japan/TKYK20856/2021	EPI_ISL_3673685	2021-08-25
hCoV-19/Japan/TKYK20958/2021	EPI_ISL_3673690	2021-08-25
hCoV-19/Japan/TKYK20959/2021	EPI_ISL_3673691	2021-08-25
hCoV-19/Japan/TKYK20964/2021	EPI_ISL_3673692	2021-08-25
hCoV-19/Japan/TKYK21063/2021	EPI_ISL_3673693	2021-08-25
hCoV-19/Japan/TKYK21064/2021	EPI_ISL_3673694	2021-08-25
hCoV-19/Japan/TKYK21231/2021	EPI_ISL_3673695	2021-08-25
hCoV-19/Japan/TKYK21527/2021	EPI_ISL_3673696	2021-08-25
hCoV-19/Japan/TKYK25111/2021	EPI_ISL_3673697	2021-08-25
hCoV-19/Japan/TKYK25338/2021	EPI_ISL_3673698	2021-08-25
hCoV-19/Japan/TKYK25470/2021	EPI_ISL_3673701	2021-08-25
hCoV-19/Japan/TKYK25471/2021	EPI_ISL_3673702	2021-08-25
hCoV-19/Japan/TKYK25497/2021	EPI_ISL_3673703	2021-08-25

virus_name	epiID	submission_date
hCoV-19/Japan/TKYK26070/2021	EPI_ISL_3673704	2021-08-25
hCoV-19/Japan/TKYS00441/2021	EPI_ISL_3673686	2021-08-25
hCoV-19/Japan/TKYS00442/2021	EPI_ISL_3673687	2021-08-25
hCoV-19/Japan/TKYS00444/2021	EPI_ISL_3673688	2021-08-25
hCoV-19/Japan/TKYS00445/2021	EPI_ISL_3673689	2021-08-25
hCoV-19/Japan/TKYS00467/2021	EPI_ISL_3673699	2021-08-25
hCoV-19/Japan/TKYS00468/2021	EPI_ISL_3673700	2021-08-25
hCoV-19/Japan/OPHO-ky11-11/2021	EPI_ISL_3707870	2021-08-26
hCoV-19/Japan/OPHO-ky11-1/2021	EPI_ISL_3707862	2021-08-26
hCoV-19/Japan/OPHO-ky11-12/2021	EPI_ISL_3707871	2021-08-26
hCoV-19/Japan/OPHO-ky11-14/2021	EPI_ISL_3707872	2021-08-26
hCoV-19/Japan/OPHO-ky11-15/2021	EPI_ISL_3707873	2021-08-26
hCoV-19/Japan/OPHO-ky11-2/2021	EPI_ISL_3707863	2021-08-26
hCoV-19/Japan/OPHO-ky11-3/2021	EPI_ISL_3707864	2021-08-26
hCoV-19/Japan/OPHO-Ky11-4/2021	EPI_ISL_3707865	2021-08-26
hCoV-19/Japan/OPHO-ky11-5/2021	EPI_ISL_3707866	2021-08-26
hCoV-19/Japan/OPHO-ky11-6/2021	EPI_ISL_3707867	2021-08-26
hCoV-19/Japan/OPHO-ky11-7/2021	EPI_ISL_3707868	2021-08-26
hCoV-19/Japan/OPHO-ky11-8/2021	EPI_ISL_3707869	2021-08-26
hCoV-19/Japan/OPHO-M20/2021	EPI_ISL_3707875	2021-08-26
hCoV-19/Japan/OPHO-M25/2021	EPI_ISL_3707876	2021-08-26
hCoV-19/Japan/OPHO-M27/2021	EPI_ISL_3707878	2021-08-26
hCoV-19/Japan/YCH0575/2021	EPI_ISL_3706138	2021-08-26
hCoV-19/Japan/YCH0576/2021	EPI_ISL_3706142	2021-08-26
hCoV-19/Japan/YCH0577/2021	EPI_ISL_3706146	2021-08-26
hCoV-19/Japan/YCH0578/2021	EPI_ISL_3706149	2021-08-26
hCoV-19/Japan/YCH0579/2021	EPI_ISL_3706153	2021-08-26
hCoV-19/Japan/YCH0580/2021	EPI_ISL_3706157	2021-08-26
hCoV-19/Japan/YCH0581/2021	EPI_ISL_3706160	2021-08-26
hCoV-19/Japan/YCH0582/2021	EPI_ISL_3706164	2021-08-26
hCoV-19/Japan/YCH0583/2021	EPI_ISL_3706168	2021-08-26
hCoV-19/Japan/YCH0584/2021	EPI_ISL_3706172	2021-08-26
hCoV-19/Japan/YCH0585/2021	EPI_ISL_3706175	2021-08-26
hCoV-19/Japan/YCH0586/2021	EPI_ISL_3706179	2021-08-26
hCoV-19/Japan/YCH0587/2021	EPI_ISL_3706183	2021-08-26
hCoV-19/Japan/YCH0588/2021	EPI_ISL_3706191	2021-08-26
hCoV-19/Japan/YCH0589/2021	EPI_ISL_3706195	2021-08-26
hCoV-19/Japan/YCH0590/2021	EPI_ISL_3706199	2021-08-26
hCoV-19/Japan/CV00022528/2021	EPI_ISL_3737277	2021-08-27
hCoV-19/Japan/CV00022529/2021	EPI_ISL_3737314	2021-08-27
hCoV-19/Japan/CV00022534/2021	EPI_ISL_3737290	2021-08-27
hCoV-19/Japan/CV00022991/2021	EPI_ISL_3737279	2021-08-27
hCoV-19/Japan/CV00025481/2021	EPI_ISL_3737309	2021-08-27
hCoV-19/Japan/CV00025492/2021	EPI_ISL_3737284	2021-08-27
hCoV-19/Japan/CV00036856/2020	EPI_ISL_3737316	2021-08-27
hCoV-19/Japan/CV00037161/2021	EPI_ISL_3737289	2021-08-27
hCoV-19/Japan/CV00037536/2021	EPI_ISL_3737291	2021-08-27
hCoV-19/Japan/CV00037537/2020	EPI_ISL_3737287	2021-08-27
hCoV-19/Japan/CV00037583/2020	EPI_ISL_3737313	2021-08-27
hCoV-19/Japan/CV00037588/2020	EPI_ISL_3737311	2021-08-27
hCoV-19/Japan/CV00040058/2021	EPI_ISL_3737271	2021-08-27
hCoV-19/Japan/CV00042270/2021	EPI_ISL_3737297	2021-08-27
hCoV-19/Japan/CV00043932/2021	EPI_ISL_3737317	2021-08-27
hCoV-19/Japan/CV00047814/2021	EPI_ISL_3737307	2021-08-27

virus_name	epiID	submission_date
hCoV-19/Japan/CV00048749/2021	EPI_ISL_3737293	2021-08-27
hCoV-19/Japan/CV00051275/2021	EPI_ISL_3737301	2021-08-27
hCoV-19/Japan/CV00051357/2021	EPI_ISL_3737312	2021-08-27
hCoV-19/Japan/CV00054437/2021	EPI_ISL_3737300	2021-08-27
hCoV-19/Japan/CV00080686/2021	EPI_ISL_3737270	2021-08-27
hCoV-19/Japan/CV00082365/2021	EPI_ISL_3737292	2021-08-27
hCoV-19/Japan/CV00082500/2021	EPI_ISL_3737320	2021-08-27
hCoV-19/Japan/CV00082526/2020	EPI_ISL_3737302	2021-08-27
hCoV-19/Japan/CV00082658/2021	EPI_ISL_3737306	2021-08-27
hCoV-19/Japan/CV00083203/2021	EPI_ISL_3737298	2021-08-27
hCoV-19/Japan/CV00083553/2021	EPI_ISL_3737278	2021-08-27
hCoV-19/Japan/CV00085310/2021	EPI_ISL_3737275	2021-08-27
hCoV-19/Japan/CV00085315/2021	EPI_ISL_3737295	2021-08-27
hCoV-19/Japan/CV00085730/2021	EPI_ISL_3737273	2021-08-27
hCoV-19/Japan/CV00085731/2021	EPI_ISL_3737305	2021-08-27
hCoV-19/Japan/CV00086923/2021	EPI_ISL_3737276	2021-08-27
hCoV-19/Japan/CV00087041/2021	EPI_ISL_3737283	2021-08-27
hCoV-19/Japan/CV00090311/2021	EPI_ISL_3737288	2021-08-27
hCoV-19/Japan/CV00090342/2020	EPI_ISL_3737280	2021-08-27
hCoV-19/Japan/CV00090757/2020	EPI_ISL_3737269	2021-08-27
hCoV-19/Japan/CV00090823/2020	EPI_ISL_3737319	2021-08-27
hCoV-19/Japan/CV00090826/2020	EPI_ISL_3737310	2021-08-27
hCoV-19/Japan/CV00090891/2020	EPI_ISL_3737318	2021-08-27
hCoV-19/Japan/CV00090894/2020	EPI_ISL_3737303	2021-08-27
hCoV-19/Japan/CV00090896/2020	EPI_ISL_3737282	2021-08-27
hCoV-19/Japan/CV00090897/2021	EPI_ISL_3737304	2021-08-27
hCoV-19/Japan/CV00091775/2021	EPI_ISL_3737272	2021-08-27
hCoV-19/Japan/CV00093172/2021	EPI_ISL_3737315	2021-08-27
hCoV-19/Japan/CV00096469/2020	EPI_ISL_3737299	2021-08-27
hCoV-19/Japan/CV00097097/2021	EPI_ISL_3737274	2021-08-27
hCoV-19/Japan/CV00097099/2021	EPI_ISL_3737281	2021-08-27
hCoV-19/Japan/CV00097174/2020	EPI_ISL_3737308	2021-08-27
hCoV-19/Japan/CV00098960/2021	EPI_ISL_3737285	2021-08-27
hCoV-19/Japan/CV00098997/2021	EPI_ISL_3737286	2021-08-27
hCoV-19/Japan/CV00099332/2021	EPI_ISL_3737296	2021-08-27
hCoV-19/Japan/CV00150713/2021	EPI_ISL_3737294	2021-08-27
hCoV-19/Japan/OPHO-M26-2/2021	EPI_ISL_3722428	2021-08-27
hCoV-19/Japan/IC-1783/2021	EPI_ISL_3769583	2021-08-30
hCoV-19/Japan/IC-1784/2021	EPI_ISL_3769584	2021-08-30
hCoV-19/Japan/IC-1785/2021	EPI_ISL_3769585	2021-08-30
hCoV-19/Japan/IC-1786/2021	EPI_ISL_3769586	2021-08-30
hCoV-19/Japan/IC-1787/2021	EPI_ISL_3769587	2021-08-30
hCoV-19/Japan/IC-1788/2021	EPI_ISL_3769588	2021-08-30
hCoV-19/Japan/IC-1789/2021	EPI_ISL_3769589	2021-08-30
hCoV-19/Japan/IC-1790/2021	EPI_ISL_3769590	2021-08-30
hCoV-19/Japan/IC-1791/2021	EPI_ISL_3769591	2021-08-30
hCoV-19/Japan/IC-1792/2021	EPI_ISL_3769592	2021-08-30
hCoV-19/Japan/IC-1793/2021	EPI_ISL_3769593	2021-08-30
hCoV-19/Japan/IC-1794/2021	EPI_ISL_3769594	2021-08-30
hCoV-19/Japan/IC-1795/2021	EPI_ISL_3769595	2021-08-30
hCoV-19/Japan/IC-1796/2021	EPI_ISL_3769596	2021-08-30
hCoV-19/Japan/IC-1797/2021	EPI_ISL_3769597	2021-08-30
hCoV-19/Japan/IC-1798/2021	EPI_ISL_3769598	2021-08-30
hCoV-19/Japan/IC-1799/2021	EPI_ISL_3769599	2021-08-30

virus_name	epiID	submission_date
hCoV-19/Japan/IC-1800/2021	EPI_ISL_3769600	2021-08-30
hCoV-19/Japan/IC-1801/2021	EPI_ISL_3769601	2021-08-30
hCoV-19/Japan/IC-1802/2021	EPI_ISL_3769602	2021-08-30
hCoV-19/Japan/IC-1803/2021	EPI_ISL_3769603	2021-08-30
hCoV-19/Japan/IC-1804/2021	EPI_ISL_3769604	2021-08-30
hCoV-19/Japan/IC-1805/2021	EPI_ISL_3769605	2021-08-30
hCoV-19/Japan/IC-1806/2021	EPI_ISL_3769606	2021-08-30
hCoV-19/Japan/IC-1807/2021	EPI_ISL_3769607	2021-08-30
hCoV-19/Japan/IC-1808/2021	EPI_ISL_3769608	2021-08-30
hCoV-19/Japan/IC-1809/2021	EPI_ISL_3769609	2021-08-30
hCoV-19/Japan/IC-1810/2021	EPI_ISL_3769610	2021-08-30
hCoV-19/Japan/IC-1811/2021	EPI_ISL_3769611	2021-08-30
hCoV-19/Japan/IC-1812/2021	EPI_ISL_3769612	2021-08-30
hCoV-19/Japan/IC-1813/2021	EPI_ISL_3769613	2021-08-30
hCoV-19/Japan/IC-1814/2021	EPI_ISL_3769614	2021-08-30
hCoV-19/Japan/IC-1815/2021	EPI_ISL_3769615	2021-08-30
hCoV-19/Japan/IC-1816/2021	EPI_ISL_3769616	2021-08-30
hCoV-19/Japan/IC-1817/2021	EPI_ISL_3769617	2021-08-30
hCoV-19/Japan/IC-1818/2021	EPI_ISL_3769618	2021-08-30
hCoV-19/Japan/IC-1819/2021	EPI_ISL_3769619	2021-08-30
hCoV-19/Japan/IC-1820/2021	EPI_ISL_3769620	2021-08-30
hCoV-19/Japan/IC-1821/2021	EPI_ISL_3769621	2021-08-30
hCoV-19/Japan/IC-1822/2021	EPI_ISL_3769622	2021-08-30
hCoV-19/Japan/IC-1823/2021	EPI_ISL_3769623	2021-08-30
hCoV-19/Japan/IC-1824/2021	EPI_ISL_3769624	2021-08-30
hCoV-19/Japan/IC-1825/2021	EPI_ISL_3769625	2021-08-30
hCoV-19/Japan/IC-1826/2021	EPI_ISL_3769626	2021-08-30
hCoV-19/Japan/IC-1827/2021	EPI_ISL_3769627	2021-08-30
hCoV-19/Japan/IC-1828/2021	EPI_ISL_3769628	2021-08-30
hCoV-19/Japan/IC-1829/2021	EPI_ISL_3769629	2021-08-30
hCoV-19/Japan/IC-1830/2021	EPI_ISL_3769630	2021-08-30
hCoV-19/Japan/IC-1831/2021	EPI_ISL_3769631	2021-08-30
hCoV-19/Japan/IC-1832/2021	EPI_ISL_3769632	2021-08-30
hCoV-19/Japan/IC-1833/2021	EPI_ISL_3769633	2021-08-30
hCoV-19/Japan/IC-1834/2021	EPI_ISL_3769634	2021-08-30
hCoV-19/Japan/IC-1835/2021	EPI_ISL_3769635	2021-08-30
hCoV-19/Japan/IC-1836/2021	EPI_ISL_3769636	2021-08-30
hCoV-19/Japan/IC-1837/2021	EPI_ISL_3769637	2021-08-30
hCoV-19/Japan/IC-1838/2021	EPI_ISL_3769638	2021-08-30
hCoV-19/Japan/IC-1839/2021	EPI_ISL_3769639	2021-08-30
hCoV-19/Japan/OPHO-hb8-62/2021	EPI_ISL_3772541	2021-08-30
hCoV-19/Japan/OPHO-hb8-64/2021	EPI_ISL_3773143	2021-08-30
hCoV-19/Japan/OPHO-hb8-66/2021	EPI_ISL_3773412	2021-08-30
hCoV-19/Japan/OPHO-hb8-67/2021	EPI_ISL_3773809	2021-08-30
hCoV-19/Japan/OPHO-hb8-69/2021	EPI_ISL_3774128	2021-08-30
hCoV-19/Japan/OPHO-hb8-71/2021	EPI_ISL_3774500	2021-08-30
hCoV-19/Japan/OPHO-hb8-73/2021	EPI_ISL_3774835	2021-08-30
hCoV-19/Japan/OPHO-hb8-74/2021	EPI_ISL_3775095	2021-08-30
hCoV-19/Japan/OPHO-hb8-76/2021	EPI_ISL_3775600	2021-08-30
hCoV-19/Japan/OPHO-hb8-77/2021	EPI_ISL_3775911	2021-08-30
hCoV-19/Japan/OPHO-hb8-78/2021	EPI_ISL_3776268	2021-08-30
hCoV-19/Japan/OPHO-hb8-79/2021	EPI_ISL_3776565	2021-08-30
hCoV-19/Japan/OPHO-hb8-80/2021	EPI_ISL_3776900	2021-08-30
hCoV-19/Japan/OPHO-hb8-81/2021	EPI_ISL_3777341	2021-08-30

virus_name	epiID	submission_date
hCoV-19/Japan/OPHO-hb8-82/2021	EPI_ISL_3777910	2021-08-30
hCoV-19/Japan/OPHO-hb8-83/2021	EPI_ISL_3778506	2021-08-30
hCoV-19/Japan/OPHO-hb8-85/2021	EPI_ISL_3778944	2021-08-30
hCoV-19/Japan/RIMD00307/2021	EPI_ISL_3799882	2021-08-30
hCoV-19/Japan/RIMD00308/2021	EPI_ISL_3799883	2021-08-30
hCoV-19/Japan/RIMD00309/2021	EPI_ISL_3799884	2021-08-30
hCoV-19/Japan/RIMD00310/2021	EPI_ISL_3799885	2021-08-30
hCoV-19/Japan/RIMD00311/2021	EPI_ISL_3799886	2021-08-30
hCoV-19/Japan/RIMD00312/2021	EPI_ISL_3799887	2021-08-30
hCoV-19/Japan/RIMD00313/2021	EPI_ISL_3799888	2021-08-30
hCoV-19/Japan/RIMD00314/2021	EPI_ISL_3799889	2021-08-30
hCoV-19/Japan/RIMD00315/2021	EPI_ISL_3799890	2021-08-30
hCoV-19/Japan/RIMD00317/2021	EPI_ISL_3799891	2021-08-30
hCoV-19/Japan/RIMD00318/2021	EPI_ISL_3799892	2021-08-30
hCoV-19/Japan/RIMD00319/2021	EPI_ISL_3799893	2021-08-30
hCoV-19/Japan/RIMD00320/2021	EPI_ISL_3799894	2021-08-30
hCoV-19/Japan/RIMD00321/2021	EPI_ISL_3799895	2021-08-30
hCoV-19/Japan/RIMD00322/2021	EPI_ISL_3799896	2021-08-30
hCoV-19/Japan/RIMD00323/2021	EPI_ISL_3799897	2021-08-30
hCoV-19/Japan/RIMD00324/2021	EPI_ISL_3799898	2021-08-30
hCoV-19/Japan/RIMD00325/2021	EPI_ISL_3799899	2021-08-30
hCoV-19/Japan/RIMD00326/2021	EPI_ISL_3799900	2021-08-30
hCoV-19/Japan/RIMD00327/2021	EPI_ISL_3799901	2021-08-30
hCoV-19/Japan/RIMD00328/2021	EPI_ISL_3799902	2021-08-30
hCoV-19/Japan/RIMD00329/2021	EPI_ISL_3799903	2021-08-30
hCoV-19/Japan/RIMD00330/2021	EPI_ISL_3799904	2021-08-30
hCoV-19/Japan/RIMD00331/2021	EPI_ISL_3799905	2021-08-30
hCoV-19/Japan/RIMD00332/2021	EPI_ISL_3799906	2021-08-30
hCoV-19/Japan/RIMD00333/2021	EPI_ISL_3799907	2021-08-30
hCoV-19/Japan/RIMD00334/2021	EPI_ISL_3799908	2021-08-30
hCoV-19/Japan/RIMD00335/2021	EPI_ISL_3799909	2021-08-30
hCoV-19/Japan/RIMD00336/2021	EPI_ISL_3799910	2021-08-30
hCoV-19/Japan/RIMD00337/2021	EPI_ISL_3799911	2021-08-30
hCoV-19/Japan/RIMD00338/2021	EPI_ISL_3799912	2021-08-30
hCoV-19/Japan/RIMD00339/2021	EPI_ISL_3799913	2021-08-30
hCoV-19/Japan/RIMD00340/2021	EPI_ISL_3799914	2021-08-30
hCoV-19/Japan/RIMD00341/2021	EPI_ISL_3799915	2021-08-30
hCoV-19/Japan/RIMD00342/2021	EPI_ISL_3799916	2021-08-30
hCoV-19/Japan/RIMD00343/2021	EPI_ISL_3799917	2021-08-30
hCoV-19/Japan/RIMD00344/2021	EPI_ISL_3799918	2021-08-30
hCoV-19/Japan/RIMD00345/2021	EPI_ISL_3799919	2021-08-30
hCoV-19/Japan/RIMD00346/2021	EPI_ISL_3799920	2021-08-30
hCoV-19/Japan/RIMD00347/2021	EPI_ISL_3799921	2021-08-30
hCoV-19/Japan/RIMD00348/2021	EPI_ISL_3799922	2021-08-30
hCoV-19/Japan/RIMD00349/2021	EPI_ISL_3799923	2021-08-30
hCoV-19/Japan/RIMD00350/2021	EPI_ISL_3799924	2021-08-30
hCoV-19/Japan/RIMD00351/2021	EPI_ISL_3799925	2021-08-30
hCoV-19/Japan/RIMD00352/2021	EPI_ISL_3799926	2021-08-30
hCoV-19/Japan/RIMD00353/2021	EPI_ISL_3799928	2021-08-30
hCoV-19/Japan/RIMD00354/2021	EPI_ISL_3799929	2021-08-30
hCoV-19/Japan/RIMD00355/2021	EPI_ISL_3799930	2021-08-30
hCoV-19/Japan/RIMD00356/2021	EPI_ISL_3799931	2021-08-30
hCoV-19/Japan/RIMD00357/2021	EPI_ISL_3799932	2021-08-30
hCoV-19/Japan/RIMD00358/2021	EPI_ISL_3799933	2021-08-30

virus_name	epiID	submission_date
hCoV-19/Japan/RIMD00359/2021	EPI_ISL_3799934	2021-08-30
hCoV-19/Japan/RIMD00360/2021	EPI_ISL_3799935	2021-08-30
hCoV-19/Japan/RIMD00361/2021	EPI_ISL_3799936	2021-08-30
hCoV-19/Japan/RIMD00362/2021	EPI_ISL_3799937	2021-08-30
hCoV-19/Japan/RIMD00363/2021	EPI_ISL_3799938	2021-08-30
hCoV-19/Japan/RIMD00364/2021	EPI_ISL_3799939	2021-08-30
hCoV-19/Japan/RIMD00365/2021	EPI_ISL_3799940	2021-08-30
hCoV-19/Japan/RIMD00366/2021	EPI_ISL_3799941	2021-08-30
hCoV-19/Japan/RIMD00367/2021	EPI_ISL_3799942	2021-08-30
hCoV-19/Japan/RIMD00368/2021	EPI_ISL_3799943	2021-08-30
hCoV-19/Japan/RIMD00369/2021	EPI_ISL_3799944	2021-08-30
hCoV-19/Japan/RIMD00370/2021	EPI_ISL_3799945	2021-08-30
hCoV-19/Japan/RIMD00371/2021	EPI_ISL_3799946	2021-08-30
hCoV-19/Japan/RIMD00372/2021	EPI_ISL_3799947	2021-08-30
hCoV-19/Japan/RIMD00373/2021	EPI_ISL_3799948	2021-08-30
hCoV-19/Japan/RIMD00374/2021	EPI_ISL_3799949	2021-08-30
hCoV-19/Japan/RIMD00375/2021	EPI_ISL_3799950	2021-08-30
hCoV-19/Japan/RIMD00376/2021	EPI_ISL_3799951	2021-08-30
hCoV-19/Japan/RIMD00377/2021	EPI_ISL_3799952	2021-08-30
hCoV-19/Japan/RIMD00378/2021	EPI_ISL_3799953	2021-08-30
hCoV-19/Japan/RIMD00379/2021	EPI_ISL_3799954	2021-08-30
hCoV-19/Japan/RIMD00380/2021	EPI_ISL_3799955	2021-08-30
hCoV-19/Japan/RIMD00381/2021	EPI_ISL_3799956	2021-08-30
hCoV-19/Japan/RIMD00382/2021	EPI_ISL_3799957	2021-08-30
hCoV-19/Japan/RIMD00383/2021	EPI_ISL_3799958	2021-08-30
hCoV-19/Japan/RIMD00384/2021	EPI_ISL_3799959	2021-08-30
hCoV-19/Japan/RIMD00385/2021	EPI_ISL_3799960	2021-08-30
hCoV-19/Japan/RIMD00386/2021	EPI_ISL_3799961	2021-08-30
hCoV-19/Japan/RIMD00387/2021	EPI_ISL_3799962	2021-08-30
hCoV-19/Japan/RIMD00388/2021	EPI_ISL_3799963	2021-08-30
hCoV-19/Japan/RIMD00389/2021	EPI_ISL_3799964	2021-08-30
hCoV-19/Japan/RIMD00390/2021	EPI_ISL_3799965	2021-08-30
hCoV-19/Japan/RIMD00391/2021	EPI_ISL_3799966	2021-08-30
hCoV-19/Japan/RIMD00392/2021	EPI_ISL_3799967	2021-08-30
hCoV-19/Japan/RIMD00393/2021	EPI_ISL_3799968	2021-08-30
hCoV-19/Japan/RIMD00394/2021	EPI_ISL_3799969	2021-08-30
hCoV-19/Japan/RIMD00395/2021	EPI_ISL_3799970	2021-08-30
hCoV-19/Japan/RIMD00396/2021	EPI_ISL_3799971	2021-08-30
hCoV-19/Japan/RIMD00397/2021	EPI_ISL_3799972	2021-08-30
hCoV-19/Japan/RIMD00398/2021	EPI_ISL_3799973	2021-08-30
hCoV-19/Japan/RIMD00399/2021	EPI_ISL_3799974	2021-08-30
hCoV-19/Japan/YCH0591/2021	EPI_ISL_3827026	2021-08-31
hCoV-19/Japan/YCH0592/2021	EPI_ISL_3827046	2021-08-31
hCoV-19/Japan/YCH0593/2021	EPI_ISL_3827036	2021-08-31
hCoV-19/Japan/YCH0594/2021	EPI_ISL_3827045	2021-08-31
hCoV-19/Japan/YCH0595/2021	EPI_ISL_3827038	2021-08-31
hCoV-19/Japan/YCH0596/2021	EPI_ISL_3827025	2021-08-31
hCoV-19/Japan/YCH0597/2021	EPI_ISL_3827020	2021-08-31
hCoV-19/Japan/YCH0598/2021	EPI_ISL_3827035	2021-08-31
hCoV-19/Japan/YCH0599/2021	EPI_ISL_3827021	2021-08-31
hCoV-19/Japan/YCH0600/2021	EPI_ISL_3827022	2021-08-31
hCoV-19/Japan/YCH0601/2021	EPI_ISL_3827023	2021-08-31
hCoV-19/Japan/YCH0602/2021	EPI_ISL_3827043	2021-08-31
hCoV-19/Japan/YCH0603/2021	EPI_ISL_3827044	2021-08-31

virus_name	epiID	submission_date
hCoV-19/Japan/YCH0604/2021	EPI_ISL_3827024	2021-08-31
hCoV-19/Japan/YCH0605/2021	EPI_ISL_3827033	2021-08-31
hCoV-19/Japan/YCH0606/2021	EPI_ISL_3827042	2021-08-31
hCoV-19/Japan/YCH0607/2021	EPI_ISL_3827034	2021-08-31
hCoV-19/Japan/YCH0608/2021	EPI_ISL_3827028	2021-08-31
hCoV-19/Japan/YCH0609/2021	EPI_ISL_3827029	2021-08-31
hCoV-19/Japan/YCH0610/2021	EPI_ISL_3827037	2021-08-31
hCoV-19/Japan/YCH0611/2021	EPI_ISL_3827030	2021-08-31
hCoV-19/Japan/YCH0612/2021	EPI_ISL_3827031	2021-08-31
hCoV-19/Japan/YCH0613/2021	EPI_ISL_3827041	2021-08-31
hCoV-19/Japan/YCH0614/2021	EPI_ISL_3827019	2021-08-31
hCoV-19/Japan/YCH0615/2021	EPI_ISL_3827032	2021-08-31
hCoV-19/Japan/YCH0616/2021	EPI_ISL_3827013	2021-08-31
hCoV-19/Japan/YCH0617/2021	EPI_ISL_3827014	2021-08-31
hCoV-19/Japan/YCH0618/2021	EPI_ISL_3827027	2021-08-31
hCoV-19/Japan/YCH0619/2021	EPI_ISL_3827015	2021-08-31
hCoV-19/Japan/YCH0620/2021	EPI_ISL_3827016	2021-08-31
hCoV-19/Japan/YCH0621/2021	EPI_ISL_3827017	2021-08-31
hCoV-19/Japan/YCH0622/2021	EPI_ISL_3827018	2021-08-31
hCoV-19/Japan/YCH0623/2021	EPI_ISL_3827039	2021-08-31
hCoV-19/Japan/YCH0624/2021	EPI_ISL_3827040	2021-08-31
hCoV-19/Japan/TY28-233-P0/2021	EPI_ISL_3869203	2021-09-01
hCoV-19/Japan/TY28-236-P0/2021	EPI_ISL_3869204	2021-09-01
hCoV-19/Japan/TY28-248-P0/2021	EPI_ISL_3869205	2021-09-01
hCoV-19/Japan/TY28-257-P0/2021	EPI_ISL_3869206	2021-09-01
hCoV-19/Japan/TY28-440-P0/2021	EPI_ISL_3869207	2021-09-01
hCoV-19/Japan/TY28-444-P0/2021	EPI_ISL_3869208	2021-09-01
hCoV-19/Japan/TY28-453-P0/2021	EPI_ISL_3869209	2021-09-01
hCoV-19/Japan/TY28-454-P0/2021	EPI_ISL_3869210	2021-09-01
hCoV-19/Japan/TY28-456-P0/2021	EPI_ISL_3869211	2021-09-01
hCoV-19/Japan/TY28-463-P0/2021	EPI_ISL_3869212	2021-09-01
hCoV-19/Japan/TY28-484-P0/2021	EPI_ISL_3869213	2021-09-01
hCoV-19/Japan/TY28-486-P0/2021	EPI_ISL_3869214	2021-09-01
hCoV-19/Japan/YCH0625/2021	EPI_ISL_3857532	2021-09-01
hCoV-19/Japan/YCH0626/2021	EPI_ISL_3857536	2021-09-01
hCoV-19/Japan/YCH0627/2021	EPI_ISL_3857540	2021-09-01
hCoV-19/Japan/YCH0628/2021	EPI_ISL_3857544	2021-09-01
hCoV-19/Japan/YCH0629/2021	EPI_ISL_3857546	2021-09-01
hCoV-19/Japan/YCH0630/2021	EPI_ISL_3857548	2021-09-01
hCoV-19/Japan/YCH0631/2021	EPI_ISL_3857550	2021-09-01
hCoV-19/Japan/YCH0632/2021	EPI_ISL_3857554	2021-09-01
hCoV-19/Japan/YCH0633/2021	EPI_ISL_3857559	2021-09-01
hCoV-19/Japan/YCH0634/2021	EPI_ISL_3857563	2021-09-01
hCoV-19/Japan/YCH0635/2021	EPI_ISL_3857568	2021-09-01
hCoV-19/Japan/YCH0636/2021	EPI_ISL_3857572	2021-09-01
hCoV-19/Japan/YCH0637/2021	EPI_ISL_3857576	2021-09-01
hCoV-19/Japan/YCH0638/2021	EPI_ISL_3857578	2021-09-01
hCoV-19/Japan/20210708-29742/2021	EPI_ISL_3908507	2021-09-02
hCoV-19/Japan/20210712-30066/2021	EPI_ISL_3908504	2021-09-02
hCoV-19/Japan/20210713-30234/2021	EPI_ISL_3908499	2021-09-02
hCoV-19/Japan/20210715-30464/2021	EPI_ISL_3908500	2021-09-02
hCoV-19/Japan/20210716-30558/2021	EPI_ISL_3908498	2021-09-02
hCoV-19/Japan/20210719-30697/2021	EPI_ISL_3908509	2021-09-02
hCoV-19/Japan/20210722-31112/2021	EPI_ISL_3908512	2021-09-02

virus_name	epiID	submission_date
hCoV-19/Japan/20210724-31258/2021	EPI_ISL_3908502	2021-09-02
hCoV-19/Japan/20210725-31263/2021	EPI_ISL_3908510	2021-09-02
hCoV-19/Japan/20210726-31399/2021	EPI_ISL_3908503	2021-09-02
hCoV-19/Japan/20210726-31400/2021	EPI_ISL_3908501	2021-09-02
hCoV-19/Japan/20210729-31764/2021	EPI_ISL_3908508	2021-09-02
hCoV-19/Japan/20210730-31868/2021	EPI_ISL_3908506	2021-09-02
hCoV-19/Japan/20210731-31928/2021	EPI_ISL_3908505	2021-09-02
hCoV-19/Japan/20210731-32008/2021	EPI_ISL_3908511	2021-09-02
hCoV-19/Japan/PG-64120/2021	EPI_ISL_3876259	2021-09-02
hCoV-19/Japan/PG-64125/2021	EPI_ISL_3876260	2021-09-02
hCoV-19/Japan/PG-64164/2021	EPI_ISL_3876261	2021-09-02
hCoV-19/Japan/PG-64176/2021	EPI_ISL_3876262	2021-09-02
hCoV-19/Japan/PG-64215/2021	EPI_ISL_3876263	2021-09-02
hCoV-19/Japan/PG-65056/2021	EPI_ISL_3871139	2021-09-02
hCoV-19/Japan/PG-65058/2021	EPI_ISL_3871140	2021-09-02
hCoV-19/Japan/PG-66342/2021	EPI_ISL_3875955	2021-09-02
hCoV-19/Japan/PG-66343/2021	EPI_ISL_3875956	2021-09-02
hCoV-19/Japan/PG-66344/2021	EPI_ISL_3875957	2021-09-02
hCoV-19/Japan/PG-66345/2021	EPI_ISL_3875958	2021-09-02
hCoV-19/Japan/PG-66375/2021	EPI_ISL_3872360	2021-09-02
hCoV-19/Japan/PG-66382/2021	EPI_ISL_3872361	2021-09-02
hCoV-19/Japan/PG-66389/2021	EPI_ISL_3871006	2021-09-02
hCoV-19/Japan/PG-66390/2021	EPI_ISL_3871007	2021-09-02
hCoV-19/Japan/PG-66391/2021	EPI_ISL_3872116	2021-09-02
hCoV-19/Japan/PG-66404/2021	EPI_ISL_3874133	2021-09-02
hCoV-19/Japan/PG-66420/2021	EPI_ISL_3876264	2021-09-02
hCoV-19/Japan/PG-66460/2021	EPI_ISL_3876265	2021-09-02
hCoV-19/Japan/PG-66461/2021	EPI_ISL_3876266	2021-09-02
hCoV-19/Japan/PG-66462/2021	EPI_ISL_3876267	2021-09-02
hCoV-19/Japan/PG-66463/2021	EPI_ISL_3876268	2021-09-02
hCoV-19/Japan/PG-66464/2021	EPI_ISL_3876269	2021-09-02
hCoV-19/Japan/PG-66465/2021	EPI_ISL_3876270	2021-09-02
hCoV-19/Japan/PG-66473/2021	EPI_ISL_3876271	2021-09-02
hCoV-19/Japan/PG-66511/2021	EPI_ISL_3876272	2021-09-02
hCoV-19/Japan/PG-66519/2021	EPI_ISL_3876273	2021-09-02
hCoV-19/Japan/PG-66520/2021	EPI_ISL_3876274	2021-09-02
hCoV-19/Japan/PG-66616/2021	EPI_ISL_3876275	2021-09-02
hCoV-19/Japan/PG-66844/2021	EPI_ISL_3874708	2021-09-02
hCoV-19/Japan/PG-66845/2021	EPI_ISL_3874710	2021-09-02
hCoV-19/Japan/PG-66846/2021	EPI_ISL_3874711	2021-09-02
hCoV-19/Japan/PG-66847/2021	EPI_ISL_3874712	2021-09-02
hCoV-19/Japan/PG-66904/2021	EPI_ISL_3871141	2021-09-02
hCoV-19/Japan/PG-66905/2021	EPI_ISL_3871142	2021-09-02
hCoV-19/Japan/PG-66906/2021	EPI_ISL_3871143	2021-09-02
hCoV-19/Japan/PG-66907/2021	EPI_ISL_3871144	2021-09-02
hCoV-19/Japan/PG-66908/2021	EPI_ISL_3871145	2021-09-02
hCoV-19/Japan/PG-66921/2021	EPI_ISL_3871146	2021-09-02
hCoV-19/Japan/PG-66925/2021	EPI_ISL_3871147	2021-09-02
hCoV-19/Japan/PG-66926/2021	EPI_ISL_3871148	2021-09-02
hCoV-19/Japan/PG-66950/2021	EPI_ISL_3872231	2021-09-02
hCoV-19/Japan/PG-66988/2021	EPI_ISL_3876276	2021-09-02
hCoV-19/Japan/PG-67100/2021	EPI_ISL_3876277	2021-09-02
hCoV-19/Japan/PG-67246/2021	EPI_ISL_3876278	2021-09-02
hCoV-19/Japan/PG-67302/2021	EPI_ISL_3876279	2021-09-02

virus_name	epiID	submission_date
hCoV-19/Japan/PG-67309/2021	EPI_ISL_3876280	2021-09-02
hCoV-19/Japan/PG-67313/2021	EPI_ISL_3876281	2021-09-02
hCoV-19/Japan/PG-67319/2021	EPI_ISL_3876282	2021-09-02
hCoV-19/Japan/PG-67702/2021	EPI_ISL_3875959	2021-09-02
hCoV-19/Japan/PG-67703/2021	EPI_ISL_3875960	2021-09-02
hCoV-19/Japan/PG-67704/2021	EPI_ISL_3875961	2021-09-02
hCoV-19/Japan/PG-67705/2021	EPI_ISL_3875962	2021-09-02
hCoV-19/Japan/PG-67706/2021	EPI_ISL_3875963	2021-09-02
hCoV-19/Japan/PG-67748/2021	EPI_ISL_3875713	2021-09-02
hCoV-19/Japan/PG-67749/2021	EPI_ISL_3875716	2021-09-02
hCoV-19/Japan/PG-67750/2021	EPI_ISL_3875719	2021-09-02
hCoV-19/Japan/PG-67751/2021	EPI_ISL_3872519	2021-09-02
hCoV-19/Japan/PG-67752/2021	EPI_ISL_3872520	2021-09-02
hCoV-19/Japan/PG-67753/2021	EPI_ISL_3872521	2021-09-02
hCoV-19/Japan/PG-67754/2021	EPI_ISL_3872522	2021-09-02
hCoV-19/Japan/PG-67755/2021	EPI_ISL_3872523	2021-09-02
hCoV-19/Japan/PG-67756/2021	EPI_ISL_3872524	2021-09-02
hCoV-19/Japan/PG-67757/2021	EPI_ISL_3872525	2021-09-02
hCoV-19/Japan/PG-67758/2021	EPI_ISL_3872526	2021-09-02
hCoV-19/Japan/PG-67759/2021	EPI_ISL_3872527	2021-09-02
hCoV-19/Japan/PG-67760/2021	EPI_ISL_3872528	2021-09-02
hCoV-19/Japan/PG-67761/2021	EPI_ISL_3872529	2021-09-02
hCoV-19/Japan/PG-67762/2021	EPI_ISL_3872530	2021-09-02
hCoV-19/Japan/PG-67763/2021	EPI_ISL_3872531	2021-09-02
hCoV-19/Japan/PG-67764/2021	EPI_ISL_3872532	2021-09-02
hCoV-19/Japan/PG-67777/2021	EPI_ISL_3872533	2021-09-02
hCoV-19/Japan/PG-67780/2021	EPI_ISL_3872534	2021-09-02
hCoV-19/Japan/PG-67781/2021	EPI_ISL_3872535	2021-09-02
hCoV-19/Japan/PG-67782/2021	EPI_ISL_3872511	2021-09-02
hCoV-19/Japan/PG-67783/2021	EPI_ISL_3872512	2021-09-02
hCoV-19/Japan/PG-67784/2021	EPI_ISL_3872513	2021-09-02
hCoV-19/Japan/PG-67785/2021	EPI_ISL_3872514	2021-09-02
hCoV-19/Japan/PG-67790/2021	EPI_ISL_3872117	2021-09-02
hCoV-19/Japan/PG-67791/2021	EPI_ISL_3872118	2021-09-02
hCoV-19/Japan/PG-67792/2021	EPI_ISL_3872119	2021-09-02
hCoV-19/Japan/PG-67793/2021	EPI_ISL_3872120	2021-09-02
hCoV-19/Japan/PG-67805/2021	EPI_ISL_3872121	2021-09-02
hCoV-19/Japan/PG-67806/2021	EPI_ISL_3872122	2021-09-02
hCoV-19/Japan/PG-67807/2021	EPI_ISL_3872123	2021-09-02
hCoV-19/Japan/PG-67808/2021	EPI_ISL_3872124	2021-09-02
hCoV-19/Japan/PG-67837/2021	EPI_ISL_3876283	2021-09-02
hCoV-19/Japan/PG-67895/2021	EPI_ISL_3876284	2021-09-02
hCoV-19/Japan/PG-68301/2021	EPI_ISL_3875964	2021-09-02
hCoV-19/Japan/PG-68302/2021	EPI_ISL_3875965	2021-09-02
hCoV-19/Japan/PG-68303/2021	EPI_ISL_3875966	2021-09-02
hCoV-19/Japan/PG-68304/2021	EPI_ISL_3875967	2021-09-02
hCoV-19/Japan/PG-68305/2021	EPI_ISL_3875968	2021-09-02
hCoV-19/Japan/PG-68306/2021	EPI_ISL_3875969	2021-09-02
hCoV-19/Japan/PG-68307/2021	EPI_ISL_3875970	2021-09-02
hCoV-19/Japan/PG-68308/2021	EPI_ISL_3875971	2021-09-02
hCoV-19/Japan/PG-68309/2021	EPI_ISL_3875972	2021-09-02
hCoV-19/Japan/PG-68310/2021	EPI_ISL_3875973	2021-09-02
hCoV-19/Japan/PG-68311/2021	EPI_ISL_3875974	2021-09-02
hCoV-19/Japan/PG-68312/2021	EPI_ISL_3875975	2021-09-02

virus_name	epiID	submission_date
hCoV-19/Japan/PG-68313/2021	EPI_ISL_3875976	2021-09-02
hCoV-19/Japan/PG-68314/2021	EPI_ISL_3875977	2021-09-02
hCoV-19/Japan/PG-68315/2021	EPI_ISL_3875978	2021-09-02
hCoV-19/Japan/PG-68316/2021	EPI_ISL_3875979	2021-09-02
hCoV-19/Japan/PG-68317/2021	EPI_ISL_3875980	2021-09-02
hCoV-19/Japan/PG-68318/2021	EPI_ISL_3875981	2021-09-02
hCoV-19/Japan/PG-68346/2021	EPI_ISL_3873293	2021-09-02
hCoV-19/Japan/PG-68349/2021	EPI_ISL_3873294	2021-09-02
hCoV-19/Japan/PG-68356/2021	EPI_ISL_3871464	2021-09-02
hCoV-19/Japan/PG-68357/2021	EPI_ISL_3871465	2021-09-02
hCoV-19/Japan/PG-68358/2021	EPI_ISL_3871466	2021-09-02
hCoV-19/Japan/PG-68359/2021	EPI_ISL_3871467	2021-09-02
hCoV-19/Japan/PG-68360/2021	EPI_ISL_3871468	2021-09-02
hCoV-19/Japan/PG-68365/2021	EPI_ISL_3871149	2021-09-02
hCoV-19/Japan/PG-68366/2021	EPI_ISL_3871150	2021-09-02
hCoV-19/Japan/PG-68367/2021	EPI_ISL_3871151	2021-09-02
hCoV-19/Japan/PG-68368/2021	EPI_ISL_3871152	2021-09-02
hCoV-19/Japan/PG-68369/2021	EPI_ISL_3871153	2021-09-02
hCoV-19/Japan/PG-68370/2021	EPI_ISL_3871154	2021-09-02
hCoV-19/Japan/PG-68371/2021	EPI_ISL_3871155	2021-09-02
hCoV-19/Japan/PG-68372/2021	EPI_ISL_3871156	2021-09-02
hCoV-19/Japan/PG-68373/2021	EPI_ISL_3871157	2021-09-02
hCoV-19/Japan/PG-68374/2021	EPI_ISL_3871158	2021-09-02
hCoV-19/Japan/PG-68375/2021	EPI_ISL_3871159	2021-09-02
hCoV-19/Japan/PG-68376/2021	EPI_ISL_3871160	2021-09-02
hCoV-19/Japan/PG-68377/2021	EPI_ISL_3871161	2021-09-02
hCoV-19/Japan/PG-68378/2021	EPI_ISL_3871162	2021-09-02
hCoV-19/Japan/PG-68379/2021	EPI_ISL_3871163	2021-09-02
hCoV-19/Japan/PG-68390/2021	EPI_ISL_3873329	2021-09-02
hCoV-19/Japan/PG-68391/2021	EPI_ISL_3873330	2021-09-02
hCoV-19/Japan/PG-68392/2021	EPI_ISL_3873331	2021-09-02
hCoV-19/Japan/PG-68393/2021	EPI_ISL_3873332	2021-09-02
hCoV-19/Japan/PG-68394/2021	EPI_ISL_3873333	2021-09-02
hCoV-19/Japan/PG-68396/2021	EPI_ISL_3873334	2021-09-02
hCoV-19/Japan/PG-68397/2021	EPI_ISL_3873335	2021-09-02
hCoV-19/Japan/PG-68398/2021	EPI_ISL_3873336	2021-09-02
hCoV-19/Japan/PG-68399/2021	EPI_ISL_3873337	2021-09-02
hCoV-19/Japan/PG-68401/2021	EPI_ISL_3874519	2021-09-02
hCoV-19/Japan/PG-68402/2021	EPI_ISL_3874520	2021-09-02
hCoV-19/Japan/PG-68403/2021	EPI_ISL_3874521	2021-09-02
hCoV-19/Japan/PG-68404/2021	EPI_ISL_3874522	2021-09-02
hCoV-19/Japan/PG-68405/2021	EPI_ISL_3874523	2021-09-02
hCoV-19/Japan/PG-68406/2021	EPI_ISL_3874524	2021-09-02
hCoV-19/Japan/PG-68407/2021	EPI_ISL_3874525	2021-09-02
hCoV-19/Japan/PG-68408/2021	EPI_ISL_3874526	2021-09-02
hCoV-19/Japan/PG-68409/2021	EPI_ISL_3874527	2021-09-02
hCoV-19/Japan/PG-68411/2021	EPI_ISL_3870954	2021-09-02
hCoV-19/Japan/PG-68412/2021	EPI_ISL_3870955	2021-09-02
hCoV-19/Japan/PG-68413/2021	EPI_ISL_3870956	2021-09-02
hCoV-19/Japan/PG-68414/2021	EPI_ISL_3870957	2021-09-02
hCoV-19/Japan/PG-68415/2021	EPI_ISL_3872232	2021-09-02
hCoV-19/Japan/PG-68416/2021	EPI_ISL_3872233	2021-09-02
hCoV-19/Japan/PG-68417/2021	EPI_ISL_3872234	2021-09-02
hCoV-19/Japan/PG-68418/2021	EPI_ISL_3872235	2021-09-02

virus_name	epiID	submission_date
hCoV-19/Japan/PG-68419/2021	EPI_ISL_3872236	2021-09-02
hCoV-19/Japan/PG-68420/2021	EPI_ISL_3872237	2021-09-02
hCoV-19/Japan/PG-68422/2021	EPI_ISL_3873525	2021-09-02
hCoV-19/Japan/PG-68423/2021	EPI_ISL_3873526	2021-09-02
hCoV-19/Japan/PG-68433/2021	EPI_ISL_3873527	2021-09-02
hCoV-19/Japan/PG-68434/2021	EPI_ISL_3873528	2021-09-02
hCoV-19/Japan/PG-68437/2021	EPI_ISL_3873529	2021-09-02
hCoV-19/Japan/PG-68454/2021	EPI_ISL_3873530	2021-09-02
hCoV-19/Japan/PG-68455/2021	EPI_ISL_3873531	2021-09-02
hCoV-19/Japan/PG-68460/2021	EPI_ISL_3873532	2021-09-02
hCoV-19/Japan/PG-68462/2021	EPI_ISL_3873533	2021-09-02
hCoV-19/Japan/PG-68464/2021	EPI_ISL_3873534	2021-09-02
hCoV-19/Japan/PG-68547/2021	EPI_ISL_3874257	2021-09-02
hCoV-19/Japan/PG-68548/2021	EPI_ISL_3874258	2021-09-02
hCoV-19/Japan/PG-68549/2021	EPI_ISL_3874259	2021-09-02
hCoV-19/Japan/PG-68550/2021	EPI_ISL_3874260	2021-09-02
hCoV-19/Japan/PG-68551/2021	EPI_ISL_3874261	2021-09-02
hCoV-19/Japan/PG-68552/2021	EPI_ISL_3874262	2021-09-02
hCoV-19/Japan/PG-68554/2021	EPI_ISL_3874263	2021-09-02
hCoV-19/Japan/PG-68575/2021	EPI_ISL_3876285	2021-09-02
hCoV-19/Japan/PG-68576/2021	EPI_ISL_3876286	2021-09-02
hCoV-19/Japan/PG-68577/2021	EPI_ISL_3876287	2021-09-02
hCoV-19/Japan/PG-68624/2021	EPI_ISL_3876288	2021-09-02
hCoV-19/Japan/PG-68661/2021	EPI_ISL_3876289	2021-09-02
hCoV-19/Japan/PG-68662/2021	EPI_ISL_3876290	2021-09-02
hCoV-19/Japan/PG-68663/2021	EPI_ISL_3876291	2021-09-02
hCoV-19/Japan/PG-68664/2021	EPI_ISL_3876292	2021-09-02
hCoV-19/Japan/PG-68665/2021	EPI_ISL_3876293	2021-09-02
hCoV-19/Japan/PG-68666/2021	EPI_ISL_3876294	2021-09-02
hCoV-19/Japan/PG-68669/2021	EPI_ISL_3876295	2021-09-02
hCoV-19/Japan/PG-68670/2021	EPI_ISL_3876296	2021-09-02
hCoV-19/Japan/PG-68671/2021	EPI_ISL_3876297	2021-09-02
hCoV-19/Japan/PG-68673/2021	EPI_ISL_3876298	2021-09-02
hCoV-19/Japan/PG-68714/2021	EPI_ISL_3876299	2021-09-02
hCoV-19/Japan/PG-68715/2021	EPI_ISL_3876300	2021-09-02
hCoV-19/Japan/PG-68716/2021	EPI_ISL_3876301	2021-09-02
hCoV-19/Japan/PG-68717/2021	EPI_ISL_3876302	2021-09-02
hCoV-19/Japan/PG-68738/2021	EPI_ISL_3876303	2021-09-02
hCoV-19/Japan/PG-68739/2021	EPI_ISL_3876304	2021-09-02
hCoV-19/Japan/PG-68740/2021	EPI_ISL_3876305	2021-09-02
hCoV-19/Japan/PG-68741/2021	EPI_ISL_3876306	2021-09-02
hCoV-19/Japan/PG-68742/2021	EPI_ISL_3876307	2021-09-02
hCoV-19/Japan/PG-68743/2021	EPI_ISL_3876308	2021-09-02
hCoV-19/Japan/PG-68744/2021	EPI_ISL_3876309	2021-09-02
hCoV-19/Japan/PG-68745/2021	EPI_ISL_3876310	2021-09-02
hCoV-19/Japan/PG-68746/2021	EPI_ISL_3876311	2021-09-02
hCoV-19/Japan/PG-68747/2021	EPI_ISL_3876312	2021-09-02
hCoV-19/Japan/PG-68748/2021	EPI_ISL_3876313	2021-09-02
hCoV-19/Japan/PG-68749/2021	EPI_ISL_3876314	2021-09-02
hCoV-19/Japan/PG-68750/2021	EPI_ISL_3876315	2021-09-02
hCoV-19/Japan/PG-68751/2021	EPI_ISL_3876316	2021-09-02
hCoV-19/Japan/PG-68752/2021	EPI_ISL_3876317	2021-09-02
hCoV-19/Japan/PG-68753/2021	EPI_ISL_3876318	2021-09-02
hCoV-19/Japan/PG-68754/2021	EPI_ISL_3876319	2021-09-02

virus_name	epiID	submission_date
hCoV-19/Japan/PG-68755/2021	EPI_ISL_3876320	2021-09-02
hCoV-19/Japan/PG-68756/2021	EPI_ISL_3876321	2021-09-02
hCoV-19/Japan/PG-68757/2021	EPI_ISL_3876322	2021-09-02
hCoV-19/Japan/PG-68758/2021	EPI_ISL_3876323	2021-09-02
hCoV-19/Japan/PG-68759/2021	EPI_ISL_3876324	2021-09-02
hCoV-19/Japan/PG-68760/2021	EPI_ISL_3876325	2021-09-02
hCoV-19/Japan/PG-68761/2021	EPI_ISL_3876326	2021-09-02
hCoV-19/Japan/PG-68762/2021	EPI_ISL_3876327	2021-09-02
hCoV-19/Japan/PG-68763/2021	EPI_ISL_3876328	2021-09-02
hCoV-19/Japan/PG-68764/2021	EPI_ISL_3876329	2021-09-02
hCoV-19/Japan/PG-68765/2021	EPI_ISL_3876330	2021-09-02
hCoV-19/Japan/PG-68766/2021	EPI_ISL_3876331	2021-09-02
hCoV-19/Japan/PG-68767/2021	EPI_ISL_3876332	2021-09-02
hCoV-19/Japan/PG-68768/2021	EPI_ISL_3876333	2021-09-02
hCoV-19/Japan/PG-68769/2021	EPI_ISL_3876334	2021-09-02
hCoV-19/Japan/PG-68770/2021	EPI_ISL_3876335	2021-09-02
hCoV-19/Japan/PG-68771/2021	EPI_ISL_3876336	2021-09-02
hCoV-19/Japan/PG-68772/2021	EPI_ISL_3876337	2021-09-02
hCoV-19/Japan/PG-68773/2021	EPI_ISL_3876338	2021-09-02
hCoV-19/Japan/PG-68774/2021	EPI_ISL_3876339	2021-09-02
hCoV-19/Japan/PG-68775/2021	EPI_ISL_3876340	2021-09-02
hCoV-19/Japan/PG-68776/2021	EPI_ISL_3876341	2021-09-02
hCoV-19/Japan/PG-68777/2021	EPI_ISL_3876342	2021-09-02
hCoV-19/Japan/PG-68778/2021	EPI_ISL_3876343	2021-09-02
hCoV-19/Japan/PG-68779/2021	EPI_ISL_3876344	2021-09-02
hCoV-19/Japan/PG-68780/2021	EPI_ISL_3876345	2021-09-02
hCoV-19/Japan/PG-68781/2021	EPI_ISL_3876346	2021-09-02
hCoV-19/Japan/PG-68782/2021	EPI_ISL_3876347	2021-09-02
hCoV-19/Japan/PG-68784/2021	EPI_ISL_3876348	2021-09-02
hCoV-19/Japan/PG-68811/2021	EPI_ISL_3876349	2021-09-02
hCoV-19/Japan/PG-68820/2021	EPI_ISL_3876350	2021-09-02
hCoV-19/Japan/PG-68821/2021	EPI_ISL_3876351	2021-09-02
hCoV-19/Japan/PG-68822/2021	EPI_ISL_3876352	2021-09-02
hCoV-19/Japan/PG-68838/2021	EPI_ISL_3876353	2021-09-02
hCoV-19/Japan/PG-68839/2021	EPI_ISL_3876354	2021-09-02
hCoV-19/Japan/PG-68840/2021	EPI_ISL_3876355	2021-09-02
hCoV-19/Japan/PG-68841/2021	EPI_ISL_3876356	2021-09-02
hCoV-19/Japan/PG-68842/2021	EPI_ISL_3876357	2021-09-02
hCoV-19/Japan/PG-68899/2021	EPI_ISL_3876358	2021-09-02
hCoV-19/Japan/PG-68905/2021	EPI_ISL_3876359	2021-09-02
hCoV-19/Japan/PG-68912/2021	EPI_ISL_3876360	2021-09-02
hCoV-19/Japan/PG-68913/2021	EPI_ISL_3876361	2021-09-02
hCoV-19/Japan/PG-68914/2021	EPI_ISL_3876362	2021-09-02
hCoV-19/Japan/PG-68915/2021	EPI_ISL_3876363	2021-09-02
hCoV-19/Japan/PG-68916/2021	EPI_ISL_3876364	2021-09-02
hCoV-19/Japan/PG-68917/2021	EPI_ISL_3876365	2021-09-02
hCoV-19/Japan/PG-68918/2021	EPI_ISL_3876366	2021-09-02
hCoV-19/Japan/PG-68919/2021	EPI_ISL_3876367	2021-09-02
hCoV-19/Japan/PG-68920/2021	EPI_ISL_3876368	2021-09-02
hCoV-19/Japan/PG-68921/2021	EPI_ISL_3876369	2021-09-02
hCoV-19/Japan/PG-68922/2021	EPI_ISL_3876370	2021-09-02
hCoV-19/Japan/PG-68923/2021	EPI_ISL_3876371	2021-09-02
hCoV-19/Japan/PG-68924/2021	EPI_ISL_3876372	2021-09-02
hCoV-19/Japan/PG-68925/2021	EPI_ISL_3876373	2021-09-02

virus_name	epiID	submission_date
hCoV-19/Japan/PG-68926/2021	EPI_ISL_3876374	2021-09-02
hCoV-19/Japan/PG-68927/2021	EPI_ISL_3876375	2021-09-02
hCoV-19/Japan/PG-68928/2021	EPI_ISL_3876376	2021-09-02
hCoV-19/Japan/PG-68929/2021	EPI_ISL_3876377	2021-09-02
hCoV-19/Japan/PG-68930/2021	EPI_ISL_3876378	2021-09-02
hCoV-19/Japan/PG-68931/2021	EPI_ISL_3876379	2021-09-02
hCoV-19/Japan/PG-68932/2021	EPI_ISL_3876380	2021-09-02
hCoV-19/Japan/PG-68933/2021	EPI_ISL_3876381	2021-09-02
hCoV-19/Japan/PG-68934/2021	EPI_ISL_3876382	2021-09-02
hCoV-19/Japan/PG-68935/2021	EPI_ISL_3876383	2021-09-02
hCoV-19/Japan/PG-68936/2021	EPI_ISL_3876384	2021-09-02
hCoV-19/Japan/PG-68937/2021	EPI_ISL_3876385	2021-09-02
hCoV-19/Japan/PG-68938/2021	EPI_ISL_3876386	2021-09-02
hCoV-19/Japan/PG-68939/2021	EPI_ISL_3876387	2021-09-02
hCoV-19/Japan/PG-68940/2021	EPI_ISL_3876388	2021-09-02
hCoV-19/Japan/PG-68941/2021	EPI_ISL_3876389	2021-09-02
hCoV-19/Japan/PG-68942/2021	EPI_ISL_3876390	2021-09-02
hCoV-19/Japan/PG-68943/2021	EPI_ISL_3876391	2021-09-02
hCoV-19/Japan/PG-68947/2021	EPI_ISL_3876392	2021-09-02
hCoV-19/Japan/PG-68950/2021	EPI_ISL_3876393	2021-09-02
hCoV-19/Japan/PG-68951/2021	EPI_ISL_3876394	2021-09-02
hCoV-19/Japan/PG-68952/2021	EPI_ISL_3876395	2021-09-02
hCoV-19/Japan/PG-68953/2021	EPI_ISL_3876396	2021-09-02
hCoV-19/Japan/PG-68961/2021	EPI_ISL_3876397	2021-09-02
hCoV-19/Japan/PG-68963/2021	EPI_ISL_3876398	2021-09-02
hCoV-19/Japan/PG-68964/2021	EPI_ISL_3876399	2021-09-02
hCoV-19/Japan/PG-68965/2021	EPI_ISL_3876400	2021-09-02
hCoV-19/Japan/PG-68966/2021	EPI_ISL_3876401	2021-09-02
hCoV-19/Japan/PG-68972/2021	EPI_ISL_3876402	2021-09-02
hCoV-19/Japan/PG-68977/2021	EPI_ISL_3876403	2021-09-02
hCoV-19/Japan/PG-68978/2021	EPI_ISL_3876404	2021-09-02
hCoV-19/Japan/PG-68979/2021	EPI_ISL_3876405	2021-09-02
hCoV-19/Japan/PG-68980/2021	EPI_ISL_3870483	2021-09-02
hCoV-19/Japan/PG-68981/2021	EPI_ISL_3870484	2021-09-02
hCoV-19/Japan/PG-68982/2021	EPI_ISL_3870485	2021-09-02
hCoV-19/Japan/PG-68983/2021	EPI_ISL_3870486	2021-09-02
hCoV-19/Japan/PG-68991/2021	EPI_ISL_3875460	2021-09-02
hCoV-19/Japan/PG-68992/2021	EPI_ISL_3875461	2021-09-02
hCoV-19/Japan/PG-68993/2021	EPI_ISL_3875462	2021-09-02
hCoV-19/Japan/PG-68994/2021	EPI_ISL_3875463	2021-09-02
hCoV-19/Japan/PG-68997/2021	EPI_ISL_3875464	2021-09-02
hCoV-19/Japan/PG-68998/2021	EPI_ISL_3875465	2021-09-02
hCoV-19/Japan/PG-68999/2021	EPI_ISL_3875466	2021-09-02
hCoV-19/Japan/PG-69000/2021	EPI_ISL_3875467	2021-09-02
hCoV-19/Japan/PG-69001/2021	EPI_ISL_3875468	2021-09-02
hCoV-19/Japan/PG-69002/2021	EPI_ISL_3875469	2021-09-02
hCoV-19/Japan/PG-69010/2021	EPI_ISL_3875470	2021-09-02
hCoV-19/Japan/PG-69011/2021	EPI_ISL_3875471	2021-09-02
hCoV-19/Japan/PG-69012/2021	EPI_ISL_3875472	2021-09-02
hCoV-19/Japan/PG-69013/2021	EPI_ISL_3875473	2021-09-02
hCoV-19/Japan/PG-69014/2021	EPI_ISL_3875474	2021-09-02
hCoV-19/Japan/PG-69015/2021	EPI_ISL_3871387	2021-09-02
hCoV-19/Japan/PG-69016/2021	EPI_ISL_3871388	2021-09-02
hCoV-19/Japan/PG-69017/2021	EPI_ISL_3871389	2021-09-02

virus_name	epiID	submission_date
hCoV-19/Japan/PG-69018/2021	EPI_ISL_3871390	2021-09-02
hCoV-19/Japan/PG-69024/2021	EPI_ISL_3871391	2021-09-02
hCoV-19/Japan/PG-69025/2021	EPI_ISL_3871392	2021-09-02
hCoV-19/Japan/PG-69026/2021	EPI_ISL_3871393	2021-09-02
hCoV-19/Japan/PG-69027/2021	EPI_ISL_3871394	2021-09-02
hCoV-19/Japan/PG-69035/2021	EPI_ISL_3871746	2021-09-02
hCoV-19/Japan/PG-69036/2021	EPI_ISL_3871747	2021-09-02
hCoV-19/Japan/PG-69037/2021	EPI_ISL_3871748	2021-09-02
hCoV-19/Japan/PG-69056/2021	EPI_ISL_3872362	2021-09-02
hCoV-19/Japan/PG-69057/2021	EPI_ISL_3872363	2021-09-02
hCoV-19/Japan/PG-69058/2021	EPI_ISL_3872364	2021-09-02
hCoV-19/Japan/PG-69059/2021	EPI_ISL_3872365	2021-09-02
hCoV-19/Japan/PG-69060/2021	EPI_ISL_3872366	2021-09-02
hCoV-19/Japan/PG-69062/2021	EPI_ISL_3872367	2021-09-02
hCoV-19/Japan/PG-69063/2021	EPI_ISL_3872368	2021-09-02
hCoV-19/Japan/PG-69064/2021	EPI_ISL_3872369	2021-09-02
hCoV-19/Japan/PG-69065/2021	EPI_ISL_3872370	2021-09-02
hCoV-19/Japan/PG-69066/2021	EPI_ISL_3872371	2021-09-02
hCoV-19/Japan/PG-69067/2021	EPI_ISL_3872372	2021-09-02
hCoV-19/Japan/PG-69068/2021	EPI_ISL_3872373	2021-09-02
hCoV-19/Japan/PG-69069/2021	EPI_ISL_3872374	2021-09-02
hCoV-19/Japan/PG-69070/2021	EPI_ISL_3872375	2021-09-02
hCoV-19/Japan/PG-69071/2021	EPI_ISL_3872376	2021-09-02
hCoV-19/Japan/PG-69072/2021	EPI_ISL_3872377	2021-09-02
hCoV-19/Japan/PG-69073/2021	EPI_ISL_3872378	2021-09-02
hCoV-19/Japan/PG-69074/2021	EPI_ISL_3872379	2021-09-02
hCoV-19/Japan/PG-69075/2021	EPI_ISL_3872380	2021-09-02
hCoV-19/Japan/PG-69076/2021	EPI_ISL_3872381	2021-09-02
hCoV-19/Japan/PG-69077/2021	EPI_ISL_3872536	2021-09-02
hCoV-19/Japan/PG-69078/2021	EPI_ISL_3872537	2021-09-02
hCoV-19/Japan/PG-69079/2021	EPI_ISL_3872538	2021-09-02
hCoV-19/Japan/PG-69080/2021	EPI_ISL_3872539	2021-09-02
hCoV-19/Japan/PG-69081/2021	EPI_ISL_3872540	2021-09-02
hCoV-19/Japan/PG-69082/2021	EPI_ISL_3872541	2021-09-02
hCoV-19/Japan/PG-69083/2021	EPI_ISL_3872542	2021-09-02
hCoV-19/Japan/PG-69084/2021	EPI_ISL_3872543	2021-09-02
hCoV-19/Japan/PG-69085/2021	EPI_ISL_3872544	2021-09-02
hCoV-19/Japan/PG-69086/2021	EPI_ISL_3872545	2021-09-02
hCoV-19/Japan/PG-69087/2021	EPI_ISL_3872546	2021-09-02
hCoV-19/Japan/PG-69088/2021	EPI_ISL_3872547	2021-09-02
hCoV-19/Japan/PG-69089/2021	EPI_ISL_3872548	2021-09-02
hCoV-19/Japan/PG-69090/2021	EPI_ISL_3872549	2021-09-02
hCoV-19/Japan/PG-69091/2021	EPI_ISL_3872550	2021-09-02
hCoV-19/Japan/PG-69092/2021	EPI_ISL_3872551	2021-09-02
hCoV-19/Japan/PG-69093/2021	EPI_ISL_3872552	2021-09-02
hCoV-19/Japan/PG-69094/2021	EPI_ISL_3872553	2021-09-02
hCoV-19/Japan/PG-69095/2021	EPI_ISL_3872554	2021-09-02
hCoV-19/Japan/PG-69096/2021	EPI_ISL_3872555	2021-09-02
hCoV-19/Japan/PG-69097/2021	EPI_ISL_3872556	2021-09-02
hCoV-19/Japan/PG-69098/2021	EPI_ISL_3872557	2021-09-02
hCoV-19/Japan/PG-69099/2021	EPI_ISL_3872558	2021-09-02
hCoV-19/Japan/PG-69100/2021	EPI_ISL_3871563	2021-09-02
hCoV-19/Japan/PG-69101/2021	EPI_ISL_3874471	2021-09-02
hCoV-19/Japan/PG-69102/2021	EPI_ISL_3874472	2021-09-02

virus_name	epiID	submission_date
hCoV-19/Japan/PG-69103/2021	EPI_ISL_3874473	2021-09-02
hCoV-19/Japan/PG-69104/2021	EPI_ISL_3874474	2021-09-02
hCoV-19/Japan/PG-69105/2021	EPI_ISL_3874475	2021-09-02
hCoV-19/Japan/PG-69106/2021	EPI_ISL_3874476	2021-09-02
hCoV-19/Japan/PG-69107/2021	EPI_ISL_3874477	2021-09-02
hCoV-19/Japan/PG-69108/2021	EPI_ISL_3874478	2021-09-02
hCoV-19/Japan/PG-69109/2021	EPI_ISL_3872515	2021-09-02
hCoV-19/Japan/PG-69115/2021	EPI_ISL_3873956	2021-09-02
hCoV-19/Japan/PG-69117/2021	EPI_ISL_3871008	2021-09-02
hCoV-19/Japan/PG-69118/2021	EPI_ISL_3871009	2021-09-02
hCoV-19/Japan/PG-69119/2021	EPI_ISL_3871010	2021-09-02
hCoV-19/Japan/PG-69120/2021	EPI_ISL_3871011	2021-09-02
hCoV-19/Japan/PG-69121/2021	EPI_ISL_3871012	2021-09-02
hCoV-19/Japan/PG-69123/2021	EPI_ISL_3871164	2021-09-02
hCoV-19/Japan/PG-69125/2021	EPI_ISL_3871165	2021-09-02
hCoV-19/Japan/PG-69129/2021	EPI_ISL_3871166	2021-09-02
hCoV-19/Japan/PG-69130/2021	EPI_ISL_3871167	2021-09-02
hCoV-19/Japan/PG-69131/2021	EPI_ISL_3871168	2021-09-02
hCoV-19/Japan/PG-69132/2021	EPI_ISL_3871169	2021-09-02
hCoV-19/Japan/PG-69133/2021	EPI_ISL_3871170	2021-09-02
hCoV-19/Japan/PG-69134/2021	EPI_ISL_3871171	2021-09-02
hCoV-19/Japan/PG-69135/2021	EPI_ISL_3871172	2021-09-02
hCoV-19/Japan/PG-69136/2021	EPI_ISL_3871173	2021-09-02
hCoV-19/Japan/PG-69137/2021	EPI_ISL_3871174	2021-09-02
hCoV-19/Japan/PG-69138/2021	EPI_ISL_3871175	2021-09-02
hCoV-19/Japan/PG-69139/2021	EPI_ISL_3871176	2021-09-02
hCoV-19/Japan/PG-69140/2021	EPI_ISL_3871177	2021-09-02
hCoV-19/Japan/PG-69141/2021	EPI_ISL_3871178	2021-09-02
hCoV-19/Japan/PG-69142/2021	EPI_ISL_3871179	2021-09-02
hCoV-19/Japan/PG-69143/2021	EPI_ISL_3871180	2021-09-02
hCoV-19/Japan/PG-69144/2021	EPI_ISL_3871181	2021-09-02
hCoV-19/Japan/PG-69145/2021	EPI_ISL_3871967	2021-09-02
hCoV-19/Japan/PG-69146/2021	EPI_ISL_3871968	2021-09-02
hCoV-19/Japan/PG-69147/2021	EPI_ISL_3871969	2021-09-02
hCoV-19/Japan/PG-69148/2021	EPI_ISL_3871970	2021-09-02
hCoV-19/Japan/PG-69149/2021	EPI_ISL_3871971	2021-09-02
hCoV-19/Japan/PG-69150/2021	EPI_ISL_3871972	2021-09-02
hCoV-19/Japan/PG-69151/2021	EPI_ISL_3871973	2021-09-02
hCoV-19/Japan/PG-69152/2021	EPI_ISL_3871974	2021-09-02
hCoV-19/Japan/PG-69153/2021	EPI_ISL_3871975	2021-09-02
hCoV-19/Japan/PG-69154/2021	EPI_ISL_3871976	2021-09-02
hCoV-19/Japan/PG-69155/2021	EPI_ISL_3871977	2021-09-02
hCoV-19/Japan/PG-69156/2021	EPI_ISL_3871978	2021-09-02
hCoV-19/Japan/PG-69157/2021	EPI_ISL_3871979	2021-09-02
hCoV-19/Japan/PG-69158/2021	EPI_ISL_3871980	2021-09-02
hCoV-19/Japan/PG-69163/2021	EPI_ISL_3873338	2021-09-02
hCoV-19/Japan/PG-69164/2021	EPI_ISL_3873339	2021-09-02
hCoV-19/Japan/PG-69165/2021	EPI_ISL_3873340	2021-09-02
hCoV-19/Japan/PG-69166/2021	EPI_ISL_3873341	2021-09-02
hCoV-19/Japan/PG-69167/2021	EPI_ISL_3873342	2021-09-02
hCoV-19/Japan/PG-69169/2021	EPI_ISL_3873535	2021-09-02
hCoV-19/Japan/PG-69170/2021	EPI_ISL_3873536	2021-09-02
hCoV-19/Japan/PG-69175/2021	EPI_ISL_3873537	2021-09-02
hCoV-19/Japan/PG-69176/2021	EPI_ISL_3873538	2021-09-02

virus_name	epiID	submission_date
hCoV-19/Japan/PG-69177/2021	EPI_ISL_3873539	2021-09-02
hCoV-19/Japan/PG-69178/2021	EPI_ISL_3873540	2021-09-02
hCoV-19/Japan/PG-69179/2021	EPI_ISL_3873541	2021-09-02
hCoV-19/Japan/PG-69180/2021	EPI_ISL_3873542	2021-09-02
hCoV-19/Japan/PG-69181/2021	EPI_ISL_3873543	2021-09-02
hCoV-19/Japan/PG-69182/2021	EPI_ISL_3873544	2021-09-02
hCoV-19/Japan/PG-69183/2021	EPI_ISL_3873545	2021-09-02
hCoV-19/Japan/PG-69184/2021	EPI_ISL_3873546	2021-09-02
hCoV-19/Japan/PG-69185/2021	EPI_ISL_3873547	2021-09-02
hCoV-19/Japan/PG-69186/2021	EPI_ISL_3873548	2021-09-02
hCoV-19/Japan/PG-69187/2021	EPI_ISL_3873549	2021-09-02
hCoV-19/Japan/PG-69188/2021	EPI_ISL_3873550	2021-09-02
hCoV-19/Japan/PG-69189/2021	EPI_ISL_3873551	2021-09-02
hCoV-19/Japan/PG-69190/2021	EPI_ISL_3873552	2021-09-02
hCoV-19/Japan/PG-69191/2021	EPI_ISL_3873553	2021-09-02
hCoV-19/Japan/PG-69192/2021	EPI_ISL_3873554	2021-09-02
hCoV-19/Japan/PG-69193/2021	EPI_ISL_3873555	2021-09-02
hCoV-19/Japan/PG-69194/2021	EPI_ISL_3873556	2021-09-02
hCoV-19/Japan/PG-69195/2021	EPI_ISL_3873557	2021-09-02
hCoV-19/Japan/PG-69207/2021	EPI_ISL_3874713	2021-09-02
hCoV-19/Japan/PG-69208/2021	EPI_ISL_3874714	2021-09-02
hCoV-19/Japan/PG-69209/2021	EPI_ISL_3874715	2021-09-02
hCoV-19/Japan/PG-69210/2021	EPI_ISL_3874716	2021-09-02
hCoV-19/Japan/PG-69211/2021	EPI_ISL_3874717	2021-09-02
hCoV-19/Japan/PG-69212/2021	EPI_ISL_3874718	2021-09-02
hCoV-19/Japan/PG-69213/2021	EPI_ISL_3874719	2021-09-02
hCoV-19/Japan/PG-69214/2021	EPI_ISL_3874720	2021-09-02
hCoV-19/Japan/PG-69215/2021	EPI_ISL_3874721	2021-09-02
hCoV-19/Japan/PG-69216/2021	EPI_ISL_3874722	2021-09-02
hCoV-19/Japan/PG-69217/2021	EPI_ISL_3874723	2021-09-02
hCoV-19/Japan/PG-69218/2021	EPI_ISL_3874724	2021-09-02
hCoV-19/Japan/PG-69219/2021	EPI_ISL_3874725	2021-09-02
hCoV-19/Japan/PG-69220/2021	EPI_ISL_3874726	2021-09-02
hCoV-19/Japan/PG-69221/2021	EPI_ISL_3874727	2021-09-02
hCoV-19/Japan/PG-69222/2021	EPI_ISL_3874728	2021-09-02
hCoV-19/Japan/PG-69223/2021	EPI_ISL_3874729	2021-09-02
hCoV-19/Japan/PG-69224/2021	EPI_ISL_3874730	2021-09-02
hCoV-19/Japan/PG-69225/2021	EPI_ISL_3874731	2021-09-02
hCoV-19/Japan/PG-69226/2021	EPI_ISL_3874732	2021-09-02
hCoV-19/Japan/PG-69227/2021	EPI_ISL_3874733	2021-09-02
hCoV-19/Japan/PG-69228/2021	EPI_ISL_3874734	2021-09-02
hCoV-19/Japan/PG-69229/2021	EPI_ISL_3874735	2021-09-02
hCoV-19/Japan/PG-69230/2021	EPI_ISL_3874736	2021-09-02
hCoV-19/Japan/PG-69231/2021	EPI_ISL_3874737	2021-09-02
hCoV-19/Japan/PG-69232/2021	EPI_ISL_3874738	2021-09-02
hCoV-19/Japan/PG-69233/2021	EPI_ISL_3874739	2021-09-02
hCoV-19/Japan/PG-69234/2021	EPI_ISL_3874740	2021-09-02
hCoV-19/Japan/PG-69309/2021	EPI_ISL_3874741	2021-09-02
hCoV-19/Japan/PG-69310/2021	EPI_ISL_3874742	2021-09-02
hCoV-19/Japan/PG-69311/2021	EPI_ISL_3874743	2021-09-02
hCoV-19/Japan/PG-69312/2021	EPI_ISL_3874744	2021-09-02
hCoV-19/Japan/PG-69313/2021	EPI_ISL_3874745	2021-09-02
hCoV-19/Japan/PG-69314/2021	EPI_ISL_3874746	2021-09-02
hCoV-19/Japan/PG-69315/2021	EPI_ISL_3874747	2021-09-02

virus_name	epiID	submission_date
hCoV-19/Japan/PG-69316/2021	EPI_ISL_3874748	2021-09-02
hCoV-19/Japan/PG-69317/2021	EPI_ISL_3874749	2021-09-02
hCoV-19/Japan/PG-69318/2021	EPI_ISL_3874750	2021-09-02
hCoV-19/Japan/PG-69319/2021	EPI_ISL_3874751	2021-09-02
hCoV-19/Japan/PG-69320/2021	EPI_ISL_3874752	2021-09-02
hCoV-19/Japan/PG-69321/2021	EPI_ISL_3874753	2021-09-02
hCoV-19/Japan/PG-69322/2021	EPI_ISL_3874754	2021-09-02
hCoV-19/Japan/PG-69323/2021	EPI_ISL_3874755	2021-09-02
hCoV-19/Japan/PG-69324/2021	EPI_ISL_3874756	2021-09-02
hCoV-19/Japan/PG-69325/2021	EPI_ISL_3874757	2021-09-02
hCoV-19/Japan/PG-69326/2021	EPI_ISL_3874758	2021-09-02
hCoV-19/Japan/PG-69327/2021	EPI_ISL_3874759	2021-09-02
hCoV-19/Japan/PG-69328/2021	EPI_ISL_3875982	2021-09-02
hCoV-19/Japan/PG-69329/2021	EPI_ISL_3875983	2021-09-02
hCoV-19/Japan/PG-69330/2021	EPI_ISL_3875984	2021-09-02
hCoV-19/Japan/PG-69331/2021	EPI_ISL_3875985	2021-09-02
hCoV-19/Japan/PG-69332/2021	EPI_ISL_3875986	2021-09-02
hCoV-19/Japan/PG-69333/2021	EPI_ISL_3875987	2021-09-02
hCoV-19/Japan/PG-69334/2021	EPI_ISL_3875988	2021-09-02
hCoV-19/Japan/PG-69335/2021	EPI_ISL_3875989	2021-09-02
hCoV-19/Japan/PG-69336/2021	EPI_ISL_3875990	2021-09-02
hCoV-19/Japan/PG-69337/2021	EPI_ISL_3875991	2021-09-02
hCoV-19/Japan/PG-69338/2021	EPI_ISL_3875992	2021-09-02
hCoV-19/Japan/PG-69339/2021	EPI_ISL_3875993	2021-09-02
hCoV-19/Japan/PG-69340/2021	EPI_ISL_3875994	2021-09-02
hCoV-19/Japan/PG-69341/2021	EPI_ISL_3875995	2021-09-02
hCoV-19/Japan/PG-69342/2021	EPI_ISL_3875996	2021-09-02
hCoV-19/Japan/PG-69346/2021	EPI_ISL_3875997	2021-09-02
hCoV-19/Japan/PG-69347/2021	EPI_ISL_3875998	2021-09-02
hCoV-19/Japan/PG-69348/2021	EPI_ISL_3875999	2021-09-02
hCoV-19/Japan/PG-69349/2021	EPI_ISL_3876000	2021-09-02
hCoV-19/Japan/PG-69352/2021	EPI_ISL_3870549	2021-09-02
hCoV-19/Japan/PG-69353/2021	EPI_ISL_3870550	2021-09-02
hCoV-19/Japan/PG-69354/2021	EPI_ISL_3870551	2021-09-02
hCoV-19/Japan/PG-69355/2021	EPI_ISL_3870552	2021-09-02
hCoV-19/Japan/PG-69358/2021	EPI_ISL_3870553	2021-09-02
hCoV-19/Japan/PG-69359/2021	EPI_ISL_3870554	2021-09-02
hCoV-19/Japan/PG-69360/2021	EPI_ISL_3870555	2021-09-02
hCoV-19/Japan/PG-69361/2021	EPI_ISL_3870556	2021-09-02
hCoV-19/Japan/PG-69362/2021	EPI_ISL_3870557	2021-09-02
hCoV-19/Japan/PG-69363/2021	EPI_ISL_3870558	2021-09-02
hCoV-19/Japan/PG-69364/2021	EPI_ISL_3870559	2021-09-02
hCoV-19/Japan/PG-69365/2021	EPI_ISL_3870560	2021-09-02
hCoV-19/Japan/PG-69366/2021	EPI_ISL_3870561	2021-09-02
hCoV-19/Japan/PG-69367/2021	EPI_ISL_3870562	2021-09-02
hCoV-19/Japan/PG-69390/2021	EPI_ISL_3871182	2021-09-02
hCoV-19/Japan/PG-69391/2021	EPI_ISL_3871183	2021-09-02
hCoV-19/Japan/PG-69392/2021	EPI_ISL_3871184	2021-09-02
hCoV-19/Japan/PG-69393/2021	EPI_ISL_3871185	2021-09-02
hCoV-19/Japan/PG-69394/2021	EPI_ISL_3871186	2021-09-02
hCoV-19/Japan/PG-69395/2021	EPI_ISL_3871187	2021-09-02
hCoV-19/Japan/PG-69396/2021	EPI_ISL_3871188	2021-09-02
hCoV-19/Japan/PG-69397/2021	EPI_ISL_3871189	2021-09-02
hCoV-19/Japan/PG-69408/2021	EPI_ISL_3874006	2021-09-02

virus_name	epiID	submission_date
hCoV-19/Japan/PG-69409/2021	EPI_ISL_3874007	2021-09-02
hCoV-19/Japan/PG-69410/2021	EPI_ISL_3874008	2021-09-02
hCoV-19/Japan/PG-69419/2021	EPI_ISL_3870463	2021-09-02
hCoV-19/Japan/PG-69420/2021	EPI_ISL_3870464	2021-09-02
hCoV-19/Japan/PG-69421/2021	EPI_ISL_3874134	2021-09-02
hCoV-19/Japan/PG-69422/2021	EPI_ISL_3874135	2021-09-02
hCoV-19/Japan/PG-69423/2021	EPI_ISL_3874136	2021-09-02
hCoV-19/Japan/PG-69424/2021	EPI_ISL_3874137	2021-09-02
hCoV-19/Japan/PG-69425/2021	EPI_ISL_3874138	2021-09-02
hCoV-19/Japan/PG-69426/2021	EPI_ISL_3874139	2021-09-02
hCoV-19/Japan/PG-69427/2021	EPI_ISL_3874140	2021-09-02
hCoV-19/Japan/PG-69428/2021	EPI_ISL_3874141	2021-09-02
hCoV-19/Japan/PG-69429/2021	EPI_ISL_3874142	2021-09-02
hCoV-19/Japan/PG-69430/2021	EPI_ISL_3874143	2021-09-02
hCoV-19/Japan/PG-69431/2021	EPI_ISL_3874144	2021-09-02
hCoV-19/Japan/PG-69432/2021	EPI_ISL_3874145	2021-09-02
hCoV-19/Japan/PG-69433/2021	EPI_ISL_3874146	2021-09-02
hCoV-19/Japan/PG-69442/2021	EPI_ISL_3876406	2021-09-02
hCoV-19/Japan/PG-69452/2021	EPI_ISL_3876407	2021-09-02
hCoV-19/Japan/PG-69454/2021	EPI_ISL_3876408	2021-09-02
hCoV-19/Japan/PG-69455/2021	EPI_ISL_3876409	2021-09-02
hCoV-19/Japan/PG-69456/2021	EPI_ISL_3876410	2021-09-02
hCoV-19/Japan/PG-69457/2021	EPI_ISL_3876411	2021-09-02
hCoV-19/Japan/PG-69458/2021	EPI_ISL_3876412	2021-09-02
hCoV-19/Japan/PG-69459/2021	EPI_ISL_3876413	2021-09-02
hCoV-19/Japan/PG-69460/2021	EPI_ISL_3876414	2021-09-02
hCoV-19/Japan/PG-69461/2021	EPI_ISL_3876415	2021-09-02
hCoV-19/Japan/PG-69462/2021	EPI_ISL_3876416	2021-09-02
hCoV-19/Japan/PG-69463/2021	EPI_ISL_3876417	2021-09-02
hCoV-19/Japan/PG-69464/2021	EPI_ISL_3876418	2021-09-02
hCoV-19/Japan/PG-69470/2021	EPI_ISL_3876419	2021-09-02
hCoV-19/Japan/PG-69471/2021	EPI_ISL_3876420	2021-09-02
hCoV-19/Japan/PG-69472/2021	EPI_ISL_3876421	2021-09-02
hCoV-19/Japan/PG-69473/2021	EPI_ISL_3876422	2021-09-02
hCoV-19/Japan/PG-69474/2021	EPI_ISL_3876423	2021-09-02
hCoV-19/Japan/PG-69475/2021	EPI_ISL_3876424	2021-09-02
hCoV-19/Japan/PG-69476/2021	EPI_ISL_3876425	2021-09-02
hCoV-19/Japan/PG-69477/2021	EPI_ISL_3876426	2021-09-02
hCoV-19/Japan/PG-69478/2021	EPI_ISL_3876427	2021-09-02
hCoV-19/Japan/PG-69479/2021	EPI_ISL_3876428	2021-09-02
hCoV-19/Japan/PG-69480/2021	EPI_ISL_3876429	2021-09-02
hCoV-19/Japan/PG-69481/2021	EPI_ISL_3876430	2021-09-02
hCoV-19/Japan/PG-69482/2021	EPI_ISL_3876431	2021-09-02
hCoV-19/Japan/PG-69483/2021	EPI_ISL_3876432	2021-09-02
hCoV-19/Japan/PG-69484/2021	EPI_ISL_3876433	2021-09-02
hCoV-19/Japan/PG-69485/2021	EPI_ISL_3876434	2021-09-02
hCoV-19/Japan/PG-69486/2021	EPI_ISL_3876435	2021-09-02
hCoV-19/Japan/PG-69487/2021	EPI_ISL_3876436	2021-09-02
hCoV-19/Japan/PG-69488/2021	EPI_ISL_3876437	2021-09-02
hCoV-19/Japan/PG-69489/2021	EPI_ISL_3876438	2021-09-02
hCoV-19/Japan/PG-69490/2021	EPI_ISL_3876439	2021-09-02
hCoV-19/Japan/PG-69498/2021	EPI_ISL_3876440	2021-09-02
hCoV-19/Japan/PG-69499/2021	EPI_ISL_3876441	2021-09-02
hCoV-19/Japan/PG-69500/2021	EPI_ISL_3876442	2021-09-02

virus_name	epiID	submission_date
hCoV-19/Japan/PG-69501/2021	EPI_ISL_3876443	2021-09-02
hCoV-19/Japan/PG-69502/2021	EPI_ISL_3876444	2021-09-02
hCoV-19/Japan/PG-69503/2021	EPI_ISL_3876445	2021-09-02
hCoV-19/Japan/PG-69504/2021	EPI_ISL_3876446	2021-09-02
hCoV-19/Japan/PG-69505/2021	EPI_ISL_3876447	2021-09-02
hCoV-19/Japan/PG-69506/2021	EPI_ISL_3876448	2021-09-02
hCoV-19/Japan/PG-69515/2021	EPI_ISL_3876449	2021-09-02
hCoV-19/Japan/PG-69535/2021	EPI_ISL_3876450	2021-09-02
hCoV-19/Japan/PG-69536/2021	EPI_ISL_3876451	2021-09-02
hCoV-19/Japan/PG-69537/2021	EPI_ISL_3876452	2021-09-02
hCoV-19/Japan/PG-69538/2021	EPI_ISL_3876453	2021-09-02
hCoV-19/Japan/PG-69539/2021	EPI_ISL_3876454	2021-09-02
hCoV-19/Japan/PG-69540/2021	EPI_ISL_3876455	2021-09-02
hCoV-19/Japan/PG-69541/2021	EPI_ISL_3876456	2021-09-02
hCoV-19/Japan/PG-69542/2021	EPI_ISL_3876457	2021-09-02
hCoV-19/Japan/PG-69543/2021	EPI_ISL_3876458	2021-09-02
hCoV-19/Japan/PG-69544/2021	EPI_ISL_3876459	2021-09-02
hCoV-19/Japan/PG-69545/2021	EPI_ISL_3876460	2021-09-02
hCoV-19/Japan/PG-69546/2021	EPI_ISL_3876461	2021-09-02
hCoV-19/Japan/PG-69547/2021	EPI_ISL_3876462	2021-09-02
hCoV-19/Japan/PG-69548/2021	EPI_ISL_3876463	2021-09-02
hCoV-19/Japan/PG-69549/2021	EPI_ISL_3876464	2021-09-02
hCoV-19/Japan/PG-69550/2021	EPI_ISL_3876465	2021-09-02
hCoV-19/Japan/PG-69562/2021	EPI_ISL_3876466	2021-09-02
hCoV-19/Japan/PG-69563/2021	EPI_ISL_3876467	2021-09-02
hCoV-19/Japan/PG-69564/2021	EPI_ISL_3870563	2021-09-02
hCoV-19/Japan/PG-69565/2021	EPI_ISL_3870564	2021-09-02
hCoV-19/Japan/PG-69566/2021	EPI_ISL_3870565	2021-09-02
hCoV-19/Japan/PG-69567/2021	EPI_ISL_3870566	2021-09-02
hCoV-19/Japan/PG-69568/2021	EPI_ISL_3870567	2021-09-02
hCoV-19/Japan/PG-69569/2021	EPI_ISL_3870568	2021-09-02
hCoV-19/Japan/PG-69570/2021	EPI_ISL_3870569	2021-09-02
hCoV-19/Japan/PG-69571/2021	EPI_ISL_3870570	2021-09-02
hCoV-19/Japan/PG-69572/2021	EPI_ISL_3870571	2021-09-02
hCoV-19/Japan/PG-69573/2021	EPI_ISL_3870572	2021-09-02
hCoV-19/Japan/PG-69574/2021	EPI_ISL_3870573	2021-09-02
hCoV-19/Japan/PG-69575/2021	EPI_ISL_3870574	2021-09-02
hCoV-19/Japan/PG-69576/2021	EPI_ISL_3870575	2021-09-02
hCoV-19/Japan/PG-69577/2021	EPI_ISL_3870576	2021-09-02
hCoV-19/Japan/PG-69578/2021	EPI_ISL_3870577	2021-09-02
hCoV-19/Japan/PG-69579/2021	EPI_ISL_3870578	2021-09-02
hCoV-19/Japan/PG-69580/2021	EPI_ISL_3870579	2021-09-02
hCoV-19/Japan/PG-69581/2021	EPI_ISL_3870580	2021-09-02
hCoV-19/Japan/PG-69582/2021	EPI_ISL_3870581	2021-09-02
hCoV-19/Japan/PG-69583/2021	EPI_ISL_3870582	2021-09-02
hCoV-19/Japan/PG-69584/2021	EPI_ISL_3870583	2021-09-02
hCoV-19/Japan/PG-69585/2021	EPI_ISL_3870584	2021-09-02
hCoV-19/Japan/PG-69586/2021	EPI_ISL_3870585	2021-09-02
hCoV-19/Japan/PG-69587/2021	EPI_ISL_3870586	2021-09-02
hCoV-19/Japan/PG-69588/2021	EPI_ISL_3870587	2021-09-02
hCoV-19/Japan/PG-69589/2021	EPI_ISL_3870588	2021-09-02
hCoV-19/Japan/PG-69590/2021	EPI_ISL_3870589	2021-09-02
hCoV-19/Japan/PG-69591/2021	EPI_ISL_3870590	2021-09-02
hCoV-19/Japan/PG-69592/2021	EPI_ISL_3870591	2021-09-02

virus_name	epiID	submission_date
hCoV-19/Japan/PG-69593/2021	EPI_ISL_3870592	2021-09-02
hCoV-19/Japan/PG-69594/2021	EPI_ISL_3870593	2021-09-02
hCoV-19/Japan/PG-69595/2021	EPI_ISL_3870594	2021-09-02
hCoV-19/Japan/PG-69596/2021	EPI_ISL_3870595	2021-09-02
hCoV-19/Japan/PG-69597/2021	EPI_ISL_3870596	2021-09-02
hCoV-19/Japan/PG-69598/2021	EPI_ISL_3870597	2021-09-02
hCoV-19/Japan/PG-69599/2021	EPI_ISL_3870598	2021-09-02
hCoV-19/Japan/PG-69600/2021	EPI_ISL_3870599	2021-09-02
hCoV-19/Japan/PG-69601/2021	EPI_ISL_3870600	2021-09-02
hCoV-19/Japan/PG-69602/2021	EPI_ISL_3870601	2021-09-02
hCoV-19/Japan/PG-69603/2021	EPI_ISL_3870602	2021-09-02
hCoV-19/Japan/PG-69604/2021	EPI_ISL_3870603	2021-09-02
hCoV-19/Japan/PG-69605/2021	EPI_ISL_3870604	2021-09-02
hCoV-19/Japan/PG-69606/2021	EPI_ISL_3870605	2021-09-02
hCoV-19/Japan/PG-69607/2021	EPI_ISL_3870606	2021-09-02
hCoV-19/Japan/PG-69610/2021	EPI_ISL_3872136	2021-09-02
hCoV-19/Japan/PG-69611/2021	EPI_ISL_3872137	2021-09-02
hCoV-19/Japan/PG-69612/2021	EPI_ISL_3872138	2021-09-02
hCoV-19/Japan/PG-69613/2021	EPI_ISL_3872139	2021-09-02
hCoV-19/Japan/PG-69614/2021	EPI_ISL_3872140	2021-09-02
hCoV-19/Japan/PG-69615/2021	EPI_ISL_3872141	2021-09-02
hCoV-19/Japan/PG-69616/2021	EPI_ISL_3872143	2021-09-02
hCoV-19/Japan/PG-69617/2021	EPI_ISL_3872144	2021-09-02
hCoV-19/Japan/PG-69618/2021	EPI_ISL_3872145	2021-09-02
hCoV-19/Japan/PG-69619/2021	EPI_ISL_3872146	2021-09-02
hCoV-19/Japan/PG-70703/2021	EPI_ISL_3876468	2021-09-02
hCoV-19/Japan/PG-70733/2021	EPI_ISL_3876469	2021-09-02
hCoV-19/Japan/PG-71301/2021	EPI_ISL_3874760	2021-09-02
hCoV-19/Japan/PG-71302/2021	EPI_ISL_3874761	2021-09-02
hCoV-19/Japan/PG-71303/2021	EPI_ISL_3874762	2021-09-02
hCoV-19/Japan/PG-71418/2021	EPI_ISL_3874763	2021-09-02
hCoV-19/Japan/PG-71419/2021	EPI_ISL_3874764	2021-09-02
hCoV-19/Japan/PG-71420/2021	EPI_ISL_3874765	2021-09-02
hCoV-19/Japan/PG-71421/2021	EPI_ISL_3874766	2021-09-02
hCoV-19/Japan/PG-71422/2021	EPI_ISL_3874767	2021-09-02
hCoV-19/Japan/PG-71423/2021	EPI_ISL_3874768	2021-09-02
hCoV-19/Japan/PG-71424/2021	EPI_ISL_3874769	2021-09-02
hCoV-19/Japan/PG-71425/2021	EPI_ISL_3875475	2021-09-02
hCoV-19/Japan/PG-71426/2021	EPI_ISL_3875476	2021-09-02
hCoV-19/Japan/PG-71427/2021	EPI_ISL_3875477	2021-09-02
hCoV-19/Japan/PG-71428/2021	EPI_ISL_3875478	2021-09-02
hCoV-19/Japan/PG-71429/2021	EPI_ISL_3875479	2021-09-02
hCoV-19/Japan/PG-71430/2021	EPI_ISL_3875480	2021-09-02
hCoV-19/Japan/PG-71431/2021	EPI_ISL_3875481	2021-09-02
hCoV-19/Japan/PG-71432/2021	EPI_ISL_3875482	2021-09-02
hCoV-19/Japan/PG-71433/2021	EPI_ISL_3875483	2021-09-02
hCoV-19/Japan/PG-71434/2021	EPI_ISL_3875484	2021-09-02
hCoV-19/Japan/PG-71435/2021	EPI_ISL_3875485	2021-09-02
hCoV-19/Japan/PG-71436/2021	EPI_ISL_3875486	2021-09-02
hCoV-19/Japan/PG-71437/2021	EPI_ISL_3875487	2021-09-02
hCoV-19/Japan/PG-71438/2021	EPI_ISL_3875488	2021-09-02
hCoV-19/Japan/PG-71439/2021	EPI_ISL_3875489	2021-09-02
hCoV-19/Japan/PG-71440/2021	EPI_ISL_3875490	2021-09-02
hCoV-19/Japan/PG-71441/2021	EPI_ISL_3875491	2021-09-02

virus_name	epiID	submission_date
hCoV-19/Japan/PG-71442/2021	EPI_ISL_3875492	2021-09-02
hCoV-19/Japan/PG-71443/2021	EPI_ISL_3875493	2021-09-02
hCoV-19/Japan/PG-71444/2021	EPI_ISL_3875494	2021-09-02
hCoV-19/Japan/PG-71445/2021	EPI_ISL_3875495	2021-09-02
hCoV-19/Japan/PG-71446/2021	EPI_ISL_3875496	2021-09-02
hCoV-19/Japan/PG-71447/2021	EPI_ISL_3875497	2021-09-02
hCoV-19/Japan/PG-71448/2021	EPI_ISL_3875498	2021-09-02
hCoV-19/Japan/PG-71449/2021	EPI_ISL_3875499	2021-09-02
hCoV-19/Japan/PG-71450/2021	EPI_ISL_3875500	2021-09-02
hCoV-19/Japan/PG-71451/2021	EPI_ISL_3875501	2021-09-02
hCoV-19/Japan/PG-71452/2021	EPI_ISL_3875502	2021-09-02
hCoV-19/Japan/PG-71453/2021	EPI_ISL_3875503	2021-09-02
hCoV-19/Japan/PG-71454/2021	EPI_ISL_3875504	2021-09-02
hCoV-19/Japan/PG-71455/2021	EPI_ISL_3875505	2021-09-02
hCoV-19/Japan/PG-71456/2021	EPI_ISL_3875506	2021-09-02
hCoV-19/Japan/PG-71457/2021	EPI_ISL_3875507	2021-09-02
hCoV-19/Japan/PG-71458/2021	EPI_ISL_3875508	2021-09-02
hCoV-19/Japan/PG-71459/2021	EPI_ISL_3875509	2021-09-02
hCoV-19/Japan/PG-71460/2021	EPI_ISL_3875510	2021-09-02
hCoV-19/Japan/PG-71461/2021	EPI_ISL_3875511	2021-09-02
hCoV-19/Japan/PG-71462/2021	EPI_ISL_3875512	2021-09-02
hCoV-19/Japan/PG-71463/2021	EPI_ISL_3875513	2021-09-02
hCoV-19/Japan/PG-71464/2021	EPI_ISL_3875514	2021-09-02
hCoV-19/Japan/PG-71465/2021	EPI_ISL_3875515	2021-09-02
hCoV-19/Japan/PG-71466/2021	EPI_ISL_3875516	2021-09-02
hCoV-19/Japan/PG-71467/2021	EPI_ISL_3875517	2021-09-02
hCoV-19/Japan/PG-71468/2021	EPI_ISL_3875518	2021-09-02
hCoV-19/Japan/PG-71469/2021	EPI_ISL_3875519	2021-09-02
hCoV-19/Japan/PG-71470/2021	EPI_ISL_3875520	2021-09-02
hCoV-19/Japan/PG-71471/2021	EPI_ISL_3875521	2021-09-02
hCoV-19/Japan/PG-71472/2021	EPI_ISL_3875522	2021-09-02
hCoV-19/Japan/PG-71473/2021	EPI_ISL_3871749	2021-09-02
hCoV-19/Japan/PG-71486/2021	EPI_ISL_3871750	2021-09-02
hCoV-19/Japan/PG-71487/2021	EPI_ISL_3871751	2021-09-02
hCoV-19/Japan/PG-71488/2021	EPI_ISL_3876001	2021-09-02
hCoV-19/Japan/PG-71489/2021	EPI_ISL_3876002	2021-09-02
hCoV-19/Japan/PG-71490/2021	EPI_ISL_3876003	2021-09-02
hCoV-19/Japan/PG-71491/2021	EPI_ISL_3876004	2021-09-02
hCoV-19/Japan/PG-71492/2021	EPI_ISL_3876005	2021-09-02
hCoV-19/Japan/PG-71493/2021	EPI_ISL_3876006	2021-09-02
hCoV-19/Japan/PG-71494/2021	EPI_ISL_3875722	2021-09-02
hCoV-19/Japan/PG-71495/2021	EPI_ISL_3875725	2021-09-02
hCoV-19/Japan/PG-71506/2021	EPI_ISL_3875729	2021-09-02
hCoV-19/Japan/PG-71507/2021	EPI_ISL_3875732	2021-09-02
hCoV-19/Japan/PG-71508/2021	EPI_ISL_3875735	2021-09-02
hCoV-19/Japan/PG-71509/2021	EPI_ISL_3875738	2021-09-02
hCoV-19/Japan/PG-71510/2021	EPI_ISL_3875741	2021-09-02
hCoV-19/Japan/PG-71511/2021	EPI_ISL_3875744	2021-09-02
hCoV-19/Japan/PG-71512/2021	EPI_ISL_3875747	2021-09-02
hCoV-19/Japan/PG-71513/2021	EPI_ISL_3875751	2021-09-02
hCoV-19/Japan/PG-71514/2021	EPI_ISL_3875754	2021-09-02
hCoV-19/Japan/PG-71515/2021	EPI_ISL_3875757	2021-09-02
hCoV-19/Japan/PG-71518/2021	EPI_ISL_3870607	2021-09-02
hCoV-19/Japan/PG-71519/2021	EPI_ISL_3870608	2021-09-02

virus_name	epiID	submission_date
hCoV-19/Japan/PG-71520/2021	EPI_ISL_3870609	2021-09-02
hCoV-19/Japan/PG-71521/2021	EPI_ISL_3870610	2021-09-02
hCoV-19/Japan/PG-71522/2021	EPI_ISL_3870611	2021-09-02
hCoV-19/Japan/PG-71523/2021	EPI_ISL_3870612	2021-09-02
hCoV-19/Japan/PG-71524/2021	EPI_ISL_3870613	2021-09-02
hCoV-19/Japan/PG-71525/2021	EPI_ISL_3870614	2021-09-02
hCoV-19/Japan/PG-71526/2021	EPI_ISL_3872559	2021-09-02
hCoV-19/Japan/PG-71527/2021	EPI_ISL_3872560	2021-09-02
hCoV-19/Japan/PG-71528/2021	EPI_ISL_3872561	2021-09-02
hCoV-19/Japan/PG-71529/2021	EPI_ISL_3872562	2021-09-02
hCoV-19/Japan/PG-71530/2021	EPI_ISL_3872563	2021-09-02
hCoV-19/Japan/PG-71531/2021	EPI_ISL_3872564	2021-09-02
hCoV-19/Japan/PG-71532/2021	EPI_ISL_3872565	2021-09-02
hCoV-19/Japan/PG-71533/2021	EPI_ISL_3872566	2021-09-02
hCoV-19/Japan/PG-71534/2021	EPI_ISL_3872567	2021-09-02
hCoV-19/Japan/PG-71535/2021	EPI_ISL_3872568	2021-09-02
hCoV-19/Japan/PG-71536/2021	EPI_ISL_3872569	2021-09-02
hCoV-19/Japan/PG-71537/2021	EPI_ISL_3872570	2021-09-02
hCoV-19/Japan/PG-71538/2021	EPI_ISL_3872571	2021-09-02
hCoV-19/Japan/PG-71539/2021	EPI_ISL_3872572	2021-09-02
hCoV-19/Japan/PG-71540/2021	EPI_ISL_3872573	2021-09-02
hCoV-19/Japan/PG-71541/2021	EPI_ISL_3872574	2021-09-02
hCoV-19/Japan/PG-71542/2021	EPI_ISL_3872575	2021-09-02
hCoV-19/Japan/PG-71543/2021	EPI_ISL_3872576	2021-09-02
hCoV-19/Japan/PG-71544/2021	EPI_ISL_3872577	2021-09-02
hCoV-19/Japan/PG-71545/2021	EPI_ISL_3872578	2021-09-02
hCoV-19/Japan/PG-71546/2021	EPI_ISL_3872579	2021-09-02
hCoV-19/Japan/PG-71550/2021	EPI_ISL_3871681	2021-09-02
hCoV-19/Japan/PG-71551/2021	EPI_ISL_3871682	2021-09-02
hCoV-19/Japan/PG-71552/2021	EPI_ISL_3874479	2021-09-02
hCoV-19/Japan/PG-71553/2021	EPI_ISL_3874480	2021-09-02
hCoV-19/Japan/PG-71554/2021	EPI_ISL_3874481	2021-09-02
hCoV-19/Japan/PG-71555/2021	EPI_ISL_3874482	2021-09-02
hCoV-19/Japan/PG-71556/2021	EPI_ISL_3874483	2021-09-02
hCoV-19/Japan/PG-71557/2021	EPI_ISL_3874484	2021-09-02
hCoV-19/Japan/PG-71558/2021	EPI_ISL_3874485	2021-09-02
hCoV-19/Japan/PG-71559/2021	EPI_ISL_3874486	2021-09-02
hCoV-19/Japan/PG-71562/2021	EPI_ISL_3873957	2021-09-02
hCoV-19/Japan/PG-71563/2021	EPI_ISL_3873958	2021-09-02
hCoV-19/Japan/PG-71564/2021	EPI_ISL_3873959	2021-09-02
hCoV-19/Japan/PG-71565/2021	EPI_ISL_3873960	2021-09-02
hCoV-19/Japan/PG-71566/2021	EPI_ISL_3873961	2021-09-02
hCoV-19/Japan/PG-71567/2021	EPI_ISL_3873962	2021-09-02
hCoV-19/Japan/PG-71568/2021	EPI_ISL_3873963	2021-09-02
hCoV-19/Japan/PG-71569/2021	EPI_ISL_3873964	2021-09-02
hCoV-19/Japan/PG-71570/2021	EPI_ISL_3873343	2021-09-02
hCoV-19/Japan/PG-71571/2021	EPI_ISL_3873344	2021-09-02
hCoV-19/Japan/PG-71572/2021	EPI_ISL_3873345	2021-09-02
hCoV-19/Japan/PG-71573/2021	EPI_ISL_3873346	2021-09-02
hCoV-19/Japan/PG-71574/2021	EPI_ISL_3873347	2021-09-02
hCoV-19/Japan/PG-71575/2021	EPI_ISL_3873348	2021-09-02
hCoV-19/Japan/PG-71576/2021	EPI_ISL_3873349	2021-09-02
hCoV-19/Japan/PG-71577/2021	EPI_ISL_3873350	2021-09-02
hCoV-19/Japan/PG-71578/2021	EPI_ISL_3873351	2021-09-02

virus_name	epiID	submission_date
hCoV-19/Japan/PG-71579/2021	EPI_ISL_3873352	2021-09-02
hCoV-19/Japan/PG-71580/2021	EPI_ISL_3873353	2021-09-02
hCoV-19/Japan/PG-71581/2021	EPI_ISL_3874399	2021-09-02
hCoV-19/Japan/PG-71586/2021	EPI_ISL_3872147	2021-09-02
hCoV-19/Japan/PG-71587/2021	EPI_ISL_3872148	2021-09-02
hCoV-19/Japan/PG-71588/2021	EPI_ISL_3872149	2021-09-02
hCoV-19/Japan/PG-71589/2021	EPI_ISL_3872150	2021-09-02
hCoV-19/Japan/PG-71590/2021	EPI_ISL_3872238	2021-09-02
hCoV-19/Japan/PG-71591/2021	EPI_ISL_3872239	2021-09-02
hCoV-19/Japan/PG-71592/2021	EPI_ISL_3872240	2021-09-02
hCoV-19/Japan/PG-71593/2021	EPI_ISL_3872241	2021-09-02
hCoV-19/Japan/PG-71594/2021	EPI_ISL_3872242	2021-09-02
hCoV-19/Japan/PG-71595/2021	EPI_ISL_3872243	2021-09-02
hCoV-19/Japan/PG-71596/2021	EPI_ISL_3872244	2021-09-02
hCoV-19/Japan/PG-71597/2021	EPI_ISL_3872245	2021-09-02
hCoV-19/Japan/PG-71598/2021	EPI_ISL_3876470	2021-09-02
hCoV-19/Japan/PG-71599/2021	EPI_ISL_3876471	2021-09-02
hCoV-19/Japan/PG-71600/2021	EPI_ISL_3876472	2021-09-02
hCoV-19/Japan/PG-71601/2021	EPI_ISL_3876473	2021-09-02
hCoV-19/Japan/PG-71602/2021	EPI_ISL_3876474	2021-09-02
hCoV-19/Japan/PG-71603/2021	EPI_ISL_3876475	2021-09-02
hCoV-19/Japan/PG-71606/2021	EPI_ISL_3876476	2021-09-02
hCoV-19/Japan/PG-71607/2021	EPI_ISL_3876477	2021-09-02
hCoV-19/Japan/PG-71608/2021	EPI_ISL_3876478	2021-09-02
hCoV-19/Japan/PG-71609/2021	EPI_ISL_3876479	2021-09-02
hCoV-19/Japan/PG-71610/2021	EPI_ISL_3876480	2021-09-02
hCoV-19/Japan/PG-71611/2021	EPI_ISL_3876483	2021-09-02
hCoV-19/Japan/PG-71612/2021	EPI_ISL_3876486	2021-09-02
hCoV-19/Japan/PG-71613/2021	EPI_ISL_3876490	2021-09-02
hCoV-19/Japan/PG-71614/2021	EPI_ISL_3876493	2021-09-02
hCoV-19/Japan/PG-71615/2021	EPI_ISL_3876494	2021-09-02
hCoV-19/Japan/PG-71616/2021	EPI_ISL_3876495	2021-09-02
hCoV-19/Japan/PG-71617/2021	EPI_ISL_3876496	2021-09-02
hCoV-19/Japan/PG-71618/2021	EPI_ISL_3876497	2021-09-02
hCoV-19/Japan/PG-71619/2021	EPI_ISL_3876498	2021-09-02
hCoV-19/Japan/PG-71620/2021	EPI_ISL_3876499	2021-09-02
hCoV-19/Japan/PG-71622/2021	EPI_ISL_3876500	2021-09-02
hCoV-19/Japan/PG-71623/2021	EPI_ISL_3876501	2021-09-02
hCoV-19/Japan/PG-71624/2021	EPI_ISL_3876502	2021-09-02
hCoV-19/Japan/PG-71625/2021	EPI_ISL_3876503	2021-09-02
hCoV-19/Japan/PG-71626/2021	EPI_ISL_3876504	2021-09-02
hCoV-19/Japan/PG-71627/2021	EPI_ISL_3876505	2021-09-02
hCoV-19/Japan/PG-71628/2021	EPI_ISL_3876506	2021-09-02
hCoV-19/Japan/PG-71629/2021	EPI_ISL_3876507	2021-09-02
hCoV-19/Japan/PG-71630/2021	EPI_ISL_3876508	2021-09-02
hCoV-19/Japan/PG-71631/2021	EPI_ISL_3876509	2021-09-02
hCoV-19/Japan/PG-71632/2021	EPI_ISL_3876510	2021-09-02
hCoV-19/Japan/PG-71633/2021	EPI_ISL_3876511	2021-09-02
hCoV-19/Japan/PG-71634/2021	EPI_ISL_3876512	2021-09-02
hCoV-19/Japan/PG-71635/2021	EPI_ISL_3876513	2021-09-02
hCoV-19/Japan/PG-71636/2021	EPI_ISL_3876514	2021-09-02
hCoV-19/Japan/PG-71637/2021	EPI_ISL_3876515	2021-09-02
hCoV-19/Japan/PG-71638/2021	EPI_ISL_3876516	2021-09-02
hCoV-19/Japan/PG-71639/2021	EPI_ISL_3876517	2021-09-02

virus_name	epiID	submission_date
hCoV-19/Japan/PG-71640/2021	EPI_ISL_3876518	2021-09-02
hCoV-19/Japan/PG-71641/2021	EPI_ISL_3876519	2021-09-02
hCoV-19/Japan/PG-71642/2021	EPI_ISL_3876520	2021-09-02
hCoV-19/Japan/PG-71643/2021	EPI_ISL_3876521	2021-09-02
hCoV-19/Japan/PG-71644/2021	EPI_ISL_3876522	2021-09-02
hCoV-19/Japan/PG-71645/2021	EPI_ISL_3876523	2021-09-02
hCoV-19/Japan/PG-71646/2021	EPI_ISL_3876524	2021-09-02
hCoV-19/Japan/PG-71647/2021	EPI_ISL_3876525	2021-09-02
hCoV-19/Japan/PG-71648/2021	EPI_ISL_3876526	2021-09-02
hCoV-19/Japan/PG-71649/2021	EPI_ISL_3876527	2021-09-02
hCoV-19/Japan/PG-71650/2021	EPI_ISL_3876528	2021-09-02
hCoV-19/Japan/PG-71651/2021	EPI_ISL_3876529	2021-09-02
hCoV-19/Japan/PG-71652/2021	EPI_ISL_3876530	2021-09-02
hCoV-19/Japan/PG-71653/2021	EPI_ISL_3876531	2021-09-02
hCoV-19/Japan/PG-71654/2021	EPI_ISL_3876532	2021-09-02
hCoV-19/Japan/PG-71655/2021	EPI_ISL_3876533	2021-09-02
hCoV-19/Japan/PG-71656/2021	EPI_ISL_3876534	2021-09-02
hCoV-19/Japan/PG-71657/2021	EPI_ISL_3876535	2021-09-02
hCoV-19/Japan/PG-71658/2021	EPI_ISL_3876536	2021-09-02
hCoV-19/Japan/PG-71659/2021	EPI_ISL_3876537	2021-09-02
hCoV-19/Japan/PG-71660/2021	EPI_ISL_3876538	2021-09-02
hCoV-19/Japan/PG-71661/2021	EPI_ISL_3876539	2021-09-02
hCoV-19/Japan/PG-71662/2021	EPI_ISL_3876540	2021-09-02
hCoV-19/Japan/PG-71663/2021	EPI_ISL_3876542	2021-09-02
hCoV-19/Japan/PG-71664/2021	EPI_ISL_3876544	2021-09-02
hCoV-19/Japan/PG-71665/2021	EPI_ISL_3876546	2021-09-02
hCoV-19/Japan/PG-71666/2021	EPI_ISL_3876548	2021-09-02
hCoV-19/Japan/PG-71667/2021	EPI_ISL_3876550	2021-09-02
hCoV-19/Japan/PG-71668/2021	EPI_ISL_3876552	2021-09-02
hCoV-19/Japan/PG-71669/2021	EPI_ISL_3876555	2021-09-02
hCoV-19/Japan/PG-71670/2021	EPI_ISL_3876557	2021-09-02
hCoV-19/Japan/PG-71671/2021	EPI_ISL_3876559	2021-09-02
hCoV-19/Japan/PG-71672/2021	EPI_ISL_3876561	2021-09-02
hCoV-19/Japan/PG-71673/2021	EPI_ISL_3876563	2021-09-02
hCoV-19/Japan/PG-71674/2021	EPI_ISL_3876565	2021-09-02
hCoV-19/Japan/PG-71675/2021	EPI_ISL_3876567	2021-09-02
hCoV-19/Japan/PG-71676/2021	EPI_ISL_3876569	2021-09-02
hCoV-19/Japan/PG-71677/2021	EPI_ISL_3876571	2021-09-02
hCoV-19/Japan/PG-71678/2021	EPI_ISL_3876574	2021-09-02
hCoV-19/Japan/PG-71679/2021	EPI_ISL_3876576	2021-09-02
hCoV-19/Japan/PG-71680/2021	EPI_ISL_3876579	2021-09-02
hCoV-19/Japan/PG-71681/2021	EPI_ISL_3876581	2021-09-02
hCoV-19/Japan/PG-71682/2021	EPI_ISL_3876583	2021-09-02
hCoV-19/Japan/PG-71683/2021	EPI_ISL_3876586	2021-09-02
hCoV-19/Japan/PG-71684/2021	EPI_ISL_3876588	2021-09-02
hCoV-19/Japan/PG-71685/2021	EPI_ISL_3876590	2021-09-02
hCoV-19/Japan/PG-71686/2021	EPI_ISL_3876592	2021-09-02
hCoV-19/Japan/PG-71687/2021	EPI_ISL_3876594	2021-09-02
hCoV-19/Japan/PG-71688/2021	EPI_ISL_3876596	2021-09-02
hCoV-19/Japan/PG-71689/2021	EPI_ISL_3876599	2021-09-02
hCoV-19/Japan/PG-71690/2021	EPI_ISL_3876601	2021-09-02
hCoV-19/Japan/PG-71691/2021	EPI_ISL_3876604	2021-09-02
hCoV-19/Japan/PG-71692/2021	EPI_ISL_3876606	2021-09-02
hCoV-19/Japan/PG-71693/2021	EPI_ISL_3876607	2021-09-02

virus_name	epiID	submission_date
hCoV-19/Japan/PG-71694/2021	EPI_ISL_3876609	2021-09-02
hCoV-19/Japan/PG-71695/2021	EPI_ISL_3876611	2021-09-02
hCoV-19/Japan/PG-71696/2021	EPI_ISL_3876613	2021-09-02
hCoV-19/Japan/PG-71697/2021	EPI_ISL_3876614	2021-09-02
hCoV-19/Japan/PG-71698/2021	EPI_ISL_3876616	2021-09-02
hCoV-19/Japan/PG-71699/2021	EPI_ISL_3876618	2021-09-02
hCoV-19/Japan/PG-71700/2021	EPI_ISL_3876620	2021-09-02
hCoV-19/Japan/PG-71701/2021	EPI_ISL_3876622	2021-09-02
hCoV-19/Japan/PG-71702/2021	EPI_ISL_3876624	2021-09-02
hCoV-19/Japan/PG-71703/2021	EPI_ISL_3876626	2021-09-02
hCoV-19/Japan/PG-71704/2021	EPI_ISL_3876629	2021-09-02
hCoV-19/Japan/PG-71705/2021	EPI_ISL_3876632	2021-09-02
hCoV-19/Japan/PG-71706/2021	EPI_ISL_3876634	2021-09-02
hCoV-19/Japan/PG-71707/2021	EPI_ISL_3876637	2021-09-02
hCoV-19/Japan/PG-71708/2021	EPI_ISL_3876639	2021-09-02
hCoV-19/Japan/PG-71709/2021	EPI_ISL_3876641	2021-09-02
hCoV-19/Japan/PG-71710/2021	EPI_ISL_3876643	2021-09-02
hCoV-19/Japan/PG-71711/2021	EPI_ISL_3876646	2021-09-02
hCoV-19/Japan/PG-71712/2021	EPI_ISL_3876649	2021-09-02
hCoV-19/Japan/PG-71713/2021	EPI_ISL_3876651	2021-09-02
hCoV-19/Japan/PG-71714/2021	EPI_ISL_3876653	2021-09-02
hCoV-19/Japan/PG-71715/2021	EPI_ISL_3876655	2021-09-02
hCoV-19/Japan/PG-71716/2021	EPI_ISL_3876657	2021-09-02
hCoV-19/Japan/PG-71717/2021	EPI_ISL_3876660	2021-09-02
hCoV-19/Japan/PG-71718/2021	EPI_ISL_3876663	2021-09-02
hCoV-19/Japan/PG-71719/2021	EPI_ISL_3876665	2021-09-02
hCoV-19/Japan/PG-71720/2021	EPI_ISL_3876668	2021-09-02
hCoV-19/Japan/PG-71721/2021	EPI_ISL_3876671	2021-09-02
hCoV-19/Japan/PG-71722/2021	EPI_ISL_3876674	2021-09-02
hCoV-19/Japan/PG-71723/2021	EPI_ISL_3876676	2021-09-02
hCoV-19/Japan/PG-71724/2021	EPI_ISL_3876679	2021-09-02
hCoV-19/Japan/PG-71725/2021	EPI_ISL_3876681	2021-09-02
hCoV-19/Japan/PG-71726/2021	EPI_ISL_3876684	2021-09-02
hCoV-19/Japan/PG-71727/2021	EPI_ISL_3876686	2021-09-02
hCoV-19/Japan/PG-71728/2021	EPI_ISL_3876688	2021-09-02
hCoV-19/Japan/PG-71729/2021	EPI_ISL_3876690	2021-09-02
hCoV-19/Japan/PG-71730/2021	EPI_ISL_3876692	2021-09-02
hCoV-19/Japan/PG-71731/2021	EPI_ISL_3876695	2021-09-02
hCoV-19/Japan/PG-71732/2021	EPI_ISL_3876697	2021-09-02
hCoV-19/Japan/PG-71733/2021	EPI_ISL_3876700	2021-09-02
hCoV-19/Japan/PG-71734/2021	EPI_ISL_3876702	2021-09-02
hCoV-19/Japan/PG-71735/2021	EPI_ISL_3876705	2021-09-02
hCoV-19/Japan/PG-71736/2021	EPI_ISL_3876708	2021-09-02
hCoV-19/Japan/PG-71737/2021	EPI_ISL_3876711	2021-09-02
hCoV-19/Japan/PG-71738/2021	EPI_ISL_3876714	2021-09-02
hCoV-19/Japan/PG-71739/2021	EPI_ISL_3876716	2021-09-02
hCoV-19/Japan/PG-71740/2021	EPI_ISL_3876718	2021-09-02
hCoV-19/Japan/PG-71741/2021	EPI_ISL_3876721	2021-09-02
hCoV-19/Japan/PG-71742/2021	EPI_ISL_3876724	2021-09-02
hCoV-19/Japan/PG-71743/2021	EPI_ISL_3876726	2021-09-02
hCoV-19/Japan/PG-71744/2021	EPI_ISL_3876728	2021-09-02
hCoV-19/Japan/PG-71745/2021	EPI_ISL_3876731	2021-09-02
hCoV-19/Japan/PG-71746/2021	EPI_ISL_3876733	2021-09-02
hCoV-19/Japan/PG-71747/2021	EPI_ISL_3876735	2021-09-02

virus_name	epiID	submission_date
hCoV-19/Japan/PG-71748/2021	EPI_ISL_3876738	2021-09-02
hCoV-19/Japan/PG-71749/2021	EPI_ISL_3876741	2021-09-02
hCoV-19/Japan/PG-71750/2021	EPI_ISL_3876744	2021-09-02
hCoV-19/Japan/PG-71751/2021	EPI_ISL_3876745	2021-09-02
hCoV-19/Japan/PG-71752/2021	EPI_ISL_3876747	2021-09-02
hCoV-19/Japan/PG-71753/2021	EPI_ISL_3876748	2021-09-02
hCoV-19/Japan/PG-71754/2021	EPI_ISL_3876751	2021-09-02
hCoV-19/Japan/PG-71755/2021	EPI_ISL_3876753	2021-09-02
hCoV-19/Japan/PG-71756/2021	EPI_ISL_3876754	2021-09-02
hCoV-19/Japan/PG-71757/2021	EPI_ISL_3876757	2021-09-02
hCoV-19/Japan/PG-71758/2021	EPI_ISL_3876759	2021-09-02
hCoV-19/Japan/PG-71759/2021	EPI_ISL_3876761	2021-09-02
hCoV-19/Japan/PG-71760/2021	EPI_ISL_3876764	2021-09-02
hCoV-19/Japan/PG-71761/2021	EPI_ISL_3876766	2021-09-02
hCoV-19/Japan/PG-71762/2021	EPI_ISL_3876769	2021-09-02
hCoV-19/Japan/PG-71763/2021	EPI_ISL_3876771	2021-09-02
hCoV-19/Japan/PG-71764/2021	EPI_ISL_3876772	2021-09-02
hCoV-19/Japan/PG-71765/2021	EPI_ISL_3876774	2021-09-02
hCoV-19/Japan/PG-71766/2021	EPI_ISL_3876776	2021-09-02
hCoV-19/Japan/PG-71767/2021	EPI_ISL_3876778	2021-09-02
hCoV-19/Japan/PG-71768/2021	EPI_ISL_3876780	2021-09-02
hCoV-19/Japan/PG-71769/2021	EPI_ISL_3876782	2021-09-02
hCoV-19/Japan/PG-71770/2021	EPI_ISL_3876784	2021-09-02
hCoV-19/Japan/PG-71771/2021	EPI_ISL_3876786	2021-09-02
hCoV-19/Japan/PG-71772/2021	EPI_ISL_3876787	2021-09-02
hCoV-19/Japan/PG-71773/2021	EPI_ISL_3876789	2021-09-02
hCoV-19/Japan/PG-71774/2021	EPI_ISL_3876791	2021-09-02
hCoV-19/Japan/PG-71775/2021	EPI_ISL_3876794	2021-09-02
hCoV-19/Japan/PG-71776/2021	EPI_ISL_3876797	2021-09-02
hCoV-19/Japan/PG-71777/2021	EPI_ISL_3876799	2021-09-02
hCoV-19/Japan/PG-71778/2021	EPI_ISL_3876802	2021-09-02
hCoV-19/Japan/PG-71779/2021	EPI_ISL_3876803	2021-09-02
hCoV-19/Japan/PG-71780/2021	EPI_ISL_3876804	2021-09-02
hCoV-19/Japan/PG-71781/2021	EPI_ISL_3876805	2021-09-02
hCoV-19/Japan/PG-71782/2021	EPI_ISL_3876808	2021-09-02
hCoV-19/Japan/PG-71783/2021	EPI_ISL_3876811	2021-09-02
hCoV-19/Japan/PG-71784/2021	EPI_ISL_3876813	2021-09-02
hCoV-19/Japan/PG-71785/2021	EPI_ISL_3876816	2021-09-02
hCoV-19/Japan/PG-71786/2021	EPI_ISL_3876818	2021-09-02
hCoV-19/Japan/PG-71787/2021	EPI_ISL_3876820	2021-09-02
hCoV-19/Japan/PG-71788/2021	EPI_ISL_3876823	2021-09-02
hCoV-19/Japan/PG-71789/2021	EPI_ISL_3876826	2021-09-02
hCoV-19/Japan/PG-71790/2021	EPI_ISL_3876827	2021-09-02
hCoV-19/Japan/PG-71791/2021	EPI_ISL_3876828	2021-09-02
hCoV-19/Japan/PG-71792/2021	EPI_ISL_3876831	2021-09-02
hCoV-19/Japan/PG-71793/2021	EPI_ISL_3876833	2021-09-02
hCoV-19/Japan/PG-71794/2021	EPI_ISL_3876835	2021-09-02
hCoV-19/Japan/PG-71795/2021	EPI_ISL_3876837	2021-09-02
hCoV-19/Japan/PG-71796/2021	EPI_ISL_3876838	2021-09-02
hCoV-19/Japan/PG-71797/2021	EPI_ISL_3876839	2021-09-02
hCoV-19/Japan/PG-71798/2021	EPI_ISL_3876840	2021-09-02
hCoV-19/Japan/PG-71799/2021	EPI_ISL_3876841	2021-09-02
hCoV-19/Japan/PG-71800/2021	EPI_ISL_3876842	2021-09-02
hCoV-19/Japan/PG-71807/2021	EPI_ISL_3876845	2021-09-02

virus_name	epiID	submission_date
hCoV-19/Japan/PG-71808/2021	EPI_ISL_3876847	2021-09-02
hCoV-19/Japan/PG-71809/2021	EPI_ISL_3876850	2021-09-02
hCoV-19/Japan/PG-71810/2021	EPI_ISL_3876853	2021-09-02
hCoV-19/Japan/PG-71811/2021	EPI_ISL_3876856	2021-09-02
hCoV-19/Japan/PG-71812/2021	EPI_ISL_3876858	2021-09-02
hCoV-19/Japan/PG-71813/2021	EPI_ISL_3876860	2021-09-02
hCoV-19/Japan/PG-71814/2021	EPI_ISL_3876862	2021-09-02
hCoV-19/Japan/PG-71821/2021	EPI_ISL_3876864	2021-09-02
hCoV-19/Japan/PG-71822/2021	EPI_ISL_3876867	2021-09-02
hCoV-19/Japan/PG-71823/2021	EPI_ISL_3876869	2021-09-02
hCoV-19/Japan/PG-71824/2021	EPI_ISL_3876872	2021-09-02
hCoV-19/Japan/PG-71825/2021	EPI_ISL_3876874	2021-09-02
hCoV-19/Japan/PG-71826/2021	EPI_ISL_3876876	2021-09-02
hCoV-19/Japan/PG-71827/2021	EPI_ISL_3876879	2021-09-02
hCoV-19/Japan/PG-71828/2021	EPI_ISL_3876881	2021-09-02
hCoV-19/Japan/PG-71829/2021	EPI_ISL_3876883	2021-09-02
hCoV-19/Japan/PG-71830/2021	EPI_ISL_3876885	2021-09-02
hCoV-19/Japan/PG-71831/2021	EPI_ISL_3876886	2021-09-02
hCoV-19/Japan/PG-71832/2021	EPI_ISL_3876889	2021-09-02
hCoV-19/Japan/PG-71833/2021	EPI_ISL_3876892	2021-09-02
hCoV-19/Japan/PG-71837/2021	EPI_ISL_3876893	2021-09-02
hCoV-19/Japan/PG-71838/2021	EPI_ISL_3876896	2021-09-02
hCoV-19/Japan/PG-71839/2021	EPI_ISL_3876899	2021-09-02
hCoV-19/Japan/PG-71840/2021	EPI_ISL_3876903	2021-09-02
hCoV-19/Japan/PG-71841/2021	EPI_ISL_3876906	2021-09-02
hCoV-19/Japan/PG-71842/2021	EPI_ISL_3876909	2021-09-02
hCoV-19/Japan/PG-71843/2021	EPI_ISL_3876911	2021-09-02
hCoV-19/Japan/PG-71845/2021	EPI_ISL_3876914	2021-09-02
hCoV-19/Japan/PG-71846/2021	EPI_ISL_3876917	2021-09-02
hCoV-19/Japan/PG-71848/2021	EPI_ISL_3876919	2021-09-02
hCoV-19/Japan/PG-71849/2021	EPI_ISL_3876921	2021-09-02
hCoV-19/Japan/PG-71850/2021	EPI_ISL_3876924	2021-09-02
hCoV-19/Japan/PG-71851/2021	EPI_ISL_3876927	2021-09-02
hCoV-19/Japan/PG-71852/2021	EPI_ISL_3876929	2021-09-02
hCoV-19/Japan/PG-71853/2021	EPI_ISL_3876931	2021-09-02
hCoV-19/Japan/PG-71854/2021	EPI_ISL_3876934	2021-09-02
hCoV-19/Japan/PG-71855/2021	EPI_ISL_3876936	2021-09-02
hCoV-19/Japan/PG-71856/2021	EPI_ISL_3876939	2021-09-02
hCoV-19/Japan/PG-71857/2021	EPI_ISL_3876941	2021-09-02
hCoV-19/Japan/PG-71858/2021	EPI_ISL_3876943	2021-09-02
hCoV-19/Japan/PG-71859/2021	EPI_ISL_3876944	2021-09-02
hCoV-19/Japan/PG-71860/2021	EPI_ISL_3876945	2021-09-02
hCoV-19/Japan/PG-71861/2021	EPI_ISL_3876946	2021-09-02
hCoV-19/Japan/PG-71862/2021	EPI_ISL_3876947	2021-09-02
hCoV-19/Japan/PG-71863/2021	EPI_ISL_3876948	2021-09-02
hCoV-19/Japan/PG-71864/2021	EPI_ISL_3876950	2021-09-02
hCoV-19/Japan/PG-71865/2021	EPI_ISL_3876952	2021-09-02
hCoV-19/Japan/PG-71866/2021	EPI_ISL_3876954	2021-09-02
hCoV-19/Japan/PG-71867/2021	EPI_ISL_3876956	2021-09-02
hCoV-19/Japan/PG-71868/2021	EPI_ISL_3876958	2021-09-02
hCoV-19/Japan/PG-71869/2021	EPI_ISL_3876961	2021-09-02
hCoV-19/Japan/PG-71870/2021	EPI_ISL_3876963	2021-09-02
hCoV-19/Japan/PG-71871/2021	EPI_ISL_3876965	2021-09-02
hCoV-19/Japan/PG-71872/2021	EPI_ISL_3876967	2021-09-02

virus_name	epiID	submission_date
hCoV-19/Japan/PG-71873/2021	EPI_ISL_3876969	2021-09-02
hCoV-19/Japan/PG-71874/2021	EPI_ISL_3876971	2021-09-02
hCoV-19/Japan/PG-71875/2021	EPI_ISL_3876973	2021-09-02
hCoV-19/Japan/PG-71876/2021	EPI_ISL_3876976	2021-09-02
hCoV-19/Japan/PG-71877/2021	EPI_ISL_3876977	2021-09-02
hCoV-19/Japan/PG-71878/2021	EPI_ISL_3876979	2021-09-02
hCoV-19/Japan/PG-71879/2021	EPI_ISL_3876981	2021-09-02
hCoV-19/Japan/PG-71880/2021	EPI_ISL_3876983	2021-09-02
hCoV-19/Japan/PG-71881/2021	EPI_ISL_3876984	2021-09-02
hCoV-19/Japan/PG-71882/2021	EPI_ISL_3876987	2021-09-02
hCoV-19/Japan/PG-71883/2021	EPI_ISL_3876989	2021-09-02
hCoV-19/Japan/PG-71884/2021	EPI_ISL_3876990	2021-09-02
hCoV-19/Japan/PG-71885/2021	EPI_ISL_3876992	2021-09-02
hCoV-19/Japan/PG-71886/2021	EPI_ISL_3876995	2021-09-02
hCoV-19/Japan/PG-71887/2021	EPI_ISL_3876998	2021-09-02
hCoV-19/Japan/PG-71888/2021	EPI_ISL_3877000	2021-09-02
hCoV-19/Japan/PG-71889/2021	EPI_ISL_3877002	2021-09-02
hCoV-19/Japan/PG-71890/2021	EPI_ISL_3877005	2021-09-02
hCoV-19/Japan/PG-71891/2021	EPI_ISL_3877008	2021-09-02
hCoV-19/Japan/PG-71892/2021	EPI_ISL_3877010	2021-09-02
hCoV-19/Japan/PG-71893/2021	EPI_ISL_3877012	2021-09-02
hCoV-19/Japan/PG-71894/2021	EPI_ISL_3877014	2021-09-02
hCoV-19/Japan/PG-71895/2021	EPI_ISL_3877016	2021-09-02
hCoV-19/Japan/PG-71896/2021	EPI_ISL_3877018	2021-09-02
hCoV-19/Japan/PG-71897/2021	EPI_ISL_3877020	2021-09-02
hCoV-19/Japan/PG-71898/2021	EPI_ISL_3877023	2021-09-02
hCoV-19/Japan/PG-71899/2021	EPI_ISL_3877025	2021-09-02
hCoV-19/Japan/PG-71953/2021	EPI_ISL_3870487	2021-09-02
hCoV-19/Japan/PG-71954/2021	EPI_ISL_3870488	2021-09-02
hCoV-19/Japan/PG-71955/2021	EPI_ISL_3870489	2021-09-02
hCoV-19/Japan/PG-71956/2021	EPI_ISL_3870490	2021-09-02
hCoV-19/Japan/PG-71957/2021	EPI_ISL_3870491	2021-09-02
hCoV-19/Japan/PG-71958/2021	EPI_ISL_3870492	2021-09-02
hCoV-19/Japan/PG-71959/2021	EPI_ISL_3870493	2021-09-02
hCoV-19/Japan/PG-71960/2021	EPI_ISL_3870494	2021-09-02
hCoV-19/Japan/PG-71961/2021	EPI_ISL_3870495	2021-09-02
hCoV-19/Japan/PG-71962/2021	EPI_ISL_3870496	2021-09-02
hCoV-19/Japan/PG-71963/2021	EPI_ISL_3870497	2021-09-02
hCoV-19/Japan/PG-71964/2021	EPI_ISL_3870498	2021-09-02
hCoV-19/Japan/PG-71965/2021	EPI_ISL_3870499	2021-09-02
hCoV-19/Japan/PG-71966/2021	EPI_ISL_3870500	2021-09-02
hCoV-19/Japan/PG-71967/2021	EPI_ISL_3870501	2021-09-02
hCoV-19/Japan/PG-71968/2021	EPI_ISL_3870502	2021-09-02
hCoV-19/Japan/PG-71969/2021	EPI_ISL_3874770	2021-09-02
hCoV-19/Japan/PG-71970/2021	EPI_ISL_3874771	2021-09-02
hCoV-19/Japan/PG-71971/2021	EPI_ISL_3874772	2021-09-02
hCoV-19/Japan/PG-71972/2021	EPI_ISL_3874773	2021-09-02
hCoV-19/Japan/PG-71973/2021	EPI_ISL_3874774	2021-09-02
hCoV-19/Japan/PG-71975/2021	EPI_ISL_3874775	2021-09-02
hCoV-19/Japan/PG-72066/2021	EPI_ISL_3874776	2021-09-02
hCoV-19/Japan/PG-72067/2021	EPI_ISL_3874777	2021-09-02
hCoV-19/Japan/PG-72068/2021	EPI_ISL_3874778	2021-09-02
hCoV-19/Japan/PG-72069/2021	EPI_ISL_3874779	2021-09-02
hCoV-19/Japan/PG-72070/2021	EPI_ISL_3874780	2021-09-02

virus_name	epiID	submission_date
hCoV-19/Japan/PG-72071/2021	EPI_ISL_3874781	2021-09-02
hCoV-19/Japan/PG-72072/2021	EPI_ISL_3874782	2021-09-02
hCoV-19/Japan/PG-72073/2021	EPI_ISL_3874783	2021-09-02
hCoV-19/Japan/PG-72074/2021	EPI_ISL_3874784	2021-09-02
hCoV-19/Japan/PG-72075/2021	EPI_ISL_3874785	2021-09-02
hCoV-19/Japan/PG-72076/2021	EPI_ISL_3876007	2021-09-02
hCoV-19/Japan/PG-72077/2021	EPI_ISL_3876008	2021-09-02
hCoV-19/Japan/PG-72078/2021	EPI_ISL_3876009	2021-09-02
hCoV-19/Japan/PG-72079/2021	EPI_ISL_3876010	2021-09-02
hCoV-19/Japan/PG-72080/2021	EPI_ISL_3876011	2021-09-02
hCoV-19/Japan/PG-72081/2021	EPI_ISL_3876012	2021-09-02
hCoV-19/Japan/PG-72082/2021	EPI_ISL_3876013	2021-09-02
hCoV-19/Japan/PG-72088/2021	EPI_ISL_3876014	2021-09-02
hCoV-19/Japan/PG-72089/2021	EPI_ISL_3876015	2021-09-02
hCoV-19/Japan/PG-72090/2021	EPI_ISL_3876016	2021-09-02
hCoV-19/Japan/PG-72091/2021	EPI_ISL_3876017	2021-09-02
hCoV-19/Japan/PG-72092/2021	EPI_ISL_3876018	2021-09-02
hCoV-19/Japan/PG-72093/2021	EPI_ISL_3876019	2021-09-02
hCoV-19/Japan/PG-72094/2021	EPI_ISL_3876020	2021-09-02
hCoV-19/Japan/PG-72095/2021	EPI_ISL_3876021	2021-09-02
hCoV-19/Japan/PG-72109/2021	EPI_ISL_3871564	2021-09-02
hCoV-19/Japan/PG-72110/2021	EPI_ISL_3871565	2021-09-02
hCoV-19/Japan/PG-72111/2021	EPI_ISL_3871566	2021-09-02
hCoV-19/Japan/PG-72112/2021	EPI_ISL_3871567	2021-09-02
hCoV-19/Japan/PG-72113/2021	EPI_ISL_3871568	2021-09-02
hCoV-19/Japan/PG-72114/2021	EPI_ISL_3871569	2021-09-02
hCoV-19/Japan/PG-72115/2021	EPI_ISL_3871570	2021-09-02
hCoV-19/Japan/PG-72116/2021	EPI_ISL_3871571	2021-09-02
hCoV-19/Japan/PG-72117/2021	EPI_ISL_3871572	2021-09-02
hCoV-19/Japan/PG-72118/2021	EPI_ISL_3871573	2021-09-02
hCoV-19/Japan/PG-72119/2021	EPI_ISL_3871469	2021-09-02
hCoV-19/Japan/PG-72120/2021	EPI_ISL_3871470	2021-09-02
hCoV-19/Japan/PG-72121/2021	EPI_ISL_3871471	2021-09-02
hCoV-19/Japan/PG-72122/2021	EPI_ISL_3871472	2021-09-02
hCoV-19/Japan/PG-72123/2021	EPI_ISL_3871473	2021-09-02
hCoV-19/Japan/PG-72124/2021	EPI_ISL_3871474	2021-09-02
hCoV-19/Japan/PG-72125/2021	EPI_ISL_3871475	2021-09-02
hCoV-19/Japan/PG-72126/2021	EPI_ISL_3871476	2021-09-02
hCoV-19/Japan/PG-72127/2021	EPI_ISL_3871477	2021-09-02
hCoV-19/Japan/PG-72128/2021	EPI_ISL_3871478	2021-09-02
hCoV-19/Japan/PG-72129/2021	EPI_ISL_3871479	2021-09-02
hCoV-19/Japan/PG-72130/2021	EPI_ISL_3871480	2021-09-02
hCoV-19/Japan/PG-72131/2021	EPI_ISL_3871481	2021-09-02
hCoV-19/Japan/PG-72132/2021	EPI_ISL_3871482	2021-09-02
hCoV-19/Japan/PG-72133/2021	EPI_ISL_3871483	2021-09-02
hCoV-19/Japan/PG-72134/2021	EPI_ISL_3871484	2021-09-02
hCoV-19/Japan/PG-72135/2021	EPI_ISL_3871485	2021-09-02
hCoV-19/Japan/PG-72136/2021	EPI_ISL_3871486	2021-09-02
hCoV-19/Japan/PG-72137/2021	EPI_ISL_3871487	2021-09-02
hCoV-19/Japan/PG-72138/2021	EPI_ISL_3871488	2021-09-02
hCoV-19/Japan/PG-72139/2021	EPI_ISL_3871489	2021-09-02
hCoV-19/Japan/PG-72140/2021	EPI_ISL_3871490	2021-09-02
hCoV-19/Japan/PG-72143/2021	EPI_ISL_3871443	2021-09-02
hCoV-19/Japan/PG-72144/2021	EPI_ISL_3871190	2021-09-02

virus_name	epiID	submission_date
hCoV-19/Japan/PG-72145/2021	EPI_ISL_3871191	2021-09-02
hCoV-19/Japan/PG-72146/2021	EPI_ISL_3871192	2021-09-02
hCoV-19/Japan/PG-72147/2021	EPI_ISL_3871193	2021-09-02
hCoV-19/Japan/PG-72148/2021	EPI_ISL_3871194	2021-09-02
hCoV-19/Japan/PG-72149/2021	EPI_ISL_3871195	2021-09-02
hCoV-19/Japan/PG-72150/2021	EPI_ISL_3871196	2021-09-02
hCoV-19/Japan/PG-72151/2021	EPI_ISL_3871197	2021-09-02
hCoV-19/Japan/PG-72152/2021	EPI_ISL_3871198	2021-09-02
hCoV-19/Japan/PG-72153/2021	EPI_ISL_3871199	2021-09-02
hCoV-19/Japan/PG-72154/2021	EPI_ISL_3871200	2021-09-02
hCoV-19/Japan/PG-72155/2021	EPI_ISL_3871201	2021-09-02
hCoV-19/Japan/PG-72156/2021	EPI_ISL_3871202	2021-09-02
hCoV-19/Japan/PG-72157/2021	EPI_ISL_3871203	2021-09-02
hCoV-19/Japan/PG-72158/2021	EPI_ISL_3871204	2021-09-02
hCoV-19/Japan/PG-72159/2021	EPI_ISL_3871205	2021-09-02
hCoV-19/Japan/PG-72160/2021	EPI_ISL_3871206	2021-09-02
hCoV-19/Japan/PG-72161/2021	EPI_ISL_3871207	2021-09-02
hCoV-19/Japan/PG-72162/2021	EPI_ISL_3871208	2021-09-02
hCoV-19/Japan/PG-72163/2021	EPI_ISL_3871209	2021-09-02
hCoV-19/Japan/PG-72164/2021	EPI_ISL_3871210	2021-09-02
hCoV-19/Japan/PG-72165/2021	EPI_ISL_3871211	2021-09-02
hCoV-19/Japan/PG-72166/2021	EPI_ISL_3871981	2021-09-02
hCoV-19/Japan/PG-72167/2021	EPI_ISL_3871982	2021-09-02
hCoV-19/Japan/PG-72168/2021	EPI_ISL_3871983	2021-09-02
hCoV-19/Japan/PG-72169/2021	EPI_ISL_3871984	2021-09-02
hCoV-19/Japan/PG-72170/2021	EPI_ISL_3871985	2021-09-02
hCoV-19/Japan/PG-72171/2021	EPI_ISL_3871986	2021-09-02
hCoV-19/Japan/PG-72172/2021	EPI_ISL_3871987	2021-09-02
hCoV-19/Japan/PG-72173/2021	EPI_ISL_3871988	2021-09-02
hCoV-19/Japan/PG-72174/2021	EPI_ISL_3871989	2021-09-02
hCoV-19/Japan/PG-72175/2021	EPI_ISL_3871990	2021-09-02
hCoV-19/Japan/PG-72176/2021	EPI_ISL_3871991	2021-09-02
hCoV-19/Japan/PG-72177/2021	EPI_ISL_3871992	2021-09-02
hCoV-19/Japan/PG-72178/2021	EPI_ISL_3871993	2021-09-02
hCoV-19/Japan/PG-72179/2021	EPI_ISL_3871994	2021-09-02
hCoV-19/Japan/PG-72180/2021	EPI_ISL_3871995	2021-09-02
hCoV-19/Japan/PG-72181/2021	EPI_ISL_3871996	2021-09-02
hCoV-19/Japan/PG-72182/2021	EPI_ISL_3874147	2021-09-02
hCoV-19/Japan/PG-72183/2021	EPI_ISL_3874148	2021-09-02
hCoV-19/Japan/PG-72184/2021	EPI_ISL_3874149	2021-09-02
hCoV-19/Japan/PG-72185/2021	EPI_ISL_3874150	2021-09-02
hCoV-19/Japan/PG-72186/2021	EPI_ISL_3874151	2021-09-02
hCoV-19/Japan/PG-72187/2021	EPI_ISL_3874152	2021-09-02
hCoV-19/Japan/PG-72188/2021	EPI_ISL_3874153	2021-09-02
hCoV-19/Japan/PG-72189/2021	EPI_ISL_3874154	2021-09-02
hCoV-19/Japan/PG-72190/2021	EPI_ISL_3874155	2021-09-02
hCoV-19/Japan/PG-72191/2021	EPI_ISL_3874156	2021-09-02
hCoV-19/Japan/PG-72192/2021	EPI_ISL_3874157	2021-09-02
hCoV-19/Japan/PG-72193/2021	EPI_ISL_3874158	2021-09-02
hCoV-19/Japan/PG-72194/2021	EPI_ISL_3874159	2021-09-02
hCoV-19/Japan/PG-72195/2021	EPI_ISL_3874160	2021-09-02
hCoV-19/Japan/PG-72196/2021	EPI_ISL_3874161	2021-09-02
hCoV-19/Japan/PG-72197/2021	EPI_ISL_3874162	2021-09-02
hCoV-19/Japan/PG-72198/2021	EPI_ISL_3874163	2021-09-02

virus_name	epiID	submission_date
hCoV-19/Japan/PG-72199/2021	EPI_ISL_3874164	2021-09-02
hCoV-19/Japan/PG-72200/2021	EPI_ISL_3874165	2021-09-02
hCoV-19/Japan/PG-72201/2021	EPI_ISL_3874166	2021-09-02
hCoV-19/Japan/PG-72202/2021	EPI_ISL_3877027	2021-09-02
hCoV-19/Japan/PG-72203/2021	EPI_ISL_3877028	2021-09-02
hCoV-19/Japan/PG-72204/2021	EPI_ISL_3877031	2021-09-02
hCoV-19/Japan/PG-72205/2021	EPI_ISL_3877034	2021-09-02
hCoV-19/Japan/PG-72206/2021	EPI_ISL_3877036	2021-09-02
hCoV-19/Japan/PG-72207/2021	EPI_ISL_3877039	2021-09-02
hCoV-19/Japan/PG-72208/2021	EPI_ISL_3877042	2021-09-02
hCoV-19/Japan/PG-72209/2021	EPI_ISL_3877043	2021-09-02
hCoV-19/Japan/PG-72210/2021	EPI_ISL_3877046	2021-09-02
hCoV-19/Japan/PG-72211/2021	EPI_ISL_3877048	2021-09-02
hCoV-19/Japan/PG-72212/2021	EPI_ISL_3877051	2021-09-02
hCoV-19/Japan/PG-72213/2021	EPI_ISL_3877054	2021-09-02
hCoV-19/Japan/PG-72214/2021	EPI_ISL_3877056	2021-09-02
hCoV-19/Japan/PG-72215/2021	EPI_ISL_3877058	2021-09-02
hCoV-19/Japan/PG-72216/2021	EPI_ISL_3877060	2021-09-02
hCoV-19/Japan/PG-72217/2021	EPI_ISL_3877063	2021-09-02
hCoV-19/Japan/PG-72218/2021	EPI_ISL_3877066	2021-09-02
hCoV-19/Japan/PG-72219/2021	EPI_ISL_3877068	2021-09-02
hCoV-19/Japan/PG-72220/2021	EPI_ISL_3877070	2021-09-02
hCoV-19/Japan/PG-72221/2021	EPI_ISL_3877072	2021-09-02
hCoV-19/Japan/PG-72222/2021	EPI_ISL_3877074	2021-09-02
hCoV-19/Japan/PG-72223/2021	EPI_ISL_3877076	2021-09-02
hCoV-19/Japan/PG-72224/2021	EPI_ISL_3877078	2021-09-02
hCoV-19/Japan/PG-72225/2021	EPI_ISL_3877080	2021-09-02
hCoV-19/Japan/PG-72226/2021	EPI_ISL_3877083	2021-09-02
hCoV-19/Japan/PG-72227/2021	EPI_ISL_3877085	2021-09-02
hCoV-19/Japan/PG-72228/2021	EPI_ISL_3877087	2021-09-02
hCoV-19/Japan/PG-72229/2021	EPI_ISL_3877095	2021-09-02
hCoV-19/Japan/PG-72230/2021	EPI_ISL_3877097	2021-09-02
hCoV-19/Japan/PG-72231/2021	EPI_ISL_3877099	2021-09-02
hCoV-19/Japan/PG-72232/2021	EPI_ISL_3877102	2021-09-02
hCoV-19/Japan/PG-72233/2021	EPI_ISL_3877105	2021-09-02
hCoV-19/Japan/PG-72234/2021	EPI_ISL_3877107	2021-09-02
hCoV-19/Japan/PG-72235/2021	EPI_ISL_3877110	2021-09-02
hCoV-19/Japan/PG-72236/2021	EPI_ISL_3877111	2021-09-02
hCoV-19/Japan/PG-72237/2021	EPI_ISL_3877112	2021-09-02
hCoV-19/Japan/PG-72238/2021	EPI_ISL_3877115	2021-09-02
hCoV-19/Japan/PG-72239/2021	EPI_ISL_3877118	2021-09-02
hCoV-19/Japan/PG-72240/2021	EPI_ISL_3877120	2021-09-02
hCoV-19/Japan/PG-72241/2021	EPI_ISL_3877122	2021-09-02
hCoV-19/Japan/PG-72242/2021	EPI_ISL_3877125	2021-09-02
hCoV-19/Japan/PG-72243/2021	EPI_ISL_3877127	2021-09-02
hCoV-19/Japan/PG-72244/2021	EPI_ISL_3877129	2021-09-02
hCoV-19/Japan/PG-72245/2021	EPI_ISL_3877131	2021-09-02
hCoV-19/Japan/PG-72246/2021	EPI_ISL_3877134	2021-09-02
hCoV-19/Japan/PG-72247/2021	EPI_ISL_3877136	2021-09-02
hCoV-19/Japan/PG-72248/2021	EPI_ISL_3877139	2021-09-02
hCoV-19/Japan/PG-72249/2021	EPI_ISL_3877141	2021-09-02
hCoV-19/Japan/PG-72250/2021	EPI_ISL_3877144	2021-09-02
hCoV-19/Japan/PG-72251/2021	EPI_ISL_3877146	2021-09-02
hCoV-19/Japan/PG-72252/2021	EPI_ISL_3877149	2021-09-02

virus_name	epiID	submission_date
hCoV-19/Japan/PG-72253/2021	EPI_ISL_3877152	2021-09-02
hCoV-19/Japan/PG-72254/2021	EPI_ISL_3877154	2021-09-02
hCoV-19/Japan/PG-72255/2021	EPI_ISL_3877157	2021-09-02
hCoV-19/Japan/PG-72256/2021	EPI_ISL_3877159	2021-09-02
hCoV-19/Japan/PG-72257/2021	EPI_ISL_3877161	2021-09-02
hCoV-19/Japan/PG-72258/2021	EPI_ISL_3877164	2021-09-02
hCoV-19/Japan/PG-72259/2021	EPI_ISL_3877165	2021-09-02
hCoV-19/Japan/PG-72260/2021	EPI_ISL_3877166	2021-09-02
hCoV-19/Japan/PG-72261/2021	EPI_ISL_3877167	2021-09-02
hCoV-19/Japan/PG-72262/2021	EPI_ISL_3877168	2021-09-02
hCoV-19/Japan/PG-72263/2021	EPI_ISL_3877169	2021-09-02
hCoV-19/Japan/PG-72264/2021	EPI_ISL_3877171	2021-09-02
hCoV-19/Japan/PG-72265/2021	EPI_ISL_3877174	2021-09-02
hCoV-19/Japan/PG-72266/2021	EPI_ISL_3877176	2021-09-02
hCoV-19/Japan/PG-72267/2021	EPI_ISL_3877180	2021-09-02
hCoV-19/Japan/PG-72268/2021	EPI_ISL_3877182	2021-09-02
hCoV-19/Japan/PG-72269/2021	EPI_ISL_3877185	2021-09-02
hCoV-19/Japan/PG-72270/2021	EPI_ISL_3877187	2021-09-02
hCoV-19/Japan/PG-72271/2021	EPI_ISL_3877190	2021-09-02
hCoV-19/Japan/PG-72272/2021	EPI_ISL_3877193	2021-09-02
hCoV-19/Japan/PG-72273/2021	EPI_ISL_3877195	2021-09-02
hCoV-19/Japan/PG-72274/2021	EPI_ISL_3877198	2021-09-02
hCoV-19/Japan/PG-72275/2021	EPI_ISL_3877200	2021-09-02
hCoV-19/Japan/PG-72276/2021	EPI_ISL_3877202	2021-09-02
hCoV-19/Japan/PG-72277/2021	EPI_ISL_3877205	2021-09-02
hCoV-19/Japan/PG-72278/2021	EPI_ISL_3877207	2021-09-02
hCoV-19/Japan/PG-72279/2021	EPI_ISL_3877210	2021-09-02
hCoV-19/Japan/PG-72280/2021	EPI_ISL_3877212	2021-09-02
hCoV-19/Japan/PG-72281/2021	EPI_ISL_3877215	2021-09-02
hCoV-19/Japan/PG-72282/2021	EPI_ISL_3877218	2021-09-02
hCoV-19/Japan/PG-72283/2021	EPI_ISL_3877221	2021-09-02
hCoV-19/Japan/PG-72284/2021	EPI_ISL_3877224	2021-09-02
hCoV-19/Japan/PG-72285/2021	EPI_ISL_3877227	2021-09-02
hCoV-19/Japan/PG-72286/2021	EPI_ISL_3877230	2021-09-02
hCoV-19/Japan/PG-72287/2021	EPI_ISL_3877233	2021-09-02
hCoV-19/Japan/PG-72288/2021	EPI_ISL_3877236	2021-09-02
hCoV-19/Japan/PG-72289/2021	EPI_ISL_3877239	2021-09-02
hCoV-19/Japan/PG-72290/2021	EPI_ISL_3877242	2021-09-02
hCoV-19/Japan/PG-72291/2021	EPI_ISL_3877246	2021-09-02
hCoV-19/Japan/PG-72292/2021	EPI_ISL_3877248	2021-09-02
hCoV-19/Japan/PG-72293/2021	EPI_ISL_3877251	2021-09-02
hCoV-19/Japan/PG-72294/2021	EPI_ISL_3877254	2021-09-02
hCoV-19/Japan/PG-72295/2021	EPI_ISL_3877256	2021-09-02
hCoV-19/Japan/PG-72296/2021	EPI_ISL_3877259	2021-09-02
hCoV-19/Japan/PG-72297/2021	EPI_ISL_3877262	2021-09-02
hCoV-19/Japan/PG-72298/2021	EPI_ISL_3877264	2021-09-02
hCoV-19/Japan/PG-72299/2021	EPI_ISL_3877266	2021-09-02
hCoV-19/Japan/PG-72300/2021	EPI_ISL_3877269	2021-09-02
hCoV-19/Japan/PG-72301/2021	EPI_ISL_3877271	2021-09-02
hCoV-19/Japan/PG-72302/2021	EPI_ISL_3877274	2021-09-02
hCoV-19/Japan/PG-72303/2021	EPI_ISL_3877277	2021-09-02
hCoV-19/Japan/PG-72304/2021	EPI_ISL_3877280	2021-09-02
hCoV-19/Japan/PG-72305/2021	EPI_ISL_3877282	2021-09-02
hCoV-19/Japan/PG-72306/2021	EPI_ISL_3877285	2021-09-02

virus_name	epiID	submission_date
hCoV-19/Japan/PG-72307/2021	EPI_ISL_3877288	2021-09-02
hCoV-19/Japan/PG-72308/2021	EPI_ISL_3877290	2021-09-02
hCoV-19/Japan/PG-72309/2021	EPI_ISL_3877293	2021-09-02
hCoV-19/Japan/PG-72310/2021	EPI_ISL_3877295	2021-09-02
hCoV-19/Japan/PG-72311/2021	EPI_ISL_3877298	2021-09-02
hCoV-19/Japan/PG-72312/2021	EPI_ISL_3877300	2021-09-02
hCoV-19/Japan/PG-72313/2021	EPI_ISL_3877303	2021-09-02
hCoV-19/Japan/PG-72314/2021	EPI_ISL_3877306	2021-09-02
hCoV-19/Japan/PG-72315/2021	EPI_ISL_3877308	2021-09-02
hCoV-19/Japan/PG-72316/2021	EPI_ISL_3877310	2021-09-02
hCoV-19/Japan/PG-72317/2021	EPI_ISL_3877313	2021-09-02
hCoV-19/Japan/PG-72318/2021	EPI_ISL_3877316	2021-09-02
hCoV-19/Japan/PG-72319/2021	EPI_ISL_3877319	2021-09-02
hCoV-19/Japan/PG-72320/2021	EPI_ISL_3877322	2021-09-02
hCoV-19/Japan/PG-72321/2021	EPI_ISL_3877324	2021-09-02
hCoV-19/Japan/PG-72322/2021	EPI_ISL_3877327	2021-09-02
hCoV-19/Japan/PG-72323/2021	EPI_ISL_3877329	2021-09-02
hCoV-19/Japan/PG-72324/2021	EPI_ISL_3877332	2021-09-02
hCoV-19/Japan/PG-72325/2021	EPI_ISL_3877334	2021-09-02
hCoV-19/Japan/PG-72326/2021	EPI_ISL_3877336	2021-09-02
hCoV-19/Japan/PG-72327/2021	EPI_ISL_3877339	2021-09-02
hCoV-19/Japan/PG-72328/2021	EPI_ISL_3877342	2021-09-02
hCoV-19/Japan/PG-72329/2021	EPI_ISL_3877345	2021-09-02
hCoV-19/Japan/PG-72330/2021	EPI_ISL_3877348	2021-09-02
hCoV-19/Japan/PG-72331/2021	EPI_ISL_3877350	2021-09-02
hCoV-19/Japan/PG-72332/2021	EPI_ISL_3877352	2021-09-02
hCoV-19/Japan/PG-72333/2021	EPI_ISL_3877355	2021-09-02
hCoV-19/Japan/PG-72334/2021	EPI_ISL_3877358	2021-09-02
hCoV-19/Japan/PG-72335/2021	EPI_ISL_3877359	2021-09-02
hCoV-19/Japan/PG-72336/2021	EPI_ISL_3877360	2021-09-02
hCoV-19/Japan/PG-72337/2021	EPI_ISL_3877361	2021-09-02
hCoV-19/Japan/PG-72338/2021	EPI_ISL_3877362	2021-09-02
hCoV-19/Japan/PG-72339/2021	EPI_ISL_3877363	2021-09-02
hCoV-19/Japan/PG-72340/2021	EPI_ISL_3877366	2021-09-02
hCoV-19/Japan/PG-72341/2021	EPI_ISL_3877369	2021-09-02
hCoV-19/Japan/PG-72342/2021	EPI_ISL_3877371	2021-09-02
hCoV-19/Japan/PG-72343/2021	EPI_ISL_3877374	2021-09-02
hCoV-19/Japan/PG-72344/2021	EPI_ISL_3877376	2021-09-02
hCoV-19/Japan/PG-72345/2021	EPI_ISL_3877378	2021-09-02
hCoV-19/Japan/PG-72346/2021	EPI_ISL_3877381	2021-09-02
hCoV-19/Japan/PG-72347/2021	EPI_ISL_3877384	2021-09-02
hCoV-19/Japan/PG-72348/2021	EPI_ISL_3877387	2021-09-02
hCoV-19/Japan/PG-72349/2021	EPI_ISL_3877390	2021-09-02
hCoV-19/Japan/PG-72350/2021	EPI_ISL_3877393	2021-09-02
hCoV-19/Japan/PG-72351/2021	EPI_ISL_3877395	2021-09-02
hCoV-19/Japan/PG-72352/2021	EPI_ISL_3877398	2021-09-02
hCoV-19/Japan/PG-72353/2021	EPI_ISL_3877401	2021-09-02
hCoV-19/Japan/PG-72354/2021	EPI_ISL_3877403	2021-09-02
hCoV-19/Japan/PG-72355/2021	EPI_ISL_3877406	2021-09-02
hCoV-19/Japan/PG-72356/2021	EPI_ISL_3877409	2021-09-02
hCoV-19/Japan/PG-72357/2021	EPI_ISL_3877410	2021-09-02
hCoV-19/Japan/PG-72358/2021	EPI_ISL_3877411	2021-09-02
hCoV-19/Japan/PG-72359/2021	EPI_ISL_3877412	2021-09-02
hCoV-19/Japan/PG-72360/2021	EPI_ISL_3877413	2021-09-02

virus_name	epiID	submission_date
hCoV-19/Japan/PG-72361/2021	EPI_ISL_3877414	2021-09-02
hCoV-19/Japan/PG-72362/2021	EPI_ISL_3877416	2021-09-02
hCoV-19/Japan/PG-72363/2021	EPI_ISL_3877419	2021-09-02
hCoV-19/Japan/PG-72364/2021	EPI_ISL_3877422	2021-09-02
hCoV-19/Japan/PG-72365/2021	EPI_ISL_3877425	2021-09-02
hCoV-19/Japan/PG-72366/2021	EPI_ISL_3877426	2021-09-02
hCoV-19/Japan/PG-72367/2021	EPI_ISL_3877428	2021-09-02
hCoV-19/Japan/PG-72368/2021	EPI_ISL_3877431	2021-09-02
hCoV-19/Japan/PG-72369/2021	EPI_ISL_3877432	2021-09-02
hCoV-19/Japan/PG-72370/2021	EPI_ISL_3877433	2021-09-02
hCoV-19/Japan/PG-72371/2021	EPI_ISL_3877435	2021-09-02
hCoV-19/Japan/PG-72372/2021	EPI_ISL_3877438	2021-09-02
hCoV-19/Japan/PG-72373/2021	EPI_ISL_3877440	2021-09-02
hCoV-19/Japan/PG-72374/2021	EPI_ISL_3877442	2021-09-02
hCoV-19/Japan/PG-72375/2021	EPI_ISL_3877445	2021-09-02
hCoV-19/Japan/PG-72376/2021	EPI_ISL_3877448	2021-09-02
hCoV-19/Japan/PG-72377/2021	EPI_ISL_3877451	2021-09-02
hCoV-19/Japan/PG-72378/2021	EPI_ISL_3877454	2021-09-02
hCoV-19/Japan/PG-72379/2021	EPI_ISL_3877457	2021-09-02
hCoV-19/Japan/PG-72380/2021	EPI_ISL_3877460	2021-09-02
hCoV-19/Japan/PG-72381/2021	EPI_ISL_3877463	2021-09-02
hCoV-19/Japan/PG-72382/2021	EPI_ISL_3877466	2021-09-02
hCoV-19/Japan/PG-72383/2021	EPI_ISL_3877469	2021-09-02
hCoV-19/Japan/PG-72384/2021	EPI_ISL_3877472	2021-09-02
hCoV-19/Japan/PG-72385/2021	EPI_ISL_3877475	2021-09-02
hCoV-19/Japan/PG-72386/2021	EPI_ISL_3877479	2021-09-02
hCoV-19/Japan/PG-72387/2021	EPI_ISL_3877482	2021-09-02
hCoV-19/Japan/PG-72388/2021	EPI_ISL_3877485	2021-09-02
hCoV-19/Japan/PG-72389/2021	EPI_ISL_3877488	2021-09-02
hCoV-19/Japan/PG-72390/2021	EPI_ISL_3877491	2021-09-02
hCoV-19/Japan/PG-72391/2021	EPI_ISL_3877493	2021-09-02
hCoV-19/Japan/PG-72392/2021	EPI_ISL_3877496	2021-09-02
hCoV-19/Japan/PG-72393/2021	EPI_ISL_3877497	2021-09-02
hCoV-19/Japan/PG-72394/2021	EPI_ISL_3877499	2021-09-02
hCoV-19/Japan/PG-72395/2021	EPI_ISL_3877502	2021-09-02
hCoV-19/Japan/PG-72396/2021	EPI_ISL_3877505	2021-09-02
hCoV-19/Japan/PG-72397/2021	EPI_ISL_3877508	2021-09-02
hCoV-19/Japan/PG-72398/2021	EPI_ISL_3877510	2021-09-02
hCoV-19/Japan/PG-72399/2021	EPI_ISL_3877512	2021-09-02
hCoV-19/Japan/PG-72400/2021	EPI_ISL_3877516	2021-09-02
hCoV-19/Japan/PG-72401/2021	EPI_ISL_3877519	2021-09-02
hCoV-19/Japan/PG-72402/2021	EPI_ISL_3877521	2021-09-02
hCoV-19/Japan/PG-72403/2021	EPI_ISL_3877524	2021-09-02
hCoV-19/Japan/PG-72404/2021	EPI_ISL_3877527	2021-09-02
hCoV-19/Japan/PG-72405/2021	EPI_ISL_3877530	2021-09-02
hCoV-19/Japan/PG-72406/2021	EPI_ISL_3877533	2021-09-02
hCoV-19/Japan/PG-72407/2021	EPI_ISL_3877536	2021-09-02
hCoV-19/Japan/PG-72408/2021	EPI_ISL_3877539	2021-09-02
hCoV-19/Japan/PG-72409/2021	EPI_ISL_3877542	2021-09-02
hCoV-19/Japan/PG-72410/2021	EPI_ISL_3877544	2021-09-02
hCoV-19/Japan/PG-72411/2021	EPI_ISL_3877547	2021-09-02
hCoV-19/Japan/PG-72412/2021	EPI_ISL_3877549	2021-09-02
hCoV-19/Japan/PG-72413/2021	EPI_ISL_3877552	2021-09-02
hCoV-19/Japan/PG-72414/2021	EPI_ISL_3877554	2021-09-02

virus_name	epiID	submission_date
hCoV-19/Japan/PG-72415/2021	EPI_ISL_3877555	2021-09-02
hCoV-19/Japan/PG-72416/2021	EPI_ISL_3877558	2021-09-02
hCoV-19/Japan/PG-72417/2021	EPI_ISL_3877561	2021-09-02
hCoV-19/Japan/PG-72418/2021	EPI_ISL_3877563	2021-09-02
hCoV-19/Japan/PG-72419/2021	EPI_ISL_3877566	2021-09-02
hCoV-19/Japan/PG-72420/2021	EPI_ISL_3877569	2021-09-02
hCoV-19/Japan/PG-72421/2021	EPI_ISL_3877571	2021-09-02
hCoV-19/Japan/PG-72422/2021	EPI_ISL_3877574	2021-09-02
hCoV-19/Japan/PG-72423/2021	EPI_ISL_3877576	2021-09-02
hCoV-19/Japan/PG-72424/2021	EPI_ISL_3877579	2021-09-02
hCoV-19/Japan/PG-72425/2021	EPI_ISL_3877581	2021-09-02
hCoV-19/Japan/PG-72426/2021	EPI_ISL_3877585	2021-09-02
hCoV-19/Japan/PG-72427/2021	EPI_ISL_3877588	2021-09-02
hCoV-19/Japan/PG-72428/2021	EPI_ISL_3877590	2021-09-02
hCoV-19/Japan/PG-72429/2021	EPI_ISL_3877593	2021-09-02
hCoV-19/Japan/PG-72430/2021	EPI_ISL_3877596	2021-09-02
hCoV-19/Japan/PG-72431/2021	EPI_ISL_3877599	2021-09-02
hCoV-19/Japan/PG-72432/2021	EPI_ISL_3877602	2021-09-02
hCoV-19/Japan/PG-72433/2021	EPI_ISL_3877604	2021-09-02
hCoV-19/Japan/PG-72434/2021	EPI_ISL_3877607	2021-09-02
hCoV-19/Japan/PG-72435/2021	EPI_ISL_3877609	2021-09-02
hCoV-19/Japan/PG-72436/2021	EPI_ISL_3877612	2021-09-02
hCoV-19/Japan/PG-72437/2021	EPI_ISL_3877615	2021-09-02
hCoV-19/Japan/PG-72438/2021	EPI_ISL_3877617	2021-09-02
hCoV-19/Japan/PG-72439/2021	EPI_ISL_3877619	2021-09-02
hCoV-19/Japan/PG-72440/2021	EPI_ISL_3877621	2021-09-02
hCoV-19/Japan/PG-72441/2021	EPI_ISL_3877622	2021-09-02
hCoV-19/Japan/PG-72442/2021	EPI_ISL_3877624	2021-09-02
hCoV-19/Japan/PG-72443/2021	EPI_ISL_3877627	2021-09-02
hCoV-19/Japan/PG-72444/2021	EPI_ISL_3877629	2021-09-02
hCoV-19/Japan/PG-72445/2021	EPI_ISL_3877632	2021-09-02
hCoV-19/Japan/PG-72446/2021	EPI_ISL_3877636	2021-09-02
hCoV-19/Japan/PG-72447/2021	EPI_ISL_3877639	2021-09-02
hCoV-19/Japan/PG-72448/2021	EPI_ISL_3877642	2021-09-02
hCoV-19/Japan/PG-72449/2021	EPI_ISL_3877645	2021-09-02
hCoV-19/Japan/PG-72450/2021	EPI_ISL_3877648	2021-09-02
hCoV-19/Japan/PG-72451/2021	EPI_ISL_3877651	2021-09-02
hCoV-19/Japan/PG-72452/2021	EPI_ISL_3877654	2021-09-02
hCoV-19/Japan/PG-72453/2021	EPI_ISL_3877658	2021-09-02
hCoV-19/Japan/PG-72454/2021	EPI_ISL_3877661	2021-09-02
hCoV-19/Japan/PG-72455/2021	EPI_ISL_3877663	2021-09-02
hCoV-19/Japan/PG-72456/2021	EPI_ISL_3877665	2021-09-02
hCoV-19/Japan/PG-72457/2021	EPI_ISL_3877667	2021-09-02
hCoV-19/Japan/PG-72458/2021	EPI_ISL_3877671	2021-09-02
hCoV-19/Japan/PG-72459/2021	EPI_ISL_3877673	2021-09-02
hCoV-19/Japan/PG-72460/2021	EPI_ISL_3877677	2021-09-02
hCoV-19/Japan/PG-72461/2021	EPI_ISL_3877680	2021-09-02
hCoV-19/Japan/PG-72462/2021	EPI_ISL_3877683	2021-09-02
hCoV-19/Japan/PG-72463/2021	EPI_ISL_3877686	2021-09-02
hCoV-19/Japan/PG-72464/2021	EPI_ISL_3877688	2021-09-02
hCoV-19/Japan/PG-72465/2021	EPI_ISL_3877691	2021-09-02
hCoV-19/Japan/PG-72466/2021	EPI_ISL_3877694	2021-09-02
hCoV-19/Japan/PG-72467/2021	EPI_ISL_3877697	2021-09-02
hCoV-19/Japan/PG-72468/2021	EPI_ISL_3877700	2021-09-02

virus_name	epiID	submission_date
hCoV-19/Japan/PG-72469/2021	EPI_ISL_3877704	2021-09-02
hCoV-19/Japan/PG-72470/2021	EPI_ISL_3877707	2021-09-02
hCoV-19/Japan/PG-72471/2021	EPI_ISL_3877709	2021-09-02
hCoV-19/Japan/PG-72472/2021	EPI_ISL_3877711	2021-09-02
hCoV-19/Japan/PG-72473/2021	EPI_ISL_3877714	2021-09-02
hCoV-19/Japan/PG-72474/2021	EPI_ISL_3877715	2021-09-02
hCoV-19/Japan/PG-72475/2021	EPI_ISL_3877717	2021-09-02
hCoV-19/Japan/PG-72476/2021	EPI_ISL_3877720	2021-09-02
hCoV-19/Japan/PG-72477/2021	EPI_ISL_3877724	2021-09-02
hCoV-19/Japan/PG-72478/2021	EPI_ISL_3877727	2021-09-02
hCoV-19/Japan/PG-72479/2021	EPI_ISL_3877730	2021-09-02
hCoV-19/Japan/PG-72480/2021	EPI_ISL_3877733	2021-09-02
hCoV-19/Japan/PG-72481/2021	EPI_ISL_3877736	2021-09-02
hCoV-19/Japan/PG-72482/2021	EPI_ISL_3877740	2021-09-02
hCoV-19/Japan/PG-72483/2021	EPI_ISL_3877742	2021-09-02
hCoV-19/Japan/PG-72484/2021	EPI_ISL_3877745	2021-09-02
hCoV-19/Japan/PG-72485/2021	EPI_ISL_3877748	2021-09-02
hCoV-19/Japan/PG-72486/2021	EPI_ISL_3877751	2021-09-02
hCoV-19/Japan/PG-72487/2021	EPI_ISL_3877754	2021-09-02
hCoV-19/Japan/PG-72488/2021	EPI_ISL_3877757	2021-09-02
hCoV-19/Japan/PG-72489/2021	EPI_ISL_3877759	2021-09-02
hCoV-19/Japan/PG-72490/2021	EPI_ISL_3877763	2021-09-02
hCoV-19/Japan/PG-72491/2021	EPI_ISL_3877766	2021-09-02
hCoV-19/Japan/PG-72492/2021	EPI_ISL_3877769	2021-09-02
hCoV-19/Japan/PG-72493/2021	EPI_ISL_3877771	2021-09-02
hCoV-19/Japan/PG-72494/2021	EPI_ISL_3877774	2021-09-02
hCoV-19/Japan/PG-72495/2021	EPI_ISL_3877777	2021-09-02
hCoV-19/Japan/PG-72496/2021	EPI_ISL_3877780	2021-09-02
hCoV-19/Japan/PG-72497/2021	EPI_ISL_3877783	2021-09-02
hCoV-19/Japan/PG-72498/2021	EPI_ISL_3877786	2021-09-02
hCoV-19/Japan/PG-72499/2021	EPI_ISL_3877790	2021-09-02
hCoV-19/Japan/PG-72500/2021	EPI_ISL_3877792	2021-09-02
hCoV-19/Japan/PG-72501/2021	EPI_ISL_3877795	2021-09-02
hCoV-19/Japan/PG-72502/2021	EPI_ISL_3877797	2021-09-02
hCoV-19/Japan/PG-72503/2021	EPI_ISL_3877800	2021-09-02
hCoV-19/Japan/PG-72504/2021	EPI_ISL_3877803	2021-09-02
hCoV-19/Japan/PG-72505/2021	EPI_ISL_3877806	2021-09-02
hCoV-19/Japan/PG-72506/2021	EPI_ISL_3877808	2021-09-02
hCoV-19/Japan/PG-72507/2021	EPI_ISL_3877811	2021-09-02
hCoV-19/Japan/PG-72508/2021	EPI_ISL_3877814	2021-09-02
hCoV-19/Japan/PG-72509/2021	EPI_ISL_3877816	2021-09-02
hCoV-19/Japan/PG-72510/2021	EPI_ISL_3877819	2021-09-02
hCoV-19/Japan/PG-72511/2021	EPI_ISL_3877822	2021-09-02
hCoV-19/Japan/PG-72512/2021	EPI_ISL_3877825	2021-09-02
hCoV-19/Japan/PG-72513/2021	EPI_ISL_3877828	2021-09-02
hCoV-19/Japan/PG-72514/2021	EPI_ISL_3877831	2021-09-02
hCoV-19/Japan/PG-72515/2021	EPI_ISL_3877834	2021-09-02
hCoV-19/Japan/PG-72516/2021	EPI_ISL_3877836	2021-09-02
hCoV-19/Japan/PG-72517/2021	EPI_ISL_3877840	2021-09-02
hCoV-19/Japan/PG-72518/2021	EPI_ISL_3877842	2021-09-02
hCoV-19/Japan/PG-72519/2021	EPI_ISL_3877844	2021-09-02
hCoV-19/Japan/PG-72520/2021	EPI_ISL_3877847	2021-09-02
hCoV-19/Japan/PG-72521/2021	EPI_ISL_3877850	2021-09-02
hCoV-19/Japan/PG-72522/2021	EPI_ISL_3877853	2021-09-02

virus_name	epiID	submission_date
hCoV-19/Japan/PG-72523/2021	EPI_ISL_3877856	2021-09-02
hCoV-19/Japan/PG-72524/2021	EPI_ISL_3877858	2021-09-02
hCoV-19/Japan/PG-72525/2021	EPI_ISL_3877861	2021-09-02
hCoV-19/Japan/PG-72526/2021	EPI_ISL_3877864	2021-09-02
hCoV-19/Japan/PG-72527/2021	EPI_ISL_3877867	2021-09-02
hCoV-19/Japan/PG-72528/2021	EPI_ISL_3877869	2021-09-02
hCoV-19/Japan/PG-72529/2021	EPI_ISL_3877873	2021-09-02
hCoV-19/Japan/PG-72530/2021	EPI_ISL_3877876	2021-09-02
hCoV-19/Japan/PG-72531/2021	EPI_ISL_3877879	2021-09-02
hCoV-19/Japan/PG-72532/2021	EPI_ISL_3877881	2021-09-02
hCoV-19/Japan/PG-72533/2021	EPI_ISL_3877883	2021-09-02
hCoV-19/Japan/PG-72534/2021	EPI_ISL_3877887	2021-09-02
hCoV-19/Japan/PG-72535/2021	EPI_ISL_3877890	2021-09-02
hCoV-19/Japan/PG-72536/2021	EPI_ISL_3877893	2021-09-02
hCoV-19/Japan/PG-72537/2021	EPI_ISL_3877895	2021-09-02
hCoV-19/Japan/PG-72538/2021	EPI_ISL_3877898	2021-09-02
hCoV-19/Japan/PG-72539/2021	EPI_ISL_3877901	2021-09-02
hCoV-19/Japan/PG-72540/2021	EPI_ISL_3877904	2021-09-02
hCoV-19/Japan/PG-72541/2021	EPI_ISL_3877907	2021-09-02
hCoV-19/Japan/PG-72542/2021	EPI_ISL_3877910	2021-09-02
hCoV-19/Japan/PG-72543/2021	EPI_ISL_3877912	2021-09-02
hCoV-19/Japan/PG-72544/2021	EPI_ISL_3877915	2021-09-02
hCoV-19/Japan/PG-72545/2021	EPI_ISL_3877918	2021-09-02
hCoV-19/Japan/PG-72546/2021	EPI_ISL_3877920	2021-09-02
hCoV-19/Japan/PG-72547/2021	EPI_ISL_3877922	2021-09-02
hCoV-19/Japan/PG-72548/2021	EPI_ISL_3877926	2021-09-02
hCoV-19/Japan/PG-72549/2021	EPI_ISL_3877927	2021-09-02
hCoV-19/Japan/PG-72550/2021	EPI_ISL_3877929	2021-09-02
hCoV-19/Japan/PG-72551/2021	EPI_ISL_3877931	2021-09-02
hCoV-19/Japan/PG-72552/2021	EPI_ISL_3877935	2021-09-02
hCoV-19/Japan/PG-72553/2021	EPI_ISL_3877937	2021-09-02
hCoV-19/Japan/PG-72554/2021	EPI_ISL_3877940	2021-09-02
hCoV-19/Japan/PG-72555/2021	EPI_ISL_3877942	2021-09-02
hCoV-19/Japan/PG-72556/2021	EPI_ISL_3877946	2021-09-02
hCoV-19/Japan/PG-72557/2021	EPI_ISL_3877948	2021-09-02
hCoV-19/Japan/PG-72558/2021	EPI_ISL_3877951	2021-09-02
hCoV-19/Japan/PG-72559/2021	EPI_ISL_3877954	2021-09-02
hCoV-19/Japan/PG-72560/2021	EPI_ISL_3877957	2021-09-02
hCoV-19/Japan/PG-72561/2021	EPI_ISL_3877960	2021-09-02
hCoV-19/Japan/PG-72562/2021	EPI_ISL_3877963	2021-09-02
hCoV-19/Japan/PG-72563/2021	EPI_ISL_3877966	2021-09-02
hCoV-19/Japan/PG-72564/2021	EPI_ISL_3877968	2021-09-02
hCoV-19/Japan/PG-72565/2021	EPI_ISL_3877971	2021-09-02
hCoV-19/Japan/PG-72566/2021	EPI_ISL_3877974	2021-09-02
hCoV-19/Japan/PG-72567/2021	EPI_ISL_3877976	2021-09-02
hCoV-19/Japan/PG-72568/2021	EPI_ISL_3877978	2021-09-02
hCoV-19/Japan/PG-72569/2021	EPI_ISL_3877981	2021-09-02
hCoV-19/Japan/PG-72570/2021	EPI_ISL_3877983	2021-09-02
hCoV-19/Japan/PG-72571/2021	EPI_ISL_3877984	2021-09-02
hCoV-19/Japan/PG-72572/2021	EPI_ISL_3877986	2021-09-02
hCoV-19/Japan/PG-72573/2021	EPI_ISL_3877989	2021-09-02
hCoV-19/Japan/PG-72574/2021	EPI_ISL_3877991	2021-09-02
hCoV-19/Japan/PG-72575/2021	EPI_ISL_3877994	2021-09-02
hCoV-19/Japan/PG-72576/2021	EPI_ISL_3877996	2021-09-02

virus_name	epiID	submission_date
hCoV-19/Japan/PG-72577/2021	EPI_ISL_3877999	2021-09-02
hCoV-19/Japan/PG-72578/2021	EPI_ISL_3878002	2021-09-02
hCoV-19/Japan/PG-72579/2021	EPI_ISL_3878004	2021-09-02
hCoV-19/Japan/PG-72580/2021	EPI_ISL_3878006	2021-09-02
hCoV-19/Japan/PG-72581/2021	EPI_ISL_3878007	2021-09-02
hCoV-19/Japan/PG-72582/2021	EPI_ISL_3878010	2021-09-02
hCoV-19/Japan/PG-72583/2021	EPI_ISL_3878012	2021-09-02
hCoV-19/Japan/PG-72584/2021	EPI_ISL_3878014	2021-09-02
hCoV-19/Japan/PG-72585/2021	EPI_ISL_3878017	2021-09-02
hCoV-19/Japan/PG-72586/2021	EPI_ISL_3878018	2021-09-02
hCoV-19/Japan/PG-72587/2021	EPI_ISL_3878019	2021-09-02
hCoV-19/Japan/PG-72588/2021	EPI_ISL_3878021	2021-09-02
hCoV-19/Japan/PG-72589/2021	EPI_ISL_3878023	2021-09-02
hCoV-19/Japan/PG-72590/2021	EPI_ISL_3878025	2021-09-02
hCoV-19/Japan/PG-72591/2021	EPI_ISL_3878028	2021-09-02
hCoV-19/Japan/PG-72592/2021	EPI_ISL_3878030	2021-09-02
hCoV-19/Japan/PG-72593/2021	EPI_ISL_3878032	2021-09-02
hCoV-19/Japan/PG-72594/2021	EPI_ISL_3878035	2021-09-02
hCoV-19/Japan/PG-72595/2021	EPI_ISL_3878038	2021-09-02
hCoV-19/Japan/PG-72596/2021	EPI_ISL_3878040	2021-09-02
hCoV-19/Japan/PG-72597/2021	EPI_ISL_3878043	2021-09-02
hCoV-19/Japan/PG-72598/2021	EPI_ISL_3878047	2021-09-02
hCoV-19/Japan/PG-72599/2021	EPI_ISL_3878052	2021-09-02
hCoV-19/Japan/PG-72600/2021	EPI_ISL_3878056	2021-09-02
hCoV-19/Japan/PG-72601/2021	EPI_ISL_3878059	2021-09-02
hCoV-19/Japan/PG-72602/2021	EPI_ISL_3878064	2021-09-02
hCoV-19/Japan/PG-72603/2021	EPI_ISL_3878069	2021-09-02
hCoV-19/Japan/PG-72604/2021	EPI_ISL_3878074	2021-09-02
hCoV-19/Japan/PG-72605/2021	EPI_ISL_3878079	2021-09-02
hCoV-19/Japan/PG-72606/2021	EPI_ISL_3878083	2021-09-02
hCoV-19/Japan/PG-72607/2021	EPI_ISL_3878087	2021-09-02
hCoV-19/Japan/PG-72608/2021	EPI_ISL_3878092	2021-09-02
hCoV-19/Japan/PG-72609/2021	EPI_ISL_3878096	2021-09-02
hCoV-19/Japan/PG-72610/2021	EPI_ISL_3878099	2021-09-02
hCoV-19/Japan/PG-72611/2021	EPI_ISL_3878103	2021-09-02
hCoV-19/Japan/PG-72612/2021	EPI_ISL_3878107	2021-09-02
hCoV-19/Japan/PG-72613/2021	EPI_ISL_3878112	2021-09-02
hCoV-19/Japan/PG-72614/2021	EPI_ISL_3878117	2021-09-02
hCoV-19/Japan/PG-72615/2021	EPI_ISL_3878121	2021-09-02
hCoV-19/Japan/PG-72616/2021	EPI_ISL_3878125	2021-09-02
hCoV-19/Japan/PG-72617/2021	EPI_ISL_3878130	2021-09-02
hCoV-19/Japan/PG-72618/2021	EPI_ISL_3878134	2021-09-02
hCoV-19/Japan/PG-72619/2021	EPI_ISL_3878138	2021-09-02
hCoV-19/Japan/PG-72620/2021	EPI_ISL_3878140	2021-09-02
hCoV-19/Japan/PG-72621/2021	EPI_ISL_3878146	2021-09-02
hCoV-19/Japan/PG-72622/2021	EPI_ISL_3878150	2021-09-02
hCoV-19/Japan/PG-72623/2021	EPI_ISL_3878153	2021-09-02
hCoV-19/Japan/PG-72624/2021	EPI_ISL_3878158	2021-09-02
hCoV-19/Japan/PG-72625/2021	EPI_ISL_3878162	2021-09-02
hCoV-19/Japan/PG-72626/2021	EPI_ISL_3878165	2021-09-02
hCoV-19/Japan/PG-72627/2021	EPI_ISL_3878169	2021-09-02
hCoV-19/Japan/PG-72628/2021	EPI_ISL_3878173	2021-09-02
hCoV-19/Japan/PG-72629/2021	EPI_ISL_3878177	2021-09-02
hCoV-19/Japan/PG-72630/2021	EPI_ISL_3878183	2021-09-02

virus_name	epiID	submission_date
hCoV-19/Japan/PG-72631/2021	EPI_ISL_3878187	2021-09-02
hCoV-19/Japan/PG-72632/2021	EPI_ISL_3878189	2021-09-02
hCoV-19/Japan/PG-72633/2021	EPI_ISL_3878192	2021-09-02
hCoV-19/Japan/PG-72634/2021	EPI_ISL_3878195	2021-09-02
hCoV-19/Japan/PG-72635/2021	EPI_ISL_3878198	2021-09-02
hCoV-19/Japan/PG-72636/2021	EPI_ISL_3878202	2021-09-02
hCoV-19/Japan/PG-72637/2021	EPI_ISL_3878204	2021-09-02
hCoV-19/Japan/PG-72638/2021	EPI_ISL_3878207	2021-09-02
hCoV-19/Japan/PG-72639/2021	EPI_ISL_3878211	2021-09-02
hCoV-19/Japan/PG-72640/2021	EPI_ISL_3878213	2021-09-02
hCoV-19/Japan/PG-72641/2021	EPI_ISL_3878216	2021-09-02
hCoV-19/Japan/PG-72642/2021	EPI_ISL_3878220	2021-09-02
hCoV-19/Japan/PG-72643/2021	EPI_ISL_3878223	2021-09-02
hCoV-19/Japan/PG-72644/2021	EPI_ISL_3878226	2021-09-02
hCoV-19/Japan/PG-72645/2021	EPI_ISL_3878229	2021-09-02
hCoV-19/Japan/PG-72646/2021	EPI_ISL_3878231	2021-09-02
hCoV-19/Japan/PG-72647/2021	EPI_ISL_3878232	2021-09-02
hCoV-19/Japan/PG-72648/2021	EPI_ISL_3878237	2021-09-02
hCoV-19/Japan/PG-72649/2021	EPI_ISL_3878241	2021-09-02
hCoV-19/Japan/PG-72650/2021	EPI_ISL_3878244	2021-09-02
hCoV-19/Japan/PG-72651/2021	EPI_ISL_3878246	2021-09-02
hCoV-19/Japan/PG-72652/2021	EPI_ISL_3878250	2021-09-02
hCoV-19/Japan/PG-72653/2021	EPI_ISL_3878253	2021-09-02
hCoV-19/Japan/PG-72654/2021	EPI_ISL_3878255	2021-09-02
hCoV-19/Japan/PG-72655/2021	EPI_ISL_3878258	2021-09-02
hCoV-19/Japan/PG-72656/2021	EPI_ISL_3878261	2021-09-02
hCoV-19/Japan/PG-72657/2021	EPI_ISL_3878264	2021-09-02
hCoV-19/Japan/PG-72658/2021	EPI_ISL_3878267	2021-09-02
hCoV-19/Japan/PG-72659/2021	EPI_ISL_3878272	2021-09-02
hCoV-19/Japan/PG-72660/2021	EPI_ISL_3878276	2021-09-02
hCoV-19/Japan/PG-72661/2021	EPI_ISL_3878280	2021-09-02
hCoV-19/Japan/PG-72662/2021	EPI_ISL_3878282	2021-09-02
hCoV-19/Japan/PG-72663/2021	EPI_ISL_3878285	2021-09-02
hCoV-19/Japan/PG-72664/2021	EPI_ISL_3878286	2021-09-02
hCoV-19/Japan/PG-72665/2021	EPI_ISL_3878289	2021-09-02
hCoV-19/Japan/PG-72666/2021	EPI_ISL_3878292	2021-09-02
hCoV-19/Japan/PG-72667/2021	EPI_ISL_3878296	2021-09-02
hCoV-19/Japan/PG-72668/2021	EPI_ISL_3878301	2021-09-02
hCoV-19/Japan/PG-72669/2021	EPI_ISL_3878303	2021-09-02
hCoV-19/Japan/PG-72670/2021	EPI_ISL_3878307	2021-09-02
hCoV-19/Japan/PG-72671/2021	EPI_ISL_3878308	2021-09-02
hCoV-19/Japan/PG-72672/2021	EPI_ISL_3878313	2021-09-02
hCoV-19/Japan/PG-72673/2021	EPI_ISL_3878316	2021-09-02
hCoV-19/Japan/PG-72674/2021	EPI_ISL_3878319	2021-09-02
hCoV-19/Japan/PG-72675/2021	EPI_ISL_3878322	2021-09-02
hCoV-19/Japan/PG-72676/2021	EPI_ISL_3878325	2021-09-02
hCoV-19/Japan/PG-72677/2021	EPI_ISL_3878328	2021-09-02
hCoV-19/Japan/PG-72678/2021	EPI_ISL_3878330	2021-09-02
hCoV-19/Japan/PG-72679/2021	EPI_ISL_3878332	2021-09-02
hCoV-19/Japan/PG-72680/2021	EPI_ISL_3878335	2021-09-02
hCoV-19/Japan/PG-72681/2021	EPI_ISL_3878340	2021-09-02
hCoV-19/Japan/PG-72682/2021	EPI_ISL_3878343	2021-09-02
hCoV-19/Japan/PG-72683/2021	EPI_ISL_3878347	2021-09-02
hCoV-19/Japan/PG-72684/2021	EPI_ISL_3878351	2021-09-02

virus_name	epiID	submission_date
hCoV-19/Japan/PG-72685/2021	EPI_ISL_3878355	2021-09-02
hCoV-19/Japan/PG-72686/2021	EPI_ISL_3878357	2021-09-02
hCoV-19/Japan/PG-72687/2021	EPI_ISL_3878361	2021-09-02
hCoV-19/Japan/PG-72688/2021	EPI_ISL_3878366	2021-09-02
hCoV-19/Japan/PG-72689/2021	EPI_ISL_3878369	2021-09-02
hCoV-19/Japan/PG-72690/2021	EPI_ISL_3878372	2021-09-02
hCoV-19/Japan/PG-72691/2021	EPI_ISL_3878374	2021-09-02
hCoV-19/Japan/PG-72692/2021	EPI_ISL_3878376	2021-09-02
hCoV-19/Japan/PG-72693/2021	EPI_ISL_3878378	2021-09-02
hCoV-19/Japan/PG-72694/2021	EPI_ISL_3878381	2021-09-02
hCoV-19/Japan/PG-72695/2021	EPI_ISL_3878382	2021-09-02
hCoV-19/Japan/PG-72696/2021	EPI_ISL_3878385	2021-09-02
hCoV-19/Japan/PG-72697/2021	EPI_ISL_3878387	2021-09-02
hCoV-19/Japan/PG-72698/2021	EPI_ISL_3878390	2021-09-02
hCoV-19/Japan/PG-72699/2021	EPI_ISL_3878394	2021-09-02
hCoV-19/Japan/PG-72700/2021	EPI_ISL_3878397	2021-09-02
hCoV-19/Japan/PG-72701/2021	EPI_ISL_3878401	2021-09-02
hCoV-19/Japan/PG-72702/2021	EPI_ISL_3878405	2021-09-02
hCoV-19/Japan/PG-72703/2021	EPI_ISL_3878408	2021-09-02
hCoV-19/Japan/PG-72704/2021	EPI_ISL_3878412	2021-09-02
hCoV-19/Japan/PG-72705/2021	EPI_ISL_3878416	2021-09-02
hCoV-19/Japan/PG-72706/2021	EPI_ISL_3878419	2021-09-02
hCoV-19/Japan/PG-72707/2021	EPI_ISL_3878423	2021-09-02
hCoV-19/Japan/PG-72708/2021	EPI_ISL_3878427	2021-09-02
hCoV-19/Japan/PG-72709/2021	EPI_ISL_3878429	2021-09-02
hCoV-19/Japan/PG-72710/2021	EPI_ISL_3878430	2021-09-02
hCoV-19/Japan/PG-72711/2021	EPI_ISL_3878433	2021-09-02
hCoV-19/Japan/PG-72712/2021	EPI_ISL_3878436	2021-09-02
hCoV-19/Japan/PG-72713/2021	EPI_ISL_3878437	2021-09-02
hCoV-19/Japan/PG-72714/2021	EPI_ISL_3878440	2021-09-02
hCoV-19/Japan/PG-72715/2021	EPI_ISL_3878441	2021-09-02
hCoV-19/Japan/PG-72716/2021	EPI_ISL_3878443	2021-09-02
hCoV-19/Japan/PG-72717/2021	EPI_ISL_3878446	2021-09-02
hCoV-19/Japan/PG-72718/2021	EPI_ISL_3878448	2021-09-02
hCoV-19/Japan/PG-72719/2021	EPI_ISL_3878453	2021-09-02
hCoV-19/Japan/PG-72720/2021	EPI_ISL_3878456	2021-09-02
hCoV-19/Japan/PG-72721/2021	EPI_ISL_3878458	2021-09-02
hCoV-19/Japan/PG-72722/2021	EPI_ISL_3878460	2021-09-02
hCoV-19/Japan/PG-72723/2021	EPI_ISL_3878463	2021-09-02
hCoV-19/Japan/PG-72724/2021	EPI_ISL_3878467	2021-09-02
hCoV-19/Japan/PG-72725/2021	EPI_ISL_3878472	2021-09-02
hCoV-19/Japan/PG-72726/2021	EPI_ISL_3878476	2021-09-02
hCoV-19/Japan/PG-72727/2021	EPI_ISL_3878480	2021-09-02
hCoV-19/Japan/PG-72728/2021	EPI_ISL_3878484	2021-09-02
hCoV-19/Japan/PG-72729/2021	EPI_ISL_3878496	2021-09-02
hCoV-19/Japan/PG-72730/2021	EPI_ISL_3878500	2021-09-02
hCoV-19/Japan/PG-72731/2021	EPI_ISL_3878502	2021-09-02
hCoV-19/Japan/PG-72732/2021	EPI_ISL_3878503	2021-09-02
hCoV-19/Japan/PG-72733/2021	EPI_ISL_3878505	2021-09-02
hCoV-19/Japan/PG-72734/2021	EPI_ISL_3878507	2021-09-02
hCoV-19/Japan/PG-72735/2021	EPI_ISL_3878508	2021-09-02
hCoV-19/Japan/PG-72736/2021	EPI_ISL_3878509	2021-09-02
hCoV-19/Japan/PG-72737/2021	EPI_ISL_3878511	2021-09-02
hCoV-19/Japan/PG-72738/2021	EPI_ISL_3878512	2021-09-02

virus_name	epiID	submission_date
hCoV-19/Japan/PG-72739/2021	EPI_ISL_3878514	2021-09-02
hCoV-19/Japan/PG-72740/2021	EPI_ISL_3878516	2021-09-02
hCoV-19/Japan/PG-72741/2021	EPI_ISL_3878518	2021-09-02
hCoV-19/Japan/PG-72742/2021	EPI_ISL_3878520	2021-09-02
hCoV-19/Japan/PG-72743/2021	EPI_ISL_3878521	2021-09-02
hCoV-19/Japan/PG-72744/2021	EPI_ISL_3878523	2021-09-02
hCoV-19/Japan/PG-72745/2021	EPI_ISL_3878524	2021-09-02
hCoV-19/Japan/PG-72746/2021	EPI_ISL_3878525	2021-09-02
hCoV-19/Japan/PG-72747/2021	EPI_ISL_3878527	2021-09-02
hCoV-19/Japan/PG-72748/2021	EPI_ISL_3878528	2021-09-02
hCoV-19/Japan/PG-72749/2021	EPI_ISL_3878529	2021-09-02
hCoV-19/Japan/PG-72750/2021	EPI_ISL_3878531	2021-09-02
hCoV-19/Japan/PG-72751/2021	EPI_ISL_3878532	2021-09-02
hCoV-19/Japan/PG-72752/2021	EPI_ISL_3878534	2021-09-02
hCoV-19/Japan/PG-72753/2021	EPI_ISL_3878536	2021-09-02
hCoV-19/Japan/PG-72754/2021	EPI_ISL_3878538	2021-09-02
hCoV-19/Japan/PG-72755/2021	EPI_ISL_3878539	2021-09-02
hCoV-19/Japan/PG-72756/2021	EPI_ISL_3878541	2021-09-02
hCoV-19/Japan/PG-72757/2021	EPI_ISL_3878543	2021-09-02
hCoV-19/Japan/PG-72758/2021	EPI_ISL_3878545	2021-09-02
hCoV-19/Japan/PG-72759/2021	EPI_ISL_3878547	2021-09-02
hCoV-19/Japan/PG-72760/2021	EPI_ISL_3878549	2021-09-02
hCoV-19/Japan/PG-72761/2021	EPI_ISL_3878551	2021-09-02
hCoV-19/Japan/PG-72762/2021	EPI_ISL_3878552	2021-09-02
hCoV-19/Japan/PG-72763/2021	EPI_ISL_3878554	2021-09-02
hCoV-19/Japan/PG-72764/2021	EPI_ISL_3878555	2021-09-02
hCoV-19/Japan/PG-72765/2021	EPI_ISL_3878556	2021-09-02
hCoV-19/Japan/PG-72766/2021	EPI_ISL_3878558	2021-09-02
hCoV-19/Japan/PG-72767/2021	EPI_ISL_3878559	2021-09-02
hCoV-19/Japan/PG-72768/2021	EPI_ISL_3878561	2021-09-02
hCoV-19/Japan/PG-72769/2021	EPI_ISL_3878562	2021-09-02
hCoV-19/Japan/PG-72770/2021	EPI_ISL_3878564	2021-09-02
hCoV-19/Japan/PG-72771/2021	EPI_ISL_3878565	2021-09-02
hCoV-19/Japan/PG-72772/2021	EPI_ISL_3878566	2021-09-02
hCoV-19/Japan/PG-72773/2021	EPI_ISL_3878568	2021-09-02
hCoV-19/Japan/PG-72774/2021	EPI_ISL_3878570	2021-09-02
hCoV-19/Japan/PG-72775/2021	EPI_ISL_3878571	2021-09-02
hCoV-19/Japan/PG-72776/2021	EPI_ISL_3878572	2021-09-02
hCoV-19/Japan/PG-72777/2021	EPI_ISL_3878573	2021-09-02
hCoV-19/Japan/PG-72778/2021	EPI_ISL_3878575	2021-09-02
hCoV-19/Japan/PG-72779/2021	EPI_ISL_3878576	2021-09-02
hCoV-19/Japan/PG-72780/2021	EPI_ISL_3878578	2021-09-02
hCoV-19/Japan/PG-72781/2021	EPI_ISL_3878580	2021-09-02
hCoV-19/Japan/PG-72782/2021	EPI_ISL_3878582	2021-09-02
hCoV-19/Japan/PG-72783/2021	EPI_ISL_3878583	2021-09-02
hCoV-19/Japan/PG-72784/2021	EPI_ISL_3878585	2021-09-02
hCoV-19/Japan/PG-72785/2021	EPI_ISL_3878587	2021-09-02
hCoV-19/Japan/PG-72786/2021	EPI_ISL_3878589	2021-09-02
hCoV-19/Japan/PG-72787/2021	EPI_ISL_3878591	2021-09-02
hCoV-19/Japan/PG-72788/2021	EPI_ISL_3878593	2021-09-02
hCoV-19/Japan/PG-72789/2021	EPI_ISL_3878594	2021-09-02
hCoV-19/Japan/PG-72790/2021	EPI_ISL_3878596	2021-09-02
hCoV-19/Japan/PG-72791/2021	EPI_ISL_3878598	2021-09-02
hCoV-19/Japan/PG-72792/2021	EPI_ISL_3878599	2021-09-02

virus_name	epiID	submission_date
hCoV-19/Japan/PG-72793/2021	EPI_ISL_3878601	2021-09-02
hCoV-19/Japan/PG-72794/2021	EPI_ISL_3878603	2021-09-02
hCoV-19/Japan/PG-72795/2021	EPI_ISL_3878605	2021-09-02
hCoV-19/Japan/PG-72796/2021	EPI_ISL_3878606	2021-09-02
hCoV-19/Japan/PG-72797/2021	EPI_ISL_3878608	2021-09-02
hCoV-19/Japan/PG-72798/2021	EPI_ISL_3878610	2021-09-02
hCoV-19/Japan/PG-72799/2021	EPI_ISL_3878611	2021-09-02
hCoV-19/Japan/PG-72800/2021	EPI_ISL_3878613	2021-09-02
hCoV-19/Japan/PG-72801/2021	EPI_ISL_3878615	2021-09-02
hCoV-19/Japan/PG-72802/2021	EPI_ISL_3878617	2021-09-02
hCoV-19/Japan/PG-72803/2021	EPI_ISL_3878619	2021-09-02
hCoV-19/Japan/PG-72804/2021	EPI_ISL_3878620	2021-09-02
hCoV-19/Japan/PG-72805/2021	EPI_ISL_3878622	2021-09-02
hCoV-19/Japan/PG-72806/2021	EPI_ISL_3878624	2021-09-02
hCoV-19/Japan/PG-72807/2021	EPI_ISL_3878626	2021-09-02
hCoV-19/Japan/PG-72808/2021	EPI_ISL_3878627	2021-09-02
hCoV-19/Japan/PG-72809/2021	EPI_ISL_3878628	2021-09-02
hCoV-19/Japan/PG-72810/2021	EPI_ISL_3878630	2021-09-02
hCoV-19/Japan/PG-72811/2021	EPI_ISL_3878631	2021-09-02
hCoV-19/Japan/PG-72813/2021	EPI_ISL_3878633	2021-09-02
hCoV-19/Japan/PG-72814/2021	EPI_ISL_3878634	2021-09-02
hCoV-19/Japan/PG-72815/2021	EPI_ISL_3878635	2021-09-02
hCoV-19/Japan/PG-72816/2021	EPI_ISL_3878637	2021-09-02
hCoV-19/Japan/PG-72817/2021	EPI_ISL_3878638	2021-09-02
hCoV-19/Japan/PG-72818/2021	EPI_ISL_3878640	2021-09-02
hCoV-19/Japan/PG-72819/2021	EPI_ISL_3878642	2021-09-02
hCoV-19/Japan/PG-72820/2021	EPI_ISL_3878644	2021-09-02
hCoV-19/Japan/PG-72821/2021	EPI_ISL_3878646	2021-09-02
hCoV-19/Japan/PG-72822/2021	EPI_ISL_3878647	2021-09-02
hCoV-19/Japan/PG-72823/2021	EPI_ISL_3878649	2021-09-02
hCoV-19/Japan/PG-72824/2021	EPI_ISL_3878650	2021-09-02
hCoV-19/Japan/PG-72825/2021	EPI_ISL_3878652	2021-09-02
hCoV-19/Japan/PG-72826/2021	EPI_ISL_3878654	2021-09-02
hCoV-19/Japan/PG-72827/2021	EPI_ISL_3878655	2021-09-02
hCoV-19/Japan/PG-72828/2021	EPI_ISL_3878656	2021-09-02
hCoV-19/Japan/PG-72829/2021	EPI_ISL_3878658	2021-09-02
hCoV-19/Japan/PG-72830/2021	EPI_ISL_3878659	2021-09-02
hCoV-19/Japan/PG-72831/2021	EPI_ISL_3878661	2021-09-02
hCoV-19/Japan/PG-72832/2021	EPI_ISL_3878662	2021-09-02
hCoV-19/Japan/PG-72833/2021	EPI_ISL_3878664	2021-09-02
hCoV-19/Japan/PG-72834/2021	EPI_ISL_3878665	2021-09-02
hCoV-19/Japan/PG-72835/2021	EPI_ISL_3878666	2021-09-02
hCoV-19/Japan/PG-72836/2021	EPI_ISL_3878668	2021-09-02
hCoV-19/Japan/PG-72837/2021	EPI_ISL_3878669	2021-09-02
hCoV-19/Japan/PG-72838/2021	EPI_ISL_3878671	2021-09-02
hCoV-19/Japan/PG-72839/2021	EPI_ISL_3878673	2021-09-02
hCoV-19/Japan/PG-72840/2021	EPI_ISL_3878674	2021-09-02
hCoV-19/Japan/PG-72841/2021	EPI_ISL_3878676	2021-09-02
hCoV-19/Japan/PG-72842/2021	EPI_ISL_3878678	2021-09-02
hCoV-19/Japan/PG-72843/2021	EPI_ISL_3878680	2021-09-02
hCoV-19/Japan/PG-72844/2021	EPI_ISL_3878682	2021-09-02
hCoV-19/Japan/PG-72845/2021	EPI_ISL_3878684	2021-09-02
hCoV-19/Japan/PG-72846/2021	EPI_ISL_3878685	2021-09-02
hCoV-19/Japan/PG-72847/2021	EPI_ISL_3878687	2021-09-02

virus_name	epiID	submission_date
hCoV-19/Japan/PG-72848/2021	EPI_ISL_3878689	2021-09-02
hCoV-19/Japan/PG-72849/2021	EPI_ISL_3878691	2021-09-02
hCoV-19/Japan/PG-72850/2021	EPI_ISL_3878692	2021-09-02
hCoV-19/Japan/PG-72851/2021	EPI_ISL_3878693	2021-09-02
hCoV-19/Japan/PG-72852/2021	EPI_ISL_3878695	2021-09-02
hCoV-19/Japan/PG-72853/2021	EPI_ISL_3878697	2021-09-02
hCoV-19/Japan/PG-72854/2021	EPI_ISL_3878698	2021-09-02
hCoV-19/Japan/PG-72855/2021	EPI_ISL_3878700	2021-09-02
hCoV-19/Japan/PG-72856/2021	EPI_ISL_3878702	2021-09-02
hCoV-19/Japan/PG-72857/2021	EPI_ISL_3878703	2021-09-02
hCoV-19/Japan/PG-72858/2021	EPI_ISL_3878705	2021-09-02
hCoV-19/Japan/PG-72859/2021	EPI_ISL_3878707	2021-09-02
hCoV-19/Japan/PG-72860/2021	EPI_ISL_3878710	2021-09-02
hCoV-19/Japan/PG-72861/2021	EPI_ISL_3878711	2021-09-02
hCoV-19/Japan/PG-72862/2021	EPI_ISL_3878713	2021-09-02
hCoV-19/Japan/PG-72863/2021	EPI_ISL_3878715	2021-09-02
hCoV-19/Japan/PG-72864/2021	EPI_ISL_3878716	2021-09-02
hCoV-19/Japan/PG-72865/2021	EPI_ISL_3878718	2021-09-02
hCoV-19/Japan/PG-72866/2021	EPI_ISL_3878720	2021-09-02
hCoV-19/Japan/PG-72867/2021	EPI_ISL_3878722	2021-09-02
hCoV-19/Japan/PG-72868/2021	EPI_ISL_3878723	2021-09-02
hCoV-19/Japan/PG-72869/2021	EPI_ISL_3878725	2021-09-02
hCoV-19/Japan/PG-72870/2021	EPI_ISL_3878727	2021-09-02
hCoV-19/Japan/PG-72871/2021	EPI_ISL_3878729	2021-09-02
hCoV-19/Japan/PG-72872/2021	EPI_ISL_3878731	2021-09-02
hCoV-19/Japan/PG-72873/2021	EPI_ISL_3878733	2021-09-02
hCoV-19/Japan/PG-72874/2021	EPI_ISL_3878734	2021-09-02
hCoV-19/Japan/PG-72875/2021	EPI_ISL_3878736	2021-09-02
hCoV-19/Japan/PG-72876/2021	EPI_ISL_3878738	2021-09-02
hCoV-19/Japan/PG-72877/2021	EPI_ISL_3878740	2021-09-02
hCoV-19/Japan/PG-72878/2021	EPI_ISL_3878742	2021-09-02
hCoV-19/Japan/PG-72879/2021	EPI_ISL_3878745	2021-09-02
hCoV-19/Japan/PG-72880/2021	EPI_ISL_3878746	2021-09-02
hCoV-19/Japan/PG-72881/2021	EPI_ISL_3878748	2021-09-02
hCoV-19/Japan/PG-72882/2021	EPI_ISL_3878750	2021-09-02
hCoV-19/Japan/PG-72883/2021	EPI_ISL_3878752	2021-09-02
hCoV-19/Japan/PG-72884/2021	EPI_ISL_3878754	2021-09-02
hCoV-19/Japan/PG-72885/2021	EPI_ISL_3878756	2021-09-02
hCoV-19/Japan/PG-72886/2021	EPI_ISL_3878758	2021-09-02
hCoV-19/Japan/PG-72887/2021	EPI_ISL_3878760	2021-09-02
hCoV-19/Japan/PG-72888/2021	EPI_ISL_3878762	2021-09-02
hCoV-19/Japan/PG-72889/2021	EPI_ISL_3878763	2021-09-02
hCoV-19/Japan/PG-72890/2021	EPI_ISL_3878765	2021-09-02
hCoV-19/Japan/PG-72891/2021	EPI_ISL_3878766	2021-09-02
hCoV-19/Japan/PG-72892/2021	EPI_ISL_3878768	2021-09-02
hCoV-19/Japan/PG-72893/2021	EPI_ISL_3878769	2021-09-02
hCoV-19/Japan/PG-72894/2021	EPI_ISL_3878771	2021-09-02
hCoV-19/Japan/PG-72895/2021	EPI_ISL_3878773	2021-09-02
hCoV-19/Japan/PG-72896/2021	EPI_ISL_3878774	2021-09-02
hCoV-19/Japan/PG-72897/2021	EPI_ISL_3878775	2021-09-02
hCoV-19/Japan/PG-72898/2021	EPI_ISL_3878777	2021-09-02
hCoV-19/Japan/PG-72899/2021	EPI_ISL_3878778	2021-09-02
hCoV-19/Japan/PG-72900/2021	EPI_ISL_3878781	2021-09-02
hCoV-19/Japan/PG-72901/2021	EPI_ISL_3878783	2021-09-02

virus_name	epiID	submission_date
hCoV-19/Japan/PG-72902/2021	EPI_ISL_3878785	2021-09-02
hCoV-19/Japan/PG-72903/2021	EPI_ISL_3878786	2021-09-02
hCoV-19/Japan/PG-72904/2021	EPI_ISL_3878789	2021-09-02
hCoV-19/Japan/PG-72905/2021	EPI_ISL_3878791	2021-09-02
hCoV-19/Japan/PG-72906/2021	EPI_ISL_3878793	2021-09-02
hCoV-19/Japan/PG-72907/2021	EPI_ISL_3874264	2021-09-02
hCoV-19/Japan/PG-72908/2021	EPI_ISL_3874265	2021-09-02
hCoV-19/Japan/PG-72909/2021	EPI_ISL_3874266	2021-09-02
hCoV-19/Japan/PG-72910/2021	EPI_ISL_3874267	2021-09-02
hCoV-19/Japan/PG-72911/2021	EPI_ISL_3874268	2021-09-02
hCoV-19/Japan/PG-72912/2021	EPI_ISL_3874269	2021-09-02
hCoV-19/Japan/PG-72913/2021	EPI_ISL_3874270	2021-09-02
hCoV-19/Japan/PG-72914/2021	EPI_ISL_3874271	2021-09-02
hCoV-19/Japan/PG-72915/2021	EPI_ISL_3874272	2021-09-02
hCoV-19/Japan/PG-72916/2021	EPI_ISL_3874273	2021-09-02
hCoV-19/Japan/PG-72917/2021	EPI_ISL_3874274	2021-09-02
hCoV-19/Japan/PG-72918/2021	EPI_ISL_3874275	2021-09-02
hCoV-19/Japan/PG-72919/2021	EPI_ISL_3874276	2021-09-02
hCoV-19/Japan/PG-72920/2021	EPI_ISL_3874277	2021-09-02
hCoV-19/Japan/PG-72921/2021	EPI_ISL_3874278	2021-09-02
hCoV-19/Japan/PG-72922/2021	EPI_ISL_3874279	2021-09-02
hCoV-19/Japan/PG-72923/2021	EPI_ISL_3874280	2021-09-02
hCoV-19/Japan/PG-72924/2021	EPI_ISL_3874281	2021-09-02
hCoV-19/Japan/PG-72925/2021	EPI_ISL_3874282	2021-09-02
hCoV-19/Japan/PG-72926/2021	EPI_ISL_3878794	2021-09-02
hCoV-19/Japan/PG-72927/2021	EPI_ISL_3878797	2021-09-02
hCoV-19/Japan/PG-72928/2021	EPI_ISL_3878798	2021-09-02
hCoV-19/Japan/PG-72929/2021	EPI_ISL_3878801	2021-09-02
hCoV-19/Japan/PG-72930/2021	EPI_ISL_3878802	2021-09-02
hCoV-19/Japan/PG-72931/2021	EPI_ISL_3878804	2021-09-02
hCoV-19/Japan/PG-72932/2021	EPI_ISL_3878806	2021-09-02
hCoV-19/Japan/PG-72933/2021	EPI_ISL_3878808	2021-09-02
hCoV-19/Japan/PG-72934/2021	EPI_ISL_3878810	2021-09-02
hCoV-19/Japan/PG-72935/2021	EPI_ISL_3878812	2021-09-02
hCoV-19/Japan/PG-72936/2021	EPI_ISL_3878814	2021-09-02
hCoV-19/Japan/PG-72937/2021	EPI_ISL_3878816	2021-09-02
hCoV-19/Japan/PG-72938/2021	EPI_ISL_3878818	2021-09-02
hCoV-19/Japan/PG-72939/2021	EPI_ISL_3878819	2021-09-02
hCoV-19/Japan/PG-72940/2021	EPI_ISL_3878821	2021-09-02
hCoV-19/Japan/PG-72941/2021	EPI_ISL_3878823	2021-09-02
hCoV-19/Japan/PG-72942/2021	EPI_ISL_3878825	2021-09-02
hCoV-19/Japan/PG-72943/2021	EPI_ISL_3878827	2021-09-02
hCoV-19/Japan/PG-72944/2021	EPI_ISL_3878828	2021-09-02
hCoV-19/Japan/PG-72945/2021	EPI_ISL_3878831	2021-09-02
hCoV-19/Japan/PG-72946/2021	EPI_ISL_3878833	2021-09-02
hCoV-19/Japan/PG-72947/2021	EPI_ISL_3878835	2021-09-02
hCoV-19/Japan/PG-72948/2021	EPI_ISL_3878837	2021-09-02
hCoV-19/Japan/PG-72949/2021	EPI_ISL_3878839	2021-09-02
hCoV-19/Japan/PG-72950/2021	EPI_ISL_3878840	2021-09-02
hCoV-19/Japan/PG-72951/2021	EPI_ISL_3878843	2021-09-02
hCoV-19/Japan/PG-72952/2021	EPI_ISL_3878844	2021-09-02
hCoV-19/Japan/PG-72953/2021	EPI_ISL_3878846	2021-09-02
hCoV-19/Japan/PG-72954/2021	EPI_ISL_3878847	2021-09-02
hCoV-19/Japan/PG-72955/2021	EPI_ISL_3878849	2021-09-02

virus_name	epiID	submission_date
hCoV-19/Japan/PG-72956/2021	EPI_ISL_3878851	2021-09-02
hCoV-19/Japan/PG-72957/2021	EPI_ISL_3878852	2021-09-02
hCoV-19/Japan/PG-72958/2021	EPI_ISL_3878854	2021-09-02
hCoV-19/Japan/PG-72959/2021	EPI_ISL_3878856	2021-09-02
hCoV-19/Japan/PG-72960/2021	EPI_ISL_3878858	2021-09-02
hCoV-19/Japan/PG-72961/2021	EPI_ISL_3878860	2021-09-02
hCoV-19/Japan/PG-72962/2021	EPI_ISL_3878861	2021-09-02
hCoV-19/Japan/PG-72963/2021	EPI_ISL_3878863	2021-09-02
hCoV-19/Japan/PG-72964/2021	EPI_ISL_3878865	2021-09-02
hCoV-19/Japan/PG-72965/2021	EPI_ISL_3878867	2021-09-02
hCoV-19/Japan/PG-72966/2021	EPI_ISL_3878869	2021-09-02
hCoV-19/Japan/PG-72967/2021	EPI_ISL_3878872	2021-09-02
hCoV-19/Japan/PG-72968/2021	EPI_ISL_3878873	2021-09-02
hCoV-19/Japan/PG-72969/2021	EPI_ISL_3878875	2021-09-02
hCoV-19/Japan/PG-72970/2021	EPI_ISL_3878876	2021-09-02
hCoV-19/Japan/PG-72971/2021	EPI_ISL_3878878	2021-09-02
hCoV-19/Japan/PG-72972/2021	EPI_ISL_3878880	2021-09-02
hCoV-19/Japan/PG-72973/2021	EPI_ISL_3878881	2021-09-02
hCoV-19/Japan/PG-72974/2021	EPI_ISL_3878883	2021-09-02
hCoV-19/Japan/PG-72975/2021	EPI_ISL_3878884	2021-09-02
hCoV-19/Japan/PG-72976/2021	EPI_ISL_3878885	2021-09-02
hCoV-19/Japan/PG-72977/2021	EPI_ISL_3878887	2021-09-02
hCoV-19/Japan/PG-72978/2021	EPI_ISL_3878889	2021-09-02
hCoV-19/Japan/PG-72979/2021	EPI_ISL_3878890	2021-09-02
hCoV-19/Japan/PG-72980/2021	EPI_ISL_3878892	2021-09-02
hCoV-19/Japan/PG-72981/2021	EPI_ISL_3878893	2021-09-02
hCoV-19/Japan/PG-72982/2021	EPI_ISL_3878894	2021-09-02
hCoV-19/Japan/PG-72983/2021	EPI_ISL_3878895	2021-09-02
hCoV-19/Japan/PG-72984/2021	EPI_ISL_3878897	2021-09-02
hCoV-19/Japan/PG-72985/2021	EPI_ISL_3878898	2021-09-02
hCoV-19/Japan/PG-72986/2021	EPI_ISL_3878899	2021-09-02
hCoV-19/Japan/PG-72987/2021	EPI_ISL_3878901	2021-09-02
hCoV-19/Japan/PG-72988/2021	EPI_ISL_3878902	2021-09-02
hCoV-19/Japan/PG-72989/2021	EPI_ISL_3878903	2021-09-02
hCoV-19/Japan/PG-72990/2021	EPI_ISL_3878905	2021-09-02
hCoV-19/Japan/PG-72991/2021	EPI_ISL_3878906	2021-09-02
hCoV-19/Japan/PG-72992/2021	EPI_ISL_3878908	2021-09-02
hCoV-19/Japan/PG-72993/2021	EPI_ISL_3878909	2021-09-02
hCoV-19/Japan/PG-72994/2021	EPI_ISL_3878910	2021-09-02
hCoV-19/Japan/PG-72995/2021	EPI_ISL_3878912	2021-09-02
hCoV-19/Japan/PG-72996/2021	EPI_ISL_3878913	2021-09-02
hCoV-19/Japan/PG-72997/2021	EPI_ISL_3878915	2021-09-02
hCoV-19/Japan/PG-72998/2021	EPI_ISL_3878916	2021-09-02
hCoV-19/Japan/PG-72999/2021	EPI_ISL_3878917	2021-09-02
hCoV-19/Japan/PG-73000/2021	EPI_ISL_3878919	2021-09-02
hCoV-19/Japan/PG-73001/2021	EPI_ISL_3878920	2021-09-02
hCoV-19/Japan/PG-73002/2021	EPI_ISL_3878922	2021-09-02
hCoV-19/Japan/PG-73003/2021	EPI_ISL_3878923	2021-09-02
hCoV-19/Japan/PG-73004/2021	EPI_ISL_3878924	2021-09-02
hCoV-19/Japan/PG-73005/2021	EPI_ISL_3878926	2021-09-02
hCoV-19/Japan/PG-73006/2021	EPI_ISL_3878927	2021-09-02
hCoV-19/Japan/PG-73007/2021	EPI_ISL_3878929	2021-09-02
hCoV-19/Japan/PG-73008/2021	EPI_ISL_3878931	2021-09-02
hCoV-19/Japan/PG-73009/2021	EPI_ISL_3878933	2021-09-02

virus_name	epiID	submission_date
hCoV-19/Japan/PG-73010/2021	EPI_ISL_3878934	2021-09-02
hCoV-19/Japan/PG-73011/2021	EPI_ISL_3878936	2021-09-02
hCoV-19/Japan/PG-73012/2021	EPI_ISL_3878938	2021-09-02
hCoV-19/Japan/PG-73013/2021	EPI_ISL_3878939	2021-09-02
hCoV-19/Japan/PG-73014/2021	EPI_ISL_3878941	2021-09-02
hCoV-19/Japan/PG-73015/2021	EPI_ISL_3878944	2021-09-02
hCoV-19/Japan/PG-73016/2021	EPI_ISL_3878946	2021-09-02
hCoV-19/Japan/PG-73017/2021	EPI_ISL_3878947	2021-09-02
hCoV-19/Japan/PG-73018/2021	EPI_ISL_3878950	2021-09-02
hCoV-19/Japan/PG-73019/2021	EPI_ISL_3878952	2021-09-02
hCoV-19/Japan/PG-73020/2021	EPI_ISL_3878953	2021-09-02
hCoV-19/Japan/PG-73021/2021	EPI_ISL_3878955	2021-09-02
hCoV-19/Japan/PG-73022/2021	EPI_ISL_3878957	2021-09-02
hCoV-19/Japan/PG-73023/2021	EPI_ISL_3878959	2021-09-02
hCoV-19/Japan/PG-73024/2021	EPI_ISL_3878960	2021-09-02
hCoV-19/Japan/PG-73025/2021	EPI_ISL_3878963	2021-09-02
hCoV-19/Japan/PG-73026/2021	EPI_ISL_3878964	2021-09-02
hCoV-19/Japan/PG-73027/2021	EPI_ISL_3878966	2021-09-02
hCoV-19/Japan/PG-73028/2021	EPI_ISL_3878967	2021-09-02
hCoV-19/Japan/PG-73029/2021	EPI_ISL_3878969	2021-09-02
hCoV-19/Japan/PG-73030/2021	EPI_ISL_3878971	2021-09-02
hCoV-19/Japan/PG-73031/2021	EPI_ISL_3878973	2021-09-02
hCoV-19/Japan/PG-73032/2021	EPI_ISL_3878975	2021-09-02
hCoV-19/Japan/PG-73033/2021	EPI_ISL_3878977	2021-09-02
hCoV-19/Japan/PG-73034/2021	EPI_ISL_3878980	2021-09-02
hCoV-19/Japan/PG-73035/2021	EPI_ISL_3878981	2021-09-02
hCoV-19/Japan/PG-73036/2021	EPI_ISL_3878983	2021-09-02
hCoV-19/Japan/PG-73037/2021	EPI_ISL_3878985	2021-09-02
hCoV-19/Japan/PG-73038/2021	EPI_ISL_3878987	2021-09-02
hCoV-19/Japan/PG-73039/2021	EPI_ISL_3878990	2021-09-02
hCoV-19/Japan/PG-73040/2021	EPI_ISL_3878991	2021-09-02
hCoV-19/Japan/PG-73041/2021	EPI_ISL_3878993	2021-09-02
hCoV-19/Japan/PG-73042/2021	EPI_ISL_3878994	2021-09-02
hCoV-19/Japan/PG-73043/2021	EPI_ISL_3878996	2021-09-02
hCoV-19/Japan/PG-73044/2021	EPI_ISL_3878998	2021-09-02
hCoV-19/Japan/PG-73045/2021	EPI_ISL_3879000	2021-09-02
hCoV-19/Japan/PG-73046/2021	EPI_ISL_3879002	2021-09-02
hCoV-19/Japan/PG-73047/2021	EPI_ISL_3879003	2021-09-02
hCoV-19/Japan/PG-73048/2021	EPI_ISL_3879005	2021-09-02
hCoV-19/Japan/PG-73049/2021	EPI_ISL_3879007	2021-09-02
hCoV-19/Japan/PG-73050/2021	EPI_ISL_3879010	2021-09-02
hCoV-19/Japan/PG-73051/2021	EPI_ISL_3879013	2021-09-02
hCoV-19/Japan/PG-73052/2021	EPI_ISL_3879015	2021-09-02
hCoV-19/Japan/PG-73053/2021	EPI_ISL_3879017	2021-09-02
hCoV-19/Japan/PG-73054/2021	EPI_ISL_3879019	2021-09-02
hCoV-19/Japan/PG-73055/2021	EPI_ISL_3879021	2021-09-02
hCoV-19/Japan/PG-73056/2021	EPI_ISL_3879023	2021-09-02
hCoV-19/Japan/PG-73057/2021	EPI_ISL_3879025	2021-09-02
hCoV-19/Japan/PG-73058/2021	EPI_ISL_3879027	2021-09-02
hCoV-19/Japan/PG-73059/2021	EPI_ISL_3879028	2021-09-02
hCoV-19/Japan/PG-73060/2021	EPI_ISL_3879030	2021-09-02
hCoV-19/Japan/PG-73061/2021	EPI_ISL_3879031	2021-09-02
hCoV-19/Japan/PG-73062/2021	EPI_ISL_3879034	2021-09-02
hCoV-19/Japan/PG-73063/2021	EPI_ISL_3879036	2021-09-02

virus_name	epiID	submission_date
hCoV-19/Japan/PG-73064/2021	EPI_ISL_3879037	2021-09-02
hCoV-19/Japan/PG-73065/2021	EPI_ISL_3879039	2021-09-02
hCoV-19/Japan/PG-73066/2021	EPI_ISL_3879042	2021-09-02
hCoV-19/Japan/PG-73067/2021	EPI_ISL_3879044	2021-09-02
hCoV-19/Japan/PG-73068/2021	EPI_ISL_3879045	2021-09-02
hCoV-19/Japan/PG-73069/2021	EPI_ISL_3879047	2021-09-02
hCoV-19/Japan/PG-73070/2021	EPI_ISL_3879050	2021-09-02
hCoV-19/Japan/PG-73071/2021	EPI_ISL_3879052	2021-09-02
hCoV-19/Japan/PG-73072/2021	EPI_ISL_3879054	2021-09-02
hCoV-19/Japan/PG-73073/2021	EPI_ISL_3879056	2021-09-02
hCoV-19/Japan/PG-73074/2021	EPI_ISL_3879058	2021-09-02
hCoV-19/Japan/PG-73075/2021	EPI_ISL_3879059	2021-09-02
hCoV-19/Japan/PG-73076/2021	EPI_ISL_3879061	2021-09-02
hCoV-19/Japan/PG-73077/2021	EPI_ISL_3879063	2021-09-02
hCoV-19/Japan/PG-73078/2021	EPI_ISL_3879065	2021-09-02
hCoV-19/Japan/PG-73079/2021	EPI_ISL_3879067	2021-09-02
hCoV-19/Japan/PG-73080/2021	EPI_ISL_3879069	2021-09-02
hCoV-19/Japan/PG-73081/2021	EPI_ISL_3879071	2021-09-02
hCoV-19/Japan/PG-73082/2021	EPI_ISL_3879073	2021-09-02
hCoV-19/Japan/PG-73083/2021	EPI_ISL_3879075	2021-09-02
hCoV-19/Japan/PG-73084/2021	EPI_ISL_3879077	2021-09-02
hCoV-19/Japan/PG-73085/2021	EPI_ISL_3879079	2021-09-02
hCoV-19/Japan/PG-73086/2021	EPI_ISL_3879081	2021-09-02
hCoV-19/Japan/PG-73087/2021	EPI_ISL_3879083	2021-09-02
hCoV-19/Japan/PG-73088/2021	EPI_ISL_3879085	2021-09-02
hCoV-19/Japan/PG-73089/2021	EPI_ISL_3879087	2021-09-02
hCoV-19/Japan/PG-73090/2021	EPI_ISL_3879088	2021-09-02
hCoV-19/Japan/PG-73091/2021	EPI_ISL_3879090	2021-09-02
hCoV-19/Japan/PG-73092/2021	EPI_ISL_3879092	2021-09-02
hCoV-19/Japan/PG-73093/2021	EPI_ISL_3879094	2021-09-02
hCoV-19/Japan/PG-73094/2021	EPI_ISL_3879096	2021-09-02
hCoV-19/Japan/PG-73095/2021	EPI_ISL_3879098	2021-09-02
hCoV-19/Japan/PG-73096/2021	EPI_ISL_3879100	2021-09-02
hCoV-19/Japan/PG-73097/2021	EPI_ISL_3879102	2021-09-02
hCoV-19/Japan/PG-73098/2021	EPI_ISL_3879105	2021-09-02
hCoV-19/Japan/PG-73099/2021	EPI_ISL_3879107	2021-09-02
hCoV-19/Japan/PG-73100/2021	EPI_ISL_3879110	2021-09-02
hCoV-19/Japan/PG-73101/2021	EPI_ISL_3879112	2021-09-02
hCoV-19/Japan/PG-73102/2021	EPI_ISL_3879114	2021-09-02
hCoV-19/Japan/PG-73103/2021	EPI_ISL_3879115	2021-09-02
hCoV-19/Japan/PG-73104/2021	EPI_ISL_3879118	2021-09-02
hCoV-19/Japan/PG-73105/2021	EPI_ISL_3879120	2021-09-02
hCoV-19/Japan/PG-73106/2021	EPI_ISL_3879122	2021-09-02
hCoV-19/Japan/PG-73107/2021	EPI_ISL_3879125	2021-09-02
hCoV-19/Japan/PG-73108/2021	EPI_ISL_3879128	2021-09-02
hCoV-19/Japan/PG-73109/2021	EPI_ISL_3879129	2021-09-02
hCoV-19/Japan/PG-73110/2021	EPI_ISL_3879132	2021-09-02
hCoV-19/Japan/PG-73111/2021	EPI_ISL_3879134	2021-09-02
hCoV-19/Japan/PG-73112/2021	EPI_ISL_3879137	2021-09-02
hCoV-19/Japan/PG-73113/2021	EPI_ISL_3879139	2021-09-02
hCoV-19/Japan/PG-73114/2021	EPI_ISL_3879142	2021-09-02
hCoV-19/Japan/PG-73115/2021	EPI_ISL_3879144	2021-09-02
hCoV-19/Japan/PG-73116/2021	EPI_ISL_3879146	2021-09-02
hCoV-19/Japan/PG-73117/2021	EPI_ISL_3879148	2021-09-02

virus_name	epiID	submission_date
hCoV-19/Japan/PG-73118/2021	EPI_ISL_3879151	2021-09-02
hCoV-19/Japan/PG-73119/2021	EPI_ISL_3879152	2021-09-02
hCoV-19/Japan/PG-73120/2021	EPI_ISL_3879154	2021-09-02
hCoV-19/Japan/PG-73121/2021	EPI_ISL_3879156	2021-09-02
hCoV-19/Japan/PG-73122/2021	EPI_ISL_3879159	2021-09-02
hCoV-19/Japan/PG-73123/2021	EPI_ISL_3879161	2021-09-02
hCoV-19/Japan/PG-73124/2021	EPI_ISL_3879163	2021-09-02
hCoV-19/Japan/PG-73125/2021	EPI_ISL_3879164	2021-09-02
hCoV-19/Japan/PG-73126/2021	EPI_ISL_3879167	2021-09-02
hCoV-19/Japan/PG-73127/2021	EPI_ISL_3879168	2021-09-02
hCoV-19/Japan/PG-73128/2021	EPI_ISL_3879171	2021-09-02
hCoV-19/Japan/PG-73129/2021	EPI_ISL_3879173	2021-09-02
hCoV-19/Japan/PG-73130/2021	EPI_ISL_3879176	2021-09-02
hCoV-19/Japan/PG-73131/2021	EPI_ISL_3879178	2021-09-02
hCoV-19/Japan/PG-73132/2021	EPI_ISL_3879180	2021-09-02
hCoV-19/Japan/PG-73133/2021	EPI_ISL_3879182	2021-09-02
hCoV-19/Japan/PG-73134/2021	EPI_ISL_3879184	2021-09-02
hCoV-19/Japan/PG-73135/2021	EPI_ISL_3879186	2021-09-02
hCoV-19/Japan/PG-73136/2021	EPI_ISL_3879188	2021-09-02
hCoV-19/Japan/PG-73137/2021	EPI_ISL_3879190	2021-09-02
hCoV-19/Japan/PG-73138/2021	EPI_ISL_3879192	2021-09-02
hCoV-19/Japan/PG-73139/2021	EPI_ISL_3879194	2021-09-02
hCoV-19/Japan/PG-73140/2021	EPI_ISL_3879196	2021-09-02
hCoV-19/Japan/PG-73141/2021	EPI_ISL_3879198	2021-09-02
hCoV-19/Japan/PG-73142/2021	EPI_ISL_3879200	2021-09-02
hCoV-19/Japan/PG-73143/2021	EPI_ISL_3879202	2021-09-02
hCoV-19/Japan/PG-73144/2021	EPI_ISL_3879205	2021-09-02
hCoV-19/Japan/PG-73145/2021	EPI_ISL_3879208	2021-09-02
hCoV-19/Japan/PG-73146/2021	EPI_ISL_3879210	2021-09-02
hCoV-19/Japan/PG-73147/2021	EPI_ISL_3879212	2021-09-02
hCoV-19/Japan/PG-73148/2021	EPI_ISL_3879213	2021-09-02
hCoV-19/Japan/PG-73149/2021	EPI_ISL_3879214	2021-09-02
hCoV-19/Japan/PG-73150/2021	EPI_ISL_3879215	2021-09-02
hCoV-19/Japan/PG-73151/2021	EPI_ISL_3879216	2021-09-02
hCoV-19/Japan/PG-73152/2021	EPI_ISL_3879217	2021-09-02
hCoV-19/Japan/PG-73153/2021	EPI_ISL_3879219	2021-09-02
hCoV-19/Japan/PG-73154/2021	EPI_ISL_3879221	2021-09-02
hCoV-19/Japan/PG-73155/2021	EPI_ISL_3879223	2021-09-02
hCoV-19/Japan/PG-73156/2021	EPI_ISL_3879225	2021-09-02
hCoV-19/Japan/PG-73157/2021	EPI_ISL_3879227	2021-09-02
hCoV-19/Japan/PG-73158/2021	EPI_ISL_3879229	2021-09-02
hCoV-19/Japan/PG-73159/2021	EPI_ISL_3879231	2021-09-02
hCoV-19/Japan/PG-73160/2021	EPI_ISL_3879233	2021-09-02
hCoV-19/Japan/PG-73161/2021	EPI_ISL_3879236	2021-09-02
hCoV-19/Japan/PG-73162/2021	EPI_ISL_3879238	2021-09-02
hCoV-19/Japan/PG-73163/2021	EPI_ISL_3879240	2021-09-02
hCoV-19/Japan/PG-73164/2021	EPI_ISL_3879241	2021-09-02
hCoV-19/Japan/PG-73165/2021	EPI_ISL_3879244	2021-09-02
hCoV-19/Japan/PG-73166/2021	EPI_ISL_3879246	2021-09-02
hCoV-19/Japan/PG-73167/2021	EPI_ISL_3879248	2021-09-02
hCoV-19/Japan/PG-73168/2021	EPI_ISL_3879251	2021-09-02
hCoV-19/Japan/PG-73169/2021	EPI_ISL_3879253	2021-09-02
hCoV-19/Japan/PG-73170/2021	EPI_ISL_3879255	2021-09-02
hCoV-19/Japan/PG-73171/2021	EPI_ISL_3879257	2021-09-02

virus_name	epiID	submission_date
hCoV-19/Japan/PG-73172/2021	EPI_ISL_3879259	2021-09-02
hCoV-19/Japan/PG-73173/2021	EPI_ISL_3879262	2021-09-02
hCoV-19/Japan/PG-73174/2021	EPI_ISL_3879265	2021-09-02
hCoV-19/Japan/PG-73175/2021	EPI_ISL_3879267	2021-09-02
hCoV-19/Japan/PG-73176/2021	EPI_ISL_3879269	2021-09-02
hCoV-19/Japan/PG-73177/2021	EPI_ISL_3879271	2021-09-02
hCoV-19/Japan/PG-73178/2021	EPI_ISL_3879273	2021-09-02
hCoV-19/Japan/PG-73179/2021	EPI_ISL_3879276	2021-09-02
hCoV-19/Japan/PG-73180/2021	EPI_ISL_3879278	2021-09-02
hCoV-19/Japan/PG-73181/2021	EPI_ISL_3879280	2021-09-02
hCoV-19/Japan/PG-73182/2021	EPI_ISL_3879282	2021-09-02
hCoV-19/Japan/PG-73183/2021	EPI_ISL_3879284	2021-09-02
hCoV-19/Japan/PG-73184/2021	EPI_ISL_3879287	2021-09-02
hCoV-19/Japan/PG-73185/2021	EPI_ISL_3879289	2021-09-02
hCoV-19/Japan/PG-73186/2021	EPI_ISL_3879291	2021-09-02
hCoV-19/Japan/PG-73187/2021	EPI_ISL_3879294	2021-09-02
hCoV-19/Japan/PG-73188/2021	EPI_ISL_3879296	2021-09-02
hCoV-19/Japan/PG-73189/2021	EPI_ISL_3873558	2021-09-02
hCoV-19/Japan/PG-73190/2021	EPI_ISL_3873559	2021-09-02
hCoV-19/Japan/PG-73191/2021	EPI_ISL_3873560	2021-09-02
hCoV-19/Japan/PG-73192/2021	EPI_ISL_3873561	2021-09-02
hCoV-19/Japan/PG-73193/2021	EPI_ISL_3873562	2021-09-02
hCoV-19/Japan/PG-73194/2021	EPI_ISL_3873563	2021-09-02
hCoV-19/Japan/PG-73195/2021	EPI_ISL_3873564	2021-09-02
hCoV-19/Japan/PG-73196/2021	EPI_ISL_3873565	2021-09-02
hCoV-19/Japan/PG-73197/2021	EPI_ISL_3873566	2021-09-02
hCoV-19/Japan/PG-73198/2021	EPI_ISL_3873567	2021-09-02
hCoV-19/Japan/PG-73199/2021	EPI_ISL_3873568	2021-09-02
hCoV-19/Japan/PG-73200/2021	EPI_ISL_3873569	2021-09-02
hCoV-19/Japan/PG-73201/2021	EPI_ISL_3873570	2021-09-02
hCoV-19/Japan/PG-73202/2021	EPI_ISL_3873571	2021-09-02
hCoV-19/Japan/PG-73203/2021	EPI_ISL_3873572	2021-09-02
hCoV-19/Japan/PG-73204/2021	EPI_ISL_3873573	2021-09-02
hCoV-19/Japan/PG-73205/2021	EPI_ISL_3873574	2021-09-02
hCoV-19/Japan/PG-73206/2021	EPI_ISL_3873575	2021-09-02
hCoV-19/Japan/PG-73207/2021	EPI_ISL_3873576	2021-09-02
hCoV-19/Japan/PG-73208/2021	EPI_ISL_3873577	2021-09-02
hCoV-19/Japan/PG-73209/2021	EPI_ISL_3873578	2021-09-02
hCoV-19/Japan/PG-73210/2021	EPI_ISL_3873579	2021-09-02
hCoV-19/Japan/PG-73211/2021	EPI_ISL_3873580	2021-09-02
hCoV-19/Japan/PG-73212/2021	EPI_ISL_3873581	2021-09-02
hCoV-19/Japan/PG-73213/2021	EPI_ISL_3873582	2021-09-02
hCoV-19/Japan/PG-73214/2021	EPI_ISL_3873583	2021-09-02
hCoV-19/Japan/PG-73215/2021	EPI_ISL_3873584	2021-09-02
hCoV-19/Japan/PG-73216/2021	EPI_ISL_3872580	2021-09-02
hCoV-19/Japan/PG-73217/2021	EPI_ISL_3872581	2021-09-02
hCoV-19/Japan/PG-73218/2021	EPI_ISL_3872582	2021-09-02
hCoV-19/Japan/PG-73219/2021	EPI_ISL_3872583	2021-09-02
hCoV-19/Japan/PG-73220/2021	EPI_ISL_3872584	2021-09-02
hCoV-19/Japan/PG-73221/2021	EPI_ISL_3872585	2021-09-02
hCoV-19/Japan/PG-73222/2021	EPI_ISL_3872586	2021-09-02
hCoV-19/Japan/PG-73223/2021	EPI_ISL_3872587	2021-09-02
hCoV-19/Japan/PG-73224/2021	EPI_ISL_3872588	2021-09-02
hCoV-19/Japan/PG-73225/2021	EPI_ISL_3872589	2021-09-02

virus_name	epiID	submission_date
hCoV-19/Japan/PG-73226/2021	EPI_ISL_3872590	2021-09-02
hCoV-19/Japan/PG-73227/2021	EPI_ISL_3872591	2021-09-02
hCoV-19/Japan/PG-73228/2021	EPI_ISL_3872592	2021-09-02
hCoV-19/Japan/PG-73229/2021	EPI_ISL_3872593	2021-09-02
hCoV-19/Japan/PG-73230/2021	EPI_ISL_3872594	2021-09-02
hCoV-19/Japan/PG-73231/2021	EPI_ISL_3872595	2021-09-02
hCoV-19/Japan/PG-73232/2021	EPI_ISL_3872596	2021-09-02
hCoV-19/Japan/PG-73233/2021	EPI_ISL_3872597	2021-09-02
hCoV-19/Japan/PG-73234/2021	EPI_ISL_3872598	2021-09-02
hCoV-19/Japan/PG-73235/2021	EPI_ISL_3872599	2021-09-02
hCoV-19/Japan/PG-73236/2021	EPI_ISL_3872600	2021-09-02
hCoV-19/Japan/PG-73237/2021	EPI_ISL_3872601	2021-09-02
hCoV-19/Japan/PG-73238/2021	EPI_ISL_3872602	2021-09-02
hCoV-19/Japan/PG-73239/2021	EPI_ISL_3872603	2021-09-02
hCoV-19/Japan/PG-73240/2021	EPI_ISL_3872604	2021-09-02
hCoV-19/Japan/PG-73241/2021	EPI_ISL_3872605	2021-09-02
hCoV-19/Japan/PG-73242/2021	EPI_ISL_3872606	2021-09-02
hCoV-19/Japan/PG-73243/2021	EPI_ISL_3872607	2021-09-02
hCoV-19/Japan/PG-73244/2021	EPI_ISL_3872608	2021-09-02
hCoV-19/Japan/PG-73245/2021	EPI_ISL_3872609	2021-09-02
hCoV-19/Japan/PG-73246/2021	EPI_ISL_3872610	2021-09-02
hCoV-19/Japan/PG-73247/2021	EPI_ISL_3872611	2021-09-02
hCoV-19/Japan/PG-73248/2021	EPI_ISL_3872612	2021-09-02
hCoV-19/Japan/PG-73249/2021	EPI_ISL_3872613	2021-09-02
hCoV-19/Japan/PG-73250/2021	EPI_ISL_3872614	2021-09-02
hCoV-19/Japan/PG-73251/2021	EPI_ISL_3872615	2021-09-02
hCoV-19/Japan/PG-73252/2021	EPI_ISL_3872616	2021-09-02
hCoV-19/Japan/PG-73253/2021	EPI_ISL_3872617	2021-09-02
hCoV-19/Japan/PG-73254/2021	EPI_ISL_3872618	2021-09-02
hCoV-19/Japan/PG-73255/2021	EPI_ISL_3872619	2021-09-02
hCoV-19/Japan/PG-73256/2021	EPI_ISL_3872620	2021-09-02
hCoV-19/Japan/PG-73257/2021	EPI_ISL_3872621	2021-09-02
hCoV-19/Japan/PG-73258/2021	EPI_ISL_3872622	2021-09-02
hCoV-19/Japan/PG-73259/2021	EPI_ISL_3872623	2021-09-02
hCoV-19/Japan/PG-73260/2021	EPI_ISL_3872624	2021-09-02
hCoV-19/Japan/PG-73261/2021	EPI_ISL_3872625	2021-09-02
hCoV-19/Japan/PG-73262/2021	EPI_ISL_3872626	2021-09-02
hCoV-19/Japan/PG-73263/2021	EPI_ISL_3872627	2021-09-02
hCoV-19/Japan/PG-73264/2021	EPI_ISL_3872628	2021-09-02
hCoV-19/Japan/PG-73265/2021	EPI_ISL_3872629	2021-09-02
hCoV-19/Japan/PG-73266/2021	EPI_ISL_3872630	2021-09-02
hCoV-19/Japan/PG-73267/2021	EPI_ISL_3872631	2021-09-02
hCoV-19/Japan/PG-73268/2021	EPI_ISL_3879298	2021-09-02
hCoV-19/Japan/PG-73269/2021	EPI_ISL_3879300	2021-09-02
hCoV-19/Japan/PG-73270/2021	EPI_ISL_3879303	2021-09-02
hCoV-19/Japan/PG-73271/2021	EPI_ISL_3879305	2021-09-02
hCoV-19/Japan/PG-73272/2021	EPI_ISL_3879307	2021-09-02
hCoV-19/Japan/PG-73273/2021	EPI_ISL_3879308	2021-09-02
hCoV-19/Japan/PG-73274/2021	EPI_ISL_3879311	2021-09-02
hCoV-19/Japan/PG-73275/2021	EPI_ISL_3879312	2021-09-02
hCoV-19/Japan/PG-73276/2021	EPI_ISL_3879313	2021-09-02
hCoV-19/Japan/PG-73277/2021	EPI_ISL_3879316	2021-09-02
hCoV-19/Japan/PG-73278/2021	EPI_ISL_3879318	2021-09-02
hCoV-19/Japan/PG-73279/2021	EPI_ISL_3879321	2021-09-02

virus_name	epiID	submission_date
hCoV-19/Japan/PG-73280/2021	EPI_ISL_3879324	2021-09-02
hCoV-19/Japan/PG-73281/2021	EPI_ISL_3879327	2021-09-02
hCoV-19/Japan/PG-73282/2021	EPI_ISL_3879330	2021-09-02
hCoV-19/Japan/PG-73283/2021	EPI_ISL_3879332	2021-09-02
hCoV-19/Japan/PG-73284/2021	EPI_ISL_3879335	2021-09-02
hCoV-19/Japan/PG-73285/2021	EPI_ISL_3879337	2021-09-02
hCoV-19/Japan/PG-73286/2021	EPI_ISL_3879339	2021-09-02
hCoV-19/Japan/PG-73287/2021	EPI_ISL_3879342	2021-09-02
hCoV-19/Japan/PG-73288/2021	EPI_ISL_3879345	2021-09-02
hCoV-19/Japan/PG-73289/2021	EPI_ISL_3879348	2021-09-02
hCoV-19/Japan/PG-73290/2021	EPI_ISL_3879350	2021-09-02
hCoV-19/Japan/PG-73291/2021	EPI_ISL_3879353	2021-09-02
hCoV-19/Japan/PG-73292/2021	EPI_ISL_3879355	2021-09-02
hCoV-19/Japan/PG-73293/2021	EPI_ISL_3879357	2021-09-02
hCoV-19/Japan/PG-73294/2021	EPI_ISL_3879359	2021-09-02
hCoV-19/Japan/PG-73295/2021	EPI_ISL_3879362	2021-09-02
hCoV-19/Japan/PG-73296/2021	EPI_ISL_3879363	2021-09-02
hCoV-19/Japan/PG-73297/2021	EPI_ISL_3879365	2021-09-02
hCoV-19/Japan/PG-73298/2021	EPI_ISL_3879367	2021-09-02
hCoV-19/Japan/PG-73299/2021	EPI_ISL_3879370	2021-09-02
hCoV-19/Japan/PG-73300/2021	EPI_ISL_3879372	2021-09-02
hCoV-19/Japan/PG-73301/2021	EPI_ISL_3879374	2021-09-02
hCoV-19/Japan/PG-73302/2021	EPI_ISL_3879376	2021-09-02
hCoV-19/Japan/PG-73303/2021	EPI_ISL_3879379	2021-09-02
hCoV-19/Japan/PG-73304/2021	EPI_ISL_3879382	2021-09-02
hCoV-19/Japan/PG-73305/2021	EPI_ISL_3879384	2021-09-02
hCoV-19/Japan/PG-73306/2021	EPI_ISL_3879386	2021-09-02
hCoV-19/Japan/PG-73307/2021	EPI_ISL_3879389	2021-09-02
hCoV-19/Japan/PG-73308/2021	EPI_ISL_3879391	2021-09-02
hCoV-19/Japan/PG-73309/2021	EPI_ISL_3879394	2021-09-02
hCoV-19/Japan/PG-73310/2021	EPI_ISL_3879396	2021-09-02
hCoV-19/Japan/PG-73311/2021	EPI_ISL_3879398	2021-09-02
hCoV-19/Japan/PG-73312/2021	EPI_ISL_3879400	2021-09-02
hCoV-19/Japan/PG-73313/2021	EPI_ISL_3879403	2021-09-02
hCoV-19/Japan/PG-73314/2021	EPI_ISL_3879405	2021-09-02
hCoV-19/Japan/PG-73315/2021	EPI_ISL_3879407	2021-09-02
hCoV-19/Japan/PG-73316/2021	EPI_ISL_3879410	2021-09-02
hCoV-19/Japan/PG-73317/2021	EPI_ISL_3879412	2021-09-02
hCoV-19/Japan/PG-73318/2021	EPI_ISL_3879415	2021-09-02
hCoV-19/Japan/PG-73319/2021	EPI_ISL_3879417	2021-09-02
hCoV-19/Japan/PG-73320/2021	EPI_ISL_3879419	2021-09-02
hCoV-19/Japan/PG-73321/2021	EPI_ISL_3879420	2021-09-02
hCoV-19/Japan/PG-73322/2021	EPI_ISL_3879422	2021-09-02
hCoV-19/Japan/PG-73323/2021	EPI_ISL_3879424	2021-09-02
hCoV-19/Japan/PG-73324/2021	EPI_ISL_3879426	2021-09-02
hCoV-19/Japan/PG-73325/2021	EPI_ISL_3879428	2021-09-02
hCoV-19/Japan/PG-73326/2021	EPI_ISL_3879430	2021-09-02
hCoV-19/Japan/PG-73327/2021	EPI_ISL_3879432	2021-09-02
hCoV-19/Japan/PG-73328/2021	EPI_ISL_3879436	2021-09-02
hCoV-19/Japan/PG-73329/2021	EPI_ISL_3879437	2021-09-02
hCoV-19/Japan/PG-73330/2021	EPI_ISL_3879439	2021-09-02
hCoV-19/Japan/PG-73331/2021	EPI_ISL_3879441	2021-09-02
hCoV-19/Japan/PG-73332/2021	EPI_ISL_3879444	2021-09-02
hCoV-19/Japan/PG-73333/2021	EPI_ISL_3879446	2021-09-02

virus_name	epiID	submission_date
hCoV-19/Japan/PG-73334/2021	EPI_ISL_3879448	2021-09-02
hCoV-19/Japan/PG-73335/2021	EPI_ISL_3879450	2021-09-02
hCoV-19/Japan/PG-73336/2021	EPI_ISL_3879452	2021-09-02
hCoV-19/Japan/PG-73337/2021	EPI_ISL_3879454	2021-09-02
hCoV-19/Japan/PG-73338/2021	EPI_ISL_3879457	2021-09-02
hCoV-19/Japan/PG-73339/2021	EPI_ISL_3879459	2021-09-02
hCoV-19/Japan/PG-73340/2021	EPI_ISL_3879461	2021-09-02
hCoV-19/Japan/PG-73341/2021	EPI_ISL_3879463	2021-09-02
hCoV-19/Japan/PG-73342/2021	EPI_ISL_3879465	2021-09-02
hCoV-19/Japan/PG-73343/2021	EPI_ISL_3879467	2021-09-02
hCoV-19/Japan/PG-73344/2021	EPI_ISL_3879469	2021-09-02
hCoV-19/Japan/PG-73345/2021	EPI_ISL_3879471	2021-09-02
hCoV-19/Japan/PG-73346/2021	EPI_ISL_3879473	2021-09-02
hCoV-19/Japan/PG-73347/2021	EPI_ISL_3879476	2021-09-02
hCoV-19/Japan/PG-73348/2021	EPI_ISL_3879478	2021-09-02
hCoV-19/Japan/PG-73349/2021	EPI_ISL_3879480	2021-09-02
hCoV-19/Japan/PG-73350/2021	EPI_ISL_3879482	2021-09-02
hCoV-19/Japan/PG-73351/2021	EPI_ISL_3879484	2021-09-02
hCoV-19/Japan/PG-73352/2021	EPI_ISL_3879486	2021-09-02
hCoV-19/Japan/PG-73353/2021	EPI_ISL_3879489	2021-09-02
hCoV-19/Japan/PG-73354/2021	EPI_ISL_3879491	2021-09-02
hCoV-19/Japan/PG-73355/2021	EPI_ISL_3879494	2021-09-02
hCoV-19/Japan/PG-73356/2021	EPI_ISL_3879497	2021-09-02
hCoV-19/Japan/PG-73357/2021	EPI_ISL_3879499	2021-09-02
hCoV-19/Japan/PG-73358/2021	EPI_ISL_3879501	2021-09-02
hCoV-19/Japan/PG-73359/2021	EPI_ISL_3879504	2021-09-02
hCoV-19/Japan/PG-73360/2021	EPI_ISL_3879506	2021-09-02
hCoV-19/Japan/PG-73361/2021	EPI_ISL_3879507	2021-09-02
hCoV-19/Japan/PG-73362/2021	EPI_ISL_3879509	2021-09-02
hCoV-19/Japan/PG-73363/2021	EPI_ISL_3879512	2021-09-02
hCoV-19/Japan/PG-73364/2021	EPI_ISL_3879514	2021-09-02
hCoV-19/Japan/PG-73365/2021	EPI_ISL_3879516	2021-09-02
hCoV-19/Japan/PG-73366/2021	EPI_ISL_3879518	2021-09-02
hCoV-19/Japan/PG-73367/2021	EPI_ISL_3879521	2021-09-02
hCoV-19/Japan/PG-73368/2021	EPI_ISL_3879522	2021-09-02
hCoV-19/Japan/PG-73369/2021	EPI_ISL_3879523	2021-09-02
hCoV-19/Japan/PG-73370/2021	EPI_ISL_3879525	2021-09-02
hCoV-19/Japan/PG-73371/2021	EPI_ISL_3879527	2021-09-02
hCoV-19/Japan/PG-73372/2021	EPI_ISL_3879530	2021-09-02
hCoV-19/Japan/PG-73373/2021	EPI_ISL_3879532	2021-09-02
hCoV-19/Japan/PG-73374/2021	EPI_ISL_3879535	2021-09-02
hCoV-19/Japan/PG-73375/2021	EPI_ISL_3879537	2021-09-02
hCoV-19/Japan/PG-73376/2021	EPI_ISL_3879540	2021-09-02
hCoV-19/Japan/PG-73377/2021	EPI_ISL_3879542	2021-09-02
hCoV-19/Japan/PG-73378/2021	EPI_ISL_3879544	2021-09-02
hCoV-19/Japan/PG-73379/2021	EPI_ISL_3879545	2021-09-02
hCoV-19/Japan/PG-73380/2021	EPI_ISL_3879547	2021-09-02
hCoV-19/Japan/PG-73381/2021	EPI_ISL_3879549	2021-09-02
hCoV-19/Japan/PG-73382/2021	EPI_ISL_3879551	2021-09-02
hCoV-19/Japan/PG-73383/2021	EPI_ISL_3879554	2021-09-02
hCoV-19/Japan/PG-73384/2021	EPI_ISL_3879556	2021-09-02
hCoV-19/Japan/PG-73385/2021	EPI_ISL_3879558	2021-09-02
hCoV-19/Japan/PG-73386/2021	EPI_ISL_3879560	2021-09-02
hCoV-19/Japan/PG-73387/2021	EPI_ISL_3879562	2021-09-02

virus_name	epiID	submission_date
hCoV-19/Japan/PG-73388/2021	EPI_ISL_3879565	2021-09-02
hCoV-19/Japan/PG-73389/2021	EPI_ISL_3879568	2021-09-02
hCoV-19/Japan/PG-73390/2021	EPI_ISL_3879570	2021-09-02
hCoV-19/Japan/PG-73391/2021	EPI_ISL_3879572	2021-09-02
hCoV-19/Japan/PG-73392/2021	EPI_ISL_3879575	2021-09-02
hCoV-19/Japan/PG-73393/2021	EPI_ISL_3879577	2021-09-02
hCoV-19/Japan/PG-73394/2021	EPI_ISL_3879579	2021-09-02
hCoV-19/Japan/PG-73395/2021	EPI_ISL_3879582	2021-09-02
hCoV-19/Japan/PG-73396/2021	EPI_ISL_3879585	2021-09-02
hCoV-19/Japan/PG-73397/2021	EPI_ISL_3879587	2021-09-02
hCoV-19/Japan/PG-73398/2021	EPI_ISL_3879590	2021-09-02
hCoV-19/Japan/PG-73399/2021	EPI_ISL_3879592	2021-09-02
hCoV-19/Japan/PG-73400/2021	EPI_ISL_3879595	2021-09-02
hCoV-19/Japan/PG-73401/2021	EPI_ISL_3879596	2021-09-02
hCoV-19/Japan/PG-73402/2021	EPI_ISL_3879599	2021-09-02
hCoV-19/Japan/PG-73403/2021	EPI_ISL_3879601	2021-09-02
hCoV-19/Japan/PG-73404/2021	EPI_ISL_3879603	2021-09-02
hCoV-19/Japan/PG-73405/2021	EPI_ISL_3879605	2021-09-02
hCoV-19/Japan/PG-73406/2021	EPI_ISL_3879608	2021-09-02
hCoV-19/Japan/PG-73407/2021	EPI_ISL_3879610	2021-09-02
hCoV-19/Japan/PG-73408/2021	EPI_ISL_3879612	2021-09-02
hCoV-19/Japan/PG-73409/2021	EPI_ISL_3879615	2021-09-02
hCoV-19/Japan/PG-73410/2021	EPI_ISL_3879617	2021-09-02
hCoV-19/Japan/PG-73411/2021	EPI_ISL_3879619	2021-09-02
hCoV-19/Japan/PG-73412/2021	EPI_ISL_3879622	2021-09-02
hCoV-19/Japan/PG-73413/2021	EPI_ISL_3879625	2021-09-02
hCoV-19/Japan/PG-73414/2021	EPI_ISL_3879627	2021-09-02
hCoV-19/Japan/PG-73415/2021	EPI_ISL_3879629	2021-09-02
hCoV-19/Japan/PG-73416/2021	EPI_ISL_3879631	2021-09-02
hCoV-19/Japan/PG-73417/2021	EPI_ISL_3879632	2021-09-02
hCoV-19/Japan/PG-73418/2021	EPI_ISL_3879635	2021-09-02
hCoV-19/Japan/PG-73419/2021	EPI_ISL_3879637	2021-09-02
hCoV-19/Japan/PG-73420/2021	EPI_ISL_3879640	2021-09-02
hCoV-19/Japan/PG-73421/2021	EPI_ISL_3879641	2021-09-02
hCoV-19/Japan/PG-73422/2021	EPI_ISL_3879643	2021-09-02
hCoV-19/Japan/PG-73423/2021	EPI_ISL_3879646	2021-09-02
hCoV-19/Japan/PG-73424/2021	EPI_ISL_3879647	2021-09-02
hCoV-19/Japan/PG-73425/2021	EPI_ISL_3879650	2021-09-02
hCoV-19/Japan/PG-73426/2021	EPI_ISL_3879652	2021-09-02
hCoV-19/Japan/PG-73427/2021	EPI_ISL_3879654	2021-09-02
hCoV-19/Japan/PG-73428/2021	EPI_ISL_3879656	2021-09-02
hCoV-19/Japan/PG-73429/2021	EPI_ISL_3879658	2021-09-02
hCoV-19/Japan/PG-73430/2021	EPI_ISL_3879660	2021-09-02
hCoV-19/Japan/PG-73431/2021	EPI_ISL_3879663	2021-09-02
hCoV-19/Japan/PG-73432/2021	EPI_ISL_3879665	2021-09-02
hCoV-19/Japan/PG-73433/2021	EPI_ISL_3879666	2021-09-02
hCoV-19/Japan/PG-73434/2021	EPI_ISL_3879668	2021-09-02
hCoV-19/Japan/PG-73435/2021	EPI_ISL_3879669	2021-09-02
hCoV-19/Japan/PG-73436/2021	EPI_ISL_3879671	2021-09-02
hCoV-19/Japan/PG-73437/2021	EPI_ISL_3879673	2021-09-02
hCoV-19/Japan/PG-73438/2021	EPI_ISL_3879676	2021-09-02
hCoV-19/Japan/PG-73439/2021	EPI_ISL_3879679	2021-09-02
hCoV-19/Japan/PG-73440/2021	EPI_ISL_3879682	2021-09-02
hCoV-19/Japan/PG-73441/2021	EPI_ISL_3879684	2021-09-02

virus_name	epiID	submission_date
hCoV-19/Japan/PG-73442/2021	EPI_ISL_3879687	2021-09-02
hCoV-19/Japan/PG-73443/2021	EPI_ISL_3879689	2021-09-02
hCoV-19/Japan/PG-73444/2021	EPI_ISL_3879691	2021-09-02
hCoV-19/Japan/PG-73445/2021	EPI_ISL_3879694	2021-09-02
hCoV-19/Japan/PG-73446/2021	EPI_ISL_3879696	2021-09-02
hCoV-19/Japan/PG-73447/2021	EPI_ISL_3879699	2021-09-02
hCoV-19/Japan/PG-73448/2021	EPI_ISL_3879701	2021-09-02
hCoV-19/Japan/PG-73449/2021	EPI_ISL_3879703	2021-09-02
hCoV-19/Japan/PG-73450/2021	EPI_ISL_3879706	2021-09-02
hCoV-19/Japan/PG-73451/2021	EPI_ISL_3879708	2021-09-02
hCoV-19/Japan/PG-73452/2021	EPI_ISL_3879710	2021-09-02
hCoV-19/Japan/PG-73453/2021	EPI_ISL_3879713	2021-09-02
hCoV-19/Japan/PG-73454/2021	EPI_ISL_3879715	2021-09-02
hCoV-19/Japan/PG-73455/2021	EPI_ISL_3879717	2021-09-02
hCoV-19/Japan/PG-73456/2021	EPI_ISL_3879719	2021-09-02
hCoV-19/Japan/PG-73457/2021	EPI_ISL_3879722	2021-09-02
hCoV-19/Japan/PG-73458/2021	EPI_ISL_3879725	2021-09-02
hCoV-19/Japan/PG-73459/2021	EPI_ISL_3879726	2021-09-02
hCoV-19/Japan/PG-73460/2021	EPI_ISL_3879729	2021-09-02
hCoV-19/Japan/PG-73461/2021	EPI_ISL_3879731	2021-09-02
hCoV-19/Japan/PG-73462/2021	EPI_ISL_3879733	2021-09-02
hCoV-19/Japan/PG-73463/2021	EPI_ISL_3879735	2021-09-02
hCoV-19/Japan/PG-73464/2021	EPI_ISL_3879738	2021-09-02
hCoV-19/Japan/PG-73465/2021	EPI_ISL_3879741	2021-09-02
hCoV-19/Japan/PG-73466/2021	EPI_ISL_3879744	2021-09-02
hCoV-19/Japan/PG-73467/2021	EPI_ISL_3879745	2021-09-02
hCoV-19/Japan/PG-73468/2021	EPI_ISL_3879748	2021-09-02
hCoV-19/Japan/PG-73469/2021	EPI_ISL_3879750	2021-09-02
hCoV-19/Japan/PG-73470/2021	EPI_ISL_3879752	2021-09-02
hCoV-19/Japan/PG-73471/2021	EPI_ISL_3879755	2021-09-02
hCoV-19/Japan/PG-73472/2021	EPI_ISL_3879757	2021-09-02
hCoV-19/Japan/PG-73473/2021	EPI_ISL_3879760	2021-09-02
hCoV-19/Japan/PG-73474/2021	EPI_ISL_3879762	2021-09-02
hCoV-19/Japan/PG-73475/2021	EPI_ISL_3879764	2021-09-02
hCoV-19/Japan/PG-73476/2021	EPI_ISL_3879766	2021-09-02
hCoV-19/Japan/PG-73477/2021	EPI_ISL_3879768	2021-09-02
hCoV-19/Japan/PG-73478/2021	EPI_ISL_3879770	2021-09-02
hCoV-19/Japan/PG-73479/2021	EPI_ISL_3879772	2021-09-02
hCoV-19/Japan/PG-73480/2021	EPI_ISL_3879775	2021-09-02
hCoV-19/Japan/PG-73481/2021	EPI_ISL_3879777	2021-09-02
hCoV-19/Japan/PG-73482/2021	EPI_ISL_3879779	2021-09-02
hCoV-19/Japan/PG-73483/2021	EPI_ISL_3879780	2021-09-02
hCoV-19/Japan/PG-73484/2021	EPI_ISL_3879782	2021-09-02
hCoV-19/Japan/PG-73485/2021	EPI_ISL_3879783	2021-09-02
hCoV-19/Japan/PG-73486/2021	EPI_ISL_3879785	2021-09-02
hCoV-19/Japan/PG-73487/2021	EPI_ISL_3879787	2021-09-02
hCoV-19/Japan/PG-73488/2021	EPI_ISL_3879789	2021-09-02
hCoV-19/Japan/PG-73489/2021	EPI_ISL_3879791	2021-09-02
hCoV-19/Japan/PG-73490/2021	EPI_ISL_3879793	2021-09-02
hCoV-19/Japan/PG-73491/2021	EPI_ISL_3879796	2021-09-02
hCoV-19/Japan/PG-73492/2021	EPI_ISL_3879798	2021-09-02
hCoV-19/Japan/PG-73493/2021	EPI_ISL_3879799	2021-09-02
hCoV-19/Japan/PG-73494/2021	EPI_ISL_3879800	2021-09-02
hCoV-19/Japan/PG-73495/2021	EPI_ISL_3879801	2021-09-02

virus_name	epiID	submission_date
hCoV-19/Japan/PG-73496/2021	EPI_ISL_3879802	2021-09-02
hCoV-19/Japan/PG-73497/2021	EPI_ISL_3879803	2021-09-02
hCoV-19/Japan/PG-73498/2021	EPI_ISL_3879804	2021-09-02
hCoV-19/Japan/PG-73499/2021	EPI_ISL_3879806	2021-09-02
hCoV-19/Japan/PG-73500/2021	EPI_ISL_3879809	2021-09-02
hCoV-19/Japan/PG-73501/2021	EPI_ISL_3879811	2021-09-02
hCoV-19/Japan/PG-73502/2021	EPI_ISL_3879813	2021-09-02
hCoV-19/Japan/PG-73503/2021	EPI_ISL_3879814	2021-09-02
hCoV-19/Japan/PG-73504/2021	EPI_ISL_3879815	2021-09-02
hCoV-19/Japan/PG-73505/2021	EPI_ISL_3879816	2021-09-02
hCoV-19/Japan/PG-73506/2021	EPI_ISL_3879817	2021-09-02
hCoV-19/Japan/PG-73507/2021	EPI_ISL_3879818	2021-09-02
hCoV-19/Japan/PG-73508/2021	EPI_ISL_3879821	2021-09-02
hCoV-19/Japan/PG-73509/2021	EPI_ISL_3879824	2021-09-02
hCoV-19/Japan/PG-73510/2021	EPI_ISL_3879827	2021-09-02
hCoV-19/Japan/PG-73511/2021	EPI_ISL_3879828	2021-09-02
hCoV-19/Japan/PG-73512/2021	EPI_ISL_3879831	2021-09-02
hCoV-19/Japan/PG-73513/2021	EPI_ISL_3879833	2021-09-02
hCoV-19/Japan/PG-73514/2021	EPI_ISL_3879836	2021-09-02
hCoV-19/Japan/PG-73515/2021	EPI_ISL_3879838	2021-09-02
hCoV-19/Japan/PG-73516/2021	EPI_ISL_3879841	2021-09-02
hCoV-19/Japan/PG-73517/2021	EPI_ISL_3879844	2021-09-02
hCoV-19/Japan/PG-73518/2021	EPI_ISL_3879846	2021-09-02
hCoV-19/Japan/PG-73519/2021	EPI_ISL_3879848	2021-09-02
hCoV-19/Japan/PG-73520/2021	EPI_ISL_3879851	2021-09-02
hCoV-19/Japan/PG-73521/2021	EPI_ISL_3879853	2021-09-02
hCoV-19/Japan/PG-73522/2021	EPI_ISL_3879855	2021-09-02
hCoV-19/Japan/PG-73523/2021	EPI_ISL_3879858	2021-09-02
hCoV-19/Japan/PG-73524/2021	EPI_ISL_3879860	2021-09-02
hCoV-19/Japan/PG-73525/2021	EPI_ISL_3879862	2021-09-02
hCoV-19/Japan/PG-73526/2021	EPI_ISL_3879863	2021-09-02
hCoV-19/Japan/PG-73527/2021	EPI_ISL_3879865	2021-09-02
hCoV-19/Japan/PG-73528/2021	EPI_ISL_3879867	2021-09-02
hCoV-19/Japan/PG-73529/2021	EPI_ISL_3879869	2021-09-02
hCoV-19/Japan/PG-73530/2021	EPI_ISL_3879871	2021-09-02
hCoV-19/Japan/PG-73531/2021	EPI_ISL_3879872	2021-09-02
hCoV-19/Japan/PG-73532/2021	EPI_ISL_3879874	2021-09-02
hCoV-19/Japan/PG-73533/2021	EPI_ISL_3879876	2021-09-02
hCoV-19/Japan/PG-73534/2021	EPI_ISL_3879878	2021-09-02
hCoV-19/Japan/PG-73535/2021	EPI_ISL_3879880	2021-09-02
hCoV-19/Japan/PG-73536/2021	EPI_ISL_3879883	2021-09-02
hCoV-19/Japan/PG-73537/2021	EPI_ISL_3879885	2021-09-02
hCoV-19/Japan/PG-73538/2021	EPI_ISL_3879887	2021-09-02
hCoV-19/Japan/PG-73539/2021	EPI_ISL_3879889	2021-09-02
hCoV-19/Japan/PG-73540/2021	EPI_ISL_3879892	2021-09-02
hCoV-19/Japan/PG-73541/2021	EPI_ISL_3879894	2021-09-02
hCoV-19/Japan/PG-73542/2021	EPI_ISL_3879895	2021-09-02
hCoV-19/Japan/PG-73543/2021	EPI_ISL_3879896	2021-09-02
hCoV-19/Japan/PG-73544/2021	EPI_ISL_3879898	2021-09-02
hCoV-19/Japan/PG-73545/2021	EPI_ISL_3879899	2021-09-02
hCoV-19/Japan/PG-73546/2021	EPI_ISL_3879901	2021-09-02
hCoV-19/Japan/PG-73547/2021	EPI_ISL_3879902	2021-09-02
hCoV-19/Japan/PG-73548/2021	EPI_ISL_3879904	2021-09-02
hCoV-19/Japan/PG-73549/2021	EPI_ISL_3879906	2021-09-02

virus_name	epiID	submission_date
hCoV-19/Japan/PG-73550/2021	EPI_ISL_3879909	2021-09-02
hCoV-19/Japan/PG-73551/2021	EPI_ISL_3879910	2021-09-02
hCoV-19/Japan/PG-73552/2021	EPI_ISL_3879912	2021-09-02
hCoV-19/Japan/PG-73553/2021	EPI_ISL_3879914	2021-09-02
hCoV-19/Japan/PG-73554/2021	EPI_ISL_3879916	2021-09-02
hCoV-19/Japan/PG-73555/2021	EPI_ISL_3879918	2021-09-02
hCoV-19/Japan/PG-73556/2021	EPI_ISL_3879921	2021-09-02
hCoV-19/Japan/PG-73557/2021	EPI_ISL_3879923	2021-09-02
hCoV-19/Japan/PG-73558/2021	EPI_ISL_3879925	2021-09-02
hCoV-19/Japan/PG-73559/2021	EPI_ISL_3879927	2021-09-02
hCoV-19/Japan/PG-73560/2021	EPI_ISL_3879930	2021-09-02
hCoV-19/Japan/PG-73561/2021	EPI_ISL_3879932	2021-09-02
hCoV-19/Japan/PG-73562/2021	EPI_ISL_3879934	2021-09-02
hCoV-19/Japan/PG-73563/2021	EPI_ISL_3879936	2021-09-02
hCoV-19/Japan/PG-73564/2021	EPI_ISL_3879938	2021-09-02
hCoV-19/Japan/PG-73565/2021	EPI_ISL_3879941	2021-09-02
hCoV-19/Japan/PG-73566/2021	EPI_ISL_3879943	2021-09-02
hCoV-19/Japan/PG-73567/2021	EPI_ISL_3879945	2021-09-02
hCoV-19/Japan/PG-73568/2021	EPI_ISL_3879948	2021-09-02
hCoV-19/Japan/PG-73569/2021	EPI_ISL_3879950	2021-09-02
hCoV-19/Japan/PG-73570/2021	EPI_ISL_3879953	2021-09-02
hCoV-19/Japan/PG-73571/2021	EPI_ISL_3879955	2021-09-02
hCoV-19/Japan/PG-73572/2021	EPI_ISL_3879957	2021-09-02
hCoV-19/Japan/PG-73573/2021	EPI_ISL_3879959	2021-09-02
hCoV-19/Japan/PG-73574/2021	EPI_ISL_3879961	2021-09-02
hCoV-19/Japan/PG-73575/2021	EPI_ISL_3879963	2021-09-02
hCoV-19/Japan/PG-73576/2021	EPI_ISL_3879965	2021-09-02
hCoV-19/Japan/PG-73577/2021	EPI_ISL_3879966	2021-09-02
hCoV-19/Japan/PG-73578/2021	EPI_ISL_3879968	2021-09-02
hCoV-19/Japan/PG-73579/2021	EPI_ISL_3879970	2021-09-02
hCoV-19/Japan/PG-73580/2021	EPI_ISL_3879972	2021-09-02
hCoV-19/Japan/PG-73581/2021	EPI_ISL_3879973	2021-09-02
hCoV-19/Japan/PG-73582/2021	EPI_ISL_3879976	2021-09-02
hCoV-19/Japan/PG-73583/2021	EPI_ISL_3879978	2021-09-02
hCoV-19/Japan/PG-73584/2021	EPI_ISL_3879980	2021-09-02
hCoV-19/Japan/PG-73585/2021	EPI_ISL_3879981	2021-09-02
hCoV-19/Japan/PG-73586/2021	EPI_ISL_3879982	2021-09-02
hCoV-19/Japan/PG-73587/2021	EPI_ISL_3879983	2021-09-02
hCoV-19/Japan/PG-73588/2021	EPI_ISL_3879985	2021-09-02
hCoV-19/Japan/PG-73589/2021	EPI_ISL_3879987	2021-09-02
hCoV-19/Japan/PG-73590/2021	EPI_ISL_3879990	2021-09-02
hCoV-19/Japan/PG-73591/2021	EPI_ISL_3879991	2021-09-02
hCoV-19/Japan/PG-73592/2021	EPI_ISL_3879993	2021-09-02
hCoV-19/Japan/PG-73593/2021	EPI_ISL_3879994	2021-09-02
hCoV-19/Japan/PG-73594/2021	EPI_ISL_3879996	2021-09-02
hCoV-19/Japan/PG-73595/2021	EPI_ISL_3879998	2021-09-02
hCoV-19/Japan/PG-73596/2021	EPI_ISL_3880000	2021-09-02
hCoV-19/Japan/PG-73597/2021	EPI_ISL_3880002	2021-09-02
hCoV-19/Japan/PG-73598/2021	EPI_ISL_3880004	2021-09-02
hCoV-19/Japan/PG-73599/2021	EPI_ISL_3880006	2021-09-02
hCoV-19/Japan/PG-73600/2021	EPI_ISL_3880008	2021-09-02
hCoV-19/Japan/PG-73601/2021	EPI_ISL_3880011	2021-09-02
hCoV-19/Japan/PG-73602/2021	EPI_ISL_3880012	2021-09-02
hCoV-19/Japan/PG-73603/2021	EPI_ISL_3880014	2021-09-02

virus_name	epiID	submission_date
hCoV-19/Japan/PG-73604/2021	EPI_ISL_3880016	2021-09-02
hCoV-19/Japan/PG-73605/2021	EPI_ISL_3880018	2021-09-02
hCoV-19/Japan/PG-73606/2021	EPI_ISL_3880019	2021-09-02
hCoV-19/Japan/PG-73607/2021	EPI_ISL_3880021	2021-09-02
hCoV-19/Japan/PG-73608/2021	EPI_ISL_3880022	2021-09-02
hCoV-19/Japan/PG-73609/2021	EPI_ISL_3880025	2021-09-02
hCoV-19/Japan/PG-73610/2021	EPI_ISL_3880027	2021-09-02
hCoV-19/Japan/PG-73611/2021	EPI_ISL_3880029	2021-09-02
hCoV-19/Japan/PG-73612/2021	EPI_ISL_3880030	2021-09-02
hCoV-19/Japan/PG-73613/2021	EPI_ISL_3880032	2021-09-02
hCoV-19/Japan/PG-73614/2021	EPI_ISL_3880034	2021-09-02
hCoV-19/Japan/PG-73615/2021	EPI_ISL_3880037	2021-09-02
hCoV-19/Japan/PG-73616/2021	EPI_ISL_3880039	2021-09-02
hCoV-19/Japan/PG-73617/2021	EPI_ISL_3880041	2021-09-02
hCoV-19/Japan/PG-73618/2021	EPI_ISL_3880044	2021-09-02
hCoV-19/Japan/PG-73619/2021	EPI_ISL_3880046	2021-09-02
hCoV-19/Japan/PG-73620/2021	EPI_ISL_3880048	2021-09-02
hCoV-19/Japan/PG-73621/2021	EPI_ISL_3880050	2021-09-02
hCoV-19/Japan/PG-73622/2021	EPI_ISL_3880052	2021-09-02
hCoV-19/Japan/PG-73623/2021	EPI_ISL_3880054	2021-09-02
hCoV-19/Japan/PG-73624/2021	EPI_ISL_3880056	2021-09-02
hCoV-19/Japan/PG-73625/2021	EPI_ISL_3880059	2021-09-02
hCoV-19/Japan/PG-73626/2021	EPI_ISL_3880060	2021-09-02
hCoV-19/Japan/PG-73627/2021	EPI_ISL_3880063	2021-09-02
hCoV-19/Japan/PG-73628/2021	EPI_ISL_3880066	2021-09-02
hCoV-19/Japan/PG-73629/2021	EPI_ISL_3880068	2021-09-02
hCoV-19/Japan/PG-73630/2021	EPI_ISL_3880070	2021-09-02
hCoV-19/Japan/PG-73631/2021	EPI_ISL_3880072	2021-09-02
hCoV-19/Japan/PG-73632/2021	EPI_ISL_3880074	2021-09-02
hCoV-19/Japan/PG-73633/2021	EPI_ISL_3880076	2021-09-02
hCoV-19/Japan/PG-73634/2021	EPI_ISL_3880078	2021-09-02
hCoV-19/Japan/PG-73635/2021	EPI_ISL_3880081	2021-09-02
hCoV-19/Japan/PG-73636/2021	EPI_ISL_3880083	2021-09-02
hCoV-19/Japan/PG-73637/2021	EPI_ISL_3880085	2021-09-02
hCoV-19/Japan/PG-73638/2021	EPI_ISL_3880087	2021-09-02
hCoV-19/Japan/PG-73639/2021	EPI_ISL_3880090	2021-09-02
hCoV-19/Japan/PG-73640/2021	EPI_ISL_3880092	2021-09-02
hCoV-19/Japan/PG-73641/2021	EPI_ISL_3880095	2021-09-02
hCoV-19/Japan/PG-73642/2021	EPI_ISL_3880098	2021-09-02
hCoV-19/Japan/PG-73643/2021	EPI_ISL_3880100	2021-09-02
hCoV-19/Japan/PG-73644/2021	EPI_ISL_3880102	2021-09-02
hCoV-19/Japan/PG-73645/2021	EPI_ISL_3880105	2021-09-02
hCoV-19/Japan/PG-73646/2021	EPI_ISL_3880107	2021-09-02
hCoV-19/Japan/PG-73647/2021	EPI_ISL_3880109	2021-09-02
hCoV-19/Japan/PG-73648/2021	EPI_ISL_3880112	2021-09-02
hCoV-19/Japan/PG-73649/2021	EPI_ISL_3880114	2021-09-02
hCoV-19/Japan/PG-73650/2021	EPI_ISL_3880116	2021-09-02
hCoV-19/Japan/PG-73651/2021	EPI_ISL_3880118	2021-09-02
hCoV-19/Japan/PG-73652/2021	EPI_ISL_3880121	2021-09-02
hCoV-19/Japan/PG-73653/2021	EPI_ISL_3880123	2021-09-02
hCoV-19/Japan/PG-73654/2021	EPI_ISL_3880125	2021-09-02
hCoV-19/Japan/PG-73655/2021	EPI_ISL_3880127	2021-09-02
hCoV-19/Japan/PG-73656/2021	EPI_ISL_3880129	2021-09-02
hCoV-19/Japan/PG-73657/2021	EPI_ISL_3880131	2021-09-02

virus_name	epiID	submission_date
hCoV-19/Japan/PG-73658/2021	EPI_ISL_3880133	2021-09-02
hCoV-19/Japan/PG-73659/2021	EPI_ISL_3880136	2021-09-02
hCoV-19/Japan/PG-73660/2021	EPI_ISL_3880138	2021-09-02
hCoV-19/Japan/PG-73661/2021	EPI_ISL_3880141	2021-09-02
hCoV-19/Japan/PG-73662/2021	EPI_ISL_3880143	2021-09-02
hCoV-19/Japan/PG-73663/2021	EPI_ISL_3880145	2021-09-02
hCoV-19/Japan/PG-73664/2021	EPI_ISL_3880147	2021-09-02
hCoV-19/Japan/PG-73665/2021	EPI_ISL_3880150	2021-09-02
hCoV-19/Japan/PG-73666/2021	EPI_ISL_3880152	2021-09-02
hCoV-19/Japan/PG-73667/2021	EPI_ISL_3880154	2021-09-02
hCoV-19/Japan/PG-73668/2021	EPI_ISL_3880156	2021-09-02
hCoV-19/Japan/PG-73669/2021	EPI_ISL_3880158	2021-09-02
hCoV-19/Japan/PG-73670/2021	EPI_ISL_3880159	2021-09-02
hCoV-19/Japan/PG-73671/2021	EPI_ISL_3880162	2021-09-02
hCoV-19/Japan/PG-73672/2021	EPI_ISL_3880164	2021-09-02
hCoV-19/Japan/PG-73673/2021	EPI_ISL_3880167	2021-09-02
hCoV-19/Japan/PG-73674/2021	EPI_ISL_3880169	2021-09-02
hCoV-19/Japan/PG-73675/2021	EPI_ISL_3880171	2021-09-02
hCoV-19/Japan/PG-73676/2021	EPI_ISL_3880173	2021-09-02
hCoV-19/Japan/PG-73677/2021	EPI_ISL_3880175	2021-09-02
hCoV-19/Japan/PG-73678/2021	EPI_ISL_3880176	2021-09-02
hCoV-19/Japan/PG-73679/2021	EPI_ISL_3880177	2021-09-02
hCoV-19/Japan/PG-73680/2021	EPI_ISL_3880178	2021-09-02
hCoV-19/Japan/PG-73681/2021	EPI_ISL_3880179	2021-09-02
hCoV-19/Japan/PG-73682/2021	EPI_ISL_3880180	2021-09-02
hCoV-19/Japan/PG-73683/2021	EPI_ISL_3880182	2021-09-02
hCoV-19/Japan/PG-73684/2021	EPI_ISL_3880184	2021-09-02
hCoV-19/Japan/PG-73685/2021	EPI_ISL_3880185	2021-09-02
hCoV-19/Japan/PG-73686/2021	EPI_ISL_3880187	2021-09-02
hCoV-19/Japan/PG-73687/2021	EPI_ISL_3880190	2021-09-02
hCoV-19/Japan/PG-73688/2021	EPI_ISL_3880192	2021-09-02
hCoV-19/Japan/PG-73689/2021	EPI_ISL_3880194	2021-09-02
hCoV-19/Japan/PG-73690/2021	EPI_ISL_3880196	2021-09-02
hCoV-19/Japan/PG-73691/2021	EPI_ISL_3880198	2021-09-02
hCoV-19/Japan/PG-73692/2021	EPI_ISL_3880201	2021-09-02
hCoV-19/Japan/PG-73693/2021	EPI_ISL_3880203	2021-09-02
hCoV-19/Japan/PG-73694/2021	EPI_ISL_3880205	2021-09-02
hCoV-19/Japan/PG-73695/2021	EPI_ISL_3880207	2021-09-02
hCoV-19/Japan/PG-73696/2021	EPI_ISL_3880210	2021-09-02
hCoV-19/Japan/PG-73697/2021	EPI_ISL_3880213	2021-09-02
hCoV-19/Japan/PG-73698/2021	EPI_ISL_3880215	2021-09-02
hCoV-19/Japan/PG-73699/2021	EPI_ISL_3880217	2021-09-02
hCoV-19/Japan/PG-73700/2021	EPI_ISL_3880220	2021-09-02
hCoV-19/Japan/PG-73701/2021	EPI_ISL_3880222	2021-09-02
hCoV-19/Japan/PG-73702/2021	EPI_ISL_3880224	2021-09-02
hCoV-19/Japan/PG-73703/2021	EPI_ISL_3880226	2021-09-02
hCoV-19/Japan/PG-73704/2021	EPI_ISL_3880229	2021-09-02
hCoV-19/Japan/PG-73705/2021	EPI_ISL_3880232	2021-09-02
hCoV-19/Japan/PG-73706/2021	EPI_ISL_3880234	2021-09-02
hCoV-19/Japan/PG-73707/2021	EPI_ISL_3880237	2021-09-02
hCoV-19/Japan/PG-73708/2021	EPI_ISL_3880239	2021-09-02
hCoV-19/Japan/PG-73709/2021	EPI_ISL_3880242	2021-09-02
hCoV-19/Japan/PG-73710/2021	EPI_ISL_3880244	2021-09-02
hCoV-19/Japan/PG-73711/2021	EPI_ISL_3880246	2021-09-02

virus_name	epiID	submission_date
hCoV-19/Japan/PG-73712/2021	EPI_ISL_3880249	2021-09-02
hCoV-19/Japan/PG-73713/2021	EPI_ISL_3880251	2021-09-02
hCoV-19/Japan/PG-73714/2021	EPI_ISL_3880254	2021-09-02
hCoV-19/Japan/PG-73715/2021	EPI_ISL_3880256	2021-09-02
hCoV-19/Japan/PG-73716/2021	EPI_ISL_3880258	2021-09-02
hCoV-19/Japan/PG-73717/2021	EPI_ISL_3880260	2021-09-02
hCoV-19/Japan/PG-73718/2021	EPI_ISL_3880262	2021-09-02
hCoV-19/Japan/PG-73719/2021	EPI_ISL_3880264	2021-09-02
hCoV-19/Japan/PG-73720/2021	EPI_ISL_3880266	2021-09-02
hCoV-19/Japan/PG-73721/2021	EPI_ISL_3880268	2021-09-02
hCoV-19/Japan/PG-73722/2021	EPI_ISL_3880270	2021-09-02
hCoV-19/Japan/PG-73723/2021	EPI_ISL_3880272	2021-09-02
hCoV-19/Japan/PG-73724/2021	EPI_ISL_3880274	2021-09-02
hCoV-19/Japan/PG-73725/2021	EPI_ISL_3880276	2021-09-02
hCoV-19/Japan/PG-73726/2021	EPI_ISL_3880279	2021-09-02
hCoV-19/Japan/PG-73727/2021	EPI_ISL_3880281	2021-09-02
hCoV-19/Japan/PG-73728/2021	EPI_ISL_3880282	2021-09-02
hCoV-19/Japan/PG-73729/2021	EPI_ISL_3880283	2021-09-02
hCoV-19/Japan/PG-73730/2021	EPI_ISL_3880284	2021-09-02
hCoV-19/Japan/PG-73731/2021	EPI_ISL_3880285	2021-09-02
hCoV-19/Japan/PG-73732/2021	EPI_ISL_3880286	2021-09-02
hCoV-19/Japan/PG-73733/2021	EPI_ISL_3880287	2021-09-02
hCoV-19/Japan/PG-73734/2021	EPI_ISL_3880289	2021-09-02
hCoV-19/Japan/PG-73735/2021	EPI_ISL_3880291	2021-09-02
hCoV-19/Japan/PG-73736/2021	EPI_ISL_3880293	2021-09-02
hCoV-19/Japan/PG-73737/2021	EPI_ISL_3880295	2021-09-02
hCoV-19/Japan/PG-73738/2021	EPI_ISL_3880298	2021-09-02
hCoV-19/Japan/PG-73739/2021	EPI_ISL_3880300	2021-09-02
hCoV-19/Japan/PG-73740/2021	EPI_ISL_3880302	2021-09-02
hCoV-19/Japan/PG-73741/2021	EPI_ISL_3880305	2021-09-02
hCoV-19/Japan/PG-73742/2021	EPI_ISL_3880308	2021-09-02
hCoV-19/Japan/PG-73743/2021	EPI_ISL_3880310	2021-09-02
hCoV-19/Japan/PG-73744/2021	EPI_ISL_3880312	2021-09-02
hCoV-19/Japan/PG-73745/2021	EPI_ISL_3880314	2021-09-02
hCoV-19/Japan/PG-73746/2021	EPI_ISL_3880317	2021-09-02
hCoV-19/Japan/PG-73747/2021	EPI_ISL_3880319	2021-09-02
hCoV-19/Japan/PG-73748/2021	EPI_ISL_3880321	2021-09-02
hCoV-19/Japan/PG-73749/2021	EPI_ISL_3880323	2021-09-02
hCoV-19/Japan/PG-73750/2021	EPI_ISL_3880325	2021-09-02
hCoV-19/Japan/PG-73751/2021	EPI_ISL_3880328	2021-09-02
hCoV-19/Japan/PG-73752/2021	EPI_ISL_3880330	2021-09-02
hCoV-19/Japan/PG-73753/2021	EPI_ISL_3880333	2021-09-02
hCoV-19/Japan/PG-73754/2021	EPI_ISL_3880334	2021-09-02
hCoV-19/Japan/PG-73755/2021	EPI_ISL_3880337	2021-09-02
hCoV-19/Japan/PG-73756/2021	EPI_ISL_3880340	2021-09-02
hCoV-19/Japan/PG-73757/2021	EPI_ISL_3880343	2021-09-02
hCoV-19/Japan/PG-73758/2021	EPI_ISL_3880345	2021-09-02
hCoV-19/Japan/PG-73759/2021	EPI_ISL_3880347	2021-09-02
hCoV-19/Japan/PG-73760/2021	EPI_ISL_3880350	2021-09-02
hCoV-19/Japan/PG-73761/2021	EPI_ISL_3880352	2021-09-02
hCoV-19/Japan/PG-73762/2021	EPI_ISL_3880355	2021-09-02
hCoV-19/Japan/PG-73763/2021	EPI_ISL_3880358	2021-09-02
hCoV-19/Japan/PG-73764/2021	EPI_ISL_3880360	2021-09-02
hCoV-19/Japan/PG-73765/2021	EPI_ISL_3880363	2021-09-02

virus_name	epiID	submission_date
hCoV-19/Japan/PG-73766/2021	EPI_ISL_3880365	2021-09-02
hCoV-19/Japan/PG-73767/2021	EPI_ISL_3880368	2021-09-02
hCoV-19/Japan/PG-73768/2021	EPI_ISL_3880371	2021-09-02
hCoV-19/Japan/PG-73769/2021	EPI_ISL_3880374	2021-09-02
hCoV-19/Japan/PG-73770/2021	EPI_ISL_3880376	2021-09-02
hCoV-19/Japan/PG-73771/2021	EPI_ISL_3880378	2021-09-02
hCoV-19/Japan/PG-73772/2021	EPI_ISL_3880379	2021-09-02
hCoV-19/Japan/PG-73773/2021	EPI_ISL_3880381	2021-09-02
hCoV-19/Japan/PG-73774/2021	EPI_ISL_3880382	2021-09-02
hCoV-19/Japan/PG-73775/2021	EPI_ISL_3880384	2021-09-02
hCoV-19/Japan/PG-73776/2021	EPI_ISL_3880387	2021-09-02
hCoV-19/Japan/PG-73777/2021	EPI_ISL_3880389	2021-09-02
hCoV-19/Japan/PG-73778/2021	EPI_ISL_3880392	2021-09-02
hCoV-19/Japan/PG-73779/2021	EPI_ISL_3880394	2021-09-02
hCoV-19/Japan/PG-73780/2021	EPI_ISL_3880397	2021-09-02
hCoV-19/Japan/PG-73781/2021	EPI_ISL_3880399	2021-09-02
hCoV-19/Japan/PG-73782/2021	EPI_ISL_3880402	2021-09-02
hCoV-19/Japan/PG-73783/2021	EPI_ISL_3880405	2021-09-02
hCoV-19/Japan/PG-73784/2021	EPI_ISL_3880406	2021-09-02
hCoV-19/Japan/PG-73785/2021	EPI_ISL_3880409	2021-09-02
hCoV-19/Japan/PG-73786/2021	EPI_ISL_3880411	2021-09-02
hCoV-19/Japan/PG-73787/2021	EPI_ISL_3880413	2021-09-02
hCoV-19/Japan/PG-73788/2021	EPI_ISL_3880416	2021-09-02
hCoV-19/Japan/PG-73789/2021	EPI_ISL_3880418	2021-09-02
hCoV-19/Japan/PG-73790/2021	EPI_ISL_3880419	2021-09-02
hCoV-19/Japan/PG-73791/2021	EPI_ISL_3880421	2021-09-02
hCoV-19/Japan/PG-73792/2021	EPI_ISL_3880424	2021-09-02
hCoV-19/Japan/PG-73793/2021	EPI_ISL_3880426	2021-09-02
hCoV-19/Japan/PG-73794/2021	EPI_ISL_3880429	2021-09-02
hCoV-19/Japan/PG-73795/2021	EPI_ISL_3880430	2021-09-02
hCoV-19/Japan/PG-73796/2021	EPI_ISL_3880433	2021-09-02
hCoV-19/Japan/PG-73797/2021	EPI_ISL_3880435	2021-09-02
hCoV-19/Japan/PG-73798/2021	EPI_ISL_3880438	2021-09-02
hCoV-19/Japan/PG-73799/2021	EPI_ISL_3880440	2021-09-02
hCoV-19/Japan/PG-73800/2021	EPI_ISL_3880443	2021-09-02
hCoV-19/Japan/PG-73801/2021	EPI_ISL_3880445	2021-09-02
hCoV-19/Japan/PG-73802/2021	EPI_ISL_3880447	2021-09-02
hCoV-19/Japan/PG-73803/2021	EPI_ISL_3880449	2021-09-02
hCoV-19/Japan/PG-73804/2021	EPI_ISL_3880451	2021-09-02
hCoV-19/Japan/PG-73805/2021	EPI_ISL_3880454	2021-09-02
hCoV-19/Japan/PG-73806/2021	EPI_ISL_3880456	2021-09-02
hCoV-19/Japan/PG-73807/2021	EPI_ISL_3880459	2021-09-02
hCoV-19/Japan/PG-73808/2021	EPI_ISL_3880461	2021-09-02
hCoV-19/Japan/PG-73809/2021	EPI_ISL_3880463	2021-09-02
hCoV-19/Japan/PG-73810/2021	EPI_ISL_3880464	2021-09-02
hCoV-19/Japan/PG-73811/2021	EPI_ISL_3880465	2021-09-02
hCoV-19/Japan/PG-73812/2021	EPI_ISL_3880468	2021-09-02
hCoV-19/Japan/PG-73813/2021	EPI_ISL_3880470	2021-09-02
hCoV-19/Japan/PG-73814/2021	EPI_ISL_3880473	2021-09-02
hCoV-19/Japan/PG-73815/2021	EPI_ISL_3880475	2021-09-02
hCoV-19/Japan/PG-73816/2021	EPI_ISL_3880477	2021-09-02
hCoV-19/Japan/PG-73817/2021	EPI_ISL_3880479	2021-09-02
hCoV-19/Japan/PG-73818/2021	EPI_ISL_3880482	2021-09-02
hCoV-19/Japan/PG-73819/2021	EPI_ISL_3880484	2021-09-02

virus_name	epiID	submission_date
hCoV-19/Japan/PG-73820/2021	EPI_ISL_3880486	2021-09-02
hCoV-19/Japan/PG-73821/2021	EPI_ISL_3880488	2021-09-02
hCoV-19/Japan/PG-73822/2021	EPI_ISL_3880490	2021-09-02
hCoV-19/Japan/PG-73823/2021	EPI_ISL_3880492	2021-09-02
hCoV-19/Japan/PG-73824/2021	EPI_ISL_3880494	2021-09-02
hCoV-19/Japan/PG-73825/2021	EPI_ISL_3880497	2021-09-02
hCoV-19/Japan/PG-73826/2021	EPI_ISL_3880499	2021-09-02
hCoV-19/Japan/PG-73827/2021	EPI_ISL_3880501	2021-09-02
hCoV-19/Japan/PG-73828/2021	EPI_ISL_3880506	2021-09-02
hCoV-19/Japan/PG-73829/2021	EPI_ISL_3880507	2021-09-02
hCoV-19/Japan/PG-73830/2021	EPI_ISL_3880509	2021-09-02
hCoV-19/Japan/PG-73831/2021	EPI_ISL_3880512	2021-09-02
hCoV-19/Japan/PG-73832/2021	EPI_ISL_3880514	2021-09-02
hCoV-19/Japan/PG-73833/2021	EPI_ISL_3880517	2021-09-02
hCoV-19/Japan/PG-73834/2021	EPI_ISL_3880519	2021-09-02
hCoV-19/Japan/PG-73835/2021	EPI_ISL_3880521	2021-09-02
hCoV-19/Japan/PG-73836/2021	EPI_ISL_3880523	2021-09-02
hCoV-19/Japan/PG-73837/2021	EPI_ISL_3880526	2021-09-02
hCoV-19/Japan/PG-73838/2021	EPI_ISL_3880528	2021-09-02
hCoV-19/Japan/PG-73839/2021	EPI_ISL_3880530	2021-09-02
hCoV-19/Japan/PG-73840/2021	EPI_ISL_3880532	2021-09-02
hCoV-19/Japan/PG-73841/2021	EPI_ISL_3880535	2021-09-02
hCoV-19/Japan/PG-73842/2021	EPI_ISL_3880538	2021-09-02
hCoV-19/Japan/PG-73843/2021	EPI_ISL_3880541	2021-09-02
hCoV-19/Japan/PG-73844/2021	EPI_ISL_3880543	2021-09-02
hCoV-19/Japan/PG-73845/2021	EPI_ISL_3880546	2021-09-02
hCoV-19/Japan/PG-73846/2021	EPI_ISL_3880548	2021-09-02
hCoV-19/Japan/PG-73847/2021	EPI_ISL_3880550	2021-09-02
hCoV-19/Japan/PG-73848/2021	EPI_ISL_3880552	2021-09-02
hCoV-19/Japan/PG-73849/2021	EPI_ISL_3880553	2021-09-02
hCoV-19/Japan/PG-73850/2021	EPI_ISL_3880556	2021-09-02
hCoV-19/Japan/PG-73851/2021	EPI_ISL_3880558	2021-09-02
hCoV-19/Japan/PG-73852/2021	EPI_ISL_3880561	2021-09-02
hCoV-19/Japan/PG-73853/2021	EPI_ISL_3880562	2021-09-02
hCoV-19/Japan/PG-73854/2021	EPI_ISL_3880564	2021-09-02
hCoV-19/Japan/PG-73855/2021	EPI_ISL_3880566	2021-09-02
hCoV-19/Japan/PG-73856/2021	EPI_ISL_3880568	2021-09-02
hCoV-19/Japan/PG-73857/2021	EPI_ISL_3880570	2021-09-02
hCoV-19/Japan/PG-73858/2021	EPI_ISL_3880571	2021-09-02
hCoV-19/Japan/PG-73859/2021	EPI_ISL_3880573	2021-09-02
hCoV-19/Japan/PG-73860/2021	EPI_ISL_3880575	2021-09-02
hCoV-19/Japan/PG-73861/2021	EPI_ISL_3880576	2021-09-02
hCoV-19/Japan/PG-73862/2021	EPI_ISL_3880578	2021-09-02
hCoV-19/Japan/PG-73863/2021	EPI_ISL_3880579	2021-09-02
hCoV-19/Japan/PG-73864/2021	EPI_ISL_3880580	2021-09-02
hCoV-19/Japan/PG-73865/2021	EPI_ISL_3880582	2021-09-02
hCoV-19/Japan/PG-73866/2021	EPI_ISL_3880583	2021-09-02
hCoV-19/Japan/PG-73867/2021	EPI_ISL_3880585	2021-09-02
hCoV-19/Japan/PG-73868/2021	EPI_ISL_3880587	2021-09-02
hCoV-19/Japan/PG-73869/2021	EPI_ISL_3880589	2021-09-02
hCoV-19/Japan/PG-73870/2021	EPI_ISL_3880591	2021-09-02
hCoV-19/Japan/PG-73871/2021	EPI_ISL_3880594	2021-09-02
hCoV-19/Japan/PG-73872/2021	EPI_ISL_3880595	2021-09-02
hCoV-19/Japan/PG-73873/2021	EPI_ISL_3880596	2021-09-02

virus_name	epiID	submission_date
hCoV-19/Japan/PG-73874/2021	EPI_ISL_3880597	2021-09-02
hCoV-19/Japan/PG-73875/2021	EPI_ISL_3880598	2021-09-02
hCoV-19/Japan/PG-73876/2021	EPI_ISL_3880600	2021-09-02
hCoV-19/Japan/PG-73877/2021	EPI_ISL_3880601	2021-09-02
hCoV-19/Japan/PG-73878/2021	EPI_ISL_3880602	2021-09-02
hCoV-19/Japan/PG-73879/2021	EPI_ISL_3880605	2021-09-02
hCoV-19/Japan/PG-73880/2021	EPI_ISL_3880607	2021-09-02
hCoV-19/Japan/PG-73881/2021	EPI_ISL_3880608	2021-09-02
hCoV-19/Japan/PG-73882/2021	EPI_ISL_3880610	2021-09-02
hCoV-19/Japan/PG-73883/2021	EPI_ISL_3880612	2021-09-02
hCoV-19/Japan/PG-73884/2021	EPI_ISL_3880614	2021-09-02
hCoV-19/Japan/PG-73885/2021	EPI_ISL_3880615	2021-09-02
hCoV-19/Japan/PG-73886/2021	EPI_ISL_3880618	2021-09-02
hCoV-19/Japan/PG-73887/2021	EPI_ISL_3880620	2021-09-02
hCoV-19/Japan/PG-73888/2021	EPI_ISL_3880622	2021-09-02
hCoV-19/Japan/PG-73889/2021	EPI_ISL_3880624	2021-09-02
hCoV-19/Japan/PG-73890/2021	EPI_ISL_3880625	2021-09-02
hCoV-19/Japan/PG-73891/2021	EPI_ISL_3880628	2021-09-02
hCoV-19/Japan/PG-73892/2021	EPI_ISL_3880631	2021-09-02
hCoV-19/Japan/PG-73893/2021	EPI_ISL_3880633	2021-09-02
hCoV-19/Japan/PG-73894/2021	EPI_ISL_3880635	2021-09-02
hCoV-19/Japan/PG-73895/2021	EPI_ISL_3880637	2021-09-02
hCoV-19/Japan/PG-73896/2021	EPI_ISL_3880638	2021-09-02
hCoV-19/Japan/PG-73897/2021	EPI_ISL_3880640	2021-09-02
hCoV-19/Japan/PG-73898/2021	EPI_ISL_3880641	2021-09-02
hCoV-19/Japan/PG-73899/2021	EPI_ISL_3880643	2021-09-02
hCoV-19/Japan/PG-73900/2021	EPI_ISL_3880646	2021-09-02
hCoV-19/Japan/PG-73901/2021	EPI_ISL_3880647	2021-09-02
hCoV-19/Japan/PG-73902/2021	EPI_ISL_3880649	2021-09-02
hCoV-19/Japan/PG-73903/2021	EPI_ISL_3880651	2021-09-02
hCoV-19/Japan/PG-73904/2021	EPI_ISL_3880653	2021-09-02
hCoV-19/Japan/PG-73905/2021	EPI_ISL_3880654	2021-09-02
hCoV-19/Japan/PG-73906/2021	EPI_ISL_3880655	2021-09-02
hCoV-19/Japan/PG-73907/2021	EPI_ISL_3880657	2021-09-02
hCoV-19/Japan/PG-73908/2021	EPI_ISL_3880659	2021-09-02
hCoV-19/Japan/PG-73909/2021	EPI_ISL_3880662	2021-09-02
hCoV-19/Japan/PG-73910/2021	EPI_ISL_3880664	2021-09-02
hCoV-19/Japan/PG-73911/2021	EPI_ISL_3880667	2021-09-02
hCoV-19/Japan/PG-73912/2021	EPI_ISL_3880669	2021-09-02
hCoV-19/Japan/PG-73913/2021	EPI_ISL_3880670	2021-09-02
hCoV-19/Japan/PG-73914/2021	EPI_ISL_3880672	2021-09-02
hCoV-19/Japan/PG-73915/2021	EPI_ISL_3880675	2021-09-02
hCoV-19/Japan/PG-73916/2021	EPI_ISL_3880677	2021-09-02
hCoV-19/Japan/PG-73917/2021	EPI_ISL_3880678	2021-09-02
hCoV-19/Japan/PG-73918/2021	EPI_ISL_3880682	2021-09-02
hCoV-19/Japan/PG-73919/2021	EPI_ISL_3880684	2021-09-02
hCoV-19/Japan/PG-73920/2021	EPI_ISL_3880686	2021-09-02
hCoV-19/Japan/PG-73921/2021	EPI_ISL_3880688	2021-09-02
hCoV-19/Japan/PG-73922/2021	EPI_ISL_3880690	2021-09-02
hCoV-19/Japan/PG-73923/2021	EPI_ISL_3880692	2021-09-02
hCoV-19/Japan/PG-73924/2021	EPI_ISL_3880695	2021-09-02
hCoV-19/Japan/PG-73925/2021	EPI_ISL_3880697	2021-09-02
hCoV-19/Japan/PG-73926/2021	EPI_ISL_3880698	2021-09-02
hCoV-19/Japan/PG-73927/2021	EPI_ISL_3880700	2021-09-02

virus_name	epiID	submission_date
hCoV-19/Japan/PG-73928/2021	EPI_ISL_3880703	2021-09-02
hCoV-19/Japan/PG-73929/2021	EPI_ISL_3880705	2021-09-02
hCoV-19/Japan/PG-73930/2021	EPI_ISL_3880707	2021-09-02
hCoV-19/Japan/PG-73931/2021	EPI_ISL_3880709	2021-09-02
hCoV-19/Japan/PG-73932/2021	EPI_ISL_3880711	2021-09-02
hCoV-19/Japan/PG-73933/2021	EPI_ISL_3880713	2021-09-02
hCoV-19/Japan/PG-73934/2021	EPI_ISL_3880715	2021-09-02
hCoV-19/Japan/PG-73935/2021	EPI_ISL_3880717	2021-09-02
hCoV-19/Japan/PG-73936/2021	EPI_ISL_3880719	2021-09-02
hCoV-19/Japan/PG-73937/2021	EPI_ISL_3880722	2021-09-02
hCoV-19/Japan/PG-73938/2021	EPI_ISL_3880724	2021-09-02
hCoV-19/Japan/PG-73939/2021	EPI_ISL_3880726	2021-09-02
hCoV-19/Japan/PG-73940/2021	EPI_ISL_3880729	2021-09-02
hCoV-19/Japan/PG-73941/2021	EPI_ISL_3880730	2021-09-02
hCoV-19/Japan/PG-73942/2021	EPI_ISL_3880733	2021-09-02
hCoV-19/Japan/PG-73943/2021	EPI_ISL_3880735	2021-09-02
hCoV-19/Japan/PG-73944/2021	EPI_ISL_3880736	2021-09-02
hCoV-19/Japan/PG-73945/2021	EPI_ISL_3880738	2021-09-02
hCoV-19/Japan/PG-73946/2021	EPI_ISL_3880740	2021-09-02
hCoV-19/Japan/PG-73947/2021	EPI_ISL_3880742	2021-09-02
hCoV-19/Japan/PG-73948/2021	EPI_ISL_3880745	2021-09-02
hCoV-19/Japan/PG-73949/2021	EPI_ISL_3880747	2021-09-02
hCoV-19/Japan/PG-73950/2021	EPI_ISL_3880749	2021-09-02
hCoV-19/Japan/PG-73951/2021	EPI_ISL_3880752	2021-09-02
hCoV-19/Japan/PG-73952/2021	EPI_ISL_3880754	2021-09-02
hCoV-19/Japan/PG-73953/2021	EPI_ISL_3880756	2021-09-02
hCoV-19/Japan/PG-73954/2021	EPI_ISL_3880758	2021-09-02
hCoV-19/Japan/PG-73955/2021	EPI_ISL_3880760	2021-09-02
hCoV-19/Japan/PG-73956/2021	EPI_ISL_3880762	2021-09-02
hCoV-19/Japan/PG-73957/2021	EPI_ISL_3880765	2021-09-02
hCoV-19/Japan/PG-73958/2021	EPI_ISL_3880767	2021-09-02
hCoV-19/Japan/PG-73959/2021	EPI_ISL_3880769	2021-09-02
hCoV-19/Japan/PG-73960/2021	EPI_ISL_3880771	2021-09-02
hCoV-19/Japan/PG-73961/2021	EPI_ISL_3880773	2021-09-02
hCoV-19/Japan/PG-73962/2021	EPI_ISL_3880774	2021-09-02
hCoV-19/Japan/PG-73963/2021	EPI_ISL_3880776	2021-09-02
hCoV-19/Japan/PG-73964/2021	EPI_ISL_3880778	2021-09-02
hCoV-19/Japan/PG-73965/2021	EPI_ISL_3880781	2021-09-02
hCoV-19/Japan/PG-73966/2021	EPI_ISL_3880783	2021-09-02
hCoV-19/Japan/PG-73967/2021	EPI_ISL_3880786	2021-09-02
hCoV-19/Japan/PG-73968/2021	EPI_ISL_3880789	2021-09-02
hCoV-19/Japan/PG-73969/2021	EPI_ISL_3880791	2021-09-02
hCoV-19/Japan/PG-73970/2021	EPI_ISL_3880794	2021-09-02
hCoV-19/Japan/PG-73971/2021	EPI_ISL_3880796	2021-09-02
hCoV-19/Japan/PG-73972/2021	EPI_ISL_3880798	2021-09-02
hCoV-19/Japan/PG-73973/2021	EPI_ISL_3880799	2021-09-02
hCoV-19/Japan/PG-73974/2021	EPI_ISL_3880801	2021-09-02
hCoV-19/Japan/PG-73975/2021	EPI_ISL_3880803	2021-09-02
hCoV-19/Japan/PG-73976/2021	EPI_ISL_3880805	2021-09-02
hCoV-19/Japan/PG-73977/2021	EPI_ISL_3880807	2021-09-02
hCoV-19/Japan/PG-73978/2021	EPI_ISL_3880809	2021-09-02
hCoV-19/Japan/PG-73979/2021	EPI_ISL_3880812	2021-09-02
hCoV-19/Japan/PG-73980/2021	EPI_ISL_3880814	2021-09-02
hCoV-19/Japan/PG-73981/2021	EPI_ISL_3880816	2021-09-02

virus_name	epiID	submission_date
hCoV-19/Japan/PG-73982/2021	EPI_ISL_3880818	2021-09-02
hCoV-19/Japan/PG-73983/2021	EPI_ISL_3880820	2021-09-02
hCoV-19/Japan/PG-73984/2021	EPI_ISL_3880822	2021-09-02
hCoV-19/Japan/PG-73985/2021	EPI_ISL_3880824	2021-09-02
hCoV-19/Japan/PG-73986/2021	EPI_ISL_3880826	2021-09-02
hCoV-19/Japan/PG-73987/2021	EPI_ISL_3880829	2021-09-02
hCoV-19/Japan/PG-73988/2021	EPI_ISL_3880831	2021-09-02
hCoV-19/Japan/PG-73989/2021	EPI_ISL_3880834	2021-09-02
hCoV-19/Japan/PG-73990/2021	EPI_ISL_3880836	2021-09-02
hCoV-19/Japan/PG-73991/2021	EPI_ISL_3880838	2021-09-02
hCoV-19/Japan/PG-73992/2021	EPI_ISL_3880841	2021-09-02
hCoV-19/Japan/PG-73993/2021	EPI_ISL_3880843	2021-09-02
hCoV-19/Japan/PG-73994/2021	EPI_ISL_3880845	2021-09-02
hCoV-19/Japan/PG-73995/2021	EPI_ISL_3880847	2021-09-02
hCoV-19/Japan/PG-73996/2021	EPI_ISL_3880849	2021-09-02
hCoV-19/Japan/PG-73997/2021	EPI_ISL_3880851	2021-09-02
hCoV-19/Japan/PG-73998/2021	EPI_ISL_3880853	2021-09-02
hCoV-19/Japan/PG-73999/2021	EPI_ISL_3880856	2021-09-02
hCoV-19/Japan/PG-74000/2021	EPI_ISL_3880858	2021-09-02
hCoV-19/Japan/PG-74001/2021	EPI_ISL_3880860	2021-09-02
hCoV-19/Japan/PG-74002/2021	EPI_ISL_3880862	2021-09-02
hCoV-19/Japan/PG-74003/2021	EPI_ISL_3880863	2021-09-02
hCoV-19/Japan/PG-74004/2021	EPI_ISL_3880866	2021-09-02
hCoV-19/Japan/PG-74005/2021	EPI_ISL_3880868	2021-09-02
hCoV-19/Japan/PG-74006/2021	EPI_ISL_3880870	2021-09-02
hCoV-19/Japan/PG-74007/2021	EPI_ISL_3880872	2021-09-02
hCoV-19/Japan/PG-74008/2021	EPI_ISL_3880874	2021-09-02
hCoV-19/Japan/PG-74009/2021	EPI_ISL_3880875	2021-09-02
hCoV-19/Japan/PG-74010/2021	EPI_ISL_3880877	2021-09-02
hCoV-19/Japan/PG-74011/2021	EPI_ISL_3880879	2021-09-02
hCoV-19/Japan/PG-74012/2021	EPI_ISL_3880881	2021-09-02
hCoV-19/Japan/PG-74013/2021	EPI_ISL_3880884	2021-09-02
hCoV-19/Japan/PG-74014/2021	EPI_ISL_3880886	2021-09-02
hCoV-19/Japan/PG-74015/2021	EPI_ISL_3880888	2021-09-02
hCoV-19/Japan/PG-74016/2021	EPI_ISL_3880890	2021-09-02
hCoV-19/Japan/PG-74017/2021	EPI_ISL_3880891	2021-09-02
hCoV-19/Japan/PG-74018/2021	EPI_ISL_3880893	2021-09-02
hCoV-19/Japan/PG-74019/2021	EPI_ISL_3880895	2021-09-02
hCoV-19/Japan/PG-74020/2021	EPI_ISL_3880898	2021-09-02
hCoV-19/Japan/PG-74021/2021	EPI_ISL_3880899	2021-09-02
hCoV-19/Japan/PG-74022/2021	EPI_ISL_3880901	2021-09-02
hCoV-19/Japan/PG-74023/2021	EPI_ISL_3880903	2021-09-02
hCoV-19/Japan/PG-74024/2021	EPI_ISL_3880905	2021-09-02
hCoV-19/Japan/PG-74025/2021	EPI_ISL_3880907	2021-09-02
hCoV-19/Japan/PG-74026/2021	EPI_ISL_3880909	2021-09-02
hCoV-19/Japan/PG-74027/2021	EPI_ISL_3880911	2021-09-02
hCoV-19/Japan/PG-74028/2021	EPI_ISL_3880914	2021-09-02
hCoV-19/Japan/PG-74029/2021	EPI_ISL_3880916	2021-09-02
hCoV-19/Japan/PG-74030/2021	EPI_ISL_3880919	2021-09-02
hCoV-19/Japan/PG-74031/2021	EPI_ISL_3880922	2021-09-02
hCoV-19/Japan/PG-74032/2021	EPI_ISL_3880924	2021-09-02
hCoV-19/Japan/PG-74033/2021	EPI_ISL_3880926	2021-09-02
hCoV-19/Japan/PG-74034/2021	EPI_ISL_3880928	2021-09-02
hCoV-19/Japan/PG-74035/2021	EPI_ISL_3880930	2021-09-02

virus_name	epiID	submission_date
hCoV-19/Japan/PG-74036/2021	EPI_ISL_3880933	2021-09-02
hCoV-19/Japan/PG-74037/2021	EPI_ISL_3880936	2021-09-02
hCoV-19/Japan/PG-74038/2021	EPI_ISL_3880938	2021-09-02
hCoV-19/Japan/PG-74039/2021	EPI_ISL_3880940	2021-09-02
hCoV-19/Japan/PG-74040/2021	EPI_ISL_3880943	2021-09-02
hCoV-19/Japan/PG-74041/2021	EPI_ISL_3880945	2021-09-02
hCoV-19/Japan/PG-74042/2021	EPI_ISL_3880948	2021-09-02
hCoV-19/Japan/PG-74043/2021	EPI_ISL_3880950	2021-09-02
hCoV-19/Japan/PG-74044/2021	EPI_ISL_3880952	2021-09-02
hCoV-19/Japan/PG-74045/2021	EPI_ISL_3880954	2021-09-02
hCoV-19/Japan/PG-74046/2021	EPI_ISL_3880957	2021-09-02
hCoV-19/Japan/PG-74047/2021	EPI_ISL_3880959	2021-09-02
hCoV-19/Japan/PG-74048/2021	EPI_ISL_3880961	2021-09-02
hCoV-19/Japan/PG-74049/2021	EPI_ISL_3880964	2021-09-02
hCoV-19/Japan/PG-74050/2021	EPI_ISL_3880967	2021-09-02
hCoV-19/Japan/PG-74051/2021	EPI_ISL_3880970	2021-09-02
hCoV-19/Japan/PG-74052/2021	EPI_ISL_3880972	2021-09-02
hCoV-19/Japan/PG-74053/2021	EPI_ISL_3880975	2021-09-02
hCoV-19/Japan/PG-74054/2021	EPI_ISL_3880977	2021-09-02
hCoV-19/Japan/PG-74055/2021	EPI_ISL_3880980	2021-09-02
hCoV-19/Japan/PG-74056/2021	EPI_ISL_3880982	2021-09-02
hCoV-19/Japan/PG-74057/2021	EPI_ISL_3880984	2021-09-02
hCoV-19/Japan/PG-74058/2021	EPI_ISL_3880987	2021-09-02
hCoV-19/Japan/PG-74059/2021	EPI_ISL_3880990	2021-09-02
hCoV-19/Japan/PG-74060/2021	EPI_ISL_3880992	2021-09-02
hCoV-19/Japan/PG-74061/2021	EPI_ISL_3880994	2021-09-02
hCoV-19/Japan/PG-74062/2021	EPI_ISL_3880996	2021-09-02
hCoV-19/Japan/PG-74063/2021	EPI_ISL_3880999	2021-09-02
hCoV-19/Japan/PG-74064/2021	EPI_ISL_3881001	2021-09-02
hCoV-19/Japan/PG-74065/2021	EPI_ISL_3881003	2021-09-02
hCoV-19/Japan/PG-74066/2021	EPI_ISL_3881006	2021-09-02
hCoV-19/Japan/PG-74067/2021	EPI_ISL_3881008	2021-09-02
hCoV-19/Japan/PG-74068/2021	EPI_ISL_3881010	2021-09-02
hCoV-19/Japan/PG-74069/2021	EPI_ISL_3881013	2021-09-02
hCoV-19/Japan/PG-74070/2021	EPI_ISL_3881015	2021-09-02
hCoV-19/Japan/PG-74071/2021	EPI_ISL_3881017	2021-09-02
hCoV-19/Japan/PG-74072/2021	EPI_ISL_3881019	2021-09-02
hCoV-19/Japan/PG-74073/2021	EPI_ISL_3881022	2021-09-02
hCoV-19/Japan/PG-74074/2021	EPI_ISL_3881024	2021-09-02
hCoV-19/Japan/PG-74075/2021	EPI_ISL_3881026	2021-09-02
hCoV-19/Japan/PG-74076/2021	EPI_ISL_3881027	2021-09-02
hCoV-19/Japan/PG-74077/2021	EPI_ISL_3881029	2021-09-02
hCoV-19/Japan/PG-74078/2021	EPI_ISL_3881032	2021-09-02
hCoV-19/Japan/PG-74079/2021	EPI_ISL_3881034	2021-09-02
hCoV-19/Japan/PG-74080/2021	EPI_ISL_3881037	2021-09-02
hCoV-19/Japan/PG-74081/2021	EPI_ISL_3881039	2021-09-02
hCoV-19/Japan/PG-74082/2021	EPI_ISL_3881042	2021-09-02
hCoV-19/Japan/PG-74083/2021	EPI_ISL_3881044	2021-09-02
hCoV-19/Japan/PG-74084/2021	EPI_ISL_3881047	2021-09-02
hCoV-19/Japan/PG-74085/2021	EPI_ISL_3881050	2021-09-02
hCoV-19/Japan/PG-74086/2021	EPI_ISL_3881052	2021-09-02
hCoV-19/Japan/PG-74087/2021	EPI_ISL_3881055	2021-09-02
hCoV-19/Japan/PG-74088/2021	EPI_ISL_3881058	2021-09-02
hCoV-19/Japan/PG-74089/2021	EPI_ISL_3881060	2021-09-02

virus_name	epiID	submission_date
hCoV-19/Japan/PG-74090/2021	EPI_ISL_3881062	2021-09-02
hCoV-19/Japan/PG-74091/2021	EPI_ISL_3881064	2021-09-02
hCoV-19/Japan/PG-74092/2021	EPI_ISL_3881066	2021-09-02
hCoV-19/Japan/PG-74093/2021	EPI_ISL_3881068	2021-09-02
hCoV-19/Japan/PG-74094/2021	EPI_ISL_3881070	2021-09-02
hCoV-19/Japan/PG-74095/2021	EPI_ISL_3881071	2021-09-02
hCoV-19/Japan/PG-74096/2021	EPI_ISL_3881073	2021-09-02
hCoV-19/Japan/PG-74097/2021	EPI_ISL_3881075	2021-09-02
hCoV-19/Japan/PG-74098/2021	EPI_ISL_3881077	2021-09-02
hCoV-19/Japan/PG-74099/2021	EPI_ISL_3881080	2021-09-02
hCoV-19/Japan/PG-74100/2021	EPI_ISL_3881082	2021-09-02
hCoV-19/Japan/PG-74101/2021	EPI_ISL_3881084	2021-09-02
hCoV-19/Japan/PG-74102/2021	EPI_ISL_3881086	2021-09-02
hCoV-19/Japan/PG-74103/2021	EPI_ISL_3881088	2021-09-02
hCoV-19/Japan/PG-74104/2021	EPI_ISL_3881091	2021-09-02
hCoV-19/Japan/PG-74105/2021	EPI_ISL_3881093	2021-09-02
hCoV-19/Japan/PG-74106/2021	EPI_ISL_3881096	2021-09-02
hCoV-19/Japan/PG-74107/2021	EPI_ISL_3881097	2021-09-02
hCoV-19/Japan/PG-74108/2021	EPI_ISL_3881098	2021-09-02
hCoV-19/Japan/PG-74109/2021	EPI_ISL_3881100	2021-09-02
hCoV-19/Japan/PG-74110/2021	EPI_ISL_3881101	2021-09-02
hCoV-19/Japan/PG-74111/2021	EPI_ISL_3881104	2021-09-02
hCoV-19/Japan/PG-74112/2021	EPI_ISL_3881107	2021-09-02
hCoV-19/Japan/PG-74113/2021	EPI_ISL_3881108	2021-09-02
hCoV-19/Japan/PG-74114/2021	EPI_ISL_3881110	2021-09-02
hCoV-19/Japan/PG-74115/2021	EPI_ISL_3881112	2021-09-02
hCoV-19/Japan/PG-74116/2021	EPI_ISL_3881113	2021-09-02
hCoV-19/Japan/PG-74117/2021	EPI_ISL_3881115	2021-09-02
hCoV-19/Japan/PG-74118/2021	EPI_ISL_3881118	2021-09-02
hCoV-19/Japan/PG-74119/2021	EPI_ISL_3881120	2021-09-02
hCoV-19/Japan/PG-74120/2021	EPI_ISL_3881122	2021-09-02
hCoV-19/Japan/PG-74121/2021	EPI_ISL_3881123	2021-09-02
hCoV-19/Japan/PG-74122/2021	EPI_ISL_3881125	2021-09-02
hCoV-19/Japan/PG-74123/2021	EPI_ISL_3881127	2021-09-02
hCoV-19/Japan/PG-74124/2021	EPI_ISL_3881128	2021-09-02
hCoV-19/Japan/PG-74125/2021	EPI_ISL_3881130	2021-09-02
hCoV-19/Japan/PG-74126/2021	EPI_ISL_3881132	2021-09-02
hCoV-19/Japan/PG-74127/2021	EPI_ISL_3881133	2021-09-02
hCoV-19/Japan/PG-74128/2021	EPI_ISL_3881135	2021-09-02
hCoV-19/Japan/PG-74129/2021	EPI_ISL_3881137	2021-09-02
hCoV-19/Japan/PG-74130/2021	EPI_ISL_3881140	2021-09-02
hCoV-19/Japan/PG-74131/2021	EPI_ISL_3881144	2021-09-02
hCoV-19/Japan/PG-74132/2021	EPI_ISL_3881146	2021-09-02
hCoV-19/Japan/PG-74133/2021	EPI_ISL_3881149	2021-09-02
hCoV-19/Japan/PG-74134/2021	EPI_ISL_3881152	2021-09-02
hCoV-19/Japan/PG-74135/2021	EPI_ISL_3881154	2021-09-02
hCoV-19/Japan/PG-74136/2021	EPI_ISL_3873585	2021-09-02
hCoV-19/Japan/PG-74137/2021	EPI_ISL_3873586	2021-09-02
hCoV-19/Japan/PG-74138/2021	EPI_ISL_3873587	2021-09-02
hCoV-19/Japan/PG-74139/2021	EPI_ISL_3873588	2021-09-02
hCoV-19/Japan/PG-74140/2021	EPI_ISL_3873589	2021-09-02
hCoV-19/Japan/PG-74141/2021	EPI_ISL_3873590	2021-09-02
hCoV-19/Japan/PG-74142/2021	EPI_ISL_3873591	2021-09-02
hCoV-19/Japan/PG-74143/2021	EPI_ISL_3873592	2021-09-02

virus_name	epiID	submission_date
hCoV-19/Japan/PG-74144/2021	EPI_ISL_3873593	2021-09-02
hCoV-19/Japan/PG-74145/2021	EPI_ISL_3873594	2021-09-02
hCoV-19/Japan/PG-74146/2021	EPI_ISL_3873595	2021-09-02
hCoV-19/Japan/PG-74147/2021	EPI_ISL_3874786	2021-09-02
hCoV-19/Japan/PG-74148/2021	EPI_ISL_3874787	2021-09-02
hCoV-19/Japan/PG-74149/2021	EPI_ISL_3874788	2021-09-02
hCoV-19/Japan/PG-74150/2021	EPI_ISL_3874789	2021-09-02
hCoV-19/Japan/PG-74151/2021	EPI_ISL_3874790	2021-09-02
hCoV-19/Japan/PG-74152/2021	EPI_ISL_3874791	2021-09-02
hCoV-19/Japan/PG-74153/2021	EPI_ISL_3874792	2021-09-02
hCoV-19/Japan/PG-74154/2021	EPI_ISL_3874793	2021-09-02
hCoV-19/Japan/PG-74155/2021	EPI_ISL_3874794	2021-09-02
hCoV-19/Japan/PG-74156/2021	EPI_ISL_3874795	2021-09-02
hCoV-19/Japan/PG-74157/2021	EPI_ISL_3874796	2021-09-02
hCoV-19/Japan/PG-74158/2021	EPI_ISL_3874797	2021-09-02
hCoV-19/Japan/PG-74159/2021	EPI_ISL_3874798	2021-09-02
hCoV-19/Japan/PG-74160/2021	EPI_ISL_3874799	2021-09-02
hCoV-19/Japan/PG-74161/2021	EPI_ISL_3874800	2021-09-02
hCoV-19/Japan/PG-74162/2021	EPI_ISL_3874801	2021-09-02
hCoV-19/Japan/PG-74163/2021	EPI_ISL_3874802	2021-09-02
hCoV-19/Japan/PG-74164/2021	EPI_ISL_3874803	2021-09-02
hCoV-19/Japan/PG-74165/2021	EPI_ISL_3874804	2021-09-02
hCoV-19/Japan/PG-74166/2021	EPI_ISL_3874805	2021-09-02
hCoV-19/Japan/PG-74167/2021	EPI_ISL_3874806	2021-09-02
hCoV-19/Japan/PG-74168/2021	EPI_ISL_3874807	2021-09-02
hCoV-19/Japan/PG-74169/2021	EPI_ISL_3874808	2021-09-02
hCoV-19/Japan/PG-74170/2021	EPI_ISL_3874809	2021-09-02
hCoV-19/Japan/PG-74171/2021	EPI_ISL_3874810	2021-09-02
hCoV-19/Japan/PG-74172/2021	EPI_ISL_3874811	2021-09-02
hCoV-19/Japan/PG-74173/2021	EPI_ISL_3874812	2021-09-02
hCoV-19/Japan/PG-74174/2021	EPI_ISL_3874813	2021-09-02
hCoV-19/Japan/PG-74175/2021	EPI_ISL_3874814	2021-09-02
hCoV-19/Japan/PG-74176/2021	EPI_ISL_3874815	2021-09-02
hCoV-19/Japan/PG-74177/2021	EPI_ISL_3874816	2021-09-02
hCoV-19/Japan/PG-74178/2021	EPI_ISL_3874817	2021-09-02
hCoV-19/Japan/PG-74179/2021	EPI_ISL_3874818	2021-09-02
hCoV-19/Japan/PG-74180/2021	EPI_ISL_3874819	2021-09-02
hCoV-19/Japan/PG-74181/2021	EPI_ISL_3874820	2021-09-02
hCoV-19/Japan/PG-74182/2021	EPI_ISL_3874821	2021-09-02
hCoV-19/Japan/PG-74183/2021	EPI_ISL_3874822	2021-09-02
hCoV-19/Japan/PG-74184/2021	EPI_ISL_3874823	2021-09-02
hCoV-19/Japan/PG-74185/2021	EPI_ISL_3874824	2021-09-02
hCoV-19/Japan/PG-74186/2021	EPI_ISL_3874825	2021-09-02
hCoV-19/Japan/PG-74187/2021	EPI_ISL_3874826	2021-09-02
hCoV-19/Japan/PG-74188/2021	EPI_ISL_3874827	2021-09-02
hCoV-19/Japan/PG-74189/2021	EPI_ISL_3874828	2021-09-02
hCoV-19/Japan/PG-74190/2021	EPI_ISL_3874829	2021-09-02
hCoV-19/Japan/PG-74191/2021	EPI_ISL_3874830	2021-09-02
hCoV-19/Japan/PG-74192/2021	EPI_ISL_3874831	2021-09-02
hCoV-19/Japan/PG-74193/2021	EPI_ISL_3874832	2021-09-02
hCoV-19/Japan/PG-74194/2021	EPI_ISL_3874833	2021-09-02
hCoV-19/Japan/PG-74195/2021	EPI_ISL_3874834	2021-09-02
hCoV-19/Japan/PG-74196/2021	EPI_ISL_3874835	2021-09-02
hCoV-19/Japan/PG-74197/2021	EPI_ISL_3874836	2021-09-02

virus_name	epiID	submission_date
hCoV-19/Japan/PG-74198/2021	EPI_ISL_3874837	2021-09-02
hCoV-19/Japan/PG-74199/2021	EPI_ISL_3874838	2021-09-02
hCoV-19/Japan/PG-74200/2021	EPI_ISL_3874839	2021-09-02
hCoV-19/Japan/PG-74201/2021	EPI_ISL_3874840	2021-09-02
hCoV-19/Japan/PG-74202/2021	EPI_ISL_3874841	2021-09-02
hCoV-19/Japan/PG-74203/2021	EPI_ISL_3874842	2021-09-02
hCoV-19/Japan/PG-74204/2021	EPI_ISL_3874843	2021-09-02
hCoV-19/Japan/PG-74205/2021	EPI_ISL_3874844	2021-09-02
hCoV-19/Japan/PG-74206/2021	EPI_ISL_3874845	2021-09-02
hCoV-19/Japan/PG-74207/2021	EPI_ISL_3874846	2021-09-02
hCoV-19/Japan/PG-74208/2021	EPI_ISL_3874847	2021-09-02
hCoV-19/Japan/PG-74209/2021	EPI_ISL_3874848	2021-09-02
hCoV-19/Japan/PG-74210/2021	EPI_ISL_3874849	2021-09-02
hCoV-19/Japan/PG-74211/2021	EPI_ISL_3874850	2021-09-02
hCoV-19/Japan/PG-74212/2021	EPI_ISL_3874851	2021-09-02
hCoV-19/Japan/PG-74213/2021	EPI_ISL_3874852	2021-09-02
hCoV-19/Japan/PG-74214/2021	EPI_ISL_3874853	2021-09-02
hCoV-19/Japan/PG-74215/2021	EPI_ISL_3874854	2021-09-02
hCoV-19/Japan/PG-74216/2021	EPI_ISL_3874855	2021-09-02
hCoV-19/Japan/PG-74217/2021	EPI_ISL_3874856	2021-09-02
hCoV-19/Japan/PG-74218/2021	EPI_ISL_3874857	2021-09-02
hCoV-19/Japan/PG-74219/2021	EPI_ISL_3874858	2021-09-02
hCoV-19/Japan/PG-74220/2021	EPI_ISL_3874859	2021-09-02
hCoV-19/Japan/PG-74221/2021	EPI_ISL_3874860	2021-09-02
hCoV-19/Japan/PG-74222/2021	EPI_ISL_3874861	2021-09-02
hCoV-19/Japan/PG-74223/2021	EPI_ISL_3874862	2021-09-02
hCoV-19/Japan/PG-74224/2021	EPI_ISL_3874863	2021-09-02
hCoV-19/Japan/PG-74225/2021	EPI_ISL_3874864	2021-09-02
hCoV-19/Japan/PG-74226/2021	EPI_ISL_3874865	2021-09-02
hCoV-19/Japan/PG-74227/2021	EPI_ISL_3874866	2021-09-02
hCoV-19/Japan/PG-74228/2021	EPI_ISL_3874867	2021-09-02
hCoV-19/Japan/PG-74229/2021	EPI_ISL_3874868	2021-09-02
hCoV-19/Japan/PG-74230/2021	EPI_ISL_3874869	2021-09-02
hCoV-19/Japan/PG-74231/2021	EPI_ISL_3874870	2021-09-02
hCoV-19/Japan/PG-74232/2021	EPI_ISL_3871688	2021-09-02
hCoV-19/Japan/PG-74233/2021	EPI_ISL_3871689	2021-09-02
hCoV-19/Japan/PG-74234/2021	EPI_ISL_3871690	2021-09-02
hCoV-19/Japan/PG-74235/2021	EPI_ISL_3871691	2021-09-02
hCoV-19/Japan/PG-74236/2021	EPI_ISL_3871692	2021-09-02
hCoV-19/Japan/PG-74237/2021	EPI_ISL_3871693	2021-09-02
hCoV-19/Japan/PG-74238/2021	EPI_ISL_3871694	2021-09-02
hCoV-19/Japan/PG-74239/2021	EPI_ISL_3871695	2021-09-02
hCoV-19/Japan/PG-74240/2021	EPI_ISL_3871696	2021-09-02
hCoV-19/Japan/PG-74241/2021	EPI_ISL_3871697	2021-09-02
hCoV-19/Japan/PG-74242/2021	EPI_ISL_3871698	2021-09-02
hCoV-19/Japan/PG-74243/2021	EPI_ISL_3871699	2021-09-02
hCoV-19/Japan/PG-74244/2021	EPI_ISL_3871700	2021-09-02
hCoV-19/Japan/PG-74245/2021	EPI_ISL_3871701	2021-09-02
hCoV-19/Japan/PG-74246/2021	EPI_ISL_3871702	2021-09-02
hCoV-19/Japan/PG-74247/2021	EPI_ISL_3871703	2021-09-02
hCoV-19/Japan/PG-74248/2021	EPI_ISL_3871704	2021-09-02
hCoV-19/Japan/PG-74249/2021	EPI_ISL_3871705	2021-09-02
hCoV-19/Japan/PG-74250/2021	EPI_ISL_3873354	2021-09-02
hCoV-19/Japan/PG-74251/2021	EPI_ISL_3873355	2021-09-02

virus_name	epiID	submission_date
hCoV-19/Japan/PG-74252/2021	EPI_ISL_3873356	2021-09-02
hCoV-19/Japan/PG-74253/2021	EPI_ISL_3873357	2021-09-02
hCoV-19/Japan/PG-74254/2021	EPI_ISL_3873358	2021-09-02
hCoV-19/Japan/PG-74255/2021	EPI_ISL_3873359	2021-09-02
hCoV-19/Japan/PG-74256/2021	EPI_ISL_3873360	2021-09-02
hCoV-19/Japan/PG-74257/2021	EPI_ISL_3873361	2021-09-02
hCoV-19/Japan/PG-74258/2021	EPI_ISL_3873362	2021-09-02
hCoV-19/Japan/PG-74259/2021	EPI_ISL_3873363	2021-09-02
hCoV-19/Japan/PG-74260/2021	EPI_ISL_3873364	2021-09-02
hCoV-19/Japan/PG-74261/2021	EPI_ISL_3881157	2021-09-02
hCoV-19/Japan/PG-74262/2021	EPI_ISL_3881160	2021-09-02
hCoV-19/Japan/PG-74263/2021	EPI_ISL_3881164	2021-09-02
hCoV-19/Japan/PG-74264/2021	EPI_ISL_3881167	2021-09-02
hCoV-19/Japan/PG-74265/2021	EPI_ISL_3881169	2021-09-02
hCoV-19/Japan/PG-74266/2021	EPI_ISL_3881173	2021-09-02
hCoV-19/Japan/PG-74267/2021	EPI_ISL_3881175	2021-09-02
hCoV-19/Japan/PG-74268/2021	EPI_ISL_3881179	2021-09-02
hCoV-19/Japan/PG-74269/2021	EPI_ISL_3881181	2021-09-02
hCoV-19/Japan/PG-74270/2021	EPI_ISL_3881185	2021-09-02
hCoV-19/Japan/PG-74271/2021	EPI_ISL_3881187	2021-09-02
hCoV-19/Japan/PG-74272/2021	EPI_ISL_3881191	2021-09-02
hCoV-19/Japan/PG-74273/2021	EPI_ISL_3881193	2021-09-02
hCoV-19/Japan/PG-74274/2021	EPI_ISL_3881194	2021-09-02
hCoV-19/Japan/PG-74275/2021	EPI_ISL_3881196	2021-09-02
hCoV-19/Japan/PG-74276/2021	EPI_ISL_3881198	2021-09-02
hCoV-19/Japan/PG-74277/2021	EPI_ISL_3881200	2021-09-02
hCoV-19/Japan/PG-74278/2021	EPI_ISL_3881203	2021-09-02
hCoV-19/Japan/PG-74279/2021	EPI_ISL_3881206	2021-09-02
hCoV-19/Japan/PG-74280/2021	EPI_ISL_3881209	2021-09-02
hCoV-19/Japan/PG-74281/2021	EPI_ISL_3881211	2021-09-02
hCoV-19/Japan/PG-74282/2021	EPI_ISL_3881215	2021-09-02
hCoV-19/Japan/PG-74283/2021	EPI_ISL_3881217	2021-09-02
hCoV-19/Japan/PG-74284/2021	EPI_ISL_3881221	2021-09-02
hCoV-19/Japan/PG-74285/2021	EPI_ISL_3881223	2021-09-02
hCoV-19/Japan/PG-74286/2021	EPI_ISL_3881227	2021-09-02
hCoV-19/Japan/PG-74287/2021	EPI_ISL_3881231	2021-09-02
hCoV-19/Japan/PG-74288/2021	EPI_ISL_3881233	2021-09-02
hCoV-19/Japan/PG-74289/2021	EPI_ISL_3881237	2021-09-02
hCoV-19/Japan/PG-74290/2021	EPI_ISL_3881239	2021-09-02
hCoV-19/Japan/PG-74291/2021	EPI_ISL_3881242	2021-09-02
hCoV-19/Japan/PG-74292/2021	EPI_ISL_3881245	2021-09-02
hCoV-19/Japan/PG-74293/2021	EPI_ISL_3881248	2021-09-02
hCoV-19/Japan/PG-74294/2021	EPI_ISL_3881251	2021-09-02
hCoV-19/Japan/PG-74295/2021	EPI_ISL_3881254	2021-09-02
hCoV-19/Japan/PG-74296/2021	EPI_ISL_3881257	2021-09-02
hCoV-19/Japan/PG-74297/2021	EPI_ISL_3881260	2021-09-02
hCoV-19/Japan/PG-74298/2021	EPI_ISL_3881263	2021-09-02
hCoV-19/Japan/PG-74299/2021	EPI_ISL_3881265	2021-09-02
hCoV-19/Japan/PG-74300/2021	EPI_ISL_3881268	2021-09-02
hCoV-19/Japan/PG-74301/2021	EPI_ISL_3881270	2021-09-02
hCoV-19/Japan/PG-74302/2021	EPI_ISL_3881272	2021-09-02
hCoV-19/Japan/PG-74303/2021	EPI_ISL_3881275	2021-09-02
hCoV-19/Japan/PG-74304/2021	EPI_ISL_3881279	2021-09-02
hCoV-19/Japan/PG-74305/2021	EPI_ISL_3881281	2021-09-02

virus_name	epiID	submission_date
hCoV-19/Japan/PG-74306/2021	EPI_ISL_3881282	2021-09-02
hCoV-19/Japan/PG-74307/2021	EPI_ISL_3881285	2021-09-02
hCoV-19/Japan/PG-74308/2021	EPI_ISL_3881287	2021-09-02
hCoV-19/Japan/PG-74309/2021	EPI_ISL_3881290	2021-09-02
hCoV-19/Japan/PG-74310/2021	EPI_ISL_3881291	2021-09-02
hCoV-19/Japan/PG-74311/2021	EPI_ISL_3881293	2021-09-02
hCoV-19/Japan/PG-74312/2021	EPI_ISL_3881294	2021-09-02
hCoV-19/Japan/PG-74313/2021	EPI_ISL_3881296	2021-09-02
hCoV-19/Japan/PG-74314/2021	EPI_ISL_3881297	2021-09-02
hCoV-19/Japan/PG-74315/2021	EPI_ISL_3881299	2021-09-02
hCoV-19/Japan/PG-74316/2021	EPI_ISL_3881302	2021-09-02
hCoV-19/Japan/PG-74317/2021	EPI_ISL_3881306	2021-09-02
hCoV-19/Japan/PG-74318/2021	EPI_ISL_3881308	2021-09-02
hCoV-19/Japan/PG-74319/2021	EPI_ISL_3881309	2021-09-02
hCoV-19/Japan/PG-74320/2021	EPI_ISL_3881312	2021-09-02
hCoV-19/Japan/PG-74321/2021	EPI_ISL_3881317	2021-09-02
hCoV-19/Japan/PG-74322/2021	EPI_ISL_3881320	2021-09-02
hCoV-19/Japan/PG-74323/2021	EPI_ISL_3881322	2021-09-02
hCoV-19/Japan/PG-74324/2021	EPI_ISL_3881325	2021-09-02
hCoV-19/Japan/PG-74325/2021	EPI_ISL_3881327	2021-09-02
hCoV-19/Japan/PG-74326/2021	EPI_ISL_3881331	2021-09-02
hCoV-19/Japan/PG-74327/2021	EPI_ISL_3881335	2021-09-02
hCoV-19/Japan/PG-74328/2021	EPI_ISL_3881339	2021-09-02
hCoV-19/Japan/PG-74329/2021	EPI_ISL_3881343	2021-09-02
hCoV-19/Japan/PG-74330/2021	EPI_ISL_3881347	2021-09-02
hCoV-19/Japan/PG-74331/2021	EPI_ISL_3881349	2021-09-02
hCoV-19/Japan/PG-74332/2021	EPI_ISL_3881352	2021-09-02
hCoV-19/Japan/PG-74333/2021	EPI_ISL_3881354	2021-09-02
hCoV-19/Japan/PG-74334/2021	EPI_ISL_3881357	2021-09-02
hCoV-19/Japan/PG-74335/2021	EPI_ISL_3881359	2021-09-02
hCoV-19/Japan/PG-74336/2021	EPI_ISL_3881361	2021-09-02
hCoV-19/Japan/PG-74337/2021	EPI_ISL_3881362	2021-09-02
hCoV-19/Japan/PG-74338/2021	EPI_ISL_3881364	2021-09-02
hCoV-19/Japan/PG-74339/2021	EPI_ISL_3881365	2021-09-02
hCoV-19/Japan/PG-74340/2021	EPI_ISL_3881367	2021-09-02
hCoV-19/Japan/PG-74341/2021	EPI_ISL_3881369	2021-09-02
hCoV-19/Japan/PG-74342/2021	EPI_ISL_3881372	2021-09-02
hCoV-19/Japan/PG-74343/2021	EPI_ISL_3881374	2021-09-02
hCoV-19/Japan/PG-74344/2021	EPI_ISL_3881377	2021-09-02
hCoV-19/Japan/PG-74345/2021	EPI_ISL_3881379	2021-09-02
hCoV-19/Japan/PG-74346/2021	EPI_ISL_3881383	2021-09-02
hCoV-19/Japan/PG-74347/2021	EPI_ISL_3881386	2021-09-02
hCoV-19/Japan/PG-74348/2021	EPI_ISL_3881387	2021-09-02
hCoV-19/Japan/PG-74349/2021	EPI_ISL_3881390	2021-09-02
hCoV-19/Japan/PG-74350/2021	EPI_ISL_3881394	2021-09-02
hCoV-19/Japan/PG-74399/2021	EPI_ISL_3881397	2021-09-02
hCoV-19/Japan/PG-74400/2021	EPI_ISL_3881399	2021-09-02
hCoV-19/Japan/PG-74401/2021	EPI_ISL_3881402	2021-09-02
hCoV-19/Japan/PG-74402/2021	EPI_ISL_3881404	2021-09-02
hCoV-19/Japan/PG-74403/2021	EPI_ISL_3881407	2021-09-02
hCoV-19/Japan/PG-74404/2021	EPI_ISL_3881409	2021-09-02
hCoV-19/Japan/PG-74405/2021	EPI_ISL_3881412	2021-09-02
hCoV-19/Japan/PG-74406/2021	EPI_ISL_3881415	2021-09-02
hCoV-19/Japan/PG-74407/2021	EPI_ISL_3881417	2021-09-02

virus_name	epiID	submission_date
hCoV-19/Japan/PG-74408/2021	EPI_ISL_3881418	2021-09-02
hCoV-19/Japan/PG-74409/2021	EPI_ISL_3881419	2021-09-02
hCoV-19/Japan/PG-74410/2021	EPI_ISL_3881421	2021-09-02
hCoV-19/Japan/PG-74411/2021	EPI_ISL_3881422	2021-09-02
hCoV-19/Japan/PG-74412/2021	EPI_ISL_3881424	2021-09-02
hCoV-19/Japan/PG-74413/2021	EPI_ISL_3881426	2021-09-02
hCoV-19/Japan/PG-74414/2021	EPI_ISL_3881429	2021-09-02
hCoV-19/Japan/PG-74415/2021	EPI_ISL_3881432	2021-09-02
hCoV-19/Japan/PG-74416/2021	EPI_ISL_3881436	2021-09-02
hCoV-19/Japan/PG-74417/2021	EPI_ISL_3881438	2021-09-02
hCoV-19/Japan/PG-74418/2021	EPI_ISL_3881442	2021-09-02
hCoV-19/Japan/PG-74419/2021	EPI_ISL_3881445	2021-09-02
hCoV-19/Japan/PG-74420/2021	EPI_ISL_3881449	2021-09-02
hCoV-19/Japan/PG-74421/2021	EPI_ISL_3881453	2021-09-02
hCoV-19/Japan/PG-74422/2021	EPI_ISL_3873596	2021-09-02
hCoV-19/Japan/PG-74423/2021	EPI_ISL_3873597	2021-09-02
hCoV-19/Japan/PG-74424/2021	EPI_ISL_3873598	2021-09-02
hCoV-19/Japan/PG-74425/2021	EPI_ISL_3873599	2021-09-02
hCoV-19/Japan/PG-74426/2021	EPI_ISL_3873600	2021-09-02
hCoV-19/Japan/PG-74427/2021	EPI_ISL_3873601	2021-09-02
hCoV-19/Japan/PG-74428/2021	EPI_ISL_3873602	2021-09-02
hCoV-19/Japan/PG-74429/2021	EPI_ISL_3873603	2021-09-02
hCoV-19/Japan/PG-74430/2021	EPI_ISL_3873604	2021-09-02
hCoV-19/Japan/PG-74431/2021	EPI_ISL_3873605	2021-09-02
hCoV-19/Japan/PG-74432/2021	EPI_ISL_3873606	2021-09-02
hCoV-19/Japan/PG-74433/2021	EPI_ISL_3873607	2021-09-02
hCoV-19/Japan/PG-74434/2021	EPI_ISL_3873608	2021-09-02
hCoV-19/Japan/PG-74435/2021	EPI_ISL_3873609	2021-09-02
hCoV-19/Japan/PG-74436/2021	EPI_ISL_3873610	2021-09-02
hCoV-19/Japan/PG-74437/2021	EPI_ISL_3873611	2021-09-02
hCoV-19/Japan/PG-74438/2021	EPI_ISL_3873612	2021-09-02
hCoV-19/Japan/PG-74439/2021	EPI_ISL_3873613	2021-09-02
hCoV-19/Japan/PG-74440/2021	EPI_ISL_3873615	2021-09-02
hCoV-19/Japan/PG-74441/2021	EPI_ISL_3873617	2021-09-02
hCoV-19/Japan/PG-74442/2021	EPI_ISL_3873619	2021-09-02
hCoV-19/Japan/PG-74443/2021	EPI_ISL_3873621	2021-09-02
hCoV-19/Japan/PG-74444/2021	EPI_ISL_3873623	2021-09-02
hCoV-19/Japan/PG-74445/2021	EPI_ISL_3873625	2021-09-02
hCoV-19/Japan/PG-74446/2021	EPI_ISL_3873628	2021-09-02
hCoV-19/Japan/PG-74447/2021	EPI_ISL_3873629	2021-09-02
hCoV-19/Japan/PG-74448/2021	EPI_ISL_3873631	2021-09-02
hCoV-19/Japan/PG-74449/2021	EPI_ISL_3873633	2021-09-02
hCoV-19/Japan/PG-74450/2021	EPI_ISL_3873634	2021-09-02
hCoV-19/Japan/PG-74451/2021	EPI_ISL_3873636	2021-09-02
hCoV-19/Japan/PG-74452/2021	EPI_ISL_3873638	2021-09-02
hCoV-19/Japan/PG-74453/2021	EPI_ISL_3873640	2021-09-02
hCoV-19/Japan/PG-74454/2021	EPI_ISL_3873642	2021-09-02
hCoV-19/Japan/PG-74455/2021	EPI_ISL_3873645	2021-09-02
hCoV-19/Japan/PG-74456/2021	EPI_ISL_3873647	2021-09-02
hCoV-19/Japan/PG-74457/2021	EPI_ISL_3873649	2021-09-02
hCoV-19/Japan/PG-74458/2021	EPI_ISL_3873651	2021-09-02
hCoV-19/Japan/PG-74459/2021	EPI_ISL_3873653	2021-09-02
hCoV-19/Japan/PG-74460/2021	EPI_ISL_3873655	2021-09-02
hCoV-19/Japan/PG-74461/2021	EPI_ISL_3873657	2021-09-02

virus_name	epiID	submission_date
hCoV-19/Japan/PG-74462/2021	EPI_ISL_3871395	2021-09-02
hCoV-19/Japan/PG-74463/2021	EPI_ISL_3871396	2021-09-02
hCoV-19/Japan/PG-74464/2021	EPI_ISL_3871397	2021-09-02
hCoV-19/Japan/PG-74465/2021	EPI_ISL_3871398	2021-09-02
hCoV-19/Japan/PG-74466/2021	EPI_ISL_3871399	2021-09-02
hCoV-19/Japan/PG-74467/2021	EPI_ISL_3871400	2021-09-02
hCoV-19/Japan/PG-74468/2021	EPI_ISL_3871401	2021-09-02
hCoV-19/Japan/PG-74469/2021	EPI_ISL_3871402	2021-09-02
hCoV-19/Japan/PG-74470/2021	EPI_ISL_3871403	2021-09-02
hCoV-19/Japan/PG-74471/2021	EPI_ISL_3871404	2021-09-02
hCoV-19/Japan/PG-74472/2021	EPI_ISL_3871405	2021-09-02
hCoV-19/Japan/PG-74473/2021	EPI_ISL_3871406	2021-09-02
hCoV-19/Japan/PG-74474/2021	EPI_ISL_3871407	2021-09-02
hCoV-19/Japan/PG-74475/2021	EPI_ISL_3871408	2021-09-02
hCoV-19/Japan/PG-74476/2021	EPI_ISL_3871409	2021-09-02
hCoV-19/Japan/PG-74477/2021	EPI_ISL_3871410	2021-09-02
hCoV-19/Japan/PG-74478/2021	EPI_ISL_3871411	2021-09-02
hCoV-19/Japan/PG-74479/2021	EPI_ISL_3871412	2021-09-02
hCoV-19/Japan/PG-74480/2021	EPI_ISL_3871413	2021-09-02
hCoV-19/Japan/PG-74481/2021	EPI_ISL_3871414	2021-09-02
hCoV-19/Japan/PG-74482/2021	EPI_ISL_3876022	2021-09-02
hCoV-19/Japan/PG-74483/2021	EPI_ISL_3876023	2021-09-02
hCoV-19/Japan/PG-74484/2021	EPI_ISL_3876024	2021-09-02
hCoV-19/Japan/PG-74485/2021	EPI_ISL_3876025	2021-09-02
hCoV-19/Japan/PG-74486/2021	EPI_ISL_3876026	2021-09-02
hCoV-19/Japan/PG-74487/2021	EPI_ISL_3876027	2021-09-02
hCoV-19/Japan/PG-74488/2021	EPI_ISL_3876028	2021-09-02
hCoV-19/Japan/PG-74489/2021	EPI_ISL_3876029	2021-09-02
hCoV-19/Japan/PG-74490/2021	EPI_ISL_3876030	2021-09-02
hCoV-19/Japan/PG-74491/2021	EPI_ISL_3876031	2021-09-02
hCoV-19/Japan/PG-74492/2021	EPI_ISL_3876032	2021-09-02
hCoV-19/Japan/PG-74493/2021	EPI_ISL_3876033	2021-09-02
hCoV-19/Japan/PG-74494/2021	EPI_ISL_3876034	2021-09-02
hCoV-19/Japan/PG-74495/2021	EPI_ISL_3876035	2021-09-02
hCoV-19/Japan/PG-74496/2021	EPI_ISL_3876036	2021-09-02
hCoV-19/Japan/PG-74497/2021	EPI_ISL_3876037	2021-09-02
hCoV-19/Japan/PG-74498/2021	EPI_ISL_3876038	2021-09-02
hCoV-19/Japan/PG-74499/2021	EPI_ISL_3876039	2021-09-02
hCoV-19/Japan/PG-74500/2021	EPI_ISL_3876040	2021-09-02
hCoV-19/Japan/PG-74501/2021	EPI_ISL_3876041	2021-09-02
hCoV-19/Japan/PG-74502/2021	EPI_ISL_3876042	2021-09-02
hCoV-19/Japan/PG-74503/2021	EPI_ISL_3876043	2021-09-02
hCoV-19/Japan/PG-74504/2021	EPI_ISL_3876044	2021-09-02
hCoV-19/Japan/PG-74505/2021	EPI_ISL_3875760	2021-09-02
hCoV-19/Japan/PG-74506/2021	EPI_ISL_3875763	2021-09-02
hCoV-19/Japan/PG-74507/2021	EPI_ISL_3875767	2021-09-02
hCoV-19/Japan/PG-74508/2021	EPI_ISL_3875770	2021-09-02
hCoV-19/Japan/PG-74509/2021	EPI_ISL_3875773	2021-09-02
hCoV-19/Japan/PG-74510/2021	EPI_ISL_3875776	2021-09-02
hCoV-19/Japan/PG-74511/2021	EPI_ISL_3875780	2021-09-02
hCoV-19/Japan/PG-74512/2021	EPI_ISL_3875783	2021-09-02
hCoV-19/Japan/PG-74513/2021	EPI_ISL_3875786	2021-09-02
hCoV-19/Japan/PG-74514/2021	EPI_ISL_3875788	2021-09-02
hCoV-19/Japan/PG-74515/2021	EPI_ISL_3875792	2021-09-02

virus_name	epiID	submission_date
hCoV-19/Japan/PG-74516/2021	EPI_ISL_3875795	2021-09-02
hCoV-19/Japan/PG-74517/2021	EPI_ISL_3875798	2021-09-02
hCoV-19/Japan/PG-74518/2021	EPI_ISL_3875801	2021-09-02
hCoV-19/Japan/PG-74519/2021	EPI_ISL_3875804	2021-09-02
hCoV-19/Japan/PG-74520/2021	EPI_ISL_3875807	2021-09-02
hCoV-19/Japan/PG-74521/2021	EPI_ISL_3875810	2021-09-02
hCoV-19/Japan/PG-74522/2021	EPI_ISL_3875814	2021-09-02
hCoV-19/Japan/PG-74523/2021	EPI_ISL_3875817	2021-09-02
hCoV-19/Japan/PG-74524/2021	EPI_ISL_3875820	2021-09-02
hCoV-19/Japan/PG-74525/2021	EPI_ISL_3875823	2021-09-02
hCoV-19/Japan/PG-74526/2021	EPI_ISL_3873423	2021-09-02
hCoV-19/Japan/PG-74527/2021	EPI_ISL_3873424	2021-09-02
hCoV-19/Japan/PG-74528/2021	EPI_ISL_3873425	2021-09-02
hCoV-19/Japan/PG-74529/2021	EPI_ISL_3873426	2021-09-02
hCoV-19/Japan/PG-74530/2021	EPI_ISL_3873427	2021-09-02
hCoV-19/Japan/PG-74531/2021	EPI_ISL_3873428	2021-09-02
hCoV-19/Japan/PG-74532/2021	EPI_ISL_3873429	2021-09-02
hCoV-19/Japan/PG-74533/2021	EPI_ISL_3873430	2021-09-02
hCoV-19/Japan/PG-74534/2021	EPI_ISL_3873431	2021-09-02
hCoV-19/Japan/PG-74535/2021	EPI_ISL_3873432	2021-09-02
hCoV-19/Japan/PG-74536/2021	EPI_ISL_3871364	2021-09-02
hCoV-19/Japan/PG-74537/2021	EPI_ISL_3871365	2021-09-02
hCoV-19/Japan/PG-74538/2021	EPI_ISL_3871366	2021-09-02
hCoV-19/Japan/PG-74539/2021	EPI_ISL_3871367	2021-09-02
hCoV-19/Japan/PG-74540/2021	EPI_ISL_3871368	2021-09-02
hCoV-19/Japan/PG-74541/2021	EPI_ISL_3871876	2021-09-02
hCoV-19/Japan/PG-74542/2021	EPI_ISL_3871877	2021-09-02
hCoV-19/Japan/PG-74543/2021	EPI_ISL_3871878	2021-09-02
hCoV-19/Japan/PG-74544/2021	EPI_ISL_3871879	2021-09-02
hCoV-19/Japan/PG-74545/2021	EPI_ISL_3871880	2021-09-02
hCoV-19/Japan/PG-74546/2021	EPI_ISL_3871881	2021-09-02
hCoV-19/Japan/PG-74547/2021	EPI_ISL_3871882	2021-09-02
hCoV-19/Japan/PG-74548/2021	EPI_ISL_3871883	2021-09-02
hCoV-19/Japan/PG-74549/2021	EPI_ISL_3871884	2021-09-02
hCoV-19/Japan/PG-74550/2021	EPI_ISL_3871885	2021-09-02
hCoV-19/Japan/PG-74551/2021	EPI_ISL_3871886	2021-09-02
hCoV-19/Japan/PG-74552/2021	EPI_ISL_3871887	2021-09-02
hCoV-19/Japan/PG-74553/2021	EPI_ISL_3871888	2021-09-02
hCoV-19/Japan/PG-74554/2021	EPI_ISL_3871889	2021-09-02
hCoV-19/Japan/PG-74555/2021	EPI_ISL_3871890	2021-09-02
hCoV-19/Japan/PG-74556/2021	EPI_ISL_3871891	2021-09-02
hCoV-19/Japan/PG-74557/2021	EPI_ISL_3871892	2021-09-02
hCoV-19/Japan/PG-74558/2021	EPI_ISL_3871893	2021-09-02
hCoV-19/Japan/PG-74559/2021	EPI_ISL_3871894	2021-09-02
hCoV-19/Japan/PG-74560/2021	EPI_ISL_3871895	2021-09-02
hCoV-19/Japan/PG-74561/2021	EPI_ISL_3871896	2021-09-02
hCoV-19/Japan/PG-74562/2021	EPI_ISL_3871897	2021-09-02
hCoV-19/Japan/PG-74563/2021	EPI_ISL_3871898	2021-09-02
hCoV-19/Japan/PG-74564/2021	EPI_ISL_3871899	2021-09-02
hCoV-19/Japan/PG-74565/2021	EPI_ISL_3871683	2021-09-02
hCoV-19/Japan/PG-74566/2021	EPI_ISL_3871684	2021-09-02
hCoV-19/Japan/PG-74567/2021	EPI_ISL_3871685	2021-09-02
hCoV-19/Japan/PG-74568/2021	EPI_ISL_3871686	2021-09-02
hCoV-19/Japan/PG-74569/2021	EPI_ISL_3871687	2021-09-02

virus_name	epiID	submission_date
hCoV-19/Japan/PG-74570/2021	EPI_ISL_3871491	2021-09-02
hCoV-19/Japan/PG-74571/2021	EPI_ISL_3871492	2021-09-02
hCoV-19/Japan/PG-74572/2021	EPI_ISL_3871493	2021-09-02
hCoV-19/Japan/PG-74573/2021	EPI_ISL_3871494	2021-09-02
hCoV-19/Japan/PG-74574/2021	EPI_ISL_3871495	2021-09-02
hCoV-19/Japan/PG-74575/2021	EPI_ISL_3871496	2021-09-02
hCoV-19/Japan/PG-74576/2021	EPI_ISL_3871497	2021-09-02
hCoV-19/Japan/PG-74577/2021	EPI_ISL_3871498	2021-09-02
hCoV-19/Japan/PG-74578/2021	EPI_ISL_3871499	2021-09-02
hCoV-19/Japan/PG-74579/2021	EPI_ISL_3871500	2021-09-02
hCoV-19/Japan/PG-74580/2021	EPI_ISL_3871501	2021-09-02
hCoV-19/Japan/PG-74581/2021	EPI_ISL_3871502	2021-09-02
hCoV-19/Japan/PG-74582/2021	EPI_ISL_3871503	2021-09-02
hCoV-19/Japan/PG-74583/2021	EPI_ISL_3871504	2021-09-02
hCoV-19/Japan/PG-74584/2021	EPI_ISL_3871505	2021-09-02
hCoV-19/Japan/PG-74585/2021	EPI_ISL_3871013	2021-09-02
hCoV-19/Japan/PG-74586/2021	EPI_ISL_3871014	2021-09-02
hCoV-19/Japan/PG-74587/2021	EPI_ISL_3871015	2021-09-02
hCoV-19/Japan/PG-74588/2021	EPI_ISL_3871016	2021-09-02
hCoV-19/Japan/PG-74589/2021	EPI_ISL_3871017	2021-09-02
hCoV-19/Japan/PG-74590/2021	EPI_ISL_3871212	2021-09-02
hCoV-19/Japan/PG-74591/2021	EPI_ISL_3871213	2021-09-02
hCoV-19/Japan/PG-74592/2021	EPI_ISL_3871214	2021-09-02
hCoV-19/Japan/PG-74593/2021	EPI_ISL_3871215	2021-09-02
hCoV-19/Japan/PG-74594/2021	EPI_ISL_3871216	2021-09-02
hCoV-19/Japan/PG-74595/2021	EPI_ISL_3871217	2021-09-02
hCoV-19/Japan/PG-74596/2021	EPI_ISL_3871218	2021-09-02
hCoV-19/Japan/PG-74597/2021	EPI_ISL_3871219	2021-09-02
hCoV-19/Japan/PG-74598/2021	EPI_ISL_3871220	2021-09-02
hCoV-19/Japan/PG-74599/2021	EPI_ISL_3871221	2021-09-02
hCoV-19/Japan/PG-74600/2021	EPI_ISL_3871222	2021-09-02
hCoV-19/Japan/PG-74601/2021	EPI_ISL_3871223	2021-09-02
hCoV-19/Japan/PG-74602/2021	EPI_ISL_3871224	2021-09-02
hCoV-19/Japan/PG-74603/2021	EPI_ISL_3871225	2021-09-02
hCoV-19/Japan/PG-74604/2021	EPI_ISL_3871226	2021-09-02
hCoV-19/Japan/PG-74605/2021	EPI_ISL_3871227	2021-09-02
hCoV-19/Japan/PG-74606/2021	EPI_ISL_3871228	2021-09-02
hCoV-19/Japan/PG-74607/2021	EPI_ISL_3871229	2021-09-02
hCoV-19/Japan/PG-74608/2021	EPI_ISL_3871230	2021-09-02
hCoV-19/Japan/PG-74609/2021	EPI_ISL_3871231	2021-09-02
hCoV-19/Japan/PG-74610/2021	EPI_ISL_3871232	2021-09-02
hCoV-19/Japan/PG-74611/2021	EPI_ISL_3871233	2021-09-02
hCoV-19/Japan/PG-74612/2021	EPI_ISL_3871234	2021-09-02
hCoV-19/Japan/PG-74613/2021	EPI_ISL_3871235	2021-09-02
hCoV-19/Japan/PG-74614/2021	EPI_ISL_3872125	2021-09-02
hCoV-19/Japan/PG-74615/2021	EPI_ISL_3872126	2021-09-02
hCoV-19/Japan/PG-74616/2021	EPI_ISL_3872127	2021-09-02
hCoV-19/Japan/PG-74617/2021	EPI_ISL_3872128	2021-09-02
hCoV-19/Japan/PG-74618/2021	EPI_ISL_3872129	2021-09-02
hCoV-19/Japan/PG-74619/2021	EPI_ISL_3872130	2021-09-02
hCoV-19/Japan/PG-74620/2021	EPI_ISL_3872131	2021-09-02
hCoV-19/Japan/PG-74621/2021	EPI_ISL_3872132	2021-09-02
hCoV-19/Japan/PG-74622/2021	EPI_ISL_3872133	2021-09-02
hCoV-19/Japan/PG-74623/2021	EPI_ISL_3872134	2021-09-02

virus_name	epiID	submission_date
hCoV-19/Japan/PG-74624/2021	EPI_ISL_3872135	2021-09-02
hCoV-19/Japan/PG-74625/2021	EPI_ISL_3874400	2021-09-02
hCoV-19/Japan/PG-74626/2021	EPI_ISL_3874401	2021-09-02
hCoV-19/Japan/PG-74627/2021	EPI_ISL_3874402	2021-09-02
hCoV-19/Japan/PG-74628/2021	EPI_ISL_3874403	2021-09-02
hCoV-19/Japan/PG-74629/2021	EPI_ISL_3874404	2021-09-02
hCoV-19/Japan/PG-74630/2021	EPI_ISL_3874405	2021-09-02
hCoV-19/Japan/PG-74631/2021	EPI_ISL_3874406	2021-09-02
hCoV-19/Japan/PG-74632/2021	EPI_ISL_3874407	2021-09-02
hCoV-19/Japan/PG-74633/2021	EPI_ISL_3874408	2021-09-02
hCoV-19/Japan/PG-74634/2021	EPI_ISL_3874409	2021-09-02
hCoV-19/Japan/PG-74635/2021	EPI_ISL_3874410	2021-09-02
hCoV-19/Japan/PG-74636/2021	EPI_ISL_3874411	2021-09-02
hCoV-19/Japan/PG-74637/2021	EPI_ISL_3874412	2021-09-02
hCoV-19/Japan/PG-74638/2021	EPI_ISL_3874413	2021-09-02
hCoV-19/Japan/PG-74639/2021	EPI_ISL_3874414	2021-09-02
hCoV-19/Japan/PG-74640/2021	EPI_ISL_3874415	2021-09-02
hCoV-19/Japan/PG-74641/2021	EPI_ISL_3874416	2021-09-02
hCoV-19/Japan/PG-74642/2021	EPI_ISL_3874417	2021-09-02
hCoV-19/Japan/PG-74643/2021	EPI_ISL_3874418	2021-09-02
hCoV-19/Japan/PG-74644/2021	EPI_ISL_3874419	2021-09-02
hCoV-19/Japan/PG-74645/2021	EPI_ISL_3874420	2021-09-02
hCoV-19/Japan/PG-74646/2021	EPI_ISL_3874421	2021-09-02
hCoV-19/Japan/PG-74647/2021	EPI_ISL_3870958	2021-09-02
hCoV-19/Japan/PG-74648/2021	EPI_ISL_3870959	2021-09-02
hCoV-19/Japan/PG-74649/2021	EPI_ISL_3870960	2021-09-02
hCoV-19/Japan/PG-74650/2021	EPI_ISL_3870961	2021-09-02
hCoV-19/Japan/PG-74651/2021	EPI_ISL_3870962	2021-09-02
hCoV-19/Japan/PG-74652/2021	EPI_ISL_3870963	2021-09-02
hCoV-19/Japan/PG-74653/2021	EPI_ISL_3872151	2021-09-02
hCoV-19/Japan/PG-74654/2021	EPI_ISL_3872152	2021-09-02
hCoV-19/Japan/PG-74655/2021	EPI_ISL_3872156	2021-09-02
hCoV-19/Japan/PG-74656/2021	EPI_ISL_3872153	2021-09-02
hCoV-19/Japan/PG-74657/2021	EPI_ISL_3872154	2021-09-02
hCoV-19/Japan/PG-74658/2021	EPI_ISL_3872155	2021-09-02
hCoV-19/Japan/PG-74661/2021	EPI_ISL_3872246	2021-09-02
hCoV-19/Japan/PG-74662/2021	EPI_ISL_3872247	2021-09-02
hCoV-19/Japan/PG-74663/2021	EPI_ISL_3872248	2021-09-02
hCoV-19/Japan/PG-74664/2021	EPI_ISL_3872249	2021-09-02
hCoV-19/Japan/PG-74665/2021	EPI_ISL_3872250	2021-09-02
hCoV-19/Japan/PG-74666/2021	EPI_ISL_3872251	2021-09-02
hCoV-19/Japan/PG-74667/2021	EPI_ISL_3872252	2021-09-02
hCoV-19/Japan/PG-74668/2021	EPI_ISL_3872253	2021-09-02
hCoV-19/Japan/PG-74669/2021	EPI_ISL_3872254	2021-09-02
hCoV-19/Japan/PG-74670/2021	EPI_ISL_3872255	2021-09-02
hCoV-19/Japan/PG-74671/2021	EPI_ISL_3872256	2021-09-02
hCoV-19/Japan/PG-74672/2021	EPI_ISL_3872257	2021-09-02
hCoV-19/Japan/PG-74673/2021	EPI_ISL_3872258	2021-09-02
hCoV-19/Japan/PG-74674/2021	EPI_ISL_3872259	2021-09-02
hCoV-19/Japan/PG-74675/2021	EPI_ISL_3881454	2021-09-02
hCoV-19/Japan/PG-74676/2021	EPI_ISL_3881456	2021-09-02
hCoV-19/Japan/PG-74677/2021	EPI_ISL_3881462	2021-09-02
hCoV-19/Japan/PG-74678/2021	EPI_ISL_3881466	2021-09-02
hCoV-19/Japan/PG-74679/2021	EPI_ISL_3881469	2021-09-02

virus_name	epiID	submission_date
hCoV-19/Japan/PG-74680/2021	EPI_ISL_3881471	2021-09-02
hCoV-19/Japan/PG-74681/2021	EPI_ISL_3881475	2021-09-02
hCoV-19/Japan/PG-74682/2021	EPI_ISL_3881480	2021-09-02
hCoV-19/Japan/PG-74683/2021	EPI_ISL_3881484	2021-09-02
hCoV-19/Japan/PG-74684/2021	EPI_ISL_3881489	2021-09-02
hCoV-19/Japan/PG-74685/2021	EPI_ISL_3881494	2021-09-02
hCoV-19/Japan/PG-74686/2021	EPI_ISL_3881498	2021-09-02
hCoV-19/Japan/PG-74687/2021	EPI_ISL_3881503	2021-09-02
hCoV-19/Japan/PG-74688/2021	EPI_ISL_3881507	2021-09-02
hCoV-19/Japan/PG-74689/2021	EPI_ISL_3881512	2021-09-02
hCoV-19/Japan/PG-74690/2021	EPI_ISL_3881515	2021-09-02
hCoV-19/Japan/PG-74691/2021	EPI_ISL_3881519	2021-09-02
hCoV-19/Japan/PG-74692/2021	EPI_ISL_3881523	2021-09-02
hCoV-19/Japan/PG-74693/2021	EPI_ISL_3881527	2021-09-02
hCoV-19/Japan/PG-74694/2021	EPI_ISL_3881530	2021-09-02
hCoV-19/Japan/PG-74695/2021	EPI_ISL_3881533	2021-09-02
hCoV-19/Japan/PG-74696/2021	EPI_ISL_3881537	2021-09-02
hCoV-19/Japan/PG-74697/2021	EPI_ISL_3881541	2021-09-02
hCoV-19/Japan/PG-74698/2021	EPI_ISL_3881546	2021-09-02
hCoV-19/Japan/PG-74699/2021	EPI_ISL_3881549	2021-09-02
hCoV-19/Japan/PG-74700/2021	EPI_ISL_3881552	2021-09-02
hCoV-19/Japan/PG-74701/2021	EPI_ISL_3881557	2021-09-02
hCoV-19/Japan/PG-74702/2021	EPI_ISL_3881561	2021-09-02
hCoV-19/Japan/PG-74703/2021	EPI_ISL_3881565	2021-09-02
hCoV-19/Japan/PG-74704/2021	EPI_ISL_3881570	2021-09-02
hCoV-19/Japan/PG-74705/2021	EPI_ISL_3881571	2021-09-02
hCoV-19/Japan/PG-74706/2021	EPI_ISL_3881573	2021-09-02
hCoV-19/Japan/PG-74707/2021	EPI_ISL_3881574	2021-09-02
hCoV-19/Japan/PG-74708/2021	EPI_ISL_3881578	2021-09-02
hCoV-19/Japan/PG-74709/2021	EPI_ISL_3881582	2021-09-02
hCoV-19/Japan/PG-74710/2021	EPI_ISL_3881585	2021-09-02
hCoV-19/Japan/PG-74711/2021	EPI_ISL_3881589	2021-09-02
hCoV-19/Japan/PG-74712/2021	EPI_ISL_3881592	2021-09-02
hCoV-19/Japan/PG-74713/2021	EPI_ISL_3881596	2021-09-02
hCoV-19/Japan/PG-74714/2021	EPI_ISL_3881600	2021-09-02
hCoV-19/Japan/PG-74715/2021	EPI_ISL_3881602	2021-09-02
hCoV-19/Japan/PG-74716/2021	EPI_ISL_3881604	2021-09-02
hCoV-19/Japan/PG-74717/2021	EPI_ISL_3881607	2021-09-02
hCoV-19/Japan/PG-74718/2021	EPI_ISL_3881612	2021-09-02
hCoV-19/Japan/PG-74719/2021	EPI_ISL_3881616	2021-09-02
hCoV-19/Japan/PG-74720/2021	EPI_ISL_3881619	2021-09-02
hCoV-19/Japan/PG-74721/2021	EPI_ISL_3881621	2021-09-02
hCoV-19/Japan/PG-74722/2021	EPI_ISL_3881623	2021-09-02
hCoV-19/Japan/PG-74723/2021	EPI_ISL_3881625	2021-09-02
hCoV-19/Japan/PG-74724/2021	EPI_ISL_3881629	2021-09-02
hCoV-19/Japan/PG-74725/2021	EPI_ISL_3881632	2021-09-02
hCoV-19/Japan/PG-74726/2021	EPI_ISL_3881634	2021-09-02
hCoV-19/Japan/PG-74727/2021	EPI_ISL_3881636	2021-09-02
hCoV-19/Japan/PG-74728/2021	EPI_ISL_3881638	2021-09-02
hCoV-19/Japan/PG-74729/2021	EPI_ISL_3881639	2021-09-02
hCoV-19/Japan/PG-74730/2021	EPI_ISL_3881642	2021-09-02
hCoV-19/Japan/PG-74731/2021	EPI_ISL_3881644	2021-09-02
hCoV-19/Japan/PG-74732/2021	EPI_ISL_3881646	2021-09-02
hCoV-19/Japan/PG-74733/2021	EPI_ISL_3881647	2021-09-02

virus_name	epiID	submission_date
hCoV-19/Japan/PG-74734/2021	EPI_ISL_3881650	2021-09-02
hCoV-19/Japan/PG-74735/2021	EPI_ISL_3881652	2021-09-02
hCoV-19/Japan/PG-74736/2021	EPI_ISL_3881657	2021-09-02
hCoV-19/Japan/PG-74737/2021	EPI_ISL_3881661	2021-09-02
hCoV-19/Japan/PG-74738/2021	EPI_ISL_3881664	2021-09-02
hCoV-19/Japan/PG-74739/2021	EPI_ISL_3881668	2021-09-02
hCoV-19/Japan/PG-74740/2021	EPI_ISL_3881672	2021-09-02
hCoV-19/Japan/PG-74741/2021	EPI_ISL_3881675	2021-09-02
hCoV-19/Japan/PG-74742/2021	EPI_ISL_3881680	2021-09-02
hCoV-19/Japan/PG-74743/2021	EPI_ISL_3881684	2021-09-02
hCoV-19/Japan/PG-74744/2021	EPI_ISL_3881687	2021-09-02
hCoV-19/Japan/PG-74745/2021	EPI_ISL_3881691	2021-09-02
hCoV-19/Japan/PG-74746/2021	EPI_ISL_3881693	2021-09-02
hCoV-19/Japan/PG-74747/2021	EPI_ISL_3881695	2021-09-02
hCoV-19/Japan/PG-74748/2021	EPI_ISL_3881700	2021-09-02
hCoV-19/Japan/PG-74749/2021	EPI_ISL_3881703	2021-09-02
hCoV-19/Japan/PG-74750/2021	EPI_ISL_3881705	2021-09-02
hCoV-19/Japan/PG-74751/2021	EPI_ISL_3881709	2021-09-02
hCoV-19/Japan/PG-74752/2021	EPI_ISL_3881714	2021-09-02
hCoV-19/Japan/PG-74753/2021	EPI_ISL_3881716	2021-09-02
hCoV-19/Japan/PG-74754/2021	EPI_ISL_3881723	2021-09-02
hCoV-19/Japan/PG-74755/2021	EPI_ISL_3881725	2021-09-02
hCoV-19/Japan/PG-74756/2021	EPI_ISL_3881726	2021-09-02
hCoV-19/Japan/PG-74757/2021	EPI_ISL_3881729	2021-09-02
hCoV-19/Japan/PG-74758/2021	EPI_ISL_3881733	2021-09-02
hCoV-19/Japan/PG-74759/2021	EPI_ISL_3881736	2021-09-02
hCoV-19/Japan/PG-74760/2021	EPI_ISL_3881740	2021-09-02
hCoV-19/Japan/PG-74761/2021	EPI_ISL_3881742	2021-09-02
hCoV-19/Japan/PG-74762/2021	EPI_ISL_3881744	2021-09-02
hCoV-19/Japan/PG-74763/2021	EPI_ISL_3881746	2021-09-02
hCoV-19/Japan/PG-74764/2021	EPI_ISL_3881750	2021-09-02
hCoV-19/Japan/PG-74765/2021	EPI_ISL_3881753	2021-09-02
hCoV-19/Japan/PG-74766/2021	EPI_ISL_3881755	2021-09-02
hCoV-19/Japan/PG-74767/2021	EPI_ISL_3881757	2021-09-02
hCoV-19/Japan/PG-74768/2021	EPI_ISL_3881761	2021-09-02
hCoV-19/Japan/PG-74769/2021	EPI_ISL_3881765	2021-09-02
hCoV-19/Japan/PG-74770/2021	EPI_ISL_3881769	2021-09-02
hCoV-19/Japan/PG-74771/2021	EPI_ISL_3881771	2021-09-02
hCoV-19/Japan/PG-74772/2021	EPI_ISL_3881773	2021-09-02
hCoV-19/Japan/PG-74773/2021	EPI_ISL_3881775	2021-09-02
hCoV-19/Japan/PG-74774/2021	EPI_ISL_3881778	2021-09-02
hCoV-19/Japan/PG-74775/2021	EPI_ISL_3881782	2021-09-02
hCoV-19/Japan/PG-74776/2021	EPI_ISL_3881786	2021-09-02
hCoV-19/Japan/PG-74777/2021	EPI_ISL_3881790	2021-09-02
hCoV-19/Japan/PG-74778/2021	EPI_ISL_3881792	2021-09-02
hCoV-19/Japan/PG-74779/2021	EPI_ISL_3881795	2021-09-02
hCoV-19/Japan/PG-74780/2021	EPI_ISL_3881798	2021-09-02
hCoV-19/Japan/PG-74781/2021	EPI_ISL_3881801	2021-09-02
hCoV-19/Japan/PG-74782/2021	EPI_ISL_3881802	2021-09-02
hCoV-19/Japan/PG-74783/2021	EPI_ISL_3881805	2021-09-02
hCoV-19/Japan/PG-74784/2021	EPI_ISL_3881806	2021-09-02
hCoV-19/Japan/PG-74785/2021	EPI_ISL_3881809	2021-09-02
hCoV-19/Japan/PG-74786/2021	EPI_ISL_3881811	2021-09-02
hCoV-19/Japan/PG-74787/2021	EPI_ISL_3881813	2021-09-02

virus_name	epiID	submission_date
hCoV-19/Japan/PG-74788/2021	EPI_ISL_3881814	2021-09-02
hCoV-19/Japan/PG-74789/2021	EPI_ISL_3881816	2021-09-02
hCoV-19/Japan/PG-74790/2021	EPI_ISL_3881818	2021-09-02
hCoV-19/Japan/PG-74791/2021	EPI_ISL_3881822	2021-09-02
hCoV-19/Japan/PG-74792/2021	EPI_ISL_3881826	2021-09-02
hCoV-19/Japan/PG-74793/2021	EPI_ISL_3881829	2021-09-02
hCoV-19/Japan/PG-74794/2021	EPI_ISL_3881831	2021-09-02
hCoV-19/Japan/PG-74795/2021	EPI_ISL_3881834	2021-09-02
hCoV-19/Japan/PG-74796/2021	EPI_ISL_3881836	2021-09-02
hCoV-19/Japan/PG-74797/2021	EPI_ISL_3881842	2021-09-02
hCoV-19/Japan/PG-74798/2021	EPI_ISL_3881845	2021-09-02
hCoV-19/Japan/PG-74799/2021	EPI_ISL_3881847	2021-09-02
hCoV-19/Japan/PG-74800/2021	EPI_ISL_3881850	2021-09-02
hCoV-19/Japan/PG-74801/2021	EPI_ISL_3881852	2021-09-02
hCoV-19/Japan/PG-74802/2021	EPI_ISL_3881853	2021-09-02
hCoV-19/Japan/PG-74803/2021	EPI_ISL_3881856	2021-09-02
hCoV-19/Japan/PG-74804/2021	EPI_ISL_3881858	2021-09-02
hCoV-19/Japan/PG-74805/2021	EPI_ISL_3881861	2021-09-02
hCoV-19/Japan/PG-74806/2021	EPI_ISL_3881864	2021-09-02
hCoV-19/Japan/PG-74807/2021	EPI_ISL_3881866	2021-09-02
hCoV-19/Japan/PG-74808/2021	EPI_ISL_3881868	2021-09-02
hCoV-19/Japan/PG-74809/2021	EPI_ISL_3881873	2021-09-02
hCoV-19/Japan/PG-74810/2021	EPI_ISL_3881876	2021-09-02
hCoV-19/Japan/PG-74811/2021	EPI_ISL_3881879	2021-09-02
hCoV-19/Japan/PG-74812/2021	EPI_ISL_3881880	2021-09-02
hCoV-19/Japan/PG-74813/2021	EPI_ISL_3881881	2021-09-02
hCoV-19/Japan/PG-74814/2021	EPI_ISL_3881882	2021-09-02
hCoV-19/Japan/PG-74815/2021	EPI_ISL_3881884	2021-09-02
hCoV-19/Japan/PG-74816/2021	EPI_ISL_3881886	2021-09-02
hCoV-19/Japan/PG-74817/2021	EPI_ISL_3881889	2021-09-02
hCoV-19/Japan/PG-74818/2021	EPI_ISL_3881891	2021-09-02
hCoV-19/Japan/PG-74819/2021	EPI_ISL_3881893	2021-09-02
hCoV-19/Japan/PG-74820/2021	EPI_ISL_3881895	2021-09-02
hCoV-19/Japan/PG-74821/2021	EPI_ISL_3881897	2021-09-02
hCoV-19/Japan/PG-74822/2021	EPI_ISL_3881899	2021-09-02
hCoV-19/Japan/PG-74823/2021	EPI_ISL_3881901	2021-09-02
hCoV-19/Japan/PG-74824/2021	EPI_ISL_3881903	2021-09-02
hCoV-19/Japan/PG-74825/2021	EPI_ISL_3881905	2021-09-02
hCoV-19/Japan/PG-74826/2021	EPI_ISL_3881907	2021-09-02
hCoV-19/Japan/PG-74827/2021	EPI_ISL_3881908	2021-09-02
hCoV-19/Japan/PG-74828/2021	EPI_ISL_3881909	2021-09-02
hCoV-19/Japan/PG-74829/2021	EPI_ISL_3881912	2021-09-02
hCoV-19/Japan/PG-74830/2021	EPI_ISL_3881913	2021-09-02
hCoV-19/Japan/PG-74831/2021	EPI_ISL_3881915	2021-09-02
hCoV-19/Japan/PG-74832/2021	EPI_ISL_3881917	2021-09-02
hCoV-19/Japan/PG-74833/2021	EPI_ISL_3881919	2021-09-02
hCoV-19/Japan/PG-74834/2021	EPI_ISL_3881922	2021-09-02
hCoV-19/Japan/PG-74835/2021	EPI_ISL_3881923	2021-09-02
hCoV-19/Japan/PG-74836/2021	EPI_ISL_3881925	2021-09-02
hCoV-19/Japan/PG-74837/2021	EPI_ISL_3881926	2021-09-02
hCoV-19/Japan/PG-74838/2021	EPI_ISL_3881928	2021-09-02
hCoV-19/Japan/PG-74839/2021	EPI_ISL_3881930	2021-09-02
hCoV-19/Japan/PG-74840/2021	EPI_ISL_3881931	2021-09-02
hCoV-19/Japan/PG-74841/2021	EPI_ISL_3881933	2021-09-02

virus_name	epiID	submission_date
hCoV-19/Japan/PG-74842/2021	EPI_ISL_3881935	2021-09-02
hCoV-19/Japan/PG-74843/2021	EPI_ISL_3881937	2021-09-02
hCoV-19/Japan/PG-74844/2021	EPI_ISL_3881939	2021-09-02
hCoV-19/Japan/PG-74845/2021	EPI_ISL_3881941	2021-09-02
hCoV-19/Japan/PG-74846/2021	EPI_ISL_3881943	2021-09-02
hCoV-19/Japan/PG-74847/2021	EPI_ISL_3881945	2021-09-02
hCoV-19/Japan/PG-74848/2021	EPI_ISL_3881948	2021-09-02
hCoV-19/Japan/PG-74849/2021	EPI_ISL_3881949	2021-09-02
hCoV-19/Japan/PG-74850/2021	EPI_ISL_3881950	2021-09-02
hCoV-19/Japan/PG-74851/2021	EPI_ISL_3881952	2021-09-02
hCoV-19/Japan/PG-74852/2021	EPI_ISL_3881953	2021-09-02
hCoV-19/Japan/PG-74853/2021	EPI_ISL_3881955	2021-09-02
hCoV-19/Japan/PG-74854/2021	EPI_ISL_3881956	2021-09-02
hCoV-19/Japan/PG-74855/2021	EPI_ISL_3881958	2021-09-02
hCoV-19/Japan/PG-74856/2021	EPI_ISL_3881959	2021-09-02
hCoV-19/Japan/PG-74857/2021	EPI_ISL_3881961	2021-09-02
hCoV-19/Japan/PG-74858/2021	EPI_ISL_3881963	2021-09-02
hCoV-19/Japan/PG-74859/2021	EPI_ISL_3881965	2021-09-02
hCoV-19/Japan/PG-74860/2021	EPI_ISL_3881966	2021-09-02
hCoV-19/Japan/PG-74861/2021	EPI_ISL_3881968	2021-09-02
hCoV-19/Japan/PG-74862/2021	EPI_ISL_3881970	2021-09-02
hCoV-19/Japan/PG-74863/2021	EPI_ISL_3881972	2021-09-02
hCoV-19/Japan/PG-74864/2021	EPI_ISL_3881974	2021-09-02
hCoV-19/Japan/PG-74865/2021	EPI_ISL_3881977	2021-09-02
hCoV-19/Japan/PG-74866/2021	EPI_ISL_3881979	2021-09-02
hCoV-19/Japan/PG-74867/2021	EPI_ISL_3881981	2021-09-02
hCoV-19/Japan/PG-74868/2021	EPI_ISL_3881982	2021-09-02
hCoV-19/Japan/PG-74869/2021	EPI_ISL_3881984	2021-09-02
hCoV-19/Japan/PG-74870/2021	EPI_ISL_3881986	2021-09-02
hCoV-19/Japan/PG-74871/2021	EPI_ISL_3881988	2021-09-02
hCoV-19/Japan/PG-74872/2021	EPI_ISL_3881989	2021-09-02
hCoV-19/Japan/PG-74873/2021	EPI_ISL_3881991	2021-09-02
hCoV-19/Japan/PG-74874/2021	EPI_ISL_3881995	2021-09-02
hCoV-19/Japan/PG-74875/2021	EPI_ISL_3882003	2021-09-02
hCoV-19/Japan/PG-74876/2021	EPI_ISL_3882012	2021-09-02
hCoV-19/Japan/PG-74877/2021	EPI_ISL_3882019	2021-09-02
hCoV-19/Japan/PG-74878/2021	EPI_ISL_3882025	2021-09-02
hCoV-19/Japan/PG-74879/2021	EPI_ISL_3882032	2021-09-02
hCoV-19/Japan/PG-74880/2021	EPI_ISL_3882039	2021-09-02
hCoV-19/Japan/PG-74881/2021	EPI_ISL_3882046	2021-09-02
hCoV-19/Japan/PG-74882/2021	EPI_ISL_3882053	2021-09-02
hCoV-19/Japan/PG-74883/2021	EPI_ISL_3882060	2021-09-02
hCoV-19/Japan/PG-74884/2021	EPI_ISL_3882068	2021-09-02
hCoV-19/Japan/PG-74885/2021	EPI_ISL_3882074	2021-09-02
hCoV-19/Japan/PG-74886/2021	EPI_ISL_3882081	2021-09-02
hCoV-19/Japan/PG-74887/2021	EPI_ISL_3882088	2021-09-02
hCoV-19/Japan/PG-74888/2021	EPI_ISL_3882097	2021-09-02
hCoV-19/Japan/PG-74889/2021	EPI_ISL_3882103	2021-09-02
hCoV-19/Japan/PG-74890/2021	EPI_ISL_3882110	2021-09-02
hCoV-19/Japan/PG-74891/2021	EPI_ISL_3882117	2021-09-02
hCoV-19/Japan/PG-74892/2021	EPI_ISL_3882125	2021-09-02
hCoV-19/Japan/PG-74893/2021	EPI_ISL_3882132	2021-09-02
hCoV-19/Japan/PG-74894/2021	EPI_ISL_3882139	2021-09-02
hCoV-19/Japan/PG-74895/2021	EPI_ISL_3882147	2021-09-02

virus_name	epiID	submission_date
hCoV-19/Japan/PG-74896/2021	EPI_ISL_3882154	2021-09-02
hCoV-19/Japan/PG-74897/2021	EPI_ISL_3882160	2021-09-02
hCoV-19/Japan/PG-74898/2021	EPI_ISL_3882168	2021-09-02
hCoV-19/Japan/PG-74899/2021	EPI_ISL_3882175	2021-09-02
hCoV-19/Japan/PG-74900/2021	EPI_ISL_3882183	2021-09-02
hCoV-19/Japan/PG-74901/2021	EPI_ISL_3882191	2021-09-02
hCoV-19/Japan/PG-74902/2021	EPI_ISL_3882199	2021-09-02
hCoV-19/Japan/PG-74903/2021	EPI_ISL_3882206	2021-09-02
hCoV-19/Japan/PG-74904/2021	EPI_ISL_3882213	2021-09-02
hCoV-19/Japan/PG-74905/2021	EPI_ISL_3882222	2021-09-02
hCoV-19/Japan/PG-74906/2021	EPI_ISL_3882229	2021-09-02
hCoV-19/Japan/PG-74907/2021	EPI_ISL_3882238	2021-09-02
hCoV-19/Japan/PG-74908/2021	EPI_ISL_3882241	2021-09-02
hCoV-19/Japan/PG-74909/2021	EPI_ISL_3882249	2021-09-02
hCoV-19/Japan/PG-74910/2021	EPI_ISL_3882257	2021-09-02
hCoV-19/Japan/PG-74911/2021	EPI_ISL_3882264	2021-09-02
hCoV-19/Japan/PG-74912/2021	EPI_ISL_3882270	2021-09-02
hCoV-19/Japan/PG-74913/2021	EPI_ISL_3882278	2021-09-02
hCoV-19/Japan/PG-74914/2021	EPI_ISL_3882285	2021-09-02
hCoV-19/Japan/PG-74915/2021	EPI_ISL_3882290	2021-09-02
hCoV-19/Japan/PG-74916/2021	EPI_ISL_3882298	2021-09-02
hCoV-19/Japan/PG-74917/2021	EPI_ISL_3882304	2021-09-02
hCoV-19/Japan/PG-74918/2021	EPI_ISL_3882313	2021-09-02
hCoV-19/Japan/PG-74919/2021	EPI_ISL_3882320	2021-09-02
hCoV-19/Japan/PG-74920/2021	EPI_ISL_3882327	2021-09-02
hCoV-19/Japan/PG-74921/2021	EPI_ISL_3882336	2021-09-02
hCoV-19/Japan/PG-74922/2021	EPI_ISL_3882342	2021-09-02
hCoV-19/Japan/PG-74923/2021	EPI_ISL_3882351	2021-09-02
hCoV-19/Japan/PG-74924/2021	EPI_ISL_3882358	2021-09-02
hCoV-19/Japan/PG-74925/2021	EPI_ISL_3882365	2021-09-02
hCoV-19/Japan/PG-74926/2021	EPI_ISL_3882373	2021-09-02
hCoV-19/Japan/PG-74927/2021	EPI_ISL_3882379	2021-09-02
hCoV-19/Japan/PG-74928/2021	EPI_ISL_3882386	2021-09-02
hCoV-19/Japan/PG-74929/2021	EPI_ISL_3882394	2021-09-02
hCoV-19/Japan/PG-74930/2021	EPI_ISL_3882400	2021-09-02
hCoV-19/Japan/PG-74931/2021	EPI_ISL_3882407	2021-09-02
hCoV-19/Japan/PG-74932/2021	EPI_ISL_3882415	2021-09-02
hCoV-19/Japan/PG-74933/2021	EPI_ISL_3882422	2021-09-02
hCoV-19/Japan/PG-74934/2021	EPI_ISL_3882426	2021-09-02
hCoV-19/Japan/PG-74935/2021	EPI_ISL_3882433	2021-09-02
hCoV-19/Japan/PG-74936/2021	EPI_ISL_3882440	2021-09-02
hCoV-19/Japan/PG-74937/2021	EPI_ISL_3882447	2021-09-02
hCoV-19/Japan/PG-74938/2021	EPI_ISL_3882454	2021-09-02
hCoV-19/Japan/PG-74939/2021	EPI_ISL_3882462	2021-09-02
hCoV-19/Japan/PG-74940/2021	EPI_ISL_3882469	2021-09-02
hCoV-19/Japan/PG-74941/2021	EPI_ISL_3882477	2021-09-02
hCoV-19/Japan/PG-74942/2021	EPI_ISL_3882486	2021-09-02
hCoV-19/Japan/PG-74943/2021	EPI_ISL_3882492	2021-09-02
hCoV-19/Japan/PG-74944/2021	EPI_ISL_3882500	2021-09-02
hCoV-19/Japan/PG-74945/2021	EPI_ISL_3882508	2021-09-02
hCoV-19/Japan/PG-74946/2021	EPI_ISL_3882514	2021-09-02
hCoV-19/Japan/PG-74947/2021	EPI_ISL_3882521	2021-09-02
hCoV-19/Japan/PG-74948/2021	EPI_ISL_3882527	2021-09-02
hCoV-19/Japan/PG-74949/2021	EPI_ISL_3882535	2021-09-02

virus_name	epiID	submission_date
hCoV-19/Japan/PG-74950/2021	EPI_ISL_3882543	2021-09-02
hCoV-19/Japan/PG-74951/2021	EPI_ISL_3882550	2021-09-02
hCoV-19/Japan/PG-74952/2021	EPI_ISL_3882557	2021-09-02
hCoV-19/Japan/PG-74953/2021	EPI_ISL_3882565	2021-09-02
hCoV-19/Japan/PG-74954/2021	EPI_ISL_3882573	2021-09-02
hCoV-19/Japan/PG-74955/2021	EPI_ISL_3882581	2021-09-02
hCoV-19/Japan/PG-74956/2021	EPI_ISL_3882588	2021-09-02
hCoV-19/Japan/PG-74957/2021	EPI_ISL_3882595	2021-09-02
hCoV-19/Japan/PG-74958/2021	EPI_ISL_3882601	2021-09-02
hCoV-19/Japan/PG-74959/2021	EPI_ISL_3882609	2021-09-02
hCoV-19/Japan/PG-74960/2021	EPI_ISL_3882617	2021-09-02
hCoV-19/Japan/PG-74961/2021	EPI_ISL_3882624	2021-09-02
hCoV-19/Japan/PG-74962/2021	EPI_ISL_3882633	2021-09-02
hCoV-19/Japan/PG-74963/2021	EPI_ISL_3882637	2021-09-02
hCoV-19/Japan/PG-74964/2021	EPI_ISL_3882638	2021-09-02
hCoV-19/Japan/PG-74965/2021	EPI_ISL_3882640	2021-09-02
hCoV-19/Japan/PG-74966/2021	EPI_ISL_3882642	2021-09-02
hCoV-19/Japan/PG-74967/2021	EPI_ISL_3882643	2021-09-02
hCoV-19/Japan/PG-74968/2021	EPI_ISL_3882645	2021-09-02
hCoV-19/Japan/PG-74969/2021	EPI_ISL_3882646	2021-09-02
hCoV-19/Japan/PG-74970/2021	EPI_ISL_3882648	2021-09-02
hCoV-19/Japan/PG-74971/2021	EPI_ISL_3882649	2021-09-02
hCoV-19/Japan/PG-74972/2021	EPI_ISL_3882651	2021-09-02
hCoV-19/Japan/PG-74973/2021	EPI_ISL_3882652	2021-09-02
hCoV-19/Japan/PG-74974/2021	EPI_ISL_3882654	2021-09-02
hCoV-19/Japan/PG-74975/2021	EPI_ISL_3882656	2021-09-02
hCoV-19/Japan/PG-74976/2021	EPI_ISL_3882658	2021-09-02
hCoV-19/Japan/PG-74977/2021	EPI_ISL_3882659	2021-09-02
hCoV-19/Japan/PG-74978/2021	EPI_ISL_3882661	2021-09-02
hCoV-19/Japan/PG-74979/2021	EPI_ISL_3882663	2021-09-02
hCoV-19/Japan/PG-74980/2021	EPI_ISL_3882664	2021-09-02
hCoV-19/Japan/PG-74981/2021	EPI_ISL_3882665	2021-09-02
hCoV-19/Japan/PG-74982/2021	EPI_ISL_3882666	2021-09-02
hCoV-19/Japan/PG-74983/2021	EPI_ISL_3882670	2021-09-02
hCoV-19/Japan/PG-74984/2021	EPI_ISL_3882676	2021-09-02
hCoV-19/Japan/PG-74985/2021	EPI_ISL_3882684	2021-09-02
hCoV-19/Japan/PG-74986/2021	EPI_ISL_3882690	2021-09-02
hCoV-19/Japan/PG-74987/2021	EPI_ISL_3882697	2021-09-02
hCoV-19/Japan/PG-74988/2021	EPI_ISL_3882704	2021-09-02
hCoV-19/Japan/PG-74989/2021	EPI_ISL_3882711	2021-09-02
hCoV-19/Japan/PG-74990/2021	EPI_ISL_3882719	2021-09-02
hCoV-19/Japan/PG-74991/2021	EPI_ISL_3882727	2021-09-02
hCoV-19/Japan/PG-74992/2021	EPI_ISL_3882734	2021-09-02
hCoV-19/Japan/PG-74993/2021	EPI_ISL_3882743	2021-09-02
hCoV-19/Japan/PG-74994/2021	EPI_ISL_3882750	2021-09-02
hCoV-19/Japan/PG-74995/2021	EPI_ISL_3882757	2021-09-02
hCoV-19/Japan/PG-74996/2021	EPI_ISL_3882764	2021-09-02
hCoV-19/Japan/PG-74997/2021	EPI_ISL_3882772	2021-09-02
hCoV-19/Japan/PG-74998/2021	EPI_ISL_3882780	2021-09-02
hCoV-19/Japan/PG-74999/2021	EPI_ISL_3882787	2021-09-02
hCoV-19/Japan/PG-75000/2021	EPI_ISL_3882795	2021-09-02
hCoV-19/Japan/PG-75001/2021	EPI_ISL_3882802	2021-09-02
hCoV-19/Japan/PG-75002/2021	EPI_ISL_3882809	2021-09-02
hCoV-19/Japan/PG-75003/2021	EPI_ISL_3882818	2021-09-02

virus_name	epiID	submission_date
hCoV-19/Japan/PG-75004/2021	EPI_ISL_3882825	2021-09-02
hCoV-19/Japan/PG-75005/2021	EPI_ISL_3882833	2021-09-02
hCoV-19/Japan/PG-75006/2021	EPI_ISL_3882842	2021-09-02
hCoV-19/Japan/PG-75007/2021	EPI_ISL_3882850	2021-09-02
hCoV-19/Japan/PG-75008/2021	EPI_ISL_3882858	2021-09-02
hCoV-19/Japan/PG-75009/2021	EPI_ISL_3882866	2021-09-02
hCoV-19/Japan/PG-75010/2021	EPI_ISL_3882874	2021-09-02
hCoV-19/Japan/PG-75011/2021	EPI_ISL_3882883	2021-09-02
hCoV-19/Japan/PG-75012/2021	EPI_ISL_3882891	2021-09-02
hCoV-19/Japan/PG-75013/2021	EPI_ISL_3882899	2021-09-02
hCoV-19/Japan/PG-75014/2021	EPI_ISL_3882907	2021-09-02
hCoV-19/Japan/PG-75015/2021	EPI_ISL_3882915	2021-09-02
hCoV-19/Japan/PG-75016/2021	EPI_ISL_3882922	2021-09-02
hCoV-19/Japan/PG-75017/2021	EPI_ISL_3882928	2021-09-02
hCoV-19/Japan/PG-75018/2021	EPI_ISL_3882936	2021-09-02
hCoV-19/Japan/PG-75019/2021	EPI_ISL_3882944	2021-09-02
hCoV-19/Japan/PG-75020/2021	EPI_ISL_3882951	2021-09-02
hCoV-19/Japan/PG-75021/2021	EPI_ISL_3882956	2021-09-02
hCoV-19/Japan/PG-75022/2021	EPI_ISL_3882962	2021-09-02
hCoV-19/Japan/PG-75023/2021	EPI_ISL_3882970	2021-09-02
hCoV-19/Japan/PG-75024/2021	EPI_ISL_3882978	2021-09-02
hCoV-19/Japan/PG-75025/2021	EPI_ISL_3882985	2021-09-02
hCoV-19/Japan/PG-75026/2021	EPI_ISL_3882993	2021-09-02
hCoV-19/Japan/PG-75027/2021	EPI_ISL_3883001	2021-09-02
hCoV-19/Japan/PG-75028/2021	EPI_ISL_3883010	2021-09-02
hCoV-19/Japan/PG-75029/2021	EPI_ISL_3883017	2021-09-02
hCoV-19/Japan/PG-75030/2021	EPI_ISL_3883025	2021-09-02
hCoV-19/Japan/PG-75031/2021	EPI_ISL_3883032	2021-09-02
hCoV-19/Japan/PG-75032/2021	EPI_ISL_3883040	2021-09-02
hCoV-19/Japan/PG-75033/2021	EPI_ISL_3883048	2021-09-02
hCoV-19/Japan/PG-75034/2021	EPI_ISL_3883057	2021-09-02
hCoV-19/Japan/PG-75035/2021	EPI_ISL_3883066	2021-09-02
hCoV-19/Japan/PG-75036/2021	EPI_ISL_3883074	2021-09-02
hCoV-19/Japan/PG-75037/2021	EPI_ISL_3883083	2021-09-02
hCoV-19/Japan/PG-75038/2021	EPI_ISL_3883091	2021-09-02
hCoV-19/Japan/PG-75039/2021	EPI_ISL_3883099	2021-09-02
hCoV-19/Japan/PG-75040/2021	EPI_ISL_3883108	2021-09-02
hCoV-19/Japan/PG-75041/2021	EPI_ISL_3883114	2021-09-02
hCoV-19/Japan/PG-75042/2021	EPI_ISL_3883121	2021-09-02
hCoV-19/Japan/PG-75043/2021	EPI_ISL_3883129	2021-09-02
hCoV-19/Japan/PG-75044/2021	EPI_ISL_3883136	2021-09-02
hCoV-19/Japan/PG-75045/2021	EPI_ISL_3883144	2021-09-02
hCoV-19/Japan/PG-75046/2021	EPI_ISL_3883152	2021-09-02
hCoV-19/Japan/PG-75047/2021	EPI_ISL_3883160	2021-09-02
hCoV-19/Japan/PG-75048/2021	EPI_ISL_3883167	2021-09-02
hCoV-19/Japan/PG-75049/2021	EPI_ISL_3883174	2021-09-02
hCoV-19/Japan/PG-75050/2021	EPI_ISL_3883181	2021-09-02
hCoV-19/Japan/PG-75051/2021	EPI_ISL_3883189	2021-09-02
hCoV-19/Japan/PG-75052/2021	EPI_ISL_3883196	2021-09-02
hCoV-19/Japan/PG-75053/2021	EPI_ISL_3883203	2021-09-02
hCoV-19/Japan/PG-75054/2021	EPI_ISL_3883210	2021-09-02
hCoV-19/Japan/PG-75055/2021	EPI_ISL_3883218	2021-09-02
hCoV-19/Japan/PG-75056/2021	EPI_ISL_3883226	2021-09-02
hCoV-19/Japan/PG-75057/2021	EPI_ISL_3883233	2021-09-02

virus_name	epiID	submission_date
hCoV-19/Japan/PG-75058/2021	EPI_ISL_3883240	2021-09-02
hCoV-19/Japan/PG-75059/2021	EPI_ISL_3883245	2021-09-02
hCoV-19/Japan/PG-75060/2021	EPI_ISL_3883253	2021-09-02
hCoV-19/Japan/PG-75061/2021	EPI_ISL_3883261	2021-09-02
hCoV-19/Japan/PG-75062/2021	EPI_ISL_3883269	2021-09-02
hCoV-19/Japan/PG-75063/2021	EPI_ISL_3883276	2021-09-02
hCoV-19/Japan/PG-75064/2021	EPI_ISL_3883284	2021-09-02
hCoV-19/Japan/PG-75065/2021	EPI_ISL_3883292	2021-09-02
hCoV-19/Japan/PG-75066/2021	EPI_ISL_3883300	2021-09-02
hCoV-19/Japan/PG-75067/2021	EPI_ISL_3883308	2021-09-02
hCoV-19/Japan/PG-75068/2021	EPI_ISL_3883315	2021-09-02
hCoV-19/Japan/PG-75069/2021	EPI_ISL_3883321	2021-09-02
hCoV-19/Japan/PG-75070/2021	EPI_ISL_3883323	2021-09-02
hCoV-19/Japan/PG-75071/2021	EPI_ISL_3883325	2021-09-02
hCoV-19/Japan/PG-75072/2021	EPI_ISL_3883326	2021-09-02
hCoV-19/Japan/PG-75073/2021	EPI_ISL_3883328	2021-09-02
hCoV-19/Japan/PG-75074/2021	EPI_ISL_3883330	2021-09-02
hCoV-19/Japan/PG-75075/2021	EPI_ISL_3883332	2021-09-02
hCoV-19/Japan/PG-75076/2021	EPI_ISL_3883333	2021-09-02
hCoV-19/Japan/PG-75077/2021	EPI_ISL_3883334	2021-09-02
hCoV-19/Japan/PG-75078/2021	EPI_ISL_3883336	2021-09-02
hCoV-19/Japan/PG-75079/2021	EPI_ISL_3883337	2021-09-02
hCoV-19/Japan/PG-75080/2021	EPI_ISL_3883339	2021-09-02
hCoV-19/Japan/PG-75081/2021	EPI_ISL_3883340	2021-09-02
hCoV-19/Japan/PG-75082/2021	EPI_ISL_3883342	2021-09-02
hCoV-19/Japan/PG-75083/2021	EPI_ISL_3883344	2021-09-02
hCoV-19/Japan/PG-75084/2021	EPI_ISL_3883346	2021-09-02
hCoV-19/Japan/PG-75085/2021	EPI_ISL_3883349	2021-09-02
hCoV-19/Japan/PG-75086/2021	EPI_ISL_3883350	2021-09-02
hCoV-19/Japan/PG-75087/2021	EPI_ISL_3883352	2021-09-02
hCoV-19/Japan/PG-75088/2021	EPI_ISL_3883354	2021-09-02
hCoV-19/Japan/PG-75089/2021	EPI_ISL_3883356	2021-09-02
hCoV-19/Japan/PG-75090/2021	EPI_ISL_3883363	2021-09-02
hCoV-19/Japan/PG-75091/2021	EPI_ISL_3883369	2021-09-02
hCoV-19/Japan/PG-75092/2021	EPI_ISL_3883378	2021-09-02
hCoV-19/Japan/PG-75093/2021	EPI_ISL_3883386	2021-09-02
hCoV-19/Japan/PG-75094/2021	EPI_ISL_3883392	2021-09-02
hCoV-19/Japan/PG-75095/2021	EPI_ISL_3883398	2021-09-02
hCoV-19/Japan/PG-75096/2021	EPI_ISL_3883407	2021-09-02
hCoV-19/Japan/PG-75097/2021	EPI_ISL_3883415	2021-09-02
hCoV-19/Japan/PG-75098/2021	EPI_ISL_3883422	2021-09-02
hCoV-19/Japan/PG-75099/2021	EPI_ISL_3883429	2021-09-02
hCoV-19/Japan/PG-75100/2021	EPI_ISL_3883437	2021-09-02
hCoV-19/Japan/PG-75101/2021	EPI_ISL_3883444	2021-09-02
hCoV-19/Japan/PG-75102/2021	EPI_ISL_3883451	2021-09-02
hCoV-19/Japan/PG-75103/2021	EPI_ISL_3883458	2021-09-02
hCoV-19/Japan/PG-75104/2021	EPI_ISL_3883466	2021-09-02
hCoV-19/Japan/PG-75105/2021	EPI_ISL_3883473	2021-09-02
hCoV-19/Japan/PG-75106/2021	EPI_ISL_3883481	2021-09-02
hCoV-19/Japan/PG-75107/2021	EPI_ISL_3883489	2021-09-02
hCoV-19/Japan/PG-75108/2021	EPI_ISL_3883496	2021-09-02
hCoV-19/Japan/PG-75109/2021	EPI_ISL_3883504	2021-09-02
hCoV-19/Japan/PG-75110/2021	EPI_ISL_3883511	2021-09-02
hCoV-19/Japan/PG-75111/2021	EPI_ISL_3883518	2021-09-02

virus_name	epiID	submission_date
hCoV-19/Japan/PG-75112/2021	EPI_ISL_3883527	2021-09-02
hCoV-19/Japan/PG-75113/2021	EPI_ISL_3883534	2021-09-02
hCoV-19/Japan/PG-75114/2021	EPI_ISL_3883542	2021-09-02
hCoV-19/Japan/PG-75115/2021	EPI_ISL_3883549	2021-09-02
hCoV-19/Japan/PG-75116/2021	EPI_ISL_3883556	2021-09-02
hCoV-19/Japan/PG-75117/2021	EPI_ISL_3883565	2021-09-02
hCoV-19/Japan/PG-75118/2021	EPI_ISL_3883573	2021-09-02
hCoV-19/Japan/PG-75119/2021	EPI_ISL_3883577	2021-09-02
hCoV-19/Japan/PG-75120/2021	EPI_ISL_3883586	2021-09-02
hCoV-19/Japan/PG-75121/2021	EPI_ISL_3883593	2021-09-02
hCoV-19/Japan/PG-75122/2021	EPI_ISL_3883599	2021-09-02
hCoV-19/Japan/PG-75123/2021	EPI_ISL_3883607	2021-09-02
hCoV-19/Japan/PG-75124/2021	EPI_ISL_3883614	2021-09-02
hCoV-19/Japan/PG-75125/2021	EPI_ISL_3883623	2021-09-02
hCoV-19/Japan/PG-75126/2021	EPI_ISL_3883632	2021-09-02
hCoV-19/Japan/PG-75127/2021	EPI_ISL_3883638	2021-09-02
hCoV-19/Japan/PG-75128/2021	EPI_ISL_3883646	2021-09-02
hCoV-19/Japan/PG-75129/2021	EPI_ISL_3883654	2021-09-02
hCoV-19/Japan/PG-75130/2021	EPI_ISL_3883660	2021-09-02
hCoV-19/Japan/PG-75131/2021	EPI_ISL_3883668	2021-09-02
hCoV-19/Japan/PG-75132/2021	EPI_ISL_3883674	2021-09-02
hCoV-19/Japan/PG-75133/2021	EPI_ISL_3883682	2021-09-02
hCoV-19/Japan/PG-75134/2021	EPI_ISL_3883690	2021-09-02
hCoV-19/Japan/PG-75135/2021	EPI_ISL_3883698	2021-09-02
hCoV-19/Japan/PG-75136/2021	EPI_ISL_3883706	2021-09-02
hCoV-19/Japan/PG-75137/2021	EPI_ISL_3883713	2021-09-02
hCoV-19/Japan/PG-75138/2021	EPI_ISL_3883721	2021-09-02
hCoV-19/Japan/PG-75139/2021	EPI_ISL_3883729	2021-09-02
hCoV-19/Japan/PG-75140/2021	EPI_ISL_3883738	2021-09-02
hCoV-19/Japan/PG-75141/2021	EPI_ISL_3883745	2021-09-02
hCoV-19/Japan/PG-75142/2021	EPI_ISL_3883753	2021-09-02
hCoV-19/Japan/PG-75143/2021	EPI_ISL_3883760	2021-09-02
hCoV-19/Japan/PG-75144/2021	EPI_ISL_3883768	2021-09-02
hCoV-19/Japan/PG-75145/2021	EPI_ISL_3883776	2021-09-02
hCoV-19/Japan/PG-75146/2021	EPI_ISL_3883784	2021-09-02
hCoV-19/Japan/PG-75147/2021	EPI_ISL_3883794	2021-09-02
hCoV-19/Japan/PG-75148/2021	EPI_ISL_3883803	2021-09-02
hCoV-19/Japan/PG-75149/2021	EPI_ISL_3883810	2021-09-02
hCoV-19/Japan/PG-75150/2021	EPI_ISL_3883818	2021-09-02
hCoV-19/Japan/PG-75151/2021	EPI_ISL_3883825	2021-09-02
hCoV-19/Japan/PG-75152/2021	EPI_ISL_3883832	2021-09-02
hCoV-19/Japan/PG-75153/2021	EPI_ISL_3883840	2021-09-02
hCoV-19/Japan/PG-75154/2021	EPI_ISL_3883848	2021-09-02
hCoV-19/Japan/PG-75155/2021	EPI_ISL_3883856	2021-09-02
hCoV-19/Japan/PG-75156/2021	EPI_ISL_3883863	2021-09-02
hCoV-19/Japan/PG-75157/2021	EPI_ISL_3883870	2021-09-02
hCoV-19/Japan/PG-75158/2021	EPI_ISL_3883877	2021-09-02
hCoV-19/Japan/PG-75159/2021	EPI_ISL_3883884	2021-09-02
hCoV-19/Japan/PG-75160/2021	EPI_ISL_3883890	2021-09-02
hCoV-19/Japan/PG-75161/2021	EPI_ISL_3883897	2021-09-02
hCoV-19/Japan/PG-75162/2021	EPI_ISL_3883904	2021-09-02
hCoV-19/Japan/PG-75163/2021	EPI_ISL_3883911	2021-09-02
hCoV-19/Japan/PG-75164/2021	EPI_ISL_3883920	2021-09-02
hCoV-19/Japan/PG-75165/2021	EPI_ISL_3883927	2021-09-02

virus_name	epiID	submission_date
hCoV-19/Japan/PG-75166/2021	EPI_ISL_3883935	2021-09-02
hCoV-19/Japan/PG-75167/2021	EPI_ISL_3883944	2021-09-02
hCoV-19/Japan/PG-75168/2021	EPI_ISL_3883951	2021-09-02
hCoV-19/Japan/PG-75169/2021	EPI_ISL_3883961	2021-09-02
hCoV-19/Japan/PG-75170/2021	EPI_ISL_3883967	2021-09-02
hCoV-19/Japan/PG-75171/2021	EPI_ISL_3883975	2021-09-02
hCoV-19/Japan/PG-75172/2021	EPI_ISL_3883983	2021-09-02
hCoV-19/Japan/PG-75173/2021	EPI_ISL_3883988	2021-09-02
hCoV-19/Japan/PG-75174/2021	EPI_ISL_3883995	2021-09-02
hCoV-19/Japan/PG-75175/2021	EPI_ISL_3883997	2021-09-02
hCoV-19/Japan/PG-75176/2021	EPI_ISL_3884001	2021-09-02
hCoV-19/Japan/PG-75177/2021	EPI_ISL_3884008	2021-09-02
hCoV-19/Japan/PG-75178/2021	EPI_ISL_3884016	2021-09-02
hCoV-19/Japan/PG-75179/2021	EPI_ISL_3884023	2021-09-02
hCoV-19/Japan/PG-75180/2021	EPI_ISL_3884030	2021-09-02
hCoV-19/Japan/PG-75181/2021	EPI_ISL_3884035	2021-09-02
hCoV-19/Japan/PG-75182/2021	EPI_ISL_3884038	2021-09-02
hCoV-19/Japan/PG-75183/2021	EPI_ISL_3884040	2021-09-02
hCoV-19/Japan/PG-75184/2021	EPI_ISL_3884043	2021-09-02
hCoV-19/Japan/PG-75185/2021	EPI_ISL_3884045	2021-09-02
hCoV-19/Japan/PG-75186/2021	EPI_ISL_3884047	2021-09-02
hCoV-19/Japan/PG-75187/2021	EPI_ISL_3884049	2021-09-02
hCoV-19/Japan/PG-75188/2021	EPI_ISL_3884052	2021-09-02
hCoV-19/Japan/PG-75189/2021	EPI_ISL_3884054	2021-09-02
hCoV-19/Japan/PG-75190/2021	EPI_ISL_3884057	2021-09-02
hCoV-19/Japan/PG-75191/2021	EPI_ISL_3884059	2021-09-02
hCoV-19/Japan/PG-75192/2021	EPI_ISL_3884062	2021-09-02
hCoV-19/Japan/PG-75193/2021	EPI_ISL_3884064	2021-09-02
hCoV-19/Japan/PG-75194/2021	EPI_ISL_3884066	2021-09-02
hCoV-19/Japan/PG-75195/2021	EPI_ISL_3884068	2021-09-02
hCoV-19/Japan/PG-75196/2021	EPI_ISL_3884071	2021-09-02
hCoV-19/Japan/PG-75197/2021	EPI_ISL_3884072	2021-09-02
hCoV-19/Japan/PG-75198/2021	EPI_ISL_3884073	2021-09-02
hCoV-19/Japan/PG-75199/2021	EPI_ISL_3884075	2021-09-02
hCoV-19/Japan/PG-75200/2021	EPI_ISL_3884078	2021-09-02
hCoV-19/Japan/PG-75201/2021	EPI_ISL_3884084	2021-09-02
hCoV-19/Japan/PG-75202/2021	EPI_ISL_3884091	2021-09-02
hCoV-19/Japan/PG-75203/2021	EPI_ISL_3884098	2021-09-02
hCoV-19/Japan/PG-75204/2021	EPI_ISL_3884106	2021-09-02
hCoV-19/Japan/PG-75205/2021	EPI_ISL_3884114	2021-09-02
hCoV-19/Japan/PG-75206/2021	EPI_ISL_3884121	2021-09-02
hCoV-19/Japan/PG-75207/2021	EPI_ISL_3884130	2021-09-02
hCoV-19/Japan/PG-75208/2021	EPI_ISL_3884137	2021-09-02
hCoV-19/Japan/PG-75209/2021	EPI_ISL_3884143	2021-09-02
hCoV-19/Japan/PG-75210/2021	EPI_ISL_3884152	2021-09-02
hCoV-19/Japan/PG-75211/2021	EPI_ISL_3884160	2021-09-02
hCoV-19/Japan/PG-75212/2021	EPI_ISL_3884168	2021-09-02
hCoV-19/Japan/PG-75213/2021	EPI_ISL_3884176	2021-09-02
hCoV-19/Japan/PG-75214/2021	EPI_ISL_3884183	2021-09-02
hCoV-19/Japan/PG-75215/2021	EPI_ISL_3884191	2021-09-02
hCoV-19/Japan/PG-75216/2021	EPI_ISL_3884197	2021-09-02
hCoV-19/Japan/PG-75217/2021	EPI_ISL_3884206	2021-09-02
hCoV-19/Japan/PG-75218/2021	EPI_ISL_3884214	2021-09-02
hCoV-19/Japan/PG-75219/2021	EPI_ISL_3884221	2021-09-02

virus_name	epiID	submission_date
hCoV-19/Japan/PG-75220/2021	EPI_ISL_3884230	2021-09-02
hCoV-19/Japan/PG-75221/2021	EPI_ISL_3884237	2021-09-02
hCoV-19/Japan/PG-75222/2021	EPI_ISL_3884244	2021-09-02
hCoV-19/Japan/PG-75223/2021	EPI_ISL_3884252	2021-09-02
hCoV-19/Japan/PG-75224/2021	EPI_ISL_3884258	2021-09-02
hCoV-19/Japan/PG-75225/2021	EPI_ISL_3884266	2021-09-02
hCoV-19/Japan/PG-75226/2021	EPI_ISL_3884275	2021-09-02
hCoV-19/Japan/PG-75227/2021	EPI_ISL_3884282	2021-09-02
hCoV-19/Japan/PG-75228/2021	EPI_ISL_3884291	2021-09-02
hCoV-19/Japan/PG-75229/2021	EPI_ISL_3884299	2021-09-02
hCoV-19/Japan/PG-75230/2021	EPI_ISL_3884306	2021-09-02
hCoV-19/Japan/PG-75231/2021	EPI_ISL_3884312	2021-09-02
hCoV-19/Japan/PG-75232/2021	EPI_ISL_3884320	2021-09-02
hCoV-19/Japan/PG-75233/2021	EPI_ISL_3884328	2021-09-02
hCoV-19/Japan/PG-75234/2021	EPI_ISL_3884335	2021-09-02
hCoV-19/Japan/PG-75235/2021	EPI_ISL_3884343	2021-09-02
hCoV-19/Japan/PG-75236/2021	EPI_ISL_3884350	2021-09-02
hCoV-19/Japan/PG-75237/2021	EPI_ISL_3873659	2021-09-02
hCoV-19/Japan/PG-75238/2021	EPI_ISL_3873662	2021-09-02
hCoV-19/Japan/PG-75239/2021	EPI_ISL_3873664	2021-09-02
hCoV-19/Japan/PG-75240/2021	EPI_ISL_3873667	2021-09-02
hCoV-19/Japan/PG-75241/2021	EPI_ISL_3873669	2021-09-02
hCoV-19/Japan/PG-75242/2021	EPI_ISL_3873671	2021-09-02
hCoV-19/Japan/PG-75243/2021	EPI_ISL_3873672	2021-09-02
hCoV-19/Japan/PG-75244/2021	EPI_ISL_3873673	2021-09-02
hCoV-19/Japan/PG-75245/2021	EPI_ISL_3873674	2021-09-02
hCoV-19/Japan/PG-75246/2021	EPI_ISL_3873675	2021-09-02
hCoV-19/Japan/PG-75247/2021	EPI_ISL_3873676	2021-09-02
hCoV-19/Japan/PG-75248/2021	EPI_ISL_3873677	2021-09-02
hCoV-19/Japan/PG-75249/2021	EPI_ISL_3873678	2021-09-02
hCoV-19/Japan/PG-75250/2021	EPI_ISL_3873679	2021-09-02
hCoV-19/Japan/PG-75251/2021	EPI_ISL_3875523	2021-09-02
hCoV-19/Japan/PG-75252/2021	EPI_ISL_3875524	2021-09-02
hCoV-19/Japan/PG-75253/2021	EPI_ISL_3875525	2021-09-02
hCoV-19/Japan/PG-75254/2021	EPI_ISL_3875526	2021-09-02
hCoV-19/Japan/PG-75255/2021	EPI_ISL_3875527	2021-09-02
hCoV-19/Japan/PG-75256/2021	EPI_ISL_3875528	2021-09-02
hCoV-19/Japan/PG-75257/2021	EPI_ISL_3875529	2021-09-02
hCoV-19/Japan/PG-75258/2021	EPI_ISL_3875530	2021-09-02
hCoV-19/Japan/PG-75259/2021	EPI_ISL_3875531	2021-09-02
hCoV-19/Japan/PG-75260/2021	EPI_ISL_3875532	2021-09-02
hCoV-19/Japan/PG-75261/2021	EPI_ISL_3875533	2021-09-02
hCoV-19/Japan/PG-75262/2021	EPI_ISL_3875534	2021-09-02
hCoV-19/Japan/PG-75263/2021	EPI_ISL_3875535	2021-09-02
hCoV-19/Japan/PG-75264/2021	EPI_ISL_3875536	2021-09-02
hCoV-19/Japan/PG-75265/2021	EPI_ISL_3875537	2021-09-02
hCoV-19/Japan/PG-75266/2021	EPI_ISL_3875538	2021-09-02
hCoV-19/Japan/PG-75267/2021	EPI_ISL_3875539	2021-09-02
hCoV-19/Japan/PG-75268/2021	EPI_ISL_3875540	2021-09-02
hCoV-19/Japan/PG-75269/2021	EPI_ISL_3875541	2021-09-02
hCoV-19/Japan/PG-75270/2021	EPI_ISL_3875542	2021-09-02
hCoV-19/Japan/PG-75271/2021	EPI_ISL_3875543	2021-09-02
hCoV-19/Japan/PG-75272/2021	EPI_ISL_3875544	2021-09-02
hCoV-19/Japan/PG-75273/2021	EPI_ISL_3875545	2021-09-02

virus_name	epiID	submission_date
hCoV-19/Japan/PG-75274/2021	EPI_ISL_3875546	2021-09-02
hCoV-19/Japan/PG-75275/2021	EPI_ISL_3875547	2021-09-02
hCoV-19/Japan/PG-75276/2021	EPI_ISL_3875548	2021-09-02
hCoV-19/Japan/PG-75277/2021	EPI_ISL_3875549	2021-09-02
hCoV-19/Japan/PG-75278/2021	EPI_ISL_3875550	2021-09-02
hCoV-19/Japan/PG-75279/2021	EPI_ISL_3875551	2021-09-02
hCoV-19/Japan/PG-75280/2021	EPI_ISL_3875824	2021-09-02
hCoV-19/Japan/PG-75281/2021	EPI_ISL_3875825	2021-09-02
hCoV-19/Japan/PG-75282/2021	EPI_ISL_3875826	2021-09-02
hCoV-19/Japan/PG-75283/2021	EPI_ISL_3875827	2021-09-02
hCoV-19/Japan/PG-75284/2021	EPI_ISL_3875828	2021-09-02
hCoV-19/Japan/PG-75285/2021	EPI_ISL_3875829	2021-09-02
hCoV-19/Japan/PG-75286/2021	EPI_ISL_3875830	2021-09-02
hCoV-19/Japan/PG-75287/2021	EPI_ISL_3875831	2021-09-02
hCoV-19/Japan/PG-75288/2021	EPI_ISL_3875832	2021-09-02
hCoV-19/Japan/PG-75289/2021	EPI_ISL_3875833	2021-09-02
hCoV-19/Japan/PG-75290/2021	EPI_ISL_3875834	2021-09-02
hCoV-19/Japan/PG-75291/2021	EPI_ISL_3875835	2021-09-02
hCoV-19/Japan/PG-75292/2021	EPI_ISL_3875836	2021-09-02
hCoV-19/Japan/PG-75293/2021	EPI_ISL_3875837	2021-09-02
hCoV-19/Japan/PG-75294/2021	EPI_ISL_3875838	2021-09-02
hCoV-19/Japan/PG-75295/2021	EPI_ISL_3870615	2021-09-02
hCoV-19/Japan/PG-75296/2021	EPI_ISL_3870616	2021-09-02
hCoV-19/Japan/PG-75297/2021	EPI_ISL_3870617	2021-09-02
hCoV-19/Japan/PG-75298/2021	EPI_ISL_3870618	2021-09-02
hCoV-19/Japan/PG-75299/2021	EPI_ISL_3870619	2021-09-02
hCoV-19/Japan/PG-75300/2021	EPI_ISL_3870620	2021-09-02
hCoV-19/Japan/PG-75301/2021	EPI_ISL_3870621	2021-09-02
hCoV-19/Japan/PG-75302/2021	EPI_ISL_3870622	2021-09-02
hCoV-19/Japan/PG-75303/2021	EPI_ISL_3870623	2021-09-02
hCoV-19/Japan/PG-75304/2021	EPI_ISL_3870624	2021-09-02
hCoV-19/Japan/PG-75305/2021	EPI_ISL_3870625	2021-09-02
hCoV-19/Japan/PG-75306/2021	EPI_ISL_3870626	2021-09-02
hCoV-19/Japan/PG-75307/2021	EPI_ISL_3870627	2021-09-02
hCoV-19/Japan/PG-75308/2021	EPI_ISL_3870628	2021-09-02
hCoV-19/Japan/PG-75309/2021	EPI_ISL_3870629	2021-09-02
hCoV-19/Japan/PG-75310/2021	EPI_ISL_3872382	2021-09-02
hCoV-19/Japan/PG-75311/2021	EPI_ISL_3872383	2021-09-02
hCoV-19/Japan/PG-75312/2021	EPI_ISL_3872384	2021-09-02
hCoV-19/Japan/PG-75313/2021	EPI_ISL_3872385	2021-09-02
hCoV-19/Japan/PG-75314/2021	EPI_ISL_3872386	2021-09-02
hCoV-19/Japan/PG-75315/2021	EPI_ISL_3872387	2021-09-02
hCoV-19/Japan/PG-75316/2021	EPI_ISL_3872388	2021-09-02
hCoV-19/Japan/PG-75317/2021	EPI_ISL_3872389	2021-09-02
hCoV-19/Japan/PG-75318/2021	EPI_ISL_3872390	2021-09-02
hCoV-19/Japan/PG-75319/2021	EPI_ISL_3872391	2021-09-02
hCoV-19/Japan/PG-75320/2021	EPI_ISL_3872392	2021-09-02
hCoV-19/Japan/PG-75321/2021	EPI_ISL_3872393	2021-09-02
hCoV-19/Japan/PG-75322/2021	EPI_ISL_3872394	2021-09-02
hCoV-19/Japan/PG-75323/2021	EPI_ISL_3872395	2021-09-02
hCoV-19/Japan/PG-75324/2021	EPI_ISL_3872396	2021-09-02
hCoV-19/Japan/PG-75325/2021	EPI_ISL_3872397	2021-09-02
hCoV-19/Japan/PG-75326/2021	EPI_ISL_3872398	2021-09-02
hCoV-19/Japan/PG-75327/2021	EPI_ISL_3872399	2021-09-02

virus_name	epiID	submission_date
hCoV-19/Japan/PG-75328/2021	EPI_ISL_3872400	2021-09-02
hCoV-19/Japan/PG-75329/2021	EPI_ISL_3872401	2021-09-02
hCoV-19/Japan/PG-75330/2021	EPI_ISL_3871574	2021-09-02
hCoV-19/Japan/PG-75331/2021	EPI_ISL_3871575	2021-09-02
hCoV-19/Japan/PG-75332/2021	EPI_ISL_3871576	2021-09-02
hCoV-19/Japan/PG-75333/2021	EPI_ISL_3871577	2021-09-02
hCoV-19/Japan/PG-75334/2021	EPI_ISL_3871578	2021-09-02
hCoV-19/Japan/PG-75335/2021	EPI_ISL_3871579	2021-09-02
hCoV-19/Japan/PG-75336/2021	EPI_ISL_3871580	2021-09-02
hCoV-19/Japan/PG-75337/2021	EPI_ISL_3871581	2021-09-02
hCoV-19/Japan/PG-75338/2021	EPI_ISL_3871582	2021-09-02
hCoV-19/Japan/PG-75339/2021	EPI_ISL_3871583	2021-09-02
hCoV-19/Japan/PG-75340/2021	EPI_ISL_3871584	2021-09-02
hCoV-19/Japan/PG-75341/2021	EPI_ISL_3871585	2021-09-02
hCoV-19/Japan/PG-75342/2021	EPI_ISL_3871444	2021-09-02
hCoV-19/Japan/PG-75343/2021	EPI_ISL_3871445	2021-09-02
hCoV-19/Japan/PG-75344/2021	EPI_ISL_3871446	2021-09-02
hCoV-19/Japan/PG-75345/2021	EPI_ISL_3871447	2021-09-02
hCoV-19/Japan/PG-75346/2021	EPI_ISL_3871448	2021-09-02
hCoV-19/Japan/PG-75347/2021	EPI_ISL_3871449	2021-09-02
hCoV-19/Japan/PG-75348/2021	EPI_ISL_3871450	2021-09-02
hCoV-19/Japan/PG-75349/2021	EPI_ISL_3871451	2021-09-02
hCoV-19/Japan/PG-75350/2021	EPI_ISL_3874487	2021-09-02
hCoV-19/Japan/PG-75351/2021	EPI_ISL_3874488	2021-09-02
hCoV-19/Japan/PG-75352/2021	EPI_ISL_3874489	2021-09-02
hCoV-19/Japan/PG-75353/2021	EPI_ISL_3874490	2021-09-02
hCoV-19/Japan/PG-75354/2021	EPI_ISL_3874491	2021-09-02
hCoV-19/Japan/PG-75355/2021	EPI_ISL_3874492	2021-09-02
hCoV-19/Japan/PG-75356/2021	EPI_ISL_3874493	2021-09-02
hCoV-19/Japan/PG-75357/2021	EPI_ISL_3874494	2021-09-02
hCoV-19/Japan/PG-75358/2021	EPI_ISL_3874495	2021-09-02
hCoV-19/Japan/PG-75359/2021	EPI_ISL_3874496	2021-09-02
hCoV-19/Japan/PG-75360/2021	EPI_ISL_3871997	2021-09-02
hCoV-19/Japan/PG-75361/2021	EPI_ISL_3871998	2021-09-02
hCoV-19/Japan/PG-75362/2021	EPI_ISL_3871999	2021-09-02
hCoV-19/Japan/PG-75363/2021	EPI_ISL_3872000	2021-09-02
hCoV-19/Japan/PG-75364/2021	EPI_ISL_3872001	2021-09-02
hCoV-19/Japan/PG-75365/2021	EPI_ISL_3872002	2021-09-02
hCoV-19/Japan/PG-75366/2021	EPI_ISL_3872003	2021-09-02
hCoV-19/Japan/PG-75367/2021	EPI_ISL_3872004	2021-09-02
hCoV-19/Japan/PG-75368/2021	EPI_ISL_3872005	2021-09-02
hCoV-19/Japan/PG-75369/2021	EPI_ISL_3872006	2021-09-02
hCoV-19/Japan/PG-75370/2021	EPI_ISL_3872007	2021-09-02
hCoV-19/Japan/PG-75371/2021	EPI_ISL_3872008	2021-09-02
hCoV-19/Japan/PG-75372/2021	EPI_ISL_3872009	2021-09-02
hCoV-19/Japan/PG-75373/2021	EPI_ISL_3872010	2021-09-02
hCoV-19/Japan/PG-75374/2021	EPI_ISL_3872011	2021-09-02
hCoV-19/Japan/PG-75375/2021	EPI_ISL_3872012	2021-09-02
hCoV-19/Japan/PG-75376/2021	EPI_ISL_3872013	2021-09-02
hCoV-19/Japan/PG-75377/2021	EPI_ISL_3872014	2021-09-02
hCoV-19/Japan/PG-75378/2021	EPI_ISL_3872015	2021-09-02
hCoV-19/Japan/PG-75379/2021	EPI_ISL_3872016	2021-09-02
hCoV-19/Japan/PG-75380/2021	EPI_ISL_3872017	2021-09-02
hCoV-19/Japan/PG-75381/2021	EPI_ISL_3872018	2021-09-02

virus_name	epiID	submission_date
hCoV-19/Japan/PG-75382/2021	EPI_ISL_3872019	2021-09-02
hCoV-19/Japan/PG-75383/2021	EPI_ISL_3872020	2021-09-02
hCoV-19/Japan/PG-75384/2021	EPI_ISL_3872021	2021-09-02
hCoV-19/Japan/PG-75385/2021	EPI_ISL_3872022	2021-09-02
hCoV-19/Japan/PG-75386/2021	EPI_ISL_3872023	2021-09-02
hCoV-19/Japan/PG-75387/2021	EPI_ISL_3872024	2021-09-02
hCoV-19/Japan/PG-75388/2021	EPI_ISL_3872025	2021-09-02
hCoV-19/Japan/PG-75389/2021	EPI_ISL_3872026	2021-09-02
hCoV-19/Japan/PG-75390/2021	EPI_ISL_3872027	2021-09-02
hCoV-19/Japan/PG-75391/2021	EPI_ISL_3872028	2021-09-02
hCoV-19/Japan/PG-75392/2021	EPI_ISL_3872029	2021-09-02
hCoV-19/Japan/PG-75393/2021	EPI_ISL_3872030	2021-09-02
hCoV-19/Japan/PG-75394/2021	EPI_ISL_3872031	2021-09-02
hCoV-19/Japan/PG-75395/2021	EPI_ISL_3872032	2021-09-02
hCoV-19/Japan/PG-75396/2021	EPI_ISL_3872033	2021-09-02
hCoV-19/Japan/PG-75397/2021	EPI_ISL_3872034	2021-09-02
hCoV-19/Japan/PG-75398/2021	EPI_ISL_3872035	2021-09-02
hCoV-19/Japan/PG-75399/2021	EPI_ISL_3872036	2021-09-02
hCoV-19/Japan/PG-75400/2021	EPI_ISL_3872037	2021-09-02
hCoV-19/Japan/PG-75401/2021	EPI_ISL_3872038	2021-09-02
hCoV-19/Japan/PG-75402/2021	EPI_ISL_3872039	2021-09-02
hCoV-19/Japan/PG-75403/2021	EPI_ISL_3872040	2021-09-02
hCoV-19/Japan/PG-75404/2021	EPI_ISL_3874283	2021-09-02
hCoV-19/Japan/PG-75405/2021	EPI_ISL_3874284	2021-09-02
hCoV-19/Japan/PG-75406/2021	EPI_ISL_3874285	2021-09-02
hCoV-19/Japan/PG-75407/2021	EPI_ISL_3874286	2021-09-02
hCoV-19/Japan/PG-75408/2021	EPI_ISL_3874287	2021-09-02
hCoV-19/Japan/PG-75409/2021	EPI_ISL_3874288	2021-09-02
hCoV-19/Japan/PG-75410/2021	EPI_ISL_3874289	2021-09-02
hCoV-19/Japan/PG-75411/2021	EPI_ISL_3874290	2021-09-02
hCoV-19/Japan/PG-75412/2021	EPI_ISL_3874291	2021-09-02
hCoV-19/Japan/PG-75413/2021	EPI_ISL_3874292	2021-09-02
hCoV-19/Japan/PG-75414/2021	EPI_ISL_3874293	2021-09-02
hCoV-19/Japan/PG-75415/2021	EPI_ISL_3874294	2021-09-02
hCoV-19/Japan/PG-75416/2021	EPI_ISL_3874295	2021-09-02
hCoV-19/Japan/PG-75417/2021	EPI_ISL_3874296	2021-09-02
hCoV-19/Japan/PG-75418/2021	EPI_ISL_3874297	2021-09-02
hCoV-19/Japan/PG-75419/2021	EPI_ISL_3874298	2021-09-02
hCoV-19/Japan/PG-75420/2021	EPI_ISL_3874299	2021-09-02
hCoV-19/Japan/PG-75421/2021	EPI_ISL_3874300	2021-09-02
hCoV-19/Japan/PG-75422/2021	EPI_ISL_3874301	2021-09-02
hCoV-19/Japan/PG-75423/2021	EPI_ISL_3874302	2021-09-02
hCoV-19/Japan/PG-75424/2021	EPI_ISL_3874303	2021-09-02
hCoV-19/Japan/PG-75425/2021	EPI_ISL_3874304	2021-09-02
hCoV-19/Japan/PG-75426/2021	EPI_ISL_3873365	2021-09-02
hCoV-19/Japan/PG-75427/2021	EPI_ISL_3873366	2021-09-02
hCoV-19/Japan/PG-75428/2021	EPI_ISL_3873367	2021-09-02
hCoV-19/Japan/PG-75429/2021	EPI_ISL_3873368	2021-09-02
hCoV-19/Japan/PG-75430/2021	EPI_ISL_3873369	2021-09-02
hCoV-19/Japan/PG-75431/2021	EPI_ISL_3873370	2021-09-02
hCoV-19/Japan/PG-75432/2021	EPI_ISL_3873371	2021-09-02
hCoV-19/Japan/PG-75433/2021	EPI_ISL_3873372	2021-09-02
hCoV-19/Japan/PG-75434/2021	EPI_ISL_3873373	2021-09-02
hCoV-19/Japan/PG-75435/2021	EPI_ISL_3873374	2021-09-02

virus_name	epiID	submission_date
hCoV-19/Japan/PG-75436/2021	EPI_ISL_3873375	2021-09-02
hCoV-19/Japan/PG-75437/2021	EPI_ISL_3873376	2021-09-02
hCoV-19/Japan/PG-75438/2021	EPI_ISL_3874528	2021-09-02
hCoV-19/Japan/PG-75439/2021	EPI_ISL_3874529	2021-09-02
hCoV-19/Japan/PG-75440/2021	EPI_ISL_3874530	2021-09-02
hCoV-19/Japan/PG-75441/2021	EPI_ISL_3874531	2021-09-02
hCoV-19/Japan/PG-75442/2021	EPI_ISL_3874532	2021-09-02
hCoV-19/Japan/PG-75443/2021	EPI_ISL_3874533	2021-09-02
hCoV-19/Japan/PG-75444/2021	EPI_ISL_3874534	2021-09-02
hCoV-19/Japan/PG-75445/2021	EPI_ISL_3874535	2021-09-02
hCoV-19/Japan/PG-75446/2021	EPI_ISL_3874536	2021-09-02
hCoV-19/Japan/PG-75447/2021	EPI_ISL_3874537	2021-09-02
hCoV-19/Japan/PG-75448/2021	EPI_ISL_3874538	2021-09-02
hCoV-19/Japan/PG-75449/2021	EPI_ISL_3874539	2021-09-02
hCoV-19/Japan/PG-75450/2021	EPI_ISL_3874540	2021-09-02
hCoV-19/Japan/PG-75451/2021	EPI_ISL_3874541	2021-09-02
hCoV-19/Japan/PG-75452/2021	EPI_ISL_3874542	2021-09-02
hCoV-19/Japan/PG-75453/2021	EPI_ISL_3874543	2021-09-02
hCoV-19/Japan/PG-75454/2021	EPI_ISL_3874544	2021-09-02
hCoV-19/Japan/PG-75455/2021	EPI_ISL_3884359	2021-09-02
hCoV-19/Japan/PG-75456/2021	EPI_ISL_3884366	2021-09-02
hCoV-19/Japan/PG-75457/2021	EPI_ISL_3884374	2021-09-02
hCoV-19/Japan/PG-75458/2021	EPI_ISL_3884382	2021-09-02
hCoV-19/Japan/PG-75459/2021	EPI_ISL_3884390	2021-09-02
hCoV-19/Japan/PG-75460/2021	EPI_ISL_3884398	2021-09-02
hCoV-19/Japan/PG-75461/2021	EPI_ISL_3884405	2021-09-02
hCoV-19/Japan/PG-75462/2021	EPI_ISL_3884412	2021-09-02
hCoV-19/Japan/PG-75463/2021	EPI_ISL_3884418	2021-09-02
hCoV-19/Japan/PG-75464/2021	EPI_ISL_3884426	2021-09-02
hCoV-19/Japan/PG-75465/2021	EPI_ISL_3884431	2021-09-02
hCoV-19/Japan/PG-75466/2021	EPI_ISL_3884440	2021-09-02
hCoV-19/Japan/PG-75467/2021	EPI_ISL_3884447	2021-09-02
hCoV-19/Japan/PG-75468/2021	EPI_ISL_3884456	2021-09-02
hCoV-19/Japan/PG-75469/2021	EPI_ISL_3884464	2021-09-02
hCoV-19/Japan/PG-75470/2021	EPI_ISL_3884471	2021-09-02
hCoV-19/Japan/PG-75471/2021	EPI_ISL_3884479	2021-09-02
hCoV-19/Japan/PG-75472/2021	EPI_ISL_3884504	2021-09-02
hCoV-19/Japan/PG-75473/2021	EPI_ISL_3884513	2021-09-02
hCoV-19/Japan/PG-75474/2021	EPI_ISL_3884519	2021-09-02
hCoV-19/Japan/PG-75475/2021	EPI_ISL_3884529	2021-09-02
hCoV-19/Japan/PG-75476/2021	EPI_ISL_3884537	2021-09-02
hCoV-19/Japan/PG-75477/2021	EPI_ISL_3884546	2021-09-02
hCoV-19/Japan/PG-75478/2021	EPI_ISL_3884552	2021-09-02
hCoV-19/Japan/PG-75479/2021	EPI_ISL_3884559	2021-09-02
hCoV-19/Japan/PG-75480/2021	EPI_ISL_3884568	2021-09-02
hCoV-19/Japan/PG-75481/2021	EPI_ISL_3884577	2021-09-02
hCoV-19/Japan/PG-75482/2021	EPI_ISL_3884585	2021-09-02
hCoV-19/Japan/PG-75483/2021	EPI_ISL_3884593	2021-09-02
hCoV-19/Japan/PG-75484/2021	EPI_ISL_3884603	2021-09-02
hCoV-19/Japan/PG-75485/2021	EPI_ISL_3884610	2021-09-02
hCoV-19/Japan/PG-75486/2021	EPI_ISL_3884619	2021-09-02
hCoV-19/Japan/PG-75487/2021	EPI_ISL_3884628	2021-09-02
hCoV-19/Japan/PG-75488/2021	EPI_ISL_3884635	2021-09-02
hCoV-19/Japan/PG-75489/2021	EPI_ISL_3884644	2021-09-02

virus_name	epiID	submission_date
hCoV-19/Japan/PG-75490/2021	EPI_ISL_3884653	2021-09-02
hCoV-19/Japan/PG-75491/2021	EPI_ISL_3884662	2021-09-02
hCoV-19/Japan/PG-75492/2021	EPI_ISL_3884670	2021-09-02
hCoV-19/Japan/PG-75493/2021	EPI_ISL_3884677	2021-09-02
hCoV-19/Japan/PG-75494/2021	EPI_ISL_3884686	2021-09-02
hCoV-19/Japan/PG-75495/2021	EPI_ISL_3884695	2021-09-02
hCoV-19/Japan/PG-75496/2021	EPI_ISL_3884704	2021-09-02
hCoV-19/Japan/PG-75497/2021	EPI_ISL_3884712	2021-09-02
hCoV-19/Japan/PG-75498/2021	EPI_ISL_3884723	2021-09-02
hCoV-19/Japan/PG-75499/2021	EPI_ISL_3884732	2021-09-02
hCoV-19/Japan/PG-75500/2021	EPI_ISL_3884739	2021-09-02
hCoV-19/Japan/PG-75501/2021	EPI_ISL_3884749	2021-09-02
hCoV-19/Japan/PG-75502/2021	EPI_ISL_3884757	2021-09-02
hCoV-19/Japan/PG-75503/2021	EPI_ISL_3884765	2021-09-02
hCoV-19/Japan/PG-75504/2021	EPI_ISL_3884771	2021-09-02
hCoV-19/Japan/PG-75505/2021	EPI_ISL_3884773	2021-09-02
hCoV-19/Japan/PG-75506/2021	EPI_ISL_3884775	2021-09-02
hCoV-19/Japan/PG-75507/2021	EPI_ISL_3884776	2021-09-02
hCoV-19/Japan/PG-75508/2021	EPI_ISL_3884777	2021-09-02
hCoV-19/Japan/PG-75509/2021	EPI_ISL_3884778	2021-09-02
hCoV-19/Japan/PG-75510/2021	EPI_ISL_3884780	2021-09-02
hCoV-19/Japan/PG-75511/2021	EPI_ISL_3884782	2021-09-02
hCoV-19/Japan/PG-75512/2021	EPI_ISL_3884785	2021-09-02
hCoV-19/Japan/PG-75513/2021	EPI_ISL_3884787	2021-09-02
hCoV-19/Japan/PG-75514/2021	EPI_ISL_3884790	2021-09-02
hCoV-19/Japan/PG-75515/2021	EPI_ISL_3884792	2021-09-02
hCoV-19/Japan/PG-75516/2021	EPI_ISL_3884794	2021-09-02
hCoV-19/Japan/PG-75517/2021	EPI_ISL_3884796	2021-09-02
hCoV-19/Japan/PG-75518/2021	EPI_ISL_3884798	2021-09-02
hCoV-19/Japan/PG-75519/2021	EPI_ISL_3884800	2021-09-02
hCoV-19/Japan/PG-75520/2021	EPI_ISL_3884802	2021-09-02
hCoV-19/Japan/PG-75521/2021	EPI_ISL_3884804	2021-09-02
hCoV-19/Japan/PG-75522/2021	EPI_ISL_3884807	2021-09-02
hCoV-19/Japan/PG-75523/2021	EPI_ISL_3884809	2021-09-02
hCoV-19/Japan/PG-75524/2021	EPI_ISL_3884812	2021-09-02
hCoV-19/Japan/PG-75525/2021	EPI_ISL_3884820	2021-09-02
hCoV-19/Japan/PG-75526/2021	EPI_ISL_3884828	2021-09-02
hCoV-19/Japan/PG-75527/2021	EPI_ISL_3884835	2021-09-02
hCoV-19/Japan/PG-75528/2021	EPI_ISL_3884843	2021-09-02
hCoV-19/Japan/PG-75529/2021	EPI_ISL_3884849	2021-09-02
hCoV-19/Japan/PG-75530/2021	EPI_ISL_3884857	2021-09-02
hCoV-19/Japan/PG-75531/2021	EPI_ISL_3884866	2021-09-02
hCoV-19/Japan/PG-75532/2021	EPI_ISL_3884875	2021-09-02
hCoV-19/Japan/PG-75533/2021	EPI_ISL_3884884	2021-09-02
hCoV-19/Japan/PG-75534/2021	EPI_ISL_3884890	2021-09-02
hCoV-19/Japan/PG-75535/2021	EPI_ISL_3884896	2021-09-02
hCoV-19/Japan/PG-75536/2021	EPI_ISL_3884904	2021-09-02
hCoV-19/Japan/PG-75537/2021	EPI_ISL_3884913	2021-09-02
hCoV-19/Japan/PG-75538/2021	EPI_ISL_3884921	2021-09-02
hCoV-19/Japan/PG-75539/2021	EPI_ISL_3884928	2021-09-02
hCoV-19/Japan/PG-75540/2021	EPI_ISL_3884935	2021-09-02
hCoV-19/Japan/PG-75541/2021	EPI_ISL_3884941	2021-09-02
hCoV-19/Japan/PG-75542/2021	EPI_ISL_3884950	2021-09-02
hCoV-19/Japan/PG-75543/2021	EPI_ISL_3884956	2021-09-02

virus_name	epiID	submission_date
hCoV-19/Japan/PG-75544/2021	EPI_ISL_3884965	2021-09-02
hCoV-19/Japan/PG-75545/2021	EPI_ISL_3884974	2021-09-02
hCoV-19/Japan/PG-75546/2021	EPI_ISL_3884983	2021-09-02
hCoV-19/Japan/PG-75547/2021	EPI_ISL_3884991	2021-09-02
hCoV-19/Japan/PG-75548/2021	EPI_ISL_3884998	2021-09-02
hCoV-19/Japan/PG-75549/2021	EPI_ISL_3885007	2021-09-02
hCoV-19/Japan/PG-75550/2021	EPI_ISL_3885016	2021-09-02
hCoV-19/Japan/PG-75551/2021	EPI_ISL_3885022	2021-09-02
hCoV-19/Japan/PG-75552/2021	EPI_ISL_3885031	2021-09-02
hCoV-19/Japan/PG-75553/2021	EPI_ISL_3885039	2021-09-02
hCoV-19/Japan/PG-75554/2021	EPI_ISL_3885047	2021-09-02
hCoV-19/Japan/PG-75555/2021	EPI_ISL_3885054	2021-09-02
hCoV-19/Japan/PG-75556/2021	EPI_ISL_3885061	2021-09-02
hCoV-19/Japan/PG-75557/2021	EPI_ISL_3885068	2021-09-02
hCoV-19/Japan/PG-75558/2021	EPI_ISL_3885075	2021-09-02
hCoV-19/Japan/PG-75559/2021	EPI_ISL_3885080	2021-09-02
hCoV-19/Japan/PG-75560/2021	EPI_ISL_3885086	2021-09-02
hCoV-19/Japan/PG-75561/2021	EPI_ISL_3885093	2021-09-02
hCoV-19/Japan/PG-75562/2021	EPI_ISL_3885101	2021-09-02
hCoV-19/Japan/PG-75563/2021	EPI_ISL_3885108	2021-09-02
hCoV-19/Japan/PG-75564/2021	EPI_ISL_3885115	2021-09-02
hCoV-19/Japan/PG-75565/2021	EPI_ISL_3885124	2021-09-02
hCoV-19/Japan/PG-75566/2021	EPI_ISL_3885131	2021-09-02
hCoV-19/Japan/PG-75567/2021	EPI_ISL_3885139	2021-09-02
hCoV-19/Japan/PG-75568/2021	EPI_ISL_3885146	2021-09-02
hCoV-19/Japan/PG-75569/2021	EPI_ISL_3885152	2021-09-02
hCoV-19/Japan/PG-75570/2021	EPI_ISL_3885160	2021-09-02
hCoV-19/Japan/PG-75571/2021	EPI_ISL_3885166	2021-09-02
hCoV-19/Japan/PG-75572/2021	EPI_ISL_3885174	2021-09-02
hCoV-19/Japan/PG-75573/2021	EPI_ISL_3885182	2021-09-02
hCoV-19/Japan/PG-75574/2021	EPI_ISL_3885189	2021-09-02
hCoV-19/Japan/PG-75575/2021	EPI_ISL_3885196	2021-09-02
hCoV-19/Japan/PG-75576/2021	EPI_ISL_3885204	2021-09-02
hCoV-19/Japan/PG-75577/2021	EPI_ISL_3885212	2021-09-02
hCoV-19/Japan/PG-75578/2021	EPI_ISL_3885219	2021-09-02
hCoV-19/Japan/PG-75579/2021	EPI_ISL_3885227	2021-09-02
hCoV-19/Japan/PG-75580/2021	EPI_ISL_3885235	2021-09-02
hCoV-19/Japan/PG-75581/2021	EPI_ISL_3885242	2021-09-02
hCoV-19/Japan/PG-75582/2021	EPI_ISL_3885251	2021-09-02
hCoV-19/Japan/PG-75583/2021	EPI_ISL_3885258	2021-09-02
hCoV-19/Japan/PG-75584/2021	EPI_ISL_3885266	2021-09-02
hCoV-19/Japan/PG-75585/2021	EPI_ISL_3885274	2021-09-02
hCoV-19/Japan/PG-75586/2021	EPI_ISL_3885283	2021-09-02
hCoV-19/Japan/PG-75587/2021	EPI_ISL_3885290	2021-09-02
hCoV-19/Japan/PG-75588/2021	EPI_ISL_3885298	2021-09-02
hCoV-19/Japan/PG-75589/2021	EPI_ISL_3885305	2021-09-02
hCoV-19/Japan/PG-75590/2021	EPI_ISL_3885313	2021-09-02
hCoV-19/Japan/PG-75591/2021	EPI_ISL_3885322	2021-09-02
hCoV-19/Japan/PG-75592/2021	EPI_ISL_3885330	2021-09-02
hCoV-19/Japan/PG-75593/2021	EPI_ISL_3885338	2021-09-02
hCoV-19/Japan/PG-75594/2021	EPI_ISL_3885345	2021-09-02
hCoV-19/Japan/PG-75595/2021	EPI_ISL_3885352	2021-09-02
hCoV-19/Japan/PG-75596/2021	EPI_ISL_3885360	2021-09-02
hCoV-19/Japan/PG-75597/2021	EPI_ISL_3885368	2021-09-02

virus_name	epiID	submission_date
hCoV-19/Japan/PG-75598/2021	EPI_ISL_3885376	2021-09-02
hCoV-19/Japan/PG-75599/2021	EPI_ISL_3885379	2021-09-02
hCoV-19/Japan/PG-75600/2021	EPI_ISL_3885382	2021-09-02
hCoV-19/Japan/PG-75601/2021	EPI_ISL_3885384	2021-09-02
hCoV-19/Japan/PG-75602/2021	EPI_ISL_3885387	2021-09-02
hCoV-19/Japan/PG-75603/2021	EPI_ISL_3885389	2021-09-02
hCoV-19/Japan/PG-75604/2021	EPI_ISL_3885391	2021-09-02
hCoV-19/Japan/PG-75605/2021	EPI_ISL_3885394	2021-09-02
hCoV-19/Japan/PG-75606/2021	EPI_ISL_3885396	2021-09-02
hCoV-19/Japan/PG-75607/2021	EPI_ISL_3885398	2021-09-02
hCoV-19/Japan/PG-75608/2021	EPI_ISL_3885400	2021-09-02
hCoV-19/Japan/PG-75609/2021	EPI_ISL_3885402	2021-09-02
hCoV-19/Japan/PG-75610/2021	EPI_ISL_3885404	2021-09-02
hCoV-19/Japan/PG-75611/2021	EPI_ISL_3885406	2021-09-02
hCoV-19/Japan/PG-75612/2021	EPI_ISL_3885408	2021-09-02
hCoV-19/Japan/PG-75613/2021	EPI_ISL_3885411	2021-09-02
hCoV-19/Japan/PG-75614/2021	EPI_ISL_3885413	2021-09-02
hCoV-19/Japan/PG-75615/2021	EPI_ISL_3885416	2021-09-02
hCoV-19/Japan/PG-75616/2021	EPI_ISL_3885417	2021-09-02
hCoV-19/Japan/PG-75617/2021	EPI_ISL_3885419	2021-09-02
hCoV-19/Japan/PG-75618/2021	EPI_ISL_3885420	2021-09-02
hCoV-19/Japan/PG-75619/2021	EPI_ISL_3885422	2021-09-02
hCoV-19/Japan/PG-75620/2021	EPI_ISL_3885425	2021-09-02
hCoV-19/Japan/PG-75621/2021	EPI_ISL_3885427	2021-09-02
hCoV-19/Japan/PG-75622/2021	EPI_ISL_3885430	2021-09-02
hCoV-19/Japan/PG-75623/2021	EPI_ISL_3885432	2021-09-02
hCoV-19/Japan/PG-75624/2021	EPI_ISL_3885434	2021-09-02
hCoV-19/Japan/PG-75625/2021	EPI_ISL_3885437	2021-09-02
hCoV-19/Japan/PG-75626/2021	EPI_ISL_3885438	2021-09-02
hCoV-19/Japan/PG-75627/2021	EPI_ISL_3885441	2021-09-02
hCoV-19/Japan/PG-75628/2021	EPI_ISL_3885443	2021-09-02
hCoV-19/Japan/PG-75629/2021	EPI_ISL_3885446	2021-09-02
hCoV-19/Japan/PG-75630/2021	EPI_ISL_3885448	2021-09-02
hCoV-19/Japan/PG-75631/2021	EPI_ISL_3885450	2021-09-02
hCoV-19/Japan/PG-75632/2021	EPI_ISL_3885453	2021-09-02
hCoV-19/Japan/PG-75633/2021	EPI_ISL_3885455	2021-09-02
hCoV-19/Japan/PG-75634/2021	EPI_ISL_3885457	2021-09-02
hCoV-19/Japan/PG-75635/2021	EPI_ISL_3885459	2021-09-02
hCoV-19/Japan/PG-75636/2021	EPI_ISL_3885460	2021-09-02
hCoV-19/Japan/PG-75637/2021	EPI_ISL_3885462	2021-09-02
hCoV-19/Japan/PG-75638/2021	EPI_ISL_3885464	2021-09-02
hCoV-19/Japan/PG-75639/2021	EPI_ISL_3885466	2021-09-02
hCoV-19/Japan/PG-75640/2021	EPI_ISL_3885468	2021-09-02
hCoV-19/Japan/PG-75641/2021	EPI_ISL_3885471	2021-09-02
hCoV-19/Japan/PG-75642/2021	EPI_ISL_3885473	2021-09-02
hCoV-19/Japan/PG-75643/2021	EPI_ISL_3885475	2021-09-02
hCoV-19/Japan/PG-75644/2021	EPI_ISL_3885477	2021-09-02
hCoV-19/Japan/PG-75645/2021	EPI_ISL_3885479	2021-09-02
hCoV-19/Japan/PG-75646/2021	EPI_ISL_3885480	2021-09-02
hCoV-19/Japan/PG-75647/2021	EPI_ISL_3885483	2021-09-02
hCoV-19/Japan/PG-75648/2021	EPI_ISL_3885484	2021-09-02
hCoV-19/Japan/PG-75649/2021	EPI_ISL_3885485	2021-09-02
hCoV-19/Japan/PG-75650/2021	EPI_ISL_3885488	2021-09-02
hCoV-19/Japan/PG-75651/2021	EPI_ISL_3885489	2021-09-02

virus_name	epiID	submission_date
hCoV-19/Japan/PG-75652/2021	EPI_ISL_3885491	2021-09-02
hCoV-19/Japan/PG-75653/2021	EPI_ISL_3885493	2021-09-02
hCoV-19/Japan/PG-75654/2021	EPI_ISL_3885495	2021-09-02
hCoV-19/Japan/PG-75655/2021	EPI_ISL_3885497	2021-09-02
hCoV-19/Japan/PG-75656/2021	EPI_ISL_3885499	2021-09-02
hCoV-19/Japan/PG-75657/2021	EPI_ISL_3885502	2021-09-02
hCoV-19/Japan/PG-75658/2021	EPI_ISL_3885505	2021-09-02
hCoV-19/Japan/PG-75659/2021	EPI_ISL_3885506	2021-09-02
hCoV-19/Japan/PG-75660/2021	EPI_ISL_3885509	2021-09-02
hCoV-19/Japan/PG-75661/2021	EPI_ISL_3885512	2021-09-02
hCoV-19/Japan/PG-75662/2021	EPI_ISL_3885513	2021-09-02
hCoV-19/Japan/PG-75663/2021	EPI_ISL_3885516	2021-09-02
hCoV-19/Japan/PG-75664/2021	EPI_ISL_3885518	2021-09-02
hCoV-19/Japan/PG-75665/2021	EPI_ISL_3885521	2021-09-02
hCoV-19/Japan/PG-75666/2021	EPI_ISL_3885524	2021-09-02
hCoV-19/Japan/PG-75667/2021	EPI_ISL_3885526	2021-09-02
hCoV-19/Japan/PG-75668/2021	EPI_ISL_3885528	2021-09-02
hCoV-19/Japan/PG-75669/2021	EPI_ISL_3885530	2021-09-02
hCoV-19/Japan/PG-75670/2021	EPI_ISL_3885533	2021-09-02
hCoV-19/Japan/PG-75671/2021	EPI_ISL_3885535	2021-09-02
hCoV-19/Japan/PG-75672/2021	EPI_ISL_3885538	2021-09-02
hCoV-19/Japan/PG-75673/2021	EPI_ISL_3885540	2021-09-02
hCoV-19/Japan/PG-75674/2021	EPI_ISL_3885542	2021-09-02
hCoV-19/Japan/PG-75675/2021	EPI_ISL_3885544	2021-09-02
hCoV-19/Japan/PG-75676/2021	EPI_ISL_3885547	2021-09-02
hCoV-19/Japan/PG-75677/2021	EPI_ISL_3885549	2021-09-02
hCoV-19/Japan/PG-75678/2021	EPI_ISL_3885551	2021-09-02
hCoV-19/Japan/PG-75679/2021	EPI_ISL_3885553	2021-09-02
hCoV-19/Japan/PG-75680/2021	EPI_ISL_3885556	2021-09-02
hCoV-19/Japan/PG-75681/2021	EPI_ISL_3885557	2021-09-02
hCoV-19/Japan/PG-75682/2021	EPI_ISL_3885558	2021-09-02
hCoV-19/Japan/PG-75683/2021	EPI_ISL_3885560	2021-09-02
hCoV-19/Japan/PG-75684/2021	EPI_ISL_3885563	2021-09-02
hCoV-19/Japan/PG-75685/2021	EPI_ISL_3885565	2021-09-02
hCoV-19/Japan/PG-75686/2021	EPI_ISL_3885566	2021-09-02
hCoV-19/Japan/PG-75687/2021	EPI_ISL_3885567	2021-09-02
hCoV-19/Japan/PG-75688/2021	EPI_ISL_3885569	2021-09-02
hCoV-19/Japan/PG-75689/2021	EPI_ISL_3885571	2021-09-02
hCoV-19/Japan/PG-75690/2021	EPI_ISL_3885573	2021-09-02
hCoV-19/Japan/PG-75691/2021	EPI_ISL_3885575	2021-09-02
hCoV-19/Japan/PG-75692/2021	EPI_ISL_3885577	2021-09-02
hCoV-19/Japan/PG-75693/2021	EPI_ISL_3885580	2021-09-02
hCoV-19/Japan/PG-75694/2021	EPI_ISL_3885583	2021-09-02
hCoV-19/Japan/PG-75695/2021	EPI_ISL_3885585	2021-09-02
hCoV-19/Japan/PG-75696/2021	EPI_ISL_3885587	2021-09-02
hCoV-19/Japan/PG-75697/2021	EPI_ISL_3885590	2021-09-02
hCoV-19/Japan/PG-75698/2021	EPI_ISL_3885593	2021-09-02
hCoV-19/Japan/PG-75699/2021	EPI_ISL_3885595	2021-09-02
hCoV-19/Japan/PG-75700/2021	EPI_ISL_3885598	2021-09-02
hCoV-19/Japan/PG-75701/2021	EPI_ISL_3885600	2021-09-02
hCoV-19/Japan/PG-75702/2021	EPI_ISL_3885602	2021-09-02
hCoV-19/Japan/PG-75703/2021	EPI_ISL_3885604	2021-09-02
hCoV-19/Japan/PG-75704/2021	EPI_ISL_3885606	2021-09-02
hCoV-19/Japan/PG-75705/2021	EPI_ISL_3885608	2021-09-02

virus_name	epiID	submission_date
hCoV-19/Japan/PG-75706/2021	EPI_ISL_3885610	2021-09-02
hCoV-19/Japan/PG-75707/2021	EPI_ISL_3885612	2021-09-02
hCoV-19/Japan/PG-75708/2021	EPI_ISL_3885614	2021-09-02
hCoV-19/Japan/PG-75709/2021	EPI_ISL_3885616	2021-09-02
hCoV-19/Japan/PG-75710/2021	EPI_ISL_3885618	2021-09-02
hCoV-19/Japan/PG-75711/2021	EPI_ISL_3885620	2021-09-02
hCoV-19/Japan/PG-75712/2021	EPI_ISL_3885623	2021-09-02
hCoV-19/Japan/PG-75713/2021	EPI_ISL_3885625	2021-09-02
hCoV-19/Japan/PG-75714/2021	EPI_ISL_3885628	2021-09-02
hCoV-19/Japan/PG-75715/2021	EPI_ISL_3885630	2021-09-02
hCoV-19/Japan/PG-75716/2021	EPI_ISL_3885632	2021-09-02
hCoV-19/Japan/PG-75717/2021	EPI_ISL_3885635	2021-09-02
hCoV-19/Japan/PG-75718/2021	EPI_ISL_3885637	2021-09-02
hCoV-19/Japan/PG-75719/2021	EPI_ISL_3885641	2021-09-02
hCoV-19/Japan/PG-75720/2021	EPI_ISL_3885645	2021-09-02
hCoV-19/Japan/PG-75721/2021	EPI_ISL_3885648	2021-09-02
hCoV-19/Japan/PG-75722/2021	EPI_ISL_3885653	2021-09-02
hCoV-19/Japan/PG-75723/2021	EPI_ISL_3885657	2021-09-02
hCoV-19/Japan/PG-75724/2021	EPI_ISL_3885661	2021-09-02
hCoV-19/Japan/PG-75725/2021	EPI_ISL_3885666	2021-09-02
hCoV-19/Japan/PG-75726/2021	EPI_ISL_3885670	2021-09-02
hCoV-19/Japan/PG-75727/2021	EPI_ISL_3885675	2021-09-02
hCoV-19/Japan/PG-75728/2021	EPI_ISL_3885678	2021-09-02
hCoV-19/Japan/PG-75729/2021	EPI_ISL_3885682	2021-09-02
hCoV-19/Japan/PG-75730/2021	EPI_ISL_3885687	2021-09-02
hCoV-19/Japan/PG-75731/2021	EPI_ISL_3885692	2021-09-02
hCoV-19/Japan/PG-75732/2021	EPI_ISL_3885697	2021-09-02
hCoV-19/Japan/PG-75733/2021	EPI_ISL_3885701	2021-09-02
hCoV-19/Japan/PG-75734/2021	EPI_ISL_3885705	2021-09-02
hCoV-19/Japan/PG-75735/2021	EPI_ISL_3885708	2021-09-02
hCoV-19/Japan/PG-75736/2021	EPI_ISL_3885710	2021-09-02
hCoV-19/Japan/PG-75737/2021	EPI_ISL_3885711	2021-09-02
hCoV-19/Japan/PG-75738/2021	EPI_ISL_3885712	2021-09-02
hCoV-19/Japan/PG-75739/2021	EPI_ISL_3885715	2021-09-02
hCoV-19/Japan/PG-75740/2021	EPI_ISL_3885718	2021-09-02
hCoV-19/Japan/PG-75741/2021	EPI_ISL_3885720	2021-09-02
hCoV-19/Japan/PG-75742/2021	EPI_ISL_3885723	2021-09-02
hCoV-19/Japan/PG-75743/2021	EPI_ISL_3885725	2021-09-02
hCoV-19/Japan/PG-75744/2021	EPI_ISL_3885727	2021-09-02
hCoV-19/Japan/PG-75745/2021	EPI_ISL_3885728	2021-09-02
hCoV-19/Japan/PG-75746/2021	EPI_ISL_3885731	2021-09-02
hCoV-19/Japan/PG-75747/2021	EPI_ISL_3885733	2021-09-02
hCoV-19/Japan/PG-75748/2021	EPI_ISL_3885736	2021-09-02
hCoV-19/Japan/PG-75749/2021	EPI_ISL_3885738	2021-09-02
hCoV-19/Japan/PG-75750/2021	EPI_ISL_3885740	2021-09-02
hCoV-19/Japan/PG-75751/2021	EPI_ISL_3885743	2021-09-02
hCoV-19/Japan/PG-75752/2021	EPI_ISL_3885744	2021-09-02
hCoV-19/Japan/PG-75753/2021	EPI_ISL_3885747	2021-09-02
hCoV-19/Japan/PG-75754/2021	EPI_ISL_3885749	2021-09-02
hCoV-19/Japan/PG-75755/2021	EPI_ISL_3885751	2021-09-02
hCoV-19/Japan/PG-75756/2021	EPI_ISL_3885754	2021-09-02
hCoV-19/Japan/PG-75757/2021	EPI_ISL_3885756	2021-09-02
hCoV-19/Japan/PG-75758/2021	EPI_ISL_3885758	2021-09-02
hCoV-19/Japan/PG-75759/2021	EPI_ISL_3885760	2021-09-02

virus_name	epiID	submission_date
hCoV-19/Japan/PG-75760/2021	EPI_ISL_3885763	2021-09-02
hCoV-19/Japan/PG-75761/2021	EPI_ISL_3885765	2021-09-02
hCoV-19/Japan/PG-75762/2021	EPI_ISL_3885766	2021-09-02
hCoV-19/Japan/PG-75763/2021	EPI_ISL_3885768	2021-09-02
hCoV-19/Japan/PG-75764/2021	EPI_ISL_3885771	2021-09-02
hCoV-19/Japan/PG-75765/2021	EPI_ISL_3885773	2021-09-02
hCoV-19/Japan/PG-75766/2021	EPI_ISL_3885776	2021-09-02
hCoV-19/Japan/PG-75767/2021	EPI_ISL_3885778	2021-09-02
hCoV-19/Japan/PG-75768/2021	EPI_ISL_3885780	2021-09-02
hCoV-19/Japan/PG-75769/2021	EPI_ISL_3885783	2021-09-02
hCoV-19/Japan/PG-75770/2021	EPI_ISL_3885785	2021-09-02
hCoV-19/Japan/PG-75771/2021	EPI_ISL_3885786	2021-09-02
hCoV-19/Japan/PG-75772/2021	EPI_ISL_3885788	2021-09-02
hCoV-19/Japan/PG-75773/2021	EPI_ISL_3885791	2021-09-02
hCoV-19/Japan/PG-75774/2021	EPI_ISL_3885792	2021-09-02
hCoV-19/Japan/PG-75775/2021	EPI_ISL_3885794	2021-09-02
hCoV-19/Japan/PG-75776/2021	EPI_ISL_3885796	2021-09-02
hCoV-19/Japan/PG-75777/2021	EPI_ISL_3885799	2021-09-02
hCoV-19/Japan/PG-75778/2021	EPI_ISL_3885802	2021-09-02
hCoV-19/Japan/PG-75779/2021	EPI_ISL_3885804	2021-09-02
hCoV-19/Japan/PG-75780/2021	EPI_ISL_3885805	2021-09-02
hCoV-19/Japan/PG-75781/2021	EPI_ISL_3885808	2021-09-02
hCoV-19/Japan/PG-75782/2021	EPI_ISL_3885810	2021-09-02
hCoV-19/Japan/PG-75783/2021	EPI_ISL_3885811	2021-09-02
hCoV-19/Japan/PG-75784/2021	EPI_ISL_3885814	2021-09-02
hCoV-19/Japan/PG-75785/2021	EPI_ISL_3885816	2021-09-02
hCoV-19/Japan/PG-75786/2021	EPI_ISL_3885819	2021-09-02
hCoV-19/Japan/PG-75787/2021	EPI_ISL_3885821	2021-09-02
hCoV-19/Japan/PG-75788/2021	EPI_ISL_3885823	2021-09-02
hCoV-19/Japan/PG-75789/2021	EPI_ISL_3885826	2021-09-02
hCoV-19/Japan/PG-75790/2021	EPI_ISL_3885827	2021-09-02
hCoV-19/Japan/PG-75791/2021	EPI_ISL_3885830	2021-09-02
hCoV-19/Japan/PG-75792/2021	EPI_ISL_3885833	2021-09-02
hCoV-19/Japan/PG-75793/2021	EPI_ISL_3885835	2021-09-02
hCoV-19/Japan/PG-75794/2021	EPI_ISL_3885837	2021-09-02
hCoV-19/Japan/PG-75795/2021	EPI_ISL_3885840	2021-09-02
hCoV-19/Japan/PG-75796/2021	EPI_ISL_3885842	2021-09-02
hCoV-19/Japan/PG-75797/2021	EPI_ISL_3885845	2021-09-02
hCoV-19/Japan/PG-75798/2021	EPI_ISL_3885847	2021-09-02
hCoV-19/Japan/PG-75799/2021	EPI_ISL_3885850	2021-09-02
hCoV-19/Japan/PG-75800/2021	EPI_ISL_3885852	2021-09-02
hCoV-19/Japan/PG-75801/2021	EPI_ISL_3885854	2021-09-02
hCoV-19/Japan/PG-75802/2021	EPI_ISL_3885856	2021-09-02
hCoV-19/Japan/PG-75803/2021	EPI_ISL_3885859	2021-09-02
hCoV-19/Japan/PG-75804/2021	EPI_ISL_3885860	2021-09-02
hCoV-19/Japan/PG-75805/2021	EPI_ISL_3885863	2021-09-02
hCoV-19/Japan/PG-75806/2021	EPI_ISL_3885865	2021-09-02
hCoV-19/Japan/PG-75807/2021	EPI_ISL_3885868	2021-09-02
hCoV-19/Japan/PG-75808/2021	EPI_ISL_3885870	2021-09-02
hCoV-19/Japan/PG-75809/2021	EPI_ISL_3885873	2021-09-02
hCoV-19/Japan/PG-75810/2021	EPI_ISL_3885875	2021-09-02
hCoV-19/Japan/PG-75811/2021	EPI_ISL_3885876	2021-09-02
hCoV-19/Japan/PG-75812/2021	EPI_ISL_3885878	2021-09-02
hCoV-19/Japan/PG-75813/2021	EPI_ISL_3885880	2021-09-02

virus_name	epiID	submission_date
hCoV-19/Japan/PG-75814/2021	EPI_ISL_3885881	2021-09-02
hCoV-19/Japan/PG-75815/2021	EPI_ISL_3885883	2021-09-02
hCoV-19/Japan/PG-75816/2021	EPI_ISL_3885885	2021-09-02
hCoV-19/Japan/PG-75817/2021	EPI_ISL_3885887	2021-09-02
hCoV-19/Japan/PG-75818/2021	EPI_ISL_3885889	2021-09-02
hCoV-19/Japan/PG-75819/2021	EPI_ISL_3885891	2021-09-02
hCoV-19/Japan/PG-75820/2021	EPI_ISL_3885893	2021-09-02
hCoV-19/Japan/PG-75821/2021	EPI_ISL_3885896	2021-09-02
hCoV-19/Japan/PG-75822/2021	EPI_ISL_3885898	2021-09-02
hCoV-19/Japan/PG-75823/2021	EPI_ISL_3885900	2021-09-02
hCoV-19/Japan/PG-75824/2021	EPI_ISL_3885902	2021-09-02
hCoV-19/Japan/PG-75825/2021	EPI_ISL_3885905	2021-09-02
hCoV-19/Japan/PG-75826/2021	EPI_ISL_3885907	2021-09-02
hCoV-19/Japan/PG-75827/2021	EPI_ISL_3885908	2021-09-02
hCoV-19/Japan/PG-75828/2021	EPI_ISL_3885909	2021-09-02
hCoV-19/Japan/PG-75829/2021	EPI_ISL_3885912	2021-09-02
hCoV-19/Japan/PG-75830/2021	EPI_ISL_3885914	2021-09-02
hCoV-19/Japan/PG-75831/2021	EPI_ISL_3885917	2021-09-02
hCoV-19/Japan/PG-75832/2021	EPI_ISL_3885918	2021-09-02
hCoV-19/Japan/PG-75833/2021	EPI_ISL_3885920	2021-09-02
hCoV-19/Japan/PG-75834/2021	EPI_ISL_3885922	2021-09-02
hCoV-19/Japan/PG-75835/2021	EPI_ISL_3885924	2021-09-02
hCoV-19/Japan/PG-75836/2021	EPI_ISL_3885927	2021-09-02
hCoV-19/Japan/PG-75837/2021	EPI_ISL_3885930	2021-09-02
hCoV-19/Japan/PG-75838/2021	EPI_ISL_3885932	2021-09-02
hCoV-19/Japan/PG-75839/2021	EPI_ISL_3885935	2021-09-02
hCoV-19/Japan/PG-75840/2021	EPI_ISL_3885937	2021-09-02
hCoV-19/Japan/PG-75841/2021	EPI_ISL_3885940	2021-09-02
hCoV-19/Japan/PG-75842/2021	EPI_ISL_3885942	2021-09-02
hCoV-19/Japan/PG-75843/2021	EPI_ISL_3885944	2021-09-02
hCoV-19/Japan/PG-75844/2021	EPI_ISL_3885947	2021-09-02
hCoV-19/Japan/PG-75845/2021	EPI_ISL_3885950	2021-09-02
hCoV-19/Japan/PG-75846/2021	EPI_ISL_3885952	2021-09-02
hCoV-19/Japan/PG-75847/2021	EPI_ISL_3885955	2021-09-02
hCoV-19/Japan/PG-75848/2021	EPI_ISL_3885958	2021-09-02
hCoV-19/Japan/PG-75849/2021	EPI_ISL_3885960	2021-09-02
hCoV-19/Japan/PG-75850/2021	EPI_ISL_3885962	2021-09-02
hCoV-19/Japan/PG-75851/2021	EPI_ISL_3885965	2021-09-02
hCoV-19/Japan/PG-75852/2021	EPI_ISL_3885968	2021-09-02
hCoV-19/Japan/PG-75853/2021	EPI_ISL_3885970	2021-09-02
hCoV-19/Japan/PG-75854/2021	EPI_ISL_3885973	2021-09-02
hCoV-19/Japan/PG-75855/2021	EPI_ISL_3885975	2021-09-02
hCoV-19/Japan/PG-75856/2021	EPI_ISL_3885977	2021-09-02
hCoV-19/Japan/PG-75857/2021	EPI_ISL_3885980	2021-09-02
hCoV-19/Japan/PG-75858/2021	EPI_ISL_3885983	2021-09-02
hCoV-19/Japan/PG-75859/2021	EPI_ISL_3885985	2021-09-02
hCoV-19/Japan/PG-75860/2021	EPI_ISL_3885987	2021-09-02
hCoV-19/Japan/PG-75861/2021	EPI_ISL_3885989	2021-09-02
hCoV-19/Japan/PG-75862/2021	EPI_ISL_3885991	2021-09-02
hCoV-19/Japan/PG-75863/2021	EPI_ISL_3885993	2021-09-02
hCoV-19/Japan/PG-75864/2021	EPI_ISL_3885994	2021-09-02
hCoV-19/Japan/PG-75865/2021	EPI_ISL_3885996	2021-09-02
hCoV-19/Japan/PG-75866/2021	EPI_ISL_3885998	2021-09-02
hCoV-19/Japan/PG-75867/2021	EPI_ISL_3886000	2021-09-02

virus_name	epiID	submission_date
hCoV-19/Japan/PG-75868/2021	EPI_ISL_3886003	2021-09-02
hCoV-19/Japan/PG-75869/2021	EPI_ISL_3886005	2021-09-02
hCoV-19/Japan/PG-75870/2021	EPI_ISL_3886007	2021-09-02
hCoV-19/Japan/PG-75871/2021	EPI_ISL_3886009	2021-09-02
hCoV-19/Japan/PG-75872/2021	EPI_ISL_3886012	2021-09-02
hCoV-19/Japan/PG-75873/2021	EPI_ISL_3886014	2021-09-02
hCoV-19/Japan/PG-75874/2021	EPI_ISL_3886017	2021-09-02
hCoV-19/Japan/PG-75875/2021	EPI_ISL_3886019	2021-09-02
hCoV-19/Japan/PG-75876/2021	EPI_ISL_3886021	2021-09-02
hCoV-19/Japan/PG-75877/2021	EPI_ISL_3886024	2021-09-02
hCoV-19/Japan/PG-75878/2021	EPI_ISL_3886026	2021-09-02
hCoV-19/Japan/PG-75879/2021	EPI_ISL_3886028	2021-09-02
hCoV-19/Japan/PG-75880/2021	EPI_ISL_3886030	2021-09-02
hCoV-19/Japan/PG-75881/2021	EPI_ISL_3886032	2021-09-02
hCoV-19/Japan/PG-75882/2021	EPI_ISL_3886035	2021-09-02
hCoV-19/Japan/PG-75883/2021	EPI_ISL_3886037	2021-09-02
hCoV-19/Japan/PG-75884/2021	EPI_ISL_3886040	2021-09-02
hCoV-19/Japan/PG-75885/2021	EPI_ISL_3886042	2021-09-02
hCoV-19/Japan/PG-75886/2021	EPI_ISL_3886045	2021-09-02
hCoV-19/Japan/PG-75887/2021	EPI_ISL_3886047	2021-09-02
hCoV-19/Japan/PG-75888/2021	EPI_ISL_3886049	2021-09-02
hCoV-19/Japan/PG-75889/2021	EPI_ISL_3886052	2021-09-02
hCoV-19/Japan/PG-75890/2021	EPI_ISL_3886055	2021-09-02
hCoV-19/Japan/PG-75891/2021	EPI_ISL_3886057	2021-09-02
hCoV-19/Japan/PG-75892/2021	EPI_ISL_3886060	2021-09-02
hCoV-19/Japan/PG-75893/2021	EPI_ISL_3886062	2021-09-02
hCoV-19/Japan/PG-75894/2021	EPI_ISL_3886064	2021-09-02
hCoV-19/Japan/PG-75895/2021	EPI_ISL_3886066	2021-09-02
hCoV-19/Japan/PG-75896/2021	EPI_ISL_3886067	2021-09-02
hCoV-19/Japan/PG-75897/2021	EPI_ISL_3886069	2021-09-02
hCoV-19/Japan/PG-75898/2021	EPI_ISL_3886071	2021-09-02
hCoV-19/Japan/PG-75899/2021	EPI_ISL_3886074	2021-09-02
hCoV-19/Japan/PG-75900/2021	EPI_ISL_3886077	2021-09-02
hCoV-19/Japan/PG-75901/2021	EPI_ISL_3886079	2021-09-02
hCoV-19/Japan/PG-75902/2021	EPI_ISL_3886080	2021-09-02
hCoV-19/Japan/PG-75903/2021	EPI_ISL_3886083	2021-09-02
hCoV-19/Japan/PG-75904/2021	EPI_ISL_3886084	2021-09-02
hCoV-19/Japan/PG-75905/2021	EPI_ISL_3886087	2021-09-02
hCoV-19/Japan/PG-75906/2021	EPI_ISL_3886088	2021-09-02
hCoV-19/Japan/PG-75907/2021	EPI_ISL_3886091	2021-09-02
hCoV-19/Japan/PG-75908/2021	EPI_ISL_3886092	2021-09-02
hCoV-19/Japan/PG-75909/2021	EPI_ISL_3886094	2021-09-02
hCoV-19/Japan/PG-75910/2021	EPI_ISL_3886096	2021-09-02
hCoV-19/Japan/PG-75911/2021	EPI_ISL_3886098	2021-09-02
hCoV-19/Japan/PG-75912/2021	EPI_ISL_3886100	2021-09-02
hCoV-19/Japan/PG-75913/2021	EPI_ISL_3886102	2021-09-02
hCoV-19/Japan/PG-75914/2021	EPI_ISL_3886104	2021-09-02
hCoV-19/Japan/PG-75915/2021	EPI_ISL_3886106	2021-09-02
hCoV-19/Japan/PG-75916/2021	EPI_ISL_3886108	2021-09-02
hCoV-19/Japan/PG-75917/2021	EPI_ISL_3886111	2021-09-02
hCoV-19/Japan/PG-75918/2021	EPI_ISL_3886112	2021-09-02
hCoV-19/Japan/PG-75919/2021	EPI_ISL_3886115	2021-09-02
hCoV-19/Japan/PG-75920/2021	EPI_ISL_3886117	2021-09-02
hCoV-19/Japan/PG-75921/2021	EPI_ISL_3886120	2021-09-02

virus_name	epiID	submission_date
hCoV-19/Japan/PG-75922/2021	EPI_ISL_3886122	2021-09-02
hCoV-19/Japan/PG-75923/2021	EPI_ISL_3886125	2021-09-02
hCoV-19/Japan/PG-75924/2021	EPI_ISL_3886127	2021-09-02
hCoV-19/Japan/PG-75925/2021	EPI_ISL_3886129	2021-09-02
hCoV-19/Japan/PG-75926/2021	EPI_ISL_3886132	2021-09-02
hCoV-19/Japan/PG-75927/2021	EPI_ISL_3886134	2021-09-02
hCoV-19/Japan/PG-75928/2021	EPI_ISL_3886136	2021-09-02
hCoV-19/Japan/PG-75929/2021	EPI_ISL_3886138	2021-09-02
hCoV-19/Japan/PG-75930/2021	EPI_ISL_3886140	2021-09-02
hCoV-19/Japan/PG-75931/2021	EPI_ISL_3886143	2021-09-02
hCoV-19/Japan/PG-75932/2021	EPI_ISL_3886145	2021-09-02
hCoV-19/Japan/PG-75933/2021	EPI_ISL_3886146	2021-09-02
hCoV-19/Japan/PG-75934/2021	EPI_ISL_3886147	2021-09-02
hCoV-19/Japan/PG-75935/2021	EPI_ISL_3886149	2021-09-02
hCoV-19/Japan/PG-75936/2021	EPI_ISL_3886151	2021-09-02
hCoV-19/Japan/PG-75937/2021	EPI_ISL_3886153	2021-09-02
hCoV-19/Japan/PG-75938/2021	EPI_ISL_3886154	2021-09-02
hCoV-19/Japan/PG-75939/2021	EPI_ISL_3886157	2021-09-02
hCoV-19/Japan/PG-75940/2021	EPI_ISL_3886159	2021-09-02
hCoV-19/Japan/PG-75941/2021	EPI_ISL_3886162	2021-09-02
hCoV-19/Japan/PG-75942/2021	EPI_ISL_3886164	2021-09-02
hCoV-19/Japan/PG-75943/2021	EPI_ISL_3886167	2021-09-02
hCoV-19/Japan/PG-75944/2021	EPI_ISL_3886168	2021-09-02
hCoV-19/Japan/PG-75945/2021	EPI_ISL_3886170	2021-09-02
hCoV-19/Japan/PG-75946/2021	EPI_ISL_3886171	2021-09-02
hCoV-19/Japan/PG-75947/2021	EPI_ISL_3886173	2021-09-02
hCoV-19/Japan/PG-75948/2021	EPI_ISL_3886176	2021-09-02
hCoV-19/Japan/PG-75949/2021	EPI_ISL_3886178	2021-09-02
hCoV-19/Japan/PG-75950/2021	EPI_ISL_3886180	2021-09-02
hCoV-19/Japan/PG-75951/2021	EPI_ISL_3886183	2021-09-02
hCoV-19/Japan/PG-75952/2021	EPI_ISL_3886185	2021-09-02
hCoV-19/Japan/PG-75953/2021	EPI_ISL_3886187	2021-09-02
hCoV-19/Japan/PG-75954/2021	EPI_ISL_3886190	2021-09-02
hCoV-19/Japan/PG-75955/2021	EPI_ISL_3886192	2021-09-02
hCoV-19/Japan/PG-75956/2021	EPI_ISL_3886195	2021-09-02
hCoV-19/Japan/PG-75957/2021	EPI_ISL_3886197	2021-09-02
hCoV-19/Japan/PG-75958/2021	EPI_ISL_3886200	2021-09-02
hCoV-19/Japan/PG-75959/2021	EPI_ISL_3886202	2021-09-02
hCoV-19/Japan/PG-75960/2021	EPI_ISL_3886204	2021-09-02
hCoV-19/Japan/PG-75961/2021	EPI_ISL_3886206	2021-09-02
hCoV-19/Japan/PG-75962/2021	EPI_ISL_3886209	2021-09-02
hCoV-19/Japan/PG-75963/2021	EPI_ISL_3886211	2021-09-02
hCoV-19/Japan/PG-75964/2021	EPI_ISL_3886214	2021-09-02
hCoV-19/Japan/PG-75965/2021	EPI_ISL_3886216	2021-09-02
hCoV-19/Japan/PG-75966/2021	EPI_ISL_3886218	2021-09-02
hCoV-19/Japan/PG-75967/2021	EPI_ISL_3886219	2021-09-02
hCoV-19/Japan/PG-75968/2021	EPI_ISL_3886222	2021-09-02
hCoV-19/Japan/PG-75969/2021	EPI_ISL_3886223	2021-09-02
hCoV-19/Japan/PG-75970/2021	EPI_ISL_3886226	2021-09-02
hCoV-19/Japan/PG-75971/2021	EPI_ISL_3886228	2021-09-02
hCoV-19/Japan/PG-75972/2021	EPI_ISL_3886234	2021-09-02
hCoV-19/Japan/PG-75973/2021	EPI_ISL_3886236	2021-09-02
hCoV-19/Japan/PG-75974/2021	EPI_ISL_3886238	2021-09-02
hCoV-19/Japan/PG-75975/2021	EPI_ISL_3886240	2021-09-02

virus_name	epiID	submission_date
hCoV-19/Japan/PG-75976/2021	EPI_ISL_3886242	2021-09-02
hCoV-19/Japan/PG-75977/2021	EPI_ISL_3886244	2021-09-02
hCoV-19/Japan/PG-75978/2021	EPI_ISL_3886246	2021-09-02
hCoV-19/Japan/PG-75979/2021	EPI_ISL_3886248	2021-09-02
hCoV-19/Japan/PG-75980/2021	EPI_ISL_3886249	2021-09-02
hCoV-19/Japan/PG-75981/2021	EPI_ISL_3886252	2021-09-02
hCoV-19/Japan/PG-75982/2021	EPI_ISL_3886254	2021-09-02
hCoV-19/Japan/PG-75983/2021	EPI_ISL_3886256	2021-09-02
hCoV-19/Japan/PG-75984/2021	EPI_ISL_3886258	2021-09-02
hCoV-19/Japan/PG-75985/2021	EPI_ISL_3886261	2021-09-02
hCoV-19/Japan/PG-75986/2021	EPI_ISL_3886263	2021-09-02
hCoV-19/Japan/PG-75987/2021	EPI_ISL_3886264	2021-09-02
hCoV-19/Japan/PG-75988/2021	EPI_ISL_3886266	2021-09-02
hCoV-19/Japan/PG-75989/2021	EPI_ISL_3886268	2021-09-02
hCoV-19/Japan/PG-75990/2021	EPI_ISL_3886270	2021-09-02
hCoV-19/Japan/PG-75991/2021	EPI_ISL_3886272	2021-09-02
hCoV-19/Japan/PG-75992/2021	EPI_ISL_3886274	2021-09-02
hCoV-19/Japan/PG-75993/2021	EPI_ISL_3886276	2021-09-02
hCoV-19/Japan/PG-75994/2021	EPI_ISL_3886278	2021-09-02
hCoV-19/Japan/PG-75995/2021	EPI_ISL_3886280	2021-09-02
hCoV-19/Japan/PG-75996/2021	EPI_ISL_3886282	2021-09-02
hCoV-19/Japan/PG-75997/2021	EPI_ISL_3886283	2021-09-02
hCoV-19/Japan/PG-75998/2021	EPI_ISL_3886286	2021-09-02
hCoV-19/Japan/PG-75999/2021	EPI_ISL_3886287	2021-09-02
hCoV-19/Japan/PG-76000/2021	EPI_ISL_3886289	2021-09-02
hCoV-19/Japan/PG-76001/2021	EPI_ISL_3886291	2021-09-02
hCoV-19/Japan/PG-76002/2021	EPI_ISL_3886293	2021-09-02
hCoV-19/Japan/PG-76003/2021	EPI_ISL_3886296	2021-09-02
hCoV-19/Japan/PG-76004/2021	EPI_ISL_3886299	2021-09-02
hCoV-19/Japan/PG-76005/2021	EPI_ISL_3886302	2021-09-02
hCoV-19/Japan/PG-76006/2021	EPI_ISL_3886304	2021-09-02
hCoV-19/Japan/PG-76007/2021	EPI_ISL_3886307	2021-09-02
hCoV-19/Japan/PG-76008/2021	EPI_ISL_3886309	2021-09-02
hCoV-19/Japan/PG-76009/2021	EPI_ISL_3886311	2021-09-02
hCoV-19/Japan/PG-76010/2021	EPI_ISL_3886313	2021-09-02
hCoV-19/Japan/PG-76011/2021	EPI_ISL_3886315	2021-09-02
hCoV-19/Japan/PG-76012/2021	EPI_ISL_3886318	2021-09-02
hCoV-19/Japan/PG-76013/2021	EPI_ISL_3886319	2021-09-02
hCoV-19/Japan/PG-76014/2021	EPI_ISL_3886320	2021-09-02
hCoV-19/Japan/PG-76015/2021	EPI_ISL_3886322	2021-09-02
hCoV-19/Japan/PG-76016/2021	EPI_ISL_3886324	2021-09-02
hCoV-19/Japan/PG-76017/2021	EPI_ISL_3886326	2021-09-02
hCoV-19/Japan/PG-76018/2021	EPI_ISL_3886328	2021-09-02
hCoV-19/Japan/PG-76019/2021	EPI_ISL_3886330	2021-09-02
hCoV-19/Japan/PG-76020/2021	EPI_ISL_3886332	2021-09-02
hCoV-19/Japan/PG-76021/2021	EPI_ISL_3886334	2021-09-02
hCoV-19/Japan/PG-76022/2021	EPI_ISL_3886336	2021-09-02
hCoV-19/Japan/PG-76023/2021	EPI_ISL_3886338	2021-09-02
hCoV-19/Japan/PG-76024/2021	EPI_ISL_3886340	2021-09-02
hCoV-19/Japan/PG-76025/2021	EPI_ISL_3886343	2021-09-02
hCoV-19/Japan/PG-76026/2021	EPI_ISL_3886345	2021-09-02
hCoV-19/Japan/PG-76027/2021	EPI_ISL_3886348	2021-09-02
hCoV-19/Japan/PG-76028/2021	EPI_ISL_3886349	2021-09-02
hCoV-19/Japan/PG-76029/2021	EPI_ISL_3886352	2021-09-02

virus_name	epiID	submission_date
hCoV-19/Japan/PG-76030/2021	EPI_ISL_3886353	2021-09-02
hCoV-19/Japan/PG-76031/2021	EPI_ISL_3886354	2021-09-02
hCoV-19/Japan/PG-76032/2021	EPI_ISL_3886355	2021-09-02
hCoV-19/Japan/PG-76033/2021	EPI_ISL_3886356	2021-09-02
hCoV-19/Japan/PG-76034/2021	EPI_ISL_3886357	2021-09-02
hCoV-19/Japan/PG-76035/2021	EPI_ISL_3886358	2021-09-02
hCoV-19/Japan/PG-76036/2021	EPI_ISL_3886361	2021-09-02
hCoV-19/Japan/PG-76037/2021	EPI_ISL_3886363	2021-09-02
hCoV-19/Japan/PG-76038/2021	EPI_ISL_3886365	2021-09-02
hCoV-19/Japan/PG-76039/2021	EPI_ISL_3886368	2021-09-02
hCoV-19/Japan/PG-76040/2021	EPI_ISL_3886370	2021-09-02
hCoV-19/Japan/PG-76041/2021	EPI_ISL_3886372	2021-09-02
hCoV-19/Japan/PG-76042/2021	EPI_ISL_3886375	2021-09-02
hCoV-19/Japan/PG-76043/2021	EPI_ISL_3886377	2021-09-02
hCoV-19/Japan/PG-76044/2021	EPI_ISL_3886380	2021-09-02
hCoV-19/Japan/PG-76045/2021	EPI_ISL_3886382	2021-09-02
hCoV-19/Japan/PG-76046/2021	EPI_ISL_3886385	2021-09-02
hCoV-19/Japan/PG-76047/2021	EPI_ISL_3886387	2021-09-02
hCoV-19/Japan/PG-76048/2021	EPI_ISL_3886389	2021-09-02
hCoV-19/Japan/PG-76049/2021	EPI_ISL_3886391	2021-09-02
hCoV-19/Japan/PG-76050/2021	EPI_ISL_3886394	2021-09-02
hCoV-19/Japan/PG-76051/2021	EPI_ISL_3886396	2021-09-02
hCoV-19/Japan/PG-76052/2021	EPI_ISL_3886398	2021-09-02
hCoV-19/Japan/PG-76053/2021	EPI_ISL_3886400	2021-09-02
hCoV-19/Japan/PG-76054/2021	EPI_ISL_3886402	2021-09-02
hCoV-19/Japan/PG-76055/2021	EPI_ISL_3886404	2021-09-02
hCoV-19/Japan/PG-76056/2021	EPI_ISL_3886407	2021-09-02
hCoV-19/Japan/PG-76057/2021	EPI_ISL_3886409	2021-09-02
hCoV-19/Japan/PG-76058/2021	EPI_ISL_3886411	2021-09-02
hCoV-19/Japan/PG-76059/2021	EPI_ISL_3886412	2021-09-02
hCoV-19/Japan/PG-76060/2021	EPI_ISL_3886414	2021-09-02
hCoV-19/Japan/PG-76061/2021	EPI_ISL_3886416	2021-09-02
hCoV-19/Japan/PG-76062/2021	EPI_ISL_3886418	2021-09-02
hCoV-19/Japan/PG-76063/2021	EPI_ISL_3886419	2021-09-02
hCoV-19/Japan/PG-76064/2021	EPI_ISL_3886420	2021-09-02
hCoV-19/Japan/PG-76065/2021	EPI_ISL_3886422	2021-09-02
hCoV-19/Japan/PG-76066/2021	EPI_ISL_3886424	2021-09-02
hCoV-19/Japan/PG-76067/2021	EPI_ISL_3886426	2021-09-02
hCoV-19/Japan/PG-76068/2021	EPI_ISL_3886428	2021-09-02
hCoV-19/Japan/PG-76069/2021	EPI_ISL_3886431	2021-09-02
hCoV-19/Japan/PG-76070/2021	EPI_ISL_3886433	2021-09-02
hCoV-19/Japan/PG-76071/2021	EPI_ISL_3886436	2021-09-02
hCoV-19/Japan/PG-76072/2021	EPI_ISL_3886437	2021-09-02
hCoV-19/Japan/PG-76073/2021	EPI_ISL_3886439	2021-09-02
hCoV-19/Japan/PG-76074/2021	EPI_ISL_3886440	2021-09-02
hCoV-19/Japan/PG-76075/2021	EPI_ISL_3886442	2021-09-02
hCoV-19/Japan/PG-76076/2021	EPI_ISL_3886444	2021-09-02
hCoV-19/Japan/PG-76077/2021	EPI_ISL_3886446	2021-09-02
hCoV-19/Japan/PG-76078/2021	EPI_ISL_3886448	2021-09-02
hCoV-19/Japan/PG-76079/2021	EPI_ISL_3886450	2021-09-02
hCoV-19/Japan/PG-76080/2021	EPI_ISL_3886451	2021-09-02
hCoV-19/Japan/PG-76081/2021	EPI_ISL_3873680	2021-09-02
hCoV-19/Japan/PG-76082/2021	EPI_ISL_3873681	2021-09-02
hCoV-19/Japan/PG-76083/2021	EPI_ISL_3873682	2021-09-02

virus_name	epiID	submission_date
hCoV-19/Japan/PG-76084/2021	EPI_ISL_3873683	2021-09-02
hCoV-19/Japan/PG-76085/2021	EPI_ISL_3873684	2021-09-02
hCoV-19/Japan/PG-76086/2021	EPI_ISL_3873685	2021-09-02
hCoV-19/Japan/PG-76087/2021	EPI_ISL_3873686	2021-09-02
hCoV-19/Japan/PG-76088/2021	EPI_ISL_3873687	2021-09-02
hCoV-19/Japan/PG-76089/2021	EPI_ISL_3873688	2021-09-02
hCoV-19/Japan/PG-76090/2021	EPI_ISL_3873689	2021-09-02
hCoV-19/Japan/PG-76091/2021	EPI_ISL_3873690	2021-09-02
hCoV-19/Japan/PG-76092/2021	EPI_ISL_3873691	2021-09-02
hCoV-19/Japan/PG-76093/2021	EPI_ISL_3873692	2021-09-02
hCoV-19/Japan/PG-76094/2021	EPI_ISL_3873693	2021-09-02
hCoV-19/Japan/PG-76095/2021	EPI_ISL_3873694	2021-09-02
hCoV-19/Japan/PG-76096/2021	EPI_ISL_3873695	2021-09-02
hCoV-19/Japan/PG-76097/2021	EPI_ISL_3873696	2021-09-02
hCoV-19/Japan/PG-76098/2021	EPI_ISL_3873697	2021-09-02
hCoV-19/Japan/PG-76099/2021	EPI_ISL_3873698	2021-09-02
hCoV-19/Japan/PG-76100/2021	EPI_ISL_3873699	2021-09-02
hCoV-19/Japan/PG-76101/2021	EPI_ISL_3873700	2021-09-02
hCoV-19/Japan/PG-76102/2021	EPI_ISL_3873701	2021-09-02
hCoV-19/Japan/PG-76103/2021	EPI_ISL_3873702	2021-09-02
hCoV-19/Japan/PG-76104/2021	EPI_ISL_3873703	2021-09-02
hCoV-19/Japan/PG-76105/2021	EPI_ISL_3873704	2021-09-02
hCoV-19/Japan/PG-76106/2021	EPI_ISL_3873705	2021-09-02
hCoV-19/Japan/PG-76107/2021	EPI_ISL_3873706	2021-09-02
hCoV-19/Japan/PG-76108/2021	EPI_ISL_3873707	2021-09-02
hCoV-19/Japan/PG-76109/2021	EPI_ISL_3873708	2021-09-02
hCoV-19/Japan/PG-76110/2021	EPI_ISL_3873709	2021-09-02
hCoV-19/Japan/PG-76111/2021	EPI_ISL_3873710	2021-09-02
hCoV-19/Japan/PG-76112/2021	EPI_ISL_3873711	2021-09-02
hCoV-19/Japan/PG-76113/2021	EPI_ISL_3873712	2021-09-02
hCoV-19/Japan/PG-76114/2021	EPI_ISL_3873713	2021-09-02
hCoV-19/Japan/PG-76115/2021	EPI_ISL_3873714	2021-09-02
hCoV-19/Japan/PG-76116/2021	EPI_ISL_3873715	2021-09-02
hCoV-19/Japan/PG-76117/2021	EPI_ISL_3873716	2021-09-02
hCoV-19/Japan/PG-76118/2021	EPI_ISL_3873717	2021-09-02
hCoV-19/Japan/PG-76119/2021	EPI_ISL_3873718	2021-09-02
hCoV-19/Japan/PG-76120/2021	EPI_ISL_3873719	2021-09-02
hCoV-19/Japan/PG-76121/2021	EPI_ISL_3873720	2021-09-02
hCoV-19/Japan/PG-76122/2021	EPI_ISL_3873721	2021-09-02
hCoV-19/Japan/PG-76123/2021	EPI_ISL_3873722	2021-09-02
hCoV-19/Japan/PG-76124/2021	EPI_ISL_3873723	2021-09-02
hCoV-19/Japan/PG-76125/2021	EPI_ISL_3870503	2021-09-02
hCoV-19/Japan/PG-76126/2021	EPI_ISL_3870504	2021-09-02
hCoV-19/Japan/PG-76127/2021	EPI_ISL_3870505	2021-09-02
hCoV-19/Japan/PG-76128/2021	EPI_ISL_3870506	2021-09-02
hCoV-19/Japan/PG-76129/2021	EPI_ISL_3870507	2021-09-02
hCoV-19/Japan/PG-76130/2021	EPI_ISL_3870508	2021-09-02
hCoV-19/Japan/PG-76131/2021	EPI_ISL_3870509	2021-09-02
hCoV-19/Japan/PG-76132/2021	EPI_ISL_3870510	2021-09-02
hCoV-19/Japan/PG-76133/2021	EPI_ISL_3870511	2021-09-02
hCoV-19/Japan/PG-76134/2021	EPI_ISL_3870512	2021-09-02
hCoV-19/Japan/PG-76135/2021	EPI_ISL_3870513	2021-09-02
hCoV-19/Japan/PG-76136/2021	EPI_ISL_3870514	2021-09-02
hCoV-19/Japan/PG-76137/2021	EPI_ISL_3870515	2021-09-02

virus_name	epiID	submission_date
hCoV-19/Japan/PG-76138/2021	EPI_ISL_3870516	2021-09-02
hCoV-19/Japan/PG-76139/2021	EPI_ISL_3870517	2021-09-02
hCoV-19/Japan/PG-76140/2021	EPI_ISL_3870518	2021-09-02
hCoV-19/Japan/PG-76141/2021	EPI_ISL_3870519	2021-09-02
hCoV-19/Japan/PG-76142/2021	EPI_ISL_3870520	2021-09-02
hCoV-19/Japan/PG-76143/2021	EPI_ISL_3870521	2021-09-02
hCoV-19/Japan/PG-76144/2021	EPI_ISL_3870522	2021-09-02
hCoV-19/Japan/PG-76145/2021	EPI_ISL_3870523	2021-09-02
hCoV-19/Japan/PG-76146/2021	EPI_ISL_3870524	2021-09-02
hCoV-19/Japan/PG-76147/2021	EPI_ISL_3870525	2021-09-02
hCoV-19/Japan/PG-76148/2021	EPI_ISL_3870526	2021-09-02
hCoV-19/Japan/PG-76149/2021	EPI_ISL_3874871	2021-09-02
hCoV-19/Japan/PG-76150/2021	EPI_ISL_3874872	2021-09-02
hCoV-19/Japan/PG-76151/2021	EPI_ISL_3874873	2021-09-02
hCoV-19/Japan/PG-76152/2021	EPI_ISL_3874874	2021-09-02
hCoV-19/Japan/PG-76153/2021	EPI_ISL_3874875	2021-09-02
hCoV-19/Japan/PG-76155/2021	EPI_ISL_3874876	2021-09-02
hCoV-19/Japan/PG-76156/2021	EPI_ISL_3874877	2021-09-02
hCoV-19/Japan/PG-76157/2021	EPI_ISL_3874878	2021-09-02
hCoV-19/Japan/PG-76158/2021	EPI_ISL_3874879	2021-09-02
hCoV-19/Japan/PG-76159/2021	EPI_ISL_3874880	2021-09-02
hCoV-19/Japan/PG-76160/2021	EPI_ISL_3874881	2021-09-02
hCoV-19/Japan/PG-76161/2021	EPI_ISL_3874882	2021-09-02
hCoV-19/Japan/PG-76162/2021	EPI_ISL_3874883	2021-09-02
hCoV-19/Japan/PG-76163/2021	EPI_ISL_3874884	2021-09-02
hCoV-19/Japan/PG-76164/2021	EPI_ISL_3874885	2021-09-02
hCoV-19/Japan/PG-76165/2021	EPI_ISL_3874886	2021-09-02
hCoV-19/Japan/PG-76166/2021	EPI_ISL_3874887	2021-09-02
hCoV-19/Japan/PG-76167/2021	EPI_ISL_3874888	2021-09-02
hCoV-19/Japan/PG-76168/2021	EPI_ISL_3874889	2021-09-02
hCoV-19/Japan/PG-76169/2021	EPI_ISL_3874890	2021-09-02
hCoV-19/Japan/PG-76170/2021	EPI_ISL_3874891	2021-09-02
hCoV-19/Japan/PG-76171/2021	EPI_ISL_3874892	2021-09-02
hCoV-19/Japan/PG-76172/2021	EPI_ISL_3874893	2021-09-02
hCoV-19/Japan/PG-76173/2021	EPI_ISL_3874894	2021-09-02
hCoV-19/Japan/PG-76174/2021	EPI_ISL_3874895	2021-09-02
hCoV-19/Japan/PG-76175/2021	EPI_ISL_3874896	2021-09-02
hCoV-19/Japan/PG-76176/2021	EPI_ISL_3874897	2021-09-02
hCoV-19/Japan/PG-76177/2021	EPI_ISL_3874898	2021-09-02
hCoV-19/Japan/PG-76178/2021	EPI_ISL_3874899	2021-09-02
hCoV-19/Japan/PG-76179/2021	EPI_ISL_3874900	2021-09-02
hCoV-19/Japan/PG-76180/2021	EPI_ISL_3874901	2021-09-02
hCoV-19/Japan/PG-76181/2021	EPI_ISL_3874902	2021-09-02
hCoV-19/Japan/PG-76182/2021	EPI_ISL_3874903	2021-09-02
hCoV-19/Japan/PG-76183/2021	EPI_ISL_3874904	2021-09-02
hCoV-19/Japan/PG-76184/2021	EPI_ISL_3874905	2021-09-02
hCoV-19/Japan/PG-76185/2021	EPI_ISL_3874906	2021-09-02
hCoV-19/Japan/PG-76186/2021	EPI_ISL_3874907	2021-09-02
hCoV-19/Japan/PG-76187/2021	EPI_ISL_3874908	2021-09-02
hCoV-19/Japan/PG-76188/2021	EPI_ISL_3874909	2021-09-02
hCoV-19/Japan/PG-76189/2021	EPI_ISL_3874910	2021-09-02
hCoV-19/Japan/PG-76190/2021	EPI_ISL_3874911	2021-09-02
hCoV-19/Japan/PG-76191/2021	EPI_ISL_3874912	2021-09-02
hCoV-19/Japan/PG-76192/2021	EPI_ISL_3874913	2021-09-02

virus_name	epiID	submission_date
hCoV-19/Japan/PG-76193/2021	EPI_ISL_3874914	2021-09-02
hCoV-19/Japan/PG-76194/2021	EPI_ISL_3874915	2021-09-02
hCoV-19/Japan/PG-76195/2021	EPI_ISL_3874916	2021-09-02
hCoV-19/Japan/PG-76196/2021	EPI_ISL_3874917	2021-09-02
hCoV-19/Japan/PG-76197/2021	EPI_ISL_3874918	2021-09-02
hCoV-19/Japan/PG-76198/2021	EPI_ISL_3874919	2021-09-02
hCoV-19/Japan/PG-76199/2021	EPI_ISL_3874920	2021-09-02
hCoV-19/Japan/PG-76200/2021	EPI_ISL_3874921	2021-09-02
hCoV-19/Japan/PG-76201/2021	EPI_ISL_3874922	2021-09-02
hCoV-19/Japan/PG-76202/2021	EPI_ISL_3874923	2021-09-02
hCoV-19/Japan/PG-76203/2021	EPI_ISL_3874924	2021-09-02
hCoV-19/Japan/PG-76204/2021	EPI_ISL_3874925	2021-09-02
hCoV-19/Japan/PG-76205/2021	EPI_ISL_3874926	2021-09-02
hCoV-19/Japan/PG-76206/2021	EPI_ISL_3874927	2021-09-02
hCoV-19/Japan/PG-76207/2021	EPI_ISL_3874928	2021-09-02
hCoV-19/Japan/PG-76208/2021	EPI_ISL_3874929	2021-09-02
hCoV-19/Japan/PG-76209/2021	EPI_ISL_3874930	2021-09-02
hCoV-19/Japan/PG-76210/2021	EPI_ISL_3874931	2021-09-02
hCoV-19/Japan/PG-76211/2021	EPI_ISL_3874932	2021-09-02
hCoV-19/Japan/PG-76212/2021	EPI_ISL_3874933	2021-09-02
hCoV-19/Japan/PG-76213/2021	EPI_ISL_3874934	2021-09-02
hCoV-19/Japan/PG-76214/2021	EPI_ISL_3874935	2021-09-02
hCoV-19/Japan/PG-76215/2021	EPI_ISL_3874936	2021-09-02
hCoV-19/Japan/PG-76216/2021	EPI_ISL_3874937	2021-09-02
hCoV-19/Japan/PG-76217/2021	EPI_ISL_3874938	2021-09-02
hCoV-19/Japan/PG-76218/2021	EPI_ISL_3874939	2021-09-02
hCoV-19/Japan/PG-76219/2021	EPI_ISL_3874940	2021-09-02
hCoV-19/Japan/PG-76220/2021	EPI_ISL_3874941	2021-09-02
hCoV-19/Japan/PG-76221/2021	EPI_ISL_3874942	2021-09-02
hCoV-19/Japan/PG-76222/2021	EPI_ISL_3874943	2021-09-02
hCoV-19/Japan/PG-76223/2021	EPI_ISL_3874944	2021-09-02
hCoV-19/Japan/PG-76224/2021	EPI_ISL_3874945	2021-09-02
hCoV-19/Japan/PG-76225/2021	EPI_ISL_3874946	2021-09-02
hCoV-19/Japan/PG-76226/2021	EPI_ISL_3874947	2021-09-02
hCoV-19/Japan/PG-76227/2021	EPI_ISL_3874948	2021-09-02
hCoV-19/Japan/PG-76228/2021	EPI_ISL_3874949	2021-09-02
hCoV-19/Japan/PG-76229/2021	EPI_ISL_3874950	2021-09-02
hCoV-19/Japan/PG-76230/2021	EPI_ISL_3874951	2021-09-02
hCoV-19/Japan/PG-76231/2021	EPI_ISL_3874952	2021-09-02
hCoV-19/Japan/PG-76232/2021	EPI_ISL_3874953	2021-09-02
hCoV-19/Japan/PG-76233/2021	EPI_ISL_3874954	2021-09-02
hCoV-19/Japan/PG-76234/2021	EPI_ISL_3874955	2021-09-02
hCoV-19/Japan/PG-76235/2021	EPI_ISL_3874956	2021-09-02
hCoV-19/Japan/PG-76236/2021	EPI_ISL_3874957	2021-09-02
hCoV-19/Japan/PG-76237/2021	EPI_ISL_3874958	2021-09-02
hCoV-19/Japan/PG-76238/2021	EPI_ISL_3874959	2021-09-02
hCoV-19/Japan/PG-76239/2021	EPI_ISL_3874960	2021-09-02
hCoV-19/Japan/PG-76240/2021	EPI_ISL_3874961	2021-09-02
hCoV-19/Japan/PG-76241/2021	EPI_ISL_3874962	2021-09-02
hCoV-19/Japan/PG-76242/2021	EPI_ISL_3874963	2021-09-02
hCoV-19/Japan/PG-76243/2021	EPI_ISL_3874964	2021-09-02
hCoV-19/Japan/PG-76244/2021	EPI_ISL_3874965	2021-09-02
hCoV-19/Japan/PG-76245/2021	EPI_ISL_3874966	2021-09-02
hCoV-19/Japan/PG-76246/2021	EPI_ISL_3874967	2021-09-02

virus_name	epiID	submission_date
hCoV-19/Japan/PG-76248/2021	EPI_ISL_3874968	2021-09-02
hCoV-19/Japan/PG-76256/2021	EPI_ISL_3874969	2021-09-02
hCoV-19/Japan/PG-76258/2021	EPI_ISL_3874970	2021-09-02
hCoV-19/Japan/PG-76260/2021	EPI_ISL_3874971	2021-09-02
hCoV-19/Japan/PG-76261/2021	EPI_ISL_3874972	2021-09-02
hCoV-19/Japan/PG-76263/2021	EPI_ISL_3874973	2021-09-02
hCoV-19/Japan/PG-76264/2021	EPI_ISL_3871752	2021-09-02
hCoV-19/Japan/PG-76265/2021	EPI_ISL_3871753	2021-09-02
hCoV-19/Japan/PG-76266/2021	EPI_ISL_3871754	2021-09-02
hCoV-19/Japan/PG-76267/2021	EPI_ISL_3871755	2021-09-02
hCoV-19/Japan/PG-76268/2021	EPI_ISL_3871756	2021-09-02
hCoV-19/Japan/PG-76269/2021	EPI_ISL_3871757	2021-09-02
hCoV-19/Japan/PG-76270/2021	EPI_ISL_3871758	2021-09-02
hCoV-19/Japan/PG-76271/2021	EPI_ISL_3871759	2021-09-02
hCoV-19/Japan/PG-76272/2021	EPI_ISL_3871760	2021-09-02
hCoV-19/Japan/PG-76273/2021	EPI_ISL_3871761	2021-09-02
hCoV-19/Japan/PG-76274/2021	EPI_ISL_3871762	2021-09-02
hCoV-19/Japan/PG-76275/2021	EPI_ISL_3871763	2021-09-02
hCoV-19/Japan/PG-76276/2021	EPI_ISL_3871764	2021-09-02
hCoV-19/Japan/PG-76277/2021	EPI_ISL_3871765	2021-09-02
hCoV-19/Japan/PG-76278/2021	EPI_ISL_3871766	2021-09-02
hCoV-19/Japan/PG-76279/2021	EPI_ISL_3871767	2021-09-02
hCoV-19/Japan/PG-76280/2021	EPI_ISL_3871768	2021-09-02
hCoV-19/Japan/PG-76281/2021	EPI_ISL_3871769	2021-09-02
hCoV-19/Japan/PG-76282/2021	EPI_ISL_3871770	2021-09-02
hCoV-19/Japan/PG-76283/2021	EPI_ISL_3871771	2021-09-02
hCoV-19/Japan/PG-76284/2021	EPI_ISL_3871772	2021-09-02
hCoV-19/Japan/PG-76285/2021	EPI_ISL_3871773	2021-09-02
hCoV-19/Japan/PG-76286/2021	EPI_ISL_3871774	2021-09-02
hCoV-19/Japan/PG-76287/2021	EPI_ISL_3876045	2021-09-02
hCoV-19/Japan/PG-76288/2021	EPI_ISL_3876046	2021-09-02
hCoV-19/Japan/PG-76289/2021	EPI_ISL_3876047	2021-09-02
hCoV-19/Japan/PG-76290/2021	EPI_ISL_3876048	2021-09-02
hCoV-19/Japan/PG-76291/2021	EPI_ISL_3876049	2021-09-02
hCoV-19/Japan/PG-76292/2021	EPI_ISL_3876050	2021-09-02
hCoV-19/Japan/PG-76293/2021	EPI_ISL_3876051	2021-09-02
hCoV-19/Japan/PG-76294/2021	EPI_ISL_3876052	2021-09-02
hCoV-19/Japan/PG-76295/2021	EPI_ISL_3876053	2021-09-02
hCoV-19/Japan/PG-76296/2021	EPI_ISL_3876054	2021-09-02
hCoV-19/Japan/PG-76297/2021	EPI_ISL_3876055	2021-09-02
hCoV-19/Japan/PG-76298/2021	EPI_ISL_3876056	2021-09-02
hCoV-19/Japan/PG-76299/2021	EPI_ISL_3876057	2021-09-02
hCoV-19/Japan/PG-76300/2021	EPI_ISL_3876058	2021-09-02
hCoV-19/Japan/PG-76301/2021	EPI_ISL_3876059	2021-09-02
hCoV-19/Japan/PG-76302/2021	EPI_ISL_3876060	2021-09-02
hCoV-19/Japan/PG-76303/2021	EPI_ISL_3876061	2021-09-02
hCoV-19/Japan/PG-76304/2021	EPI_ISL_3876062	2021-09-02
hCoV-19/Japan/PG-76305/2021	EPI_ISL_3876063	2021-09-02
hCoV-19/Japan/PG-76306/2021	EPI_ISL_3876064	2021-09-02
hCoV-19/Japan/PG-76307/2021	EPI_ISL_3876065	2021-09-02
hCoV-19/Japan/PG-76308/2021	EPI_ISL_3876066	2021-09-02
hCoV-19/Japan/PG-76309/2021	EPI_ISL_3876067	2021-09-02
hCoV-19/Japan/PG-76310/2021	EPI_ISL_3876068	2021-09-02
hCoV-19/Japan/PG-76311/2021	EPI_ISL_3876069	2021-09-02

virus_name	epiID	submission_date
hCoV-19/Japan/PG-76312/2021	EPI_ISL_3876070	2021-09-02
hCoV-19/Japan/PG-76313/2021	EPI_ISL_3876071	2021-09-02
hCoV-19/Japan/PG-76314/2021	EPI_ISL_3875839	2021-09-02
hCoV-19/Japan/PG-76315/2021	EPI_ISL_3875840	2021-09-02
hCoV-19/Japan/PG-76316/2021	EPI_ISL_3875841	2021-09-02
hCoV-19/Japan/PG-76317/2021	EPI_ISL_3875842	2021-09-02
hCoV-19/Japan/PG-76318/2021	EPI_ISL_3875843	2021-09-02
hCoV-19/Japan/PG-76319/2021	EPI_ISL_3875844	2021-09-02
hCoV-19/Japan/PG-76320/2021	EPI_ISL_3875845	2021-09-02
hCoV-19/Japan/PG-76321/2021	EPI_ISL_3875846	2021-09-02
hCoV-19/Japan/PG-76322/2021	EPI_ISL_3870630	2021-09-02
hCoV-19/Japan/PG-76323/2021	EPI_ISL_3870631	2021-09-02
hCoV-19/Japan/PG-76324/2021	EPI_ISL_3870632	2021-09-02
hCoV-19/Japan/PG-76325/2021	EPI_ISL_3870633	2021-09-02
hCoV-19/Japan/PG-76326/2021	EPI_ISL_3870634	2021-09-02
hCoV-19/Japan/PG-76327/2021	EPI_ISL_3870635	2021-09-02
hCoV-19/Japan/PG-76328/2021	EPI_ISL_3870636	2021-09-02
hCoV-19/Japan/PG-76329/2021	EPI_ISL_3870637	2021-09-02
hCoV-19/Japan/PG-76330/2021	EPI_ISL_3870638	2021-09-02
hCoV-19/Japan/PG-76331/2021	EPI_ISL_3870639	2021-09-02
hCoV-19/Japan/PG-76332/2021	EPI_ISL_3870640	2021-09-02
hCoV-19/Japan/PG-76333/2021	EPI_ISL_3870641	2021-09-02
hCoV-19/Japan/PG-76334/2021	EPI_ISL_3870642	2021-09-02
hCoV-19/Japan/PG-76335/2021	EPI_ISL_3870643	2021-09-02
hCoV-19/Japan/PG-76336/2021	EPI_ISL_3870644	2021-09-02
hCoV-19/Japan/PG-76337/2021	EPI_ISL_3870645	2021-09-02
hCoV-19/Japan/PG-76338/2021	EPI_ISL_3870646	2021-09-02
hCoV-19/Japan/PG-76339/2021	EPI_ISL_3870647	2021-09-02
hCoV-19/Japan/PG-76340/2021	EPI_ISL_3873433	2021-09-02
hCoV-19/Japan/PG-76341/2021	EPI_ISL_3873434	2021-09-02
hCoV-19/Japan/PG-76342/2021	EPI_ISL_3873435	2021-09-02
hCoV-19/Japan/PG-76343/2021	EPI_ISL_3873436	2021-09-02
hCoV-19/Japan/PG-76344/2021	EPI_ISL_3873437	2021-09-02
hCoV-19/Japan/PG-76345/2021	EPI_ISL_3873438	2021-09-02
hCoV-19/Japan/PG-76346/2021	EPI_ISL_3873439	2021-09-02
hCoV-19/Japan/PG-76347/2021	EPI_ISL_3873440	2021-09-02
hCoV-19/Japan/PG-76348/2021	EPI_ISL_3873441	2021-09-02
hCoV-19/Japan/PG-76349/2021	EPI_ISL_3873442	2021-09-02
hCoV-19/Japan/PG-76350/2021	EPI_ISL_3873443	2021-09-02
hCoV-19/Japan/PG-76351/2021	EPI_ISL_3873444	2021-09-02
hCoV-19/Japan/PG-76352/2021	EPI_ISL_3873445	2021-09-02
hCoV-19/Japan/PG-76353/2021	EPI_ISL_3873446	2021-09-02
hCoV-19/Japan/PG-76354/2021	EPI_ISL_3872632	2021-09-02
hCoV-19/Japan/PG-76355/2021	EPI_ISL_3872633	2021-09-02
hCoV-19/Japan/PG-76356/2021	EPI_ISL_3872634	2021-09-02
hCoV-19/Japan/PG-76357/2021	EPI_ISL_3872635	2021-09-02
hCoV-19/Japan/PG-76358/2021	EPI_ISL_3872636	2021-09-02
hCoV-19/Japan/PG-76359/2021	EPI_ISL_3872637	2021-09-02
hCoV-19/Japan/PG-76360/2021	EPI_ISL_3872638	2021-09-02
hCoV-19/Japan/PG-76361/2021	EPI_ISL_3872639	2021-09-02
hCoV-19/Japan/PG-76362/2021	EPI_ISL_3872640	2021-09-02
hCoV-19/Japan/PG-76363/2021	EPI_ISL_3872641	2021-09-02
hCoV-19/Japan/PG-76364/2021	EPI_ISL_3872642	2021-09-02
hCoV-19/Japan/PG-76365/2021	EPI_ISL_3872643	2021-09-02

virus_name	epiID	submission_date
hCoV-19/Japan/PG-76366/2021	EPI_ISL_3872644	2021-09-02
hCoV-19/Japan/PG-76367/2021	EPI_ISL_3872645	2021-09-02
hCoV-19/Japan/PG-76368/2021	EPI_ISL_3872646	2021-09-02
hCoV-19/Japan/PG-76369/2021	EPI_ISL_3872647	2021-09-02
hCoV-19/Japan/PG-76370/2021	EPI_ISL_3872648	2021-09-02
hCoV-19/Japan/PG-76371/2021	EPI_ISL_3872649	2021-09-02
hCoV-19/Japan/PG-76372/2021	EPI_ISL_3872650	2021-09-02
hCoV-19/Japan/PG-76373/2021	EPI_ISL_3872651	2021-09-02
hCoV-19/Japan/PG-76374/2021	EPI_ISL_3872652	2021-09-02
hCoV-19/Japan/PG-76375/2021	EPI_ISL_3872653	2021-09-02
hCoV-19/Japan/PG-76376/2021	EPI_ISL_3872654	2021-09-02
hCoV-19/Japan/PG-76377/2021	EPI_ISL_3872655	2021-09-02
hCoV-19/Japan/PG-76378/2021	EPI_ISL_3872656	2021-09-02
hCoV-19/Japan/PG-76379/2021	EPI_ISL_3872657	2021-09-02
hCoV-19/Japan/PG-76380/2021	EPI_ISL_3872658	2021-09-02
hCoV-19/Japan/PG-76381/2021	EPI_ISL_3872659	2021-09-02
hCoV-19/Japan/PG-76382/2021	EPI_ISL_3872660	2021-09-02
hCoV-19/Japan/PG-76383/2021	EPI_ISL_3872661	2021-09-02
hCoV-19/Japan/PG-76384/2021	EPI_ISL_3872662	2021-09-02
hCoV-19/Japan/PG-76385/2021	EPI_ISL_3872663	2021-09-02
hCoV-19/Japan/PG-76386/2021	EPI_ISL_3872664	2021-09-02
hCoV-19/Japan/PG-76387/2021	EPI_ISL_3872665	2021-09-02
hCoV-19/Japan/PG-76388/2021	EPI_ISL_3872666	2021-09-02
hCoV-19/Japan/PG-76389/2021	EPI_ISL_3872667	2021-09-02
hCoV-19/Japan/PG-76390/2021	EPI_ISL_3872668	2021-09-02
hCoV-19/Japan/PG-76391/2021	EPI_ISL_3872669	2021-09-02
hCoV-19/Japan/PG-76392/2021	EPI_ISL_3872670	2021-09-02
hCoV-19/Japan/PG-76393/2021	EPI_ISL_3872671	2021-09-02
hCoV-19/Japan/PG-76394/2021	EPI_ISL_3872672	2021-09-02
hCoV-19/Japan/PG-76395/2021	EPI_ISL_3872673	2021-09-02
hCoV-19/Japan/PG-76396/2021	EPI_ISL_3872674	2021-09-02
hCoV-19/Japan/PG-76397/2021	EPI_ISL_3872675	2021-09-02
hCoV-19/Japan/PG-76398/2021	EPI_ISL_3872676	2021-09-02
hCoV-19/Japan/PG-76399/2021	EPI_ISL_3872677	2021-09-02
hCoV-19/Japan/PG-76400/2021	EPI_ISL_3872678	2021-09-02
hCoV-19/Japan/PG-76401/2021	EPI_ISL_3872679	2021-09-02
hCoV-19/Japan/PG-76402/2021	EPI_ISL_3872680	2021-09-02
hCoV-19/Japan/PG-76403/2021	EPI_ISL_3872681	2021-09-02
hCoV-19/Japan/PG-76404/2021	EPI_ISL_3872682	2021-09-02
hCoV-19/Japan/PG-76405/2021	EPI_ISL_3872683	2021-09-02
hCoV-19/Japan/PG-76406/2021	EPI_ISL_3872684	2021-09-02
hCoV-19/Japan/PG-76407/2021	EPI_ISL_3872685	2021-09-02
hCoV-19/Japan/PG-76408/2021	EPI_ISL_3872686	2021-09-02
hCoV-19/Japan/PG-76409/2021	EPI_ISL_3872687	2021-09-02
hCoV-19/Japan/PG-76410/2021	EPI_ISL_3872688	2021-09-02
hCoV-19/Japan/PG-76411/2021	EPI_ISL_3872689	2021-09-02
hCoV-19/Japan/PG-76412/2021	EPI_ISL_3872690	2021-09-02
hCoV-19/Japan/PG-76413/2021	EPI_ISL_3872691	2021-09-02
hCoV-19/Japan/PG-76414/2021	EPI_ISL_3872692	2021-09-02
hCoV-19/Japan/PG-76415/2021	EPI_ISL_3872693	2021-09-02
hCoV-19/Japan/PG-76416/2021	EPI_ISL_3872694	2021-09-02
hCoV-19/Japan/PG-76417/2021	EPI_ISL_3872695	2021-09-02
hCoV-19/Japan/PG-76418/2021	EPI_ISL_3872696	2021-09-02
hCoV-19/Japan/PG-76419/2021	EPI_ISL_3872697	2021-09-02

virus_name	epiID	submission_date
hCoV-19/Japan/PG-76420/2021	EPI_ISL_3872698	2021-09-02
hCoV-19/Japan/PG-76421/2021	EPI_ISL_3872699	2021-09-02
hCoV-19/Japan/PG-76422/2021	EPI_ISL_3872700	2021-09-02
hCoV-19/Japan/PG-76423/2021	EPI_ISL_3872701	2021-09-02
hCoV-19/Japan/PG-76424/2021	EPI_ISL_3872702	2021-09-02
hCoV-19/Japan/PG-76425/2021	EPI_ISL_3872703	2021-09-02
hCoV-19/Japan/PG-76426/2021	EPI_ISL_3872704	2021-09-02
hCoV-19/Japan/PG-76427/2021	EPI_ISL_3872705	2021-09-02
hCoV-19/Japan/PG-76428/2021	EPI_ISL_3872706	2021-09-02
hCoV-19/Japan/PG-76429/2021	EPI_ISL_3872707	2021-09-02
hCoV-19/Japan/PG-76430/2021	EPI_ISL_3872708	2021-09-02
hCoV-19/Japan/PG-76431/2021	EPI_ISL_3872709	2021-09-02
hCoV-19/Japan/PG-76432/2021	EPI_ISL_3871586	2021-09-02
hCoV-19/Japan/PG-76433/2021	EPI_ISL_3871587	2021-09-02
hCoV-19/Japan/PG-76434/2021	EPI_ISL_3871588	2021-09-02
hCoV-19/Japan/PG-76435/2021	EPI_ISL_3871589	2021-09-02
hCoV-19/Japan/PG-76436/2021	EPI_ISL_3871590	2021-09-02
hCoV-19/Japan/PG-76437/2021	EPI_ISL_3871591	2021-09-02
hCoV-19/Japan/PG-76438/2021	EPI_ISL_3871592	2021-09-02
hCoV-19/Japan/PG-76439/2021	EPI_ISL_3871593	2021-09-02
hCoV-19/Japan/PG-76440/2021	EPI_ISL_3871594	2021-09-02
hCoV-19/Japan/PG-76441/2021	EPI_ISL_3871595	2021-09-02
hCoV-19/Japan/PG-76442/2021	EPI_ISL_3871596	2021-09-02
hCoV-19/Japan/PG-76443/2021	EPI_ISL_3871597	2021-09-02
hCoV-19/Japan/PG-76444/2021	EPI_ISL_3871598	2021-09-02
hCoV-19/Japan/PG-76445/2021	EPI_ISL_3871599	2021-09-02
hCoV-19/Japan/PG-76446/2021	EPI_ISL_3871600	2021-09-02
hCoV-19/Japan/PG-76447/2021	EPI_ISL_3871601	2021-09-02
hCoV-19/Japan/PG-76448/2021	EPI_ISL_3871602	2021-09-02
hCoV-19/Japan/PG-76449/2021	EPI_ISL_3871603	2021-09-02
hCoV-19/Japan/PG-76450/2021	EPI_ISL_3871604	2021-09-02
hCoV-19/Japan/PG-76451/2021	EPI_ISL_3871605	2021-09-02
hCoV-19/Japan/PG-76452/2021	EPI_ISL_3871606	2021-09-02
hCoV-19/Japan/PG-76453/2021	EPI_ISL_3871607	2021-09-02
hCoV-19/Japan/PG-76454/2021	EPI_ISL_3871608	2021-09-02
hCoV-19/Japan/PG-76455/2021	EPI_ISL_3871609	2021-09-02
hCoV-19/Japan/PG-76456/2021	EPI_ISL_3871018	2021-09-02
hCoV-19/Japan/PG-76457/2021	EPI_ISL_3871019	2021-09-02
hCoV-19/Japan/PG-76458/2021	EPI_ISL_3871020	2021-09-02
hCoV-19/Japan/PG-76459/2021	EPI_ISL_3871021	2021-09-02
hCoV-19/Japan/PG-76460/2021	EPI_ISL_3871022	2021-09-02
hCoV-19/Japan/PG-76461/2021	EPI_ISL_3871023	2021-09-02
hCoV-19/Japan/PG-76462/2021	EPI_ISL_3871024	2021-09-02
hCoV-19/Japan/PG-76463/2021	EPI_ISL_3871025	2021-09-02
hCoV-19/Japan/PG-76464/2021	EPI_ISL_3871026	2021-09-02
hCoV-19/Japan/PG-76465/2021	EPI_ISL_3871027	2021-09-02
hCoV-19/Japan/PG-76466/2021	EPI_ISL_3871028	2021-09-02
hCoV-19/Japan/PG-76467/2021	EPI_ISL_3871029	2021-09-02
hCoV-19/Japan/PG-76468/2021	EPI_ISL_3871030	2021-09-02
hCoV-19/Japan/PG-76469/2021	EPI_ISL_3871032	2021-09-02
hCoV-19/Japan/PG-76470/2021	EPI_ISL_3871034	2021-09-02
hCoV-19/Japan/PG-76471/2021	EPI_ISL_3871036	2021-09-02
hCoV-19/Japan/PG-76472/2021	EPI_ISL_3871038	2021-09-02
hCoV-19/Japan/PG-76473/2021	EPI_ISL_3871040	2021-09-02

virus_name	epiID	submission_date
hCoV-19/Japan/PG-76474/2021	EPI_ISL_3871042	2021-09-02
hCoV-19/Japan/PG-76475/2021	EPI_ISL_3871044	2021-09-02
hCoV-19/Japan/PG-76476/2021	EPI_ISL_3871046	2021-09-02
hCoV-19/Japan/PG-76477/2021	EPI_ISL_3871047	2021-09-02
hCoV-19/Japan/PG-76478/2021	EPI_ISL_3871049	2021-09-02
hCoV-19/Japan/PG-76479/2021	EPI_ISL_3871051	2021-09-02
hCoV-19/Japan/PG-76480/2021	EPI_ISL_3871236	2021-09-02
hCoV-19/Japan/PG-76481/2021	EPI_ISL_3871237	2021-09-02
hCoV-19/Japan/PG-76482/2021	EPI_ISL_3871238	2021-09-02
hCoV-19/Japan/PG-76483/2021	EPI_ISL_3871239	2021-09-02
hCoV-19/Japan/PG-76484/2021	EPI_ISL_3871240	2021-09-02
hCoV-19/Japan/PG-76485/2021	EPI_ISL_3871241	2021-09-02
hCoV-19/Japan/PG-76486/2021	EPI_ISL_3871242	2021-09-02
hCoV-19/Japan/PG-76487/2021	EPI_ISL_3871243	2021-09-02
hCoV-19/Japan/PG-76488/2021	EPI_ISL_3871244	2021-09-02
hCoV-19/Japan/PG-76489/2021	EPI_ISL_3871245	2021-09-02
hCoV-19/Japan/PG-76490/2021	EPI_ISL_3871246	2021-09-02
hCoV-19/Japan/PG-76491/2021	EPI_ISL_3871247	2021-09-02
hCoV-19/Japan/PG-76492/2021	EPI_ISL_3871248	2021-09-02
hCoV-19/Japan/PG-76493/2021	EPI_ISL_3871249	2021-09-02
hCoV-19/Japan/PG-76494/2021	EPI_ISL_3871250	2021-09-02
hCoV-19/Japan/PG-76495/2021	EPI_ISL_3871251	2021-09-02
hCoV-19/Japan/PG-76496/2021	EPI_ISL_3871252	2021-09-02
hCoV-19/Japan/PG-76497/2021	EPI_ISL_3871253	2021-09-02
hCoV-19/Japan/PG-76498/2021	EPI_ISL_3871254	2021-09-02
hCoV-19/Japan/PG-76499/2021	EPI_ISL_3871255	2021-09-02
hCoV-19/Japan/PG-76500/2021	EPI_ISL_3871256	2021-09-02
hCoV-19/Japan/PG-76501/2021	EPI_ISL_3871257	2021-09-02
hCoV-19/Japan/PG-76502/2021	EPI_ISL_3871258	2021-09-02
hCoV-19/Japan/PG-76503/2021	EPI_ISL_3871259	2021-09-02
hCoV-19/Japan/PG-76504/2021	EPI_ISL_3874009	2021-09-02
hCoV-19/Japan/PG-76505/2021	EPI_ISL_3874010	2021-09-02
hCoV-19/Japan/PG-76506/2021	EPI_ISL_3874011	2021-09-02
hCoV-19/Japan/PG-76507/2021	EPI_ISL_3874012	2021-09-02
hCoV-19/Japan/PG-76508/2021	EPI_ISL_3874013	2021-09-02
hCoV-19/Japan/PG-76509/2021	EPI_ISL_3874014	2021-09-02
hCoV-19/Japan/PG-76510/2021	EPI_ISL_3874015	2021-09-02
hCoV-19/Japan/PG-76511/2021	EPI_ISL_3874016	2021-09-02
hCoV-19/Japan/PG-76512/2021	EPI_ISL_3874017	2021-09-02
hCoV-19/Japan/PG-76513/2021	EPI_ISL_3874018	2021-09-02
hCoV-19/Japan/PG-76517/2021	EPI_ISL_3874019	2021-09-02
hCoV-19/Japan/PG-76518/2021	EPI_ISL_3874020	2021-09-02
hCoV-19/Japan/PG-76519/2021	EPI_ISL_3874021	2021-09-02
hCoV-19/Japan/PG-76520/2021	EPI_ISL_3874022	2021-09-02
hCoV-19/Japan/PG-76521/2021	EPI_ISL_3874023	2021-09-02
hCoV-19/Japan/PG-76522/2021	EPI_ISL_3874024	2021-09-02
hCoV-19/Japan/PG-76523/2021	EPI_ISL_3874025	2021-09-02
hCoV-19/Japan/PG-76524/2021	EPI_ISL_3874026	2021-09-02
hCoV-19/Japan/PG-76525/2021	EPI_ISL_3874027	2021-09-02
hCoV-19/Japan/PG-76526/2021	EPI_ISL_3874305	2021-09-02
hCoV-19/Japan/PG-76527/2021	EPI_ISL_3874306	2021-09-02
hCoV-19/Japan/PG-76528/2021	EPI_ISL_3874307	2021-09-02
hCoV-19/Japan/PG-76529/2021	EPI_ISL_3874167	2021-09-02
hCoV-19/Japan/PG-76530/2021	EPI_ISL_3874168	2021-09-02

virus_name	epiID	submission_date
hCoV-19/Japan/PG-76531/2021	EPI_ISL_3874169	2021-09-02
hCoV-19/Japan/PG-76532/2021	EPI_ISL_3874170	2021-09-02
hCoV-19/Japan/PG-76533/2021	EPI_ISL_3874171	2021-09-02
hCoV-19/Japan/PG-76534/2021	EPI_ISL_3874172	2021-09-02
hCoV-19/Japan/PG-76535/2021	EPI_ISL_3874173	2021-09-02
hCoV-19/Japan/PG-76536/2021	EPI_ISL_3874174	2021-09-02
hCoV-19/Japan/PG-76537/2021	EPI_ISL_3874175	2021-09-02
hCoV-19/Japan/PG-76538/2021	EPI_ISL_3874176	2021-09-02
hCoV-19/Japan/PG-76539/2021	EPI_ISL_3874177	2021-09-02
hCoV-19/Japan/PG-76540/2021	EPI_ISL_3874178	2021-09-02
hCoV-19/Japan/PG-76541/2021	EPI_ISL_3874179	2021-09-02
hCoV-19/Japan/PG-76542/2021	EPI_ISL_3874180	2021-09-02
hCoV-19/Japan/PG-76543/2021	EPI_ISL_3874181	2021-09-02
hCoV-19/Japan/PG-76544/2021	EPI_ISL_3886453	2021-09-02
hCoV-19/Japan/PG-76545/2021	EPI_ISL_3886455	2021-09-02
hCoV-19/Japan/PG-76546/2021	EPI_ISL_3886457	2021-09-02
hCoV-19/Japan/PG-76547/2021	EPI_ISL_3886459	2021-09-02
hCoV-19/Japan/PG-76548/2021	EPI_ISL_3886461	2021-09-02
hCoV-19/Japan/PG-76549/2021	EPI_ISL_3886463	2021-09-02
hCoV-19/Japan/PG-76550/2021	EPI_ISL_3886464	2021-09-02
hCoV-19/Japan/PG-76551/2021	EPI_ISL_3886467	2021-09-02
hCoV-19/Japan/PG-76552/2021	EPI_ISL_3886469	2021-09-02
hCoV-19/Japan/PG-76553/2021	EPI_ISL_3886471	2021-09-02
hCoV-19/Japan/PG-76554/2021	EPI_ISL_3886474	2021-09-02
hCoV-19/Japan/PG-76555/2021	EPI_ISL_3886476	2021-09-02
hCoV-19/Japan/PG-76556/2021	EPI_ISL_3886478	2021-09-02
hCoV-19/Japan/PG-76557/2021	EPI_ISL_3886481	2021-09-02
hCoV-19/Japan/PG-76558/2021	EPI_ISL_3886483	2021-09-02
hCoV-19/Japan/PG-76559/2021	EPI_ISL_3886485	2021-09-02
hCoV-19/Japan/PG-76560/2021	EPI_ISL_3886487	2021-09-02
hCoV-19/Japan/PG-76561/2021	EPI_ISL_3886489	2021-09-02
hCoV-19/Japan/PG-76562/2021	EPI_ISL_3886491	2021-09-02
hCoV-19/Japan/PG-76563/2021	EPI_ISL_3886493	2021-09-02
hCoV-19/Japan/PG-76564/2021	EPI_ISL_3886495	2021-09-02
hCoV-19/Japan/PG-76565/2021	EPI_ISL_3886497	2021-09-02
hCoV-19/Japan/PG-76566/2021	EPI_ISL_3886499	2021-09-02
hCoV-19/Japan/PG-76567/2021	EPI_ISL_3886501	2021-09-02
hCoV-19/Japan/PG-76568/2021	EPI_ISL_3886503	2021-09-02
hCoV-19/Japan/PG-76569/2021	EPI_ISL_3886505	2021-09-02
hCoV-19/Japan/PG-76570/2021	EPI_ISL_3886507	2021-09-02
hCoV-19/Japan/PG-76571/2021	EPI_ISL_3886509	2021-09-02
hCoV-19/Japan/PG-76572/2021	EPI_ISL_3886511	2021-09-02
hCoV-19/Japan/PG-76573/2021	EPI_ISL_3886513	2021-09-02
hCoV-19/Japan/PG-76574/2021	EPI_ISL_3886515	2021-09-02
hCoV-19/Japan/PG-76575/2021	EPI_ISL_3886518	2021-09-02
hCoV-19/Japan/PG-76576/2021	EPI_ISL_3886520	2021-09-02
hCoV-19/Japan/PG-76577/2021	EPI_ISL_3886522	2021-09-02
hCoV-19/Japan/PG-76578/2021	EPI_ISL_3886524	2021-09-02
hCoV-19/Japan/PG-76579/2021	EPI_ISL_3886527	2021-09-02
hCoV-19/Japan/PG-76580/2021	EPI_ISL_3886529	2021-09-02
hCoV-19/Japan/PG-76581/2021	EPI_ISL_3886531	2021-09-02
hCoV-19/Japan/PG-76582/2021	EPI_ISL_3886533	2021-09-02
hCoV-19/Japan/PG-76583/2021	EPI_ISL_3886534	2021-09-02
hCoV-19/Japan/PG-76584/2021	EPI_ISL_3886537	2021-09-02

virus_name	epiID	submission_date
hCoV-19/Japan/PG-76585/2021	EPI_ISL_3886539	2021-09-02
hCoV-19/Japan/PG-76586/2021	EPI_ISL_3886541	2021-09-02
hCoV-19/Japan/PG-76587/2021	EPI_ISL_3886544	2021-09-02
hCoV-19/Japan/PG-76588/2021	EPI_ISL_3886545	2021-09-02
hCoV-19/Japan/PG-76589/2021	EPI_ISL_3886548	2021-09-02
hCoV-19/Japan/PG-76590/2021	EPI_ISL_3886551	2021-09-02
hCoV-19/Japan/PG-76591/2021	EPI_ISL_3886555	2021-09-02
hCoV-19/Japan/PG-76592/2021	EPI_ISL_3886559	2021-09-02
hCoV-19/Japan/PG-76593/2021	EPI_ISL_3886563	2021-09-02
hCoV-19/Japan/PG-76594/2021	EPI_ISL_3886567	2021-09-02
hCoV-19/Japan/PG-76595/2021	EPI_ISL_3886570	2021-09-02
hCoV-19/Japan/PG-76596/2021	EPI_ISL_3886572	2021-09-02
hCoV-19/Japan/PG-76597/2021	EPI_ISL_3886575	2021-09-02
hCoV-19/Japan/PG-76598/2021	EPI_ISL_3886577	2021-09-02
hCoV-19/Japan/PG-76599/2021	EPI_ISL_3886580	2021-09-02
hCoV-19/Japan/PG-76600/2021	EPI_ISL_3886583	2021-09-02
hCoV-19/Japan/PG-76601/2021	EPI_ISL_3886585	2021-09-02
hCoV-19/Japan/PG-76602/2021	EPI_ISL_3886588	2021-09-02
hCoV-19/Japan/PG-76603/2021	EPI_ISL_3886589	2021-09-02
hCoV-19/Japan/PG-76604/2021	EPI_ISL_3886592	2021-09-02
hCoV-19/Japan/PG-76605/2021	EPI_ISL_3886594	2021-09-02
hCoV-19/Japan/PG-76606/2021	EPI_ISL_3886596	2021-09-02
hCoV-19/Japan/PG-76607/2021	EPI_ISL_3886599	2021-09-02
hCoV-19/Japan/PG-76608/2021	EPI_ISL_3886601	2021-09-02
hCoV-19/Japan/PG-76609/2021	EPI_ISL_3886602	2021-09-02
hCoV-19/Japan/PG-76610/2021	EPI_ISL_3886605	2021-09-02
hCoV-19/Japan/PG-76611/2021	EPI_ISL_3886608	2021-09-02
hCoV-19/Japan/PG-76612/2021	EPI_ISL_3886610	2021-09-02
hCoV-19/Japan/PG-76613/2021	EPI_ISL_3886612	2021-09-02
hCoV-19/Japan/PG-76614/2021	EPI_ISL_3886614	2021-09-02
hCoV-19/Japan/PG-76615/2021	EPI_ISL_3886616	2021-09-02
hCoV-19/Japan/PG-76616/2021	EPI_ISL_3886618	2021-09-02
hCoV-19/Japan/PG-76617/2021	EPI_ISL_3886620	2021-09-02
hCoV-19/Japan/PG-76618/2021	EPI_ISL_3886622	2021-09-02
hCoV-19/Japan/PG-76619/2021	EPI_ISL_3886625	2021-09-02
hCoV-19/Japan/PG-76620/2021	EPI_ISL_3886626	2021-09-02
hCoV-19/Japan/PG-76621/2021	EPI_ISL_3886628	2021-09-02
hCoV-19/Japan/PG-76622/2021	EPI_ISL_3886630	2021-09-02
hCoV-19/Japan/PG-76623/2021	EPI_ISL_3886633	2021-09-02
hCoV-19/Japan/PG-76624/2021	EPI_ISL_3886636	2021-09-02
hCoV-19/Japan/PG-76625/2021	EPI_ISL_3886638	2021-09-02
hCoV-19/Japan/PG-76626/2021	EPI_ISL_3886640	2021-09-02
hCoV-19/Japan/PG-76627/2021	EPI_ISL_3886642	2021-09-02
hCoV-19/Japan/PG-76628/2021	EPI_ISL_3886644	2021-09-02
hCoV-19/Japan/PG-76629/2021	EPI_ISL_3886645	2021-09-02
hCoV-19/Japan/PG-76630/2021	EPI_ISL_3886647	2021-09-02
hCoV-19/Japan/PG-76631/2021	EPI_ISL_3886649	2021-09-02
hCoV-19/Japan/PG-76632/2021	EPI_ISL_3886651	2021-09-02
hCoV-19/Japan/PG-76633/2021	EPI_ISL_3886653	2021-09-02
hCoV-19/Japan/PG-76634/2021	EPI_ISL_3886655	2021-09-02
hCoV-19/Japan/PG-76635/2021	EPI_ISL_3886658	2021-09-02
hCoV-19/Japan/PG-76636/2021	EPI_ISL_3886660	2021-09-02
hCoV-19/Japan/PG-76637/2021	EPI_ISL_3886661	2021-09-02
hCoV-19/Japan/PG-76638/2021	EPI_ISL_3886663	2021-09-02

virus_name	epiID	submission_date
hCoV-19/Japan/PG-76639/2021	EPI_ISL_3886664	2021-09-02
hCoV-19/Japan/PG-76640/2021	EPI_ISL_3886666	2021-09-02
hCoV-19/Japan/PG-76641/2021	EPI_ISL_3886668	2021-09-02
hCoV-19/Japan/PG-76642/2021	EPI_ISL_3886670	2021-09-02
hCoV-19/Japan/PG-76643/2021	EPI_ISL_3886672	2021-09-02
hCoV-19/Japan/PG-76644/2021	EPI_ISL_3886674	2021-09-02
hCoV-19/Japan/PG-76645/2021	EPI_ISL_3886677	2021-09-02
hCoV-19/Japan/PG-76646/2021	EPI_ISL_3886679	2021-09-02
hCoV-19/Japan/PG-76647/2021	EPI_ISL_3886681	2021-09-02
hCoV-19/Japan/PG-76648/2021	EPI_ISL_3886683	2021-09-02
hCoV-19/Japan/PG-76649/2021	EPI_ISL_3886686	2021-09-02
hCoV-19/Japan/PG-76650/2021	EPI_ISL_3886688	2021-09-02
hCoV-19/Japan/PG-76651/2021	EPI_ISL_3886691	2021-09-02
hCoV-19/Japan/PG-76652/2021	EPI_ISL_3886693	2021-09-02
hCoV-19/Japan/PG-76653/2021	EPI_ISL_3886695	2021-09-02
hCoV-19/Japan/PG-76654/2021	EPI_ISL_3886698	2021-09-02
hCoV-19/Japan/PG-76655/2021	EPI_ISL_3886700	2021-09-02
hCoV-19/Japan/PG-76656/2021	EPI_ISL_3886702	2021-09-02
hCoV-19/Japan/PG-76657/2021	EPI_ISL_3886705	2021-09-02
hCoV-19/Japan/PG-76658/2021	EPI_ISL_3886707	2021-09-02
hCoV-19/Japan/PG-76659/2021	EPI_ISL_3886710	2021-09-02
hCoV-19/Japan/PG-76660/2021	EPI_ISL_3886711	2021-09-02
hCoV-19/Japan/PG-76661/2021	EPI_ISL_3886713	2021-09-02
hCoV-19/Japan/PG-76662/2021	EPI_ISL_3886714	2021-09-02
hCoV-19/Japan/PG-76663/2021	EPI_ISL_3886717	2021-09-02
hCoV-19/Japan/PG-76664/2021	EPI_ISL_3886718	2021-09-02
hCoV-19/Japan/PG-76665/2021	EPI_ISL_3886719	2021-09-02
hCoV-19/Japan/PG-76666/2021	EPI_ISL_3886722	2021-09-02
hCoV-19/Japan/PG-76667/2021	EPI_ISL_3886723	2021-09-02
hCoV-19/Japan/PG-76668/2021	EPI_ISL_3886726	2021-09-02
hCoV-19/Japan/PG-76669/2021	EPI_ISL_3886728	2021-09-02
hCoV-19/Japan/PG-76670/2021	EPI_ISL_3886730	2021-09-02
hCoV-19/Japan/PG-76671/2021	EPI_ISL_3886732	2021-09-02
hCoV-19/Japan/PG-76672/2021	EPI_ISL_3886734	2021-09-02
hCoV-19/Japan/PG-76673/2021	EPI_ISL_3886736	2021-09-02
hCoV-19/Japan/PG-76674/2021	EPI_ISL_3886738	2021-09-02
hCoV-19/Japan/PG-76675/2021	EPI_ISL_3886740	2021-09-02
hCoV-19/Japan/PG-76676/2021	EPI_ISL_3886742	2021-09-02
hCoV-19/Japan/PG-76677/2021	EPI_ISL_3886744	2021-09-02
hCoV-19/Japan/PG-76678/2021	EPI_ISL_3886746	2021-09-02
hCoV-19/Japan/PG-76679/2021	EPI_ISL_3886748	2021-09-02
hCoV-19/Japan/PG-76680/2021	EPI_ISL_3886750	2021-09-02
hCoV-19/Japan/PG-76681/2021	EPI_ISL_3886751	2021-09-02
hCoV-19/Japan/PG-76682/2021	EPI_ISL_3886753	2021-09-02
hCoV-19/Japan/PG-76683/2021	EPI_ISL_3886755	2021-09-02
hCoV-19/Japan/PG-76684/2021	EPI_ISL_3886756	2021-09-02
hCoV-19/Japan/PG-76685/2021	EPI_ISL_3886757	2021-09-02
hCoV-19/Japan/PG-76686/2021	EPI_ISL_3886759	2021-09-02
hCoV-19/Japan/PG-76687/2021	EPI_ISL_3886762	2021-09-02
hCoV-19/Japan/PG-76688/2021	EPI_ISL_3886763	2021-09-02
hCoV-19/Japan/PG-76689/2021	EPI_ISL_3886766	2021-09-02
hCoV-19/Japan/PG-76690/2021	EPI_ISL_3886768	2021-09-02
hCoV-19/Japan/PG-76691/2021	EPI_ISL_3886771	2021-09-02
hCoV-19/Japan/PG-76692/2021	EPI_ISL_3886773	2021-09-02

virus_name	epiID	submission_date
hCoV-19/Japan/PG-76693/2021	EPI_ISL_3886775	2021-09-02
hCoV-19/Japan/PG-76694/2021	EPI_ISL_3886777	2021-09-02
hCoV-19/Japan/PG-76695/2021	EPI_ISL_3886779	2021-09-02
hCoV-19/Japan/PG-76696/2021	EPI_ISL_3886781	2021-09-02
hCoV-19/Japan/PG-76697/2021	EPI_ISL_3886783	2021-09-02
hCoV-19/Japan/PG-76698/2021	EPI_ISL_3886785	2021-09-02
hCoV-19/Japan/PG-76699/2021	EPI_ISL_3886788	2021-09-02
hCoV-19/Japan/PG-76700/2021	EPI_ISL_3886790	2021-09-02
hCoV-19/Japan/PG-76701/2021	EPI_ISL_3886792	2021-09-02
hCoV-19/Japan/PG-76702/2021	EPI_ISL_3886794	2021-09-02
hCoV-19/Japan/PG-76703/2021	EPI_ISL_3886795	2021-09-02
hCoV-19/Japan/PG-76704/2021	EPI_ISL_3886796	2021-09-02
hCoV-19/Japan/PG-76705/2021	EPI_ISL_3886798	2021-09-02
hCoV-19/Japan/PG-76706/2021	EPI_ISL_3886799	2021-09-02
hCoV-19/Japan/PG-76707/2021	EPI_ISL_3886801	2021-09-02
hCoV-19/Japan/PG-76708/2021	EPI_ISL_3886804	2021-09-02
hCoV-19/Japan/PG-76709/2021	EPI_ISL_3886806	2021-09-02
hCoV-19/Japan/PG-76710/2021	EPI_ISL_3886808	2021-09-02
hCoV-19/Japan/PG-76711/2021	EPI_ISL_3886811	2021-09-02
hCoV-19/Japan/PG-76712/2021	EPI_ISL_3886813	2021-09-02
hCoV-19/Japan/PG-76713/2021	EPI_ISL_3886816	2021-09-02
hCoV-19/Japan/PG-76714/2021	EPI_ISL_3886817	2021-09-02
hCoV-19/Japan/PG-76715/2021	EPI_ISL_3886820	2021-09-02
hCoV-19/Japan/PG-76716/2021	EPI_ISL_3886822	2021-09-02
hCoV-19/Japan/PG-76717/2021	EPI_ISL_3886824	2021-09-02
hCoV-19/Japan/PG-76718/2021	EPI_ISL_3886826	2021-09-02
hCoV-19/Japan/PG-76719/2021	EPI_ISL_3886828	2021-09-02
hCoV-19/Japan/PG-76720/2021	EPI_ISL_3886830	2021-09-02
hCoV-19/Japan/PG-76721/2021	EPI_ISL_3886832	2021-09-02
hCoV-19/Japan/PG-76722/2021	EPI_ISL_3886834	2021-09-02
hCoV-19/Japan/PG-76723/2021	EPI_ISL_3886836	2021-09-02
hCoV-19/Japan/PG-76724/2021	EPI_ISL_3886838	2021-09-02
hCoV-19/Japan/PG-76725/2021	EPI_ISL_3886840	2021-09-02
hCoV-19/Japan/PG-76726/2021	EPI_ISL_3886842	2021-09-02
hCoV-19/Japan/PG-76727/2021	EPI_ISL_3886845	2021-09-02
hCoV-19/Japan/PG-76728/2021	EPI_ISL_3886847	2021-09-02
hCoV-19/Japan/PG-76729/2021	EPI_ISL_3886849	2021-09-02
hCoV-19/Japan/PG-76730/2021	EPI_ISL_3886851	2021-09-02
hCoV-19/Japan/PG-76731/2021	EPI_ISL_3886853	2021-09-02
hCoV-19/Japan/PG-76732/2021	EPI_ISL_3886855	2021-09-02
hCoV-19/Japan/PG-76733/2021	EPI_ISL_3886858	2021-09-02
hCoV-19/Japan/PG-76734/2021	EPI_ISL_3886860	2021-09-02
hCoV-19/Japan/PG-76735/2021	EPI_ISL_3886862	2021-09-02
hCoV-19/Japan/PG-76736/2021	EPI_ISL_3886865	2021-09-02
hCoV-19/Japan/PG-76737/2021	EPI_ISL_3886867	2021-09-02
hCoV-19/Japan/PG-76738/2021	EPI_ISL_3886870	2021-09-02
hCoV-19/Japan/PG-76739/2021	EPI_ISL_3886872	2021-09-02
hCoV-19/Japan/PG-76740/2021	EPI_ISL_3886874	2021-09-02
hCoV-19/Japan/PG-76741/2021	EPI_ISL_3886876	2021-09-02
hCoV-19/Japan/PG-76742/2021	EPI_ISL_3886878	2021-09-02
hCoV-19/Japan/PG-76743/2021	EPI_ISL_3886879	2021-09-02
hCoV-19/Japan/PG-76744/2021	EPI_ISL_3886882	2021-09-02
hCoV-19/Japan/PG-76745/2021	EPI_ISL_3886884	2021-09-02
hCoV-19/Japan/PG-76746/2021	EPI_ISL_3886885	2021-09-02

virus_name	epiID	submission_date
hCoV-19/Japan/PG-76747/2021	EPI_ISL_3886886	2021-09-02
hCoV-19/Japan/PG-76748/2021	EPI_ISL_3886888	2021-09-02
hCoV-19/Japan/PG-76749/2021	EPI_ISL_3886890	2021-09-02
hCoV-19/Japan/PG-76750/2021	EPI_ISL_3886892	2021-09-02
hCoV-19/Japan/PG-76751/2021	EPI_ISL_3886893	2021-09-02
hCoV-19/Japan/PG-76752/2021	EPI_ISL_3886895	2021-09-02
hCoV-19/Japan/PG-76753/2021	EPI_ISL_3886897	2021-09-02
hCoV-19/Japan/PG-76754/2021	EPI_ISL_3886898	2021-09-02
hCoV-19/Japan/PG-76755/2021	EPI_ISL_3886900	2021-09-02
hCoV-19/Japan/PG-76756/2021	EPI_ISL_3886901	2021-09-02
hCoV-19/Japan/PG-76757/2021	EPI_ISL_3886903	2021-09-02
hCoV-19/Japan/PG-76758/2021	EPI_ISL_3886905	2021-09-02
hCoV-19/Japan/PG-76759/2021	EPI_ISL_3886907	2021-09-02
hCoV-19/Japan/PG-76760/2021	EPI_ISL_3886909	2021-09-02
hCoV-19/Japan/PG-76761/2021	EPI_ISL_3886912	2021-09-02
hCoV-19/Japan/PG-76762/2021	EPI_ISL_3886913	2021-09-02
hCoV-19/Japan/PG-76763/2021	EPI_ISL_3886915	2021-09-02
hCoV-19/Japan/PG-76764/2021	EPI_ISL_3886918	2021-09-02
hCoV-19/Japan/PG-76765/2021	EPI_ISL_3886920	2021-09-02
hCoV-19/Japan/PG-76766/2021	EPI_ISL_3886922	2021-09-02
hCoV-19/Japan/PG-76767/2021	EPI_ISL_3886924	2021-09-02
hCoV-19/Japan/PG-76768/2021	EPI_ISL_3886925	2021-09-02
hCoV-19/Japan/PG-76769/2021	EPI_ISL_3886926	2021-09-02
hCoV-19/Japan/PG-76770/2021	EPI_ISL_3886927	2021-09-02
hCoV-19/Japan/PG-76771/2021	EPI_ISL_3886928	2021-09-02
hCoV-19/Japan/PG-76772/2021	EPI_ISL_3886929	2021-09-02
hCoV-19/Japan/PG-76773/2021	EPI_ISL_3886931	2021-09-02
hCoV-19/Japan/PG-76774/2021	EPI_ISL_3886933	2021-09-02
hCoV-19/Japan/PG-76775/2021	EPI_ISL_3886935	2021-09-02
hCoV-19/Japan/PG-76776/2021	EPI_ISL_3886938	2021-09-02
hCoV-19/Japan/PG-76777/2021	EPI_ISL_3886940	2021-09-02
hCoV-19/Japan/PG-76778/2021	EPI_ISL_3886942	2021-09-02
hCoV-19/Japan/PG-76779/2021	EPI_ISL_3886944	2021-09-02
hCoV-19/Japan/PG-76780/2021	EPI_ISL_3886945	2021-09-02
hCoV-19/Japan/PG-76781/2021	EPI_ISL_3886948	2021-09-02
hCoV-19/Japan/PG-76782/2021	EPI_ISL_3886950	2021-09-02
hCoV-19/Japan/PG-76783/2021	EPI_ISL_3886952	2021-09-02
hCoV-19/Japan/PG-76784/2021	EPI_ISL_3886954	2021-09-02
hCoV-19/Japan/PG-76785/2021	EPI_ISL_3886955	2021-09-02
hCoV-19/Japan/PG-76786/2021	EPI_ISL_3886956	2021-09-02
hCoV-19/Japan/PG-76787/2021	EPI_ISL_3886957	2021-09-02
hCoV-19/Japan/PG-76788/2021	EPI_ISL_3886958	2021-09-02
hCoV-19/Japan/PG-76789/2021	EPI_ISL_3886959	2021-09-02
hCoV-19/Japan/PG-76790/2021	EPI_ISL_3886961	2021-09-02
hCoV-19/Japan/PG-76791/2021	EPI_ISL_3886962	2021-09-02
hCoV-19/Japan/PG-76792/2021	EPI_ISL_3886964	2021-09-02
hCoV-19/Japan/PG-76793/2021	EPI_ISL_3886966	2021-09-02
hCoV-19/Japan/PG-76794/2021	EPI_ISL_3886967	2021-09-02
hCoV-19/Japan/PG-76795/2021	EPI_ISL_3886969	2021-09-02
hCoV-19/Japan/PG-76796/2021	EPI_ISL_3886970	2021-09-02
hCoV-19/Japan/PG-76797/2021	EPI_ISL_3886972	2021-09-02
hCoV-19/Japan/PG-76798/2021	EPI_ISL_3886973	2021-09-02
hCoV-19/Japan/PG-76799/2021	EPI_ISL_3886975	2021-09-02
hCoV-19/Japan/PG-76800/2021	EPI_ISL_3886978	2021-09-02

virus_name	epiID	submission_date
hCoV-19/Japan/PG-76801/2021	EPI_ISL_3886980	2021-09-02
hCoV-19/Japan/PG-76802/2021	EPI_ISL_3886982	2021-09-02
hCoV-19/Japan/PG-76803/2021	EPI_ISL_3886984	2021-09-02
hCoV-19/Japan/PG-76804/2021	EPI_ISL_3886986	2021-09-02
hCoV-19/Japan/PG-76805/2021	EPI_ISL_3886988	2021-09-02
hCoV-19/Japan/PG-76806/2021	EPI_ISL_3886990	2021-09-02
hCoV-19/Japan/PG-76807/2021	EPI_ISL_3886992	2021-09-02
hCoV-19/Japan/PG-76808/2021	EPI_ISL_3886993	2021-09-02
hCoV-19/Japan/PG-76809/2021	EPI_ISL_3886995	2021-09-02
hCoV-19/Japan/PG-76810/2021	EPI_ISL_3886998	2021-09-02
hCoV-19/Japan/PG-76811/2021	EPI_ISL_3886999	2021-09-02
hCoV-19/Japan/PG-76812/2021	EPI_ISL_3887000	2021-09-02
hCoV-19/Japan/PG-76813/2021	EPI_ISL_3887001	2021-09-02
hCoV-19/Japan/PG-76814/2021	EPI_ISL_3887003	2021-09-02
hCoV-19/Japan/PG-76815/2021	EPI_ISL_3887005	2021-09-02
hCoV-19/Japan/PG-76816/2021	EPI_ISL_3887007	2021-09-02
hCoV-19/Japan/PG-76817/2021	EPI_ISL_3887009	2021-09-02
hCoV-19/Japan/PG-76818/2021	EPI_ISL_3887012	2021-09-02
hCoV-19/Japan/PG-76819/2021	EPI_ISL_3887014	2021-09-02
hCoV-19/Japan/PG-76820/2021	EPI_ISL_3887017	2021-09-02
hCoV-19/Japan/PG-76821/2021	EPI_ISL_3887019	2021-09-02
hCoV-19/Japan/PG-76822/2021	EPI_ISL_3887021	2021-09-02
hCoV-19/Japan/PG-76823/2021	EPI_ISL_3887023	2021-09-02
hCoV-19/Japan/PG-76824/2021	EPI_ISL_3887026	2021-09-02
hCoV-19/Japan/PG-76825/2021	EPI_ISL_3887029	2021-09-02
hCoV-19/Japan/PG-76826/2021	EPI_ISL_3887031	2021-09-02
hCoV-19/Japan/PG-76827/2021	EPI_ISL_3887034	2021-09-02
hCoV-19/Japan/PG-76828/2021	EPI_ISL_3887036	2021-09-02
hCoV-19/Japan/PG-76829/2021	EPI_ISL_3887038	2021-09-02
hCoV-19/Japan/PG-76830/2021	EPI_ISL_3887040	2021-09-02
hCoV-19/Japan/PG-76831/2021	EPI_ISL_3887042	2021-09-02
hCoV-19/Japan/PG-76832/2021	EPI_ISL_3887044	2021-09-02
hCoV-19/Japan/PG-76833/2021	EPI_ISL_3887046	2021-09-02
hCoV-19/Japan/PG-76834/2021	EPI_ISL_3887048	2021-09-02
hCoV-19/Japan/PG-76835/2021	EPI_ISL_3887050	2021-09-02
hCoV-19/Japan/PG-76836/2021	EPI_ISL_3887052	2021-09-02
hCoV-19/Japan/PG-76837/2021	EPI_ISL_3887054	2021-09-02
hCoV-19/Japan/PG-76838/2021	EPI_ISL_3887057	2021-09-02
hCoV-19/Japan/PG-76839/2021	EPI_ISL_3887059	2021-09-02
hCoV-19/Japan/PG-76840/2021	EPI_ISL_3887060	2021-09-02
hCoV-19/Japan/PG-76841/2021	EPI_ISL_3887061	2021-09-02
hCoV-19/Japan/PG-76842/2021	EPI_ISL_3887063	2021-09-02
hCoV-19/Japan/PG-76843/2021	EPI_ISL_3887066	2021-09-02
hCoV-19/Japan/PG-76844/2021	EPI_ISL_3887068	2021-09-02
hCoV-19/Japan/PG-76845/2021	EPI_ISL_3887070	2021-09-02
hCoV-19/Japan/PG-76846/2021	EPI_ISL_3887072	2021-09-02
hCoV-19/Japan/PG-76847/2021	EPI_ISL_3887073	2021-09-02
hCoV-19/Japan/PG-76848/2021	EPI_ISL_3887075	2021-09-02
hCoV-19/Japan/PG-76849/2021	EPI_ISL_3887078	2021-09-02
hCoV-19/Japan/PG-76850/2021	EPI_ISL_3887079	2021-09-02
hCoV-19/Japan/PG-76851/2021	EPI_ISL_3887082	2021-09-02
hCoV-19/Japan/PG-76852/2021	EPI_ISL_3887083	2021-09-02
hCoV-19/Japan/PG-76853/2021	EPI_ISL_3887085	2021-09-02
hCoV-19/Japan/PG-76854/2021	EPI_ISL_3887087	2021-09-02

virus_name	epiID	submission_date
hCoV-19/Japan/PG-76855/2021	EPI_ISL_3887090	2021-09-02
hCoV-19/Japan/PG-76856/2021	EPI_ISL_3887092	2021-09-02
hCoV-19/Japan/PG-76857/2021	EPI_ISL_3887094	2021-09-02
hCoV-19/Japan/PG-76858/2021	EPI_ISL_3887096	2021-09-02
hCoV-19/Japan/PG-76859/2021	EPI_ISL_3887099	2021-09-02
hCoV-19/Japan/PG-76860/2021	EPI_ISL_3887100	2021-09-02
hCoV-19/Japan/PG-76861/2021	EPI_ISL_3887102	2021-09-02
hCoV-19/Japan/PG-76862/2021	EPI_ISL_3887104	2021-09-02
hCoV-19/Japan/PG-76863/2021	EPI_ISL_3887106	2021-09-02
hCoV-19/Japan/PG-76864/2021	EPI_ISL_3887109	2021-09-02
hCoV-19/Japan/PG-76865/2021	EPI_ISL_3887111	2021-09-02
hCoV-19/Japan/PG-76866/2021	EPI_ISL_3887113	2021-09-02
hCoV-19/Japan/PG-76867/2021	EPI_ISL_3887115	2021-09-02
hCoV-19/Japan/PG-76868/2021	EPI_ISL_3887117	2021-09-02
hCoV-19/Japan/PG-76869/2021	EPI_ISL_3887120	2021-09-02
hCoV-19/Japan/PG-76870/2021	EPI_ISL_3887121	2021-09-02
hCoV-19/Japan/PG-76871/2021	EPI_ISL_3887123	2021-09-02
hCoV-19/Japan/PG-76872/2021	EPI_ISL_3887125	2021-09-02
hCoV-19/Japan/PG-76873/2021	EPI_ISL_3887127	2021-09-02
hCoV-19/Japan/PG-76874/2021	EPI_ISL_3887129	2021-09-02
hCoV-19/Japan/PG-76875/2021	EPI_ISL_3887130	2021-09-02
hCoV-19/Japan/PG-76876/2021	EPI_ISL_3887133	2021-09-02
hCoV-19/Japan/PG-76877/2021	EPI_ISL_3887135	2021-09-02
hCoV-19/Japan/PG-76878/2021	EPI_ISL_3887138	2021-09-02
hCoV-19/Japan/PG-76879/2021	EPI_ISL_3887139	2021-09-02
hCoV-19/Japan/PG-76880/2021	EPI_ISL_3887140	2021-09-02
hCoV-19/Japan/PG-76881/2021	EPI_ISL_3887141	2021-09-02
hCoV-19/Japan/PG-76882/2021	EPI_ISL_3887143	2021-09-02
hCoV-19/Japan/PG-76883/2021	EPI_ISL_3887145	2021-09-02
hCoV-19/Japan/PG-76884/2021	EPI_ISL_3887148	2021-09-02
hCoV-19/Japan/PG-76885/2021	EPI_ISL_3887150	2021-09-02
hCoV-19/Japan/PG-76886/2021	EPI_ISL_3887152	2021-09-02
hCoV-19/Japan/PG-76887/2021	EPI_ISL_3887154	2021-09-02
hCoV-19/Japan/PG-76888/2021	EPI_ISL_3887156	2021-09-02
hCoV-19/Japan/PG-76889/2021	EPI_ISL_3887158	2021-09-02
hCoV-19/Japan/PG-76890/2021	EPI_ISL_3887161	2021-09-02
hCoV-19/Japan/PG-76891/2021	EPI_ISL_3887163	2021-09-02
hCoV-19/Japan/PG-76892/2021	EPI_ISL_3887166	2021-09-02
hCoV-19/Japan/PG-76893/2021	EPI_ISL_3887167	2021-09-02
hCoV-19/Japan/PG-76894/2021	EPI_ISL_3887169	2021-09-02
hCoV-19/Japan/PG-76895/2021	EPI_ISL_3887172	2021-09-02
hCoV-19/Japan/PG-76896/2021	EPI_ISL_3887174	2021-09-02
hCoV-19/Japan/PG-76897/2021	EPI_ISL_3887176	2021-09-02
hCoV-19/Japan/PG-76898/2021	EPI_ISL_3887178	2021-09-02
hCoV-19/Japan/PG-76899/2021	EPI_ISL_3887180	2021-09-02
hCoV-19/Japan/PG-76900/2021	EPI_ISL_3887182	2021-09-02
hCoV-19/Japan/PG-76901/2021	EPI_ISL_3887184	2021-09-02
hCoV-19/Japan/PG-76902/2021	EPI_ISL_3887187	2021-09-02
hCoV-19/Japan/PG-76903/2021	EPI_ISL_3887189	2021-09-02
hCoV-19/Japan/PG-76904/2021	EPI_ISL_3887191	2021-09-02
hCoV-19/Japan/PG-76905/2021	EPI_ISL_3887193	2021-09-02
hCoV-19/Japan/PG-76906/2021	EPI_ISL_3887195	2021-09-02
hCoV-19/Japan/PG-76907/2021	EPI_ISL_3887197	2021-09-02
hCoV-19/Japan/PG-76908/2021	EPI_ISL_3887199	2021-09-02

virus_name	epiID	submission_date
hCoV-19/Japan/PG-76909/2021	EPI_ISL_3887201	2021-09-02
hCoV-19/Japan/PG-76910/2021	EPI_ISL_3887204	2021-09-02
hCoV-19/Japan/PG-76911/2021	EPI_ISL_3887206	2021-09-02
hCoV-19/Japan/PG-76912/2021	EPI_ISL_3887207	2021-09-02
hCoV-19/Japan/PG-76913/2021	EPI_ISL_3887209	2021-09-02
hCoV-19/Japan/PG-76914/2021	EPI_ISL_3887211	2021-09-02
hCoV-19/Japan/PG-76915/2021	EPI_ISL_3887212	2021-09-02
hCoV-19/Japan/PG-76916/2021	EPI_ISL_3887214	2021-09-02
hCoV-19/Japan/PG-76917/2021	EPI_ISL_3887216	2021-09-02
hCoV-19/Japan/PG-76918/2021	EPI_ISL_3887218	2021-09-02
hCoV-19/Japan/PG-76919/2021	EPI_ISL_3887220	2021-09-02
hCoV-19/Japan/PG-76920/2021	EPI_ISL_3887222	2021-09-02
hCoV-19/Japan/PG-76921/2021	EPI_ISL_3887225	2021-09-02
hCoV-19/Japan/PG-76922/2021	EPI_ISL_3887227	2021-09-02
hCoV-19/Japan/PG-76923/2021	EPI_ISL_3887229	2021-09-02
hCoV-19/Japan/PG-76924/2021	EPI_ISL_3887231	2021-09-02
hCoV-19/Japan/PG-76925/2021	EPI_ISL_3887233	2021-09-02
hCoV-19/Japan/PG-76926/2021	EPI_ISL_3887236	2021-09-02
hCoV-19/Japan/PG-76927/2021	EPI_ISL_3887237	2021-09-02
hCoV-19/Japan/PG-76928/2021	EPI_ISL_3887238	2021-09-02
hCoV-19/Japan/PG-76929/2021	EPI_ISL_3887240	2021-09-02
hCoV-19/Japan/PG-76930/2021	EPI_ISL_3887242	2021-09-02
hCoV-19/Japan/PG-76931/2021	EPI_ISL_3887245	2021-09-02
hCoV-19/Japan/PG-76932/2021	EPI_ISL_3887247	2021-09-02
hCoV-19/Japan/PG-76933/2021	EPI_ISL_3887249	2021-09-02
hCoV-19/Japan/PG-76934/2021	EPI_ISL_3887251	2021-09-02
hCoV-19/Japan/PG-76935/2021	EPI_ISL_3887257	2021-09-02
hCoV-19/Japan/PG-76936/2021	EPI_ISL_3887259	2021-09-02
hCoV-19/Japan/PG-76937/2021	EPI_ISL_3887261	2021-09-02
hCoV-19/Japan/PG-76938/2021	EPI_ISL_3887262	2021-09-02
hCoV-19/Japan/PG-76939/2021	EPI_ISL_3887264	2021-09-02
hCoV-19/Japan/PG-76940/2021	EPI_ISL_3887266	2021-09-02
hCoV-19/Japan/PG-76941/2021	EPI_ISL_3887268	2021-09-02
hCoV-19/Japan/PG-76942/2021	EPI_ISL_3887271	2021-09-02
hCoV-19/Japan/PG-76943/2021	EPI_ISL_3887273	2021-09-02
hCoV-19/Japan/PG-76944/2021	EPI_ISL_3887275	2021-09-02
hCoV-19/Japan/PG-76945/2021	EPI_ISL_3887276	2021-09-02
hCoV-19/Japan/PG-76946/2021	EPI_ISL_3887278	2021-09-02
hCoV-19/Japan/PG-76947/2021	EPI_ISL_3887281	2021-09-02
hCoV-19/Japan/PG-76948/2021	EPI_ISL_3887283	2021-09-02
hCoV-19/Japan/PG-76949/2021	EPI_ISL_3887285	2021-09-02
hCoV-19/Japan/PG-76950/2021	EPI_ISL_3887287	2021-09-02
hCoV-19/Japan/PG-76951/2021	EPI_ISL_3887289	2021-09-02
hCoV-19/Japan/PG-76952/2021	EPI_ISL_3887291	2021-09-02
hCoV-19/Japan/PG-76953/2021	EPI_ISL_3887292	2021-09-02
hCoV-19/Japan/PG-76954/2021	EPI_ISL_3887293	2021-09-02
hCoV-19/Japan/PG-76955/2021	EPI_ISL_3887295	2021-09-02
hCoV-19/Japan/PG-76956/2021	EPI_ISL_3887296	2021-09-02
hCoV-19/Japan/PG-76957/2021	EPI_ISL_3887298	2021-09-02
hCoV-19/Japan/PG-76958/2021	EPI_ISL_3887300	2021-09-02
hCoV-19/Japan/PG-76959/2021	EPI_ISL_3887301	2021-09-02
hCoV-19/Japan/PG-76960/2021	EPI_ISL_3887304	2021-09-02
hCoV-19/Japan/PG-76961/2021	EPI_ISL_3887305	2021-09-02
hCoV-19/Japan/PG-76962/2021	EPI_ISL_3887308	2021-09-02

virus_name	epiID	submission_date
hCoV-19/Japan/PG-76963/2021	EPI_ISL_3887309	2021-09-02
hCoV-19/Japan/PG-76964/2021	EPI_ISL_3887311	2021-09-02
hCoV-19/Japan/PG-76965/2021	EPI_ISL_3887312	2021-09-02
hCoV-19/Japan/PG-76966/2021	EPI_ISL_3887314	2021-09-02
hCoV-19/Japan/PG-76967/2021	EPI_ISL_3887316	2021-09-02
hCoV-19/Japan/PG-76968/2021	EPI_ISL_3887318	2021-09-02
hCoV-19/Japan/PG-76969/2021	EPI_ISL_3887320	2021-09-02
hCoV-19/Japan/PG-76970/2021	EPI_ISL_3887322	2021-09-02
hCoV-19/Japan/PG-76971/2021	EPI_ISL_3887323	2021-09-02
hCoV-19/Japan/PG-76972/2021	EPI_ISL_3887325	2021-09-02
hCoV-19/Japan/PG-76973/2021	EPI_ISL_3887326	2021-09-02
hCoV-19/Japan/PG-76974/2021	EPI_ISL_3887327	2021-09-02
hCoV-19/Japan/PG-76975/2021	EPI_ISL_3887328	2021-09-02
hCoV-19/Japan/PG-76976/2021	EPI_ISL_3887330	2021-09-02
hCoV-19/Japan/PG-76977/2021	EPI_ISL_3887332	2021-09-02
hCoV-19/Japan/PG-76978/2021	EPI_ISL_3887333	2021-09-02
hCoV-19/Japan/PG-76979/2021	EPI_ISL_3887335	2021-09-02
hCoV-19/Japan/PG-76980/2021	EPI_ISL_3887336	2021-09-02
hCoV-19/Japan/PG-76981/2021	EPI_ISL_3887337	2021-09-02
hCoV-19/Japan/PG-76982/2021	EPI_ISL_3887338	2021-09-02
hCoV-19/Japan/PG-76983/2021	EPI_ISL_3887339	2021-09-02
hCoV-19/Japan/PG-76984/2021	EPI_ISL_3887341	2021-09-02
hCoV-19/Japan/PG-76985/2021	EPI_ISL_3887342	2021-09-02
hCoV-19/Japan/PG-76986/2021	EPI_ISL_3887344	2021-09-02
hCoV-19/Japan/PG-76987/2021	EPI_ISL_3887346	2021-09-02
hCoV-19/Japan/PG-76988/2021	EPI_ISL_3887347	2021-09-02
hCoV-19/Japan/PG-76989/2021	EPI_ISL_3887348	2021-09-02
hCoV-19/Japan/PG-76990/2021	EPI_ISL_3887349	2021-09-02
hCoV-19/Japan/PG-76991/2021	EPI_ISL_3887350	2021-09-02
hCoV-19/Japan/PG-76992/2021	EPI_ISL_3887351	2021-09-02
hCoV-19/Japan/PG-76993/2021	EPI_ISL_3887352	2021-09-02
hCoV-19/Japan/PG-76994/2021	EPI_ISL_3887354	2021-09-02
hCoV-19/Japan/PG-76995/2021	EPI_ISL_3887356	2021-09-02
hCoV-19/Japan/PG-76996/2021	EPI_ISL_3887357	2021-09-02
hCoV-19/Japan/PG-76997/2021	EPI_ISL_3887359	2021-09-02
hCoV-19/Japan/PG-76998/2021	EPI_ISL_3887361	2021-09-02
hCoV-19/Japan/PG-76999/2021	EPI_ISL_3887363	2021-09-02
hCoV-19/Japan/PG-77000/2021	EPI_ISL_3887364	2021-09-02
hCoV-19/Japan/PG-77001/2021	EPI_ISL_3887366	2021-09-02
hCoV-19/Japan/PG-77002/2021	EPI_ISL_3887368	2021-09-02
hCoV-19/Japan/PG-77003/2021	EPI_ISL_3887369	2021-09-02
hCoV-19/Japan/PG-77004/2021	EPI_ISL_3887370	2021-09-02
hCoV-19/Japan/PG-77005/2021	EPI_ISL_3887372	2021-09-02
hCoV-19/Japan/PG-77006/2021	EPI_ISL_3887374	2021-09-02
hCoV-19/Japan/PG-77007/2021	EPI_ISL_3887376	2021-09-02
hCoV-19/Japan/PG-77008/2021	EPI_ISL_3887377	2021-09-02
hCoV-19/Japan/PG-77009/2021	EPI_ISL_3887380	2021-09-02
hCoV-19/Japan/PG-77010/2021	EPI_ISL_3887381	2021-09-02
hCoV-19/Japan/PG-77011/2021	EPI_ISL_3887383	2021-09-02
hCoV-19/Japan/PG-77012/2021	EPI_ISL_3887385	2021-09-02
hCoV-19/Japan/PG-77013/2021	EPI_ISL_3887386	2021-09-02
hCoV-19/Japan/PG-77014/2021	EPI_ISL_3887388	2021-09-02
hCoV-19/Japan/PG-77015/2021	EPI_ISL_3887389	2021-09-02
hCoV-19/Japan/PG-77016/2021	EPI_ISL_3887391	2021-09-02

virus_name	epiID	submission_date
hCoV-19/Japan/PG-77017/2021	EPI_ISL_3887392	2021-09-02
hCoV-19/Japan/PG-77018/2021	EPI_ISL_3887394	2021-09-02
hCoV-19/Japan/PG-77019/2021	EPI_ISL_3887396	2021-09-02
hCoV-19/Japan/PG-77020/2021	EPI_ISL_3887398	2021-09-02
hCoV-19/Japan/PG-77021/2021	EPI_ISL_3887399	2021-09-02
hCoV-19/Japan/PG-77022/2021	EPI_ISL_3887401	2021-09-02
hCoV-19/Japan/PG-77023/2021	EPI_ISL_3887403	2021-09-02
hCoV-19/Japan/PG-77024/2021	EPI_ISL_3887405	2021-09-02
hCoV-19/Japan/PG-77025/2021	EPI_ISL_3887406	2021-09-02
hCoV-19/Japan/PG-77026/2021	EPI_ISL_3887407	2021-09-02
hCoV-19/Japan/PG-77027/2021	EPI_ISL_3887408	2021-09-02
hCoV-19/Japan/PG-77028/2021	EPI_ISL_3887409	2021-09-02
hCoV-19/Japan/PG-77029/2021	EPI_ISL_3887410	2021-09-02
hCoV-19/Japan/PG-77030/2021	EPI_ISL_3887411	2021-09-02
hCoV-19/Japan/PG-77031/2021	EPI_ISL_3887412	2021-09-02
hCoV-19/Japan/PG-77032/2021	EPI_ISL_3887414	2021-09-02
hCoV-19/Japan/PG-77033/2021	EPI_ISL_3887415	2021-09-02
hCoV-19/Japan/PG-77034/2021	EPI_ISL_3887417	2021-09-02
hCoV-19/Japan/PG-77035/2021	EPI_ISL_3887419	2021-09-02
hCoV-19/Japan/PG-77036/2021	EPI_ISL_3887420	2021-09-02
hCoV-19/Japan/PG-77037/2021	EPI_ISL_3887422	2021-09-02
hCoV-19/Japan/PG-77038/2021	EPI_ISL_3887424	2021-09-02
hCoV-19/Japan/PG-77039/2021	EPI_ISL_3887425	2021-09-02
hCoV-19/Japan/PG-77040/2021	EPI_ISL_3887426	2021-09-02
hCoV-19/Japan/PG-77041/2021	EPI_ISL_3887428	2021-09-02
hCoV-19/Japan/PG-77042/2021	EPI_ISL_3887430	2021-09-02
hCoV-19/Japan/PG-77043/2021	EPI_ISL_3887431	2021-09-02
hCoV-19/Japan/PG-77044/2021	EPI_ISL_3887432	2021-09-02
hCoV-19/Japan/PG-77045/2021	EPI_ISL_3887434	2021-09-02
hCoV-19/Japan/PG-77046/2021	EPI_ISL_3887435	2021-09-02
hCoV-19/Japan/PG-77047/2021	EPI_ISL_3887437	2021-09-02
hCoV-19/Japan/PG-77048/2021	EPI_ISL_3887439	2021-09-02
hCoV-19/Japan/PG-77049/2021	EPI_ISL_3887441	2021-09-02
hCoV-19/Japan/PG-77050/2021	EPI_ISL_3887442	2021-09-02
hCoV-19/Japan/PG-77051/2021	EPI_ISL_3887444	2021-09-02
hCoV-19/Japan/PG-77052/2021	EPI_ISL_3887446	2021-09-02
hCoV-19/Japan/PG-77053/2021	EPI_ISL_3887448	2021-09-02
hCoV-19/Japan/PG-77054/2021	EPI_ISL_3887449	2021-09-02
hCoV-19/Japan/PG-77055/2021	EPI_ISL_3887451	2021-09-02
hCoV-19/Japan/PG-77056/2021	EPI_ISL_3887453	2021-09-02
hCoV-19/Japan/PG-77057/2021	EPI_ISL_3887455	2021-09-02
hCoV-19/Japan/PG-77058/2021	EPI_ISL_3887456	2021-09-02
hCoV-19/Japan/PG-77059/2021	EPI_ISL_3887458	2021-09-02
hCoV-19/Japan/PG-77060/2021	EPI_ISL_3887459	2021-09-02
hCoV-19/Japan/PG-77061/2021	EPI_ISL_3887460	2021-09-02
hCoV-19/Japan/PG-77062/2021	EPI_ISL_3887461	2021-09-02
hCoV-19/Japan/PG-77063/2021	EPI_ISL_3887463	2021-09-02
hCoV-19/Japan/PG-77064/2021	EPI_ISL_3887464	2021-09-02
hCoV-19/Japan/PG-77065/2021	EPI_ISL_3887466	2021-09-02
hCoV-19/Japan/PG-77066/2021	EPI_ISL_3887468	2021-09-02
hCoV-19/Japan/PG-77067/2021	EPI_ISL_3887470	2021-09-02
hCoV-19/Japan/PG-77068/2021	EPI_ISL_3887472	2021-09-02
hCoV-19/Japan/PG-77069/2021	EPI_ISL_3887473	2021-09-02
hCoV-19/Japan/PG-77070/2021	EPI_ISL_3887475	2021-09-02

virus_name	epiID	submission_date
hCoV-19/Japan/PG-77071/2021	EPI_ISL_3887477	2021-09-02
hCoV-19/Japan/PG-77072/2021	EPI_ISL_3887480	2021-09-02
hCoV-19/Japan/PG-77073/2021	EPI_ISL_3887481	2021-09-02
hCoV-19/Japan/PG-77074/2021	EPI_ISL_3887482	2021-09-02
hCoV-19/Japan/PG-77075/2021	EPI_ISL_3887483	2021-09-02
hCoV-19/Japan/PG-77076/2021	EPI_ISL_3887484	2021-09-02
hCoV-19/Japan/PG-77077/2021	EPI_ISL_3887485	2021-09-02
hCoV-19/Japan/PG-77078/2021	EPI_ISL_3887487	2021-09-02
hCoV-19/Japan/PG-77079/2021	EPI_ISL_3887488	2021-09-02
hCoV-19/Japan/PG-77080/2021	EPI_ISL_3887490	2021-09-02
hCoV-19/Japan/PG-77081/2021	EPI_ISL_3887492	2021-09-02
hCoV-19/Japan/PG-77082/2021	EPI_ISL_3887494	2021-09-02
hCoV-19/Japan/PG-77083/2021	EPI_ISL_3887496	2021-09-02
hCoV-19/Japan/PG-77084/2021	EPI_ISL_3887498	2021-09-02
hCoV-19/Japan/PG-77085/2021	EPI_ISL_3887499	2021-09-02
hCoV-19/Japan/PG-77086/2021	EPI_ISL_3887500	2021-09-02
hCoV-19/Japan/PG-77087/2021	EPI_ISL_3887502	2021-09-02
hCoV-19/Japan/PG-77088/2021	EPI_ISL_3887503	2021-09-02
hCoV-19/Japan/PG-77089/2021	EPI_ISL_3887504	2021-09-02
hCoV-19/Japan/PG-77090/2021	EPI_ISL_3887506	2021-09-02
hCoV-19/Japan/PG-77091/2021	EPI_ISL_3887507	2021-09-02
hCoV-19/Japan/PG-77092/2021	EPI_ISL_3887509	2021-09-02
hCoV-19/Japan/PG-77093/2021	EPI_ISL_3887511	2021-09-02
hCoV-19/Japan/PG-77094/2021	EPI_ISL_3887512	2021-09-02
hCoV-19/Japan/PG-77095/2021	EPI_ISL_3887514	2021-09-02
hCoV-19/Japan/PG-77096/2021	EPI_ISL_3887516	2021-09-02
hCoV-19/Japan/PG-77097/2021	EPI_ISL_3887517	2021-09-02
hCoV-19/Japan/PG-77098/2021	EPI_ISL_3887520	2021-09-02
hCoV-19/Japan/PG-77099/2021	EPI_ISL_3887522	2021-09-02
hCoV-19/Japan/PG-77100/2021	EPI_ISL_3887524	2021-09-02
hCoV-19/Japan/PG-77101/2021	EPI_ISL_3887526	2021-09-02
hCoV-19/Japan/PG-77102/2021	EPI_ISL_3887528	2021-09-02
hCoV-19/Japan/PG-77103/2021	EPI_ISL_3887530	2021-09-02
hCoV-19/Japan/PG-77104/2021	EPI_ISL_3887531	2021-09-02
hCoV-19/Japan/PG-77105/2021	EPI_ISL_3887532	2021-09-02
hCoV-19/Japan/PG-77106/2021	EPI_ISL_3887534	2021-09-02
hCoV-19/Japan/PG-77107/2021	EPI_ISL_3887536	2021-09-02
hCoV-19/Japan/PG-77108/2021	EPI_ISL_3887538	2021-09-02
hCoV-19/Japan/PG-77109/2021	EPI_ISL_3887540	2021-09-02
hCoV-19/Japan/PG-77110/2021	EPI_ISL_3887543	2021-09-02
hCoV-19/Japan/PG-77111/2021	EPI_ISL_3887544	2021-09-02
hCoV-19/Japan/PG-77112/2021	EPI_ISL_3887546	2021-09-02
hCoV-19/Japan/PG-77113/2021	EPI_ISL_3887548	2021-09-02
hCoV-19/Japan/PG-77114/2021	EPI_ISL_3887550	2021-09-02
hCoV-19/Japan/PG-77115/2021	EPI_ISL_3887552	2021-09-02
hCoV-19/Japan/PG-77116/2021	EPI_ISL_3887554	2021-09-02
hCoV-19/Japan/PG-77117/2021	EPI_ISL_3887556	2021-09-02
hCoV-19/Japan/PG-77118/2021	EPI_ISL_3887557	2021-09-02
hCoV-19/Japan/PG-77119/2021	EPI_ISL_3887559	2021-09-02
hCoV-19/Japan/PG-77120/2021	EPI_ISL_3887561	2021-09-02
hCoV-19/Japan/PG-77121/2021	EPI_ISL_3887564	2021-09-02
hCoV-19/Japan/PG-77122/2021	EPI_ISL_3887566	2021-09-02
hCoV-19/Japan/PG-77123/2021	EPI_ISL_3887569	2021-09-02
hCoV-19/Japan/PG-77124/2021	EPI_ISL_3887571	2021-09-02

virus_name	epiID	submission_date
hCoV-19/Japan/PG-77125/2021	EPI_ISL_3887573	2021-09-02
hCoV-19/Japan/PG-77126/2021	EPI_ISL_3887575	2021-09-02
hCoV-19/Japan/PG-77127/2021	EPI_ISL_3887577	2021-09-02
hCoV-19/Japan/PG-77128/2021	EPI_ISL_3887579	2021-09-02
hCoV-19/Japan/PG-77129/2021	EPI_ISL_3887581	2021-09-02
hCoV-19/Japan/PG-77130/2021	EPI_ISL_3887583	2021-09-02
hCoV-19/Japan/PG-77131/2021	EPI_ISL_3887585	2021-09-02
hCoV-19/Japan/PG-77132/2021	EPI_ISL_3887586	2021-09-02
hCoV-19/Japan/PG-77133/2021	EPI_ISL_3887589	2021-09-02
hCoV-19/Japan/PG-77134/2021	EPI_ISL_3887590	2021-09-02
hCoV-19/Japan/PG-77135/2021	EPI_ISL_3887592	2021-09-02
hCoV-19/Japan/PG-77136/2021	EPI_ISL_3887594	2021-09-02
hCoV-19/Japan/PG-77137/2021	EPI_ISL_3887597	2021-09-02
hCoV-19/Japan/PG-77138/2021	EPI_ISL_3887599	2021-09-02
hCoV-19/Japan/PG-77139/2021	EPI_ISL_3887601	2021-09-02
hCoV-19/Japan/PG-77140/2021	EPI_ISL_3887603	2021-09-02
hCoV-19/Japan/PG-77141/2021	EPI_ISL_3887605	2021-09-02
hCoV-19/Japan/PG-77142/2021	EPI_ISL_3887608	2021-09-02
hCoV-19/Japan/PG-77143/2021	EPI_ISL_3887610	2021-09-02
hCoV-19/Japan/PG-77144/2021	EPI_ISL_3887612	2021-09-02
hCoV-19/Japan/PG-77145/2021	EPI_ISL_3887614	2021-09-02
hCoV-19/Japan/PG-77146/2021	EPI_ISL_3887615	2021-09-02
hCoV-19/Japan/PG-77147/2021	EPI_ISL_3887617	2021-09-02
hCoV-19/Japan/PG-77148/2021	EPI_ISL_3887619	2021-09-02
hCoV-19/Japan/PG-77149/2021	EPI_ISL_3887621	2021-09-02
hCoV-19/Japan/PG-77150/2021	EPI_ISL_3887623	2021-09-02
hCoV-19/Japan/PG-77151/2021	EPI_ISL_3887624	2021-09-02
hCoV-19/Japan/PG-77152/2021	EPI_ISL_3887626	2021-09-02
hCoV-19/Japan/PG-77153/2021	EPI_ISL_3887627	2021-09-02
hCoV-19/Japan/PG-77154/2021	EPI_ISL_3887629	2021-09-02
hCoV-19/Japan/PG-77155/2021	EPI_ISL_3887630	2021-09-02
hCoV-19/Japan/PG-77156/2021	EPI_ISL_3887632	2021-09-02
hCoV-19/Japan/PG-77157/2021	EPI_ISL_3887634	2021-09-02
hCoV-19/Japan/PG-77158/2021	EPI_ISL_3887635	2021-09-02
hCoV-19/Japan/PG-77159/2021	EPI_ISL_3887636	2021-09-02
hCoV-19/Japan/PG-77160/2021	EPI_ISL_3887638	2021-09-02
hCoV-19/Japan/PG-77161/2021	EPI_ISL_3887640	2021-09-02
hCoV-19/Japan/PG-77162/2021	EPI_ISL_3887642	2021-09-02
hCoV-19/Japan/PG-77163/2021	EPI_ISL_3887643	2021-09-02
hCoV-19/Japan/PG-77164/2021	EPI_ISL_3887644	2021-09-02
hCoV-19/Japan/PG-77165/2021	EPI_ISL_3887646	2021-09-02
hCoV-19/Japan/PG-77166/2021	EPI_ISL_3887648	2021-09-02
hCoV-19/Japan/PG-77167/2021	EPI_ISL_3887649	2021-09-02
hCoV-19/Japan/PG-77168/2021	EPI_ISL_3887651	2021-09-02
hCoV-19/Japan/PG-77169/2021	EPI_ISL_3887652	2021-09-02
hCoV-19/Japan/PG-77170/2021	EPI_ISL_3887655	2021-09-02
hCoV-19/Japan/PG-77171/2021	EPI_ISL_3887657	2021-09-02
hCoV-19/Japan/PG-77172/2021	EPI_ISL_3887659	2021-09-02
hCoV-19/Japan/PG-77173/2021	EPI_ISL_3887661	2021-09-02
hCoV-19/Japan/PG-77174/2021	EPI_ISL_3887663	2021-09-02
hCoV-19/Japan/PG-77175/2021	EPI_ISL_3887665	2021-09-02
hCoV-19/Japan/PG-77176/2021	EPI_ISL_3887666	2021-09-02
hCoV-19/Japan/PG-77177/2021	EPI_ISL_3887668	2021-09-02
hCoV-19/Japan/PG-77178/2021	EPI_ISL_3887670	2021-09-02

virus_name	epiID	submission_date
hCoV-19/Japan/PG-77179/2021	EPI_ISL_3887672	2021-09-02
hCoV-19/Japan/PG-77180/2021	EPI_ISL_3887674	2021-09-02
hCoV-19/Japan/PG-77181/2021	EPI_ISL_3887675	2021-09-02
hCoV-19/Japan/PG-77182/2021	EPI_ISL_3887677	2021-09-02
hCoV-19/Japan/PG-77183/2021	EPI_ISL_3887679	2021-09-02
hCoV-19/Japan/PG-77184/2021	EPI_ISL_3887680	2021-09-02
hCoV-19/Japan/PG-77185/2021	EPI_ISL_3887681	2021-09-02
hCoV-19/Japan/PG-77186/2021	EPI_ISL_3887683	2021-09-02
hCoV-19/Japan/PG-77187/2021	EPI_ISL_3887685	2021-09-02
hCoV-19/Japan/PG-77188/2021	EPI_ISL_3887686	2021-09-02
hCoV-19/Japan/PG-77189/2021	EPI_ISL_3887688	2021-09-02
hCoV-19/Japan/PG-77190/2021	EPI_ISL_3887689	2021-09-02
hCoV-19/Japan/PG-77191/2021	EPI_ISL_3887691	2021-09-02
hCoV-19/Japan/PG-77192/2021	EPI_ISL_3887692	2021-09-02
hCoV-19/Japan/PG-77193/2021	EPI_ISL_3887694	2021-09-02
hCoV-19/Japan/PG-77194/2021	EPI_ISL_3887697	2021-09-02
hCoV-19/Japan/PG-77195/2021	EPI_ISL_3887698	2021-09-02
hCoV-19/Japan/PG-77196/2021	EPI_ISL_3887700	2021-09-02
hCoV-19/Japan/PG-77197/2021	EPI_ISL_3887702	2021-09-02
hCoV-19/Japan/PG-77198/2021	EPI_ISL_3887703	2021-09-02
hCoV-19/Japan/PG-77199/2021	EPI_ISL_3887705	2021-09-02
hCoV-19/Japan/PG-77200/2021	EPI_ISL_3887707	2021-09-02
hCoV-19/Japan/PG-77201/2021	EPI_ISL_3887708	2021-09-02
hCoV-19/Japan/PG-77202/2021	EPI_ISL_3887710	2021-09-02
hCoV-19/Japan/PG-77203/2021	EPI_ISL_3887711	2021-09-02
hCoV-19/Japan/PG-77204/2021	EPI_ISL_3887713	2021-09-02
hCoV-19/Japan/PG-77205/2021	EPI_ISL_3887715	2021-09-02
hCoV-19/Japan/PG-77206/2021	EPI_ISL_3887718	2021-09-02
hCoV-19/Japan/PG-77207/2021	EPI_ISL_3887720	2021-09-02
hCoV-19/Japan/PG-77208/2021	EPI_ISL_3887722	2021-09-02
hCoV-19/Japan/PG-77209/2021	EPI_ISL_3887724	2021-09-02
hCoV-19/Japan/PG-77210/2021	EPI_ISL_3887726	2021-09-02
hCoV-19/Japan/PG-77211/2021	EPI_ISL_3887728	2021-09-02
hCoV-19/Japan/PG-77212/2021	EPI_ISL_3887730	2021-09-02
hCoV-19/Japan/PG-77213/2021	EPI_ISL_3887731	2021-09-02
hCoV-19/Japan/PG-77214/2021	EPI_ISL_3887733	2021-09-02
hCoV-19/Japan/PG-77215/2021	EPI_ISL_3887735	2021-09-02
hCoV-19/Japan/PG-77216/2021	EPI_ISL_3887737	2021-09-02
hCoV-19/Japan/PG-77217/2021	EPI_ISL_3887739	2021-09-02
hCoV-19/Japan/PG-77218/2021	EPI_ISL_3887740	2021-09-02
hCoV-19/Japan/PG-77219/2021	EPI_ISL_3887742	2021-09-02
hCoV-19/Japan/PG-77220/2021	EPI_ISL_3887744	2021-09-02
hCoV-19/Japan/PG-77221/2021	EPI_ISL_3887746	2021-09-02
hCoV-19/Japan/PG-77223/2021	EPI_ISL_3887749	2021-09-02
hCoV-19/Japan/PG-77224/2021	EPI_ISL_3887751	2021-09-02
hCoV-19/Japan/PG-77225/2021	EPI_ISL_3887752	2021-09-02
hCoV-19/Japan/PG-77226/2021	EPI_ISL_3887755	2021-09-02
hCoV-19/Japan/PG-77227/2021	EPI_ISL_3887757	2021-09-02
hCoV-19/Japan/PG-77228/2021	EPI_ISL_3887759	2021-09-02
hCoV-19/Japan/PG-77229/2021	EPI_ISL_3887761	2021-09-02
hCoV-19/Japan/PG-77230/2021	EPI_ISL_3887763	2021-09-02
hCoV-19/Japan/PG-77231/2021	EPI_ISL_3887765	2021-09-02
hCoV-19/Japan/PG-77232/2021	EPI_ISL_3887766	2021-09-02
hCoV-19/Japan/PG-77233/2021	EPI_ISL_3887768	2021-09-02

virus_name	epiID	submission_date
hCoV-19/Japan/PG-77234/2021	EPI_ISL_3887770	2021-09-02
hCoV-19/Japan/PG-77235/2021	EPI_ISL_3887772	2021-09-02
hCoV-19/Japan/PG-77236/2021	EPI_ISL_3887773	2021-09-02
hCoV-19/Japan/PG-77237/2021	EPI_ISL_3887775	2021-09-02
hCoV-19/Japan/PG-77238/2021	EPI_ISL_3887777	2021-09-02
hCoV-19/Japan/PG-77239/2021	EPI_ISL_3887778	2021-09-02
hCoV-19/Japan/PG-77240/2021	EPI_ISL_3887781	2021-09-02
hCoV-19/Japan/PG-77241/2021	EPI_ISL_3887783	2021-09-02
hCoV-19/Japan/PG-77242/2021	EPI_ISL_3887785	2021-09-02
hCoV-19/Japan/PG-77243/2021	EPI_ISL_3887787	2021-09-02
hCoV-19/Japan/PG-77244/2021	EPI_ISL_3887789	2021-09-02
hCoV-19/Japan/PG-77245/2021	EPI_ISL_3887791	2021-09-02
hCoV-19/Japan/PG-77246/2021	EPI_ISL_3887793	2021-09-02
hCoV-19/Japan/PG-77247/2021	EPI_ISL_3887795	2021-09-02
hCoV-19/Japan/PG-77248/2021	EPI_ISL_3887797	2021-09-02
hCoV-19/Japan/PG-77249/2021	EPI_ISL_3887798	2021-09-02
hCoV-19/Japan/PG-77250/2021	EPI_ISL_3887800	2021-09-02
hCoV-19/Japan/PG-77251/2021	EPI_ISL_3887802	2021-09-02
hCoV-19/Japan/PG-77252/2021	EPI_ISL_3887804	2021-09-02
hCoV-19/Japan/PG-77253/2021	EPI_ISL_3887806	2021-09-02
hCoV-19/Japan/PG-77254/2021	EPI_ISL_3887807	2021-09-02
hCoV-19/Japan/PG-77255/2021	EPI_ISL_3887809	2021-09-02
hCoV-19/Japan/PG-77256/2021	EPI_ISL_3887811	2021-09-02
hCoV-19/Japan/PG-77257/2021	EPI_ISL_3887813	2021-09-02
hCoV-19/Japan/PG-77258/2021	EPI_ISL_3887815	2021-09-02
hCoV-19/Japan/PG-77259/2021	EPI_ISL_3887816	2021-09-02
hCoV-19/Japan/PG-77260/2021	EPI_ISL_3887819	2021-09-02
hCoV-19/Japan/PG-77261/2021	EPI_ISL_3887821	2021-09-02
hCoV-19/Japan/PG-77262/2021	EPI_ISL_3887823	2021-09-02
hCoV-19/Japan/PG-77263/2021	EPI_ISL_3887826	2021-09-02
hCoV-19/Japan/PG-77264/2021	EPI_ISL_3887828	2021-09-02
hCoV-19/Japan/PG-77265/2021	EPI_ISL_3887829	2021-09-02
hCoV-19/Japan/PG-77266/2021	EPI_ISL_3887832	2021-09-02
hCoV-19/Japan/PG-77267/2021	EPI_ISL_3887833	2021-09-02
hCoV-19/Japan/PG-77268/2021	EPI_ISL_3887835	2021-09-02
hCoV-19/Japan/PG-77269/2021	EPI_ISL_3887837	2021-09-02
hCoV-19/Japan/PG-77270/2021	EPI_ISL_3887839	2021-09-02
hCoV-19/Japan/PG-77271/2021	EPI_ISL_3887842	2021-09-02
hCoV-19/Japan/PG-77272/2021	EPI_ISL_3887844	2021-09-02
hCoV-19/Japan/PG-77273/2021	EPI_ISL_3887846	2021-09-02
hCoV-19/Japan/PG-77274/2021	EPI_ISL_3887848	2021-09-02
hCoV-19/Japan/PG-77275/2021	EPI_ISL_3887849	2021-09-02
hCoV-19/Japan/PG-77276/2021	EPI_ISL_3887851	2021-09-02
hCoV-19/Japan/PG-77277/2021	EPI_ISL_3887853	2021-09-02
hCoV-19/Japan/PG-77278/2021	EPI_ISL_3887855	2021-09-02
hCoV-19/Japan/PG-77279/2021	EPI_ISL_3887856	2021-09-02
hCoV-19/Japan/PG-77280/2021	EPI_ISL_3887858	2021-09-02
hCoV-19/Japan/PG-77281/2021	EPI_ISL_3887860	2021-09-02
hCoV-19/Japan/PG-77282/2021	EPI_ISL_3887862	2021-09-02
hCoV-19/Japan/PG-77283/2021	EPI_ISL_3887864	2021-09-02
hCoV-19/Japan/PG-77284/2021	EPI_ISL_3887866	2021-09-02
hCoV-19/Japan/PG-77285/2021	EPI_ISL_3887869	2021-09-02
hCoV-19/Japan/PG-77286/2021	EPI_ISL_3887871	2021-09-02
hCoV-19/Japan/PG-77287/2021	EPI_ISL_3887873	2021-09-02

virus_name	epiID	submission_date
hCoV-19/Japan/PG-77288/2021	EPI_ISL_3887875	2021-09-02
hCoV-19/Japan/PG-77289/2021	EPI_ISL_3887877	2021-09-02
hCoV-19/Japan/PG-77290/2021	EPI_ISL_3887879	2021-09-02
hCoV-19/Japan/PG-77291/2021	EPI_ISL_3887881	2021-09-02
hCoV-19/Japan/PG-77292/2021	EPI_ISL_3887883	2021-09-02
hCoV-19/Japan/PG-77293/2021	EPI_ISL_3887885	2021-09-02
hCoV-19/Japan/PG-77294/2021	EPI_ISL_3887886	2021-09-02
hCoV-19/Japan/PG-77295/2021	EPI_ISL_3887889	2021-09-02
hCoV-19/Japan/PG-77296/2021	EPI_ISL_3887891	2021-09-02
hCoV-19/Japan/PG-77297/2021	EPI_ISL_3887893	2021-09-02
hCoV-19/Japan/PG-77298/2021	EPI_ISL_3887895	2021-09-02
hCoV-19/Japan/PG-77299/2021	EPI_ISL_3887897	2021-09-02
hCoV-19/Japan/PG-77300/2021	EPI_ISL_3887899	2021-09-02
hCoV-19/Japan/PG-77301/2021	EPI_ISL_3887901	2021-09-02
hCoV-19/Japan/PG-77302/2021	EPI_ISL_3887903	2021-09-02
hCoV-19/Japan/PG-77303/2021	EPI_ISL_3887905	2021-09-02
hCoV-19/Japan/PG-77304/2021	EPI_ISL_3887907	2021-09-02
hCoV-19/Japan/PG-77305/2021	EPI_ISL_3887909	2021-09-02
hCoV-19/Japan/PG-77306/2021	EPI_ISL_3887911	2021-09-02
hCoV-19/Japan/PG-77307/2021	EPI_ISL_3887913	2021-09-02
hCoV-19/Japan/PG-77308/2021	EPI_ISL_3887915	2021-09-02
hCoV-19/Japan/PG-77309/2021	EPI_ISL_3887917	2021-09-02
hCoV-19/Japan/PG-77310/2021	EPI_ISL_3887919	2021-09-02
hCoV-19/Japan/PG-77311/2021	EPI_ISL_3887921	2021-09-02
hCoV-19/Japan/PG-77312/2021	EPI_ISL_3887923	2021-09-02
hCoV-19/Japan/PG-77313/2021	EPI_ISL_3887925	2021-09-02
hCoV-19/Japan/PG-77314/2021	EPI_ISL_3887927	2021-09-02
hCoV-19/Japan/PG-77315/2021	EPI_ISL_3887929	2021-09-02
hCoV-19/Japan/PG-77316/2021	EPI_ISL_3887931	2021-09-02
hCoV-19/Japan/PG-77317/2021	EPI_ISL_3887932	2021-09-02
hCoV-19/Japan/PG-77318/2021	EPI_ISL_3887934	2021-09-02
hCoV-19/Japan/PG-77319/2021	EPI_ISL_3887935	2021-09-02
hCoV-19/Japan/PG-77320/2021	EPI_ISL_3887937	2021-09-02
hCoV-19/Japan/PG-77321/2021	EPI_ISL_3887938	2021-09-02
hCoV-19/Japan/PG-77322/2021	EPI_ISL_3887940	2021-09-02
hCoV-19/Japan/PG-77323/2021	EPI_ISL_3887941	2021-09-02
hCoV-19/Japan/PG-77324/2021	EPI_ISL_3887943	2021-09-02
hCoV-19/Japan/PG-77325/2021	EPI_ISL_3887944	2021-09-02
hCoV-19/Japan/PG-77326/2021	EPI_ISL_3887946	2021-09-02
hCoV-19/Japan/PG-77327/2021	EPI_ISL_3887947	2021-09-02
hCoV-19/Japan/PG-77328/2021	EPI_ISL_3887948	2021-09-02
hCoV-19/Japan/PG-77329/2021	EPI_ISL_3887949	2021-09-02
hCoV-19/Japan/PG-77330/2021	EPI_ISL_3887951	2021-09-02
hCoV-19/Japan/PG-77331/2021	EPI_ISL_3887953	2021-09-02
hCoV-19/Japan/PG-77332/2021	EPI_ISL_3887954	2021-09-02
hCoV-19/Japan/PG-77333/2021	EPI_ISL_3887956	2021-09-02
hCoV-19/Japan/PG-77334/2021	EPI_ISL_3887958	2021-09-02
hCoV-19/Japan/PG-77335/2021	EPI_ISL_3887960	2021-09-02
hCoV-19/Japan/PG-77336/2021	EPI_ISL_3887962	2021-09-02
hCoV-19/Japan/PG-77337/2021	EPI_ISL_3887964	2021-09-02
hCoV-19/Japan/PG-77338/2021	EPI_ISL_3887967	2021-09-02
hCoV-19/Japan/PG-77339/2021	EPI_ISL_3887969	2021-09-02
hCoV-19/Japan/PG-77340/2021	EPI_ISL_3887972	2021-09-02
hCoV-19/Japan/PG-77341/2021	EPI_ISL_3887974	2021-09-02

virus_name	epiID	submission_date
hCoV-19/Japan/PG-77342/2021	EPI_ISL_3887976	2021-09-02
hCoV-19/Japan/PG-77343/2021	EPI_ISL_3887978	2021-09-02
hCoV-19/Japan/PG-77344/2021	EPI_ISL_3887980	2021-09-02
hCoV-19/Japan/PG-77345/2021	EPI_ISL_3887982	2021-09-02
hCoV-19/Japan/PG-77346/2021	EPI_ISL_3887984	2021-09-02
hCoV-19/Japan/PG-77347/2021	EPI_ISL_3887986	2021-09-02
hCoV-19/Japan/PG-77348/2021	EPI_ISL_3887988	2021-09-02
hCoV-19/Japan/PG-77349/2021	EPI_ISL_3887990	2021-09-02
hCoV-19/Japan/PG-77350/2021	EPI_ISL_3887992	2021-09-02
hCoV-19/Japan/PG-77351/2021	EPI_ISL_3887994	2021-09-02
hCoV-19/Japan/PG-77352/2021	EPI_ISL_3887996	2021-09-02
hCoV-19/Japan/PG-77353/2021	EPI_ISL_3887998	2021-09-02
hCoV-19/Japan/PG-77354/2021	EPI_ISL_3888000	2021-09-02
hCoV-19/Japan/PG-77355/2021	EPI_ISL_3888001	2021-09-02
hCoV-19/Japan/PG-77356/2021	EPI_ISL_3888003	2021-09-02
hCoV-19/Japan/PG-77357/2021	EPI_ISL_3888005	2021-09-02
hCoV-19/Japan/PG-77358/2021	EPI_ISL_3888007	2021-09-02
hCoV-19/Japan/PG-77359/2021	EPI_ISL_3888009	2021-09-02
hCoV-19/Japan/PG-77360/2021	EPI_ISL_3888011	2021-09-02
hCoV-19/Japan/PG-77361/2021	EPI_ISL_3888013	2021-09-02
hCoV-19/Japan/PG-77362/2021	EPI_ISL_3888015	2021-09-02
hCoV-19/Japan/PG-77363/2021	EPI_ISL_3888018	2021-09-02
hCoV-19/Japan/PG-77364/2021	EPI_ISL_3888020	2021-09-02
hCoV-19/Japan/PG-77365/2021	EPI_ISL_3888022	2021-09-02
hCoV-19/Japan/PG-77366/2021	EPI_ISL_3888025	2021-09-02
hCoV-19/Japan/PG-77367/2021	EPI_ISL_3888027	2021-09-02
hCoV-19/Japan/PG-77368/2021	EPI_ISL_3888029	2021-09-02
hCoV-19/Japan/PG-77369/2021	EPI_ISL_3888030	2021-09-02
hCoV-19/Japan/PG-77370/2021	EPI_ISL_3888032	2021-09-02
hCoV-19/Japan/PG-77371/2021	EPI_ISL_3888035	2021-09-02
hCoV-19/Japan/PG-77372/2021	EPI_ISL_3888037	2021-09-02
hCoV-19/Japan/PG-77373/2021	EPI_ISL_3888039	2021-09-02
hCoV-19/Japan/PG-77374/2021	EPI_ISL_3888040	2021-09-02
hCoV-19/Japan/PG-77375/2021	EPI_ISL_3888042	2021-09-02
hCoV-19/Japan/PG-77376/2021	EPI_ISL_3888045	2021-09-02
hCoV-19/Japan/PG-77377/2021	EPI_ISL_3888047	2021-09-02
hCoV-19/Japan/PG-77378/2021	EPI_ISL_3888048	2021-09-02
hCoV-19/Japan/PG-77379/2021	EPI_ISL_3888051	2021-09-02
hCoV-19/Japan/PG-77380/2021	EPI_ISL_3888053	2021-09-02
hCoV-19/Japan/PG-77381/2021	EPI_ISL_3888054	2021-09-02
hCoV-19/Japan/PG-77382/2021	EPI_ISL_3888056	2021-09-02
hCoV-19/Japan/PG-77383/2021	EPI_ISL_3888057	2021-09-02
hCoV-19/Japan/PG-77384/2021	EPI_ISL_3888059	2021-09-02
hCoV-19/Japan/PG-77385/2021	EPI_ISL_3888061	2021-09-02
hCoV-19/Japan/PG-77386/2021	EPI_ISL_3888064	2021-09-02
hCoV-19/Japan/PG-77387/2021	EPI_ISL_3888066	2021-09-02
hCoV-19/Japan/PG-77388/2021	EPI_ISL_3888068	2021-09-02
hCoV-19/Japan/PG-77389/2021	EPI_ISL_3888070	2021-09-02
hCoV-19/Japan/PG-77390/2021	EPI_ISL_3888072	2021-09-02
hCoV-19/Japan/PG-77391/2021	EPI_ISL_3888074	2021-09-02
hCoV-19/Japan/PG-77392/2021	EPI_ISL_3888076	2021-09-02
hCoV-19/Japan/PG-77393/2021	EPI_ISL_3888078	2021-09-02
hCoV-19/Japan/PG-77394/2021	EPI_ISL_3888080	2021-09-02
hCoV-19/Japan/PG-77395/2021	EPI_ISL_3888082	2021-09-02

virus_name	epiID	submission_date
hCoV-19/Japan/PG-77396/2021	EPI_ISL_3888084	2021-09-02
hCoV-19/Japan/PG-77397/2021	EPI_ISL_3888086	2021-09-02
hCoV-19/Japan/PG-77398/2021	EPI_ISL_3888088	2021-09-02
hCoV-19/Japan/PG-77399/2021	EPI_ISL_3888089	2021-09-02
hCoV-19/Japan/PG-77400/2021	EPI_ISL_3888090	2021-09-02
hCoV-19/Japan/PG-77401/2021	EPI_ISL_3888092	2021-09-02
hCoV-19/Japan/PG-77402/2021	EPI_ISL_3888095	2021-09-02
hCoV-19/Japan/PG-77403/2021	EPI_ISL_3888097	2021-09-02
hCoV-19/Japan/PG-77404/2021	EPI_ISL_3888098	2021-09-02
hCoV-19/Japan/PG-77405/2021	EPI_ISL_3888100	2021-09-02
hCoV-19/Japan/PG-77406/2021	EPI_ISL_3888101	2021-09-02
hCoV-19/Japan/PG-77408/2021	EPI_ISL_3888103	2021-09-02
hCoV-19/Japan/PG-77409/2021	EPI_ISL_3888105	2021-09-02
hCoV-19/Japan/PG-77410/2021	EPI_ISL_3888107	2021-09-02
hCoV-19/Japan/PG-77411/2021	EPI_ISL_3888109	2021-09-02
hCoV-19/Japan/PG-77412/2021	EPI_ISL_3888112	2021-09-02
hCoV-19/Japan/PG-77413/2021	EPI_ISL_3888113	2021-09-02
hCoV-19/Japan/PG-77414/2021	EPI_ISL_3888115	2021-09-02
hCoV-19/Japan/PG-77415/2021	EPI_ISL_3888117	2021-09-02
hCoV-19/Japan/PG-77416/2021	EPI_ISL_3888118	2021-09-02
hCoV-19/Japan/PG-77417/2021	EPI_ISL_3888119	2021-09-02
hCoV-19/Japan/PG-77418/2021	EPI_ISL_3888121	2021-09-02
hCoV-19/Japan/PG-77419/2021	EPI_ISL_3888122	2021-09-02
hCoV-19/Japan/PG-77420/2021	EPI_ISL_3888124	2021-09-02
hCoV-19/Japan/PG-77421/2021	EPI_ISL_3888125	2021-09-02
hCoV-19/Japan/PG-77422/2021	EPI_ISL_3888127	2021-09-02
hCoV-19/Japan/PG-77423/2021	EPI_ISL_3888129	2021-09-02
hCoV-19/Japan/PG-77424/2021	EPI_ISL_3888130	2021-09-02
hCoV-19/Japan/PG-77425/2021	EPI_ISL_3888132	2021-09-02
hCoV-19/Japan/PG-77426/2021	EPI_ISL_3888133	2021-09-02
hCoV-19/Japan/PG-77427/2021	EPI_ISL_3888134	2021-09-02
hCoV-19/Japan/PG-77428/2021	EPI_ISL_3888136	2021-09-02
hCoV-19/Japan/PG-77429/2021	EPI_ISL_3888138	2021-09-02
hCoV-19/Japan/PG-77430/2021	EPI_ISL_3888140	2021-09-02
hCoV-19/Japan/PG-77431/2021	EPI_ISL_3888141	2021-09-02
hCoV-19/Japan/PG-77432/2021	EPI_ISL_3888142	2021-09-02
hCoV-19/Japan/PG-77433/2021	EPI_ISL_3888145	2021-09-02
hCoV-19/Japan/PG-77434/2021	EPI_ISL_3888147	2021-09-02
hCoV-19/Japan/PG-77435/2021	EPI_ISL_3888149	2021-09-02
hCoV-19/Japan/PG-77436/2021	EPI_ISL_3888151	2021-09-02
hCoV-19/Japan/PG-77437/2021	EPI_ISL_3888155	2021-09-02
hCoV-19/Japan/PG-77438/2021	EPI_ISL_3888157	2021-09-02
hCoV-19/Japan/PG-77439/2021	EPI_ISL_3888159	2021-09-02
hCoV-19/Japan/PG-77440/2021	EPI_ISL_3888161	2021-09-02
hCoV-19/Japan/PG-77441/2021	EPI_ISL_3888164	2021-09-02
hCoV-19/Japan/PG-77442/2021	EPI_ISL_3888166	2021-09-02
hCoV-19/Japan/PG-77443/2021	EPI_ISL_3888168	2021-09-02
hCoV-19/Japan/PG-77444/2021	EPI_ISL_3888170	2021-09-02
hCoV-19/Japan/PG-77445/2021	EPI_ISL_3888172	2021-09-02
hCoV-19/Japan/PG-77446/2021	EPI_ISL_3888174	2021-09-02
hCoV-19/Japan/PG-77447/2021	EPI_ISL_3888177	2021-09-02
hCoV-19/Japan/PG-77448/2021	EPI_ISL_3888179	2021-09-02
hCoV-19/Japan/PG-77449/2021	EPI_ISL_3888180	2021-09-02
hCoV-19/Japan/PG-77450/2021	EPI_ISL_3888181	2021-09-02

virus_name	epiID	submission_date
hCoV-19/Japan/PG-77451/2021	EPI_ISL_3888183	2021-09-02
hCoV-19/Japan/PG-77452/2021	EPI_ISL_3888185	2021-09-02
hCoV-19/Japan/PG-77453/2021	EPI_ISL_3888187	2021-09-02
hCoV-19/Japan/PG-77454/2021	EPI_ISL_3888190	2021-09-02
hCoV-19/Japan/PG-77455/2021	EPI_ISL_3888192	2021-09-02
hCoV-19/Japan/PG-77456/2021	EPI_ISL_3888194	2021-09-02
hCoV-19/Japan/PG-77457/2021	EPI_ISL_3888196	2021-09-02
hCoV-19/Japan/PG-77458/2021	EPI_ISL_3888197	2021-09-02
hCoV-19/Japan/PG-77459/2021	EPI_ISL_3888199	2021-09-02
hCoV-19/Japan/PG-77460/2021	EPI_ISL_3888201	2021-09-02
hCoV-19/Japan/PG-77461/2021	EPI_ISL_3888202	2021-09-02
hCoV-19/Japan/PG-77462/2021	EPI_ISL_3888204	2021-09-02
hCoV-19/Japan/PG-77463/2021	EPI_ISL_3888206	2021-09-02
hCoV-19/Japan/PG-77464/2021	EPI_ISL_3888207	2021-09-02
hCoV-19/Japan/PG-77465/2021	EPI_ISL_3888209	2021-09-02
hCoV-19/Japan/PG-77466/2021	EPI_ISL_3888211	2021-09-02
hCoV-19/Japan/PG-77467/2021	EPI_ISL_3888213	2021-09-02
hCoV-19/Japan/PG-77468/2021	EPI_ISL_3888214	2021-09-02
hCoV-19/Japan/PG-77469/2021	EPI_ISL_3888215	2021-09-02
hCoV-19/Japan/PG-77470/2021	EPI_ISL_3888217	2021-09-02
hCoV-19/Japan/PG-77471/2021	EPI_ISL_3888219	2021-09-02
hCoV-19/Japan/PG-77472/2021	EPI_ISL_3888221	2021-09-02
hCoV-19/Japan/PG-77473/2021	EPI_ISL_3888223	2021-09-02
hCoV-19/Japan/PG-77474/2021	EPI_ISL_3888225	2021-09-02
hCoV-19/Japan/PG-77475/2021	EPI_ISL_3888227	2021-09-02
hCoV-19/Japan/PG-77476/2021	EPI_ISL_3888229	2021-09-02
hCoV-19/Japan/PG-77477/2021	EPI_ISL_3888230	2021-09-02
hCoV-19/Japan/PG-77478/2021	EPI_ISL_3888232	2021-09-02
hCoV-19/Japan/PG-77479/2021	EPI_ISL_3888234	2021-09-02
hCoV-19/Japan/PG-77480/2021	EPI_ISL_3888236	2021-09-02
hCoV-19/Japan/PG-77481/2021	EPI_ISL_3888238	2021-09-02
hCoV-19/Japan/PG-77482/2021	EPI_ISL_3888240	2021-09-02
hCoV-19/Japan/PG-77483/2021	EPI_ISL_3888241	2021-09-02
hCoV-19/Japan/PG-77484/2021	EPI_ISL_3888242	2021-09-02
hCoV-19/Japan/PG-77485/2021	EPI_ISL_3888244	2021-09-02
hCoV-19/Japan/PG-77486/2021	EPI_ISL_3888247	2021-09-02
hCoV-19/Japan/PG-77487/2021	EPI_ISL_3888249	2021-09-02
hCoV-19/Japan/PG-77488/2021	EPI_ISL_3888251	2021-09-02
hCoV-19/Japan/PG-77489/2021	EPI_ISL_3888253	2021-09-02
hCoV-19/Japan/PG-77490/2021	EPI_ISL_3888255	2021-09-02
hCoV-19/Japan/PG-77491/2021	EPI_ISL_3888257	2021-09-02
hCoV-19/Japan/PG-77492/2021	EPI_ISL_3888260	2021-09-02
hCoV-19/Japan/PG-77493/2021	EPI_ISL_3888262	2021-09-02
hCoV-19/Japan/PG-77494/2021	EPI_ISL_3888263	2021-09-02
hCoV-19/Japan/PG-77495/2021	EPI_ISL_3888265	2021-09-02
hCoV-19/Japan/PG-77496/2021	EPI_ISL_3888267	2021-09-02
hCoV-19/Japan/PG-77497/2021	EPI_ISL_3888269	2021-09-02
hCoV-19/Japan/PG-77498/2021	EPI_ISL_3888271	2021-09-02
hCoV-19/Japan/PG-77499/2021	EPI_ISL_3888274	2021-09-02
hCoV-19/Japan/PG-77500/2021	EPI_ISL_3888276	2021-09-02
hCoV-19/Japan/PG-77501/2021	EPI_ISL_3888279	2021-09-02
hCoV-19/Japan/PG-77502/2021	EPI_ISL_3888281	2021-09-02
hCoV-19/Japan/PG-77503/2021	EPI_ISL_3888283	2021-09-02
hCoV-19/Japan/PG-77504/2021	EPI_ISL_3888286	2021-09-02

virus_name	epiID	submission_date
hCoV-19/Japan/PG-77505/2021	EPI_ISL_3888288	2021-09-02
hCoV-19/Japan/PG-77506/2021	EPI_ISL_3888291	2021-09-02
hCoV-19/Japan/PG-77507/2021	EPI_ISL_3888293	2021-09-02
hCoV-19/Japan/PG-77508/2021	EPI_ISL_3888295	2021-09-02
hCoV-19/Japan/PG-77509/2021	EPI_ISL_3888298	2021-09-02
hCoV-19/Japan/PG-77510/2021	EPI_ISL_3888300	2021-09-02
hCoV-19/Japan/PG-77511/2021	EPI_ISL_3888302	2021-09-02
hCoV-19/Japan/PG-77512/2021	EPI_ISL_3888304	2021-09-02
hCoV-19/Japan/PG-77513/2021	EPI_ISL_3888306	2021-09-02
hCoV-19/Japan/PG-77514/2021	EPI_ISL_3888308	2021-09-02
hCoV-19/Japan/PG-77515/2021	EPI_ISL_3888310	2021-09-02
hCoV-19/Japan/PG-77516/2021	EPI_ISL_3888311	2021-09-02
hCoV-19/Japan/PG-77517/2021	EPI_ISL_3888313	2021-09-02
hCoV-19/Japan/PG-77518/2021	EPI_ISL_3888315	2021-09-02
hCoV-19/Japan/PG-77519/2021	EPI_ISL_3888316	2021-09-02
hCoV-19/Japan/PG-77520/2021	EPI_ISL_3888318	2021-09-02
hCoV-19/Japan/PG-77521/2021	EPI_ISL_3888320	2021-09-02
hCoV-19/Japan/PG-77522/2021	EPI_ISL_3888322	2021-09-02
hCoV-19/Japan/PG-77523/2021	EPI_ISL_3888324	2021-09-02
hCoV-19/Japan/PG-77524/2021	EPI_ISL_3888327	2021-09-02
hCoV-19/Japan/PG-77525/2021	EPI_ISL_3888329	2021-09-02
hCoV-19/Japan/PG-77526/2021	EPI_ISL_3888332	2021-09-02
hCoV-19/Japan/PG-77527/2021	EPI_ISL_3888334	2021-09-02
hCoV-19/Japan/PG-77528/2021	EPI_ISL_3888336	2021-09-02
hCoV-19/Japan/PG-77529/2021	EPI_ISL_3888339	2021-09-02
hCoV-19/Japan/PG-77530/2021	EPI_ISL_3888340	2021-09-02
hCoV-19/Japan/PG-77531/2021	EPI_ISL_3888343	2021-09-02
hCoV-19/Japan/PG-77532/2021	EPI_ISL_3888346	2021-09-02
hCoV-19/Japan/PG-77533/2021	EPI_ISL_3888348	2021-09-02
hCoV-19/Japan/PG-77534/2021	EPI_ISL_3888349	2021-09-02
hCoV-19/Japan/PG-77535/2021	EPI_ISL_3888350	2021-09-02
hCoV-19/Japan/PG-77536/2021	EPI_ISL_3888352	2021-09-02
hCoV-19/Japan/PG-77537/2021	EPI_ISL_3888354	2021-09-02
hCoV-19/Japan/PG-77538/2021	EPI_ISL_3888355	2021-09-02
hCoV-19/Japan/PG-77539/2021	EPI_ISL_3888358	2021-09-02
hCoV-19/Japan/PG-77540/2021	EPI_ISL_3888360	2021-09-02
hCoV-19/Japan/PG-77541/2021	EPI_ISL_3888362	2021-09-02
hCoV-19/Japan/PG-77542/2021	EPI_ISL_3888363	2021-09-02
hCoV-19/Japan/PG-77543/2021	EPI_ISL_3888364	2021-09-02
hCoV-19/Japan/PG-77544/2021	EPI_ISL_3888366	2021-09-02
hCoV-19/Japan/PG-77545/2021	EPI_ISL_3888368	2021-09-02
hCoV-19/Japan/PG-77546/2021	EPI_ISL_3888370	2021-09-02
hCoV-19/Japan/PG-77547/2021	EPI_ISL_3888372	2021-09-02
hCoV-19/Japan/PG-77548/2021	EPI_ISL_3888374	2021-09-02
hCoV-19/Japan/PG-77549/2021	EPI_ISL_3888376	2021-09-02
hCoV-19/Japan/PG-77550/2021	EPI_ISL_3888378	2021-09-02
hCoV-19/Japan/PG-77551/2021	EPI_ISL_3888380	2021-09-02
hCoV-19/Japan/PG-77552/2021	EPI_ISL_3888382	2021-09-02
hCoV-19/Japan/PG-77553/2021	EPI_ISL_3888384	2021-09-02
hCoV-19/Japan/PG-77554/2021	EPI_ISL_3888386	2021-09-02
hCoV-19/Japan/PG-77555/2021	EPI_ISL_3888388	2021-09-02
hCoV-19/Japan/PG-77556/2021	EPI_ISL_3888389	2021-09-02
hCoV-19/Japan/PG-77557/2021	EPI_ISL_3888390	2021-09-02
hCoV-19/Japan/PG-77558/2021	EPI_ISL_3888391	2021-09-02

virus_name	epiID	submission_date
hCoV-19/Japan/PG-77559/2021	EPI_ISL_3888392	2021-09-02
hCoV-19/Japan/PG-77560/2021	EPI_ISL_3888393	2021-09-02
hCoV-19/Japan/PG-77561/2021	EPI_ISL_3888394	2021-09-02
hCoV-19/Japan/PG-77562/2021	EPI_ISL_3888395	2021-09-02
hCoV-19/Japan/PG-77563/2021	EPI_ISL_3888396	2021-09-02
hCoV-19/Japan/PG-77564/2021	EPI_ISL_3888397	2021-09-02
hCoV-19/Japan/PG-77565/2021	EPI_ISL_3888398	2021-09-02
hCoV-19/Japan/PG-77566/2021	EPI_ISL_3888399	2021-09-02
hCoV-19/Japan/PG-77567/2021	EPI_ISL_3888400	2021-09-02
hCoV-19/Japan/PG-77568/2021	EPI_ISL_3888401	2021-09-02
hCoV-19/Japan/PG-77569/2021	EPI_ISL_3888402	2021-09-02
hCoV-19/Japan/PG-77570/2021	EPI_ISL_3888403	2021-09-02
hCoV-19/Japan/PG-77571/2021	EPI_ISL_3888404	2021-09-02
hCoV-19/Japan/PG-77572/2021	EPI_ISL_3888406	2021-09-02
hCoV-19/Japan/PG-77573/2021	EPI_ISL_3888409	2021-09-02
hCoV-19/Japan/PG-77574/2021	EPI_ISL_3888411	2021-09-02
hCoV-19/Japan/PG-77575/2021	EPI_ISL_3888412	2021-09-02
hCoV-19/Japan/PG-77576/2021	EPI_ISL_3888415	2021-09-02
hCoV-19/Japan/PG-77577/2021	EPI_ISL_3888417	2021-09-02
hCoV-19/Japan/PG-77578/2021	EPI_ISL_3888419	2021-09-02
hCoV-19/Japan/PG-77579/2021	EPI_ISL_3888421	2021-09-02
hCoV-19/Japan/PG-77580/2021	EPI_ISL_3888423	2021-09-02
hCoV-19/Japan/PG-77581/2021	EPI_ISL_3888425	2021-09-02
hCoV-19/Japan/PG-77582/2021	EPI_ISL_3888427	2021-09-02
hCoV-19/Japan/PG-77583/2021	EPI_ISL_3888430	2021-09-02
hCoV-19/Japan/PG-77584/2021	EPI_ISL_3888432	2021-09-02
hCoV-19/Japan/PG-77585/2021	EPI_ISL_3888434	2021-09-02
hCoV-19/Japan/PG-77586/2021	EPI_ISL_3888435	2021-09-02
hCoV-19/Japan/PG-77587/2021	EPI_ISL_3888438	2021-09-02
hCoV-19/Japan/PG-77588/2021	EPI_ISL_3888440	2021-09-02
hCoV-19/Japan/PG-77589/2021	EPI_ISL_3888442	2021-09-02
hCoV-19/Japan/PG-77590/2021	EPI_ISL_3888444	2021-09-02
hCoV-19/Japan/PG-77591/2021	EPI_ISL_3888445	2021-09-02
hCoV-19/Japan/PG-77592/2021	EPI_ISL_3888446	2021-09-02
hCoV-19/Japan/PG-77593/2021	EPI_ISL_3888447	2021-09-02
hCoV-19/Japan/PG-77594/2021	EPI_ISL_3888448	2021-09-02
hCoV-19/Japan/PG-77595/2021	EPI_ISL_3888449	2021-09-02
hCoV-19/Japan/PG-77596/2021	EPI_ISL_3888452	2021-09-02
hCoV-19/Japan/PG-77597/2021	EPI_ISL_3888454	2021-09-02
hCoV-19/Japan/PG-77598/2021	EPI_ISL_3888457	2021-09-02
hCoV-19/Japan/PG-77599/2021	EPI_ISL_3888459	2021-09-02
hCoV-19/Japan/PG-77600/2021	EPI_ISL_3888461	2021-09-02
hCoV-19/Japan/PG-77601/2021	EPI_ISL_3888464	2021-09-02
hCoV-19/Japan/PG-77602/2021	EPI_ISL_3888467	2021-09-02
hCoV-19/Japan/PG-77603/2021	EPI_ISL_3888470	2021-09-02
hCoV-19/Japan/PG-77604/2021	EPI_ISL_3888473	2021-09-02
hCoV-19/Japan/PG-77605/2021	EPI_ISL_3888475	2021-09-02
hCoV-19/Japan/PG-77606/2021	EPI_ISL_3888478	2021-09-02
hCoV-19/Japan/PG-77607/2021	EPI_ISL_3888479	2021-09-02
hCoV-19/Japan/PG-77608/2021	EPI_ISL_3888481	2021-09-02
hCoV-19/Japan/PG-77609/2021	EPI_ISL_3888484	2021-09-02
hCoV-19/Japan/PG-77610/2021	EPI_ISL_3888485	2021-09-02
hCoV-19/Japan/PG-77611/2021	EPI_ISL_3888488	2021-09-02
hCoV-19/Japan/PG-77612/2021	EPI_ISL_3888491	2021-09-02

virus_name	epiID	submission_date
hCoV-19/Japan/PG-77613/2021	EPI_ISL_3888493	2021-09-02
hCoV-19/Japan/PG-77614/2021	EPI_ISL_3888496	2021-09-02
hCoV-19/Japan/PG-77615/2021	EPI_ISL_3888499	2021-09-02
hCoV-19/Japan/PG-77616/2021	EPI_ISL_3888501	2021-09-02
hCoV-19/Japan/PG-77617/2021	EPI_ISL_3888503	2021-09-02
hCoV-19/Japan/PG-77618/2021	EPI_ISL_3888504	2021-09-02
hCoV-19/Japan/PG-77619/2021	EPI_ISL_3888506	2021-09-02
hCoV-19/Japan/PG-77620/2021	EPI_ISL_3888508	2021-09-02
hCoV-19/Japan/PG-77621/2021	EPI_ISL_3888511	2021-09-02
hCoV-19/Japan/PG-77622/2021	EPI_ISL_3888513	2021-09-02
hCoV-19/Japan/PG-77623/2021	EPI_ISL_3888514	2021-09-02
hCoV-19/Japan/PG-77624/2021	EPI_ISL_3888515	2021-09-02
hCoV-19/Japan/PG-77625/2021	EPI_ISL_3888516	2021-09-02
hCoV-19/Japan/PG-77626/2021	EPI_ISL_3888517	2021-09-02
hCoV-19/Japan/PG-77627/2021	EPI_ISL_3888518	2021-09-02
hCoV-19/Japan/PG-77628/2021	EPI_ISL_3888519	2021-09-02
hCoV-19/Japan/PG-77629/2021	EPI_ISL_3888522	2021-09-02
hCoV-19/Japan/PG-77630/2021	EPI_ISL_3888524	2021-09-02
hCoV-19/Japan/PG-77631/2021	EPI_ISL_3888526	2021-09-02
hCoV-19/Japan/PG-77632/2021	EPI_ISL_3888528	2021-09-02
hCoV-19/Japan/PG-77633/2021	EPI_ISL_3888531	2021-09-02
hCoV-19/Japan/PG-77634/2021	EPI_ISL_3888534	2021-09-02
hCoV-19/Japan/PG-77635/2021	EPI_ISL_3888537	2021-09-02
hCoV-19/Japan/PG-77636/2021	EPI_ISL_3888539	2021-09-02
hCoV-19/Japan/PG-77637/2021	EPI_ISL_3888541	2021-09-02
hCoV-19/Japan/PG-77638/2021	EPI_ISL_3888544	2021-09-02
hCoV-19/Japan/PG-77639/2021	EPI_ISL_3888546	2021-09-02
hCoV-19/Japan/PG-77640/2021	EPI_ISL_3888549	2021-09-02
hCoV-19/Japan/PG-77641/2021	EPI_ISL_3888551	2021-09-02
hCoV-19/Japan/PG-77642/2021	EPI_ISL_3888554	2021-09-02
hCoV-19/Japan/PG-77643/2021	EPI_ISL_3888556	2021-09-02
hCoV-19/Japan/PG-77644/2021	EPI_ISL_3888558	2021-09-02
hCoV-19/Japan/PG-77645/2021	EPI_ISL_3888561	2021-09-02
hCoV-19/Japan/PG-77646/2021	EPI_ISL_3888563	2021-09-02
hCoV-19/Japan/PG-77647/2021	EPI_ISL_3888565	2021-09-02
hCoV-19/Japan/PG-77648/2021	EPI_ISL_3888566	2021-09-02
hCoV-19/Japan/PG-77649/2021	EPI_ISL_3888569	2021-09-02
hCoV-19/Japan/PG-77650/2021	EPI_ISL_3888571	2021-09-02
hCoV-19/Japan/PG-77651/2021	EPI_ISL_3888574	2021-09-02
hCoV-19/Japan/PG-77652/2021	EPI_ISL_3888577	2021-09-02
hCoV-19/Japan/PG-77653/2021	EPI_ISL_3888579	2021-09-02
hCoV-19/Japan/PG-77654/2021	EPI_ISL_3888581	2021-09-02
hCoV-19/Japan/PG-77655/2021	EPI_ISL_3888583	2021-09-02
hCoV-19/Japan/PG-77656/2021	EPI_ISL_3888586	2021-09-02
hCoV-19/Japan/PG-77657/2021	EPI_ISL_3888587	2021-09-02
hCoV-19/Japan/PG-77658/2021	EPI_ISL_3888590	2021-09-02
hCoV-19/Japan/PG-77659/2021	EPI_ISL_3888593	2021-09-02
hCoV-19/Japan/PG-77660/2021	EPI_ISL_3888595	2021-09-02
hCoV-19/Japan/PG-77661/2021	EPI_ISL_3888597	2021-09-02
hCoV-19/Japan/PG-77662/2021	EPI_ISL_3888599	2021-09-02
hCoV-19/Japan/PG-77663/2021	EPI_ISL_3888601	2021-09-02
hCoV-19/Japan/PG-77664/2021	EPI_ISL_3888604	2021-09-02
hCoV-19/Japan/PG-77665/2021	EPI_ISL_3888606	2021-09-02
hCoV-19/Japan/PG-77666/2021	EPI_ISL_3888607	2021-09-02

virus_name	epiID	submission_date
hCoV-19/Japan/PG-77667/2021	EPI_ISL_3888609	2021-09-02
hCoV-19/Japan/PG-77668/2021	EPI_ISL_3888612	2021-09-02
hCoV-19/Japan/PG-77669/2021	EPI_ISL_3888614	2021-09-02
hCoV-19/Japan/PG-77670/2021	EPI_ISL_3888617	2021-09-02
hCoV-19/Japan/PG-77671/2021	EPI_ISL_3888620	2021-09-02
hCoV-19/Japan/PG-77672/2021	EPI_ISL_3888622	2021-09-02
hCoV-19/Japan/PG-77673/2021	EPI_ISL_3888624	2021-09-02
hCoV-19/Japan/PG-77674/2021	EPI_ISL_3888626	2021-09-02
hCoV-19/Japan/PG-77675/2021	EPI_ISL_3888628	2021-09-02
hCoV-19/Japan/PG-77676/2021	EPI_ISL_3888630	2021-09-02
hCoV-19/Japan/PG-77677/2021	EPI_ISL_3888632	2021-09-02
hCoV-19/Japan/PG-77678/2021	EPI_ISL_3888634	2021-09-02
hCoV-19/Japan/PG-77679/2021	EPI_ISL_3888636	2021-09-02
hCoV-19/Japan/PG-77680/2021	EPI_ISL_3888638	2021-09-02
hCoV-19/Japan/PG-77681/2021	EPI_ISL_3888639	2021-09-02
hCoV-19/Japan/PG-77682/2021	EPI_ISL_3888641	2021-09-02
hCoV-19/Japan/PG-77683/2021	EPI_ISL_3888643	2021-09-02
hCoV-19/Japan/PG-77684/2021	EPI_ISL_3888646	2021-09-02
hCoV-19/Japan/PG-77685/2021	EPI_ISL_3888647	2021-09-02
hCoV-19/Japan/PG-77686/2021	EPI_ISL_3888649	2021-09-02
hCoV-19/Japan/PG-77687/2021	EPI_ISL_3888651	2021-09-02
hCoV-19/Japan/PG-77688/2021	EPI_ISL_3888653	2021-09-02
hCoV-19/Japan/PG-77689/2021	EPI_ISL_3888655	2021-09-02
hCoV-19/Japan/PG-77690/2021	EPI_ISL_3888657	2021-09-02
hCoV-19/Japan/PG-77691/2021	EPI_ISL_3888659	2021-09-02
hCoV-19/Japan/PG-77692/2021	EPI_ISL_3888662	2021-09-02
hCoV-19/Japan/PG-77693/2021	EPI_ISL_3888663	2021-09-02
hCoV-19/Japan/PG-77694/2021	EPI_ISL_3888665	2021-09-02
hCoV-19/Japan/PG-77695/2021	EPI_ISL_3888668	2021-09-02
hCoV-19/Japan/PG-77696/2021	EPI_ISL_3888670	2021-09-02
hCoV-19/Japan/PG-77697/2021	EPI_ISL_3888673	2021-09-02
hCoV-19/Japan/PG-77698/2021	EPI_ISL_3888675	2021-09-02
hCoV-19/Japan/PG-77699/2021	EPI_ISL_3888676	2021-09-02
hCoV-19/Japan/PG-77700/2021	EPI_ISL_3888679	2021-09-02
hCoV-19/Japan/PG-77701/2021	EPI_ISL_3888681	2021-09-02
hCoV-19/Japan/PG-77702/2021	EPI_ISL_3888684	2021-09-02
hCoV-19/Japan/PG-77703/2021	EPI_ISL_3888686	2021-09-02
hCoV-19/Japan/PG-77704/2021	EPI_ISL_3888688	2021-09-02
hCoV-19/Japan/PG-77705/2021	EPI_ISL_3888690	2021-09-02
hCoV-19/Japan/PG-77706/2021	EPI_ISL_3888692	2021-09-02
hCoV-19/Japan/PG-77707/2021	EPI_ISL_3888694	2021-09-02
hCoV-19/Japan/PG-77708/2021	EPI_ISL_3888696	2021-09-02
hCoV-19/Japan/PG-77709/2021	EPI_ISL_3888698	2021-09-02
hCoV-19/Japan/PG-77710/2021	EPI_ISL_3888701	2021-09-02
hCoV-19/Japan/PG-77711/2021	EPI_ISL_3888703	2021-09-02
hCoV-19/Japan/PG-77712/2021	EPI_ISL_3888705	2021-09-02
hCoV-19/Japan/PG-77713/2021	EPI_ISL_3888707	2021-09-02
hCoV-19/Japan/PG-77714/2021	EPI_ISL_3888709	2021-09-02
hCoV-19/Japan/PG-77715/2021	EPI_ISL_3888711	2021-09-02
hCoV-19/Japan/PG-77716/2021	EPI_ISL_3888713	2021-09-02
hCoV-19/Japan/PG-77717/2021	EPI_ISL_3888715	2021-09-02
hCoV-19/Japan/PG-77718/2021	EPI_ISL_3888717	2021-09-02
hCoV-19/Japan/PG-77719/2021	EPI_ISL_3888719	2021-09-02
hCoV-19/Japan/PG-77720/2021	EPI_ISL_3888721	2021-09-02

virus_name	epiID	submission_date
hCoV-19/Japan/PG-77721/2021	EPI_ISL_3888722	2021-09-02
hCoV-19/Japan/PG-77722/2021	EPI_ISL_3888724	2021-09-02
hCoV-19/Japan/PG-77723/2021	EPI_ISL_3888727	2021-09-02
hCoV-19/Japan/PG-77724/2021	EPI_ISL_3888729	2021-09-02
hCoV-19/Japan/PG-77725/2021	EPI_ISL_3888731	2021-09-02
hCoV-19/Japan/PG-77726/2021	EPI_ISL_3888733	2021-09-02
hCoV-19/Japan/PG-77727/2021	EPI_ISL_3888736	2021-09-02
hCoV-19/Japan/PG-77728/2021	EPI_ISL_3888738	2021-09-02
hCoV-19/Japan/PG-77729/2021	EPI_ISL_3888741	2021-09-02
hCoV-19/Japan/PG-77730/2021	EPI_ISL_3888743	2021-09-02
hCoV-19/Japan/PG-77731/2021	EPI_ISL_3888745	2021-09-02
hCoV-19/Japan/PG-77732/2021	EPI_ISL_3888747	2021-09-02
hCoV-19/Japan/PG-77733/2021	EPI_ISL_3888749	2021-09-02
hCoV-19/Japan/PG-77734/2021	EPI_ISL_3888751	2021-09-02
hCoV-19/Japan/PG-77735/2021	EPI_ISL_3888752	2021-09-02
hCoV-19/Japan/PG-77736/2021	EPI_ISL_3888754	2021-09-02
hCoV-19/Japan/PG-77737/2021	EPI_ISL_3888757	2021-09-02
hCoV-19/Japan/PG-77738/2021	EPI_ISL_3888759	2021-09-02
hCoV-19/Japan/PG-77739/2021	EPI_ISL_3888761	2021-09-02
hCoV-19/Japan/PG-77740/2021	EPI_ISL_3888763	2021-09-02
hCoV-19/Japan/PG-77741/2021	EPI_ISL_3888764	2021-09-02
hCoV-19/Japan/PG-77742/2021	EPI_ISL_3888767	2021-09-02
hCoV-19/Japan/PG-77743/2021	EPI_ISL_3888769	2021-09-02
hCoV-19/Japan/PG-77744/2021	EPI_ISL_3888771	2021-09-02
hCoV-19/Japan/PG-77745/2021	EPI_ISL_3888773	2021-09-02
hCoV-19/Japan/PG-77746/2021	EPI_ISL_3888776	2021-09-02
hCoV-19/Japan/PG-77747/2021	EPI_ISL_3888778	2021-09-02
hCoV-19/Japan/PG-77748/2021	EPI_ISL_3888780	2021-09-02
hCoV-19/Japan/PG-77749/2021	EPI_ISL_3888783	2021-09-02
hCoV-19/Japan/PG-77750/2021	EPI_ISL_3888785	2021-09-02
hCoV-19/Japan/PG-77751/2021	EPI_ISL_3888788	2021-09-02
hCoV-19/Japan/PG-77752/2021	EPI_ISL_3888790	2021-09-02
hCoV-19/Japan/PG-77753/2021	EPI_ISL_3888792	2021-09-02
hCoV-19/Japan/PG-77754/2021	EPI_ISL_3888794	2021-09-02
hCoV-19/Japan/PG-77755/2021	EPI_ISL_3888795	2021-09-02
hCoV-19/Japan/PG-77756/2021	EPI_ISL_3888797	2021-09-02
hCoV-19/Japan/PG-77757/2021	EPI_ISL_3888800	2021-09-02
hCoV-19/Japan/PG-77758/2021	EPI_ISL_3888803	2021-09-02
hCoV-19/Japan/PG-77759/2021	EPI_ISL_3888804	2021-09-02
hCoV-19/Japan/PG-77760/2021	EPI_ISL_3888806	2021-09-02
hCoV-19/Japan/PG-77761/2021	EPI_ISL_3888808	2021-09-02
hCoV-19/Japan/PG-77762/2021	EPI_ISL_3888811	2021-09-02
hCoV-19/Japan/PG-77763/2021	EPI_ISL_3888813	2021-09-02
hCoV-19/Japan/PG-77764/2021	EPI_ISL_3888815	2021-09-02
hCoV-19/Japan/PG-77765/2021	EPI_ISL_3888817	2021-09-02
hCoV-19/Japan/PG-77766/2021	EPI_ISL_3888819	2021-09-02
hCoV-19/Japan/PG-77767/2021	EPI_ISL_3888822	2021-09-02
hCoV-19/Japan/PG-77768/2021	EPI_ISL_3888824	2021-09-02
hCoV-19/Japan/PG-77769/2021	EPI_ISL_3888826	2021-09-02
hCoV-19/Japan/PG-77770/2021	EPI_ISL_3888829	2021-09-02
hCoV-19/Japan/PG-77771/2021	EPI_ISL_3888831	2021-09-02
hCoV-19/Japan/PG-77772/2021	EPI_ISL_3888832	2021-09-02
hCoV-19/Japan/PG-77773/2021	EPI_ISL_3888834	2021-09-02
hCoV-19/Japan/PG-77774/2021	EPI_ISL_3888837	2021-09-02

virus_name	epiID	submission_date
hCoV-19/Japan/PG-77775/2021	EPI_ISL_3888839	2021-09-02
hCoV-19/Japan/PG-77776/2021	EPI_ISL_3888841	2021-09-02
hCoV-19/Japan/PG-77777/2021	EPI_ISL_3888844	2021-09-02
hCoV-19/Japan/PG-77778/2021	EPI_ISL_3888845	2021-09-02
hCoV-19/Japan/PG-77779/2021	EPI_ISL_3888848	2021-09-02
hCoV-19/Japan/PG-77780/2021	EPI_ISL_3888850	2021-09-02
hCoV-19/Japan/PG-77781/2021	EPI_ISL_3888852	2021-09-02
hCoV-19/Japan/PG-77782/2021	EPI_ISL_3888854	2021-09-02
hCoV-19/Japan/PG-77783/2021	EPI_ISL_3888856	2021-09-02
hCoV-19/Japan/PG-77784/2021	EPI_ISL_3888858	2021-09-02
hCoV-19/Japan/PG-77785/2021	EPI_ISL_3888860	2021-09-02
hCoV-19/Japan/PG-77786/2021	EPI_ISL_3888862	2021-09-02
hCoV-19/Japan/PG-77787/2021	EPI_ISL_3888865	2021-09-02
hCoV-19/Japan/PG-77788/2021	EPI_ISL_3888867	2021-09-02
hCoV-19/Japan/PG-77789/2021	EPI_ISL_3888868	2021-09-02
hCoV-19/Japan/PG-77790/2021	EPI_ISL_3888869	2021-09-02
hCoV-19/Japan/PG-77791/2021	EPI_ISL_3888871	2021-09-02
hCoV-19/Japan/PG-77792/2021	EPI_ISL_3888873	2021-09-02
hCoV-19/Japan/PG-77793/2021	EPI_ISL_3888876	2021-09-02
hCoV-19/Japan/PG-77794/2021	EPI_ISL_3888878	2021-09-02
hCoV-19/Japan/PG-77795/2021	EPI_ISL_3888880	2021-09-02
hCoV-19/Japan/PG-77796/2021	EPI_ISL_3888883	2021-09-02
hCoV-19/Japan/PG-77797/2021	EPI_ISL_3888885	2021-09-02
hCoV-19/Japan/PG-77798/2021	EPI_ISL_3888888	2021-09-02
hCoV-19/Japan/PG-77799/2021	EPI_ISL_3888890	2021-09-02
hCoV-19/Japan/PG-77800/2021	EPI_ISL_3888893	2021-09-02
hCoV-19/Japan/PG-77801/2021	EPI_ISL_3888895	2021-09-02
hCoV-19/Japan/PG-77802/2021	EPI_ISL_3888897	2021-09-02
hCoV-19/Japan/PG-77803/2021	EPI_ISL_3888900	2021-09-02
hCoV-19/Japan/PG-77804/2021	EPI_ISL_3888901	2021-09-02
hCoV-19/Japan/PG-77805/2021	EPI_ISL_3888904	2021-09-02
hCoV-19/Japan/PG-77806/2021	EPI_ISL_3888906	2021-09-02
hCoV-19/Japan/PG-77807/2021	EPI_ISL_3888908	2021-09-02
hCoV-19/Japan/PG-77808/2021	EPI_ISL_3888911	2021-09-02
hCoV-19/Japan/PG-77809/2021	EPI_ISL_3888913	2021-09-02
hCoV-19/Japan/PG-77810/2021	EPI_ISL_3888914	2021-09-02
hCoV-19/Japan/PG-77811/2021	EPI_ISL_3888916	2021-09-02
hCoV-19/Japan/PG-77812/2021	EPI_ISL_3888918	2021-09-02
hCoV-19/Japan/PG-77813/2021	EPI_ISL_3888920	2021-09-02
hCoV-19/Japan/PG-77814/2021	EPI_ISL_3888923	2021-09-02
hCoV-19/Japan/PG-77815/2021	EPI_ISL_3888925	2021-09-02
hCoV-19/Japan/PG-77816/2021	EPI_ISL_3888928	2021-09-02
hCoV-19/Japan/PG-77817/2021	EPI_ISL_3888930	2021-09-02
hCoV-19/Japan/PG-77818/2021	EPI_ISL_3888932	2021-09-02
hCoV-19/Japan/PG-77819/2021	EPI_ISL_3888934	2021-09-02
hCoV-19/Japan/PG-77820/2021	EPI_ISL_3888937	2021-09-02
hCoV-19/Japan/PG-77821/2021	EPI_ISL_3888939	2021-09-02
hCoV-19/Japan/PG-77822/2021	EPI_ISL_3888941	2021-09-02
hCoV-19/Japan/PG-77823/2021	EPI_ISL_3888943	2021-09-02
hCoV-19/Japan/PG-77824/2021	EPI_ISL_3888946	2021-09-02
hCoV-19/Japan/PG-77825/2021	EPI_ISL_3888949	2021-09-02
hCoV-19/Japan/PG-77826/2021	EPI_ISL_3888951	2021-09-02
hCoV-19/Japan/PG-77827/2021	EPI_ISL_3888953	2021-09-02
hCoV-19/Japan/PG-77828/2021	EPI_ISL_3870465	2021-09-02

virus_name	epiID	submission_date
hCoV-19/Japan/PG-77829/2021	EPI_ISL_3870466	2021-09-02
hCoV-19/Japan/PG-77830/2021	EPI_ISL_3870467	2021-09-02
hCoV-19/Japan/PG-77831/2021	EPI_ISL_3874308	2021-09-02
hCoV-19/Japan/PG-77832/2021	EPI_ISL_3874309	2021-09-02
hCoV-19/Japan/PG-77833/2021	EPI_ISL_3874310	2021-09-02
hCoV-19/Japan/PG-77834/2021	EPI_ISL_3874311	2021-09-02
hCoV-19/Japan/PG-77835/2021	EPI_ISL_3874312	2021-09-02
hCoV-19/Japan/PG-77836/2021	EPI_ISL_3874313	2021-09-02
hCoV-19/Japan/PG-77837/2021	EPI_ISL_3874314	2021-09-02
hCoV-19/Japan/PG-77838/2021	EPI_ISL_3874315	2021-09-02
hCoV-19/Japan/PG-77839/2021	EPI_ISL_3874316	2021-09-02
hCoV-19/Japan/PG-77840/2021	EPI_ISL_3874317	2021-09-02
hCoV-19/Japan/PG-77841/2021	EPI_ISL_3874318	2021-09-02
hCoV-19/Japan/PG-77842/2021	EPI_ISL_3874319	2021-09-02
hCoV-19/Japan/PG-77843/2021	EPI_ISL_3874320	2021-09-02
hCoV-19/Japan/PG-77844/2021	EPI_ISL_3874321	2021-09-02
hCoV-19/Japan/PG-77845/2021	EPI_ISL_3874322	2021-09-02
hCoV-19/Japan/PG-77846/2021	EPI_ISL_3874182	2021-09-02
hCoV-19/Japan/PG-77847/2021	EPI_ISL_3874183	2021-09-02
hCoV-19/Japan/PG-77848/2021	EPI_ISL_3874184	2021-09-02
hCoV-19/Japan/PG-77849/2021	EPI_ISL_3874185	2021-09-02
hCoV-19/Japan/PG-77850/2021	EPI_ISL_3874186	2021-09-02
hCoV-19/Japan/PG-77851/2021	EPI_ISL_3874187	2021-09-02
hCoV-19/Japan/PG-77852/2021	EPI_ISL_3874188	2021-09-02
hCoV-19/Japan/PG-77853/2021	EPI_ISL_3874189	2021-09-02
hCoV-19/Japan/PG-77854/2021	EPI_ISL_3872402	2021-09-02
hCoV-19/Japan/PG-77858/2021	EPI_ISL_3871260	2021-09-02
hCoV-19/Japan/PG-77859/2021	EPI_ISL_3871261	2021-09-02
hCoV-19/Japan/PG-77860/2021	EPI_ISL_3871262	2021-09-02
hCoV-19/Japan/PG-77861/2021	EPI_ISL_3871263	2021-09-02
hCoV-19/Japan/PG-77862/2021	EPI_ISL_3871264	2021-09-02
hCoV-19/Japan/PG-77863/2021	EPI_ISL_3871265	2021-09-02
hCoV-19/Japan/PG-77864/2021	EPI_ISL_3871266	2021-09-02
hCoV-19/Japan/PG-77865/2021	EPI_ISL_3871267	2021-09-02
hCoV-19/Japan/PG-77866/2021	EPI_ISL_3871268	2021-09-02
hCoV-19/Japan/PG-77867/2021	EPI_ISL_3871269	2021-09-02
hCoV-19/Japan/PG-77868/2021	EPI_ISL_3871270	2021-09-02
hCoV-19/Japan/PG-77869/2021	EPI_ISL_3871271	2021-09-02
hCoV-19/Japan/PG-77870/2021	EPI_ISL_3871272	2021-09-02
hCoV-19/Japan/PG-77871/2021	EPI_ISL_3871273	2021-09-02
hCoV-19/Japan/PG-77872/2021	EPI_ISL_3871274	2021-09-02
hCoV-19/Japan/PG-77873/2021	EPI_ISL_3871275	2021-09-02
hCoV-19/Japan/PG-77874/2021	EPI_ISL_3871276	2021-09-02
hCoV-19/Japan/PG-77875/2021	EPI_ISL_3871277	2021-09-02
hCoV-19/Japan/PG-77876/2021	EPI_ISL_3871278	2021-09-02
hCoV-19/Japan/PG-77877/2021	EPI_ISL_3871279	2021-09-02
hCoV-19/Japan/PG-77878/2021	EPI_ISL_3871280	2021-09-02
hCoV-19/Japan/PG-77879/2021	EPI_ISL_3871281	2021-09-02
hCoV-19/Japan/PG-77880/2021	EPI_ISL_3871282	2021-09-02
hCoV-19/Japan/PG-77881/2021	EPI_ISL_3873724	2021-09-02
hCoV-19/Japan/PG-77882/2021	EPI_ISL_3873725	2021-09-02
hCoV-19/Japan/PG-77883/2021	EPI_ISL_3873726	2021-09-02
hCoV-19/Japan/PG-77884/2021	EPI_ISL_3873727	2021-09-02
hCoV-19/Japan/PG-77885/2021	EPI_ISL_3873728	2021-09-02

virus_name	epiID	submission_date
hCoV-19/Japan/PG-77886/2021	EPI_ISL_3873729	2021-09-02
hCoV-19/Japan/PG-77887/2021	EPI_ISL_3873730	2021-09-02
hCoV-19/Japan/PG-77888/2021	EPI_ISL_3873731	2021-09-02
hCoV-19/Japan/PG-77889/2021	EPI_ISL_3873732	2021-09-02
hCoV-19/Japan/PG-77890/2021	EPI_ISL_3873733	2021-09-02
hCoV-19/Japan/PG-77891/2021	EPI_ISL_3873734	2021-09-02
hCoV-19/Japan/PG-77892/2021	EPI_ISL_3873735	2021-09-02
hCoV-19/Japan/PG-77893/2021	EPI_ISL_3873736	2021-09-02
hCoV-19/Japan/PG-77894/2021	EPI_ISL_3873737	2021-09-02
hCoV-19/Japan/PG-77895/2021	EPI_ISL_3873738	2021-09-02
hCoV-19/Japan/PG-77896/2021	EPI_ISL_3873739	2021-09-02
hCoV-19/Japan/PG-77897/2021	EPI_ISL_3873740	2021-09-02
hCoV-19/Japan/PG-77898/2021	EPI_ISL_3873741	2021-09-02
hCoV-19/Japan/PG-77899/2021	EPI_ISL_3873742	2021-09-02
hCoV-19/Japan/PG-77900/2021	EPI_ISL_3873743	2021-09-02
hCoV-19/Japan/PG-77901/2021	EPI_ISL_3873744	2021-09-02
hCoV-19/Japan/PG-77902/2021	EPI_ISL_3873745	2021-09-02
hCoV-19/Japan/PG-77903/2021	EPI_ISL_3873746	2021-09-02
hCoV-19/Japan/PG-77904/2021	EPI_ISL_3873747	2021-09-02
hCoV-19/Japan/PG-77905/2021	EPI_ISL_3873748	2021-09-02
hCoV-19/Japan/PG-77906/2021	EPI_ISL_3873749	2021-09-02
hCoV-19/Japan/PG-77907/2021	EPI_ISL_3873750	2021-09-02
hCoV-19/Japan/PG-77908/2021	EPI_ISL_3873751	2021-09-02
hCoV-19/Japan/PG-77909/2021	EPI_ISL_3873752	2021-09-02
hCoV-19/Japan/PG-77910/2021	EPI_ISL_3873753	2021-09-02
hCoV-19/Japan/PG-77911/2021	EPI_ISL_3873754	2021-09-02
hCoV-19/Japan/PG-77912/2021	EPI_ISL_3873755	2021-09-02
hCoV-19/Japan/PG-77913/2021	EPI_ISL_3873756	2021-09-02
hCoV-19/Japan/PG-77914/2021	EPI_ISL_3873757	2021-09-02
hCoV-19/Japan/PG-77915/2021	EPI_ISL_3873758	2021-09-02
hCoV-19/Japan/PG-77916/2021	EPI_ISL_3873759	2021-09-02
hCoV-19/Japan/PG-77917/2021	EPI_ISL_3873760	2021-09-02
hCoV-19/Japan/PG-77918/2021	EPI_ISL_3873761	2021-09-02
hCoV-19/Japan/PG-77919/2021	EPI_ISL_3873762	2021-09-02
hCoV-19/Japan/PG-77920/2021	EPI_ISL_3873763	2021-09-02
hCoV-19/Japan/PG-77921/2021	EPI_ISL_3873764	2021-09-02
hCoV-19/Japan/PG-77922/2021	EPI_ISL_3873765	2021-09-02
hCoV-19/Japan/PG-77923/2021	EPI_ISL_3873766	2021-09-02
hCoV-19/Japan/PG-77924/2021	EPI_ISL_3873767	2021-09-02
hCoV-19/Japan/PG-77925/2021	EPI_ISL_3873768	2021-09-02
hCoV-19/Japan/PG-77926/2021	EPI_ISL_3875552	2021-09-02
hCoV-19/Japan/PG-77927/2021	EPI_ISL_3875553	2021-09-02
hCoV-19/Japan/PG-77928/2021	EPI_ISL_3875554	2021-09-02
hCoV-19/Japan/PG-77929/2021	EPI_ISL_3875555	2021-09-02
hCoV-19/Japan/PG-77930/2021	EPI_ISL_3875556	2021-09-02
hCoV-19/Japan/PG-77931/2021	EPI_ISL_3875557	2021-09-02
hCoV-19/Japan/PG-77932/2021	EPI_ISL_3875558	2021-09-02
hCoV-19/Japan/PG-77933/2021	EPI_ISL_3875559	2021-09-02
hCoV-19/Japan/PG-77934/2021	EPI_ISL_3875560	2021-09-02
hCoV-19/Japan/PG-77935/2021	EPI_ISL_3875561	2021-09-02
hCoV-19/Japan/PG-77936/2021	EPI_ISL_3875562	2021-09-02
hCoV-19/Japan/PG-77937/2021	EPI_ISL_3875563	2021-09-02
hCoV-19/Japan/PG-77938/2021	EPI_ISL_3875564	2021-09-02
hCoV-19/Japan/PG-77939/2021	EPI_ISL_3875565	2021-09-02

virus_name	epiID	submission_date
hCoV-19/Japan/PG-77940/2021	EPI_ISL_3875566	2021-09-02
hCoV-19/Japan/PG-77941/2021	EPI_ISL_3875567	2021-09-02
hCoV-19/Japan/PG-77942/2021	EPI_ISL_3875568	2021-09-02
hCoV-19/Japan/PG-77943/2021	EPI_ISL_3875569	2021-09-02
hCoV-19/Japan/PG-77944/2021	EPI_ISL_3875570	2021-09-02
hCoV-19/Japan/PG-77945/2021	EPI_ISL_3875571	2021-09-02
hCoV-19/Japan/PG-77946/2021	EPI_ISL_3875572	2021-09-02
hCoV-19/Japan/PG-77947/2021	EPI_ISL_3875573	2021-09-02
hCoV-19/Japan/PG-77948/2021	EPI_ISL_3875574	2021-09-02
hCoV-19/Japan/PG-77949/2021	EPI_ISL_3875575	2021-09-02
hCoV-19/Japan/PG-77950/2021	EPI_ISL_3875576	2021-09-02
hCoV-19/Japan/PG-77951/2021	EPI_ISL_3875577	2021-09-02
hCoV-19/Japan/PG-77952/2021	EPI_ISL_3875578	2021-09-02
hCoV-19/Japan/PG-77953/2021	EPI_ISL_3875579	2021-09-02
hCoV-19/Japan/PG-77954/2021	EPI_ISL_3875580	2021-09-02
hCoV-19/Japan/PG-77955/2021	EPI_ISL_3875581	2021-09-02
hCoV-19/Japan/PG-77956/2021	EPI_ISL_3875582	2021-09-02
hCoV-19/Japan/PG-77957/2021	EPI_ISL_3875583	2021-09-02
hCoV-19/Japan/PG-77958/2021	EPI_ISL_3875584	2021-09-02
hCoV-19/Japan/PG-77959/2021	EPI_ISL_3875585	2021-09-02
hCoV-19/Japan/PG-77960/2021	EPI_ISL_3875586	2021-09-02
hCoV-19/Japan/PG-77961/2021	EPI_ISL_3875587	2021-09-02
hCoV-19/Japan/PG-77962/2021	EPI_ISL_3875588	2021-09-02
hCoV-19/Japan/PG-77963/2021	EPI_ISL_3875589	2021-09-02
hCoV-19/Japan/PG-77964/2021	EPI_ISL_3875590	2021-09-02
hCoV-19/Japan/PG-77965/2021	EPI_ISL_3875591	2021-09-02
hCoV-19/Japan/PG-77966/2021	EPI_ISL_3875592	2021-09-02
hCoV-19/Japan/PG-77967/2021	EPI_ISL_3875593	2021-09-02
hCoV-19/Japan/PG-77968/2021	EPI_ISL_3875594	2021-09-02
hCoV-19/Japan/PG-77969/2021	EPI_ISL_3875595	2021-09-02
hCoV-19/Japan/PG-77970/2021	EPI_ISL_3875596	2021-09-02
hCoV-19/Japan/PG-77971/2021	EPI_ISL_3875597	2021-09-02
hCoV-19/Japan/PG-77972/2021	EPI_ISL_3871775	2021-09-02
hCoV-19/Japan/PG-77973/2021	EPI_ISL_3871776	2021-09-02
hCoV-19/Japan/PG-77974/2021	EPI_ISL_3871777	2021-09-02
hCoV-19/Japan/PG-77975/2021	EPI_ISL_3871778	2021-09-02
hCoV-19/Japan/PG-77976/2021	EPI_ISL_3871779	2021-09-02
hCoV-19/Japan/PG-77977/2021	EPI_ISL_3871780	2021-09-02
hCoV-19/Japan/PG-77978/2021	EPI_ISL_3871781	2021-09-02
hCoV-19/Japan/PG-77979/2021	EPI_ISL_3871782	2021-09-02
hCoV-19/Japan/PG-77980/2021	EPI_ISL_3871783	2021-09-02
hCoV-19/Japan/PG-77981/2021	EPI_ISL_3871784	2021-09-02
hCoV-19/Japan/PG-77982/2021	EPI_ISL_3871785	2021-09-02
hCoV-19/Japan/PG-77983/2021	EPI_ISL_3871786	2021-09-02
hCoV-19/Japan/PG-77984/2021	EPI_ISL_3871787	2021-09-02
hCoV-19/Japan/PG-77985/2021	EPI_ISL_3871788	2021-09-02
hCoV-19/Japan/PG-77986/2021	EPI_ISL_3871789	2021-09-02
hCoV-19/Japan/PG-77987/2021	EPI_ISL_3871790	2021-09-02
hCoV-19/Japan/PG-77988/2021	EPI_ISL_3871791	2021-09-02
hCoV-19/Japan/PG-77989/2021	EPI_ISL_3871792	2021-09-02
hCoV-19/Japan/PG-77990/2021	EPI_ISL_3871793	2021-09-02
hCoV-19/Japan/PG-77991/2021	EPI_ISL_3871794	2021-09-02
hCoV-19/Japan/PG-77992/2021	EPI_ISL_3871795	2021-09-02
hCoV-19/Japan/PG-77993/2021	EPI_ISL_3871796	2021-09-02

virus_name	epiID	submission_date
hCoV-19/Japan/PG-77994/2021	EPI_ISL_3871797	2021-09-02
hCoV-19/Japan/PG-77995/2021	EPI_ISL_3876072	2021-09-02
hCoV-19/Japan/PG-77996/2021	EPI_ISL_3876073	2021-09-02
hCoV-19/Japan/PG-77997/2021	EPI_ISL_3876074	2021-09-02
hCoV-19/Japan/PG-77998/2021	EPI_ISL_3876075	2021-09-02
hCoV-19/Japan/PG-77999/2021	EPI_ISL_3876076	2021-09-02
hCoV-19/Japan/PG-78000/2021	EPI_ISL_3876077	2021-09-02
hCoV-19/Japan/PG-78001/2021	EPI_ISL_3876078	2021-09-02
hCoV-19/Japan/PG-78002/2021	EPI_ISL_3876079	2021-09-02
hCoV-19/Japan/PG-78003/2021	EPI_ISL_3876080	2021-09-02
hCoV-19/Japan/PG-78004/2021	EPI_ISL_3876081	2021-09-02
hCoV-19/Japan/PG-78005/2021	EPI_ISL_3876082	2021-09-02
hCoV-19/Japan/PG-78006/2021	EPI_ISL_3876083	2021-09-02
hCoV-19/Japan/PG-78007/2021	EPI_ISL_3876084	2021-09-02
hCoV-19/Japan/PG-78008/2021	EPI_ISL_3876085	2021-09-02
hCoV-19/Japan/PG-78009/2021	EPI_ISL_3876086	2021-09-02
hCoV-19/Japan/PG-78010/2021	EPI_ISL_3876087	2021-09-02
hCoV-19/Japan/PG-78011/2021	EPI_ISL_3876088	2021-09-02
hCoV-19/Japan/PG-78012/2021	EPI_ISL_3876089	2021-09-02
hCoV-19/Japan/PG-78013/2021	EPI_ISL_3876090	2021-09-02
hCoV-19/Japan/PG-78014/2021	EPI_ISL_3876091	2021-09-02
hCoV-19/Japan/PG-78015/2021	EPI_ISL_3876092	2021-09-02
hCoV-19/Japan/PG-78016/2021	EPI_ISL_3876093	2021-09-02
hCoV-19/Japan/PG-78017/2021	EPI_ISL_3876094	2021-09-02
hCoV-19/Japan/PG-78018/2021	EPI_ISL_3876095	2021-09-02
hCoV-19/Japan/PG-78019/2021	EPI_ISL_3876096	2021-09-02
hCoV-19/Japan/PG-78020/2021	EPI_ISL_3876097	2021-09-02
hCoV-19/Japan/PG-78021/2021	EPI_ISL_3876098	2021-09-02
hCoV-19/Japan/PG-78022/2021	EPI_ISL_3876099	2021-09-02
hCoV-19/Japan/PG-78023/2021	EPI_ISL_3876100	2021-09-02
hCoV-19/Japan/PG-78024/2021	EPI_ISL_3876101	2021-09-02
hCoV-19/Japan/PG-78025/2021	EPI_ISL_3876102	2021-09-02
hCoV-19/Japan/PG-78026/2021	EPI_ISL_3876103	2021-09-02
hCoV-19/Japan/PG-78027/2021	EPI_ISL_3875847	2021-09-02
hCoV-19/Japan/PG-78028/2021	EPI_ISL_3875848	2021-09-02
hCoV-19/Japan/PG-78029/2021	EPI_ISL_3875849	2021-09-02
hCoV-19/Japan/PG-78030/2021	EPI_ISL_3875850	2021-09-02
hCoV-19/Japan/PG-78031/2021	EPI_ISL_3875851	2021-09-02
hCoV-19/Japan/PG-78032/2021	EPI_ISL_3875852	2021-09-02
hCoV-19/Japan/PG-78033/2021	EPI_ISL_3875853	2021-09-02
hCoV-19/Japan/PG-78034/2021	EPI_ISL_3875854	2021-09-02
hCoV-19/Japan/PG-78035/2021	EPI_ISL_3875855	2021-09-02
hCoV-19/Japan/PG-78036/2021	EPI_ISL_3875856	2021-09-02
hCoV-19/Japan/PG-78037/2021	EPI_ISL_3875857	2021-09-02
hCoV-19/Japan/PG-78038/2021	EPI_ISL_3875858	2021-09-02
hCoV-19/Japan/PG-78039/2021	EPI_ISL_3875859	2021-09-02
hCoV-19/Japan/PG-78040/2021	EPI_ISL_3875860	2021-09-02
hCoV-19/Japan/PG-78041/2021	EPI_ISL_3875861	2021-09-02
hCoV-19/Japan/PG-78042/2021	EPI_ISL_3875862	2021-09-02
hCoV-19/Japan/PG-78043/2021	EPI_ISL_3875863	2021-09-02
hCoV-19/Japan/PG-78044/2021	EPI_ISL_3875864	2021-09-02
hCoV-19/Japan/PG-78045/2021	EPI_ISL_3875865	2021-09-02
hCoV-19/Japan/PG-78046/2021	EPI_ISL_3875866	2021-09-02
hCoV-19/Japan/PG-78047/2021	EPI_ISL_3871369	2021-09-02

virus_name	epiID	submission_date
hCoV-19/Japan/PG-78048/2021	EPI_ISL_3871370	2021-09-02
hCoV-19/Japan/PG-78049/2021	EPI_ISL_3871371	2021-09-02
hCoV-19/Japan/PG-78050/2021	EPI_ISL_3871372	2021-09-02
hCoV-19/Japan/PG-78051/2021	EPI_ISL_3871373	2021-09-02
hCoV-19/Japan/PG-78052/2021	EPI_ISL_3871374	2021-09-02
hCoV-19/Japan/PG-78053/2021	EPI_ISL_3872403	2021-09-02
hCoV-19/Japan/PG-78054/2021	EPI_ISL_3872404	2021-09-02
hCoV-19/Japan/PG-78055/2021	EPI_ISL_3872405	2021-09-02
hCoV-19/Japan/PG-78056/2021	EPI_ISL_3872406	2021-09-02
hCoV-19/Japan/PG-78057/2021	EPI_ISL_3872407	2021-09-02
hCoV-19/Japan/PG-78058/2021	EPI_ISL_3872408	2021-09-02
hCoV-19/Japan/PG-78059/2021	EPI_ISL_3872409	2021-09-02
hCoV-19/Japan/PG-78060/2021	EPI_ISL_3872410	2021-09-02
hCoV-19/Japan/PG-78061/2021	EPI_ISL_3872411	2021-09-02
hCoV-19/Japan/PG-78062/2021	EPI_ISL_3872412	2021-09-02
hCoV-19/Japan/PG-78063/2021	EPI_ISL_3872413	2021-09-02
hCoV-19/Japan/PG-78064/2021	EPI_ISL_3872414	2021-09-02
hCoV-19/Japan/PG-78065/2021	EPI_ISL_3872415	2021-09-02
hCoV-19/Japan/PG-78066/2021	EPI_ISL_3872416	2021-09-02
hCoV-19/Japan/PG-78067/2021	EPI_ISL_3872417	2021-09-02
hCoV-19/Japan/PG-78068/2021	EPI_ISL_3872418	2021-09-02
hCoV-19/Japan/PG-78069/2021	EPI_ISL_3872419	2021-09-02
hCoV-19/Japan/PG-78070/2021	EPI_ISL_3872420	2021-09-02
hCoV-19/Japan/PG-78071/2021	EPI_ISL_3872421	2021-09-02
hCoV-19/Japan/PG-78072/2021	EPI_ISL_3872422	2021-09-02
hCoV-19/Japan/PG-78073/2021	EPI_ISL_3872423	2021-09-02
hCoV-19/Japan/PG-78074/2021	EPI_ISL_3872424	2021-09-02
hCoV-19/Japan/PG-78075/2021	EPI_ISL_3872425	2021-09-02
hCoV-19/Japan/PG-78076/2021	EPI_ISL_3872426	2021-09-02
hCoV-19/Japan/PG-78077/2021	EPI_ISL_3871948	2021-09-02
hCoV-19/Japan/PG-78078/2021	EPI_ISL_3871949	2021-09-02
hCoV-19/Japan/PG-78079/2021	EPI_ISL_3871950	2021-09-02
hCoV-19/Japan/PG-78080/2021	EPI_ISL_3871951	2021-09-02
hCoV-19/Japan/PG-78081/2021	EPI_ISL_3871952	2021-09-02
hCoV-19/Japan/PG-78082/2021	EPI_ISL_3871953	2021-09-02
hCoV-19/Japan/PG-78083/2021	EPI_ISL_3871452	2021-09-02
hCoV-19/Japan/PG-78084/2021	EPI_ISL_3871453	2021-09-02
hCoV-19/Japan/PG-78085/2021	EPI_ISL_3871454	2021-09-02
hCoV-19/Japan/PG-78086/2021	EPI_ISL_3871455	2021-09-02
hCoV-19/Japan/PG-78087/2021	EPI_ISL_3871456	2021-09-02
hCoV-19/Japan/PG-78088/2021	EPI_ISL_3874076	2021-09-02
hCoV-19/Japan/PG-78089/2021	EPI_ISL_3874077	2021-09-02
hCoV-19/Japan/PG-78090/2021	EPI_ISL_3874078	2021-09-02
hCoV-19/Japan/PG-78091/2021	EPI_ISL_3874079	2021-09-02
hCoV-19/Japan/PG-78092/2021	EPI_ISL_3874080	2021-09-02
hCoV-19/Japan/PG-78093/2021	EPI_ISL_3874081	2021-09-02
hCoV-19/Japan/PG-78094/2021	EPI_ISL_3874082	2021-09-02
hCoV-19/Japan/PG-78095/2021	EPI_ISL_3874083	2021-09-02
hCoV-19/Japan/PG-78096/2021	EPI_ISL_3874084	2021-09-02
hCoV-19/Japan/PG-78097/2021	EPI_ISL_3874086	2021-09-02
hCoV-19/Japan/PG-78098/2021	EPI_ISL_3874497	2021-09-02
hCoV-19/Japan/PG-78099/2021	EPI_ISL_3874498	2021-09-02
hCoV-19/Japan/PG-78100/2021	EPI_ISL_3874499	2021-09-02
hCoV-19/Japan/PG-78101/2021	EPI_ISL_3874500	2021-09-02

virus_name	epiID	submission_date
hCoV-19/Japan/PG-78102/2021	EPI_ISL_3874501	2021-09-02
hCoV-19/Japan/PG-78103/2021	EPI_ISL_3874502	2021-09-02
hCoV-19/Japan/PG-78104/2021	EPI_ISL_3874503	2021-09-02
hCoV-19/Japan/PG-78105/2021	EPI_ISL_3874504	2021-09-02
hCoV-19/Japan/PG-78106/2021	EPI_ISL_3874505	2021-09-02
hCoV-19/Japan/PG-78107/2021	EPI_ISL_3874506	2021-09-02
hCoV-19/Japan/PG-78108/2021	EPI_ISL_3874507	2021-09-02
hCoV-19/Japan/PG-78109/2021	EPI_ISL_3874508	2021-09-02
hCoV-19/Japan/PG-78110/2021	EPI_ISL_3873965	2021-09-02
hCoV-19/Japan/PG-78111/2021	EPI_ISL_3873966	2021-09-02
hCoV-19/Japan/PG-78112/2021	EPI_ISL_3873967	2021-09-02
hCoV-19/Japan/PG-78113/2021	EPI_ISL_3873968	2021-09-02
hCoV-19/Japan/PG-78114/2021	EPI_ISL_3873969	2021-09-02
hCoV-19/Japan/PG-78115/2021	EPI_ISL_3873970	2021-09-02
hCoV-19/Japan/PG-78116/2021	EPI_ISL_3873971	2021-09-02
hCoV-19/Japan/PG-78117/2021	EPI_ISL_3873972	2021-09-02
hCoV-19/Japan/PG-78118/2021	EPI_ISL_3873973	2021-09-02
hCoV-19/Japan/PG-78119/2021	EPI_ISL_3873974	2021-09-02
hCoV-19/Japan/PG-78120/2021	EPI_ISL_3873975	2021-09-02
hCoV-19/Japan/PG-78121/2021	EPI_ISL_3873976	2021-09-02
hCoV-19/Japan/PG-78122/2021	EPI_ISL_3873977	2021-09-02
hCoV-19/Japan/PG-78123/2021	EPI_ISL_3873978	2021-09-02
hCoV-19/Japan/PG-78124/2021	EPI_ISL_3873979	2021-09-02
hCoV-19/Japan/PG-78125/2021	EPI_ISL_3873980	2021-09-02
hCoV-19/Japan/PG-78126/2021	EPI_ISL_3873981	2021-09-02
hCoV-19/Japan/PG-78127/2021	EPI_ISL_3873982	2021-09-02
hCoV-19/Japan/PG-78128/2021	EPI_ISL_3873983	2021-09-02
hCoV-19/Japan/PG-78129/2021	EPI_ISL_3871052	2021-09-02
hCoV-19/Japan/PG-78130/2021	EPI_ISL_3871053	2021-09-02
hCoV-19/Japan/PG-78131/2021	EPI_ISL_3871054	2021-09-02
hCoV-19/Japan/PG-78132/2021	EPI_ISL_3871055	2021-09-02
hCoV-19/Japan/PG-78133/2021	EPI_ISL_3871056	2021-09-02
hCoV-19/Japan/PG-78134/2021	EPI_ISL_3871057	2021-09-02
hCoV-19/Japan/PG-78135/2021	EPI_ISL_3871058	2021-09-02
hCoV-19/Japan/PG-78136/2021	EPI_ISL_3871059	2021-09-02
hCoV-19/Japan/PG-78137/2021	EPI_ISL_3871060	2021-09-02
hCoV-19/Japan/PG-78138/2021	EPI_ISL_3871061	2021-09-02
hCoV-19/Japan/PG-78139/2021	EPI_ISL_3871062	2021-09-02
hCoV-19/Japan/PG-78140/2021	EPI_ISL_3871063	2021-09-02
hCoV-19/Japan/PG-78141/2021	EPI_ISL_3871064	2021-09-02
hCoV-19/Japan/PG-78142/2021	EPI_ISL_3871065	2021-09-02
hCoV-19/Japan/PG-78143/2021	EPI_ISL_3871066	2021-09-02
hCoV-19/Japan/PG-78144/2021	EPI_ISL_3871067	2021-09-02
hCoV-19/Japan/PG-78145/2021	EPI_ISL_3871068	2021-09-02
hCoV-19/Japan/PG-78146/2021	EPI_ISL_3871069	2021-09-02
hCoV-19/Japan/PG-78147/2021	EPI_ISL_3871070	2021-09-02
hCoV-19/Japan/PG-78148/2021	EPI_ISL_3871071	2021-09-02
hCoV-19/Japan/PG-78149/2021	EPI_ISL_3871072	2021-09-02
hCoV-19/Japan/PG-78150/2021	EPI_ISL_3871073	2021-09-02
hCoV-19/Japan/PG-78151/2021	EPI_ISL_3874323	2021-09-02
hCoV-19/Japan/PG-78152/2021	EPI_ISL_3874324	2021-09-02
hCoV-19/Japan/PG-78153/2021	EPI_ISL_3874325	2021-09-02
hCoV-19/Japan/PG-78154/2021	EPI_ISL_3874326	2021-09-02
hCoV-19/Japan/PG-78155/2021	EPI_ISL_3874327	2021-09-02

virus_name	epiID	submission_date
hCoV-19/Japan/PG-78156/2021	EPI_ISL_3874328	2021-09-02
hCoV-19/Japan/PG-78157/2021	EPI_ISL_3874329	2021-09-02
hCoV-19/Japan/PG-78158/2021	EPI_ISL_3874330	2021-09-02
hCoV-19/Japan/PG-78159/2021	EPI_ISL_3874331	2021-09-02
hCoV-19/Japan/PG-78160/2021	EPI_ISL_3874332	2021-09-02
hCoV-19/Japan/PG-78161/2021	EPI_ISL_3873377	2021-09-02
hCoV-19/Japan/PG-78162/2021	EPI_ISL_3873378	2021-09-02
hCoV-19/Japan/PG-78163/2021	EPI_ISL_3873379	2021-09-02
hCoV-19/Japan/PG-78164/2021	EPI_ISL_3873380	2021-09-02
hCoV-19/Japan/PG-78165/2021	EPI_ISL_3873381	2021-09-02
hCoV-19/Japan/PG-78166/2021	EPI_ISL_3873382	2021-09-02
hCoV-19/Japan/PG-78167/2021	EPI_ISL_3873383	2021-09-02
hCoV-19/Japan/PG-78168/2021	EPI_ISL_3873384	2021-09-02
hCoV-19/Japan/PG-78169/2021	EPI_ISL_3873385	2021-09-02
hCoV-19/Japan/PG-78170/2021	EPI_ISL_3873386	2021-09-02
hCoV-19/Japan/PG-78171/2021	EPI_ISL_3873387	2021-09-02
hCoV-19/Japan/PG-78172/2021	EPI_ISL_3873388	2021-09-02
hCoV-19/Japan/PG-78173/2021	EPI_ISL_3873389	2021-09-02
hCoV-19/Japan/PG-78174/2021	EPI_ISL_3873390	2021-09-02
hCoV-19/Japan/PG-78175/2021	EPI_ISL_3873391	2021-09-02
hCoV-19/Japan/PG-78176/2021	EPI_ISL_3873392	2021-09-02
hCoV-19/Japan/PG-78177/2021	EPI_ISL_3874190	2021-09-02
hCoV-19/Japan/PG-78178/2021	EPI_ISL_3874191	2021-09-02
hCoV-19/Japan/PG-78179/2021	EPI_ISL_3874192	2021-09-02
hCoV-19/Japan/PG-78180/2021	EPI_ISL_3874193	2021-09-02
hCoV-19/Japan/PG-78181/2021	EPI_ISL_3874194	2021-09-02
hCoV-19/Japan/PG-78182/2021	EPI_ISL_3874195	2021-09-02
hCoV-19/Japan/PG-78183/2021	EPI_ISL_3874196	2021-09-02
hCoV-19/Japan/PG-78184/2021	EPI_ISL_3874197	2021-09-02
hCoV-19/Japan/PG-78185/2021	EPI_ISL_3874198	2021-09-02
hCoV-19/Japan/PG-78186/2021	EPI_ISL_3874199	2021-09-02
hCoV-19/Japan/PG-78187/2021	EPI_ISL_3874200	2021-09-02
hCoV-19/Japan/PG-78188/2021	EPI_ISL_3874201	2021-09-02
hCoV-19/Japan/PG-78189/2021	EPI_ISL_3874202	2021-09-02
hCoV-19/Japan/PG-78190/2021	EPI_ISL_3874203	2021-09-02
hCoV-19/Japan/PG-78191/2021	EPI_ISL_3874204	2021-09-02
hCoV-19/Japan/PG-78192/2021	EPI_ISL_3874205	2021-09-02
hCoV-19/Japan/PG-78193/2021	EPI_ISL_3874206	2021-09-02
hCoV-19/Japan/PG-78194/2021	EPI_ISL_3874207	2021-09-02
hCoV-19/Japan/PG-78195/2021	EPI_ISL_3874208	2021-09-02
hCoV-19/Japan/PG-78196/2021	EPI_ISL_3874209	2021-09-02
hCoV-19/Japan/PG-78197/2021	EPI_ISL_3874210	2021-09-02
hCoV-19/Japan/PG-78198/2021	EPI_ISL_3874211	2021-09-02
hCoV-19/Japan/PG-78199/2021	EPI_ISL_3874212	2021-09-02
hCoV-19/Japan/PG-78200/2021	EPI_ISL_3874545	2021-09-02
hCoV-19/Japan/PG-78201/2021	EPI_ISL_3874546	2021-09-02
hCoV-19/Japan/PG-78202/2021	EPI_ISL_3874547	2021-09-02
hCoV-19/Japan/PG-78203/2021	EPI_ISL_3874548	2021-09-02
hCoV-19/Japan/PG-78204/2021	EPI_ISL_3874549	2021-09-02
hCoV-19/Japan/PG-78205/2021	EPI_ISL_3874550	2021-09-02
hCoV-19/Japan/PG-78206/2021	EPI_ISL_3874551	2021-09-02
hCoV-19/Japan/PG-78207/2021	EPI_ISL_3874552	2021-09-02
hCoV-19/Japan/PG-78208/2021	EPI_ISL_3874553	2021-09-02
hCoV-19/Japan/PG-78209/2021	EPI_ISL_3874554	2021-09-02

virus_name	epiID	submission_date
hCoV-19/Japan/PG-78210/2021	EPI_ISL_3874555	2021-09-02
hCoV-19/Japan/PG-78211/2021	EPI_ISL_3874556	2021-09-02
hCoV-19/Japan/PG-78212/2021	EPI_ISL_3874557	2021-09-02
hCoV-19/Japan/PG-78213/2021	EPI_ISL_3874558	2021-09-02
hCoV-19/Japan/PG-78214/2021	EPI_ISL_3874559	2021-09-02
hCoV-19/Japan/PG-78215/2021	EPI_ISL_3874560	2021-09-02
hCoV-19/Japan/PG-78216/2021	EPI_ISL_3874561	2021-09-02
hCoV-19/Japan/PG-78217/2021	EPI_ISL_3874562	2021-09-02
hCoV-19/Japan/PG-78218/2021	EPI_ISL_3874563	2021-09-02
hCoV-19/Japan/PG-78219/2021	EPI_ISL_3874564	2021-09-02
hCoV-19/Japan/PG-78220/2021	EPI_ISL_3874565	2021-09-02
hCoV-19/Japan/PG-78221/2021	EPI_ISL_3874566	2021-09-02
hCoV-19/Japan/PG-78222/2021	EPI_ISL_3874567	2021-09-02
hCoV-19/Japan/PG-78223/2021	EPI_ISL_3870964	2021-09-02
hCoV-19/Japan/PG-78224/2021	EPI_ISL_3870965	2021-09-02
hCoV-19/Japan/PG-78225/2021	EPI_ISL_3870966	2021-09-02
hCoV-19/Japan/PG-78226/2021	EPI_ISL_3870967	2021-09-02
hCoV-19/Japan/PG-78227/2021	EPI_ISL_3870968	2021-09-02
hCoV-19/Japan/PG-78228/2021	EPI_ISL_3870969	2021-09-02
hCoV-19/Japan/PG-78229/2021	EPI_ISL_3870970	2021-09-02
hCoV-19/Japan/PG-78230/2021	EPI_ISL_3870971	2021-09-02
hCoV-19/Japan/PG-78231/2021	EPI_ISL_3870972	2021-09-02
hCoV-19/Japan/PG-78232/2021	EPI_ISL_3870973	2021-09-02
hCoV-19/Japan/PG-78233/2021	EPI_ISL_3870974	2021-09-02
hCoV-19/Japan/PG-78234/2021	EPI_ISL_3870975	2021-09-02
hCoV-19/Japan/PG-78235/2021	EPI_ISL_3870976	2021-09-02
hCoV-19/Japan/PG-78236/2021	EPI_ISL_3870977	2021-09-02
hCoV-19/Japan/PG-78237/2021	EPI_ISL_3870978	2021-09-02
hCoV-19/Japan/PG-78238/2021	EPI_ISL_3870979	2021-09-02
hCoV-19/Japan/PG-78239/2021	EPI_ISL_3870980	2021-09-02
hCoV-19/Japan/PG-78240/2021	EPI_ISL_3872157	2021-09-02
hCoV-19/Japan/PG-78241/2021	EPI_ISL_3872158	2021-09-02
hCoV-19/Japan/PG-78242/2021	EPI_ISL_3872159	2021-09-02
hCoV-19/Japan/PG-78243/2021	EPI_ISL_3872160	2021-09-02
hCoV-19/Japan/PG-78244/2021	EPI_ISL_3872161	2021-09-02
hCoV-19/Japan/PG-78245/2021	EPI_ISL_3872162	2021-09-02
hCoV-19/Japan/PG-78246/2021	EPI_ISL_3872163	2021-09-02
hCoV-19/Japan/PG-78247/2021	EPI_ISL_3872164	2021-09-02
hCoV-19/Japan/PG-78248/2021	EPI_ISL_3872165	2021-09-02
hCoV-19/Japan/PG-78249/2021	EPI_ISL_3872166	2021-09-02
hCoV-19/Japan/PG-78250/2021	EPI_ISL_3872167	2021-09-02
hCoV-19/Japan/PG-78251/2021	EPI_ISL_3872168	2021-09-02
hCoV-19/Japan/PG-78252/2021	EPI_ISL_3872169	2021-09-02
hCoV-19/Japan/PG-78253/2021	EPI_ISL_3872170	2021-09-02
hCoV-19/Japan/PG-78254/2021	EPI_ISL_3872171	2021-09-02
hCoV-19/Japan/PG-78255/2021	EPI_ISL_3872172	2021-09-02
hCoV-19/Japan/PG-78256/2021	EPI_ISL_3872173	2021-09-02
hCoV-19/Japan/PG-78257/2021	EPI_ISL_3872260	2021-09-02
hCoV-19/Japan/PG-78258/2021	EPI_ISL_3872261	2021-09-02
hCoV-19/Japan/PG-78259/2021	EPI_ISL_3872262	2021-09-02
hCoV-19/Japan/PG-78260/2021	EPI_ISL_3872263	2021-09-02
hCoV-19/Japan/PG-78261/2021	EPI_ISL_3872264	2021-09-02
hCoV-19/Japan/PG-78262/2021	EPI_ISL_3872265	2021-09-02
hCoV-19/Japan/PG-78263/2021	EPI_ISL_3872266	2021-09-02

virus_name	epiID	submission_date
hCoV-19/Japan/PG-78264/2021	EPI_ISL_3872267	2021-09-02
hCoV-19/Japan/PG-78265/2021	EPI_ISL_3872268	2021-09-02
hCoV-19/Japan/PG-78266/2021	EPI_ISL_3872269	2021-09-02
hCoV-19/Japan/PG-78267/2021	EPI_ISL_3872270	2021-09-02
hCoV-19/Japan/PG-78268/2021	EPI_ISL_3872271	2021-09-02
hCoV-19/Japan/PG-78269/2021	EPI_ISL_3872272	2021-09-02
hCoV-19/Japan/PG-78270/2021	EPI_ISL_3872273	2021-09-02
hCoV-19/Japan/PG-78271/2021	EPI_ISL_3872274	2021-09-02
hCoV-19/Japan/PG-78272/2021	EPI_ISL_3872275	2021-09-02
hCoV-19/Japan/PG-78273/2021	EPI_ISL_3872276	2021-09-02
hCoV-19/Japan/PG-78274/2021	EPI_ISL_3872277	2021-09-02
hCoV-19/Japan/PG-78275/2021	EPI_ISL_3872278	2021-09-02
hCoV-19/Japan/PG-78276/2021	EPI_ISL_3872279	2021-09-02
hCoV-19/Japan/PG-78277/2021	EPI_ISL_3872280	2021-09-02
hCoV-19/Japan/PG-78278/2021	EPI_ISL_3872281	2021-09-02
hCoV-19/Japan/PG-78279/2021	EPI_ISL_3872282	2021-09-02
hCoV-19/Japan/PG-78280/2021	EPI_ISL_3872283	2021-09-02
hCoV-19/Japan/PG-78281/2021	EPI_ISL_3888955	2021-09-02
hCoV-19/Japan/PG-78282/2021	EPI_ISL_3888958	2021-09-02
hCoV-19/Japan/PG-78283/2021	EPI_ISL_3888959	2021-09-02
hCoV-19/Japan/PG-78284/2021	EPI_ISL_3888960	2021-09-02
hCoV-19/Japan/PG-78285/2021	EPI_ISL_3888962	2021-09-02
hCoV-19/Japan/PG-78286/2021	EPI_ISL_3888964	2021-09-02
hCoV-19/Japan/PG-78287/2021	EPI_ISL_3888967	2021-09-02
hCoV-19/Japan/PG-78288/2021	EPI_ISL_3888969	2021-09-02
hCoV-19/Japan/PG-78289/2021	EPI_ISL_3888971	2021-09-02
hCoV-19/Japan/PG-78290/2021	EPI_ISL_3888973	2021-09-02
hCoV-19/Japan/PG-78291/2021	EPI_ISL_3888975	2021-09-02
hCoV-19/Japan/PG-78292/2021	EPI_ISL_3888978	2021-09-02
hCoV-19/Japan/PG-78293/2021	EPI_ISL_3888981	2021-09-02
hCoV-19/Japan/PG-78294/2021	EPI_ISL_3888983	2021-09-02
hCoV-19/Japan/PG-78295/2021	EPI_ISL_3888985	2021-09-02
hCoV-19/Japan/PG-78296/2021	EPI_ISL_3888986	2021-09-02
hCoV-19/Japan/PG-78297/2021	EPI_ISL_3888988	2021-09-02
hCoV-19/Japan/PG-78298/2021	EPI_ISL_3888990	2021-09-02
hCoV-19/Japan/PG-78299/2021	EPI_ISL_3888992	2021-09-02
hCoV-19/Japan/PG-78300/2021	EPI_ISL_3888994	2021-09-02
hCoV-19/Japan/PG-78301/2021	EPI_ISL_3888996	2021-09-02
hCoV-19/Japan/PG-78302/2021	EPI_ISL_3888998	2021-09-02
hCoV-19/Japan/PG-78303/2021	EPI_ISL_3889001	2021-09-02
hCoV-19/Japan/PG-78304/2021	EPI_ISL_3889003	2021-09-02
hCoV-19/Japan/PG-78305/2021	EPI_ISL_3889005	2021-09-02
hCoV-19/Japan/PG-78306/2021	EPI_ISL_3889008	2021-09-02
hCoV-19/Japan/PG-78307/2021	EPI_ISL_3889010	2021-09-02
hCoV-19/Japan/PG-78308/2021	EPI_ISL_3889013	2021-09-02
hCoV-19/Japan/PG-78309/2021	EPI_ISL_3889015	2021-09-02
hCoV-19/Japan/PG-78310/2021	EPI_ISL_3889018	2021-09-02
hCoV-19/Japan/PG-78311/2021	EPI_ISL_3889021	2021-09-02
hCoV-19/Japan/PG-78312/2021	EPI_ISL_3889024	2021-09-02
hCoV-19/Japan/PG-78313/2021	EPI_ISL_3889026	2021-09-02
hCoV-19/Japan/PG-78314/2021	EPI_ISL_3889029	2021-09-02
hCoV-19/Japan/PG-78315/2021	EPI_ISL_3889031	2021-09-02
hCoV-19/Japan/PG-78316/2021	EPI_ISL_3889033	2021-09-02
hCoV-19/Japan/PG-78317/2021	EPI_ISL_3889035	2021-09-02

virus_name	epiID	submission_date
hCoV-19/Japan/PG-78318/2021	EPI_ISL_3889038	2021-09-02
hCoV-19/Japan/PG-78319/2021	EPI_ISL_3889040	2021-09-02
hCoV-19/Japan/PG-78320/2021	EPI_ISL_3889043	2021-09-02
hCoV-19/Japan/PG-78321/2021	EPI_ISL_3889046	2021-09-02
hCoV-19/Japan/PG-78322/2021	EPI_ISL_3889048	2021-09-02
hCoV-19/Japan/PG-78323/2021	EPI_ISL_3889051	2021-09-02
hCoV-19/Japan/PG-78324/2021	EPI_ISL_3889052	2021-09-02
hCoV-19/Japan/PG-78325/2021	EPI_ISL_3889054	2021-09-02
hCoV-19/Japan/PG-78326/2021	EPI_ISL_3889056	2021-09-02
hCoV-19/Japan/PG-78327/2021	EPI_ISL_3889057	2021-09-02
hCoV-19/Japan/PG-78328/2021	EPI_ISL_3889061	2021-09-02
hCoV-19/Japan/PG-78329/2021	EPI_ISL_3889064	2021-09-02
hCoV-19/Japan/PG-78330/2021	EPI_ISL_3889066	2021-09-02
hCoV-19/Japan/PG-78331/2021	EPI_ISL_3889068	2021-09-02
hCoV-19/Japan/PG-78332/2021	EPI_ISL_3889070	2021-09-02
hCoV-19/Japan/PG-78333/2021	EPI_ISL_3889073	2021-09-02
hCoV-19/Japan/PG-78334/2021	EPI_ISL_3889075	2021-09-02
hCoV-19/Japan/PG-78335/2021	EPI_ISL_3889078	2021-09-02
hCoV-19/Japan/PG-78336/2021	EPI_ISL_3889080	2021-09-02
hCoV-19/Japan/PG-78337/2021	EPI_ISL_3889083	2021-09-02
hCoV-19/Japan/PG-78338/2021	EPI_ISL_3889085	2021-09-02
hCoV-19/Japan/PG-78339/2021	EPI_ISL_3889088	2021-09-02
hCoV-19/Japan/PG-78340/2021	EPI_ISL_3889090	2021-09-02
hCoV-19/Japan/PG-78341/2021	EPI_ISL_3889092	2021-09-02
hCoV-19/Japan/PG-78342/2021	EPI_ISL_3889095	2021-09-02
hCoV-19/Japan/PG-78343/2021	EPI_ISL_3889097	2021-09-02
hCoV-19/Japan/PG-78344/2021	EPI_ISL_3889099	2021-09-02
hCoV-19/Japan/PG-78345/2021	EPI_ISL_3889101	2021-09-02
hCoV-19/Japan/PG-78346/2021	EPI_ISL_3889102	2021-09-02
hCoV-19/Japan/PG-78347/2021	EPI_ISL_3889104	2021-09-02
hCoV-19/Japan/PG-78348/2021	EPI_ISL_3889107	2021-09-02
hCoV-19/Japan/PG-78349/2021	EPI_ISL_3889109	2021-09-02
hCoV-19/Japan/PG-78350/2021	EPI_ISL_3889112	2021-09-02
hCoV-19/Japan/PG-78351/2021	EPI_ISL_3889114	2021-09-02
hCoV-19/Japan/PG-78352/2021	EPI_ISL_3889116	2021-09-02
hCoV-19/Japan/PG-78353/2021	EPI_ISL_3889118	2021-09-02
hCoV-19/Japan/PG-78354/2021	EPI_ISL_3889121	2021-09-02
hCoV-19/Japan/PG-78355/2021	EPI_ISL_3889123	2021-09-02
hCoV-19/Japan/PG-78356/2021	EPI_ISL_3889125	2021-09-02
hCoV-19/Japan/PG-78357/2021	EPI_ISL_3889128	2021-09-02
hCoV-19/Japan/PG-78358/2021	EPI_ISL_3889131	2021-09-02
hCoV-19/Japan/PG-78359/2021	EPI_ISL_3889133	2021-09-02
hCoV-19/Japan/PG-78360/2021	EPI_ISL_3889135	2021-09-02
hCoV-19/Japan/PG-78361/2021	EPI_ISL_3889137	2021-09-02
hCoV-19/Japan/PG-78362/2021	EPI_ISL_3889141	2021-09-02
hCoV-19/Japan/PG-78363/2021	EPI_ISL_3889143	2021-09-02
hCoV-19/Japan/PG-78364/2021	EPI_ISL_3889145	2021-09-02
hCoV-19/Japan/PG-78365/2021	EPI_ISL_3889147	2021-09-02
hCoV-19/Japan/PG-78366/2021	EPI_ISL_3889149	2021-09-02
hCoV-19/Japan/PG-78367/2021	EPI_ISL_3889152	2021-09-02
hCoV-19/Japan/PG-78368/2021	EPI_ISL_3889154	2021-09-02
hCoV-19/Japan/PG-78369/2021	EPI_ISL_3889156	2021-09-02
hCoV-19/Japan/PG-78370/2021	EPI_ISL_3889157	2021-09-02
hCoV-19/Japan/PG-78371/2021	EPI_ISL_3889159	2021-09-02

virus_name	epiID	submission_date
hCoV-19/Japan/PG-78372/2021	EPI_ISL_3889161	2021-09-02
hCoV-19/Japan/PG-78373/2021	EPI_ISL_3889164	2021-09-02
hCoV-19/Japan/PG-78374/2021	EPI_ISL_3889166	2021-09-02
hCoV-19/Japan/PG-78375/2021	EPI_ISL_3889168	2021-09-02
hCoV-19/Japan/PG-78376/2021	EPI_ISL_3889170	2021-09-02
hCoV-19/Japan/PG-78377/2021	EPI_ISL_3889172	2021-09-02
hCoV-19/Japan/PG-78378/2021	EPI_ISL_3889173	2021-09-02
hCoV-19/Japan/PG-78379/2021	EPI_ISL_3889175	2021-09-02
hCoV-19/Japan/PG-78380/2021	EPI_ISL_3889178	2021-09-02
hCoV-19/Japan/PG-78381/2021	EPI_ISL_3889180	2021-09-02
hCoV-19/Japan/PG-78382/2021	EPI_ISL_3889181	2021-09-02
hCoV-19/Japan/PG-78383/2021	EPI_ISL_3889183	2021-09-02
hCoV-19/Japan/PG-78384/2021	EPI_ISL_3889185	2021-09-02
hCoV-19/Japan/PG-78385/2021	EPI_ISL_3889188	2021-09-02
hCoV-19/Japan/PG-78386/2021	EPI_ISL_3889189	2021-09-02
hCoV-19/Japan/PG-78387/2021	EPI_ISL_3889191	2021-09-02
hCoV-19/Japan/PG-78388/2021	EPI_ISL_3889193	2021-09-02
hCoV-19/Japan/PG-78389/2021	EPI_ISL_3889194	2021-09-02
hCoV-19/Japan/PG-78390/2021	EPI_ISL_3889199	2021-09-02
hCoV-19/Japan/PG-78391/2021	EPI_ISL_3889201	2021-09-02
hCoV-19/Japan/PG-78392/2021	EPI_ISL_3889203	2021-09-02
hCoV-19/Japan/PG-78393/2021	EPI_ISL_3889206	2021-09-02
hCoV-19/Japan/PG-78394/2021	EPI_ISL_3889208	2021-09-02
hCoV-19/Japan/PG-78395/2021	EPI_ISL_3889210	2021-09-02
hCoV-19/Japan/PG-78396/2021	EPI_ISL_3889212	2021-09-02
hCoV-19/Japan/PG-78397/2021	EPI_ISL_3889215	2021-09-02
hCoV-19/Japan/PG-78398/2021	EPI_ISL_3889217	2021-09-02
hCoV-19/Japan/PG-78399/2021	EPI_ISL_3889220	2021-09-02
hCoV-19/Japan/PG-78400/2021	EPI_ISL_3889223	2021-09-02
hCoV-19/Japan/PG-78401/2021	EPI_ISL_3889225	2021-09-02
hCoV-19/Japan/PG-78402/2021	EPI_ISL_3889228	2021-09-02
hCoV-19/Japan/PG-78403/2021	EPI_ISL_3889229	2021-09-02
hCoV-19/Japan/PG-78404/2021	EPI_ISL_3889232	2021-09-02
hCoV-19/Japan/PG-78405/2021	EPI_ISL_3889234	2021-09-02
hCoV-19/Japan/PG-78406/2021	EPI_ISL_3889237	2021-09-02
hCoV-19/Japan/PG-78407/2021	EPI_ISL_3889240	2021-09-02
hCoV-19/Japan/PG-78408/2021	EPI_ISL_3889242	2021-09-02
hCoV-19/Japan/PG-78409/2021	EPI_ISL_3889245	2021-09-02
hCoV-19/Japan/PG-78410/2021	EPI_ISL_3889247	2021-09-02
hCoV-19/Japan/PG-78411/2021	EPI_ISL_3889250	2021-09-02
hCoV-19/Japan/PG-78412/2021	EPI_ISL_3889253	2021-09-02
hCoV-19/Japan/PG-78413/2021	EPI_ISL_3889255	2021-09-02
hCoV-19/Japan/PG-78414/2021	EPI_ISL_3889258	2021-09-02
hCoV-19/Japan/PG-78415/2021	EPI_ISL_3889260	2021-09-02
hCoV-19/Japan/PG-78416/2021	EPI_ISL_3889262	2021-09-02
hCoV-19/Japan/PG-78417/2021	EPI_ISL_3889265	2021-09-02
hCoV-19/Japan/PG-78418/2021	EPI_ISL_3889268	2021-09-02
hCoV-19/Japan/PG-78419/2021	EPI_ISL_3889271	2021-09-02
hCoV-19/Japan/PG-78420/2021	EPI_ISL_3889273	2021-09-02
hCoV-19/Japan/PG-78421/2021	EPI_ISL_3889276	2021-09-02
hCoV-19/Japan/PG-78422/2021	EPI_ISL_3889279	2021-09-02
hCoV-19/Japan/PG-78423/2021	EPI_ISL_3889282	2021-09-02
hCoV-19/Japan/PG-78424/2021	EPI_ISL_3889285	2021-09-02
hCoV-19/Japan/PG-78425/2021	EPI_ISL_3889287	2021-09-02

virus_name	epiID	submission_date
hCoV-19/Japan/PG-78426/2021	EPI_ISL_3889290	2021-09-02
hCoV-19/Japan/PG-78427/2021	EPI_ISL_3889292	2021-09-02
hCoV-19/Japan/PG-78428/2021	EPI_ISL_3889295	2021-09-02
hCoV-19/Japan/PG-78429/2021	EPI_ISL_3889297	2021-09-02
hCoV-19/Japan/PG-78430/2021	EPI_ISL_3889300	2021-09-02
hCoV-19/Japan/PG-78431/2021	EPI_ISL_3889303	2021-09-02
hCoV-19/Japan/PG-78432/2021	EPI_ISL_3889305	2021-09-02
hCoV-19/Japan/PG-78433/2021	EPI_ISL_3889308	2021-09-02
hCoV-19/Japan/PG-78434/2021	EPI_ISL_3889309	2021-09-02
hCoV-19/Japan/PG-78435/2021	EPI_ISL_3889312	2021-09-02
hCoV-19/Japan/PG-78436/2021	EPI_ISL_3889314	2021-09-02
hCoV-19/Japan/PG-78437/2021	EPI_ISL_3889316	2021-09-02
hCoV-19/Japan/PG-78438/2021	EPI_ISL_3889317	2021-09-02
hCoV-19/Japan/PG-78439/2021	EPI_ISL_3889319	2021-09-02
hCoV-19/Japan/PG-78440/2021	EPI_ISL_3889322	2021-09-02
hCoV-19/Japan/PG-78441/2021	EPI_ISL_3889325	2021-09-02
hCoV-19/Japan/PG-78442/2021	EPI_ISL_3889328	2021-09-02
hCoV-19/Japan/PG-78443/2021	EPI_ISL_3889330	2021-09-02
hCoV-19/Japan/PG-78444/2021	EPI_ISL_3889333	2021-09-02
hCoV-19/Japan/PG-78445/2021	EPI_ISL_3889335	2021-09-02
hCoV-19/Japan/PG-78446/2021	EPI_ISL_3889337	2021-09-02
hCoV-19/Japan/PG-78447/2021	EPI_ISL_3889340	2021-09-02
hCoV-19/Japan/PG-78448/2021	EPI_ISL_3889343	2021-09-02
hCoV-19/Japan/PG-78449/2021	EPI_ISL_3889345	2021-09-02
hCoV-19/Japan/PG-78450/2021	EPI_ISL_3889348	2021-09-02
hCoV-19/Japan/PG-78451/2021	EPI_ISL_3889351	2021-09-02
hCoV-19/Japan/PG-78452/2021	EPI_ISL_3889353	2021-09-02
hCoV-19/Japan/PG-78453/2021	EPI_ISL_3889356	2021-09-02
hCoV-19/Japan/PG-78454/2021	EPI_ISL_3889358	2021-09-02
hCoV-19/Japan/PG-78455/2021	EPI_ISL_3889360	2021-09-02
hCoV-19/Japan/PG-78456/2021	EPI_ISL_3889363	2021-09-02
hCoV-19/Japan/PG-78457/2021	EPI_ISL_3889365	2021-09-02
hCoV-19/Japan/PG-78458/2021	EPI_ISL_3889368	2021-09-02
hCoV-19/Japan/PG-78459/2021	EPI_ISL_3889370	2021-09-02
hCoV-19/Japan/PG-78460/2021	EPI_ISL_3889373	2021-09-02
hCoV-19/Japan/PG-78461/2021	EPI_ISL_3889376	2021-09-02
hCoV-19/Japan/PG-78462/2021	EPI_ISL_3889379	2021-09-02
hCoV-19/Japan/PG-78463/2021	EPI_ISL_3889381	2021-09-02
hCoV-19/Japan/PG-78464/2021	EPI_ISL_3889384	2021-09-02
hCoV-19/Japan/PG-78465/2021	EPI_ISL_3889386	2021-09-02
hCoV-19/Japan/PG-78466/2021	EPI_ISL_3889388	2021-09-02
hCoV-19/Japan/PG-78467/2021	EPI_ISL_3889391	2021-09-02
hCoV-19/Japan/PG-78468/2021	EPI_ISL_3889394	2021-09-02
hCoV-19/Japan/PG-78469/2021	EPI_ISL_3889396	2021-09-02
hCoV-19/Japan/PG-78470/2021	EPI_ISL_3889398	2021-09-02
hCoV-19/Japan/PG-78471/2021	EPI_ISL_3889401	2021-09-02
hCoV-19/Japan/PG-78472/2021	EPI_ISL_3889404	2021-09-02
hCoV-19/Japan/PG-78473/2021	EPI_ISL_3889407	2021-09-02
hCoV-19/Japan/PG-78474/2021	EPI_ISL_3889410	2021-09-02
hCoV-19/Japan/PG-78475/2021	EPI_ISL_3889412	2021-09-02
hCoV-19/Japan/PG-78476/2021	EPI_ISL_3889415	2021-09-02
hCoV-19/Japan/PG-78477/2021	EPI_ISL_3889417	2021-09-02
hCoV-19/Japan/PG-78478/2021	EPI_ISL_3889420	2021-09-02
hCoV-19/Japan/PG-78479/2021	EPI_ISL_3889423	2021-09-02

virus_name	epiID	submission_date
hCoV-19/Japan/PG-78480/2021	EPI_ISL_3889426	2021-09-02
hCoV-19/Japan/PG-78481/2021	EPI_ISL_3889428	2021-09-02
hCoV-19/Japan/PG-78482/2021	EPI_ISL_3889431	2021-09-02
hCoV-19/Japan/PG-78483/2021	EPI_ISL_3889434	2021-09-02
hCoV-19/Japan/PG-78484/2021	EPI_ISL_3889437	2021-09-02
hCoV-19/Japan/PG-78485/2021	EPI_ISL_3889439	2021-09-02
hCoV-19/Japan/PG-78514/2021	EPI_ISL_3889442	2021-09-02
hCoV-19/Japan/PG-78515/2021	EPI_ISL_3889444	2021-09-02
hCoV-19/Japan/PG-78516/2021	EPI_ISL_3889447	2021-09-02
hCoV-19/Japan/PG-78517/2021	EPI_ISL_3889450	2021-09-02
hCoV-19/Japan/PG-78518/2021	EPI_ISL_3889452	2021-09-02
hCoV-19/Japan/PG-78519/2021	EPI_ISL_3889455	2021-09-02
hCoV-19/Japan/PG-78520/2021	EPI_ISL_3889457	2021-09-02
hCoV-19/Japan/PG-78521/2021	EPI_ISL_3889460	2021-09-02
hCoV-19/Japan/PG-78522/2021	EPI_ISL_3889463	2021-09-02
hCoV-19/Japan/PG-78523/2021	EPI_ISL_3889466	2021-09-02
hCoV-19/Japan/PG-78524/2021	EPI_ISL_3889469	2021-09-02
hCoV-19/Japan/PG-78525/2021	EPI_ISL_3889472	2021-09-02
hCoV-19/Japan/PG-78526/2021	EPI_ISL_3889475	2021-09-02
hCoV-19/Japan/PG-78527/2021	EPI_ISL_3889477	2021-09-02
hCoV-19/Japan/PG-78528/2021	EPI_ISL_3889480	2021-09-02
hCoV-19/Japan/PG-78529/2021	EPI_ISL_3889483	2021-09-02
hCoV-19/Japan/PG-78530/2021	EPI_ISL_3889485	2021-09-02
hCoV-19/Japan/PG-78531/2021	EPI_ISL_3889487	2021-09-02
hCoV-19/Japan/PG-78532/2021	EPI_ISL_3889489	2021-09-02
hCoV-19/Japan/PG-78533/2021	EPI_ISL_3889492	2021-09-02
hCoV-19/Japan/PG-78534/2021	EPI_ISL_3889494	2021-09-02
hCoV-19/Japan/PG-78535/2021	EPI_ISL_3889495	2021-09-02
hCoV-19/Japan/PG-78536/2021	EPI_ISL_3889498	2021-09-02
hCoV-19/Japan/PG-78537/2021	EPI_ISL_3889499	2021-09-02
hCoV-19/Japan/PG-78538/2021	EPI_ISL_3889500	2021-09-02
hCoV-19/Japan/PG-78539/2021	EPI_ISL_3889501	2021-09-02
hCoV-19/Japan/PG-78540/2021	EPI_ISL_3889502	2021-09-02
hCoV-19/Japan/PG-78541/2021	EPI_ISL_3889503	2021-09-02
hCoV-19/Japan/PG-78542/2021	EPI_ISL_3889505	2021-09-02
hCoV-19/Japan/PG-78543/2021	EPI_ISL_3889508	2021-09-02
hCoV-19/Japan/PG-78544/2021	EPI_ISL_3889511	2021-09-02
hCoV-19/Japan/PG-78545/2021	EPI_ISL_3889513	2021-09-02
hCoV-19/Japan/PG-78546/2021	EPI_ISL_3889516	2021-09-02
hCoV-19/Japan/PG-78547/2021	EPI_ISL_3889518	2021-09-02
hCoV-19/Japan/PG-78548/2021	EPI_ISL_3889521	2021-09-02
hCoV-19/Japan/PG-78549/2021	EPI_ISL_3889523	2021-09-02
hCoV-19/Japan/PG-78550/2021	EPI_ISL_3889525	2021-09-02
hCoV-19/Japan/PG-78551/2021	EPI_ISL_3889527	2021-09-02
hCoV-19/Japan/PG-78552/2021	EPI_ISL_3889529	2021-09-02
hCoV-19/Japan/PG-78553/2021	EPI_ISL_3889532	2021-09-02
hCoV-19/Japan/PG-78554/2021	EPI_ISL_3889535	2021-09-02
hCoV-19/Japan/PG-78555/2021	EPI_ISL_3889538	2021-09-02
hCoV-19/Japan/PG-78556/2021	EPI_ISL_3889540	2021-09-02
hCoV-19/Japan/PG-78557/2021	EPI_ISL_3889543	2021-09-02
hCoV-19/Japan/PG-78558/2021	EPI_ISL_3889547	2021-09-02
hCoV-19/Japan/PG-78559/2021	EPI_ISL_3889549	2021-09-02
hCoV-19/Japan/PG-78560/2021	EPI_ISL_3889553	2021-09-02
hCoV-19/Japan/PG-78561/2021	EPI_ISL_3889554	2021-09-02

virus_name	epiID	submission_date
hCoV-19/Japan/PG-78562/2021	EPI_ISL_3889556	2021-09-02
hCoV-19/Japan/PG-78563/2021	EPI_ISL_3889558	2021-09-02
hCoV-19/Japan/PG-78564/2021	EPI_ISL_3889561	2021-09-02
hCoV-19/Japan/PG-78565/2021	EPI_ISL_3889564	2021-09-02
hCoV-19/Japan/PG-78566/2021	EPI_ISL_3889566	2021-09-02
hCoV-19/Japan/PG-78567/2021	EPI_ISL_3889569	2021-09-02
hCoV-19/Japan/PG-78568/2021	EPI_ISL_3889572	2021-09-02
hCoV-19/Japan/PG-78569/2021	EPI_ISL_3889575	2021-09-02
hCoV-19/Japan/PG-78570/2021	EPI_ISL_3889577	2021-09-02
hCoV-19/Japan/PG-78571/2021	EPI_ISL_3889580	2021-09-02
hCoV-19/Japan/PG-78572/2021	EPI_ISL_3889582	2021-09-02
hCoV-19/Japan/PG-78573/2021	EPI_ISL_3889584	2021-09-02
hCoV-19/Japan/PG-78574/2021	EPI_ISL_3889587	2021-09-02
hCoV-19/Japan/PG-78575/2021	EPI_ISL_3889590	2021-09-02
hCoV-19/Japan/PG-78576/2021	EPI_ISL_3889593	2021-09-02
hCoV-19/Japan/PG-78577/2021	EPI_ISL_3889595	2021-09-02
hCoV-19/Japan/PG-78578/2021	EPI_ISL_3889597	2021-09-02
hCoV-19/Japan/PG-78579/2021	EPI_ISL_3889600	2021-09-02
hCoV-19/Japan/PG-78580/2021	EPI_ISL_3889603	2021-09-02
hCoV-19/Japan/PG-78581/2021	EPI_ISL_3889605	2021-09-02
hCoV-19/Japan/PG-78582/2021	EPI_ISL_3889607	2021-09-02
hCoV-19/Japan/PG-78583/2021	EPI_ISL_3889610	2021-09-02
hCoV-19/Japan/PG-78584/2021	EPI_ISL_3889613	2021-09-02
hCoV-19/Japan/PG-78585/2021	EPI_ISL_3889615	2021-09-02
hCoV-19/Japan/PG-78586/2021	EPI_ISL_3889617	2021-09-02
hCoV-19/Japan/PG-78587/2021	EPI_ISL_3889619	2021-09-02
hCoV-19/Japan/PG-78588/2021	EPI_ISL_3889621	2021-09-02
hCoV-19/Japan/PG-78589/2021	EPI_ISL_3889624	2021-09-02
hCoV-19/Japan/PG-78590/2021	EPI_ISL_3889626	2021-09-02
hCoV-19/Japan/PG-78591/2021	EPI_ISL_3889628	2021-09-02
hCoV-19/Japan/PG-78592/2021	EPI_ISL_3889630	2021-09-02
hCoV-19/Japan/PG-78593/2021	EPI_ISL_3889632	2021-09-02
hCoV-19/Japan/PG-78594/2021	EPI_ISL_3889635	2021-09-02
hCoV-19/Japan/PG-78595/2021	EPI_ISL_3889637	2021-09-02
hCoV-19/Japan/PG-78596/2021	EPI_ISL_3889639	2021-09-02
hCoV-19/Japan/PG-78597/2021	EPI_ISL_3889642	2021-09-02
hCoV-19/Japan/PG-78598/2021	EPI_ISL_3889645	2021-09-02
hCoV-19/Japan/PG-78599/2021	EPI_ISL_3889646	2021-09-02
hCoV-19/Japan/PG-78600/2021	EPI_ISL_3889647	2021-09-02
hCoV-19/Japan/PG-78601/2021	EPI_ISL_3889648	2021-09-02
hCoV-19/Japan/PG-78602/2021	EPI_ISL_3889649	2021-09-02
hCoV-19/Japan/PG-78603/2021	EPI_ISL_3889650	2021-09-02
hCoV-19/Japan/PG-78604/2021	EPI_ISL_3889651	2021-09-02
hCoV-19/Japan/PG-78605/2021	EPI_ISL_3889655	2021-09-02
hCoV-19/Japan/PG-78606/2021	EPI_ISL_3889657	2021-09-02
hCoV-19/Japan/PG-78607/2021	EPI_ISL_3889659	2021-09-02
hCoV-19/Japan/PG-78608/2021	EPI_ISL_3889661	2021-09-02
hCoV-19/Japan/PG-78609/2021	EPI_ISL_3889665	2021-09-02
hCoV-19/Japan/PG-78610/2021	EPI_ISL_3889667	2021-09-02
hCoV-19/Japan/PG-78611/2021	EPI_ISL_3889670	2021-09-02
hCoV-19/Japan/PG-78612/2021	EPI_ISL_3889672	2021-09-02
hCoV-19/Japan/PG-78613/2021	EPI_ISL_3889676	2021-09-02
hCoV-19/Japan/PG-78614/2021	EPI_ISL_3889678	2021-09-02
hCoV-19/Japan/PG-78615/2021	EPI_ISL_3889681	2021-09-02

virus_name	epiID	submission_date
hCoV-19/Japan/PG-78616/2021	EPI_ISL_3889684	2021-09-02
hCoV-19/Japan/PG-78617/2021	EPI_ISL_3889687	2021-09-02
hCoV-19/Japan/PG-78618/2021	EPI_ISL_3889690	2021-09-02
hCoV-19/Japan/PG-78619/2021	EPI_ISL_3889693	2021-09-02
hCoV-19/Japan/PG-78620/2021	EPI_ISL_3889696	2021-09-02
hCoV-19/Japan/PG-78621/2021	EPI_ISL_3889698	2021-09-02
hCoV-19/Japan/PG-78622/2021	EPI_ISL_3889702	2021-09-02
hCoV-19/Japan/PG-78623/2021	EPI_ISL_3889703	2021-09-02
hCoV-19/Japan/PG-78624/2021	EPI_ISL_3889705	2021-09-02
hCoV-19/Japan/PG-78625/2021	EPI_ISL_3889708	2021-09-02
hCoV-19/Japan/PG-78626/2021	EPI_ISL_3889710	2021-09-02
hCoV-19/Japan/PG-78627/2021	EPI_ISL_3889713	2021-09-02
hCoV-19/Japan/PG-78628/2021	EPI_ISL_3889717	2021-09-02
hCoV-19/Japan/PG-78629/2021	EPI_ISL_3889719	2021-09-02
hCoV-19/Japan/PG-78630/2021	EPI_ISL_3889722	2021-09-02
hCoV-19/Japan/PG-78631/2021	EPI_ISL_3889725	2021-09-02
hCoV-19/Japan/PG-78632/2021	EPI_ISL_3889728	2021-09-02
hCoV-19/Japan/PG-78633/2021	EPI_ISL_3889731	2021-09-02
hCoV-19/Japan/PG-78634/2021	EPI_ISL_3889734	2021-09-02
hCoV-19/Japan/PG-78635/2021	EPI_ISL_3889736	2021-09-02
hCoV-19/Japan/PG-78636/2021	EPI_ISL_3889739	2021-09-02
hCoV-19/Japan/PG-78637/2021	EPI_ISL_3889742	2021-09-02
hCoV-19/Japan/PG-78638/2021	EPI_ISL_3889744	2021-09-02
hCoV-19/Japan/PG-78639/2021	EPI_ISL_3889747	2021-09-02
hCoV-19/Japan/PG-78640/2021	EPI_ISL_3889750	2021-09-02
hCoV-19/Japan/PG-78641/2021	EPI_ISL_3889753	2021-09-02
hCoV-19/Japan/PG-78642/2021	EPI_ISL_3889756	2021-09-02
hCoV-19/Japan/PG-78643/2021	EPI_ISL_3889758	2021-09-02
hCoV-19/Japan/PG-78644/2021	EPI_ISL_3889762	2021-09-02
hCoV-19/Japan/PG-78645/2021	EPI_ISL_3889764	2021-09-02
hCoV-19/Japan/PG-78646/2021	EPI_ISL_3889767	2021-09-02
hCoV-19/Japan/PG-78647/2021	EPI_ISL_3889770	2021-09-02
hCoV-19/Japan/PG-78648/2021	EPI_ISL_3889773	2021-09-02
hCoV-19/Japan/PG-78649/2021	EPI_ISL_3889775	2021-09-02
hCoV-19/Japan/PG-78650/2021	EPI_ISL_3889778	2021-09-02
hCoV-19/Japan/PG-78651/2021	EPI_ISL_3889781	2021-09-02
hCoV-19/Japan/PG-78652/2021	EPI_ISL_3889783	2021-09-02
hCoV-19/Japan/PG-78653/2021	EPI_ISL_3889785	2021-09-02
hCoV-19/Japan/PG-78654/2021	EPI_ISL_3889788	2021-09-02
hCoV-19/Japan/PG-78655/2021	EPI_ISL_3889790	2021-09-02
hCoV-19/Japan/PG-78656/2021	EPI_ISL_3889792	2021-09-02
hCoV-19/Japan/PG-78657/2021	EPI_ISL_3889794	2021-09-02
hCoV-19/Japan/PG-78658/2021	EPI_ISL_3889796	2021-09-02
hCoV-19/Japan/PG-78659/2021	EPI_ISL_3889798	2021-09-02
hCoV-19/Japan/PG-78660/2021	EPI_ISL_3889801	2021-09-02
hCoV-19/Japan/PG-78661/2021	EPI_ISL_3889803	2021-09-02
hCoV-19/Japan/PG-78662/2021	EPI_ISL_3889806	2021-09-02
hCoV-19/Japan/PG-78663/2021	EPI_ISL_3889808	2021-09-02
hCoV-19/Japan/PG-78664/2021	EPI_ISL_3889811	2021-09-02
hCoV-19/Japan/PG-78665/2021	EPI_ISL_3889813	2021-09-02
hCoV-19/Japan/PG-78666/2021	EPI_ISL_3889815	2021-09-02
hCoV-19/Japan/PG-78667/2021	EPI_ISL_3889817	2021-09-02
hCoV-19/Japan/PG-78668/2021	EPI_ISL_3889819	2021-09-02
hCoV-19/Japan/PG-78669/2021	EPI_ISL_3889822	2021-09-02

virus_name	epiID	submission_date
hCoV-19/Japan/PG-78670/2021	EPI_ISL_3889824	2021-09-02
hCoV-19/Japan/PG-78671/2021	EPI_ISL_3889827	2021-09-02
hCoV-19/Japan/PG-78672/2021	EPI_ISL_3889829	2021-09-02
hCoV-19/Japan/PG-78673/2021	EPI_ISL_3889832	2021-09-02
hCoV-19/Japan/PG-78674/2021	EPI_ISL_3889833	2021-09-02
hCoV-19/Japan/PG-78675/2021	EPI_ISL_3889837	2021-09-02
hCoV-19/Japan/PG-78676/2021	EPI_ISL_3889839	2021-09-02
hCoV-19/Japan/PG-78677/2021	EPI_ISL_3889842	2021-09-02
hCoV-19/Japan/PG-78678/2021	EPI_ISL_3889845	2021-09-02
hCoV-19/Japan/PG-78679/2021	EPI_ISL_3889848	2021-09-02
hCoV-19/Japan/PG-78680/2021	EPI_ISL_3889850	2021-09-02
hCoV-19/Japan/PG-78681/2021	EPI_ISL_3889852	2021-09-02
hCoV-19/Japan/PG-78682/2021	EPI_ISL_3889855	2021-09-02
hCoV-19/Japan/PG-78683/2021	EPI_ISL_3889858	2021-09-02
hCoV-19/Japan/PG-78684/2021	EPI_ISL_3889860	2021-09-02
hCoV-19/Japan/PG-78685/2021	EPI_ISL_3889861	2021-09-02
hCoV-19/Japan/PG-78686/2021	EPI_ISL_3889862	2021-09-02
hCoV-19/Japan/PG-78687/2021	EPI_ISL_3889863	2021-09-02
hCoV-19/Japan/PG-78688/2021	EPI_ISL_3889864	2021-09-02
hCoV-19/Japan/PG-78689/2021	EPI_ISL_3889865	2021-09-02
hCoV-19/Japan/PG-78690/2021	EPI_ISL_3889866	2021-09-02
hCoV-19/Japan/PG-78691/2021	EPI_ISL_3889869	2021-09-02
hCoV-19/Japan/PG-78692/2021	EPI_ISL_3889872	2021-09-02
hCoV-19/Japan/PG-78693/2021	EPI_ISL_3889874	2021-09-02
hCoV-19/Japan/PG-78694/2021	EPI_ISL_3889876	2021-09-02
hCoV-19/Japan/PG-78695/2021	EPI_ISL_3889879	2021-09-02
hCoV-19/Japan/PG-78696/2021	EPI_ISL_3889881	2021-09-02
hCoV-19/Japan/PG-78697/2021	EPI_ISL_3889884	2021-09-02
hCoV-19/Japan/PG-78698/2021	EPI_ISL_3889886	2021-09-02
hCoV-19/Japan/PG-78699/2021	EPI_ISL_3889889	2021-09-02
hCoV-19/Japan/PG-78700/2021	EPI_ISL_3889891	2021-09-02
hCoV-19/Japan/PG-78701/2021	EPI_ISL_3889893	2021-09-02
hCoV-19/Japan/PG-78702/2021	EPI_ISL_3889895	2021-09-02
hCoV-19/Japan/PG-78703/2021	EPI_ISL_3889897	2021-09-02
hCoV-19/Japan/PG-78704/2021	EPI_ISL_3889900	2021-09-02
hCoV-19/Japan/PG-78705/2021	EPI_ISL_3889903	2021-09-02
hCoV-19/Japan/PG-78706/2021	EPI_ISL_3889905	2021-09-02
hCoV-19/Japan/PG-78707/2021	EPI_ISL_3889908	2021-09-02
hCoV-19/Japan/PG-78708/2021	EPI_ISL_3889910	2021-09-02
hCoV-19/Japan/PG-78709/2021	EPI_ISL_3889914	2021-09-02
hCoV-19/Japan/PG-78710/2021	EPI_ISL_3889916	2021-09-02
hCoV-19/Japan/PG-78711/2021	EPI_ISL_3889919	2021-09-02
hCoV-19/Japan/PG-78712/2021	EPI_ISL_3889922	2021-09-02
hCoV-19/Japan/PG-78713/2021	EPI_ISL_3889925	2021-09-02
hCoV-19/Japan/PG-78714/2021	EPI_ISL_3889928	2021-09-02
hCoV-19/Japan/PG-78715/2021	EPI_ISL_3889930	2021-09-02
hCoV-19/Japan/PG-78716/2021	EPI_ISL_3889933	2021-09-02
hCoV-19/Japan/PG-78717/2021	EPI_ISL_3889936	2021-09-02
hCoV-19/Japan/PG-78718/2021	EPI_ISL_3889939	2021-09-02
hCoV-19/Japan/PG-78719/2021	EPI_ISL_3889940	2021-09-02
hCoV-19/Japan/PG-78720/2021	EPI_ISL_3889943	2021-09-02
hCoV-19/Japan/PG-78721/2021	EPI_ISL_3889945	2021-09-02
hCoV-19/Japan/PG-78722/2021	EPI_ISL_3889948	2021-09-02
hCoV-19/Japan/PG-78723/2021	EPI_ISL_3889950	2021-09-02

virus_name	epiID	submission_date
hCoV-19/Japan/PG-78724/2021	EPI_ISL_3889953	2021-09-02
hCoV-19/Japan/PG-78725/2021	EPI_ISL_3889956	2021-09-02
hCoV-19/Japan/PG-78726/2021	EPI_ISL_3889958	2021-09-02
hCoV-19/Japan/PG-78727/2021	EPI_ISL_3889961	2021-09-02
hCoV-19/Japan/PG-78728/2021	EPI_ISL_3889964	2021-09-02
hCoV-19/Japan/PG-78729/2021	EPI_ISL_3889966	2021-09-02
hCoV-19/Japan/PG-78730/2021	EPI_ISL_3889969	2021-09-02
hCoV-19/Japan/PG-78731/2021	EPI_ISL_3889972	2021-09-02
hCoV-19/Japan/PG-78732/2021	EPI_ISL_3889974	2021-09-02
hCoV-19/Japan/PG-78733/2021	EPI_ISL_3889977	2021-09-02
hCoV-19/Japan/PG-78734/2021	EPI_ISL_3889979	2021-09-02
hCoV-19/Japan/PG-78735/2021	EPI_ISL_3889983	2021-09-02
hCoV-19/Japan/PG-78736/2021	EPI_ISL_3889986	2021-09-02
hCoV-19/Japan/PG-78737/2021	EPI_ISL_3889989	2021-09-02
hCoV-19/Japan/PG-78738/2021	EPI_ISL_3889991	2021-09-02
hCoV-19/Japan/PG-78739/2021	EPI_ISL_3889993	2021-09-02
hCoV-19/Japan/PG-78740/2021	EPI_ISL_3889995	2021-09-02
hCoV-19/Japan/PG-78741/2021	EPI_ISL_3889998	2021-09-02
hCoV-19/Japan/PG-78742/2021	EPI_ISL_3890000	2021-09-02
hCoV-19/Japan/PG-78743/2021	EPI_ISL_3890003	2021-09-02
hCoV-19/Japan/PG-78744/2021	EPI_ISL_3890006	2021-09-02
hCoV-19/Japan/PG-78745/2021	EPI_ISL_3890009	2021-09-02
hCoV-19/Japan/PG-78746/2021	EPI_ISL_3890012	2021-09-02
hCoV-19/Japan/PG-78747/2021	EPI_ISL_3890014	2021-09-02
hCoV-19/Japan/PG-78748/2021	EPI_ISL_3890017	2021-09-02
hCoV-19/Japan/PG-78749/2021	EPI_ISL_3890020	2021-09-02
hCoV-19/Japan/PG-78750/2021	EPI_ISL_3890022	2021-09-02
hCoV-19/Japan/PG-78751/2021	EPI_ISL_3890025	2021-09-02
hCoV-19/Japan/PG-78752/2021	EPI_ISL_3890027	2021-09-02
hCoV-19/Japan/PG-78753/2021	EPI_ISL_3890029	2021-09-02
hCoV-19/Japan/PG-78754/2021	EPI_ISL_3890032	2021-09-02
hCoV-19/Japan/PG-78755/2021	EPI_ISL_3890033	2021-09-02
hCoV-19/Japan/PG-78756/2021	EPI_ISL_3890034	2021-09-02
hCoV-19/Japan/PG-78757/2021	EPI_ISL_3890036	2021-09-02
hCoV-19/Japan/PG-78758/2021	EPI_ISL_3890039	2021-09-02
hCoV-19/Japan/PG-78759/2021	EPI_ISL_3890041	2021-09-02
hCoV-19/Japan/PG-78760/2021	EPI_ISL_3890044	2021-09-02
hCoV-19/Japan/PG-78761/2021	EPI_ISL_3890047	2021-09-02
hCoV-19/Japan/PG-78762/2021	EPI_ISL_3890050	2021-09-02
hCoV-19/Japan/PG-78763/2021	EPI_ISL_3890053	2021-09-02
hCoV-19/Japan/PG-78764/2021	EPI_ISL_3890055	2021-09-02
hCoV-19/Japan/PG-78765/2021	EPI_ISL_3890057	2021-09-02
hCoV-19/Japan/PG-78766/2021	EPI_ISL_3890059	2021-09-02
hCoV-19/Japan/PG-78767/2021	EPI_ISL_3890061	2021-09-02
hCoV-19/Japan/PG-78768/2021	EPI_ISL_3890064	2021-09-02
hCoV-19/Japan/PG-78769/2021	EPI_ISL_3890066	2021-09-02
hCoV-19/Japan/PG-78770/2021	EPI_ISL_3890068	2021-09-02
hCoV-19/Japan/PG-78771/2021	EPI_ISL_3890071	2021-09-02
hCoV-19/Japan/PG-78772/2021	EPI_ISL_3890074	2021-09-02
hCoV-19/Japan/PG-78773/2021	EPI_ISL_3890076	2021-09-02
hCoV-19/Japan/PG-78774/2021	EPI_ISL_3890079	2021-09-02
hCoV-19/Japan/PG-78775/2021	EPI_ISL_3890081	2021-09-02
hCoV-19/Japan/PG-78776/2021	EPI_ISL_3890084	2021-09-02
hCoV-19/Japan/PG-78777/2021	EPI_ISL_3890087	2021-09-02

virus_name	epiID	submission_date
hCoV-19/Japan/PG-78778/2021	EPI_ISL_3890089	2021-09-02
hCoV-19/Japan/PG-78779/2021	EPI_ISL_3890092	2021-09-02
hCoV-19/Japan/PG-78780/2021	EPI_ISL_3890094	2021-09-02
hCoV-19/Japan/PG-78781/2021	EPI_ISL_3890098	2021-09-02
hCoV-19/Japan/PG-78782/2021	EPI_ISL_3890101	2021-09-02
hCoV-19/Japan/PG-78783/2021	EPI_ISL_3890103	2021-09-02
hCoV-19/Japan/PG-78784/2021	EPI_ISL_3890106	2021-09-02
hCoV-19/Japan/PG-78785/2021	EPI_ISL_3890109	2021-09-02
hCoV-19/Japan/PG-78786/2021	EPI_ISL_3890111	2021-09-02
hCoV-19/Japan/PG-78787/2021	EPI_ISL_3890114	2021-09-02
hCoV-19/Japan/PG-78788/2021	EPI_ISL_3890116	2021-09-02
hCoV-19/Japan/PG-78789/2021	EPI_ISL_3890119	2021-09-02
hCoV-19/Japan/PG-78790/2021	EPI_ISL_3890122	2021-09-02
hCoV-19/Japan/PG-78791/2021	EPI_ISL_3890125	2021-09-02
hCoV-19/Japan/PG-78792/2021	EPI_ISL_3890128	2021-09-02
hCoV-19/Japan/PG-78793/2021	EPI_ISL_3890131	2021-09-02
hCoV-19/Japan/PG-78794/2021	EPI_ISL_3890133	2021-09-02
hCoV-19/Japan/PG-78795/2021	EPI_ISL_3890135	2021-09-02
hCoV-19/Japan/PG-78796/2021	EPI_ISL_3890138	2021-09-02
hCoV-19/Japan/PG-78797/2021	EPI_ISL_3890140	2021-09-02
hCoV-19/Japan/PG-78798/2021	EPI_ISL_3890143	2021-09-02
hCoV-19/Japan/PG-78799/2021	EPI_ISL_3890146	2021-09-02
hCoV-19/Japan/PG-78800/2021	EPI_ISL_3890148	2021-09-02
hCoV-19/Japan/PG-78801/2021	EPI_ISL_3890151	2021-09-02
hCoV-19/Japan/PG-78802/2021	EPI_ISL_3890154	2021-09-02
hCoV-19/Japan/PG-78803/2021	EPI_ISL_3890156	2021-09-02
hCoV-19/Japan/PG-78804/2021	EPI_ISL_3890159	2021-09-02
hCoV-19/Japan/PG-78805/2021	EPI_ISL_3890162	2021-09-02
hCoV-19/Japan/PG-78806/2021	EPI_ISL_3890165	2021-09-02
hCoV-19/Japan/PG-78807/2021	EPI_ISL_3890167	2021-09-02
hCoV-19/Japan/PG-78808/2021	EPI_ISL_3890170	2021-09-02
hCoV-19/Japan/PG-78809/2021	EPI_ISL_3890173	2021-09-02
hCoV-19/Japan/PG-78810/2021	EPI_ISL_3890175	2021-09-02
hCoV-19/Japan/PG-78811/2021	EPI_ISL_3890177	2021-09-02
hCoV-19/Japan/PG-78812/2021	EPI_ISL_3890179	2021-09-02
hCoV-19/Japan/PG-78813/2021	EPI_ISL_3890182	2021-09-02
hCoV-19/Japan/PG-78814/2021	EPI_ISL_3890184	2021-09-02
hCoV-19/Japan/PG-78815/2021	EPI_ISL_3890187	2021-09-02
hCoV-19/Japan/PG-78816/2021	EPI_ISL_3890190	2021-09-02
hCoV-19/Japan/PG-78817/2021	EPI_ISL_3890192	2021-09-02
hCoV-19/Japan/PG-78818/2021	EPI_ISL_3890194	2021-09-02
hCoV-19/Japan/PG-78819/2021	EPI_ISL_3890197	2021-09-02
hCoV-19/Japan/PG-78820/2021	EPI_ISL_3890199	2021-09-02
hCoV-19/Japan/PG-78821/2021	EPI_ISL_3890202	2021-09-02
hCoV-19/Japan/PG-78822/2021	EPI_ISL_3890203	2021-09-02
hCoV-19/Japan/PG-78823/2021	EPI_ISL_3890204	2021-09-02
hCoV-19/Japan/PG-78824/2021	EPI_ISL_3890206	2021-09-02
hCoV-19/Japan/PG-78825/2021	EPI_ISL_3890209	2021-09-02
hCoV-19/Japan/PG-78826/2021	EPI_ISL_3890213	2021-09-02
hCoV-19/Japan/PG-78827/2021	EPI_ISL_3890215	2021-09-02
hCoV-19/Japan/PG-78828/2021	EPI_ISL_3890218	2021-09-02
hCoV-19/Japan/PG-78829/2021	EPI_ISL_3890220	2021-09-02
hCoV-19/Japan/PG-78830/2021	EPI_ISL_3890223	2021-09-02
hCoV-19/Japan/PG-78831/2021	EPI_ISL_3890225	2021-09-02

virus_name	epiID	submission_date
hCoV-19/Japan/PG-78832/2021	EPI_ISL_3890228	2021-09-02
hCoV-19/Japan/PG-78833/2021	EPI_ISL_3890230	2021-09-02
hCoV-19/Japan/PG-78834/2021	EPI_ISL_3890232	2021-09-02
hCoV-19/Japan/PG-78835/2021	EPI_ISL_3890236	2021-09-02
hCoV-19/Japan/PG-78836/2021	EPI_ISL_3890238	2021-09-02
hCoV-19/Japan/PG-78837/2021	EPI_ISL_3890240	2021-09-02
hCoV-19/Japan/PG-78838/2021	EPI_ISL_3890243	2021-09-02
hCoV-19/Japan/PG-78839/2021	EPI_ISL_3890246	2021-09-02
hCoV-19/Japan/PG-78840/2021	EPI_ISL_3890249	2021-09-02
hCoV-19/Japan/PG-78841/2021	EPI_ISL_3890252	2021-09-02
hCoV-19/Japan/PG-78842/2021	EPI_ISL_3890255	2021-09-02
hCoV-19/Japan/PG-78843/2021	EPI_ISL_3890259	2021-09-02
hCoV-19/Japan/PG-78844/2021	EPI_ISL_3890261	2021-09-02
hCoV-19/Japan/PG-78845/2021	EPI_ISL_3890264	2021-09-02
hCoV-19/Japan/PG-78846/2021	EPI_ISL_3890267	2021-09-02
hCoV-19/Japan/PG-78847/2021	EPI_ISL_3890270	2021-09-02
hCoV-19/Japan/PG-78848/2021	EPI_ISL_3890273	2021-09-02
hCoV-19/Japan/PG-78849/2021	EPI_ISL_3890275	2021-09-02
hCoV-19/Japan/PG-78850/2021	EPI_ISL_3890278	2021-09-02
hCoV-19/Japan/PG-78851/2021	EPI_ISL_3890280	2021-09-02
hCoV-19/Japan/PG-78852/2021	EPI_ISL_3890283	2021-09-02
hCoV-19/Japan/PG-78853/2021	EPI_ISL_3890285	2021-09-02
hCoV-19/Japan/PG-78854/2021	EPI_ISL_3890288	2021-09-02
hCoV-19/Japan/PG-78855/2021	EPI_ISL_3890291	2021-09-02
hCoV-19/Japan/PG-78856/2021	EPI_ISL_3890293	2021-09-02
hCoV-19/Japan/PG-78857/2021	EPI_ISL_3890294	2021-09-02
hCoV-19/Japan/PG-78858/2021	EPI_ISL_3890296	2021-09-02
hCoV-19/Japan/PG-78859/2021	EPI_ISL_3890299	2021-09-02
hCoV-19/Japan/PG-78860/2021	EPI_ISL_3890301	2021-09-02
hCoV-19/Japan/PG-78861/2021	EPI_ISL_3890302	2021-09-02
hCoV-19/Japan/PG-78862/2021	EPI_ISL_3890305	2021-09-02
hCoV-19/Japan/PG-78863/2021	EPI_ISL_3904455	2021-09-02
hCoV-19/Japan/PG-78864/2021	EPI_ISL_3904458	2021-09-02
hCoV-19/Japan/PG-78865/2021	EPI_ISL_3904462	2021-09-02
hCoV-19/Japan/PG-78866/2021	EPI_ISL_3904465	2021-09-02
hCoV-19/Japan/PG-78867/2021	EPI_ISL_3904467	2021-09-02
hCoV-19/Japan/PG-78868/2021	EPI_ISL_3904468	2021-09-02
hCoV-19/Japan/PG-78869/2021	EPI_ISL_3904469	2021-09-02
hCoV-19/Japan/PG-78870/2021	EPI_ISL_3904470	2021-09-02
hCoV-19/Japan/PG-78871/2021	EPI_ISL_3904471	2021-09-02
hCoV-19/Japan/PG-78872/2021	EPI_ISL_3904472	2021-09-02
hCoV-19/Japan/PG-78873/2021	EPI_ISL_3904473	2021-09-02
hCoV-19/Japan/PG-78874/2021	EPI_ISL_3904474	2021-09-02
hCoV-19/Japan/PG-78875/2021	EPI_ISL_3904475	2021-09-02
hCoV-19/Japan/PG-78876/2021	EPI_ISL_3904476	2021-09-02
hCoV-19/Japan/PG-78877/2021	EPI_ISL_3904477	2021-09-02
hCoV-19/Japan/PG-78878/2021	EPI_ISL_3904478	2021-09-02
hCoV-19/Japan/PG-78879/2021	EPI_ISL_3904479	2021-09-02
hCoV-19/Japan/PG-78880/2021	EPI_ISL_3904480	2021-09-02
hCoV-19/Japan/PG-78881/2021	EPI_ISL_3904481	2021-09-02
hCoV-19/Japan/PG-78882/2021	EPI_ISL_3904482	2021-09-02
hCoV-19/Japan/PG-78883/2021	EPI_ISL_3904483	2021-09-02
hCoV-19/Japan/PG-78884/2021	EPI_ISL_3904484	2021-09-02
hCoV-19/Japan/PG-78885/2021	EPI_ISL_3904485	2021-09-02

virus_name	epiID	submission_date
hCoV-19/Japan/PG-78886/2021	EPI_ISL_3904486	2021-09-02
hCoV-19/Japan/PG-78887/2021	EPI_ISL_3904487	2021-09-02
hCoV-19/Japan/PG-78888/2021	EPI_ISL_3904488	2021-09-02
hCoV-19/Japan/PG-78889/2021	EPI_ISL_3904489	2021-09-02
hCoV-19/Japan/PG-78890/2021	EPI_ISL_3904490	2021-09-02
hCoV-19/Japan/PG-78891/2021	EPI_ISL_3904491	2021-09-02
hCoV-19/Japan/PG-78892/2021	EPI_ISL_3904492	2021-09-02
hCoV-19/Japan/PG-78893/2021	EPI_ISL_3904493	2021-09-02
hCoV-19/Japan/PG-78894/2021	EPI_ISL_3904494	2021-09-02
hCoV-19/Japan/PG-78895/2021	EPI_ISL_3904495	2021-09-02
hCoV-19/Japan/PG-78896/2021	EPI_ISL_3904496	2021-09-02
hCoV-19/Japan/PG-78897/2021	EPI_ISL_3904497	2021-09-02
hCoV-19/Japan/PG-78898/2021	EPI_ISL_3904498	2021-09-02
hCoV-19/Japan/PG-78899/2021	EPI_ISL_3904499	2021-09-02
hCoV-19/Japan/PG-78900/2021	EPI_ISL_3904500	2021-09-02
hCoV-19/Japan/PG-78901/2021	EPI_ISL_3904501	2021-09-02
hCoV-19/Japan/PG-78902/2021	EPI_ISL_3904502	2021-09-02
hCoV-19/Japan/PG-78903/2021	EPI_ISL_3904503	2021-09-02
hCoV-19/Japan/PG-78904/2021	EPI_ISL_3904504	2021-09-02
hCoV-19/Japan/PG-78905/2021	EPI_ISL_3904505	2021-09-02
hCoV-19/Japan/PG-78906/2021	EPI_ISL_3904506	2021-09-02
hCoV-19/Japan/PG-78907/2021	EPI_ISL_3904507	2021-09-02
hCoV-19/Japan/PG-78908/2021	EPI_ISL_3904508	2021-09-02
hCoV-19/Japan/PG-78909/2021	EPI_ISL_3904509	2021-09-02
hCoV-19/Japan/PG-78910/2021	EPI_ISL_3904510	2021-09-02
hCoV-19/Japan/PG-78911/2021	EPI_ISL_3904511	2021-09-02
hCoV-19/Japan/PG-78912/2021	EPI_ISL_3904512	2021-09-02
hCoV-19/Japan/PG-78913/2021	EPI_ISL_3904513	2021-09-02
hCoV-19/Japan/PG-78914/2021	EPI_ISL_3904514	2021-09-02
hCoV-19/Japan/PG-78915/2021	EPI_ISL_3904515	2021-09-02
hCoV-19/Japan/PG-78916/2021	EPI_ISL_3904516	2021-09-02
hCoV-19/Japan/PG-78917/2021	EPI_ISL_3904517	2021-09-02
hCoV-19/Japan/PG-78918/2021	EPI_ISL_3890309	2021-09-02
hCoV-19/Japan/PG-78919/2021	EPI_ISL_3890311	2021-09-02
hCoV-19/Japan/PG-78920/2021	EPI_ISL_3890314	2021-09-02
hCoV-19/Japan/PG-78921/2021	EPI_ISL_3890316	2021-09-02
hCoV-19/Japan/PG-78922/2021	EPI_ISL_3890319	2021-09-02
hCoV-19/Japan/PG-78923/2021	EPI_ISL_3890321	2021-09-02
hCoV-19/Japan/PG-78924/2021	EPI_ISL_3890324	2021-09-02
hCoV-19/Japan/PG-78925/2021	EPI_ISL_3890326	2021-09-02
hCoV-19/Japan/PG-78926/2021	EPI_ISL_3890329	2021-09-02
hCoV-19/Japan/PG-78927/2021	EPI_ISL_3890332	2021-09-02
hCoV-19/Japan/PG-78928/2021	EPI_ISL_3890334	2021-09-02
hCoV-19/Japan/PG-78929/2021	EPI_ISL_3890337	2021-09-02
hCoV-19/Japan/PG-78930/2021	EPI_ISL_3890339	2021-09-02
hCoV-19/Japan/PG-78931/2021	EPI_ISL_3890341	2021-09-02
hCoV-19/Japan/PG-78932/2021	EPI_ISL_3890343	2021-09-02
hCoV-19/Japan/PG-78933/2021	EPI_ISL_3890345	2021-09-02
hCoV-19/Japan/PG-78934/2021	EPI_ISL_3890348	2021-09-02
hCoV-19/Japan/PG-78935/2021	EPI_ISL_3890352	2021-09-02
hCoV-19/Japan/PG-78936/2021	EPI_ISL_3890354	2021-09-02
hCoV-19/Japan/PG-78937/2021	EPI_ISL_3890355	2021-09-02
hCoV-19/Japan/PG-78938/2021	EPI_ISL_3890357	2021-09-02
hCoV-19/Japan/PG-78939/2021	EPI_ISL_3890359	2021-09-02

virus_name	epiID	submission_date
hCoV-19/Japan/PG-78940/2021	EPI_ISL_3890362	2021-09-02
hCoV-19/Japan/PG-78941/2021	EPI_ISL_3890365	2021-09-02
hCoV-19/Japan/PG-78942/2021	EPI_ISL_3890368	2021-09-02
hCoV-19/Japan/PG-78943/2021	EPI_ISL_3890370	2021-09-02
hCoV-19/Japan/PG-78944/2021	EPI_ISL_3890373	2021-09-02
hCoV-19/Japan/PG-78945/2021	EPI_ISL_3890375	2021-09-02
hCoV-19/Japan/PG-78946/2021	EPI_ISL_3890378	2021-09-02
hCoV-19/Japan/PG-78947/2021	EPI_ISL_3890381	2021-09-02
hCoV-19/Japan/PG-78948/2021	EPI_ISL_3890384	2021-09-02
hCoV-19/Japan/PG-78949/2021	EPI_ISL_3890386	2021-09-02
hCoV-19/Japan/PG-78950/2021	EPI_ISL_3890388	2021-09-02
hCoV-19/Japan/PG-78951/2021	EPI_ISL_3890390	2021-09-02
hCoV-19/Japan/PG-78952/2021	EPI_ISL_3890392	2021-09-02
hCoV-19/Japan/PG-78953/2021	EPI_ISL_3890395	2021-09-02
hCoV-19/Japan/PG-78954/2021	EPI_ISL_3890398	2021-09-02
hCoV-19/Japan/PG-78955/2021	EPI_ISL_3890401	2021-09-02
hCoV-19/Japan/PG-78956/2021	EPI_ISL_3890403	2021-09-02
hCoV-19/Japan/PG-78957/2021	EPI_ISL_3890405	2021-09-02
hCoV-19/Japan/PG-78958/2021	EPI_ISL_3890408	2021-09-02
hCoV-19/Japan/PG-78959/2021	EPI_ISL_3890410	2021-09-02
hCoV-19/Japan/PG-78960/2021	EPI_ISL_3890411	2021-09-02
hCoV-19/Japan/PG-78961/2021	EPI_ISL_3890414	2021-09-02
hCoV-19/Japan/PG-78962/2021	EPI_ISL_3890417	2021-09-02
hCoV-19/Japan/PG-78963/2021	EPI_ISL_3890419	2021-09-02
hCoV-19/Japan/PG-78964/2021	EPI_ISL_3890422	2021-09-02
hCoV-19/Japan/PG-78965/2021	EPI_ISL_3890425	2021-09-02
hCoV-19/Japan/PG-78966/2021	EPI_ISL_3890428	2021-09-02
hCoV-19/Japan/PG-78967/2021	EPI_ISL_3890430	2021-09-02
hCoV-19/Japan/PG-78968/2021	EPI_ISL_3890433	2021-09-02
hCoV-19/Japan/PG-78969/2021	EPI_ISL_3890436	2021-09-02
hCoV-19/Japan/PG-78970/2021	EPI_ISL_3890439	2021-09-02
hCoV-19/Japan/PG-78971/2021	EPI_ISL_3890442	2021-09-02
hCoV-19/Japan/PG-78972/2021	EPI_ISL_3890443	2021-09-02
hCoV-19/Japan/PG-78973/2021	EPI_ISL_3890445	2021-09-02
hCoV-19/Japan/PG-78974/2021	EPI_ISL_3890448	2021-09-02
hCoV-19/Japan/PG-78975/2021	EPI_ISL_3890450	2021-09-02
hCoV-19/Japan/PG-78976/2021	EPI_ISL_3890453	2021-09-02
hCoV-19/Japan/PG-78977/2021	EPI_ISL_3890456	2021-09-02
hCoV-19/Japan/PG-78978/2021	EPI_ISL_3890459	2021-09-02
hCoV-19/Japan/PG-78979/2021	EPI_ISL_3890462	2021-09-02
hCoV-19/Japan/PG-78980/2021	EPI_ISL_3890464	2021-09-02
hCoV-19/Japan/PG-78981/2021	EPI_ISL_3890467	2021-09-02
hCoV-19/Japan/PG-78982/2021	EPI_ISL_3890470	2021-09-02
hCoV-19/Japan/PG-78983/2021	EPI_ISL_3890472	2021-09-02
hCoV-19/Japan/PG-78984/2021	EPI_ISL_3890475	2021-09-02
hCoV-19/Japan/PG-78985/2021	EPI_ISL_3890478	2021-09-02
hCoV-19/Japan/PG-78986/2021	EPI_ISL_3890480	2021-09-02
hCoV-19/Japan/PG-78987/2021	EPI_ISL_3890483	2021-09-02
hCoV-19/Japan/PG-78988/2021	EPI_ISL_3890486	2021-09-02
hCoV-19/Japan/PG-78989/2021	EPI_ISL_3890489	2021-09-02
hCoV-19/Japan/PG-78990/2021	EPI_ISL_3890491	2021-09-02
hCoV-19/Japan/PG-78991/2021	EPI_ISL_3890492	2021-09-02
hCoV-19/Japan/PG-78992/2021	EPI_ISL_3890495	2021-09-02
hCoV-19/Japan/PG-78993/2021	EPI_ISL_3890496	2021-09-02

virus_name	epiID	submission_date
hCoV-19/Japan/PG-78994/2021	EPI_ISL_3890499	2021-09-02
hCoV-19/Japan/PG-78995/2021	EPI_ISL_3890502	2021-09-02
hCoV-19/Japan/PG-78996/2021	EPI_ISL_3890504	2021-09-02
hCoV-19/Japan/PG-78997/2021	EPI_ISL_3890507	2021-09-02
hCoV-19/Japan/PG-78998/2021	EPI_ISL_3890508	2021-09-02
hCoV-19/Japan/PG-78999/2021	EPI_ISL_3890511	2021-09-02
hCoV-19/Japan/PG-79000/2021	EPI_ISL_3890513	2021-09-02
hCoV-19/Japan/PG-79001/2021	EPI_ISL_3890515	2021-09-02
hCoV-19/Japan/PG-79002/2021	EPI_ISL_3890518	2021-09-02
hCoV-19/Japan/PG-79003/2021	EPI_ISL_3890520	2021-09-02
hCoV-19/Japan/PG-79004/2021	EPI_ISL_3890523	2021-09-02
hCoV-19/Japan/PG-79005/2021	EPI_ISL_3890525	2021-09-02
hCoV-19/Japan/PG-79006/2021	EPI_ISL_3890528	2021-09-02
hCoV-19/Japan/PG-79007/2021	EPI_ISL_3890530	2021-09-02
hCoV-19/Japan/PG-79008/2021	EPI_ISL_3890532	2021-09-02
hCoV-19/Japan/PG-79009/2021	EPI_ISL_3890535	2021-09-02
hCoV-19/Japan/PG-79010/2021	EPI_ISL_3890537	2021-09-02
hCoV-19/Japan/PG-79011/2021	EPI_ISL_3890539	2021-09-02
hCoV-19/Japan/PG-79012/2021	EPI_ISL_3890542	2021-09-02
hCoV-19/Japan/PG-79013/2021	EPI_ISL_3890544	2021-09-02
hCoV-19/Japan/PG-79014/2021	EPI_ISL_3890547	2021-09-02
hCoV-19/Japan/PG-79015/2021	EPI_ISL_3890549	2021-09-02
hCoV-19/Japan/PG-79016/2021	EPI_ISL_3890552	2021-09-02
hCoV-19/Japan/PG-79017/2021	EPI_ISL_3890554	2021-09-02
hCoV-19/Japan/PG-79018/2021	EPI_ISL_3890557	2021-09-02
hCoV-19/Japan/PG-79019/2021	EPI_ISL_3890558	2021-09-02
hCoV-19/Japan/PG-79020/2021	EPI_ISL_3890559	2021-09-02
hCoV-19/Japan/PG-79021/2021	EPI_ISL_3890560	2021-09-02
hCoV-19/Japan/PG-79022/2021	EPI_ISL_3890563	2021-09-02
hCoV-19/Japan/PG-79023/2021	EPI_ISL_3890565	2021-09-02
hCoV-19/Japan/PG-79024/2021	EPI_ISL_3890568	2021-09-02
hCoV-19/Japan/PG-79025/2021	EPI_ISL_3890571	2021-09-02
hCoV-19/Japan/PG-79026/2021	EPI_ISL_3890573	2021-09-02
hCoV-19/Japan/PG-79027/2021	EPI_ISL_3890576	2021-09-02
hCoV-19/Japan/PG-79028/2021	EPI_ISL_3890579	2021-09-02
hCoV-19/Japan/PG-79029/2021	EPI_ISL_3890581	2021-09-02
hCoV-19/Japan/PG-79030/2021	EPI_ISL_3890584	2021-09-02
hCoV-19/Japan/PG-79031/2021	EPI_ISL_3890587	2021-09-02
hCoV-19/Japan/PG-79032/2021	EPI_ISL_3890590	2021-09-02
hCoV-19/Japan/PG-79033/2021	EPI_ISL_3890592	2021-09-02
hCoV-19/Japan/PG-79034/2021	EPI_ISL_3890595	2021-09-02
hCoV-19/Japan/PG-79035/2021	EPI_ISL_3890597	2021-09-02
hCoV-19/Japan/PG-79036/2021	EPI_ISL_3890600	2021-09-02
hCoV-19/Japan/PG-79037/2021	EPI_ISL_3890602	2021-09-02
hCoV-19/Japan/PG-79038/2021	EPI_ISL_3890605	2021-09-02
hCoV-19/Japan/PG-79039/2021	EPI_ISL_3890607	2021-09-02
hCoV-19/Japan/PG-79040/2021	EPI_ISL_3890609	2021-09-02
hCoV-19/Japan/PG-79041/2021	EPI_ISL_3890612	2021-09-02
hCoV-19/Japan/PG-79042/2021	EPI_ISL_3890614	2021-09-02
hCoV-19/Japan/PG-79043/2021	EPI_ISL_3890616	2021-09-02
hCoV-19/Japan/PG-79044/2021	EPI_ISL_3890619	2021-09-02
hCoV-19/Japan/PG-79045/2021	EPI_ISL_3890622	2021-09-02
hCoV-19/Japan/PG-79046/2021	EPI_ISL_3890624	2021-09-02
hCoV-19/Japan/PG-79047/2021	EPI_ISL_3890627	2021-09-02

virus_name	epiID	submission_date
hCoV-19/Japan/PG-79048/2021	EPI_ISL_3890630	2021-09-02
hCoV-19/Japan/PG-79049/2021	EPI_ISL_3890632	2021-09-02
hCoV-19/Japan/PG-79050/2021	EPI_ISL_3890633	2021-09-02
hCoV-19/Japan/PG-79051/2021	EPI_ISL_3890634	2021-09-02
hCoV-19/Japan/PG-79052/2021	EPI_ISL_3890635	2021-09-02
hCoV-19/Japan/PG-79053/2021	EPI_ISL_3890636	2021-09-02
hCoV-19/Japan/PG-79054/2021	EPI_ISL_3890638	2021-09-02
hCoV-19/Japan/PG-79055/2021	EPI_ISL_3890640	2021-09-02
hCoV-19/Japan/PG-79056/2021	EPI_ISL_3890643	2021-09-02
hCoV-19/Japan/PG-79057/2021	EPI_ISL_3890645	2021-09-02
hCoV-19/Japan/PG-79058/2021	EPI_ISL_3890648	2021-09-02
hCoV-19/Japan/PG-79059/2021	EPI_ISL_3890650	2021-09-02
hCoV-19/Japan/PG-79060/2021	EPI_ISL_3890653	2021-09-02
hCoV-19/Japan/PG-79061/2021	EPI_ISL_3890657	2021-09-02
hCoV-19/Japan/PG-79062/2021	EPI_ISL_3890659	2021-09-02
hCoV-19/Japan/PG-79063/2021	EPI_ISL_3890662	2021-09-02
hCoV-19/Japan/PG-79064/2021	EPI_ISL_3890664	2021-09-02
hCoV-19/Japan/PG-79065/2021	EPI_ISL_3890667	2021-09-02
hCoV-19/Japan/PG-79066/2021	EPI_ISL_3890670	2021-09-02
hCoV-19/Japan/PG-79067/2021	EPI_ISL_3890672	2021-09-02
hCoV-19/Japan/PG-79068/2021	EPI_ISL_3890675	2021-09-02
hCoV-19/Japan/PG-79069/2021	EPI_ISL_3890678	2021-09-02
hCoV-19/Japan/PG-79070/2021	EPI_ISL_3890680	2021-09-02
hCoV-19/Japan/PG-79071/2021	EPI_ISL_3890684	2021-09-02
hCoV-19/Japan/PG-79072/2021	EPI_ISL_3890686	2021-09-02
hCoV-19/Japan/PG-79073/2021	EPI_ISL_3890689	2021-09-02
hCoV-19/Japan/PG-79074/2021	EPI_ISL_3890691	2021-09-02
hCoV-19/Japan/PG-79075/2021	EPI_ISL_3890694	2021-09-02
hCoV-19/Japan/PG-79076/2021	EPI_ISL_3890697	2021-09-02
hCoV-19/Japan/PG-79077/2021	EPI_ISL_3890699	2021-09-02
hCoV-19/Japan/PG-79078/2021	EPI_ISL_3890702	2021-09-02
hCoV-19/Japan/PG-79079/2021	EPI_ISL_3890704	2021-09-02
hCoV-19/Japan/PG-79080/2021	EPI_ISL_3890707	2021-09-02
hCoV-19/Japan/PG-79081/2021	EPI_ISL_3890709	2021-09-02
hCoV-19/Japan/PG-79082/2021	EPI_ISL_3890713	2021-09-02
hCoV-19/Japan/PG-79083/2021	EPI_ISL_3890715	2021-09-02
hCoV-19/Japan/PG-79084/2021	EPI_ISL_3890718	2021-09-02
hCoV-19/Japan/PG-79085/2021	EPI_ISL_3890721	2021-09-02
hCoV-19/Japan/PG-79086/2021	EPI_ISL_3890722	2021-09-02
hCoV-19/Japan/PG-79087/2021	EPI_ISL_3890725	2021-09-02
hCoV-19/Japan/PG-79088/2021	EPI_ISL_3890727	2021-09-02
hCoV-19/Japan/PG-79089/2021	EPI_ISL_3890729	2021-09-02
hCoV-19/Japan/PG-79090/2021	EPI_ISL_3890732	2021-09-02
hCoV-19/Japan/PG-79091/2021	EPI_ISL_3890733	2021-09-02
hCoV-19/Japan/PG-79092/2021	EPI_ISL_3890734	2021-09-02
hCoV-19/Japan/PG-79093/2021	EPI_ISL_3890735	2021-09-02
hCoV-19/Japan/PG-79094/2021	EPI_ISL_3890739	2021-09-02
hCoV-19/Japan/PG-79095/2021	EPI_ISL_3890742	2021-09-02
hCoV-19/Japan/PG-79096/2021	EPI_ISL_3890745	2021-09-02
hCoV-19/Japan/PG-79097/2021	EPI_ISL_3890748	2021-09-02
hCoV-19/Japan/PG-79098/2021	EPI_ISL_3890750	2021-09-02
hCoV-19/Japan/PG-79099/2021	EPI_ISL_3890753	2021-09-02
hCoV-19/Japan/PG-79100/2021	EPI_ISL_3890755	2021-09-02
hCoV-19/Japan/PG-79101/2021	EPI_ISL_3890757	2021-09-02

virus_name	epiID	submission_date
hCoV-19/Japan/PG-79102/2021	EPI_ISL_3890760	2021-09-02
hCoV-19/Japan/PG-79103/2021	EPI_ISL_3890762	2021-09-02
hCoV-19/Japan/PG-79104/2021	EPI_ISL_3890765	2021-09-02
hCoV-19/Japan/PG-79105/2021	EPI_ISL_3890767	2021-09-02
hCoV-19/Japan/PG-79106/2021	EPI_ISL_3890768	2021-09-02
hCoV-19/Japan/PG-79107/2021	EPI_ISL_3890771	2021-09-02
hCoV-19/Japan/PG-79108/2021	EPI_ISL_3890773	2021-09-02
hCoV-19/Japan/PG-79109/2021	EPI_ISL_3890776	2021-09-02
hCoV-19/Japan/PG-79110/2021	EPI_ISL_3890778	2021-09-02
hCoV-19/Japan/PG-79111/2021	EPI_ISL_3890779	2021-09-02
hCoV-19/Japan/PG-79112/2021	EPI_ISL_3890782	2021-09-02
hCoV-19/Japan/PG-79113/2021	EPI_ISL_3890784	2021-09-02
hCoV-19/Japan/PG-79114/2021	EPI_ISL_3890787	2021-09-02
hCoV-19/Japan/PG-79115/2021	EPI_ISL_3890789	2021-09-02
hCoV-19/Japan/PG-79116/2021	EPI_ISL_3890792	2021-09-02
hCoV-19/Japan/PG-79117/2021	EPI_ISL_3890794	2021-09-02
hCoV-19/Japan/PG-79118/2021	EPI_ISL_3890796	2021-09-02
hCoV-19/Japan/PG-79119/2021	EPI_ISL_3890799	2021-09-02
hCoV-19/Japan/PG-79120/2021	EPI_ISL_3890802	2021-09-02
hCoV-19/Japan/PG-79121/2021	EPI_ISL_3890804	2021-09-02
hCoV-19/Japan/PG-79122/2021	EPI_ISL_3890807	2021-09-02
hCoV-19/Japan/PG-79123/2021	EPI_ISL_3890809	2021-09-02
hCoV-19/Japan/PG-79124/2021	EPI_ISL_3890812	2021-09-02
hCoV-19/Japan/PG-79125/2021	EPI_ISL_3890815	2021-09-02
hCoV-19/Japan/PG-79126/2021	EPI_ISL_3890816	2021-09-02
hCoV-19/Japan/PG-79127/2021	EPI_ISL_3890819	2021-09-02
hCoV-19/Japan/PG-79128/2021	EPI_ISL_3890822	2021-09-02
hCoV-19/Japan/PG-79129/2021	EPI_ISL_3890824	2021-09-02
hCoV-19/Japan/PG-79130/2021	EPI_ISL_3890827	2021-09-02
hCoV-19/Japan/PG-79131/2021	EPI_ISL_3890830	2021-09-02
hCoV-19/Japan/PG-79132/2021	EPI_ISL_3890833	2021-09-02
hCoV-19/Japan/PG-79133/2021	EPI_ISL_3890836	2021-09-02
hCoV-19/Japan/PG-79134/2021	EPI_ISL_3890839	2021-09-02
hCoV-19/Japan/PG-79135/2021	EPI_ISL_3890842	2021-09-02
hCoV-19/Japan/PG-79136/2021	EPI_ISL_3890845	2021-09-02
hCoV-19/Japan/PG-79137/2021	EPI_ISL_3890847	2021-09-02
hCoV-19/Japan/PG-79138/2021	EPI_ISL_3890850	2021-09-02
hCoV-19/Japan/PG-79139/2021	EPI_ISL_3890853	2021-09-02
hCoV-19/Japan/PG-79140/2021	EPI_ISL_3890856	2021-09-02
hCoV-19/Japan/PG-79141/2021	EPI_ISL_3890858	2021-09-02
hCoV-19/Japan/PG-79142/2021	EPI_ISL_3890861	2021-09-02
hCoV-19/Japan/PG-79143/2021	EPI_ISL_3890864	2021-09-02
hCoV-19/Japan/PG-79144/2021	EPI_ISL_3890867	2021-09-02
hCoV-19/Japan/PG-79145/2021	EPI_ISL_3890870	2021-09-02
hCoV-19/Japan/PG-79146/2021	EPI_ISL_3890873	2021-09-02
hCoV-19/Japan/PG-79147/2021	EPI_ISL_3890876	2021-09-02
hCoV-19/Japan/PG-79148/2021	EPI_ISL_3890879	2021-09-02
hCoV-19/Japan/PG-79149/2021	EPI_ISL_3890882	2021-09-02
hCoV-19/Japan/PG-79150/2021	EPI_ISL_3890884	2021-09-02
hCoV-19/Japan/PG-79151/2021	EPI_ISL_3890886	2021-09-02
hCoV-19/Japan/PG-79152/2021	EPI_ISL_3890888	2021-09-02
hCoV-19/Japan/PG-79153/2021	EPI_ISL_3890891	2021-09-02
hCoV-19/Japan/PG-79154/2021	EPI_ISL_3890893	2021-09-02
hCoV-19/Japan/PG-79155/2021	EPI_ISL_3890896	2021-09-02

virus_name	epiID	submission_date
hCoV-19/Japan/PG-79156/2021	EPI_ISL_3890898	2021-09-02
hCoV-19/Japan/PG-79157/2021	EPI_ISL_3890899	2021-09-02
hCoV-19/Japan/PG-79158/2021	EPI_ISL_3890902	2021-09-02
hCoV-19/Japan/PG-79159/2021	EPI_ISL_3890905	2021-09-02
hCoV-19/Japan/PG-79160/2021	EPI_ISL_3890908	2021-09-02
hCoV-19/Japan/PG-79161/2021	EPI_ISL_3890911	2021-09-02
hCoV-19/Japan/PG-79162/2021	EPI_ISL_3890914	2021-09-02
hCoV-19/Japan/PG-79163/2021	EPI_ISL_3890917	2021-09-02
hCoV-19/Japan/PG-79164/2021	EPI_ISL_3890920	2021-09-02
hCoV-19/Japan/PG-79165/2021	EPI_ISL_3890923	2021-09-02
hCoV-19/Japan/PG-79166/2021	EPI_ISL_3890927	2021-09-02
hCoV-19/Japan/PG-79167/2021	EPI_ISL_3890930	2021-09-02
hCoV-19/Japan/PG-79168/2021	EPI_ISL_3890933	2021-09-02
hCoV-19/Japan/PG-79169/2021	EPI_ISL_3890936	2021-09-02
hCoV-19/Japan/PG-79170/2021	EPI_ISL_3890939	2021-09-02
hCoV-19/Japan/PG-79171/2021	EPI_ISL_3890942	2021-09-02
hCoV-19/Japan/PG-79172/2021	EPI_ISL_3890945	2021-09-02
hCoV-19/Japan/PG-79173/2021	EPI_ISL_3890948	2021-09-02
hCoV-19/Japan/PG-79174/2021	EPI_ISL_3890950	2021-09-02
hCoV-19/Japan/PG-79175/2021	EPI_ISL_3890951	2021-09-02
hCoV-19/Japan/PG-79176/2021	EPI_ISL_3890952	2021-09-02
hCoV-19/Japan/PG-79177/2021	EPI_ISL_3890953	2021-09-02
hCoV-19/Japan/PG-79178/2021	EPI_ISL_3890954	2021-09-02
hCoV-19/Japan/PG-79179/2021	EPI_ISL_3890955	2021-09-02
hCoV-19/Japan/PG-79180/2021	EPI_ISL_3890957	2021-09-02
hCoV-19/Japan/PG-79181/2021	EPI_ISL_3890959	2021-09-02
hCoV-19/Japan/PG-79182/2021	EPI_ISL_3890961	2021-09-02
hCoV-19/Japan/PG-79183/2021	EPI_ISL_3890963	2021-09-02
hCoV-19/Japan/PG-79184/2021	EPI_ISL_3890966	2021-09-02
hCoV-19/Japan/PG-79185/2021	EPI_ISL_3890969	2021-09-02
hCoV-19/Japan/PG-79186/2021	EPI_ISL_3890971	2021-09-02
hCoV-19/Japan/PG-79187/2021	EPI_ISL_3890973	2021-09-02
hCoV-19/Japan/PG-79188/2021	EPI_ISL_3890976	2021-09-02
hCoV-19/Japan/PG-79189/2021	EPI_ISL_3890978	2021-09-02
hCoV-19/Japan/PG-79190/2021	EPI_ISL_3890981	2021-09-02
hCoV-19/Japan/PG-79191/2021	EPI_ISL_3890984	2021-09-02
hCoV-19/Japan/PG-79192/2021	EPI_ISL_3890987	2021-09-02
hCoV-19/Japan/PG-79193/2021	EPI_ISL_3890990	2021-09-02
hCoV-19/Japan/PG-79194/2021	EPI_ISL_3890992	2021-09-02
hCoV-19/Japan/PG-79195/2021	EPI_ISL_3890995	2021-09-02
hCoV-19/Japan/PG-79196/2021	EPI_ISL_3890998	2021-09-02
hCoV-19/Japan/PG-79197/2021	EPI_ISL_3891001	2021-09-02
hCoV-19/Japan/PG-79198/2021	EPI_ISL_3891002	2021-09-02
hCoV-19/Japan/PG-79199/2021	EPI_ISL_3891003	2021-09-02
hCoV-19/Japan/PG-79200/2021	EPI_ISL_3891004	2021-09-02
hCoV-19/Japan/PG-79201/2021	EPI_ISL_3891005	2021-09-02
hCoV-19/Japan/PG-79202/2021	EPI_ISL_3891006	2021-09-02
hCoV-19/Japan/PG-79203/2021	EPI_ISL_3891007	2021-09-02
hCoV-19/Japan/PG-79204/2021	EPI_ISL_3891009	2021-09-02
hCoV-19/Japan/PG-79205/2021	EPI_ISL_3891012	2021-09-02
hCoV-19/Japan/PG-79206/2021	EPI_ISL_3891014	2021-09-02
hCoV-19/Japan/PG-79207/2021	EPI_ISL_3891015	2021-09-02
hCoV-19/Japan/PG-79208/2021	EPI_ISL_3891016	2021-09-02
hCoV-19/Japan/PG-79209/2021	EPI_ISL_3891017	2021-09-02

virus_name	epiID	submission_date
hCoV-19/Japan/PG-79210/2021	EPI_ISL_3891018	2021-09-02
hCoV-19/Japan/PG-79211/2021	EPI_ISL_3891019	2021-09-02
hCoV-19/Japan/PG-79212/2021	EPI_ISL_3891022	2021-09-02
hCoV-19/Japan/PG-79213/2021	EPI_ISL_3891025	2021-09-02
hCoV-19/Japan/PG-79214/2021	EPI_ISL_3891028	2021-09-02
hCoV-19/Japan/PG-79215/2021	EPI_ISL_3891030	2021-09-02
hCoV-19/Japan/PG-79216/2021	EPI_ISL_3891033	2021-09-02
hCoV-19/Japan/PG-79217/2021	EPI_ISL_3891035	2021-09-02
hCoV-19/Japan/PG-79218/2021	EPI_ISL_3891038	2021-09-02
hCoV-19/Japan/PG-79219/2021	EPI_ISL_3891041	2021-09-02
hCoV-19/Japan/PG-79220/2021	EPI_ISL_3891044	2021-09-02
hCoV-19/Japan/PG-79221/2021	EPI_ISL_3891046	2021-09-02
hCoV-19/Japan/PG-79222/2021	EPI_ISL_3891048	2021-09-02
hCoV-19/Japan/PG-79223/2021	EPI_ISL_3891050	2021-09-02
hCoV-19/Japan/PG-79224/2021	EPI_ISL_3891053	2021-09-02
hCoV-19/Japan/PG-79225/2021	EPI_ISL_3891056	2021-09-02
hCoV-19/Japan/PG-79226/2021	EPI_ISL_3891059	2021-09-02
hCoV-19/Japan/PG-79227/2021	EPI_ISL_3891061	2021-09-02
hCoV-19/Japan/PG-79228/2021	EPI_ISL_3891064	2021-09-02
hCoV-19/Japan/PG-79229/2021	EPI_ISL_3891066	2021-09-02
hCoV-19/Japan/PG-79230/2021	EPI_ISL_3891069	2021-09-02
hCoV-19/Japan/PG-79231/2021	EPI_ISL_3891071	2021-09-02
hCoV-19/Japan/PG-79232/2021	EPI_ISL_3891074	2021-09-02
hCoV-19/Japan/PG-79233/2021	EPI_ISL_3891076	2021-09-02
hCoV-19/Japan/PG-79234/2021	EPI_ISL_3891079	2021-09-02
hCoV-19/Japan/PG-79235/2021	EPI_ISL_3891081	2021-09-02
hCoV-19/Japan/PG-79236/2021	EPI_ISL_3891085	2021-09-02
hCoV-19/Japan/PG-79237/2021	EPI_ISL_3891087	2021-09-02
hCoV-19/Japan/PG-79238/2021	EPI_ISL_3891090	2021-09-02
hCoV-19/Japan/PG-79239/2021	EPI_ISL_3891093	2021-09-02
hCoV-19/Japan/PG-79240/2021	EPI_ISL_3891094	2021-09-02
hCoV-19/Japan/PG-79241/2021	EPI_ISL_3891096	2021-09-02
hCoV-19/Japan/PG-79242/2021	EPI_ISL_3891099	2021-09-02
hCoV-19/Japan/PG-79243/2021	EPI_ISL_3891100	2021-09-02
hCoV-19/Japan/PG-79244/2021	EPI_ISL_3891103	2021-09-02
hCoV-19/Japan/PG-79245/2021	EPI_ISL_3891106	2021-09-02
hCoV-19/Japan/PG-79246/2021	EPI_ISL_3891108	2021-09-02
hCoV-19/Japan/PG-79247/2021	EPI_ISL_3891110	2021-09-02
hCoV-19/Japan/PG-79248/2021	EPI_ISL_3891113	2021-09-02
hCoV-19/Japan/PG-79249/2021	EPI_ISL_3891115	2021-09-02
hCoV-19/Japan/PG-79250/2021	EPI_ISL_3891117	2021-09-02
hCoV-19/Japan/PG-79251/2021	EPI_ISL_3891119	2021-09-02
hCoV-19/Japan/PG-79252/2021	EPI_ISL_3891121	2021-09-02
hCoV-19/Japan/PG-79253/2021	EPI_ISL_3891123	2021-09-02
hCoV-19/Japan/PG-79254/2021	EPI_ISL_3891126	2021-09-02
hCoV-19/Japan/PG-79255/2021	EPI_ISL_3891128	2021-09-02
hCoV-19/Japan/PG-79256/2021	EPI_ISL_3891130	2021-09-02
hCoV-19/Japan/PG-79257/2021	EPI_ISL_3891132	2021-09-02
hCoV-19/Japan/PG-79258/2021	EPI_ISL_3891134	2021-09-02
hCoV-19/Japan/PG-79259/2021	EPI_ISL_3891137	2021-09-02
hCoV-19/Japan/PG-79260/2021	EPI_ISL_3891139	2021-09-02
hCoV-19/Japan/PG-79261/2021	EPI_ISL_3891141	2021-09-02
hCoV-19/Japan/PG-79262/2021	EPI_ISL_3891144	2021-09-02
hCoV-19/Japan/PG-79263/2021	EPI_ISL_3891147	2021-09-02

virus_name	epiID	submission_date
hCoV-19/Japan/PG-79264/2021	EPI_ISL_3891149	2021-09-02
hCoV-19/Japan/PG-79265/2021	EPI_ISL_3891152	2021-09-02
hCoV-19/Japan/PG-79266/2021	EPI_ISL_3891155	2021-09-02
hCoV-19/Japan/PG-79267/2021	EPI_ISL_3891158	2021-09-02
hCoV-19/Japan/PG-79268/2021	EPI_ISL_3891161	2021-09-02
hCoV-19/Japan/PG-79269/2021	EPI_ISL_3891163	2021-09-02
hCoV-19/Japan/PG-79270/2021	EPI_ISL_3891167	2021-09-02
hCoV-19/Japan/PG-79271/2021	EPI_ISL_3891169	2021-09-02
hCoV-19/Japan/PG-79272/2021	EPI_ISL_3891172	2021-09-02
hCoV-19/Japan/PG-79273/2021	EPI_ISL_3891175	2021-09-02
hCoV-19/Japan/PG-79274/2021	EPI_ISL_3891178	2021-09-02
hCoV-19/Japan/PG-79275/2021	EPI_ISL_3891180	2021-09-02
hCoV-19/Japan/PG-79276/2021	EPI_ISL_3891183	2021-09-02
hCoV-19/Japan/PG-79277/2021	EPI_ISL_3891186	2021-09-02
hCoV-19/Japan/PG-79278/2021	EPI_ISL_3891189	2021-09-02
hCoV-19/Japan/PG-79279/2021	EPI_ISL_3891191	2021-09-02
hCoV-19/Japan/PG-79280/2021	EPI_ISL_3891193	2021-09-02
hCoV-19/Japan/PG-79281/2021	EPI_ISL_3891195	2021-09-02
hCoV-19/Japan/PG-79282/2021	EPI_ISL_3891198	2021-09-02
hCoV-19/Japan/PG-79283/2021	EPI_ISL_3873313	2021-09-02
hCoV-19/Japan/PG-79284/2021	EPI_ISL_3873314	2021-09-02
hCoV-19/Japan/PG-79285/2021	EPI_ISL_3873315	2021-09-02
hCoV-19/Japan/PG-79286/2021	EPI_ISL_3873316	2021-09-02
hCoV-19/Japan/PG-79287/2021	EPI_ISL_3873317	2021-09-02
hCoV-19/Japan/PG-79288/2021	EPI_ISL_3873318	2021-09-02
hCoV-19/Japan/PG-79289/2021	EPI_ISL_3873319	2021-09-02
hCoV-19/Japan/PG-79290/2021	EPI_ISL_3873320	2021-09-02
hCoV-19/Japan/PG-79291/2021	EPI_ISL_3873321	2021-09-02
hCoV-19/Japan/PG-79292/2021	EPI_ISL_3873322	2021-09-02
hCoV-19/Japan/PG-79293/2021	EPI_ISL_3873323	2021-09-02
hCoV-19/Japan/PG-79294/2021	EPI_ISL_3873324	2021-09-02
hCoV-19/Japan/PG-79295/2021	EPI_ISL_3873325	2021-09-02
hCoV-19/Japan/PG-79296/2021	EPI_ISL_3873326	2021-09-02
hCoV-19/Japan/PG-79297/2021	EPI_ISL_3873327	2021-09-02
hCoV-19/Japan/PG-79298/2021	EPI_ISL_3873328	2021-09-02
hCoV-19/Japan/PG-79299/2021	EPI_ISL_3870527	2021-09-02
hCoV-19/Japan/PG-79300/2021	EPI_ISL_3870528	2021-09-02
hCoV-19/Japan/PG-79301/2021	EPI_ISL_3870529	2021-09-02
hCoV-19/Japan/PG-79302/2021	EPI_ISL_3870530	2021-09-02
hCoV-19/Japan/PG-79303/2021	EPI_ISL_3870531	2021-09-02
hCoV-19/Japan/PG-79304/2021	EPI_ISL_3870532	2021-09-02
hCoV-19/Japan/PG-79305/2021	EPI_ISL_3870533	2021-09-02
hCoV-19/Japan/PG-79306/2021	EPI_ISL_3870534	2021-09-02
hCoV-19/Japan/PG-79307/2021	EPI_ISL_3870535	2021-09-02
hCoV-19/Japan/PG-79308/2021	EPI_ISL_3870536	2021-09-02
hCoV-19/Japan/PG-79309/2021	EPI_ISL_3870537	2021-09-02
hCoV-19/Japan/PG-79310/2021	EPI_ISL_3870538	2021-09-02
hCoV-19/Japan/PG-79311/2021	EPI_ISL_3870539	2021-09-02
hCoV-19/Japan/PG-79312/2021	EPI_ISL_3870540	2021-09-02
hCoV-19/Japan/PG-79313/2021	EPI_ISL_3870541	2021-09-02
hCoV-19/Japan/PG-79314/2021	EPI_ISL_3870542	2021-09-02
hCoV-19/Japan/PG-79315/2021	EPI_ISL_3870543	2021-09-02
hCoV-19/Japan/PG-79316/2021	EPI_ISL_3870544	2021-09-02
hCoV-19/Japan/PG-79317/2021	EPI_ISL_3870545	2021-09-02

virus_name	epiID	submission_date
hCoV-19/Japan/PG-79318/2021	EPI_ISL_3870546	2021-09-02
hCoV-19/Japan/PG-79319/2021	EPI_ISL_3870547	2021-09-02
hCoV-19/Japan/PG-79320/2021	EPI_ISL_3870548	2021-09-02
hCoV-19/Japan/PG-79321/2021	EPI_ISL_3870648	2021-09-02
hCoV-19/Japan/PG-79322/2021	EPI_ISL_3870649	2021-09-02
hCoV-19/Japan/PG-79323/2021	EPI_ISL_3870650	2021-09-02
hCoV-19/Japan/PG-79324/2021	EPI_ISL_3870651	2021-09-02
hCoV-19/Japan/PG-79325/2021	EPI_ISL_3870652	2021-09-02
hCoV-19/Japan/PG-79326/2021	EPI_ISL_3870653	2021-09-02
hCoV-19/Japan/PG-79327/2021	EPI_ISL_3870654	2021-09-02
hCoV-19/Japan/PG-79328/2021	EPI_ISL_3870655	2021-09-02
hCoV-19/Japan/PG-79329/2021	EPI_ISL_3870656	2021-09-02
hCoV-19/Japan/PG-79330/2021	EPI_ISL_3870657	2021-09-02
hCoV-19/Japan/PG-79331/2021	EPI_ISL_3870658	2021-09-02
hCoV-19/Japan/PG-79332/2021	EPI_ISL_3870659	2021-09-02
hCoV-19/Japan/PG-79333/2021	EPI_ISL_3870660	2021-09-02
hCoV-19/Japan/PG-79334/2021	EPI_ISL_3870661	2021-09-02
hCoV-19/Japan/PG-79335/2021	EPI_ISL_3870662	2021-09-02
hCoV-19/Japan/PG-79336/2021	EPI_ISL_3870663	2021-09-02
hCoV-19/Japan/PG-79337/2021	EPI_ISL_3870664	2021-09-02
hCoV-19/Japan/PG-79338/2021	EPI_ISL_3870665	2021-09-02
hCoV-19/Japan/PG-79339/2021	EPI_ISL_3870666	2021-09-02
hCoV-19/Japan/PG-79340/2021	EPI_ISL_3870667	2021-09-02
hCoV-19/Japan/PG-79341/2021	EPI_ISL_3870668	2021-09-02
hCoV-19/Japan/PG-79342/2021	EPI_ISL_3870669	2021-09-02
hCoV-19/Japan/PG-79343/2021	EPI_ISL_3870670	2021-09-02
hCoV-19/Japan/PG-79344/2021	EPI_ISL_3870671	2021-09-02
hCoV-19/Japan/PG-79345/2021	EPI_ISL_3870672	2021-09-02
hCoV-19/Japan/PG-79346/2021	EPI_ISL_3870673	2021-09-02
hCoV-19/Japan/PG-79347/2021	EPI_ISL_3870674	2021-09-02
hCoV-19/Japan/PG-79348/2021	EPI_ISL_3870675	2021-09-02
hCoV-19/Japan/PG-79349/2021	EPI_ISL_3870676	2021-09-02
hCoV-19/Japan/PG-79350/2021	EPI_ISL_3870677	2021-09-02
hCoV-19/Japan/PG-79351/2021	EPI_ISL_3870678	2021-09-02
hCoV-19/Japan/PG-79352/2021	EPI_ISL_3870679	2021-09-02
hCoV-19/Japan/PG-79353/2021	EPI_ISL_3870680	2021-09-02
hCoV-19/Japan/PG-79354/2021	EPI_ISL_3870681	2021-09-02
hCoV-19/Japan/PG-79355/2021	EPI_ISL_3870682	2021-09-02
hCoV-19/Japan/PG-79356/2021	EPI_ISL_3870683	2021-09-02
hCoV-19/Japan/PG-79357/2021	EPI_ISL_3870684	2021-09-02
hCoV-19/Japan/PG-79358/2021	EPI_ISL_3870685	2021-09-02
hCoV-19/Japan/PG-79359/2021	EPI_ISL_3870686	2021-09-02
hCoV-19/Japan/PG-79360/2021	EPI_ISL_3870687	2021-09-02
hCoV-19/Japan/PG-79361/2021	EPI_ISL_3870688	2021-09-02
hCoV-19/Japan/PG-79362/2021	EPI_ISL_3870689	2021-09-02
hCoV-19/Japan/PG-79363/2021	EPI_ISL_3870690	2021-09-02
hCoV-19/Japan/PG-79364/2021	EPI_ISL_3870691	2021-09-02
hCoV-19/Japan/PG-79365/2021	EPI_ISL_3870692	2021-09-02
hCoV-19/Japan/PG-79366/2021	EPI_ISL_3870693	2021-09-02
hCoV-19/Japan/PG-79367/2021	EPI_ISL_3870694	2021-09-02
hCoV-19/Japan/PG-79368/2021	EPI_ISL_3870695	2021-09-02
hCoV-19/Japan/PG-79369/2021	EPI_ISL_3870696	2021-09-02
hCoV-19/Japan/PG-79370/2021	EPI_ISL_3870697	2021-09-02
hCoV-19/Japan/PG-79371/2021	EPI_ISL_3870698	2021-09-02

virus_name	epiID	submission_date
hCoV-19/Japan/PG-79372/2021	EPI_ISL_3870699	2021-09-02
hCoV-19/Japan/PG-79373/2021	EPI_ISL_3870700	2021-09-02
hCoV-19/Japan/PG-79374/2021	EPI_ISL_3870701	2021-09-02
hCoV-19/Japan/PG-79375/2021	EPI_ISL_3870702	2021-09-02
hCoV-19/Japan/PG-79376/2021	EPI_ISL_3870703	2021-09-02
hCoV-19/Japan/PG-79377/2021	EPI_ISL_3870704	2021-09-02
hCoV-19/Japan/PG-79378/2021	EPI_ISL_3870705	2021-09-02
hCoV-19/Japan/PG-79379/2021	EPI_ISL_3870706	2021-09-02
hCoV-19/Japan/PG-79380/2021	EPI_ISL_3870707	2021-09-02
hCoV-19/Japan/PG-79381/2021	EPI_ISL_3870708	2021-09-02
hCoV-19/Japan/PG-79382/2021	EPI_ISL_3870709	2021-09-02
hCoV-19/Japan/PG-79383/2021	EPI_ISL_3873295	2021-09-02
hCoV-19/Japan/PG-79384/2021	EPI_ISL_3873296	2021-09-02
hCoV-19/Japan/PG-79385/2021	EPI_ISL_3873297	2021-09-02
hCoV-19/Japan/PG-79386/2021	EPI_ISL_3873298	2021-09-02
hCoV-19/Japan/PG-79387/2021	EPI_ISL_3873299	2021-09-02
hCoV-19/Japan/PG-79388/2021	EPI_ISL_3873300	2021-09-02
hCoV-19/Japan/PG-79389/2021	EPI_ISL_3873301	2021-09-02
hCoV-19/Japan/PG-79390/2021	EPI_ISL_3873302	2021-09-02
hCoV-19/Japan/PG-79391/2021	EPI_ISL_3873303	2021-09-02
hCoV-19/Japan/PG-79392/2021	EPI_ISL_3873304	2021-09-02
hCoV-19/Japan/PG-79393/2021	EPI_ISL_3873305	2021-09-02
hCoV-19/Japan/PG-79394/2021	EPI_ISL_3873306	2021-09-02
hCoV-19/Japan/PG-79395/2021	EPI_ISL_3873307	2021-09-02
hCoV-19/Japan/PG-79396/2021	EPI_ISL_3873308	2021-09-02
hCoV-19/Japan/PG-79397/2021	EPI_ISL_3873309	2021-09-02
hCoV-19/Japan/PG-79398/2021	EPI_ISL_3873310	2021-09-02
hCoV-19/Japan/PG-79399/2021	EPI_ISL_3873311	2021-09-02
hCoV-19/Japan/PG-79400/2021	EPI_ISL_3873312	2021-09-02
hCoV-19/Japan/PG-79401/2021	EPI_ISL_3872710	2021-09-02
hCoV-19/Japan/PG-79402/2021	EPI_ISL_3872711	2021-09-02
hCoV-19/Japan/PG-79403/2021	EPI_ISL_3872712	2021-09-02
hCoV-19/Japan/PG-79404/2021	EPI_ISL_3872713	2021-09-02
hCoV-19/Japan/PG-79405/2021	EPI_ISL_3872714	2021-09-02
hCoV-19/Japan/PG-79406/2021	EPI_ISL_3872715	2021-09-02
hCoV-19/Japan/PG-79407/2021	EPI_ISL_3872716	2021-09-02
hCoV-19/Japan/PG-79408/2021	EPI_ISL_3872717	2021-09-02
hCoV-19/Japan/PG-79409/2021	EPI_ISL_3872718	2021-09-02
hCoV-19/Japan/PG-79410/2021	EPI_ISL_3872719	2021-09-02
hCoV-19/Japan/PG-79411/2021	EPI_ISL_3872720	2021-09-02
hCoV-19/Japan/PG-79412/2021	EPI_ISL_3872721	2021-09-02
hCoV-19/Japan/PG-79413/2021	EPI_ISL_3872722	2021-09-02
hCoV-19/Japan/PG-79414/2021	EPI_ISL_3872723	2021-09-02
hCoV-19/Japan/PG-79415/2021	EPI_ISL_3872724	2021-09-02
hCoV-19/Japan/PG-79416/2021	EPI_ISL_3872725	2021-09-02
hCoV-19/Japan/PG-79417/2021	EPI_ISL_3872726	2021-09-02
hCoV-19/Japan/PG-79418/2021	EPI_ISL_3872727	2021-09-02
hCoV-19/Japan/PG-79419/2021	EPI_ISL_3872728	2021-09-02
hCoV-19/Japan/PG-79420/2021	EPI_ISL_3872729	2021-09-02
hCoV-19/Japan/PG-79421/2021	EPI_ISL_3872730	2021-09-02
hCoV-19/Japan/PG-79422/2021	EPI_ISL_3872731	2021-09-02
hCoV-19/Japan/PG-79423/2021	EPI_ISL_3872732	2021-09-02
hCoV-19/Japan/PG-79424/2021	EPI_ISL_3872733	2021-09-02
hCoV-19/Japan/PG-79425/2021	EPI_ISL_3872734	2021-09-02

virus_name	epiID	submission_date
hCoV-19/Japan/PG-79426/2021	EPI_ISL_3872735	2021-09-02
hCoV-19/Japan/PG-79427/2021	EPI_ISL_3872736	2021-09-02
hCoV-19/Japan/PG-79428/2021	EPI_ISL_3872737	2021-09-02
hCoV-19/Japan/PG-79429/2021	EPI_ISL_3872738	2021-09-02
hCoV-19/Japan/PG-79430/2021	EPI_ISL_3872739	2021-09-02
hCoV-19/Japan/PG-79431/2021	EPI_ISL_3872740	2021-09-02
hCoV-19/Japan/PG-79432/2021	EPI_ISL_3872741	2021-09-02
hCoV-19/Japan/PG-79433/2021	EPI_ISL_3872742	2021-09-02
hCoV-19/Japan/PG-79434/2021	EPI_ISL_3872743	2021-09-02
hCoV-19/Japan/PG-79435/2021	EPI_ISL_3872744	2021-09-02
hCoV-19/Japan/PG-79436/2021	EPI_ISL_3872745	2021-09-02
hCoV-19/Japan/PG-79437/2021	EPI_ISL_3872746	2021-09-02
hCoV-19/Japan/PG-79438/2021	EPI_ISL_3872747	2021-09-02
hCoV-19/Japan/PG-79439/2021	EPI_ISL_3872748	2021-09-02
hCoV-19/Japan/PG-79440/2021	EPI_ISL_3872749	2021-09-02
hCoV-19/Japan/PG-79441/2021	EPI_ISL_3872750	2021-09-02
hCoV-19/Japan/PG-79442/2021	EPI_ISL_3872751	2021-09-02
hCoV-19/Japan/PG-79443/2021	EPI_ISL_3872752	2021-09-02
hCoV-19/Japan/PG-79444/2021	EPI_ISL_3872753	2021-09-02
hCoV-19/Japan/PG-79445/2021	EPI_ISL_3872754	2021-09-02
hCoV-19/Japan/PG-79446/2021	EPI_ISL_3872755	2021-09-02
hCoV-19/Japan/PG-79447/2021	EPI_ISL_3872756	2021-09-02
hCoV-19/Japan/PG-79448/2021	EPI_ISL_3872757	2021-09-02
hCoV-19/Japan/PG-79449/2021	EPI_ISL_3872758	2021-09-02
hCoV-19/Japan/PG-79450/2021	EPI_ISL_3872759	2021-09-02
hCoV-19/Japan/PG-79451/2021	EPI_ISL_3872760	2021-09-02
hCoV-19/Japan/PG-79452/2021	EPI_ISL_3872761	2021-09-02
hCoV-19/Japan/PG-79453/2021	EPI_ISL_3872762	2021-09-02
hCoV-19/Japan/PG-79454/2021	EPI_ISL_3872763	2021-09-02
hCoV-19/Japan/PG-79455/2021	EPI_ISL_3872764	2021-09-02
hCoV-19/Japan/PG-79456/2021	EPI_ISL_3872765	2021-09-02
hCoV-19/Japan/PG-79457/2021	EPI_ISL_3872766	2021-09-02
hCoV-19/Japan/PG-79458/2021	EPI_ISL_3872767	2021-09-02
hCoV-19/Japan/PG-79459/2021	EPI_ISL_3872768	2021-09-02
hCoV-19/Japan/PG-79460/2021	EPI_ISL_3872769	2021-09-02
hCoV-19/Japan/PG-79461/2021	EPI_ISL_3872770	2021-09-02
hCoV-19/Japan/PG-79462/2021	EPI_ISL_3872771	2021-09-02
hCoV-19/Japan/PG-79463/2021	EPI_ISL_3872772	2021-09-02
hCoV-19/Japan/PG-79464/2021	EPI_ISL_3872773	2021-09-02
hCoV-19/Japan/PG-79465/2021	EPI_ISL_3872774	2021-09-02
hCoV-19/Japan/PG-79466/2021	EPI_ISL_3871610	2021-09-02
hCoV-19/Japan/PG-79467/2021	EPI_ISL_3871611	2021-09-02
hCoV-19/Japan/PG-79468/2021	EPI_ISL_3871612	2021-09-02
hCoV-19/Japan/PG-79469/2021	EPI_ISL_3871613	2021-09-02
hCoV-19/Japan/PG-79470/2021	EPI_ISL_3871506	2021-09-02
hCoV-19/Japan/PG-79471/2021	EPI_ISL_3871507	2021-09-02
hCoV-19/Japan/PG-79472/2021	EPI_ISL_3871508	2021-09-02
hCoV-19/Japan/PG-79473/2021	EPI_ISL_3871509	2021-09-02
hCoV-19/Japan/PG-79474/2021	EPI_ISL_3871510	2021-09-02
hCoV-19/Japan/PG-79475/2021	EPI_ISL_3871511	2021-09-02
hCoV-19/Japan/PG-79476/2021	EPI_ISL_3871512	2021-09-02
hCoV-19/Japan/PG-79477/2021	EPI_ISL_3871513	2021-09-02
hCoV-19/Japan/PG-79478/2021	EPI_ISL_3871514	2021-09-02
hCoV-19/Japan/PG-79479/2021	EPI_ISL_3871515	2021-09-02

virus_name	epiID	submission_date
hCoV-19/Japan/PG-79480/2021	EPI_ISL_3871516	2021-09-02
hCoV-19/Japan/PG-79481/2021	EPI_ISL_3871517	2021-09-02
hCoV-19/Japan/PG-79482/2021	EPI_ISL_3871518	2021-09-02
hCoV-19/Japan/PG-79483/2021	EPI_ISL_3871519	2021-09-02
hCoV-19/Japan/PG-79484/2021	EPI_ISL_3871520	2021-09-02
hCoV-19/Japan/PG-79485/2021	EPI_ISL_3871521	2021-09-02
hCoV-19/Japan/PG-79486/2021	EPI_ISL_3871522	2021-09-02
hCoV-19/Japan/PG-79487/2021	EPI_ISL_3872516	2021-09-02
hCoV-19/Japan/PG-79488/2021	EPI_ISL_3872517	2021-09-02
hCoV-19/Japan/PG-79489/2021	EPI_ISL_3872518	2021-09-02
hCoV-19/Japan/PG-79490/2021	EPI_ISL_3874333	2021-09-02
hCoV-19/Japan/PG-79491/2021	EPI_ISL_3874334	2021-09-02
hCoV-19/Japan/PG-79492/2021	EPI_ISL_3874335	2021-09-02
hCoV-19/Japan/PG-79493/2021	EPI_ISL_3874336	2021-09-02
hCoV-19/Japan/PG-79494/2021	EPI_ISL_3874337	2021-09-02
hCoV-19/Japan/PG-79495/2021	EPI_ISL_3874338	2021-09-02
hCoV-19/Japan/PG-79496/2021	EPI_ISL_3874339	2021-09-02
hCoV-19/Japan/PG-79497/2021	EPI_ISL_3874340	2021-09-02
hCoV-19/Japan/PG-79498/2021	EPI_ISL_3874341	2021-09-02
hCoV-19/Japan/PG-79499/2021	EPI_ISL_3874342	2021-09-02
hCoV-19/Japan/PG-79500/2021	EPI_ISL_3874343	2021-09-02
hCoV-19/Japan/PG-79501/2021	EPI_ISL_3874344	2021-09-02
hCoV-19/Japan/PG-79502/2021	EPI_ISL_3874345	2021-09-02
hCoV-19/Japan/PG-79503/2021	EPI_ISL_3874346	2021-09-02
hCoV-19/Japan/PG-79504/2021	EPI_ISL_3874422	2021-09-02
hCoV-19/Japan/PG-79505/2021	EPI_ISL_3874423	2021-09-02
hCoV-19/Japan/PG-79506/2021	EPI_ISL_3874424	2021-09-02
hCoV-19/Japan/PG-79507/2021	EPI_ISL_3874425	2021-09-02
hCoV-19/Japan/PG-79508/2021	EPI_ISL_3874426	2021-09-02
hCoV-19/Japan/PG-79509/2021	EPI_ISL_3874427	2021-09-02
hCoV-19/Japan/PG-79510/2021	EPI_ISL_3874428	2021-09-02
hCoV-19/Japan/PG-79511/2021	EPI_ISL_3874429	2021-09-02
hCoV-19/Japan/PG-79512/2021	EPI_ISL_3874430	2021-09-02
hCoV-19/Japan/PG-79513/2021	EPI_ISL_3874431	2021-09-02
hCoV-19/Japan/PG-79514/2021	EPI_ISL_3874432	2021-09-02
hCoV-19/Japan/PG-79515/2021	EPI_ISL_3874433	2021-09-02
hCoV-19/Japan/PG-79516/2021	EPI_ISL_3874434	2021-09-02
hCoV-19/Japan/PG-79517/2021	EPI_ISL_3874435	2021-09-02
hCoV-19/Japan/PG-79518/2021	EPI_ISL_3874436	2021-09-02
hCoV-19/Japan/PG-79519/2021	EPI_ISL_3874437	2021-09-02
hCoV-19/Japan/PG-79520/2021	EPI_ISL_3874438	2021-09-02
hCoV-19/Japan/PG-79521/2021	EPI_ISL_3874568	2021-09-02
hCoV-19/Japan/PG-79522/2021	EPI_ISL_3872284	2021-09-02
hCoV-19/Japan/PG-79523/2021	EPI_ISL_3872285	2021-09-02
hCoV-19/Japan/PG-79524/2021	EPI_ISL_3872286	2021-09-02
hCoV-19/Japan/PG-79525/2021	EPI_ISL_3872287	2021-09-02
hCoV-19/Japan/PG-79526/2021	EPI_ISL_3872288	2021-09-02
hCoV-19/Japan/PG-79527/2021	EPI_ISL_3872289	2021-09-02
hCoV-19/Japan/PG-79528/2021	EPI_ISL_3872290	2021-09-02
hCoV-19/Japan/PG-79529/2021	EPI_ISL_3872291	2021-09-02
hCoV-19/Japan/PG-79530/2021	EPI_ISL_3872292	2021-09-02
hCoV-19/Japan/PG-79531/2021	EPI_ISL_3872293	2021-09-02
hCoV-19/Japan/PG-79532/2021	EPI_ISL_3872294	2021-09-02
hCoV-19/Japan/PG-79533/2021	EPI_ISL_3872295	2021-09-02

virus_name	epiID	submission_date
hCoV-19/Japan/PG-79534/2021	EPI_ISL_3872296	2021-09-02
hCoV-19/Japan/PG-79535/2021	EPI_ISL_3872297	2021-09-02
hCoV-19/Japan/PG-79536/2021	EPI_ISL_3891200	2021-09-02
hCoV-19/Japan/PG-79538/2021	EPI_ISL_3891203	2021-09-02
hCoV-19/Japan/PG-79539/2021	EPI_ISL_3891205	2021-09-02
hCoV-19/Japan/PG-79540/2021	EPI_ISL_3891208	2021-09-02
hCoV-19/Japan/PG-79541/2021	EPI_ISL_3891210	2021-09-02
hCoV-19/Japan/PG-79542/2021	EPI_ISL_3891212	2021-09-02
hCoV-19/Japan/PG-79543/2021	EPI_ISL_3891215	2021-09-02
hCoV-19/Japan/PG-79544/2021	EPI_ISL_3891218	2021-09-02
hCoV-19/Japan/PG-79545/2021	EPI_ISL_3891220	2021-09-02
hCoV-19/Japan/PG-79546/2021	EPI_ISL_3891223	2021-09-02
hCoV-19/Japan/PG-79547/2021	EPI_ISL_3891226	2021-09-02
hCoV-19/Japan/PG-79548/2021	EPI_ISL_3891227	2021-09-02
hCoV-19/Japan/PG-79549/2021	EPI_ISL_3891228	2021-09-02
hCoV-19/Japan/PG-79550/2021	EPI_ISL_3891231	2021-09-02
hCoV-19/Japan/PG-79551/2021	EPI_ISL_3891234	2021-09-02
hCoV-19/Japan/PG-79552/2021	EPI_ISL_3891235	2021-09-02
hCoV-19/Japan/PG-79553/2021	EPI_ISL_3891238	2021-09-02
hCoV-19/Japan/PG-79554/2021	EPI_ISL_3891241	2021-09-02
hCoV-19/Japan/PG-79555/2021	EPI_ISL_3891244	2021-09-02
hCoV-19/Japan/PG-79556/2021	EPI_ISL_3891247	2021-09-02
hCoV-19/Japan/PG-79557/2021	EPI_ISL_3891249	2021-09-02
hCoV-19/Japan/PG-79558/2021	EPI_ISL_3891252	2021-09-02
hCoV-19/Japan/PG-79559/2021	EPI_ISL_3891254	2021-09-02
hCoV-19/Japan/PG-79560/2021	EPI_ISL_3891257	2021-09-02
hCoV-19/Japan/PG-79561/2021	EPI_ISL_3891259	2021-09-02
hCoV-19/Japan/PG-79562/2021	EPI_ISL_3891263	2021-09-02
hCoV-19/Japan/PG-79563/2021	EPI_ISL_3891265	2021-09-02
hCoV-19/Japan/PG-79564/2021	EPI_ISL_3891268	2021-09-02
hCoV-19/Japan/PG-79565/2021	EPI_ISL_3891271	2021-09-02
hCoV-19/Japan/PG-79566/2021	EPI_ISL_3891273	2021-09-02
hCoV-19/Japan/PG-79567/2021	EPI_ISL_3891276	2021-09-02
hCoV-19/Japan/PG-79568/2021	EPI_ISL_3891278	2021-09-02
hCoV-19/Japan/PG-79569/2021	EPI_ISL_3891281	2021-09-02
hCoV-19/Japan/PG-79570/2021	EPI_ISL_3891283	2021-09-02
hCoV-19/Japan/PG-79571/2021	EPI_ISL_3891286	2021-09-02
hCoV-19/Japan/PG-79572/2021	EPI_ISL_3891289	2021-09-02
hCoV-19/Japan/PG-79573/2021	EPI_ISL_3891291	2021-09-02
hCoV-19/Japan/PG-79574/2021	EPI_ISL_3891293	2021-09-02
hCoV-19/Japan/PG-79575/2021	EPI_ISL_3891296	2021-09-02
hCoV-19/Japan/PG-79576/2021	EPI_ISL_3891297	2021-09-02
hCoV-19/Japan/PG-79577/2021	EPI_ISL_3891300	2021-09-02
hCoV-19/Japan/PG-79578/2021	EPI_ISL_3891302	2021-09-02
hCoV-19/Japan/PG-79579/2021	EPI_ISL_3891304	2021-09-02
hCoV-19/Japan/PG-79580/2021	EPI_ISL_3891306	2021-09-02
hCoV-19/Japan/PG-79581/2021	EPI_ISL_3891308	2021-09-02
hCoV-19/Japan/PG-79582/2021	EPI_ISL_3891310	2021-09-02
hCoV-19/Japan/PG-79583/2021	EPI_ISL_3891313	2021-09-02
hCoV-19/Japan/PG-79584/2021	EPI_ISL_3891316	2021-09-02
hCoV-19/Japan/PG-79585/2021	EPI_ISL_3891319	2021-09-02
hCoV-19/Japan/PG-79586/2021	EPI_ISL_3891322	2021-09-02
hCoV-19/Japan/PG-79587/2021	EPI_ISL_3891325	2021-09-02
hCoV-19/Japan/PG-79588/2021	EPI_ISL_3891328	2021-09-02

virus_name	epiID	submission_date
hCoV-19/Japan/PG-79589/2021	EPI_ISL_3891331	2021-09-02
hCoV-19/Japan/PG-79590/2021	EPI_ISL_3891334	2021-09-02
hCoV-19/Japan/PG-79591/2021	EPI_ISL_3891335	2021-09-02
hCoV-19/Japan/PG-79592/2021	EPI_ISL_3891337	2021-09-02
hCoV-19/Japan/PG-79593/2021	EPI_ISL_3891340	2021-09-02
hCoV-19/Japan/PG-79594/2021	EPI_ISL_3891342	2021-09-02
hCoV-19/Japan/PG-79595/2021	EPI_ISL_3891345	2021-09-02
hCoV-19/Japan/PG-79596/2021	EPI_ISL_3891348	2021-09-02
hCoV-19/Japan/PG-79597/2021	EPI_ISL_3891350	2021-09-02
hCoV-19/Japan/PG-79598/2021	EPI_ISL_3891353	2021-09-02
hCoV-19/Japan/PG-79599/2021	EPI_ISL_3891356	2021-09-02
hCoV-19/Japan/PG-79600/2021	EPI_ISL_3891359	2021-09-02
hCoV-19/Japan/PG-79601/2021	EPI_ISL_3891362	2021-09-02
hCoV-19/Japan/PG-79602/2021	EPI_ISL_3891365	2021-09-02
hCoV-19/Japan/PG-79603/2021	EPI_ISL_3891367	2021-09-02
hCoV-19/Japan/PG-79604/2021	EPI_ISL_3891370	2021-09-02
hCoV-19/Japan/PG-79605/2021	EPI_ISL_3891372	2021-09-02
hCoV-19/Japan/PG-79606/2021	EPI_ISL_3891374	2021-09-02
hCoV-19/Japan/PG-79607/2021	EPI_ISL_3891377	2021-09-02
hCoV-19/Japan/PG-79608/2021	EPI_ISL_3891380	2021-09-02
hCoV-19/Japan/PG-79609/2021	EPI_ISL_3891383	2021-09-02
hCoV-19/Japan/PG-79610/2021	EPI_ISL_3891384	2021-09-02
hCoV-19/Japan/PG-79611/2021	EPI_ISL_3891385	2021-09-02
hCoV-19/Japan/PG-79612/2021	EPI_ISL_3891387	2021-09-02
hCoV-19/Japan/PG-79613/2021	EPI_ISL_3891390	2021-09-02
hCoV-19/Japan/PG-79614/2021	EPI_ISL_3891392	2021-09-02
hCoV-19/Japan/PG-79615/2021	EPI_ISL_3891395	2021-09-02
hCoV-19/Japan/PG-79616/2021	EPI_ISL_3891396	2021-09-02
hCoV-19/Japan/PG-79617/2021	EPI_ISL_3891397	2021-09-02
hCoV-19/Japan/PG-79618/2021	EPI_ISL_3891400	2021-09-02
hCoV-19/Japan/PG-79619/2021	EPI_ISL_3891403	2021-09-02
hCoV-19/Japan/PG-79620/2021	EPI_ISL_3891406	2021-09-02
hCoV-19/Japan/PG-79621/2021	EPI_ISL_3891408	2021-09-02
hCoV-19/Japan/PG-79622/2021	EPI_ISL_3891409	2021-09-02
hCoV-19/Japan/PG-79623/2021	EPI_ISL_3891412	2021-09-02
hCoV-19/Japan/PG-79624/2021	EPI_ISL_3891414	2021-09-02
hCoV-19/Japan/PG-79625/2021	EPI_ISL_3891416	2021-09-02
hCoV-19/Japan/PG-79626/2021	EPI_ISL_3891418	2021-09-02
hCoV-19/Japan/PG-79627/2021	EPI_ISL_3891421	2021-09-02
hCoV-19/Japan/PG-79628/2021	EPI_ISL_3891422	2021-09-02
hCoV-19/Japan/PG-79629/2021	EPI_ISL_3891425	2021-09-02
hCoV-19/Japan/PG-79630/2021	EPI_ISL_3891427	2021-09-02
hCoV-19/Japan/PG-79631/2021	EPI_ISL_3891429	2021-09-02
hCoV-19/Japan/PG-79632/2021	EPI_ISL_3891432	2021-09-02
hCoV-19/Japan/PG-79633/2021	EPI_ISL_3891435	2021-09-02
hCoV-19/Japan/PG-79634/2021	EPI_ISL_3891439	2021-09-02
hCoV-19/Japan/PG-79635/2021	EPI_ISL_3891441	2021-09-02
hCoV-19/Japan/PG-79636/2021	EPI_ISL_3891444	2021-09-02
hCoV-19/Japan/PG-79637/2021	EPI_ISL_3891447	2021-09-02
hCoV-19/Japan/PG-79638/2021	EPI_ISL_3891450	2021-09-02
hCoV-19/Japan/PG-79639/2021	EPI_ISL_3891453	2021-09-02
hCoV-19/Japan/PG-79640/2021	EPI_ISL_3891454	2021-09-02
hCoV-19/Japan/PG-79641/2021	EPI_ISL_3891456	2021-09-02
hCoV-19/Japan/PG-79642/2021	EPI_ISL_3891459	2021-09-02

virus_name	epiID	submission_date
hCoV-19/Japan/PG-79643/2021	EPI_ISL_3891461	2021-09-02
hCoV-19/Japan/PG-79644/2021	EPI_ISL_3891464	2021-09-02
hCoV-19/Japan/PG-79645/2021	EPI_ISL_3891466	2021-09-02
hCoV-19/Japan/PG-79646/2021	EPI_ISL_3891467	2021-09-02
hCoV-19/Japan/PG-79647/2021	EPI_ISL_3891470	2021-09-02
hCoV-19/Japan/PG-79648/2021	EPI_ISL_3891472	2021-09-02
hCoV-19/Japan/PG-79649/2021	EPI_ISL_3891474	2021-09-02
hCoV-19/Japan/PG-79650/2021	EPI_ISL_3891477	2021-09-02
hCoV-19/Japan/PG-79651/2021	EPI_ISL_3891480	2021-09-02
hCoV-19/Japan/PG-79652/2021	EPI_ISL_3891482	2021-09-02
hCoV-19/Japan/PG-79653/2021	EPI_ISL_3891485	2021-09-02
hCoV-19/Japan/PG-79654/2021	EPI_ISL_3891488	2021-09-02
hCoV-19/Japan/PG-79655/2021	EPI_ISL_3891490	2021-09-02
hCoV-19/Japan/PG-79656/2021	EPI_ISL_3891493	2021-09-02
hCoV-19/Japan/PG-79657/2021	EPI_ISL_3891496	2021-09-02
hCoV-19/Japan/PG-79658/2021	EPI_ISL_3891498	2021-09-02
hCoV-19/Japan/PG-79659/2021	EPI_ISL_3891501	2021-09-02
hCoV-19/Japan/PG-79660/2021	EPI_ISL_3891503	2021-09-02
hCoV-19/Japan/PG-79661/2021	EPI_ISL_3891505	2021-09-02
hCoV-19/Japan/PG-79662/2021	EPI_ISL_3891508	2021-09-02
hCoV-19/Japan/PG-79663/2021	EPI_ISL_3891510	2021-09-02
hCoV-19/Japan/PG-79664/2021	EPI_ISL_3891513	2021-09-02
hCoV-19/Japan/PG-79665/2021	EPI_ISL_3891515	2021-09-02
hCoV-19/Japan/PG-79666/2021	EPI_ISL_3891518	2021-09-02
hCoV-19/Japan/PG-79667/2021	EPI_ISL_3891521	2021-09-02
hCoV-19/Japan/PG-79668/2021	EPI_ISL_3891522	2021-09-02
hCoV-19/Japan/PG-79669/2021	EPI_ISL_3891525	2021-09-02
hCoV-19/Japan/PG-79670/2021	EPI_ISL_3891528	2021-09-02
hCoV-19/Japan/PG-79671/2021	EPI_ISL_3891531	2021-09-02
hCoV-19/Japan/PG-79672/2021	EPI_ISL_3891538	2021-09-02
hCoV-19/Japan/PG-79673/2021	EPI_ISL_3891541	2021-09-02
hCoV-19/Japan/PG-79674/2021	EPI_ISL_3891544	2021-09-02
hCoV-19/Japan/PG-79675/2021	EPI_ISL_3891546	2021-09-02
hCoV-19/Japan/PG-79676/2021	EPI_ISL_3891547	2021-09-02
hCoV-19/Japan/PG-79677/2021	EPI_ISL_3891549	2021-09-02
hCoV-19/Japan/PG-79678/2021	EPI_ISL_3891552	2021-09-02
hCoV-19/Japan/PG-79679/2021	EPI_ISL_3891554	2021-09-02
hCoV-19/Japan/PG-79680/2021	EPI_ISL_3891556	2021-09-02
hCoV-19/Japan/PG-79681/2021	EPI_ISL_3891558	2021-09-02
hCoV-19/Japan/PG-79682/2021	EPI_ISL_3891561	2021-09-02
hCoV-19/Japan/PG-79683/2021	EPI_ISL_3891563	2021-09-02
hCoV-19/Japan/PG-79684/2021	EPI_ISL_3891565	2021-09-02
hCoV-19/Japan/PG-79685/2021	EPI_ISL_3891568	2021-09-02
hCoV-19/Japan/PG-79686/2021	EPI_ISL_3891571	2021-09-02
hCoV-19/Japan/PG-79687/2021	EPI_ISL_3891573	2021-09-02
hCoV-19/Japan/PG-79688/2021	EPI_ISL_3891576	2021-09-02
hCoV-19/Japan/PG-79689/2021	EPI_ISL_3891577	2021-09-02
hCoV-19/Japan/PG-79690/2021	EPI_ISL_3891578	2021-09-02
hCoV-19/Japan/PG-79691/2021	EPI_ISL_3891581	2021-09-02
hCoV-19/Japan/PG-79692/2021	EPI_ISL_3891583	2021-09-02
hCoV-19/Japan/PG-79693/2021	EPI_ISL_3891586	2021-09-02
hCoV-19/Japan/PG-79694/2021	EPI_ISL_3891588	2021-09-02
hCoV-19/Japan/PG-79695/2021	EPI_ISL_3891591	2021-09-02
hCoV-19/Japan/PG-79696/2021	EPI_ISL_3891594	2021-09-02

virus_name	epiID	submission_date
hCoV-19/Japan/PG-79697/2021	EPI_ISL_3891596	2021-09-02
hCoV-19/Japan/PG-79698/2021	EPI_ISL_3891599	2021-09-02
hCoV-19/Japan/PG-79699/2021	EPI_ISL_3891602	2021-09-02
hCoV-19/Japan/PG-79700/2021	EPI_ISL_3891604	2021-09-02
hCoV-19/Japan/PG-79701/2021	EPI_ISL_3891607	2021-09-02
hCoV-19/Japan/PG-79702/2021	EPI_ISL_3891610	2021-09-02
hCoV-19/Japan/PG-79703/2021	EPI_ISL_3891612	2021-09-02
hCoV-19/Japan/PG-79704/2021	EPI_ISL_3891615	2021-09-02
hCoV-19/Japan/PG-79705/2021	EPI_ISL_3891617	2021-09-02
hCoV-19/Japan/PG-79706/2021	EPI_ISL_3891618	2021-09-02
hCoV-19/Japan/PG-79707/2021	EPI_ISL_3891619	2021-09-02
hCoV-19/Japan/PG-79708/2021	EPI_ISL_3891621	2021-09-02
hCoV-19/Japan/PG-79709/2021	EPI_ISL_3891623	2021-09-02
hCoV-19/Japan/PG-79710/2021	EPI_ISL_3891626	2021-09-02
hCoV-19/Japan/PG-79711/2021	EPI_ISL_3891628	2021-09-02
hCoV-19/Japan/PG-79712/2021	EPI_ISL_3891630	2021-09-02
hCoV-19/Japan/PG-79713/2021	EPI_ISL_3891632	2021-09-02
hCoV-19/Japan/PG-79714/2021	EPI_ISL_3891636	2021-09-02
hCoV-19/Japan/PG-79715/2021	EPI_ISL_3891638	2021-09-02
hCoV-19/Japan/PG-79716/2021	EPI_ISL_3891641	2021-09-02
hCoV-19/Japan/PG-79717/2021	EPI_ISL_3891644	2021-09-02
hCoV-19/Japan/PG-79718/2021	EPI_ISL_3891647	2021-09-02
hCoV-19/Japan/PG-79719/2021	EPI_ISL_3891650	2021-09-02
hCoV-19/Japan/PG-79720/2021	EPI_ISL_3891653	2021-09-02
hCoV-19/Japan/PG-79721/2021	EPI_ISL_3891655	2021-09-02
hCoV-19/Japan/PG-79722/2021	EPI_ISL_3891658	2021-09-02
hCoV-19/Japan/PG-79723/2021	EPI_ISL_3891661	2021-09-02
hCoV-19/Japan/PG-79724/2021	EPI_ISL_3891662	2021-09-02
hCoV-19/Japan/PG-79725/2021	EPI_ISL_3891664	2021-09-02
hCoV-19/Japan/PG-79726/2021	EPI_ISL_3891666	2021-09-02
hCoV-19/Japan/PG-79727/2021	EPI_ISL_3891668	2021-09-02
hCoV-19/Japan/PG-79728/2021	EPI_ISL_3891670	2021-09-02
hCoV-19/Japan/PG-79729/2021	EPI_ISL_3891673	2021-09-02
hCoV-19/Japan/PG-79730/2021	EPI_ISL_3891675	2021-09-02
hCoV-19/Japan/PG-79731/2021	EPI_ISL_3891678	2021-09-02
hCoV-19/Japan/PG-79732/2021	EPI_ISL_3891680	2021-09-02
hCoV-19/Japan/PG-79733/2021	EPI_ISL_3891682	2021-09-02
hCoV-19/Japan/PG-79734/2021	EPI_ISL_3891685	2021-09-02
hCoV-19/Japan/PG-79735/2021	EPI_ISL_3891687	2021-09-02
hCoV-19/Japan/PG-79736/2021	EPI_ISL_3891690	2021-09-02
hCoV-19/Japan/PG-79737/2021	EPI_ISL_3891692	2021-09-02
hCoV-19/Japan/PG-79738/2021	EPI_ISL_3891694	2021-09-02
hCoV-19/Japan/PG-79739/2021	EPI_ISL_3891696	2021-09-02
hCoV-19/Japan/PG-79740/2021	EPI_ISL_3891698	2021-09-02
hCoV-19/Japan/PG-79741/2021	EPI_ISL_3891700	2021-09-02
hCoV-19/Japan/PG-79742/2021	EPI_ISL_3891703	2021-09-02
hCoV-19/Japan/PG-79743/2021	EPI_ISL_3891707	2021-09-02
hCoV-19/Japan/PG-79744/2021	EPI_ISL_3891710	2021-09-02
hCoV-19/Japan/PG-79745/2021	EPI_ISL_3891713	2021-09-02
hCoV-19/Japan/PG-79746/2021	EPI_ISL_3891716	2021-09-02
hCoV-19/Japan/PG-79747/2021	EPI_ISL_3891719	2021-09-02
hCoV-19/Japan/PG-79748/2021	EPI_ISL_3891722	2021-09-02
hCoV-19/Japan/PG-79749/2021	EPI_ISL_3891724	2021-09-02
hCoV-19/Japan/PG-79750/2021	EPI_ISL_3891727	2021-09-02

virus_name	epiID	submission_date
hCoV-19/Japan/PG-79751/2021	EPI_ISL_3891730	2021-09-02
hCoV-19/Japan/PG-79752/2021	EPI_ISL_3891733	2021-09-02
hCoV-19/Japan/PG-79753/2021	EPI_ISL_3891737	2021-09-02
hCoV-19/Japan/PG-79754/2021	EPI_ISL_3891740	2021-09-02
hCoV-19/Japan/PG-79755/2021	EPI_ISL_3891742	2021-09-02
hCoV-19/Japan/PG-79756/2021	EPI_ISL_3891746	2021-09-02
hCoV-19/Japan/PG-79757/2021	EPI_ISL_3891748	2021-09-02
hCoV-19/Japan/PG-79758/2021	EPI_ISL_3891751	2021-09-02
hCoV-19/Japan/PG-79759/2021	EPI_ISL_3891752	2021-09-02
hCoV-19/Japan/PG-79760/2021	EPI_ISL_3891754	2021-09-02
hCoV-19/Japan/PG-79761/2021	EPI_ISL_3891757	2021-09-02
hCoV-19/Japan/PG-79762/2021	EPI_ISL_3891760	2021-09-02
hCoV-19/Japan/PG-79763/2021	EPI_ISL_3891763	2021-09-02
hCoV-19/Japan/PG-79764/2021	EPI_ISL_3891766	2021-09-02
hCoV-19/Japan/PG-79765/2021	EPI_ISL_3891769	2021-09-02
hCoV-19/Japan/PG-79766/2021	EPI_ISL_3891771	2021-09-02
hCoV-19/Japan/PG-79767/2021	EPI_ISL_3891774	2021-09-02
hCoV-19/Japan/PG-79768/2021	EPI_ISL_3891776	2021-09-02
hCoV-19/Japan/PG-79769/2021	EPI_ISL_3891777	2021-09-02
hCoV-19/Japan/PG-79770/2021	EPI_ISL_3891780	2021-09-02
hCoV-19/Japan/PG-79771/2021	EPI_ISL_3891783	2021-09-02
hCoV-19/Japan/PG-79772/2021	EPI_ISL_3891786	2021-09-02
hCoV-19/Japan/PG-79773/2021	EPI_ISL_3891788	2021-09-02
hCoV-19/Japan/PG-79774/2021	EPI_ISL_3891791	2021-09-02
hCoV-19/Japan/PG-79775/2021	EPI_ISL_3891795	2021-09-02
hCoV-19/Japan/PG-79776/2021	EPI_ISL_3891797	2021-09-02
hCoV-19/Japan/PG-79777/2021	EPI_ISL_3891800	2021-09-02
hCoV-19/Japan/PG-79778/2021	EPI_ISL_3891803	2021-09-02
hCoV-19/Japan/PG-79779/2021	EPI_ISL_3891805	2021-09-02
hCoV-19/Japan/PG-79780/2021	EPI_ISL_3891807	2021-09-02
hCoV-19/Japan/PG-79781/2021	EPI_ISL_3891808	2021-09-02
hCoV-19/Japan/PG-79782/2021	EPI_ISL_3891809	2021-09-02
hCoV-19/Japan/PG-79783/2021	EPI_ISL_3891812	2021-09-02
hCoV-19/Japan/PG-79784/2021	EPI_ISL_3891814	2021-09-02
hCoV-19/Japan/PG-79785/2021	EPI_ISL_3891816	2021-09-02
hCoV-19/Japan/PG-79786/2021	EPI_ISL_3891820	2021-09-02
hCoV-19/Japan/PG-79787/2021	EPI_ISL_3891821	2021-09-02
hCoV-19/Japan/PG-79788/2021	EPI_ISL_3891823	2021-09-02
hCoV-19/Japan/PG-79789/2021	EPI_ISL_3891826	2021-09-02
hCoV-19/Japan/PG-79790/2021	EPI_ISL_3891828	2021-09-02
hCoV-19/Japan/PG-79791/2021	EPI_ISL_3891831	2021-09-02
hCoV-19/Japan/PG-79792/2021	EPI_ISL_3891833	2021-09-02
hCoV-19/Japan/PG-79793/2021	EPI_ISL_3891836	2021-09-02
hCoV-19/Japan/PG-79794/2021	EPI_ISL_3891838	2021-09-02
hCoV-19/Japan/PG-79795/2021	EPI_ISL_3891841	2021-09-02
hCoV-19/Japan/PG-79796/2021	EPI_ISL_3891842	2021-09-02
hCoV-19/Japan/PG-79797/2021	EPI_ISL_3891844	2021-09-02
hCoV-19/Japan/PG-79798/2021	EPI_ISL_3891847	2021-09-02
hCoV-19/Japan/PG-79799/2021	EPI_ISL_3891850	2021-09-02
hCoV-19/Japan/PG-79800/2021	EPI_ISL_3891852	2021-09-02
hCoV-19/Japan/PG-79801/2021	EPI_ISL_3891854	2021-09-02
hCoV-19/Japan/PG-79802/2021	EPI_ISL_3891856	2021-09-02
hCoV-19/Japan/PG-79803/2021	EPI_ISL_3891859	2021-09-02
hCoV-19/Japan/PG-79804/2021	EPI_ISL_3891862	2021-09-02

virus_name	epiID	submission_date
hCoV-19/Japan/PG-79805/2021	EPI_ISL_3891864	2021-09-02
hCoV-19/Japan/PG-79806/2021	EPI_ISL_3891867	2021-09-02
hCoV-19/Japan/PG-79807/2021	EPI_ISL_3891870	2021-09-02
hCoV-19/Japan/PG-79808/2021	EPI_ISL_3891872	2021-09-02
hCoV-19/Japan/PG-79809/2021	EPI_ISL_3891874	2021-09-02
hCoV-19/Japan/PG-79810/2021	EPI_ISL_3891876	2021-09-02
hCoV-19/Japan/PG-79811/2021	EPI_ISL_3891879	2021-09-02
hCoV-19/Japan/PG-79812/2021	EPI_ISL_3891881	2021-09-02
hCoV-19/Japan/PG-79813/2021	EPI_ISL_3891884	2021-09-02
hCoV-19/Japan/PG-79814/2021	EPI_ISL_3891886	2021-09-02
hCoV-19/Japan/PG-79815/2021	EPI_ISL_3891889	2021-09-02
hCoV-19/Japan/PG-79816/2021	EPI_ISL_3891892	2021-09-02
hCoV-19/Japan/PG-79817/2021	EPI_ISL_3891894	2021-09-02
hCoV-19/Japan/PG-79818/2021	EPI_ISL_3891898	2021-09-02
hCoV-19/Japan/PG-79819/2021	EPI_ISL_3891901	2021-09-02
hCoV-19/Japan/PG-79820/2021	EPI_ISL_3891904	2021-09-02
hCoV-19/Japan/PG-79821/2021	EPI_ISL_3891906	2021-09-02
hCoV-19/Japan/PG-79822/2021	EPI_ISL_3891909	2021-09-02
hCoV-19/Japan/PG-79823/2021	EPI_ISL_3891912	2021-09-02
hCoV-19/Japan/PG-79824/2021	EPI_ISL_3891915	2021-09-02
hCoV-19/Japan/PG-79825/2021	EPI_ISL_3891917	2021-09-02
hCoV-19/Japan/PG-79826/2021	EPI_ISL_3891920	2021-09-02
hCoV-19/Japan/PG-79827/2021	EPI_ISL_3891923	2021-09-02
hCoV-19/Japan/PG-79828/2021	EPI_ISL_3891926	2021-09-02
hCoV-19/Japan/PG-79829/2021	EPI_ISL_3891928	2021-09-02
hCoV-19/Japan/PG-79830/2021	EPI_ISL_3891931	2021-09-02
hCoV-19/Japan/PG-79831/2021	EPI_ISL_3891934	2021-09-02
hCoV-19/Japan/PG-79832/2021	EPI_ISL_3891937	2021-09-02
hCoV-19/Japan/PG-79833/2021	EPI_ISL_3891939	2021-09-02
hCoV-19/Japan/PG-79834/2021	EPI_ISL_3891941	2021-09-02
hCoV-19/Japan/PG-79835/2021	EPI_ISL_3891943	2021-09-02
hCoV-19/Japan/PG-79836/2021	EPI_ISL_3891946	2021-09-02
hCoV-19/Japan/PG-79837/2021	EPI_ISL_3891949	2021-09-02
hCoV-19/Japan/PG-79838/2021	EPI_ISL_3891951	2021-09-02
hCoV-19/Japan/PG-79839/2021	EPI_ISL_3891954	2021-09-02
hCoV-19/Japan/PG-79840/2021	EPI_ISL_3891957	2021-09-02
hCoV-19/Japan/PG-79841/2021	EPI_ISL_3891960	2021-09-02
hCoV-19/Japan/PG-79842/2021	EPI_ISL_3891962	2021-09-02
hCoV-19/Japan/PG-79843/2021	EPI_ISL_3891965	2021-09-02
hCoV-19/Japan/PG-79844/2021	EPI_ISL_3891967	2021-09-02
hCoV-19/Japan/PG-79845/2021	EPI_ISL_3891969	2021-09-02
hCoV-19/Japan/PG-79846/2021	EPI_ISL_3891971	2021-09-02
hCoV-19/Japan/PG-79847/2021	EPI_ISL_3891973	2021-09-02
hCoV-19/Japan/PG-79848/2021	EPI_ISL_3891976	2021-09-02
hCoV-19/Japan/PG-79849/2021	EPI_ISL_3891978	2021-09-02
hCoV-19/Japan/PG-79850/2021	EPI_ISL_3891980	2021-09-02
hCoV-19/Japan/PG-79851/2021	EPI_ISL_3891982	2021-09-02
hCoV-19/Japan/PG-79852/2021	EPI_ISL_3891984	2021-09-02
hCoV-19/Japan/PG-79853/2021	EPI_ISL_3891986	2021-09-02
hCoV-19/Japan/PG-79854/2021	EPI_ISL_3891988	2021-09-02
hCoV-19/Japan/PG-79855/2021	EPI_ISL_3891989	2021-09-02
hCoV-19/Japan/PG-79856/2021	EPI_ISL_3891991	2021-09-02
hCoV-19/Japan/PG-79857/2021	EPI_ISL_3891993	2021-09-02
hCoV-19/Japan/PG-79858/2021	EPI_ISL_3891995	2021-09-02

virus_name	epiID	submission_date
hCoV-19/Japan/PG-79859/2021	EPI_ISL_3891998	2021-09-02
hCoV-19/Japan/PG-79860/2021	EPI_ISL_3892000	2021-09-02
hCoV-19/Japan/PG-79861/2021	EPI_ISL_3892003	2021-09-02
hCoV-19/Japan/PG-79862/2021	EPI_ISL_3892005	2021-09-02
hCoV-19/Japan/PG-79863/2021	EPI_ISL_3892007	2021-09-02
hCoV-19/Japan/PG-79864/2021	EPI_ISL_3892010	2021-09-02
hCoV-19/Japan/PG-79865/2021	EPI_ISL_3892013	2021-09-02
hCoV-19/Japan/PG-79866/2021	EPI_ISL_3892015	2021-09-02
hCoV-19/Japan/PG-79867/2021	EPI_ISL_3892017	2021-09-02
hCoV-19/Japan/PG-79868/2021	EPI_ISL_3892019	2021-09-02
hCoV-19/Japan/PG-79869/2021	EPI_ISL_3892021	2021-09-02
hCoV-19/Japan/PG-79870/2021	EPI_ISL_3892024	2021-09-02
hCoV-19/Japan/PG-79871/2021	EPI_ISL_3892027	2021-09-02
hCoV-19/Japan/PG-79872/2021	EPI_ISL_3892029	2021-09-02
hCoV-19/Japan/PG-79873/2021	EPI_ISL_3892032	2021-09-02
hCoV-19/Japan/PG-79874/2021	EPI_ISL_3892034	2021-09-02
hCoV-19/Japan/PG-79875/2021	EPI_ISL_3892036	2021-09-02
hCoV-19/Japan/PG-79876/2021	EPI_ISL_3892038	2021-09-02
hCoV-19/Japan/PG-79877/2021	EPI_ISL_3892040	2021-09-02
hCoV-19/Japan/PG-79878/2021	EPI_ISL_3892042	2021-09-02
hCoV-19/Japan/PG-79879/2021	EPI_ISL_3892047	2021-09-02
hCoV-19/Japan/PG-79880/2021	EPI_ISL_3892050	2021-09-02
hCoV-19/Japan/PG-79881/2021	EPI_ISL_3892054	2021-09-02
hCoV-19/Japan/PG-79882/2021	EPI_ISL_3892059	2021-09-02
hCoV-19/Japan/PG-79883/2021	EPI_ISL_3892064	2021-09-02
hCoV-19/Japan/PG-79884/2021	EPI_ISL_3892068	2021-09-02
hCoV-19/Japan/PG-79885/2021	EPI_ISL_3892072	2021-09-02
hCoV-19/Japan/PG-79886/2021	EPI_ISL_3892074	2021-09-02
hCoV-19/Japan/PG-79887/2021	EPI_ISL_3892077	2021-09-02
hCoV-19/Japan/PG-79888/2021	EPI_ISL_3892080	2021-09-02
hCoV-19/Japan/PG-79889/2021	EPI_ISL_3892082	2021-09-02
hCoV-19/Japan/PG-79890/2021	EPI_ISL_3892087	2021-09-02
hCoV-19/Japan/PG-79891/2021	EPI_ISL_3892091	2021-09-02
hCoV-19/Japan/PG-79892/2021	EPI_ISL_3892096	2021-09-02
hCoV-19/Japan/PG-79893/2021	EPI_ISL_3892099	2021-09-02
hCoV-19/Japan/PG-79894/2021	EPI_ISL_3892103	2021-09-02
hCoV-19/Japan/PG-79895/2021	EPI_ISL_3892105	2021-09-02
hCoV-19/Japan/PG-79896/2021	EPI_ISL_3892110	2021-09-02
hCoV-19/Japan/PG-79897/2021	EPI_ISL_3892114	2021-09-02
hCoV-19/Japan/PG-79898/2021	EPI_ISL_3892117	2021-09-02
hCoV-19/Japan/PG-79899/2021	EPI_ISL_3892118	2021-09-02
hCoV-19/Japan/PG-79900/2021	EPI_ISL_3892122	2021-09-02
hCoV-19/Japan/PG-79901/2021	EPI_ISL_3892125	2021-09-02
hCoV-19/Japan/PG-79902/2021	EPI_ISL_3892128	2021-09-02
hCoV-19/Japan/PG-79903/2021	EPI_ISL_3892131	2021-09-02
hCoV-19/Japan/PG-79904/2021	EPI_ISL_3892133	2021-09-02
hCoV-19/Japan/PG-79905/2021	EPI_ISL_3892137	2021-09-02
hCoV-19/Japan/PG-79906/2021	EPI_ISL_3892143	2021-09-02
hCoV-19/Japan/PG-79907/2021	EPI_ISL_3892147	2021-09-02
hCoV-19/Japan/PG-79908/2021	EPI_ISL_3892152	2021-09-02
hCoV-19/Japan/PG-79909/2021	EPI_ISL_3892156	2021-09-02
hCoV-19/Japan/PG-79910/2021	EPI_ISL_3892159	2021-09-02
hCoV-19/Japan/PG-79911/2021	EPI_ISL_3892164	2021-09-02
hCoV-19/Japan/PG-79912/2021	EPI_ISL_3892169	2021-09-02

virus_name	epiID	submission_date
hCoV-19/Japan/PG-79913/2021	EPI_ISL_3892174	2021-09-02
hCoV-19/Japan/PG-79914/2021	EPI_ISL_3892179	2021-09-02
hCoV-19/Japan/PG-79915/2021	EPI_ISL_3873769	2021-09-02
hCoV-19/Japan/PG-79916/2021	EPI_ISL_3873770	2021-09-02
hCoV-19/Japan/PG-79917/2021	EPI_ISL_3873771	2021-09-02
hCoV-19/Japan/PG-79918/2021	EPI_ISL_3873772	2021-09-02
hCoV-19/Japan/PG-79919/2021	EPI_ISL_3873773	2021-09-02
hCoV-19/Japan/PG-79920/2021	EPI_ISL_3873774	2021-09-02
hCoV-19/Japan/PG-79921/2021	EPI_ISL_3873775	2021-09-02
hCoV-19/Japan/PG-79922/2021	EPI_ISL_3873776	2021-09-02
hCoV-19/Japan/PG-79923/2021	EPI_ISL_3873777	2021-09-02
hCoV-19/Japan/PG-79924/2021	EPI_ISL_3873778	2021-09-02
hCoV-19/Japan/PG-79925/2021	EPI_ISL_3873779	2021-09-02
hCoV-19/Japan/PG-79926/2021	EPI_ISL_3873780	2021-09-02
hCoV-19/Japan/PG-79927/2021	EPI_ISL_3873781	2021-09-02
hCoV-19/Japan/PG-79928/2021	EPI_ISL_3873782	2021-09-02
hCoV-19/Japan/PG-79929/2021	EPI_ISL_3873783	2021-09-02
hCoV-19/Japan/PG-79930/2021	EPI_ISL_3873784	2021-09-02
hCoV-19/Japan/PG-79931/2021	EPI_ISL_3873785	2021-09-02
hCoV-19/Japan/PG-79932/2021	EPI_ISL_3873786	2021-09-02
hCoV-19/Japan/PG-79933/2021	EPI_ISL_3873787	2021-09-02
hCoV-19/Japan/PG-79934/2021	EPI_ISL_3873788	2021-09-02
hCoV-19/Japan/PG-79935/2021	EPI_ISL_3873789	2021-09-02
hCoV-19/Japan/PG-79936/2021	EPI_ISL_3873790	2021-09-02
hCoV-19/Japan/PG-79937/2021	EPI_ISL_3873791	2021-09-02
hCoV-19/Japan/PG-79938/2021	EPI_ISL_3873792	2021-09-02
hCoV-19/Japan/PG-79939/2021	EPI_ISL_3873793	2021-09-02
hCoV-19/Japan/PG-79940/2021	EPI_ISL_3873794	2021-09-02
hCoV-19/Japan/PG-79941/2021	EPI_ISL_3873795	2021-09-02
hCoV-19/Japan/PG-79942/2021	EPI_ISL_3873796	2021-09-02
hCoV-19/Japan/PG-79943/2021	EPI_ISL_3873797	2021-09-02
hCoV-19/Japan/PG-79944/2021	EPI_ISL_3873798	2021-09-02
hCoV-19/Japan/PG-79945/2021	EPI_ISL_3874627	2021-09-02
hCoV-19/Japan/PG-79946/2021	EPI_ISL_3874630	2021-09-02
hCoV-19/Japan/PG-79947/2021	EPI_ISL_3874631	2021-09-02
hCoV-19/Japan/PG-79948/2021	EPI_ISL_3874634	2021-09-02
hCoV-19/Japan/PG-79949/2021	EPI_ISL_3874637	2021-09-02
hCoV-19/Japan/PG-79950/2021	EPI_ISL_3874640	2021-09-02
hCoV-19/Japan/PG-79951/2021	EPI_ISL_3874643	2021-09-02
hCoV-19/Japan/PG-79952/2021	EPI_ISL_3874645	2021-09-02
hCoV-19/Japan/PG-79953/2021	EPI_ISL_3874648	2021-09-02
hCoV-19/Japan/PG-79954/2021	EPI_ISL_3874651	2021-09-02
hCoV-19/Japan/PG-79955/2021	EPI_ISL_3874653	2021-09-02
hCoV-19/Japan/PG-79956/2021	EPI_ISL_3874657	2021-09-02
hCoV-19/Japan/PG-79957/2021	EPI_ISL_3874660	2021-09-02
hCoV-19/Japan/PG-79958/2021	EPI_ISL_3874662	2021-09-02
hCoV-19/Japan/PG-79959/2021	EPI_ISL_3874665	2021-09-02
hCoV-19/Japan/PG-79960/2021	EPI_ISL_3874668	2021-09-02
hCoV-19/Japan/PG-79961/2021	EPI_ISL_3874670	2021-09-02
hCoV-19/Japan/PG-79962/2021	EPI_ISL_3874673	2021-09-02
hCoV-19/Japan/PG-79963/2021	EPI_ISL_3874676	2021-09-02
hCoV-19/Japan/PG-79964/2021	EPI_ISL_3874679	2021-09-02
hCoV-19/Japan/PG-79965/2021	EPI_ISL_3874974	2021-09-02
hCoV-19/Japan/PG-79966/2021	EPI_ISL_3874975	2021-09-02

virus_name	epiID	submission_date
hCoV-19/Japan/PG-79967/2021	EPI_ISL_3874976	2021-09-02
hCoV-19/Japan/PG-79968/2021	EPI_ISL_3874977	2021-09-02
hCoV-19/Japan/PG-79969/2021	EPI_ISL_3874978	2021-09-02
hCoV-19/Japan/PG-79973/2021	EPI_ISL_3874979	2021-09-02
hCoV-19/Japan/PG-79974/2021	EPI_ISL_3874980	2021-09-02
hCoV-19/Japan/PG-79975/2021	EPI_ISL_3874981	2021-09-02
hCoV-19/Japan/PG-79976/2021	EPI_ISL_3874982	2021-09-02
hCoV-19/Japan/PG-79978/2021	EPI_ISL_3874983	2021-09-02
hCoV-19/Japan/PG-79981/2021	EPI_ISL_3874984	2021-09-02
hCoV-19/Japan/PG-79982/2021	EPI_ISL_3874985	2021-09-02
hCoV-19/Japan/PG-79983/2021	EPI_ISL_3874986	2021-09-02
hCoV-19/Japan/PG-79984/2021	EPI_ISL_3874987	2021-09-02
hCoV-19/Japan/PG-79985/2021	EPI_ISL_3874988	2021-09-02
hCoV-19/Japan/PG-79986/2021	EPI_ISL_3874989	2021-09-02
hCoV-19/Japan/PG-79987/2021	EPI_ISL_3874990	2021-09-02
hCoV-19/Japan/PG-79988/2021	EPI_ISL_3874991	2021-09-02
hCoV-19/Japan/PG-79990/2021	EPI_ISL_3874992	2021-09-02
hCoV-19/Japan/PG-79991/2021	EPI_ISL_3874993	2021-09-02
hCoV-19/Japan/PG-79992/2021	EPI_ISL_3874994	2021-09-02
hCoV-19/Japan/PG-79993/2021	EPI_ISL_3874995	2021-09-02
hCoV-19/Japan/PG-79994/2021	EPI_ISL_3874996	2021-09-02
hCoV-19/Japan/PG-79995/2021	EPI_ISL_3874997	2021-09-02
hCoV-19/Japan/PG-79996/2021	EPI_ISL_3874998	2021-09-02
hCoV-19/Japan/PG-79997/2021	EPI_ISL_3874999	2021-09-02
hCoV-19/Japan/PG-79998/2021	EPI_ISL_3875000	2021-09-02
hCoV-19/Japan/PG-79999/2021	EPI_ISL_3875001	2021-09-02
hCoV-19/Japan/PG-80000/2021	EPI_ISL_3875002	2021-09-02
hCoV-19/Japan/PG-80001/2021	EPI_ISL_3875003	2021-09-02
hCoV-19/Japan/PG-80002/2021	EPI_ISL_3875004	2021-09-02
hCoV-19/Japan/PG-80003/2021	EPI_ISL_3875005	2021-09-02
hCoV-19/Japan/PG-80004/2021	EPI_ISL_3875006	2021-09-02
hCoV-19/Japan/PG-80005/2021	EPI_ISL_3875007	2021-09-02
hCoV-19/Japan/PG-80006/2021	EPI_ISL_3875008	2021-09-02
hCoV-19/Japan/PG-80008/2021	EPI_ISL_3875009	2021-09-02
hCoV-19/Japan/PG-80010/2021	EPI_ISL_3875010	2021-09-02
hCoV-19/Japan/PG-80011/2021	EPI_ISL_3875011	2021-09-02
hCoV-19/Japan/PG-80012/2021	EPI_ISL_3875012	2021-09-02
hCoV-19/Japan/PG-80013/2021	EPI_ISL_3875013	2021-09-02
hCoV-19/Japan/PG-80017/2021	EPI_ISL_3875014	2021-09-02
hCoV-19/Japan/PG-80018/2021	EPI_ISL_3875015	2021-09-02
hCoV-19/Japan/PG-80019/2021	EPI_ISL_3875016	2021-09-02
hCoV-19/Japan/PG-80020/2021	EPI_ISL_3875017	2021-09-02
hCoV-19/Japan/PG-80021/2021	EPI_ISL_3875018	2021-09-02
hCoV-19/Japan/PG-80022/2021	EPI_ISL_3875019	2021-09-02
hCoV-19/Japan/PG-80023/2021	EPI_ISL_3875020	2021-09-02
hCoV-19/Japan/PG-80024/2021	EPI_ISL_3875021	2021-09-02
hCoV-19/Japan/PG-80025/2021	EPI_ISL_3875022	2021-09-02
hCoV-19/Japan/PG-80026/2021	EPI_ISL_3875023	2021-09-02
hCoV-19/Japan/PG-80027/2021	EPI_ISL_3875024	2021-09-02
hCoV-19/Japan/PG-80028/2021	EPI_ISL_3875025	2021-09-02
hCoV-19/Japan/PG-80029/2021	EPI_ISL_3875026	2021-09-02
hCoV-19/Japan/PG-80030/2021	EPI_ISL_3875027	2021-09-02
hCoV-19/Japan/PG-80032/2021	EPI_ISL_3875028	2021-09-02
hCoV-19/Japan/PG-80033/2021	EPI_ISL_3875029	2021-09-02

virus_name	epiID	submission_date
hCoV-19/Japan/PG-80034/2021	EPI_ISL_3875030	2021-09-02
hCoV-19/Japan/PG-80035/2021	EPI_ISL_3875031	2021-09-02
hCoV-19/Japan/PG-80036/2021	EPI_ISL_3875032	2021-09-02
hCoV-19/Japan/PG-80037/2021	EPI_ISL_3875033	2021-09-02
hCoV-19/Japan/PG-80038/2021	EPI_ISL_3875034	2021-09-02
hCoV-19/Japan/PG-80039/2021	EPI_ISL_3875035	2021-09-02
hCoV-19/Japan/PG-80040/2021	EPI_ISL_3875036	2021-09-02
hCoV-19/Japan/PG-80041/2021	EPI_ISL_3875037	2021-09-02
hCoV-19/Japan/PG-80043/2021	EPI_ISL_3875038	2021-09-02
hCoV-19/Japan/PG-80046/2021	EPI_ISL_3875039	2021-09-02
hCoV-19/Japan/PG-80047/2021	EPI_ISL_3875040	2021-09-02
hCoV-19/Japan/PG-80048/2021	EPI_ISL_3875041	2021-09-02
hCoV-19/Japan/PG-80049/2021	EPI_ISL_3875042	2021-09-02
hCoV-19/Japan/PG-80050/2021	EPI_ISL_3875043	2021-09-02
hCoV-19/Japan/PG-80051/2021	EPI_ISL_3875044	2021-09-02
hCoV-19/Japan/PG-80052/2021	EPI_ISL_3875045	2021-09-02
hCoV-19/Japan/PG-80053/2021	EPI_ISL_3875046	2021-09-02
hCoV-19/Japan/PG-80054/2021	EPI_ISL_3875047	2021-09-02
hCoV-19/Japan/PG-80055/2021	EPI_ISL_3875048	2021-09-02
hCoV-19/Japan/PG-80065/2021	EPI_ISL_3875049	2021-09-02
hCoV-19/Japan/PG-80067/2021	EPI_ISL_3875050	2021-09-02
hCoV-19/Japan/PG-80068/2021	EPI_ISL_3875051	2021-09-02
hCoV-19/Japan/PG-80069/2021	EPI_ISL_3875052	2021-09-02
hCoV-19/Japan/PG-80071/2021	EPI_ISL_3875053	2021-09-02
hCoV-19/Japan/PG-80072/2021	EPI_ISL_3875054	2021-09-02
hCoV-19/Japan/PG-80073/2021	EPI_ISL_3875055	2021-09-02
hCoV-19/Japan/PG-80076/2021	EPI_ISL_3875056	2021-09-02
hCoV-19/Japan/PG-80077/2021	EPI_ISL_3875057	2021-09-02
hCoV-19/Japan/PG-80078/2021	EPI_ISL_3875058	2021-09-02
hCoV-19/Japan/PG-80080/2021	EPI_ISL_3875059	2021-09-02
hCoV-19/Japan/PG-80081/2021	EPI_ISL_3875060	2021-09-02
hCoV-19/Japan/PG-80082/2021	EPI_ISL_3875061	2021-09-02
hCoV-19/Japan/PG-80083/2021	EPI_ISL_3875062	2021-09-02
hCoV-19/Japan/PG-80086/2021	EPI_ISL_3875063	2021-09-02
hCoV-19/Japan/PG-80087/2021	EPI_ISL_3875064	2021-09-02
hCoV-19/Japan/PG-80088/2021	EPI_ISL_3875065	2021-09-02
hCoV-19/Japan/PG-80089/2021	EPI_ISL_3875066	2021-09-02
hCoV-19/Japan/PG-80090/2021	EPI_ISL_3875067	2021-09-02
hCoV-19/Japan/PG-80093/2021	EPI_ISL_3875068	2021-09-02
hCoV-19/Japan/PG-80094/2021	EPI_ISL_3875069	2021-09-02
hCoV-19/Japan/PG-80095/2021	EPI_ISL_3875070	2021-09-02
hCoV-19/Japan/PG-80096/2021	EPI_ISL_3875071	2021-09-02
hCoV-19/Japan/PG-80097/2021	EPI_ISL_3875072	2021-09-02
hCoV-19/Japan/PG-80098/2021	EPI_ISL_3875073	2021-09-02
hCoV-19/Japan/PG-80099/2021	EPI_ISL_3875074	2021-09-02
hCoV-19/Japan/PG-80100/2021	EPI_ISL_3875075	2021-09-02
hCoV-19/Japan/PG-80101/2021	EPI_ISL_3875076	2021-09-02
hCoV-19/Japan/PG-80102/2021	EPI_ISL_3875077	2021-09-02
hCoV-19/Japan/PG-80103/2021	EPI_ISL_3875078	2021-09-02
hCoV-19/Japan/PG-80104/2021	EPI_ISL_3875079	2021-09-02
hCoV-19/Japan/PG-80105/2021	EPI_ISL_3875080	2021-09-02
hCoV-19/Japan/PG-80106/2021	EPI_ISL_3875081	2021-09-02
hCoV-19/Japan/PG-80107/2021	EPI_ISL_3875082	2021-09-02
hCoV-19/Japan/PG-80108/2021	EPI_ISL_3875083	2021-09-02

virus_name	epiID	submission_date
hCoV-19/Japan/PG-80109/2021	EPI_ISL_3875084	2021-09-02
hCoV-19/Japan/PG-80111/2021	EPI_ISL_3875085	2021-09-02
hCoV-19/Japan/PG-80112/2021	EPI_ISL_3875086	2021-09-02
hCoV-19/Japan/PG-80113/2021	EPI_ISL_3875087	2021-09-02
hCoV-19/Japan/PG-80115/2021	EPI_ISL_3875088	2021-09-02
hCoV-19/Japan/PG-80116/2021	EPI_ISL_3875089	2021-09-02
hCoV-19/Japan/PG-80118/2021	EPI_ISL_3875090	2021-09-02
hCoV-19/Japan/PG-80119/2021	EPI_ISL_3875091	2021-09-02
hCoV-19/Japan/PG-80121/2021	EPI_ISL_3875092	2021-09-02
hCoV-19/Japan/PG-80122/2021	EPI_ISL_3875093	2021-09-02
hCoV-19/Japan/PG-80123/2021	EPI_ISL_3875094	2021-09-02
hCoV-19/Japan/PG-80125/2021	EPI_ISL_3875095	2021-09-02
hCoV-19/Japan/PG-80126/2021	EPI_ISL_3875096	2021-09-02
hCoV-19/Japan/PG-80127/2021	EPI_ISL_3875097	2021-09-02
hCoV-19/Japan/PG-80128/2021	EPI_ISL_3875098	2021-09-02
hCoV-19/Japan/PG-80129/2021	EPI_ISL_3875099	2021-09-02
hCoV-19/Japan/PG-80130/2021	EPI_ISL_3875100	2021-09-02
hCoV-19/Japan/PG-80131/2021	EPI_ISL_3875101	2021-09-02
hCoV-19/Japan/PG-80132/2021	EPI_ISL_3875102	2021-09-02
hCoV-19/Japan/PG-80133/2021	EPI_ISL_3875103	2021-09-02
hCoV-19/Japan/PG-80134/2021	EPI_ISL_3875104	2021-09-02
hCoV-19/Japan/PG-80135/2021	EPI_ISL_3875105	2021-09-02
hCoV-19/Japan/PG-80136/2021	EPI_ISL_3875106	2021-09-02
hCoV-19/Japan/PG-80137/2021	EPI_ISL_3875107	2021-09-02
hCoV-19/Japan/PG-80138/2021	EPI_ISL_3871798	2021-09-02
hCoV-19/Japan/PG-80139/2021	EPI_ISL_3871799	2021-09-02
hCoV-19/Japan/PG-80140/2021	EPI_ISL_3871800	2021-09-02
hCoV-19/Japan/PG-80141/2021	EPI_ISL_3871801	2021-09-02
hCoV-19/Japan/PG-80142/2021	EPI_ISL_3871802	2021-09-02
hCoV-19/Japan/PG-80143/2021	EPI_ISL_3871803	2021-09-02
hCoV-19/Japan/PG-80144/2021	EPI_ISL_3871804	2021-09-02
hCoV-19/Japan/PG-80145/2021	EPI_ISL_3871805	2021-09-02
hCoV-19/Japan/PG-80146/2021	EPI_ISL_3871806	2021-09-02
hCoV-19/Japan/PG-80147/2021	EPI_ISL_3871807	2021-09-02
hCoV-19/Japan/PG-80148/2021	EPI_ISL_3871808	2021-09-02
hCoV-19/Japan/PG-80149/2021	EPI_ISL_3871809	2021-09-02
hCoV-19/Japan/PG-80150/2021	EPI_ISL_3871810	2021-09-02
hCoV-19/Japan/PG-80151/2021	EPI_ISL_3871811	2021-09-02
hCoV-19/Japan/PG-80152/2021	EPI_ISL_3871812	2021-09-02
hCoV-19/Japan/PG-80153/2021	EPI_ISL_3871813	2021-09-02
hCoV-19/Japan/PG-80154/2021	EPI_ISL_3871814	2021-09-02
hCoV-19/Japan/PG-80155/2021	EPI_ISL_3871815	2021-09-02
hCoV-19/Japan/PG-80156/2021	EPI_ISL_3871816	2021-09-02
hCoV-19/Japan/PG-80157/2021	EPI_ISL_3871817	2021-09-02
hCoV-19/Japan/PG-80158/2021	EPI_ISL_3871818	2021-09-02
hCoV-19/Japan/PG-80159/2021	EPI_ISL_3871819	2021-09-02
hCoV-19/Japan/PG-80160/2021	EPI_ISL_3871820	2021-09-02
hCoV-19/Japan/PG-80161/2021	EPI_ISL_3871821	2021-09-02
hCoV-19/Japan/PG-80162/2021	EPI_ISL_3871822	2021-09-02
hCoV-19/Japan/PG-80163/2021	EPI_ISL_3876104	2021-09-02
hCoV-19/Japan/PG-80164/2021	EPI_ISL_3876105	2021-09-02
hCoV-19/Japan/PG-80165/2021	EPI_ISL_3876106	2021-09-02
hCoV-19/Japan/PG-80166/2021	EPI_ISL_3876107	2021-09-02
hCoV-19/Japan/PG-80167/2021	EPI_ISL_3876108	2021-09-02

virus_name	epiID	submission_date
hCoV-19/Japan/PG-80168/2021	EPI_ISL_3876109	2021-09-02
hCoV-19/Japan/PG-80169/2021	EPI_ISL_3876110	2021-09-02
hCoV-19/Japan/PG-80170/2021	EPI_ISL_3876111	2021-09-02
hCoV-19/Japan/PG-80171/2021	EPI_ISL_3876112	2021-09-02
hCoV-19/Japan/PG-80172/2021	EPI_ISL_3876113	2021-09-02
hCoV-19/Japan/PG-80173/2021	EPI_ISL_3876114	2021-09-02
hCoV-19/Japan/PG-80174/2021	EPI_ISL_3876115	2021-09-02
hCoV-19/Japan/PG-80175/2021	EPI_ISL_3876116	2021-09-02
hCoV-19/Japan/PG-80176/2021	EPI_ISL_3876117	2021-09-02
hCoV-19/Japan/PG-80177/2021	EPI_ISL_3876118	2021-09-02
hCoV-19/Japan/PG-80178/2021	EPI_ISL_3876119	2021-09-02
hCoV-19/Japan/PG-80179/2021	EPI_ISL_3876120	2021-09-02
hCoV-19/Japan/PG-80180/2021	EPI_ISL_3876121	2021-09-02
hCoV-19/Japan/PG-80181/2021	EPI_ISL_3876122	2021-09-02
hCoV-19/Japan/PG-80182/2021	EPI_ISL_3876123	2021-09-02
hCoV-19/Japan/PG-80183/2021	EPI_ISL_3876124	2021-09-02
hCoV-19/Japan/PG-80184/2021	EPI_ISL_3876125	2021-09-02
hCoV-19/Japan/PG-80185/2021	EPI_ISL_3876126	2021-09-02
hCoV-19/Japan/PG-80186/2021	EPI_ISL_3876127	2021-09-02
hCoV-19/Japan/PG-80187/2021	EPI_ISL_3876128	2021-09-02
hCoV-19/Japan/PG-80188/2021	EPI_ISL_3876129	2021-09-02
hCoV-19/Japan/PG-80189/2021	EPI_ISL_3876130	2021-09-02
hCoV-19/Japan/PG-80190/2021	EPI_ISL_3876131	2021-09-02
hCoV-19/Japan/PG-80191/2021	EPI_ISL_3876132	2021-09-02
hCoV-19/Japan/PG-80192/2021	EPI_ISL_3876133	2021-09-02
hCoV-19/Japan/PG-80193/2021	EPI_ISL_3876134	2021-09-02
hCoV-19/Japan/PG-80194/2021	EPI_ISL_3876135	2021-09-02
hCoV-19/Japan/PG-80195/2021	EPI_ISL_3876136	2021-09-02
hCoV-19/Japan/PG-80196/2021	EPI_ISL_3876137	2021-09-02
hCoV-19/Japan/PG-80197/2021	EPI_ISL_3876138	2021-09-02
hCoV-19/Japan/PG-80198/2021	EPI_ISL_3876139	2021-09-02
hCoV-19/Japan/PG-80199/2021	EPI_ISL_3876140	2021-09-02
hCoV-19/Japan/PG-80200/2021	EPI_ISL_3876141	2021-09-02
hCoV-19/Japan/PG-80201/2021	EPI_ISL_3876142	2021-09-02
hCoV-19/Japan/PG-80202/2021	EPI_ISL_3876143	2021-09-02
hCoV-19/Japan/PG-80203/2021	EPI_ISL_3876144	2021-09-02
hCoV-19/Japan/PG-80204/2021	EPI_ISL_3876145	2021-09-02
hCoV-19/Japan/PG-80205/2021	EPI_ISL_3876146	2021-09-02
hCoV-19/Japan/PG-80206/2021	EPI_ISL_3876147	2021-09-02
hCoV-19/Japan/PG-80207/2021	EPI_ISL_3876148	2021-09-02
hCoV-19/Japan/PG-80208/2021	EPI_ISL_3876149	2021-09-02
hCoV-19/Japan/PG-80209/2021	EPI_ISL_3876150	2021-09-02
hCoV-19/Japan/PG-80210/2021	EPI_ISL_3876151	2021-09-02
hCoV-19/Japan/PG-80211/2021	EPI_ISL_3876152	2021-09-02
hCoV-19/Japan/PG-80212/2021	EPI_ISL_3876153	2021-09-02
hCoV-19/Japan/PG-80213/2021	EPI_ISL_3876154	2021-09-02
hCoV-19/Japan/PG-80214/2021	EPI_ISL_3876155	2021-09-02
hCoV-19/Japan/PG-80215/2021	EPI_ISL_3876156	2021-09-02
hCoV-19/Japan/PG-80216/2021	EPI_ISL_3876157	2021-09-02
hCoV-19/Japan/PG-80217/2021	EPI_ISL_3876158	2021-09-02
hCoV-19/Japan/PG-80218/2021	EPI_ISL_3876159	2021-09-02
hCoV-19/Japan/PG-80219/2021	EPI_ISL_3876160	2021-09-02
hCoV-19/Japan/PG-80220/2021	EPI_ISL_3876161	2021-09-02
hCoV-19/Japan/PG-80221/2021	EPI_ISL_3876162	2021-09-02

virus_name	epiID	submission_date
hCoV-19/Japan/PG-80222/2021	EPI_ISL_3876163	2021-09-02
hCoV-19/Japan/PG-80223/2021	EPI_ISL_3876164	2021-09-02
hCoV-19/Japan/PG-80224/2021	EPI_ISL_3876165	2021-09-02
hCoV-19/Japan/PG-80225/2021	EPI_ISL_3876166	2021-09-02
hCoV-19/Japan/PG-80226/2021	EPI_ISL_3876167	2021-09-02
hCoV-19/Japan/PG-80227/2021	EPI_ISL_3875867	2021-09-02
hCoV-19/Japan/PG-80228/2021	EPI_ISL_3875868	2021-09-02
hCoV-19/Japan/PG-80229/2021	EPI_ISL_3875869	2021-09-02
hCoV-19/Japan/PG-80230/2021	EPI_ISL_3875870	2021-09-02
hCoV-19/Japan/PG-80231/2021	EPI_ISL_3875871	2021-09-02
hCoV-19/Japan/PG-80232/2021	EPI_ISL_3875872	2021-09-02
hCoV-19/Japan/PG-80233/2021	EPI_ISL_3875873	2021-09-02
hCoV-19/Japan/PG-80234/2021	EPI_ISL_3875874	2021-09-02
hCoV-19/Japan/PG-80235/2021	EPI_ISL_3875875	2021-09-02
hCoV-19/Japan/PG-80236/2021	EPI_ISL_3875876	2021-09-02
hCoV-19/Japan/PG-80237/2021	EPI_ISL_3875877	2021-09-02
hCoV-19/Japan/PG-80238/2021	EPI_ISL_3875878	2021-09-02
hCoV-19/Japan/PG-80239/2021	EPI_ISL_3875879	2021-09-02
hCoV-19/Japan/PG-80240/2021	EPI_ISL_3875880	2021-09-02
hCoV-19/Japan/PG-80241/2021	EPI_ISL_3875881	2021-09-02
hCoV-19/Japan/PG-80242/2021	EPI_ISL_3875882	2021-09-02
hCoV-19/Japan/PG-80243/2021	EPI_ISL_3870710	2021-09-02
hCoV-19/Japan/PG-80244/2021	EPI_ISL_3870711	2021-09-02
hCoV-19/Japan/PG-80245/2021	EPI_ISL_3870712	2021-09-02
hCoV-19/Japan/PG-80246/2021	EPI_ISL_3870713	2021-09-02
hCoV-19/Japan/PG-80247/2021	EPI_ISL_3870714	2021-09-02
hCoV-19/Japan/PG-80248/2021	EPI_ISL_3870715	2021-09-02
hCoV-19/Japan/PG-80249/2021	EPI_ISL_3870716	2021-09-02
hCoV-19/Japan/PG-80250/2021	EPI_ISL_3870717	2021-09-02
hCoV-19/Japan/PG-80251/2021	EPI_ISL_3870718	2021-09-02
hCoV-19/Japan/PG-80252/2021	EPI_ISL_3870719	2021-09-02
hCoV-19/Japan/PG-80253/2021	EPI_ISL_3870720	2021-09-02
hCoV-19/Japan/PG-80254/2021	EPI_ISL_3870721	2021-09-02
hCoV-19/Japan/PG-80255/2021	EPI_ISL_3870722	2021-09-02
hCoV-19/Japan/PG-80256/2021	EPI_ISL_3870723	2021-09-02
hCoV-19/Japan/PG-80257/2021	EPI_ISL_3870724	2021-09-02
hCoV-19/Japan/PG-80258/2021	EPI_ISL_3870725	2021-09-02
hCoV-19/Japan/PG-80259/2021	EPI_ISL_3870726	2021-09-02
hCoV-19/Japan/PG-80260/2021	EPI_ISL_3870727	2021-09-02
hCoV-19/Japan/PG-80261/2021	EPI_ISL_3870728	2021-09-02
hCoV-19/Japan/PG-80262/2021	EPI_ISL_3870729	2021-09-02
hCoV-19/Japan/PG-80263/2021	EPI_ISL_3870730	2021-09-02
hCoV-19/Japan/PG-80264/2021	EPI_ISL_3870731	2021-09-02
hCoV-19/Japan/PG-80265/2021	EPI_ISL_3870732	2021-09-02
hCoV-19/Japan/PG-80266/2021	EPI_ISL_3870733	2021-09-02
hCoV-19/Japan/PG-80267/2021	EPI_ISL_3870734	2021-09-02
hCoV-19/Japan/PG-80268/2021	EPI_ISL_3870735	2021-09-02
hCoV-19/Japan/PG-80269/2021	EPI_ISL_3870736	2021-09-02
hCoV-19/Japan/PG-80270/2021	EPI_ISL_3870737	2021-09-02
hCoV-19/Japan/PG-80271/2021	EPI_ISL_3870738	2021-09-02
hCoV-19/Japan/PG-80272/2021	EPI_ISL_3870739	2021-09-02
hCoV-19/Japan/PG-80273/2021	EPI_ISL_3870740	2021-09-02
hCoV-19/Japan/PG-80274/2021	EPI_ISL_3870741	2021-09-02
hCoV-19/Japan/PG-80275/2021	EPI_ISL_3870742	2021-09-02

virus_name	epiID	submission_date
hCoV-19/Japan/PG-80276/2021	EPI_ISL_3870743	2021-09-02
hCoV-19/Japan/PG-80277/2021	EPI_ISL_3870744	2021-09-02
hCoV-19/Japan/PG-80278/2021	EPI_ISL_3870745	2021-09-02
hCoV-19/Japan/PG-80279/2021	EPI_ISL_3870746	2021-09-02
hCoV-19/Japan/PG-80280/2021	EPI_ISL_3870747	2021-09-02
hCoV-19/Japan/PG-80281/2021	EPI_ISL_3870748	2021-09-02
hCoV-19/Japan/PG-80282/2021	EPI_ISL_3870749	2021-09-02
hCoV-19/Japan/PG-80283/2021	EPI_ISL_3870750	2021-09-02
hCoV-19/Japan/PG-80284/2021	EPI_ISL_3870751	2021-09-02
hCoV-19/Japan/PG-80285/2021	EPI_ISL_3870752	2021-09-02
hCoV-19/Japan/PG-80286/2021	EPI_ISL_3870753	2021-09-02
hCoV-19/Japan/PG-80287/2021	EPI_ISL_3870754	2021-09-02
hCoV-19/Japan/PG-80288/2021	EPI_ISL_3870755	2021-09-02
hCoV-19/Japan/PG-80289/2021	EPI_ISL_3870756	2021-09-02
hCoV-19/Japan/PG-80290/2021	EPI_ISL_3870757	2021-09-02
hCoV-19/Japan/PG-80291/2021	EPI_ISL_3870758	2021-09-02
hCoV-19/Japan/PG-80292/2021	EPI_ISL_3873447	2021-09-02
hCoV-19/Japan/PG-80293/2021	EPI_ISL_3873448	2021-09-02
hCoV-19/Japan/PG-80294/2021	EPI_ISL_3873449	2021-09-02
hCoV-19/Japan/PG-80295/2021	EPI_ISL_3873450	2021-09-02
hCoV-19/Japan/PG-80296/2021	EPI_ISL_3873451	2021-09-02
hCoV-19/Japan/PG-80297/2021	EPI_ISL_3873452	2021-09-02
hCoV-19/Japan/PG-80298/2021	EPI_ISL_3873453	2021-09-02
hCoV-19/Japan/PG-80299/2021	EPI_ISL_3873454	2021-09-02
hCoV-19/Japan/PG-80300/2021	EPI_ISL_3873455	2021-09-02
hCoV-19/Japan/PG-80301/2021	EPI_ISL_3873456	2021-09-02
hCoV-19/Japan/PG-80302/2021	EPI_ISL_3873457	2021-09-02
hCoV-19/Japan/PG-80303/2021	EPI_ISL_3873458	2021-09-02
hCoV-19/Japan/PG-80304/2021	EPI_ISL_3873459	2021-09-02
hCoV-19/Japan/PG-80305/2021	EPI_ISL_3873460	2021-09-02
hCoV-19/Japan/PG-80306/2021	EPI_ISL_3873461	2021-09-02
hCoV-19/Japan/PG-80307/2021	EPI_ISL_3873462	2021-09-02
hCoV-19/Japan/PG-80308/2021	EPI_ISL_3873463	2021-09-02
hCoV-19/Japan/PG-80309/2021	EPI_ISL_3873464	2021-09-02
hCoV-19/Japan/PG-80310/2021	EPI_ISL_3873465	2021-09-02
hCoV-19/Japan/PG-80311/2021	EPI_ISL_3873466	2021-09-02
hCoV-19/Japan/PG-80312/2021	EPI_ISL_3873467	2021-09-02
hCoV-19/Japan/PG-80313/2021	EPI_ISL_3872427	2021-09-02
hCoV-19/Japan/PG-80314/2021	EPI_ISL_3872428	2021-09-02
hCoV-19/Japan/PG-80315/2021	EPI_ISL_3872429	2021-09-02
hCoV-19/Japan/PG-80316/2021	EPI_ISL_3872430	2021-09-02
hCoV-19/Japan/PG-80317/2021	EPI_ISL_3872431	2021-09-02
hCoV-19/Japan/PG-80318/2021	EPI_ISL_3872432	2021-09-02
hCoV-19/Japan/PG-80319/2021	EPI_ISL_3872433	2021-09-02
hCoV-19/Japan/PG-80320/2021	EPI_ISL_3872434	2021-09-02
hCoV-19/Japan/PG-80321/2021	EPI_ISL_3872435	2021-09-02
hCoV-19/Japan/PG-80322/2021	EPI_ISL_3872436	2021-09-02
hCoV-19/Japan/PG-80323/2021	EPI_ISL_3872437	2021-09-02
hCoV-19/Japan/PG-80324/2021	EPI_ISL_3872438	2021-09-02
hCoV-19/Japan/PG-80325/2021	EPI_ISL_3872439	2021-09-02
hCoV-19/Japan/PG-80326/2021	EPI_ISL_3872440	2021-09-02
hCoV-19/Japan/PG-80327/2021	EPI_ISL_3872441	2021-09-02
hCoV-19/Japan/PG-80328/2021	EPI_ISL_3872442	2021-09-02
hCoV-19/Japan/PG-80329/2021	EPI_ISL_3872443	2021-09-02

virus_name	epiID	submission_date
hCoV-19/Japan/PG-80330/2021	EPI_ISL_3872444	2021-09-02
hCoV-19/Japan/PG-80331/2021	EPI_ISL_3872445	2021-09-02
hCoV-19/Japan/PG-80332/2021	EPI_ISL_3872446	2021-09-02
hCoV-19/Japan/PG-80333/2021	EPI_ISL_3872447	2021-09-02
hCoV-19/Japan/PG-80334/2021	EPI_ISL_3872448	2021-09-02
hCoV-19/Japan/PG-80335/2021	EPI_ISL_3872449	2021-09-02
hCoV-19/Japan/PG-80336/2021	EPI_ISL_3872775	2021-09-02
hCoV-19/Japan/PG-80337/2021	EPI_ISL_3872776	2021-09-02
hCoV-19/Japan/PG-80338/2021	EPI_ISL_3872777	2021-09-02
hCoV-19/Japan/PG-80339/2021	EPI_ISL_3872778	2021-09-02
hCoV-19/Japan/PG-80340/2021	EPI_ISL_3872779	2021-09-02
hCoV-19/Japan/PG-80341/2021	EPI_ISL_3872780	2021-09-02
hCoV-19/Japan/PG-80342/2021	EPI_ISL_3872781	2021-09-02
hCoV-19/Japan/PG-80343/2021	EPI_ISL_3872782	2021-09-02
hCoV-19/Japan/PG-80344/2021	EPI_ISL_3872783	2021-09-02
hCoV-19/Japan/PG-80345/2021	EPI_ISL_3872784	2021-09-02
hCoV-19/Japan/PG-80346/2021	EPI_ISL_3872785	2021-09-02
hCoV-19/Japan/PG-80347/2021	EPI_ISL_3872786	2021-09-02
hCoV-19/Japan/PG-80348/2021	EPI_ISL_3872787	2021-09-02
hCoV-19/Japan/PG-80349/2021	EPI_ISL_3872788	2021-09-02
hCoV-19/Japan/PG-80350/2021	EPI_ISL_3872789	2021-09-02
hCoV-19/Japan/PG-80351/2021	EPI_ISL_3872790	2021-09-02
hCoV-19/Japan/PG-80352/2021	EPI_ISL_3872791	2021-09-02
hCoV-19/Japan/PG-80353/2021	EPI_ISL_3872792	2021-09-02
hCoV-19/Japan/PG-80354/2021	EPI_ISL_3872793	2021-09-02
hCoV-19/Japan/PG-80355/2021	EPI_ISL_3872794	2021-09-02
hCoV-19/Japan/PG-80356/2021	EPI_ISL_3872795	2021-09-02
hCoV-19/Japan/PG-80357/2021	EPI_ISL_3872796	2021-09-02
hCoV-19/Japan/PG-80358/2021	EPI_ISL_3872797	2021-09-02
hCoV-19/Japan/PG-80359/2021	EPI_ISL_3872798	2021-09-02
hCoV-19/Japan/PG-80360/2021	EPI_ISL_3872799	2021-09-02
hCoV-19/Japan/PG-80361/2021	EPI_ISL_3872800	2021-09-02
hCoV-19/Japan/PG-80362/2021	EPI_ISL_3872801	2021-09-02
hCoV-19/Japan/PG-80363/2021	EPI_ISL_3872802	2021-09-02
hCoV-19/Japan/PG-80364/2021	EPI_ISL_3872803	2021-09-02
hCoV-19/Japan/PG-80365/2021	EPI_ISL_3872804	2021-09-02
hCoV-19/Japan/PG-80366/2021	EPI_ISL_3872805	2021-09-02
hCoV-19/Japan/PG-80367/2021	EPI_ISL_3872806	2021-09-02
hCoV-19/Japan/PG-80368/2021	EPI_ISL_3872807	2021-09-02
hCoV-19/Japan/PG-80369/2021	EPI_ISL_3872808	2021-09-02
hCoV-19/Japan/PG-80370/2021	EPI_ISL_3872809	2021-09-02
hCoV-19/Japan/PG-80371/2021	EPI_ISL_3872810	2021-09-02
hCoV-19/Japan/PG-80372/2021	EPI_ISL_3872811	2021-09-02
hCoV-19/Japan/PG-80373/2021	EPI_ISL_3872812	2021-09-02
hCoV-19/Japan/PG-80374/2021	EPI_ISL_3872813	2021-09-02
hCoV-19/Japan/PG-80375/2021	EPI_ISL_3872814	2021-09-02
hCoV-19/Japan/PG-80376/2021	EPI_ISL_3872815	2021-09-02
hCoV-19/Japan/PG-80377/2021	EPI_ISL_3872816	2021-09-02
hCoV-19/Japan/PG-80378/2021	EPI_ISL_3872817	2021-09-02
hCoV-19/Japan/PG-80379/2021	EPI_ISL_3872818	2021-09-02
hCoV-19/Japan/PG-80380/2021	EPI_ISL_3872819	2021-09-02
hCoV-19/Japan/PG-80381/2021	EPI_ISL_3872820	2021-09-02
hCoV-19/Japan/PG-80382/2021	EPI_ISL_3872821	2021-09-02
hCoV-19/Japan/PG-80383/2021	EPI_ISL_3872822	2021-09-02

virus_name	epiID	submission_date
hCoV-19/Japan/PG-80384/2021	EPI_ISL_3872823	2021-09-02
hCoV-19/Japan/PG-80385/2021	EPI_ISL_3872824	2021-09-02
hCoV-19/Japan/PG-80386/2021	EPI_ISL_3872825	2021-09-02
hCoV-19/Japan/PG-80387/2021	EPI_ISL_3872826	2021-09-02
hCoV-19/Japan/PG-80388/2021	EPI_ISL_3872827	2021-09-02
hCoV-19/Japan/PG-80389/2021	EPI_ISL_3872828	2021-09-02
hCoV-19/Japan/PG-80390/2021	EPI_ISL_3872829	2021-09-02
hCoV-19/Japan/PG-80391/2021	EPI_ISL_3872830	2021-09-02
hCoV-19/Japan/PG-80392/2021	EPI_ISL_3872831	2021-09-02
hCoV-19/Japan/PG-80393/2021	EPI_ISL_3872832	2021-09-02
hCoV-19/Japan/PG-80394/2021	EPI_ISL_3872833	2021-09-02
hCoV-19/Japan/PG-80395/2021	EPI_ISL_3872834	2021-09-02
hCoV-19/Japan/PG-80396/2021	EPI_ISL_3872835	2021-09-02
hCoV-19/Japan/PG-80397/2021	EPI_ISL_3872836	2021-09-02
hCoV-19/Japan/PG-80398/2021	EPI_ISL_3872837	2021-09-02
hCoV-19/Japan/PG-80399/2021	EPI_ISL_3872838	2021-09-02
hCoV-19/Japan/PG-80400/2021	EPI_ISL_3872839	2021-09-02
hCoV-19/Japan/PG-80401/2021	EPI_ISL_3872840	2021-09-02
hCoV-19/Japan/PG-80402/2021	EPI_ISL_3872841	2021-09-02
hCoV-19/Japan/PG-80403/2021	EPI_ISL_3872842	2021-09-02
hCoV-19/Japan/PG-80404/2021	EPI_ISL_3872843	2021-09-02
hCoV-19/Japan/PG-80405/2021	EPI_ISL_3872844	2021-09-02
hCoV-19/Japan/PG-80406/2021	EPI_ISL_3872845	2021-09-02
hCoV-19/Japan/PG-80407/2021	EPI_ISL_3872846	2021-09-02
hCoV-19/Japan/PG-80408/2021	EPI_ISL_3872847	2021-09-02
hCoV-19/Japan/PG-80409/2021	EPI_ISL_3872848	2021-09-02
hCoV-19/Japan/PG-80410/2021	EPI_ISL_3872849	2021-09-02
hCoV-19/Japan/PG-80411/2021	EPI_ISL_3872850	2021-09-02
hCoV-19/Japan/PG-80412/2021	EPI_ISL_3872851	2021-09-02
hCoV-19/Japan/PG-80413/2021	EPI_ISL_3872852	2021-09-02
hCoV-19/Japan/PG-80414/2021	EPI_ISL_3872853	2021-09-02
hCoV-19/Japan/PG-80415/2021	EPI_ISL_3872854	2021-09-02
hCoV-19/Japan/PG-80416/2021	EPI_ISL_3872855	2021-09-02
hCoV-19/Japan/PG-80417/2021	EPI_ISL_3872856	2021-09-02
hCoV-19/Japan/PG-80418/2021	EPI_ISL_3872857	2021-09-02
hCoV-19/Japan/PG-80419/2021	EPI_ISL_3872858	2021-09-02
hCoV-19/Japan/PG-80420/2021	EPI_ISL_3872859	2021-09-02
hCoV-19/Japan/PG-80421/2021	EPI_ISL_3871614	2021-09-02
hCoV-19/Japan/PG-80422/2021	EPI_ISL_3871615	2021-09-02
hCoV-19/Japan/PG-80423/2021	EPI_ISL_3871616	2021-09-02
hCoV-19/Japan/PG-80424/2021	EPI_ISL_3871617	2021-09-02
hCoV-19/Japan/PG-80425/2021	EPI_ISL_3871618	2021-09-02
hCoV-19/Japan/PG-80426/2021	EPI_ISL_3871619	2021-09-02
hCoV-19/Japan/PG-80427/2021	EPI_ISL_3871620	2021-09-02
hCoV-19/Japan/PG-80428/2021	EPI_ISL_3871621	2021-09-02
hCoV-19/Japan/PG-80429/2021	EPI_ISL_3871622	2021-09-02
hCoV-19/Japan/PG-80430/2021	EPI_ISL_3871623	2021-09-02
hCoV-19/Japan/PG-80431/2021	EPI_ISL_3871624	2021-09-02
hCoV-19/Japan/PG-80432/2021	EPI_ISL_3871625	2021-09-02
hCoV-19/Japan/PG-80433/2021	EPI_ISL_3871626	2021-09-02
hCoV-19/Japan/PG-80434/2021	EPI_ISL_3871627	2021-09-02
hCoV-19/Japan/PG-80435/2021	EPI_ISL_3871628	2021-09-02
hCoV-19/Japan/PG-80436/2021	EPI_ISL_3871629	2021-09-02
hCoV-19/Japan/PG-80437/2021	EPI_ISL_3871630	2021-09-02

virus_name	epiID	submission_date
hCoV-19/Japan/PG-80438/2021	EPI_ISL_3871631	2021-09-02
hCoV-19/Japan/PG-80439/2021	EPI_ISL_3871632	2021-09-02
hCoV-19/Japan/PG-80440/2021	EPI_ISL_3871633	2021-09-02
hCoV-19/Japan/PG-80441/2021	EPI_ISL_3892182	2021-09-02
hCoV-19/Japan/PG-80442/2021	EPI_ISL_3892184	2021-09-02
hCoV-19/Japan/PG-80443/2021	EPI_ISL_3892186	2021-09-02
hCoV-19/Japan/PG-80444/2021	EPI_ISL_3892189	2021-09-02
hCoV-19/Japan/PG-80445/2021	EPI_ISL_3892191	2021-09-02
hCoV-19/Japan/PG-80446/2021	EPI_ISL_3892193	2021-09-02
hCoV-19/Japan/PG-80447/2021	EPI_ISL_3892196	2021-09-02
hCoV-19/Japan/PG-80448/2021	EPI_ISL_3892197	2021-09-02
hCoV-19/Japan/PG-80449/2021	EPI_ISL_3871283	2021-09-02
hCoV-19/Japan/PG-80450/2021	EPI_ISL_3871284	2021-09-02
hCoV-19/Japan/PG-80451/2021	EPI_ISL_3871285	2021-09-02
hCoV-19/Japan/PG-80452/2021	EPI_ISL_3871286	2021-09-02
hCoV-19/Japan/PG-80453/2021	EPI_ISL_3871287	2021-09-02
hCoV-19/Japan/PG-80454/2021	EPI_ISL_3871288	2021-09-02
hCoV-19/Japan/PG-80455/2021	EPI_ISL_3871289	2021-09-02
hCoV-19/Japan/PG-80456/2021	EPI_ISL_3871290	2021-09-02
hCoV-19/Japan/PG-80463/2021	EPI_ISL_3874028	2021-09-02
hCoV-19/Japan/PG-80466/2021	EPI_ISL_3874029	2021-09-02
hCoV-19/Japan/PG-80467/2021	EPI_ISL_3874030	2021-09-02
hCoV-19/Japan/PG-80473/2021	EPI_ISL_3874031	2021-09-02
hCoV-19/Japan/PG-80474/2021	EPI_ISL_3874032	2021-09-02
hCoV-19/Japan/PG-80475/2021	EPI_ISL_3874033	2021-09-02
hCoV-19/Japan/PG-80476/2021	EPI_ISL_3874034	2021-09-02
hCoV-19/Japan/PG-80477/2021	EPI_ISL_3874035	2021-09-02
hCoV-19/Japan/PG-80478/2021	EPI_ISL_3874036	2021-09-02
hCoV-19/Japan/PG-80479/2021	EPI_ISL_3872041	2021-09-02
hCoV-19/Japan/PG-80480/2021	EPI_ISL_3872042	2021-09-02
hCoV-19/Japan/PG-80481/2021	EPI_ISL_3872043	2021-09-02
hCoV-19/Japan/PG-80482/2021	EPI_ISL_3872044	2021-09-02
hCoV-19/Japan/PG-80483/2021	EPI_ISL_3872045	2021-09-02
hCoV-19/Japan/PG-80484/2021	EPI_ISL_3872046	2021-09-02
hCoV-19/Japan/PG-80485/2021	EPI_ISL_3872047	2021-09-02
hCoV-19/Japan/PG-80486/2021	EPI_ISL_3872048	2021-09-02
hCoV-19/Japan/PG-80487/2021	EPI_ISL_3872049	2021-09-02
hCoV-19/Japan/PG-80488/2021	EPI_ISL_3872050	2021-09-02
hCoV-19/Japan/PG-80489/2021	EPI_ISL_3872051	2021-09-02
hCoV-19/Japan/PG-80490/2021	EPI_ISL_3872052	2021-09-02
hCoV-19/Japan/PG-80491/2021	EPI_ISL_3872053	2021-09-02
hCoV-19/Japan/PG-80492/2021	EPI_ISL_3872054	2021-09-02
hCoV-19/Japan/PG-80493/2021	EPI_ISL_3872055	2021-09-02
hCoV-19/Japan/PG-80494/2021	EPI_ISL_3872056	2021-09-02
hCoV-19/Japan/PG-80495/2021	EPI_ISL_3872057	2021-09-02
hCoV-19/Japan/PG-80496/2021	EPI_ISL_3872058	2021-09-02
hCoV-19/Japan/PG-80497/2021	EPI_ISL_3872059	2021-09-02
hCoV-19/Japan/PG-80498/2021	EPI_ISL_3872060	2021-09-02
hCoV-19/Japan/PG-80499/2021	EPI_ISL_3872061	2021-09-02
hCoV-19/Japan/PG-80500/2021	EPI_ISL_3872062	2021-09-02
hCoV-19/Japan/PG-80501/2021	EPI_ISL_3872063	2021-09-02
hCoV-19/Japan/PG-80502/2021	EPI_ISL_3872064	2021-09-02
hCoV-19/Japan/PG-80503/2021	EPI_ISL_3872065	2021-09-02
hCoV-19/Japan/PG-80504/2021	EPI_ISL_3872066	2021-09-02

virus_name	epiID	submission_date
hCoV-19/Japan/PG-80505/2021	EPI_ISL_3872067	2021-09-02
hCoV-19/Japan/PG-80506/2021	EPI_ISL_3872068	2021-09-02
hCoV-19/Japan/PG-80507/2021	EPI_ISL_3872069	2021-09-02
hCoV-19/Japan/PG-80508/2021	EPI_ISL_3872070	2021-09-02
hCoV-19/Japan/PG-80509/2021	EPI_ISL_3872071	2021-09-02
hCoV-19/Japan/PG-80510/2021	EPI_ISL_3872072	2021-09-02
hCoV-19/Japan/PG-80511/2021	EPI_ISL_3872073	2021-09-02
hCoV-19/Japan/PG-80512/2021	EPI_ISL_3872074	2021-09-02
hCoV-19/Japan/PG-80513/2021	EPI_ISL_3872075	2021-09-02
hCoV-19/Japan/PG-80514/2021	EPI_ISL_3872076	2021-09-02
hCoV-19/Japan/PG-80515/2021	EPI_ISL_3872077	2021-09-02
hCoV-19/Japan/PG-80516/2021	EPI_ISL_3872078	2021-09-02
hCoV-19/Japan/PG-80517/2021	EPI_ISL_3872079	2021-09-02
hCoV-19/Japan/PG-80518/2021	EPI_ISL_3872080	2021-09-02
hCoV-19/Japan/PG-80519/2021	EPI_ISL_3872081	2021-09-02
hCoV-19/Japan/PG-80520/2021	EPI_ISL_3872082	2021-09-02
hCoV-19/Japan/PG-80521/2021	EPI_ISL_3872083	2021-09-02
hCoV-19/Japan/PG-80522/2021	EPI_ISL_3872356	2021-09-02
hCoV-19/Japan/PG-80523/2021	EPI_ISL_3872357	2021-09-02
hCoV-19/Japan/PG-80524/2021	EPI_ISL_3872358	2021-09-02
hCoV-19/Japan/PG-80525/2021	EPI_ISL_3872359	2021-09-02
hCoV-19/Japan/PG-80528/2021	EPI_ISL_3874347	2021-09-02
hCoV-19/Japan/PG-80529/2021	EPI_ISL_3874348	2021-09-02
hCoV-19/Japan/PG-80530/2021	EPI_ISL_3874349	2021-09-02
hCoV-19/Japan/PG-80531/2021	EPI_ISL_3874350	2021-09-02
hCoV-19/Japan/PG-80532/2021	EPI_ISL_3874351	2021-09-02
hCoV-19/Japan/PG-80533/2021	EPI_ISL_3874352	2021-09-02
hCoV-19/Japan/PG-80534/2021	EPI_ISL_3874353	2021-09-02
hCoV-19/Japan/PG-80535/2021	EPI_ISL_3874354	2021-09-02
hCoV-19/Japan/PG-80536/2021	EPI_ISL_3874355	2021-09-02
hCoV-19/Japan/PG-80537/2021	EPI_ISL_3874356	2021-09-02
hCoV-19/Japan/PG-80538/2021	EPI_ISL_3874357	2021-09-02
hCoV-19/Japan/PG-80539/2021	EPI_ISL_3874358	2021-09-02
hCoV-19/Japan/PG-80540/2021	EPI_ISL_3874359	2021-09-02
hCoV-19/Japan/PG-80541/2021	EPI_ISL_3874360	2021-09-02
hCoV-19/Japan/PG-80542/2021	EPI_ISL_3874361	2021-09-02
hCoV-19/Japan/PG-80543/2021	EPI_ISL_3874362	2021-09-02
hCoV-19/Japan/PG-80544/2021	EPI_ISL_3874363	2021-09-02
hCoV-19/Japan/PG-80545/2021	EPI_ISL_3874364	2021-09-02
hCoV-19/Japan/PG-80546/2021	EPI_ISL_3874365	2021-09-02
hCoV-19/Japan/PG-80547/2021	EPI_ISL_3874366	2021-09-02
hCoV-19/Japan/PG-80548/2021	EPI_ISL_3874367	2021-09-02
hCoV-19/Japan/PG-80549/2021	EPI_ISL_3874368	2021-09-02
hCoV-19/Japan/PG-80550/2021	EPI_ISL_3874369	2021-09-02
hCoV-19/Japan/PG-80551/2021	EPI_ISL_3874370	2021-09-02
hCoV-19/Japan/PG-80573/2021	EPI_ISL_3872174	2021-09-02
hCoV-19/Japan/PG-80574/2021	EPI_ISL_3872175	2021-09-02
hCoV-19/Japan/PG-80575/2021	EPI_ISL_3872176	2021-09-02
hCoV-19/Japan/PG-80576/2021	EPI_ISL_3872177	2021-09-02
hCoV-19/Japan/PG-80577/2021	EPI_ISL_3872178	2021-09-02
hCoV-19/Japan/PG-80578/2021	EPI_ISL_3872179	2021-09-02
hCoV-19/Japan/PG-80579/2021	EPI_ISL_3872180	2021-09-02
hCoV-19/Japan/PG-80580/2021	EPI_ISL_3872181	2021-09-02
hCoV-19/Japan/PG-80581/2021	EPI_ISL_3872182	2021-09-02

virus_name	epiID	submission_date
hCoV-19/Japan/PG-80582/2021	EPI_ISL_3872183	2021-09-02
hCoV-19/Japan/PG-80583/2021	EPI_ISL_3872184	2021-09-02
hCoV-19/Japan/PG-80584/2021	EPI_ISL_3872185	2021-09-02
hCoV-19/Japan/PG-80585/2021	EPI_ISL_3872186	2021-09-02
hCoV-19/Japan/PG-80586/2021	EPI_ISL_3872187	2021-09-02
hCoV-19/Japan/PG-80587/2021	EPI_ISL_3872188	2021-09-02
hCoV-19/Japan/PG-80588/2021	EPI_ISL_3872189	2021-09-02
hCoV-19/Japan/PG-80589/2021	EPI_ISL_3872190	2021-09-02
hCoV-19/Japan/PG-80590/2021	EPI_ISL_3872191	2021-09-02
hCoV-19/Japan/PG-80591/2021	EPI_ISL_3872192	2021-09-02
hCoV-19/Japan/PG-80592/2021	EPI_ISL_3872193	2021-09-02
hCoV-19/Japan/PG-80593/2021	EPI_ISL_3872194	2021-09-02
hCoV-19/Japan/PG-80594/2021	EPI_ISL_3872195	2021-09-02
hCoV-19/Japan/PG-80595/2021	EPI_ISL_3872196	2021-09-02
hCoV-19/Japan/PG-80596/2021	EPI_ISL_3872298	2021-09-02
hCoV-19/Japan/PG-80597/2021	EPI_ISL_3872299	2021-09-02
hCoV-19/Japan/PG-80598/2021	EPI_ISL_3872300	2021-09-02
hCoV-19/Japan/PG-80599/2021	EPI_ISL_3872301	2021-09-02
hCoV-19/Japan/PG-80600/2021	EPI_ISL_3892200	2021-09-02
hCoV-19/Japan/PG-80601/2021	EPI_ISL_3892202	2021-09-02
hCoV-19/Japan/PG-80602/2021	EPI_ISL_3892205	2021-09-02
hCoV-19/Japan/PG-80603/2021	EPI_ISL_3892208	2021-09-02
hCoV-19/Japan/PG-80604/2021	EPI_ISL_3892211	2021-09-02
hCoV-19/Japan/PG-80605/2021	EPI_ISL_3892213	2021-09-02
hCoV-19/Japan/PG-80606/2021	EPI_ISL_3892216	2021-09-02
hCoV-19/Japan/PG-80607/2021	EPI_ISL_3892218	2021-09-02
hCoV-19/Japan/PG-80608/2021	EPI_ISL_3892221	2021-09-02
hCoV-19/Japan/PG-80609/2021	EPI_ISL_3892224	2021-09-02
hCoV-19/Japan/PG-80610/2021	EPI_ISL_3892226	2021-09-02
hCoV-19/Japan/PG-80611/2021	EPI_ISL_3892229	2021-09-02
hCoV-19/Japan/PG-80612/2021	EPI_ISL_3892232	2021-09-02
hCoV-19/Japan/PG-80613/2021	EPI_ISL_3892235	2021-09-02
hCoV-19/Japan/PG-80614/2021	EPI_ISL_3892238	2021-09-02
hCoV-19/Japan/PG-80615/2021	EPI_ISL_3892240	2021-09-02
hCoV-19/Japan/PG-80616/2021	EPI_ISL_3892243	2021-09-02
hCoV-19/Japan/PG-80617/2021	EPI_ISL_3892246	2021-09-02
hCoV-19/Japan/PG-80618/2021	EPI_ISL_3892249	2021-09-02
hCoV-19/Japan/PG-80619/2021	EPI_ISL_3892251	2021-09-02
hCoV-19/Japan/PG-80620/2021	EPI_ISL_3892253	2021-09-02
hCoV-19/Japan/PG-80621/2021	EPI_ISL_3892256	2021-09-02
hCoV-19/Japan/PG-80622/2021	EPI_ISL_3892257	2021-09-02
hCoV-19/Japan/PG-80623/2021	EPI_ISL_3892260	2021-09-02
hCoV-19/Japan/PG-80624/2021	EPI_ISL_3892262	2021-09-02
hCoV-19/Japan/PG-80625/2021	EPI_ISL_3892263	2021-09-02
hCoV-19/Japan/PG-80626/2021	EPI_ISL_3892265	2021-09-02
hCoV-19/Japan/PG-80627/2021	EPI_ISL_3892267	2021-09-02
hCoV-19/Japan/PG-80628/2021	EPI_ISL_3892270	2021-09-02
hCoV-19/Japan/PG-80629/2021	EPI_ISL_3892272	2021-09-02
hCoV-19/Japan/PG-80630/2021	EPI_ISL_3892274	2021-09-02
hCoV-19/Japan/PG-80631/2021	EPI_ISL_3892277	2021-09-02
hCoV-19/Japan/PG-80632/2021	EPI_ISL_3892280	2021-09-02
hCoV-19/Japan/PG-80633/2021	EPI_ISL_3892282	2021-09-02
hCoV-19/Japan/PG-80634/2021	EPI_ISL_3892283	2021-09-02
hCoV-19/Japan/PG-80635/2021	EPI_ISL_3892285	2021-09-02

virus_name	epiID	submission_date
hCoV-19/Japan/PG-80636/2021	EPI_ISL_3892287	2021-09-02
hCoV-19/Japan/PG-80637/2021	EPI_ISL_3892290	2021-09-02
hCoV-19/Japan/PG-80638/2021	EPI_ISL_3892292	2021-09-02
hCoV-19/Japan/PG-80639/2021	EPI_ISL_3892295	2021-09-02
hCoV-19/Japan/PG-80640/2021	EPI_ISL_3892298	2021-09-02
hCoV-19/Japan/PG-80641/2021	EPI_ISL_3892300	2021-09-02
hCoV-19/Japan/PG-80642/2021	EPI_ISL_3892303	2021-09-02
hCoV-19/Japan/PG-80643/2021	EPI_ISL_3892305	2021-09-02
hCoV-19/Japan/PG-80644/2021	EPI_ISL_3892307	2021-09-02
hCoV-19/Japan/PG-80645/2021	EPI_ISL_3892309	2021-09-02
hCoV-19/Japan/PG-80646/2021	EPI_ISL_3892312	2021-09-02
hCoV-19/Japan/PG-80647/2021	EPI_ISL_3892314	2021-09-02
hCoV-19/Japan/PG-80648/2021	EPI_ISL_3892317	2021-09-02
hCoV-19/Japan/PG-80649/2021	EPI_ISL_3892319	2021-09-02
hCoV-19/Japan/PG-80650/2021	EPI_ISL_3892320	2021-09-02
hCoV-19/Japan/PG-80651/2021	EPI_ISL_3892323	2021-09-02
hCoV-19/Japan/PG-80652/2021	EPI_ISL_3892326	2021-09-02
hCoV-19/Japan/PG-80653/2021	EPI_ISL_3892329	2021-09-02
hCoV-19/Japan/PG-80654/2021	EPI_ISL_3892332	2021-09-02
hCoV-19/Japan/PG-80655/2021	EPI_ISL_3892335	2021-09-02
hCoV-19/Japan/PG-80656/2021	EPI_ISL_3892337	2021-09-02
hCoV-19/Japan/PG-80657/2021	EPI_ISL_3892340	2021-09-02
hCoV-19/Japan/PG-80658/2021	EPI_ISL_3892342	2021-09-02
hCoV-19/Japan/PG-80659/2021	EPI_ISL_3892345	2021-09-02
hCoV-19/Japan/PG-80660/2021	EPI_ISL_3892348	2021-09-02
hCoV-19/Japan/PG-80661/2021	EPI_ISL_3892350	2021-09-02
hCoV-19/Japan/PG-80662/2021	EPI_ISL_3892353	2021-09-02
hCoV-19/Japan/PG-80663/2021	EPI_ISL_3892354	2021-09-02
hCoV-19/Japan/PG-80664/2021	EPI_ISL_3892357	2021-09-02
hCoV-19/Japan/PG-80665/2021	EPI_ISL_3892359	2021-09-02
hCoV-19/Japan/PG-80666/2021	EPI_ISL_3892361	2021-09-02
hCoV-19/Japan/PG-80667/2021	EPI_ISL_3892365	2021-09-02
hCoV-19/Japan/PG-80668/2021	EPI_ISL_3892367	2021-09-02
hCoV-19/Japan/PG-80669/2021	EPI_ISL_3892370	2021-09-02
hCoV-19/Japan/PG-80670/2021	EPI_ISL_3892372	2021-09-02
hCoV-19/Japan/PG-80671/2021	EPI_ISL_3892374	2021-09-02
hCoV-19/Japan/PG-80672/2021	EPI_ISL_3892375	2021-09-02
hCoV-19/Japan/PG-80673/2021	EPI_ISL_3892376	2021-09-02
hCoV-19/Japan/PG-80674/2021	EPI_ISL_3892378	2021-09-02
hCoV-19/Japan/PG-80675/2021	EPI_ISL_3892380	2021-09-02
hCoV-19/Japan/PG-80676/2021	EPI_ISL_3892383	2021-09-02
hCoV-19/Japan/PG-80677/2021	EPI_ISL_3892385	2021-09-02
hCoV-19/Japan/PG-80678/2021	EPI_ISL_3892387	2021-09-02
hCoV-19/Japan/PG-80679/2021	EPI_ISL_3892388	2021-09-02
hCoV-19/Japan/PG-80680/2021	EPI_ISL_3892389	2021-09-02
hCoV-19/Japan/PG-80681/2021	EPI_ISL_3892390	2021-09-02
hCoV-19/Japan/PG-80682/2021	EPI_ISL_3892391	2021-09-02
hCoV-19/Japan/PG-80683/2021	EPI_ISL_3892392	2021-09-02
hCoV-19/Japan/PG-80684/2021	EPI_ISL_3892393	2021-09-02
hCoV-19/Japan/PG-80685/2021	EPI_ISL_3892396	2021-09-02
hCoV-19/Japan/PG-80686/2021	EPI_ISL_3892397	2021-09-02
hCoV-19/Japan/PG-80687/2021	EPI_ISL_3892399	2021-09-02
hCoV-19/Japan/PG-80688/2021	EPI_ISL_3892401	2021-09-02
hCoV-19/Japan/PG-80689/2021	EPI_ISL_3892404	2021-09-02

virus_name	epiID	submission_date
hCoV-19/Japan/PG-80690/2021	EPI_ISL_3892407	2021-09-02
hCoV-19/Japan/PG-80691/2021	EPI_ISL_3892410	2021-09-02
hCoV-19/Japan/PG-80692/2021	EPI_ISL_3892412	2021-09-02
hCoV-19/Japan/PG-80693/2021	EPI_ISL_3892415	2021-09-02
hCoV-19/Japan/PG-80694/2021	EPI_ISL_3892418	2021-09-02
hCoV-19/Japan/PG-80695/2021	EPI_ISL_3892420	2021-09-02
hCoV-19/Japan/PG-80696/2021	EPI_ISL_3892423	2021-09-02
hCoV-19/Japan/PG-80697/2021	EPI_ISL_3892426	2021-09-02
hCoV-19/Japan/PG-80698/2021	EPI_ISL_3892429	2021-09-02
hCoV-19/Japan/PG-80699/2021	EPI_ISL_3892431	2021-09-02
hCoV-19/Japan/PG-80700/2021	EPI_ISL_3892433	2021-09-02
hCoV-19/Japan/PG-80701/2021	EPI_ISL_3892435	2021-09-02
hCoV-19/Japan/PG-80702/2021	EPI_ISL_3892436	2021-09-02
hCoV-19/Japan/PG-80703/2021	EPI_ISL_3892437	2021-09-02
hCoV-19/Japan/PG-80704/2021	EPI_ISL_3892439	2021-09-02
hCoV-19/Japan/PG-80705/2021	EPI_ISL_3892442	2021-09-02
hCoV-19/Japan/PG-80706/2021	EPI_ISL_3892445	2021-09-02
hCoV-19/Japan/PG-80707/2021	EPI_ISL_3892448	2021-09-02
hCoV-19/Japan/PG-80708/2021	EPI_ISL_3892450	2021-09-02
hCoV-19/Japan/PG-80709/2021	EPI_ISL_3892453	2021-09-02
hCoV-19/Japan/PG-80710/2021	EPI_ISL_3892455	2021-09-02
hCoV-19/Japan/PG-80711/2021	EPI_ISL_3892458	2021-09-02
hCoV-19/Japan/PG-80712/2021	EPI_ISL_3892460	2021-09-02
hCoV-19/Japan/PG-80713/2021	EPI_ISL_3892462	2021-09-02
hCoV-19/Japan/PG-80714/2021	EPI_ISL_3892465	2021-09-02
hCoV-19/Japan/PG-80715/2021	EPI_ISL_3892468	2021-09-02
hCoV-19/Japan/PG-80716/2021	EPI_ISL_3892470	2021-09-02
hCoV-19/Japan/PG-80717/2021	EPI_ISL_3892472	2021-09-02
hCoV-19/Japan/PG-80718/2021	EPI_ISL_3892475	2021-09-02
hCoV-19/Japan/PG-80719/2021	EPI_ISL_3892478	2021-09-02
hCoV-19/Japan/PG-80720/2021	EPI_ISL_3892480	2021-09-02
hCoV-19/Japan/PG-80721/2021	EPI_ISL_3892481	2021-09-02
hCoV-19/Japan/PG-80722/2021	EPI_ISL_3892483	2021-09-02
hCoV-19/Japan/PG-80723/2021	EPI_ISL_3892486	2021-09-02
hCoV-19/Japan/PG-80724/2021	EPI_ISL_3892488	2021-09-02
hCoV-19/Japan/PG-80725/2021	EPI_ISL_3892491	2021-09-02
hCoV-19/Japan/PG-80726/2021	EPI_ISL_3892493	2021-09-02
hCoV-19/Japan/PG-80727/2021	EPI_ISL_3892494	2021-09-02
hCoV-19/Japan/PG-80728/2021	EPI_ISL_3892495	2021-09-02
hCoV-19/Japan/PG-80729/2021	EPI_ISL_3892498	2021-09-02
hCoV-19/Japan/PG-80730/2021	EPI_ISL_3892500	2021-09-02
hCoV-19/Japan/PG-80731/2021	EPI_ISL_3892504	2021-09-02
hCoV-19/Japan/PG-80732/2021	EPI_ISL_3892506	2021-09-02
hCoV-19/Japan/PG-80733/2021	EPI_ISL_3892509	2021-09-02
hCoV-19/Japan/PG-80734/2021	EPI_ISL_3892511	2021-09-02
hCoV-19/Japan/PG-80735/2021	EPI_ISL_3892514	2021-09-02
hCoV-19/Japan/PG-80736/2021	EPI_ISL_3892516	2021-09-02
hCoV-19/Japan/PG-80737/2021	EPI_ISL_3892518	2021-09-02
hCoV-19/Japan/PG-80738/2021	EPI_ISL_3892521	2021-09-02
hCoV-19/Japan/PG-80739/2021	EPI_ISL_3892524	2021-09-02
hCoV-19/Japan/PG-80740/2021	EPI_ISL_3892525	2021-09-02
hCoV-19/Japan/PG-80741/2021	EPI_ISL_3892528	2021-09-02
hCoV-19/Japan/PG-80742/2021	EPI_ISL_3892531	2021-09-02
hCoV-19/Japan/PG-80743/2021	EPI_ISL_3892533	2021-09-02

virus_name	epiID	submission_date
hCoV-19/Japan/PG-80744/2021	EPI_ISL_3892535	2021-09-02
hCoV-19/Japan/PG-80745/2021	EPI_ISL_3892536	2021-09-02
hCoV-19/Japan/PG-80746/2021	EPI_ISL_3892538	2021-09-02
hCoV-19/Japan/PG-80747/2021	EPI_ISL_3892539	2021-09-02
hCoV-19/Japan/PG-80748/2021	EPI_ISL_3892541	2021-09-02
hCoV-19/Japan/PG-80749/2021	EPI_ISL_3892543	2021-09-02
hCoV-19/Japan/PG-80750/2021	EPI_ISL_3892545	2021-09-02
hCoV-19/Japan/PG-80751/2021	EPI_ISL_3892548	2021-09-02
hCoV-19/Japan/PG-80752/2021	EPI_ISL_3892550	2021-09-02
hCoV-19/Japan/PG-80753/2021	EPI_ISL_3892552	2021-09-02
hCoV-19/Japan/PG-80754/2021	EPI_ISL_3892554	2021-09-02
hCoV-19/Japan/PG-80755/2021	EPI_ISL_3892555	2021-09-02
hCoV-19/Japan/PG-80756/2021	EPI_ISL_3892556	2021-09-02
hCoV-19/Japan/PG-80757/2021	EPI_ISL_3892557	2021-09-02
hCoV-19/Japan/PG-80758/2021	EPI_ISL_3892558	2021-09-02
hCoV-19/Japan/PG-80759/2021	EPI_ISL_3892559	2021-09-02
hCoV-19/Japan/PG-80760/2021	EPI_ISL_3892562	2021-09-02
hCoV-19/Japan/PG-80761/2021	EPI_ISL_3892563	2021-09-02
hCoV-19/Japan/PG-80762/2021	EPI_ISL_3892564	2021-09-02
hCoV-19/Japan/PG-80763/2021	EPI_ISL_3892567	2021-09-02
hCoV-19/Japan/PG-80764/2021	EPI_ISL_3892570	2021-09-02
hCoV-19/Japan/PG-80765/2021	EPI_ISL_3892572	2021-09-02
hCoV-19/Japan/PG-80766/2021	EPI_ISL_3892575	2021-09-02
hCoV-19/Japan/PG-80767/2021	EPI_ISL_3892578	2021-09-02
hCoV-19/Japan/PG-80768/2021	EPI_ISL_3892580	2021-09-02
hCoV-19/Japan/PG-80769/2021	EPI_ISL_3892582	2021-09-02
hCoV-19/Japan/PG-80770/2021	EPI_ISL_3892584	2021-09-02
hCoV-19/Japan/PG-80771/2021	EPI_ISL_3892587	2021-09-02
hCoV-19/Japan/PG-80772/2021	EPI_ISL_3892590	2021-09-02
hCoV-19/Japan/PG-80773/2021	EPI_ISL_3892592	2021-09-02
hCoV-19/Japan/PG-80774/2021	EPI_ISL_3892593	2021-09-02
hCoV-19/Japan/PG-80775/2021	EPI_ISL_3892595	2021-09-02
hCoV-19/Japan/PG-80776/2021	EPI_ISL_3892597	2021-09-02
hCoV-19/Japan/PG-80777/2021	EPI_ISL_3892600	2021-09-02
hCoV-19/Japan/PG-80778/2021	EPI_ISL_3892603	2021-09-02
hCoV-19/Japan/PG-80779/2021	EPI_ISL_3892605	2021-09-02
hCoV-19/Japan/PG-80780/2021	EPI_ISL_3892607	2021-09-02
hCoV-19/Japan/PG-80781/2021	EPI_ISL_3892609	2021-09-02
hCoV-19/Japan/PG-80782/2021	EPI_ISL_3892612	2021-09-02
hCoV-19/Japan/PG-80783/2021	EPI_ISL_3892614	2021-09-02
hCoV-19/Japan/PG-80784/2021	EPI_ISL_3892617	2021-09-02
hCoV-19/Japan/PG-80785/2021	EPI_ISL_3892618	2021-09-02
hCoV-19/Japan/PG-80786/2021	EPI_ISL_3892621	2021-09-02
hCoV-19/Japan/PG-80787/2021	EPI_ISL_3892624	2021-09-02
hCoV-19/Japan/PG-80788/2021	EPI_ISL_3892626	2021-09-02
hCoV-19/Japan/PG-80789/2021	EPI_ISL_3892628	2021-09-02
hCoV-19/Japan/PG-80790/2021	EPI_ISL_3892630	2021-09-02
hCoV-19/Japan/PG-80791/2021	EPI_ISL_3892632	2021-09-02
hCoV-19/Japan/PG-80792/2021	EPI_ISL_3892635	2021-09-02
hCoV-19/Japan/PG-80793/2021	EPI_ISL_3892637	2021-09-02
hCoV-19/Japan/PG-80794/2021	EPI_ISL_3892639	2021-09-02
hCoV-19/Japan/PG-80795/2021	EPI_ISL_3892642	2021-09-02
hCoV-19/Japan/PG-80796/2021	EPI_ISL_3892645	2021-09-02
hCoV-19/Japan/PG-80797/2021	EPI_ISL_3892647	2021-09-02

virus_name	epiID	submission_date
hCoV-19/Japan/PG-80798/2021	EPI_ISL_3892650	2021-09-02
hCoV-19/Japan/PG-80799/2021	EPI_ISL_3892652	2021-09-02
hCoV-19/Japan/PG-80800/2021	EPI_ISL_3892655	2021-09-02
hCoV-19/Japan/PG-80801/2021	EPI_ISL_3892658	2021-09-02
hCoV-19/Japan/PG-80802/2021	EPI_ISL_3892660	2021-09-02
hCoV-19/Japan/PG-80803/2021	EPI_ISL_3892662	2021-09-02
hCoV-19/Japan/PG-80804/2021	EPI_ISL_3892665	2021-09-02
hCoV-19/Japan/PG-80805/2021	EPI_ISL_3892666	2021-09-02
hCoV-19/Japan/PG-80806/2021	EPI_ISL_3892669	2021-09-02
hCoV-19/Japan/PG-80807/2021	EPI_ISL_3892671	2021-09-02
hCoV-19/Japan/PG-80808/2021	EPI_ISL_3892674	2021-09-02
hCoV-19/Japan/PG-80809/2021	EPI_ISL_3892676	2021-09-02
hCoV-19/Japan/PG-80810/2021	EPI_ISL_3892679	2021-09-02
hCoV-19/Japan/PG-80811/2021	EPI_ISL_3892682	2021-09-02
hCoV-19/Japan/PG-80812/2021	EPI_ISL_3892684	2021-09-02
hCoV-19/Japan/PG-80813/2021	EPI_ISL_3892687	2021-09-02
hCoV-19/Japan/PG-80814/2021	EPI_ISL_3892690	2021-09-02
hCoV-19/Japan/PG-80815/2021	EPI_ISL_3892693	2021-09-02
hCoV-19/Japan/PG-80816/2021	EPI_ISL_3892696	2021-09-02
hCoV-19/Japan/PG-80817/2021	EPI_ISL_3892698	2021-09-02
hCoV-19/Japan/PG-80818/2021	EPI_ISL_3892701	2021-09-02
hCoV-19/Japan/PG-80819/2021	EPI_ISL_3892704	2021-09-02
hCoV-19/Japan/PG-80820/2021	EPI_ISL_3892706	2021-09-02
hCoV-19/Japan/PG-80821/2021	EPI_ISL_3892709	2021-09-02
hCoV-19/Japan/PG-80822/2021	EPI_ISL_3892712	2021-09-02
hCoV-19/Japan/PG-80823/2021	EPI_ISL_3892714	2021-09-02
hCoV-19/Japan/PG-80824/2021	EPI_ISL_3892715	2021-09-02
hCoV-19/Japan/PG-80825/2021	EPI_ISL_3892717	2021-09-02
hCoV-19/Japan/PG-80826/2021	EPI_ISL_3892719	2021-09-02
hCoV-19/Japan/PG-80827/2021	EPI_ISL_3892721	2021-09-02
hCoV-19/Japan/PG-80828/2021	EPI_ISL_3892724	2021-09-02
hCoV-19/Japan/PG-80829/2021	EPI_ISL_3892727	2021-09-02
hCoV-19/Japan/PG-80830/2021	EPI_ISL_3892730	2021-09-02
hCoV-19/Japan/PG-80831/2021	EPI_ISL_3892733	2021-09-02
hCoV-19/Japan/PG-80832/2021	EPI_ISL_3892736	2021-09-02
hCoV-19/Japan/PG-80833/2021	EPI_ISL_3892738	2021-09-02
hCoV-19/Japan/PG-80834/2021	EPI_ISL_3892741	2021-09-02
hCoV-19/Japan/PG-80835/2021	EPI_ISL_3892744	2021-09-02
hCoV-19/Japan/PG-80836/2021	EPI_ISL_3892747	2021-09-02
hCoV-19/Japan/PG-80837/2021	EPI_ISL_3892750	2021-09-02
hCoV-19/Japan/PG-80838/2021	EPI_ISL_3892753	2021-09-02
hCoV-19/Japan/PG-80839/2021	EPI_ISL_3892756	2021-09-02
hCoV-19/Japan/PG-80840/2021	EPI_ISL_3892759	2021-09-02
hCoV-19/Japan/PG-80841/2021	EPI_ISL_3892762	2021-09-02
hCoV-19/Japan/PG-80842/2021	EPI_ISL_3892765	2021-09-02
hCoV-19/Japan/PG-80843/2021	EPI_ISL_3892767	2021-09-02
hCoV-19/Japan/PG-80844/2021	EPI_ISL_3892770	2021-09-02
hCoV-19/Japan/PG-80845/2021	EPI_ISL_3892772	2021-09-02
hCoV-19/Japan/PG-80846/2021	EPI_ISL_3892775	2021-09-02
hCoV-19/Japan/PG-80847/2021	EPI_ISL_3892778	2021-09-02
hCoV-19/Japan/PG-80848/2021	EPI_ISL_3892781	2021-09-02
hCoV-19/Japan/PG-80849/2021	EPI_ISL_3892786	2021-09-02
hCoV-19/Japan/PG-80850/2021	EPI_ISL_3892789	2021-09-02
hCoV-19/Japan/PG-80851/2021	EPI_ISL_3892792	2021-09-02

virus_name	epiID	submission_date
hCoV-19/Japan/PG-80852/2021	EPI_ISL_3892795	2021-09-02
hCoV-19/Japan/PG-80853/2021	EPI_ISL_3892798	2021-09-02
hCoV-19/Japan/PG-80854/2021	EPI_ISL_3892800	2021-09-02
hCoV-19/Japan/PG-80855/2021	EPI_ISL_3892803	2021-09-02
hCoV-19/Japan/PG-80856/2021	EPI_ISL_3892806	2021-09-02
hCoV-19/Japan/PG-80857/2021	EPI_ISL_3892809	2021-09-02
hCoV-19/Japan/PG-80858/2021	EPI_ISL_3892812	2021-09-02
hCoV-19/Japan/PG-80859/2021	EPI_ISL_3892815	2021-09-02
hCoV-19/Japan/PG-80860/2021	EPI_ISL_3892818	2021-09-02
hCoV-19/Japan/PG-80861/2021	EPI_ISL_3892822	2021-09-02
hCoV-19/Japan/PG-80862/2021	EPI_ISL_3892824	2021-09-02
hCoV-19/Japan/PG-80863/2021	EPI_ISL_3892826	2021-09-02
hCoV-19/Japan/PG-80864/2021	EPI_ISL_3892827	2021-09-02
hCoV-19/Japan/PG-80865/2021	EPI_ISL_3892828	2021-09-02
hCoV-19/Japan/PG-80866/2021	EPI_ISL_3892830	2021-09-02
hCoV-19/Japan/PG-80867/2021	EPI_ISL_3892832	2021-09-02
hCoV-19/Japan/PG-80868/2021	EPI_ISL_3892833	2021-09-02
hCoV-19/Japan/PG-80869/2021	EPI_ISL_3892835	2021-09-02
hCoV-19/Japan/PG-80870/2021	EPI_ISL_3892838	2021-09-02
hCoV-19/Japan/PG-80871/2021	EPI_ISL_3892841	2021-09-02
hCoV-19/Japan/PG-80872/2021	EPI_ISL_3892844	2021-09-02
hCoV-19/Japan/PG-80873/2021	EPI_ISL_3892845	2021-09-02
hCoV-19/Japan/PG-80874/2021	EPI_ISL_3892847	2021-09-02
hCoV-19/Japan/PG-80875/2021	EPI_ISL_3892849	2021-09-02
hCoV-19/Japan/PG-80876/2021	EPI_ISL_3892852	2021-09-02
hCoV-19/Japan/PG-80877/2021	EPI_ISL_3892855	2021-09-02
hCoV-19/Japan/PG-80878/2021	EPI_ISL_3892857	2021-09-02
hCoV-19/Japan/PG-80879/2021	EPI_ISL_3892860	2021-09-02
hCoV-19/Japan/PG-80880/2021	EPI_ISL_3892862	2021-09-02
hCoV-19/Japan/PG-80881/2021	EPI_ISL_3892864	2021-09-02
hCoV-19/Japan/PG-80882/2021	EPI_ISL_3892866	2021-09-02
hCoV-19/Japan/PG-80883/2021	EPI_ISL_3892869	2021-09-02
hCoV-19/Japan/PG-80884/2021	EPI_ISL_3892871	2021-09-02
hCoV-19/Japan/PG-80885/2021	EPI_ISL_3892874	2021-09-02
hCoV-19/Japan/PG-80886/2021	EPI_ISL_3892876	2021-09-02
hCoV-19/Japan/PG-80887/2021	EPI_ISL_3892879	2021-09-02
hCoV-19/Japan/PG-80888/2021	EPI_ISL_3892882	2021-09-02
hCoV-19/Japan/PG-80889/2021	EPI_ISL_3892885	2021-09-02
hCoV-19/Japan/PG-80890/2021	EPI_ISL_3892887	2021-09-02
hCoV-19/Japan/PG-80891/2021	EPI_ISL_3892889	2021-09-02
hCoV-19/Japan/PG-80892/2021	EPI_ISL_3892891	2021-09-02
hCoV-19/Japan/PG-80893/2021	EPI_ISL_3892892	2021-09-02
hCoV-19/Japan/PG-80894/2021	EPI_ISL_3892893	2021-09-02
hCoV-19/Japan/PG-80895/2021	EPI_ISL_3892894	2021-09-02
hCoV-19/Japan/PG-80896/2021	EPI_ISL_3892895	2021-09-02
hCoV-19/Japan/PG-80897/2021	EPI_ISL_3892897	2021-09-02
hCoV-19/Japan/PG-80898/2021	EPI_ISL_3892899	2021-09-02
hCoV-19/Japan/PG-80899/2021	EPI_ISL_3892901	2021-09-02
hCoV-19/Japan/PG-80900/2021	EPI_ISL_3892904	2021-09-02
hCoV-19/Japan/PG-80901/2021	EPI_ISL_3892906	2021-09-02
hCoV-19/Japan/PG-80902/2021	EPI_ISL_3892908	2021-09-02
hCoV-19/Japan/PG-80903/2021	EPI_ISL_3892911	2021-09-02
hCoV-19/Japan/PG-80904/2021	EPI_ISL_3892913	2021-09-02
hCoV-19/Japan/PG-80905/2021	EPI_ISL_3892916	2021-09-02

virus_name	epiID	submission_date
hCoV-19/Japan/PG-80906/2021	EPI_ISL_3892918	2021-09-02
hCoV-19/Japan/PG-80907/2021	EPI_ISL_3892920	2021-09-02
hCoV-19/Japan/PG-80908/2021	EPI_ISL_3892922	2021-09-02
hCoV-19/Japan/PG-80909/2021	EPI_ISL_3892924	2021-09-02
hCoV-19/Japan/PG-80910/2021	EPI_ISL_3892927	2021-09-02
hCoV-19/Japan/PG-80911/2021	EPI_ISL_3892929	2021-09-02
hCoV-19/Japan/PG-80912/2021	EPI_ISL_3892932	2021-09-02
hCoV-19/Japan/PG-80913/2021	EPI_ISL_3892935	2021-09-02
hCoV-19/Japan/PG-80914/2021	EPI_ISL_3892939	2021-09-02
hCoV-19/Japan/PG-80915/2021	EPI_ISL_3892941	2021-09-02
hCoV-19/Japan/PG-80916/2021	EPI_ISL_3892944	2021-09-02
hCoV-19/Japan/PG-80917/2021	EPI_ISL_3892947	2021-09-02
hCoV-19/Japan/PG-80918/2021	EPI_ISL_3892950	2021-09-02
hCoV-19/Japan/PG-80919/2021	EPI_ISL_3892953	2021-09-02
hCoV-19/Japan/PG-80920/2021	EPI_ISL_3892957	2021-09-02
hCoV-19/Japan/PG-80921/2021	EPI_ISL_3892960	2021-09-02
hCoV-19/Japan/PG-80922/2021	EPI_ISL_3892963	2021-09-02
hCoV-19/Japan/PG-80923/2021	EPI_ISL_3892967	2021-09-02
hCoV-19/Japan/PG-80924/2021	EPI_ISL_3892969	2021-09-02
hCoV-19/Japan/PG-80925/2021	EPI_ISL_3892971	2021-09-02
hCoV-19/Japan/PG-80926/2021	EPI_ISL_3892972	2021-09-02
hCoV-19/Japan/PG-80927/2021	EPI_ISL_3892975	2021-09-02
hCoV-19/Japan/PG-80928/2021	EPI_ISL_3892977	2021-09-02
hCoV-19/Japan/PG-80929/2021	EPI_ISL_3892980	2021-09-02
hCoV-19/Japan/PG-80930/2021	EPI_ISL_3892982	2021-09-02
hCoV-19/Japan/PG-80931/2021	EPI_ISL_3892985	2021-09-02
hCoV-19/Japan/PG-80932/2021	EPI_ISL_3892987	2021-09-02
hCoV-19/Japan/PG-80933/2021	EPI_ISL_3892990	2021-09-02
hCoV-19/Japan/PG-80934/2021	EPI_ISL_3892993	2021-09-02
hCoV-19/Japan/PG-80935/2021	EPI_ISL_3892996	2021-09-02
hCoV-19/Japan/PG-80936/2021	EPI_ISL_3892999	2021-09-02
hCoV-19/Japan/PG-80937/2021	EPI_ISL_3893002	2021-09-02
hCoV-19/Japan/PG-80938/2021	EPI_ISL_3893004	2021-09-02
hCoV-19/Japan/PG-80939/2021	EPI_ISL_3893007	2021-09-02
hCoV-19/Japan/PG-80940/2021	EPI_ISL_3893010	2021-09-02
hCoV-19/Japan/PG-80941/2021	EPI_ISL_3893012	2021-09-02
hCoV-19/Japan/PG-80942/2021	EPI_ISL_3893014	2021-09-02
hCoV-19/Japan/PG-80943/2021	EPI_ISL_3893017	2021-09-02
hCoV-19/Japan/PG-80944/2021	EPI_ISL_3893019	2021-09-02
hCoV-19/Japan/PG-80945/2021	EPI_ISL_3893021	2021-09-02
hCoV-19/Japan/PG-80946/2021	EPI_ISL_3893024	2021-09-02
hCoV-19/Japan/PG-80947/2021	EPI_ISL_3893026	2021-09-02
hCoV-19/Japan/PG-80948/2021	EPI_ISL_3893029	2021-09-02
hCoV-19/Japan/PG-80949/2021	EPI_ISL_3893032	2021-09-02
hCoV-19/Japan/PG-80950/2021	EPI_ISL_3893035	2021-09-02
hCoV-19/Japan/PG-80951/2021	EPI_ISL_3893037	2021-09-02
hCoV-19/Japan/PG-80952/2021	EPI_ISL_3893040	2021-09-02
hCoV-19/Japan/PG-80953/2021	EPI_ISL_3893042	2021-09-02
hCoV-19/Japan/PG-80954/2021	EPI_ISL_3893044	2021-09-02
hCoV-19/Japan/PG-80955/2021	EPI_ISL_3893047	2021-09-02
hCoV-19/Japan/PG-80956/2021	EPI_ISL_3893050	2021-09-02
hCoV-19/Japan/PG-80957/2021	EPI_ISL_3893052	2021-09-02
hCoV-19/Japan/PG-80958/2021	EPI_ISL_3893055	2021-09-02
hCoV-19/Japan/PG-80959/2021	EPI_ISL_3893058	2021-09-02

virus_name	epiID	submission_date
hCoV-19/Japan/PG-80960/2021	EPI_ISL_3893060	2021-09-02
hCoV-19/Japan/PG-80961/2021	EPI_ISL_3893063	2021-09-02
hCoV-19/Japan/PG-80962/2021	EPI_ISL_3893065	2021-09-02
hCoV-19/Japan/PG-80963/2021	EPI_ISL_3893068	2021-09-02
hCoV-19/Japan/PG-80964/2021	EPI_ISL_3893072	2021-09-02
hCoV-19/Japan/PG-80965/2021	EPI_ISL_3893074	2021-09-02
hCoV-19/Japan/PG-80966/2021	EPI_ISL_3893078	2021-09-02
hCoV-19/Japan/PG-80967/2021	EPI_ISL_3893080	2021-09-02
hCoV-19/Japan/PG-80968/2021	EPI_ISL_3893083	2021-09-02
hCoV-19/Japan/PG-80969/2021	EPI_ISL_3893087	2021-09-02
hCoV-19/Japan/PG-80970/2021	EPI_ISL_3893090	2021-09-02
hCoV-19/Japan/PG-80971/2021	EPI_ISL_3893093	2021-09-02
hCoV-19/Japan/PG-80972/2021	EPI_ISL_3893096	2021-09-02
hCoV-19/Japan/PG-80973/2021	EPI_ISL_3893098	2021-09-02
hCoV-19/Japan/PG-80974/2021	EPI_ISL_3893102	2021-09-02
hCoV-19/Japan/PG-80975/2021	EPI_ISL_3893105	2021-09-02
hCoV-19/Japan/PG-80976/2021	EPI_ISL_3893108	2021-09-02
hCoV-19/Japan/PG-80977/2021	EPI_ISL_3893111	2021-09-02
hCoV-19/Japan/PG-80978/2021	EPI_ISL_3893113	2021-09-02
hCoV-19/Japan/PG-80979/2021	EPI_ISL_3893116	2021-09-02
hCoV-19/Japan/PG-80980/2021	EPI_ISL_3893119	2021-09-02
hCoV-19/Japan/PG-80981/2021	EPI_ISL_3893121	2021-09-02
hCoV-19/Japan/PG-80982/2021	EPI_ISL_3893124	2021-09-02
hCoV-19/Japan/PG-80983/2021	EPI_ISL_3893127	2021-09-02
hCoV-19/Japan/PG-80984/2021	EPI_ISL_3893129	2021-09-02
hCoV-19/Japan/PG-80985/2021	EPI_ISL_3893132	2021-09-02
hCoV-19/Japan/PG-80986/2021	EPI_ISL_3893135	2021-09-02
hCoV-19/Japan/PG-80987/2021	EPI_ISL_3893138	2021-09-02
hCoV-19/Japan/PG-80988/2021	EPI_ISL_3893140	2021-09-02
hCoV-19/Japan/PG-80989/2021	EPI_ISL_3893143	2021-09-02
hCoV-19/Japan/PG-80990/2021	EPI_ISL_3893146	2021-09-02
hCoV-19/Japan/PG-80991/2021	EPI_ISL_3893148	2021-09-02
hCoV-19/Japan/PG-80992/2021	EPI_ISL_3893151	2021-09-02
hCoV-19/Japan/PG-80993/2021	EPI_ISL_3893155	2021-09-02
hCoV-19/Japan/PG-80994/2021	EPI_ISL_3893158	2021-09-02
hCoV-19/Japan/PG-80995/2021	EPI_ISL_3893161	2021-09-02
hCoV-19/Japan/PG-80996/2021	EPI_ISL_3893163	2021-09-02
hCoV-19/Japan/PG-80997/2021	EPI_ISL_3893166	2021-09-02
hCoV-19/Japan/PG-80998/2021	EPI_ISL_3893169	2021-09-02
hCoV-19/Japan/PG-80999/2021	EPI_ISL_3893172	2021-09-02
hCoV-19/Japan/PG-81000/2021	EPI_ISL_3893175	2021-09-02
hCoV-19/Japan/PG-81001/2021	EPI_ISL_3893177	2021-09-02
hCoV-19/Japan/PG-81002/2021	EPI_ISL_3893180	2021-09-02
hCoV-19/Japan/PG-81003/2021	EPI_ISL_3893183	2021-09-02
hCoV-19/Japan/PG-81004/2021	EPI_ISL_3893184	2021-09-02
hCoV-19/Japan/PG-81005/2021	EPI_ISL_3893185	2021-09-02
hCoV-19/Japan/PG-81006/2021	EPI_ISL_3893187	2021-09-02
hCoV-19/Japan/PG-81007/2021	EPI_ISL_3893189	2021-09-02
hCoV-19/Japan/PG-81008/2021	EPI_ISL_3893192	2021-09-02
hCoV-19/Japan/PG-81009/2021	EPI_ISL_3893195	2021-09-02
hCoV-19/Japan/PG-81010/2021	EPI_ISL_3893198	2021-09-02
hCoV-19/Japan/PG-81011/2021	EPI_ISL_3893200	2021-09-02
hCoV-19/Japan/PG-81012/2021	EPI_ISL_3893203	2021-09-02
hCoV-19/Japan/PG-81013/2021	EPI_ISL_3893206	2021-09-02

virus_name	epiID	submission_date
hCoV-19/Japan/PG-81014/2021	EPI_ISL_3893209	2021-09-02
hCoV-19/Japan/PG-81015/2021	EPI_ISL_3893211	2021-09-02
hCoV-19/Japan/PG-81016/2021	EPI_ISL_3893214	2021-09-02
hCoV-19/Japan/PG-81017/2021	EPI_ISL_3893217	2021-09-02
hCoV-19/Japan/PG-81018/2021	EPI_ISL_3893222	2021-09-02
hCoV-19/Japan/PG-81019/2021	EPI_ISL_3893227	2021-09-02
hCoV-19/Japan/PG-81020/2021	EPI_ISL_3893230	2021-09-02
hCoV-19/Japan/PG-81021/2021	EPI_ISL_3893234	2021-09-02
hCoV-19/Japan/PG-81022/2021	EPI_ISL_3893238	2021-09-02
hCoV-19/Japan/PG-81023/2021	EPI_ISL_3893244	2021-09-02
hCoV-19/Japan/PG-81024/2021	EPI_ISL_3893249	2021-09-02
hCoV-19/Japan/PG-81025/2021	EPI_ISL_3893254	2021-09-02
hCoV-19/Japan/PG-81026/2021	EPI_ISL_3893260	2021-09-02
hCoV-19/Japan/PG-81027/2021	EPI_ISL_3893264	2021-09-02
hCoV-19/Japan/PG-81028/2021	EPI_ISL_3893269	2021-09-02
hCoV-19/Japan/PG-81029/2021	EPI_ISL_3893273	2021-09-02
hCoV-19/Japan/PG-81030/2021	EPI_ISL_3893276	2021-09-02
hCoV-19/Japan/PG-81031/2021	EPI_ISL_3893278	2021-09-02
hCoV-19/Japan/PG-81032/2021	EPI_ISL_3893280	2021-09-02
hCoV-19/Japan/PG-81033/2021	EPI_ISL_3893283	2021-09-02
hCoV-19/Japan/PG-81034/2021	EPI_ISL_3893285	2021-09-02
hCoV-19/Japan/PG-81035/2021	EPI_ISL_3893288	2021-09-02
hCoV-19/Japan/PG-81036/2021	EPI_ISL_3893291	2021-09-02
hCoV-19/Japan/PG-81037/2021	EPI_ISL_3893293	2021-09-02
hCoV-19/Japan/PG-81038/2021	EPI_ISL_3893296	2021-09-02
hCoV-19/Japan/PG-81039/2021	EPI_ISL_3893299	2021-09-02
hCoV-19/Japan/PG-81040/2021	EPI_ISL_3893302	2021-09-02
hCoV-19/Japan/PG-81041/2021	EPI_ISL_3893305	2021-09-02
hCoV-19/Japan/PG-81042/2021	EPI_ISL_3893308	2021-09-02
hCoV-19/Japan/PG-81043/2021	EPI_ISL_3893310	2021-09-02
hCoV-19/Japan/PG-81044/2021	EPI_ISL_3893313	2021-09-02
hCoV-19/Japan/PG-81045/2021	EPI_ISL_3893316	2021-09-02
hCoV-19/Japan/PG-81046/2021	EPI_ISL_3893318	2021-09-02
hCoV-19/Japan/PG-81047/2021	EPI_ISL_3893321	2021-09-02
hCoV-19/Japan/PG-81048/2021	EPI_ISL_3893324	2021-09-02
hCoV-19/Japan/PG-81049/2021	EPI_ISL_3893327	2021-09-02
hCoV-19/Japan/PG-81050/2021	EPI_ISL_3893330	2021-09-02
hCoV-19/Japan/PG-81051/2021	EPI_ISL_3893333	2021-09-02
hCoV-19/Japan/PG-81052/2021	EPI_ISL_3893335	2021-09-02
hCoV-19/Japan/PG-81053/2021	EPI_ISL_3893338	2021-09-02
hCoV-19/Japan/PG-81054/2021	EPI_ISL_3893341	2021-09-02
hCoV-19/Japan/PG-81055/2021	EPI_ISL_3893343	2021-09-02
hCoV-19/Japan/PG-81056/2021	EPI_ISL_3893345	2021-09-02
hCoV-19/Japan/PG-81057/2021	EPI_ISL_3893346	2021-09-02
hCoV-19/Japan/PG-81058/2021	EPI_ISL_3893349	2021-09-02
hCoV-19/Japan/PG-81059/2021	EPI_ISL_3893350	2021-09-02
hCoV-19/Japan/PG-81060/2021	EPI_ISL_3893351	2021-09-02
hCoV-19/Japan/PG-81061/2021	EPI_ISL_3893353	2021-09-02
hCoV-19/Japan/PG-81062/2021	EPI_ISL_3893355	2021-09-02
hCoV-19/Japan/PG-81063/2021	EPI_ISL_3893358	2021-09-02
hCoV-19/Japan/PG-81064/2021	EPI_ISL_3893360	2021-09-02
hCoV-19/Japan/PG-81065/2021	EPI_ISL_3893363	2021-09-02
hCoV-19/Japan/PG-81066/2021	EPI_ISL_3893366	2021-09-02
hCoV-19/Japan/PG-81067/2021	EPI_ISL_3893369	2021-09-02

virus_name	epiID	submission_date
hCoV-19/Japan/PG-81068/2021	EPI_ISL_3893372	2021-09-02
hCoV-19/Japan/PG-81069/2021	EPI_ISL_3893374	2021-09-02
hCoV-19/Japan/PG-81070/2021	EPI_ISL_3893377	2021-09-02
hCoV-19/Japan/PG-81071/2021	EPI_ISL_3893379	2021-09-02
hCoV-19/Japan/PG-81072/2021	EPI_ISL_3893383	2021-09-02
hCoV-19/Japan/PG-81073/2021	EPI_ISL_3893385	2021-09-02
hCoV-19/Japan/PG-81074/2021	EPI_ISL_3893388	2021-09-02
hCoV-19/Japan/PG-81075/2021	EPI_ISL_3893391	2021-09-02
hCoV-19/Japan/PG-81076/2021	EPI_ISL_3893394	2021-09-02
hCoV-19/Japan/PG-81077/2021	EPI_ISL_3893397	2021-09-02
hCoV-19/Japan/PG-81078/2021	EPI_ISL_3893399	2021-09-02
hCoV-19/Japan/PG-81079/2021	EPI_ISL_3893401	2021-09-02
hCoV-19/Japan/PG-81080/2021	EPI_ISL_3893404	2021-09-02
hCoV-19/Japan/PG-81081/2021	EPI_ISL_3893406	2021-09-02
hCoV-19/Japan/PG-81082/2021	EPI_ISL_3893409	2021-09-02
hCoV-19/Japan/PG-81083/2021	EPI_ISL_3893412	2021-09-02
hCoV-19/Japan/PG-81084/2021	EPI_ISL_3893413	2021-09-02
hCoV-19/Japan/PG-81085/2021	EPI_ISL_3893416	2021-09-02
hCoV-19/Japan/PG-81086/2021	EPI_ISL_3893419	2021-09-02
hCoV-19/Japan/PG-81087/2021	EPI_ISL_3893421	2021-09-02
hCoV-19/Japan/PG-81088/2021	EPI_ISL_3893425	2021-09-02
hCoV-19/Japan/PG-81089/2021	EPI_ISL_3893427	2021-09-02
hCoV-19/Japan/PG-81090/2021	EPI_ISL_3893430	2021-09-02
hCoV-19/Japan/PG-81091/2021	EPI_ISL_3893432	2021-09-02
hCoV-19/Japan/PG-81092/2021	EPI_ISL_3893435	2021-09-02
hCoV-19/Japan/PG-81093/2021	EPI_ISL_3893437	2021-09-02
hCoV-19/Japan/PG-81094/2021	EPI_ISL_3893439	2021-09-02
hCoV-19/Japan/PG-81095/2021	EPI_ISL_3893442	2021-09-02
hCoV-19/Japan/PG-81096/2021	EPI_ISL_3893445	2021-09-02
hCoV-19/Japan/PG-81097/2021	EPI_ISL_3893447	2021-09-02
hCoV-19/Japan/PG-81098/2021	EPI_ISL_3893450	2021-09-02
hCoV-19/Japan/PG-81099/2021	EPI_ISL_3893453	2021-09-02
hCoV-19/Japan/PG-81100/2021	EPI_ISL_3893456	2021-09-02
hCoV-19/Japan/PG-81101/2021	EPI_ISL_3893459	2021-09-02
hCoV-19/Japan/PG-81102/2021	EPI_ISL_3893461	2021-09-02
hCoV-19/Japan/PG-81103/2021	EPI_ISL_3893465	2021-09-02
hCoV-19/Japan/PG-81104/2021	EPI_ISL_3893467	2021-09-02
hCoV-19/Japan/PG-81105/2021	EPI_ISL_3893470	2021-09-02
hCoV-19/Japan/PG-81106/2021	EPI_ISL_3893473	2021-09-02
hCoV-19/Japan/PG-81107/2021	EPI_ISL_3893476	2021-09-02
hCoV-19/Japan/PG-81108/2021	EPI_ISL_3893479	2021-09-02
hCoV-19/Japan/PG-81109/2021	EPI_ISL_3893482	2021-09-02
hCoV-19/Japan/PG-81110/2021	EPI_ISL_3893485	2021-09-02
hCoV-19/Japan/PG-81111/2021	EPI_ISL_3893488	2021-09-02
hCoV-19/Japan/PG-81112/2021	EPI_ISL_3893490	2021-09-02
hCoV-19/Japan/PG-81113/2021	EPI_ISL_3893493	2021-09-02
hCoV-19/Japan/PG-81114/2021	EPI_ISL_3893496	2021-09-02
hCoV-19/Japan/PG-81115/2021	EPI_ISL_3893499	2021-09-02
hCoV-19/Japan/PG-81116/2021	EPI_ISL_3893502	2021-09-02
hCoV-19/Japan/PG-81117/2021	EPI_ISL_3893504	2021-09-02
hCoV-19/Japan/PG-81118/2021	EPI_ISL_3893507	2021-09-02
hCoV-19/Japan/PG-81119/2021	EPI_ISL_3893509	2021-09-02
hCoV-19/Japan/PG-81120/2021	EPI_ISL_3893512	2021-09-02
hCoV-19/Japan/PG-81121/2021	EPI_ISL_3893516	2021-09-02

virus_name	epiID	submission_date
hCoV-19/Japan/PG-81122/2021	EPI_ISL_3893518	2021-09-02
hCoV-19/Japan/PG-81123/2021	EPI_ISL_3893521	2021-09-02
hCoV-19/Japan/PG-81124/2021	EPI_ISL_3893525	2021-09-02
hCoV-19/Japan/PG-81125/2021	EPI_ISL_3893527	2021-09-02
hCoV-19/Japan/PG-81126/2021	EPI_ISL_3893532	2021-09-02
hCoV-19/Japan/PG-81127/2021	EPI_ISL_3893534	2021-09-02
hCoV-19/Japan/PG-81128/2021	EPI_ISL_3893538	2021-09-02
hCoV-19/Japan/PG-81129/2021	EPI_ISL_3893540	2021-09-02
hCoV-19/Japan/PG-81130/2021	EPI_ISL_3893544	2021-09-02
hCoV-19/Japan/PG-81131/2021	EPI_ISL_3893547	2021-09-02
hCoV-19/Japan/PG-81132/2021	EPI_ISL_3893550	2021-09-02
hCoV-19/Japan/PG-81133/2021	EPI_ISL_3893553	2021-09-02
hCoV-19/Japan/PG-81134/2021	EPI_ISL_3893556	2021-09-02
hCoV-19/Japan/PG-81135/2021	EPI_ISL_3893561	2021-09-02
hCoV-19/Japan/PG-81136/2021	EPI_ISL_3893562	2021-09-02
hCoV-19/Japan/PG-81137/2021	EPI_ISL_3893565	2021-09-02
hCoV-19/Japan/PG-81138/2021	EPI_ISL_3893568	2021-09-02
hCoV-19/Japan/PG-81139/2021	EPI_ISL_3893571	2021-09-02
hCoV-19/Japan/PG-81140/2021	EPI_ISL_3893575	2021-09-02
hCoV-19/Japan/PG-81141/2021	EPI_ISL_3893578	2021-09-02
hCoV-19/Japan/PG-81142/2021	EPI_ISL_3893583	2021-09-02
hCoV-19/Japan/PG-81143/2021	EPI_ISL_3893588	2021-09-02
hCoV-19/Japan/PG-81144/2021	EPI_ISL_3893592	2021-09-02
hCoV-19/Japan/PG-81145/2021	EPI_ISL_3893597	2021-09-02
hCoV-19/Japan/PG-81146/2021	EPI_ISL_3893600	2021-09-02
hCoV-19/Japan/PG-81147/2021	EPI_ISL_3893603	2021-09-02
hCoV-19/Japan/PG-81148/2021	EPI_ISL_3893607	2021-09-02
hCoV-19/Japan/PG-81149/2021	EPI_ISL_3893610	2021-09-02
hCoV-19/Japan/PG-81150/2021	EPI_ISL_3893614	2021-09-02
hCoV-19/Japan/PG-81151/2021	EPI_ISL_3893617	2021-09-02
hCoV-19/Japan/PG-81152/2021	EPI_ISL_3893622	2021-09-02
hCoV-19/Japan/PG-81153/2021	EPI_ISL_3893627	2021-09-02
hCoV-19/Japan/PG-81154/2021	EPI_ISL_3893631	2021-09-02
hCoV-19/Japan/PG-81155/2021	EPI_ISL_3893636	2021-09-02
hCoV-19/Japan/PG-81156/2021	EPI_ISL_3893641	2021-09-02
hCoV-19/Japan/PG-81157/2021	EPI_ISL_3893647	2021-09-02
hCoV-19/Japan/PG-81158/2021	EPI_ISL_3893652	2021-09-02
hCoV-19/Japan/PG-81159/2021	EPI_ISL_3893655	2021-09-02
hCoV-19/Japan/PG-81160/2021	EPI_ISL_3893658	2021-09-02
hCoV-19/Japan/PG-81161/2021	EPI_ISL_3893662	2021-09-02
hCoV-19/Japan/PG-81162/2021	EPI_ISL_3893665	2021-09-02
hCoV-19/Japan/PG-81163/2021	EPI_ISL_3893668	2021-09-02
hCoV-19/Japan/PG-81164/2021	EPI_ISL_3893670	2021-09-02
hCoV-19/Japan/PG-81165/2021	EPI_ISL_3893673	2021-09-02
hCoV-19/Japan/PG-81166/2021	EPI_ISL_3893676	2021-09-02
hCoV-19/Japan/PG-81167/2021	EPI_ISL_3893678	2021-09-02
hCoV-19/Japan/PG-81168/2021	EPI_ISL_3893681	2021-09-02
hCoV-19/Japan/PG-81169/2021	EPI_ISL_3893684	2021-09-02
hCoV-19/Japan/PG-81170/2021	EPI_ISL_3893687	2021-09-02
hCoV-19/Japan/PG-81171/2021	EPI_ISL_3893690	2021-09-02
hCoV-19/Japan/PG-81172/2021	EPI_ISL_3893692	2021-09-02
hCoV-19/Japan/PG-81173/2021	EPI_ISL_3893696	2021-09-02
hCoV-19/Japan/PG-81174/2021	EPI_ISL_3893698	2021-09-02
hCoV-19/Japan/PG-81175/2021	EPI_ISL_3893701	2021-09-02

virus_name	epiID	submission_date
hCoV-19/Japan/PG-81176/2021	EPI_ISL_3893704	2021-09-02
hCoV-19/Japan/PG-81177/2021	EPI_ISL_3893707	2021-09-02
hCoV-19/Japan/PG-81178/2021	EPI_ISL_3893709	2021-09-02
hCoV-19/Japan/PG-81179/2021	EPI_ISL_3893712	2021-09-02
hCoV-19/Japan/PG-81180/2021	EPI_ISL_3893713	2021-09-02
hCoV-19/Japan/PG-81181/2021	EPI_ISL_3893714	2021-09-02
hCoV-19/Japan/PG-81182/2021	EPI_ISL_3893715	2021-09-02
hCoV-19/Japan/PG-81183/2021	EPI_ISL_3893716	2021-09-02
hCoV-19/Japan/PG-81184/2021	EPI_ISL_3893717	2021-09-02
hCoV-19/Japan/PG-81185/2021	EPI_ISL_3893718	2021-09-02
hCoV-19/Japan/PG-81186/2021	EPI_ISL_3893719	2021-09-02
hCoV-19/Japan/PG-81187/2021	EPI_ISL_3893720	2021-09-02
hCoV-19/Japan/PG-81188/2021	EPI_ISL_3893721	2021-09-02
hCoV-19/Japan/PG-81189/2021	EPI_ISL_3893722	2021-09-02
hCoV-19/Japan/PG-81190/2021	EPI_ISL_3893723	2021-09-02
hCoV-19/Japan/PG-81191/2021	EPI_ISL_3893724	2021-09-02
hCoV-19/Japan/PG-81192/2021	EPI_ISL_3893725	2021-09-02
hCoV-19/Japan/PG-81193/2021	EPI_ISL_3893726	2021-09-02
hCoV-19/Japan/PG-81194/2021	EPI_ISL_3893727	2021-09-02
hCoV-19/Japan/PG-81195/2021	EPI_ISL_3893728	2021-09-02
hCoV-19/Japan/PG-81196/2021	EPI_ISL_3893729	2021-09-02
hCoV-19/Japan/PG-81197/2021	EPI_ISL_3893730	2021-09-02
hCoV-19/Japan/PG-81198/2021	EPI_ISL_3893731	2021-09-02
hCoV-19/Japan/PG-81199/2021	EPI_ISL_3893732	2021-09-02
hCoV-19/Japan/PG-81200/2021	EPI_ISL_3893733	2021-09-02
hCoV-19/Japan/PG-81201/2021	EPI_ISL_3893734	2021-09-02
hCoV-19/Japan/PG-81202/2021	EPI_ISL_3893735	2021-09-02
hCoV-19/Japan/PG-81203/2021	EPI_ISL_3893736	2021-09-02
hCoV-19/Japan/PG-81204/2021	EPI_ISL_3893737	2021-09-02
hCoV-19/Japan/PG-81205/2021	EPI_ISL_3893738	2021-09-02
hCoV-19/Japan/PG-81206/2021	EPI_ISL_3893739	2021-09-02
hCoV-19/Japan/PG-81207/2021	EPI_ISL_3893740	2021-09-02
hCoV-19/Japan/PG-81208/2021	EPI_ISL_3893741	2021-09-02
hCoV-19/Japan/PG-81209/2021	EPI_ISL_3893742	2021-09-02
hCoV-19/Japan/PG-81210/2021	EPI_ISL_3893743	2021-09-02
hCoV-19/Japan/PG-81211/2021	EPI_ISL_3893745	2021-09-02
hCoV-19/Japan/PG-81212/2021	EPI_ISL_3893746	2021-09-02
hCoV-19/Japan/PG-81213/2021	EPI_ISL_3893747	2021-09-02
hCoV-19/Japan/PG-81214/2021	EPI_ISL_3893749	2021-09-02
hCoV-19/Japan/PG-81215/2021	EPI_ISL_3893750	2021-09-02
hCoV-19/Japan/PG-81216/2021	EPI_ISL_3893752	2021-09-02
hCoV-19/Japan/PG-81217/2021	EPI_ISL_3893753	2021-09-02
hCoV-19/Japan/PG-81218/2021	EPI_ISL_3893754	2021-09-02
hCoV-19/Japan/PG-81219/2021	EPI_ISL_3873799	2021-09-02
hCoV-19/Japan/PG-81220/2021	EPI_ISL_3873800	2021-09-02
hCoV-19/Japan/PG-81221/2021	EPI_ISL_3873801	2021-09-02
hCoV-19/Japan/PG-81222/2021	EPI_ISL_3873802	2021-09-02
hCoV-19/Japan/PG-81223/2021	EPI_ISL_3873803	2021-09-02
hCoV-19/Japan/PG-81224/2021	EPI_ISL_3873804	2021-09-02
hCoV-19/Japan/PG-81225/2021	EPI_ISL_3873805	2021-09-02
hCoV-19/Japan/PG-81226/2021	EPI_ISL_3875883	2021-09-02
hCoV-19/Japan/PG-81227/2021	EPI_ISL_3875884	2021-09-02
hCoV-19/Japan/PG-81228/2021	EPI_ISL_3875885	2021-09-02
hCoV-19/Japan/PG-81229/2021	EPI_ISL_3875886	2021-09-02

virus_name	epiID	submission_date
hCoV-19/Japan/PG-81230/2021	EPI_ISL_3875887	2021-09-02
hCoV-19/Japan/PG-81231/2021	EPI_ISL_3875888	2021-09-02
hCoV-19/Japan/PG-81232/2021	EPI_ISL_3875889	2021-09-02
hCoV-19/Japan/PG-81233/2021	EPI_ISL_3875890	2021-09-02
hCoV-19/Japan/PG-81234/2021	EPI_ISL_3875891	2021-09-02
hCoV-19/Japan/PG-81235/2021	EPI_ISL_3875892	2021-09-02
hCoV-19/Japan/PG-81236/2021	EPI_ISL_3875893	2021-09-02
hCoV-19/Japan/PG-81237/2021	EPI_ISL_3875894	2021-09-02
hCoV-19/Japan/PG-81238/2021	EPI_ISL_3875895	2021-09-02
hCoV-19/Japan/PG-81239/2021	EPI_ISL_3875896	2021-09-02
hCoV-19/Japan/PG-81240/2021	EPI_ISL_3875897	2021-09-02
hCoV-19/Japan/PG-81241/2021	EPI_ISL_3875898	2021-09-02
hCoV-19/Japan/PG-81242/2021	EPI_ISL_3875899	2021-09-02
hCoV-19/Japan/PG-81243/2021	EPI_ISL_3875900	2021-09-02
hCoV-19/Japan/PG-81244/2021	EPI_ISL_3875901	2021-09-02
hCoV-19/Japan/PG-81245/2021	EPI_ISL_3875902	2021-09-02
hCoV-19/Japan/PG-81246/2021	EPI_ISL_3875903	2021-09-02
hCoV-19/Japan/PG-81247/2021	EPI_ISL_3875904	2021-09-02
hCoV-19/Japan/PG-81248/2021	EPI_ISL_3875905	2021-09-02
hCoV-19/Japan/PG-81249/2021	EPI_ISL_3870759	2021-09-02
hCoV-19/Japan/PG-81250/2021	EPI_ISL_3870760	2021-09-02
hCoV-19/Japan/PG-81251/2021	EPI_ISL_3870761	2021-09-02
hCoV-19/Japan/PG-81252/2021	EPI_ISL_3870762	2021-09-02
hCoV-19/Japan/PG-81253/2021	EPI_ISL_3870763	2021-09-02
hCoV-19/Japan/PG-81254/2021	EPI_ISL_3870764	2021-09-02
hCoV-19/Japan/PG-81255/2021	EPI_ISL_3870765	2021-09-02
hCoV-19/Japan/PG-81256/2021	EPI_ISL_3870766	2021-09-02
hCoV-19/Japan/PG-81257/2021	EPI_ISL_3870767	2021-09-02
hCoV-19/Japan/PG-81258/2021	EPI_ISL_3870768	2021-09-02
hCoV-19/Japan/PG-81259/2021	EPI_ISL_3870769	2021-09-02
hCoV-19/Japan/PG-81260/2021	EPI_ISL_3870770	2021-09-02
hCoV-19/Japan/PG-81261/2021	EPI_ISL_3870771	2021-09-02
hCoV-19/Japan/PG-81262/2021	EPI_ISL_3870772	2021-09-02
hCoV-19/Japan/PG-81263/2021	EPI_ISL_3870773	2021-09-02
hCoV-19/Japan/PG-81264/2021	EPI_ISL_3870774	2021-09-02
hCoV-19/Japan/PG-81265/2021	EPI_ISL_3870775	2021-09-02
hCoV-19/Japan/PG-81266/2021	EPI_ISL_3870776	2021-09-02
hCoV-19/Japan/PG-81267/2021	EPI_ISL_3870777	2021-09-02
hCoV-19/Japan/PG-81268/2021	EPI_ISL_3870778	2021-09-02
hCoV-19/Japan/PG-81269/2021	EPI_ISL_3870779	2021-09-02
hCoV-19/Japan/PG-81270/2021	EPI_ISL_3870780	2021-09-02
hCoV-19/Japan/PG-81271/2021	EPI_ISL_3870781	2021-09-02
hCoV-19/Japan/PG-81272/2021	EPI_ISL_3873468	2021-09-02
hCoV-19/Japan/PG-81273/2021	EPI_ISL_3873469	2021-09-02
hCoV-19/Japan/PG-81274/2021	EPI_ISL_3873470	2021-09-02
hCoV-19/Japan/PG-81275/2021	EPI_ISL_3873471	2021-09-02
hCoV-19/Japan/PG-81276/2021	EPI_ISL_3873472	2021-09-02
hCoV-19/Japan/PG-81277/2021	EPI_ISL_3873473	2021-09-02
hCoV-19/Japan/PG-81278/2021	EPI_ISL_3873474	2021-09-02
hCoV-19/Japan/PG-81279/2021	EPI_ISL_3873475	2021-09-02
hCoV-19/Japan/PG-81280/2021	EPI_ISL_3873476	2021-09-02
hCoV-19/Japan/PG-81281/2021	EPI_ISL_3873477	2021-09-02
hCoV-19/Japan/PG-81282/2021	EPI_ISL_3873478	2021-09-02
hCoV-19/Japan/PG-81283/2021	EPI_ISL_3873479	2021-09-02

virus_name	epiID	submission_date
hCoV-19/Japan/PG-81284/2021	EPI_ISL_3873480	2021-09-02
hCoV-19/Japan/PG-81285/2021	EPI_ISL_3873481	2021-09-02
hCoV-19/Japan/PG-81286/2021	EPI_ISL_3873482	2021-09-02
hCoV-19/Japan/PG-81287/2021	EPI_ISL_3873483	2021-09-02
hCoV-19/Japan/PG-81288/2021	EPI_ISL_3873484	2021-09-02
hCoV-19/Japan/PG-81289/2021	EPI_ISL_3873485	2021-09-02
hCoV-19/Japan/PG-81290/2021	EPI_ISL_3873486	2021-09-02
hCoV-19/Japan/PG-81291/2021	EPI_ISL_3872860	2021-09-02
hCoV-19/Japan/PG-81292/2021	EPI_ISL_3872861	2021-09-02
hCoV-19/Japan/PG-81293/2021	EPI_ISL_3872862	2021-09-02
hCoV-19/Japan/PG-81294/2021	EPI_ISL_3872863	2021-09-02
hCoV-19/Japan/PG-81295/2021	EPI_ISL_3872864	2021-09-02
hCoV-19/Japan/PG-81296/2021	EPI_ISL_3872865	2021-09-02
hCoV-19/Japan/PG-81297/2021	EPI_ISL_3872866	2021-09-02
hCoV-19/Japan/PG-81298/2021	EPI_ISL_3872867	2021-09-02
hCoV-19/Japan/PG-81299/2021	EPI_ISL_3872868	2021-09-02
hCoV-19/Japan/PG-81300/2021	EPI_ISL_3872869	2021-09-02
hCoV-19/Japan/PG-81301/2021	EPI_ISL_3872870	2021-09-02
hCoV-19/Japan/PG-81302/2021	EPI_ISL_3872871	2021-09-02
hCoV-19/Japan/PG-81303/2021	EPI_ISL_3872872	2021-09-02
hCoV-19/Japan/PG-81304/2021	EPI_ISL_3872873	2021-09-02
hCoV-19/Japan/PG-81305/2021	EPI_ISL_3872874	2021-09-02
hCoV-19/Japan/PG-81306/2021	EPI_ISL_3872875	2021-09-02
hCoV-19/Japan/PG-81307/2021	EPI_ISL_3872876	2021-09-02
hCoV-19/Japan/PG-81308/2021	EPI_ISL_3872877	2021-09-02
hCoV-19/Japan/PG-81309/2021	EPI_ISL_3872878	2021-09-02
hCoV-19/Japan/PG-81310/2021	EPI_ISL_3872879	2021-09-02
hCoV-19/Japan/PG-81311/2021	EPI_ISL_3872880	2021-09-02
hCoV-19/Japan/PG-81312/2021	EPI_ISL_3872881	2021-09-02
hCoV-19/Japan/PG-81313/2021	EPI_ISL_3872882	2021-09-02
hCoV-19/Japan/PG-81314/2021	EPI_ISL_3872883	2021-09-02
hCoV-19/Japan/PG-81315/2021	EPI_ISL_3872884	2021-09-02
hCoV-19/Japan/PG-81316/2021	EPI_ISL_3872885	2021-09-02
hCoV-19/Japan/PG-81317/2021	EPI_ISL_3872886	2021-09-02
hCoV-19/Japan/PG-81318/2021	EPI_ISL_3872887	2021-09-02
hCoV-19/Japan/PG-81319/2021	EPI_ISL_3872888	2021-09-02
hCoV-19/Japan/PG-81320/2021	EPI_ISL_3872889	2021-09-02
hCoV-19/Japan/PG-81321/2021	EPI_ISL_3872890	2021-09-02
hCoV-19/Japan/PG-81322/2021	EPI_ISL_3872891	2021-09-02
hCoV-19/Japan/PG-81323/2021	EPI_ISL_3872892	2021-09-02
hCoV-19/Japan/PG-81324/2021	EPI_ISL_3872893	2021-09-02
hCoV-19/Japan/PG-81325/2021	EPI_ISL_3872894	2021-09-02
hCoV-19/Japan/PG-81326/2021	EPI_ISL_3872895	2021-09-02
hCoV-19/Japan/PG-81327/2021	EPI_ISL_3872896	2021-09-02
hCoV-19/Japan/PG-81328/2021	EPI_ISL_3872897	2021-09-02
hCoV-19/Japan/PG-81329/2021	EPI_ISL_3872898	2021-09-02
hCoV-19/Japan/PG-81330/2021	EPI_ISL_3872899	2021-09-02
hCoV-19/Japan/PG-81331/2021	EPI_ISL_3872900	2021-09-02
hCoV-19/Japan/PG-81332/2021	EPI_ISL_3872901	2021-09-02
hCoV-19/Japan/PG-81333/2021	EPI_ISL_3872902	2021-09-02
hCoV-19/Japan/PG-81334/2021	EPI_ISL_3872903	2021-09-02
hCoV-19/Japan/PG-81335/2021	EPI_ISL_3872904	2021-09-02
hCoV-19/Japan/PG-81336/2021	EPI_ISL_3872905	2021-09-02
hCoV-19/Japan/PG-81337/2021	EPI_ISL_3872906	2021-09-02

virus_name	epiID	submission_date
hCoV-19/Japan/PG-81338/2021	EPI_ISL_3872907	2021-09-02
hCoV-19/Japan/PG-81339/2021	EPI_ISL_3872908	2021-09-02
hCoV-19/Japan/PG-81340/2021	EPI_ISL_3872909	2021-09-02
hCoV-19/Japan/PG-81341/2021	EPI_ISL_3872910	2021-09-02
hCoV-19/Japan/PG-81342/2021	EPI_ISL_3872911	2021-09-02
hCoV-19/Japan/PG-81343/2021	EPI_ISL_3872912	2021-09-02
hCoV-19/Japan/PG-81344/2021	EPI_ISL_3872913	2021-09-02
hCoV-19/Japan/PG-81345/2021	EPI_ISL_3872914	2021-09-02
hCoV-19/Japan/PG-81346/2021	EPI_ISL_3872915	2021-09-02
hCoV-19/Japan/PG-81347/2021	EPI_ISL_3872916	2021-09-02
hCoV-19/Japan/PG-81348/2021	EPI_ISL_3872917	2021-09-02
hCoV-19/Japan/PG-81349/2021	EPI_ISL_3872918	2021-09-02
hCoV-19/Japan/PG-81350/2021	EPI_ISL_3872919	2021-09-02
hCoV-19/Japan/PG-81351/2021	EPI_ISL_3872920	2021-09-02
hCoV-19/Japan/PG-81352/2021	EPI_ISL_3872921	2021-09-02
hCoV-19/Japan/PG-81353/2021	EPI_ISL_3872922	2021-09-02
hCoV-19/Japan/PG-81354/2021	EPI_ISL_3872923	2021-09-02
hCoV-19/Japan/PG-81355/2021	EPI_ISL_3872924	2021-09-02
hCoV-19/Japan/PG-81356/2021	EPI_ISL_3872925	2021-09-02
hCoV-19/Japan/PG-81357/2021	EPI_ISL_3872926	2021-09-02
hCoV-19/Japan/PG-81358/2021	EPI_ISL_3872927	2021-09-02
hCoV-19/Japan/PG-81359/2021	EPI_ISL_3872928	2021-09-02
hCoV-19/Japan/PG-81360/2021	EPI_ISL_3872929	2021-09-02
hCoV-19/Japan/PG-81361/2021	EPI_ISL_3872930	2021-09-02
hCoV-19/Japan/PG-81362/2021	EPI_ISL_3872931	2021-09-02
hCoV-19/Japan/PG-81363/2021	EPI_ISL_3872932	2021-09-02
hCoV-19/Japan/PG-81364/2021	EPI_ISL_3872933	2021-09-02
hCoV-19/Japan/PG-81365/2021	EPI_ISL_3872934	2021-09-02
hCoV-19/Japan/PG-81366/2021	EPI_ISL_3872935	2021-09-02
hCoV-19/Japan/PG-81367/2021	EPI_ISL_3872936	2021-09-02
hCoV-19/Japan/PG-81368/2021	EPI_ISL_3872937	2021-09-02
hCoV-19/Japan/PG-81369/2021	EPI_ISL_3872938	2021-09-02
hCoV-19/Japan/PG-81370/2021	EPI_ISL_3872939	2021-09-02
hCoV-19/Japan/PG-81371/2021	EPI_ISL_3872940	2021-09-02
hCoV-19/Japan/PG-81372/2021	EPI_ISL_3872941	2021-09-02
hCoV-19/Japan/PG-81373/2021	EPI_ISL_3871634	2021-09-02
hCoV-19/Japan/PG-81374/2021	EPI_ISL_3871635	2021-09-02
hCoV-19/Japan/PG-81375/2021	EPI_ISL_3871636	2021-09-02
hCoV-19/Japan/PG-81376/2021	EPI_ISL_3871637	2021-09-02
hCoV-19/Japan/PG-81377/2021	EPI_ISL_3871638	2021-09-02
hCoV-19/Japan/PG-81378/2021	EPI_ISL_3871639	2021-09-02
hCoV-19/Japan/PG-81379/2021	EPI_ISL_3871640	2021-09-02
hCoV-19/Japan/PG-81384/2021	EPI_ISL_3872302	2021-09-02
hCoV-19/Japan/PG-81385/2021	EPI_ISL_3872303	2021-09-02
hCoV-19/Japan/PG-81386/2021	EPI_ISL_3872304	2021-09-02
hCoV-19/Japan/PG-81387/2021	EPI_ISL_3872305	2021-09-02
hCoV-19/Japan/PG-81388/2021	EPI_ISL_3872306	2021-09-02
hCoV-19/Japan/PG-81389/2021	EPI_ISL_3872307	2021-09-02
hCoV-19/Japan/PG-81390/2021	EPI_ISL_3872308	2021-09-02
hCoV-19/Japan/PG-81391/2021	EPI_ISL_3872309	2021-09-02
hCoV-19/Japan/PG-81392/2021	EPI_ISL_3872310	2021-09-02
hCoV-19/Japan/PG-81393/2021	EPI_ISL_3872311	2021-09-02
hCoV-19/Japan/PG-81394/2021	EPI_ISL_3893756	2021-09-02
hCoV-19/Japan/PG-81395/2021	EPI_ISL_3893757	2021-09-02

virus_name	epiID	submission_date
hCoV-19/Japan/PG-81396/2021	EPI_ISL_3893758	2021-09-02
hCoV-19/Japan/PG-81397/2021	EPI_ISL_3893759	2021-09-02
hCoV-19/Japan/PG-81398/2021	EPI_ISL_3893760	2021-09-02
hCoV-19/Japan/PG-81399/2021	EPI_ISL_3893761	2021-09-02
hCoV-19/Japan/PG-81400/2021	EPI_ISL_3893762	2021-09-02
hCoV-19/Japan/PG-81401/2021	EPI_ISL_3893763	2021-09-02
hCoV-19/Japan/PG-81402/2021	EPI_ISL_3893764	2021-09-02
hCoV-19/Japan/PG-81403/2021	EPI_ISL_3893765	2021-09-02
hCoV-19/Japan/PG-81404/2021	EPI_ISL_3893766	2021-09-02
hCoV-19/Japan/PG-81405/2021	EPI_ISL_3893767	2021-09-02
hCoV-19/Japan/PG-81406/2021	EPI_ISL_3893768	2021-09-02
hCoV-19/Japan/PG-81407/2021	EPI_ISL_3893769	2021-09-02
hCoV-19/Japan/PG-81408/2021	EPI_ISL_3893770	2021-09-02
hCoV-19/Japan/PG-81409/2021	EPI_ISL_3893771	2021-09-02
hCoV-19/Japan/PG-81410/2021	EPI_ISL_3893772	2021-09-02
hCoV-19/Japan/PG-81411/2021	EPI_ISL_3893773	2021-09-02
hCoV-19/Japan/PG-81412/2021	EPI_ISL_3893774	2021-09-02
hCoV-19/Japan/PG-81413/2021	EPI_ISL_3893775	2021-09-02
hCoV-19/Japan/PG-81414/2021	EPI_ISL_3893776	2021-09-02
hCoV-19/Japan/PG-81415/2021	EPI_ISL_3893777	2021-09-02
hCoV-19/Japan/PG-81416/2021	EPI_ISL_3893778	2021-09-02
hCoV-19/Japan/PG-81417/2021	EPI_ISL_3893779	2021-09-02
hCoV-19/Japan/PG-81418/2021	EPI_ISL_3893780	2021-09-02
hCoV-19/Japan/PG-81419/2021	EPI_ISL_3893781	2021-09-02
hCoV-19/Japan/PG-81420/2021	EPI_ISL_3893782	2021-09-02
hCoV-19/Japan/PG-81421/2021	EPI_ISL_3893783	2021-09-02
hCoV-19/Japan/PG-81422/2021	EPI_ISL_3893784	2021-09-02
hCoV-19/Japan/PG-81423/2021	EPI_ISL_3893785	2021-09-02
hCoV-19/Japan/PG-81424/2021	EPI_ISL_3893786	2021-09-02
hCoV-19/Japan/PG-81425/2021	EPI_ISL_3893787	2021-09-02
hCoV-19/Japan/PG-81426/2021	EPI_ISL_3893788	2021-09-02
hCoV-19/Japan/PG-81427/2021	EPI_ISL_3893789	2021-09-02
hCoV-19/Japan/PG-81428/2021	EPI_ISL_3893790	2021-09-02
hCoV-19/Japan/PG-81429/2021	EPI_ISL_3893791	2021-09-02
hCoV-19/Japan/PG-81430/2021	EPI_ISL_3893792	2021-09-02
hCoV-19/Japan/PG-81431/2021	EPI_ISL_3893793	2021-09-02
hCoV-19/Japan/PG-81432/2021	EPI_ISL_3893794	2021-09-02
hCoV-19/Japan/PG-81433/2021	EPI_ISL_3893795	2021-09-02
hCoV-19/Japan/PG-81434/2021	EPI_ISL_3893796	2021-09-02
hCoV-19/Japan/PG-81435/2021	EPI_ISL_3893797	2021-09-02
hCoV-19/Japan/PG-81436/2021	EPI_ISL_3893798	2021-09-02
hCoV-19/Japan/PG-81437/2021	EPI_ISL_3893799	2021-09-02
hCoV-19/Japan/PG-81438/2021	EPI_ISL_3893800	2021-09-02
hCoV-19/Japan/PG-81439/2021	EPI_ISL_3893801	2021-09-02
hCoV-19/Japan/PG-81440/2021	EPI_ISL_3893802	2021-09-02
hCoV-19/Japan/PG-81441/2021	EPI_ISL_3893803	2021-09-02
hCoV-19/Japan/PG-81442/2021	EPI_ISL_3893804	2021-09-02
hCoV-19/Japan/PG-81443/2021	EPI_ISL_3893805	2021-09-02
hCoV-19/Japan/PG-81444/2021	EPI_ISL_3893806	2021-09-02
hCoV-19/Japan/PG-81445/2021	EPI_ISL_3893807	2021-09-02
hCoV-19/Japan/PG-81446/2021	EPI_ISL_3893808	2021-09-02
hCoV-19/Japan/PG-81447/2021	EPI_ISL_3893809	2021-09-02
hCoV-19/Japan/PG-81448/2021	EPI_ISL_3893810	2021-09-02
hCoV-19/Japan/PG-81449/2021	EPI_ISL_3893811	2021-09-02

virus_name	epiID	submission_date
hCoV-19/Japan/PG-81450/2021	EPI_ISL_3893812	2021-09-02
hCoV-19/Japan/PG-81451/2021	EPI_ISL_3893813	2021-09-02
hCoV-19/Japan/PG-81452/2021	EPI_ISL_3893814	2021-09-02
hCoV-19/Japan/PG-81453/2021	EPI_ISL_3893815	2021-09-02
hCoV-19/Japan/PG-81454/2021	EPI_ISL_3893816	2021-09-02
hCoV-19/Japan/PG-81455/2021	EPI_ISL_3893817	2021-09-02
hCoV-19/Japan/PG-81456/2021	EPI_ISL_3893818	2021-09-02
hCoV-19/Japan/PG-81457/2021	EPI_ISL_3893819	2021-09-02
hCoV-19/Japan/PG-81458/2021	EPI_ISL_3893820	2021-09-02
hCoV-19/Japan/PG-81459/2021	EPI_ISL_3893821	2021-09-02
hCoV-19/Japan/PG-81460/2021	EPI_ISL_3893822	2021-09-02
hCoV-19/Japan/PG-81461/2021	EPI_ISL_3893823	2021-09-02
hCoV-19/Japan/PG-81462/2021	EPI_ISL_3893824	2021-09-02
hCoV-19/Japan/PG-81463/2021	EPI_ISL_3893825	2021-09-02
hCoV-19/Japan/PG-81464/2021	EPI_ISL_3893826	2021-09-02
hCoV-19/Japan/PG-81465/2021	EPI_ISL_3893827	2021-09-02
hCoV-19/Japan/PG-81466/2021	EPI_ISL_3893828	2021-09-02
hCoV-19/Japan/PG-81467/2021	EPI_ISL_3893829	2021-09-02
hCoV-19/Japan/PG-81468/2021	EPI_ISL_3893830	2021-09-02
hCoV-19/Japan/PG-81469/2021	EPI_ISL_3893831	2021-09-02
hCoV-19/Japan/PG-81470/2021	EPI_ISL_3893832	2021-09-02
hCoV-19/Japan/PG-81471/2021	EPI_ISL_3893833	2021-09-02
hCoV-19/Japan/PG-81472/2021	EPI_ISL_3893834	2021-09-02
hCoV-19/Japan/PG-81473/2021	EPI_ISL_3893835	2021-09-02
hCoV-19/Japan/PG-81474/2021	EPI_ISL_3893836	2021-09-02
hCoV-19/Japan/PG-81475/2021	EPI_ISL_3893837	2021-09-02
hCoV-19/Japan/PG-81476/2021	EPI_ISL_3893838	2021-09-02
hCoV-19/Japan/PG-81477/2021	EPI_ISL_3893839	2021-09-02
hCoV-19/Japan/PG-81478/2021	EPI_ISL_3893840	2021-09-02
hCoV-19/Japan/PG-81479/2021	EPI_ISL_3893841	2021-09-02
hCoV-19/Japan/PG-81480/2021	EPI_ISL_3893842	2021-09-02
hCoV-19/Japan/PG-81481/2021	EPI_ISL_3893843	2021-09-02
hCoV-19/Japan/PG-81482/2021	EPI_ISL_3893844	2021-09-02
hCoV-19/Japan/PG-81483/2021	EPI_ISL_3893845	2021-09-02
hCoV-19/Japan/PG-81484/2021	EPI_ISL_3893846	2021-09-02
hCoV-19/Japan/PG-81485/2021	EPI_ISL_3893847	2021-09-02
hCoV-19/Japan/PG-81486/2021	EPI_ISL_3893848	2021-09-02
hCoV-19/Japan/PG-81487/2021	EPI_ISL_3893849	2021-09-02
hCoV-19/Japan/PG-81488/2021	EPI_ISL_3893850	2021-09-02
hCoV-19/Japan/PG-81489/2021	EPI_ISL_3893851	2021-09-02
hCoV-19/Japan/PG-81490/2021	EPI_ISL_3893852	2021-09-02
hCoV-19/Japan/PG-81491/2021	EPI_ISL_3893853	2021-09-02
hCoV-19/Japan/PG-81492/2021	EPI_ISL_3893854	2021-09-02
hCoV-19/Japan/PG-81493/2021	EPI_ISL_3893855	2021-09-02
hCoV-19/Japan/PG-81494/2021	EPI_ISL_3893856	2021-09-02
hCoV-19/Japan/PG-81495/2021	EPI_ISL_3893857	2021-09-02
hCoV-19/Japan/PG-81496/2021	EPI_ISL_3893858	2021-09-02
hCoV-19/Japan/PG-81497/2021	EPI_ISL_3893859	2021-09-02
hCoV-19/Japan/PG-81498/2021	EPI_ISL_3893860	2021-09-02
hCoV-19/Japan/PG-81499/2021	EPI_ISL_3893861	2021-09-02
hCoV-19/Japan/PG-81500/2021	EPI_ISL_3893862	2021-09-02
hCoV-19/Japan/PG-81501/2021	EPI_ISL_3893863	2021-09-02
hCoV-19/Japan/PG-81502/2021	EPI_ISL_3893864	2021-09-02
hCoV-19/Japan/PG-81503/2021	EPI_ISL_3893865	2021-09-02

virus_name	epiID	submission_date
hCoV-19/Japan/PG-81504/2021	EPI_ISL_3893866	2021-09-02
hCoV-19/Japan/PG-81505/2021	EPI_ISL_3893867	2021-09-02
hCoV-19/Japan/PG-81506/2021	EPI_ISL_3893868	2021-09-02
hCoV-19/Japan/PG-81507/2021	EPI_ISL_3893869	2021-09-02
hCoV-19/Japan/PG-81508/2021	EPI_ISL_3893870	2021-09-02
hCoV-19/Japan/PG-81509/2021	EPI_ISL_3893871	2021-09-02
hCoV-19/Japan/PG-81510/2021	EPI_ISL_3893872	2021-09-02
hCoV-19/Japan/PG-81511/2021	EPI_ISL_3893873	2021-09-02
hCoV-19/Japan/PG-81512/2021	EPI_ISL_3893874	2021-09-02
hCoV-19/Japan/PG-81513/2021	EPI_ISL_3893875	2021-09-02
hCoV-19/Japan/PG-81514/2021	EPI_ISL_3893876	2021-09-02
hCoV-19/Japan/PG-81515/2021	EPI_ISL_3893877	2021-09-02
hCoV-19/Japan/PG-81516/2021	EPI_ISL_3893878	2021-09-02
hCoV-19/Japan/PG-81517/2021	EPI_ISL_3893879	2021-09-02
hCoV-19/Japan/PG-81518/2021	EPI_ISL_3893880	2021-09-02
hCoV-19/Japan/PG-81519/2021	EPI_ISL_3893881	2021-09-02
hCoV-19/Japan/PG-81520/2021	EPI_ISL_3893882	2021-09-02
hCoV-19/Japan/PG-81521/2021	EPI_ISL_3893883	2021-09-02
hCoV-19/Japan/PG-81522/2021	EPI_ISL_3893884	2021-09-02
hCoV-19/Japan/PG-81523/2021	EPI_ISL_3893885	2021-09-02
hCoV-19/Japan/PG-81524/2021	EPI_ISL_3893886	2021-09-02
hCoV-19/Japan/PG-81525/2021	EPI_ISL_3893887	2021-09-02
hCoV-19/Japan/PG-81526/2021	EPI_ISL_3893888	2021-09-02
hCoV-19/Japan/PG-81527/2021	EPI_ISL_3893889	2021-09-02
hCoV-19/Japan/PG-81528/2021	EPI_ISL_3893890	2021-09-02
hCoV-19/Japan/PG-81529/2021	EPI_ISL_3893891	2021-09-02
hCoV-19/Japan/PG-81530/2021	EPI_ISL_3893892	2021-09-02
hCoV-19/Japan/PG-81531/2021	EPI_ISL_3893893	2021-09-02
hCoV-19/Japan/PG-81532/2021	EPI_ISL_3893894	2021-09-02
hCoV-19/Japan/PG-81533/2021	EPI_ISL_3893895	2021-09-02
hCoV-19/Japan/PG-81534/2021	EPI_ISL_3893896	2021-09-02
hCoV-19/Japan/PG-81535/2021	EPI_ISL_3893897	2021-09-02
hCoV-19/Japan/PG-81536/2021	EPI_ISL_3893898	2021-09-02
hCoV-19/Japan/PG-81537/2021	EPI_ISL_3893899	2021-09-02
hCoV-19/Japan/PG-81538/2021	EPI_ISL_3893900	2021-09-02
hCoV-19/Japan/PG-81539/2021	EPI_ISL_3893901	2021-09-02
hCoV-19/Japan/PG-81540/2021	EPI_ISL_3893902	2021-09-02
hCoV-19/Japan/PG-81541/2021	EPI_ISL_3893904	2021-09-02
hCoV-19/Japan/PG-81542/2021	EPI_ISL_3893905	2021-09-02
hCoV-19/Japan/PG-81543/2021	EPI_ISL_3893906	2021-09-02
hCoV-19/Japan/PG-81544/2021	EPI_ISL_3893907	2021-09-02
hCoV-19/Japan/PG-81545/2021	EPI_ISL_3893908	2021-09-02
hCoV-19/Japan/PG-81546/2021	EPI_ISL_3893909	2021-09-02
hCoV-19/Japan/PG-81547/2021	EPI_ISL_3893910	2021-09-02
hCoV-19/Japan/PG-81548/2021	EPI_ISL_3893911	2021-09-02
hCoV-19/Japan/PG-81549/2021	EPI_ISL_3893912	2021-09-02
hCoV-19/Japan/PG-81550/2021	EPI_ISL_3893913	2021-09-02
hCoV-19/Japan/PG-81551/2021	EPI_ISL_3893914	2021-09-02
hCoV-19/Japan/PG-81552/2021	EPI_ISL_3893915	2021-09-02
hCoV-19/Japan/PG-81553/2021	EPI_ISL_3893916	2021-09-02
hCoV-19/Japan/PG-81554/2021	EPI_ISL_3893917	2021-09-02
hCoV-19/Japan/PG-81555/2021	EPI_ISL_3893918	2021-09-02
hCoV-19/Japan/PG-81556/2021	EPI_ISL_3893919	2021-09-02
hCoV-19/Japan/PG-81557/2021	EPI_ISL_3893920	2021-09-02

virus_name	epiID	submission_date
hCoV-19/Japan/PG-81558/2021	EPI_ISL_3893921	2021-09-02
hCoV-19/Japan/PG-81559/2021	EPI_ISL_3893922	2021-09-02
hCoV-19/Japan/PG-81560/2021	EPI_ISL_3893923	2021-09-02
hCoV-19/Japan/PG-81561/2021	EPI_ISL_3893924	2021-09-02
hCoV-19/Japan/PG-81562/2021	EPI_ISL_3893925	2021-09-02
hCoV-19/Japan/PG-81563/2021	EPI_ISL_3893926	2021-09-02
hCoV-19/Japan/PG-81564/2021	EPI_ISL_3893927	2021-09-02
hCoV-19/Japan/PG-81565/2021	EPI_ISL_3893928	2021-09-02
hCoV-19/Japan/PG-81566/2021	EPI_ISL_3893929	2021-09-02
hCoV-19/Japan/PG-81567/2021	EPI_ISL_3893930	2021-09-02
hCoV-19/Japan/PG-81568/2021	EPI_ISL_3893931	2021-09-02
hCoV-19/Japan/PG-81569/2021	EPI_ISL_3893932	2021-09-02
hCoV-19/Japan/PG-81570/2021	EPI_ISL_3893933	2021-09-02
hCoV-19/Japan/PG-81571/2021	EPI_ISL_3893934	2021-09-02
hCoV-19/Japan/PG-81572/2021	EPI_ISL_3893935	2021-09-02
hCoV-19/Japan/PG-81573/2021	EPI_ISL_3893936	2021-09-02
hCoV-19/Japan/PG-81574/2021	EPI_ISL_3893937	2021-09-02
hCoV-19/Japan/PG-81575/2021	EPI_ISL_3893938	2021-09-02
hCoV-19/Japan/PG-81576/2021	EPI_ISL_3893939	2021-09-02
hCoV-19/Japan/PG-81577/2021	EPI_ISL_3893940	2021-09-02
hCoV-19/Japan/PG-81578/2021	EPI_ISL_3893941	2021-09-02
hCoV-19/Japan/PG-81579/2021	EPI_ISL_3893942	2021-09-02
hCoV-19/Japan/PG-81580/2021	EPI_ISL_3893943	2021-09-02
hCoV-19/Japan/PG-81581/2021	EPI_ISL_3893944	2021-09-02
hCoV-19/Japan/PG-81582/2021	EPI_ISL_3893945	2021-09-02
hCoV-19/Japan/PG-81583/2021	EPI_ISL_3893946	2021-09-02
hCoV-19/Japan/PG-81584/2021	EPI_ISL_3893947	2021-09-02
hCoV-19/Japan/PG-81585/2021	EPI_ISL_3893948	2021-09-02
hCoV-19/Japan/PG-81586/2021	EPI_ISL_3893949	2021-09-02
hCoV-19/Japan/PG-81587/2021	EPI_ISL_3893950	2021-09-02
hCoV-19/Japan/PG-81588/2021	EPI_ISL_3893951	2021-09-02
hCoV-19/Japan/PG-81589/2021	EPI_ISL_3893952	2021-09-02
hCoV-19/Japan/PG-81590/2021	EPI_ISL_3893953	2021-09-02
hCoV-19/Japan/PG-81591/2021	EPI_ISL_3893954	2021-09-02
hCoV-19/Japan/PG-81592/2021	EPI_ISL_3893955	2021-09-02
hCoV-19/Japan/PG-81593/2021	EPI_ISL_3893956	2021-09-02
hCoV-19/Japan/PG-81594/2021	EPI_ISL_3893957	2021-09-02
hCoV-19/Japan/PG-81595/2021	EPI_ISL_3893958	2021-09-02
hCoV-19/Japan/PG-81596/2021	EPI_ISL_3893959	2021-09-02
hCoV-19/Japan/PG-81597/2021	EPI_ISL_3893960	2021-09-02
hCoV-19/Japan/PG-81598/2021	EPI_ISL_3893961	2021-09-02
hCoV-19/Japan/PG-81599/2021	EPI_ISL_3893962	2021-09-02
hCoV-19/Japan/PG-81600/2021	EPI_ISL_3893963	2021-09-02
hCoV-19/Japan/PG-81601/2021	EPI_ISL_3893964	2021-09-02
hCoV-19/Japan/PG-81602/2021	EPI_ISL_3893965	2021-09-02
hCoV-19/Japan/PG-81603/2021	EPI_ISL_3893966	2021-09-02
hCoV-19/Japan/PG-81604/2021	EPI_ISL_3893967	2021-09-02
hCoV-19/Japan/PG-81605/2021	EPI_ISL_3893968	2021-09-02
hCoV-19/Japan/PG-81606/2021	EPI_ISL_3893969	2021-09-02
hCoV-19/Japan/PG-81607/2021	EPI_ISL_3893970	2021-09-02
hCoV-19/Japan/PG-81608/2021	EPI_ISL_3893971	2021-09-02
hCoV-19/Japan/PG-81609/2021	EPI_ISL_3893972	2021-09-02
hCoV-19/Japan/PG-81610/2021	EPI_ISL_3893973	2021-09-02
hCoV-19/Japan/PG-81611/2021	EPI_ISL_3893974	2021-09-02

virus_name	epiID	submission_date
hCoV-19/Japan/PG-81612/2021	EPI_ISL_3893975	2021-09-02
hCoV-19/Japan/PG-81613/2021	EPI_ISL_3893976	2021-09-02
hCoV-19/Japan/PG-81614/2021	EPI_ISL_3893977	2021-09-02
hCoV-19/Japan/PG-81615/2021	EPI_ISL_3893978	2021-09-02
hCoV-19/Japan/PG-81616/2021	EPI_ISL_3893979	2021-09-02
hCoV-19/Japan/PG-81617/2021	EPI_ISL_3893980	2021-09-02
hCoV-19/Japan/PG-81618/2021	EPI_ISL_3893981	2021-09-02
hCoV-19/Japan/PG-81619/2021	EPI_ISL_3893982	2021-09-02
hCoV-19/Japan/PG-81620/2021	EPI_ISL_3893983	2021-09-02
hCoV-19/Japan/PG-81621/2021	EPI_ISL_3893984	2021-09-02
hCoV-19/Japan/PG-81622/2021	EPI_ISL_3893985	2021-09-02
hCoV-19/Japan/PG-81623/2021	EPI_ISL_3893986	2021-09-02
hCoV-19/Japan/PG-81624/2021	EPI_ISL_3893987	2021-09-02
hCoV-19/Japan/PG-81625/2021	EPI_ISL_3893988	2021-09-02
hCoV-19/Japan/PG-81626/2021	EPI_ISL_3893989	2021-09-02
hCoV-19/Japan/PG-81627/2021	EPI_ISL_3893990	2021-09-02
hCoV-19/Japan/PG-81628/2021	EPI_ISL_3893991	2021-09-02
hCoV-19/Japan/PG-81629/2021	EPI_ISL_3893992	2021-09-02
hCoV-19/Japan/PG-81630/2021	EPI_ISL_3893993	2021-09-02
hCoV-19/Japan/PG-81631/2021	EPI_ISL_3893994	2021-09-02
hCoV-19/Japan/PG-81632/2021	EPI_ISL_3893995	2021-09-02
hCoV-19/Japan/PG-81633/2021	EPI_ISL_3893996	2021-09-02
hCoV-19/Japan/PG-81634/2021	EPI_ISL_3893997	2021-09-02
hCoV-19/Japan/PG-81635/2021	EPI_ISL_3893998	2021-09-02
hCoV-19/Japan/PG-81636/2021	EPI_ISL_3893999	2021-09-02
hCoV-19/Japan/PG-81637/2021	EPI_ISL_3894000	2021-09-02
hCoV-19/Japan/PG-81638/2021	EPI_ISL_3894001	2021-09-02
hCoV-19/Japan/PG-81639/2021	EPI_ISL_3894002	2021-09-02
hCoV-19/Japan/PG-81640/2021	EPI_ISL_3894003	2021-09-02
hCoV-19/Japan/PG-81641/2021	EPI_ISL_3894004	2021-09-02
hCoV-19/Japan/PG-81642/2021	EPI_ISL_3894005	2021-09-02
hCoV-19/Japan/PG-81643/2021	EPI_ISL_3894006	2021-09-02
hCoV-19/Japan/PG-81644/2021	EPI_ISL_3894007	2021-09-02
hCoV-19/Japan/PG-81645/2021	EPI_ISL_3894008	2021-09-02
hCoV-19/Japan/PG-81646/2021	EPI_ISL_3894009	2021-09-02
hCoV-19/Japan/PG-81647/2021	EPI_ISL_3894010	2021-09-02
hCoV-19/Japan/PG-81648/2021	EPI_ISL_3894011	2021-09-02
hCoV-19/Japan/PG-81649/2021	EPI_ISL_3894012	2021-09-02
hCoV-19/Japan/PG-81650/2021	EPI_ISL_3894013	2021-09-02
hCoV-19/Japan/PG-81651/2021	EPI_ISL_3894014	2021-09-02
hCoV-19/Japan/PG-81652/2021	EPI_ISL_3894015	2021-09-02
hCoV-19/Japan/PG-81653/2021	EPI_ISL_3894016	2021-09-02
hCoV-19/Japan/PG-81654/2021	EPI_ISL_3894017	2021-09-02
hCoV-19/Japan/PG-81655/2021	EPI_ISL_3894018	2021-09-02
hCoV-19/Japan/PG-81656/2021	EPI_ISL_3894019	2021-09-02
hCoV-19/Japan/PG-81657/2021	EPI_ISL_3894020	2021-09-02
hCoV-19/Japan/PG-81658/2021	EPI_ISL_3894021	2021-09-02
hCoV-19/Japan/PG-81659/2021	EPI_ISL_3894022	2021-09-02
hCoV-19/Japan/PG-81660/2021	EPI_ISL_3894023	2021-09-02
hCoV-19/Japan/PG-81661/2021	EPI_ISL_3894024	2021-09-02
hCoV-19/Japan/PG-81662/2021	EPI_ISL_3894025	2021-09-02
hCoV-19/Japan/PG-81663/2021	EPI_ISL_3894026	2021-09-02
hCoV-19/Japan/PG-81664/2021	EPI_ISL_3894027	2021-09-02
hCoV-19/Japan/PG-81665/2021	EPI_ISL_3894028	2021-09-02

virus_name	epiID	submission_date
hCoV-19/Japan/PG-81666/2021	EPI_ISL_3894029	2021-09-02
hCoV-19/Japan/PG-81667/2021	EPI_ISL_3894030	2021-09-02
hCoV-19/Japan/PG-81668/2021	EPI_ISL_3894031	2021-09-02
hCoV-19/Japan/PG-81669/2021	EPI_ISL_3894032	2021-09-02
hCoV-19/Japan/PG-81670/2021	EPI_ISL_3894033	2021-09-02
hCoV-19/Japan/PG-81671/2021	EPI_ISL_3894034	2021-09-02
hCoV-19/Japan/PG-81672/2021	EPI_ISL_3894036	2021-09-02
hCoV-19/Japan/PG-81673/2021	EPI_ISL_3894037	2021-09-02
hCoV-19/Japan/PG-81674/2021	EPI_ISL_3894038	2021-09-02
hCoV-19/Japan/PG-81675/2021	EPI_ISL_3894039	2021-09-02
hCoV-19/Japan/PG-81676/2021	EPI_ISL_3894040	2021-09-02
hCoV-19/Japan/PG-81677/2021	EPI_ISL_3894041	2021-09-02
hCoV-19/Japan/PG-81678/2021	EPI_ISL_3894042	2021-09-02
hCoV-19/Japan/PG-81679/2021	EPI_ISL_3894043	2021-09-02
hCoV-19/Japan/PG-81680/2021	EPI_ISL_3894044	2021-09-02
hCoV-19/Japan/PG-81681/2021	EPI_ISL_3894045	2021-09-02
hCoV-19/Japan/PG-81682/2021	EPI_ISL_3894046	2021-09-02
hCoV-19/Japan/PG-81683/2021	EPI_ISL_3894047	2021-09-02
hCoV-19/Japan/PG-81684/2021	EPI_ISL_3894048	2021-09-02
hCoV-19/Japan/PG-81685/2021	EPI_ISL_3894049	2021-09-02
hCoV-19/Japan/PG-81686/2021	EPI_ISL_3894050	2021-09-02
hCoV-19/Japan/PG-81687/2021	EPI_ISL_3894051	2021-09-02
hCoV-19/Japan/PG-81688/2021	EPI_ISL_3894052	2021-09-02
hCoV-19/Japan/PG-81689/2021	EPI_ISL_3894053	2021-09-02
hCoV-19/Japan/PG-81690/2021	EPI_ISL_3894054	2021-09-02
hCoV-19/Japan/PG-81691/2021	EPI_ISL_3894055	2021-09-02
hCoV-19/Japan/PG-81692/2021	EPI_ISL_3894056	2021-09-02
hCoV-19/Japan/PG-81693/2021	EPI_ISL_3894057	2021-09-02
hCoV-19/Japan/PG-81694/2021	EPI_ISL_3894058	2021-09-02
hCoV-19/Japan/PG-81695/2021	EPI_ISL_3894059	2021-09-02
hCoV-19/Japan/PG-81696/2021	EPI_ISL_3894060	2021-09-02
hCoV-19/Japan/PG-81697/2021	EPI_ISL_3894061	2021-09-02
hCoV-19/Japan/PG-81698/2021	EPI_ISL_3894062	2021-09-02
hCoV-19/Japan/PG-81699/2021	EPI_ISL_3894063	2021-09-02
hCoV-19/Japan/PG-81700/2021	EPI_ISL_3894064	2021-09-02
hCoV-19/Japan/PG-81701/2021	EPI_ISL_3894065	2021-09-02
hCoV-19/Japan/PG-81702/2021	EPI_ISL_3894066	2021-09-02
hCoV-19/Japan/PG-81703/2021	EPI_ISL_3894067	2021-09-02
hCoV-19/Japan/PG-81704/2021	EPI_ISL_3894068	2021-09-02
hCoV-19/Japan/PG-81705/2021	EPI_ISL_3894069	2021-09-02
hCoV-19/Japan/PG-81706/2021	EPI_ISL_3894070	2021-09-02
hCoV-19/Japan/PG-81707/2021	EPI_ISL_3894071	2021-09-02
hCoV-19/Japan/PG-81708/2021	EPI_ISL_3894072	2021-09-02
hCoV-19/Japan/PG-81709/2021	EPI_ISL_3894073	2021-09-02
hCoV-19/Japan/PG-81710/2021	EPI_ISL_3894074	2021-09-02
hCoV-19/Japan/PG-81711/2021	EPI_ISL_3894075	2021-09-02
hCoV-19/Japan/PG-81712/2021	EPI_ISL_3894076	2021-09-02
hCoV-19/Japan/PG-81713/2021	EPI_ISL_3894077	2021-09-02
hCoV-19/Japan/PG-81714/2021	EPI_ISL_3894078	2021-09-02
hCoV-19/Japan/PG-81715/2021	EPI_ISL_3894079	2021-09-02
hCoV-19/Japan/PG-81716/2021	EPI_ISL_3894080	2021-09-02
hCoV-19/Japan/PG-81717/2021	EPI_ISL_3894081	2021-09-02
hCoV-19/Japan/PG-81718/2021	EPI_ISL_3894082	2021-09-02
hCoV-19/Japan/PG-81719/2021	EPI_ISL_3894083	2021-09-02

virus_name	epiID	submission_date
hCoV-19/Japan/PG-81720/2021	EPI_ISL_3894084	2021-09-02
hCoV-19/Japan/PG-81721/2021	EPI_ISL_3894085	2021-09-02
hCoV-19/Japan/PG-81722/2021	EPI_ISL_3894086	2021-09-02
hCoV-19/Japan/PG-81723/2021	EPI_ISL_3894087	2021-09-02
hCoV-19/Japan/PG-81724/2021	EPI_ISL_3894088	2021-09-02
hCoV-19/Japan/PG-81725/2021	EPI_ISL_3894089	2021-09-02
hCoV-19/Japan/PG-81726/2021	EPI_ISL_3894090	2021-09-02
hCoV-19/Japan/PG-81727/2021	EPI_ISL_3894091	2021-09-02
hCoV-19/Japan/PG-81728/2021	EPI_ISL_3894092	2021-09-02
hCoV-19/Japan/PG-81729/2021	EPI_ISL_3894093	2021-09-02
hCoV-19/Japan/PG-81730/2021	EPI_ISL_3894094	2021-09-02
hCoV-19/Japan/PG-81731/2021	EPI_ISL_3894095	2021-09-02
hCoV-19/Japan/PG-81732/2021	EPI_ISL_3894096	2021-09-02
hCoV-19/Japan/PG-81733/2021	EPI_ISL_3894097	2021-09-02
hCoV-19/Japan/PG-81734/2021	EPI_ISL_3894098	2021-09-02
hCoV-19/Japan/PG-81735/2021	EPI_ISL_3894099	2021-09-02
hCoV-19/Japan/PG-81736/2021	EPI_ISL_3894100	2021-09-02
hCoV-19/Japan/PG-81737/2021	EPI_ISL_3894101	2021-09-02
hCoV-19/Japan/PG-81738/2021	EPI_ISL_3894102	2021-09-02
hCoV-19/Japan/PG-81739/2021	EPI_ISL_3894103	2021-09-02
hCoV-19/Japan/PG-81740/2021	EPI_ISL_3894104	2021-09-02
hCoV-19/Japan/PG-81741/2021	EPI_ISL_3894105	2021-09-02
hCoV-19/Japan/PG-81742/2021	EPI_ISL_3894106	2021-09-02
hCoV-19/Japan/PG-81743/2021	EPI_ISL_3894107	2021-09-02
hCoV-19/Japan/PG-81744/2021	EPI_ISL_3894108	2021-09-02
hCoV-19/Japan/PG-81745/2021	EPI_ISL_3894109	2021-09-02
hCoV-19/Japan/PG-81746/2021	EPI_ISL_3894110	2021-09-02
hCoV-19/Japan/PG-81747/2021	EPI_ISL_3894111	2021-09-02
hCoV-19/Japan/PG-81748/2021	EPI_ISL_3894112	2021-09-02
hCoV-19/Japan/PG-81749/2021	EPI_ISL_3894113	2021-09-02
hCoV-19/Japan/PG-81750/2021	EPI_ISL_3894114	2021-09-02
hCoV-19/Japan/PG-81751/2021	EPI_ISL_3894115	2021-09-02
hCoV-19/Japan/PG-81752/2021	EPI_ISL_3894116	2021-09-02
hCoV-19/Japan/PG-81753/2021	EPI_ISL_3894117	2021-09-02
hCoV-19/Japan/PG-81754/2021	EPI_ISL_3894118	2021-09-02
hCoV-19/Japan/PG-81755/2021	EPI_ISL_3894119	2021-09-02
hCoV-19/Japan/PG-81756/2021	EPI_ISL_3894120	2021-09-02
hCoV-19/Japan/PG-81757/2021	EPI_ISL_3894121	2021-09-02
hCoV-19/Japan/PG-81758/2021	EPI_ISL_3894122	2021-09-02
hCoV-19/Japan/PG-81759/2021	EPI_ISL_3894123	2021-09-02
hCoV-19/Japan/PG-81760/2021	EPI_ISL_3894124	2021-09-02
hCoV-19/Japan/PG-81761/2021	EPI_ISL_3894125	2021-09-02
hCoV-19/Japan/PG-81762/2021	EPI_ISL_3894126	2021-09-02
hCoV-19/Japan/PG-81763/2021	EPI_ISL_3894127	2021-09-02
hCoV-19/Japan/PG-81764/2021	EPI_ISL_3894128	2021-09-02
hCoV-19/Japan/PG-81765/2021	EPI_ISL_3894129	2021-09-02
hCoV-19/Japan/PG-81766/2021	EPI_ISL_3894130	2021-09-02
hCoV-19/Japan/PG-81767/2021	EPI_ISL_3894131	2021-09-02
hCoV-19/Japan/PG-81768/2021	EPI_ISL_3894132	2021-09-02
hCoV-19/Japan/PG-81769/2021	EPI_ISL_3894133	2021-09-02
hCoV-19/Japan/PG-81770/2021	EPI_ISL_3894134	2021-09-02
hCoV-19/Japan/PG-81771/2021	EPI_ISL_3894135	2021-09-02
hCoV-19/Japan/PG-81772/2021	EPI_ISL_3894136	2021-09-02
hCoV-19/Japan/PG-81773/2021	EPI_ISL_3894137	2021-09-02

virus_name	epiID	submission_date
hCoV-19/Japan/PG-81774/2021	EPI_ISL_3894138	2021-09-02
hCoV-19/Japan/PG-81775/2021	EPI_ISL_3894139	2021-09-02
hCoV-19/Japan/PG-81776/2021	EPI_ISL_3894140	2021-09-02
hCoV-19/Japan/PG-81777/2021	EPI_ISL_3894141	2021-09-02
hCoV-19/Japan/PG-81778/2021	EPI_ISL_3894142	2021-09-02
hCoV-19/Japan/PG-81779/2021	EPI_ISL_3894143	2021-09-02
hCoV-19/Japan/PG-81780/2021	EPI_ISL_3894144	2021-09-02
hCoV-19/Japan/PG-81781/2021	EPI_ISL_3894145	2021-09-02
hCoV-19/Japan/PG-81782/2021	EPI_ISL_3894146	2021-09-02
hCoV-19/Japan/PG-81783/2021	EPI_ISL_3894147	2021-09-02
hCoV-19/Japan/PG-81784/2021	EPI_ISL_3894148	2021-09-02
hCoV-19/Japan/PG-81785/2021	EPI_ISL_3894149	2021-09-02
hCoV-19/Japan/PG-81786/2021	EPI_ISL_3894150	2021-09-02
hCoV-19/Japan/PG-81787/2021	EPI_ISL_3894151	2021-09-02
hCoV-19/Japan/PG-81788/2021	EPI_ISL_3894152	2021-09-02
hCoV-19/Japan/PG-81789/2021	EPI_ISL_3894153	2021-09-02
hCoV-19/Japan/PG-81790/2021	EPI_ISL_3894154	2021-09-02
hCoV-19/Japan/PG-81791/2021	EPI_ISL_3894155	2021-09-02
hCoV-19/Japan/PG-81792/2021	EPI_ISL_3894156	2021-09-02
hCoV-19/Japan/PG-81793/2021	EPI_ISL_3894157	2021-09-02
hCoV-19/Japan/PG-81794/2021	EPI_ISL_3894158	2021-09-02
hCoV-19/Japan/PG-81795/2021	EPI_ISL_3894159	2021-09-02
hCoV-19/Japan/PG-81796/2021	EPI_ISL_3894160	2021-09-02
hCoV-19/Japan/PG-81797/2021	EPI_ISL_3894161	2021-09-02
hCoV-19/Japan/PG-81798/2021	EPI_ISL_3894162	2021-09-02
hCoV-19/Japan/PG-81799/2021	EPI_ISL_3894163	2021-09-02
hCoV-19/Japan/PG-81800/2021	EPI_ISL_3894164	2021-09-02
hCoV-19/Japan/PG-81801/2021	EPI_ISL_3894165	2021-09-02
hCoV-19/Japan/PG-81802/2021	EPI_ISL_3894166	2021-09-02
hCoV-19/Japan/PG-81803/2021	EPI_ISL_3894167	2021-09-02
hCoV-19/Japan/PG-81804/2021	EPI_ISL_3894168	2021-09-02
hCoV-19/Japan/PG-81805/2021	EPI_ISL_3894169	2021-09-02
hCoV-19/Japan/PG-81806/2021	EPI_ISL_3894170	2021-09-02
hCoV-19/Japan/PG-81807/2021	EPI_ISL_3894171	2021-09-02
hCoV-19/Japan/PG-81808/2021	EPI_ISL_3894172	2021-09-02
hCoV-19/Japan/PG-81809/2021	EPI_ISL_3894173	2021-09-02
hCoV-19/Japan/PG-81810/2021	EPI_ISL_3894174	2021-09-02
hCoV-19/Japan/PG-81811/2021	EPI_ISL_3894175	2021-09-02
hCoV-19/Japan/PG-81812/2021	EPI_ISL_3894176	2021-09-02
hCoV-19/Japan/PG-81813/2021	EPI_ISL_3894177	2021-09-02
hCoV-19/Japan/PG-81814/2021	EPI_ISL_3894178	2021-09-02
hCoV-19/Japan/PG-81815/2021	EPI_ISL_3894179	2021-09-02
hCoV-19/Japan/PG-81816/2021	EPI_ISL_3894180	2021-09-02
hCoV-19/Japan/PG-81817/2021	EPI_ISL_3894181	2021-09-02
hCoV-19/Japan/PG-81818/2021	EPI_ISL_3894182	2021-09-02
hCoV-19/Japan/PG-81819/2021	EPI_ISL_3894183	2021-09-02
hCoV-19/Japan/PG-81820/2021	EPI_ISL_3894184	2021-09-02
hCoV-19/Japan/PG-81821/2021	EPI_ISL_3894185	2021-09-02
hCoV-19/Japan/PG-81822/2021	EPI_ISL_3894186	2021-09-02
hCoV-19/Japan/PG-81823/2021	EPI_ISL_3894187	2021-09-02
hCoV-19/Japan/PG-81824/2021	EPI_ISL_3894188	2021-09-02
hCoV-19/Japan/PG-81825/2021	EPI_ISL_3894189	2021-09-02
hCoV-19/Japan/PG-81826/2021	EPI_ISL_3894190	2021-09-02
hCoV-19/Japan/PG-81827/2021	EPI_ISL_3894191	2021-09-02

virus_name	epiID	submission_date
hCoV-19/Japan/PG-81828/2021	EPI_ISL_3894192	2021-09-02
hCoV-19/Japan/PG-81829/2021	EPI_ISL_3894193	2021-09-02
hCoV-19/Japan/PG-81830/2021	EPI_ISL_3894194	2021-09-02
hCoV-19/Japan/PG-81831/2021	EPI_ISL_3894195	2021-09-02
hCoV-19/Japan/PG-81832/2021	EPI_ISL_3894196	2021-09-02
hCoV-19/Japan/PG-81833/2021	EPI_ISL_3894197	2021-09-02
hCoV-19/Japan/PG-81834/2021	EPI_ISL_3894198	2021-09-02
hCoV-19/Japan/PG-81835/2021	EPI_ISL_3894199	2021-09-02
hCoV-19/Japan/PG-81836/2021	EPI_ISL_3894200	2021-09-02
hCoV-19/Japan/PG-81837/2021	EPI_ISL_3894201	2021-09-02
hCoV-19/Japan/PG-81838/2021	EPI_ISL_3894202	2021-09-02
hCoV-19/Japan/PG-81839/2021	EPI_ISL_3894203	2021-09-02
hCoV-19/Japan/PG-81840/2021	EPI_ISL_3894204	2021-09-02
hCoV-19/Japan/PG-81841/2021	EPI_ISL_3894205	2021-09-02
hCoV-19/Japan/PG-81842/2021	EPI_ISL_3894206	2021-09-02
hCoV-19/Japan/PG-81843/2021	EPI_ISL_3894207	2021-09-02
hCoV-19/Japan/PG-81844/2021	EPI_ISL_3894208	2021-09-02
hCoV-19/Japan/PG-81845/2021	EPI_ISL_3894209	2021-09-02
hCoV-19/Japan/PG-81846/2021	EPI_ISL_3894210	2021-09-02
hCoV-19/Japan/PG-81847/2021	EPI_ISL_3894211	2021-09-02
hCoV-19/Japan/PG-81848/2021	EPI_ISL_3894212	2021-09-02
hCoV-19/Japan/PG-81849/2021	EPI_ISL_3894213	2021-09-02
hCoV-19/Japan/PG-81850/2021	EPI_ISL_3894214	2021-09-02
hCoV-19/Japan/PG-81851/2021	EPI_ISL_3894215	2021-09-02
hCoV-19/Japan/PG-81852/2021	EPI_ISL_3894216	2021-09-02
hCoV-19/Japan/PG-81853/2021	EPI_ISL_3894217	2021-09-02
hCoV-19/Japan/PG-81854/2021	EPI_ISL_3894218	2021-09-02
hCoV-19/Japan/PG-81855/2021	EPI_ISL_3894219	2021-09-02
hCoV-19/Japan/PG-81856/2021	EPI_ISL_3894220	2021-09-02
hCoV-19/Japan/PG-81857/2021	EPI_ISL_3894221	2021-09-02
hCoV-19/Japan/PG-81858/2021	EPI_ISL_3894222	2021-09-02
hCoV-19/Japan/PG-81859/2021	EPI_ISL_3894223	2021-09-02
hCoV-19/Japan/PG-81860/2021	EPI_ISL_3894224	2021-09-02
hCoV-19/Japan/PG-81861/2021	EPI_ISL_3894225	2021-09-02
hCoV-19/Japan/PG-81862/2021	EPI_ISL_3894226	2021-09-02
hCoV-19/Japan/PG-81863/2021	EPI_ISL_3894227	2021-09-02
hCoV-19/Japan/PG-81864/2021	EPI_ISL_3894228	2021-09-02
hCoV-19/Japan/PG-81865/2021	EPI_ISL_3894229	2021-09-02
hCoV-19/Japan/PG-81866/2021	EPI_ISL_3894230	2021-09-02
hCoV-19/Japan/PG-81867/2021	EPI_ISL_3894231	2021-09-02
hCoV-19/Japan/PG-81868/2021	EPI_ISL_3894232	2021-09-02
hCoV-19/Japan/PG-81869/2021	EPI_ISL_3894233	2021-09-02
hCoV-19/Japan/PG-81870/2021	EPI_ISL_3894234	2021-09-02
hCoV-19/Japan/PG-81871/2021	EPI_ISL_3894235	2021-09-02
hCoV-19/Japan/PG-81872/2021	EPI_ISL_3894236	2021-09-02
hCoV-19/Japan/PG-81873/2021	EPI_ISL_3894237	2021-09-02
hCoV-19/Japan/PG-81874/2021	EPI_ISL_3894238	2021-09-02
hCoV-19/Japan/PG-81875/2021	EPI_ISL_3894239	2021-09-02
hCoV-19/Japan/PG-81876/2021	EPI_ISL_3894240	2021-09-02
hCoV-19/Japan/PG-81877/2021	EPI_ISL_3894241	2021-09-02
hCoV-19/Japan/PG-81878/2021	EPI_ISL_3894242	2021-09-02
hCoV-19/Japan/PG-81879/2021	EPI_ISL_3894243	2021-09-02
hCoV-19/Japan/PG-81880/2021	EPI_ISL_3894244	2021-09-02
hCoV-19/Japan/PG-81881/2021	EPI_ISL_3894245	2021-09-02

virus_name	epiID	submission_date
hCoV-19/Japan/PG-81882/2021	EPI_ISL_3894246	2021-09-02
hCoV-19/Japan/PG-81883/2021	EPI_ISL_3894247	2021-09-02
hCoV-19/Japan/PG-81884/2021	EPI_ISL_3894248	2021-09-02
hCoV-19/Japan/PG-81885/2021	EPI_ISL_3894249	2021-09-02
hCoV-19/Japan/PG-81886/2021	EPI_ISL_3894250	2021-09-02
hCoV-19/Japan/PG-81887/2021	EPI_ISL_3894251	2021-09-02
hCoV-19/Japan/PG-81888/2021	EPI_ISL_3894252	2021-09-02
hCoV-19/Japan/PG-81889/2021	EPI_ISL_3894253	2021-09-02
hCoV-19/Japan/PG-81890/2021	EPI_ISL_3894254	2021-09-02
hCoV-19/Japan/PG-81891/2021	EPI_ISL_3894255	2021-09-02
hCoV-19/Japan/PG-81892/2021	EPI_ISL_3894256	2021-09-02
hCoV-19/Japan/PG-81893/2021	EPI_ISL_3894257	2021-09-02
hCoV-19/Japan/PG-81894/2021	EPI_ISL_3894258	2021-09-02
hCoV-19/Japan/PG-81895/2021	EPI_ISL_3894259	2021-09-02
hCoV-19/Japan/PG-81896/2021	EPI_ISL_3894260	2021-09-02
hCoV-19/Japan/PG-81897/2021	EPI_ISL_3894261	2021-09-02
hCoV-19/Japan/PG-81898/2021	EPI_ISL_3894262	2021-09-02
hCoV-19/Japan/PG-81899/2021	EPI_ISL_3894263	2021-09-02
hCoV-19/Japan/PG-81900/2021	EPI_ISL_3894264	2021-09-02
hCoV-19/Japan/PG-81901/2021	EPI_ISL_3894265	2021-09-02
hCoV-19/Japan/PG-81902/2021	EPI_ISL_3894266	2021-09-02
hCoV-19/Japan/PG-81903/2021	EPI_ISL_3894267	2021-09-02
hCoV-19/Japan/PG-81904/2021	EPI_ISL_3894268	2021-09-02
hCoV-19/Japan/PG-81905/2021	EPI_ISL_3894269	2021-09-02
hCoV-19/Japan/PG-81906/2021	EPI_ISL_3894270	2021-09-02
hCoV-19/Japan/PG-81907/2021	EPI_ISL_3894271	2021-09-02
hCoV-19/Japan/PG-81908/2021	EPI_ISL_3894272	2021-09-02
hCoV-19/Japan/PG-81909/2021	EPI_ISL_3894273	2021-09-02
hCoV-19/Japan/PG-81910/2021	EPI_ISL_3894274	2021-09-02
hCoV-19/Japan/PG-81911/2021	EPI_ISL_3894275	2021-09-02
hCoV-19/Japan/PG-81912/2021	EPI_ISL_3894276	2021-09-02
hCoV-19/Japan/PG-81913/2021	EPI_ISL_3894277	2021-09-02
hCoV-19/Japan/PG-81914/2021	EPI_ISL_3894278	2021-09-02
hCoV-19/Japan/PG-81915/2021	EPI_ISL_3894279	2021-09-02
hCoV-19/Japan/PG-81916/2021	EPI_ISL_3894280	2021-09-02
hCoV-19/Japan/PG-81917/2021	EPI_ISL_3894281	2021-09-02
hCoV-19/Japan/PG-81918/2021	EPI_ISL_3894282	2021-09-02
hCoV-19/Japan/PG-81919/2021	EPI_ISL_3894283	2021-09-02
hCoV-19/Japan/PG-81920/2021	EPI_ISL_3894284	2021-09-02
hCoV-19/Japan/PG-81921/2021	EPI_ISL_3894285	2021-09-02
hCoV-19/Japan/PG-81922/2021	EPI_ISL_3894286	2021-09-02
hCoV-19/Japan/PG-81923/2021	EPI_ISL_3894287	2021-09-02
hCoV-19/Japan/PG-81924/2021	EPI_ISL_3894290	2021-09-02
hCoV-19/Japan/PG-81925/2021	EPI_ISL_3894293	2021-09-02
hCoV-19/Japan/PG-81926/2021	EPI_ISL_3894297	2021-09-02
hCoV-19/Japan/PG-81927/2021	EPI_ISL_3894300	2021-09-02
hCoV-19/Japan/PG-81928/2021	EPI_ISL_3894303	2021-09-02
hCoV-19/Japan/PG-81929/2021	EPI_ISL_3894306	2021-09-02
hCoV-19/Japan/PG-81930/2021	EPI_ISL_3894310	2021-09-02
hCoV-19/Japan/PG-81931/2021	EPI_ISL_3894313	2021-09-02
hCoV-19/Japan/PG-81932/2021	EPI_ISL_3894317	2021-09-02
hCoV-19/Japan/PG-81933/2021	EPI_ISL_3894320	2021-09-02
hCoV-19/Japan/PG-81934/2021	EPI_ISL_3894323	2021-09-02
hCoV-19/Japan/PG-81935/2021	EPI_ISL_3894327	2021-09-02

virus_name	epiID	submission_date
hCoV-19/Japan/PG-81936/2021	EPI_ISL_3894330	2021-09-02
hCoV-19/Japan/PG-81937/2021	EPI_ISL_3894332	2021-09-02
hCoV-19/Japan/PG-81938/2021	EPI_ISL_3894336	2021-09-02
hCoV-19/Japan/PG-81939/2021	EPI_ISL_3894339	2021-09-02
hCoV-19/Japan/PG-81940/2021	EPI_ISL_3894340	2021-09-02
hCoV-19/Japan/PG-81941/2021	EPI_ISL_3894342	2021-09-02
hCoV-19/Japan/PG-81942/2021	EPI_ISL_3894346	2021-09-02
hCoV-19/Japan/PG-81943/2021	EPI_ISL_3894349	2021-09-02
hCoV-19/Japan/PG-81944/2021	EPI_ISL_3894353	2021-09-02
hCoV-19/Japan/PG-81945/2021	EPI_ISL_3894356	2021-09-02
hCoV-19/Japan/PG-81946/2021	EPI_ISL_3894358	2021-09-02
hCoV-19/Japan/PG-81947/2021	EPI_ISL_3894360	2021-09-02
hCoV-19/Japan/PG-81948/2021	EPI_ISL_3894361	2021-09-02
hCoV-19/Japan/PG-81949/2021	EPI_ISL_3894362	2021-09-02
hCoV-19/Japan/PG-81950/2021	EPI_ISL_3894363	2021-09-02
hCoV-19/Japan/PG-81951/2021	EPI_ISL_3894364	2021-09-02
hCoV-19/Japan/PG-81952/2021	EPI_ISL_3894365	2021-09-02
hCoV-19/Japan/PG-81953/2021	EPI_ISL_3894366	2021-09-02
hCoV-19/Japan/PG-81954/2021	EPI_ISL_3894367	2021-09-02
hCoV-19/Japan/PG-81955/2021	EPI_ISL_3894368	2021-09-02
hCoV-19/Japan/PG-81956/2021	EPI_ISL_3894369	2021-09-02
hCoV-19/Japan/PG-81957/2021	EPI_ISL_3894370	2021-09-02
hCoV-19/Japan/PG-81958/2021	EPI_ISL_3894371	2021-09-02
hCoV-19/Japan/PG-81959/2021	EPI_ISL_3894372	2021-09-02
hCoV-19/Japan/PG-81960/2021	EPI_ISL_3894373	2021-09-02
hCoV-19/Japan/PG-81961/2021	EPI_ISL_3894374	2021-09-02
hCoV-19/Japan/PG-81962/2021	EPI_ISL_3894375	2021-09-02
hCoV-19/Japan/PG-81963/2021	EPI_ISL_3894376	2021-09-02
hCoV-19/Japan/PG-81964/2021	EPI_ISL_3894377	2021-09-02
hCoV-19/Japan/PG-81965/2021	EPI_ISL_3894378	2021-09-02
hCoV-19/Japan/PG-81966/2021	EPI_ISL_3894379	2021-09-02
hCoV-19/Japan/PG-81967/2021	EPI_ISL_3894380	2021-09-02
hCoV-19/Japan/PG-81968/2021	EPI_ISL_3894381	2021-09-02
hCoV-19/Japan/PG-81969/2021	EPI_ISL_3894382	2021-09-02
hCoV-19/Japan/PG-81970/2021	EPI_ISL_3894383	2021-09-02
hCoV-19/Japan/PG-81971/2021	EPI_ISL_3894384	2021-09-02
hCoV-19/Japan/PG-81972/2021	EPI_ISL_3894385	2021-09-02
hCoV-19/Japan/PG-81973/2021	EPI_ISL_3894386	2021-09-02
hCoV-19/Japan/PG-81974/2021	EPI_ISL_3894387	2021-09-02
hCoV-19/Japan/PG-81975/2021	EPI_ISL_3894388	2021-09-02
hCoV-19/Japan/PG-81976/2021	EPI_ISL_3894389	2021-09-02
hCoV-19/Japan/PG-81977/2021	EPI_ISL_3894390	2021-09-02
hCoV-19/Japan/PG-81978/2021	EPI_ISL_3894391	2021-09-02
hCoV-19/Japan/PG-81979/2021	EPI_ISL_3894392	2021-09-02
hCoV-19/Japan/PG-81980/2021	EPI_ISL_3894393	2021-09-02
hCoV-19/Japan/PG-81981/2021	EPI_ISL_3894394	2021-09-02
hCoV-19/Japan/PG-81982/2021	EPI_ISL_3894395	2021-09-02
hCoV-19/Japan/PG-81983/2021	EPI_ISL_3894396	2021-09-02
hCoV-19/Japan/PG-81984/2021	EPI_ISL_3894397	2021-09-02
hCoV-19/Japan/PG-81985/2021	EPI_ISL_3894398	2021-09-02
hCoV-19/Japan/PG-81986/2021	EPI_ISL_3894399	2021-09-02
hCoV-19/Japan/PG-81987/2021	EPI_ISL_3894400	2021-09-02
hCoV-19/Japan/PG-81988/2021	EPI_ISL_3894403	2021-09-02
hCoV-19/Japan/PG-81989/2021	EPI_ISL_3894405	2021-09-02

virus_name	epiID	submission_date
hCoV-19/Japan/PG-81990/2021	EPI_ISL_3894406	2021-09-02
hCoV-19/Japan/PG-81991/2021	EPI_ISL_3894408	2021-09-02
hCoV-19/Japan/PG-81992/2021	EPI_ISL_3894409	2021-09-02
hCoV-19/Japan/PG-81993/2021	EPI_ISL_3894410	2021-09-02
hCoV-19/Japan/PG-81994/2021	EPI_ISL_3894414	2021-09-02
hCoV-19/Japan/PG-81995/2021	EPI_ISL_3894417	2021-09-02
hCoV-19/Japan/PG-81996/2021	EPI_ISL_3894420	2021-09-02
hCoV-19/Japan/PG-81997/2021	EPI_ISL_3894424	2021-09-02
hCoV-19/Japan/PG-81998/2021	EPI_ISL_3894425	2021-09-02
hCoV-19/Japan/PG-81999/2021	EPI_ISL_3894426	2021-09-02
hCoV-19/Japan/PG-82000/2021	EPI_ISL_3894428	2021-09-02
hCoV-19/Japan/PG-82001/2021	EPI_ISL_3894429	2021-09-02
hCoV-19/Japan/PG-82002/2021	EPI_ISL_3894431	2021-09-02
hCoV-19/Japan/PG-82003/2021	EPI_ISL_3894432	2021-09-02
hCoV-19/Japan/PG-82004/2021	EPI_ISL_3894434	2021-09-02
hCoV-19/Japan/PG-82005/2021	EPI_ISL_3894435	2021-09-02
hCoV-19/Japan/PG-82006/2021	EPI_ISL_3894437	2021-09-02
hCoV-19/Japan/PG-82007/2021	EPI_ISL_3894438	2021-09-02
hCoV-19/Japan/PG-82008/2021	EPI_ISL_3894440	2021-09-02
hCoV-19/Japan/PG-82009/2021	EPI_ISL_3894444	2021-09-02
hCoV-19/Japan/PG-82010/2021	EPI_ISL_3894447	2021-09-02
hCoV-19/Japan/PG-82011/2021	EPI_ISL_3894451	2021-09-02
hCoV-19/Japan/PG-82012/2021	EPI_ISL_3894454	2021-09-02
hCoV-19/Japan/PG-82013/2021	EPI_ISL_3894457	2021-09-02
hCoV-19/Japan/PG-82014/2021	EPI_ISL_3894460	2021-09-02
hCoV-19/Japan/PG-82015/2021	EPI_ISL_3894461	2021-09-02
hCoV-19/Japan/PG-82016/2021	EPI_ISL_3894463	2021-09-02
hCoV-19/Japan/PG-82017/2021	EPI_ISL_3894465	2021-09-02
hCoV-19/Japan/PG-82018/2021	EPI_ISL_3894467	2021-09-02
hCoV-19/Japan/PG-82019/2021	EPI_ISL_3894470	2021-09-02
hCoV-19/Japan/PG-82020/2021	EPI_ISL_3894471	2021-09-02
hCoV-19/Japan/PG-82021/2021	EPI_ISL_3894472	2021-09-02
hCoV-19/Japan/PG-82022/2021	EPI_ISL_3894474	2021-09-02
hCoV-19/Japan/PG-82023/2021	EPI_ISL_3894475	2021-09-02
hCoV-19/Japan/PG-82024/2021	EPI_ISL_3894478	2021-09-02
hCoV-19/Japan/PG-82025/2021	EPI_ISL_3894479	2021-09-02
hCoV-19/Japan/PG-82026/2021	EPI_ISL_3894481	2021-09-02
hCoV-19/Japan/PG-82027/2021	EPI_ISL_3894482	2021-09-02
hCoV-19/Japan/PG-82028/2021	EPI_ISL_3894484	2021-09-02
hCoV-19/Japan/PG-82029/2021	EPI_ISL_3894487	2021-09-02
hCoV-19/Japan/PG-82030/2021	EPI_ISL_3894490	2021-09-02
hCoV-19/Japan/PG-82031/2021	EPI_ISL_3894492	2021-09-02
hCoV-19/Japan/PG-82032/2021	EPI_ISL_3894493	2021-09-02
hCoV-19/Japan/PG-82033/2021	EPI_ISL_3894495	2021-09-02
hCoV-19/Japan/PG-82034/2021	EPI_ISL_3894496	2021-09-02
hCoV-19/Japan/PG-82035/2021	EPI_ISL_3894498	2021-09-02
hCoV-19/Japan/PG-82036/2021	EPI_ISL_3894499	2021-09-02
hCoV-19/Japan/PG-82037/2021	EPI_ISL_3894501	2021-09-02
hCoV-19/Japan/PG-82038/2021	EPI_ISL_3894502	2021-09-02
hCoV-19/Japan/PG-82039/2021	EPI_ISL_3894503	2021-09-02
hCoV-19/Japan/PG-82040/2021	EPI_ISL_3894505	2021-09-02
hCoV-19/Japan/PG-82041/2021	EPI_ISL_3894506	2021-09-02
hCoV-19/Japan/PG-82042/2021	EPI_ISL_3894508	2021-09-02
hCoV-19/Japan/PG-82043/2021	EPI_ISL_3894509	2021-09-02

virus_name	epiID	submission_date
hCoV-19/Japan/PG-82044/2021	EPI_ISL_3894511	2021-09-02
hCoV-19/Japan/PG-82045/2021	EPI_ISL_3894512	2021-09-02
hCoV-19/Japan/PG-82046/2021	EPI_ISL_3894513	2021-09-02
hCoV-19/Japan/PG-82047/2021	EPI_ISL_3894515	2021-09-02
hCoV-19/Japan/PG-82048/2021	EPI_ISL_3894516	2021-09-02
hCoV-19/Japan/PG-82049/2021	EPI_ISL_3894518	2021-09-02
hCoV-19/Japan/PG-82050/2021	EPI_ISL_3894519	2021-09-02
hCoV-19/Japan/PG-82051/2021	EPI_ISL_3894520	2021-09-02
hCoV-19/Japan/PG-82052/2021	EPI_ISL_3894522	2021-09-02
hCoV-19/Japan/PG-82053/2021	EPI_ISL_3894523	2021-09-02
hCoV-19/Japan/PG-82054/2021	EPI_ISL_3894525	2021-09-02
hCoV-19/Japan/PG-82055/2021	EPI_ISL_3894526	2021-09-02
hCoV-19/Japan/PG-82056/2021	EPI_ISL_3894528	2021-09-02
hCoV-19/Japan/PG-82057/2021	EPI_ISL_3894529	2021-09-02
hCoV-19/Japan/PG-82058/2021	EPI_ISL_3894530	2021-09-02
hCoV-19/Japan/PG-82059/2021	EPI_ISL_3894533	2021-09-02
hCoV-19/Japan/PG-82060/2021	EPI_ISL_3894537	2021-09-02
hCoV-19/Japan/PG-82061/2021	EPI_ISL_3894540	2021-09-02
hCoV-19/Japan/PG-82062/2021	EPI_ISL_3894543	2021-09-02
hCoV-19/Japan/PG-82063/2021	EPI_ISL_3894547	2021-09-02
hCoV-19/Japan/PG-82064/2021	EPI_ISL_3894550	2021-09-02
hCoV-19/Japan/PG-82065/2021	EPI_ISL_3894554	2021-09-02
hCoV-19/Japan/PG-82066/2021	EPI_ISL_3894557	2021-09-02
hCoV-19/Japan/PG-82067/2021	EPI_ISL_3894561	2021-09-02
hCoV-19/Japan/PG-82068/2021	EPI_ISL_3894564	2021-09-02
hCoV-19/Japan/PG-82069/2021	EPI_ISL_3894568	2021-09-02
hCoV-19/Japan/PG-82070/2021	EPI_ISL_3894571	2021-09-02
hCoV-19/Japan/PG-82071/2021	EPI_ISL_3894573	2021-09-02
hCoV-19/Japan/PG-82072/2021	EPI_ISL_3904518	2021-09-02
hCoV-19/Japan/PG-82073/2021	EPI_ISL_3904519	2021-09-02
hCoV-19/Japan/PG-82074/2021	EPI_ISL_3904520	2021-09-02
hCoV-19/Japan/PG-82075/2021	EPI_ISL_3904521	2021-09-02
hCoV-19/Japan/PG-82076/2021	EPI_ISL_3904522	2021-09-02
hCoV-19/Japan/PG-82077/2021	EPI_ISL_3904523	2021-09-02
hCoV-19/Japan/PG-82078/2021	EPI_ISL_3904524	2021-09-02
hCoV-19/Japan/PG-82079/2021	EPI_ISL_3904525	2021-09-02
hCoV-19/Japan/PG-82080/2021	EPI_ISL_3904526	2021-09-02
hCoV-19/Japan/PG-82081/2021	EPI_ISL_3904527	2021-09-02
hCoV-19/Japan/PG-82082/2021	EPI_ISL_3904528	2021-09-02
hCoV-19/Japan/PG-82083/2021	EPI_ISL_3904529	2021-09-02
hCoV-19/Japan/PG-82084/2021	EPI_ISL_3904530	2021-09-02
hCoV-19/Japan/PG-82085/2021	EPI_ISL_3904531	2021-09-02
hCoV-19/Japan/PG-82086/2021	EPI_ISL_3904532	2021-09-02
hCoV-19/Japan/PG-82087/2021	EPI_ISL_3904533	2021-09-02
hCoV-19/Japan/PG-82088/2021	EPI_ISL_3904534	2021-09-02
hCoV-19/Japan/PG-82089/2021	EPI_ISL_3904535	2021-09-02
hCoV-19/Japan/PG-82090/2021	EPI_ISL_3904536	2021-09-02
hCoV-19/Japan/PG-82091/2021	EPI_ISL_3904537	2021-09-02
hCoV-19/Japan/PG-82092/2021	EPI_ISL_3904538	2021-09-02
hCoV-19/Japan/PG-82093/2021	EPI_ISL_3904539	2021-09-02
hCoV-19/Japan/PG-82094/2021	EPI_ISL_3904540	2021-09-02
hCoV-19/Japan/PG-82095/2021	EPI_ISL_3904541	2021-09-02
hCoV-19/Japan/PG-82096/2021	EPI_ISL_3904542	2021-09-02
hCoV-19/Japan/PG-82097/2021	EPI_ISL_3904543	2021-09-02

virus_name	epiID	submission_date
hCoV-19/Japan/PG-82098/2021	EPI_ISL_3904544	2021-09-02
hCoV-19/Japan/PG-82099/2021	EPI_ISL_3904545	2021-09-02
hCoV-19/Japan/PG-82100/2021	EPI_ISL_3894574	2021-09-02
hCoV-19/Japan/PG-82101/2021	EPI_ISL_3894575	2021-09-02
hCoV-19/Japan/PG-82102/2021	EPI_ISL_3894576	2021-09-02
hCoV-19/Japan/PG-82103/2021	EPI_ISL_3894577	2021-09-02
hCoV-19/Japan/PG-82104/2021	EPI_ISL_3894578	2021-09-02
hCoV-19/Japan/PG-82105/2021	EPI_ISL_3894579	2021-09-02
hCoV-19/Japan/PG-82106/2021	EPI_ISL_3894580	2021-09-02
hCoV-19/Japan/PG-82107/2021	EPI_ISL_3894581	2021-09-02
hCoV-19/Japan/PG-82108/2021	EPI_ISL_3894582	2021-09-02
hCoV-19/Japan/PG-82109/2021	EPI_ISL_3894583	2021-09-02
hCoV-19/Japan/PG-82110/2021	EPI_ISL_3894584	2021-09-02
hCoV-19/Japan/PG-82111/2021	EPI_ISL_3894585	2021-09-02
hCoV-19/Japan/PG-82112/2021	EPI_ISL_3894586	2021-09-02
hCoV-19/Japan/PG-82113/2021	EPI_ISL_3894587	2021-09-02
hCoV-19/Japan/PG-82114/2021	EPI_ISL_3894588	2021-09-02
hCoV-19/Japan/PG-82115/2021	EPI_ISL_3894589	2021-09-02
hCoV-19/Japan/PG-82116/2021	EPI_ISL_3894590	2021-09-02
hCoV-19/Japan/PG-82117/2021	EPI_ISL_3894591	2021-09-02
hCoV-19/Japan/PG-82118/2021	EPI_ISL_3894592	2021-09-02
hCoV-19/Japan/PG-82119/2021	EPI_ISL_3894593	2021-09-02
hCoV-19/Japan/PG-82120/2021	EPI_ISL_3894594	2021-09-02
hCoV-19/Japan/PG-82121/2021	EPI_ISL_3894595	2021-09-02
hCoV-19/Japan/PG-82122/2021	EPI_ISL_3894596	2021-09-02
hCoV-19/Japan/PG-82123/2021	EPI_ISL_3894597	2021-09-02
hCoV-19/Japan/PG-82124/2021	EPI_ISL_3894598	2021-09-02
hCoV-19/Japan/PG-82125/2021	EPI_ISL_3894599	2021-09-02
hCoV-19/Japan/PG-82126/2021	EPI_ISL_3894600	2021-09-02
hCoV-19/Japan/PG-82127/2021	EPI_ISL_3894601	2021-09-02
hCoV-19/Japan/PG-82128/2021	EPI_ISL_3894602	2021-09-02
hCoV-19/Japan/PG-82129/2021	EPI_ISL_3894603	2021-09-02
hCoV-19/Japan/PG-82130/2021	EPI_ISL_3894604	2021-09-02
hCoV-19/Japan/PG-82131/2021	EPI_ISL_3894605	2021-09-02
hCoV-19/Japan/PG-82132/2021	EPI_ISL_3894606	2021-09-02
hCoV-19/Japan/PG-82133/2021	EPI_ISL_3894607	2021-09-02
hCoV-19/Japan/PG-82134/2021	EPI_ISL_3894608	2021-09-02
hCoV-19/Japan/PG-82135/2021	EPI_ISL_3894609	2021-09-02
hCoV-19/Japan/PG-82136/2021	EPI_ISL_3894610	2021-09-02
hCoV-19/Japan/PG-82137/2021	EPI_ISL_3894611	2021-09-02
hCoV-19/Japan/PG-82138/2021	EPI_ISL_3894612	2021-09-02
hCoV-19/Japan/PG-82139/2021	EPI_ISL_3894613	2021-09-02
hCoV-19/Japan/PG-82140/2021	EPI_ISL_3894614	2021-09-02
hCoV-19/Japan/PG-82141/2021	EPI_ISL_3894615	2021-09-02
hCoV-19/Japan/PG-82142/2021	EPI_ISL_3894616	2021-09-02
hCoV-19/Japan/PG-82143/2021	EPI_ISL_3894617	2021-09-02
hCoV-19/Japan/PG-82144/2021	EPI_ISL_3894618	2021-09-02
hCoV-19/Japan/PG-82145/2021	EPI_ISL_3894619	2021-09-02
hCoV-19/Japan/PG-82146/2021	EPI_ISL_3894620	2021-09-02
hCoV-19/Japan/PG-82147/2021	EPI_ISL_3894621	2021-09-02
hCoV-19/Japan/PG-82148/2021	EPI_ISL_3894622	2021-09-02
hCoV-19/Japan/PG-82149/2021	EPI_ISL_3894623	2021-09-02
hCoV-19/Japan/PG-82150/2021	EPI_ISL_3894624	2021-09-02
hCoV-19/Japan/PG-82151/2021	EPI_ISL_3894625	2021-09-02

virus_name	epiID	submission_date
hCoV-19/Japan/PG-82152/2021	EPI_ISL_3894626	2021-09-02
hCoV-19/Japan/PG-82153/2021	EPI_ISL_3894627	2021-09-02
hCoV-19/Japan/PG-82154/2021	EPI_ISL_3894628	2021-09-02
hCoV-19/Japan/PG-82155/2021	EPI_ISL_3894629	2021-09-02
hCoV-19/Japan/PG-82156/2021	EPI_ISL_3894630	2021-09-02
hCoV-19/Japan/PG-82157/2021	EPI_ISL_3894631	2021-09-02
hCoV-19/Japan/PG-82158/2021	EPI_ISL_3894632	2021-09-02
hCoV-19/Japan/PG-82159/2021	EPI_ISL_3894633	2021-09-02
hCoV-19/Japan/PG-82160/2021	EPI_ISL_3894634	2021-09-02
hCoV-19/Japan/PG-82161/2021	EPI_ISL_3894635	2021-09-02
hCoV-19/Japan/PG-82162/2021	EPI_ISL_3894636	2021-09-02
hCoV-19/Japan/PG-82163/2021	EPI_ISL_3894637	2021-09-02
hCoV-19/Japan/PG-82164/2021	EPI_ISL_3894638	2021-09-02
hCoV-19/Japan/PG-82165/2021	EPI_ISL_3894639	2021-09-02
hCoV-19/Japan/PG-82166/2021	EPI_ISL_3894640	2021-09-02
hCoV-19/Japan/PG-82167/2021	EPI_ISL_3894641	2021-09-02
hCoV-19/Japan/PG-82168/2021	EPI_ISL_3894642	2021-09-02
hCoV-19/Japan/PG-82169/2021	EPI_ISL_3894643	2021-09-02
hCoV-19/Japan/PG-82170/2021	EPI_ISL_3894644	2021-09-02
hCoV-19/Japan/PG-82171/2021	EPI_ISL_3894645	2021-09-02
hCoV-19/Japan/PG-82172/2021	EPI_ISL_3894646	2021-09-02
hCoV-19/Japan/PG-82173/2021	EPI_ISL_3894647	2021-09-02
hCoV-19/Japan/PG-82174/2021	EPI_ISL_3894648	2021-09-02
hCoV-19/Japan/PG-82175/2021	EPI_ISL_3894649	2021-09-02
hCoV-19/Japan/PG-82176/2021	EPI_ISL_3894650	2021-09-02
hCoV-19/Japan/PG-82177/2021	EPI_ISL_3894651	2021-09-02
hCoV-19/Japan/PG-82178/2021	EPI_ISL_3894652	2021-09-02
hCoV-19/Japan/PG-82179/2021	EPI_ISL_3894653	2021-09-02
hCoV-19/Japan/PG-82180/2021	EPI_ISL_3894654	2021-09-02
hCoV-19/Japan/PG-82181/2021	EPI_ISL_3894655	2021-09-02
hCoV-19/Japan/PG-82182/2021	EPI_ISL_3894656	2021-09-02
hCoV-19/Japan/PG-82183/2021	EPI_ISL_3894657	2021-09-02
hCoV-19/Japan/PG-82184/2021	EPI_ISL_3894658	2021-09-02
hCoV-19/Japan/PG-82185/2021	EPI_ISL_3894659	2021-09-02
hCoV-19/Japan/PG-82186/2021	EPI_ISL_3894660	2021-09-02
hCoV-19/Japan/PG-82187/2021	EPI_ISL_3894661	2021-09-02
hCoV-19/Japan/PG-82188/2021	EPI_ISL_3894662	2021-09-02
hCoV-19/Japan/PG-82189/2021	EPI_ISL_3894663	2021-09-02
hCoV-19/Japan/PG-82190/2021	EPI_ISL_3894664	2021-09-02
hCoV-19/Japan/PG-82191/2021	EPI_ISL_3894665	2021-09-02
hCoV-19/Japan/PG-82192/2021	EPI_ISL_3894666	2021-09-02
hCoV-19/Japan/PG-82193/2021	EPI_ISL_3894667	2021-09-02
hCoV-19/Japan/PG-82194/2021	EPI_ISL_3894668	2021-09-02
hCoV-19/Japan/PG-82195/2021	EPI_ISL_3894669	2021-09-02
hCoV-19/Japan/PG-82196/2021	EPI_ISL_3894670	2021-09-02
hCoV-19/Japan/PG-82197/2021	EPI_ISL_3894671	2021-09-02
hCoV-19/Japan/PG-82198/2021	EPI_ISL_3894672	2021-09-02
hCoV-19/Japan/PG-82199/2021	EPI_ISL_3894673	2021-09-02
hCoV-19/Japan/PG-82200/2021	EPI_ISL_3894674	2021-09-02
hCoV-19/Japan/PG-82201/2021	EPI_ISL_3894675	2021-09-02
hCoV-19/Japan/PG-82202/2021	EPI_ISL_3894676	2021-09-02
hCoV-19/Japan/PG-82203/2021	EPI_ISL_3894677	2021-09-02
hCoV-19/Japan/PG-82204/2021	EPI_ISL_3894678	2021-09-02
hCoV-19/Japan/PG-82205/2021	EPI_ISL_3894679	2021-09-02

virus_name	epiID	submission_date
hCoV-19/Japan/PG-82206/2021	EPI_ISL_3894680	2021-09-02
hCoV-19/Japan/PG-82207/2021	EPI_ISL_3894681	2021-09-02
hCoV-19/Japan/PG-82208/2021	EPI_ISL_3894682	2021-09-02
hCoV-19/Japan/PG-82209/2021	EPI_ISL_3894683	2021-09-02
hCoV-19/Japan/PG-82210/2021	EPI_ISL_3894684	2021-09-02
hCoV-19/Japan/PG-82211/2021	EPI_ISL_3894685	2021-09-02
hCoV-19/Japan/PG-82212/2021	EPI_ISL_3894686	2021-09-02
hCoV-19/Japan/PG-82213/2021	EPI_ISL_3894687	2021-09-02
hCoV-19/Japan/PG-82214/2021	EPI_ISL_3894688	2021-09-02
hCoV-19/Japan/PG-82215/2021	EPI_ISL_3894689	2021-09-02
hCoV-19/Japan/PG-82216/2021	EPI_ISL_3894690	2021-09-02
hCoV-19/Japan/PG-82217/2021	EPI_ISL_3894691	2021-09-02
hCoV-19/Japan/PG-82218/2021	EPI_ISL_3894692	2021-09-02
hCoV-19/Japan/PG-82219/2021	EPI_ISL_3894693	2021-09-02
hCoV-19/Japan/PG-82220/2021	EPI_ISL_3894694	2021-09-02
hCoV-19/Japan/PG-82221/2021	EPI_ISL_3894695	2021-09-02
hCoV-19/Japan/PG-82222/2021	EPI_ISL_3894696	2021-09-02
hCoV-19/Japan/PG-82223/2021	EPI_ISL_3894697	2021-09-02
hCoV-19/Japan/PG-82224/2021	EPI_ISL_3894698	2021-09-02
hCoV-19/Japan/PG-82225/2021	EPI_ISL_3894699	2021-09-02
hCoV-19/Japan/PG-82226/2021	EPI_ISL_3894700	2021-09-02
hCoV-19/Japan/PG-82227/2021	EPI_ISL_3894701	2021-09-02
hCoV-19/Japan/PG-82228/2021	EPI_ISL_3894702	2021-09-02
hCoV-19/Japan/PG-82229/2021	EPI_ISL_3894703	2021-09-02
hCoV-19/Japan/PG-82230/2021	EPI_ISL_3894704	2021-09-02
hCoV-19/Japan/PG-82231/2021	EPI_ISL_3894705	2021-09-02
hCoV-19/Japan/PG-82232/2021	EPI_ISL_3894706	2021-09-02
hCoV-19/Japan/PG-82233/2021	EPI_ISL_3894707	2021-09-02
hCoV-19/Japan/PG-82234/2021	EPI_ISL_3894708	2021-09-02
hCoV-19/Japan/PG-82235/2021	EPI_ISL_3894709	2021-09-02
hCoV-19/Japan/PG-82236/2021	EPI_ISL_3894710	2021-09-02
hCoV-19/Japan/PG-82237/2021	EPI_ISL_3894711	2021-09-02
hCoV-19/Japan/PG-82238/2021	EPI_ISL_3894712	2021-09-02
hCoV-19/Japan/PG-82239/2021	EPI_ISL_3894713	2021-09-02
hCoV-19/Japan/PG-82240/2021	EPI_ISL_3894714	2021-09-02
hCoV-19/Japan/PG-82241/2021	EPI_ISL_3894715	2021-09-02
hCoV-19/Japan/PG-82242/2021	EPI_ISL_3894716	2021-09-02
hCoV-19/Japan/PG-82243/2021	EPI_ISL_3894717	2021-09-02
hCoV-19/Japan/PG-82244/2021	EPI_ISL_3894718	2021-09-02
hCoV-19/Japan/PG-82245/2021	EPI_ISL_3894719	2021-09-02
hCoV-19/Japan/PG-82246/2021	EPI_ISL_3894720	2021-09-02
hCoV-19/Japan/PG-82247/2021	EPI_ISL_3894721	2021-09-02
hCoV-19/Japan/PG-82248/2021	EPI_ISL_3894722	2021-09-02
hCoV-19/Japan/PG-82249/2021	EPI_ISL_3894723	2021-09-02
hCoV-19/Japan/PG-82250/2021	EPI_ISL_3894724	2021-09-02
hCoV-19/Japan/PG-82251/2021	EPI_ISL_3894725	2021-09-02
hCoV-19/Japan/PG-82252/2021	EPI_ISL_3894726	2021-09-02
hCoV-19/Japan/PG-82253/2021	EPI_ISL_3894727	2021-09-02
hCoV-19/Japan/PG-82254/2021	EPI_ISL_3894728	2021-09-02
hCoV-19/Japan/PG-82255/2021	EPI_ISL_3894729	2021-09-02
hCoV-19/Japan/PG-82256/2021	EPI_ISL_3894730	2021-09-02
hCoV-19/Japan/PG-82257/2021	EPI_ISL_3894731	2021-09-02
hCoV-19/Japan/PG-82258/2021	EPI_ISL_3894732	2021-09-02
hCoV-19/Japan/PG-82259/2021	EPI_ISL_3894733	2021-09-02

virus_name	epiID	submission_date
hCoV-19/Japan/PG-82260/2021	EPI_ISL_3894734	2021-09-02
hCoV-19/Japan/PG-82261/2021	EPI_ISL_3894735	2021-09-02
hCoV-19/Japan/PG-82262/2021	EPI_ISL_3894736	2021-09-02
hCoV-19/Japan/PG-82263/2021	EPI_ISL_3894737	2021-09-02
hCoV-19/Japan/PG-82264/2021	EPI_ISL_3894738	2021-09-02
hCoV-19/Japan/PG-82265/2021	EPI_ISL_3894739	2021-09-02
hCoV-19/Japan/PG-82266/2021	EPI_ISL_3894740	2021-09-02
hCoV-19/Japan/PG-82267/2021	EPI_ISL_3894741	2021-09-02
hCoV-19/Japan/PG-82268/2021	EPI_ISL_3894742	2021-09-02
hCoV-19/Japan/PG-82269/2021	EPI_ISL_3894743	2021-09-02
hCoV-19/Japan/PG-82270/2021	EPI_ISL_3894744	2021-09-02
hCoV-19/Japan/PG-82271/2021	EPI_ISL_3894745	2021-09-02
hCoV-19/Japan/PG-82272/2021	EPI_ISL_3894746	2021-09-02
hCoV-19/Japan/PG-82273/2021	EPI_ISL_3894747	2021-09-02
hCoV-19/Japan/PG-82274/2021	EPI_ISL_3894748	2021-09-02
hCoV-19/Japan/PG-82275/2021	EPI_ISL_3894749	2021-09-02
hCoV-19/Japan/PG-82276/2021	EPI_ISL_3894750	2021-09-02
hCoV-19/Japan/PG-82277/2021	EPI_ISL_3894751	2021-09-02
hCoV-19/Japan/PG-82278/2021	EPI_ISL_3894752	2021-09-02
hCoV-19/Japan/PG-82279/2021	EPI_ISL_3894753	2021-09-02
hCoV-19/Japan/PG-82280/2021	EPI_ISL_3894754	2021-09-02
hCoV-19/Japan/PG-82281/2021	EPI_ISL_3894755	2021-09-02
hCoV-19/Japan/PG-82282/2021	EPI_ISL_3894756	2021-09-02
hCoV-19/Japan/PG-82283/2021	EPI_ISL_3894757	2021-09-02
hCoV-19/Japan/PG-82284/2021	EPI_ISL_3894758	2021-09-02
hCoV-19/Japan/PG-82285/2021	EPI_ISL_3894759	2021-09-02
hCoV-19/Japan/PG-82286/2021	EPI_ISL_3894760	2021-09-02
hCoV-19/Japan/PG-82287/2021	EPI_ISL_3894761	2021-09-02
hCoV-19/Japan/PG-82288/2021	EPI_ISL_3894762	2021-09-02
hCoV-19/Japan/PG-82289/2021	EPI_ISL_3894763	2021-09-02
hCoV-19/Japan/PG-82290/2021	EPI_ISL_3894764	2021-09-02
hCoV-19/Japan/PG-82291/2021	EPI_ISL_3894765	2021-09-02
hCoV-19/Japan/PG-82292/2021	EPI_ISL_3894766	2021-09-02
hCoV-19/Japan/PG-82293/2021	EPI_ISL_3894767	2021-09-02
hCoV-19/Japan/PG-82294/2021	EPI_ISL_3894768	2021-09-02
hCoV-19/Japan/PG-82295/2021	EPI_ISL_3894769	2021-09-02
hCoV-19/Japan/PG-82296/2021	EPI_ISL_3894770	2021-09-02
hCoV-19/Japan/PG-82297/2021	EPI_ISL_3894771	2021-09-02
hCoV-19/Japan/PG-82298/2021	EPI_ISL_3894772	2021-09-02
hCoV-19/Japan/PG-82299/2021	EPI_ISL_3894773	2021-09-02
hCoV-19/Japan/PG-82300/2021	EPI_ISL_3894774	2021-09-02
hCoV-19/Japan/PG-82301/2021	EPI_ISL_3894775	2021-09-02
hCoV-19/Japan/PG-82302/2021	EPI_ISL_3894776	2021-09-02
hCoV-19/Japan/PG-82303/2021	EPI_ISL_3894777	2021-09-02
hCoV-19/Japan/PG-82304/2021	EPI_ISL_3894778	2021-09-02
hCoV-19/Japan/PG-82305/2021	EPI_ISL_3894779	2021-09-02
hCoV-19/Japan/PG-82306/2021	EPI_ISL_3894780	2021-09-02
hCoV-19/Japan/PG-82307/2021	EPI_ISL_3894781	2021-09-02
hCoV-19/Japan/PG-82308/2021	EPI_ISL_3894782	2021-09-02
hCoV-19/Japan/PG-82309/2021	EPI_ISL_3894783	2021-09-02
hCoV-19/Japan/PG-82310/2021	EPI_ISL_3894784	2021-09-02
hCoV-19/Japan/PG-82311/2021	EPI_ISL_3894785	2021-09-02
hCoV-19/Japan/PG-82312/2021	EPI_ISL_3894786	2021-09-02
hCoV-19/Japan/PG-82313/2021	EPI_ISL_3894787	2021-09-02

virus_name	epiID	submission_date
hCoV-19/Japan/PG-82314/2021	EPI_ISL_3894788	2021-09-02
hCoV-19/Japan/PG-82315/2021	EPI_ISL_3894789	2021-09-02
hCoV-19/Japan/PG-82316/2021	EPI_ISL_3894790	2021-09-02
hCoV-19/Japan/PG-82317/2021	EPI_ISL_3894791	2021-09-02
hCoV-19/Japan/PG-82318/2021	EPI_ISL_3894792	2021-09-02
hCoV-19/Japan/PG-82319/2021	EPI_ISL_3894793	2021-09-02
hCoV-19/Japan/PG-82320/2021	EPI_ISL_3894794	2021-09-02
hCoV-19/Japan/PG-82321/2021	EPI_ISL_3894795	2021-09-02
hCoV-19/Japan/PG-82322/2021	EPI_ISL_3894796	2021-09-02
hCoV-19/Japan/PG-82323/2021	EPI_ISL_3894797	2021-09-02
hCoV-19/Japan/PG-82324/2021	EPI_ISL_3894798	2021-09-02
hCoV-19/Japan/PG-82325/2021	EPI_ISL_3894799	2021-09-02
hCoV-19/Japan/PG-82326/2021	EPI_ISL_3894800	2021-09-02
hCoV-19/Japan/PG-82327/2021	EPI_ISL_3894801	2021-09-02
hCoV-19/Japan/PG-82328/2021	EPI_ISL_3894802	2021-09-02
hCoV-19/Japan/PG-82329/2021	EPI_ISL_3894803	2021-09-02
hCoV-19/Japan/PG-82330/2021	EPI_ISL_3894804	2021-09-02
hCoV-19/Japan/PG-82331/2021	EPI_ISL_3894805	2021-09-02
hCoV-19/Japan/PG-82332/2021	EPI_ISL_3894806	2021-09-02
hCoV-19/Japan/PG-82333/2021	EPI_ISL_3894807	2021-09-02
hCoV-19/Japan/PG-82334/2021	EPI_ISL_3894808	2021-09-02
hCoV-19/Japan/PG-82335/2021	EPI_ISL_3894809	2021-09-02
hCoV-19/Japan/PG-82336/2021	EPI_ISL_3894810	2021-09-02
hCoV-19/Japan/PG-82337/2021	EPI_ISL_3894811	2021-09-02
hCoV-19/Japan/PG-82338/2021	EPI_ISL_3894812	2021-09-02
hCoV-19/Japan/PG-82339/2021	EPI_ISL_3894813	2021-09-02
hCoV-19/Japan/PG-82340/2021	EPI_ISL_3894814	2021-09-02
hCoV-19/Japan/PG-82341/2021	EPI_ISL_3894815	2021-09-02
hCoV-19/Japan/PG-82342/2021	EPI_ISL_3894816	2021-09-02
hCoV-19/Japan/PG-82343/2021	EPI_ISL_3894817	2021-09-02
hCoV-19/Japan/PG-82344/2021	EPI_ISL_3894818	2021-09-02
hCoV-19/Japan/PG-82345/2021	EPI_ISL_3894819	2021-09-02
hCoV-19/Japan/PG-82346/2021	EPI_ISL_3894820	2021-09-02
hCoV-19/Japan/PG-82347/2021	EPI_ISL_3894821	2021-09-02
hCoV-19/Japan/PG-82348/2021	EPI_ISL_3894822	2021-09-02
hCoV-19/Japan/PG-82349/2021	EPI_ISL_3894823	2021-09-02
hCoV-19/Japan/PG-82350/2021	EPI_ISL_3894824	2021-09-02
hCoV-19/Japan/PG-82351/2021	EPI_ISL_3894825	2021-09-02
hCoV-19/Japan/PG-82352/2021	EPI_ISL_3894826	2021-09-02
hCoV-19/Japan/PG-82353/2021	EPI_ISL_3894827	2021-09-02
hCoV-19/Japan/PG-82354/2021	EPI_ISL_3894828	2021-09-02
hCoV-19/Japan/PG-82355/2021	EPI_ISL_3894829	2021-09-02
hCoV-19/Japan/PG-82356/2021	EPI_ISL_3894830	2021-09-02
hCoV-19/Japan/PG-82357/2021	EPI_ISL_3894831	2021-09-02
hCoV-19/Japan/PG-82358/2021	EPI_ISL_3894832	2021-09-02
hCoV-19/Japan/PG-82359/2021	EPI_ISL_3894833	2021-09-02
hCoV-19/Japan/PG-82360/2021	EPI_ISL_3894834	2021-09-02
hCoV-19/Japan/PG-82361/2021	EPI_ISL_3894835	2021-09-02
hCoV-19/Japan/PG-82362/2021	EPI_ISL_3894836	2021-09-02
hCoV-19/Japan/PG-82363/2021	EPI_ISL_3894837	2021-09-02
hCoV-19/Japan/PG-82364/2021	EPI_ISL_3894838	2021-09-02
hCoV-19/Japan/PG-82365/2021	EPI_ISL_3894839	2021-09-02
hCoV-19/Japan/PG-82366/2021	EPI_ISL_3894840	2021-09-02
hCoV-19/Japan/PG-82367/2021	EPI_ISL_3894841	2021-09-02

virus_name	epiID	submission_date
hCoV-19/Japan/PG-82368/2021	EPI_ISL_3894842	2021-09-02
hCoV-19/Japan/PG-82369/2021	EPI_ISL_3894843	2021-09-02
hCoV-19/Japan/PG-82370/2021	EPI_ISL_3894844	2021-09-02
hCoV-19/Japan/PG-82371/2021	EPI_ISL_3894845	2021-09-02
hCoV-19/Japan/PG-82372/2021	EPI_ISL_3894846	2021-09-02
hCoV-19/Japan/PG-82373/2021	EPI_ISL_3894847	2021-09-02
hCoV-19/Japan/PG-82374/2021	EPI_ISL_3894848	2021-09-02
hCoV-19/Japan/PG-82375/2021	EPI_ISL_3894849	2021-09-02
hCoV-19/Japan/PG-82376/2021	EPI_ISL_3894850	2021-09-02
hCoV-19/Japan/PG-82377/2021	EPI_ISL_3894851	2021-09-02
hCoV-19/Japan/PG-82378/2021	EPI_ISL_3894852	2021-09-02
hCoV-19/Japan/PG-82379/2021	EPI_ISL_3894853	2021-09-02
hCoV-19/Japan/PG-82380/2021	EPI_ISL_3894854	2021-09-02
hCoV-19/Japan/PG-82381/2021	EPI_ISL_3894855	2021-09-02
hCoV-19/Japan/PG-82382/2021	EPI_ISL_3894856	2021-09-02
hCoV-19/Japan/PG-82383/2021	EPI_ISL_3894857	2021-09-02
hCoV-19/Japan/PG-82384/2021	EPI_ISL_3894858	2021-09-02
hCoV-19/Japan/PG-82385/2021	EPI_ISL_3894859	2021-09-02
hCoV-19/Japan/PG-82386/2021	EPI_ISL_3894860	2021-09-02
hCoV-19/Japan/PG-82387/2021	EPI_ISL_3894861	2021-09-02
hCoV-19/Japan/PG-82388/2021	EPI_ISL_3894862	2021-09-02
hCoV-19/Japan/PG-82389/2021	EPI_ISL_3894863	2021-09-02
hCoV-19/Japan/PG-82390/2021	EPI_ISL_3894864	2021-09-02
hCoV-19/Japan/PG-82391/2021	EPI_ISL_3894865	2021-09-02
hCoV-19/Japan/PG-82392/2021	EPI_ISL_3894866	2021-09-02
hCoV-19/Japan/PG-82393/2021	EPI_ISL_3894867	2021-09-02
hCoV-19/Japan/PG-82394/2021	EPI_ISL_3894868	2021-09-02
hCoV-19/Japan/PG-82395/2021	EPI_ISL_3894869	2021-09-02
hCoV-19/Japan/PG-82396/2021	EPI_ISL_3894870	2021-09-02
hCoV-19/Japan/PG-82397/2021	EPI_ISL_3894871	2021-09-02
hCoV-19/Japan/PG-82398/2021	EPI_ISL_3894872	2021-09-02
hCoV-19/Japan/PG-82399/2021	EPI_ISL_3894873	2021-09-02
hCoV-19/Japan/PG-82400/2021	EPI_ISL_3894874	2021-09-02
hCoV-19/Japan/PG-82401/2021	EPI_ISL_3894875	2021-09-02
hCoV-19/Japan/PG-82402/2021	EPI_ISL_3894876	2021-09-02
hCoV-19/Japan/PG-82403/2021	EPI_ISL_3894877	2021-09-02
hCoV-19/Japan/PG-82404/2021	EPI_ISL_3894878	2021-09-02
hCoV-19/Japan/PG-82405/2021	EPI_ISL_3894879	2021-09-02
hCoV-19/Japan/PG-82406/2021	EPI_ISL_3894880	2021-09-02
hCoV-19/Japan/PG-82407/2021	EPI_ISL_3873806	2021-09-02
hCoV-19/Japan/PG-82408/2021	EPI_ISL_3873807	2021-09-02
hCoV-19/Japan/PG-82409/2021	EPI_ISL_3873808	2021-09-02
hCoV-19/Japan/PG-82410/2021	EPI_ISL_3873809	2021-09-02
hCoV-19/Japan/PG-82411/2021	EPI_ISL_3873810	2021-09-02
hCoV-19/Japan/PG-82412/2021	EPI_ISL_3873811	2021-09-02
hCoV-19/Japan/PG-82413/2021	EPI_ISL_3873812	2021-09-02
hCoV-19/Japan/PG-82414/2021	EPI_ISL_3873813	2021-09-02
hCoV-19/Japan/PG-82415/2021	EPI_ISL_3873814	2021-09-02
hCoV-19/Japan/PG-82416/2021	EPI_ISL_3873815	2021-09-02
hCoV-19/Japan/PG-82417/2021	EPI_ISL_3873816	2021-09-02
hCoV-19/Japan/PG-82418/2021	EPI_ISL_3873817	2021-09-02
hCoV-19/Japan/PG-82419/2021	EPI_ISL_3873818	2021-09-02
hCoV-19/Japan/PG-82420/2021	EPI_ISL_3873819	2021-09-02
hCoV-19/Japan/PG-82421/2021	EPI_ISL_3873820	2021-09-02

virus_name	epiID	submission_date
hCoV-19/Japan/PG-82422/2021	EPI_ISL_3873821	2021-09-02
hCoV-19/Japan/PG-82423/2021	EPI_ISL_3873822	2021-09-02
hCoV-19/Japan/PG-82424/2021	EPI_ISL_3873823	2021-09-02
hCoV-19/Japan/PG-82425/2021	EPI_ISL_3873824	2021-09-02
hCoV-19/Japan/PG-82426/2021	EPI_ISL_3873825	2021-09-02
hCoV-19/Japan/PG-82427/2021	EPI_ISL_3873826	2021-09-02
hCoV-19/Japan/PG-82428/2021	EPI_ISL_3873827	2021-09-02
hCoV-19/Japan/PG-82429/2021	EPI_ISL_3873828	2021-09-02
hCoV-19/Japan/PG-82430/2021	EPI_ISL_3873829	2021-09-02
hCoV-19/Japan/PG-82431/2021	EPI_ISL_3873830	2021-09-02
hCoV-19/Japan/PG-82432/2021	EPI_ISL_3873831	2021-09-02
hCoV-19/Japan/PG-82433/2021	EPI_ISL_3873832	2021-09-02
hCoV-19/Japan/PG-82434/2021	EPI_ISL_3873833	2021-09-02
hCoV-19/Japan/PG-82435/2021	EPI_ISL_3873834	2021-09-02
hCoV-19/Japan/PG-82436/2021	EPI_ISL_3873835	2021-09-02
hCoV-19/Japan/PG-82437/2021	EPI_ISL_3873836	2021-09-02
hCoV-19/Japan/PG-82438/2021	EPI_ISL_3873837	2021-09-02
hCoV-19/Japan/PG-82439/2021	EPI_ISL_3871074	2021-09-02
hCoV-19/Japan/PG-82440/2021	EPI_ISL_3871075	2021-09-02
hCoV-19/Japan/PG-82441/2021	EPI_ISL_3871076	2021-09-02
hCoV-19/Japan/PG-82442/2021	EPI_ISL_3871077	2021-09-02
hCoV-19/Japan/PG-82443/2021	EPI_ISL_3871078	2021-09-02
hCoV-19/Japan/PG-82444/2021	EPI_ISL_3871079	2021-09-02
hCoV-19/Japan/PG-82445/2021	EPI_ISL_3871080	2021-09-02
hCoV-19/Japan/PG-82446/2021	EPI_ISL_3871081	2021-09-02
hCoV-19/Japan/PG-82447/2021	EPI_ISL_3871082	2021-09-02
hCoV-19/Japan/PG-82448/2021	EPI_ISL_3871083	2021-09-02
hCoV-19/Japan/PG-82449/2021	EPI_ISL_3871084	2021-09-02
hCoV-19/Japan/PG-82450/2021	EPI_ISL_3871085	2021-09-02
hCoV-19/Japan/PG-82451/2021	EPI_ISL_3871086	2021-09-02
hCoV-19/Japan/PG-82452/2021	EPI_ISL_3871087	2021-09-02
hCoV-19/Japan/PG-82453/2021	EPI_ISL_3871088	2021-09-02
hCoV-19/Japan/PG-82454/2021	EPI_ISL_3871089	2021-09-02
hCoV-19/Japan/PG-82455/2021	EPI_ISL_3873393	2021-09-02
hCoV-19/Japan/PG-82456/2021	EPI_ISL_3873394	2021-09-02
hCoV-19/Japan/PG-82457/2021	EPI_ISL_3873395	2021-09-02
hCoV-19/Japan/PG-82458/2021	EPI_ISL_3873396	2021-09-02
hCoV-19/Japan/PG-82459/2021	EPI_ISL_3873397	2021-09-02
hCoV-19/Japan/PG-82460/2021	EPI_ISL_3873398	2021-09-02
hCoV-19/Japan/PG-82461/2021	EPI_ISL_3873399	2021-09-02
hCoV-19/Japan/PG-82462/2021	EPI_ISL_3873400	2021-09-02
hCoV-19/Japan/PG-82463/2021	EPI_ISL_3873401	2021-09-02
hCoV-19/Japan/PG-82464/2021	EPI_ISL_3873402	2021-09-02
hCoV-19/Japan/PG-82465/2021	EPI_ISL_3873403	2021-09-02
hCoV-19/Japan/PG-82466/2021	EPI_ISL_3873404	2021-09-02
hCoV-19/Japan/PG-82467/2021	EPI_ISL_3873405	2021-09-02
hCoV-19/Japan/PG-82468/2021	EPI_ISL_3873406	2021-09-02
hCoV-19/Japan/PG-82469/2021	EPI_ISL_3873407	2021-09-02
hCoV-19/Japan/PG-82470/2021	EPI_ISL_3873408	2021-09-02
hCoV-19/Japan/PG-82471/2021	EPI_ISL_3873409	2021-09-02
hCoV-19/Japan/PG-82472/2021	EPI_ISL_3873410	2021-09-02
hCoV-19/Japan/PG-82473/2021	EPI_ISL_3873411	2021-09-02
hCoV-19/Japan/PG-82474/2021	EPI_ISL_3873412	2021-09-02
hCoV-19/Japan/PG-82475/2021	EPI_ISL_3873413	2021-09-02

virus_name	epiID	submission_date
hCoV-19/Japan/PG-82476/2021	EPI_ISL_3873414	2021-09-02
hCoV-19/Japan/PG-82477/2021	EPI_ISL_3894881	2021-09-02
hCoV-19/Japan/PG-82478/2021	EPI_ISL_3894882	2021-09-02
hCoV-19/Japan/PG-82479/2021	EPI_ISL_3894883	2021-09-02
hCoV-19/Japan/PG-82480/2021	EPI_ISL_3894884	2021-09-02
hCoV-19/Japan/PG-82481/2021	EPI_ISL_3894885	2021-09-02
hCoV-19/Japan/PG-82482/2021	EPI_ISL_3894886	2021-09-02
hCoV-19/Japan/PG-82483/2021	EPI_ISL_3894887	2021-09-02
hCoV-19/Japan/PG-82484/2021	EPI_ISL_3894888	2021-09-02
hCoV-19/Japan/PG-82485/2021	EPI_ISL_3894889	2021-09-02
hCoV-19/Japan/PG-82486/2021	EPI_ISL_3894890	2021-09-02
hCoV-19/Japan/PG-82487/2021	EPI_ISL_3894891	2021-09-02
hCoV-19/Japan/PG-82488/2021	EPI_ISL_3894892	2021-09-02
hCoV-19/Japan/PG-82489/2021	EPI_ISL_3894893	2021-09-02
hCoV-19/Japan/PG-82490/2021	EPI_ISL_3894894	2021-09-02
hCoV-19/Japan/PG-82491/2021	EPI_ISL_3894895	2021-09-02
hCoV-19/Japan/PG-82492/2021	EPI_ISL_3894896	2021-09-02
hCoV-19/Japan/PG-82493/2021	EPI_ISL_3894897	2021-09-02
hCoV-19/Japan/PG-82494/2021	EPI_ISL_3894898	2021-09-02
hCoV-19/Japan/PG-82495/2021	EPI_ISL_3894899	2021-09-02
hCoV-19/Japan/PG-82496/2021	EPI_ISL_3894900	2021-09-02
hCoV-19/Japan/PG-82497/2021	EPI_ISL_3894901	2021-09-02
hCoV-19/Japan/PG-82498/2021	EPI_ISL_3894902	2021-09-02
hCoV-19/Japan/PG-82499/2021	EPI_ISL_3894903	2021-09-02
hCoV-19/Japan/PG-82500/2021	EPI_ISL_3894904	2021-09-02
hCoV-19/Japan/PG-82501/2021	EPI_ISL_3894905	2021-09-02
hCoV-19/Japan/PG-82502/2021	EPI_ISL_3894906	2021-09-02
hCoV-19/Japan/PG-82503/2021	EPI_ISL_3894907	2021-09-02
hCoV-19/Japan/PG-82504/2021	EPI_ISL_3894908	2021-09-02
hCoV-19/Japan/PG-82505/2021	EPI_ISL_3894909	2021-09-02
hCoV-19/Japan/PG-82506/2021	EPI_ISL_3894910	2021-09-02
hCoV-19/Japan/PG-82507/2021	EPI_ISL_3894911	2021-09-02
hCoV-19/Japan/PG-82508/2021	EPI_ISL_3894912	2021-09-02
hCoV-19/Japan/PG-82509/2021	EPI_ISL_3894913	2021-09-02
hCoV-19/Japan/PG-82510/2021	EPI_ISL_3894914	2021-09-02
hCoV-19/Japan/PG-82511/2021	EPI_ISL_3894915	2021-09-02
hCoV-19/Japan/PG-82512/2021	EPI_ISL_3894916	2021-09-02
hCoV-19/Japan/PG-82513/2021	EPI_ISL_3894917	2021-09-02
hCoV-19/Japan/PG-82514/2021	EPI_ISL_3894918	2021-09-02
hCoV-19/Japan/PG-82515/2021	EPI_ISL_3894919	2021-09-02
hCoV-19/Japan/PG-82516/2021	EPI_ISL_3894920	2021-09-02
hCoV-19/Japan/PG-82517/2021	EPI_ISL_3894921	2021-09-02
hCoV-19/Japan/PG-82518/2021	EPI_ISL_3894922	2021-09-02
hCoV-19/Japan/PG-82519/2021	EPI_ISL_3894923	2021-09-02
hCoV-19/Japan/PG-82520/2021	EPI_ISL_3894924	2021-09-02
hCoV-19/Japan/PG-82521/2021	EPI_ISL_3894925	2021-09-02
hCoV-19/Japan/PG-82522/2021	EPI_ISL_3894926	2021-09-02
hCoV-19/Japan/PG-82523/2021	EPI_ISL_3894927	2021-09-02
hCoV-19/Japan/PG-82524/2021	EPI_ISL_3894928	2021-09-02
hCoV-19/Japan/PG-82525/2021	EPI_ISL_3894929	2021-09-02
hCoV-19/Japan/PG-82526/2021	EPI_ISL_3894930	2021-09-02
hCoV-19/Japan/PG-82527/2021	EPI_ISL_3894931	2021-09-02
hCoV-19/Japan/PG-82528/2021	EPI_ISL_3894932	2021-09-02
hCoV-19/Japan/PG-82529/2021	EPI_ISL_3894933	2021-09-02

virus_name	epiID	submission_date
hCoV-19/Japan/PG-82530/2021	EPI_ISL_3894934	2021-09-02
hCoV-19/Japan/PG-82531/2021	EPI_ISL_3894935	2021-09-02
hCoV-19/Japan/PG-82532/2021	EPI_ISL_3894936	2021-09-02
hCoV-19/Japan/PG-82533/2021	EPI_ISL_3894937	2021-09-02
hCoV-19/Japan/PG-82534/2021	EPI_ISL_3894938	2021-09-02
hCoV-19/Japan/PG-82535/2021	EPI_ISL_3894939	2021-09-02
hCoV-19/Japan/PG-82536/2021	EPI_ISL_3894940	2021-09-02
hCoV-19/Japan/PG-82537/2021	EPI_ISL_3894941	2021-09-02
hCoV-19/Japan/PG-82538/2021	EPI_ISL_3894942	2021-09-02
hCoV-19/Japan/PG-82539/2021	EPI_ISL_3894943	2021-09-02
hCoV-19/Japan/PG-82540/2021	EPI_ISL_3894944	2021-09-02
hCoV-19/Japan/PG-82541/2021	EPI_ISL_3894945	2021-09-02
hCoV-19/Japan/PG-82542/2021	EPI_ISL_3894946	2021-09-02
hCoV-19/Japan/PG-82543/2021	EPI_ISL_3894947	2021-09-02
hCoV-19/Japan/PG-82544/2021	EPI_ISL_3894948	2021-09-02
hCoV-19/Japan/PG-82545/2021	EPI_ISL_3894949	2021-09-02
hCoV-19/Japan/PG-82546/2021	EPI_ISL_3894950	2021-09-02
hCoV-19/Japan/PG-82547/2021	EPI_ISL_3894951	2021-09-02
hCoV-19/Japan/PG-82548/2021	EPI_ISL_3894952	2021-09-02
hCoV-19/Japan/PG-82549/2021	EPI_ISL_3894953	2021-09-02
hCoV-19/Japan/PG-82550/2021	EPI_ISL_3894954	2021-09-02
hCoV-19/Japan/PG-82551/2021	EPI_ISL_3894955	2021-09-02
hCoV-19/Japan/PG-82552/2021	EPI_ISL_3894956	2021-09-02
hCoV-19/Japan/PG-82553/2021	EPI_ISL_3894957	2021-09-02
hCoV-19/Japan/PG-82554/2021	EPI_ISL_3894958	2021-09-02
hCoV-19/Japan/PG-82555/2021	EPI_ISL_3894959	2021-09-02
hCoV-19/Japan/PG-82556/2021	EPI_ISL_3894960	2021-09-02
hCoV-19/Japan/PG-82557/2021	EPI_ISL_3894961	2021-09-02
hCoV-19/Japan/PG-82558/2021	EPI_ISL_3894962	2021-09-02
hCoV-19/Japan/PG-82559/2021	EPI_ISL_3894963	2021-09-02
hCoV-19/Japan/PG-82560/2021	EPI_ISL_3894964	2021-09-02
hCoV-19/Japan/PG-82561/2021	EPI_ISL_3894965	2021-09-02
hCoV-19/Japan/PG-82562/2021	EPI_ISL_3894966	2021-09-02
hCoV-19/Japan/PG-82563/2021	EPI_ISL_3894967	2021-09-02
hCoV-19/Japan/PG-82564/2021	EPI_ISL_3894968	2021-09-02
hCoV-19/Japan/PG-82565/2021	EPI_ISL_3894969	2021-09-02
hCoV-19/Japan/PG-82566/2021	EPI_ISL_3894970	2021-09-02
hCoV-19/Japan/PG-82567/2021	EPI_ISL_3894971	2021-09-02
hCoV-19/Japan/PG-82568/2021	EPI_ISL_3894972	2021-09-02
hCoV-19/Japan/PG-82569/2021	EPI_ISL_3894973	2021-09-02
hCoV-19/Japan/PG-82570/2021	EPI_ISL_3894974	2021-09-02
hCoV-19/Japan/PG-82571/2021	EPI_ISL_3894975	2021-09-02
hCoV-19/Japan/PG-82572/2021	EPI_ISL_3894976	2021-09-02
hCoV-19/Japan/PG-82573/2021	EPI_ISL_3894977	2021-09-02
hCoV-19/Japan/PG-82574/2021	EPI_ISL_3894978	2021-09-02
hCoV-19/Japan/PG-82575/2021	EPI_ISL_3894979	2021-09-02
hCoV-19/Japan/PG-82576/2021	EPI_ISL_3894980	2021-09-02
hCoV-19/Japan/PG-82577/2021	EPI_ISL_3894981	2021-09-02
hCoV-19/Japan/PG-82578/2021	EPI_ISL_3894982	2021-09-02
hCoV-19/Japan/PG-82579/2021	EPI_ISL_3894983	2021-09-02
hCoV-19/Japan/PG-82580/2021	EPI_ISL_3894984	2021-09-02
hCoV-19/Japan/PG-82581/2021	EPI_ISL_3894985	2021-09-02
hCoV-19/Japan/PG-82582/2021	EPI_ISL_3894986	2021-09-02
hCoV-19/Japan/PG-82583/2021	EPI_ISL_3894987	2021-09-02

virus_name	epiID	submission_date
hCoV-19/Japan/PG-82584/2021	EPI_ISL_3894988	2021-09-02
hCoV-19/Japan/PG-82585/2021	EPI_ISL_3894989	2021-09-02
hCoV-19/Japan/PG-82586/2021	EPI_ISL_3894990	2021-09-02
hCoV-19/Japan/PG-82587/2021	EPI_ISL_3894991	2021-09-02
hCoV-19/Japan/PG-82588/2021	EPI_ISL_3894992	2021-09-02
hCoV-19/Japan/PG-82589/2021	EPI_ISL_3894993	2021-09-02
hCoV-19/Japan/PG-82590/2021	EPI_ISL_3894994	2021-09-02
hCoV-19/Japan/PG-82591/2021	EPI_ISL_3894995	2021-09-02
hCoV-19/Japan/PG-82592/2021	EPI_ISL_3894996	2021-09-02
hCoV-19/Japan/PG-82593/2021	EPI_ISL_3894997	2021-09-02
hCoV-19/Japan/PG-82594/2021	EPI_ISL_3894998	2021-09-02
hCoV-19/Japan/PG-82595/2021	EPI_ISL_3894999	2021-09-02
hCoV-19/Japan/PG-82596/2021	EPI_ISL_3895000	2021-09-02
hCoV-19/Japan/PG-82597/2021	EPI_ISL_3895001	2021-09-02
hCoV-19/Japan/PG-82598/2021	EPI_ISL_3895002	2021-09-02
hCoV-19/Japan/PG-82599/2021	EPI_ISL_3895003	2021-09-02
hCoV-19/Japan/PG-82600/2021	EPI_ISL_3895004	2021-09-02
hCoV-19/Japan/PG-82601/2021	EPI_ISL_3895005	2021-09-02
hCoV-19/Japan/PG-82602/2021	EPI_ISL_3895006	2021-09-02
hCoV-19/Japan/PG-82603/2021	EPI_ISL_3895007	2021-09-02
hCoV-19/Japan/PG-82604/2021	EPI_ISL_3895008	2021-09-02
hCoV-19/Japan/PG-82605/2021	EPI_ISL_3895009	2021-09-02
hCoV-19/Japan/PG-82606/2021	EPI_ISL_3895010	2021-09-02
hCoV-19/Japan/PG-82607/2021	EPI_ISL_3895011	2021-09-02
hCoV-19/Japan/PG-82608/2021	EPI_ISL_3895012	2021-09-02
hCoV-19/Japan/PG-82609/2021	EPI_ISL_3895013	2021-09-02
hCoV-19/Japan/PG-82610/2021	EPI_ISL_3895014	2021-09-02
hCoV-19/Japan/PG-82611/2021	EPI_ISL_3895015	2021-09-02
hCoV-19/Japan/PG-82612/2021	EPI_ISL_3895016	2021-09-02
hCoV-19/Japan/PG-82613/2021	EPI_ISL_3895017	2021-09-02
hCoV-19/Japan/PG-82614/2021	EPI_ISL_3895018	2021-09-02
hCoV-19/Japan/PG-82615/2021	EPI_ISL_3895019	2021-09-02
hCoV-19/Japan/PG-82616/2021	EPI_ISL_3895020	2021-09-02
hCoV-19/Japan/PG-82617/2021	EPI_ISL_3895021	2021-09-02
hCoV-19/Japan/PG-82618/2021	EPI_ISL_3895022	2021-09-02
hCoV-19/Japan/PG-82619/2021	EPI_ISL_3895023	2021-09-02
hCoV-19/Japan/PG-82620/2021	EPI_ISL_3895024	2021-09-02
hCoV-19/Japan/PG-82621/2021	EPI_ISL_3895025	2021-09-02
hCoV-19/Japan/PG-82622/2021	EPI_ISL_3895026	2021-09-02
hCoV-19/Japan/PG-82623/2021	EPI_ISL_3895027	2021-09-02
hCoV-19/Japan/PG-82624/2021	EPI_ISL_3895028	2021-09-02
hCoV-19/Japan/PG-82625/2021	EPI_ISL_3895029	2021-09-02
hCoV-19/Japan/PG-82626/2021	EPI_ISL_3895030	2021-09-02
hCoV-19/Japan/PG-82627/2021	EPI_ISL_3895031	2021-09-02
hCoV-19/Japan/PG-82628/2021	EPI_ISL_3895032	2021-09-02
hCoV-19/Japan/PG-82629/2021	EPI_ISL_3895033	2021-09-02
hCoV-19/Japan/PG-82630/2021	EPI_ISL_3895034	2021-09-02
hCoV-19/Japan/PG-82631/2021	EPI_ISL_3895035	2021-09-02
hCoV-19/Japan/PG-82632/2021	EPI_ISL_3895036	2021-09-02
hCoV-19/Japan/PG-82633/2021	EPI_ISL_3895037	2021-09-02
hCoV-19/Japan/PG-82634/2021	EPI_ISL_3895038	2021-09-02
hCoV-19/Japan/PG-82635/2021	EPI_ISL_3895039	2021-09-02
hCoV-19/Japan/PG-82636/2021	EPI_ISL_3895040	2021-09-02
hCoV-19/Japan/PG-82637/2021	EPI_ISL_3895041	2021-09-02

virus_name	epiID	submission_date
hCoV-19/Japan/PG-82638/2021	EPI_ISL_3895042	2021-09-02
hCoV-19/Japan/PG-82639/2021	EPI_ISL_3895043	2021-09-02
hCoV-19/Japan/PG-82640/2021	EPI_ISL_3895044	2021-09-02
hCoV-19/Japan/PG-82641/2021	EPI_ISL_3895045	2021-09-02
hCoV-19/Japan/PG-82642/2021	EPI_ISL_3895046	2021-09-02
hCoV-19/Japan/PG-82643/2021	EPI_ISL_3895047	2021-09-02
hCoV-19/Japan/PG-82644/2021	EPI_ISL_3895048	2021-09-02
hCoV-19/Japan/PG-82645/2021	EPI_ISL_3895049	2021-09-02
hCoV-19/Japan/PG-82646/2021	EPI_ISL_3895050	2021-09-02
hCoV-19/Japan/PG-82647/2021	EPI_ISL_3895051	2021-09-02
hCoV-19/Japan/PG-82648/2021	EPI_ISL_3895052	2021-09-02
hCoV-19/Japan/PG-82649/2021	EPI_ISL_3895053	2021-09-02
hCoV-19/Japan/PG-82650/2021	EPI_ISL_3895054	2021-09-02
hCoV-19/Japan/PG-82651/2021	EPI_ISL_3895055	2021-09-02
hCoV-19/Japan/PG-82652/2021	EPI_ISL_3895056	2021-09-02
hCoV-19/Japan/PG-82653/2021	EPI_ISL_3895057	2021-09-02
hCoV-19/Japan/PG-82654/2021	EPI_ISL_3895058	2021-09-02
hCoV-19/Japan/PG-82655/2021	EPI_ISL_3895059	2021-09-02
hCoV-19/Japan/PG-82656/2021	EPI_ISL_3895060	2021-09-02
hCoV-19/Japan/PG-82657/2021	EPI_ISL_3895061	2021-09-02
hCoV-19/Japan/PG-82658/2021	EPI_ISL_3895062	2021-09-02
hCoV-19/Japan/PG-82659/2021	EPI_ISL_3895063	2021-09-02
hCoV-19/Japan/PG-82660/2021	EPI_ISL_3895064	2021-09-02
hCoV-19/Japan/PG-82661/2021	EPI_ISL_3895065	2021-09-02
hCoV-19/Japan/PG-82662/2021	EPI_ISL_3895066	2021-09-02
hCoV-19/Japan/PG-82663/2021	EPI_ISL_3895067	2021-09-02
hCoV-19/Japan/PG-82664/2021	EPI_ISL_3895068	2021-09-02
hCoV-19/Japan/PG-82665/2021	EPI_ISL_3895069	2021-09-02
hCoV-19/Japan/PG-82666/2021	EPI_ISL_3895070	2021-09-02
hCoV-19/Japan/PG-82667/2021	EPI_ISL_3895071	2021-09-02
hCoV-19/Japan/PG-82668/2021	EPI_ISL_3895072	2021-09-02
hCoV-19/Japan/PG-82669/2021	EPI_ISL_3895073	2021-09-02
hCoV-19/Japan/PG-82670/2021	EPI_ISL_3895074	2021-09-02
hCoV-19/Japan/PG-82671/2021	EPI_ISL_3895075	2021-09-02
hCoV-19/Japan/PG-82672/2021	EPI_ISL_3895076	2021-09-02
hCoV-19/Japan/PG-82673/2021	EPI_ISL_3895077	2021-09-02
hCoV-19/Japan/PG-82674/2021	EPI_ISL_3895078	2021-09-02
hCoV-19/Japan/PG-82675/2021	EPI_ISL_3895079	2021-09-02
hCoV-19/Japan/PG-82676/2021	EPI_ISL_3895080	2021-09-02
hCoV-19/Japan/PG-82677/2021	EPI_ISL_3895081	2021-09-02
hCoV-19/Japan/PG-82678/2021	EPI_ISL_3895082	2021-09-02
hCoV-19/Japan/PG-82679/2021	EPI_ISL_3895083	2021-09-02
hCoV-19/Japan/PG-82680/2021	EPI_ISL_3895084	2021-09-02
hCoV-19/Japan/PG-82681/2021	EPI_ISL_3895085	2021-09-02
hCoV-19/Japan/PG-82682/2021	EPI_ISL_3895086	2021-09-02
hCoV-19/Japan/PG-82683/2021	EPI_ISL_3895087	2021-09-02
hCoV-19/Japan/PG-82684/2021	EPI_ISL_3895088	2021-09-02
hCoV-19/Japan/PG-82685/2021	EPI_ISL_3895089	2021-09-02
hCoV-19/Japan/PG-82686/2021	EPI_ISL_3895090	2021-09-02
hCoV-19/Japan/PG-82687/2021	EPI_ISL_3895091	2021-09-02
hCoV-19/Japan/PG-82688/2021	EPI_ISL_3895092	2021-09-02
hCoV-19/Japan/PG-82689/2021	EPI_ISL_3895093	2021-09-02
hCoV-19/Japan/PG-82690/2021	EPI_ISL_3895094	2021-09-02
hCoV-19/Japan/PG-82691/2021	EPI_ISL_3895095	2021-09-02

virus_name	epiID	submission_date
hCoV-19/Japan/PG-82692/2021	EPI_ISL_3895096	2021-09-02
hCoV-19/Japan/PG-82693/2021	EPI_ISL_3895097	2021-09-02
hCoV-19/Japan/PG-82694/2021	EPI_ISL_3895098	2021-09-02
hCoV-19/Japan/PG-82695/2021	EPI_ISL_3895099	2021-09-02
hCoV-19/Japan/PG-82696/2021	EPI_ISL_3895100	2021-09-02
hCoV-19/Japan/PG-82697/2021	EPI_ISL_3895101	2021-09-02
hCoV-19/Japan/PG-82698/2021	EPI_ISL_3895102	2021-09-02
hCoV-19/Japan/PG-82699/2021	EPI_ISL_3895103	2021-09-02
hCoV-19/Japan/PG-82700/2021	EPI_ISL_3895104	2021-09-02
hCoV-19/Japan/PG-82701/2021	EPI_ISL_3895105	2021-09-02
hCoV-19/Japan/PG-82702/2021	EPI_ISL_3895106	2021-09-02
hCoV-19/Japan/PG-82703/2021	EPI_ISL_3895107	2021-09-02
hCoV-19/Japan/PG-82704/2021	EPI_ISL_3895108	2021-09-02
hCoV-19/Japan/PG-82705/2021	EPI_ISL_3895109	2021-09-02
hCoV-19/Japan/PG-82706/2021	EPI_ISL_3895110	2021-09-02
hCoV-19/Japan/PG-82707/2021	EPI_ISL_3895111	2021-09-02
hCoV-19/Japan/PG-82708/2021	EPI_ISL_3895112	2021-09-02
hCoV-19/Japan/PG-82709/2021	EPI_ISL_3895113	2021-09-02
hCoV-19/Japan/PG-82710/2021	EPI_ISL_3895114	2021-09-02
hCoV-19/Japan/PG-82711/2021	EPI_ISL_3895115	2021-09-02
hCoV-19/Japan/PG-82712/2021	EPI_ISL_3895116	2021-09-02
hCoV-19/Japan/PG-82713/2021	EPI_ISL_3895117	2021-09-02
hCoV-19/Japan/PG-82714/2021	EPI_ISL_3895118	2021-09-02
hCoV-19/Japan/PG-82715/2021	EPI_ISL_3895119	2021-09-02
hCoV-19/Japan/PG-82716/2021	EPI_ISL_3895120	2021-09-02
hCoV-19/Japan/PG-82717/2021	EPI_ISL_3895121	2021-09-02
hCoV-19/Japan/PG-82718/2021	EPI_ISL_3895122	2021-09-02
hCoV-19/Japan/PG-82719/2021	EPI_ISL_3895123	2021-09-02
hCoV-19/Japan/PG-82720/2021	EPI_ISL_3895124	2021-09-02
hCoV-19/Japan/PG-82721/2021	EPI_ISL_3895125	2021-09-02
hCoV-19/Japan/PG-82722/2021	EPI_ISL_3895126	2021-09-02
hCoV-19/Japan/PG-82723/2021	EPI_ISL_3895127	2021-09-02
hCoV-19/Japan/PG-82724/2021	EPI_ISL_3895128	2021-09-02
hCoV-19/Japan/PG-82725/2021	EPI_ISL_3895129	2021-09-02
hCoV-19/Japan/PG-82726/2021	EPI_ISL_3895130	2021-09-02
hCoV-19/Japan/PG-82727/2021	EPI_ISL_3895131	2021-09-02
hCoV-19/Japan/PG-82728/2021	EPI_ISL_3895132	2021-09-02
hCoV-19/Japan/PG-82729/2021	EPI_ISL_3895133	2021-09-02
hCoV-19/Japan/PG-82730/2021	EPI_ISL_3895134	2021-09-02
hCoV-19/Japan/PG-82731/2021	EPI_ISL_3895135	2021-09-02
hCoV-19/Japan/PG-82732/2021	EPI_ISL_3895136	2021-09-02
hCoV-19/Japan/PG-82733/2021	EPI_ISL_3895137	2021-09-02
hCoV-19/Japan/PG-82734/2021	EPI_ISL_3895138	2021-09-02
hCoV-19/Japan/PG-82735/2021	EPI_ISL_3895139	2021-09-02
hCoV-19/Japan/PG-82736/2021	EPI_ISL_3895140	2021-09-02
hCoV-19/Japan/PG-82737/2021	EPI_ISL_3895141	2021-09-02
hCoV-19/Japan/PG-82738/2021	EPI_ISL_3895142	2021-09-02
hCoV-19/Japan/PG-82739/2021	EPI_ISL_3895143	2021-09-02
hCoV-19/Japan/PG-82740/2021	EPI_ISL_3895145	2021-09-02
hCoV-19/Japan/PG-82741/2021	EPI_ISL_3895147	2021-09-02
hCoV-19/Japan/PG-82742/2021	EPI_ISL_3895152	2021-09-02
hCoV-19/Japan/PG-82743/2021	EPI_ISL_3895157	2021-09-02
hCoV-19/Japan/PG-82744/2021	EPI_ISL_3895161	2021-09-02
hCoV-19/Japan/PG-82745/2021	EPI_ISL_3895167	2021-09-02

virus_name	epiID	submission_date
hCoV-19/Japan/PG-82746/2021	EPI_ISL_3895172	2021-09-02
hCoV-19/Japan/PG-82747/2021	EPI_ISL_3895177	2021-09-02
hCoV-19/Japan/PG-82748/2021	EPI_ISL_3895181	2021-09-02
hCoV-19/Japan/PG-82749/2021	EPI_ISL_3895186	2021-09-02
hCoV-19/Japan/PG-82750/2021	EPI_ISL_3895191	2021-09-02
hCoV-19/Japan/PG-82751/2021	EPI_ISL_3895196	2021-09-02
hCoV-19/Japan/PG-82752/2021	EPI_ISL_3895202	2021-09-02
hCoV-19/Japan/PG-82753/2021	EPI_ISL_3895206	2021-09-02
hCoV-19/Japan/PG-82754/2021	EPI_ISL_3895211	2021-09-02
hCoV-19/Japan/PG-82755/2021	EPI_ISL_3895216	2021-09-02
hCoV-19/Japan/PG-82756/2021	EPI_ISL_3895221	2021-09-02
hCoV-19/Japan/PG-82757/2021	EPI_ISL_3895226	2021-09-02
hCoV-19/Japan/PG-82758/2021	EPI_ISL_3895230	2021-09-02
hCoV-19/Japan/PG-82759/2021	EPI_ISL_3895235	2021-09-02
hCoV-19/Japan/PG-82760/2021	EPI_ISL_3895240	2021-09-02
hCoV-19/Japan/PG-82761/2021	EPI_ISL_3895244	2021-09-02
hCoV-19/Japan/PG-82762/2021	EPI_ISL_3895249	2021-09-02
hCoV-19/Japan/PG-82763/2021	EPI_ISL_3895254	2021-09-02
hCoV-19/Japan/PG-82764/2021	EPI_ISL_3895257	2021-09-02
hCoV-19/Japan/PG-82765/2021	EPI_ISL_3895259	2021-09-02
hCoV-19/Japan/PG-82766/2021	EPI_ISL_3895264	2021-09-02
hCoV-19/Japan/PG-82767/2021	EPI_ISL_3895269	2021-09-02
hCoV-19/Japan/PG-82768/2021	EPI_ISL_3895273	2021-09-02
hCoV-19/Japan/PG-82769/2021	EPI_ISL_3895279	2021-09-02
hCoV-19/Japan/PG-82770/2021	EPI_ISL_3895284	2021-09-02
hCoV-19/Japan/PG-82771/2021	EPI_ISL_3895289	2021-09-02
hCoV-19/Japan/PG-82772/2021	EPI_ISL_3895294	2021-09-02
hCoV-19/Japan/PG-82773/2021	EPI_ISL_3895298	2021-09-02
hCoV-19/Japan/PG-82774/2021	EPI_ISL_3895302	2021-09-02
hCoV-19/Japan/PG-82775/2021	EPI_ISL_3895307	2021-09-02
hCoV-19/Japan/PG-82776/2021	EPI_ISL_3895312	2021-09-02
hCoV-19/Japan/PG-82777/2021	EPI_ISL_3895317	2021-09-02
hCoV-19/Japan/PG-82778/2021	EPI_ISL_3895321	2021-09-02
hCoV-19/Japan/PG-82779/2021	EPI_ISL_3895326	2021-09-02
hCoV-19/Japan/PG-82780/2021	EPI_ISL_3895332	2021-09-02
hCoV-19/Japan/PG-82781/2021	EPI_ISL_3895337	2021-09-02
hCoV-19/Japan/PG-82782/2021	EPI_ISL_3895342	2021-09-02
hCoV-19/Japan/PG-82783/2021	EPI_ISL_3895347	2021-09-02
hCoV-19/Japan/PG-82784/2021	EPI_ISL_3895352	2021-09-02
hCoV-19/Japan/PG-82785/2021	EPI_ISL_3895357	2021-09-02
hCoV-19/Japan/PG-82786/2021	EPI_ISL_3895363	2021-09-02
hCoV-19/Japan/PG-82787/2021	EPI_ISL_3895368	2021-09-02
hCoV-19/Japan/PG-82788/2021	EPI_ISL_3895374	2021-09-02
hCoV-19/Japan/PG-82789/2021	EPI_ISL_3895378	2021-09-02
hCoV-19/Japan/PG-82790/2021	EPI_ISL_3895384	2021-09-02
hCoV-19/Japan/PG-82791/2021	EPI_ISL_3895389	2021-09-02
hCoV-19/Japan/PG-82792/2021	EPI_ISL_3895394	2021-09-02
hCoV-19/Japan/PG-82793/2021	EPI_ISL_3895399	2021-09-02
hCoV-19/Japan/PG-82794/2021	EPI_ISL_3895404	2021-09-02
hCoV-19/Japan/PG-82795/2021	EPI_ISL_3895409	2021-09-02
hCoV-19/Japan/PG-82796/2021	EPI_ISL_3895414	2021-09-02
hCoV-19/Japan/PG-82797/2021	EPI_ISL_3895419	2021-09-02
hCoV-19/Japan/PG-82798/2021	EPI_ISL_3895424	2021-09-02
hCoV-19/Japan/PG-82799/2021	EPI_ISL_3895429	2021-09-02

virus_name	epiID	submission_date
hCoV-19/Japan/PG-82800/2021	EPI_ISL_3895434	2021-09-02
hCoV-19/Japan/PG-82801/2021	EPI_ISL_3895439	2021-09-02
hCoV-19/Japan/PG-82802/2021	EPI_ISL_3895443	2021-09-02
hCoV-19/Japan/PG-82803/2021	EPI_ISL_3895448	2021-09-02
hCoV-19/Japan/PG-82804/2021	EPI_ISL_3895453	2021-09-02
hCoV-19/Japan/PG-82805/2021	EPI_ISL_3895456	2021-09-02
hCoV-19/Japan/PG-82806/2021	EPI_ISL_3895457	2021-09-02
hCoV-19/Japan/PG-82807/2021	EPI_ISL_3895458	2021-09-02
hCoV-19/Japan/PG-82808/2021	EPI_ISL_3895459	2021-09-02
hCoV-19/Japan/PG-82809/2021	EPI_ISL_3895460	2021-09-02
hCoV-19/Japan/PG-82810/2021	EPI_ISL_3895461	2021-09-02
hCoV-19/Japan/PG-82811/2021	EPI_ISL_3895462	2021-09-02
hCoV-19/Japan/PG-82812/2021	EPI_ISL_3895463	2021-09-02
hCoV-19/Japan/PG-82813/2021	EPI_ISL_3895464	2021-09-02
hCoV-19/Japan/PG-82814/2021	EPI_ISL_3895465	2021-09-02
hCoV-19/Japan/PG-82815/2021	EPI_ISL_3895466	2021-09-02
hCoV-19/Japan/PG-82816/2021	EPI_ISL_3895467	2021-09-02
hCoV-19/Japan/PG-82817/2021	EPI_ISL_3895468	2021-09-02
hCoV-19/Japan/PG-82818/2021	EPI_ISL_3895469	2021-09-02
hCoV-19/Japan/PG-82819/2021	EPI_ISL_3895470	2021-09-02
hCoV-19/Japan/PG-82820/2021	EPI_ISL_3895471	2021-09-02
hCoV-19/Japan/PG-82821/2021	EPI_ISL_3895472	2021-09-02
hCoV-19/Japan/PG-82822/2021	EPI_ISL_3895473	2021-09-02
hCoV-19/Japan/PG-82823/2021	EPI_ISL_3895474	2021-09-02
hCoV-19/Japan/PG-82824/2021	EPI_ISL_3895475	2021-09-02
hCoV-19/Japan/PG-82825/2021	EPI_ISL_3895476	2021-09-02
hCoV-19/Japan/PG-82826/2021	EPI_ISL_3895477	2021-09-02
hCoV-19/Japan/PG-82827/2021	EPI_ISL_3895478	2021-09-02
hCoV-19/Japan/PG-82828/2021	EPI_ISL_3895479	2021-09-02
hCoV-19/Japan/PG-82829/2021	EPI_ISL_3895480	2021-09-02
hCoV-19/Japan/PG-82830/2021	EPI_ISL_3895481	2021-09-02
hCoV-19/Japan/PG-82831/2021	EPI_ISL_3895482	2021-09-02
hCoV-19/Japan/PG-82832/2021	EPI_ISL_3895483	2021-09-02
hCoV-19/Japan/PG-82833/2021	EPI_ISL_3895484	2021-09-02
hCoV-19/Japan/PG-82834/2021	EPI_ISL_3895485	2021-09-02
hCoV-19/Japan/PG-82835/2021	EPI_ISL_3895486	2021-09-02
hCoV-19/Japan/PG-82836/2021	EPI_ISL_3895487	2021-09-02
hCoV-19/Japan/PG-82837/2021	EPI_ISL_3895488	2021-09-02
hCoV-19/Japan/PG-82838/2021	EPI_ISL_3895489	2021-09-02
hCoV-19/Japan/PG-82839/2021	EPI_ISL_3895490	2021-09-02
hCoV-19/Japan/PG-82840/2021	EPI_ISL_3895491	2021-09-02
hCoV-19/Japan/PG-82841/2021	EPI_ISL_3895492	2021-09-02
hCoV-19/Japan/PG-82842/2021	EPI_ISL_3895493	2021-09-02
hCoV-19/Japan/PG-82843/2021	EPI_ISL_3895494	2021-09-02
hCoV-19/Japan/PG-82844/2021	EPI_ISL_3895495	2021-09-02
hCoV-19/Japan/PG-82845/2021	EPI_ISL_3895496	2021-09-02
hCoV-19/Japan/PG-82846/2021	EPI_ISL_3895497	2021-09-02
hCoV-19/Japan/PG-82847/2021	EPI_ISL_3895498	2021-09-02
hCoV-19/Japan/PG-82848/2021	EPI_ISL_3895499	2021-09-02
hCoV-19/Japan/PG-82849/2021	EPI_ISL_3895500	2021-09-02
hCoV-19/Japan/PG-82850/2021	EPI_ISL_3895501	2021-09-02
hCoV-19/Japan/PG-82851/2021	EPI_ISL_3895502	2021-09-02
hCoV-19/Japan/PG-82852/2021	EPI_ISL_3895503	2021-09-02
hCoV-19/Japan/PG-82853/2021	EPI_ISL_3895504	2021-09-02

virus_name	epiID	submission_date
hCoV-19/Japan/PG-82854/2021	EPI_ISL_3895505	2021-09-02
hCoV-19/Japan/PG-82855/2021	EPI_ISL_3895506	2021-09-02
hCoV-19/Japan/PG-82856/2021	EPI_ISL_3895507	2021-09-02
hCoV-19/Japan/PG-82857/2021	EPI_ISL_3895508	2021-09-02
hCoV-19/Japan/PG-82858/2021	EPI_ISL_3895509	2021-09-02
hCoV-19/Japan/PG-82859/2021	EPI_ISL_3895510	2021-09-02
hCoV-19/Japan/PG-82860/2021	EPI_ISL_3895511	2021-09-02
hCoV-19/Japan/PG-82861/2021	EPI_ISL_3895512	2021-09-02
hCoV-19/Japan/PG-82862/2021	EPI_ISL_3895513	2021-09-02
hCoV-19/Japan/PG-82863/2021	EPI_ISL_3895514	2021-09-02
hCoV-19/Japan/PG-82864/2021	EPI_ISL_3895515	2021-09-02
hCoV-19/Japan/PG-82865/2021	EPI_ISL_3895516	2021-09-02
hCoV-19/Japan/PG-82866/2021	EPI_ISL_3895517	2021-09-02
hCoV-19/Japan/PG-82867/2021	EPI_ISL_3895518	2021-09-02
hCoV-19/Japan/PG-82868/2021	EPI_ISL_3895519	2021-09-02
hCoV-19/Japan/PG-82869/2021	EPI_ISL_3895520	2021-09-02
hCoV-19/Japan/PG-82870/2021	EPI_ISL_3895521	2021-09-02
hCoV-19/Japan/PG-82871/2021	EPI_ISL_3895522	2021-09-02
hCoV-19/Japan/PG-82872/2021	EPI_ISL_3895523	2021-09-02
hCoV-19/Japan/PG-82873/2021	EPI_ISL_3895524	2021-09-02
hCoV-19/Japan/PG-82874/2021	EPI_ISL_3895525	2021-09-02
hCoV-19/Japan/PG-82875/2021	EPI_ISL_3895526	2021-09-02
hCoV-19/Japan/PG-82876/2021	EPI_ISL_3895527	2021-09-02
hCoV-19/Japan/PG-82877/2021	EPI_ISL_3895528	2021-09-02
hCoV-19/Japan/PG-82878/2021	EPI_ISL_3895529	2021-09-02
hCoV-19/Japan/PG-82879/2021	EPI_ISL_3895530	2021-09-02
hCoV-19/Japan/PG-82880/2021	EPI_ISL_3895531	2021-09-02
hCoV-19/Japan/PG-82881/2021	EPI_ISL_3895532	2021-09-02
hCoV-19/Japan/PG-82882/2021	EPI_ISL_3895533	2021-09-02
hCoV-19/Japan/PG-82883/2021	EPI_ISL_3895534	2021-09-02
hCoV-19/Japan/PG-82884/2021	EPI_ISL_3895535	2021-09-02
hCoV-19/Japan/PG-82885/2021	EPI_ISL_3895536	2021-09-02
hCoV-19/Japan/PG-82886/2021	EPI_ISL_3895537	2021-09-02
hCoV-19/Japan/PG-82887/2021	EPI_ISL_3895538	2021-09-02
hCoV-19/Japan/PG-82888/2021	EPI_ISL_3895539	2021-09-02
hCoV-19/Japan/PG-82889/2021	EPI_ISL_3895540	2021-09-02
hCoV-19/Japan/PG-82890/2021	EPI_ISL_3895541	2021-09-02
hCoV-19/Japan/PG-82891/2021	EPI_ISL_3895542	2021-09-02
hCoV-19/Japan/PG-82892/2021	EPI_ISL_3895543	2021-09-02
hCoV-19/Japan/PG-82893/2021	EPI_ISL_3895544	2021-09-02
hCoV-19/Japan/PG-82894/2021	EPI_ISL_3895545	2021-09-02
hCoV-19/Japan/PG-82895/2021	EPI_ISL_3895546	2021-09-02
hCoV-19/Japan/PG-82896/2021	EPI_ISL_3895547	2021-09-02
hCoV-19/Japan/PG-82897/2021	EPI_ISL_3895548	2021-09-02
hCoV-19/Japan/PG-82898/2021	EPI_ISL_3895549	2021-09-02
hCoV-19/Japan/PG-82899/2021	EPI_ISL_3895550	2021-09-02
hCoV-19/Japan/PG-82900/2021	EPI_ISL_3895551	2021-09-02
hCoV-19/Japan/PG-82901/2021	EPI_ISL_3895552	2021-09-02
hCoV-19/Japan/PG-82902/2021	EPI_ISL_3895553	2021-09-02
hCoV-19/Japan/PG-82903/2021	EPI_ISL_3895554	2021-09-02
hCoV-19/Japan/PG-82904/2021	EPI_ISL_3895555	2021-09-02
hCoV-19/Japan/PG-82905/2021	EPI_ISL_3895556	2021-09-02
hCoV-19/Japan/PG-82906/2021	EPI_ISL_3895557	2021-09-02
hCoV-19/Japan/PG-82907/2021	EPI_ISL_3895558	2021-09-02

virus_name	epiID	submission_date
hCoV-19/Japan/PG-82908/2021	EPI_ISL_3895559	2021-09-02
hCoV-19/Japan/PG-82909/2021	EPI_ISL_3895560	2021-09-02
hCoV-19/Japan/PG-82910/2021	EPI_ISL_3895561	2021-09-02
hCoV-19/Japan/PG-82911/2021	EPI_ISL_3895562	2021-09-02
hCoV-19/Japan/PG-82912/2021	EPI_ISL_3895563	2021-09-02
hCoV-19/Japan/PG-82913/2021	EPI_ISL_3895564	2021-09-02
hCoV-19/Japan/PG-82914/2021	EPI_ISL_3895565	2021-09-02
hCoV-19/Japan/PG-82915/2021	EPI_ISL_3895566	2021-09-02
hCoV-19/Japan/PG-82916/2021	EPI_ISL_3895567	2021-09-02
hCoV-19/Japan/PG-82917/2021	EPI_ISL_3895568	2021-09-02
hCoV-19/Japan/PG-82918/2021	EPI_ISL_3895569	2021-09-02
hCoV-19/Japan/PG-82919/2021	EPI_ISL_3895570	2021-09-02
hCoV-19/Japan/PG-82920/2021	EPI_ISL_3895571	2021-09-02
hCoV-19/Japan/PG-82921/2021	EPI_ISL_3895572	2021-09-02
hCoV-19/Japan/PG-82922/2021	EPI_ISL_3895573	2021-09-02
hCoV-19/Japan/PG-82923/2021	EPI_ISL_3895574	2021-09-02
hCoV-19/Japan/PG-82924/2021	EPI_ISL_3895575	2021-09-02
hCoV-19/Japan/PG-82925/2021	EPI_ISL_3895576	2021-09-02
hCoV-19/Japan/PG-82926/2021	EPI_ISL_3895577	2021-09-02
hCoV-19/Japan/PG-82927/2021	EPI_ISL_3895578	2021-09-02
hCoV-19/Japan/PG-82928/2021	EPI_ISL_3895579	2021-09-02
hCoV-19/Japan/PG-82929/2021	EPI_ISL_3895580	2021-09-02
hCoV-19/Japan/PG-82930/2021	EPI_ISL_3895581	2021-09-02
hCoV-19/Japan/PG-82931/2021	EPI_ISL_3895582	2021-09-02
hCoV-19/Japan/PG-82932/2021	EPI_ISL_3895583	2021-09-02
hCoV-19/Japan/PG-82933/2021	EPI_ISL_3895584	2021-09-02
hCoV-19/Japan/PG-82934/2021	EPI_ISL_3895585	2021-09-02
hCoV-19/Japan/PG-82935/2021	EPI_ISL_3895586	2021-09-02
hCoV-19/Japan/PG-82936/2021	EPI_ISL_3895587	2021-09-02
hCoV-19/Japan/PG-82937/2021	EPI_ISL_3895588	2021-09-02
hCoV-19/Japan/PG-82938/2021	EPI_ISL_3895589	2021-09-02
hCoV-19/Japan/PG-82939/2021	EPI_ISL_3895590	2021-09-02
hCoV-19/Japan/PG-82940/2021	EPI_ISL_3895591	2021-09-02
hCoV-19/Japan/PG-82941/2021	EPI_ISL_3895592	2021-09-02
hCoV-19/Japan/PG-82942/2021	EPI_ISL_3895593	2021-09-02
hCoV-19/Japan/PG-82943/2021	EPI_ISL_3895594	2021-09-02
hCoV-19/Japan/PG-82944/2021	EPI_ISL_3895595	2021-09-02
hCoV-19/Japan/PG-82945/2021	EPI_ISL_3895596	2021-09-02
hCoV-19/Japan/PG-82946/2021	EPI_ISL_3895597	2021-09-02
hCoV-19/Japan/PG-82947/2021	EPI_ISL_3895598	2021-09-02
hCoV-19/Japan/PG-82948/2021	EPI_ISL_3895599	2021-09-02
hCoV-19/Japan/PG-82949/2021	EPI_ISL_3895600	2021-09-02
hCoV-19/Japan/PG-82950/2021	EPI_ISL_3895601	2021-09-02
hCoV-19/Japan/PG-82951/2021	EPI_ISL_3895602	2021-09-02
hCoV-19/Japan/PG-82952/2021	EPI_ISL_3895603	2021-09-02
hCoV-19/Japan/PG-82953/2021	EPI_ISL_3895604	2021-09-02
hCoV-19/Japan/PG-82954/2021	EPI_ISL_3895605	2021-09-02
hCoV-19/Japan/PG-82955/2021	EPI_ISL_3895606	2021-09-02
hCoV-19/Japan/PG-82956/2021	EPI_ISL_3895607	2021-09-02
hCoV-19/Japan/PG-82957/2021	EPI_ISL_3895608	2021-09-02
hCoV-19/Japan/PG-82958/2021	EPI_ISL_3895609	2021-09-02
hCoV-19/Japan/PG-82959/2021	EPI_ISL_3895610	2021-09-02
hCoV-19/Japan/PG-82960/2021	EPI_ISL_3895611	2021-09-02
hCoV-19/Japan/PG-82961/2021	EPI_ISL_3895612	2021-09-02

virus_name	epiID	submission_date
hCoV-19/Japan/PG-82962/2021	EPI_ISL_3895613	2021-09-02
hCoV-19/Japan/PG-82963/2021	EPI_ISL_3895614	2021-09-02
hCoV-19/Japan/PG-82964/2021	EPI_ISL_3895615	2021-09-02
hCoV-19/Japan/PG-82965/2021	EPI_ISL_3895616	2021-09-02
hCoV-19/Japan/PG-82966/2021	EPI_ISL_3895617	2021-09-02
hCoV-19/Japan/PG-82967/2021	EPI_ISL_3895618	2021-09-02
hCoV-19/Japan/PG-82968/2021	EPI_ISL_3895619	2021-09-02
hCoV-19/Japan/PG-82969/2021	EPI_ISL_3895620	2021-09-02
hCoV-19/Japan/PG-82970/2021	EPI_ISL_3895621	2021-09-02
hCoV-19/Japan/PG-82971/2021	EPI_ISL_3895622	2021-09-02
hCoV-19/Japan/PG-82972/2021	EPI_ISL_3895623	2021-09-02
hCoV-19/Japan/PG-82973/2021	EPI_ISL_3895624	2021-09-02
hCoV-19/Japan/PG-82974/2021	EPI_ISL_3895625	2021-09-02
hCoV-19/Japan/PG-82975/2021	EPI_ISL_3895626	2021-09-02
hCoV-19/Japan/PG-82976/2021	EPI_ISL_3895627	2021-09-02
hCoV-19/Japan/PG-82977/2021	EPI_ISL_3895628	2021-09-02
hCoV-19/Japan/PG-82978/2021	EPI_ISL_3895629	2021-09-02
hCoV-19/Japan/PG-82979/2021	EPI_ISL_3895630	2021-09-02
hCoV-19/Japan/PG-82980/2021	EPI_ISL_3895631	2021-09-02
hCoV-19/Japan/PG-82981/2021	EPI_ISL_3895632	2021-09-02
hCoV-19/Japan/PG-82982/2021	EPI_ISL_3895633	2021-09-02
hCoV-19/Japan/PG-82983/2021	EPI_ISL_3895634	2021-09-02
hCoV-19/Japan/PG-82984/2021	EPI_ISL_3895635	2021-09-02
hCoV-19/Japan/PG-82985/2021	EPI_ISL_3895636	2021-09-02
hCoV-19/Japan/PG-82986/2021	EPI_ISL_3895637	2021-09-02
hCoV-19/Japan/PG-82987/2021	EPI_ISL_3895638	2021-09-02
hCoV-19/Japan/PG-82988/2021	EPI_ISL_3895639	2021-09-02
hCoV-19/Japan/PG-82989/2021	EPI_ISL_3895640	2021-09-02
hCoV-19/Japan/PG-82990/2021	EPI_ISL_3895641	2021-09-02
hCoV-19/Japan/PG-82991/2021	EPI_ISL_3895642	2021-09-02
hCoV-19/Japan/PG-82992/2021	EPI_ISL_3895643	2021-09-02
hCoV-19/Japan/PG-82993/2021	EPI_ISL_3895644	2021-09-02
hCoV-19/Japan/PG-82994/2021	EPI_ISL_3895645	2021-09-02
hCoV-19/Japan/PG-82995/2021	EPI_ISL_3895646	2021-09-02
hCoV-19/Japan/PG-82996/2021	EPI_ISL_3895647	2021-09-02
hCoV-19/Japan/PG-82997/2021	EPI_ISL_3895648	2021-09-02
hCoV-19/Japan/PG-82998/2021	EPI_ISL_3895649	2021-09-02
hCoV-19/Japan/PG-82999/2021	EPI_ISL_3895650	2021-09-02
hCoV-19/Japan/PG-83000/2021	EPI_ISL_3895651	2021-09-02
hCoV-19/Japan/PG-83001/2021	EPI_ISL_3895652	2021-09-02
hCoV-19/Japan/PG-83002/2021	EPI_ISL_3895653	2021-09-02
hCoV-19/Japan/PG-83003/2021	EPI_ISL_3895654	2021-09-02
hCoV-19/Japan/PG-83004/2021	EPI_ISL_3895655	2021-09-02
hCoV-19/Japan/PG-83005/2021	EPI_ISL_3895656	2021-09-02
hCoV-19/Japan/PG-83006/2021	EPI_ISL_3895657	2021-09-02
hCoV-19/Japan/PG-83007/2021	EPI_ISL_3895658	2021-09-02
hCoV-19/Japan/PG-83008/2021	EPI_ISL_3895659	2021-09-02
hCoV-19/Japan/PG-83009/2021	EPI_ISL_3895660	2021-09-02
hCoV-19/Japan/PG-83010/2021	EPI_ISL_3895661	2021-09-02
hCoV-19/Japan/PG-83011/2021	EPI_ISL_3895662	2021-09-02
hCoV-19/Japan/PG-83012/2021	EPI_ISL_3895663	2021-09-02
hCoV-19/Japan/PG-83013/2021	EPI_ISL_3895664	2021-09-02
hCoV-19/Japan/PG-83014/2021	EPI_ISL_3895665	2021-09-02
hCoV-19/Japan/PG-83015/2021	EPI_ISL_3895666	2021-09-02

virus_name	epiID	submission_date
hCoV-19/Japan/PG-83016/2021	EPI_ISL_3895667	2021-09-02
hCoV-19/Japan/PG-83017/2021	EPI_ISL_3895668	2021-09-02
hCoV-19/Japan/PG-83018/2021	EPI_ISL_3895669	2021-09-02
hCoV-19/Japan/PG-83019/2021	EPI_ISL_3895670	2021-09-02
hCoV-19/Japan/PG-83020/2021	EPI_ISL_3895671	2021-09-02
hCoV-19/Japan/PG-83021/2021	EPI_ISL_3895672	2021-09-02
hCoV-19/Japan/PG-83022/2021	EPI_ISL_3895673	2021-09-02
hCoV-19/Japan/PG-83023/2021	EPI_ISL_3895674	2021-09-02
hCoV-19/Japan/PG-83024/2021	EPI_ISL_3895675	2021-09-02
hCoV-19/Japan/PG-83025/2021	EPI_ISL_3895676	2021-09-02
hCoV-19/Japan/PG-83026/2021	EPI_ISL_3895677	2021-09-02
hCoV-19/Japan/PG-83027/2021	EPI_ISL_3895678	2021-09-02
hCoV-19/Japan/PG-83028/2021	EPI_ISL_3895679	2021-09-02
hCoV-19/Japan/PG-83029/2021	EPI_ISL_3895680	2021-09-02
hCoV-19/Japan/PG-83030/2021	EPI_ISL_3895681	2021-09-02
hCoV-19/Japan/PG-83031/2021	EPI_ISL_3895682	2021-09-02
hCoV-19/Japan/PG-83032/2021	EPI_ISL_3895683	2021-09-02
hCoV-19/Japan/PG-83033/2021	EPI_ISL_3895684	2021-09-02
hCoV-19/Japan/PG-83034/2021	EPI_ISL_3895685	2021-09-02
hCoV-19/Japan/PG-83035/2021	EPI_ISL_3895686	2021-09-02
hCoV-19/Japan/PG-83036/2021	EPI_ISL_3895687	2021-09-02
hCoV-19/Japan/PG-83037/2021	EPI_ISL_3895688	2021-09-02
hCoV-19/Japan/PG-83038/2021	EPI_ISL_3895689	2021-09-02
hCoV-19/Japan/PG-83039/2021	EPI_ISL_3895690	2021-09-02
hCoV-19/Japan/PG-83040/2021	EPI_ISL_3895691	2021-09-02
hCoV-19/Japan/PG-83041/2021	EPI_ISL_3895692	2021-09-02
hCoV-19/Japan/PG-83042/2021	EPI_ISL_3895693	2021-09-02
hCoV-19/Japan/PG-83043/2021	EPI_ISL_3895694	2021-09-02
hCoV-19/Japan/PG-83044/2021	EPI_ISL_3895695	2021-09-02
hCoV-19/Japan/PG-83045/2021	EPI_ISL_3895696	2021-09-02
hCoV-19/Japan/PG-83046/2021	EPI_ISL_3895697	2021-09-02
hCoV-19/Japan/PG-83047/2021	EPI_ISL_3895698	2021-09-02
hCoV-19/Japan/PG-83048/2021	EPI_ISL_3895699	2021-09-02
hCoV-19/Japan/PG-83049/2021	EPI_ISL_3895700	2021-09-02
hCoV-19/Japan/PG-83050/2021	EPI_ISL_3895701	2021-09-02
hCoV-19/Japan/PG-83051/2021	EPI_ISL_3895702	2021-09-02
hCoV-19/Japan/PG-83052/2021	EPI_ISL_3895703	2021-09-02
hCoV-19/Japan/PG-83053/2021	EPI_ISL_3895704	2021-09-02
hCoV-19/Japan/PG-83054/2021	EPI_ISL_3895705	2021-09-02
hCoV-19/Japan/PG-83055/2021	EPI_ISL_3895706	2021-09-02
hCoV-19/Japan/PG-83056/2021	EPI_ISL_3895707	2021-09-02
hCoV-19/Japan/PG-83057/2021	EPI_ISL_3895708	2021-09-02
hCoV-19/Japan/PG-83058/2021	EPI_ISL_3895709	2021-09-02
hCoV-19/Japan/PG-83059/2021	EPI_ISL_3895710	2021-09-02
hCoV-19/Japan/PG-83060/2021	EPI_ISL_3895711	2021-09-02
hCoV-19/Japan/PG-83061/2021	EPI_ISL_3895712	2021-09-02
hCoV-19/Japan/PG-83062/2021	EPI_ISL_3895713	2021-09-02
hCoV-19/Japan/PG-83063/2021	EPI_ISL_3895714	2021-09-02
hCoV-19/Japan/PG-83064/2021	EPI_ISL_3895715	2021-09-02
hCoV-19/Japan/PG-83065/2021	EPI_ISL_3895716	2021-09-02
hCoV-19/Japan/PG-83066/2021	EPI_ISL_3895717	2021-09-02
hCoV-19/Japan/PG-83067/2021	EPI_ISL_3895718	2021-09-02
hCoV-19/Japan/PG-83068/2021	EPI_ISL_3895719	2021-09-02
hCoV-19/Japan/PG-83069/2021	EPI_ISL_3895720	2021-09-02

virus_name	epiID	submission_date
hCoV-19/Japan/PG-83070/2021	EPI_ISL_3895721	2021-09-02
hCoV-19/Japan/PG-83071/2021	EPI_ISL_3895722	2021-09-02
hCoV-19/Japan/PG-83072/2021	EPI_ISL_3895723	2021-09-02
hCoV-19/Japan/PG-83073/2021	EPI_ISL_3895724	2021-09-02
hCoV-19/Japan/PG-83074/2021	EPI_ISL_3895725	2021-09-02
hCoV-19/Japan/PG-83075/2021	EPI_ISL_3895726	2021-09-02
hCoV-19/Japan/PG-83076/2021	EPI_ISL_3895727	2021-09-02
hCoV-19/Japan/PG-83077/2021	EPI_ISL_3895728	2021-09-02
hCoV-19/Japan/PG-83078/2021	EPI_ISL_3895729	2021-09-02
hCoV-19/Japan/PG-83079/2021	EPI_ISL_3895730	2021-09-02
hCoV-19/Japan/PG-83080/2021	EPI_ISL_3895731	2021-09-02
hCoV-19/Japan/PG-83081/2021	EPI_ISL_3895732	2021-09-02
hCoV-19/Japan/PG-83082/2021	EPI_ISL_3895733	2021-09-02
hCoV-19/Japan/PG-83083/2021	EPI_ISL_3895734	2021-09-02
hCoV-19/Japan/PG-83084/2021	EPI_ISL_3895735	2021-09-02
hCoV-19/Japan/PG-83085/2021	EPI_ISL_3895736	2021-09-02
hCoV-19/Japan/PG-83086/2021	EPI_ISL_3895737	2021-09-02
hCoV-19/Japan/PG-83087/2021	EPI_ISL_3895738	2021-09-02
hCoV-19/Japan/PG-83088/2021	EPI_ISL_3895739	2021-09-02
hCoV-19/Japan/PG-83089/2021	EPI_ISL_3895740	2021-09-02
hCoV-19/Japan/PG-83090/2021	EPI_ISL_3895741	2021-09-02
hCoV-19/Japan/PG-83091/2021	EPI_ISL_3895742	2021-09-02
hCoV-19/Japan/PG-83092/2021	EPI_ISL_3895743	2021-09-02
hCoV-19/Japan/PG-83093/2021	EPI_ISL_3895744	2021-09-02
hCoV-19/Japan/PG-83094/2021	EPI_ISL_3895745	2021-09-02
hCoV-19/Japan/PG-83095/2021	EPI_ISL_3895746	2021-09-02
hCoV-19/Japan/PG-83096/2021	EPI_ISL_3895747	2021-09-02
hCoV-19/Japan/PG-83097/2021	EPI_ISL_3895748	2021-09-02
hCoV-19/Japan/PG-83098/2021	EPI_ISL_3895749	2021-09-02
hCoV-19/Japan/PG-83099/2021	EPI_ISL_3895750	2021-09-02
hCoV-19/Japan/PG-83100/2021	EPI_ISL_3895751	2021-09-02
hCoV-19/Japan/PG-83101/2021	EPI_ISL_3895752	2021-09-02
hCoV-19/Japan/PG-83102/2021	EPI_ISL_3895753	2021-09-02
hCoV-19/Japan/PG-83103/2021	EPI_ISL_3895754	2021-09-02
hCoV-19/Japan/PG-83104/2021	EPI_ISL_3895755	2021-09-02
hCoV-19/Japan/PG-83105/2021	EPI_ISL_3895756	2021-09-02
hCoV-19/Japan/PG-83106/2021	EPI_ISL_3895757	2021-09-02
hCoV-19/Japan/PG-83107/2021	EPI_ISL_3895758	2021-09-02
hCoV-19/Japan/PG-83108/2021	EPI_ISL_3895759	2021-09-02
hCoV-19/Japan/PG-83109/2021	EPI_ISL_3895760	2021-09-02
hCoV-19/Japan/PG-83110/2021	EPI_ISL_3895761	2021-09-02
hCoV-19/Japan/PG-83111/2021	EPI_ISL_3895762	2021-09-02
hCoV-19/Japan/PG-83112/2021	EPI_ISL_3895763	2021-09-02
hCoV-19/Japan/PG-83113/2021	EPI_ISL_3895764	2021-09-02
hCoV-19/Japan/PG-83114/2021	EPI_ISL_3895765	2021-09-02
hCoV-19/Japan/PG-83115/2021	EPI_ISL_3895766	2021-09-02
hCoV-19/Japan/PG-83116/2021	EPI_ISL_3895767	2021-09-02
hCoV-19/Japan/PG-83117/2021	EPI_ISL_3895768	2021-09-02
hCoV-19/Japan/PG-83118/2021	EPI_ISL_3895769	2021-09-02
hCoV-19/Japan/PG-83119/2021	EPI_ISL_3895770	2021-09-02
hCoV-19/Japan/PG-83120/2021	EPI_ISL_3895771	2021-09-02
hCoV-19/Japan/PG-83121/2021	EPI_ISL_3895772	2021-09-02
hCoV-19/Japan/PG-83122/2021	EPI_ISL_3895773	2021-09-02
hCoV-19/Japan/PG-83123/2021	EPI_ISL_3895774	2021-09-02

virus_name	epiID	submission_date
hCoV-19/Japan/PG-83124/2021	EPI_ISL_3895775	2021-09-02
hCoV-19/Japan/PG-83125/2021	EPI_ISL_3895776	2021-09-02
hCoV-19/Japan/PG-83126/2021	EPI_ISL_3895777	2021-09-02
hCoV-19/Japan/PG-83127/2021	EPI_ISL_3895778	2021-09-02
hCoV-19/Japan/PG-83128/2021	EPI_ISL_3895779	2021-09-02
hCoV-19/Japan/PG-83129/2021	EPI_ISL_3895780	2021-09-02
hCoV-19/Japan/PG-83130/2021	EPI_ISL_3895781	2021-09-02
hCoV-19/Japan/PG-83131/2021	EPI_ISL_3895782	2021-09-02
hCoV-19/Japan/PG-83132/2021	EPI_ISL_3895783	2021-09-02
hCoV-19/Japan/PG-83133/2021	EPI_ISL_3895784	2021-09-02
hCoV-19/Japan/PG-83134/2021	EPI_ISL_3895785	2021-09-02
hCoV-19/Japan/PG-83135/2021	EPI_ISL_3895786	2021-09-02
hCoV-19/Japan/PG-83136/2021	EPI_ISL_3895787	2021-09-02
hCoV-19/Japan/PG-83137/2021	EPI_ISL_3895788	2021-09-02
hCoV-19/Japan/PG-83138/2021	EPI_ISL_3895789	2021-09-02
hCoV-19/Japan/PG-83139/2021	EPI_ISL_3895790	2021-09-02
hCoV-19/Japan/PG-83140/2021	EPI_ISL_3895791	2021-09-02
hCoV-19/Japan/PG-83141/2021	EPI_ISL_3895792	2021-09-02
hCoV-19/Japan/PG-83142/2021	EPI_ISL_3895793	2021-09-02
hCoV-19/Japan/PG-83143/2021	EPI_ISL_3895794	2021-09-02
hCoV-19/Japan/PG-83144/2021	EPI_ISL_3895795	2021-09-02
hCoV-19/Japan/PG-83145/2021	EPI_ISL_3895796	2021-09-02
hCoV-19/Japan/PG-83146/2021	EPI_ISL_3895797	2021-09-02
hCoV-19/Japan/PG-83147/2021	EPI_ISL_3895798	2021-09-02
hCoV-19/Japan/PG-83148/2021	EPI_ISL_3895799	2021-09-02
hCoV-19/Japan/PG-83149/2021	EPI_ISL_3895800	2021-09-02
hCoV-19/Japan/PG-83150/2021	EPI_ISL_3895801	2021-09-02
hCoV-19/Japan/PG-83151/2021	EPI_ISL_3895802	2021-09-02
hCoV-19/Japan/PG-83152/2021	EPI_ISL_3895803	2021-09-02
hCoV-19/Japan/PG-83153/2021	EPI_ISL_3895804	2021-09-02
hCoV-19/Japan/PG-83154/2021	EPI_ISL_3895805	2021-09-02
hCoV-19/Japan/PG-83155/2021	EPI_ISL_3895806	2021-09-02
hCoV-19/Japan/PG-83156/2021	EPI_ISL_3895807	2021-09-02
hCoV-19/Japan/PG-83157/2021	EPI_ISL_3895808	2021-09-02
hCoV-19/Japan/PG-83158/2021	EPI_ISL_3895809	2021-09-02
hCoV-19/Japan/PG-83159/2021	EPI_ISL_3895810	2021-09-02
hCoV-19/Japan/PG-83160/2021	EPI_ISL_3895811	2021-09-02
hCoV-19/Japan/PG-83161/2021	EPI_ISL_3895812	2021-09-02
hCoV-19/Japan/PG-83162/2021	EPI_ISL_3895813	2021-09-02
hCoV-19/Japan/PG-83163/2021	EPI_ISL_3895814	2021-09-02
hCoV-19/Japan/PG-83164/2021	EPI_ISL_3895815	2021-09-02
hCoV-19/Japan/PG-83165/2021	EPI_ISL_3895816	2021-09-02
hCoV-19/Japan/PG-83166/2021	EPI_ISL_3895817	2021-09-02
hCoV-19/Japan/PG-83167/2021	EPI_ISL_3895818	2021-09-02
hCoV-19/Japan/PG-83168/2021	EPI_ISL_3895819	2021-09-02
hCoV-19/Japan/PG-83169/2021	EPI_ISL_3895820	2021-09-02
hCoV-19/Japan/PG-83170/2021	EPI_ISL_3895821	2021-09-02
hCoV-19/Japan/PG-83171/2021	EPI_ISL_3895822	2021-09-02
hCoV-19/Japan/PG-83172/2021	EPI_ISL_3895823	2021-09-02
hCoV-19/Japan/PG-83173/2021	EPI_ISL_3895824	2021-09-02
hCoV-19/Japan/PG-83174/2021	EPI_ISL_3895825	2021-09-02
hCoV-19/Japan/PG-83175/2021	EPI_ISL_3895826	2021-09-02
hCoV-19/Japan/PG-83176/2021	EPI_ISL_3895827	2021-09-02
hCoV-19/Japan/PG-83177/2021	EPI_ISL_3895828	2021-09-02

virus_name	epiID	submission_date
hCoV-19/Japan/PG-83178/2021	EPI_ISL_3895829	2021-09-02
hCoV-19/Japan/PG-83179/2021	EPI_ISL_3895830	2021-09-02
hCoV-19/Japan/PG-83180/2021	EPI_ISL_3895831	2021-09-02
hCoV-19/Japan/PG-83181/2021	EPI_ISL_3895832	2021-09-02
hCoV-19/Japan/PG-83182/2021	EPI_ISL_3895833	2021-09-02
hCoV-19/Japan/PG-83183/2021	EPI_ISL_3895834	2021-09-02
hCoV-19/Japan/PG-83184/2021	EPI_ISL_3895835	2021-09-02
hCoV-19/Japan/PG-83185/2021	EPI_ISL_3895836	2021-09-02
hCoV-19/Japan/PG-83186/2021	EPI_ISL_3895837	2021-09-02
hCoV-19/Japan/PG-83187/2021	EPI_ISL_3895838	2021-09-02
hCoV-19/Japan/PG-83188/2021	EPI_ISL_3895839	2021-09-02
hCoV-19/Japan/PG-83189/2021	EPI_ISL_3895840	2021-09-02
hCoV-19/Japan/PG-83190/2021	EPI_ISL_3895841	2021-09-02
hCoV-19/Japan/PG-83191/2021	EPI_ISL_3895842	2021-09-02
hCoV-19/Japan/PG-83192/2021	EPI_ISL_3895843	2021-09-02
hCoV-19/Japan/PG-83193/2021	EPI_ISL_3895844	2021-09-02
hCoV-19/Japan/PG-83194/2021	EPI_ISL_3895845	2021-09-02
hCoV-19/Japan/PG-83195/2021	EPI_ISL_3895846	2021-09-02
hCoV-19/Japan/PG-83196/2021	EPI_ISL_3895847	2021-09-02
hCoV-19/Japan/PG-83197/2021	EPI_ISL_3895848	2021-09-02
hCoV-19/Japan/PG-83198/2021	EPI_ISL_3895850	2021-09-02
hCoV-19/Japan/PG-83199/2021	EPI_ISL_3895851	2021-09-02
hCoV-19/Japan/PG-83200/2021	EPI_ISL_3895852	2021-09-02
hCoV-19/Japan/PG-83201/2021	EPI_ISL_3895853	2021-09-02
hCoV-19/Japan/PG-83202/2021	EPI_ISL_3895854	2021-09-02
hCoV-19/Japan/PG-83203/2021	EPI_ISL_3895855	2021-09-02
hCoV-19/Japan/PG-83204/2021	EPI_ISL_3895856	2021-09-02
hCoV-19/Japan/PG-83205/2021	EPI_ISL_3895857	2021-09-02
hCoV-19/Japan/PG-83206/2021	EPI_ISL_3895858	2021-09-02
hCoV-19/Japan/PG-83207/2021	EPI_ISL_3895859	2021-09-02
hCoV-19/Japan/PG-83208/2021	EPI_ISL_3895860	2021-09-02
hCoV-19/Japan/PG-83209/2021	EPI_ISL_3895861	2021-09-02
hCoV-19/Japan/PG-83210/2021	EPI_ISL_3895862	2021-09-02
hCoV-19/Japan/PG-83211/2021	EPI_ISL_3895863	2021-09-02
hCoV-19/Japan/PG-83212/2021	EPI_ISL_3895864	2021-09-02
hCoV-19/Japan/PG-83213/2021	EPI_ISL_3895865	2021-09-02
hCoV-19/Japan/PG-83214/2021	EPI_ISL_3895866	2021-09-02
hCoV-19/Japan/PG-83215/2021	EPI_ISL_3895867	2021-09-02
hCoV-19/Japan/PG-83216/2021	EPI_ISL_3895868	2021-09-02
hCoV-19/Japan/PG-83217/2021	EPI_ISL_3895869	2021-09-02
hCoV-19/Japan/PG-83218/2021	EPI_ISL_3895870	2021-09-02
hCoV-19/Japan/PG-83219/2021	EPI_ISL_3895871	2021-09-02
hCoV-19/Japan/PG-83220/2021	EPI_ISL_3895872	2021-09-02
hCoV-19/Japan/PG-83221/2021	EPI_ISL_3895873	2021-09-02
hCoV-19/Japan/PG-83222/2021	EPI_ISL_3895874	2021-09-02
hCoV-19/Japan/PG-83223/2021	EPI_ISL_3895875	2021-09-02
hCoV-19/Japan/PG-83224/2021	EPI_ISL_3895876	2021-09-02
hCoV-19/Japan/PG-83225/2021	EPI_ISL_3895877	2021-09-02
hCoV-19/Japan/PG-83226/2021	EPI_ISL_3895878	2021-09-02
hCoV-19/Japan/PG-83227/2021	EPI_ISL_3895879	2021-09-02
hCoV-19/Japan/PG-83228/2021	EPI_ISL_3895880	2021-09-02
hCoV-19/Japan/PG-83229/2021	EPI_ISL_3895881	2021-09-02
hCoV-19/Japan/PG-83230/2021	EPI_ISL_3895882	2021-09-02
hCoV-19/Japan/PG-83231/2021	EPI_ISL_3895883	2021-09-02

virus_name	epiID	submission_date
hCoV-19/Japan/PG-83232/2021	EPI_ISL_3895884	2021-09-02
hCoV-19/Japan/PG-83233/2021	EPI_ISL_3895885	2021-09-02
hCoV-19/Japan/PG-83234/2021	EPI_ISL_3895886	2021-09-02
hCoV-19/Japan/PG-83235/2021	EPI_ISL_3895887	2021-09-02
hCoV-19/Japan/PG-83236/2021	EPI_ISL_3895888	2021-09-02
hCoV-19/Japan/PG-83237/2021	EPI_ISL_3895889	2021-09-02
hCoV-19/Japan/PG-83238/2021	EPI_ISL_3895890	2021-09-02
hCoV-19/Japan/PG-83239/2021	EPI_ISL_3895891	2021-09-02
hCoV-19/Japan/PG-83240/2021	EPI_ISL_3895892	2021-09-02
hCoV-19/Japan/PG-83241/2021	EPI_ISL_3895893	2021-09-02
hCoV-19/Japan/PG-83242/2021	EPI_ISL_3895894	2021-09-02
hCoV-19/Japan/PG-83243/2021	EPI_ISL_3895895	2021-09-02
hCoV-19/Japan/PG-83244/2021	EPI_ISL_3895896	2021-09-02
hCoV-19/Japan/PG-83245/2021	EPI_ISL_3895897	2021-09-02
hCoV-19/Japan/PG-83246/2021	EPI_ISL_3895898	2021-09-02
hCoV-19/Japan/PG-83247/2021	EPI_ISL_3895899	2021-09-02
hCoV-19/Japan/PG-83248/2021	EPI_ISL_3895900	2021-09-02
hCoV-19/Japan/PG-83249/2021	EPI_ISL_3895901	2021-09-02
hCoV-19/Japan/PG-83250/2021	EPI_ISL_3895902	2021-09-02
hCoV-19/Japan/PG-83251/2021	EPI_ISL_3895903	2021-09-02
hCoV-19/Japan/PG-83252/2021	EPI_ISL_3895904	2021-09-02
hCoV-19/Japan/PG-83253/2021	EPI_ISL_3895905	2021-09-02
hCoV-19/Japan/PG-83254/2021	EPI_ISL_3895906	2021-09-02
hCoV-19/Japan/PG-83255/2021	EPI_ISL_3895907	2021-09-02
hCoV-19/Japan/PG-83256/2021	EPI_ISL_3895908	2021-09-02
hCoV-19/Japan/PG-83257/2021	EPI_ISL_3895909	2021-09-02
hCoV-19/Japan/PG-83258/2021	EPI_ISL_3895910	2021-09-02
hCoV-19/Japan/PG-83259/2021	EPI_ISL_3895911	2021-09-02
hCoV-19/Japan/PG-83260/2021	EPI_ISL_3895912	2021-09-02
hCoV-19/Japan/PG-83261/2021	EPI_ISL_3895913	2021-09-02
hCoV-19/Japan/PG-83262/2021	EPI_ISL_3895914	2021-09-02
hCoV-19/Japan/PG-83263/2021	EPI_ISL_3895915	2021-09-02
hCoV-19/Japan/PG-83264/2021	EPI_ISL_3895916	2021-09-02
hCoV-19/Japan/PG-83265/2021	EPI_ISL_3895917	2021-09-02
hCoV-19/Japan/PG-83266/2021	EPI_ISL_3895918	2021-09-02
hCoV-19/Japan/PG-83267/2021	EPI_ISL_3895919	2021-09-02
hCoV-19/Japan/PG-83268/2021	EPI_ISL_3895920	2021-09-02
hCoV-19/Japan/PG-83269/2021	EPI_ISL_3895921	2021-09-02
hCoV-19/Japan/PG-83270/2021	EPI_ISL_3895922	2021-09-02
hCoV-19/Japan/PG-83271/2021	EPI_ISL_3895923	2021-09-02
hCoV-19/Japan/PG-83272/2021	EPI_ISL_3895924	2021-09-02
hCoV-19/Japan/PG-83273/2021	EPI_ISL_3895925	2021-09-02
hCoV-19/Japan/PG-83274/2021	EPI_ISL_3895926	2021-09-02
hCoV-19/Japan/PG-83275/2021	EPI_ISL_3895927	2021-09-02
hCoV-19/Japan/PG-83276/2021	EPI_ISL_3895928	2021-09-02
hCoV-19/Japan/PG-83277/2021	EPI_ISL_3895929	2021-09-02
hCoV-19/Japan/PG-83278/2021	EPI_ISL_3895930	2021-09-02
hCoV-19/Japan/PG-83279/2021	EPI_ISL_3895931	2021-09-02
hCoV-19/Japan/PG-83280/2021	EPI_ISL_3895932	2021-09-02
hCoV-19/Japan/PG-83281/2021	EPI_ISL_3895933	2021-09-02
hCoV-19/Japan/PG-83282/2021	EPI_ISL_3895934	2021-09-02
hCoV-19/Japan/PG-83283/2021	EPI_ISL_3895935	2021-09-02
hCoV-19/Japan/PG-83284/2021	EPI_ISL_3895936	2021-09-02
hCoV-19/Japan/PG-83285/2021	EPI_ISL_3895937	2021-09-02

virus_name	epiID	submission_date
hCoV-19/Japan/PG-83286/2021	EPI_ISL_3895938	2021-09-02
hCoV-19/Japan/PG-83287/2021	EPI_ISL_3895939	2021-09-02
hCoV-19/Japan/PG-83288/2021	EPI_ISL_3895940	2021-09-02
hCoV-19/Japan/PG-83289/2021	EPI_ISL_3895941	2021-09-02
hCoV-19/Japan/PG-83290/2021	EPI_ISL_3895942	2021-09-02
hCoV-19/Japan/PG-83291/2021	EPI_ISL_3895943	2021-09-02
hCoV-19/Japan/PG-83292/2021	EPI_ISL_3895944	2021-09-02
hCoV-19/Japan/PG-83293/2021	EPI_ISL_3895945	2021-09-02
hCoV-19/Japan/PG-83294/2021	EPI_ISL_3895946	2021-09-02
hCoV-19/Japan/PG-83295/2021	EPI_ISL_3895947	2021-09-02
hCoV-19/Japan/PG-83296/2021	EPI_ISL_3895948	2021-09-02
hCoV-19/Japan/PG-83297/2021	EPI_ISL_3895949	2021-09-02
hCoV-19/Japan/PG-83298/2021	EPI_ISL_3895950	2021-09-02
hCoV-19/Japan/PG-83299/2021	EPI_ISL_3895951	2021-09-02
hCoV-19/Japan/PG-83300/2021	EPI_ISL_3895952	2021-09-02
hCoV-19/Japan/PG-83301/2021	EPI_ISL_3895953	2021-09-02
hCoV-19/Japan/PG-83302/2021	EPI_ISL_3895954	2021-09-02
hCoV-19/Japan/PG-83303/2021	EPI_ISL_3895955	2021-09-02
hCoV-19/Japan/PG-83304/2021	EPI_ISL_3895956	2021-09-02
hCoV-19/Japan/PG-83305/2021	EPI_ISL_3895957	2021-09-02
hCoV-19/Japan/PG-83306/2021	EPI_ISL_3895958	2021-09-02
hCoV-19/Japan/PG-83307/2021	EPI_ISL_3895959	2021-09-02
hCoV-19/Japan/PG-83308/2021	EPI_ISL_3895960	2021-09-02
hCoV-19/Japan/PG-83309/2021	EPI_ISL_3895961	2021-09-02
hCoV-19/Japan/PG-83310/2021	EPI_ISL_3895962	2021-09-02
hCoV-19/Japan/PG-83311/2021	EPI_ISL_3895963	2021-09-02
hCoV-19/Japan/PG-83312/2021	EPI_ISL_3895964	2021-09-02
hCoV-19/Japan/PG-83313/2021	EPI_ISL_3895965	2021-09-02
hCoV-19/Japan/PG-83314/2021	EPI_ISL_3895966	2021-09-02
hCoV-19/Japan/PG-83315/2021	EPI_ISL_3895967	2021-09-02
hCoV-19/Japan/PG-83316/2021	EPI_ISL_3895968	2021-09-02
hCoV-19/Japan/PG-83317/2021	EPI_ISL_3895969	2021-09-02
hCoV-19/Japan/PG-83318/2021	EPI_ISL_3895970	2021-09-02
hCoV-19/Japan/PG-83319/2021	EPI_ISL_3895971	2021-09-02
hCoV-19/Japan/PG-83320/2021	EPI_ISL_3895972	2021-09-02
hCoV-19/Japan/PG-83321/2021	EPI_ISL_3895973	2021-09-02
hCoV-19/Japan/PG-83322/2021	EPI_ISL_3895974	2021-09-02
hCoV-19/Japan/PG-83323/2021	EPI_ISL_3895975	2021-09-02
hCoV-19/Japan/PG-83324/2021	EPI_ISL_3895976	2021-09-02
hCoV-19/Japan/PG-83325/2021	EPI_ISL_3895977	2021-09-02
hCoV-19/Japan/PG-83326/2021	EPI_ISL_3895978	2021-09-02
hCoV-19/Japan/PG-83327/2021	EPI_ISL_3895979	2021-09-02
hCoV-19/Japan/PG-83328/2021	EPI_ISL_3895980	2021-09-02
hCoV-19/Japan/PG-83329/2021	EPI_ISL_3895981	2021-09-02
hCoV-19/Japan/PG-83330/2021	EPI_ISL_3895982	2021-09-02
hCoV-19/Japan/PG-83331/2021	EPI_ISL_3895983	2021-09-02
hCoV-19/Japan/PG-83332/2021	EPI_ISL_3895984	2021-09-02
hCoV-19/Japan/PG-83333/2021	EPI_ISL_3895985	2021-09-02
hCoV-19/Japan/PG-83334/2021	EPI_ISL_3895986	2021-09-02
hCoV-19/Japan/PG-83335/2021	EPI_ISL_3895987	2021-09-02
hCoV-19/Japan/PG-83336/2021	EPI_ISL_3895988	2021-09-02
hCoV-19/Japan/PG-83337/2021	EPI_ISL_3895989	2021-09-02
hCoV-19/Japan/PG-83338/2021	EPI_ISL_3895990	2021-09-02
hCoV-19/Japan/PG-83339/2021	EPI_ISL_3895991	2021-09-02

virus_name	epiID	submission_date
hCoV-19/Japan/PG-83340/2021	EPI_ISL_3895992	2021-09-02
hCoV-19/Japan/PG-83341/2021	EPI_ISL_3895993	2021-09-02
hCoV-19/Japan/PG-83342/2021	EPI_ISL_3895994	2021-09-02
hCoV-19/Japan/PG-83343/2021	EPI_ISL_3895995	2021-09-02
hCoV-19/Japan/PG-83344/2021	EPI_ISL_3895996	2021-09-02
hCoV-19/Japan/PG-83345/2021	EPI_ISL_3895997	2021-09-02
hCoV-19/Japan/PG-83346/2021	EPI_ISL_3895998	2021-09-02
hCoV-19/Japan/PG-83347/2021	EPI_ISL_3895999	2021-09-02
hCoV-19/Japan/PG-83348/2021	EPI_ISL_3896000	2021-09-02
hCoV-19/Japan/PG-83349/2021	EPI_ISL_3896001	2021-09-02
hCoV-19/Japan/PG-83350/2021	EPI_ISL_3896002	2021-09-02
hCoV-19/Japan/PG-83351/2021	EPI_ISL_3896003	2021-09-02
hCoV-19/Japan/PG-83352/2021	EPI_ISL_3896004	2021-09-02
hCoV-19/Japan/PG-83353/2021	EPI_ISL_3896005	2021-09-02
hCoV-19/Japan/PG-83354/2021	EPI_ISL_3896006	2021-09-02
hCoV-19/Japan/PG-83355/2021	EPI_ISL_3896007	2021-09-02
hCoV-19/Japan/PG-83356/2021	EPI_ISL_3896008	2021-09-02
hCoV-19/Japan/PG-83357/2021	EPI_ISL_3896009	2021-09-02
hCoV-19/Japan/PG-83358/2021	EPI_ISL_3896010	2021-09-02
hCoV-19/Japan/PG-83359/2021	EPI_ISL_3896011	2021-09-02
hCoV-19/Japan/PG-83360/2021	EPI_ISL_3896012	2021-09-02
hCoV-19/Japan/PG-83361/2021	EPI_ISL_3896013	2021-09-02
hCoV-19/Japan/PG-83362/2021	EPI_ISL_3896014	2021-09-02
hCoV-19/Japan/PG-83363/2021	EPI_ISL_3896015	2021-09-02
hCoV-19/Japan/PG-83364/2021	EPI_ISL_3896016	2021-09-02
hCoV-19/Japan/PG-83365/2021	EPI_ISL_3896017	2021-09-02
hCoV-19/Japan/PG-83366/2021	EPI_ISL_3896018	2021-09-02
hCoV-19/Japan/PG-83367/2021	EPI_ISL_3896019	2021-09-02
hCoV-19/Japan/PG-83368/2021	EPI_ISL_3896020	2021-09-02
hCoV-19/Japan/PG-83369/2021	EPI_ISL_3896021	2021-09-02
hCoV-19/Japan/PG-83370/2021	EPI_ISL_3896022	2021-09-02
hCoV-19/Japan/PG-83371/2021	EPI_ISL_3896023	2021-09-02
hCoV-19/Japan/PG-83372/2021	EPI_ISL_3896024	2021-09-02
hCoV-19/Japan/PG-83373/2021	EPI_ISL_3896025	2021-09-02
hCoV-19/Japan/PG-83374/2021	EPI_ISL_3896026	2021-09-02
hCoV-19/Japan/PG-83375/2021	EPI_ISL_3896027	2021-09-02
hCoV-19/Japan/PG-83376/2021	EPI_ISL_3896028	2021-09-02
hCoV-19/Japan/PG-83377/2021	EPI_ISL_3896029	2021-09-02
hCoV-19/Japan/PG-83378/2021	EPI_ISL_3896030	2021-09-02
hCoV-19/Japan/PG-83379/2021	EPI_ISL_3896031	2021-09-02
hCoV-19/Japan/PG-83380/2021	EPI_ISL_3896032	2021-09-02
hCoV-19/Japan/PG-83381/2021	EPI_ISL_3896033	2021-09-02
hCoV-19/Japan/PG-83382/2021	EPI_ISL_3896034	2021-09-02
hCoV-19/Japan/PG-83383/2021	EPI_ISL_3896035	2021-09-02
hCoV-19/Japan/PG-83384/2021	EPI_ISL_3896036	2021-09-02
hCoV-19/Japan/PG-83385/2021	EPI_ISL_3896037	2021-09-02
hCoV-19/Japan/PG-83386/2021	EPI_ISL_3896038	2021-09-02
hCoV-19/Japan/PG-83387/2021	EPI_ISL_3896039	2021-09-02
hCoV-19/Japan/PG-83388/2021	EPI_ISL_3896040	2021-09-02
hCoV-19/Japan/PG-83389/2021	EPI_ISL_3896041	2021-09-02
hCoV-19/Japan/PG-83390/2021	EPI_ISL_3896042	2021-09-02
hCoV-19/Japan/PG-83391/2021	EPI_ISL_3896043	2021-09-02
hCoV-19/Japan/PG-83392/2021	EPI_ISL_3896044	2021-09-02
hCoV-19/Japan/PG-83393/2021	EPI_ISL_3896045	2021-09-02

virus_name	epiID	submission_date
hCoV-19/Japan/PG-83394/2021	EPI_ISL_3896046	2021-09-02
hCoV-19/Japan/PG-83395/2021	EPI_ISL_3896047	2021-09-02
hCoV-19/Japan/PG-83396/2021	EPI_ISL_3896048	2021-09-02
hCoV-19/Japan/PG-83397/2021	EPI_ISL_3896049	2021-09-02
hCoV-19/Japan/PG-83398/2021	EPI_ISL_3896050	2021-09-02
hCoV-19/Japan/PG-83399/2021	EPI_ISL_3896051	2021-09-02
hCoV-19/Japan/PG-83400/2021	EPI_ISL_3896052	2021-09-02
hCoV-19/Japan/PG-83401/2021	EPI_ISL_3896053	2021-09-02
hCoV-19/Japan/PG-83402/2021	EPI_ISL_3896054	2021-09-02
hCoV-19/Japan/PG-83403/2021	EPI_ISL_3896055	2021-09-02
hCoV-19/Japan/PG-83404/2021	EPI_ISL_3896056	2021-09-02
hCoV-19/Japan/PG-83405/2021	EPI_ISL_3896057	2021-09-02
hCoV-19/Japan/PG-83406/2021	EPI_ISL_3896058	2021-09-02
hCoV-19/Japan/PG-83407/2021	EPI_ISL_3896059	2021-09-02
hCoV-19/Japan/PG-83408/2021	EPI_ISL_3896060	2021-09-02
hCoV-19/Japan/PG-83409/2021	EPI_ISL_3896061	2021-09-02
hCoV-19/Japan/PG-83410/2021	EPI_ISL_3896062	2021-09-02
hCoV-19/Japan/PG-83411/2021	EPI_ISL_3896063	2021-09-02
hCoV-19/Japan/PG-83412/2021	EPI_ISL_3896064	2021-09-02
hCoV-19/Japan/PG-83413/2021	EPI_ISL_3896065	2021-09-02
hCoV-19/Japan/PG-83414/2021	EPI_ISL_3896066	2021-09-02
hCoV-19/Japan/PG-83415/2021	EPI_ISL_3896067	2021-09-02
hCoV-19/Japan/PG-83416/2021	EPI_ISL_3896068	2021-09-02
hCoV-19/Japan/PG-83417/2021	EPI_ISL_3896069	2021-09-02
hCoV-19/Japan/PG-83418/2021	EPI_ISL_3896070	2021-09-02
hCoV-19/Japan/PG-83419/2021	EPI_ISL_3896071	2021-09-02
hCoV-19/Japan/PG-83420/2021	EPI_ISL_3896072	2021-09-02
hCoV-19/Japan/PG-83421/2021	EPI_ISL_3896073	2021-09-02
hCoV-19/Japan/PG-83422/2021	EPI_ISL_3896074	2021-09-02
hCoV-19/Japan/PG-83423/2021	EPI_ISL_3896075	2021-09-02
hCoV-19/Japan/PG-83424/2021	EPI_ISL_3896076	2021-09-02
hCoV-19/Japan/PG-83425/2021	EPI_ISL_3896077	2021-09-02
hCoV-19/Japan/PG-83426/2021	EPI_ISL_3896078	2021-09-02
hCoV-19/Japan/PG-83427/2021	EPI_ISL_3896079	2021-09-02
hCoV-19/Japan/PG-83428/2021	EPI_ISL_3896080	2021-09-02
hCoV-19/Japan/PG-83429/2021	EPI_ISL_3896081	2021-09-02
hCoV-19/Japan/PG-83430/2021	EPI_ISL_3896082	2021-09-02
hCoV-19/Japan/PG-83431/2021	EPI_ISL_3896083	2021-09-02
hCoV-19/Japan/PG-83432/2021	EPI_ISL_3896084	2021-09-02
hCoV-19/Japan/PG-83433/2021	EPI_ISL_3896085	2021-09-02
hCoV-19/Japan/PG-83434/2021	EPI_ISL_3896086	2021-09-02
hCoV-19/Japan/PG-83435/2021	EPI_ISL_3896087	2021-09-02
hCoV-19/Japan/PG-83436/2021	EPI_ISL_3896088	2021-09-02
hCoV-19/Japan/PG-83437/2021	EPI_ISL_3896089	2021-09-02
hCoV-19/Japan/PG-83438/2021	EPI_ISL_3896090	2021-09-02
hCoV-19/Japan/PG-83439/2021	EPI_ISL_3896091	2021-09-02
hCoV-19/Japan/PG-83440/2021	EPI_ISL_3896092	2021-09-02
hCoV-19/Japan/PG-83441/2021	EPI_ISL_3896093	2021-09-02
hCoV-19/Japan/PG-83442/2021	EPI_ISL_3896094	2021-09-02
hCoV-19/Japan/PG-83443/2021	EPI_ISL_3896095	2021-09-02
hCoV-19/Japan/PG-83444/2021	EPI_ISL_3896096	2021-09-02
hCoV-19/Japan/PG-83445/2021	EPI_ISL_3896097	2021-09-02
hCoV-19/Japan/PG-83446/2021	EPI_ISL_3896098	2021-09-02
hCoV-19/Japan/PG-83447/2021	EPI_ISL_3896099	2021-09-02

virus_name	epiID	submission_date
hCoV-19/Japan/PG-83448/2021	EPI_ISL_3896100	2021-09-02
hCoV-19/Japan/PG-83449/2021	EPI_ISL_3896101	2021-09-02
hCoV-19/Japan/PG-83450/2021	EPI_ISL_3896102	2021-09-02
hCoV-19/Japan/PG-83451/2021	EPI_ISL_3896103	2021-09-02
hCoV-19/Japan/PG-83452/2021	EPI_ISL_3896104	2021-09-02
hCoV-19/Japan/PG-83453/2021	EPI_ISL_3896105	2021-09-02
hCoV-19/Japan/PG-83454/2021	EPI_ISL_3896106	2021-09-02
hCoV-19/Japan/PG-83455/2021	EPI_ISL_3896107	2021-09-02
hCoV-19/Japan/PG-83456/2021	EPI_ISL_3896108	2021-09-02
hCoV-19/Japan/PG-83457/2021	EPI_ISL_3896109	2021-09-02
hCoV-19/Japan/PG-83458/2021	EPI_ISL_3896110	2021-09-02
hCoV-19/Japan/PG-83459/2021	EPI_ISL_3896111	2021-09-02
hCoV-19/Japan/PG-83460/2021	EPI_ISL_3896112	2021-09-02
hCoV-19/Japan/PG-83461/2021	EPI_ISL_3896113	2021-09-02
hCoV-19/Japan/PG-83462/2021	EPI_ISL_3896114	2021-09-02
hCoV-19/Japan/PG-83463/2021	EPI_ISL_3896115	2021-09-02
hCoV-19/Japan/PG-83464/2021	EPI_ISL_3896116	2021-09-02
hCoV-19/Japan/PG-83465/2021	EPI_ISL_3896117	2021-09-02
hCoV-19/Japan/PG-83466/2021	EPI_ISL_3896118	2021-09-02
hCoV-19/Japan/PG-83467/2021	EPI_ISL_3896119	2021-09-02
hCoV-19/Japan/PG-83468/2021	EPI_ISL_3896120	2021-09-02
hCoV-19/Japan/PG-83469/2021	EPI_ISL_3896121	2021-09-02
hCoV-19/Japan/PG-83470/2021	EPI_ISL_3896122	2021-09-02
hCoV-19/Japan/PG-83471/2021	EPI_ISL_3896123	2021-09-02
hCoV-19/Japan/PG-83472/2021	EPI_ISL_3896124	2021-09-02
hCoV-19/Japan/PG-83473/2021	EPI_ISL_3896125	2021-09-02
hCoV-19/Japan/PG-83474/2021	EPI_ISL_3896126	2021-09-02
hCoV-19/Japan/PG-83475/2021	EPI_ISL_3896127	2021-09-02
hCoV-19/Japan/PG-83476/2021	EPI_ISL_3896128	2021-09-02
hCoV-19/Japan/PG-83477/2021	EPI_ISL_3896129	2021-09-02
hCoV-19/Japan/PG-83478/2021	EPI_ISL_3896130	2021-09-02
hCoV-19/Japan/PG-83479/2021	EPI_ISL_3896131	2021-09-02
hCoV-19/Japan/PG-83480/2021	EPI_ISL_3896132	2021-09-02
hCoV-19/Japan/PG-83481/2021	EPI_ISL_3896133	2021-09-02
hCoV-19/Japan/PG-83482/2021	EPI_ISL_3896134	2021-09-02
hCoV-19/Japan/PG-83483/2021	EPI_ISL_3896135	2021-09-02
hCoV-19/Japan/PG-83484/2021	EPI_ISL_3896136	2021-09-02
hCoV-19/Japan/PG-83485/2021	EPI_ISL_3896137	2021-09-02
hCoV-19/Japan/PG-83486/2021	EPI_ISL_3896138	2021-09-02
hCoV-19/Japan/PG-83487/2021	EPI_ISL_3896139	2021-09-02
hCoV-19/Japan/PG-83488/2021	EPI_ISL_3896140	2021-09-02
hCoV-19/Japan/PG-83489/2021	EPI_ISL_3896141	2021-09-02
hCoV-19/Japan/PG-83490/2021	EPI_ISL_3896142	2021-09-02
hCoV-19/Japan/PG-83491/2021	EPI_ISL_3896143	2021-09-02
hCoV-19/Japan/PG-83492/2021	EPI_ISL_3896144	2021-09-02
hCoV-19/Japan/PG-83493/2021	EPI_ISL_3896145	2021-09-02
hCoV-19/Japan/PG-83494/2021	EPI_ISL_3896146	2021-09-02
hCoV-19/Japan/PG-83495/2021	EPI_ISL_3896147	2021-09-02
hCoV-19/Japan/PG-83496/2021	EPI_ISL_3896148	2021-09-02
hCoV-19/Japan/PG-83497/2021	EPI_ISL_3896149	2021-09-02
hCoV-19/Japan/PG-83498/2021	EPI_ISL_3896150	2021-09-02
hCoV-19/Japan/PG-83499/2021	EPI_ISL_3896151	2021-09-02
hCoV-19/Japan/PG-83500/2021	EPI_ISL_3896152	2021-09-02
hCoV-19/Japan/PG-83501/2021	EPI_ISL_3896153	2021-09-02

virus_name	epiID	submission_date
hCoV-19/Japan/PG-83502/2021	EPI_ISL_3896154	2021-09-02
hCoV-19/Japan/PG-83503/2021	EPI_ISL_3896155	2021-09-02
hCoV-19/Japan/PG-83504/2021	EPI_ISL_3896156	2021-09-02
hCoV-19/Japan/PG-83505/2021	EPI_ISL_3896157	2021-09-02
hCoV-19/Japan/PG-83506/2021	EPI_ISL_3896158	2021-09-02
hCoV-19/Japan/PG-83507/2021	EPI_ISL_3896159	2021-09-02
hCoV-19/Japan/PG-83508/2021	EPI_ISL_3896160	2021-09-02
hCoV-19/Japan/PG-83509/2021	EPI_ISL_3896161	2021-09-02
hCoV-19/Japan/PG-83510/2021	EPI_ISL_3896162	2021-09-02
hCoV-19/Japan/PG-83511/2021	EPI_ISL_3896163	2021-09-02
hCoV-19/Japan/PG-83512/2021	EPI_ISL_3896164	2021-09-02
hCoV-19/Japan/PG-83513/2021	EPI_ISL_3896165	2021-09-02
hCoV-19/Japan/PG-83514/2021	EPI_ISL_3896166	2021-09-02
hCoV-19/Japan/PG-83515/2021	EPI_ISL_3896167	2021-09-02
hCoV-19/Japan/PG-83516/2021	EPI_ISL_3896168	2021-09-02
hCoV-19/Japan/PG-83517/2021	EPI_ISL_3896169	2021-09-02
hCoV-19/Japan/PG-83518/2021	EPI_ISL_3896170	2021-09-02
hCoV-19/Japan/PG-83519/2021	EPI_ISL_3896171	2021-09-02
hCoV-19/Japan/PG-83520/2021	EPI_ISL_3896172	2021-09-02
hCoV-19/Japan/PG-83521/2021	EPI_ISL_3896173	2021-09-02
hCoV-19/Japan/PG-83522/2021	EPI_ISL_3896174	2021-09-02
hCoV-19/Japan/PG-83523/2021	EPI_ISL_3896175	2021-09-02
hCoV-19/Japan/PG-83524/2021	EPI_ISL_3896176	2021-09-02
hCoV-19/Japan/PG-83525/2021	EPI_ISL_3896177	2021-09-02
hCoV-19/Japan/PG-83526/2021	EPI_ISL_3896178	2021-09-02
hCoV-19/Japan/PG-83527/2021	EPI_ISL_3896179	2021-09-02
hCoV-19/Japan/PG-83528/2021	EPI_ISL_3896180	2021-09-02
hCoV-19/Japan/PG-83529/2021	EPI_ISL_3896181	2021-09-02
hCoV-19/Japan/PG-83530/2021	EPI_ISL_3896182	2021-09-02
hCoV-19/Japan/PG-83531/2021	EPI_ISL_3896183	2021-09-02
hCoV-19/Japan/PG-83532/2021	EPI_ISL_3896184	2021-09-02
hCoV-19/Japan/PG-83533/2021	EPI_ISL_3896185	2021-09-02
hCoV-19/Japan/PG-83534/2021	EPI_ISL_3896186	2021-09-02
hCoV-19/Japan/PG-83535/2021	EPI_ISL_3896187	2021-09-02
hCoV-19/Japan/PG-83536/2021	EPI_ISL_3896188	2021-09-02
hCoV-19/Japan/PG-83537/2021	EPI_ISL_3896189	2021-09-02
hCoV-19/Japan/PG-83538/2021	EPI_ISL_3896190	2021-09-02
hCoV-19/Japan/PG-83539/2021	EPI_ISL_3896191	2021-09-02
hCoV-19/Japan/PG-83540/2021	EPI_ISL_3896192	2021-09-02
hCoV-19/Japan/PG-83541/2021	EPI_ISL_3896193	2021-09-02
hCoV-19/Japan/PG-83542/2021	EPI_ISL_3896194	2021-09-02
hCoV-19/Japan/PG-83543/2021	EPI_ISL_3896195	2021-09-02
hCoV-19/Japan/PG-83544/2021	EPI_ISL_3896196	2021-09-02
hCoV-19/Japan/PG-83545/2021	EPI_ISL_3896197	2021-09-02
hCoV-19/Japan/PG-83546/2021	EPI_ISL_3896198	2021-09-02
hCoV-19/Japan/PG-83547/2021	EPI_ISL_3896199	2021-09-02
hCoV-19/Japan/PG-83548/2021	EPI_ISL_3896200	2021-09-02
hCoV-19/Japan/PG-83549/2021	EPI_ISL_3896201	2021-09-02
hCoV-19/Japan/PG-83550/2021	EPI_ISL_3896202	2021-09-02
hCoV-19/Japan/PG-83551/2021	EPI_ISL_3896203	2021-09-02
hCoV-19/Japan/PG-83552/2021	EPI_ISL_3896204	2021-09-02
hCoV-19/Japan/PG-83553/2021	EPI_ISL_3896205	2021-09-02
hCoV-19/Japan/PG-83554/2021	EPI_ISL_3896206	2021-09-02
hCoV-19/Japan/PG-83555/2021	EPI_ISL_3896207	2021-09-02

virus_name	epiID	submission_date
hCoV-19/Japan/PG-83556/2021	EPI_ISL_3896208	2021-09-02
hCoV-19/Japan/PG-83557/2021	EPI_ISL_3896209	2021-09-02
hCoV-19/Japan/PG-83558/2021	EPI_ISL_3896210	2021-09-02
hCoV-19/Japan/PG-83559/2021	EPI_ISL_3896211	2021-09-02
hCoV-19/Japan/PG-83560/2021	EPI_ISL_3896212	2021-09-02
hCoV-19/Japan/PG-83561/2021	EPI_ISL_3896213	2021-09-02
hCoV-19/Japan/PG-83562/2021	EPI_ISL_3896214	2021-09-02
hCoV-19/Japan/PG-83563/2021	EPI_ISL_3896215	2021-09-02
hCoV-19/Japan/PG-83564/2021	EPI_ISL_3896216	2021-09-02
hCoV-19/Japan/PG-83565/2021	EPI_ISL_3896217	2021-09-02
hCoV-19/Japan/PG-83566/2021	EPI_ISL_3896218	2021-09-02
hCoV-19/Japan/PG-83567/2021	EPI_ISL_3896219	2021-09-02
hCoV-19/Japan/PG-83568/2021	EPI_ISL_3896220	2021-09-02
hCoV-19/Japan/PG-83569/2021	EPI_ISL_3896221	2021-09-02
hCoV-19/Japan/PG-83570/2021	EPI_ISL_3896222	2021-09-02
hCoV-19/Japan/PG-83571/2021	EPI_ISL_3896223	2021-09-02
hCoV-19/Japan/PG-83572/2021	EPI_ISL_3896224	2021-09-02
hCoV-19/Japan/PG-83573/2021	EPI_ISL_3896225	2021-09-02
hCoV-19/Japan/PG-83574/2021	EPI_ISL_3896226	2021-09-02
hCoV-19/Japan/PG-83575/2021	EPI_ISL_3896227	2021-09-02
hCoV-19/Japan/PG-83576/2021	EPI_ISL_3896228	2021-09-02
hCoV-19/Japan/PG-83577/2021	EPI_ISL_3896229	2021-09-02
hCoV-19/Japan/PG-83578/2021	EPI_ISL_3896230	2021-09-02
hCoV-19/Japan/PG-83579/2021	EPI_ISL_3896231	2021-09-02
hCoV-19/Japan/PG-83580/2021	EPI_ISL_3896232	2021-09-02
hCoV-19/Japan/PG-83581/2021	EPI_ISL_3896233	2021-09-02
hCoV-19/Japan/PG-83582/2021	EPI_ISL_3896234	2021-09-02
hCoV-19/Japan/PG-83583/2021	EPI_ISL_3896235	2021-09-02
hCoV-19/Japan/PG-83584/2021	EPI_ISL_3896236	2021-09-02
hCoV-19/Japan/PG-83585/2021	EPI_ISL_3896237	2021-09-02
hCoV-19/Japan/PG-83586/2021	EPI_ISL_3896238	2021-09-02
hCoV-19/Japan/PG-83587/2021	EPI_ISL_3896239	2021-09-02
hCoV-19/Japan/PG-83588/2021	EPI_ISL_3896241	2021-09-02
hCoV-19/Japan/PG-83589/2021	EPI_ISL_3896242	2021-09-02
hCoV-19/Japan/PG-83590/2021	EPI_ISL_3896243	2021-09-02
hCoV-19/Japan/PG-83591/2021	EPI_ISL_3896244	2021-09-02
hCoV-19/Japan/PG-83592/2021	EPI_ISL_3896245	2021-09-02
hCoV-19/Japan/PG-83593/2021	EPI_ISL_3896246	2021-09-02
hCoV-19/Japan/PG-83594/2021	EPI_ISL_3896247	2021-09-02
hCoV-19/Japan/PG-83595/2021	EPI_ISL_3896248	2021-09-02
hCoV-19/Japan/PG-83596/2021	EPI_ISL_3896249	2021-09-02
hCoV-19/Japan/PG-83597/2021	EPI_ISL_3896250	2021-09-02
hCoV-19/Japan/PG-83598/2021	EPI_ISL_3896251	2021-09-02
hCoV-19/Japan/PG-83599/2021	EPI_ISL_3896252	2021-09-02
hCoV-19/Japan/PG-83600/2021	EPI_ISL_3896253	2021-09-02
hCoV-19/Japan/PG-83601/2021	EPI_ISL_3896254	2021-09-02
hCoV-19/Japan/PG-83602/2021	EPI_ISL_3896255	2021-09-02
hCoV-19/Japan/PG-83603/2021	EPI_ISL_3896256	2021-09-02
hCoV-19/Japan/PG-83604/2021	EPI_ISL_3896257	2021-09-02
hCoV-19/Japan/PG-83605/2021	EPI_ISL_3896258	2021-09-02
hCoV-19/Japan/PG-83606/2021	EPI_ISL_3896259	2021-09-02
hCoV-19/Japan/PG-83607/2021	EPI_ISL_3896260	2021-09-02
hCoV-19/Japan/PG-83608/2021	EPI_ISL_3896261	2021-09-02
hCoV-19/Japan/PG-83609/2021	EPI_ISL_3896262	2021-09-02

virus_name	epiID	submission_date
hCoV-19/Japan/PG-83610/2021	EPI_ISL_3896263	2021-09-02
hCoV-19/Japan/PG-83611/2021	EPI_ISL_3896264	2021-09-02
hCoV-19/Japan/PG-83612/2021	EPI_ISL_3896265	2021-09-02
hCoV-19/Japan/PG-83613/2021	EPI_ISL_3896266	2021-09-02
hCoV-19/Japan/PG-83614/2021	EPI_ISL_3896267	2021-09-02
hCoV-19/Japan/PG-83615/2021	EPI_ISL_3896268	2021-09-02
hCoV-19/Japan/PG-83616/2021	EPI_ISL_3896269	2021-09-02
hCoV-19/Japan/PG-83617/2021	EPI_ISL_3896270	2021-09-02
hCoV-19/Japan/PG-83618/2021	EPI_ISL_3896271	2021-09-02
hCoV-19/Japan/PG-83619/2021	EPI_ISL_3896272	2021-09-02
hCoV-19/Japan/PG-83620/2021	EPI_ISL_3896273	2021-09-02
hCoV-19/Japan/PG-83621/2021	EPI_ISL_3896274	2021-09-02
hCoV-19/Japan/PG-83622/2021	EPI_ISL_3896275	2021-09-02
hCoV-19/Japan/PG-83623/2021	EPI_ISL_3896276	2021-09-02
hCoV-19/Japan/PG-83624/2021	EPI_ISL_3896277	2021-09-02
hCoV-19/Japan/PG-83625/2021	EPI_ISL_3896278	2021-09-02
hCoV-19/Japan/PG-83626/2021	EPI_ISL_3896279	2021-09-02
hCoV-19/Japan/PG-83627/2021	EPI_ISL_3896280	2021-09-02
hCoV-19/Japan/PG-83628/2021	EPI_ISL_3896281	2021-09-02
hCoV-19/Japan/PG-83629/2021	EPI_ISL_3896282	2021-09-02
hCoV-19/Japan/PG-83630/2021	EPI_ISL_3896283	2021-09-02
hCoV-19/Japan/PG-83631/2021	EPI_ISL_3896284	2021-09-02
hCoV-19/Japan/PG-83632/2021	EPI_ISL_3896285	2021-09-02
hCoV-19/Japan/PG-83633/2021	EPI_ISL_3896286	2021-09-02
hCoV-19/Japan/PG-83634/2021	EPI_ISL_3896287	2021-09-02
hCoV-19/Japan/PG-83635/2021	EPI_ISL_3896288	2021-09-02
hCoV-19/Japan/PG-83636/2021	EPI_ISL_3896289	2021-09-02
hCoV-19/Japan/PG-83637/2021	EPI_ISL_3896290	2021-09-02
hCoV-19/Japan/PG-83638/2021	EPI_ISL_3896291	2021-09-02
hCoV-19/Japan/PG-83639/2021	EPI_ISL_3896292	2021-09-02
hCoV-19/Japan/PG-83640/2021	EPI_ISL_3896293	2021-09-02
hCoV-19/Japan/PG-83641/2021	EPI_ISL_3896294	2021-09-02
hCoV-19/Japan/PG-83642/2021	EPI_ISL_3896295	2021-09-02
hCoV-19/Japan/PG-83643/2021	EPI_ISL_3896296	2021-09-02
hCoV-19/Japan/PG-83644/2021	EPI_ISL_3896297	2021-09-02
hCoV-19/Japan/PG-83645/2021	EPI_ISL_3896298	2021-09-02
hCoV-19/Japan/PG-83646/2021	EPI_ISL_3896299	2021-09-02
hCoV-19/Japan/PG-83647/2021	EPI_ISL_3896300	2021-09-02
hCoV-19/Japan/PG-83648/2021	EPI_ISL_3896301	2021-09-02
hCoV-19/Japan/PG-83649/2021	EPI_ISL_3896302	2021-09-02
hCoV-19/Japan/PG-83650/2021	EPI_ISL_3896303	2021-09-02
hCoV-19/Japan/PG-83651/2021	EPI_ISL_3896304	2021-09-02
hCoV-19/Japan/PG-83652/2021	EPI_ISL_3896305	2021-09-02
hCoV-19/Japan/PG-83653/2021	EPI_ISL_3896306	2021-09-02
hCoV-19/Japan/PG-83654/2021	EPI_ISL_3896307	2021-09-02
hCoV-19/Japan/PG-83655/2021	EPI_ISL_3896308	2021-09-02
hCoV-19/Japan/PG-83656/2021	EPI_ISL_3896309	2021-09-02
hCoV-19/Japan/PG-83657/2021	EPI_ISL_3896310	2021-09-02
hCoV-19/Japan/PG-83658/2021	EPI_ISL_3896311	2021-09-02
hCoV-19/Japan/PG-83659/2021	EPI_ISL_3896312	2021-09-02
hCoV-19/Japan/PG-83660/2021	EPI_ISL_3896313	2021-09-02
hCoV-19/Japan/PG-83661/2021	EPI_ISL_3896314	2021-09-02
hCoV-19/Japan/PG-83662/2021	EPI_ISL_3896315	2021-09-02
hCoV-19/Japan/PG-83663/2021	EPI_ISL_3896316	2021-09-02

virus_name	epiID	submission_date
hCoV-19/Japan/PG-83664/2021	EPI_ISL_3896317	2021-09-02
hCoV-19/Japan/PG-83665/2021	EPI_ISL_3896318	2021-09-02
hCoV-19/Japan/PG-83666/2021	EPI_ISL_3896319	2021-09-02
hCoV-19/Japan/PG-83667/2021	EPI_ISL_3896320	2021-09-02
hCoV-19/Japan/PG-83668/2021	EPI_ISL_3896321	2021-09-02
hCoV-19/Japan/PG-83669/2021	EPI_ISL_3896322	2021-09-02
hCoV-19/Japan/PG-83670/2021	EPI_ISL_3896323	2021-09-02
hCoV-19/Japan/PG-83671/2021	EPI_ISL_3896324	2021-09-02
hCoV-19/Japan/PG-83672/2021	EPI_ISL_3896325	2021-09-02
hCoV-19/Japan/PG-83673/2021	EPI_ISL_3896326	2021-09-02
hCoV-19/Japan/PG-83674/2021	EPI_ISL_3896327	2021-09-02
hCoV-19/Japan/PG-83675/2021	EPI_ISL_3896328	2021-09-02
hCoV-19/Japan/PG-83676/2021	EPI_ISL_3896329	2021-09-02
hCoV-19/Japan/PG-83677/2021	EPI_ISL_3896330	2021-09-02
hCoV-19/Japan/PG-83678/2021	EPI_ISL_3896331	2021-09-02
hCoV-19/Japan/PG-83679/2021	EPI_ISL_3896332	2021-09-02
hCoV-19/Japan/PG-83680/2021	EPI_ISL_3896333	2021-09-02
hCoV-19/Japan/PG-83681/2021	EPI_ISL_3896334	2021-09-02
hCoV-19/Japan/PG-83682/2021	EPI_ISL_3896335	2021-09-02
hCoV-19/Japan/PG-83683/2021	EPI_ISL_3896336	2021-09-02
hCoV-19/Japan/PG-83684/2021	EPI_ISL_3896337	2021-09-02
hCoV-19/Japan/PG-83685/2021	EPI_ISL_3896338	2021-09-02
hCoV-19/Japan/PG-83686/2021	EPI_ISL_3896339	2021-09-02
hCoV-19/Japan/PG-83687/2021	EPI_ISL_3896340	2021-09-02
hCoV-19/Japan/PG-83688/2021	EPI_ISL_3896341	2021-09-02
hCoV-19/Japan/PG-83689/2021	EPI_ISL_3896342	2021-09-02
hCoV-19/Japan/PG-83690/2021	EPI_ISL_3896343	2021-09-02
hCoV-19/Japan/PG-83691/2021	EPI_ISL_3896344	2021-09-02
hCoV-19/Japan/PG-83692/2021	EPI_ISL_3896345	2021-09-02
hCoV-19/Japan/PG-83693/2021	EPI_ISL_3896346	2021-09-02
hCoV-19/Japan/PG-83694/2021	EPI_ISL_3896347	2021-09-02
hCoV-19/Japan/PG-83695/2021	EPI_ISL_3896348	2021-09-02
hCoV-19/Japan/PG-83696/2021	EPI_ISL_3896349	2021-09-02
hCoV-19/Japan/PG-83697/2021	EPI_ISL_3896350	2021-09-02
hCoV-19/Japan/PG-83698/2021	EPI_ISL_3896351	2021-09-02
hCoV-19/Japan/PG-83699/2021	EPI_ISL_3896352	2021-09-02
hCoV-19/Japan/PG-83700/2021	EPI_ISL_3896353	2021-09-02
hCoV-19/Japan/PG-83701/2021	EPI_ISL_3896354	2021-09-02
hCoV-19/Japan/PG-83702/2021	EPI_ISL_3896355	2021-09-02
hCoV-19/Japan/PG-83703/2021	EPI_ISL_3896356	2021-09-02
hCoV-19/Japan/PG-83704/2021	EPI_ISL_3896357	2021-09-02
hCoV-19/Japan/PG-83705/2021	EPI_ISL_3896358	2021-09-02
hCoV-19/Japan/PG-83706/2021	EPI_ISL_3896359	2021-09-02
hCoV-19/Japan/PG-83707/2021	EPI_ISL_3896360	2021-09-02
hCoV-19/Japan/PG-83708/2021	EPI_ISL_3896361	2021-09-02
hCoV-19/Japan/PG-83709/2021	EPI_ISL_3896362	2021-09-02
hCoV-19/Japan/PG-83710/2021	EPI_ISL_3896363	2021-09-02
hCoV-19/Japan/PG-83711/2021	EPI_ISL_3896364	2021-09-02
hCoV-19/Japan/PG-83712/2021	EPI_ISL_3896365	2021-09-02
hCoV-19/Japan/PG-83713/2021	EPI_ISL_3896366	2021-09-02
hCoV-19/Japan/PG-83714/2021	EPI_ISL_3896367	2021-09-02
hCoV-19/Japan/PG-83715/2021	EPI_ISL_3896368	2021-09-02
hCoV-19/Japan/PG-83716/2021	EPI_ISL_3896369	2021-09-02
hCoV-19/Japan/PG-83717/2021	EPI_ISL_3896370	2021-09-02

virus_name	epiID	submission_date
hCoV-19/Japan/PG-83718/2021	EPI_ISL_3896371	2021-09-02
hCoV-19/Japan/PG-83719/2021	EPI_ISL_3896372	2021-09-02
hCoV-19/Japan/PG-83720/2021	EPI_ISL_3896373	2021-09-02
hCoV-19/Japan/PG-83721/2021	EPI_ISL_3896374	2021-09-02
hCoV-19/Japan/PG-83722/2021	EPI_ISL_3896375	2021-09-02
hCoV-19/Japan/PG-83723/2021	EPI_ISL_3896376	2021-09-02
hCoV-19/Japan/PG-83724/2021	EPI_ISL_3896377	2021-09-02
hCoV-19/Japan/PG-83725/2021	EPI_ISL_3896378	2021-09-02
hCoV-19/Japan/PG-83726/2021	EPI_ISL_3896379	2021-09-02
hCoV-19/Japan/PG-83727/2021	EPI_ISL_3896380	2021-09-02
hCoV-19/Japan/PG-83728/2021	EPI_ISL_3896381	2021-09-02
hCoV-19/Japan/PG-83729/2021	EPI_ISL_3896382	2021-09-02
hCoV-19/Japan/PG-83730/2021	EPI_ISL_3896383	2021-09-02
hCoV-19/Japan/PG-83731/2021	EPI_ISL_3896384	2021-09-02
hCoV-19/Japan/PG-83732/2021	EPI_ISL_3896385	2021-09-02
hCoV-19/Japan/PG-83733/2021	EPI_ISL_3896386	2021-09-02
hCoV-19/Japan/PG-83734/2021	EPI_ISL_3896387	2021-09-02
hCoV-19/Japan/PG-83735/2021	EPI_ISL_3896388	2021-09-02
hCoV-19/Japan/PG-83736/2021	EPI_ISL_3896389	2021-09-02
hCoV-19/Japan/PG-83737/2021	EPI_ISL_3896390	2021-09-02
hCoV-19/Japan/PG-83738/2021	EPI_ISL_3896391	2021-09-02
hCoV-19/Japan/PG-83739/2021	EPI_ISL_3896392	2021-09-02
hCoV-19/Japan/PG-83740/2021	EPI_ISL_3896393	2021-09-02
hCoV-19/Japan/PG-83741/2021	EPI_ISL_3896394	2021-09-02
hCoV-19/Japan/PG-83742/2021	EPI_ISL_3896395	2021-09-02
hCoV-19/Japan/PG-83743/2021	EPI_ISL_3896396	2021-09-02
hCoV-19/Japan/PG-83744/2021	EPI_ISL_3896397	2021-09-02
hCoV-19/Japan/PG-83745/2021	EPI_ISL_3896398	2021-09-02
hCoV-19/Japan/PG-83746/2021	EPI_ISL_3896399	2021-09-02
hCoV-19/Japan/PG-83747/2021	EPI_ISL_3896400	2021-09-02
hCoV-19/Japan/PG-83748/2021	EPI_ISL_3896401	2021-09-02
hCoV-19/Japan/PG-83749/2021	EPI_ISL_3896402	2021-09-02
hCoV-19/Japan/PG-83750/2021	EPI_ISL_3896403	2021-09-02
hCoV-19/Japan/PG-83751/2021	EPI_ISL_3896404	2021-09-02
hCoV-19/Japan/PG-83752/2021	EPI_ISL_3896405	2021-09-02
hCoV-19/Japan/PG-83753/2021	EPI_ISL_3896406	2021-09-02
hCoV-19/Japan/PG-83754/2021	EPI_ISL_3896407	2021-09-02
hCoV-19/Japan/PG-83755/2021	EPI_ISL_3896408	2021-09-02
hCoV-19/Japan/PG-83756/2021	EPI_ISL_3896409	2021-09-02
hCoV-19/Japan/PG-83757/2021	EPI_ISL_3896410	2021-09-02
hCoV-19/Japan/PG-83758/2021	EPI_ISL_3896411	2021-09-02
hCoV-19/Japan/PG-83759/2021	EPI_ISL_3896412	2021-09-02
hCoV-19/Japan/PG-83760/2021	EPI_ISL_3896413	2021-09-02
hCoV-19/Japan/PG-83761/2021	EPI_ISL_3896414	2021-09-02
hCoV-19/Japan/PG-83762/2021	EPI_ISL_3896415	2021-09-02
hCoV-19/Japan/PG-83763/2021	EPI_ISL_3896416	2021-09-02
hCoV-19/Japan/PG-83764/2021	EPI_ISL_3896417	2021-09-02
hCoV-19/Japan/PG-83765/2021	EPI_ISL_3896418	2021-09-02
hCoV-19/Japan/PG-83766/2021	EPI_ISL_3896419	2021-09-02
hCoV-19/Japan/PG-83767/2021	EPI_ISL_3896420	2021-09-02
hCoV-19/Japan/PG-83768/2021	EPI_ISL_3896421	2021-09-02
hCoV-19/Japan/PG-83769/2021	EPI_ISL_3896422	2021-09-02
hCoV-19/Japan/PG-83770/2021	EPI_ISL_3896423	2021-09-02
hCoV-19/Japan/PG-83771/2021	EPI_ISL_3896424	2021-09-02

virus_name	epiID	submission_date
hCoV-19/Japan/PG-83772/2021	EPI_ISL_3896425	2021-09-02
hCoV-19/Japan/PG-83773/2021	EPI_ISL_3896426	2021-09-02
hCoV-19/Japan/PG-83774/2021	EPI_ISL_3896427	2021-09-02
hCoV-19/Japan/PG-83775/2021	EPI_ISL_3896428	2021-09-02
hCoV-19/Japan/PG-83776/2021	EPI_ISL_3896429	2021-09-02
hCoV-19/Japan/PG-83777/2021	EPI_ISL_3896430	2021-09-02
hCoV-19/Japan/PG-83778/2021	EPI_ISL_3896431	2021-09-02
hCoV-19/Japan/PG-83779/2021	EPI_ISL_3896432	2021-09-02
hCoV-19/Japan/PG-83780/2021	EPI_ISL_3896433	2021-09-02
hCoV-19/Japan/PG-83781/2021	EPI_ISL_3896434	2021-09-02
hCoV-19/Japan/PG-83782/2021	EPI_ISL_3896435	2021-09-02
hCoV-19/Japan/PG-83783/2021	EPI_ISL_3896436	2021-09-02
hCoV-19/Japan/PG-83784/2021	EPI_ISL_3896437	2021-09-02
hCoV-19/Japan/PG-83785/2021	EPI_ISL_3896438	2021-09-02
hCoV-19/Japan/PG-83786/2021	EPI_ISL_3896439	2021-09-02
hCoV-19/Japan/PG-83787/2021	EPI_ISL_3896440	2021-09-02
hCoV-19/Japan/PG-83788/2021	EPI_ISL_3896441	2021-09-02
hCoV-19/Japan/PG-83789/2021	EPI_ISL_3896442	2021-09-02
hCoV-19/Japan/PG-83790/2021	EPI_ISL_3896443	2021-09-02
hCoV-19/Japan/PG-83791/2021	EPI_ISL_3896444	2021-09-02
hCoV-19/Japan/PG-83792/2021	EPI_ISL_3896445	2021-09-02
hCoV-19/Japan/PG-83793/2021	EPI_ISL_3896446	2021-09-02
hCoV-19/Japan/PG-83794/2021	EPI_ISL_3896447	2021-09-02
hCoV-19/Japan/PG-83795/2021	EPI_ISL_3896448	2021-09-02
hCoV-19/Japan/PG-83796/2021	EPI_ISL_3896449	2021-09-02
hCoV-19/Japan/PG-83797/2021	EPI_ISL_3896450	2021-09-02
hCoV-19/Japan/PG-83798/2021	EPI_ISL_3896451	2021-09-02
hCoV-19/Japan/PG-83799/2021	EPI_ISL_3896452	2021-09-02
hCoV-19/Japan/PG-83800/2021	EPI_ISL_3896453	2021-09-02
hCoV-19/Japan/PG-83801/2021	EPI_ISL_3896454	2021-09-02
hCoV-19/Japan/PG-83802/2021	EPI_ISL_3896455	2021-09-02
hCoV-19/Japan/PG-83803/2021	EPI_ISL_3896457	2021-09-02
hCoV-19/Japan/PG-83804/2021	EPI_ISL_3896458	2021-09-02
hCoV-19/Japan/PG-83805/2021	EPI_ISL_3896459	2021-09-02
hCoV-19/Japan/PG-83806/2021	EPI_ISL_3896460	2021-09-02
hCoV-19/Japan/PG-83807/2021	EPI_ISL_3896461	2021-09-02
hCoV-19/Japan/PG-83808/2021	EPI_ISL_3896462	2021-09-02
hCoV-19/Japan/PG-83809/2021	EPI_ISL_3896463	2021-09-02
hCoV-19/Japan/PG-83810/2021	EPI_ISL_3896464	2021-09-02
hCoV-19/Japan/PG-83811/2021	EPI_ISL_3896465	2021-09-02
hCoV-19/Japan/PG-83812/2021	EPI_ISL_3896466	2021-09-02
hCoV-19/Japan/PG-83813/2021	EPI_ISL_3896467	2021-09-02
hCoV-19/Japan/PG-83814/2021	EPI_ISL_3896468	2021-09-02
hCoV-19/Japan/PG-83815/2021	EPI_ISL_3896469	2021-09-02
hCoV-19/Japan/PG-83816/2021	EPI_ISL_3896470	2021-09-02
hCoV-19/Japan/PG-83817/2021	EPI_ISL_3896471	2021-09-02
hCoV-19/Japan/PG-83818/2021	EPI_ISL_3896472	2021-09-02
hCoV-19/Japan/PG-83819/2021	EPI_ISL_3896473	2021-09-02
hCoV-19/Japan/PG-83820/2021	EPI_ISL_3896474	2021-09-02
hCoV-19/Japan/PG-83821/2021	EPI_ISL_3896475	2021-09-02
hCoV-19/Japan/PG-83822/2021	EPI_ISL_3896476	2021-09-02
hCoV-19/Japan/PG-83823/2021	EPI_ISL_3896477	2021-09-02
hCoV-19/Japan/PG-83824/2021	EPI_ISL_3896478	2021-09-02
hCoV-19/Japan/PG-83825/2021	EPI_ISL_3896479	2021-09-02

virus_name	epiID	submission_date
hCoV-19/Japan/PG-83826/2021	EPI_ISL_3896480	2021-09-02
hCoV-19/Japan/PG-83827/2021	EPI_ISL_3896481	2021-09-02
hCoV-19/Japan/PG-83828/2021	EPI_ISL_3896482	2021-09-02
hCoV-19/Japan/PG-83829/2021	EPI_ISL_3896483	2021-09-02
hCoV-19/Japan/PG-83830/2021	EPI_ISL_3896484	2021-09-02
hCoV-19/Japan/PG-83831/2021	EPI_ISL_3896485	2021-09-02
hCoV-19/Japan/PG-83832/2021	EPI_ISL_3896486	2021-09-02
hCoV-19/Japan/PG-83833/2021	EPI_ISL_3896487	2021-09-02
hCoV-19/Japan/PG-83834/2021	EPI_ISL_3896488	2021-09-02
hCoV-19/Japan/PG-83835/2021	EPI_ISL_3896489	2021-09-02
hCoV-19/Japan/PG-83836/2021	EPI_ISL_3896490	2021-09-02
hCoV-19/Japan/PG-83837/2021	EPI_ISL_3896491	2021-09-02
hCoV-19/Japan/PG-83838/2021	EPI_ISL_3896492	2021-09-02
hCoV-19/Japan/PG-83839/2021	EPI_ISL_3896493	2021-09-02
hCoV-19/Japan/PG-83840/2021	EPI_ISL_3896494	2021-09-02
hCoV-19/Japan/PG-83841/2021	EPI_ISL_3896495	2021-09-02
hCoV-19/Japan/PG-83842/2021	EPI_ISL_3896496	2021-09-02
hCoV-19/Japan/PG-83843/2021	EPI_ISL_3896497	2021-09-02
hCoV-19/Japan/PG-83844/2021	EPI_ISL_3896498	2021-09-02
hCoV-19/Japan/PG-83845/2021	EPI_ISL_3896499	2021-09-02
hCoV-19/Japan/PG-83846/2021	EPI_ISL_3896500	2021-09-02
hCoV-19/Japan/PG-83847/2021	EPI_ISL_3896501	2021-09-02
hCoV-19/Japan/PG-83848/2021	EPI_ISL_3896502	2021-09-02
hCoV-19/Japan/PG-83849/2021	EPI_ISL_3896503	2021-09-02
hCoV-19/Japan/PG-83850/2021	EPI_ISL_3896504	2021-09-02
hCoV-19/Japan/PG-83851/2021	EPI_ISL_3896505	2021-09-02
hCoV-19/Japan/PG-83852/2021	EPI_ISL_3896506	2021-09-02
hCoV-19/Japan/PG-83853/2021	EPI_ISL_3896507	2021-09-02
hCoV-19/Japan/PG-83854/2021	EPI_ISL_3896508	2021-09-02
hCoV-19/Japan/PG-83855/2021	EPI_ISL_3896509	2021-09-02
hCoV-19/Japan/PG-83856/2021	EPI_ISL_3896510	2021-09-02
hCoV-19/Japan/PG-83857/2021	EPI_ISL_3896511	2021-09-02
hCoV-19/Japan/PG-83858/2021	EPI_ISL_3896512	2021-09-02
hCoV-19/Japan/PG-83859/2021	EPI_ISL_3896513	2021-09-02
hCoV-19/Japan/PG-83860/2021	EPI_ISL_3896514	2021-09-02
hCoV-19/Japan/PG-83861/2021	EPI_ISL_3896515	2021-09-02
hCoV-19/Japan/PG-83862/2021	EPI_ISL_3896516	2021-09-02
hCoV-19/Japan/PG-83863/2021	EPI_ISL_3896517	2021-09-02
hCoV-19/Japan/PG-83864/2021	EPI_ISL_3896518	2021-09-02
hCoV-19/Japan/PG-83865/2021	EPI_ISL_3896519	2021-09-02
hCoV-19/Japan/PG-83866/2021	EPI_ISL_3896520	2021-09-02
hCoV-19/Japan/PG-83867/2021	EPI_ISL_3896521	2021-09-02
hCoV-19/Japan/PG-83868/2021	EPI_ISL_3896522	2021-09-02
hCoV-19/Japan/PG-83869/2021	EPI_ISL_3896523	2021-09-02
hCoV-19/Japan/PG-83870/2021	EPI_ISL_3896524	2021-09-02
hCoV-19/Japan/PG-83871/2021	EPI_ISL_3896525	2021-09-02
hCoV-19/Japan/PG-83872/2021	EPI_ISL_3896526	2021-09-02
hCoV-19/Japan/PG-83873/2021	EPI_ISL_3896527	2021-09-02
hCoV-19/Japan/PG-83874/2021	EPI_ISL_3896528	2021-09-02
hCoV-19/Japan/PG-83875/2021	EPI_ISL_3896529	2021-09-02
hCoV-19/Japan/PG-83876/2021	EPI_ISL_3896530	2021-09-02
hCoV-19/Japan/PG-83877/2021	EPI_ISL_3896531	2021-09-02
hCoV-19/Japan/PG-83878/2021	EPI_ISL_3896532	2021-09-02
hCoV-19/Japan/PG-83879/2021	EPI_ISL_3896533	2021-09-02

virus_name	epiID	submission_date
hCoV-19/Japan/PG-83880/2021	EPI_ISL_3896534	2021-09-02
hCoV-19/Japan/PG-83881/2021	EPI_ISL_3896535	2021-09-02
hCoV-19/Japan/PG-83882/2021	EPI_ISL_3896536	2021-09-02
hCoV-19/Japan/PG-83883/2021	EPI_ISL_3896537	2021-09-02
hCoV-19/Japan/PG-83884/2021	EPI_ISL_3896538	2021-09-02
hCoV-19/Japan/PG-83885/2021	EPI_ISL_3896539	2021-09-02
hCoV-19/Japan/PG-83886/2021	EPI_ISL_3896540	2021-09-02
hCoV-19/Japan/PG-83887/2021	EPI_ISL_3896541	2021-09-02
hCoV-19/Japan/PG-83888/2021	EPI_ISL_3896542	2021-09-02
hCoV-19/Japan/PG-83889/2021	EPI_ISL_3896543	2021-09-02
hCoV-19/Japan/PG-83890/2021	EPI_ISL_3896544	2021-09-02
hCoV-19/Japan/PG-83891/2021	EPI_ISL_3896545	2021-09-02
hCoV-19/Japan/PG-83892/2021	EPI_ISL_3896546	2021-09-02
hCoV-19/Japan/PG-83893/2021	EPI_ISL_3896547	2021-09-02
hCoV-19/Japan/PG-83894/2021	EPI_ISL_3896548	2021-09-02
hCoV-19/Japan/PG-83895/2021	EPI_ISL_3896549	2021-09-02
hCoV-19/Japan/PG-83896/2021	EPI_ISL_3896550	2021-09-02
hCoV-19/Japan/PG-83897/2021	EPI_ISL_3896551	2021-09-02
hCoV-19/Japan/PG-83898/2021	EPI_ISL_3896552	2021-09-02
hCoV-19/Japan/PG-83899/2021	EPI_ISL_3896553	2021-09-02
hCoV-19/Japan/PG-83900/2021	EPI_ISL_3896554	2021-09-02
hCoV-19/Japan/PG-83901/2021	EPI_ISL_3896555	2021-09-02
hCoV-19/Japan/PG-83902/2021	EPI_ISL_3896556	2021-09-02
hCoV-19/Japan/PG-83903/2021	EPI_ISL_3896557	2021-09-02
hCoV-19/Japan/PG-83904/2021	EPI_ISL_3896558	2021-09-02
hCoV-19/Japan/PG-83905/2021	EPI_ISL_3896559	2021-09-02
hCoV-19/Japan/PG-83906/2021	EPI_ISL_3896560	2021-09-02
hCoV-19/Japan/PG-83907/2021	EPI_ISL_3896561	2021-09-02
hCoV-19/Japan/PG-83908/2021	EPI_ISL_3896562	2021-09-02
hCoV-19/Japan/PG-83909/2021	EPI_ISL_3896563	2021-09-02
hCoV-19/Japan/PG-83910/2021	EPI_ISL_3896564	2021-09-02
hCoV-19/Japan/PG-83911/2021	EPI_ISL_3896565	2021-09-02
hCoV-19/Japan/PG-83912/2021	EPI_ISL_3896566	2021-09-02
hCoV-19/Japan/PG-83913/2021	EPI_ISL_3896567	2021-09-02
hCoV-19/Japan/PG-83914/2021	EPI_ISL_3896568	2021-09-02
hCoV-19/Japan/PG-83915/2021	EPI_ISL_3896569	2021-09-02
hCoV-19/Japan/PG-83916/2021	EPI_ISL_3896570	2021-09-02
hCoV-19/Japan/PG-83917/2021	EPI_ISL_3896571	2021-09-02
hCoV-19/Japan/PG-83918/2021	EPI_ISL_3896572	2021-09-02
hCoV-19/Japan/PG-83919/2021	EPI_ISL_3896573	2021-09-02
hCoV-19/Japan/PG-83920/2021	EPI_ISL_3896574	2021-09-02
hCoV-19/Japan/PG-83921/2021	EPI_ISL_3896575	2021-09-02
hCoV-19/Japan/PG-83922/2021	EPI_ISL_3896576	2021-09-02
hCoV-19/Japan/PG-83923/2021	EPI_ISL_3896577	2021-09-02
hCoV-19/Japan/PG-83924/2021	EPI_ISL_3896578	2021-09-02
hCoV-19/Japan/PG-83925/2021	EPI_ISL_3896579	2021-09-02
hCoV-19/Japan/PG-83926/2021	EPI_ISL_3896580	2021-09-02
hCoV-19/Japan/PG-83927/2021	EPI_ISL_3896581	2021-09-02
hCoV-19/Japan/PG-83928/2021	EPI_ISL_3896582	2021-09-02
hCoV-19/Japan/PG-83929/2021	EPI_ISL_3896583	2021-09-02
hCoV-19/Japan/PG-83930/2021	EPI_ISL_3896584	2021-09-02
hCoV-19/Japan/PG-83931/2021	EPI_ISL_3896585	2021-09-02
hCoV-19/Japan/PG-83932/2021	EPI_ISL_3896586	2021-09-02
hCoV-19/Japan/PG-83933/2021	EPI_ISL_3896587	2021-09-02

virus_name	epiID	submission_date
hCoV-19/Japan/PG-83934/2021	EPI_ISL_3896588	2021-09-02
hCoV-19/Japan/PG-83935/2021	EPI_ISL_3896589	2021-09-02
hCoV-19/Japan/PG-83936/2021	EPI_ISL_3896590	2021-09-02
hCoV-19/Japan/PG-83937/2021	EPI_ISL_3896591	2021-09-02
hCoV-19/Japan/PG-83938/2021	EPI_ISL_3896592	2021-09-02
hCoV-19/Japan/PG-83939/2021	EPI_ISL_3896593	2021-09-02
hCoV-19/Japan/PG-83940/2021	EPI_ISL_3896594	2021-09-02
hCoV-19/Japan/PG-83941/2021	EPI_ISL_3896595	2021-09-02
hCoV-19/Japan/PG-83942/2021	EPI_ISL_3896596	2021-09-02
hCoV-19/Japan/PG-83943/2021	EPI_ISL_3896597	2021-09-02
hCoV-19/Japan/PG-83944/2021	EPI_ISL_3896598	2021-09-02
hCoV-19/Japan/PG-83945/2021	EPI_ISL_3896599	2021-09-02
hCoV-19/Japan/PG-83946/2021	EPI_ISL_3896600	2021-09-02
hCoV-19/Japan/PG-83947/2021	EPI_ISL_3896601	2021-09-02
hCoV-19/Japan/PG-83948/2021	EPI_ISL_3896602	2021-09-02
hCoV-19/Japan/PG-83949/2021	EPI_ISL_3896603	2021-09-02
hCoV-19/Japan/PG-83950/2021	EPI_ISL_3896604	2021-09-02
hCoV-19/Japan/PG-83951/2021	EPI_ISL_3896605	2021-09-02
hCoV-19/Japan/PG-83952/2021	EPI_ISL_3896606	2021-09-02
hCoV-19/Japan/PG-83953/2021	EPI_ISL_3896607	2021-09-02
hCoV-19/Japan/PG-83954/2021	EPI_ISL_3896608	2021-09-02
hCoV-19/Japan/PG-83955/2021	EPI_ISL_3896609	2021-09-02
hCoV-19/Japan/PG-83956/2021	EPI_ISL_3896610	2021-09-02
hCoV-19/Japan/PG-83957/2021	EPI_ISL_3896611	2021-09-02
hCoV-19/Japan/PG-83958/2021	EPI_ISL_3896612	2021-09-02
hCoV-19/Japan/PG-83959/2021	EPI_ISL_3896613	2021-09-02
hCoV-19/Japan/PG-83960/2021	EPI_ISL_3896614	2021-09-02
hCoV-19/Japan/PG-83961/2021	EPI_ISL_3896615	2021-09-02
hCoV-19/Japan/PG-83962/2021	EPI_ISL_3896616	2021-09-02
hCoV-19/Japan/PG-83963/2021	EPI_ISL_3896617	2021-09-02
hCoV-19/Japan/PG-83964/2021	EPI_ISL_3896618	2021-09-02
hCoV-19/Japan/PG-83965/2021	EPI_ISL_3896619	2021-09-02
hCoV-19/Japan/PG-83966/2021	EPI_ISL_3896620	2021-09-02
hCoV-19/Japan/PG-83967/2021	EPI_ISL_3896621	2021-09-02
hCoV-19/Japan/PG-83968/2021	EPI_ISL_3896622	2021-09-02
hCoV-19/Japan/PG-83969/2021	EPI_ISL_3896623	2021-09-02
hCoV-19/Japan/PG-83970/2021	EPI_ISL_3896624	2021-09-02
hCoV-19/Japan/PG-83971/2021	EPI_ISL_3896625	2021-09-02
hCoV-19/Japan/PG-83972/2021	EPI_ISL_3896626	2021-09-02
hCoV-19/Japan/PG-83973/2021	EPI_ISL_3896627	2021-09-02
hCoV-19/Japan/PG-83974/2021	EPI_ISL_3896628	2021-09-02
hCoV-19/Japan/PG-83975/2021	EPI_ISL_3896629	2021-09-02
hCoV-19/Japan/PG-83976/2021	EPI_ISL_3896630	2021-09-02
hCoV-19/Japan/PG-83977/2021	EPI_ISL_3896631	2021-09-02
hCoV-19/Japan/PG-83978/2021	EPI_ISL_3896632	2021-09-02
hCoV-19/Japan/PG-83979/2021	EPI_ISL_3896633	2021-09-02
hCoV-19/Japan/PG-83980/2021	EPI_ISL_3896634	2021-09-02
hCoV-19/Japan/PG-83981/2021	EPI_ISL_3896635	2021-09-02
hCoV-19/Japan/PG-83982/2021	EPI_ISL_3896636	2021-09-02
hCoV-19/Japan/PG-83983/2021	EPI_ISL_3896637	2021-09-02
hCoV-19/Japan/PG-83984/2021	EPI_ISL_3896638	2021-09-02
hCoV-19/Japan/PG-83985/2021	EPI_ISL_3896639	2021-09-02
hCoV-19/Japan/PG-83986/2021	EPI_ISL_3896641	2021-09-02
hCoV-19/Japan/PG-83987/2021	EPI_ISL_3896642	2021-09-02

virus_name	epiID	submission_date
hCoV-19/Japan/PG-83988/2021	EPI_ISL_3896643	2021-09-02
hCoV-19/Japan/PG-83989/2021	EPI_ISL_3896644	2021-09-02
hCoV-19/Japan/PG-83990/2021	EPI_ISL_3896645	2021-09-02
hCoV-19/Japan/PG-83991/2021	EPI_ISL_3896646	2021-09-02
hCoV-19/Japan/PG-83992/2021	EPI_ISL_3896647	2021-09-02
hCoV-19/Japan/PG-83993/2021	EPI_ISL_3896648	2021-09-02
hCoV-19/Japan/PG-83994/2021	EPI_ISL_3896649	2021-09-02
hCoV-19/Japan/PG-83995/2021	EPI_ISL_3896650	2021-09-02
hCoV-19/Japan/PG-83996/2021	EPI_ISL_3896651	2021-09-02
hCoV-19/Japan/PG-83997/2021	EPI_ISL_3896652	2021-09-02
hCoV-19/Japan/PG-83998/2021	EPI_ISL_3896653	2021-09-02
hCoV-19/Japan/PG-83999/2021	EPI_ISL_3896654	2021-09-02
hCoV-19/Japan/PG-84000/2021	EPI_ISL_3896655	2021-09-02
hCoV-19/Japan/PG-84001/2021	EPI_ISL_3896656	2021-09-02
hCoV-19/Japan/PG-84002/2021	EPI_ISL_3896657	2021-09-02
hCoV-19/Japan/PG-84003/2021	EPI_ISL_3896658	2021-09-02
hCoV-19/Japan/PG-84004/2021	EPI_ISL_3896659	2021-09-02
hCoV-19/Japan/PG-84005/2021	EPI_ISL_3896660	2021-09-02
hCoV-19/Japan/PG-84006/2021	EPI_ISL_3896661	2021-09-02
hCoV-19/Japan/PG-84007/2021	EPI_ISL_3896662	2021-09-02
hCoV-19/Japan/PG-84008/2021	EPI_ISL_3896663	2021-09-02
hCoV-19/Japan/PG-84009/2021	EPI_ISL_3896664	2021-09-02
hCoV-19/Japan/PG-84010/2021	EPI_ISL_3896665	2021-09-02
hCoV-19/Japan/PG-84011/2021	EPI_ISL_3896666	2021-09-02
hCoV-19/Japan/PG-84012/2021	EPI_ISL_3896667	2021-09-02
hCoV-19/Japan/PG-84013/2021	EPI_ISL_3896668	2021-09-02
hCoV-19/Japan/PG-84014/2021	EPI_ISL_3896669	2021-09-02
hCoV-19/Japan/PG-84015/2021	EPI_ISL_3896670	2021-09-02
hCoV-19/Japan/PG-84016/2021	EPI_ISL_3896671	2021-09-02
hCoV-19/Japan/PG-84017/2021	EPI_ISL_3896672	2021-09-02
hCoV-19/Japan/PG-84018/2021	EPI_ISL_3896673	2021-09-02
hCoV-19/Japan/PG-84019/2021	EPI_ISL_3896674	2021-09-02
hCoV-19/Japan/PG-84020/2021	EPI_ISL_3896675	2021-09-02
hCoV-19/Japan/PG-84021/2021	EPI_ISL_3896676	2021-09-02
hCoV-19/Japan/PG-84022/2021	EPI_ISL_3896677	2021-09-02
hCoV-19/Japan/PG-84023/2021	EPI_ISL_3896678	2021-09-02
hCoV-19/Japan/PG-84024/2021	EPI_ISL_3896679	2021-09-02
hCoV-19/Japan/PG-84025/2021	EPI_ISL_3896680	2021-09-02
hCoV-19/Japan/PG-84026/2021	EPI_ISL_3896681	2021-09-02
hCoV-19/Japan/PG-84027/2021	EPI_ISL_3896682	2021-09-02
hCoV-19/Japan/PG-84028/2021	EPI_ISL_3896683	2021-09-02
hCoV-19/Japan/PG-84029/2021	EPI_ISL_3896684	2021-09-02
hCoV-19/Japan/PG-84030/2021	EPI_ISL_3896685	2021-09-02
hCoV-19/Japan/PG-84031/2021	EPI_ISL_3896686	2021-09-02
hCoV-19/Japan/PG-84032/2021	EPI_ISL_3896687	2021-09-02
hCoV-19/Japan/PG-84033/2021	EPI_ISL_3896688	2021-09-02
hCoV-19/Japan/PG-84034/2021	EPI_ISL_3896689	2021-09-02
hCoV-19/Japan/PG-84035/2021	EPI_ISL_3896690	2021-09-02
hCoV-19/Japan/PG-84036/2021	EPI_ISL_3896691	2021-09-02
hCoV-19/Japan/PG-84037/2021	EPI_ISL_3896692	2021-09-02
hCoV-19/Japan/PG-84038/2021	EPI_ISL_3896693	2021-09-02
hCoV-19/Japan/PG-84039/2021	EPI_ISL_3896694	2021-09-02
hCoV-19/Japan/PG-84040/2021	EPI_ISL_3896695	2021-09-02
hCoV-19/Japan/PG-84041/2021	EPI_ISL_3896696	2021-09-02

virus_name	epiID	submission_date
hCoV-19/Japan/PG-84042/2021	EPI_ISL_3896697	2021-09-02
hCoV-19/Japan/PG-84043/2021	EPI_ISL_3896698	2021-09-02
hCoV-19/Japan/PG-84044/2021	EPI_ISL_3896699	2021-09-02
hCoV-19/Japan/PG-84045/2021	EPI_ISL_3896700	2021-09-02
hCoV-19/Japan/PG-84046/2021	EPI_ISL_3896701	2021-09-02
hCoV-19/Japan/PG-84047/2021	EPI_ISL_3896702	2021-09-02
hCoV-19/Japan/PG-84048/2021	EPI_ISL_3896703	2021-09-02
hCoV-19/Japan/PG-84049/2021	EPI_ISL_3896704	2021-09-02
hCoV-19/Japan/PG-84050/2021	EPI_ISL_3896705	2021-09-02
hCoV-19/Japan/PG-84051/2021	EPI_ISL_3896706	2021-09-02
hCoV-19/Japan/PG-84052/2021	EPI_ISL_3896707	2021-09-02
hCoV-19/Japan/PG-84053/2021	EPI_ISL_3896708	2021-09-02
hCoV-19/Japan/PG-84054/2021	EPI_ISL_3896709	2021-09-02
hCoV-19/Japan/PG-84055/2021	EPI_ISL_3896710	2021-09-02
hCoV-19/Japan/PG-84056/2021	EPI_ISL_3896711	2021-09-02
hCoV-19/Japan/PG-84057/2021	EPI_ISL_3896712	2021-09-02
hCoV-19/Japan/PG-84058/2021	EPI_ISL_3896713	2021-09-02
hCoV-19/Japan/PG-84059/2021	EPI_ISL_3896714	2021-09-02
hCoV-19/Japan/PG-84060/2021	EPI_ISL_3896715	2021-09-02
hCoV-19/Japan/PG-84061/2021	EPI_ISL_3896716	2021-09-02
hCoV-19/Japan/PG-84062/2021	EPI_ISL_3896717	2021-09-02
hCoV-19/Japan/PG-84063/2021	EPI_ISL_3896718	2021-09-02
hCoV-19/Japan/PG-84064/2021	EPI_ISL_3896719	2021-09-02
hCoV-19/Japan/PG-84065/2021	EPI_ISL_3896720	2021-09-02
hCoV-19/Japan/PG-84066/2021	EPI_ISL_3896721	2021-09-02
hCoV-19/Japan/PG-84067/2021	EPI_ISL_3896722	2021-09-02
hCoV-19/Japan/PG-84068/2021	EPI_ISL_3896723	2021-09-02
hCoV-19/Japan/PG-84069/2021	EPI_ISL_3896724	2021-09-02
hCoV-19/Japan/PG-84070/2021	EPI_ISL_3896725	2021-09-02
hCoV-19/Japan/PG-84071/2021	EPI_ISL_3896726	2021-09-02
hCoV-19/Japan/PG-84072/2021	EPI_ISL_3896727	2021-09-02
hCoV-19/Japan/PG-84073/2021	EPI_ISL_3896728	2021-09-02
hCoV-19/Japan/PG-84074/2021	EPI_ISL_3896729	2021-09-02
hCoV-19/Japan/PG-84075/2021	EPI_ISL_3896730	2021-09-02
hCoV-19/Japan/PG-84076/2021	EPI_ISL_3896731	2021-09-02
hCoV-19/Japan/PG-84077/2021	EPI_ISL_3896732	2021-09-02
hCoV-19/Japan/PG-84078/2021	EPI_ISL_3896733	2021-09-02
hCoV-19/Japan/PG-84079/2021	EPI_ISL_3896734	2021-09-02
hCoV-19/Japan/PG-84080/2021	EPI_ISL_3896735	2021-09-02
hCoV-19/Japan/PG-84081/2021	EPI_ISL_3896736	2021-09-02
hCoV-19/Japan/PG-84082/2021	EPI_ISL_3896737	2021-09-02
hCoV-19/Japan/PG-84083/2021	EPI_ISL_3896738	2021-09-02
hCoV-19/Japan/PG-84084/2021	EPI_ISL_3896739	2021-09-02
hCoV-19/Japan/PG-84085/2021	EPI_ISL_3896740	2021-09-02
hCoV-19/Japan/PG-84086/2021	EPI_ISL_3896741	2021-09-02
hCoV-19/Japan/PG-84087/2021	EPI_ISL_3896742	2021-09-02
hCoV-19/Japan/PG-84088/2021	EPI_ISL_3896743	2021-09-02
hCoV-19/Japan/PG-84089/2021	EPI_ISL_3896744	2021-09-02
hCoV-19/Japan/PG-84090/2021	EPI_ISL_3896745	2021-09-02
hCoV-19/Japan/PG-84091/2021	EPI_ISL_3896746	2021-09-02
hCoV-19/Japan/PG-84092/2021	EPI_ISL_3896747	2021-09-02
hCoV-19/Japan/PG-84093/2021	EPI_ISL_3896748	2021-09-02
hCoV-19/Japan/PG-84094/2021	EPI_ISL_3896749	2021-09-02
hCoV-19/Japan/PG-84095/2021	EPI_ISL_3896750	2021-09-02

virus_name	epiID	submission_date
hCoV-19/Japan/PG-84096/2021	EPI_ISL_3896751	2021-09-02
hCoV-19/Japan/PG-84097/2021	EPI_ISL_3896752	2021-09-02
hCoV-19/Japan/PG-84098/2021	EPI_ISL_3896753	2021-09-02
hCoV-19/Japan/PG-84099/2021	EPI_ISL_3896754	2021-09-02
hCoV-19/Japan/PG-84100/2021	EPI_ISL_3896755	2021-09-02
hCoV-19/Japan/PG-84101/2021	EPI_ISL_3896756	2021-09-02
hCoV-19/Japan/PG-84102/2021	EPI_ISL_3896757	2021-09-02
hCoV-19/Japan/PG-84103/2021	EPI_ISL_3896758	2021-09-02
hCoV-19/Japan/PG-84104/2021	EPI_ISL_3896759	2021-09-02
hCoV-19/Japan/PG-84105/2021	EPI_ISL_3896760	2021-09-02
hCoV-19/Japan/PG-84106/2021	EPI_ISL_3896761	2021-09-02
hCoV-19/Japan/PG-84107/2021	EPI_ISL_3896762	2021-09-02
hCoV-19/Japan/PG-84108/2021	EPI_ISL_3896763	2021-09-02
hCoV-19/Japan/PG-84109/2021	EPI_ISL_3896764	2021-09-02
hCoV-19/Japan/PG-84110/2021	EPI_ISL_3896765	2021-09-02
hCoV-19/Japan/PG-84111/2021	EPI_ISL_3896766	2021-09-02
hCoV-19/Japan/PG-84112/2021	EPI_ISL_3896767	2021-09-02
hCoV-19/Japan/PG-84113/2021	EPI_ISL_3896768	2021-09-02
hCoV-19/Japan/PG-84114/2021	EPI_ISL_3896769	2021-09-02
hCoV-19/Japan/PG-84115/2021	EPI_ISL_3896770	2021-09-02
hCoV-19/Japan/PG-84116/2021	EPI_ISL_3896771	2021-09-02
hCoV-19/Japan/PG-84117/2021	EPI_ISL_3896772	2021-09-02
hCoV-19/Japan/PG-84118/2021	EPI_ISL_3896773	2021-09-02
hCoV-19/Japan/PG-84119/2021	EPI_ISL_3896774	2021-09-02
hCoV-19/Japan/PG-84120/2021	EPI_ISL_3896775	2021-09-02
hCoV-19/Japan/PG-84121/2021	EPI_ISL_3896776	2021-09-02
hCoV-19/Japan/PG-84122/2021	EPI_ISL_3896777	2021-09-02
hCoV-19/Japan/PG-84123/2021	EPI_ISL_3896778	2021-09-02
hCoV-19/Japan/PG-84124/2021	EPI_ISL_3896779	2021-09-02
hCoV-19/Japan/PG-84125/2021	EPI_ISL_3896780	2021-09-02
hCoV-19/Japan/PG-84126/2021	EPI_ISL_3896781	2021-09-02
hCoV-19/Japan/PG-84127/2021	EPI_ISL_3896782	2021-09-02
hCoV-19/Japan/PG-84128/2021	EPI_ISL_3896783	2021-09-02
hCoV-19/Japan/PG-84129/2021	EPI_ISL_3896784	2021-09-02
hCoV-19/Japan/PG-84130/2021	EPI_ISL_3896785	2021-09-02
hCoV-19/Japan/PG-84131/2021	EPI_ISL_3896786	2021-09-02
hCoV-19/Japan/PG-84132/2021	EPI_ISL_3896787	2021-09-02
hCoV-19/Japan/PG-84133/2021	EPI_ISL_3896788	2021-09-02
hCoV-19/Japan/PG-84134/2021	EPI_ISL_3896789	2021-09-02
hCoV-19/Japan/PG-84135/2021	EPI_ISL_3896790	2021-09-02
hCoV-19/Japan/PG-84136/2021	EPI_ISL_3896791	2021-09-02
hCoV-19/Japan/PG-84137/2021	EPI_ISL_3896792	2021-09-02
hCoV-19/Japan/PG-84138/2021	EPI_ISL_3896793	2021-09-02
hCoV-19/Japan/PG-84139/2021	EPI_ISL_3896794	2021-09-02
hCoV-19/Japan/PG-84140/2021	EPI_ISL_3896795	2021-09-02
hCoV-19/Japan/PG-84141/2021	EPI_ISL_3896796	2021-09-02
hCoV-19/Japan/PG-84142/2021	EPI_ISL_3896797	2021-09-02
hCoV-19/Japan/PG-84143/2021	EPI_ISL_3896798	2021-09-02
hCoV-19/Japan/PG-84144/2021	EPI_ISL_3896799	2021-09-02
hCoV-19/Japan/PG-84145/2021	EPI_ISL_3896800	2021-09-02
hCoV-19/Japan/PG-84146/2021	EPI_ISL_3896801	2021-09-02
hCoV-19/Japan/PG-84147/2021	EPI_ISL_3896802	2021-09-02
hCoV-19/Japan/PG-84148/2021	EPI_ISL_3896803	2021-09-02
hCoV-19/Japan/PG-84149/2021	EPI_ISL_3896804	2021-09-02

virus_name	epiID	submission_date
hCoV-19/Japan/PG-84150/2021	EPI_ISL_3896805	2021-09-02
hCoV-19/Japan/PG-84151/2021	EPI_ISL_3896806	2021-09-02
hCoV-19/Japan/PG-84152/2021	EPI_ISL_3896807	2021-09-02
hCoV-19/Japan/PG-84153/2021	EPI_ISL_3896808	2021-09-02
hCoV-19/Japan/PG-84154/2021	EPI_ISL_3896809	2021-09-02
hCoV-19/Japan/PG-84155/2021	EPI_ISL_3896810	2021-09-02
hCoV-19/Japan/PG-84156/2021	EPI_ISL_3896811	2021-09-02
hCoV-19/Japan/PG-84157/2021	EPI_ISL_3896812	2021-09-02
hCoV-19/Japan/PG-84158/2021	EPI_ISL_3896813	2021-09-02
hCoV-19/Japan/PG-84159/2021	EPI_ISL_3896814	2021-09-02
hCoV-19/Japan/PG-84160/2021	EPI_ISL_3896815	2021-09-02
hCoV-19/Japan/PG-84161/2021	EPI_ISL_3896816	2021-09-02
hCoV-19/Japan/PG-84162/2021	EPI_ISL_3896817	2021-09-02
hCoV-19/Japan/PG-84163/2021	EPI_ISL_3896818	2021-09-02
hCoV-19/Japan/PG-84164/2021	EPI_ISL_3896819	2021-09-02
hCoV-19/Japan/PG-84165/2021	EPI_ISL_3896820	2021-09-02
hCoV-19/Japan/PG-84166/2021	EPI_ISL_3896821	2021-09-02
hCoV-19/Japan/PG-84167/2021	EPI_ISL_3896822	2021-09-02
hCoV-19/Japan/PG-84168/2021	EPI_ISL_3896823	2021-09-02
hCoV-19/Japan/PG-84169/2021	EPI_ISL_3896824	2021-09-02
hCoV-19/Japan/PG-84170/2021	EPI_ISL_3896825	2021-09-02
hCoV-19/Japan/PG-84171/2021	EPI_ISL_3896826	2021-09-02
hCoV-19/Japan/PG-84172/2021	EPI_ISL_3896827	2021-09-02
hCoV-19/Japan/PG-84173/2021	EPI_ISL_3896828	2021-09-02
hCoV-19/Japan/PG-84174/2021	EPI_ISL_3896829	2021-09-02
hCoV-19/Japan/PG-84175/2021	EPI_ISL_3896830	2021-09-02
hCoV-19/Japan/PG-84176/2021	EPI_ISL_3896831	2021-09-02
hCoV-19/Japan/PG-84177/2021	EPI_ISL_3896832	2021-09-02
hCoV-19/Japan/PG-84178/2021	EPI_ISL_3896833	2021-09-02
hCoV-19/Japan/PG-84179/2021	EPI_ISL_3896834	2021-09-02
hCoV-19/Japan/PG-84180/2021	EPI_ISL_3896835	2021-09-02
hCoV-19/Japan/PG-84181/2021	EPI_ISL_3896836	2021-09-02
hCoV-19/Japan/PG-84182/2021	EPI_ISL_3896837	2021-09-02
hCoV-19/Japan/PG-84183/2021	EPI_ISL_3896838	2021-09-02
hCoV-19/Japan/PG-84184/2021	EPI_ISL_3896839	2021-09-02
hCoV-19/Japan/PG-84185/2021	EPI_ISL_3896840	2021-09-02
hCoV-19/Japan/PG-84186/2021	EPI_ISL_3896841	2021-09-02
hCoV-19/Japan/PG-84187/2021	EPI_ISL_3896842	2021-09-02
hCoV-19/Japan/PG-84188/2021	EPI_ISL_3896843	2021-09-02
hCoV-19/Japan/PG-84189/2021	EPI_ISL_3896844	2021-09-02
hCoV-19/Japan/PG-84190/2021	EPI_ISL_3896845	2021-09-02
hCoV-19/Japan/PG-84191/2021	EPI_ISL_3896846	2021-09-02
hCoV-19/Japan/PG-84192/2021	EPI_ISL_3896847	2021-09-02
hCoV-19/Japan/PG-84193/2021	EPI_ISL_3896848	2021-09-02
hCoV-19/Japan/PG-84194/2021	EPI_ISL_3896849	2021-09-02
hCoV-19/Japan/PG-84195/2021	EPI_ISL_3896850	2021-09-02
hCoV-19/Japan/PG-84196/2021	EPI_ISL_3896851	2021-09-02
hCoV-19/Japan/PG-84197/2021	EPI_ISL_3896852	2021-09-02
hCoV-19/Japan/PG-84198/2021	EPI_ISL_3896853	2021-09-02
hCoV-19/Japan/PG-84199/2021	EPI_ISL_3896854	2021-09-02
hCoV-19/Japan/PG-84200/2021	EPI_ISL_3896855	2021-09-02
hCoV-19/Japan/PG-84201/2021	EPI_ISL_3896856	2021-09-02
hCoV-19/Japan/PG-84202/2021	EPI_ISL_3896857	2021-09-02
hCoV-19/Japan/PG-84203/2021	EPI_ISL_3896858	2021-09-02

virus_name	epiID	submission_date
hCoV-19/Japan/PG-84204/2021	EPI_ISL_3896859	2021-09-02
hCoV-19/Japan/PG-84205/2021	EPI_ISL_3896860	2021-09-02
hCoV-19/Japan/PG-84206/2021	EPI_ISL_3896861	2021-09-02
hCoV-19/Japan/PG-84207/2021	EPI_ISL_3896862	2021-09-02
hCoV-19/Japan/PG-84208/2021	EPI_ISL_3896863	2021-09-02
hCoV-19/Japan/PG-84209/2021	EPI_ISL_3896864	2021-09-02
hCoV-19/Japan/PG-84210/2021	EPI_ISL_3896865	2021-09-02
hCoV-19/Japan/PG-84211/2021	EPI_ISL_3896866	2021-09-02
hCoV-19/Japan/PG-84212/2021	EPI_ISL_3896867	2021-09-02
hCoV-19/Japan/PG-84213/2021	EPI_ISL_3896868	2021-09-02
hCoV-19/Japan/PG-84214/2021	EPI_ISL_3896869	2021-09-02
hCoV-19/Japan/PG-84215/2021	EPI_ISL_3896870	2021-09-02
hCoV-19/Japan/PG-84216/2021	EPI_ISL_3896871	2021-09-02
hCoV-19/Japan/PG-84217/2021	EPI_ISL_3896872	2021-09-02
hCoV-19/Japan/PG-84218/2021	EPI_ISL_3896873	2021-09-02
hCoV-19/Japan/PG-84219/2021	EPI_ISL_3896874	2021-09-02
hCoV-19/Japan/PG-84220/2021	EPI_ISL_3896875	2021-09-02
hCoV-19/Japan/PG-84221/2021	EPI_ISL_3896876	2021-09-02
hCoV-19/Japan/PG-84222/2021	EPI_ISL_3896877	2021-09-02
hCoV-19/Japan/PG-84223/2021	EPI_ISL_3896878	2021-09-02
hCoV-19/Japan/PG-84224/2021	EPI_ISL_3896879	2021-09-02
hCoV-19/Japan/PG-84225/2021	EPI_ISL_3896880	2021-09-02
hCoV-19/Japan/PG-84226/2021	EPI_ISL_3896881	2021-09-02
hCoV-19/Japan/PG-84227/2021	EPI_ISL_3896882	2021-09-02
hCoV-19/Japan/PG-84228/2021	EPI_ISL_3896883	2021-09-02
hCoV-19/Japan/PG-84229/2021	EPI_ISL_3896884	2021-09-02
hCoV-19/Japan/PG-84230/2021	EPI_ISL_3896885	2021-09-02
hCoV-19/Japan/PG-84231/2021	EPI_ISL_3896886	2021-09-02
hCoV-19/Japan/PG-84232/2021	EPI_ISL_3896887	2021-09-02
hCoV-19/Japan/PG-84233/2021	EPI_ISL_3896888	2021-09-02
hCoV-19/Japan/PG-84234/2021	EPI_ISL_3896889	2021-09-02
hCoV-19/Japan/PG-84235/2021	EPI_ISL_3896890	2021-09-02
hCoV-19/Japan/PG-84236/2021	EPI_ISL_3896891	2021-09-02
hCoV-19/Japan/PG-84237/2021	EPI_ISL_3896892	2021-09-02
hCoV-19/Japan/PG-84238/2021	EPI_ISL_3896893	2021-09-02
hCoV-19/Japan/PG-84239/2021	EPI_ISL_3896894	2021-09-02
hCoV-19/Japan/PG-84240/2021	EPI_ISL_3896895	2021-09-02
hCoV-19/Japan/PG-84241/2021	EPI_ISL_3896896	2021-09-02
hCoV-19/Japan/PG-84242/2021	EPI_ISL_3896897	2021-09-02
hCoV-19/Japan/PG-84243/2021	EPI_ISL_3896898	2021-09-02
hCoV-19/Japan/PG-84244/2021	EPI_ISL_3896899	2021-09-02
hCoV-19/Japan/PG-84245/2021	EPI_ISL_3896900	2021-09-02
hCoV-19/Japan/PG-84246/2021	EPI_ISL_3896901	2021-09-02
hCoV-19/Japan/PG-84247/2021	EPI_ISL_3896902	2021-09-02
hCoV-19/Japan/PG-84248/2021	EPI_ISL_3896903	2021-09-02
hCoV-19/Japan/PG-84249/2021	EPI_ISL_3896904	2021-09-02
hCoV-19/Japan/PG-84250/2021	EPI_ISL_3896905	2021-09-02
hCoV-19/Japan/PG-84251/2021	EPI_ISL_3896906	2021-09-02
hCoV-19/Japan/PG-84252/2021	EPI_ISL_3896907	2021-09-02
hCoV-19/Japan/PG-84253/2021	EPI_ISL_3896908	2021-09-02
hCoV-19/Japan/PG-84254/2021	EPI_ISL_3896909	2021-09-02
hCoV-19/Japan/PG-84255/2021	EPI_ISL_3896910	2021-09-02
hCoV-19/Japan/PG-84256/2021	EPI_ISL_3896911	2021-09-02
hCoV-19/Japan/PG-84257/2021	EPI_ISL_3896912	2021-09-02

virus_name	epiID	submission_date
hCoV-19/Japan/PG-84258/2021	EPI_ISL_3896913	2021-09-02
hCoV-19/Japan/PG-84259/2021	EPI_ISL_3896914	2021-09-02
hCoV-19/Japan/PG-84260/2021	EPI_ISL_3896915	2021-09-02
hCoV-19/Japan/PG-84261/2021	EPI_ISL_3896916	2021-09-02
hCoV-19/Japan/PG-84262/2021	EPI_ISL_3896917	2021-09-02
hCoV-19/Japan/PG-84263/2021	EPI_ISL_3896918	2021-09-02
hCoV-19/Japan/PG-84264/2021	EPI_ISL_3896919	2021-09-02
hCoV-19/Japan/PG-84265/2021	EPI_ISL_3896920	2021-09-02
hCoV-19/Japan/PG-84266/2021	EPI_ISL_3896921	2021-09-02
hCoV-19/Japan/PG-84267/2021	EPI_ISL_3896922	2021-09-02
hCoV-19/Japan/PG-84268/2021	EPI_ISL_3896923	2021-09-02
hCoV-19/Japan/PG-84269/2021	EPI_ISL_3896924	2021-09-02
hCoV-19/Japan/PG-84270/2021	EPI_ISL_3896925	2021-09-02
hCoV-19/Japan/PG-84271/2021	EPI_ISL_3896926	2021-09-02
hCoV-19/Japan/PG-84272/2021	EPI_ISL_3896927	2021-09-02
hCoV-19/Japan/PG-84273/2021	EPI_ISL_3896928	2021-09-02
hCoV-19/Japan/PG-84274/2021	EPI_ISL_3896929	2021-09-02
hCoV-19/Japan/PG-84275/2021	EPI_ISL_3896930	2021-09-02
hCoV-19/Japan/PG-84276/2021	EPI_ISL_3896931	2021-09-02
hCoV-19/Japan/PG-84277/2021	EPI_ISL_3896932	2021-09-02
hCoV-19/Japan/PG-84278/2021	EPI_ISL_3896933	2021-09-02
hCoV-19/Japan/PG-84279/2021	EPI_ISL_3896934	2021-09-02
hCoV-19/Japan/PG-84280/2021	EPI_ISL_3896935	2021-09-02
hCoV-19/Japan/PG-84281/2021	EPI_ISL_3896936	2021-09-02
hCoV-19/Japan/PG-84282/2021	EPI_ISL_3896937	2021-09-02
hCoV-19/Japan/PG-84283/2021	EPI_ISL_3896938	2021-09-02
hCoV-19/Japan/PG-84284/2021	EPI_ISL_3896939	2021-09-02
hCoV-19/Japan/PG-84285/2021	EPI_ISL_3896940	2021-09-02
hCoV-19/Japan/PG-84286/2021	EPI_ISL_3896941	2021-09-02
hCoV-19/Japan/PG-84287/2021	EPI_ISL_3896942	2021-09-02
hCoV-19/Japan/PG-84288/2021	EPI_ISL_3896943	2021-09-02
hCoV-19/Japan/PG-84289/2021	EPI_ISL_3896944	2021-09-02
hCoV-19/Japan/PG-84290/2021	EPI_ISL_3896945	2021-09-02
hCoV-19/Japan/PG-84291/2021	EPI_ISL_3896946	2021-09-02
hCoV-19/Japan/PG-84292/2021	EPI_ISL_3896947	2021-09-02
hCoV-19/Japan/PG-84293/2021	EPI_ISL_3896948	2021-09-02
hCoV-19/Japan/PG-84294/2021	EPI_ISL_3896949	2021-09-02
hCoV-19/Japan/PG-84295/2021	EPI_ISL_3896950	2021-09-02
hCoV-19/Japan/PG-84296/2021	EPI_ISL_3896951	2021-09-02
hCoV-19/Japan/PG-84297/2021	EPI_ISL_3896952	2021-09-02
hCoV-19/Japan/PG-84298/2021	EPI_ISL_3896953	2021-09-02
hCoV-19/Japan/PG-84299/2021	EPI_ISL_3896954	2021-09-02
hCoV-19/Japan/PG-84300/2021	EPI_ISL_3896955	2021-09-02
hCoV-19/Japan/PG-84301/2021	EPI_ISL_3896956	2021-09-02
hCoV-19/Japan/PG-84302/2021	EPI_ISL_3896957	2021-09-02
hCoV-19/Japan/PG-84303/2021	EPI_ISL_3896958	2021-09-02
hCoV-19/Japan/PG-84304/2021	EPI_ISL_3896959	2021-09-02
hCoV-19/Japan/PG-84305/2021	EPI_ISL_3896960	2021-09-02
hCoV-19/Japan/PG-84306/2021	EPI_ISL_3896961	2021-09-02
hCoV-19/Japan/PG-84307/2021	EPI_ISL_3896962	2021-09-02
hCoV-19/Japan/PG-84308/2021	EPI_ISL_3896963	2021-09-02
hCoV-19/Japan/PG-84309/2021	EPI_ISL_3896964	2021-09-02
hCoV-19/Japan/PG-84310/2021	EPI_ISL_3896965	2021-09-02
hCoV-19/Japan/PG-84311/2021	EPI_ISL_3896966	2021-09-02

virus_name	epiID	submission_date
hCoV-19/Japan/PG-84312/2021	EPI_ISL_3896967	2021-09-02
hCoV-19/Japan/PG-84313/2021	EPI_ISL_3896968	2021-09-02
hCoV-19/Japan/PG-84314/2021	EPI_ISL_3896969	2021-09-02
hCoV-19/Japan/PG-84315/2021	EPI_ISL_3896970	2021-09-02
hCoV-19/Japan/PG-84316/2021	EPI_ISL_3896971	2021-09-02
hCoV-19/Japan/PG-84317/2021	EPI_ISL_3896972	2021-09-02
hCoV-19/Japan/PG-84318/2021	EPI_ISL_3896973	2021-09-02
hCoV-19/Japan/PG-84319/2021	EPI_ISL_3896974	2021-09-02
hCoV-19/Japan/PG-84320/2021	EPI_ISL_3896975	2021-09-02
hCoV-19/Japan/PG-84321/2021	EPI_ISL_3896976	2021-09-02
hCoV-19/Japan/PG-84322/2021	EPI_ISL_3896977	2021-09-02
hCoV-19/Japan/PG-84323/2021	EPI_ISL_3896978	2021-09-02
hCoV-19/Japan/PG-84324/2021	EPI_ISL_3896979	2021-09-02
hCoV-19/Japan/PG-84325/2021	EPI_ISL_3896980	2021-09-02
hCoV-19/Japan/PG-84326/2021	EPI_ISL_3896981	2021-09-02
hCoV-19/Japan/PG-84327/2021	EPI_ISL_3896982	2021-09-02
hCoV-19/Japan/PG-84328/2021	EPI_ISL_3896983	2021-09-02
hCoV-19/Japan/PG-84329/2021	EPI_ISL_3896984	2021-09-02
hCoV-19/Japan/PG-84330/2021	EPI_ISL_3896985	2021-09-02
hCoV-19/Japan/PG-84331/2021	EPI_ISL_3896986	2021-09-02
hCoV-19/Japan/PG-84332/2021	EPI_ISL_3896987	2021-09-02
hCoV-19/Japan/PG-84333/2021	EPI_ISL_3896988	2021-09-02
hCoV-19/Japan/PG-84334/2021	EPI_ISL_3896989	2021-09-02
hCoV-19/Japan/PG-84335/2021	EPI_ISL_3896990	2021-09-02
hCoV-19/Japan/PG-84336/2021	EPI_ISL_3896991	2021-09-02
hCoV-19/Japan/PG-84337/2021	EPI_ISL_3896992	2021-09-02
hCoV-19/Japan/PG-84338/2021	EPI_ISL_3896993	2021-09-02
hCoV-19/Japan/PG-84339/2021	EPI_ISL_3896994	2021-09-02
hCoV-19/Japan/PG-84340/2021	EPI_ISL_3896995	2021-09-02
hCoV-19/Japan/PG-84341/2021	EPI_ISL_3896996	2021-09-02
hCoV-19/Japan/PG-84342/2021	EPI_ISL_3896997	2021-09-02
hCoV-19/Japan/PG-84343/2021	EPI_ISL_3896998	2021-09-02
hCoV-19/Japan/PG-84344/2021	EPI_ISL_3896999	2021-09-02
hCoV-19/Japan/PG-84345/2021	EPI_ISL_3897000	2021-09-02
hCoV-19/Japan/PG-84346/2021	EPI_ISL_3897001	2021-09-02
hCoV-19/Japan/PG-84347/2021	EPI_ISL_3897002	2021-09-02
hCoV-19/Japan/PG-84348/2021	EPI_ISL_3897004	2021-09-02
hCoV-19/Japan/PG-84349/2021	EPI_ISL_3897005	2021-09-02
hCoV-19/Japan/PG-84350/2021	EPI_ISL_3897006	2021-09-02
hCoV-19/Japan/PG-84351/2021	EPI_ISL_3897007	2021-09-02
hCoV-19/Japan/PG-84352/2021	EPI_ISL_3897008	2021-09-02
hCoV-19/Japan/PG-84353/2021	EPI_ISL_3897009	2021-09-02
hCoV-19/Japan/PG-84354/2021	EPI_ISL_3897010	2021-09-02
hCoV-19/Japan/PG-84355/2021	EPI_ISL_3897011	2021-09-02
hCoV-19/Japan/PG-84356/2021	EPI_ISL_3897012	2021-09-02
hCoV-19/Japan/PG-84357/2021	EPI_ISL_3897013	2021-09-02
hCoV-19/Japan/PG-84358/2021	EPI_ISL_3897014	2021-09-02
hCoV-19/Japan/PG-84359/2021	EPI_ISL_3897015	2021-09-02
hCoV-19/Japan/PG-84360/2021	EPI_ISL_3897016	2021-09-02
hCoV-19/Japan/PG-84361/2021	EPI_ISL_3897017	2021-09-02
hCoV-19/Japan/PG-84362/2021	EPI_ISL_3897018	2021-09-02
hCoV-19/Japan/PG-84363/2021	EPI_ISL_3897019	2021-09-02
hCoV-19/Japan/PG-84364/2021	EPI_ISL_3897020	2021-09-02
hCoV-19/Japan/PG-84365/2021	EPI_ISL_3897021	2021-09-02

virus_name	epiID	submission_date
hCoV-19/Japan/PG-84366/2021	EPI_ISL_3897022	2021-09-02
hCoV-19/Japan/PG-84367/2021	EPI_ISL_3897023	2021-09-02
hCoV-19/Japan/PG-84368/2021	EPI_ISL_3897024	2021-09-02
hCoV-19/Japan/PG-84369/2021	EPI_ISL_3897025	2021-09-02
hCoV-19/Japan/PG-84370/2021	EPI_ISL_3897026	2021-09-02
hCoV-19/Japan/PG-84371/2021	EPI_ISL_3897027	2021-09-02
hCoV-19/Japan/PG-84372/2021	EPI_ISL_3897028	2021-09-02
hCoV-19/Japan/PG-84373/2021	EPI_ISL_3897029	2021-09-02
hCoV-19/Japan/PG-84374/2021	EPI_ISL_3897030	2021-09-02
hCoV-19/Japan/PG-84375/2021	EPI_ISL_3897031	2021-09-02
hCoV-19/Japan/PG-84376/2021	EPI_ISL_3897032	2021-09-02
hCoV-19/Japan/PG-84377/2021	EPI_ISL_3897033	2021-09-02
hCoV-19/Japan/PG-84378/2021	EPI_ISL_3897034	2021-09-02
hCoV-19/Japan/PG-84379/2021	EPI_ISL_3897035	2021-09-02
hCoV-19/Japan/PG-84380/2021	EPI_ISL_3897036	2021-09-02
hCoV-19/Japan/PG-84381/2021	EPI_ISL_3897037	2021-09-02
hCoV-19/Japan/PG-84382/2021	EPI_ISL_3897038	2021-09-02
hCoV-19/Japan/PG-84383/2021	EPI_ISL_3897039	2021-09-02
hCoV-19/Japan/PG-84384/2021	EPI_ISL_3897040	2021-09-02
hCoV-19/Japan/PG-84385/2021	EPI_ISL_3897041	2021-09-02
hCoV-19/Japan/PG-84386/2021	EPI_ISL_3897042	2021-09-02
hCoV-19/Japan/PG-84387/2021	EPI_ISL_3897043	2021-09-02
hCoV-19/Japan/PG-84388/2021	EPI_ISL_3897044	2021-09-02
hCoV-19/Japan/PG-84389/2021	EPI_ISL_3897045	2021-09-02
hCoV-19/Japan/PG-84390/2021	EPI_ISL_3897046	2021-09-02
hCoV-19/Japan/PG-84391/2021	EPI_ISL_3897047	2021-09-02
hCoV-19/Japan/PG-84392/2021	EPI_ISL_3897048	2021-09-02
hCoV-19/Japan/PG-84393/2021	EPI_ISL_3897049	2021-09-02
hCoV-19/Japan/PG-84394/2021	EPI_ISL_3897050	2021-09-02
hCoV-19/Japan/PG-84395/2021	EPI_ISL_3897051	2021-09-02
hCoV-19/Japan/PG-84396/2021	EPI_ISL_3897052	2021-09-02
hCoV-19/Japan/PG-84397/2021	EPI_ISL_3897053	2021-09-02
hCoV-19/Japan/PG-84398/2021	EPI_ISL_3897054	2021-09-02
hCoV-19/Japan/PG-84399/2021	EPI_ISL_3897055	2021-09-02
hCoV-19/Japan/PG-84400/2021	EPI_ISL_3897056	2021-09-02
hCoV-19/Japan/PG-84401/2021	EPI_ISL_3897057	2021-09-02
hCoV-19/Japan/PG-84402/2021	EPI_ISL_3897058	2021-09-02
hCoV-19/Japan/PG-84403/2021	EPI_ISL_3897059	2021-09-02
hCoV-19/Japan/PG-84404/2021	EPI_ISL_3897060	2021-09-02
hCoV-19/Japan/PG-84405/2021	EPI_ISL_3897061	2021-09-02
hCoV-19/Japan/PG-84406/2021	EPI_ISL_3897062	2021-09-02
hCoV-19/Japan/PG-84407/2021	EPI_ISL_3897063	2021-09-02
hCoV-19/Japan/PG-84408/2021	EPI_ISL_3897064	2021-09-02
hCoV-19/Japan/PG-84409/2021	EPI_ISL_3897065	2021-09-02
hCoV-19/Japan/PG-84410/2021	EPI_ISL_3897066	2021-09-02
hCoV-19/Japan/PG-84411/2021	EPI_ISL_3897067	2021-09-02
hCoV-19/Japan/PG-84412/2021	EPI_ISL_3897068	2021-09-02
hCoV-19/Japan/PG-84413/2021	EPI_ISL_3897069	2021-09-02
hCoV-19/Japan/PG-84414/2021	EPI_ISL_3897070	2021-09-02
hCoV-19/Japan/PG-84415/2021	EPI_ISL_3897071	2021-09-02
hCoV-19/Japan/PG-84416/2021	EPI_ISL_3897072	2021-09-02
hCoV-19/Japan/PG-84417/2021	EPI_ISL_3897073	2021-09-02
hCoV-19/Japan/PG-84418/2021	EPI_ISL_3897074	2021-09-02
hCoV-19/Japan/PG-84419/2021	EPI_ISL_3897075	2021-09-02

virus_name	epiID	submission_date
hCoV-19/Japan/PG-84420/2021	EPI_ISL_3897076	2021-09-02
hCoV-19/Japan/PG-84421/2021	EPI_ISL_3897077	2021-09-02
hCoV-19/Japan/PG-84422/2021	EPI_ISL_3897078	2021-09-02
hCoV-19/Japan/PG-84423/2021	EPI_ISL_3897079	2021-09-02
hCoV-19/Japan/PG-84424/2021	EPI_ISL_3897080	2021-09-02
hCoV-19/Japan/PG-84425/2021	EPI_ISL_3897081	2021-09-02
hCoV-19/Japan/PG-84426/2021	EPI_ISL_3897082	2021-09-02
hCoV-19/Japan/PG-84427/2021	EPI_ISL_3897083	2021-09-02
hCoV-19/Japan/PG-84428/2021	EPI_ISL_3897084	2021-09-02
hCoV-19/Japan/PG-84429/2021	EPI_ISL_3897085	2021-09-02
hCoV-19/Japan/PG-84430/2021	EPI_ISL_3897086	2021-09-02
hCoV-19/Japan/PG-84431/2021	EPI_ISL_3897087	2021-09-02
hCoV-19/Japan/PG-84432/2021	EPI_ISL_3897088	2021-09-02
hCoV-19/Japan/PG-84433/2021	EPI_ISL_3897089	2021-09-02
hCoV-19/Japan/PG-84434/2021	EPI_ISL_3897090	2021-09-02
hCoV-19/Japan/PG-84435/2021	EPI_ISL_3897091	2021-09-02
hCoV-19/Japan/PG-84436/2021	EPI_ISL_3897092	2021-09-02
hCoV-19/Japan/PG-84437/2021	EPI_ISL_3897093	2021-09-02
hCoV-19/Japan/PG-84438/2021	EPI_ISL_3897094	2021-09-02
hCoV-19/Japan/PG-84439/2021	EPI_ISL_3897095	2021-09-02
hCoV-19/Japan/PG-84440/2021	EPI_ISL_3897096	2021-09-02
hCoV-19/Japan/PG-84441/2021	EPI_ISL_3897097	2021-09-02
hCoV-19/Japan/PG-84442/2021	EPI_ISL_3897098	2021-09-02
hCoV-19/Japan/PG-84443/2021	EPI_ISL_3897099	2021-09-02
hCoV-19/Japan/PG-84444/2021	EPI_ISL_3897100	2021-09-02
hCoV-19/Japan/PG-84445/2021	EPI_ISL_3897101	2021-09-02
hCoV-19/Japan/PG-84446/2021	EPI_ISL_3897102	2021-09-02
hCoV-19/Japan/PG-84447/2021	EPI_ISL_3897103	2021-09-02
hCoV-19/Japan/PG-84448/2021	EPI_ISL_3897104	2021-09-02
hCoV-19/Japan/PG-84449/2021	EPI_ISL_3897105	2021-09-02
hCoV-19/Japan/PG-84450/2021	EPI_ISL_3897106	2021-09-02
hCoV-19/Japan/PG-84451/2021	EPI_ISL_3897107	2021-09-02
hCoV-19/Japan/PG-84452/2021	EPI_ISL_3897108	2021-09-02
hCoV-19/Japan/PG-84453/2021	EPI_ISL_3897109	2021-09-02
hCoV-19/Japan/PG-84454/2021	EPI_ISL_3897110	2021-09-02
hCoV-19/Japan/PG-84455/2021	EPI_ISL_3897111	2021-09-02
hCoV-19/Japan/PG-84456/2021	EPI_ISL_3897112	2021-09-02
hCoV-19/Japan/PG-84457/2021	EPI_ISL_3897113	2021-09-02
hCoV-19/Japan/PG-84458/2021	EPI_ISL_3897114	2021-09-02
hCoV-19/Japan/PG-84459/2021	EPI_ISL_3897115	2021-09-02
hCoV-19/Japan/PG-84460/2021	EPI_ISL_3897116	2021-09-02
hCoV-19/Japan/PG-84461/2021	EPI_ISL_3897117	2021-09-02
hCoV-19/Japan/PG-84462/2021	EPI_ISL_3897118	2021-09-02
hCoV-19/Japan/PG-84463/2021	EPI_ISL_3897119	2021-09-02
hCoV-19/Japan/PG-84464/2021	EPI_ISL_3897120	2021-09-02
hCoV-19/Japan/PG-84465/2021	EPI_ISL_3897121	2021-09-02
hCoV-19/Japan/PG-84466/2021	EPI_ISL_3897122	2021-09-02
hCoV-19/Japan/PG-84467/2021	EPI_ISL_3897123	2021-09-02
hCoV-19/Japan/PG-84468/2021	EPI_ISL_3897124	2021-09-02
hCoV-19/Japan/PG-84469/2021	EPI_ISL_3897125	2021-09-02
hCoV-19/Japan/PG-84470/2021	EPI_ISL_3897126	2021-09-02
hCoV-19/Japan/PG-84471/2021	EPI_ISL_3897127	2021-09-02
hCoV-19/Japan/PG-84472/2021	EPI_ISL_3897128	2021-09-02
hCoV-19/Japan/PG-84473/2021	EPI_ISL_3897129	2021-09-02

virus_name	epiID	submission_date
hCoV-19/Japan/PG-84474/2021	EPI_ISL_3897130	2021-09-02
hCoV-19/Japan/PG-84475/2021	EPI_ISL_3897131	2021-09-02
hCoV-19/Japan/PG-84476/2021	EPI_ISL_3897132	2021-09-02
hCoV-19/Japan/PG-84477/2021	EPI_ISL_3897133	2021-09-02
hCoV-19/Japan/PG-84478/2021	EPI_ISL_3897134	2021-09-02
hCoV-19/Japan/PG-84479/2021	EPI_ISL_3897135	2021-09-02
hCoV-19/Japan/PG-84480/2021	EPI_ISL_3897136	2021-09-02
hCoV-19/Japan/PG-84481/2021	EPI_ISL_3897137	2021-09-02
hCoV-19/Japan/PG-84482/2021	EPI_ISL_3897138	2021-09-02
hCoV-19/Japan/PG-84483/2021	EPI_ISL_3897139	2021-09-02
hCoV-19/Japan/PG-84484/2021	EPI_ISL_3897140	2021-09-02
hCoV-19/Japan/PG-84485/2021	EPI_ISL_3897141	2021-09-02
hCoV-19/Japan/PG-84486/2021	EPI_ISL_3897142	2021-09-02
hCoV-19/Japan/PG-84487/2021	EPI_ISL_3897143	2021-09-02
hCoV-19/Japan/PG-84488/2021	EPI_ISL_3897144	2021-09-02
hCoV-19/Japan/PG-84489/2021	EPI_ISL_3897145	2021-09-02
hCoV-19/Japan/PG-84490/2021	EPI_ISL_3897146	2021-09-02
hCoV-19/Japan/PG-84491/2021	EPI_ISL_3897147	2021-09-02
hCoV-19/Japan/PG-84492/2021	EPI_ISL_3897148	2021-09-02
hCoV-19/Japan/PG-84493/2021	EPI_ISL_3897149	2021-09-02
hCoV-19/Japan/PG-84494/2021	EPI_ISL_3897150	2021-09-02
hCoV-19/Japan/PG-84495/2021	EPI_ISL_3897151	2021-09-02
hCoV-19/Japan/PG-84496/2021	EPI_ISL_3897152	2021-09-02
hCoV-19/Japan/PG-84497/2021	EPI_ISL_3897153	2021-09-02
hCoV-19/Japan/PG-84498/2021	EPI_ISL_3897154	2021-09-02
hCoV-19/Japan/PG-84499/2021	EPI_ISL_3897155	2021-09-02
hCoV-19/Japan/PG-84500/2021	EPI_ISL_3897156	2021-09-02
hCoV-19/Japan/PG-84501/2021	EPI_ISL_3897157	2021-09-02
hCoV-19/Japan/PG-84502/2021	EPI_ISL_3897158	2021-09-02
hCoV-19/Japan/PG-84503/2021	EPI_ISL_3897159	2021-09-02
hCoV-19/Japan/PG-84504/2021	EPI_ISL_3897160	2021-09-02
hCoV-19/Japan/PG-84505/2021	EPI_ISL_3897161	2021-09-02
hCoV-19/Japan/PG-84506/2021	EPI_ISL_3897162	2021-09-02
hCoV-19/Japan/PG-84507/2021	EPI_ISL_3897163	2021-09-02
hCoV-19/Japan/PG-84508/2021	EPI_ISL_3897164	2021-09-02
hCoV-19/Japan/PG-84509/2021	EPI_ISL_3897165	2021-09-02
hCoV-19/Japan/PG-84510/2021	EPI_ISL_3897166	2021-09-02
hCoV-19/Japan/PG-84511/2021	EPI_ISL_3897167	2021-09-02
hCoV-19/Japan/PG-84512/2021	EPI_ISL_3897168	2021-09-02
hCoV-19/Japan/PG-84513/2021	EPI_ISL_3897169	2021-09-02
hCoV-19/Japan/PG-84514/2021	EPI_ISL_3897170	2021-09-02
hCoV-19/Japan/PG-84515/2021	EPI_ISL_3897171	2021-09-02
hCoV-19/Japan/PG-84516/2021	EPI_ISL_3897172	2021-09-02
hCoV-19/Japan/PG-84517/2021	EPI_ISL_3897173	2021-09-02
hCoV-19/Japan/PG-84518/2021	EPI_ISL_3897174	2021-09-02
hCoV-19/Japan/PG-84519/2021	EPI_ISL_3897175	2021-09-02
hCoV-19/Japan/PG-84520/2021	EPI_ISL_3897176	2021-09-02
hCoV-19/Japan/PG-84521/2021	EPI_ISL_3897177	2021-09-02
hCoV-19/Japan/PG-84522/2021	EPI_ISL_3897178	2021-09-02
hCoV-19/Japan/PG-84523/2021	EPI_ISL_3897179	2021-09-02
hCoV-19/Japan/PG-84524/2021	EPI_ISL_3897180	2021-09-02
hCoV-19/Japan/PG-84525/2021	EPI_ISL_3897181	2021-09-02
hCoV-19/Japan/PG-84526/2021	EPI_ISL_3897182	2021-09-02
hCoV-19/Japan/PG-84527/2021	EPI_ISL_3897183	2021-09-02

virus_name	epiID	submission_date
hCoV-19/Japan/PG-84528/2021	EPI_ISL_3897184	2021-09-02
hCoV-19/Japan/PG-84529/2021	EPI_ISL_3897185	2021-09-02
hCoV-19/Japan/PG-84530/2021	EPI_ISL_3897186	2021-09-02
hCoV-19/Japan/PG-84531/2021	EPI_ISL_3897187	2021-09-02
hCoV-19/Japan/PG-84532/2021	EPI_ISL_3897188	2021-09-02
hCoV-19/Japan/PG-84533/2021	EPI_ISL_3897189	2021-09-02
hCoV-19/Japan/PG-84534/2021	EPI_ISL_3897190	2021-09-02
hCoV-19/Japan/PG-84535/2021	EPI_ISL_3897191	2021-09-02
hCoV-19/Japan/PG-84536/2021	EPI_ISL_3897192	2021-09-02
hCoV-19/Japan/PG-84537/2021	EPI_ISL_3897193	2021-09-02
hCoV-19/Japan/PG-84538/2021	EPI_ISL_3897194	2021-09-02
hCoV-19/Japan/PG-84539/2021	EPI_ISL_3897195	2021-09-02
hCoV-19/Japan/PG-84540/2021	EPI_ISL_3897196	2021-09-02
hCoV-19/Japan/PG-84541/2021	EPI_ISL_3897197	2021-09-02
hCoV-19/Japan/PG-84542/2021	EPI_ISL_3897198	2021-09-02
hCoV-19/Japan/PG-84543/2021	EPI_ISL_3897199	2021-09-02
hCoV-19/Japan/PG-84544/2021	EPI_ISL_3897200	2021-09-02
hCoV-19/Japan/PG-84545/2021	EPI_ISL_3897201	2021-09-02
hCoV-19/Japan/PG-84546/2021	EPI_ISL_3897202	2021-09-02
hCoV-19/Japan/PG-84547/2021	EPI_ISL_3897203	2021-09-02
hCoV-19/Japan/PG-84548/2021	EPI_ISL_3897204	2021-09-02
hCoV-19/Japan/PG-84549/2021	EPI_ISL_3897205	2021-09-02
hCoV-19/Japan/PG-84550/2021	EPI_ISL_3897206	2021-09-02
hCoV-19/Japan/PG-84551/2021	EPI_ISL_3897207	2021-09-02
hCoV-19/Japan/PG-84552/2021	EPI_ISL_3897208	2021-09-02
hCoV-19/Japan/PG-84553/2021	EPI_ISL_3897209	2021-09-02
hCoV-19/Japan/PG-84554/2021	EPI_ISL_3897210	2021-09-02
hCoV-19/Japan/PG-84555/2021	EPI_ISL_3897211	2021-09-02
hCoV-19/Japan/PG-84556/2021	EPI_ISL_3897212	2021-09-02
hCoV-19/Japan/PG-84557/2021	EPI_ISL_3897213	2021-09-02
hCoV-19/Japan/PG-84558/2021	EPI_ISL_3897214	2021-09-02
hCoV-19/Japan/PG-84559/2021	EPI_ISL_3897215	2021-09-02
hCoV-19/Japan/PG-84560/2021	EPI_ISL_3897216	2021-09-02
hCoV-19/Japan/PG-84561/2021	EPI_ISL_3897217	2021-09-02
hCoV-19/Japan/PG-84562/2021	EPI_ISL_3897218	2021-09-02
hCoV-19/Japan/PG-84563/2021	EPI_ISL_3897219	2021-09-02
hCoV-19/Japan/PG-84564/2021	EPI_ISL_3897220	2021-09-02
hCoV-19/Japan/PG-84565/2021	EPI_ISL_3897221	2021-09-02
hCoV-19/Japan/PG-84566/2021	EPI_ISL_3897222	2021-09-02
hCoV-19/Japan/PG-84567/2021	EPI_ISL_3897223	2021-09-02
hCoV-19/Japan/PG-84568/2021	EPI_ISL_3897224	2021-09-02
hCoV-19/Japan/PG-84569/2021	EPI_ISL_3897225	2021-09-02
hCoV-19/Japan/PG-84570/2021	EPI_ISL_3897226	2021-09-02
hCoV-19/Japan/PG-84571/2021	EPI_ISL_3897227	2021-09-02
hCoV-19/Japan/PG-84572/2021	EPI_ISL_3897228	2021-09-02
hCoV-19/Japan/PG-84573/2021	EPI_ISL_3897229	2021-09-02
hCoV-19/Japan/PG-84574/2021	EPI_ISL_3897230	2021-09-02
hCoV-19/Japan/PG-84575/2021	EPI_ISL_3897231	2021-09-02
hCoV-19/Japan/PG-84576/2021	EPI_ISL_3897232	2021-09-02
hCoV-19/Japan/PG-84577/2021	EPI_ISL_3897233	2021-09-02
hCoV-19/Japan/PG-84578/2021	EPI_ISL_3897234	2021-09-02
hCoV-19/Japan/PG-84579/2021	EPI_ISL_3897235	2021-09-02
hCoV-19/Japan/PG-84580/2021	EPI_ISL_3897236	2021-09-02
hCoV-19/Japan/PG-84581/2021	EPI_ISL_3897237	2021-09-02

virus_name	epiID	submission_date
hCoV-19/Japan/PG-84582/2021	EPI_ISL_3897238	2021-09-02
hCoV-19/Japan/PG-84583/2021	EPI_ISL_3897239	2021-09-02
hCoV-19/Japan/PG-84584/2021	EPI_ISL_3897240	2021-09-02
hCoV-19/Japan/PG-84585/2021	EPI_ISL_3897241	2021-09-02
hCoV-19/Japan/PG-84586/2021	EPI_ISL_3897242	2021-09-02
hCoV-19/Japan/PG-84587/2021	EPI_ISL_3897243	2021-09-02
hCoV-19/Japan/PG-84588/2021	EPI_ISL_3897244	2021-09-02
hCoV-19/Japan/PG-84589/2021	EPI_ISL_3897245	2021-09-02
hCoV-19/Japan/PG-84590/2021	EPI_ISL_3897246	2021-09-02
hCoV-19/Japan/PG-84591/2021	EPI_ISL_3897247	2021-09-02
hCoV-19/Japan/PG-84592/2021	EPI_ISL_3897248	2021-09-02
hCoV-19/Japan/PG-84593/2021	EPI_ISL_3897249	2021-09-02
hCoV-19/Japan/PG-84594/2021	EPI_ISL_3897250	2021-09-02
hCoV-19/Japan/PG-84595/2021	EPI_ISL_3897251	2021-09-02
hCoV-19/Japan/PG-84596/2021	EPI_ISL_3897252	2021-09-02
hCoV-19/Japan/PG-84597/2021	EPI_ISL_3873838	2021-09-02
hCoV-19/Japan/PG-84598/2021	EPI_ISL_3873839	2021-09-02
hCoV-19/Japan/PG-84599/2021	EPI_ISL_3873840	2021-09-02
hCoV-19/Japan/PG-84600/2021	EPI_ISL_3873841	2021-09-02
hCoV-19/Japan/PG-84601/2021	EPI_ISL_3873842	2021-09-02
hCoV-19/Japan/PG-84602/2021	EPI_ISL_3873843	2021-09-02
hCoV-19/Japan/PG-84603/2021	EPI_ISL_3873844	2021-09-02
hCoV-19/Japan/PG-84604/2021	EPI_ISL_3873845	2021-09-02
hCoV-19/Japan/PG-84605/2021	EPI_ISL_3873846	2021-09-02
hCoV-19/Japan/PG-84606/2021	EPI_ISL_3873847	2021-09-02
hCoV-19/Japan/PG-84607/2021	EPI_ISL_3873848	2021-09-02
hCoV-19/Japan/PG-84608/2021	EPI_ISL_3873849	2021-09-02
hCoV-19/Japan/PG-84609/2021	EPI_ISL_3873850	2021-09-02
hCoV-19/Japan/PG-84610/2021	EPI_ISL_3873851	2021-09-02
hCoV-19/Japan/PG-84611/2021	EPI_ISL_3873852	2021-09-02
hCoV-19/Japan/PG-84612/2021	EPI_ISL_3873853	2021-09-02
hCoV-19/Japan/PG-84613/2021	EPI_ISL_3873854	2021-09-02
hCoV-19/Japan/PG-84614/2021	EPI_ISL_3873855	2021-09-02
hCoV-19/Japan/PG-84615/2021	EPI_ISL_3873856	2021-09-02
hCoV-19/Japan/PG-84616/2021	EPI_ISL_3873857	2021-09-02
hCoV-19/Japan/PG-84617/2021	EPI_ISL_3873858	2021-09-02
hCoV-19/Japan/PG-84618/2021	EPI_ISL_3873859	2021-09-02
hCoV-19/Japan/PG-84619/2021	EPI_ISL_3873860	2021-09-02
hCoV-19/Japan/PG-84620/2021	EPI_ISL_3873861	2021-09-02
hCoV-19/Japan/PG-84621/2021	EPI_ISL_3873862	2021-09-02
hCoV-19/Japan/PG-84622/2021	EPI_ISL_3873863	2021-09-02
hCoV-19/Japan/PG-84623/2021	EPI_ISL_3873864	2021-09-02
hCoV-19/Japan/PG-84624/2021	EPI_ISL_3873865	2021-09-02
hCoV-19/Japan/PG-84625/2021	EPI_ISL_3873866	2021-09-02
hCoV-19/Japan/PG-84626/2021	EPI_ISL_3873867	2021-09-02
hCoV-19/Japan/PG-84627/2021	EPI_ISL_3873868	2021-09-02
hCoV-19/Japan/PG-84628/2021	EPI_ISL_3873869	2021-09-02
hCoV-19/Japan/PG-84629/2021	EPI_ISL_3873870	2021-09-02
hCoV-19/Japan/PG-84630/2021	EPI_ISL_3873871	2021-09-02
hCoV-19/Japan/PG-84631/2021	EPI_ISL_3873872	2021-09-02
hCoV-19/Japan/PG-84632/2021	EPI_ISL_3873873	2021-09-02
hCoV-19/Japan/PG-84633/2021	EPI_ISL_3873874	2021-09-02
hCoV-19/Japan/PG-84634/2021	EPI_ISL_3873875	2021-09-02
hCoV-19/Japan/PG-84635/2021	EPI_ISL_3873876	2021-09-02

virus_name	epiID	submission_date
hCoV-19/Japan/PG-84636/2021	EPI_ISL_3873877	2021-09-02
hCoV-19/Japan/PG-84637/2021	EPI_ISL_3873878	2021-09-02
hCoV-19/Japan/PG-84638/2021	EPI_ISL_3873879	2021-09-02
hCoV-19/Japan/PG-84639/2021	EPI_ISL_3873880	2021-09-02
hCoV-19/Japan/PG-84640/2021	EPI_ISL_3873881	2021-09-02
hCoV-19/Japan/PG-84641/2021	EPI_ISL_3875598	2021-09-02
hCoV-19/Japan/PG-84642/2021	EPI_ISL_3875599	2021-09-02
hCoV-19/Japan/PG-84643/2021	EPI_ISL_3875600	2021-09-02
hCoV-19/Japan/PG-84644/2021	EPI_ISL_3875601	2021-09-02
hCoV-19/Japan/PG-84645/2021	EPI_ISL_3875602	2021-09-02
hCoV-19/Japan/PG-84646/2021	EPI_ISL_3875603	2021-09-02
hCoV-19/Japan/PG-84647/2021	EPI_ISL_3875604	2021-09-02
hCoV-19/Japan/PG-84648/2021	EPI_ISL_3875605	2021-09-02
hCoV-19/Japan/PG-84649/2021	EPI_ISL_3875606	2021-09-02
hCoV-19/Japan/PG-84650/2021	EPI_ISL_3875607	2021-09-02
hCoV-19/Japan/PG-84651/2021	EPI_ISL_3875608	2021-09-02
hCoV-19/Japan/PG-84652/2021	EPI_ISL_3875609	2021-09-02
hCoV-19/Japan/PG-84653/2021	EPI_ISL_3875610	2021-09-02
hCoV-19/Japan/PG-84654/2021	EPI_ISL_3875611	2021-09-02
hCoV-19/Japan/PG-84655/2021	EPI_ISL_3875612	2021-09-02
hCoV-19/Japan/PG-84656/2021	EPI_ISL_3875613	2021-09-02
hCoV-19/Japan/PG-84657/2021	EPI_ISL_3875614	2021-09-02
hCoV-19/Japan/PG-84658/2021	EPI_ISL_3875615	2021-09-02
hCoV-19/Japan/PG-84659/2021	EPI_ISL_3875616	2021-09-02
hCoV-19/Japan/PG-84660/2021	EPI_ISL_3875617	2021-09-02
hCoV-19/Japan/PG-84661/2021	EPI_ISL_3875618	2021-09-02
hCoV-19/Japan/PG-84662/2021	EPI_ISL_3875619	2021-09-02
hCoV-19/Japan/PG-84663/2021	EPI_ISL_3875620	2021-09-02
hCoV-19/Japan/PG-84664/2021	EPI_ISL_3875621	2021-09-02
hCoV-19/Japan/PG-84665/2021	EPI_ISL_3875622	2021-09-02
hCoV-19/Japan/PG-84667/2021	EPI_ISL_3875623	2021-09-02
hCoV-19/Japan/PG-84668/2021	EPI_ISL_3875624	2021-09-02
hCoV-19/Japan/PG-84669/2021	EPI_ISL_3875625	2021-09-02
hCoV-19/Japan/PG-84670/2021	EPI_ISL_3875626	2021-09-02
hCoV-19/Japan/PG-84671/2021	EPI_ISL_3875627	2021-09-02
hCoV-19/Japan/PG-84672/2021	EPI_ISL_3875628	2021-09-02
hCoV-19/Japan/PG-84673/2021	EPI_ISL_3875629	2021-09-02
hCoV-19/Japan/PG-84674/2021	EPI_ISL_3875630	2021-09-02
hCoV-19/Japan/PG-84675/2021	EPI_ISL_3875631	2021-09-02
hCoV-19/Japan/PG-84676/2021	EPI_ISL_3875632	2021-09-02
hCoV-19/Japan/PG-84677/2021	EPI_ISL_3875633	2021-09-02
hCoV-19/Japan/PG-84678/2021	EPI_ISL_3875634	2021-09-02
hCoV-19/Japan/PG-84679/2021	EPI_ISL_3875635	2021-09-02
hCoV-19/Japan/PG-84680/2021	EPI_ISL_3875636	2021-09-02
hCoV-19/Japan/PG-84681/2021	EPI_ISL_3875637	2021-09-02
hCoV-19/Japan/PG-84682/2021	EPI_ISL_3875638	2021-09-02
hCoV-19/Japan/PG-84683/2021	EPI_ISL_3875639	2021-09-02
hCoV-19/Japan/PG-84684/2021	EPI_ISL_3875640	2021-09-02
hCoV-19/Japan/PG-84685/2021	EPI_ISL_3875641	2021-09-02
hCoV-19/Japan/PG-84686/2021	EPI_ISL_3875642	2021-09-02
hCoV-19/Japan/PG-84687/2021	EPI_ISL_3875643	2021-09-02
hCoV-19/Japan/PG-84688/2021	EPI_ISL_3875644	2021-09-02
hCoV-19/Japan/PG-84689/2021	EPI_ISL_3875645	2021-09-02
hCoV-19/Japan/PG-84690/2021	EPI_ISL_3875647	2021-09-02

virus_name	epiID	submission_date
hCoV-19/Japan/PG-84691/2021	EPI_ISL_3875649	2021-09-02
hCoV-19/Japan/PG-84692/2021	EPI_ISL_3875653	2021-09-02
hCoV-19/Japan/PG-84693/2021	EPI_ISL_3875656	2021-09-02
hCoV-19/Japan/PG-84694/2021	EPI_ISL_3875659	2021-09-02
hCoV-19/Japan/PG-84695/2021	EPI_ISL_3871415	2021-09-02
hCoV-19/Japan/PG-84696/2021	EPI_ISL_3871416	2021-09-02
hCoV-19/Japan/PG-84697/2021	EPI_ISL_3871417	2021-09-02
hCoV-19/Japan/PG-84698/2021	EPI_ISL_3871418	2021-09-02
hCoV-19/Japan/PG-84699/2021	EPI_ISL_3871419	2021-09-02
hCoV-19/Japan/PG-84700/2021	EPI_ISL_3871420	2021-09-02
hCoV-19/Japan/PG-84701/2021	EPI_ISL_3871421	2021-09-02
hCoV-19/Japan/PG-84702/2021	EPI_ISL_3871422	2021-09-02
hCoV-19/Japan/PG-84703/2021	EPI_ISL_3871423	2021-09-02
hCoV-19/Japan/PG-84704/2021	EPI_ISL_3871424	2021-09-02
hCoV-19/Japan/PG-84705/2021	EPI_ISL_3871425	2021-09-02
hCoV-19/Japan/PG-84706/2021	EPI_ISL_3871426	2021-09-02
hCoV-19/Japan/PG-84707/2021	EPI_ISL_3871427	2021-09-02
hCoV-19/Japan/PG-84708/2021	EPI_ISL_3871428	2021-09-02
hCoV-19/Japan/PG-84709/2021	EPI_ISL_3871429	2021-09-02
hCoV-19/Japan/PG-84710/2021	EPI_ISL_3871823	2021-09-02
hCoV-19/Japan/PG-84711/2021	EPI_ISL_3871824	2021-09-02
hCoV-19/Japan/PG-84712/2021	EPI_ISL_3871825	2021-09-02
hCoV-19/Japan/PG-84713/2021	EPI_ISL_3871826	2021-09-02
hCoV-19/Japan/PG-84714/2021	EPI_ISL_3871827	2021-09-02
hCoV-19/Japan/PG-84715/2021	EPI_ISL_3871828	2021-09-02
hCoV-19/Japan/PG-84716/2021	EPI_ISL_3871829	2021-09-02
hCoV-19/Japan/PG-84717/2021	EPI_ISL_3871830	2021-09-02
hCoV-19/Japan/PG-84718/2021	EPI_ISL_3871831	2021-09-02
hCoV-19/Japan/PG-84719/2021	EPI_ISL_3871832	2021-09-02
hCoV-19/Japan/PG-84720/2021	EPI_ISL_3871833	2021-09-02
hCoV-19/Japan/PG-84721/2021	EPI_ISL_3871834	2021-09-02
hCoV-19/Japan/PG-84722/2021	EPI_ISL_3871835	2021-09-02
hCoV-19/Japan/PG-84723/2021	EPI_ISL_3871836	2021-09-02
hCoV-19/Japan/PG-84724/2021	EPI_ISL_3871837	2021-09-02
hCoV-19/Japan/PG-84725/2021	EPI_ISL_3871838	2021-09-02
hCoV-19/Japan/PG-84726/2021	EPI_ISL_3871839	2021-09-02
hCoV-19/Japan/PG-84727/2021	EPI_ISL_3871840	2021-09-02
hCoV-19/Japan/PG-84728/2021	EPI_ISL_3871841	2021-09-02
hCoV-19/Japan/PG-84729/2021	EPI_ISL_3871842	2021-09-02
hCoV-19/Japan/PG-84730/2021	EPI_ISL_3871843	2021-09-02
hCoV-19/Japan/PG-84731/2021	EPI_ISL_3871844	2021-09-02
hCoV-19/Japan/PG-84732/2021	EPI_ISL_3871845	2021-09-02
hCoV-19/Japan/PG-84733/2021	EPI_ISL_3876168	2021-09-02
hCoV-19/Japan/PG-84734/2021	EPI_ISL_3876169	2021-09-02
hCoV-19/Japan/PG-84735/2021	EPI_ISL_3876170	2021-09-02
hCoV-19/Japan/PG-84736/2021	EPI_ISL_3876171	2021-09-02
hCoV-19/Japan/PG-84737/2021	EPI_ISL_3876172	2021-09-02
hCoV-19/Japan/PG-84738/2021	EPI_ISL_3876173	2021-09-02
hCoV-19/Japan/PG-84739/2021	EPI_ISL_3876174	2021-09-02
hCoV-19/Japan/PG-84740/2021	EPI_ISL_3876175	2021-09-02
hCoV-19/Japan/PG-84741/2021	EPI_ISL_3876176	2021-09-02
hCoV-19/Japan/PG-84742/2021	EPI_ISL_3876177	2021-09-02
hCoV-19/Japan/PG-84743/2021	EPI_ISL_3876178	2021-09-02
hCoV-19/Japan/PG-84744/2021	EPI_ISL_3876179	2021-09-02

virus_name	epiID	submission_date
hCoV-19/Japan/PG-84745/2021	EPI_ISL_3876180	2021-09-02
hCoV-19/Japan/PG-84746/2021	EPI_ISL_3876181	2021-09-02
hCoV-19/Japan/PG-84747/2021	EPI_ISL_3876182	2021-09-02
hCoV-19/Japan/PG-84748/2021	EPI_ISL_3876183	2021-09-02
hCoV-19/Japan/PG-84749/2021	EPI_ISL_3876184	2021-09-02
hCoV-19/Japan/PG-84750/2021	EPI_ISL_3876185	2021-09-02
hCoV-19/Japan/PG-84751/2021	EPI_ISL_3876186	2021-09-02
hCoV-19/Japan/PG-84752/2021	EPI_ISL_3876187	2021-09-02
hCoV-19/Japan/PG-84753/2021	EPI_ISL_3876188	2021-09-02
hCoV-19/Japan/PG-84754/2021	EPI_ISL_3876189	2021-09-02
hCoV-19/Japan/PG-84759/2021	EPI_ISL_3876190	2021-09-02
hCoV-19/Japan/PG-84760/2021	EPI_ISL_3876191	2021-09-02
hCoV-19/Japan/PG-84761/2021	EPI_ISL_3876192	2021-09-02
hCoV-19/Japan/PG-84762/2021	EPI_ISL_3876193	2021-09-02
hCoV-19/Japan/PG-84763/2021	EPI_ISL_3876194	2021-09-02
hCoV-19/Japan/PG-84765/2021	EPI_ISL_3870782	2021-09-02
hCoV-19/Japan/PG-84766/2021	EPI_ISL_3870783	2021-09-02
hCoV-19/Japan/PG-84767/2021	EPI_ISL_3870784	2021-09-02
hCoV-19/Japan/PG-84768/2021	EPI_ISL_3870785	2021-09-02
hCoV-19/Japan/PG-84769/2021	EPI_ISL_3870786	2021-09-02
hCoV-19/Japan/PG-84770/2021	EPI_ISL_3870787	2021-09-02
hCoV-19/Japan/PG-84771/2021	EPI_ISL_3870788	2021-09-02
hCoV-19/Japan/PG-84772/2021	EPI_ISL_3870789	2021-09-02
hCoV-19/Japan/PG-84773/2021	EPI_ISL_3870790	2021-09-02
hCoV-19/Japan/PG-84774/2021	EPI_ISL_3870791	2021-09-02
hCoV-19/Japan/PG-84775/2021	EPI_ISL_3870792	2021-09-02
hCoV-19/Japan/PG-84776/2021	EPI_ISL_3870793	2021-09-02
hCoV-19/Japan/PG-84777/2021	EPI_ISL_3870794	2021-09-02
hCoV-19/Japan/PG-84778/2021	EPI_ISL_3870795	2021-09-02
hCoV-19/Japan/PG-84779/2021	EPI_ISL_3870796	2021-09-02
hCoV-19/Japan/PG-84780/2021	EPI_ISL_3870797	2021-09-02
hCoV-19/Japan/PG-84781/2021	EPI_ISL_3870798	2021-09-02
hCoV-19/Japan/PG-84782/2021	EPI_ISL_3870799	2021-09-02
hCoV-19/Japan/PG-84783/2021	EPI_ISL_3870800	2021-09-02
hCoV-19/Japan/PG-84784/2021	EPI_ISL_3870801	2021-09-02
hCoV-19/Japan/PG-84785/2021	EPI_ISL_3870802	2021-09-02
hCoV-19/Japan/PG-84786/2021	EPI_ISL_3870803	2021-09-02
hCoV-19/Japan/PG-84787/2021	EPI_ISL_3870804	2021-09-02
hCoV-19/Japan/PG-84788/2021	EPI_ISL_3870805	2021-09-02
hCoV-19/Japan/PG-84789/2021	EPI_ISL_3870806	2021-09-02
hCoV-19/Japan/PG-84790/2021	EPI_ISL_3870807	2021-09-02
hCoV-19/Japan/PG-84791/2021	EPI_ISL_3870808	2021-09-02
hCoV-19/Japan/PG-84792/2021	EPI_ISL_3870809	2021-09-02
hCoV-19/Japan/PG-84793/2021	EPI_ISL_3870810	2021-09-02
hCoV-19/Japan/PG-84794/2021	EPI_ISL_3870811	2021-09-02
hCoV-19/Japan/PG-84795/2021	EPI_ISL_3870812	2021-09-02
hCoV-19/Japan/PG-84796/2021	EPI_ISL_3870813	2021-09-02
hCoV-19/Japan/PG-84797/2021	EPI_ISL_3870814	2021-09-02
hCoV-19/Japan/PG-84798/2021	EPI_ISL_3870815	2021-09-02
hCoV-19/Japan/PG-84799/2021	EPI_ISL_3870816	2021-09-02
hCoV-19/Japan/PG-84800/2021	EPI_ISL_3870817	2021-09-02
hCoV-19/Japan/PG-84801/2021	EPI_ISL_3870818	2021-09-02
hCoV-19/Japan/PG-84802/2021	EPI_ISL_3870819	2021-09-02
hCoV-19/Japan/PG-84803/2021	EPI_ISL_3870820	2021-09-02

virus_name	epiID	submission_date
hCoV-19/Japan/PG-84804/2021	EPI_ISL_3870821	2021-09-02
hCoV-19/Japan/PG-84805/2021	EPI_ISL_3870822	2021-09-02
hCoV-19/Japan/PG-84806/2021	EPI_ISL_3870823	2021-09-02
hCoV-19/Japan/PG-84807/2021	EPI_ISL_3871375	2021-09-02
hCoV-19/Japan/PG-84808/2021	EPI_ISL_3871376	2021-09-02
hCoV-19/Japan/PG-84809/2021	EPI_ISL_3871377	2021-09-02
hCoV-19/Japan/PG-84810/2021	EPI_ISL_3871378	2021-09-02
hCoV-19/Japan/PG-84811/2021	EPI_ISL_3871379	2021-09-02
hCoV-19/Japan/PG-84812/2021	EPI_ISL_3872450	2021-09-02
hCoV-19/Japan/PG-84813/2021	EPI_ISL_3872451	2021-09-02
hCoV-19/Japan/PG-84814/2021	EPI_ISL_3872452	2021-09-02
hCoV-19/Japan/PG-84815/2021	EPI_ISL_3872453	2021-09-02
hCoV-19/Japan/PG-84816/2021	EPI_ISL_3872454	2021-09-02
hCoV-19/Japan/PG-84817/2021	EPI_ISL_3872455	2021-09-02
hCoV-19/Japan/PG-84818/2021	EPI_ISL_3872456	2021-09-02
hCoV-19/Japan/PG-84819/2021	EPI_ISL_3872457	2021-09-02
hCoV-19/Japan/PG-84820/2021	EPI_ISL_3872458	2021-09-02
hCoV-19/Japan/PG-84821/2021	EPI_ISL_3872459	2021-09-02
hCoV-19/Japan/PG-84823/2021	EPI_ISL_3872460	2021-09-02
hCoV-19/Japan/PG-84824/2021	EPI_ISL_3872461	2021-09-02
hCoV-19/Japan/PG-84825/2021	EPI_ISL_3872462	2021-09-02
hCoV-19/Japan/PG-84826/2021	EPI_ISL_3872463	2021-09-02
hCoV-19/Japan/PG-84827/2021	EPI_ISL_3872464	2021-09-02
hCoV-19/Japan/PG-84828/2021	EPI_ISL_3872465	2021-09-02
hCoV-19/Japan/PG-84829/2021	EPI_ISL_3872466	2021-09-02
hCoV-19/Japan/PG-84830/2021	EPI_ISL_3872467	2021-09-02
hCoV-19/Japan/PG-84831/2021	EPI_ISL_3872468	2021-09-02
hCoV-19/Japan/PG-84832/2021	EPI_ISL_3872469	2021-09-02
hCoV-19/Japan/PG-84833/2021	EPI_ISL_3872470	2021-09-02
hCoV-19/Japan/PG-84834/2021	EPI_ISL_3872471	2021-09-02
hCoV-19/Japan/PG-84835/2021	EPI_ISL_3872472	2021-09-02
hCoV-19/Japan/PG-84836/2021	EPI_ISL_3872473	2021-09-02
hCoV-19/Japan/PG-84837/2021	EPI_ISL_3872474	2021-09-02
hCoV-19/Japan/PG-84838/2021	EPI_ISL_3872475	2021-09-02
hCoV-19/Japan/PG-84839/2021	EPI_ISL_3872476	2021-09-02
hCoV-19/Japan/PG-84840/2021	EPI_ISL_3872477	2021-09-02
hCoV-19/Japan/PG-84841/2021	EPI_ISL_3872478	2021-09-02
hCoV-19/Japan/PG-84842/2021	EPI_ISL_3872479	2021-09-02
hCoV-19/Japan/PG-84843/2021	EPI_ISL_3872480	2021-09-02
hCoV-19/Japan/PG-84844/2021	EPI_ISL_3872481	2021-09-02
hCoV-19/Japan/PG-84845/2021	EPI_ISL_3872482	2021-09-02
hCoV-19/Japan/PG-84846/2021	EPI_ISL_3872483	2021-09-02
hCoV-19/Japan/PG-84847/2021	EPI_ISL_3872484	2021-09-02
hCoV-19/Japan/PG-84848/2021	EPI_ISL_3872485	2021-09-02
hCoV-19/Japan/PG-84849/2021	EPI_ISL_3872486	2021-09-02
hCoV-19/Japan/PG-84850/2021	EPI_ISL_3872487	2021-09-02
hCoV-19/Japan/PG-84851/2021	EPI_ISL_3872488	2021-09-02
hCoV-19/Japan/PG-84852/2021	EPI_ISL_3872489	2021-09-02
hCoV-19/Japan/PG-84853/2021	EPI_ISL_3872490	2021-09-02
hCoV-19/Japan/PG-84854/2021	EPI_ISL_3872491	2021-09-02
hCoV-19/Japan/PG-84855/2021	EPI_ISL_3872492	2021-09-02
hCoV-19/Japan/PG-84856/2021	EPI_ISL_3872493	2021-09-02
hCoV-19/Japan/PG-84857/2021	EPI_ISL_3872494	2021-09-02
hCoV-19/Japan/PG-84858/2021	EPI_ISL_3872495	2021-09-02

virus_name	epiID	submission_date
hCoV-19/Japan/PG-84859/2021	EPI_ISL_3872496	2021-09-02
hCoV-19/Japan/PG-84860/2021	EPI_ISL_3872942	2021-09-02
hCoV-19/Japan/PG-84861/2021	EPI_ISL_3872943	2021-09-02
hCoV-19/Japan/PG-84862/2021	EPI_ISL_3872944	2021-09-02
hCoV-19/Japan/PG-84863/2021	EPI_ISL_3872945	2021-09-02
hCoV-19/Japan/PG-84864/2021	EPI_ISL_3872946	2021-09-02
hCoV-19/Japan/PG-84865/2021	EPI_ISL_3872947	2021-09-02
hCoV-19/Japan/PG-84866/2021	EPI_ISL_3872948	2021-09-02
hCoV-19/Japan/PG-84867/2021	EPI_ISL_3872949	2021-09-02
hCoV-19/Japan/PG-84868/2021	EPI_ISL_3872950	2021-09-02
hCoV-19/Japan/PG-84869/2021	EPI_ISL_3872951	2021-09-02
hCoV-19/Japan/PG-84870/2021	EPI_ISL_3872952	2021-09-02
hCoV-19/Japan/PG-84871/2021	EPI_ISL_3872953	2021-09-02
hCoV-19/Japan/PG-84872/2021	EPI_ISL_3872954	2021-09-02
hCoV-19/Japan/PG-84873/2021	EPI_ISL_3872955	2021-09-02
hCoV-19/Japan/PG-84874/2021	EPI_ISL_3872956	2021-09-02
hCoV-19/Japan/PG-84875/2021	EPI_ISL_3872957	2021-09-02
hCoV-19/Japan/PG-84876/2021	EPI_ISL_3872958	2021-09-02
hCoV-19/Japan/PG-84877/2021	EPI_ISL_3872959	2021-09-02
hCoV-19/Japan/PG-84878/2021	EPI_ISL_3872960	2021-09-02
hCoV-19/Japan/PG-84879/2021	EPI_ISL_3872961	2021-09-02
hCoV-19/Japan/PG-84880/2021	EPI_ISL_3872962	2021-09-02
hCoV-19/Japan/PG-84881/2021	EPI_ISL_3872963	2021-09-02
hCoV-19/Japan/PG-84882/2021	EPI_ISL_3872964	2021-09-02
hCoV-19/Japan/PG-84883/2021	EPI_ISL_3872965	2021-09-02
hCoV-19/Japan/PG-84884/2021	EPI_ISL_3872966	2021-09-02
hCoV-19/Japan/PG-84885/2021	EPI_ISL_3872967	2021-09-02
hCoV-19/Japan/PG-84886/2021	EPI_ISL_3872968	2021-09-02
hCoV-19/Japan/PG-84887/2021	EPI_ISL_3872969	2021-09-02
hCoV-19/Japan/PG-84888/2021	EPI_ISL_3872970	2021-09-02
hCoV-19/Japan/PG-84889/2021	EPI_ISL_3872971	2021-09-02
hCoV-19/Japan/PG-84890/2021	EPI_ISL_3872972	2021-09-02
hCoV-19/Japan/PG-84891/2021	EPI_ISL_3872973	2021-09-02
hCoV-19/Japan/PG-84892/2021	EPI_ISL_3872974	2021-09-02
hCoV-19/Japan/PG-84893/2021	EPI_ISL_3872975	2021-09-02
hCoV-19/Japan/PG-84894/2021	EPI_ISL_3872976	2021-09-02
hCoV-19/Japan/PG-84895/2021	EPI_ISL_3872977	2021-09-02
hCoV-19/Japan/PG-84896/2021	EPI_ISL_3872978	2021-09-02
hCoV-19/Japan/PG-84897/2021	EPI_ISL_3872979	2021-09-02
hCoV-19/Japan/PG-84898/2021	EPI_ISL_3872980	2021-09-02
hCoV-19/Japan/PG-84899/2021	EPI_ISL_3872981	2021-09-02
hCoV-19/Japan/PG-84900/2021	EPI_ISL_3872982	2021-09-02
hCoV-19/Japan/PG-84901/2021	EPI_ISL_3872983	2021-09-02
hCoV-19/Japan/PG-84902/2021	EPI_ISL_3872984	2021-09-02
hCoV-19/Japan/PG-84903/2021	EPI_ISL_3872985	2021-09-02
hCoV-19/Japan/PG-84904/2021	EPI_ISL_3872986	2021-09-02
hCoV-19/Japan/PG-84905/2021	EPI_ISL_3872987	2021-09-02
hCoV-19/Japan/PG-84906/2021	EPI_ISL_3872988	2021-09-02
hCoV-19/Japan/PG-84907/2021	EPI_ISL_3872989	2021-09-02
hCoV-19/Japan/PG-84908/2021	EPI_ISL_3872990	2021-09-02
hCoV-19/Japan/PG-84909/2021	EPI_ISL_3872991	2021-09-02
hCoV-19/Japan/PG-84910/2021	EPI_ISL_3872992	2021-09-02
hCoV-19/Japan/PG-84911/2021	EPI_ISL_3872993	2021-09-02
hCoV-19/Japan/PG-84912/2021	EPI_ISL_3872994	2021-09-02

virus_name	epiID	submission_date
hCoV-19/Japan/PG-84913/2021	EPI_ISL_3872995	2021-09-02
hCoV-19/Japan/PG-84914/2021	EPI_ISL_3872996	2021-09-02
hCoV-19/Japan/PG-84915/2021	EPI_ISL_3872997	2021-09-02
hCoV-19/Japan/PG-84916/2021	EPI_ISL_3872998	2021-09-02
hCoV-19/Japan/PG-84917/2021	EPI_ISL_3872999	2021-09-02
hCoV-19/Japan/PG-84918/2021	EPI_ISL_3873000	2021-09-02
hCoV-19/Japan/PG-84919/2021	EPI_ISL_3873001	2021-09-02
hCoV-19/Japan/PG-84920/2021	EPI_ISL_3873002	2021-09-02
hCoV-19/Japan/PG-84921/2021	EPI_ISL_3873003	2021-09-02
hCoV-19/Japan/PG-84922/2021	EPI_ISL_3873004	2021-09-02
hCoV-19/Japan/PG-84923/2021	EPI_ISL_3873005	2021-09-02
hCoV-19/Japan/PG-84924/2021	EPI_ISL_3873006	2021-09-02
hCoV-19/Japan/PG-84925/2021	EPI_ISL_3873007	2021-09-02
hCoV-19/Japan/PG-84926/2021	EPI_ISL_3873008	2021-09-02
hCoV-19/Japan/PG-84927/2021	EPI_ISL_3873009	2021-09-02
hCoV-19/Japan/PG-84928/2021	EPI_ISL_3873010	2021-09-02
hCoV-19/Japan/PG-84929/2021	EPI_ISL_3873011	2021-09-02
hCoV-19/Japan/PG-84930/2021	EPI_ISL_3873012	2021-09-02
hCoV-19/Japan/PG-84931/2021	EPI_ISL_3873013	2021-09-02
hCoV-19/Japan/PG-84932/2021	EPI_ISL_3873014	2021-09-02
hCoV-19/Japan/PG-84933/2021	EPI_ISL_3873015	2021-09-02
hCoV-19/Japan/PG-84934/2021	EPI_ISL_3873016	2021-09-02
hCoV-19/Japan/PG-84935/2021	EPI_ISL_3873017	2021-09-02
hCoV-19/Japan/PG-84936/2021	EPI_ISL_3873018	2021-09-02
hCoV-19/Japan/PG-84937/2021	EPI_ISL_3873019	2021-09-02
hCoV-19/Japan/PG-84938/2021	EPI_ISL_3873020	2021-09-02
hCoV-19/Japan/PG-84939/2021	EPI_ISL_3873021	2021-09-02
hCoV-19/Japan/PG-84940/2021	EPI_ISL_3873022	2021-09-02
hCoV-19/Japan/PG-84941/2021	EPI_ISL_3873023	2021-09-02
hCoV-19/Japan/PG-84959/2021	EPI_ISL_3871641	2021-09-02
hCoV-19/Japan/PG-84960/2021	EPI_ISL_3871642	2021-09-02
hCoV-19/Japan/PG-84961/2021	EPI_ISL_3871643	2021-09-02
hCoV-19/Japan/PG-84962/2021	EPI_ISL_3871644	2021-09-02
hCoV-19/Japan/PG-84963/2021	EPI_ISL_3871645	2021-09-02
hCoV-19/Japan/PG-84964/2021	EPI_ISL_3871646	2021-09-02
hCoV-19/Japan/PG-84965/2021	EPI_ISL_3871647	2021-09-02
hCoV-19/Japan/PG-84966/2021	EPI_ISL_3871648	2021-09-02
hCoV-19/Japan/PG-84967/2021	EPI_ISL_3871649	2021-09-02
hCoV-19/Japan/PG-84968/2021	EPI_ISL_3871650	2021-09-02
hCoV-19/Japan/PG-84969/2021	EPI_ISL_3871651	2021-09-02
hCoV-19/Japan/PG-84970/2021	EPI_ISL_3871652	2021-09-02
hCoV-19/Japan/PG-84971/2021	EPI_ISL_3871653	2021-09-02
hCoV-19/Japan/PG-84972/2021	EPI_ISL_3871654	2021-09-02
hCoV-19/Japan/PG-84973/2021	EPI_ISL_3871655	2021-09-02
hCoV-19/Japan/PG-84974/2021	EPI_ISL_3871656	2021-09-02
hCoV-19/Japan/PG-84975/2021	EPI_ISL_3871657	2021-09-02
hCoV-19/Japan/PG-84976/2021	EPI_ISL_3874509	2021-09-02
hCoV-19/Japan/PG-84977/2021	EPI_ISL_3874510	2021-09-02
hCoV-19/Japan/PG-84978/2021	EPI_ISL_3874511	2021-09-02
hCoV-19/Japan/PG-84979/2021	EPI_ISL_3874512	2021-09-02
hCoV-19/Japan/PG-84980/2021	EPI_ISL_3874513	2021-09-02
hCoV-19/Japan/PG-84981/2021	EPI_ISL_3874514	2021-09-02
hCoV-19/Japan/PG-84982/2021	EPI_ISL_3874515	2021-09-02
hCoV-19/Japan/PG-84983/2021	EPI_ISL_3874516	2021-09-02

virus_name	epiID	submission_date
hCoV-19/Japan/PG-84984/2021	EPI_ISL_3874517	2021-09-02
hCoV-19/Japan/PG-84985/2021	EPI_ISL_3873984	2021-09-02
hCoV-19/Japan/PG-84986/2021	EPI_ISL_3873985	2021-09-02
hCoV-19/Japan/PG-84987/2021	EPI_ISL_3873986	2021-09-02
hCoV-19/Japan/PG-84988/2021	EPI_ISL_3873987	2021-09-02
hCoV-19/Japan/PG-84989/2021	EPI_ISL_3873988	2021-09-02
hCoV-19/Japan/PG-84990/2021	EPI_ISL_3873989	2021-09-02
hCoV-19/Japan/PG-84991/2021	EPI_ISL_3873990	2021-09-02
hCoV-19/Japan/PG-84992/2021	EPI_ISL_3873991	2021-09-02
hCoV-19/Japan/PG-84993/2021	EPI_ISL_3873992	2021-09-02
hCoV-19/Japan/PG-84994/2021	EPI_ISL_3873993	2021-09-02
hCoV-19/Japan/PG-84995/2021	EPI_ISL_3873994	2021-09-02
hCoV-19/Japan/PG-84996/2021	EPI_ISL_3873995	2021-09-02
hCoV-19/Japan/PG-84997/2021	EPI_ISL_3873996	2021-09-02
hCoV-19/Japan/PG-84998/2021	EPI_ISL_3873997	2021-09-02
hCoV-19/Japan/PG-84999/2021	EPI_ISL_3873998	2021-09-02
hCoV-19/Japan/PG-85000/2021	EPI_ISL_3873999	2021-09-02
hCoV-19/Japan/PG-85001/2021	EPI_ISL_3874000	2021-09-02
hCoV-19/Japan/PG-85002/2021	EPI_ISL_3874001	2021-09-02
hCoV-19/Japan/PG-85003/2021	EPI_ISL_3874002	2021-09-02
hCoV-19/Japan/PG-85004/2021	EPI_ISL_3874003	2021-09-02
hCoV-19/Japan/PG-85005/2021	EPI_ISL_3874004	2021-09-02
hCoV-19/Japan/PG-85006/2021	EPI_ISL_3874005	2021-09-02
hCoV-19/Japan/PG-85007/2021	EPI_ISL_3871291	2021-09-02
hCoV-19/Japan/PG-85008/2021	EPI_ISL_3871292	2021-09-02
hCoV-19/Japan/PG-85009/2021	EPI_ISL_3871293	2021-09-02
hCoV-19/Japan/PG-85010/2021	EPI_ISL_3871294	2021-09-02
hCoV-19/Japan/PG-85011/2021	EPI_ISL_3871295	2021-09-02
hCoV-19/Japan/PG-85012/2021	EPI_ISL_3871296	2021-09-02
hCoV-19/Japan/PG-85013/2021	EPI_ISL_3871297	2021-09-02
hCoV-19/Japan/PG-85014/2021	EPI_ISL_3871298	2021-09-02
hCoV-19/Japan/PG-85015/2021	EPI_ISL_3871299	2021-09-02
hCoV-19/Japan/PG-85016/2021	EPI_ISL_3871300	2021-09-02
hCoV-19/Japan/PG-85017/2021	EPI_ISL_3871301	2021-09-02
hCoV-19/Japan/PG-85018/2021	EPI_ISL_3871302	2021-09-02
hCoV-19/Japan/PG-85019/2021	EPI_ISL_3871303	2021-09-02
hCoV-19/Japan/PG-85020/2021	EPI_ISL_3871304	2021-09-02
hCoV-19/Japan/PG-85021/2021	EPI_ISL_3871305	2021-09-02
hCoV-19/Japan/PG-85022/2021	EPI_ISL_3871306	2021-09-02
hCoV-19/Japan/PG-85023/2021	EPI_ISL_3871307	2021-09-02
hCoV-19/Japan/PG-85024/2021	EPI_ISL_3871308	2021-09-02
hCoV-19/Japan/PG-85025/2021	EPI_ISL_3871309	2021-09-02
hCoV-19/Japan/PG-85026/2021	EPI_ISL_3871310	2021-09-02
hCoV-19/Japan/PG-85027/2021	EPI_ISL_3871311	2021-09-02
hCoV-19/Japan/PG-85028/2021	EPI_ISL_3871312	2021-09-02
hCoV-19/Japan/PG-85029/2021	EPI_ISL_3871313	2021-09-02
hCoV-19/Japan/PG-85030/2021	EPI_ISL_3872084	2021-09-02
hCoV-19/Japan/PG-85031/2021	EPI_ISL_3872085	2021-09-02
hCoV-19/Japan/PG-85032/2021	EPI_ISL_3872086	2021-09-02
hCoV-19/Japan/PG-85033/2021	EPI_ISL_3872087	2021-09-02
hCoV-19/Japan/PG-85034/2021	EPI_ISL_3872088	2021-09-02
hCoV-19/Japan/PG-85035/2021	EPI_ISL_3872089	2021-09-02
hCoV-19/Japan/PG-85036/2021	EPI_ISL_3872090	2021-09-02
hCoV-19/Japan/PG-85037/2021	EPI_ISL_3872091	2021-09-02

virus_name	epiID	submission_date
hCoV-19/Japan/PG-85038/2021	EPI_ISL_3872092	2021-09-02
hCoV-19/Japan/PG-85039/2021	EPI_ISL_3872093	2021-09-02
hCoV-19/Japan/PG-85040/2021	EPI_ISL_3872094	2021-09-02
hCoV-19/Japan/PG-85041/2021	EPI_ISL_3872095	2021-09-02
hCoV-19/Japan/PG-85042/2021	EPI_ISL_3872096	2021-09-02
hCoV-19/Japan/PG-85043/2021	EPI_ISL_3872097	2021-09-02
hCoV-19/Japan/PG-85044/2021	EPI_ISL_3872098	2021-09-02
hCoV-19/Japan/PG-85045/2021	EPI_ISL_3872099	2021-09-02
hCoV-19/Japan/PG-85046/2021	EPI_ISL_3872100	2021-09-02
hCoV-19/Japan/PG-85047/2021	EPI_ISL_3872101	2021-09-02
hCoV-19/Japan/PG-85048/2021	EPI_ISL_3872102	2021-09-02
hCoV-19/Japan/PG-85049/2021	EPI_ISL_3872103	2021-09-02
hCoV-19/Japan/PG-85050/2021	EPI_ISL_3872104	2021-09-02
hCoV-19/Japan/PG-85051/2021	EPI_ISL_3872105	2021-09-02
hCoV-19/Japan/PG-85052/2021	EPI_ISL_3872106	2021-09-02
hCoV-19/Japan/PG-85053/2021	EPI_ISL_3872107	2021-09-02
hCoV-19/Japan/PG-85054/2021	EPI_ISL_3872108	2021-09-02
hCoV-19/Japan/PG-85055/2021	EPI_ISL_3872109	2021-09-02
hCoV-19/Japan/PG-85056/2021	EPI_ISL_3872110	2021-09-02
hCoV-19/Japan/PG-85057/2021	EPI_ISL_3872111	2021-09-02
hCoV-19/Japan/PG-85058/2021	EPI_ISL_3872112	2021-09-02
hCoV-19/Japan/PG-85059/2021	EPI_ISL_3872113	2021-09-02
hCoV-19/Japan/PG-85060/2021	EPI_ISL_3872114	2021-09-02
hCoV-19/Japan/PG-85061/2021	EPI_ISL_3872115	2021-09-02
hCoV-19/Japan/PG-85063/2021	EPI_ISL_3874371	2021-09-02
hCoV-19/Japan/PG-85064/2021	EPI_ISL_3874372	2021-09-02
hCoV-19/Japan/PG-85065/2021	EPI_ISL_3874373	2021-09-02
hCoV-19/Japan/PG-85066/2021	EPI_ISL_3874374	2021-09-02
hCoV-19/Japan/PG-85067/2021	EPI_ISL_3874375	2021-09-02
hCoV-19/Japan/PG-85068/2021	EPI_ISL_3874376	2021-09-02
hCoV-19/Japan/PG-85069/2021	EPI_ISL_3874377	2021-09-02
hCoV-19/Japan/PG-85070/2021	EPI_ISL_3874378	2021-09-02
hCoV-19/Japan/PG-85071/2021	EPI_ISL_3874379	2021-09-02
hCoV-19/Japan/PG-85072/2021	EPI_ISL_3874380	2021-09-02
hCoV-19/Japan/PG-85073/2021	EPI_ISL_3874381	2021-09-02
hCoV-19/Japan/PG-85074/2021	EPI_ISL_3874382	2021-09-02
hCoV-19/Japan/PG-85075/2021	EPI_ISL_3874383	2021-09-02
hCoV-19/Japan/PG-85076/2021	EPI_ISL_3874384	2021-09-02
hCoV-19/Japan/PG-85077/2021	EPI_ISL_3874385	2021-09-02
hCoV-19/Japan/PG-85078/2021	EPI_ISL_3874386	2021-09-02
hCoV-19/Japan/PG-85079/2021	EPI_ISL_3874387	2021-09-02
hCoV-19/Japan/PG-85080/2021	EPI_ISL_3874388	2021-09-02
hCoV-19/Japan/PG-85081/2021	EPI_ISL_3874389	2021-09-02
hCoV-19/Japan/PG-85082/2021	EPI_ISL_3874390	2021-09-02
hCoV-19/Japan/PG-85083/2021	EPI_ISL_3874391	2021-09-02
hCoV-19/Japan/PG-85084/2021	EPI_ISL_3874392	2021-09-02
hCoV-19/Japan/PG-85085/2021	EPI_ISL_3874393	2021-09-02
hCoV-19/Japan/PG-85086/2021	EPI_ISL_3874394	2021-09-02
hCoV-19/Japan/PG-85087/2021	EPI_ISL_3874439	2021-09-02
hCoV-19/Japan/PG-85088/2021	EPI_ISL_3874440	2021-09-02
hCoV-19/Japan/PG-85089/2021	EPI_ISL_3874441	2021-09-02
hCoV-19/Japan/PG-85090/2021	EPI_ISL_3874442	2021-09-02
hCoV-19/Japan/PG-85091/2021	EPI_ISL_3874443	2021-09-02
hCoV-19/Japan/PG-85092/2021	EPI_ISL_3874444	2021-09-02

virus_name	epiID	submission_date
hCoV-19/Japan/PG-85093/2021	EPI_ISL_3874445	2021-09-02
hCoV-19/Japan/PG-85094/2021	EPI_ISL_3874446	2021-09-02
hCoV-19/Japan/PG-85095/2021	EPI_ISL_3874447	2021-09-02
hCoV-19/Japan/PG-85096/2021	EPI_ISL_3874448	2021-09-02
hCoV-19/Japan/PG-85097/2021	EPI_ISL_3874449	2021-09-02
hCoV-19/Japan/PG-85098/2021	EPI_ISL_3874450	2021-09-02
hCoV-19/Japan/PG-85099/2021	EPI_ISL_3874451	2021-09-02
hCoV-19/Japan/PG-85100/2021	EPI_ISL_3874452	2021-09-02
hCoV-19/Japan/PG-85101/2021	EPI_ISL_3874453	2021-09-02
hCoV-19/Japan/PG-85102/2021	EPI_ISL_3874454	2021-09-02
hCoV-19/Japan/PG-85103/2021	EPI_ISL_3874455	2021-09-02
hCoV-19/Japan/PG-85104/2021	EPI_ISL_3874456	2021-09-02
hCoV-19/Japan/PG-85105/2021	EPI_ISL_3874457	2021-09-02
hCoV-19/Japan/PG-85106/2021	EPI_ISL_3874458	2021-09-02
hCoV-19/Japan/PG-85107/2021	EPI_ISL_3874459	2021-09-02
hCoV-19/Japan/PG-85108/2021	EPI_ISL_3874460	2021-09-02
hCoV-19/Japan/PG-85109/2021	EPI_ISL_3874461	2021-09-02
hCoV-19/Japan/PG-85110/2021	EPI_ISL_3874213	2021-09-02
hCoV-19/Japan/PG-85111/2021	EPI_ISL_3874214	2021-09-02
hCoV-19/Japan/PG-85112/2021	EPI_ISL_3874215	2021-09-02
hCoV-19/Japan/PG-85113/2021	EPI_ISL_3874216	2021-09-02
hCoV-19/Japan/PG-85114/2021	EPI_ISL_3874217	2021-09-02
hCoV-19/Japan/PG-85115/2021	EPI_ISL_3874218	2021-09-02
hCoV-19/Japan/PG-85116/2021	EPI_ISL_3874219	2021-09-02
hCoV-19/Japan/PG-85117/2021	EPI_ISL_3874220	2021-09-02
hCoV-19/Japan/PG-85118/2021	EPI_ISL_3874221	2021-09-02
hCoV-19/Japan/PG-85119/2021	EPI_ISL_3874222	2021-09-02
hCoV-19/Japan/PG-85120/2021	EPI_ISL_3874223	2021-09-02
hCoV-19/Japan/PG-85121/2021	EPI_ISL_3874224	2021-09-02
hCoV-19/Japan/PG-85122/2021	EPI_ISL_3874225	2021-09-02
hCoV-19/Japan/PG-85123/2021	EPI_ISL_3874226	2021-09-02
hCoV-19/Japan/PG-85124/2021	EPI_ISL_3874227	2021-09-02
hCoV-19/Japan/PG-85125/2021	EPI_ISL_3874228	2021-09-02
hCoV-19/Japan/PG-85126/2021	EPI_ISL_3874229	2021-09-02
hCoV-19/Japan/PG-85127/2021	EPI_ISL_3874230	2021-09-02
hCoV-19/Japan/PG-85128/2021	EPI_ISL_3874231	2021-09-02
hCoV-19/Japan/PG-85129/2021	EPI_ISL_3874232	2021-09-02
hCoV-19/Japan/PG-85130/2021	EPI_ISL_3874233	2021-09-02
hCoV-19/Japan/PG-85131/2021	EPI_ISL_3874234	2021-09-02
hCoV-19/Japan/PG-85132/2021	EPI_ISL_3870981	2021-09-02
hCoV-19/Japan/PG-85133/2021	EPI_ISL_3870982	2021-09-02
hCoV-19/Japan/PG-85134/2021	EPI_ISL_3870983	2021-09-02
hCoV-19/Japan/PG-85135/2021	EPI_ISL_3870984	2021-09-02
hCoV-19/Japan/PG-85136/2021	EPI_ISL_3870985	2021-09-02
hCoV-19/Japan/PG-85137/2021	EPI_ISL_3870986	2021-09-02
hCoV-19/Japan/PG-85138/2021	EPI_ISL_3870987	2021-09-02
hCoV-19/Japan/PG-85139/2021	EPI_ISL_3870988	2021-09-02
hCoV-19/Japan/PG-85140/2021	EPI_ISL_3870989	2021-09-02
hCoV-19/Japan/PG-85141/2021	EPI_ISL_3870990	2021-09-02
hCoV-19/Japan/PG-85142/2021	EPI_ISL_3870991	2021-09-02
hCoV-19/Japan/PG-85143/2021	EPI_ISL_3870992	2021-09-02
hCoV-19/Japan/PG-85144/2021	EPI_ISL_3870993	2021-09-02
hCoV-19/Japan/PG-85145/2021	EPI_ISL_3870994	2021-09-02
hCoV-19/Japan/PG-85146/2021	EPI_ISL_3897253	2021-09-02

virus_name	epiID	submission_date
hCoV-19/Japan/PG-85147/2021	EPI_ISL_3897254	2021-09-02
hCoV-19/Japan/PG-85148/2021	EPI_ISL_3897255	2021-09-02
hCoV-19/Japan/PG-85149/2021	EPI_ISL_3897256	2021-09-02
hCoV-19/Japan/PG-85150/2021	EPI_ISL_3897257	2021-09-02
hCoV-19/Japan/PG-85151/2021	EPI_ISL_3897258	2021-09-02
hCoV-19/Japan/PG-85152/2021	EPI_ISL_3897259	2021-09-02
hCoV-19/Japan/PG-85153/2021	EPI_ISL_3897260	2021-09-02
hCoV-19/Japan/PG-85154/2021	EPI_ISL_3897261	2021-09-02
hCoV-19/Japan/PG-85155/2021	EPI_ISL_3897262	2021-09-02
hCoV-19/Japan/PG-85156/2021	EPI_ISL_3897263	2021-09-02
hCoV-19/Japan/PG-85157/2021	EPI_ISL_3897264	2021-09-02
hCoV-19/Japan/PG-85158/2021	EPI_ISL_3897265	2021-09-02
hCoV-19/Japan/PG-85159/2021	EPI_ISL_3897266	2021-09-02
hCoV-19/Japan/PG-85160/2021	EPI_ISL_3897267	2021-09-02
hCoV-19/Japan/PG-85161/2021	EPI_ISL_3897268	2021-09-02
hCoV-19/Japan/PG-85162/2021	EPI_ISL_3897269	2021-09-02
hCoV-19/Japan/PG-85163/2021	EPI_ISL_3897270	2021-09-02
hCoV-19/Japan/PG-85164/2021	EPI_ISL_3897271	2021-09-02
hCoV-19/Japan/PG-85165/2021	EPI_ISL_3897272	2021-09-02
hCoV-19/Japan/PG-85166/2021	EPI_ISL_3897273	2021-09-02
hCoV-19/Japan/PG-85167/2021	EPI_ISL_3897274	2021-09-02
hCoV-19/Japan/PG-85168/2021	EPI_ISL_3897275	2021-09-02
hCoV-19/Japan/PG-85169/2021	EPI_ISL_3897276	2021-09-02
hCoV-19/Japan/PG-85170/2021	EPI_ISL_3897277	2021-09-02
hCoV-19/Japan/PG-85171/2021	EPI_ISL_3897278	2021-09-02
hCoV-19/Japan/PG-85172/2021	EPI_ISL_3897279	2021-09-02
hCoV-19/Japan/PG-85173/2021	EPI_ISL_3897280	2021-09-02
hCoV-19/Japan/PG-85174/2021	EPI_ISL_3897281	2021-09-02
hCoV-19/Japan/PG-85175/2021	EPI_ISL_3897282	2021-09-02
hCoV-19/Japan/PG-85176/2021	EPI_ISL_3897283	2021-09-02
hCoV-19/Japan/PG-85177/2021	EPI_ISL_3897284	2021-09-02
hCoV-19/Japan/PG-85178/2021	EPI_ISL_3897285	2021-09-02
hCoV-19/Japan/PG-85179/2021	EPI_ISL_3897286	2021-09-02
hCoV-19/Japan/PG-85180/2021	EPI_ISL_3897287	2021-09-02
hCoV-19/Japan/PG-85181/2021	EPI_ISL_3897288	2021-09-02
hCoV-19/Japan/PG-85182/2021	EPI_ISL_3897289	2021-09-02
hCoV-19/Japan/PG-85183/2021	EPI_ISL_3897290	2021-09-02
hCoV-19/Japan/PG-85184/2021	EPI_ISL_3897291	2021-09-02
hCoV-19/Japan/PG-85185/2021	EPI_ISL_3897292	2021-09-02
hCoV-19/Japan/PG-85186/2021	EPI_ISL_3897293	2021-09-02
hCoV-19/Japan/PG-85187/2021	EPI_ISL_3897294	2021-09-02
hCoV-19/Japan/PG-85188/2021	EPI_ISL_3897295	2021-09-02
hCoV-19/Japan/PG-85189/2021	EPI_ISL_3897296	2021-09-02
hCoV-19/Japan/PG-85190/2021	EPI_ISL_3897297	2021-09-02
hCoV-19/Japan/PG-85191/2021	EPI_ISL_3897298	2021-09-02
hCoV-19/Japan/PG-85192/2021	EPI_ISL_3897299	2021-09-02
hCoV-19/Japan/PG-85193/2021	EPI_ISL_3897300	2021-09-02
hCoV-19/Japan/PG-85194/2021	EPI_ISL_3897301	2021-09-02
hCoV-19/Japan/PG-85195/2021	EPI_ISL_3897302	2021-09-02
hCoV-19/Japan/PG-85196/2021	EPI_ISL_3897303	2021-09-02
hCoV-19/Japan/PG-85197/2021	EPI_ISL_3897304	2021-09-02
hCoV-19/Japan/PG-85198/2021	EPI_ISL_3897305	2021-09-02
hCoV-19/Japan/PG-85199/2021	EPI_ISL_3897306	2021-09-02
hCoV-19/Japan/PG-85200/2021	EPI_ISL_3897307	2021-09-02

virus_name	epiID	submission_date
hCoV-19/Japan/PG-85201/2021	EPI_ISL_3897308	2021-09-02
hCoV-19/Japan/PG-85202/2021	EPI_ISL_3897309	2021-09-02
hCoV-19/Japan/PG-85203/2021	EPI_ISL_3897310	2021-09-02
hCoV-19/Japan/PG-85204/2021	EPI_ISL_3897311	2021-09-02
hCoV-19/Japan/PG-85205/2021	EPI_ISL_3897312	2021-09-02
hCoV-19/Japan/PG-85206/2021	EPI_ISL_3897313	2021-09-02
hCoV-19/Japan/PG-85207/2021	EPI_ISL_3897314	2021-09-02
hCoV-19/Japan/PG-85208/2021	EPI_ISL_3897315	2021-09-02
hCoV-19/Japan/PG-85209/2021	EPI_ISL_3897316	2021-09-02
hCoV-19/Japan/PG-85210/2021	EPI_ISL_3897317	2021-09-02
hCoV-19/Japan/PG-85211/2021	EPI_ISL_3897318	2021-09-02
hCoV-19/Japan/PG-85212/2021	EPI_ISL_3897319	2021-09-02
hCoV-19/Japan/PG-85213/2021	EPI_ISL_3897320	2021-09-02
hCoV-19/Japan/PG-85214/2021	EPI_ISL_3897321	2021-09-02
hCoV-19/Japan/PG-85215/2021	EPI_ISL_3897322	2021-09-02
hCoV-19/Japan/PG-85216/2021	EPI_ISL_3897323	2021-09-02
hCoV-19/Japan/PG-85217/2021	EPI_ISL_3897324	2021-09-02
hCoV-19/Japan/PG-85218/2021	EPI_ISL_3897325	2021-09-02
hCoV-19/Japan/PG-85219/2021	EPI_ISL_3897326	2021-09-02
hCoV-19/Japan/PG-85220/2021	EPI_ISL_3897327	2021-09-02
hCoV-19/Japan/PG-85221/2021	EPI_ISL_3897328	2021-09-02
hCoV-19/Japan/PG-85222/2021	EPI_ISL_3897329	2021-09-02
hCoV-19/Japan/PG-85223/2021	EPI_ISL_3897330	2021-09-02
hCoV-19/Japan/PG-85224/2021	EPI_ISL_3897331	2021-09-02
hCoV-19/Japan/PG-85225/2021	EPI_ISL_3897332	2021-09-02
hCoV-19/Japan/PG-85226/2021	EPI_ISL_3897333	2021-09-02
hCoV-19/Japan/PG-85227/2021	EPI_ISL_3897334	2021-09-02
hCoV-19/Japan/PG-85228/2021	EPI_ISL_3897335	2021-09-02
hCoV-19/Japan/PG-85229/2021	EPI_ISL_3897336	2021-09-02
hCoV-19/Japan/PG-85230/2021	EPI_ISL_3897337	2021-09-02
hCoV-19/Japan/PG-85231/2021	EPI_ISL_3897338	2021-09-02
hCoV-19/Japan/PG-85232/2021	EPI_ISL_3897339	2021-09-02
hCoV-19/Japan/PG-85233/2021	EPI_ISL_3897340	2021-09-02
hCoV-19/Japan/PG-85234/2021	EPI_ISL_3897341	2021-09-02
hCoV-19/Japan/PG-85235/2021	EPI_ISL_3897342	2021-09-02
hCoV-19/Japan/PG-85236/2021	EPI_ISL_3897343	2021-09-02
hCoV-19/Japan/PG-85237/2021	EPI_ISL_3897344	2021-09-02
hCoV-19/Japan/PG-85238/2021	EPI_ISL_3897345	2021-09-02
hCoV-19/Japan/PG-85239/2021	EPI_ISL_3897346	2021-09-02
hCoV-19/Japan/PG-85240/2021	EPI_ISL_3897347	2021-09-02
hCoV-19/Japan/PG-85241/2021	EPI_ISL_3897348	2021-09-02
hCoV-19/Japan/PG-85242/2021	EPI_ISL_3897349	2021-09-02
hCoV-19/Japan/PG-85243/2021	EPI_ISL_3897350	2021-09-02
hCoV-19/Japan/PG-85244/2021	EPI_ISL_3897351	2021-09-02
hCoV-19/Japan/PG-85245/2021	EPI_ISL_3897352	2021-09-02
hCoV-19/Japan/PG-85246/2021	EPI_ISL_3897353	2021-09-02
hCoV-19/Japan/PG-85247/2021	EPI_ISL_3897354	2021-09-02
hCoV-19/Japan/PG-85248/2021	EPI_ISL_3897355	2021-09-02
hCoV-19/Japan/PG-85249/2021	EPI_ISL_3897356	2021-09-02
hCoV-19/Japan/PG-85250/2021	EPI_ISL_3897357	2021-09-02
hCoV-19/Japan/PG-85251/2021	EPI_ISL_3897358	2021-09-02
hCoV-19/Japan/PG-85252/2021	EPI_ISL_3897359	2021-09-02
hCoV-19/Japan/PG-85253/2021	EPI_ISL_3897360	2021-09-02
hCoV-19/Japan/PG-85254/2021	EPI_ISL_3897362	2021-09-02

virus_name	epiID	submission_date
hCoV-19/Japan/PG-85255/2021	EPI_ISL_3897363	2021-09-02
hCoV-19/Japan/PG-85256/2021	EPI_ISL_3897364	2021-09-02
hCoV-19/Japan/PG-85257/2021	EPI_ISL_3897365	2021-09-02
hCoV-19/Japan/PG-85258/2021	EPI_ISL_3897366	2021-09-02
hCoV-19/Japan/PG-85259/2021	EPI_ISL_3897367	2021-09-02
hCoV-19/Japan/PG-85260/2021	EPI_ISL_3897368	2021-09-02
hCoV-19/Japan/PG-85261/2021	EPI_ISL_3897369	2021-09-02
hCoV-19/Japan/PG-85262/2021	EPI_ISL_3897370	2021-09-02
hCoV-19/Japan/PG-85263/2021	EPI_ISL_3897371	2021-09-02
hCoV-19/Japan/PG-85264/2021	EPI_ISL_3897372	2021-09-02
hCoV-19/Japan/PG-85265/2021	EPI_ISL_3897373	2021-09-02
hCoV-19/Japan/PG-85266/2021	EPI_ISL_3897374	2021-09-02
hCoV-19/Japan/PG-85267/2021	EPI_ISL_3897375	2021-09-02
hCoV-19/Japan/PG-85268/2021	EPI_ISL_3897376	2021-09-02
hCoV-19/Japan/PG-85269/2021	EPI_ISL_3897377	2021-09-02
hCoV-19/Japan/PG-85270/2021	EPI_ISL_3897378	2021-09-02
hCoV-19/Japan/PG-85271/2021	EPI_ISL_3897379	2021-09-02
hCoV-19/Japan/PG-85272/2021	EPI_ISL_3897380	2021-09-02
hCoV-19/Japan/PG-85273/2021	EPI_ISL_3897381	2021-09-02
hCoV-19/Japan/PG-85274/2021	EPI_ISL_3897382	2021-09-02
hCoV-19/Japan/PG-85275/2021	EPI_ISL_3897383	2021-09-02
hCoV-19/Japan/PG-85276/2021	EPI_ISL_3897384	2021-09-02
hCoV-19/Japan/PG-85277/2021	EPI_ISL_3897385	2021-09-02
hCoV-19/Japan/PG-85278/2021	EPI_ISL_3897386	2021-09-02
hCoV-19/Japan/PG-85279/2021	EPI_ISL_3897387	2021-09-02
hCoV-19/Japan/PG-85280/2021	EPI_ISL_3897388	2021-09-02
hCoV-19/Japan/PG-85281/2021	EPI_ISL_3897389	2021-09-02
hCoV-19/Japan/PG-85282/2021	EPI_ISL_3897392	2021-09-02
hCoV-19/Japan/PG-85283/2021	EPI_ISL_3897395	2021-09-02
hCoV-19/Japan/PG-85284/2021	EPI_ISL_3897398	2021-09-02
hCoV-19/Japan/PG-85285/2021	EPI_ISL_3897401	2021-09-02
hCoV-19/Japan/PG-85286/2021	EPI_ISL_3897405	2021-09-02
hCoV-19/Japan/PG-85287/2021	EPI_ISL_3897407	2021-09-02
hCoV-19/Japan/PG-85288/2021	EPI_ISL_3897409	2021-09-02
hCoV-19/Japan/PG-85289/2021	EPI_ISL_3897410	2021-09-02
hCoV-19/Japan/PG-85290/2021	EPI_ISL_3897412	2021-09-02
hCoV-19/Japan/PG-85291/2021	EPI_ISL_3897413	2021-09-02
hCoV-19/Japan/PG-85292/2021	EPI_ISL_3897415	2021-09-02
hCoV-19/Japan/PG-85293/2021	EPI_ISL_3897416	2021-09-02
hCoV-19/Japan/PG-85294/2021	EPI_ISL_3897417	2021-09-02
hCoV-19/Japan/PG-85295/2021	EPI_ISL_3897419	2021-09-02
hCoV-19/Japan/PG-85296/2021	EPI_ISL_3897420	2021-09-02
hCoV-19/Japan/PG-85297/2021	EPI_ISL_3897422	2021-09-02
hCoV-19/Japan/PG-85298/2021	EPI_ISL_3897423	2021-09-02
hCoV-19/Japan/PG-85299/2021	EPI_ISL_3897424	2021-09-02
hCoV-19/Japan/PG-85300/2021	EPI_ISL_3897426	2021-09-02
hCoV-19/Japan/PG-85301/2021	EPI_ISL_3897427	2021-09-02
hCoV-19/Japan/PG-85302/2021	EPI_ISL_3897429	2021-09-02
hCoV-19/Japan/PG-85303/2021	EPI_ISL_3897430	2021-09-02
hCoV-19/Japan/PG-85304/2021	EPI_ISL_3897431	2021-09-02
hCoV-19/Japan/PG-85305/2021	EPI_ISL_3897434	2021-09-02
hCoV-19/Japan/PG-85306/2021	EPI_ISL_3897437	2021-09-02
hCoV-19/Japan/PG-85307/2021	EPI_ISL_3897441	2021-09-02
hCoV-19/Japan/PG-85308/2021	EPI_ISL_3897444	2021-09-02

virus_name	epiID	submission_date
hCoV-19/Japan/PG-85309/2021	EPI_ISL_3897447	2021-09-02
hCoV-19/Japan/PG-85310/2021	EPI_ISL_3897449	2021-09-02
hCoV-19/Japan/PG-85311/2021	EPI_ISL_3897450	2021-09-02
hCoV-19/Japan/PG-85312/2021	EPI_ISL_3897452	2021-09-02
hCoV-19/Japan/PG-85313/2021	EPI_ISL_3897453	2021-09-02
hCoV-19/Japan/PG-85314/2021	EPI_ISL_3897455	2021-09-02
hCoV-19/Japan/PG-85315/2021	EPI_ISL_3897456	2021-09-02
hCoV-19/Japan/PG-85316/2021	EPI_ISL_3897457	2021-09-02
hCoV-19/Japan/PG-85317/2021	EPI_ISL_3897459	2021-09-02
hCoV-19/Japan/PG-85318/2021	EPI_ISL_3897460	2021-09-02
hCoV-19/Japan/PG-85319/2021	EPI_ISL_3897462	2021-09-02
hCoV-19/Japan/PG-85320/2021	EPI_ISL_3897463	2021-09-02
hCoV-19/Japan/PG-85321/2021	EPI_ISL_3897465	2021-09-02
hCoV-19/Japan/PG-85322/2021	EPI_ISL_3897466	2021-09-02
hCoV-19/Japan/PG-85323/2021	EPI_ISL_3897467	2021-09-02
hCoV-19/Japan/PG-85324/2021	EPI_ISL_3897469	2021-09-02
hCoV-19/Japan/PG-85325/2021	EPI_ISL_3897470	2021-09-02
hCoV-19/Japan/PG-85326/2021	EPI_ISL_3897472	2021-09-02
hCoV-19/Japan/PG-85327/2021	EPI_ISL_3897473	2021-09-02
hCoV-19/Japan/PG-85328/2021	EPI_ISL_3897475	2021-09-02
hCoV-19/Japan/PG-85329/2021	EPI_ISL_3897476	2021-09-02
hCoV-19/Japan/PG-85330/2021	EPI_ISL_3897477	2021-09-02
hCoV-19/Japan/PG-85331/2021	EPI_ISL_3897479	2021-09-02
hCoV-19/Japan/PG-85332/2021	EPI_ISL_3897480	2021-09-02
hCoV-19/Japan/PG-85333/2021	EPI_ISL_3897482	2021-09-02
hCoV-19/Japan/PG-85334/2021	EPI_ISL_3897483	2021-09-02
hCoV-19/Japan/PG-85335/2021	EPI_ISL_3897484	2021-09-02
hCoV-19/Japan/PG-85336/2021	EPI_ISL_3897486	2021-09-02
hCoV-19/Japan/PG-85337/2021	EPI_ISL_3897487	2021-09-02
hCoV-19/Japan/PG-85338/2021	EPI_ISL_3897489	2021-09-02
hCoV-19/Japan/PG-85339/2021	EPI_ISL_3897490	2021-09-02
hCoV-19/Japan/PG-85340/2021	EPI_ISL_3897492	2021-09-02
hCoV-19/Japan/PG-85341/2021	EPI_ISL_3897493	2021-09-02
hCoV-19/Japan/PG-85342/2021	EPI_ISL_3897495	2021-09-02
hCoV-19/Japan/PG-85343/2021	EPI_ISL_3897496	2021-09-02
hCoV-19/Japan/PG-85344/2021	EPI_ISL_3897498	2021-09-02
hCoV-19/Japan/PG-85345/2021	EPI_ISL_3897501	2021-09-02
hCoV-19/Japan/PG-85346/2021	EPI_ISL_3897504	2021-09-02
hCoV-19/Japan/PG-85347/2021	EPI_ISL_3897506	2021-09-02
hCoV-19/Japan/PG-85348/2021	EPI_ISL_3897507	2021-09-02
hCoV-19/Japan/PG-85349/2021	EPI_ISL_3897509	2021-09-02
hCoV-19/Japan/PG-85350/2021	EPI_ISL_3897510	2021-09-02
hCoV-19/Japan/PG-85351/2021	EPI_ISL_3897512	2021-09-02
hCoV-19/Japan/PG-85352/2021	EPI_ISL_3897513	2021-09-02
hCoV-19/Japan/PG-85353/2021	EPI_ISL_3897515	2021-09-02
hCoV-19/Japan/PG-85354/2021	EPI_ISL_3897516	2021-09-02
hCoV-19/Japan/PG-85355/2021	EPI_ISL_3897518	2021-09-02
hCoV-19/Japan/PG-85356/2021	EPI_ISL_3897519	2021-09-02
hCoV-19/Japan/PG-85357/2021	EPI_ISL_3897521	2021-09-02
hCoV-19/Japan/PG-85358/2021	EPI_ISL_3897522	2021-09-02
hCoV-19/Japan/PG-85359/2021	EPI_ISL_3897524	2021-09-02
hCoV-19/Japan/PG-85360/2021	EPI_ISL_3897527	2021-09-02
hCoV-19/Japan/PG-85361/2021	EPI_ISL_3897531	2021-09-02
hCoV-19/Japan/PG-85362/2021	EPI_ISL_3897534	2021-09-02

virus_name	epiID	submission_date
hCoV-19/Japan/PG-85363/2021	EPI_ISL_3897537	2021-09-02
hCoV-19/Japan/PG-85364/2021	EPI_ISL_3897540	2021-09-02
hCoV-19/Japan/PG-85365/2021	EPI_ISL_3897541	2021-09-02
hCoV-19/Japan/PG-85366/2021	EPI_ISL_3897542	2021-09-02
hCoV-19/Japan/PG-85367/2021	EPI_ISL_3897544	2021-09-02
hCoV-19/Japan/PG-85368/2021	EPI_ISL_3897545	2021-09-02
hCoV-19/Japan/PG-85369/2021	EPI_ISL_3897547	2021-09-02
hCoV-19/Japan/PG-85370/2021	EPI_ISL_3897548	2021-09-02
hCoV-19/Japan/PG-85371/2021	EPI_ISL_3897550	2021-09-02
hCoV-19/Japan/PG-85372/2021	EPI_ISL_3897551	2021-09-02
hCoV-19/Japan/PG-85373/2021	EPI_ISL_3897553	2021-09-02
hCoV-19/Japan/PG-85374/2021	EPI_ISL_3897554	2021-09-02
hCoV-19/Japan/PG-85375/2021	EPI_ISL_3897555	2021-09-02
hCoV-19/Japan/PG-85376/2021	EPI_ISL_3897557	2021-09-02
hCoV-19/Japan/PG-85377/2021	EPI_ISL_3897558	2021-09-02
hCoV-19/Japan/PG-85378/2021	EPI_ISL_3897560	2021-09-02
hCoV-19/Japan/PG-85379/2021	EPI_ISL_3897561	2021-09-02
hCoV-19/Japan/PG-85380/2021	EPI_ISL_3897563	2021-09-02
hCoV-19/Japan/PG-85381/2021	EPI_ISL_3897564	2021-09-02
hCoV-19/Japan/PG-85382/2021	EPI_ISL_3897566	2021-09-02
hCoV-19/Japan/PG-85383/2021	EPI_ISL_3897567	2021-09-02
hCoV-19/Japan/PG-85384/2021	EPI_ISL_3897569	2021-09-02
hCoV-19/Japan/PG-85385/2021	EPI_ISL_3897570	2021-09-02
hCoV-19/Japan/PG-85386/2021	EPI_ISL_3897572	2021-09-02
hCoV-19/Japan/PG-85387/2021	EPI_ISL_3897573	2021-09-02
hCoV-19/Japan/PG-85388/2021	EPI_ISL_3897575	2021-09-02
hCoV-19/Japan/PG-85389/2021	EPI_ISL_3897576	2021-09-02
hCoV-19/Japan/PG-85390/2021	EPI_ISL_3897579	2021-09-02
hCoV-19/Japan/PG-85391/2021	EPI_ISL_3897582	2021-09-02
hCoV-19/Japan/PG-85392/2021	EPI_ISL_3897585	2021-09-02
hCoV-19/Japan/PG-85393/2021	EPI_ISL_3897589	2021-09-02
hCoV-19/Japan/PG-85394/2021	EPI_ISL_3897592	2021-09-02
hCoV-19/Japan/PG-85395/2021	EPI_ISL_3897593	2021-09-02
hCoV-19/Japan/PG-85396/2021	EPI_ISL_3897595	2021-09-02
hCoV-19/Japan/PG-85397/2021	EPI_ISL_3897596	2021-09-02
hCoV-19/Japan/PG-85398/2021	EPI_ISL_3897599	2021-09-02
hCoV-19/Japan/PG-85399/2021	EPI_ISL_3897602	2021-09-02
hCoV-19/Japan/PG-85400/2021	EPI_ISL_3897606	2021-09-02
hCoV-19/Japan/PG-85401/2021	EPI_ISL_3897609	2021-09-02
hCoV-19/Japan/PG-85402/2021	EPI_ISL_3897613	2021-09-02
hCoV-19/Japan/PG-85403/2021	EPI_ISL_3897616	2021-09-02
hCoV-19/Japan/PG-85404/2021	EPI_ISL_3897620	2021-09-02
hCoV-19/Japan/PG-85405/2021	EPI_ISL_3897623	2021-09-02
hCoV-19/Japan/PG-85406/2021	EPI_ISL_3897627	2021-09-02
hCoV-19/Japan/PG-85407/2021	EPI_ISL_3897628	2021-09-02
hCoV-19/Japan/PG-85408/2021	EPI_ISL_3897629	2021-09-02
hCoV-19/Japan/PG-85409/2021	EPI_ISL_3897631	2021-09-02
hCoV-19/Japan/PG-85410/2021	EPI_ISL_3897632	2021-09-02
hCoV-19/Japan/PG-85411/2021	EPI_ISL_3897635	2021-09-02
hCoV-19/Japan/PG-85412/2021	EPI_ISL_3897638	2021-09-02
hCoV-19/Japan/PG-85413/2021	EPI_ISL_3897641	2021-09-02
hCoV-19/Japan/PG-85414/2021	EPI_ISL_3897643	2021-09-02
hCoV-19/Japan/PG-85415/2021	EPI_ISL_3897644	2021-09-02
hCoV-19/Japan/PG-85416/2021	EPI_ISL_3897646	2021-09-02

virus_name	epiID	submission_date
hCoV-19/Japan/PG-85417/2021	EPI_ISL_3897647	2021-09-02
hCoV-19/Japan/PG-85418/2021	EPI_ISL_3897648	2021-09-02
hCoV-19/Japan/PG-85419/2021	EPI_ISL_3897650	2021-09-02
hCoV-19/Japan/PG-85420/2021	EPI_ISL_3897651	2021-09-02
hCoV-19/Japan/PG-85421/2021	EPI_ISL_3897653	2021-09-02
hCoV-19/Japan/PG-85422/2021	EPI_ISL_3897654	2021-09-02
hCoV-19/Japan/PG-85423/2021	EPI_ISL_3897655	2021-09-02
hCoV-19/Japan/PG-85424/2021	EPI_ISL_3897659	2021-09-02
hCoV-19/Japan/PG-85425/2021	EPI_ISL_3897662	2021-09-02
hCoV-19/Japan/PG-85426/2021	EPI_ISL_3897665	2021-09-02
hCoV-19/Japan/PG-85427/2021	EPI_ISL_3897669	2021-09-02
hCoV-19/Japan/PG-85428/2021	EPI_ISL_3897672	2021-09-02
hCoV-19/Japan/PG-85429/2021	EPI_ISL_3897676	2021-09-02
hCoV-19/Japan/PG-85430/2021	EPI_ISL_3897679	2021-09-02
hCoV-19/Japan/PG-85431/2021	EPI_ISL_3897683	2021-09-02
hCoV-19/Japan/PG-85432/2021	EPI_ISL_3897686	2021-09-02
hCoV-19/Japan/PG-85433/2021	EPI_ISL_3897689	2021-09-02
hCoV-19/Japan/PG-85434/2021	EPI_ISL_3897692	2021-09-02
hCoV-19/Japan/PG-85435/2021	EPI_ISL_3897696	2021-09-02
hCoV-19/Japan/PG-85436/2021	EPI_ISL_3897699	2021-09-02
hCoV-19/Japan/PG-85437/2021	EPI_ISL_3897703	2021-09-02
hCoV-19/Japan/PG-85438/2021	EPI_ISL_3897706	2021-09-02
hCoV-19/Japan/PG-85439/2021	EPI_ISL_3897710	2021-09-02
hCoV-19/Japan/PG-85440/2021	EPI_ISL_3897713	2021-09-02
hCoV-19/Japan/PG-85441/2021	EPI_ISL_3897717	2021-09-02
hCoV-19/Japan/PG-85442/2021	EPI_ISL_3897718	2021-09-02
hCoV-19/Japan/PG-85443/2021	EPI_ISL_3897720	2021-09-02
hCoV-19/Japan/PG-85444/2021	EPI_ISL_3897721	2021-09-02
hCoV-19/Japan/PG-85445/2021	EPI_ISL_3897722	2021-09-02
hCoV-19/Japan/PG-85446/2021	EPI_ISL_3897724	2021-09-02
hCoV-19/Japan/PG-85447/2021	EPI_ISL_3897725	2021-09-02
hCoV-19/Japan/PG-85448/2021	EPI_ISL_3897727	2021-09-02
hCoV-19/Japan/PG-85449/2021	EPI_ISL_3897728	2021-09-02
hCoV-19/Japan/PG-85450/2021	EPI_ISL_3897729	2021-09-02
hCoV-19/Japan/PG-85451/2021	EPI_ISL_3897731	2021-09-02
hCoV-19/Japan/PG-85452/2021	EPI_ISL_3897732	2021-09-02
hCoV-19/Japan/PG-85453/2021	EPI_ISL_3897733	2021-09-02
hCoV-19/Japan/PG-85454/2021	EPI_ISL_3897736	2021-09-02
hCoV-19/Japan/PG-85455/2021	EPI_ISL_3897739	2021-09-02
hCoV-19/Japan/PG-85456/2021	EPI_ISL_3897742	2021-09-02
hCoV-19/Japan/PG-85457/2021	EPI_ISL_3897743	2021-09-02
hCoV-19/Japan/PG-85458/2021	EPI_ISL_3897744	2021-09-02
hCoV-19/Japan/PG-85459/2021	EPI_ISL_3897747	2021-09-02
hCoV-19/Japan/PG-85460/2021	EPI_ISL_3897751	2021-09-02
hCoV-19/Japan/PG-85461/2021	EPI_ISL_3897752	2021-09-02
hCoV-19/Japan/PG-85462/2021	EPI_ISL_3897753	2021-09-02
hCoV-19/Japan/PG-85463/2021	EPI_ISL_3897754	2021-09-02
hCoV-19/Japan/PG-85464/2021	EPI_ISL_3897755	2021-09-02
hCoV-19/Japan/PG-85465/2021	EPI_ISL_3897756	2021-09-02
hCoV-19/Japan/PG-85466/2021	EPI_ISL_3897757	2021-09-02
hCoV-19/Japan/PG-85467/2021	EPI_ISL_3897758	2021-09-02
hCoV-19/Japan/PG-85468/2021	EPI_ISL_3897759	2021-09-02
hCoV-19/Japan/PG-85469/2021	EPI_ISL_3897760	2021-09-02
hCoV-19/Japan/PG-85470/2021	EPI_ISL_3897761	2021-09-02

virus_name	epiID	submission_date
hCoV-19/Japan/PG-85471/2021	EPI_ISL_3897762	2021-09-02
hCoV-19/Japan/PG-85472/2021	EPI_ISL_3897763	2021-09-02
hCoV-19/Japan/PG-85473/2021	EPI_ISL_3897764	2021-09-02
hCoV-19/Japan/PG-85474/2021	EPI_ISL_3897765	2021-09-02
hCoV-19/Japan/PG-85475/2021	EPI_ISL_3897766	2021-09-02
hCoV-19/Japan/PG-85476/2021	EPI_ISL_3897767	2021-09-02
hCoV-19/Japan/PG-85477/2021	EPI_ISL_3897768	2021-09-02
hCoV-19/Japan/PG-85478/2021	EPI_ISL_3897769	2021-09-02
hCoV-19/Japan/PG-85479/2021	EPI_ISL_3897770	2021-09-02
hCoV-19/Japan/PG-85480/2021	EPI_ISL_3897771	2021-09-02
hCoV-19/Japan/PG-85481/2021	EPI_ISL_3897772	2021-09-02
hCoV-19/Japan/PG-85482/2021	EPI_ISL_3897773	2021-09-02
hCoV-19/Japan/PG-85483/2021	EPI_ISL_3897774	2021-09-02
hCoV-19/Japan/PG-85484/2021	EPI_ISL_3897775	2021-09-02
hCoV-19/Japan/PG-85485/2021	EPI_ISL_3897776	2021-09-02
hCoV-19/Japan/PG-85486/2021	EPI_ISL_3897777	2021-09-02
hCoV-19/Japan/PG-85487/2021	EPI_ISL_3897778	2021-09-02
hCoV-19/Japan/PG-85488/2021	EPI_ISL_3897779	2021-09-02
hCoV-19/Japan/PG-85489/2021	EPI_ISL_3897780	2021-09-02
hCoV-19/Japan/PG-85490/2021	EPI_ISL_3897781	2021-09-02
hCoV-19/Japan/PG-85491/2021	EPI_ISL_3897782	2021-09-02
hCoV-19/Japan/PG-85492/2021	EPI_ISL_3897783	2021-09-02
hCoV-19/Japan/PG-85493/2021	EPI_ISL_3897784	2021-09-02
hCoV-19/Japan/PG-85494/2021	EPI_ISL_3897785	2021-09-02
hCoV-19/Japan/PG-85495/2021	EPI_ISL_3897786	2021-09-02
hCoV-19/Japan/PG-85496/2021	EPI_ISL_3897787	2021-09-02
hCoV-19/Japan/PG-85497/2021	EPI_ISL_3897788	2021-09-02
hCoV-19/Japan/PG-85498/2021	EPI_ISL_3897789	2021-09-02
hCoV-19/Japan/PG-85499/2021	EPI_ISL_3897790	2021-09-02
hCoV-19/Japan/PG-85500/2021	EPI_ISL_3897791	2021-09-02
hCoV-19/Japan/PG-85501/2021	EPI_ISL_3897792	2021-09-02
hCoV-19/Japan/PG-85502/2021	EPI_ISL_3897793	2021-09-02
hCoV-19/Japan/PG-85503/2021	EPI_ISL_3897794	2021-09-02
hCoV-19/Japan/PG-85504/2021	EPI_ISL_3897795	2021-09-02
hCoV-19/Japan/PG-85505/2021	EPI_ISL_3897796	2021-09-02
hCoV-19/Japan/PG-85506/2021	EPI_ISL_3897797	2021-09-02
hCoV-19/Japan/PG-85507/2021	EPI_ISL_3897798	2021-09-02
hCoV-19/Japan/PG-85508/2021	EPI_ISL_3897799	2021-09-02
hCoV-19/Japan/PG-85509/2021	EPI_ISL_3897800	2021-09-02
hCoV-19/Japan/PG-85510/2021	EPI_ISL_3897801	2021-09-02
hCoV-19/Japan/PG-85511/2021	EPI_ISL_3897802	2021-09-02
hCoV-19/Japan/PG-85512/2021	EPI_ISL_3897803	2021-09-02
hCoV-19/Japan/PG-85513/2021	EPI_ISL_3897804	2021-09-02
hCoV-19/Japan/PG-85514/2021	EPI_ISL_3897805	2021-09-02
hCoV-19/Japan/PG-85515/2021	EPI_ISL_3897806	2021-09-02
hCoV-19/Japan/PG-85516/2021	EPI_ISL_3897807	2021-09-02
hCoV-19/Japan/PG-85517/2021	EPI_ISL_3897808	2021-09-02
hCoV-19/Japan/PG-85518/2021	EPI_ISL_3897809	2021-09-02
hCoV-19/Japan/PG-85519/2021	EPI_ISL_3897810	2021-09-02
hCoV-19/Japan/PG-85520/2021	EPI_ISL_3897811	2021-09-02
hCoV-19/Japan/PG-85521/2021	EPI_ISL_3897812	2021-09-02
hCoV-19/Japan/PG-85522/2021	EPI_ISL_3897813	2021-09-02
hCoV-19/Japan/PG-85523/2021	EPI_ISL_3897814	2021-09-02
hCoV-19/Japan/PG-85524/2021	EPI_ISL_3897815	2021-09-02

virus_name	epiID	submission_date
hCoV-19/Japan/PG-85525/2021	EPI_ISL_3897816	2021-09-02
hCoV-19/Japan/PG-85526/2021	EPI_ISL_3897817	2021-09-02
hCoV-19/Japan/PG-85527/2021	EPI_ISL_3897818	2021-09-02
hCoV-19/Japan/PG-85528/2021	EPI_ISL_3897819	2021-09-02
hCoV-19/Japan/PG-85529/2021	EPI_ISL_3897820	2021-09-02
hCoV-19/Japan/PG-85530/2021	EPI_ISL_3897821	2021-09-02
hCoV-19/Japan/PG-85531/2021	EPI_ISL_3897822	2021-09-02
hCoV-19/Japan/PG-85532/2021	EPI_ISL_3897823	2021-09-02
hCoV-19/Japan/PG-85533/2021	EPI_ISL_3897824	2021-09-02
hCoV-19/Japan/PG-85534/2021	EPI_ISL_3897825	2021-09-02
hCoV-19/Japan/PG-85535/2021	EPI_ISL_3897826	2021-09-02
hCoV-19/Japan/PG-85536/2021	EPI_ISL_3897827	2021-09-02
hCoV-19/Japan/PG-85537/2021	EPI_ISL_3897828	2021-09-02
hCoV-19/Japan/PG-85538/2021	EPI_ISL_3897829	2021-09-02
hCoV-19/Japan/PG-85539/2021	EPI_ISL_3897830	2021-09-02
hCoV-19/Japan/PG-85540/2021	EPI_ISL_3897831	2021-09-02
hCoV-19/Japan/PG-85541/2021	EPI_ISL_3897832	2021-09-02
hCoV-19/Japan/PG-85542/2021	EPI_ISL_3897833	2021-09-02
hCoV-19/Japan/PG-85543/2021	EPI_ISL_3897834	2021-09-02
hCoV-19/Japan/PG-85544/2021	EPI_ISL_3897835	2021-09-02
hCoV-19/Japan/PG-85545/2021	EPI_ISL_3897836	2021-09-02
hCoV-19/Japan/PG-85546/2021	EPI_ISL_3897837	2021-09-02
hCoV-19/Japan/PG-85547/2021	EPI_ISL_3897838	2021-09-02
hCoV-19/Japan/PG-85548/2021	EPI_ISL_3897839	2021-09-02
hCoV-19/Japan/PG-85549/2021	EPI_ISL_3897840	2021-09-02
hCoV-19/Japan/PG-85550/2021	EPI_ISL_3897841	2021-09-02
hCoV-19/Japan/PG-85551/2021	EPI_ISL_3897842	2021-09-02
hCoV-19/Japan/PG-85552/2021	EPI_ISL_3897843	2021-09-02
hCoV-19/Japan/PG-85553/2021	EPI_ISL_3897844	2021-09-02
hCoV-19/Japan/PG-85554/2021	EPI_ISL_3897845	2021-09-02
hCoV-19/Japan/PG-85555/2021	EPI_ISL_3897846	2021-09-02
hCoV-19/Japan/PG-85556/2021	EPI_ISL_3897847	2021-09-02
hCoV-19/Japan/PG-85557/2021	EPI_ISL_3897848	2021-09-02
hCoV-19/Japan/PG-85558/2021	EPI_ISL_3897849	2021-09-02
hCoV-19/Japan/PG-85559/2021	EPI_ISL_3897850	2021-09-02
hCoV-19/Japan/PG-85560/2021	EPI_ISL_3897851	2021-09-02
hCoV-19/Japan/PG-85561/2021	EPI_ISL_3897852	2021-09-02
hCoV-19/Japan/PG-85562/2021	EPI_ISL_3897853	2021-09-02
hCoV-19/Japan/PG-85563/2021	EPI_ISL_3897854	2021-09-02
hCoV-19/Japan/PG-85564/2021	EPI_ISL_3897855	2021-09-02
hCoV-19/Japan/PG-85565/2021	EPI_ISL_3897856	2021-09-02
hCoV-19/Japan/PG-85566/2021	EPI_ISL_3897857	2021-09-02
hCoV-19/Japan/PG-85567/2021	EPI_ISL_3897858	2021-09-02
hCoV-19/Japan/PG-85568/2021	EPI_ISL_3897859	2021-09-02
hCoV-19/Japan/PG-85569/2021	EPI_ISL_3897860	2021-09-02
hCoV-19/Japan/PG-85570/2021	EPI_ISL_3897861	2021-09-02
hCoV-19/Japan/PG-85571/2021	EPI_ISL_3897862	2021-09-02
hCoV-19/Japan/PG-85572/2021	EPI_ISL_3897863	2021-09-02
hCoV-19/Japan/PG-85573/2021	EPI_ISL_3897864	2021-09-02
hCoV-19/Japan/PG-85574/2021	EPI_ISL_3897865	2021-09-02
hCoV-19/Japan/PG-85575/2021	EPI_ISL_3897866	2021-09-02
hCoV-19/Japan/PG-85576/2021	EPI_ISL_3897867	2021-09-02
hCoV-19/Japan/PG-85577/2021	EPI_ISL_3897868	2021-09-02
hCoV-19/Japan/PG-85578/2021	EPI_ISL_3897869	2021-09-02

virus_name	epiID	submission_date
hCoV-19/Japan/PG-85579/2021	EPI_ISL_3897870	2021-09-02
hCoV-19/Japan/PG-85580/2021	EPI_ISL_3897871	2021-09-02
hCoV-19/Japan/PG-85581/2021	EPI_ISL_3897872	2021-09-02
hCoV-19/Japan/PG-85582/2021	EPI_ISL_3897873	2021-09-02
hCoV-19/Japan/PG-85583/2021	EPI_ISL_3897874	2021-09-02
hCoV-19/Japan/PG-85584/2021	EPI_ISL_3897875	2021-09-02
hCoV-19/Japan/PG-85585/2021	EPI_ISL_3897876	2021-09-02
hCoV-19/Japan/PG-85586/2021	EPI_ISL_3897877	2021-09-02
hCoV-19/Japan/PG-85587/2021	EPI_ISL_3897878	2021-09-02
hCoV-19/Japan/PG-85588/2021	EPI_ISL_3897879	2021-09-02
hCoV-19/Japan/PG-85589/2021	EPI_ISL_3897880	2021-09-02
hCoV-19/Japan/PG-85590/2021	EPI_ISL_3897881	2021-09-02
hCoV-19/Japan/PG-85591/2021	EPI_ISL_3897882	2021-09-02
hCoV-19/Japan/PG-85592/2021	EPI_ISL_3897883	2021-09-02
hCoV-19/Japan/PG-85593/2021	EPI_ISL_3897884	2021-09-02
hCoV-19/Japan/PG-85594/2021	EPI_ISL_3897885	2021-09-02
hCoV-19/Japan/PG-85595/2021	EPI_ISL_3897886	2021-09-02
hCoV-19/Japan/PG-85596/2021	EPI_ISL_3897887	2021-09-02
hCoV-19/Japan/PG-85597/2021	EPI_ISL_3897888	2021-09-02
hCoV-19/Japan/PG-85598/2021	EPI_ISL_3897889	2021-09-02
hCoV-19/Japan/PG-85599/2021	EPI_ISL_3897890	2021-09-02
hCoV-19/Japan/PG-85600/2021	EPI_ISL_3897891	2021-09-02
hCoV-19/Japan/PG-85601/2021	EPI_ISL_3897892	2021-09-02
hCoV-19/Japan/PG-85602/2021	EPI_ISL_3897893	2021-09-02
hCoV-19/Japan/PG-85603/2021	EPI_ISL_3897894	2021-09-02
hCoV-19/Japan/PG-85604/2021	EPI_ISL_3897895	2021-09-02
hCoV-19/Japan/PG-85605/2021	EPI_ISL_3897896	2021-09-02
hCoV-19/Japan/PG-85606/2021	EPI_ISL_3897897	2021-09-02
hCoV-19/Japan/PG-85607/2021	EPI_ISL_3897898	2021-09-02
hCoV-19/Japan/PG-85608/2021	EPI_ISL_3897899	2021-09-02
hCoV-19/Japan/PG-85609/2021	EPI_ISL_3897900	2021-09-02
hCoV-19/Japan/PG-85610/2021	EPI_ISL_3897901	2021-09-02
hCoV-19/Japan/PG-85611/2021	EPI_ISL_3897902	2021-09-02
hCoV-19/Japan/PG-85612/2021	EPI_ISL_3897903	2021-09-02
hCoV-19/Japan/PG-85613/2021	EPI_ISL_3897904	2021-09-02
hCoV-19/Japan/PG-85614/2021	EPI_ISL_3897905	2021-09-02
hCoV-19/Japan/PG-85615/2021	EPI_ISL_3897906	2021-09-02
hCoV-19/Japan/PG-85616/2021	EPI_ISL_3897907	2021-09-02
hCoV-19/Japan/PG-85617/2021	EPI_ISL_3897908	2021-09-02
hCoV-19/Japan/PG-85618/2021	EPI_ISL_3897909	2021-09-02
hCoV-19/Japan/PG-85619/2021	EPI_ISL_3897910	2021-09-02
hCoV-19/Japan/PG-85620/2021	EPI_ISL_3897911	2021-09-02
hCoV-19/Japan/PG-85621/2021	EPI_ISL_3897912	2021-09-02
hCoV-19/Japan/PG-85622/2021	EPI_ISL_3897913	2021-09-02
hCoV-19/Japan/PG-85623/2021	EPI_ISL_3897914	2021-09-02
hCoV-19/Japan/PG-85624/2021	EPI_ISL_3897915	2021-09-02
hCoV-19/Japan/PG-85625/2021	EPI_ISL_3897916	2021-09-02
hCoV-19/Japan/PG-85626/2021	EPI_ISL_3897917	2021-09-02
hCoV-19/Japan/PG-85627/2021	EPI_ISL_3897918	2021-09-02
hCoV-19/Japan/PG-85628/2021	EPI_ISL_3897919	2021-09-02
hCoV-19/Japan/PG-85629/2021	EPI_ISL_3897920	2021-09-02
hCoV-19/Japan/PG-85630/2021	EPI_ISL_3897921	2021-09-02
hCoV-19/Japan/PG-85631/2021	EPI_ISL_3897922	2021-09-02
hCoV-19/Japan/PG-85632/2021	EPI_ISL_3897923	2021-09-02

virus_name	epiID	submission_date
hCoV-19/Japan/PG-85633/2021	EPI_ISL_3897924	2021-09-02
hCoV-19/Japan/PG-85634/2021	EPI_ISL_3897925	2021-09-02
hCoV-19/Japan/PG-85635/2021	EPI_ISL_3897926	2021-09-02
hCoV-19/Japan/PG-85636/2021	EPI_ISL_3897927	2021-09-02
hCoV-19/Japan/PG-85637/2021	EPI_ISL_3897928	2021-09-02
hCoV-19/Japan/PG-85638/2021	EPI_ISL_3897929	2021-09-02
hCoV-19/Japan/PG-85639/2021	EPI_ISL_3897930	2021-09-02
hCoV-19/Japan/PG-85640/2021	EPI_ISL_3897931	2021-09-02
hCoV-19/Japan/PG-85641/2021	EPI_ISL_3897932	2021-09-02
hCoV-19/Japan/PG-85642/2021	EPI_ISL_3897933	2021-09-02
hCoV-19/Japan/PG-85643/2021	EPI_ISL_3897934	2021-09-02
hCoV-19/Japan/PG-85644/2021	EPI_ISL_3897935	2021-09-02
hCoV-19/Japan/PG-85645/2021	EPI_ISL_3897936	2021-09-02
hCoV-19/Japan/PG-85646/2021	EPI_ISL_3897937	2021-09-02
hCoV-19/Japan/PG-85647/2021	EPI_ISL_3897938	2021-09-02
hCoV-19/Japan/PG-85648/2021	EPI_ISL_3897939	2021-09-02
hCoV-19/Japan/PG-85649/2021	EPI_ISL_3897940	2021-09-02
hCoV-19/Japan/PG-85650/2021	EPI_ISL_3897941	2021-09-02
hCoV-19/Japan/PG-85651/2021	EPI_ISL_3897942	2021-09-02
hCoV-19/Japan/PG-85652/2021	EPI_ISL_3897943	2021-09-02
hCoV-19/Japan/PG-85653/2021	EPI_ISL_3897944	2021-09-02
hCoV-19/Japan/PG-85654/2021	EPI_ISL_3897945	2021-09-02
hCoV-19/Japan/PG-85655/2021	EPI_ISL_3897946	2021-09-02
hCoV-19/Japan/PG-85656/2021	EPI_ISL_3897947	2021-09-02
hCoV-19/Japan/PG-85657/2021	EPI_ISL_3897948	2021-09-02
hCoV-19/Japan/PG-85658/2021	EPI_ISL_3897949	2021-09-02
hCoV-19/Japan/PG-85659/2021	EPI_ISL_3897950	2021-09-02
hCoV-19/Japan/PG-85660/2021	EPI_ISL_3897951	2021-09-02
hCoV-19/Japan/PG-85661/2021	EPI_ISL_3897952	2021-09-02
hCoV-19/Japan/PG-85662/2021	EPI_ISL_3897953	2021-09-02
hCoV-19/Japan/PG-85663/2021	EPI_ISL_3897954	2021-09-02
hCoV-19/Japan/PG-85664/2021	EPI_ISL_3897955	2021-09-02
hCoV-19/Japan/PG-85665/2021	EPI_ISL_3897956	2021-09-02
hCoV-19/Japan/PG-85666/2021	EPI_ISL_3897957	2021-09-02
hCoV-19/Japan/PG-85667/2021	EPI_ISL_3897958	2021-09-02
hCoV-19/Japan/PG-85668/2021	EPI_ISL_3897959	2021-09-02
hCoV-19/Japan/PG-85669/2021	EPI_ISL_3897960	2021-09-02
hCoV-19/Japan/PG-85670/2021	EPI_ISL_3897961	2021-09-02
hCoV-19/Japan/PG-85671/2021	EPI_ISL_3897962	2021-09-02
hCoV-19/Japan/PG-85672/2021	EPI_ISL_3897963	2021-09-02
hCoV-19/Japan/PG-85673/2021	EPI_ISL_3897964	2021-09-02
hCoV-19/Japan/PG-85674/2021	EPI_ISL_3897965	2021-09-02
hCoV-19/Japan/PG-85675/2021	EPI_ISL_3897966	2021-09-02
hCoV-19/Japan/PG-85676/2021	EPI_ISL_3897967	2021-09-02
hCoV-19/Japan/PG-85677/2021	EPI_ISL_3897968	2021-09-02
hCoV-19/Japan/PG-85678/2021	EPI_ISL_3897969	2021-09-02
hCoV-19/Japan/PG-85679/2021	EPI_ISL_3897970	2021-09-02
hCoV-19/Japan/PG-85680/2021	EPI_ISL_3897971	2021-09-02
hCoV-19/Japan/PG-85681/2021	EPI_ISL_3897972	2021-09-02
hCoV-19/Japan/PG-85682/2021	EPI_ISL_3897973	2021-09-02
hCoV-19/Japan/PG-85683/2021	EPI_ISL_3897974	2021-09-02
hCoV-19/Japan/PG-85684/2021	EPI_ISL_3897975	2021-09-02
hCoV-19/Japan/PG-85685/2021	EPI_ISL_3897976	2021-09-02
hCoV-19/Japan/PG-85686/2021	EPI_ISL_3897977	2021-09-02

virus_name	epiID	submission_date
hCoV-19/Japan/PG-85687/2021	EPI_ISL_3897978	2021-09-02
hCoV-19/Japan/PG-85688/2021	EPI_ISL_3897979	2021-09-02
hCoV-19/Japan/PG-85689/2021	EPI_ISL_3897980	2021-09-02
hCoV-19/Japan/PG-85690/2021	EPI_ISL_3897981	2021-09-02
hCoV-19/Japan/PG-85691/2021	EPI_ISL_3897982	2021-09-02
hCoV-19/Japan/PG-85692/2021	EPI_ISL_3897983	2021-09-02
hCoV-19/Japan/PG-85693/2021	EPI_ISL_3897984	2021-09-02
hCoV-19/Japan/PG-85694/2021	EPI_ISL_3897985	2021-09-02
hCoV-19/Japan/PG-85695/2021	EPI_ISL_3897986	2021-09-02
hCoV-19/Japan/PG-85696/2021	EPI_ISL_3897987	2021-09-02
hCoV-19/Japan/PG-85697/2021	EPI_ISL_3897988	2021-09-02
hCoV-19/Japan/PG-85698/2021	EPI_ISL_3897989	2021-09-02
hCoV-19/Japan/PG-85699/2021	EPI_ISL_3897990	2021-09-02
hCoV-19/Japan/PG-85700/2021	EPI_ISL_3897991	2021-09-02
hCoV-19/Japan/PG-85701/2021	EPI_ISL_3897992	2021-09-02
hCoV-19/Japan/PG-85702/2021	EPI_ISL_3897993	2021-09-02
hCoV-19/Japan/PG-85703/2021	EPI_ISL_3897994	2021-09-02
hCoV-19/Japan/PG-85704/2021	EPI_ISL_3897995	2021-09-02
hCoV-19/Japan/PG-85705/2021	EPI_ISL_3897996	2021-09-02
hCoV-19/Japan/PG-85706/2021	EPI_ISL_3897997	2021-09-02
hCoV-19/Japan/PG-85709/2021	EPI_ISL_3897998	2021-09-02
hCoV-19/Japan/PG-85710/2021	EPI_ISL_3897999	2021-09-02
hCoV-19/Japan/PG-85711/2021	EPI_ISL_3898000	2021-09-02
hCoV-19/Japan/PG-85712/2021	EPI_ISL_3898001	2021-09-02
hCoV-19/Japan/PG-85713/2021	EPI_ISL_3898002	2021-09-02
hCoV-19/Japan/PG-85714/2021	EPI_ISL_3898003	2021-09-02
hCoV-19/Japan/PG-85716/2021	EPI_ISL_3898004	2021-09-02
hCoV-19/Japan/PG-85717/2021	EPI_ISL_3898005	2021-09-02
hCoV-19/Japan/PG-85718/2021	EPI_ISL_3898006	2021-09-02
hCoV-19/Japan/PG-85719/2021	EPI_ISL_3898007	2021-09-02
hCoV-19/Japan/PG-85720/2021	EPI_ISL_3898008	2021-09-02
hCoV-19/Japan/PG-85721/2021	EPI_ISL_3898009	2021-09-02
hCoV-19/Japan/PG-85722/2021	EPI_ISL_3898010	2021-09-02
hCoV-19/Japan/PG-85723/2021	EPI_ISL_3898011	2021-09-02
hCoV-19/Japan/PG-85724/2021	EPI_ISL_3898012	2021-09-02
hCoV-19/Japan/PG-85725/2021	EPI_ISL_3898013	2021-09-02
hCoV-19/Japan/PG-85726/2021	EPI_ISL_3898014	2021-09-02
hCoV-19/Japan/PG-85727/2021	EPI_ISL_3898015	2021-09-02
hCoV-19/Japan/PG-85728/2021	EPI_ISL_3898016	2021-09-02
hCoV-19/Japan/PG-85729/2021	EPI_ISL_3898017	2021-09-02
hCoV-19/Japan/PG-85730/2021	EPI_ISL_3898018	2021-09-02
hCoV-19/Japan/PG-85731/2021	EPI_ISL_3898019	2021-09-02
hCoV-19/Japan/PG-85732/2021	EPI_ISL_3898020	2021-09-02
hCoV-19/Japan/PG-85733/2021	EPI_ISL_3898021	2021-09-02
hCoV-19/Japan/PG-85734/2021	EPI_ISL_3898022	2021-09-02
hCoV-19/Japan/PG-85742/2021	EPI_ISL_3898023	2021-09-02
hCoV-19/Japan/PG-85743/2021	EPI_ISL_3898024	2021-09-02
hCoV-19/Japan/PG-85744/2021	EPI_ISL_3898025	2021-09-02
hCoV-19/Japan/PG-85745/2021	EPI_ISL_3898026	2021-09-02
hCoV-19/Japan/PG-85746/2021	EPI_ISL_3898027	2021-09-02
hCoV-19/Japan/PG-85747/2021	EPI_ISL_3898028	2021-09-02
hCoV-19/Japan/PG-85748/2021	EPI_ISL_3898029	2021-09-02
hCoV-19/Japan/PG-85749/2021	EPI_ISL_3898030	2021-09-02
hCoV-19/Japan/PG-85750/2021	EPI_ISL_3898031	2021-09-02

virus_name	epiID	submission_date
hCoV-19/Japan/PG-85751/2021	EPI_ISL_3898032	2021-09-02
hCoV-19/Japan/PG-85752/2021	EPI_ISL_3898033	2021-09-02
hCoV-19/Japan/PG-85753/2021	EPI_ISL_3898034	2021-09-02
hCoV-19/Japan/PG-85754/2021	EPI_ISL_3898035	2021-09-02
hCoV-19/Japan/PG-85755/2021	EPI_ISL_3898036	2021-09-02
hCoV-19/Japan/PG-85756/2021	EPI_ISL_3898037	2021-09-02
hCoV-19/Japan/PG-85757/2021	EPI_ISL_3898038	2021-09-02
hCoV-19/Japan/PG-85758/2021	EPI_ISL_3898039	2021-09-02
hCoV-19/Japan/PG-85761/2021	EPI_ISL_3898040	2021-09-02
hCoV-19/Japan/PG-85762/2021	EPI_ISL_3898041	2021-09-02
hCoV-19/Japan/PG-85763/2021	EPI_ISL_3898042	2021-09-02
hCoV-19/Japan/PG-85764/2021	EPI_ISL_3898043	2021-09-02
hCoV-19/Japan/PG-85765/2021	EPI_ISL_3898044	2021-09-02
hCoV-19/Japan/PG-85766/2021	EPI_ISL_3898045	2021-09-02
hCoV-19/Japan/PG-85767/2021	EPI_ISL_3898046	2021-09-02
hCoV-19/Japan/PG-85768/2021	EPI_ISL_3898047	2021-09-02
hCoV-19/Japan/PG-85769/2021	EPI_ISL_3898048	2021-09-02
hCoV-19/Japan/PG-85770/2021	EPI_ISL_3898049	2021-09-02
hCoV-19/Japan/PG-85771/2021	EPI_ISL_3898050	2021-09-02
hCoV-19/Japan/PG-85772/2021	EPI_ISL_3898051	2021-09-02
hCoV-19/Japan/PG-85773/2021	EPI_ISL_3898052	2021-09-02
hCoV-19/Japan/PG-85774/2021	EPI_ISL_3898053	2021-09-02
hCoV-19/Japan/PG-85775/2021	EPI_ISL_3898054	2021-09-02
hCoV-19/Japan/PG-85776/2021	EPI_ISL_3898055	2021-09-02
hCoV-19/Japan/PG-85777/2021	EPI_ISL_3898056	2021-09-02
hCoV-19/Japan/PG-85778/2021	EPI_ISL_3898057	2021-09-02
hCoV-19/Japan/PG-85779/2021	EPI_ISL_3898058	2021-09-02
hCoV-19/Japan/PG-85780/2021	EPI_ISL_3898059	2021-09-02
hCoV-19/Japan/PG-85781/2021	EPI_ISL_3898060	2021-09-02
hCoV-19/Japan/PG-85782/2021	EPI_ISL_3898061	2021-09-02
hCoV-19/Japan/PG-85783/2021	EPI_ISL_3898062	2021-09-02
hCoV-19/Japan/PG-85784/2021	EPI_ISL_3898063	2021-09-02
hCoV-19/Japan/PG-85785/2021	EPI_ISL_3898064	2021-09-02
hCoV-19/Japan/PG-85786/2021	EPI_ISL_3898065	2021-09-02
hCoV-19/Japan/PG-85787/2021	EPI_ISL_3898066	2021-09-02
hCoV-19/Japan/PG-85788/2021	EPI_ISL_3898067	2021-09-02
hCoV-19/Japan/PG-85789/2021	EPI_ISL_3898068	2021-09-02
hCoV-19/Japan/PG-85790/2021	EPI_ISL_3898069	2021-09-02
hCoV-19/Japan/PG-85791/2021	EPI_ISL_3898070	2021-09-02
hCoV-19/Japan/PG-85792/2021	EPI_ISL_3898071	2021-09-02
hCoV-19/Japan/PG-85793/2021	EPI_ISL_3898072	2021-09-02
hCoV-19/Japan/PG-85794/2021	EPI_ISL_3898073	2021-09-02
hCoV-19/Japan/PG-85795/2021	EPI_ISL_3898074	2021-09-02
hCoV-19/Japan/PG-85796/2021	EPI_ISL_3898075	2021-09-02
hCoV-19/Japan/PG-85797/2021	EPI_ISL_3898076	2021-09-02
hCoV-19/Japan/PG-85798/2021	EPI_ISL_3898077	2021-09-02
hCoV-19/Japan/PG-85799/2021	EPI_ISL_3898078	2021-09-02
hCoV-19/Japan/PG-85800/2021	EPI_ISL_3898079	2021-09-02
hCoV-19/Japan/PG-85801/2021	EPI_ISL_3898080	2021-09-02
hCoV-19/Japan/PG-85802/2021	EPI_ISL_3898081	2021-09-02
hCoV-19/Japan/PG-85803/2021	EPI_ISL_3898082	2021-09-02
hCoV-19/Japan/PG-85804/2021	EPI_ISL_3898083	2021-09-02
hCoV-19/Japan/PG-85805/2021	EPI_ISL_3898084	2021-09-02
hCoV-19/Japan/PG-85806/2021	EPI_ISL_3898085	2021-09-02

virus_name	epiID	submission_date
hCoV-19/Japan/PG-85807/2021	EPI_ISL_3898086	2021-09-02
hCoV-19/Japan/PG-85808/2021	EPI_ISL_3898087	2021-09-02
hCoV-19/Japan/PG-85809/2021	EPI_ISL_3898088	2021-09-02
hCoV-19/Japan/PG-85810/2021	EPI_ISL_3898089	2021-09-02
hCoV-19/Japan/PG-85811/2021	EPI_ISL_3898090	2021-09-02
hCoV-19/Japan/PG-85812/2021	EPI_ISL_3898091	2021-09-02
hCoV-19/Japan/PG-85813/2021	EPI_ISL_3898092	2021-09-02
hCoV-19/Japan/PG-85814/2021	EPI_ISL_3898093	2021-09-02
hCoV-19/Japan/PG-85815/2021	EPI_ISL_3898094	2021-09-02
hCoV-19/Japan/PG-85816/2021	EPI_ISL_3898095	2021-09-02
hCoV-19/Japan/PG-85817/2021	EPI_ISL_3898096	2021-09-02
hCoV-19/Japan/PG-85818/2021	EPI_ISL_3898097	2021-09-02
hCoV-19/Japan/PG-85819/2021	EPI_ISL_3898098	2021-09-02
hCoV-19/Japan/PG-85820/2021	EPI_ISL_3898099	2021-09-02
hCoV-19/Japan/PG-85821/2021	EPI_ISL_3898100	2021-09-02
hCoV-19/Japan/PG-85822/2021	EPI_ISL_3898101	2021-09-02
hCoV-19/Japan/PG-85823/2021	EPI_ISL_3898102	2021-09-02
hCoV-19/Japan/PG-85824/2021	EPI_ISL_3898103	2021-09-02
hCoV-19/Japan/PG-85825/2021	EPI_ISL_3898104	2021-09-02
hCoV-19/Japan/PG-85826/2021	EPI_ISL_3898105	2021-09-02
hCoV-19/Japan/PG-85827/2021	EPI_ISL_3875108	2021-09-02
hCoV-19/Japan/PG-85828/2021	EPI_ISL_3875109	2021-09-02
hCoV-19/Japan/PG-85829/2021	EPI_ISL_3875110	2021-09-02
hCoV-19/Japan/PG-85830/2021	EPI_ISL_3875111	2021-09-02
hCoV-19/Japan/PG-85831/2021	EPI_ISL_3875112	2021-09-02
hCoV-19/Japan/PG-85832/2021	EPI_ISL_3875113	2021-09-02
hCoV-19/Japan/PG-85833/2021	EPI_ISL_3875114	2021-09-02
hCoV-19/Japan/PG-85834/2021	EPI_ISL_3875115	2021-09-02
hCoV-19/Japan/PG-85835/2021	EPI_ISL_3875116	2021-09-02
hCoV-19/Japan/PG-85836/2021	EPI_ISL_3875117	2021-09-02
hCoV-19/Japan/PG-85837/2021	EPI_ISL_3875118	2021-09-02
hCoV-19/Japan/PG-85838/2021	EPI_ISL_3875119	2021-09-02
hCoV-19/Japan/PG-85839/2021	EPI_ISL_3875120	2021-09-02
hCoV-19/Japan/PG-85840/2021	EPI_ISL_3875121	2021-09-02
hCoV-19/Japan/PG-85841/2021	EPI_ISL_3875122	2021-09-02
hCoV-19/Japan/PG-85842/2021	EPI_ISL_3875123	2021-09-02
hCoV-19/Japan/PG-85843/2021	EPI_ISL_3875124	2021-09-02
hCoV-19/Japan/PG-85844/2021	EPI_ISL_3875125	2021-09-02
hCoV-19/Japan/PG-85845/2021	EPI_ISL_3875126	2021-09-02
hCoV-19/Japan/PG-85846/2021	EPI_ISL_3875127	2021-09-02
hCoV-19/Japan/PG-85847/2021	EPI_ISL_3875128	2021-09-02
hCoV-19/Japan/PG-85848/2021	EPI_ISL_3875129	2021-09-02
hCoV-19/Japan/PG-85849/2021	EPI_ISL_3875130	2021-09-02
hCoV-19/Japan/PG-85850/2021	EPI_ISL_3875131	2021-09-02
hCoV-19/Japan/PG-85851/2021	EPI_ISL_3875132	2021-09-02
hCoV-19/Japan/PG-85852/2021	EPI_ISL_3875133	2021-09-02
hCoV-19/Japan/PG-85853/2021	EPI_ISL_3875134	2021-09-02
hCoV-19/Japan/PG-85854/2021	EPI_ISL_3875135	2021-09-02
hCoV-19/Japan/PG-85855/2021	EPI_ISL_3875136	2021-09-02
hCoV-19/Japan/PG-85856/2021	EPI_ISL_3875137	2021-09-02
hCoV-19/Japan/PG-85857/2021	EPI_ISL_3875138	2021-09-02
hCoV-19/Japan/PG-85858/2021	EPI_ISL_3875139	2021-09-02
hCoV-19/Japan/PG-85859/2021	EPI_ISL_3875140	2021-09-02
hCoV-19/Japan/PG-85860/2021	EPI_ISL_3875141	2021-09-02

virus_name	epiID	submission_date
hCoV-19/Japan/PG-85861/2021	EPI_ISL_3875142	2021-09-02
hCoV-19/Japan/PG-85862/2021	EPI_ISL_3875143	2021-09-02
hCoV-19/Japan/PG-85863/2021	EPI_ISL_3875144	2021-09-02
hCoV-19/Japan/PG-85864/2021	EPI_ISL_3875145	2021-09-02
hCoV-19/Japan/PG-85865/2021	EPI_ISL_3875146	2021-09-02
hCoV-19/Japan/PG-85866/2021	EPI_ISL_3875147	2021-09-02
hCoV-19/Japan/PG-85867/2021	EPI_ISL_3875148	2021-09-02
hCoV-19/Japan/PG-85868/2021	EPI_ISL_3875149	2021-09-02
hCoV-19/Japan/PG-85869/2021	EPI_ISL_3875150	2021-09-02
hCoV-19/Japan/PG-85870/2021	EPI_ISL_3875151	2021-09-02
hCoV-19/Japan/PG-85871/2021	EPI_ISL_3875152	2021-09-02
hCoV-19/Japan/PG-85872/2021	EPI_ISL_3875153	2021-09-02
hCoV-19/Japan/PG-85873/2021	EPI_ISL_3875154	2021-09-02
hCoV-19/Japan/PG-85874/2021	EPI_ISL_3875155	2021-09-02
hCoV-19/Japan/PG-85875/2021	EPI_ISL_3875156	2021-09-02
hCoV-19/Japan/PG-85876/2021	EPI_ISL_3875157	2021-09-02
hCoV-19/Japan/PG-85877/2021	EPI_ISL_3875158	2021-09-02
hCoV-19/Japan/PG-85878/2021	EPI_ISL_3875159	2021-09-02
hCoV-19/Japan/PG-85879/2021	EPI_ISL_3875160	2021-09-02
hCoV-19/Japan/PG-85880/2021	EPI_ISL_3875161	2021-09-02
hCoV-19/Japan/PG-85881/2021	EPI_ISL_3875162	2021-09-02
hCoV-19/Japan/PG-85882/2021	EPI_ISL_3875163	2021-09-02
hCoV-19/Japan/PG-85883/2021	EPI_ISL_3875164	2021-09-02
hCoV-19/Japan/PG-85884/2021	EPI_ISL_3875165	2021-09-02
hCoV-19/Japan/PG-85885/2021	EPI_ISL_3875166	2021-09-02
hCoV-19/Japan/PG-85886/2021	EPI_ISL_3875167	2021-09-02
hCoV-19/Japan/PG-85887/2021	EPI_ISL_3875168	2021-09-02
hCoV-19/Japan/PG-85888/2021	EPI_ISL_3875169	2021-09-02
hCoV-19/Japan/PG-85889/2021	EPI_ISL_3875170	2021-09-02
hCoV-19/Japan/PG-85890/2021	EPI_ISL_3875171	2021-09-02
hCoV-19/Japan/PG-85891/2021	EPI_ISL_3875172	2021-09-02
hCoV-19/Japan/PG-85892/2021	EPI_ISL_3875173	2021-09-02
hCoV-19/Japan/PG-85893/2021	EPI_ISL_3875174	2021-09-02
hCoV-19/Japan/PG-85894/2021	EPI_ISL_3875175	2021-09-02
hCoV-19/Japan/PG-85895/2021	EPI_ISL_3875176	2021-09-02
hCoV-19/Japan/PG-85896/2021	EPI_ISL_3875177	2021-09-02
hCoV-19/Japan/PG-85897/2021	EPI_ISL_3875178	2021-09-02
hCoV-19/Japan/PG-85898/2021	EPI_ISL_3875179	2021-09-02
hCoV-19/Japan/PG-85899/2021	EPI_ISL_3875180	2021-09-02
hCoV-19/Japan/PG-85900/2021	EPI_ISL_3875181	2021-09-02
hCoV-19/Japan/PG-85901/2021	EPI_ISL_3875182	2021-09-02
hCoV-19/Japan/PG-85902/2021	EPI_ISL_3875183	2021-09-02
hCoV-19/Japan/PG-85903/2021	EPI_ISL_3875184	2021-09-02
hCoV-19/Japan/PG-85904/2021	EPI_ISL_3875185	2021-09-02
hCoV-19/Japan/PG-85905/2021	EPI_ISL_3875186	2021-09-02
hCoV-19/Japan/PG-85906/2021	EPI_ISL_3875187	2021-09-02
hCoV-19/Japan/PG-85907/2021	EPI_ISL_3875188	2021-09-02
hCoV-19/Japan/PG-85908/2021	EPI_ISL_3875189	2021-09-02
hCoV-19/Japan/PG-85909/2021	EPI_ISL_3875190	2021-09-02
hCoV-19/Japan/PG-85910/2021	EPI_ISL_3875191	2021-09-02
hCoV-19/Japan/PG-85911/2021	EPI_ISL_3875192	2021-09-02
hCoV-19/Japan/PG-85912/2021	EPI_ISL_3875193	2021-09-02
hCoV-19/Japan/PG-85913/2021	EPI_ISL_3875194	2021-09-02
hCoV-19/Japan/PG-85914/2021	EPI_ISL_3875195	2021-09-02

virus_name	epiID	submission_date
hCoV-19/Japan/PG-85915/2021	EPI_ISL_3875906	2021-09-02
hCoV-19/Japan/PG-85916/2021	EPI_ISL_3875907	2021-09-02
hCoV-19/Japan/PG-85917/2021	EPI_ISL_3875908	2021-09-02
hCoV-19/Japan/PG-85918/2021	EPI_ISL_3875909	2021-09-02
hCoV-19/Japan/PG-85919/2021	EPI_ISL_3875910	2021-09-02
hCoV-19/Japan/PG-85920/2021	EPI_ISL_3875911	2021-09-02
hCoV-19/Japan/PG-85921/2021	EPI_ISL_3875912	2021-09-02
hCoV-19/Japan/PG-85922/2021	EPI_ISL_3875913	2021-09-02
hCoV-19/Japan/PG-85923/2021	EPI_ISL_3875914	2021-09-02
hCoV-19/Japan/PG-85924/2021	EPI_ISL_3875915	2021-09-02
hCoV-19/Japan/PG-85925/2021	EPI_ISL_3875916	2021-09-02
hCoV-19/Japan/PG-85926/2021	EPI_ISL_3875917	2021-09-02
hCoV-19/Japan/PG-85927/2021	EPI_ISL_3875918	2021-09-02
hCoV-19/Japan/PG-85928/2021	EPI_ISL_3875919	2021-09-02
hCoV-19/Japan/PG-85929/2021	EPI_ISL_3875920	2021-09-02
hCoV-19/Japan/PG-85930/2021	EPI_ISL_3875921	2021-09-02
hCoV-19/Japan/PG-85931/2021	EPI_ISL_3875922	2021-09-02
hCoV-19/Japan/PG-85932/2021	EPI_ISL_3875923	2021-09-02
hCoV-19/Japan/PG-85933/2021	EPI_ISL_3875924	2021-09-02
hCoV-19/Japan/PG-85934/2021	EPI_ISL_3875925	2021-09-02
hCoV-19/Japan/PG-85935/2021	EPI_ISL_3870824	2021-09-02
hCoV-19/Japan/PG-85936/2021	EPI_ISL_3870825	2021-09-02
hCoV-19/Japan/PG-85937/2021	EPI_ISL_3870826	2021-09-02
hCoV-19/Japan/PG-85938/2021	EPI_ISL_3870827	2021-09-02
hCoV-19/Japan/PG-85939/2021	EPI_ISL_3870828	2021-09-02
hCoV-19/Japan/PG-85940/2021	EPI_ISL_3870829	2021-09-02
hCoV-19/Japan/PG-85941/2021	EPI_ISL_3870830	2021-09-02
hCoV-19/Japan/PG-85942/2021	EPI_ISL_3870831	2021-09-02
hCoV-19/Japan/PG-85943/2021	EPI_ISL_3870832	2021-09-02
hCoV-19/Japan/PG-85944/2021	EPI_ISL_3870833	2021-09-02
hCoV-19/Japan/PG-85945/2021	EPI_ISL_3870834	2021-09-02
hCoV-19/Japan/PG-85946/2021	EPI_ISL_3870835	2021-09-02
hCoV-19/Japan/PG-85947/2021	EPI_ISL_3870836	2021-09-02
hCoV-19/Japan/PG-85948/2021	EPI_ISL_3870837	2021-09-02
hCoV-19/Japan/PG-85949/2021	EPI_ISL_3870838	2021-09-02
hCoV-19/Japan/PG-85950/2021	EPI_ISL_3870839	2021-09-02
hCoV-19/Japan/PG-85951/2021	EPI_ISL_3870840	2021-09-02
hCoV-19/Japan/PG-85952/2021	EPI_ISL_3870841	2021-09-02
hCoV-19/Japan/PG-85953/2021	EPI_ISL_3873024	2021-09-02
hCoV-19/Japan/PG-85954/2021	EPI_ISL_3873025	2021-09-02
hCoV-19/Japan/PG-85955/2021	EPI_ISL_3873026	2021-09-02
hCoV-19/Japan/PG-85956/2021	EPI_ISL_3873027	2021-09-02
hCoV-19/Japan/PG-85957/2021	EPI_ISL_3873028	2021-09-02
hCoV-19/Japan/PG-85958/2021	EPI_ISL_3873029	2021-09-02
hCoV-19/Japan/PG-85959/2021	EPI_ISL_3873030	2021-09-02
hCoV-19/Japan/PG-85960/2021	EPI_ISL_3873031	2021-09-02
hCoV-19/Japan/PG-85961/2021	EPI_ISL_3873032	2021-09-02
hCoV-19/Japan/PG-85962/2021	EPI_ISL_3873033	2021-09-02
hCoV-19/Japan/PG-85963/2021	EPI_ISL_3873034	2021-09-02
hCoV-19/Japan/PG-85964/2021	EPI_ISL_3873035	2021-09-02
hCoV-19/Japan/PG-85965/2021	EPI_ISL_3873036	2021-09-02
hCoV-19/Japan/PG-85966/2021	EPI_ISL_3873037	2021-09-02
hCoV-19/Japan/PG-85967/2021	EPI_ISL_3873038	2021-09-02
hCoV-19/Japan/PG-85968/2021	EPI_ISL_3873039	2021-09-02

virus_name	epiID	submission_date
hCoV-19/Japan/PG-86006/2021	EPI_ISL_3873040	2021-09-02
hCoV-19/Japan/PG-86007/2021	EPI_ISL_3873041	2021-09-02
hCoV-19/Japan/PG-86008/2021	EPI_ISL_3873042	2021-09-02
hCoV-19/Japan/PG-86009/2021	EPI_ISL_3873043	2021-09-02
hCoV-19/Japan/PG-86010/2021	EPI_ISL_3873044	2021-09-02
hCoV-19/Japan/PG-86011/2021	EPI_ISL_3873045	2021-09-02
hCoV-19/Japan/PG-86012/2021	EPI_ISL_3873046	2021-09-02
hCoV-19/Japan/PG-86013/2021	EPI_ISL_3873047	2021-09-02
hCoV-19/Japan/PG-86014/2021	EPI_ISL_3873048	2021-09-02
hCoV-19/Japan/PG-86015/2021	EPI_ISL_3873049	2021-09-02
hCoV-19/Japan/PG-86016/2021	EPI_ISL_3873050	2021-09-02
hCoV-19/Japan/PG-86017/2021	EPI_ISL_3873051	2021-09-02
hCoV-19/Japan/PG-86018/2021	EPI_ISL_3873052	2021-09-02
hCoV-19/Japan/PG-86019/2021	EPI_ISL_3873053	2021-09-02
hCoV-19/Japan/PG-86020/2021	EPI_ISL_3873054	2021-09-02
hCoV-19/Japan/PG-86021/2021	EPI_ISL_3873055	2021-09-02
hCoV-19/Japan/PG-86022/2021	EPI_ISL_3873056	2021-09-02
hCoV-19/Japan/PG-86023/2021	EPI_ISL_3873057	2021-09-02
hCoV-19/Japan/PG-86024/2021	EPI_ISL_3873058	2021-09-02
hCoV-19/Japan/PG-86025/2021	EPI_ISL_3873059	2021-09-02
hCoV-19/Japan/PG-86026/2021	EPI_ISL_3873060	2021-09-02
hCoV-19/Japan/PG-86027/2021	EPI_ISL_3873061	2021-09-02
hCoV-19/Japan/PG-86028/2021	EPI_ISL_3873062	2021-09-02
hCoV-19/Japan/PG-86029/2021	EPI_ISL_3873063	2021-09-02
hCoV-19/Japan/PG-86030/2021	EPI_ISL_3873064	2021-09-02
hCoV-19/Japan/PG-86031/2021	EPI_ISL_3873065	2021-09-02
hCoV-19/Japan/PG-86032/2021	EPI_ISL_3873066	2021-09-02
hCoV-19/Japan/PG-86033/2021	EPI_ISL_3873067	2021-09-02
hCoV-19/Japan/PG-86034/2021	EPI_ISL_3873068	2021-09-02
hCoV-19/Japan/PG-86035/2021	EPI_ISL_3873069	2021-09-02
hCoV-19/Japan/PG-86036/2021	EPI_ISL_3873070	2021-09-02
hCoV-19/Japan/PG-86037/2021	EPI_ISL_3873071	2021-09-02
hCoV-19/Japan/PG-86038/2021	EPI_ISL_3873072	2021-09-02
hCoV-19/Japan/PG-86039/2021	EPI_ISL_3873073	2021-09-02
hCoV-19/Japan/PG-86040/2021	EPI_ISL_3873074	2021-09-02
hCoV-19/Japan/PG-86041/2021	EPI_ISL_3873075	2021-09-02
hCoV-19/Japan/PG-86042/2021	EPI_ISL_3873076	2021-09-02
hCoV-19/Japan/PG-86043/2021	EPI_ISL_3873077	2021-09-02
hCoV-19/Japan/PG-86044/2021	EPI_ISL_3873078	2021-09-02
hCoV-19/Japan/PG-86045/2021	EPI_ISL_3873079	2021-09-02
hCoV-19/Japan/PG-86046/2021	EPI_ISL_3873080	2021-09-02
hCoV-19/Japan/PG-86047/2021	EPI_ISL_3873081	2021-09-02
hCoV-19/Japan/PG-86048/2021	EPI_ISL_3873082	2021-09-02
hCoV-19/Japan/PG-86049/2021	EPI_ISL_3873083	2021-09-02
hCoV-19/Japan/PG-86050/2021	EPI_ISL_3873084	2021-09-02
hCoV-19/Japan/PG-86051/2021	EPI_ISL_3873085	2021-09-02
hCoV-19/Japan/PG-86052/2021	EPI_ISL_3873086	2021-09-02
hCoV-19/Japan/PG-86053/2021	EPI_ISL_3873087	2021-09-02
hCoV-19/Japan/PG-86054/2021	EPI_ISL_3873088	2021-09-02
hCoV-19/Japan/PG-86055/2021	EPI_ISL_3873089	2021-09-02
hCoV-19/Japan/PG-86056/2021	EPI_ISL_3873090	2021-09-02
hCoV-19/Japan/PG-86057/2021	EPI_ISL_3873091	2021-09-02
hCoV-19/Japan/PG-86058/2021	EPI_ISL_3873092	2021-09-02
hCoV-19/Japan/PG-86059/2021	EPI_ISL_3873093	2021-09-02

virus_name	epiID	submission_date
hCoV-19/Japan/PG-86060/2021	EPI_ISL_3873094	2021-09-02
hCoV-19/Japan/PG-86061/2021	EPI_ISL_3873095	2021-09-02
hCoV-19/Japan/PG-86062/2021	EPI_ISL_3873096	2021-09-02
hCoV-19/Japan/PG-86063/2021	EPI_ISL_3873097	2021-09-02
hCoV-19/Japan/PG-86064/2021	EPI_ISL_3873098	2021-09-02
hCoV-19/Japan/PG-86065/2021	EPI_ISL_3873099	2021-09-02
hCoV-19/Japan/PG-86066/2021	EPI_ISL_3873100	2021-09-02
hCoV-19/Japan/PG-86067/2021	EPI_ISL_3873101	2021-09-02
hCoV-19/Japan/PG-86068/2021	EPI_ISL_3873102	2021-09-02
hCoV-19/Japan/PG-86069/2021	EPI_ISL_3873103	2021-09-02
hCoV-19/Japan/PG-86070/2021	EPI_ISL_3873104	2021-09-02
hCoV-19/Japan/PG-86071/2021	EPI_ISL_3873105	2021-09-02
hCoV-19/Japan/PG-86072/2021	EPI_ISL_3873106	2021-09-02
hCoV-19/Japan/PG-86073/2021	EPI_ISL_3873107	2021-09-02
hCoV-19/Japan/PG-86074/2021	EPI_ISL_3873108	2021-09-02
hCoV-19/Japan/PG-86075/2021	EPI_ISL_3873109	2021-09-02
hCoV-19/Japan/PG-86076/2021	EPI_ISL_3873110	2021-09-02
hCoV-19/Japan/PG-86077/2021	EPI_ISL_3873111	2021-09-02
hCoV-19/Japan/PG-86078/2021	EPI_ISL_3873112	2021-09-02
hCoV-19/Japan/PG-86079/2021	EPI_ISL_3873113	2021-09-02
hCoV-19/Japan/PG-86080/2021	EPI_ISL_3873114	2021-09-02
hCoV-19/Japan/PG-86081/2021	EPI_ISL_3873115	2021-09-02
hCoV-19/Japan/PG-86082/2021	EPI_ISL_3873116	2021-09-02
hCoV-19/Japan/PG-86083/2021	EPI_ISL_3873117	2021-09-02
hCoV-19/Japan/PG-86084/2021	EPI_ISL_3873118	2021-09-02
hCoV-19/Japan/PG-86085/2021	EPI_ISL_3873119	2021-09-02
hCoV-19/Japan/PG-86086/2021	EPI_ISL_3873120	2021-09-02
hCoV-19/Japan/PG-86087/2021	EPI_ISL_3873121	2021-09-02
hCoV-19/Japan/PG-86088/2021	EPI_ISL_3873122	2021-09-02
hCoV-19/Japan/PG-86089/2021	EPI_ISL_3873123	2021-09-02
hCoV-19/Japan/PG-86090/2021	EPI_ISL_3873124	2021-09-02
hCoV-19/Japan/PG-86091/2021	EPI_ISL_3873125	2021-09-02
hCoV-19/Japan/PG-86092/2021	EPI_ISL_3873126	2021-09-02
hCoV-19/Japan/PG-86093/2021	EPI_ISL_3873127	2021-09-02
hCoV-19/Japan/PG-86094/2021	EPI_ISL_3873128	2021-09-02
hCoV-19/Japan/PG-86095/2021	EPI_ISL_3873129	2021-09-02
hCoV-19/Japan/PG-86096/2021	EPI_ISL_3873130	2021-09-02
hCoV-19/Japan/PG-86097/2021	EPI_ISL_3873131	2021-09-02
hCoV-19/Japan/PG-86098/2021	EPI_ISL_3873132	2021-09-02
hCoV-19/Japan/PG-86099/2021	EPI_ISL_3873133	2021-09-02
hCoV-19/Japan/PG-86100/2021	EPI_ISL_3873134	2021-09-02
hCoV-19/Japan/PG-86101/2021	EPI_ISL_3873135	2021-09-02
hCoV-19/Japan/PG-86102/2021	EPI_ISL_3873136	2021-09-02
hCoV-19/Japan/PG-86103/2021	EPI_ISL_3873137	2021-09-02
hCoV-19/Japan/PG-86104/2021	EPI_ISL_3873138	2021-09-02
hCoV-19/Japan/PG-86105/2021	EPI_ISL_3873139	2021-09-02
hCoV-19/Japan/PG-86106/2021	EPI_ISL_3873140	2021-09-02
hCoV-19/Japan/PG-86107/2021	EPI_ISL_3873141	2021-09-02
hCoV-19/Japan/PG-86108/2021	EPI_ISL_3873142	2021-09-02
hCoV-19/Japan/PG-86109/2021	EPI_ISL_3873143	2021-09-02
hCoV-19/Japan/PG-86110/2021	EPI_ISL_3873144	2021-09-02
hCoV-19/Japan/PG-86111/2021	EPI_ISL_3873145	2021-09-02
hCoV-19/Japan/PG-86112/2021	EPI_ISL_3873146	2021-09-02
hCoV-19/Japan/PG-86113/2021	EPI_ISL_3873147	2021-09-02

virus_name	epiID	submission_date
hCoV-19/Japan/PG-86114/2021	EPI_ISL_3873148	2021-09-02
hCoV-19/Japan/PG-86115/2021	EPI_ISL_3873149	2021-09-02
hCoV-19/Japan/PG-86116/2021	EPI_ISL_3873150	2021-09-02
hCoV-19/Japan/PG-86117/2021	EPI_ISL_3873151	2021-09-02
hCoV-19/Japan/PG-86118/2021	EPI_ISL_3873152	2021-09-02
hCoV-19/Japan/PG-86119/2021	EPI_ISL_3873153	2021-09-02
hCoV-19/Japan/PG-86120/2021	EPI_ISL_3871523	2021-09-02
hCoV-19/Japan/PG-86121/2021	EPI_ISL_3871524	2021-09-02
hCoV-19/Japan/PG-86122/2021	EPI_ISL_3871525	2021-09-02
hCoV-19/Japan/PG-86123/2021	EPI_ISL_3871526	2021-09-02
hCoV-19/Japan/PG-86124/2021	EPI_ISL_3871527	2021-09-02
hCoV-19/Japan/PG-86125/2021	EPI_ISL_3871528	2021-09-02
hCoV-19/Japan/PG-86126/2021	EPI_ISL_3871529	2021-09-02
hCoV-19/Japan/PG-86127/2021	EPI_ISL_3871530	2021-09-02
hCoV-19/Japan/PG-86128/2021	EPI_ISL_3871531	2021-09-02
hCoV-19/Japan/PG-86129/2021	EPI_ISL_3871532	2021-09-02
hCoV-19/Japan/PG-86130/2021	EPI_ISL_3871533	2021-09-02
hCoV-19/Japan/PG-86131/2021	EPI_ISL_3871534	2021-09-02
hCoV-19/Japan/PG-86132/2021	EPI_ISL_3871535	2021-09-02
hCoV-19/Japan/PG-86133/2021	EPI_ISL_3871536	2021-09-02
hCoV-19/Japan/PG-86134/2021	EPI_ISL_3871537	2021-09-02
hCoV-19/Japan/PG-86135/2021	EPI_ISL_3871538	2021-09-02
hCoV-19/Japan/PG-86136/2021	EPI_ISL_3871539	2021-09-02
hCoV-19/Japan/PG-86137/2021	EPI_ISL_3871540	2021-09-02
hCoV-19/Japan/PG-86138/2021	EPI_ISL_3871541	2021-09-02
hCoV-19/Japan/PG-86139/2021	EPI_ISL_3871542	2021-09-02
hCoV-19/Japan/PG-86140/2021	EPI_ISL_3871543	2021-09-02
hCoV-19/Japan/PG-86141/2021	EPI_ISL_3871090	2021-09-02
hCoV-19/Japan/PG-86142/2021	EPI_ISL_3871091	2021-09-02
hCoV-19/Japan/PG-86143/2021	EPI_ISL_3871092	2021-09-02
hCoV-19/Japan/PG-86144/2021	EPI_ISL_3871093	2021-09-02
hCoV-19/Japan/PG-86145/2021	EPI_ISL_3871094	2021-09-02
hCoV-19/Japan/PG-86146/2021	EPI_ISL_3871095	2021-09-02
hCoV-19/Japan/PG-86147/2021	EPI_ISL_3871096	2021-09-02
hCoV-19/Japan/PG-86148/2021	EPI_ISL_3871097	2021-09-02
hCoV-19/Japan/PG-86149/2021	EPI_ISL_3871098	2021-09-02
hCoV-19/Japan/PG-86150/2021	EPI_ISL_3871099	2021-09-02
hCoV-19/Japan/PG-86151/2021	EPI_ISL_3871100	2021-09-02
hCoV-19/Japan/PG-86152/2021	EPI_ISL_3871101	2021-09-02
hCoV-19/Japan/PG-86153/2021	EPI_ISL_3871102	2021-09-02
hCoV-19/Japan/PG-86154/2021	EPI_ISL_3871103	2021-09-02
hCoV-19/Japan/PG-86155/2021	EPI_ISL_3871104	2021-09-02
hCoV-19/Japan/PG-86156/2021	EPI_ISL_3871105	2021-09-02
hCoV-19/Japan/PG-86157/2021	EPI_ISL_3871106	2021-09-02
hCoV-19/Japan/PG-86158/2021	EPI_ISL_3871107	2021-09-02
hCoV-19/Japan/PG-86159/2021	EPI_ISL_3871108	2021-09-02
hCoV-19/Japan/PG-86160/2021	EPI_ISL_3871109	2021-09-02
hCoV-19/Japan/PG-86161/2021	EPI_ISL_3871314	2021-09-02
hCoV-19/Japan/PG-86162/2021	EPI_ISL_3871315	2021-09-02
hCoV-19/Japan/PG-86163/2021	EPI_ISL_3871316	2021-09-02
hCoV-19/Japan/PG-86164/2021	EPI_ISL_3871317	2021-09-02
hCoV-19/Japan/PG-86165/2021	EPI_ISL_3871318	2021-09-02
hCoV-19/Japan/PG-86166/2021	EPI_ISL_3871319	2021-09-02
hCoV-19/Japan/PG-86167/2021	EPI_ISL_3871320	2021-09-02

virus_name	epiID	submission_date
hCoV-19/Japan/PG-86168/2021	EPI_ISL_3871321	2021-09-02
hCoV-19/Japan/PG-86169/2021	EPI_ISL_3871322	2021-09-02
hCoV-19/Japan/PG-86170/2021	EPI_ISL_3871323	2021-09-02
hCoV-19/Japan/PG-86171/2021	EPI_ISL_3871324	2021-09-02
hCoV-19/Japan/PG-86172/2021	EPI_ISL_3871325	2021-09-02
hCoV-19/Japan/PG-86173/2021	EPI_ISL_3871326	2021-09-02
hCoV-19/Japan/PG-86174/2021	EPI_ISL_3871327	2021-09-02
hCoV-19/Japan/PG-86175/2021	EPI_ISL_3871328	2021-09-02
hCoV-19/Japan/PG-86176/2021	EPI_ISL_3871329	2021-09-02
hCoV-19/Japan/PG-86177/2021	EPI_ISL_3871330	2021-09-02
hCoV-19/Japan/PG-86178/2021	EPI_ISL_3871331	2021-09-02
hCoV-19/Japan/PG-86179/2021	EPI_ISL_3871332	2021-09-02
hCoV-19/Japan/PG-86180/2021	EPI_ISL_3871333	2021-09-02
hCoV-19/Japan/PG-86181/2021	EPI_ISL_3871334	2021-09-02
hCoV-19/Japan/PG-86182/2021	EPI_ISL_3871335	2021-09-02
hCoV-19/Japan/PG-86183/2021	EPI_ISL_3874037	2021-09-02
hCoV-19/Japan/PG-86184/2021	EPI_ISL_3874038	2021-09-02
hCoV-19/Japan/PG-86185/2021	EPI_ISL_3874039	2021-09-02
hCoV-19/Japan/PG-86186/2021	EPI_ISL_3874040	2021-09-02
hCoV-19/Japan/PG-86187/2021	EPI_ISL_3874041	2021-09-02
hCoV-19/Japan/PG-86188/2021	EPI_ISL_3874042	2021-09-02
hCoV-19/Japan/PG-86189/2021	EPI_ISL_3874043	2021-09-02
hCoV-19/Japan/PG-86190/2021	EPI_ISL_3874044	2021-09-02
hCoV-19/Japan/PG-86191/2021	EPI_ISL_3874045	2021-09-02
hCoV-19/Japan/PG-86192/2021	EPI_ISL_3874046	2021-09-02
hCoV-19/Japan/PG-86193/2021	EPI_ISL_3874047	2021-09-02
hCoV-19/Japan/PG-86194/2021	EPI_ISL_3874048	2021-09-02
hCoV-19/Japan/PG-86195/2021	EPI_ISL_3874049	2021-09-02
hCoV-19/Japan/PG-86196/2021	EPI_ISL_3874050	2021-09-02
hCoV-19/Japan/PG-86197/2021	EPI_ISL_3874051	2021-09-02
hCoV-19/Japan/PG-86198/2021	EPI_ISL_3874052	2021-09-02
hCoV-19/Japan/PG-86199/2021	EPI_ISL_3874053	2021-09-02
hCoV-19/Japan/PG-86200/2021	EPI_ISL_3874054	2021-09-02
hCoV-19/Japan/PG-86201/2021	EPI_ISL_3874055	2021-09-02
hCoV-19/Japan/PG-86202/2021	EPI_ISL_3874056	2021-09-02
hCoV-19/Japan/PG-86203/2021	EPI_ISL_3874057	2021-09-02
hCoV-19/Japan/PG-86204/2021	EPI_ISL_3874058	2021-09-02
hCoV-19/Japan/PG-86205/2021	EPI_ISL_3874059	2021-09-02
hCoV-19/Japan/PG-86206/2021	EPI_ISL_3874060	2021-09-02
hCoV-19/Japan/PG-86207/2021	EPI_ISL_3874061	2021-09-02
hCoV-19/Japan/PG-86208/2021	EPI_ISL_3874062	2021-09-02
hCoV-19/Japan/PG-86209/2021	EPI_ISL_3874063	2021-09-02
hCoV-19/Japan/PG-86210/2021	EPI_ISL_3874064	2021-09-02
hCoV-19/Japan/PG-86212/2021	EPI_ISL_3874065	2021-09-02
hCoV-19/Japan/PG-86213/2021	EPI_ISL_3870468	2021-09-02
hCoV-19/Japan/PG-86214/2021	EPI_ISL_3870469	2021-09-02
hCoV-19/Japan/PG-86215/2021	EPI_ISL_3870470	2021-09-02
hCoV-19/Japan/PG-86216/2021	EPI_ISL_3870471	2021-09-02
hCoV-19/Japan/PG-86217/2021	EPI_ISL_3870472	2021-09-02
hCoV-19/Japan/PG-86218/2021	EPI_ISL_3870473	2021-09-02
hCoV-19/Japan/PG-86219/2021	EPI_ISL_3870474	2021-09-02
hCoV-19/Japan/PG-86220/2021	EPI_ISL_3870475	2021-09-02
hCoV-19/Japan/PG-86221/2021	EPI_ISL_3870476	2021-09-02
hCoV-19/Japan/PG-86222/2021	EPI_ISL_3870477	2021-09-02

virus_name	epiID	submission_date
hCoV-19/Japan/PG-86223/2021	EPI_ISL_3870478	2021-09-02
hCoV-19/Japan/PG-86224/2021	EPI_ISL_3870479	2021-09-02
hCoV-19/Japan/PG-86225/2021	EPI_ISL_3870480	2021-09-02
hCoV-19/Japan/PG-86226/2021	EPI_ISL_3870481	2021-09-02
hCoV-19/Japan/PG-86227/2021	EPI_ISL_3870482	2021-09-02
hCoV-19/Japan/PG-86232/2021	EPI_ISL_3874235	2021-09-02
hCoV-19/Japan/PG-86233/2021	EPI_ISL_3874236	2021-09-02
hCoV-19/Japan/PG-86234/2021	EPI_ISL_3874237	2021-09-02
hCoV-19/Japan/PG-86236/2021	EPI_ISL_3874238	2021-09-02
hCoV-19/Japan/PG-86237/2021	EPI_ISL_3874239	2021-09-02
hCoV-19/Japan/PG-86238/2021	EPI_ISL_3874240	2021-09-02
hCoV-19/Japan/PG-86239/2021	EPI_ISL_3874241	2021-09-02
hCoV-19/Japan/PG-86240/2021	EPI_ISL_3874242	2021-09-02
hCoV-19/Japan/PG-86241/2021	EPI_ISL_3874243	2021-09-02
hCoV-19/Japan/PG-86243/2021	EPI_ISL_3874244	2021-09-02
hCoV-19/Japan/PG-86244/2021	EPI_ISL_3874245	2021-09-02
hCoV-19/Japan/PG-86245/2021	EPI_ISL_3874246	2021-09-02
hCoV-19/Japan/PG-86246/2021	EPI_ISL_3874247	2021-09-02
hCoV-19/Japan/PG-86247/2021	EPI_ISL_3874248	2021-09-02
hCoV-19/Japan/PG-86248/2021	EPI_ISL_3874249	2021-09-02
hCoV-19/Japan/PG-86249/2021	EPI_ISL_3872197	2021-09-02
hCoV-19/Japan/PG-86251/2021	EPI_ISL_3872198	2021-09-02
hCoV-19/Japan/PG-86252/2021	EPI_ISL_3872199	2021-09-02
hCoV-19/Japan/PG-86253/2021	EPI_ISL_3872200	2021-09-02
hCoV-19/Japan/PG-86254/2021	EPI_ISL_3872201	2021-09-02
hCoV-19/Japan/PG-86255/2021	EPI_ISL_3872202	2021-09-02
hCoV-19/Japan/PG-86256/2021	EPI_ISL_3872203	2021-09-02
hCoV-19/Japan/PG-86258/2021	EPI_ISL_3872204	2021-09-02
hCoV-19/Japan/PG-86259/2021	EPI_ISL_3872205	2021-09-02
hCoV-19/Japan/PG-86260/2021	EPI_ISL_3872206	2021-09-02
hCoV-19/Japan/PG-86261/2021	EPI_ISL_3872207	2021-09-02
hCoV-19/Japan/PG-86262/2021	EPI_ISL_3872208	2021-09-02
hCoV-19/Japan/PG-86263/2021	EPI_ISL_3872209	2021-09-02
hCoV-19/Japan/PG-86264/2021	EPI_ISL_3872210	2021-09-02
hCoV-19/Japan/PG-86265/2021	EPI_ISL_3872211	2021-09-02
hCoV-19/Japan/PG-86266/2021	EPI_ISL_3872212	2021-09-02
hCoV-19/Japan/PG-86267/2021	EPI_ISL_3872213	2021-09-02
hCoV-19/Japan/PG-86268/2021	EPI_ISL_3872214	2021-09-02
hCoV-19/Japan/PG-86269/2021	EPI_ISL_3872215	2021-09-02
hCoV-19/Japan/PG-86270/2021	EPI_ISL_3872216	2021-09-02
hCoV-19/Japan/PG-86273/2021	EPI_ISL_3872312	2021-09-02
hCoV-19/Japan/PG-86274/2021	EPI_ISL_3872313	2021-09-02
hCoV-19/Japan/PG-86275/2021	EPI_ISL_3872314	2021-09-02
hCoV-19/Japan/PG-86276/2021	EPI_ISL_3872315	2021-09-02
hCoV-19/Japan/PG-86277/2021	EPI_ISL_3872316	2021-09-02
hCoV-19/Japan/PG-86278/2021	EPI_ISL_3872317	2021-09-02
hCoV-19/Japan/PG-86279/2021	EPI_ISL_3872318	2021-09-02
hCoV-19/Japan/PG-86280/2021	EPI_ISL_3872319	2021-09-02
hCoV-19/Japan/PG-86281/2021	EPI_ISL_3872320	2021-09-02
hCoV-19/Japan/PG-86282/2021	EPI_ISL_3872321	2021-09-02
hCoV-19/Japan/PG-86283/2021	EPI_ISL_3872322	2021-09-02
hCoV-19/Japan/PG-86284/2021	EPI_ISL_3872323	2021-09-02
hCoV-19/Japan/PG-86285/2021	EPI_ISL_3872324	2021-09-02
hCoV-19/Japan/PG-86286/2021	EPI_ISL_3872325	2021-09-02

virus_name	epiID	submission_date
hCoV-19/Japan/PG-86287/2021	EPI_ISL_3872326	2021-09-02
hCoV-19/Japan/PG-86288/2021	EPI_ISL_3872327	2021-09-02
hCoV-19/Japan/PG-86289/2021	EPI_ISL_3872328	2021-09-02
hCoV-19/Japan/PG-86290/2021	EPI_ISL_3871846	2021-09-02
hCoV-19/Japan/PG-86291/2021	EPI_ISL_3871847	2021-09-02
hCoV-19/Japan/PG-86292/2021	EPI_ISL_3871848	2021-09-02
hCoV-19/Japan/PG-86293/2021	EPI_ISL_3871849	2021-09-02
hCoV-19/Japan/PG-86294/2021	EPI_ISL_3871850	2021-09-02
hCoV-19/Japan/PG-86295/2021	EPI_ISL_3871851	2021-09-02
hCoV-19/Japan/PG-86298/2021	EPI_ISL_3871852	2021-09-02
hCoV-19/Japan/PG-86299/2021	EPI_ISL_3871853	2021-09-02
hCoV-19/Japan/PG-86300/2021	EPI_ISL_3871854	2021-09-02
hCoV-19/Japan/PG-86301/2021	EPI_ISL_3871855	2021-09-02
hCoV-19/Japan/PG-86302/2021	EPI_ISL_3871856	2021-09-02
hCoV-19/Japan/PG-86303/2021	EPI_ISL_3871857	2021-09-02
hCoV-19/Japan/PG-86304/2021	EPI_ISL_3871858	2021-09-02
hCoV-19/Japan/PG-86305/2021	EPI_ISL_3871859	2021-09-02
hCoV-19/Japan/PG-86306/2021	EPI_ISL_3871860	2021-09-02
hCoV-19/Japan/PG-86307/2021	EPI_ISL_3871861	2021-09-02
hCoV-19/Japan/PG-86308/2021	EPI_ISL_3871862	2021-09-02
hCoV-19/Japan/PG-86309/2021	EPI_ISL_3871863	2021-09-02
hCoV-19/Japan/PG-86310/2021	EPI_ISL_3871864	2021-09-02
hCoV-19/Japan/PG-86311/2021	EPI_ISL_3871865	2021-09-02
hCoV-19/Japan/PG-86312/2021	EPI_ISL_3871866	2021-09-02
hCoV-19/Japan/PG-86314/2021	EPI_ISL_3871900	2021-09-02
hCoV-19/Japan/PG-86315/2021	EPI_ISL_3871901	2021-09-02
hCoV-19/Japan/PG-86316/2021	EPI_ISL_3871902	2021-09-02
hCoV-19/Japan/PG-86317/2021	EPI_ISL_3871903	2021-09-02
hCoV-19/Japan/PG-86318/2021	EPI_ISL_3871904	2021-09-02
hCoV-19/Japan/PG-86319/2021	EPI_ISL_3871905	2021-09-02
hCoV-19/Japan/PG-86320/2021	EPI_ISL_3871906	2021-09-02
hCoV-19/Japan/PG-86321/2021	EPI_ISL_3871907	2021-09-02
hCoV-19/Japan/PG-86322/2021	EPI_ISL_3871908	2021-09-02
hCoV-19/Japan/PG-86323/2021	EPI_ISL_3871909	2021-09-02
hCoV-19/Japan/PG-86324/2021	EPI_ISL_3871910	2021-09-02
hCoV-19/Japan/PG-86325/2021	EPI_ISL_3871911	2021-09-02
hCoV-19/Japan/PG-86326/2021	EPI_ISL_3871912	2021-09-02
hCoV-19/Japan/PG-86327/2021	EPI_ISL_3871913	2021-09-02
hCoV-19/Japan/PG-86328/2021	EPI_ISL_3871914	2021-09-02
hCoV-19/Japan/PG-86329/2021	EPI_ISL_3871915	2021-09-02
hCoV-19/Japan/PG-86330/2021	EPI_ISL_3871916	2021-09-02
hCoV-19/Japan/PG-86331/2021	EPI_ISL_3871917	2021-09-02
hCoV-19/Japan/PG-86332/2021	EPI_ISL_3871918	2021-09-02
hCoV-19/Japan/PG-86333/2021	EPI_ISL_3871919	2021-09-02
hCoV-19/Japan/PG-86334/2021	EPI_ISL_3871920	2021-09-02
hCoV-19/Japan/PG-86335/2021	EPI_ISL_3871921	2021-09-02
hCoV-19/Japan/PG-86336/2021	EPI_ISL_3871922	2021-09-02
hCoV-19/Japan/PG-86337/2021	EPI_ISL_3871923	2021-09-02
hCoV-19/Japan/PG-86338/2021	EPI_ISL_3871924	2021-09-02
hCoV-19/Japan/PG-86339/2021	EPI_ISL_3871925	2021-09-02
hCoV-19/Japan/PG-86340/2021	EPI_ISL_3871926	2021-09-02
hCoV-19/Japan/PG-86341/2021	EPI_ISL_3871927	2021-09-02
hCoV-19/Japan/PG-86342/2021	EPI_ISL_3871928	2021-09-02
hCoV-19/Japan/PG-86343/2021	EPI_ISL_3871929	2021-09-02

virus_name	epiID	submission_date
hCoV-19/Japan/PG-86344/2021	EPI_ISL_3871930	2021-09-02
hCoV-19/Japan/PG-86345/2021	EPI_ISL_3871931	2021-09-02
hCoV-19/Japan/PG-86346/2021	EPI_ISL_3871932	2021-09-02
hCoV-19/Japan/PG-86347/2021	EPI_ISL_3871933	2021-09-02
hCoV-19/Japan/PG-86348/2021	EPI_ISL_3871934	2021-09-02
hCoV-19/Japan/PG-86349/2021	EPI_ISL_3871935	2021-09-02
hCoV-19/Japan/PG-86350/2021	EPI_ISL_3871936	2021-09-02
hCoV-19/Japan/PG-86351/2021	EPI_ISL_3871937	2021-09-02
hCoV-19/Japan/PG-86352/2021	EPI_ISL_3871938	2021-09-02
hCoV-19/Japan/PG-86353/2021	EPI_ISL_3871939	2021-09-02
hCoV-19/Japan/PG-86354/2021	EPI_ISL_3871940	2021-09-02
hCoV-19/Japan/PG-86355/2021	EPI_ISL_3871941	2021-09-02
hCoV-19/Japan/PG-86356/2021	EPI_ISL_3871942	2021-09-02
hCoV-19/Japan/PG-86357/2021	EPI_ISL_3871943	2021-09-02
hCoV-19/Japan/PG-86358/2021	EPI_ISL_3871944	2021-09-02
hCoV-19/Japan/PG-86359/2021	EPI_ISL_3871945	2021-09-02
hCoV-19/Japan/PG-86360/2021	EPI_ISL_3871946	2021-09-02
hCoV-19/Japan/PG-86361/2021	EPI_ISL_3871947	2021-09-02
hCoV-19/Japan/PG-86362/2021	EPI_ISL_3873415	2021-09-02
hCoV-19/Japan/PG-86363/2021	EPI_ISL_3873416	2021-09-02
hCoV-19/Japan/PG-86364/2021	EPI_ISL_3873417	2021-09-02
hCoV-19/Japan/PG-86376/2021	EPI_ISL_3873418	2021-09-02
hCoV-19/Japan/PG-86377/2021	EPI_ISL_3873419	2021-09-02
hCoV-19/Japan/PG-86378/2021	EPI_ISL_3873420	2021-09-02
hCoV-19/Japan/PG-86379/2021	EPI_ISL_3873421	2021-09-02
hCoV-19/Japan/PG-86380/2021	EPI_ISL_3873422	2021-09-02
hCoV-19/Japan/PG-86385/2021	EPI_ISL_3874250	2021-09-02
hCoV-19/Japan/PG-86386/2021	EPI_ISL_3872329	2021-09-02
hCoV-19/Japan/PG-86387/2021	EPI_ISL_3872330	2021-09-02
hCoV-19/Japan/PG-86388/2021	EPI_ISL_3872331	2021-09-02
hCoV-19/Japan/PG-86389/2021	EPI_ISL_3872332	2021-09-02
hCoV-19/Japan/PG-86390/2021	EPI_ISL_3872333	2021-09-02
hCoV-19/Japan/PG-86391/2021	EPI_ISL_3898106	2021-09-02
hCoV-19/Japan/PG-86392/2021	EPI_ISL_3898107	2021-09-02
hCoV-19/Japan/PG-86393/2021	EPI_ISL_3898108	2021-09-02
hCoV-19/Japan/PG-86394/2021	EPI_ISL_3898109	2021-09-02
hCoV-19/Japan/PG-86395/2021	EPI_ISL_3898110	2021-09-02
hCoV-19/Japan/PG-86396/2021	EPI_ISL_3898111	2021-09-02
hCoV-19/Japan/PG-86397/2021	EPI_ISL_3898112	2021-09-02
hCoV-19/Japan/PG-86398/2021	EPI_ISL_3898113	2021-09-02
hCoV-19/Japan/PG-86399/2021	EPI_ISL_3898114	2021-09-02
hCoV-19/Japan/PG-86400/2021	EPI_ISL_3898115	2021-09-02
hCoV-19/Japan/PG-86401/2021	EPI_ISL_3898116	2021-09-02
hCoV-19/Japan/PG-86402/2021	EPI_ISL_3898117	2021-09-02
hCoV-19/Japan/PG-86403/2021	EPI_ISL_3898118	2021-09-02
hCoV-19/Japan/PG-86404/2021	EPI_ISL_3898119	2021-09-02
hCoV-19/Japan/PG-86405/2021	EPI_ISL_3898120	2021-09-02
hCoV-19/Japan/PG-86406/2021	EPI_ISL_3898121	2021-09-02
hCoV-19/Japan/PG-86407/2021	EPI_ISL_3898122	2021-09-02
hCoV-19/Japan/PG-86408/2021	EPI_ISL_3898123	2021-09-02
hCoV-19/Japan/PG-86409/2021	EPI_ISL_3898124	2021-09-02
hCoV-19/Japan/PG-86410/2021	EPI_ISL_3898125	2021-09-02
hCoV-19/Japan/PG-86411/2021	EPI_ISL_3898126	2021-09-02
hCoV-19/Japan/PG-86412/2021	EPI_ISL_3898127	2021-09-02

virus_name	epiID	submission_date
hCoV-19/Japan/PG-86413/2021	EPI_ISL_3898128	2021-09-02
hCoV-19/Japan/PG-86414/2021	EPI_ISL_3898129	2021-09-02
hCoV-19/Japan/PG-86415/2021	EPI_ISL_3898130	2021-09-02
hCoV-19/Japan/PG-86416/2021	EPI_ISL_3898131	2021-09-02
hCoV-19/Japan/PG-86417/2021	EPI_ISL_3898132	2021-09-02
hCoV-19/Japan/PG-86418/2021	EPI_ISL_3898133	2021-09-02
hCoV-19/Japan/PG-86419/2021	EPI_ISL_3898134	2021-09-02
hCoV-19/Japan/PG-86420/2021	EPI_ISL_3898135	2021-09-02
hCoV-19/Japan/PG-86421/2021	EPI_ISL_3898136	2021-09-02
hCoV-19/Japan/PG-86422/2021	EPI_ISL_3898137	2021-09-02
hCoV-19/Japan/PG-86423/2021	EPI_ISL_3898138	2021-09-02
hCoV-19/Japan/PG-86424/2021	EPI_ISL_3898139	2021-09-02
hCoV-19/Japan/PG-86425/2021	EPI_ISL_3898140	2021-09-02
hCoV-19/Japan/PG-86426/2021	EPI_ISL_3898141	2021-09-02
hCoV-19/Japan/PG-86427/2021	EPI_ISL_3898142	2021-09-02
hCoV-19/Japan/PG-86428/2021	EPI_ISL_3898143	2021-09-02
hCoV-19/Japan/PG-86429/2021	EPI_ISL_3898144	2021-09-02
hCoV-19/Japan/PG-86430/2021	EPI_ISL_3898145	2021-09-02
hCoV-19/Japan/PG-86431/2021	EPI_ISL_3898146	2021-09-02
hCoV-19/Japan/PG-86432/2021	EPI_ISL_3898147	2021-09-02
hCoV-19/Japan/PG-86433/2021	EPI_ISL_3898148	2021-09-02
hCoV-19/Japan/PG-86434/2021	EPI_ISL_3898149	2021-09-02
hCoV-19/Japan/PG-86435/2021	EPI_ISL_3898150	2021-09-02
hCoV-19/Japan/PG-86436/2021	EPI_ISL_3898151	2021-09-02
hCoV-19/Japan/PG-86437/2021	EPI_ISL_3898152	2021-09-02
hCoV-19/Japan/PG-86438/2021	EPI_ISL_3898153	2021-09-02
hCoV-19/Japan/PG-86439/2021	EPI_ISL_3898154	2021-09-02
hCoV-19/Japan/PG-86440/2021	EPI_ISL_3898155	2021-09-02
hCoV-19/Japan/PG-86441/2021	EPI_ISL_3898156	2021-09-02
hCoV-19/Japan/PG-86442/2021	EPI_ISL_3898157	2021-09-02
hCoV-19/Japan/PG-86443/2021	EPI_ISL_3898158	2021-09-02
hCoV-19/Japan/PG-86444/2021	EPI_ISL_3898159	2021-09-02
hCoV-19/Japan/PG-86445/2021	EPI_ISL_3898160	2021-09-02
hCoV-19/Japan/PG-86446/2021	EPI_ISL_3898161	2021-09-02
hCoV-19/Japan/PG-86447/2021	EPI_ISL_3898162	2021-09-02
hCoV-19/Japan/PG-86448/2021	EPI_ISL_3898163	2021-09-02
hCoV-19/Japan/PG-86449/2021	EPI_ISL_3898164	2021-09-02
hCoV-19/Japan/PG-86450/2021	EPI_ISL_3898165	2021-09-02
hCoV-19/Japan/PG-86451/2021	EPI_ISL_3898166	2021-09-02
hCoV-19/Japan/PG-86452/2021	EPI_ISL_3898167	2021-09-02
hCoV-19/Japan/PG-86453/2021	EPI_ISL_3898168	2021-09-02
hCoV-19/Japan/PG-86454/2021	EPI_ISL_3898169	2021-09-02
hCoV-19/Japan/PG-86455/2021	EPI_ISL_3898170	2021-09-02
hCoV-19/Japan/PG-86456/2021	EPI_ISL_3898171	2021-09-02
hCoV-19/Japan/PG-86457/2021	EPI_ISL_3898172	2021-09-02
hCoV-19/Japan/PG-86458/2021	EPI_ISL_3898173	2021-09-02
hCoV-19/Japan/PG-86459/2021	EPI_ISL_3898174	2021-09-02
hCoV-19/Japan/PG-86460/2021	EPI_ISL_3898175	2021-09-02
hCoV-19/Japan/PG-86461/2021	EPI_ISL_3898176	2021-09-02
hCoV-19/Japan/PG-86462/2021	EPI_ISL_3898177	2021-09-02
hCoV-19/Japan/PG-86463/2021	EPI_ISL_3898178	2021-09-02
hCoV-19/Japan/PG-86464/2021	EPI_ISL_3898179	2021-09-02
hCoV-19/Japan/PG-86465/2021	EPI_ISL_3898180	2021-09-02
hCoV-19/Japan/PG-86466/2021	EPI_ISL_3898181	2021-09-02

virus_name	epiID	submission_date
hCoV-19/Japan/PG-86467/2021	EPI_ISL_3898182	2021-09-02
hCoV-19/Japan/PG-86468/2021	EPI_ISL_3898183	2021-09-02
hCoV-19/Japan/PG-86469/2021	EPI_ISL_3898184	2021-09-02
hCoV-19/Japan/PG-86470/2021	EPI_ISL_3898185	2021-09-02
hCoV-19/Japan/PG-86471/2021	EPI_ISL_3898186	2021-09-02
hCoV-19/Japan/PG-86472/2021	EPI_ISL_3898187	2021-09-02
hCoV-19/Japan/PG-86473/2021	EPI_ISL_3898188	2021-09-02
hCoV-19/Japan/PG-86474/2021	EPI_ISL_3898189	2021-09-02
hCoV-19/Japan/PG-86475/2021	EPI_ISL_3898190	2021-09-02
hCoV-19/Japan/PG-86476/2021	EPI_ISL_3898191	2021-09-02
hCoV-19/Japan/PG-86477/2021	EPI_ISL_3898192	2021-09-02
hCoV-19/Japan/PG-86478/2021	EPI_ISL_3898193	2021-09-02
hCoV-19/Japan/PG-86479/2021	EPI_ISL_3898194	2021-09-02
hCoV-19/Japan/PG-86480/2021	EPI_ISL_3898195	2021-09-02
hCoV-19/Japan/PG-86481/2021	EPI_ISL_3898196	2021-09-02
hCoV-19/Japan/PG-86482/2021	EPI_ISL_3898197	2021-09-02
hCoV-19/Japan/PG-86483/2021	EPI_ISL_3898198	2021-09-02
hCoV-19/Japan/PG-86484/2021	EPI_ISL_3898199	2021-09-02
hCoV-19/Japan/PG-86485/2021	EPI_ISL_3898200	2021-09-02
hCoV-19/Japan/PG-86486/2021	EPI_ISL_3898201	2021-09-02
hCoV-19/Japan/PG-86487/2021	EPI_ISL_3898202	2021-09-02
hCoV-19/Japan/PG-86488/2021	EPI_ISL_3898203	2021-09-02
hCoV-19/Japan/PG-86489/2021	EPI_ISL_3898204	2021-09-02
hCoV-19/Japan/PG-86490/2021	EPI_ISL_3898205	2021-09-02
hCoV-19/Japan/PG-86491/2021	EPI_ISL_3898206	2021-09-02
hCoV-19/Japan/PG-86492/2021	EPI_ISL_3898207	2021-09-02
hCoV-19/Japan/PG-86493/2021	EPI_ISL_3898208	2021-09-02
hCoV-19/Japan/PG-86494/2021	EPI_ISL_3898209	2021-09-02
hCoV-19/Japan/PG-86495/2021	EPI_ISL_3898210	2021-09-02
hCoV-19/Japan/PG-86496/2021	EPI_ISL_3898211	2021-09-02
hCoV-19/Japan/PG-86497/2021	EPI_ISL_3898212	2021-09-02
hCoV-19/Japan/PG-86498/2021	EPI_ISL_3898213	2021-09-02
hCoV-19/Japan/PG-86499/2021	EPI_ISL_3898214	2021-09-02
hCoV-19/Japan/PG-86500/2021	EPI_ISL_3898215	2021-09-02
hCoV-19/Japan/PG-86501/2021	EPI_ISL_3898216	2021-09-02
hCoV-19/Japan/PG-86502/2021	EPI_ISL_3898217	2021-09-02
hCoV-19/Japan/PG-86503/2021	EPI_ISL_3898218	2021-09-02
hCoV-19/Japan/PG-86504/2021	EPI_ISL_3898219	2021-09-02
hCoV-19/Japan/PG-86505/2021	EPI_ISL_3898220	2021-09-02
hCoV-19/Japan/PG-86506/2021	EPI_ISL_3898221	2021-09-02
hCoV-19/Japan/PG-86507/2021	EPI_ISL_3898222	2021-09-02
hCoV-19/Japan/PG-86508/2021	EPI_ISL_3898223	2021-09-02
hCoV-19/Japan/PG-86509/2021	EPI_ISL_3898224	2021-09-02
hCoV-19/Japan/PG-86510/2021	EPI_ISL_3898225	2021-09-02
hCoV-19/Japan/PG-86511/2021	EPI_ISL_3898226	2021-09-02
hCoV-19/Japan/PG-86512/2021	EPI_ISL_3898227	2021-09-02
hCoV-19/Japan/PG-86513/2021	EPI_ISL_3898228	2021-09-02
hCoV-19/Japan/PG-86514/2021	EPI_ISL_3898229	2021-09-02
hCoV-19/Japan/PG-86515/2021	EPI_ISL_3898230	2021-09-02
hCoV-19/Japan/PG-86516/2021	EPI_ISL_3898231	2021-09-02
hCoV-19/Japan/PG-86517/2021	EPI_ISL_3898232	2021-09-02
hCoV-19/Japan/PG-86518/2021	EPI_ISL_3898233	2021-09-02
hCoV-19/Japan/PG-86519/2021	EPI_ISL_3898234	2021-09-02
hCoV-19/Japan/PG-86520/2021	EPI_ISL_3898235	2021-09-02

virus_name	epiID	submission_date
hCoV-19/Japan/PG-86521/2021	EPI_ISL_3898236	2021-09-02
hCoV-19/Japan/PG-86522/2021	EPI_ISL_3898237	2021-09-02
hCoV-19/Japan/PG-86523/2021	EPI_ISL_3898238	2021-09-02
hCoV-19/Japan/PG-86524/2021	EPI_ISL_3898239	2021-09-02
hCoV-19/Japan/PG-86525/2021	EPI_ISL_3898240	2021-09-02
hCoV-19/Japan/PG-86526/2021	EPI_ISL_3898241	2021-09-02
hCoV-19/Japan/PG-86527/2021	EPI_ISL_3898242	2021-09-02
hCoV-19/Japan/PG-86528/2021	EPI_ISL_3898243	2021-09-02
hCoV-19/Japan/PG-86529/2021	EPI_ISL_3898244	2021-09-02
hCoV-19/Japan/PG-86530/2021	EPI_ISL_3898245	2021-09-02
hCoV-19/Japan/PG-86531/2021	EPI_ISL_3898246	2021-09-02
hCoV-19/Japan/PG-86532/2021	EPI_ISL_3898247	2021-09-02
hCoV-19/Japan/PG-86533/2021	EPI_ISL_3898248	2021-09-02
hCoV-19/Japan/PG-86534/2021	EPI_ISL_3898249	2021-09-02
hCoV-19/Japan/PG-86535/2021	EPI_ISL_3898250	2021-09-02
hCoV-19/Japan/PG-86536/2021	EPI_ISL_3898251	2021-09-02
hCoV-19/Japan/PG-86537/2021	EPI_ISL_3898252	2021-09-02
hCoV-19/Japan/PG-86538/2021	EPI_ISL_3898253	2021-09-02
hCoV-19/Japan/PG-86539/2021	EPI_ISL_3898254	2021-09-02
hCoV-19/Japan/PG-86540/2021	EPI_ISL_3898255	2021-09-02
hCoV-19/Japan/PG-86541/2021	EPI_ISL_3898256	2021-09-02
hCoV-19/Japan/PG-86542/2021	EPI_ISL_3898257	2021-09-02
hCoV-19/Japan/PG-86543/2021	EPI_ISL_3898258	2021-09-02
hCoV-19/Japan/PG-86544/2021	EPI_ISL_3898259	2021-09-02
hCoV-19/Japan/PG-86545/2021	EPI_ISL_3898260	2021-09-02
hCoV-19/Japan/PG-86546/2021	EPI_ISL_3898261	2021-09-02
hCoV-19/Japan/PG-86547/2021	EPI_ISL_3898262	2021-09-02
hCoV-19/Japan/PG-86548/2021	EPI_ISL_3898263	2021-09-02
hCoV-19/Japan/PG-86549/2021	EPI_ISL_3898264	2021-09-02
hCoV-19/Japan/PG-86550/2021	EPI_ISL_3898265	2021-09-02
hCoV-19/Japan/PG-86551/2021	EPI_ISL_3898266	2021-09-02
hCoV-19/Japan/PG-86552/2021	EPI_ISL_3898267	2021-09-02
hCoV-19/Japan/PG-86553/2021	EPI_ISL_3898268	2021-09-02
hCoV-19/Japan/PG-86554/2021	EPI_ISL_3898269	2021-09-02
hCoV-19/Japan/PG-86555/2021	EPI_ISL_3898270	2021-09-02
hCoV-19/Japan/PG-86556/2021	EPI_ISL_3898271	2021-09-02
hCoV-19/Japan/PG-86557/2021	EPI_ISL_3898272	2021-09-02
hCoV-19/Japan/PG-86558/2021	EPI_ISL_3898273	2021-09-02
hCoV-19/Japan/PG-86559/2021	EPI_ISL_3898274	2021-09-02
hCoV-19/Japan/PG-86560/2021	EPI_ISL_3898275	2021-09-02
hCoV-19/Japan/PG-86561/2021	EPI_ISL_3898276	2021-09-02
hCoV-19/Japan/PG-86562/2021	EPI_ISL_3898277	2021-09-02
hCoV-19/Japan/PG-86563/2021	EPI_ISL_3898278	2021-09-02
hCoV-19/Japan/PG-86564/2021	EPI_ISL_3898279	2021-09-02
hCoV-19/Japan/PG-86565/2021	EPI_ISL_3898280	2021-09-02
hCoV-19/Japan/PG-86566/2021	EPI_ISL_3898281	2021-09-02
hCoV-19/Japan/PG-86567/2021	EPI_ISL_3898282	2021-09-02
hCoV-19/Japan/PG-86568/2021	EPI_ISL_3898283	2021-09-02
hCoV-19/Japan/PG-86569/2021	EPI_ISL_3898284	2021-09-02
hCoV-19/Japan/PG-86570/2021	EPI_ISL_3898285	2021-09-02
hCoV-19/Japan/PG-86571/2021	EPI_ISL_3898286	2021-09-02
hCoV-19/Japan/PG-86572/2021	EPI_ISL_3898287	2021-09-02
hCoV-19/Japan/PG-86573/2021	EPI_ISL_3898288	2021-09-02
hCoV-19/Japan/PG-86574/2021	EPI_ISL_3898289	2021-09-02

virus_name	epiID	submission_date
hCoV-19/Japan/PG-86575/2021	EPI_ISL_3898290	2021-09-02
hCoV-19/Japan/PG-86576/2021	EPI_ISL_3898291	2021-09-02
hCoV-19/Japan/PG-86577/2021	EPI_ISL_3898292	2021-09-02
hCoV-19/Japan/PG-86578/2021	EPI_ISL_3898293	2021-09-02
hCoV-19/Japan/PG-86579/2021	EPI_ISL_3898294	2021-09-02
hCoV-19/Japan/PG-86580/2021	EPI_ISL_3898295	2021-09-02
hCoV-19/Japan/PG-86581/2021	EPI_ISL_3898296	2021-09-02
hCoV-19/Japan/PG-86582/2021	EPI_ISL_3898297	2021-09-02
hCoV-19/Japan/PG-86583/2021	EPI_ISL_3898298	2021-09-02
hCoV-19/Japan/PG-86584/2021	EPI_ISL_3898299	2021-09-02
hCoV-19/Japan/PG-86585/2021	EPI_ISL_3898300	2021-09-02
hCoV-19/Japan/PG-86586/2021	EPI_ISL_3898301	2021-09-02
hCoV-19/Japan/PG-86587/2021	EPI_ISL_3898302	2021-09-02
hCoV-19/Japan/PG-86588/2021	EPI_ISL_3898303	2021-09-02
hCoV-19/Japan/PG-86589/2021	EPI_ISL_3898304	2021-09-02
hCoV-19/Japan/PG-86590/2021	EPI_ISL_3898305	2021-09-02
hCoV-19/Japan/PG-86591/2021	EPI_ISL_3898306	2021-09-02
hCoV-19/Japan/PG-86592/2021	EPI_ISL_3898307	2021-09-02
hCoV-19/Japan/PG-86593/2021	EPI_ISL_3898308	2021-09-02
hCoV-19/Japan/PG-86594/2021	EPI_ISL_3898309	2021-09-02
hCoV-19/Japan/PG-86595/2021	EPI_ISL_3898310	2021-09-02
hCoV-19/Japan/PG-86596/2021	EPI_ISL_3898311	2021-09-02
hCoV-19/Japan/PG-86597/2021	EPI_ISL_3898312	2021-09-02
hCoV-19/Japan/PG-86598/2021	EPI_ISL_3898313	2021-09-02
hCoV-19/Japan/PG-86599/2021	EPI_ISL_3898314	2021-09-02
hCoV-19/Japan/PG-86600/2021	EPI_ISL_3898315	2021-09-02
hCoV-19/Japan/PG-86601/2021	EPI_ISL_3898316	2021-09-02
hCoV-19/Japan/PG-86602/2021	EPI_ISL_3898317	2021-09-02
hCoV-19/Japan/PG-86603/2021	EPI_ISL_3898318	2021-09-02
hCoV-19/Japan/PG-86604/2021	EPI_ISL_3898319	2021-09-02
hCoV-19/Japan/PG-86605/2021	EPI_ISL_3898320	2021-09-02
hCoV-19/Japan/PG-86606/2021	EPI_ISL_3898321	2021-09-02
hCoV-19/Japan/PG-86607/2021	EPI_ISL_3898322	2021-09-02
hCoV-19/Japan/PG-86608/2021	EPI_ISL_3898323	2021-09-02
hCoV-19/Japan/PG-86609/2021	EPI_ISL_3898324	2021-09-02
hCoV-19/Japan/PG-86610/2021	EPI_ISL_3898325	2021-09-02
hCoV-19/Japan/PG-86611/2021	EPI_ISL_3898326	2021-09-02
hCoV-19/Japan/PG-86612/2021	EPI_ISL_3898327	2021-09-02
hCoV-19/Japan/PG-86613/2021	EPI_ISL_3898328	2021-09-02
hCoV-19/Japan/PG-86614/2021	EPI_ISL_3898329	2021-09-02
hCoV-19/Japan/PG-86615/2021	EPI_ISL_3898330	2021-09-02
hCoV-19/Japan/PG-86616/2021	EPI_ISL_3898331	2021-09-02
hCoV-19/Japan/PG-86617/2021	EPI_ISL_3898332	2021-09-02
hCoV-19/Japan/PG-86618/2021	EPI_ISL_3898333	2021-09-02
hCoV-19/Japan/PG-86619/2021	EPI_ISL_3898334	2021-09-02
hCoV-19/Japan/PG-86620/2021	EPI_ISL_3898335	2021-09-02
hCoV-19/Japan/PG-86621/2021	EPI_ISL_3898336	2021-09-02
hCoV-19/Japan/PG-86622/2021	EPI_ISL_3898337	2021-09-02
hCoV-19/Japan/PG-86623/2021	EPI_ISL_3898338	2021-09-02
hCoV-19/Japan/PG-86624/2021	EPI_ISL_3898339	2021-09-02
hCoV-19/Japan/PG-86625/2021	EPI_ISL_3898340	2021-09-02
hCoV-19/Japan/PG-86626/2021	EPI_ISL_3898341	2021-09-02
hCoV-19/Japan/PG-86627/2021	EPI_ISL_3898342	2021-09-02
hCoV-19/Japan/PG-86628/2021	EPI_ISL_3898343	2021-09-02

virus_name	epiID	submission_date
hCoV-19/Japan/PG-86629/2021	EPI_ISL_3898344	2021-09-02
hCoV-19/Japan/PG-86630/2021	EPI_ISL_3898345	2021-09-02
hCoV-19/Japan/PG-86631/2021	EPI_ISL_3898346	2021-09-02
hCoV-19/Japan/PG-86632/2021	EPI_ISL_3898347	2021-09-02
hCoV-19/Japan/PG-86633/2021	EPI_ISL_3898348	2021-09-02
hCoV-19/Japan/PG-86634/2021	EPI_ISL_3898349	2021-09-02
hCoV-19/Japan/PG-86635/2021	EPI_ISL_3898350	2021-09-02
hCoV-19/Japan/PG-86636/2021	EPI_ISL_3898351	2021-09-02
hCoV-19/Japan/PG-86637/2021	EPI_ISL_3898352	2021-09-02
hCoV-19/Japan/PG-86638/2021	EPI_ISL_3898353	2021-09-02
hCoV-19/Japan/PG-86639/2021	EPI_ISL_3898354	2021-09-02
hCoV-19/Japan/PG-86640/2021	EPI_ISL_3898355	2021-09-02
hCoV-19/Japan/PG-86641/2021	EPI_ISL_3898356	2021-09-02
hCoV-19/Japan/PG-86642/2021	EPI_ISL_3898357	2021-09-02
hCoV-19/Japan/PG-86643/2021	EPI_ISL_3898358	2021-09-02
hCoV-19/Japan/PG-86644/2021	EPI_ISL_3898359	2021-09-02
hCoV-19/Japan/PG-86645/2021	EPI_ISL_3898360	2021-09-02
hCoV-19/Japan/PG-86646/2021	EPI_ISL_3898361	2021-09-02
hCoV-19/Japan/PG-86647/2021	EPI_ISL_3898362	2021-09-02
hCoV-19/Japan/PG-86648/2021	EPI_ISL_3898363	2021-09-02
hCoV-19/Japan/PG-86649/2021	EPI_ISL_3898364	2021-09-02
hCoV-19/Japan/PG-86650/2021	EPI_ISL_3898365	2021-09-02
hCoV-19/Japan/PG-86651/2021	EPI_ISL_3898366	2021-09-02
hCoV-19/Japan/PG-86652/2021	EPI_ISL_3898367	2021-09-02
hCoV-19/Japan/PG-86653/2021	EPI_ISL_3898368	2021-09-02
hCoV-19/Japan/PG-86654/2021	EPI_ISL_3898369	2021-09-02
hCoV-19/Japan/PG-86655/2021	EPI_ISL_3898370	2021-09-02
hCoV-19/Japan/PG-86656/2021	EPI_ISL_3898371	2021-09-02
hCoV-19/Japan/PG-86657/2021	EPI_ISL_3898372	2021-09-02
hCoV-19/Japan/PG-86658/2021	EPI_ISL_3898373	2021-09-02
hCoV-19/Japan/PG-86659/2021	EPI_ISL_3898374	2021-09-02
hCoV-19/Japan/PG-86660/2021	EPI_ISL_3898375	2021-09-02
hCoV-19/Japan/PG-86661/2021	EPI_ISL_3898376	2021-09-02
hCoV-19/Japan/PG-86662/2021	EPI_ISL_3898377	2021-09-02
hCoV-19/Japan/PG-86663/2021	EPI_ISL_3898378	2021-09-02
hCoV-19/Japan/PG-86664/2021	EPI_ISL_3898379	2021-09-02
hCoV-19/Japan/PG-86665/2021	EPI_ISL_3898380	2021-09-02
hCoV-19/Japan/PG-86666/2021	EPI_ISL_3898381	2021-09-02
hCoV-19/Japan/PG-86667/2021	EPI_ISL_3898382	2021-09-02
hCoV-19/Japan/PG-86668/2021	EPI_ISL_3898383	2021-09-02
hCoV-19/Japan/PG-86669/2021	EPI_ISL_3898384	2021-09-02
hCoV-19/Japan/PG-86670/2021	EPI_ISL_3898385	2021-09-02
hCoV-19/Japan/PG-86671/2021	EPI_ISL_3898386	2021-09-02
hCoV-19/Japan/PG-86672/2021	EPI_ISL_3898387	2021-09-02
hCoV-19/Japan/PG-86673/2021	EPI_ISL_3898388	2021-09-02
hCoV-19/Japan/PG-86674/2021	EPI_ISL_3898389	2021-09-02
hCoV-19/Japan/PG-86675/2021	EPI_ISL_3898390	2021-09-02
hCoV-19/Japan/PG-86676/2021	EPI_ISL_3898391	2021-09-02
hCoV-19/Japan/PG-86677/2021	EPI_ISL_3898392	2021-09-02
hCoV-19/Japan/PG-86678/2021	EPI_ISL_3898393	2021-09-02
hCoV-19/Japan/PG-86679/2021	EPI_ISL_3898394	2021-09-02
hCoV-19/Japan/PG-86680/2021	EPI_ISL_3898395	2021-09-02
hCoV-19/Japan/PG-86681/2021	EPI_ISL_3898396	2021-09-02
hCoV-19/Japan/PG-86682/2021	EPI_ISL_3898397	2021-09-02

virus_name	epiID	submission_date
hCoV-19/Japan/PG-86683/2021	EPI_ISL_3898398	2021-09-02
hCoV-19/Japan/PG-86684/2021	EPI_ISL_3898399	2021-09-02
hCoV-19/Japan/PG-86685/2021	EPI_ISL_3898400	2021-09-02
hCoV-19/Japan/PG-86686/2021	EPI_ISL_3898401	2021-09-02
hCoV-19/Japan/PG-86687/2021	EPI_ISL_3898402	2021-09-02
hCoV-19/Japan/PG-86688/2021	EPI_ISL_3898403	2021-09-02
hCoV-19/Japan/PG-86689/2021	EPI_ISL_3898404	2021-09-02
hCoV-19/Japan/PG-86690/2021	EPI_ISL_3898405	2021-09-02
hCoV-19/Japan/PG-86691/2021	EPI_ISL_3898406	2021-09-02
hCoV-19/Japan/PG-86692/2021	EPI_ISL_3898407	2021-09-02
hCoV-19/Japan/PG-86693/2021	EPI_ISL_3898408	2021-09-02
hCoV-19/Japan/PG-86694/2021	EPI_ISL_3898409	2021-09-02
hCoV-19/Japan/PG-86695/2021	EPI_ISL_3898410	2021-09-02
hCoV-19/Japan/PG-86696/2021	EPI_ISL_3898411	2021-09-02
hCoV-19/Japan/PG-86697/2021	EPI_ISL_3898412	2021-09-02
hCoV-19/Japan/PG-86698/2021	EPI_ISL_3898413	2021-09-02
hCoV-19/Japan/PG-86699/2021	EPI_ISL_3898414	2021-09-02
hCoV-19/Japan/PG-86700/2021	EPI_ISL_3898415	2021-09-02
hCoV-19/Japan/PG-86701/2021	EPI_ISL_3898416	2021-09-02
hCoV-19/Japan/PG-86702/2021	EPI_ISL_3898417	2021-09-02
hCoV-19/Japan/PG-86703/2021	EPI_ISL_3898418	2021-09-02
hCoV-19/Japan/PG-86704/2021	EPI_ISL_3898419	2021-09-02
hCoV-19/Japan/PG-86705/2021	EPI_ISL_3898420	2021-09-02
hCoV-19/Japan/PG-86706/2021	EPI_ISL_3898421	2021-09-02
hCoV-19/Japan/PG-86707/2021	EPI_ISL_3898422	2021-09-02
hCoV-19/Japan/PG-86708/2021	EPI_ISL_3898423	2021-09-02
hCoV-19/Japan/PG-86709/2021	EPI_ISL_3898424	2021-09-02
hCoV-19/Japan/PG-86710/2021	EPI_ISL_3898425	2021-09-02
hCoV-19/Japan/PG-86711/2021	EPI_ISL_3898426	2021-09-02
hCoV-19/Japan/PG-86712/2021	EPI_ISL_3898427	2021-09-02
hCoV-19/Japan/PG-86713/2021	EPI_ISL_3898428	2021-09-02
hCoV-19/Japan/PG-86714/2021	EPI_ISL_3898429	2021-09-02
hCoV-19/Japan/PG-86715/2021	EPI_ISL_3898430	2021-09-02
hCoV-19/Japan/PG-86716/2021	EPI_ISL_3898431	2021-09-02
hCoV-19/Japan/PG-86717/2021	EPI_ISL_3898432	2021-09-02
hCoV-19/Japan/PG-86718/2021	EPI_ISL_3898433	2021-09-02
hCoV-19/Japan/PG-86719/2021	EPI_ISL_3898434	2021-09-02
hCoV-19/Japan/PG-86720/2021	EPI_ISL_3898435	2021-09-02
hCoV-19/Japan/PG-86721/2021	EPI_ISL_3898436	2021-09-02
hCoV-19/Japan/PG-86722/2021	EPI_ISL_3898437	2021-09-02
hCoV-19/Japan/PG-86723/2021	EPI_ISL_3898438	2021-09-02
hCoV-19/Japan/PG-86724/2021	EPI_ISL_3898439	2021-09-02
hCoV-19/Japan/PG-86725/2021	EPI_ISL_3898440	2021-09-02
hCoV-19/Japan/PG-86726/2021	EPI_ISL_3898441	2021-09-02
hCoV-19/Japan/PG-86727/2021	EPI_ISL_3898442	2021-09-02
hCoV-19/Japan/PG-86728/2021	EPI_ISL_3898443	2021-09-02
hCoV-19/Japan/PG-86729/2021	EPI_ISL_3898444	2021-09-02
hCoV-19/Japan/PG-86730/2021	EPI_ISL_3898445	2021-09-02
hCoV-19/Japan/PG-86731/2021	EPI_ISL_3898446	2021-09-02
hCoV-19/Japan/PG-86732/2021	EPI_ISL_3898447	2021-09-02
hCoV-19/Japan/PG-86733/2021	EPI_ISL_3898448	2021-09-02
hCoV-19/Japan/PG-86734/2021	EPI_ISL_3898449	2021-09-02
hCoV-19/Japan/PG-86735/2021	EPI_ISL_3898450	2021-09-02
hCoV-19/Japan/PG-86736/2021	EPI_ISL_3898451	2021-09-02

virus_name	epiID	submission_date
hCoV-19/Japan/PG-86737/2021	EPI_ISL_3898452	2021-09-02
hCoV-19/Japan/PG-86738/2021	EPI_ISL_3898453	2021-09-02
hCoV-19/Japan/PG-86739/2021	EPI_ISL_3898454	2021-09-02
hCoV-19/Japan/PG-86740/2021	EPI_ISL_3898455	2021-09-02
hCoV-19/Japan/PG-86741/2021	EPI_ISL_3898456	2021-09-02
hCoV-19/Japan/PG-86742/2021	EPI_ISL_3898457	2021-09-02
hCoV-19/Japan/PG-86743/2021	EPI_ISL_3898458	2021-09-02
hCoV-19/Japan/PG-86744/2021	EPI_ISL_3898459	2021-09-02
hCoV-19/Japan/PG-86745/2021	EPI_ISL_3898460	2021-09-02
hCoV-19/Japan/PG-86746/2021	EPI_ISL_3898461	2021-09-02
hCoV-19/Japan/PG-86747/2021	EPI_ISL_3898462	2021-09-02
hCoV-19/Japan/PG-86748/2021	EPI_ISL_3898463	2021-09-02
hCoV-19/Japan/PG-86749/2021	EPI_ISL_3898464	2021-09-02
hCoV-19/Japan/PG-86750/2021	EPI_ISL_3898465	2021-09-02
hCoV-19/Japan/PG-86751/2021	EPI_ISL_3898466	2021-09-02
hCoV-19/Japan/PG-86752/2021	EPI_ISL_3898467	2021-09-02
hCoV-19/Japan/PG-86753/2021	EPI_ISL_3898468	2021-09-02
hCoV-19/Japan/PG-86754/2021	EPI_ISL_3898469	2021-09-02
hCoV-19/Japan/PG-86755/2021	EPI_ISL_3898470	2021-09-02
hCoV-19/Japan/PG-86756/2021	EPI_ISL_3898471	2021-09-02
hCoV-19/Japan/PG-86757/2021	EPI_ISL_3898472	2021-09-02
hCoV-19/Japan/PG-86758/2021	EPI_ISL_3898473	2021-09-02
hCoV-19/Japan/PG-86759/2021	EPI_ISL_3898474	2021-09-02
hCoV-19/Japan/PG-86760/2021	EPI_ISL_3898475	2021-09-02
hCoV-19/Japan/PG-86761/2021	EPI_ISL_3898476	2021-09-02
hCoV-19/Japan/PG-86762/2021	EPI_ISL_3898477	2021-09-02
hCoV-19/Japan/PG-86763/2021	EPI_ISL_3898478	2021-09-02
hCoV-19/Japan/PG-86764/2021	EPI_ISL_3898479	2021-09-02
hCoV-19/Japan/PG-86765/2021	EPI_ISL_3898480	2021-09-02
hCoV-19/Japan/PG-86766/2021	EPI_ISL_3898481	2021-09-02
hCoV-19/Japan/PG-86767/2021	EPI_ISL_3898482	2021-09-02
hCoV-19/Japan/PG-86768/2021	EPI_ISL_3898483	2021-09-02
hCoV-19/Japan/PG-86769/2021	EPI_ISL_3898484	2021-09-02
hCoV-19/Japan/PG-86770/2021	EPI_ISL_3898485	2021-09-02
hCoV-19/Japan/PG-86771/2021	EPI_ISL_3898486	2021-09-02
hCoV-19/Japan/PG-86772/2021	EPI_ISL_3898487	2021-09-02
hCoV-19/Japan/PG-86773/2021	EPI_ISL_3898488	2021-09-02
hCoV-19/Japan/PG-86774/2021	EPI_ISL_3898489	2021-09-02
hCoV-19/Japan/PG-86775/2021	EPI_ISL_3898490	2021-09-02
hCoV-19/Japan/PG-86776/2021	EPI_ISL_3898491	2021-09-02
hCoV-19/Japan/PG-86777/2021	EPI_ISL_3898492	2021-09-02
hCoV-19/Japan/PG-86778/2021	EPI_ISL_3898493	2021-09-02
hCoV-19/Japan/PG-86779/2021	EPI_ISL_3898494	2021-09-02
hCoV-19/Japan/PG-86780/2021	EPI_ISL_3898495	2021-09-02
hCoV-19/Japan/PG-86781/2021	EPI_ISL_3898496	2021-09-02
hCoV-19/Japan/PG-86782/2021	EPI_ISL_3898497	2021-09-02
hCoV-19/Japan/PG-86783/2021	EPI_ISL_3898498	2021-09-02
hCoV-19/Japan/PG-86784/2021	EPI_ISL_3898499	2021-09-02
hCoV-19/Japan/PG-86785/2021	EPI_ISL_3898500	2021-09-02
hCoV-19/Japan/PG-86786/2021	EPI_ISL_3898501	2021-09-02
hCoV-19/Japan/PG-86787/2021	EPI_ISL_3898502	2021-09-02
hCoV-19/Japan/PG-86788/2021	EPI_ISL_3898503	2021-09-02
hCoV-19/Japan/PG-86789/2021	EPI_ISL_3898504	2021-09-02
hCoV-19/Japan/PG-86790/2021	EPI_ISL_3898505	2021-09-02

virus_name	epiID	submission_date
hCoV-19/Japan/PG-86791/2021	EPI_ISL_3898506	2021-09-02
hCoV-19/Japan/PG-86792/2021	EPI_ISL_3898507	2021-09-02
hCoV-19/Japan/PG-86793/2021	EPI_ISL_3898508	2021-09-02
hCoV-19/Japan/PG-86794/2021	EPI_ISL_3898509	2021-09-02
hCoV-19/Japan/PG-86795/2021	EPI_ISL_3898510	2021-09-02
hCoV-19/Japan/PG-86796/2021	EPI_ISL_3898511	2021-09-02
hCoV-19/Japan/PG-86797/2021	EPI_ISL_3898512	2021-09-02
hCoV-19/Japan/PG-86798/2021	EPI_ISL_3898513	2021-09-02
hCoV-19/Japan/PG-86799/2021	EPI_ISL_3898514	2021-09-02
hCoV-19/Japan/PG-86800/2021	EPI_ISL_3898515	2021-09-02
hCoV-19/Japan/PG-86801/2021	EPI_ISL_3898516	2021-09-02
hCoV-19/Japan/PG-86802/2021	EPI_ISL_3898517	2021-09-02
hCoV-19/Japan/PG-86803/2021	EPI_ISL_3898518	2021-09-02
hCoV-19/Japan/PG-86804/2021	EPI_ISL_3898519	2021-09-02
hCoV-19/Japan/PG-86805/2021	EPI_ISL_3898520	2021-09-02
hCoV-19/Japan/PG-86806/2021	EPI_ISL_3898521	2021-09-02
hCoV-19/Japan/PG-86807/2021	EPI_ISL_3898522	2021-09-02
hCoV-19/Japan/PG-86808/2021	EPI_ISL_3898523	2021-09-02
hCoV-19/Japan/PG-86809/2021	EPI_ISL_3898524	2021-09-02
hCoV-19/Japan/PG-86810/2021	EPI_ISL_3898525	2021-09-02
hCoV-19/Japan/PG-86811/2021	EPI_ISL_3898526	2021-09-02
hCoV-19/Japan/PG-86812/2021	EPI_ISL_3898527	2021-09-02
hCoV-19/Japan/PG-86813/2021	EPI_ISL_3898528	2021-09-02
hCoV-19/Japan/PG-86814/2021	EPI_ISL_3898529	2021-09-02
hCoV-19/Japan/PG-86815/2021	EPI_ISL_3898530	2021-09-02
hCoV-19/Japan/PG-86816/2021	EPI_ISL_3898531	2021-09-02
hCoV-19/Japan/PG-86817/2021	EPI_ISL_3898532	2021-09-02
hCoV-19/Japan/PG-86818/2021	EPI_ISL_3898533	2021-09-02
hCoV-19/Japan/PG-86819/2021	EPI_ISL_3898534	2021-09-02
hCoV-19/Japan/PG-86820/2021	EPI_ISL_3898535	2021-09-02
hCoV-19/Japan/PG-86821/2021	EPI_ISL_3898536	2021-09-02
hCoV-19/Japan/PG-86822/2021	EPI_ISL_3898537	2021-09-02
hCoV-19/Japan/PG-86823/2021	EPI_ISL_3898538	2021-09-02
hCoV-19/Japan/PG-86824/2021	EPI_ISL_3898539	2021-09-02
hCoV-19/Japan/PG-86825/2021	EPI_ISL_3898540	2021-09-02
hCoV-19/Japan/PG-86826/2021	EPI_ISL_3898541	2021-09-02
hCoV-19/Japan/PG-86827/2021	EPI_ISL_3898542	2021-09-02
hCoV-19/Japan/PG-86828/2021	EPI_ISL_3898543	2021-09-02
hCoV-19/Japan/PG-86829/2021	EPI_ISL_3898544	2021-09-02
hCoV-19/Japan/PG-86830/2021	EPI_ISL_3898545	2021-09-02
hCoV-19/Japan/PG-86831/2021	EPI_ISL_3898546	2021-09-02
hCoV-19/Japan/PG-86832/2021	EPI_ISL_3898547	2021-09-02
hCoV-19/Japan/PG-86833/2021	EPI_ISL_3898548	2021-09-02
hCoV-19/Japan/PG-86834/2021	EPI_ISL_3898549	2021-09-02
hCoV-19/Japan/PG-86835/2021	EPI_ISL_3898550	2021-09-02
hCoV-19/Japan/PG-86836/2021	EPI_ISL_3898551	2021-09-02
hCoV-19/Japan/PG-86837/2021	EPI_ISL_3898552	2021-09-02
hCoV-19/Japan/PG-86838/2021	EPI_ISL_3898553	2021-09-02
hCoV-19/Japan/PG-86839/2021	EPI_ISL_3898554	2021-09-02
hCoV-19/Japan/PG-86840/2021	EPI_ISL_3898555	2021-09-02
hCoV-19/Japan/PG-86841/2021	EPI_ISL_3898556	2021-09-02
hCoV-19/Japan/PG-86842/2021	EPI_ISL_3898557	2021-09-02
hCoV-19/Japan/PG-86843/2021	EPI_ISL_3898558	2021-09-02
hCoV-19/Japan/PG-86844/2021	EPI_ISL_3898559	2021-09-02

virus_name	epiID	submission_date
hCoV-19/Japan/PG-86845/2021	EPI_ISL_3898560	2021-09-02
hCoV-19/Japan/PG-86846/2021	EPI_ISL_3898561	2021-09-02
hCoV-19/Japan/PG-86847/2021	EPI_ISL_3898562	2021-09-02
hCoV-19/Japan/PG-86848/2021	EPI_ISL_3898563	2021-09-02
hCoV-19/Japan/PG-86849/2021	EPI_ISL_3898564	2021-09-02
hCoV-19/Japan/PG-86850/2021	EPI_ISL_3898565	2021-09-02
hCoV-19/Japan/PG-86851/2021	EPI_ISL_3898566	2021-09-02
hCoV-19/Japan/PG-86852/2021	EPI_ISL_3898567	2021-09-02
hCoV-19/Japan/PG-86853/2021	EPI_ISL_3898568	2021-09-02
hCoV-19/Japan/PG-86854/2021	EPI_ISL_3898569	2021-09-02
hCoV-19/Japan/PG-86855/2021	EPI_ISL_3898570	2021-09-02
hCoV-19/Japan/PG-86856/2021	EPI_ISL_3898571	2021-09-02
hCoV-19/Japan/PG-86857/2021	EPI_ISL_3898572	2021-09-02
hCoV-19/Japan/PG-86858/2021	EPI_ISL_3898573	2021-09-02
hCoV-19/Japan/PG-86859/2021	EPI_ISL_3898574	2021-09-02
hCoV-19/Japan/PG-86860/2021	EPI_ISL_3898575	2021-09-02
hCoV-19/Japan/PG-86861/2021	EPI_ISL_3898576	2021-09-02
hCoV-19/Japan/PG-86862/2021	EPI_ISL_3898577	2021-09-02
hCoV-19/Japan/PG-86863/2021	EPI_ISL_3898578	2021-09-02
hCoV-19/Japan/PG-86864/2021	EPI_ISL_3898579	2021-09-02
hCoV-19/Japan/PG-86865/2021	EPI_ISL_3898580	2021-09-02
hCoV-19/Japan/PG-86866/2021	EPI_ISL_3898581	2021-09-02
hCoV-19/Japan/PG-86867/2021	EPI_ISL_3898582	2021-09-02
hCoV-19/Japan/PG-86868/2021	EPI_ISL_3898583	2021-09-02
hCoV-19/Japan/PG-86869/2021	EPI_ISL_3898584	2021-09-02
hCoV-19/Japan/PG-86870/2021	EPI_ISL_3898585	2021-09-02
hCoV-19/Japan/PG-86871/2021	EPI_ISL_3898586	2021-09-02
hCoV-19/Japan/PG-86872/2021	EPI_ISL_3898587	2021-09-02
hCoV-19/Japan/PG-86873/2021	EPI_ISL_3898588	2021-09-02
hCoV-19/Japan/PG-86874/2021	EPI_ISL_3898589	2021-09-02
hCoV-19/Japan/PG-86875/2021	EPI_ISL_3898590	2021-09-02
hCoV-19/Japan/PG-86876/2021	EPI_ISL_3898591	2021-09-02
hCoV-19/Japan/PG-86877/2021	EPI_ISL_3898592	2021-09-02
hCoV-19/Japan/PG-86878/2021	EPI_ISL_3898593	2021-09-02
hCoV-19/Japan/PG-86879/2021	EPI_ISL_3898594	2021-09-02
hCoV-19/Japan/PG-86880/2021	EPI_ISL_3898595	2021-09-02
hCoV-19/Japan/PG-86881/2021	EPI_ISL_3898596	2021-09-02
hCoV-19/Japan/PG-86882/2021	EPI_ISL_3898597	2021-09-02
hCoV-19/Japan/PG-86883/2021	EPI_ISL_3898598	2021-09-02
hCoV-19/Japan/PG-86884/2021	EPI_ISL_3898599	2021-09-02
hCoV-19/Japan/PG-86885/2021	EPI_ISL_3898600	2021-09-02
hCoV-19/Japan/PG-86886/2021	EPI_ISL_3898601	2021-09-02
hCoV-19/Japan/PG-86887/2021	EPI_ISL_3898602	2021-09-02
hCoV-19/Japan/PG-86888/2021	EPI_ISL_3898603	2021-09-02
hCoV-19/Japan/PG-86889/2021	EPI_ISL_3898604	2021-09-02
hCoV-19/Japan/PG-86890/2021	EPI_ISL_3898605	2021-09-02
hCoV-19/Japan/PG-86891/2021	EPI_ISL_3898606	2021-09-02
hCoV-19/Japan/PG-86892/2021	EPI_ISL_3898607	2021-09-02
hCoV-19/Japan/PG-86893/2021	EPI_ISL_3898608	2021-09-02
hCoV-19/Japan/PG-86894/2021	EPI_ISL_3898609	2021-09-02
hCoV-19/Japan/PG-86895/2021	EPI_ISL_3898610	2021-09-02
hCoV-19/Japan/PG-86896/2021	EPI_ISL_3898611	2021-09-02
hCoV-19/Japan/PG-86897/2021	EPI_ISL_3898612	2021-09-02
hCoV-19/Japan/PG-86898/2021	EPI_ISL_3898613	2021-09-02

virus_name	epiID	submission_date
hCoV-19/Japan/PG-86899/2021	EPI_ISL_3898614	2021-09-02
hCoV-19/Japan/PG-86900/2021	EPI_ISL_3898615	2021-09-02
hCoV-19/Japan/PG-86901/2021	EPI_ISL_3898616	2021-09-02
hCoV-19/Japan/PG-86902/2021	EPI_ISL_3898617	2021-09-02
hCoV-19/Japan/PG-86903/2021	EPI_ISL_3898618	2021-09-02
hCoV-19/Japan/PG-86904/2021	EPI_ISL_3898619	2021-09-02
hCoV-19/Japan/PG-86905/2021	EPI_ISL_3898620	2021-09-02
hCoV-19/Japan/PG-86906/2021	EPI_ISL_3898621	2021-09-02
hCoV-19/Japan/PG-86907/2021	EPI_ISL_3898622	2021-09-02
hCoV-19/Japan/PG-86908/2021	EPI_ISL_3898623	2021-09-02
hCoV-19/Japan/PG-86909/2021	EPI_ISL_3898624	2021-09-02
hCoV-19/Japan/PG-86910/2021	EPI_ISL_3898625	2021-09-02
hCoV-19/Japan/PG-86911/2021	EPI_ISL_3898626	2021-09-02
hCoV-19/Japan/PG-86912/2021	EPI_ISL_3898627	2021-09-02
hCoV-19/Japan/PG-86913/2021	EPI_ISL_3898628	2021-09-02
hCoV-19/Japan/PG-86914/2021	EPI_ISL_3898629	2021-09-02
hCoV-19/Japan/PG-86915/2021	EPI_ISL_3898630	2021-09-02
hCoV-19/Japan/PG-86916/2021	EPI_ISL_3898631	2021-09-02
hCoV-19/Japan/PG-86917/2021	EPI_ISL_3898632	2021-09-02
hCoV-19/Japan/PG-86918/2021	EPI_ISL_3898633	2021-09-02
hCoV-19/Japan/PG-86919/2021	EPI_ISL_3898634	2021-09-02
hCoV-19/Japan/PG-86920/2021	EPI_ISL_3898635	2021-09-02
hCoV-19/Japan/PG-86921/2021	EPI_ISL_3898636	2021-09-02
hCoV-19/Japan/PG-86922/2021	EPI_ISL_3898637	2021-09-02
hCoV-19/Japan/PG-86923/2021	EPI_ISL_3898638	2021-09-02
hCoV-19/Japan/PG-86924/2021	EPI_ISL_3898639	2021-09-02
hCoV-19/Japan/PG-86925/2021	EPI_ISL_3898640	2021-09-02
hCoV-19/Japan/PG-86926/2021	EPI_ISL_3898641	2021-09-02
hCoV-19/Japan/PG-86927/2021	EPI_ISL_3898642	2021-09-02
hCoV-19/Japan/PG-86928/2021	EPI_ISL_3898643	2021-09-02
hCoV-19/Japan/PG-86929/2021	EPI_ISL_3898644	2021-09-02
hCoV-19/Japan/PG-86930/2021	EPI_ISL_3898645	2021-09-02
hCoV-19/Japan/PG-86931/2021	EPI_ISL_3898646	2021-09-02
hCoV-19/Japan/PG-86932/2021	EPI_ISL_3898647	2021-09-02
hCoV-19/Japan/PG-86933/2021	EPI_ISL_3898648	2021-09-02
hCoV-19/Japan/PG-86934/2021	EPI_ISL_3898649	2021-09-02
hCoV-19/Japan/PG-86935/2021	EPI_ISL_3898650	2021-09-02
hCoV-19/Japan/PG-86936/2021	EPI_ISL_3898651	2021-09-02
hCoV-19/Japan/PG-86937/2021	EPI_ISL_3898652	2021-09-02
hCoV-19/Japan/PG-86938/2021	EPI_ISL_3898653	2021-09-02
hCoV-19/Japan/PG-86939/2021	EPI_ISL_3898654	2021-09-02
hCoV-19/Japan/PG-86940/2021	EPI_ISL_3898655	2021-09-02
hCoV-19/Japan/PG-86941/2021	EPI_ISL_3898656	2021-09-02
hCoV-19/Japan/PG-86942/2021	EPI_ISL_3898657	2021-09-02
hCoV-19/Japan/PG-86943/2021	EPI_ISL_3898658	2021-09-02
hCoV-19/Japan/PG-86944/2021	EPI_ISL_3898659	2021-09-02
hCoV-19/Japan/PG-86945/2021	EPI_ISL_3898660	2021-09-02
hCoV-19/Japan/PG-86946/2021	EPI_ISL_3898661	2021-09-02
hCoV-19/Japan/PG-86947/2021	EPI_ISL_3898662	2021-09-02
hCoV-19/Japan/PG-86948/2021	EPI_ISL_3898663	2021-09-02
hCoV-19/Japan/PG-86949/2021	EPI_ISL_3898664	2021-09-02
hCoV-19/Japan/PG-86950/2021	EPI_ISL_3898665	2021-09-02
hCoV-19/Japan/PG-86951/2021	EPI_ISL_3898666	2021-09-02
hCoV-19/Japan/PG-86952/2021	EPI_ISL_3898667	2021-09-02

virus_name	epiID	submission_date
hCoV-19/Japan/PG-86953/2021	EPI_ISL_3898668	2021-09-02
hCoV-19/Japan/PG-86954/2021	EPI_ISL_3898669	2021-09-02
hCoV-19/Japan/PG-86955/2021	EPI_ISL_3898670	2021-09-02
hCoV-19/Japan/PG-86956/2021	EPI_ISL_3898671	2021-09-02
hCoV-19/Japan/PG-86957/2021	EPI_ISL_3898672	2021-09-02
hCoV-19/Japan/PG-86958/2021	EPI_ISL_3898673	2021-09-02
hCoV-19/Japan/PG-86959/2021	EPI_ISL_3898674	2021-09-02
hCoV-19/Japan/PG-86960/2021	EPI_ISL_3898675	2021-09-02
hCoV-19/Japan/PG-86961/2021	EPI_ISL_3898676	2021-09-02
hCoV-19/Japan/PG-86962/2021	EPI_ISL_3898677	2021-09-02
hCoV-19/Japan/PG-86963/2021	EPI_ISL_3898678	2021-09-02
hCoV-19/Japan/PG-86964/2021	EPI_ISL_3898679	2021-09-02
hCoV-19/Japan/PG-86965/2021	EPI_ISL_3898680	2021-09-02
hCoV-19/Japan/PG-86966/2021	EPI_ISL_3898681	2021-09-02
hCoV-19/Japan/PG-86967/2021	EPI_ISL_3898682	2021-09-02
hCoV-19/Japan/PG-86968/2021	EPI_ISL_3898683	2021-09-02
hCoV-19/Japan/PG-86969/2021	EPI_ISL_3898684	2021-09-02
hCoV-19/Japan/PG-86970/2021	EPI_ISL_3898685	2021-09-02
hCoV-19/Japan/PG-86971/2021	EPI_ISL_3898686	2021-09-02
hCoV-19/Japan/PG-86972/2021	EPI_ISL_3898687	2021-09-02
hCoV-19/Japan/PG-86973/2021	EPI_ISL_3898688	2021-09-02
hCoV-19/Japan/PG-86974/2021	EPI_ISL_3898689	2021-09-02
hCoV-19/Japan/PG-86975/2021	EPI_ISL_3898690	2021-09-02
hCoV-19/Japan/PG-86976/2021	EPI_ISL_3898691	2021-09-02
hCoV-19/Japan/PG-86977/2021	EPI_ISL_3898692	2021-09-02
hCoV-19/Japan/PG-86978/2021	EPI_ISL_3898693	2021-09-02
hCoV-19/Japan/PG-86979/2021	EPI_ISL_3898694	2021-09-02
hCoV-19/Japan/PG-86980/2021	EPI_ISL_3898695	2021-09-02
hCoV-19/Japan/PG-86981/2021	EPI_ISL_3898696	2021-09-02
hCoV-19/Japan/PG-86982/2021	EPI_ISL_3898697	2021-09-02
hCoV-19/Japan/PG-86983/2021	EPI_ISL_3898698	2021-09-02
hCoV-19/Japan/PG-86984/2021	EPI_ISL_3898699	2021-09-02
hCoV-19/Japan/PG-86985/2021	EPI_ISL_3898700	2021-09-02
hCoV-19/Japan/PG-86986/2021	EPI_ISL_3898701	2021-09-02
hCoV-19/Japan/PG-86987/2021	EPI_ISL_3898702	2021-09-02
hCoV-19/Japan/PG-86988/2021	EPI_ISL_3898703	2021-09-02
hCoV-19/Japan/PG-86989/2021	EPI_ISL_3898704	2021-09-02
hCoV-19/Japan/PG-86990/2021	EPI_ISL_3898705	2021-09-02
hCoV-19/Japan/PG-86991/2021	EPI_ISL_3898706	2021-09-02
hCoV-19/Japan/PG-86992/2021	EPI_ISL_3898707	2021-09-02
hCoV-19/Japan/PG-86993/2021	EPI_ISL_3898708	2021-09-02
hCoV-19/Japan/PG-86994/2021	EPI_ISL_3898709	2021-09-02
hCoV-19/Japan/PG-86995/2021	EPI_ISL_3898710	2021-09-02
hCoV-19/Japan/PG-86996/2021	EPI_ISL_3898711	2021-09-02
hCoV-19/Japan/PG-86997/2021	EPI_ISL_3898712	2021-09-02
hCoV-19/Japan/PG-86998/2021	EPI_ISL_3898713	2021-09-02
hCoV-19/Japan/PG-86999/2021	EPI_ISL_3898714	2021-09-02
hCoV-19/Japan/PG-87000/2021	EPI_ISL_3898715	2021-09-02
hCoV-19/Japan/PG-87001/2021	EPI_ISL_3898716	2021-09-02
hCoV-19/Japan/PG-87002/2021	EPI_ISL_3898717	2021-09-02
hCoV-19/Japan/PG-87003/2021	EPI_ISL_3898718	2021-09-02
hCoV-19/Japan/PG-87004/2021	EPI_ISL_3898719	2021-09-02
hCoV-19/Japan/PG-87005/2021	EPI_ISL_3898720	2021-09-02
hCoV-19/Japan/PG-87006/2021	EPI_ISL_3898721	2021-09-02

virus_name	epiID	submission_date
hCoV-19/Japan/PG-87007/2021	EPI_ISL_3898722	2021-09-02
hCoV-19/Japan/PG-87008/2021	EPI_ISL_3898723	2021-09-02
hCoV-19/Japan/PG-87009/2021	EPI_ISL_3898724	2021-09-02
hCoV-19/Japan/PG-87010/2021	EPI_ISL_3898725	2021-09-02
hCoV-19/Japan/PG-87011/2021	EPI_ISL_3898726	2021-09-02
hCoV-19/Japan/PG-87012/2021	EPI_ISL_3898727	2021-09-02
hCoV-19/Japan/PG-87013/2021	EPI_ISL_3898728	2021-09-02
hCoV-19/Japan/PG-87014/2021	EPI_ISL_3898729	2021-09-02
hCoV-19/Japan/PG-87015/2021	EPI_ISL_3898730	2021-09-02
hCoV-19/Japan/PG-87016/2021	EPI_ISL_3898731	2021-09-02
hCoV-19/Japan/PG-87017/2021	EPI_ISL_3898732	2021-09-02
hCoV-19/Japan/PG-87018/2021	EPI_ISL_3898733	2021-09-02
hCoV-19/Japan/PG-87019/2021	EPI_ISL_3898734	2021-09-02
hCoV-19/Japan/PG-87020/2021	EPI_ISL_3898735	2021-09-02
hCoV-19/Japan/PG-87021/2021	EPI_ISL_3898736	2021-09-02
hCoV-19/Japan/PG-87022/2021	EPI_ISL_3898737	2021-09-02
hCoV-19/Japan/PG-87023/2021	EPI_ISL_3898738	2021-09-02
hCoV-19/Japan/PG-87024/2021	EPI_ISL_3898739	2021-09-02
hCoV-19/Japan/PG-87025/2021	EPI_ISL_3898740	2021-09-02
hCoV-19/Japan/PG-87026/2021	EPI_ISL_3898741	2021-09-02
hCoV-19/Japan/PG-87027/2021	EPI_ISL_3898742	2021-09-02
hCoV-19/Japan/PG-87028/2021	EPI_ISL_3898743	2021-09-02
hCoV-19/Japan/PG-87029/2021	EPI_ISL_3898744	2021-09-02
hCoV-19/Japan/PG-87030/2021	EPI_ISL_3898745	2021-09-02
hCoV-19/Japan/PG-87031/2021	EPI_ISL_3898746	2021-09-02
hCoV-19/Japan/PG-87032/2021	EPI_ISL_3898747	2021-09-02
hCoV-19/Japan/PG-87033/2021	EPI_ISL_3898748	2021-09-02
hCoV-19/Japan/PG-87034/2021	EPI_ISL_3898749	2021-09-02
hCoV-19/Japan/PG-87035/2021	EPI_ISL_3898750	2021-09-02
hCoV-19/Japan/PG-87036/2021	EPI_ISL_3898751	2021-09-02
hCoV-19/Japan/PG-87037/2021	EPI_ISL_3898752	2021-09-02
hCoV-19/Japan/PG-87038/2021	EPI_ISL_3898753	2021-09-02
hCoV-19/Japan/PG-87039/2021	EPI_ISL_3898754	2021-09-02
hCoV-19/Japan/PG-87040/2021	EPI_ISL_3898755	2021-09-02
hCoV-19/Japan/PG-87041/2021	EPI_ISL_3898756	2021-09-02
hCoV-19/Japan/PG-87042/2021	EPI_ISL_3898757	2021-09-02
hCoV-19/Japan/PG-87043/2021	EPI_ISL_3898758	2021-09-02
hCoV-19/Japan/PG-87044/2021	EPI_ISL_3898759	2021-09-02
hCoV-19/Japan/PG-87045/2021	EPI_ISL_3898760	2021-09-02
hCoV-19/Japan/PG-87046/2021	EPI_ISL_3898761	2021-09-02
hCoV-19/Japan/PG-87047/2021	EPI_ISL_3898762	2021-09-02
hCoV-19/Japan/PG-87048/2021	EPI_ISL_3898763	2021-09-02
hCoV-19/Japan/PG-87049/2021	EPI_ISL_3898764	2021-09-02
hCoV-19/Japan/PG-87050/2021	EPI_ISL_3898765	2021-09-02
hCoV-19/Japan/PG-87051/2021	EPI_ISL_3898766	2021-09-02
hCoV-19/Japan/PG-87052/2021	EPI_ISL_3898767	2021-09-02
hCoV-19/Japan/PG-87053/2021	EPI_ISL_3898768	2021-09-02
hCoV-19/Japan/PG-87054/2021	EPI_ISL_3898769	2021-09-02
hCoV-19/Japan/PG-87055/2021	EPI_ISL_3898770	2021-09-02
hCoV-19/Japan/PG-87056/2021	EPI_ISL_3898771	2021-09-02
hCoV-19/Japan/PG-87057/2021	EPI_ISL_3898772	2021-09-02
hCoV-19/Japan/PG-87058/2021	EPI_ISL_3898773	2021-09-02
hCoV-19/Japan/PG-87059/2021	EPI_ISL_3898774	2021-09-02
hCoV-19/Japan/PG-87060/2021	EPI_ISL_3898775	2021-09-02

virus_name	epiID	submission_date
hCoV-19/Japan/PG-87061/2021	EPI_ISL_3898776	2021-09-02
hCoV-19/Japan/PG-87062/2021	EPI_ISL_3898777	2021-09-02
hCoV-19/Japan/PG-87063/2021	EPI_ISL_3898778	2021-09-02
hCoV-19/Japan/PG-87064/2021	EPI_ISL_3898779	2021-09-02
hCoV-19/Japan/PG-87065/2021	EPI_ISL_3898780	2021-09-02
hCoV-19/Japan/PG-87066/2021	EPI_ISL_3898781	2021-09-02
hCoV-19/Japan/PG-87067/2021	EPI_ISL_3898782	2021-09-02
hCoV-19/Japan/PG-87068/2021	EPI_ISL_3898783	2021-09-02
hCoV-19/Japan/PG-87069/2021	EPI_ISL_3898784	2021-09-02
hCoV-19/Japan/PG-87070/2021	EPI_ISL_3898785	2021-09-02
hCoV-19/Japan/PG-87071/2021	EPI_ISL_3898786	2021-09-02
hCoV-19/Japan/PG-87072/2021	EPI_ISL_3898787	2021-09-02
hCoV-19/Japan/PG-87073/2021	EPI_ISL_3898788	2021-09-02
hCoV-19/Japan/PG-87074/2021	EPI_ISL_3898789	2021-09-02
hCoV-19/Japan/PG-87075/2021	EPI_ISL_3898790	2021-09-02
hCoV-19/Japan/PG-87076/2021	EPI_ISL_3898791	2021-09-02
hCoV-19/Japan/PG-87077/2021	EPI_ISL_3898792	2021-09-02
hCoV-19/Japan/PG-87078/2021	EPI_ISL_3898793	2021-09-02
hCoV-19/Japan/PG-87079/2021	EPI_ISL_3898794	2021-09-02
hCoV-19/Japan/PG-87080/2021	EPI_ISL_3898795	2021-09-02
hCoV-19/Japan/PG-87081/2021	EPI_ISL_3898796	2021-09-02
hCoV-19/Japan/PG-87082/2021	EPI_ISL_3898797	2021-09-02
hCoV-19/Japan/PG-87083/2021	EPI_ISL_3898798	2021-09-02
hCoV-19/Japan/PG-87084/2021	EPI_ISL_3898799	2021-09-02
hCoV-19/Japan/PG-87085/2021	EPI_ISL_3898800	2021-09-02
hCoV-19/Japan/PG-87086/2021	EPI_ISL_3898801	2021-09-02
hCoV-19/Japan/PG-87087/2021	EPI_ISL_3898802	2021-09-02
hCoV-19/Japan/PG-87088/2021	EPI_ISL_3898803	2021-09-02
hCoV-19/Japan/PG-87089/2021	EPI_ISL_3898804	2021-09-02
hCoV-19/Japan/PG-87090/2021	EPI_ISL_3898805	2021-09-02
hCoV-19/Japan/PG-87091/2021	EPI_ISL_3898806	2021-09-02
hCoV-19/Japan/PG-87092/2021	EPI_ISL_3898807	2021-09-02
hCoV-19/Japan/PG-87093/2021	EPI_ISL_3898808	2021-09-02
hCoV-19/Japan/PG-87094/2021	EPI_ISL_3898809	2021-09-02
hCoV-19/Japan/PG-87095/2021	EPI_ISL_3898810	2021-09-02
hCoV-19/Japan/PG-87096/2021	EPI_ISL_3898811	2021-09-02
hCoV-19/Japan/PG-87097/2021	EPI_ISL_3898812	2021-09-02
hCoV-19/Japan/PG-87098/2021	EPI_ISL_3898813	2021-09-02
hCoV-19/Japan/PG-87099/2021	EPI_ISL_3898814	2021-09-02
hCoV-19/Japan/PG-87100/2021	EPI_ISL_3898815	2021-09-02
hCoV-19/Japan/PG-87101/2021	EPI_ISL_3898816	2021-09-02
hCoV-19/Japan/PG-87102/2021	EPI_ISL_3898817	2021-09-02
hCoV-19/Japan/PG-87103/2021	EPI_ISL_3898818	2021-09-02
hCoV-19/Japan/PG-87104/2021	EPI_ISL_3898819	2021-09-02
hCoV-19/Japan/PG-87105/2021	EPI_ISL_3898820	2021-09-02
hCoV-19/Japan/PG-87106/2021	EPI_ISL_3898821	2021-09-02
hCoV-19/Japan/PG-87107/2021	EPI_ISL_3898822	2021-09-02
hCoV-19/Japan/PG-87108/2021	EPI_ISL_3898823	2021-09-02
hCoV-19/Japan/PG-87109/2021	EPI_ISL_3898824	2021-09-02
hCoV-19/Japan/PG-87110/2021	EPI_ISL_3898825	2021-09-02
hCoV-19/Japan/PG-87111/2021	EPI_ISL_3898826	2021-09-02
hCoV-19/Japan/PG-87112/2021	EPI_ISL_3898827	2021-09-02
hCoV-19/Japan/PG-87113/2021	EPI_ISL_3898828	2021-09-02
hCoV-19/Japan/PG-87114/2021	EPI_ISL_3898829	2021-09-02

virus_name	epiID	submission_date
hCoV-19/Japan/PG-87115/2021	EPI_ISL_3898830	2021-09-02
hCoV-19/Japan/PG-87116/2021	EPI_ISL_3898831	2021-09-02
hCoV-19/Japan/PG-87117/2021	EPI_ISL_3898832	2021-09-02
hCoV-19/Japan/PG-87118/2021	EPI_ISL_3898833	2021-09-02
hCoV-19/Japan/PG-87119/2021	EPI_ISL_3898834	2021-09-02
hCoV-19/Japan/PG-87120/2021	EPI_ISL_3898835	2021-09-02
hCoV-19/Japan/PG-87121/2021	EPI_ISL_3898836	2021-09-02
hCoV-19/Japan/PG-87122/2021	EPI_ISL_3898837	2021-09-02
hCoV-19/Japan/PG-87123/2021	EPI_ISL_3898838	2021-09-02
hCoV-19/Japan/PG-87124/2021	EPI_ISL_3898839	2021-09-02
hCoV-19/Japan/PG-87125/2021	EPI_ISL_3898840	2021-09-02
hCoV-19/Japan/PG-87126/2021	EPI_ISL_3898841	2021-09-02
hCoV-19/Japan/PG-87127/2021	EPI_ISL_3898842	2021-09-02
hCoV-19/Japan/PG-87128/2021	EPI_ISL_3898843	2021-09-02
hCoV-19/Japan/PG-87129/2021	EPI_ISL_3898844	2021-09-02
hCoV-19/Japan/PG-87130/2021	EPI_ISL_3898845	2021-09-02
hCoV-19/Japan/PG-87131/2021	EPI_ISL_3898846	2021-09-02
hCoV-19/Japan/PG-87132/2021	EPI_ISL_3898847	2021-09-02
hCoV-19/Japan/PG-87133/2021	EPI_ISL_3898848	2021-09-02
hCoV-19/Japan/PG-87134/2021	EPI_ISL_3898849	2021-09-02
hCoV-19/Japan/PG-87135/2021	EPI_ISL_3898850	2021-09-02
hCoV-19/Japan/PG-87136/2021	EPI_ISL_3898851	2021-09-02
hCoV-19/Japan/PG-87137/2021	EPI_ISL_3898852	2021-09-02
hCoV-19/Japan/PG-87138/2021	EPI_ISL_3898853	2021-09-02
hCoV-19/Japan/PG-87139/2021	EPI_ISL_3898854	2021-09-02
hCoV-19/Japan/PG-87140/2021	EPI_ISL_3898855	2021-09-02
hCoV-19/Japan/PG-87141/2021	EPI_ISL_3898856	2021-09-02
hCoV-19/Japan/PG-87142/2021	EPI_ISL_3898857	2021-09-02
hCoV-19/Japan/PG-87143/2021	EPI_ISL_3898858	2021-09-02
hCoV-19/Japan/PG-87144/2021	EPI_ISL_3898859	2021-09-02
hCoV-19/Japan/PG-87145/2021	EPI_ISL_3898860	2021-09-02
hCoV-19/Japan/PG-87146/2021	EPI_ISL_3898861	2021-09-02
hCoV-19/Japan/PG-87147/2021	EPI_ISL_3898862	2021-09-02
hCoV-19/Japan/PG-87148/2021	EPI_ISL_3898863	2021-09-02
hCoV-19/Japan/PG-87149/2021	EPI_ISL_3898864	2021-09-02
hCoV-19/Japan/PG-87150/2021	EPI_ISL_3898865	2021-09-02
hCoV-19/Japan/PG-87151/2021	EPI_ISL_3898866	2021-09-02
hCoV-19/Japan/PG-87152/2021	EPI_ISL_3898867	2021-09-02
hCoV-19/Japan/PG-87153/2021	EPI_ISL_3898868	2021-09-02
hCoV-19/Japan/PG-87154/2021	EPI_ISL_3898869	2021-09-02
hCoV-19/Japan/PG-87155/2021	EPI_ISL_3898870	2021-09-02
hCoV-19/Japan/PG-87156/2021	EPI_ISL_3898871	2021-09-02
hCoV-19/Japan/PG-87157/2021	EPI_ISL_3898872	2021-09-02
hCoV-19/Japan/PG-87158/2021	EPI_ISL_3898873	2021-09-02
hCoV-19/Japan/PG-87159/2021	EPI_ISL_3898874	2021-09-02
hCoV-19/Japan/PG-87160/2021	EPI_ISL_3898875	2021-09-02
hCoV-19/Japan/PG-87161/2021	EPI_ISL_3898876	2021-09-02
hCoV-19/Japan/PG-87162/2021	EPI_ISL_3898877	2021-09-02
hCoV-19/Japan/PG-87163/2021	EPI_ISL_3898878	2021-09-02
hCoV-19/Japan/PG-87164/2021	EPI_ISL_3898879	2021-09-02
hCoV-19/Japan/PG-87165/2021	EPI_ISL_3898880	2021-09-02
hCoV-19/Japan/PG-87166/2021	EPI_ISL_3898881	2021-09-02
hCoV-19/Japan/PG-87167/2021	EPI_ISL_3898882	2021-09-02
hCoV-19/Japan/PG-87168/2021	EPI_ISL_3898883	2021-09-02

virus_name	epiID	submission_date
hCoV-19/Japan/PG-87169/2021	EPI_ISL_3898884	2021-09-02
hCoV-19/Japan/PG-87170/2021	EPI_ISL_3898885	2021-09-02
hCoV-19/Japan/PG-87171/2021	EPI_ISL_3898886	2021-09-02
hCoV-19/Japan/PG-87172/2021	EPI_ISL_3898887	2021-09-02
hCoV-19/Japan/PG-87173/2021	EPI_ISL_3898888	2021-09-02
hCoV-19/Japan/PG-87174/2021	EPI_ISL_3898889	2021-09-02
hCoV-19/Japan/PG-87175/2021	EPI_ISL_3898890	2021-09-02
hCoV-19/Japan/PG-87176/2021	EPI_ISL_3898891	2021-09-02
hCoV-19/Japan/PG-87177/2021	EPI_ISL_3898892	2021-09-02
hCoV-19/Japan/PG-87178/2021	EPI_ISL_3898893	2021-09-02
hCoV-19/Japan/PG-87179/2021	EPI_ISL_3898894	2021-09-02
hCoV-19/Japan/PG-87180/2021	EPI_ISL_3898895	2021-09-02
hCoV-19/Japan/PG-87181/2021	EPI_ISL_3898896	2021-09-02
hCoV-19/Japan/PG-87182/2021	EPI_ISL_3898897	2021-09-02
hCoV-19/Japan/PG-87183/2021	EPI_ISL_3898898	2021-09-02
hCoV-19/Japan/PG-87184/2021	EPI_ISL_3898899	2021-09-02
hCoV-19/Japan/PG-87185/2021	EPI_ISL_3898900	2021-09-02
hCoV-19/Japan/PG-87186/2021	EPI_ISL_3898901	2021-09-02
hCoV-19/Japan/PG-87187/2021	EPI_ISL_3898902	2021-09-02
hCoV-19/Japan/PG-87188/2021	EPI_ISL_3898903	2021-09-02
hCoV-19/Japan/PG-87189/2021	EPI_ISL_3898904	2021-09-02
hCoV-19/Japan/PG-87190/2021	EPI_ISL_3898905	2021-09-02
hCoV-19/Japan/PG-87191/2021	EPI_ISL_3898906	2021-09-02
hCoV-19/Japan/PG-87192/2021	EPI_ISL_3898907	2021-09-02
hCoV-19/Japan/PG-87193/2021	EPI_ISL_3898908	2021-09-02
hCoV-19/Japan/PG-87194/2021	EPI_ISL_3898909	2021-09-02
hCoV-19/Japan/PG-87195/2021	EPI_ISL_3898910	2021-09-02
hCoV-19/Japan/PG-87196/2021	EPI_ISL_3898911	2021-09-02
hCoV-19/Japan/PG-87197/2021	EPI_ISL_3898912	2021-09-02
hCoV-19/Japan/PG-87198/2021	EPI_ISL_3898913	2021-09-02
hCoV-19/Japan/PG-87199/2021	EPI_ISL_3898914	2021-09-02
hCoV-19/Japan/PG-87200/2021	EPI_ISL_3898915	2021-09-02
hCoV-19/Japan/PG-87201/2021	EPI_ISL_3898916	2021-09-02
hCoV-19/Japan/PG-87202/2021	EPI_ISL_3898917	2021-09-02
hCoV-19/Japan/PG-87203/2021	EPI_ISL_3898918	2021-09-02
hCoV-19/Japan/PG-87204/2021	EPI_ISL_3898919	2021-09-02
hCoV-19/Japan/PG-87205/2021	EPI_ISL_3898920	2021-09-02
hCoV-19/Japan/PG-87206/2021	EPI_ISL_3898921	2021-09-02
hCoV-19/Japan/PG-87207/2021	EPI_ISL_3898922	2021-09-02
hCoV-19/Japan/PG-87208/2021	EPI_ISL_3898923	2021-09-02
hCoV-19/Japan/PG-87209/2021	EPI_ISL_3898924	2021-09-02
hCoV-19/Japan/PG-87210/2021	EPI_ISL_3898925	2021-09-02
hCoV-19/Japan/PG-87211/2021	EPI_ISL_3898926	2021-09-02
hCoV-19/Japan/PG-87212/2021	EPI_ISL_3898927	2021-09-02
hCoV-19/Japan/PG-87213/2021	EPI_ISL_3898928	2021-09-02
hCoV-19/Japan/PG-87214/2021	EPI_ISL_3898929	2021-09-02
hCoV-19/Japan/PG-87215/2021	EPI_ISL_3898930	2021-09-02
hCoV-19/Japan/PG-87216/2021	EPI_ISL_3898931	2021-09-02
hCoV-19/Japan/PG-87217/2021	EPI_ISL_3898932	2021-09-02
hCoV-19/Japan/PG-87218/2021	EPI_ISL_3898933	2021-09-02
hCoV-19/Japan/PG-87219/2021	EPI_ISL_3898934	2021-09-02
hCoV-19/Japan/PG-87220/2021	EPI_ISL_3898935	2021-09-02
hCoV-19/Japan/PG-87221/2021	EPI_ISL_3898936	2021-09-02
hCoV-19/Japan/PG-87222/2021	EPI_ISL_3898937	2021-09-02

virus_name	epiID	submission_date
hCoV-19/Japan/PG-87223/2021	EPI_ISL_3898938	2021-09-02
hCoV-19/Japan/PG-87224/2021	EPI_ISL_3898939	2021-09-02
hCoV-19/Japan/PG-87225/2021	EPI_ISL_3898940	2021-09-02
hCoV-19/Japan/PG-87226/2021	EPI_ISL_3898941	2021-09-02
hCoV-19/Japan/PG-87227/2021	EPI_ISL_3898942	2021-09-02
hCoV-19/Japan/PG-87228/2021	EPI_ISL_3898943	2021-09-02
hCoV-19/Japan/PG-87229/2021	EPI_ISL_3898944	2021-09-02
hCoV-19/Japan/PG-87230/2021	EPI_ISL_3898945	2021-09-02
hCoV-19/Japan/PG-87231/2021	EPI_ISL_3898946	2021-09-02
hCoV-19/Japan/PG-87232/2021	EPI_ISL_3898947	2021-09-02
hCoV-19/Japan/PG-87233/2021	EPI_ISL_3898948	2021-09-02
hCoV-19/Japan/PG-87234/2021	EPI_ISL_3898949	2021-09-02
hCoV-19/Japan/PG-87235/2021	EPI_ISL_3898950	2021-09-02
hCoV-19/Japan/PG-87236/2021	EPI_ISL_3898952	2021-09-02
hCoV-19/Japan/PG-87237/2021	EPI_ISL_3898954	2021-09-02
hCoV-19/Japan/PG-87238/2021	EPI_ISL_3898956	2021-09-02
hCoV-19/Japan/PG-87239/2021	EPI_ISL_3898957	2021-09-02
hCoV-19/Japan/PG-87240/2021	EPI_ISL_3898958	2021-09-02
hCoV-19/Japan/PG-87241/2021	EPI_ISL_3898959	2021-09-02
hCoV-19/Japan/PG-87242/2021	EPI_ISL_3898960	2021-09-02
hCoV-19/Japan/PG-87243/2021	EPI_ISL_3898961	2021-09-02
hCoV-19/Japan/PG-87244/2021	EPI_ISL_3898962	2021-09-02
hCoV-19/Japan/PG-87245/2021	EPI_ISL_3898963	2021-09-02
hCoV-19/Japan/PG-87246/2021	EPI_ISL_3898964	2021-09-02
hCoV-19/Japan/PG-87247/2021	EPI_ISL_3898965	2021-09-02
hCoV-19/Japan/PG-87248/2021	EPI_ISL_3898966	2021-09-02
hCoV-19/Japan/PG-87249/2021	EPI_ISL_3898967	2021-09-02
hCoV-19/Japan/PG-87250/2021	EPI_ISL_3898968	2021-09-02
hCoV-19/Japan/PG-87251/2021	EPI_ISL_3898969	2021-09-02
hCoV-19/Japan/PG-87252/2021	EPI_ISL_3898970	2021-09-02
hCoV-19/Japan/PG-87253/2021	EPI_ISL_3898971	2021-09-02
hCoV-19/Japan/PG-87254/2021	EPI_ISL_3898972	2021-09-02
hCoV-19/Japan/PG-87255/2021	EPI_ISL_3898973	2021-09-02
hCoV-19/Japan/PG-87256/2021	EPI_ISL_3898974	2021-09-02
hCoV-19/Japan/PG-87257/2021	EPI_ISL_3898975	2021-09-02
hCoV-19/Japan/PG-87258/2021	EPI_ISL_3898976	2021-09-02
hCoV-19/Japan/PG-87259/2021	EPI_ISL_3898977	2021-09-02
hCoV-19/Japan/PG-87260/2021	EPI_ISL_3898978	2021-09-02
hCoV-19/Japan/PG-87261/2021	EPI_ISL_3898979	2021-09-02
hCoV-19/Japan/PG-87262/2021	EPI_ISL_3898980	2021-09-02
hCoV-19/Japan/PG-87263/2021	EPI_ISL_3898981	2021-09-02
hCoV-19/Japan/PG-87264/2021	EPI_ISL_3898982	2021-09-02
hCoV-19/Japan/PG-87265/2021	EPI_ISL_3898983	2021-09-02
hCoV-19/Japan/PG-87266/2021	EPI_ISL_3898984	2021-09-02
hCoV-19/Japan/PG-87267/2021	EPI_ISL_3898985	2021-09-02
hCoV-19/Japan/PG-87268/2021	EPI_ISL_3898986	2021-09-02
hCoV-19/Japan/PG-87269/2021	EPI_ISL_3898987	2021-09-02
hCoV-19/Japan/PG-87270/2021	EPI_ISL_3898988	2021-09-02
hCoV-19/Japan/PG-87271/2021	EPI_ISL_3898989	2021-09-02
hCoV-19/Japan/PG-87272/2021	EPI_ISL_3898990	2021-09-02
hCoV-19/Japan/PG-87273/2021	EPI_ISL_3898991	2021-09-02
hCoV-19/Japan/PG-87274/2021	EPI_ISL_3898992	2021-09-02
hCoV-19/Japan/PG-87275/2021	EPI_ISL_3898993	2021-09-02
hCoV-19/Japan/PG-87276/2021	EPI_ISL_3898994	2021-09-02

virus_name	epiID	submission_date
hCoV-19/Japan/PG-87277/2021	EPI_ISL_3898995	2021-09-02
hCoV-19/Japan/PG-87278/2021	EPI_ISL_3898996	2021-09-02
hCoV-19/Japan/PG-87279/2021	EPI_ISL_3898997	2021-09-02
hCoV-19/Japan/PG-87280/2021	EPI_ISL_3898998	2021-09-02
hCoV-19/Japan/PG-87281/2021	EPI_ISL_3898999	2021-09-02
hCoV-19/Japan/PG-87282/2021	EPI_ISL_3899000	2021-09-02
hCoV-19/Japan/PG-87283/2021	EPI_ISL_3899001	2021-09-02
hCoV-19/Japan/PG-87284/2021	EPI_ISL_3899002	2021-09-02
hCoV-19/Japan/PG-87285/2021	EPI_ISL_3899003	2021-09-02
hCoV-19/Japan/PG-87286/2021	EPI_ISL_3899004	2021-09-02
hCoV-19/Japan/PG-87287/2021	EPI_ISL_3899005	2021-09-02
hCoV-19/Japan/PG-87288/2021	EPI_ISL_3899006	2021-09-02
hCoV-19/Japan/PG-87289/2021	EPI_ISL_3899007	2021-09-02
hCoV-19/Japan/PG-87290/2021	EPI_ISL_3899008	2021-09-02
hCoV-19/Japan/PG-87291/2021	EPI_ISL_3899009	2021-09-02
hCoV-19/Japan/PG-87292/2021	EPI_ISL_3899010	2021-09-02
hCoV-19/Japan/PG-87293/2021	EPI_ISL_3899011	2021-09-02
hCoV-19/Japan/PG-87294/2021	EPI_ISL_3899012	2021-09-02
hCoV-19/Japan/PG-87295/2021	EPI_ISL_3899013	2021-09-02
hCoV-19/Japan/PG-87296/2021	EPI_ISL_3899014	2021-09-02
hCoV-19/Japan/PG-87297/2021	EPI_ISL_3899015	2021-09-02
hCoV-19/Japan/PG-87298/2021	EPI_ISL_3899016	2021-09-02
hCoV-19/Japan/PG-87299/2021	EPI_ISL_3899017	2021-09-02
hCoV-19/Japan/PG-87300/2021	EPI_ISL_3899018	2021-09-02
hCoV-19/Japan/PG-87301/2021	EPI_ISL_3899019	2021-09-02
hCoV-19/Japan/PG-87302/2021	EPI_ISL_3899020	2021-09-02
hCoV-19/Japan/PG-87303/2021	EPI_ISL_3899021	2021-09-02
hCoV-19/Japan/PG-87304/2021	EPI_ISL_3899022	2021-09-02
hCoV-19/Japan/PG-87305/2021	EPI_ISL_3899023	2021-09-02
hCoV-19/Japan/PG-87306/2021	EPI_ISL_3899024	2021-09-02
hCoV-19/Japan/PG-87307/2021	EPI_ISL_3899025	2021-09-02
hCoV-19/Japan/PG-87308/2021	EPI_ISL_3899026	2021-09-02
hCoV-19/Japan/PG-87309/2021	EPI_ISL_3899027	2021-09-02
hCoV-19/Japan/PG-87310/2021	EPI_ISL_3899028	2021-09-02
hCoV-19/Japan/PG-87311/2021	EPI_ISL_3899029	2021-09-02
hCoV-19/Japan/PG-87312/2021	EPI_ISL_3899030	2021-09-02
hCoV-19/Japan/PG-87313/2021	EPI_ISL_3899031	2021-09-02
hCoV-19/Japan/PG-87314/2021	EPI_ISL_3899032	2021-09-02
hCoV-19/Japan/PG-87315/2021	EPI_ISL_3899033	2021-09-02
hCoV-19/Japan/PG-87316/2021	EPI_ISL_3899034	2021-09-02
hCoV-19/Japan/PG-87317/2021	EPI_ISL_3899035	2021-09-02
hCoV-19/Japan/PG-87318/2021	EPI_ISL_3899036	2021-09-02
hCoV-19/Japan/PG-87319/2021	EPI_ISL_3899037	2021-09-02
hCoV-19/Japan/PG-87320/2021	EPI_ISL_3899038	2021-09-02
hCoV-19/Japan/PG-87321/2021	EPI_ISL_3899039	2021-09-02
hCoV-19/Japan/PG-87322/2021	EPI_ISL_3899040	2021-09-02
hCoV-19/Japan/PG-87323/2021	EPI_ISL_3899041	2021-09-02
hCoV-19/Japan/PG-87324/2021	EPI_ISL_3899042	2021-09-02
hCoV-19/Japan/PG-87325/2021	EPI_ISL_3899043	2021-09-02
hCoV-19/Japan/PG-87326/2021	EPI_ISL_3899044	2021-09-02
hCoV-19/Japan/PG-87327/2021	EPI_ISL_3899045	2021-09-02
hCoV-19/Japan/PG-87328/2021	EPI_ISL_3899046	2021-09-02
hCoV-19/Japan/PG-87329/2021	EPI_ISL_3899047	2021-09-02
hCoV-19/Japan/PG-87330/2021	EPI_ISL_3899048	2021-09-02

virus_name	epiID	submission_date
hCoV-19/Japan/PG-87331/2021	EPI_ISL_3899049	2021-09-02
hCoV-19/Japan/PG-87332/2021	EPI_ISL_3899050	2021-09-02
hCoV-19/Japan/PG-87333/2021	EPI_ISL_3899051	2021-09-02
hCoV-19/Japan/PG-87334/2021	EPI_ISL_3899052	2021-09-02
hCoV-19/Japan/PG-87335/2021	EPI_ISL_3899053	2021-09-02
hCoV-19/Japan/PG-87336/2021	EPI_ISL_3899054	2021-09-02
hCoV-19/Japan/PG-87337/2021	EPI_ISL_3899055	2021-09-02
hCoV-19/Japan/PG-87338/2021	EPI_ISL_3899056	2021-09-02
hCoV-19/Japan/PG-87339/2021	EPI_ISL_3899057	2021-09-02
hCoV-19/Japan/PG-87340/2021	EPI_ISL_3899058	2021-09-02
hCoV-19/Japan/PG-87341/2021	EPI_ISL_3899059	2021-09-02
hCoV-19/Japan/PG-87342/2021	EPI_ISL_3899060	2021-09-02
hCoV-19/Japan/PG-87343/2021	EPI_ISL_3899061	2021-09-02
hCoV-19/Japan/PG-87344/2021	EPI_ISL_3899062	2021-09-02
hCoV-19/Japan/PG-87345/2021	EPI_ISL_3899063	2021-09-02
hCoV-19/Japan/PG-87346/2021	EPI_ISL_3899064	2021-09-02
hCoV-19/Japan/PG-87347/2021	EPI_ISL_3899065	2021-09-02
hCoV-19/Japan/PG-87348/2021	EPI_ISL_3899066	2021-09-02
hCoV-19/Japan/PG-87349/2021	EPI_ISL_3899067	2021-09-02
hCoV-19/Japan/PG-87350/2021	EPI_ISL_3899068	2021-09-02
hCoV-19/Japan/PG-87351/2021	EPI_ISL_3899069	2021-09-02
hCoV-19/Japan/PG-87352/2021	EPI_ISL_3899070	2021-09-02
hCoV-19/Japan/PG-87353/2021	EPI_ISL_3899071	2021-09-02
hCoV-19/Japan/PG-87354/2021	EPI_ISL_3899072	2021-09-02
hCoV-19/Japan/PG-87355/2021	EPI_ISL_3899073	2021-09-02
hCoV-19/Japan/PG-87356/2021	EPI_ISL_3899074	2021-09-02
hCoV-19/Japan/PG-87357/2021	EPI_ISL_3899075	2021-09-02
hCoV-19/Japan/PG-87358/2021	EPI_ISL_3899076	2021-09-02
hCoV-19/Japan/PG-87359/2021	EPI_ISL_3899077	2021-09-02
hCoV-19/Japan/PG-87360/2021	EPI_ISL_3899078	2021-09-02
hCoV-19/Japan/PG-87361/2021	EPI_ISL_3899079	2021-09-02
hCoV-19/Japan/PG-87362/2021	EPI_ISL_3899080	2021-09-02
hCoV-19/Japan/PG-87363/2021	EPI_ISL_3899081	2021-09-02
hCoV-19/Japan/PG-87364/2021	EPI_ISL_3899082	2021-09-02
hCoV-19/Japan/PG-87365/2021	EPI_ISL_3899083	2021-09-02
hCoV-19/Japan/PG-87366/2021	EPI_ISL_3899084	2021-09-02
hCoV-19/Japan/PG-87367/2021	EPI_ISL_3899085	2021-09-02
hCoV-19/Japan/PG-87368/2021	EPI_ISL_3899086	2021-09-02
hCoV-19/Japan/PG-87369/2021	EPI_ISL_3899087	2021-09-02
hCoV-19/Japan/PG-87370/2021	EPI_ISL_3899088	2021-09-02
hCoV-19/Japan/PG-87371/2021	EPI_ISL_3899089	2021-09-02
hCoV-19/Japan/PG-87372/2021	EPI_ISL_3899090	2021-09-02
hCoV-19/Japan/PG-87373/2021	EPI_ISL_3899091	2021-09-02
hCoV-19/Japan/PG-87374/2021	EPI_ISL_3899092	2021-09-02
hCoV-19/Japan/PG-87375/2021	EPI_ISL_3899093	2021-09-02
hCoV-19/Japan/PG-87376/2021	EPI_ISL_3899094	2021-09-02
hCoV-19/Japan/PG-87377/2021	EPI_ISL_3899095	2021-09-02
hCoV-19/Japan/PG-87378/2021	EPI_ISL_3899096	2021-09-02
hCoV-19/Japan/PG-87379/2021	EPI_ISL_3899097	2021-09-02
hCoV-19/Japan/PG-87380/2021	EPI_ISL_3899098	2021-09-02
hCoV-19/Japan/PG-87381/2021	EPI_ISL_3899099	2021-09-02
hCoV-19/Japan/PG-87382/2021	EPI_ISL_3899100	2021-09-02
hCoV-19/Japan/PG-87383/2021	EPI_ISL_3899101	2021-09-02
hCoV-19/Japan/PG-87384/2021	EPI_ISL_3899102	2021-09-02

virus_name	epiID	submission_date
hCoV-19/Japan/PG-87385/2021	EPI_ISL_3899103	2021-09-02
hCoV-19/Japan/PG-87386/2021	EPI_ISL_3899104	2021-09-02
hCoV-19/Japan/PG-87387/2021	EPI_ISL_3899105	2021-09-02
hCoV-19/Japan/PG-87388/2021	EPI_ISL_3899106	2021-09-02
hCoV-19/Japan/PG-87389/2021	EPI_ISL_3899107	2021-09-02
hCoV-19/Japan/PG-87390/2021	EPI_ISL_3899108	2021-09-02
hCoV-19/Japan/PG-87391/2021	EPI_ISL_3899109	2021-09-02
hCoV-19/Japan/PG-87392/2021	EPI_ISL_3899110	2021-09-02
hCoV-19/Japan/PG-87393/2021	EPI_ISL_3899111	2021-09-02
hCoV-19/Japan/PG-87394/2021	EPI_ISL_3899112	2021-09-02
hCoV-19/Japan/PG-87395/2021	EPI_ISL_3899113	2021-09-02
hCoV-19/Japan/PG-87396/2021	EPI_ISL_3899114	2021-09-02
hCoV-19/Japan/PG-87397/2021	EPI_ISL_3899115	2021-09-02
hCoV-19/Japan/PG-87398/2021	EPI_ISL_3899116	2021-09-02
hCoV-19/Japan/PG-87399/2021	EPI_ISL_3899117	2021-09-02
hCoV-19/Japan/PG-87400/2021	EPI_ISL_3899118	2021-09-02
hCoV-19/Japan/PG-87401/2021	EPI_ISL_3899119	2021-09-02
hCoV-19/Japan/PG-87402/2021	EPI_ISL_3899120	2021-09-02
hCoV-19/Japan/PG-87403/2021	EPI_ISL_3899121	2021-09-02
hCoV-19/Japan/PG-87404/2021	EPI_ISL_3899122	2021-09-02
hCoV-19/Japan/PG-87405/2021	EPI_ISL_3899123	2021-09-02
hCoV-19/Japan/PG-87406/2021	EPI_ISL_3899124	2021-09-02
hCoV-19/Japan/PG-87407/2021	EPI_ISL_3899125	2021-09-02
hCoV-19/Japan/PG-87408/2021	EPI_ISL_3899126	2021-09-02
hCoV-19/Japan/PG-87409/2021	EPI_ISL_3899127	2021-09-02
hCoV-19/Japan/PG-87410/2021	EPI_ISL_3899128	2021-09-02
hCoV-19/Japan/PG-87411/2021	EPI_ISL_3899129	2021-09-02
hCoV-19/Japan/PG-87412/2021	EPI_ISL_3899130	2021-09-02
hCoV-19/Japan/PG-87413/2021	EPI_ISL_3899131	2021-09-02
hCoV-19/Japan/PG-87414/2021	EPI_ISL_3899132	2021-09-02
hCoV-19/Japan/PG-87415/2021	EPI_ISL_3899133	2021-09-02
hCoV-19/Japan/PG-87416/2021	EPI_ISL_3899134	2021-09-02
hCoV-19/Japan/PG-87417/2021	EPI_ISL_3899135	2021-09-02
hCoV-19/Japan/PG-87418/2021	EPI_ISL_3899136	2021-09-02
hCoV-19/Japan/PG-87419/2021	EPI_ISL_3899137	2021-09-02
hCoV-19/Japan/PG-87420/2021	EPI_ISL_3899138	2021-09-02
hCoV-19/Japan/PG-87421/2021	EPI_ISL_3899139	2021-09-02
hCoV-19/Japan/PG-87422/2021	EPI_ISL_3899140	2021-09-02
hCoV-19/Japan/PG-87423/2021	EPI_ISL_3899141	2021-09-02
hCoV-19/Japan/PG-87424/2021	EPI_ISL_3899142	2021-09-02
hCoV-19/Japan/PG-87425/2021	EPI_ISL_3899143	2021-09-02
hCoV-19/Japan/PG-87426/2021	EPI_ISL_3899144	2021-09-02
hCoV-19/Japan/PG-87427/2021	EPI_ISL_3899145	2021-09-02
hCoV-19/Japan/PG-87428/2021	EPI_ISL_3899146	2021-09-02
hCoV-19/Japan/PG-87429/2021	EPI_ISL_3899147	2021-09-02
hCoV-19/Japan/PG-87430/2021	EPI_ISL_3899148	2021-09-02
hCoV-19/Japan/PG-87431/2021	EPI_ISL_3899149	2021-09-02
hCoV-19/Japan/PG-87432/2021	EPI_ISL_3899150	2021-09-02
hCoV-19/Japan/PG-87433/2021	EPI_ISL_3899151	2021-09-02
hCoV-19/Japan/PG-87434/2021	EPI_ISL_3899152	2021-09-02
hCoV-19/Japan/PG-87435/2021	EPI_ISL_3899153	2021-09-02
hCoV-19/Japan/PG-87436/2021	EPI_ISL_3899154	2021-09-02
hCoV-19/Japan/PG-87437/2021	EPI_ISL_3899155	2021-09-02
hCoV-19/Japan/PG-87438/2021	EPI_ISL_3899156	2021-09-02

virus_name	epiID	submission_date
hCoV-19/Japan/PG-87439/2021	EPI_ISL_3899157	2021-09-02
hCoV-19/Japan/PG-87440/2021	EPI_ISL_3899158	2021-09-02
hCoV-19/Japan/PG-87441/2021	EPI_ISL_3899159	2021-09-02
hCoV-19/Japan/PG-87442/2021	EPI_ISL_3899160	2021-09-02
hCoV-19/Japan/PG-87443/2021	EPI_ISL_3899161	2021-09-02
hCoV-19/Japan/PG-87444/2021	EPI_ISL_3899162	2021-09-02
hCoV-19/Japan/PG-87445/2021	EPI_ISL_3899163	2021-09-02
hCoV-19/Japan/PG-87446/2021	EPI_ISL_3899164	2021-09-02
hCoV-19/Japan/PG-87447/2021	EPI_ISL_3899165	2021-09-02
hCoV-19/Japan/PG-87448/2021	EPI_ISL_3899166	2021-09-02
hCoV-19/Japan/PG-87449/2021	EPI_ISL_3899167	2021-09-02
hCoV-19/Japan/PG-87450/2021	EPI_ISL_3899169	2021-09-02
hCoV-19/Japan/PG-87451/2021	EPI_ISL_3899171	2021-09-02
hCoV-19/Japan/PG-87452/2021	EPI_ISL_3899174	2021-09-02
hCoV-19/Japan/PG-87453/2021	EPI_ISL_3899176	2021-09-02
hCoV-19/Japan/PG-87454/2021	EPI_ISL_3899179	2021-09-02
hCoV-19/Japan/PG-87455/2021	EPI_ISL_3899182	2021-09-02
hCoV-19/Japan/PG-87456/2021	EPI_ISL_3899185	2021-09-02
hCoV-19/Japan/PG-87457/2021	EPI_ISL_3899188	2021-09-02
hCoV-19/Japan/PG-87458/2021	EPI_ISL_3899191	2021-09-02
hCoV-19/Japan/PG-87459/2021	EPI_ISL_3899194	2021-09-02
hCoV-19/Japan/PG-87460/2021	EPI_ISL_3899197	2021-09-02
hCoV-19/Japan/PG-87461/2021	EPI_ISL_3899201	2021-09-02
hCoV-19/Japan/PG-87462/2021	EPI_ISL_3899204	2021-09-02
hCoV-19/Japan/PG-87463/2021	EPI_ISL_3899207	2021-09-02
hCoV-19/Japan/PG-87464/2021	EPI_ISL_3899210	2021-09-02
hCoV-19/Japan/PG-87465/2021	EPI_ISL_3899213	2021-09-02
hCoV-19/Japan/PG-87466/2021	EPI_ISL_3899214	2021-09-02
hCoV-19/Japan/PG-87467/2021	EPI_ISL_3899215	2021-09-02
hCoV-19/Japan/PG-87468/2021	EPI_ISL_3899216	2021-09-02
hCoV-19/Japan/PG-87469/2021	EPI_ISL_3899217	2021-09-02
hCoV-19/Japan/PG-87470/2021	EPI_ISL_3899218	2021-09-02
hCoV-19/Japan/PG-87471/2021	EPI_ISL_3899219	2021-09-02
hCoV-19/Japan/PG-87472/2021	EPI_ISL_3899220	2021-09-02
hCoV-19/Japan/PG-87473/2021	EPI_ISL_3899221	2021-09-02
hCoV-19/Japan/PG-87474/2021	EPI_ISL_3899222	2021-09-02
hCoV-19/Japan/PG-87475/2021	EPI_ISL_3899223	2021-09-02
hCoV-19/Japan/PG-87476/2021	EPI_ISL_3899224	2021-09-02
hCoV-19/Japan/PG-87477/2021	EPI_ISL_3899225	2021-09-02
hCoV-19/Japan/PG-87478/2021	EPI_ISL_3899226	2021-09-02
hCoV-19/Japan/PG-87479/2021	EPI_ISL_3899227	2021-09-02
hCoV-19/Japan/PG-87480/2021	EPI_ISL_3899228	2021-09-02
hCoV-19/Japan/PG-87481/2021	EPI_ISL_3899229	2021-09-02
hCoV-19/Japan/PG-87482/2021	EPI_ISL_3899230	2021-09-02
hCoV-19/Japan/PG-87483/2021	EPI_ISL_3899231	2021-09-02
hCoV-19/Japan/PG-87484/2021	EPI_ISL_3899232	2021-09-02
hCoV-19/Japan/PG-87485/2021	EPI_ISL_3899233	2021-09-02
hCoV-19/Japan/PG-87486/2021	EPI_ISL_3899234	2021-09-02
hCoV-19/Japan/PG-87487/2021	EPI_ISL_3899235	2021-09-02
hCoV-19/Japan/PG-87488/2021	EPI_ISL_3899236	2021-09-02
hCoV-19/Japan/PG-87489/2021	EPI_ISL_3899237	2021-09-02
hCoV-19/Japan/PG-87490/2021	EPI_ISL_3899238	2021-09-02
hCoV-19/Japan/PG-87491/2021	EPI_ISL_3899239	2021-09-02
hCoV-19/Japan/PG-87492/2021	EPI_ISL_3899240	2021-09-02

virus_name	epiID	submission_date
hCoV-19/Japan/PG-87493/2021	EPI_ISL_3899241	2021-09-02
hCoV-19/Japan/PG-87494/2021	EPI_ISL_3899244	2021-09-02
hCoV-19/Japan/PG-87495/2021	EPI_ISL_3899247	2021-09-02
hCoV-19/Japan/PG-87496/2021	EPI_ISL_3899249	2021-09-02
hCoV-19/Japan/PG-87497/2021	EPI_ISL_3899250	2021-09-02
hCoV-19/Japan/PG-87498/2021	EPI_ISL_3899252	2021-09-02
hCoV-19/Japan/PG-87499/2021	EPI_ISL_3899255	2021-09-02
hCoV-19/Japan/PG-87500/2021	EPI_ISL_3899257	2021-09-02
hCoV-19/Japan/PG-87501/2021	EPI_ISL_3899260	2021-09-02
hCoV-19/Japan/PG-87502/2021	EPI_ISL_3899261	2021-09-02
hCoV-19/Japan/PG-87503/2021	EPI_ISL_3899262	2021-09-02
hCoV-19/Japan/PG-87504/2021	EPI_ISL_3899264	2021-09-02
hCoV-19/Japan/PG-87505/2021	EPI_ISL_3899267	2021-09-02
hCoV-19/Japan/PG-87506/2021	EPI_ISL_3899270	2021-09-02
hCoV-19/Japan/PG-87507/2021	EPI_ISL_3899273	2021-09-02
hCoV-19/Japan/PG-87508/2021	EPI_ISL_3899276	2021-09-02
hCoV-19/Japan/PG-87509/2021	EPI_ISL_3899279	2021-09-02
hCoV-19/Japan/PG-87510/2021	EPI_ISL_3899283	2021-09-02
hCoV-19/Japan/PG-87511/2021	EPI_ISL_3899286	2021-09-02
hCoV-19/Japan/PG-87512/2021	EPI_ISL_3899288	2021-09-02
hCoV-19/Japan/PG-87513/2021	EPI_ISL_3899292	2021-09-02
hCoV-19/Japan/PG-87514/2021	EPI_ISL_3899295	2021-09-02
hCoV-19/Japan/PG-87515/2021	EPI_ISL_3899298	2021-09-02
hCoV-19/Japan/PG-87516/2021	EPI_ISL_3899302	2021-09-02
hCoV-19/Japan/PG-87517/2021	EPI_ISL_3899303	2021-09-02
hCoV-19/Japan/PG-87518/2021	EPI_ISL_3899304	2021-09-02
hCoV-19/Japan/PG-87519/2021	EPI_ISL_3899305	2021-09-02
hCoV-19/Japan/PG-87520/2021	EPI_ISL_3899306	2021-09-02
hCoV-19/Japan/PG-87521/2021	EPI_ISL_3899307	2021-09-02
hCoV-19/Japan/PG-87522/2021	EPI_ISL_3899308	2021-09-02
hCoV-19/Japan/PG-87523/2021	EPI_ISL_3899309	2021-09-02
hCoV-19/Japan/PG-87524/2021	EPI_ISL_3899310	2021-09-02
hCoV-19/Japan/PG-87525/2021	EPI_ISL_3899311	2021-09-02
hCoV-19/Japan/PG-87526/2021	EPI_ISL_3899312	2021-09-02
hCoV-19/Japan/PG-87527/2021	EPI_ISL_3899313	2021-09-02
hCoV-19/Japan/PG-87528/2021	EPI_ISL_3899314	2021-09-02
hCoV-19/Japan/PG-87529/2021	EPI_ISL_3899315	2021-09-02
hCoV-19/Japan/PG-87530/2021	EPI_ISL_3899316	2021-09-02
hCoV-19/Japan/PG-87531/2021	EPI_ISL_3899317	2021-09-02
hCoV-19/Japan/PG-87532/2021	EPI_ISL_3899318	2021-09-02
hCoV-19/Japan/PG-87533/2021	EPI_ISL_3899319	2021-09-02
hCoV-19/Japan/PG-87534/2021	EPI_ISL_3899320	2021-09-02
hCoV-19/Japan/PG-87535/2021	EPI_ISL_3899321	2021-09-02
hCoV-19/Japan/PG-87536/2021	EPI_ISL_3899322	2021-09-02
hCoV-19/Japan/PG-87537/2021	EPI_ISL_3899323	2021-09-02
hCoV-19/Japan/PG-87538/2021	EPI_ISL_3899324	2021-09-02
hCoV-19/Japan/PG-87539/2021	EPI_ISL_3899325	2021-09-02
hCoV-19/Japan/PG-87540/2021	EPI_ISL_3899326	2021-09-02
hCoV-19/Japan/PG-87541/2021	EPI_ISL_3899327	2021-09-02
hCoV-19/Japan/PG-87542/2021	EPI_ISL_3899328	2021-09-02
hCoV-19/Japan/PG-87543/2021	EPI_ISL_3899329	2021-09-02
hCoV-19/Japan/PG-87544/2021	EPI_ISL_3899330	2021-09-02
hCoV-19/Japan/PG-87545/2021	EPI_ISL_3899331	2021-09-02
hCoV-19/Japan/PG-87546/2021	EPI_ISL_3899332	2021-09-02

virus_name	epiID	submission_date
hCoV-19/Japan/PG-87547/2021	EPI_ISL_3899333	2021-09-02
hCoV-19/Japan/PG-87548/2021	EPI_ISL_3899334	2021-09-02
hCoV-19/Japan/PG-87549/2021	EPI_ISL_3899335	2021-09-02
hCoV-19/Japan/PG-87550/2021	EPI_ISL_3899336	2021-09-02
hCoV-19/Japan/PG-87551/2021	EPI_ISL_3899337	2021-09-02
hCoV-19/Japan/PG-87552/2021	EPI_ISL_3899338	2021-09-02
hCoV-19/Japan/PG-87553/2021	EPI_ISL_3899339	2021-09-02
hCoV-19/Japan/PG-87554/2021	EPI_ISL_3899340	2021-09-02
hCoV-19/Japan/PG-87555/2021	EPI_ISL_3899341	2021-09-02
hCoV-19/Japan/PG-87556/2021	EPI_ISL_3899342	2021-09-02
hCoV-19/Japan/PG-87557/2021	EPI_ISL_3899343	2021-09-02
hCoV-19/Japan/PG-87558/2021	EPI_ISL_3899344	2021-09-02
hCoV-19/Japan/PG-87559/2021	EPI_ISL_3899345	2021-09-02
hCoV-19/Japan/PG-87560/2021	EPI_ISL_3899346	2021-09-02
hCoV-19/Japan/PG-87561/2021	EPI_ISL_3899347	2021-09-02
hCoV-19/Japan/PG-87562/2021	EPI_ISL_3899348	2021-09-02
hCoV-19/Japan/PG-87563/2021	EPI_ISL_3899349	2021-09-02
hCoV-19/Japan/PG-87564/2021	EPI_ISL_3899350	2021-09-02
hCoV-19/Japan/PG-87565/2021	EPI_ISL_3899351	2021-09-02
hCoV-19/Japan/PG-87566/2021	EPI_ISL_3899352	2021-09-02
hCoV-19/Japan/PG-87567/2021	EPI_ISL_3899353	2021-09-02
hCoV-19/Japan/PG-87568/2021	EPI_ISL_3899354	2021-09-02
hCoV-19/Japan/PG-87569/2021	EPI_ISL_3899355	2021-09-02
hCoV-19/Japan/PG-87570/2021	EPI_ISL_3899356	2021-09-02
hCoV-19/Japan/PG-87571/2021	EPI_ISL_3899357	2021-09-02
hCoV-19/Japan/PG-87572/2021	EPI_ISL_3899358	2021-09-02
hCoV-19/Japan/PG-87573/2021	EPI_ISL_3899359	2021-09-02
hCoV-19/Japan/PG-87574/2021	EPI_ISL_3899360	2021-09-02
hCoV-19/Japan/PG-87575/2021	EPI_ISL_3899361	2021-09-02
hCoV-19/Japan/PG-87576/2021	EPI_ISL_3899362	2021-09-02
hCoV-19/Japan/PG-87577/2021	EPI_ISL_3899363	2021-09-02
hCoV-19/Japan/PG-87578/2021	EPI_ISL_3899364	2021-09-02
hCoV-19/Japan/PG-87579/2021	EPI_ISL_3899365	2021-09-02
hCoV-19/Japan/PG-87580/2021	EPI_ISL_3899366	2021-09-02
hCoV-19/Japan/PG-87581/2021	EPI_ISL_3899367	2021-09-02
hCoV-19/Japan/PG-87582/2021	EPI_ISL_3899368	2021-09-02
hCoV-19/Japan/PG-87583/2021	EPI_ISL_3899369	2021-09-02
hCoV-19/Japan/PG-87584/2021	EPI_ISL_3899370	2021-09-02
hCoV-19/Japan/PG-87585/2021	EPI_ISL_3899371	2021-09-02
hCoV-19/Japan/PG-87586/2021	EPI_ISL_3899372	2021-09-02
hCoV-19/Japan/PG-87587/2021	EPI_ISL_3899373	2021-09-02
hCoV-19/Japan/PG-87588/2021	EPI_ISL_3899374	2021-09-02
hCoV-19/Japan/PG-87589/2021	EPI_ISL_3899375	2021-09-02
hCoV-19/Japan/PG-87590/2021	EPI_ISL_3899376	2021-09-02
hCoV-19/Japan/PG-87591/2021	EPI_ISL_3899377	2021-09-02
hCoV-19/Japan/PG-87592/2021	EPI_ISL_3899378	2021-09-02
hCoV-19/Japan/PG-87593/2021	EPI_ISL_3899379	2021-09-02
hCoV-19/Japan/PG-87594/2021	EPI_ISL_3899380	2021-09-02
hCoV-19/Japan/PG-87595/2021	EPI_ISL_3899381	2021-09-02
hCoV-19/Japan/PG-87596/2021	EPI_ISL_3899382	2021-09-02
hCoV-19/Japan/PG-87597/2021	EPI_ISL_3899383	2021-09-02
hCoV-19/Japan/PG-87766/2021	EPI_ISL_3899384	2021-09-02
hCoV-19/Japan/PG-87767/2021	EPI_ISL_3899385	2021-09-02
hCoV-19/Japan/PG-87768/2021	EPI_ISL_3899386	2021-09-02

virus_name	epiID	submission_date
hCoV-19/Japan/PG-87769/2021	EPI_ISL_3899387	2021-09-02
hCoV-19/Japan/PG-87770/2021	EPI_ISL_3899388	2021-09-02
hCoV-19/Japan/PG-87771/2021	EPI_ISL_3899389	2021-09-02
hCoV-19/Japan/PG-87772/2021	EPI_ISL_3899390	2021-09-02
hCoV-19/Japan/PG-87773/2021	EPI_ISL_3899391	2021-09-02
hCoV-19/Japan/PG-87774/2021	EPI_ISL_3899392	2021-09-02
hCoV-19/Japan/PG-87775/2021	EPI_ISL_3899393	2021-09-02
hCoV-19/Japan/PG-87776/2021	EPI_ISL_3899394	2021-09-02
hCoV-19/Japan/PG-87777/2021	EPI_ISL_3899395	2021-09-02
hCoV-19/Japan/PG-87778/2021	EPI_ISL_3899396	2021-09-02
hCoV-19/Japan/PG-87779/2021	EPI_ISL_3899397	2021-09-02
hCoV-19/Japan/PG-87780/2021	EPI_ISL_3899398	2021-09-02
hCoV-19/Japan/PG-87781/2021	EPI_ISL_3899399	2021-09-02
hCoV-19/Japan/PG-87782/2021	EPI_ISL_3899400	2021-09-02
hCoV-19/Japan/PG-87783/2021	EPI_ISL_3899401	2021-09-02
hCoV-19/Japan/PG-87784/2021	EPI_ISL_3899402	2021-09-02
hCoV-19/Japan/PG-87785/2021	EPI_ISL_3899403	2021-09-02
hCoV-19/Japan/PG-87786/2021	EPI_ISL_3899404	2021-09-02
hCoV-19/Japan/PG-87787/2021	EPI_ISL_3899405	2021-09-02
hCoV-19/Japan/PG-87788/2021	EPI_ISL_3899406	2021-09-02
hCoV-19/Japan/PG-87789/2021	EPI_ISL_3899407	2021-09-02
hCoV-19/Japan/PG-87790/2021	EPI_ISL_3899408	2021-09-02
hCoV-19/Japan/PG-87791/2021	EPI_ISL_3899409	2021-09-02
hCoV-19/Japan/PG-87792/2021	EPI_ISL_3899410	2021-09-02
hCoV-19/Japan/PG-87793/2021	EPI_ISL_3899411	2021-09-02
hCoV-19/Japan/PG-87794/2021	EPI_ISL_3899412	2021-09-02
hCoV-19/Japan/PG-87795/2021	EPI_ISL_3899413	2021-09-02
hCoV-19/Japan/PG-87796/2021	EPI_ISL_3899414	2021-09-02
hCoV-19/Japan/PG-87797/2021	EPI_ISL_3899416	2021-09-02
hCoV-19/Japan/PG-87798/2021	EPI_ISL_3899417	2021-09-02
hCoV-19/Japan/PG-87799/2021	EPI_ISL_3899418	2021-09-02
hCoV-19/Japan/PG-87800/2021	EPI_ISL_3899419	2021-09-02
hCoV-19/Japan/PG-87801/2021	EPI_ISL_3899420	2021-09-02
hCoV-19/Japan/PG-87802/2021	EPI_ISL_3899421	2021-09-02
hCoV-19/Japan/PG-87803/2021	EPI_ISL_3899422	2021-09-02
hCoV-19/Japan/PG-87804/2021	EPI_ISL_3899423	2021-09-02
hCoV-19/Japan/PG-87805/2021	EPI_ISL_3899424	2021-09-02
hCoV-19/Japan/PG-87806/2021	EPI_ISL_3899425	2021-09-02
hCoV-19/Japan/PG-87807/2021	EPI_ISL_3899426	2021-09-02
hCoV-19/Japan/PG-87808/2021	EPI_ISL_3899427	2021-09-02
hCoV-19/Japan/PG-87809/2021	EPI_ISL_3899428	2021-09-02
hCoV-19/Japan/PG-87810/2021	EPI_ISL_3899429	2021-09-02
hCoV-19/Japan/PG-87811/2021	EPI_ISL_3899430	2021-09-02
hCoV-19/Japan/PG-87812/2021	EPI_ISL_3899431	2021-09-02
hCoV-19/Japan/PG-87813/2021	EPI_ISL_3899432	2021-09-02
hCoV-19/Japan/PG-87814/2021	EPI_ISL_3899433	2021-09-02
hCoV-19/Japan/PG-87815/2021	EPI_ISL_3899434	2021-09-02
hCoV-19/Japan/PG-87816/2021	EPI_ISL_3899435	2021-09-02
hCoV-19/Japan/PG-87817/2021	EPI_ISL_3899436	2021-09-02
hCoV-19/Japan/PG-87818/2021	EPI_ISL_3899437	2021-09-02
hCoV-19/Japan/PG-87819/2021	EPI_ISL_3899438	2021-09-02
hCoV-19/Japan/PG-87820/2021	EPI_ISL_3899440	2021-09-02
hCoV-19/Japan/PG-87821/2021	EPI_ISL_3899442	2021-09-02
hCoV-19/Japan/PG-87822/2021	EPI_ISL_3899444	2021-09-02

virus_name	epiID	submission_date
hCoV-19/Japan/PG-87823/2021	EPI_ISL_3899445	2021-09-02
hCoV-19/Japan/PG-87824/2021	EPI_ISL_3899447	2021-09-02
hCoV-19/Japan/PG-87825/2021	EPI_ISL_3899449	2021-09-02
hCoV-19/Japan/PG-87826/2021	EPI_ISL_3899452	2021-09-02
hCoV-19/Japan/PG-87827/2021	EPI_ISL_3899455	2021-09-02
hCoV-19/Japan/PG-87828/2021	EPI_ISL_3899458	2021-09-02
hCoV-19/Japan/PG-87829/2021	EPI_ISL_3899460	2021-09-02
hCoV-19/Japan/PG-87830/2021	EPI_ISL_3899461	2021-09-02
hCoV-19/Japan/PG-87831/2021	EPI_ISL_3899462	2021-09-02
hCoV-19/Japan/PG-87832/2021	EPI_ISL_3899463	2021-09-02
hCoV-19/Japan/PG-87833/2021	EPI_ISL_3899467	2021-09-02
hCoV-19/Japan/PG-87834/2021	EPI_ISL_3899470	2021-09-02
hCoV-19/Japan/PG-87835/2021	EPI_ISL_3899473	2021-09-02
hCoV-19/Japan/PG-87836/2021	EPI_ISL_3899474	2021-09-02
hCoV-19/Japan/PG-87837/2021	EPI_ISL_3899475	2021-09-02
hCoV-19/Japan/PG-87838/2021	EPI_ISL_3899477	2021-09-02
hCoV-19/Japan/PG-87839/2021	EPI_ISL_3899480	2021-09-02
hCoV-19/Japan/PG-87840/2021	EPI_ISL_3899483	2021-09-02
hCoV-19/Japan/PG-87841/2021	EPI_ISL_3899485	2021-09-02
hCoV-19/Japan/PG-87842/2021	EPI_ISL_3899486	2021-09-02
hCoV-19/Japan/PG-87843/2021	EPI_ISL_3899487	2021-09-02
hCoV-19/Japan/PG-87844/2021	EPI_ISL_3899490	2021-09-02
hCoV-19/Japan/PG-87845/2021	EPI_ISL_3899492	2021-09-02
hCoV-19/Japan/PG-87846/2021	EPI_ISL_3899494	2021-09-02
hCoV-19/Japan/PG-87847/2021	EPI_ISL_3899496	2021-09-02
hCoV-19/Japan/PG-87848/2021	EPI_ISL_3899499	2021-09-02
hCoV-19/Japan/PG-87849/2021	EPI_ISL_3899502	2021-09-02
hCoV-19/Japan/PG-87850/2021	EPI_ISL_3899505	2021-09-02
hCoV-19/Japan/PG-87851/2021	EPI_ISL_3899508	2021-09-02
hCoV-19/Japan/PG-87852/2021	EPI_ISL_3899511	2021-09-02
hCoV-19/Japan/PG-87853/2021	EPI_ISL_3899512	2021-09-02
hCoV-19/Japan/PG-87854/2021	EPI_ISL_3899513	2021-09-02
hCoV-19/Japan/PG-87855/2021	EPI_ISL_3899515	2021-09-02
hCoV-19/Japan/PG-87856/2021	EPI_ISL_3899516	2021-09-02
hCoV-19/Japan/PG-87857/2021	EPI_ISL_3899517	2021-09-02
hCoV-19/Japan/PG-87858/2021	EPI_ISL_3899520	2021-09-02
hCoV-19/Japan/PG-87859/2021	EPI_ISL_3899523	2021-09-02
hCoV-19/Japan/PG-87860/2021	EPI_ISL_3899527	2021-09-02
hCoV-19/Japan/PG-87861/2021	EPI_ISL_3899530	2021-09-02
hCoV-19/Japan/PG-87862/2021	EPI_ISL_3899533	2021-09-02
hCoV-19/Japan/PG-87863/2021	EPI_ISL_3899536	2021-09-02
hCoV-19/Japan/PG-87864/2021	EPI_ISL_3899539	2021-09-02
hCoV-19/Japan/PG-87865/2021	EPI_ISL_3899541	2021-09-02
hCoV-19/Japan/PG-87866/2021	EPI_ISL_3899543	2021-09-02
hCoV-19/Japan/PG-87867/2021	EPI_ISL_3899544	2021-09-02
hCoV-19/Japan/PG-87868/2021	EPI_ISL_3899546	2021-09-02
hCoV-19/Japan/PG-87869/2021	EPI_ISL_3899547	2021-09-02
hCoV-19/Japan/PG-87870/2021	EPI_ISL_3899548	2021-09-02
hCoV-19/Japan/PG-87871/2021	EPI_ISL_3899549	2021-09-02
hCoV-19/Japan/PG-87872/2021	EPI_ISL_3899551	2021-09-02
hCoV-19/Japan/PG-87873/2021	EPI_ISL_3899554	2021-09-02
hCoV-19/Japan/PG-87874/2021	EPI_ISL_3899556	2021-09-02
hCoV-19/Japan/PG-87875/2021	EPI_ISL_3899559	2021-09-02
hCoV-19/Japan/PG-87876/2021	EPI_ISL_3899560	2021-09-02

virus_name	epiID	submission_date
hCoV-19/Japan/PG-87877/2021	EPI_ISL_3899561	2021-09-02
hCoV-19/Japan/PG-87878/2021	EPI_ISL_3899563	2021-09-02
hCoV-19/Japan/PG-87879/2021	EPI_ISL_3899566	2021-09-02
hCoV-19/Japan/PG-87880/2021	EPI_ISL_3899569	2021-09-02
hCoV-19/Japan/PG-87881/2021	EPI_ISL_3899572	2021-09-02
hCoV-19/Japan/PG-87882/2021	EPI_ISL_3899573	2021-09-02
hCoV-19/Japan/PG-87883/2021	EPI_ISL_3899574	2021-09-02
hCoV-19/Japan/PG-87884/2021	EPI_ISL_3899575	2021-09-02
hCoV-19/Japan/PG-87885/2021	EPI_ISL_3899576	2021-09-02
hCoV-19/Japan/PG-87886/2021	EPI_ISL_3899577	2021-09-02
hCoV-19/Japan/PG-87887/2021	EPI_ISL_3899578	2021-09-02
hCoV-19/Japan/PG-87888/2021	EPI_ISL_3899579	2021-09-02
hCoV-19/Japan/PG-87889/2021	EPI_ISL_3899580	2021-09-02
hCoV-19/Japan/PG-87890/2021	EPI_ISL_3899581	2021-09-02
hCoV-19/Japan/PG-87891/2021	EPI_ISL_3899582	2021-09-02
hCoV-19/Japan/PG-87892/2021	EPI_ISL_3899583	2021-09-02
hCoV-19/Japan/PG-87893/2021	EPI_ISL_3899584	2021-09-02
hCoV-19/Japan/PG-87894/2021	EPI_ISL_3899585	2021-09-02
hCoV-19/Japan/PG-87895/2021	EPI_ISL_3899586	2021-09-02
hCoV-19/Japan/PG-87896/2021	EPI_ISL_3899587	2021-09-02
hCoV-19/Japan/PG-87897/2021	EPI_ISL_3899588	2021-09-02
hCoV-19/Japan/PG-87898/2021	EPI_ISL_3899589	2021-09-02
hCoV-19/Japan/PG-87899/2021	EPI_ISL_3899590	2021-09-02
hCoV-19/Japan/PG-87900/2021	EPI_ISL_3899591	2021-09-02
hCoV-19/Japan/PG-87901/2021	EPI_ISL_3899592	2021-09-02
hCoV-19/Japan/PG-87902/2021	EPI_ISL_3899593	2021-09-02
hCoV-19/Japan/PG-87903/2021	EPI_ISL_3899594	2021-09-02
hCoV-19/Japan/PG-87904/2021	EPI_ISL_3899595	2021-09-02
hCoV-19/Japan/PG-87905/2021	EPI_ISL_3899596	2021-09-02
hCoV-19/Japan/PG-87906/2021	EPI_ISL_3899597	2021-09-02
hCoV-19/Japan/PG-87907/2021	EPI_ISL_3899598	2021-09-02
hCoV-19/Japan/PG-87908/2021	EPI_ISL_3899599	2021-09-02
hCoV-19/Japan/PG-87909/2021	EPI_ISL_3899600	2021-09-02
hCoV-19/Japan/PG-87910/2021	EPI_ISL_3899601	2021-09-02
hCoV-19/Japan/PG-87911/2021	EPI_ISL_3899602	2021-09-02
hCoV-19/Japan/PG-87912/2021	EPI_ISL_3899603	2021-09-02
hCoV-19/Japan/PG-87913/2021	EPI_ISL_3899604	2021-09-02
hCoV-19/Japan/PG-87914/2021	EPI_ISL_3899605	2021-09-02
hCoV-19/Japan/PG-87915/2021	EPI_ISL_3899606	2021-09-02
hCoV-19/Japan/PG-87916/2021	EPI_ISL_3899607	2021-09-02
hCoV-19/Japan/PG-87917/2021	EPI_ISL_3899608	2021-09-02
hCoV-19/Japan/PG-87918/2021	EPI_ISL_3899609	2021-09-02
hCoV-19/Japan/PG-87919/2021	EPI_ISL_3899610	2021-09-02
hCoV-19/Japan/PG-87920/2021	EPI_ISL_3899611	2021-09-02
hCoV-19/Japan/PG-87921/2021	EPI_ISL_3899612	2021-09-02
hCoV-19/Japan/PG-87922/2021	EPI_ISL_3899613	2021-09-02
hCoV-19/Japan/PG-87923/2021	EPI_ISL_3899614	2021-09-02
hCoV-19/Japan/PG-87924/2021	EPI_ISL_3899615	2021-09-02
hCoV-19/Japan/PG-87925/2021	EPI_ISL_3899616	2021-09-02
hCoV-19/Japan/PG-87926/2021	EPI_ISL_3899617	2021-09-02
hCoV-19/Japan/PG-87927/2021	EPI_ISL_3899618	2021-09-02
hCoV-19/Japan/PG-87928/2021	EPI_ISL_3899619	2021-09-02
hCoV-19/Japan/PG-87929/2021	EPI_ISL_3899620	2021-09-02
hCoV-19/Japan/PG-87930/2021	EPI_ISL_3899621	2021-09-02

virus_name	epiID	submission_date
hCoV-19/Japan/PG-87931/2021	EPI_ISL_3899622	2021-09-02
hCoV-19/Japan/PG-87932/2021	EPI_ISL_3899623	2021-09-02
hCoV-19/Japan/PG-87933/2021	EPI_ISL_3899624	2021-09-02
hCoV-19/Japan/PG-87934/2021	EPI_ISL_3899625	2021-09-02
hCoV-19/Japan/PG-87935/2021	EPI_ISL_3899626	2021-09-02
hCoV-19/Japan/PG-87936/2021	EPI_ISL_3899627	2021-09-02
hCoV-19/Japan/PG-87937/2021	EPI_ISL_3899628	2021-09-02
hCoV-19/Japan/PG-87938/2021	EPI_ISL_3899629	2021-09-02
hCoV-19/Japan/PG-87939/2021	EPI_ISL_3899630	2021-09-02
hCoV-19/Japan/PG-87940/2021	EPI_ISL_3899631	2021-09-02
hCoV-19/Japan/PG-87941/2021	EPI_ISL_3899632	2021-09-02
hCoV-19/Japan/PG-87942/2021	EPI_ISL_3899633	2021-09-02
hCoV-19/Japan/PG-87943/2021	EPI_ISL_3899634	2021-09-02
hCoV-19/Japan/PG-87944/2021	EPI_ISL_3899635	2021-09-02
hCoV-19/Japan/PG-87945/2021	EPI_ISL_3899636	2021-09-02
hCoV-19/Japan/PG-87946/2021	EPI_ISL_3899637	2021-09-02
hCoV-19/Japan/PG-87947/2021	EPI_ISL_3899638	2021-09-02
hCoV-19/Japan/PG-87948/2021	EPI_ISL_3899639	2021-09-02
hCoV-19/Japan/PG-87949/2021	EPI_ISL_3899640	2021-09-02
hCoV-19/Japan/PG-87950/2021	EPI_ISL_3899641	2021-09-02
hCoV-19/Japan/PG-87951/2021	EPI_ISL_3899642	2021-09-02
hCoV-19/Japan/PG-87952/2021	EPI_ISL_3899643	2021-09-02
hCoV-19/Japan/PG-87953/2021	EPI_ISL_3899644	2021-09-02
hCoV-19/Japan/PG-87954/2021	EPI_ISL_3899645	2021-09-02
hCoV-19/Japan/PG-87955/2021	EPI_ISL_3899646	2021-09-02
hCoV-19/Japan/PG-87956/2021	EPI_ISL_3899647	2021-09-02
hCoV-19/Japan/PG-87957/2021	EPI_ISL_3899648	2021-09-02
hCoV-19/Japan/PG-87958/2021	EPI_ISL_3899649	2021-09-02
hCoV-19/Japan/PG-87959/2021	EPI_ISL_3899650	2021-09-02
hCoV-19/Japan/PG-87960/2021	EPI_ISL_3899651	2021-09-02
hCoV-19/Japan/PG-87961/2021	EPI_ISL_3899652	2021-09-02
hCoV-19/Japan/PG-87962/2021	EPI_ISL_3899653	2021-09-02
hCoV-19/Japan/PG-87963/2021	EPI_ISL_3899654	2021-09-02
hCoV-19/Japan/PG-87964/2021	EPI_ISL_3899655	2021-09-02
hCoV-19/Japan/PG-87965/2021	EPI_ISL_3899656	2021-09-02
hCoV-19/Japan/PG-87966/2021	EPI_ISL_3899657	2021-09-02
hCoV-19/Japan/PG-87967/2021	EPI_ISL_3899658	2021-09-02
hCoV-19/Japan/PG-87968/2021	EPI_ISL_3899659	2021-09-02
hCoV-19/Japan/PG-87969/2021	EPI_ISL_3899660	2021-09-02
hCoV-19/Japan/PG-87970/2021	EPI_ISL_3899661	2021-09-02
hCoV-19/Japan/PG-87971/2021	EPI_ISL_3899662	2021-09-02
hCoV-19/Japan/PG-87972/2021	EPI_ISL_3899663	2021-09-02
hCoV-19/Japan/PG-87973/2021	EPI_ISL_3899664	2021-09-02
hCoV-19/Japan/PG-87974/2021	EPI_ISL_3899665	2021-09-02
hCoV-19/Japan/PG-87975/2021	EPI_ISL_3899666	2021-09-02
hCoV-19/Japan/PG-87976/2021	EPI_ISL_3899667	2021-09-02
hCoV-19/Japan/PG-87977/2021	EPI_ISL_3899668	2021-09-02
hCoV-19/Japan/PG-87978/2021	EPI_ISL_3899669	2021-09-02
hCoV-19/Japan/PG-87979/2021	EPI_ISL_3899670	2021-09-02
hCoV-19/Japan/PG-87980/2021	EPI_ISL_3899671	2021-09-02
hCoV-19/Japan/PG-87981/2021	EPI_ISL_3899672	2021-09-02
hCoV-19/Japan/PG-87982/2021	EPI_ISL_3899673	2021-09-02
hCoV-19/Japan/PG-87983/2021	EPI_ISL_3899674	2021-09-02
hCoV-19/Japan/PG-87984/2021	EPI_ISL_3899675	2021-09-02

virus_name	epiID	submission_date
hCoV-19/Japan/PG-87985/2021	EPI_ISL_3899676	2021-09-02
hCoV-19/Japan/PG-87986/2021	EPI_ISL_3899677	2021-09-02
hCoV-19/Japan/PG-87987/2021	EPI_ISL_3899678	2021-09-02
hCoV-19/Japan/PG-87988/2021	EPI_ISL_3899679	2021-09-02
hCoV-19/Japan/PG-87989/2021	EPI_ISL_3899680	2021-09-02
hCoV-19/Japan/PG-87990/2021	EPI_ISL_3899681	2021-09-02
hCoV-19/Japan/PG-87991/2021	EPI_ISL_3899682	2021-09-02
hCoV-19/Japan/PG-87992/2021	EPI_ISL_3899683	2021-09-02
hCoV-19/Japan/PG-87993/2021	EPI_ISL_3899684	2021-09-02
hCoV-19/Japan/PG-87994/2021	EPI_ISL_3899685	2021-09-02
hCoV-19/Japan/PG-87995/2021	EPI_ISL_3899686	2021-09-02
hCoV-19/Japan/PG-87996/2021	EPI_ISL_3899687	2021-09-02
hCoV-19/Japan/PG-87997/2021	EPI_ISL_3899688	2021-09-02
hCoV-19/Japan/PG-87998/2021	EPI_ISL_3899689	2021-09-02
hCoV-19/Japan/PG-87999/2021	EPI_ISL_3899690	2021-09-02
hCoV-19/Japan/PG-88000/2021	EPI_ISL_3899691	2021-09-02
hCoV-19/Japan/PG-88001/2021	EPI_ISL_3899692	2021-09-02
hCoV-19/Japan/PG-88002/2021	EPI_ISL_3899693	2021-09-02
hCoV-19/Japan/PG-88003/2021	EPI_ISL_3899694	2021-09-02
hCoV-19/Japan/PG-88004/2021	EPI_ISL_3899695	2021-09-02
hCoV-19/Japan/PG-88005/2021	EPI_ISL_3899696	2021-09-02
hCoV-19/Japan/PG-88006/2021	EPI_ISL_3899698	2021-09-02
hCoV-19/Japan/PG-88007/2021	EPI_ISL_3899699	2021-09-02
hCoV-19/Japan/PG-88008/2021	EPI_ISL_3899702	2021-09-02
hCoV-19/Japan/PG-88009/2021	EPI_ISL_3899705	2021-09-02
hCoV-19/Japan/PG-88010/2021	EPI_ISL_3899708	2021-09-02
hCoV-19/Japan/PG-88011/2021	EPI_ISL_3899712	2021-09-02
hCoV-19/Japan/PG-88012/2021	EPI_ISL_3899714	2021-09-02
hCoV-19/Japan/PG-88013/2021	EPI_ISL_3899715	2021-09-02
hCoV-19/Japan/PG-88014/2021	EPI_ISL_3899716	2021-09-02
hCoV-19/Japan/PG-88015/2021	EPI_ISL_3899720	2021-09-02
hCoV-19/Japan/PG-88016/2021	EPI_ISL_3899723	2021-09-02
hCoV-19/Japan/PG-88017/2021	EPI_ISL_3899726	2021-09-02
hCoV-19/Japan/PG-88018/2021	EPI_ISL_3899727	2021-09-02
hCoV-19/Japan/PG-88019/2021	EPI_ISL_3899729	2021-09-02
hCoV-19/Japan/PG-88020/2021	EPI_ISL_3899732	2021-09-02
hCoV-19/Japan/PG-88021/2021	EPI_ISL_3899736	2021-09-02
hCoV-19/Japan/PG-88022/2021	EPI_ISL_3899738	2021-09-02
hCoV-19/Japan/PG-88023/2021	EPI_ISL_3899742	2021-09-02
hCoV-19/Japan/PG-88024/2021	EPI_ISL_3899745	2021-09-02
hCoV-19/Japan/PG-88025/2021	EPI_ISL_3899748	2021-09-02
hCoV-19/Japan/PG-88026/2021	EPI_ISL_3899751	2021-09-02
hCoV-19/Japan/PG-88027/2021	EPI_ISL_3899754	2021-09-02
hCoV-19/Japan/PG-88028/2021	EPI_ISL_3899758	2021-09-02
hCoV-19/Japan/PG-88029/2021	EPI_ISL_3899761	2021-09-02
hCoV-19/Japan/PG-88030/2021	EPI_ISL_3899763	2021-09-02
hCoV-19/Japan/PG-88031/2021	EPI_ISL_3899764	2021-09-02
hCoV-19/Japan/PG-88032/2021	EPI_ISL_3899765	2021-09-02
hCoV-19/Japan/PG-88033/2021	EPI_ISL_3899768	2021-09-02
hCoV-19/Japan/PG-88034/2021	EPI_ISL_3899772	2021-09-02
hCoV-19/Japan/PG-88035/2021	EPI_ISL_3899775	2021-09-02
hCoV-19/Japan/PG-88036/2021	EPI_ISL_3899778	2021-09-02
hCoV-19/Japan/PG-88037/2021	EPI_ISL_3899781	2021-09-02
hCoV-19/Japan/PG-88038/2021	EPI_ISL_3899784	2021-09-02

virus_name	epiID	submission_date
hCoV-19/Japan/PG-88039/2021	EPI_ISL_3870842	2021-09-02
hCoV-19/Japan/PG-88040/2021	EPI_ISL_3870843	2021-09-02
hCoV-19/Japan/PG-88041/2021	EPI_ISL_3870844	2021-09-02
hCoV-19/Japan/PG-88042/2021	EPI_ISL_3870845	2021-09-02
hCoV-19/Japan/PG-88043/2021	EPI_ISL_3870846	2021-09-02
hCoV-19/Japan/PG-88044/2021	EPI_ISL_3870847	2021-09-02
hCoV-19/Japan/PG-88045/2021	EPI_ISL_3870848	2021-09-02
hCoV-19/Japan/PG-88046/2021	EPI_ISL_3870849	2021-09-02
hCoV-19/Japan/PG-88047/2021	EPI_ISL_3870850	2021-09-02
hCoV-19/Japan/PG-88048/2021	EPI_ISL_3870851	2021-09-02
hCoV-19/Japan/PG-88049/2021	EPI_ISL_3870852	2021-09-02
hCoV-19/Japan/PG-88050/2021	EPI_ISL_3870853	2021-09-02
hCoV-19/Japan/PG-88051/2021	EPI_ISL_3870854	2021-09-02
hCoV-19/Japan/PG-88052/2021	EPI_ISL_3870855	2021-09-02
hCoV-19/Japan/PG-88053/2021	EPI_ISL_3870857	2021-09-02
hCoV-19/Japan/PG-88054/2021	EPI_ISL_3870859	2021-09-02
hCoV-19/Japan/PG-88055/2021	EPI_ISL_3873154	2021-09-02
hCoV-19/Japan/PG-88056/2021	EPI_ISL_3873155	2021-09-02
hCoV-19/Japan/PG-88057/2021	EPI_ISL_3873156	2021-09-02
hCoV-19/Japan/PG-88058/2021	EPI_ISL_3873157	2021-09-02
hCoV-19/Japan/PG-88059/2021	EPI_ISL_3873158	2021-09-02
hCoV-19/Japan/PG-88060/2021	EPI_ISL_3873159	2021-09-02
hCoV-19/Japan/PG-88061/2021	EPI_ISL_3873160	2021-09-02
hCoV-19/Japan/PG-88062/2021	EPI_ISL_3873161	2021-09-02
hCoV-19/Japan/PG-88063/2021	EPI_ISL_3873162	2021-09-02
hCoV-19/Japan/PG-88064/2021	EPI_ISL_3873163	2021-09-02
hCoV-19/Japan/PG-88065/2021	EPI_ISL_3873164	2021-09-02
hCoV-19/Japan/PG-88066/2021	EPI_ISL_3873165	2021-09-02
hCoV-19/Japan/PG-88067/2021	EPI_ISL_3873166	2021-09-02
hCoV-19/Japan/PG-88068/2021	EPI_ISL_3873167	2021-09-02
hCoV-19/Japan/PG-88069/2021	EPI_ISL_3873168	2021-09-02
hCoV-19/Japan/PG-88070/2021	EPI_ISL_3873169	2021-09-02
hCoV-19/Japan/PG-88071/2021	EPI_ISL_3873170	2021-09-02
hCoV-19/Japan/PG-88072/2021	EPI_ISL_3873171	2021-09-02
hCoV-19/Japan/PG-88073/2021	EPI_ISL_3873172	2021-09-02
hCoV-19/Japan/PG-88074/2021	EPI_ISL_3873173	2021-09-02
hCoV-19/Japan/PG-88075/2021	EPI_ISL_3873174	2021-09-02
hCoV-19/Japan/PG-88076/2021	EPI_ISL_3873175	2021-09-02
hCoV-19/Japan/PG-88077/2021	EPI_ISL_3873176	2021-09-02
hCoV-19/Japan/PG-88078/2021	EPI_ISL_3873177	2021-09-02
hCoV-19/Japan/PG-88079/2021	EPI_ISL_3873178	2021-09-02
hCoV-19/Japan/PG-88080/2021	EPI_ISL_3873179	2021-09-02
hCoV-19/Japan/PG-88081/2021	EPI_ISL_3873180	2021-09-02
hCoV-19/Japan/PG-88082/2021	EPI_ISL_3873181	2021-09-02
hCoV-19/Japan/PG-88083/2021	EPI_ISL_3873182	2021-09-02
hCoV-19/Japan/PG-88084/2021	EPI_ISL_3873183	2021-09-02
hCoV-19/Japan/PG-88085/2021	EPI_ISL_3873184	2021-09-02
hCoV-19/Japan/PG-88086/2021	EPI_ISL_3873185	2021-09-02
hCoV-19/Japan/PG-88087/2021	EPI_ISL_3873186	2021-09-02
hCoV-19/Japan/PG-88088/2021	EPI_ISL_3873187	2021-09-02
hCoV-19/Japan/PG-88089/2021	EPI_ISL_3873188	2021-09-02
hCoV-19/Japan/PG-88090/2021	EPI_ISL_3873189	2021-09-02
hCoV-19/Japan/PG-88091/2021	EPI_ISL_3873190	2021-09-02
hCoV-19/Japan/PG-88092/2021	EPI_ISL_3873191	2021-09-02

virus_name	epiID	submission_date
hCoV-19/Japan/PG-88093/2021	EPI_ISL_3873192	2021-09-02
hCoV-19/Japan/PG-88094/2021	EPI_ISL_3873193	2021-09-02
hCoV-19/Japan/PG-88095/2021	EPI_ISL_3873194	2021-09-02
hCoV-19/Japan/PG-88096/2021	EPI_ISL_3873195	2021-09-02
hCoV-19/Japan/PG-88097/2021	EPI_ISL_3873196	2021-09-02
hCoV-19/Japan/PG-88098/2021	EPI_ISL_3873197	2021-09-02
hCoV-19/Japan/PG-88099/2021	EPI_ISL_3873198	2021-09-02
hCoV-19/Japan/PG-88100/2021	EPI_ISL_3873199	2021-09-02
hCoV-19/Japan/PG-88101/2021	EPI_ISL_3873200	2021-09-02
hCoV-19/Japan/PG-88102/2021	EPI_ISL_3873201	2021-09-02
hCoV-19/Japan/PG-88103/2021	EPI_ISL_3873202	2021-09-02
hCoV-19/Japan/PG-88104/2021	EPI_ISL_3873203	2021-09-02
hCoV-19/Japan/PG-88105/2021	EPI_ISL_3873204	2021-09-02
hCoV-19/Japan/PG-88106/2021	EPI_ISL_3873205	2021-09-02
hCoV-19/Japan/PG-88107/2021	EPI_ISL_3873206	2021-09-02
hCoV-19/Japan/PG-88108/2021	EPI_ISL_3873207	2021-09-02
hCoV-19/Japan/PG-88109/2021	EPI_ISL_3873208	2021-09-02
hCoV-19/Japan/PG-88110/2021	EPI_ISL_3873209	2021-09-02
hCoV-19/Japan/PG-88111/2021	EPI_ISL_3873210	2021-09-02
hCoV-19/Japan/PG-88112/2021	EPI_ISL_3873211	2021-09-02
hCoV-19/Japan/PG-88113/2021	EPI_ISL_3873212	2021-09-02
hCoV-19/Japan/PG-88114/2021	EPI_ISL_3873213	2021-09-02
hCoV-19/Japan/PG-88115/2021	EPI_ISL_3873214	2021-09-02
hCoV-19/Japan/PG-88116/2021	EPI_ISL_3873215	2021-09-02
hCoV-19/Japan/PG-88117/2021	EPI_ISL_3873216	2021-09-02
hCoV-19/Japan/PG-88118/2021	EPI_ISL_3873217	2021-09-02
hCoV-19/Japan/PG-88119/2021	EPI_ISL_3873218	2021-09-02
hCoV-19/Japan/PG-88120/2021	EPI_ISL_3873219	2021-09-02
hCoV-19/Japan/PG-88121/2021	EPI_ISL_3873220	2021-09-02
hCoV-19/Japan/PG-88122/2021	EPI_ISL_3873221	2021-09-02
hCoV-19/Japan/PG-88123/2021	EPI_ISL_3873222	2021-09-02
hCoV-19/Japan/PG-88124/2021	EPI_ISL_3873223	2021-09-02
hCoV-19/Japan/PG-88125/2021	EPI_ISL_3873224	2021-09-02
hCoV-19/Japan/PG-88126/2021	EPI_ISL_3873225	2021-09-02
hCoV-19/Japan/PG-88130/2021	EPI_ISL_3873226	2021-09-02
hCoV-19/Japan/PG-88131/2021	EPI_ISL_3873227	2021-09-02
hCoV-19/Japan/PG-88132/2021	EPI_ISL_3873228	2021-09-02
hCoV-19/Japan/PG-88133/2021	EPI_ISL_3873229	2021-09-02
hCoV-19/Japan/PG-88134/2021	EPI_ISL_3873230	2021-09-02
hCoV-19/Japan/PG-88135/2021	EPI_ISL_3873231	2021-09-02
hCoV-19/Japan/PG-88136/2021	EPI_ISL_3871954	2021-09-02
hCoV-19/Japan/PG-88137/2021	EPI_ISL_3871955	2021-09-02
hCoV-19/Japan/PG-88138/2021	EPI_ISL_3871956	2021-09-02
hCoV-19/Japan/PG-88139/2021	EPI_ISL_3871957	2021-09-02
hCoV-19/Japan/PG-88140/2021	EPI_ISL_3871958	2021-09-02
hCoV-19/Japan/PG-88141/2021	EPI_ISL_3871959	2021-09-02
hCoV-19/Japan/PG-88142/2021	EPI_ISL_3871960	2021-09-02
hCoV-19/Japan/PG-88143/2021	EPI_ISL_3871961	2021-09-02
hCoV-19/Japan/PG-88144/2021	EPI_ISL_3871962	2021-09-02
hCoV-19/Japan/PG-88145/2021	EPI_ISL_3871963	2021-09-02
hCoV-19/Japan/PG-88146/2021	EPI_ISL_3871964	2021-09-02
hCoV-19/Japan/PG-88147/2021	EPI_ISL_3871965	2021-09-02
hCoV-19/Japan/PG-88148/2021	EPI_ISL_3871966	2021-09-02
hCoV-19/Japan/PG-88149/2021	EPI_ISL_3871110	2021-09-02

virus_name	epiID	submission_date
hCoV-19/Japan/PG-88150/2021	EPI_ISL_3871111	2021-09-02
hCoV-19/Japan/PG-88151/2021	EPI_ISL_3871112	2021-09-02
hCoV-19/Japan/PG-88152/2021	EPI_ISL_3871113	2021-09-02
hCoV-19/Japan/PG-88153/2021	EPI_ISL_3871114	2021-09-02
hCoV-19/Japan/PG-88154/2021	EPI_ISL_3871115	2021-09-02
hCoV-19/Japan/PG-88155/2021	EPI_ISL_3871116	2021-09-02
hCoV-19/Japan/PG-88156/2021	EPI_ISL_3871117	2021-09-02
hCoV-19/Japan/PG-88157/2021	EPI_ISL_3871118	2021-09-02
hCoV-19/Japan/PG-88158/2021	EPI_ISL_3871119	2021-09-02
hCoV-19/Japan/PG-88159/2021	EPI_ISL_3899788	2021-09-02
hCoV-19/Japan/PG-88160/2021	EPI_ISL_3899791	2021-09-02
hCoV-19/Japan/PG-88161/2021	EPI_ISL_3899794	2021-09-02
hCoV-19/Japan/PG-88162/2021	EPI_ISL_3899797	2021-09-02
hCoV-19/Japan/PG-88163/2021	EPI_ISL_3899800	2021-09-02
hCoV-19/Japan/PG-88164/2021	EPI_ISL_3899801	2021-09-02
hCoV-19/Japan/PG-88165/2021	EPI_ISL_3899802	2021-09-02
hCoV-19/Japan/PG-88166/2021	EPI_ISL_3899805	2021-09-02
hCoV-19/Japan/PG-88167/2021	EPI_ISL_3899808	2021-09-02
hCoV-19/Japan/PG-88168/2021	EPI_ISL_3899811	2021-09-02
hCoV-19/Japan/PG-88169/2021	EPI_ISL_3899813	2021-09-02
hCoV-19/Japan/PG-88170/2021	EPI_ISL_3899814	2021-09-02
hCoV-19/Japan/PG-88171/2021	EPI_ISL_3899816	2021-09-02
hCoV-19/Japan/PG-88172/2021	EPI_ISL_3899819	2021-09-02
hCoV-19/Japan/PG-88173/2021	EPI_ISL_3899821	2021-09-02
hCoV-19/Japan/PG-88174/2021	EPI_ISL_3899822	2021-09-02
hCoV-19/Japan/PG-88175/2021	EPI_ISL_3899823	2021-09-02
hCoV-19/Japan/PG-88176/2021	EPI_ISL_3899824	2021-09-02
hCoV-19/Japan/PG-88177/2021	EPI_ISL_3899825	2021-09-02
hCoV-19/Japan/PG-88178/2021	EPI_ISL_3899826	2021-09-02
hCoV-19/Japan/PG-88179/2021	EPI_ISL_3899827	2021-09-02
hCoV-19/Japan/PG-88180/2021	EPI_ISL_3899828	2021-09-02
hCoV-19/Japan/PG-88181/2021	EPI_ISL_3899829	2021-09-02
hCoV-19/Japan/PG-88182/2021	EPI_ISL_3899830	2021-09-02
hCoV-19/Japan/PG-88183/2021	EPI_ISL_3899831	2021-09-02
hCoV-19/Japan/PG-88184/2021	EPI_ISL_3899832	2021-09-02
hCoV-19/Japan/PG-88185/2021	EPI_ISL_3899833	2021-09-02
hCoV-19/Japan/PG-88186/2021	EPI_ISL_3899834	2021-09-02
hCoV-19/Japan/PG-88187/2021	EPI_ISL_3899835	2021-09-02
hCoV-19/Japan/PG-88188/2021	EPI_ISL_3899836	2021-09-02
hCoV-19/Japan/PG-88189/2021	EPI_ISL_3899837	2021-09-02
hCoV-19/Japan/PG-88190/2021	EPI_ISL_3899838	2021-09-02
hCoV-19/Japan/PG-88191/2021	EPI_ISL_3899839	2021-09-02
hCoV-19/Japan/PG-88192/2021	EPI_ISL_3899840	2021-09-02
hCoV-19/Japan/PG-88193/2021	EPI_ISL_3899841	2021-09-02
hCoV-19/Japan/PG-88194/2021	EPI_ISL_3899842	2021-09-02
hCoV-19/Japan/PG-88195/2021	EPI_ISL_3899843	2021-09-02
hCoV-19/Japan/PG-88196/2021	EPI_ISL_3899844	2021-09-02
hCoV-19/Japan/PG-88197/2021	EPI_ISL_3899845	2021-09-02
hCoV-19/Japan/PG-88198/2021	EPI_ISL_3899846	2021-09-02
hCoV-19/Japan/PG-88199/2021	EPI_ISL_3899847	2021-09-02
hCoV-19/Japan/PG-88200/2021	EPI_ISL_3899848	2021-09-02
hCoV-19/Japan/PG-88201/2021	EPI_ISL_3899849	2021-09-02
hCoV-19/Japan/PG-88202/2021	EPI_ISL_3899850	2021-09-02
hCoV-19/Japan/PG-88203/2021	EPI_ISL_3899851	2021-09-02

virus_name	epiID	submission_date
hCoV-19/Japan/PG-88204/2021	EPI_ISL_3899853	2021-09-02
hCoV-19/Japan/PG-88205/2021	EPI_ISL_3899856	2021-09-02
hCoV-19/Japan/PG-88206/2021	EPI_ISL_3899859	2021-09-02
hCoV-19/Japan/PG-88207/2021	EPI_ISL_3899862	2021-09-02
hCoV-19/Japan/PG-88208/2021	EPI_ISL_3899865	2021-09-02
hCoV-19/Japan/PG-88209/2021	EPI_ISL_3899868	2021-09-02
hCoV-19/Japan/PG-88210/2021	EPI_ISL_3899872	2021-09-02
hCoV-19/Japan/PG-88211/2021	EPI_ISL_3899875	2021-09-02
hCoV-19/Japan/PG-88212/2021	EPI_ISL_3899878	2021-09-02
hCoV-19/Japan/PG-88213/2021	EPI_ISL_3899881	2021-09-02
hCoV-19/Japan/PG-88214/2021	EPI_ISL_3899884	2021-09-02
hCoV-19/Japan/PG-88215/2021	EPI_ISL_3899887	2021-09-02
hCoV-19/Japan/PG-88216/2021	EPI_ISL_3899891	2021-09-02
hCoV-19/Japan/PG-88217/2021	EPI_ISL_3899894	2021-09-02
hCoV-19/Japan/PG-88218/2021	EPI_ISL_3899898	2021-09-02
hCoV-19/Japan/PG-88219/2021	EPI_ISL_3899901	2021-09-02
hCoV-19/Japan/PG-88220/2021	EPI_ISL_3899904	2021-09-02
hCoV-19/Japan/PG-88221/2021	EPI_ISL_3899907	2021-09-02
hCoV-19/Japan/PG-88222/2021	EPI_ISL_3899910	2021-09-02
hCoV-19/Japan/PG-88223/2021	EPI_ISL_3899914	2021-09-02
hCoV-19/Japan/PG-88224/2021	EPI_ISL_3899917	2021-09-02
hCoV-19/Japan/PG-88225/2021	EPI_ISL_3899920	2021-09-02
hCoV-19/Japan/PG-88226/2021	EPI_ISL_3899923	2021-09-02
hCoV-19/Japan/PG-88227/2021	EPI_ISL_3899925	2021-09-02
hCoV-19/Japan/PG-88228/2021	EPI_ISL_3899929	2021-09-02
hCoV-19/Japan/PG-88229/2021	EPI_ISL_3899931	2021-09-02
hCoV-19/Japan/PG-88230/2021	EPI_ISL_3899935	2021-09-02
hCoV-19/Japan/PG-88231/2021	EPI_ISL_3899938	2021-09-02
hCoV-19/Japan/PG-88232/2021	EPI_ISL_3899941	2021-09-02
hCoV-19/Japan/PG-88233/2021	EPI_ISL_3899945	2021-09-02
hCoV-19/Japan/PG-88234/2021	EPI_ISL_3899948	2021-09-02
hCoV-19/Japan/PG-88235/2021	EPI_ISL_3899951	2021-09-02
hCoV-19/Japan/PG-88236/2021	EPI_ISL_3899954	2021-09-02
hCoV-19/Japan/PG-88237/2021	EPI_ISL_3899957	2021-09-02
hCoV-19/Japan/PG-88238/2021	EPI_ISL_3899958	2021-09-02
hCoV-19/Japan/PG-88239/2021	EPI_ISL_3899959	2021-09-02
hCoV-19/Japan/PG-88240/2021	EPI_ISL_3899960	2021-09-02
hCoV-19/Japan/PG-88241/2021	EPI_ISL_3899961	2021-09-02
hCoV-19/Japan/PG-88242/2021	EPI_ISL_3899963	2021-09-02
hCoV-19/Japan/PG-88243/2021	EPI_ISL_3899964	2021-09-02
hCoV-19/Japan/PG-88244/2021	EPI_ISL_3899965	2021-09-02
hCoV-19/Japan/PG-88245/2021	EPI_ISL_3899966	2021-09-02
hCoV-19/Japan/PG-88246/2021	EPI_ISL_3899967	2021-09-02
hCoV-19/Japan/PG-88247/2021	EPI_ISL_3899968	2021-09-02
hCoV-19/Japan/PG-88248/2021	EPI_ISL_3899969	2021-09-02
hCoV-19/Japan/PG-88249/2021	EPI_ISL_3899970	2021-09-02
hCoV-19/Japan/PG-88250/2021	EPI_ISL_3899971	2021-09-02
hCoV-19/Japan/PG-88251/2021	EPI_ISL_3899972	2021-09-02
hCoV-19/Japan/PG-88252/2021	EPI_ISL_3899973	2021-09-02
hCoV-19/Japan/PG-88253/2021	EPI_ISL_3899974	2021-09-02
hCoV-19/Japan/PG-88254/2021	EPI_ISL_3899975	2021-09-02
hCoV-19/Japan/PG-88255/2021	EPI_ISL_3899976	2021-09-02
hCoV-19/Japan/PG-88256/2021	EPI_ISL_3899977	2021-09-02
hCoV-19/Japan/PG-88257/2021	EPI_ISL_3899978	2021-09-02

virus_name	epiID	submission_date
hCoV-19/Japan/PG-88258/2021	EPI_ISL_3899979	2021-09-02
hCoV-19/Japan/PG-88259/2021	EPI_ISL_3899980	2021-09-02
hCoV-19/Japan/PG-88260/2021	EPI_ISL_3899981	2021-09-02
hCoV-19/Japan/PG-88261/2021	EPI_ISL_3899982	2021-09-02
hCoV-19/Japan/PG-88262/2021	EPI_ISL_3899983	2021-09-02
hCoV-19/Japan/PG-88263/2021	EPI_ISL_3899984	2021-09-02
hCoV-19/Japan/PG-88264/2021	EPI_ISL_3899985	2021-09-02
hCoV-19/Japan/PG-88265/2021	EPI_ISL_3899986	2021-09-02
hCoV-19/Japan/PG-88266/2021	EPI_ISL_3899987	2021-09-02
hCoV-19/Japan/PG-88267/2021	EPI_ISL_3899988	2021-09-02
hCoV-19/Japan/PG-88268/2021	EPI_ISL_3899989	2021-09-02
hCoV-19/Japan/PG-88269/2021	EPI_ISL_3899990	2021-09-02
hCoV-19/Japan/PG-88270/2021	EPI_ISL_3899991	2021-09-02
hCoV-19/Japan/PG-88271/2021	EPI_ISL_3899992	2021-09-02
hCoV-19/Japan/PG-88272/2021	EPI_ISL_3899993	2021-09-02
hCoV-19/Japan/PG-88273/2021	EPI_ISL_3899994	2021-09-02
hCoV-19/Japan/PG-88274/2021	EPI_ISL_3899995	2021-09-02
hCoV-19/Japan/PG-88275/2021	EPI_ISL_3899996	2021-09-02
hCoV-19/Japan/PG-88276/2021	EPI_ISL_3899997	2021-09-02
hCoV-19/Japan/PG-88277/2021	EPI_ISL_3899998	2021-09-02
hCoV-19/Japan/PG-88278/2021	EPI_ISL_3899999	2021-09-02
hCoV-19/Japan/PG-88279/2021	EPI_ISL_3900000	2021-09-02
hCoV-19/Japan/PG-88280/2021	EPI_ISL_3900001	2021-09-02
hCoV-19/Japan/PG-88281/2021	EPI_ISL_3900002	2021-09-02
hCoV-19/Japan/PG-88282/2021	EPI_ISL_3900003	2021-09-02
hCoV-19/Japan/PG-88283/2021	EPI_ISL_3900004	2021-09-02
hCoV-19/Japan/PG-88284/2021	EPI_ISL_3900005	2021-09-02
hCoV-19/Japan/PG-88285/2021	EPI_ISL_3900006	2021-09-02
hCoV-19/Japan/PG-88286/2021	EPI_ISL_3900007	2021-09-02
hCoV-19/Japan/PG-88287/2021	EPI_ISL_3900008	2021-09-02
hCoV-19/Japan/PG-88288/2021	EPI_ISL_3900009	2021-09-02
hCoV-19/Japan/PG-88289/2021	EPI_ISL_3900010	2021-09-02
hCoV-19/Japan/PG-88290/2021	EPI_ISL_3900011	2021-09-02
hCoV-19/Japan/PG-88291/2021	EPI_ISL_3900012	2021-09-02
hCoV-19/Japan/PG-88292/2021	EPI_ISL_3900013	2021-09-02
hCoV-19/Japan/PG-88293/2021	EPI_ISL_3900014	2021-09-02
hCoV-19/Japan/PG-88294/2021	EPI_ISL_3900015	2021-09-02
hCoV-19/Japan/PG-88295/2021	EPI_ISL_3900016	2021-09-02
hCoV-19/Japan/PG-88296/2021	EPI_ISL_3900017	2021-09-02
hCoV-19/Japan/PG-88297/2021	EPI_ISL_3900018	2021-09-02
hCoV-19/Japan/PG-88298/2021	EPI_ISL_3900019	2021-09-02
hCoV-19/Japan/PG-88299/2021	EPI_ISL_3900020	2021-09-02
hCoV-19/Japan/PG-88300/2021	EPI_ISL_3900021	2021-09-02
hCoV-19/Japan/PG-88301/2021	EPI_ISL_3900022	2021-09-02
hCoV-19/Japan/PG-88302/2021	EPI_ISL_3900023	2021-09-02
hCoV-19/Japan/PG-88303/2021	EPI_ISL_3900024	2021-09-02
hCoV-19/Japan/PG-88304/2021	EPI_ISL_3900025	2021-09-02
hCoV-19/Japan/PG-88305/2021	EPI_ISL_3900026	2021-09-02
hCoV-19/Japan/PG-88306/2021	EPI_ISL_3900027	2021-09-02
hCoV-19/Japan/PG-88307/2021	EPI_ISL_3900028	2021-09-02
hCoV-19/Japan/PG-88308/2021	EPI_ISL_3900029	2021-09-02
hCoV-19/Japan/PG-88309/2021	EPI_ISL_3900030	2021-09-02
hCoV-19/Japan/PG-88310/2021	EPI_ISL_3900031	2021-09-02
hCoV-19/Japan/PG-88311/2021	EPI_ISL_3900032	2021-09-02

virus_name	epiID	submission_date
hCoV-19/Japan/PG-88312/2021	EPI_ISL_3900033	2021-09-02
hCoV-19/Japan/PG-88313/2021	EPI_ISL_3900034	2021-09-02
hCoV-19/Japan/PG-88314/2021	EPI_ISL_3900035	2021-09-02
hCoV-19/Japan/PG-88315/2021	EPI_ISL_3900036	2021-09-02
hCoV-19/Japan/PG-88316/2021	EPI_ISL_3900037	2021-09-02
hCoV-19/Japan/PG-88317/2021	EPI_ISL_3900038	2021-09-02
hCoV-19/Japan/PG-88318/2021	EPI_ISL_3900039	2021-09-02
hCoV-19/Japan/PG-88319/2021	EPI_ISL_3900040	2021-09-02
hCoV-19/Japan/PG-88320/2021	EPI_ISL_3900041	2021-09-02
hCoV-19/Japan/PG-88321/2021	EPI_ISL_3900042	2021-09-02
hCoV-19/Japan/PG-88322/2021	EPI_ISL_3900043	2021-09-02
hCoV-19/Japan/PG-88323/2021	EPI_ISL_3900044	2021-09-02
hCoV-19/Japan/PG-88324/2021	EPI_ISL_3900045	2021-09-02
hCoV-19/Japan/PG-88325/2021	EPI_ISL_3900046	2021-09-02
hCoV-19/Japan/PG-88326/2021	EPI_ISL_3900047	2021-09-02
hCoV-19/Japan/PG-88327/2021	EPI_ISL_3900048	2021-09-02
hCoV-19/Japan/PG-88328/2021	EPI_ISL_3900049	2021-09-02
hCoV-19/Japan/PG-88329/2021	EPI_ISL_3900050	2021-09-02
hCoV-19/Japan/PG-88330/2021	EPI_ISL_3900051	2021-09-02
hCoV-19/Japan/PG-88331/2021	EPI_ISL_3900052	2021-09-02
hCoV-19/Japan/PG-88332/2021	EPI_ISL_3900053	2021-09-02
hCoV-19/Japan/PG-88333/2021	EPI_ISL_3900054	2021-09-02
hCoV-19/Japan/PG-88334/2021	EPI_ISL_3900055	2021-09-02
hCoV-19/Japan/PG-88335/2021	EPI_ISL_3900056	2021-09-02
hCoV-19/Japan/PG-88336/2021	EPI_ISL_3900057	2021-09-02
hCoV-19/Japan/PG-88337/2021	EPI_ISL_3900058	2021-09-02
hCoV-19/Japan/PG-88338/2021	EPI_ISL_3900059	2021-09-02
hCoV-19/Japan/PG-88339/2021	EPI_ISL_3900060	2021-09-02
hCoV-19/Japan/PG-88340/2021	EPI_ISL_3900061	2021-09-02
hCoV-19/Japan/PG-88341/2021	EPI_ISL_3900062	2021-09-02
hCoV-19/Japan/PG-88342/2021	EPI_ISL_3900063	2021-09-02
hCoV-19/Japan/PG-88343/2021	EPI_ISL_3900064	2021-09-02
hCoV-19/Japan/PG-88344/2021	EPI_ISL_3900065	2021-09-02
hCoV-19/Japan/PG-88345/2021	EPI_ISL_3900066	2021-09-02
hCoV-19/Japan/PG-88346/2021	EPI_ISL_3900067	2021-09-02
hCoV-19/Japan/PG-88347/2021	EPI_ISL_3900068	2021-09-02
hCoV-19/Japan/PG-88348/2021	EPI_ISL_3900069	2021-09-02
hCoV-19/Japan/PG-88349/2021	EPI_ISL_3900070	2021-09-02
hCoV-19/Japan/PG-88350/2021	EPI_ISL_3900071	2021-09-02
hCoV-19/Japan/PG-88351/2021	EPI_ISL_3900072	2021-09-02
hCoV-19/Japan/PG-88352/2021	EPI_ISL_3900073	2021-09-02
hCoV-19/Japan/PG-88353/2021	EPI_ISL_3900074	2021-09-02
hCoV-19/Japan/PG-88354/2021	EPI_ISL_3900075	2021-09-02
hCoV-19/Japan/PG-88355/2021	EPI_ISL_3900076	2021-09-02
hCoV-19/Japan/PG-88356/2021	EPI_ISL_3900077	2021-09-02
hCoV-19/Japan/PG-88357/2021	EPI_ISL_3900078	2021-09-02
hCoV-19/Japan/PG-88358/2021	EPI_ISL_3900079	2021-09-02
hCoV-19/Japan/PG-88359/2021	EPI_ISL_3900080	2021-09-02
hCoV-19/Japan/PG-88360/2021	EPI_ISL_3900081	2021-09-02
hCoV-19/Japan/PG-88361/2021	EPI_ISL_3900082	2021-09-02
hCoV-19/Japan/PG-88362/2021	EPI_ISL_3900083	2021-09-02
hCoV-19/Japan/PG-88363/2021	EPI_ISL_3900084	2021-09-02
hCoV-19/Japan/PG-88364/2021	EPI_ISL_3900085	2021-09-02
hCoV-19/Japan/PG-88365/2021	EPI_ISL_3900086	2021-09-02

virus_name	epiID	submission_date
hCoV-19/Japan/PG-88366/2021	EPI_ISL_3900087	2021-09-02
hCoV-19/Japan/PG-88367/2021	EPI_ISL_3900088	2021-09-02
hCoV-19/Japan/PG-88368/2021	EPI_ISL_3900089	2021-09-02
hCoV-19/Japan/PG-88369/2021	EPI_ISL_3900090	2021-09-02
hCoV-19/Japan/PG-88370/2021	EPI_ISL_3900091	2021-09-02
hCoV-19/Japan/PG-88371/2021	EPI_ISL_3900092	2021-09-02
hCoV-19/Japan/PG-88372/2021	EPI_ISL_3900093	2021-09-02
hCoV-19/Japan/PG-88373/2021	EPI_ISL_3900094	2021-09-02
hCoV-19/Japan/PG-88374/2021	EPI_ISL_3900095	2021-09-02
hCoV-19/Japan/PG-88375/2021	EPI_ISL_3900096	2021-09-02
hCoV-19/Japan/PG-88376/2021	EPI_ISL_3900097	2021-09-02
hCoV-19/Japan/PG-88377/2021	EPI_ISL_3900098	2021-09-02
hCoV-19/Japan/PG-88378/2021	EPI_ISL_3900099	2021-09-02
hCoV-19/Japan/PG-88379/2021	EPI_ISL_3900100	2021-09-02
hCoV-19/Japan/PG-88380/2021	EPI_ISL_3900101	2021-09-02
hCoV-19/Japan/PG-88381/2021	EPI_ISL_3900102	2021-09-02
hCoV-19/Japan/PG-88382/2021	EPI_ISL_3900103	2021-09-02
hCoV-19/Japan/PG-88383/2021	EPI_ISL_3900104	2021-09-02
hCoV-19/Japan/PG-88384/2021	EPI_ISL_3900105	2021-09-02
hCoV-19/Japan/PG-88385/2021	EPI_ISL_3900106	2021-09-02
hCoV-19/Japan/PG-88386/2021	EPI_ISL_3900107	2021-09-02
hCoV-19/Japan/PG-88387/2021	EPI_ISL_3900108	2021-09-02
hCoV-19/Japan/PG-88388/2021	EPI_ISL_3900109	2021-09-02
hCoV-19/Japan/PG-88389/2021	EPI_ISL_3900110	2021-09-02
hCoV-19/Japan/PG-88390/2021	EPI_ISL_3900111	2021-09-02
hCoV-19/Japan/PG-88391/2021	EPI_ISL_3900112	2021-09-02
hCoV-19/Japan/PG-88392/2021	EPI_ISL_3900113	2021-09-02
hCoV-19/Japan/PG-88393/2021	EPI_ISL_3900114	2021-09-02
hCoV-19/Japan/PG-88394/2021	EPI_ISL_3900115	2021-09-02
hCoV-19/Japan/PG-88395/2021	EPI_ISL_3900116	2021-09-02
hCoV-19/Japan/PG-88396/2021	EPI_ISL_3900117	2021-09-02
hCoV-19/Japan/PG-88397/2021	EPI_ISL_3900118	2021-09-02
hCoV-19/Japan/PG-88398/2021	EPI_ISL_3900119	2021-09-02
hCoV-19/Japan/PG-88399/2021	EPI_ISL_3900120	2021-09-02
hCoV-19/Japan/PG-88400/2021	EPI_ISL_3900121	2021-09-02
hCoV-19/Japan/PG-88401/2021	EPI_ISL_3900122	2021-09-02
hCoV-19/Japan/PG-88402/2021	EPI_ISL_3900123	2021-09-02
hCoV-19/Japan/PG-88403/2021	EPI_ISL_3900124	2021-09-02
hCoV-19/Japan/PG-88404/2021	EPI_ISL_3900125	2021-09-02
hCoV-19/Japan/PG-88405/2021	EPI_ISL_3900126	2021-09-02
hCoV-19/Japan/PG-88406/2021	EPI_ISL_3900127	2021-09-02
hCoV-19/Japan/PG-88407/2021	EPI_ISL_3900128	2021-09-02
hCoV-19/Japan/PG-88408/2021	EPI_ISL_3900129	2021-09-02
hCoV-19/Japan/PG-88409/2021	EPI_ISL_3900130	2021-09-02
hCoV-19/Japan/PG-88410/2021	EPI_ISL_3900131	2021-09-02
hCoV-19/Japan/PG-88411/2021	EPI_ISL_3900132	2021-09-02
hCoV-19/Japan/PG-88412/2021	EPI_ISL_3900133	2021-09-02
hCoV-19/Japan/PG-88413/2021	EPI_ISL_3900134	2021-09-02
hCoV-19/Japan/PG-88414/2021	EPI_ISL_3900135	2021-09-02
hCoV-19/Japan/PG-88415/2021	EPI_ISL_3900136	2021-09-02
hCoV-19/Japan/PG-88416/2021	EPI_ISL_3900137	2021-09-02
hCoV-19/Japan/PG-88417/2021	EPI_ISL_3900138	2021-09-02
hCoV-19/Japan/PG-88418/2021	EPI_ISL_3900139	2021-09-02
hCoV-19/Japan/PG-88419/2021	EPI_ISL_3900140	2021-09-02

virus_name	epiID	submission_date
hCoV-19/Japan/PG-88420/2021	EPI_ISL_3900141	2021-09-02
hCoV-19/Japan/PG-88421/2021	EPI_ISL_3900142	2021-09-02
hCoV-19/Japan/PG-88422/2021	EPI_ISL_3900144	2021-09-02
hCoV-19/Japan/PG-88423/2021	EPI_ISL_3900145	2021-09-02
hCoV-19/Japan/PG-88424/2021	EPI_ISL_3900146	2021-09-02
hCoV-19/Japan/PG-88425/2021	EPI_ISL_3900147	2021-09-02
hCoV-19/Japan/PG-88426/2021	EPI_ISL_3900148	2021-09-02
hCoV-19/Japan/PG-88427/2021	EPI_ISL_3900149	2021-09-02
hCoV-19/Japan/PG-88428/2021	EPI_ISL_3900150	2021-09-02
hCoV-19/Japan/PG-88429/2021	EPI_ISL_3900151	2021-09-02
hCoV-19/Japan/PG-88430/2021	EPI_ISL_3900152	2021-09-02
hCoV-19/Japan/PG-88431/2021	EPI_ISL_3900153	2021-09-02
hCoV-19/Japan/PG-88432/2021	EPI_ISL_3900154	2021-09-02
hCoV-19/Japan/PG-88433/2021	EPI_ISL_3900155	2021-09-02
hCoV-19/Japan/PG-88434/2021	EPI_ISL_3900156	2021-09-02
hCoV-19/Japan/PG-88435/2021	EPI_ISL_3900157	2021-09-02
hCoV-19/Japan/PG-88436/2021	EPI_ISL_3900158	2021-09-02
hCoV-19/Japan/PG-88437/2021	EPI_ISL_3900159	2021-09-02
hCoV-19/Japan/PG-88438/2021	EPI_ISL_3900160	2021-09-02
hCoV-19/Japan/PG-88439/2021	EPI_ISL_3900161	2021-09-02
hCoV-19/Japan/PG-88440/2021	EPI_ISL_3900162	2021-09-02
hCoV-19/Japan/PG-88441/2021	EPI_ISL_3900163	2021-09-02
hCoV-19/Japan/PG-88442/2021	EPI_ISL_3900164	2021-09-02
hCoV-19/Japan/PG-88443/2021	EPI_ISL_3900165	2021-09-02
hCoV-19/Japan/PG-88444/2021	EPI_ISL_3900166	2021-09-02
hCoV-19/Japan/PG-88445/2021	EPI_ISL_3900167	2021-09-02
hCoV-19/Japan/PG-88446/2021	EPI_ISL_3900168	2021-09-02
hCoV-19/Japan/PG-88447/2021	EPI_ISL_3900169	2021-09-02
hCoV-19/Japan/PG-88448/2021	EPI_ISL_3900170	2021-09-02
hCoV-19/Japan/PG-88449/2021	EPI_ISL_3900171	2021-09-02
hCoV-19/Japan/PG-88450/2021	EPI_ISL_3900172	2021-09-02
hCoV-19/Japan/PG-88451/2021	EPI_ISL_3900173	2021-09-02
hCoV-19/Japan/PG-88452/2021	EPI_ISL_3900174	2021-09-02
hCoV-19/Japan/PG-88453/2021	EPI_ISL_3900175	2021-09-02
hCoV-19/Japan/PG-88454/2021	EPI_ISL_3900176	2021-09-02
hCoV-19/Japan/PG-88455/2021	EPI_ISL_3900177	2021-09-02
hCoV-19/Japan/PG-88456/2021	EPI_ISL_3900178	2021-09-02
hCoV-19/Japan/PG-88457/2021	EPI_ISL_3900179	2021-09-02
hCoV-19/Japan/PG-88458/2021	EPI_ISL_3900180	2021-09-02
hCoV-19/Japan/PG-88459/2021	EPI_ISL_3900181	2021-09-02
hCoV-19/Japan/PG-88460/2021	EPI_ISL_3900182	2021-09-02
hCoV-19/Japan/PG-88461/2021	EPI_ISL_3900183	2021-09-02
hCoV-19/Japan/PG-88462/2021	EPI_ISL_3900184	2021-09-02
hCoV-19/Japan/PG-88463/2021	EPI_ISL_3900185	2021-09-02
hCoV-19/Japan/PG-88464/2021	EPI_ISL_3900186	2021-09-02
hCoV-19/Japan/PG-88465/2021	EPI_ISL_3900187	2021-09-02
hCoV-19/Japan/PG-88466/2021	EPI_ISL_3900188	2021-09-02
hCoV-19/Japan/PG-88467/2021	EPI_ISL_3900189	2021-09-02
hCoV-19/Japan/PG-88468/2021	EPI_ISL_3900190	2021-09-02
hCoV-19/Japan/PG-88469/2021	EPI_ISL_3900191	2021-09-02
hCoV-19/Japan/PG-88470/2021	EPI_ISL_3900192	2021-09-02
hCoV-19/Japan/PG-88471/2021	EPI_ISL_3900193	2021-09-02
hCoV-19/Japan/PG-88472/2021	EPI_ISL_3900194	2021-09-02
hCoV-19/Japan/PG-88473/2021	EPI_ISL_3900195	2021-09-02

virus_name	epiID	submission_date
hCoV-19/Japan/PG-88474/2021	EPI_ISL_3900196	2021-09-02
hCoV-19/Japan/PG-88475/2021	EPI_ISL_3900197	2021-09-02
hCoV-19/Japan/PG-88476/2021	EPI_ISL_3900198	2021-09-02
hCoV-19/Japan/PG-88477/2021	EPI_ISL_3900199	2021-09-02
hCoV-19/Japan/PG-88478/2021	EPI_ISL_3900200	2021-09-02
hCoV-19/Japan/PG-88479/2021	EPI_ISL_3900201	2021-09-02
hCoV-19/Japan/PG-88480/2021	EPI_ISL_3900202	2021-09-02
hCoV-19/Japan/PG-88481/2021	EPI_ISL_3900203	2021-09-02
hCoV-19/Japan/PG-88482/2021	EPI_ISL_3900204	2021-09-02
hCoV-19/Japan/PG-88483/2021	EPI_ISL_3900205	2021-09-02
hCoV-19/Japan/PG-88484/2021	EPI_ISL_3900206	2021-09-02
hCoV-19/Japan/PG-88485/2021	EPI_ISL_3900207	2021-09-02
hCoV-19/Japan/PG-88486/2021	EPI_ISL_3900208	2021-09-02
hCoV-19/Japan/PG-88487/2021	EPI_ISL_3900209	2021-09-02
hCoV-19/Japan/PG-88488/2021	EPI_ISL_3900210	2021-09-02
hCoV-19/Japan/PG-88489/2021	EPI_ISL_3900211	2021-09-02
hCoV-19/Japan/PG-88490/2021	EPI_ISL_3900212	2021-09-02
hCoV-19/Japan/PG-88491/2021	EPI_ISL_3900213	2021-09-02
hCoV-19/Japan/PG-88492/2021	EPI_ISL_3900214	2021-09-02
hCoV-19/Japan/PG-88493/2021	EPI_ISL_3900215	2021-09-02
hCoV-19/Japan/PG-88494/2021	EPI_ISL_3900216	2021-09-02
hCoV-19/Japan/PG-88495/2021	EPI_ISL_3900217	2021-09-02
hCoV-19/Japan/PG-88496/2021	EPI_ISL_3900218	2021-09-02
hCoV-19/Japan/PG-88497/2021	EPI_ISL_3900219	2021-09-02
hCoV-19/Japan/PG-88498/2021	EPI_ISL_3900220	2021-09-02
hCoV-19/Japan/PG-88499/2021	EPI_ISL_3900221	2021-09-02
hCoV-19/Japan/PG-88500/2021	EPI_ISL_3900222	2021-09-02
hCoV-19/Japan/PG-88501/2021	EPI_ISL_3900223	2021-09-02
hCoV-19/Japan/PG-88502/2021	EPI_ISL_3900224	2021-09-02
hCoV-19/Japan/PG-88503/2021	EPI_ISL_3900225	2021-09-02
hCoV-19/Japan/PG-88504/2021	EPI_ISL_3900226	2021-09-02
hCoV-19/Japan/PG-88505/2021	EPI_ISL_3900227	2021-09-02
hCoV-19/Japan/PG-88506/2021	EPI_ISL_3900228	2021-09-02
hCoV-19/Japan/PG-88507/2021	EPI_ISL_3900229	2021-09-02
hCoV-19/Japan/PG-88508/2021	EPI_ISL_3900230	2021-09-02
hCoV-19/Japan/PG-88509/2021	EPI_ISL_3900231	2021-09-02
hCoV-19/Japan/PG-88510/2021	EPI_ISL_3900232	2021-09-02
hCoV-19/Japan/PG-88511/2021	EPI_ISL_3900233	2021-09-02
hCoV-19/Japan/PG-88512/2021	EPI_ISL_3900234	2021-09-02
hCoV-19/Japan/PG-88513/2021	EPI_ISL_3900235	2021-09-02
hCoV-19/Japan/PG-88514/2021	EPI_ISL_3900236	2021-09-02
hCoV-19/Japan/PG-88515/2021	EPI_ISL_3900237	2021-09-02
hCoV-19/Japan/PG-88516/2021	EPI_ISL_3900238	2021-09-02
hCoV-19/Japan/PG-88517/2021	EPI_ISL_3900239	2021-09-02
hCoV-19/Japan/PG-88518/2021	EPI_ISL_3900240	2021-09-02
hCoV-19/Japan/PG-88519/2021	EPI_ISL_3900241	2021-09-02
hCoV-19/Japan/PG-88520/2021	EPI_ISL_3900242	2021-09-02
hCoV-19/Japan/PG-88521/2021	EPI_ISL_3900243	2021-09-02
hCoV-19/Japan/PG-88522/2021	EPI_ISL_3900244	2021-09-02
hCoV-19/Japan/PG-88523/2021	EPI_ISL_3900245	2021-09-02
hCoV-19/Japan/PG-88524/2021	EPI_ISL_3900246	2021-09-02
hCoV-19/Japan/PG-88525/2021	EPI_ISL_3900247	2021-09-02
hCoV-19/Japan/PG-88526/2021	EPI_ISL_3900248	2021-09-02
hCoV-19/Japan/PG-88527/2021	EPI_ISL_3900249	2021-09-02

virus_name	epiID	submission_date
hCoV-19/Japan/PG-88528/2021	EPI_ISL_3900250	2021-09-02
hCoV-19/Japan/PG-88529/2021	EPI_ISL_3900251	2021-09-02
hCoV-19/Japan/PG-88530/2021	EPI_ISL_3900252	2021-09-02
hCoV-19/Japan/PG-88531/2021	EPI_ISL_3900253	2021-09-02
hCoV-19/Japan/PG-88532/2021	EPI_ISL_3900254	2021-09-02
hCoV-19/Japan/PG-88533/2021	EPI_ISL_3900255	2021-09-02
hCoV-19/Japan/PG-88534/2021	EPI_ISL_3900256	2021-09-02
hCoV-19/Japan/PG-88535/2021	EPI_ISL_3900257	2021-09-02
hCoV-19/Japan/PG-88536/2021	EPI_ISL_3900258	2021-09-02
hCoV-19/Japan/PG-88537/2021	EPI_ISL_3900259	2021-09-02
hCoV-19/Japan/PG-88538/2021	EPI_ISL_3900260	2021-09-02
hCoV-19/Japan/PG-88539/2021	EPI_ISL_3900261	2021-09-02
hCoV-19/Japan/PG-88540/2021	EPI_ISL_3900262	2021-09-02
hCoV-19/Japan/PG-88541/2021	EPI_ISL_3900263	2021-09-02
hCoV-19/Japan/PG-88542/2021	EPI_ISL_3900264	2021-09-02
hCoV-19/Japan/PG-88543/2021	EPI_ISL_3900265	2021-09-02
hCoV-19/Japan/PG-88544/2021	EPI_ISL_3900266	2021-09-02
hCoV-19/Japan/PG-88545/2021	EPI_ISL_3900267	2021-09-02
hCoV-19/Japan/PG-88546/2021	EPI_ISL_3900268	2021-09-02
hCoV-19/Japan/PG-88547/2021	EPI_ISL_3900269	2021-09-02
hCoV-19/Japan/PG-88548/2021	EPI_ISL_3900270	2021-09-02
hCoV-19/Japan/PG-88549/2021	EPI_ISL_3900271	2021-09-02
hCoV-19/Japan/PG-88550/2021	EPI_ISL_3900272	2021-09-02
hCoV-19/Japan/PG-88551/2021	EPI_ISL_3900273	2021-09-02
hCoV-19/Japan/PG-88552/2021	EPI_ISL_3900274	2021-09-02
hCoV-19/Japan/PG-88553/2021	EPI_ISL_3900275	2021-09-02
hCoV-19/Japan/PG-88554/2021	EPI_ISL_3900276	2021-09-02
hCoV-19/Japan/PG-88555/2021	EPI_ISL_3900277	2021-09-02
hCoV-19/Japan/PG-88556/2021	EPI_ISL_3900278	2021-09-02
hCoV-19/Japan/PG-88557/2021	EPI_ISL_3900279	2021-09-02
hCoV-19/Japan/PG-88558/2021	EPI_ISL_3900280	2021-09-02
hCoV-19/Japan/PG-88559/2021	EPI_ISL_3900281	2021-09-02
hCoV-19/Japan/PG-88560/2021	EPI_ISL_3900282	2021-09-02
hCoV-19/Japan/PG-88561/2021	EPI_ISL_3900283	2021-09-02
hCoV-19/Japan/PG-88562/2021	EPI_ISL_3900284	2021-09-02
hCoV-19/Japan/PG-88563/2021	EPI_ISL_3900285	2021-09-02
hCoV-19/Japan/PG-88564/2021	EPI_ISL_3900286	2021-09-02
hCoV-19/Japan/PG-88565/2021	EPI_ISL_3900287	2021-09-02
hCoV-19/Japan/PG-88566/2021	EPI_ISL_3900288	2021-09-02
hCoV-19/Japan/PG-88567/2021	EPI_ISL_3900289	2021-09-02
hCoV-19/Japan/PG-88568/2021	EPI_ISL_3900290	2021-09-02
hCoV-19/Japan/PG-88569/2021	EPI_ISL_3900291	2021-09-02
hCoV-19/Japan/PG-88570/2021	EPI_ISL_3900292	2021-09-02
hCoV-19/Japan/PG-88571/2021	EPI_ISL_3900293	2021-09-02
hCoV-19/Japan/PG-88572/2021	EPI_ISL_3900294	2021-09-02
hCoV-19/Japan/PG-88573/2021	EPI_ISL_3900295	2021-09-02
hCoV-19/Japan/PG-88574/2021	EPI_ISL_3900296	2021-09-02
hCoV-19/Japan/PG-88575/2021	EPI_ISL_3900297	2021-09-02
hCoV-19/Japan/PG-88576/2021	EPI_ISL_3900298	2021-09-02
hCoV-19/Japan/PG-88577/2021	EPI_ISL_3900299	2021-09-02
hCoV-19/Japan/PG-88578/2021	EPI_ISL_3900300	2021-09-02
hCoV-19/Japan/PG-88579/2021	EPI_ISL_3900301	2021-09-02
hCoV-19/Japan/PG-88580/2021	EPI_ISL_3900302	2021-09-02
hCoV-19/Japan/PG-88581/2021	EPI_ISL_3900303	2021-09-02

virus_name	epiID	submission_date
hCoV-19/Japan/PG-88582/2021	EPI_ISL_3900304	2021-09-02
hCoV-19/Japan/PG-88583/2021	EPI_ISL_3900305	2021-09-02
hCoV-19/Japan/PG-88584/2021	EPI_ISL_3900306	2021-09-02
hCoV-19/Japan/PG-88585/2021	EPI_ISL_3900307	2021-09-02
hCoV-19/Japan/PG-88586/2021	EPI_ISL_3900308	2021-09-02
hCoV-19/Japan/PG-88587/2021	EPI_ISL_3900309	2021-09-02
hCoV-19/Japan/PG-88588/2021	EPI_ISL_3900310	2021-09-02
hCoV-19/Japan/PG-88589/2021	EPI_ISL_3900311	2021-09-02
hCoV-19/Japan/PG-88590/2021	EPI_ISL_3900312	2021-09-02
hCoV-19/Japan/PG-88591/2021	EPI_ISL_3900313	2021-09-02
hCoV-19/Japan/PG-88592/2021	EPI_ISL_3900314	2021-09-02
hCoV-19/Japan/PG-88593/2021	EPI_ISL_3900315	2021-09-02
hCoV-19/Japan/PG-88594/2021	EPI_ISL_3900316	2021-09-02
hCoV-19/Japan/PG-88595/2021	EPI_ISL_3900317	2021-09-02
hCoV-19/Japan/PG-88596/2021	EPI_ISL_3900318	2021-09-02
hCoV-19/Japan/PG-88597/2021	EPI_ISL_3900319	2021-09-02
hCoV-19/Japan/PG-88598/2021	EPI_ISL_3900320	2021-09-02
hCoV-19/Japan/PG-88599/2021	EPI_ISL_3900321	2021-09-02
hCoV-19/Japan/PG-88600/2021	EPI_ISL_3900322	2021-09-02
hCoV-19/Japan/PG-88601/2021	EPI_ISL_3900323	2021-09-02
hCoV-19/Japan/PG-88602/2021	EPI_ISL_3900324	2021-09-02
hCoV-19/Japan/PG-88603/2021	EPI_ISL_3900325	2021-09-02
hCoV-19/Japan/PG-88604/2021	EPI_ISL_3900326	2021-09-02
hCoV-19/Japan/PG-88605/2021	EPI_ISL_3900327	2021-09-02
hCoV-19/Japan/PG-88606/2021	EPI_ISL_3900328	2021-09-02
hCoV-19/Japan/PG-88607/2021	EPI_ISL_3900329	2021-09-02
hCoV-19/Japan/PG-88608/2021	EPI_ISL_3900330	2021-09-02
hCoV-19/Japan/PG-88609/2021	EPI_ISL_3900331	2021-09-02
hCoV-19/Japan/PG-88610/2021	EPI_ISL_3900332	2021-09-02
hCoV-19/Japan/PG-88611/2021	EPI_ISL_3900333	2021-09-02
hCoV-19/Japan/PG-88612/2021	EPI_ISL_3900334	2021-09-02
hCoV-19/Japan/PG-88613/2021	EPI_ISL_3900335	2021-09-02
hCoV-19/Japan/PG-88614/2021	EPI_ISL_3900336	2021-09-02
hCoV-19/Japan/PG-88615/2021	EPI_ISL_3900337	2021-09-02
hCoV-19/Japan/PG-88616/2021	EPI_ISL_3900338	2021-09-02
hCoV-19/Japan/PG-88617/2021	EPI_ISL_3900339	2021-09-02
hCoV-19/Japan/PG-88618/2021	EPI_ISL_3900340	2021-09-02
hCoV-19/Japan/PG-88619/2021	EPI_ISL_3900341	2021-09-02
hCoV-19/Japan/PG-88620/2021	EPI_ISL_3900342	2021-09-02
hCoV-19/Japan/PG-88621/2021	EPI_ISL_3900343	2021-09-02
hCoV-19/Japan/PG-88622/2021	EPI_ISL_3900344	2021-09-02
hCoV-19/Japan/PG-88623/2021	EPI_ISL_3900345	2021-09-02
hCoV-19/Japan/PG-88624/2021	EPI_ISL_3900346	2021-09-02
hCoV-19/Japan/PG-88625/2021	EPI_ISL_3900347	2021-09-02
hCoV-19/Japan/PG-88626/2021	EPI_ISL_3900348	2021-09-02
hCoV-19/Japan/PG-88627/2021	EPI_ISL_3900349	2021-09-02
hCoV-19/Japan/PG-88628/2021	EPI_ISL_3900350	2021-09-02
hCoV-19/Japan/PG-88629/2021	EPI_ISL_3900351	2021-09-02
hCoV-19/Japan/PG-88630/2021	EPI_ISL_3900352	2021-09-02
hCoV-19/Japan/PG-88631/2021	EPI_ISL_3900353	2021-09-02
hCoV-19/Japan/PG-88632/2021	EPI_ISL_3900354	2021-09-02
hCoV-19/Japan/PG-88633/2021	EPI_ISL_3900355	2021-09-02
hCoV-19/Japan/PG-88634/2021	EPI_ISL_3900356	2021-09-02
hCoV-19/Japan/PG-88635/2021	EPI_ISL_3900357	2021-09-02

virus_name	epiID	submission_date
hCoV-19/Japan/PG-88636/2021	EPI_ISL_3900358	2021-09-02
hCoV-19/Japan/PG-88637/2021	EPI_ISL_3900359	2021-09-02
hCoV-19/Japan/PG-88638/2021	EPI_ISL_3900360	2021-09-02
hCoV-19/Japan/PG-88639/2021	EPI_ISL_3900361	2021-09-02
hCoV-19/Japan/PG-88640/2021	EPI_ISL_3900362	2021-09-02
hCoV-19/Japan/PG-88641/2021	EPI_ISL_3900363	2021-09-02
hCoV-19/Japan/PG-88642/2021	EPI_ISL_3900364	2021-09-02
hCoV-19/Japan/PG-88643/2021	EPI_ISL_3900365	2021-09-02
hCoV-19/Japan/PG-88644/2021	EPI_ISL_3900366	2021-09-02
hCoV-19/Japan/PG-88645/2021	EPI_ISL_3900367	2021-09-02
hCoV-19/Japan/PG-88646/2021	EPI_ISL_3900368	2021-09-02
hCoV-19/Japan/PG-88647/2021	EPI_ISL_3900369	2021-09-02
hCoV-19/Japan/PG-88648/2021	EPI_ISL_3900370	2021-09-02
hCoV-19/Japan/PG-88649/2021	EPI_ISL_3900371	2021-09-02
hCoV-19/Japan/PG-88650/2021	EPI_ISL_3900372	2021-09-02
hCoV-19/Japan/PG-88651/2021	EPI_ISL_3900373	2021-09-02
hCoV-19/Japan/PG-88652/2021	EPI_ISL_3900374	2021-09-02
hCoV-19/Japan/PG-88653/2021	EPI_ISL_3900375	2021-09-02
hCoV-19/Japan/PG-88654/2021	EPI_ISL_3900376	2021-09-02
hCoV-19/Japan/PG-88655/2021	EPI_ISL_3900377	2021-09-02
hCoV-19/Japan/PG-88656/2021	EPI_ISL_3900378	2021-09-02
hCoV-19/Japan/PG-88657/2021	EPI_ISL_3900379	2021-09-02
hCoV-19/Japan/PG-88658/2021	EPI_ISL_3900380	2021-09-02
hCoV-19/Japan/PG-88659/2021	EPI_ISL_3900381	2021-09-02
hCoV-19/Japan/PG-88660/2021	EPI_ISL_3900382	2021-09-02
hCoV-19/Japan/PG-88661/2021	EPI_ISL_3900383	2021-09-02
hCoV-19/Japan/PG-88662/2021	EPI_ISL_3900384	2021-09-02
hCoV-19/Japan/PG-88663/2021	EPI_ISL_3900385	2021-09-02
hCoV-19/Japan/PG-88664/2021	EPI_ISL_3900386	2021-09-02
hCoV-19/Japan/PG-88665/2021	EPI_ISL_3900387	2021-09-02
hCoV-19/Japan/PG-88666/2021	EPI_ISL_3900388	2021-09-02
hCoV-19/Japan/PG-88667/2021	EPI_ISL_3900389	2021-09-02
hCoV-19/Japan/PG-88668/2021	EPI_ISL_3900390	2021-09-02
hCoV-19/Japan/PG-88669/2021	EPI_ISL_3900391	2021-09-02
hCoV-19/Japan/PG-88670/2021	EPI_ISL_3900392	2021-09-02
hCoV-19/Japan/PG-88671/2021	EPI_ISL_3900393	2021-09-02
hCoV-19/Japan/PG-88672/2021	EPI_ISL_3900394	2021-09-02
hCoV-19/Japan/PG-88673/2021	EPI_ISL_3900395	2021-09-02
hCoV-19/Japan/PG-88674/2021	EPI_ISL_3900396	2021-09-02
hCoV-19/Japan/PG-88675/2021	EPI_ISL_3900397	2021-09-02
hCoV-19/Japan/PG-88676/2021	EPI_ISL_3900398	2021-09-02
hCoV-19/Japan/PG-88677/2021	EPI_ISL_3900399	2021-09-02
hCoV-19/Japan/PG-88678/2021	EPI_ISL_3900400	2021-09-02
hCoV-19/Japan/PG-88679/2021	EPI_ISL_3900401	2021-09-02
hCoV-19/Japan/PG-88680/2021	EPI_ISL_3900402	2021-09-02
hCoV-19/Japan/PG-88681/2021	EPI_ISL_3900403	2021-09-02
hCoV-19/Japan/PG-88682/2021	EPI_ISL_3900404	2021-09-02
hCoV-19/Japan/PG-88683/2021	EPI_ISL_3900405	2021-09-02
hCoV-19/Japan/PG-88684/2021	EPI_ISL_3900406	2021-09-02
hCoV-19/Japan/PG-88685/2021	EPI_ISL_3900407	2021-09-02
hCoV-19/Japan/PG-88686/2021	EPI_ISL_3900408	2021-09-02
hCoV-19/Japan/PG-88687/2021	EPI_ISL_3900409	2021-09-02
hCoV-19/Japan/PG-88688/2021	EPI_ISL_3900410	2021-09-02
hCoV-19/Japan/PG-88689/2021	EPI_ISL_3900411	2021-09-02

virus_name	epiID	submission_date
hCoV-19/Japan/PG-88690/2021	EPI_ISL_3900412	2021-09-02
hCoV-19/Japan/PG-88691/2021	EPI_ISL_3900413	2021-09-02
hCoV-19/Japan/PG-88692/2021	EPI_ISL_3900414	2021-09-02
hCoV-19/Japan/PG-88693/2021	EPI_ISL_3900415	2021-09-02
hCoV-19/Japan/PG-88694/2021	EPI_ISL_3900416	2021-09-02
hCoV-19/Japan/PG-88695/2021	EPI_ISL_3900417	2021-09-02
hCoV-19/Japan/PG-88696/2021	EPI_ISL_3900418	2021-09-02
hCoV-19/Japan/PG-88697/2021	EPI_ISL_3900419	2021-09-02
hCoV-19/Japan/PG-88698/2021	EPI_ISL_3900420	2021-09-02
hCoV-19/Japan/PG-88699/2021	EPI_ISL_3900421	2021-09-02
hCoV-19/Japan/PG-88700/2021	EPI_ISL_3900422	2021-09-02
hCoV-19/Japan/PG-88701/2021	EPI_ISL_3900423	2021-09-02
hCoV-19/Japan/PG-88702/2021	EPI_ISL_3900424	2021-09-02
hCoV-19/Japan/PG-88703/2021	EPI_ISL_3900425	2021-09-02
hCoV-19/Japan/PG-88704/2021	EPI_ISL_3900426	2021-09-02
hCoV-19/Japan/PG-88705/2021	EPI_ISL_3900427	2021-09-02
hCoV-19/Japan/PG-88706/2021	EPI_ISL_3900428	2021-09-02
hCoV-19/Japan/PG-88707/2021	EPI_ISL_3900429	2021-09-02
hCoV-19/Japan/PG-88708/2021	EPI_ISL_3900430	2021-09-02
hCoV-19/Japan/PG-88709/2021	EPI_ISL_3900431	2021-09-02
hCoV-19/Japan/PG-88710/2021	EPI_ISL_3900432	2021-09-02
hCoV-19/Japan/PG-88711/2021	EPI_ISL_3900433	2021-09-02
hCoV-19/Japan/PG-88712/2021	EPI_ISL_3900434	2021-09-02
hCoV-19/Japan/PG-88713/2021	EPI_ISL_3900435	2021-09-02
hCoV-19/Japan/PG-88714/2021	EPI_ISL_3900436	2021-09-02
hCoV-19/Japan/PG-88715/2021	EPI_ISL_3900437	2021-09-02
hCoV-19/Japan/PG-88716/2021	EPI_ISL_3900438	2021-09-02
hCoV-19/Japan/PG-88717/2021	EPI_ISL_3900439	2021-09-02
hCoV-19/Japan/PG-88718/2021	EPI_ISL_3900440	2021-09-02
hCoV-19/Japan/PG-88719/2021	EPI_ISL_3900441	2021-09-02
hCoV-19/Japan/PG-88720/2021	EPI_ISL_3900442	2021-09-02
hCoV-19/Japan/PG-88721/2021	EPI_ISL_3900443	2021-09-02
hCoV-19/Japan/PG-88722/2021	EPI_ISL_3900444	2021-09-02
hCoV-19/Japan/PG-88723/2021	EPI_ISL_3900445	2021-09-02
hCoV-19/Japan/PG-88724/2021	EPI_ISL_3900446	2021-09-02
hCoV-19/Japan/PG-88725/2021	EPI_ISL_3900447	2021-09-02
hCoV-19/Japan/PG-88726/2021	EPI_ISL_3900448	2021-09-02
hCoV-19/Japan/PG-88727/2021	EPI_ISL_3900449	2021-09-02
hCoV-19/Japan/PG-88728/2021	EPI_ISL_3900450	2021-09-02
hCoV-19/Japan/PG-88729/2021	EPI_ISL_3900451	2021-09-02
hCoV-19/Japan/PG-88730/2021	EPI_ISL_3900452	2021-09-02
hCoV-19/Japan/PG-88731/2021	EPI_ISL_3900453	2021-09-02
hCoV-19/Japan/PG-88732/2021	EPI_ISL_3900454	2021-09-02
hCoV-19/Japan/PG-88733/2021	EPI_ISL_3900455	2021-09-02
hCoV-19/Japan/PG-88734/2021	EPI_ISL_3900456	2021-09-02
hCoV-19/Japan/PG-88735/2021	EPI_ISL_3900457	2021-09-02
hCoV-19/Japan/PG-88736/2021	EPI_ISL_3900458	2021-09-02
hCoV-19/Japan/PG-88737/2021	EPI_ISL_3900459	2021-09-02
hCoV-19/Japan/PG-88738/2021	EPI_ISL_3900460	2021-09-02
hCoV-19/Japan/PG-88739/2021	EPI_ISL_3900461	2021-09-02
hCoV-19/Japan/PG-88740/2021	EPI_ISL_3900462	2021-09-02
hCoV-19/Japan/PG-88741/2021	EPI_ISL_3900463	2021-09-02
hCoV-19/Japan/PG-88742/2021	EPI_ISL_3900464	2021-09-02
hCoV-19/Japan/PG-88743/2021	EPI_ISL_3900465	2021-09-02

virus_name	epiID	submission_date
hCoV-19/Japan/PG-88744/2021	EPI_ISL_3900466	2021-09-02
hCoV-19/Japan/PG-88745/2021	EPI_ISL_3900467	2021-09-02
hCoV-19/Japan/PG-88746/2021	EPI_ISL_3900468	2021-09-02
hCoV-19/Japan/PG-88747/2021	EPI_ISL_3900469	2021-09-02
hCoV-19/Japan/PG-88748/2021	EPI_ISL_3900470	2021-09-02
hCoV-19/Japan/PG-88749/2021	EPI_ISL_3900471	2021-09-02
hCoV-19/Japan/PG-88750/2021	EPI_ISL_3900472	2021-09-02
hCoV-19/Japan/PG-88751/2021	EPI_ISL_3900473	2021-09-02
hCoV-19/Japan/PG-88752/2021	EPI_ISL_3900474	2021-09-02
hCoV-19/Japan/PG-88753/2021	EPI_ISL_3900475	2021-09-02
hCoV-19/Japan/PG-88754/2021	EPI_ISL_3900476	2021-09-02
hCoV-19/Japan/PG-88755/2021	EPI_ISL_3900477	2021-09-02
hCoV-19/Japan/PG-88756/2021	EPI_ISL_3900478	2021-09-02
hCoV-19/Japan/PG-88757/2021	EPI_ISL_3900479	2021-09-02
hCoV-19/Japan/PG-88758/2021	EPI_ISL_3900480	2021-09-02
hCoV-19/Japan/PG-88759/2021	EPI_ISL_3900481	2021-09-02
hCoV-19/Japan/PG-88760/2021	EPI_ISL_3900482	2021-09-02
hCoV-19/Japan/PG-88761/2021	EPI_ISL_3900483	2021-09-02
hCoV-19/Japan/PG-88762/2021	EPI_ISL_3900484	2021-09-02
hCoV-19/Japan/PG-88763/2021	EPI_ISL_3900485	2021-09-02
hCoV-19/Japan/PG-88764/2021	EPI_ISL_3900486	2021-09-02
hCoV-19/Japan/PG-88765/2021	EPI_ISL_3900487	2021-09-02
hCoV-19/Japan/PG-88766/2021	EPI_ISL_3900488	2021-09-02
hCoV-19/Japan/PG-88767/2021	EPI_ISL_3900489	2021-09-02
hCoV-19/Japan/PG-88768/2021	EPI_ISL_3900490	2021-09-02
hCoV-19/Japan/PG-88769/2021	EPI_ISL_3900491	2021-09-02
hCoV-19/Japan/PG-88770/2021	EPI_ISL_3900492	2021-09-02
hCoV-19/Japan/PG-88771/2021	EPI_ISL_3900493	2021-09-02
hCoV-19/Japan/PG-88772/2021	EPI_ISL_3900494	2021-09-02
hCoV-19/Japan/PG-88773/2021	EPI_ISL_3900495	2021-09-02
hCoV-19/Japan/PG-88774/2021	EPI_ISL_3900496	2021-09-02
hCoV-19/Japan/PG-88775/2021	EPI_ISL_3900497	2021-09-02
hCoV-19/Japan/PG-88776/2021	EPI_ISL_3900498	2021-09-02
hCoV-19/Japan/PG-88777/2021	EPI_ISL_3900499	2021-09-02
hCoV-19/Japan/PG-88778/2021	EPI_ISL_3900500	2021-09-02
hCoV-19/Japan/PG-88779/2021	EPI_ISL_3900501	2021-09-02
hCoV-19/Japan/PG-88780/2021	EPI_ISL_3900502	2021-09-02
hCoV-19/Japan/PG-88781/2021	EPI_ISL_3900503	2021-09-02
hCoV-19/Japan/PG-88782/2021	EPI_ISL_3900504	2021-09-02
hCoV-19/Japan/PG-88783/2021	EPI_ISL_3900505	2021-09-02
hCoV-19/Japan/PG-88784/2021	EPI_ISL_3900506	2021-09-02
hCoV-19/Japan/PG-88785/2021	EPI_ISL_3900507	2021-09-02
hCoV-19/Japan/PG-88786/2021	EPI_ISL_3900508	2021-09-02
hCoV-19/Japan/PG-88787/2021	EPI_ISL_3900509	2021-09-02
hCoV-19/Japan/PG-88788/2021	EPI_ISL_3900510	2021-09-02
hCoV-19/Japan/PG-88789/2021	EPI_ISL_3900511	2021-09-02
hCoV-19/Japan/PG-88790/2021	EPI_ISL_3900512	2021-09-02
hCoV-19/Japan/PG-88791/2021	EPI_ISL_3900513	2021-09-02
hCoV-19/Japan/PG-88792/2021	EPI_ISL_3900514	2021-09-02
hCoV-19/Japan/PG-88793/2021	EPI_ISL_3900515	2021-09-02
hCoV-19/Japan/PG-88794/2021	EPI_ISL_3900516	2021-09-02
hCoV-19/Japan/PG-88795/2021	EPI_ISL_3900517	2021-09-02
hCoV-19/Japan/PG-88796/2021	EPI_ISL_3900518	2021-09-02
hCoV-19/Japan/PG-88797/2021	EPI_ISL_3900519	2021-09-02

virus_name	epiID	submission_date
hCoV-19/Japan/PG-88798/2021	EPI_ISL_3900520	2021-09-02
hCoV-19/Japan/PG-88799/2021	EPI_ISL_3900521	2021-09-02
hCoV-19/Japan/PG-88800/2021	EPI_ISL_3900522	2021-09-02
hCoV-19/Japan/PG-88801/2021	EPI_ISL_3900523	2021-09-02
hCoV-19/Japan/PG-88802/2021	EPI_ISL_3900524	2021-09-02
hCoV-19/Japan/PG-88803/2021	EPI_ISL_3900525	2021-09-02
hCoV-19/Japan/PG-88804/2021	EPI_ISL_3900526	2021-09-02
hCoV-19/Japan/PG-88805/2021	EPI_ISL_3900527	2021-09-02
hCoV-19/Japan/PG-88806/2021	EPI_ISL_3900528	2021-09-02
hCoV-19/Japan/PG-88807/2021	EPI_ISL_3900529	2021-09-02
hCoV-19/Japan/PG-88808/2021	EPI_ISL_3900530	2021-09-02
hCoV-19/Japan/PG-88809/2021	EPI_ISL_3900531	2021-09-02
hCoV-19/Japan/PG-88810/2021	EPI_ISL_3900532	2021-09-02
hCoV-19/Japan/PG-88811/2021	EPI_ISL_3900533	2021-09-02
hCoV-19/Japan/PG-88812/2021	EPI_ISL_3900534	2021-09-02
hCoV-19/Japan/PG-88813/2021	EPI_ISL_3900535	2021-09-02
hCoV-19/Japan/PG-88814/2021	EPI_ISL_3900536	2021-09-02
hCoV-19/Japan/PG-88815/2021	EPI_ISL_3900537	2021-09-02
hCoV-19/Japan/PG-88816/2021	EPI_ISL_3900538	2021-09-02
hCoV-19/Japan/PG-88817/2021	EPI_ISL_3900539	2021-09-02
hCoV-19/Japan/PG-88818/2021	EPI_ISL_3900540	2021-09-02
hCoV-19/Japan/PG-88819/2021	EPI_ISL_3900541	2021-09-02
hCoV-19/Japan/PG-88820/2021	EPI_ISL_3900542	2021-09-02
hCoV-19/Japan/PG-88821/2021	EPI_ISL_3900543	2021-09-02
hCoV-19/Japan/PG-88822/2021	EPI_ISL_3900544	2021-09-02
hCoV-19/Japan/PG-88823/2021	EPI_ISL_3900545	2021-09-02
hCoV-19/Japan/PG-88824/2021	EPI_ISL_3900546	2021-09-02
hCoV-19/Japan/PG-88825/2021	EPI_ISL_3900547	2021-09-02
hCoV-19/Japan/PG-88826/2021	EPI_ISL_3900548	2021-09-02
hCoV-19/Japan/PG-88827/2021	EPI_ISL_3900549	2021-09-02
hCoV-19/Japan/PG-88828/2021	EPI_ISL_3900550	2021-09-02
hCoV-19/Japan/PG-88829/2021	EPI_ISL_3900551	2021-09-02
hCoV-19/Japan/PG-88830/2021	EPI_ISL_3900552	2021-09-02
hCoV-19/Japan/PG-88831/2021	EPI_ISL_3900553	2021-09-02
hCoV-19/Japan/PG-88832/2021	EPI_ISL_3900554	2021-09-02
hCoV-19/Japan/PG-88833/2021	EPI_ISL_3900555	2021-09-02
hCoV-19/Japan/PG-89248/2021	EPI_ISL_3900556	2021-09-02
hCoV-19/Japan/PG-89249/2021	EPI_ISL_3900557	2021-09-02
hCoV-19/Japan/PG-89250/2021	EPI_ISL_3900558	2021-09-02
hCoV-19/Japan/PG-89251/2021	EPI_ISL_3900559	2021-09-02
hCoV-19/Japan/PG-89252/2021	EPI_ISL_3900560	2021-09-02
hCoV-19/Japan/PG-89254/2021	EPI_ISL_3900561	2021-09-02
hCoV-19/Japan/PG-89255/2021	EPI_ISL_3900562	2021-09-02
hCoV-19/Japan/PG-89256/2021	EPI_ISL_3900563	2021-09-02
hCoV-19/Japan/PG-89257/2021	EPI_ISL_3900564	2021-09-02
hCoV-19/Japan/PG-89258/2021	EPI_ISL_3900565	2021-09-02
hCoV-19/Japan/PG-89259/2021	EPI_ISL_3900566	2021-09-02
hCoV-19/Japan/PG-89260/2021	EPI_ISL_3900567	2021-09-02
hCoV-19/Japan/PG-89261/2021	EPI_ISL_3900568	2021-09-02
hCoV-19/Japan/PG-89262/2021	EPI_ISL_3900569	2021-09-02
hCoV-19/Japan/PG-89263/2021	EPI_ISL_3900570	2021-09-02
hCoV-19/Japan/PG-89264/2021	EPI_ISL_3900571	2021-09-02
hCoV-19/Japan/PG-89265/2021	EPI_ISL_3900572	2021-09-02
hCoV-19/Japan/PG-89266/2021	EPI_ISL_3900573	2021-09-02

virus_name	epiID	submission_date
hCoV-19/Japan/PG-89267/2021	EPI_ISL_3900574	2021-09-02
hCoV-19/Japan/PG-89268/2021	EPI_ISL_3900575	2021-09-02
hCoV-19/Japan/PG-89269/2021	EPI_ISL_3900576	2021-09-02
hCoV-19/Japan/PG-89270/2021	EPI_ISL_3900577	2021-09-02
hCoV-19/Japan/PG-89271/2021	EPI_ISL_3900578	2021-09-02
hCoV-19/Japan/PG-89272/2021	EPI_ISL_3900579	2021-09-02
hCoV-19/Japan/PG-89273/2021	EPI_ISL_3900580	2021-09-02
hCoV-19/Japan/PG-89274/2021	EPI_ISL_3900581	2021-09-02
hCoV-19/Japan/PG-89275/2021	EPI_ISL_3900582	2021-09-02
hCoV-19/Japan/PG-89276/2021	EPI_ISL_3900583	2021-09-02
hCoV-19/Japan/PG-89277/2021	EPI_ISL_3900584	2021-09-02
hCoV-19/Japan/PG-89278/2021	EPI_ISL_3900585	2021-09-02
hCoV-19/Japan/PG-89279/2021	EPI_ISL_3900586	2021-09-02
hCoV-19/Japan/PG-89280/2021	EPI_ISL_3900587	2021-09-02
hCoV-19/Japan/PG-89281/2021	EPI_ISL_3900588	2021-09-02
hCoV-19/Japan/PG-89282/2021	EPI_ISL_3900589	2021-09-02
hCoV-19/Japan/PG-89283/2021	EPI_ISL_3900590	2021-09-02
hCoV-19/Japan/PG-89284/2021	EPI_ISL_3900591	2021-09-02
hCoV-19/Japan/PG-89285/2021	EPI_ISL_3900592	2021-09-02
hCoV-19/Japan/PG-89286/2021	EPI_ISL_3900593	2021-09-02
hCoV-19/Japan/PG-89287/2021	EPI_ISL_3900594	2021-09-02
hCoV-19/Japan/PG-89288/2021	EPI_ISL_3900595	2021-09-02
hCoV-19/Japan/PG-89290/2021	EPI_ISL_3900596	2021-09-02
hCoV-19/Japan/PG-89291/2021	EPI_ISL_3900597	2021-09-02
hCoV-19/Japan/PG-89292/2021	EPI_ISL_3900598	2021-09-02
hCoV-19/Japan/PG-89293/2021	EPI_ISL_3900599	2021-09-02
hCoV-19/Japan/PG-89294/2021	EPI_ISL_3900600	2021-09-02
hCoV-19/Japan/PG-89295/2021	EPI_ISL_3900601	2021-09-02
hCoV-19/Japan/PG-89296/2021	EPI_ISL_3900602	2021-09-02
hCoV-19/Japan/PG-89297/2021	EPI_ISL_3900603	2021-09-02
hCoV-19/Japan/PG-89298/2021	EPI_ISL_3900604	2021-09-02
hCoV-19/Japan/PG-89299/2021	EPI_ISL_3900605	2021-09-02
hCoV-19/Japan/PG-89300/2021	EPI_ISL_3900606	2021-09-02
hCoV-19/Japan/PG-89301/2021	EPI_ISL_3900607	2021-09-02
hCoV-19/Japan/PG-89302/2021	EPI_ISL_3900608	2021-09-02
hCoV-19/Japan/PG-89303/2021	EPI_ISL_3873882	2021-09-02
hCoV-19/Japan/PG-89304/2021	EPI_ISL_3873883	2021-09-02
hCoV-19/Japan/PG-89305/2021	EPI_ISL_3873884	2021-09-02
hCoV-19/Japan/PG-89306/2021	EPI_ISL_3873885	2021-09-02
hCoV-19/Japan/PG-89307/2021	EPI_ISL_3873886	2021-09-02
hCoV-19/Japan/PG-89308/2021	EPI_ISL_3873887	2021-09-02
hCoV-19/Japan/PG-89309/2021	EPI_ISL_3873888	2021-09-02
hCoV-19/Japan/PG-89310/2021	EPI_ISL_3873889	2021-09-02
hCoV-19/Japan/PG-89311/2021	EPI_ISL_3873890	2021-09-02
hCoV-19/Japan/PG-89312/2021	EPI_ISL_3873891	2021-09-02
hCoV-19/Japan/PG-89313/2021	EPI_ISL_3873892	2021-09-02
hCoV-19/Japan/PG-89314/2021	EPI_ISL_3873893	2021-09-02
hCoV-19/Japan/PG-89315/2021	EPI_ISL_3873894	2021-09-02
hCoV-19/Japan/PG-89316/2021	EPI_ISL_3873895	2021-09-02
hCoV-19/Japan/PG-89317/2021	EPI_ISL_3873896	2021-09-02
hCoV-19/Japan/PG-89318/2021	EPI_ISL_3873897	2021-09-02
hCoV-19/Japan/PG-89319/2021	EPI_ISL_3873898	2021-09-02
hCoV-19/Japan/PG-89320/2021	EPI_ISL_3873899	2021-09-02
hCoV-19/Japan/PG-89321/2021	EPI_ISL_3873900	2021-09-02

virus_name	epiID	submission_date
hCoV-19/Japan/PG-89322/2021	EPI_ISL_3873901	2021-09-02
hCoV-19/Japan/PG-89323/2021	EPI_ISL_3873902	2021-09-02
hCoV-19/Japan/PG-89324/2021	EPI_ISL_3873903	2021-09-02
hCoV-19/Japan/PG-89325/2021	EPI_ISL_3873904	2021-09-02
hCoV-19/Japan/PG-89326/2021	EPI_ISL_3873905	2021-09-02
hCoV-19/Japan/PG-89327/2021	EPI_ISL_3873906	2021-09-02
hCoV-19/Japan/PG-89328/2021	EPI_ISL_3873907	2021-09-02
hCoV-19/Japan/PG-89329/2021	EPI_ISL_3873908	2021-09-02
hCoV-19/Japan/PG-89330/2021	EPI_ISL_3873909	2021-09-02
hCoV-19/Japan/PG-89331/2021	EPI_ISL_3873910	2021-09-02
hCoV-19/Japan/PG-89332/2021	EPI_ISL_3873911	2021-09-02
hCoV-19/Japan/PG-89333/2021	EPI_ISL_3873912	2021-09-02
hCoV-19/Japan/PG-89334/2021	EPI_ISL_3873913	2021-09-02
hCoV-19/Japan/PG-89335/2021	EPI_ISL_3873914	2021-09-02
hCoV-19/Japan/PG-89336/2021	EPI_ISL_3873915	2021-09-02
hCoV-19/Japan/PG-89337/2021	EPI_ISL_3873916	2021-09-02
hCoV-19/Japan/PG-89338/2021	EPI_ISL_3873917	2021-09-02
hCoV-19/Japan/PG-89339/2021	EPI_ISL_3873918	2021-09-02
hCoV-19/Japan/PG-89340/2021	EPI_ISL_3873919	2021-09-02
hCoV-19/Japan/PG-89341/2021	EPI_ISL_3873920	2021-09-02
hCoV-19/Japan/PG-89342/2021	EPI_ISL_3873921	2021-09-02
hCoV-19/Japan/PG-89343/2021	EPI_ISL_3873922	2021-09-02
hCoV-19/Japan/PG-89344/2021	EPI_ISL_3873923	2021-09-02
hCoV-19/Japan/PG-89345/2021	EPI_ISL_3873924	2021-09-02
hCoV-19/Japan/PG-89346/2021	EPI_ISL_3873925	2021-09-02
hCoV-19/Japan/PG-89347/2021	EPI_ISL_3873926	2021-09-02
hCoV-19/Japan/PG-89348/2021	EPI_ISL_3873927	2021-09-02
hCoV-19/Japan/PG-89349/2021	EPI_ISL_3873928	2021-09-02
hCoV-19/Japan/PG-89350/2021	EPI_ISL_3873929	2021-09-02
hCoV-19/Japan/PG-89351/2021	EPI_ISL_3873930	2021-09-02
hCoV-19/Japan/PG-89352/2021	EPI_ISL_3873931	2021-09-02
hCoV-19/Japan/PG-89356/2021	EPI_ISL_3873932	2021-09-02
hCoV-19/Japan/PG-89357/2021	EPI_ISL_3873933	2021-09-02
hCoV-19/Japan/PG-89358/2021	EPI_ISL_3873934	2021-09-02
hCoV-19/Japan/PG-89359/2021	EPI_ISL_3873935	2021-09-02
hCoV-19/Japan/PG-89360/2021	EPI_ISL_3873936	2021-09-02
hCoV-19/Japan/PG-89361/2021	EPI_ISL_3873937	2021-09-02
hCoV-19/Japan/PG-89362/2021	EPI_ISL_3873938	2021-09-02
hCoV-19/Japan/PG-89363/2021	EPI_ISL_3873939	2021-09-02
hCoV-19/Japan/PG-89364/2021	EPI_ISL_3873940	2021-09-02
hCoV-19/Japan/PG-89365/2021	EPI_ISL_3873941	2021-09-02
hCoV-19/Japan/PG-89366/2021	EPI_ISL_3873942	2021-09-02
hCoV-19/Japan/PG-89367/2021	EPI_ISL_3873943	2021-09-02
hCoV-19/Japan/PG-89368/2021	EPI_ISL_3873944	2021-09-02
hCoV-19/Japan/PG-89369/2021	EPI_ISL_3873945	2021-09-02
hCoV-19/Japan/PG-89370/2021	EPI_ISL_3873946	2021-09-02
hCoV-19/Japan/PG-89371/2021	EPI_ISL_3873947	2021-09-02
hCoV-19/Japan/PG-89372/2021	EPI_ISL_3873948	2021-09-02
hCoV-19/Japan/PG-89373/2021	EPI_ISL_3873949	2021-09-02
hCoV-19/Japan/PG-89374/2021	EPI_ISL_3873950	2021-09-02
hCoV-19/Japan/PG-89375/2021	EPI_ISL_3873951	2021-09-02
hCoV-19/Japan/PG-89377/2021	EPI_ISL_3875661	2021-09-02
hCoV-19/Japan/PG-89378/2021	EPI_ISL_3875665	2021-09-02
hCoV-19/Japan/PG-89379/2021	EPI_ISL_3875668	2021-09-02

virus_name	epiID	submission_date
hCoV-19/Japan/PG-89390/2021	EPI_ISL_3875671	2021-09-02
hCoV-19/Japan/PG-89391/2021	EPI_ISL_3875674	2021-09-02
hCoV-19/Japan/PG-89392/2021	EPI_ISL_3875676	2021-09-02
hCoV-19/Japan/PG-89393/2021	EPI_ISL_3875680	2021-09-02
hCoV-19/Japan/PG-89394/2021	EPI_ISL_3875683	2021-09-02
hCoV-19/Japan/PG-89395/2021	EPI_ISL_3875686	2021-09-02
hCoV-19/Japan/PG-89396/2021	EPI_ISL_3875688	2021-09-02
hCoV-19/Japan/PG-89397/2021	EPI_ISL_3875690	2021-09-02
hCoV-19/Japan/PG-89398/2021	EPI_ISL_3875691	2021-09-02
hCoV-19/Japan/PG-89399/2021	EPI_ISL_3875694	2021-09-02
hCoV-19/Japan/PG-89400/2021	EPI_ISL_3875697	2021-09-02
hCoV-19/Japan/PG-89401/2021	EPI_ISL_3875700	2021-09-02
hCoV-19/Japan/PG-89402/2021	EPI_ISL_3875703	2021-09-02
hCoV-19/Japan/PG-89403/2021	EPI_ISL_3875707	2021-09-02
hCoV-19/Japan/PG-89404/2021	EPI_ISL_3875710	2021-09-02
hCoV-19/Japan/PG-89405/2021	EPI_ISL_3871867	2021-09-02
hCoV-19/Japan/PG-89417/2021	EPI_ISL_3871868	2021-09-02
hCoV-19/Japan/PG-89418/2021	EPI_ISL_3871869	2021-09-02
hCoV-19/Japan/PG-89419/2021	EPI_ISL_3871870	2021-09-02
hCoV-19/Japan/PG-89421/2021	EPI_ISL_3871871	2021-09-02
hCoV-19/Japan/PG-89422/2021	EPI_ISL_3871872	2021-09-02
hCoV-19/Japan/PG-89423/2021	EPI_ISL_3871873	2021-09-02
hCoV-19/Japan/PG-89427/2021	EPI_ISL_3871874	2021-09-02
hCoV-19/Japan/PG-89428/2021	EPI_ISL_3871875	2021-09-02
hCoV-19/Japan/PG-89429/2021	EPI_ISL_3871380	2021-09-02
hCoV-19/Japan/PG-89430/2021	EPI_ISL_3871381	2021-09-02
hCoV-19/Japan/PG-89431/2021	EPI_ISL_3871382	2021-09-02
hCoV-19/Japan/PG-89432/2021	EPI_ISL_3871383	2021-09-02
hCoV-19/Japan/PG-89433/2021	EPI_ISL_3871384	2021-09-02
hCoV-19/Japan/PG-89434/2021	EPI_ISL_3871385	2021-09-02
hCoV-19/Japan/PG-89435/2021	EPI_ISL_3871386	2021-09-02
hCoV-19/Japan/PG-89436/2021	EPI_ISL_3871658	2021-09-02
hCoV-19/Japan/PG-89437/2021	EPI_ISL_3871659	2021-09-02
hCoV-19/Japan/PG-89438/2021	EPI_ISL_3871660	2021-09-02
hCoV-19/Japan/PG-89439/2021	EPI_ISL_3871661	2021-09-02
hCoV-19/Japan/PG-89440/2021	EPI_ISL_3871662	2021-09-02
hCoV-19/Japan/PG-89441/2021	EPI_ISL_3871663	2021-09-02
hCoV-19/Japan/PG-89442/2021	EPI_ISL_3871664	2021-09-02
hCoV-19/Japan/PG-89443/2021	EPI_ISL_3871665	2021-09-02
hCoV-19/Japan/PG-89444/2021	EPI_ISL_3871666	2021-09-02
hCoV-19/Japan/PG-89445/2021	EPI_ISL_3871667	2021-09-02
hCoV-19/Japan/PG-89446/2021	EPI_ISL_3871457	2021-09-02
hCoV-19/Japan/PG-89447/2021	EPI_ISL_3871458	2021-09-02
hCoV-19/Japan/PG-89448/2021	EPI_ISL_3871459	2021-09-02
hCoV-19/Japan/PG-89449/2021	EPI_ISL_3871460	2021-09-02
hCoV-19/Japan/PG-89450/2021	EPI_ISL_3871461	2021-09-02
hCoV-19/Japan/PG-89451/2021	EPI_ISL_3871462	2021-09-02
hCoV-19/Japan/PG-89452/2021	EPI_ISL_3871463	2021-09-02
hCoV-19/Japan/PG-89453/2021	EPI_ISL_3871336	2021-09-02
hCoV-19/Japan/PG-89454/2021	EPI_ISL_3871337	2021-09-02
hCoV-19/Japan/PG-89455/2021	EPI_ISL_3871338	2021-09-02
hCoV-19/Japan/PG-89456/2021	EPI_ISL_3871339	2021-09-02
hCoV-19/Japan/PG-89457/2021	EPI_ISL_3871340	2021-09-02
hCoV-19/Japan/PG-89458/2021	EPI_ISL_3871341	2021-09-02

virus_name	epiID	submission_date
hCoV-19/Japan/PG-89459/2021	EPI_ISL_3871342	2021-09-02
hCoV-19/Japan/PG-89460/2021	EPI_ISL_3871343	2021-09-02
hCoV-19/Japan/PG-89461/2021	EPI_ISL_3871344	2021-09-02
hCoV-19/Japan/PG-89462/2021	EPI_ISL_3871345	2021-09-02
hCoV-19/Japan/PG-89463/2021	EPI_ISL_3871346	2021-09-02
hCoV-19/Japan/PG-89464/2021	EPI_ISL_3871347	2021-09-02
hCoV-19/Japan/PG-89465/2021	EPI_ISL_3871348	2021-09-02
hCoV-19/Japan/PG-89466/2021	EPI_ISL_3871349	2021-09-02
hCoV-19/Japan/PG-89467/2021	EPI_ISL_3871350	2021-09-02
hCoV-19/Japan/PG-89468/2021	EPI_ISL_3871351	2021-09-02
hCoV-19/Japan/PG-89469/2021	EPI_ISL_3871352	2021-09-02
hCoV-19/Japan/PG-89470/2021	EPI_ISL_3871353	2021-09-02
hCoV-19/Japan/PG-89471/2021	EPI_ISL_3871354	2021-09-02
hCoV-19/Japan/PG-89472/2021	EPI_ISL_3871355	2021-09-02
hCoV-19/Japan/PG-89473/2021	EPI_ISL_3871356	2021-09-02
hCoV-19/Japan/PG-89474/2021	EPI_ISL_3871357	2021-09-02
hCoV-19/Japan/PG-89475/2021	EPI_ISL_3871358	2021-09-02
hCoV-19/Japan/PG-89493/2021	EPI_ISL_3873502	2021-09-02
hCoV-19/Japan/PG-89494/2021	EPI_ISL_3873503	2021-09-02
hCoV-19/Japan/PG-89495/2021	EPI_ISL_3873504	2021-09-02
hCoV-19/Japan/PG-89496/2021	EPI_ISL_3873505	2021-09-02
hCoV-19/Japan/PG-89497/2021	EPI_ISL_3873506	2021-09-02
hCoV-19/Japan/PG-89498/2021	EPI_ISL_3873507	2021-09-02
hCoV-19/Japan/PG-89499/2021	EPI_ISL_3873508	2021-09-02
hCoV-19/Japan/PG-89500/2021	EPI_ISL_3873509	2021-09-02
hCoV-19/Japan/PG-89501/2021	EPI_ISL_3873510	2021-09-02
hCoV-19/Japan/PG-89502/2021	EPI_ISL_3873511	2021-09-02
hCoV-19/Japan/PG-89503/2021	EPI_ISL_3873512	2021-09-02
hCoV-19/Japan/PG-89504/2021	EPI_ISL_3873513	2021-09-02
hCoV-19/Japan/PG-89505/2021	EPI_ISL_3873514	2021-09-02
hCoV-19/Japan/PG-89506/2021	EPI_ISL_3873515	2021-09-02
hCoV-19/Japan/PG-89507/2021	EPI_ISL_3873516	2021-09-02
hCoV-19/Japan/PG-89508/2021	EPI_ISL_3873517	2021-09-02
hCoV-19/Japan/PG-89509/2021	EPI_ISL_3873518	2021-09-02
hCoV-19/Japan/PG-89510/2021	EPI_ISL_3873519	2021-09-02
hCoV-19/Japan/PG-89511/2021	EPI_ISL_3873520	2021-09-02
hCoV-19/Japan/PG-89512/2021	EPI_ISL_3873521	2021-09-02
hCoV-19/Japan/PG-89513/2021	EPI_ISL_3873522	2021-09-02
hCoV-19/Japan/PG-89514/2021	EPI_ISL_3873523	2021-09-02
hCoV-19/Japan/PG-89515/2021	EPI_ISL_3873524	2021-09-02
hCoV-19/Japan/PG-89516/2021	EPI_ISL_3874570	2021-09-02
hCoV-19/Japan/PG-89517/2021	EPI_ISL_3874571	2021-09-02
hCoV-19/Japan/PG-89518/2021	EPI_ISL_3874574	2021-09-02
hCoV-19/Japan/PG-89519/2021	EPI_ISL_3874577	2021-09-02
hCoV-19/Japan/PG-89520/2021	EPI_ISL_3874579	2021-09-02
hCoV-19/Japan/PG-89521/2021	EPI_ISL_3874582	2021-09-02
hCoV-19/Japan/PG-89522/2021	EPI_ISL_3874585	2021-09-02
hCoV-19/Japan/PG-89523/2021	EPI_ISL_3874587	2021-09-02
hCoV-19/Japan/PG-89524/2021	EPI_ISL_3874589	2021-09-02
hCoV-19/Japan/PG-89525/2021	EPI_ISL_3874592	2021-09-02
hCoV-19/Japan/PG-89526/2021	EPI_ISL_3874594	2021-09-02
hCoV-19/Japan/PG-89527/2021	EPI_ISL_3874597	2021-09-02
hCoV-19/Japan/PG-89528/2021	EPI_ISL_3874599	2021-09-02
hCoV-19/Japan/PG-89529/2021	EPI_ISL_3874601	2021-09-02

virus_name	epiID	submission_date
hCoV-19/Japan/PG-89530/2021	EPI_ISL_3874603	2021-09-02
hCoV-19/Japan/PG-89531/2021	EPI_ISL_3874606	2021-09-02
hCoV-19/Japan/PG-89532/2021	EPI_ISL_3874609	2021-09-02
hCoV-19/Japan/PG-89533/2021	EPI_ISL_3874611	2021-09-02
hCoV-19/Japan/PG-89534/2021	EPI_ISL_3874614	2021-09-02
hCoV-19/Japan/PG-89535/2021	EPI_ISL_3874615	2021-09-02
hCoV-19/Japan/PG-89536/2021	EPI_ISL_3874617	2021-09-02
hCoV-19/Japan/PG-89537/2021	EPI_ISL_3874619	2021-09-02
hCoV-19/Japan/PG-89538/2021	EPI_ISL_3874622	2021-09-02
hCoV-19/Japan/PG-89539/2021	EPI_ISL_3874624	2021-09-02
hCoV-19/Japan/PG-89540/2021	EPI_ISL_3872334	2021-09-02
hCoV-19/Japan/PG-89541/2021	EPI_ISL_3872335	2021-09-02
hCoV-19/Japan/PG-89542/2021	EPI_ISL_3872336	2021-09-02
hCoV-19/Japan/PG-89543/2021	EPI_ISL_3872337	2021-09-02
hCoV-19/Japan/PG-89544/2021	EPI_ISL_3872338	2021-09-02
hCoV-19/Japan/PG-89545/2021	EPI_ISL_3872339	2021-09-02
hCoV-19/Japan/PG-89546/2021	EPI_ISL_3872340	2021-09-02
hCoV-19/Japan/PG-89547/2021	EPI_ISL_3872341	2021-09-02
hCoV-19/Japan/PG-89548/2021	EPI_ISL_3872342	2021-09-02
hCoV-19/Japan/PG-89549/2021	EPI_ISL_3872343	2021-09-02
hCoV-19/Japan/PG-89550/2021	EPI_ISL_3872344	2021-09-02
hCoV-19/Japan/PG-89551/2021	EPI_ISL_3872345	2021-09-02
hCoV-19/Japan/PG-89552/2021	EPI_ISL_3872346	2021-09-02
hCoV-19/Japan/PG-89553/2021	EPI_ISL_3900609	2021-09-02
hCoV-19/Japan/PG-89554/2021	EPI_ISL_3900610	2021-09-02
hCoV-19/Japan/PG-89555/2021	EPI_ISL_3900611	2021-09-02
hCoV-19/Japan/PG-89556/2021	EPI_ISL_3900612	2021-09-02
hCoV-19/Japan/PG-89557/2021	EPI_ISL_3900613	2021-09-02
hCoV-19/Japan/PG-89558/2021	EPI_ISL_3900614	2021-09-02
hCoV-19/Japan/PG-89559/2021	EPI_ISL_3900615	2021-09-02
hCoV-19/Japan/PG-89560/2021	EPI_ISL_3900616	2021-09-02
hCoV-19/Japan/PG-89561/2021	EPI_ISL_3900617	2021-09-02
hCoV-19/Japan/PG-89562/2021	EPI_ISL_3900618	2021-09-02
hCoV-19/Japan/PG-89563/2021	EPI_ISL_3900619	2021-09-02
hCoV-19/Japan/PG-89564/2021	EPI_ISL_3900620	2021-09-02
hCoV-19/Japan/PG-89565/2021	EPI_ISL_3900621	2021-09-02
hCoV-19/Japan/PG-89566/2021	EPI_ISL_3900622	2021-09-02
hCoV-19/Japan/PG-89567/2021	EPI_ISL_3900623	2021-09-02
hCoV-19/Japan/PG-89568/2021	EPI_ISL_3900624	2021-09-02
hCoV-19/Japan/PG-89569/2021	EPI_ISL_3900625	2021-09-02
hCoV-19/Japan/PG-89570/2021	EPI_ISL_3900626	2021-09-02
hCoV-19/Japan/PG-89571/2021	EPI_ISL_3900627	2021-09-02
hCoV-19/Japan/PG-89572/2021	EPI_ISL_3900628	2021-09-02
hCoV-19/Japan/PG-89573/2021	EPI_ISL_3900629	2021-09-02
hCoV-19/Japan/PG-89574/2021	EPI_ISL_3900630	2021-09-02
hCoV-19/Japan/PG-89575/2021	EPI_ISL_3900631	2021-09-02
hCoV-19/Japan/PG-89576/2021	EPI_ISL_3900632	2021-09-02
hCoV-19/Japan/PG-89577/2021	EPI_ISL_3900633	2021-09-02
hCoV-19/Japan/PG-89578/2021	EPI_ISL_3900634	2021-09-02
hCoV-19/Japan/PG-89579/2021	EPI_ISL_3900635	2021-09-02
hCoV-19/Japan/PG-89580/2021	EPI_ISL_3900636	2021-09-02
hCoV-19/Japan/PG-89581/2021	EPI_ISL_3900637	2021-09-02
hCoV-19/Japan/PG-89582/2021	EPI_ISL_3900638	2021-09-02
hCoV-19/Japan/PG-89583/2021	EPI_ISL_3900639	2021-09-02

virus_name	epiID	submission_date
hCoV-19/Japan/PG-89584/2021	EPI_ISL_3900640	2021-09-02
hCoV-19/Japan/PG-89585/2021	EPI_ISL_3900641	2021-09-02
hCoV-19/Japan/PG-89586/2021	EPI_ISL_3900642	2021-09-02
hCoV-19/Japan/PG-89587/2021	EPI_ISL_3900643	2021-09-02
hCoV-19/Japan/PG-89588/2021	EPI_ISL_3900644	2021-09-02
hCoV-19/Japan/PG-89589/2021	EPI_ISL_3900645	2021-09-02
hCoV-19/Japan/PG-89590/2021	EPI_ISL_3900646	2021-09-02
hCoV-19/Japan/PG-89591/2021	EPI_ISL_3900647	2021-09-02
hCoV-19/Japan/PG-89592/2021	EPI_ISL_3900648	2021-09-02
hCoV-19/Japan/PG-89593/2021	EPI_ISL_3900649	2021-09-02
hCoV-19/Japan/PG-89594/2021	EPI_ISL_3900650	2021-09-02
hCoV-19/Japan/PG-89595/2021	EPI_ISL_3900651	2021-09-02
hCoV-19/Japan/PG-89596/2021	EPI_ISL_3900652	2021-09-02
hCoV-19/Japan/PG-89597/2021	EPI_ISL_3900653	2021-09-02
hCoV-19/Japan/PG-89598/2021	EPI_ISL_3900654	2021-09-02
hCoV-19/Japan/PG-89599/2021	EPI_ISL_3900655	2021-09-02
hCoV-19/Japan/PG-89600/2021	EPI_ISL_3900656	2021-09-02
hCoV-19/Japan/PG-89601/2021	EPI_ISL_3900657	2021-09-02
hCoV-19/Japan/PG-89602/2021	EPI_ISL_3900658	2021-09-02
hCoV-19/Japan/PG-89603/2021	EPI_ISL_3900659	2021-09-02
hCoV-19/Japan/PG-89604/2021	EPI_ISL_3900661	2021-09-02
hCoV-19/Japan/PG-89605/2021	EPI_ISL_3900663	2021-09-02
hCoV-19/Japan/PG-89606/2021	EPI_ISL_3900666	2021-09-02
hCoV-19/Japan/PG-89607/2021	EPI_ISL_3900669	2021-09-02
hCoV-19/Japan/PG-89608/2021	EPI_ISL_3900673	2021-09-02
hCoV-19/Japan/PG-89609/2021	EPI_ISL_3900676	2021-09-02
hCoV-19/Japan/PG-89610/2021	EPI_ISL_3900679	2021-09-02
hCoV-19/Japan/PG-89611/2021	EPI_ISL_3900683	2021-09-02
hCoV-19/Japan/PG-89612/2021	EPI_ISL_3900686	2021-09-02
hCoV-19/Japan/PG-89613/2021	EPI_ISL_3900689	2021-09-02
hCoV-19/Japan/PG-89614/2021	EPI_ISL_3900692	2021-09-02
hCoV-19/Japan/PG-89615/2021	EPI_ISL_3900695	2021-09-02
hCoV-19/Japan/PG-89616/2021	EPI_ISL_3900699	2021-09-02
hCoV-19/Japan/PG-89617/2021	EPI_ISL_3900702	2021-09-02
hCoV-19/Japan/PG-89618/2021	EPI_ISL_3900705	2021-09-02
hCoV-19/Japan/PG-89619/2021	EPI_ISL_3900708	2021-09-02
hCoV-19/Japan/PG-89620/2021	EPI_ISL_3900711	2021-09-02
hCoV-19/Japan/PG-89621/2021	EPI_ISL_3900714	2021-09-02
hCoV-19/Japan/PG-89622/2021	EPI_ISL_3900717	2021-09-02
hCoV-19/Japan/PG-89623/2021	EPI_ISL_3900720	2021-09-02
hCoV-19/Japan/PG-89624/2021	EPI_ISL_3900723	2021-09-02
hCoV-19/Japan/PG-89625/2021	EPI_ISL_3900726	2021-09-02
hCoV-19/Japan/PG-89626/2021	EPI_ISL_3900729	2021-09-02
hCoV-19/Japan/PG-89627/2021	EPI_ISL_3900732	2021-09-02
hCoV-19/Japan/PG-89628/2021	EPI_ISL_3900734	2021-09-02
hCoV-19/Japan/PG-89629/2021	EPI_ISL_3900736	2021-09-02
hCoV-19/Japan/PG-89630/2021	EPI_ISL_3900739	2021-09-02
hCoV-19/Japan/PG-89631/2021	EPI_ISL_3900742	2021-09-02
hCoV-19/Japan/PG-89632/2021	EPI_ISL_3900744	2021-09-02
hCoV-19/Japan/PG-89633/2021	EPI_ISL_3900745	2021-09-02
hCoV-19/Japan/PG-89634/2021	EPI_ISL_3900746	2021-09-02
hCoV-19/Japan/PG-89635/2021	EPI_ISL_3900747	2021-09-02
hCoV-19/Japan/PG-89636/2021	EPI_ISL_3900748	2021-09-02
hCoV-19/Japan/PG-89637/2021	EPI_ISL_3900749	2021-09-02

virus_name	epiID	submission_date
hCoV-19/Japan/PG-89638/2021	EPI_ISL_3900750	2021-09-02
hCoV-19/Japan/PG-89639/2021	EPI_ISL_3900751	2021-09-02
hCoV-19/Japan/PG-89640/2021	EPI_ISL_3900752	2021-09-02
hCoV-19/Japan/PG-89641/2021	EPI_ISL_3900753	2021-09-02
hCoV-19/Japan/PG-89642/2021	EPI_ISL_3900754	2021-09-02
hCoV-19/Japan/PG-89643/2021	EPI_ISL_3900755	2021-09-02
hCoV-19/Japan/PG-89644/2021	EPI_ISL_3900756	2021-09-02
hCoV-19/Japan/PG-89645/2021	EPI_ISL_3900757	2021-09-02
hCoV-19/Japan/PG-89646/2021	EPI_ISL_3900758	2021-09-02
hCoV-19/Japan/PG-89647/2021	EPI_ISL_3900759	2021-09-02
hCoV-19/Japan/PG-89648/2021	EPI_ISL_3900760	2021-09-02
hCoV-19/Japan/PG-89649/2021	EPI_ISL_3900761	2021-09-02
hCoV-19/Japan/PG-89650/2021	EPI_ISL_3900762	2021-09-02
hCoV-19/Japan/PG-89651/2021	EPI_ISL_3900763	2021-09-02
hCoV-19/Japan/PG-89652/2021	EPI_ISL_3900764	2021-09-02
hCoV-19/Japan/PG-89653/2021	EPI_ISL_3900765	2021-09-02
hCoV-19/Japan/PG-89654/2021	EPI_ISL_3900766	2021-09-02
hCoV-19/Japan/PG-89655/2021	EPI_ISL_3900767	2021-09-02
hCoV-19/Japan/PG-89656/2021	EPI_ISL_3900768	2021-09-02
hCoV-19/Japan/PG-89657/2021	EPI_ISL_3900769	2021-09-02
hCoV-19/Japan/PG-89658/2021	EPI_ISL_3900770	2021-09-02
hCoV-19/Japan/PG-89659/2021	EPI_ISL_3900771	2021-09-02
hCoV-19/Japan/PG-89660/2021	EPI_ISL_3900772	2021-09-02
hCoV-19/Japan/PG-89661/2021	EPI_ISL_3900773	2021-09-02
hCoV-19/Japan/PG-89662/2021	EPI_ISL_3900774	2021-09-02
hCoV-19/Japan/PG-89663/2021	EPI_ISL_3900775	2021-09-02
hCoV-19/Japan/PG-89664/2021	EPI_ISL_3900776	2021-09-02
hCoV-19/Japan/PG-89665/2021	EPI_ISL_3900777	2021-09-02
hCoV-19/Japan/PG-89666/2021	EPI_ISL_3900778	2021-09-02
hCoV-19/Japan/PG-89667/2021	EPI_ISL_3900779	2021-09-02
hCoV-19/Japan/PG-89668/2021	EPI_ISL_3900780	2021-09-02
hCoV-19/Japan/PG-89669/2021	EPI_ISL_3900781	2021-09-02
hCoV-19/Japan/PG-89670/2021	EPI_ISL_3900782	2021-09-02
hCoV-19/Japan/PG-89671/2021	EPI_ISL_3900783	2021-09-02
hCoV-19/Japan/PG-89672/2021	EPI_ISL_3900784	2021-09-02
hCoV-19/Japan/PG-89673/2021	EPI_ISL_3900785	2021-09-02
hCoV-19/Japan/PG-89674/2021	EPI_ISL_3900786	2021-09-02
hCoV-19/Japan/PG-89675/2021	EPI_ISL_3900787	2021-09-02
hCoV-19/Japan/PG-89676/2021	EPI_ISL_3900788	2021-09-02
hCoV-19/Japan/PG-89677/2021	EPI_ISL_3900789	2021-09-02
hCoV-19/Japan/PG-89678/2021	EPI_ISL_3900790	2021-09-02
hCoV-19/Japan/PG-89679/2021	EPI_ISL_3900791	2021-09-02
hCoV-19/Japan/PG-89680/2021	EPI_ISL_3900792	2021-09-02
hCoV-19/Japan/PG-89681/2021	EPI_ISL_3900793	2021-09-02
hCoV-19/Japan/PG-89682/2021	EPI_ISL_3900794	2021-09-02
hCoV-19/Japan/PG-89683/2021	EPI_ISL_3900795	2021-09-02
hCoV-19/Japan/PG-89684/2021	EPI_ISL_3900796	2021-09-02
hCoV-19/Japan/PG-89685/2021	EPI_ISL_3900797	2021-09-02
hCoV-19/Japan/PG-89686/2021	EPI_ISL_3900798	2021-09-02
hCoV-19/Japan/PG-89687/2021	EPI_ISL_3900799	2021-09-02
hCoV-19/Japan/PG-89688/2021	EPI_ISL_3900800	2021-09-02
hCoV-19/Japan/PG-89689/2021	EPI_ISL_3900801	2021-09-02
hCoV-19/Japan/PG-89690/2021	EPI_ISL_3900802	2021-09-02
hCoV-19/Japan/PG-89691/2021	EPI_ISL_3900803	2021-09-02

virus_name	epiID	submission_date
hCoV-19/Japan/PG-89692/2021	EPI_ISL_3900804	2021-09-02
hCoV-19/Japan/PG-89693/2021	EPI_ISL_3900805	2021-09-02
hCoV-19/Japan/PG-89694/2021	EPI_ISL_3900806	2021-09-02
hCoV-19/Japan/PG-89695/2021	EPI_ISL_3900807	2021-09-02
hCoV-19/Japan/PG-89696/2021	EPI_ISL_3900808	2021-09-02
hCoV-19/Japan/PG-89697/2021	EPI_ISL_3900809	2021-09-02
hCoV-19/Japan/PG-89698/2021	EPI_ISL_3900810	2021-09-02
hCoV-19/Japan/PG-89699/2021	EPI_ISL_3900811	2021-09-02
hCoV-19/Japan/PG-89700/2021	EPI_ISL_3900812	2021-09-02
hCoV-19/Japan/PG-89701/2021	EPI_ISL_3900813	2021-09-02
hCoV-19/Japan/PG-89702/2021	EPI_ISL_3900814	2021-09-02
hCoV-19/Japan/PG-89703/2021	EPI_ISL_3900815	2021-09-02
hCoV-19/Japan/PG-89704/2021	EPI_ISL_3900816	2021-09-02
hCoV-19/Japan/PG-89705/2021	EPI_ISL_3900817	2021-09-02
hCoV-19/Japan/PG-89706/2021	EPI_ISL_3900818	2021-09-02
hCoV-19/Japan/PG-89707/2021	EPI_ISL_3900819	2021-09-02
hCoV-19/Japan/PG-89708/2021	EPI_ISL_3900820	2021-09-02
hCoV-19/Japan/PG-89709/2021	EPI_ISL_3900821	2021-09-02
hCoV-19/Japan/PG-89710/2021	EPI_ISL_3900822	2021-09-02
hCoV-19/Japan/PG-89711/2021	EPI_ISL_3900823	2021-09-02
hCoV-19/Japan/PG-89712/2021	EPI_ISL_3900824	2021-09-02
hCoV-19/Japan/PG-89713/2021	EPI_ISL_3900825	2021-09-02
hCoV-19/Japan/PG-89714/2021	EPI_ISL_3900826	2021-09-02
hCoV-19/Japan/PG-89715/2021	EPI_ISL_3900827	2021-09-02
hCoV-19/Japan/PG-89716/2021	EPI_ISL_3900828	2021-09-02
hCoV-19/Japan/PG-89717/2021	EPI_ISL_3900829	2021-09-02
hCoV-19/Japan/PG-89718/2021	EPI_ISL_3900830	2021-09-02
hCoV-19/Japan/PG-89719/2021	EPI_ISL_3900831	2021-09-02
hCoV-19/Japan/PG-89720/2021	EPI_ISL_3900832	2021-09-02
hCoV-19/Japan/PG-89721/2021	EPI_ISL_3900833	2021-09-02
hCoV-19/Japan/PG-89722/2021	EPI_ISL_3900834	2021-09-02
hCoV-19/Japan/PG-89723/2021	EPI_ISL_3900835	2021-09-02
hCoV-19/Japan/PG-89724/2021	EPI_ISL_3900836	2021-09-02
hCoV-19/Japan/PG-89725/2021	EPI_ISL_3900837	2021-09-02
hCoV-19/Japan/PG-89726/2021	EPI_ISL_3900838	2021-09-02
hCoV-19/Japan/PG-89727/2021	EPI_ISL_3900839	2021-09-02
hCoV-19/Japan/PG-89728/2021	EPI_ISL_3900840	2021-09-02
hCoV-19/Japan/PG-89729/2021	EPI_ISL_3900841	2021-09-02
hCoV-19/Japan/PG-89730/2021	EPI_ISL_3900842	2021-09-02
hCoV-19/Japan/PG-89731/2021	EPI_ISL_3900843	2021-09-02
hCoV-19/Japan/PG-89732/2021	EPI_ISL_3900844	2021-09-02
hCoV-19/Japan/PG-89733/2021	EPI_ISL_3900845	2021-09-02
hCoV-19/Japan/PG-89734/2021	EPI_ISL_3900846	2021-09-02
hCoV-19/Japan/PG-89735/2021	EPI_ISL_3900847	2021-09-02
hCoV-19/Japan/PG-89736/2021	EPI_ISL_3900848	2021-09-02
hCoV-19/Japan/PG-89737/2021	EPI_ISL_3900849	2021-09-02
hCoV-19/Japan/PG-89738/2021	EPI_ISL_3900850	2021-09-02
hCoV-19/Japan/PG-89739/2021	EPI_ISL_3900851	2021-09-02
hCoV-19/Japan/PG-89740/2021	EPI_ISL_3900852	2021-09-02
hCoV-19/Japan/PG-89741/2021	EPI_ISL_3872217	2021-09-02
hCoV-19/Japan/PG-89742/2021	EPI_ISL_3872218	2021-09-02
hCoV-19/Japan/PG-89743/2021	EPI_ISL_3872219	2021-09-02
hCoV-19/Japan/PG-89744/2021	EPI_ISL_3872220	2021-09-02
hCoV-19/Japan/PG-89745/2021	EPI_ISL_3872221	2021-09-02

virus_name	epiID	submission_date
hCoV-19/Japan/PG-89746/2021	EPI_ISL_3873952	2021-09-02
hCoV-19/Japan/PG-89747/2021	EPI_ISL_3873953	2021-09-02
hCoV-19/Japan/PG-89748/2021	EPI_ISL_3873954	2021-09-02
hCoV-19/Japan/PG-89749/2021	EPI_ISL_3873955	2021-09-02
hCoV-19/Japan/PG-89750/2021	EPI_ISL_3874683	2021-09-02
hCoV-19/Japan/PG-89752/2021	EPI_ISL_3874685	2021-09-02
hCoV-19/Japan/PG-89753/2021	EPI_ISL_3874686	2021-09-02
hCoV-19/Japan/PG-89754/2021	EPI_ISL_3874687	2021-09-02
hCoV-19/Japan/PG-89755/2021	EPI_ISL_3874688	2021-09-02
hCoV-19/Japan/PG-89756/2021	EPI_ISL_3874689	2021-09-02
hCoV-19/Japan/PG-89757/2021	EPI_ISL_3874691	2021-09-02
hCoV-19/Japan/PG-89762/2021	EPI_ISL_3874693	2021-09-02
hCoV-19/Japan/PG-89764/2021	EPI_ISL_3874695	2021-09-02
hCoV-19/Japan/PG-89765/2021	EPI_ISL_3874696	2021-09-02
hCoV-19/Japan/PG-89767/2021	EPI_ISL_3874698	2021-09-02
hCoV-19/Japan/PG-89768/2021	EPI_ISL_3874700	2021-09-02
hCoV-19/Japan/PG-89769/2021	EPI_ISL_3874702	2021-09-02
hCoV-19/Japan/PG-89770/2021	EPI_ISL_3874703	2021-09-02
hCoV-19/Japan/PG-89771/2021	EPI_ISL_3874705	2021-09-02
hCoV-19/Japan/PG-89772/2021	EPI_ISL_3874707	2021-09-02
hCoV-19/Japan/PG-89773/2021	EPI_ISL_3875196	2021-09-02
hCoV-19/Japan/PG-89774/2021	EPI_ISL_3875197	2021-09-02
hCoV-19/Japan/PG-89775/2021	EPI_ISL_3875198	2021-09-02
hCoV-19/Japan/PG-89776/2021	EPI_ISL_3875199	2021-09-02
hCoV-19/Japan/PG-89777/2021	EPI_ISL_3875200	2021-09-02
hCoV-19/Japan/PG-89778/2021	EPI_ISL_3875201	2021-09-02
hCoV-19/Japan/PG-89779/2021	EPI_ISL_3875202	2021-09-02
hCoV-19/Japan/PG-89780/2021	EPI_ISL_3875203	2021-09-02
hCoV-19/Japan/PG-89781/2021	EPI_ISL_3875204	2021-09-02
hCoV-19/Japan/PG-89782/2021	EPI_ISL_3875205	2021-09-02
hCoV-19/Japan/PG-89783/2021	EPI_ISL_3875206	2021-09-02
hCoV-19/Japan/PG-89784/2021	EPI_ISL_3875207	2021-09-02
hCoV-19/Japan/PG-89785/2021	EPI_ISL_3875208	2021-09-02
hCoV-19/Japan/PG-89786/2021	EPI_ISL_3875209	2021-09-02
hCoV-19/Japan/PG-89787/2021	EPI_ISL_3875210	2021-09-02
hCoV-19/Japan/PG-89788/2021	EPI_ISL_3875211	2021-09-02
hCoV-19/Japan/PG-89789/2021	EPI_ISL_3875212	2021-09-02
hCoV-19/Japan/PG-89790/2021	EPI_ISL_3875213	2021-09-02
hCoV-19/Japan/PG-89791/2021	EPI_ISL_3875214	2021-09-02
hCoV-19/Japan/PG-89792/2021	EPI_ISL_3875215	2021-09-02
hCoV-19/Japan/PG-89793/2021	EPI_ISL_3875216	2021-09-02
hCoV-19/Japan/PG-89794/2021	EPI_ISL_3875217	2021-09-02
hCoV-19/Japan/PG-89795/2021	EPI_ISL_3875218	2021-09-02
hCoV-19/Japan/PG-89796/2021	EPI_ISL_3875219	2021-09-02
hCoV-19/Japan/PG-89797/2021	EPI_ISL_3875220	2021-09-02
hCoV-19/Japan/PG-89798/2021	EPI_ISL_3875221	2021-09-02
hCoV-19/Japan/PG-89799/2021	EPI_ISL_3875222	2021-09-02
hCoV-19/Japan/PG-89800/2021	EPI_ISL_3875223	2021-09-02
hCoV-19/Japan/PG-89801/2021	EPI_ISL_3875224	2021-09-02
hCoV-19/Japan/PG-89802/2021	EPI_ISL_3875225	2021-09-02
hCoV-19/Japan/PG-89803/2021	EPI_ISL_3875226	2021-09-02
hCoV-19/Japan/PG-89804/2021	EPI_ISL_3875227	2021-09-02
hCoV-19/Japan/PG-89805/2021	EPI_ISL_3875228	2021-09-02
hCoV-19/Japan/PG-89806/2021	EPI_ISL_3875229	2021-09-02

virus_name	epiID	submission_date
hCoV-19/Japan/PG-89807/2021	EPI_ISL_3875230	2021-09-02
hCoV-19/Japan/PG-89808/2021	EPI_ISL_3875231	2021-09-02
hCoV-19/Japan/PG-89809/2021	EPI_ISL_3875232	2021-09-02
hCoV-19/Japan/PG-89810/2021	EPI_ISL_3875233	2021-09-02
hCoV-19/Japan/PG-89811/2021	EPI_ISL_3875234	2021-09-02
hCoV-19/Japan/PG-89812/2021	EPI_ISL_3875235	2021-09-02
hCoV-19/Japan/PG-89813/2021	EPI_ISL_3875236	2021-09-02
hCoV-19/Japan/PG-89814/2021	EPI_ISL_3875237	2021-09-02
hCoV-19/Japan/PG-89815/2021	EPI_ISL_3875238	2021-09-02
hCoV-19/Japan/PG-89816/2021	EPI_ISL_3875239	2021-09-02
hCoV-19/Japan/PG-89817/2021	EPI_ISL_3875240	2021-09-02
hCoV-19/Japan/PG-89818/2021	EPI_ISL_3875241	2021-09-02
hCoV-19/Japan/PG-89819/2021	EPI_ISL_3875242	2021-09-02
hCoV-19/Japan/PG-89820/2021	EPI_ISL_3875243	2021-09-02
hCoV-19/Japan/PG-89821/2021	EPI_ISL_3875244	2021-09-02
hCoV-19/Japan/PG-89822/2021	EPI_ISL_3875245	2021-09-02
hCoV-19/Japan/PG-89847/2021	EPI_ISL_3875246	2021-09-02
hCoV-19/Japan/PG-89848/2021	EPI_ISL_3875247	2021-09-02
hCoV-19/Japan/PG-89849/2021	EPI_ISL_3875248	2021-09-02
hCoV-19/Japan/PG-89850/2021	EPI_ISL_3875249	2021-09-02
hCoV-19/Japan/PG-89851/2021	EPI_ISL_3875250	2021-09-02
hCoV-19/Japan/PG-89852/2021	EPI_ISL_3875251	2021-09-02
hCoV-19/Japan/PG-89853/2021	EPI_ISL_3875252	2021-09-02
hCoV-19/Japan/PG-89854/2021	EPI_ISL_3875253	2021-09-02
hCoV-19/Japan/PG-89855/2021	EPI_ISL_3875254	2021-09-02
hCoV-19/Japan/PG-89856/2021	EPI_ISL_3875255	2021-09-02
hCoV-19/Japan/PG-89857/2021	EPI_ISL_3875256	2021-09-02
hCoV-19/Japan/PG-89858/2021	EPI_ISL_3875257	2021-09-02
hCoV-19/Japan/PG-89859/2021	EPI_ISL_3875258	2021-09-02
hCoV-19/Japan/PG-89860/2021	EPI_ISL_3875259	2021-09-02
hCoV-19/Japan/PG-89861/2021	EPI_ISL_3875260	2021-09-02
hCoV-19/Japan/PG-89862/2021	EPI_ISL_3875261	2021-09-02
hCoV-19/Japan/PG-89863/2021	EPI_ISL_3875262	2021-09-02
hCoV-19/Japan/PG-89864/2021	EPI_ISL_3875263	2021-09-02
hCoV-19/Japan/PG-89865/2021	EPI_ISL_3875264	2021-09-02
hCoV-19/Japan/PG-89866/2021	EPI_ISL_3875265	2021-09-02
hCoV-19/Japan/PG-89867/2021	EPI_ISL_3875266	2021-09-02
hCoV-19/Japan/PG-89868/2021	EPI_ISL_3875267	2021-09-02
hCoV-19/Japan/PG-89869/2021	EPI_ISL_3875268	2021-09-02
hCoV-19/Japan/PG-89870/2021	EPI_ISL_3875269	2021-09-02
hCoV-19/Japan/PG-89871/2021	EPI_ISL_3875270	2021-09-02
hCoV-19/Japan/PG-89872/2021	EPI_ISL_3875271	2021-09-02
hCoV-19/Japan/PG-89873/2021	EPI_ISL_3875272	2021-09-02
hCoV-19/Japan/PG-89874/2021	EPI_ISL_3875273	2021-09-02
hCoV-19/Japan/PG-89875/2021	EPI_ISL_3875274	2021-09-02
hCoV-19/Japan/PG-89876/2021	EPI_ISL_3875275	2021-09-02
hCoV-19/Japan/PG-89877/2021	EPI_ISL_3875276	2021-09-02
hCoV-19/Japan/PG-89878/2021	EPI_ISL_3871430	2021-09-02
hCoV-19/Japan/PG-89879/2021	EPI_ISL_3871431	2021-09-02
hCoV-19/Japan/PG-89880/2021	EPI_ISL_3871432	2021-09-02
hCoV-19/Japan/PG-89881/2021	EPI_ISL_3871433	2021-09-02
hCoV-19/Japan/PG-89882/2021	EPI_ISL_3871434	2021-09-02
hCoV-19/Japan/PG-89883/2021	EPI_ISL_3871435	2021-09-02
hCoV-19/Japan/PG-89884/2021	EPI_ISL_3871436	2021-09-02

virus_name	epiID	submission_date
hCoV-19/Japan/PG-89885/2021	EPI_ISL_3871437	2021-09-02
hCoV-19/Japan/PG-89886/2021	EPI_ISL_3871438	2021-09-02
hCoV-19/Japan/PG-89887/2021	EPI_ISL_3871439	2021-09-02
hCoV-19/Japan/PG-89888/2021	EPI_ISL_3871440	2021-09-02
hCoV-19/Japan/PG-89889/2021	EPI_ISL_3871441	2021-09-02
hCoV-19/Japan/PG-89890/2021	EPI_ISL_3871442	2021-09-02
hCoV-19/Japan/PG-89900/2021	EPI_ISL_3871706	2021-09-02
hCoV-19/Japan/PG-89901/2021	EPI_ISL_3876195	2021-09-02
hCoV-19/Japan/PG-89902/2021	EPI_ISL_3876196	2021-09-02
hCoV-19/Japan/PG-89904/2021	EPI_ISL_3876197	2021-09-02
hCoV-19/Japan/PG-89906/2021	EPI_ISL_3876198	2021-09-02
hCoV-19/Japan/PG-89907/2021	EPI_ISL_3876199	2021-09-02
hCoV-19/Japan/PG-89908/2021	EPI_ISL_3876200	2021-09-02
hCoV-19/Japan/PG-89909/2021	EPI_ISL_3876202	2021-09-02
hCoV-19/Japan/PG-89910/2021	EPI_ISL_3876206	2021-09-02
hCoV-19/Japan/PG-89912/2021	EPI_ISL_3876209	2021-09-02
hCoV-19/Japan/PG-89913/2021	EPI_ISL_3876212	2021-09-02
hCoV-19/Japan/PG-89914/2021	EPI_ISL_3876215	2021-09-02
hCoV-19/Japan/PG-89915/2021	EPI_ISL_3876219	2021-09-02
hCoV-19/Japan/PG-89916/2021	EPI_ISL_3876221	2021-09-02
hCoV-19/Japan/PG-89917/2021	EPI_ISL_3876225	2021-09-02
hCoV-19/Japan/PG-89918/2021	EPI_ISL_3876228	2021-09-02
hCoV-19/Japan/PG-89919/2021	EPI_ISL_3876231	2021-09-02
hCoV-19/Japan/PG-89920/2021	EPI_ISL_3876235	2021-09-02
hCoV-19/Japan/PG-89921/2021	EPI_ISL_3876238	2021-09-02
hCoV-19/Japan/PG-89925/2021	EPI_ISL_3876241	2021-09-02
hCoV-19/Japan/PG-89926/2021	EPI_ISL_3876243	2021-09-02
hCoV-19/Japan/PG-89927/2021	EPI_ISL_3876244	2021-09-02
hCoV-19/Japan/PG-89928/2021	EPI_ISL_3876245	2021-09-02
hCoV-19/Japan/PG-89929/2021	EPI_ISL_3876246	2021-09-02
hCoV-19/Japan/PG-89930/2021	EPI_ISL_3876247	2021-09-02
hCoV-19/Japan/PG-89931/2021	EPI_ISL_3876248	2021-09-02
hCoV-19/Japan/PG-89932/2021	EPI_ISL_3876249	2021-09-02
hCoV-19/Japan/PG-89933/2021	EPI_ISL_3876250	2021-09-02
hCoV-19/Japan/PG-89935/2021	EPI_ISL_3876251	2021-09-02
hCoV-19/Japan/PG-89936/2021	EPI_ISL_3876252	2021-09-02
hCoV-19/Japan/PG-89937/2021	EPI_ISL_3876253	2021-09-02
hCoV-19/Japan/PG-89938/2021	EPI_ISL_3876254	2021-09-02
hCoV-19/Japan/PG-89939/2021	EPI_ISL_3876255	2021-09-02
hCoV-19/Japan/PG-89970/2021	EPI_ISL_3873487	2021-09-02
hCoV-19/Japan/PG-89971/2021	EPI_ISL_3873488	2021-09-02
hCoV-19/Japan/PG-89972/2021	EPI_ISL_3873489	2021-09-02
hCoV-19/Japan/PG-89973/2021	EPI_ISL_3873490	2021-09-02
hCoV-19/Japan/PG-89974/2021	EPI_ISL_3873491	2021-09-02
hCoV-19/Japan/PG-89975/2021	EPI_ISL_3873492	2021-09-02
hCoV-19/Japan/PG-89976/2021	EPI_ISL_3873493	2021-09-02
hCoV-19/Japan/PG-89977/2021	EPI_ISL_3873494	2021-09-02
hCoV-19/Japan/PG-89978/2021	EPI_ISL_3873495	2021-09-02
hCoV-19/Japan/PG-89979/2021	EPI_ISL_3873496	2021-09-02
hCoV-19/Japan/PG-89980/2021	EPI_ISL_3873497	2021-09-02
hCoV-19/Japan/PG-89981/2021	EPI_ISL_3873498	2021-09-02
hCoV-19/Japan/PG-89982/2021	EPI_ISL_3873499	2021-09-02
hCoV-19/Japan/PG-89983/2021	EPI_ISL_3873500	2021-09-02
hCoV-19/Japan/PG-89984/2021	EPI_ISL_3873501	2021-09-02

virus_name	epiID	submission_date
hCoV-19/Japan/PG-89989/2021	EPI_ISL_3872497	2021-09-02
hCoV-19/Japan/PG-89990/2021	EPI_ISL_3872498	2021-09-02
hCoV-19/Japan/PG-89991/2021	EPI_ISL_3872499	2021-09-02
hCoV-19/Japan/PG-89992/2021	EPI_ISL_3872500	2021-09-02
hCoV-19/Japan/PG-89993/2021	EPI_ISL_3872501	2021-09-02
hCoV-19/Japan/PG-89995/2021	EPI_ISL_3872502	2021-09-02
hCoV-19/Japan/PG-89996/2021	EPI_ISL_3872503	2021-09-02
hCoV-19/Japan/PG-89997/2021	EPI_ISL_3872504	2021-09-02
hCoV-19/Japan/PG-89998/2021	EPI_ISL_3872505	2021-09-02
hCoV-19/Japan/PG-89999/2021	EPI_ISL_3872506	2021-09-02
hCoV-19/Japan/PG-90001/2021	EPI_ISL_3872507	2021-09-02
hCoV-19/Japan/PG-90002/2021	EPI_ISL_3872508	2021-09-02
hCoV-19/Japan/PG-90005/2021	EPI_ISL_3872509	2021-09-02
hCoV-19/Japan/PG-90009/2021	EPI_ISL_3872510	2021-09-02
hCoV-19/Japan/PG-90039/2021	EPI_ISL_3873232	2021-09-02
hCoV-19/Japan/PG-90040/2021	EPI_ISL_3873233	2021-09-02
hCoV-19/Japan/PG-90041/2021	EPI_ISL_3873234	2021-09-02
hCoV-19/Japan/PG-90042/2021	EPI_ISL_3873235	2021-09-02
hCoV-19/Japan/PG-90043/2021	EPI_ISL_3873236	2021-09-02
hCoV-19/Japan/PG-90044/2021	EPI_ISL_3873237	2021-09-02
hCoV-19/Japan/PG-90045/2021	EPI_ISL_3873238	2021-09-02
hCoV-19/Japan/PG-90046/2021	EPI_ISL_3873239	2021-09-02
hCoV-19/Japan/PG-90047/2021	EPI_ISL_3873240	2021-09-02
hCoV-19/Japan/PG-90048/2021	EPI_ISL_3873241	2021-09-02
hCoV-19/Japan/PG-90049/2021	EPI_ISL_3873242	2021-09-02
hCoV-19/Japan/PG-90050/2021	EPI_ISL_3873243	2021-09-02
hCoV-19/Japan/PG-90051/2021	EPI_ISL_3873244	2021-09-02
hCoV-19/Japan/PG-90052/2021	EPI_ISL_3873245	2021-09-02
hCoV-19/Japan/PG-90053/2021	EPI_ISL_3873246	2021-09-02
hCoV-19/Japan/PG-90054/2021	EPI_ISL_3873247	2021-09-02
hCoV-19/Japan/PG-90055/2021	EPI_ISL_3873248	2021-09-02
hCoV-19/Japan/PG-90056/2021	EPI_ISL_3873249	2021-09-02
hCoV-19/Japan/PG-90057/2021	EPI_ISL_3873250	2021-09-02
hCoV-19/Japan/PG-90058/2021	EPI_ISL_3873251	2021-09-02
hCoV-19/Japan/PG-90059/2021	EPI_ISL_3873252	2021-09-02
hCoV-19/Japan/PG-90060/2021	EPI_ISL_3873253	2021-09-02
hCoV-19/Japan/PG-90070/2021	EPI_ISL_3873254	2021-09-02
hCoV-19/Japan/PG-90071/2021	EPI_ISL_3873255	2021-09-02
hCoV-19/Japan/PG-90072/2021	EPI_ISL_3873256	2021-09-02
hCoV-19/Japan/PG-90073/2021	EPI_ISL_3873257	2021-09-02
hCoV-19/Japan/PG-90074/2021	EPI_ISL_3873258	2021-09-02
hCoV-19/Japan/PG-90075/2021	EPI_ISL_3873259	2021-09-02
hCoV-19/Japan/PG-90076/2021	EPI_ISL_3873260	2021-09-02
hCoV-19/Japan/PG-90077/2021	EPI_ISL_3873261	2021-09-02
hCoV-19/Japan/PG-90078/2021	EPI_ISL_3873262	2021-09-02
hCoV-19/Japan/PG-90079/2021	EPI_ISL_3873263	2021-09-02
hCoV-19/Japan/PG-90080/2021	EPI_ISL_3873264	2021-09-02
hCoV-19/Japan/PG-90081/2021	EPI_ISL_3873265	2021-09-02
hCoV-19/Japan/PG-90082/2021	EPI_ISL_3873266	2021-09-02
hCoV-19/Japan/PG-90083/2021	EPI_ISL_3873267	2021-09-02
hCoV-19/Japan/PG-90101/2021	EPI_ISL_3873268	2021-09-02
hCoV-19/Japan/PG-90102/2021	EPI_ISL_3873269	2021-09-02
hCoV-19/Japan/PG-90103/2021	EPI_ISL_3873270	2021-09-02
hCoV-19/Japan/PG-90104/2021	EPI_ISL_3873271	2021-09-02

virus_name	epiID	submission_date
hCoV-19/Japan/PG-90108/2021	EPI_ISL_3873272	2021-09-02
hCoV-19/Japan/PG-90109/2021	EPI_ISL_3873273	2021-09-02
hCoV-19/Japan/PG-90110/2021	EPI_ISL_3871668	2021-09-02
hCoV-19/Japan/PG-90111/2021	EPI_ISL_3871669	2021-09-02
hCoV-19/Japan/PG-90112/2021	EPI_ISL_3871670	2021-09-02
hCoV-19/Japan/PG-90113/2021	EPI_ISL_3871671	2021-09-02
hCoV-19/Japan/PG-90114/2021	EPI_ISL_3871672	2021-09-02
hCoV-19/Japan/PG-90115/2021	EPI_ISL_3871673	2021-09-02
hCoV-19/Japan/PG-90116/2021	EPI_ISL_3871674	2021-09-02
hCoV-19/Japan/PG-90117/2021	EPI_ISL_3871675	2021-09-02
hCoV-19/Japan/PG-90118/2021	EPI_ISL_3871676	2021-09-02
hCoV-19/Japan/PG-90119/2021	EPI_ISL_3871677	2021-09-02
hCoV-19/Japan/PG-90120/2021	EPI_ISL_3871678	2021-09-02
hCoV-19/Japan/PG-90121/2021	EPI_ISL_3871679	2021-09-02
hCoV-19/Japan/PG-90122/2021	EPI_ISL_3871680	2021-09-02
hCoV-19/Japan/PG-90123/2021	EPI_ISL_3871544	2021-09-02
hCoV-19/Japan/PG-90124/2021	EPI_ISL_3871545	2021-09-02
hCoV-19/Japan/PG-90125/2021	EPI_ISL_3871546	2021-09-02
hCoV-19/Japan/PG-90126/2021	EPI_ISL_3871547	2021-09-02
hCoV-19/Japan/PG-90127/2021	EPI_ISL_3871548	2021-09-02
hCoV-19/Japan/PG-90128/2021	EPI_ISL_3871549	2021-09-02
hCoV-19/Japan/PG-90129/2021	EPI_ISL_3871550	2021-09-02
hCoV-19/Japan/PG-90130/2021	EPI_ISL_3871551	2021-09-02
hCoV-19/Japan/PG-90131/2021	EPI_ISL_3871552	2021-09-02
hCoV-19/Japan/PG-90132/2021	EPI_ISL_3871553	2021-09-02
hCoV-19/Japan/PG-90133/2021	EPI_ISL_3871554	2021-09-02
hCoV-19/Japan/PG-90134/2021	EPI_ISL_3871555	2021-09-02
hCoV-19/Japan/PG-90135/2021	EPI_ISL_3871556	2021-09-02
hCoV-19/Japan/PG-90136/2021	EPI_ISL_3871557	2021-09-02
hCoV-19/Japan/PG-90137/2021	EPI_ISL_3871558	2021-09-02
hCoV-19/Japan/PG-90138/2021	EPI_ISL_3871559	2021-09-02
hCoV-19/Japan/PG-90139/2021	EPI_ISL_3871560	2021-09-02
hCoV-19/Japan/PG-90140/2021	EPI_ISL_3871561	2021-09-02
hCoV-19/Japan/PG-90141/2021	EPI_ISL_3871562	2021-09-02
hCoV-19/Japan/PG-90142/2021	EPI_ISL_3874518	2021-09-02
hCoV-19/Japan/PG-90154/2021	EPI_ISL_3871120	2021-09-02
hCoV-19/Japan/PG-90155/2021	EPI_ISL_3871121	2021-09-02
hCoV-19/Japan/PG-90156/2021	EPI_ISL_3871122	2021-09-02
hCoV-19/Japan/PG-90157/2021	EPI_ISL_3871123	2021-09-02
hCoV-19/Japan/PG-90158/2021	EPI_ISL_3871124	2021-09-02
hCoV-19/Japan/PG-90159/2021	EPI_ISL_3871125	2021-09-02
hCoV-19/Japan/PG-90162/2021	EPI_ISL_3871126	2021-09-02
hCoV-19/Japan/PG-90164/2021	EPI_ISL_3871127	2021-09-02
hCoV-19/Japan/PG-90166/2021	EPI_ISL_3871128	2021-09-02
hCoV-19/Japan/PG-90167/2021	EPI_ISL_3871129	2021-09-02
hCoV-19/Japan/PG-90168/2021	EPI_ISL_3871130	2021-09-02
hCoV-19/Japan/PG-90169/2021	EPI_ISL_3871131	2021-09-02
hCoV-19/Japan/PG-90170/2021	EPI_ISL_3871132	2021-09-02
hCoV-19/Japan/PG-90171/2021	EPI_ISL_3871133	2021-09-02
hCoV-19/Japan/PG-90172/2021	EPI_ISL_3871134	2021-09-02
hCoV-19/Japan/PG-90173/2021	EPI_ISL_3871135	2021-09-02
hCoV-19/Japan/PG-90174/2021	EPI_ISL_3871136	2021-09-02
hCoV-19/Japan/PG-90175/2021	EPI_ISL_3871137	2021-09-02
hCoV-19/Japan/PG-90176/2021	EPI_ISL_3871138	2021-09-02

virus_name	epiID	submission_date
hCoV-19/Japan/PG-90203/2021	EPI_ISL_3874395	2021-09-02
hCoV-19/Japan/PG-90204/2021	EPI_ISL_3874396	2021-09-02
hCoV-19/Japan/PG-90218/2021	EPI_ISL_3874397	2021-09-02
hCoV-19/Japan/PG-90220/2021	EPI_ISL_3874398	2021-09-02
hCoV-19/Japan/PG-90224/2021	EPI_ISL_3872222	2021-09-02
hCoV-19/Japan/PG-90225/2021	EPI_ISL_3872223	2021-09-02
hCoV-19/Japan/PG-90226/2021	EPI_ISL_3872224	2021-09-02
hCoV-19/Japan/PG-90227/2021	EPI_ISL_3872225	2021-09-02
hCoV-19/Japan/PG-90228/2021	EPI_ISL_3872226	2021-09-02
hCoV-19/Japan/PG-90230/2021	EPI_ISL_3872227	2021-09-02
hCoV-19/Japan/PG-90231/2021	EPI_ISL_3872228	2021-09-02
hCoV-19/Japan/PG-90233/2021	EPI_ISL_3872229	2021-09-02
hCoV-19/Japan/PG-90234/2021	EPI_ISL_3872230	2021-09-02
hCoV-19/Japan/PG-90248/2021	EPI_ISL_3900853	2021-09-02
hCoV-19/Japan/PG-90249/2021	EPI_ISL_3900854	2021-09-02
hCoV-19/Japan/PG-90250/2021	EPI_ISL_3900855	2021-09-02
hCoV-19/Japan/PG-90251/2021	EPI_ISL_3900856	2021-09-02
hCoV-19/Japan/PG-90252/2021	EPI_ISL_3900857	2021-09-02
hCoV-19/Japan/PG-90253/2021	EPI_ISL_3900858	2021-09-02
hCoV-19/Japan/PG-90254/2021	EPI_ISL_3900859	2021-09-02
hCoV-19/Japan/PG-90255/2021	EPI_ISL_3900860	2021-09-02
hCoV-19/Japan/PG-90256/2021	EPI_ISL_3900861	2021-09-02
hCoV-19/Japan/PG-90257/2021	EPI_ISL_3900862	2021-09-02
hCoV-19/Japan/PG-90258/2021	EPI_ISL_3900863	2021-09-02
hCoV-19/Japan/PG-90259/2021	EPI_ISL_3900864	2021-09-02
hCoV-19/Japan/PG-90260/2021	EPI_ISL_3900865	2021-09-02
hCoV-19/Japan/PG-90261/2021	EPI_ISL_3900866	2021-09-02
hCoV-19/Japan/PG-90262/2021	EPI_ISL_3900867	2021-09-02
hCoV-19/Japan/PG-90263/2021	EPI_ISL_3900868	2021-09-02
hCoV-19/Japan/PG-90264/2021	EPI_ISL_3900869	2021-09-02
hCoV-19/Japan/PG-90265/2021	EPI_ISL_3900870	2021-09-02
hCoV-19/Japan/PG-90266/2021	EPI_ISL_3900871	2021-09-02
hCoV-19/Japan/PG-90267/2021	EPI_ISL_3900872	2021-09-02
hCoV-19/Japan/PG-90268/2021	EPI_ISL_3900873	2021-09-02
hCoV-19/Japan/PG-90269/2021	EPI_ISL_3900874	2021-09-02
hCoV-19/Japan/PG-90270/2021	EPI_ISL_3900875	2021-09-02
hCoV-19/Japan/PG-90271/2021	EPI_ISL_3900876	2021-09-02
hCoV-19/Japan/PG-90272/2021	EPI_ISL_3900877	2021-09-02
hCoV-19/Japan/PG-90273/2021	EPI_ISL_3900878	2021-09-02
hCoV-19/Japan/PG-90274/2021	EPI_ISL_3900879	2021-09-02
hCoV-19/Japan/PG-90275/2021	EPI_ISL_3900880	2021-09-02
hCoV-19/Japan/PG-90276/2021	EPI_ISL_3900881	2021-09-02
hCoV-19/Japan/PG-90277/2021	EPI_ISL_3900882	2021-09-02
hCoV-19/Japan/PG-90278/2021	EPI_ISL_3900883	2021-09-02
hCoV-19/Japan/PG-90279/2021	EPI_ISL_3900884	2021-09-02
hCoV-19/Japan/PG-90280/2021	EPI_ISL_3900885	2021-09-02
hCoV-19/Japan/PG-90281/2021	EPI_ISL_3900886	2021-09-02
hCoV-19/Japan/PG-90282/2021	EPI_ISL_3900887	2021-09-02
hCoV-19/Japan/PG-90283/2021	EPI_ISL_3900888	2021-09-02
hCoV-19/Japan/PG-90284/2021	EPI_ISL_3900889	2021-09-02
hCoV-19/Japan/PG-90285/2021	EPI_ISL_3900890	2021-09-02
hCoV-19/Japan/PG-90286/2021	EPI_ISL_3900891	2021-09-02
hCoV-19/Japan/PG-90287/2021	EPI_ISL_3900892	2021-09-02
hCoV-19/Japan/PG-90288/2021	EPI_ISL_3900893	2021-09-02

virus_name	epiID	submission_date
hCoV-19/Japan/PG-90289/2021	EPI_ISL_3900894	2021-09-02
hCoV-19/Japan/PG-90290/2021	EPI_ISL_3900895	2021-09-02
hCoV-19/Japan/PG-90291/2021	EPI_ISL_3900896	2021-09-02
hCoV-19/Japan/PG-90292/2021	EPI_ISL_3900897	2021-09-02
hCoV-19/Japan/PG-90293/2021	EPI_ISL_3900898	2021-09-02
hCoV-19/Japan/PG-90294/2021	EPI_ISL_3900899	2021-09-02
hCoV-19/Japan/PG-90295/2021	EPI_ISL_3900900	2021-09-02
hCoV-19/Japan/PG-90296/2021	EPI_ISL_3900901	2021-09-02
hCoV-19/Japan/PG-90297/2021	EPI_ISL_3900902	2021-09-02
hCoV-19/Japan/PG-90298/2021	EPI_ISL_3900903	2021-09-02
hCoV-19/Japan/PG-90299/2021	EPI_ISL_3900904	2021-09-02
hCoV-19/Japan/PG-90300/2021	EPI_ISL_3900905	2021-09-02
hCoV-19/Japan/PG-90301/2021	EPI_ISL_3900906	2021-09-02
hCoV-19/Japan/PG-90302/2021	EPI_ISL_3900907	2021-09-02
hCoV-19/Japan/PG-90303/2021	EPI_ISL_3900908	2021-09-02
hCoV-19/Japan/PG-90304/2021	EPI_ISL_3900909	2021-09-02
hCoV-19/Japan/PG-90305/2021	EPI_ISL_3900910	2021-09-02
hCoV-19/Japan/PG-90306/2021	EPI_ISL_3900911	2021-09-02
hCoV-19/Japan/PG-90307/2021	EPI_ISL_3900912	2021-09-02
hCoV-19/Japan/PG-90308/2021	EPI_ISL_3900913	2021-09-02
hCoV-19/Japan/PG-90309/2021	EPI_ISL_3900914	2021-09-02
hCoV-19/Japan/PG-90310/2021	EPI_ISL_3900915	2021-09-02
hCoV-19/Japan/PG-90311/2021	EPI_ISL_3900916	2021-09-02
hCoV-19/Japan/PG-90312/2021	EPI_ISL_3900917	2021-09-02
hCoV-19/Japan/PG-90313/2021	EPI_ISL_3900918	2021-09-02
hCoV-19/Japan/PG-90314/2021	EPI_ISL_3900919	2021-09-02
hCoV-19/Japan/PG-90315/2021	EPI_ISL_3900920	2021-09-02
hCoV-19/Japan/PG-90316/2021	EPI_ISL_3900921	2021-09-02
hCoV-19/Japan/PG-90317/2021	EPI_ISL_3900922	2021-09-02
hCoV-19/Japan/PG-90318/2021	EPI_ISL_3900923	2021-09-02
hCoV-19/Japan/PG-90319/2021	EPI_ISL_3900924	2021-09-02
hCoV-19/Japan/PG-90320/2021	EPI_ISL_3900925	2021-09-02
hCoV-19/Japan/PG-90321/2021	EPI_ISL_3900926	2021-09-02
hCoV-19/Japan/PG-90322/2021	EPI_ISL_3900927	2021-09-02
hCoV-19/Japan/PG-90323/2021	EPI_ISL_3900928	2021-09-02
hCoV-19/Japan/PG-90324/2021	EPI_ISL_3900929	2021-09-02
hCoV-19/Japan/PG-90325/2021	EPI_ISL_3900930	2021-09-02
hCoV-19/Japan/PG-90326/2021	EPI_ISL_3900931	2021-09-02
hCoV-19/Japan/PG-90327/2021	EPI_ISL_3900932	2021-09-02
hCoV-19/Japan/PG-90328/2021	EPI_ISL_3900933	2021-09-02
hCoV-19/Japan/PG-90329/2021	EPI_ISL_3900934	2021-09-02
hCoV-19/Japan/PG-90330/2021	EPI_ISL_3900935	2021-09-02
hCoV-19/Japan/PG-90331/2021	EPI_ISL_3900936	2021-09-02
hCoV-19/Japan/PG-90332/2021	EPI_ISL_3900937	2021-09-02
hCoV-19/Japan/PG-90333/2021	EPI_ISL_3900938	2021-09-02
hCoV-19/Japan/PG-90334/2021	EPI_ISL_3900939	2021-09-02
hCoV-19/Japan/PG-90335/2021	EPI_ISL_3900940	2021-09-02
hCoV-19/Japan/PG-90336/2021	EPI_ISL_3900941	2021-09-02
hCoV-19/Japan/PG-90337/2021	EPI_ISL_3900942	2021-09-02
hCoV-19/Japan/PG-90338/2021	EPI_ISL_3900943	2021-09-02
hCoV-19/Japan/PG-90339/2021	EPI_ISL_3900944	2021-09-02
hCoV-19/Japan/PG-90340/2021	EPI_ISL_3900945	2021-09-02
hCoV-19/Japan/PG-90341/2021	EPI_ISL_3900946	2021-09-02
hCoV-19/Japan/PG-90342/2021	EPI_ISL_3900947	2021-09-02

virus_name	epiID	submission_date
hCoV-19/Japan/PG-90343/2021	EPI_ISL_3900948	2021-09-02
hCoV-19/Japan/PG-90344/2021	EPI_ISL_3900949	2021-09-02
hCoV-19/Japan/PG-90345/2021	EPI_ISL_3900950	2021-09-02
hCoV-19/Japan/PG-90346/2021	EPI_ISL_3900951	2021-09-02
hCoV-19/Japan/PG-90347/2021	EPI_ISL_3900952	2021-09-02
hCoV-19/Japan/PG-90348/2021	EPI_ISL_3900953	2021-09-02
hCoV-19/Japan/PG-90349/2021	EPI_ISL_3900954	2021-09-02
hCoV-19/Japan/PG-90350/2021	EPI_ISL_3900955	2021-09-02
hCoV-19/Japan/PG-90351/2021	EPI_ISL_3900956	2021-09-02
hCoV-19/Japan/PG-90352/2021	EPI_ISL_3900957	2021-09-02
hCoV-19/Japan/PG-90353/2021	EPI_ISL_3900958	2021-09-02
hCoV-19/Japan/PG-90354/2021	EPI_ISL_3900959	2021-09-02
hCoV-19/Japan/PG-90355/2021	EPI_ISL_3900960	2021-09-02
hCoV-19/Japan/PG-90356/2021	EPI_ISL_3900961	2021-09-02
hCoV-19/Japan/PG-90357/2021	EPI_ISL_3900962	2021-09-02
hCoV-19/Japan/PG-90358/2021	EPI_ISL_3900963	2021-09-02
hCoV-19/Japan/PG-90359/2021	EPI_ISL_3900964	2021-09-02
hCoV-19/Japan/PG-90360/2021	EPI_ISL_3900965	2021-09-02
hCoV-19/Japan/PG-90361/2021	EPI_ISL_3900966	2021-09-02
hCoV-19/Japan/PG-90362/2021	EPI_ISL_3900967	2021-09-02
hCoV-19/Japan/PG-90363/2021	EPI_ISL_3900968	2021-09-02
hCoV-19/Japan/PG-90364/2021	EPI_ISL_3900969	2021-09-02
hCoV-19/Japan/PG-90365/2021	EPI_ISL_3900970	2021-09-02
hCoV-19/Japan/PG-90366/2021	EPI_ISL_3900971	2021-09-02
hCoV-19/Japan/PG-90367/2021	EPI_ISL_3900972	2021-09-02
hCoV-19/Japan/PG-90368/2021	EPI_ISL_3900973	2021-09-02
hCoV-19/Japan/PG-90369/2021	EPI_ISL_3900974	2021-09-02
hCoV-19/Japan/PG-90370/2021	EPI_ISL_3900975	2021-09-02
hCoV-19/Japan/PG-90371/2021	EPI_ISL_3900976	2021-09-02
hCoV-19/Japan/PG-90372/2021	EPI_ISL_3900977	2021-09-02
hCoV-19/Japan/PG-90373/2021	EPI_ISL_3900978	2021-09-02
hCoV-19/Japan/PG-90374/2021	EPI_ISL_3900979	2021-09-02
hCoV-19/Japan/PG-90375/2021	EPI_ISL_3900980	2021-09-02
hCoV-19/Japan/PG-90376/2021	EPI_ISL_3900981	2021-09-02
hCoV-19/Japan/PG-90377/2021	EPI_ISL_3900982	2021-09-02
hCoV-19/Japan/PG-90378/2021	EPI_ISL_3900983	2021-09-02
hCoV-19/Japan/PG-90379/2021	EPI_ISL_3900984	2021-09-02
hCoV-19/Japan/PG-90380/2021	EPI_ISL_3900985	2021-09-02
hCoV-19/Japan/PG-90384/2021	EPI_ISL_3900986	2021-09-02
hCoV-19/Japan/PG-90385/2021	EPI_ISL_3900987	2021-09-02
hCoV-19/Japan/PG-90386/2021	EPI_ISL_3900988	2021-09-02
hCoV-19/Japan/PG-90387/2021	EPI_ISL_3900989	2021-09-02
hCoV-19/Japan/PG-90388/2021	EPI_ISL_3900990	2021-09-02
hCoV-19/Japan/PG-90389/2021	EPI_ISL_3900991	2021-09-02
hCoV-19/Japan/PG-90390/2021	EPI_ISL_3900992	2021-09-02
hCoV-19/Japan/PG-90391/2021	EPI_ISL_3900993	2021-09-02
hCoV-19/Japan/PG-90392/2021	EPI_ISL_3900994	2021-09-02
hCoV-19/Japan/PG-90393/2021	EPI_ISL_3900995	2021-09-02
hCoV-19/Japan/PG-90394/2021	EPI_ISL_3900996	2021-09-02
hCoV-19/Japan/PG-90395/2021	EPI_ISL_3900997	2021-09-02
hCoV-19/Japan/PG-90396/2021	EPI_ISL_3900998	2021-09-02
hCoV-19/Japan/PG-90397/2021	EPI_ISL_3900999	2021-09-02
hCoV-19/Japan/PG-90398/2021	EPI_ISL_3901000	2021-09-02
hCoV-19/Japan/PG-90399/2021	EPI_ISL_3901001	2021-09-02

virus_name	epiID	submission_date
hCoV-19/Japan/PG-90400/2021	EPI_ISL_3901002	2021-09-02
hCoV-19/Japan/PG-90401/2021	EPI_ISL_3901003	2021-09-02
hCoV-19/Japan/PG-90402/2021	EPI_ISL_3901004	2021-09-02
hCoV-19/Japan/PG-90406/2021	EPI_ISL_3901005	2021-09-02
hCoV-19/Japan/PG-90407/2021	EPI_ISL_3901006	2021-09-02
hCoV-19/Japan/PG-90408/2021	EPI_ISL_3901007	2021-09-02
hCoV-19/Japan/PG-90409/2021	EPI_ISL_3901008	2021-09-02
hCoV-19/Japan/PG-90410/2021	EPI_ISL_3901009	2021-09-02
hCoV-19/Japan/PG-90411/2021	EPI_ISL_3901010	2021-09-02
hCoV-19/Japan/PG-90412/2021	EPI_ISL_3901011	2021-09-02
hCoV-19/Japan/PG-90413/2021	EPI_ISL_3901012	2021-09-02
hCoV-19/Japan/PG-90419/2021	EPI_ISL_3901013	2021-09-02
hCoV-19/Japan/PG-90420/2021	EPI_ISL_3901014	2021-09-02
hCoV-19/Japan/PG-90421/2021	EPI_ISL_3901015	2021-09-02
hCoV-19/Japan/PG-90422/2021	EPI_ISL_3901016	2021-09-02
hCoV-19/Japan/PG-90423/2021	EPI_ISL_3901017	2021-09-02
hCoV-19/Japan/PG-90424/2021	EPI_ISL_3901018	2021-09-02
hCoV-19/Japan/PG-90425/2021	EPI_ISL_3901019	2021-09-02
hCoV-19/Japan/PG-90426/2021	EPI_ISL_3901020	2021-09-02
hCoV-19/Japan/PG-90427/2021	EPI_ISL_3901021	2021-09-02
hCoV-19/Japan/PG-90428/2021	EPI_ISL_3901022	2021-09-02
hCoV-19/Japan/PG-90429/2021	EPI_ISL_3901023	2021-09-02
hCoV-19/Japan/PG-90430/2021	EPI_ISL_3901024	2021-09-02
hCoV-19/Japan/PG-90431/2021	EPI_ISL_3901025	2021-09-02
hCoV-19/Japan/PG-90432/2021	EPI_ISL_3901026	2021-09-02
hCoV-19/Japan/PG-90433/2021	EPI_ISL_3901027	2021-09-02
hCoV-19/Japan/PG-90434/2021	EPI_ISL_3901028	2021-09-02
hCoV-19/Japan/PG-90435/2021	EPI_ISL_3901029	2021-09-02
hCoV-19/Japan/PG-90436/2021	EPI_ISL_3901030	2021-09-02
hCoV-19/Japan/PG-90437/2021	EPI_ISL_3901031	2021-09-02
hCoV-19/Japan/PG-90438/2021	EPI_ISL_3901032	2021-09-02
hCoV-19/Japan/PG-90441/2021	EPI_ISL_3901033	2021-09-02
hCoV-19/Japan/PG-90442/2021	EPI_ISL_3901034	2021-09-02
hCoV-19/Japan/PG-90443/2021	EPI_ISL_3901035	2021-09-02
hCoV-19/Japan/PG-90444/2021	EPI_ISL_3901036	2021-09-02
hCoV-19/Japan/PG-90445/2021	EPI_ISL_3901037	2021-09-02
hCoV-19/Japan/PG-90446/2021	EPI_ISL_3901038	2021-09-02
hCoV-19/Japan/PG-90447/2021	EPI_ISL_3901039	2021-09-02
hCoV-19/Japan/PG-90448/2021	EPI_ISL_3901040	2021-09-02
hCoV-19/Japan/PG-90449/2021	EPI_ISL_3901041	2021-09-02
hCoV-19/Japan/PG-90450/2021	EPI_ISL_3901042	2021-09-02
hCoV-19/Japan/PG-90451/2021	EPI_ISL_3901043	2021-09-02
hCoV-19/Japan/PG-90452/2021	EPI_ISL_3901044	2021-09-02
hCoV-19/Japan/PG-90453/2021	EPI_ISL_3901045	2021-09-02
hCoV-19/Japan/PG-90489/2021	EPI_ISL_3901046	2021-09-02
hCoV-19/Japan/PG-90490/2021	EPI_ISL_3901047	2021-09-02
hCoV-19/Japan/PG-90491/2021	EPI_ISL_3901048	2021-09-02
hCoV-19/Japan/PG-90492/2021	EPI_ISL_3901049	2021-09-02
hCoV-19/Japan/PG-90493/2021	EPI_ISL_3901050	2021-09-02
hCoV-19/Japan/PG-90494/2021	EPI_ISL_3901051	2021-09-02
hCoV-19/Japan/PG-90495/2021	EPI_ISL_3901052	2021-09-02
hCoV-19/Japan/PG-90496/2021	EPI_ISL_3901053	2021-09-02
hCoV-19/Japan/PG-90497/2021	EPI_ISL_3901054	2021-09-02
hCoV-19/Japan/PG-90498/2021	EPI_ISL_3901055	2021-09-02

virus_name	epiID	submission_date
hCoV-19/Japan/PG-90499/2021	EPI_ISL_3901056	2021-09-02
hCoV-19/Japan/PG-90500/2021	EPI_ISL_3901057	2021-09-02
hCoV-19/Japan/PG-90501/2021	EPI_ISL_3901058	2021-09-02
hCoV-19/Japan/PG-90502/2021	EPI_ISL_3901059	2021-09-02
hCoV-19/Japan/PG-90503/2021	EPI_ISL_3901060	2021-09-02
hCoV-19/Japan/PG-90504/2021	EPI_ISL_3901061	2021-09-02
hCoV-19/Japan/PG-90505/2021	EPI_ISL_3901062	2021-09-02
hCoV-19/Japan/PG-90506/2021	EPI_ISL_3901063	2021-09-02
hCoV-19/Japan/PG-90507/2021	EPI_ISL_3901064	2021-09-02
hCoV-19/Japan/PG-90508/2021	EPI_ISL_3901065	2021-09-02
hCoV-19/Japan/PG-90509/2021	EPI_ISL_3901066	2021-09-02
hCoV-19/Japan/PG-90510/2021	EPI_ISL_3901067	2021-09-02
hCoV-19/Japan/PG-90511/2021	EPI_ISL_3901068	2021-09-02
hCoV-19/Japan/PG-90512/2021	EPI_ISL_3901069	2021-09-02
hCoV-19/Japan/PG-90513/2021	EPI_ISL_3901070	2021-09-02
hCoV-19/Japan/PG-90514/2021	EPI_ISL_3901071	2021-09-02
hCoV-19/Japan/PG-90515/2021	EPI_ISL_3901072	2021-09-02
hCoV-19/Japan/PG-90516/2021	EPI_ISL_3901073	2021-09-02
hCoV-19/Japan/PG-90517/2021	EPI_ISL_3901074	2021-09-02
hCoV-19/Japan/PG-90518/2021	EPI_ISL_3901075	2021-09-02
hCoV-19/Japan/PG-90519/2021	EPI_ISL_3901076	2021-09-02
hCoV-19/Japan/PG-90520/2021	EPI_ISL_3901077	2021-09-02
hCoV-19/Japan/PG-90521/2021	EPI_ISL_3901078	2021-09-02
hCoV-19/Japan/PG-90522/2021	EPI_ISL_3901079	2021-09-02
hCoV-19/Japan/PG-90523/2021	EPI_ISL_3901080	2021-09-02
hCoV-19/Japan/PG-90524/2021	EPI_ISL_3901081	2021-09-02
hCoV-19/Japan/PG-90525/2021	EPI_ISL_3901082	2021-09-02
hCoV-19/Japan/PG-90526/2021	EPI_ISL_3901083	2021-09-02
hCoV-19/Japan/PG-90527/2021	EPI_ISL_3901084	2021-09-02
hCoV-19/Japan/PG-90528/2021	EPI_ISL_3901085	2021-09-02
hCoV-19/Japan/PG-90529/2021	EPI_ISL_3901086	2021-09-02
hCoV-19/Japan/PG-90530/2021	EPI_ISL_3901087	2021-09-02
hCoV-19/Japan/PG-90531/2021	EPI_ISL_3901088	2021-09-02
hCoV-19/Japan/PG-90532/2021	EPI_ISL_3901089	2021-09-02
hCoV-19/Japan/PG-90533/2021	EPI_ISL_3901090	2021-09-02
hCoV-19/Japan/PG-90534/2021	EPI_ISL_3901091	2021-09-02
hCoV-19/Japan/PG-90535/2021	EPI_ISL_3901092	2021-09-02
hCoV-19/Japan/PG-90536/2021	EPI_ISL_3901093	2021-09-02
hCoV-19/Japan/PG-90537/2021	EPI_ISL_3901094	2021-09-02
hCoV-19/Japan/PG-90538/2021	EPI_ISL_3901095	2021-09-02
hCoV-19/Japan/PG-90539/2021	EPI_ISL_3901096	2021-09-02
hCoV-19/Japan/PG-90540/2021	EPI_ISL_3901097	2021-09-02
hCoV-19/Japan/PG-90541/2021	EPI_ISL_3901098	2021-09-02
hCoV-19/Japan/PG-90542/2021	EPI_ISL_3901099	2021-09-02
hCoV-19/Japan/PG-90543/2021	EPI_ISL_3901100	2021-09-02
hCoV-19/Japan/PG-90544/2021	EPI_ISL_3901101	2021-09-02
hCoV-19/Japan/PG-90545/2021	EPI_ISL_3901102	2021-09-02
hCoV-19/Japan/PG-90546/2021	EPI_ISL_3901103	2021-09-02
hCoV-19/Japan/PG-90547/2021	EPI_ISL_3901104	2021-09-02
hCoV-19/Japan/PG-90548/2021	EPI_ISL_3901105	2021-09-02
hCoV-19/Japan/PG-90549/2021	EPI_ISL_3901106	2021-09-02
hCoV-19/Japan/PG-90550/2021	EPI_ISL_3901107	2021-09-02
hCoV-19/Japan/PG-90551/2021	EPI_ISL_3901108	2021-09-02
hCoV-19/Japan/PG-90552/2021	EPI_ISL_3901109	2021-09-02

virus_name	epiID	submission_date
hCoV-19/Japan/PG-90553/2021	EPI_ISL_3901110	2021-09-02
hCoV-19/Japan/PG-90554/2021	EPI_ISL_3901111	2021-09-02
hCoV-19/Japan/PG-90555/2021	EPI_ISL_3901112	2021-09-02
hCoV-19/Japan/PG-90556/2021	EPI_ISL_3901113	2021-09-02
hCoV-19/Japan/PG-90557/2021	EPI_ISL_3901114	2021-09-02
hCoV-19/Japan/PG-90558/2021	EPI_ISL_3901115	2021-09-02
hCoV-19/Japan/PG-90559/2021	EPI_ISL_3901116	2021-09-02
hCoV-19/Japan/PG-90560/2021	EPI_ISL_3901117	2021-09-02
hCoV-19/Japan/PG-90561/2021	EPI_ISL_3901118	2021-09-02
hCoV-19/Japan/PG-90562/2021	EPI_ISL_3901119	2021-09-02
hCoV-19/Japan/PG-90563/2021	EPI_ISL_3901120	2021-09-02
hCoV-19/Japan/PG-90564/2021	EPI_ISL_3901121	2021-09-02
hCoV-19/Japan/PG-90565/2021	EPI_ISL_3901122	2021-09-02
hCoV-19/Japan/PG-90566/2021	EPI_ISL_3901123	2021-09-02
hCoV-19/Japan/PG-90567/2021	EPI_ISL_3901124	2021-09-02
hCoV-19/Japan/PG-90568/2021	EPI_ISL_3901125	2021-09-02
hCoV-19/Japan/PG-90569/2021	EPI_ISL_3901126	2021-09-02
hCoV-19/Japan/PG-90570/2021	EPI_ISL_3901127	2021-09-02
hCoV-19/Japan/PG-90571/2021	EPI_ISL_3901128	2021-09-02
hCoV-19/Japan/PG-90572/2021	EPI_ISL_3901129	2021-09-02
hCoV-19/Japan/PG-90573/2021	EPI_ISL_3901130	2021-09-02
hCoV-19/Japan/PG-90574/2021	EPI_ISL_3901131	2021-09-02
hCoV-19/Japan/PG-90575/2021	EPI_ISL_3901132	2021-09-02
hCoV-19/Japan/PG-90576/2021	EPI_ISL_3901133	2021-09-02
hCoV-19/Japan/PG-90577/2021	EPI_ISL_3901134	2021-09-02
hCoV-19/Japan/PG-90578/2021	EPI_ISL_3901135	2021-09-02
hCoV-19/Japan/PG-90579/2021	EPI_ISL_3901136	2021-09-02
hCoV-19/Japan/PG-90580/2021	EPI_ISL_3901137	2021-09-02
hCoV-19/Japan/PG-90581/2021	EPI_ISL_3901138	2021-09-02
hCoV-19/Japan/PG-90582/2021	EPI_ISL_3901139	2021-09-02
hCoV-19/Japan/PG-90583/2021	EPI_ISL_3901140	2021-09-02
hCoV-19/Japan/PG-90584/2021	EPI_ISL_3901141	2021-09-02
hCoV-19/Japan/PG-90585/2021	EPI_ISL_3901142	2021-09-02
hCoV-19/Japan/PG-90586/2021	EPI_ISL_3901143	2021-09-02
hCoV-19/Japan/PG-90587/2021	EPI_ISL_3901144	2021-09-02
hCoV-19/Japan/PG-90588/2021	EPI_ISL_3901145	2021-09-02
hCoV-19/Japan/PG-90589/2021	EPI_ISL_3901146	2021-09-02
hCoV-19/Japan/PG-90590/2021	EPI_ISL_3901147	2021-09-02
hCoV-19/Japan/PG-90591/2021	EPI_ISL_3901148	2021-09-02
hCoV-19/Japan/PG-90592/2021	EPI_ISL_3901149	2021-09-02
hCoV-19/Japan/PG-90593/2021	EPI_ISL_3901150	2021-09-02
hCoV-19/Japan/PG-90594/2021	EPI_ISL_3901151	2021-09-02
hCoV-19/Japan/PG-90595/2021	EPI_ISL_3901152	2021-09-02
hCoV-19/Japan/PG-90596/2021	EPI_ISL_3901153	2021-09-02
hCoV-19/Japan/PG-90597/2021	EPI_ISL_3901154	2021-09-02
hCoV-19/Japan/PG-90598/2021	EPI_ISL_3901155	2021-09-02
hCoV-19/Japan/PG-90599/2021	EPI_ISL_3901156	2021-09-02
hCoV-19/Japan/PG-90600/2021	EPI_ISL_3901157	2021-09-02
hCoV-19/Japan/PG-90601/2021	EPI_ISL_3901158	2021-09-02
hCoV-19/Japan/PG-90602/2021	EPI_ISL_3901159	2021-09-02
hCoV-19/Japan/PG-90603/2021	EPI_ISL_3901160	2021-09-02
hCoV-19/Japan/PG-90604/2021	EPI_ISL_3901161	2021-09-02
hCoV-19/Japan/PG-90605/2021	EPI_ISL_3901162	2021-09-02
hCoV-19/Japan/PG-90606/2021	EPI_ISL_3901163	2021-09-02

virus_name	epiID	submission_date
hCoV-19/Japan/PG-90607/2021	EPI_ISL_3901164	2021-09-02
hCoV-19/Japan/PG-90608/2021	EPI_ISL_3901165	2021-09-02
hCoV-19/Japan/PG-90609/2021	EPI_ISL_3901166	2021-09-02
hCoV-19/Japan/PG-90610/2021	EPI_ISL_3901167	2021-09-02
hCoV-19/Japan/PG-90611/2021	EPI_ISL_3901168	2021-09-02
hCoV-19/Japan/PG-90612/2021	EPI_ISL_3901169	2021-09-02
hCoV-19/Japan/PG-90613/2021	EPI_ISL_3901170	2021-09-02
hCoV-19/Japan/PG-90614/2021	EPI_ISL_3901171	2021-09-02
hCoV-19/Japan/PG-90615/2021	EPI_ISL_3901172	2021-09-02
hCoV-19/Japan/PG-90616/2021	EPI_ISL_3901173	2021-09-02
hCoV-19/Japan/PG-90617/2021	EPI_ISL_3901174	2021-09-02
hCoV-19/Japan/PG-90618/2021	EPI_ISL_3901175	2021-09-02
hCoV-19/Japan/PG-90619/2021	EPI_ISL_3901176	2021-09-02
hCoV-19/Japan/PG-90620/2021	EPI_ISL_3901177	2021-09-02
hCoV-19/Japan/PG-90621/2021	EPI_ISL_3901178	2021-09-02
hCoV-19/Japan/PG-90622/2021	EPI_ISL_3901179	2021-09-02
hCoV-19/Japan/PG-90623/2021	EPI_ISL_3901180	2021-09-02
hCoV-19/Japan/PG-90624/2021	EPI_ISL_3901181	2021-09-02
hCoV-19/Japan/PG-90625/2021	EPI_ISL_3901182	2021-09-02
hCoV-19/Japan/PG-90626/2021	EPI_ISL_3901183	2021-09-02
hCoV-19/Japan/PG-90627/2021	EPI_ISL_3901184	2021-09-02
hCoV-19/Japan/PG-90628/2021	EPI_ISL_3901185	2021-09-02
hCoV-19/Japan/PG-90629/2021	EPI_ISL_3901186	2021-09-02
hCoV-19/Japan/PG-90630/2021	EPI_ISL_3901187	2021-09-02
hCoV-19/Japan/PG-90631/2021	EPI_ISL_3901188	2021-09-02
hCoV-19/Japan/PG-90632/2021	EPI_ISL_3901189	2021-09-02
hCoV-19/Japan/PG-90633/2021	EPI_ISL_3901190	2021-09-02
hCoV-19/Japan/PG-90634/2021	EPI_ISL_3901191	2021-09-02
hCoV-19/Japan/PG-90635/2021	EPI_ISL_3901192	2021-09-02
hCoV-19/Japan/PG-90636/2021	EPI_ISL_3901193	2021-09-02
hCoV-19/Japan/PG-90637/2021	EPI_ISL_3901194	2021-09-02
hCoV-19/Japan/PG-90638/2021	EPI_ISL_3901195	2021-09-02
hCoV-19/Japan/PG-90639/2021	EPI_ISL_3901196	2021-09-02
hCoV-19/Japan/PG-90640/2021	EPI_ISL_3901197	2021-09-02
hCoV-19/Japan/PG-90641/2021	EPI_ISL_3901198	2021-09-02
hCoV-19/Japan/PG-90642/2021	EPI_ISL_3901199	2021-09-02
hCoV-19/Japan/PG-90643/2021	EPI_ISL_3901200	2021-09-02
hCoV-19/Japan/PG-90644/2021	EPI_ISL_3901201	2021-09-02
hCoV-19/Japan/PG-90645/2021	EPI_ISL_3901202	2021-09-02
hCoV-19/Japan/PG-90646/2021	EPI_ISL_3901203	2021-09-02
hCoV-19/Japan/PG-90647/2021	EPI_ISL_3901204	2021-09-02
hCoV-19/Japan/PG-90648/2021	EPI_ISL_3901205	2021-09-02
hCoV-19/Japan/PG-90649/2021	EPI_ISL_3901206	2021-09-02
hCoV-19/Japan/PG-90650/2021	EPI_ISL_3901207	2021-09-02
hCoV-19/Japan/PG-90651/2021	EPI_ISL_3901208	2021-09-02
hCoV-19/Japan/PG-90652/2021	EPI_ISL_3901209	2021-09-02
hCoV-19/Japan/PG-90653/2021	EPI_ISL_3901210	2021-09-02
hCoV-19/Japan/PG-90654/2021	EPI_ISL_3901211	2021-09-02
hCoV-19/Japan/PG-90655/2021	EPI_ISL_3901212	2021-09-02
hCoV-19/Japan/PG-90656/2021	EPI_ISL_3901213	2021-09-02
hCoV-19/Japan/PG-90657/2021	EPI_ISL_3901214	2021-09-02
hCoV-19/Japan/PG-90658/2021	EPI_ISL_3901215	2021-09-02
hCoV-19/Japan/PG-90659/2021	EPI_ISL_3901216	2021-09-02
hCoV-19/Japan/PG-90660/2021	EPI_ISL_3901217	2021-09-02

virus_name	epiID	submission_date
hCoV-19/Japan/PG-90661/2021	EPI_ISL_3901218	2021-09-02
hCoV-19/Japan/PG-90662/2021	EPI_ISL_3901219	2021-09-02
hCoV-19/Japan/PG-90663/2021	EPI_ISL_3901220	2021-09-02
hCoV-19/Japan/PG-90664/2021	EPI_ISL_3901221	2021-09-02
hCoV-19/Japan/PG-90665/2021	EPI_ISL_3901222	2021-09-02
hCoV-19/Japan/PG-90666/2021	EPI_ISL_3901223	2021-09-02
hCoV-19/Japan/PG-90667/2021	EPI_ISL_3901224	2021-09-02
hCoV-19/Japan/PG-90668/2021	EPI_ISL_3901225	2021-09-02
hCoV-19/Japan/PG-90669/2021	EPI_ISL_3901226	2021-09-02
hCoV-19/Japan/PG-90670/2021	EPI_ISL_3901227	2021-09-02
hCoV-19/Japan/PG-90671/2021	EPI_ISL_3901228	2021-09-02
hCoV-19/Japan/PG-90672/2021	EPI_ISL_3901229	2021-09-02
hCoV-19/Japan/PG-90673/2021	EPI_ISL_3901230	2021-09-02
hCoV-19/Japan/PG-90674/2021	EPI_ISL_3901231	2021-09-02
hCoV-19/Japan/PG-90675/2021	EPI_ISL_3901232	2021-09-02
hCoV-19/Japan/PG-90676/2021	EPI_ISL_3901233	2021-09-02
hCoV-19/Japan/PG-90677/2021	EPI_ISL_3901234	2021-09-02
hCoV-19/Japan/PG-90678/2021	EPI_ISL_3901235	2021-09-02
hCoV-19/Japan/PG-90679/2021	EPI_ISL_3901236	2021-09-02
hCoV-19/Japan/PG-90680/2021	EPI_ISL_3901237	2021-09-02
hCoV-19/Japan/PG-90681/2021	EPI_ISL_3901238	2021-09-02
hCoV-19/Japan/PG-90682/2021	EPI_ISL_3901239	2021-09-02
hCoV-19/Japan/PG-90683/2021	EPI_ISL_3901240	2021-09-02
hCoV-19/Japan/PG-90684/2021	EPI_ISL_3901241	2021-09-02
hCoV-19/Japan/PG-90685/2021	EPI_ISL_3901242	2021-09-02
hCoV-19/Japan/PG-90686/2021	EPI_ISL_3901243	2021-09-02
hCoV-19/Japan/PG-90687/2021	EPI_ISL_3901244	2021-09-02
hCoV-19/Japan/PG-90688/2021	EPI_ISL_3901245	2021-09-02
hCoV-19/Japan/PG-90689/2021	EPI_ISL_3901246	2021-09-02
hCoV-19/Japan/PG-90690/2021	EPI_ISL_3901247	2021-09-02
hCoV-19/Japan/PG-90691/2021	EPI_ISL_3901248	2021-09-02
hCoV-19/Japan/PG-90692/2021	EPI_ISL_3901249	2021-09-02
hCoV-19/Japan/PG-90693/2021	EPI_ISL_3901250	2021-09-02
hCoV-19/Japan/PG-90694/2021	EPI_ISL_3901251	2021-09-02
hCoV-19/Japan/PG-90695/2021	EPI_ISL_3901252	2021-09-02
hCoV-19/Japan/PG-90696/2021	EPI_ISL_3901253	2021-09-02
hCoV-19/Japan/PG-90697/2021	EPI_ISL_3901254	2021-09-02
hCoV-19/Japan/PG-90698/2021	EPI_ISL_3901255	2021-09-02
hCoV-19/Japan/PG-90699/2021	EPI_ISL_3901256	2021-09-02
hCoV-19/Japan/PG-90700/2021	EPI_ISL_3901257	2021-09-02
hCoV-19/Japan/PG-90701/2021	EPI_ISL_3901258	2021-09-02
hCoV-19/Japan/PG-90702/2021	EPI_ISL_3901259	2021-09-02
hCoV-19/Japan/PG-90703/2021	EPI_ISL_3901260	2021-09-02
hCoV-19/Japan/PG-90704/2021	EPI_ISL_3901261	2021-09-02
hCoV-19/Japan/PG-90705/2021	EPI_ISL_3901262	2021-09-02
hCoV-19/Japan/PG-90706/2021	EPI_ISL_3901263	2021-09-02
hCoV-19/Japan/PG-90707/2021	EPI_ISL_3901264	2021-09-02
hCoV-19/Japan/PG-90708/2021	EPI_ISL_3901265	2021-09-02
hCoV-19/Japan/PG-90709/2021	EPI_ISL_3901266	2021-09-02
hCoV-19/Japan/PG-90710/2021	EPI_ISL_3901267	2021-09-02
hCoV-19/Japan/PG-90711/2021	EPI_ISL_3901268	2021-09-02
hCoV-19/Japan/PG-90712/2021	EPI_ISL_3901269	2021-09-02
hCoV-19/Japan/PG-90713/2021	EPI_ISL_3901270	2021-09-02
hCoV-19/Japan/PG-90714/2021	EPI_ISL_3901271	2021-09-02

virus_name	epiID	submission_date
hCoV-19/Japan/PG-90715/2021	EPI_ISL_3901272	2021-09-02
hCoV-19/Japan/PG-90716/2021	EPI_ISL_3901273	2021-09-02
hCoV-19/Japan/PG-90717/2021	EPI_ISL_3901274	2021-09-02
hCoV-19/Japan/PG-90718/2021	EPI_ISL_3901275	2021-09-02
hCoV-19/Japan/PG-90719/2021	EPI_ISL_3901276	2021-09-02
hCoV-19/Japan/PG-90720/2021	EPI_ISL_3901277	2021-09-02
hCoV-19/Japan/PG-90721/2021	EPI_ISL_3901278	2021-09-02
hCoV-19/Japan/PG-90722/2021	EPI_ISL_3901279	2021-09-02
hCoV-19/Japan/PG-90723/2021	EPI_ISL_3901280	2021-09-02
hCoV-19/Japan/PG-90724/2021	EPI_ISL_3901281	2021-09-02
hCoV-19/Japan/PG-90725/2021	EPI_ISL_3901282	2021-09-02
hCoV-19/Japan/PG-90726/2021	EPI_ISL_3901283	2021-09-02
hCoV-19/Japan/PG-90727/2021	EPI_ISL_3901284	2021-09-02
hCoV-19/Japan/PG-90728/2021	EPI_ISL_3901285	2021-09-02
hCoV-19/Japan/PG-90729/2021	EPI_ISL_3901286	2021-09-02
hCoV-19/Japan/PG-90730/2021	EPI_ISL_3901287	2021-09-02
hCoV-19/Japan/PG-90731/2021	EPI_ISL_3901288	2021-09-02
hCoV-19/Japan/PG-90732/2021	EPI_ISL_3901289	2021-09-02
hCoV-19/Japan/PG-90733/2021	EPI_ISL_3901290	2021-09-02
hCoV-19/Japan/PG-90734/2021	EPI_ISL_3901291	2021-09-02
hCoV-19/Japan/PG-90735/2021	EPI_ISL_3901292	2021-09-02
hCoV-19/Japan/PG-90736/2021	EPI_ISL_3901293	2021-09-02
hCoV-19/Japan/PG-90737/2021	EPI_ISL_3901294	2021-09-02
hCoV-19/Japan/PG-90738/2021	EPI_ISL_3901295	2021-09-02
hCoV-19/Japan/PG-90739/2021	EPI_ISL_3901296	2021-09-02
hCoV-19/Japan/PG-90740/2021	EPI_ISL_3901297	2021-09-02
hCoV-19/Japan/PG-90741/2021	EPI_ISL_3901298	2021-09-02
hCoV-19/Japan/PG-90742/2021	EPI_ISL_3901299	2021-09-02
hCoV-19/Japan/PG-90743/2021	EPI_ISL_3901300	2021-09-02
hCoV-19/Japan/PG-90744/2021	EPI_ISL_3901301	2021-09-02
hCoV-19/Japan/PG-90745/2021	EPI_ISL_3901302	2021-09-02
hCoV-19/Japan/PG-90746/2021	EPI_ISL_3901303	2021-09-02
hCoV-19/Japan/PG-90747/2021	EPI_ISL_3901304	2021-09-02
hCoV-19/Japan/PG-90748/2021	EPI_ISL_3901305	2021-09-02
hCoV-19/Japan/PG-90749/2021	EPI_ISL_3901306	2021-09-02
hCoV-19/Japan/PG-90750/2021	EPI_ISL_3901307	2021-09-02
hCoV-19/Japan/PG-90751/2021	EPI_ISL_3901308	2021-09-02
hCoV-19/Japan/PG-90752/2021	EPI_ISL_3901309	2021-09-02
hCoV-19/Japan/PG-90753/2021	EPI_ISL_3901310	2021-09-02
hCoV-19/Japan/PG-90754/2021	EPI_ISL_3901311	2021-09-02
hCoV-19/Japan/PG-90755/2021	EPI_ISL_3901312	2021-09-02
hCoV-19/Japan/PG-90756/2021	EPI_ISL_3901313	2021-09-02
hCoV-19/Japan/PG-90757/2021	EPI_ISL_3901314	2021-09-02
hCoV-19/Japan/PG-90758/2021	EPI_ISL_3901315	2021-09-02
hCoV-19/Japan/PG-90759/2021	EPI_ISL_3901316	2021-09-02
hCoV-19/Japan/PG-90760/2021	EPI_ISL_3901317	2021-09-02
hCoV-19/Japan/PG-90761/2021	EPI_ISL_3901318	2021-09-02
hCoV-19/Japan/PG-90762/2021	EPI_ISL_3901319	2021-09-02
hCoV-19/Japan/PG-90763/2021	EPI_ISL_3901320	2021-09-02
hCoV-19/Japan/PG-90764/2021	EPI_ISL_3901321	2021-09-02
hCoV-19/Japan/PG-90765/2021	EPI_ISL_3901322	2021-09-02
hCoV-19/Japan/PG-90766/2021	EPI_ISL_3901323	2021-09-02
hCoV-19/Japan/PG-90767/2021	EPI_ISL_3901324	2021-09-02
hCoV-19/Japan/PG-90768/2021	EPI_ISL_3901325	2021-09-02

virus_name	epiID	submission_date
hCoV-19/Japan/PG-90769/2021	EPI_ISL_3901326	2021-09-02
hCoV-19/Japan/PG-90770/2021	EPI_ISL_3901327	2021-09-02
hCoV-19/Japan/PG-90771/2021	EPI_ISL_3901328	2021-09-02
hCoV-19/Japan/PG-90772/2021	EPI_ISL_3901329	2021-09-02
hCoV-19/Japan/PG-90773/2021	EPI_ISL_3901330	2021-09-02
hCoV-19/Japan/PG-90774/2021	EPI_ISL_3901331	2021-09-02
hCoV-19/Japan/PG-90775/2021	EPI_ISL_3901332	2021-09-02
hCoV-19/Japan/PG-90776/2021	EPI_ISL_3901333	2021-09-02
hCoV-19/Japan/PG-90777/2021	EPI_ISL_3901334	2021-09-02
hCoV-19/Japan/PG-90778/2021	EPI_ISL_3901335	2021-09-02
hCoV-19/Japan/PG-90779/2021	EPI_ISL_3901336	2021-09-02
hCoV-19/Japan/PG-90780/2021	EPI_ISL_3901337	2021-09-02
hCoV-19/Japan/PG-90781/2021	EPI_ISL_3901338	2021-09-02
hCoV-19/Japan/PG-90782/2021	EPI_ISL_3901339	2021-09-02
hCoV-19/Japan/PG-90783/2021	EPI_ISL_3901340	2021-09-02
hCoV-19/Japan/PG-90784/2021	EPI_ISL_3901341	2021-09-02
hCoV-19/Japan/PG-90785/2021	EPI_ISL_3901342	2021-09-02
hCoV-19/Japan/PG-90786/2021	EPI_ISL_3901343	2021-09-02
hCoV-19/Japan/PG-90787/2021	EPI_ISL_3901344	2021-09-02
hCoV-19/Japan/PG-90788/2021	EPI_ISL_3901345	2021-09-02
hCoV-19/Japan/PG-90789/2021	EPI_ISL_3901346	2021-09-02
hCoV-19/Japan/PG-90790/2021	EPI_ISL_3901347	2021-09-02
hCoV-19/Japan/PG-90791/2021	EPI_ISL_3901348	2021-09-02
hCoV-19/Japan/PG-90792/2021	EPI_ISL_3901349	2021-09-02
hCoV-19/Japan/PG-90793/2021	EPI_ISL_3901350	2021-09-02
hCoV-19/Japan/PG-90794/2021	EPI_ISL_3901351	2021-09-02
hCoV-19/Japan/PG-90795/2021	EPI_ISL_3901352	2021-09-02
hCoV-19/Japan/PG-90796/2021	EPI_ISL_3901353	2021-09-02
hCoV-19/Japan/PG-90797/2021	EPI_ISL_3901354	2021-09-02
hCoV-19/Japan/PG-90798/2021	EPI_ISL_3901355	2021-09-02
hCoV-19/Japan/PG-90799/2021	EPI_ISL_3901356	2021-09-02
hCoV-19/Japan/PG-90800/2021	EPI_ISL_3901357	2021-09-02
hCoV-19/Japan/PG-90801/2021	EPI_ISL_3901358	2021-09-02
hCoV-19/Japan/PG-90802/2021	EPI_ISL_3901359	2021-09-02
hCoV-19/Japan/PG-90803/2021	EPI_ISL_3901360	2021-09-02
hCoV-19/Japan/PG-90804/2021	EPI_ISL_3901361	2021-09-02
hCoV-19/Japan/PG-90805/2021	EPI_ISL_3901362	2021-09-02
hCoV-19/Japan/PG-90806/2021	EPI_ISL_3901363	2021-09-02
hCoV-19/Japan/PG-90807/2021	EPI_ISL_3901364	2021-09-02
hCoV-19/Japan/PG-90808/2021	EPI_ISL_3901365	2021-09-02
hCoV-19/Japan/PG-90809/2021	EPI_ISL_3901366	2021-09-02
hCoV-19/Japan/PG-90810/2021	EPI_ISL_3901367	2021-09-02
hCoV-19/Japan/PG-90811/2021	EPI_ISL_3901368	2021-09-02
hCoV-19/Japan/PG-90812/2021	EPI_ISL_3901369	2021-09-02
hCoV-19/Japan/PG-90813/2021	EPI_ISL_3901370	2021-09-02
hCoV-19/Japan/PG-90814/2021	EPI_ISL_3901371	2021-09-02
hCoV-19/Japan/PG-90815/2021	EPI_ISL_3901372	2021-09-02
hCoV-19/Japan/PG-90816/2021	EPI_ISL_3901373	2021-09-02
hCoV-19/Japan/PG-90817/2021	EPI_ISL_3901374	2021-09-02
hCoV-19/Japan/PG-90818/2021	EPI_ISL_3901375	2021-09-02
hCoV-19/Japan/PG-90819/2021	EPI_ISL_3901376	2021-09-02
hCoV-19/Japan/PG-90820/2021	EPI_ISL_3901377	2021-09-02
hCoV-19/Japan/PG-90821/2021	EPI_ISL_3901378	2021-09-02
hCoV-19/Japan/PG-90822/2021	EPI_ISL_3901379	2021-09-02

virus_name	epiID	submission_date
hCoV-19/Japan/PG-90823/2021	EPI_ISL_3901380	2021-09-02
hCoV-19/Japan/PG-90824/2021	EPI_ISL_3901381	2021-09-02
hCoV-19/Japan/PG-90825/2021	EPI_ISL_3901382	2021-09-02
hCoV-19/Japan/PG-90826/2021	EPI_ISL_3901383	2021-09-02
hCoV-19/Japan/PG-90827/2021	EPI_ISL_3901384	2021-09-02
hCoV-19/Japan/PG-90828/2021	EPI_ISL_3901385	2021-09-02
hCoV-19/Japan/PG-90829/2021	EPI_ISL_3901386	2021-09-02
hCoV-19/Japan/PG-90830/2021	EPI_ISL_3901387	2021-09-02
hCoV-19/Japan/PG-90831/2021	EPI_ISL_3901388	2021-09-02
hCoV-19/Japan/PG-90832/2021	EPI_ISL_3901389	2021-09-02
hCoV-19/Japan/PG-90833/2021	EPI_ISL_3901390	2021-09-02
hCoV-19/Japan/PG-90834/2021	EPI_ISL_3901391	2021-09-02
hCoV-19/Japan/PG-90835/2021	EPI_ISL_3901392	2021-09-02
hCoV-19/Japan/PG-90836/2021	EPI_ISL_3901393	2021-09-02
hCoV-19/Japan/PG-90837/2021	EPI_ISL_3901394	2021-09-02
hCoV-19/Japan/PG-90838/2021	EPI_ISL_3901395	2021-09-02
hCoV-19/Japan/PG-90839/2021	EPI_ISL_3901396	2021-09-02
hCoV-19/Japan/PG-90840/2021	EPI_ISL_3901397	2021-09-02
hCoV-19/Japan/PG-90841/2021	EPI_ISL_3901398	2021-09-02
hCoV-19/Japan/PG-90842/2021	EPI_ISL_3901399	2021-09-02
hCoV-19/Japan/PG-90843/2021	EPI_ISL_3901400	2021-09-02
hCoV-19/Japan/PG-90844/2021	EPI_ISL_3901401	2021-09-02
hCoV-19/Japan/PG-90845/2021	EPI_ISL_3901402	2021-09-02
hCoV-19/Japan/PG-90846/2021	EPI_ISL_3901403	2021-09-02
hCoV-19/Japan/PG-90847/2021	EPI_ISL_3901404	2021-09-02
hCoV-19/Japan/PG-90848/2021	EPI_ISL_3901405	2021-09-02
hCoV-19/Japan/PG-90849/2021	EPI_ISL_3901406	2021-09-02
hCoV-19/Japan/PG-90850/2021	EPI_ISL_3901407	2021-09-02
hCoV-19/Japan/PG-90851/2021	EPI_ISL_3901408	2021-09-02
hCoV-19/Japan/PG-90852/2021	EPI_ISL_3901409	2021-09-02
hCoV-19/Japan/PG-90853/2021	EPI_ISL_3901410	2021-09-02
hCoV-19/Japan/PG-90854/2021	EPI_ISL_3901411	2021-09-02
hCoV-19/Japan/PG-90855/2021	EPI_ISL_3901412	2021-09-02
hCoV-19/Japan/PG-90856/2021	EPI_ISL_3901413	2021-09-02
hCoV-19/Japan/PG-90857/2021	EPI_ISL_3901414	2021-09-02
hCoV-19/Japan/PG-90858/2021	EPI_ISL_3901415	2021-09-02
hCoV-19/Japan/PG-90859/2021	EPI_ISL_3901416	2021-09-02
hCoV-19/Japan/PG-90860/2021	EPI_ISL_3901417	2021-09-02
hCoV-19/Japan/PG-90861/2021	EPI_ISL_3901418	2021-09-02
hCoV-19/Japan/PG-90862/2021	EPI_ISL_3901419	2021-09-02
hCoV-19/Japan/PG-90863/2021	EPI_ISL_3901420	2021-09-02
hCoV-19/Japan/PG-90864/2021	EPI_ISL_3901421	2021-09-02
hCoV-19/Japan/PG-90865/2021	EPI_ISL_3901422	2021-09-02
hCoV-19/Japan/PG-90866/2021	EPI_ISL_3901423	2021-09-02
hCoV-19/Japan/PG-90867/2021	EPI_ISL_3901424	2021-09-02
hCoV-19/Japan/PG-90868/2021	EPI_ISL_3901425	2021-09-02
hCoV-19/Japan/PG-90869/2021	EPI_ISL_3901426	2021-09-02
hCoV-19/Japan/PG-90870/2021	EPI_ISL_3901427	2021-09-02
hCoV-19/Japan/PG-90871/2021	EPI_ISL_3901428	2021-09-02
hCoV-19/Japan/PG-90872/2021	EPI_ISL_3901429	2021-09-02
hCoV-19/Japan/PG-90873/2021	EPI_ISL_3901430	2021-09-02
hCoV-19/Japan/PG-90874/2021	EPI_ISL_3901431	2021-09-02
hCoV-19/Japan/PG-90875/2021	EPI_ISL_3901432	2021-09-02
hCoV-19/Japan/PG-90876/2021	EPI_ISL_3901433	2021-09-02

virus_name	epiID	submission_date
hCoV-19/Japan/PG-90877/2021	EPI_ISL_3901434	2021-09-02
hCoV-19/Japan/PG-90878/2021	EPI_ISL_3901435	2021-09-02
hCoV-19/Japan/PG-90879/2021	EPI_ISL_3901436	2021-09-02
hCoV-19/Japan/PG-90880/2021	EPI_ISL_3901437	2021-09-02
hCoV-19/Japan/PG-90881/2021	EPI_ISL_3901438	2021-09-02
hCoV-19/Japan/PG-90882/2021	EPI_ISL_3901439	2021-09-02
hCoV-19/Japan/PG-90883/2021	EPI_ISL_3901440	2021-09-02
hCoV-19/Japan/PG-90884/2021	EPI_ISL_3901441	2021-09-02
hCoV-19/Japan/PG-90885/2021	EPI_ISL_3901442	2021-09-02
hCoV-19/Japan/PG-90886/2021	EPI_ISL_3901443	2021-09-02
hCoV-19/Japan/PG-90887/2021	EPI_ISL_3901444	2021-09-02
hCoV-19/Japan/PG-90888/2021	EPI_ISL_3901445	2021-09-02
hCoV-19/Japan/PG-90889/2021	EPI_ISL_3901446	2021-09-02
hCoV-19/Japan/PG-90890/2021	EPI_ISL_3901447	2021-09-02
hCoV-19/Japan/PG-90891/2021	EPI_ISL_3901448	2021-09-02
hCoV-19/Japan/PG-90892/2021	EPI_ISL_3901449	2021-09-02
hCoV-19/Japan/PG-90893/2021	EPI_ISL_3901450	2021-09-02
hCoV-19/Japan/PG-90894/2021	EPI_ISL_3901451	2021-09-02
hCoV-19/Japan/PG-90895/2021	EPI_ISL_3901452	2021-09-02
hCoV-19/Japan/PG-90896/2021	EPI_ISL_3901453	2021-09-02
hCoV-19/Japan/PG-90897/2021	EPI_ISL_3901454	2021-09-02
hCoV-19/Japan/PG-90898/2021	EPI_ISL_3901455	2021-09-02
hCoV-19/Japan/PG-90899/2021	EPI_ISL_3901456	2021-09-02
hCoV-19/Japan/PG-90900/2021	EPI_ISL_3901457	2021-09-02
hCoV-19/Japan/PG-90901/2021	EPI_ISL_3901458	2021-09-02
hCoV-19/Japan/PG-90902/2021	EPI_ISL_3901459	2021-09-02
hCoV-19/Japan/PG-90903/2021	EPI_ISL_3901460	2021-09-02
hCoV-19/Japan/PG-90904/2021	EPI_ISL_3901461	2021-09-02
hCoV-19/Japan/PG-90905/2021	EPI_ISL_3901462	2021-09-02
hCoV-19/Japan/PG-90906/2021	EPI_ISL_3901463	2021-09-02
hCoV-19/Japan/PG-90907/2021	EPI_ISL_3901464	2021-09-02
hCoV-19/Japan/PG-90908/2021	EPI_ISL_3901465	2021-09-02
hCoV-19/Japan/PG-90909/2021	EPI_ISL_3901466	2021-09-02
hCoV-19/Japan/PG-90910/2021	EPI_ISL_3901467	2021-09-02
hCoV-19/Japan/PG-90911/2021	EPI_ISL_3901468	2021-09-02
hCoV-19/Japan/PG-90912/2021	EPI_ISL_3901469	2021-09-02
hCoV-19/Japan/PG-90913/2021	EPI_ISL_3901470	2021-09-02
hCoV-19/Japan/PG-90914/2021	EPI_ISL_3901471	2021-09-02
hCoV-19/Japan/PG-90915/2021	EPI_ISL_3901472	2021-09-02
hCoV-19/Japan/PG-90916/2021	EPI_ISL_3901473	2021-09-02
hCoV-19/Japan/PG-90917/2021	EPI_ISL_3901474	2021-09-02
hCoV-19/Japan/PG-90918/2021	EPI_ISL_3901475	2021-09-02
hCoV-19/Japan/PG-90919/2021	EPI_ISL_3901476	2021-09-02
hCoV-19/Japan/PG-90920/2021	EPI_ISL_3901477	2021-09-02
hCoV-19/Japan/PG-90921/2021	EPI_ISL_3901478	2021-09-02
hCoV-19/Japan/PG-90922/2021	EPI_ISL_3901479	2021-09-02
hCoV-19/Japan/PG-90923/2021	EPI_ISL_3901480	2021-09-02
hCoV-19/Japan/PG-90924/2021	EPI_ISL_3901481	2021-09-02
hCoV-19/Japan/PG-90925/2021	EPI_ISL_3901482	2021-09-02
hCoV-19/Japan/PG-90926/2021	EPI_ISL_3901483	2021-09-02
hCoV-19/Japan/PG-90927/2021	EPI_ISL_3901484	2021-09-02
hCoV-19/Japan/PG-90928/2021	EPI_ISL_3901485	2021-09-02
hCoV-19/Japan/PG-90929/2021	EPI_ISL_3901486	2021-09-02
hCoV-19/Japan/PG-90930/2021	EPI_ISL_3901487	2021-09-02

virus_name	epiID	submission_date
hCoV-19/Japan/PG-90931/2021	EPI_ISL_3901488	2021-09-02
hCoV-19/Japan/PG-90932/2021	EPI_ISL_3901489	2021-09-02
hCoV-19/Japan/PG-90933/2021	EPI_ISL_3901490	2021-09-02
hCoV-19/Japan/PG-90934/2021	EPI_ISL_3901491	2021-09-02
hCoV-19/Japan/PG-90935/2021	EPI_ISL_3901492	2021-09-02
hCoV-19/Japan/PG-90936/2021	EPI_ISL_3901493	2021-09-02
hCoV-19/Japan/PG-90937/2021	EPI_ISL_3901494	2021-09-02
hCoV-19/Japan/PG-90938/2021	EPI_ISL_3901495	2021-09-02
hCoV-19/Japan/PG-90939/2021	EPI_ISL_3901496	2021-09-02
hCoV-19/Japan/PG-90940/2021	EPI_ISL_3901497	2021-09-02
hCoV-19/Japan/PG-90941/2021	EPI_ISL_3901498	2021-09-02
hCoV-19/Japan/PG-90942/2021	EPI_ISL_3901499	2021-09-02
hCoV-19/Japan/PG-90943/2021	EPI_ISL_3901500	2021-09-02
hCoV-19/Japan/PG-90944/2021	EPI_ISL_3901501	2021-09-02
hCoV-19/Japan/PG-90945/2021	EPI_ISL_3901502	2021-09-02
hCoV-19/Japan/PG-90946/2021	EPI_ISL_3901503	2021-09-02
hCoV-19/Japan/PG-90947/2021	EPI_ISL_3901504	2021-09-02
hCoV-19/Japan/PG-90948/2021	EPI_ISL_3901505	2021-09-02
hCoV-19/Japan/PG-90949/2021	EPI_ISL_3901506	2021-09-02
hCoV-19/Japan/PG-90950/2021	EPI_ISL_3901507	2021-09-02
hCoV-19/Japan/PG-90951/2021	EPI_ISL_3901508	2021-09-02
hCoV-19/Japan/PG-90952/2021	EPI_ISL_3901509	2021-09-02
hCoV-19/Japan/PG-90953/2021	EPI_ISL_3901510	2021-09-02
hCoV-19/Japan/PG-90954/2021	EPI_ISL_3901511	2021-09-02
hCoV-19/Japan/PG-90955/2021	EPI_ISL_3901512	2021-09-02
hCoV-19/Japan/PG-90956/2021	EPI_ISL_3901513	2021-09-02
hCoV-19/Japan/PG-90957/2021	EPI_ISL_3901514	2021-09-02
hCoV-19/Japan/PG-90958/2021	EPI_ISL_3901515	2021-09-02
hCoV-19/Japan/PG-90959/2021	EPI_ISL_3901516	2021-09-02
hCoV-19/Japan/PG-90960/2021	EPI_ISL_3901517	2021-09-02
hCoV-19/Japan/PG-90961/2021	EPI_ISL_3901518	2021-09-02
hCoV-19/Japan/PG-90962/2021	EPI_ISL_3901519	2021-09-02
hCoV-19/Japan/PG-90963/2021	EPI_ISL_3901520	2021-09-02
hCoV-19/Japan/PG-90964/2021	EPI_ISL_3901521	2021-09-02
hCoV-19/Japan/PG-90965/2021	EPI_ISL_3901522	2021-09-02
hCoV-19/Japan/PG-90966/2021	EPI_ISL_3901523	2021-09-02
hCoV-19/Japan/PG-90967/2021	EPI_ISL_3901524	2021-09-02
hCoV-19/Japan/PG-90968/2021	EPI_ISL_3901525	2021-09-02
hCoV-19/Japan/PG-90969/2021	EPI_ISL_3901526	2021-09-02
hCoV-19/Japan/PG-90970/2021	EPI_ISL_3901527	2021-09-02
hCoV-19/Japan/PG-90971/2021	EPI_ISL_3901528	2021-09-02
hCoV-19/Japan/PG-90972/2021	EPI_ISL_3901529	2021-09-02
hCoV-19/Japan/PG-90973/2021	EPI_ISL_3901530	2021-09-02
hCoV-19/Japan/PG-90974/2021	EPI_ISL_3901531	2021-09-02
hCoV-19/Japan/PG-90975/2021	EPI_ISL_3901532	2021-09-02
hCoV-19/Japan/PG-90976/2021	EPI_ISL_3901533	2021-09-02
hCoV-19/Japan/PG-90977/2021	EPI_ISL_3901534	2021-09-02
hCoV-19/Japan/PG-90978/2021	EPI_ISL_3901535	2021-09-02
hCoV-19/Japan/PG-90979/2021	EPI_ISL_3901536	2021-09-02
hCoV-19/Japan/PG-90980/2021	EPI_ISL_3901537	2021-09-02
hCoV-19/Japan/PG-90981/2021	EPI_ISL_3901538	2021-09-02
hCoV-19/Japan/PG-90982/2021	EPI_ISL_3901539	2021-09-02
hCoV-19/Japan/PG-90983/2021	EPI_ISL_3901540	2021-09-02
hCoV-19/Japan/PG-90984/2021	EPI_ISL_3901541	2021-09-02

virus_name	epiID	submission_date
hCoV-19/Japan/PG-90985/2021	EPI_ISL_3901542	2021-09-02
hCoV-19/Japan/PG-90986/2021	EPI_ISL_3901543	2021-09-02
hCoV-19/Japan/PG-90987/2021	EPI_ISL_3901544	2021-09-02
hCoV-19/Japan/PG-90988/2021	EPI_ISL_3901545	2021-09-02
hCoV-19/Japan/PG-90989/2021	EPI_ISL_3901546	2021-09-02
hCoV-19/Japan/PG-90990/2021	EPI_ISL_3901547	2021-09-02
hCoV-19/Japan/PG-90991/2021	EPI_ISL_3901548	2021-09-02
hCoV-19/Japan/PG-90992/2021	EPI_ISL_3901549	2021-09-02
hCoV-19/Japan/PG-90993/2021	EPI_ISL_3901550	2021-09-02
hCoV-19/Japan/PG-90994/2021	EPI_ISL_3901551	2021-09-02
hCoV-19/Japan/PG-90995/2021	EPI_ISL_3901552	2021-09-02
hCoV-19/Japan/PG-90996/2021	EPI_ISL_3901553	2021-09-02
hCoV-19/Japan/PG-90997/2021	EPI_ISL_3901554	2021-09-02
hCoV-19/Japan/PG-90998/2021	EPI_ISL_3901555	2021-09-02
hCoV-19/Japan/PG-90999/2021	EPI_ISL_3901556	2021-09-02
hCoV-19/Japan/PG-91000/2021	EPI_ISL_3901557	2021-09-02
hCoV-19/Japan/PG-91001/2021	EPI_ISL_3901558	2021-09-02
hCoV-19/Japan/PG-91002/2021	EPI_ISL_3901559	2021-09-02
hCoV-19/Japan/PG-91003/2021	EPI_ISL_3901560	2021-09-02
hCoV-19/Japan/PG-91004/2021	EPI_ISL_3901562	2021-09-02
hCoV-19/Japan/PG-91005/2021	EPI_ISL_3901563	2021-09-02
hCoV-19/Japan/PG-91006/2021	EPI_ISL_3901564	2021-09-02
hCoV-19/Japan/PG-91007/2021	EPI_ISL_3901565	2021-09-02
hCoV-19/Japan/PG-91008/2021	EPI_ISL_3901566	2021-09-02
hCoV-19/Japan/PG-91009/2021	EPI_ISL_3901567	2021-09-02
hCoV-19/Japan/PG-91010/2021	EPI_ISL_3901568	2021-09-02
hCoV-19/Japan/PG-91011/2021	EPI_ISL_3901569	2021-09-02
hCoV-19/Japan/PG-91012/2021	EPI_ISL_3901570	2021-09-02
hCoV-19/Japan/PG-91013/2021	EPI_ISL_3901571	2021-09-02
hCoV-19/Japan/PG-91014/2021	EPI_ISL_3901572	2021-09-02
hCoV-19/Japan/PG-91015/2021	EPI_ISL_3901573	2021-09-02
hCoV-19/Japan/PG-91016/2021	EPI_ISL_3901574	2021-09-02
hCoV-19/Japan/PG-91017/2021	EPI_ISL_3901575	2021-09-02
hCoV-19/Japan/PG-91018/2021	EPI_ISL_3901576	2021-09-02
hCoV-19/Japan/PG-91019/2021	EPI_ISL_3901577	2021-09-02
hCoV-19/Japan/PG-91020/2021	EPI_ISL_3901578	2021-09-02
hCoV-19/Japan/PG-91021/2021	EPI_ISL_3901579	2021-09-02
hCoV-19/Japan/PG-91022/2021	EPI_ISL_3901580	2021-09-02
hCoV-19/Japan/PG-91023/2021	EPI_ISL_3901581	2021-09-02
hCoV-19/Japan/PG-91024/2021	EPI_ISL_3901582	2021-09-02
hCoV-19/Japan/PG-91025/2021	EPI_ISL_3901583	2021-09-02
hCoV-19/Japan/PG-91026/2021	EPI_ISL_3901584	2021-09-02
hCoV-19/Japan/PG-91027/2021	EPI_ISL_3901585	2021-09-02
hCoV-19/Japan/PG-91028/2021	EPI_ISL_3901586	2021-09-02
hCoV-19/Japan/PG-91029/2021	EPI_ISL_3901587	2021-09-02
hCoV-19/Japan/PG-91030/2021	EPI_ISL_3901588	2021-09-02
hCoV-19/Japan/PG-91031/2021	EPI_ISL_3901589	2021-09-02
hCoV-19/Japan/PG-91032/2021	EPI_ISL_3901590	2021-09-02
hCoV-19/Japan/PG-91033/2021	EPI_ISL_3901591	2021-09-02
hCoV-19/Japan/PG-91034/2021	EPI_ISL_3901592	2021-09-02
hCoV-19/Japan/PG-91035/2021	EPI_ISL_3901593	2021-09-02
hCoV-19/Japan/PG-91036/2021	EPI_ISL_3901594	2021-09-02
hCoV-19/Japan/PG-91037/2021	EPI_ISL_3901595	2021-09-02
hCoV-19/Japan/PG-91038/2021	EPI_ISL_3901596	2021-09-02

virus_name	epiID	submission_date
hCoV-19/Japan/PG-91039/2021	EPI_ISL_3901597	2021-09-02
hCoV-19/Japan/PG-91040/2021	EPI_ISL_3901598	2021-09-02
hCoV-19/Japan/PG-91041/2021	EPI_ISL_3901599	2021-09-02
hCoV-19/Japan/PG-91042/2021	EPI_ISL_3901600	2021-09-02
hCoV-19/Japan/PG-91043/2021	EPI_ISL_3901601	2021-09-02
hCoV-19/Japan/PG-91044/2021	EPI_ISL_3901602	2021-09-02
hCoV-19/Japan/PG-91045/2021	EPI_ISL_3901603	2021-09-02
hCoV-19/Japan/PG-91046/2021	EPI_ISL_3901604	2021-09-02
hCoV-19/Japan/PG-91047/2021	EPI_ISL_3901605	2021-09-02
hCoV-19/Japan/PG-91048/2021	EPI_ISL_3901606	2021-09-02
hCoV-19/Japan/PG-91049/2021	EPI_ISL_3901607	2021-09-02
hCoV-19/Japan/PG-91050/2021	EPI_ISL_3901608	2021-09-02
hCoV-19/Japan/PG-91051/2021	EPI_ISL_3901609	2021-09-02
hCoV-19/Japan/PG-91052/2021	EPI_ISL_3901610	2021-09-02
hCoV-19/Japan/PG-91053/2021	EPI_ISL_3901612	2021-09-02
hCoV-19/Japan/PG-91054/2021	EPI_ISL_3901613	2021-09-02
hCoV-19/Japan/PG-91055/2021	EPI_ISL_3901614	2021-09-02
hCoV-19/Japan/PG-91056/2021	EPI_ISL_3901617	2021-09-02
hCoV-19/Japan/PG-91057/2021	EPI_ISL_3901620	2021-09-02
hCoV-19/Japan/PG-91058/2021	EPI_ISL_3901623	2021-09-02
hCoV-19/Japan/PG-91059/2021	EPI_ISL_3901626	2021-09-02
hCoV-19/Japan/PG-91060/2021	EPI_ISL_3901629	2021-09-02
hCoV-19/Japan/PG-91061/2021	EPI_ISL_3901633	2021-09-02
hCoV-19/Japan/PG-91062/2021	EPI_ISL_3901636	2021-09-02
hCoV-19/Japan/PG-91063/2021	EPI_ISL_3901639	2021-09-02
hCoV-19/Japan/PG-91064/2021	EPI_ISL_3901642	2021-09-02
hCoV-19/Japan/PG-91065/2021	EPI_ISL_3901645	2021-09-02
hCoV-19/Japan/PG-91066/2021	EPI_ISL_3901648	2021-09-02
hCoV-19/Japan/PG-91067/2021	EPI_ISL_3901649	2021-09-02
hCoV-19/Japan/PG-91068/2021	EPI_ISL_3901650	2021-09-02
hCoV-19/Japan/PG-91069/2021	EPI_ISL_3901652	2021-09-02
hCoV-19/Japan/PG-91070/2021	EPI_ISL_3901653	2021-09-02
hCoV-19/Japan/PG-91071/2021	EPI_ISL_3901654	2021-09-02
hCoV-19/Japan/PG-91072/2021	EPI_ISL_3901655	2021-09-02
hCoV-19/Japan/PG-91073/2021	EPI_ISL_3901657	2021-09-02
hCoV-19/Japan/PG-91074/2021	EPI_ISL_3901658	2021-09-02
hCoV-19/Japan/PG-91075/2021	EPI_ISL_3901659	2021-09-02
hCoV-19/Japan/PG-91076/2021	EPI_ISL_3901662	2021-09-02
hCoV-19/Japan/PG-91077/2021	EPI_ISL_3901665	2021-09-02
hCoV-19/Japan/PG-91078/2021	EPI_ISL_3901668	2021-09-02
hCoV-19/Japan/PG-91079/2021	EPI_ISL_3901670	2021-09-02
hCoV-19/Japan/PG-91080/2021	EPI_ISL_3901671	2021-09-02
hCoV-19/Japan/PG-91081/2021	EPI_ISL_3901672	2021-09-02
hCoV-19/Japan/PG-91082/2021	EPI_ISL_3901674	2021-09-02
hCoV-19/Japan/PG-91083/2021	EPI_ISL_3901675	2021-09-02
hCoV-19/Japan/PG-91084/2021	EPI_ISL_3901676	2021-09-02
hCoV-19/Japan/PG-91085/2021	EPI_ISL_3901678	2021-09-02
hCoV-19/Japan/PG-91086/2021	EPI_ISL_3901679	2021-09-02
hCoV-19/Japan/PG-91087/2021	EPI_ISL_3901680	2021-09-02
hCoV-19/Japan/PG-91088/2021	EPI_ISL_3901682	2021-09-02
hCoV-19/Japan/PG-91089/2021	EPI_ISL_3901683	2021-09-02
hCoV-19/Japan/PG-91090/2021	EPI_ISL_3901684	2021-09-02
hCoV-19/Japan/PG-91091/2021	EPI_ISL_3901686	2021-09-02
hCoV-19/Japan/PG-91092/2021	EPI_ISL_3901687	2021-09-02

virus_name	epiID	submission_date
hCoV-19/Japan/PG-91093/2021	EPI_ISL_3901688	2021-09-02
hCoV-19/Japan/PG-91094/2021	EPI_ISL_3901690	2021-09-02
hCoV-19/Japan/PG-91095/2021	EPI_ISL_3901691	2021-09-02
hCoV-19/Japan/PG-91096/2021	EPI_ISL_3901692	2021-09-02
hCoV-19/Japan/PG-91097/2021	EPI_ISL_3901694	2021-09-02
hCoV-19/Japan/PG-91098/2021	EPI_ISL_3901695	2021-09-02
hCoV-19/Japan/PG-91099/2021	EPI_ISL_3901696	2021-09-02
hCoV-19/Japan/PG-91100/2021	EPI_ISL_3901698	2021-09-02
hCoV-19/Japan/PG-91101/2021	EPI_ISL_3901699	2021-09-02
hCoV-19/Japan/PG-91102/2021	EPI_ISL_3901701	2021-09-02
hCoV-19/Japan/PG-91103/2021	EPI_ISL_3901702	2021-09-02
hCoV-19/Japan/PG-91104/2021	EPI_ISL_3901703	2021-09-02
hCoV-19/Japan/PG-91105/2021	EPI_ISL_3901705	2021-09-02
hCoV-19/Japan/PG-91106/2021	EPI_ISL_3901706	2021-09-02
hCoV-19/Japan/PG-91107/2021	EPI_ISL_3901707	2021-09-02
hCoV-19/Japan/PG-91108/2021	EPI_ISL_3901709	2021-09-02
hCoV-19/Japan/PG-91109/2021	EPI_ISL_3901710	2021-09-02
hCoV-19/Japan/PG-91110/2021	EPI_ISL_3901711	2021-09-02
hCoV-19/Japan/PG-91111/2021	EPI_ISL_3901713	2021-09-02
hCoV-19/Japan/PG-91112/2021	EPI_ISL_3901714	2021-09-02
hCoV-19/Japan/PG-91113/2021	EPI_ISL_3901715	2021-09-02
hCoV-19/Japan/PG-91114/2021	EPI_ISL_3901718	2021-09-02
hCoV-19/Japan/PG-91115/2021	EPI_ISL_3901721	2021-09-02
hCoV-19/Japan/PG-91116/2021	EPI_ISL_3901724	2021-09-02
hCoV-19/Japan/PG-91117/2021	EPI_ISL_3901726	2021-09-02
hCoV-19/Japan/PG-91118/2021	EPI_ISL_3901727	2021-09-02
hCoV-19/Japan/PG-91119/2021	EPI_ISL_3901728	2021-09-02
hCoV-19/Japan/PG-91120/2021	EPI_ISL_3901731	2021-09-02
hCoV-19/Japan/PG-91121/2021	EPI_ISL_3901734	2021-09-02
hCoV-19/Japan/PG-91122/2021	EPI_ISL_3901737	2021-09-02
hCoV-19/Japan/PG-91123/2021	EPI_ISL_3901739	2021-09-02
hCoV-19/Japan/PG-91124/2021	EPI_ISL_3901740	2021-09-02
hCoV-19/Japan/PG-91125/2021	EPI_ISL_3901741	2021-09-02
hCoV-19/Japan/PG-91126/2021	EPI_ISL_3901745	2021-09-02
hCoV-19/Japan/PG-91127/2021	EPI_ISL_3901748	2021-09-02
hCoV-19/Japan/PG-91128/2021	EPI_ISL_3901751	2021-09-02
hCoV-19/Japan/PG-91129/2021	EPI_ISL_3901752	2021-09-02
hCoV-19/Japan/PG-91130/2021	EPI_ISL_3901753	2021-09-02
hCoV-19/Japan/PG-91131/2021	EPI_ISL_3901755	2021-09-02
hCoV-19/Japan/PG-91132/2021	EPI_ISL_3901758	2021-09-02
hCoV-19/Japan/PG-91133/2021	EPI_ISL_3901761	2021-09-02
hCoV-19/Japan/PG-91134/2021	EPI_ISL_3901763	2021-09-02
hCoV-19/Japan/PG-91135/2021	EPI_ISL_3901764	2021-09-02
hCoV-19/Japan/PG-91136/2021	EPI_ISL_3901766	2021-09-02
hCoV-19/Japan/PG-91137/2021	EPI_ISL_3901769	2021-09-02
hCoV-19/Japan/PG-91138/2021	EPI_ISL_3901773	2021-09-02
hCoV-19/Japan/PG-91139/2021	EPI_ISL_3901776	2021-09-02
hCoV-19/Japan/PG-91140/2021	EPI_ISL_3901779	2021-09-02
hCoV-19/Japan/PG-91141/2021	EPI_ISL_3901782	2021-09-02
hCoV-19/Japan/PG-91142/2021	EPI_ISL_3901785	2021-09-02
hCoV-19/Japan/PG-91143/2021	EPI_ISL_3901788	2021-09-02
hCoV-19/Japan/PG-91144/2021	EPI_ISL_3901792	2021-09-02
hCoV-19/Japan/PG-91145/2021	EPI_ISL_3901795	2021-09-02
hCoV-19/Japan/PG-91146/2021	EPI_ISL_3901798	2021-09-02

virus_name	epiID	submission_date
hCoV-19/Japan/PG-91147/2021	EPI_ISL_3901801	2021-09-02
hCoV-19/Japan/PG-91148/2021	EPI_ISL_3901805	2021-09-02
hCoV-19/Japan/PG-91149/2021	EPI_ISL_3901806	2021-09-02
hCoV-19/Japan/PG-91150/2021	EPI_ISL_3901807	2021-09-02
hCoV-19/Japan/PG-91151/2021	EPI_ISL_3901809	2021-09-02
hCoV-19/Japan/PG-91152/2021	EPI_ISL_3901812	2021-09-02
hCoV-19/Japan/PG-91153/2021	EPI_ISL_3901815	2021-09-02
hCoV-19/Japan/PG-91154/2021	EPI_ISL_3901818	2021-09-02
hCoV-19/Japan/PG-91155/2021	EPI_ISL_3901820	2021-09-02
hCoV-19/Japan/PG-91156/2021	EPI_ISL_3901821	2021-09-02
hCoV-19/Japan/PG-91157/2021	EPI_ISL_3901823	2021-09-02
hCoV-19/Japan/PG-91158/2021	EPI_ISL_3901826	2021-09-02
hCoV-19/Japan/PG-91159/2021	EPI_ISL_3901829	2021-09-02
hCoV-19/Japan/PG-91160/2021	EPI_ISL_3901832	2021-09-02
hCoV-19/Japan/PG-91161/2021	EPI_ISL_3901835	2021-09-02
hCoV-19/Japan/PG-91162/2021	EPI_ISL_3901839	2021-09-02
hCoV-19/Japan/PG-91163/2021	EPI_ISL_3901842	2021-09-02
hCoV-19/Japan/PG-91164/2021	EPI_ISL_3901843	2021-09-02
hCoV-19/Japan/PG-91165/2021	EPI_ISL_3901844	2021-09-02
hCoV-19/Japan/PG-91166/2021	EPI_ISL_3901846	2021-09-02
hCoV-19/Japan/PG-91167/2021	EPI_ISL_3901847	2021-09-02
hCoV-19/Japan/PG-91168/2021	EPI_ISL_3901848	2021-09-02
hCoV-19/Japan/PG-91169/2021	EPI_ISL_3901850	2021-09-02
hCoV-19/Japan/PG-91170/2021	EPI_ISL_3901851	2021-09-02
hCoV-19/Japan/PG-91171/2021	EPI_ISL_3901852	2021-09-02
hCoV-19/Japan/PG-91172/2021	EPI_ISL_3901854	2021-09-02
hCoV-19/Japan/PG-91173/2021	EPI_ISL_3901855	2021-09-02
hCoV-19/Japan/PG-91174/2021	EPI_ISL_3901856	2021-09-02
hCoV-19/Japan/PG-91175/2021	EPI_ISL_3901858	2021-09-02
hCoV-19/Japan/PG-91176/2021	EPI_ISL_3901859	2021-09-02
hCoV-19/Japan/PG-91177/2021	EPI_ISL_3901860	2021-09-02
hCoV-19/Japan/PG-91178/2021	EPI_ISL_3901861	2021-09-02
hCoV-19/Japan/PG-91179/2021	EPI_ISL_3901864	2021-09-02
hCoV-19/Japan/PG-91180/2021	EPI_ISL_3901868	2021-09-02
hCoV-19/Japan/PG-91181/2021	EPI_ISL_3901870	2021-09-02
hCoV-19/Japan/PG-91182/2021	EPI_ISL_3901873	2021-09-02
hCoV-19/Japan/PG-91183/2021	EPI_ISL_3901876	2021-09-02
hCoV-19/Japan/PG-91184/2021	EPI_ISL_3901880	2021-09-02
hCoV-19/Japan/PG-91185/2021	EPI_ISL_3901883	2021-09-02
hCoV-19/Japan/PG-91186/2021	EPI_ISL_3901886	2021-09-02
hCoV-19/Japan/PG-91187/2021	EPI_ISL_3901889	2021-09-02
hCoV-19/Japan/PG-91188/2021	EPI_ISL_3901892	2021-09-02
hCoV-19/Japan/PG-91189/2021	EPI_ISL_3901893	2021-09-02
hCoV-19/Japan/PG-91190/2021	EPI_ISL_3901894	2021-09-02
hCoV-19/Japan/PG-91191/2021	EPI_ISL_3901897	2021-09-02
hCoV-19/Japan/PG-91192/2021	EPI_ISL_3901900	2021-09-02
hCoV-19/Japan/PG-91193/2021	EPI_ISL_3901903	2021-09-02
hCoV-19/Japan/PG-91194/2021	EPI_ISL_3901906	2021-09-02
hCoV-19/Japan/PG-91195/2021	EPI_ISL_3901910	2021-09-02
hCoV-19/Japan/PG-91196/2021	EPI_ISL_3901913	2021-09-02
hCoV-19/Japan/PG-91197/2021	EPI_ISL_3901916	2021-09-02
hCoV-19/Japan/PG-91198/2021	EPI_ISL_3901919	2021-09-02
hCoV-19/Japan/PG-91199/2021	EPI_ISL_3901921	2021-09-02
hCoV-19/Japan/PG-91200/2021	EPI_ISL_3901922	2021-09-02

virus_name	epiID	submission_date
hCoV-19/Japan/PG-91201/2021	EPI_ISL_3901924	2021-09-02
hCoV-19/Japan/PG-91202/2021	EPI_ISL_3901928	2021-09-02
hCoV-19/Japan/PG-91203/2021	EPI_ISL_3901931	2021-09-02
hCoV-19/Japan/PG-91204/2021	EPI_ISL_3901934	2021-09-02
hCoV-19/Japan/PG-91205/2021	EPI_ISL_3901937	2021-09-02
hCoV-19/Japan/PG-91206/2021	EPI_ISL_3901940	2021-09-02
hCoV-19/Japan/PG-91207/2021	EPI_ISL_3901944	2021-09-02
hCoV-19/Japan/PG-91208/2021	EPI_ISL_3901947	2021-09-02
hCoV-19/Japan/PG-91209/2021	EPI_ISL_3901950	2021-09-02
hCoV-19/Japan/PG-91210/2021	EPI_ISL_3901953	2021-09-02
hCoV-19/Japan/PG-91211/2021	EPI_ISL_3901956	2021-09-02
hCoV-19/Japan/PG-91212/2021	EPI_ISL_3901960	2021-09-02
hCoV-19/Japan/PG-91213/2021	EPI_ISL_3901963	2021-09-02
hCoV-19/Japan/PG-91214/2021	EPI_ISL_3901966	2021-09-02
hCoV-19/Japan/PG-91215/2021	EPI_ISL_3901969	2021-09-02
hCoV-19/Japan/PG-91216/2021	EPI_ISL_3901973	2021-09-02
hCoV-19/Japan/PG-91217/2021	EPI_ISL_3901974	2021-09-02
hCoV-19/Japan/PG-91218/2021	EPI_ISL_3901975	2021-09-02
hCoV-19/Japan/PG-91219/2021	EPI_ISL_3901977	2021-09-02
hCoV-19/Japan/PG-91220/2021	EPI_ISL_3901981	2021-09-02
hCoV-19/Japan/PG-91221/2021	EPI_ISL_3901984	2021-09-02
hCoV-19/Japan/PG-91222/2021	EPI_ISL_3901987	2021-09-02
hCoV-19/Japan/PG-91223/2021	EPI_ISL_3901990	2021-09-02
hCoV-19/Japan/PG-91224/2021	EPI_ISL_3901993	2021-09-02
hCoV-19/Japan/PG-91225/2021	EPI_ISL_3901995	2021-09-02
hCoV-19/Japan/PG-91226/2021	EPI_ISL_3901996	2021-09-02
hCoV-19/Japan/PG-91227/2021	EPI_ISL_3901998	2021-09-02
hCoV-19/Japan/PG-91228/2021	EPI_ISL_3902000	2021-09-02
hCoV-19/Japan/PG-91229/2021	EPI_ISL_3902001	2021-09-02
hCoV-19/Japan/PG-91230/2021	EPI_ISL_3902002	2021-09-02
hCoV-19/Japan/PG-91231/2021	EPI_ISL_3902004	2021-09-02
hCoV-19/Japan/PG-91232/2021	EPI_ISL_3902005	2021-09-02
hCoV-19/Japan/PG-91233/2021	EPI_ISL_3902006	2021-09-02
hCoV-19/Japan/PG-91234/2021	EPI_ISL_3902008	2021-09-02
hCoV-19/Japan/PG-91235/2021	EPI_ISL_3902009	2021-09-02
hCoV-19/Japan/PG-91236/2021	EPI_ISL_3902010	2021-09-02
hCoV-19/Japan/PG-91237/2021	EPI_ISL_3902012	2021-09-02
hCoV-19/Japan/PG-91238/2021	EPI_ISL_3902013	2021-09-02
hCoV-19/Japan/PG-91239/2021	EPI_ISL_3902015	2021-09-02
hCoV-19/Japan/PG-91240/2021	EPI_ISL_3902016	2021-09-02
hCoV-19/Japan/PG-91241/2021	EPI_ISL_3902017	2021-09-02
hCoV-19/Japan/PG-91242/2021	EPI_ISL_3902019	2021-09-02
hCoV-19/Japan/PG-91243/2021	EPI_ISL_3902020	2021-09-02
hCoV-19/Japan/PG-91244/2021	EPI_ISL_3902021	2021-09-02
hCoV-19/Japan/PG-91245/2021	EPI_ISL_3902023	2021-09-02
hCoV-19/Japan/PG-91246/2021	EPI_ISL_3902024	2021-09-02
hCoV-19/Japan/PG-91247/2021	EPI_ISL_3902025	2021-09-02
hCoV-19/Japan/PG-91248/2021	EPI_ISL_3902028	2021-09-02
hCoV-19/Japan/PG-91249/2021	EPI_ISL_3902031	2021-09-02
hCoV-19/Japan/PG-91250/2021	EPI_ISL_3902035	2021-09-02
hCoV-19/Japan/PG-91251/2021	EPI_ISL_3902036	2021-09-02
hCoV-19/Japan/PG-91252/2021	EPI_ISL_3902037	2021-09-02
hCoV-19/Japan/PG-91253/2021	EPI_ISL_3902039	2021-09-02
hCoV-19/Japan/PG-91254/2021	EPI_ISL_3902040	2021-09-02

virus_name	epiID	submission_date
hCoV-19/Japan/PG-91255/2021	EPI_ISL_3902042	2021-09-02
hCoV-19/Japan/PG-91256/2021	EPI_ISL_3902043	2021-09-02
hCoV-19/Japan/PG-91257/2021	EPI_ISL_3902044	2021-09-02
hCoV-19/Japan/PG-91258/2021	EPI_ISL_3902046	2021-09-02
hCoV-19/Japan/PG-91259/2021	EPI_ISL_3902047	2021-09-02
hCoV-19/Japan/PG-91260/2021	EPI_ISL_3902048	2021-09-02
hCoV-19/Japan/PG-91261/2021	EPI_ISL_3902050	2021-09-02
hCoV-19/Japan/PG-91262/2021	EPI_ISL_3902051	2021-09-02
hCoV-19/Japan/PG-91263/2021	EPI_ISL_3902052	2021-09-02
hCoV-19/Japan/PG-91264/2021	EPI_ISL_3902054	2021-09-02
hCoV-19/Japan/PG-91265/2021	EPI_ISL_3902055	2021-09-02
hCoV-19/Japan/PG-91266/2021	EPI_ISL_3902057	2021-09-02
hCoV-19/Japan/PG-91267/2021	EPI_ISL_3902058	2021-09-02
hCoV-19/Japan/PG-91268/2021	EPI_ISL_3902059	2021-09-02
hCoV-19/Japan/PG-91269/2021	EPI_ISL_3902061	2021-09-02
hCoV-19/Japan/PG-91270/2021	EPI_ISL_3902062	2021-09-02
hCoV-19/Japan/PG-91271/2021	EPI_ISL_3902063	2021-09-02
hCoV-19/Japan/PG-91272/2021	EPI_ISL_3902065	2021-09-02
hCoV-19/Japan/PG-91273/2021	EPI_ISL_3902066	2021-09-02
hCoV-19/Japan/PG-91274/2021	EPI_ISL_3902067	2021-09-02
hCoV-19/Japan/PG-91275/2021	EPI_ISL_3902069	2021-09-02
hCoV-19/Japan/PG-91276/2021	EPI_ISL_3902070	2021-09-02
hCoV-19/Japan/PG-91277/2021	EPI_ISL_3902071	2021-09-02
hCoV-19/Japan/PG-91278/2021	EPI_ISL_3902073	2021-09-02
hCoV-19/Japan/PG-91279/2021	EPI_ISL_3902074	2021-09-02
hCoV-19/Japan/PG-91280/2021	EPI_ISL_3902075	2021-09-02
hCoV-19/Japan/PG-91281/2021	EPI_ISL_3902077	2021-09-02
hCoV-19/Japan/PG-91282/2021	EPI_ISL_3902078	2021-09-02
hCoV-19/Japan/PG-91283/2021	EPI_ISL_3902080	2021-09-02
hCoV-19/Japan/PG-91284/2021	EPI_ISL_3902083	2021-09-02
hCoV-19/Japan/PG-91285/2021	EPI_ISL_3875277	2021-09-02
hCoV-19/Japan/PG-91286/2021	EPI_ISL_3875278	2021-09-02
hCoV-19/Japan/PG-91287/2021	EPI_ISL_3875279	2021-09-02
hCoV-19/Japan/PG-91290/2021	EPI_ISL_3875280	2021-09-02
hCoV-19/Japan/PG-91291/2021	EPI_ISL_3875281	2021-09-02
hCoV-19/Japan/PG-91292/2021	EPI_ISL_3875282	2021-09-02
hCoV-19/Japan/PG-91293/2021	EPI_ISL_3875283	2021-09-02
hCoV-19/Japan/PG-91294/2021	EPI_ISL_3875284	2021-09-02
hCoV-19/Japan/PG-91295/2021	EPI_ISL_3875285	2021-09-02
hCoV-19/Japan/PG-91296/2021	EPI_ISL_3875286	2021-09-02
hCoV-19/Japan/PG-91297/2021	EPI_ISL_3875287	2021-09-02
hCoV-19/Japan/PG-91298/2021	EPI_ISL_3875288	2021-09-02
hCoV-19/Japan/PG-91299/2021	EPI_ISL_3875289	2021-09-02
hCoV-19/Japan/PG-91300/2021	EPI_ISL_3875290	2021-09-02
hCoV-19/Japan/PG-91301/2021	EPI_ISL_3875291	2021-09-02
hCoV-19/Japan/PG-91302/2021	EPI_ISL_3875292	2021-09-02
hCoV-19/Japan/PG-91303/2021	EPI_ISL_3875293	2021-09-02
hCoV-19/Japan/PG-91304/2021	EPI_ISL_3875294	2021-09-02
hCoV-19/Japan/PG-91305/2021	EPI_ISL_3875295	2021-09-02
hCoV-19/Japan/PG-91306/2021	EPI_ISL_3875296	2021-09-02
hCoV-19/Japan/PG-91307/2021	EPI_ISL_3875297	2021-09-02
hCoV-19/Japan/PG-91308/2021	EPI_ISL_3875298	2021-09-02
hCoV-19/Japan/PG-91309/2021	EPI_ISL_3875299	2021-09-02
hCoV-19/Japan/PG-91310/2021	EPI_ISL_3875300	2021-09-02

virus_name	epiID	submission_date
hCoV-19/Japan/PG-91311/2021	EPI_ISL_3875301	2021-09-02
hCoV-19/Japan/PG-91312/2021	EPI_ISL_3875302	2021-09-02
hCoV-19/Japan/PG-91313/2021	EPI_ISL_3875303	2021-09-02
hCoV-19/Japan/PG-91314/2021	EPI_ISL_3875304	2021-09-02
hCoV-19/Japan/PG-91315/2021	EPI_ISL_3875305	2021-09-02
hCoV-19/Japan/PG-91316/2021	EPI_ISL_3875306	2021-09-02
hCoV-19/Japan/PG-91317/2021	EPI_ISL_3875307	2021-09-02
hCoV-19/Japan/PG-91318/2021	EPI_ISL_3875308	2021-09-02
hCoV-19/Japan/PG-91319/2021	EPI_ISL_3875309	2021-09-02
hCoV-19/Japan/PG-91320/2021	EPI_ISL_3875310	2021-09-02
hCoV-19/Japan/PG-91321/2021	EPI_ISL_3875311	2021-09-02
hCoV-19/Japan/PG-91322/2021	EPI_ISL_3875312	2021-09-02
hCoV-19/Japan/PG-91323/2021	EPI_ISL_3875313	2021-09-02
hCoV-19/Japan/PG-91324/2021	EPI_ISL_3875314	2021-09-02
hCoV-19/Japan/PG-91325/2021	EPI_ISL_3875315	2021-09-02
hCoV-19/Japan/PG-91326/2021	EPI_ISL_3875316	2021-09-02
hCoV-19/Japan/PG-91327/2021	EPI_ISL_3875317	2021-09-02
hCoV-19/Japan/PG-91328/2021	EPI_ISL_3875318	2021-09-02
hCoV-19/Japan/PG-91329/2021	EPI_ISL_3875319	2021-09-02
hCoV-19/Japan/PG-91330/2021	EPI_ISL_3875320	2021-09-02
hCoV-19/Japan/PG-91331/2021	EPI_ISL_3875321	2021-09-02
hCoV-19/Japan/PG-91332/2021	EPI_ISL_3875322	2021-09-02
hCoV-19/Japan/PG-91333/2021	EPI_ISL_3875323	2021-09-02
hCoV-19/Japan/PG-91334/2021	EPI_ISL_3875324	2021-09-02
hCoV-19/Japan/PG-91335/2021	EPI_ISL_3875325	2021-09-02
hCoV-19/Japan/PG-91336/2021	EPI_ISL_3875326	2021-09-02
hCoV-19/Japan/PG-91337/2021	EPI_ISL_3875327	2021-09-02
hCoV-19/Japan/PG-91338/2021	EPI_ISL_3875328	2021-09-02
hCoV-19/Japan/PG-91339/2021	EPI_ISL_3875329	2021-09-02
hCoV-19/Japan/PG-91340/2021	EPI_ISL_3875330	2021-09-02
hCoV-19/Japan/PG-91341/2021	EPI_ISL_3875331	2021-09-02
hCoV-19/Japan/PG-91342/2021	EPI_ISL_3875332	2021-09-02
hCoV-19/Japan/PG-91343/2021	EPI_ISL_3875333	2021-09-02
hCoV-19/Japan/PG-91344/2021	EPI_ISL_3875334	2021-09-02
hCoV-19/Japan/PG-91345/2021	EPI_ISL_3875335	2021-09-02
hCoV-19/Japan/PG-91346/2021	EPI_ISL_3875336	2021-09-02
hCoV-19/Japan/PG-91347/2021	EPI_ISL_3875337	2021-09-02
hCoV-19/Japan/PG-91348/2021	EPI_ISL_3875338	2021-09-02
hCoV-19/Japan/PG-91349/2021	EPI_ISL_3875339	2021-09-02
hCoV-19/Japan/PG-91350/2021	EPI_ISL_3875340	2021-09-02
hCoV-19/Japan/PG-91351/2021	EPI_ISL_3875341	2021-09-02
hCoV-19/Japan/PG-91352/2021	EPI_ISL_3875342	2021-09-02
hCoV-19/Japan/PG-91353/2021	EPI_ISL_3875343	2021-09-02
hCoV-19/Japan/PG-91354/2021	EPI_ISL_3875344	2021-09-02
hCoV-19/Japan/PG-91355/2021	EPI_ISL_3875345	2021-09-02
hCoV-19/Japan/PG-91356/2021	EPI_ISL_3875346	2021-09-02
hCoV-19/Japan/PG-91357/2021	EPI_ISL_3875347	2021-09-02
hCoV-19/Japan/PG-91358/2021	EPI_ISL_3875348	2021-09-02
hCoV-19/Japan/PG-91359/2021	EPI_ISL_3875349	2021-09-02
hCoV-19/Japan/PG-91360/2021	EPI_ISL_3875350	2021-09-02
hCoV-19/Japan/PG-91361/2021	EPI_ISL_3875351	2021-09-02
hCoV-19/Japan/PG-91362/2021	EPI_ISL_3875352	2021-09-02
hCoV-19/Japan/PG-91363/2021	EPI_ISL_3875353	2021-09-02
hCoV-19/Japan/PG-91364/2021	EPI_ISL_3875354	2021-09-02

virus_name	epiID	submission_date
hCoV-19/Japan/PG-91365/2021	EPI_ISL_3875355	2021-09-02
hCoV-19/Japan/PG-91366/2021	EPI_ISL_3875356	2021-09-02
hCoV-19/Japan/PG-91367/2021	EPI_ISL_3875357	2021-09-02
hCoV-19/Japan/PG-91368/2021	EPI_ISL_3875358	2021-09-02
hCoV-19/Japan/PG-91369/2021	EPI_ISL_3875359	2021-09-02
hCoV-19/Japan/PG-91370/2021	EPI_ISL_3875360	2021-09-02
hCoV-19/Japan/PG-91371/2021	EPI_ISL_3875361	2021-09-02
hCoV-19/Japan/PG-91372/2021	EPI_ISL_3875362	2021-09-02
hCoV-19/Japan/PG-91373/2021	EPI_ISL_3875363	2021-09-02
hCoV-19/Japan/PG-91374/2021	EPI_ISL_3875364	2021-09-02
hCoV-19/Japan/PG-91375/2021	EPI_ISL_3875365	2021-09-02
hCoV-19/Japan/PG-91376/2021	EPI_ISL_3875366	2021-09-02
hCoV-19/Japan/PG-91377/2021	EPI_ISL_3875367	2021-09-02
hCoV-19/Japan/PG-91378/2021	EPI_ISL_3875368	2021-09-02
hCoV-19/Japan/PG-91379/2021	EPI_ISL_3875369	2021-09-02
hCoV-19/Japan/PG-91380/2021	EPI_ISL_3875370	2021-09-02
hCoV-19/Japan/PG-91381/2021	EPI_ISL_3875371	2021-09-02
hCoV-19/Japan/PG-91382/2021	EPI_ISL_3875372	2021-09-02
hCoV-19/Japan/PG-91383/2021	EPI_ISL_3875373	2021-09-02
hCoV-19/Japan/PG-91384/2021	EPI_ISL_3875374	2021-09-02
hCoV-19/Japan/PG-91385/2021	EPI_ISL_3875375	2021-09-02
hCoV-19/Japan/PG-91386/2021	EPI_ISL_3875376	2021-09-02
hCoV-19/Japan/PG-91387/2021	EPI_ISL_3875377	2021-09-02
hCoV-19/Japan/PG-91388/2021	EPI_ISL_3875378	2021-09-02
hCoV-19/Japan/PG-91389/2021	EPI_ISL_3875379	2021-09-02
hCoV-19/Japan/PG-91390/2021	EPI_ISL_3875380	2021-09-02
hCoV-19/Japan/PG-91393/2021	EPI_ISL_3875381	2021-09-02
hCoV-19/Japan/PG-91394/2021	EPI_ISL_3875382	2021-09-02
hCoV-19/Japan/PG-91395/2021	EPI_ISL_3875383	2021-09-02
hCoV-19/Japan/PG-91396/2021	EPI_ISL_3875384	2021-09-02
hCoV-19/Japan/PG-91397/2021	EPI_ISL_3875385	2021-09-02
hCoV-19/Japan/PG-91398/2021	EPI_ISL_3875386	2021-09-02
hCoV-19/Japan/PG-91399/2021	EPI_ISL_3875387	2021-09-02
hCoV-19/Japan/PG-91400/2021	EPI_ISL_3875388	2021-09-02
hCoV-19/Japan/PG-91401/2021	EPI_ISL_3875389	2021-09-02
hCoV-19/Japan/PG-91402/2021	EPI_ISL_3875390	2021-09-02
hCoV-19/Japan/PG-91403/2021	EPI_ISL_3875391	2021-09-02
hCoV-19/Japan/PG-91404/2021	EPI_ISL_3875392	2021-09-02
hCoV-19/Japan/PG-91405/2021	EPI_ISL_3875393	2021-09-02
hCoV-19/Japan/PG-91406/2021	EPI_ISL_3875394	2021-09-02
hCoV-19/Japan/PG-91407/2021	EPI_ISL_3875395	2021-09-02
hCoV-19/Japan/PG-91408/2021	EPI_ISL_3875396	2021-09-02
hCoV-19/Japan/PG-91409/2021	EPI_ISL_3875397	2021-09-02
hCoV-19/Japan/PG-91410/2021	EPI_ISL_3875398	2021-09-02
hCoV-19/Japan/PG-91411/2021	EPI_ISL_3875399	2021-09-02
hCoV-19/Japan/PG-91412/2021	EPI_ISL_3875400	2021-09-02
hCoV-19/Japan/PG-91413/2021	EPI_ISL_3875401	2021-09-02
hCoV-19/Japan/PG-91414/2021	EPI_ISL_3875402	2021-09-02
hCoV-19/Japan/PG-91415/2021	EPI_ISL_3875403	2021-09-02
hCoV-19/Japan/PG-91416/2021	EPI_ISL_3875404	2021-09-02
hCoV-19/Japan/PG-91417/2021	EPI_ISL_3875405	2021-09-02
hCoV-19/Japan/PG-91418/2021	EPI_ISL_3875406	2021-09-02
hCoV-19/Japan/PG-91419/2021	EPI_ISL_3875407	2021-09-02
hCoV-19/Japan/PG-91420/2021	EPI_ISL_3875408	2021-09-02

virus_name	epiID	submission_date
hCoV-19/Japan/PG-91421/2021	EPI_ISL_3875409	2021-09-02
hCoV-19/Japan/PG-91422/2021	EPI_ISL_3875410	2021-09-02
hCoV-19/Japan/PG-91423/2021	EPI_ISL_3875411	2021-09-02
hCoV-19/Japan/PG-91424/2021	EPI_ISL_3875412	2021-09-02
hCoV-19/Japan/PG-91425/2021	EPI_ISL_3875413	2021-09-02
hCoV-19/Japan/PG-91426/2021	EPI_ISL_3875414	2021-09-02
hCoV-19/Japan/PG-91427/2021	EPI_ISL_3875416	2021-09-02
hCoV-19/Japan/PG-91428/2021	EPI_ISL_3875418	2021-09-02
hCoV-19/Japan/PG-91429/2021	EPI_ISL_3875421	2021-09-02
hCoV-19/Japan/PG-91430/2021	EPI_ISL_3875425	2021-09-02
hCoV-19/Japan/PG-91431/2021	EPI_ISL_3875428	2021-09-02
hCoV-19/Japan/PG-91432/2021	EPI_ISL_3875432	2021-09-02
hCoV-19/Japan/PG-91433/2021	EPI_ISL_3875436	2021-09-02
hCoV-19/Japan/PG-91434/2021	EPI_ISL_3875439	2021-09-02
hCoV-19/Japan/PG-91435/2021	EPI_ISL_3875443	2021-09-02
hCoV-19/Japan/PG-91436/2021	EPI_ISL_3875446	2021-09-02
hCoV-19/Japan/PG-91437/2021	EPI_ISL_3875450	2021-09-02
hCoV-19/Japan/PG-91438/2021	EPI_ISL_3875453	2021-09-02
hCoV-19/Japan/PG-91439/2021	EPI_ISL_3875456	2021-09-02
hCoV-19/Japan/PG-91440/2021	EPI_ISL_3875457	2021-09-02
hCoV-19/Japan/PG-91441/2021	EPI_ISL_3875458	2021-09-02
hCoV-19/Japan/PG-91442/2021	EPI_ISL_3875459	2021-09-02
hCoV-19/Japan/PG-91443/2021	EPI_ISL_3871707	2021-09-02
hCoV-19/Japan/PG-91444/2021	EPI_ISL_3871708	2021-09-02
hCoV-19/Japan/PG-91445/2021	EPI_ISL_3871709	2021-09-02
hCoV-19/Japan/PG-91446/2021	EPI_ISL_3871710	2021-09-02
hCoV-19/Japan/PG-91447/2021	EPI_ISL_3871711	2021-09-02
hCoV-19/Japan/PG-91448/2021	EPI_ISL_3871712	2021-09-02
hCoV-19/Japan/PG-91449/2021	EPI_ISL_3871713	2021-09-02
hCoV-19/Japan/PG-91450/2021	EPI_ISL_3871714	2021-09-02
hCoV-19/Japan/PG-91451/2021	EPI_ISL_3871715	2021-09-02
hCoV-19/Japan/PG-91452/2021	EPI_ISL_3871716	2021-09-02
hCoV-19/Japan/PG-91453/2021	EPI_ISL_3871717	2021-09-02
hCoV-19/Japan/PG-91454/2021	EPI_ISL_3871718	2021-09-02
hCoV-19/Japan/PG-91455/2021	EPI_ISL_3871719	2021-09-02
hCoV-19/Japan/PG-91456/2021	EPI_ISL_3871720	2021-09-02
hCoV-19/Japan/PG-91457/2021	EPI_ISL_3871721	2021-09-02
hCoV-19/Japan/PG-91458/2021	EPI_ISL_3871722	2021-09-02
hCoV-19/Japan/PG-91459/2021	EPI_ISL_3871723	2021-09-02
hCoV-19/Japan/PG-91460/2021	EPI_ISL_3871724	2021-09-02
hCoV-19/Japan/PG-91461/2021	EPI_ISL_3871725	2021-09-02
hCoV-19/Japan/PG-91462/2021	EPI_ISL_3871726	2021-09-02
hCoV-19/Japan/PG-91463/2021	EPI_ISL_3871727	2021-09-02
hCoV-19/Japan/PG-91464/2021	EPI_ISL_3871728	2021-09-02
hCoV-19/Japan/PG-91465/2021	EPI_ISL_3871729	2021-09-02
hCoV-19/Japan/PG-91466/2021	EPI_ISL_3871730	2021-09-02
hCoV-19/Japan/PG-91467/2021	EPI_ISL_3871731	2021-09-02
hCoV-19/Japan/PG-91468/2021	EPI_ISL_3871732	2021-09-02
hCoV-19/Japan/PG-91469/2021	EPI_ISL_3871733	2021-09-02
hCoV-19/Japan/PG-91470/2021	EPI_ISL_3871734	2021-09-02
hCoV-19/Japan/PG-91471/2021	EPI_ISL_3871735	2021-09-02
hCoV-19/Japan/PG-91472/2021	EPI_ISL_3871736	2021-09-02
hCoV-19/Japan/PG-91473/2021	EPI_ISL_3871737	2021-09-02
hCoV-19/Japan/PG-91474/2021	EPI_ISL_3871738	2021-09-02

virus_name	epiID	submission_date
hCoV-19/Japan/PG-91475/2021	EPI_ISL_3871739	2021-09-02
hCoV-19/Japan/PG-91476/2021	EPI_ISL_3871740	2021-09-02
hCoV-19/Japan/PG-91477/2021	EPI_ISL_3871741	2021-09-02
hCoV-19/Japan/PG-91478/2021	EPI_ISL_3871742	2021-09-02
hCoV-19/Japan/PG-91479/2021	EPI_ISL_3871743	2021-09-02
hCoV-19/Japan/PG-91480/2021	EPI_ISL_3871744	2021-09-02
hCoV-19/Japan/PG-91481/2021	EPI_ISL_3871745	2021-09-02
hCoV-19/Japan/PG-91496/2021	EPI_ISL_3876256	2021-09-02
hCoV-19/Japan/PG-91497/2021	EPI_ISL_3876257	2021-09-02
hCoV-19/Japan/PG-91498/2021	EPI_ISL_3876258	2021-09-02
hCoV-19/Japan/PG-91513/2021	EPI_ISL_3875926	2021-09-02
hCoV-19/Japan/PG-91514/2021	EPI_ISL_3875927	2021-09-02
hCoV-19/Japan/PG-91515/2021	EPI_ISL_3875928	2021-09-02
hCoV-19/Japan/PG-91516/2021	EPI_ISL_3875929	2021-09-02
hCoV-19/Japan/PG-91517/2021	EPI_ISL_3875930	2021-09-02
hCoV-19/Japan/PG-91518/2021	EPI_ISL_3875931	2021-09-02
hCoV-19/Japan/PG-91519/2021	EPI_ISL_3875932	2021-09-02
hCoV-19/Japan/PG-91520/2021	EPI_ISL_3875933	2021-09-02
hCoV-19/Japan/PG-91521/2021	EPI_ISL_3875934	2021-09-02
hCoV-19/Japan/PG-91522/2021	EPI_ISL_3875935	2021-09-02
hCoV-19/Japan/PG-91523/2021	EPI_ISL_3875936	2021-09-02
hCoV-19/Japan/PG-91524/2021	EPI_ISL_3875937	2021-09-02
hCoV-19/Japan/PG-91525/2021	EPI_ISL_3875938	2021-09-02
hCoV-19/Japan/PG-91526/2021	EPI_ISL_3875939	2021-09-02
hCoV-19/Japan/PG-91527/2021	EPI_ISL_3875940	2021-09-02
hCoV-19/Japan/PG-91528/2021	EPI_ISL_3875941	2021-09-02
hCoV-19/Japan/PG-91529/2021	EPI_ISL_3875942	2021-09-02
hCoV-19/Japan/PG-91530/2021	EPI_ISL_3875943	2021-09-02
hCoV-19/Japan/PG-91531/2021	EPI_ISL_3875944	2021-09-02
hCoV-19/Japan/PG-91532/2021	EPI_ISL_3875945	2021-09-02
hCoV-19/Japan/PG-91533/2021	EPI_ISL_3875946	2021-09-02
hCoV-19/Japan/PG-91534/2021	EPI_ISL_3875947	2021-09-02
hCoV-19/Japan/PG-91535/2021	EPI_ISL_3875948	2021-09-02
hCoV-19/Japan/PG-91536/2021	EPI_ISL_3875949	2021-09-02
hCoV-19/Japan/PG-91537/2021	EPI_ISL_3875950	2021-09-02
hCoV-19/Japan/PG-91538/2021	EPI_ISL_3875951	2021-09-02
hCoV-19/Japan/PG-91539/2021	EPI_ISL_3875952	2021-09-02
hCoV-19/Japan/PG-91540/2021	EPI_ISL_3875953	2021-09-02
hCoV-19/Japan/PG-91545/2021	EPI_ISL_3875954	2021-09-02
hCoV-19/Japan/PG-91628/2021	EPI_ISL_3873274	2021-09-02
hCoV-19/Japan/PG-91629/2021	EPI_ISL_3873275	2021-09-02
hCoV-19/Japan/PG-91630/2021	EPI_ISL_3873276	2021-09-02
hCoV-19/Japan/PG-91631/2021	EPI_ISL_3873277	2021-09-02
hCoV-19/Japan/PG-91632/2021	EPI_ISL_3873278	2021-09-02
hCoV-19/Japan/PG-91633/2021	EPI_ISL_3873279	2021-09-02
hCoV-19/Japan/PG-91634/2021	EPI_ISL_3873280	2021-09-02
hCoV-19/Japan/PG-91642/2021	EPI_ISL_3873281	2021-09-02
hCoV-19/Japan/PG-91643/2021	EPI_ISL_3873282	2021-09-02
hCoV-19/Japan/PG-91644/2021	EPI_ISL_3873283	2021-09-02
hCoV-19/Japan/PG-91645/2021	EPI_ISL_3873284	2021-09-02
hCoV-19/Japan/PG-91663/2021	EPI_ISL_3873285	2021-09-02
hCoV-19/Japan/PG-91664/2021	EPI_ISL_3873286	2021-09-02
hCoV-19/Japan/PG-91665/2021	EPI_ISL_3873287	2021-09-02
hCoV-19/Japan/PG-91666/2021	EPI_ISL_3873288	2021-09-02

virus_name	epiID	submission_date
hCoV-19/Japan/PG-91667/2021	EPI_ISL_3873289	2021-09-02
hCoV-19/Japan/PG-91668/2021	EPI_ISL_3873290	2021-09-02
hCoV-19/Japan/PG-91704/2021	EPI_ISL_3873291	2021-09-02
hCoV-19/Japan/PG-91705/2021	EPI_ISL_3873292	2021-09-02
hCoV-19/Japan/PG-91741/2021	EPI_ISL_3874087	2021-09-02
hCoV-19/Japan/PG-91742/2021	EPI_ISL_3874088	2021-09-02
hCoV-19/Japan/PG-91743/2021	EPI_ISL_3874089	2021-09-02
hCoV-19/Japan/PG-91744/2021	EPI_ISL_3874090	2021-09-02
hCoV-19/Japan/PG-91745/2021	EPI_ISL_3874091	2021-09-02
hCoV-19/Japan/PG-91746/2021	EPI_ISL_3874092	2021-09-02
hCoV-19/Japan/PG-91747/2021	EPI_ISL_3874093	2021-09-02
hCoV-19/Japan/PG-91748/2021	EPI_ISL_3874094	2021-09-02
hCoV-19/Japan/PG-91749/2021	EPI_ISL_3874097	2021-09-02
hCoV-19/Japan/PG-91750/2021	EPI_ISL_3874100	2021-09-02
hCoV-19/Japan/PG-91751/2021	EPI_ISL_3874103	2021-09-02
hCoV-19/Japan/PG-91752/2021	EPI_ISL_3874105	2021-09-02
hCoV-19/Japan/PG-91753/2021	EPI_ISL_3874108	2021-09-02
hCoV-19/Japan/PG-91754/2021	EPI_ISL_3874111	2021-09-02
hCoV-19/Japan/PG-91755/2021	EPI_ISL_3874113	2021-09-02
hCoV-19/Japan/PG-91756/2021	EPI_ISL_3874116	2021-09-02
hCoV-19/Japan/PG-91757/2021	EPI_ISL_3874118	2021-09-02
hCoV-19/Japan/PG-91758/2021	EPI_ISL_3874119	2021-09-02
hCoV-19/Japan/PG-91759/2021	EPI_ISL_3874120	2021-09-02
hCoV-19/Japan/PG-91760/2021	EPI_ISL_3874121	2021-09-02
hCoV-19/Japan/PG-91761/2021	EPI_ISL_3874122	2021-09-02
hCoV-19/Japan/PG-91762/2021	EPI_ISL_3874123	2021-09-02
hCoV-19/Japan/PG-91763/2021	EPI_ISL_3874124	2021-09-02
hCoV-19/Japan/PG-91764/2021	EPI_ISL_3874125	2021-09-02
hCoV-19/Japan/PG-91765/2021	EPI_ISL_3874126	2021-09-02
hCoV-19/Japan/PG-91766/2021	EPI_ISL_3874127	2021-09-02
hCoV-19/Japan/PG-91767/2021	EPI_ISL_3874128	2021-09-02
hCoV-19/Japan/PG-91768/2021	EPI_ISL_3874129	2021-09-02
hCoV-19/Japan/PG-91769/2021	EPI_ISL_3874130	2021-09-02
hCoV-19/Japan/PG-91770/2021	EPI_ISL_3874131	2021-09-02
hCoV-19/Japan/PG-91771/2021	EPI_ISL_3874132	2021-09-02
hCoV-19/Japan/PG-91785/2021	EPI_ISL_3871359	2021-09-02
hCoV-19/Japan/PG-91791/2021	EPI_ISL_3871360	2021-09-02
hCoV-19/Japan/PG-91792/2021	EPI_ISL_3871361	2021-09-02
hCoV-19/Japan/PG-91794/2021	EPI_ISL_3871362	2021-09-02
hCoV-19/Japan/PG-91795/2021	EPI_ISL_3871363	2021-09-02
hCoV-19/Japan/PG-91796/2021	EPI_ISL_3874066	2021-09-02
hCoV-19/Japan/PG-91797/2021	EPI_ISL_3874067	2021-09-02
hCoV-19/Japan/PG-91798/2021	EPI_ISL_3874068	2021-09-02
hCoV-19/Japan/PG-91799/2021	EPI_ISL_3874069	2021-09-02
hCoV-19/Japan/PG-91800/2021	EPI_ISL_3874070	2021-09-02
hCoV-19/Japan/PG-91801/2021	EPI_ISL_3874071	2021-09-02
hCoV-19/Japan/PG-91802/2021	EPI_ISL_3874072	2021-09-02
hCoV-19/Japan/PG-91803/2021	EPI_ISL_3874073	2021-09-02
hCoV-19/Japan/PG-91815/2021	EPI_ISL_3874074	2021-09-02
hCoV-19/Japan/PG-91816/2021	EPI_ISL_3874075	2021-09-02
hCoV-19/Japan/PG-91882/2021	EPI_ISL_3874251	2021-09-02
hCoV-19/Japan/PG-91883/2021	EPI_ISL_3874252	2021-09-02
hCoV-19/Japan/PG-91884/2021	EPI_ISL_3874253	2021-09-02
hCoV-19/Japan/PG-91885/2021	EPI_ISL_3874254	2021-09-02

virus_name	epiID	submission_date
hCoV-19/Japan/PG-91886/2021	EPI_ISL_3874255	2021-09-02
hCoV-19/Japan/PG-91887/2021	EPI_ISL_3874256	2021-09-02
hCoV-19/Japan/PG-91891/2021	EPI_ISL_3872347	2021-09-02
hCoV-19/Japan/PG-91898/2021	EPI_ISL_3872348	2021-09-02
hCoV-19/Japan/PG-91901/2021	EPI_ISL_3872349	2021-09-02
hCoV-19/Japan/PG-91905/2021	EPI_ISL_3872350	2021-09-02
hCoV-19/Japan/PG-91907/2021	EPI_ISL_3872351	2021-09-02
hCoV-19/Japan/PG-91908/2021	EPI_ISL_3872352	2021-09-02
hCoV-19/Japan/PG-91910/2021	EPI_ISL_3872353	2021-09-02
hCoV-19/Japan/PG-91917/2021	EPI_ISL_3872354	2021-09-02
hCoV-19/Japan/PG-91918/2021	EPI_ISL_3872355	2021-09-02
hCoV-19/Japan/PG-91919/2021	EPI_ISL_3902086	2021-09-02
hCoV-19/Japan/PG-91920/2021	EPI_ISL_3902090	2021-09-02
hCoV-19/Japan/PG-91921/2021	EPI_ISL_3902093	2021-09-02
hCoV-19/Japan/PG-91922/2021	EPI_ISL_3902096	2021-09-02
hCoV-19/Japan/PG-91923/2021	EPI_ISL_3902100	2021-09-02
hCoV-19/Japan/PG-91924/2021	EPI_ISL_3902103	2021-09-02
hCoV-19/Japan/PG-91925/2021	EPI_ISL_3902106	2021-09-02
hCoV-19/Japan/PG-91926/2021	EPI_ISL_3902107	2021-09-02
hCoV-19/Japan/PG-91928/2021	EPI_ISL_3902108	2021-09-02
hCoV-19/Japan/PG-91931/2021	EPI_ISL_3902109	2021-09-02
hCoV-19/Japan/PG-91932/2021	EPI_ISL_3902111	2021-09-02
hCoV-19/Japan/PG-91933/2021	EPI_ISL_3902112	2021-09-02
hCoV-19/Japan/PG-91934/2021	EPI_ISL_3902113	2021-09-02
hCoV-19/Japan/PG-91935/2021	EPI_ISL_3902116	2021-09-02
hCoV-19/Japan/PG-91936/2021	EPI_ISL_3902119	2021-09-02
hCoV-19/Japan/PG-91937/2021	EPI_ISL_3902122	2021-09-02
hCoV-19/Japan/PG-91938/2021	EPI_ISL_3902125	2021-09-02
hCoV-19/Japan/PG-91939/2021	EPI_ISL_3902128	2021-09-02
hCoV-19/Japan/PG-91940/2021	EPI_ISL_3902130	2021-09-02
hCoV-19/Japan/PG-91941/2021	EPI_ISL_3902133	2021-09-02
hCoV-19/Japan/PG-91942/2021	EPI_ISL_3902136	2021-09-02
hCoV-19/Japan/PG-91943/2021	EPI_ISL_3902139	2021-09-02
hCoV-19/Japan/PG-91944/2021	EPI_ISL_3902142	2021-09-02
hCoV-19/Japan/PG-91945/2021	EPI_ISL_3902146	2021-09-02
hCoV-19/Japan/PG-91946/2021	EPI_ISL_3902148	2021-09-02
hCoV-19/Japan/PG-91947/2021	EPI_ISL_3902151	2021-09-02
hCoV-19/Japan/PG-91948/2021	EPI_ISL_3902154	2021-09-02
hCoV-19/Japan/PG-91949/2021	EPI_ISL_3902157	2021-09-02
hCoV-19/Japan/PG-91950/2021	EPI_ISL_3902161	2021-09-02
hCoV-19/Japan/PG-91951/2021	EPI_ISL_3902163	2021-09-02
hCoV-19/Japan/PG-91952/2021	EPI_ISL_3902167	2021-09-02
hCoV-19/Japan/PG-91953/2021	EPI_ISL_3902170	2021-09-02
hCoV-19/Japan/PG-91954/2021	EPI_ISL_3902171	2021-09-02
hCoV-19/Japan/PG-91955/2021	EPI_ISL_3902172	2021-09-02
hCoV-19/Japan/PG-91956/2021	EPI_ISL_3902174	2021-09-02
hCoV-19/Japan/PG-91957/2021	EPI_ISL_3902177	2021-09-02
hCoV-19/Japan/PG-91958/2021	EPI_ISL_3902180	2021-09-02
hCoV-19/Japan/PG-91966/2021	EPI_ISL_3902183	2021-09-02
hCoV-19/Japan/PG-91967/2021	EPI_ISL_3902186	2021-09-02
hCoV-19/Japan/PG-91968/2021	EPI_ISL_3902189	2021-09-02
hCoV-19/Japan/PG-91969/2021	EPI_ISL_3902192	2021-09-02
hCoV-19/Japan/PG-91970/2021	EPI_ISL_3902195	2021-09-02
hCoV-19/Japan/PG-91971/2021	EPI_ISL_3902199	2021-09-02

virus_name	epiID	submission_date
hCoV-19/Japan/PG-91972/2021	EPI_ISL_3902202	2021-09-02
hCoV-19/Japan/PG-91973/2021	EPI_ISL_3902205	2021-09-02
hCoV-19/Japan/PG-91974/2021	EPI_ISL_3902209	2021-09-02
hCoV-19/Japan/PG-91975/2021	EPI_ISL_3902212	2021-09-02
hCoV-19/Japan/PG-91976/2021	EPI_ISL_3902216	2021-09-02
hCoV-19/Japan/PG-91977/2021	EPI_ISL_3902217	2021-09-02
hCoV-19/Japan/PG-91978/2021	EPI_ISL_3902218	2021-09-02
hCoV-19/Japan/PG-91979/2021	EPI_ISL_3902220	2021-09-02
hCoV-19/Japan/PG-91980/2021	EPI_ISL_3902221	2021-09-02
hCoV-19/Japan/PG-91981/2021	EPI_ISL_3902222	2021-09-02
hCoV-19/Japan/PG-91982/2021	EPI_ISL_3902224	2021-09-02
hCoV-19/Japan/PG-91983/2021	EPI_ISL_3902225	2021-09-02
hCoV-19/Japan/PG-91984/2021	EPI_ISL_3902227	2021-09-02
hCoV-19/Japan/PG-91985/2021	EPI_ISL_3902228	2021-09-02
hCoV-19/Japan/PG-91986/2021	EPI_ISL_3902229	2021-09-02
hCoV-19/Japan/PG-91987/2021	EPI_ISL_3902232	2021-09-02
hCoV-19/Japan/PG-91988/2021	EPI_ISL_3902234	2021-09-02
hCoV-19/Japan/PG-91989/2021	EPI_ISL_3902238	2021-09-02
hCoV-19/Japan/PG-91990/2021	EPI_ISL_3902241	2021-09-02
hCoV-19/Japan/PG-91991/2021	EPI_ISL_3902245	2021-09-02
hCoV-19/Japan/PG-91992/2021	EPI_ISL_3902248	2021-09-02
hCoV-19/Japan/PG-91993/2021	EPI_ISL_3902251	2021-09-02
hCoV-19/Japan/PG-91994/2021	EPI_ISL_3902255	2021-09-02
hCoV-19/Japan/PG-91995/2021	EPI_ISL_3902258	2021-09-02
hCoV-19/Japan/PG-91996/2021	EPI_ISL_3902262	2021-09-02
hCoV-19/Japan/PG-91997/2021	EPI_ISL_3902265	2021-09-02
hCoV-19/Japan/PG-91998/2021	EPI_ISL_3902269	2021-09-02
hCoV-19/Japan/PG-91999/2021	EPI_ISL_3902272	2021-09-02
hCoV-19/Japan/PG-92000/2021	EPI_ISL_3902276	2021-09-02
hCoV-19/Japan/PG-92001/2021	EPI_ISL_3902279	2021-09-02
hCoV-19/Japan/PG-92002/2021	EPI_ISL_3902283	2021-09-02
hCoV-19/Japan/PG-92003/2021	EPI_ISL_3902286	2021-09-02
hCoV-19/Japan/PG-92004/2021	EPI_ISL_3902287	2021-09-02
hCoV-19/Japan/PG-92005/2021	EPI_ISL_3902288	2021-09-02
hCoV-19/Japan/PG-92006/2021	EPI_ISL_3902290	2021-09-02
hCoV-19/Japan/PG-92007/2021	EPI_ISL_3902291	2021-09-02
hCoV-19/Japan/PG-92008/2021	EPI_ISL_3902292	2021-09-02
hCoV-19/Japan/PG-92009/2021	EPI_ISL_3902294	2021-09-02
hCoV-19/Japan/PG-92010/2021	EPI_ISL_3902295	2021-09-02
hCoV-19/Japan/PG-92011/2021	EPI_ISL_3902296	2021-09-02
hCoV-19/Japan/PG-92012/2021	EPI_ISL_3902299	2021-09-02
hCoV-19/Japan/PG-92013/2021	EPI_ISL_3902302	2021-09-02
hCoV-19/Japan/PG-92014/2021	EPI_ISL_3902305	2021-09-02
hCoV-19/Japan/PG-92015/2021	EPI_ISL_3902309	2021-09-02
hCoV-19/Japan/PG-92016/2021	EPI_ISL_3902311	2021-09-02
hCoV-19/Japan/PG-92017/2021	EPI_ISL_3902313	2021-09-02
hCoV-19/Japan/PG-92018/2021	EPI_ISL_3902316	2021-09-02
hCoV-19/Japan/PG-92019/2021	EPI_ISL_3902319	2021-09-02
hCoV-19/Japan/PG-92020/2021	EPI_ISL_3902322	2021-09-02
hCoV-19/Japan/PG-92021/2021	EPI_ISL_3902325	2021-09-02
hCoV-19/Japan/PG-92022/2021	EPI_ISL_3902328	2021-09-02
hCoV-19/Japan/PG-92023/2021	EPI_ISL_3902329	2021-09-02
hCoV-19/Japan/PG-92024/2021	EPI_ISL_3902330	2021-09-02
hCoV-19/Japan/PG-92025/2021	EPI_ISL_3902332	2021-09-02

virus_name	epiID	submission_date
hCoV-19/Japan/PG-92026/2021	EPI_ISL_3902333	2021-09-02
hCoV-19/Japan/PG-92027/2021	EPI_ISL_3902334	2021-09-02
hCoV-19/Japan/PG-92028/2021	EPI_ISL_3902336	2021-09-02
hCoV-19/Japan/PG-92052/2021	EPI_ISL_3902337	2021-09-02
hCoV-19/Japan/PG-92053/2021	EPI_ISL_3902338	2021-09-02
hCoV-19/Japan/PG-92054/2021	EPI_ISL_3902340	2021-09-02
hCoV-19/Japan/PG-92055/2021	EPI_ISL_3902341	2021-09-02
hCoV-19/Japan/PG-92056/2021	EPI_ISL_3902342	2021-09-02
hCoV-19/Japan/PG-92057/2021	EPI_ISL_3902345	2021-09-02
hCoV-19/Japan/PG-92058/2021	EPI_ISL_3902348	2021-09-02
hCoV-19/Japan/PG-92059/2021	EPI_ISL_3902351	2021-09-02
hCoV-19/Japan/PG-92060/2021	EPI_ISL_3902354	2021-09-02
hCoV-19/Japan/PG-92061/2021	EPI_ISL_3902358	2021-09-02
hCoV-19/Japan/PG-92062/2021	EPI_ISL_3902361	2021-09-02
hCoV-19/Japan/PG-92063/2021	EPI_ISL_3902364	2021-09-02
hCoV-19/Japan/PG-92064/2021	EPI_ISL_3902367	2021-09-02
hCoV-19/Japan/PG-92065/2021	EPI_ISL_3902370	2021-09-02
hCoV-19/Japan/PG-92066/2021	EPI_ISL_3902373	2021-09-02
hCoV-19/Japan/PG-92067/2021	EPI_ISL_3902376	2021-09-02
hCoV-19/Japan/PG-92068/2021	EPI_ISL_3902379	2021-09-02
hCoV-19/Japan/PG-92069/2021	EPI_ISL_3902383	2021-09-02
hCoV-19/Japan/PG-92070/2021	EPI_ISL_3902386	2021-09-02
hCoV-19/Japan/PG-92071/2021	EPI_ISL_3902389	2021-09-02
hCoV-19/Japan/PG-92072/2021	EPI_ISL_3902392	2021-09-02
hCoV-19/Japan/PG-92073/2021	EPI_ISL_3902395	2021-09-02
hCoV-19/Japan/PG-92074/2021	EPI_ISL_3902398	2021-09-02
hCoV-19/Japan/PG-92075/2021	EPI_ISL_3902402	2021-09-02
hCoV-19/Japan/PG-92076/2021	EPI_ISL_3902404	2021-09-02
hCoV-19/Japan/PG-92077/2021	EPI_ISL_3902408	2021-09-02
hCoV-19/Japan/PG-92078/2021	EPI_ISL_3902410	2021-09-02
hCoV-19/Japan/PG-92079/2021	EPI_ISL_3902414	2021-09-02
hCoV-19/Japan/PG-92080/2021	EPI_ISL_3902416	2021-09-02
hCoV-19/Japan/PG-92081/2021	EPI_ISL_3902420	2021-09-02
hCoV-19/Japan/PG-92082/2021	EPI_ISL_3902423	2021-09-02
hCoV-19/Japan/PG-92083/2021	EPI_ISL_3902426	2021-09-02
hCoV-19/Japan/PG-92084/2021	EPI_ISL_3902429	2021-09-02
hCoV-19/Japan/PG-92085/2021	EPI_ISL_3902432	2021-09-02
hCoV-19/Japan/PG-92086/2021	EPI_ISL_3902436	2021-09-02
hCoV-19/Japan/PG-92087/2021	EPI_ISL_3902439	2021-09-02
hCoV-19/Japan/PG-92088/2021	EPI_ISL_3902442	2021-09-02
hCoV-19/Japan/PG-92089/2021	EPI_ISL_3902446	2021-09-02
hCoV-19/Japan/PG-92090/2021	EPI_ISL_3902449	2021-09-02
hCoV-19/Japan/PG-92091/2021	EPI_ISL_3902452	2021-09-02
hCoV-19/Japan/PG-92092/2021	EPI_ISL_3902455	2021-09-02
hCoV-19/Japan/PG-92093/2021	EPI_ISL_3902458	2021-09-02
hCoV-19/Japan/PG-92094/2021	EPI_ISL_3902462	2021-09-02
hCoV-19/Japan/PG-92095/2021	EPI_ISL_3902465	2021-09-02
hCoV-19/Japan/PG-92096/2021	EPI_ISL_3902468	2021-09-02
hCoV-19/Japan/PG-92097/2021	EPI_ISL_3902471	2021-09-02
hCoV-19/Japan/PG-92113/2021	EPI_ISL_3902475	2021-09-02
hCoV-19/Japan/PG-92114/2021	EPI_ISL_3902478	2021-09-02
hCoV-19/Japan/PG-92115/2021	EPI_ISL_3902481	2021-09-02
hCoV-19/Japan/PG-92116/2021	EPI_ISL_3902484	2021-09-02
hCoV-19/Japan/PG-92117/2021	EPI_ISL_3902487	2021-09-02

virus_name	epiID	submission_date
hCoV-19/Japan/PG-92118/2021	EPI_ISL_3902491	2021-09-02
hCoV-19/Japan/PG-92119/2021	EPI_ISL_3902494	2021-09-02
hCoV-19/Japan/PG-92120/2021	EPI_ISL_3902497	2021-09-02
hCoV-19/Japan/PG-92121/2021	EPI_ISL_3902500	2021-09-02
hCoV-19/Japan/PG-92122/2021	EPI_ISL_3902504	2021-09-02
hCoV-19/Japan/PG-92123/2021	EPI_ISL_3902507	2021-09-02
hCoV-19/Japan/PG-92124/2021	EPI_ISL_3902510	2021-09-02
hCoV-19/Japan/PG-92127/2021	EPI_ISL_3902514	2021-09-02
hCoV-19/Japan/PG-92128/2021	EPI_ISL_3902517	2021-09-02
hCoV-19/Japan/PG-92129/2021	EPI_ISL_3902520	2021-09-02
hCoV-19/Japan/PG-92130/2021	EPI_ISL_3902523	2021-09-02
hCoV-19/Japan/PG-92131/2021	EPI_ISL_3902526	2021-09-02
hCoV-19/Japan/PG-92132/2021	EPI_ISL_3902529	2021-09-02
hCoV-19/Japan/PG-92133/2021	EPI_ISL_3902532	2021-09-02
hCoV-19/Japan/PG-92134/2021	EPI_ISL_3902535	2021-09-02
hCoV-19/Japan/PG-92135/2021	EPI_ISL_3902539	2021-09-02
hCoV-19/Japan/PG-92136/2021	EPI_ISL_3902542	2021-09-02
hCoV-19/Japan/PG-92137/2021	EPI_ISL_3902545	2021-09-02
hCoV-19/Japan/PG-92138/2021	EPI_ISL_3902548	2021-09-02
hCoV-19/Japan/PG-92139/2021	EPI_ISL_3902551	2021-09-02
hCoV-19/Japan/PG-92140/2021	EPI_ISL_3902553	2021-09-02
hCoV-19/Japan/PG-92141/2021	EPI_ISL_3902557	2021-09-02
hCoV-19/Japan/PG-92142/2021	EPI_ISL_3902558	2021-09-02
hCoV-19/Japan/PG-92168/2021	EPI_ISL_3902562	2021-09-02
hCoV-19/Japan/PG-92169/2021	EPI_ISL_3902565	2021-09-02
hCoV-19/Japan/PG-92170/2021	EPI_ISL_3902568	2021-09-02
hCoV-19/Japan/PG-92171/2021	EPI_ISL_3902571	2021-09-02
hCoV-19/Japan/PG-92172/2021	EPI_ISL_3902575	2021-09-02
hCoV-19/Japan/PG-92173/2021	EPI_ISL_3902577	2021-09-02
hCoV-19/Japan/PG-92174/2021	EPI_ISL_3902581	2021-09-02
hCoV-19/Japan/PG-92175/2021	EPI_ISL_3902584	2021-09-02
hCoV-19/Japan/PG-92176/2021	EPI_ISL_3902587	2021-09-02
hCoV-19/Japan/PG-92177/2021	EPI_ISL_3902590	2021-09-02
hCoV-19/Japan/PG-92178/2021	EPI_ISL_3902593	2021-09-02
hCoV-19/Japan/PG-92179/2021	EPI_ISL_3902596	2021-09-02
hCoV-19/Japan/PG-92180/2021	EPI_ISL_3902598	2021-09-02
hCoV-19/Japan/PG-92181/2021	EPI_ISL_3902602	2021-09-02
hCoV-19/Japan/PG-92182/2021	EPI_ISL_3902605	2021-09-02
hCoV-19/Japan/PG-92183/2021	EPI_ISL_3902608	2021-09-02
hCoV-19/Japan/PG-92184/2021	EPI_ISL_3902610	2021-09-02
hCoV-19/Japan/PG-92185/2021	EPI_ISL_3902614	2021-09-02
hCoV-19/Japan/PG-92186/2021	EPI_ISL_3902616	2021-09-02
hCoV-19/Japan/PG-92187/2021	EPI_ISL_3902619	2021-09-02
hCoV-19/Japan/PG-92188/2021	EPI_ISL_3902623	2021-09-02
hCoV-19/Japan/PG-92189/2021	EPI_ISL_3902626	2021-09-02
hCoV-19/Japan/PG-92190/2021	EPI_ISL_3902629	2021-09-02
hCoV-19/Japan/PG-92191/2021	EPI_ISL_3902632	2021-09-02
hCoV-19/Japan/PG-92192/2021	EPI_ISL_3902635	2021-09-02
hCoV-19/Japan/PG-92193/2021	EPI_ISL_3902638	2021-09-02
hCoV-19/Japan/PG-92194/2021	EPI_ISL_3902641	2021-09-02
hCoV-19/Japan/PG-92195/2021	EPI_ISL_3902645	2021-09-02
hCoV-19/Japan/PG-92196/2021	EPI_ISL_3902648	2021-09-02
hCoV-19/Japan/PG-92197/2021	EPI_ISL_3902651	2021-09-02
hCoV-19/Japan/PG-92198/2021	EPI_ISL_3902654	2021-09-02

virus_name	epiID	submission_date
hCoV-19/Japan/PG-92199/2021	EPI_ISL_3902657	2021-09-02
hCoV-19/Japan/PG-92200/2021	EPI_ISL_3902661	2021-09-02
hCoV-19/Japan/PG-92201/2021	EPI_ISL_3902664	2021-09-02
hCoV-19/Japan/PG-92202/2021	EPI_ISL_3902667	2021-09-02
hCoV-19/Japan/PG-92203/2021	EPI_ISL_3902670	2021-09-02
hCoV-19/Japan/PG-92204/2021	EPI_ISL_3902673	2021-09-02
hCoV-19/Japan/PG-92205/2021	EPI_ISL_3902676	2021-09-02
hCoV-19/Japan/PG-92206/2021	EPI_ISL_3902680	2021-09-02
hCoV-19/Japan/PG-92207/2021	EPI_ISL_3902682	2021-09-02
hCoV-19/Japan/PG-92208/2021	EPI_ISL_3902686	2021-09-02
hCoV-19/Japan/PG-92209/2021	EPI_ISL_3902689	2021-09-02
hCoV-19/Japan/PG-92210/2021	EPI_ISL_3902692	2021-09-02
hCoV-19/Japan/PG-92211/2021	EPI_ISL_3902695	2021-09-02
hCoV-19/Japan/PG-92212/2021	EPI_ISL_3902699	2021-09-02
hCoV-19/Japan/PG-92213/2021	EPI_ISL_3902702	2021-09-02
hCoV-19/Japan/PG-92214/2021	EPI_ISL_3902705	2021-09-02
hCoV-19/Japan/PG-92215/2021	EPI_ISL_3902709	2021-09-02
hCoV-19/Japan/PG-92216/2021	EPI_ISL_3902712	2021-09-02
hCoV-19/Japan/PG-92217/2021	EPI_ISL_3902715	2021-09-02
hCoV-19/Japan/PG-92218/2021	EPI_ISL_3902718	2021-09-02
hCoV-19/Japan/PG-92219/2021	EPI_ISL_3902722	2021-09-02
hCoV-19/Japan/PG-92220/2021	EPI_ISL_3902725	2021-09-02
hCoV-19/Japan/PG-92221/2021	EPI_ISL_3902728	2021-09-02
hCoV-19/Japan/PG-92222/2021	EPI_ISL_3902731	2021-09-02
hCoV-19/Japan/PG-92223/2021	EPI_ISL_3902733	2021-09-02
hCoV-19/Japan/PG-92224/2021	EPI_ISL_3902734	2021-09-02
hCoV-19/Japan/PG-92225/2021	EPI_ISL_3902735	2021-09-02
hCoV-19/Japan/PG-92226/2021	EPI_ISL_3902736	2021-09-02
hCoV-19/Japan/PG-92227/2021	EPI_ISL_3902737	2021-09-02
hCoV-19/Japan/PG-92228/2021	EPI_ISL_3902738	2021-09-02
hCoV-19/Japan/PG-92229/2021	EPI_ISL_3902739	2021-09-02
hCoV-19/Japan/PG-92230/2021	EPI_ISL_3902740	2021-09-02
hCoV-19/Japan/PG-92231/2021	EPI_ISL_3902741	2021-09-02
hCoV-19/Japan/PG-92232/2021	EPI_ISL_3902742	2021-09-02
hCoV-19/Japan/PG-92233/2021	EPI_ISL_3902743	2021-09-02
hCoV-19/Japan/PG-92234/2021	EPI_ISL_3902744	2021-09-02
hCoV-19/Japan/PG-92235/2021	EPI_ISL_3902745	2021-09-02
hCoV-19/Japan/PG-92236/2021	EPI_ISL_3902746	2021-09-02
hCoV-19/Japan/PG-92237/2021	EPI_ISL_3902747	2021-09-02
hCoV-19/Japan/PG-92238/2021	EPI_ISL_3902748	2021-09-02
hCoV-19/Japan/PG-92239/2021	EPI_ISL_3902749	2021-09-02
hCoV-19/Japan/PG-92240/2021	EPI_ISL_3902750	2021-09-02
hCoV-19/Japan/PG-92241/2021	EPI_ISL_3902751	2021-09-02
hCoV-19/Japan/PG-92242/2021	EPI_ISL_3902752	2021-09-02
hCoV-19/Japan/PG-92243/2021	EPI_ISL_3902753	2021-09-02
hCoV-19/Japan/PG-92244/2021	EPI_ISL_3902754	2021-09-02
hCoV-19/Japan/PG-92245/2021	EPI_ISL_3902755	2021-09-02
hCoV-19/Japan/PG-92246/2021	EPI_ISL_3902756	2021-09-02
hCoV-19/Japan/PG-92247/2021	EPI_ISL_3902757	2021-09-02
hCoV-19/Japan/PG-92248/2021	EPI_ISL_3902758	2021-09-02
hCoV-19/Japan/PG-92249/2021	EPI_ISL_3902759	2021-09-02
hCoV-19/Japan/PG-92250/2021	EPI_ISL_3902760	2021-09-02
hCoV-19/Japan/PG-92251/2021	EPI_ISL_3902761	2021-09-02
hCoV-19/Japan/PG-92252/2021	EPI_ISL_3902762	2021-09-02

virus_name	epiID	submission_date
hCoV-19/Japan/PG-92253/2021	EPI_ISL_3902763	2021-09-02
hCoV-19/Japan/PG-92254/2021	EPI_ISL_3902764	2021-09-02
hCoV-19/Japan/PG-92255/2021	EPI_ISL_3902765	2021-09-02
hCoV-19/Japan/PG-92256/2021	EPI_ISL_3902766	2021-09-02
hCoV-19/Japan/PG-92257/2021	EPI_ISL_3902767	2021-09-02
hCoV-19/Japan/PG-92258/2021	EPI_ISL_3902768	2021-09-02
hCoV-19/Japan/PG-92259/2021	EPI_ISL_3902769	2021-09-02
hCoV-19/Japan/PG-92260/2021	EPI_ISL_3902770	2021-09-02
hCoV-19/Japan/PG-92261/2021	EPI_ISL_3902771	2021-09-02
hCoV-19/Japan/PG-92262/2021	EPI_ISL_3902772	2021-09-02
hCoV-19/Japan/PG-92263/2021	EPI_ISL_3902773	2021-09-02
hCoV-19/Japan/PG-92264/2021	EPI_ISL_3902774	2021-09-02
hCoV-19/Japan/PG-92265/2021	EPI_ISL_3902775	2021-09-02
hCoV-19/Japan/PG-92266/2021	EPI_ISL_3902776	2021-09-02
hCoV-19/Japan/PG-92267/2021	EPI_ISL_3902777	2021-09-02
hCoV-19/Japan/PG-92268/2021	EPI_ISL_3902778	2021-09-02
hCoV-19/Japan/PG-92269/2021	EPI_ISL_3902779	2021-09-02
hCoV-19/Japan/PG-92270/2021	EPI_ISL_3902780	2021-09-02
hCoV-19/Japan/PG-92271/2021	EPI_ISL_3902781	2021-09-02
hCoV-19/Japan/PG-92272/2021	EPI_ISL_3902782	2021-09-02
hCoV-19/Japan/PG-92273/2021	EPI_ISL_3902783	2021-09-02
hCoV-19/Japan/PG-92274/2021	EPI_ISL_3902784	2021-09-02
hCoV-19/Japan/PG-92275/2021	EPI_ISL_3902785	2021-09-02
hCoV-19/Japan/PG-92276/2021	EPI_ISL_3902786	2021-09-02
hCoV-19/Japan/PG-92277/2021	EPI_ISL_3902787	2021-09-02
hCoV-19/Japan/PG-92278/2021	EPI_ISL_3902788	2021-09-02
hCoV-19/Japan/PG-92279/2021	EPI_ISL_3902789	2021-09-02
hCoV-19/Japan/PG-92280/2021	EPI_ISL_3902790	2021-09-02
hCoV-19/Japan/PG-92281/2021	EPI_ISL_3902791	2021-09-02
hCoV-19/Japan/PG-92282/2021	EPI_ISL_3902792	2021-09-02
hCoV-19/Japan/PG-92283/2021	EPI_ISL_3902793	2021-09-02
hCoV-19/Japan/PG-92284/2021	EPI_ISL_3902794	2021-09-02
hCoV-19/Japan/PG-92285/2021	EPI_ISL_3902795	2021-09-02
hCoV-19/Japan/PG-92286/2021	EPI_ISL_3902796	2021-09-02
hCoV-19/Japan/PG-92287/2021	EPI_ISL_3902797	2021-09-02
hCoV-19/Japan/PG-92288/2021	EPI_ISL_3902798	2021-09-02
hCoV-19/Japan/PG-92289/2021	EPI_ISL_3902799	2021-09-02
hCoV-19/Japan/PG-92290/2021	EPI_ISL_3902800	2021-09-02
hCoV-19/Japan/PG-92291/2021	EPI_ISL_3902801	2021-09-02
hCoV-19/Japan/PG-92292/2021	EPI_ISL_3902802	2021-09-02
hCoV-19/Japan/PG-92293/2021	EPI_ISL_3902803	2021-09-02
hCoV-19/Japan/PG-92294/2021	EPI_ISL_3902804	2021-09-02
hCoV-19/Japan/PG-92295/2021	EPI_ISL_3902805	2021-09-02
hCoV-19/Japan/PG-92296/2021	EPI_ISL_3902806	2021-09-02
hCoV-19/Japan/PG-92297/2021	EPI_ISL_3902807	2021-09-02
hCoV-19/Japan/PG-92298/2021	EPI_ISL_3902808	2021-09-02
hCoV-19/Japan/PG-92299/2021	EPI_ISL_3902809	2021-09-02
hCoV-19/Japan/PG-92300/2021	EPI_ISL_3902810	2021-09-02
hCoV-19/Japan/PG-92301/2021	EPI_ISL_3902811	2021-09-02
hCoV-19/Japan/PG-92302/2021	EPI_ISL_3902812	2021-09-02
hCoV-19/Japan/PG-92303/2021	EPI_ISL_3902813	2021-09-02
hCoV-19/Japan/PG-92304/2021	EPI_ISL_3902814	2021-09-02
hCoV-19/Japan/PG-92305/2021	EPI_ISL_3902815	2021-09-02
hCoV-19/Japan/PG-92306/2021	EPI_ISL_3904546	2021-09-02

virus_name	epiID	submission_date
hCoV-19/Japan/PG-92307/2021	EPI_ISL_3902817	2021-09-02
hCoV-19/Japan/PG-92308/2021	EPI_ISL_3902820	2021-09-02
hCoV-19/Japan/PG-92309/2021	EPI_ISL_3902823	2021-09-02
hCoV-19/Japan/PG-92310/2021	EPI_ISL_3902826	2021-09-02
hCoV-19/Japan/PG-92311/2021	EPI_ISL_3902830	2021-09-02
hCoV-19/Japan/PG-92312/2021	EPI_ISL_3902833	2021-09-02
hCoV-19/Japan/PG-92313/2021	EPI_ISL_3902836	2021-09-02
hCoV-19/Japan/PG-92314/2021	EPI_ISL_3902840	2021-09-02
hCoV-19/Japan/PG-92315/2021	EPI_ISL_3902843	2021-09-02
hCoV-19/Japan/PG-92316/2021	EPI_ISL_3902847	2021-09-02
hCoV-19/Japan/PG-92317/2021	EPI_ISL_3902850	2021-09-02
hCoV-19/Japan/PG-92318/2021	EPI_ISL_3902853	2021-09-02
hCoV-19/Japan/PG-92319/2021	EPI_ISL_3902856	2021-09-02
hCoV-19/Japan/PG-92320/2021	EPI_ISL_3902859	2021-09-02
hCoV-19/Japan/PG-92321/2021	EPI_ISL_3902863	2021-09-02
hCoV-19/Japan/PG-92322/2021	EPI_ISL_3902866	2021-09-02
hCoV-19/Japan/PG-92323/2021	EPI_ISL_3902869	2021-09-02
hCoV-19/Japan/PG-92324/2021	EPI_ISL_3902872	2021-09-02
hCoV-19/Japan/PG-92325/2021	EPI_ISL_3902874	2021-09-02
hCoV-19/Japan/PG-92326/2021	EPI_ISL_3902877	2021-09-02
hCoV-19/Japan/PG-92327/2021	EPI_ISL_3902881	2021-09-02
hCoV-19/Japan/PG-92328/2021	EPI_ISL_3902884	2021-09-02
hCoV-19/Japan/PG-92329/2021	EPI_ISL_3902887	2021-09-02
hCoV-19/Japan/PG-92330/2021	EPI_ISL_3904547	2021-09-02
hCoV-19/Japan/PG-92331/2021	EPI_ISL_3902893	2021-09-02
hCoV-19/Japan/PG-92332/2021	EPI_ISL_3902896	2021-09-02
hCoV-19/Japan/PG-92333/2021	EPI_ISL_3902900	2021-09-02
hCoV-19/Japan/PG-92334/2021	EPI_ISL_3902903	2021-09-02
hCoV-19/Japan/PG-92335/2021	EPI_ISL_3902907	2021-09-02
hCoV-19/Japan/PG-92336/2021	EPI_ISL_3902910	2021-09-02
hCoV-19/Japan/PG-92337/2021	EPI_ISL_3902913	2021-09-02
hCoV-19/Japan/PG-92338/2021	EPI_ISL_3902916	2021-09-02
hCoV-19/Japan/PG-92339/2021	EPI_ISL_3902920	2021-09-02
hCoV-19/Japan/PG-92340/2021	EPI_ISL_3902922	2021-09-02
hCoV-19/Japan/PG-92341/2021	EPI_ISL_3902926	2021-09-02
hCoV-19/Japan/PG-92342/2021	EPI_ISL_3902929	2021-09-02
hCoV-19/Japan/PG-92343/2021	EPI_ISL_3902931	2021-09-02
hCoV-19/Japan/PG-92344/2021	EPI_ISL_3902935	2021-09-02
hCoV-19/Japan/PG-92345/2021	EPI_ISL_3904548	2021-09-02
hCoV-19/Japan/PG-92346/2021	EPI_ISL_3902940	2021-09-02
hCoV-19/Japan/PG-92347/2021	EPI_ISL_3902944	2021-09-02
hCoV-19/Japan/PG-92348/2021	EPI_ISL_3902947	2021-09-02
hCoV-19/Japan/PG-92349/2021	EPI_ISL_3902951	2021-09-02
hCoV-19/Japan/PG-92350/2021	EPI_ISL_3902954	2021-09-02
hCoV-19/Japan/PG-92351/2021	EPI_ISL_3902958	2021-09-02
hCoV-19/Japan/PG-92352/2021	EPI_ISL_3902961	2021-09-02
hCoV-19/Japan/PG-92353/2021	EPI_ISL_3902965	2021-09-02
hCoV-19/Japan/PG-92354/2021	EPI_ISL_3902968	2021-09-02
hCoV-19/Japan/PG-92355/2021	EPI_ISL_3902971	2021-09-02
hCoV-19/Japan/PG-92356/2021	EPI_ISL_3902974	2021-09-02
hCoV-19/Japan/PG-92357/2021	EPI_ISL_3902978	2021-09-02
hCoV-19/Japan/PG-92358/2021	EPI_ISL_3902981	2021-09-02
hCoV-19/Japan/PG-92359/2021	EPI_ISL_3902984	2021-09-02
hCoV-19/Japan/PG-92360/2021	EPI_ISL_3902988	2021-09-02

virus_name	epiID	submission_date
hCoV-19/Japan/PG-92361/2021	EPI_ISL_3902991	2021-09-02
hCoV-19/Japan/PG-92362/2021	EPI_ISL_3902994	2021-09-02
hCoV-19/Japan/PG-92363/2021	EPI_ISL_3902997	2021-09-02
hCoV-19/Japan/PG-92364/2021	EPI_ISL_3903000	2021-09-02
hCoV-19/Japan/PG-92365/2021	EPI_ISL_3903004	2021-09-02
hCoV-19/Japan/PG-92366/2021	EPI_ISL_3903007	2021-09-02
hCoV-19/Japan/PG-92367/2021	EPI_ISL_3903010	2021-09-02
hCoV-19/Japan/PG-92368/2021	EPI_ISL_3903013	2021-09-02
hCoV-19/Japan/PG-92369/2021	EPI_ISL_3903016	2021-09-02
hCoV-19/Japan/PG-92370/2021	EPI_ISL_3903019	2021-09-02
hCoV-19/Japan/PG-92371/2021	EPI_ISL_3903022	2021-09-02
hCoV-19/Japan/PG-92372/2021	EPI_ISL_3903025	2021-09-02
hCoV-19/Japan/PG-92373/2021	EPI_ISL_3903028	2021-09-02
hCoV-19/Japan/PG-92374/2021	EPI_ISL_3903032	2021-09-02
hCoV-19/Japan/PG-92375/2021	EPI_ISL_3903035	2021-09-02
hCoV-19/Japan/PG-92376/2021	EPI_ISL_3903039	2021-09-02
hCoV-19/Japan/PG-92377/2021	EPI_ISL_3903042	2021-09-02
hCoV-19/Japan/PG-92378/2021	EPI_ISL_3903046	2021-09-02
hCoV-19/Japan/PG-92379/2021	EPI_ISL_3903049	2021-09-02
hCoV-19/Japan/PG-92380/2021	EPI_ISL_3903053	2021-09-02
hCoV-19/Japan/PG-92381/2021	EPI_ISL_3903056	2021-09-02
hCoV-19/Japan/PG-92382/2021	EPI_ISL_3903060	2021-09-02
hCoV-19/Japan/PG-92383/2021	EPI_ISL_3903063	2021-09-02
hCoV-19/Japan/PG-92384/2021	EPI_ISL_3903066	2021-09-02
hCoV-19/Japan/PG-92385/2021	EPI_ISL_3903069	2021-09-02
hCoV-19/Japan/PG-92386/2021	EPI_ISL_3903073	2021-09-02
hCoV-19/Japan/PG-92387/2021	EPI_ISL_3903076	2021-09-02
hCoV-19/Japan/PG-92388/2021	EPI_ISL_3903079	2021-09-02
hCoV-19/Japan/PG-92389/2021	EPI_ISL_3903082	2021-09-02
hCoV-19/Japan/PG-92390/2021	EPI_ISL_3903085	2021-09-02
hCoV-19/Japan/PG-92391/2021	EPI_ISL_3903088	2021-09-02
hCoV-19/Japan/PG-92392/2021	EPI_ISL_3903092	2021-09-02
hCoV-19/Japan/PG-92393/2021	EPI_ISL_3903094	2021-09-02
hCoV-19/Japan/PG-92394/2021	EPI_ISL_3903098	2021-09-02
hCoV-19/Japan/PG-92395/2021	EPI_ISL_3903101	2021-09-02
hCoV-19/Japan/PG-92396/2021	EPI_ISL_3903104	2021-09-02
hCoV-19/Japan/PG-92397/2021	EPI_ISL_3903108	2021-09-02
hCoV-19/Japan/PG-92398/2021	EPI_ISL_3903111	2021-09-02
hCoV-19/Japan/PG-92399/2021	EPI_ISL_3903114	2021-09-02
hCoV-19/Japan/PG-92400/2021	EPI_ISL_3903118	2021-09-02
hCoV-19/Japan/PG-92401/2021	EPI_ISL_3903121	2021-09-02
hCoV-19/Japan/PG-92402/2021	EPI_ISL_3903125	2021-09-02
hCoV-19/Japan/PG-92403/2021	EPI_ISL_3903128	2021-09-02
hCoV-19/Japan/PG-92404/2021	EPI_ISL_3903131	2021-09-02
hCoV-19/Japan/PG-92405/2021	EPI_ISL_3903133	2021-09-02
hCoV-19/Japan/PG-92406/2021	EPI_ISL_3904549	2021-09-02
hCoV-19/Japan/PG-92407/2021	EPI_ISL_3903139	2021-09-02
hCoV-19/Japan/PG-92408/2021	EPI_ISL_3903142	2021-09-02
hCoV-19/Japan/PG-92409/2021	EPI_ISL_3903144	2021-09-02
hCoV-19/Japan/PG-92410/2021	EPI_ISL_3903147	2021-09-02
hCoV-19/Japan/PG-92411/2021	EPI_ISL_3903151	2021-09-02
hCoV-19/Japan/PG-92412/2021	EPI_ISL_3903154	2021-09-02
hCoV-19/Japan/PG-92413/2021	EPI_ISL_3903157	2021-09-02
hCoV-19/Japan/PG-92414/2021	EPI_ISL_3903161	2021-09-02

virus_name	epiID	submission_date
hCoV-19/Japan/PG-92415/2021	EPI_ISL_3903164	2021-09-02
hCoV-19/Japan/PG-92416/2021	EPI_ISL_3903168	2021-09-02
hCoV-19/Japan/PG-92417/2021	EPI_ISL_3903171	2021-09-02
hCoV-19/Japan/PG-92418/2021	EPI_ISL_3903174	2021-09-02
hCoV-19/Japan/PG-92419/2021	EPI_ISL_3903178	2021-09-02
hCoV-19/Japan/PG-92420/2021	EPI_ISL_3903180	2021-09-02
hCoV-19/Japan/PG-92421/2021	EPI_ISL_3903184	2021-09-02
hCoV-19/Japan/PG-92422/2021	EPI_ISL_3903187	2021-09-02
hCoV-19/Japan/PG-92425/2021	EPI_ISL_3903189	2021-09-02
hCoV-19/Japan/PG-92426/2021	EPI_ISL_3903193	2021-09-02
hCoV-19/Japan/PG-92427/2021	EPI_ISL_3903195	2021-09-02
hCoV-19/Japan/PG-92428/2021	EPI_ISL_3903198	2021-09-02
hCoV-19/Japan/PG-92429/2021	EPI_ISL_3903201	2021-09-02
hCoV-19/Japan/PG-92430/2021	EPI_ISL_3903205	2021-09-02
hCoV-19/Japan/PG-92431/2021	EPI_ISL_3903208	2021-09-02
hCoV-19/Japan/PG-92432/2021	EPI_ISL_3903211	2021-09-02
hCoV-19/Japan/PG-92433/2021	EPI_ISL_3903215	2021-09-02
hCoV-19/Japan/PG-92434/2021	EPI_ISL_3903218	2021-09-02
hCoV-19/Japan/PG-92435/2021	EPI_ISL_3903222	2021-09-02
hCoV-19/Japan/PG-92436/2021	EPI_ISL_3903225	2021-09-02
hCoV-19/Japan/PG-92437/2021	EPI_ISL_3903228	2021-09-02
hCoV-19/Japan/PG-92438/2021	EPI_ISL_3903232	2021-09-02
hCoV-19/Japan/PG-92439/2021	EPI_ISL_3903241	2021-09-02
hCoV-19/Japan/PG-92440/2021	EPI_ISL_3903244	2021-09-02
hCoV-19/Japan/PG-92441/2021	EPI_ISL_3903248	2021-09-02
hCoV-19/Japan/PG-92442/2021	EPI_ISL_3903251	2021-09-02
hCoV-19/Japan/PG-92443/2021	EPI_ISL_3903255	2021-09-02
hCoV-19/Japan/PG-92444/2021	EPI_ISL_3903259	2021-09-02
hCoV-19/Japan/PG-92445/2021	EPI_ISL_3903262	2021-09-02
hCoV-19/Japan/PG-92446/2021	EPI_ISL_3903265	2021-09-02
hCoV-19/Japan/PG-92447/2021	EPI_ISL_3903268	2021-09-02
hCoV-19/Japan/PG-92448/2021	EPI_ISL_3903272	2021-09-02
hCoV-19/Japan/PG-92449/2021	EPI_ISL_3903275	2021-09-02
hCoV-19/Japan/PG-92450/2021	EPI_ISL_3903278	2021-09-02
hCoV-19/Japan/PG-92451/2021	EPI_ISL_3903281	2021-09-02
hCoV-19/Japan/PG-92452/2021	EPI_ISL_3903284	2021-09-02
hCoV-19/Japan/PG-92453/2021	EPI_ISL_3903288	2021-09-02
hCoV-19/Japan/PG-92454/2021	EPI_ISL_3903291	2021-09-02
hCoV-19/Japan/PG-92455/2021	EPI_ISL_3903294	2021-09-02
hCoV-19/Japan/PG-92456/2021	EPI_ISL_3903297	2021-09-02
hCoV-19/Japan/PG-92457/2021	EPI_ISL_3903301	2021-09-02
hCoV-19/Japan/PG-92458/2021	EPI_ISL_3903304	2021-09-02
hCoV-19/Japan/PG-92459/2021	EPI_ISL_3903308	2021-09-02
hCoV-19/Japan/PG-92460/2021	EPI_ISL_3903311	2021-09-02
hCoV-19/Japan/PG-92461/2021	EPI_ISL_3903314	2021-09-02
hCoV-19/Japan/PG-92462/2021	EPI_ISL_3903318	2021-09-02
hCoV-19/Japan/PG-92463/2021	EPI_ISL_3903321	2021-09-02
hCoV-19/Japan/PG-92464/2021	EPI_ISL_3903324	2021-09-02
hCoV-19/Japan/PG-92465/2021	EPI_ISL_3903327	2021-09-02
hCoV-19/Japan/PG-92466/2021	EPI_ISL_3903330	2021-09-02
hCoV-19/Japan/PG-92467/2021	EPI_ISL_3903334	2021-09-02
hCoV-19/Japan/PG-92468/2021	EPI_ISL_3903337	2021-09-02
hCoV-19/Japan/PG-92469/2021	EPI_ISL_3903338	2021-09-02
hCoV-19/Japan/PG-92470/2021	EPI_ISL_3903339	2021-09-02

virus_name	epiID	submission_date
hCoV-19/Japan/PG-92471/2021	EPI_ISL_3903341	2021-09-02
hCoV-19/Japan/PG-92472/2021	EPI_ISL_3903342	2021-09-02
hCoV-19/Japan/PG-92473/2021	EPI_ISL_3903343	2021-09-02
hCoV-19/Japan/PG-92474/2021	EPI_ISL_3903345	2021-09-02
hCoV-19/Japan/PG-92475/2021	EPI_ISL_3903346	2021-09-02
hCoV-19/Japan/PG-92476/2021	EPI_ISL_3903347	2021-09-02
hCoV-19/Japan/PG-92477/2021	EPI_ISL_3903349	2021-09-02
hCoV-19/Japan/PG-92478/2021	EPI_ISL_3903350	2021-09-02
hCoV-19/Japan/PG-92479/2021	EPI_ISL_3903351	2021-09-02
hCoV-19/Japan/PG-92480/2021	EPI_ISL_3903352	2021-09-02
hCoV-19/Japan/PG-92481/2021	EPI_ISL_3903354	2021-09-02
hCoV-19/Japan/PG-92482/2021	EPI_ISL_3903355	2021-09-02
hCoV-19/Japan/PG-92483/2021	EPI_ISL_3903356	2021-09-02
hCoV-19/Japan/PG-92484/2021	EPI_ISL_3903358	2021-09-02
hCoV-19/Japan/PG-92485/2021	EPI_ISL_3903359	2021-09-02
hCoV-19/Japan/PG-92486/2021	EPI_ISL_3903360	2021-09-02
hCoV-19/Japan/PG-92487/2021	EPI_ISL_3903362	2021-09-02
hCoV-19/Japan/PG-92488/2021	EPI_ISL_3903363	2021-09-02
hCoV-19/Japan/PG-92489/2021	EPI_ISL_3903364	2021-09-02
hCoV-19/Japan/PG-92490/2021	EPI_ISL_3903366	2021-09-02
hCoV-19/Japan/PG-92491/2021	EPI_ISL_3903367	2021-09-02
hCoV-19/Japan/PG-92492/2021	EPI_ISL_3903368	2021-09-02
hCoV-19/Japan/PG-92493/2021	EPI_ISL_3903369	2021-09-02
hCoV-19/Japan/PG-92494/2021	EPI_ISL_3903371	2021-09-02
hCoV-19/Japan/PG-92495/2021	EPI_ISL_3903372	2021-09-02
hCoV-19/Japan/PG-92496/2021	EPI_ISL_3903373	2021-09-02
hCoV-19/Japan/PG-92505/2021	EPI_ISL_3870860	2021-09-02
hCoV-19/Japan/PG-92506/2021	EPI_ISL_3870861	2021-09-02
hCoV-19/Japan/PG-92507/2021	EPI_ISL_3870862	2021-09-02
hCoV-19/Japan/PG-92508/2021	EPI_ISL_3870863	2021-09-02
hCoV-19/Japan/PG-92509/2021	EPI_ISL_3870864	2021-09-02
hCoV-19/Japan/PG-92510/2021	EPI_ISL_3870865	2021-09-02
hCoV-19/Japan/PG-92511/2021	EPI_ISL_3870866	2021-09-02
hCoV-19/Japan/PG-92512/2021	EPI_ISL_3870867	2021-09-02
hCoV-19/Japan/PG-92513/2021	EPI_ISL_3870868	2021-09-02
hCoV-19/Japan/PG-92514/2021	EPI_ISL_3870869	2021-09-02
hCoV-19/Japan/PG-92515/2021	EPI_ISL_3870870	2021-09-02
hCoV-19/Japan/PG-92516/2021	EPI_ISL_3870871	2021-09-02
hCoV-19/Japan/PG-92517/2021	EPI_ISL_3870872	2021-09-02
hCoV-19/Japan/PG-92518/2021	EPI_ISL_3870873	2021-09-02
hCoV-19/Japan/PG-92519/2021	EPI_ISL_3870874	2021-09-02
hCoV-19/Japan/PG-92520/2021	EPI_ISL_3870875	2021-09-02
hCoV-19/Japan/PG-92521/2021	EPI_ISL_3870876	2021-09-02
hCoV-19/Japan/PG-92522/2021	EPI_ISL_3870877	2021-09-02
hCoV-19/Japan/PG-92523/2021	EPI_ISL_3870878	2021-09-02
hCoV-19/Japan/PG-92524/2021	EPI_ISL_3870879	2021-09-02
hCoV-19/Japan/PG-92525/2021	EPI_ISL_3870880	2021-09-02
hCoV-19/Japan/PG-92526/2021	EPI_ISL_3870881	2021-09-02
hCoV-19/Japan/PG-92527/2021	EPI_ISL_3870882	2021-09-02
hCoV-19/Japan/PG-92528/2021	EPI_ISL_3870883	2021-09-02
hCoV-19/Japan/PG-92529/2021	EPI_ISL_3870884	2021-09-02
hCoV-19/Japan/PG-92530/2021	EPI_ISL_3870885	2021-09-02
hCoV-19/Japan/PG-92531/2021	EPI_ISL_3870886	2021-09-02
hCoV-19/Japan/PG-92532/2021	EPI_ISL_3870887	2021-09-02

virus_name	epiID	submission_date
hCoV-19/Japan/PG-92533/2021	EPI_ISL_3870888	2021-09-02
hCoV-19/Japan/PG-92534/2021	EPI_ISL_3870889	2021-09-02
hCoV-19/Japan/PG-92535/2021	EPI_ISL_3870890	2021-09-02
hCoV-19/Japan/PG-92536/2021	EPI_ISL_3870891	2021-09-02
hCoV-19/Japan/PG-92537/2021	EPI_ISL_3870892	2021-09-02
hCoV-19/Japan/PG-92538/2021	EPI_ISL_3870893	2021-09-02
hCoV-19/Japan/PG-92539/2021	EPI_ISL_3870894	2021-09-02
hCoV-19/Japan/PG-92540/2021	EPI_ISL_3870895	2021-09-02
hCoV-19/Japan/PG-92541/2021	EPI_ISL_3870896	2021-09-02
hCoV-19/Japan/PG-92542/2021	EPI_ISL_3870897	2021-09-02
hCoV-19/Japan/PG-92543/2021	EPI_ISL_3870898	2021-09-02
hCoV-19/Japan/PG-92544/2021	EPI_ISL_3870899	2021-09-02
hCoV-19/Japan/PG-92545/2021	EPI_ISL_3870900	2021-09-02
hCoV-19/Japan/PG-92546/2021	EPI_ISL_3870901	2021-09-02
hCoV-19/Japan/PG-92547/2021	EPI_ISL_3870902	2021-09-02
hCoV-19/Japan/PG-92548/2021	EPI_ISL_3870903	2021-09-02
hCoV-19/Japan/PG-92549/2021	EPI_ISL_3870904	2021-09-02
hCoV-19/Japan/PG-92550/2021	EPI_ISL_3870905	2021-09-02
hCoV-19/Japan/PG-92551/2021	EPI_ISL_3870906	2021-09-02
hCoV-19/Japan/PG-92552/2021	EPI_ISL_3870907	2021-09-02
hCoV-19/Japan/PG-92553/2021	EPI_ISL_3870908	2021-09-02
hCoV-19/Japan/PG-92554/2021	EPI_ISL_3870909	2021-09-02
hCoV-19/Japan/PG-92555/2021	EPI_ISL_3870910	2021-09-02
hCoV-19/Japan/PG-92556/2021	EPI_ISL_3870911	2021-09-02
hCoV-19/Japan/PG-92557/2021	EPI_ISL_3870912	2021-09-02
hCoV-19/Japan/PG-92558/2021	EPI_ISL_3870913	2021-09-02
hCoV-19/Japan/PG-92559/2021	EPI_ISL_3870914	2021-09-02
hCoV-19/Japan/PG-92560/2021	EPI_ISL_3870915	2021-09-02
hCoV-19/Japan/PG-92561/2021	EPI_ISL_3870916	2021-09-02
hCoV-19/Japan/PG-92562/2021	EPI_ISL_3870917	2021-09-02
hCoV-19/Japan/PG-92563/2021	EPI_ISL_3870918	2021-09-02
hCoV-19/Japan/PG-92564/2021	EPI_ISL_3870919	2021-09-02
hCoV-19/Japan/PG-92565/2021	EPI_ISL_3870920	2021-09-02
hCoV-19/Japan/PG-92566/2021	EPI_ISL_3870921	2021-09-02
hCoV-19/Japan/PG-92567/2021	EPI_ISL_3870922	2021-09-02
hCoV-19/Japan/PG-92568/2021	EPI_ISL_3870923	2021-09-02
hCoV-19/Japan/PG-92569/2021	EPI_ISL_3870924	2021-09-02
hCoV-19/Japan/PG-92570/2021	EPI_ISL_3870925	2021-09-02
hCoV-19/Japan/PG-92571/2021	EPI_ISL_3870926	2021-09-02
hCoV-19/Japan/PG-92572/2021	EPI_ISL_3870927	2021-09-02
hCoV-19/Japan/PG-92573/2021	EPI_ISL_3870928	2021-09-02
hCoV-19/Japan/PG-92574/2021	EPI_ISL_3870929	2021-09-02
hCoV-19/Japan/PG-92575/2021	EPI_ISL_3870930	2021-09-02
hCoV-19/Japan/PG-92576/2021	EPI_ISL_3870931	2021-09-02
hCoV-19/Japan/PG-92577/2021	EPI_ISL_3870932	2021-09-02
hCoV-19/Japan/PG-92578/2021	EPI_ISL_3870933	2021-09-02
hCoV-19/Japan/PG-92579/2021	EPI_ISL_3870934	2021-09-02
hCoV-19/Japan/PG-92580/2021	EPI_ISL_3870935	2021-09-02
hCoV-19/Japan/PG-92581/2021	EPI_ISL_3870936	2021-09-02
hCoV-19/Japan/PG-92582/2021	EPI_ISL_3870937	2021-09-02
hCoV-19/Japan/PG-92583/2021	EPI_ISL_3870938	2021-09-02
hCoV-19/Japan/PG-92584/2021	EPI_ISL_3870939	2021-09-02
hCoV-19/Japan/PG-92585/2021	EPI_ISL_3870940	2021-09-02
hCoV-19/Japan/PG-92586/2021	EPI_ISL_3870941	2021-09-02

virus_name	epiID	submission_date
hCoV-19/Japan/PG-92587/2021	EPI_ISL_3870942	2021-09-02
hCoV-19/Japan/PG-92588/2021	EPI_ISL_3870943	2021-09-02
hCoV-19/Japan/PG-92589/2021	EPI_ISL_3870944	2021-09-02
hCoV-19/Japan/PG-92590/2021	EPI_ISL_3870945	2021-09-02
hCoV-19/Japan/PG-92591/2021	EPI_ISL_3870946	2021-09-02
hCoV-19/Japan/PG-92592/2021	EPI_ISL_3870947	2021-09-02
hCoV-19/Japan/PG-92593/2021	EPI_ISL_3870948	2021-09-02
hCoV-19/Japan/PG-92594/2021	EPI_ISL_3870949	2021-09-02
hCoV-19/Japan/PG-92595/2021	EPI_ISL_3870950	2021-09-02
hCoV-19/Japan/PG-92596/2021	EPI_ISL_3870951	2021-09-02
hCoV-19/Japan/PG-92597/2021	EPI_ISL_3870952	2021-09-02
hCoV-19/Japan/PG-92598/2021	EPI_ISL_3870953	2021-09-02
hCoV-19/Japan/PG-92706/2021	EPI_ISL_3874462	2021-09-02
hCoV-19/Japan/PG-92707/2021	EPI_ISL_3874463	2021-09-02
hCoV-19/Japan/PG-92708/2021	EPI_ISL_3874464	2021-09-02
hCoV-19/Japan/PG-92709/2021	EPI_ISL_3874465	2021-09-02
hCoV-19/Japan/PG-92710/2021	EPI_ISL_3874466	2021-09-02
hCoV-19/Japan/PG-92711/2021	EPI_ISL_3874467	2021-09-02
hCoV-19/Japan/PG-92712/2021	EPI_ISL_3874468	2021-09-02
hCoV-19/Japan/PG-92713/2021	EPI_ISL_3874469	2021-09-02
hCoV-19/Japan/PG-92714/2021	EPI_ISL_3874470	2021-09-02
hCoV-19/Japan/PG-92715/2021	EPI_ISL_3870995	2021-09-02
hCoV-19/Japan/PG-92716/2021	EPI_ISL_3870996	2021-09-02
hCoV-19/Japan/PG-92717/2021	EPI_ISL_3870997	2021-09-02
hCoV-19/Japan/PG-92718/2021	EPI_ISL_3870998	2021-09-02
hCoV-19/Japan/PG-92719/2021	EPI_ISL_3870999	2021-09-02
hCoV-19/Japan/PG-92720/2021	EPI_ISL_3871000	2021-09-02
hCoV-19/Japan/PG-92721/2021	EPI_ISL_3871001	2021-09-02
hCoV-19/Japan/PG-92722/2021	EPI_ISL_3871002	2021-09-02
hCoV-19/Japan/PG-92723/2021	EPI_ISL_3871003	2021-09-02
hCoV-19/Japan/PG-92724/2021	EPI_ISL_3871004	2021-09-02
hCoV-19/Japan/PG-92725/2021	EPI_ISL_3871005	2021-09-02
hCoV-19/Japan/TKYK26491/2021	EPI_ISL_3907913	2021-09-02
hCoV-19/Japan/TKYK26496/2021	EPI_ISL_3907914	2021-09-02
hCoV-19/Japan/TKYK26530/2021	EPI_ISL_3907915	2021-09-02
hCoV-19/Japan/TKYK26635/2021	EPI_ISL_3907916	2021-09-02
hCoV-19/Japan/TKYK26816/2021	EPI_ISL_3907917	2021-09-02
hCoV-19/Japan/TKYK26869/2021	EPI_ISL_3907918	2021-09-02
hCoV-19/Japan/TKYK26935/2021	EPI_ISL_3907919	2021-09-02
hCoV-19/Japan/TKYK27003/2021	EPI_ISL_3907925	2021-09-02
hCoV-19/Japan/TKYK27011/2021	EPI_ISL_3907926	2021-09-02
hCoV-19/Japan/TKYK27013/2021	EPI_ISL_3907927	2021-09-02
hCoV-19/Japan/TKYK27382/2021	EPI_ISL_3907928	2021-09-02
hCoV-19/Japan/TKYK27393/2021	EPI_ISL_3907929	2021-09-02
hCoV-19/Japan/TKYK27738/2021	EPI_ISL_3907930	2021-09-02
hCoV-19/Japan/TKYK27762/2021	EPI_ISL_3907931	2021-09-02
hCoV-19/Japan/TKYK28006/2021	EPI_ISL_3907932	2021-09-02
hCoV-19/Japan/TKYS00490/2021	EPI_ISL_3907920	2021-09-02
hCoV-19/Japan/TKYS00493/2021	EPI_ISL_3907921	2021-09-02
hCoV-19/Japan/TKYS00521/2021	EPI_ISL_3907922	2021-09-02
hCoV-19/Japan/TKYS00524/2021	EPI_ISL_3907923	2021-09-02
hCoV-19/Japan/TKYS00534/2021	EPI_ISL_3907924	2021-09-02
hCoV-19/Japan/YCH0639/2021	EPI_ISL_3909431	2021-09-02
hCoV-19/Japan/YCH0640/2021	EPI_ISL_3909432	2021-09-02

virus_name	epiID	submission_date
hCoV-19/Japan/YCH0641/2021	EPI_ISL_3909433	2021-09-02
hCoV-19/Japan/YCH0642/2021	EPI_ISL_3909434	2021-09-02
hCoV-19/Japan/YCH0643/2021	EPI_ISL_3909435	2021-09-02
hCoV-19/Japan/YCH0644/2021	EPI_ISL_3909436	2021-09-02
hCoV-19/Japan/YCH0645/2021	EPI_ISL_3909437	2021-09-02
hCoV-19/Japan/YCH0646/2021	EPI_ISL_3909438	2021-09-02
hCoV-19/Japan/YCH0647/2021	EPI_ISL_3909439	2021-09-02
hCoV-19/Japan/YCH0648/2021	EPI_ISL_3909440	2021-09-02
hCoV-19/Japan/YCH0649/2021	EPI_ISL_3909441	2021-09-02
hCoV-19/Japan/YCH0650/2021	EPI_ISL_3909442	2021-09-02
hCoV-19/Japan/YCH0651/2021	EPI_ISL_3909443	2021-09-02
hCoV-19/Japan/YCH0652/2021	EPI_ISL_3909444	2021-09-02
hCoV-19/Japan/YCH0653/2021	EPI_ISL_3909445	2021-09-02
hCoV-19/Japan/YCH0654/2021	EPI_ISL_3909446	2021-09-02
